# Variation in selection constraints on teleost TLRs with emphasis on their repertoire in the Walking catfish, *Clarias batrachus*

**DOI:** 10.1038/s41598-020-78347-6

**Published:** 2020-12-07

**Authors:** Manisha Priyam, Sanjay K. Gupta, Biplab Sarkar, T. R. Sharma, A. Pattanayak

**Affiliations:** School of Molecular Diagnostics and Prophylactics, ICAR-Indian Institute of Agricultural Biotechnology, Ranchi, Jharkhand 834 010 India

**Keywords:** Innate immunity, Toll-like receptors, Phylogenetics

## Abstract

The high degree of conservation of toll-like receptors (TLRs), and yet their subtle variations for better adaptation of species in the host–pathogen arms race make them worthy candidates for understanding evolution. We have attempted to track the trend of TLR evolution in the most diverse vertebrate group—teleosts, where *Clarias batrachus * was given emphasis, considering its traits for terrestrial adaptation. Eleven *C. batrachus* TLRs (TLR1, 2, 3, 5, 7, 8 9, 13, 22, 25, 26) were identified in this study which clustered in proximity to its *Siluriformes* relative orthologues in the phylogenetic analysis of 228 TLRs from 25 teleosts. Ten TLRs (TLR1, 2, 3, 5, 7, 8 9, 13, 21, 22) with at least 15 member orthologues for each alignment were processed for selection pressure and coevolutionary analysis. TLR1, 7, 8 and 9 were found to be under positive selection in the alignment-wide test. TLR1 also showed maximum episodic diversification in its clades while the teleost group *Eupercaria* showed the maximum divergence in their TLR repertoire. Episodic diversification was evident in *C. batrachus* TLR1 and 7 alignments. These results present a strong evidence of a divergent TLR repertoire in teleosts which may be contributing towards species-specific variation in TLR functions.

## Introduction

At the molecular level, the immune system was steadily shaped by the local pathogen pressure that has led to a wide range of variation in immune responses, even within the organisms of the same vertebrate class. The toll-like receptors (TLRs) are such ancient sentinels of innate immunity that bind to pathogen-associated molecular patterns (PAMPs) and danger-associated molecular patterns (DAMPs) to provide protection against pathogenic infections and endogenous damage. They serve as excellent models for gaining an insight into host–pathogen interaction along the evolutionary timeline. TLR is comprised of three domains—an extracellular ligand recognition domain, a transmembrane domain and an intracellular toll/ interleukin-1 receptor (TIR) domain. The extracellular domain of each TLR is constituted of leucine-rich repeat (LRR) motifs which determine the ligand specificity of the TLR. However, despite high degree of structural conservation in the receptors, there are numerous reports on species-specific ligand recognition with respect to TLRs. This observation highlights the significance of extrinsic factors (ecological niche, feeding habits, microbial milieu of its environment, host genetics) that guide the selection constraints on the host receptor for its adaptation in the given environment. Phylogenetic analyses also reflect the evolutionary course of changes in immunity in response to surrounding microorganisms^1^.

Based on the number of cysteine clusters in their ectodomain, the TLRs are divided into multiple-cysteine cluster TLRs (mccTLRs) and single-cysteine cluster TLRs (sccTLRs)^2^. The former seems to have emerged in phylum Cnidaria while the presence of the latter in molluscs and vertebrates suggests that sccTLRs predate the origin of bilaterians. It is hypothesised that these prototype TLRs arose by domain fusion of LRR only and TIR only genes^3^. The evaluation of architecture in vertebrate TLR extracellular domain divided them into three types—three-domain TLRs (TLR family 1 and 4) that recognise proteins and nucleic acids, trans-three-domain TLRs (TLR family 11) for recognising proteins and single-domain TLRs (TLR family 3, 5, 7, 13) for recognition of hydrophobic ligands. Despite being a component of non-specific immunity, it is interesting to note the architectural nuances in the ectodomains of various TLRs that define their precision for ligand recognition^4^. Differential evolution of TLRs has been reported both, within the receptor as well as between the receptors. The TIR domain shows a higher degree of conservation in comparison to the extracellular ligand recognition domain in species from various vertebrate classes^5–8^.

As lower vertebrates, the fish acquired a robust innate immune system during the timeline of immune evolution, however, due to the later origin of adaptive immunity, this response in fishes remains less sophisticated. The teleost lineage is the most diverse among vertebrates in terms of species richness across its phylogenetic clades. Cutting edge advances in genome deep sequencing have been catalytic to generate information on identification of TLRs across multiple teleost species. The TLR family in teleosts consists of more than 16 members with maximum number of non-mammalian TLRs (TLR19, 20, 21, 22, 23, 27) in this class^9^. Despite the considerable similarity in the TLR network in fishes and mammals, their contrasting habitats and taxonomic diversity has resulted in disparity in pathogen diversity and load which has imprinted distinct features in the both the systems. The divergence of TLR genes is said to have occurred both between mammalian and fish systems as well as within the fish lineage^10^. The diversification of TLR family within the teleost lineage is often attributed to fish-specific genome duplications and single gene duplication events^9,11^. Considering the above-said information, it would be apt to suggest that the investigation of the evolutionary course of teleost TLRs would be unique among vertebrate classes. Despite multiple reports on the evolution of this gene family in the vertebrate or mammalian lineage, very few studies have been undertaken to analyse the selection constraints on TLRs within the teleost lineage. Tong et al.’s^12^ work on *Gymnocypris przewalskii* (Tibet fish) hypothesised that TLRs of all the species except TLR4, had undergone purifying selection. *Boleophthalmus pectinirostris* (mudskipper) TLR11 paralogs exhibited purifying selection courtesy of the functional constraints^11^. In a similar study on *Gadus morhua* (Atlantic cod), TLR8, 9, 21, 22 and 25 paralogs were seen to be under diversifying selection. The afore-mentioned reports deduced intra-species analyses and hence do not reflect the selection constraints active on teleost TLRs during evolution.

*Clarias batrachus* is an air-breathing catfish from the Indian sub-continent, belonging to the order *Siluriformes*. Despite its high economic value, it is enlisted as an endangered species on the IUCN red list^13^. Though the immune network of its close relative *Ictalurus punctatus* has been widely explored, there is a void with respect to information on *C. batrachus* immunity^14,15^. With the recent availability of its genome sequence, its adaptations for aerial respiration and high tolerance to hypoxia have been brought into highlight^16^. The exposure of *C. batrachus* to diverse pathogens in both aquatic and terrestrial habitats broadens the scope for finding signatures of selection in its TLR repertoire.

In the present study, the *C. batrachus* TLRs were identified and analysed for phylogenetic proximity to their teleost orthologues. We attempted to assess the selection constraints on 10 teleost TLRs across 25 teleost species by performing alignment-wide and site-based selection tests. Further, the divergent branches in each of the TLR phylogenies were identified followed by prediction of co-evolving clusters in the TLRs to complement the selection pressure analysis. The domain mapping of residues undergoing co-evolution and positive and negative selection was performed on *C. batrachus* TLR1 and 7 to gauge the extent of adaptive evolution in the species.

## Results

In silico identification of eleven *C. batrachus* TLRs (TLR1, 2, 3, 5, 7, 8, 9, 13, 22, 25 and 26) was achieved in this study, using TLR sequences of other *Siluriformes* species as queries in BLAST homology search against its reference genome. *C. batrachus* TLR4 could not be identified using the above approach, suggesting a possible loss of the receptor in the species. The sequence of *C. batrachus* TLR21 was already available on the NCBI database and it was included in further phylogenetic and selection pressure analyses for the selected teleost TLRs.

### Phylogenetic analysis

**Figure 1:**
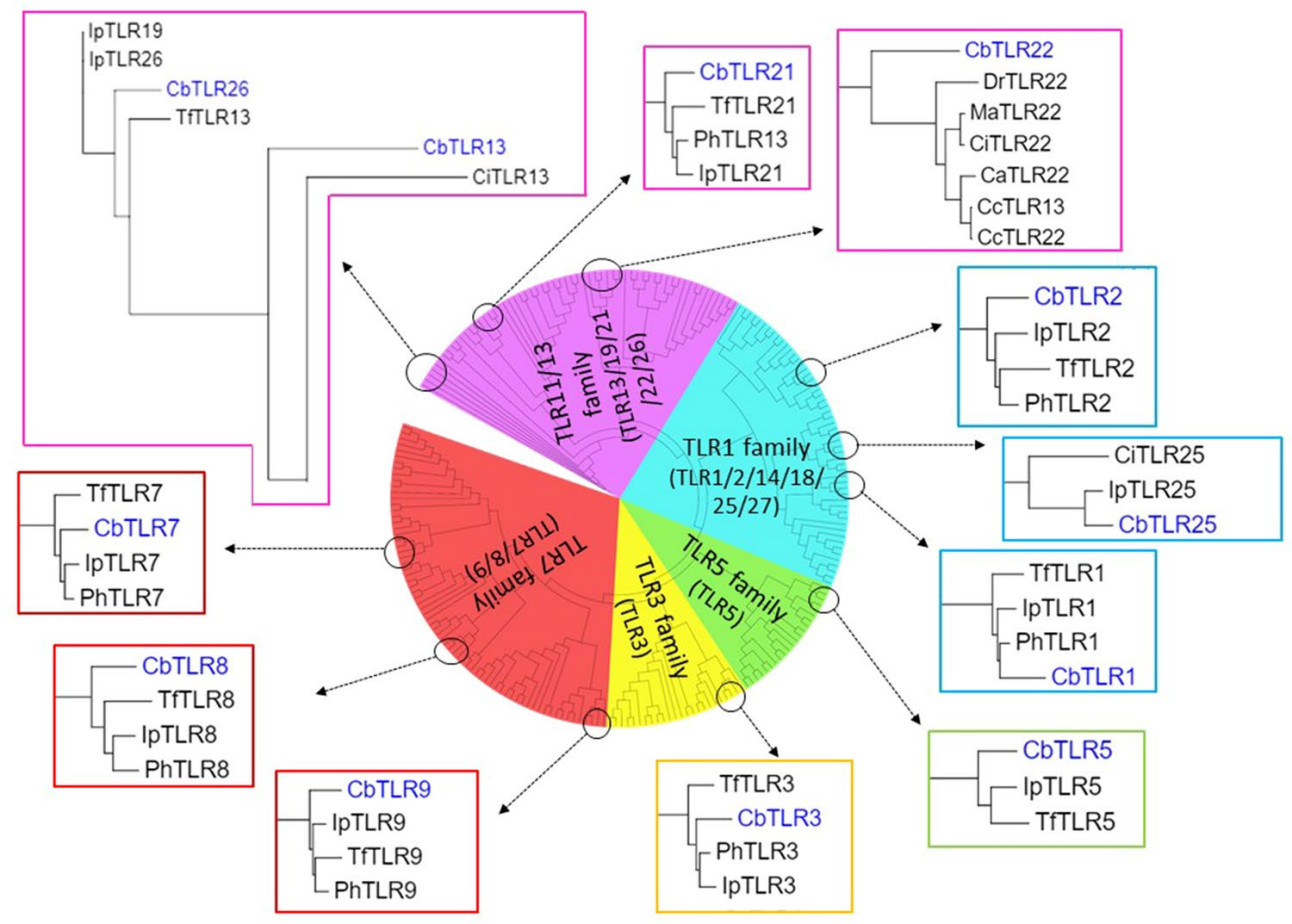
Schematic representation of the radial dendrogram inferred from the phylogenetic analyses of full-length teleost TLR sequences. The dendrogram is comprised of five sectors, each representing the clustering of teleost TLRs from 5 TLR families (**a**) red—TLR7 family with TLR7, 8 and 9 orthologues, (**b**) yellow—TLR3 family with TLR3 orthologues, (**c**) green—TLR5 family with TLR5 orthologues, (**d**) blue—TLR1 family with TLR1, 2, 14, 18, 25 and 27 orthologues and (**e**) pink—TLR11/13 family with TLR13, 19, 21, 22, 26 orthologues. Since *C. batrachus* TLR4 could not be detected in this study, this family was excluded from the analysis. The black circles on the dendrogram have been used to magnify the clades consisting of *C. batrachus* TLR in each sector. The phylogenetic positioning of all *C. batrachus* TLRs is seen to be proximal to its *Siluriformes* counterparts (*I. punctatus, T. fulvidraco, P. hypophthalamus*), except for *C. batrachus* TLR13 and 22, which are to cluster with their respective orthologues from *C. idella* and *D.rerio*. The full-length tree for this analysis with bootstrap values can be found in Supplementary Data 27. The species are denoted with two letters comprised of the starting alphabets of the genus and species names (the abbreviation key can be found in Supplementary data 1).

Phylogenetic analysis of teleost TLRs in this study showed distinct clustering of TLR orthologues from various species across the five TLR families (TLR1, 3, 5, 7 and 13 families) (Fig. 1). Since *C. batrachus TLR*4 was not identified in the study, this family was not included in any of the analyses. All *C. batrachus* TLRs clustered with their respective *I. punctatus, P. hypophthalmus and T. fulvidraco* orthologues. The fish-specific *C. batrachus* TLR25 and *C. batrachus* TLR26 clustered within the clades of TLR1 and 13 families. The clustering pattern of clades within the TLR1 family showed a higher proximity of fish-specific TLRs (TLR 14, 18, 25 and 27) to TLR1 than TLR2. The TLR orthologues from the species of the orders *Cypriniformes* (*D. rerio, C. carpio, C. auratus, C. idella, M. amblycephala*) and *Salmoniformes* (*S. salar, S. trutta, O.* mykiss) clustered in their separate clades. The clade of *Cypriniformes* TLRs also shared a common node of origin with the *Siluriformes* TLR orthologues throughout the tree with strong support of bootstrap values. The trees inferred using both maximum likelihood and neighbour joining methods showed congruency of phylogenetic inference (Supplementary data 27).

### Selection pressure analysis

Alignment-wide selection test (PARRIS) for the 10 TLRs predicted an evidence of positive selection for TLR1, 7, 8 and 9 codon alignments at p < 0.05. Further, BUSTED, which allows the variation of ω along the branches, was employed to detect the gene-wide evidence of positive selection acting on a subset of sites in a subset of branches of a phylogeny. Interestingly, BUSTED detected an evidence of episodic diversifying selection for all the 10 TLRs in this study (Supporting data 25).

**Figure 2:**
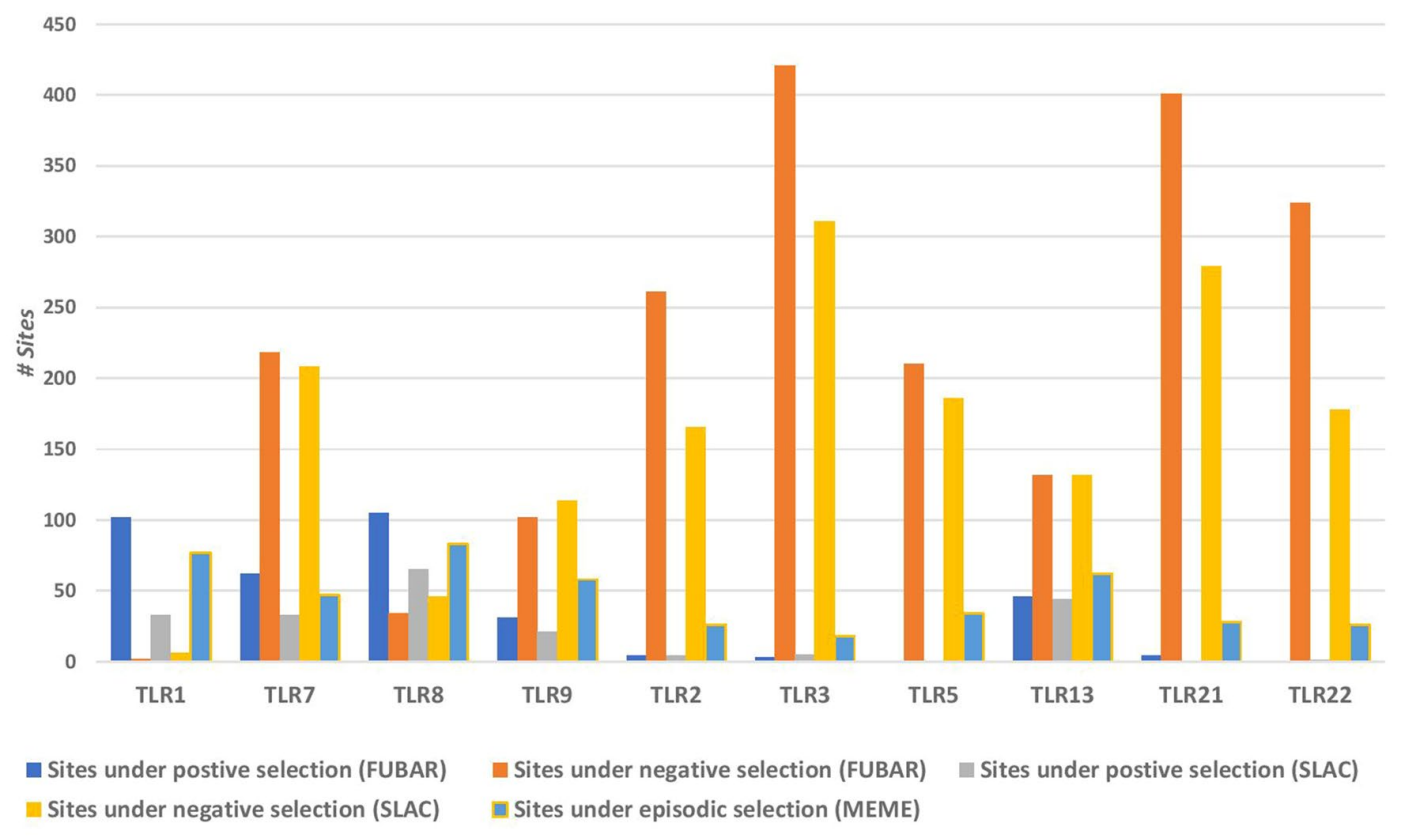
Bar graph representing the number of residues experiencing negative and positive selection for each of the selected TLR lineages. FUBAR and SLAC have been used to detect the sites under negative selection and pervasive positive selection and MEME has been used to detect the sites under positive episodic selection. TLR1, 7, 8 and 9 show a higher number of positively selected sites as compared to the negatively selected ones, while a vice-versa trend is seen for TLR2, 3, 5, 13, 21 and 22.

The site-based selection tests used in this study were SLAC, FUBAR and MEME (Fig. 2). While SLAC enhances the stringency of the test by integration of maximum likelihood (ML) and counting approaches to derive the rates of synonymous (dS) and non-synonymous (dN) substitution at a site, FUBAR enhances the power of detection by employing Bayesian algorithm for the deduction of the dN and dS of sites in a codon alignment^17^. MEME, on the other hand, detects the sites undergoing episodic positive selection by using mixed effects ML test^18^. The graph in Fig. 2 shows the distribution of positively and negatively selected sites for the teleost TLRs being studied. It depicts the contrast in the proportion of positively selected sites for TLRs 1, 7, 8 and 9 (with evidence of alignment-wide positive selection—deduced by PARRIS) versus TLR2, 3, 5, 13, 21 and 22 (with no evidence of alignment-wide positive selection—deduced by PARRIS). While the former category showed a higher proportion of positively selected sites than the negatively selected sites, the latter category showed a *vice-versa* trend. For enhancing the confidence intervals of the deduced sites undergoing selection, only those sites were considered significant that were deduced by at least two approaches. Table 1 enlists the number of positively and negatively selected sites deduced to be significant after screening.

**Table 1:** The TLRs in the study have been divided into two parts—(A) and (B) in the table, based on the results of alignment-wide selection analysis via PARRIS.

Site-specific selection→	No. of sites under positive selection	No. of sites under purifying selection
**(A) TLRs showing an evidence of alignment-wide positive selection**		
TLR1	53	1
TLR7	44	198
TLR8	73	28
TLR9	20	96
**(B) TLRs showing no evidence of alignment-wide positive selection**		
TLR2	1	158
TLR3	1	311
TLR5	0	176
TLR13	45	125
TLR21	3	270
TLR22	1	79

The site-based selection methods (SLAC, FUBAR, MEME) have been used to deduce the sites under positive and purifying selection for each of the TLRs. To maintain the stringency of parameters, the sites in the table have been deduced only if they were detected via two or more analysis methods.

**Figure 3:**
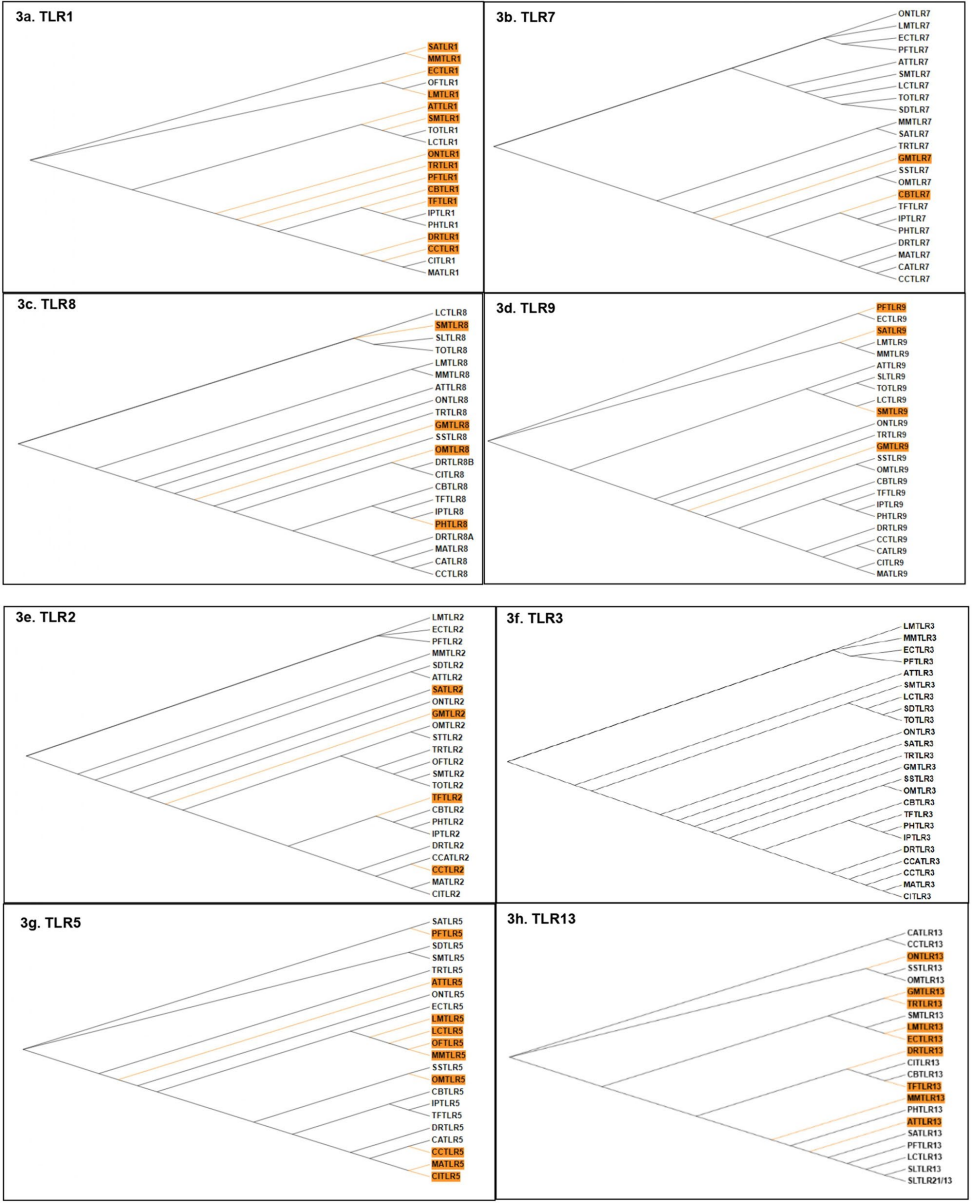

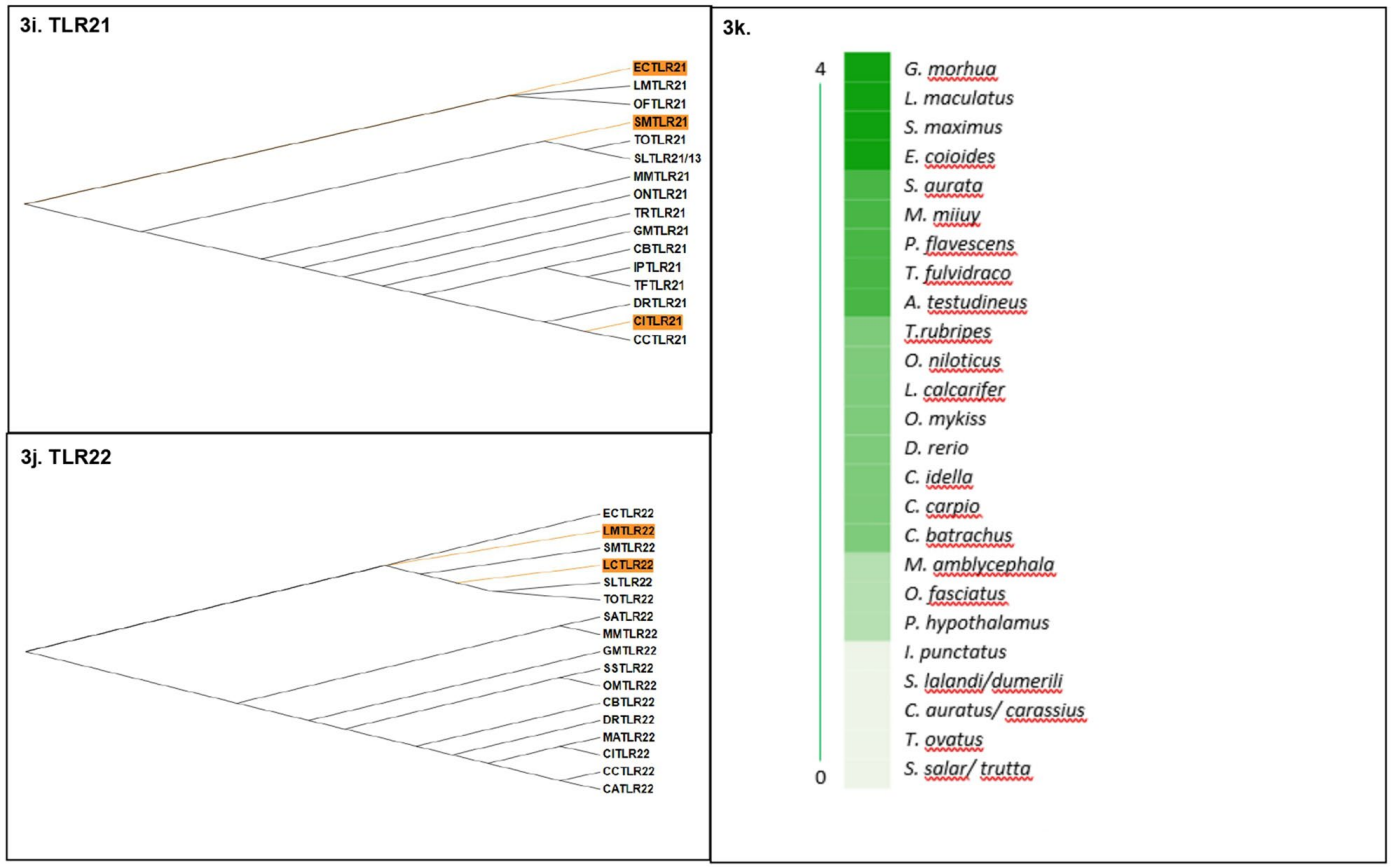
(**a**–**k**) Detection of the divergent branches (highlighted in orange) on selected TLR1 (**a**), TLR2 (**b**), TLR3 (**c**), TLR5 (**d**), TLR7 (**e**), TLR8 (**f**), TLR9 (**g**), TLR13 (**h**), TLR21 (**i**), TLR22 (**j**) lineages via aBSREL. The clades showing significant divergence for TLR phylogeny are highlighted in orange (p < 0.05). The species are denoted with two letters comprised of the starting alphabets of the genus and species names. (The abbreviation key can be found in Supplementary data 1) TLR1, 5 and 13 have the maximum number of divergent nodes while TLR3 shows none of its clades undergoing divergence. (**k**) Scale of divergence shows *G. morhua, L. maculatus, S. maximus* and *E. coioides* to bear the most divergent profile of TLRs, among the teleost TLRs analysed.

Based on the evidence of gene-wide episodic divergence deduced by BUSTED for all TLRs, the branches of TLR phylogenies undergoing episodic diversification were predicted by aBSREL (Fig. 3). The maximum number of nodes undergoing significant diversification were identified for TLR1 phylogeny (Fig. 3a), followed by TLR5 (Fig. 3g) and 13 (Fig. 3h). In contrast, TLR3 showed no evidence of episodic diversification in any of its leaf nodes (Fig. 3f). The analysis of nodes under episodic selection deduced by aBSREL identified *G. morhua*, *L. maculatus, S. maximus* and *E. coioides* as the species with the most divergent TLR repertoire while *S. salar, T. ovatus, C. auratus, S. lalandi and I. punctatus* TLR orthologues were the least divergent (Fig. 3k). With *C. batrachus* TLR1 and 7 predicted as diversifying nodes in their respective phylogenies, *C. batrachus* was in mid-way of this divergence scale.

### Comparative domain analysis

**Figure 4:**
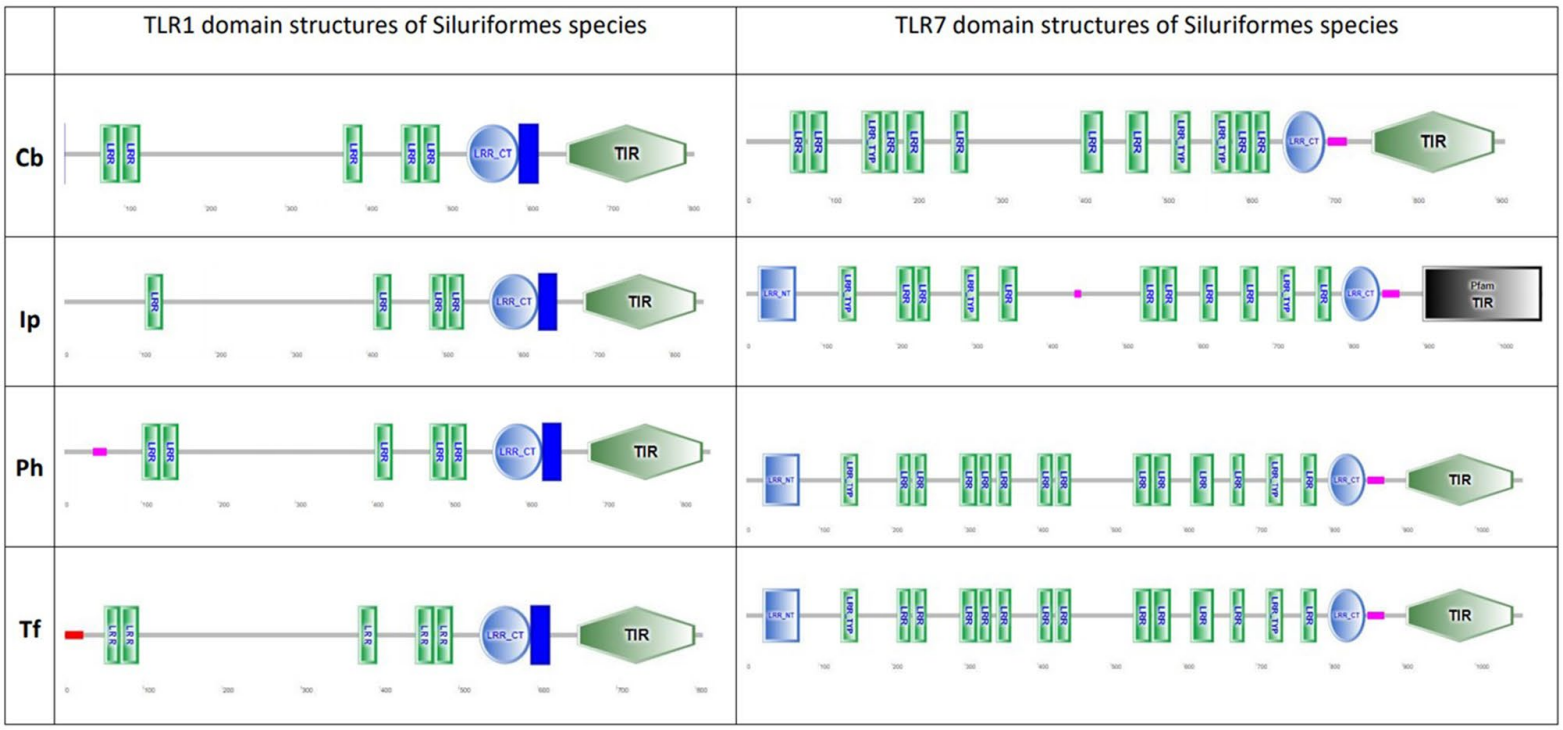
Comparative domain analysis of TLR1 and 7 of *Siluriformes* species (*Clarias batrachus (Cb), Ictalurus punctatus (Ip), Tachysurus fulvidraco (Tf), Pangasianodon hypophthalmus (Ph))*. (Domains predicted using SMART). The four domains detected in this analysis are (**a**) LRR—Leucine-rich repeats (**b**) LRR-CT—LRR-C-terminal (**c**) LRR-N-terminal and (**d**) TIR—Toll/interleukin-1 receptor. The comparative analysis shows a high degree of similarity in the organization of TLR1 and 7 domains of *C. batrachus* and its *Siluriformes* relatives.

Based on the results from aBSREL, domain prediction was carried out for *C. batrachus TLR* 1 and 7 and their orthologues from *Siluriformes* members *I. punctatus, P. hypophthalamus and T. fulvidraco* (Fig. 4). Comparative domain analysis showed a slight variation in the number of LRRs in the extracellular domain of the TLR 1 and 7 orthologues with a high degree of conservation in the TIR domain. *P. hypophthalamus and T. fulvidraco* orthologues showed a highly similar LRR domain distribution with 5 and 14 LRRs in TLR1 and 7, respectively, while *I. punctatus* orthologues showed a lower number of LRRs for both TLRs.

### Co-evolution analysis

**Figure 5:**
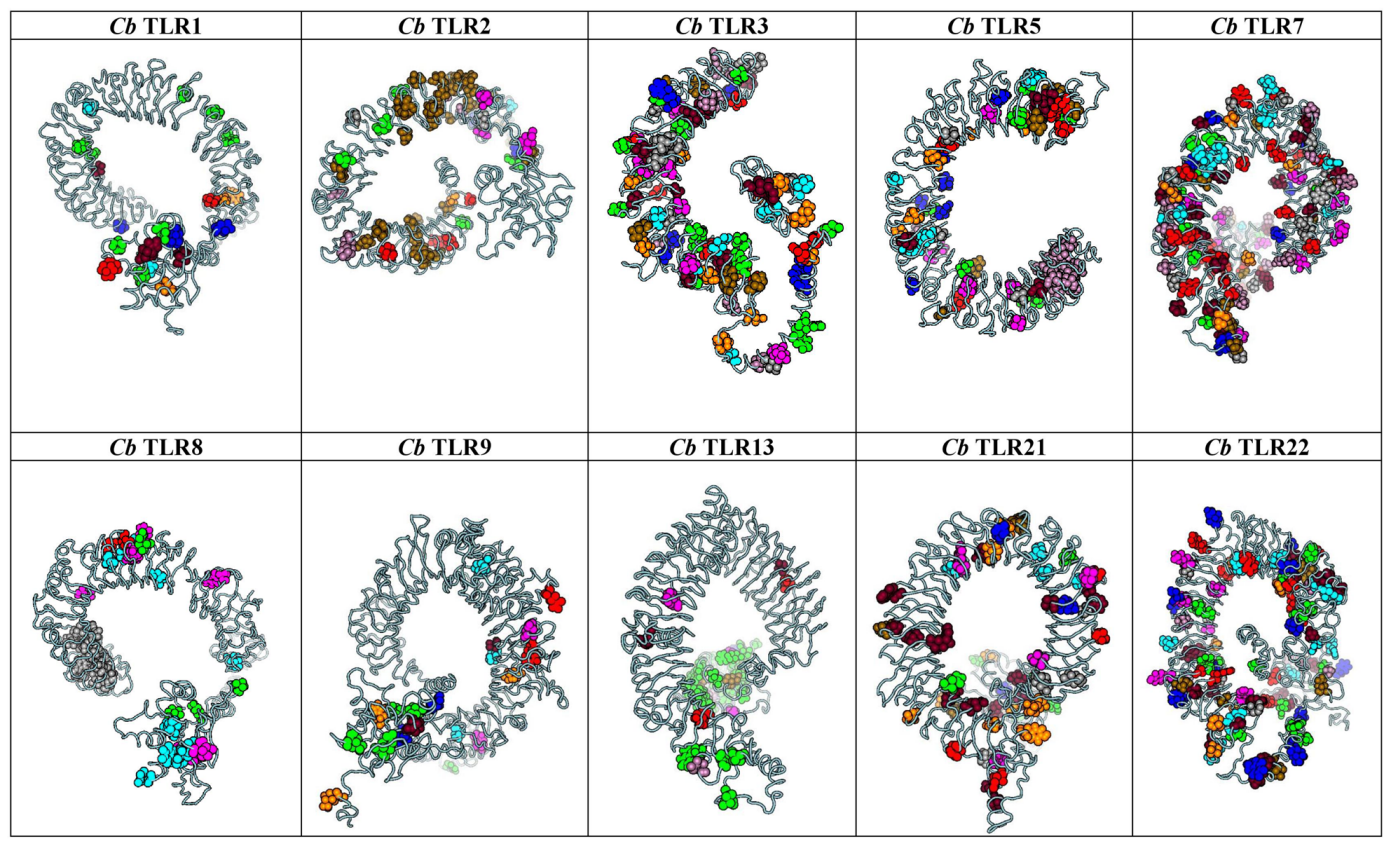
Mapping of predicted co-evolved clusters (by BIS2 analyzer) to the 3D structures of *Clarias batrachus (Cb) TLRs* (predicted by I-TASSER). A cluster of residues under co-evolution in each TLR are denoted in the same color. Maximum number of co-evolving clusters are seen in *C. batrachus* TLR 3 and 5, while *C. batrachus* TLR1 and 8 have the lowest number of these clusters.

BIS2Analyser predicted the maximum number of co-evolving clusters in TLR3 and 5 while the number of clusters was the lowest for TLR1 and 8 at p < 0.05 (Supporting Data 25). Despite both teleost TLR1 and 7 being under positive selection, there is a noticeable difference in their number of co-evolved clusters. This is evident in their mapping on the I-TASSER predicted structures of *C. batrachus* TLR1 and 7 (Fig. 5). The mapping of co-evolved residues and positively and negatively selected sites (from the above results) to the individual domains of *C. batrachus* TLRs showed TLR1 with maximum positively selected sites in its extracellular domain, followed by the members of TLR7 family (Supporting Data 26, Fig. 6). TLR1, 7, 8 and 9 also showed a significant number of sites under positive selection in their TIR domain. Interestingly, the proportion of co-evolved sites and negatively selected sites in ECD of *C. batrachus* TLR2, 3, 5 and 7 was similar. The mapping of the residues under positive selection and coevolution to *C. batrachus TLR*1 and 7 protein sequences depicted their localisation within the functional domains (LRR and TIR) (Fig. 7a,b). An overlap was also seen for 4 residues under both positive selection and coevolution in *C. batrachus* TLR7 (Fig. 7b).

**Figure 6:**
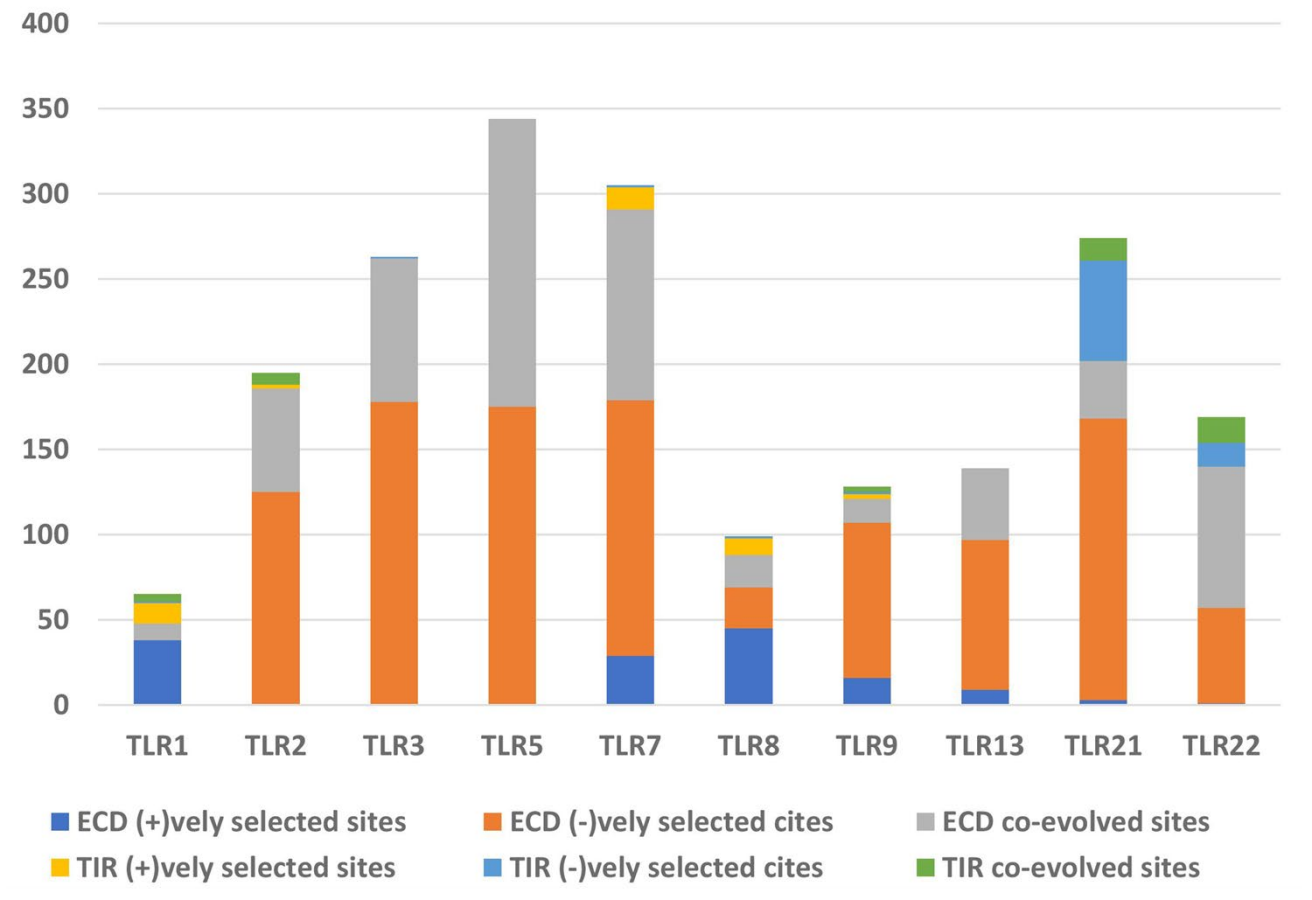
Graph depicting the localization of positively selected, negatively selected and co-evolved residues in the extracellular (ECD) and toll/interleukin-1 (TIR) domain of *Clarias batrachus* TLRs. The figure shows the localization of a higher number of positively selected sites to the extracellular domain (ECD) of TLR1, 7, 8 and 9, in comparison to their mapping on the Toll/Interleukin-1 receptor (TIR) domain. The ECDs of TLR2, 3, 5, 13, 21 and 22 are dominated by negatively selected and co-evolved residues.

**Figure 7:**
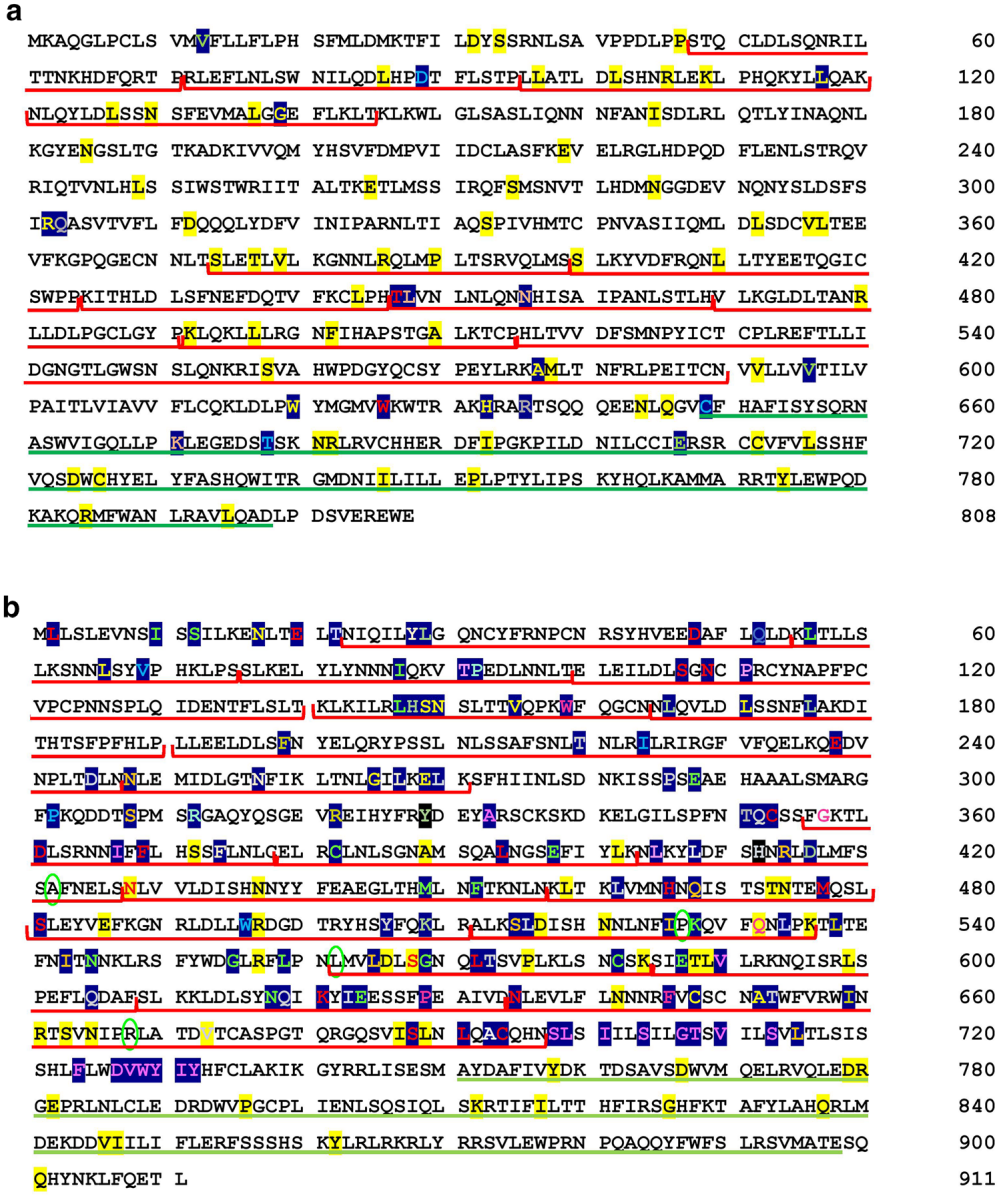
Representation of positively selected and co-evolved sites on the *C. batrachus* TLR1 (**a**) and TLR7 (**b**) amino acid sequences. The LRR domains detected via LRRfinder (http://www.lrrfinder.com/lrrfinder.php) are demarcated with red square brackets under the sequence and the TIR domain has been underlined in green. The residues under positive selection and co-evolution have been highlighted in yellow and blue, respectively. The various colored alphabets highlighted in blue denote the co-evolving residues from the same cluster. The residues experiencing both positive selection and coevolution are encircled in green. The figure depicts the nestling of positively selected and co-evolved sites within the functional domains (LRR, TIR) of the receptors.

## Discussion

The present study aimed to study the adaptive evolution of teleost TLRs and consequently gain a deeper insight into the evolutionary trend of TLRs in *Siluriformes* species, *C. batrachus*. Phylogenetic analysis of the teleost TLRs depicted clustering of the 12 *C. batrachus* TLRs (TLR1, 2, 3, 5, 7, 8, 9, 13, 21, 22, 25, 26) with their respective orthologues in the five TLR subfamilies, thereby suggesting a high sequence-level conservation of teleost TLRs. The phylogenetic clustering of the *Siluriformes* and *Cypriniformes* TLRs in the inferred trees is in congruence with the taxonomic proximity between the two groups. Despite that TLR4 has been identified in *Siluriformes I. punctatus, P. hypophthalamus* and *T. fulvidraco,* we did not detect a corresponding orthologue in *C. batrachus* via in silico approach^14,19^. Loss of TLR4 has been reported in several teleosts while its co-stimulatory molecules (CD14 and MD2) involved in activation of lipopolysaccharide (LPS) recognition pathway remain absent across all teleost genomes^9^. Alternate pathways for LPS recognition, mediated by other pathogen recognition receptors, have also been reported in some teleosts^20^. Though this evidence seems to justify the loss of TLR4 in some fish, yet, in order to get a complete picture of its evolution, further exploration is required to unveil the function of TLR4 orthologues in other teleost species.

Divergence, a well-cited teleost trait, is vividly reflected at the level of molecular evolution in TLRs^21^. Earlier, similar studies have suggested the occurrence of adaptive evolution in teleost V1R1 receptors, insulin genes and TUDOR domain containing protein 7 (Tdrd7)^22,23^. The evaluation of selection constraints on one of the most conserved vertebrate gene families, TLRs, indicates the role of both pervasive and episodic positive selection in shaping their current day repertoire. This is evident from the results of alignment-wide selection tests which detected episodic divergence in all the TLR alignments and a trend of positive selection for TLR1, 7, 8 and 9. Site-based selection also corroborated these results showing a higher number of positively selected codons for TLR1, 7, 8 and 9. While the divergent nature of TLR1 has been demonstrated in multiple vertebrate groups, the adaptive constraints on TLR7 family members vary widely^24–26^. Kloch et al. identified contrasting pressure of selection on rodent TLR1 *versus* TLR7 and 9^27^. A report on avian TLR3 and 7 detected purifying selection acting on both these genes^28^. Nonetheless, Park et al. and Areal et al. identified signatures of positive selection in TLR7 family members in mammals^5,29^. The relaxed selection constraints on TLR9 in teleosts and its subclade, *Perciformes*, has previously been reported by Chen et al. and Zhu et al., respectively^30,31^ . Our findings showed a stringency in selection constraints on nucleic acid sensing TLR3, 13, 21 and 22. This is in contrast with the results from a previous study in teleosts, where TLR21 and 22 seem to have evolved under positive selection^32^. This variation in findings may be due to the lower number of species orthologues included in this study. The constitution of species and their phylogenetic proximity is a critical factor in computation of dN/dS ratios. It is noteworthy that the nucleic acid sensing TLRs (TLR7, 8, 9) with a pan-vertebrate presence depict a trend of positive selection while those (TLR13, 21, 22) that have suffered species-specific loss along the vertebrate evolutionary timeline are under purifying selection. Interestingly, despite showing no alignment-wide evidence of positive selection, TLR13 in teleosts has a considerably high number of positively selected sites; which indicates a higher degree of episodic selection in TLR13. The deduction of multiple divergent leaf nodes via aBSREL analysis also corroborates this hypothesis. Considering that TLR13 was split from the TLR11 family due to its scc architecture to form a subfamily that also includes its paralogs TLR21, 22 and 23^4,11^; it may be suggested that TLR13 may be a hotspot for duplication in teleosts, wherein partitioning of functions from the parent gene may have led to neofunctionalization of the duplicated TLR along the evolutionary timeline^33^. The variation in selection constraints of the teleost TLRs may be endowed to their diverse habitats ranging from marine, fresh water, estuarine to terrestrial. The degree of exposure to microorganisms is also enhanced due to ingestion of the surrounding water along with feed.

Episodic diversifying selection refers to the trend of positive selection limited to only a subset of clades in a phylogeny. Since it usually occurs in bursts or episodes along the evolutionary timeline, its transient nature results in divergence of those lineages in the phylogeny. The analysis of diversifying branches for each TLR phylogeny reiterated the trend of divergence in teleost TLR1. Among the selected teleost species, the supraordinal group *Eupercaria* comprising of *G. morhua*, *L. maculatus*, *S. maximus* and *E. coioides* constituted the most divergent species. This is in agreement with the study on *G. morhua* where the authors have reported an extreme TLR repertoire in the species in comparison to other teleosts, guided by gene expansions and losses^34^. *C. batrachus* orthologues of TLR1 and 7 showed an evidence of episodic diversification. This was further endorsed through comparative domain analysis showing a slight variation in LRR numbers across the *Siluriformes* TLR1 and 7 orthologues.

The above-said data was complemented with the results of coevolutionary analysis where TLR 3 and 5 showed the highest number of co-evolving clusters, while TLR 1 and 8 had the lowest number of clusters. Co-evolution at molecular level, occurs at one site of a protein to compensate for an erroneous substitution at another site of the protein. The complexity of a coevolutionary network/ cluster is directly proportional to the stringency of selection constraints acting on its residues which prevents a dramatic change in the structural and functional parameters of the protein^35^. The localisation of the positively selected and coevolved residues to *C. batrachus TLR*1 and 7 amino acid sequences showed their nestling in the LRR and TIR domains. This proximity of co-evolved residues and positively selected residues within the functional domains of TLR suggests that the coevolved sites may be imparting functional and structural stability to the receptor consequent to divergence. Four residues in teleost TLR7 were under simultaneous coevolution and positive selection, a pattern which has also been reported in the evolutionary analysis of the highly conserved plant enzyme, Rubisco^36^. This finding reiterates the hypothesis that a fitness enhancing mutation may be preceded by neutral substitutions^37^.

The difference in the functioning of mammalian and fish TLRs is already established^9^. Nonetheless, the divergence of teleost TLRs at molecular level may also be contributing to functional divergence of these receptors in this vertebrate group. Studies in teleosts have suggested species-specific variation in ligand recognition by TLRs^38^. While TLR2 homodimers have been demonstrated to recognise the conventional ligands (lipoteichoic acid and peptidoglycan) in *C. carpio*, its heterodimer with TLR1 was seen to be upregulated in response to the conventional TLR3 and 4 ligands—polyI:C and lipopolysaccharide, respectively^39,40^. PolyI:C challenge in *T. ovatus* also showed upregulation of TLR7 and 8, however TLR7 and 8a1 in *O. mykiss* did not respond to either to polyI:C or R848^41,42^. This is justified at molecular level by our results indicating the diversification of teleost TLRs. Comparative genomics studies have attributed this trend of divergence in teleost lineage to enhanced molecular evolutionary rates of nucleotide and protein coding sequences and higher rate of gain and loss of cis-regulatory elements that consequently alters morphology or function^43^. The variation in the selection constraints across the TLR families may be endowed upon the variability in pathogen repertoire in various aquatic ecosystems. Thus, it may be suggested that ligand promiscuity in teleost TLRs may have evolved over time as an adaptation to enhance their fitness in the host–pathogen dynamic of their respective ecosystem. This highlights the need for functional validation experiments for enhancing the confidence intervals on the suggested hypothesis. To the best of our knowledge, this is the first study reporting the evolutionary relationship of *C. batrachus TLRs* with their teleost orthologues. Commercially prized fish like *C. batrachus* often succumb to microbial infections which is detrimental to both natural diversity and aquaculture economy. Understanding the molecular evolution of immune sentinels in fish would garner an insight into their disease resistance mechanisms.

## Methods

### Data retrieval

The sequences of 228 TLRs from a total of 25 teleost species were analysed in this study (Supporting Data 1). The NCBI nucleotide database was used for the retrieval of TLR sequences from 24 species including *Sparus Aurata, Ictalurus punctatus, Takifugu rubripes, Seriola lalandi/dumerii, Gadus morhua, Oreochromis niloticus, Carassius auratus/ carassius, Lateolabrax maculatus, Miichthys miiuy, Lates calcarifer, Trachinotus ovatus, Scophthalmus maximus, Epinephelus coioides, Salmo salar/trutta, Oncorhynchus mykiss, Danio rerio, Perca flavescens, Megalobroma amblycephala, Ctenopharyngodon Idella, Cyprinus carpio, Tachysurus fulvidraco, Oplegnathus fasciatus , Anabas testudineus and Pangasianodon hypophthalmus*. The sequences and the accession numbers of TLR 1, 2, 3, 5, 7, 8, 9, 13, 21, 22 and fifteen fish-specific TLRs forming the miscellaneous category are listed in Supplementary Data 2–12. The sequences for *C. batrachus* TLRs were identified by conducting BLAST homology search against its reference genome (Assembly version—GCA_003987875.1) using the TLR sequences of its closest *Siluriformes* relatives, *I. punctatus*, *T. fulvidraco* and *P. hypophthalamus* as queries. Stringent cut-offs of E-value (< 1 × 10^–5^), percentage identity (> 80%) and query coverage (> 95%) were maintained to ensure the identification of *C. batrachus* TLR orthologues with potential full-length coding sequences. ORF finder from NCBI was used to detect the open reading frames and corresponding amino acid sequences from the identified *C. batrachus* scaffolds (Scaffold ids, ORFs and amino acid sequences listed in Supporting Data 13–24). Eleven *C. batrachus* TLRs with potential full-length coding sequences were identified by this approach including *C. batrachus* TLR1, 2, 3, 5, 7, 8, 9, 13, 22, 25 and 26. The sequence of *C. batrachus* TLR21 used in this analysis was extracted from NCBI Accession no. AGM39445.1.

### Phylogeny analysis

The 228 TLR full-length sequences from 25 teleost species were aligned using online version of MAFFT and processed for assessment of phylogeny using PhyML 3.0 where approximate Likelihood-Ratio Test (aLRT) sh-like algorithm for Branches was implemented for tree construction^44,45^. Annotation of tree was carried out using Interactive Tree of Life (iTOL) v4 (https://itol.embl.de/). In order to check the congruency of phylogeny inference, a tree was also constructed via neighbour-joining method with the same data using MEGA6 (ref).

### Selection pressure analysis

The tools from the Datamonkey webserver were used to conduct evolutionary analysis of the teleost TLRs. The TLRs (TLR1, 2, 3, 5, 7, 8, 9, 13, 21 and 22) with more than 15 orthologues from the selected teleost species were processed for evolutionary analysis to ensure meaningful and reliable inference (https://www.datamonkey.org/). Partitioning approach for robust inference of selection (PARRIS)^46^ was used to infer the evidence of alignment-wide positive selection for each of the ten TLRs at a significance level of p < 0.05. Branch-site Unrestricted Statistical Test for Episodic Diversification (BUSTED)^47^ was used to detect the significant stochastic variation of selection pressure over the branches in the entire TLR phylogeny at p < 0.05. Further, three site-based selection tests—single likelihood ancestor counting (SLAC), fast, unconstrained Bayesian approximation (FUBAR) and mixed effects model selection (MEME) were executed to detect the instances of positive and purifying selection in the ten TLR codon alignments^17,18,48^. The significance levels of SLAC and MEME were at p < 0.05 while for FUBAR the posterior probability was cut off at 0.95. In order to maintain the degree of reliability of results, the sites were deduced to be negatively or positively selected only if inferred by at least two methods. In order to detect the divergent clades in each of the 10 TLR phylogenies, adaptive branch-site random effects likelihood test (aBSREL) was used (p < 0.05)^49^ . The raw data composed of sequence files and the results achieved by each of the tools in the workflow for each TLR are listed in Supporting data 25.

### Comparative domain analysis

The identification of the three TLR domains (extracellular domain (ECD), transmembrane (TM) domain and toll/ interleukin-1 receptor domain) for the divergent TLRs (TLR1, 7) deduced for *C. batrachus* was carried out using Simple Modular Architecture Research Tool (SMART) (http://smart.embl-heidelberg.de/). Comparative domain analysis was conducted among the TLR1 and 7 orthologues of the *Siluriformes* relatives of *C. batrachus – I. punctatus, P. hypophthalamus, T. fulvidraco*.

### Co-evolution analysis

The identification of co-evolving sites along the amino acid alignments of the 10 TLRs was carried out using Blocks in Sequences (BIS)2 Analyzer at a significance level of p < 0.05^50^.

A comparative analysis of the number of residues (co-evolving, positively selected and negatively selected) localised in the extracellular domain and TIR domain of each *C. batrachus* TLR was carried out. The LRR domains in *C. batrachus* TLR1 and 7 sequences were also detected using LRRfinder (http://www.lrrfinder.com/lrrfinder.php) with the lower and upper boundary limits demarcated at 90% and 95%, respectively. The search was carried out twice (a) against tLRRdb and (b) TLR1/7-specific databases and only the LRRs deduced via both searches were depicted on the respective *C. batrachus* amino acid sequences. The localised residues of the most divergent TLRs (TLR1, 7) detected at the *C. batrachus* node were mapped on to their I-TASSER^51^ predicted structures as well as amino acid sequences.

## Supplementary Information

Supplementary Information 1.

Supplementary Information 2.

Supplementary Information 3.

Supplementary Information 4.

Supplementary Information 5.

Supplementary Information 6.

Supplementary Information 7.

Supplementary Information 8.

Supplementary Information 9.

Supplementary Information 10.

Supplementary Information 11.

Supplementary Information 12.

Supplementary Information 13.

Supplementary Information 14.

Supplementary Information 15.

Supplementary Information 16.

Supplementary Information 17.

Supplementary Information 18.

Supplementary Information 19.

Supplementary Information 20.

Supplementary Information 21.

Supplementary Information 22.

Supplementary Information 23.

Supplementary Information 24.

Supplementary Information 25.

Supplementary Information 26.

Supplementary Information 27.

Supplementary Information 28.

Supplementary Information 29.

Supplementary Information 30.

Supplementary Information 31.

Supplementary Information 32.

Supplementary Information 33.

Supplementary Information 34.

Supplementary Information 35.

Supplementary Information 36.

Supplementary Information 37.

## References

[1] Bagheri M, Zahmatkesh A (2018). Evolution and species-specific conservation of toll-like receptors in terrestrial vertebrates. Int. Rev. Immunol..

[2] Leulier F, Lemaitre B (2008). Toll-like receptors - Taking an evolutionary approach. Nat. Rev. Genet..

[3] Brennan JJ, Gilmore TD (2018). Evolutionary origins of toll-like receptor signaling. Mol. Biol. Evol..

[4] Wang J, Zhang Z, Liu J, Zhao J, Yin D (2016). Ectodomain architecture affects sequence and functional evolution of vertebrate toll-like receptors. Sci. Rep..

[5] Areal H, Abrantes J, Esteves PJ (2011). Signatures of positive selection in Toll-like receptor (TLR) genes in mammals. BMC Evol. Biol..

[6] Vinkler M, Bainova H, Bryja J (2014). Protein evolution of Toll-like receptors 4, 5 and 7 within Galloanserae birds. Genet. Sel. Evol..

[7] Priyam M, Tripathy M, Rai U, Ghorai SM (2018). Divergence of protein sensing (TLR 4, 5) and nucleic acid sensing (TLR 3, 7) within the reptilian lineage. Mol. Phylogenet. Evol..

[8] Mikami T, Miyashita H, Takatsuka S, Kuroki Y, Matsushima N (2012). Molecular evolution of vertebrate Toll-like receptors: Evolutionary rate difference between their leucine-rich repeats and their TIR domains. Gene.

[9] Palti Y (2011). Toll-like receptors in bony fish: From genomics to function. Dev. Comp. Immunol..

[10] Takano T (2011). Diseases in Asian Aquaculture VII Toll-like receptors in teleosts..

[11] Qiu HT (2019). Paralogues from the expanded Tlr11 gene family in Mudskipper (Boleophthalmus pectinirostris) are under positive selection and respond differently to LPS/Poly(I:C) challenge. Front. Immunol..

[12] Tong C (2015). Transcriptome-wide identification, molecular evolution and expression analysis of Toll-like receptor family in a Tibet fish Gymnocypris przewalskii. Fish Shellfish Immunol..

[13] Khedkar GD (2016). Genetic structure of populations and conservation issues relating to an endangered catfish, Clarias batrachus India. Mitochondrial DNA.

[14] Quiniou SMA, Boudinot P, Bengtén E (2013). Comprehensive survey and genomic characterization of Toll-like receptors (TLRs) in channel catfish, Ictalurus punctatus: identification of novel fish TLRs. Immunogenetics.

[15] Zhao F (2013). Expression profiles of toll-like receptors in channel catfish (Ictalurus punctatus) after infection with Ichthyophthirius multifiliis. Fish Shellfish Immunol..

[16] Li N (2018). Genome sequence of walking catfish (Clarias batrachus) provides insights into terrestrial adaptation. BMC Genomics.

[17] Murrell B (2013). FUBAR: A fast, unconstrained bayesian AppRoximation for inferring selection. Mol. Biol. Evol..

[18] Murrell, B. *et al.* Detecting individual sites subject to episodic diversifying selection. *PLoS Genet.* **8**, (2012). 10.1371/journal.pgen.1002764 PMC3395634 22807683

[19] Ghode, G. & Prasad, K. P. wagner. (2018).

[20] Lu XJ, Ning YJ, Liu H, Nie L, Chen J (2018). A novel lipopolysaccharide recognition mechanism mediated by internalization in teleost macrophages. Front. Immunol..

[21] Ravi V, Venkatesh B (2008). Rapidly evolving fish genomes and teleost diversity. Curr. Opin. Genet. Dev..

[22] Pfister P, Randall J, Montoya-Burgos JI, Rodriguez I (2007). Divergent evolution among teleost V1r receptor genes. PLoS ONE.

[23] Wang, B. *et al.* Comparative studies on duplicated tdrd7 paralogs in teleosts: Molecular evolution caused neo-functionalization. *Comp. Biochem. Physiol. Part D Genomics Proteomics* **30**, 347–357 (2019). 10.1016/j.cbd.2019.04.006 31059868

[24] Wlasiuk G, Nachman MW (2010). Adaptation and constraint at toll-like receptors in primates. Mol. Biol. Evol..

[25] Huang Y (2011). Molecular evolution of the vertebrate TLR1 gene family: a complex history of gene duplication, gene conversion, positive selection and co-evolution. BMC Evol. Biol..

[26] Heffelfinger C (2014). Haplotype structure and positive selection at TLR1. Eur. J. Hum. Genet..

[27] Kloch A (2018). Signatures of balancing selection in toll-like receptor (TLRs) genes: novel insights from a free-living rodent. Sci. Rep..

[28] Raven N (2017). Purifying selection and concerted evolution of RNA-sensing toll-like receptors in migratory waders. Infect. Genet. Evol..

[29] Park SG, Park D, Jung YJ, Chung E, Choi SS (2010). Positive selection signatures in the TLR7 family. Genes Genomics.

[30] Chen JSC, Wang TY, Tzeng TD, Wang CY, Wang D (2008). Evidence for positive selection in the TLR9 gene of teleosts. Fish Shellfish Immunol..

[31] Zhu Z, Sun Y, Wang R, Xu T (2013). Evolutionary analysis of TLR9 genes reveals the positive selection of extant teleosts in Perciformes. Fish Shellfish Immunol..

[32] Sundaram AYM, Consuegra S, Kiron V, Fernandes JMO (2012). Positive selection pressure within teleost toll-like receptors tlr21 and tlr22 subfamilies and their response to temperature stress and microbial components in zebrafish. Mol. Biol. Rep..

[33] Pietretti D, Wiegertjes GF (2014). Ligand specificities of Toll-like receptors in fish: Indications from infection studies. Dev. Comp. Immunol..

[34] Solbakken, M. H. *et al.* Evolutionary redesign of the Atlantic cod (Gadus morhua L.) Toll-like receptor repertoire by gene losses and expansions. *Sci. Rep.* **6**, 1–14 (2016). 10.1038/srep25211 PMC4850435 27126702

[35] Fares MA, Travers SAA (2006). A novel method for detecting intramolecular coevolution: adding a further dimension to selective constraints analyses. Genetics.

[36] Wang M, Kapralov MV, Anisimova M (2011). Coevolution of amino acid residues in the key photosynthetic enzyme Rubisco. BMC Evol. Biol..

[37] Wagner, A. Perspective in evolution.

[38] Zhang J (2014). Toll-like receptor recognition of bacteria in fish: Ligand specificity and signal pathways. Fish Shellfish Immunol..

[39] Wei YC (2011). Cloning and expression of Toll-like receptors 1 and 2 from a teleost fish, the orange-spotted grouper Epinephelus coioides. Vet. Immunol. Immunopathol..

[40] Ribeiro CMS, Hermsen T, Taverne-Thiele AJ, Savelkoul HFJ, Wiegertjes GF (2010). Evolution of recognition of ligands from gram-positive bacteria: similarities and differences in the TLR2-mediated response between mammalian vertebrates and teleost fish. J. Immunol..

[41] Palti Y (2010). Identification, characterization and genetic mapping of TLR7, TLR8a1 and TLR8a2 genes in rainbow trout (Oncorhynchus mykiss). Dev. Comp. Immunol..

[42] Wei Y (2017). Molecular cloning and expression analysis of toll-like receptor genes (TLR7, TLR8 and TLR9) of golden pompano (Trachinotus ovatus). Fish Shellfish Immunol..

[43] Ravi V, Venkatesh B (2018). The divergent genomes of teleosts. Annu. Rev. Anim. Biosci..

[44] Katoh K, Rozewicki J, Yamada KD (2018). MAFFT online service: Multiple sequence alignment, interactive sequence choice and visualization. Brief. Bioinform..

[45] Anisimova M, Gascuel O (2006). Approximate likelihood-ratio test for branches: a fast, accurate, and powerful alternative. Syst. Biol..

[46] Scheffler, K. & Seoighe, C. PARRIS : a PARtitioning approach for Robust Inference of Selection . PARRIS features. 6–7 (2015).

[47] Murrell B (2015). Gene-wide identification of episodic selection. Mol. Biol. Evol..

[48] Kosakovsky Pond, S. L. & Frost, S. D. W. Datamonkey: Rapid detection of selective pressure on individual sites of codon alignments. *Bioinformatics* **21**, 2531–2533 (2005). 10.1093/bioinformatics/bti320 15713735

[49] Smith MD (2015). Less is more: an adaptive branch-site random effects model for efficient detection of episodic diversifying selection. Mol. Biol. Evol..

[50] Oteri F, Nadalin F, Champeimont R, Carbone A (2017). BIS2 analyzer: a server for co-evolution analysis of conserved protein families. Nucleic Acids Res..

[51] Yang J (2014). The I-TASSER suite: Protein structure and function prediction. Nat. Methods.

