# Supplementary material for: Variation in selection constraints on teleost TLRs with emphasis on their repertoire in the Walking catfish, Clarias batrachus
Source: Sci Rep. 2020 Dec 7;10:21394. doi: 10.1038/s41598-020-78347-6 (PMC7721727; doi:10.1038/s41598-020-78347-6)
Supplement: Supplementary file 1 — Supplementary Information 1. [file 41598_2020_78347_MOESM1_ESM.pdf]

Supplementary Table1: Listing of TLRs from the selected species used in the study

Species	Abbv.	Misc. TLRs	TLR22	TLR21	TLR13	TLR9	TLR8	TLR7	TLR5	TLR3	TLR2	TLR1
Sparus Aurata	Sa	nil	*	nil	*	*	nil	*	*	*	*	*
Ictalurus Punctatus	Ip	18,19,25,26	*	*	*	*	*	*	*	*	*	*
Takifugu rubripes	Tr	nil	*	*	*	*	*	*	*	*	*	*
Seriola lalandi/dumerii	Sl/ Sd	nil	*	*	*	*	*	*	*	*	*	nil
Gadus morhua	Gm	nil	*	*	*	*	*	*	nil	*	*	nil
Oreochromis niloticus	On	nil	nil	*	*	*	*	*	*	*	*	*
Carassius auratus/ carassius	Ca/Cca	nil	*	nil	*	*	*	*	*	*	*	nil
Lateolabrax maculatus	Lm	14	*	*	*	*	*	*	*	*	*	*
Miichthys miiuy	Mm	nil	*	*	*	*	*	*	*	*	*	*
Lates calcarifer	Lc	nil	*	nil	*	*	*	*	*	*	nil	*
Trachinotus ovatus	To	14	*	*	*	*	*	*	nil	*	*	*
Scophthalmus maximus	Sm	nil	*	*	*	*	*	*	*	*	*	*
Epinephelus coioides	Ec	nil	*	*	*	*	nil	*	*	*	*	*
Salmo salar/trutta	Ss/St	nil	*	nil	*	*	*	*	*	*	*	nil
Oncorhynchus mykiss	Om	nil	*	nil	*	*	*	*	*	*	*	nil
Danio rerio	Dr	19	*	*	*	*	*	*	*	*	*	*
Perca flavescens	Pf	nil	nil	nil	*	*	nil	*	*	*	*	*
Megalobroma amblycephala	Ma	18,19	*	nil	*	*	*	*	*	*	*	*
Ctenopharyngodon idella	Ci	18,19,20,25	*	*	*	*	*	nil	*	*	*	*
Cyprinus carpio	Cc	nil	*	*	*	*	*	*	*	*	*	*
Tachysurus fulvidraco	Tf	nil	nil	*	*	*	*	*	*	*	*	*
Oplegnathus fasciatus	Of	nil	nil	*	nil	nil	nil	nil	*	nil	*	*
Anabas testudineus	At	nil	nil	nil	*	*	*	*	*	*	*	*
Pangasianodon hypophthalmus	Ph	nil	nil	nil	*	*	*	*	nil	*	*	*
Clarias batrachus	Cb	25,26	*	*	*	*	*	*	*	*	*	*

* → TLRs from the given species detected in the NCBI database

Nil → TLRs for the given species not found in the database

Abbv. → Abbreviations used for the given species in the study
