# Supplementary material for: Variation in selection constraints on teleost TLRs with emphasis on their repertoire in the Walking catfish, Clarias batrachus
Source: Sci Rep. 2020 Dec 7;10:21394. doi: 10.1038/s41598-020-78347-6 (PMC7721727; doi:10.1038/s41598-020-78347-6)
Supplement: Supplementary file 25 — Supplementary Information 25. [file 41598_2020_78347_MOESM25_ESM.zip › T1/BIS2/summary/PF00000-NONREDUNDANT-5DD-dim0-table.html]

BIS cluster table


Clusters with env. score >= 0.5 and sym. score >= 0.5 :

| Dim | Cluster | Sym | Env | Pvalue | Hit patterns and blocks |
| --- | --- | --- | --- | --- | --- |
| 0 | 5 | 1 | 1 | 2.268114e-07 | Hit patterns:   |  |  |  | | --- | --- | --- | | Positions: | 593 | 773 | | 12 sequences: | T | I | | 4 sequences: | V | L | | 4 sequences: | D | V |  All positions in cluster: 593-594 773 |
| 0 | 2 | 1 | 1 | 5.953799e-06 | Hit patterns:   |  |  |  |  | | --- | --- | --- | --- | | Positions: | 164 | 796 | 822 | | 11 sequences: | N | K | V | | 9 sequences: | D | Q | I |  All positions in cluster: 163-165 796-797 822 |
| 0 | 4 | 1 | 1 | 7.938398e-06 | Hit patterns:   |  |  |  |  |  |  |  |  |  | | --- | --- | --- | --- | --- | --- | --- | --- | --- | | Positions: | 191 | 216 | 428 | 465 | 509 | 734 | 767 | 780 | | 12 sequences: | G | L | D | S | L | D | V | V | | 8 sequences: | S | I | E | T | F | N | I | I |  All positions in cluster: 191 216 428 465 509 734 766-767 780 |
| 0 | 1 | 1 | 1 | 0.0002063983 | Hit patterns:   |  |  |  |  | | --- | --- | --- | --- | | Positions: | 84 | 742 | 854 | | 16 sequences: | P | L | D | | 4 sequences: | K | F | E |  All positions in cluster: 84 742 854 |
| 0 | 6 | 1 | 1 | 0.0002063983 | Hit patterns:   |  |  |  |  | | --- | --- | --- | --- | | Positions: | 594 | 601 | 818 | | 16 sequences: | L | F | S | | 4 sequences: | V | Y | N |  All positions in cluster: 594 601 818 |
| 0 | 3 | 1 | 1 | 0.005263158 | Hit patterns:   |  |  |  | | --- | --- | --- | | Positions: | 304 | 783 | | 18 sequences: | L | S | | 2 sequences: | F | C |  All positions in cluster: 304 783 |
| 0 | 7 | 1 | 1 | 1 | All positions in cluster: 72 75-76 79-80 91 93 95 97 111 114 121 129 132 135 138 141 143 145 160 163 165 168 179 182 185 188 203 211 225 230 260 369 375 404 419 422 431 451 453 456 471 474 478 482 493 495-496 504 507 514 526 545 550 553 558-559 565 567 570 575 585 591 599 603 605 610 636-637 640 642 645 653 663 691 694-695 698 702 706 725-727 729-731 737-738 746-747 762 766 769 775-776 788-791 793-794 797 799-800 802-804 806 809 824-826 828 833 839 847-848 850-852 857 861-863 865 869 |

Table created with bis2html version 8.
