# Supplementary material for: Variation in selection constraints on teleost TLRs with emphasis on their repertoire in the Walking catfish, Clarias batrachus
Source: Sci Rep. 2020 Dec 7;10:21394. doi: 10.1038/s41598-020-78347-6 (PMC7721727; doi:10.1038/s41598-020-78347-6)
Supplement: Supplementary file 25 — Supplementary Information 25. [file 41598_2020_78347_MOESM25_ESM.zip › T1/BIS2/summary/PF00000-NONREDUNDANT-5DD-dim1.html]

Alignment and BIS clusters


|  |  |  |  |  |  |  |  |  |  |  |  |  |  |  |  |  |  |  |  |  |  |  |  |  |  |  |  |  |  |  |  |  |  |  |  |  |  |  |  |  |  |  |  |  |  |  |  |  |  |  |  |  |  |  |  |  |  |  |  |  |  |  |  |  |  |  |  |  |  |  |  |  |  |  |  |  |  |  |  |  |  |  |  |  |  |  |  |  |  |  |  |  |  |  |  |  |  |  |  |  |  |  |  |  |  |  |  |  |  |  |  |  |  |  |  |  |  |  |  |  |  |  |  |  |  |  |  |  |  |  |  |  |  |  |  |  |  |  |  |  |  |  |  |  |  |  |  |  |  |  |  |  |  |  |  |  |  |  |  |  |  |  |  |  |  |  |  |  |  |  |  |  |  |  |  |  |  |  |  |  |  |  |  |  |  |  |  |  |  |  |  |  |  |  |  |  |  |  |  |  |  |  |  |  |  |  |  |  |  |  |  |  |  |  |  |  |  |  |  |  |  |  |  |  |  |  |  |  |  |  |  |  |  |  |  |  |  |  |  |  |  |  |  |  |  |  |  |  |  |  |  |  |  |  |  |  |  |  |  |  |  |  |  |  |  |  |  |  |  |  |  |  |  |  |  |  |  |  |  |  |  |  |  |  |  |  |  |  |  |  |  |  |  |  |  |  |  |  |  |  |  |  |  |  |  |  |  |  |  |  |  |  |  |  |  |  |  |  |  |  |  |  |  |  |  |  |  |  |  |  |  |  |  |  |  |  |  |  |  |  |  |  |  |  |  |  |  |  |  |  |  |  |  |  |  |  |  |  |  |  |  |  |  |  |  |  |  |  |  |  |  |  |  |  |  |  |  |  |  |  |  |  |  |  |  |  |  |  |  |  |  |  |  |  |  |  |  |  |  |  |  |  |  |  |  |  |  |  |  |  |  |  |  |  |  |  |  |  |  |  |  |  |  |  |  |  |  |  |  |  |  |  |  |  |  |  |  |  |  |  |  |  |  |  |  |  |  |  |  |  |  |  |  |  |  |  |  |  |  |  |  |  |  |  |  |  |  |  |  |  |  |  |  |  |  |  |  |  |  |  |  |  |  |  |  |  |  |  |  |  |  |  |  |  |  |  |  |  |  |  |  |  |  |  |  |  |  |  |  |  |  |  |  |  |  |  |  |  |  |  |  |  |  |  |  |  |  |  |  |  |  |  |  |  |  |  |  |  |  |  |  |  |  |  |  |  |  |  |  |  |  |  |  |  |  |  |  |  |  |  |  |  |  |  |  |  |  |  |  |  |  |  |  |  |  |  |  |  |  |  |  |  |  |  |  |  |  |  |  |  |  |  |  |  |  |  |  |  |  |  |  |  |  |  |  |  |  |  |  |  |  |  |  |  |  |  |  |  |  |  |  |  |  |  |  |  |  |  |  |  |  |  |  |  |  |  |  |  |  |  |  |  |  |  |  |  |  |  |  |  |  |  |  |  |  |  |  |  |  |  |  |  |  |  |  |  |  |  |  |  |  |  |  |  |  |  |  |  |  |  |  |  |  |  |  |  |  |  |  |  |  |  |  |  |  |  |  |  |  |  |  |  |  |  |  |  |  |  |  |  |  |  |  |  |  |  |  |  |  |  |  |  |  |  |  |  |  |  |  |  |  |  |  |  |  |  |  |  |  |  |  |  |  |  |  |  |  |  |  |  |  |  |  |  |  |  |  |  |  |  |  |  |  |  |  |  |  |  |  |  |  |  |  |  |  |  |  |  |  |  |  |  |  |  |  |  |  |  |  |  |  |  |  |  |  |  |  |  |  |  |  |  |  |  |  |  |  |  |  |  |  |  |  |  |  |  |  |  |  |  |  |  |  |  |  |  |  |  |  |  |  |  |  |  |  |  |  |  |  |  |  |  |  |  |  |  |  |  |  |  |  |  |  |  |  |  |  |  |  |  |  |  |  |  |  |  |  |  |  |  |  |  |  |  |  |  |  |  |  |  |  |  |  |  |  |  |  |  |  |  |  |  |  |  |  |  |  |  |  |  |  |  |  |  |  |  |  |  |  |  |  |  |  |  |  |  |  |  |  |  |  |  |  |  |  |  |  |  |  |  |  |  |  |  |  |  |  |
| --- | --- | --- | --- | --- | --- | --- | --- | --- | --- | --- | --- | --- | --- | --- | --- | --- | --- | --- | --- | --- | --- | --- | --- | --- | --- | --- | --- | --- | --- | --- | --- | --- | --- | --- | --- | --- | --- | --- | --- | --- | --- | --- | --- | --- | --- | --- | --- | --- | --- | --- | --- | --- | --- | --- | --- | --- | --- | --- | --- | --- | --- | --- | --- | --- | --- | --- | --- | --- | --- | --- | --- | --- | --- | --- | --- | --- | --- | --- | --- | --- | --- | --- | --- | --- | --- | --- | --- | --- | --- | --- | --- | --- | --- | --- | --- | --- | --- | --- | --- | --- | --- | --- | --- | --- | --- | --- | --- | --- | --- | --- | --- | --- | --- | --- | --- | --- | --- | --- | --- | --- | --- | --- | --- | --- | --- | --- | --- | --- | --- | --- | --- | --- | --- | --- | --- | --- | --- | --- | --- | --- | --- | --- | --- | --- | --- | --- | --- | --- | --- | --- | --- | --- | --- | --- | --- | --- | --- | --- | --- | --- | --- | --- | --- | --- | --- | --- | --- | --- | --- | --- | --- | --- | --- | --- | --- | --- | --- | --- | --- | --- | --- | --- | --- | --- | --- | --- | --- | --- | --- | --- | --- | --- | --- | --- | --- | --- | --- | --- | --- | --- | --- | --- | --- | --- | --- | --- | --- | --- | --- | --- | --- | --- | --- | --- | --- | --- | --- | --- | --- | --- | --- | --- | --- | --- | --- | --- | --- | --- | --- | --- | --- | --- | --- | --- | --- | --- | --- | --- | --- | --- | --- | --- | --- | --- | --- | --- | --- | --- | --- | --- | --- | --- | --- | --- | --- | --- | --- | --- | --- | --- | --- | --- | --- | --- | --- | --- | --- | --- | --- | --- | --- | --- | --- | --- | --- | --- | --- | --- | --- | --- | --- | --- | --- | --- | --- | --- | --- | --- | --- | --- | --- | --- | --- | --- | --- | --- | --- | --- | --- | --- | --- | --- | --- | --- | --- | --- | --- | --- | --- | --- | --- | --- | --- | --- | --- | --- | --- | --- | --- | --- | --- | --- | --- | --- | --- | --- | --- | --- | --- | --- | --- | --- | --- | --- | --- | --- | --- | --- | --- | --- | --- | --- | --- | --- | --- | --- | --- | --- | --- | --- | --- | --- | --- | --- | --- | --- | --- | --- | --- | --- | --- | --- | --- | --- | --- | --- | --- | --- | --- | --- | --- | --- | --- | --- | --- | --- | --- | --- | --- | --- | --- | --- | --- | --- | --- | --- | --- | --- | --- | --- | --- | --- | --- | --- | --- | --- | --- | --- | --- | --- | --- | --- | --- | --- | --- | --- | --- | --- | --- | --- | --- | --- | --- | --- | --- | --- | --- | --- | --- | --- | --- | --- | --- | --- | --- | --- | --- | --- | --- | --- | --- | --- | --- | --- | --- | --- | --- | --- | --- | --- | --- | --- | --- | --- | --- | --- | --- | --- | --- | --- | --- | --- | --- | --- | --- | --- | --- | --- | --- | --- | --- | --- | --- | --- | --- | --- | --- | --- | --- | --- | --- | --- | --- | --- | --- | --- | --- | --- | --- | --- | --- | --- | --- | --- | --- | --- | --- | --- | --- | --- | --- | --- | --- | --- | --- | --- | --- | --- | --- | --- | --- | --- | --- | --- | --- | --- | --- | --- | --- | --- | --- | --- | --- | --- | --- | --- | --- | --- | --- | --- | --- | --- | --- | --- | --- | --- | --- | --- | --- | --- | --- | --- | --- | --- | --- | --- | --- | --- | --- | --- | --- | --- | --- | --- | --- | --- | --- | --- | --- | --- | --- | --- | --- | --- | --- | --- | --- | --- | --- | --- | --- | --- | --- | --- | --- | --- | --- | --- | --- | --- | --- | --- | --- | --- | --- | --- | --- | --- | --- | --- | --- | --- | --- | --- | --- | --- | --- | --- | --- | --- | --- | --- | --- | --- | --- | --- | --- | --- | --- | --- | --- | --- | --- | --- | --- | --- | --- | --- | --- | --- | --- | --- | --- | --- | --- | --- | --- | --- | --- | --- | --- | --- | --- | --- | --- | --- | --- | --- | --- | --- | --- | --- | --- | --- | --- | --- | --- | --- | --- | --- | --- | --- | --- | --- | --- | --- | --- | --- | --- | --- | --- | --- | --- | --- | --- | --- | --- | --- | --- | --- | --- | --- | --- | --- | --- | --- | --- | --- | --- | --- | --- | --- | --- | --- | --- | --- | --- | --- | --- | --- | --- | --- | --- | --- | --- | --- | --- | --- | --- | --- | --- | --- | --- | --- | --- | --- | --- | --- | --- | --- | --- | --- | --- | --- | --- | --- | --- | --- | --- | --- | --- | --- | --- | --- | --- | --- | --- | --- | --- | --- | --- | --- | --- | --- | --- | --- | --- | --- | --- | --- | --- | --- | --- | --- | --- | --- | --- | --- | --- | --- | --- | --- | --- | --- | --- | --- | --- | --- | --- | --- | --- | --- | --- | --- | --- | --- | --- | --- | --- | --- | --- | --- | --- | --- | --- | --- | --- | --- | --- | --- | --- | --- | --- | --- | --- | --- | --- | --- | --- | --- | --- | --- | --- | --- | --- | --- | --- | --- | --- | --- | --- | --- | --- | --- | --- | --- | --- | --- | --- | --- | --- | --- | --- | --- | --- | --- | --- | --- | --- | --- | --- | --- | --- | --- | --- | --- | --- | --- | --- | --- | --- | --- | --- | --- | --- | --- | --- | --- | --- | --- | --- | --- | --- | --- | --- | --- | --- | --- | --- | --- | --- | --- | --- | --- | --- | --- | --- | --- | --- | --- | --- | --- | --- | --- | --- | --- | --- | --- | --- | --- | --- | --- | --- | --- | --- | --- | --- | --- | --- | --- | --- | --- | --- | --- | --- | --- | --- | --- | --- | --- | --- | --- | --- | --- | --- | --- | --- | --- | --- | --- | --- | --- | --- | --- | --- | --- | --- | --- | --- | --- | --- | --- | --- | --- | --- | --- | --- | --- | --- | --- | --- | --- | --- | --- | --- | --- | --- | --- | --- | --- | --- | --- | --- | --- | --- | --- | --- | --- | --- | --- | --- | --- | --- | --- | --- | --- | --- |
|  |  |  |  |  |  |  |  | 1 | 0 |  |  |  |  |  |  |  |  | 2 | 0 |  |  |  |  |  |  |  |  | 3 | 0 |  |  |  |  |  |  |  |  | 4 | 0 |  |  |  |  |  |  |  |  | 5 | 0 |  |  |  |  |  |  |  |  | 6 | 0 |  |  |  |  |  |  |  |  | 7 | 0 |  |  |  |  |  |  |  |  | 8 | 0 |  |  |  |  |  |  |  |  | 9 | 0 |  |  |  |  |  |  |  | 1 | 0 | 0 |  |  |  |  |  |  |  | 1 | 1 | 0 |  |  |  |  |  |  |  | 1 | 2 | 0 |  |  |  |  |  |  |  | 1 | 3 | 0 |  |  |  |  |  |  |  | 1 | 4 | 0 |  |  |  |  |  |  |  | 1 | 5 | 0 |  |  |  |  |  |  |  | 1 | 6 | 0 |  |  |  |  |  |  |  | 1 | 7 | 0 |  |  |  |  |  |  |  | 1 | 8 | 0 |  |  |  |  |  |  |  | 1 | 9 | 0 |  |  |  |  |  |  |  | 2 | 0 | 0 |  |  |  |  |  |  |  | 2 | 1 | 0 |  |  |  |  |  |  |  | 2 | 2 | 0 |  |  |  |  |  |  |  | 2 | 3 | 0 |  |  |  |  |  |  |  | 2 | 4 | 0 |  |  |  |  |  |  |  | 2 | 5 | 0 |  |  |  |  |  |  |  | 2 | 6 | 0 |  |  |  |  |  |  |  | 2 | 7 | 0 |  |  |  |  |  |  |  | 2 | 8 | 0 |  |  |  |  |  |  |  | 2 | 9 | 0 |  |  |  |  |  |  |  | 3 | 0 | 0 |  |  |  |  |  |  |  | 3 | 1 | 0 |  |  |  |  |  |  |  | 3 | 2 | 0 |  |  |  |  |  |  |  | 3 | 3 | 0 |  |  |  |  |  |  |  | 3 | 4 | 0 |  |  |  |  |  |  |  | 3 | 5 | 0 |  |  |  |  |  |  |  | 3 | 6 | 0 |  |  |  |  |  |  |  | 3 | 7 | 0 |  |  |  |  |  |  |  | 3 | 8 | 0 |  |  |  |  |  |  |  | 3 | 9 | 0 |  |  |  |  |  |  |  | 4 | 0 | 0 |  |  |  |  |  |  |  | 4 | 1 | 0 |  |  |  |  |  |  |  | 4 | 2 | 0 |  |  |  |  |  |  |  | 4 | 3 | 0 |  |  |  |  |  |  |  | 4 | 4 | 0 |  |  |  |  |  |  |  | 4 | 5 | 0 |  |  |  |  |  |  |  | 4 | 6 | 0 |  |  |  |  |  |  |  | 4 | 7 | 0 |  |  |  |  |  |  |  | 4 | 8 | 0 |  |  |  |  |  |  |  | 4 | 9 | 0 |  |  |  |  |  |  |  | 5 | 0 | 0 |  |  |  |  |  |  |  | 5 | 1 | 0 |  |  |  |  |  |  |  | 5 | 2 | 0 |  |  |  |  |  |  |  | 5 | 3 | 0 |  |  |  |  |  |  |  | 5 | 4 | 0 |  |  |  |  |  |  |  | 5 | 5 | 0 |  |  |  |  |  |  |  | 5 | 6 | 0 |  |  |  |  |  |  |  | 5 | 7 | 0 |  |  |  |  |  |  |  | 5 | 8 | 0 |  |  |  |  |  |  |  | 5 | 9 | 0 |  |  |  |  |  |  |  | 6 | 0 | 0 |  |  |  |  |  |  |  | 6 | 1 | 0 |  |  |  |  |  |  |  | 6 | 2 | 0 |  |  |  |  |  |  |  | 6 | 3 | 0 |  |  |  |  |  |  |  | 6 | 4 | 0 |  |  |  |  |  |  |  | 6 | 5 | 0 |  |  |  |  |  |  |  | 6 | 6 | 0 |  |  |  |  |  |  |  | 6 | 7 | 0 |  |  |  |  |  |  |  | 6 | 8 | 0 |  |  |  |  |  |  |  | 6 | 9 | 0 |  |  |  |  |  |  |  | 7 | 0 | 0 |  |  |  |  |  |  |  | 7 | 1 | 0 |  |  |  |  |  |  |  | 7 | 2 | 0 |  |  |  |  |  |  |  | 7 | 3 | 0 |  |  |  |  |  |  |  | 7 | 4 | 0 |  |  |  |  |  |  |  | 7 | 5 | 0 |  |  |  |  |  |  |  | 7 | 6 | 0 |  |  |  |  |  |  |  | 7 | 7 | 0 |  |  |  |  |  |  |  | 7 | 8 | 0 |  |  |  |  |  |  |  | 7 | 9 | 0 |  |  |  |  |  |  |  | 8 | 0 | 0 |  |  |  |  |  |  |  | 8 | 1 | 0 |  |  |  |  |  |  |  | 8 | 2 | 0 |  |  |  |  |  |  |  | 8 | 3 | 0 |  |  |  |  |  |  |  | 8 | 4 | 0 |  |  |  |  |  |  |  | 8 | 5 | 0 |  |  |  |  |  |  |  | 8 | 6 | 0 |  |  |  |  |  |  |  | 8 | 7 | 0 |  |  |  |  |  |  |  | 8 | 8 | 0 |  |  |  |  |  |  |  | 8 | 9 | 0 |  |  |  |  |  |  |  | 9 | 0 | 0 |  |  |  |  |  |  |  | 9 | 1 | 0 |  |  |  |  |  |  |  | 9 | 2 | 0 |  |  |  |  |  |  |  | 9 | 3 | 0 |  |  |  |  |  |  |  |  |
|  |  |  |  |  |  |  |  |  | | |  |  |  |  |  |  |  |  |  | | |  |  |  |  |  |  |  |  |  | | |  |  |  |  |  |  |  |  |  | | |  |  |  |  |  |  |  |  |  | | |  |  |  |  |  |  |  |  |  | | |  |  |  |  |  |  |  |  |  | | |  |  |  |  |  |  |  |  |  | | |  |  |  |  |  |  |  |  |  | | |  |  |  |  |  |  |  |  |  | | |  |  |  |  |  |  |  |  |  | | |  |  |  |  |  |  |  |  |  | | |  |  |  |  |  |  |  |  |  | | |  |  |  |  |  |  |  |  |  | | |  |  |  |  |  |  |  |  |  | | |  |  |  |  |  |  |  |  |  | | |  |  |  |  |  |  |  |  |  | | |  |  |  |  |  |  |  |  |  | | |  |  |  |  |  |  |  |  |  | | |  |  |  |  |  |  |  |  |  | | |  |  |  |  |  |  |  |  |  | | |  |  |  |  |  |  |  |  |  | | |  |  |  |  |  |  |  |  |  | | |  |  |  |  |  |  |  |  |  | | |  |  |  |  |  |  |  |  |  | | |  |  |  |  |  |  |  |  |  | | |  |  |  |  |  |  |  |  |  | | |  |  |  |  |  |  |  |  |  | | |  |  |  |  |  |  |  |  |  | | |  |  |  |  |  |  |  |  |  | | |  |  |  |  |  |  |  |  |  | | |  |  |  |  |  |  |  |  |  | | |  |  |  |  |  |  |  |  |  | | |  |  |  |  |  |  |  |  |  | | |  |  |  |  |  |  |  |  |  | | |  |  |  |  |  |  |  |  |  | | |  |  |  |  |  |  |  |  |  | | |  |  |  |  |  |  |  |  |  | | |  |  |  |  |  |  |  |  |  | | |  |  |  |  |  |  |  |  |  | | |  |  |  |  |  |  |  |  |  | | |  |  |  |  |  |  |  |  |  | | |  |  |  |  |  |  |  |  |  | | |  |  |  |  |  |  |  |  |  | | |  |  |  |  |  |  |  |  |  | | |  |  |  |  |  |  |  |  |  | | |  |  |  |  |  |  |  |  |  | | |  |  |  |  |  |  |  |  |  | | |  |  |  |  |  |  |  |  |  | | |  |  |  |  |  |  |  |  |  | | |  |  |  |  |  |  |  |  |  | | |  |  |  |  |  |  |  |  |  | | |  |  |  |  |  |  |  |  |  | | |  |  |  |  |  |  |  |  |  | | |  |  |  |  |  |  |  |  |  | | |  |  |  |  |  |  |  |  |  | | |  |  |  |  |  |  |  |  |  | | |  |  |  |  |  |  |  |  |  | | |  |  |  |  |  |  |  |  |  | | |  |  |  |  |  |  |  |  |  | | |  |  |  |  |  |  |  |  |  | | |  |  |  |  |  |  |  |  |  | | |  |  |  |  |  |  |  |  |  | | |  |  |  |  |  |  |  |  |  | | |  |  |  |  |  |  |  |  |  | | |  |  |  |  |  |  |  |  |  | | |  |  |  |  |  |  |  |  |  | | |  |  |  |  |  |  |  |  |  | | |  |  |  |  |  |  |  |  |  | | |  |  |  |  |  |  |  |  |  | | |  |  |  |  |  |  |  |  |  | | |  |  |  |  |  |  |  |  |  | | |  |  |  |  |  |  |  |  |  | | |  |  |  |  |  |  |  |  |  | | |  |  |  |  |  |  |  |  |  | | |  |  |  |  |  |  |  |  |  | | |  |  |  |  |  |  |  |  |  | | |  |  |  |  |  |  |  |  |  | | |  |  |  |  |  |  |  |  |  | | |  |  |  |  |  |  |  |  |  | | |  |  |  |  |  |  |  |  |  | | |  |  |  |  |  |  |  |  |  | | |  |  |  |  |  |  |  |  |  | | |  |  |  |  |  |  |  |  |  | | |  |  |  |  |  |  |  |  |  | | |  |  |  |  |  |  |  |  |  | | |  |  |  |  |  |  |  |  |  | | |  |  |  |  |  |  |  |  |  | | |  |  |  |  |  |  |  |  |  | | |  |  |  |  |  |  |  |  |  | | |  |  |  |  |  |  |  |  |  | | |  |  |  |  |  |  |  |  |  | | |  |  |  |  |  |  |  |  |  | | |  |  |  |  |  |  |  |  |
| PFTLR1 | - | - | - | - | - | - | - | - | - | - | - | - | - | - | - | - | - | - | - | - | - | - | - | - | - | - | - | - | - | - | - | - | - | - | - | - | - | M | K | P | D | W | V | A | G | R | K | D | E | R | G | F | K | L | C | V | - | - | - | - | - | - | T | S | L | Q | R | M | K | D | L | S | H | Q | N | L | T | T | V | P | L | N | L | P | K | D | T | E | Y | L | D | I | S | H | N | S | I | Q | R | L | S | G | A | P | F | S | R | L | S | R | L | C | C | L | K | V | T | H | C | G | L | Q | N | I | S | P | A | V | F | A | H | T | P | A | L | K | V | L | N | I | S | Y | N | D | L | A | L | I | P | D | I | P | - | - | - | - | L | K | Q | L | K | I | L | D | L | A | N | N | L | Y | S | S | Y | S | I | P | A | S | F | Q | K | L | T | N | L | D | V | L | S | L | G | S | T | A | A | P | S | V | N | L | H | D | F | D | P | L | A | N | V | S | L | H | H | L | V | L | G | A | G | I | G | W | Q | K | Y | D | A | G | A | L | A | K | L | K | S | L | Q | T | F | S | L | F | A | S | - | - | - | - | - | - | - | - | F | C | A | A | P | G | M | F | K | N | V | L | V | D | L | N | A | T | R | A | T | S | L | R | F | I | T | P | F | P | E | K | C | S | V | T | G | E | M | F | N | N | L | R | T | M | P | F | I | R | N | L | T | V | E | N | T | W | I | N | S | S | F | M | E | G | F | F | K | N | V | W | L | S | H | L | Y | D | L | S | F | V | N | I | T | Y | N | E | D | T | P | D | G | F | Q | F | - | - | - | - | - | - | R | T | I | N | - | H | T | V | N | L | C | S | I | T | F | N | G | V | H | H | Y | Q | Y | R | Y | P | T | I | N | M | S | L | E | A | I | S | N | L | T | Y | L | K | F | S | R | S | G | M | N | I | I | P | C | N | L | F | S | V | L | P | A | L | E | T | L | D | L | S | D | N | L | L | T | D | A | G | F | W | W | F | - | - | - | K | C | T | Q | T | S | V | F | P | K | L | R | R | L | S | L | S | K | N | R | F | S | S | L | S | F | I | S | E | Q | T | N | Q | M | K | T | L | E | S | L | D | L | S | F | N | S | I | Y | L | D | R | - | - | - | E | C | S | W | P | A | Q | L | T | E | L | R | L | G | N | N | N | L | G | N | S | V | F | E | Y | L | S | S | H | F | E | R | I | D | L | S | K | T | G | I | T | A | V | T | Q | E | T | L | S | R | F | P | K | L | T | H | L | Q | L | S | F | N | S | I | Q | V | L | P | A | D | L | S | A | P | A | L | L | S | L | Y | V | D | Q | N | A | I | T | S | V | S | R | E | V | L | A | G | L | P | G | L | Q | T | L | K | A | G | N | N | P | F | V | C | S | C | D | S | Y | W | F | V | T | T | L | N | K | - | - | - | - | - | - | - | - | - | - | - | - | - | - | S | L | L | P | D | W | P | L | D | Y | T | C | S | T | P | P | S | F | A | G | L | S | L | S | E | Y | K | T | S | E | L | S | C | E | M | W | L | Q | A | A | V | A | V | P | V | V | I | M | I | S | A | A | M | G | L | L | F | Y | K | C | D | G | V | W | Y | T | K | M | L | W | V | W | I | R | V | K | R | R | G | R | K | R | S | H | M | L | K | N | L | - | S | C | S | Y | H | A | F | I | S | Y | S | H | Q | D | S | G | W | V | G | S | Q | L | V | P | T | L | E | G | A | R | - | - | - | - | - | - | F | S | L | C | V | H | E | R | D | F | V | P | G | E | W | I | I | D | N | I | I | N | C | V | E | S | S | Y | K | T | L | F | V | L | S | K | H | F | V | Q | S | E | W | C | N | Y | E | L | F | F | A | Q | H | R | A | I | S | V | Q | Q | D | S | L | V | F | I | L | L | E | P | I | P | T | N | S | L | P | K | K | F | L | R | L | R | S | L | L | R | Q | Q | T | Y | L | E | W | P | K | D | E | R | K | Q | Q | V | F | W | A | S | L | K | S | M | L | H | M | A | D | K | S | M | V | L | K | D | V | A | L | A | L | S | D | T | A | P | L | V | T | D | Q | E | X | X | X | X | X | X | X | X | X | X | X | X | X | X | X | X | X | X | X | X | X | X | X | X | X | X | X | X | X | X | X | X | X | X | X | X | - | X | X | - | - | - |
| TRTLR1 | - | - | - | - | - | - | - | - | - | - | - | - | - | - | - | - | - | - | - | - | - | - | - | - | - | - | - | - | - | - | - | - | - | - | - | - | - | M | M | G | S | T | I | A | I | F | C | A | A | A | M | L | M | P | Q | L | - | S | V | S | Y | - | - | - | L | R | N | Y | I | D | L | S | S | R | N | L | S | S | V | P | G | D | L | P | K | E | A | E | Y | I | D | L | S | L | N | H | I | R | Q | L | K | R | G | D | F | R | N | T | P | I | L | R | F | L | N | I | S | G | N | C | L | D | S | I | H | P | E | T | F | L | H | T | P | L | L | E | D | L | D | L | S | H | N | E | L | K | D | L | M | D | Q | P | Y | L | Q | H | T | E | N | L | V | A | L | N | L | A | H | N | K | F | L | T | M | T | L | A | R | E | F | G | S | L | V | K | L | Q | R | L | T | L | G | G | - | - | - | S | T | I | S | V | G | D | F | R | N | I | A | D | V | E | L | R | V | M | S | L | F | L | E | - | G | V | L | V | Y | E | P | G | S | L | Q | D | V | Y | A | R | R | L | Q | V | K | F | N | - | - | N | K | F | P | H | D | L | M | E | D | A | L | S | F | F | P | E | V | E | L | L | - | - | E | - | - | - | - | - | - | - | L | S | R | G | Y | Q | - | - | - | - | E | L | S | K | Q | L | - | S | Q | R | S | E | I | Y | T | S | C | L | Y | L | T | H | I | S | I | N | W | P | D | F | T | Q | Y | V | H | V | A | L | N | T | T | V | T | H | L | G | V | S | G | V | T | M | Y | R | L | P | Q | N | A | T | P | V | A | E | T | S | R | V | K | S | L | T | F | R | R | A | V | V | R | S | F | L | F | S | Q | E | A | V | Y | N | F | F | - | - | - | I | N | M | P | V | E | N | L | - | - | - | - | - | - | A | L | T | E | - | - | T | S | I | I | H | M | T | C | P | K | S | E | S | P | I | T | Q | L | N | F | S | Y | C | S | M | T | D | T | I | F | S | R | V | E | G | L | I | T | V | E | C | K | T | L | G | N | V | R | T | L | A | L | A | G | N | N | L | K | S | L | K | S | L | S | I | R | M | Q | Y | M | K | S | L | Q | D | L | D | L | S | L | N | L | L | V | Y | D | G | - | Q | K | G | C | V | W | P | Q | N | I | S | S | M | S | L | S | S | N | G | L | T | D | S | V | F | Q | C | L | P | E | N | V | E | R | L | D | L | Q | N | N | Q | I | S | A | V | S | S | S | - | T | L | G | L | V | R | L | W | Y | L | N | L | N | A | N | K | L | L | D | L | P | V | C | H | N | F | P | L | L | Q | E | L | L | L | K | S | N | S | L | H | T | P | S | V | D | R | L | K | S | C | R | S | L | K | T | L | D | A | S | S | N | P | F | T | C | T | C | D | L | I | G | F | I | Q | L | G | L | K | F | E | K | - | - | - | - | - | G | - | - | G | A | A | V | T | F | L | Q | W | P | H | G | Y | Y | C | S | Y | P | E | A | V | R | D | S | N | L | N | N | F | W | I | P | E | I | S | C | N | S | Y | L | L | A | A | A | I | L | C | P | A | V | V | V | M | T | V | V | L | T | L | C | R | H | F | D | I | P | W | Y | L | G | M | I | W | Q | W | M | Q | A | K | H | R | A | R | Q | R | Q | P | R | P | E | V | L | L | G | I | E | F | H | A | F | V | S | Y | S | Q | K | D | A | A | W | V | F | D | H | L | L | P | N | L | E | S | P | A | - | - | - | - | G | G | L | R | I | C | H | H | G | K | N | F | V | P | G | K | P | I | I | N | N | I | M | T | C | V | E | K | S | R | R | C | V | F | V | L | S | A | H | F | V | K | S | D | W | C | H | Y | E | L | Y | F | T | S | H | Q | H | L | A | L | G | S | D | S | V | V | L | V | L | L | E | P | V | P | Q | Y | L | I | P | S | K | Y | Y | Q | L | K | F | M | M | A | R | H | T | Y | L | E | W | P | Q | D | R | A | K | Q | R | L | F | W | A | N | L | R | A | A | L | Q | A | D | L | P | H | L | T | V | T | E | I | E | E | X | X | X | X | X | X | X | - | X | X | X | X | X | X | X | X | X | X | X | X | X | X | X | X | X | X | X | X | X | X | X | X | X | - | - | - | - | - | - | - | X | X | X | X | X | X | X | X | X | X | X | X | X | X | X |
| ONTLR1 | - | - | - | - | - | - | - | - | - | - | - | - | - | - | - | - | - | - | - | - | - | - | - | - | - | - | - | - | - | - | - | - | - | - | - | - | - | M | R | V | T | A | A | V | L | W | T | A | A | V | L | V | G | L | Q | K | - | S | A | S | S | - | - | - | P | D | N | F | V | D | R | S | S | Q | N | L | S | S | V | P | T | D | L | P | Q | T | T | E | F | L | D | L | S | R | N | Y | I | H | Q | L | H | N | G | D | F | E | K | T | T | H | L | R | F | L | N | V | S | W | N | G | L | E | E | I | D | P | Q | T | F | L | D | T | P | L | L | E | H | L | D | L | S | H | N | N | L | K | N | L | S | G | Q | Q | Y | L | L | H | T | V | N | L | L | V | L | N | L | A | F | N | R | F | L | T | M | T | L | G | S | E | F | S | S | L | V | K | L | E | R | L | K | V | G | A | - | - | - | K | N | I | S | V | G | D | F | K | N | I | A | K | V | K | L | R | T | L | T | L | L | L | E | - | D | E | L | Y | Y | E | A | G | S | L | E | D | V | H | A | Q | R | L | Q | I | A | F | G | H | N | Q | K | I | D | R | N | L | T | A | D | A | L | S | L | F | V | E | V | E | M | M | - | - | N | - | - | - | - | - | - | - | M | K | D | G | Y | K | - | - | - | - | D | L | S | K | Q | L | - | R | E | T | V | K | I | H | T | T | S | F | Y | L | T | N | I | T | I | K | W | Q | D | L | T | E | Y | V | N | V | A | L | N | T | T | L | K | H | L | D | A | S | D | V | R | I | M | Q | P | P | R | N | E | T | E | V | I | K | T | S | Q | V | I | S | F | T | A | R | Q | A | V | V | T | T | F | F | F | S | Q | E | A | L | Y | N | F | F | - | - | - | I | S | M | P | V | E | R | V | - | - | - | - | - | - | A | I | T | D | - | - | T | P | I | I | H | M | T | C | P | K | S | Q | S | P | I | R | Q | L | D | F | S | N | C | A | M | S | D | T | I | F | S | S | V | V | D | D | K | F | V | E | C | Q | N | L | R | N | L | K | N | L | T | L | V | G | N | S | L | K | K | L | E | I | L | S | K | R | F | Q | Y | M | S | S | L | Q | H | L | D | L | S | V | N | S | I | G | Y | D | Q | - | S | L | E | C | I | W | P | A | S | I | T | N | I | N | L | S | S | N | S | L | T | D | S | V | F | Q | C | L | P | N | G | T | Q | I | L | D | L | Q | N | N | Q | I | S | V | V | P | Q | S | - | I | F | K | L | R | N | L | S | T | L | N | L | N | A | N | R | L | R | D | L | P | L | C | D | T | F | P | S | L | H | K | L | L | L | R | S | N | S | L | H | A | P | S | V | N | I | L | E | S | C | P | E | L | R | T | L | D | V | S | F | N | P | F | T | C | I | C | T | L | T | S | F | I | K | L | A | I | N | S | E | K | - | - | - | - | - | N | N | S | Q | S | G | I | E | L | L | N | W | P | Q | G | Y | Y | C | T | Y | P | E | D | V | R | N | S | T | L | R | D | I | S | I | P | E | V | S | C | S | I | G | L | L | V | V | A | I | L | V | P | A | G | T | L | I | I | S | V | V | I | L | C | H | C | L | D | V | P | W | Y | M | G | M | I | W | Q | W | T | R | A | K | H | R | A | R | M | R | Q | A | R | P | E | D | L | V | G | L | E | F | H | A | F | V | S | Y | S | Q | H | D | V | D | W | V | R | N | S | L | L | P | N | L | E | G | P | A | - | - | - | - | G | G | L | R | I | C | H | H | E | Q | H | F | V | P | G | K | T | I | I | E | N | I | I | S | C | V | E | K | C | R | R | S | V | F | V | L | S | A | H | F | V | K | S | E | W | C | H | Y | E | L | Y | F | A | A | H | Q | R | L | T | L | G | S | D | S | I | V | L | V | L | L | E | P | L | P | Q | Y | L | I | P | S | K | Y | Y | Q | L | K | S | M | M | N | R | H | T | Y | L | E | W | P | Q | D | R | A | K | Q | R | L | F | W | A | N | L | R | A | A | L | Q | T | D | L | P | N | A | P | V | T | Q | V | Q | E | H | L | A | E | E | R | L | - | E | E | G | Q | R | L | I | G | X | X | X | X | X | X | X | X | X | X | X | X | X | X | X | X | X | - | - | - | - | - | - | - | X | X | X | X | X | X | X | X | X | X | X | - | - | - | - |
| ECTLR1 | - | - | - | - | - | - | - | - | - | - | - | - | - | - | - | - | - | - | - | - | - | - | - | - | - | - | - | - | - | - | - | - | - | - | - | - | - | M | R | P | V | N | A | A | L | W | A | A | A | I | L | M | G | L | Q | L | - | S | S | S | S | - | - | - | - | P | N | F | V | D | L | S | S | K | N | L | S | S | V | P | S | D | L | P | E | T | V | E | F | L | D | L | S | C | N | H | I | Q | Q | L | H | Q | G | D | F | K | S | T | P | L | L | R | F | L | N | I | S | W | N | N | L | E | G | I | D | P | E | T | F | L | D | T | S | L | L | E | D | L | D | L | S | H | N | R | L | T | N | L | S | G | Q | R | Y | L | L | H | T | E | N | L | R | V | L | N | L | T | C | N | N | F | L | T | M | T | L | G | S | E | F | S | F | L | V | K | L | E | S | L | A | V | G | A | - | - | - | P | N | I | S | V | G | D | F | K | N | I | A | Q | V | K | L | R | T | L | T | L | S | L | E | - | D | Q | L | N | Y | T | P | G | S | L | K | D | V | N | A | K | R | L | Q | I | T | F | S | R | H | Q | K | I | D | L | H | V | I | D | D | A | L | S | L | F | A | E | V | E | L | M | - | - | D | - | - | - | - | - | - | - | L | T | H | D | Y | S | - | - | - | - | N | L | S | Q | Q | L | - | S | E | R | K | E | I | L | T | S | H | L | Y | L | T | N | I | S | I | E | W | S | S | L | T | H | F | V | K | A | V | L | R | T | S | I | R | H | L | S | A | S | D | V | A | M | S | K | L | P | Y | F | D | T | V | V | T | Y | T | S | Q | M | E | S | F | T | A | S | R | A | V | V | T | T | F | F | F | S | Q | E | A | V | Y | N | F | F | - | - | - | I | N | L | P | V | K | R | L | - | - | - | - | - | - | V | I | N | E | - | - | T | S | I | I | H | M | T | C | P | G | S | Q | S | P | I | R | E | L | D | F | S | Y | C | A | M | S | D | S | I | F | T | T | A | D | A | T | G | I | K | E | C | M | N | L | G | N | L | T | K | L | N | L | A | G | N | S | L | K | S | L | Q | R | L | S | Q | R | V | Q | Y | M | T | S | L | Q | D | V | D | L | S | F | N | S | L | T | Y | D | G | - | L | T | E | C | I | W | P | P | K | I | T | N | M | T | L | A | S | N | S | L | T | D | S | V | F | K | C | L | P | K | E | T | E | T | L | D | L | Q | N | N | Q | I | S | V | I | P | S | S | - | I | L | K | L | E | N | L | K | S | L | N | L | N | S | N | R | L | R | D | L | P | A | C | N | G | F | P | I | L | S | E | L | L | L | R | S | N | S | L | N | T | P | S | V | N | N | L | E | S | C | A | K | L | K | T | L | D | V | S | N | N | P | F | A | C | T | C | A | L | K | N | F | I | S | L | G | V | R | S | E | T | - | - | - | - | - | K | K | S | L | T | G | I | E | L | V | S | W | P | L | D | Y | C | C | S | Y | P | D | N | V | R | D | S | T | L | K | D | T | Q | I | P | E | I | S | C | N | V | G | L | L | A | T | T | I | L | C | P | A | I | A | V | I | I | V | V | V | T | A | C | H | R | L | D | V | P | W | Y | M | G | M | I | W | Q | W | T | R | A | K | H | R | A | R | M | R | Q | V | R | P | E | D | L | A | G | V | E | F | H | A | F | V | S | Y | S | Q | H | D | A | D | W | V | H | N | S | L | L | P | N | L | E | G | P | A | - | - | - | - | G | G | L | R | I | C | H | H | E | K | N | F | V | P | G | K | T | I | I | E | N | I | I | G | C | V | E | K | S | R | R | S | V | F | V | L | S | A | H | F | V | K | S | E | W | C | H | Y | E | L | Y | F | A | S | H | Q | R | L | A | W | G | S | D | S | I | V | L | V | L | L | E | P | L | P | Q | Y | L | I | P | S | K | Y | Y | Q | L | K | S | M | M | G | R | H | T | Y | L | E | W | P | Q | D | T | K | K | H | R | L | F | W | A | N | L | R | A | A | L | Q | T | D | L | P | D | A | P | V | T | G | L | E | E | X | X | X | X | X | X | X | - | X | X | X | X | X | X | X | X | X | X | X | X | X | X | X | X | X | X | X | X | X | X | X | X | X | - | - | - | - | - | - | - | X | X | X | X | X | X | X | X | X | X | X | X | - | - | - |
| ATTLR1 | - | - | - | - | - | - | - | - | - | - | - | - | - | - | - | - | - | - | - | - | - | - | - | - | - | - | - | - | - | - | - | - | - | - | - | - | - | M | K | P | - | T | T | A | L | W | T | A | A | M | L | V | G | L | Q | L | - | I | A | S | S | - | - | - | S | D | N | I | V | D | R | S | S | R | N | L | S | S | V | P | R | D | L | P | Q | K | V | E | F | L | D | L | S | C | N | N | I | Q | R | L | H | S | G | D | F | K | N | T | T | L | L | K | F | L | N | I | S | W | N | Q | L | E | H | I | D | P | E | T | F | L | D | T | P | Q | L | E | D | L | D | L | S | H | N | R | L | M | I | L | L | G | Q | Q | Y | L | L | H | A | G | N | L | K | M | L | N | L | A | Y | N | E | F | L | N | M | T | L | G | T | E | F | T | F | L | V | K | L | E | R | L | A | L | G | A | - | - | - | K | N | V | S | I | G | D | F | K | N | I | A | K | L | K | L | R | S | L | T | L | C | L | E | - | D | G | F | S | Y | E | T | G | S | L | Q | A | L | H | A | Q | K | L | Q | I | V | A | T | S | N | E | A | I | N | F | N | L | T | A | D | A | L | L | L | F | D | E | V | E | L | M | - | - | H | - | - | - | - | - | - | - | L | T | H | G | Y | R | - | - | - | - | Q | L | S | D | Q | L | - | L | - | K | I | K | I | N | T | S | H | L | Y | L | T | N | I | A | I | E | W | K | D | L | T | H | Y | V | N | T | V | L | S | T | S | I | R | H | L | S | A | S | D | V | A | I | Y | K | P | P | Y | I | D | T | P | V | T | S | K | S | I | M | K | S | F | S | A | K | R | A | V | V | M | S | F | F | F | S | Q | N | A | V | Y | N | F | F | - | - | - | I | N | M | P | V | E | S | L | - | - | - | - | - | - | A | I | T | E | - | - | T | S | I | I | H | M | T | C | P | K | S | Q | S | P | I | L | Q | L | D | F | S | Y | C | A | L | S | D | T | I | F | S | R | V | E | G | Q | E | T | I | E | C | T | N | L | G | N | L | T | R | L | I | L | L | G | N | N | L | K | S | L | Q | L | L | S | K | R | M | Q | F | M | K | S | L | Q | H | L | D | L | S | L | N | T | L | V | Y | D | G | - | M | V | E | C | V | W | P | P | N | I | T | N | M | I | L | S | S | N | S | L | T | D | S | A | F | K | C | L | P | R | G | I | S | T | L | D | L | Q | N | N | Q | I | S | A | V | P | S | S | - | I | L | K | L | E | N | L | S | S | L | N | L | N | A | N | R | L | R | D | L | P | V | C | N | G | F | P | I | L | Q | E | L | L | L | K | S | N | S | I | H | A | P | S | V | N | Q | L | T | S | C | P | K | L | N | T | L | D | V | S | L | N | P | F | T | C | T | C | V | L | R | S | F | I | S | L | G | V | K | S | E | K | - | - | - | - | - | K | H | S | H | T | G | F | Q | L | L | A | W | P | L | D | Y | Y | C | S | Y | P | E | S | V | R | D | T | L | L | E | H | I | S | I | Q | E | V | S | C | N | V | G | I | L | A | A | S | I | L | C | P | T | V | V | V | I | I | A | I | V | T | L | C | N | H | L | D | I | P | W | Y | M | R | M | I | C | Q | W | T | R | A | K | H | R | A | R | T | R | Q | L | R | P | E | D | L | V | G | V | Q | F | H | A | F | V | S | Y | S | Q | H | D | A | D | W | V | H | D | T | L | L | P | N | L | E | G | P | A | - | - | - | - | G | G | L | R | I | C | Q | H | E | K | N | F | V | P | G | K | T | I | I | E | N | I | I | N | C | V | E | K | S | R | R | S | V | F | V | L | S | A | H | F | V | K | S | E | W | C | H | Y | E | L | Y | F | A | S | H | Q | R | L | T | R | G | S | D | S | I | V | L | V | L | L | E | P | L | P | Q | Y | L | I | P | S | K | Y | Y | Q | L | K | S | M | M | K | R | H | T | Y | L | E | W | P | Q | D | R | A | K | Q | R | L | F | W | A | N | L | R | A | A | L | Q | A | H | L | P | N | A | P | V | V | E | X | X | X | X | X | X | X | X | X | X | - | X | X | X | X | X | X | X | X | X | X | X | X | X | X | X | X | X | X | X | X | X | X | X | X | X | - | - | - | - | - | - | - | X | X | X | X | X | X | X | X | X | X | X | X | X | - | - |
| SATLR1 | - | - | - | - | - | - | - | - | - | - | - | - | - | - | - | - | - | - | - | - | - | - | - | - | - | - | - | - | - | - | - | - | - | - | - | - | - | M | R | P | V | Y | A | A | I | W | A | A | A | I | L | V | R | L | Q | L | - | S | A | S | S | - | - | - | S | E | T | N | V | N | R | S | S | Q | N | L | S | S | V | P | G | D | L | P | E | T | V | E | V | L | D | L | S | C | N | R | I | Q | Q | L | H | Q | G | D | F | N | N | T | I | H | L | K | S | L | N | I | S | W | N | S | L | E | V | I | D | P | D | T | F | V | N | T | P | L | L | Q | D | L | D | L | S | H | N | R | L | K | N | L | S | G | Q | R | Y | L | L | H | T | K | N | L | Q | V | L | N | L | T | C | N | D | F | L | K | M | T | L | G | S | E | F | S | S | L | V | K | L | E | W | L | A | L | G | A | - | - | - | E | E | I | H | L | G | D | F | R | N | I | A | E | V | K | L | L | T | L | T | L | S | L | E | - | G | H | L | V | Y | E | A | G | S | L | K | D | V | Q | A | Q | Q | L | Q | I | A | M | T | R | N | Q | I | L | D | H | D | L | F | H | D | A | L | S | H | F | V | E | V | E | L | M | - | - | N | - | - | - | - | - | - | - | V | T | G | G | F | D | - | - | - | - | S | L | S | K | Q | L | - | S | E | R | A | K | I | K | T | S | Y | L | Y | L | T | N | I | T | I | S | W | H | D | L | T | D | F | V | N | V | V | L | G | T | S | I | S | H | M | G | A | S | D | V | A | M | H | N | L | P | L | E | E | S | P | M | P | K | T | S | K | I | K | S | F | T | A | R | R | A | R | V | M | S | F | I | F | S | Q | E | V | V | Y | D | F | F | - | - | - | I | N | L | R | V | E | S | L | - | - | - | - | - | - | A | I | L | E | - | - | T | S | I | I | H | M | T | C | P | K | S | Q | S | E | L | L | Q | L | D | F | S | L | C | A | L | S | D | S | I | F | S | R | V | E | G | Q | E | L | I | E | C | E | N | L | A | N | L | R | K | L | I | L | V | G | N | N | L | K | N | F | Q | L | L | S | K | R | M | Q | H | M | K | S | L | Q | H | L | D | L | S | V | N | Q | L | M | Y | D | G | - | V | E | E | C | V | W | P | P | N | I | T | N | M | S | L | S | S | N | R | L | T | D | S | V | F | K | C | V | P | K | G | M | E | T | L | D | L | Q | N | N | Q | V | S | V | V | P | S | S | - | I | M | N | L | K | N | L | S | S | L | N | L | N | A | N | R | L | R | D | L | P | V | C | N | G | L | P | I | L | N | V | L | L | L | K | S | N | S | I | H | A | P | S | V | N | K | L | E | S | C | P | K | L | K | T | L | D | V | S | H | N | P | F | T | C | T | C | S | L | R | S | F | I | S | L | G | I | K | T | E | K | - | - | - | - | - | K | R | S | D | T | G | I | E | L | L | S | W | P | L | G | Y | Y | C | I | Y | P | E | T | I | K | D | S | T | L | N | K | I | W | I | P | V | V | S | C | N | V | G | L | L | A | A | T | I | L | C | P | A | V | F | L | I | L | V | V | L | T | L | C | H | R | L | D | I | P | W | Y | M | G | M | I | W | Q | W | T | R | A | K | H | R | A | R | R | R | Q | L | R | P | E | D | L | V | G | V | E | F | H | A | F | V | S | Y | S | Q | H | D | A | D | W | V | H | N | S | L | L | P | N | L | E | G | P | A | - | - | - | - | G | G | L | R | I | C | H | H | E | K | H | F | V | P | G | K | T | I | I | E | N | I | M | S | C | V | E | K | S | R | R | A | M | F | V | L | S | A | H | F | V | K | S | D | W | C | H | Y | E | L | Y | F | A | T | H | H | R | L | G | H | G | S | D | S | I | V | L | V | L | L | E | P | L | P | Q | Y | L | I | P | S | K | Y | N | Q | L | K | S | M | M | S | R | H | T | Y | L | E | W | P | Q | D | R | A | K | Q | R | L | F | W | A | N | L | R | A | A | L | Q | V | D | L | P | N | A | P | V | T | E | L | E | E | X | X | X | X | X | X | X | - | X | X | X | X | X | X | X | X | X | X | X | X | X | X | X | X | X | X | X | X | X | X | X | X | X | - | - | - | - | - | - | - | X | X | X | X | X | X | X | X | X | X | X | - | - | - | - |
| MMTLR1 | - | - | - | - | - | - | - | - | - | - | - | - | - | - | - | - | - | - | - | - | - | - | - | - | - | - | - | - | - | - | - | - | - | - | - | - | - | M | R | P | - | T | I | A | L | W | A | A | V | L | F | L | G | L | Q | L | - | S | A | S | F | - | - | - | S | D | S | I | V | D | R | S | S | Q | N | L | S | S | V | P | R | D | L | P | H | T | T | E | S | L | D | L | S | C | N | H | I | Q | K | L | H | R | G | D | F | K | N | T | T | L | L | R | I | L | N | I | S | W | N | S | L | E | E | I | D | P | E | T | F | L | D | T | P | L | L | E | D | L | D | L | S | H | N | M | L | K | N | L | L | D | Q | R | Y | L | L | H | A | D | N | L | L | V | L | N | L | A | C | N | K | F | L | T | M | T | L | G | S | E | F | S | S | L | V | K | L | E | R | L | A | L | G | A | - | - | - | K | N | I | S | V | G | D | F | K | N | I | A | E | V | K | L | H | T | L | T | L | S | L | E | - | D | D | L | G | Y | E | A | G | S | L | K | D | V | H | A | Q | K | L | Q | I | D | L | T | R | N | Q | I | I | V | H | D | L | L | A | D | G | L | S | L | F | A | E | L | E | L | I | - | - | N | - | - | - | - | - | - | - | L | R | K | G | Y | K | - | - | - | - | D | L | S | D | Q | L | - | K | A | R | K | E | I | Y | T | S | S | L | Y | L | T | N | I | S | I | K | W | P | D | L | T | H | F | V | K | V | V | L | N | T | S | I | A | H | L | Y | T | A | D | V | V | L | N | D | L | P | Q | T | D | T | E | G | E | T | A | S | K | M | K | S | F | R | V | R | R | V | V | V | K | S | F | F | F | S | Q | E | A | V | Y | N | F | F | - | - | - | I | N | T | P | V | D | T | L | - | - | - | - | - | - | A | I | I | D | - | - | S | P | I | I | Y | M | T | C | P | K | S | Q | S | R | V | L | H | L | D | F | S | Y | C | A | L | S | D | S | I | F | S | R | V | V | G | Q | E | T | I | E | C | E | N | L | D | N | L | R | N | L | D | L | V | G | N | N | L | K | S | L | H | L | L | S | K | R | T | H | N | M | K | S | L | R | H | L | D | L | G | L | N | S | L | V | Y | D | G | - | V | E | E | C | F | W | P | P | N | I | T | N | M | S | L | Y | S | N | G | L | T | D | S | V | F | K | C | L | P | K | G | T | E | I | L | D | L | Q | N | N | Q | I | S | V | I | P | S | T | - | I | L | K | L | E | N | L | S | S | L | N | L | N | A | N | R | L | R | D | L | P | G | C | N | G | F | P | I | L | N | A | L | F | L | K | S | N | S | L | H | T | P | S | V | N | K | L | K | S | C | S | R | L | K | T | L | D | V | S | S | N | P | F | T | C | T | C | G | L | K | S | F | I | N | L | G | I | K | S | E | N | - | - | - | - | - | Q | E | S | D | T | G | I | E | L | L | S | W | P | S | D | Y | R | C | A | Y | P | E | V | N | R | E | S | T | L | N | T | I | L | I | P | E | V | S | C | N | V | G | L | L | A | T | T | I | L | C | P | A | V | A | M | I | I | L | V | M | V | L | C | H | R | L | D | V | P | W | Y | M | G | M | I | W | K | W | T | R | A | K | H | R | A | R | M | R | Q | V | R | P | E | D | L | V | G | I | E | F | H | A | F | V | S | Y | S | Q | H | D | A | D | W | V | H | N | S | L | I | P | N | L | E | G | P | S | - | - | - | - | G | G | L | R | I | C | H | H | E | K | H | F | V | A | G | K | S | I | I | E | N | I | I | T | S | V | E | K | S | R | R | S | L | F | V | L | S | A | H | F | V | K | S | E | W | C | H | Y | E | L | Y | F | A | S | H | Q | H | L | T | Q | G | S | D | S | V | V | L | V | L | L | E | P | L | P | Q | Y | L | I | P | S | K | Y | Y | Q | L | K | S | M | M | S | R | H | T | Y | I | E | W | P | Q | D | R | A | K | H | R | L | F | W | A | N | L | R | A | A | L | Q | A | D | L | P | N | A | P | V | T | E | L | E | E | X | X | X | X | X | X | X | - | X | X | X | X | X | X | X | X | X | X | X | X | X | X | X | X | X | X | X | X | X | X | X | X | X | - | - | - | - | - | - | - | X | X | X | X | X | X | X | X | X | X | X | X | - | - | - |
| SMTLR1 | - | - | - | - | - | - | - | - | - | - | - | - | - | - | - | - | - | - | - | - | - | - | - | - | - | - | - | - | - | - | - | - | - | - | - | - | - | M | R | P | T | T | A | A | L | W | A | A | V | M | L | V | G | L | Q | Q | - | L | A | S | L | A | A | S | P | D | S | R | V | D | C | S | S | K | N | L | S | S | V | P | R | D | L | P | Q | E | V | E | F | L | D | L | S | C | N | H | I | K | Q | L | H | R | G | D | F | R | N | T | T | L | L | R | S | L | N | V | S | W | N | G | L | E | E | I | D | P | G | T | F | S | D | T | P | L | L | E | V | L | D | L | S | H | N | R | L | K | N | L | S | G | Q | R | Y | L | L | H | T | G | N | L | L | T | L | N | L | T | W | N | S | F | L | T | M | T | L | G | S | A | F | T | S | L | V | R | L | E | R | L | A | L | G | A | - | - | - | K | N | I | S | M | D | D | F | K | N | I | A | G | A | K | L | Q | T | L | T | L | C | L | G | - | D | E | L | G | Y | E | A | G | S | L | K | D | V | H | A | L | R | L | Q | I | A | S | T | S | G | K | L | V | D | D | Y | L | Y | A | D | A | L | S | L | F | D | E | V | E | L | M | - | - | N | - | - | - | - | - | - | - | M | T | G | G | Y | R | - | - | - | - | E | L | S | E | Q | L | - | S | Q | R | I | E | I | H | T | S | H | L | Y | L | T | N | I | L | I | H | W | Q | D | L | T | H | Y | V | N | V | I | L | R | T | S | I | T | H | L | C | F | S | D | M | A | M | S | R | L | P | Q | V | D | T | D | V | T | K | K | S | K | M | K | S | F | S | G | R | R | V | V | V | K | S | F | L | F | S | Q | E | A | V | Y | N | F | F | - | - | - | I | N | M | P | V | E | S | L | - | - | - | - | - | - | A | I | T | E | - | - | T | S | I | I | H | M | T | C | P | K | S | Q | S | P | I | L | Q | L | D | F | S | Y | C | A | L | S | D | S | I | F | S | R | V | E | G | Q | Q | T | L | E | C | Q | N | L | G | N | V | R | K | L | I | L | V | G | N | N | L | K | N | L | Q | F | L | S | K | R | V | Q | Y | M | K | S | L | Q | H | L | D | L | S | L | N | S | L | I | Y | E | G | - | L | E | E | C | V | W | P | P | N | I | T | N | V | S | L | S | S | N | R | L | T | H | T | V | F | K | C | L | T | K | G | I | E | T | L | D | L | Q | N | N | Q | V | S | V | I | P | S | S | - | I | L | K | L | G | N | L | S | S | L | N | L | N | A | N | R | L | R | D | L | P | V | C | D | G | F | P | I | L | N | Q | L | L | L | R | S | N | S | L | H | A | P | F | V | N | K | L | E | S | C | P | K | L | K | T | L | D | V | G | N | N | P | F | T | C | T | C | A | L | R | S | F | I | H | L | G | I | K | S | E | R | - | - | - | - | - | - | K | S | H | P | G | I | E | L | L | G | W | P | L | D | Y | Y | C | T | Y | P | E | D | V | R | D | T | I | L | K | D | I | L | I | P | D | V | T | C | N | V | A | I | L | A | V | T | I | L | C | P | A | V | V | V | I | V | A | V | V | I | L | C | H | R | F | D | V | P | W | Y | M | G | M | I | W | Q | W | T | R | A | K | H | R | A | R | T | R | Q | V | R | P | E | D | L | V | G | V | E | F | H | A | F | V | S | Y | S | Q | H | D | T | D | W | V | H | N | S | L | L | P | N | L | E | G | P | A | - | - | - | - | G | G | L | R | I | C | L | H | E | K | S | F | V | A | G | K | T | V | I | E | N | I | M | G | C | V | G | K | S | R | R | S | L | F | V | L | S | A | H | F | V | K | S | E | W | C | H | Y | E | L | Y | F | A | S | H | Q | Q | L | A | L | G | S | D | S | I | V | L | V | L | L | E | P | L | P | Q | Y | T | I | P | S | K | Y | N | Q | L | K | S | M | M | G | R | H | T | Y | L | E | W | P | Q | D | M | A | K | H | R | L | F | W | A | N | L | R | A | A | L | Q | A | D | L | P | D | V | P | V | T | E | I | E | E | X | X | X | X | X | X | X | - | X | X | X | X | X | X | X | X | X | X | X | X | X | X | X | X | X | X | X | X | X | X | X | X | X | - | - | - | - | - | - | - | X | X | X | X | X | X | X | X | X | - | - | - | - | - | - |
| TOTLR1 | - | - | - | - | - | - | - | - | - | - | - | - | - | - | - | - | - | - | - | - | - | - | - | - | - | - | - | - | - | - | - | - | - | - | - | - | - | M | R | V | M | N | A | A | L | W | V | A | V | M | I | V | V | L | Q | Q | - | I | N | S | S | - | - | P | - | D | G | I | E | D | R | S | S | Q | N | L | S | S | V | P | R | D | L | P | Q | T | V | K | F | L | D | L | S | S | N | H | I | K | Q | L | H | K | G | D | F | Q | N | T | T | L | L | K | F | L | N | V | S | W | N | S | L | E | E | I | D | P | E | T | F | L | S | T | P | L | L | E | Y | L | D | L | S | H | N | W | L | K | N | L | S | G | Q | Q | Y | L | L | H | T | G | N | L | L | M | L | N | L | A | C | N | M | F | P | S | M | T | L | G | S | A | F | C | A | L | V | K | L | E | R | L | A | L | G | A | - | - | - | K | N | I | S | M | G | D | F | K | N | I | A | G | V | K | L | R | T | L | T | L | S | M | G | - | D | E | L | G | Y | E | A | G | S | L | K | E | V | Q | A | Q | K | L | Q | I | I | L | S | T | N | Q | I | V | G | R | D | L | V | E | D | A | L | S | L | F | V | Q | V | E | L | M | - | - | N | - | - | - | - | - | - | - | L | T | G | G | Y | R | - | - | - | - | E | L | T | E | Q | L | - | R | Q | R | T | K | I | Y | T | S | H | L | Y | L | T | N | I | K | I | Q | W | E | D | L | T | H | Y | V | N | M | V | L | Q | T | S | I | T | H | L | D | A | S | D | V | V | L | T | Q | L | P | Y | H | D | T | D | V | T | K | I | S | K | M | K | S | F | S | A | R | R | V | V | V | T | T | F | F | F | S | Q | E | S | V | Y | N | Y | F | - | - | - | M | D | M | P | V | E | R | M | - | - | - | - | - | - | A | I | T | E | - | - | T | P | I | I | H | M | T | C | P | H | S | Q | S | R | I | L | E | L | D | F | S | Y | C | S | I | S | D | S | I | F | S | I | V | E | K | Q | E | I | L | E | C | K | N | L | G | N | L | R | K | L | I | L | T | S | N | S | F | K | N | L | Q | V | L | S | K | R | L | Q | Y | M | K | S | L | Q | H | L | D | L | S | L | N | S | L | V | Y | E | S | - | L | E | E | C | L | W | P | P | N | I | I | N | M | T | L | T | S | N | R | L | T | D | S | V | F | K | C | L | P | K | G | T | E | T | L | S | L | E | N | N | Q | I | S | V | V | P | S | S | - | I | L | K | M | E | N | L | S | S | L | N | L | N | A | N | R | L | R | D | L | P | V | C | D | G | F | P | I | L | N | E | L | L | L | R | A | N | S | L | H | A | P | S | L | N | K | L | E | S | C | P | K | L | K | T | L | D | V | S | Y | N | P | F | T | C | T | C | A | L | R | G | F | I | S | L | G | I | K | S | E | K | - | - | - | - | - | - | D | S | N | T | G | I | K | L | L | S | W | P | L | D | Y | S | C | T | Y | P | E | D | V | R | D | S | T | L | N | G | I | Q | I | Q | E | V | S | C | N | V | G | I | L | A | A | T | I | L | C | P | A | V | I | V | I | V | L | T | M | T | L | C | H | H | L | D | V | P | W | Y | M | G | M | I | W | Q | W | T | R | A | K | H | R | A | R | T | Q | H | I | R | P | E | D | L | M | G | V | E | F | H | A | F | V | S | Y | S | Q | H | D | A | G | W | V | H | N | S | L | L | P | N | L | E | G | P | A | - | - | - | - | G | G | L | R | I | S | H | H | E | K | N | F | V | P | G | K | T | I | I | E | N | I | I | S | C | V | E | K | S | R | R | S | M | F | V | L | S | A | H | F | V | K | S | E | W | C | H | Y | E | L | Y | F | A | S | H | Q | R | L | S | R | G | S | D | S | I | V | L | V | L | L | E | P | L | P | E | Y | T | I | P | S | K | Y | Y | Q | L | K | S | M | M | G | R | H | T | Y | L | E | W | P | Q | D | R | A | K | H | R | L | F | W | A | N | L | R | A | A | L | Q | A | D | L | P | N | A | P | V | I | E | I | E | E | X | X | X | X | X | X | X | - | X | X | X | X | X | X | X | X | X | X | X | X | X | X | X | X | X | X | X | X | X | X | X | X | X | - | - | - | - | - | - | - | X | X | X | X | X | X | X | X | X | X | X | X | - | - | - |
| LCTLR1 | - | - | - | - | - | - | - | - | - | - | - | - | - | - | - | - | - | - | - | - | - | - | - | - | - | - | - | - | - | - | - | - | - | - | - | - | - | M | R | P | M | T | A | T | L | W | A | A | I | M | F | V | G | L | K | L | - | I | T | A | S | - | - | P | N | I | S | I | V | D | R | S | S | Q | N | L | S | S | V | P | R | D | L | P | Q | T | V | E | F | L | D | L | S | C | N | H | I | K | Q | L | H | R | E | D | F | K | N | T | P | L | L | T | F | L | N | V | S | W | N | S | L | E | G | I | D | P | E | T | F | L | D | T | P | L | L | E | N | L | D | L | S | H | N | R | L | R | N | L | S | G | Q | Q | Y | L | L | H | T | G | N | L | L | M | L | N | L | A | W | N | P | F | L | T | M | T | L | G | S | A | F | S | S | L | V | K | L | E | R | L | A | L | G | A | - | - | - | K | N | I | S | M | G | D | F | K | N | I | A | G | V | K | L | R | T | L | T | L | C | V | G | - | P | E | L | G | Y | E | A | G | S | L | M | D | V | H | T | Q | R | L | Q | V | A | F | S | R | N | P | M | I | N | R | Y | L | V | G | D | A | L | S | L | F | V | E | V | E | F | M | - | - | I | - | - | - | - | - | - | - | M | T | G | G | Y | R | - | - | - | - | E | L | S | E | Q | L | - | S | E | R | M | E | I | R | T | S | H | L | Y | L | T | N | I | S | I | R | W | P | D | L | T | H | F | V | N | V | A | L | L | T | S | I | T | H | L | S | A | S | D | V | V | I | S | N | L | P | R | K | D | T | N | V | P | K | T | S | K | M | K | S | F | S | A | S | R | A | V | V | N | S | F | F | F | S | Q | E | C | V | Y | N | Y | F | - | - | - | I | S | L | P | V | E | S | M | - | - | - | - | - | - | A | I | T | E | - | - | T | S | I | V | H | M | T | C | P | K | S | P | S | Q | I | L | Q | L | D | F | S | N | C | A | L | S | D | T | I | F | S | R | V | E | G | A | K | T | L | E | C | E | N | L | G | N | V | K | K | L | I | L | V | G | N | N | L | K | S | L | K | L | L | S | Q | R | V | Q | Y | M | K | S | L | Q | E | L | D | L | S | L | N | T | L | V | Y | D | G | - | L | E | E | C | V | W | P | P | N | I | T | T | M | T | L | S | S | N | S | L | T | D | S | V | F | K | C | L | P | K | G | T | E | T | L | D | L | Q | N | N | Q | I | S | V | V | P | P | S | - | I | L | K | L | E | N | L | S | S | L | N | L | N | A | N | R | L | R | D | L | P | V | C | N | G | F | P | I | L | N | K | L | L | L | K | S | N | S | L | H | A | P | S | V | N | K | L | E | S | C | P | K | L | K | T | L | D | V | S | Y | N | P | F | T | C | T | C | A | L | R | G | F | I | S | L | G | I | K | S | E | K | - | - | - | - | - | - | N | S | H | T | G | I | E | L | L | N | W | P | L | D | Y | Y | C | T | Y | P | D | D | V | R | D | S | T | L | K | S | I | S | I | P | L | V | S | C | D | V | G | V | L | A | A | T | I | L | C | P | A | V | V | V | I | V | A | V | V | T | L | C | H | R | L | D | A | P | W | Y | M | G | M | I | W | K | W | T | R | A | K | H | R | A | R | T | R | H | L | R | P | E | D | L | V | G | V | E | F | H | A | F | V | S | Y | S | Q | H | D | A | D | W | V | R | D | S | L | L | P | N | L | E | G | S | A | - | - | - | - | G | G | L | R | I | C | H | H | E | K | H | F | V | P | G | K | T | I | I | E | N | I | I | G | C | V | E | K | S | R | R | S | V | F | V | L | S | A | H | F | V | K | S | D | W | C | H | Y | E | L | Y | F | A | S | H | Q | R | L | S | R | G | S | D | S | I | V | L | V | L | L | E | P | L | P | Q | Y | T | I | P | S | K | Y | Y | Q | L | K | S | M | M | S | R | H | T | Y | L | E | W | P | Q | D | R | A | K | H | R | L | F | W | A | N | L | R | A | A | L | Q | A | D | L | P | S | A | P | E | I | E | E | X | X | X | X | X | X | X | X | X | - | X | X | X | X | X | X | X | X | X | X | X | X | X | X | X | X | X | X | X | X | X | X | X | X | X | - | - | - | - | - | - | - | X | X | X | X | X | X | X | X | X | X | X | - | - | - | - |
| OFTLR1 | - | - | - | - | - | - | - | - | - | - | - | - | - | - | - | - | - | - | - | - | - | - | - | - | - | - | - | - | - | - | - | - | - | - | - | - | - | M | R | P | V | T | A | A | L | W | A | A | A | M | W | V | G | L | Q | L | - | S | A | S | S | - | - | - | S | D | I | Y | V | D | R | S | S | K | N | L | S | S | V | P | R | D | L | P | Q | S | V | E | C | L | D | L | S | R | N | H | I | R | Q | L | H | Q | G | D | F | K | N | T | T | V | L | R | F | L | N | I | S | W | N | I | L | E | E | I | D | T | E | T | F | L | D | T | P | H | L | E | D | L | D | L | S | H | N | R | L | E | N | L | S | G | Q | W | Y | L | L | H | T | E | N | L | Q | V | L | N | L | T | C | N | N | F | L | T | M | T | L | G | R | E | F | T | S | L | V | K | L | K | S | L | A | L | G | A | - | - | - | K | N | I | S | M | G | D | F | K | N | I | A | K | V | K | L | R | T | L | T | L | S | L | E | - | D | E | L | H | Y | E | A | G | S | L | K | D | V | H | A | Q | R | L | Q | I | A | L | T | S | N | R | I | I | D | P | D | L | F | A | D | A | L | L | L | F | A | E | V | E | L | M | - | - | D | - | - | - | - | - | - | - | V | T | N | G | Y | R | - | - | - | - | E | Q | S | E | E | L | - | S | K | R | A | E | I | H | T | S | H | L | Y | L | T | N | I | S | I | D | W | S | D | L | T | R | Y | V | N | A | V | L | G | S | S | I | S | H | L | T | A | S | D | V | V | M | Q | S | L | P | Y | T | D | T | P | V | I | G | T | S | K | L | K | S | F | T | A | I | R | V | V | V | T | S | F | F | F | S | Q | E | A | V | Y | N | F | F | - | - | - | I | N | M | P | V | E | S | L | - | - | - | - | - | - | A | I | V | E | - | - | T | S | I | I | H | M | T | C | P | K | S | Q | S | R | I | L | Q | L | D | F | S | Y | C | A | L | S | D | S | I | F | S | R | V | E | G | Q | E | T | L | E | C | R | N | L | S | N | I | R | K | L | T | L | V | G | N | N | L | K | S | L | Q | L | L | S | K | R | V | Q | H | M | K | S | L | K | H | L | D | L | S | L | N | S | L | V | Y | D | G | - | L | E | E | C | V | W | P | S | N | I | T | N | M | T | L | S | S | N | G | L | T | D | S | V | F | K | C | L | P | S | G | T | E | T | L | D | L | Q | N | N | Q | V | S | V | V | P | S | S | - | M | F | K | L | E | K | L | L | S | L | N | L | N | A | N | R | L | R | D | L | P | V | C | N | G | F | P | I | L | N | E | L | L | L | K | L | N | S | L | H | A | P | S | V | Y | K | L | E | S | C | P | K | L | K | T | L | D | V | S | Y | N | P | F | T | C | T | C | S | L | R | C | F | I | S | L | G | V | K | S | E | K | - | - | - | - | - | T | K | S | N | T | G | I | E | L | L | S | W | P | S | D | Y | Y | C | T | Y | P | E | A | V | R | D | S | K | L | K | H | I | L | I | P | E | V | S | C | N | V | G | L | L | A | A | T | I | L | C | P | A | V | I | V | I | I | A | V | V | T | L | C | H | Q | L | D | V | P | W | Y | M | G | M | I | W | Q | W | T | R | A | K | H | R | A | R | T | R | Q | V | R | P | E | D | L | V | G | V | E | F | H | A | F | V | S | Y | S | Q | H | D | A | D | W | V | H | N | S | L | L | S | N | L | E | G | P | A | - | - | - | - | G | G | L | R | I | C | H | H | E | K | N | F | V | P | G | K | T | I | I | E | N | I | I | T | C | V | E | K | S | R | R | S | V | F | V | L | S | A | H | F | V | K | S | E | W | C | H | Y | E | L | Y | F | A | S | H | Q | R | L | A | Q | G | S | D | S | V | V | L | V | L | L | E | P | L | P | Q | Y | L | I | P | S | K | Y | Y | Q | L | K | S | M | M | G | R | H | T | Y | L | E | W | P | Q | D | R | A | K | H | R | L | F | W | A | N | L | R | A | A | L | Q | A | D | L | P | N | A | P | V | T | E | L | E | E | X | X | X | X | X | X | X | - | X | X | X | X | X | X | X | X | X | X | X | X | X | X | X | X | X | X | X | X | X | X | X | X | X | - | - | - | - | - | - | - | X | X | X | X | X | X | X | X | X | X | X | - | - | - | - |
| LMTLR1 | - | - | - | - | - | - | - | - | - | - | - | - | - | - | - | - | - | - | - | - | - | - | - | - | - | - | - | - | - | - | - | - | - | - | - | - | - | M | R | P | V | T | A | V | L | W | A | A | A | M | L | V | G | L | Q | P | - | S | A | S | S | - | - | - | L | D | S | I | V | D | R | S | S | K | N | L | S | S | V | P | G | D | L | P | Q | T | V | E | F | L | D | L | S | C | N | H | I | Q | Q | L | H | R | G | D | F | K | N | T | T | L | L | R | F | L | N | I | S | W | N | S | L | E | D | I | D | P | E | T | F | V | D | T | P | L | L | E | E | L | D | L | S | Y | N | S | L | K | N | L | S | G | Q | R | Y | L | V | H | T | E | N | L | W | A | L | N | L | A | C | N | K | F | H | K | M | T | L | G | S | E | F | S | S | L | V | K | L | E | R | L | K | L | G | A | - | - | - | K | N | I | S | M | G | D | F | K | N | I | A | N | V | N | L | S | A | L | T | L | S | L | E | - | D | E | L | G | Y | E | A | G | S | L | K | D | V | N | A | Q | R | L | Q | I | V | L | T | R | N | R | I | M | D | P | A | L | F | A | D | A | L | S | L | F | V | E | V | E | L | M | - | - | N | - | - | - | - | - | - | - | L | T | R | G | Y | K | - | - | - | - | D | L | S | E | Q | L | - | H | K | R | V | E | I | H | T | S | H | F | Y | L | T | N | I | S | I | D | W | R | D | L | T | L | Y | V | N | T | V | L | L | T | S | I | A | H | L | S | A | S | D | V | N | I | Y | N | L | P | Y | A | D | T | K | V | T | A | T | S | K | M | K | S | F | T | A | R | R | A | V | V | N | S | F | F | F | S | Q | E | A | V | Y | N | Y | F | - | - | - | I | N | M | P | V | E | S | L | - | - | - | - | - | - | A | I | V | E | - | - | T | S | I | I | H | M | T | C | P | K | S | Q | S | R | V | L | Q | L | D | F | S | Y | C | A | L | S | D | S | I | F | S | S | V | S | G | Q | K | T | I | E | C | E | N | L | V | N | V | R | K | L | I | L | V | G | N | N | L | K | S | L | Q | L | L | S | K | R | V | Q | Y | M | T | S | L | Q | Y | L | D | L | T | L | N | S | L | V | Y | D | G | - | L | E | E | C | T | W | P | P | N | I | T | N | M | V | L | S | S | N | G | L | T | D | S | V | F | K | C | L | P | K | G | T | E | T | L | D | L | Q | N | N | Q | I | F | T | V | P | S | S | - | I | L | K | L | E | N | L | L | S | L | N | L | N | A | N | R | L | R | D | L | P | V | C | N | G | F | P | R | L | N | D | L | L | L | K | S | N | S | L | H | A | P | S | V | N | K | L | E | S | C | P | E | L | K | T | L | D | V | S | N | N | P | F | T | C | T | C | T | L | R | S | F | I | N | L | G | I | K | S | E | K | - | - | - | - | - | K | K | S | H | T | G | I | E | L | L | S | W | P | L | D | Y | Y | C | T | Y | P | A | A | D | R | D | S | T | L | K | D | I | Q | I | A | E | V | S | C | N | A | G | L | L | A | A | S | I | L | C | P | A | V | I | V | I | I | A | V | V | T | L | C | H | R | L | D | V | P | W | Y | M | G | M | I | W | Q | W | A | R | A | K | H | R | A | R | T | R | Q | V | R | P | E | D | L | A | G | V | E | F | H | A | F | V | S | Y | S | Q | H | D | A | D | W | V | H | N | S | L | L | P | S | L | E | G | P | A | - | - | - | - | G | G | L | R | I | C | H | H | E | K | N | F | V | P | G | K | T | I | I | E | N | I | I | T | C | V | E | K | C | R | R | S | V | F | V | L | S | A | H | F | V | K | S | D | W | C | H | Y | E | L | Y | F | A | S | H | Q | R | L | A | Q | G | S | D | S | V | V | L | V | M | L | E | P | L | P | Q | Y | L | I | P | S | K | Y | Y | Q | L | K | S | M | M | G | R | H | T | Y | L | E | W | P | Q | D | R | A | K | H | R | L | F | W | A | N | L | R | A | A | L | Q | A | D | L | P | N | A | P | V | T | E | L | Q | X | X | X | X | X | X | X | X | - | X | X | X | X | X | X | X | X | X | X | X | X | X | X | X | X | X | X | X | X | X | X | X | X | X | - | - | - | - | - | - | - | X | X | X | X | X | X | X | X | X | X | X | - | - | - | - |
| TFTLR1 | - | - | - | - | - | - | - | - | - | - | - | - | - | - | - | - | - | - | - | - | - | - | - | - | - | - | - | - | - | - | - | - | - | - | - | - | - | M | K | A | Q | G | L | P | S | C | L | S | L | V | V | L | L | M | S | L | P | P | S | F | L | L | D | M | E | I | L | I | L | D | Y | S | S | R | N | L | S | A | V | P | T | D | I | P | P | F | T | Q | S | L | D | L | S | Q | N | R | I | W | T | L | K | K | Y | D | F | H | G | T | P | R | L | H | F | L | N | L | S | W | N | I | L | K | D | I | H | P | D | T | F | I | S | T | P | L | L | T | T | L | D | L | S | H | N | N | L | E | M | P | S | H | Q | Q | Y | L | V | R | A | Q | N | L | Q | Y | L | D | L | S | S | N | V | L | A | V | I | A | L | G | G | E | F | T | K | L | T | K | L | Q | W | L | G | L | S | A | - | - | - | S | S | I | E | N | N | S | F | A | N | I | S | G | L | H | L | Q | I | L | F | I | Q | A | Q | - | N | L | N | S | Y | E | N | G | S | L | T | G | T | K | T | N | K | I | V | V | Q | M | P | N | - | N | A | Y | D | L | P | I | I | V | D | A | L | T | S | F | R | E | V | E | L | R | - | - | G | - | - | - | - | - | - | - | L | H | N | P | E | D | - | - | - | - | F | F | R | N | P | M | - | V | R | Q | V | H | I | Q | T | V | H | L | H | L | S | F | I | F | T | T | W | E | V | I | T | S | L | T | S | G | A | L | I | S | S | I | R | Q | F | S | L | S | Q | L | T | L | N | S | M | K | - | G | L | Y | F | V | I | Q | G | Y | S | L | D | S | F | S | I | R | Q | A | S | V | T | V | F | L | F | D | Q | Q | N | L | Y | D | Y | I | - | - | - | I | N | I | P | T | K | N | L | - | - | - | - | - | - | T | F | A | Q | - | - | S | P | I | V | H | M | T | C | P | R | V | V | S | M | I | Q | M | L | D | L | S | D | C | V | L | T | E | K | V | F | K | - | G | - | - | - | P | E | G | E | C | K | T | L | I | N | L | E | I | L | L | L | K | G | N | N | L | R | H | L | M | P | L | T | S | R | V | Q | L | M | S | S | L | R | Y | V | D | F | S | Q | N | S | L | T | Y | K | E | T | Q | G | R | C | T | W | P | S | K | I | T | H | L | D | L | S | F | N | E | F | D | Q | T | V | F | K | C | L | P | N | N | L | V | N | L | N | L | Q | S | N | H | I | S | A | I | S | V | N | - | I | S | G | L | N | F | L | K | V | L | D | L | T | A | N | R | L | V | D | L | P | D | C | L | G | Y | P | K | L | Q | K | L | V | L | R | G | N | F | L | H | A | P | S | T | R | T | L | K | T | C | S | H | L | S | V | V | D | I | S | S | N | P | Y | I | C | T | C | P | L | R | E | F | T | N | L | I | D | D | K | G | T | L | W | G | S | K | S | S | K | N | R | R | I | T | M | D | R | W | P | D | G | Y | Q | C | S | Y | P | E | Y | W | K | K | T | M | L | K | N | F | R | L | L | E | I | T | C | N | A | G | L | L | A | V | T | I | L | V | P | A | I | I | L | A | V | A | V | G | I | L | C | Q | Q | L | D | L | P | W | Y | M | S | M | I | W | K | W | T | R | A | K | H | R | A | Q | R | S | Q | Q | R | A | E | D | L | Q | G | V | C | F | H | A | F | I | S | Y | S | Q | R | N | A | S | W | V | I | G | Q | L | L | P | K | L | E | G | E | D | S | A | S | K | N | R | L | R | V | C | H | H | E | R | D | F | I | P | G | R | P | I | L | D | N | I | L | R | C | I | E | Q | S | R | C | C | V | F | V | L | S | S | H | F | V | Q | S | E | W | C | H | Y | E | L | Y | F | A | S | H | E | L | I | T | R | G | T | N | N | I | I | L | I | L | L | E | P | L | P | A | Y | L | I | P | S | N | Y | N | Q | L | K | A | M | L | A | R | R | T | Y | L | E | W | P | Q | D | K | A | K | Q | R | M | F | W | A | N | L | R | A | A | L | E | A | D | L | P | E | S | V | E | R | P | W | E | X | X | X | X | X | X | X | X | - | X | X | X | X | X | X | X | X | X | X | X | X | X | X | X | X | X | X | X | X | X | X | X | X | X | X | X | X | - | - | - | - | - | - | - | - | - | - | - | - | - | - | - | - | - | - | - |
| CBTLR1 | - | - | - | - | - | - | - | - | - | - | - | - | - | - | - | - | - | - | - | - | - | - | - | - | - | - | - | - | - | - | - | - | - | - | - | - | - | M | K | A | Q | G | L | P | - | C | L | S | V | M | V | F | L | L | F | L | P | H | S | F | M | L | D | M | K | T | F | I | L | D | Y | S | S | R | N | L | S | A | V | P | P | D | L | P | P | S | T | Q | C | L | D | L | S | Q | N | R | I | L | T | T | N | K | H | D | F | Q | R | T | P | R | L | E | F | L | N | L | S | W | N | I | L | Q | D | L | H | P | D | T | F | L | S | T | P | L | L | A | T | L | D | L | S | H | N | R | L | E | K | L | P | H | Q | K | Y | L | L | Q | A | K | N | L | Q | Y | L | D | L | S | S | N | S | F | E | V | M | A | L | G | G | E | F | L | K | L | T | K | L | K | W | L | G | L | S | A | - | - | - | S | L | I | Q | N | N | N | F | A | N | I | S | D | L | R | L | Q | T | L | Y | I | N | A | Q | - | N | L | K | G | Y | E | N | G | S | L | T | G | T | K | A | D | K | I | V | V | Q | M | Y | H | - | S | V | F | D | M | P | V | I | I | D | C | L | A | S | F | K | E | V | E | L | R | - | - | G | - | - | - | - | - | - | - | L | H | D | P | Q | D | - | - | - | - | F | L | E | N | L | S | - | T | R | Q | V | R | I | Q | T | V | N | L | H | L | S | S | I | W | S | T | W | R | I | I | T | A | L | T | K | E | T | L | M | S | S | I | R | Q | F | S | M | S | N | V | T | L | H | D | M | N | - | G | G | D | E | V | N | Q | N | Y | S | L | D | S | F | S | I | R | Q | A | S | V | T | V | F | L | F | D | Q | Q | Q | L | Y | D | F | V | - | - | - | I | N | I | P | A | R | N | L | - | - | - | - | - | - | T | I | A | Q | - | - | S | P | I | V | H | M | T | C | P | N | V | A | S | I | I | Q | M | L | D | L | S | D | C | V | L | T | E | E | V | F | K | - | G | - | - | - | P | Q | G | E | C | N | N | L | T | S | L | E | T | L | V | L | K | G | N | N | L | R | Q | L | M | P | L | T | S | R | V | Q | L | M | S | S | L | K | Y | V | D | F | R | Q | N | L | L | T | Y | E | E | T | Q | G | I | C | S | W | P | P | K | I | T | H | L | D | L | S | F | N | E | F | D | Q | T | V | F | K | C | L | P | H | T | L | V | N | L | N | L | Q | N | N | H | I | S | A | I | P | A | N | - | L | S | T | L | H | V | L | K | G | L | D | L | T | A | N | R | L | L | D | L | P | G | C | L | G | Y | P | K | L | Q | K | L | L | L | R | G | N | F | I | H | A | P | S | T | G | A | L | K | T | C | P | H | L | T | V | V | D | F | S | M | N | P | Y | I | C | T | C | P | L | R | E | F | T | L | L | I | D | G | N | G | T | L | G | W | S | N | S | L | Q | N | K | R | I | S | V | A | H | W | P | D | G | Y | Q | C | S | Y | P | E | Y | L | R | K | A | M | L | T | N | F | R | L | P | E | I | T | C | N | V | V | L | L | V | V | T | I | L | V | P | A | I | T | L | V | I | A | V | V | F | L | C | Q | K | L | D | L | P | W | Y | M | G | M | V | W | K | W | T | R | A | K | H | R | A | R | T | S | Q | Q | Q | E | E | N | L | Q | G | V | C | F | H | A | F | I | S | Y | S | Q | R | N | A | S | W | V | I | G | Q | L | L | P | K | L | E | G | E | D | S | T | S | K | N | R | L | R | V | C | H | H | E | R | D | F | I | P | G | K | P | I | L | D | N | I | L | C | C | I | E | R | S | R | C | C | V | F | V | L | S | S | H | F | V | Q | S | D | W | C | H | Y | E | L | Y | F | A | S | H | Q | W | I | T | R | G | M | D | N | I | I | L | I | L | L | E | P | L | P | T | Y | L | I | P | S | K | Y | H | Q | L | K | A | M | M | A | R | R | T | Y | L | E | W | P | Q | D | K | A | K | Q | R | M | F | W | A | N | L | R | A | V | L | Q | A | D | L | P | D | S | V | E | R | E | W | E | X | X | X | X | X | X | X | X | - | X | X | X | X | X | X | X | X | X | X | X | X | X | X | X | X | X | X | X | X | X | X | X | X | X | X | X | X | X | - | - | - | - | - | - | - | - | - | - | - | - | - | - | - | - | - | - |
| IPTLR1 | M | L | H | L | N | N | M | R | C | A | T | V | N | P | H | H | P | L | S | R | T | F | S | K | Q | I | T | Q | N | S | S | L | I | I | L | A | L | M | K | A | Q | G | L | P | - | W | L | S | V | V | A | L | L | V | S | L | P | Y | S | S | L | L | N | M | E | T | F | I | L | D | Y | S | S | R | N | L | S | A | V | P | P | D | L | P | P | S | V | Q | C | L | D | V | S | Q | N | R | I | W | T | L | K | K | H | D | F | H | R | T | P | R | L | H | F | L | N | L | S | W | N | I | L | E | D | I | H | P | D | T | F | I | S | T | P | L | L | A | T | L | D | L | S | H | N | S | L | K | N | L | S | H | Q | Q | Y | L | V | K | A | Q | N | L | Q | Y | L | D | L | S | S | N | L | F | A | V | M | A | L | G | Y | E | F | S | K | L | M | K | L | K | W | L | G | L | S | A | - | - | - | R | I | I | Q | N | N | N | F | V | N | V | S | D | L | H | L | Q | T | L | F | I | Q | A | Q | - | D | L | T | V | Y | E | N | G | S | L | T | G | A | K | S | D | K | I | V | I | L | M | P | S | - | N | V | F | D | L | P | I | I | V | D | A | L | T | S | F | K | Q | V | E | L | R | - | - | G | - | - | - | - | - | - | - | L | Y | N | P | E | D | - | - | - | - | F | L | G | I | L | V | - | T | R | K | V | R | I | Q | T | V | N | L | H | L | S | S | V | W | S | T | W | K | V | I | T | A | L | T | N | S | A | L | R | S | T | I | C | Q | F | S | M | S | N | L | T | L | Y | D | M | Y | - | G | D | Y | F | V | I | Q | G | Y | S | L | D | S | F | S | I | R | Q | A | S | V | T | V | F | I | F | N | Q | L | S | L | Y | D | F | I | - | - | - | I | N | I | P | A | R | N | L | - | - | - | - | - | - | T | F | A | Q | - | - | S | P | I | V | H | M | T | C | P | K | V | V | S | M | I | Q | M | L | D | L | S | D | C | V | L | T | E | N | V | F | K | - | D | - | - | - | P | L | G | E | C | N | T | L | S | N | L | E | I | L | V | L | K | R | N | N | L | R | Q | L | M | P | L | T | S | R | V | Q | L | M | S | S | L | R | H | V | D | F | S | Q | N | S | L | T | Y | E | E | T | Q | G | R | C | N | W | P | S | K | I | S | H | L | D | L | S | F | N | E | F | E | Q | T | V | F | K | C | L | P | T | A | L | V | N | L | N | L | Q | N | N | H | I | S | A | I | P | A | N | - | I | S | G | L | D | S | L | K | V | L | D | L | T | A | N | R | L | L | D | L | P | D | C | L | G | Y | P | K | L | Q | K | L | V | L | R | G | N | F | L | H | A | P | S | T | G | S | L | K | T | C | S | H | L | T | V | V | D | M | S | M | N | P | Y | I | C | T | C | P | L | R | E | F | T | N | L | I | D | E | K | G | T | L | G | G | S | N | S | W | K | Y | Q | R | I | T | V | A | H | W | P | D | G | Y | R | C | S | Y | P | E | Y | W | R | K | A | M | L | K | N | F | S | M | L | E | I | T | C | N | A | G | L | L | A | V | T | I | L | V | P | A | I | I | L | I | I | A | V | G | I | L | C | Q | Q | L | D | L | P | W | Y | I | S | M | I | W | R | W | T | R | A | K | H | H | A | R | S | S | Q | Q | R | Q | E | D | L | Q | G | V | H | F | H | A | F | I | S | Y | S | Q | R | N | A | N | W | V | I | G | Q | L | L | P | K | L | E | G | E | D | S | S | T | Q | N | G | L | R | V | C | H | H | E | R | D | F | I | P | G | R | P | I | L | D | N | I | L | H | C | I | E | Q | S | R | C | C | V | F | V | L | S | S | H | F | V | Q | S | D | W | C | H | Y | E | L | Y | F | A | S | H | Q | W | I | T | R | G | M | D | N | I | I | L | I | L | L | E | P | L | P | T | Y | L | I | P | S | K | Y | Y | Q | L | K | A | M | M | A | R | R | T | Y | L | E | W | P | Q | D | T | A | K | Q | R | L | F | W | A | N | L | R | A | A | L | Q | A | D | L | P | D | C | G | E | R | E | W | E | - | - | - | - | - | - | - | - | - | - | - | - | - | - | - | - | - | - | - | - | - | - | - | - | - | - | - | - | - | - | - | - | - | - | - | - | - | - | - | - | - | - | - | - | - | - | - | - | - | - | - | - | - | - | - | - |
| PHTLR1 | - | - | - | - | - | - | M | R | C | A | T | V | S | H | C | R | P | F | C | R | T | F | S | G | T | D | N | A | G | F | F | P | I | I | L | A | L | M | Q | A | Q | E | L | P | - | W | L | S | V | V | V | L | L | V | S | L | P | H | S | F | L | L | D | M | E | T | F | I | L | D | Y | S | S | R | N | L | S | A | V | P | P | D | L | P | P | S | I | Q | C | L | D | L | S | Q | N | R | I | L | T | L | N | K | H | D | F | H | R | T | P | R | L | H | F | L | N | L | S | W | N | I | L | E | D | I | H | Q | D | T | F | I | S | T | P | L | L | V | T | L | D | L | S | H | N | R | L | K | K | L | S | H | Q | Q | Y | L | L | Q | A | Q | N | L | Q | Y | L | D | L | S | S | N | L | F | A | V | M | A | L | G | G | E | F | P | K | L | T | K | L | K | W | L | G | L | S | A | - | - | - | R | T | I | Q | N | N | N | F | A | N | I | S | D | L | H | L | Q | T | L | F | I | Q | A | Q | - | N | L | M | G | Y | E | N | G | S | L | T | G | A | K | A | D | K | I | V | V | L | M | S | N | - | N | V | F | D | L | P | V | I | A | D | A | L | A | S | F | K | E | V | E | L | R | - | - | G | - | - | - | - | - | - | - | L | H | D | P | E | D | - | - | - | - | F | L | G | I | L | A | - | T | R | Q | V | R | I | Q | T | V | N | L | H | L | S | S | V | W | S | T | W | K | V | I | T | A | L | T | N | R | T | L | M | S | S | I | R | Q | F | S | L | S | N | L | T | L | H | D | M | M | - | G | R | Y | F | V | V | Q | G | Y | S | L | D | S | F | S | I | R | Q | A | S | V | T | V | F | L | F | D | Q | Q | S | L | Y | D | F | I | - | - | - | I | S | I | P | A | R | N | L | - | - | - | - | - | - | T | F | A | Q | - | - | T | P | I | V | H | M | T | C | P | K | V | V | S | M | I | Q | M | L | D | L | S | D | C | V | L | T | E | K | V | F | K | - | G | - | - | - | L | H | G | E | C | N | T | L | I | N | L | E | V | L | V | L | K | G | N | N | L | R | Q | L | M | P | L | T | S | R | V | Q | L | M | S | S | L | R | H | V | D | F | S | Q | N | S | L | T | Y | E | E | T | Q | G | G | C | T | W | P | S | K | I | T | H | L | D | L | S | F | N | E | F | D | Q | T | V | F | K | C | L | P | N | A | L | V | N | L | N | L | Q | N | N | H | I | S | A | I | P | A | N | - | I | S | G | L | D | S | L | K | I | L | D | L | T | A | N | R | L | L | D | L | P | D | C | L | G | Y | P | K | L | Q | K | L | V | L | R | G | N | F | L | H | A | P | S | T | G | S | L | K | T | C | S | H | L | T | V | V | D | I | S | M | N | P | Y | I | C | T | C | P | L | R | E | F | T | N | L | I | D | D | K | G | T | L | G | G | S | N | S | W | K | N | Q | R | I | T | V | A | H | W | P | D | G | Y | Q | C | S | Y | P | E | Y | W | R | K | A | M | L | K | N | F | S | L | L | E | I | T | C | N | A | G | L | L | A | V | T | I | L | L | P | A | I | I | L | V | I | T | V | G | I | L | C | Q | Q | L | D | L | P | W | Y | M | G | M | I | W | K | W | T | R | A | K | H | R | A | R | S | S | Q | Q | R | P | E | D | L | E | G | V | R | Y | H | A | F | I | S | Y | S | Q | R | N | A | N | W | V | I | D | Q | L | L | P | K | L | E | G | E | D | S | T | T | Q | N | G | L | R | V | C | H | H | E | R | D | F | I | P | G | R | P | I | L | D | N | I | L | R | C | I | E | Q | S | R | C | C | V | F | V | L | S | S | H | F | V | Q | S | D | W | C | H | Y | E | L | Y | F | A | S | H | Q | W | I | T | R | G | M | D | N | I | I | L | I | L | L | E | P | L | P | T | Y | L | I | P | S | K | Y | H | Q | L | K | A | M | M | A | R | R | T | Y | L | E | W | P | Q | D | K | A | K | Q | R | M | F | W | A | N | L | R | A | A | L | Q | A | D | L | P | H | S | V | E | R | E | W | E | X | X | X | X | X | X | - | - | - | - | - | - | - | - | - | - | - | - | - | - | - | - | - | - | - | - | - | - | - | - | - | - | - | - | - | - | - | - | - | - | - | - | - | - | - | - | - | - | - | - | - | - | - | - | - | - |
| DRTLR1 | - | - | - | - | - | - | - | - | - | - | - | - | - | - | - | - | - | - | - | - | - | - | - | - | - | - | - | - | - | - | - | - | - | - | - | - | M | K | P | - | - | - | S | S | G | W | W | L | V | S | V | Y | L | T | C | F | H | P | S | L | I | P | A | I | Q | R | I | I | V | N | Y | S | S | Q | N | L | S | S | V | P | D | D | L | K | P | S | T | E | D | L | D | L | S | L | N | H | I | Q | S | L | N | C | R | D | F | N | T | T | P | R | L | R | F | L | N | L | S | W | N | I | L | E | N | I | D | R | D | T | F | T | S | T | P | A | L | E | M | L | D | L | S | H | N | G | L | Q | N | L | S | E | Q | P | Y | L | L | H | L | G | C | L | E | L | L | D | L | S | S | N | R | F | S | A | M | A | L | G | E | E | F | S | M | L | K | R | L | Q | W | L | G | L | S | A | - | - | - | K | S | I | S | I | Q | D | F | T | H | I | S | N | L | T | L | R | T | L | F | I | N | A | D | - | G | L | L | T | Y | E | G | N | S | L | D | D | V | H | A | E | K | A | V | I | A | L | S | S | - | T | N | V | D | I | A | I | A | N | D | V | F | A | R | F | K | E | V | E | F | T | - | - | K | - | - | - | - | - | - | - | V | D | G | K | M | E | - | - | - | - | V | V | Q | Q | M | - | - | R | S | R | A | L | M | R | T | V | R | L | E | I | S | N | V | K | T | T | W | E | F | L | T | S | S | V | N | T | I | L | S | S | T | I | R | E | L | S | L | T | D | L | T | L | T | E | M | K | - | D | G | A | N | Q | S | S | T | H | I | L | E | S | F | S | T | K | R | A | S | V | T | T | F | I | F | D | Q | K | M | L | Y | D | F | F | - | - | - | I | N | T | P | A | R | K | V | - | - | - | - | - | - | S | L | T | E | - | - | S | P | I | I | F | M | T | C | P | G | T | I | S | K | I | Q | E | L | D | L | S | D | C | A | L | T | E | K | I | F | S | V | N | - | - | - | P | E | T | E | C | G | T | L | V | N | L | T | R | L | V | L | R | G | N | N | L | K | H | L | S | P | L | T | S | R | I | N | L | M | D | S | L | Q | Y | I | D | L | S | Q | N | T | L | T | Y | S | E | N | Q | G | R | C | F | W | P | P | R | V | L | H | V | D | L | S | R | N | G | F | D | E | V | V | F | K | C | L | P | D | S | V | Q | V | L | N | L | R | H | N | R | V | S | T | V | P | A | D | - | I | H | T | F | D | T | L | Q | V | I | D | L | T | F | N | R | L | L | D | L | P | T | C | R | S | F | P | S | L | Q | K | L | L | I | R | S | N | S | I | H | S | P | V | P | G | S | L | K | T | C | Q | H | L | Q | D | L | D | L | S | H | N | P | F | I | C | T | C | A | L | R | D | F | A | S | L | I | N | A | Q | G | I | K | - | - | - | - | - | - | - | T | F | K | S | T | L | K | H | W | P | D | G | Y | R | C | S | Y | P | E | S | W | S | N | S | T | L | E | D | F | Y | L | P | E | I | S | C | N | A | W | I | L | A | I | T | I | L | I | P | T | I | S | L | I | V | A | V | S | L | L | C | N | R | L | D | I | P | W | Y | V | R | M | M | W | K | W | T | R | A | K | H | Y | S | I | T | S | Q | L | K | E | E | D | V | E | R | L | H | F | H | A | F | V | S | Y | S | Q | K | N | A | G | W | V | K | S | Q | F | L | P | K | L | E | G | D | - | - | - | - | - | C | G | L | R | M | C | H | H | E | R | D | F | I | P | G | K | T | V | V | Q | N | I | L | R | C | I | E | Q | S | R | R | C | V | F | V | L | S | S | H | F | V | Q | S | E | W | C | H | Y | E | L | Y | F | A | N | H | Q | K | L | T | R | G | M | D | S | I | L | L | I | L | L | E | P | L | P | L | Y | L | I | P | S | K | Y | Y | Q | L | K | T | M | M | S | R | R | T | Y | L | E | W | P | Q | E | G | A | K | Q | K | L | F | W | A | N | L | R | A | A | L | Q | A | E | L | P | N | T | P | D | R | E | E | E | X | X | X | X | X | X | X | X | - | X | X | X | X | X | X | X | X | X | X | X | X | X | X | X | X | X | X | X | X | X | X | X | X | X | X | X | X | X | X | X | X | X | X | X | X | X | X | X | X | X | - | X | - | - | - | - |
| CCTLR1 | - | - | - | - | - | - | - | - | - | - | - | - | - | - | - | - | - | - | - | - | - | - | - | - | - | - | - | - | - | - | - | - | - | - | - | - | - | M | K | - | - | - | P | L | G | W | W | L | L | F | V | Y | V | T | C | F | H | T | S | P | I | L | A | I | K | R | I | I | V | N | Y | S | S | Q | N | L | S | S | V | P | P | G | L | K | P | S | T | E | D | L | D | L | S | L | N | H | I | Q | A | L | S | A | K | D | L | S | T | T | P | R | L | H | F | L | N | L | S | W | N | I | L | E | N | I | D | T | D | A | F | N | S | T | P | A | L | E | I | L | D | L | S | H | N | R | L | Q | N | L | S | N | Q | P | Y | L | L | K | T | G | R | L | Q | L | L | D | L | S | S | N | L | F | S | I | M | A | L | G | R | E | F | S | T | L | K | H | L | Q | W | L | G | L | S | A | - | - | - | K | S | I | S | N | Q | D | F | T | Y | V | A | N | L | T | L | K | T | L | F | I | N | A | N | - | S | L | Q | T | Y | Q | E | N | S | L | T | E | V | R | S | E | K | A | I | I | A | L | S | K | - | S | D | L | D | I | A | I | A | V | D | V | F | A | A | F | K | E | V | E | F | T | - | - | L | - | - | - | - | - | - | - | V | D | S | E | M | K | - | - | - | - | V | I | Q | Q | I | - | - | H | R | R | G | I | L | R | T | V | S | L | E | I | S | K | V | E | T | T | W | H | V | L | T | S | C | A | N | T | I | L | Q | S | T | I | R | Q | L | S | F | S | D | L | T | M | T | K | M | E | - | N | G | T | L | L | S | T | S | R | M | L | D | S | F | S | T | T | R | A | S | V | T | E | F | I | F | N | Q | K | E | L | Y | D | F | F | - | - | - | I | N | M | P | A | R | N | I | - | - | - | - | - | - | S | L | T | Q | - | - | T | P | I | I | F | M | T | C | P | L | T | V | S | Q | I | E | V | L | D | L | S | D | C | A | L | T | E | N | V | F | S | V | D | - | - | - | P | D | T | E | C | S | T | L | T | N | L | V | K | L | V | L | K | G | N | N | L | K | L | L | R | P | L | T | S | R | I | H | L | M | D | S | L | Q | Y | I | D | L | G | Q | N | T | L | T | Y | S | E | E | Q | G | K | C | L | W | P | P | K | V | V | Q | L | D | L | S | S | N | G | F | D | Q | S | V | F | K | C | L | P | D | S | I | R | I | L | N | L | R | N | N | R | V | T | A | V | P | S | E | - | L | Q | V | L | D | D | L | R | V | L | D | L | M | D | N | R | L | L | D | L | P | T | C | Q | A | F | P | N | L | Q | K | L | S | V | R | S | N | S | I | H | S | P | L | P | G | A | L | E | T | C | P | H | L | E | D | L | D | L | S | R | N | S | F | I | C | T | C | A | L | R | E | F | T | T | L | I | K | D | R | V | T | R | P | R | G | E | - | - | - | T | L | G | L | T | L | G | H | W | P | E | G | Y | R | C | S | Y | P | E | S | W | S | N | S | L | L | E | D | F | Y | L | P | E | I | S | C | N | A | W | I | L | A | I | T | I | L | I | P | T | I | T | L | I | V | A | I | S | L | L | C | I | R | L | D | V | P | W | Y | L | K | M | I | W | K | W | T | R | A | K | H | N | A | I | T | S | Q | R | K | S | E | D | L | E | G | L | R | F | H | A | F | V | S | Y | S | Q | K | N | A | D | W | V | K | S | Q | F | L | P | K | L | E | G | D | - | - | - | - | - | Y | G | L | R | V | C | H | H | E | R | D | F | I | P | G | K | T | I | V | Q | N | I | L | L | C | I | E | Q | S | R | K | C | V | F | V | L | S | S | H | F | V | Q | S | E | W | C | H | Y | E | L | Y | F | A | N | H | Q | R | V | T | R | G | M | D | S | I | I | L | I | L | L | E | P | L | P | L | Y | L | I | P | S | K | Y | Y | Q | L | K | A | M | M | S | R | R | T | Y | L | E | W | P | P | E | G | A | K | Q | K | L | F | W | A | N | L | R | A | A | L | Q | A | N | L | P | S | P | P | E | R | E | E | X | X | X | X | X | X | X | X | X | - | X | X | X | X | X | X | X | X | X | X | X | X | X | X | X | X | X | X | X | X | X | X | X | X | X | X | X | X | X | X | X | X | X | X | X | X | X | X | X | - | - | - | - | - | - | - | - |
| CITLR1 | - | - | - | - | - | - | - | - | - | - | - | - | - | - | - | - | - | - | - | - | - | - | - | - | - | - | - | - | - | - | - | - | - | - | - | - | - | M | K | - | - | - | P | S | C | W | W | L | M | S | V | Y | V | T | C | L | H | P | S | L | V | L | A | I | K | R | I | I | V | N | Y | S | S | Q | N | L | S | S | V | P | P | G | L | K | P | S | T | E | D | L | D | L | S | L | N | H | I | Q | T | L | S | G | K | D | F | S | T | T | P | R | L | H | F | L | N | L | S | W | N | I | L | E | N | I | D | M | D | T | F | N | S | T | P | A | L | E | I | L | D | L | S | H | N | R | L | Q | N | L | S | D | Q | P | Y | L | L | K | A | G | N | L | Q | F | L | D | L | S | S | N | M | F | S | V | M | A | L | G | K | E | F | S | T | L | K | H | L | Q | W | L | G | L | S | A | - | - | - | I | S | I | G | N | E | D | F | T | Y | I | A | N | L | T | L | K | T | L | F | I | N | T | D | - | K | L | Q | K | Y | E | E | R | S | L | M | G | A | H | A | E | K | A | V | I | A | L | S | N | - | K | D | L | D | I | A | V | A | D | D | V | F | A | S | F | K | E | V | E | F | T | - | - | K | - | - | - | - | - | - | - | M | D | S | E | M | K | - | - | - | - | V | L | Q | Q | I | - | - | C | Q | R | G | T | V | R | T | I | S | L | E | I | S | K | V | K | T | T | W | E | V | L | T | G | C | V | N | T | I | L | S | S | A | I | Q | Q | L | S | F | S | D | L | T | M | T | K | M | I | - | D | G | R | P | V | S | S | S | R | L | L | E | S | F | S | T | R | R | A | S | V | T | E | F | I | F | N | Q | T | E | L | Y | D | F | F | - | - | - | I | S | M | P | A | R | K | V | - | - | - | - | - | - | S | L | T | Q | - | - | T | P | I | I | F | M | T | C | P | Q | T | V | S | K | I | E | V | L | D | L | S | D | C | A | L | T | E | N | I | F | S | V | G | - | - | - | P | D | T | E | C | S | T | L | T | N | L | E | S | L | V | L | R | G | N | N | L | K | H | L | G | L | L | T | S | R | I | H | L | M | S | S | L | K | Y | A | D | F | S | Q | N | K | L | T | Y | S | E | E | Q | G | K | C | V | W | P | R | K | V | A | H | V | D | L | S | S | N | G | F | D | Q | S | I | F | K | C | L | P | D | S | I | R | I | L | N | L | R | N | N | R | V | T | T | V | P | A | D | - | V | G | V | L | E | D | L | S | I | L | D | L | A | D | N | R | L | L | D | L | P | T | C | Q | A | F | P | N | L | Q | K | L | L | V | R | T | N | S | I | H | S | P | L | P | G | T | L | E | T | C | P | H | L | K | D | L | D | L | S | R | N | P | F | I | C | T | C | A | L | R | E | F | A | A | L | I | K | N | Q | A | T | Q | - | - | - | - | - | - | - | T | P | G | V | T | L | G | H | W | P | E | G | Y | Q | C | S | Y | P | E | S | R | S | N | T | M | L | K | D | F | Y | L | P | E | I | S | C | D | G | W | I | L | A | V | T | I | L | I | P | T | I | T | L | V | V | A | I | S | L | L | C | H | R | L | D | V | P | W | Y | L | K | M | M | W | K | W | T | R | A | K | H | H | A | I | T | S | Q | K | K | T | E | D | M | E | G | L | R | F | H | A | F | V | S | Y | S | Q | K | N | A | D | W | V | K | A | Q | F | L | P | K | L | E | G | D | - | - | - | - | - | C | G | L | R | V | C | H | H | E | R | D | F | I | P | G | K | T | I | V | Q | N | I | L | R | C | I | E | H | S | R | R | C | V | F | V | L | S | S | H | F | I | Q | S | E | W | C | H | Y | E | L | Y | F | A | N | H | Q | R | V | M | R | G | M | D | S | I | L | L | I | L | L | E | P | L | P | L | Y | L | I | P | S | K | Y | Y | Q | L | K | A | M | M | S | R | R | T | Y | L | E | W | P | Q | E | G | A | K | Q | K | L | F | W | A | N | L | R | A | A | L | Q | A | N | L | P | I | P | S | E | R | E | E | E | X | X | X | X | X | X | X | X | - | X | X | X | X | X | X | X | X | X | X | X | X | X | X | X | X | X | X | X | X | X | X | X | X | X | X | X | X | X | X | X | X | X | X | X | X | X | X | X | X | X | X | X | - | - | - | - |
| MATLR1 | - | - | - | - | - | - | - | - | - | - | - | - | - | - | - | - | - | - | - | - | - | - | - | - | - | - | - | - | - | - | - | - | - | - | - | - | - | M | K | - | - | - | P | S | C | W | W | L | V | F | V | Y | V | T | C | L | H | P | S | L | I | L | A | I | K | R | I | I | V | N | Y | S | S | Q | N | L | S | S | V | P | P | G | L | K | P | S | T | E | D | L | D | L | S | L | N | H | I | Q | M | L | S | G | K | D | F | S | T | T | P | R | L | H | F | L | N | L | S | W | N | I | L | E | N | I | D | M | D | A | F | N | S | T | S | A | L | E | I | L | D | L | S | H | N | R | L | Q | N | L | S | D | Q | P | Y | L | L | K | V | G | N | L | Q | F | L | D | L | S | S | N | M | F | S | V | M | A | L | G | K | E | F | S | T | L | K | H | L | Q | W | L | G | L | S | A | - | - | - | I | S | I | G | N | E | D | F | T | Y | I | A | N | L | T | L | K | T | L | F | I | N | T | D | - | K | L | Q | K | Y | E | E | R | S | L | T | G | V | H | A | E | K | A | V | I | A | L | S | N | - | K | D | L | D | I | A | I | A | D | D | V | F | A | S | F | K | E | V | E | F | T | - | - | K | - | - | - | - | - | - | - | V | D | S | E | M | K | - | - | - | - | V | L | Q | Q | I | - | - | R | Q | R | G | T | V | R | T | V | S | L | E | I | S | K | V | K | T | T | W | E | V | L | T | G | C | V | N | T | I | L | S | S | A | I | Q | Q | L | S | F | S | E | L | T | L | T | K | M | V | - | N | G | R | P | V | T | S | S | R | V | L | E | S | F | S | I | R | R | A | T | V | T | E | F | I | F | N | Q | T | E | L | Y | D | F | F | - | - | - | I | S | M | P | A | R | K | V | - | - | - | - | - | - | I | L | T | Q | - | - | T | P | I | I | F | M | T | C | P | Q | T | V | S | K | I | E | V | L | D | F | S | D | C | A | L | T | E | N | I | F | S | V | G | - | - | - | P | D | T | E | C | S | T | L | T | N | L | K | K | L | V | L | R | G | N | N | L | K | H | L | G | P | L | T | S | R | I | N | L | M | S | S | L | Q | Y | V | D | L | S | Q | N | T | L | T | Y | S | E | E | Q | G | K | C | V | W | P | R | K | V | A | R | V | D | L | S | S | N | V | F | D | Q | S | I | F | K | C | L | P | D | S | I | R | I | L | N | L | R | N | N | R | V | T | T | V | P | A | D | - | V | G | V | L | K | D | L | N | I | L | D | L | M | D | N | R | L | L | D | L | P | T | C | Q | A | F | P | N | L | Q | K | L | L | V | R | T | N | S | I | H | S | P | L | P | G | T | L | E | T | C | P | H | L | K | D | L | D | L | S | R | N | P | F | I | C | T | C | A | L | R | E | F | A | A | L | I | N | N | R | V | T | Q | - | - | - | - | - | - | - | T | P | G | V | T | L | G | H | W | P | E | G | Y | R | C | S | Y | P | E | S | R | S | N | T | T | L | K | D | F | Y | L | P | E | I | S | C | N | G | W | I | L | A | V | T | I | L | I | P | T | I | T | L | V | V | A | V | S | L | L | C | H | R | L | D | V | P | W | Y | L | K | M | M | W | K | W | T | R | A | K | H | H | A | I | T | S | Q | K | K | A | E | D | M | E | G | L | R | F | H | A | F | V | S | Y | S | Q | K | N | A | D | W | V | K | A | Q | F | L | P | K | L | E | G | D | - | - | - | - | - | C | G | L | R | I | C | H | H | E | R | D | F | I | P | G | K | T | I | V | Q | N | I | L | R | C | I | E | Q | S | R | R | C | V | F | V | L | S | S | H | F | V | Q | S | E | W | C | H | Y | E | L | Y | F | A | N | H | Q | R | V | M | R | G | M | D | S | I | L | L | I | L | L | E | P | L | P | L | Y | L | I | P | S | K | Y | Y | Q | L | K | A | M | M | S | R | R | T | Y | L | E | W | P | Q | E | G | A | K | Q | K | L | F | W | A | N | L | R | A | A | L | Q | A | N | L | P | I | P | S | E | R | E | E | E | X | X | X | X | X | X | X | X | - | X | X | X | X | X | X | X | X | X | X | X | X | X | X | X | X | X | X | X | X | X | X | X | X | X | X | X | X | X | X | X | X | X | X | X | X | X | X | X | X | X | X | X | - | - | - | - |
| conservation |  |  |  |  |  |  |  |  |  |  |  |  |  |  |  |  |  |  |  |  |  |  |  |  |  |  |  |  |  |  |  |  |  |  |  |  |  |  |  |  |  |  |  |  |  |  |  |  |  |  |  |  |  |  |  |  |  |  |  |  |  |  |  |  |  |  |  |  |  |  |  |  |  |  |  |  |  |  |  |  |  |  |  |  |  |  |  |  |  |  |  |  |  |  |  |  |  |  |  |  |  |  |  |  |  |  |  |  |  |  |  |  |  |  |  |  |  |  |  |  |  |  |  |  |  |  |  |  |  |  |  |  |  |  |  |  |  |  |  |  |  |  |  |  |  |  |  |  |  |  |  |  |  |  |  |  |  |  |  |  |  |  |  |  |  |  |  |  |  |  |  |  |  |  |  |  |  |  |  |  |  |  |  |  |  |  |  |  |  |  |  |  |  |  |  |  |  |  |  |  |  |  |  |  |  |  |  |  |  |  |  |  |  |  |  |  |  |  |  |  |  |  |  |  |  |  |  |  |  |  |  |  |  |  |  |  |  |  |  |  |  |  |  |  |  |  |  |  |  |  |  |  |  |  |  |  |  |  |  |  |  |  |  |  |  |  |  |  |  |  |  |  |  |  |  |  |  |  |  |  |  |  |  |  |  |  |  |  |  |  |  |  |  |  |  |  |  |  |  |  |  |  |  |  |  |  |  |  |  |  |  |  |  |  |  |  |  |  |  |  |  |  |  |  |  |  |  |  |  |  |  |  |  |  |  |  |  |  |  |  |  |  |  |  |  |  |  |  |  |  |  |  |  |  |  |  |  |  |  |  |  |  |  |  |  |  |  |  |  |  |  |  |  |  |  |  |  |  |  |  |  |  |  |  |  |  |  |  |  |  |  |  |  |  |  |  |  |  |  |  |  |  |  |  |  |  |  |  |  |  |  |  |  |  |  |  |  |  |  |  |  |  |  |  |  |  |  |  |  |  |  |  |  |  |  |  |  |  |  |  |  |  |  |  |  |  |  |  |  |  |  |  |  |  |  |  |  |  |  |  |  |  |  |  |  |  |  |  |  |  |  |  |  |  |  |  |  |  |  |  |  |  |  |  |  |  |  |  |  |  |  |  |  |  |  |  |  |  |  |  |  |  |  |  |  |  |  |  |  |  |  |  |  |  |  |  |  |  |  |  |  |  |  |  |  |  |  |  |  |  |  |  |  |  |  |  |  |  |  |  |  |  |  |  |  |  |  |  |  |  |  |  |  |  |  |  |  |  |  |  |  |  |  |  |  |  |  |  |  |  |  |  |  |  |  |  |  |  |  |  |  |  |  |  |  |  |  |  |  |  |  |  |  |  |  |  |  |  |  |  |  |  |  |  |  |  |  |  |  |  |  |  |  |  |  |  |  |  |  |  |  |  |  |  |  |  |  |  |  |  |  |  |  |  |  |  |  |  |  |  |  |  |  |  |  |  |  |  |  |  |  |  |  |  |  |  |  |  |  |  |  |  |  |  |  |  |  |  |  |  |  |  |  |  |  |  |  |  |  |  |  |  |  |  |  |  |  |  |  |  |  |  |  |  |  |  |  |  |  |  |  |  |  |  |  |  |  |  |  |  |  |  |  |  |  |  |  |  |  |  |  |  |  |  |  |  |  |  |  |  |  |  |  |  |  |  |  |  |  |  |  |  |  |  |  |  |  |  |  |  |  |  |  |  |  |  |  |  |  |  |  |  |  |  |  |  |  |  |  |  |  |  |  |  |  |  |  |  |  |  |  |  |  |  |  |  |  |  |  |  |  |  |  |  |  |  |  |  |  |  |  |  |  |  |  |  |  |  |  |  |  |  |  |  |  |  |  |  |  |  |  |  |  |  |  |  |  |  |  |  |  |  |  |  |  |  |  |  |  |  |  |  |  |  |  |  |  |  |  |  |  |  |  |  |  |  |  |  |  |  |  |  |  |  |  |  |  |  |  |  |  |  |  |  |  |  |  |  |  |  |  |  |  |  |  |  |  |  |  |  |  |  |  |  |  |  |  |  |  |  |  |  |  |  |  |  |  |  |  |  |  |  |  |  |  |  |  |  |  |  |  |  |  |  |  |  |  |  |  |  |  |  |  |  |  |  |  |  |
|  |  |  |  |  |  |  |  |  |  |  |  |  |  |  |  |  |  |  |  |  |  |  |  |  |  |  |  |  |  |  |  |  |  |  |  |  |  |  |  |  |  |  |  |  |  |  |  |  |  |  |  |  |  |  |  |  |  |  |  |  |  |  |  |  |  |  |  |  |  |  |  |  |  |  |  |  |  |  |  |  |  |  |  |  |  |  |  |  |  |  |  |  |  |  |  |  |  |  |  |  |  |  |  |  |  |  |  |  |  |  |  |  |  |  |  |  |  |  |  |  |  |  |  |  |  |  |  |  |  |  |  |  |  |  |  |  |  |  |  |  |  |  |  |  |  |  |  |  |  |  |  |  |  |  |  |  |  |  |  |  |  |  |  |  |  |  |  |  |  |  |  |  |  |  |  |  |  |  |  |  |  |  |  |  |  |  |  |  |  |  |  |  |  |  |  |  |  |  |  |  |  |  |  |  |  |  |  |  |  |  |  |  |  |  |  |  |  |  |  |  |  |  |  |  |  |  |  |  |  |  |  |  |  |  |  |  |  |  |  |  |  |  |  |  |  |  |  |  |  |  |  |  |  |  |  |  |  |  |  |  |  |  |  |  |  |  |  |  |  |  |  |  |  |  |  |  |  |  |  |  |  |  |  |  |  |  |  |  |  |  |  |  |  |  |  |  |  |  |  |  |  |  |  |  |  |  |  |  |  |  |  |  |  |  |  |  |  |  |  |  |  |  |  |  |  |  |  |  |  |  |  |  |  |  |  |  |  |  |  |  |  |  |  |  |  |  |  |  |  |  |  |  |  |  |  |  |  |  |  |  |  |  |  |  |  |  |  |  |  |  |  |  |  |  |  |  |  |  |  |  |  |  |  |  |  |  |  |  |  |  |  |  |  |  |  |  |  |  |  |  |  |  |  |  |  |  |  |  |  |  |  |  |  |  |  |  |  |  |  |  |  |  |  |  |  |  |  |  |  |  |  |  |  |  |  |  |  |  |  |  |  |  |  |  |  |  |  |  |  |  |  |  |  |  |  |  |  |  |  |  |  |  |  |  |  |  |  |  |  |  |  |  |  |  |  |  |  |  |  |  |  |  |  |  |  |  |  |  |  |  |  |  |  |  |  |  |  |  |  |  |  |  |  |  |  |  |  |  |  |  |  |  |  |  |  |  |  |  |  |  |  |  |  |  |  |  |  |  |  |  |  |  |  |  |  |  |  |  |  |  |  |  |  |  |  |  |  |  |  |  |  |  |  |  |  |  |  |  |  |  |  |  |  |  |  |  |  |  |  |  |  |  |  |  |  |  |  |  |  |  |  |  |  |  |  |  |  |  |  |  |  |  |  |  |  |  |  |  |  |  |  |  |  |  |  |  |  |  |  |  |  |  |  |  |  |  |  |  |  |  |  |  |  |  |  |  |  |  |  |  |  |  |  |  |  |  |  |  |  |  |  |  |  |  |  |  |  |  |  |  |  |  |  |  |  |  |  |  |  |  |  |  |  |  |  |  |  |  |  |  |  |  |  |  |  |  |  |  |  |  |  |  |  |  |  |  |  |  |  |  |  |  |  |  |  |  |  |  |  |  |  |  |  |  |  |  |  |  |  |  |  |  |  |  |  |  |  |  |  |  |  |  |  |  |  |  |  |  |  |  |  |  |  |  |  |  |  |  |  |  |  |  |  |  |  |  |  |  |  |  |  |  |  |  |  |  |  |  |  |  |  |  |  |  |  |  |  |  |  |  |  |  |  |  |  |  |  |  |  |  |  |  |  |  |  |  |  |  |  |  |  |  |  |  |  |  |  |  |  |  |  |  |  |  |  |  |  |  |  |  |  |  |  |  |  |  |  |  |  |  |  |  |  |  |  |  |  |  |  |  |  |  |  |  |  |  |  |  |  |  |  |  |  |  |  |  |  |  |  |  |  |  |  |  |  |  |  |  |  |  |  |  |  |  |  |  |  |  |  |  |  |  |  |  |  |  |  |  |  |  |  |  |  |  |  |  |  |  |  |  |  |  |  |  |  |  |  |  |  |  |  |  |  |  |  |  |  |  |  |  |  |  |  |  |  |  |  |  |  |  |  |  |  |  |  |  |  |  |  |  |  |  |  |  |  |  |  |  |
|  |  |  |  |  |  |  |  |  |  |  |  |  |  |  |  |  |  |  |  |  |  |  |  |  |  |  |  |  |  |  |  |  |  |  |  |  |  |  |  |  |  |  |  |  |  |  |  |  |  |  |  |  |  |  |  |  |  |  |  |  |  |  |  |  |  |  |  |  |  |  |  |  |  |  |  |  |  |  |  |  |  |  |  |  |  |  |  |  |  |  |  |  |  |  |  |  |  |  |  |  |  |  |  |  |  |  |  |  |  |  |  |  |  |  |  |  |  |  |  |  |  |  |  |  |  |  |  |  |  |  |  |  |  |  |  |  |  |  |  |  |  |  |  |  |  |  |  |  |  |  |  |  |  |  |  |  |  |  |  |  |  |  |  |  |  |  |  |  |  |  |  |  |  |  |  |  |  |  |  |  |  |  |  |  |  |  |  |  |  |  |  |  |  |  |  |  |  |  |  |  |  |  |  |  |  |  |  |  |  |  |  |  |  |  |  |  |  |  |  |  |  |  |  |  |  |  |  |  |  |  |  |  |  |  |  |  |  |  |  |  |  |  |  |  |  |  |  |  |  |  |  |  |  |  |  |  |  |  |  |  |  |  |  |  |  |  |  |  |  |  |  |  |  |  |  |  |  |  |  |  |  |  |  |  |  |  |  |  |  |  |  |  |  |  |  |  |  |  |  |  |  |  |  |  |  |  |  |  |  |  |  |  |  |  |  |  |  |  |  |  |  |  |  |  |  |  |  |  |  |  |  |  |  |  |  |  |  |  |  |  |  |  |  |  |  |  |  |  |  |  |  |  |  |  |  |  |  |  |  |  |  |  |  |  |  |  |  |  |  |  |  |  |  |  |  |  |  |  |  |  |  |  |  |  |  |  |  |  |  |  |  |  |  |  |  |  |  |  |  |  |  |  |  |  |  |  |  |  |  |  |  |  |  |  |  |  |  |  |  |  |  |  |  |  |  |  |  |  |  |  |  |  |  |  |  |  |  |  |  |  |  |  |  |  |  |  |  |  |  |  |  |  |  |  |  |  |  |  |  |  |  |  |  |  |  |  |  |  |  |  |  |  |  |  |  |  |  |  |  |  |  |  |  |  |  |  |  |  |  |  |  |  |  |  |  |  |  |  |  |  |  |  |  |  |  |  |  |  |  |  |  |  |  |  |  |  |  |  |  |  |  |  |  |  |  |  |  |  |  |  |  |  |  |  |  |  |  |  |  |  |  |  |  |  |  |  |  |  |  |  |  |  |  |  |  |  |  |  |  |  |  |  |  |  |  |  |  |  |  |  |  |  |  |  |  |  |  |  |  |  |  |  |  |  |  |  |  |  |  |  |  |  |  |  |  |  |  |  |  |  |  |  |  |  |  |  |  |  |  |  |  |  |  |  |  |  |  |  |  |  |  |  |  |  |  |  |  |  |  |  |  |  |  |  |  |  |  |  |  |  |  |  |  |  |  |  |  |  |  |  |  |  |  |  |  |  |  |  |  |  |  |  |  |  |  |  |  |  |  |  |  |  |  |  |  |  |  |  |  |  |  |  |  |  |  |  |  |  |  |  |  |  |  |  |  |  |  |  |  |  |  |  |  |  |  |  |  |  |  |  |  |  |  |  |  |  |  |  |  |  |  |  |  |  |  |  |  |  |  |  |  |  |  |  |  |  |  |  |  |  |  |  |  |  |  |  |  |  |  |  |  |  |  |  |  |  |  |  |  |  |  |  |  |  |  |  |  |  |  |  |  |  |  |  |  |  |  |  |  |  |  |  |  |  |  |  |  |  |  |  |  |  |  |  |  |  |  |  |  |  |  |  |  |  |  |  |  |  |  |  |  |  |  |  |  |  |  |  |  |  |  |  |  |  |  |  |  |  |  |  |  |  |  |  |  |  |  |  |  |  |  |  |  |  |  |  |  |  |  |  |  |  |  |  |  |  |  |  |  |  |  |  |  |  |  |  |  |  |  |  |  |  |  |  |  |  |  |  |  |  |  |  |  |  |  |  |  |  |  |  |  |  |  |  |  |  |  |  |  |  |  |  |  |  |  |  |  |  |  |  |  |  |  |  |  |  |  |  |  |  |  |  |  |  |  |  |  |  |  |  |  |  |  |  |  |  |  |  |
|  |  |  |  |  |  |  |  |  |  |  |  |  |  |  |  |  |  |  |  |  |  |  |  |  |  |  |  |  |  |  |  |  |  |  |  |  |  |  |  |  |  |  |  |  |  |  |  |  |  |  |  |  |  |  |  |  |  |  |  |  |  |  |  |  |  |  |  |  |  |  |  |  |  |  |  |  |  |  |  |  |  |  |  |  |  |  |  |  |  |  |  |  |  |  |  |  |  |  |  |  |  |  |  |  |  |  |  |  |  |  |  |  |  |  |  |  |  |  |  |  |  |  |  |  |  |  |  |  |  |  |  |  |  |  |  |  |  |  |  |  |  |  |  |  |  |  |  |  |  |  |  |  |  |  |  |  |  |  |  |  |  |  |  |  |  |  |  |  |  |  |  |  |  |  |  |  |  |  |  |  |  |  |  |  |  |  |  |  |  |  |  |  |  |  |  |  |  |  |  |  |  |  |  |  |  |  |  |  |  |  |  |  |  |  |  |  |  |  |  |  |  |  |  |  |  |  |  |  |  |  |  |  |  |  |  |  |  |  |  |  |  |  |  |  |  |  |  |  |  |  |  |  |  |  |  |  |  |  |  |  |  |  |  |  |  |  |  |  |  |  |  |  |  |  |  |  |  |  |  |  |  |  |  |  |  |  |  |  |  |  |  |  |  |  |  |  |  |  |  |  |  |  |  |  |  |  |  |  |  |  |  |  |  |  |  |  |  |  |  |  |  |  |  |  |  |  |  |  |  |  |  |  |  |  |  |  |  |  |  |  |  |  |  |  |  |  |  |  |  |  |  |  |  |  |  |  |  |  |  |  |  |  |  |  |  |  |  |  |  |  |  |  |  |  |  |  |  |  |  |  |  |  |  |  |  |  |  |  |  |  |  |  |  |  |  |  |  |  |  |  |  |  |  |  |  |  |  |  |  |  |  |  |  |  |  |  |  |  |  |  |  |  |  |  |  |  |  |  |  |  |  |  |  |  |  |  |  |  |  |  |  |  |  |  |  |  |  |  |  |  |  |  |  |  |  |  |  |  |  |  |  |  |  |  |  |  |  |  |  |  |  |  |  |  |  |  |  |  |  |  |  |  |  |  |  |  |  |  |  |  |  |  |  |  |  |  |  |  |  |  |  |  |  |  |  |  |  |  |  |  |  |  |  |  |  |  |  |  |  |  |  |  |  |  |  |  |  |  |  |  |  |  |  |  |  |  |  |  |  |  |  |  |  |  |  |  |  |  |  |  |  |  |  |  |  |  |  |  |  |  |  |  |  |  |  |  |  |  |  |  |  |  |  |  |  |  |  |  |  |  |  |  |  |  |  |  |  |  |  |  |  |  |  |  |  |  |  |  |  |  |  |  |  |  |  |  |  |  |  |  |  |  |  |  |  |  |  |  |  |  |  |  |  |  |  |  |  |  |  |  |  |  |  |  |  |  |  |  |  |  |  |  |  |  |  |  |  |  |  |  |  |  |  |  |  |  |  |  |  |  |  |  |  |  |  |  |  |  |  |  |  |  |  |  |  |  |  |  |  |  |  |  |  |  |  |  |  |  |  |  |  |  |  |  |  |  |  |  |  |  |  |  |  |  |  |  |  |  |  |  |  |  |  |  |  |  |  |  |  |  |  |  |  |  |  |  |  |  |  |  |  |  |  |  |  |  |  |  |  |  |  |  |  |  |  |  |  |  |  |  |  |  |  |  |  |  |  |  |  |  |  |  |  |  |  |  |  |  |  |  |  |  |  |  |  |  |  |  |  |  |  |  |  |  |  |  |  |  |  |  |  |  |  |  |  |  |  |  |  |  |  |  |  |  |  |  |  |  |  |  |  |  |  |  |  |  |  |  |  |  |  |  |  |  |  |  |  |  |  |  |  |  |  |  |  |  |  |  |  |  |  |  |  |  |  |  |  |  |  |  |  |  |  |  |  |  |  |  |  |  |  |  |  |  |  |  |  |  |  |  |  |  |  |  |  |  |  |  |  |  |  |  |  |  |  |  |  |  |  |  |  |  |  |  |  |  |  |  |  |  |  |  |  |  |  |  |  |  |  |  |  |  |  |  |  |  |  |  |  |  |  |  |  |  |  |  |  |  |  |  |  |  |  |  |  |  |  |  |
|  |  |  |  |  |  |  |  |  |  |  |  |  |  |  |  |  |  |  |  |  |  |  |  |  |  |  |  |  |  |  |  |  |  |  |  |  |  |  |  |  |  |  |  |  |  |  |  |  |  |  |  |  |  |  |  |  |  |  |  |  |  |  |  |  |  |  |  |  |  |  |  |  |  |  |  |  |  |  |  |  |  |  |  |  |  |  |  |  |  |  |  |  |  |  |  |  |  |  |  |  |  |  |  |  |  |  |  |  |  |  |  |  |  |  |  |  |  |  |  |  |  |  |  |  |  |  |  |  |  |  |  |  |  |  |  |  |  |  |  |  |  |  |  |  |  |  |  |  |  |  |  |  |  |  |  |  |  |  |  |  |  |  |  |  |  |  |  |  |  |  |  |  |  |  |  |  |  |  |  |  |  |  |  |  |  |  |  |  |  |  |  |  |  |  |  |  |  |  |  |  |  |  |  |  |  |  |  |  |  |  |  |  |  |  |  |  |  |  |  |  |  |  |  |  |  |  |  |  |  |  |  |  |  |  |  |  |  |  |  |  |  |  |  |  |  |  |  |  |  |  |  |  |  |  |  |  |  |  |  |  |  |  |  |  |  |  |  |  |  |  |  |  |  |  |  |  |  |  |  |  |  |  |  |  |  |  |  |  |  |  |  |  |  |  |  |  |  |  |  |  |  |  |  |  |  |  |  |  |  |  |  |  |  |  |  |  |  |  |  |  |  |  |  |  |  |  |  |  |  |  |  |  |  |  |  |  |  |  |  |  |  |  |  |  |  |  |  |  |  |  |  |  |  |  |  |  |  |  |  |  |  |  |  |  |  |  |  |  |  |  |  |  |  |  |  |  |  |  |  |  |  |  |  |  |  |  |  |  |  |  |  |  |  |  |  |  |  |  |  |  |  |  |  |  |  |  |  |  |  |  |  |  |  |  |  |  |  |  |  |  |  |  |  |  |  |  |  |  |  |  |  |  |  |  |  |  |  |  |  |  |  |  |  |  |  |  |  |  |  |  |  |  |  |  |  |  |  |  |  |  |  |  |  |  |  |  |  |  |  |  |  |  |  |  |  |  |  |  |  |  |  |  |  |  |  |  |  |  |  |  |  |  |  |  |  |  |  |  |  |  |  |  |  |  |  |  |  |  |  |  |  |  |  |  |  |  |  |  |  |  |  |  |  |  |  |  |  |  |  |  |  |  |  |  |  |  |  |  |  |  |  |  |  |  |  |  |  |  |  |  |  |  |  |  |  |  |  |  |  |  |  |  |  |  |  |  |  |  |  |  |  |  |  |  |  |  |  |  |  |  |  |  |  |  |  |  |  |  |  |  |  |  |  |  |  |  |  |  |  |  |  |  |  |  |  |  |  |  |  |  |  |  |  |  |  |  |  |  |  |  |  |  |  |  |  |  |  |  |  |  |  |  |  |  |  |  |  |  |  |  |  |  |  |  |  |  |  |  |  |  |  |  |  |  |  |  |  |  |  |  |  |  |  |  |  |  |  |  |  |  |  |  |  |  |  |  |  |  |  |  |  |  |  |  |  |  |  |  |  |  |  |  |  |  |  |  |  |  |  |  |  |  |  |  |  |  |  |  |  |  |  |  |  |  |  |  |  |  |  |  |  |  |  |  |  |  |  |  |  |  |  |  |  |  |  |  |  |  |  |  |  |  |  |  |  |  |  |  |  |  |  |  |  |  |  |  |  |  |  |  |  |  |  |  |  |  |  |  |  |  |  |  |  |  |  |  |  |  |  |  |  |  |  |  |  |  |  |  |  |  |  |  |  |  |  |  |  |  |  |  |  |  |  |  |  |  |  |  |  |  |  |  |  |  |  |  |  |  |  |  |  |  |  |  |  |  |  |  |  |  |  |  |  |  |  |  |  |  |  |  |  |  |  |  |  |  |  |  |  |  |  |  |  |  |  |  |  |  |  |  |  |  |  |  |  |  |  |  |  |  |  |  |  |  |  |  |  |  |  |  |  |  |  |  |  |  |  |  |  |  |  |  |  |  |  |  |  |  |  |  |  |  |  |  |  |  |  |  |  |  |  |  |  |  |  |  |  |  |  |  |  |  |  |  |  |  |  |  |  |  |  |  |  |  |  |  |  |  |
|  |  |  |  |  |  |  |  |  |  |  |  |  |  |  |  |  |  |  |  |  |  |  |  |  |  |  |  |  |  |  |  |  |  |  |  |  |  |  |  |  |  |  |  |  |  |  |  |  |  |  |  |  |  |  |  |  |  |  |  |  |  |  |  |  |  |  |  |  |  |  |  |  |  |  |  |  |  |  |  |  |  |  |  |  |  |  |  |  |  |  |  |  |  |  |  |  |  |  |  |  |  |  |  |  |  |  |  |  |  |  |  |  |  |  |  |  |  |  |  |  |  |  |  |  |  |  |  |  |  |  |  |  |  |  |  |  |  |  |  |  |  |  |  |  |  |  |  |  |  |  |  |  |  |  |  |  |  |  |  |  |  |  |  |  |  |  |  |  |  |  |  |  |  |  |  |  |  |  |  |  |  |  |  |  |  |  |  |  |  |  |  |  |  |  |  |  |  |  |  |  |  |  |  |  |  |  |  |  |  |  |  |  |  |  |  |  |  |  |  |  |  |  |  |  |  |  |  |  |  |  |  |  |  |  |  |  |  |  |  |  |  |  |  |  |  |  |  |  |  |  |  |  |  |  |  |  |  |  |  |  |  |  |  |  |  |  |  |  |  |  |  |  |  |  |  |  |  |  |  |  |  |  |  |  |  |  |  |  |  |  |  |  |  |  |  |  |  |  |  |  |  |  |  |  |  |  |  |  |  |  |  |  |  |  |  |  |  |  |  |  |  |  |  |  |  |  |  |  |  |  |  |  |  |  |  |  |  |  |  |  |  |  |  |  |  |  |  |  |  |  |  |  |  |  |  |  |  |  |  |  |  |  |  |  |  |  |  |  |  |  |  |  |  |  |  |  |  |  |  |  |  |  |  |  |  |  |  |  |  |  |  |  |  |  |  |  |  |  |  |  |  |  |  |  |  |  |  |  |  |  |  |  |  |  |  |  |  |  |  |  |  |  |  |  |  |  |  |  |  |  |  |  |  |  |  |  |  |  |  |  |  |  |  |  |  |  |  |  |  |  |  |  |  |  |  |  |  |  |  |  |  |  |  |  |  |  |  |  |  |  |  |  |  |  |  |  |  |  |  |  |  |  |  |  |  |  |  |  |  |  |  |  |  |  |  |  |  |  |  |  |  |  |  |  |  |  |  |  |  |  |  |  |  |  |  |  |  |  |  |  |  |  |  |  |  |  |  |  |  |  |  |  |  |  |  |  |  |  |  |  |  |  |  |  |  |  |  |  |  |  |  |  |  |  |  |  |  |  |  |  |  |  |  |  |  |  |  |  |  |  |  |  |  |  |  |  |  |  |  |  |  |  |  |  |  |  |  |  |  |  |  |  |  |  |  |  |  |  |  |  |  |  |  |  |  |  |  |  |  |  |  |  |  |  |  |  |  |  |  |  |  |  |  |  |  |  |  |  |  |  |  |  |  |  |  |  |  |  |  |  |  |  |  |  |  |  |  |  |  |  |  |  |  |  |  |  |  |  |  |  |  |  |  |  |  |  |  |  |  |  |  |  |  |  |  |  |  |  |  |  |  |  |  |  |  |  |  |  |  |  |  |  |  |  |  |  |  |  |  |  |  |  |  |  |  |  |  |  |  |  |  |  |  |  |  |  |  |  |  |  |  |  |  |  |  |  |  |  |  |  |  |  |  |  |  |  |  |  |  |  |  |  |  |  |  |  |  |  |  |  |  |  |  |  |  |  |  |  |  |  |  |  |  |  |  |  |  |  |  |  |  |  |  |  |  |  |  |  |  |  |  |  |  |  |  |  |  |  |  |  |  |  |  |  |  |  |  |  |  |  |  |  |  |  |  |  |  |  |  |  |  |  |  |  |  |  |  |  |  |  |  |  |  |  |  |  |  |  |  |  |  |  |  |  |  |  |  |  |  |  |  |  |  |  |  |  |  |  |  |  |  |  |  |  |  |  |  |  |  |  |  |  |  |  |  |  |  |  |  |  |  |  |  |  |  |  |  |  |  |  |  |  |  |  |  |  |  |  |  |  |  |  |  |  |  |  |  |  |  |  |  |  |  |  |  |  |  |  |  |  |  |  |  |  |  |  |  |  |  |  |  |  |  |  |  |  |  |  |  |  |  |  |  |  |  |  |  |  |
|  |  |  |  |  |  |  |  |  |  |  |  |  |  |  |  |  |  |  |  |  |  |  |  |  |  |  |  |  |  |  |  |  |  |  |  |  |  |  |  |  |  |  |  |  |  |  |  |  |  |  |  |  |  |  |  |  |  |  |  |  |  |  |  |  |  |  |  |  |  |  |  |  |  |  |  |  |  |  |  |  |  |  |  |  |  |  |  |  |  |  |  |  |  |  |  |  |  |  |  |  |  |  |  |  |  |  |  |  |  |  |  |  |  |  |  |  |  |  |  |  |  |  |  |  |  |  |  |  |  |  |  |  |  |  |  |  |  |  |  |  |  |  |  |  |  |  |  |  |  |  |  |  |  |  |  |  |  |  |  |  |  |  |  |  |  |  |  |  |  |  |  |  |  |  |  |  |  |  |  |  |  |  |  |  |  |  |  |  |  |  |  |  |  |  |  |  |  |  |  |  |  |  |  |  |  |  |  |  |  |  |  |  |  |  |  |  |  |  |  |  |  |  |  |  |  |  |  |  |  |  |  |  |  |  |  |  |  |  |  |  |  |  |  |  |  |  |  |  |  |  |  |  |  |  |  |  |  |  |  |  |  |  |  |  |  |  |  |  |  |  |  |  |  |  |  |  |  |  |  |  |  |  |  |  |  |  |  |  |  |  |  |  |  |  |  |  |  |  |  |  |  |  |  |  |  |  |  |  |  |  |  |  |  |  |  |  |  |  |  |  |  |  |  |  |  |  |  |  |  |  |  |  |  |  |  |  |  |  |  |  |  |  |  |  |  |  |  |  |  |  |  |  |  |  |  |  |  |  |  |  |  |  |  |  |  |  |  |  |  |  |  |  |  |  |  |  |  |  |  |  |  |  |  |  |  |  |  |  |  |  |  |  |  |  |  |  |  |  |  |  |  |  |  |  |  |  |  |  |  |  |  |  |  |  |  |  |  |  |  |  |  |  |  |  |  |  |  |  |  |  |  |  |  |  |  |  |  |  |  |  |  |  |  |  |  |  |  |  |  |  |  |  |  |  |  |  |  |  |  |  |  |  |  |  |  |  |  |  |  |  |  |  |  |  |  |  |  |  |  |  |  |  |  |  |  |  |  |  |  |  |  |  |  |  |  |  |  |  |  |  |  |  |  |  |  |  |  |  |  |  |  |  |  |  |  |  |  |  |  |  |  |  |  |  |  |  |  |  |  |  |  |  |  |  |  |  |  |  |  |  |  |  |  |  |  |  |  |  |  |  |  |  |  |  |  |  |  |  |  |  |  |  |  |  |  |  |  |  |  |  |  |  |  |  |  |  |  |  |  |  |  |  |  |  |  |  |  |  |  |  |  |  |  |  |  |  |  |  |  |  |  |  |  |  |  |  |  |  |  |  |  |  |  |  |  |  |  |  |  |  |  |  |  |  |  |  |  |  |  |  |  |  |  |  |  |  |  |  |  |  |  |  |  |  |  |  |  |  |  |  |  |  |  |  |  |  |  |  |  |  |  |  |  |  |  |  |  |  |  |  |  |  |  |  |  |  |  |  |  |  |  |  |  |  |  |  |  |  |  |  |  |  |  |  |  |  |  |  |  |  |  |  |  |  |  |  |  |  |  |  |  |  |  |  |  |  |  |  |  |  |  |  |  |  |  |  |  |  |  |  |  |  |  |  |  |  |  |  |  |  |  |  |  |  |  |  |  |  |  |  |  |  |  |  |  |  |  |  |  |  |  |  |  |  |  |  |  |  |  |  |  |  |  |  |  |  |  |  |  |  |  |  |  |  |  |  |  |  |  |  |  |  |  |  |  |  |  |  |  |  |  |  |  |  |  |  |  |  |  |  |  |  |  |  |  |  |  |  |  |  |  |  |  |  |  |  |  |  |  |  |  |  |  |  |  |  |  |  |  |  |  |  |  |  |  |  |  |  |  |  |  |  |  |  |  |  |  |  |  |  |  |  |  |  |  |  |  |  |  |  |  |  |  |  |  |  |  |  |  |  |  |  |  |  |  |  |  |  |  |  |  |  |  |  |  |  |  |  |  |  |  |  |  |  |  |  |  |  |  |  |  |  |  |  |  |  |  |  |  |  |  |  |  |  |  |  |  |  |  |  |  |  |  |  |  |  |  |  |
|  |  |  |  |  |  |  |  |  |  |  |  |  |  |  |  |  |  |  |  |  |  |  |  |  |  |  |  |  |  |  |  |  |  |  |  |  |  |  |  |  |  |  |  |  |  |  |  |  |  |  |  |  |  |  |  |  |  |  |  |  |  |  |  |  |  |  |  |  |  |  |  |  |  |  |  |  |  |  |  |  |  |  |  |  |  |  |  |  |  |  |  |  |  |  |  |  |  |  |  |  |  |  |  |  |  |  |  |  |  |  |  |  |  |  |  |  |  |  |  |  |  |  |  |  |  |  |  |  |  |  |  |  |  |  |  |  |  |  |  |  |  |  |  |  |  |  |  |  |  |  |  |  |  |  |  |  |  |  |  |  |  |  |  |  |  |  |  |  |  |  |  |  |  |  |  |  |  |  |  |  |  |  |  |  |  |  |  |  |  |  |  |  |  |  |  |  |  |  |  |  |  |  |  |  |  |  |  |  |  |  |  |  |  |  |  |  |  |  |  |  |  |  |  |  |  |  |  |  |  |  |  |  |  |  |  |  |  |  |  |  |  |  |  |  |  |  |  |  |  |  |  |  |  |  |  |  |  |  |  |  |  |  |  |  |  |  |  |  |  |  |  |  |  |  |  |  |  |  |  |  |  |  |  |  |  |  |  |  |  |  |  |  |  |  |  |  |  |  |  |  |  |  |  |  |  |  |  |  |  |  |  |  |  |  |  |  |  |  |  |  |  |  |  |  |  |  |  |  |  |  |  |  |  |  |  |  |  |  |  |  |  |  |  |  |  |  |  |  |  |  |  |  |  |  |  |  |  |  |  |  |  |  |  |  |  |  |  |  |  |  |  |  |  |  |  |  |  |  |  |  |  |  |  |  |  |  |  |  |  |  |  |  |  |  |  |  |  |  |  |  |  |  |  |  |  |  |  |  |  |  |  |  |  |  |  |  |  |  |  |  |  |  |  |  |  |  |  |  |  |  |  |  |  |  |  |  |  |  |  |  |  |  |  |  |  |  |  |  |  |  |  |  |  |  |  |  |  |  |  |  |  |  |  |  |  |  |  |  |  |  |  |  |  |  |  |  |  |  |  |  |  |  |  |  |  |  |  |  |  |  |  |  |  |  |  |  |  |  |  |  |  |  |  |  |  |  |  |  |  |  |  |  |  |  |  |  |  |  |  |  |  |  |  |  |  |  |  |  |  |  |  |  |  |  |  |  |  |  |  |  |  |  |  |  |  |  |  |  |  |  |  |  |  |  |  |  |  |  |  |  |  |  |  |  |  |  |  |  |  |  |  |  |  |  |  |  |  |  |  |  |  |  |  |  |  |  |  |  |  |  |  |  |  |  |  |  |  |  |  |  |  |  |  |  |  |  |  |  |  |  |  |  |  |  |  |  |  |  |  |  |  |  |  |  |  |  |  |  |  |  |  |  |  |  |  |  |  |  |  |  |  |  |  |  |  |  |  |  |  |  |  |  |  |  |  |  |  |  |  |  |  |  |  |  |  |  |  |  |  |  |  |  |  |  |  |  |  |  |  |  |  |  |  |  |  |  |  |  |  |  |  |  |  |  |  |  |  |  |  |  |  |  |  |  |  |  |  |  |  |  |  |  |  |  |  |  |  |  |  |  |  |  |  |  |  |  |  |  |  |  |  |  |  |  |  |  |  |  |  |  |  |  |  |  |  |  |  |  |  |  |  |  |  |  |  |  |  |  |  |  |  |  |  |  |  |  |  |  |  |  |  |  |  |  |  |  |  |  |  |  |  |  |  |  |  |  |  |  |  |  |  |  |  |  |  |  |  |  |  |  |  |  |  |  |  |  |  |  |  |  |  |  |  |  |  |  |  |  |  |  |  |  |  |  |  |  |  |  |  |  |  |  |  |  |  |  |  |  |  |  |  |  |  |  |  |  |  |  |  |  |  |  |  |  |  |  |  |  |  |  |  |  |  |  |  |  |  |  |  |  |  |  |  |  |  |  |  |  |  |  |  |  |  |  |  |  |  |  |  |  |  |  |  |  |  |  |  |  |  |  |  |  |  |  |  |  |  |  |  |  |  |  |  |  |  |  |  |  |  |  |  |  |  |  |  |  |  |  |  |  |  |  |  |  |  |  |  |  |
|  |  |  |  |  |  |  |  |  |  |  |  |  |  |  |  |  |  |  |  |  |  |  |  |  |  |  |  |  |  |  |  |  |  |  |  |  |  |  |  |  |  |  |  |  |  |  |  |  |  |  |  |  |  |  |  |  |  |  |  |  |  |  |  |  |  |  |  |  |  |  |  |  |  |  |  |  |  |  |  |  |  |  |  |  |  |  |  |  |  |  |  |  |  |  |  |  |  |  |  |  |  |  |  |  |  |  |  |  |  |  |  |  |  |  |  |  |  |  |  |  |  |  |  |  |  |  |  |  |  |  |  |  |  |  |  |  |  |  |  |  |  |  |  |  |  |  |  |  |  |  |  |  |  |  |  |  |  |  |  |  |  |  |  |  |  |  |  |  |  |  |  |  |  |  |  |  |  |  |  |  |  |  |  |  |  |  |  |  |  |  |  |  |  |  |  |  |  |  |  |  |  |  |  |  |  |  |  |  |  |  |  |  |  |  |  |  |  |  |  |  |  |  |  |  |  |  |  |  |  |  |  |  |  |  |  |  |  |  |  |  |  |  |  |  |  |  |  |  |  |  |  |  |  |  |  |  |  |  |  |  |  |  |  |  |  |  |  |  |  |  |  |  |  |  |  |  |  |  |  |  |  |  |  |  |  |  |  |  |  |  |  |  |  |  |  |  |  |  |  |  |  |  |  |  |  |  |  |  |  |  |  |  |  |  |  |  |  |  |  |  |  |  |  |  |  |  |  |  |  |  |  |  |  |  |  |  |  |  |  |  |  |  |  |  |  |  |  |  |  |  |  |  |  |  |  |  |  |  |  |  |  |  |  |  |  |  |  |  |  |  |  |  |  |  |  |  |  |  |  |  |  |  |  |  |  |  |  |  |  |  |  |  |  |  |  |  |  |  |  |  |  |  |  |  |  |  |  |  |  |  |  |  |  |  |  |  |  |  |  |  |  |  |  |  |  |  |  |  |  |  |  |  |  |  |  |  |  |  |  |  |  |  |  |  |  |  |  |  |  |  |  |  |  |  |  |  |  |  |  |  |  |  |  |  |  |  |  |  |  |  |  |  |  |  |  |  |  |  |  |  |  |  |  |  |  |  |  |  |  |  |  |  |  |  |  |  |  |  |  |  |  |  |  |  |  |  |  |  |  |  |  |  |  |  |  |  |  |  |  |  |  |  |  |  |  |  |  |  |  |  |  |  |  |  |  |  |  |  |  |  |  |  |  |  |  |  |  |  |  |  |  |  |  |  |  |  |  |  |  |  |  |  |  |  |  |  |  |  |  |  |  |  |  |  |  |  |  |  |  |  |  |  |  |  |  |  |  |  |  |  |  |  |  |  |  |  |  |  |  |  |  |  |  |  |  |  |  |  |  |  |  |  |  |  |  |  |  |  |  |  |  |  |  |  |  |  |  |  |  |  |  |  |  |  |  |  |  |  |  |  |  |  |  |  |  |  |  |  |  |  |  |  |  |  |  |  |  |  |  |  |  |  |  |  |  |  |  |  |  |  |  |  |  |  |  |  |  |  |  |  |  |  |  |  |  |  |  |  |  |  |  |  |  |  |  |  |  |  |  |  |  |  |  |  |  |  |  |  |  |  |  |  |  |  |  |  |  |  |  |  |  |  |  |  |  |  |  |  |  |  |  |  |  |  |  |  |  |  |  |  |  |  |  |  |  |  |  |  |  |  |  |  |  |  |  |  |  |  |  |  |  |  |  |  |  |  |  |  |  |  |  |  |  |  |  |  |  |  |  |  |  |  |  |  |  |  |  |  |  |  |  |  |  |  |  |  |  |  |  |  |  |  |  |  |  |  |  |  |  |  |  |  |  |  |  |  |  |  |  |  |  |  |  |  |  |  |  |  |  |  |  |  |  |  |  |  |  |  |  |  |  |  |  |  |  |  |  |  |  |  |  |  |  |  |  |  |  |  |  |  |  |  |  |  |  |  |  |  |  |  |  |  |  |  |  |  |  |  |  |  |  |  |  |  |  |  |  |  |  |  |  |  |  |  |  |  |  |  |  |  |  |  |  |  |  |  |  |  |  |  |  |  |  |  |  |  |  |  |  |  |  |  |  |  |  |  |  |  |  |  |  |  |  |  |  |  |  |  |
|  |  |  |  |  |  |  |  |  |  |  |  |  |  |  |  |  |  |  |  |  |  |  |  |  |  |  |  |  |  |  |  |  |  |  |  |  |  |  |  |  |  |  |  |  |  |  |  |  |  |  |  |  |  |  |  |  |  |  |  |  |  |  |  |  |  |  |  |  |  |  |  |  |  |  |  |  |  |  |  |  |  |  |  |  |  |  |  |  |  |  |  |  |  |  |  |  |  |  |  |  |  |  |  |  |  |  |  |  |  |  |  |  |  |  |  |  |  |  |  |  |  |  |  |  |  |  |  |  |  |  |  |  |  |  |  |  |  |  |  |  |  |  |  |  |  |  |  |  |  |  |  |  |  |  |  |  |  |  |  |  |  |  |  |  |  |  |  |  |  |  |  |  |  |  |  |  |  |  |  |  |  |  |  |  |  |  |  |  |  |  |  |  |  |  |  |  |  |  |  |  |  |  |  |  |  |  |  |  |  |  |  |  |  |  |  |  |  |  |  |  |  |  |  |  |  |  |  |  |  |  |  |  |  |  |  |  |  |  |  |  |  |  |  |  |  |  |  |  |  |  |  |  |  |  |  |  |  |  |  |  |  |  |  |  |  |  |  |  |  |  |  |  |  |  |  |  |  |  |  |  |  |  |  |  |  |  |  |  |  |  |  |  |  |  |  |  |  |  |  |  |  |  |  |  |  |  |  |  |  |  |  |  |  |  |  |  |  |  |  |  |  |  |  |  |  |  |  |  |  |  |  |  |  |  |  |  |  |  |  |  |  |  |  |  |  |  |  |  |  |  |  |  |  |  |  |  |  |  |  |  |  |  |  |  |  |  |  |  |  |  |  |  |  |  |  |  |  |  |  |  |  |  |  |  |  |  |  |  |  |  |  |  |  |  |  |  |  |  |  |  |  |  |  |  |  |  |  |  |  |  |  |  |  |  |  |  |  |  |  |  |  |  |  |  |  |  |  |  |  |  |  |  |  |  |  |  |  |  |  |  |  |  |  |  |  |  |  |  |  |  |  |  |  |  |  |  |  |  |  |  |  |  |  |  |  |  |  |  |  |  |  |  |  |  |  |  |  |  |  |  |  |  |  |  |  |  |  |  |  |  |  |  |  |  |  |  |  |  |  |  |  |  |  |  |  |  |  |  |  |  |  |  |  |  |  |  |  |  |  |  |  |  |  |  |  |  |  |  |  |  |  |  |  |  |  |  |  |  |  |  |  |  |  |  |  |  |  |  |  |  |  |  |  |  |  |  |  |  |  |  |  |  |  |  |  |  |  |  |  |  |  |  |  |  |  |  |  |  |  |  |  |  |  |  |  |  |  |  |  |  |  |  |  |  |  |  |  |  |  |  |  |  |  |  |  |  |  |  |  |  |  |  |  |  |  |  |  |  |  |  |  |  |  |  |  |  |  |  |  |  |  |  |  |  |  |  |  |  |  |  |  |  |  |  |  |  |  |  |  |  |  |  |  |  |  |  |  |  |  |  |  |  |  |  |  |  |  |  |  |  |  |  |  |  |  |  |  |  |  |  |  |  |  |  |  |  |  |  |  |  |  |  |  |  |  |  |  |  |  |  |  |  |  |  |  |  |  |  |  |  |  |  |  |  |  |  |  |  |  |  |  |  |  |  |  |  |  |  |  |  |  |  |  |  |  |  |  |  |  |  |  |  |  |  |  |  |  |  |  |  |  |  |  |  |  |  |  |  |  |  |  |  |  |  |  |  |  |  |  |  |  |  |  |  |  |  |  |  |  |  |  |  |  |  |  |  |  |  |  |  |  |  |  |  |  |  |  |  |  |  |  |  |  |  |  |  |  |  |  |  |  |  |  |  |  |  |  |  |  |  |  |  |  |  |  |  |  |  |  |  |  |  |  |  |  |  |  |  |  |  |  |  |  |  |  |  |  |  |  |  |  |  |  |  |  |  |  |  |  |  |  |  |  |  |  |  |  |  |  |  |  |  |  |  |  |  |  |  |  |  |  |  |  |  |  |  |  |  |  |  |  |  |  |  |  |  |  |  |  |  |  |  |  |  |  |  |  |  |  |  |  |  |  |  |  |  |  |  |  |  |  |  |  |  |  |  |  |  |  |  |  |  |  |  |  |  |  |  |
|  |  |  |  |  |  |  |  |  |  |  |  |  |  |  |  |  |  |  |  |  |  |  |  |  |  |  |  |  |  |  |  |  |  |  |  |  |  |  |  |  |  |  |  |  |  |  |  |  |  |  |  |  |  |  |  |  |  |  |  |  |  |  |  |  |  |  |  |  |  |  |  |  |  |  |  |  |  |  |  |  |  |  |  |  |  |  |  |  |  |  |  |  |  |  |  |  |  |  |  |  |  |  |  |  |  |  |  |  |  |  |  |  |  |  |  |  |  |  |  |  |  |  |  |  |  |  |  |  |  |  |  |  |  |  |  |  |  |  |  |  |  |  |  |  |  |  |  |  |  |  |  |  |  |  |  |  |  |  |  |  |  |  |  |  |  |  |  |  |  |  |  |  |  |  |  |  |  |  |  |  |  |  |  |  |  |  |  |  |  |  |  |  |  |  |  |  |  |  |  |  |  |  |  |  |  |  |  |  |  |  |  |  |  |  |  |  |  |  |  |  |  |  |  |  |  |  |  |  |  |  |  |  |  |  |  |  |  |  |  |  |  |  |  |  |  |  |  |  |  |  |  |  |  |  |  |  |  |  |  |  |  |  |  |  |  |  |  |  |  |  |  |  |  |  |  |  |  |  |  |  |  |  |  |  |  |  |  |  |  |  |  |  |  |  |  |  |  |  |  |  |  |  |  |  |  |  |  |  |  |  |  |  |  |  |  |  |  |  |  |  |  |  |  |  |  |  |  |  |  |  |  |  |  |  |  |  |  |  |  |  |  |  |  |  |  |  |  |  |  |  |  |  |  |  |  |  |  |  |  |  |  |  |  |  |  |  |  |  |  |  |  |  |  |  |  |  |  |  |  |  |  |  |  |  |  |  |  |  |  |  |  |  |  |  |  |  |  |  |  |  |  |  |  |  |  |  |  |  |  |  |  |  |  |  |  |  |  |  |  |  |  |  |  |  |  |  |  |  |  |  |  |  |  |  |  |  |  |  |  |  |  |  |  |  |  |  |  |  |  |  |  |  |  |  |  |  |  |  |  |  |  |  |  |  |  |  |  |  |  |  |  |  |  |  |  |  |  |  |  |  |  |  |  |  |  |  |  |  |  |  |  |  |  |  |  |  |  |  |  |  |  |  |  |  |  |  |  |  |  |  |  |  |  |  |  |  |  |  |  |  |  |  |  |  |  |  |  |  |  |  |  |  |  |  |  |  |  |  |  |  |  |  |  |  |  |  |  |  |  |  |  |  |  |  |  |  |  |  |  |  |  |  |  |  |  |  |  |  |  |  |  |  |  |  |  |  |  |  |  |  |  |  |  |  |  |  |  |  |  |  |  |  |  |  |  |  |  |  |  |  |  |  |  |  |  |  |  |  |  |  |  |  |  |  |  |  |  |  |  |  |  |  |  |  |  |  |  |  |  |  |  |  |  |  |  |  |  |  |  |  |  |  |  |  |  |  |  |  |  |  |  |  |  |  |  |  |  |  |  |  |  |  |  |  |  |  |  |  |  |  |  |  |  |  |  |  |  |  |  |  |  |  |  |  |  |  |  |  |  |  |  |  |  |  |  |  |  |  |  |  |  |  |  |  |  |  |  |  |  |  |  |  |  |  |  |  |  |  |  |  |  |  |  |  |  |  |  |  |  |  |  |  |  |  |  |  |  |  |  |  |  |  |  |  |  |  |  |  |  |  |  |  |  |  |  |  |  |  |  |  |  |  |  |  |  |  |  |  |  |  |  |  |  |  |  |  |  |  |  |  |  |  |  |  |  |  |  |  |  |  |  |  |  |  |  |  |  |  |  |  |  |  |  |  |  |  |  |  |  |  |  |  |  |  |  |  |  |  |  |  |  |  |  |  |  |  |  |  |  |  |  |  |  |  |  |  |  |  |  |  |  |  |  |  |  |  |  |  |  |  |  |  |  |  |  |  |  |  |  |  |  |  |  |  |  |  |  |  |  |  |  |  |  |  |  |  |  |  |  |  |  |  |  |  |  |  |  |  |  |  |  |  |  |  |  |  |  |  |  |  |  |  |  |  |  |  |  |  |  |  |  |  |  |  |  |  |  |  |  |  |  |  |  |  |  |  |  |  |  |  |  |  |  |  |  |  |  |  |
|  |  |  |  |  |  |  |  |  |  |  |  |  |  |  |  |  |  |  |  |  |  |  |  |  |  |  |  |  |  |  |  |  |  |  |  |  |  |  |  |  |  |  |  |  |  |  |  |  |  |  |  |  |  |  |  |  |  |  |  |  |  |  |  |  |  |  |  |  |  |  |  |  |  |  |  |  |  |  |  |  |  |  |  |  |  |  |  |  |  |  |  |  |  |  |  |  |  |  |  |  |  |  |  |  |  |  |  |  |  |  |  |  |  |  |  |  |  |  |  |  |  |  |  |  |  |  |  |  |  |  |  |  |  |  |  |  |  |  |  |  |  |  |  |  |  |  |  |  |  |  |  |  |  |  |  |  |  |  |  |  |  |  |  |  |  |  |  |  |  |  |  |  |  |  |  |  |  |  |  |  |  |  |  |  |  |  |  |  |  |  |  |  |  |  |  |  |  |  |  |  |  |  |  |  |  |  |  |  |  |  |  |  |  |  |  |  |  |  |  |  |  |  |  |  |  |  |  |  |  |  |  |  |  |  |  |  |  |  |  |  |  |  |  |  |  |  |  |  |  |  |  |  |  |  |  |  |  |  |  |  |  |  |  |  |  |  |  |  |  |  |  |  |  |  |  |  |  |  |  |  |  |  |  |  |  |  |  |  |  |  |  |  |  |  |  |  |  |  |  |  |  |  |  |  |  |  |  |  |  |  |  |  |  |  |  |  |  |  |  |  |  |  |  |  |  |  |  |  |  |  |  |  |  |  |  |  |  |  |  |  |  |  |  |  |  |  |  |  |  |  |  |  |  |  |  |  |  |  |  |  |  |  |  |  |  |  |  |  |  |  |  |  |  |  |  |  |  |  |  |  |  |  |  |  |  |  |  |  |  |  |  |  |  |  |  |  |  |  |  |  |  |  |  |  |  |  |  |  |  |  |  |  |  |  |  |  |  |  |  |  |  |  |  |  |  |  |  |  |  |  |  |  |  |  |  |  |  |  |  |  |  |  |  |  |  |  |  |  |  |  |  |  |  |  |  |  |  |  |  |  |  |  |  |  |  |  |  |  |  |  |  |  |  |  |  |  |  |  |  |  |  |  |  |  |  |  |  |  |  |  |  |  |  |  |  |  |  |  |  |  |  |  |  |  |  |  |  |  |  |  |  |  |  |  |  |  |  |  |  |  |  |  |  |  |  |  |  |  |  |  |  |  |  |  |  |  |  |  |  |  |  |  |  |  |  |  |  |  |  |  |  |  |  |  |  |  |  |  |  |  |  |  |  |  |  |  |  |  |  |  |  |  |  |  |  |  |  |  |  |  |  |  |  |  |  |  |  |  |  |  |  |  |  |  |  |  |  |  |  |  |  |  |  |  |  |  |  |  |  |  |  |  |  |  |  |  |  |  |  |  |  |  |  |  |  |  |  |  |  |  |  |  |  |  |  |  |  |  |  |  |  |  |  |  |  |  |  |  |  |  |  |  |  |  |  |  |  |  |  |  |  |  |  |  |  |  |  |  |  |  |  |  |  |  |  |  |  |  |  |  |  |  |  |  |  |  |  |  |  |  |  |  |  |  |  |  |  |  |  |  |  |  |  |  |  |  |  |  |  |  |  |  |  |  |  |  |  |  |  |  |  |  |  |  |  |  |  |  |  |  |  |  |  |  |  |  |  |  |  |  |  |  |  |  |  |  |  |  |  |  |  |  |  |  |  |  |  |  |  |  |  |  |  |  |  |  |  |  |  |  |  |  |  |  |  |  |  |  |  |  |  |  |  |  |  |  |  |  |  |  |  |  |  |  |  |  |  |  |  |  |  |  |  |  |  |  |  |  |  |  |  |  |  |  |  |  |  |  |  |  |  |  |  |  |  |  |  |  |  |  |  |  |  |  |  |  |  |  |  |  |  |  |  |  |  |  |  |  |  |  |  |  |  |  |  |  |  |  |  |  |  |  |  |  |  |  |  |  |  |  |  |  |  |  |  |  |  |  |  |  |  |  |  |  |  |  |  |  |  |  |  |  |  |  |  |  |  |  |  |  |  |  |  |  |  |  |  |  |  |  |  |  |  |  |  |  |  |  |  |  |  |  |  |  |  |  |  |  |  |  |  |  |  |  |  |  |  |  |
|  |  |  |  |  |  |  |  |  |  |  |  |  |  |  |  |  |  |  |  |  |  |  |  |  |  |  |  |  |  |  |  |  |  |  |  |  |  |  |  |  |  |  |  |  |  |  |  |  |  |  |  |  |  |  |  |  |  |  |  |  |  |  |  |  |  |  |  |  |  |  |  |  |  |  |  |  |  |  |  |  |  |  |  |  |  |  |  |  |  |  |  |  |  |  |  |  |  |  |  |  |  |  |  |  |  |  |  |  |  |  |  |  |  |  |  |  |  |  |  |  |  |  |  |  |  |  |  |  |  |  |  |  |  |  |  |  |  |  |  |  |  |  |  |  |  |  |  |  |  |  |  |  |  |  |  |  |  |  |  |  |  |  |  |  |  |  |  |  |  |  |  |  |  |  |  |  |  |  |  |  |  |  |  |  |  |  |  |  |  |  |  |  |  |  |  |  |  |  |  |  |  |  |  |  |  |  |  |  |  |  |  |  |  |  |  |  |  |  |  |  |  |  |  |  |  |  |  |  |  |  |  |  |  |  |  |  |  |  |  |  |  |  |  |  |  |  |  |  |  |  |  |  |  |  |  |  |  |  |  |  |  |  |  |  |  |  |  |  |  |  |  |  |  |  |  |  |  |  |  |  |  |  |  |  |  |  |  |  |  |  |  |  |  |  |  |  |  |  |  |  |  |  |  |  |  |  |  |  |  |  |  |  |  |  |  |  |  |  |  |  |  |  |  |  |  |  |  |  |  |  |  |  |  |  |  |  |  |  |  |  |  |  |  |  |  |  |  |  |  |  |  |  |  |  |  |  |  |  |  |  |  |  |  |  |  |  |  |  |  |  |  |  |  |  |  |  |  |  |  |  |  |  |  |  |  |  |  |  |  |  |  |  |  |  |  |  |  |  |  |  |  |  |  |  |  |  |  |  |  |  |  |  |  |  |  |  |  |  |  |  |  |  |  |  |  |  |  |  |  |  |  |  |  |  |  |  |  |  |  |  |  |  |  |  |  |  |  |  |  |  |  |  |  |  |  |  |  |  |  |  |  |  |  |  |  |  |  |  |  |  |  |  |  |  |  |  |  |  |  |  |  |  |  |  |  |  |  |  |  |  |  |  |  |  |  |  |  |  |  |  |  |  |  |  |  |  |  |  |  |  |  |  |  |  |  |  |  |  |  |  |  |  |  |  |  |  |  |  |  |  |  |  |  |  |  |  |  |  |  |  |  |  |  |  |  |  |  |  |  |  |  |  |  |  |  |  |  |  |  |  |  |  |  |  |  |  |  |  |  |  |  |  |  |  |  |  |  |  |  |  |  |  |  |  |  |  |  |  |  |  |  |  |  |  |  |  |  |  |  |  |  |  |  |  |  |  |  |  |  |  |  |  |  |  |  |  |  |  |  |  |  |  |  |  |  |  |  |  |  |  |  |  |  |  |  |  |  |  |  |  |  |  |  |  |  |  |  |  |  |  |  |  |  |  |  |  |  |  |  |  |  |  |  |  |  |  |  |  |  |  |  |  |  |  |  |  |  |  |  |  |  |  |  |  |  |  |  |  |  |  |  |  |  |  |  |  |  |  |  |  |  |  |  |  |  |  |  |  |  |  |  |  |  |  |  |  |  |  |  |  |  |  |  |  |  |  |  |  |  |  |  |  |  |  |  |  |  |  |  |  |  |  |  |  |  |  |  |  |  |  |  |  |  |  |  |  |  |  |  |  |  |  |  |  |  |  |  |  |  |  |  |  |  |  |  |  |  |  |  |  |  |  |  |  |  |  |  |  |  |  |  |  |  |  |  |  |  |  |  |  |  |  |  |  |  |  |  |  |  |  |  |  |  |  |  |  |  |  |  |  |  |  |  |  |  |  |  |  |  |  |  |  |  |  |  |  |  |  |  |  |  |  |  |  |  |  |  |  |  |  |  |  |  |  |  |  |  |  |  |  |  |  |  |  |  |  |  |  |  |  |  |  |  |  |  |  |  |  |  |  |  |  |  |  |  |  |  |  |  |  |  |  |  |  |  |  |  |  |  |  |  |  |  |  |  |  |  |  |  |  |  |  |  |  |  |  |  |  |  |  |  |  |  |  |  |  |  |  |  |  |  |  |  |  |  |  |  |  |
|  |  |  |  |  |  |  |  |  |  |  |  |  |  |  |  |  |  |  |  |  |  |  |  |  |  |  |  |  |  |  |  |  |  |  |  |  |  |  |  |  |  |  |  |  |  |  |  |  |  |  |  |  |  |  |  |  |  |  |  |  |  |  |  |  |  |  |  |  |  |  |  |  |  |  |  |  |  |  |  |  |  |  |  |  |  |  |  |  |  |  |  |  |  |  |  |  |  |  |  |  |  |  |  |  |  |  |  |  |  |  |  |  |  |  |  |  |  |  |  |  |  |  |  |  |  |  |  |  |  |  |  |  |  |  |  |  |  |  |  |  |  |  |  |  |  |  |  |  |  |  |  |  |  |  |  |  |  |  |  |  |  |  |  |  |  |  |  |  |  |  |  |  |  |  |  |  |  |  |  |  |  |  |  |  |  |  |  |  |  |  |  |  |  |  |  |  |  |  |  |  |  |  |  |  |  |  |  |  |  |  |  |  |  |  |  |  |  |  |  |  |  |  |  |  |  |  |  |  |  |  |  |  |  |  |  |  |  |  |  |  |  |  |  |  |  |  |  |  |  |  |  |  |  |  |  |  |  |  |  |  |  |  |  |  |  |  |  |  |  |  |  |  |  |  |  |  |  |  |  |  |  |  |  |  |  |  |  |  |  |  |  |  |  |  |  |  |  |  |  |  |  |  |  |  |  |  |  |  |  |  |  |  |  |  |  |  |  |  |  |  |  |  |  |  |  |  |  |  |  |  |  |  |  |  |  |  |  |  |  |  |  |  |  |  |  |  |  |  |  |  |  |  |  |  |  |  |  |  |  |  |  |  |  |  |  |  |  |  |  |  |  |  |  |  |  |  |  |  |  |  |  |  |  |  |  |  |  |  |  |  |  |  |  |  |  |  |  |  |  |  |  |  |  |  |  |  |  |  |  |  |  |  |  |  |  |  |  |  |  |  |  |  |  |  |  |  |  |  |  |  |  |  |  |  |  |  |  |  |  |  |  |  |  |  |  |  |  |  |  |  |  |  |  |  |  |  |  |  |  |  |  |  |  |  |  |  |  |  |  |  |  |  |  |  |  |  |  |  |  |  |  |  |  |  |  |  |  |  |  |  |  |  |  |  |  |  |  |  |  |  |  |  |  |  |  |  |  |  |  |  |  |  |  |  |  |  |  |  |  |  |  |  |  |  |  |  |  |  |  |  |  |  |  |  |  |  |  |  |  |  |  |  |  |  |  |  |  |  |  |  |  |  |  |  |  |  |  |  |  |  |  |  |  |  |  |  |  |  |  |  |  |  |  |  |  |  |  |  |  |  |  |  |  |  |  |  |  |  |  |  |  |  |  |  |  |  |  |  |  |  |  |  |  |  |  |  |  |  |  |  |  |  |  |  |  |  |  |  |  |  |  |  |  |  |  |  |  |  |  |  |  |  |  |  |  |  |  |  |  |  |  |  |  |  |  |  |  |  |  |  |  |  |  |  |  |  |  |  |  |  |  |  |  |  |  |  |  |  |  |  |  |  |  |  |  |  |  |  |  |  |  |  |  |  |  |  |  |  |  |  |  |  |  |  |  |  |  |  |  |  |  |  |  |  |  |  |  |  |  |  |  |  |  |  |  |  |  |  |  |  |  |  |  |  |  |  |  |  |  |  |  |  |  |  |  |  |  |  |  |  |  |  |  |  |  |  |  |  |  |  |  |  |  |  |  |  |  |  |  |  |  |  |  |  |  |  |  |  |  |  |  |  |  |  |  |  |  |  |  |  |  |  |  |  |  |  |  |  |  |  |  |  |  |  |  |  |  |  |  |  |  |  |  |  |  |  |  |  |  |  |  |  |  |  |  |  |  |  |  |  |  |  |  |  |  |  |  |  |  |  |  |  |  |  |  |  |  |  |  |  |  |  |  |  |  |  |  |  |  |  |  |  |  |  |  |  |  |  |  |  |  |  |  |  |  |  |  |  |  |  |  |  |  |  |  |  |  |  |  |  |  |  |  |  |  |  |  |  |  |  |  |  |  |  |  |  |  |  |  |  |  |  |  |  |  |  |  |  |  |  |  |  |  |  |  |  |  |  |  |  |  |  |  |  |  |  |  |  |  |  |  |  |  |  |  |  |  |  |
|  |  |  |  |  |  |  |  |  |  |  |  |  |  |  |  |  |  |  |  |  |  |  |  |  |  |  |  |  |  |  |  |  |  |  |  |  |  |  |  |  |  |  |  |  |  |  |  |  |  |  |  |  |  |  |  |  |  |  |  |  |  |  |  |  |  |  |  |  |  |  |  |  |  |  |  |  |  |  |  |  |  |  |  |  |  |  |  |  |  |  |  |  |  |  |  |  |  |  |  |  |  |  |  |  |  |  |  |  |  |  |  |  |  |  |  |  |  |  |  |  |  |  |  |  |  |  |  |  |  |  |  |  |  |  |  |  |  |  |  |  |  |  |  |  |  |  |  |  |  |  |  |  |  |  |  |  |  |  |  |  |  |  |  |  |  |  |  |  |  |  |  |  |  |  |  |  |  |  |  |  |  |  |  |  |  |  |  |  |  |  |  |  |  |  |  |  |  |  |  |  |  |  |  |  |  |  |  |  |  |  |  |  |  |  |  |  |  |  |  |  |  |  |  |  |  |  |  |  |  |  |  |  |  |  |  |  |  |  |  |  |  |  |  |  |  |  |  |  |  |  |  |  |  |  |  |  |  |  |  |  |  |  |  |  |  |  |  |  |  |  |  |  |  |  |  |  |  |  |  |  |  |  |  |  |  |  |  |  |  |  |  |  |  |  |  |  |  |  |  |  |  |  |  |  |  |  |  |  |  |  |  |  |  |  |  |  |  |  |  |  |  |  |  |  |  |  |  |  |  |  |  |  |  |  |  |  |  |  |  |  |  |  |  |  |  |  |  |  |  |  |  |  |  |  |  |  |  |  |  |  |  |  |  |  |  |  |  |  |  |  |  |  |  |  |  |  |  |  |  |  |  |  |  |  |  |  |  |  |  |  |  |  |  |  |  |  |  |  |  |  |  |  |  |  |  |  |  |  |  |  |  |  |  |  |  |  |  |  |  |  |  |  |  |  |  |  |  |  |  |  |  |  |  |  |  |  |  |  |  |  |  |  |  |  |  |  |  |  |  |  |  |  |  |  |  |  |  |  |  |  |  |  |  |  |  |  |  |  |  |  |  |  |  |  |  |  |  |  |  |  |  |  |  |  |  |  |  |  |  |  |  |  |  |  |  |  |  |  |  |  |  |  |  |  |  |  |  |  |  |  |  |  |  |  |  |  |  |  |  |  |  |  |  |  |  |  |  |  |  |  |  |  |  |  |  |  |  |  |  |  |  |  |  |  |  |  |  |  |  |  |  |  |  |  |  |  |  |  |  |  |  |  |  |  |  |  |  |  |  |  |  |  |  |  |  |  |  |  |  |  |  |  |  |  |  |  |  |  |  |  |  |  |  |  |  |  |  |  |  |  |  |  |  |  |  |  |  |  |  |  |  |  |  |  |  |  |  |  |  |  |  |  |  |  |  |  |  |  |  |  |  |  |  |  |  |  |  |  |  |  |  |  |  |  |  |  |  |  |  |  |  |  |  |  |  |  |  |  |  |  |  |  |  |  |  |  |  |  |  |  |  |  |  |  |  |  |  |  |  |  |  |  |  |  |  |  |  |  |  |  |  |  |  |  |  |  |  |  |  |  |  |  |  |  |  |  |  |  |  |  |  |  |  |  |  |  |  |  |  |  |  |  |  |  |  |  |  |  |  |  |  |  |  |  |  |  |  |  |  |  |  |  |  |  |  |  |  |  |  |  |  |  |  |  |  |  |  |  |  |  |  |  |  |  |  |  |  |  |  |  |  |  |  |  |  |  |  |  |  |  |  |  |  |  |  |  |  |  |  |  |  |  |  |  |  |  |  |  |  |  |  |  |  |  |  |  |  |  |  |  |  |  |  |  |  |  |  |  |  |  |  |  |  |  |  |  |  |  |  |  |  |  |  |  |  |  |  |  |  |  |  |  |  |  |  |  |  |  |  |  |  |  |  |  |  |  |  |  |  |  |  |  |  |  |  |  |  |  |  |  |  |  |  |  |  |  |  |  |  |  |  |  |  |  |  |  |  |  |  |  |  |  |  |  |  |  |  |  |  |  |  |  |  |  |  |  |  |  |  |  |  |  |  |  |  |  |  |  |  |  |  |  |  |  |  |  |  |  |  |  |  |  |  |  |  |  |  |  |
|  |  |  |  |  |  |  |  |  |  |  |  |  |  |  |  |  |  |  |  |  |  |  |  |  |  |  |  |  |  |  |  |  |  |  |  |  |  |  |  |  |  |  |  |  |  |  |  |  |  |  |  |  |  |  |  |  |  |  |  |  |  |  |  |  |  |  |  |  |  |  |  |  |  |  |  |  |  |  |  |  |  |  |  |  |  |  |  |  |  |  |  |  |  |  |  |  |  |  |  |  |  |  |  |  |  |  |  |  |  |  |  |  |  |  |  |  |  |  |  |  |  |  |  |  |  |  |  |  |  |  |  |  |  |  |  |  |  |  |  |  |  |  |  |  |  |  |  |  |  |  |  |  |  |  |  |  |  |  |  |  |  |  |  |  |  |  |  |  |  |  |  |  |  |  |  |  |  |  |  |  |  |  |  |  |  |  |  |  |  |  |  |  |  |  |  |  |  |  |  |  |  |  |  |  |  |  |  |  |  |  |  |  |  |  |  |  |  |  |  |  |  |  |  |  |  |  |  |  |  |  |  |  |  |  |  |  |  |  |  |  |  |  |  |  |  |  |  |  |  |  |  |  |  |  |  |  |  |  |  |  |  |  |  |  |  |  |  |  |  |  |  |  |  |  |  |  |  |  |  |  |  |  |  |  |  |  |  |  |  |  |  |  |  |  |  |  |  |  |  |  |  |  |  |  |  |  |  |  |  |  |  |  |  |  |  |  |  |  |  |  |  |  |  |  |  |  |  |  |  |  |  |  |  |  |  |  |  |  |  |  |  |  |  |  |  |  |  |  |  |  |  |  |  |  |  |  |  |  |  |  |  |  |  |  |  |  |  |  |  |  |  |  |  |  |  |  |  |  |  |  |  |  |  |  |  |  |  |  |  |  |  |  |  |  |  |  |  |  |  |  |  |  |  |  |  |  |  |  |  |  |  |  |  |  |  |  |  |  |  |  |  |  |  |  |  |  |  |  |  |  |  |  |  |  |  |  |  |  |  |  |  |  |  |  |  |  |  |  |  |  |  |  |  |  |  |  |  |  |  |  |  |  |  |  |  |  |  |  |  |  |  |  |  |  |  |  |  |  |  |  |  |  |  |  |  |  |  |  |  |  |  |  |  |  |  |  |  |  |  |  |  |  |  |  |  |  |  |  |  |  |  |  |  |  |  |  |  |  |  |  |  |  |  |  |  |  |  |  |  |  |  |  |  |  |  |  |  |  |  |  |  |  |  |  |  |  |  |  |  |  |  |  |  |  |  |  |  |  |  |  |  |  |  |  |  |  |  |  |  |  |  |  |  |  |  |  |  |  |  |  |  |  |  |  |  |  |  |  |  |  |  |  |  |  |  |  |  |  |  |  |  |  |  |  |  |  |  |  |  |  |  |  |  |  |  |  |  |  |  |  |  |  |  |  |  |  |  |  |  |  |  |  |  |  |  |  |  |  |  |  |  |  |  |  |  |  |  |  |  |  |  |  |  |  |  |  |  |  |  |  |  |  |  |  |  |  |  |  |  |  |  |  |  |  |  |  |  |  |  |  |  |  |  |  |  |  |  |  |  |  |  |  |  |  |  |  |  |  |  |  |  |  |  |  |  |  |  |  |  |  |  |  |  |  |  |  |  |  |  |  |  |  |  |  |  |  |  |  |  |  |  |  |  |  |  |  |  |  |  |  |  |  |  |  |  |  |  |  |  |  |  |  |  |  |  |  |  |  |  |  |  |  |  |  |  |  |  |  |  |  |  |  |  |  |  |  |  |  |  |  |  |  |  |  |  |  |  |  |  |  |  |  |  |  |  |  |  |  |  |  |  |  |  |  |  |  |  |  |  |  |  |  |  |  |  |  |  |  |  |  |  |  |  |  |  |  |  |  |  |  |  |  |  |  |  |  |  |  |  |  |  |  |  |  |  |  |  |  |  |  |  |  |  |  |  |  |  |  |  |  |  |  |  |  |  |  |  |  |  |  |  |  |  |  |  |  |  |  |  |  |  |  |  |  |  |  |  |  |  |  |  |  |  |  |  |  |  |  |  |  |  |  |  |  |  |  |  |  |  |  |  |  |  |  |  |  |  |  |  |  |  |  |  |  |  |  |  |  |  |  |  |  |  |  |  |  |  |  |
|  |  |  |  |  |  |  |  |  |  |  |  |  |  |  |  |  |  |  |  |  |  |  |  |  |  |  |  |  |  |  |  |  |  |  |  |  |  |  |  |  |  |  |  |  |  |  |  |  |  |  |  |  |  |  |  |  |  |  |  |  |  |  |  |  |  |  |  |  |  |  |  |  |  |  |  |  |  |  |  |  |  |  |  |  |  |  |  |  |  |  |  |  |  |  |  |  |  |  |  |  |  |  |  |  |  |  |  |  |  |  |  |  |  |  |  |  |  |  |  |  |  |  |  |  |  |  |  |  |  |  |  |  |  |  |  |  |  |  |  |  |  |  |  |  |  |  |  |  |  |  |  |  |  |  |  |  |  |  |  |  |  |  |  |  |  |  |  |  |  |  |  |  |  |  |  |  |  |  |  |  |  |  |  |  |  |  |  |  |  |  |  |  |  |  |  |  |  |  |  |  |  |  |  |  |  |  |  |  |  |  |  |  |  |  |  |  |  |  |  |  |  |  |  |  |  |  |  |  |  |  |  |  |  |  |  |  |  |  |  |  |  |  |  |  |  |  |  |  |  |  |  |  |  |  |  |  |  |  |  |  |  |  |  |  |  |  |  |  |  |  |  |  |  |  |  |  |  |  |  |  |  |  |  |  |  |  |  |  |  |  |  |  |  |  |  |  |  |  |  |  |  |  |  |  |  |  |  |  |  |  |  |  |  |  |  |  |  |  |  |  |  |  |  |  |  |  |  |  |  |  |  |  |  |  |  |  |  |  |  |  |  |  |  |  |  |  |  |  |  |  |  |  |  |  |  |  |  |  |  |  |  |  |  |  |  |  |  |  |  |  |  |  |  |  |  |  |  |  |  |  |  |  |  |  |  |  |  |  |  |  |  |  |  |  |  |  |  |  |  |  |  |  |  |  |  |  |  |  |  |  |  |  |  |  |  |  |  |  |  |  |  |  |  |  |  |  |  |  |  |  |  |  |  |  |  |  |  |  |  |  |  |  |  |  |  |  |  |  |  |  |  |  |  |  |  |  |  |  |  |  |  |  |  |  |  |  |  |  |  |  |  |  |  |  |  |  |  |  |  |  |  |  |  |  |  |  |  |  |  |  |  |  |  |  |  |  |  |  |  |  |  |  |  |  |  |  |  |  |  |  |  |  |  |  |  |  |  |  |  |  |  |  |  |  |  |  |  |  |  |  |  |  |  |  |  |  |  |  |  |  |  |  |  |  |  |  |  |  |  |  |  |  |  |  |  |  |  |  |  |  |  |  |  |  |  |  |  |  |  |  |  |  |  |  |  |  |  |  |  |  |  |  |  |  |  |  |  |  |  |  |  |  |  |  |  |  |  |  |  |  |  |  |  |  |  |  |  |  |  |  |  |  |  |  |  |  |  |  |  |  |  |  |  |  |  |  |  |  |  |  |  |  |  |  |  |  |  |  |  |  |  |  |  |  |  |  |  |  |  |  |  |  |  |  |  |  |  |  |  |  |  |  |  |  |  |  |  |  |  |  |  |  |  |  |  |  |  |  |  |  |  |  |  |  |  |  |  |  |  |  |  |  |  |  |  |  |  |  |  |  |  |  |  |  |  |  |  |  |  |  |  |  |  |  |  |  |  |  |  |  |  |  |  |  |  |  |  |  |  |  |  |  |  |  |  |  |  |  |  |  |  |  |  |  |  |  |  |  |  |  |  |  |  |  |  |  |  |  |  |  |  |  |  |  |  |  |  |  |  |  |  |  |  |  |  |  |  |  |  |  |  |  |  |  |  |  |  |  |  |  |  |  |  |  |  |  |  |  |  |  |  |  |  |  |  |  |  |  |  |  |  |  |  |  |  |  |  |  |  |  |  |  |  |  |  |  |  |  |  |  |  |  |  |  |  |  |  |  |  |  |  |  |  |  |  |  |  |  |  |  |  |  |  |  |  |  |  |  |  |  |  |  |  |  |  |  |  |  |  |  |  |  |  |  |  |  |  |  |  |  |  |  |  |  |  |  |  |  |  |  |  |  |  |  |  |  |  |  |  |  |  |  |  |  |  |  |  |  |  |  |  |  |  |  |  |  |  |  |  |  |  |  |  |  |  |  |  |  |  |  |  |  |  |  |  |  |  |  |
|  |  |  |  |  |  |  |  |  |  |  |  |  |  |  |  |  |  |  |  |  |  |  |  |  |  |  |  |  |  |  |  |  |  |  |  |  |  |  |  |  |  |  |  |  |  |  |  |  |  |  |  |  |  |  |  |  |  |  |  |  |  |  |  |  |  |  |  |  |  |  |  |  |  |  |  |  |  |  |  |  |  |  |  |  |  |  |  |  |  |  |  |  |  |  |  |  |  |  |  |  |  |  |  |  |  |  |  |  |  |  |  |  |  |  |  |  |  |  |  |  |  |  |  |  |  |  |  |  |  |  |  |  |  |  |  |  |  |  |  |  |  |  |  |  |  |  |  |  |  |  |  |  |  |  |  |  |  |  |  |  |  |  |  |  |  |  |  |  |  |  |  |  |  |  |  |  |  |  |  |  |  |  |  |  |  |  |  |  |  |  |  |  |  |  |  |  |  |  |  |  |  |  |  |  |  |  |  |  |  |  |  |  |  |  |  |  |  |  |  |  |  |  |  |  |  |  |  |  |  |  |  |  |  |  |  |  |  |  |  |  |  |  |  |  |  |  |  |  |  |  |  |  |  |  |  |  |  |  |  |  |  |  |  |  |  |  |  |  |  |  |  |  |  |  |  |  |  |  |  |  |  |  |  |  |  |  |  |  |  |  |  |  |  |  |  |  |  |  |  |  |  |  |  |  |  |  |  |  |  |  |  |  |  |  |  |  |  |  |  |  |  |  |  |  |  |  |  |  |  |  |  |  |  |  |  |  |  |  |  |  |  |  |  |  |  |  |  |  |  |  |  |  |  |  |  |  |  |  |  |  |  |  |  |  |  |  |  |  |  |  |  |  |  |  |  |  |  |  |  |  |  |  |  |  |  |  |  |  |  |  |  |  |  |  |  |  |  |  |  |  |  |  |  |  |  |  |  |  |  |  |  |  |  |  |  |  |  |  |  |  |  |  |  |  |  |  |  |  |  |  |  |  |  |  |  |  |  |  |  |  |  |  |  |  |  |  |  |  |  |  |  |  |  |  |  |  |  |  |  |  |  |  |  |  |  |  |  |  |  |  |  |  |  |  |  |  |  |  |  |  |  |  |  |  |  |  |  |  |  |  |  |  |  |  |  |  |  |  |  |  |  |  |  |  |  |  |  |  |  |  |  |  |  |  |  |  |  |  |  |  |  |  |  |  |  |  |  |  |  |  |  |  |  |  |  |  |  |  |  |  |  |  |  |  |  |  |  |  |  |  |  |  |  |  |  |  |  |  |  |  |  |  |  |  |  |  |  |  |  |  |  |  |  |  |  |  |  |  |  |  |  |  |  |  |  |  |  |  |  |  |  |  |  |  |  |  |  |  |  |  |  |  |  |  |  |  |  |  |  |  |  |  |  |  |  |  |  |  |  |  |  |  |  |  |  |  |  |  |  |  |  |  |  |  |  |  |  |  |  |  |  |  |  |  |  |  |  |  |  |  |  |  |  |  |  |  |  |  |  |  |  |  |  |  |  |  |  |  |  |  |  |  |  |  |  |  |  |  |  |  |  |  |  |  |  |  |  |  |  |  |  |  |  |  |  |  |  |  |  |  |  |  |  |  |  |  |  |  |  |  |  |  |  |  |  |  |  |  |  |  |  |  |  |  |  |  |  |  |  |  |  |  |  |  |  |  |  |  |  |  |  |  |  |  |  |  |  |  |  |  |  |  |  |  |  |  |  |  |  |  |  |  |  |  |  |  |  |  |  |  |  |  |  |  |  |  |  |  |  |  |  |  |  |  |  |  |  |  |  |  |  |  |  |  |  |  |  |  |  |  |  |  |  |  |  |  |  |  |  |  |  |  |  |  |  |  |  |  |  |  |  |  |  |  |  |  |  |  |  |  |  |  |  |  |  |  |  |  |  |  |  |  |  |  |  |  |  |  |  |  |  |  |  |  |  |  |  |  |  |  |  |  |  |  |  |  |  |  |  |  |  |  |  |  |  |  |  |  |  |  |  |  |  |  |  |  |  |  |  |  |  |  |  |  |  |  |  |  |  |  |  |  |  |  |  |  |  |  |  |  |  |  |  |  |  |  |  |  |  |  |  |  |  |  |  |  |  |  |  |  |  |  |  |  |  |  |  |  |
|  |  |  |  |  |  |  |  |  |  |  |  |  |  |  |  |  |  |  |  |  |  |  |  |  |  |  |  |  |  |  |  |  |  |  |  |  |  |  |  |  |  |  |  |  |  |  |  |  |  |  |  |  |  |  |  |  |  |  |  |  |  |  |  |  |  |  |  |  |  |  |  |  |  |  |  |  |  |  |  |  |  |  |  |  |  |  |  |  |  |  |  |  |  |  |  |  |  |  |  |  |  |  |  |  |  |  |  |  |  |  |  |  |  |  |  |  |  |  |  |  |  |  |  |  |  |  |  |  |  |  |  |  |  |  |  |  |  |  |  |  |  |  |  |  |  |  |  |  |  |  |  |  |  |  |  |  |  |  |  |  |  |  |  |  |  |  |  |  |  |  |  |  |  |  |  |  |  |  |  |  |  |  |  |  |  |  |  |  |  |  |  |  |  |  |  |  |  |  |  |  |  |  |  |  |  |  |  |  |  |  |  |  |  |  |  |  |  |  |  |  |  |  |  |  |  |  |  |  |  |  |  |  |  |  |  |  |  |  |  |  |  |  |  |  |  |  |  |  |  |  |  |  |  |  |  |  |  |  |  |  |  |  |  |  |  |  |  |  |  |  |  |  |  |  |  |  |  |  |  |  |  |  |  |  |  |  |  |  |  |  |  |  |  |  |  |  |  |  |  |  |  |  |  |  |  |  |  |  |  |  |  |  |  |  |  |  |  |  |  |  |  |  |  |  |  |  |  |  |  |  |  |  |  |  |  |  |  |  |  |  |  |  |  |  |  |  |  |  |  |  |  |  |  |  |  |  |  |  |  |  |  |  |  |  |  |  |  |  |  |  |  |  |  |  |  |  |  |  |  |  |  |  |  |  |  |  |  |  |  |  |  |  |  |  |  |  |  |  |  |  |  |  |  |  |  |  |  |  |  |  |  |  |  |  |  |  |  |  |  |  |  |  |  |  |  |  |  |  |  |  |  |  |  |  |  |  |  |  |  |  |  |  |  |  |  |  |  |  |  |  |  |  |  |  |  |  |  |  |  |  |  |  |  |  |  |  |  |  |  |  |  |  |  |  |  |  |  |  |  |  |  |  |  |  |  |  |  |  |  |  |  |  |  |  |  |  |  |  |  |  |  |  |  |  |  |  |  |  |  |  |  |  |  |  |  |  |  |  |  |  |  |  |  |  |  |  |  |  |  |  |  |  |  |  |  |  |  |  |  |  |  |  |  |  |  |  |  |  |  |  |  |  |  |  |  |  |  |  |  |  |  |  |  |  |  |  |  |  |  |  |  |  |  |  |  |  |  |  |  |  |  |  |  |  |  |  |  |  |  |  |  |  |  |  |  |  |  |  |  |  |  |  |  |  |  |  |  |  |  |  |  |  |  |  |  |  |  |  |  |  |  |  |  |  |  |  |  |  |  |  |  |  |  |  |  |  |  |  |  |  |  |  |  |  |  |  |  |  |  |  |  |  |  |  |  |  |  |  |  |  |  |  |  |  |  |  |  |  |  |  |  |  |  |  |  |  |  |  |  |  |  |  |  |  |  |  |  |  |  |  |  |  |  |  |  |  |  |  |  |  |  |  |  |  |  |  |  |  |  |  |  |  |  |  |  |  |  |  |  |  |  |  |  |  |  |  |  |  |  |  |  |  |  |  |  |  |  |  |  |  |  |  |  |  |  |  |  |  |  |  |  |  |  |  |  |  |  |  |  |  |  |  |  |  |  |  |  |  |  |  |  |  |  |  |  |  |  |  |  |  |  |  |  |  |  |  |  |  |  |  |  |  |  |  |  |  |  |  |  |  |  |  |  |  |  |  |  |  |  |  |  |  |  |  |  |  |  |  |  |  |  |  |  |  |  |  |  |  |  |  |  |  |  |  |  |  |  |  |  |  |  |  |  |  |  |  |  |  |  |  |  |  |  |  |  |  |  |  |  |  |  |  |  |  |  |  |  |  |  |  |  |  |  |  |  |  |  |  |  |  |  |  |  |  |  |  |  |  |  |  |  |  |  |  |  |  |  |  |  |  |  |  |  |  |  |  |  |  |  |  |  |  |  |  |  |  |  |  |  |  |  |  |  |  |  |  |  |  |  |  |  |  |  |  |  |  |  |  |
|  |  |  |  |  |  |  |  |  |  |  |  |  |  |  |  |  |  |  |  |  |  |  |  |  |  |  |  |  |  |  |  |  |  |  |  |  |  |  |  |  |  |  |  |  |  |  |  |  |  |  |  |  |  |  |  |  |  |  |  |  |  |  |  |  |  |  |  |  |  |  |  |  |  |  |  |  |  |  |  |  |  |  |  |  |  |  |  |  |  |  |  |  |  |  |  |  |  |  |  |  |  |  |  |  |  |  |  |  |  |  |  |  |  |  |  |  |  |  |  |  |  |  |  |  |  |  |  |  |  |  |  |  |  |  |  |  |  |  |  |  |  |  |  |  |  |  |  |  |  |  |  |  |  |  |  |  |  |  |  |  |  |  |  |  |  |  |  |  |  |  |  |  |  |  |  |  |  |  |  |  |  |  |  |  |  |  |  |  |  |  |  |  |  |  |  |  |  |  |  |  |  |  |  |  |  |  |  |  |  |  |  |  |  |  |  |  |  |  |  |  |  |  |  |  |  |  |  |  |  |  |  |  |  |  |  |  |  |  |  |  |  |  |  |  |  |  |  |  |  |  |  |  |  |  |  |  |  |  |  |  |  |  |  |  |  |  |  |  |  |  |  |  |  |  |  |  |  |  |  |  |  |  |  |  |  |  |  |  |  |  |  |  |  |  |  |  |  |  |  |  |  |  |  |  |  |  |  |  |  |  |  |  |  |  |  |  |  |  |  |  |  |  |  |  |  |  |  |  |  |  |  |  |  |  |  |  |  |  |  |  |  |  |  |  |  |  |  |  |  |  |  |  |  |  |  |  |  |  |  |  |  |  |  |  |  |  |  |  |  |  |  |  |  |  |  |  |  |  |  |  |  |  |  |  |  |  |  |  |  |  |  |  |  |  |  |  |  |  |  |  |  |  |  |  |  |  |  |  |  |  |  |  |  |  |  |  |  |  |  |  |  |  |  |  |  |  |  |  |  |  |  |  |  |  |  |  |  |  |  |  |  |  |  |  |  |  |  |  |  |  |  |  |  |  |  |  |  |  |  |  |  |  |  |  |  |  |  |  |  |  |  |  |  |  |  |  |  |  |  |  |  |  |  |  |  |  |  |  |  |  |  |  |  |  |  |  |  |  |  |  |  |  |  |  |  |  |  |  |  |  |  |  |  |  |  |  |  |  |  |  |  |  |  |  |  |  |  |  |  |  |  |  |  |  |  |  |  |  |  |  |  |  |  |  |  |  |  |  |  |  |  |  |  |  |  |  |  |  |  |  |  |  |  |  |  |  |  |  |  |  |  |  |  |  |  |  |  |  |  |  |  |  |  |  |  |  |  |  |  |  |  |  |  |  |  |  |  |  |  |  |  |  |  |  |  |  |  |  |  |  |  |  |  |  |  |  |  |  |  |  |  |  |  |  |  |  |  |  |  |  |  |  |  |  |  |  |  |  |  |  |  |  |  |  |  |  |  |  |  |  |  |  |  |  |  |  |  |  |  |  |  |  |  |  |  |  |  |  |  |  |  |  |  |  |  |  |  |  |  |  |  |  |  |  |  |  |  |  |  |  |  |  |  |  |  |  |  |  |  |  |  |  |  |  |  |  |  |  |  |  |  |  |  |  |  |  |  |  |  |  |  |  |  |  |  |  |  |  |  |  |  |  |  |  |  |  |  |  |  |  |  |  |  |  |  |  |  |  |  |  |  |  |  |  |  |  |  |  |  |  |  |  |  |  |  |  |  |  |  |  |  |  |  |  |  |  |  |  |  |  |  |  |  |  |  |  |  |  |  |  |  |  |  |  |  |  |  |  |  |  |  |  |  |  |  |  |  |  |  |  |  |  |  |  |  |  |  |  |  |  |  |  |  |  |  |  |  |  |  |  |  |  |  |  |  |  |  |  |  |  |  |  |  |  |  |  |  |  |  |  |  |  |  |  |  |  |  |  |  |  |  |  |  |  |  |  |  |  |  |  |  |  |  |  |  |  |  |  |  |  |  |  |  |  |  |  |  |  |  |  |  |  |  |  |  |  |  |  |  |  |  |  |  |  |  |  |  |  |  |  |  |  |  |  |  |  |  |  |  |  |  |  |  |  |  |  |  |  |  |  |  |  |  |  |  |  |  |  |
|  |  |  |  |  |  |  |  |  |  |  |  |  |  |  |  |  |  |  |  |  |  |  |  |  |  |  |  |  |  |  |  |  |  |  |  |  |  |  |  |  |  |  |  |  |  |  |  |  |  |  |  |  |  |  |  |  |  |  |  |  |  |  |  |  |  |  |  |  |  |  |  |  |  |  |  |  |  |  |  |  |  |  |  |  |  |  |  |  |  |  |  |  |  |  |  |  |  |  |  |  |  |  |  |  |  |  |  |  |  |  |  |  |  |  |  |  |  |  |  |  |  |  |  |  |  |  |  |  |  |  |  |  |  |  |  |  |  |  |  |  |  |  |  |  |  |  |  |  |  |  |  |  |  |  |  |  |  |  |  |  |  |  |  |  |  |  |  |  |  |  |  |  |  |  |  |  |  |  |  |  |  |  |  |  |  |  |  |  |  |  |  |  |  |  |  |  |  |  |  |  |  |  |  |  |  |  |  |  |  |  |  |  |  |  |  |  |  |  |  |  |  |  |  |  |  |  |  |  |  |  |  |  |  |  |  |  |  |  |  |  |  |  |  |  |  |  |  |  |  |  |  |  |  |  |  |  |  |  |  |  |  |  |  |  |  |  |  |  |  |  |  |  |  |  |  |  |  |  |  |  |  |  |  |  |  |  |  |  |  |  |  |  |  |  |  |  |  |  |  |  |  |  |  |  |  |  |  |  |  |  |  |  |  |  |  |  |  |  |  |  |  |  |  |  |  |  |  |  |  |  |  |  |  |  |  |  |  |  |  |  |  |  |  |  |  |  |  |  |  |  |  |  |  |  |  |  |  |  |  |  |  |  |  |  |  |  |  |  |  |  |  |  |  |  |  |  |  |  |  |  |  |  |  |  |  |  |  |  |  |  |  |  |  |  |  |  |  |  |  |  |  |  |  |  |  |  |  |  |  |  |  |  |  |  |  |  |  |  |  |  |  |  |  |  |  |  |  |  |  |  |  |  |  |  |  |  |  |  |  |  |  |  |  |  |  |  |  |  |  |  |  |  |  |  |  |  |  |  |  |  |  |  |  |  |  |  |  |  |  |  |  |  |  |  |  |  |  |  |  |  |  |  |  |  |  |  |  |  |  |  |  |  |  |  |  |  |  |  |  |  |  |  |  |  |  |  |  |  |  |  |  |  |  |  |  |  |  |  |  |  |  |  |  |  |  |  |  |  |  |  |  |  |  |  |  |  |  |  |  |  |  |  |  |  |  |  |  |  |  |  |  |  |  |  |  |  |  |  |  |  |  |  |  |  |  |  |  |  |  |  |  |  |  |  |  |  |  |  |  |  |  |  |  |  |  |  |  |  |  |  |  |  |  |  |  |  |  |  |  |  |  |  |  |  |  |  |  |  |  |  |  |  |  |  |  |  |  |  |  |  |  |  |  |  |  |  |  |  |  |  |  |  |  |  |  |  |  |  |  |  |  |  |  |  |  |  |  |  |  |  |  |  |  |  |  |  |  |  |  |  |  |  |  |  |  |  |  |  |  |  |  |  |  |  |  |  |  |  |  |  |  |  |  |  |  |  |  |  |  |  |  |  |  |  |  |  |  |  |  |  |  |  |  |  |  |  |  |  |  |  |  |  |  |  |  |  |  |  |  |  |  |  |  |  |  |  |  |  |  |  |  |  |  |  |  |  |  |  |  |  |  |  |  |  |  |  |  |  |  |  |  |  |  |  |  |  |  |  |  |  |  |  |  |  |  |  |  |  |  |  |  |  |  |  |  |  |  |  |  |  |  |  |  |  |  |  |  |  |  |  |  |  |  |  |  |  |  |  |  |  |  |  |  |  |  |  |  |  |  |  |  |  |  |  |  |  |  |  |  |  |  |  |  |  |  |  |  |  |  |  |  |  |  |  |  |  |  |  |  |  |  |  |  |  |  |  |  |  |  |  |  |  |  |  |  |  |  |  |  |  |  |  |  |  |  |  |  |  |  |  |  |  |  |  |  |  |  |  |  |  |  |  |  |  |  |  |  |  |  |  |  |  |  |  |  |  |  |  |  |  |  |  |  |  |  |  |  |  |  |  |  |  |  |  |  |  |  |  |  |  |  |  |  |  |  |  |  |  |  |  |  |  |  |  |  |  |  |  |
