# Supplementary figures and images for: Variation in selection constraints on teleost TLRs with emphasis on their repertoire in the Walking catfish, Clarias batrachus (part 1 of 3)
Source: Sci Rep. 2020 Dec 7;10:21394. doi: 10.1038/s41598-020-78347-6 (PMC7721727; doi:10.1038/s41598-020-78347-6)

Tree scale: 0.1

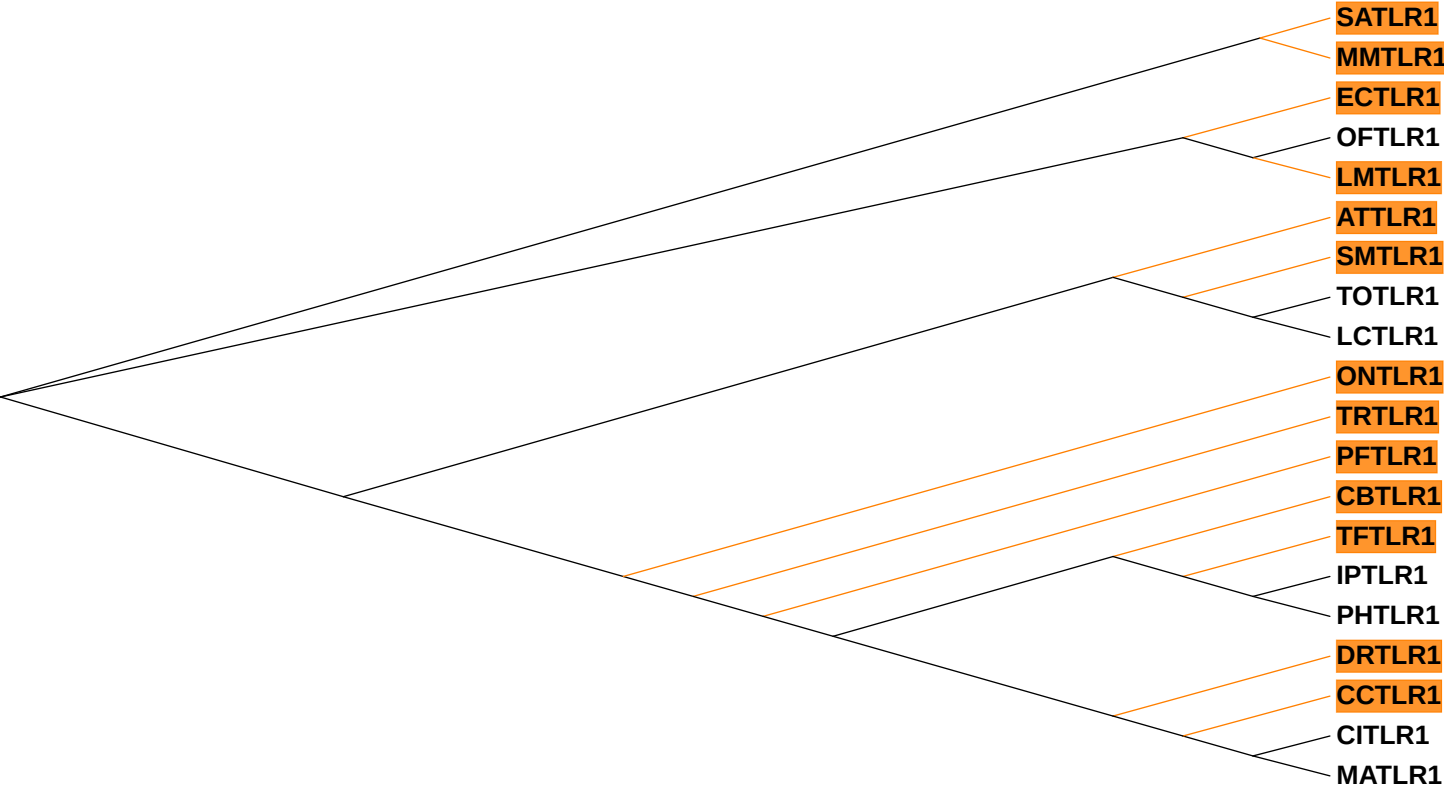

Supplement: Supplementary file 25 — Supplementary Information 25. [file 41598_2020_78347_MOESM25_ESM.zip › T1/absrel/labelledtree.pdf]

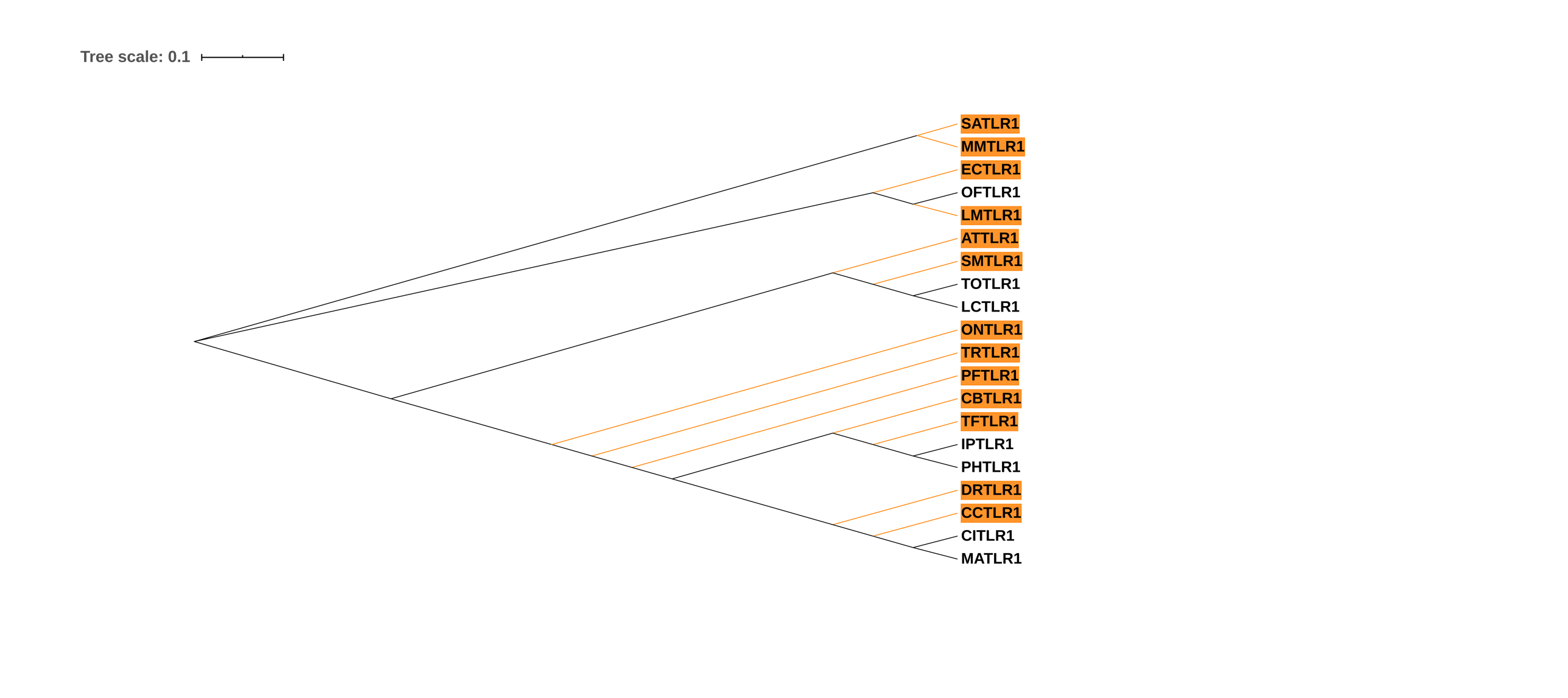

Supplement: Supplementary file 25 — Supplementary Information 25. [file 41598_2020_78347_MOESM25_ESM.zip › T1/absrel/labelledtree.png]

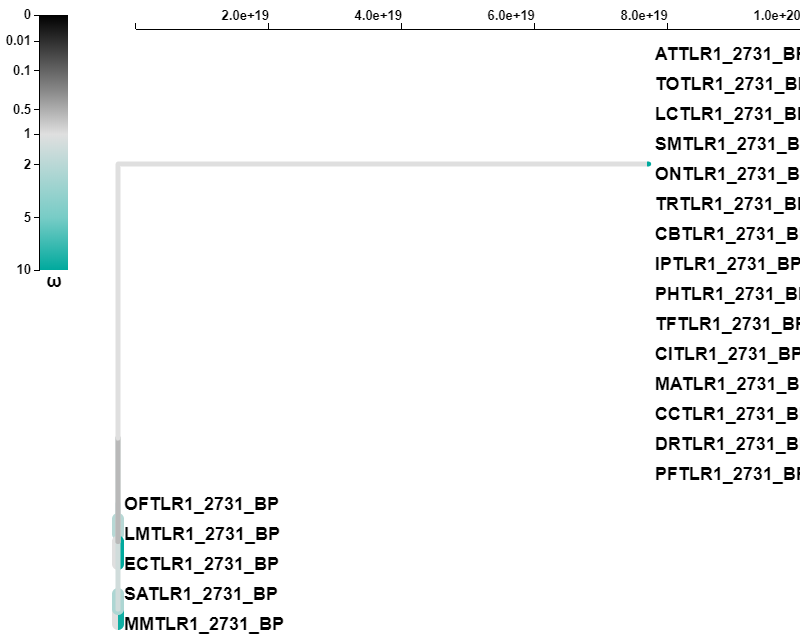

Supplement: Supplementary file 25 — Supplementary Information 25. [file 41598_2020_78347_MOESM25_ESM.zip › T1/absrel/tree.png]

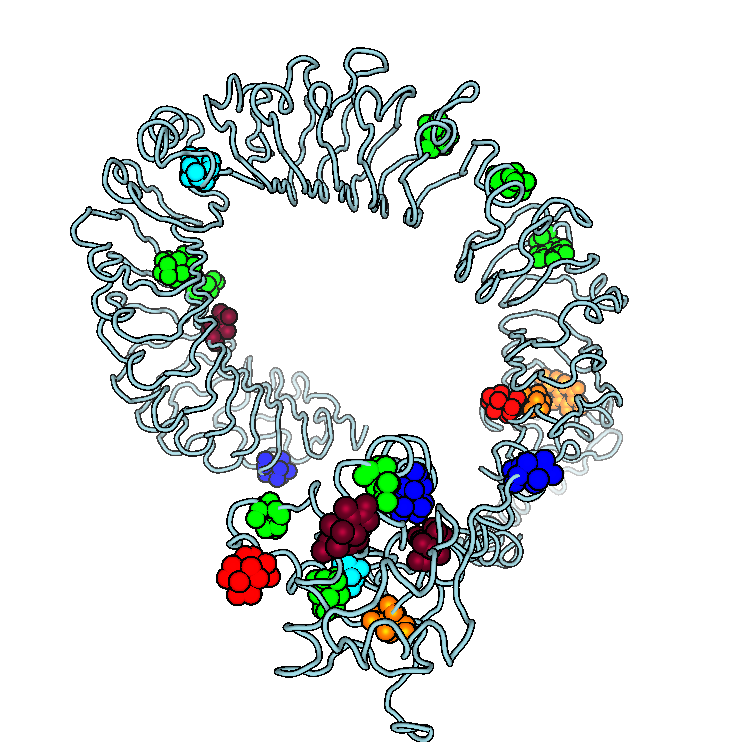

Supplement: Supplementary file 25 — Supplementary Information 25. [file 41598_2020_78347_MOESM25_ESM.zip › T1/BIS2/T1.png]

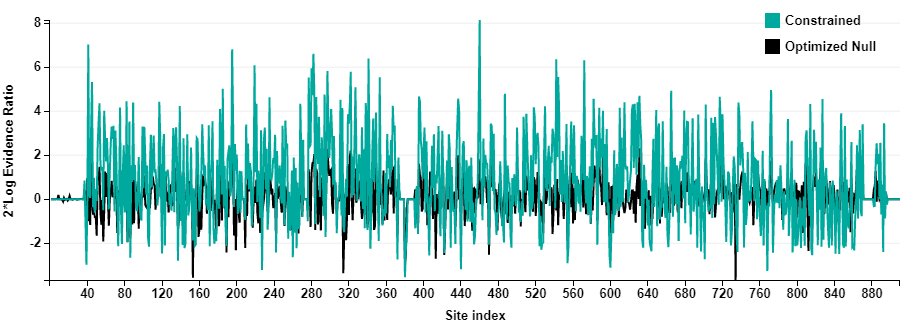

Supplement: Supplementary file 25 — Supplementary Information 25. [file 41598_2020_78347_MOESM25_ESM.zip › T1/busted/busted-chart (1).png]

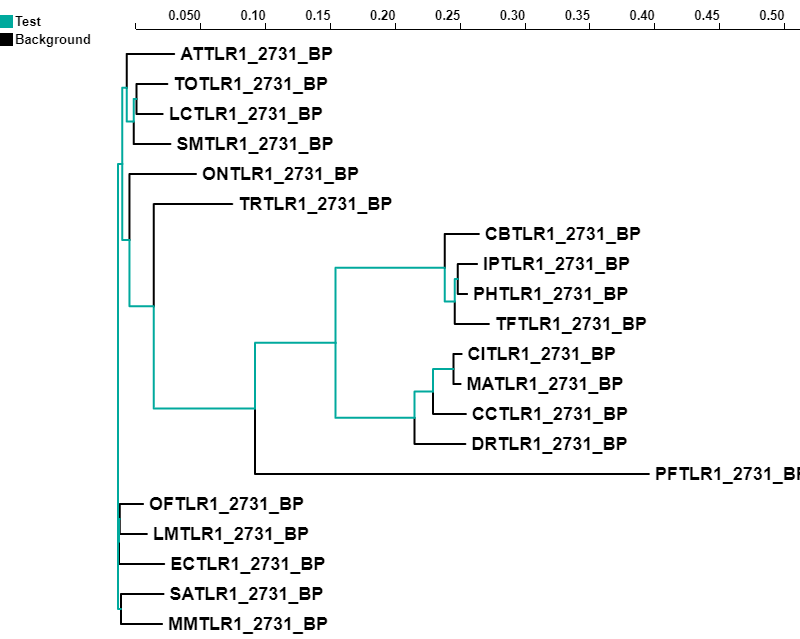

Supplement: Supplementary file 25 — Supplementary Information 25. [file 41598_2020_78347_MOESM25_ESM.zip › T1/busted/tree.png]

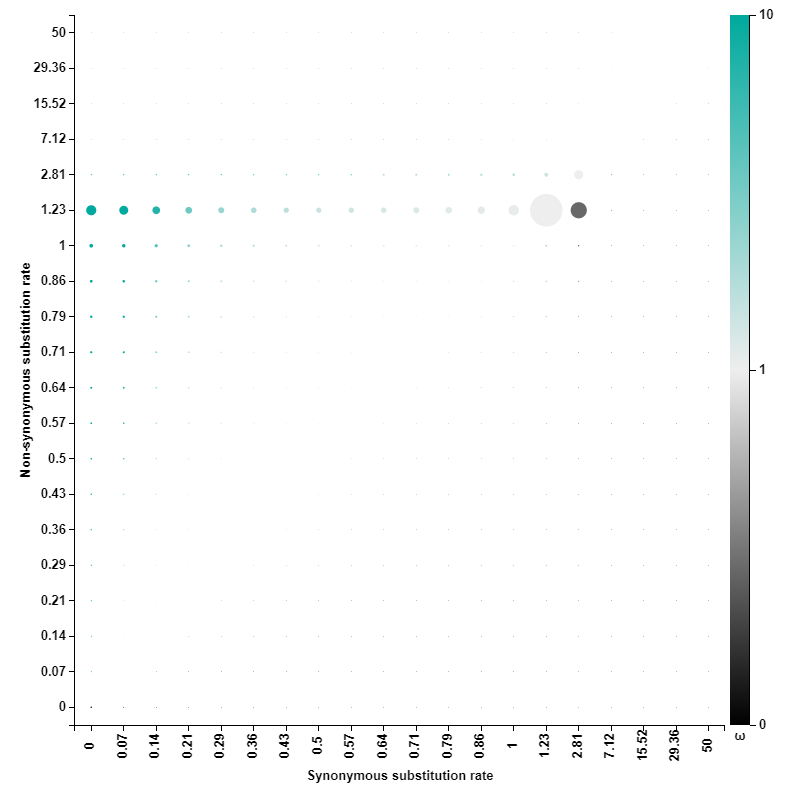

Supplement: Supplementary file 25 — Supplementary Information 25. [file 41598_2020_78347_MOESM25_ESM.zip › T1/fubar/datamonkey-chart.png]

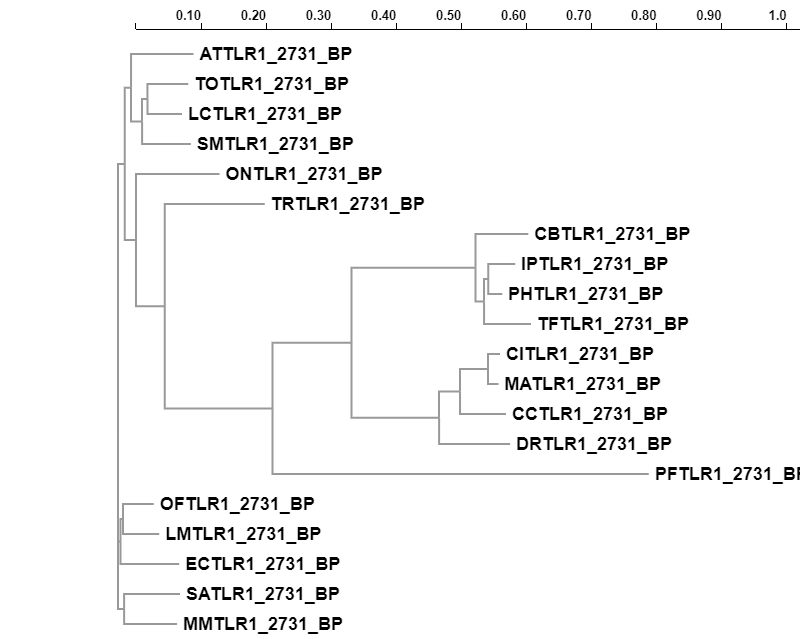

Supplement: Supplementary file 25 — Supplementary Information 25. [file 41598_2020_78347_MOESM25_ESM.zip › T1/fubar/tree.png]

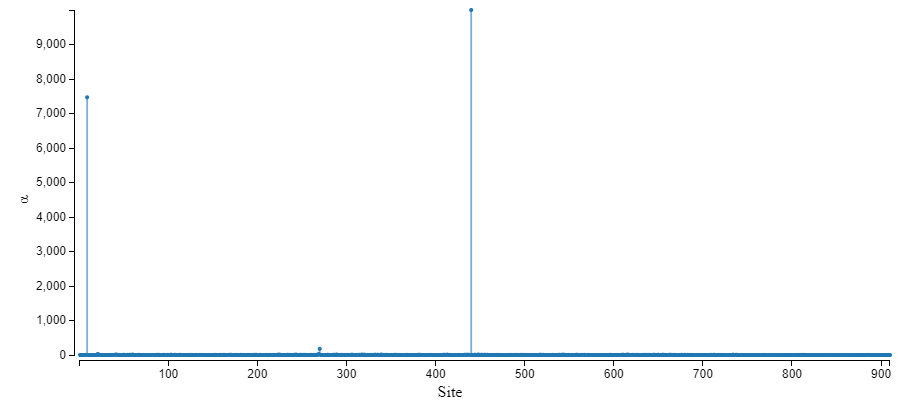

Supplement: Supplementary file 25 — Supplementary Information 25. [file 41598_2020_78347_MOESM25_ESM.zip › T1/meme/datamonkey-chart.png]

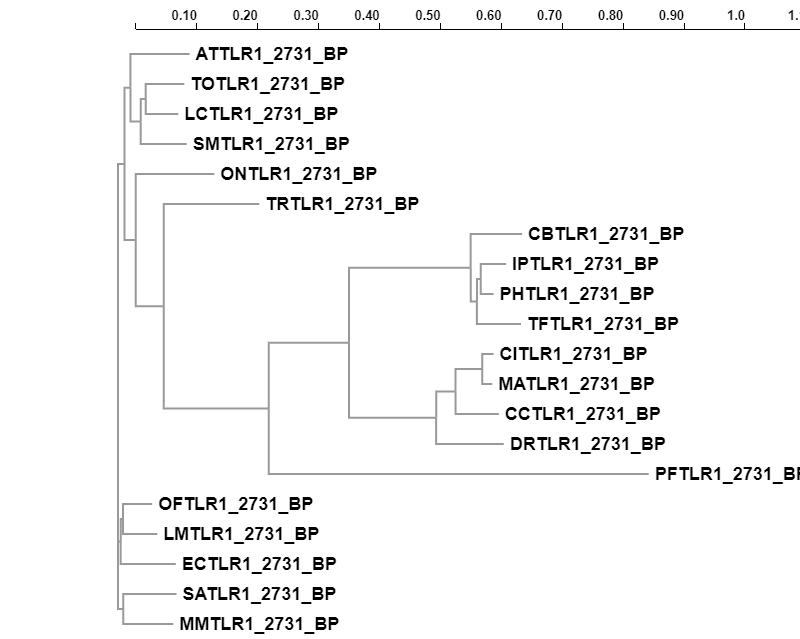

Supplement: Supplementary file 25 — Supplementary Information 25. [file 41598_2020_78347_MOESM25_ESM.zip › T1/meme/tree.png]

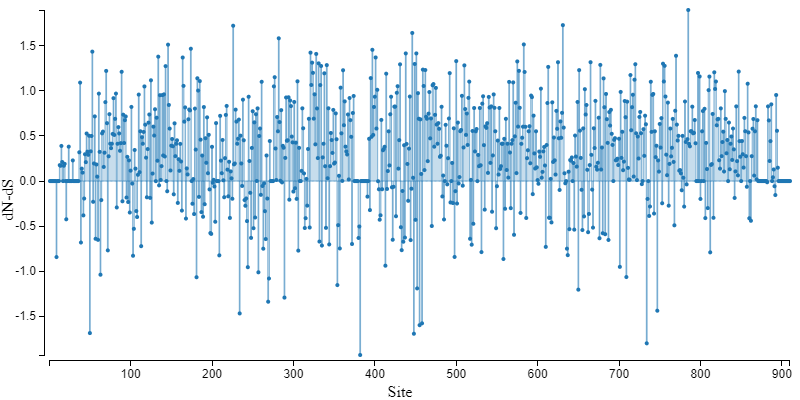

Supplement: Supplementary file 25 — Supplementary Information 25. [file 41598_2020_78347_MOESM25_ESM.zip › T1/slac/datamonkey-chart.png]

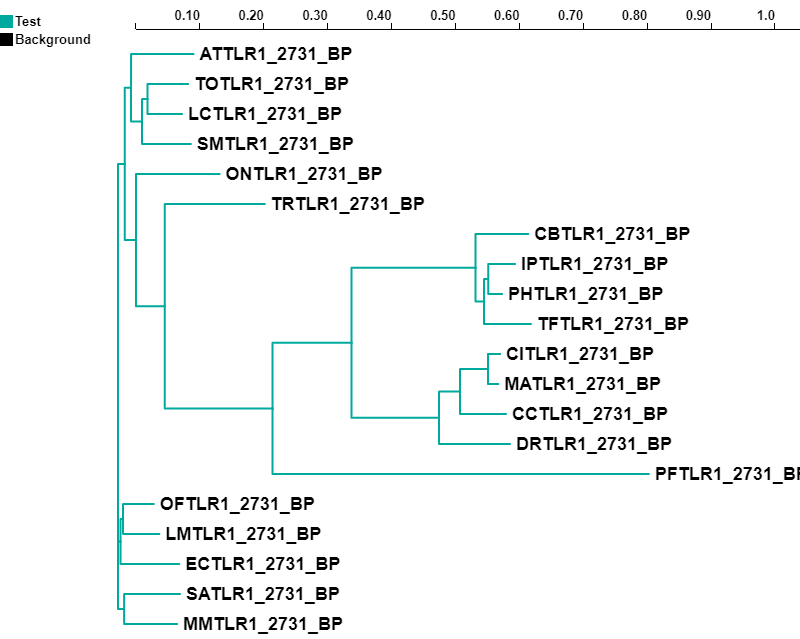

Supplement: Supplementary file 25 — Supplementary Information 25. [file 41598_2020_78347_MOESM25_ESM.zip › T1/slac/tree.png]

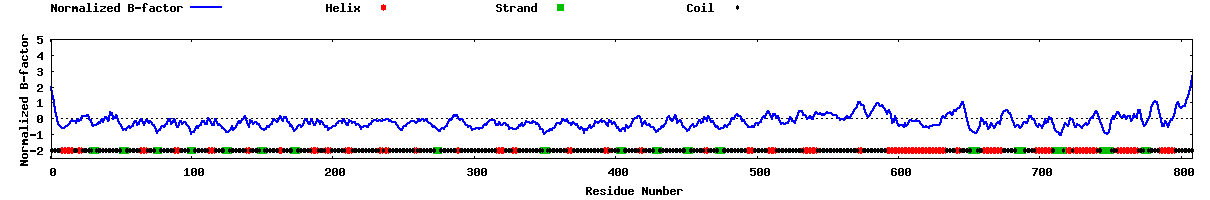

Supplement: Supplementary file 25 — Supplementary Information 25. [file 41598_2020_78347_MOESM25_ESM.zip › T1/struct/S502869_results.tar/BFP.png]

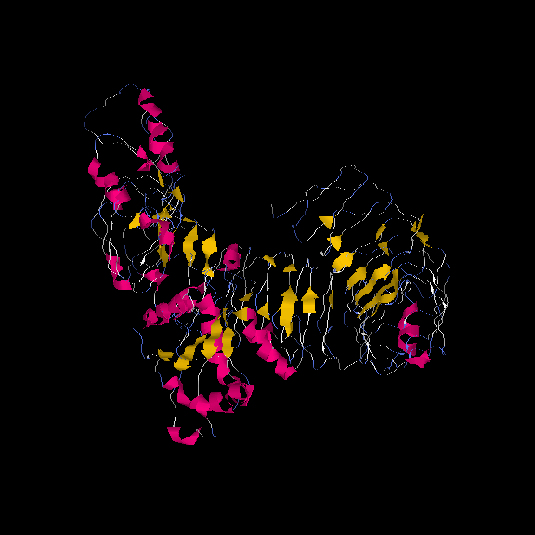

Supplement: Supplementary file 25 — Supplementary Information 25. [file 41598_2020_78347_MOESM25_ESM.zip › T1/struct/S502869_results.tar/model1.gif]

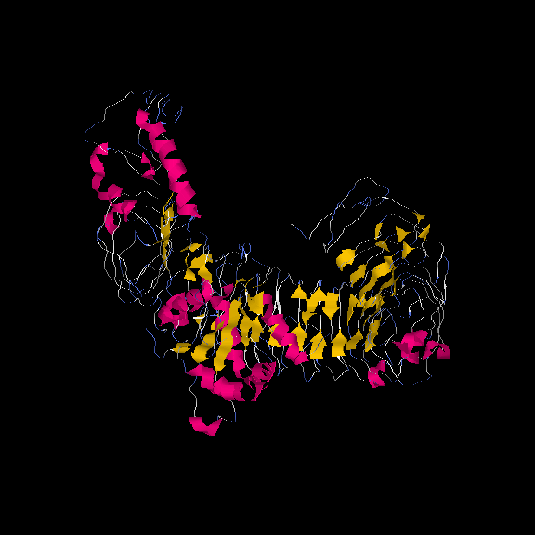

Supplement: Supplementary file 25 — Supplementary Information 25. [file 41598_2020_78347_MOESM25_ESM.zip › T1/struct/S502869_results.tar/model2.gif]

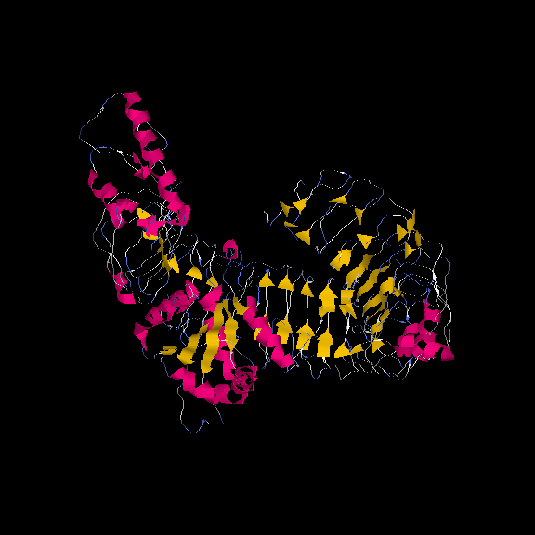

Supplement: Supplementary file 25 — Supplementary Information 25. [file 41598_2020_78347_MOESM25_ESM.zip › T1/struct/S502869_results.tar/model3.gif]

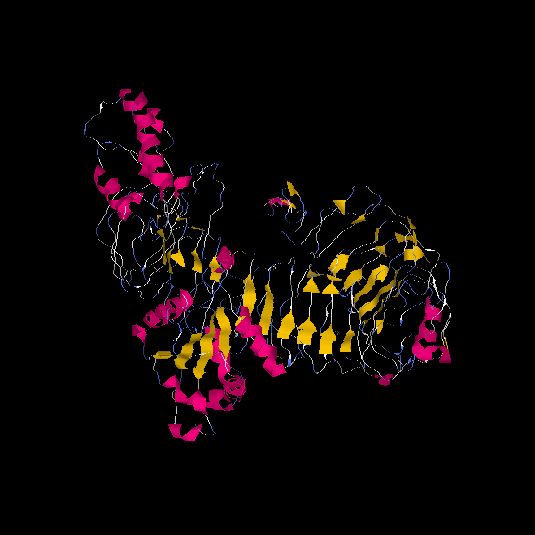

Supplement: Supplementary file 25 — Supplementary Information 25. [file 41598_2020_78347_MOESM25_ESM.zip › T1/struct/S502869_results.tar/model4.gif]

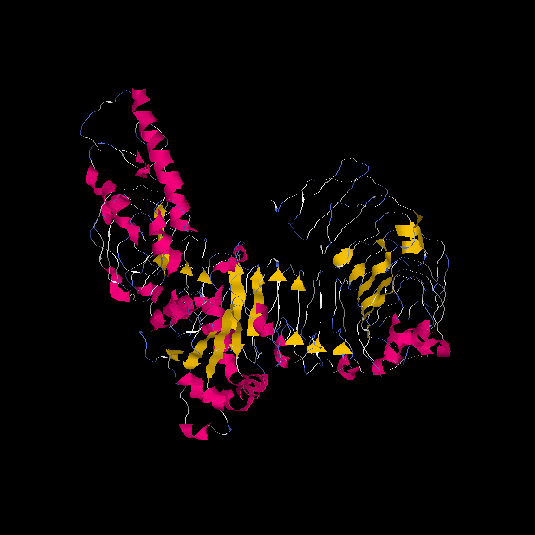

Supplement: Supplementary file 25 — Supplementary Information 25. [file 41598_2020_78347_MOESM25_ESM.zip › T1/struct/S502869_results.tar/model5.gif]

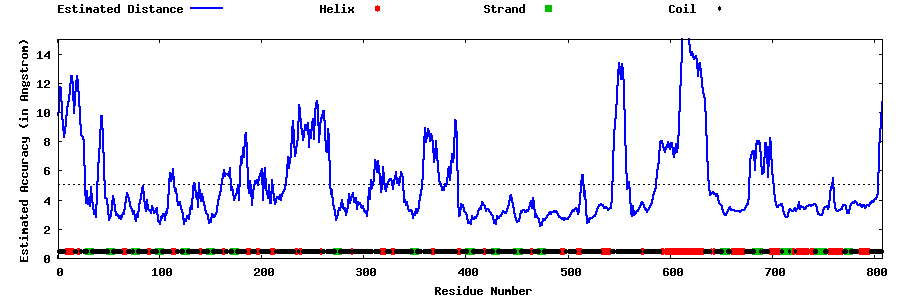

Supplement: Supplementary file 25 — Supplementary Information 25. [file 41598_2020_78347_MOESM25_ESM.zip › T1/struct/S502869_results.tar/RSQ_1.png]

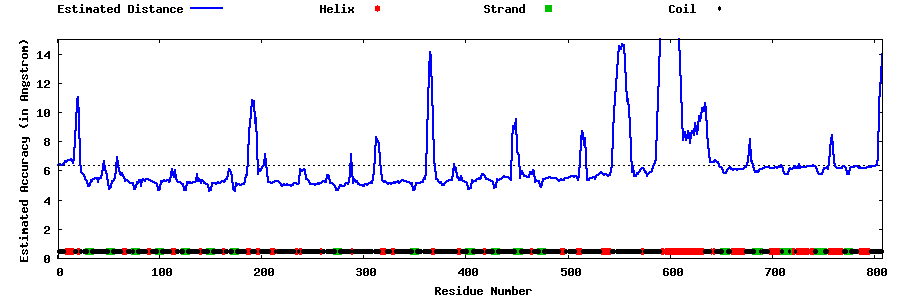

Supplement: Supplementary file 25 — Supplementary Information 25. [file 41598_2020_78347_MOESM25_ESM.zip › T1/struct/S502869_results.tar/RSQ_2.png]

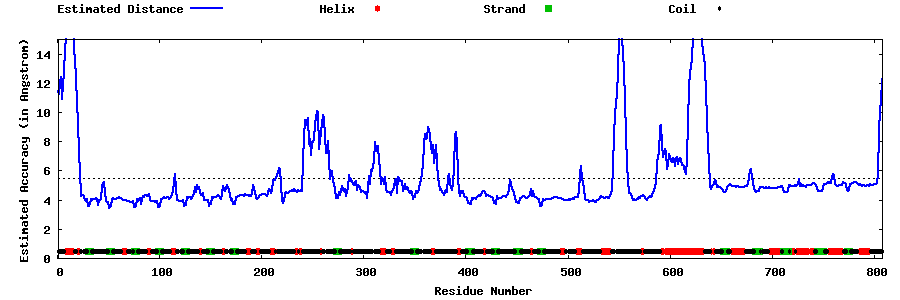

Supplement: Supplementary file 25 — Supplementary Information 25. [file 41598_2020_78347_MOESM25_ESM.zip › T1/struct/S502869_results.tar/RSQ_3.png]

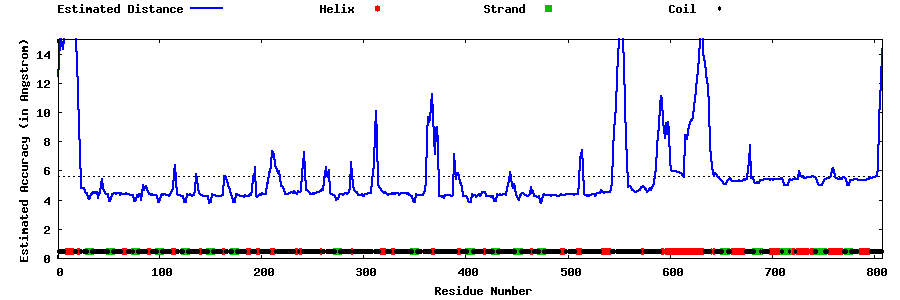

Supplement: Supplementary file 25 — Supplementary Information 25. [file 41598_2020_78347_MOESM25_ESM.zip › T1/struct/S502869_results.tar/RSQ_4.png]

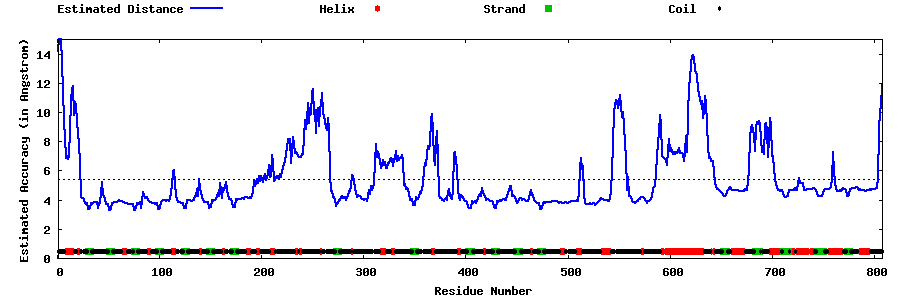

Supplement: Supplementary file 25 — Supplementary Information 25. [file 41598_2020_78347_MOESM25_ESM.zip › T1/struct/S502869_results.tar/RSQ_5.png]

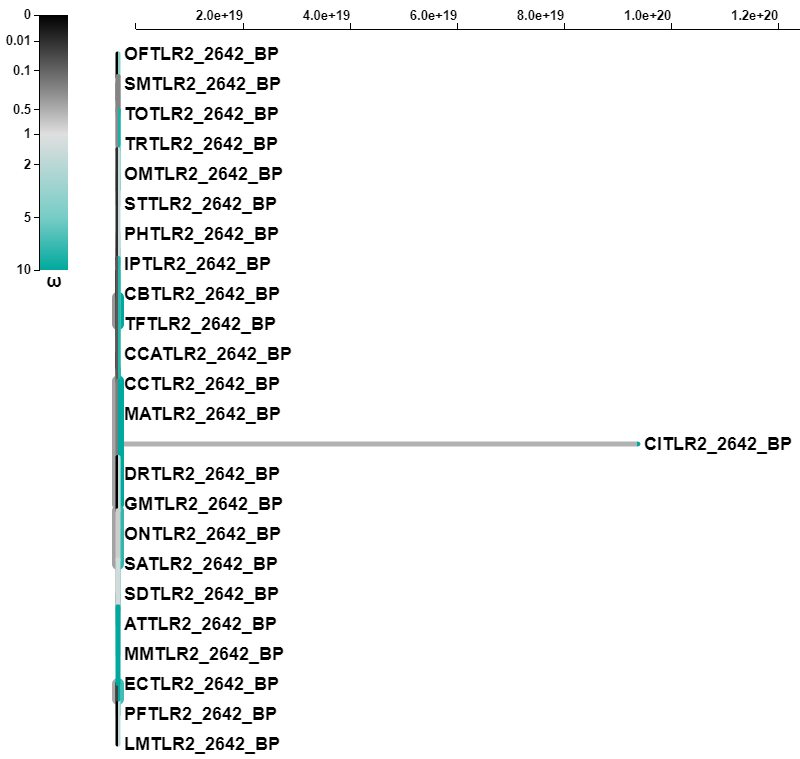

Supplement: Supplementary file 26 — Supplementary Information 26. [file 41598_2020_78347_MOESM26_ESM.zip › T2/ABSREL/tree.png]

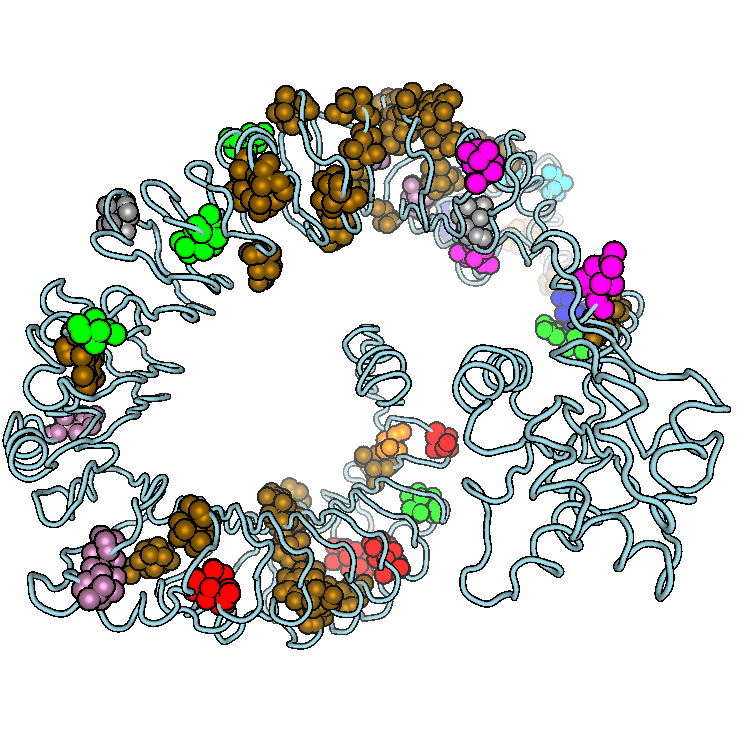

Supplement: Supplementary file 26 — Supplementary Information 26. [file 41598_2020_78347_MOESM26_ESM.zip › T2/BIS2/t2.png]

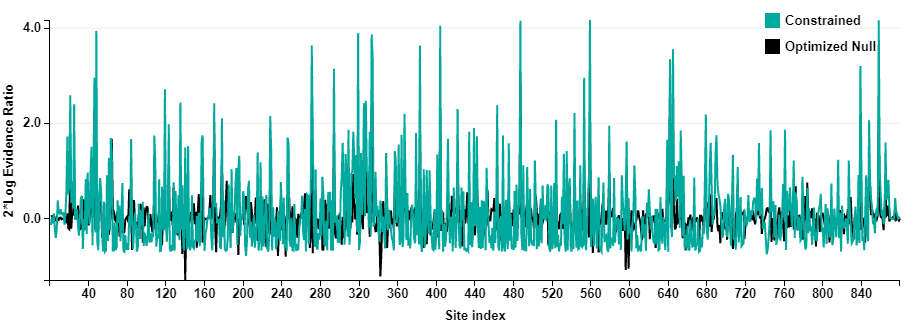

Supplement: Supplementary file 26 — Supplementary Information 26. [file 41598_2020_78347_MOESM26_ESM.zip › T2/busted/busted-chart (1).png]

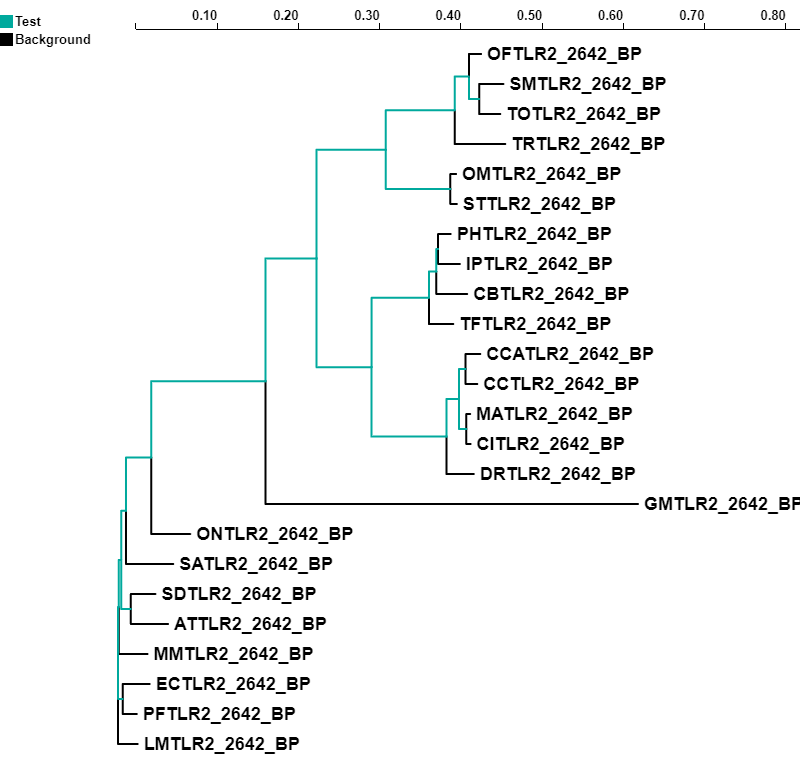

Supplement: Supplementary file 26 — Supplementary Information 26. [file 41598_2020_78347_MOESM26_ESM.zip › T2/busted/tree.png]

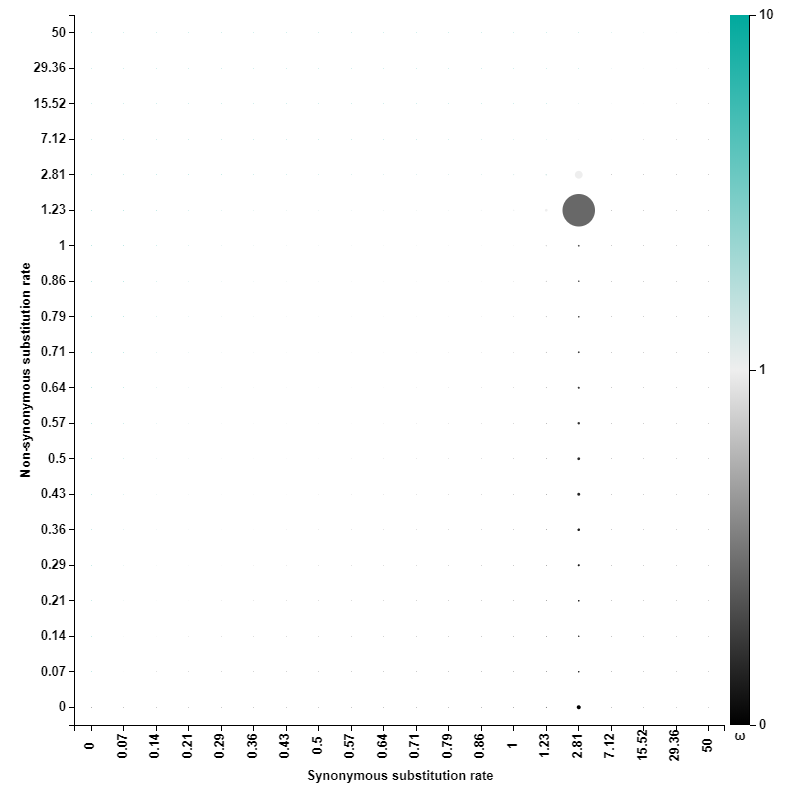

Supplement: Supplementary file 26 — Supplementary Information 26. [file 41598_2020_78347_MOESM26_ESM.zip › T2/FUBAR/datamonkey-chart.png]

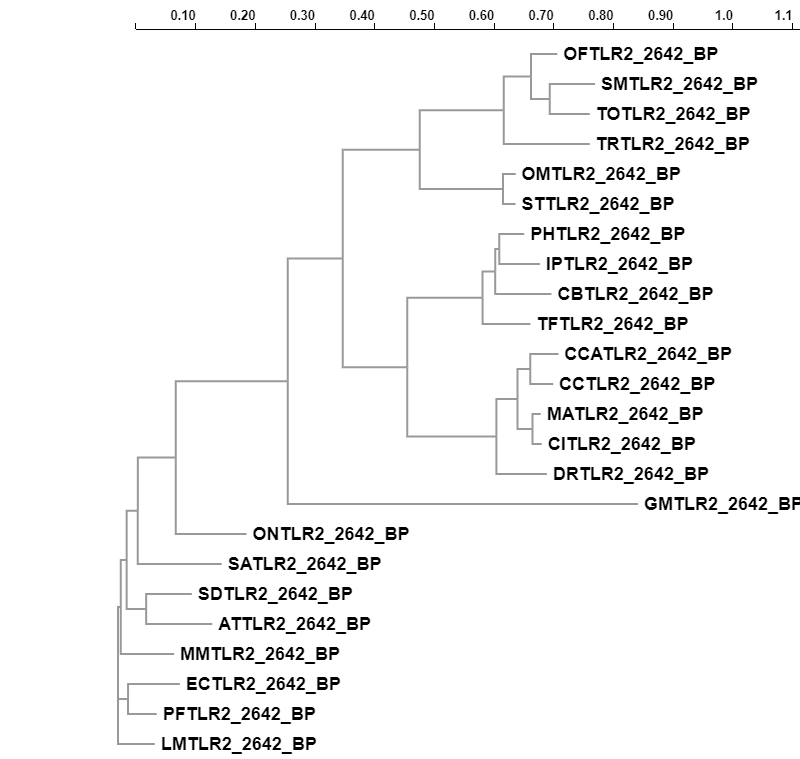

Supplement: Supplementary file 26 — Supplementary Information 26. [file 41598_2020_78347_MOESM26_ESM.zip › T2/FUBAR/tree.png]

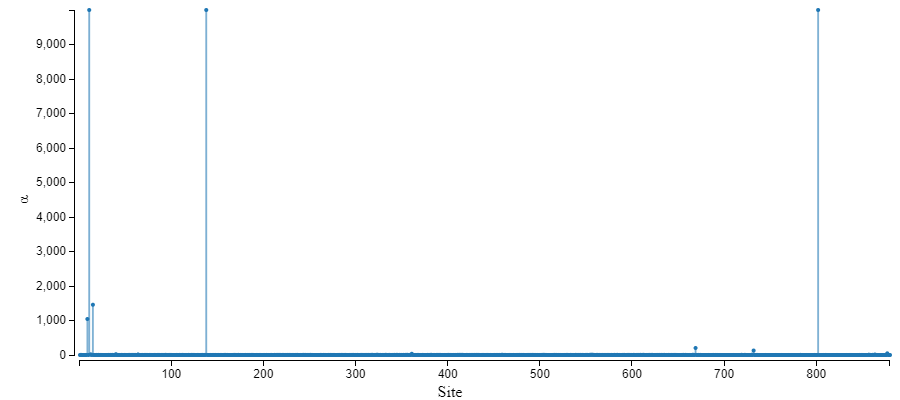

Supplement: Supplementary file 26 — Supplementary Information 26. [file 41598_2020_78347_MOESM26_ESM.zip › T2/MEME/datamonkey-chart.png]

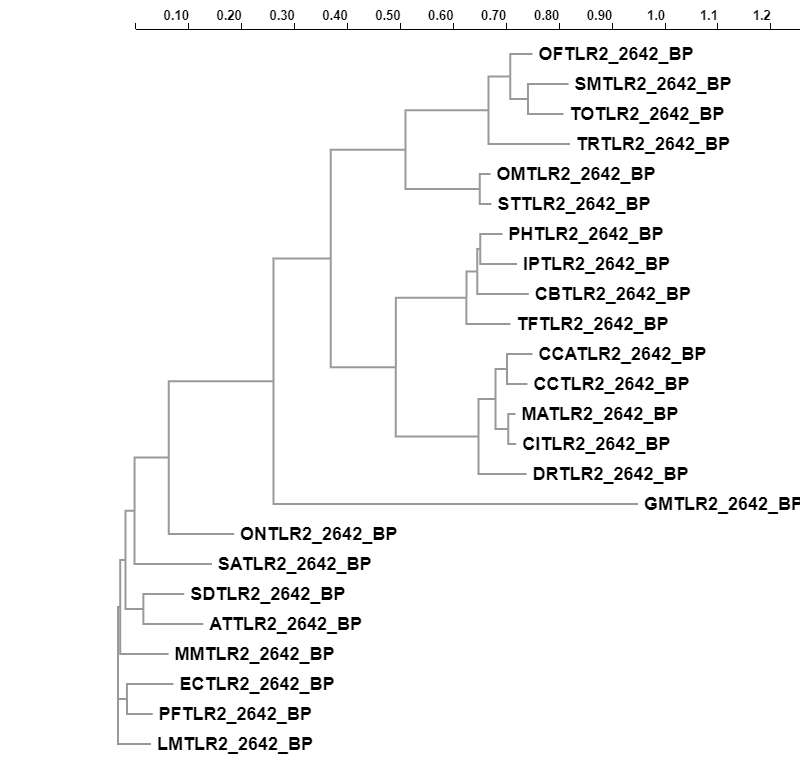

Supplement: Supplementary file 26 — Supplementary Information 26. [file 41598_2020_78347_MOESM26_ESM.zip › T2/MEME/tree.png]

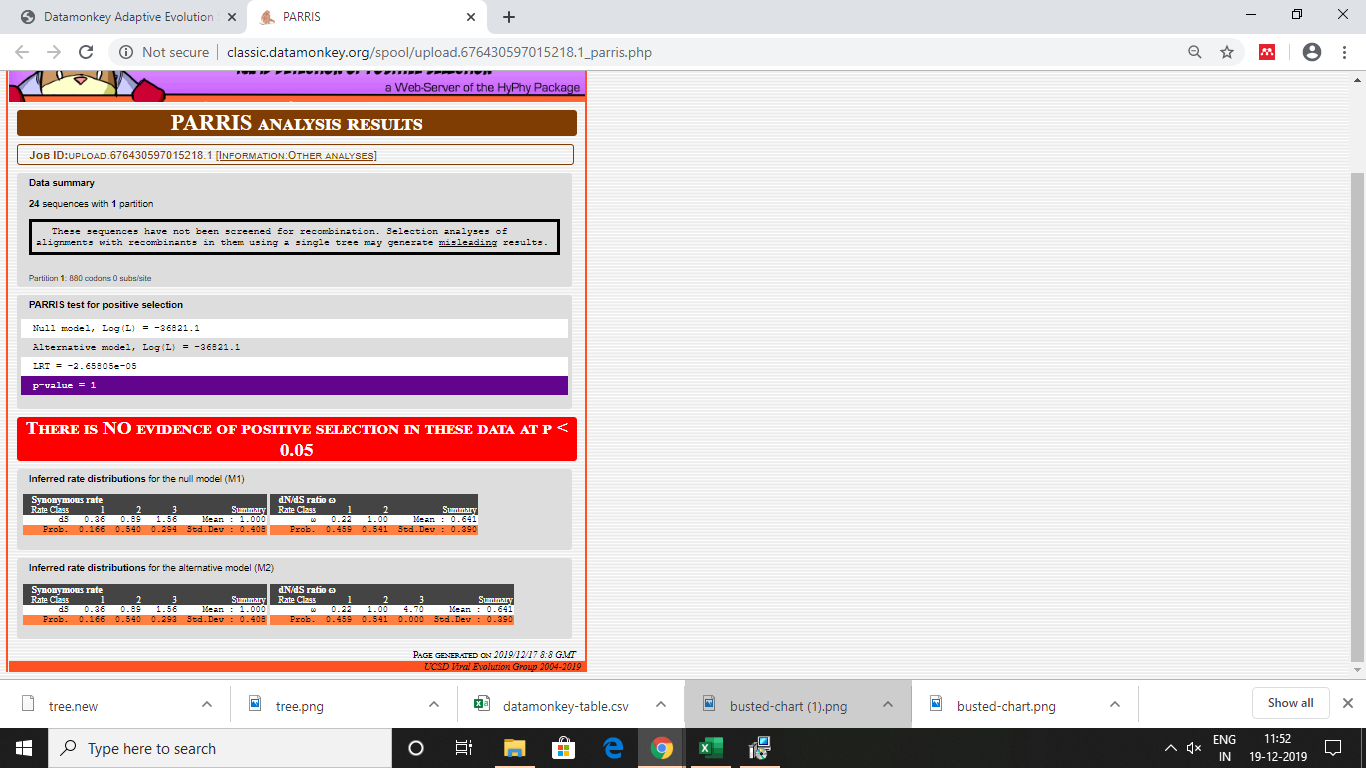

Supplement: Supplementary file 26 — Supplementary Information 26. [file 41598_2020_78347_MOESM26_ESM.zip › T2/parris/parris.docx]

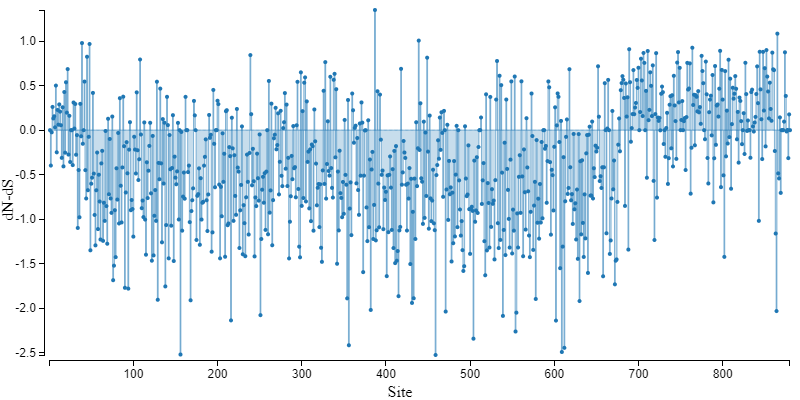

Supplement: Supplementary file 26 — Supplementary Information 26. [file 41598_2020_78347_MOESM26_ESM.zip › T2/slac/datamonkey-chart.png]

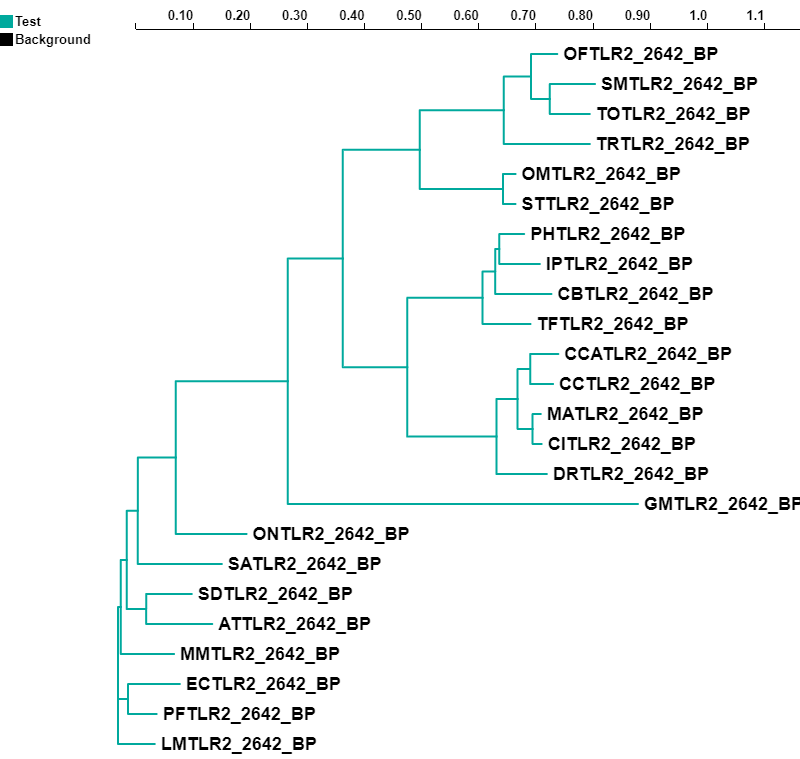

Supplement: Supplementary file 26 — Supplementary Information 26. [file 41598_2020_78347_MOESM26_ESM.zip › T2/slac/tree.png]

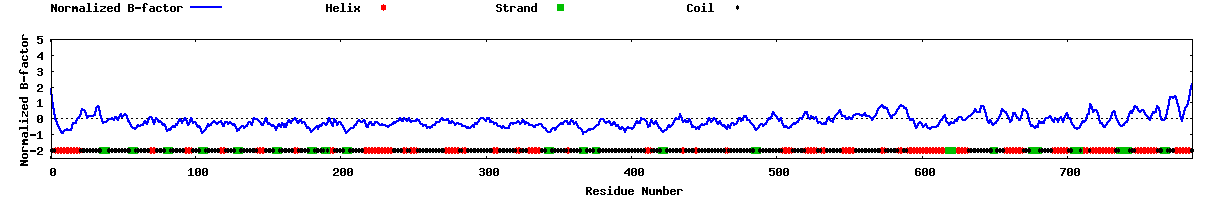

Supplement: Supplementary file 26 — Supplementary Information 26. [file 41598_2020_78347_MOESM26_ESM.zip › T2/struct/S503516_results/BFP.png]

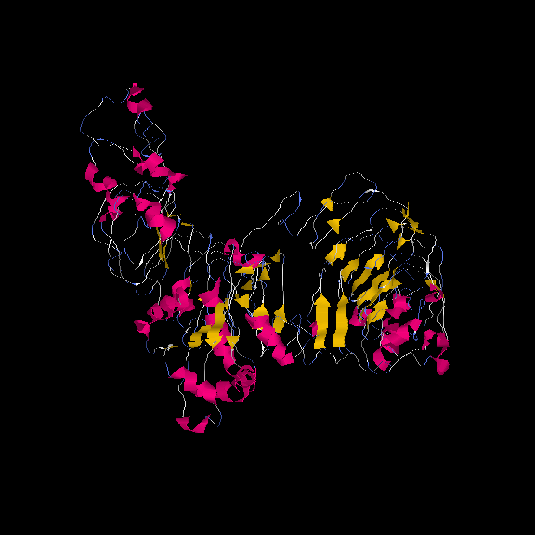

Supplement: Supplementary file 26 — Supplementary Information 26. [file 41598_2020_78347_MOESM26_ESM.zip › T2/struct/S503516_results/model1.gif]

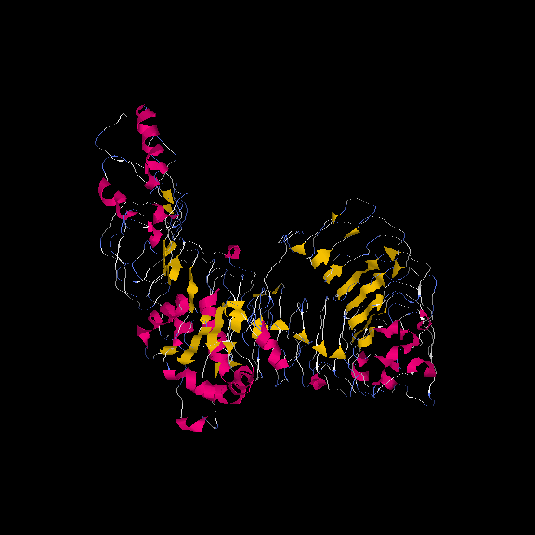

Supplement: Supplementary file 26 — Supplementary Information 26. [file 41598_2020_78347_MOESM26_ESM.zip › T2/struct/S503516_results/model2.gif]

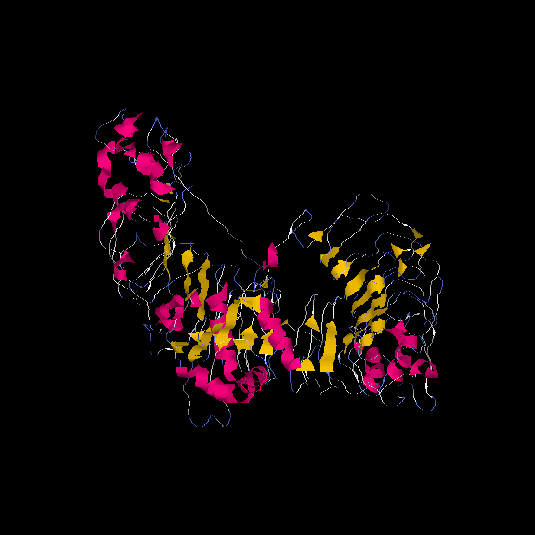

Supplement: Supplementary file 26 — Supplementary Information 26. [file 41598_2020_78347_MOESM26_ESM.zip › T2/struct/S503516_results/model3.gif]

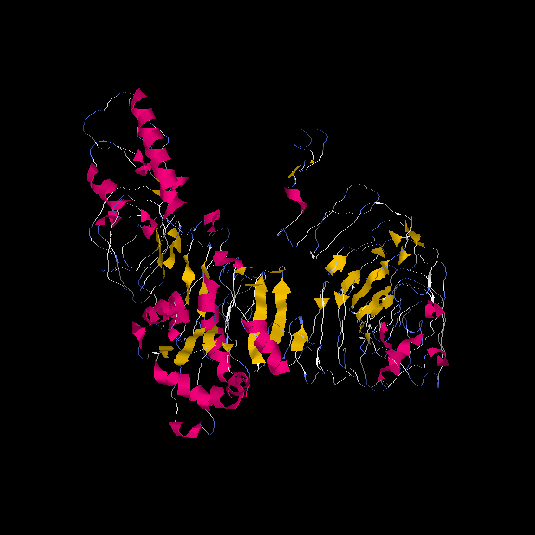

Supplement: Supplementary file 26 — Supplementary Information 26. [file 41598_2020_78347_MOESM26_ESM.zip › T2/struct/S503516_results/model4.gif]

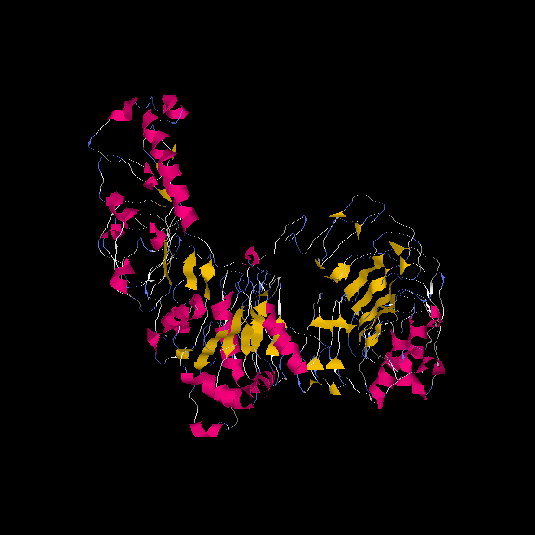

Supplement: Supplementary file 26 — Supplementary Information 26. [file 41598_2020_78347_MOESM26_ESM.zip › T2/struct/S503516_results/model5.gif]

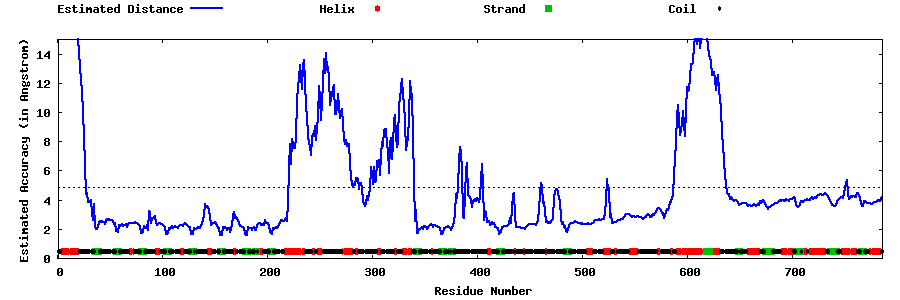

Supplement: Supplementary file 26 — Supplementary Information 26. [file 41598_2020_78347_MOESM26_ESM.zip › T2/struct/S503516_results/RSQ_1.png]

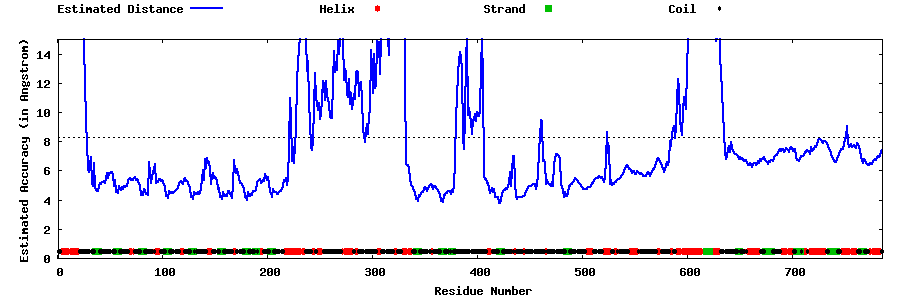

Supplement: Supplementary file 26 — Supplementary Information 26. [file 41598_2020_78347_MOESM26_ESM.zip › T2/struct/S503516_results/RSQ_2.png]

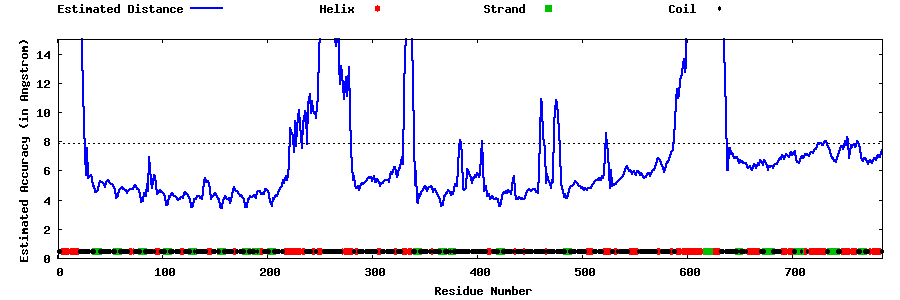

Supplement: Supplementary file 26 — Supplementary Information 26. [file 41598_2020_78347_MOESM26_ESM.zip › T2/struct/S503516_results/RSQ_3.png]

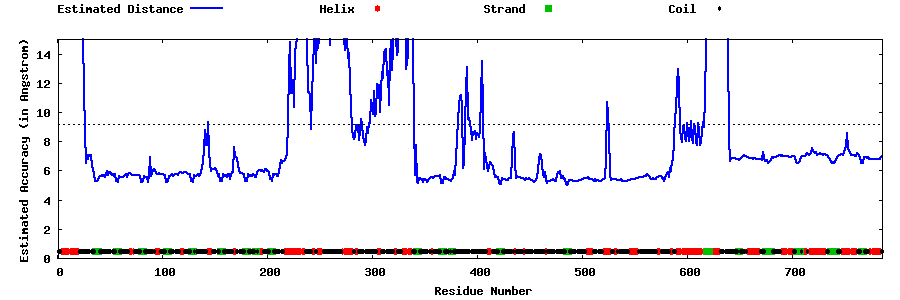

Supplement: Supplementary file 26 — Supplementary Information 26. [file 41598_2020_78347_MOESM26_ESM.zip › T2/struct/S503516_results/RSQ_4.png]

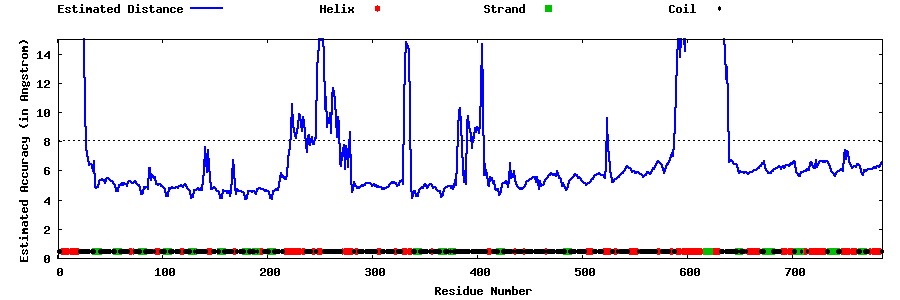

Supplement: Supplementary file 26 — Supplementary Information 26. [file 41598_2020_78347_MOESM26_ESM.zip › T2/struct/S503516_results/RSQ_5.png]

Tree scale: 0.1

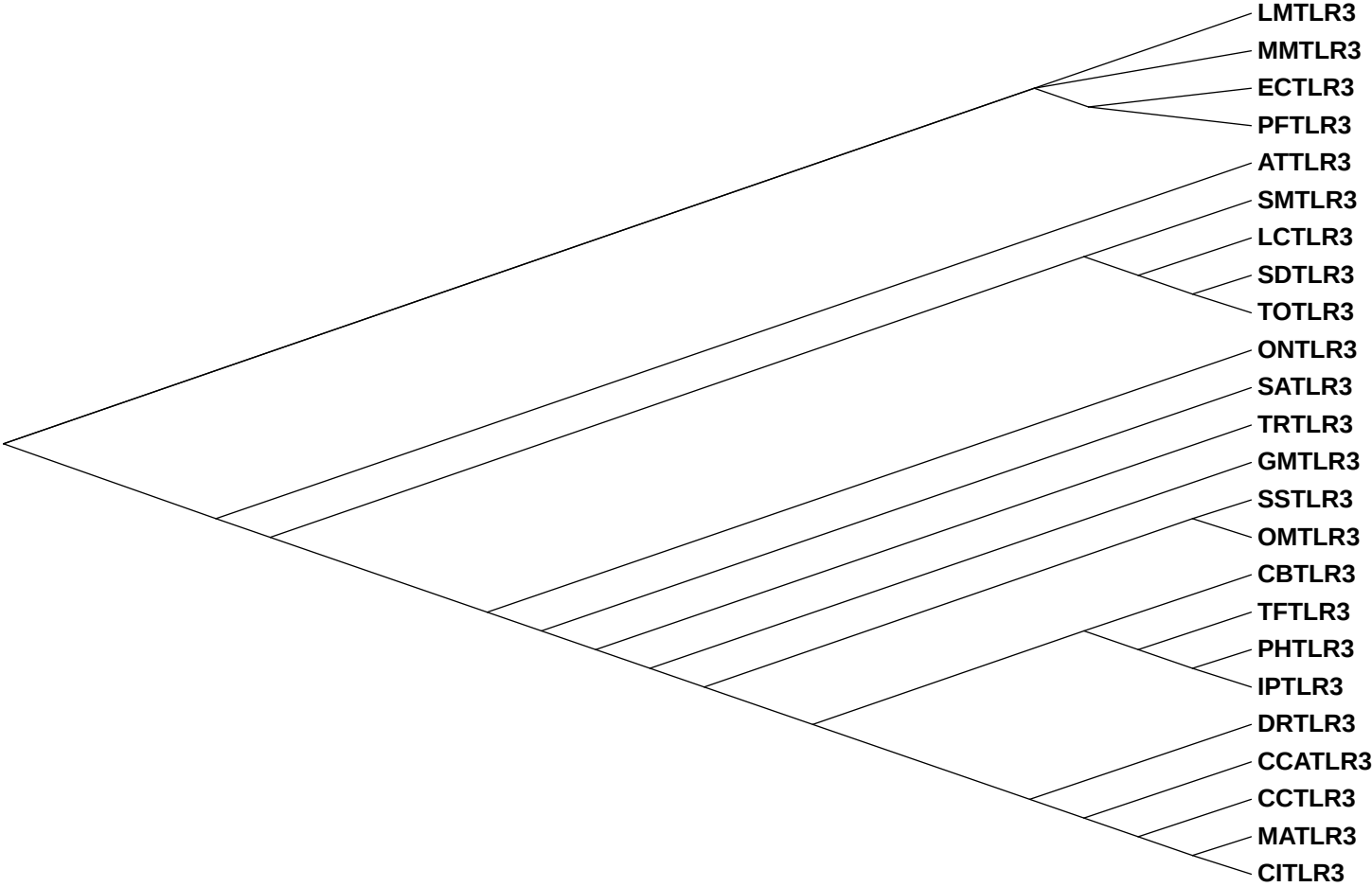

Supplement: Supplementary file 27 — Supplementary Information 27. [file 41598_2020_78347_MOESM27_ESM.zip › T3/absrel/labelledtree.pdf]

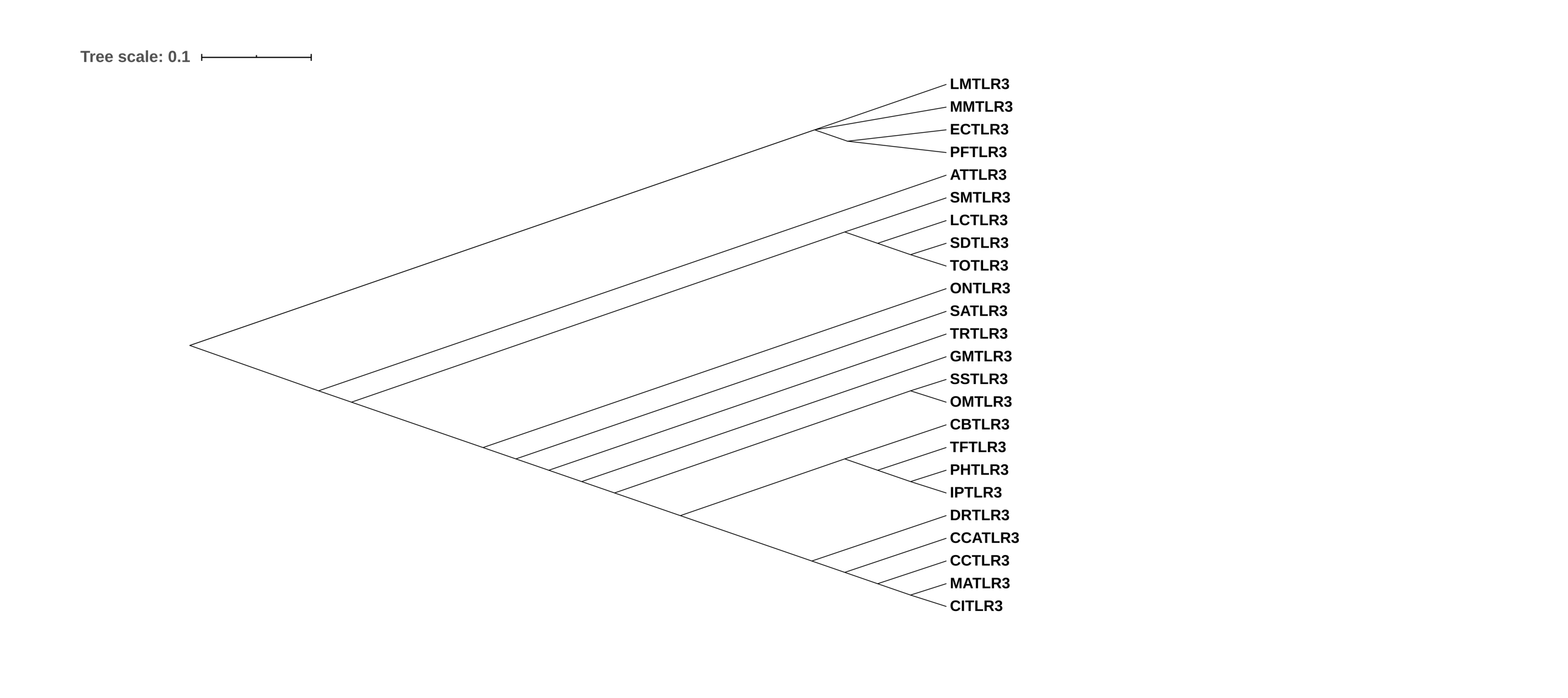

Supplement: Supplementary file 27 — Supplementary Information 27. [file 41598_2020_78347_MOESM27_ESM.zip › T3/absrel/labelledtree.png]

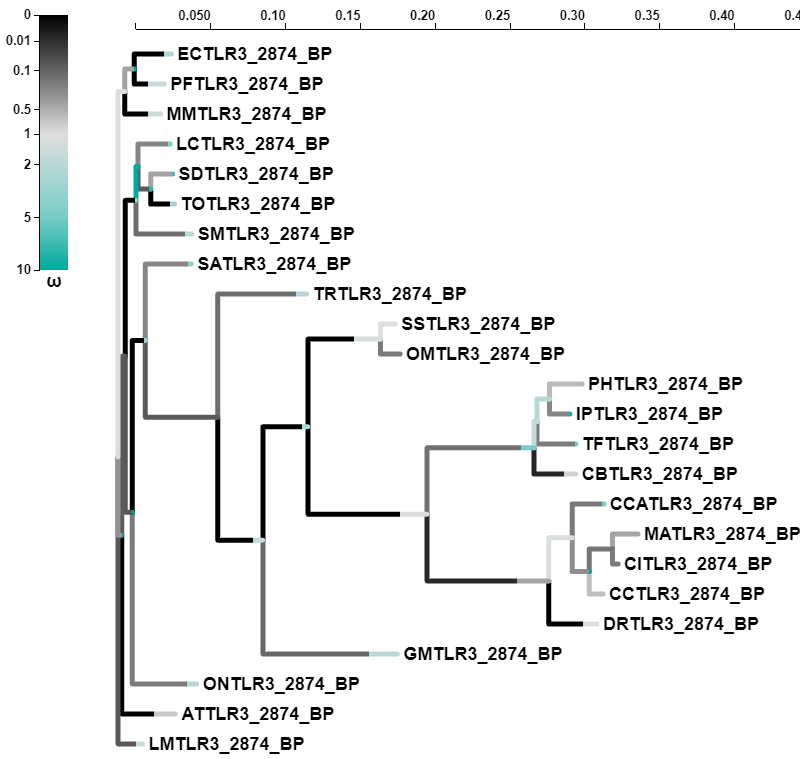

Supplement: Supplementary file 27 — Supplementary Information 27. [file 41598_2020_78347_MOESM27_ESM.zip › T3/absrel/tree.png]

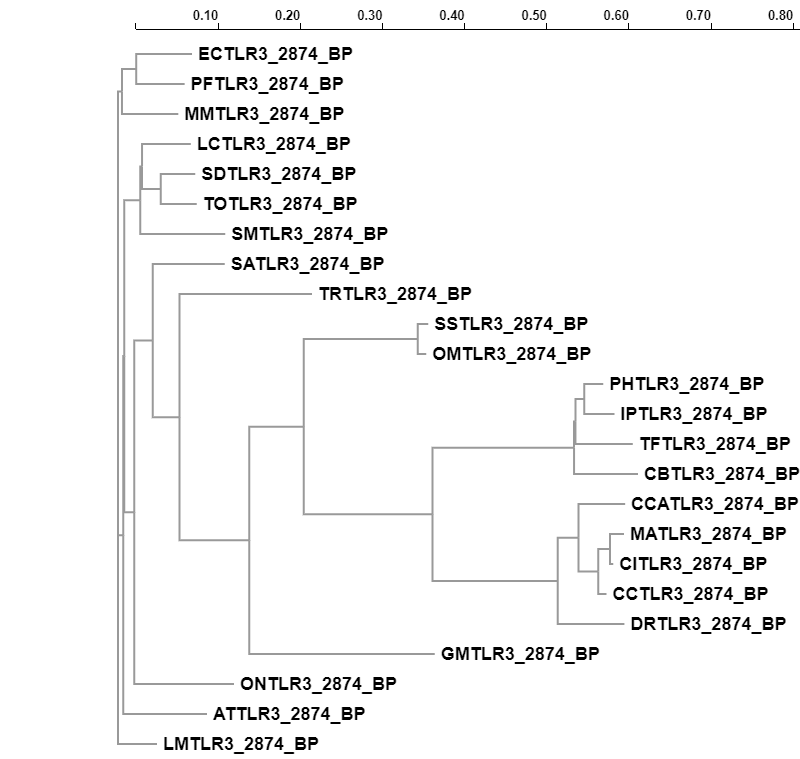

Supplement: Supplementary file 27 — Supplementary Information 27. [file 41598_2020_78347_MOESM27_ESM.zip › T3/bgm/tree.png]

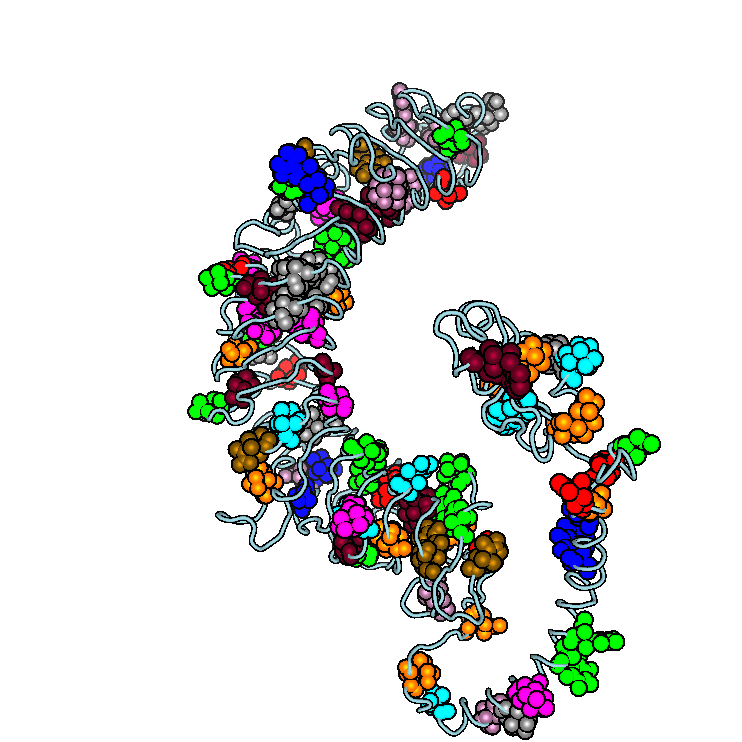

Supplement: Supplementary file 27 — Supplementary Information 27. [file 41598_2020_78347_MOESM27_ESM.zip › T3/bis2/t3.png]

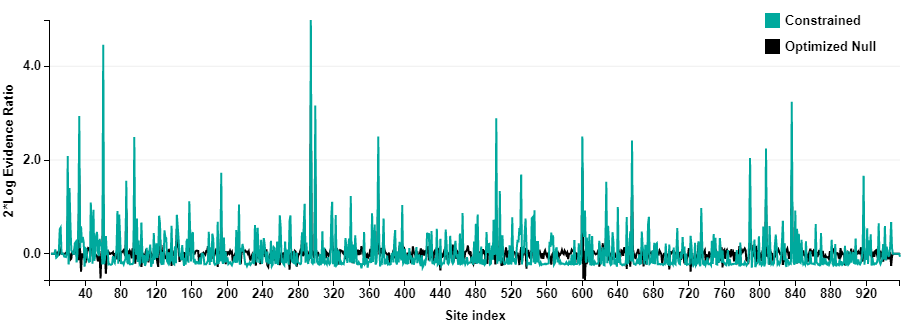

Supplement: Supplementary file 27 — Supplementary Information 27. [file 41598_2020_78347_MOESM27_ESM.zip › T3/busted/busted-chart (1).png]

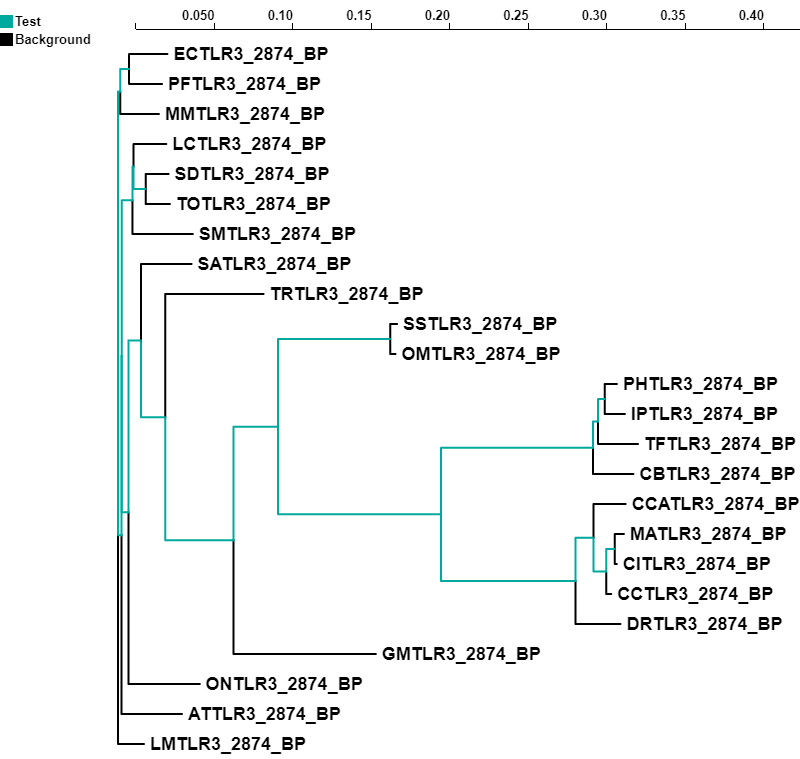

Supplement: Supplementary file 27 — Supplementary Information 27. [file 41598_2020_78347_MOESM27_ESM.zip › T3/busted/tree.png]

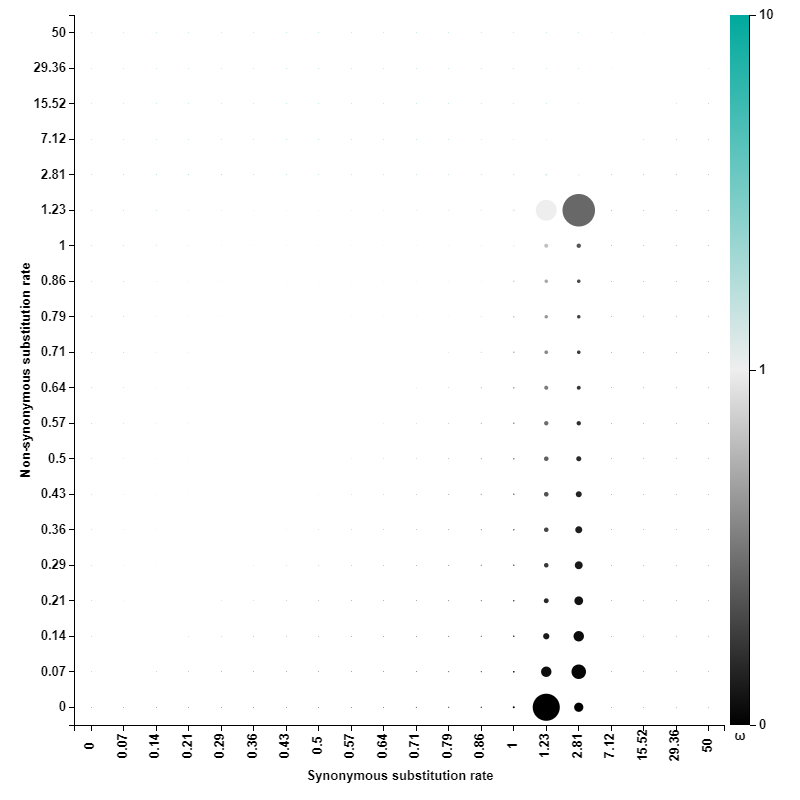

Supplement: Supplementary file 27 — Supplementary Information 27. [file 41598_2020_78347_MOESM27_ESM.zip › T3/fubar/datamonkey-chart.png]

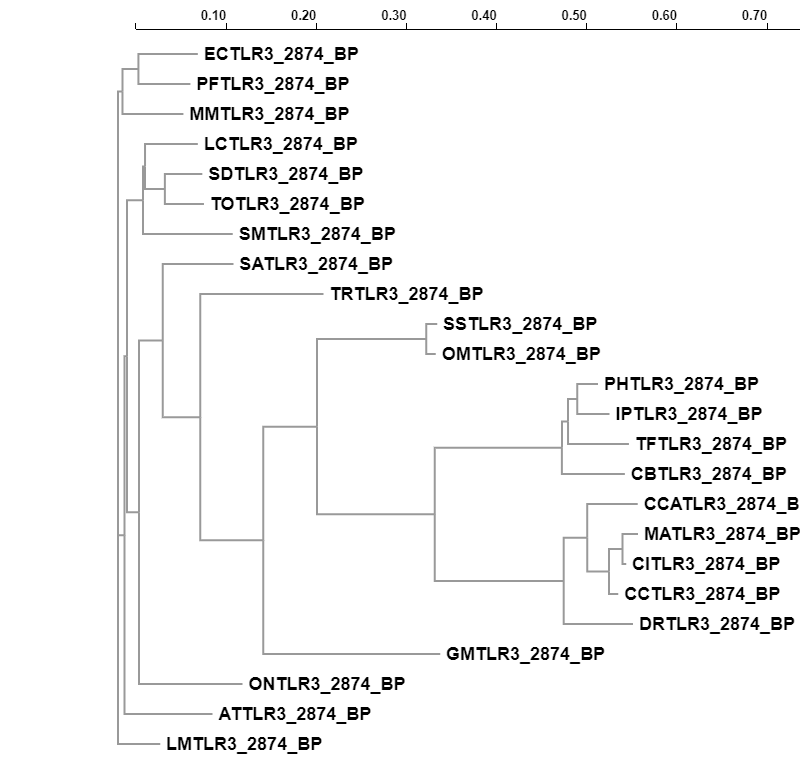

Supplement: Supplementary file 27 — Supplementary Information 27. [file 41598_2020_78347_MOESM27_ESM.zip › T3/fubar/tree.png]

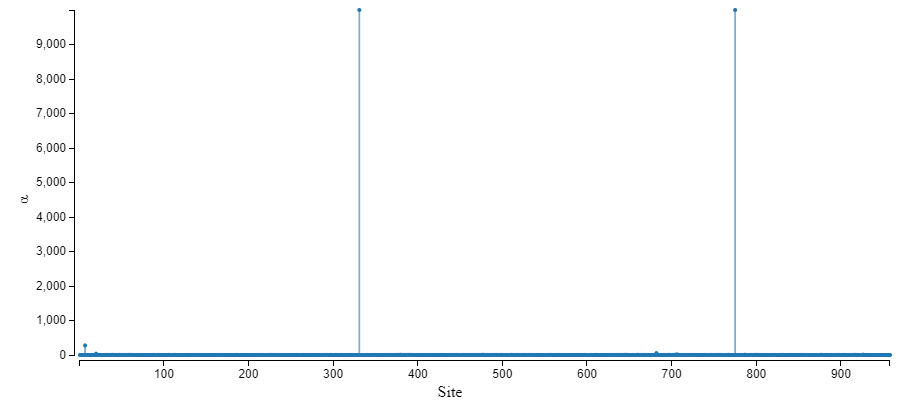

Supplement: Supplementary file 27 — Supplementary Information 27. [file 41598_2020_78347_MOESM27_ESM.zip › T3/meme/datamonkey-chart.png]

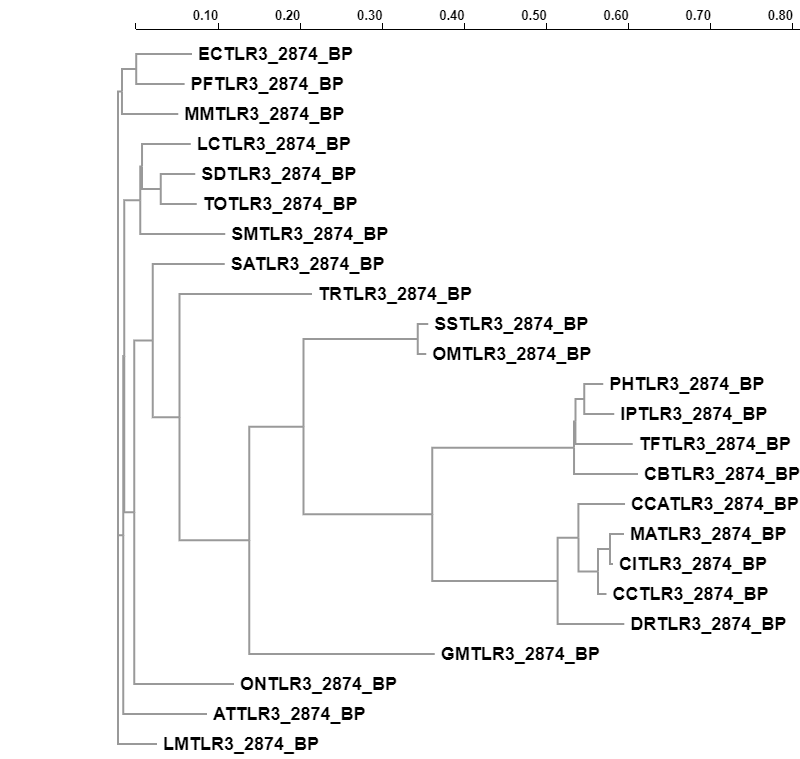

Supplement: Supplementary file 27 — Supplementary Information 27. [file 41598_2020_78347_MOESM27_ESM.zip › T3/meme/tree.png]

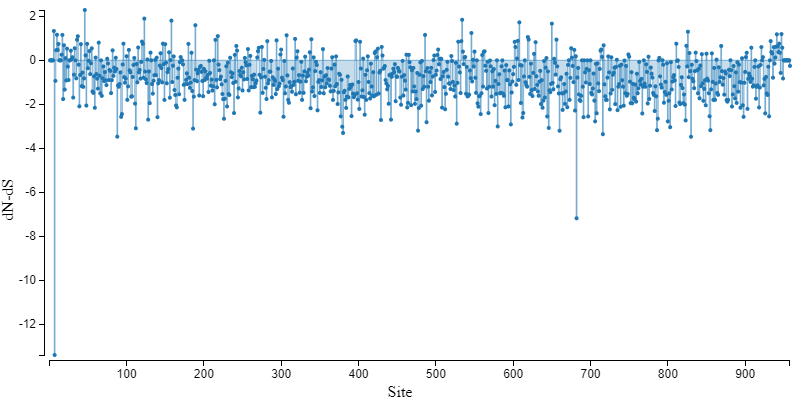

Supplement: Supplementary file 27 — Supplementary Information 27. [file 41598_2020_78347_MOESM27_ESM.zip › T3/slac/datamonkey-chart.png]

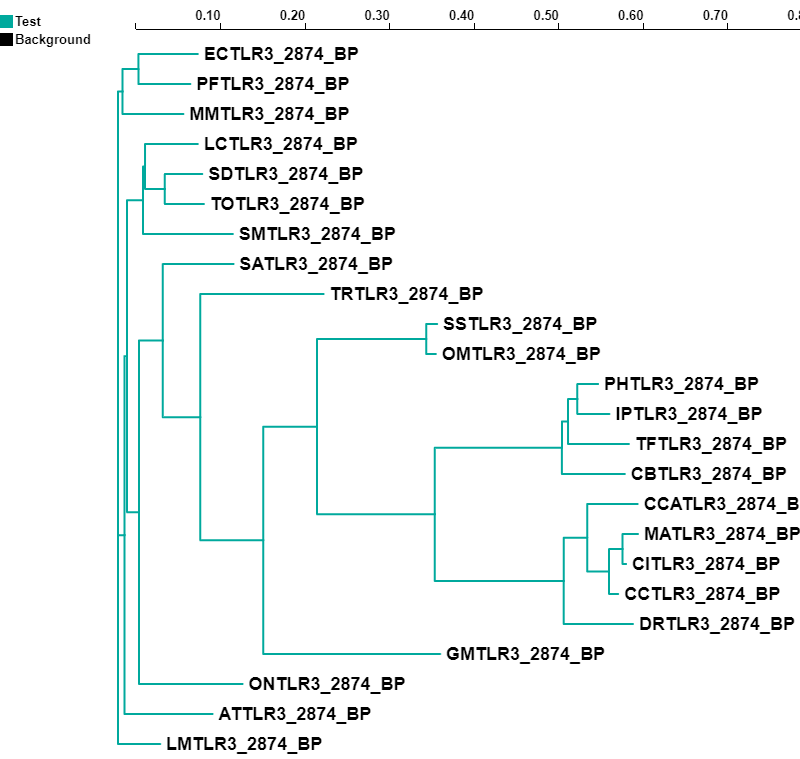

Supplement: Supplementary file 27 — Supplementary Information 27. [file 41598_2020_78347_MOESM27_ESM.zip › T3/slac/tree.png]

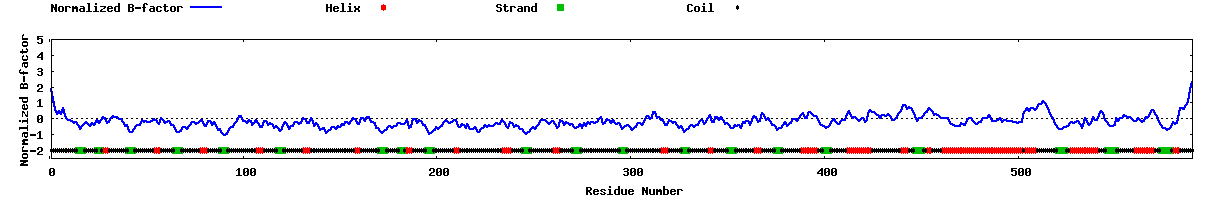

Supplement: Supplementary file 27 — Supplementary Information 27. [file 41598_2020_78347_MOESM27_ESM.zip › T3/struct/S506035_results/BFP.png]

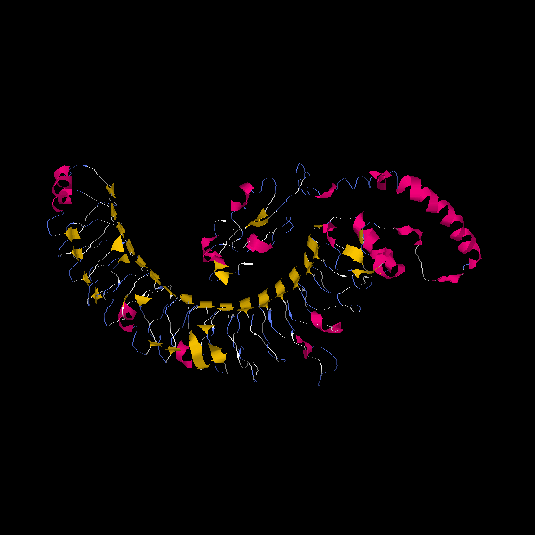

Supplement: Supplementary file 27 — Supplementary Information 27. [file 41598_2020_78347_MOESM27_ESM.zip › T3/struct/S506035_results/model1.gif]

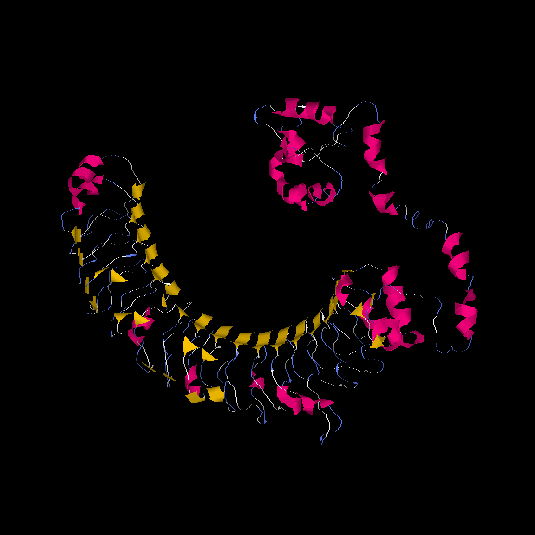

Supplement: Supplementary file 27 — Supplementary Information 27. [file 41598_2020_78347_MOESM27_ESM.zip › T3/struct/S506035_results/model2.gif]

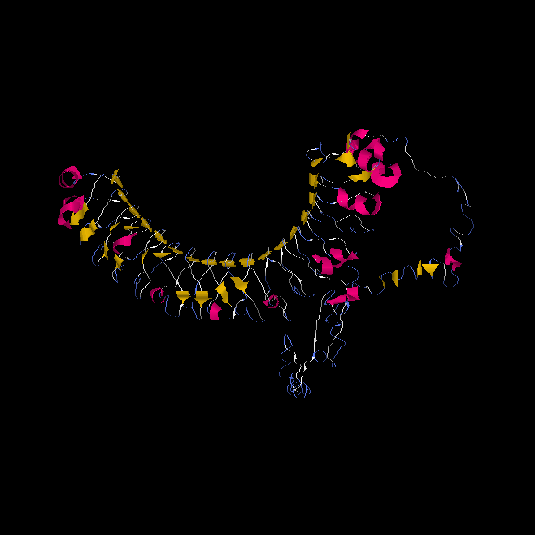

Supplement: Supplementary file 27 — Supplementary Information 27. [file 41598_2020_78347_MOESM27_ESM.zip › T3/struct/S506035_results/model3.gif]

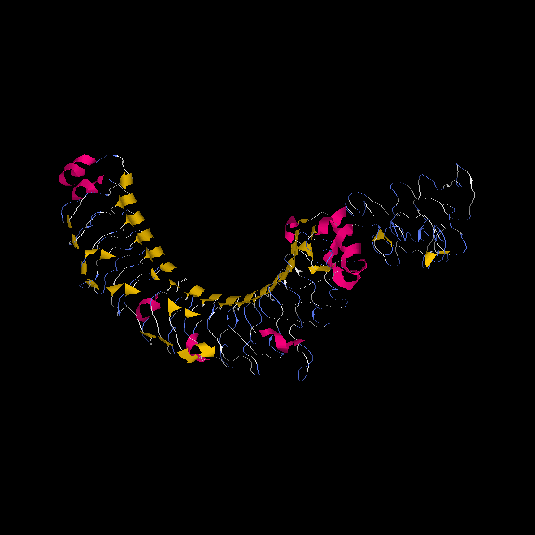

Supplement: Supplementary file 27 — Supplementary Information 27. [file 41598_2020_78347_MOESM27_ESM.zip › T3/struct/S506035_results/model4.gif]

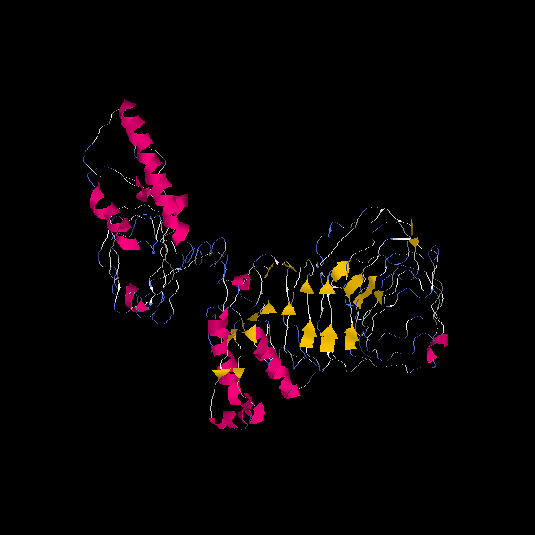

Supplement: Supplementary file 27 — Supplementary Information 27. [file 41598_2020_78347_MOESM27_ESM.zip › T3/struct/S506035_results/model5.gif]

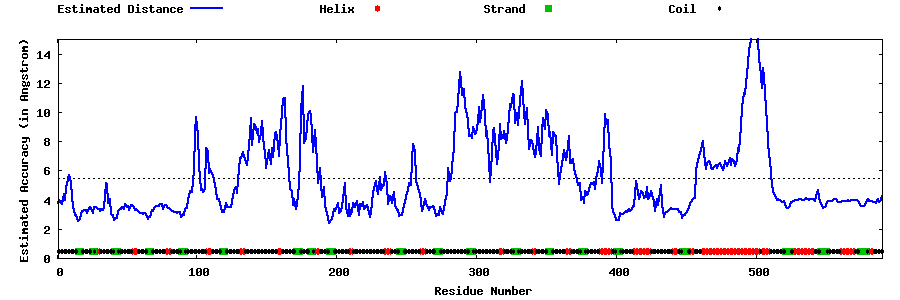

Supplement: Supplementary file 27 — Supplementary Information 27. [file 41598_2020_78347_MOESM27_ESM.zip › T3/struct/S506035_results/RSQ_1.png]

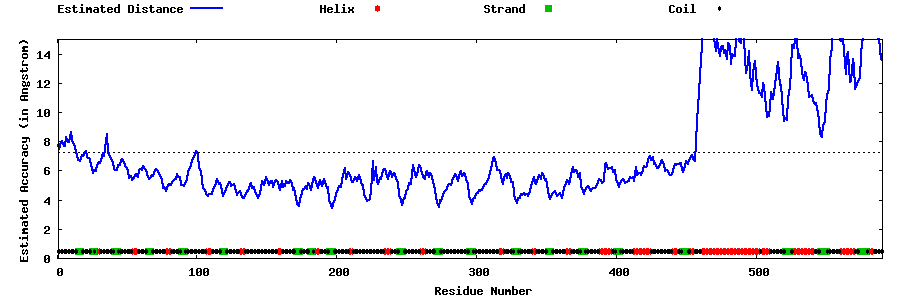

Supplement: Supplementary file 27 — Supplementary Information 27. [file 41598_2020_78347_MOESM27_ESM.zip › T3/struct/S506035_results/RSQ_2.png]

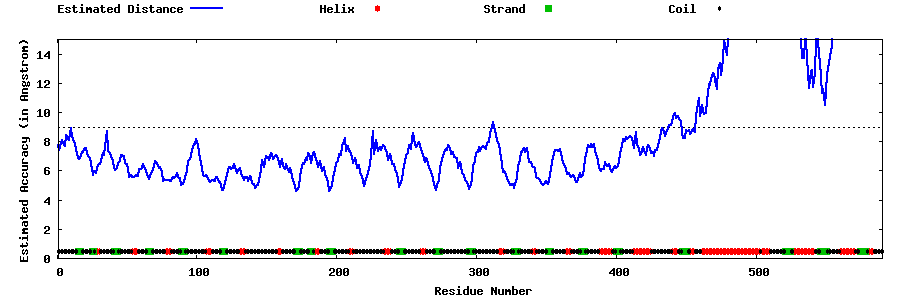

Supplement: Supplementary file 27 — Supplementary Information 27. [file 41598_2020_78347_MOESM27_ESM.zip › T3/struct/S506035_results/RSQ_3.png]

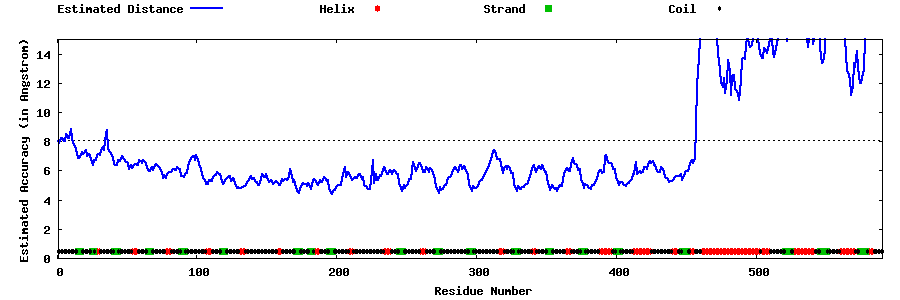

Supplement: Supplementary file 27 — Supplementary Information 27. [file 41598_2020_78347_MOESM27_ESM.zip › T3/struct/S506035_results/RSQ_4.png]

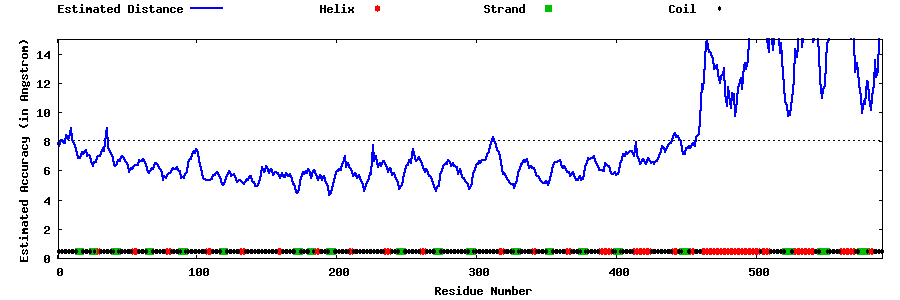

Supplement: Supplementary file 27 — Supplementary Information 27. [file 41598_2020_78347_MOESM27_ESM.zip › T3/struct/S506035_results/RSQ_5.png]

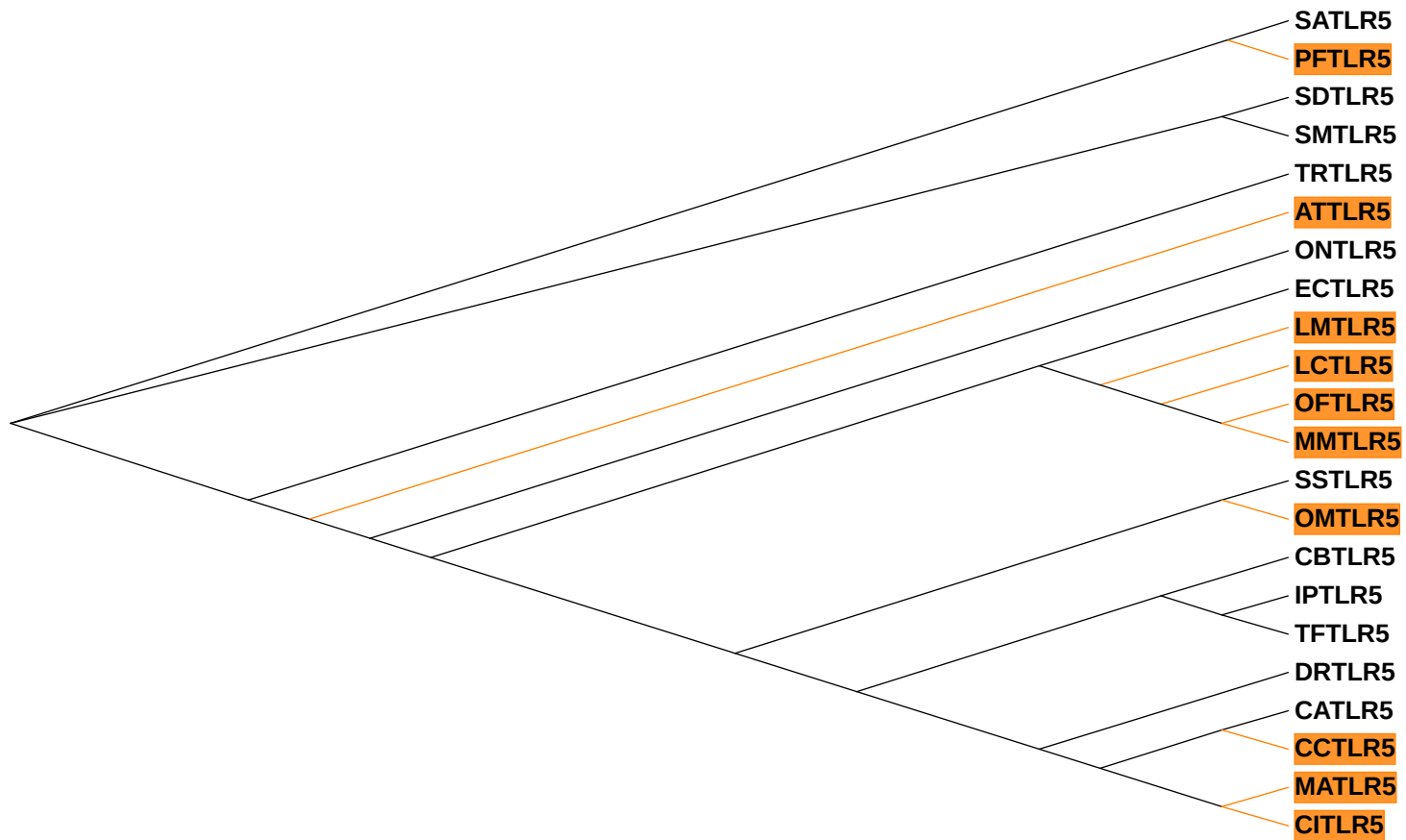

Supplement: Supplementary file 28 — Supplementary Information 28. [file 41598_2020_78347_MOESM28_ESM.zip › T5/absrel/labelledtree.pdf]

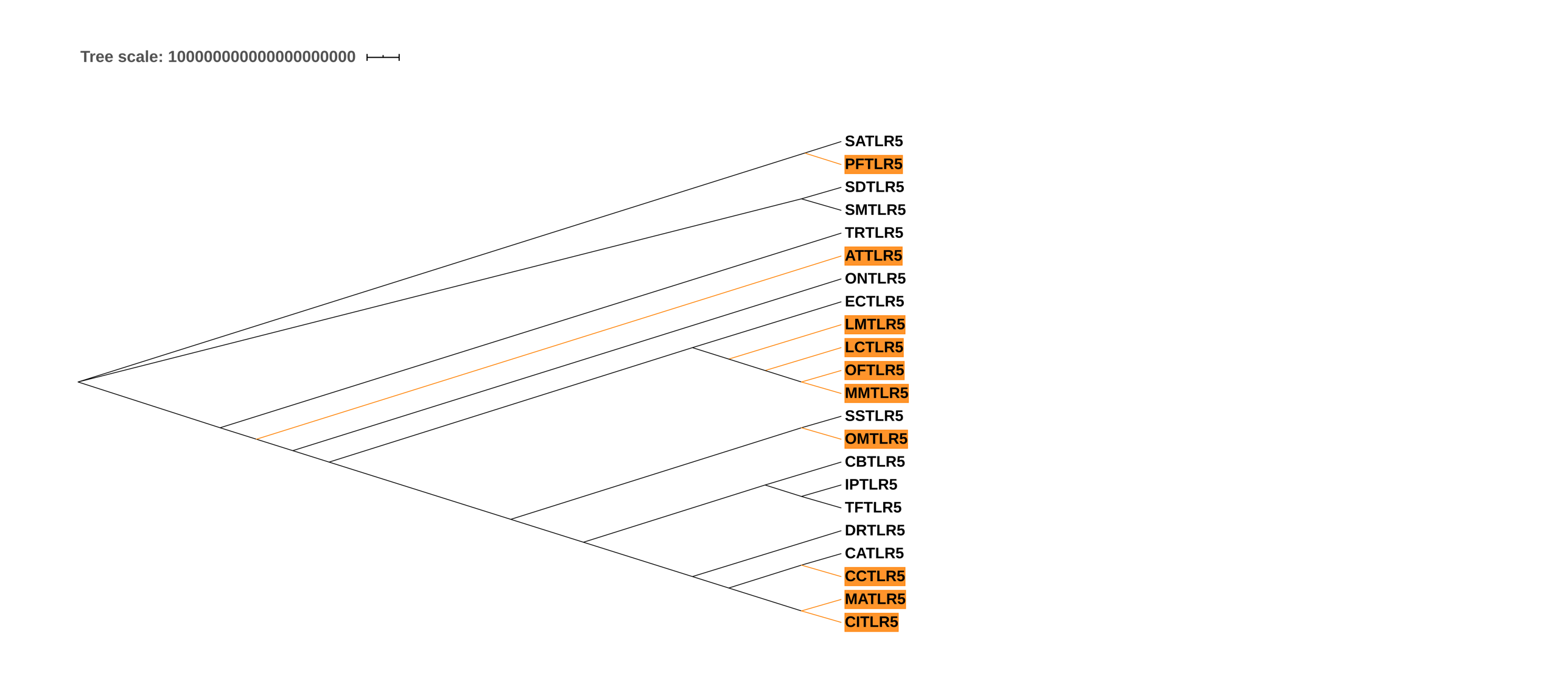

Supplement: Supplementary file 28 — Supplementary Information 28. [file 41598_2020_78347_MOESM28_ESM.zip › T5/absrel/labelledtree.png]

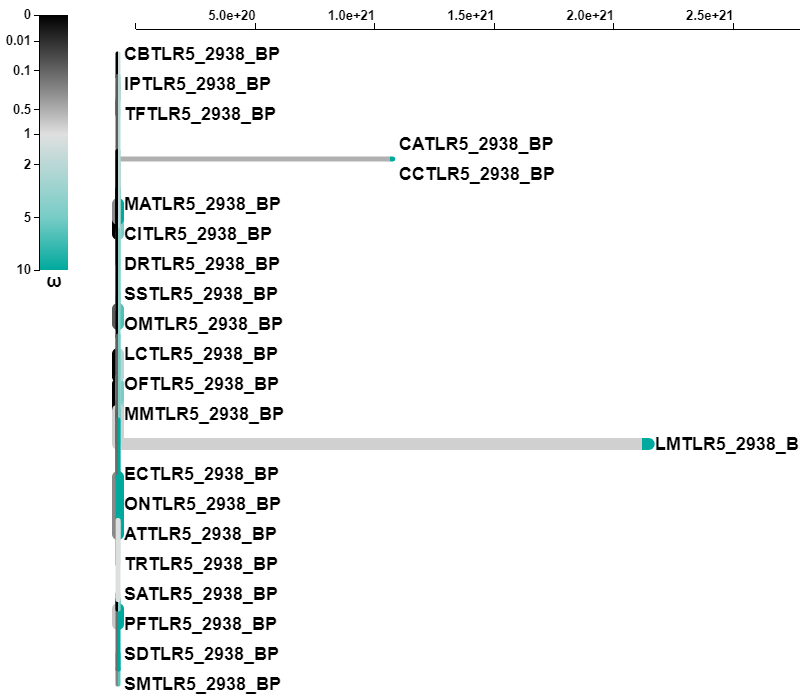

Supplement: Supplementary file 28 — Supplementary Information 28. [file 41598_2020_78347_MOESM28_ESM.zip › T5/absrel/tree.png]

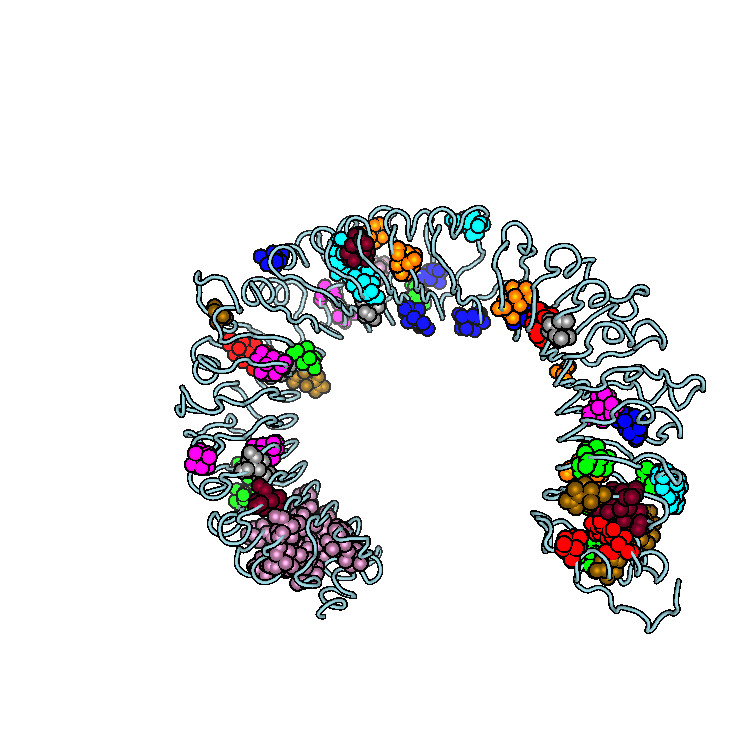

Supplement: Supplementary file 28 — Supplementary Information 28. [file 41598_2020_78347_MOESM28_ESM.zip › T5/BIS2/download.png]

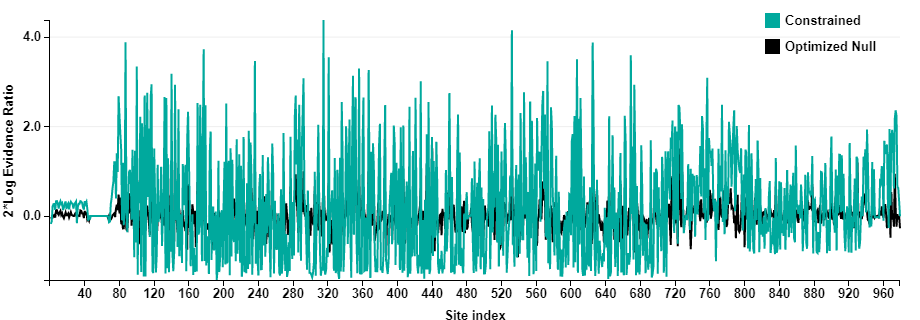

Supplement: Supplementary file 28 — Supplementary Information 28. [file 41598_2020_78347_MOESM28_ESM.zip › T5/busted/busted-chart (1).png]

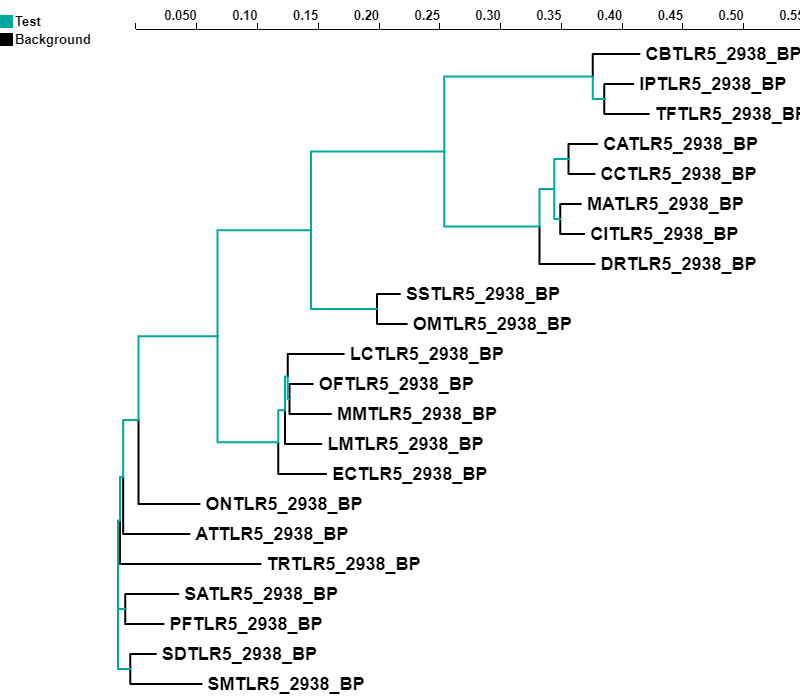

Supplement: Supplementary file 28 — Supplementary Information 28. [file 41598_2020_78347_MOESM28_ESM.zip › T5/busted/tree.png]

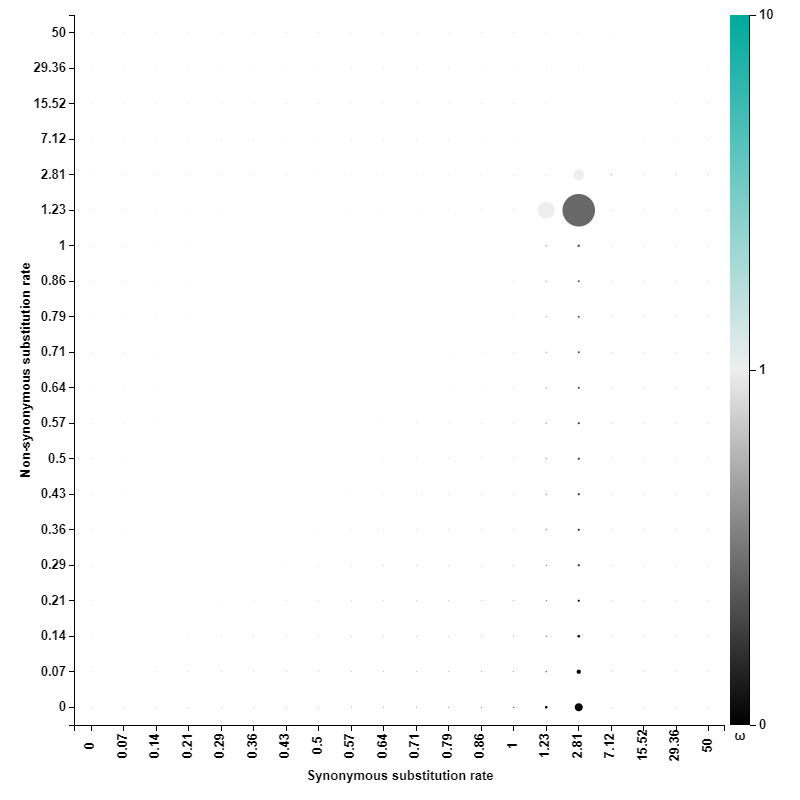

Supplement: Supplementary file 28 — Supplementary Information 28. [file 41598_2020_78347_MOESM28_ESM.zip › T5/fubar/datamonkey-chart.png]

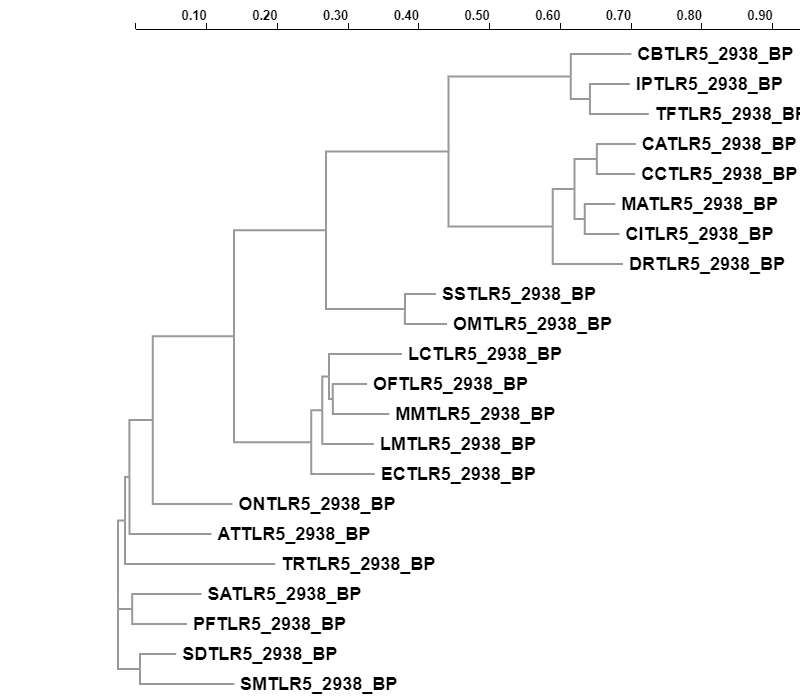

Supplement: Supplementary file 28 — Supplementary Information 28. [file 41598_2020_78347_MOESM28_ESM.zip › T5/fubar/tree.png]

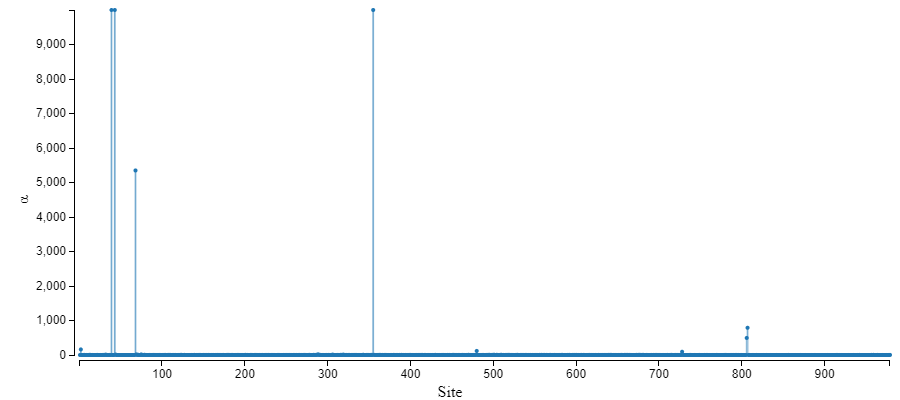

Supplement: Supplementary file 28 — Supplementary Information 28. [file 41598_2020_78347_MOESM28_ESM.zip › T5/meme/datamonkey-chart.png]

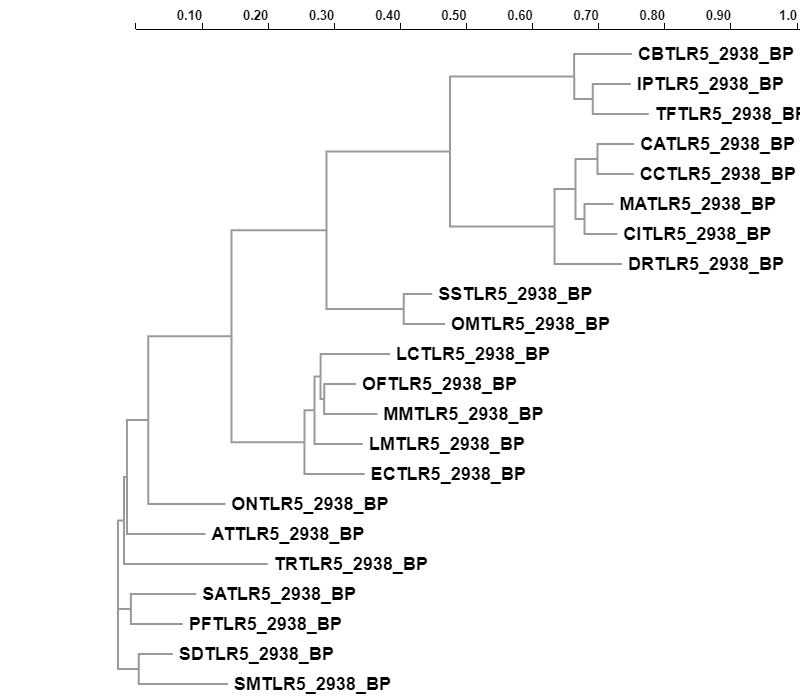

Supplement: Supplementary file 28 — Supplementary Information 28. [file 41598_2020_78347_MOESM28_ESM.zip › T5/meme/tree.png]

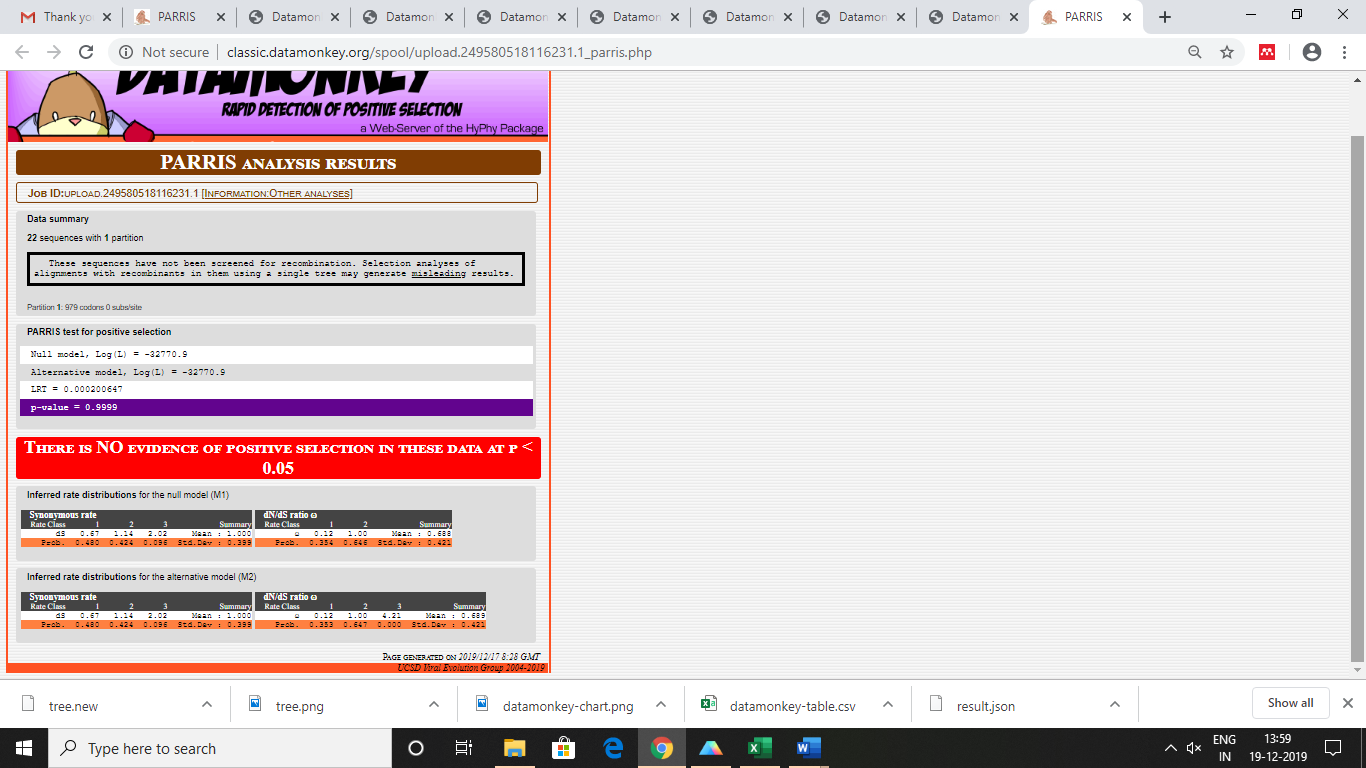

Supplement: Supplementary file 28 — Supplementary Information 28. [file 41598_2020_78347_MOESM28_ESM.zip › T5/parris.docx]

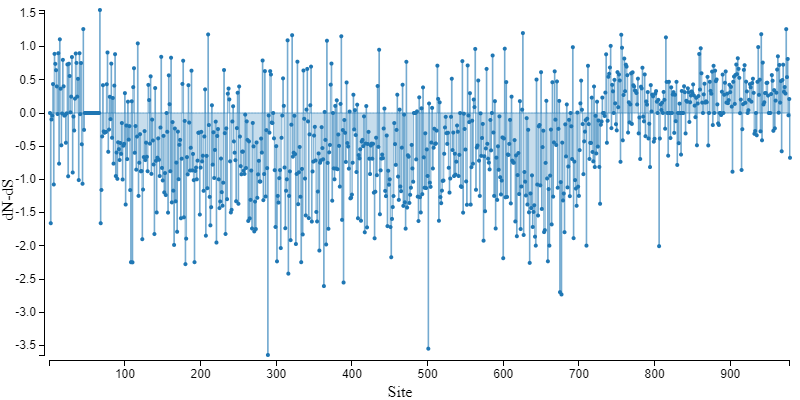

Supplement: Supplementary file 28 — Supplementary Information 28. [file 41598_2020_78347_MOESM28_ESM.zip › T5/slac/datamonkey-chart.png]

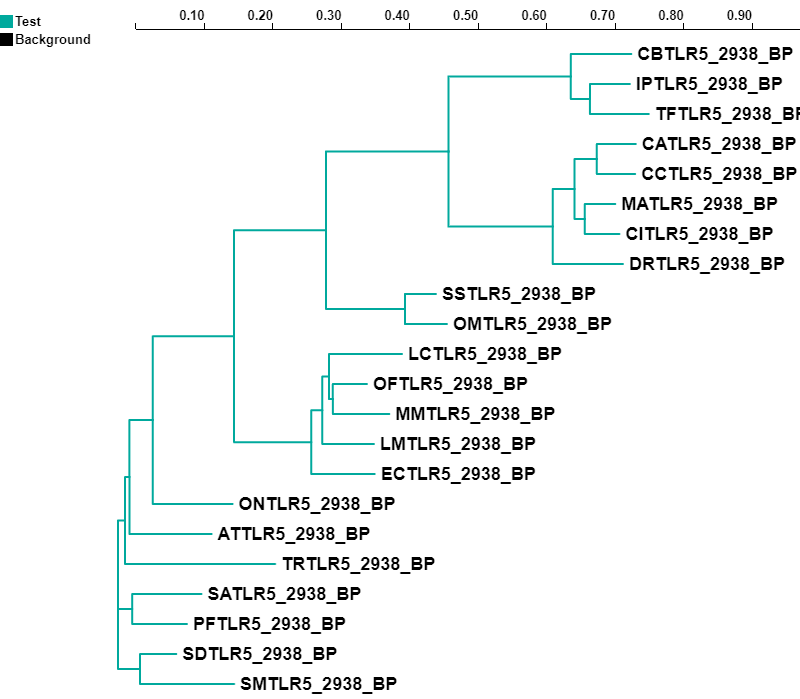

Supplement: Supplementary file 28 — Supplementary Information 28. [file 41598_2020_78347_MOESM28_ESM.zip › T5/slac/tree.png]

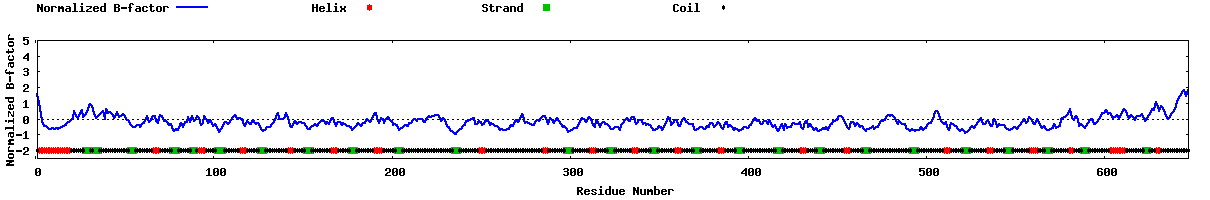

Supplement: Supplementary file 28 — Supplementary Information 28. [file 41598_2020_78347_MOESM28_ESM.zip › T5/struct/S508525_results/BFP.png]

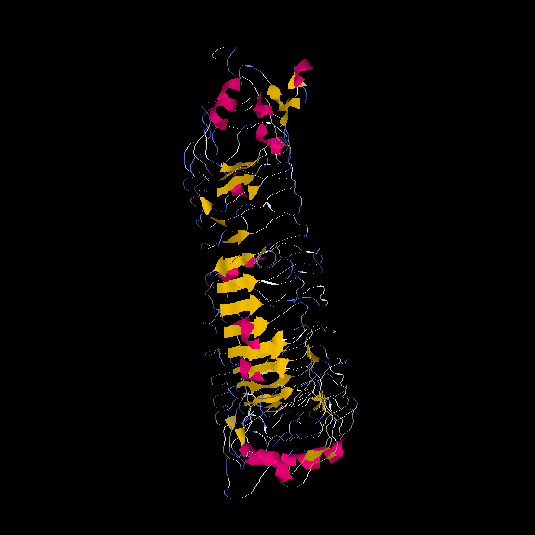

Supplement: Supplementary file 28 — Supplementary Information 28. [file 41598_2020_78347_MOESM28_ESM.zip › T5/struct/S508525_results/model1.gif]

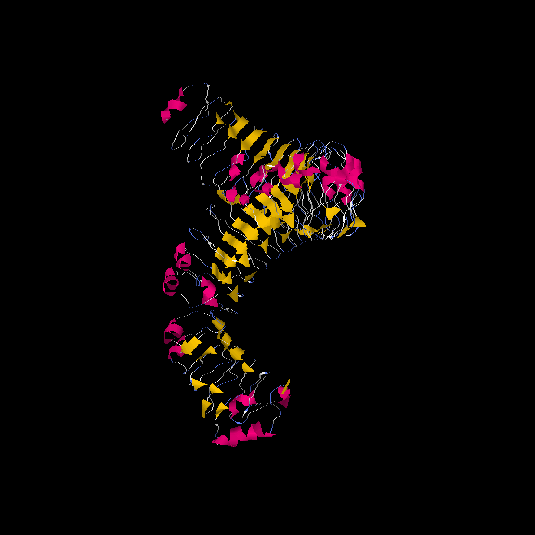

Supplement: Supplementary file 28 — Supplementary Information 28. [file 41598_2020_78347_MOESM28_ESM.zip › T5/struct/S508525_results/model2.gif]

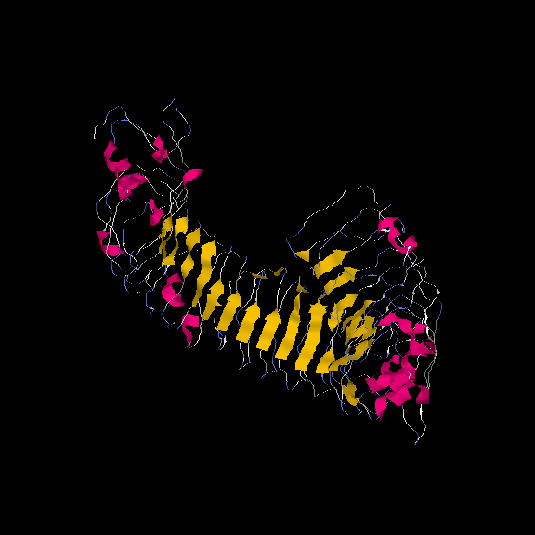

Supplement: Supplementary file 28 — Supplementary Information 28. [file 41598_2020_78347_MOESM28_ESM.zip › T5/struct/S508525_results/model3.gif]

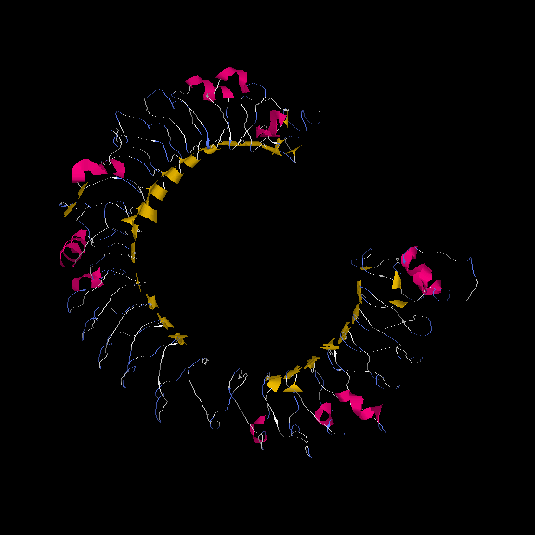

Supplement: Supplementary file 28 — Supplementary Information 28. [file 41598_2020_78347_MOESM28_ESM.zip › T5/struct/S508525_results/model4.gif]

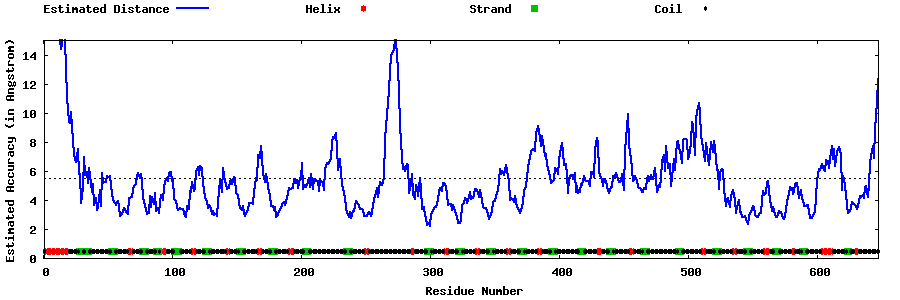

Supplement: Supplementary file 28 — Supplementary Information 28. [file 41598_2020_78347_MOESM28_ESM.zip › T5/struct/S508525_results/RSQ_1.png]

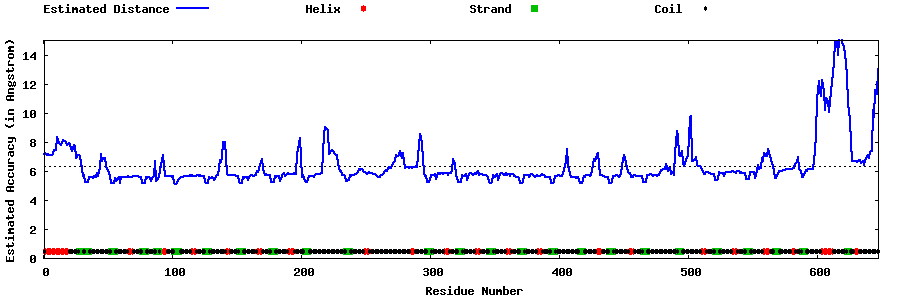

Supplement: Supplementary file 28 — Supplementary Information 28. [file 41598_2020_78347_MOESM28_ESM.zip › T5/struct/S508525_results/RSQ_2.png]

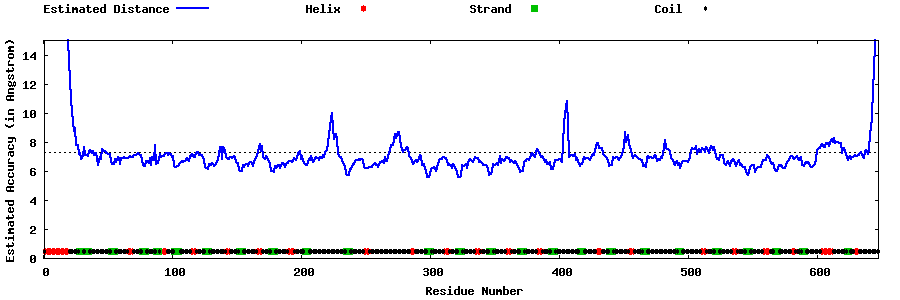

Supplement: Supplementary file 28 — Supplementary Information 28. [file 41598_2020_78347_MOESM28_ESM.zip › T5/struct/S508525_results/RSQ_3.png]

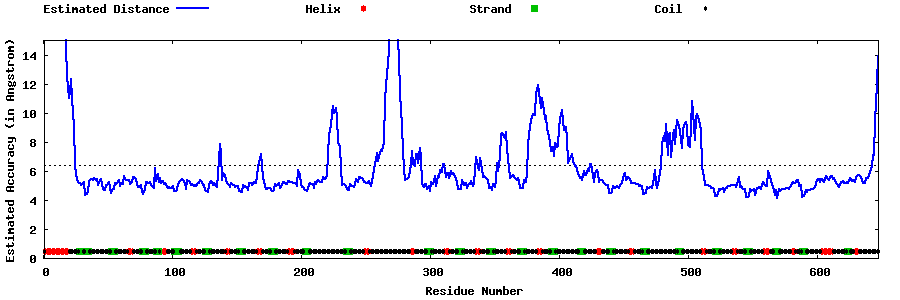

Supplement: Supplementary file 28 — Supplementary Information 28. [file 41598_2020_78347_MOESM28_ESM.zip › T5/struct/S508525_results/RSQ_4.png]

Tree scale: 0.01

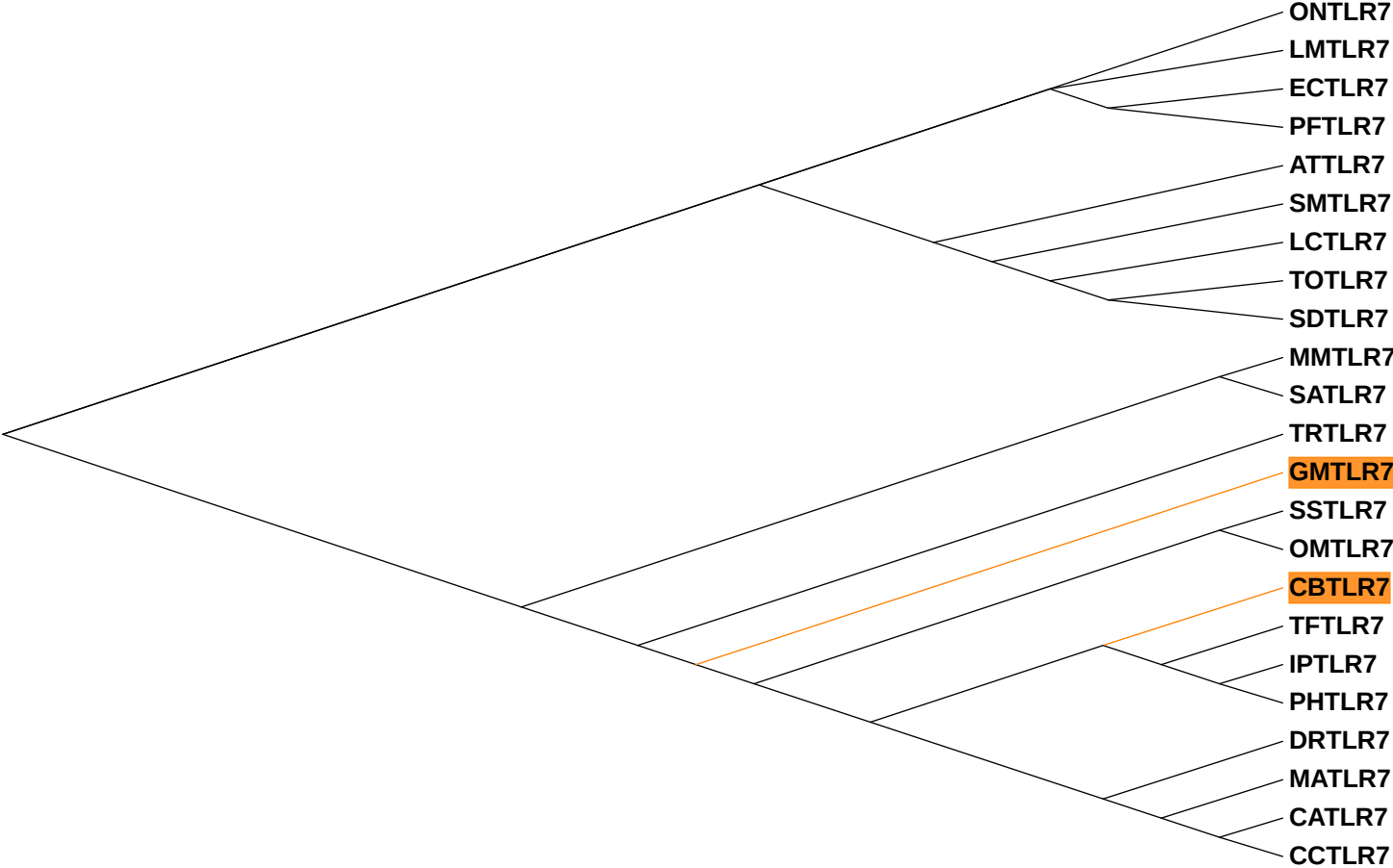

Supplement: Supplementary file 29 — Supplementary Information 29. [file 41598_2020_78347_MOESM29_ESM.zip › T7/absrel/labelledtree.pdf]

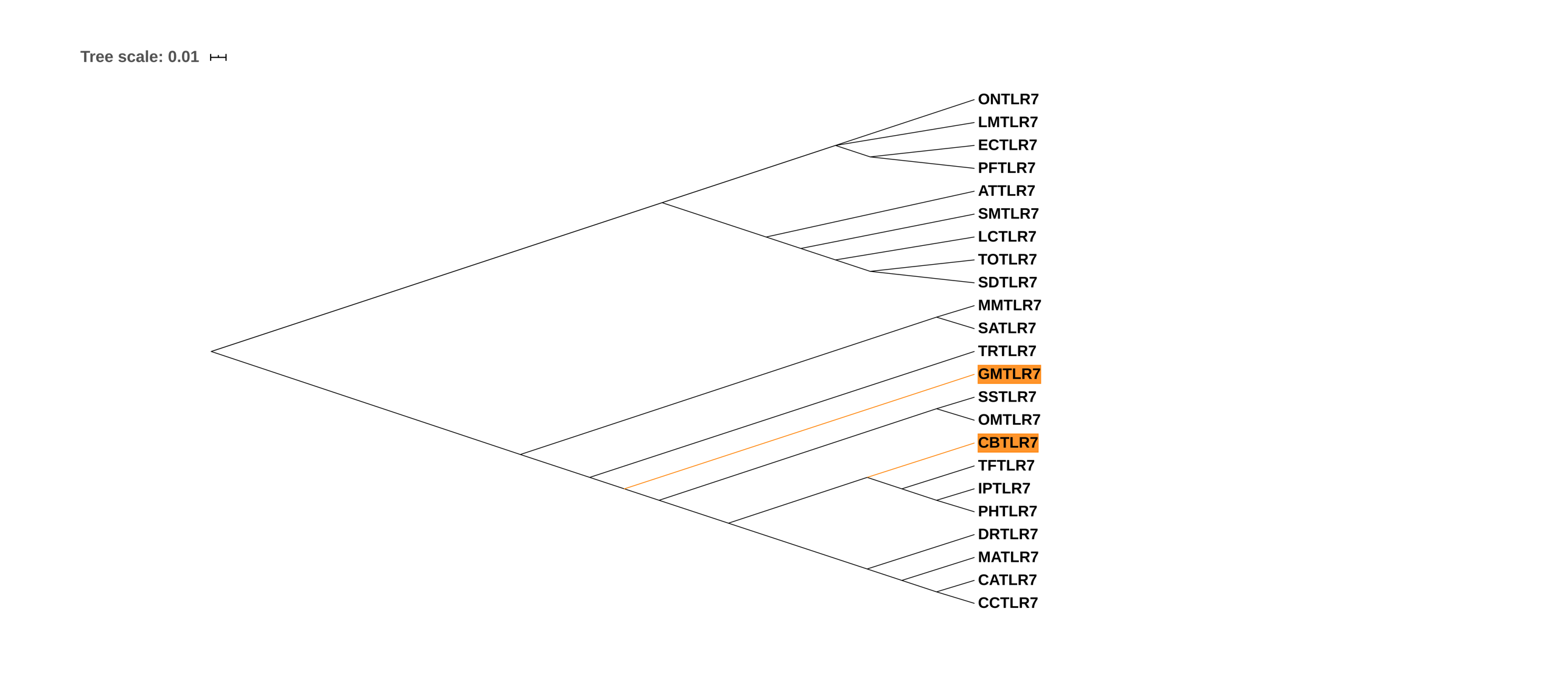

Supplement: Supplementary file 29 — Supplementary Information 29. [file 41598_2020_78347_MOESM29_ESM.zip › T7/absrel/labelledtree.png]

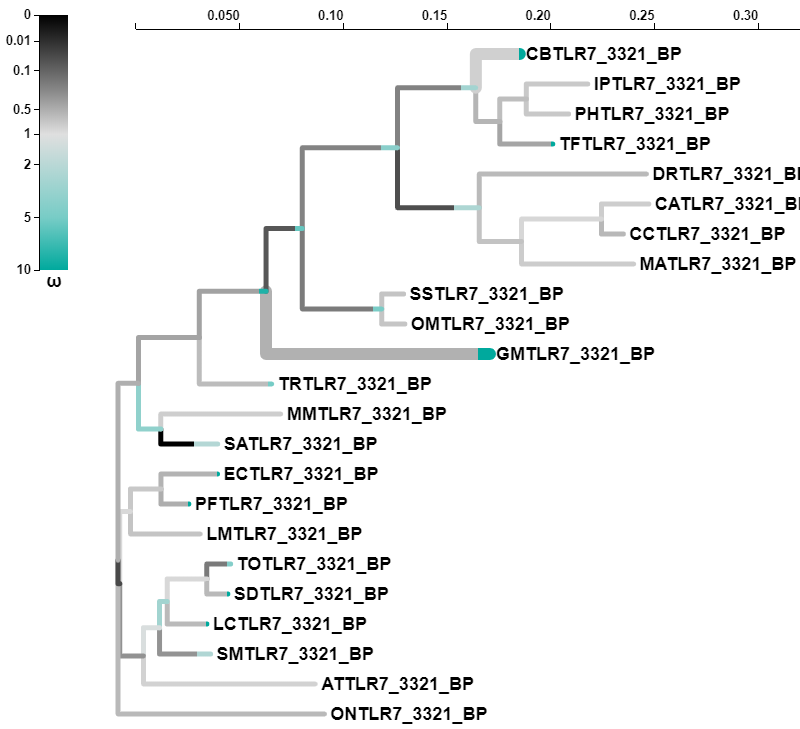

Supplement: Supplementary file 29 — Supplementary Information 29. [file 41598_2020_78347_MOESM29_ESM.zip › T7/absrel/tree.png]

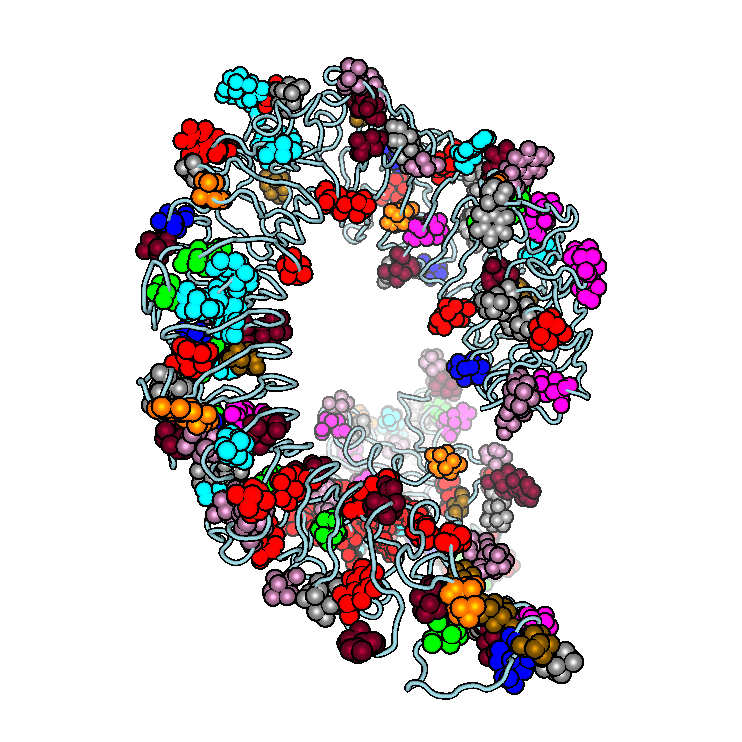

Supplement: Supplementary file 29 — Supplementary Information 29. [file 41598_2020_78347_MOESM29_ESM.zip › T7/bis2/download (1).png]

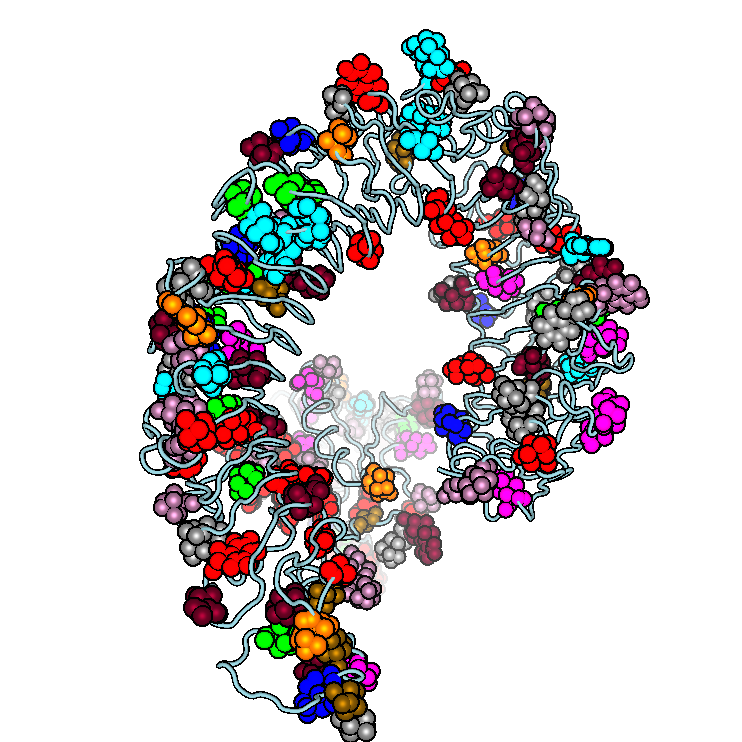

Supplement: Supplementary file 29 — Supplementary Information 29. [file 41598_2020_78347_MOESM29_ESM.zip › T7/bis2/download.png]

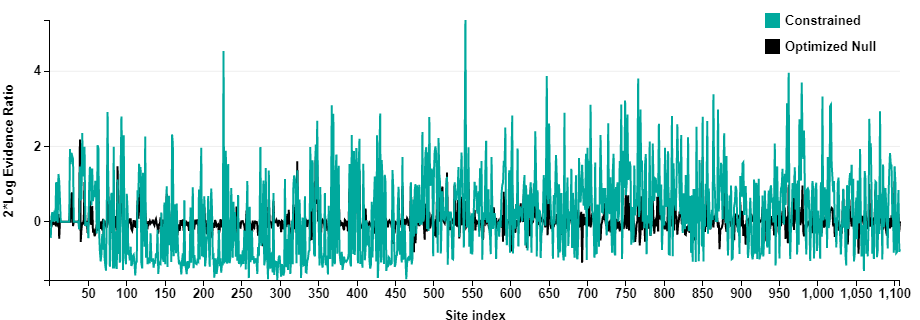

Supplement: Supplementary file 29 — Supplementary Information 29. [file 41598_2020_78347_MOESM29_ESM.zip › T7/busted/busted-chart (1).png]

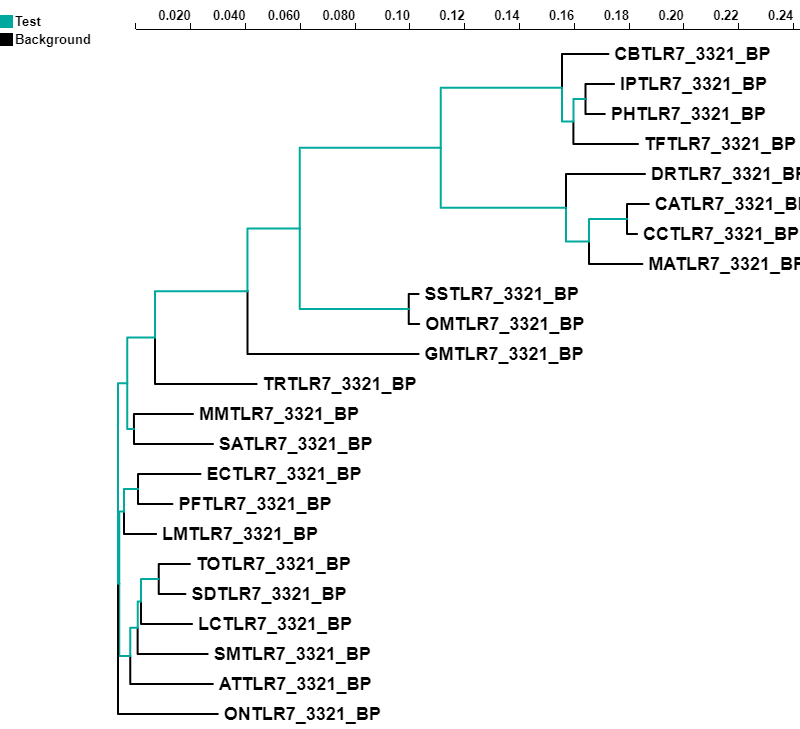

Supplement: Supplementary file 29 — Supplementary Information 29. [file 41598_2020_78347_MOESM29_ESM.zip › T7/busted/tree.png]

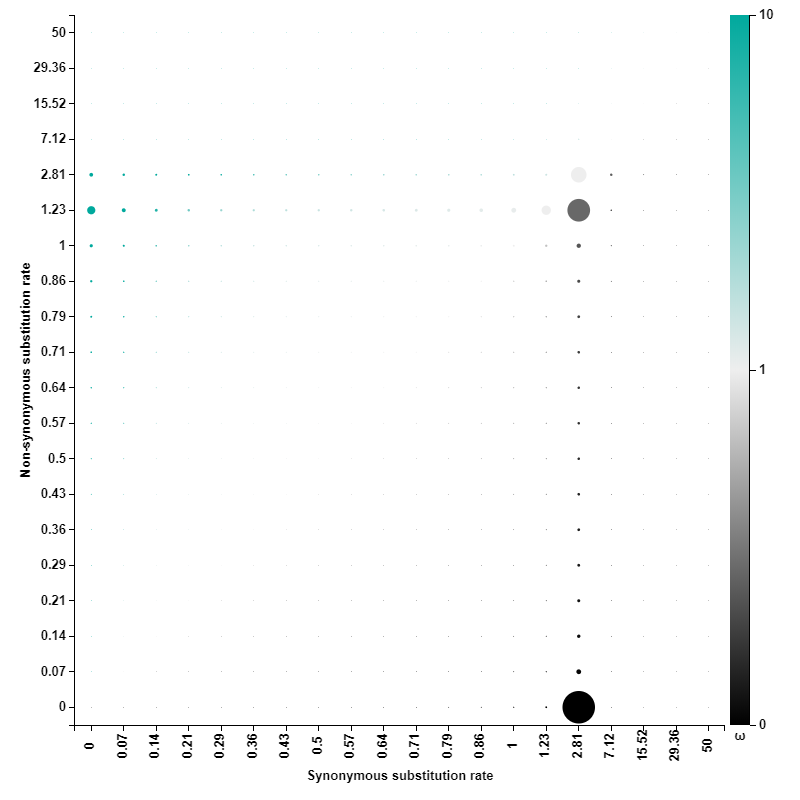

Supplement: Supplementary file 29 — Supplementary Information 29. [file 41598_2020_78347_MOESM29_ESM.zip › T7/fubar/datamonkey-chart.png]

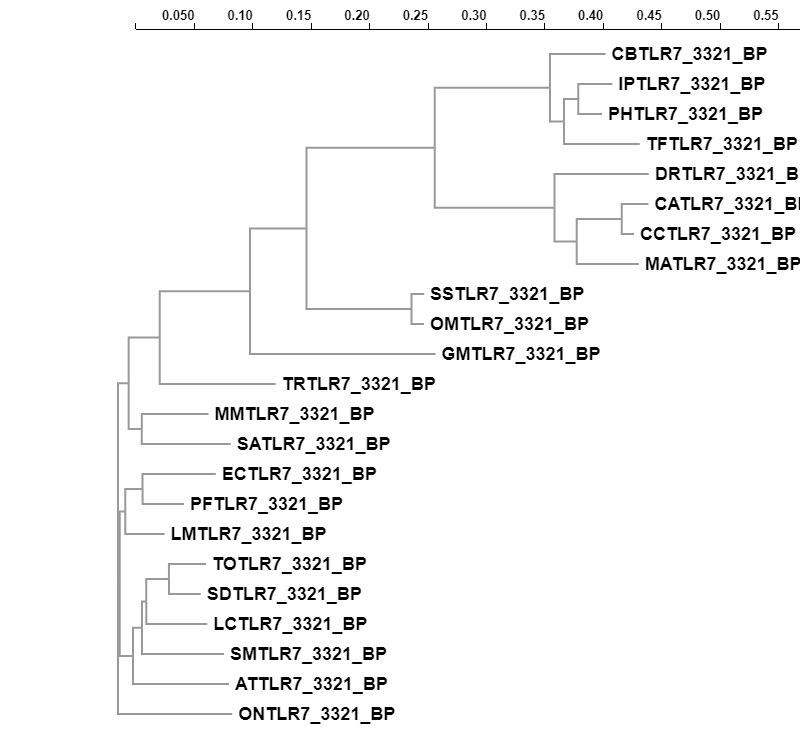

Supplement: Supplementary file 29 — Supplementary Information 29. [file 41598_2020_78347_MOESM29_ESM.zip › T7/fubar/tree.png]
