# Supplementary material for: Variation in selection constraints on teleost TLRs with emphasis on their repertoire in the Walking catfish, Clarias batrachus
Source: Sci Rep. 2020 Dec 7;10:21394. doi: 10.1038/s41598-020-78347-6 (PMC7721727; doi:10.1038/s41598-020-78347-6)
Supplement: Supplementary file 26 — Supplementary Information 26. [file 41598_2020_78347_MOESM26_ESM.zip › T2/BIS2/summary/PF00000-NONREDUNDANT-5DD-dim0.html]

Alignment and BIS clusters


|  |  |  |  |  |  |  |  |  |  |  |  |  |  |  |  |  |  |  |  |  |  |  |  |  |  |  |  |  |  |  |  |  |  |  |  |  |  |  |  |  |  |  |  |  |  |  |  |  |  |  |  |  |  |  |  |  |  |  |  |  |  |  |  |  |  |  |  |  |  |  |  |  |  |  |  |  |  |  |  |  |  |  |  |  |  |  |  |  |  |  |  |  |  |  |  |  |  |  |  |  |  |  |  |  |  |  |  |  |  |  |  |  |  |  |  |  |  |  |  |  |  |  |  |  |  |  |  |  |  |  |  |  |  |  |  |  |  |  |  |  |  |  |  |  |  |  |  |  |  |  |  |  |  |  |  |  |  |  |  |  |  |  |  |  |  |  |  |  |  |  |  |  |  |  |  |  |  |  |  |  |  |  |  |  |  |  |  |  |  |  |  |  |  |  |  |  |  |  |  |  |  |  |  |  |  |  |  |  |  |  |  |  |  |  |  |  |  |  |  |  |  |  |  |  |  |  |  |  |  |  |  |  |  |  |  |  |  |  |  |  |  |  |  |  |  |  |  |  |  |  |  |  |  |  |  |  |  |  |  |  |  |  |  |  |  |  |  |  |  |  |  |  |  |  |  |  |  |  |  |  |  |  |  |  |  |  |  |  |  |  |  |  |  |  |  |  |  |  |  |  |  |  |  |  |  |  |  |  |  |  |  |  |  |  |  |  |  |  |  |  |  |  |  |  |  |  |  |  |  |  |  |  |  |  |  |  |  |  |  |  |  |  |  |  |  |  |  |  |  |  |  |  |  |  |  |  |  |  |  |  |  |  |  |  |  |  |  |  |  |  |  |  |  |  |  |  |  |  |  |  |  |  |  |  |  |  |  |  |  |  |  |  |  |  |  |  |  |  |  |  |  |  |  |  |  |  |  |  |  |  |  |  |  |  |  |  |  |  |  |  |  |  |  |  |  |  |  |  |  |  |  |  |  |  |  |  |  |  |  |  |  |  |  |  |  |  |  |  |  |  |  |  |  |  |  |  |  |  |  |  |  |  |  |  |  |  |  |  |  |  |  |  |  |  |  |  |  |  |  |  |  |  |  |  |  |  |  |  |  |  |  |  |  |  |  |  |  |  |  |  |  |  |  |  |  |  |  |  |  |  |  |  |  |  |  |  |  |  |  |  |  |  |  |  |  |  |  |  |  |  |  |  |  |  |  |  |  |  |  |  |  |  |  |  |  |  |  |  |  |  |  |  |  |  |  |  |  |  |  |  |  |  |  |  |  |  |  |  |  |  |  |  |  |  |  |  |  |  |  |  |  |  |  |  |  |  |  |  |  |  |  |  |  |  |  |  |  |  |  |  |  |  |  |  |  |  |  |  |  |  |  |  |  |  |  |  |  |  |  |  |  |  |  |  |  |  |  |  |  |  |  |  |  |  |  |  |  |  |  |  |  |  |  |  |  |  |  |  |  |  |  |  |  |  |  |  |  |  |  |  |  |  |  |  |  |  |  |  |  |  |  |  |  |  |  |  |  |  |  |  |  |  |  |  |  |  |  |  |  |  |  |  |  |  |  |  |  |  |  |  |  |  |  |  |  |  |  |  |  |  |  |  |  |  |  |  |  |  |  |  |  |  |  |  |  |  |  |  |  |  |  |  |  |  |  |  |  |  |  |  |  |  |  |  |  |  |  |  |  |  |  |  |  |  |  |  |  |  |  |  |  |  |  |  |  |  |  |  |  |  |  |  |  |  |  |  |  |  |  |  |  |  |  |  |  |  |  |  |  |  |  |  |  |  |  |  |  |  |  |  |  |  |  |  |  |  |  |  |  |  |  |  |  |  |  |  |  |  |  |  |  |  |  |  |  |  |  |  |  |  |  |  |  |  |  |  |  |  |  |  |  |  |  |  |  |  |  |  |  |  |  |  |  |  |  |  |  |  |  |  |  |  |  |  |  |  |  |  |  |  |  |  |  |  |  |  |  |  |  |  |  |  |  |  |  |  |  |  |  |  |  |  |  |  |  |  |  |  |  |  |  |  |  |  |  |  |  |  |  |  |  |  |
| --- | --- | --- | --- | --- | --- | --- | --- | --- | --- | --- | --- | --- | --- | --- | --- | --- | --- | --- | --- | --- | --- | --- | --- | --- | --- | --- | --- | --- | --- | --- | --- | --- | --- | --- | --- | --- | --- | --- | --- | --- | --- | --- | --- | --- | --- | --- | --- | --- | --- | --- | --- | --- | --- | --- | --- | --- | --- | --- | --- | --- | --- | --- | --- | --- | --- | --- | --- | --- | --- | --- | --- | --- | --- | --- | --- | --- | --- | --- | --- | --- | --- | --- | --- | --- | --- | --- | --- | --- | --- | --- | --- | --- | --- | --- | --- | --- | --- | --- | --- | --- | --- | --- | --- | --- | --- | --- | --- | --- | --- | --- | --- | --- | --- | --- | --- | --- | --- | --- | --- | --- | --- | --- | --- | --- | --- | --- | --- | --- | --- | --- | --- | --- | --- | --- | --- | --- | --- | --- | --- | --- | --- | --- | --- | --- | --- | --- | --- | --- | --- | --- | --- | --- | --- | --- | --- | --- | --- | --- | --- | --- | --- | --- | --- | --- | --- | --- | --- | --- | --- | --- | --- | --- | --- | --- | --- | --- | --- | --- | --- | --- | --- | --- | --- | --- | --- | --- | --- | --- | --- | --- | --- | --- | --- | --- | --- | --- | --- | --- | --- | --- | --- | --- | --- | --- | --- | --- | --- | --- | --- | --- | --- | --- | --- | --- | --- | --- | --- | --- | --- | --- | --- | --- | --- | --- | --- | --- | --- | --- | --- | --- | --- | --- | --- | --- | --- | --- | --- | --- | --- | --- | --- | --- | --- | --- | --- | --- | --- | --- | --- | --- | --- | --- | --- | --- | --- | --- | --- | --- | --- | --- | --- | --- | --- | --- | --- | --- | --- | --- | --- | --- | --- | --- | --- | --- | --- | --- | --- | --- | --- | --- | --- | --- | --- | --- | --- | --- | --- | --- | --- | --- | --- | --- | --- | --- | --- | --- | --- | --- | --- | --- | --- | --- | --- | --- | --- | --- | --- | --- | --- | --- | --- | --- | --- | --- | --- | --- | --- | --- | --- | --- | --- | --- | --- | --- | --- | --- | --- | --- | --- | --- | --- | --- | --- | --- | --- | --- | --- | --- | --- | --- | --- | --- | --- | --- | --- | --- | --- | --- | --- | --- | --- | --- | --- | --- | --- | --- | --- | --- | --- | --- | --- | --- | --- | --- | --- | --- | --- | --- | --- | --- | --- | --- | --- | --- | --- | --- | --- | --- | --- | --- | --- | --- | --- | --- | --- | --- | --- | --- | --- | --- | --- | --- | --- | --- | --- | --- | --- | --- | --- | --- | --- | --- | --- | --- | --- | --- | --- | --- | --- | --- | --- | --- | --- | --- | --- | --- | --- | --- | --- | --- | --- | --- | --- | --- | --- | --- | --- | --- | --- | --- | --- | --- | --- | --- | --- | --- | --- | --- | --- | --- | --- | --- | --- | --- | --- | --- | --- | --- | --- | --- | --- | --- | --- | --- | --- | --- | --- | --- | --- | --- | --- | --- | --- | --- | --- | --- | --- | --- | --- | --- | --- | --- | --- | --- | --- | --- | --- | --- | --- | --- | --- | --- | --- | --- | --- | --- | --- | --- | --- | --- | --- | --- | --- | --- | --- | --- | --- | --- | --- | --- | --- | --- | --- | --- | --- | --- | --- | --- | --- | --- | --- | --- | --- | --- | --- | --- | --- | --- | --- | --- | --- | --- | --- | --- | --- | --- | --- | --- | --- | --- | --- | --- | --- | --- | --- | --- | --- | --- | --- | --- | --- | --- | --- | --- | --- | --- | --- | --- | --- | --- | --- | --- | --- | --- | --- | --- | --- | --- | --- | --- | --- | --- | --- | --- | --- | --- | --- | --- | --- | --- | --- | --- | --- | --- | --- | --- | --- | --- | --- | --- | --- | --- | --- | --- | --- | --- | --- | --- | --- | --- | --- | --- | --- | --- | --- | --- | --- | --- | --- | --- | --- | --- | --- | --- | --- | --- | --- | --- | --- | --- | --- | --- | --- | --- | --- | --- | --- | --- | --- | --- | --- | --- | --- | --- | --- | --- | --- | --- | --- | --- | --- | --- | --- | --- | --- | --- | --- | --- | --- | --- | --- | --- | --- | --- | --- | --- | --- | --- | --- | --- | --- | --- | --- | --- | --- | --- | --- | --- | --- | --- | --- | --- | --- | --- | --- | --- | --- | --- | --- | --- | --- | --- | --- | --- | --- | --- | --- | --- | --- | --- | --- | --- | --- | --- | --- | --- | --- | --- | --- | --- | --- | --- | --- | --- | --- | --- | --- | --- | --- | --- | --- | --- | --- | --- | --- | --- | --- | --- | --- | --- | --- | --- | --- | --- | --- | --- | --- | --- | --- | --- | --- | --- | --- | --- | --- | --- | --- | --- | --- | --- | --- | --- | --- | --- | --- | --- | --- | --- | --- | --- | --- | --- | --- | --- | --- | --- | --- | --- | --- | --- | --- | --- | --- | --- | --- | --- | --- | --- | --- | --- | --- | --- | --- | --- | --- | --- | --- | --- | --- | --- | --- | --- | --- | --- | --- | --- | --- | --- | --- | --- | --- | --- | --- | --- | --- | --- | --- | --- | --- | --- | --- | --- | --- | --- | --- | --- | --- | --- | --- | --- | --- | --- | --- | --- | --- | --- | --- | --- | --- | --- | --- | --- | --- | --- | --- | --- | --- | --- | --- | --- | --- | --- | --- | --- | --- | --- | --- | --- | --- | --- | --- | --- | --- | --- | --- | --- | --- | --- | --- | --- | --- | --- | --- | --- | --- | --- | --- | --- | --- | --- | --- | --- | --- | --- | --- | --- | --- | --- | --- | --- | --- | --- | --- | --- | --- | --- | --- | --- | --- | --- | --- | --- | --- | --- | --- | --- | --- | --- | --- | --- | --- | --- | --- | --- | --- | --- | --- | --- | --- | --- | --- | --- | --- | --- | --- | --- | --- | --- | --- | --- | --- | --- | --- | --- | --- | --- | --- | --- | --- | --- | --- | --- |
|  |  |  |  |  |  |  |  | 1 | 0 |  |  |  |  |  |  |  |  | 2 | 0 |  |  |  |  |  |  |  |  | 3 | 0 |  |  |  |  |  |  |  |  | 4 | 0 |  |  |  |  |  |  |  |  | 5 | 0 |  |  |  |  |  |  |  |  | 6 | 0 |  |  |  |  |  |  |  |  | 7 | 0 |  |  |  |  |  |  |  |  | 8 | 0 |  |  |  |  |  |  |  |  | 9 | 0 |  |  |  |  |  |  |  | 1 | 0 | 0 |  |  |  |  |  |  |  | 1 | 1 | 0 |  |  |  |  |  |  |  | 1 | 2 | 0 |  |  |  |  |  |  |  | 1 | 3 | 0 |  |  |  |  |  |  |  | 1 | 4 | 0 |  |  |  |  |  |  |  | 1 | 5 | 0 |  |  |  |  |  |  |  | 1 | 6 | 0 |  |  |  |  |  |  |  | 1 | 7 | 0 |  |  |  |  |  |  |  | 1 | 8 | 0 |  |  |  |  |  |  |  | 1 | 9 | 0 |  |  |  |  |  |  |  | 2 | 0 | 0 |  |  |  |  |  |  |  | 2 | 1 | 0 |  |  |  |  |  |  |  | 2 | 2 | 0 |  |  |  |  |  |  |  | 2 | 3 | 0 |  |  |  |  |  |  |  | 2 | 4 | 0 |  |  |  |  |  |  |  | 2 | 5 | 0 |  |  |  |  |  |  |  | 2 | 6 | 0 |  |  |  |  |  |  |  | 2 | 7 | 0 |  |  |  |  |  |  |  | 2 | 8 | 0 |  |  |  |  |  |  |  | 2 | 9 | 0 |  |  |  |  |  |  |  | 3 | 0 | 0 |  |  |  |  |  |  |  | 3 | 1 | 0 |  |  |  |  |  |  |  | 3 | 2 | 0 |  |  |  |  |  |  |  | 3 | 3 | 0 |  |  |  |  |  |  |  | 3 | 4 | 0 |  |  |  |  |  |  |  | 3 | 5 | 0 |  |  |  |  |  |  |  | 3 | 6 | 0 |  |  |  |  |  |  |  | 3 | 7 | 0 |  |  |  |  |  |  |  | 3 | 8 | 0 |  |  |  |  |  |  |  | 3 | 9 | 0 |  |  |  |  |  |  |  | 4 | 0 | 0 |  |  |  |  |  |  |  | 4 | 1 | 0 |  |  |  |  |  |  |  | 4 | 2 | 0 |  |  |  |  |  |  |  | 4 | 3 | 0 |  |  |  |  |  |  |  | 4 | 4 | 0 |  |  |  |  |  |  |  | 4 | 5 | 0 |  |  |  |  |  |  |  | 4 | 6 | 0 |  |  |  |  |  |  |  | 4 | 7 | 0 |  |  |  |  |  |  |  | 4 | 8 | 0 |  |  |  |  |  |  |  | 4 | 9 | 0 |  |  |  |  |  |  |  | 5 | 0 | 0 |  |  |  |  |  |  |  | 5 | 1 | 0 |  |  |  |  |  |  |  | 5 | 2 | 0 |  |  |  |  |  |  |  | 5 | 3 | 0 |  |  |  |  |  |  |  | 5 | 4 | 0 |  |  |  |  |  |  |  | 5 | 5 | 0 |  |  |  |  |  |  |  | 5 | 6 | 0 |  |  |  |  |  |  |  | 5 | 7 | 0 |  |  |  |  |  |  |  | 5 | 8 | 0 |  |  |  |  |  |  |  | 5 | 9 | 0 |  |  |  |  |  |  |  | 6 | 0 | 0 |  |  |  |  |  |  |  | 6 | 1 | 0 |  |  |  |  |  |  |  | 6 | 2 | 0 |  |  |  |  |  |  |  | 6 | 3 | 0 |  |  |  |  |  |  |  | 6 | 4 | 0 |  |  |  |  |  |  |  | 6 | 5 | 0 |  |  |  |  |  |  |  | 6 | 6 | 0 |  |  |  |  |  |  |  | 6 | 7 | 0 |  |  |  |  |  |  |  | 6 | 8 | 0 |  |  |  |  |  |  |  | 6 | 9 | 0 |  |  |  |  |  |  |  | 7 | 0 | 0 |  |  |  |  |  |  |  | 7 | 1 | 0 |  |  |  |  |  |  |  | 7 | 2 | 0 |  |  |  |  |  |  |  | 7 | 3 | 0 |  |  |  |  |  |  |  | 7 | 4 | 0 |  |  |  |  |  |  |  | 7 | 5 | 0 |  |  |  |  |  |  |  | 7 | 6 | 0 |  |  |  |  |  |  |  | 7 | 7 | 0 |  |  |  |  |  |  |  | 7 | 8 | 0 |  |  |  |  |  |  |  | 7 | 9 | 0 |  |  |  |  |  |  |  | 8 | 0 | 0 |  |  |  |  |  |  |  | 8 | 1 | 0 |  |  |  |  |  |  |  | 8 | 2 | 0 |  |  |  |  |  |  |  | 8 | 3 | 0 |  |  |  |  |  |  |  | 8 | 4 | 0 |  |  |  |  |  |  |  | 8 | 5 | 0 |  |  |  |  |  |  |  | 8 | 6 | 0 |  |  |  |  |  |  |  | 8 | 7 | 0 |  |  |  |  |  |  |  | 8 | 8 | 0 |  |  |  |  |  |  |  | 8 | 9 | 0 |  |  |  |  |  |  |  | 9 | 0 | 0 |  |  |  |  |  |  |  | 9 | 1 | 0 |  |  |  |
|  |  |  |  |  |  |  |  |  | | |  |  |  |  |  |  |  |  |  | | |  |  |  |  |  |  |  |  |  | | |  |  |  |  |  |  |  |  |  | | |  |  |  |  |  |  |  |  |  | | |  |  |  |  |  |  |  |  |  | | |  |  |  |  |  |  |  |  |  | | |  |  |  |  |  |  |  |  |  | | |  |  |  |  |  |  |  |  |  | | |  |  |  |  |  |  |  |  |  | | |  |  |  |  |  |  |  |  |  | | |  |  |  |  |  |  |  |  |  | | |  |  |  |  |  |  |  |  |  | | |  |  |  |  |  |  |  |  |  | | |  |  |  |  |  |  |  |  |  | | |  |  |  |  |  |  |  |  |  | | |  |  |  |  |  |  |  |  |  | | |  |  |  |  |  |  |  |  |  | | |  |  |  |  |  |  |  |  |  | | |  |  |  |  |  |  |  |  |  | | |  |  |  |  |  |  |  |  |  | | |  |  |  |  |  |  |  |  |  | | |  |  |  |  |  |  |  |  |  | | |  |  |  |  |  |  |  |  |  | | |  |  |  |  |  |  |  |  |  | | |  |  |  |  |  |  |  |  |  | | |  |  |  |  |  |  |  |  |  | | |  |  |  |  |  |  |  |  |  | | |  |  |  |  |  |  |  |  |  | | |  |  |  |  |  |  |  |  |  | | |  |  |  |  |  |  |  |  |  | | |  |  |  |  |  |  |  |  |  | | |  |  |  |  |  |  |  |  |  | | |  |  |  |  |  |  |  |  |  | | |  |  |  |  |  |  |  |  |  | | |  |  |  |  |  |  |  |  |  | | |  |  |  |  |  |  |  |  |  | | |  |  |  |  |  |  |  |  |  | | |  |  |  |  |  |  |  |  |  | | |  |  |  |  |  |  |  |  |  | | |  |  |  |  |  |  |  |  |  | | |  |  |  |  |  |  |  |  |  | | |  |  |  |  |  |  |  |  |  | | |  |  |  |  |  |  |  |  |  | | |  |  |  |  |  |  |  |  |  | | |  |  |  |  |  |  |  |  |  | | |  |  |  |  |  |  |  |  |  | | |  |  |  |  |  |  |  |  |  | | |  |  |  |  |  |  |  |  |  | | |  |  |  |  |  |  |  |  |  | | |  |  |  |  |  |  |  |  |  | | |  |  |  |  |  |  |  |  |  | | |  |  |  |  |  |  |  |  |  | | |  |  |  |  |  |  |  |  |  | | |  |  |  |  |  |  |  |  |  | | |  |  |  |  |  |  |  |  |  | | |  |  |  |  |  |  |  |  |  | | |  |  |  |  |  |  |  |  |  | | |  |  |  |  |  |  |  |  |  | | |  |  |  |  |  |  |  |  |  | | |  |  |  |  |  |  |  |  |  | | |  |  |  |  |  |  |  |  |  | | |  |  |  |  |  |  |  |  |  | | |  |  |  |  |  |  |  |  |  | | |  |  |  |  |  |  |  |  |  | | |  |  |  |  |  |  |  |  |  | | |  |  |  |  |  |  |  |  |  | | |  |  |  |  |  |  |  |  |  | | |  |  |  |  |  |  |  |  |  | | |  |  |  |  |  |  |  |  |  | | |  |  |  |  |  |  |  |  |  | | |  |  |  |  |  |  |  |  |  | | |  |  |  |  |  |  |  |  |  | | |  |  |  |  |  |  |  |  |  | | |  |  |  |  |  |  |  |  |  | | |  |  |  |  |  |  |  |  |  | | |  |  |  |  |  |  |  |  |  | | |  |  |  |  |  |  |  |  |  | | |  |  |  |  |  |  |  |  |  | | |  |  |  |  |  |  |  |  |  | | |  |  |  |  |  |  |  |  |  | | |  |  |  |  |  |  |  |  |  | | |  |  |  |  |  |  |  |  |  | | |  |  |  |  |  |  |  |  |  | | |  |  |  |  |  |  |  |  |  | | |  |  |  |  |  |  |  |  |  | | |  |  |  |  |  |  |  |  |  | | |  |  |  |  |  |  |  |  |  | | |  |  |  |  |  |  |  |  |  | | |  |  |  |  |  |  |  |  |  | | |  |  |  |  |  |  |  |  |  | | |  |  |  |
| GMTLR2\_2642\_BP | - | - | - | - | - | - | - | - | - | - | - | - | M | A | S | K | S | V | Q | V | V | - | - | - | - | - | L | L | L | W | T | T | T | V | L | S | W | G | T | F | G | L | X | X | A | E | L | I | E | D | Q | G | Y | X | X | X | X | X | X | X | X | S | C | V | T | S | X | X | R | R | V | Q | N | L | S | G | Q | N | L | T | N | V | P | L | D | L | N | N | D | M | Q | Y | L | D | L | S | H | N | P | L | V | K | L | Q | A | A | P | F | Q | R | L | S | Q | L | C | F | L | K | A | T | S | C | G | L | R | L | I | Y | P | G | L | F | F | H | T | Q | K | L | K | F | L | N | I | S | H | N | L | L | D | Y | I | P | D | L | S | X | X | X | L | P | L | L | K | I | L | D | L | A | G | N | Q | X | X | D | S | Y | Q | L | X | X | X | X | G | T | F | Q | M | L | A | X | X | X | L | S | A | L | Y | L | G | S | V | N | A | L | Q | V | D | F | N | D | F | D | S | L | S | D | S | S | L | H | H | L | I | L | G | G | G | V | E | W | Q | R | Y | D | Q | G | S | L | A | K | M | K | S | L | K | K | I | X | X | X | L | K | V | P | F | C | E | N | L | D | L | F | E | N | L | L | E | D | V | N | K | T | Q | T | T | N | L | E | L | V | K | I | F | P | D | L | C | N | V | T | G | D | P | F | R | N | L | R | T | M | P | I | V | K | N | V | T | I | L | N | T | W | I | N | S | S | F | M | M | I | F | M | K | N | V | F | L | S | N | V | V | T | I | T | F | E | N | I | T | Y | N | X | X | D | T | P | E | G | L | K | F | P | T | L | N | H | T | M | V | I | S | S | V | I | F | D | R | V | K | - | H | Y | Q | Y | K | Y | P | T | F | E | M | S | V | D | I | Y | - | - | S | K | L | E | F | V | K | F | S | G | T | G | M | N | I | V | P | C | N | T | M | A | S | L | P | S | L | E | T | L | D | L | S | N | N | L | L | S | D | L | G | F | W | W | P | G | C | S | Y | X | X | S | V | F | P | K | L | R | H | L | S | L | S | K | N | R | F | V | D | L | S | F | I | A | Q | Q | T | H | Q | M | K | Y | L | E | S | L | D | L | S | F | N | X | S | I | V | L | D | K | P | C | F | W | P | I | H | I | T | S | L | S | L | S | N | N | N | L | G | N | K | V | F | S | F | L | S | Q | Y | L | Q | R | I | D | L | S | K | T | G | I | I | A | L | T | R | E | D | L | L | Q | F | P | M | L | T | H | L | F | L | S | S | N | S | I | K | V | I | X | X | T | I | L | S | P | T | L | V | S | L | Y | I | D | Q | N | S | I | T | S | I | S | R | E | S | L | A | G | L | P | S | L | R | T | L | K | A | G | N | N | T | Y | V | C | S | C | D | S | Y | W | F | I | X | X | N | X | X | X | X | X | M | N | K | S | F | L | P | D | W | P | L | D | Y | T | C | N | T | P | P | S | V | A | D | L | P | M | S | E | Y | R | T | S | R | M | S | C | E | A | W | L | Q | A | A | V | A | L | P | V | T | M | L | I | G | L | A | F | C | S | A | F | Y | A | C | D | G | V | W | Y | T | K | M | L | W | M | W | I | H | V | K | R | R | G | Q | K | Q | A | N | L | L | X | X | X | X | X | T | T | R | R | F | G | T | T | R | S | S | P | T | A | T | K | T | P | P | G | W | R | A | S | W | C | R | P | W | K | G | P | X | X | X | X | X | X | X | X | X | X | X | X | X | X | X | S | P | C | A | S | T | S | E | T | L | C | P | A | S | G | S | W | T | T | S | L | T | A | W | R | R | A | T | R | R | C | L | C | C | P | T | T | L | C | R | V | N | G | V | T | T | N | S | S | L | P | S | I | E | L | L | T | P | S | X | X | X | T | P | W | S | S | S | C | W | S | L | F | R | P | T | L | C | P | R | S | S | X | N | X | G | D | C | X | C | S | R | R | T | W | S | G | P | W | M | S | E | S | S | R | C | S | G | S | T | S | G | L | C | Y | R | W | R | T | T | G | S | - | - | - | - | - | - | - | - | - | - | - | - | - | X | R | I | W | T | X | S | X | R | K | A | L | L | X | S | Q | T | I | W | F | L | C | X |
| ONTLR2\_2642\_BP | X | X | X | X | X | X | X | X | X | X | X | X | X | X | X | X | X | X | X | M | M | - | - | - | - | - | F | L | V | F | X | X | - | - | - | - | - | - | - | - | - | - | - | - | V | X | L | L | V | S | Q | C | S | S | L | X | X | X | X | S | Q | Q | C | H | H | C | X | X | Q | T | S | C | D | C | S | S | Q | N | F | R | E | V | P | A | A | A | S | K | L | I | T | E | L | D | L | S | F | N | A | L | E | T | I | M | K | D | D | F | L | S | Y | A | A | L | R | A | L | F | V | N | N | N | R | I | K | T | I | H | E | D | A | F | H | P | L | I | D | L | E | K | L | D | L | S | S | N | Q | L | E | T | L | S | S | G | W | F | Q | N | L | I | S | L | H | Y | L | N | L | L | G | N | K | X | X | S | T | L | G | Q | X | X | X | X | N | L | F | L | P | L | R | X | X | X | L | K | T | L | H | F | G | G | P | F | L | Q | S | V | S | K | R | D | F | S | G | L | X | X | F | G | L | E | E | L | A | F | E | G | X | X | H | L | Q | V | Y | E | N | G | S | L | R | Q | I | G | P | I | S | H | V | T | L | S | L | N | G | V | F | H | R | D | L | R | V | V | L | A | I | L | L | D | V | V | H | P | N | T | X | X | X | V | T | F | T | D | T | Q | F | N | K | S | L | Q | M | F | P | V | Y | X | X | V | I | K | R | G | A | T | G | L | S | F | K | N | V | N | M | S | V | S | A | C | V | Q | L | V | N | I | L | S | G | S | D | L | T | F | V | A | I | E | D | S | Q | I | F | K | N | E | E | K | V | R | L | L | N | I | I | L | N | S | L | D | V | - | - | - | I | V | F | K | N | I | D | I | Y | S | F | Y | N | F | P | A | L | N | F | I | N | S | M | M | K | L | V | R | R | A | S | M | X | X | X | N | S | K | L | F | A | I | P | C | Q | S | I | I | H | F | S | D | L | E | F | L | D | I | S | D | N | I | I | S | D | F | A | L | M | E | M | M | C | Y | G | K | E | D | V | L | L | N | L | R | T | L | N | I | S | K | N | I | L | S | X | X | X | I | N | S | E | L | F | T | K | L | V | K | L | E | N | L | D | M | S | G | N | S | F | D | S | M | P | L | T | C | S | W | P | A | S | L | K | F | L | N | L | S | S | T | S | X | X | P | E | V | T | S | C | L | P | Q | S | L | Q | I | L | D | L | S | R | N | K | L | T | V | F | X | X | X | X | X | I | E | L | P | L | L | K | E | L | Y | I | S | G | N | K | L | G | N | L | P | D | G | H | L | Y | L | S | L | A | V | L | S | I | Q | D | N | N | L | A | K | F | N | N | K | N | L | H | D | Y | Q | S | L | R | V | L | E | A | A | G | N | P | Y | I | C | S | C | D | F | V | D | F | V | X | X | T | N | D | L | X | X | N | H | E | T | V | V | R | G | D | L | K | S | Y | I | C | D | S | P | D | A | V | R | G | E | R | V | S | D | V | K | P | S | V | F | E | C | H | T | A | L | A | I | S | L | L | C | L | G | I | L | V | L | C | V | L | I | A | G | L | C | Y | K | F | S | V | V | W | Y | M | K | M | T | W | A | W | L | K | A | K | R | K | X | Q | S | X | R | X | X | X | X | X | X | X | X | X | X | E | C | S | S | T | M | P | L | C | P | T | V | K | W | T | L | A | G | W | R | R | I | X | S | R | P | S | S | S | R | X | X | X | X | X | X | X | X | X | X | X | X | X | R | P | S | D | S | A | S | T | R | E | T | L | F | P | E | D | G | S | X | T | I | S | W | T | L | X | R | R | V | T | E | R | S | S | S | S | P | R | T | L | S | E | A | S | G | A | S | T | S | L | T | T | P | I | L | D | C | L | I | K | T | X | X | X | T | Q | L | C | X | F | Y | W | S | P | L | T | K | R | T | S | P | K | S | S | S | G | C | G | K | X | X | T | P | G | R | T | W | S | G | L | M | M | K | I | R | S | Q | R | S | G | K | V | X | E | Q | R | L | K | H | L | K | L | T | I | Q | X | X | X | X | X | I | X | X | X | X | X | X | X | X | X | X | X | X | X | X | X | X | - | - | - | - | - | - | - | - | - | - | - | - | - |
| SATLR2\_2642\_BP | X | X | X | X | X | X | X | X | X | X | X | X | X | X | X | X | M | T | N | L | T | - | - | - | - | - | L | L | T | F | X | X | - | - | - | - | - | - | - | - | - | - | - | - | V | L | L | L | T | N | P | S | F | S | L | X | X | X | X | R | P | R | C | R | R | C | X | X | Q | T | S | C | D | C | S | K | Q | N | L | R | D | V | P | A | A | P | S | K | L | I | T | K | L | D | L | S | F | N | H | L | S | T | I | T | K | D | D | F | V | A | Y | A | S | L | Q | S | L | I | V | N | N | N | M | I | G | T | I | Q | E | Q | A | F | D | P | L | T | N | L | T | E | L | D | L | S | S | N | Q | L | D | S | L | S | A | E | W | F | E | S | L | V | S | L | Q | H | L | N | L | L | G | N | R | X | X | E | T | L | G | Q | X | X | X | X | N | L | F | Q | P | L | K | X | X | X | L | K | T | L | Q | F | G | G | L | D | L | R | S | V | R | K | S | D | F | S | G | L | X | X | A | S | L | E | E | V | V | F | D | G | X | X | N | L | Q | A | Y | A | E | G | S | L | R | R | V | R | P | L | K | H | V | A | L | S | L | R | G | P | F | K | K | N | L | A | L | V | E | A | V | L | S | D | V | A | H | P | N | T | X | X | X | L | T | F | T | N | T | S | F | F | R | N | R | Q | I | N | P | F | R | X | X | L | K | E | G | G | T | T | D | F | T | F | K | N | V | N | I | T | L | G | A | V | V | A | F | F | N | T | M | S | D | S | N | I | T | K | F | S | V | I | D | T | K | I | F | L | S | F | A | S | N | L | P | E | N | Q | H | F | R | G | W | E | E | - | - | - | I | V | L | M | N | V | D | I | P | Q | F | Y | N | F | P | A | L | F | F | L | E | P | L | L | N | V | V | R | R | V | S | L | X | X | X | N | V | K | L | Y | N | V | V | C | E | N | S | R | D | F | S | R | L | E | Y | L | D | V | S | D | N | M | L | N | D | D | T | L | G | E | M | M | C | N | G | X | X | L | D | F | S | G | L | Q | T | L | N | L | S | W | N | N | L | H | X | X | X | I | N | S | R | L | F | T | K | L | H | E | L | E | N | I | D | L | S | G | N | A | V | Y | R | M | P | E | T | C | H | W | P | P | R | L | Q | F | L | N | L | S | S | A | L | X | X | I | E | V | T | A | C | L | P | K | S | L | R | I | L | D | L | S | H | N | A | L | T | V | F | X | X | X | X | X | I | Q | L | P | Y | L | T | K | L | Y | I | S | G | N | K | L | S | I | L | P | E | A | S | L | L | P | R | L | S | F | L | F | V | E | N | N | D | L | Q | T | L | G | S | T | V | L | N | V | Y | N | N | L | T | T | I | E | A | G | A | Q | S | Y | E | C | S | C | D | F | V | A | F | M | X | X | T | S | D | L | X | X | R | Q | R | V | T | I | A | D | E | I | E | S | Y | V | C | D | S | P | D | A | M | R | G | K | R | V | T | D | A | R | L | S | V | F | E | C | Q | M | A | L | A | L | S | V | L | C | A | G | I | L | A | V | I | L | L | V | V | G | L | C | H | K | F | S | V | P | W | Y | V | R | M | M | W | A | W | L | R | A | K | R | K | X | Q | S | X | K | X | X | X | X | X | X | X | X | X | X | E | S | W | S | T | T | P | L | C | L | T | A | R | W | T | P | V | G | W | K | H | I | X | S | R | S | S | S | R | T | X | X | X | X | X | X | X | X | X | X | X | X | X | L | L | S | G | S | A | S | T | R | E | T | S | F | Q | E | A | G | S | W | T | T | S | W | R | P | S | R | R | V | T | E | L | C | L | S | S | L | S | T | S | S | I | V | N | G | A | S | T | S | W | T | T | P | I | S | D | C | L | T | T | T | X | X | X | T | R | S | C | X | F | C | W | S | P | S | T | K | T | Q | S | Q | K | S | S | A | S | C | G | E | S | X | T | P | G | R | T | W | S | G | L | M | T | T | I | R | S | P | G | S | G | K | V | X | D | Q | R | S | K | D | L | R | M | V | K | E | X | X | X | X | X | T | L | X | X | K | X | X | X | X | X | X | T | X | X | X | X | X | - | - | - | - | - | - | - | - | - | - | - | - | - |
| LMTLR2\_2642\_BP | X | X | X | X | X | X | X | X | X | X | X | X | X | X | X | X | M | R | I | L | T | - | - | - | - | - | V | V | T | F | X | X | - | - | - | - | - | - | - | - | - | - | - | - | V | L | L | L | M | H | Q | S | F | S | L | X | X | X | X | R | P | Q | C | R | S | C | X | X | Q | T | S | C | D | C | S | R | Q | N | L | R | E | V | P | A | A | P | S | K | P | I | T | E | L | D | L | S | F | N | R | L | K | A | I | M | K | K | D | F | A | A | Y | A | S | L | Q | S | L | I | M | N | N | N | R | I | E | T | I | Q | E | Q | A | F | V | P | L | T | N | L | E | K | L | D | L | S | F | N | K | L | D | T | L | S | A | G | W | F | E | N | L | F | S | L | Q | H | L | N | L | L | G | N | T | X | X | K | M | L | G | Q | X | X | X | X | N | L | F | Q | P | L | K | X | X | X | L | K | T | L | H | F | G | G | P | N | L | Q | S | V | R | I | S | D | F | S | G | L | X | X | S | G | L | E | E | V | H | F | D | G | X | X | N | L | Q | A | Y | A | R | G | S | L | R | Q | I | G | S | I | K | S | V | T | L | G | L | N | X | X | X | X | X | X | X | X | X | X | X | X | X | X | X | X | X | X | X | X | X | X | X | X | X | X | X | X | X | X | X | X | X | X | X | X | X | X | X | X | X | X | X | X | X | X | X | X | X | X | X | X | X | X | X | X | X | X | X | X | X | X | X | X | X | X | X | X | X | X | X | X | X | X | X | X | X | X | X | X | X | X | X | X | X | T | K | F | F | L | T | F | G | D | X | X | X | N | P | P | N | M | D | H | L | E | V | - | - | - | V | V | L | K | N | I | E | V | P | Q | F | Y | S | F | P | A | L | F | F | L | E | P | L | L | K | A | V | R | R | L | T | V | X | X | X | N | C | K | L | F | A | I | P | C | V | T | S | A | R | L | S | K | L | E | Y | M | D | I | S | D | N | I | F | T | D | L | A | F | S | Q | M | M | C | D | G | X | X | G | G | P | L | G | L | Q | T | I | N | I | S | R | N | H | L | Q | X | X | X | I | N | S | Q | L | F | T | K | L | D | K | L | K | N | I | D | L | S | G | N | M | F | H | S | M | P | E | T | C | Y | W | P | P | T | L | Q | F | L | N | L | S | S | T | H | X | X | R | K | V | T | T | C | L | P | V | S | L | R | I | L | D | V | S | D | N | A | L | T | V | F | X | X | X | X | X | I | H | L | P | F | L | T | E | L | Y | I | S | G | N | K | L | I | R | L | P | D | G | G | L | Y | P | R | L | T | F | L | F | I | Q | N | C | G | L | Q | T | L | S | S | T | D | L | N | Y | Y | N | D | L | K | S | L | E | A | G | G | N | T | Y | V | C | S | C | D | F | V | A | F | M | X | X | T | S | D | L | X | X | N | H | R | V | T | V | G | D | D | F | K | S | Y | I | C | D | S | P | D | A | L | R | G | T | S | V | A | E | A | T | L | S | V | F | E | C | H | T | S | L | A | F | S | V | L | C | S | G | I | L | A | V | V | L | L | V | A | G | L | C | H | K | F | S | V | L | W | Y | V | K | M | T | W | A | W | L | R | A | K | R | K | X | Q | R | X | R | X | X | X | X | X | X | X | X | X | X | E | S | S | S | M | T | P | S | C | P | T | A | K | W | T | P | V | G | W | K | H | I | W | S | R | S | S | S | R | P | X | X | X | X | X | X | X | X | X | X | X | X | X | L | L | S | G | S | A | S | T | R | E | T | S | C | L | E | A | G | S | W | T | T | S | W | T | P | X | T | R | V | T | E | L | C | L | C | S | L | S | I | L | S | G | V | N | G | A | S | T | S | W | I | T | P | I | F | D | C | L | T | I | M | X | X | X | T | Q | L | C | X | F | C | W | S | P | S | T | K | R | P | S | P | K | S | S | V | S | C | G | E | S | X | T | P | G | R | T | W | S | G | L | M | M | T | T | R | S | P | G | S | G | K | A | X | G | Q | L | L | K | D | L | R | L | I | M | E | X | X | X | X | X | V | X | X | X | S | X | X | X | X | X | W | A | X | K | F | X | X | - | - | - | - | - | - | - | - | - | - | - | - | - |
| ATTLR2\_2642\_BP | X | X | X | X | X | X | X | X | X | X | X | X | X | X | X | X | M | R | I | Q | T | - | - | - | - | - | Y | L | T | F | X | X | - | - | - | - | - | - | - | - | - | - | - | - | I | L | L | L | M | H | Q | N | F | K | M | X | X | X | X | R | P | Q | C | Y | S | C | X | X | K | T | S | C | N | C | S | R | Q | N | L | K | E | V | P | P | A | P | S | K | L | V | T | E | L | D | L | S | F | N | R | L | E | M | I | M | D | A | D | F | I | T | Y | G | S | L | K | S | L | I | M | S | N | N | K | I | K | T | I | E | E | Q | A | F | V | P | L | T | P | L | E | K | L | D | L | S | S | N | E | L | E | T | L | S | A | G | W | F | K | N | L | S | S | L | Q | S | L | N | L | L | G | N | K | X | X | E | T | L | G | P | X | X | X | X | H | L | F | H | P | L | K | X | X | X | L | R | A | L | Y | F | G | G | S | Y | F | Q | S | V | R | K | T | D | F | S | G | L | X | X | S | C | L | E | Q | L | F | F | D | G | X | X | N | L | H | D | Y | A | E | G | C | F | R | Q | I | G | P | I | S | H | V | T | L | G | L | N | G | L | F | G | R | N | Q | A | L | V | K | V | I | L | S | D | V | V | H | P | N | T | X | X | X | L | T | L | T | D | T | Q | F | T | T | A | H | Q | M | F | L | L | N | X | X | A | V | K | A | G | T | T | S | V | I | L | K | N | V | I | M | T | A | E | A | C | F | A | L | L | L | L | L | S | P | S | G | V | T | M | L | A | L | E | D | V | K | X | X | I | H | S | V | T | V | F | W | Y | I | P | D | M | K | H | V | N | V | - | - | - | V | F | L | K | N | I | E | V | P | Q | F | Y | S | F | P | A | L | L | F | L | L | P | L | L | R | V | M | K | K | M | S | L | X | X | X | N | S | M | L | F | A | I | P | C | K | S | S | S | S | M | S | K | L | E | Y | M | D | I | S | D | N | I | F | S | D | I | A | L | S | E | M | M | C | D | G | X | X | G | V | L | W | N | L | Q | T | I | N | V | S | R | N | H | L | L | X | X | X | I | N | S | Q | L | F | T | K | L | A | K | L | R | N | I | D | M | S | G | N | V | F | Q | S | M | P | E | T | C | H | W | P | P | S | L | R | Y | L | N | L | S | S | T | H | X | X | T | K | V | T | S | C | L | P | E | S | L | H | I | L | D | L | S | N | N | F | L | T | V | F | X | X | X | X | X | I | K | L | P | F | L | T | E | L | Y | I | S | G | N | K | I | S | S | F | P | H | G | G | L | Y | P | S | L | T | S | L | S | I | Q | D | N | N | L | H | T | F | S | S | N | N | L | N | D | F | N | N | L | M | S | L | K | A | A | S | N | T | Y | V | C | S | C | D | F | V | A | F | M | X | X | T | S | D | V | X | X | K | H | R | V | K | I | G | D | E | F | R | M | Y | I | C | D | S | P | F | A | M | R | G | T | S | V | V | D | V | R | L | S | V | F | E | C | H | T | A | L | A | F | S | L | L | C | S | A | I | L | A | V | F | L | L | F | V | I | L | C | H | K | F | S | V | L | W | Y | L | K | M | T | W | A | W | V | R | A | K | R | K | X | Q | S | X | L | X | X | X | X | X | X | X | X | X | X | Q | S | L | H | M | M | P | L | C | P | T | V | R | W | T | L | V | G | W | K | H | I | W | S | Q | S | L | S | S | R | X | X | X | X | X | X | X | X | X | X | X | X | X | L | L | S | G | F | A | S | T | R | E | I | L | F | L | G | A | G | S | W | T | T | S | W | T | A | L | K | R | V | T | K | H | S | S | Y | F | L | S | I | L | S | G | A | N | G | A | S | T | S | W | T | I | P | I | L | D | Y | L | T | K | M | X | X | X | I | Q | L | C | X | F | C | W | S | P | L | I | R | R | L | Y | P | K | S | S | V | S | Y | E | E | S | X | T | P | E | H | T | W | S | G | P | M | M | T | T | R | S | P | G | S | G | T | V | X | K | Q | P | L | I | D | R | X | L | L | M | M | E | X | T | Y | S | I | Q | X | L | X | X | X | X | X | X | X | X | X | X | X | X | X | - | - | - | - | - | - | - | - | - | - | - | - | - |
| MMTLR2\_2642\_BP | X | X | X | X | X | X | X | X | X | X | X | X | X | X | X | X | X | X | X | X | M | - | - | - | - | - | F | L | M | F | X | X | - | - | - | - | - | - | - | - | - | - | - | - | V | L | L | Q | T | R | Q | S | F | X | X | X | X | X | X | X | X | Q | C | Q | S | C | X | X | Q | T | S | C | D | C | S | Q | Q | N | L | R | Q | V | P | A | A | P | S | K | P | I | T | E | L | D | L | S | F | N | R | L | K | K | I | N | K | N | D | F | V | A | Y | S | S | L | R | S | L | I | I | N | N | N | I | I | K | M | I | Q | E | Q | A | F | V | P | L | T | N | L | V | K | L | D | L | S | S | N | R | L | E | T | L | S | A | E | W | F | K | N | L | L | S | L | Q | H | L | N | L | W | G | N | K | X | X | K | M | L | G | Q | X | X | X | X | N | L | F | Q | P | L | K | X | X | X | L | K | T | L | H | L | G | G | P | Y | L | E | S | V | R | K | G | D | F | S | G | L | X | X | S | A | L | D | E | V | I | F | D | G | X | X | N | L | R | V | Y | A | E | G | S | L | K | E | I | G | P | I | K | H | V | A | L | S | L | N | S | P | F | W | R | N | H | E | L | V | E | A | I | L | S | D | V | V | H | P | N | S | X | X | X | L | T | F | T | D | T | F | F | T | T | E | S | Q | T | S | P | F | K | X | X | V | N | D | G | G | T | R | H | L | I | F | K | N | V | S | L | T | I | G | A | C | M | A | V | L | S | S | L | S | D | S | N | I | T | M | L | G | L | E | E | T | K | F | F | L | S | H | F | S | G | S | I | D | S | P | N | M | K | H | L | Q | E | - | - | - | I | V | W | K | N | I | D | I | P | Q | F | Y | R | F | P | A | L | F | F | L | Q | P | L | L | K | E | V | R | R | V | S | V | X | X | X | N | C | K | L | F | A | Y | P | C | E | S | S | A | G | L | S | K | V | Q | Y | M | D | I | S | A | N | I | L | S | D | M | S | F | S | N | M | M | C | Y | G | X | X | G | S | L | G | S | L | R | T | I | N | I | S | R | N | H | L | K | X | X | X | I | N | S | K | L | F | T | K | L | D | K | L | E | N | I | D | M | S | M | N | V | F | Q | S | M | P | E | T | C | Y | W | P | P | R | L | K | F | L | N | L | S | S | A | H | X | X | R | K | V | T | T | C | L | P | M | S | L | Q | V | L | D | L | S | D | N | A | L | T | V | F | X | X | X | X | X | V | E | L | P | S | L | T | E | L | Y | I | S | G | N | R | L | S | T | L | P | E | G | R | L | Y | P | H | L | T | F | L | S | I | E | N | N | N | L | Q | T | L | S | S | K | N | L | N | N | Y | N | D | X | X | S | L | E | A | G | T | D | T | Y | V | C | S | C | D | F | V | A | L | M | X | X | T | R | D | L | X | X | Y | H | R | V | T | I | G | D | K | S | K | S | Y | I | C | D | S | P | D | S | V | R | G | K | S | V | E | D | A | R | L | S | V | F | E | C | H | T | A | L | A | F | S | L | L | C | L | G | I | L | A | V | F | L | L | V | A | G | L | C | H | K | F | S | F | L | W | Y | I | K | M | T | W | A | W | V | R | A | K | R | K | X | Q | S | X | K | X | X | X | X | X | X | X | X | X | X | E | S | L | S | M | M | P | L | C | P | T | V | K | W | T | L | V | G | W | K | H | I | W | S | Q | S | W | S | R | A | X | X | X | X | X | X | X | X | X | X | X | X | X | L | L | C | S | S | A | S | T | R | E | T | L | F | P | E | A | G | S | W | T | T | S | X | T | L | X | R | R | V | T | K | P | C | L | S | F | L | S | I | L | L | G | V | N | G | A | N | T | S | W | I | T | P | I | L | D | C | L | T | K | T | X | X | X | T | Q | S | C | X | F | C | W | S | P | L | T | Q | R | R | S | Q | K | S | S | A | S | C | E | D | S | X | T | P | G | R | T | X | S | G | L | M | M | T | T | R | S | P | G | F | G | K | I | X | E | Q | L | L | K | D | L | E | L | M | M | E | X | X | X | X | X | M | X | X | X | A | X | X | X | X | X | R | T | Y | S | C | R | I | - | - | - | - | - | - | - | - | - | - | - | - | - |
| SDTLR2\_2642\_BP | X | X | X | X | X | X | X | X | X | X | X | X | X | X | X | X | M | K | I | L | T | - | - | - | - | - | S | L | M | F | X | X | - | - | - | - | - | - | - | - | - | - | - | - | I | L | L | L | M | H | Q | S | F | S | L | X | X | X | X | T | P | Q | C | H | M | C | X | X | Q | T | S | C | N | C | S | G | Q | N | L | Q | K | V | P | A | A | P | S | K | L | I | T | E | L | D | I | S | F | N | R | L | T | T | I | L | K | D | D | F | L | A | Y | A | S | L | R | S | L | I | M | T | S | N | N | I | K | T | I | Q | E | Q | A | F | D | P | L | I | N | L | E | K | L | D | L | S | L | N | Q | L | D | T | L | S | A | G | W | F | K | N | L | S | T | L | R | Y | L | N | L | L | G | N | K | X | X | V | T | L | G | Q | X | X | X | X | N | L | F | Q | P | L | K | X | X | X | L | K | T | L | H | F | G | G | P | Q | L | Q | S | V | R | K | N | D | F | S | G | L | X | X | S | G | L | E | E | L | F | F | D | G | X | X | N | L | L | Q | Y | A | I | G | G | L | R | E | I | G | P | I | S | H | V | T | L | G | L | N | G | P | F | M | T | N | L | K | L | V | I | D | V | V | S | D | V | V | H | P | N | T | X | X | X | L | T | F | T | D | T | W | L | L | L | E | Y | Q | I | S | P | F | K | X | X | A | Y | N | R | G | T | T | G | L | I | F | K | N | V | T | M | T | V | A | A | C | I | A | L | L | N | L | L | P | D | S | N | I | T | M | L | A | L | E | D | T | T | F | F | L | T | S | L | S | H | F | T | S | I | P | D | K | Y | R | L | E | V | - | - | - | V | V | I | K | N | V | E | V | P | Q | F | Y | N | F | P | A | I | T | F | L | E | P | L | L | K | V | V | R | R | V | S | L | X | X | X | N | S | K | L | F | A | I | P | C | E | S | S | A | D | F | S | Q | L | E | Y | L | D | I | S | D | N | I | F | S | D | L | T | L | R | E | M | M | C | D | G | X | X | G | V | L | W | S | L | Q | T | I | N | I | S | R | N | D | L | R | X | X | X | I | N | S | Q | L | F | T | K | L | E | N | L | K | N | I | D | M | S | G | N | V | F | H | R | I | P | E | T | C | K | W | P | P | N | L | R | F | L | N | L | S | S | T | H | X | X | T | K | V | T | S | C | L | P | E | S | L | H | I | L | D | L | S | D | N | K | L | S | V | F | X | X | X | X | X | I | K | L | P | F | L | T | E | L | Y | I | S | G | N | K | I | G | S | L | P | D | S | S | L | Y | P | R | L | E | S | I | S | I | Q | N | N | D | L | Q | T | F | S | G | N | N | L | N | D | Y | K | N | L | K | R | L | E | A | A | A | N | T | Y | V | C | S | C | E | F | V | A | F | M | X | X | T | S | D | L | X | X | K | H | R | V | R | I | R | D | E | F | T | L | Y | I | C | D | S | P | D | A | V | R | G | K | S | V | A | D | V | R | L | S | V | F | E | C | H | R | A | L | S | F | S | L | L | C | S | G | I | L | V | M | F | L | L | F | L | H | L | C | H | K | Y | S | V | V | W | Y | I | K | M | T | L | A | Y | L | K | A | K | R | K | X | Q | S | X | K | X | X | X | X | X | X | X | X | X | X | E | S | L | S | M | T | P | L | C | P | T | V | R | W | T | L | V | G | W | K | P | I | W | F | L | S | S | S | S | A | X | X | X | X | X | X | X | X | X | X | X | X | X | P | L | S | D | S | A | F | T | K | E | T | L | Y | L | G | D | G | L | X | T | T | S | W | T | P | L | R | R | V | T | K | H | S | S | S | F | L | S | I | L | S | G | V | N | G | A | S | T | S | W | T | T | P | I | S | D | C | L | T | K | T | X | X | X | T | Q | L | F | X | F | C | W | S | P | L | T | K | T | P | S | Q | K | S | S | V | S | C | G | E | S | X | T | P | G | R | T | W | S | G | L | M | M | T | T | R | L | P | G | S | G | R | V | X | E | Q | L | L | K | D | L | X | L | I | M | M | E | S | I | Y | S | I | K | X | X | V | X | X | X | X | X | W | C | X | X | X | X | X | - | - | - | - | - | - | - | - | - | - | - | - | - |
| ECTLR2\_2642\_BP | X | X | X | X | X | X | X | X | X | X | X | X | X | X | X | X | M | R | I | F | T | - | - | - | - | - | F | L | T | F | X | X | - | - | - | - | - | - | - | - | - | - | - | - | V | L | L | L | I | H | Q | S | F | S | L | X | X | X | X | R | P | Q | C | H | S | C | X | X | Q | T | S | C | N | C | S | G | Q | N | L | T | T | V | P | A | A | P | S | K | L | I | T | Q | L | D | L | S | C | N | E | L | R | T | I | M | K | D | D | F | A | A | F | A | S | L | R | S | L | I | M | N | N | N | S | I | Q | K | I | H | E | Q | A | F | V | P | L | T | N | L | E | K | L | D | L | S | F | N | K | L | E | M | L | S | A | G | W | F | E | N | L | V | S | L | Q | H | L | N | L | L | G | N | K | X | X | K | M | L | G | Q | X | X | X | X | N | L | F | Q | P | L | K | X | X | X | L | K | T | L | Y | F | G | G | P | E | L | Q | S | V | R | K | S | D | F | S | G | L | X | X | S | G | L | E | E | V | V | F | D | G | X | X | N | L | K | D | Y | K | R | G | S | L | R | E | V | G | P | V | K | Y | V | T | L | G | L | N | G | P | F | K | E | N | Q | V | L | A | E | T | I | L | S | D | V | V | H | P | N | T | X | X | X | L | T | F | T | D | T | R | F | T | K | D | T | Y | M | S | P | F | R | X | X | A | Y | H | G | G | T | T | S | V | I | F | K | N | V | V | M | T | I | S | A | F | L | A | F | M | N | W | M | S | D | S | R | L | S | M | V | T | L | E | D | S | K | L | V | L | R | L | S | G | G | M | S | F | P | S | S | M | G | H | L | E | A | - | - | - | I | V | V | K | N | V | D | V | P | A | F | Y | L | F | P | A | L | I | G | D | E | S | L | L | P | V | L | R | R | V | T | V | X | X | X | K | C | K | V | Y | L | L | P | C | E | S | A | A | T | L | T | R | L | E | Y | L | D | F | S | D | N | L | I | T | D | M | A | L | S | N | I | M | C | N | G | X | X | S | D | F | R | L | L | Q | T | I | N | I | S | R | N | Y | L | H | X | X | X | I | N | S | Q | L | F | T | K | L | N | E | L | K | N | I | D | M | S | G | N | I | F | H | K | M | P | D | I | C | Y | W | P | P | S | L | H | F | L | N | L | S | S | T | R | X | X | T | K | V | T | T | C | L | P | V | S | L | R | V | L | D | L | S | D | N | D | L | T | V | F | X | X | X | X | X | I | E | L | P | S | L | T | D | L | Y | I | C | G | N | K | I | S | H | L | P | D | G | G | L | Y | P | S | V | V | F | V | S | I | Q | N | N | N | L | H | T | I | S | S | K | S | L | N | D | Y | N | S | L | T | S | L | E | A | G | A | N | T | F | V | C | S | C | D | F | I | A | F | M | X | X | T | T | G | W | X | X | N | H | R | V | T | I | G | D | E | F | K | S | Y | I | C | E | S | P | D | S | T | R | G | K | S | A | L | D | A | R | L | S | V | F | E | C | H | T | A | L | A | F | S | L | L | C | S | G | I | L | L | V | F | L | L | V | A | G | L | C | H | K | F | S | V | L | W | Y | M | K | M | T | W | A | W | L | R | A | K | R | K | X | Q | S | X | K | X | X | X | X | X | X | X | X | X | X | E | S | L | S | M | T | P | S | C | P | T | V | T | W | T | L | A | G | L | K | H | I | W | S | Q | S | W | S | R | P | X | X | X | X | X | X | X | X | X | X | X | X | X | L | L | S | S | S | A | S | T | R | E | T | L | F | P | V | A | G | S | W | T | T | S | W | T | P | S | R | R | V | T | E | L | C | L | S | F | L | S | I | S | S | G | V | S | G | A | S | T | S | W | I | T | P | I | L | D | C | L | T | K | M | X | X | X | T | Q | L | C | X | F | C | W | S | L | L | T | K | T | P | S | P | K | S | S | A | S | C | G | E | S | X | T | P | G | R | T | W | S | G | L | M | M | T | A | R | F | P | G | S | G | T | A | X | E | Q | L | L | K | D | L | K | L | I | T | K | X | X | X | X | X | I | X | S | L | T | X | X | X | X | X | R | T | X | X | X | X | X | - | - | - | - | - | - | - | - | - | - | - | - | - |
| PFTLR2\_2642\_BP | X | X | X | X | X | X | X | X | X | X | X | X | X | X | X | X | M | R | I | L | T | - | - | - | - | - | F | L | T | F | X | X | - | - | - | - | - | - | - | - | - | - | - | - | V | D | L | L | M | H | Q | S | L | S | L | X | X | X | X | R | P | Q | C | N | L | C | X | X | Q | T | V | C | N | C | S | R | Q | N | L | N | M | V | P | T | A | P | S | K | L | I | T | E | L | D | L | S | F | N | R | L | Q | T | I | M | N | N | D | F | V | A | F | A | S | L | Q | T | L | I | M | N | N | N | R | I | Q | K | I | Q | E | Q | A | F | V | S | L | T | N | L | E | K | L | D | L | S | L | N | R | L | D | T | L | S | A | G | W | F | E | N | L | F | S | L | Q | H | L | N | L | L | G | N | K | X | X | K | M | L | G | Q | X | X | X | X | N | L | F | K | P | L | K | X | X | X | L | K | T | L | H | F | G | G | P | D | L | H | S | I | R | K | S | D | F | S | G | L | X | X | S | R | L | E | K | V | V | L | D | G | X | X | N | L | Q | V | Y | A | E | G | S | L | Q | Q | I | E | T | I | K | Y | V | T | L | G | L | N | G | P | F | L | I | N | Q | A | L | V | G | A | I | L | S | D | V | A | H | P | N | T | X | X | X | L | T | F | V | D | T | W | F | S | A | E | Y | Q | M | L | P | F | K | X | X | T | R | D | R | G | T | T | R | V | I | F | K | N | V | N | M | T | V | L | A | C | L | A | F | L | N | L | L | S | D | S | N | L | T | M | L | A | M | E | D | T | Q | L | I | L | N | S | V | D | G | I | A | Y | P | P | H | M | D | R | L | E | A | - | - | - | I | V | L | K | N | V | D | V | P | R | F | Y | N | F | P | A | L | F | F | L | Q | P | L | L | N | V | V | R | R | V | S | V | X | X | X | N | C | K | V | F | L | I | P | C | E | S | S | V | D | F | S | K | L | E | Y | M | D | V | S | D | N | L | F | S | D | L | A | L | S | Q | M | M | C | D | G | X | X | G | V | L | W | S | L | Q | T | F | N | I | S | R | N | Y | L | H | X | X | X | I | N | S | Q | L | F | T | I | L | D | K | L | K | N | I | D | M | S | G | N | V | F | R | S | M | P | E | T | C | Y | W | P | P | S | L | Q | F | L | N | L | S | S | T | R | X | X | A | K | V | T | T | C | L | P | M | S | L | H | I | L | D | L | S | D | N | D | L | T | V | F | X | X | X | X | X | I | E | L | P | F | L | T | E | L | Y | I | S | G | N | K | I | N | S | L | P | D | G | G | L | Y | P | C | L | V | F | L | S | I | Q | N | N | N | L | Q | T | F | S | S | N | N | L | N | G | Y | S | N | L | T | S | L | E | A | G | S | D | T | Y | V | C | S | C | G | F | V | A | L | M | X | X | T | S | D | W | X | X | N | H | R | I | T | F | G | D | E | F | K | S | Y | I | C | D | S | P | D | A | V | R | G | E | S | A | A | D | V | T | L | S | V | F | K | C | H | T | A | L | A | F | S | V | L | C | S | G | I | L | L | V | F | L | L | I | A | G | L | C | H | K | F | S | V | V | W | Y | M | K | M | T | W | A | W | L | R | A | K | R | K | X | Q | S | X | K | X | X | X | X | X | X | X | X | X | X | E | S | S | S | M | T | P | L | C | P | T | V | K | W | T | L | V | G | W | K | H | I | W | S | R | S | S | S | R | P | X | X | X | X | X | X | X | X | X | X | X | X | X | L | L | F | G | S | A | S | T | R | E | T | L | F | P | E | A | G | S | W | T | T | S | X | T | P | S | R | R | V | T | E | L | C | L | S | F | L | S | I | L | S | X | V | N | G | A | S | T | S | W | I | T | P | I | L | G | C | L | T | K | T | X | X | X | T | R | L | C | X | F | C | W | S | P | L | K | K | R | P | S | P | R | S | S | A | I | C | G | E | S | X | T | P | G | R | T | W | S | G | P | M | M | R | A | X | F | P | G | S | G | K | A | X | E | R | L | L | K | H | L | I | L | M | M | E | X | X | X | X | X | V | Y | S | L | T | X | X | X | X | X | W | T | X | X | X | X | X | - | - | - | - | - | - | - | - | - | - | - | - | - |
| DRTLR2\_2642\_BP | - | - | - | - | - | - | - | - | - | - | - | X | X | X | X | X | M | R | L | X | X | X | X | X | X | X | X | V | G | T | M | T | A | I | I | L | - | - | - | - | - | - | X | X | I | M | F | I | L | A | Q | G | L | X | X | X | X | X | X | C | S | Q | T | C | K | C | D | Q | M | Y | F | C | N | C | S | S | N | N | L | H | Q | V | P | T | V | P | X | X | D | V | L | G | L | D | L | S | F | N | Q | I | E | S | I | N | M | T | D | L | S | S | Y | N | E | L | I | I | L | N | L | H | K | N | K | L | R | H | I | H | R | D | A | F | K | S | Q | H | N | L | E | V | L | D | L | S | L | N | N | L | N | N | L | S | P | S | W | F | H | K | L | K | S | L | Q | Q | L | N | L | V | G | N | P | X | X | S | T | V | G | P | X | X | X | X | P | I | F | S | S | L | V | X | X | X | L | R | T | L | H | L | G | S | P | S | L | R | E | L | H | K | N | G | L | D | V | L | X | X | T | H | L | D | E | M | T | F | F | G | X | X | N | L | R | S | Y | E | N | G | S | L | K | A | A | R | P | I | G | S | V | S | L | S | L | Q | N | L | F | E | S | D | P | E | L | V | S | K | V | L | Q | D | V | S | H | P | E | T | X | X | X | L | I | I | K | D | V | T | M | K | T | N | T | S | T | E | P | F | K | X | X | V | K | E | G | G | T | K | S | L | T | F | Q | N | S | S | T | T | D | Q | A | L | T | S | F | L | E | F | M | D | G | S | P | L | S | F | I | G | L | E | D | I | Q | F | V | G | I | G | E | W | Q | K | A | K | Y | T | H | H | D | S | L | R | T | - | - | - | A | Y | L | R | N | I | E | I | E | G | F | F | G | F | S | S | M | I | E | L | G | F | L | L | K | H | F | H | N | V | S | V | X | X | X | N | A | T | V | F | V | I | P | K | E | T | T | F | L | L | K | N | L | E | Y | M | D | L | S | Q | N | L | L | T | D | L | T | I | Q | P | T | L | Y | T | G | X | X | G | A | Y | Q | N | L | N | M | L | N | V | S | Q | N | V | L | K | S | L | G | L | M | S | R | L | V | T | N | L | K | K | L | K | Y | L | D | L | S | Y | N | S | F | V | S | M | P | E | K | C | S | W | P | V | T | L | R | F | L | N | L | S | S | T | K | X | X | S | T | L | T | P | C | L | P | S | S | L | T | V | L | D | L | S | E | N | D | L | K | A | F | X | X | X | X | X | Q | R | F | P | H | L | T | T | L | I | L | T | G | N | R | L | M | K | L | P | D | G | K | L | F | P | S | L | N | T | L | L | I | Q | R | N | A | L | R | M | F | N | Q | S | S | L | R | S | F | K | T | L | L | Y | L | E | A | G | A | N | N | F | V | C | S | C | K | F | V | S | F | F | X | X | K | K | D | X | X | X | E | D | L | I | T | L | Q | D | G | R | Q | N | Y | V | C | N | T | P | F | T | L | R | G | N | A | I | D | S | V | R | L | S | V | F | E | C | Y | M | I | P | A | V | S | V | L | C | F | G | I | I | T | A | L | G | L | V | V | L | T | C | H | K | L | H | V | I | W | Y | L | Q | M | T | K | A | W | I | Q | A | K | R | K | X | L | L | S | V | X | X | X | X | X | X | X | X | Y | L | K | S | S | A | M | M | L | L | C | L | T | A | N | M | T | L | N | G | S | R | R | F | L | L | L | S | L | K | T | L | X | X | X | X | X | X | X | X | X | X | X | X | X | L | R | F | P | C | V | C | T | N | G | T | S | D | R | A | A | G | S | W | T | T | S | L | T | Q | L | K | R | A | I | E | L | F | L | F | C | R | S | T | L | X | A | V | N | G | A | V | T | S | W | T | S | H | I | S | A | S | W | M | N | T | X | X | X | T | P | L | S | W | C | F | W | N | Q | X | R | R | R | R | F | P | N | A | S | A | S | X | E | R | L | X | T | P | G | R | I | W | S | G | L | R | M | R | T | R | E | M | S | F | G | A | I | X | E | P | L | Y | R | E | T | S | X | X | X | X | X | X | X | X | X | X | X | X | X | X | X | X | X | X | X | X | X | X | X | X | X | X | - | - | - | - | - | - | - | - | - | - | - | - | - |
| MATLR2\_2642\_BP | - | - | - | - | - | - | - | - | - | - | - | X | X | X | X | X | M | R | L | X | X | X | X | X | X | X | X | L | G | A | X | X | S | I | I | L | - | - | - | - | - | - | X | X | F | I | L | I | L | A | Q | G | S | X | X | X | X | X | X | S | S | R | T | C | K | C | D | Q | Q | Y | F | C | N | C | S | S | N | H | L | Q | Q | V | P | K | V | P | X | X | N | A | L | G | L | D | L | S | F | N | Q | I | E | S | I | N | I | N | D | L | S | P | Y | S | E | L | K | T | L | N | L | H | K | N | K | L | S | F | I | H | K | E | A | F | K | S | Q | R | N | L | K | A | L | D | L | S | L | N | N | L | K | E | L | V | S | S | W | F | H | E | L | K | S | L | Q | H | L | N | L | V | G | N | P | X | X | S | T | L | G | P | X | X | X | X | S | I | F | Q | S | L | V | X | X | X | L | R | T | L | Q | F | G | S | P | S | L | R | V | V | H | K | N | G | L | D | G | L | X | X | T | H | L | D | E | M | T | F | I | G | X | X | N | L | R | S | Y | E | N | G | S | L | K | A | A | R | P | I | G | L | V | S | L | S | L | Q | N | L | L | Q | N | D | P | E | L | V | S | K | V | L | Q | D | V | S | H | P | E | T | X | X | X | L | T | I | K | D | A | T | L | R | T | N | T | S | T | E | P | F | K | X | X | A | K | E | G | G | T | K | S | L | S | F | Q | N | V | T | T | S | D | E | A | I | T | S | L | L | Q | V | M | D | G | S | P | L | S | F | L | G | L | T | D | V | H | L | F | G | Q | G | W | W | Q | K | A | S | Y | T | H | Y | E | N | L | H | T | - | - | - | A | Y | I | R | N | L | D | I | Q | G | F | F | E | F | S | S | M | I | Q | L | G | F | L | L | V | H | L | H | K | V | S | V | X | X | X | N | G | T | V | F | V | I | P | R | E | T | T | L | L | L | R | N | L | E | Y | L | D | L | S | Q | N | L | L | S | D | L | T | I | E | P | S | L | F | T | G | X | X | G | A | Y | Q | N | L | N | T | L | N | V | S | Q | N | I | L | K | S | L | G | L | M | S | R | L | V | T | N | L | K | R | L | I | Y | L | D | L | S | H | N | S | F | V | S | M | P | E | K | C | S | W | P | A | T | L | R | F | L | N | L | S | S | T | K | X | X | R | T | M | T | A | C | L | P | S | T | L | T | V | L | D | L | S | E | N | D | L | M | V | F | X | X | X | X | X | Q | R | F | P | Q | L | T | T | L | I | L | T | G | N | R | F | M | E | L | P | Q | G | E | L | F | P | R | L | C | T | L | L | I | Q | R | N | A | L | R | M | F | N | G | S | S | L | G | R | F | K | N | L | Q | Y | L | E | A | G | N | N | N | F | V | C | S | C | E | F | V | S | F | F | X | X | K | Q | D | X | X | X | D | R | F | I | T | L | R | D | G | R | C | N | Y | V | C | D | T | P | F | A | L | R | G | D | A | I | E | S | V | R | L | S | V | F | E | C | Y | M | I | P | A | V | S | V | L | C | S | V | I | I | I | V | L | G | L | I | V | V | T | C | H | K | L | H | V | I | W | Y | L | Q | M | T | K | A | W | I | Q | A | K | R | K | X | L | Q | L | V | X | X | X | X | X | X | X | X | W | P | K | S | S | A | M | M | L | L | Y | P | T | V | N | M | T | L | S | G | S | R | R | S | L | F | Q | S | W | R | A | L | X | X | X | X | X | X | X | X | X | X | X | X | X | L | R | S | P | C | V | C | T | N | G | T | S | G | R | A | V | G | L | W | T | T | X | S | T | R | L | R | R | A | I | G | L | S | L | F | C | L | S | T | L | L | P | A | N | G | V | A | M | S | W | T | S | R | I | S | A | X | S | M | S | T | X | X | X | T | P | P | S | X | C | F | X | S | Q | S | R | R | R | P | F | L | S | V | S | A | S | C | G | R | L | X | T | P | G | R | I | W | S | G | P | R | K | R | K | R | E | A | S | S | G | A | I | X | E | P | P | Y | R | E | M | N | V | Q | R | T | P | X | X | X | X | X | X | X | X | X | X | X | X | X | X | X | X | X | X | X | X | X | - | - | - | - | - | - | - | - | - | - | - | - | - |
| CITLR2\_2642\_BP | - | - | - | - | - | - | - | - | - | - | - | X | X | X | X | X | M | R | L | X | X | X | X | X | X | X | X | L | G | A | K | E | S | I | I | L | - | - | - | - | - | - | X | X | F | I | L | I | L | A | Q | G | F | X | X | X | X | X | X | Y | S | R | T | C | K | C | D | Q | Q | Y | F | C | N | C | A | S | N | H | L | Q | Q | V | P | K | V | P | X | X | N | A | L | G | L | D | L | S | F | N | Q | I | E | S | I | N | I | N | D | L | S | P | Y | S | E | L | K | T | L | N | L | H | K | N | K | L | S | F | I | H | K | E | A | F | K | S | Q | C | N | L | K | V | L | D | L | S | L | N | N | L | N | K | L | F | S | S | W | F | H | E | L | K | S | L | Q | H | L | N | L | V | G | N | P | X | X | S | T | L | G | P | X | X | X | X | S | I | F | Q | S | L | V | X | X | X | L | R | T | L | Q | F | G | S | P | S | L | R | V | V | H | K | N | G | L | D | G | L | X | X | T | H | L | D | E | M | T | F | I | G | X | X | N | L | R | S | Y | E | S | G | S | L | K | A | A | R | P | I | G | S | V | S | L | S | L | Q | N | L | F | Q | N | D | P | E | L | V | S | K | V | L | Q | D | V | S | H | P | E | T | X | X | X | L | T | I | K | D | A | T | L | R | T | N | T | S | T | E | P | F | K | X | X | A | K | E | G | G | T | K | S | L | S | F | Q | N | V | T | T | S | D | E | A | I | T | S | L | L | Q | V | M | N | G | S | P | L | S | Y | I | G | L | T | D | V | H | L | I | G | Q | G | W | W | Q | K | A | S | Y | T | H | Y | E | N | L | Y | T | - | - | - | A | Y | I | R | N | L | D | I | Q | G | F | F | E | F | S | S | M | M | Q | L | G | F | L | L | M | H | L | Y | K | V | S | V | X | X | X | N | G | T | V | F | V | I | P | Q | Q | T | T | P | L | L | K | N | L | E | Y | L | D | L | S | Q | N | L | L | S | D | L | T | I | E | P | T | L | F | T | G | X | X | G | S | Y | Q | N | L | N | T | L | N | V | S | Q | N | I | L | K | S | L | G | L | M | S | R | L | V | T | N | L | K | R | L | I | Y | L | D | L | S | H | N | S | F | V | S | M | P | E | K | C | S | W | P | A | T | L | R | F | L | N | L | S | S | T | K | X | X | R | T | M | T | A | C | L | P | S | T | L | T | V | L | D | L | S | E | N | D | L | T | A | F | X | X | X | X | X | Q | R | F | P | Q | L | T | T | L | I | L | T | G | N | R | F | M | K | L | P | Q | G | E | L | F | P | R | L | H | M | L | L | I | Q | R | N | A | L | R | M | F | N | E | S | S | L | R | S | F | T | N | L | Q | Y | L | E | A | G | N | N | N | F | V | C | S | C | E | F | V | S | F | F | X | X | K | Q | D | X | X | X | D | R | F | I | T | L | L | D | D | R | C | N | Y | V | C | D | T | P | F | T | L | R | G | D | A | I | E | S | V | R | L | S | V | F | E | C | Y | M | I | P | A | V | S | V | L | C | S | V | I | I | I | V | L | G | L | I | A | V | T | C | H | K | L | H | V | I | W | Y | L | Q | M | T | K | A | W | I | Q | A | K | R | K | X | L | Q | L | V | X | X | X | X | X | X | X | X | L | P | K | S | S | A | M | M | L | L | Y | P | T | V | N | M | T | L | S | G | S | R | R | S | L | F | Q | S | W | R | A | L | X | X | X | X | X | X | X | X | X | X | X | X | X | L | H | S | P | C | V | C | T | N | G | T | S | G | R | A | V | G | L | W | T | T | X | S | T | R | L | R | R | A | I | G | L | S | L | F | C | L | S | T | L | L | P | A | N | G | V | A | T | S | W | T | S | H | I | S | A | X | S | M | S | T | X | X | X | T | P | L | S | X | Y | F | X | S | R | S | R | R | R | P | F | P | S | V | S | A | S | C | G | R | L | X | T | P | G | R | I | W | S | G | P | R | M | R | K | R | E | A | S | S | G | A | I | X | E | P | P | Y | R | G | X | X | X | X | X | X | X | X | X | X | X | X | X | X | X | X | X | X | X | X | X | X | X | X | X | X | X | X | - | - | - | - | - | - | - | - | - | - | - | - | - |
| CCATLR2\_2642\_BP | - | - | - | - | - | - | - | - | - | - | - | X | M | V | G | R | M | R | L | X | X | X | X | X | X | X | X | L | G | T | E | E | S | I | T | I | - | - | - | - | - | - | X | X | F | I | L | I | L | A | Q | G | F | X | X | X | X | X | X | Y | S | R | T | C | V | C | D | Q | Q | Y | F | C | N | C | S | S | N | H | L | Q | Q | V | P | K | V | P | X | X | N | A | L | G | L | D | L | S | F | N | Q | I | E | S | I | D | M | N | D | L | S | P | Y | S | E | L | K | T | L | N | L | H | K | N | K | L | K | F | I | H | K | E | A | F | K | S | Q | H | K | L | E | V | L | D | L | S | L | N | N | L | E | K | L | S | S | S | W | F | H | E | L | N | S | L | Q | Q | L | N | L | V | G | N | P | X | X | T | T | V | G | P | X | X | X | X | P | I | F | Q | S | L | F | X | X | X | L | R | T | L | Q | F | G | S | P | S | L | R | E | V | Y | K | N | G | L | D | G | L | X | X | T | H | L | D | E | M | T | F | I | G | X | X | N | L | K | S | Y | E | N | G | S | F | K | A | A | R | P | I | G | S | V | S | L | S | L | Q | S | P | F | H | N | D | P | E | L | V | S | K | V | L | K | D | V | S | H | P | E | T | X | X | X | L | T | I | K | N | V | E | L | R | T | N | T | S | T | E | S | F | K | X | X | A | R | E | G | G | T | E | S | L | S | F | H | N | C | T | T | T | D | E | A | F | T | S | F | L | V | V | M | D | N | S | S | L | S | Y | I | G | L | E | E | V | H | L | I | G | Q | G | W | W | Q | K | A | S | Y | T | H | Y | E | H | L | H | T | - | - | - | A | Y | I | R | N | L | D | I | Q | G | F | F | E | F | S | S | M | K | Q | L | G | F | L | L | V | N | L | H | K | V | S | V | X | X | X | N | G | T | V | F | V | I | P | P | I | T | T | V | L | L | K | K | L | E | Y | L | D | L | S | Q | N | L | L | S | D | L | T | I | A | P | T | L | S | T | S | X | X | G | A | Y | Q | N | L | L | T | L | N | V | S | Q | N | V | L | K | S | L | G | L | M | S | Q | L | T | T | N | L | K | S | L | T | D | L | D | L | S | H | N | S | F | V | S | M | P | E | K | C | S | W | P | A | T | L | R | F | L | N | L | S | S | T | K | X | X | R | K | M | T | P | C | L | P | S | S | L | T | V | L | D | L | S | E | N | D | L | M | V | F | X | X | X | X | X | Q | R | F | P | Q | L | I | T | L | I | L | T | G | N | R | F | K | K | L | P | Q | G | E | L | F | P | T | L | Q | T | L | L | I | Q | R | N | A | L | R | M | F | N | S | S | D | L | K | R | F | K | N | L | Q | Y | L | E | A | G | D | N | N | F | V | C | S | C | E | F | V | S | F | F | X | X | K | R | D | X | X | X | K | L | F | I | T | L | R | D | S | R | R | S | Y | V | C | D | T | P | F | T | L | R | G | D | S | I | D | S | V | R | L | S | V | F | E | C | Y | M | I | P | A | V | S | V | L | C | F | V | I | I | I | A | L | G | L | I | V | V | T | C | H | K | L | H | V | I | W | Y | L | Q | M | T | K | A | W | I | Q | A | K | R | K | X | L | Q | L | V | X | X | X | X | X | X | X | X | W | P | M | S | S | A | M | M | L | L | Y | L | T | V | N | M | T | L | S | G | S | R | K | S | L | F | Q | N | X | R | A | L | X | X | X | X | X | X | X | X | X | X | X | X | X | L | H | L | P | C | V | C | T | N | G | T | S | S | R | A | A | G | S | W | T | T | S | S | T | R | L | R | K | A | I | G | L | S | L | F | C | L | S | T | L | L | P | A | N | G | A | V | T | S | W | T | S | H | I | S | A | X | S | M | N | T | X | X | X | T | L | P | S | W | F | S | L | S | Q | S | R | R | R | P | F | P | N | V | S | A | S | Y | G | R | L | X | T | P | G | R | I | W | S | G | P | K | T | R | K | R | E | E | S | S | G | A | T | X | K | L | P | Y | K | K | M | X | X | X | X | X | X | X | X | X | X | X | X | X | X | X | X | X | X | X | X | X | X | X | X | X | X | X | - | - | - | - | - | - | - | - | - | - | - | - | - |
| CCTLR2\_2642\_BP | - | - | - | - | - | - | - | - | - | - | - | X | M | A | V | R | I | E | F | X | X | X | X | X | X | X | X | L | G | R | E | A | S | I | I | I | - | - | - | - | - | - | X | X | F | I | L | I | L | A | Q | G | F | X | X | X | X | X | X | Y | S | R | T | C | D | C | D | Q | Q | Y | F | C | N | C | S | S | N | H | L | Q | Q | V | P | K | V | P | X | X | N | A | L | G | L | D | L | S | F | N | Q | I | E | S | I | Y | M | S | D | L | S | P | Y | S | E | L | K | T | L | N | L | H | K | N | K | L | K | F | I | H | K | D | A | F | K | S | Q | H | K | L | E | V | L | D | L | S | L | N | N | L | K | N | L | S | S | S | W | F | Q | E | L | N | S | L | Q | Q | L | N | L | V | G | N | P | X | X | T | T | L | G | P | X | X | X | X | P | I | F | Q | S | L | L | X | X | X | L | R | M | L | R | F | G | S | P | S | L | R | E | V | Y | K | N | G | L | D | G | L | X | X | T | H | L | D | E | L | T | F | V | G | X | X | N | L | R | L | Y | E | N | G | S | L | K | A | A | R | P | I | G | L | V | S | L | S | L | Q | N | L | F | Q | N | N | P | E | L | V | S | K | V | L | Q | D | V | S | H | P | E | T | X | X | X | L | I | I | K | D | V | A | L | R | T | N | T | S | T | E | P | F | K | X | X | A | R | E | G | G | T | K | S | L | S | F | Q | N | S | T | T | T | D | E | A | M | T | S | L | L | M | V | M | D | G | S | S | L | S | Y | I | G | L | E | D | V | H | L | I | G | Q | G | W | W | Q | K | A | S | Y | T | H | Y | E | S | L | H | T | - | - | - | A | Y | I | R | N | L | D | I | Q | G | F | F | R | F | S | S | M | M | Q | L | G | F | L | L | V | H | L | H | K | V | S | V | X | X | X | N | G | T | V | F | V | I | P | R | E | T | T | P | L | L | K | N | L | E | Y | L | D | L | S | Q | N | L | L | S | D | L | T | I | E | P | T | L | Y | T | G | X | X | G | A | Y | Q | N | L | N | T | L | N | V | S | Q | N | V | I | K | S | L | G | L | M | S | R | L | A | T | K | L | K | S | L | T | Y | L | D | L | S | H | N | S | F | V | S | M | P | E | N | C | S | W | P | A | T | L | R | F | L | N | L | S | S | T | K | X | X | R | K | L | T | P | C | L | P | S | S | L | T | A | L | D | L | S | E | N | D | L | M | V | F | X | X | X | X | X | Q | R | F | P | Q | L | T | T | L | I | L | T | G | N | R | F | M | K | L | P | Q | G | E | L | F | P | R | L | Q | T | L | L | I | Q | R | N | A | L | R | M | F | N | G | N | D | L | R | R | F | K | T | L | Q | Y | L | E | A | S | N | N | N | F | V | C | S | C | E | F | V | S | F | F | X | X | R | H | D | X | X | X | D | H | F | I | T | I | R | D | N | R | H | D | Y | V | C | D | T | P | F | T | L | R | G | D | A | V | D | S | V | R | L | S | V | F | E | C | Y | M | I | P | A | V | L | V | L | C | S | L | I | I | I | V | L | G | L | I | V | F | T | C | Y | K | F | H | I | I | W | Y | L | H | M | T | K | A | W | I | Q | A | K | R | K | X | L | Q | L | V | X | X | X | X | X | X | X | X | W | L | K | S | S | A | M | T | L | S | Y | P | T | V | N | M | M | L | S | G | S | R | R | S | L | F | Q | S | W | R | A | L | X | X | X | X | X | X | X | X | X | X | X | X | X | H | H | L | P | C | V | C | T | N | G | T | S | S | R | A | A | G | S | W | T | T | S | L | T | Q | L | K | R | A | I | G | L | S | L | F | C | L | S | T | L | L | P | A | N | G | A | G | M | S | W | T | S | H | I | S | A | X | S | M | S | T | X | X | X | T | P | P | S | W | Y | F | L | S | Q | S | R | R | R | P | F | P | N | V | S | A | S | Y | G | R | L | X | T | P | G | R | I | W | S | G | P | K | M | R | K | R | E | V | N | S | G | A | I | X | E | L | P | Y | K | E | M | X | X | X | X | X | X | X | X | X | X | X | X | X | X | X | X | X | X | X | X | X | X | X | X | X | X | X | - | - | - | - | - | - | - | - | - | - | - | - | - |
| CBTLR2\_2642\_BP | - | - | - | - | - | - | - | - | - | - | - | X | X | X | X | X | M | K | V | X | X | X | X | X | X | - | - | X | X | P | L | G | L | C | I | C | - | - | - | F | - | S | L | T | L | T | L | T | L | T | L | T | S | X | X | X | X | X | X | R | P | T | C | N | D | C | D | E | D | H | V | C | N | C | R | A | K | N | L | H | G | V | P | I | V | P | X | X | D | V | L | Y | L | D | V | S | F | N | E | I | E | S | I | T | Q | R | D | L | T | C | Y | A | E | L | R | T | L | K | L | Q | K | N | K | L | R | T | I | H | K | E | A | F | H | S | Q | S | K | L | E | E | L | D | L | S | F | N | N | L | K | N | I | S | C | Q | W | F | S | N | L | R | S | L | K | H | L | N | I | L | G | N | Q | X | X | T | T | L | G | S | X | X | X | X | A | L | F | Q | F | V | E | N | P | A | L | R | T | L | Q | F | G | N | L | W | I | K | D | V | K | Q | N | L | L | R | N | I | X | X | R | Q | L | D | E | L | T | F | V | G | X | X | V | L | R | S | Y | E | N | G | S | F | Q | T | I | Q | P | I | R | A | V | S | V | S | L | S | R | L | F | Q | D | D | P | A | L | V | S | K | I | L | R | D | V | S | H | P | E | T | X | X | X | L | T | I | R | D | V | S | L | E | T | Q | E | L | L | E | P | L | K | X | X | V | R | E | G | G | T | R | S | L | T | F | Q | N | I | I | T | T | D | E | A | V | S | R | L | L | E | V | L | D | G | S | P | V | S | Y | I | G | L | E | D | I | R | L | I | G | Q | G | W | W | E | K | A | S | R | T | H | L | D | N | L | H | T | - | - | - | I | H | V | R | N | I | E | I | Q | G | F | F | K | F | S | S | M | L | Q | L | A | F | L | L | K | H | L | T | K | I | S | V | X | X | X | N | C | T | V | F | V | I | P | C | L | T | S | C | F | L | E | K | V | E | Y | L | D | L | S | Q | N | L | L | S | D | I | T | M | Q | E | S | L | C | N | G | X | X | S | K | M | R | N | I | N | T | L | N | V | S | H | N | S | L | K | S | L | Q | L | M | S | H | L | V | T | G | L | D | R | L | T | S | L | D | M | S | H | N | N | F | V | K | M | P | Q | S | C | S | W | P | A | S | L | R | F | M | N | L | S | T | T | K | X | X | H | R | V | T | P | C | L | P | L | S | L | T | A | L | D | L | S | Q | N | F | L | T | E | F | X | X | X | X | X | L | H | L | P | N | L | A | E | L | W | L | T | G | N | R | I | L | S | L | P | E | G | G | H | F | P | S | L | R | M | L | F | I | Q | S | N | T | L | N | M | F | N | K | S | D | L | M | A | F | Q | S | L | Q | V | L | E | A | G | H | N | N | F | F | C | S | C | D | F | V | G | F | F | X | X | K | G | S | X | X | X | D | H | L | I | T | L | G | D | G | H | R | S | Y | T | C | D | S | P | F | T | L | R | G | L | N | I | D | T | A | Q | P | S | V | F | E | C | Y | M | I | L | L | V | S | V | I | C | S | V | T | V | I | G | V | I | A | I | G | L | T | C | Y | K | F | H | I | L | W | Y | L | Q | M | M | T | A | W | L | K | A | K | S | K | X | H | P | C | K | X | X | X | X | X | X | X | X | A | A | L | L | F | D | T | M | L | S | F | R | T | A | S | T | M | R | S | G | W | R | R | S | S | C | Q | S | X | K | A | P | X | X | X | X | X | X | X | X | X | X | X | X | X | L | R | S | L | C | V | C | T | S | G | T | S | S | Q | A | A | G | S | P | T | T | S | S | S | P | S | K | A | A | I | G | P | S | L | S | C | R | R | T | S | X | R | A | S | G | A | D | T | S | X | T | F | R | I | F | G | S | S | T | S | A | X | X | X | I | R | P | S | W | S | C | W | S | P | S | P | R | R | R | F | P | S | A | S | A | N | C | A | R | X | X | T | P | G | R | T | S | S | G | L | R | T | K | K | S | E | R | N | S | G | T | I | S | E | L | H | X | X | X | X | X | X | X | X | X | X | X | X | X | X | X | X | X | X | X | X | X | X | X | X | X | X | X | X | X | X | X | - | - | - | - | - | - | - | - | - | - | - | - | - |
| TFTLR2\_2642\_BP | - | - | - | - | - | - | - | - | - | - | - | X | X | X | X | X | M | K | A | X | X | X | X | X | X | - | - | X | X | F | L | S | V | V | I | C | - | - | - | L | - | S | X | X | L | A | W | T | R | A | V | A | S | X | X | X | X | X | X | R | R | K | A | R | R | I | D | E | P | P | S | C | T | K | S | A | K | T | L | H | R | V | P | I | V | P | X | X | S | V | L | S | L | D | V | S | F | N | E | I | E | S | I | Y | Q | E | G | L | T | A | Y | T | E | L | R | T | L | K | L | R | N | N | K | L | S | M | I | H | Q | E | A | F | D | S | Q | H | K | L | E | E | L | D | L | S | Y | N | E | L | E | N | I | Y | S | V | W | F | S | H | L | R | S | L | K | H | L | N | I | L | G | N | Q | X | X | T | T | L | G | S | X | X | X | X | A | L | F | P | F | L | K | N | P | A | L | K | K | L | Q | F | G | N | T | F | I | K | D | V | K | Q | N | V | L | R | N | I | X | X | A | Q | L | D | E | L | T | F | V | G | X | X | N | L | R | S | Y | E | N | G | S | F | Q | M | A | Q | P | I | R | V | V | T | L | S | L | Q | G | L | F | Q | E | N | P | A | L | V | S | K | I | L | R | D | V | S | H | P | E | T | X | X | X | L | I | I | R | D | T | S | L | K | T | N | E | P | I | Q | T | F | K | X | X | I | R | Q | G | C | T | R | R | L | S | F | Q | N | C | S | T | T | D | E | G | V | T | L | I | L | K | V | L | D | G | S | P | V | S | Y | I | G | L | E | D | I | Y | L | I | G | R | G | W | W | Q | K | A | K | W | T | H | L | E | N | L | H | T | - | - | - | I | F | M | R | N | I | E | I | Q | G | F | I | R | F | S | S | M | L | Q | L | V | F | L | L | K | H | L | T | K | I | S | V | X | X | X | N | C | T | V | F | V | I | P | C | I | T | T | Y | F | L | R | H | V | E | Y | L | D | L | S | Q | N | L | L | S | D | I | T | M | Q | E | T | L | C | N | G | X | X | C | N | M | H | N | L | N | M | L | N | V | S | H | N | S | L | K | S | L | Q | L | I | A | S | L | V | S | H | L | Q | K | L | T | A | L | D | V | S | H | N | D | F | L | K | M | P | Q | V | C | D | W | P | A | S | L | R | F | L | N | L | S | A | T | K | X | X | H | K | L | T | P | C | I | P | L | S | L | T | A | L | D | L | S | Q | N | Y | L | T | A | F | X | X | X | X | X | L | H | L | P | K | L | M | E | L | W | L | T | G | N | R | F | I | S | L | P | E | G | G | Q | F | P | S | L | Q | M | L | F | I | Q | R | N | T | L | N | M | F | N | K | S | D | L | M | A | F | Q | S | L | Q | F | L | E | A | G | Q | N | N | F | V | C | S | C | E | F | V | E | F | F | X | X | K | G | H | X | X | X | D | H | L | I | T | L | R | D | G | H | H | S | Y | V | C | D | S | P | P | S | L | R | D | L | T | I | D | N | A | Q | L | S | V | F | K | C | H | M | I | L | S | V | S | V | V | C | S | V | T | V | V | V | L | I | A | A | V | I | A | C | Y | K | L | H | V | F | W | Y | L | Q | M | T | V | A | W | L | K | A | K | G | K | X | R | L | Y | A | X | X | X | X | X | X | X | X | R | A | L | V | Y | A | T | M | L | L | Y | H | T | V | S | T | M | Q | S | G | S | K | K | S | S | C | Q | S | X | K | A | L | X | X | X | X | X | X | X | X | X | X | X | X | X | L | R | L | L | C | V | C | T | R | E | T | S | F | Q | A | A | G | S | S | T | T | S | S | S | P | L | K | V | A | T | A | L | S | S | S | C | Q | R | T | L | X | R | A | S | G | A | A | T | S | W | T | S | H | I | S | G | S | W | T | R | T | X | X | X | T | R | P | S | X | C | C | W | S | R | S | P | T | R | R | F | L | S | V | S | A | N | S | A | K | X | X | T | P | G | R | T | S | S | G | L | R | T | K | K | G | E | E | N | S | G | T | T | S | E | L | Y | X | R | E | R | X | X | X | X | X | X | X | X | X | X | X | X | X | X | X | X | X | X | X | X | X | X | X | X | X | X | X | - | - | - | - | - | - | - | - | - | - | - | - | - |
| PHTLR2\_2642\_BP | - | - | - | - | - | - | - | - | - | - | - | M | T | A | I | R | M | K | V | X | X | X | X | X | X | - | - | X | X | S | L | S | I | F | L | S | - | - | - | F | - | S | X | X | L | T | W | I | H | T | Q | T | S | X | X | X | X | X | X | R | P | N | C | Y | E | C | D | E | D | H | F | C | N | C | S | A | K | N | L | H | R | V | P | I | V | P | X | X | N | V | L | S | L | D | V | S | F | N | E | I | E | S | I | T | Q | K | D | L | T | V | Y | T | E | L | R | T | L | K | L | Q | N | N | K | L | S | M | I | H | N | T | A | F | N | S | Q | S | K | L | E | E | L | D | L | S | F | N | K | L | E | N | I | S | S | L | W | F | S | K | L | R | S | L | K | H | L | N | I | L | G | N | Q | X | X | T | T | L | G | S | X | X | X | X | A | L | F | Q | F | A | N | N | P | A | L | R | T | L | K | F | G | N | P | L | I | K | D | V | K | R | N | M | L | G | K | I | X | X | R | Q | L | D | E | L | T | F | V | C | X | X | N | L | T | S | Y | E | N | G | S | F | Q | T | A | H | P | I | R | A | V | S | V | S | L | Q | E | L | F | Q | D | D | P | E | L | V | S | K | I | L | R | D | V | S | H | P | E | T | X | X | X | L | T | I | R | D | V | S | L | E | T | R | E | P | I | Q | A | F | K | X | X | V | R | K | G | C | T | R | R | L | T | I | Q | N | V | S | T | T | D | E | G | V | T | H | F | L | E | V | L | D | G | S | P | M | S | Y | I | G | L | E | D | I | H | L | R | G | R | G | W | W | Q | R | A | Q | W | S | H | Y | E | N | L | H | T | - | - | - | T | F | I | R | N | L | E | I | Q | G | F | F | T | F | S | S | M | I | Q | L | A | F | L | L | K | R | L | N | N | I | S | V | X | X | X | N | G | T | V | F | V | I | P | C | N | T | T | Y | F | L | R | K | V | E | Y | L | D | L | S | Q | N | L | L | S | D | I | T | M | Q | E | S | L | C | N | G | X | X | C | I | M | R | N | L | N | T | L | N | V | S | H | N | S | L | K | S | L | H | L | M | S | R | L | V | T | C | L | H | R | L | T | S | L | D | V | S | H | N | N | F | L | K | M | P | Q | I | C | N | W | P | A | S | L | R | F | M | N | L | S | A | T | K | X | X | H | R | I | T | P | C | L | P | L | S | L | T | V | L | D | L | S | Q | N | Y | L | T | E | F | X | X | X | X | X | H | H | L | P | N | L | M | E | L | W | L | T | G | N | R | F | I | S | L | P | E | G | G | R | F | P | S | L | R | I | L | L | I | Q | S | N | T | L | N | V | F | N | K | S | D | L | M | A | F | Q | S | L | Q | F | L | E | A | G | Q | N | N | F | V | C | S | C | D | F | V | E | F | F | X | X | K | G | H | X | X | X | D | Q | L | I | T | L | R | D | G | H | R | S | Y | V | C | D | S | P | F | T | L | R | G | R | T | V | D | N | A | Q | L | S | V | F | E | C | H | M | I | L | S | V | S | I | L | C | S | V | I | V | L | V | L | I | T | I | G | V | T | C | Y | K | L | H | V | L | W | Y | L | Q | M | T | M | A | W | L | K | A | K | G | K | X | Q | P | Y | T | X | X | X | X | X | X | X | X | K | M | L | F | F | A | T | M | L | S | Y | H | T | A | S | T | M | Q | S | G | S | K | K | S | X | C | R | S | X | K | A | L | X | X | X | X | X | X | X | X | X | X | X | X | X | L | R | L | L | C | V | C | T | C | E | T | S | F | Q | A | A | G | S | S | T | T | S | S | S | P | S | K | V | A | I | G | L | S | S | S | C | R | R | T | S | X | R | V | S | G | A | A | T | S | X | T | F | R | I | F | G | S | W | T | S | T | X | X | X | I | R | P | S | X | S | C | W | S | L | S | P | K | R | R | F | L | S | V | S | A | N | C | A | K | X | X | T | P | G | R | T | S | S | G | L | R | T | K | K | G | E | R | S | S | G | T | I | S | E | L | C | L | R | G | R | T | X | X | X | X | X | X | X | X | X | X | X | X | X | X | X | X | X | X | X | X | X | X | X | X | X | X | - | - | - | - | - | - | - | - | - | - | - | - | - |
| IPTLR2\_2642\_BP | - | - | - | - | - | - | - | - | - | - | - | X | X | X | X | X | M | K | L | X | X | X | X | X | X | - | - | X | X | S | L | S | V | V | I | C | - | - | - | L | - | S | X | X | L | A | W | T | R | A | Q | T | S | X | X | X | X | X | X | R | P | N | C | Y | E | C | D | E | D | H | F | C | N | C | S | A | K | T | L | H | R | V | P | I | V | P | X | X | S | V | L | S | L | D | V | S | F | N | E | I | E | S | V | T | K | K | D | L | A | A | Y | T | A | L | R | T | L | K | L | Q | E | N | E | L | S | A | I | H | K | D | A | F | H | S | Q | S | K | L | E | E | L | D | L | S | F | N | K | L | E | N | I | S | S | L | W | F | S | S | L | R | S | L | K | H | L | N | I | L | G | N | R | X | X | T | T | L | G | A | X | X | X | X | G | L | F | Q | F | V | E | D | P | A | L | R | T | L | R | F | G | N | P | S | I | E | D | V | R | R | N | M | L | N | K | I | X | X | R | Q | L | D | E | L | T | F | V | G | X | X | K | L | S | S | Y | E | S | G | S | F | K | T | A | Q | P | I | R | V | V | S | L | S | L | Q | G | L | F | Q | D | D | P | A | L | V | S | K | I | L | R | D | V | S | H | P | E | T | X | X | X | L | T | V | R | D | V | S | L | E | T | R | E | P | I | Q | S | F | K | X | X | V | R | E | G | C | T | R | R | L | T | F | Q | N | A | S | T | T | D | E | G | V | T | H | L | L | E | V | L | D | G | S | P | M | S | Y | L | G | L | E | D | I | H | L | V | G | T | G | S | W | E | K | A | R | W | T | H | F | E | N | L | H | T | - | - | - | L | F | L | R | N | V | E | I | Q | G | F | F | R | F | S | S | M | I | Q | L | A | F | L | L | K | H | L | T | K | I | S | V | X | X | X | N | A | T | V | F | V | I | P | C | S | T | T | Y | F | L | Q | K | V | E | Y | L | D | L | S | Q | N | L | L | S | D | I | T | I | Q | E | S | L | C | T | V | X | X | A | R | M | R | D | L | H | T | L | N | V | S | H | N | S | L | K | S | L | Q | L | V | S | R | L | V | T | R | L | H | R | L | T | S | L | D | V | S | H | N | D | F | L | K | M | P | E | S | C | S | W | P | A | S | L | R | F | M | N | L | S | A | T | K | X | X | H | R | I | T | P | C | L | P | V | S | L | T | V | L | D | L | S | Q | N | Y | L | T | E | F | X | X | X | X | X | Q | H | L | P | N | L | M | E | L | W | L | T | G | N | R | F | I | S | L | P | E | G | G | W | F | P | S | L | R | T | L | L | I | Q | S | N | T | L | N | M | F | N | K | S | D | L | M | A | F | Q | S | L | Q | V | L | E | A | G | R | N | N | F | V | C | S | C | D | F | V | E | F | F | X | X | T | G | R | X | X | X | D | H | L | I | T | L | R | D | G | H | R | S | Y | V | C | D | S | P | F | T | L | R | G | R | A | V | D | N | A | R | L | S | V | F | E | C | H | M | I | L | S | V | S | V | L | C | S | A | V | V | L | V | L | I | A | I | G | V | T | C | Y | K | L | H | V | L | W | Y | L | Q | M | T | L | A | W | L | R | A | K | S | K | X | R | P | Y | A | X | X | X | X | X | X | X | X | E | A | L | I | S | A | T | M | L | S | Y | R | T | A | S | T | T | R | S | G | S | K | K | S | X | C | R | S | X | R | V | R | X | X | X | X | X | X | X | X | X | X | X | X | X | L | R | S | L | C | V | F | T | S | E | T | S | F | P | A | A | G | S | S | T | T | S | S | S | P | X | K | V | A | I | G | L | S | S | S | C | R | R | T | S | X | R | A | S | G | A | A | T | S | W | T | S | R | I | S | G | S | S | T | S | T | X | X | X | I | R | P | S | X | S | C | W | S | R | S | P | R | R | R | F | P | S | V | S | A | N | C | A | K | S | X | T | P | P | R | T | S | S | G | L | T | T | K | K | S | E | R | C | S | G | R | T | S | E | L | R | S | R | G | S | T | X | X | X | X | X | X | X | X | X | X | X | X | X | X | X | X | X | X | X | X | X | X | X | X | X | X | - | - | - | - | - | - | - | - | - | - | - | - | - |
| OMTLR2\_2642\_BP | - | - | - | - | - | - | - | - | - | - | - | X | X | X | X | X | X | X | X | X | X | X | X | X | X | X | X | M | G | S | L | A | M | L | V | L | - | - | - | - | - | - | X | X | L | F | F | S | Q | G | Q | S | S | T | P | E | V | E | K | R | S | T | C | D | Q | C | D | E | R | L | F | C | N | C | S | S | R | N | Y | R | H | V | P | T | V | T | V | T | E | V | V | T | L | D | L | S | F | N | D | I | T | S | V | A | E | D | D | L | R | E | Y | T | W | L | R | T | L | D | L | H | S | N | R | I | E | S | I | H | E | W | A | F | H | L | L | Q | G | L | E | S | L | D | L | S | Y | N | Q | L | A | A | L | N | P | A | W | F | S | K | L | L | S | L | Q | H | L | N | L | L | G | S | C | X | X | R | T | L | G | P | G | G | T | L | S | L | F | R | S | L | T | X | X | X | L | R | F | L | R | F | G | N | P | A | L | E | E | V | R | R | G | D | L | A | G | V | X | X | R | Q | L | D | Q | L | E | V | Y | G | X | X | N | L | K | R | Y | D | P | G | S | L | G | D | L | W | P | L | G | V | V | T | L | R | L | R | G | P | F | Q | D | N | A | T | L | V | S | S | I | I | H | D | V | S | Y | S | E | T | X | X | X | L | V | V | A | D | V | L | L | S | E | K | T | S | V | A | P | F | R | X | X | T | N | R | R | R | V | R | S | I | M | F | Q | N | A | T | L | T | D | E | A | I | V | H | F | M | E | V | M | D | G | A | S | L | E | F | L | G | V | E | D | A | T | F | H | G | T | G | S | W | T | P | A | R | R | T | H | L | D | N | L | D | T | - | - | - | V | Y | F | R | N | I | E | V | M | N | V | Y | K | F | T | S | F | L | P | V | G | F | L | L | K | N | L | R | Q | I | S | I | X | X | X | N | C | K | V | Y | V | M | P | C | L | T | S | L | L | L | T | Q | M | V | Y | L | D | L | S | G | N | L | L | T | D | L | T | L | T | E | S | L | C | H | G | X | X | G | I | L | R | N | L | R | V | I | N | V | S | D | N | A | L | K | S | I | S | L | I | S | K | L | V | T | R | L | D | K | L | V | H | L | D | I | S | Q | N | A | Y | T | S | M | P | P | T | C | S | W | P | Q | I | L | T | H | L | N | L | S | R | A | K | X | X | R | R | V | T | P | C | L | P | P | T | L | E | I | L | D | L | G | H | N | D | L | T | A | F | X | X | X | X | S | V | A | L | P | A | L | R | E | L | H | L | S | G | N | K | L | M | S | L | P | P | G | W | L | F | P | S | L | E | V | L | L | I | Q | S | N | T | L | N | M | F | G | P | L | D | L | Q | L | Y | R | R | L | R | A | L | Q | A | G | Q | N | R | F | V | C | S | C | E | F | V | N | F | M | V | W | R | W | G | G | A | V | E | G | S | V | E | L | T | D | R | W | D | N | Y | L | C | D | S | P | L | P | L | Q | G | Q | R | V | D | R | V | Q | L | S | P | F | Q | C | H | R | I | L | L | V | S | S | L | C | G | C | M | L | A | S | G | I | L | F | V | I | L | L | W | K | I | H | A | V | W | Y | V | R | M | M | W | A | W | L | K | A | K | R | N | X | P | G | T | R | S | A | G | M | P | Q | G | X | X | P | T | S | S | A | M | T | R | S | C | R | T | A | S | K | T | Q | S | G | S | K | I | S | W | F | Q | S | W | R | I | P | X | X | X | X | X | X | X | X | X | X | X | X | X | L | R | S | P | S | A | S | T | R | E | T | S | C | Q | A | T | G | S | S | T | T | L | S | M | Q | W | N | A | V | D | A | P | S | S | S | C | Q | N | T | S | S | I | L | S | G | A | G | T | S | W | T | F | P | T | S | V | C | L | T | G | T | X | X | X | R | R | P | S | W | C | F | W | N | P | S | P | R | T | T | F | P | N | A | S | A | S | S | A | S | X | X | A | P | V | P | T | W | S | G | R | R | R | T | R | G | G | Q | N | S | G | T | T | S | V | W | P | X | G | A | E | M | S | X | X | X | X | X | X | X | X | X | X | X | X | X | X | X | X | X | X | X | X | X | X | X | X | X | - | - | - | - | - | - | - | - | - | - | - | - | - |
| STTLR2\_2642\_BP | - | - | - | - | - | - | - | - | - | - | - | X | X | X | X | X | X | X | X | X | X | X | X | X | X | X | X | M | E | S | L | A | M | L | V | L | - | - | - | - | - | - | X | X | L | F | F | S | Q | G | Q | S | S | T | P | E | V | E | K | R | S | T | C | D | R | C | D | E | R | L | F | C | D | C | S | S | R | N | Y | R | H | V | P | T | V | T | V | T | E | V | V | T | L | D | L | S | F | N | D | I | T | T | V | A | E | D | D | L | R | E | Y | T | W | L | R | T | L | D | L | R | S | N | R | I | Q | S | I | H | E | W | A | F | H | L | L | Q | G | L | E | S | L | D | L | S | Y | N | Q | L | A | A | L | N | P | V | W | F | S | K | L | L | S | L | Q | H | L | N | L | L | G | N | C | X | X | R | T | L | G | P | G | G | T | L | S | L | F | R | S | L | T | X | X | X | L | R | F | L | R | F | G | N | P | A | L | E | E | V | R | R | G | D | L | A | G | V | X | X | R | Q | L | D | Q | L | E | V | Y | G | X | X | N | L | K | R | Y | D | P | G | S | L | G | D | L | W | P | L | R | V | V | T | L | R | L | R | G | P | F | Q | D | N | L | T | L | V | S | S | I | F | H | D | V | S | Y | S | E | T | X | X | X | L | V | V | A | D | V | L | L | S | E | K | T | S | A | A | P | F | S | X | X | I | N | R | R | R | V | R | S | I | L | F | Q | N | A | T | L | T | D | E | A | M | I | H | V | L | E | V | M | D | G | A | S | L | E | S | F | G | L | E | D | A | T | F | Y | G | T | G | A | W | K | P | A | R | R | T | H | L | V | N | M | D | T | - | - | - | V | Y | F | R | N | I | E | V | M | E | V | F | K | F | T | S | F | L | N | L | G | F | L | L | K | Y | L | R | Q | I | S | I | X | X | X | N | C | K | V | Y | V | M | P | C | L | T | S | H | L | L | T | Q | L | E | Y | L | D | L | S | S | N | L | L | T | D | L | T | L | T | E | S | L | C | H | G | X | X | G | I | L | R | N | L | R | V | I | N | V | S | D | N | A | L | K | S | L | S | I | V | S | K | L | V | T | R | L | D | K | L | I | H | L | D | I | S | Q | N | A | Y | T | S | M | P | P | T | C | S | W | P | Q | T | L | T | H | L | N | I | S | W | A | K | X | X | Q | R | V | T | H | C | L | P | L | T | L | E | I | L | D | L | G | H | N | D | L | T | A | F | X | X | X | X | S | V | A | L | P | V | L | R | E | L | H | L | S | G | N | K | L | R | R | L | P | P | G | W | L | F | P | S | L | E | V | L | L | I | Q | S | N | T | L | N | M | F | G | P | M | D | L | Q | L | Y | R | K | L | R | A | L | Q | A | G | Q | N | R | F | V | C | S | C | E | F | V | Y | F | M | V | W | R | W | G | G | A | V | E | G | S | V | E | L | T | D | R | W | D | N | Y | L | C | D | S | P | L | P | L | Q | G | Q | R | V | D | R | V | R | L | S | P | F | Q | C | H | R | I | L | V | V | S | A | L | C | G | C | V | L | A | S | G | I | L | F | V | I | L | L | W | K | L | H | V | V | W | Y | V | R | M | M | W | A | W | L | K | A | K | R | N | X | P | G | T | R | G | A | G | M | P | Q | E | X | X | P | S | P | S | A | M | T | R | S | C | R | T | A | S | K | T | Q | S | G | S | K | N | S | W | F | Q | S | W | R | I | P | X | X | X | X | X | X | X | X | X | X | X | X | X | L | R | S | P | S | A | S | T | R | E | T | S | C | Q | A | T | G | S | S | T | T | L | S | M | Q | W | N | A | A | D | V | H | S | S | S | C | R | N | T | S | S | T | P | T | G | A | G | M | S | W | T | F | P | T | S | V | C | L | T | G | T | X | X | X | R | R | P | S | W | C | F | W | N | P | S | P | R | T | T | F | P | N | A | S | A | S | S | A | S | X | X | A | L | V | P | T | W | S | G | R | R | R | T | R | G | G | Q | N | S | G | A | T | S | V | W | P | X | G | G | E | M | S | X | X | X | X | X | X | X | X | X | X | X | X | X | X | X | X | X | X | X | X | X | X | X | X | X | - | - | - | - | - | - | - | - | - | - | - | - | - |
| TRTLR2\_2642\_BP | - | - | - | - | - | - | - | - | - | - | - | X | X | X | X | X | X | X | X | X | X | X | X | X | X | X | X | X | M | F | F | T | L | L | F | T | - | - | - | - | - | - | X | X | L | S | F | C | E | G | Q | G | S | I | V | K | V | X | X | R | P | S | C | D | R | C | D | H | S | F | F | C | N | C | S | F | A | G | L | T | C | V | P | V | V | T | X | X | Q | A | L | S | L | D | L | S | F | N | N | I | T | V | V | T | A | E | D | L | R | D | H | R | R | L | A | A | L | N | L | C | C | N | R | L | A | V | I | H | Q | S | A | F | E | P | L | W | N | L | E | D | L | N | L | S | N | N | Q | L | T | A | L | N | H | R | W | F | H | K | L | E | A | L | R | V | L | N | L | L | H | N | P | X | X | S | C | L | G | S | X | X | X | X | P | A | F | Q | R | L | L | X | X | X | L | R | R | L | R | F | G | G | P | A | L | E | E | L | K | R | G | D | L | A | G | I | X | X | T | H | L | E | E | L | T | V | H | A | X | X | N | L | R | R | Y | E | S | G | A | L | A | T | I | W | P | L | G | R | V | I | L | S | L | H | G | P | F | L | T | N | D | D | V | A | S | T | M | L | G | D | V | S | Y | P | E | T | X | X | X | M | I | L | R | D | L | N | L | T | W | I | Q | S | V | H | N | L | R | X | X | A | A | R | R | R | I | R | H | L | S | F | Q | N | L | S | L | S | D | M | A | T | V | E | F | L | V | V | F | D | G | V | P | L | T | H | I | S | V | E | G | V | T | L | K | G | V | G | R | W | E | K | A | N | K | T | E | H | K | G | I | D | E | - | - | - | I | F | V | R | D | V | E | V | L | D | I | Y | K | F | A | S | L | I | S | C | G | F | L | L | Q | Y | P | R | K | V | S | I | X | X | X | N | A | K | V | F | V | M | P | C | F | T | S | H | L | L | K | N | L | Q | Y | L | D | L | S | N | N | L | L | T | D | M | T | L | A | E | T | L | C | D | G | X | X | S | P | L | K | D | L | R | V | L | N | I | S | G | N | A | L | K | S | L | S | T | T | S | R | L | V | G | K | L | L | R | L | T | H | L | D | V | S | R | N | G | Y | S | S | M | P | L | G | C | S | W | P | S | S | L | R | Y | L | N | M | S | R | T | R | X | X | A | S | I | S | P | C | L | P | A | A | L | E | V | L | D | L | S | N | N | D | L | K | D | F | X | X | X | X | X | L | V | L | P | T | L | R | E | L | H | L | S | G | N | K | L | L | R | L | P | P | G | W | F | F | P | N | L | N | T | L | T | I | Q | S | N | S | L | S | M | F | G | P | S | E | L | R | T | Y | S | R | L | Q | S | L | Q | A | G | W | N | K | F | V | C | T | C | D | F | V | G | L | F | X | X | Q | S | G | V | X | X | V | G | S | V | Q | L | T | D | R | E | D | D | Y | I | C | D | A | P | L | R | L | Q | G | A | L | V | A | R | V | R | L | P | L | V | H | C | H | P | V | Q | V | V | S | A | S | C | V | V | A | L | L | A | T | A | A | L | G | T | L | L | W | H | V | H | A | F | W | Y | L | R | M | M | W | A | W | L | K | A | K | H | S | X | P | G | S | G | E | D | X | T | S | E | A | A | L | A | S | R | T | T | L | M | P | L | C | P | T | A | T | E | T | L | A | G | W | R | T | F | W | C | L | N | L | S | S | P | G | R | M | T | K | R | R | S | E | X | X | X | X | D | P | X | P | S | A | C | T | S | G | T | S | F | L | D | T | G | F | X | T | T | S | X | V | P | W | N | A | A | G | E | P | S | S | S | S | R | R | T | S | S | G | Q | T | G | A | A | T | S | W | T | S | H | T | F | G | F | L | M | V | L | X | X | E | N | Q | P | S | X | S | S | W | N | R | X | T | R | T | T | F | P | D | A | S | A | N | C | A | S | S | X | A | P | P | P | T | W | S | G | L | T | G | R | R | R | L | A | S | S | G | R | P | F | A | R | L | X | E | E | K | K | K | M | G | G | X | X | X | X | X | X | X | X | X | X | X | X | X | X | X | X | X | X | X | X | X | X | - | - | - | - | - | - | - | - | - | - | - | - | - |
| TOTLR2\_2642\_BP | - | - | - | - | - | - | - | - | - | - | - | X | X | X | X | X | M | R | E | L | T | N | C | H | F | T | F | F | L | C | I | C | L | L | L | L | - | - | - | - | - | - | X | X | L | S | P | C | W | G | Q | R | S | S | P | D | P | X | X | R | P | S | C | N | G | C | D | L | R | L | S | C | D | C | S | H | K | G | F | T | R | V | P | M | V | T | X | X | H | A | L | T | L | D | L | S | L | N | N | I | S | V | V | T | D | D | D | L | T | G | H | M | Q | L | R | A | L | S | L | H | G | N | X | X | A | M | I | H | Q | S | A | F | D | S | L | W | S | L | E | N | L | D | L | S | D | N | Q | L | T | A | L | N | P | K | W | F | S | E | L | G | A | L | Q | Q | L | N | L | L | N | N | P | Y | S | S | C | V | G | S | X | X | X | X | P | V | F | E | G | L | V | X | X | X | L | R | R | L | A | F | G | G | P | A | L | K | E | L | K | R | G | A | L | S | G | V | X | X | T | Q | L | D | E | L | T | V | H | A | X | X | N | L | T | R | Y | E | S | G | A | L | A | Y | V | W | P | L | G | H | V | S | L | S | L | H | G | P | F | L | T | N | T | A | L | A | S | A | V | L | R | D | V | S | Y | P | E | T | X | X | X | I | I | L | E | D | L | H | L | I | G | N | Q | S | V | Q | P | L | R | X | X | A | A | K | K | R | L | R | H | I | T | F | R | N | L | S | I | S | D | E | A | T | V | N | L | L | K | V | F | D | G | V | P | L | T | S | L | S | V | D | G | V | T | L | T | G | E | G | R | W | E | A | A | S | L | T | D | Q | K | S | I | D | E | - | - | - | F | F | L | R | N | A | V | V | L | D | V | F | K | F | V | S | F | L | Q | L | K | F | L | L | Q | Y | P | R | K | V | S | V | X | X | X | N | A | K | V | F | V | M | P | C | P | T | S | G | L | L | V | N | L | Q | Y | L | D | M | S | D | N | L | L | T | D | M | T | L | S | E | T | L | C | D | G | X | X | S | P | L | K | D | L | R | V | L | N | I | S | G | N | A | L | K | S | V | S | M | M | S | R | L | V | A | K | L | H | K | L | T | H | V | D | I | S | R | N | S | Y | T | S | M | P | Q | S | C | S | W | P | S | T | L | R | Y | L | N | I | S | G | A | K | X | X | T | S | I | T | S | C | L | P | A | S | L | E | V | L | D | L | S | S | N | D | L | R | D | F | X | X | X | X | X | V | A | L | P | A | L | R | E | L | H | L | S | G | N | K | F | Q | R | L | P | S | G | I | L | F | P | N | L | Q | T | L | T | I | Q | S | N | T | L | N | M | F | S | R | S | D | L | Q | S | Y | K | R | I | Q | N | L | Q | A | G | Q | N | K | F | V | C | S | C | D | F | V | A | F | L | X | X | Q | S | G | I | X | X | D | G | D | V | H | L | T | D | E | E | D | S | Y | I | C | D | S | P | L | Y | L | Q | G | K | S | V | G | Q | V | H | L | S | V | V | V | C | H | R | V | L | F | V | S | V | S | C | G | V | A | L | C | V | W | V | L | V | C | V | L | L | W | R | L | H | A | I | W | Y | L | K | M | M | W | A | W | L | R | A | K | H | S | X | Q | G | A | G | G | V | S | E | M | E | K | I | Q | N | H | C | Y | P | L | M | P | L | F | P | T | V | K | E | T | R | A | G | W | K | P | T | W | F | L | S | W | K | S | Q | G | R | L | M | K | V | L | R | I | T | E | L | P | G | R | X | P | C | V | S | T | S | G | T | S | F | L | D | S | G | L | W | T | T | S | X | V | P | X | S | A | V | G | G | P | S | S | S | S | P | R | I | L | S | S | L | I | G | V | A | T | S | W | T | S | P | T | S | G | F | L | M | G | M | P | V | E | S | Q | P | S | X | S | C | L | S | R | C | P | R | M | T | S | P | N | A | F | A | N | C | A | N | S | X | A | P | P | H | I | X | S | G | L | R | R | R | T | G | G | R | S | F | G | G | V | F | A | M | L | X | E | E | K | E | R | R | M | X | X | X | X | X | X | X | X | X | X | X | X | X | X | X | X | X | X | X | X | X | X | X | - | - | - | - | - | - | - | - | - | - | - | - | - |
| OFTLR2\_2642\_BP | - | - | - | - | - | - | - | - | - | - | - | X | X | X | X | X | M | G | Q | P | T | X | X | X | X | X | X | X | X | X | L | Y | F | I | L | L | - | - | - | - | - | - | X | X | V | S | L | C | R | G | Q | G | I | N | P | D | G | X | X | R | S | P | C | D | R | C | D | L | R | L | S | C | N | C | S | Y | G | G | F | T | H | V | P | T | V | T | X | X | R | A | L | T | L | D | L | S | F | N | N | I | T | V | L | T | V | D | D | L | T | G | H | M | R | L | R | A | L | S | L | H | G | N | R | L | A | V | I | H | P | S | A | F | D | S | L | W | S | L | E | K | L | D | L | S | D | N | Q | L | T | A | L | N | H | K | W | F | S | K | L | G | A | L | Q | Q | L | N | L | L | N | N | P | X | X | S | C | L | G | S | X | X | X | X | P | V | F | Q | S | L | V | X | X | X | L | R | R | L | G | F | G | G | P | A | L | E | E | M | K | R | G | D | L | S | G | V | X | X | T | Q | L | E | E | L | T | V | H | A | X | X | N | L | S | R | Y | E | S | G | T | L | A | D | I | W | P | L | G | C | V | T | L | S | L | H | G | P | F | X | T | N | T | A | L | A | S | A | V | L | A | D | V | S | Y | P | E | T | X | X | X | I | I | L | E | D | L | H | L | I | G | N | Q | S | V | Q | P | F | R | X | X | P | A | R | R | R | V | R | Y | I | T | F | H | N | L | S | V | S | D | E | A | V | V | D | L | L | E | V | L | D | G | A | P | L | T | S | L | S | M | D | G | V | T | L | T | G | E | G | R | W | E | R | A | S | W | T | D | H | K | S | F | D | E | - | - | - | F | F | I | R | N | I | V | A | L | D | V | F | K | F | T | S | F | L | Q | L | G | F | L | L | Q | Y | P | R | K | V | S | V | X | X | X | N | A | R | V | F | V | M | P | C | P | T | S | R | L | L | V | N | L | Q | Y | L | D | L | S | D | N | L | L | T | D | M | T | L | A | E | T | L | C | N | G | X | X | G | T | L | K | D | L | R | V | L | N | I | S | G | N | A | L | K | S | F | S | T | V | T | R | L | I | A | K | L | S | K | L | T | H | L | D | I | S | R | T | G | Y | I | S | M | P | Q | S | C | S | W | P | S | T | L | R | Y | L | N | I | S | R | A | K | X | X | T | T | I | T | P | C | L | P | A | T | L | E | V | L | D | L | S | N | N | D | L | K | D | F | X | X | X | X | X | L | I | L | P | A | L | R | E | L | H | L | S | G | N | K | I | L | R | L | P | V | G | G | L | F | P | N | L | Q | T | L | T | I | Q | A | N | T | L | N | M | F | S | R | S | D | L | Q | S | Y | R | R | L | Q | S | L | Q | A | G | H | N | K | F | V | C | S | C | G | F | V | T | F | L | X | X | Q | S | A | I | K | G | D | G | D | V | Q | L | V | D | G | E | E | S | Y | V | C | D | S | P | L | H | R | Q | G | E | P | V | G | Q | V | R | L | S | I | V | E | C | H | R | V | L | F | V | S | S | S | C | G | V | A | L | F | V | A | I | L | V | C | V | L | L | W | R | L | H | A | F | W | Y | L | K | M | T | W | A | W | L | K | A | K | R | S | X | P | G | N | G | G | D | T | E | I | E | R | V | Q | K | R | C | Y | P | L | T | P | L | C | P | T | A | R | E | M | L | A | G | W | K | T | S | W | Y | L | S | W | R | S | Q | G | R | T | M | K | T | L | R | M | P | E | A | L | G | L | X | P | C | V | S | T | N | G | T | S | F | L | D | T | G | L | W | T | T | S | X | A | L | W | S | A | A | G | G | P | S | S | S | S | Q | R | T | L | S | S | P | T | G | A | A | T | S | W | T | S | P | T | S | G | F | S | T | G | I | L | T | E | T | R | P | S | X | S | C | W | N | R | C | P | R | T | T | S | P | N | A | S | A | N | C | A | S | S | X | V | P | P | P | T | W | S | G | L | R | R | R | R | G | L | G | S | S | G | G | V | C | A | T | L | X | G | X | K | K | R | R | M | X | X | X | X | X | X | X | X | X | X | X | X | X | X | X | X | X | X | X | X | X | X | X | - | - | - | - | - | - | - | - | - | - | - | - | - |
| SMTLR2\_2642\_BP | - | - | - | - | - | - | - | - | - | - | - | X | X | X | X | X | M | G | Q | L | T | X | X | X | X | X | X | X | X | X | L | Y | V | A | V | L | - | - | - | - | - | - | X | X | L | S | L | C | W | G | Q | W | S | E | P | D | A | X | X | R | P | S | C | D | R | C | D | L | R | L | S | C | D | C | S | R | G | G | F | T | R | V | P | A | V | T | X | X | R | A | L | T | L | D | L | S | F | N | N | I | T | A | V | T | N | V | D | L | M | G | H | V | E | L | R | A | L | N | L | H | G | N | R | L | A | V | I | H | P | S | A | F | D | S | L | W | S | L | E | E | L | D | L | S | D | N | Q | L | T | A | L | N | H | D | W | F | G | E | L | G | A | L | L | Q | L | N | L | L | D | N | P | X | X | S | C | V | G | S | X | X | X | X | P | V | F | L | G | L | V | X | X | X | L | R | R | L | A | F | G | G | P | A | L | V | E | I | K | R | G | A | L | S | G | V | X | X | M | E | L | E | E | L | T | V | H | A | X | X | N | L | E | R | Y | E | S | G | A | L | A | Y | V | W | P | L | G | R | V | S | L | S | L | H | G | P | F | L | R | D | V | A | L | A | S | A | V | V | G | D | V | S | Y | P | E | T | X | X | X | V | T | L | E | D | L | H | L | I | G | N | E | S | V | Q | P | F | R | X | X | A | A | R | R | R | V | R | N | M | A | F | R | N | L | S | V | S | D | E | A | T | V | N | M | L | M | I | S | D | G | I | P | F | T | T | F | T | F | D | G | V | T | L | T | G | E | G | R | W | E | R | A | S | W | T | D | Q | R | S | I | D | E | - | - | - | F | F | I | R | N | A | V | V | L | D | V | F | K | F | V | S | F | L | Q | L | G | F | L | L | Q | Y | P | R | K | L | S | V | X | X | X | N | A | K | V | F | V | V | P | C | A | T | T | G | L | L | L | N | L | Q | Y | L | D | L | S | D | N | L | L | T | D | L | T | L | A | E | T | L | C | H | G | X | X | G | T | L | K | D | L | R | V | L | N | I | S | G | N | A | L | K | S | L | S | T | L | R | R | L | V | A | K | L | Y | K | L | T | H | L | D | I | S | R | N | R | Y | S | S | M | P | D | S | C | S | W | P | S | T | L | R | H | L | N | I | S | G | A | K | X | X | T | T | I | T | P | C | L | P | T | T | L | E | V | L | D | L | S | S | N | D | L | K | G | F | X | X | X | X | X | V | A | L | P | V | L | R | E | L | H | L | S | G | N | K | F | L | R | L | P | A | G | S | L | F | P | N | L | Q | T | L | S | A | Q | S | N | T | L | N | M | F | G | H | S | D | L | Q | S | Y | R | R | L | Q | S | L | Q | A | G | R | N | K | F | V | C | S | C | G | F | V | A | F | L | X | X | R | S | S | I | E | G | D | G | V | V | R | L | T | D | G | E | E | S | Y | I | C | D | S | P | L | Y | L | Q | G | E | P | V | G | R | V | R | L | S | V | V | V | C | H | R | V | L | F | V | S | V | S | C | G | V | A | L | L | V | G | I | L | V | S | V | L | L | W | R | L | H | A | L | W | Y | L | R | M | T | W | A | W | L | R | A | K | H | S | X | L | G | A | G | G | S | S | E | M | X | P | V | Q | K | H | Y | C | P | L | M | P | L | F | P | T | V | K | K | T | L | A | G | W | K | P | S | W | Y | L | S | W | R | S | Q | G | R | P | M | R | T | M | X | T | T | E | L | P | G | R | X | P | C | V | S | T | S | G | T | S | F | P | D | T | G | F | W | T | T | S | X | A | P | W | S | A | A | G | G | P | S | S | S | S | P | R | T | L | C | S | P | T | G | V | A | M | S | W | T | S | P | T | S | G | F | S | M | A | T | L | A | K | S | R | P | S | S | S | C | W | S | R | C | P | R | T | T | S | P | T | A | S | V | N | C | A | N | S | X | A | P | P | R | T | W | S | G | L | R | R | R | R | G | S | R | S | S | G | R | D | S | A | T | L | X | Q | E | R | M | M | X | X | X | X | X | X | X | X | X | X | X | X | X | X | X | X | X | X | X | X | X | X | X | X | X | - | - | - | - | - | - | - | - | - | - | - | - | - |
| conservation |  |  |  |  |  |  |  |  |  |  |  |  |  |  |  |  |  |  |  |  |  |  |  |  |  |  |  |  |  |  |  |  |  |  |  |  |  |  |  |  |  |  |  |  |  |  |  |  |  |  |  |  |  |  |  |  |  |  |  |  |  |  |  |  |  |  |  |  |  |  |  |  |  |  |  |  |  |  |  |  |  |  |  |  |  |  |  |  |  |  |  |  |  |  |  |  |  |  |  |  |  |  |  |  |  |  |  |  |  |  |  |  |  |  |  |  |  |  |  |  |  |  |  |  |  |  |  |  |  |  |  |  |  |  |  |  |  |  |  |  |  |  |  |  |  |  |  |  |  |  |  |  |  |  |  |  |  |  |  |  |  |  |  |  |  |  |  |  |  |  |  |  |  |  |  |  |  |  |  |  |  |  |  |  |  |  |  |  |  |  |  |  |  |  |  |  |  |  |  |  |  |  |  |  |  |  |  |  |  |  |  |  |  |  |  |  |  |  |  |  |  |  |  |  |  |  |  |  |  |  |  |  |  |  |  |  |  |  |  |  |  |  |  |  |  |  |  |  |  |  |  |  |  |  |  |  |  |  |  |  |  |  |  |  |  |  |  |  |  |  |  |  |  |  |  |  |  |  |  |  |  |  |  |  |  |  |  |  |  |  |  |  |  |  |  |  |  |  |  |  |  |  |  |  |  |  |  |  |  |  |  |  |  |  |  |  |  |  |  |  |  |  |  |  |  |  |  |  |  |  |  |  |  |  |  |  |  |  |  |  |  |  |  |  |  |  |  |  |  |  |  |  |  |  |  |  |  |  |  |  |  |  |  |  |  |  |  |  |  |  |  |  |  |  |  |  |  |  |  |  |  |  |  |  |  |  |  |  |  |  |  |  |  |  |  |  |  |  |  |  |  |  |  |  |  |  |  |  |  |  |  |  |  |  |  |  |  |  |  |  |  |  |  |  |  |  |  |  |  |  |  |  |  |  |  |  |  |  |  |  |  |  |  |  |  |  |  |  |  |  |  |  |  |  |  |  |  |  |  |  |  |  |  |  |  |  |  |  |  |  |  |  |  |  |  |  |  |  |  |  |  |  |  |  |  |  |  |  |  |  |  |  |  |  |  |  |  |  |  |  |  |  |  |  |  |  |  |  |  |  |  |  |  |  |  |  |  |  |  |  |  |  |  |  |  |  |  |  |  |  |  |  |  |  |  |  |  |  |  |  |  |  |  |  |  |  |  |  |  |  |  |  |  |  |  |  |  |  |  |  |  |  |  |  |  |  |  |  |  |  |  |  |  |  |  |  |  |  |  |  |  |  |  |  |  |  |  |  |  |  |  |  |  |  |  |  |  |  |  |  |  |  |  |  |  |  |  |  |  |  |  |  |  |  |  |  |  |  |  |  |  |  |  |  |  |  |  |  |  |  |  |  |  |  |  |  |  |  |  |  |  |  |  |  |  |  |  |  |  |  |  |  |  |  |  |  |  |  |  |  |  |  |  |  |  |  |  |  |  |  |  |  |  |  |  |  |  |  |  |  |  |  |  |  |  |  |  |  |  |  |  |  |  |  |  |  |  |  |  |  |  |  |  |  |  |  |  |  |  |  |  |  |  |  |  |  |  |  |  |  |  |  |  |  |  |  |  |  |  |  |  |  |  |  |  |  |  |  |  |  |  |  |  |  |  |  |  |  |  |  |  |  |  |  |  |  |  |  |  |  |  |  |  |  |  |  |  |  |  |  |  |  |  |  |  |  |  |  |  |  |  |  |  |  |  |  |  |  |  |  |  |  |  |  |  |  |  |  |  |  |  |  |  |  |  |  |  |  |  |  |  |  |  |  |  |  |  |  |  |  |  |  |  |  |  |  |  |  |  |  |  |  |  |  |  |  |  |  |  |  |  |  |  |  |  |  |  |  |  |  |  |  |  |  |  |  |  |  |  |  |  |  |  |  |  |  |  |  |  |  |  |  |  |  |  |  |  |  |  |  |  |  |  |  |  |  |  |  |  |  |  |  |  |  |  |  |  |  |  |  |  |  |  |  |  |  |  |  |  |  |  |  |  |
|  |  |  |  |  |  |  |  |  |  |  |  |  |  |  |  |  |  |  |  |  |  |  |  |  |  |  |  |  |  |  |  |  |  |  |  |  |  |  |  |  |  |  |  |  |  |  |  |  |  |  |  |  |  |  |  |  |  |  |  |  |  |  |  |  |  |  |  |  |  |  |  |  |  |  |  |  |  |  |  |  |  |  |  |  |  |  |  |  |  |  |  |  |  |  |  |  |  |  |  |  |  |  |  |  |  |  |  |  |  |  |  |  |  |  |  |  |  |  |  |  |  |  |  |  |  |  |  |  |  |  |  |  |  |  |  |  |  |  |  |  |  |  |  |  |  |  |  |  |  |  |  |  |  |  |  |  |  |  |  |  |  |  |  |  |  |  |  |  |  |  |  |  |  |  |  |  |  |  |  |  |  |  |  |  |  |  |  |  |  |  |  |  |  |  |  |  |  |  |  |  |  |  |  |  |  |  |  |  |  |  |  |  |  |  |  |  |  |  |  |  |  |  |  |  |  |  |  |  |  |  |  |  |  |  |  |  |  |  |  |  |  |  |  |  |  |  |  |  |  |  |  |  |  |  |  |  |  |  |  |  |  |  |  |  |  |  |  |  |  |  |  |  |  |  |  |  |  |  |  |  |  |  |  |  |  |  |  |  |  |  |  |  |  |  |  |  |  |  |  |  |  |  |  |  |  |  |  |  |  |  |  |  |  |  |  |  |  |  |  |  |  |  |  |  |  |  |  |  |  |  |  |  |  |  |  |  |  |  |  |  |  |  |  |  |  |  |  |  |  |  |  |  |  |  |  |  |  |  |  |  |  |  |  |  |  |  |  |  |  |  |  |  |  |  |  |  |  |  |  |  |  |  |  |  |  |  |  |  |  |  |  |  |  |  |  |  |  |  |  |  |  |  |  |  |  |  |  |  |  |  |  |  |  |  |  |  |  |  |  |  |  |  |  |  |  |  |  |  |  |  |  |  |  |  |  |  |  |  |  |  |  |  |  |  |  |  |  |  |  |  |  |  |  |  |  |  |  |  |  |  |  |  |  |  |  |  |  |  |  |  |  |  |  |  |  |  |  |  |  |  |  |  |  |  |  |  |  |  |  |  |  |  |  |  |  |  |  |  |  |  |  |  |  |  |  |  |  |  |  |  |  |  |  |  |  |  |  |  |  |  |  |  |  |  |  |  |  |  |  |  |  |  |  |  |  |  |  |  |  |  |  |  |  |  |  |  |  |  |  |  |  |  |  |  |  |  |  |  |  |  |  |  |  |  |  |  |  |  |  |  |  |  |  |  |  |  |  |  |  |  |  |  |  |  |  |  |  |  |  |  |  |  |  |  |  |  |  |  |  |  |  |  |  |  |  |  |  |  |  |  |  |  |  |  |  |  |  |  |  |  |  |  |  |  |  |  |  |  |  |  |  |  |  |  |  |  |  |  |  |  |  |  |  |  |  |  |  |  |  |  |  |  |  |  |  |  |  |  |  |  |  |  |  |  |  |  |  |  |  |  |  |  |  |  |  |  |  |  |  |  |  |  |  |  |  |  |  |  |  |  |  |  |  |  |  |  |  |  |  |  |  |  |  |  |  |  |  |  |  |  |  |  |  |  |  |  |  |  |  |  |  |  |  |  |  |  |  |  |  |  |  |  |  |  |  |  |  |  |  |  |  |  |  |  |  |  |  |  |  |  |  |  |  |  |  |  |  |  |  |  |  |  |  |  |  |  |  |  |  |  |  |  |  |  |  |  |  |  |  |  |  |  |  |  |  |  |  |  |  |  |  |  |  |  |  |  |  |  |  |  |  |  |  |  |  |  |  |  |  |  |  |  |  |  |  |  |  |  |  |  |  |  |  |  |  |  |  |  |  |  |  |  |  |  |  |  |  |  |  |  |  |  |  |  |  |  |  |  |  |  |  |  |  |  |  |  |  |  |  |  |  |  |  |  |  |  |  |  |  |  |  |  |  |  |  |  |  |  |  |  |  |  |  |  |  |  |  |  |  |  |  |  |  |  |  |  |  |  |  |  |  |  |  |  |  |  |  |  |  |  |  |  |  |
|  |  |  |  |  |  |  |  |  |  |  |  |  |  |  |  |  |  |  |  |  |  |  |  |  |  |  |  |  |  |  |  |  |  |  |  |  |  |  |  |  |  |  |  |  |  |  |  |  |  |  |  |  |  |  |  |  |  |  |  |  |  |  |  |  |  |  |  |  |  |  |  |  |  |  |  |  |  |  |  |  |  |  |  |  |  |  |  |  |  |  |  |  |  |  |  |  |  |  |  |  |  |  |  |  |  |  |  |  |  |  |  |  |  |  |  |  |  |  |  |  |  |  |  |  |  |  |  |  |  |  |  |  |  |  |  |  |  |  |  |  |  |  |  |  |  |  |  |  |  |  |  |  |  |  |  |  |  |  |  |  |  |  |  |  |  |  |  |  |  |  |  |  |  |  |  |  |  |  |  |  |  |  |  |  |  |  |  |  |  |  |  |  |  |  |  |  |  |  |  |  |  |  |  |  |  |  |  |  |  |  |  |  |  |  |  |  |  |  |  |  |  |  |  |  |  |  |  |  |  |  |  |  |  |  |  |  |  |  |  |  |  |  |  |  |  |  |  |  |  |  |  |  |  |  |  |  |  |  |  |  |  |  |  |  |  |  |  |  |  |  |  |  |  |  |  |  |  |  |  |  |  |  |  |  |  |  |  |  |  |  |  |  |  |  |  |  |  |  |  |  |  |  |  |  |  |  |  |  |  |  |  |  |  |  |  |  |  |  |  |  |  |  |  |  |  |  |  |  |  |  |  |  |  |  |  |  |  |  |  |  |  |  |  |  |  |  |  |  |  |  |  |  |  |  |  |  |  |  |  |  |  |  |  |  |  |  |  |  |  |  |  |  |  |  |  |  |  |  |  |  |  |  |  |  |  |  |  |  |  |  |  |  |  |  |  |  |  |  |  |  |  |  |  |  |  |  |  |  |  |  |  |  |  |  |  |  |  |  |  |  |  |  |  |  |  |  |  |  |  |  |  |  |  |  |  |  |  |  |  |  |  |  |  |  |  |  |  |  |  |  |  |  |  |  |  |  |  |  |  |  |  |  |  |  |  |  |  |  |  |  |  |  |  |  |  |  |  |  |  |  |  |  |  |  |  |  |  |  |  |  |  |  |  |  |  |  |  |  |  |  |  |  |  |  |  |  |  |  |  |  |  |  |  |  |  |  |  |  |  |  |  |  |  |  |  |  |  |  |  |  |  |  |  |  |  |  |  |  |  |  |  |  |  |  |  |  |  |  |  |  |  |  |  |  |  |  |  |  |  |  |  |  |  |  |  |  |  |  |  |  |  |  |  |  |  |  |  |  |  |  |  |  |  |  |  |  |  |  |  |  |  |  |  |  |  |  |  |  |  |  |  |  |  |  |  |  |  |  |  |  |  |  |  |  |  |  |  |  |  |  |  |  |  |  |  |  |  |  |  |  |  |  |  |  |  |  |  |  |  |  |  |  |  |  |  |  |  |  |  |  |  |  |  |  |  |  |  |  |  |  |  |  |  |  |  |  |  |  |  |  |  |  |  |  |  |  |  |  |  |  |  |  |  |  |  |  |  |  |  |  |  |  |  |  |  |  |  |  |  |  |  |  |  |  |  |  |  |  |  |  |  |  |  |  |  |  |  |  |  |  |  |  |  |  |  |  |  |  |  |  |  |  |  |  |  |  |  |  |  |  |  |  |  |  |  |  |  |  |  |  |  |  |  |  |  |  |  |  |  |  |  |  |  |  |  |  |  |  |  |  |  |  |  |  |  |  |  |  |  |  |  |  |  |  |  |  |  |  |  |  |  |  |  |  |  |  |  |  |  |  |  |  |  |  |  |  |  |  |  |  |  |  |  |  |  |  |  |  |  |  |  |  |  |  |  |  |  |  |  |  |  |  |  |  |  |  |  |  |  |  |  |  |  |  |  |  |  |  |  |  |  |  |  |  |  |  |  |  |  |  |  |  |  |  |  |  |  |  |  |  |  |  |  |  |  |  |  |  |  |  |  |  |  |  |  |  |  |  |  |  |  |  |  |  |  |  |  |  |  |  |  |  |  |  |  |  |  |  |  |  |  |  |  |
|  |  |  |  |  |  |  |  |  |  |  |  |  |  |  |  |  |  |  |  |  |  |  |  |  |  |  |  |  |  |  |  |  |  |  |  |  |  |  |  |  |  |  |  |  |  |  |  |  |  |  |  |  |  |  |  |  |  |  |  |  |  |  |  |  |  |  |  |  |  |  |  |  |  |  |  |  |  |  |  |  |  |  |  |  |  |  |  |  |  |  |  |  |  |  |  |  |  |  |  |  |  |  |  |  |  |  |  |  |  |  |  |  |  |  |  |  |  |  |  |  |  |  |  |  |  |  |  |  |  |  |  |  |  |  |  |  |  |  |  |  |  |  |  |  |  |  |  |  |  |  |  |  |  |  |  |  |  |  |  |  |  |  |  |  |  |  |  |  |  |  |  |  |  |  |  |  |  |  |  |  |  |  |  |  |  |  |  |  |  |  |  |  |  |  |  |  |  |  |  |  |  |  |  |  |  |  |  |  |  |  |  |  |  |  |  |  |  |  |  |  |  |  |  |  |  |  |  |  |  |  |  |  |  |  |  |  |  |  |  |  |  |  |  |  |  |  |  |  |  |  |  |  |  |  |  |  |  |  |  |  |  |  |  |  |  |  |  |  |  |  |  |  |  |  |  |  |  |  |  |  |  |  |  |  |  |  |  |  |  |  |  |  |  |  |  |  |  |  |  |  |  |  |  |  |  |  |  |  |  |  |  |  |  |  |  |  |  |  |  |  |  |  |  |  |  |  |  |  |  |  |  |  |  |  |  |  |  |  |  |  |  |  |  |  |  |  |  |  |  |  |  |  |  |  |  |  |  |  |  |  |  |  |  |  |  |  |  |  |  |  |  |  |  |  |  |  |  |  |  |  |  |  |  |  |  |  |  |  |  |  |  |  |  |  |  |  |  |  |  |  |  |  |  |  |  |  |  |  |  |  |  |  |  |  |  |  |  |  |  |  |  |  |  |  |  |  |  |  |  |  |  |  |  |  |  |  |  |  |  |  |  |  |  |  |  |  |  |  |  |  |  |  |  |  |  |  |  |  |  |  |  |  |  |  |  |  |  |  |  |  |  |  |  |  |  |  |  |  |  |  |  |  |  |  |  |  |  |  |  |  |  |  |  |  |  |  |  |  |  |  |  |  |  |  |  |  |  |  |  |  |  |  |  |  |  |  |  |  |  |  |  |  |  |  |  |  |  |  |  |  |  |  |  |  |  |  |  |  |  |  |  |  |  |  |  |  |  |  |  |  |  |  |  |  |  |  |  |  |  |  |  |  |  |  |  |  |  |  |  |  |  |  |  |  |  |  |  |  |  |  |  |  |  |  |  |  |  |  |  |  |  |  |  |  |  |  |  |  |  |  |  |  |  |  |  |  |  |  |  |  |  |  |  |  |  |  |  |  |  |  |  |  |  |  |  |  |  |  |  |  |  |  |  |  |  |  |  |  |  |  |  |  |  |  |  |  |  |  |  |  |  |  |  |  |  |  |  |  |  |  |  |  |  |  |  |  |  |  |  |  |  |  |  |  |  |  |  |  |  |  |  |  |  |  |  |  |  |  |  |  |  |  |  |  |  |  |  |  |  |  |  |  |  |  |  |  |  |  |  |  |  |  |  |  |  |  |  |  |  |  |  |  |  |  |  |  |  |  |  |  |  |  |  |  |  |  |  |  |  |  |  |  |  |  |  |  |  |  |  |  |  |  |  |  |  |  |  |  |  |  |  |  |  |  |  |  |  |  |  |  |  |  |  |  |  |  |  |  |  |  |  |  |  |  |  |  |  |  |  |  |  |  |  |  |  |  |  |  |  |  |  |  |  |  |  |  |  |  |  |  |  |  |  |  |  |  |  |  |  |  |  |  |  |  |  |  |  |  |  |  |  |  |  |  |  |  |  |  |  |  |  |  |  |  |  |  |  |  |  |  |  |  |  |  |  |  |  |  |  |  |  |  |  |  |  |  |  |  |  |  |  |  |  |  |  |  |  |  |  |  |  |  |  |  |  |  |  |  |  |  |  |  |  |  |  |  |  |  |  |  |  |  |  |  |  |  |  |  |  |  |  |  |  |
|  |  |  |  |  |  |  |  |  |  |  |  |  |  |  |  |  |  |  |  |  |  |  |  |  |  |  |  |  |  |  |  |  |  |  |  |  |  |  |  |  |  |  |  |  |  |  |  |  |  |  |  |  |  |  |  |  |  |  |  |  |  |  |  |  |  |  |  |  |  |  |  |  |  |  |  |  |  |  |  |  |  |  |  |  |  |  |  |  |  |  |  |  |  |  |  |  |  |  |  |  |  |  |  |  |  |  |  |  |  |  |  |  |  |  |  |  |  |  |  |  |  |  |  |  |  |  |  |  |  |  |  |  |  |  |  |  |  |  |  |  |  |  |  |  |  |  |  |  |  |  |  |  |  |  |  |  |  |  |  |  |  |  |  |  |  |  |  |  |  |  |  |  |  |  |  |  |  |  |  |  |  |  |  |  |  |  |  |  |  |  |  |  |  |  |  |  |  |  |  |  |  |  |  |  |  |  |  |  |  |  |  |  |  |  |  |  |  |  |  |  |  |  |  |  |  |  |  |  |  |  |  |  |  |  |  |  |  |  |  |  |  |  |  |  |  |  |  |  |  |  |  |  |  |  |  |  |  |  |  |  |  |  |  |  |  |  |  |  |  |  |  |  |  |  |  |  |  |  |  |  |  |  |  |  |  |  |  |  |  |  |  |  |  |  |  |  |  |  |  |  |  |  |  |  |  |  |  |  |  |  |  |  |  |  |  |  |  |  |  |  |  |  |  |  |  |  |  |  |  |  |  |  |  |  |  |  |  |  |  |  |  |  |  |  |  |  |  |  |  |  |  |  |  |  |  |  |  |  |  |  |  |  |  |  |  |  |  |  |  |  |  |  |  |  |  |  |  |  |  |  |  |  |  |  |  |  |  |  |  |  |  |  |  |  |  |  |  |  |  |  |  |  |  |  |  |  |  |  |  |  |  |  |  |  |  |  |  |  |  |  |  |  |  |  |  |  |  |  |  |  |  |  |  |  |  |  |  |  |  |  |  |  |  |  |  |  |  |  |  |  |  |  |  |  |  |  |  |  |  |  |  |  |  |  |  |  |  |  |  |  |  |  |  |  |  |  |  |  |  |  |  |  |  |  |  |  |  |  |  |  |  |  |  |  |  |  |  |  |  |  |  |  |  |  |  |  |  |  |  |  |  |  |  |  |  |  |  |  |  |  |  |  |  |  |  |  |  |  |  |  |  |  |  |  |  |  |  |  |  |  |  |  |  |  |  |  |  |  |  |  |  |  |  |  |  |  |  |  |  |  |  |  |  |  |  |  |  |  |  |  |  |  |  |  |  |  |  |  |  |  |  |  |  |  |  |  |  |  |  |  |  |  |  |  |  |  |  |  |  |  |  |  |  |  |  |  |  |  |  |  |  |  |  |  |  |  |  |  |  |  |  |  |  |  |  |  |  |  |  |  |  |  |  |  |  |  |  |  |  |  |  |  |  |  |  |  |  |  |  |  |  |  |  |  |  |  |  |  |  |  |  |  |  |  |  |  |  |  |  |  |  |  |  |  |  |  |  |  |  |  |  |  |  |  |  |  |  |  |  |  |  |  |  |  |  |  |  |  |  |  |  |  |  |  |  |  |  |  |  |  |  |  |  |  |  |  |  |  |  |  |  |  |  |  |  |  |  |  |  |  |  |  |  |  |  |  |  |  |  |  |  |  |  |  |  |  |  |  |  |  |  |  |  |  |  |  |  |  |  |  |  |  |  |  |  |  |  |  |  |  |  |  |  |  |  |  |  |  |  |  |  |  |  |  |  |  |  |  |  |  |  |  |  |  |  |  |  |  |  |  |  |  |  |  |  |  |  |  |  |  |  |  |  |  |  |  |  |  |  |  |  |  |  |  |  |  |  |  |  |  |  |  |  |  |  |  |  |  |  |  |  |  |  |  |  |  |  |  |  |  |  |  |  |  |  |  |  |  |  |  |  |  |  |  |  |  |  |  |  |  |  |  |  |  |  |  |  |  |  |  |  |  |  |  |  |  |  |  |  |  |  |  |  |  |  |  |  |  |  |  |  |  |  |  |  |  |  |  |  |  |  |  |  |
|  |  |  |  |  |  |  |  |  |  |  |  |  |  |  |  |  |  |  |  |  |  |  |  |  |  |  |  |  |  |  |  |  |  |  |  |  |  |  |  |  |  |  |  |  |  |  |  |  |  |  |  |  |  |  |  |  |  |  |  |  |  |  |  |  |  |  |  |  |  |  |  |  |  |  |  |  |  |  |  |  |  |  |  |  |  |  |  |  |  |  |  |  |  |  |  |  |  |  |  |  |  |  |  |  |  |  |  |  |  |  |  |  |  |  |  |  |  |  |  |  |  |  |  |  |  |  |  |  |  |  |  |  |  |  |  |  |  |  |  |  |  |  |  |  |  |  |  |  |  |  |  |  |  |  |  |  |  |  |  |  |  |  |  |  |  |  |  |  |  |  |  |  |  |  |  |  |  |  |  |  |  |  |  |  |  |  |  |  |  |  |  |  |  |  |  |  |  |  |  |  |  |  |  |  |  |  |  |  |  |  |  |  |  |  |  |  |  |  |  |  |  |  |  |  |  |  |  |  |  |  |  |  |  |  |  |  |  |  |  |  |  |  |  |  |  |  |  |  |  |  |  |  |  |  |  |  |  |  |  |  |  |  |  |  |  |  |  |  |  |  |  |  |  |  |  |  |  |  |  |  |  |  |  |  |  |  |  |  |  |  |  |  |  |  |  |  |  |  |  |  |  |  |  |  |  |  |  |  |  |  |  |  |  |  |  |  |  |  |  |  |  |  |  |  |  |  |  |  |  |  |  |  |  |  |  |  |  |  |  |  |  |  |  |  |  |  |  |  |  |  |  |  |  |  |  |  |  |  |  |  |  |  |  |  |  |  |  |  |  |  |  |  |  |  |  |  |  |  |  |  |  |  |  |  |  |  |  |  |  |  |  |  |  |  |  |  |  |  |  |  |  |  |  |  |  |  |  |  |  |  |  |  |  |  |  |  |  |  |  |  |  |  |  |  |  |  |  |  |  |  |  |  |  |  |  |  |  |  |  |  |  |  |  |  |  |  |  |  |  |  |  |  |  |  |  |  |  |  |  |  |  |  |  |  |  |  |  |  |  |  |  |  |  |  |  |  |  |  |  |  |  |  |  |  |  |  |  |  |  |  |  |  |  |  |  |  |  |  |  |  |  |  |  |  |  |  |  |  |  |  |  |  |  |  |  |  |  |  |  |  |  |  |  |  |  |  |  |  |  |  |  |  |  |  |  |  |  |  |  |  |  |  |  |  |  |  |  |  |  |  |  |  |  |  |  |  |  |  |  |  |  |  |  |  |  |  |  |  |  |  |  |  |  |  |  |  |  |  |  |  |  |  |  |  |  |  |  |  |  |  |  |  |  |  |  |  |  |  |  |  |  |  |  |  |  |  |  |  |  |  |  |  |  |  |  |  |  |  |  |  |  |  |  |  |  |  |  |  |  |  |  |  |  |  |  |  |  |  |  |  |  |  |  |  |  |  |  |  |  |  |  |  |  |  |  |  |  |  |  |  |  |  |  |  |  |  |  |  |  |  |  |  |  |  |  |  |  |  |  |  |  |  |  |  |  |  |  |  |  |  |  |  |  |  |  |  |  |  |  |  |  |  |  |  |  |  |  |  |  |  |  |  |  |  |  |  |  |  |  |  |  |  |  |  |  |  |  |  |  |  |  |  |  |  |  |  |  |  |  |  |  |  |  |  |  |  |  |  |  |  |  |  |  |  |  |  |  |  |  |  |  |  |  |  |  |  |  |  |  |  |  |  |  |  |  |  |  |  |  |  |  |  |  |  |  |  |  |  |  |  |  |  |  |  |  |  |  |  |  |  |  |  |  |  |  |  |  |  |  |  |  |  |  |  |  |  |  |  |  |  |  |  |  |  |  |  |  |  |  |  |  |  |  |  |  |  |  |  |  |  |  |  |  |  |  |  |  |  |  |  |  |  |  |  |  |  |  |  |  |  |  |  |  |  |  |  |  |  |  |  |  |  |  |  |  |  |  |  |  |  |  |  |  |  |  |  |  |  |  |  |  |  |  |  |  |  |  |  |  |  |  |  |  |  |  |  |  |  |  |  |  |  |  |
|  |  |  |  |  |  |  |  |  |  |  |  |  |  |  |  |  |  |  |  |  |  |  |  |  |  |  |  |  |  |  |  |  |  |  |  |  |  |  |  |  |  |  |  |  |  |  |  |  |  |  |  |  |  |  |  |  |  |  |  |  |  |  |  |  |  |  |  |  |  |  |  |  |  |  |  |  |  |  |  |  |  |  |  |  |  |  |  |  |  |  |  |  |  |  |  |  |  |  |  |  |  |  |  |  |  |  |  |  |  |  |  |  |  |  |  |  |  |  |  |  |  |  |  |  |  |  |  |  |  |  |  |  |  |  |  |  |  |  |  |  |  |  |  |  |  |  |  |  |  |  |  |  |  |  |  |  |  |  |  |  |  |  |  |  |  |  |  |  |  |  |  |  |  |  |  |  |  |  |  |  |  |  |  |  |  |  |  |  |  |  |  |  |  |  |  |  |  |  |  |  |  |  |  |  |  |  |  |  |  |  |  |  |  |  |  |  |  |  |  |  |  |  |  |  |  |  |  |  |  |  |  |  |  |  |  |  |  |  |  |  |  |  |  |  |  |  |  |  |  |  |  |  |  |  |  |  |  |  |  |  |  |  |  |  |  |  |  |  |  |  |  |  |  |  |  |  |  |  |  |  |  |  |  |  |  |  |  |  |  |  |  |  |  |  |  |  |  |  |  |  |  |  |  |  |  |  |  |  |  |  |  |  |  |  |  |  |  |  |  |  |  |  |  |  |  |  |  |  |  |  |  |  |  |  |  |  |  |  |  |  |  |  |  |  |  |  |  |  |  |  |  |  |  |  |  |  |  |  |  |  |  |  |  |  |  |  |  |  |  |  |  |  |  |  |  |  |  |  |  |  |  |  |  |  |  |  |  |  |  |  |  |  |  |  |  |  |  |  |  |  |  |  |  |  |  |  |  |  |  |  |  |  |  |  |  |  |  |  |  |  |  |  |  |  |  |  |  |  |  |  |  |  |  |  |  |  |  |  |  |  |  |  |  |  |  |  |  |  |  |  |  |  |  |  |  |  |  |  |  |  |  |  |  |  |  |  |  |  |  |  |  |  |  |  |  |  |  |  |  |  |  |  |  |  |  |  |  |  |  |  |  |  |  |  |  |  |  |  |  |  |  |  |  |  |  |  |  |  |  |  |  |  |  |  |  |  |  |  |  |  |  |  |  |  |  |  |  |  |  |  |  |  |  |  |  |  |  |  |  |  |  |  |  |  |  |  |  |  |  |  |  |  |  |  |  |  |  |  |  |  |  |  |  |  |  |  |  |  |  |  |  |  |  |  |  |  |  |  |  |  |  |  |  |  |  |  |  |  |  |  |  |  |  |  |  |  |  |  |  |  |  |  |  |  |  |  |  |  |  |  |  |  |  |  |  |  |  |  |  |  |  |  |  |  |  |  |  |  |  |  |  |  |  |  |  |  |  |  |  |  |  |  |  |  |  |  |  |  |  |  |  |  |  |  |  |  |  |  |  |  |  |  |  |  |  |  |  |  |  |  |  |  |  |  |  |  |  |  |  |  |  |  |  |  |  |  |  |  |  |  |  |  |  |  |  |  |  |  |  |  |  |  |  |  |  |  |  |  |  |  |  |  |  |  |  |  |  |  |  |  |  |  |  |  |  |  |  |  |  |  |  |  |  |  |  |  |  |  |  |  |  |  |  |  |  |  |  |  |  |  |  |  |  |  |  |  |  |  |  |  |  |  |  |  |  |  |  |  |  |  |  |  |  |  |  |  |  |  |  |  |  |  |  |  |  |  |  |  |  |  |  |  |  |  |  |  |  |  |  |  |  |  |  |  |  |  |  |  |  |  |  |  |  |  |  |  |  |  |  |  |  |  |  |  |  |  |  |  |  |  |  |  |  |  |  |  |  |  |  |  |  |  |  |  |  |  |  |  |  |  |  |  |  |  |  |  |  |  |  |  |  |  |  |  |  |  |  |  |  |  |  |  |  |  |  |  |  |  |  |  |  |  |  |  |  |  |  |  |  |  |  |  |  |  |  |  |  |  |  |  |  |  |  |  |  |  |  |  |  |  |  |  |  |
|  |  |  |  |  |  |  |  |  |  |  |  |  |  |  |  |  |  |  |  |  |  |  |  |  |  |  |  |  |  |  |  |  |  |  |  |  |  |  |  |  |  |  |  |  |  |  |  |  |  |  |  |  |  |  |  |  |  |  |  |  |  |  |  |  |  |  |  |  |  |  |  |  |  |  |  |  |  |  |  |  |  |  |  |  |  |  |  |  |  |  |  |  |  |  |  |  |  |  |  |  |  |  |  |  |  |  |  |  |  |  |  |  |  |  |  |  |  |  |  |  |  |  |  |  |  |  |  |  |  |  |  |  |  |  |  |  |  |  |  |  |  |  |  |  |  |  |  |  |  |  |  |  |  |  |  |  |  |  |  |  |  |  |  |  |  |  |  |  |  |  |  |  |  |  |  |  |  |  |  |  |  |  |  |  |  |  |  |  |  |  |  |  |  |  |  |  |  |  |  |  |  |  |  |  |  |  |  |  |  |  |  |  |  |  |  |  |  |  |  |  |  |  |  |  |  |  |  |  |  |  |  |  |  |  |  |  |  |  |  |  |  |  |  |  |  |  |  |  |  |  |  |  |  |  |  |  |  |  |  |  |  |  |  |  |  |  |  |  |  |  |  |  |  |  |  |  |  |  |  |  |  |  |  |  |  |  |  |  |  |  |  |  |  |  |  |  |  |  |  |  |  |  |  |  |  |  |  |  |  |  |  |  |  |  |  |  |  |  |  |  |  |  |  |  |  |  |  |  |  |  |  |  |  |  |  |  |  |  |  |  |  |  |  |  |  |  |  |  |  |  |  |  |  |  |  |  |  |  |  |  |  |  |  |  |  |  |  |  |  |  |  |  |  |  |  |  |  |  |  |  |  |  |  |  |  |  |  |  |  |  |  |  |  |  |  |  |  |  |  |  |  |  |  |  |  |  |  |  |  |  |  |  |  |  |  |  |  |  |  |  |  |  |  |  |  |  |  |  |  |  |  |  |  |  |  |  |  |  |  |  |  |  |  |  |  |  |  |  |  |  |  |  |  |  |  |  |  |  |  |  |  |  |  |  |  |  |  |  |  |  |  |  |  |  |  |  |  |  |  |  |  |  |  |  |  |  |  |  |  |  |  |  |  |  |  |  |  |  |  |  |  |  |  |  |  |  |  |  |  |  |  |  |  |  |  |  |  |  |  |  |  |  |  |  |  |  |  |  |  |  |  |  |  |  |  |  |  |  |  |  |  |  |  |  |  |  |  |  |  |  |  |  |  |  |  |  |  |  |  |  |  |  |  |  |  |  |  |  |  |  |  |  |  |  |  |  |  |  |  |  |  |  |  |  |  |  |  |  |  |  |  |  |  |  |  |  |  |  |  |  |  |  |  |  |  |  |  |  |  |  |  |  |  |  |  |  |  |  |  |  |  |  |  |  |  |  |  |  |  |  |  |  |  |  |  |  |  |  |  |  |  |  |  |  |  |  |  |  |  |  |  |  |  |  |  |  |  |  |  |  |  |  |  |  |  |  |  |  |  |  |  |  |  |  |  |  |  |  |  |  |  |  |  |  |  |  |  |  |  |  |  |  |  |  |  |  |  |  |  |  |  |  |  |  |  |  |  |  |  |  |  |  |  |  |  |  |  |  |  |  |  |  |  |  |  |  |  |  |  |  |  |  |  |  |  |  |  |  |  |  |  |  |  |  |  |  |  |  |  |  |  |  |  |  |  |  |  |  |  |  |  |  |  |  |  |  |  |  |  |  |  |  |  |  |  |  |  |  |  |  |  |  |  |  |  |  |  |  |  |  |  |  |  |  |  |  |  |  |  |  |  |  |  |  |  |  |  |  |  |  |  |  |  |  |  |  |  |  |  |  |  |  |  |  |  |  |  |  |  |  |  |  |  |  |  |  |  |  |  |  |  |  |  |  |  |  |  |  |  |  |  |  |  |  |  |  |  |  |  |  |  |  |  |  |  |  |  |  |  |  |  |  |  |  |  |  |  |  |  |  |  |  |  |  |  |  |  |  |  |  |  |  |  |  |  |  |  |  |  |  |  |  |  |  |  |  |  |  |  |  |  |  |  |
|  |  |  |  |  |  |  |  |  |  |  |  |  |  |  |  |  |  |  |  |  |  |  |  |  |  |  |  |  |  |  |  |  |  |  |  |  |  |  |  |  |  |  |  |  |  |  |  |  |  |  |  |  |  |  |  |  |  |  |  |  |  |  |  |  |  |  |  |  |  |  |  |  |  |  |  |  |  |  |  |  |  |  |  |  |  |  |  |  |  |  |  |  |  |  |  |  |  |  |  |  |  |  |  |  |  |  |  |  |  |  |  |  |  |  |  |  |  |  |  |  |  |  |  |  |  |  |  |  |  |  |  |  |  |  |  |  |  |  |  |  |  |  |  |  |  |  |  |  |  |  |  |  |  |  |  |  |  |  |  |  |  |  |  |  |  |  |  |  |  |  |  |  |  |  |  |  |  |  |  |  |  |  |  |  |  |  |  |  |  |  |  |  |  |  |  |  |  |  |  |  |  |  |  |  |  |  |  |  |  |  |  |  |  |  |  |  |  |  |  |  |  |  |  |  |  |  |  |  |  |  |  |  |  |  |  |  |  |  |  |  |  |  |  |  |  |  |  |  |  |  |  |  |  |  |  |  |  |  |  |  |  |  |  |  |  |  |  |  |  |  |  |  |  |  |  |  |  |  |  |  |  |  |  |  |  |  |  |  |  |  |  |  |  |  |  |  |  |  |  |  |  |  |  |  |  |  |  |  |  |  |  |  |  |  |  |  |  |  |  |  |  |  |  |  |  |  |  |  |  |  |  |  |  |  |  |  |  |  |  |  |  |  |  |  |  |  |  |  |  |  |  |  |  |  |  |  |  |  |  |  |  |  |  |  |  |  |  |  |  |  |  |  |  |  |  |  |  |  |  |  |  |  |  |  |  |  |  |  |  |  |  |  |  |  |  |  |  |  |  |  |  |  |  |  |  |  |  |  |  |  |  |  |  |  |  |  |  |  |  |  |  |  |  |  |  |  |  |  |  |  |  |  |  |  |  |  |  |  |  |  |  |  |  |  |  |  |  |  |  |  |  |  |  |  |  |  |  |  |  |  |  |  |  |  |  |  |  |  |  |  |  |  |  |  |  |  |  |  |  |  |  |  |  |  |  |  |  |  |  |  |  |  |  |  |  |  |  |  |  |  |  |  |  |  |  |  |  |  |  |  |  |  |  |  |  |  |  |  |  |  |  |  |  |  |  |  |  |  |  |  |  |  |  |  |  |  |  |  |  |  |  |  |  |  |  |  |  |  |  |  |  |  |  |  |  |  |  |  |  |  |  |  |  |  |  |  |  |  |  |  |  |  |  |  |  |  |  |  |  |  |  |  |  |  |  |  |  |  |  |  |  |  |  |  |  |  |  |  |  |  |  |  |  |  |  |  |  |  |  |  |  |  |  |  |  |  |  |  |  |  |  |  |  |  |  |  |  |  |  |  |  |  |  |  |  |  |  |  |  |  |  |  |  |  |  |  |  |  |  |  |  |  |  |  |  |  |  |  |  |  |  |  |  |  |  |  |  |  |  |  |  |  |  |  |  |  |  |  |  |  |  |  |  |  |  |  |  |  |  |  |  |  |  |  |  |  |  |  |  |  |  |  |  |  |  |  |  |  |  |  |  |  |  |  |  |  |  |  |  |  |  |  |  |  |  |  |  |  |  |  |  |  |  |  |  |  |  |  |  |  |  |  |  |  |  |  |  |  |  |  |  |  |  |  |  |  |  |  |  |  |  |  |  |  |  |  |  |  |  |  |  |  |  |  |  |  |  |  |  |  |  |  |  |  |  |  |  |  |  |  |  |  |  |  |  |  |  |  |  |  |  |  |  |  |  |  |  |  |  |  |  |  |  |  |  |  |  |  |  |  |  |  |  |  |  |  |  |  |  |  |  |  |  |  |  |  |  |  |  |  |  |  |  |  |  |  |  |  |  |  |  |  |  |  |  |  |  |  |  |  |  |  |  |  |  |  |  |  |  |  |  |  |  |  |  |  |  |  |  |  |  |  |  |  |  |  |  |  |  |  |  |  |  |  |  |  |  |  |  |  |  |  |  |  |  |  |  |  |  |  |  |  |  |
|  |  |  |  |  |  |  |  |  |  |  |  |  |  |  |  |  |  |  |  |  |  |  |  |  |  |  |  |  |  |  |  |  |  |  |  |  |  |  |  |  |  |  |  |  |  |  |  |  |  |  |  |  |  |  |  |  |  |  |  |  |  |  |  |  |  |  |  |  |  |  |  |  |  |  |  |  |  |  |  |  |  |  |  |  |  |  |  |  |  |  |  |  |  |  |  |  |  |  |  |  |  |  |  |  |  |  |  |  |  |  |  |  |  |  |  |  |  |  |  |  |  |  |  |  |  |  |  |  |  |  |  |  |  |  |  |  |  |  |  |  |  |  |  |  |  |  |  |  |  |  |  |  |  |  |  |  |  |  |  |  |  |  |  |  |  |  |  |  |  |  |  |  |  |  |  |  |  |  |  |  |  |  |  |  |  |  |  |  |  |  |  |  |  |  |  |  |  |  |  |  |  |  |  |  |  |  |  |  |  |  |  |  |  |  |  |  |  |  |  |  |  |  |  |  |  |  |  |  |  |  |  |  |  |  |  |  |  |  |  |  |  |  |  |  |  |  |  |  |  |  |  |  |  |  |  |  |  |  |  |  |  |  |  |  |  |  |  |  |  |  |  |  |  |  |  |  |  |  |  |  |  |  |  |  |  |  |  |  |  |  |  |  |  |  |  |  |  |  |  |  |  |  |  |  |  |  |  |  |  |  |  |  |  |  |  |  |  |  |  |  |  |  |  |  |  |  |  |  |  |  |  |  |  |  |  |  |  |  |  |  |  |  |  |  |  |  |  |  |  |  |  |  |  |  |  |  |  |  |  |  |  |  |  |  |  |  |  |  |  |  |  |  |  |  |  |  |  |  |  |  |  |  |  |  |  |  |  |  |  |  |  |  |  |  |  |  |  |  |  |  |  |  |  |  |  |  |  |  |  |  |  |  |  |  |  |  |  |  |  |  |  |  |  |  |  |  |  |  |  |  |  |  |  |  |  |  |  |  |  |  |  |  |  |  |  |  |  |  |  |  |  |  |  |  |  |  |  |  |  |  |  |  |  |  |  |  |  |  |  |  |  |  |  |  |  |  |  |  |  |  |  |  |  |  |  |  |  |  |  |  |  |  |  |  |  |  |  |  |  |  |  |  |  |  |  |  |  |  |  |  |  |  |  |  |  |  |  |  |  |  |  |  |  |  |  |  |  |  |  |  |  |  |  |  |  |  |  |  |  |  |  |  |  |  |  |  |  |  |  |  |  |  |  |  |  |  |  |  |  |  |  |  |  |  |  |  |  |  |  |  |  |  |  |  |  |  |  |  |  |  |  |  |  |  |  |  |  |  |  |  |  |  |  |  |  |  |  |  |  |  |  |  |  |  |  |  |  |  |  |  |  |  |  |  |  |  |  |  |  |  |  |  |  |  |  |  |  |  |  |  |  |  |  |  |  |  |  |  |  |  |  |  |  |  |  |  |  |  |  |  |  |  |  |  |  |  |  |  |  |  |  |  |  |  |  |  |  |  |  |  |  |  |  |  |  |  |  |  |  |  |  |  |  |  |  |  |  |  |  |  |  |  |  |  |  |  |  |  |  |  |  |  |  |  |  |  |  |  |  |  |  |  |  |  |  |  |  |  |  |  |  |  |  |  |  |  |  |  |  |  |  |  |  |  |  |  |  |  |  |  |  |  |  |  |  |  |  |  |  |  |  |  |  |  |  |  |  |  |  |  |  |  |  |  |  |  |  |  |  |  |  |  |  |  |  |  |  |  |  |  |  |  |  |  |  |  |  |  |  |  |  |  |  |  |  |  |  |  |  |  |  |  |  |  |  |  |  |  |  |  |  |  |  |  |  |  |  |  |  |  |  |  |  |  |  |  |  |  |  |  |  |  |  |  |  |  |  |  |  |  |  |  |  |  |  |  |  |  |  |  |  |  |  |  |  |  |  |  |  |  |  |  |  |  |  |  |  |  |  |  |  |  |  |  |  |  |  |  |  |  |  |  |  |  |  |  |  |  |  |  |  |  |  |  |  |  |  |  |  |  |  |  |  |  |  |  |  |  |  |  |  |  |  |
|  |  |  |  |  |  |  |  |  |  |  |  |  |  |  |  |  |  |  |  |  |  |  |  |  |  |  |  |  |  |  |  |  |  |  |  |  |  |  |  |  |  |  |  |  |  |  |  |  |  |  |  |  |  |  |  |  |  |  |  |  |  |  |  |  |  |  |  |  |  |  |  |  |  |  |  |  |  |  |  |  |  |  |  |  |  |  |  |  |  |  |  |  |  |  |  |  |  |  |  |  |  |  |  |  |  |  |  |  |  |  |  |  |  |  |  |  |  |  |  |  |  |  |  |  |  |  |  |  |  |  |  |  |  |  |  |  |  |  |  |  |  |  |  |  |  |  |  |  |  |  |  |  |  |  |  |  |  |  |  |  |  |  |  |  |  |  |  |  |  |  |  |  |  |  |  |  |  |  |  |  |  |  |  |  |  |  |  |  |  |  |  |  |  |  |  |  |  |  |  |  |  |  |  |  |  |  |  |  |  |  |  |  |  |  |  |  |  |  |  |  |  |  |  |  |  |  |  |  |  |  |  |  |  |  |  |  |  |  |  |  |  |  |  |  |  |  |  |  |  |  |  |  |  |  |  |  |  |  |  |  |  |  |  |  |  |  |  |  |  |  |  |  |  |  |  |  |  |  |  |  |  |  |  |  |  |  |  |  |  |  |  |  |  |  |  |  |  |  |  |  |  |  |  |  |  |  |  |  |  |  |  |  |  |  |  |  |  |  |  |  |  |  |  |  |  |  |  |  |  |  |  |  |  |  |  |  |  |  |  |  |  |  |  |  |  |  |  |  |  |  |  |  |  |  |  |  |  |  |  |  |  |  |  |  |  |  |  |  |  |  |  |  |  |  |  |  |  |  |  |  |  |  |  |  |  |  |  |  |  |  |  |  |  |  |  |  |  |  |  |  |  |  |  |  |  |  |  |  |  |  |  |  |  |  |  |  |  |  |  |  |  |  |  |  |  |  |  |  |  |  |  |  |  |  |  |  |  |  |  |  |  |  |  |  |  |  |  |  |  |  |  |  |  |  |  |  |  |  |  |  |  |  |  |  |  |  |  |  |  |  |  |  |  |  |  |  |  |  |  |  |  |  |  |  |  |  |  |  |  |  |  |  |  |  |  |  |  |  |  |  |  |  |  |  |  |  |  |  |  |  |  |  |  |  |  |  |  |  |  |  |  |  |  |  |  |  |  |  |  |  |  |  |  |  |  |  |  |  |  |  |  |  |  |  |  |  |  |  |  |  |  |  |  |  |  |  |  |  |  |  |  |  |  |  |  |  |  |  |  |  |  |  |  |  |  |  |  |  |  |  |  |  |  |  |  |  |  |  |  |  |  |  |  |  |  |  |  |  |  |  |  |  |  |  |  |  |  |  |  |  |  |  |  |  |  |  |  |  |  |  |  |  |  |  |  |  |  |  |  |  |  |  |  |  |  |  |  |  |  |  |  |  |  |  |  |  |  |  |  |  |  |  |  |  |  |  |  |  |  |  |  |  |  |  |  |  |  |  |  |  |  |  |  |  |  |  |  |  |  |  |  |  |  |  |  |  |  |  |  |  |  |  |  |  |  |  |  |  |  |  |  |  |  |  |  |  |  |  |  |  |  |  |  |  |  |  |  |  |  |  |  |  |  |  |  |  |  |  |  |  |  |  |  |  |  |  |  |  |  |  |  |  |  |  |  |  |  |  |  |  |  |  |  |  |  |  |  |  |  |  |  |  |  |  |  |  |  |  |  |  |  |  |  |  |  |  |  |  |  |  |  |  |  |  |  |  |  |  |  |  |  |  |  |  |  |  |  |  |  |  |  |  |  |  |  |  |  |  |  |  |  |  |  |  |  |  |  |  |  |  |  |  |  |  |  |  |  |  |  |  |  |  |  |  |  |  |  |  |  |  |  |  |  |  |  |  |  |  |  |  |  |  |  |  |  |  |  |  |  |  |  |  |  |  |  |  |  |  |  |  |  |  |  |  |  |  |  |  |  |  |  |  |  |  |  |  |  |  |  |  |  |  |  |  |  |  |  |  |  |  |  |  |  |  |  |  |  |  |  |  |  |  |  |
|  |  |  |  |  |  |  |  |  |  |  |  |  |  |  |  |  |  |  |  |  |  |  |  |  |  |  |  |  |  |  |  |  |  |  |  |  |  |  |  |  |  |  |  |  |  |  |  |  |  |  |  |  |  |  |  |  |  |  |  |  |  |  |  |  |  |  |  |  |  |  |  |  |  |  |  |  |  |  |  |  |  |  |  |  |  |  |  |  |  |  |  |  |  |  |  |  |  |  |  |  |  |  |  |  |  |  |  |  |  |  |  |  |  |  |  |  |  |  |  |  |  |  |  |  |  |  |  |  |  |  |  |  |  |  |  |  |  |  |  |  |  |  |  |  |  |  |  |  |  |  |  |  |  |  |  |  |  |  |  |  |  |  |  |  |  |  |  |  |  |  |  |  |  |  |  |  |  |  |  |  |  |  |  |  |  |  |  |  |  |  |  |  |  |  |  |  |  |  |  |  |  |  |  |  |  |  |  |  |  |  |  |  |  |  |  |  |  |  |  |  |  |  |  |  |  |  |  |  |  |  |  |  |  |  |  |  |  |  |  |  |  |  |  |  |  |  |  |  |  |  |  |  |  |  |  |  |  |  |  |  |  |  |  |  |  |  |  |  |  |  |  |  |  |  |  |  |  |  |  |  |  |  |  |  |  |  |  |  |  |  |  |  |  |  |  |  |  |  |  |  |  |  |  |  |  |  |  |  |  |  |  |  |  |  |  |  |  |  |  |  |  |  |  |  |  |  |  |  |  |  |  |  |  |  |  |  |  |  |  |  |  |  |  |  |  |  |  |  |  |  |  |  |  |  |  |  |  |  |  |  |  |  |  |  |  |  |  |  |  |  |  |  |  |  |  |  |  |  |  |  |  |  |  |  |  |  |  |  |  |  |  |  |  |  |  |  |  |  |  |  |  |  |  |  |  |  |  |  |  |  |  |  |  |  |  |  |  |  |  |  |  |  |  |  |  |  |  |  |  |  |  |  |  |  |  |  |  |  |  |  |  |  |  |  |  |  |  |  |  |  |  |  |  |  |  |  |  |  |  |  |  |  |  |  |  |  |  |  |  |  |  |  |  |  |  |  |  |  |  |  |  |  |  |  |  |  |  |  |  |  |  |  |  |  |  |  |  |  |  |  |  |  |  |  |  |  |  |  |  |  |  |  |  |  |  |  |  |  |  |  |  |  |  |  |  |  |  |  |  |  |  |  |  |  |  |  |  |  |  |  |  |  |  |  |  |  |  |  |  |  |  |  |  |  |  |  |  |  |  |  |  |  |  |  |  |  |  |  |  |  |  |  |  |  |  |  |  |  |  |  |  |  |  |  |  |  |  |  |  |  |  |  |  |  |  |  |  |  |  |  |  |  |  |  |  |  |  |  |  |  |  |  |  |  |  |  |  |  |  |  |  |  |  |  |  |  |  |  |  |  |  |  |  |  |  |  |  |  |  |  |  |  |  |  |  |  |  |  |  |  |  |  |  |  |  |  |  |  |  |  |  |  |  |  |  |  |  |  |  |  |  |  |  |  |  |  |  |  |  |  |  |  |  |  |  |  |  |  |  |  |  |  |  |  |  |  |  |  |  |  |  |  |  |  |  |  |  |  |  |  |  |  |  |  |  |  |  |  |  |  |  |  |  |  |  |  |  |  |  |  |  |  |  |  |  |  |  |  |  |  |  |  |  |  |  |  |  |  |  |  |  |  |  |  |  |  |  |  |  |  |  |  |  |  |  |  |  |  |  |  |  |  |  |  |  |  |  |  |  |  |  |  |  |  |  |  |  |  |  |  |  |  |  |  |  |  |  |  |  |  |  |  |  |  |  |  |  |  |  |  |  |  |  |  |  |  |  |  |  |  |  |  |  |  |  |  |  |  |  |  |  |  |  |  |  |  |  |  |  |  |  |  |  |  |  |  |  |  |  |  |  |  |  |  |  |  |  |  |  |  |  |  |  |  |  |  |  |  |  |  |  |  |  |  |  |  |  |  |  |  |  |  |  |  |  |  |  |  |  |  |  |  |  |  |  |  |  |  |  |  |  |  |  |  |  |  |  |  |  |  |  |  |  |
|  |  |  |  |  |  |  |  |  |  |  |  |  |  |  |  |  |  |  |  |  |  |  |  |  |  |  |  |  |  |  |  |  |  |  |  |  |  |  |  |  |  |  |  |  |  |  |  |  |  |  |  |  |  |  |  |  |  |  |  |  |  |  |  |  |  |  |  |  |  |  |  |  |  |  |  |  |  |  |  |  |  |  |  |  |  |  |  |  |  |  |  |  |  |  |  |  |  |  |  |  |  |  |  |  |  |  |  |  |  |  |  |  |  |  |  |  |  |  |  |  |  |  |  |  |  |  |  |  |  |  |  |  |  |  |  |  |  |  |  |  |  |  |  |  |  |  |  |  |  |  |  |  |  |  |  |  |  |  |  |  |  |  |  |  |  |  |  |  |  |  |  |  |  |  |  |  |  |  |  |  |  |  |  |  |  |  |  |  |  |  |  |  |  |  |  |  |  |  |  |  |  |  |  |  |  |  |  |  |  |  |  |  |  |  |  |  |  |  |  |  |  |  |  |  |  |  |  |  |  |  |  |  |  |  |  |  |  |  |  |  |  |  |  |  |  |  |  |  |  |  |  |  |  |  |  |  |  |  |  |  |  |  |  |  |  |  |  |  |  |  |  |  |  |  |  |  |  |  |  |  |  |  |  |  |  |  |  |  |  |  |  |  |  |  |  |  |  |  |  |  |  |  |  |  |  |  |  |  |  |  |  |  |  |  |  |  |  |  |  |  |  |  |  |  |  |  |  |  |  |  |  |  |  |  |  |  |  |  |  |  |  |  |  |  |  |  |  |  |  |  |  |  |  |  |  |  |  |  |  |  |  |  |  |  |  |  |  |  |  |  |  |  |  |  |  |  |  |  |  |  |  |  |  |  |  |  |  |  |  |  |  |  |  |  |  |  |  |  |  |  |  |  |  |  |  |  |  |  |  |  |  |  |  |  |  |  |  |  |  |  |  |  |  |  |  |  |  |  |  |  |  |  |  |  |  |  |  |  |  |  |  |  |  |  |  |  |  |  |  |  |  |  |  |  |  |  |  |  |  |  |  |  |  |  |  |  |  |  |  |  |  |  |  |  |  |  |  |  |  |  |  |  |  |  |  |  |  |  |  |  |  |  |  |  |  |  |  |  |  |  |  |  |  |  |  |  |  |  |  |  |  |  |  |  |  |  |  |  |  |  |  |  |  |  |  |  |  |  |  |  |  |  |  |  |  |  |  |  |  |  |  |  |  |  |  |  |  |  |  |  |  |  |  |  |  |  |  |  |  |  |  |  |  |  |  |  |  |  |  |  |  |  |  |  |  |  |  |  |  |  |  |  |  |  |  |  |  |  |  |  |  |  |  |  |  |  |  |  |  |  |  |  |  |  |  |  |  |  |  |  |  |  |  |  |  |  |  |  |  |  |  |  |  |  |  |  |  |  |  |  |  |  |  |  |  |  |  |  |  |  |  |  |  |  |  |  |  |  |  |  |  |  |  |  |  |  |  |  |  |  |  |  |  |  |  |  |  |  |  |  |  |  |  |  |  |  |  |  |  |  |  |  |  |  |  |  |  |  |  |  |  |  |  |  |  |  |  |  |  |  |  |  |  |  |  |  |  |  |  |  |  |  |  |  |  |  |  |  |  |  |  |  |  |  |  |  |  |  |  |  |  |  |  |  |  |  |  |  |  |  |  |  |  |  |  |  |  |  |  |  |  |  |  |  |  |  |  |  |  |  |  |  |  |  |  |  |  |  |  |  |  |  |  |  |  |  |  |  |  |  |  |  |  |  |  |  |  |  |  |  |  |  |  |  |  |  |  |  |  |  |  |  |  |  |  |  |  |  |  |  |  |  |  |  |  |  |  |  |  |  |  |  |  |  |  |  |  |  |  |  |  |  |  |  |  |  |  |  |  |  |  |  |  |  |  |  |  |  |  |  |  |  |  |  |  |  |  |  |  |  |  |  |  |  |  |  |  |  |  |  |  |  |  |  |  |  |  |  |  |  |  |  |  |  |  |  |  |  |  |  |  |  |  |  |  |  |  |  |  |  |  |  |  |  |  |  |  |  |  |  |  |  |  |
|  |  |  |  |  |  |  |  |  |  |  |  |  |  |  |  |  |  |  |  |  |  |  |  |  |  |  |  |  |  |  |  |  |  |  |  |  |  |  |  |  |  |  |  |  |  |  |  |  |  |  |  |  |  |  |  |  |  |  |  |  |  |  |  |  |  |  |  |  |  |  |  |  |  |  |  |  |  |  |  |  |  |  |  |  |  |  |  |  |  |  |  |  |  |  |  |  |  |  |  |  |  |  |  |  |  |  |  |  |  |  |  |  |  |  |  |  |  |  |  |  |  |  |  |  |  |  |  |  |  |  |  |  |  |  |  |  |  |  |  |  |  |  |  |  |  |  |  |  |  |  |  |  |  |  |  |  |  |  |  |  |  |  |  |  |  |  |  |  |  |  |  |  |  |  |  |  |  |  |  |  |  |  |  |  |  |  |  |  |  |  |  |  |  |  |  |  |  |  |  |  |  |  |  |  |  |  |  |  |  |  |  |  |  |  |  |  |  |  |  |  |  |  |  |  |  |  |  |  |  |  |  |  |  |  |  |  |  |  |  |  |  |  |  |  |  |  |  |  |  |  |  |  |  |  |  |  |  |  |  |  |  |  |  |  |  |  |  |  |  |  |  |  |  |  |  |  |  |  |  |  |  |  |  |  |  |  |  |  |  |  |  |  |  |  |  |  |  |  |  |  |  |  |  |  |  |  |  |  |  |  |  |  |  |  |  |  |  |  |  |  |  |  |  |  |  |  |  |  |  |  |  |  |  |  |  |  |  |  |  |  |  |  |  |  |  |  |  |  |  |  |  |  |  |  |  |  |  |  |  |  |  |  |  |  |  |  |  |  |  |  |  |  |  |  |  |  |  |  |  |  |  |  |  |  |  |  |  |  |  |  |  |  |  |  |  |  |  |  |  |  |  |  |  |  |  |  |  |  |  |  |  |  |  |  |  |  |  |  |  |  |  |  |  |  |  |  |  |  |  |  |  |  |  |  |  |  |  |  |  |  |  |  |  |  |  |  |  |  |  |  |  |  |  |  |  |  |  |  |  |  |  |  |  |  |  |  |  |  |  |  |  |  |  |  |  |  |  |  |  |  |  |  |  |  |  |  |  |  |  |  |  |  |  |  |  |  |  |  |  |  |  |  |  |  |  |  |  |  |  |  |  |  |  |  |  |  |  |  |  |  |  |  |  |  |  |  |  |  |  |  |  |  |  |  |  |  |  |  |  |  |  |  |  |  |  |  |  |  |  |  |  |  |  |  |  |  |  |  |  |  |  |  |  |  |  |  |  |  |  |  |  |  |  |  |  |  |  |  |  |  |  |  |  |  |  |  |  |  |  |  |  |  |  |  |  |  |  |  |  |  |  |  |  |  |  |  |  |  |  |  |  |  |  |  |  |  |  |  |  |  |  |  |  |  |  |  |  |  |  |  |  |  |  |  |  |  |  |  |  |  |  |  |  |  |  |  |  |  |  |  |  |  |  |  |  |  |  |  |  |  |  |  |  |  |  |  |  |  |  |  |  |  |  |  |  |  |  |  |  |  |  |  |  |  |  |  |  |  |  |  |  |  |  |  |  |  |  |  |  |  |  |  |  |  |  |  |  |  |  |  |  |  |  |  |  |  |  |  |  |  |  |  |  |  |  |  |  |  |  |  |  |  |  |  |  |  |  |  |  |  |  |  |  |  |  |  |  |  |  |  |  |  |  |  |  |  |  |  |  |  |  |  |  |  |  |  |  |  |  |  |  |  |  |  |  |  |  |  |  |  |  |  |  |  |  |  |  |  |  |  |  |  |  |  |  |  |  |  |  |  |  |  |  |  |  |  |  |  |  |  |  |  |  |  |  |  |  |  |  |  |  |  |  |  |  |  |  |  |  |  |  |  |  |  |  |  |  |  |  |  |  |  |  |  |  |  |  |  |  |  |  |  |  |  |  |  |  |  |  |  |  |  |  |  |  |  |  |  |  |  |  |  |  |  |  |  |  |  |  |  |  |  |  |  |  |  |  |  |  |  |  |  |  |  |  |  |  |  |  |  |  |  |  |  |  |  |  |  |  |  |  |  |  |
|  |  |  |  |  |  |  |  |  |  |  |  |  |  |  |  |  |  |  |  |  |  |  |  |  |  |  |  |  |  |  |  |  |  |  |  |  |  |  |  |  |  |  |  |  |  |  |  |  |  |  |  |  |  |  |  |  |  |  |  |  |  |  |  |  |  |  |  |  |  |  |  |  |  |  |  |  |  |  |  |  |  |  |  |  |  |  |  |  |  |  |  |  |  |  |  |  |  |  |  |  |  |  |  |  |  |  |  |  |  |  |  |  |  |  |  |  |  |  |  |  |  |  |  |  |  |  |  |  |  |  |  |  |  |  |  |  |  |  |  |  |  |  |  |  |  |  |  |  |  |  |  |  |  |  |  |  |  |  |  |  |  |  |  |  |  |  |  |  |  |  |  |  |  |  |  |  |  |  |  |  |  |  |  |  |  |  |  |  |  |  |  |  |  |  |  |  |  |  |  |  |  |  |  |  |  |  |  |  |  |  |  |  |  |  |  |  |  |  |  |  |  |  |  |  |  |  |  |  |  |  |  |  |  |  |  |  |  |  |  |  |  |  |  |  |  |  |  |  |  |  |  |  |  |  |  |  |  |  |  |  |  |  |  |  |  |  |  |  |  |  |  |  |  |  |  |  |  |  |  |  |  |  |  |  |  |  |  |  |  |  |  |  |  |  |  |  |  |  |  |  |  |  |  |  |  |  |  |  |  |  |  |  |  |  |  |  |  |  |  |  |  |  |  |  |  |  |  |  |  |  |  |  |  |  |  |  |  |  |  |  |  |  |  |  |  |  |  |  |  |  |  |  |  |  |  |  |  |  |  |  |  |  |  |  |  |  |  |  |  |  |  |  |  |  |  |  |  |  |  |  |  |  |  |  |  |  |  |  |  |  |  |  |  |  |  |  |  |  |  |  |  |  |  |  |  |  |  |  |  |  |  |  |  |  |  |  |  |  |  |  |  |  |  |  |  |  |  |  |  |  |  |  |  |  |  |  |  |  |  |  |  |  |  |  |  |  |  |  |  |  |  |  |  |  |  |  |  |  |  |  |  |  |  |  |  |  |  |  |  |  |  |  |  |  |  |  |  |  |  |  |  |  |  |  |  |  |  |  |  |  |  |  |  |  |  |  |  |  |  |  |  |  |  |  |  |  |  |  |  |  |  |  |  |  |  |  |  |  |  |  |  |  |  |  |  |  |  |  |  |  |  |  |  |  |  |  |  |  |  |  |  |  |  |  |  |  |  |  |  |  |  |  |  |  |  |  |  |  |  |  |  |  |  |  |  |  |  |  |  |  |  |  |  |  |  |  |  |  |  |  |  |  |  |  |  |  |  |  |  |  |  |  |  |  |  |  |  |  |  |  |  |  |  |  |  |  |  |  |  |  |  |  |  |  |  |  |  |  |  |  |  |  |  |  |  |  |  |  |  |  |  |  |  |  |  |  |  |  |  |  |  |  |  |  |  |  |  |  |  |  |  |  |  |  |  |  |  |  |  |  |  |  |  |  |  |  |  |  |  |  |  |  |  |  |  |  |  |  |  |  |  |  |  |  |  |  |  |  |  |  |  |  |  |  |  |  |  |  |  |  |  |  |  |  |  |  |  |  |  |  |  |  |  |  |  |  |  |  |  |  |  |  |  |  |  |  |  |  |  |  |  |  |  |  |  |  |  |  |  |  |  |  |  |  |  |  |  |  |  |  |  |  |  |  |  |  |  |  |  |  |  |  |  |  |  |  |  |  |  |  |  |  |  |  |  |  |  |  |  |  |  |  |  |  |  |  |  |  |  |  |  |  |  |  |  |  |  |  |  |  |  |  |  |  |  |  |  |  |  |  |  |  |  |  |  |  |  |  |  |  |  |  |  |  |  |  |  |  |  |  |  |  |  |  |  |  |  |  |  |  |  |  |  |  |  |  |  |  |  |  |  |  |  |  |  |  |  |  |  |  |  |  |  |  |  |  |  |  |  |  |  |  |  |  |  |  |  |  |  |  |  |  |  |  |  |  |  |  |  |  |  |  |  |  |  |  |  |  |  |  |  |  |  |  |  |  |  |  |  |  |  |  |  |
|  |  |  |  |  |  |  |  |  |  |  |  |  |  |  |  |  |  |  |  |  |  |  |  |  |  |  |  |  |  |  |  |  |  |  |  |  |  |  |  |  |  |  |  |  |  |  |  |  |  |  |  |  |  |  |  |  |  |  |  |  |  |  |  |  |  |  |  |  |  |  |  |  |  |  |  |  |  |  |  |  |  |  |  |  |  |  |  |  |  |  |  |  |  |  |  |  |  |  |  |  |  |  |  |  |  |  |  |  |  |  |  |  |  |  |  |  |  |  |  |  |  |  |  |  |  |  |  |  |  |  |  |  |  |  |  |  |  |  |  |  |  |  |  |  |  |  |  |  |  |  |  |  |  |  |  |  |  |  |  |  |  |  |  |  |  |  |  |  |  |  |  |  |  |  |  |  |  |  |  |  |  |  |  |  |  |  |  |  |  |  |  |  |  |  |  |  |  |  |  |  |  |  |  |  |  |  |  |  |  |  |  |  |  |  |  |  |  |  |  |  |  |  |  |  |  |  |  |  |  |  |  |  |  |  |  |  |  |  |  |  |  |  |  |  |  |  |  |  |  |  |  |  |  |  |  |  |  |  |  |  |  |  |  |  |  |  |  |  |  |  |  |  |  |  |  |  |  |  |  |  |  |  |  |  |  |  |  |  |  |  |  |  |  |  |  |  |  |  |  |  |  |  |  |  |  |  |  |  |  |  |  |  |  |  |  |  |  |  |  |  |  |  |  |  |  |  |  |  |  |  |  |  |  |  |  |  |  |  |  |  |  |  |  |  |  |  |  |  |  |  |  |  |  |  |  |  |  |  |  |  |  |  |  |  |  |  |  |  |  |  |  |  |  |  |  |  |  |  |  |  |  |  |  |  |  |  |  |  |  |  |  |  |  |  |  |  |  |  |  |  |  |  |  |  |  |  |  |  |  |  |  |  |  |  |  |  |  |  |  |  |  |  |  |  |  |  |  |  |  |  |  |  |  |  |  |  |  |  |  |  |  |  |  |  |  |  |  |  |  |  |  |  |  |  |  |  |  |  |  |  |  |  |  |  |  |  |  |  |  |  |  |  |  |  |  |  |  |  |  |  |  |  |  |  |  |  |  |  |  |  |  |  |  |  |  |  |  |  |  |  |  |  |  |  |  |  |  |  |  |  |  |  |  |  |  |  |  |  |  |  |  |  |  |  |  |  |  |  |  |  |  |  |  |  |  |  |  |  |  |  |  |  |  |  |  |  |  |  |  |  |  |  |  |  |  |  |  |  |  |  |  |  |  |  |  |  |  |  |  |  |  |  |  |  |  |  |  |  |  |  |  |  |  |  |  |  |  |  |  |  |  |  |  |  |  |  |  |  |  |  |  |  |  |  |  |  |  |  |  |  |  |  |  |  |  |  |  |  |  |  |  |  |  |  |  |  |  |  |  |  |  |  |  |  |  |  |  |  |  |  |  |  |  |  |  |  |  |  |  |  |  |  |  |  |  |  |  |  |  |  |  |  |  |  |  |  |  |  |  |  |  |  |  |  |  |  |  |  |  |  |  |  |  |  |  |  |  |  |  |  |  |  |  |  |  |  |  |  |  |  |  |  |  |  |  |  |  |  |  |  |  |  |  |  |  |  |  |  |  |  |  |  |  |  |  |  |  |  |  |  |  |  |  |  |  |  |  |  |  |  |  |  |  |  |  |  |  |  |  |  |  |  |  |  |  |  |  |  |  |  |  |  |  |  |  |  |  |  |  |  |  |  |  |  |  |  |  |  |  |  |  |  |  |  |  |  |  |  |  |  |  |  |  |  |  |  |  |  |  |  |  |  |  |  |  |  |  |  |  |  |  |  |  |  |  |  |  |  |  |  |  |  |  |  |  |  |  |  |  |  |  |  |  |  |  |  |  |  |  |  |  |  |  |  |  |  |  |  |  |  |  |  |  |  |  |  |  |  |  |  |  |  |  |  |  |  |  |  |  |  |  |  |  |  |  |  |  |  |  |  |  |  |  |  |  |  |  |  |  |  |  |  |  |  |  |  |  |  |  |  |  |  |  |  |  |  |  |  |  |  |  |  |  |
|  |  |  |  |  |  |  |  |  |  |  |  |  |  |  |  |  |  |  |  |  |  |  |  |  |  |  |  |  |  |  |  |  |  |  |  |  |  |  |  |  |  |  |  |  |  |  |  |  |  |  |  |  |  |  |  |  |  |  |  |  |  |  |  |  |  |  |  |  |  |  |  |  |  |  |  |  |  |  |  |  |  |  |  |  |  |  |  |  |  |  |  |  |  |  |  |  |  |  |  |  |  |  |  |  |  |  |  |  |  |  |  |  |  |  |  |  |  |  |  |  |  |  |  |  |  |  |  |  |  |  |  |  |  |  |  |  |  |  |  |  |  |  |  |  |  |  |  |  |  |  |  |  |  |  |  |  |  |  |  |  |  |  |  |  |  |  |  |  |  |  |  |  |  |  |  |  |  |  |  |  |  |  |  |  |  |  |  |  |  |  |  |  |  |  |  |  |  |  |  |  |  |  |  |  |  |  |  |  |  |  |  |  |  |  |  |  |  |  |  |  |  |  |  |  |  |  |  |  |  |  |  |  |  |  |  |  |  |  |  |  |  |  |  |  |  |  |  |  |  |  |  |  |  |  |  |  |  |  |  |  |  |  |  |  |  |  |  |  |  |  |  |  |  |  |  |  |  |  |  |  |  |  |  |  |  |  |  |  |  |  |  |  |  |  |  |  |  |  |  |  |  |  |  |  |  |  |  |  |  |  |  |  |  |  |  |  |  |  |  |  |  |  |  |  |  |  |  |  |  |  |  |  |  |  |  |  |  |  |  |  |  |  |  |  |  |  |  |  |  |  |  |  |  |  |  |  |  |  |  |  |  |  |  |  |  |  |  |  |  |  |  |  |  |  |  |  |  |  |  |  |  |  |  |  |  |  |  |  |  |  |  |  |  |  |  |  |  |  |  |  |  |  |  |  |  |  |  |  |  |  |  |  |  |  |  |  |  |  |  |  |  |  |  |  |  |  |  |  |  |  |  |  |  |  |  |  |  |  |  |  |  |  |  |  |  |  |  |  |  |  |  |  |  |  |  |  |  |  |  |  |  |  |  |  |  |  |  |  |  |  |  |  |  |  |  |  |  |  |  |  |  |  |  |  |  |  |  |  |  |  |  |  |  |  |  |  |  |  |  |  |  |  |  |  |  |  |  |  |  |  |  |  |  |  |  |  |  |  |  |  |  |  |  |  |  |  |  |  |  |  |  |  |  |  |  |  |  |  |  |  |  |  |  |  |  |  |  |  |  |  |  |  |  |  |  |  |  |  |  |  |  |  |  |  |  |  |  |  |  |  |  |  |  |  |  |  |  |  |  |  |  |  |  |  |  |  |  |  |  |  |  |  |  |  |  |  |  |  |  |  |  |  |  |  |  |  |  |  |  |  |  |  |  |  |  |  |  |  |  |  |  |  |  |  |  |  |  |  |  |  |  |  |  |  |  |  |  |  |  |  |  |  |  |  |  |  |  |  |  |  |  |  |  |  |  |  |  |  |  |  |  |  |  |  |  |  |  |  |  |  |  |  |  |  |  |  |  |  |  |  |  |  |  |  |  |  |  |  |  |  |  |  |  |  |  |  |  |  |  |  |  |  |  |  |  |  |  |  |  |  |  |  |  |  |  |  |  |  |  |  |  |  |  |  |  |  |  |  |  |  |  |  |  |  |  |  |  |  |  |  |  |  |  |  |  |  |  |  |  |  |  |  |  |  |  |  |  |  |  |  |  |  |  |  |  |  |  |  |  |  |  |  |  |  |  |  |  |  |  |  |  |  |  |  |  |  |  |  |  |  |  |  |  |  |  |  |  |  |  |  |  |  |  |  |  |  |  |  |  |  |  |  |  |  |  |  |  |  |  |  |  |  |  |  |  |  |  |  |  |  |  |  |  |  |  |  |  |  |  |  |  |  |  |  |  |  |  |  |  |  |  |  |  |  |  |  |  |  |  |  |  |  |  |  |  |  |  |  |  |  |  |  |  |  |  |  |  |  |  |  |  |  |  |  |  |  |  |  |  |  |  |  |  |  |  |  |  |  |  |  |  |  |  |  |  |  |  |  |  |  |  |  |  |
|  |  |  |  |  |  |  |  |  |  |  |  |  |  |  |  |  |  |  |  |  |  |  |  |  |  |  |  |  |  |  |  |  |  |  |  |  |  |  |  |  |  |  |  |  |  |  |  |  |  |  |  |  |  |  |  |  |  |  |  |  |  |  |  |  |  |  |  |  |  |  |  |  |  |  |  |  |  |  |  |  |  |  |  |  |  |  |  |  |  |  |  |  |  |  |  |  |  |  |  |  |  |  |  |  |  |  |  |  |  |  |  |  |  |  |  |  |  |  |  |  |  |  |  |  |  |  |  |  |  |  |  |  |  |  |  |  |  |  |  |  |  |  |  |  |  |  |  |  |  |  |  |  |  |  |  |  |  |  |  |  |  |  |  |  |  |  |  |  |  |  |  |  |  |  |  |  |  |  |  |  |  |  |  |  |  |  |  |  |  |  |  |  |  |  |  |  |  |  |  |  |  |  |  |  |  |  |  |  |  |  |  |  |  |  |  |  |  |  |  |  |  |  |  |  |  |  |  |  |  |  |  |  |  |  |  |  |  |  |  |  |  |  |  |  |  |  |  |  |  |  |  |  |  |  |  |  |  |  |  |  |  |  |  |  |  |  |  |  |  |  |  |  |  |  |  |  |  |  |  |  |  |  |  |  |  |  |  |  |  |  |  |  |  |  |  |  |  |  |  |  |  |  |  |  |  |  |  |  |  |  |  |  |  |  |  |  |  |  |  |  |  |  |  |  |  |  |  |  |  |  |  |  |  |  |  |  |  |  |  |  |  |  |  |  |  |  |  |  |  |  |  |  |  |  |  |  |  |  |  |  |  |  |  |  |  |  |  |  |  |  |  |  |  |  |  |  |  |  |  |  |  |  |  |  |  |  |  |  |  |  |  |  |  |  |  |  |  |  |  |  |  |  |  |  |  |  |  |  |  |  |  |  |  |  |  |  |  |  |  |  |  |  |  |  |  |  |  |  |  |  |  |  |  |  |  |  |  |  |  |  |  |  |  |  |  |  |  |  |  |  |  |  |  |  |  |  |  |  |  |  |  |  |  |  |  |  |  |  |  |  |  |  |  |  |  |  |  |  |  |  |  |  |  |  |  |  |  |  |  |  |  |  |  |  |  |  |  |  |  |  |  |  |  |  |  |  |  |  |  |  |  |  |  |  |  |  |  |  |  |  |  |  |  |  |  |  |  |  |  |  |  |  |  |  |  |  |  |  |  |  |  |  |  |  |  |  |  |  |  |  |  |  |  |  |  |  |  |  |  |  |  |  |  |  |  |  |  |  |  |  |  |  |  |  |  |  |  |  |  |  |  |  |  |  |  |  |  |  |  |  |  |  |  |  |  |  |  |  |  |  |  |  |  |  |  |  |  |  |  |  |  |  |  |  |  |  |  |  |  |  |  |  |  |  |  |  |  |  |  |  |  |  |  |  |  |  |  |  |  |  |  |  |  |  |  |  |  |  |  |  |  |  |  |  |  |  |  |  |  |  |  |  |  |  |  |  |  |  |  |  |  |  |  |  |  |  |  |  |  |  |  |  |  |  |  |  |  |  |  |  |  |  |  |  |  |  |  |  |  |  |  |  |  |  |  |  |  |  |  |  |  |  |  |  |  |  |  |  |  |  |  |  |  |  |  |  |  |  |  |  |  |  |  |  |  |  |  |  |  |  |  |  |  |  |  |  |  |  |  |  |  |  |  |  |  |  |  |  |  |  |  |  |  |  |  |  |  |  |  |  |  |  |  |  |  |  |  |  |  |  |  |  |  |  |  |  |  |  |  |  |  |  |  |  |  |  |  |  |  |  |  |  |  |  |  |  |  |  |  |  |  |  |  |  |  |  |  |  |  |  |  |  |  |  |  |  |  |  |  |  |  |  |  |  |  |  |  |  |  |  |  |  |  |  |  |  |  |  |  |  |  |  |  |  |  |  |  |  |  |  |  |  |  |  |  |  |  |  |  |  |  |  |  |  |  |  |  |  |  |  |  |  |  |  |  |  |  |  |  |  |  |  |  |  |  |  |  |  |  |  |  |  |  |  |  |  |  |  |  |  |  |  |  |
|  |  |  |  |  |  |  |  |  |  |  |  |  |  |  |  |  |  |  |  |  |  |  |  |  |  |  |  |  |  |  |  |  |  |  |  |  |  |  |  |  |  |  |  |  |  |  |  |  |  |  |  |  |  |  |  |  |  |  |  |  |  |  |  |  |  |  |  |  |  |  |  |  |  |  |  |  |  |  |  |  |  |  |  |  |  |  |  |  |  |  |  |  |  |  |  |  |  |  |  |  |  |  |  |  |  |  |  |  |  |  |  |  |  |  |  |  |  |  |  |  |  |  |  |  |  |  |  |  |  |  |  |  |  |  |  |  |  |  |  |  |  |  |  |  |  |  |  |  |  |  |  |  |  |  |  |  |  |  |  |  |  |  |  |  |  |  |  |  |  |  |  |  |  |  |  |  |  |  |  |  |  |  |  |  |  |  |  |  |  |  |  |  |  |  |  |  |  |  |  |  |  |  |  |  |  |  |  |  |  |  |  |  |  |  |  |  |  |  |  |  |  |  |  |  |  |  |  |  |  |  |  |  |  |  |  |  |  |  |  |  |  |  |  |  |  |  |  |  |  |  |  |  |  |  |  |  |  |  |  |  |  |  |  |  |  |  |  |  |  |  |  |  |  |  |  |  |  |  |  |  |  |  |  |  |  |  |  |  |  |  |  |  |  |  |  |  |  |  |  |  |  |  |  |  |  |  |  |  |  |  |  |  |  |  |  |  |  |  |  |  |  |  |  |  |  |  |  |  |  |  |  |  |  |  |  |  |  |  |  |  |  |  |  |  |  |  |  |  |  |  |  |  |  |  |  |  |  |  |  |  |  |  |  |  |  |  |  |  |  |  |  |  |  |  |  |  |  |  |  |  |  |  |  |  |  |  |  |  |  |  |  |  |  |  |  |  |  |  |  |  |  |  |  |  |  |  |  |  |  |  |  |  |  |  |  |  |  |  |  |  |  |  |  |  |  |  |  |  |  |  |  |  |  |  |  |  |  |  |  |  |  |  |  |  |  |  |  |  |  |  |  |  |  |  |  |  |  |  |  |  |  |  |  |  |  |  |  |  |  |  |  |  |  |  |  |  |  |  |  |  |  |  |  |  |  |  |  |  |  |  |  |  |  |  |  |  |  |  |  |  |  |  |  |  |  |  |  |  |  |  |  |  |  |  |  |  |  |  |  |  |  |  |  |  |  |  |  |  |  |  |  |  |  |  |  |  |  |  |  |  |  |  |  |  |  |  |  |  |  |  |  |  |  |  |  |  |  |  |  |  |  |  |  |  |  |  |  |  |  |  |  |  |  |  |  |  |  |  |  |  |  |  |  |  |  |  |  |  |  |  |  |  |  |  |  |  |  |  |  |  |  |  |  |  |  |  |  |  |  |  |  |  |  |  |  |  |  |  |  |  |  |  |  |  |  |  |  |  |  |  |  |  |  |  |  |  |  |  |  |  |  |  |  |  |  |  |  |  |  |  |  |  |  |  |  |  |  |  |  |  |  |  |  |  |  |  |  |  |  |  |  |  |  |  |  |  |  |  |  |  |  |  |  |  |  |  |  |  |  |  |  |  |  |  |  |  |  |  |  |  |  |  |  |  |  |  |  |  |  |  |  |  |  |  |  |  |  |  |  |  |  |  |  |  |  |  |  |  |  |  |  |  |  |  |  |  |  |  |  |  |  |  |  |  |  |  |  |  |  |  |  |  |  |  |  |  |  |  |  |  |  |  |  |  |  |  |  |  |  |  |  |  |  |  |  |  |  |  |  |  |  |  |  |  |  |  |  |  |  |  |  |  |  |  |  |  |  |  |  |  |  |  |  |  |  |  |  |  |  |  |  |  |  |  |  |  |  |  |  |  |  |  |  |  |  |  |  |  |  |  |  |  |  |  |  |  |  |  |  |  |  |  |  |  |  |  |  |  |  |  |  |  |  |  |  |  |  |  |  |  |  |  |  |  |  |  |  |  |  |  |  |  |  |  |  |  |  |  |  |  |  |  |  |  |  |  |  |  |  |  |  |  |  |  |  |  |  |  |  |  |  |  |  |  |  |  |  |  |  |  |  |  |  |
|  |  |  |  |  |  |  |  |  |  |  |  |  |  |  |  |  |  |  |  |  |  |  |  |  |  |  |  |  |  |  |  |  |  |  |  |  |  |  |  |  |  |  |  |  |  |  |  |  |  |  |  |  |  |  |  |  |  |  |  |  |  |  |  |  |  |  |  |  |  |  |  |  |  |  |  |  |  |  |  |  |  |  |  |  |  |  |  |  |  |  |  |  |  |  |  |  |  |  |  |  |  |  |  |  |  |  |  |  |  |  |  |  |  |  |  |  |  |  |  |  |  |  |  |  |  |  |  |  |  |  |  |  |  |  |  |  |  |  |  |  |  |  |  |  |  |  |  |  |  |  |  |  |  |  |  |  |  |  |  |  |  |  |  |  |  |  |  |  |  |  |  |  |  |  |  |  |  |  |  |  |  |  |  |  |  |  |  |  |  |  |  |  |  |  |  |  |  |  |  |  |  |  |  |  |  |  |  |  |  |  |  |  |  |  |  |  |  |  |  |  |  |  |  |  |  |  |  |  |  |  |  |  |  |  |  |  |  |  |  |  |  |  |  |  |  |  |  |  |  |  |  |  |  |  |  |  |  |  |  |  |  |  |  |  |  |  |  |  |  |  |  |  |  |  |  |  |  |  |  |  |  |  |  |  |  |  |  |  |  |  |  |  |  |  |  |  |  |  |  |  |  |  |  |  |  |  |  |  |  |  |  |  |  |  |  |  |  |  |  |  |  |  |  |  |  |  |  |  |  |  |  |  |  |  |  |  |  |  |  |  |  |  |  |  |  |  |  |  |  |  |  |  |  |  |  |  |  |  |  |  |  |  |  |  |  |  |  |  |  |  |  |  |  |  |  |  |  |  |  |  |  |  |  |  |  |  |  |  |  |  |  |  |  |  |  |  |  |  |  |  |  |  |  |  |  |  |  |  |  |  |  |  |  |  |  |  |  |  |  |  |  |  |  |  |  |  |  |  |  |  |  |  |  |  |  |  |  |  |  |  |  |  |  |  |  |  |  |  |  |  |  |  |  |  |  |  |  |  |  |  |  |  |  |  |  |  |  |  |  |  |  |  |  |  |  |  |  |  |  |  |  |  |  |  |  |  |  |  |  |  |  |  |  |  |  |  |  |  |  |  |  |  |  |  |  |  |  |  |  |  |  |  |  |  |  |  |  |  |  |  |  |  |  |  |  |  |  |  |  |  |  |  |  |  |  |  |  |  |  |  |  |  |  |  |  |  |  |  |  |  |  |  |  |  |  |  |  |  |  |  |  |  |  |  |  |  |  |  |  |  |  |  |  |  |  |  |  |  |  |  |  |  |  |  |  |  |  |  |  |  |  |  |  |  |  |  |  |  |  |  |  |  |  |  |  |  |  |  |  |  |  |  |  |  |  |  |  |  |  |  |  |  |  |  |  |  |  |  |  |  |  |  |  |  |  |  |  |  |  |  |  |  |  |  |  |  |  |  |  |  |  |  |  |  |  |  |  |  |  |  |  |  |  |  |  |  |  |  |  |  |  |  |  |  |  |  |  |  |  |  |  |  |  |  |  |  |  |  |  |  |  |  |  |  |  |  |  |  |  |  |  |  |  |  |  |  |  |  |  |  |  |  |  |  |  |  |  |  |  |  |  |  |  |  |  |  |  |  |  |  |  |  |  |  |  |  |  |  |  |  |  |  |  |  |  |  |  |  |  |  |  |  |  |  |  |  |  |  |  |  |  |  |  |  |  |  |  |  |  |  |  |  |  |  |  |  |  |  |  |  |  |  |  |  |  |  |  |  |  |  |  |  |  |  |  |  |  |  |  |  |  |  |  |  |  |  |  |  |  |  |  |  |  |  |  |  |  |  |  |  |  |  |  |  |  |  |  |  |  |  |  |  |  |  |  |  |  |  |  |  |  |  |  |  |  |  |  |  |  |  |  |  |  |  |  |  |  |  |  |  |  |  |  |  |  |  |  |  |  |  |  |  |  |  |  |  |  |  |  |  |  |  |  |  |  |  |  |  |  |  |  |  |  |  |  |  |  |  |  |  |  |  |  |  |  |  |  |  |  |  |  |  |  |
|  |  |  |  |  |  |  |  |  |  |  |  |  |  |  |  |  |  |  |  |  |  |  |  |  |  |  |  |  |  |  |  |  |  |  |  |  |  |  |  |  |  |  |  |  |  |  |  |  |  |  |  |  |  |  |  |  |  |  |  |  |  |  |  |  |  |  |  |  |  |  |  |  |  |  |  |  |  |  |  |  |  |  |  |  |  |  |  |  |  |  |  |  |  |  |  |  |  |  |  |  |  |  |  |  |  |  |  |  |  |  |  |  |  |  |  |  |  |  |  |  |  |  |  |  |  |  |  |  |  |  |  |  |  |  |  |  |  |  |  |  |  |  |  |  |  |  |  |  |  |  |  |  |  |  |  |  |  |  |  |  |  |  |  |  |  |  |  |  |  |  |  |  |  |  |  |  |  |  |  |  |  |  |  |  |  |  |  |  |  |  |  |  |  |  |  |  |  |  |  |  |  |  |  |  |  |  |  |  |  |  |  |  |  |  |  |  |  |  |  |  |  |  |  |  |  |  |  |  |  |  |  |  |  |  |  |  |  |  |  |  |  |  |  |  |  |  |  |  |  |  |  |  |  |  |  |  |  |  |  |  |  |  |  |  |  |  |  |  |  |  |  |  |  |  |  |  |  |  |  |  |  |  |  |  |  |  |  |  |  |  |  |  |  |  |  |  |  |  |  |  |  |  |  |  |  |  |  |  |  |  |  |  |  |  |  |  |  |  |  |  |  |  |  |  |  |  |  |  |  |  |  |  |  |  |  |  |  |  |  |  |  |  |  |  |  |  |  |  |  |  |  |  |  |  |  |  |  |  |  |  |  |  |  |  |  |  |  |  |  |  |  |  |  |  |  |  |  |  |  |  |  |  |  |  |  |  |  |  |  |  |  |  |  |  |  |  |  |  |  |  |  |  |  |  |  |  |  |  |  |  |  |  |  |  |  |  |  |  |  |  |  |  |  |  |  |  |  |  |  |  |  |  |  |  |  |  |  |  |  |  |  |  |  |  |  |  |  |  |  |  |  |  |  |  |  |  |  |  |  |  |  |  |  |  |  |  |  |  |  |  |  |  |  |  |  |  |  |  |  |  |  |  |  |  |  |  |  |  |  |  |  |  |  |  |  |  |  |  |  |  |  |  |  |  |  |  |  |  |  |  |  |  |  |  |  |  |  |  |  |  |  |  |  |  |  |  |  |  |  |  |  |  |  |  |  |  |  |  |  |  |  |  |  |  |  |  |  |  |  |  |  |  |  |  |  |  |  |  |  |  |  |  |  |  |  |  |  |  |  |  |  |  |  |  |  |  |  |  |  |  |  |  |  |  |  |  |  |  |  |  |  |  |  |  |  |  |  |  |  |  |  |  |  |  |  |  |  |  |  |  |  |  |  |  |  |  |  |  |  |  |  |  |  |  |  |  |  |  |  |  |  |  |  |  |  |  |  |  |  |  |  |  |  |  |  |  |  |  |  |  |  |  |  |  |  |  |  |  |  |  |  |  |  |  |  |  |  |  |  |  |  |  |  |  |  |  |  |  |  |  |  |  |  |  |  |  |  |  |  |  |  |  |  |  |  |  |  |  |  |  |  |  |  |  |  |  |  |  |  |  |  |  |  |  |  |  |  |  |  |  |  |  |  |  |  |  |  |  |  |  |  |  |  |  |  |  |  |  |  |  |  |  |  |  |  |  |  |  |  |  |  |  |  |  |  |  |  |  |  |  |  |  |  |  |  |  |  |  |  |  |  |  |  |  |  |  |  |  |  |  |  |  |  |  |  |  |  |  |  |  |  |  |  |  |  |  |  |  |  |  |  |  |  |  |  |  |  |  |  |  |  |  |  |  |  |  |  |  |  |  |  |  |  |  |  |  |  |  |  |  |  |  |  |  |  |  |  |  |  |  |  |  |  |  |  |  |  |  |  |  |  |  |  |  |  |  |  |  |  |  |  |  |  |  |  |  |  |  |  |  |  |  |  |  |  |  |  |  |  |  |  |  |  |  |  |  |  |  |  |  |  |  |  |  |  |  |  |  |  |  |  |  |  |  |  |  |  |  |  |  |  |  |  |
| BIS d=0 cl.2 sym=1 env=1 | - | - | - | - | - | - | - | - | - | - | - | - | - | - | - | - | - | - | - | - | - | - | - | - | - | - | - | - | - | - | - | - | - | - | - | - | - | - | - | - | - | - | - | - | - | - | - | - | - | - | - | - | - | - | - | - | - | - | - | - | - | - | - | - | - | - | H | x | x | x | x | x | x | x | x | x | x | x | x | x | x | x | x | x | x | x | x | x | x | x | x | x | x | x | x | x | x | x | x | H | x | x | x | x | x | x | x | H | x | x | x | x | x | x | x | x | x | x | x | x | x | x | x | x | x | x | x | x | x | x | x | x | x | x | x | x | x | x | x | x | x | x | x | x | x | x | x | x | x | x | x | x | x | x | x | x | x | x | x | x | x | x | x | x | x | x | x | x | x | x | x | x | x | x | x | x | x | x | x | x | x | x | x | x | x | x | x | x | x | x | x | x | x | x | x | x | x | x | x | x | x | x | x | x | x | x | x | x | x | x | H | - | - | - | - | - | - | - | - | - | - | - | - | - | - | - | - | - | - | - | - | - | - | - | - | - | - | - | - | - | - | - | - | - | - | - | - | - | - | - | - | - | - | - | - | - | - | - | - | - | - | - | - | - | - | - | - | - | - | - | - | - | - | - | - | - | - | - | - | - | - | - | - | - | - | - | - | - | - | - | - | - | - | - | - | - | - | - | - | - | - | - | - | - | - | - | - | - | - | - | - | - | - | - | - | - | - | - | - | - | - | - | - | - | - | - | - | - | - | - | - | - | - | - | - | - | - | - | - | - | - | - | - | - | - | - | - | - | - | - | - | - | - | - | - | - | - | - | - | - | - | - | - | - | - | - | - | - | - | - | - | - | - | - | - | - | - | - | - | - | - | - | - | - | - | - | - | - | - | - | - | - | - | - | - | - | - | - | - | - | - | - | - | - | - | - | - | - | - | - | - | - | - | - | - | - | - | - | - | - | - | - | - | - | - | - | - | - | - | - | - | - | - | - | - | - | - | - | - | - | - | - | - | - | - | - | - | - | - | - | - | - | - | - | - | - | - | - | - | - | - | - | - | - | - | - | - | - | - | - | - | - | - | - | - | - | - | - | - | - | - | - | - | - | - | - | - | - | - | - | - | - | - | - | - | - | - | - | - | - | - | - | - | - | - | - | - | - | - | - | - | - | - | - | - | - | - | - | - | - | - | - | - | - | - | - | - | - | - | - | - | - | - | - | - | - | - | - | - | - | - | - | - | - | - | - | - | - | - | - | - | - | - | - | - | - | - | - | - | - | - | - | - | - | - | - | - | - | - | - | - | - | - | - | - | - | - | - | - | - | - | - | - | - | - | - | - | - | - | - | - | - | - | - | - | - | - | - | - | - | - | - | - | - | - | - | - | - | - | - | - | - | - | - | - | - | - | - | - | - | - | - | - | - | - | - | - | - | - | - | - | - | - | - | - | - | - | - | - | - | - | - | - | - | - | - | - | - | - | - | - | - | - | - | - | - | - | - | - | - | - | - | - | - | - | - | - | - | - | - | - | - | - | - | - | - | - | - | - | - | - | - | - | - | - | - | - | - | - | - | - | - | - | - | - | - | - | - | - | - | - | - | - | - | - | - | - | - | - | - | - | - | - | - | - | - | - | - | - | - | - | - | - | - | - | - | - | - | - | - | - | - | - | - | - | - | - | - | - | - | - | - | - | - | - | - | - | - | - | - | - | - | - | - | - | - | - | - | - | - | - | - | - | - | - | - | - | - | - | - | - | - | - | - | - | - | - | - | - | - | - | - | - | - | - | - | - | - | - | - | - | - | - | - | - | - | - | - | - | - | - | - | - | - | - | - | - | - | - | - | - | - | - | - | - | - | - | - | - | - | - | - | - | - | - | - | - | - | - | - | - | - | - | - | - | - | - | - | - | - | - | - | - | - | - | - | - | - | - | - | - | - | - | - | - | - | - | - | - | - | - | - | - | - | - | - | - | - | - | - | - | - | - | - | - | - | - | - | - | - | - | - | - | - | - | - | - | - | - | - | - | - | - | - | - | - | - | - | - | - | - | - | - | - | - | - | - | - | - | - | - | - | - |
| BIS d=0 cl.1 sym=1 env=1 | - | - | - | - | - | - | - | - | - | - | - | - | - | - | - | - | - | - | - | - | - | - | - | - | - | - | - | - | - | - | - | - | - | - | - | - | - | - | - | - | - | - | - | - | - | - | - | - | - | - | - | - | - | - | - | - | - | - | - | - | - | - | - | - | - | - | - | - | - | - | - | - | - | - | - | - | - | - | - | - | - | H | H | x | x | x | x | x | x | x | x | x | H | H | x | H | x | H | x | x | x | x | x | x | x | x | x | x | x | x | x | x | x | H | x | x | H | x | x | x | x | x | x | x | x | x | H | x | x | x | x | H | x | x | x | x | x | H | x | x | H | x | x | H | x | H | x | H | x | x | x | x | x | x | x | x | x | x | H | x | x | H | x | x | H | x | x | x | x | x | x | x | x | x | x | x | x | x | x | x | x | x | x | x | H | x | x | x | x | x | x | x | H | x | x | H | x | x | H | x | x | x | x | x | x | x | x | x | x | x | x | x | x | x | x | x | x | x | H | x | x | x | x | x | x | x | x | x | x | x | x | x | H | x | x | H | x | x | x | x | x | x | x | x | x | x | x | x | x | x | H | x | x | x | x | x | x | x | x | x | x | x | x | x | x | x | x | x | x | x | x | x | x | x | x | x | x | x | x | x | x | x | x | x | x | x | x | x | x | x | x | x | x | x | x | x | x | x | x | x | x | x | x | x | x | x | x | x | x | x | x | x | x | x | x | x | x | x | x | x | x | x | x | x | x | x | x | x | x | x | x | x | x | x | x | x | x | x | x | x | x | x | x | x | x | x | x | x | x | x | x | x | x | x | x | x | x | x | x | x | x | x | x | x | x | x | x | x | x | x | x | x | x | x | x | x | x | x | x | x | x | x | x | x | x | x | x | x | x | x | x | x | x | x | x | x | x | x | x | x | x | x | x | x | x | x | x | x | x | x | x | x | x | x | x | x | x | x | H | x | H | x | H | x | x | x | H | x | x | x | x | x | x | x | x | x | x | x | x | x | x | x | x | x | x | x | x | x | x | x | H | x | H | x | x | x | x | x | x | x | x | x | x | x | x | x | x | x | x | x | H | x | x | x | H | x | H | x | x | x | x | x | x | x | x | x | x | H | x | H | H | x | x | x | x | x | x | x | x | H | x | x | x | x | x | x | x | x | x | x | x | x | x | x | x | H | x | x | x | H | x | x | x | x | x | x | x | x | x | x | x | x | x | x | x | x | x | H | x | H | x | x | H | x | x | x | x | H | x | x | x | x | x | x | x | x | x | x | x | x | x | x | x | x | x | x | x | x | x | x | x | x | x | x | x | x | x | x | x | H | x | x | x | x | x | x | x | x | x | x | H | x | x | x | x | x | x | H | x | H | x | x | x | x | x | x | x | x | x | x | x | x | x | x | x | x | x | x | x | x | x | x | x | x | x | x | H | x | H | x | x | H | x | x | x | x | x | x | x | x | x | x | x | x | x | x | x | x | x | H | x | x | x | x | x | x | x | x | x | x | x | x | x | x | x | x | x | x | x | x | x | x | x | x | x | x | x | x | x | x | H | H | x | x | H | x | x | x | x | x | x | x | H | x | x | x | x | x | x | x | x | x | x | x | x | x | x | x | x | x | x | x | x | x | x | x | x | x | x | x | H | x | x | x | x | x | x | H | x | x | x | x | x | x | x | x | x | x | x | x | x | x | x | x | x | x | x | x | x | x | x | x | x | x | x | x | x | x | x | x | H | x | x | x | x | x | x | x | x | H | x | x | H | x | x | x | x | x | x | x | x | x | x | x | x | x | x | x | x | x | x | x | x | x | x | x | x | H | x | x | x | x | x | x | x | x | x | x | x | x | x | x | x | x | x | x | x | x | x | x | x | x | x | x | x | x | x | x | x | x | x | x | x | x | x | x | x | x | x | x | x | x | x | x | x | x | x | x | x | x | H | H | x | x | x | x | x | x | x | x | x | x | H | - | - | - | - | - | - | - | - | - | - | - | - | - | - | - | - | - | - | - | - | - | - | - | - | - | - | - | - | - | - | - | - | - | - | - | - | - | - | - | - | - | - | - | - | - | - | - | - | - | - |
