# Supplementary material for: Variation in selection constraints on teleost TLRs with emphasis on their repertoire in the Walking catfish, Clarias batrachus
Source: Sci Rep. 2020 Dec 7;10:21394. doi: 10.1038/s41598-020-78347-6 (PMC7721727; doi:10.1038/s41598-020-78347-6)
Supplement: Supplementary file 26 — Supplementary Information 26. [file 41598_2020_78347_MOESM26_ESM.zip › T2/BIS2/summary/PF00000-NONREDUNDANT-5DD-dim0-table.html]

BIS cluster table


Clusters with env. score >= 0.5 and sym. score >= 0.5 :

| Dim | Cluster | Sym | Env | Pvalue | Hit patterns and blocks |
| --- | --- | --- | --- | --- | --- |
| 0 | 2 | 1 | 1 | 7.64816e-07 | Hit patterns:   |  |  |  |  |  | | --- | --- | --- | --- | --- | | Positions: | 67 | 100 | 108 | 211 | | 15 sequences: | D | I | L | L | | 9 sequences: | X | L | F | F |  All positions in cluster: 67 100 108 211 |
| 0 | 1 | 1 | 1 | 1 | All positions in cluster: 82-83 93-94 96 98 114 117 127 132 138 141 144 146 148 159 162 165 185 193 196 199 219 233 236 251 419 421 423 427 451 453 471 475 477 488 490-491 500 516 520 538 540 543 548 580 591 598 600 627 629 632 650 681-682 685 693 721 728 761 770 773 798 851-852 863 |

Table created with bis2html version 8.
