# Supplementary material for: Variation in selection constraints on teleost TLRs with emphasis on their repertoire in the Walking catfish, Clarias batrachus
Source: Sci Rep. 2020 Dec 7;10:21394. doi: 10.1038/s41598-020-78347-6 (PMC7721727; doi:10.1038/s41598-020-78347-6)
Supplement: Supplementary file 26 — Supplementary Information 26. [file 41598_2020_78347_MOESM26_ESM.zip › T2/BIS2/summary/PF00000-NONREDUNDANT-5DD-dim1-table.html]

BIS cluster table


Clusters with env. score >= 0.5 and sym. score >= 0.5 :

| Dim | Cluster | Sym | Env | Pvalue | Hit patterns and blocks |
| --- | --- | --- | --- | --- | --- |
| 1 | 6 | 1 | 1 | 8.497956e-08 | Hit patterns:   |  |  |  |  |  |  | | --- | --- | --- | --- | --- | --- | | Positions: | 85 | 378 | 434 | 457 | 678 | | 15 sequences: | V | S | L | S | H | | 8 sequences: | A | A | M | X | S | | 1 sequence: | D | T | G | D | D |  All positions in cluster: 85 378 434 457 678 |
| 1 | 1 | 1 | 1 | 4.127578e-07 | Hit patterns:   |  |  |  |  | | --- | --- | --- | --- | | Positions: | 636 | 674 | 807 | | 17 sequences: | R | C | I | | 6 sequences: | Q | L | T | | 1 sequence: | A | F | S |  All positions in cluster: 636 674 807 |
| 1 | 8 | 0.5 | 0.910053 | 3.797372e-05 | Hit patterns:   |  |  |  | | --- | --- | --- | | Positions: | 63 | 654 | | 17 sequences: | C | L | | 5 sequences: | T | P | | 1 sequence: | A | L | | 1 sequence: | C | Q |  All positions in cluster: 63 654 |
| 1 | 2 | 1 | 1 | 0.04166667 | All positions in cluster: 598-600 646 |
| 1 | 3 | 1 | 1 | 0.04166667 | All positions in cluster: 560 622 773-774 |
| 1 | 4 | 1 | 1 | 0.04166667 | All positions in cluster: 405 589 |
| 1 | 5 | 1 | 1 | 0.04166667 | All positions in cluster: 268-269 310 520-521 570 |
| 1 | 7 | 1 | 1 | 0.04166667 | All positions in cluster: 72 122 131-132 143-144 155-156 165-166 177 230 247 376 449 464 468 485 494 498 511 513 519-520 524 526 529 547-548 554 572 602 660 688 692-693 802 |
| 1 | 9 | 0.5 | 0.888889 | 1 | Hit patterns:   |  |  |  | | --- | --- | --- | | Positions: | 479 | 512 | | 22 sequences: | N | L | | 1 sequence: | N | I | | 1 sequence: | T | L |  All positions in cluster: 479 512 |

Table created with bis2html version 8.
