# Supplementary material for: Variation in selection constraints on teleost TLRs with emphasis on their repertoire in the Walking catfish, Clarias batrachus
Source: Sci Rep. 2020 Dec 7;10:21394. doi: 10.1038/s41598-020-78347-6 (PMC7721727; doi:10.1038/s41598-020-78347-6)
Supplement: Supplementary file 27 — Supplementary Information 27. [file 41598_2020_78347_MOESM27_ESM.zip › T3/bis2/summary/PF00000-NONREDUNDANT-5DD-dim0-table.html]

BIS cluster table


Clusters with env. score >= 0.5 and sym. score >= 0.5 :

| Dim | Cluster | Sym | Env | Pvalue | Hit patterns and blocks |
| --- | --- | --- | --- | --- | --- |
| 0 | 1 | 1 | 1 | 6.069968e-09 | Hit patterns:   |  |  |  | | --- | --- | --- | | Positions: | 786 | 796 | | 15 sequences: | S | R | | 5 sequences: | X | G | | 4 sequences: | N | C |  All positions in cluster: 786 795-796 |
| 0 | 7 | 1 | 1 | 4.006179e-07 | Hit patterns:   |  |  |  | | --- | --- | --- | | Positions: | 395 | 458 | | 13 sequences: | L | I | | 11 sequences: | M | L |  All positions in cluster: 395 458 |
| 0 | 3 | 1 | 1 | 7.64816e-07 | Hit patterns:   |  |  |  |  |  |  | | --- | --- | --- | --- | --- | --- | | Positions: | 295 | 669 | 762 | 765 | 795 | | 15 sequences: | L | V | L | R | G | | 9 sequences: | Q | T | F | H | E |  All positions in cluster: 295 669-670 762 765 795 |
| 0 | 9 | 1 | 1 | 2.35272e-05 | Hit patterns:   |  |  |  |  | | --- | --- | --- | --- | | Positions: | 311 | 625 | 782 | | 19 sequences: | L | L | T | | 5 sequences: | I | F | M |  All positions in cluster: 311 625 781-782 |
| 0 | 2 | 1 | 1 | 9.410879e-05 | Hit patterns:   |  |  |  |  |  |  |  |  | | --- | --- | --- | --- | --- | --- | --- | --- | | Positions: | 473 | 662 | 735 | 741 | 784 | 801 | 805 | | 20 sequences: | L | I | L | T | G | D | I | | 4 sequences: | V | L | E | V | D | K | V |  All positions in cluster: 472-473 662-663 735-737 741-743 784 801-802 805 |
| 0 | 4 | 1 | 1 | 0.003623188 | Hit patterns:   |  |  |  | | --- | --- | --- | | Positions: | 658 | 745 | | 22 sequences: | Q | A | | 2 sequences: | E | T |  All positions in cluster: 658-660 745 |
| 0 | 6 | 1 | 1 | 0.003623188 | Hit patterns:   |  |  |  | | --- | --- | --- | | Positions: | 502 | 637 | | 22 sequences: | N | L | | 2 sequences: | G | I |  All positions in cluster: 502 636-637 |
| 0 | 8 | 1 | 1 | 0.003623188 | Hit patterns:   |  |  |  | | --- | --- | --- | | Positions: | 279 | 455 | | 22 sequences: | E | I | | 2 sequences: | Q | V |  All positions in cluster: 279-280 455 |
| 0 | 5 | 1 | 1 | 1 | All positions in cluster: 280 288 302-303 305 314 316-317 319 324 327 329 335 340 343 348-349 355-356 359 362 367 379 384 387 390-391 393 398 408 410 414 435 439 442 463 469 472 477 481 483 485 487 490 508 513 519 522 524 540 542 545 551-553 555-557 559 561 568-569 572 574 576 578-581 583-584 586-587 590 592-594 598 604 607 612-613 615-618 628 636 646 649 652 659-660 663 665 670 677 680 685-686 688-693 696 700 702 704 718-721 724 732 736-737 739 742-743 747 768-770 773 781 802 810 813-814 816 822 829 834-835 839 846 849 853-855 858-859 861-862 864-865 867 870 |

Table created with bis2html version 8.
