# Supplementary material for: Variation in selection constraints on teleost TLRs with emphasis on their repertoire in the Walking catfish, Clarias batrachus
Source: Sci Rep. 2020 Dec 7;10:21394. doi: 10.1038/s41598-020-78347-6 (PMC7721727; doi:10.1038/s41598-020-78347-6)
Supplement: Supplementary file 27 — Supplementary Information 27. [file 41598_2020_78347_MOESM27_ESM.zip › T3/bis2/summary/PF00000-NONREDUNDANT-5DD-dim1-table.html]

BIS cluster table


Clusters with env. score >= 0.5 and sym. score >= 0.5 :

| Dim | Cluster | Sym | Env | Pvalue | Hit patterns and blocks |
| --- | --- | --- | --- | --- | --- |
| 1 | 3 | 1 | 1 | 5.098773e-08 | Hit patterns:   |  |  |  | | --- | --- | --- | | Positions: | 71 | 883 | | 14 sequences: | L | I | | 9 sequences: | M | M | | 1 sequence: | X | X |  All positions in cluster: 71 883-884 |
| 1 | 2 | 1 | 1 | 8.497956e-08 | Hit patterns:   |  |  |  | | --- | --- | --- | | Positions: | 233 | 890 | | 15 sequences: | L | V | | 8 sequences: | F | L | | 1 sequence: | X | X |  All positions in cluster: 233 890 |
| 1 | 1 | 1 | 1 | 2.35272e-05 | Hit patterns:   |  |  |  | | --- | --- | --- | | Positions: | 179 | 911 | | 20 sequences: | L | R | | 3 sequences: | T | K | | 1 sequence: | X | X |  All positions in cluster: 179 910-914 |
| 1 | 17 | 1 | 1 | 0.0001646904 | Hit patterns:   |  |  |  | | --- | --- | --- | | Positions: | 404 | 539 | | 21 sequences: | T | P | | 2 sequences: | S | T | | 1 sequence: | G | H |  All positions in cluster: 404 539-540 |
| 1 | 23 | 1 | 1 | 0.0001646904 | Hit patterns:   |  |  |  | | --- | --- | --- | | Positions: | 245 | 250 | | 21 sequences: | K | G | | 2 sequences: | A | D | | 1 sequence: | X | X |  All positions in cluster: 245 250-251 |
| 1 | 4 | 1 | 1 | 0.04166667 | All positions in cluster: 700-702 813-816 |
| 1 | 5 | 1 | 1 | 0.04166667 | All positions in cluster: 802-803 821-822 |
| 1 | 6 | 1 | 1 | 0.04166667 | All positions in cluster: 410-411 463-464 757 |
| 1 | 7 | 1 | 1 | 0.04166667 | All positions in cluster: 450 479 651-652 754 |
| 1 | 8 | 1 | 1 | 0.04166667 | All positions in cluster: 59 66-68 73 77 80-81 87-88 90-92 94 102 105 108 116 126 132 140 142 153 156 164 175-176 182 185-186 188 192 202-203 206 214 223-224 226-227 230 236 239 251 261 305-306 338 624 695-696 751 873 878 884 887-888 892 894-895 897 900 903 907 910 912-914 918 922 928-929 |
| 1 | 9 | 1 | 1 | 0.04166667 | All positions in cluster: 381 468-469 685-693 696-697 |
| 1 | 10 | 1 | 1 | 0.04166667 | All positions in cluster: 547 655 |
| 1 | 11 | 1 | 1 | 0.04166667 | All positions in cluster: 364 540-542 641 |
| 1 | 12 | 1 | 1 | 0.04166667 | All positions in cluster: 417 448 460 516 610 631-632 710 716 |
| 1 | 13 | 1 | 1 | 0.04166667 | All positions in cluster: 401 621 775 |
| 1 | 14 | 1 | 1 | 0.04166667 | All positions in cluster: 512-513 592-595 |
| 1 | 15 | 1 | 1 | 0.04166667 | All positions in cluster: 555-559 638 |
| 1 | 16 | 1 | 1 | 0.04166667 | All positions in cluster: 487-488 551-557 |
| 1 | 18 | 1 | 1 | 0.04166667 | All positions in cluster: 277 438-439 474 517 574-576 820 |
| 1 | 19 | 1 | 1 | 0.04166667 | All positions in cluster: 331 367-368 |
| 1 | 20 | 1 | 1 | 0.04166667 | All positions in cluster: 353 559-561 726 |
| 1 | 21 | 1 | 1 | 0.04166667 | All positions in cluster: 314-317 461 728 |
| 1 | 22 | 1 | 1 | 0.04166667 | All positions in cluster: 283 524-525 682 870-871 |
| 1 | 24 | 0.521739 | 0.987903 | 1 | Hit patterns:   |  |  |  |  |  |  | | --- | --- | --- | --- | --- | --- | | Positions: | 443 | 597 | 648 | 752 | 783 | | 22 sequences: | P | F | D | T | L | | 1 sequence: | P | F | D | T | I | | 1 sequence: | S | I | N | S | L |  All positions in cluster: 442-443 597-598 648-649 752 783 |

Table created with bis2html version 8.
