# Supplementary material for: Variation in selection constraints on teleost TLRs with emphasis on their repertoire in the Walking catfish, Clarias batrachus
Source: Sci Rep. 2020 Dec 7;10:21394. doi: 10.1038/s41598-020-78347-6 (PMC7721727; doi:10.1038/s41598-020-78347-6)
Supplement: Supplementary file 27 — Supplementary Information 27. [file 41598_2020_78347_MOESM27_ESM.zip › T3/bis2/summary/PF00000-NONREDUNDANT-5DD-dim2.html]

Alignment and BIS clusters


|  |  |  |  |  |  |  |  |  |  |  |  |  |  |  |  |  |  |  |  |  |  |  |  |  |  |  |  |  |  |  |  |  |  |  |  |  |  |  |  |  |  |  |  |  |  |  |  |  |  |  |  |  |  |  |  |  |  |  |  |  |  |  |  |  |  |  |  |  |  |  |  |  |  |  |  |  |  |  |  |  |  |  |  |  |  |  |  |  |  |  |  |  |  |  |  |  |  |  |  |  |  |  |  |  |  |  |  |  |  |  |  |  |  |  |  |  |  |  |  |  |  |  |  |  |  |  |  |  |  |  |  |  |  |  |  |  |  |  |  |  |  |  |  |  |  |  |  |  |  |  |  |  |  |  |  |  |  |  |  |  |  |  |  |  |  |  |  |  |  |  |  |  |  |  |  |  |  |  |  |  |  |  |  |  |  |  |  |  |  |  |  |  |  |  |  |  |  |  |  |  |  |  |  |  |  |  |  |  |  |  |  |  |  |  |  |  |  |  |  |  |  |  |  |  |  |  |  |  |  |  |  |  |  |  |  |  |  |  |  |  |  |  |  |  |  |  |  |  |  |  |  |  |  |  |  |  |  |  |  |  |  |  |  |  |  |  |  |  |  |  |  |  |  |  |  |  |  |  |  |  |  |  |  |  |  |  |  |  |  |  |  |  |  |  |  |  |  |  |  |  |  |  |  |  |  |  |  |  |  |  |  |  |  |  |  |  |  |  |  |  |  |  |  |  |  |  |  |  |  |  |  |  |  |  |  |  |  |  |  |  |  |  |  |  |  |  |  |  |  |  |  |  |  |  |  |  |  |  |  |  |  |  |  |  |  |  |  |  |  |  |  |  |  |  |  |  |  |  |  |  |  |  |  |  |  |  |  |  |  |  |  |  |  |  |  |  |  |  |  |  |  |  |  |  |  |  |  |  |  |  |  |  |  |  |  |  |  |  |  |  |  |  |  |  |  |  |  |  |  |  |  |  |  |  |  |  |  |  |  |  |  |  |  |  |  |  |  |  |  |  |  |  |  |  |  |  |  |  |  |  |  |  |  |  |  |  |  |  |  |  |  |  |  |  |  |  |  |  |  |  |  |  |  |  |  |  |  |  |  |  |  |  |  |  |  |  |  |  |  |  |  |  |  |  |  |  |  |  |  |  |  |  |  |  |  |  |  |  |  |  |  |  |  |  |  |  |  |  |  |  |  |  |  |  |  |  |  |  |  |  |  |  |  |  |  |  |  |  |  |  |  |  |  |  |  |  |  |  |  |  |  |  |  |  |  |  |  |  |  |  |  |  |  |  |  |  |  |  |  |  |  |  |  |  |  |  |  |  |  |  |  |  |  |  |  |  |  |  |  |  |  |  |  |  |  |  |  |  |  |  |  |  |  |  |  |  |  |  |  |  |  |  |  |  |  |  |  |  |  |  |  |  |  |  |  |  |  |  |  |  |  |  |  |  |  |  |  |  |  |  |  |  |  |  |  |  |  |  |  |  |  |  |  |  |  |  |  |  |  |  |  |  |  |  |  |  |  |  |  |  |  |  |  |  |  |  |  |  |  |  |  |  |  |  |  |  |  |  |  |  |  |  |  |  |  |  |  |  |  |  |  |  |  |  |  |  |  |  |  |  |  |  |  |  |  |  |  |  |  |  |  |  |  |  |  |  |  |  |  |  |  |  |  |  |  |  |  |  |  |  |  |  |  |  |  |  |  |  |  |  |  |  |  |  |  |  |  |  |  |  |  |  |  |  |  |  |  |  |  |  |  |  |  |  |  |  |  |  |  |  |  |  |  |  |  |  |  |  |  |  |  |  |  |  |  |  |  |  |  |  |  |  |  |  |  |  |  |  |  |  |  |  |  |  |  |  |  |  |  |  |  |  |  |  |  |  |  |  |  |  |  |  |  |  |  |  |  |  |  |  |  |  |  |  |  |  |  |  |  |  |  |  |  |  |  |  |  |  |  |  |  |  |  |  |  |  |  |  |  |  |  |  |  |  |  |  |  |  |  |  |  |  |  |  |  |  |  |  |  |  |  |  |  |  |  |  |  |  |  |  |  |  |  |  |  |  |  |  |  |  |  |  |  |  |  |  |  |  |  |  |  |  |  |  |  |  |  |  |  |  |  |  |  |  |  |  |  |  |  |  |  |  |  |  |  |  |  |
| --- | --- | --- | --- | --- | --- | --- | --- | --- | --- | --- | --- | --- | --- | --- | --- | --- | --- | --- | --- | --- | --- | --- | --- | --- | --- | --- | --- | --- | --- | --- | --- | --- | --- | --- | --- | --- | --- | --- | --- | --- | --- | --- | --- | --- | --- | --- | --- | --- | --- | --- | --- | --- | --- | --- | --- | --- | --- | --- | --- | --- | --- | --- | --- | --- | --- | --- | --- | --- | --- | --- | --- | --- | --- | --- | --- | --- | --- | --- | --- | --- | --- | --- | --- | --- | --- | --- | --- | --- | --- | --- | --- | --- | --- | --- | --- | --- | --- | --- | --- | --- | --- | --- | --- | --- | --- | --- | --- | --- | --- | --- | --- | --- | --- | --- | --- | --- | --- | --- | --- | --- | --- | --- | --- | --- | --- | --- | --- | --- | --- | --- | --- | --- | --- | --- | --- | --- | --- | --- | --- | --- | --- | --- | --- | --- | --- | --- | --- | --- | --- | --- | --- | --- | --- | --- | --- | --- | --- | --- | --- | --- | --- | --- | --- | --- | --- | --- | --- | --- | --- | --- | --- | --- | --- | --- | --- | --- | --- | --- | --- | --- | --- | --- | --- | --- | --- | --- | --- | --- | --- | --- | --- | --- | --- | --- | --- | --- | --- | --- | --- | --- | --- | --- | --- | --- | --- | --- | --- | --- | --- | --- | --- | --- | --- | --- | --- | --- | --- | --- | --- | --- | --- | --- | --- | --- | --- | --- | --- | --- | --- | --- | --- | --- | --- | --- | --- | --- | --- | --- | --- | --- | --- | --- | --- | --- | --- | --- | --- | --- | --- | --- | --- | --- | --- | --- | --- | --- | --- | --- | --- | --- | --- | --- | --- | --- | --- | --- | --- | --- | --- | --- | --- | --- | --- | --- | --- | --- | --- | --- | --- | --- | --- | --- | --- | --- | --- | --- | --- | --- | --- | --- | --- | --- | --- | --- | --- | --- | --- | --- | --- | --- | --- | --- | --- | --- | --- | --- | --- | --- | --- | --- | --- | --- | --- | --- | --- | --- | --- | --- | --- | --- | --- | --- | --- | --- | --- | --- | --- | --- | --- | --- | --- | --- | --- | --- | --- | --- | --- | --- | --- | --- | --- | --- | --- | --- | --- | --- | --- | --- | --- | --- | --- | --- | --- | --- | --- | --- | --- | --- | --- | --- | --- | --- | --- | --- | --- | --- | --- | --- | --- | --- | --- | --- | --- | --- | --- | --- | --- | --- | --- | --- | --- | --- | --- | --- | --- | --- | --- | --- | --- | --- | --- | --- | --- | --- | --- | --- | --- | --- | --- | --- | --- | --- | --- | --- | --- | --- | --- | --- | --- | --- | --- | --- | --- | --- | --- | --- | --- | --- | --- | --- | --- | --- | --- | --- | --- | --- | --- | --- | --- | --- | --- | --- | --- | --- | --- | --- | --- | --- | --- | --- | --- | --- | --- | --- | --- | --- | --- | --- | --- | --- | --- | --- | --- | --- | --- | --- | --- | --- | --- | --- | --- | --- | --- | --- | --- | --- | --- | --- | --- | --- | --- | --- | --- | --- | --- | --- | --- | --- | --- | --- | --- | --- | --- | --- | --- | --- | --- | --- | --- | --- | --- | --- | --- | --- | --- | --- | --- | --- | --- | --- | --- | --- | --- | --- | --- | --- | --- | --- | --- | --- | --- | --- | --- | --- | --- | --- | --- | --- | --- | --- | --- | --- | --- | --- | --- | --- | --- | --- | --- | --- | --- | --- | --- | --- | --- | --- | --- | --- | --- | --- | --- | --- | --- | --- | --- | --- | --- | --- | --- | --- | --- | --- | --- | --- | --- | --- | --- | --- | --- | --- | --- | --- | --- | --- | --- | --- | --- | --- | --- | --- | --- | --- | --- | --- | --- | --- | --- | --- | --- | --- | --- | --- | --- | --- | --- | --- | --- | --- | --- | --- | --- | --- | --- | --- | --- | --- | --- | --- | --- | --- | --- | --- | --- | --- | --- | --- | --- | --- | --- | --- | --- | --- | --- | --- | --- | --- | --- | --- | --- | --- | --- | --- | --- | --- | --- | --- | --- | --- | --- | --- | --- | --- | --- | --- | --- | --- | --- | --- | --- | --- | --- | --- | --- | --- | --- | --- | --- | --- | --- | --- | --- | --- | --- | --- | --- | --- | --- | --- | --- | --- | --- | --- | --- | --- | --- | --- | --- | --- | --- | --- | --- | --- | --- | --- | --- | --- | --- | --- | --- | --- | --- | --- | --- | --- | --- | --- | --- | --- | --- | --- | --- | --- | --- | --- | --- | --- | --- | --- | --- | --- | --- | --- | --- | --- | --- | --- | --- | --- | --- | --- | --- | --- | --- | --- | --- | --- | --- | --- | --- | --- | --- | --- | --- | --- | --- | --- | --- | --- | --- | --- | --- | --- | --- | --- | --- | --- | --- | --- | --- | --- | --- | --- | --- | --- | --- | --- | --- | --- | --- | --- | --- | --- | --- | --- | --- | --- | --- | --- | --- | --- | --- | --- | --- | --- | --- | --- | --- | --- | --- | --- | --- | --- | --- | --- | --- | --- | --- | --- | --- | --- | --- | --- | --- | --- | --- | --- | --- | --- | --- | --- | --- | --- | --- | --- | --- | --- | --- | --- | --- | --- | --- | --- | --- | --- | --- | --- | --- | --- | --- | --- | --- | --- | --- | --- | --- | --- | --- | --- | --- | --- | --- | --- | --- | --- | --- | --- | --- | --- | --- | --- | --- | --- | --- | --- | --- | --- | --- | --- | --- | --- | --- | --- | --- | --- | --- | --- | --- | --- | --- | --- | --- | --- | --- | --- | --- | --- | --- | --- | --- | --- | --- | --- | --- | --- | --- | --- | --- | --- | --- | --- | --- | --- | --- | --- | --- | --- | --- | --- | --- | --- | --- | --- | --- | --- | --- | --- | --- | --- | --- | --- | --- | --- | --- | --- | --- | --- | --- | --- | --- | --- | --- | --- | --- | --- | --- | --- | --- | --- | --- | --- | --- | --- | --- | --- | --- | --- | --- | --- | --- | --- | --- | --- | --- | --- | --- | --- | --- | --- | --- | --- | --- | --- | --- | --- | --- | --- | --- | --- | --- | --- | --- | --- | --- | --- | --- | --- | --- | --- | --- | --- | --- | --- | --- | --- | --- | --- | --- |
|  |  |  |  |  |  |  |  | 1 | 0 |  |  |  |  |  |  |  |  | 2 | 0 |  |  |  |  |  |  |  |  | 3 | 0 |  |  |  |  |  |  |  |  | 4 | 0 |  |  |  |  |  |  |  |  | 5 | 0 |  |  |  |  |  |  |  |  | 6 | 0 |  |  |  |  |  |  |  |  | 7 | 0 |  |  |  |  |  |  |  |  | 8 | 0 |  |  |  |  |  |  |  |  | 9 | 0 |  |  |  |  |  |  |  | 1 | 0 | 0 |  |  |  |  |  |  |  | 1 | 1 | 0 |  |  |  |  |  |  |  | 1 | 2 | 0 |  |  |  |  |  |  |  | 1 | 3 | 0 |  |  |  |  |  |  |  | 1 | 4 | 0 |  |  |  |  |  |  |  | 1 | 5 | 0 |  |  |  |  |  |  |  | 1 | 6 | 0 |  |  |  |  |  |  |  | 1 | 7 | 0 |  |  |  |  |  |  |  | 1 | 8 | 0 |  |  |  |  |  |  |  | 1 | 9 | 0 |  |  |  |  |  |  |  | 2 | 0 | 0 |  |  |  |  |  |  |  | 2 | 1 | 0 |  |  |  |  |  |  |  | 2 | 2 | 0 |  |  |  |  |  |  |  | 2 | 3 | 0 |  |  |  |  |  |  |  | 2 | 4 | 0 |  |  |  |  |  |  |  | 2 | 5 | 0 |  |  |  |  |  |  |  | 2 | 6 | 0 |  |  |  |  |  |  |  | 2 | 7 | 0 |  |  |  |  |  |  |  | 2 | 8 | 0 |  |  |  |  |  |  |  | 2 | 9 | 0 |  |  |  |  |  |  |  | 3 | 0 | 0 |  |  |  |  |  |  |  | 3 | 1 | 0 |  |  |  |  |  |  |  | 3 | 2 | 0 |  |  |  |  |  |  |  | 3 | 3 | 0 |  |  |  |  |  |  |  | 3 | 4 | 0 |  |  |  |  |  |  |  | 3 | 5 | 0 |  |  |  |  |  |  |  | 3 | 6 | 0 |  |  |  |  |  |  |  | 3 | 7 | 0 |  |  |  |  |  |  |  | 3 | 8 | 0 |  |  |  |  |  |  |  | 3 | 9 | 0 |  |  |  |  |  |  |  | 4 | 0 | 0 |  |  |  |  |  |  |  | 4 | 1 | 0 |  |  |  |  |  |  |  | 4 | 2 | 0 |  |  |  |  |  |  |  | 4 | 3 | 0 |  |  |  |  |  |  |  | 4 | 4 | 0 |  |  |  |  |  |  |  | 4 | 5 | 0 |  |  |  |  |  |  |  | 4 | 6 | 0 |  |  |  |  |  |  |  | 4 | 7 | 0 |  |  |  |  |  |  |  | 4 | 8 | 0 |  |  |  |  |  |  |  | 4 | 9 | 0 |  |  |  |  |  |  |  | 5 | 0 | 0 |  |  |  |  |  |  |  | 5 | 1 | 0 |  |  |  |  |  |  |  | 5 | 2 | 0 |  |  |  |  |  |  |  | 5 | 3 | 0 |  |  |  |  |  |  |  | 5 | 4 | 0 |  |  |  |  |  |  |  | 5 | 5 | 0 |  |  |  |  |  |  |  | 5 | 6 | 0 |  |  |  |  |  |  |  | 5 | 7 | 0 |  |  |  |  |  |  |  | 5 | 8 | 0 |  |  |  |  |  |  |  | 5 | 9 | 0 |  |  |  |  |  |  |  | 6 | 0 | 0 |  |  |  |  |  |  |  | 6 | 1 | 0 |  |  |  |  |  |  |  | 6 | 2 | 0 |  |  |  |  |  |  |  | 6 | 3 | 0 |  |  |  |  |  |  |  | 6 | 4 | 0 |  |  |  |  |  |  |  | 6 | 5 | 0 |  |  |  |  |  |  |  | 6 | 6 | 0 |  |  |  |  |  |  |  | 6 | 7 | 0 |  |  |  |  |  |  |  | 6 | 8 | 0 |  |  |  |  |  |  |  | 6 | 9 | 0 |  |  |  |  |  |  |  | 7 | 0 | 0 |  |  |  |  |  |  |  | 7 | 1 | 0 |  |  |  |  |  |  |  | 7 | 2 | 0 |  |  |  |  |  |  |  | 7 | 3 | 0 |  |  |  |  |  |  |  | 7 | 4 | 0 |  |  |  |  |  |  |  | 7 | 5 | 0 |  |  |  |  |  |  |  | 7 | 6 | 0 |  |  |  |  |  |  |  | 7 | 7 | 0 |  |  |  |  |  |  |  | 7 | 8 | 0 |  |  |  |  |  |  |  | 7 | 9 | 0 |  |  |  |  |  |  |  | 8 | 0 | 0 |  |  |  |  |  |  |  | 8 | 1 | 0 |  |  |  |  |  |  |  | 8 | 2 | 0 |  |  |  |  |  |  |  | 8 | 3 | 0 |  |  |  |  |  |  |  | 8 | 4 | 0 |  |  |  |  |  |  |  | 8 | 5 | 0 |  |  |  |  |  |  |  | 8 | 6 | 0 |  |  |  |  |  |  |  | 8 | 7 | 0 |  |  |  |  |  |  |  | 8 | 8 | 0 |  |  |  |  |  |  |  | 8 | 9 | 0 |  |  |  |  |  |  |  | 9 | 0 | 0 |  |  |  |  |  |  |  | 9 | 1 | 0 |  |  |  |  |  |  |  | 9 | 2 | 0 |  |  |  |  |  |  |  | 9 | 3 | 0 |  |  |  |  |  |  |  | 9 | 4 | 0 |  |  |  |  |  |  |  | 9 | 5 | 0 |  |  |  |  |  |  |  |  |
|  |  |  |  |  |  |  |  |  | | |  |  |  |  |  |  |  |  |  | | |  |  |  |  |  |  |  |  |  | | |  |  |  |  |  |  |  |  |  | | |  |  |  |  |  |  |  |  |  | | |  |  |  |  |  |  |  |  |  | | |  |  |  |  |  |  |  |  |  | | |  |  |  |  |  |  |  |  |  | | |  |  |  |  |  |  |  |  |  | | |  |  |  |  |  |  |  |  |  | | |  |  |  |  |  |  |  |  |  | | |  |  |  |  |  |  |  |  |  | | |  |  |  |  |  |  |  |  |  | | |  |  |  |  |  |  |  |  |  | | |  |  |  |  |  |  |  |  |  | | |  |  |  |  |  |  |  |  |  | | |  |  |  |  |  |  |  |  |  | | |  |  |  |  |  |  |  |  |  | | |  |  |  |  |  |  |  |  |  | | |  |  |  |  |  |  |  |  |  | | |  |  |  |  |  |  |  |  |  | | |  |  |  |  |  |  |  |  |  | | |  |  |  |  |  |  |  |  |  | | |  |  |  |  |  |  |  |  |  | | |  |  |  |  |  |  |  |  |  | | |  |  |  |  |  |  |  |  |  | | |  |  |  |  |  |  |  |  |  | | |  |  |  |  |  |  |  |  |  | | |  |  |  |  |  |  |  |  |  | | |  |  |  |  |  |  |  |  |  | | |  |  |  |  |  |  |  |  |  | | |  |  |  |  |  |  |  |  |  | | |  |  |  |  |  |  |  |  |  | | |  |  |  |  |  |  |  |  |  | | |  |  |  |  |  |  |  |  |  | | |  |  |  |  |  |  |  |  |  | | |  |  |  |  |  |  |  |  |  | | |  |  |  |  |  |  |  |  |  | | |  |  |  |  |  |  |  |  |  | | |  |  |  |  |  |  |  |  |  | | |  |  |  |  |  |  |  |  |  | | |  |  |  |  |  |  |  |  |  | | |  |  |  |  |  |  |  |  |  | | |  |  |  |  |  |  |  |  |  | | |  |  |  |  |  |  |  |  |  | | |  |  |  |  |  |  |  |  |  | | |  |  |  |  |  |  |  |  |  | | |  |  |  |  |  |  |  |  |  | | |  |  |  |  |  |  |  |  |  | | |  |  |  |  |  |  |  |  |  | | |  |  |  |  |  |  |  |  |  | | |  |  |  |  |  |  |  |  |  | | |  |  |  |  |  |  |  |  |  | | |  |  |  |  |  |  |  |  |  | | |  |  |  |  |  |  |  |  |  | | |  |  |  |  |  |  |  |  |  | | |  |  |  |  |  |  |  |  |  | | |  |  |  |  |  |  |  |  |  | | |  |  |  |  |  |  |  |  |  | | |  |  |  |  |  |  |  |  |  | | |  |  |  |  |  |  |  |  |  | | |  |  |  |  |  |  |  |  |  | | |  |  |  |  |  |  |  |  |  | | |  |  |  |  |  |  |  |  |  | | |  |  |  |  |  |  |  |  |  | | |  |  |  |  |  |  |  |  |  | | |  |  |  |  |  |  |  |  |  | | |  |  |  |  |  |  |  |  |  | | |  |  |  |  |  |  |  |  |  | | |  |  |  |  |  |  |  |  |  | | |  |  |  |  |  |  |  |  |  | | |  |  |  |  |  |  |  |  |  | | |  |  |  |  |  |  |  |  |  | | |  |  |  |  |  |  |  |  |  | | |  |  |  |  |  |  |  |  |  | | |  |  |  |  |  |  |  |  |  | | |  |  |  |  |  |  |  |  |  | | |  |  |  |  |  |  |  |  |  | | |  |  |  |  |  |  |  |  |  | | |  |  |  |  |  |  |  |  |  | | |  |  |  |  |  |  |  |  |  | | |  |  |  |  |  |  |  |  |  | | |  |  |  |  |  |  |  |  |  | | |  |  |  |  |  |  |  |  |  | | |  |  |  |  |  |  |  |  |  | | |  |  |  |  |  |  |  |  |  | | |  |  |  |  |  |  |  |  |  | | |  |  |  |  |  |  |  |  |  | | |  |  |  |  |  |  |  |  |  | | |  |  |  |  |  |  |  |  |  | | |  |  |  |  |  |  |  |  |  | | |  |  |  |  |  |  |  |  |  | | |  |  |  |  |  |  |  |  |  | | |  |  |  |  |  |  |  |  |  | | |  |  |  |  |  |  |  |  |  | | |  |  |  |  |  |  |  |  |
| CBTLR3\_2874\_BP | X | X | X | X | X | X | X | X | X | X | X | X | X | X | X | X | X | X | X | X | X | X | X | X | X | X | X | X | X | X | X | X | X | X | X | X | X | X | X | X | X | X | X | X | X | X | X | X | X | X | X | X | X | X | X | X | X | X | X | X | X | X | X | X | X | X | X | X | X | X | X | X | X | X | X | X | X | X | X | X | X | X | X | X | X | X | X | X | X | X | X | X | X | X | X | X | X | X | X | X | X | X | X | X | X | X | X | X | X | X | X | X | X | X | X | X | X | X | X | X | X | X | X | X | X | X | X | X | X | X | X | X | X | X | X | X | X | X | X | X | X | X | X | X | X | X | X | X | X | X | X | X | X | X | X | X | X | X | X | X | X | X | X | X | X | X | X | X | X | X | X | X | X | X | X | X | X | X | X | X | X | X | X | X | X | X | X | X | X | X | X | X | X | X | X | X | X | X | X | X | X | X | X | X | X | X | X | X | X | X | X | X | X | X | X | X | X | X | X | X | X | X | X | X | X | X | X | X | X | X | X | X | X | X | X | X | X | X | X | X | X | X | X | X | X | X | X | X | X | X | X | X | X | X | X | X | X | X | X | X | X | X | X | X | X | X | X | X | X | X | X | X | M | S | R | L | C | E | E | L | S | D | T | S | L | K | N | L | S | M | Q | N | T | Q | Q | L | N | L | L | I | S | T | F | K | G | F | D | K | T | N | L | T | I | L | D | L | S | N | N | D | M | S | R | I | A | D | G | A | F | Q | W | F | P | M | L | X | D | X | S | L | E | H | N | N | I | K | Q | L | T | N | G | T | F | S | G | L | K | S | L | K | K | L | N | L | Q | L | A | L | L | K | S | H | A | X | X | L | P | V | I | D | D | F | S | F | Q | P | L | V | Q | L | E | Y | L | Y | M | E | R | T | S | F | R | E | I | T | E | N | I | F | Y | G | L | P | C | L | R | E | L | N | L | G | W | S | X | X | X | T | G | L | K | T | V | S | N | T | T | F | A | S | L | K | G | S | P | L | L | Q | I | L | N | L | T | G | M | G | I | K | A | L | G | P | G | A | F | S | S | L | G | N | L | K | V | L | V | L | S | H | N | F | I | N | Q | Q | L | T | G | E | E | F | Q | G | L | X | S | I | K | E | M | H | L | S | Y | N | Q | Q | M | I | N | L | T | S | M | S | F | K | Y | V | P | T | L | R | T | L | L | L | G | H | G | L | X | X | P | N | X | X | X | K | M | D | P | S | P | F | Q | T | L | V | N | L | V | V | L | D | L | S | N | N | N | I | A | N | I | K | N | G | W | L | D | G | L | Q | H | L | S | V | L | K | L | Q | H | N | N | L | A | R | V | W | K | N | A | N | P | G | G | P | V | L | F | L | R | D | A | Q | N | L | X | V | L | E | L | D | Y | N | G | L | D | E | I | P | T | M | A | F | X | D | L | S | Q | L | R | Y | L | S | I | S | G | N | L | L | N | F | L | H | D | S | I | F | D | D | L | R | S | L | R | Y | L | R | L | Q | K | N | L | L | T | S | V | R | P | E | T | F | K | V | P | L | S | X | L | T | D | L | Y | M | D | H | N | P | F | D | C | T | C | E | S | I | M | W | F | H | K | W | L | N | T | T | N | X | T | X | V | P | X | X | V | E | S | Y | I | C | N | T | P | S | L | Y | F | N | R | S | V | L | D | F | N | P | E | S | C | K | D | L | V | P | F | Q | A | A | Y | I | I | S | N | T | L | V | L | G | L | M | V | I | A | F | L | V | H | Y | Q | G | W | R | I | H | F | Y | W | N | I | L | V | S | R | T | L | D | I | N | E | F | R | Y | K | R | L | S | E | C | R | Y | K | Y | K | A | Y | I | V | H | S | Q | K | D | I | L | W | V | E | R | S | L | L | P | L | E | D | X | X | D | F | R | F | F | L | E | D | R | D | A | M | P | G | C | S | T | L | E | T | I | V | D | N | M | R | M | S | R | K | I | V | F | V | V | T | E | A | L | L | N | D | P | W | C | R | Q | X | X | X | X | X | X | X | X | X | X | X | X | X | X | X | X | X | X | X | X | X | X | X | X | X | X | X | X | X | X | X | X | X | X | X | X | X | X | X | X | X | X | X | X | X | X | X | X | X | X | X | X | X | X | X | X | X | X | X | X | X | X | X | X | X | X | X | X | X | X | X | X | X | X | X | X | X | X | X | X | X | X | X | X | X | X |
| TFTLR3\_2874\_BP | X | X | X | X | X | X | X | X | X | X | X | X | X | X | X | X | X | X | X | X | M | E | I | W | N | X | X | T | V | L | L | L | L | X | F | L | C | M | W | I | L | M | Q | T | X | X | X | X | X | X | X | X | X | A | Y | K | N | E | C | R | V | E | L | R | K | A | D | C | S | H | M | N | L | K | T | I | P | Q | D | L | P | E | D | I | R | T | L | D | V | S | H | N | C | L | V | E | L | K | P | S | S | L | T | R | Y | H | S | L | E | Q | L | D | V | S | Y | N | S | L | K | G | V | P | A | G | L | C | Q | A | M | P | E | L | W | R | L | T | L | R | H | N | E | V | H | L | L | Q | V | R | D | M | R | N | C | T | R | L | T | H | L | D | L | S | D | N | R | L | R | L | N | X | X | X | X | D | P | F | S | G | T | E | N | L | A | W | L | D | V | S | R | N | K | L | T | T | A | R | L | G | S | R | P | Q | L | P | S | L | E | T | L | I | L | T | G | N | D | I | T | E | L | K | K | D | D | F | Y | F | L | S | Q | S | L | S | F | R | T | L | S | L | S | X | S | L | P | L | K | K | V | E | H | G | C | F | K | P | L | A | Q | L | Q | D | L | V | L | D | G | C | K | L | N | P | T | F | M | S | T | L | C | K | E | L | S | D | T | S | L | R | N | L | S | V | Q | D | T | Q | Q | V | M | L | T | N | S | T | F | M | G | L | G | K | T | N | L | T | I | L | D | L | S | N | N | K | M | N | Q | I | S | N | G | A | F | Q | W | F | P | M | L | V | Y | L | S | L | E | Y | N | N | L | K | H | L | T | N | G | T | F | M | G | L | K | S | L | R | K | L | K | L | Q | M | A | L | T | K | S | H | T | S | A | L | P | V | I | D | D | F | S | F | Q | P | L | V | Q | L | E | Y | L | Y | M | E | R | T | A | F | R | E | I | T | E | N | I | F | Y | G | L | L | C | L | R | E | L | N | L | G | W | S | X | X | X | T | G | L | K | I | I | S | N | T | T | F | A | S | L | K | G | S | P | L | L | Q | T | L | N | L | S | G | M | G | I | K | G | L | G | P | G | A | F | S | S | L | G | N | L | K | T | L | V | L | S | H | N | F | I | N | Q | N | L | T | G | K | E | F | E | G | L | N | S | I | K | E | L | H | L | S | F | N | Q | Q | M | I | T | L | M | S | T | A | F | F | H | V | P | T | L | S | T | L | M | L | G | H | A | L | X | X | P | N | X | X | X | D | V | E | P | S | P | F | R | P | L | V | N | L | M | M | L | D | L | S | N | N | N | I | A | N | I | R | N | G | L | L | E | G | L | D | H | L | S | V | L | K | L | Q | H | N | N | L | A | R | V | W | K | N | A | N | P | G | G | P | M | L | F | L | S | D | A | Q | N | L | T | V | L | Q | L | D | Y | N | G | L | D | E | I | P | R | M | A | F | Q | G | L | S | Q | L | K | Y | L | S | I | S | G | N | L | L | N | F | L | H | D | S | I | F | D | D | L | R | S | L | R | F | L | Q | L | Q | K | N | L | L | T | S | V | R | R | E | T | F | R | V | P | L | S | N | L | T | E | L | I | M | D | H | N | P | F | D | C | T | C | E | S | M | L | W | F | H | T | W | L | N | T | T | N | X | T | S | V | P | D | R | D | K | S | Y | I | C | N | T | P | S | M | Y | F | N | C | S | V | M | D | F | N | P | E | S | C | K | D | M | V | P | F | K | A | A | Y | I | I | T | S | T | L | V | L | G | L | M | A | I | A | F | L | V | H | Y | Q | G | W | R | I | Q | F | Y | W | N | I | V | V | N | R | T | L | D | I | N | D | S | R | Y | R | R | V | T | E | C | R | Y | E | Y | K | A | Y | I | V | H | A | Q | K | D | K | L | W | V | E | R | S | L | L | P | L | E | D | X | X | N | F | R | F | F | L | E | D | R | D | A | M | P | G | C | S | T | L | E | T | I | V | D | N | M | R | M | S | R | K | V | V | F | V | V | T | E | A | L | L | N | D | P | W | C | R | K | F | K | A | H | Q | A | L | H | Q | V | M | E | E | N | R | D | S | L | V | L | I | F | L | E | D | V | A | D | Y | L | L | T | Q | S | L | L | L | R | K | G | M | L | K | R | R | C | M | V | H | W | X | X | L | Q | K | E | R | I | P | X | I | S | P | E | T | A | A | S | I | S | F | K | Q | P | S | Q | X | X | X | X | X | X | X | X | X | X |
| PHTLR3\_2874\_BP | X | X | X | X | X | X | X | X | X | X | X | X | X | X | X | X | X | X | X | X | M | A | I | W | N | X | X | T | T | L | R | L | L | X | F | F | C | M | W | V | L | V | P | S | X | X | X | X | X | X | X | X | X | A | Y | R | S | E | C | R | L | E | L | R | K | A | D | C | S | H | M | N | L | N | T | I | P | Q | D | L | P | E | D | I | S | V | L | D | V | S | H | N | R | L | V | E | L | K | P | S | S | L | T | R | Y | H | S | L | E | Q | L | D | A | S | Y | N | N | L | K | V | V | P | A | G | L | C | Q | A | T | P | E | L | W | R | L | T | L | R | H | N | E | V | H | L | L | Q | D | R | D | L | R | N | C | T | H | L | I | H | L | D | L | S | D | N | R | L | R | L | N | X | X | X | X | E | P | F | S | G | T | E | N | L | A | W | L | D | V | S | R | N | K | L | T | T | A | R | L | G | S | R | S | Q | L | P | R | L | E | T | L | V | L | A | G | N | D | I | T | D | L | K | M | D | D | F | N | F | L | S | E | S | S | S | F | R | T | L | A | L | S | X | S | L | P | L | K | K | V | E | R | G | C | F | K | P | I | A | G | L | Q | D | L | V | L | N | G | C | K | L | N | P | T | L | M | A | S | L | C | E | E | L | S | E | T | S | L | R | N | L | S | V | Q | N | T | Q | Q | V | I | L | L | T | S | T | F | K | G | L | D | K | T | N | L | T | I | L | D | L | S | N | N | N | M | Y | R | I | T | D | G | A | F | Q | W | F | P | M | L | E | Y | L | S | L | E | Q | N | N | I | K | H | L | T | N | S | T | F | L | G | L | K | S | L | R | K | L | N | L | Q | S | A | L | A | K | G | H | T | S | P | L | P | V | I | D | D | F | S | F | Q | L | L | V | K | L | E | Y | L | Y | M | E | K | T | S | F | R | E | I | T | E | N | I | F | Y | G | L | P | C | L | R | E | L | N | L | G | W | S | X | X | X | T | G | L | K | T | V | S | N | T | T | F | A | S | L | K | G | S | P | L | L | Q | T | L | N | L | T | G | M | G | I | K | T | L | G | P | G | A | F | S | S | M | G | N | L | K | T | L | V | L | S | H | N | Y | I | N | Q | Y | L | T | G | E | E | F | Q | G | L | N | S | I | Q | E | I | H | L | S | F | N | Q | Q | M | I | T | L | M | S | T | S | F | I | H | V | P | T | L | R | I | L | M | L | G | H | A | L | X | X | P | N | X | X | X | E | M | E | P | S | P | F | R | L | L | V | N | L | T | V | L | D | L | S | N | N | N | I | A | N | I | K | N | G | L | L | D | G | L | Q | H | L | S | V | L | K | L | Q | H | N | N | L | A | R | V | W | K | N | A | N | P | G | G | P | V | L | F | L | R | N | A | Q | N | L | T | V | L | E | L | D | Y | N | G | L | D | E | I | P | T | M | A | F | Q | G | L | S | Q | L | K | Y | L | S | I | S | G | N | L | L | N | F | L | H | D | S | I | F | D | D | L | W | S | L | R | F | L | R | L | Q | K | N | L | L | T | S | V | R | Q | E | T | F | K | V | P | L | S | N | L | T | E | L | F | M | D | H | N | P | F | D | C | T | C | E | S | I | L | W | F | Y | S | W | L | N | T | T | N | X | T | S | V | P | D | R | A | K | I | Y | I | C | N | T | P | S | M | Y | F | N | R | S | V | L | D | F | N | P | E | S | C | K | D | L | V | P | F | Q | A | V | Y | V | I | T | S | T | L | V | L | G | L | M | V | I | A | F | L | L | H | F | Q | G | W | R | I | Q | F | Y | W | N | I | L | V | N | R | T | I | D | I | N | D | S | R | F | K | R | L | V | E | C | R | Y | E | Y | K | A | Y | V | V | H | A | Q | Q | D | K | L | W | V | E | R | S | L | L | P | L | E | D | X | X | D | F | K | F | F | L | E | D | R | D | A | M | P | G | C | S | T | L | E | T | I | V | D | N | M | R | M | S | R | K | I | I | F | V | V | T | E | A | L | L | N | D | P | W | C | R | Q | F | K | A | H | H | A | L | H | Q | V | M | E | E | N | R | D | S | L | V | L | I | F | L | E | D | V | A | D | Y | R | L | T | Q | S | L | M | L | R | K | G | M | L | K | R | H | C | M | V | Q | W | X | X | L | Q | K | E | R | I | P | X | L | P | P | E | A | T | A | S | V | S | L | K | Q | P | G | Q | L | K | P | T | I | R | A | E | P | G |
| IPTLR3\_2874\_BP | X | X | X | X | X | X | X | X | X | X | X | X | X | X | X | X | X | X | X | X | M | A | I | W | N | X | X | T | T | L | P | L | L | X | F | L | C | T | W | V | L | V | P | S | X | X | X | X | X | X | X | X | X | A | Y | R | S | E | C | R | V | E | L | R | K | A | D | C | S | H | M | N | L | N | T | I | P | Q | D | L | P | E | D | I | S | V | L | D | V | S | H | N | R | L | V | E | L | K | P | S | S | L | T | R | Y | R | S | L | E | H | I | D | A | S | Y | N | S | L | K | A | V | P | A | G | L | C | Q | A | T | P | E | L | W | W | L | T | L | R | H | N | E | V | H | L | L | Q | D | R | D | L | R | N | C | T | R | L | T | H | L | D | L | S | D | N | R | L | R | L | T | X | X | X | X | E | P | F | S | G | T | E | N | L | A | F | L | D | V | S | R | N | K | L | T | T | A | R | L | G | S | R | P | Q | L | P | S | L | Q | T | L | V | V | A | G | N | D | I | A | D | L | K | K | D | D | F | Y | F | L | N | E | S | S | L | F | R | T | L | S | L | S | X | S | L | P | L | K | K | V | D | R | G | C | F | K | P | I | A | G | L | Q | D | L | V | L | D | G | C | K | L | N | P | S | F | M | S | R | L | C | E | E | L | S | D | T | S | L | R | N | L | S | I | Q | F | T | Q | Q | V | M | L | N | N | S | T | F | K | G | L | G | K | T | N | L | T | I | L | D | L | S | N | N | N | M | A | R | I | T | D | G | A | F | Q | W | F | P | M | L | E | Y | L | S | L | E | H | N | N | L | K | H | L | T | K | G | T | F | L | G | L | K | S | L | R | K | L | N | L | Q | S | A | L | A | K | G | H | A | S | P | L | P | V | I | D | D | F | S | F | Q | P | L | V | K | L | E | Y | L | Y | M | E | K | T | S | F | R | E | I | T | E | N | I | F | Y | G | L | P | C | L | R | E | L | N | L | G | W | S | X | X | X | T | G | L | K | I | V | S | N | T | T | F | A | S | L | N | G | S | S | L | L | Q | M | L | N | L | T | G | M | G | I | K | T | L | E | P | G | A | F | S | S | L | G | N | L | E | I | L | V | L | S | H | N | Y | I | N | Q | Q | L | T | G | E | E | F | Q | G | L | H | S | I | R | E | I | H | L | S | F | N | Q | Q | M | I | T | L | M | S | T | S | F | I | H | V | P | T | L | R | T | L | M | L | G | H | A | L | X | X | S | N | X | X | X | E | V | E | P | S | P | F | R | L | L | V | N | L | T | L | L | D | L | S | N | N | N | I | A | N | I | K | N | G | L | L | D | G | L | H | H | L | S | V | L | K | L | Q | H | N | N | L | A | R | V | W | K | N | A | N | P | G | G | P | V | L | I | L | R | D | A | Q | N | L | T | V | L | E | L | D | Y | N | G | L | D | E | I | P | M | K | A | F | Q | G | L | S | K | L | K | Y | L | S | I | S | G | N | L | L | N | F | L | H | D | S | I | F | D | N | L | R | S | L | R | F | L | R | L | Q | K | N | L | L | T | S | V | R | R | E | T | F | G | V | P | L | S | N | L | T | E | L | F | M | D | H | N | P | F | D | C | T | C | E | S | I | L | W | F | N | K | W | L | N | T | T | N | X | T | S | V | P | D | R | A | K | S | Y | V | C | N | T | P | S | M | Y | F | N | R | S | V | L | D | F | N | P | E | S | C | K | D | L | V | P | F | Q | A | L | Y | V | I | T | S | S | L | V | L | G | L | M | V | L | A | F | L | V | H | F | Q | G | W | R | I | Q | F | Y | W | N | I | L | V | N | R | T | L | D | I | N | D | S | R | Y | K | R | V | A | E | C | R | Y | E | Y | K | A | Y | V | V | H | A | Q | E | D | R | L | W | V | E | R | S | L | L | P | L | E | D | X | X | D | F | R | F | F | L | E | D | R | D | A | M | P | G | S | S | R | L | E | T | I | V | D | N | M | R | I | S | R | K | I | L | F | V | V | T | E | A | L | L | N | D | P | W | C | R | Q | F | K | V | H | H | A | L | H | Q | L | M | E | E | N | R | D | S | L | V | L | I | F | L | E | D | V | A | D | Y | R | L | T | Q | S | L | M | L | R | K | G | M | L | K | H | R | C | M | L | Q | W | X | X | L | Q | K | E | R | I | P | X | F | P | P | E | A | T | A | S | V | S | L | E | X | P | G | Q | X | X | X | X | X | X | X | X | X | X |
| DRTLR3\_2874\_BP | X | X | X | X | X | X | X | X | X | X | X | X | X | X | X | X | X | X | X | X | M | D | L | M | K | X | X | X | X | L | I | L | L | P | L | F | Y | A | C | F | S | X | X | X | X | X | H | C | A | G | T | T | N | A | R | K | S | A | C | M | I | K | N | A | K | A | D | C | S | H | M | N | L | D | A | I | P | T | D | L | P | T | N | I | T | T | L | D | V | S | H | N | R | L | K | T | L | X | X | X | S | L | H | M | Y | T | N | L | V | N | I | D | A | S | Y | N | S | L | A | G | I | E | K | D | L | C | L | S | L | P | H | L | Q | F | L | N | A | Q | H | N | Q | V | Y | L | I | S | E | K | D | L | K | N | C | F | H | L | T | Q | L | D | L | S | D | N | K | L | K | L | Q | X | X | X | X | E | P | F | S | L | L | K | N | L | T | W | L | D | V | S | R | N | K | L | T | S | A | K | L | G | T | E | P | Q | L | P | N | L | V | T | L | V | L | S | G | N | N | I | N | I | L | Q | K | N | D | F | S | F | L | S | N | S | S | S | F | R | V | L | I | L | S | X | S | L | I | L | K | K | V | E | N | G | C | F | K | A | I | D | T | L | Y | D | L | V | L | D | S | S | K | L | T | S | Q | F | T | T | S | L | F | E | E | L | A | D | T | A | L | R | N | L | S | L | K | S | T | E | Q | V | T | L | S | N | T | T | F | Q | G | L | E | K | T | K | I | T | V | L | D | L | S | E | N | R | I | S | K | I | V | D | G | A | F | Q | W | L | P | Q | L | E | F | L | S | L | E | H | N | T | I | R | H | L | T | N | D | S | F | S | G | L | G | N | L | R | Q | L | N | L | R | K | A | L | I | K | S | H | X | X | S | L | P | V | I | D | D | F | S | F | Q | H | L | V | Q | L | E | Y | L | C | M | A | N | T | A | F | R | E | L | T | E | Q | I | F | S | G | L | R | N | L | K | T | L | D | L | S | W | S | X | X | X | T | G | I | K | T | V | T | N | K | T | F | A | G | L | K | E | S | P | L | L | Q | T | L | N | L | T | G | M | G | I | N | K | L | E | P | G | A | F | S | S | F | G | N | L | S | N | L | L | M | S | R | N | F | I | N | Q | Q | L | Q | G | N | E | F | K | G | L | S | N | I | K | E | I | D | M | S | I | N | Q | Q | S | I | S | L | T | N | A | S | F | V | H | V | S | T | L | R | I | L | K | L | G | R | A | L | X | X | G | T | X | X | X | D | M | E | P | S | P | F | R | P | L | V | N | L | T | I | L | D | L | S | N | N | N | I | A | N | I | N | G | D | L | L | E | G | L | Y | N | L | K | V | V | K | M | Q | H | N | N | L | A | R | L | W | K | M | A | N | P | G | G | P | V | L | F | L | K | D | A | T | N | L | S | Y | L | N | L | D | Y | N | G | I | D | E | I | P | P | N | A | F | R | G | F | S | E | L | H | E | L | S | L | R | G | N | L | L | D | Q | L | H | A | S | V | F | D | D | L | N | S | L | K | Y | L | H | L | Q | K | N | L | I | T | S | V | Q | R | A | T | F | G | V | P | L | S | K | L | K | E | L | Y | M | D | H | N | P | F | D | C | T | C | E | S | I | L | W | F | S | E | W | L | N | S | T | N | X | T | S | V | P | G | F | P | Q | S | Y | I | C | N | T | P | N | A | Y | F | N | H | S | V | M | N | F | D | P | L | S | C | K | D | M | T | P | F | K | A | L | Y | I | L | S | S | T | A | V | L | M | L | L | F | I | S | F | L | V | H | F | Q | G | W | R | I | Q | F | Y | W | N | I | M | A | N | R | M | L | G | S | X | X | L | K | D | A | K | V | T | E | G | R | F | Q | F | D | A | Y | I | I | H | A | G | E | D | K | S | W | V | E | R | S | L | L | S | L | E | D | X | X | D | L | N | F | F | Y | E | Q | R | D | S | T | P | G | H | S | R | L | K | T | I | V | D | N | M | V | H | S | R | K | I | T | F | V | I | T | E | M | L | L | K | D | P | W | C | R | Q | F | K | A | H | H | A | L | H | H | V | M | E | D | N | R | D | S | L | I | L | I | F | L | E | D | V | T | D | Y | N | L | N | R | S | L | H | L | R | R | G | M | L | K | P | K | C | V | L | Y | W | X | X | L | H | K | E | R | I | P | X | I | S | S | E | T | P | L | S | I | S | L | Y | K | Q | G | Q | X | X | X | X | X | X | X | X | X | X |
| CCATLR3\_2874\_BP | X | X | X | X | X | X | X | X | X | X | X | X | X | X | X | X | X | X | X | X | M | E | R | V | K | X | X | X | X | L | I | L | L | P | L | L | C | I | C | F | H | X | X | X | X | X | H | C | A | G | T | A | Y | P | H | K | S | K | C | T | I | G | N | N | K | A | D | C | S | L | M | N | L | D | A | V | P | T | D | L | P | K | N | I | T | T | L | D | V | S | H | N | R | L | K | N | L | X | X | X | S | L | H | L | Y | S | N | L | M | N | I | D | A | S | Y | N | S | L | K | I | I | E | E | D | L | C | I | S | L | P | H | L | Q | S | F | N | M | Q | H | N | E | V | H | L | L | N | E | K | D | L | K | N | C | S | H | L | T | R | L | D | L | S | Y | N | R | L | K | L | I | X | X | X | X | E | P | F | S | V | L | K | S | L | T | W | L | D | V | S | W | N | T | L | K | S | A | K | S | G | T | Q | P | Q | L | P | N | L | V | T | L | A | L | S | G | N | E | I | S | E | L | Q | K | N | D | F | S | F | L | S | N | S | S | V | F | R | V | L | I | L | S | X | S | L | S | L | K | K | V | E | N | G | C | F | Q | T | I | A | R | L | T | D | L | V | L | D | Y | S | K | I | S | L | Q | L | T | P | S | L | C | E | E | L | A | G | T | A | L | R | N | L | S | L | K | N | T | Q | Q | V | T | L | S | N | T | T | F | Q | G | L | D | K | T | N | I | T | V | L | D | L | S | S | N | T | M | T | K | I | A | N | G | T | F | Q | W | F | P | R | L | E | S | L | S | L | G | H | N | S | L | K | H | L | T | K | D | T | F | L | G | L | G | N | L | R | Q | L | N | L | Q | K | A | L | I | K | S | H | S | S | S | L | P | I | I | E | D | F | S | F | S | H | L | V | Q | L | E | H | L | C | M | T | D | T | A | F | R | E | I | T | E | H | I | F | S | G | L | L | N | L | K | T | L | D | L | S | W | S | X | X | X | T | G | L | K | T | V | T | N | T | T | F | A | S | L | Q | E | S | P | H | L | Q | T | L | N | L | T | A | T | G | I | N | K | L | A | P | G | A | F | S | S | L | G | N | L | T | T | L | L | L | S | H | N | F | I | N | Q | Q | L | K | G | H | E | L | E | G | L | S | N | I | K | E | L | D | M | S | E | N | Q | Q | S | I | S | L | T | N | T | S | F | I | H | V | P | T | L | R | T | L | K | L | G | R | A | V | X | X | G | T | X | X | X | D | I | E | P | S | P | F | R | P | L | V | N | L | T | I | L | D | L | N | N | N | N | I | A | N | I | N | A | G | L | L | K | G | L | Y | N | L | K | V | L | K | M | Q | H | N | N | L | A | R | L | W | K | T | A | N | P | G | G | P | V | L | F | L | K | D | A | T | K | L | S | A | L | D | L | D | Y | N | G | L | D | E | I | P | L | N | A | L | R | G | F | F | E | L | R | E | L | S | L | R | G | N | L | L | D | Q | L | H | A | S | V | F | D | D | L | Q | S | L | K | Y | L | H | L | Q | K | N | L | I | T | S | V | L | R | V | T | F | G | V | P | L | S | N | L | T | E | L | Y | M | D | H | N | P | F | D | C | T | C | E | S | I | L | W | F | S | E | W | L | N | S | T | N | X | V | S | V | P | G | F | P | Q | S | Y | I | C | N | T | P | N | A | Y | F | N | R | S | V | M | D | F | D | P | L | S | C | K | D | M | T | P | F | K | A | L | Y | I | M | S | S | T | A | V | L | M | L | L | F | T | A | F | L | L | H | F | Q | G | W | R | I | Q | F | F | W | S | I | M | V | N | R | M | L | G | S | X | X | L | K | D | E | R | I | T | E | G | R | Y | A | Y | D | A | Y | I | I | H | S | A | E | D | R | P | W | V | E | R | S | L | L | P | L | E | D | X | X | K | L | I | F | F | L | E | D | R | D | A | V | P | G | V | S | Q | L | D | A | I | V | E | N | M | G | R | S | R | K | I | I | F | V | I | T | E | M | L | L | K | D | P | W | C | R | Q | F | K | A | H | H | A | H | H | Q | V | M | E | D | N | R | D | S | L | I | L | I | F | L | Q | D | V | T | D | Y | N | L | N | R | S | L | Y | L | R | R | G | M | L | K | P | H | C | V | L | N | W | X | X | L | H | R | E | R | I | P | X | F | P | X | E | T | P | L | S | T | S | F | Y | Q | Q | S | Q | X | X | X | X | X | X | X | X | X | X |
| CCTLR3\_2874\_BP | X | X | X | X | X | X | X | X | X | X | X | X | X | X | X | X | X | X | X | X | M | E | L | M | K | X | X | X | X | L | I | L | L | P | L | F | Y | T | C | F | H | X | X | X | X | X | H | C | A | G | S | A | Y | P | H | R | S | T | C | T | I | E | N | A | K | A | D | C | S | H | M | N | L | D | V | V | P | T | N | L | P | K | N | I | T | T | L | D | V | S | H | N | R | L | K | N | L | X | X | X | S | L | L | L | Y | S | N | L | V | N | V | D | A | S | Y | N | S | L | T | A | I | E | K | D | L | C | L | S | L | P | H | L | Q | I | L | N | V | Q | H | N | E | V | Y | L | M | N | E | K | V | L | K | N | C | F | H | L | M | R | L | D | L | S | G | N | R | L | K | L | Q | X | X | X | X | E | P | F | S | V | L | K | N | L | T | W | L | D | V | S | R | Y | K | L | K | S | A | K | L | G | T | Q | P | Q | L | P | N | L | V | T | L | I | L | S | G | N | E | I | S | A | L | Q | K | N | D | F | S | F | L | S | N | S | S | A | F | R | V | L | I | L | S | X | S | L | S | L | K | K | V | E | N | G | C | F | Q | A | I | S | R | L | S | D | L | V | L | D | W | S | R | L | S | T | Q | F | T | T | N | L | C | E | E | L | A | G | T | A | L | R | N | L | S | L | K | N | T | F | Q | V | T | L | S | D | T | T | F | K | G | L | D | K | T | N | I | T | V | L | D | L | S | Y | N | T | M | S | K | I | A | D | G | A | F | Q | W | L | P | R | L | E | F | L | S | L | E | H | N | S | L | R | H | L | T | K | D | T | F | S | G | L | G | N | L | R | Q | L | N | L | Q | K | A | L | I | K | S | H | T | S | S | L | P | I | I | E | D | F | S | F | H | H | L | V | Q | L | E | H | L | C | M | A | N | T | A | F | R | E | I | T | E | H | T | F | S | G | L | L | H | L | E | T | L | D | L | S | W | S | X | X | X | T | G | L | K | T | V | T | N | K | T | F | A | S | L | Q | E | S | P | L | L | E | T | L | N | F | T | A | M | G | I | N | K | L | G | P | G | A | F | S | S | L | G | N | L | T | T | L | L | L | G | R | N | F | V | N | Q | Q | L | R | G | D | E | F | E | G | L | T | S | I | K | E | I | D | M | S | I | N | Q | Q | S | I | S | L | T | N | T | S | F | I | H | V | P | T | L | R | T | L | K | L | G | R | A | L | X | X | G | T | X | X | X | D | I | E | P | S | P | F | R | P | L | V | N | I | T | V | L | D | L | S | N | N | N | I | A | N | I | N | D | G | M | L | R | G | L | H | H | L | K | V | L | K | M | Q | H | N | N | L | A | R | L | W | K | T | A | N | P | G | G | P | V | F | F | L | K | D | A | T | K | L | S | V | L | D | L | D | Y | N | G | L | D | E | I | P | L | D | A | L | R | G | F | S | E | L | Q | E | L | S | L | R | G | N | L | L | D | Q | L | H | A | P | V | F | D | D | L | Q | T | L | K | Y | L | H | L | Q | K | N | L | I | T | S | V | Q | R | V | T | F | G | V | P | L | S | N | L | T | D | L | Y | M | D | H | N | P | F | D | C | T | C | E | S | I | L | W | F | S | E | W | L | N | S | T | N | X | V | S | V | P | G | F | P | Q | S | Y | M | C | N | T | P | N | A | Y | F | N | R | S | V | M | D | F | D | P | L | S | C | K | D | M | T | P | F | K | A | L | Y | I | L | S | S | T | A | M | L | M | L | L | F | T | A | F | L | L | H | F | Q | G | W | R | I | Q | F | F | W | S | I | M | V | N | R | M | L | G | S | X | X | L | K | D | E | S | I | T | E | G | R | Y | A | Y | D | A | Y | I | I | H | S | A | E | D | R | P | W | V | E | R | S | L | L | P | L | E | D | X | X | K | F | N | F | F | L | E | D | R | D | A | V | P | G | F | S | Q | L | D | T | I | V | E | N | M | G | Q | S | R | K | I | I | F | V | I | T | E | M | L | L | K | D | P | W | C | R | R | F | K | A | H | H | A | L | H | Q | V | M | E | D | N | R | D | S | L | I | L | I | F | L | Q | D | V | T | D | Y | N | L | N | R | S | L | Y | L | R | R | G | M | L | K | P | H | C | V | L | N | W | X | X | L | H | R | E | R | I | P | X | F | P | S | E | T | L | L | S | T | N | F | Y | Q | Q | G | Q | X | X | X | X | X | X | X | X | X | X |
| MATLR3\_2874\_BP | X | X | X | X | X | X | X | X | X | X | X | X | X | X | X | X | X | X | X | X | M | E | L | M | K | X | X | X | X | L | I | L | L | P | L | F | Y | T | C | F | H | X | X | X | X | X | H | C | A | D | S | A | Y | P | H | K | S | T | C | T | I | E | N | A | K | A | D | C | S | H | M | N | L | D | V | V | P | T | N | L | P | R | N | I | T | T | L | D | V | S | H | N | R | L | K | N | L | X | X | X | S | L | H | L | Y | S | N | L | V | N | V | D | A | S | Y | N | S | L | T | A | I | E | K | D | L | C | L | S | L | P | H | L | Q | I | L | N | L | Q | H | N | E | V | Y | L | M | S | E | K | V | L | K | N | C | F | H | L | K | R | L | D | L | S | V | N | R | L | K | L | Q | X | X | X | X | E | P | F | S | V | L | K | N | L | T | W | L | D | V | S | R | N | K | L | K | S | A | K | L | G | T | Q | P | Q | L | P | N | L | V | T | L | I | L | S | G | N | E | I | S | V | L | Q | K | N | D | F | S | F | L | S | N | S | S | A | F | R | V | L | I | L | S | X | S | L | S | L | K | K | I | E | N | G | C | F | R | A | I | A | R | L | S | D | L | V | L | D | W | S | K | L | S | T | Q | F | T | T | D | L | C | E | E | L | A | S | T | A | V | R | N | L | S | L | K | N | T | L | Q | V | T | L | S | N | M | T | F | K | G | L | D | K | T | N | I | T | V | L | D | L | S | Y | N | T | M | S | K | I | V | D | G | A | F | Q | W | F | P | R | L | E | F | L | S | L | E | H | N | S | L | R | H | L | T | K | D | T | F | S | G | L | G | N | L | R | Q | L | N | L | Q | K | A | L | I | K | S | H | T | S | S | L | P | I | I | E | D | F | S | F | H | H | L | V | Q | L | E | H | L | C | M | A | D | T | A | F | R | E | I | T | E | H | I | F | S | G | L | L | H | L | K | T | L | D | L | S | W | S | X | X | X | T | G | L | K | T | V | T | N | K | T | F | A | S | L | Q | E | S | P | L | L | E | T | L | N | L | S | A | M | G | I | N | K | L | G | P | G | A | F | S | S | L | G | N | L | T | T | L | L | L | G | R | N | F | I | N | Q | Q | L | K | G | D | E | F | E | G | L | T | S | I | K | E | I | D | M | S | I | N | Q | Q | S | I | S | L | T | N | T | S | F | I | H | V | P | T | L | R | T | L | K | L | G | R | A | L | X | X | G | T | X | X | X | D | I | E | P | S | P | F | R | P | L | V | N | L | T | V | L | D | L | S | N | N | N | I | A | N | I | N | A | G | M | L | R | G | L | Y | H | L | K | V | L | K | M | Q | H | N | N | L | A | R | L | W | K | T | A | N | P | G | G | P | V | F | F | L | K | D | A | T | K | L | S | V | L | D | L | D | Y | N | G | L | D | E | I | P | L | D | A | L | R | G | F | S | E | L | H | E | L | S | L | R | G | N | L | L | N | Q | L | H | A | S | V | F | D | D | L | Q | S | L | K | Y | L | H | L | Q | K | N | L | I | T | S | V | Q | R | A | T | F | G | V | P | L | S | N | L | T | E | L | D | M | D | R | N | P | F | D | C | T | C | E | S | I | L | W | F | S | E | W | L | N | S | T | N | X | T | S | V | P | G | F | P | K | S | Y | I | C | N | T | P | N | A | Y | F | N | R | S | V | M | Y | F | D | P | L | S | C | K | D | M | T | P | F | K | A | L | Y | I | L | T | S | T | A | V | L | M | L | L | F | T | A | F | L | V | H | F | Q | G | W | R | I | Q | F | F | W | N | I | I | V | N | R | M | L | G | S | X | X | L | K | D | E | S | V | T | E | G | R | Y | V | Y | D | A | Y | I | I | H | T | A | K | D | R | P | W | V | E | R | S | L | L | P | L | E | D | X | X | K | F | N | F | F | L | E | D | R | D | A | I | P | G | F | S | Q | L | N | T | I | I | E | N | M | G | Q | S | R | K | I | I | F | V | I | T | E | M | L | L | K | D | P | W | C | R | Q | F | K | A | H | H | A | L | H | Q | V | M | E | N | N | R | D | S | L | I | L | I | F | L | Q | D | V | T | D | Y | N | L | N | R | S | L | Y | L | R | R | G | M | L | K | P | R | C | V | L | Y | W | X | X | L | H | K | E | R | I | P | X | F | S | X | E | T | P | L | S | I | S | L | Y | Q | Q | S | X | X | X | X | X | X | X | X | X | X | X |
| CITLR3\_2874\_BP | X | X | X | X | X | X | X | X | X | X | X | X | X | X | X | X | X | X | X | X | M | E | L | M | K | X | X | X | X | L | I | L | L | P | L | F | Y | T | C | F | H | X | X | X | X | X | H | C | A | G | S | A | Y | P | H | R | S | T | C | T | I | E | N | A | K | A | D | C | S | H | M | N | L | D | V | V | P | T | N | L | P | K | N | I | T | T | L | D | V | S | H | N | R | L | K | N | L | X | X | X | S | L | L | L | Y | S | N | L | V | N | V | D | A | S | Y | N | S | L | T | A | I | E | K | D | L | C | L | S | L | P | H | L | Q | I | L | N | V | Q | H | N | E | V | Y | L | M | N | E | K | V | L | K | N | C | F | H | L | M | R | L | D | L | S | G | N | R | L | K | L | Q | X | X | X | X | E | P | F | S | V | L | K | N | L | T | W | L | D | V | S | R | N | K | L | K | S | A | K | L | G | T | Q | P | Q | L | P | N | L | V | T | L | I | L | S | G | N | E | I | S | A | L | Q | K | N | D | F | S | F | L | S | N | S | S | A | F | R | V | L | I | L | S | X | S | L | S | L | K | K | V | E | N | G | C | F | Q | A | I | S | R | L | S | D | L | V | L | D | W | S | R | L | S | T | Q | F | T | T | N | L | C | E | E | L | A | G | T | A | L | R | N | L | S | L | K | N | T | F | Q | V | T | L | S | D | T | T | F | K | G | L | D | K | T | N | I | T | V | L | D | L | S | Y | N | T | M | S | K | I | A | D | G | A | F | Q | W | F | P | R | L | E | F | L | S | L | E | H | N | S | L | R | H | L | T | K | D | T | F | S | G | L | G | N | L | R | Q | L | N | L | Q | K | A | L | I | K | S | H | T | S | S | L | P | I | I | E | D | F | S | F | H | H | L | V | Q | L | E | H | L | C | M | A | N | T | A | F | R | E | I | T | E | H | T | F | S | G | L | L | H | L | K | T | L | D | L | S | W | S | X | X | X | T | G | L | K | T | V | T | N | K | T | F | A | S | L | Q | E | S | P | L | L | E | T | L | N | L | T | A | M | G | I | N | K | L | G | P | G | A | F | S | S | L | G | N | L | T | T | L | L | L | G | R | N | F | I | N | Q | Q | L | R | G | D | E | F | E | G | L | T | S | I | K | E | I | D | M | S | I | N | Q | Q | S | I | S | L | T | N | T | S | F | I | H | V | P | T | L | R | T | L | K | L | G | R | A | L | X | X | G | T | X | X | X | D | I | E | P | S | P | F | R | P | L | V | N | I | T | V | L | D | L | S | N | N | N | I | A | N | I | N | D | G | M | L | R | G | L | Y | H | L | K | V | L | K | M | Q | H | N | N | L | A | R | L | W | K | T | A | N | P | G | G | P | V | F | F | L | K | D | A | T | K | L | S | V | L | D | L | D | Y | N | G | L | D | E | I | P | L | D | A | L | R | G | F | S | E | L | H | E | L | S | L | R | G | N | L | L | D | Q | L | H | A | S | V | F | D | D | L | Q | S | L | K | Y | L | H | L | Q | K | N | L | I | T | S | V | Q | R | A | T | F | G | V | P | L | S | N | L | T | E | L | Y | M | D | R | N | P | F | D | C | T | C | E | S | I | L | W | F | S | E | W | L | N | S | T | N | X | A | S | V | P | G | F | P | Q | S | Y | I | C | N | T | P | N | A | Y | F | N | R | S | V | M | Y | F | D | P | L | S | C | K | D | M | T | P | F | K | A | L | Y | I | M | T | S | T | A | V | L | M | L | L | F | T | A | F | L | V | H | F | Q | G | W | R | I | Q | F | F | W | N | I | I | V | N | R | M | L | G | S | X | X | P | K | D | E | S | V | T | E | G | R | Y | V | Y | D | A | Y | I | I | H | T | A | K | D | R | P | W | V | E | R | S | L | L | P | L | E | D | X | X | N | F | N | F | F | L | E | D | R | D | A | I | P | G | F | S | Q | L | N | T | I | I | E | N | M | G | Q | S | R | K | I | I | F | V | I | T | E | M | L | L | K | D | P | W | C | R | Q | F | K | A | H | H | A | L | H | Q | V | M | E | N | N | R | D | S | L | I | L | I | F | L | Q | D | V | T | D | Y | N | L | N | R | S | L | Y | L | R | R | G | M | L | K | P | R | C | V | L | Y | W | X | X | L | H | R | E | R | I | P | X | F | S | S | E | T | P | L | S | I | S | L | F | Q | Q | G | X | X | X | X | X | X | X | X | X | X | X |
| GMTLR3\_2874\_BP | X | X | X | X | X | X | X | X | X | X | X | X | X | X | X | X | X | X | X | X | M | P | L | P | H | G | A | N | R | L | L | C | W | L | A | L | F | A | H | L | T | A | A | X | X | X | H | G | R | T | A | S | E | P | K | K | T | A | C | V | V | R | G | A | S | A | D | C | S | H | M | N | L | N | V | I | P | Q | D | L | P | Q | D | I | T | R | L | D | V | S | H | N | R | L | T | G | L | P | G | A | N | L | S | R | Y | A | G | L | L | D | L | D | V | T | F | N | S | L | T | K | L | D | P | S | L | C | Q | T | L | S | R | L | R | T | L | N | L | E | R | N | E | V | H | V | L | R | D | Q | D | L | S | G | C | V | Q | L | T | V | L | N | M | A | S | N | R | L | A | F | K | A | K | W | P | D | P | F | A | A | L | E | N | L | T | T | L | D | V | S | N | N | M | L | L | T | A | K | L | G | S | K | P | Q | L | P | R | L | V | A | L | T | L | S | H | N | A | I | S | T | L | K | K | E | D | F | Y | F | L | S | R | S | P | S | L | F | V | L | N | L | S | X | Y | L | P | L | K | K | F | E | D | G | C | F | Q | Q | I | A | N | I | S | F | L | K | M | D | G | C | N | L | A | S | Q | I | X | X | K | L | C | T | E | L | S | D | T | A | I | H | T | L | S | L | Q | D | A | T | L | L | K | L | T | N | I | T | F | K | G | L | H | K | T | N | L | T | I | L | D | L | S | S | N | K | M | S | A | I | E | K | G | A | F | Q | W | L | P | T | L | K | T | L | H | L | E | R | N | N | F | K | H | L | T | K | D | T | F | V | G | L | E | N | L | K | E | L | N | L | R | Q | A | L | V | K | S | Q | T | S | S | T | S | I | I | D | D | Y | T | F | S | P | L | V | A | L | E | T | L | I | L | E | H | T | A | I | R | S | I | T | E | N | T | F | T | G | L | T | S | L | Q | Y | F | Y | I | G | W | S | S | X | X | T | S | L | K | S | L | T | E | K | T | F | V | S | L | A | A | S | P | L | X | R | L | V | N | L | T | A | T | A | I | T | S | I | G | N | G | S | F | S | A | F | P | N | L | T | T | L | L | L | D | F | N | F | I | S | Q | T | L | Q | G | A | E | F | E | G | L | G | Q | L | E | Q | L | F | F | S | N | N | R | Q | K | V | E | L | R | S | L | S | F | V | Q | L | P | K | L | S | L | L | T | L | G | R | T | L | X | X | G | S | X | X | X | D | L | V | P | S | P | F | A | P | L | K | N | L | K | V | L | D | L | S | N | N | N | I | A | N | I | N | A | R | M | L | E | G | L | E | N | L | M | V | L | K | M | Q | H | N | N | L | A | R | V | W | K | S | A | N | V | G | G | P | V | L | F | L | K | D | L | G | N | L | T | V | L | N | M | E | Y | N | G | L | D | E | I | P | T | D | A | L | T | G | L | K | K | L | K | E | M | R | F | T | G | N | L | L | N | N | L | K | D | S | V | F | E | D | L | L | S | L | Q | T | L | S | I | Q | K | N | L | I | T | S | V | R | P | Q | V | F | K | P | A | M | E | N | L | T | L | L | D | M | G | R | N | P | F | D | C | T | C | E | S | I | L | W | F | V | T | W | L | N | T | T | K | X | A | S | L | T | G | G | K | D | Q | F | T | C | N | T | P | L | A | Y | F | N | R | S | V | M | D | F | D | L | L | S | C | K | D | S | T | P | F | Q | A | L | Y | I | L | S | S | T | I | V | I | A | L | M | L | G | A | L | L | V | R | F | Q | G | W | R | I | Q | F | Y | W | N | V | L | I | N | R | T | L | G | F | S | D | A | K | V | E | E | X | X | G | R | E | F | E | F | D | A | Y | V | I | H | A | D | E | D | Q | K | W | V | E | R | S | L | L | P | L | E | T | X | X | R | Y | V | F | C | M | E | G | R | D | F | L | P | G | T | S | R | I | E | A | I | V | D | N | M | R | R | S | R | K | Y | L | F | V | V | T | E | S | L | L | N | D | H | W | C | R | G | F | K | A | H | H | A | L | H | R | V | M | E | E | R | R | D | A | V | I | L | V | F | L | Q | D | I | N | D | Y | R | L | S | R | S | L | F | L | R | R | G | M | L | Q | L | R | C | L | L | N | W | X | X | V | H | K | E | R | I | P | X | L | P | R | E | A | A | Y | R | T | G | D | D | Q | H | A | E | X | X | X | X | X | X | X | X | X | P |
| SSTLR3\_2874\_BP | X | X | X | X | X | X | X | X | X | X | X | X | X | X | X | X | X | X | X | X | M | N | W | P | D | X | X | I | I | L | I | L | L | X | A | V | N | L | G | D | L | I | T | G | P | T | L | C | H | A | S | Q | K | H | Q | K | S | E | C | Q | V | R | N | G | W | A | D | C | R | H | L | R | L | K | E | I | P | P | N | L | P | W | N | I | T | G | L | D | V | S | H | N | R | L | V | E | L | P | P | A | S | L | A | T | Y | P | G | L | V | H | L | D | V | G | F | N | S | L | T | K | L | E | D | S | L | C | Q | T | L | G | L | L | R | T | L | T | V | Q | H | N | E | V | H | G | L | T | E | E | D | L | S | N | C | T | N | L | T | E | L | N | L | A | G | N | R | L | K | L | R | X | X | X | X | D | P | F | A | A | L | Q | S | L | T | L | L | D | V | S | K | N | D | L | K | T | A | K | L | G | T | R | L | Q | L | P | S | L | V | T | L | I | L | S | S | N | S | I | S | T | I | K | K | D | D | F | S | F | L | R | N | S | S | S | L | R | V | L | H | L | S | D | L | I | T | Q | A | K | F | E | P | D | C | L | K | P | I | A | S | I | Y | E | L | V | M | N | G | S | K | L | G | P | S | L | T | S | K | L | C | T | E | L | S | G | T | A | I | R | S | L | S | L | Q | K | T | Q | L | V | T | L | D | N | T | T | F | K | G | L | G | K | T | H | L | T | T | L | D | L | S | H | N | S | I | A | K | I | G | D | G | S | F | Q | W | L | P | M | L | E | V | L | S | L | E | Q | N | N | L | K | H | L | T | K | N | T | F | H | G | L | G | N | L | T | R | L | N | L | N | M | A | L | V | K | S | R | T | S | S | Y | P | I | I | D | D | F | S | F | Q | P | L | G | A | L | E | S | L | S | M | E | N | T | A | F | R | N | I | S | V | F | T | F | A | G | L | M | S | L | R | Q | L | H | L | S | G | T | S | X | X | M | A | L | R | I | I | T | N | Q | T | F | V | S | L | A | D | S | P | L | X | L | T | L | K | L | T | R | T | A | I | S | R | L | D | P | G | A | F | S | S | L | G | N | L | T | T | L | L | L | G | N | N | S | I | S | Q | T | L | T | G | K | E | F | Q | G | L | G | Q | L | Q | E | I | Y | L | S | N | G | N | Q | K | L | I | L | S | P | M | S | F | V | H | V | P | A | L | R | T | L | M | L | G | R | A | L | X | X | S | T | X | X | X | Y | M | N | T | S | P | F | K | P | L | S | N | L | T | I | L | D | L | S | N | N | N | I | A | N | I | K | I | D | L | L | D | G | L | E | N | L | K | V | L | K | L | Q | H | N | N | L | A | R | L | W | K | S | A | N | P | G | G | P | V | L | F | L | R | G | L | R | S | L | V | A | L | E | M | D | F | N | G | L | D | E | I | P | E | E | A | L | H | G | L | T | N | L | Q | E | L | S | L | S | G | N | I | L | N | Q | L | K | D | S | V | F | N | D | L | G | S | L | R | V | L | R | L | Q | K | N | L | I | T | S | V | R | K | E | V | F | G | P | A | L | A | N | L | S | Q | L | V | M | E | K | N | P | F | D | C | T | C | E | S | I | L | W | F | V | A | W | L | N | G | T | N | X | A | S | V | P | G | I | R | D | E | Y | V | C | N | T | P | Q | A | Y | Y | N | R | S | I | M | E | F | D | R | L | S | C | L | D | M | T | P | F | Q | A | L | Y | V | L | T | S | T | A | V | L | T | L | M | V | T | S | L | L | V | R | F | Q | G | W | R | I | Q | F | Y | W | N | V | L | I | N | R | T | L | G | L | S | D | A | S | S | R | E | X | X | G | R | E | F | N | Y | D | A | F | V | I | H | A | A | K | D | K | T | W | V | E | R | S | L | L | P | I | E | D | E | Q | G | Y | R | F | Y | L | L | D | R | D | A | V | P | G | D | L | R | L | E | S | I | V | E | N | M | R | R | S | R | K | I | L | F | V | V | T | E | T | L | L | E | D | S | M | C | R | Q | F | M | A | H | H | A | L | H | Q | V | I | E | D | S | R | D | S | V | V | L | V | F | L | E | D | V | Q | D | Y | K | L | S | R | C | L | L | L | R | R | G | M | L | R | P | H | C | L | L | N | W | X | X | L | Q | R | E | R | V | P | X | L | P | P | E | A | P | H | R | P | G | H | H | Q | P | S | A | X | X | X | X | X | X | X | X | X | X |
| OMTLR3\_2874\_BP | X | X | X | X | X | X | X | X | X | X | X | X | X | X | X | X | X | X | X | X | M | N | W | P | D | X | X | I | I | L | I | I | L | X | A | V | N | L | G | N | L | I | T | G | P | T | L | C | H | A | S | Q | K | R | Q | K | S | E | C | Q | V | R | N | G | W | A | D | C | S | H | L | R | L | K | E | I | P | P | N | L | P | W | N | I | T | G | L | D | V | S | H | N | R | L | V | E | L | P | P | A | S | L | A | T | Y | P | G | L | V | H | L | D | V | G | F | N | S | L | T | K | L | E | D | S | L | C | Q | T | L | G | L | L | R | T | L | T | V | Q | H | N | E | V | H | W | L | R | E | K | D | L | S | N | C | T | N | L | T | E | L | N | L | A | G | N | R | L | K | L | R | X | X | X | X | E | P | F | A | A | L | Q | S | L | T | L | L | D | V | S | K | N | D | L | K | T | A | N | L | G | T | R | L | Q | L | P | S | L | V | T | L | I | L | S | S | N | S | I | S | T | F | K | K | D | D | F | Y | F | L | S | N | S | S | S | L | R | V | L | H | L | S | N | L | T | T | L | A | K | F | E | P | D | C | L | K | P | I | A | S | I | Y | E | L | V | M | N | G | S | K | L | G | P | S | L | T | S | K | L | C | T | E | L | S | G | T | A | I | R | S | L | S | L | Q | K | T | Q | L | V | T | L | D | N | T | T | F | K | G | L | G | K | T | H | L | T | T | L | D | L | S | H | N | S | I | A | K | I | G | D | G | S | F | Q | W | L | S | M | L | E | V | L | S | L | E | Q | N | N | L | K | R | L | T | K | N | T | F | N | G | L | G | N | L | T | R | L | N | L | N | M | A | L | V | K | S | R | T | S | S | Y | P | I | I | D | D | F | S | F | Q | P | L | R | A | L | E | S | L | S | M | E | N | T | A | F | R | N | I | S | V | L | T | F | A | G | L | M | S | L | R | Q | L | H | L | S | R | T | S | X | X | M | S | L | K | I | I | T | N | Q | T | F | V | S | L | A | G | S | P | L | X | L | M | L | K | L | T | S | T | A | I | S | R | L | D | P | G | A | F | S | S | L | G | N | L | T | T | L | L | L | G | N | N | S | I | S | Q | T | L | T | G | K | E | F | Q | G | L | G | Q | L | Q | E | I | Y | L | S | N | G | N | Q | K | L | I | L | S | P | M | S | F | V | H | V | P | A | L | R | T | L | M | L | G | R | A | L | X | X | S | T | X | X | X | Y | M | N | T | S | P | F | K | P | L | S | N | L | T | I | L | D | L | S | N | N | N | I | A | N | I | K | I | D | L | L | D | G | L | E | N | L | K | V | L | K | F | Q | H | N | N | L | A | R | L | W | K | S | A | N | P | G | G | P | V | L | F | L | R | G | L | R | S | L | V | A | L | E | M | D | F | N | G | L | D | E | I | P | D | E | A | L | H | G | L | T | N | L | Q | E | L | S | L | S | G | N | I | L | N | Q | L | R | D | S | V | F | N | D | L | G | S | L | R | V | L | R | L | Q | K | N | L | I | T | S | V | R | K | E | V | F | G | P | A | L | A | N | L | S | Q | L | V | M | E | K | N | P | F | D | C | T | C | E | S | I | L | W | F | V | A | W | L | N | R | T | N | X | A | S | V | P | G | L | R | D | E | Y | V | C | N | T | P | Q | A | Y | Y | N | R | S | I | M | E | F | D | R | L | S | C | Q | D | M | T | P | F | Q | A | L | Y | V | L | T | S | T | A | V | L | T | L | M | V | T | S | L | L | V | R | F | Q | G | W | R | I | Q | F | Y | W | N | V | L | I | N | R | T | L | G | L | S | D | A | S | S | G | E | X | X | G | R | E | F | N | Y | D | A | Y | V | I | H | A | A | K | D | K | T | W | V | E | R | S | L | L | P | L | E | D | E | Q | G | Y | T | F | Y | L | Q | D | R | D | A | V | P | G | D | S | R | L | E | S | I | V | E | N | M | R | R | S | R | K | I | L | F | V | V | T | E | T | L | L | E | D | S | M | C | R | Q | F | M | A | H | H | A | L | H | Q | V | I | E | D | S | R | D | S | V | V | L | V | F | L | E | D | V | Q | D | Y | K | L | S | R | C | L | L | L | R | R | G | M | L | R | P | N | C | L | L | N | W | X | X | L | Q | R | E | R | V | P | X | L | P | P | E | A | P | H | R | P | G | H | H | Q | P | S | A | X | X | X | X | X | X | X | X | X | X |
| TRTLR3\_2874\_BP | X | X | X | X | X | X | X | X | X | X | X | X | X | X | X | X | X | X | X | M | M | L | G | G | P | X | X | S | L | F | L | F | S | X | L | L | I | T | W | C | C | M | T | G | R | R | H | A | V | Y | G | X | X | Q | K | K | T | S | C | V | V | Q | G | S | S | A | D | C | S | H | L | S | L | S | S | I | P | P | D | L | P | R | N | L | T | S | L | D | V | S | H | N | R | L | R | G | I | P | P | E | S | L | R | P | Y | P | G | L | L | H | L | S | V | S | Y | N | T | I | A | K | L | D | G | R | L | C | E | T | L | P | R | L | Q | T | L | D | V | A | H | N | Q | V | L | A | L | R | E | E | D | L | S | R | C | S | G | L | T | A | L | I | L | R | S | N | R | L | K | L | Q | X | X | X | X | E | P | F | S | G | L | Q | K | L | T | Y | L | D | V | S | L | N | N | L | Q | S | A | R | L | G | S | R | P | Q | L | P | A | L | V | N | L | S | L | A | Q | N | G | I | T | V | L | K | R | D | D | F | S | F | L | N | H | S | S | S | L | R | V | L | N | L | S | X | S | V | P | L | K | T | L | E | P | G | C | L | K | P | I | S | G | L | S | T | L | I | L | D | G | S | N | M | G | A | L | V | I | A | G | I | C | S | Q | L | S | G | T | A | I | S | S | L | S | L | R | N | M | K | L | A | S | L | P | N | T | T | F | A | G | L | Q | Q | T | N | L | S | C | L | D | L | S | R | N | G | L | G | K | I | E | N | G | S | F | Q | W | L | P | R | L | E | T | L | I | L | M | E | N | N | I | K | H | L | T | R | D | T | F | Q | G | L | Q | S | L | K | T | L | Q | L | T | K | A | L | V | K | G | H | T | S | A | T | P | I | I | D | N | F | S | F | Q | P | L | T | T | L | E | S | L | V | L | Q | K | T | A | I | R | E | I | T | T | Q | T | F | T | G | L | T | S | L | K | E | L | D | L | S | W | S | S | X | X | L | S | L | K | T | I | T | N | E | T | F | L | S | L | A | A | S | P | L | X | R | K | L | N | L | T | G | A | A | V | V | Q | I | S | P | G | G | F | S | T | L | K | S | L | T | V | L | L | L | D | S | N | F | I | K | Q | T | L | T | G | R | E | F | E | G | L | G | Q | L | E | E | I | H | M | S | L | N | F | Q | K | V | N | L | S | S | A | S | F | A | A | V | P | R | L | K | V | L | T | L | G | K | S | L | T | S | T | A | X | X | X | N | V | D | P | S | P | F | S | P | L | V | N | L | T | F | L | D | L | S | N | N | N | I | A | N | I | R | R | T | L | L | K | G | L | V | N | L | R | V | L | K | L | Q | H | N | N | F | A | R | L | W | K | N | N | N | V | G | G | P | V | M | F | L | Q | D | T | L | K | L | K | T | L | L | M | D | S | N | G | L | D | E | I | P | A | G | A | L | R | G | L | R | E | L | Q | E | L | S | L | G | N | N | L | L | N | S | L | R | P | S | V | F | D | D | L | I | S | L | R | A | L | F | L | Q | K | N | V | I | T | S | V | R | P | E | V | F | E | T | P | L | S | N | L | S | L | L | V | M | G | K | N | P | Y | D | C | T | C | E | S | I | L | W | Y | A | T | W | L | N | N | T | N | T | T | S | V | P | D | L | A | E | Q | Y | T | C | N | T | P | L | T | Y | F | N | R | S | I | M | T | F | D | P | L | S | C | K | D | M | T | P | F | R | A | L | Y | V | V | S | S | T | T | V | I | L | L | L | T | V | A | L | F | M | R | F | H | G | W | R | I | R | F | Y | W | N | I | V | V | N | R | T | L | G | F | S | D | A | T | V | E | E | X | X | G | R | Q | F | E | Y | D | A | Y | V | I | H | A | E | D | D | G | G | W | V | E | R | R | L | L | P | L | E | N | X | X | R | C | K | F | C | L | E | V | R | D | S | N | L | G | M | S | Q | L | E | S | I | V | N | N | I | R | N | S | R | K | I | L | F | V | V | T | E | T | L | L | M | D | P | W | C | R | R | F | T | A | H | Q | A | L | H | Q | V | I | E | A | S | R | D | S | V | V | L | V | F | L | Q | D | V | H | D | Y | K | L | S | R | T | L | F | L | R | R | G | M | L | R | S | C | C | V | L | Y | W | X | X | V | H | K | E | R | V | P | X | V | P | P | K | A | P | H | S | A | R | H | D | Q | P | L | A | X | X | X | X | X | X | X | X | X | D |
| ONTLR3\_2874\_BP | X | X | X | X | X | X | X | X | X | X | X | X | X | X | X | X | X | X | X | M | M | C | A | H | H | X | X | A | R | L | L | S | W | X | I | L | V | T | C | C | V | L | T | G | P | Y | H | C | D | A | S | X | X | E | K | K | T | S | C | Y | V | E | D | G | R | A | D | C | S | R | L | R | L | S | A | I | P | S | D | L | P | R | N | I | T | S | L | D | V | S | H | N | I | L | R | G | I | T | P | P | S | L | S | P | Y | P | G | L | L | H | L | D | A | G | Y | N | S | I | T | K | V | D | A | G | L | C | Q | T | L | P | L | L | Q | T | L | N | L | E | H | N | Q | V | H | L | L | K | T | G | D | M | S | H | C | T | R | L | T | S | L | D | L | A | S | N | R | L | K | L | Q | X | X | X | X | E | P | F | S | S | L | Q | N | L | T | F | L | D | V | S | K | N | K | L | Q | S | P | K | L | G | S | Q | P | Q | L | P | S | L | V | N | F | S | L | A | F | N | E | F | T | T | L | K | K | D | D | F | S | F | L | D | H | S | S | S | L | Q | V | L | N | L | S | X | S | V | P | L | K | T | L | E | P | G | C | F | Q | A | I | S | G | L | R | T | L | I | M | D | G | G | N | L | G | T | L | M | V | S | K | L | C | S | E | L | S | G | T | A | I | D | A | L | S | L | R | K | M | N | L | V | M | V | T | N | K | T | F | T | G | L | Q | K | T | N | L | T | F | L | D | L | S | S | N | A | M | G | Q | I | E | E | G | S | F | Q | W | L | S | N | L | Q | T | L | N | L | S | H | N | N | I | K | H | L | T | N | R | T | F | Q | G | L | S | R | L | K | K | L | A | L | T | E | A | L | V | K | G | R | T | S | X | X | P | V | I | D | D | F | A | F | Q | P | L | G | M | L | E | S | L | M | L | Q | R | T | A | I | R | E | I | G | E | H | T | F | A | G | L | K | S | L | K | K | L | D | I | S | W | I | N | X | X | P | S | L | R | N | I | T | N | K | T | L | A | S | L | A | D | S | P | V | X | R | W | L | N | L | I | N | T | N | I | A | Q | I | N | P | G | S | F | S | A | L | R | N | L | T | V | L | L | L | D | Y | N | H | I | K | Q | T | L | T | G | R | E | F | E | G | L | D | Q | V | Q | E | I | H | M | S | N | N | F | Q | S | I | D | L | S | S | S | S | F | V | N | V | P | N | L | R | V | L | T | L | G | R | S | L | K | A | L | A | X | X | X | N | L | D | H | S | P | F | K | P | L | T | N | L | S | V | L | D | L | S | N | N | N | V | A | N | I | K | D | N | L | L | E | G | L | A | N | L | K | V | L | K | L | Q | H | N | N | L | A | R | L | W | K | S | A | N | L | G | G | P | V | L | F | L | K | D | A | Q | R | L | E | S | L | Q | M | D | Y | N | G | L | D | E | I | P | L | K | A | L | K | G | L | T | H | L | R | E | L | S | L | S | N | N | L | I | N | S | L | K | D | S | V | F | D | D | L | K | S | L | Q | V | L | R | F | E | K | N | S | I | T | S | V | R | P | E | V | F | R | T | P | M | S | N | L | T | Q | L | I | M | G | R | N | P | F | D | C | T | C | E | S | I | L | W | F | V | T | W | L | N | T | T | N | T | T | S | V | P | N | V | R | D | E | Y | V | C | N | T | P | R | A | Y | F | N | H | S | I | M | D | F | D | P | L | S | C | K | D | M | T | P | F | Q | T | L | Y | I | V | S | S | T | A | V | I | M | L | I | V | T | A | L | T | V | R | F | H | G | W | R | I | H | F | Y | W | N | I | M | I | N | R | T | L | G | F | S | D | A | K | V | D | E | X | X | G | R | E | Y | E | Y | D | A | Y | V | I | R | A | E | E | D | S | S | W | V | E | R | R | L | V | P | L | E | N | X | X | K | C | Q | F | C | L | E | D | R | D | S | V | A | G | M | S | Q | V | E | S | I | V | T | N | M | K | K | S | R | K | I | M | F | V | V | T | E | S | L | L | K | D | P | W | C | R | R | F | K | V | Y | H | A | L | Q | Q | V | I | E | E | S | R | D | S | V | I | L | V | F | L | Q | D | V | H | D | H | K | L | F | H | S | L | F | L | R | R | G | M | L | R | P | R | C | I | L | D | W | X | X | V | H | K | E | R | I | A | X | I | S | P | E | A | S | H | C | A | R | L | D | X | S | A | E | X | X | X | X | X | X | X | X | X | D |
| PFTLR3\_2874\_BP | X | X | X | X | X | X | X | X | X | X | X | X | X | X | X | X | X | X | X | X | M | R | A | P | R | X | X | S | L | L | X | X | L | X | V | I | T | I | C | Y | F | V | V | G | P | H | N | C | V | A | F | X | X | P | K | R | S | S | C | Y | V | Q | D | G | K | A | D | C | R | H | L | S | L | S | E | V | P | Q | D | L | P | Q | D | I | T | S | L | D | M | S | H | N | R | L | K | E | I | H | P | V | T | L | T | R | Y | P | G | L | I | H | L | N | I | G | Y | N | S | I | S | K | V | D | P | G | L | C | R | T | L | P | L | L | Q | T | L | N | M | E | H | N | Q | V | F | V | L | K | E | E | D | M | S | H | C | T | N | L | T | R | L | F | M | A | S | N | K | I | K | L | Q | X | X | X | X | E | P | F | S | A | L | Q | S | L | E | V | L | D | V | S | M | N | K | L | Q | S | A | K | L | S | S | Q | P | Q | L | P | S | L | V | N | L | S | L | A | F | N | N | I | G | T | L | K | K | D | D | F | S | F | L | N | Q | S | X | F | L | Q | F | L | N | M | S | X | S | V | P | L | K | T | L | E | P | G | C | F | T | P | I | S | S | L | R | T | L | I | M | D | G | S | S | M | D | T | L | V | I | S | K | L | C | S | E | L | S | G | S | S | I | D | V | L | F | L | R | K | M | K | L | I | K | L | T | N | T | T | F | S | G | L | Q | K | T | N | L | T | F | L | D | L | S | R | N | G | M | V | E | I | E | P | G | S | F | Q | W | L | S | R | L | L | T | L | L | L | T | D | N | N | I | K | L | L | T | K | D | T | F | Q | G | L | K | S | L | K | K | L | Q | L | T | K | A | L | G | S | G | H | X | X | H | T | P | V | I | G | D | F | S | F | Q | P | L | S | A | L | E | S | L | I | L | Q | R | T | G | V | R | A | I | T | Q | Y | T | F | T | G | L | T | S | L | K | E | L | D | M | S | W | S | S | S | L | T | S | L | R | N | I | T | S | K | T | F | V | S | L | T | Q | S | P | L | X | R | K | L | N | L | T | G | T | S | I | A | Q | I | N | P | G | C | F | S | V | L | R | N | L | T | T | L | L | L | E | F | N | F | I | K | Q | N | L | T | G | K | E | F | E | G | L | G | Q | V | E | E | I | Y | M | S | N | N | Q | Q | T | V | N | L | G | S | M | S | F | V | N | V | P | K | L | R | F | L | T | L | A | R | S | L | R | A | E | A | X | X | X | N | L | D | P | S | P | F | R | H | L | P | N | L | T | F | L | D | L | S | N | N | N | I | A | N | I | R | E | N | M | L | E | G | L | V | N | L | K | V | L | K | L | Q | H | N | N | F | A | R | L | W | K | S | A | N | L | G | G | P | V | L | F | L | K | H | T | R | R | L | T | T | L | E | M | D | N | N | G | M | D | E | I | P | V | E | A | L | R | G | L | S | D | L | R | Q | L | S | L | A | N | N | L | L | N | R | L | K | D | S | V | F | D | D | L | N | S | L | Q | V | L | N | L | Q | K | N | L | I | T | A | V | R | P | E | V | F | K | T | P | L | S | N | L | R | R | L | V | L | D | K | N | P | F | D | C | T | C | E | S | M | L | W | F | V | T | W | L | N | D | T | N | G | T | S | V | P | G | L | R | D | Q | Y | M | C | N | T | P | L | G | Y | F | N | H | S | I | L | D | F | D | G | L | S | C | K | D | M | T | P | F | Q | A | L | Y | I | L | S | S | T | A | V | L | M | L | L | V | T | A | L | L | V | R | F | Q | G | W | R | I | Q | F | Y | W | N | I | L | I | N | R | T | L | G | F | S | D | A | T | V | E | E | X | X | G | R | E | F | E | Y | D | A | Y | I | I | H | S | D | K | D | S | S | W | V | E | R | R | M | V | P | L | E | N | X | X | Q | C | R | F | C | L | D | D | R | D | A | V | P | G | T | S | Q | L | Q | S | I | V | E | N | M | R | N | S | R | K | I | V | F | V | V | T | E | T | L | L | N | D | A | L | C | S | R | F | K | A | H | H | A | L | H | Q | V | I | E | A | S | R | D | S | V | V | L | V | F | L | Q | D | V | H | D | Y | K | L | S | Q | S | L | L | L | R | R | G | M | L | R | P | C | C | I | L | D | W | X | X | V | H | K | E | R | V | P | X | L | S | P | E | T | P | H | S | T | W | H | D | X | S | I | A | X | X | X | X | X | X | X | X | X | E |
| SATLR3\_2874\_BP | X | X | X | X | X | X | M | Q | R | K | Y | X | X | X | X | X | V | F | K | M | M | H | A | P | R | X | X | Y | T | L | L | L | A | X | L | T | T | V | C | H | V | M | M | G | P | S | S | C | V | A | A | X | X | Q | K | K | T | S | C | V | V | E | D | G | R | A | D | C | S | H | L | S | L | R | E | V | P | P | N | L | P | G | N | L | T | S | L | D | M | S | H | N | R | L | G | G | I | P | P | T | S | L | T | P | Y | P | G | L | L | H | L | D | V | S | Y | N | S | I | T | G | L | V | G | G | L | C | Q | T | L | P | L | L | Q | T | L | N | V | K | H | N | Q | V | L | Y | L | K | K | E | D | L | S | H | C | T | S | L | T | Q | L | N | L | A | S | N | R | L | R | L | Q | X | X | X | X | E | P | F | I | A | L | Q | S | L | T | F | L | D | V | S | L | N | K | L | K | S | A | K | L | G | S | Q | P | Q | L | P | S | L | V | N | L | S | L | A | L | N | D | F | T | T | L | K | N | D | D | F | S | F | L | N | Q | S | S | F | L | Q | V | L | N | L | S | X | A | V | S | L | K | T | L | E | P | G | C | F | K | P | I | S | R | I | R | T | L | I | M | D | G | S | K | T | G | T | L | I | I | A | K | L | C | S | Q | L | S | G | T | A | I | D | V | L | S | L | R | N | M | R | L | V | T | L | T | N | T | T | F | T | G | L | Q | E | T | N | L | T | S | L | D | L | S | G | N | G | M | G | K | I | E | K | G | S | F | K | W | L | S | E | L | Q | S | L | V | L | A | E | N | N | I | K | H | L | T | K | D | I | F | Q | G | L | T | S | L | K | R | L | N | L | T | L | A | L | V | K | S | H | T | S | A | V | P | I | I | D | D | Y | S | F | Q | P | L | N | A | L | E | T | L | I | L | K | K | T | A | V | R | E | I | T | E | N | A | F | T | G | L | T | S | L | K | E | L | D | M | S | W | T | T | X | X | T | D | L | K | N | I | T | N | K | T | F | V | S | L | A | R | S | P | L | X | R | K | L | N | L | I | G | A | D | V | K | Q | I | N | P | G | C | F | S | V | F | R | N | L | T | T | L | L | L | D | H | N | Y | I | K | Q | T | L | T | G | R | E | F | E | G | L | D | R | I | Q | E | I | Y | M | S | D | N | F | Q | K | V | N | L | S | S | T | S | F | V | N | V | P | N | L | R | V | L | T | L | G | K | S | L | I | A | T | A | X | X | X | D | R | D | P | S | P | F | S | P | L | S | N | L | T | F | L | D | L | S | N | N | N | I | A | N | I | R | G | N | M | L | E | G | L | V | N | L | K | V | L | K | L | Q | H | N | N | L | A | R | V | W | K | S | A | N | Q | G | G | P | V | L | F | L | K | G | A | Q | K | L | T | S | L | L | M | D | S | N | G | L | D | E | I | P | V | D | A | L | R | G | L | T | E | L | R | E | L | S | L | G | N | N | L | L | N | S | L | K | D | T | I | F | E | D | L | N | S | L | R | A | L | Y | L | Q | K | N | L | I | T | T | V | R | P | E | V | F | Q | T | P | M | S | N | L | S | L | L | V | M | G | K | N | P | F | D | C | T | C | E | S | I | L | W | F | M | T | W | M | N | K | T | N | M | T | S | V | P | G | L | R | E | Q | Y | T | C | N | T | P | L | T | Y | F | N | R | S | V | M | D | F | D | A | L | S | C | K | D | M | T | P | F | Q | A | L | Y | I | L | S | S | T | A | V | L | M | L | M | V | T | A | L | L | V | R | F | H | G | W | R | F | E | F | Y | W | N | I | L | I | N | R | T | L | G | F | S | D | A | S | V | E | E | X | X | G | R | E | F | E | Y | D | A | Y | V | I | C | A | E | D | D | G | S | W | V | D | R | R | L | V | P | L | E | K | X | X | N | C | K | F | C | L | E | D | R | D | A | V | A | G | M | S | V | L | Q | S | I | V | D | N | I | R | K | S | R | K | I | L | F | V | V | T | E | A | L | L | R | D | P | W | C | R | R | F | T | A | H | Q | A | L | H | Q | V | I | E | A | S | R | D | S | V | V | L | V | F | L | Q | D | V | H | D | Y | R | L | S | Q | S | L | F | L | R | R | G | M | L | R | P | C | C | V | L | D | W | X | X | V | H | K | E | R | V | P | X | L | S | P | E | A | P | H | C | T | W | H | D | Q | S | I | S | X | X | X | X | X | X | X | X | X | E |
| ATTLR3\_2874\_BP | M | Q | Q | I | P | N | M | Q | R | H | R | X | X | X | X | X | V | F | E | A | M | C | V | H | R | X | X | S | L | L | L | T | W | X | V | I | I | I | C | S | F | M | T | R | X | X | X | C | V | A | S | X | X | Q | K | K | T | S | C | Q | V | Q | D | G | R | A | D | C | S | H | L | S | L | R | A | V | P | P | D | L | P | R | N | I | T | S | L | D | M | S | H | N | R | L | I | G | I | A | A | L | S | L | K | P | Y | P | R | L | I | H | L | D | V | S | F | N | S | I | T | K | L | E | D | S | L | C | Q | T | L | P | F | L | Q | T | L | N | M | Q | H | N | E | V | H | F | L | K | K | E | D | L | N | H | C | T | N | L | T | L | L | N | L | A | S | N | R | L | K | L | H | X | X | X | X | E | P | F | S | V | L | Q | N | L | K | F | L | D | V | S | A | N | K | L | P | S | P | R | L | G | S | Q | P | Q | L | P | S | L | V | T | L | N | L | A | F | N | E | I | T | A | L | K | K | D | D | F | S | F | L | N | R | S | S | F | L | Q | V | L | N | L | S | X | S | V | S | L | K | T | L | D | P | G | C | F | K | P | I | S | G | L | R | T | L | I | M | D | M | S | N | M | G | T | L | A | I | S | K | L | C | S | E | L | A | G | T | S | I | D | A | L | S | L | R | K | M | K | L | V | T | V | T | N | T | T | F | T | G | L | H | K | T | N | L | T | F | L | D | L | S | Y | N | G | L | G | K | I | E | G | G | S | F | Q | W | L | S | R | L | Q | T | L | V | L | A | H | N | N | I | K | H | L | T | K | E | T | F | E | G | L | K | G | L | K | K | L | Q | L | T | R | A | L | V | K | S | H | T | S | S | T | Q | I | I | N | D | F | S | F | Q | P | L | S | S | L | E | S | L | I | L | Q | N | T | A | V | R | N | L | T | E | H | T | F | T | G | L | T | S | L | K | E | L | D | M | S | W | S | S | X | X | T | V | L | R | N | I | T | S | K | T | L | V | S | L | A | E | S | P | L | X | I | K | L | N | L | A | G | T | A | I | S | Q | I | N | P | G | S | F | S | V | F | K | N | L | T | T | L | L | L | D | N | N | H | I | K | Q | T | L | T | G | R | E | F | E | G | L | G | Q | V | Q | E | I | H | M | A | N | N | Q | Q | T | V | N | L | S | S | T | S | F | V | Y | V | P | N | L | R | V | L | T | L | G | K | S | L | R | S | T | A | X | X | X | N | L | D | P | S | P | F | R | P | L | S | K | L | T | V | L | D | L | S | N | N | N | I | A | N | I | R | E | N | M | L | E | G | L | V | N | L | K | V | L | K | L | Q | H | N | N | L | A | R | L | W | K | S | A | N | L | G | G | P | V | L | F | L | K | G | A | Q | S | L | T | T | L | E | L | D | S | N | G | L | D | E | I | P | I | D | A | L | R | G | L | S | N | L | S | E | L | S | L | G | E | N | L | L | N | N | L | K | D | S | I | F | D | D | L | T | S | L | Q | I | F | R | L | Q | K | N | L | I | T | T | V | R | P | E | V | F | K | T | P | L | S | N | L | S | L | L | V | M | D | K | N | P | F | D | C | T | C | E | S | I | L | W | F | V | T | W | L | N | N | T | K | M | T | S | V | P | A | L | R | D | Q | Y | R | C | N | T | P | L | A | Y | F | N | R | S | I | A | D | F | D | T | L | S | C | K | D | M | T | P | F | Q | A | L | Y | I | L | S | S | T | V | V | I | M | L | M | V | T | A | L | L | V | R | F | H | G | W | R | I | E | F | Y | W | T | I | L | I | N | R | T | L | G | F | S | D | A | K | V | E | E | X | X | G | R | E | F | E | Y | D | A | Y | V | I | H | A | E | Q | D | A | R | W | V | E | R | R | L | E | P | L | E | N | X | X | N | C | K | F | C | L | Q | D | R | D | S | V | I | G | T | P | Y | L | Q | S | I | V | D | N | M | R | K | S | R | K | I | L | F | V | V | T | E | S | L | L | N | D | P | C | C | R | R | F | K | A | H | H | A | Y | H | Q | V | I | E | A | N | R | D | S | V | V | L | I | F | L | D | D | V | H | D | Y | K | L | S | S | A | L | L | L | R | R | G | M | L | R | P | C | C | I | L | D | W | X | X | V | Q | R | E | R | V | P | X | L | S | P | E | T | P | H | S | P | W | H | D | X | S | I | A | X | X | X | X | X | X | X | X | X | E |
| SMTLR3\_2874\_BP | X | X | X | X | X | X | M | L | W | T | H | X | X | X | X | X | R | L | E | A | M | R | T | P | R | X | X | S | P | P | L | T | W | V | V | V | V | A | C | C | V | L | A | G | P | P | Q | S | G | A | S | X | X | P | K | K | T | S | C | R | V | Q | D | G | R | A | D | C | S | H | L | S | L | R | A | I | P | P | N | L | P | R | N | I | S | S | L | D | M | S | H | N | R | L | V | G | I | P | P | A | P | L | N | L | Y | P | G | L | L | R | L | D | V | S | Y | N | S | V | T | K | L | D | G | H | L | C | Q | T | L | P | L | L | Q | T | L | S | V | A | H | N | E | V | H | L | L | K | R | E | D | L | S | H | C | T | G | L | V | L | L | N | M | A | S | N | R | L | K | L | Q | X | X | X | X | E | P | F | S | A | L | K | N | L | K | S | L | D | V | S | M | N | K | L | A | S | A | Q | L | S | S | Q | P | Q | L | P | S | L | V | N | L | N | L | A | F | N | D | F | T | A | L | K | K | D | D | F | S | F | L | D | N | S | S | F | L | Q | L | L | N | L | S | X | A | V | S | L | K | T | L | E | P | G | C | L | R | P | I | S | G | L | R | T | L | I | M | D | G | S | N | M | G | I | L | V | L | F | K | L | C | T | E | L | S | R | T | A | I | D | A | L | S | L | R | K | M | K | L | V | T | L | T | N | T | T | F | T | G | L | Q | R | A | N | L | T | F | L | D | L | S | H | N | G | M | G | K | I | E | E | G | S | F | Q | W | L | S | R | L | Q | T | L | I | L | A | D | N | N | L | K | H | L | T | K | G | T | F | Q | G | L | K | A | L | K | E | L | Q | L | T | K | A | L | V | K | S | H | T | S | S | T | P | I | I | D | D | F | S | F | E | P | L | S | A | L | E | S | L | S | L | R | Q | T | S | V | R | E | I | T | G | H | T | F | T | G | M | T | S | L | K | E | L | D | M | S | W | S | S | X | X | T | S | L | R | I | I | T | K | Q | T | L | V | S | L | A | G | S | P | L | X | V | K | L | N | L | T | G | T | A | I | T | Q | I | N | P | G | S | F | A | C | M | R | N | L | T | T | L | L | L | D | F | N | F | I | Q | Q | T | L | T | G | K | E | F | E | G | L | G | E | V | Q | E | L | H | M | T | N | N | H | W | K | V | R | L | S | S | T | S | F | V | N | V | P | N | L | R | V | L | T | L | G | K | S | L | N | N | T | A | L | N | V | D | V | D | P | S | P | F | K | P | L | C | N | L | T | F | L | D | L | S | N | N | N | I | A | N | I | R | G | S | V | L | E | G | L | V | N | L | K | V | L | K | L | Q | H | N | N | L | A | R | L | W | K | S | A | N | P | G | G | P | V | L | F | L | K | G | A | P | N | L | V | T | L | Q | M | D | S | N | G | L | D | E | I | P | E | G | A | L | R | G | L | S | S | L | W | E | L | S | L | A | T | N | L | L | N | N | L | R | Y | S | V | F | D | D | L | K | S | L | R | V | L | R | L | Q | K | N | L | I | T | S | V | N | P | E | V | F | K | T | P | M | S | N | L | S | L | L | V | M | D | K | N | P | F | D | C | T | C | E | S | I | L | W | F | V | T | W | L | N | N | T | N | T | T | F | V | P | D | L | G | D | Q | Y | I | C | N | T | P | L | A | Y | Y | N | H | S | I | M | D | F | E | T | L | S | C | K | D | M | T | P | F | Q | A | L | Y | I | L | T | S | T | T | V | M | A | V | I | V | T | A | L | L | V | R | F | Q | G | W | R | I | Q | F | Y | W | N | I | L | I | S | R | T | L | G | F | S | D | A | N | I | D | E | X | X | G | R | H | F | E | Y | D | A | Y | I | I | H | A | E | E | D | S | R | W | V | E | R | M | M | V | P | L | E | N | X | X | N | C | R | F | Y | L | E | D | R | D | A | V | P | G | V | A | L | L | E | S | I | I | D | N | M | R | R | S | R | K | I | L | F | V | V | T | E | R | L | L | K | D | P | W | C | R | R | F | K | A | H | Q | A | L | H | Q | V | I | E | A | S | R | D | S | V | V | L | V | F | L | Q | D | V | H | D | Y | K | L | S | R | S | L | Y | L | R | R | G | M | L | R | R | C | C | I | V | E | W | H | G | G | H | K | E | R | V | A | X | L | P | P | E | A | P | H | S | T | R | H | D | Q | S | I | D | X | X | X | X | X | X | X | X | X | E |
| SDTLR3\_2874\_BP | X | X | X | X | X | X | M | Q | R | Q | R | X | X | X | X | X | V | S | E | A | M | R | S | P | R | X | X | S | L | L | L | T | W | X | V | I | V | I | C | Y | F | M | T | G | P | Y | H | C | M | A | S | X | X | Q | K | K | T | S | C | S | V | Q | D | G | R | A | D | C | S | H | L | S | L | N | A | T | P | Q | N | L | P | R | N | I | T | S | L | D | M | S | H | N | R | L | V | G | I | S | P | E | S | L | S | P | Y | N | G | L | L | H | L | D | V | S | Y | N | S | I | T | K | L | D | V | R | L | C | Q | T | L | P | Q | L | Q | T | L | I | I | E | H | N | E | V | H | L | L | K | K | D | D | L | S | H | C | T | S | L | T | R | L | N | M | A | S | N | R | L | K | L | Q | X | X | X | X | E | P | F | S | S | L | K | N | L | Q | F | L | D | V | S | T | N | K | L | P | S | A | K | L | S | S | Q | P | Q | L | P | S | L | V | N | L | N | L | A | F | N | D | F | S | T | L | E | K | D | D | F | S | F | L | N | H | S | P | F | L | R | V | L | N | L | S | X | T | L | P | L | K | K | L | E | P | G | C | L | D | P | I | S | G | L | H | T | L | I | M | D | G | S | N | M | D | S | Q | V | I | S | K | L | C | L | E | L | S | G | T | S | I | E | T | L | S | L | Q | K | V | K | L | V | T | L | L | N | T | T | F | T | G | L | Q | K | A | N | L | T | F | L | D | L | S | H | N | G | M | G | K | I | E | E | G | S | F | Q | W | L | P | R | L | Q | I | L | I | L | A | N | N | N | I | K | H | L | T | K | D | T | F | Q | G | L | K | S | L | K | K | L | Q | M | T | K | A | L | V | R | G | H | T | S | S | T | P | I | I | D | D | F | S | F | Q | P | L | S | A | L | E | S | L | T | L | Q | Q | T | A | I | R | E | I | T | A | N | T | F | T | G | L | T | S | L | Q | E | L | D | M | S | W | S | T | X | X | A | S | L | R | S | I | S | N | K | T | L | V | S | L | A | G | S | P | L | X | R | Q | L | N | L | R | G | T | A | I | G | E | I | N | P | G | S | F | S | V | L | G | N | L | T | I | L | L | L | D | H | N | F | I | S | Q | I | L | T | G | E | E | F | K | G | L | G | Q | V | Q | E | L | H | M | S | D | N | Y | Q | K | V | K | L | S | S | T | S | F | V | K | V | P | N | L | R | V | L | T | L | G | K | S | I | N | M | T | A | X | X | X | K | L | D | P | S | L | F | K | P | L | S | N | L | T | V | L | D | L | S | N | N | N | I | A | N | I | I | E | N | T | F | E | G | L | V | N | L | K | V | L | K | L | Q | H | N | N | L | A | R | L | W | K | S | A | N | L | G | G | P | V | L | F | L | K | G | V | Q | N | L | I | T | L | Q | M | D | S | N | G | L | D | E | I | P | E | E | A | L | R | G | L | S | N | L | S | E | L | S | L | S | S | N | L | L | N | S | F | K | D | S | V | F | D | D | L | T | S | L | R | V | L | R | L | Q | K | N | L | I | T | T | V | R | P | E | V | F | K | T | P | M | R | N | L | S | L | L | V | M | D | K | N | P | F | D | C | T | C | E | S | I | L | W | F | V | R | W | L | N | N | T | N | V | T | R | L | P | N | L | R | D | Q | Y | T | C | N | T | P | R | A | Y | F | N | Q | S | V | M | D | F | D | T | L | S | C | K | D | M | T | P | F | Q | A | L | Y | V | L | S | S | T | V | V | L | M | L | M | V | T | A | L | V | V | R | F | H | G | W | R | I | Q | F | Y | W | N | I | L | I | N | R | T | L | G | F | S | D | A | S | V | E | E | X | X | G | R | E | F | E | Y | D | A | Y | I | I | H | A | E | E | D | S | R | W | V | E | R | M | M | V | P | L | E | N | X | X | R | C | R | F | C | L | Q | D | R | D | G | V | P | G | M | P | L | L | E | S | I | V | D | N | M | K | M | S | R | K | I | L | F | V | V | T | E | S | L | L | K | D | P | W | C | R | R | F | K | A | H | H | A | L | N | E | V | I | E | A | S | R | D | S | V | V | L | V | F | L | Q | D | V | H | D | Y | K | L | S | R | S | L | F | L | R | R | G | M | L | R | S | R | C | I | L | D | W | X | X | C | H | K | E | R | V | P | X | L | S | P | E | A | P | H | C | T | W | H | D | K | S | I | E | X | X | X | X | X | X | X | X | X | E |
| TOTLR3\_2874\_BP | X | X | X | X | X | X | M | Q | I | L | L | C | L | F | V | K | A | F | E | A | R | C | T | P | R | X | X | S | L | L | L | T | W | X | V | L | V | V | C | Y | F | M | T | G | P | Y | N | C | M | A | S | X | X | Q | K | K | T | S | C | T | V | Q | D | G | R | A | D | C | S | H | L | S | L | N | S | T | P | Q | N | L | P | R | N | I | T | S | L | D | M | S | H | N | R | L | V | V | I | S | P | V | S | L | N | P | Y | P | G | L | L | H | L | D | V | G | Y | N | S | I | T | K | M | D | E | R | L | C | Q | T | L | P | L | L | Q | T | L | N | I | E | H | N | E | V | H | L | L | K | K | E | D | V | S | H | C | T | N | L | T | W | L | K | M | A | S | N | R | L | K | L | Q | X | X | X | X | E | P | F | S | A | L | Q | N | L | K | F | L | D | V | S | T | N | K | L | L | S | A | K | L | S | S | Q | P | Q | L | P | S | L | V | N | L | N | L | A | F | N | D | F | S | T | L | K | N | D | D | F | S | F | L | S | H | S | P | L | L | R | V | L | N | L | S | X | S | V | P | L | K | T | L | E | P | G | C | L | K | P | I | S | G | L | H | V | L | I | M | D | G | S | N | M | G | S | E | V | I | S | K | L | C | S | E | L | S | E | T | S | I | E | A | L | S | L | R | K | M | S | L | V | T | L | F | N | T | T | F | S | G | L | Q | K | A | N | L | T | F | L | D | L | S | H | N | G | M | G | K | I | E | E | G | S | F | Q | C | L | P | R | L | Q | T | L | I | L | A | G | N | K | I | K | H | L | T | K | D | T | F | Q | G | L | K | S | L | K | T | L | Q | L | T | K | A | V | A | K | S | X | X | X | X | A | L | I | I | D | D | F | S | F | Q | P | L | S | A | L | E | S | L | I | L | A | Q | T | A | V | R | E | I | T | A | H | T | F | T | G | L | T | S | L | K | E | L | D | V | S | W | K | S | X | X | A | S | L | R | I | V | T | D | K | T | F | V | S | L | A | G | S | P | L | X | R | Q | L | N | L | R | G | T | A | I | T | Q | I | N | P | G | S | F | S | V | L | R | N | L | T | T | L | L | L | D | Y | N | F | I | N | Q | T | L | T | G | K | E | F | E | G | L | N | Q | V | Q | E | I | H | M | S | N | N | Y | Q | K | V | T | L | R | A | T | S | F | V | N | V | P | N | L | R | V | L | M | L | G | K | S | I | Y | T | S | A | X | X | X | Q | L | D | P | S | P | F | K | P | L | S | N | L | T | V | L | D | L | S | N | N | N | I | A | N | I | R | E | S | T | F | E | G | L | V | N | L | K | V | L | K | L | Q | H | N | N | L | A | R | L | W | K | D | A | N | L | G | G | P | V | L | F | L | K | G | L | V | N | L | I | T | L | Q | M | D | S | N | G | L | D | E | I | P | E | E | A | L | R | G | L | S | N | L | S | E | L | S | L | A | N | N | L | L | N | S | L | K | D | S | V | F | N | D | L | T | S | L | Q | V | L | R | L | Q | K | N | L | I | T | T | V | R | P | E | V | F | K | S | P | L | S | N | L | S | L | L | V | M | D | K | N | P | F | D | C | T | C | E | S | I | L | W | F | V | T | W | L | N | S | T | N | T | T | A | V | P | D | L | R | D | Q | Y | M | C | N | T | P | R | A | Y | F | N | H | S | I | M | D | F | D | T | L | S | C | K | D | M | T | P | F | Q | A | L | Y | V | L | S | S | T | A | V | M | M | L | I | V | T | A | L | L | V | R | F | H | G | W | R | I | Q | F | Y | W | N | I | L | I | N | R | T | L | G | F | S | D | A | S | V | E | E | X | X | G | R | E | F | E | Y | D | A | Y | I | I | H | A | E | E | D | S | S | W | V | E | R | M | M | V | P | L | E | N | X | X | R | C | R | F | C | F | E | D | R | D | G | V | P | G | M | S | L | L | E | S | I | V | D | N | M | R | K | S | R | K | I | L | F | V | I | T | E | R | L | L | N | D | P | W | C | R | R | F | K | A | H | Q | A | L | Y | E | V | I | E | A | S | R | D | S | V | V | L | V | F | L | Q | D | V | H | D | Y | K | L | S | R | S | L | F | L | R | R | G | M | L | R | S | R | C | I | L | D | W | X | X | G | H | K | E | R | V | P | X | L | P | P | E | A | P | H | S | T | W | H | D | X | S | I | E | X | X | X | X | X | X | X | X | X | E |
| MMTLR3\_2874\_BP | X | X | X | X | X | X | X | X | X | X | X | X | X | X | X | X | X | X | X | X | M | C | V | L | R | X | X | P | L | L | L | S | V | X | V | I | I | I | C | Y | F | V | T | X | X | S | N | C | V | T | A | X | X | V | K | K | T | S | C | Q | V | Q | D | G | R | A | D | C | S | H | L | S | L | S | E | V | P | Q | N | L | P | R | N | L | T | S | L | D | M | S | H | N | R | L | R | G | I | P | P | V | S | L | T | P | Y | P | G | L | L | H | L | D | V | S | Y | N | S | I | T | K | V | D | G | G | L | C | Q | T | L | P | L | L | Q | T | L | N | M | E | H | N | E | V | F | V | L | K | K | E | D | V | S | H | C | T | K | L | T | R | L | I | M | A | S | N | R | L | K | L | Q | X | X | X | X | E | P | F | S | A | L | Q | T | L | K | F | L | D | V | S | I | N | K | L | K | S | A | K | L | G | T | Q | P | Q | L | P | N | L | V | N | L | N | L | A | S | N | D | F | T | T | L | K | K | D | D | F | S | F | L | N | H | S | T | N | L | Q | V | L | N | L | S | X | S | V | P | L | K | T | L | E | P | G | C | F | K | P | I | S | R | L | R | T | L | I | M | D | K | S | T | M | G | T | L | V | I | A | K | L | C | K | E | L | S | G | T | A | I | D | A | L | S | L | R | N | I | N | L | V | T | L | T | N | T | T | F | A | G | L | Q | K | T | S | L | S | F | L | D | L | S | H | N | G | M | D | K | I | E | Q | G | S | F | Q | W | L | T | R | L | Q | I | L | I | L | E | D | N | K | I | K | H | L | T | K | D | T | F | Q | G | L | K | S | L | K | K | L | Q | L | T | K | A | L | G | K | S | H | T | S | X | X | X | I | I | D | D | F | S | F | Q | P | L | S | A | L | E | S | L | I | L | Q | N | T | A | V | H | D | I | T | K | H | T | F | T | G | L | T | S | L | K | E | L | D | V | S | W | S | S | X | X | T | S | L | K | N | I | S | N | E | T | L | A | S | L | A | G | S | P | L | X | R | T | L | N | L | T | G | T | A | I | T | L | I | N | P | G | A | F | S | V | F | R | N | L | T | T | L | L | L | D | H | N | F | I | S | Q | I | L | T | G | K | E | F | E | G | L | D | E | V | Q | E | L | H | M | A | N | N | Y | R | K | V | N | L | S | S | T | S | F | V | N | V | P | K | L | R | V | L | T | L | G | K | S | L | T | V | K | A | X | X | X | D | L | H | P | S | P | F | K | P | L | P | N | L | T | I | L | D | L | S | N | N | N | I | A | N | I | R | E | D | L | L | E | G | L | V | N | L | K | V | L | K | L | Q | H | N | N | L | A | R | L | W | K | N | A | N | L | G | G | P | V | L | F | L | K | G | A | P | N | L | K | T | L | L | M | D | S | N | G | L | D | E | I | P | A | G | G | L | R | G | L | H | D | L | R | E | L | S | L | G | Y | N | L | L | N | N | L | K | D | S | V | F | D | D | L | N | S | L | Q | A | L | F | L | E | K | N | M | I | T | T | V | R | P | E | V | F | K | T | P | M | S | N | L | S | L | L | I | M | G | K | N | P | F | D | C | T | C | E | S | I | L | W | F | V | T | W | L | N | N | T | N | M | T | N | V | P | G | V | R | E | Q | Y | M | C | N | T | P | L | V | Y | F | N | H | S | I | M | D | F | D | G | L | S | C | K | D | M | T | P | F | Q | T | L | Y | I | L | S | S | T | A | V | I | M | L | I | V | T | A | L | L | V | R | F | H | G | W | R | I | Q | F | Y | C | N | I | L | I | N | R | T | L | G | F | S | D | A | K | A | E | E | X | X | G | R | Q | F | K | Y | D | A | Y | V | I | H | A | E | E | D | G | I | W | V | E | R | S | M | A | P | L | E | N | X | X | N | C | K | F | C | L | E | D | R | D | S | V | P | G | M | S | Q | L | E | S | I | V | D | N | M | R | R | S | R | K | I | L | F | V | I | T | E | S | L | L | R | D | P | W | C | R | R | F | T | A | H | H | A | L | H | Q | V | I | E | A | S | R | D | S | V | V | L | V | F | L | Q | D | V | H | D | Y | K | L | S | R | S | L | F | L | R | R | G | M | L | R | P | C | C | I | L | D | W | X | X | I | H | K | E | R | I | A | X | L | S | P | E | A | P | H | S | T | W | H | D | X | S | I | A | X | X | X | X | X | X | X | X | X | E |
| LCTLR3\_2874\_BP | X | X | X | X | X | M | Q | H | W | Q | H | X | X | X | X | X | V | F | G | K | M | C | A | P | R | X | X | S | L | L | L | P | W | X | V | I | I | I | C | Y | F | X | X | X | X | C | H | C | V | A | S | X | X | I | K | K | T | S | C | H | V | Q | D | G | R | A | D | C | S | H | L | S | L | S | M | I | P | P | N | L | P | R | N | I | T | I | L | D | M | S | H | N | R | L | K | E | V | L | P | V | S | L | N | P | Y | P | G | L | L | H | L | D | V | S | Y | N | S | I | T | K | L | D | A | H | L | C | Q | T | L | P | L | L | Q | A | L | Y | L | G | H | N | E | V | H | L | L | K | K | E | D | L | S | H | C | T | G | L | I | R | L | N | M | A | S | N | R | L | K | L | Q | X | X | X | X | E | P | F | S | G | L | Q | N | L | Q | F | L | D | V | S | M | N | K | L | L | S | A | K | L | G | S | Q | P | Q | L | P | S | L | V | N | L | N | L | G | F | N | D | F | T | A | L | R | K | D | D | F | S | F | L | G | H | S | S | S | L | Q | V | L | S | L | S | X | S | V | P | L | K | T | L | E | P | G | C | F | K | P | I | S | G | L | H | T | L | I | M | D | G | S | K | M | G | T | Q | D | I | S | K | I | C | L | E | L | S | G | T | S | I | D | A | L | S | L | R | N | M | K | L | V | T | L | T | N | K | T | F | A | G | L | Q | M | T | N | L | T | F | L | D | L | S | H | N | G | M | G | K | I | E | E | G | S | F | K | W | L | S | K | L | Q | T | L | T | L | A | N | N | N | I | K | H | L | T | K | D | T | F | Q | G | L | K | S | L | K | K | L | Q | L | T | K | A | L | V | K | S | H | T | S | S | T | P | I | I | D | D | F | S | F | Q | P | L | S | A | L | E | I | L | M | L | N | E | T | A | V | R | E | I | T | E | H | T | F | T | G | L | K | N | L | Q | E | L | D | M | S | W | S | S | X | X | A | S | F | R | I | I | T | N | K | T | F | V | S | L | A | G | S | P | L | X | R | I | L | N | L | T | A | T | A | I | T | Q | I | N | P | G | S | F | S | L | L | R | N | L | T | T | L | L | L | D | Y | N | F | I | D | Q | I | L | T | G | K | E | F | E | G | M | G | Q | V | E | E | I | H | M | T | N | N | H | Q | K | V | R | L | S | T | T | S | F | V | N | V | P | N | L | R | V | L | T | L | G | K | S | L | N | T | T | A | E | G | L | D | V | D | P | S | P | F | K | P | L | L | N | L | T | F | L | D | L | S | N | N | N | I | A | N | I | R | E | S | M | L | E | G | L | V | N | L | K | V | L | K | L | Q | H | N | N | L | A | R | L | W | K | S | V | N | P | G | G | P | V | L | F | L | K | G | A | Q | N | L | M | S | L | Q | M | D | S | N | G | L | D | E | I | P | D | G | A | L | R | G | L | S | N | L | S | Q | L | S | L | G | Y | N | L | L | N | S | L | K | G | S | V | F | N | D | L | K | S | L | Q | V | L | R | L | Q | K | N | L | I | T | T | V | R | P | E | V | F | K | T | V | M | S | N | L | S | L | L | V | M | D | K | N | P | F | D | C | T | C | E | S | I | L | W | F | V | T | W | L | N | N | T | N | M | T | T | V | P | D | L | R | D | Q | Y | R | C | N | T | P | L | A | Y | F | N | R | S | I | M | D | F | D | T | L | S | C | K | D | M | T | P | F | Q | A | L | Y | I | L | S | S | T | A | V | M | M | L | I | A | T | A | L | L | V | R | F | Q | G | W | R | I | Q | F | Y | W | N | I | L | I | N | R | T | L | G | F | S | D | A | K | V | E | E | X | X | G | R | E | F | T | Y | D | A | Y | I | I | H | A | E | E | D | S | S | W | V | E | R | M | V | I | P | L | E | N | X | X | K | C | T | F | Y | L | E | D | R | D | S | V | P | G | M | S | Q | L | E | S | I | V | D | N | M | R | K | S | R | K | I | L | F | V | I | T | E | S | L | L | N | D | P | W | C | R | R | F | K | A | H | H | A | L | H | Q | V | I | E | A | S | R | D | S | V | V | L | V | F | L | Q | D | V | H | D | Y | K | L | S | R | S | L | F | I | R | R | G | M | L | R | K | R | C | I | L | E | W | X | X | G | H | K | E | R | V | P | X | L | S | P | G | A | P | H | S | T | W | H | D | X | S | I | E | X | X | X | X | X | X | X | X | X | D |
| ECTLR3\_2874\_BP | X | X | X | X | X | X | M | Q | R | P | N | X | X | X | X | X | V | F | E | T | M | R | A | P | Y | X | X | S | L | L | V | S | A | X | V | I | V | I | C | Y | C | M | T | G | P | H | N | C | V | T | S | X | X | Q | K | K | T | S | C | E | V | Q | N | G | Q | A | D | C | S | H | L | S | L | S | A | V | P | Q | D | L | P | R | N | I | T | S | L | D | M | S | H | N | R | L | T | K | F | P | P | G | S | L | T | P | Y | P | G | L | L | H | L | N | I | S | H | N | S | I | T | K | L | D | Q | G | V | C | Q | T | L | P | L | L | K | T | L | N | M | G | H | N | E | V | Y | V | L | K | K | E | E | L | S | H | C | T | N | L | T | W | L | I | M | A | G | N | I | L | K | L | Q | X | X | X | X | E | P | F | S | A | L | Q | S | L | R | V | L | D | V | S | L | N | K | L | K | S | A | K | L | G | S | Q | P | Q | L | P | N | L | V | S | L | N | L | A | H | N | D | F | T | T | L | E | T | D | D | F | L | F | L | N | H | S | P | S | L | Q | V | L | N | M | S | X | H | V | S | L | K | T | L | E | P | G | C | F | Q | P | I | S | G | L | R | T | L | V | M | D | G | S | N | M | G | P | Q | V | I | S | K | L | C | S | E | L | S | Q | T | A | I | D | A | L | F | L | R | K | M | K | L | V | T | L | T | N | T | T | F | T | G | L | Q | K | T | S | L | T | F | L | E | L | S | G | N | G | M | V | R | I | E | E | G | S | F | R | W | L | S | R | L | Q | T | L | I | L | V | D | N | N | I | K | H | L | T | K | D | T | F | Q | G | L | K | S | L | E | K | L | Q | L | T | N | A | L | A | S | S | H | X | X | P | I | P | I | I | D | D | F | S | F | Q | P | L | S | A | L | E | S | L | I | L | Q | R | T | A | V | R | N | I | T | E | H | T | F | T | G | L | T | S | L | K | E | L | D | M | S | W | S | S | X | X | T | S | L | R | N | I | S | N | K | T | F | I | S | L | A | G | S | P | L | X | R | K | L | N | L | T | G | T | A | I | A | Q | I | D | P | R | S | F | S | V | L | R | N | L | T | T | L | L | L | D | Y | N | F | I | K | Q | N | L | T | G | E | E | F | E | G | L | D | Q | V | E | Q | I | Y | M | S | N | N | H | Q | K | V | N | L | S | S | D | S | F | V | N | V | P | S | L | R | V | L | T | L | G | R | S | L | I | A | E | A | X | X | X | N | C | D | P | S | P | F | R | H | L | P | N | L | T | Y | L | D | L | S | N | N | N | I | A | N | I | R | E | N | T | F | E | G | L | V | N | L | K | V | L | K | L | Q | H | N | N | L | A | R | L | W | K | S | A | N | L | G | G | P | V | L | F | L | K | N | T | P | R | L | L | I | L | D | L | D | S | N | G | L | D | E | I | P | A | E | A | L | R | G | L | S | D | L | H | N | L | S | L | A | S | N | L | L | N | S | L | Q | E | F | I | F | D | D | L | K | S | L | R | F | L | N | L | Q | K | N | L | I | T | T | V | R | P | Q | V | F | K | T | P | L | S | N | L | S | L | L | I | M | D | K | N | P | F | D | C | T | C | E | S | M | L | W | F | V | T | W | L | N | N | T | N | M | T | T | V | P | G | L | R | D | Q | Y | T | C | N | T | P | L | A | Y | Y | N | H | P | I | M | K | F | D | A | L | S | C | K | D | M | T | P | F | Q | A | L | Y | I | L | S | S | T | A | V | I | M | L | M | V | T | A | L | M | V | R | F | H | G | W | R | I | Q | F | Y | W | N | I | L | I | N | R | T | L | G | F | S | D | A | T | V | E | E | X | X | G | R | E | F | E | Y | D | A | Y | V | I | Y | A | E | E | D | S | S | W | V | E | R | R | M | V | P | L | E | N | X | X | N | C | R | F | C | L | E | D | R | D | S | V | P | G | M | S | Q | L | E | S | I | V | D | N | M | R | R | S | R | K | I | L | F | V | V | T | E | T | L | L | K | D | P | W | C | R | R | F | K | A | H | H | A | L | H | Q | V | I | E | A | S | R | D | S | V | V | L | V | F | L | Q | D | V | H | D | Y | K | L | S | H | S | L | F | L | R | R | G | M | L | R | P | C | C | V | L | D | W | X | X | V | E | R | E | R | V | P | X | L | S | P | E | A | P | H | S | T | W | H | D | X | S | F | K | X | X | X | X | X | X | X | X | X | E |
| LMTLR3\_2874\_BP | X | X | X | X | X | X | M | Q | R | Q | N | X | X | X | X | X | V | F | E | T | M | C | A | P | R | X | X | Y | F | I | L | P | A | X | V | I | I | I | C | Y | F | M | T | G | P | H | N | C | V | V | C | X | X | Q | K | K | T | A | C | N | V | Q | Y | G | R | A | D | C | S | H | L | S | L | S | A | V | P | P | D | L | P | R | N | I | T | G | L | D | M | S | H | N | R | L | K | G | I | P | P | V | S | L | A | P | Y | P | G | L | L | H | L | D | A | S | Y | N | S | I | T | K | L | D | E | G | L | C | Q | T | L | P | L | L | Q | A | L | N | V | G | H | N | E | V | H | L | L | K | K | E | D | V | S | H | C | T | S | L | K | Q | F | I | M | A | S | N | R | L | K | L | Q | X | X | X | X | E | P | F | S | A | L | Q | N | L | E | C | L | D | I | S | I | N | K | L | K | T | A | K | L | G | S | Q | P | Q | L | Q | S | L | V | N | L | N | L | A | F | N | D | F | T | T | L | K | K | D | D | F | S | F | L | D | H | S | P | F | L | Q | V | L | N | L | S | X | S | V | S | L | K | T | L | E | P | G | C | F | K | P | I | S | G | L | R | T | L | I | M | D | R | S | N | M | G | T | S | V | I | S | K | L | C | S | E | L | S | G | T | A | I | D | I | L | S | L | R | K | M | N | L | V | T | L | T | N | A | T | F | T | G | L | Q | K | T | N | L | T | S | L | D | L | S | Y | N | G | M | G | K | I | E | E | G | S | F | Q | W | L | P | R | L | Q | T | L | I | L | A | H | N | N | I | K | H | L | T | K | D | T | L | Q | G | L | K | S | L | K | K | L | Q | L | T | E | A | L | V | K | S | H | T | S | S | T | P | I | I | D | D | F | S | F | Q | P | L | S | A | L | E | S | L | I | L | Q | R | T | A | V | R | E | L | T | E | H | T | F | T | G | L | T | S | L | K | E | L | D | L | S | W | S | S | X | X | T | S | L | R | N | I | T | N | K | T | L | V | S | L | A | G | S | P | L | X | R | K | L | D | L | T | G | T | A | I | I | Q | I | N | P | G | S | F | S | S | L | R | N | L | S | I | L | L | L | D | F | N | F | I | K | Q | T | L | T | G | K | E | F | E | G | L | D | Q | I | Q | E | I | H | M | S | N | N | H | Q | S | V | S | L | S | S | L | S | F | V | N | V | P | N | L | K | V | L | I | L | G | K | S | L | K | A | T | A | X | X | X | N | L | D | P | S | P | F | S | P | L | S | N | L | T | F | L | D | L | S | N | N | N | I | A | Q | I | R | E | S | M | V | K | G | L | R | N | L | K | V | L | K | L | Q | H | N | N | L | A | R | F | W | K | S | A | N | L | G | G | P | V | L | F | L | K | G | A | Q | S | L | T | T | L | Q | M | D | S | N | G | L | D | E | I | P | A | E | A | L | R | G | L | S | N | L | R | E | L | S | L | A | N | N | L | L | N | N | L | K | D | S | I | F | D | D | L | I | S | L | R | V | L | R | L | Q | K | N | L | I | T | A | V | R | P | E | V | F | K | T | P | M | S | N | L | S | L | L | V | M | D | K | N | P | F | D | C | T | C | E | S | I | L | W | F | V | T | W | L | N | N | T | N | M | S | S | L | P | G | I | R | E | Q | Y | M | C | N | T | P | L | A | Y | F | D | H | S | V | M | D | F | D | A | L | S | C | K | D | M | T | P | F | Q | A | L | Y | I | L | S | S | T | A | V | M | M | L | T | V | T | A | L | L | V | R | F | H | G | W | R | V | Q | F | Y | W | N | I | L | I | N | R | T | L | G | F | S | D | S | T | V | E | E | X | X | G | R | E | F | K | Y | D | A | Y | V | I | H | A | E | E | D | S | S | W | V | E | R | R | M | V | P | L | E | N | X | X | K | W | K | F | C | L | E | D | R | D | S | V | P | G | M | S | Q | L | E | S | I | V | D | N | I | R | K | S | R | K | I | L | F | V | V | T | E | T | L | L | N | D | P | W | C | R | R | F | K | A | Y | H | A | L | H | Q | V | I | E | A | S | R | D | S | V | V | L | V | F | L | Q | D | V | H | D | Y | K | L | S | R | S | L | F | L | R | R | G | M | L | R | P | C | C | I | L | D | W | X | X | V | H | K | E | R | V | P | X | L | S | P | E | A | P | H | S | T | W | H | D | X | S | I | A | X | X | X | X | X | X | X | X | X | E |
| conservation |  |  |  |  |  |  |  |  |  |  |  |  |  |  |  |  |  |  |  |  |  |  |  |  |  |  |  |  |  |  |  |  |  |  |  |  |  |  |  |  |  |  |  |  |  |  |  |  |  |  |  |  |  |  |  |  |  |  |  |  |  |  |  |  |  |  |  |  |  |  |  |  |  |  |  |  |  |  |  |  |  |  |  |  |  |  |  |  |  |  |  |  |  |  |  |  |  |  |  |  |  |  |  |  |  |  |  |  |  |  |  |  |  |  |  |  |  |  |  |  |  |  |  |  |  |  |  |  |  |  |  |  |  |  |  |  |  |  |  |  |  |  |  |  |  |  |  |  |  |  |  |  |  |  |  |  |  |  |  |  |  |  |  |  |  |  |  |  |  |  |  |  |  |  |  |  |  |  |  |  |  |  |  |  |  |  |  |  |  |  |  |  |  |  |  |  |  |  |  |  |  |  |  |  |  |  |  |  |  |  |  |  |  |  |  |  |  |  |  |  |  |  |  |  |  |  |  |  |  |  |  |  |  |  |  |  |  |  |  |  |  |  |  |  |  |  |  |  |  |  |  |  |  |  |  |  |  |  |  |  |  |  |  |  |  |  |  |  |  |  |  |  |  |  |  |  |  |  |  |  |  |  |  |  |  |  |  |  |  |  |  |  |  |  |  |  |  |  |  |  |  |  |  |  |  |  |  |  |  |  |  |  |  |  |  |  |  |  |  |  |  |  |  |  |  |  |  |  |  |  |  |  |  |  |  |  |  |  |  |  |  |  |  |  |  |  |  |  |  |  |  |  |  |  |  |  |  |  |  |  |  |  |  |  |  |  |  |  |  |  |  |  |  |  |  |  |  |  |  |  |  |  |  |  |  |  |  |  |  |  |  |  |  |  |  |  |  |  |  |  |  |  |  |  |  |  |  |  |  |  |  |  |  |  |  |  |  |  |  |  |  |  |  |  |  |  |  |  |  |  |  |  |  |  |  |  |  |  |  |  |  |  |  |  |  |  |  |  |  |  |  |  |  |  |  |  |  |  |  |  |  |  |  |  |  |  |  |  |  |  |  |  |  |  |  |  |  |  |  |  |  |  |  |  |  |  |  |  |  |  |  |  |  |  |  |  |  |  |  |  |  |  |  |  |  |  |  |  |  |  |  |  |  |  |  |  |  |  |  |  |  |  |  |  |  |  |  |  |  |  |  |  |  |  |  |  |  |  |  |  |  |  |  |  |  |  |  |  |  |  |  |  |  |  |  |  |  |  |  |  |  |  |  |  |  |  |  |  |  |  |  |  |  |  |  |  |  |  |  |  |  |  |  |  |  |  |  |  |  |  |  |  |  |  |  |  |  |  |  |  |  |  |  |  |  |  |  |  |  |  |  |  |  |  |  |  |  |  |  |  |  |  |  |  |  |  |  |  |  |  |  |  |  |  |  |  |  |  |  |  |  |  |  |  |  |  |  |  |  |  |  |  |  |  |  |  |  |  |  |  |  |  |  |  |  |  |  |  |  |  |  |  |  |  |  |  |  |  |  |  |  |  |  |  |  |  |  |  |  |  |  |  |  |  |  |  |  |  |  |  |  |  |  |  |  |  |  |  |  |  |  |  |  |  |  |  |  |  |  |  |  |  |  |  |  |  |  |  |  |  |  |  |  |  |  |  |  |  |  |  |  |  |  |  |  |  |  |  |  |  |  |  |  |  |  |  |  |  |  |  |  |  |  |  |  |  |  |  |  |  |  |  |  |  |  |  |  |  |  |  |  |  |  |  |  |  |  |  |  |  |  |  |  |  |  |  |  |  |  |  |  |  |  |  |  |  |  |  |  |  |  |  |  |  |  |  |  |  |  |  |  |  |  |  |  |  |  |  |  |  |  |  |  |  |  |  |  |  |  |  |  |  |  |  |  |  |  |  |  |  |  |  |  |  |  |  |  |  |  |  |  |  |  |  |  |  |  |  |  |  |  |  |  |  |  |  |  |  |  |  |  |  |  |  |  |  |  |  |  |  |  |  |  |  |  |  |  |  |  |  |  |  |  |  |  |  |  |  |  |  |  |  |  |  |  |  |  |  |  |  |  |  |  |  |  |  |  |  |  |  |  |  |  |  |  |  |  |  |  |  |  |  |  |  |  |  |  |  |  |  |  |  |  |  |  |  |  |  |
|  |  |  |  |  |  |  |  |  |  |  |  |  |  |  |  |  |  |  |  |  |  |  |  |  |  |  |  |  |  |  |  |  |  |  |  |  |  |  |  |  |  |  |  |  |  |  |  |  |  |  |  |  |  |  |  |  |  |  |  |  |  |  |  |  |  |  |  |  |  |  |  |  |  |  |  |  |  |  |  |  |  |  |  |  |  |  |  |  |  |  |  |  |  |  |  |  |  |  |  |  |  |  |  |  |  |  |  |  |  |  |  |  |  |  |  |  |  |  |  |  |  |  |  |  |  |  |  |  |  |  |  |  |  |  |  |  |  |  |  |  |  |  |  |  |  |  |  |  |  |  |  |  |  |  |  |  |  |  |  |  |  |  |  |  |  |  |  |  |  |  |  |  |  |  |  |  |  |  |  |  |  |  |  |  |  |  |  |  |  |  |  |  |  |  |  |  |  |  |  |  |  |  |  |  |  |  |  |  |  |  |  |  |  |  |  |  |  |  |  |  |  |  |  |  |  |  |  |  |  |  |  |  |  |  |  |  |  |  |  |  |  |  |  |  |  |  |  |  |  |  |  |  |  |  |  |  |  |  |  |  |  |  |  |  |  |  |  |  |  |  |  |  |  |  |  |  |  |  |  |  |  |  |  |  |  |  |  |  |  |  |  |  |  |  |  |  |  |  |  |  |  |  |  |  |  |  |  |  |  |  |  |  |  |  |  |  |  |  |  |  |  |  |  |  |  |  |  |  |  |  |  |  |  |  |  |  |  |  |  |  |  |  |  |  |  |  |  |  |  |  |  |  |  |  |  |  |  |  |  |  |  |  |  |  |  |  |  |  |  |  |  |  |  |  |  |  |  |  |  |  |  |  |  |  |  |  |  |  |  |  |  |  |  |  |  |  |  |  |  |  |  |  |  |  |  |  |  |  |  |  |  |  |  |  |  |  |  |  |  |  |  |  |  |  |  |  |  |  |  |  |  |  |  |  |  |  |  |  |  |  |  |  |  |  |  |  |  |  |  |  |  |  |  |  |  |  |  |  |  |  |  |  |  |  |  |  |  |  |  |  |  |  |  |  |  |  |  |  |  |  |  |  |  |  |  |  |  |  |  |  |  |  |  |  |  |  |  |  |  |  |  |  |  |  |  |  |  |  |  |  |  |  |  |  |  |  |  |  |  |  |  |  |  |  |  |  |  |  |  |  |  |  |  |  |  |  |  |  |  |  |  |  |  |  |  |  |  |  |  |  |  |  |  |  |  |  |  |  |  |  |  |  |  |  |  |  |  |  |  |  |  |  |  |  |  |  |  |  |  |  |  |  |  |  |  |  |  |  |  |  |  |  |  |  |  |  |  |  |  |  |  |  |  |  |  |  |  |  |  |  |  |  |  |  |  |  |  |  |  |  |  |  |  |  |  |  |  |  |  |  |  |  |  |  |  |  |  |  |  |  |  |  |  |  |  |  |  |  |  |  |  |  |  |  |  |  |  |  |  |  |  |  |  |  |  |  |  |  |  |  |  |  |  |  |  |  |  |  |  |  |  |  |  |  |  |  |  |  |  |  |  |  |  |  |  |  |  |  |  |  |  |  |  |  |  |  |  |  |  |  |  |  |  |  |  |  |  |  |  |  |  |  |  |  |  |  |  |  |  |  |  |  |  |  |  |  |  |  |  |  |  |  |  |  |  |  |  |  |  |  |  |  |  |  |  |  |  |  |  |  |  |  |  |  |  |  |  |  |  |  |  |  |  |  |  |  |  |  |  |  |  |  |  |  |  |  |  |  |  |  |  |  |  |  |  |  |  |  |  |  |  |  |  |  |  |  |  |  |  |  |  |  |  |  |  |  |  |  |  |  |  |  |  |  |  |  |  |  |  |  |  |  |  |  |  |  |  |  |  |  |  |  |  |  |  |  |  |  |  |  |  |  |  |  |  |  |  |  |  |  |  |  |  |  |  |  |  |  |  |  |  |  |  |  |  |  |  |  |  |  |  |  |  |  |  |  |  |  |  |  |  |  |  |  |  |  |  |  |  |  |  |  |  |  |  |  |  |  |  |  |  |  |  |  |  |  |  |  |  |  |  |  |  |  |  |  |  |  |  |  |  |  |  |  |  |  |  |  |  |  |  |  |  |  |  |  |  |  |  |  |  |  |  |  |  |  |  |  |
|  |  |  |  |  |  |  |  |  |  |  |  |  |  |  |  |  |  |  |  |  |  |  |  |  |  |  |  |  |  |  |  |  |  |  |  |  |  |  |  |  |  |  |  |  |  |  |  |  |  |  |  |  |  |  |  |  |  |  |  |  |  |  |  |  |  |  |  |  |  |  |  |  |  |  |  |  |  |  |  |  |  |  |  |  |  |  |  |  |  |  |  |  |  |  |  |  |  |  |  |  |  |  |  |  |  |  |  |  |  |  |  |  |  |  |  |  |  |  |  |  |  |  |  |  |  |  |  |  |  |  |  |  |  |  |  |  |  |  |  |  |  |  |  |  |  |  |  |  |  |  |  |  |  |  |  |  |  |  |  |  |  |  |  |  |  |  |  |  |  |  |  |  |  |  |  |  |  |  |  |  |  |  |  |  |  |  |  |  |  |  |  |  |  |  |  |  |  |  |  |  |  |  |  |  |  |  |  |  |  |  |  |  |  |  |  |  |  |  |  |  |  |  |  |  |  |  |  |  |  |  |  |  |  |  |  |  |  |  |  |  |  |  |  |  |  |  |  |  |  |  |  |  |  |  |  |  |  |  |  |  |  |  |  |  |  |  |  |  |  |  |  |  |  |  |  |  |  |  |  |  |  |  |  |  |  |  |  |  |  |  |  |  |  |  |  |  |  |  |  |  |  |  |  |  |  |  |  |  |  |  |  |  |  |  |  |  |  |  |  |  |  |  |  |  |  |  |  |  |  |  |  |  |  |  |  |  |  |  |  |  |  |  |  |  |  |  |  |  |  |  |  |  |  |  |  |  |  |  |  |  |  |  |  |  |  |  |  |  |  |  |  |  |  |  |  |  |  |  |  |  |  |  |  |  |  |  |  |  |  |  |  |  |  |  |  |  |  |  |  |  |  |  |  |  |  |  |  |  |  |  |  |  |  |  |  |  |  |  |  |  |  |  |  |  |  |  |  |  |  |  |  |  |  |  |  |  |  |  |  |  |  |  |  |  |  |  |  |  |  |  |  |  |  |  |  |  |  |  |  |  |  |  |  |  |  |  |  |  |  |  |  |  |  |  |  |  |  |  |  |  |  |  |  |  |  |  |  |  |  |  |  |  |  |  |  |  |  |  |  |  |  |  |  |  |  |  |  |  |  |  |  |  |  |  |  |  |  |  |  |  |  |  |  |  |  |  |  |  |  |  |  |  |  |  |  |  |  |  |  |  |  |  |  |  |  |  |  |  |  |  |  |  |  |  |  |  |  |  |  |  |  |  |  |  |  |  |  |  |  |  |  |  |  |  |  |  |  |  |  |  |  |  |  |  |  |  |  |  |  |  |  |  |  |  |  |  |  |  |  |  |  |  |  |  |  |  |  |  |  |  |  |  |  |  |  |  |  |  |  |  |  |  |  |  |  |  |  |  |  |  |  |  |  |  |  |  |  |  |  |  |  |  |  |  |  |  |  |  |  |  |  |  |  |  |  |  |  |  |  |  |  |  |  |  |  |  |  |  |  |  |  |  |  |  |  |  |  |  |  |  |  |  |  |  |  |  |  |  |  |  |  |  |  |  |  |  |  |  |  |  |  |  |  |  |  |  |  |  |  |  |  |  |  |  |  |  |  |  |  |  |  |  |  |  |  |  |  |  |  |  |  |  |  |  |  |  |  |  |  |  |  |  |  |  |  |  |  |  |  |  |  |  |  |  |  |  |  |  |  |  |  |  |  |  |  |  |  |  |  |  |  |  |  |  |  |  |  |  |  |  |  |  |  |  |  |  |  |  |  |  |  |  |  |  |  |  |  |  |  |  |  |  |  |  |  |  |  |  |  |  |  |  |  |  |  |  |  |  |  |  |  |  |  |  |  |  |  |  |  |  |  |  |  |  |  |  |  |  |  |  |  |  |  |  |  |  |  |  |  |  |  |  |  |  |  |  |  |  |  |  |  |  |  |  |  |  |  |  |  |  |  |  |  |  |  |  |  |  |  |  |  |  |  |  |  |  |  |  |  |  |  |  |  |  |  |  |  |  |  |  |  |  |  |  |  |  |  |  |  |  |  |  |  |  |  |  |  |  |  |  |  |  |  |  |  |  |  |  |  |  |  |  |  |  |  |  |  |  |  |  |  |  |  |  |  |  |  |  |  |  |  |  |  |  |  |  |  |  |
|  |  |  |  |  |  |  |  |  |  |  |  |  |  |  |  |  |  |  |  |  |  |  |  |  |  |  |  |  |  |  |  |  |  |  |  |  |  |  |  |  |  |  |  |  |  |  |  |  |  |  |  |  |  |  |  |  |  |  |  |  |  |  |  |  |  |  |  |  |  |  |  |  |  |  |  |  |  |  |  |  |  |  |  |  |  |  |  |  |  |  |  |  |  |  |  |  |  |  |  |  |  |  |  |  |  |  |  |  |  |  |  |  |  |  |  |  |  |  |  |  |  |  |  |  |  |  |  |  |  |  |  |  |  |  |  |  |  |  |  |  |  |  |  |  |  |  |  |  |  |  |  |  |  |  |  |  |  |  |  |  |  |  |  |  |  |  |  |  |  |  |  |  |  |  |  |  |  |  |  |  |  |  |  |  |  |  |  |  |  |  |  |  |  |  |  |  |  |  |  |  |  |  |  |  |  |  |  |  |  |  |  |  |  |  |  |  |  |  |  |  |  |  |  |  |  |  |  |  |  |  |  |  |  |  |  |  |  |  |  |  |  |  |  |  |  |  |  |  |  |  |  |  |  |  |  |  |  |  |  |  |  |  |  |  |  |  |  |  |  |  |  |  |  |  |  |  |  |  |  |  |  |  |  |  |  |  |  |  |  |  |  |  |  |  |  |  |  |  |  |  |  |  |  |  |  |  |  |  |  |  |  |  |  |  |  |  |  |  |  |  |  |  |  |  |  |  |  |  |  |  |  |  |  |  |  |  |  |  |  |  |  |  |  |  |  |  |  |  |  |  |  |  |  |  |  |  |  |  |  |  |  |  |  |  |  |  |  |  |  |  |  |  |  |  |  |  |  |  |  |  |  |  |  |  |  |  |  |  |  |  |  |  |  |  |  |  |  |  |  |  |  |  |  |  |  |  |  |  |  |  |  |  |  |  |  |  |  |  |  |  |  |  |  |  |  |  |  |  |  |  |  |  |  |  |  |  |  |  |  |  |  |  |  |  |  |  |  |  |  |  |  |  |  |  |  |  |  |  |  |  |  |  |  |  |  |  |  |  |  |  |  |  |  |  |  |  |  |  |  |  |  |  |  |  |  |  |  |  |  |  |  |  |  |  |  |  |  |  |  |  |  |  |  |  |  |  |  |  |  |  |  |  |  |  |  |  |  |  |  |  |  |  |  |  |  |  |  |  |  |  |  |  |  |  |  |  |  |  |  |  |  |  |  |  |  |  |  |  |  |  |  |  |  |  |  |  |  |  |  |  |  |  |  |  |  |  |  |  |  |  |  |  |  |  |  |  |  |  |  |  |  |  |  |  |  |  |  |  |  |  |  |  |  |  |  |  |  |  |  |  |  |  |  |  |  |  |  |  |  |  |  |  |  |  |  |  |  |  |  |  |  |  |  |  |  |  |  |  |  |  |  |  |  |  |  |  |  |  |  |  |  |  |  |  |  |  |  |  |  |  |  |  |  |  |  |  |  |  |  |  |  |  |  |  |  |  |  |  |  |  |  |  |  |  |  |  |  |  |  |  |  |  |  |  |  |  |  |  |  |  |  |  |  |  |  |  |  |  |  |  |  |  |  |  |  |  |  |  |  |  |  |  |  |  |  |  |  |  |  |  |  |  |  |  |  |  |  |  |  |  |  |  |  |  |  |  |  |  |  |  |  |  |  |  |  |  |  |  |  |  |  |  |  |  |  |  |  |  |  |  |  |  |  |  |  |  |  |  |  |  |  |  |  |  |  |  |  |  |  |  |  |  |  |  |  |  |  |  |  |  |  |  |  |  |  |  |  |  |  |  |  |  |  |  |  |  |  |  |  |  |  |  |  |  |  |  |  |  |  |  |  |  |  |  |  |  |  |  |  |  |  |  |  |  |  |  |  |  |  |  |  |  |  |  |  |  |  |  |  |  |  |  |  |  |  |  |  |  |  |  |  |  |  |  |  |  |  |  |  |  |  |  |  |  |  |  |  |  |  |  |  |  |  |  |  |  |  |  |  |  |  |  |  |  |  |  |  |  |  |  |  |  |  |  |  |  |  |  |  |  |  |  |  |  |  |  |  |  |  |  |  |  |  |  |  |  |  |  |  |  |  |  |  |  |  |  |  |  |  |  |  |  |  |  |  |  |  |  |  |  |  |  |  |  |  |  |  |  |
|  |  |  |  |  |  |  |  |  |  |  |  |  |  |  |  |  |  |  |  |  |  |  |  |  |  |  |  |  |  |  |  |  |  |  |  |  |  |  |  |  |  |  |  |  |  |  |  |  |  |  |  |  |  |  |  |  |  |  |  |  |  |  |  |  |  |  |  |  |  |  |  |  |  |  |  |  |  |  |  |  |  |  |  |  |  |  |  |  |  |  |  |  |  |  |  |  |  |  |  |  |  |  |  |  |  |  |  |  |  |  |  |  |  |  |  |  |  |  |  |  |  |  |  |  |  |  |  |  |  |  |  |  |  |  |  |  |  |  |  |  |  |  |  |  |  |  |  |  |  |  |  |  |  |  |  |  |  |  |  |  |  |  |  |  |  |  |  |  |  |  |  |  |  |  |  |  |  |  |  |  |  |  |  |  |  |  |  |  |  |  |  |  |  |  |  |  |  |  |  |  |  |  |  |  |  |  |  |  |  |  |  |  |  |  |  |  |  |  |  |  |  |  |  |  |  |  |  |  |  |  |  |  |  |  |  |  |  |  |  |  |  |  |  |  |  |  |  |  |  |  |  |  |  |  |  |  |  |  |  |  |  |  |  |  |  |  |  |  |  |  |  |  |  |  |  |  |  |  |  |  |  |  |  |  |  |  |  |  |  |  |  |  |  |  |  |  |  |  |  |  |  |  |  |  |  |  |  |  |  |  |  |  |  |  |  |  |  |  |  |  |  |  |  |  |  |  |  |  |  |  |  |  |  |  |  |  |  |  |  |  |  |  |  |  |  |  |  |  |  |  |  |  |  |  |  |  |  |  |  |  |  |  |  |  |  |  |  |  |  |  |  |  |  |  |  |  |  |  |  |  |  |  |  |  |  |  |  |  |  |  |  |  |  |  |  |  |  |  |  |  |  |  |  |  |  |  |  |  |  |  |  |  |  |  |  |  |  |  |  |  |  |  |  |  |  |  |  |  |  |  |  |  |  |  |  |  |  |  |  |  |  |  |  |  |  |  |  |  |  |  |  |  |  |  |  |  |  |  |  |  |  |  |  |  |  |  |  |  |  |  |  |  |  |  |  |  |  |  |  |  |  |  |  |  |  |  |  |  |  |  |  |  |  |  |  |  |  |  |  |  |  |  |  |  |  |  |  |  |  |  |  |  |  |  |  |  |  |  |  |  |  |  |  |  |  |  |  |  |  |  |  |  |  |  |  |  |  |  |  |  |  |  |  |  |  |  |  |  |  |  |  |  |  |  |  |  |  |  |  |  |  |  |  |  |  |  |  |  |  |  |  |  |  |  |  |  |  |  |  |  |  |  |  |  |  |  |  |  |  |  |  |  |  |  |  |  |  |  |  |  |  |  |  |  |  |  |  |  |  |  |  |  |  |  |  |  |  |  |  |  |  |  |  |  |  |  |  |  |  |  |  |  |  |  |  |  |  |  |  |  |  |  |  |  |  |  |  |  |  |  |  |  |  |  |  |  |  |  |  |  |  |  |  |  |  |  |  |  |  |  |  |  |  |  |  |  |  |  |  |  |  |  |  |  |  |  |  |  |  |  |  |  |  |  |  |  |  |  |  |  |  |  |  |  |  |  |  |  |  |  |  |  |  |  |  |  |  |  |  |  |  |  |  |  |  |  |  |  |  |  |  |  |  |  |  |  |  |  |  |  |  |  |  |  |  |  |  |  |  |  |  |  |  |  |  |  |  |  |  |  |  |  |  |  |  |  |  |  |  |  |  |  |  |  |  |  |  |  |  |  |  |  |  |  |  |  |  |  |  |  |  |  |  |  |  |  |  |  |  |  |  |  |  |  |  |  |  |  |  |  |  |  |  |  |  |  |  |  |  |  |  |  |  |  |  |  |  |  |  |  |  |  |  |  |  |  |  |  |  |  |  |  |  |  |  |  |  |  |  |  |  |  |  |  |  |  |  |  |  |  |  |  |  |  |  |  |  |  |  |  |  |  |  |  |  |  |  |  |  |  |  |  |  |  |  |  |  |  |  |  |  |  |  |  |  |  |  |  |  |  |  |  |  |  |  |  |  |  |  |  |  |  |  |  |  |  |  |  |  |  |  |  |  |  |  |  |  |  |  |  |  |  |  |  |  |  |  |  |  |  |  |  |  |  |  |  |  |  |  |  |  |  |  |  |  |  |  |  |
|  |  |  |  |  |  |  |  |  |  |  |  |  |  |  |  |  |  |  |  |  |  |  |  |  |  |  |  |  |  |  |  |  |  |  |  |  |  |  |  |  |  |  |  |  |  |  |  |  |  |  |  |  |  |  |  |  |  |  |  |  |  |  |  |  |  |  |  |  |  |  |  |  |  |  |  |  |  |  |  |  |  |  |  |  |  |  |  |  |  |  |  |  |  |  |  |  |  |  |  |  |  |  |  |  |  |  |  |  |  |  |  |  |  |  |  |  |  |  |  |  |  |  |  |  |  |  |  |  |  |  |  |  |  |  |  |  |  |  |  |  |  |  |  |  |  |  |  |  |  |  |  |  |  |  |  |  |  |  |  |  |  |  |  |  |  |  |  |  |  |  |  |  |  |  |  |  |  |  |  |  |  |  |  |  |  |  |  |  |  |  |  |  |  |  |  |  |  |  |  |  |  |  |  |  |  |  |  |  |  |  |  |  |  |  |  |  |  |  |  |  |  |  |  |  |  |  |  |  |  |  |  |  |  |  |  |  |  |  |  |  |  |  |  |  |  |  |  |  |  |  |  |  |  |  |  |  |  |  |  |  |  |  |  |  |  |  |  |  |  |  |  |  |  |  |  |  |  |  |  |  |  |  |  |  |  |  |  |  |  |  |  |  |  |  |  |  |  |  |  |  |  |  |  |  |  |  |  |  |  |  |  |  |  |  |  |  |  |  |  |  |  |  |  |  |  |  |  |  |  |  |  |  |  |  |  |  |  |  |  |  |  |  |  |  |  |  |  |  |  |  |  |  |  |  |  |  |  |  |  |  |  |  |  |  |  |  |  |  |  |  |  |  |  |  |  |  |  |  |  |  |  |  |  |  |  |  |  |  |  |  |  |  |  |  |  |  |  |  |  |  |  |  |  |  |  |  |  |  |  |  |  |  |  |  |  |  |  |  |  |  |  |  |  |  |  |  |  |  |  |  |  |  |  |  |  |  |  |  |  |  |  |  |  |  |  |  |  |  |  |  |  |  |  |  |  |  |  |  |  |  |  |  |  |  |  |  |  |  |  |  |  |  |  |  |  |  |  |  |  |  |  |  |  |  |  |  |  |  |  |  |  |  |  |  |  |  |  |  |  |  |  |  |  |  |  |  |  |  |  |  |  |  |  |  |  |  |  |  |  |  |  |  |  |  |  |  |  |  |  |  |  |  |  |  |  |  |  |  |  |  |  |  |  |  |  |  |  |  |  |  |  |  |  |  |  |  |  |  |  |  |  |  |  |  |  |  |  |  |  |  |  |  |  |  |  |  |  |  |  |  |  |  |  |  |  |  |  |  |  |  |  |  |  |  |  |  |  |  |  |  |  |  |  |  |  |  |  |  |  |  |  |  |  |  |  |  |  |  |  |  |  |  |  |  |  |  |  |  |  |  |  |  |  |  |  |  |  |  |  |  |  |  |  |  |  |  |  |  |  |  |  |  |  |  |  |  |  |  |  |  |  |  |  |  |  |  |  |  |  |  |  |  |  |  |  |  |  |  |  |  |  |  |  |  |  |  |  |  |  |  |  |  |  |  |  |  |  |  |  |  |  |  |  |  |  |  |  |  |  |  |  |  |  |  |  |  |  |  |  |  |  |  |  |  |  |  |  |  |  |  |  |  |  |  |  |  |  |  |  |  |  |  |  |  |  |  |  |  |  |  |  |  |  |  |  |  |  |  |  |  |  |  |  |  |  |  |  |  |  |  |  |  |  |  |  |  |  |  |  |  |  |  |  |  |  |  |  |  |  |  |  |  |  |  |  |  |  |  |  |  |  |  |  |  |  |  |  |  |  |  |  |  |  |  |  |  |  |  |  |  |  |  |  |  |  |  |  |  |  |  |  |  |  |  |  |  |  |  |  |  |  |  |  |  |  |  |  |  |  |  |  |  |  |  |  |  |  |  |  |  |  |  |  |  |  |  |  |  |  |  |  |  |  |  |  |  |  |  |  |  |  |  |  |  |  |  |  |  |  |  |  |  |  |  |  |  |  |  |  |  |  |  |  |  |  |  |  |  |  |  |  |  |  |  |  |  |  |  |  |  |  |  |  |  |  |  |  |  |  |  |  |  |  |  |  |  |  |  |  |  |  |  |  |  |  |  |  |  |  |  |  |  |  |  |  |  |  |  |
|  |  |  |  |  |  |  |  |  |  |  |  |  |  |  |  |  |  |  |  |  |  |  |  |  |  |  |  |  |  |  |  |  |  |  |  |  |  |  |  |  |  |  |  |  |  |  |  |  |  |  |  |  |  |  |  |  |  |  |  |  |  |  |  |  |  |  |  |  |  |  |  |  |  |  |  |  |  |  |  |  |  |  |  |  |  |  |  |  |  |  |  |  |  |  |  |  |  |  |  |  |  |  |  |  |  |  |  |  |  |  |  |  |  |  |  |  |  |  |  |  |  |  |  |  |  |  |  |  |  |  |  |  |  |  |  |  |  |  |  |  |  |  |  |  |  |  |  |  |  |  |  |  |  |  |  |  |  |  |  |  |  |  |  |  |  |  |  |  |  |  |  |  |  |  |  |  |  |  |  |  |  |  |  |  |  |  |  |  |  |  |  |  |  |  |  |  |  |  |  |  |  |  |  |  |  |  |  |  |  |  |  |  |  |  |  |  |  |  |  |  |  |  |  |  |  |  |  |  |  |  |  |  |  |  |  |  |  |  |  |  |  |  |  |  |  |  |  |  |  |  |  |  |  |  |  |  |  |  |  |  |  |  |  |  |  |  |  |  |  |  |  |  |  |  |  |  |  |  |  |  |  |  |  |  |  |  |  |  |  |  |  |  |  |  |  |  |  |  |  |  |  |  |  |  |  |  |  |  |  |  |  |  |  |  |  |  |  |  |  |  |  |  |  |  |  |  |  |  |  |  |  |  |  |  |  |  |  |  |  |  |  |  |  |  |  |  |  |  |  |  |  |  |  |  |  |  |  |  |  |  |  |  |  |  |  |  |  |  |  |  |  |  |  |  |  |  |  |  |  |  |  |  |  |  |  |  |  |  |  |  |  |  |  |  |  |  |  |  |  |  |  |  |  |  |  |  |  |  |  |  |  |  |  |  |  |  |  |  |  |  |  |  |  |  |  |  |  |  |  |  |  |  |  |  |  |  |  |  |  |  |  |  |  |  |  |  |  |  |  |  |  |  |  |  |  |  |  |  |  |  |  |  |  |  |  |  |  |  |  |  |  |  |  |  |  |  |  |  |  |  |  |  |  |  |  |  |  |  |  |  |  |  |  |  |  |  |  |  |  |  |  |  |  |  |  |  |  |  |  |  |  |  |  |  |  |  |  |  |  |  |  |  |  |  |  |  |  |  |  |  |  |  |  |  |  |  |  |  |  |  |  |  |  |  |  |  |  |  |  |  |  |  |  |  |  |  |  |  |  |  |  |  |  |  |  |  |  |  |  |  |  |  |  |  |  |  |  |  |  |  |  |  |  |  |  |  |  |  |  |  |  |  |  |  |  |  |  |  |  |  |  |  |  |  |  |  |  |  |  |  |  |  |  |  |  |  |  |  |  |  |  |  |  |  |  |  |  |  |  |  |  |  |  |  |  |  |  |  |  |  |  |  |  |  |  |  |  |  |  |  |  |  |  |  |  |  |  |  |  |  |  |  |  |  |  |  |  |  |  |  |  |  |  |  |  |  |  |  |  |  |  |  |  |  |  |  |  |  |  |  |  |  |  |  |  |  |  |  |  |  |  |  |  |  |  |  |  |  |  |  |  |  |  |  |  |  |  |  |  |  |  |  |  |  |  |  |  |  |  |  |  |  |  |  |  |  |  |  |  |  |  |  |  |  |  |  |  |  |  |  |  |  |  |  |  |  |  |  |  |  |  |  |  |  |  |  |  |  |  |  |  |  |  |  |  |  |  |  |  |  |  |  |  |  |  |  |  |  |  |  |  |  |  |  |  |  |  |  |  |  |  |  |  |  |  |  |  |  |  |  |  |  |  |  |  |  |  |  |  |  |  |  |  |  |  |  |  |  |  |  |  |  |  |  |  |  |  |  |  |  |  |  |  |  |  |  |  |  |  |  |  |  |  |  |  |  |  |  |  |  |  |  |  |  |  |  |  |  |  |  |  |  |  |  |  |  |  |  |  |  |  |  |  |  |  |  |  |  |  |  |  |  |  |  |  |  |  |  |  |  |  |  |  |  |  |  |  |  |  |  |  |  |  |  |  |  |  |  |  |  |  |  |  |  |  |  |  |  |  |  |  |  |  |  |  |  |  |  |  |  |  |  |  |  |  |  |  |  |  |  |  |  |  |  |  |  |  |  |
|  |  |  |  |  |  |  |  |  |  |  |  |  |  |  |  |  |  |  |  |  |  |  |  |  |  |  |  |  |  |  |  |  |  |  |  |  |  |  |  |  |  |  |  |  |  |  |  |  |  |  |  |  |  |  |  |  |  |  |  |  |  |  |  |  |  |  |  |  |  |  |  |  |  |  |  |  |  |  |  |  |  |  |  |  |  |  |  |  |  |  |  |  |  |  |  |  |  |  |  |  |  |  |  |  |  |  |  |  |  |  |  |  |  |  |  |  |  |  |  |  |  |  |  |  |  |  |  |  |  |  |  |  |  |  |  |  |  |  |  |  |  |  |  |  |  |  |  |  |  |  |  |  |  |  |  |  |  |  |  |  |  |  |  |  |  |  |  |  |  |  |  |  |  |  |  |  |  |  |  |  |  |  |  |  |  |  |  |  |  |  |  |  |  |  |  |  |  |  |  |  |  |  |  |  |  |  |  |  |  |  |  |  |  |  |  |  |  |  |  |  |  |  |  |  |  |  |  |  |  |  |  |  |  |  |  |  |  |  |  |  |  |  |  |  |  |  |  |  |  |  |  |  |  |  |  |  |  |  |  |  |  |  |  |  |  |  |  |  |  |  |  |  |  |  |  |  |  |  |  |  |  |  |  |  |  |  |  |  |  |  |  |  |  |  |  |  |  |  |  |  |  |  |  |  |  |  |  |  |  |  |  |  |  |  |  |  |  |  |  |  |  |  |  |  |  |  |  |  |  |  |  |  |  |  |  |  |  |  |  |  |  |  |  |  |  |  |  |  |  |  |  |  |  |  |  |  |  |  |  |  |  |  |  |  |  |  |  |  |  |  |  |  |  |  |  |  |  |  |  |  |  |  |  |  |  |  |  |  |  |  |  |  |  |  |  |  |  |  |  |  |  |  |  |  |  |  |  |  |  |  |  |  |  |  |  |  |  |  |  |  |  |  |  |  |  |  |  |  |  |  |  |  |  |  |  |  |  |  |  |  |  |  |  |  |  |  |  |  |  |  |  |  |  |  |  |  |  |  |  |  |  |  |  |  |  |  |  |  |  |  |  |  |  |  |  |  |  |  |  |  |  |  |  |  |  |  |  |  |  |  |  |  |  |  |  |  |  |  |  |  |  |  |  |  |  |  |  |  |  |  |  |  |  |  |  |  |  |  |  |  |  |  |  |  |  |  |  |  |  |  |  |  |  |  |  |  |  |  |  |  |  |  |  |  |  |  |  |  |  |  |  |  |  |  |  |  |  |  |  |  |  |  |  |  |  |  |  |  |  |  |  |  |  |  |  |  |  |  |  |  |  |  |  |  |  |  |  |  |  |  |  |  |  |  |  |  |  |  |  |  |  |  |  |  |  |  |  |  |  |  |  |  |  |  |  |  |  |  |  |  |  |  |  |  |  |  |  |  |  |  |  |  |  |  |  |  |  |  |  |  |  |  |  |  |  |  |  |  |  |  |  |  |  |  |  |  |  |  |  |  |  |  |  |  |  |  |  |  |  |  |  |  |  |  |  |  |  |  |  |  |  |  |  |  |  |  |  |  |  |  |  |  |  |  |  |  |  |  |  |  |  |  |  |  |  |  |  |  |  |  |  |  |  |  |  |  |  |  |  |  |  |  |  |  |  |  |  |  |  |  |  |  |  |  |  |  |  |  |  |  |  |  |  |  |  |  |  |  |  |  |  |  |  |  |  |  |  |  |  |  |  |  |  |  |  |  |  |  |  |  |  |  |  |  |  |  |  |  |  |  |  |  |  |  |  |  |  |  |  |  |  |  |  |  |  |  |  |  |  |  |  |  |  |  |  |  |  |  |  |  |  |  |  |  |  |  |  |  |  |  |  |  |  |  |  |  |  |  |  |  |  |  |  |  |  |  |  |  |  |  |  |  |  |  |  |  |  |  |  |  |  |  |  |  |  |  |  |  |  |  |  |  |  |  |  |  |  |  |  |  |  |  |  |  |  |  |  |  |  |  |  |  |  |  |  |  |  |  |  |  |  |  |  |  |  |  |  |  |  |  |  |  |  |  |  |  |  |  |  |  |  |  |  |  |  |  |  |  |  |  |  |  |  |  |  |  |  |  |  |  |  |  |  |  |  |  |  |  |  |  |  |  |  |  |  |  |  |  |  |  |  |  |  |  |  |  |  |  |
|  |  |  |  |  |  |  |  |  |  |  |  |  |  |  |  |  |  |  |  |  |  |  |  |  |  |  |  |  |  |  |  |  |  |  |  |  |  |  |  |  |  |  |  |  |  |  |  |  |  |  |  |  |  |  |  |  |  |  |  |  |  |  |  |  |  |  |  |  |  |  |  |  |  |  |  |  |  |  |  |  |  |  |  |  |  |  |  |  |  |  |  |  |  |  |  |  |  |  |  |  |  |  |  |  |  |  |  |  |  |  |  |  |  |  |  |  |  |  |  |  |  |  |  |  |  |  |  |  |  |  |  |  |  |  |  |  |  |  |  |  |  |  |  |  |  |  |  |  |  |  |  |  |  |  |  |  |  |  |  |  |  |  |  |  |  |  |  |  |  |  |  |  |  |  |  |  |  |  |  |  |  |  |  |  |  |  |  |  |  |  |  |  |  |  |  |  |  |  |  |  |  |  |  |  |  |  |  |  |  |  |  |  |  |  |  |  |  |  |  |  |  |  |  |  |  |  |  |  |  |  |  |  |  |  |  |  |  |  |  |  |  |  |  |  |  |  |  |  |  |  |  |  |  |  |  |  |  |  |  |  |  |  |  |  |  |  |  |  |  |  |  |  |  |  |  |  |  |  |  |  |  |  |  |  |  |  |  |  |  |  |  |  |  |  |  |  |  |  |  |  |  |  |  |  |  |  |  |  |  |  |  |  |  |  |  |  |  |  |  |  |  |  |  |  |  |  |  |  |  |  |  |  |  |  |  |  |  |  |  |  |  |  |  |  |  |  |  |  |  |  |  |  |  |  |  |  |  |  |  |  |  |  |  |  |  |  |  |  |  |  |  |  |  |  |  |  |  |  |  |  |  |  |  |  |  |  |  |  |  |  |  |  |  |  |  |  |  |  |  |  |  |  |  |  |  |  |  |  |  |  |  |  |  |  |  |  |  |  |  |  |  |  |  |  |  |  |  |  |  |  |  |  |  |  |  |  |  |  |  |  |  |  |  |  |  |  |  |  |  |  |  |  |  |  |  |  |  |  |  |  |  |  |  |  |  |  |  |  |  |  |  |  |  |  |  |  |  |  |  |  |  |  |  |  |  |  |  |  |  |  |  |  |  |  |  |  |  |  |  |  |  |  |  |  |  |  |  |  |  |  |  |  |  |  |  |  |  |  |  |  |  |  |  |  |  |  |  |  |  |  |  |  |  |  |  |  |  |  |  |  |  |  |  |  |  |  |  |  |  |  |  |  |  |  |  |  |  |  |  |  |  |  |  |  |  |  |  |  |  |  |  |  |  |  |  |  |  |  |  |  |  |  |  |  |  |  |  |  |  |  |  |  |  |  |  |  |  |  |  |  |  |  |  |  |  |  |  |  |  |  |  |  |  |  |  |  |  |  |  |  |  |  |  |  |  |  |  |  |  |  |  |  |  |  |  |  |  |  |  |  |  |  |  |  |  |  |  |  |  |  |  |  |  |  |  |  |  |  |  |  |  |  |  |  |  |  |  |  |  |  |  |  |  |  |  |  |  |  |  |  |  |  |  |  |  |  |  |  |  |  |  |  |  |  |  |  |  |  |  |  |  |  |  |  |  |  |  |  |  |  |  |  |  |  |  |  |  |  |  |  |  |  |  |  |  |  |  |  |  |  |  |  |  |  |  |  |  |  |  |  |  |  |  |  |  |  |  |  |  |  |  |  |  |  |  |  |  |  |  |  |  |  |  |  |  |  |  |  |  |  |  |  |  |  |  |  |  |  |  |  |  |  |  |  |  |  |  |  |  |  |  |  |  |  |  |  |  |  |  |  |  |  |  |  |  |  |  |  |  |  |  |  |  |  |  |  |  |  |  |  |  |  |  |  |  |  |  |  |  |  |  |  |  |  |  |  |  |  |  |  |  |  |  |  |  |  |  |  |  |  |  |  |  |  |  |  |  |  |  |  |  |  |  |  |  |  |  |  |  |  |  |  |  |  |  |  |  |  |  |  |  |  |  |  |  |  |  |  |  |  |  |  |  |  |  |  |  |  |  |  |  |  |  |  |  |  |  |  |  |  |  |  |  |  |  |  |  |  |  |  |  |  |  |  |  |  |  |  |  |  |  |  |  |  |  |  |  |  |  |  |  |  |  |  |  |  |  |  |  |  |  |  |  |  |  |  |  |  |
|  |  |  |  |  |  |  |  |  |  |  |  |  |  |  |  |  |  |  |  |  |  |  |  |  |  |  |  |  |  |  |  |  |  |  |  |  |  |  |  |  |  |  |  |  |  |  |  |  |  |  |  |  |  |  |  |  |  |  |  |  |  |  |  |  |  |  |  |  |  |  |  |  |  |  |  |  |  |  |  |  |  |  |  |  |  |  |  |  |  |  |  |  |  |  |  |  |  |  |  |  |  |  |  |  |  |  |  |  |  |  |  |  |  |  |  |  |  |  |  |  |  |  |  |  |  |  |  |  |  |  |  |  |  |  |  |  |  |  |  |  |  |  |  |  |  |  |  |  |  |  |  |  |  |  |  |  |  |  |  |  |  |  |  |  |  |  |  |  |  |  |  |  |  |  |  |  |  |  |  |  |  |  |  |  |  |  |  |  |  |  |  |  |  |  |  |  |  |  |  |  |  |  |  |  |  |  |  |  |  |  |  |  |  |  |  |  |  |  |  |  |  |  |  |  |  |  |  |  |  |  |  |  |  |  |  |  |  |  |  |  |  |  |  |  |  |  |  |  |  |  |  |  |  |  |  |  |  |  |  |  |  |  |  |  |  |  |  |  |  |  |  |  |  |  |  |  |  |  |  |  |  |  |  |  |  |  |  |  |  |  |  |  |  |  |  |  |  |  |  |  |  |  |  |  |  |  |  |  |  |  |  |  |  |  |  |  |  |  |  |  |  |  |  |  |  |  |  |  |  |  |  |  |  |  |  |  |  |  |  |  |  |  |  |  |  |  |  |  |  |  |  |  |  |  |  |  |  |  |  |  |  |  |  |  |  |  |  |  |  |  |  |  |  |  |  |  |  |  |  |  |  |  |  |  |  |  |  |  |  |  |  |  |  |  |  |  |  |  |  |  |  |  |  |  |  |  |  |  |  |  |  |  |  |  |  |  |  |  |  |  |  |  |  |  |  |  |  |  |  |  |  |  |  |  |  |  |  |  |  |  |  |  |  |  |  |  |  |  |  |  |  |  |  |  |  |  |  |  |  |  |  |  |  |  |  |  |  |  |  |  |  |  |  |  |  |  |  |  |  |  |  |  |  |  |  |  |  |  |  |  |  |  |  |  |  |  |  |  |  |  |  |  |  |  |  |  |  |  |  |  |  |  |  |  |  |  |  |  |  |  |  |  |  |  |  |  |  |  |  |  |  |  |  |  |  |  |  |  |  |  |  |  |  |  |  |  |  |  |  |  |  |  |  |  |  |  |  |  |  |  |  |  |  |  |  |  |  |  |  |  |  |  |  |  |  |  |  |  |  |  |  |  |  |  |  |  |  |  |  |  |  |  |  |  |  |  |  |  |  |  |  |  |  |  |  |  |  |  |  |  |  |  |  |  |  |  |  |  |  |  |  |  |  |  |  |  |  |  |  |  |  |  |  |  |  |  |  |  |  |  |  |  |  |  |  |  |  |  |  |  |  |  |  |  |  |  |  |  |  |  |  |  |  |  |  |  |  |  |  |  |  |  |  |  |  |  |  |  |  |  |  |  |  |  |  |  |  |  |  |  |  |  |  |  |  |  |  |  |  |  |  |  |  |  |  |  |  |  |  |  |  |  |  |  |  |  |  |  |  |  |  |  |  |  |  |  |  |  |  |  |  |  |  |  |  |  |  |  |  |  |  |  |  |  |  |  |  |  |  |  |  |  |  |  |  |  |  |  |  |  |  |  |  |  |  |  |  |  |  |  |  |  |  |  |  |  |  |  |  |  |  |  |  |  |  |  |  |  |  |  |  |  |  |  |  |  |  |  |  |  |  |  |  |  |  |  |  |  |  |  |  |  |  |  |  |  |  |  |  |  |  |  |  |  |  |  |  |  |  |  |  |  |  |  |  |  |  |  |  |  |  |  |  |  |  |  |  |  |  |  |  |  |  |  |  |  |  |  |  |  |  |  |  |  |  |  |  |  |  |  |  |  |  |  |  |  |  |  |  |  |  |  |  |  |  |  |  |  |  |  |  |  |  |  |  |  |  |  |  |  |  |  |  |  |  |  |  |  |  |  |  |  |  |  |  |  |  |  |  |  |  |  |  |  |  |  |  |  |  |  |  |  |  |  |  |  |  |  |  |  |  |  |  |  |  |  |  |  |  |  |  |  |  |  |  |  |  |  |
|  |  |  |  |  |  |  |  |  |  |  |  |  |  |  |  |  |  |  |  |  |  |  |  |  |  |  |  |  |  |  |  |  |  |  |  |  |  |  |  |  |  |  |  |  |  |  |  |  |  |  |  |  |  |  |  |  |  |  |  |  |  |  |  |  |  |  |  |  |  |  |  |  |  |  |  |  |  |  |  |  |  |  |  |  |  |  |  |  |  |  |  |  |  |  |  |  |  |  |  |  |  |  |  |  |  |  |  |  |  |  |  |  |  |  |  |  |  |  |  |  |  |  |  |  |  |  |  |  |  |  |  |  |  |  |  |  |  |  |  |  |  |  |  |  |  |  |  |  |  |  |  |  |  |  |  |  |  |  |  |  |  |  |  |  |  |  |  |  |  |  |  |  |  |  |  |  |  |  |  |  |  |  |  |  |  |  |  |  |  |  |  |  |  |  |  |  |  |  |  |  |  |  |  |  |  |  |  |  |  |  |  |  |  |  |  |  |  |  |  |  |  |  |  |  |  |  |  |  |  |  |  |  |  |  |  |  |  |  |  |  |  |  |  |  |  |  |  |  |  |  |  |  |  |  |  |  |  |  |  |  |  |  |  |  |  |  |  |  |  |  |  |  |  |  |  |  |  |  |  |  |  |  |  |  |  |  |  |  |  |  |  |  |  |  |  |  |  |  |  |  |  |  |  |  |  |  |  |  |  |  |  |  |  |  |  |  |  |  |  |  |  |  |  |  |  |  |  |  |  |  |  |  |  |  |  |  |  |  |  |  |  |  |  |  |  |  |  |  |  |  |  |  |  |  |  |  |  |  |  |  |  |  |  |  |  |  |  |  |  |  |  |  |  |  |  |  |  |  |  |  |  |  |  |  |  |  |  |  |  |  |  |  |  |  |  |  |  |  |  |  |  |  |  |  |  |  |  |  |  |  |  |  |  |  |  |  |  |  |  |  |  |  |  |  |  |  |  |  |  |  |  |  |  |  |  |  |  |  |  |  |  |  |  |  |  |  |  |  |  |  |  |  |  |  |  |  |  |  |  |  |  |  |  |  |  |  |  |  |  |  |  |  |  |  |  |  |  |  |  |  |  |  |  |  |  |  |  |  |  |  |  |  |  |  |  |  |  |  |  |  |  |  |  |  |  |  |  |  |  |  |  |  |  |  |  |  |  |  |  |  |  |  |  |  |  |  |  |  |  |  |  |  |  |  |  |  |  |  |  |  |  |  |  |  |  |  |  |  |  |  |  |  |  |  |  |  |  |  |  |  |  |  |  |  |  |  |  |  |  |  |  |  |  |  |  |  |  |  |  |  |  |  |  |  |  |  |  |  |  |  |  |  |  |  |  |  |  |  |  |  |  |  |  |  |  |  |  |  |  |  |  |  |  |  |  |  |  |  |  |  |  |  |  |  |  |  |  |  |  |  |  |  |  |  |  |  |  |  |  |  |  |  |  |  |  |  |  |  |  |  |  |  |  |  |  |  |  |  |  |  |  |  |  |  |  |  |  |  |  |  |  |  |  |  |  |  |  |  |  |  |  |  |  |  |  |  |  |  |  |  |  |  |  |  |  |  |  |  |  |  |  |  |  |  |  |  |  |  |  |  |  |  |  |  |  |  |  |  |  |  |  |  |  |  |  |  |  |  |  |  |  |  |  |  |  |  |  |  |  |  |  |  |  |  |  |  |  |  |  |  |  |  |  |  |  |  |  |  |  |  |  |  |  |  |  |  |  |  |  |  |  |  |  |  |  |  |  |  |  |  |  |  |  |  |  |  |  |  |  |  |  |  |  |  |  |  |  |  |  |  |  |  |  |  |  |  |  |  |  |  |  |  |  |  |  |  |  |  |  |  |  |  |  |  |  |  |  |  |  |  |  |  |  |  |  |  |  |  |  |  |  |  |  |  |  |  |  |  |  |  |  |  |  |  |  |  |  |  |  |  |  |  |  |  |  |  |  |  |  |  |  |  |  |  |  |  |  |  |  |  |  |  |  |  |  |  |  |  |  |  |  |  |  |  |  |  |  |  |  |  |  |  |  |  |  |  |  |  |  |  |  |  |  |  |  |  |  |  |  |  |  |  |  |  |  |  |  |  |  |  |  |  |  |  |  |  |  |  |  |  |  |  |  |  |  |  |  |  |  |  |  |  |  |  |  |  |  |  |
|  |  |  |  |  |  |  |  |  |  |  |  |  |  |  |  |  |  |  |  |  |  |  |  |  |  |  |  |  |  |  |  |  |  |  |  |  |  |  |  |  |  |  |  |  |  |  |  |  |  |  |  |  |  |  |  |  |  |  |  |  |  |  |  |  |  |  |  |  |  |  |  |  |  |  |  |  |  |  |  |  |  |  |  |  |  |  |  |  |  |  |  |  |  |  |  |  |  |  |  |  |  |  |  |  |  |  |  |  |  |  |  |  |  |  |  |  |  |  |  |  |  |  |  |  |  |  |  |  |  |  |  |  |  |  |  |  |  |  |  |  |  |  |  |  |  |  |  |  |  |  |  |  |  |  |  |  |  |  |  |  |  |  |  |  |  |  |  |  |  |  |  |  |  |  |  |  |  |  |  |  |  |  |  |  |  |  |  |  |  |  |  |  |  |  |  |  |  |  |  |  |  |  |  |  |  |  |  |  |  |  |  |  |  |  |  |  |  |  |  |  |  |  |  |  |  |  |  |  |  |  |  |  |  |  |  |  |  |  |  |  |  |  |  |  |  |  |  |  |  |  |  |  |  |  |  |  |  |  |  |  |  |  |  |  |  |  |  |  |  |  |  |  |  |  |  |  |  |  |  |  |  |  |  |  |  |  |  |  |  |  |  |  |  |  |  |  |  |  |  |  |  |  |  |  |  |  |  |  |  |  |  |  |  |  |  |  |  |  |  |  |  |  |  |  |  |  |  |  |  |  |  |  |  |  |  |  |  |  |  |  |  |  |  |  |  |  |  |  |  |  |  |  |  |  |  |  |  |  |  |  |  |  |  |  |  |  |  |  |  |  |  |  |  |  |  |  |  |  |  |  |  |  |  |  |  |  |  |  |  |  |  |  |  |  |  |  |  |  |  |  |  |  |  |  |  |  |  |  |  |  |  |  |  |  |  |  |  |  |  |  |  |  |  |  |  |  |  |  |  |  |  |  |  |  |  |  |  |  |  |  |  |  |  |  |  |  |  |  |  |  |  |  |  |  |  |  |  |  |  |  |  |  |  |  |  |  |  |  |  |  |  |  |  |  |  |  |  |  |  |  |  |  |  |  |  |  |  |  |  |  |  |  |  |  |  |  |  |  |  |  |  |  |  |  |  |  |  |  |  |  |  |  |  |  |  |  |  |  |  |  |  |  |  |  |  |  |  |  |  |  |  |  |  |  |  |  |  |  |  |  |  |  |  |  |  |  |  |  |  |  |  |  |  |  |  |  |  |  |  |  |  |  |  |  |  |  |  |  |  |  |  |  |  |  |  |  |  |  |  |  |  |  |  |  |  |  |  |  |  |  |  |  |  |  |  |  |  |  |  |  |  |  |  |  |  |  |  |  |  |  |  |  |  |  |  |  |  |  |  |  |  |  |  |  |  |  |  |  |  |  |  |  |  |  |  |  |  |  |  |  |  |  |  |  |  |  |  |  |  |  |  |  |  |  |  |  |  |  |  |  |  |  |  |  |  |  |  |  |  |  |  |  |  |  |  |  |  |  |  |  |  |  |  |  |  |  |  |  |  |  |  |  |  |  |  |  |  |  |  |  |  |  |  |  |  |  |  |  |  |  |  |  |  |  |  |  |  |  |  |  |  |  |  |  |  |  |  |  |  |  |  |  |  |  |  |  |  |  |  |  |  |  |  |  |  |  |  |  |  |  |  |  |  |  |  |  |  |  |  |  |  |  |  |  |  |  |  |  |  |  |  |  |  |  |  |  |  |  |  |  |  |  |  |  |  |  |  |  |  |  |  |  |  |  |  |  |  |  |  |  |  |  |  |  |  |  |  |  |  |  |  |  |  |  |  |  |  |  |  |  |  |  |  |  |  |  |  |  |  |  |  |  |  |  |  |  |  |  |  |  |  |  |  |  |  |  |  |  |  |  |  |  |  |  |  |  |  |  |  |  |  |  |  |  |  |  |  |  |  |  |  |  |  |  |  |  |  |  |  |  |  |  |  |  |  |  |  |  |  |  |  |  |  |  |  |  |  |  |  |  |  |  |  |  |  |  |  |  |  |  |  |  |  |  |  |  |  |  |  |  |  |  |  |  |  |  |  |  |  |  |  |  |  |  |  |  |  |  |  |  |  |  |  |  |  |  |  |  |  |  |  |  |  |  |  |  |  |  |
|  |  |  |  |  |  |  |  |  |  |  |  |  |  |  |  |  |  |  |  |  |  |  |  |  |  |  |  |  |  |  |  |  |  |  |  |  |  |  |  |  |  |  |  |  |  |  |  |  |  |  |  |  |  |  |  |  |  |  |  |  |  |  |  |  |  |  |  |  |  |  |  |  |  |  |  |  |  |  |  |  |  |  |  |  |  |  |  |  |  |  |  |  |  |  |  |  |  |  |  |  |  |  |  |  |  |  |  |  |  |  |  |  |  |  |  |  |  |  |  |  |  |  |  |  |  |  |  |  |  |  |  |  |  |  |  |  |  |  |  |  |  |  |  |  |  |  |  |  |  |  |  |  |  |  |  |  |  |  |  |  |  |  |  |  |  |  |  |  |  |  |  |  |  |  |  |  |  |  |  |  |  |  |  |  |  |  |  |  |  |  |  |  |  |  |  |  |  |  |  |  |  |  |  |  |  |  |  |  |  |  |  |  |  |  |  |  |  |  |  |  |  |  |  |  |  |  |  |  |  |  |  |  |  |  |  |  |  |  |  |  |  |  |  |  |  |  |  |  |  |  |  |  |  |  |  |  |  |  |  |  |  |  |  |  |  |  |  |  |  |  |  |  |  |  |  |  |  |  |  |  |  |  |  |  |  |  |  |  |  |  |  |  |  |  |  |  |  |  |  |  |  |  |  |  |  |  |  |  |  |  |  |  |  |  |  |  |  |  |  |  |  |  |  |  |  |  |  |  |  |  |  |  |  |  |  |  |  |  |  |  |  |  |  |  |  |  |  |  |  |  |  |  |  |  |  |  |  |  |  |  |  |  |  |  |  |  |  |  |  |  |  |  |  |  |  |  |  |  |  |  |  |  |  |  |  |  |  |  |  |  |  |  |  |  |  |  |  |  |  |  |  |  |  |  |  |  |  |  |  |  |  |  |  |  |  |  |  |  |  |  |  |  |  |  |  |  |  |  |  |  |  |  |  |  |  |  |  |  |  |  |  |  |  |  |  |  |  |  |  |  |  |  |  |  |  |  |  |  |  |  |  |  |  |  |  |  |  |  |  |  |  |  |  |  |  |  |  |  |  |  |  |  |  |  |  |  |  |  |  |  |  |  |  |  |  |  |  |  |  |  |  |  |  |  |  |  |  |  |  |  |  |  |  |  |  |  |  |  |  |  |  |  |  |  |  |  |  |  |  |  |  |  |  |  |  |  |  |  |  |  |  |  |  |  |  |  |  |  |  |  |  |  |  |  |  |  |  |  |  |  |  |  |  |  |  |  |  |  |  |  |  |  |  |  |  |  |  |  |  |  |  |  |  |  |  |  |  |  |  |  |  |  |  |  |  |  |  |  |  |  |  |  |  |  |  |  |  |  |  |  |  |  |  |  |  |  |  |  |  |  |  |  |  |  |  |  |  |  |  |  |  |  |  |  |  |  |  |  |  |  |  |  |  |  |  |  |  |  |  |  |  |  |  |  |  |  |  |  |  |  |  |  |  |  |  |  |  |  |  |  |  |  |  |  |  |  |  |  |  |  |  |  |  |  |  |  |  |  |  |  |  |  |  |  |  |  |  |  |  |  |  |  |  |  |  |  |  |  |  |  |  |  |  |  |  |  |  |  |  |  |  |  |  |  |  |  |  |  |  |  |  |  |  |  |  |  |  |  |  |  |  |  |  |  |  |  |  |  |  |  |  |  |  |  |  |  |  |  |  |  |  |  |  |  |  |  |  |  |  |  |  |  |  |  |  |  |  |  |  |  |  |  |  |  |  |  |  |  |  |  |  |  |  |  |  |  |  |  |  |  |  |  |  |  |  |  |  |  |  |  |  |  |  |  |  |  |  |  |  |  |  |  |  |  |  |  |  |  |  |  |  |  |  |  |  |  |  |  |  |  |  |  |  |  |  |  |  |  |  |  |  |  |  |  |  |  |  |  |  |  |  |  |  |  |  |  |  |  |  |  |  |  |  |  |  |  |  |  |  |  |  |  |  |  |  |  |  |  |  |  |  |  |  |  |  |  |  |  |  |  |  |  |  |  |  |  |  |  |  |  |  |  |  |  |  |  |  |  |  |  |  |  |  |  |  |  |  |  |  |  |  |  |  |  |  |  |  |  |  |  |  |  |  |  |  |  |  |  |  |  |  |  |  |  |  |  |  |  |
|  |  |  |  |  |  |  |  |  |  |  |  |  |  |  |  |  |  |  |  |  |  |  |  |  |  |  |  |  |  |  |  |  |  |  |  |  |  |  |  |  |  |  |  |  |  |  |  |  |  |  |  |  |  |  |  |  |  |  |  |  |  |  |  |  |  |  |  |  |  |  |  |  |  |  |  |  |  |  |  |  |  |  |  |  |  |  |  |  |  |  |  |  |  |  |  |  |  |  |  |  |  |  |  |  |  |  |  |  |  |  |  |  |  |  |  |  |  |  |  |  |  |  |  |  |  |  |  |  |  |  |  |  |  |  |  |  |  |  |  |  |  |  |  |  |  |  |  |  |  |  |  |  |  |  |  |  |  |  |  |  |  |  |  |  |  |  |  |  |  |  |  |  |  |  |  |  |  |  |  |  |  |  |  |  |  |  |  |  |  |  |  |  |  |  |  |  |  |  |  |  |  |  |  |  |  |  |  |  |  |  |  |  |  |  |  |  |  |  |  |  |  |  |  |  |  |  |  |  |  |  |  |  |  |  |  |  |  |  |  |  |  |  |  |  |  |  |  |  |  |  |  |  |  |  |  |  |  |  |  |  |  |  |  |  |  |  |  |  |  |  |  |  |  |  |  |  |  |  |  |  |  |  |  |  |  |  |  |  |  |  |  |  |  |  |  |  |  |  |  |  |  |  |  |  |  |  |  |  |  |  |  |  |  |  |  |  |  |  |  |  |  |  |  |  |  |  |  |  |  |  |  |  |  |  |  |  |  |  |  |  |  |  |  |  |  |  |  |  |  |  |  |  |  |  |  |  |  |  |  |  |  |  |  |  |  |  |  |  |  |  |  |  |  |  |  |  |  |  |  |  |  |  |  |  |  |  |  |  |  |  |  |  |  |  |  |  |  |  |  |  |  |  |  |  |  |  |  |  |  |  |  |  |  |  |  |  |  |  |  |  |  |  |  |  |  |  |  |  |  |  |  |  |  |  |  |  |  |  |  |  |  |  |  |  |  |  |  |  |  |  |  |  |  |  |  |  |  |  |  |  |  |  |  |  |  |  |  |  |  |  |  |  |  |  |  |  |  |  |  |  |  |  |  |  |  |  |  |  |  |  |  |  |  |  |  |  |  |  |  |  |  |  |  |  |  |  |  |  |  |  |  |  |  |  |  |  |  |  |  |  |  |  |  |  |  |  |  |  |  |  |  |  |  |  |  |  |  |  |  |  |  |  |  |  |  |  |  |  |  |  |  |  |  |  |  |  |  |  |  |  |  |  |  |  |  |  |  |  |  |  |  |  |  |  |  |  |  |  |  |  |  |  |  |  |  |  |  |  |  |  |  |  |  |  |  |  |  |  |  |  |  |  |  |  |  |  |  |  |  |  |  |  |  |  |  |  |  |  |  |  |  |  |  |  |  |  |  |  |  |  |  |  |  |  |  |  |  |  |  |  |  |  |  |  |  |  |  |  |  |  |  |  |  |  |  |  |  |  |  |  |  |  |  |  |  |  |  |  |  |  |  |  |  |  |  |  |  |  |  |  |  |  |  |  |  |  |  |  |  |  |  |  |  |  |  |  |  |  |  |  |  |  |  |  |  |  |  |  |  |  |  |  |  |  |  |  |  |  |  |  |  |  |  |  |  |  |  |  |  |  |  |  |  |  |  |  |  |  |  |  |  |  |  |  |  |  |  |  |  |  |  |  |  |  |  |  |  |  |  |  |  |  |  |  |  |  |  |  |  |  |  |  |  |  |  |  |  |  |  |  |  |  |  |  |  |  |  |  |  |  |  |  |  |  |  |  |  |  |  |  |  |  |  |  |  |  |  |  |  |  |  |  |  |  |  |  |  |  |  |  |  |  |  |  |  |  |  |  |  |  |  |  |  |  |  |  |  |  |  |  |  |  |  |  |  |  |  |  |  |  |  |  |  |  |  |  |  |  |  |  |  |  |  |  |  |  |  |  |  |  |  |  |  |  |  |  |  |  |  |  |  |  |  |  |  |  |  |  |  |  |  |  |  |  |  |  |  |  |  |  |  |  |  |  |  |  |  |  |  |  |  |  |  |  |  |  |  |  |  |  |  |  |  |  |  |  |  |  |  |  |  |  |  |  |  |  |  |  |  |  |  |  |  |  |  |  |  |  |  |  |  |  |  |  |  |  |  |  |
|  |  |  |  |  |  |  |  |  |  |  |  |  |  |  |  |  |  |  |  |  |  |  |  |  |  |  |  |  |  |  |  |  |  |  |  |  |  |  |  |  |  |  |  |  |  |  |  |  |  |  |  |  |  |  |  |  |  |  |  |  |  |  |  |  |  |  |  |  |  |  |  |  |  |  |  |  |  |  |  |  |  |  |  |  |  |  |  |  |  |  |  |  |  |  |  |  |  |  |  |  |  |  |  |  |  |  |  |  |  |  |  |  |  |  |  |  |  |  |  |  |  |  |  |  |  |  |  |  |  |  |  |  |  |  |  |  |  |  |  |  |  |  |  |  |  |  |  |  |  |  |  |  |  |  |  |  |  |  |  |  |  |  |  |  |  |  |  |  |  |  |  |  |  |  |  |  |  |  |  |  |  |  |  |  |  |  |  |  |  |  |  |  |  |  |  |  |  |  |  |  |  |  |  |  |  |  |  |  |  |  |  |  |  |  |  |  |  |  |  |  |  |  |  |  |  |  |  |  |  |  |  |  |  |  |  |  |  |  |  |  |  |  |  |  |  |  |  |  |  |  |  |  |  |  |  |  |  |  |  |  |  |  |  |  |  |  |  |  |  |  |  |  |  |  |  |  |  |  |  |  |  |  |  |  |  |  |  |  |  |  |  |  |  |  |  |  |  |  |  |  |  |  |  |  |  |  |  |  |  |  |  |  |  |  |  |  |  |  |  |  |  |  |  |  |  |  |  |  |  |  |  |  |  |  |  |  |  |  |  |  |  |  |  |  |  |  |  |  |  |  |  |  |  |  |  |  |  |  |  |  |  |  |  |  |  |  |  |  |  |  |  |  |  |  |  |  |  |  |  |  |  |  |  |  |  |  |  |  |  |  |  |  |  |  |  |  |  |  |  |  |  |  |  |  |  |  |  |  |  |  |  |  |  |  |  |  |  |  |  |  |  |  |  |  |  |  |  |  |  |  |  |  |  |  |  |  |  |  |  |  |  |  |  |  |  |  |  |  |  |  |  |  |  |  |  |  |  |  |  |  |  |  |  |  |  |  |  |  |  |  |  |  |  |  |  |  |  |  |  |  |  |  |  |  |  |  |  |  |  |  |  |  |  |  |  |  |  |  |  |  |  |  |  |  |  |  |  |  |  |  |  |  |  |  |  |  |  |  |  |  |  |  |  |  |  |  |  |  |  |  |  |  |  |  |  |  |  |  |  |  |  |  |  |  |  |  |  |  |  |  |  |  |  |  |  |  |  |  |  |  |  |  |  |  |  |  |  |  |  |  |  |  |  |  |  |  |  |  |  |  |  |  |  |  |  |  |  |  |  |  |  |  |  |  |  |  |  |  |  |  |  |  |  |  |  |  |  |  |  |  |  |  |  |  |  |  |  |  |  |  |  |  |  |  |  |  |  |  |  |  |  |  |  |  |  |  |  |  |  |  |  |  |  |  |  |  |  |  |  |  |  |  |  |  |  |  |  |  |  |  |  |  |  |  |  |  |  |  |  |  |  |  |  |  |  |  |  |  |  |  |  |  |  |  |  |  |  |  |  |  |  |  |  |  |  |  |  |  |  |  |  |  |  |  |  |  |  |  |  |  |  |  |  |  |  |  |  |  |  |  |  |  |  |  |  |  |  |  |  |  |  |  |  |  |  |  |  |  |  |  |  |  |  |  |  |  |  |  |  |  |  |  |  |  |  |  |  |  |  |  |  |  |  |  |  |  |  |  |  |  |  |  |  |  |  |  |  |  |  |  |  |  |  |  |  |  |  |  |  |  |  |  |  |  |  |  |  |  |  |  |  |  |  |  |  |  |  |  |  |  |  |  |  |  |  |  |  |  |  |  |  |  |  |  |  |  |  |  |  |  |  |  |  |  |  |  |  |  |  |  |  |  |  |  |  |  |  |  |  |  |  |  |  |  |  |  |  |  |  |  |  |  |  |  |  |  |  |  |  |  |  |  |  |  |  |  |  |  |  |  |  |  |  |  |  |  |  |  |  |  |  |  |  |  |  |  |  |  |  |  |  |  |  |  |  |  |  |  |  |  |  |  |  |  |  |  |  |  |  |  |  |  |  |  |  |  |  |  |  |  |  |  |  |  |  |  |  |  |  |  |  |  |  |  |  |  |  |  |  |  |  |  |  |  |  |  |  |  |
|  |  |  |  |  |  |  |  |  |  |  |  |  |  |  |  |  |  |  |  |  |  |  |  |  |  |  |  |  |  |  |  |  |  |  |  |  |  |  |  |  |  |  |  |  |  |  |  |  |  |  |  |  |  |  |  |  |  |  |  |  |  |  |  |  |  |  |  |  |  |  |  |  |  |  |  |  |  |  |  |  |  |  |  |  |  |  |  |  |  |  |  |  |  |  |  |  |  |  |  |  |  |  |  |  |  |  |  |  |  |  |  |  |  |  |  |  |  |  |  |  |  |  |  |  |  |  |  |  |  |  |  |  |  |  |  |  |  |  |  |  |  |  |  |  |  |  |  |  |  |  |  |  |  |  |  |  |  |  |  |  |  |  |  |  |  |  |  |  |  |  |  |  |  |  |  |  |  |  |  |  |  |  |  |  |  |  |  |  |  |  |  |  |  |  |  |  |  |  |  |  |  |  |  |  |  |  |  |  |  |  |  |  |  |  |  |  |  |  |  |  |  |  |  |  |  |  |  |  |  |  |  |  |  |  |  |  |  |  |  |  |  |  |  |  |  |  |  |  |  |  |  |  |  |  |  |  |  |  |  |  |  |  |  |  |  |  |  |  |  |  |  |  |  |  |  |  |  |  |  |  |  |  |  |  |  |  |  |  |  |  |  |  |  |  |  |  |  |  |  |  |  |  |  |  |  |  |  |  |  |  |  |  |  |  |  |  |  |  |  |  |  |  |  |  |  |  |  |  |  |  |  |  |  |  |  |  |  |  |  |  |  |  |  |  |  |  |  |  |  |  |  |  |  |  |  |  |  |  |  |  |  |  |  |  |  |  |  |  |  |  |  |  |  |  |  |  |  |  |  |  |  |  |  |  |  |  |  |  |  |  |  |  |  |  |  |  |  |  |  |  |  |  |  |  |  |  |  |  |  |  |  |  |  |  |  |  |  |  |  |  |  |  |  |  |  |  |  |  |  |  |  |  |  |  |  |  |  |  |  |  |  |  |  |  |  |  |  |  |  |  |  |  |  |  |  |  |  |  |  |  |  |  |  |  |  |  |  |  |  |  |  |  |  |  |  |  |  |  |  |  |  |  |  |  |  |  |  |  |  |  |  |  |  |  |  |  |  |  |  |  |  |  |  |  |  |  |  |  |  |  |  |  |  |  |  |  |  |  |  |  |  |  |  |  |  |  |  |  |  |  |  |  |  |  |  |  |  |  |  |  |  |  |  |  |  |  |  |  |  |  |  |  |  |  |  |  |  |  |  |  |  |  |  |  |  |  |  |  |  |  |  |  |  |  |  |  |  |  |  |  |  |  |  |  |  |  |  |  |  |  |  |  |  |  |  |  |  |  |  |  |  |  |  |  |  |  |  |  |  |  |  |  |  |  |  |  |  |  |  |  |  |  |  |  |  |  |  |  |  |  |  |  |  |  |  |  |  |  |  |  |  |  |  |  |  |  |  |  |  |  |  |  |  |  |  |  |  |  |  |  |  |  |  |  |  |  |  |  |  |  |  |  |  |  |  |  |  |  |  |  |  |  |  |  |  |  |  |  |  |  |  |  |  |  |  |  |  |  |  |  |  |  |  |  |  |  |  |  |  |  |  |  |  |  |  |  |  |  |  |  |  |  |  |  |  |  |  |  |  |  |  |  |  |  |  |  |  |  |  |  |  |  |  |  |  |  |  |  |  |  |  |  |  |  |  |  |  |  |  |  |  |  |  |  |  |  |  |  |  |  |  |  |  |  |  |  |  |  |  |  |  |  |  |  |  |  |  |  |  |  |  |  |  |  |  |  |  |  |  |  |  |  |  |  |  |  |  |  |  |  |  |  |  |  |  |  |  |  |  |  |  |  |  |  |  |  |  |  |  |  |  |  |  |  |  |  |  |  |  |  |  |  |  |  |  |  |  |  |  |  |  |  |  |  |  |  |  |  |  |  |  |  |  |  |  |  |  |  |  |  |  |  |  |  |  |  |  |  |  |  |  |  |  |  |  |  |  |  |  |  |  |  |  |  |  |  |  |  |  |  |  |  |  |  |  |  |  |  |  |  |  |  |  |  |  |  |  |  |  |  |  |  |  |  |  |  |  |  |  |  |  |  |  |  |  |  |  |  |  |  |  |  |  |  |  |  |  |  |  |  |  |  |  |  |  |  |  |  |
|  |  |  |  |  |  |  |  |  |  |  |  |  |  |  |  |  |  |  |  |  |  |  |  |  |  |  |  |  |  |  |  |  |  |  |  |  |  |  |  |  |  |  |  |  |  |  |  |  |  |  |  |  |  |  |  |  |  |  |  |  |  |  |  |  |  |  |  |  |  |  |  |  |  |  |  |  |  |  |  |  |  |  |  |  |  |  |  |  |  |  |  |  |  |  |  |  |  |  |  |  |  |  |  |  |  |  |  |  |  |  |  |  |  |  |  |  |  |  |  |  |  |  |  |  |  |  |  |  |  |  |  |  |  |  |  |  |  |  |  |  |  |  |  |  |  |  |  |  |  |  |  |  |  |  |  |  |  |  |  |  |  |  |  |  |  |  |  |  |  |  |  |  |  |  |  |  |  |  |  |  |  |  |  |  |  |  |  |  |  |  |  |  |  |  |  |  |  |  |  |  |  |  |  |  |  |  |  |  |  |  |  |  |  |  |  |  |  |  |  |  |  |  |  |  |  |  |  |  |  |  |  |  |  |  |  |  |  |  |  |  |  |  |  |  |  |  |  |  |  |  |  |  |  |  |  |  |  |  |  |  |  |  |  |  |  |  |  |  |  |  |  |  |  |  |  |  |  |  |  |  |  |  |  |  |  |  |  |  |  |  |  |  |  |  |  |  |  |  |  |  |  |  |  |  |  |  |  |  |  |  |  |  |  |  |  |  |  |  |  |  |  |  |  |  |  |  |  |  |  |  |  |  |  |  |  |  |  |  |  |  |  |  |  |  |  |  |  |  |  |  |  |  |  |  |  |  |  |  |  |  |  |  |  |  |  |  |  |  |  |  |  |  |  |  |  |  |  |  |  |  |  |  |  |  |  |  |  |  |  |  |  |  |  |  |  |  |  |  |  |  |  |  |  |  |  |  |  |  |  |  |  |  |  |  |  |  |  |  |  |  |  |  |  |  |  |  |  |  |  |  |  |  |  |  |  |  |  |  |  |  |  |  |  |  |  |  |  |  |  |  |  |  |  |  |  |  |  |  |  |  |  |  |  |  |  |  |  |  |  |  |  |  |  |  |  |  |  |  |  |  |  |  |  |  |  |  |  |  |  |  |  |  |  |  |  |  |  |  |  |  |  |  |  |  |  |  |  |  |  |  |  |  |  |  |  |  |  |  |  |  |  |  |  |  |  |  |  |  |  |  |  |  |  |  |  |  |  |  |  |  |  |  |  |  |  |  |  |  |  |  |  |  |  |  |  |  |  |  |  |  |  |  |  |  |  |  |  |  |  |  |  |  |  |  |  |  |  |  |  |  |  |  |  |  |  |  |  |  |  |  |  |  |  |  |  |  |  |  |  |  |  |  |  |  |  |  |  |  |  |  |  |  |  |  |  |  |  |  |  |  |  |  |  |  |  |  |  |  |  |  |  |  |  |  |  |  |  |  |  |  |  |  |  |  |  |  |  |  |  |  |  |  |  |  |  |  |  |  |  |  |  |  |  |  |  |  |  |  |  |  |  |  |  |  |  |  |  |  |  |  |  |  |  |  |  |  |  |  |  |  |  |  |  |  |  |  |  |  |  |  |  |  |  |  |  |  |  |  |  |  |  |  |  |  |  |  |  |  |  |  |  |  |  |  |  |  |  |  |  |  |  |  |  |  |  |  |  |  |  |  |  |  |  |  |  |  |  |  |  |  |  |  |  |  |  |  |  |  |  |  |  |  |  |  |  |  |  |  |  |  |  |  |  |  |  |  |  |  |  |  |  |  |  |  |  |  |  |  |  |  |  |  |  |  |  |  |  |  |  |  |  |  |  |  |  |  |  |  |  |  |  |  |  |  |  |  |  |  |  |  |  |  |  |  |  |  |  |  |  |  |  |  |  |  |  |  |  |  |  |  |  |  |  |  |  |  |  |  |  |  |  |  |  |  |  |  |  |  |  |  |  |  |  |  |  |  |  |  |  |  |  |  |  |  |  |  |  |  |  |  |  |  |  |  |  |  |  |  |  |  |  |  |  |  |  |  |  |  |  |  |  |  |  |  |  |  |  |  |  |  |  |  |  |  |  |  |  |  |  |  |  |  |  |  |  |  |  |  |  |  |  |  |  |  |  |  |  |  |  |  |  |  |  |  |  |  |  |  |  |  |  |  |  |  |  |  |  |  |
|  |  |  |  |  |  |  |  |  |  |  |  |  |  |  |  |  |  |  |  |  |  |  |  |  |  |  |  |  |  |  |  |  |  |  |  |  |  |  |  |  |  |  |  |  |  |  |  |  |  |  |  |  |  |  |  |  |  |  |  |  |  |  |  |  |  |  |  |  |  |  |  |  |  |  |  |  |  |  |  |  |  |  |  |  |  |  |  |  |  |  |  |  |  |  |  |  |  |  |  |  |  |  |  |  |  |  |  |  |  |  |  |  |  |  |  |  |  |  |  |  |  |  |  |  |  |  |  |  |  |  |  |  |  |  |  |  |  |  |  |  |  |  |  |  |  |  |  |  |  |  |  |  |  |  |  |  |  |  |  |  |  |  |  |  |  |  |  |  |  |  |  |  |  |  |  |  |  |  |  |  |  |  |  |  |  |  |  |  |  |  |  |  |  |  |  |  |  |  |  |  |  |  |  |  |  |  |  |  |  |  |  |  |  |  |  |  |  |  |  |  |  |  |  |  |  |  |  |  |  |  |  |  |  |  |  |  |  |  |  |  |  |  |  |  |  |  |  |  |  |  |  |  |  |  |  |  |  |  |  |  |  |  |  |  |  |  |  |  |  |  |  |  |  |  |  |  |  |  |  |  |  |  |  |  |  |  |  |  |  |  |  |  |  |  |  |  |  |  |  |  |  |  |  |  |  |  |  |  |  |  |  |  |  |  |  |  |  |  |  |  |  |  |  |  |  |  |  |  |  |  |  |  |  |  |  |  |  |  |  |  |  |  |  |  |  |  |  |  |  |  |  |  |  |  |  |  |  |  |  |  |  |  |  |  |  |  |  |  |  |  |  |  |  |  |  |  |  |  |  |  |  |  |  |  |  |  |  |  |  |  |  |  |  |  |  |  |  |  |  |  |  |  |  |  |  |  |  |  |  |  |  |  |  |  |  |  |  |  |  |  |  |  |  |  |  |  |  |  |  |  |  |  |  |  |  |  |  |  |  |  |  |  |  |  |  |  |  |  |  |  |  |  |  |  |  |  |  |  |  |  |  |  |  |  |  |  |  |  |  |  |  |  |  |  |  |  |  |  |  |  |  |  |  |  |  |  |  |  |  |  |  |  |  |  |  |  |  |  |  |  |  |  |  |  |  |  |  |  |  |  |  |  |  |  |  |  |  |  |  |  |  |  |  |  |  |  |  |  |  |  |  |  |  |  |  |  |  |  |  |  |  |  |  |  |  |  |  |  |  |  |  |  |  |  |  |  |  |  |  |  |  |  |  |  |  |  |  |  |  |  |  |  |  |  |  |  |  |  |  |  |  |  |  |  |  |  |  |  |  |  |  |  |  |  |  |  |  |  |  |  |  |  |  |  |  |  |  |  |  |  |  |  |  |  |  |  |  |  |  |  |  |  |  |  |  |  |  |  |  |  |  |  |  |  |  |  |  |  |  |  |  |  |  |  |  |  |  |  |  |  |  |  |  |  |  |  |  |  |  |  |  |  |  |  |  |  |  |  |  |  |  |  |  |  |  |  |  |  |  |  |  |  |  |  |  |  |  |  |  |  |  |  |  |  |  |  |  |  |  |  |  |  |  |  |  |  |  |  |  |  |  |  |  |  |  |  |  |  |  |  |  |  |  |  |  |  |  |  |  |  |  |  |  |  |  |  |  |  |  |  |  |  |  |  |  |  |  |  |  |  |  |  |  |  |  |  |  |  |  |  |  |  |  |  |  |  |  |  |  |  |  |  |  |  |  |  |  |  |  |  |  |  |  |  |  |  |  |  |  |  |  |  |  |  |  |  |  |  |  |  |  |  |  |  |  |  |  |  |  |  |  |  |  |  |  |  |  |  |  |  |  |  |  |  |  |  |  |  |  |  |  |  |  |  |  |  |  |  |  |  |  |  |  |  |  |  |  |  |  |  |  |  |  |  |  |  |  |  |  |  |  |  |  |  |  |  |  |  |  |  |  |  |  |  |  |  |  |  |  |  |  |  |  |  |  |  |  |  |  |  |  |  |  |  |  |  |  |  |  |  |  |  |  |  |  |  |  |  |  |  |  |  |  |  |  |  |  |  |  |  |  |  |  |  |  |  |  |  |  |  |  |  |  |  |  |  |  |  |  |  |  |  |  |  |  |  |  |  |  |  |  |  |  |  |  |  |  |  |
|  |  |  |  |  |  |  |  |  |  |  |  |  |  |  |  |  |  |  |  |  |  |  |  |  |  |  |  |  |  |  |  |  |  |  |  |  |  |  |  |  |  |  |  |  |  |  |  |  |  |  |  |  |  |  |  |  |  |  |  |  |  |  |  |  |  |  |  |  |  |  |  |  |  |  |  |  |  |  |  |  |  |  |  |  |  |  |  |  |  |  |  |  |  |  |  |  |  |  |  |  |  |  |  |  |  |  |  |  |  |  |  |  |  |  |  |  |  |  |  |  |  |  |  |  |  |  |  |  |  |  |  |  |  |  |  |  |  |  |  |  |  |  |  |  |  |  |  |  |  |  |  |  |  |  |  |  |  |  |  |  |  |  |  |  |  |  |  |  |  |  |  |  |  |  |  |  |  |  |  |  |  |  |  |  |  |  |  |  |  |  |  |  |  |  |  |  |  |  |  |  |  |  |  |  |  |  |  |  |  |  |  |  |  |  |  |  |  |  |  |  |  |  |  |  |  |  |  |  |  |  |  |  |  |  |  |  |  |  |  |  |  |  |  |  |  |  |  |  |  |  |  |  |  |  |  |  |  |  |  |  |  |  |  |  |  |  |  |  |  |  |  |  |  |  |  |  |  |  |  |  |  |  |  |  |  |  |  |  |  |  |  |  |  |  |  |  |  |  |  |  |  |  |  |  |  |  |  |  |  |  |  |  |  |  |  |  |  |  |  |  |  |  |  |  |  |  |  |  |  |  |  |  |  |  |  |  |  |  |  |  |  |  |  |  |  |  |  |  |  |  |  |  |  |  |  |  |  |  |  |  |  |  |  |  |  |  |  |  |  |  |  |  |  |  |  |  |  |  |  |  |  |  |  |  |  |  |  |  |  |  |  |  |  |  |  |  |  |  |  |  |  |  |  |  |  |  |  |  |  |  |  |  |  |  |  |  |  |  |  |  |  |  |  |  |  |  |  |  |  |  |  |  |  |  |  |  |  |  |  |  |  |  |  |  |  |  |  |  |  |  |  |  |  |  |  |  |  |  |  |  |  |  |  |  |  |  |  |  |  |  |  |  |  |  |  |  |  |  |  |  |  |  |  |  |  |  |  |  |  |  |  |  |  |  |  |  |  |  |  |  |  |  |  |  |  |  |  |  |  |  |  |  |  |  |  |  |  |  |  |  |  |  |  |  |  |  |  |  |  |  |  |  |  |  |  |  |  |  |  |  |  |  |  |  |  |  |  |  |  |  |  |  |  |  |  |  |  |  |  |  |  |  |  |  |  |  |  |  |  |  |  |  |  |  |  |  |  |  |  |  |  |  |  |  |  |  |  |  |  |  |  |  |  |  |  |  |  |  |  |  |  |  |  |  |  |  |  |  |  |  |  |  |  |  |  |  |  |  |  |  |  |  |  |  |  |  |  |  |  |  |  |  |  |  |  |  |  |  |  |  |  |  |  |  |  |  |  |  |  |  |  |  |  |  |  |  |  |  |  |  |  |  |  |  |  |  |  |  |  |  |  |  |  |  |  |  |  |  |  |  |  |  |  |  |  |  |  |  |  |  |  |  |  |  |  |  |  |  |  |  |  |  |  |  |  |  |  |  |  |  |  |  |  |  |  |  |  |  |  |  |  |  |  |  |  |  |  |  |  |  |  |  |  |  |  |  |  |  |  |  |  |  |  |  |  |  |  |  |  |  |  |  |  |  |  |  |  |  |  |  |  |  |  |  |  |  |  |  |  |  |  |  |  |  |  |  |  |  |  |  |  |  |  |  |  |  |  |  |  |  |  |  |  |  |  |  |  |  |  |  |  |  |  |  |  |  |  |  |  |  |  |  |  |  |  |  |  |  |  |  |  |  |  |  |  |  |  |  |  |  |  |  |  |  |  |  |  |  |  |  |  |  |  |  |  |  |  |  |  |  |  |  |  |  |  |  |  |  |  |  |  |  |  |  |  |  |  |  |  |  |  |  |  |  |  |  |  |  |  |  |  |  |  |  |  |  |  |  |  |  |  |  |  |  |  |  |  |  |  |  |  |  |  |  |  |  |  |  |  |  |  |  |  |  |  |  |  |  |  |  |  |  |  |  |  |  |  |  |  |  |  |  |  |  |  |  |  |  |  |  |  |  |  |  |  |  |  |  |  |  |  |  |  |  |  |  |  |  |
|  |  |  |  |  |  |  |  |  |  |  |  |  |  |  |  |  |  |  |  |  |  |  |  |  |  |  |  |  |  |  |  |  |  |  |  |  |  |  |  |  |  |  |  |  |  |  |  |  |  |  |  |  |  |  |  |  |  |  |  |  |  |  |  |  |  |  |  |  |  |  |  |  |  |  |  |  |  |  |  |  |  |  |  |  |  |  |  |  |  |  |  |  |  |  |  |  |  |  |  |  |  |  |  |  |  |  |  |  |  |  |  |  |  |  |  |  |  |  |  |  |  |  |  |  |  |  |  |  |  |  |  |  |  |  |  |  |  |  |  |  |  |  |  |  |  |  |  |  |  |  |  |  |  |  |  |  |  |  |  |  |  |  |  |  |  |  |  |  |  |  |  |  |  |  |  |  |  |  |  |  |  |  |  |  |  |  |  |  |  |  |  |  |  |  |  |  |  |  |  |  |  |  |  |  |  |  |  |  |  |  |  |  |  |  |  |  |  |  |  |  |  |  |  |  |  |  |  |  |  |  |  |  |  |  |  |  |  |  |  |  |  |  |  |  |  |  |  |  |  |  |  |  |  |  |  |  |  |  |  |  |  |  |  |  |  |  |  |  |  |  |  |  |  |  |  |  |  |  |  |  |  |  |  |  |  |  |  |  |  |  |  |  |  |  |  |  |  |  |  |  |  |  |  |  |  |  |  |  |  |  |  |  |  |  |  |  |  |  |  |  |  |  |  |  |  |  |  |  |  |  |  |  |  |  |  |  |  |  |  |  |  |  |  |  |  |  |  |  |  |  |  |  |  |  |  |  |  |  |  |  |  |  |  |  |  |  |  |  |  |  |  |  |  |  |  |  |  |  |  |  |  |  |  |  |  |  |  |  |  |  |  |  |  |  |  |  |  |  |  |  |  |  |  |  |  |  |  |  |  |  |  |  |  |  |  |  |  |  |  |  |  |  |  |  |  |  |  |  |  |  |  |  |  |  |  |  |  |  |  |  |  |  |  |  |  |  |  |  |  |  |  |  |  |  |  |  |  |  |  |  |  |  |  |  |  |  |  |  |  |  |  |  |  |  |  |  |  |  |  |  |  |  |  |  |  |  |  |  |  |  |  |  |  |  |  |  |  |  |  |  |  |  |  |  |  |  |  |  |  |  |  |  |  |  |  |  |  |  |  |  |  |  |  |  |  |  |  |  |  |  |  |  |  |  |  |  |  |  |  |  |  |  |  |  |  |  |  |  |  |  |  |  |  |  |  |  |  |  |  |  |  |  |  |  |  |  |  |  |  |  |  |  |  |  |  |  |  |  |  |  |  |  |  |  |  |  |  |  |  |  |  |  |  |  |  |  |  |  |  |  |  |  |  |  |  |  |  |  |  |  |  |  |  |  |  |  |  |  |  |  |  |  |  |  |  |  |  |  |  |  |  |  |  |  |  |  |  |  |  |  |  |  |  |  |  |  |  |  |  |  |  |  |  |  |  |  |  |  |  |  |  |  |  |  |  |  |  |  |  |  |  |  |  |  |  |  |  |  |  |  |  |  |  |  |  |  |  |  |  |  |  |  |  |  |  |  |  |  |  |  |  |  |  |  |  |  |  |  |  |  |  |  |  |  |  |  |  |  |  |  |  |  |  |  |  |  |  |  |  |  |  |  |  |  |  |  |  |  |  |  |  |  |  |  |  |  |  |  |  |  |  |  |  |  |  |  |  |  |  |  |  |  |  |  |  |  |  |  |  |  |  |  |  |  |  |  |  |  |  |  |  |  |  |  |  |  |  |  |  |  |  |  |  |  |  |  |  |  |  |  |  |  |  |  |  |  |  |  |  |  |  |  |  |  |  |  |  |  |  |  |  |  |  |  |  |  |  |  |  |  |  |  |  |  |  |  |  |  |  |  |  |  |  |  |  |  |  |  |  |  |  |  |  |  |  |  |  |  |  |  |  |  |  |  |  |  |  |  |  |  |  |  |  |  |  |  |  |  |  |  |  |  |  |  |  |  |  |  |  |  |  |  |  |  |  |  |  |  |  |  |  |  |  |  |  |  |  |  |  |  |  |  |  |  |  |  |  |  |  |  |  |  |  |  |  |  |  |  |  |  |  |  |  |  |  |  |  |  |  |  |  |  |  |  |  |  |  |  |  |  |  |  |  |  |  |  |  |  |
|  |  |  |  |  |  |  |  |  |  |  |  |  |  |  |  |  |  |  |  |  |  |  |  |  |  |  |  |  |  |  |  |  |  |  |  |  |  |  |  |  |  |  |  |  |  |  |  |  |  |  |  |  |  |  |  |  |  |  |  |  |  |  |  |  |  |  |  |  |  |  |  |  |  |  |  |  |  |  |  |  |  |  |  |  |  |  |  |  |  |  |  |  |  |  |  |  |  |  |  |  |  |  |  |  |  |  |  |  |  |  |  |  |  |  |  |  |  |  |  |  |  |  |  |  |  |  |  |  |  |  |  |  |  |  |  |  |  |  |  |  |  |  |  |  |  |  |  |  |  |  |  |  |  |  |  |  |  |  |  |  |  |  |  |  |  |  |  |  |  |  |  |  |  |  |  |  |  |  |  |  |  |  |  |  |  |  |  |  |  |  |  |  |  |  |  |  |  |  |  |  |  |  |  |  |  |  |  |  |  |  |  |  |  |  |  |  |  |  |  |  |  |  |  |  |  |  |  |  |  |  |  |  |  |  |  |  |  |  |  |  |  |  |  |  |  |  |  |  |  |  |  |  |  |  |  |  |  |  |  |  |  |  |  |  |  |  |  |  |  |  |  |  |  |  |  |  |  |  |  |  |  |  |  |  |  |  |  |  |  |  |  |  |  |  |  |  |  |  |  |  |  |  |  |  |  |  |  |  |  |  |  |  |  |  |  |  |  |  |  |  |  |  |  |  |  |  |  |  |  |  |  |  |  |  |  |  |  |  |  |  |  |  |  |  |  |  |  |  |  |  |  |  |  |  |  |  |  |  |  |  |  |  |  |  |  |  |  |  |  |  |  |  |  |  |  |  |  |  |  |  |  |  |  |  |  |  |  |  |  |  |  |  |  |  |  |  |  |  |  |  |  |  |  |  |  |  |  |  |  |  |  |  |  |  |  |  |  |  |  |  |  |  |  |  |  |  |  |  |  |  |  |  |  |  |  |  |  |  |  |  |  |  |  |  |  |  |  |  |  |  |  |  |  |  |  |  |  |  |  |  |  |  |  |  |  |  |  |  |  |  |  |  |  |  |  |  |  |  |  |  |  |  |  |  |  |  |  |  |  |  |  |  |  |  |  |  |  |  |  |  |  |  |  |  |  |  |  |  |  |  |  |  |  |  |  |  |  |  |  |  |  |  |  |  |  |  |  |  |  |  |  |  |  |  |  |  |  |  |  |  |  |  |  |  |  |  |  |  |  |  |  |  |  |  |  |  |  |  |  |  |  |  |  |  |  |  |  |  |  |  |  |  |  |  |  |  |  |  |  |  |  |  |  |  |  |  |  |  |  |  |  |  |  |  |  |  |  |  |  |  |  |  |  |  |  |  |  |  |  |  |  |  |  |  |  |  |  |  |  |  |  |  |  |  |  |  |  |  |  |  |  |  |  |  |  |  |  |  |  |  |  |  |  |  |  |  |  |  |  |  |  |  |  |  |  |  |  |  |  |  |  |  |  |  |  |  |  |  |  |  |  |  |  |  |  |  |  |  |  |  |  |  |  |  |  |  |  |  |  |  |  |  |  |  |  |  |  |  |  |  |  |  |  |  |  |  |  |  |  |  |  |  |  |  |  |  |  |  |  |  |  |  |  |  |  |  |  |  |  |  |  |  |  |  |  |  |  |  |  |  |  |  |  |  |  |  |  |  |  |  |  |  |  |  |  |  |  |  |  |  |  |  |  |  |  |  |  |  |  |  |  |  |  |  |  |  |  |  |  |  |  |  |  |  |  |  |  |  |  |  |  |  |  |  |  |  |  |  |  |  |  |  |  |  |  |  |  |  |  |  |  |  |  |  |  |  |  |  |  |  |  |  |  |  |  |  |  |  |  |  |  |  |  |  |  |  |  |  |  |  |  |  |  |  |  |  |  |  |  |  |  |  |  |  |  |  |  |  |  |  |  |  |  |  |  |  |  |  |  |  |  |  |  |  |  |  |  |  |  |  |  |  |  |  |  |  |  |  |  |  |  |  |  |  |  |  |  |  |  |  |  |  |  |  |  |  |  |  |  |  |  |  |  |  |  |  |  |  |  |  |  |  |  |  |  |  |  |  |  |  |  |  |  |  |  |  |  |  |  |  |  |  |  |  |  |  |  |  |  |  |  |  |  |  |  |  |  |  |
