# Supplementary figures and images for: Variation in selection constraints on teleost TLRs with emphasis on their repertoire in the Walking catfish, Clarias batrachus (part 2 of 3)
Source: Sci Rep. 2020 Dec 7;10:21394. doi: 10.1038/s41598-020-78347-6 (PMC7721727; doi:10.1038/s41598-020-78347-6)

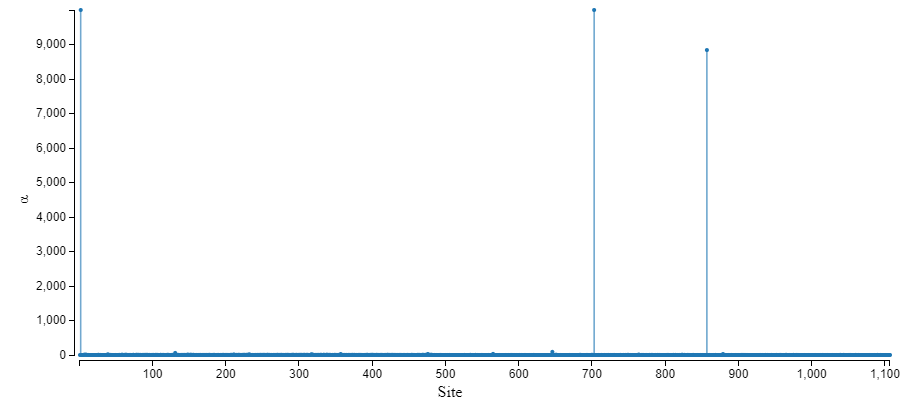

Supplement: Supplementary file 29 — Supplementary Information 29. [file 41598_2020_78347_MOESM29_ESM.zip › T7/meme/datamonkey-chart.png]

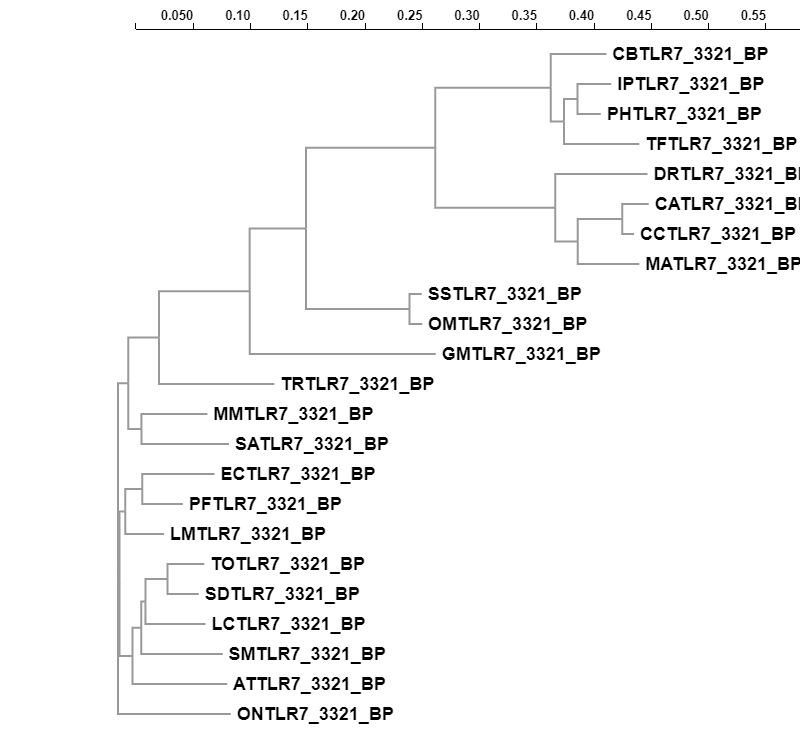

Supplement: Supplementary file 29 — Supplementary Information 29. [file 41598_2020_78347_MOESM29_ESM.zip › T7/meme/tree.png]

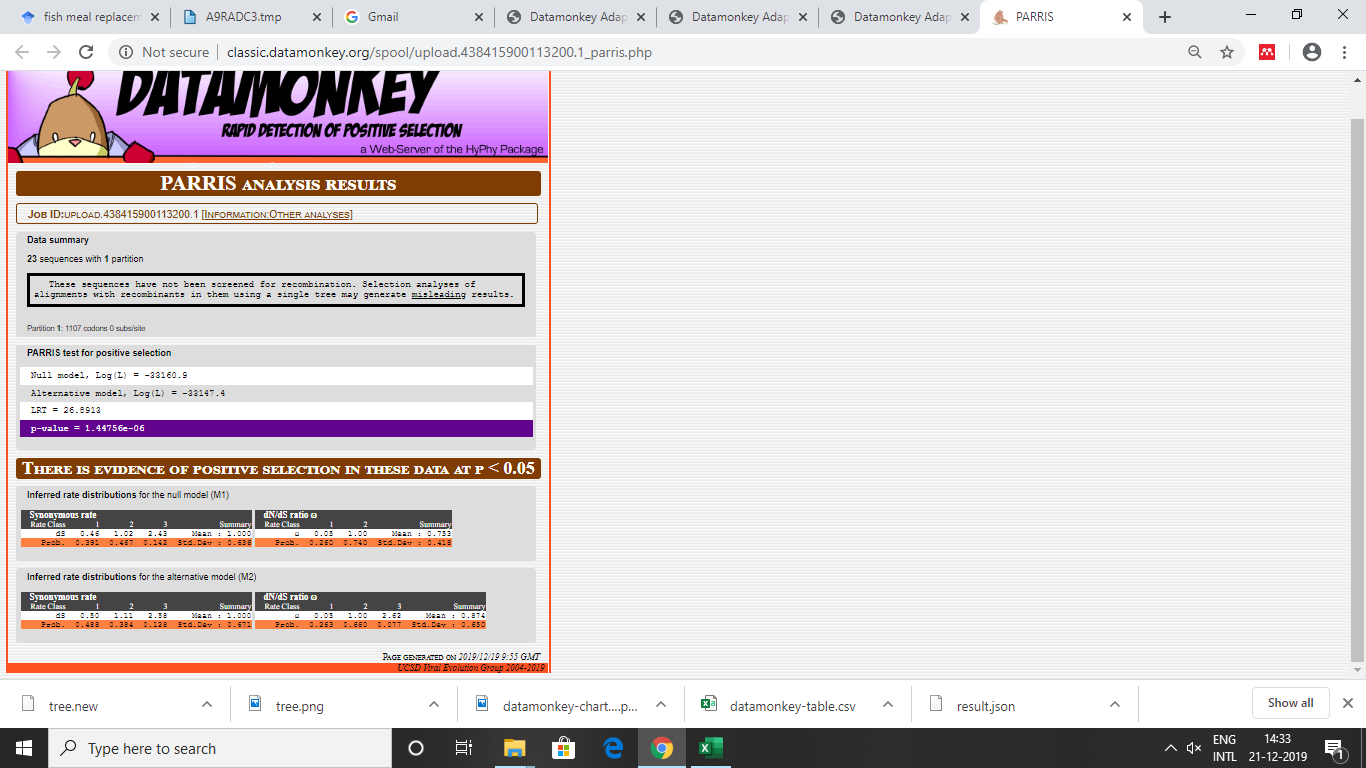

Supplement: Supplementary file 29 — Supplementary Information 29. [file 41598_2020_78347_MOESM29_ESM.zip › T7/parris.docx]

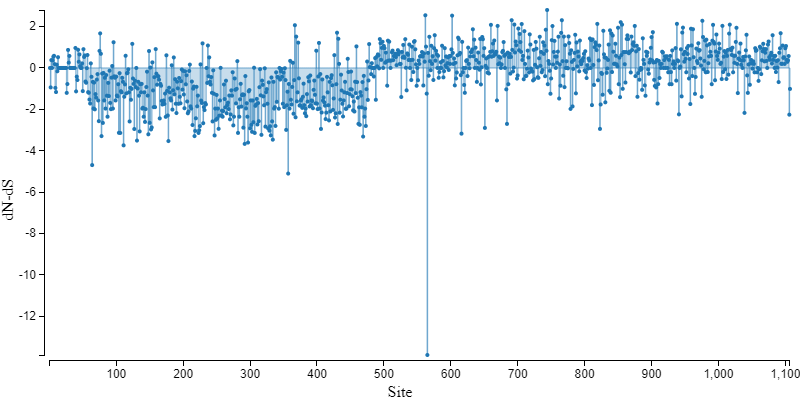

Supplement: Supplementary file 29 — Supplementary Information 29. [file 41598_2020_78347_MOESM29_ESM.zip › T7/slac/datamonkey-chart (1).png]

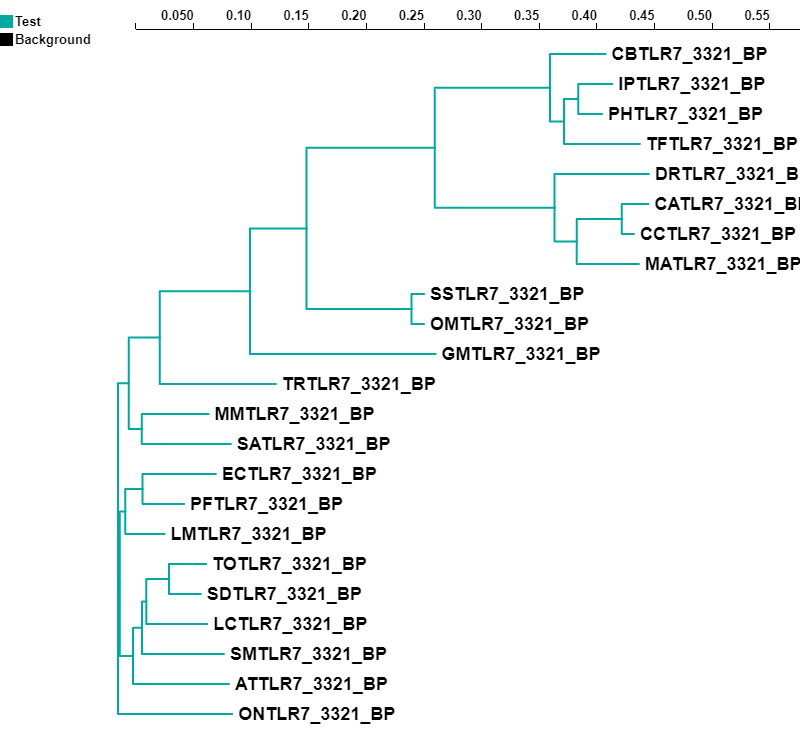

Supplement: Supplementary file 29 — Supplementary Information 29. [file 41598_2020_78347_MOESM29_ESM.zip › T7/slac/tree.png]

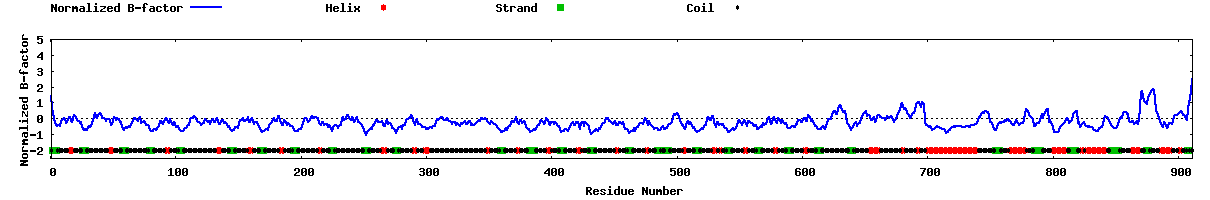

Supplement: Supplementary file 29 — Supplementary Information 29. [file 41598_2020_78347_MOESM29_ESM.zip › T7/struct/S507412_results/BFP.png]

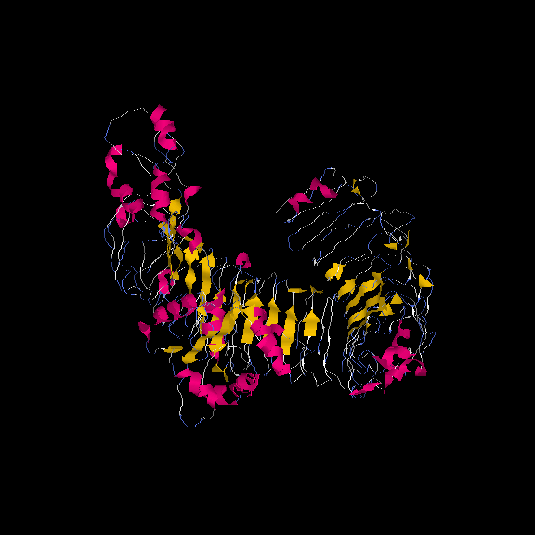

Supplement: Supplementary file 29 — Supplementary Information 29. [file 41598_2020_78347_MOESM29_ESM.zip › T7/struct/S507412_results/model1.gif]

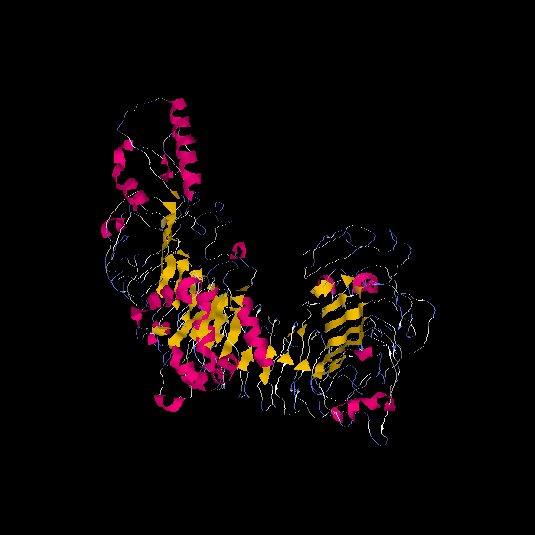

Supplement: Supplementary file 29 — Supplementary Information 29. [file 41598_2020_78347_MOESM29_ESM.zip › T7/struct/S507412_results/model2.gif]

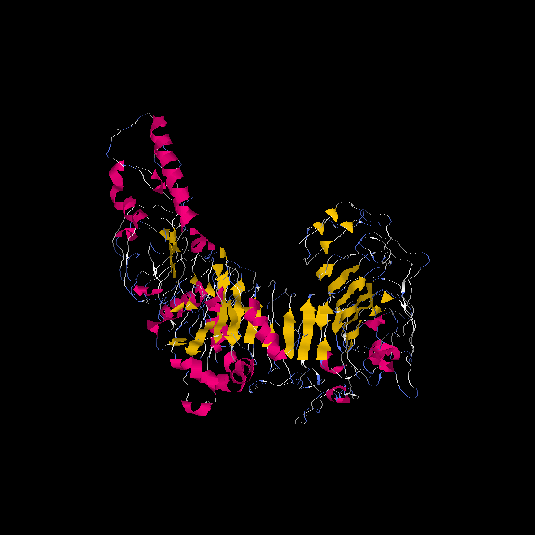

Supplement: Supplementary file 29 — Supplementary Information 29. [file 41598_2020_78347_MOESM29_ESM.zip › T7/struct/S507412_results/model3.gif]

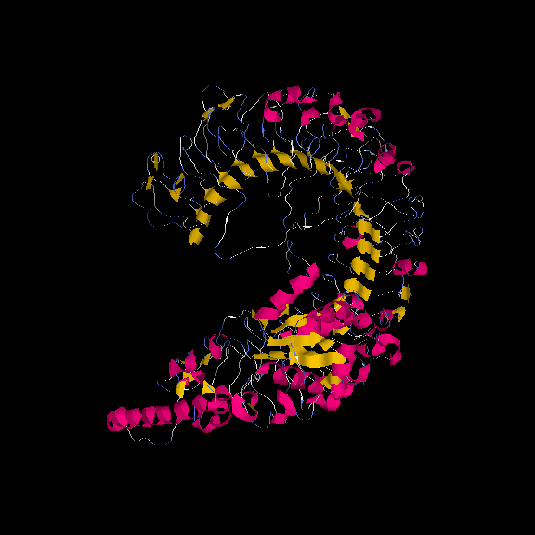

Supplement: Supplementary file 29 — Supplementary Information 29. [file 41598_2020_78347_MOESM29_ESM.zip › T7/struct/S507412_results/model4.gif]

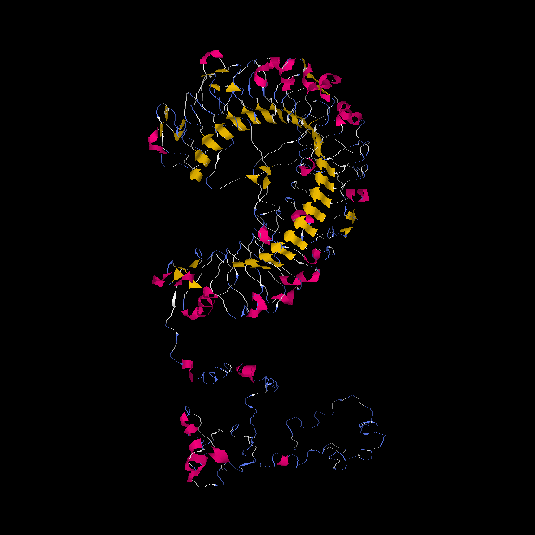

Supplement: Supplementary file 29 — Supplementary Information 29. [file 41598_2020_78347_MOESM29_ESM.zip › T7/struct/S507412_results/model5.gif]

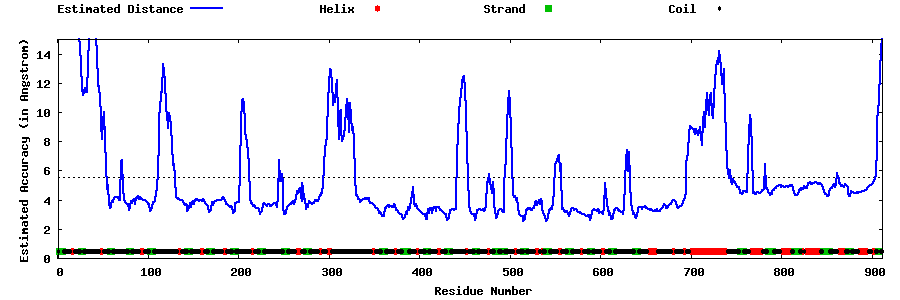

Supplement: Supplementary file 29 — Supplementary Information 29. [file 41598_2020_78347_MOESM29_ESM.zip › T7/struct/S507412_results/RSQ_1.png]

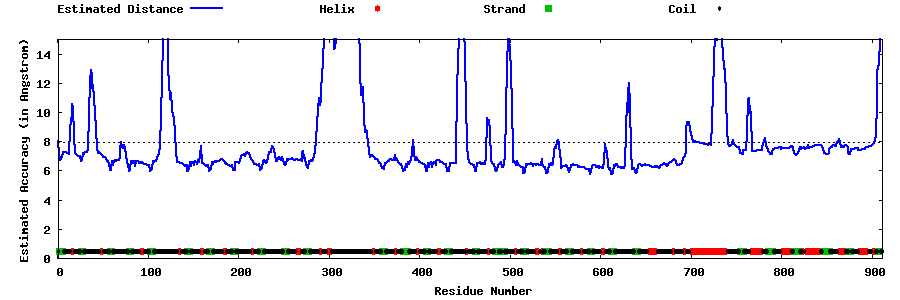

Supplement: Supplementary file 29 — Supplementary Information 29. [file 41598_2020_78347_MOESM29_ESM.zip › T7/struct/S507412_results/RSQ_2.png]

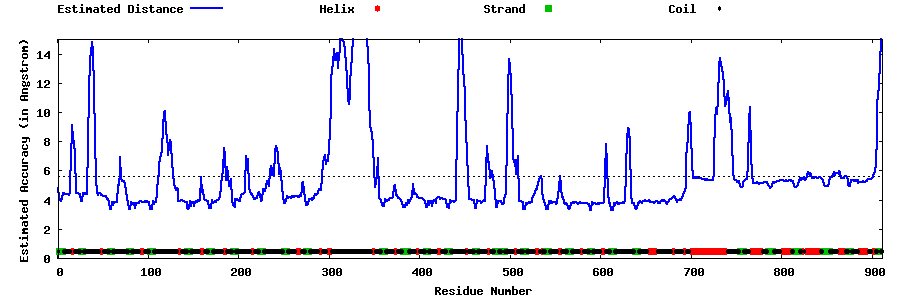

Supplement: Supplementary file 29 — Supplementary Information 29. [file 41598_2020_78347_MOESM29_ESM.zip › T7/struct/S507412_results/RSQ_3.png]

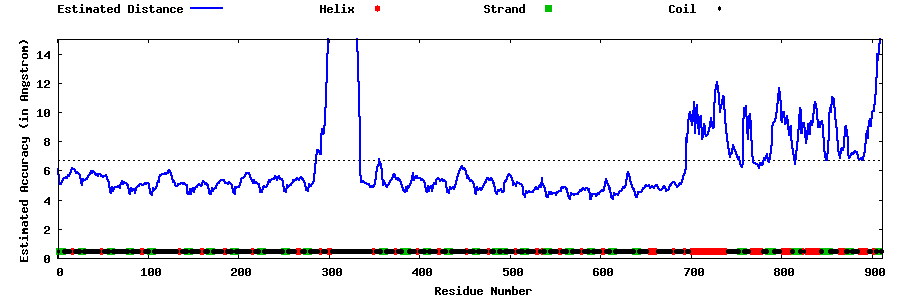

Supplement: Supplementary file 29 — Supplementary Information 29. [file 41598_2020_78347_MOESM29_ESM.zip › T7/struct/S507412_results/RSQ_4.png]

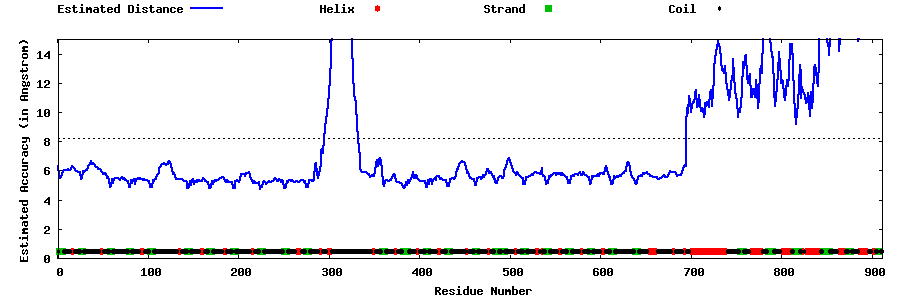

Supplement: Supplementary file 29 — Supplementary Information 29. [file 41598_2020_78347_MOESM29_ESM.zip › T7/struct/S507412_results/RSQ_5.png]

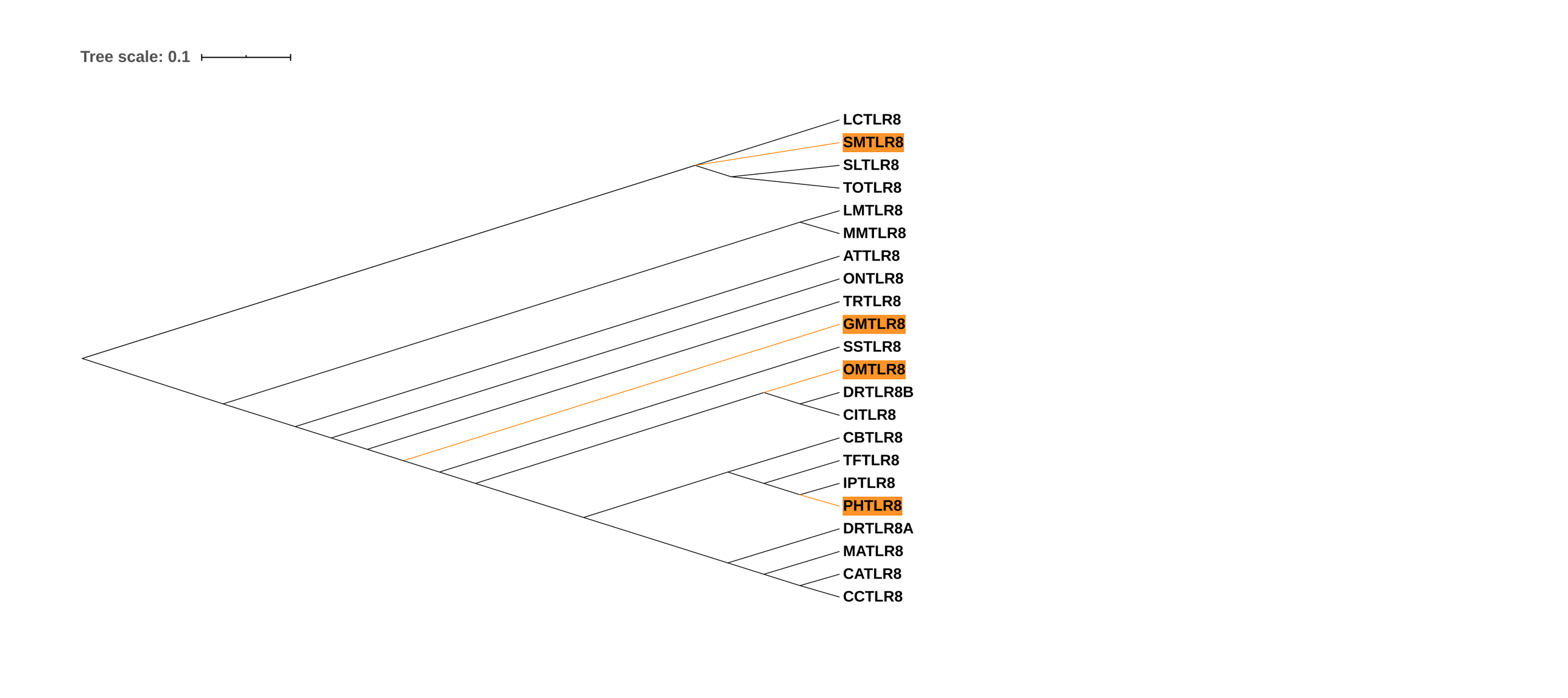

Supplement: Supplementary file 30 — Supplementary Information 30. [file 41598_2020_78347_MOESM30_ESM.zip › T8/absrel/labelled tree.png]

Tree scale: 0.1

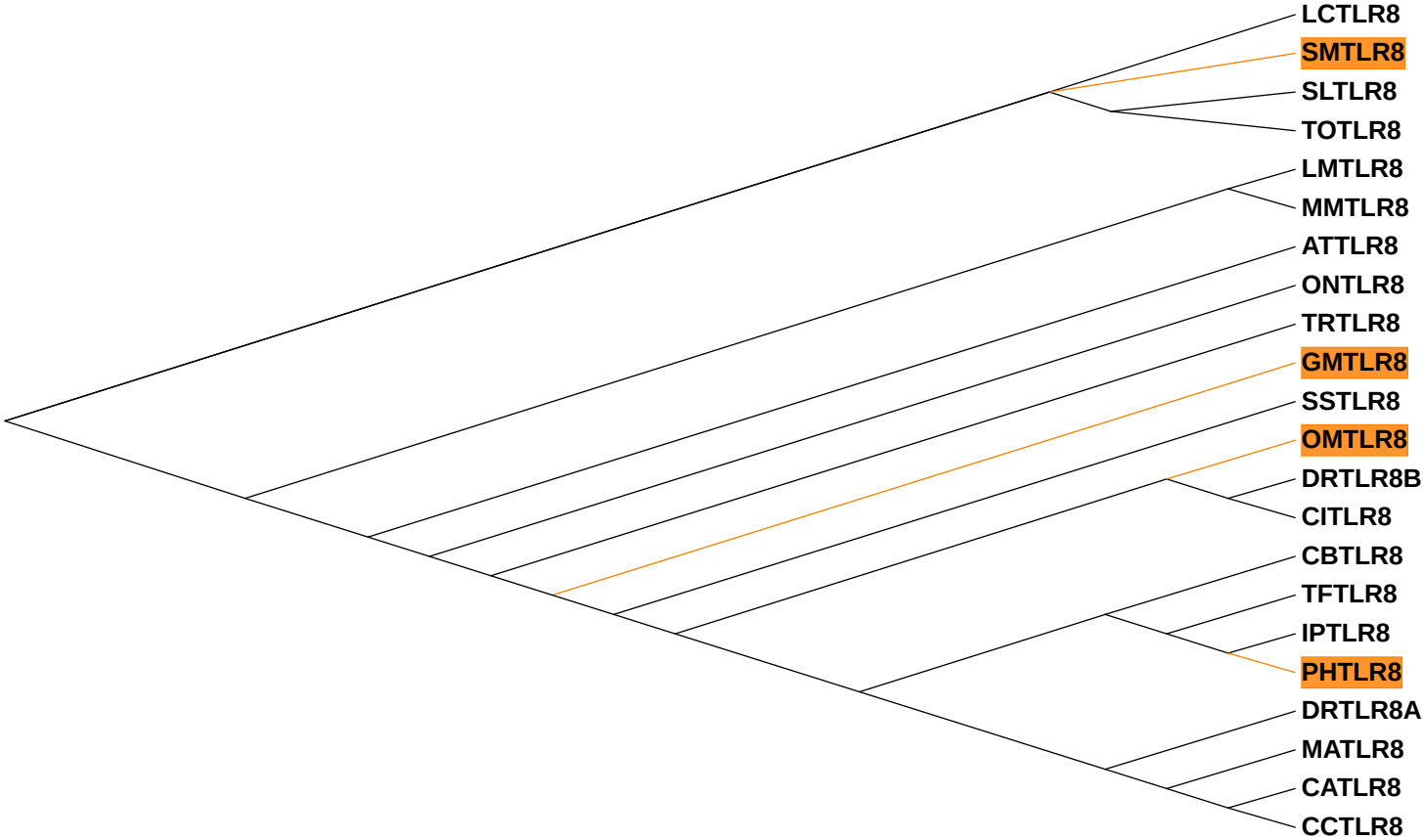

Supplement: Supplementary file 30 — Supplementary Information 30. [file 41598_2020_78347_MOESM30_ESM.zip › T8/absrel/labelledtree.pdf]

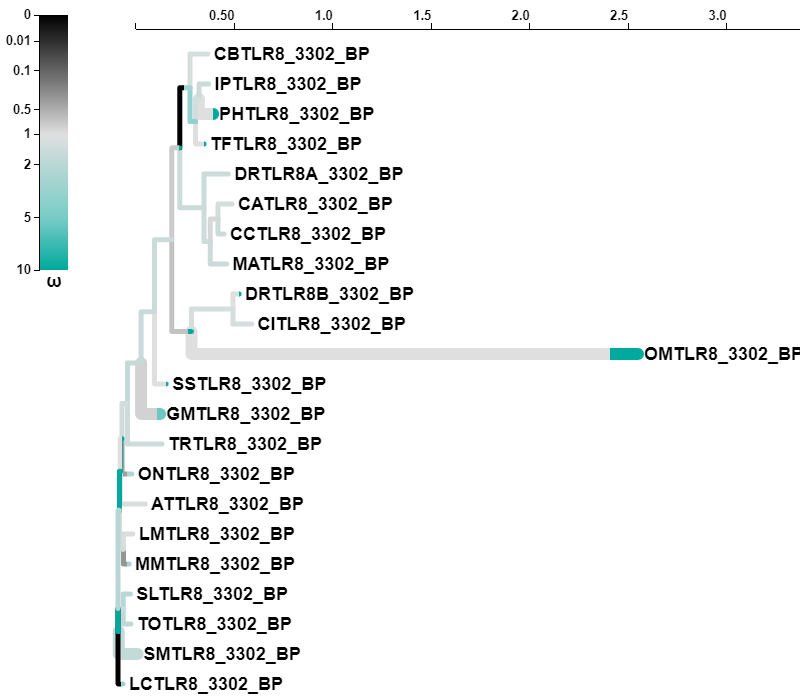

Supplement: Supplementary file 30 — Supplementary Information 30. [file 41598_2020_78347_MOESM30_ESM.zip › T8/absrel/tree.png]

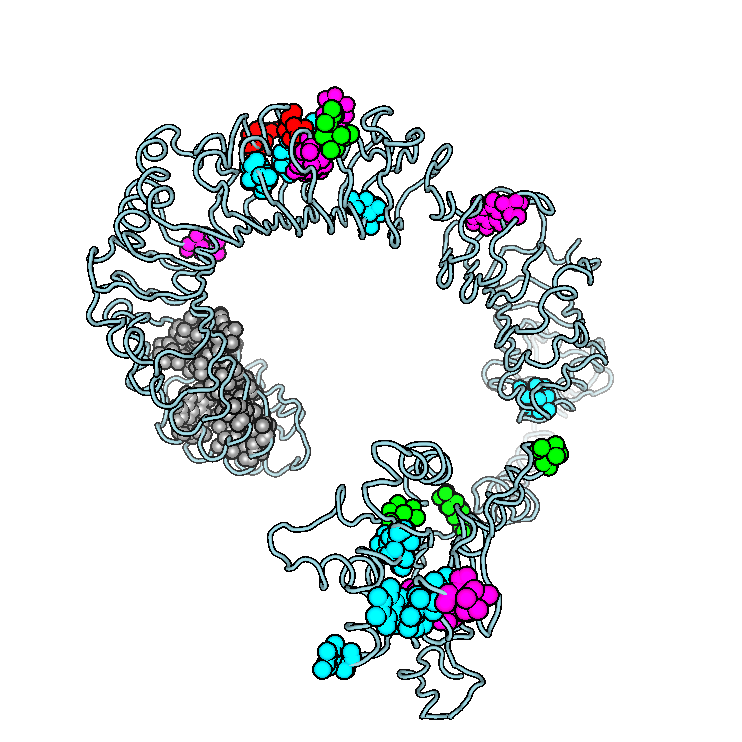

Supplement: Supplementary file 30 — Supplementary Information 30. [file 41598_2020_78347_MOESM30_ESM.zip › T8/bis2/T8.png]

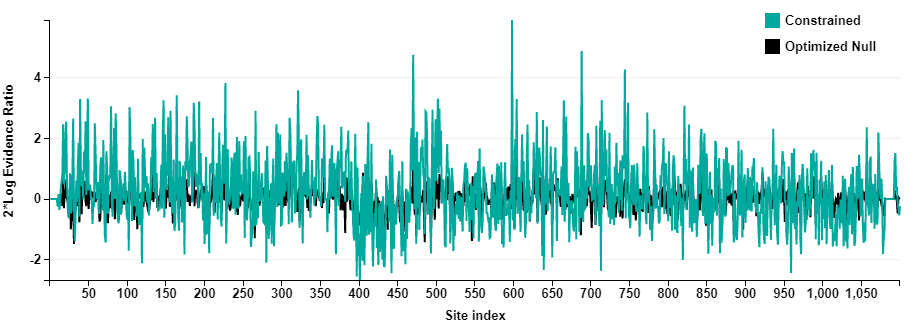

Supplement: Supplementary file 30 — Supplementary Information 30. [file 41598_2020_78347_MOESM30_ESM.zip › T8/busted/busted-chart (1).png]

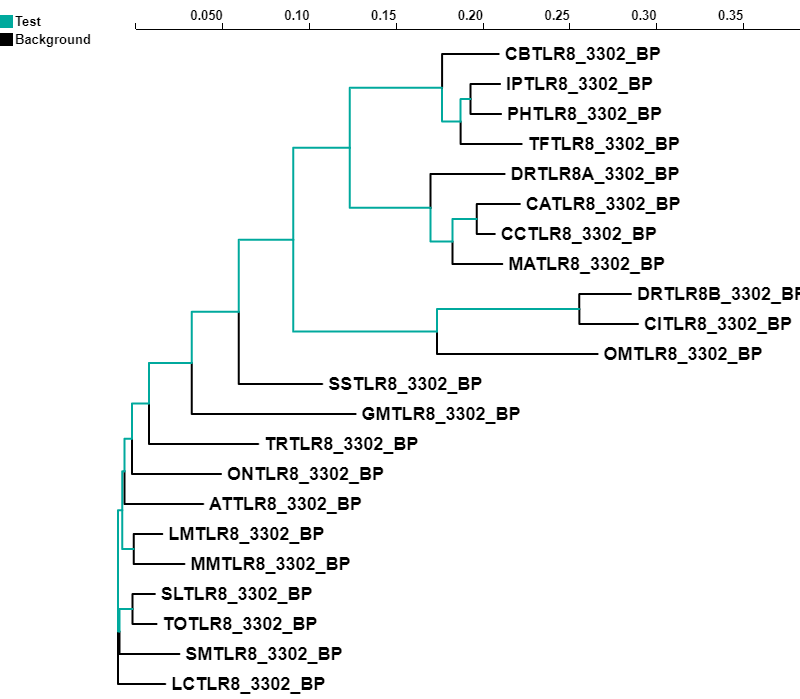

Supplement: Supplementary file 30 — Supplementary Information 30. [file 41598_2020_78347_MOESM30_ESM.zip › T8/busted/tree.png]

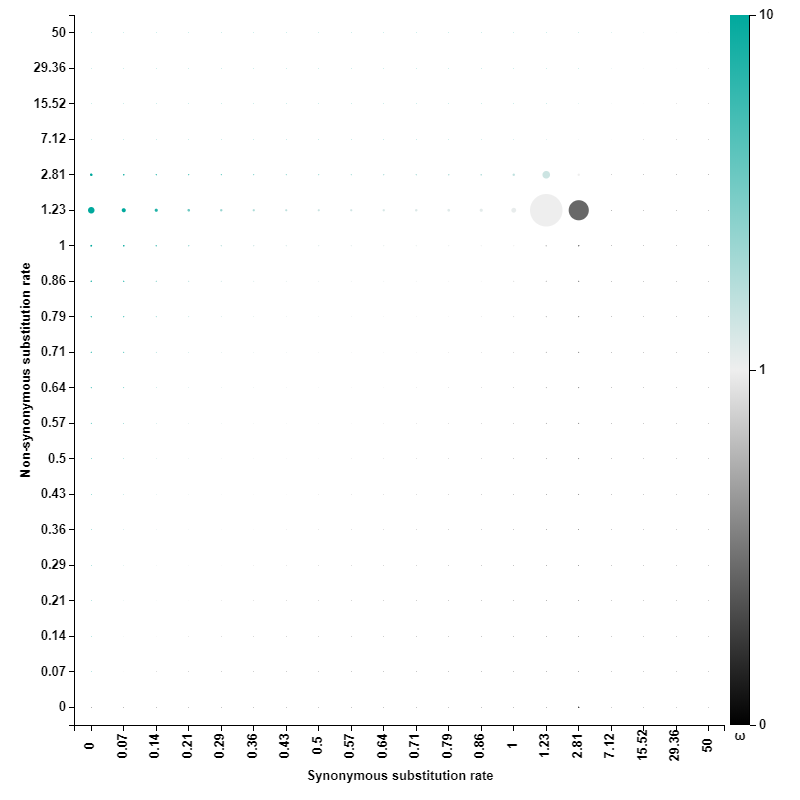

Supplement: Supplementary file 30 — Supplementary Information 30. [file 41598_2020_78347_MOESM30_ESM.zip › T8/fubar/datamonkey-chart.png]

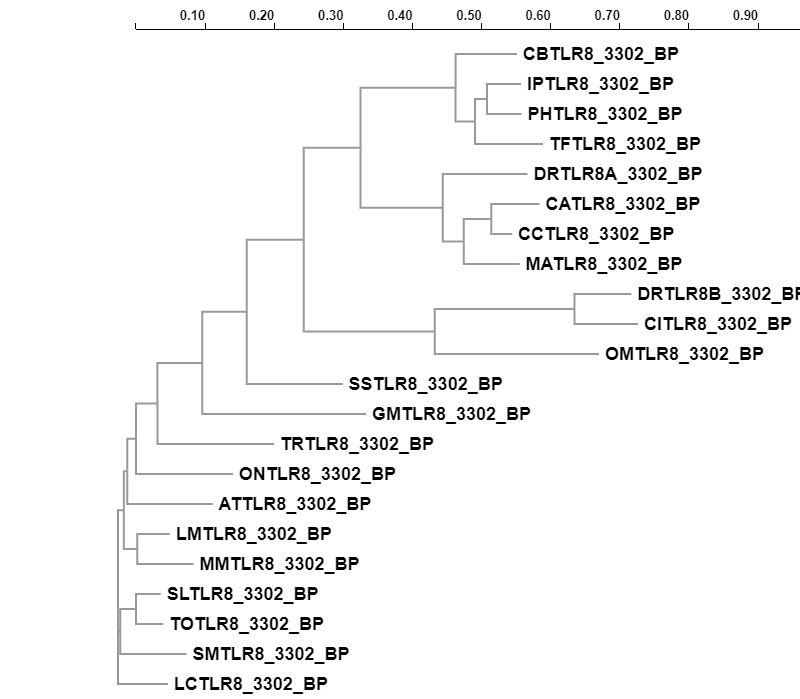

Supplement: Supplementary file 30 — Supplementary Information 30. [file 41598_2020_78347_MOESM30_ESM.zip › T8/fubar/tree.png]

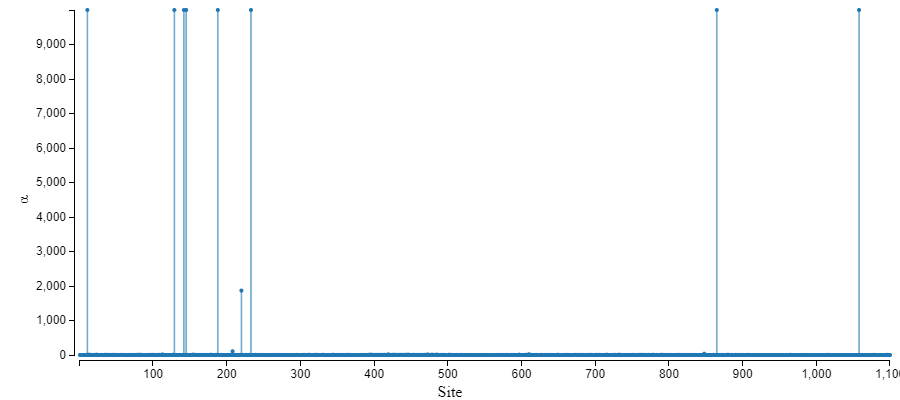

Supplement: Supplementary file 30 — Supplementary Information 30. [file 41598_2020_78347_MOESM30_ESM.zip › T8/meme/datamonkey-chart.png]

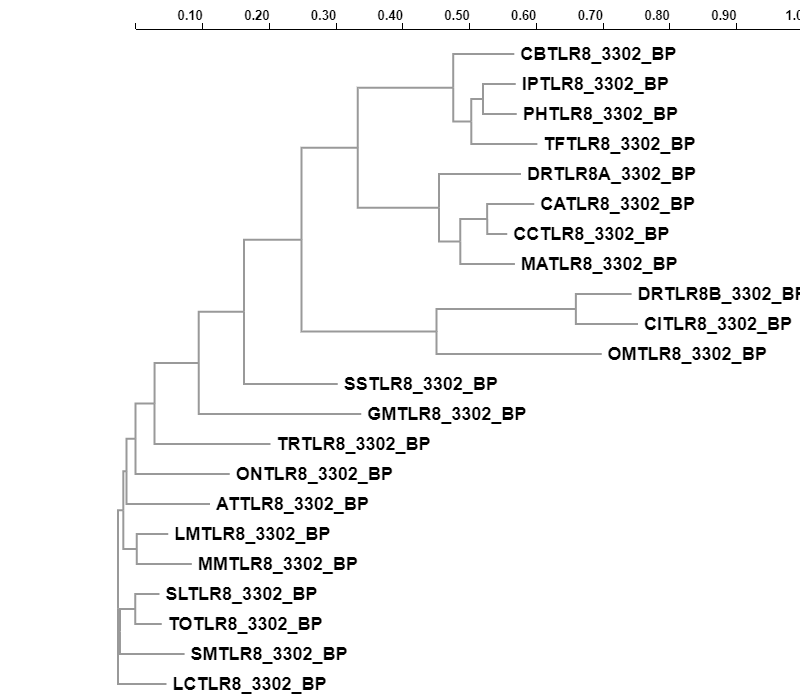

Supplement: Supplementary file 30 — Supplementary Information 30. [file 41598_2020_78347_MOESM30_ESM.zip › T8/meme/tree.png]

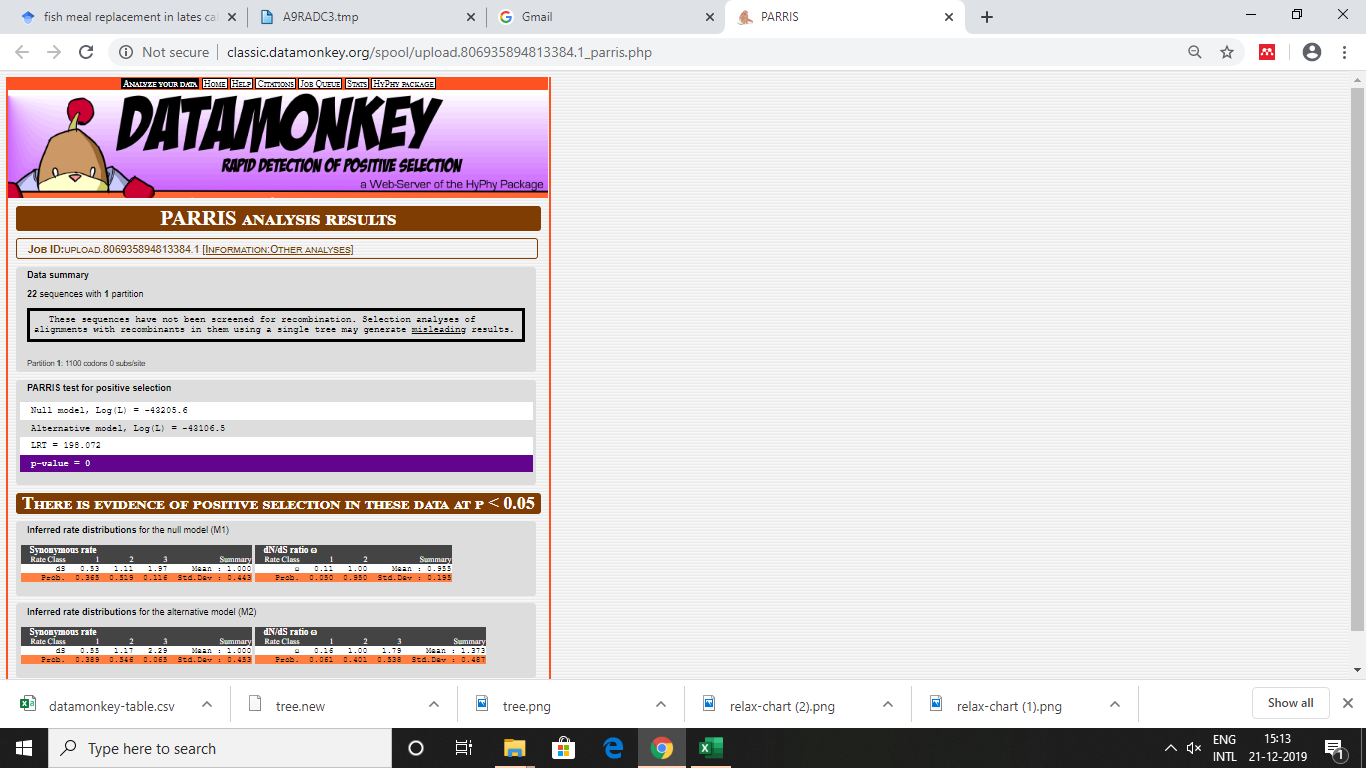

Supplement: Supplementary file 30 — Supplementary Information 30. [file 41598_2020_78347_MOESM30_ESM.zip › T8/parris.docx]

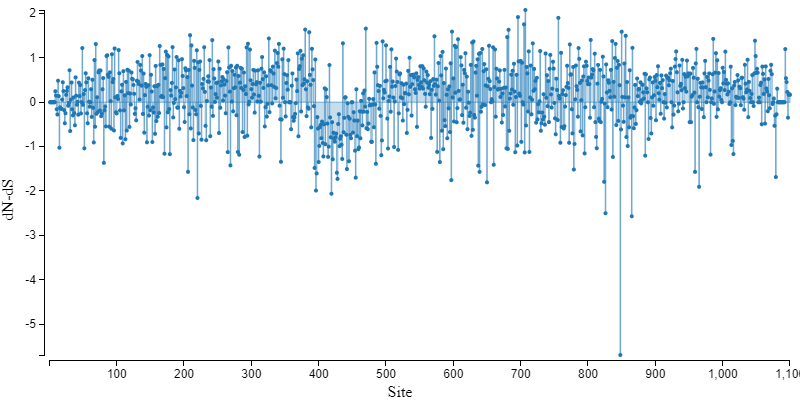

Supplement: Supplementary file 30 — Supplementary Information 30. [file 41598_2020_78347_MOESM30_ESM.zip › T8/slac/datamonkey-chart.png]

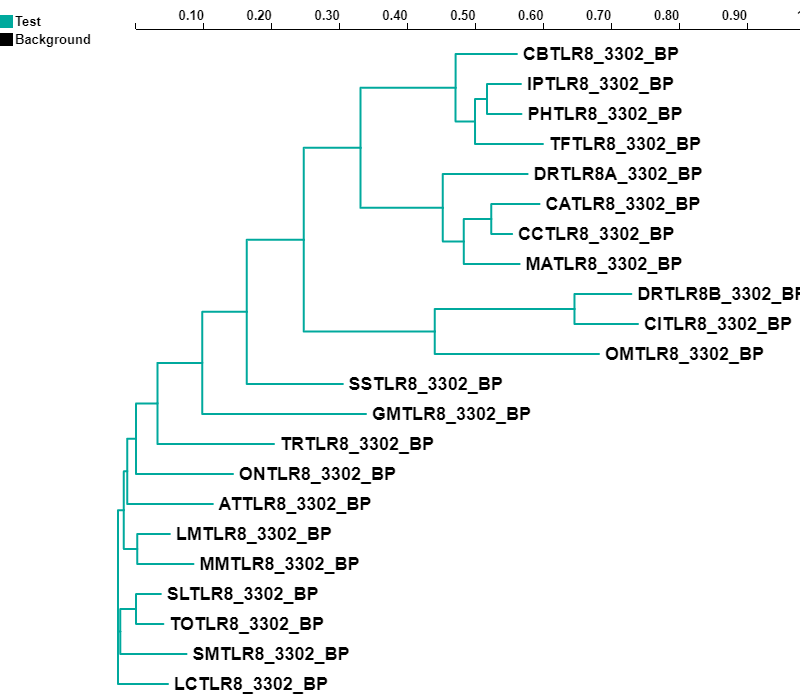

Supplement: Supplementary file 30 — Supplementary Information 30. [file 41598_2020_78347_MOESM30_ESM.zip › T8/slac/tree.png]

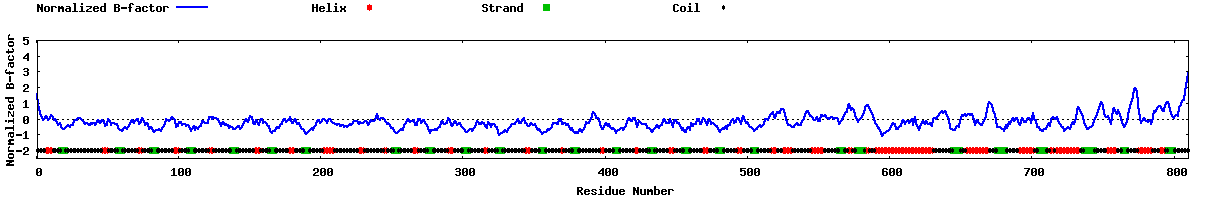

Supplement: Supplementary file 30 — Supplementary Information 30. [file 41598_2020_78347_MOESM30_ESM.zip › T8/struct/S514316_results/BFP.png]

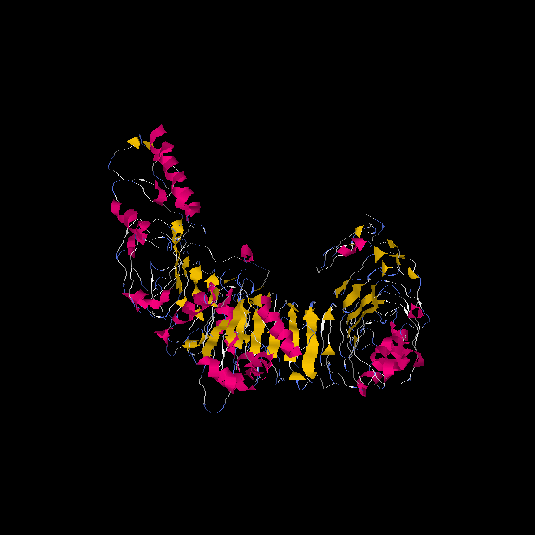

Supplement: Supplementary file 30 — Supplementary Information 30. [file 41598_2020_78347_MOESM30_ESM.zip › T8/struct/S514316_results/model1.gif]

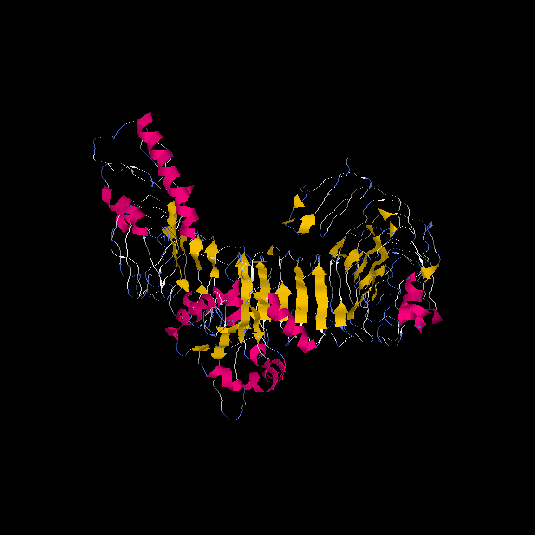

Supplement: Supplementary file 30 — Supplementary Information 30. [file 41598_2020_78347_MOESM30_ESM.zip › T8/struct/S514316_results/model2.gif]

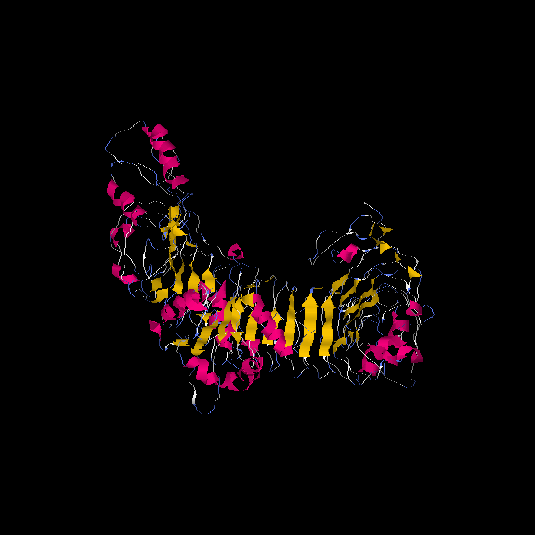

Supplement: Supplementary file 30 — Supplementary Information 30. [file 41598_2020_78347_MOESM30_ESM.zip › T8/struct/S514316_results/model3.gif]

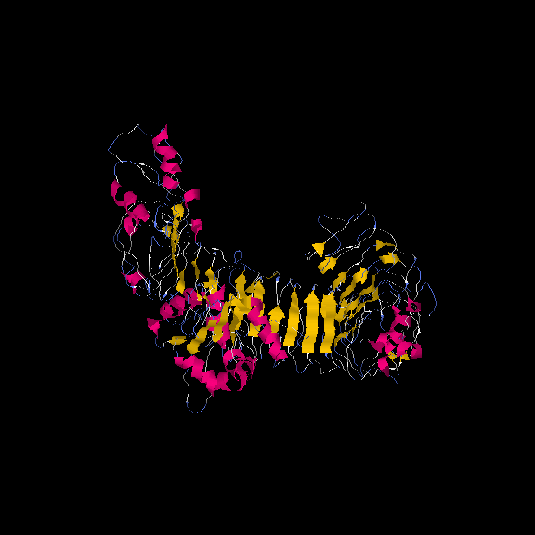

Supplement: Supplementary file 30 — Supplementary Information 30. [file 41598_2020_78347_MOESM30_ESM.zip › T8/struct/S514316_results/model4.gif]

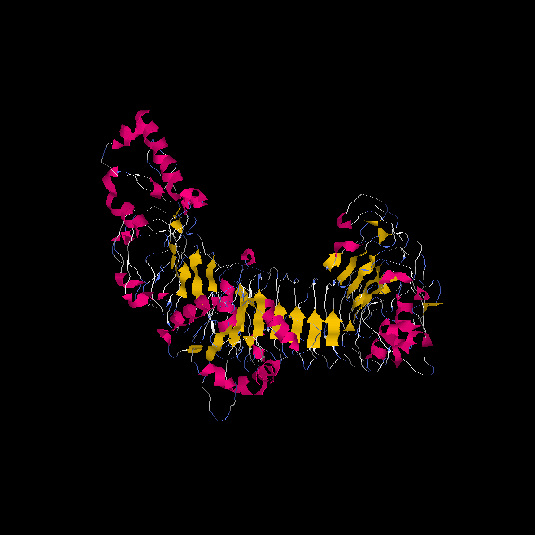

Supplement: Supplementary file 30 — Supplementary Information 30. [file 41598_2020_78347_MOESM30_ESM.zip › T8/struct/S514316_results/model5.gif]

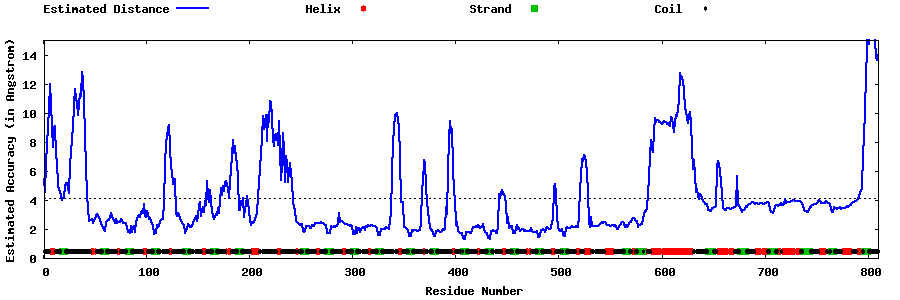

Supplement: Supplementary file 30 — Supplementary Information 30. [file 41598_2020_78347_MOESM30_ESM.zip › T8/struct/S514316_results/RSQ_1.png]

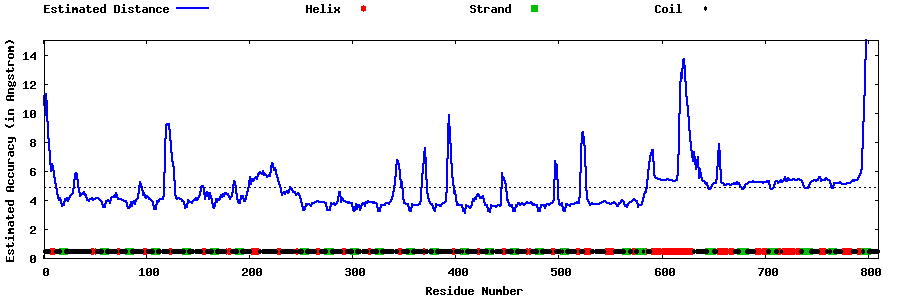

Supplement: Supplementary file 30 — Supplementary Information 30. [file 41598_2020_78347_MOESM30_ESM.zip › T8/struct/S514316_results/RSQ_2.png]

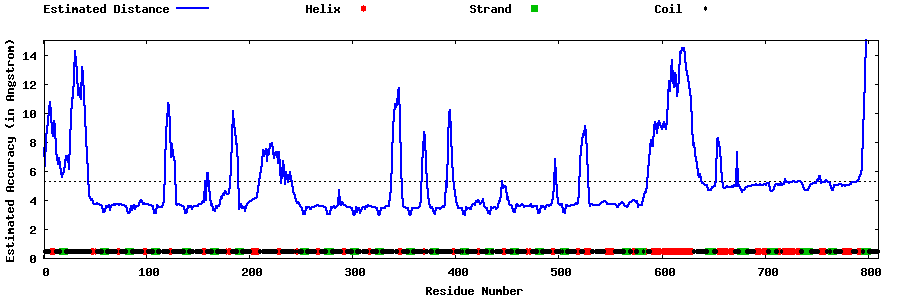

Supplement: Supplementary file 30 — Supplementary Information 30. [file 41598_2020_78347_MOESM30_ESM.zip › T8/struct/S514316_results/RSQ_3.png]

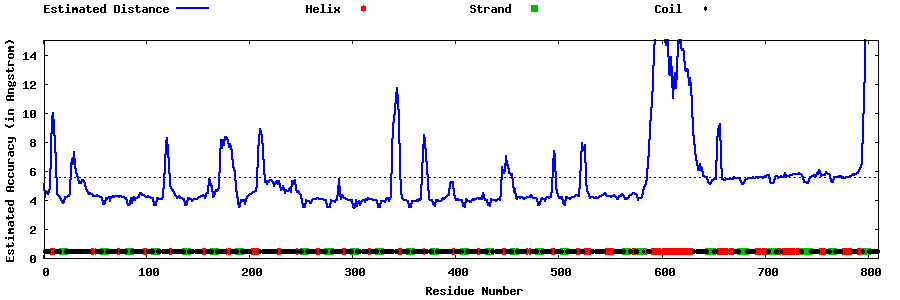

Supplement: Supplementary file 30 — Supplementary Information 30. [file 41598_2020_78347_MOESM30_ESM.zip › T8/struct/S514316_results/RSQ_4.png]

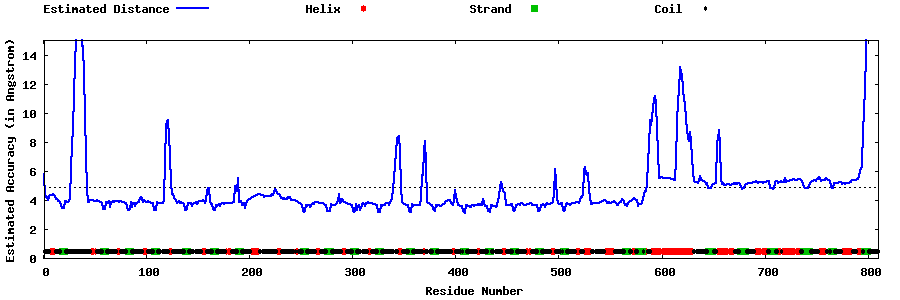

Supplement: Supplementary file 30 — Supplementary Information 30. [file 41598_2020_78347_MOESM30_ESM.zip › T8/struct/S514316_results/RSQ_5.png]

Tree scale: 0.1

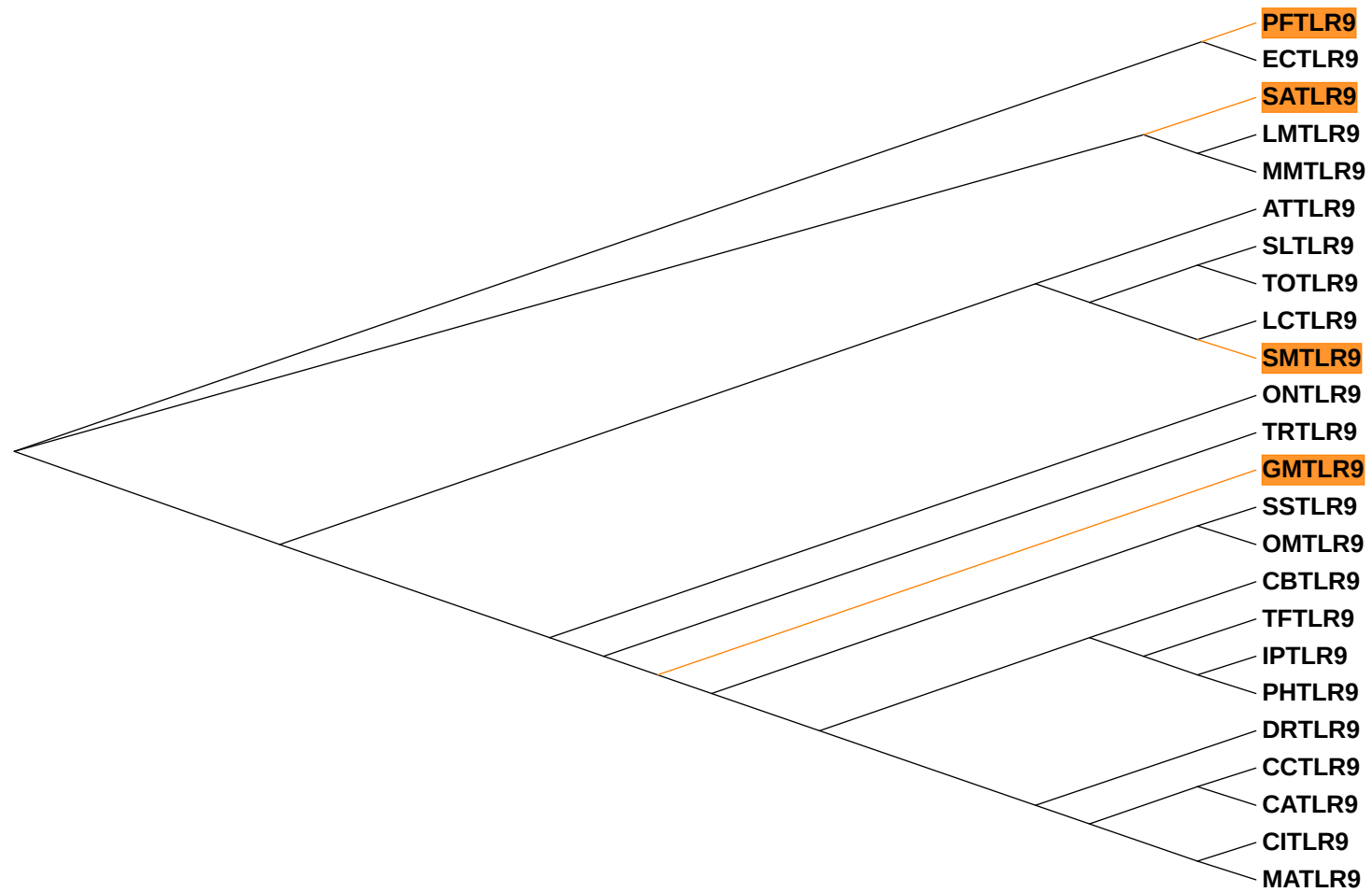

Supplement: Supplementary file 31 — Supplementary Information 31. [file 41598_2020_78347_MOESM31_ESM.zip › T9/ABSREL/LABELLEDTREE.pdf]

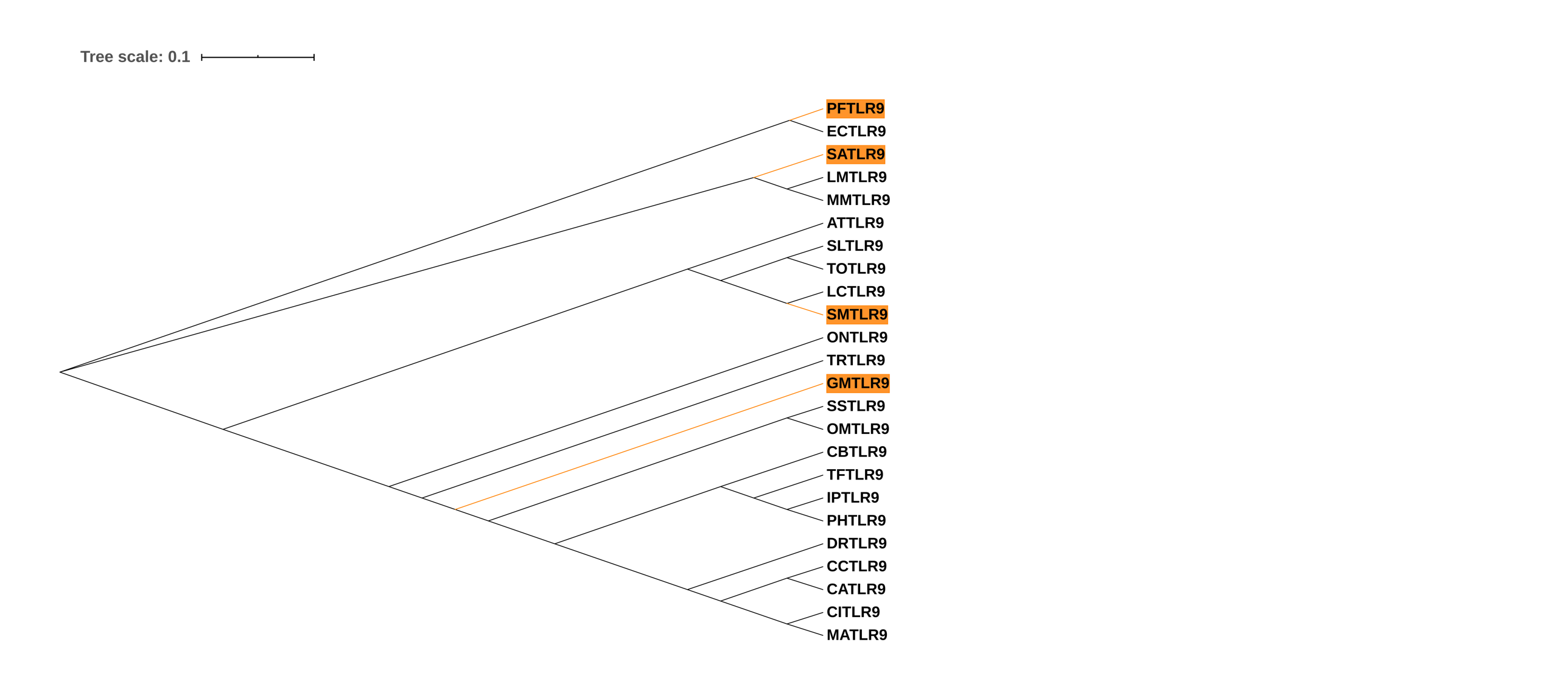

Supplement: Supplementary file 31 — Supplementary Information 31. [file 41598_2020_78347_MOESM31_ESM.zip › T9/ABSREL/LABELLEDTREE.png]

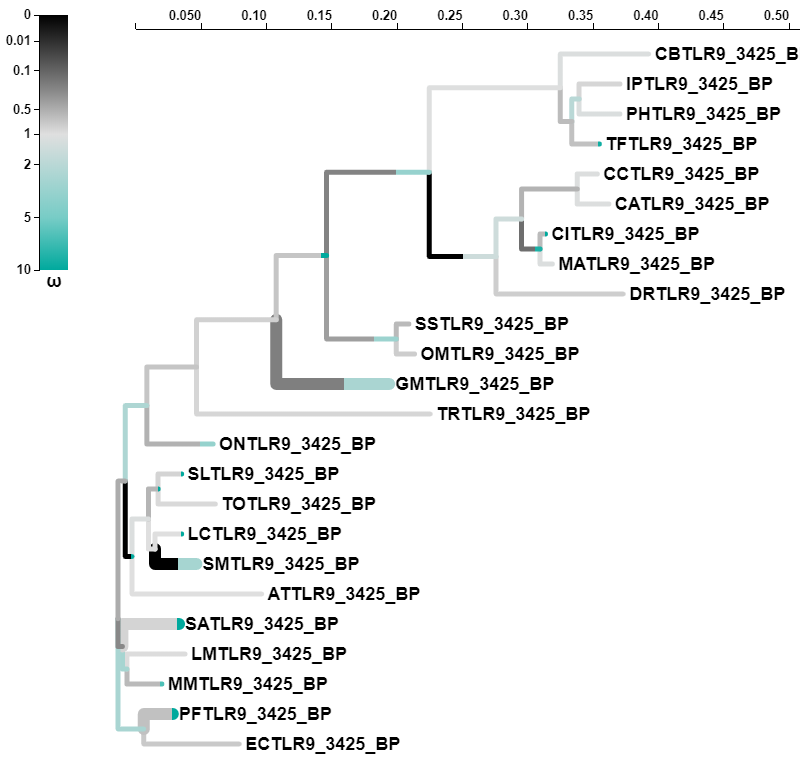

Supplement: Supplementary file 31 — Supplementary Information 31. [file 41598_2020_78347_MOESM31_ESM.zip › T9/ABSREL/tree.png]

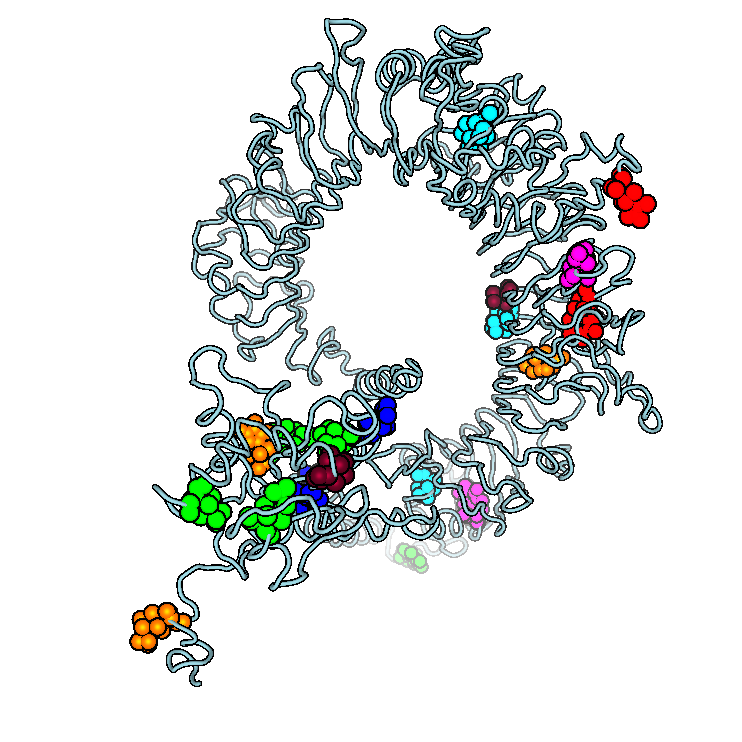

Supplement: Supplementary file 31 — Supplementary Information 31. [file 41598_2020_78347_MOESM31_ESM.zip › T9/BIS2/T9.png]

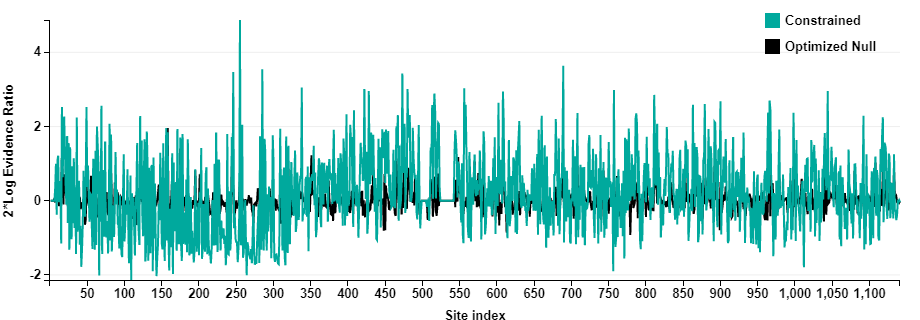

Supplement: Supplementary file 31 — Supplementary Information 31. [file 41598_2020_78347_MOESM31_ESM.zip › T9/BUSTED/busted-chart (1).png]

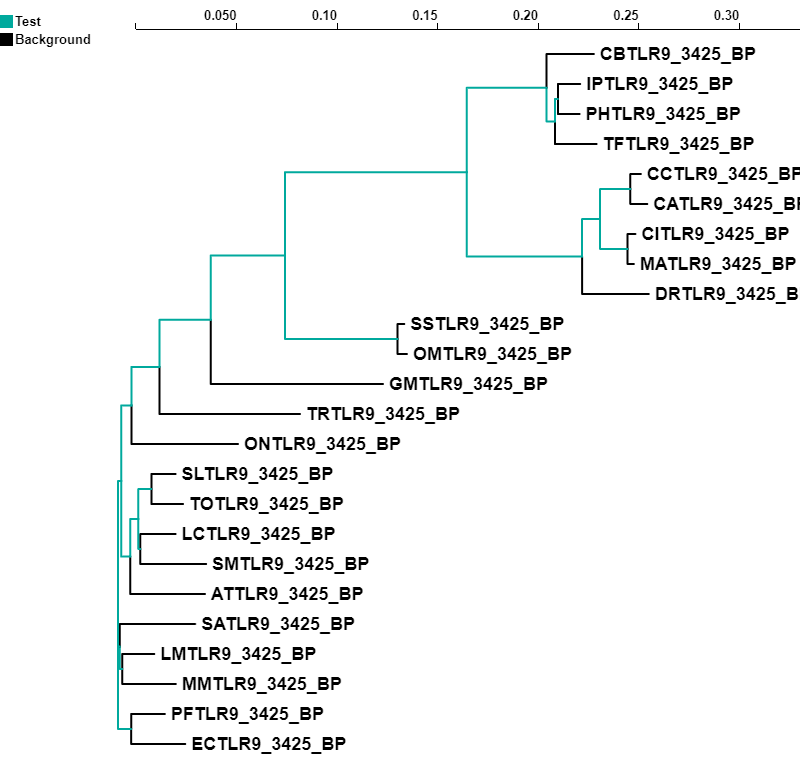

Supplement: Supplementary file 31 — Supplementary Information 31. [file 41598_2020_78347_MOESM31_ESM.zip › T9/BUSTED/tree.png]

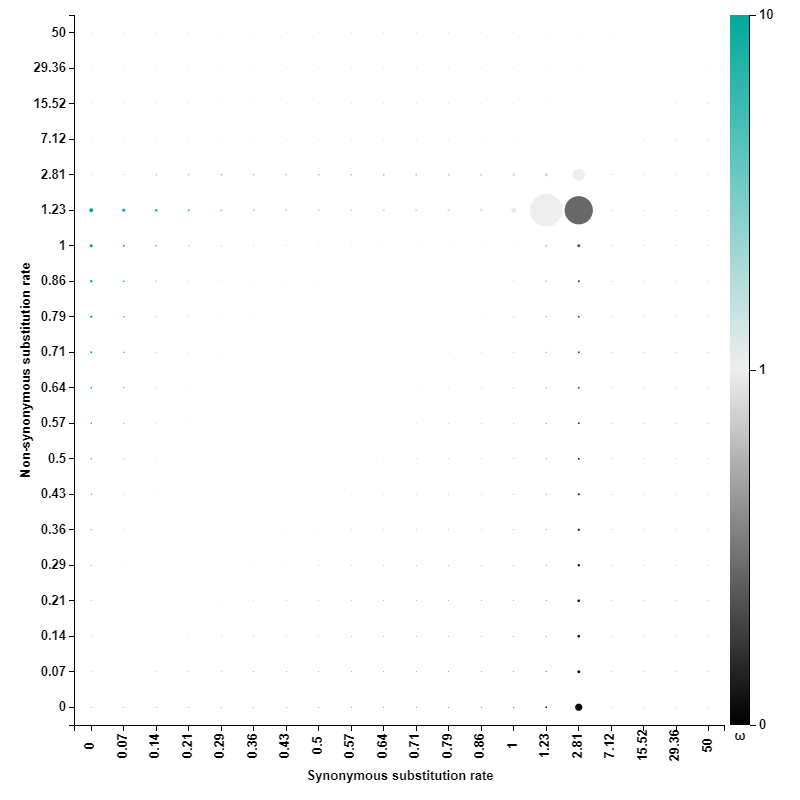

Supplement: Supplementary file 31 — Supplementary Information 31. [file 41598_2020_78347_MOESM31_ESM.zip › T9/FUBAR/datamonkey-chart.png]

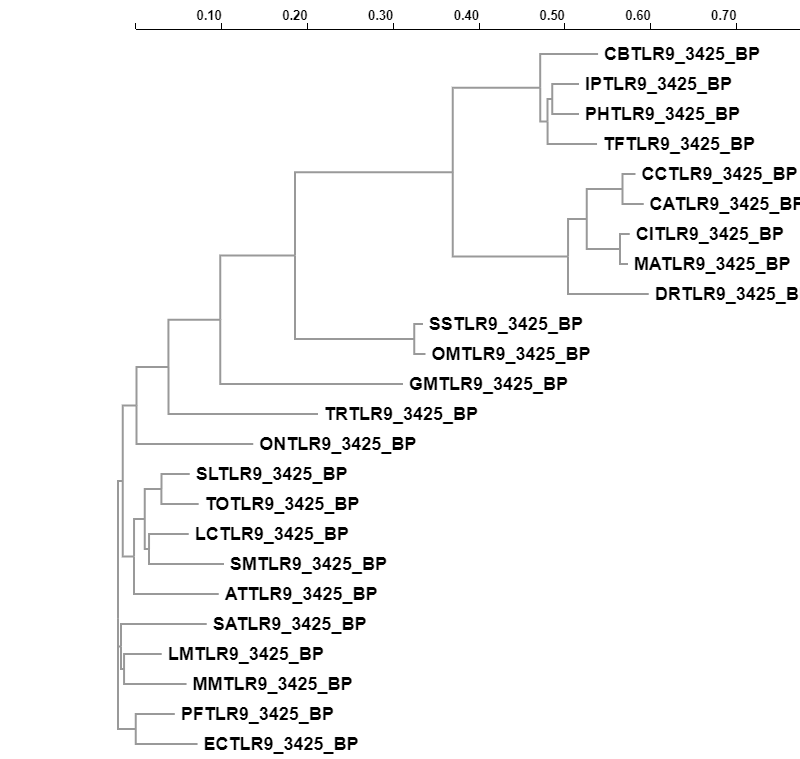

Supplement: Supplementary file 31 — Supplementary Information 31. [file 41598_2020_78347_MOESM31_ESM.zip › T9/FUBAR/tree.png]

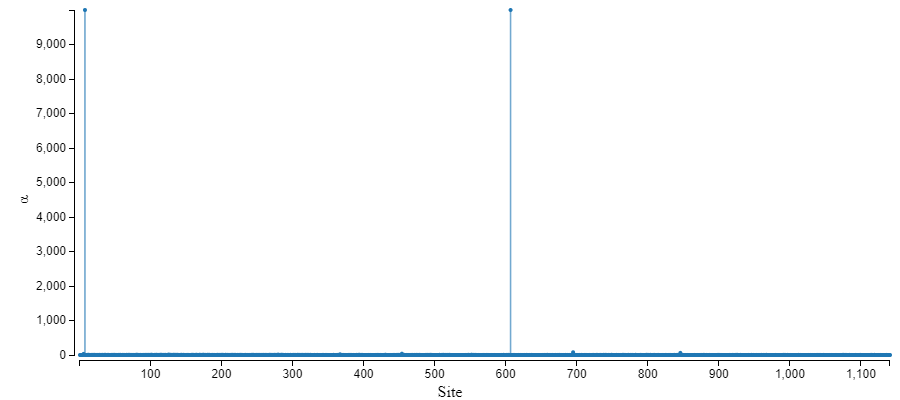

Supplement: Supplementary file 31 — Supplementary Information 31. [file 41598_2020_78347_MOESM31_ESM.zip › T9/MEME/datamonkey-chart.png]

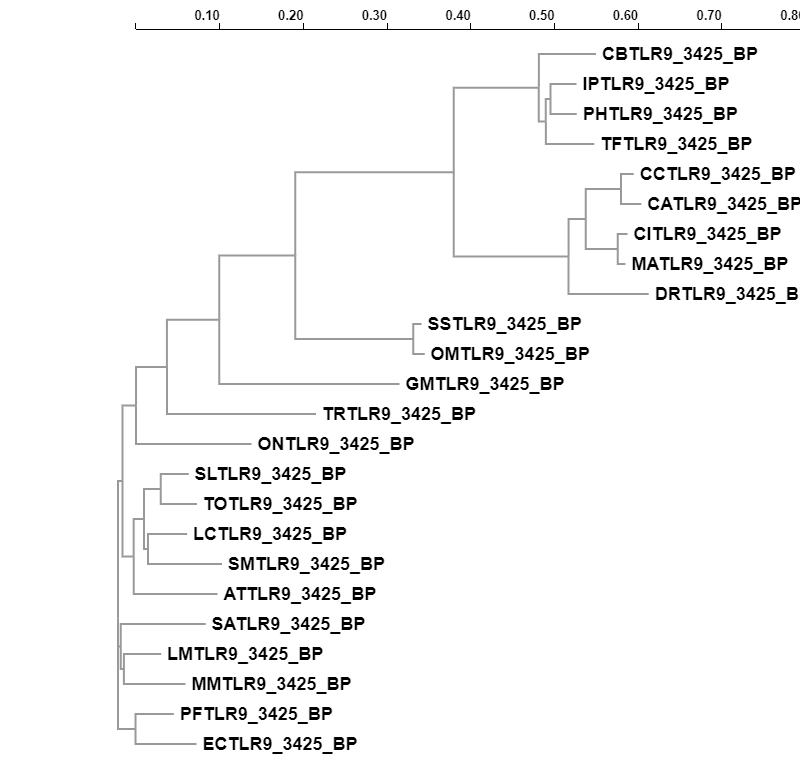

Supplement: Supplementary file 31 — Supplementary Information 31. [file 41598_2020_78347_MOESM31_ESM.zip › T9/MEME/tree.png]

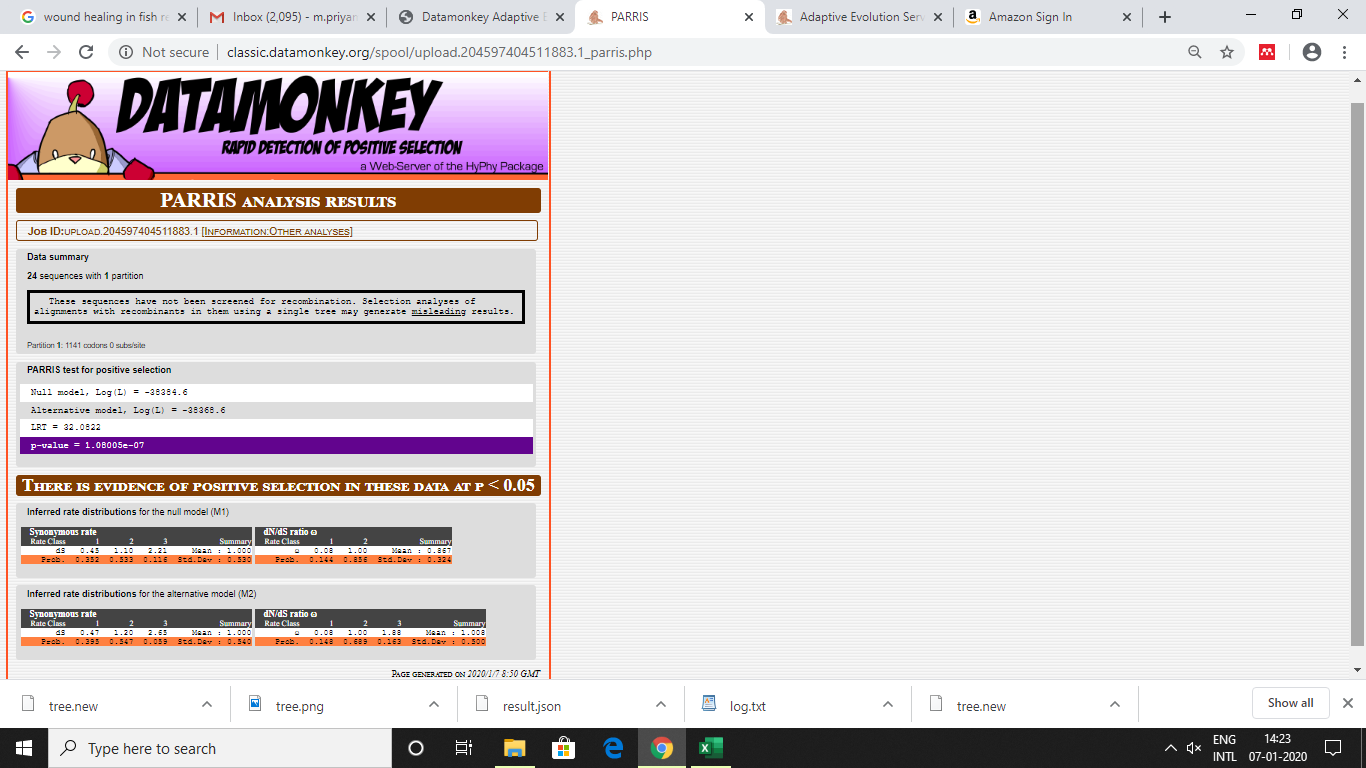

Supplement: Supplementary file 31 — Supplementary Information 31. [file 41598_2020_78347_MOESM31_ESM.zip › T9/PARRIS/PARRIS.png]

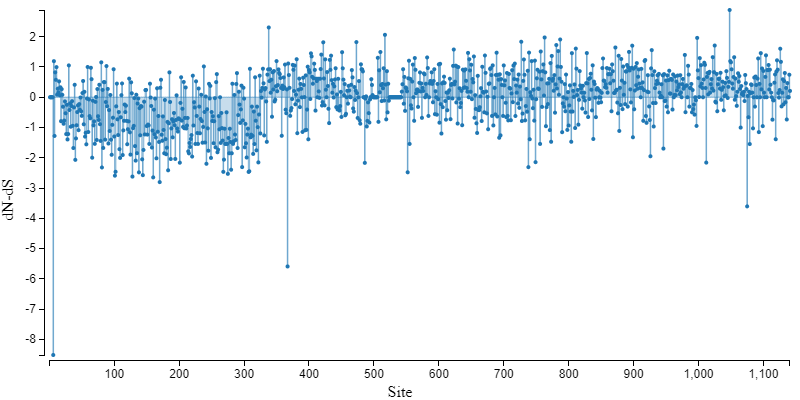

Supplement: Supplementary file 31 — Supplementary Information 31. [file 41598_2020_78347_MOESM31_ESM.zip › T9/slac/datamonkey-chart.png]

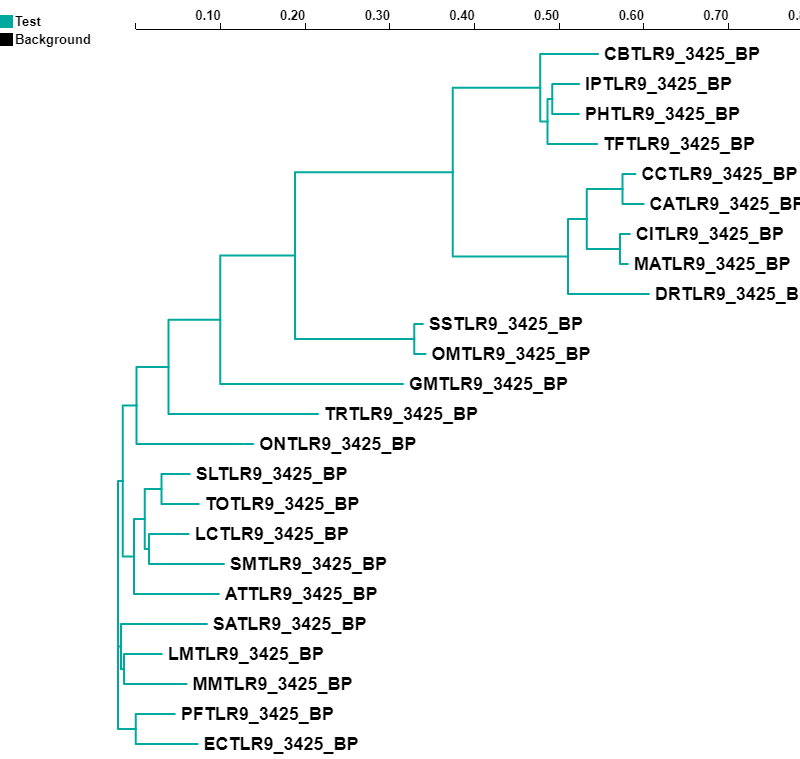

Supplement: Supplementary file 31 — Supplementary Information 31. [file 41598_2020_78347_MOESM31_ESM.zip › T9/slac/tree.png]

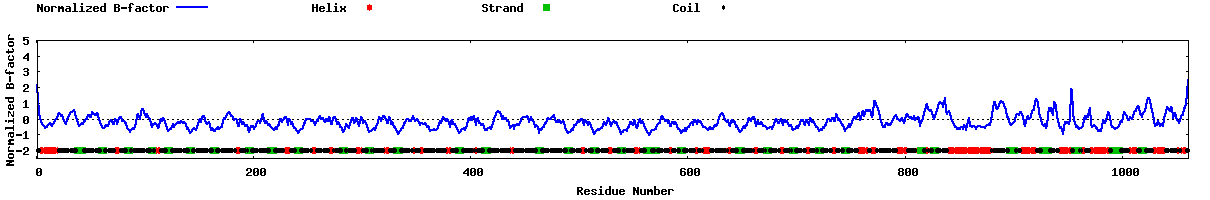

Supplement: Supplementary file 31 — Supplementary Information 31. [file 41598_2020_78347_MOESM31_ESM.zip › T9/struct/S514930_results/BFP.png]

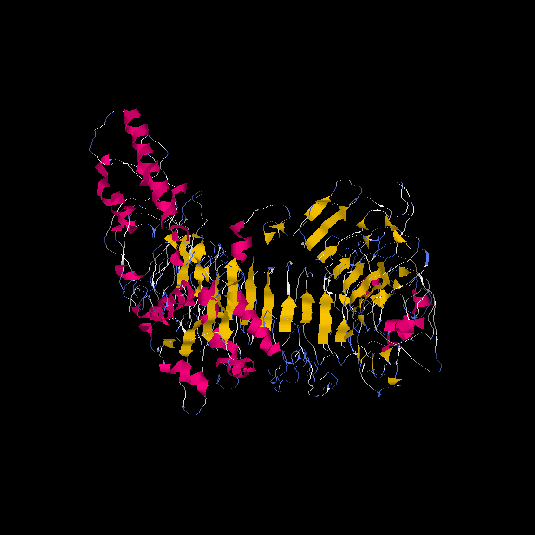

Supplement: Supplementary file 31 — Supplementary Information 31. [file 41598_2020_78347_MOESM31_ESM.zip › T9/struct/S514930_results/model1.gif]

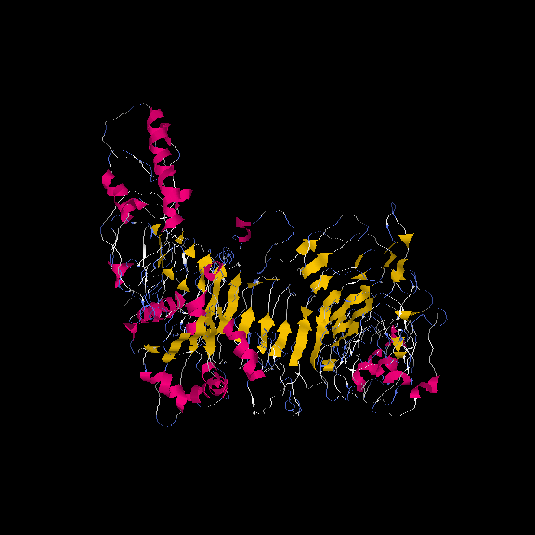

Supplement: Supplementary file 31 — Supplementary Information 31. [file 41598_2020_78347_MOESM31_ESM.zip › T9/struct/S514930_results/model2.gif]

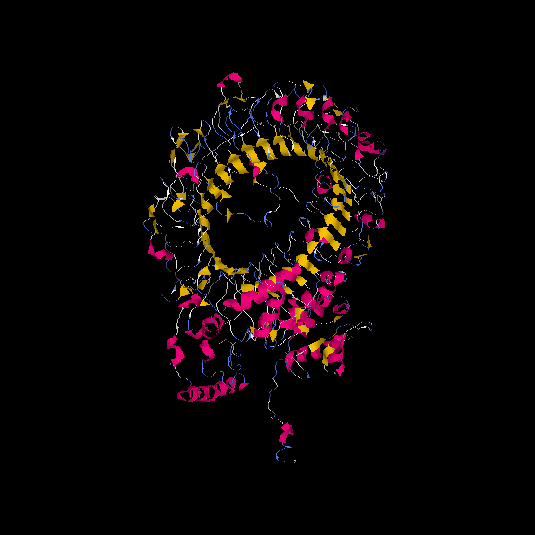

Supplement: Supplementary file 31 — Supplementary Information 31. [file 41598_2020_78347_MOESM31_ESM.zip › T9/struct/S514930_results/model3.gif]

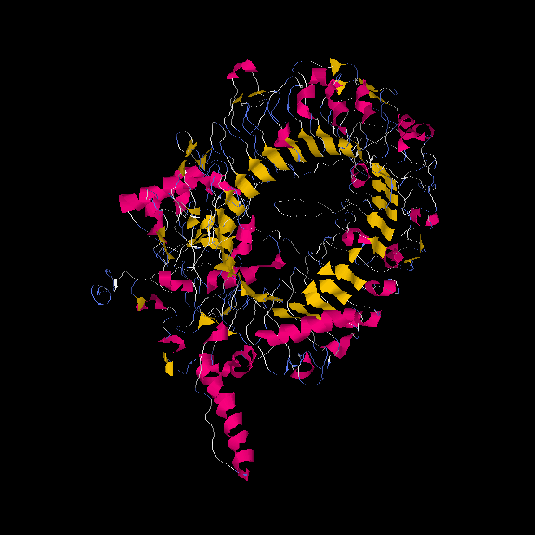

Supplement: Supplementary file 31 — Supplementary Information 31. [file 41598_2020_78347_MOESM31_ESM.zip › T9/struct/S514930_results/model4.gif]

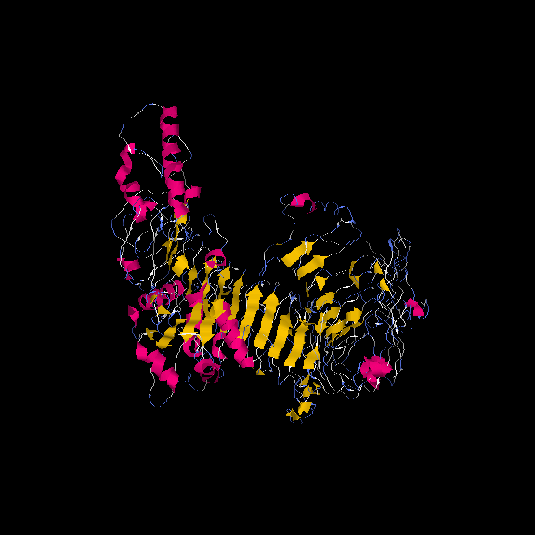

Supplement: Supplementary file 31 — Supplementary Information 31. [file 41598_2020_78347_MOESM31_ESM.zip › T9/struct/S514930_results/model5.gif]

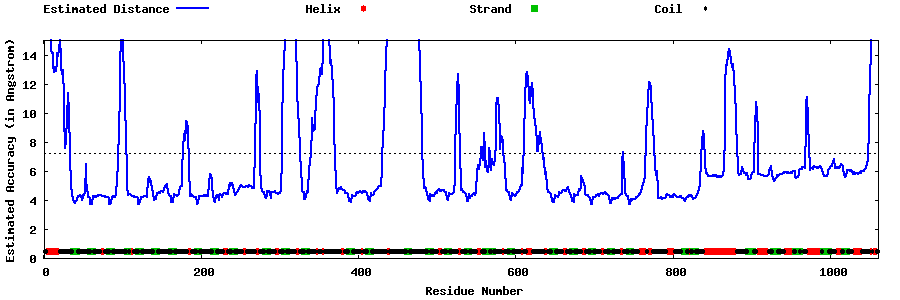

Supplement: Supplementary file 31 — Supplementary Information 31. [file 41598_2020_78347_MOESM31_ESM.zip › T9/struct/S514930_results/RSQ_1.png]

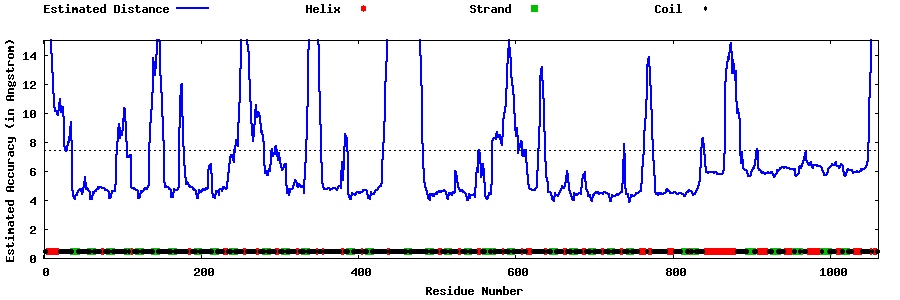

Supplement: Supplementary file 31 — Supplementary Information 31. [file 41598_2020_78347_MOESM31_ESM.zip › T9/struct/S514930_results/RSQ_2.png]

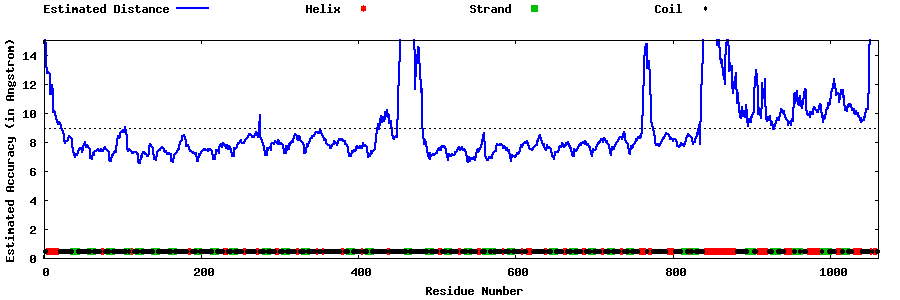

Supplement: Supplementary file 31 — Supplementary Information 31. [file 41598_2020_78347_MOESM31_ESM.zip › T9/struct/S514930_results/RSQ_3.png]

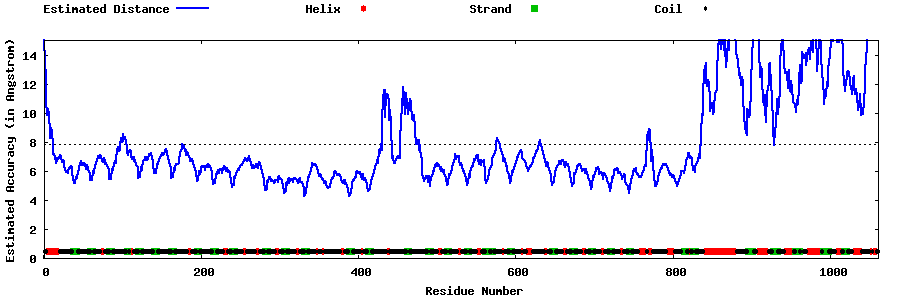

Supplement: Supplementary file 31 — Supplementary Information 31. [file 41598_2020_78347_MOESM31_ESM.zip › T9/struct/S514930_results/RSQ_4.png]

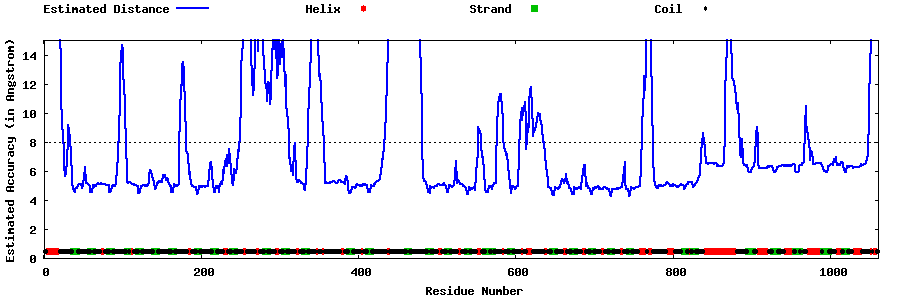

Supplement: Supplementary file 31 — Supplementary Information 31. [file 41598_2020_78347_MOESM31_ESM.zip › T9/struct/S514930_results/RSQ_5.png]

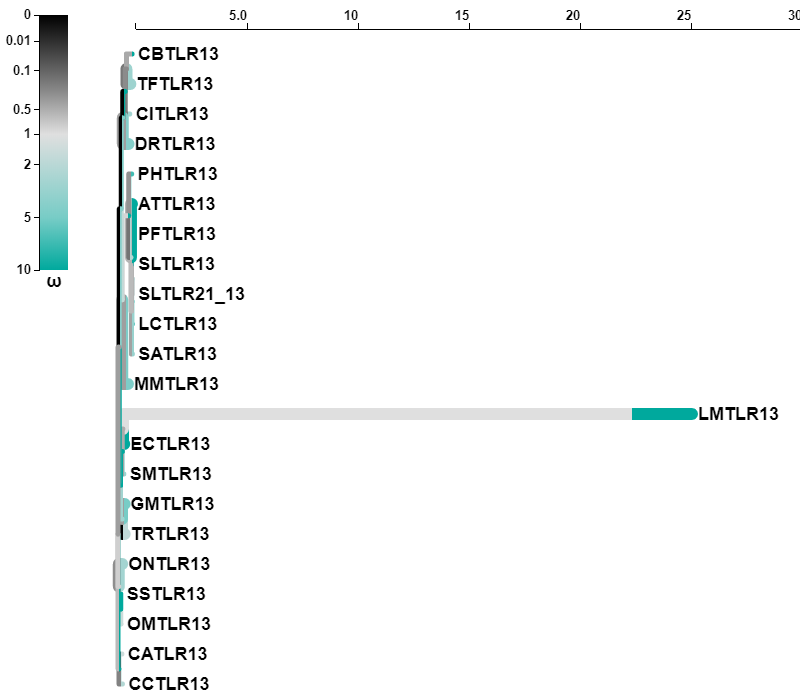

Supplement: Supplementary file 32 — Supplementary Information 32. [file 41598_2020_78347_MOESM32_ESM.zip › T13/absrel/tree.png]

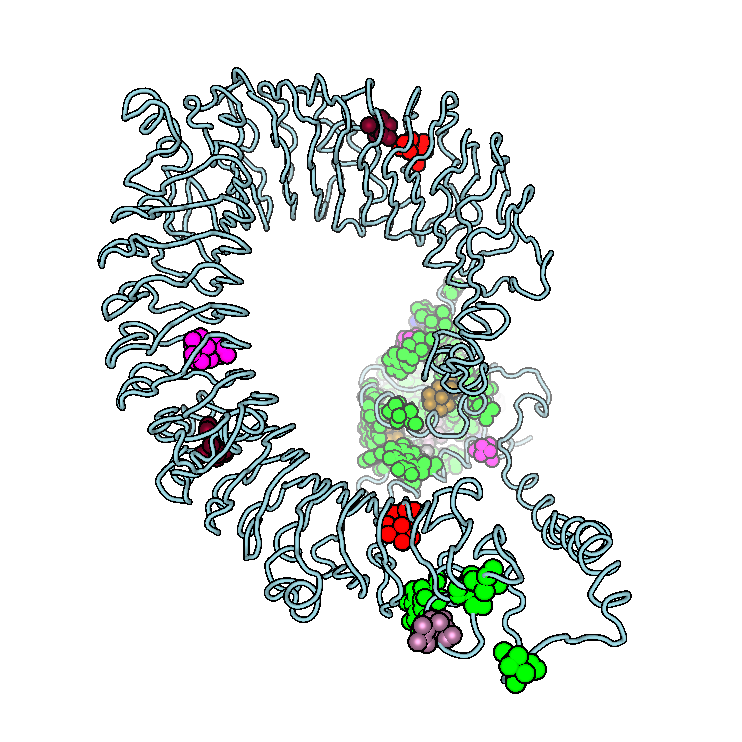

Supplement: Supplementary file 32 — Supplementary Information 32. [file 41598_2020_78347_MOESM32_ESM.zip › T13/BIS2/download (1).png]

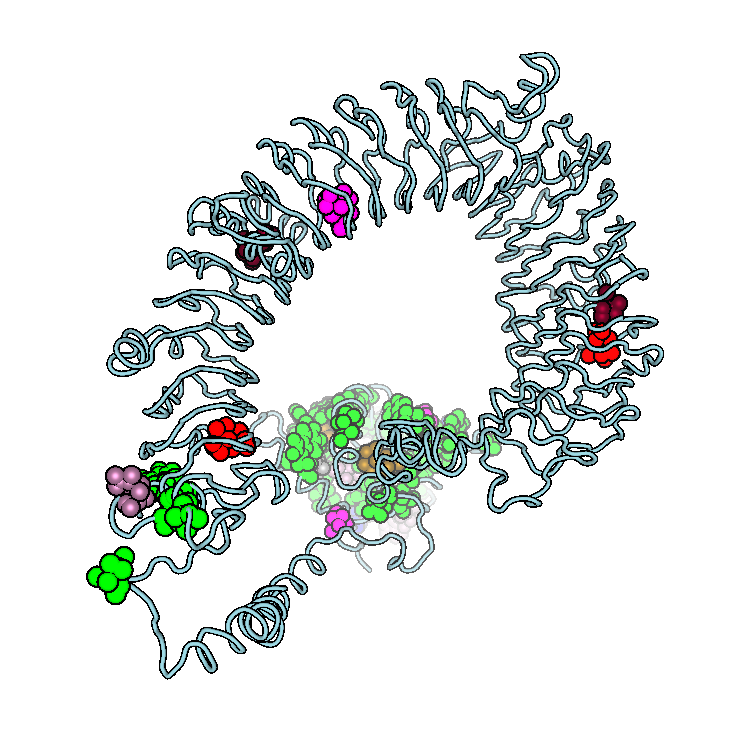

Supplement: Supplementary file 32 — Supplementary Information 32. [file 41598_2020_78347_MOESM32_ESM.zip › T13/BIS2/download (2).png]

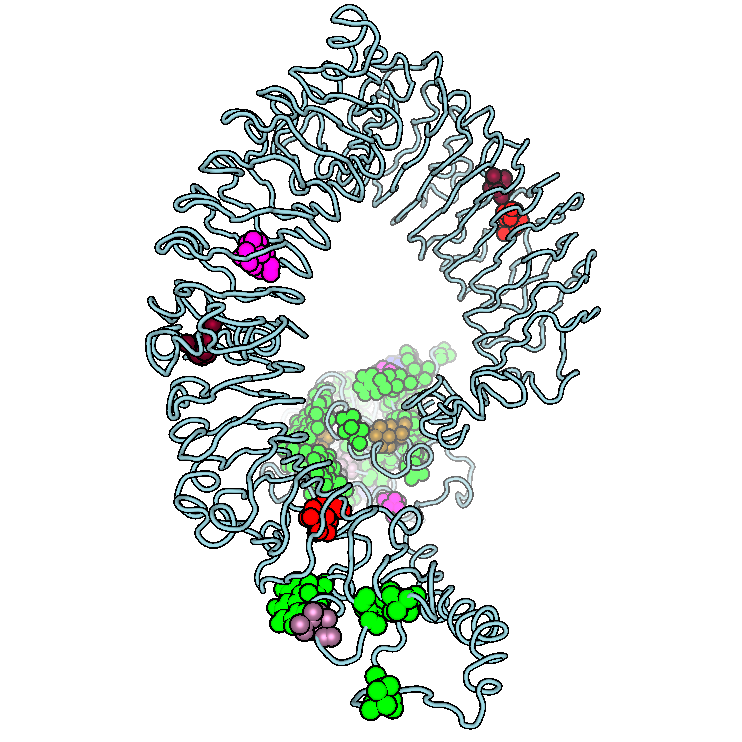

Supplement: Supplementary file 32 — Supplementary Information 32. [file 41598_2020_78347_MOESM32_ESM.zip › T13/BIS2/download.png]

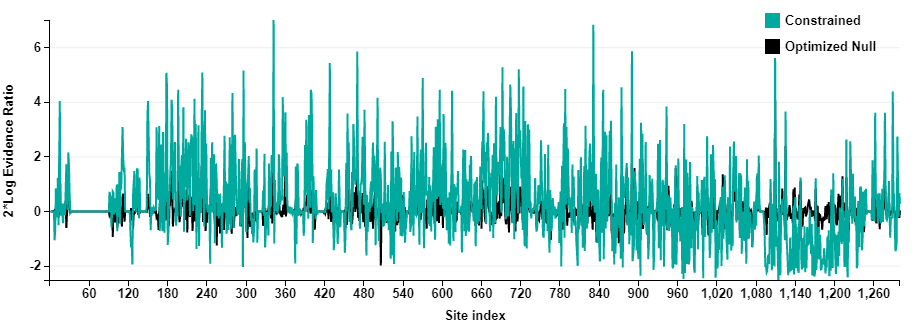

Supplement: Supplementary file 32 — Supplementary Information 32. [file 41598_2020_78347_MOESM32_ESM.zip › T13/busted/busted-chart (1).png]

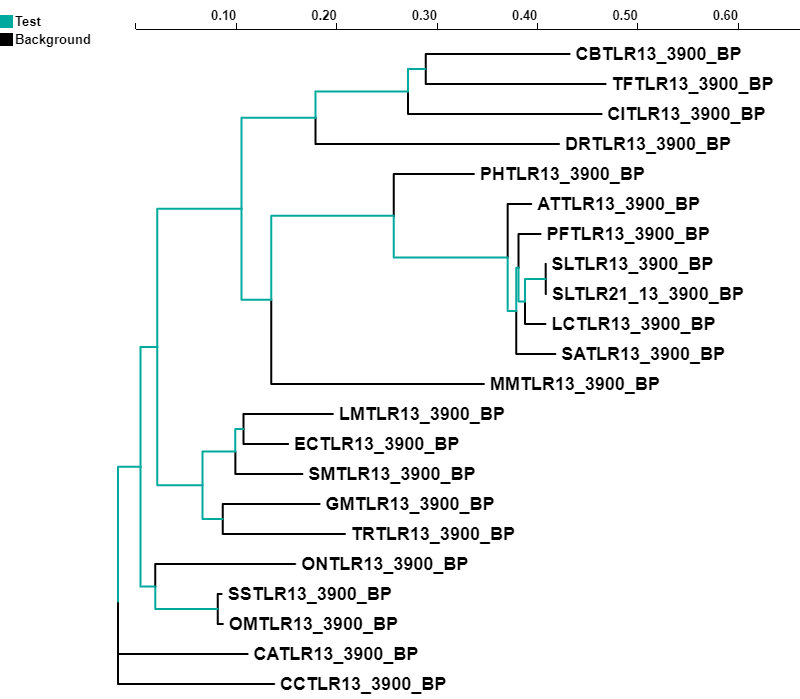

Supplement: Supplementary file 32 — Supplementary Information 32. [file 41598_2020_78347_MOESM32_ESM.zip › T13/busted/tree.png]

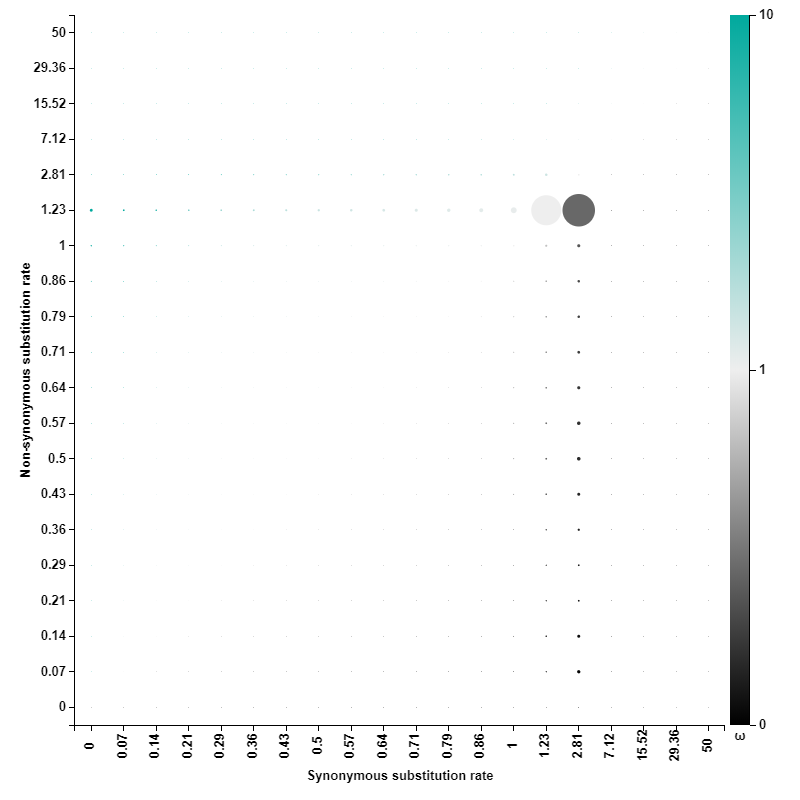

Supplement: Supplementary file 32 — Supplementary Information 32. [file 41598_2020_78347_MOESM32_ESM.zip › T13/fubar/datamonkey-chart.png]

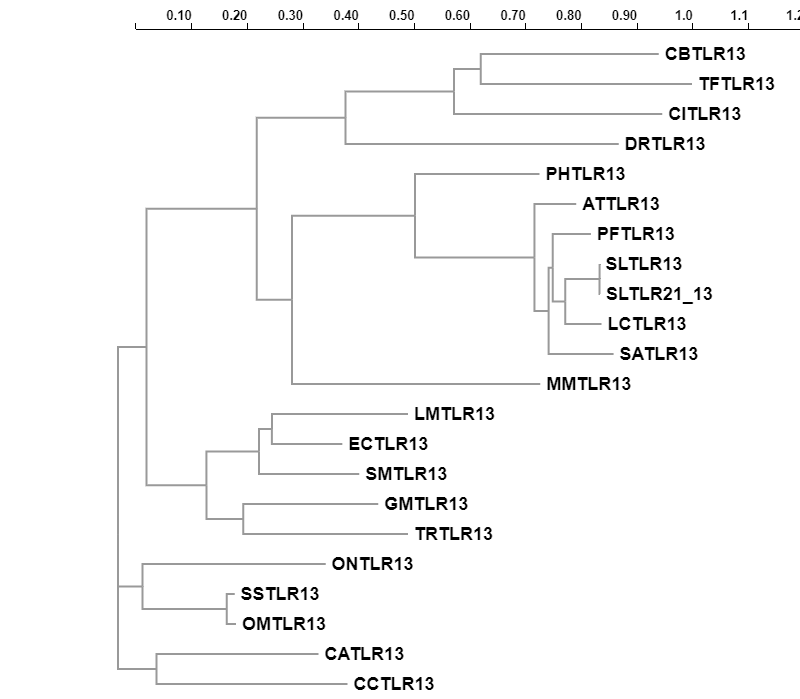

Supplement: Supplementary file 32 — Supplementary Information 32. [file 41598_2020_78347_MOESM32_ESM.zip › T13/fubar/tree.png]

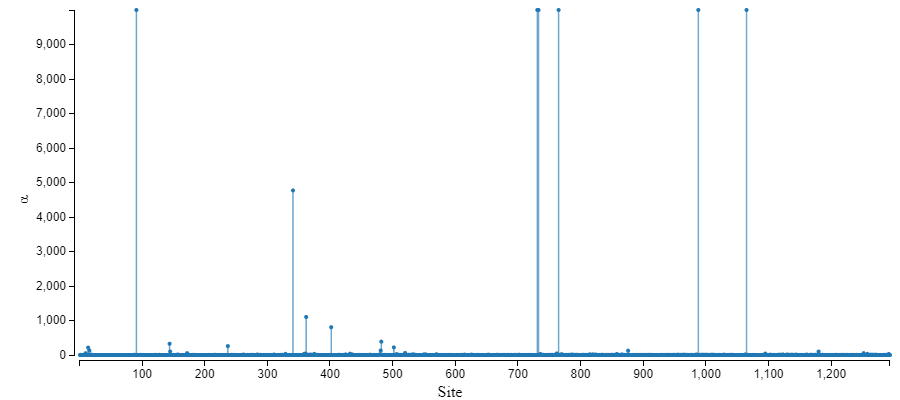

Supplement: Supplementary file 32 — Supplementary Information 32. [file 41598_2020_78347_MOESM32_ESM.zip › T13/meme/datamonkey-chart.png]

Tree scale: 0.1

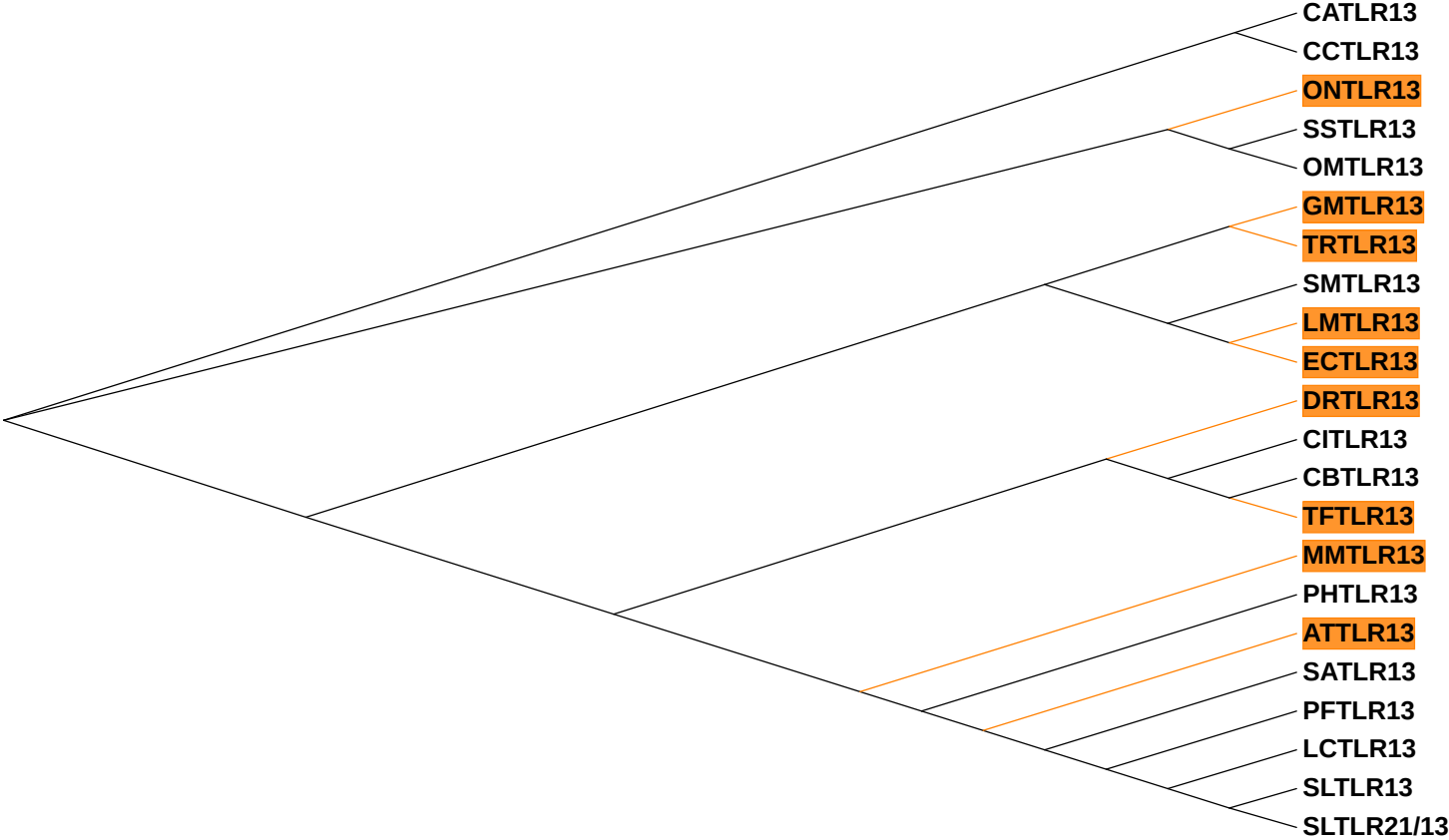

Supplement: Supplementary file 32 — Supplementary Information 32. [file 41598_2020_78347_MOESM32_ESM.zip › T13/meme/labelledtree.pdf]

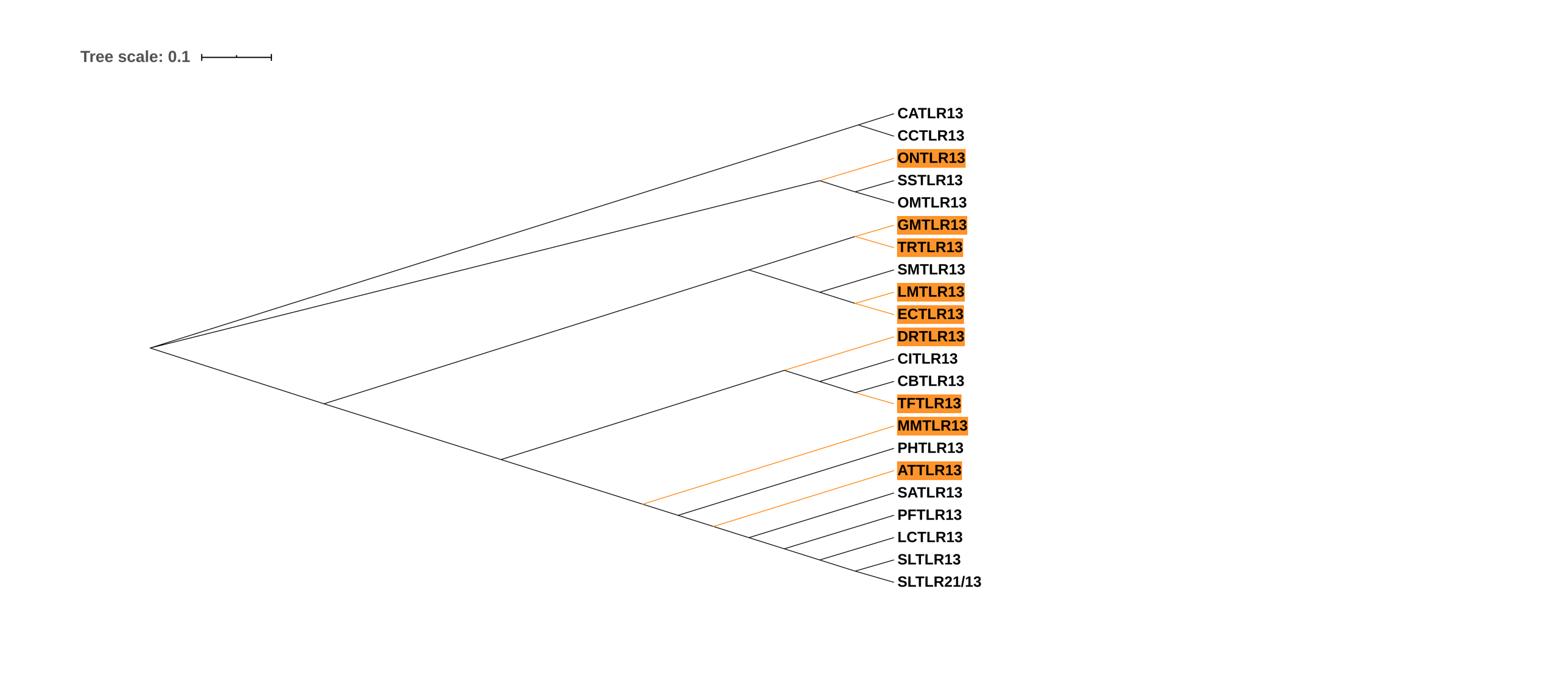

Supplement: Supplementary file 32 — Supplementary Information 32. [file 41598_2020_78347_MOESM32_ESM.zip › T13/meme/labelledtree.png]

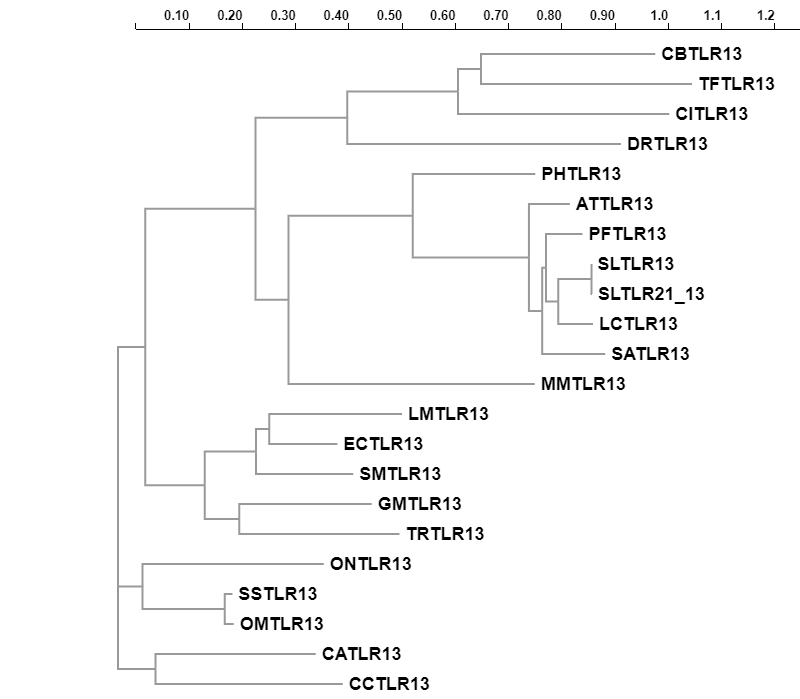

Supplement: Supplementary file 32 — Supplementary Information 32. [file 41598_2020_78347_MOESM32_ESM.zip › T13/meme/tree.png]

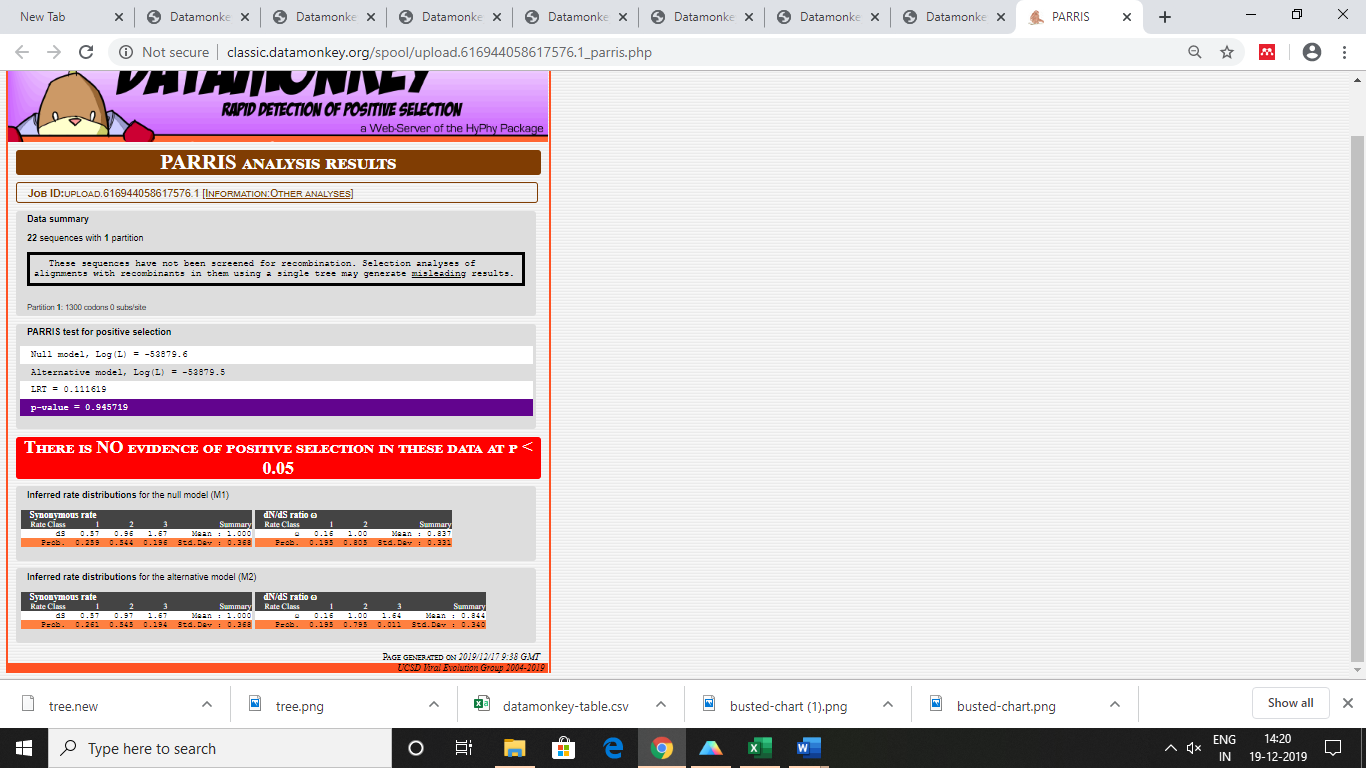

Supplement: Supplementary file 32 — Supplementary Information 32. [file 41598_2020_78347_MOESM32_ESM.zip › T13/parris.docx]

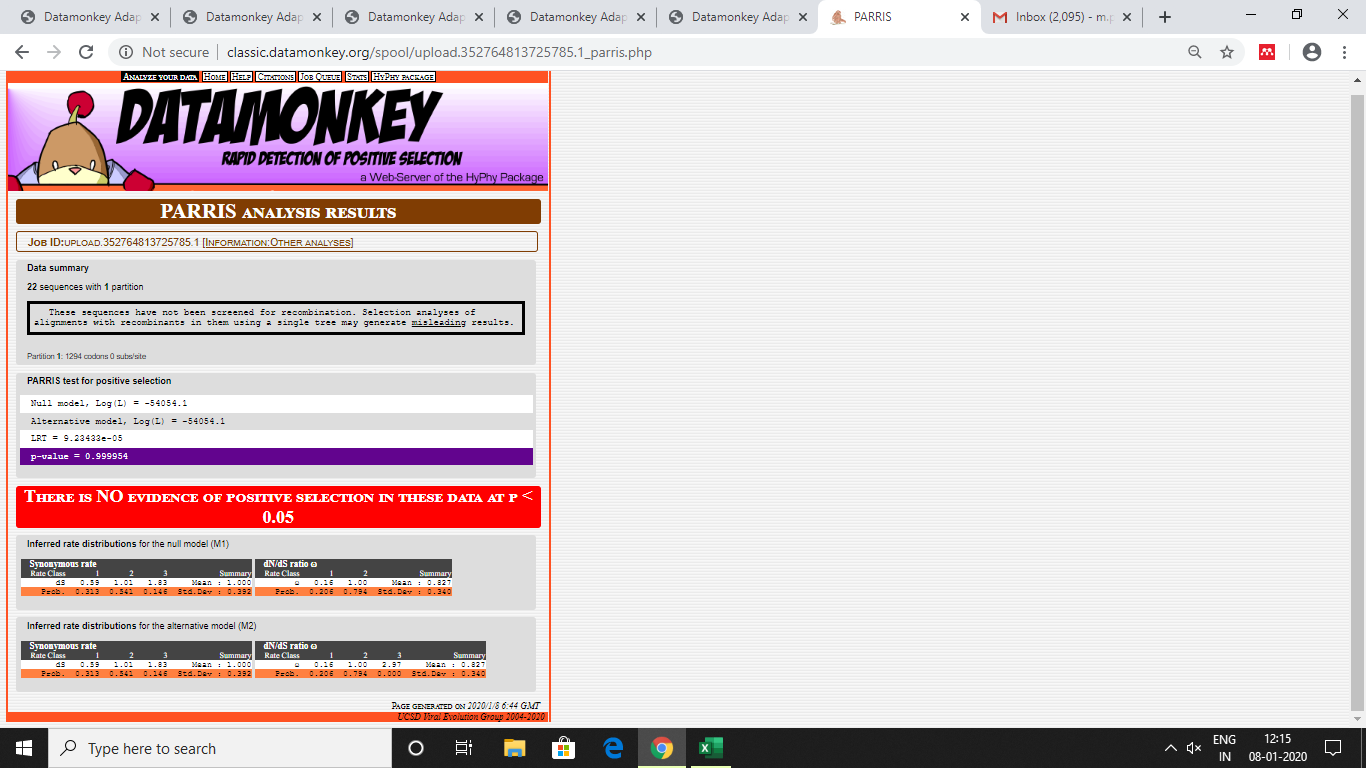

Supplement: Supplementary file 32 — Supplementary Information 32. [file 41598_2020_78347_MOESM32_ESM.zip › T13/parris/parris.png]

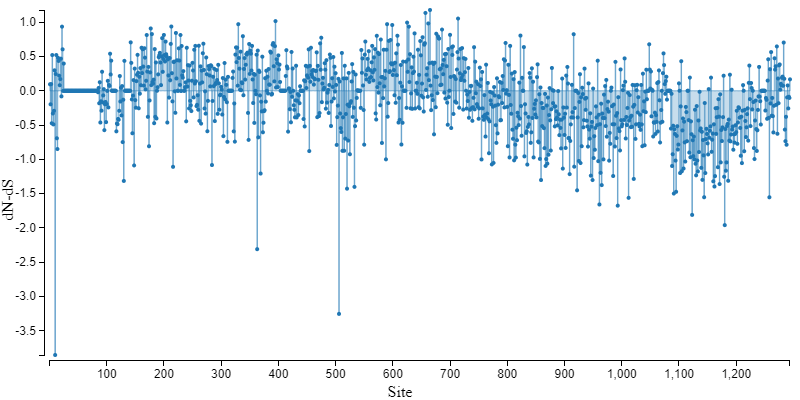

Supplement: Supplementary file 32 — Supplementary Information 32. [file 41598_2020_78347_MOESM32_ESM.zip › T13/slac/datamonkey-chart.png]

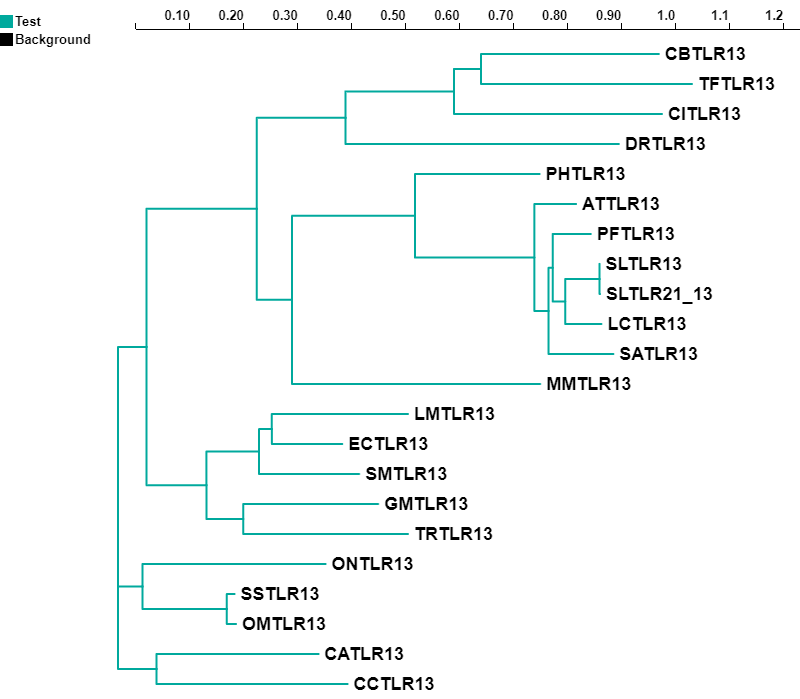

Supplement: Supplementary file 32 — Supplementary Information 32. [file 41598_2020_78347_MOESM32_ESM.zip › T13/slac/tree.png]

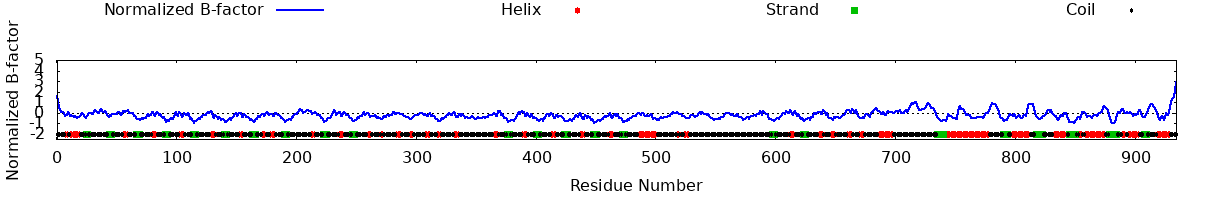

Supplement: Supplementary file 32 — Supplementary Information 32. [file 41598_2020_78347_MOESM32_ESM.zip › T13/struct/S509363_results/BFP.png]

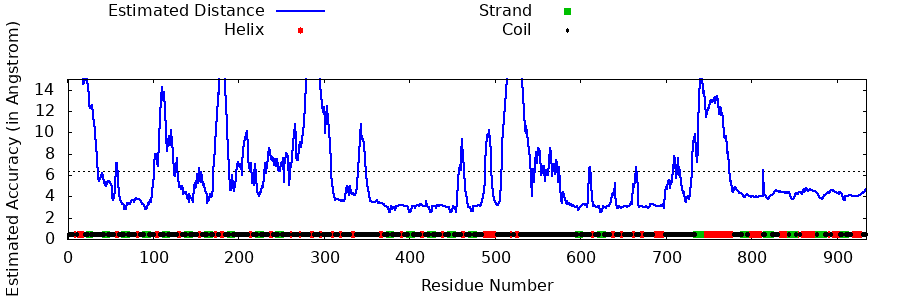

Supplement: Supplementary file 32 — Supplementary Information 32. [file 41598_2020_78347_MOESM32_ESM.zip › T13/struct/S509363_results/RSQ_1.png]

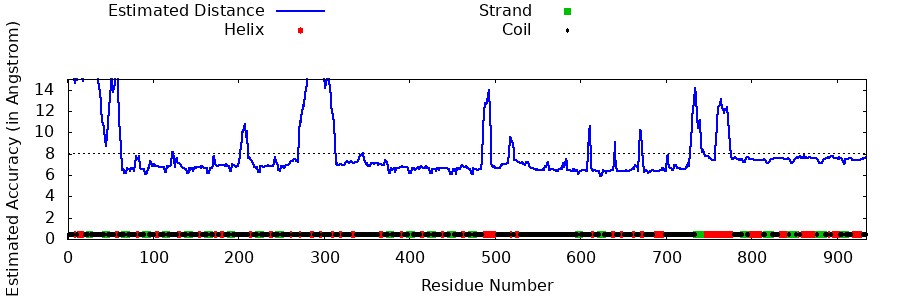

Supplement: Supplementary file 32 — Supplementary Information 32. [file 41598_2020_78347_MOESM32_ESM.zip › T13/struct/S509363_results/RSQ_2.png]

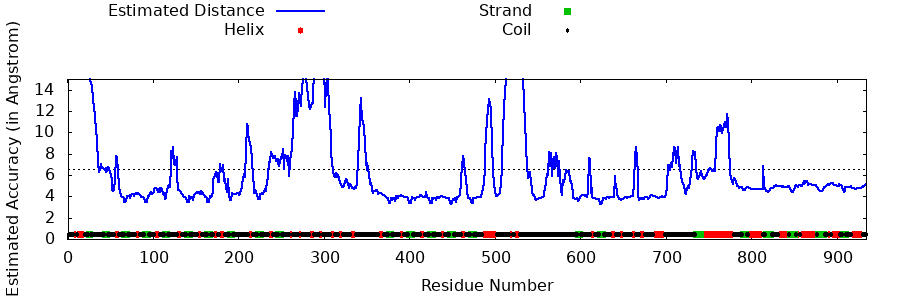

Supplement: Supplementary file 32 — Supplementary Information 32. [file 41598_2020_78347_MOESM32_ESM.zip › T13/struct/S509363_results/RSQ_3.png]

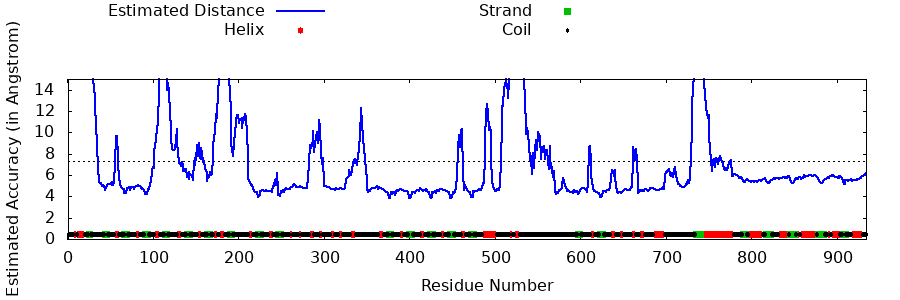

Supplement: Supplementary file 32 — Supplementary Information 32. [file 41598_2020_78347_MOESM32_ESM.zip › T13/struct/S509363_results/RSQ_4.png]

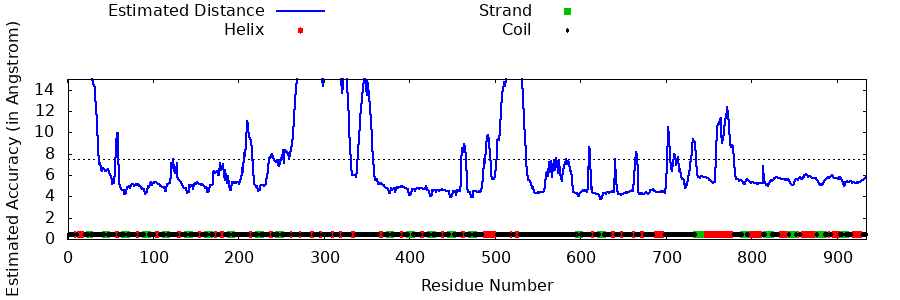

Supplement: Supplementary file 32 — Supplementary Information 32. [file 41598_2020_78347_MOESM32_ESM.zip › T13/struct/S509363_results/RSQ_5.png]

Tree scale: 0.01

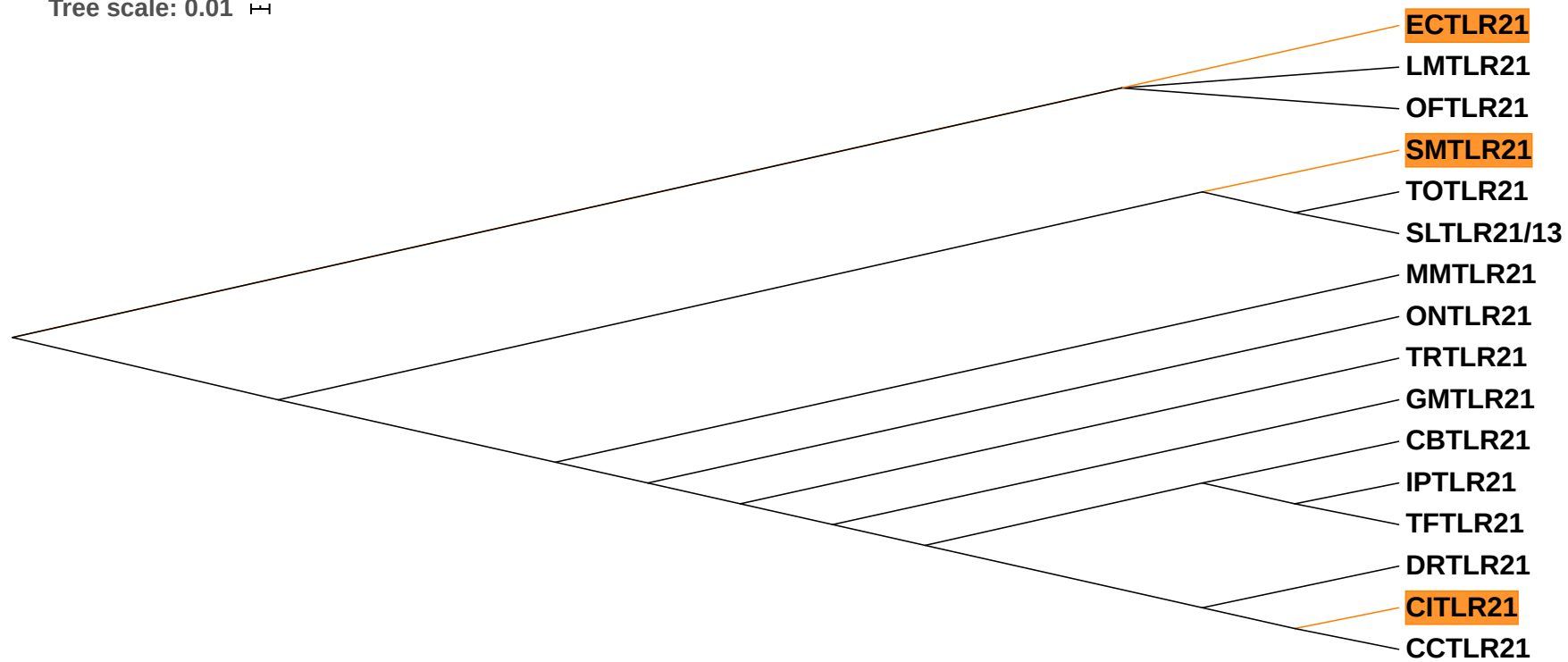

Supplement: Supplementary file 33 — Supplementary Information 33. [file 41598_2020_78347_MOESM33_ESM.zip › T21/absrel/labelledtree.pdf]

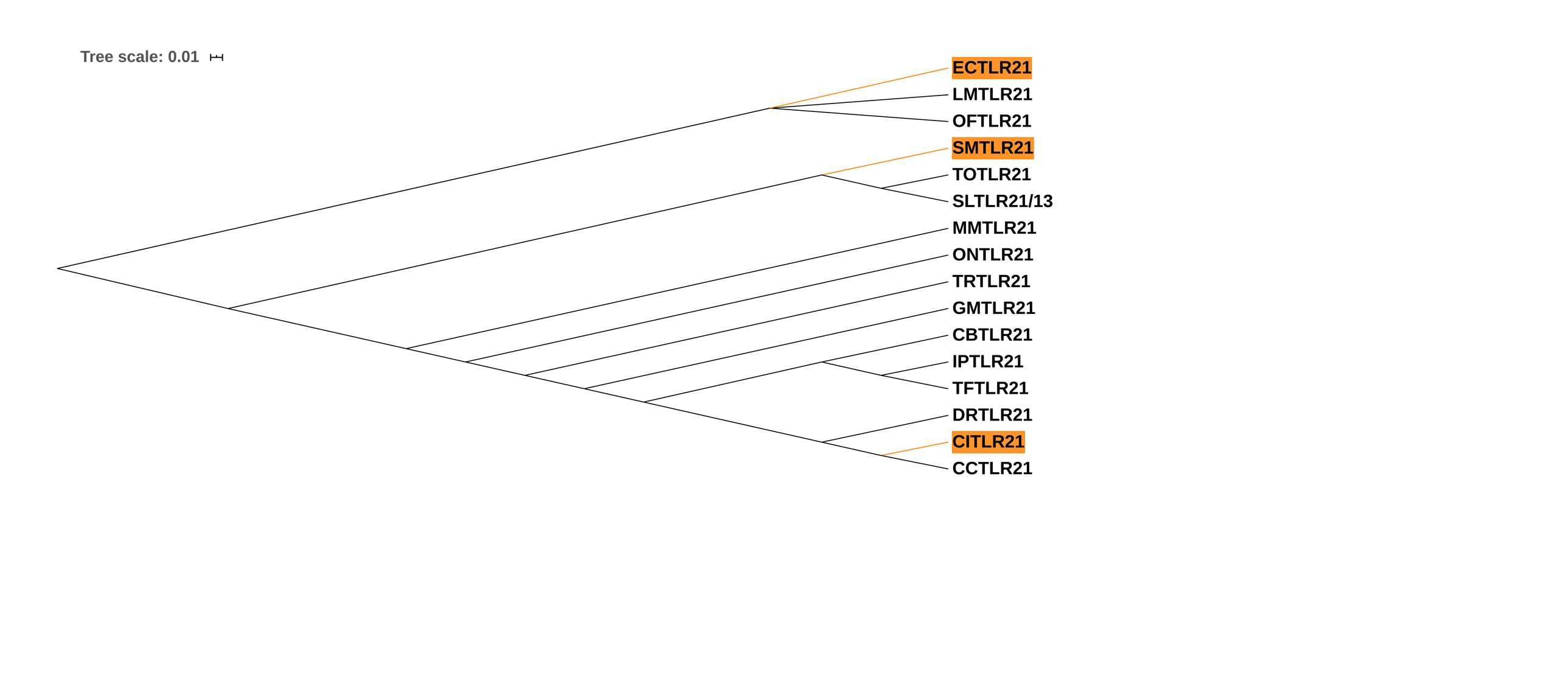

Supplement: Supplementary file 33 — Supplementary Information 33. [file 41598_2020_78347_MOESM33_ESM.zip › T21/absrel/labelledtree.png]

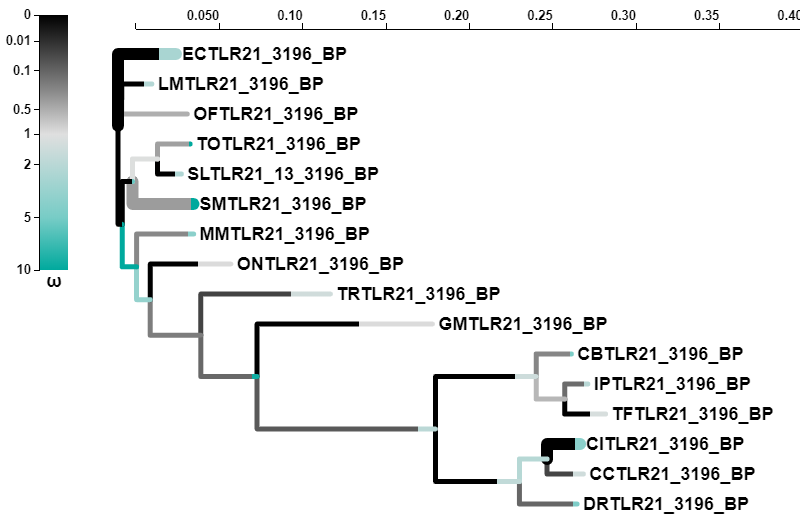

Supplement: Supplementary file 33 — Supplementary Information 33. [file 41598_2020_78347_MOESM33_ESM.zip › T21/absrel/tree.png]

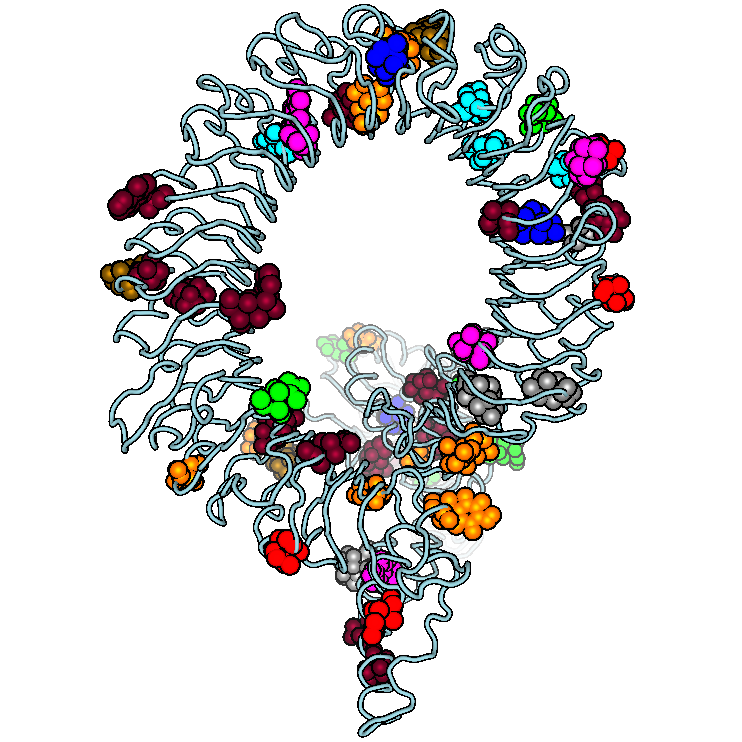

Supplement: Supplementary file 33 — Supplementary Information 33. [file 41598_2020_78347_MOESM33_ESM.zip › T21/bis2/download (1).png]

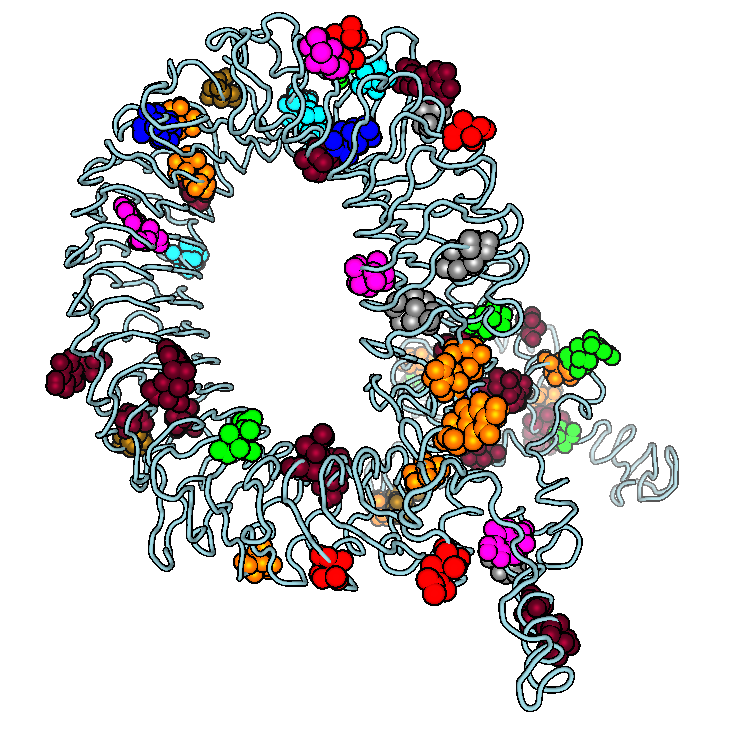

Supplement: Supplementary file 33 — Supplementary Information 33. [file 41598_2020_78347_MOESM33_ESM.zip › T21/bis2/download.png]

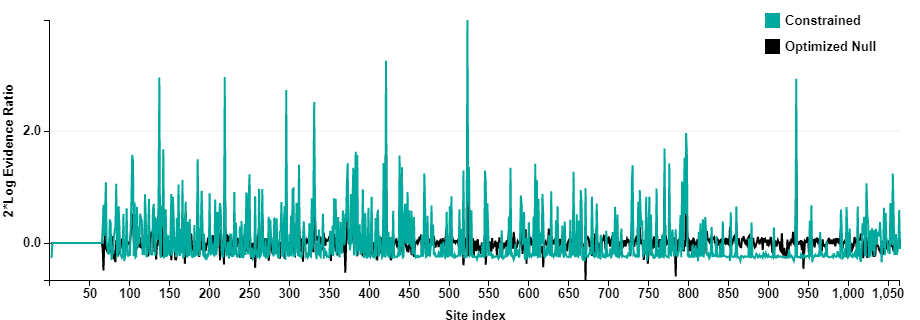

Supplement: Supplementary file 33 — Supplementary Information 33. [file 41598_2020_78347_MOESM33_ESM.zip › T21/busted/busted-chart (1).png]

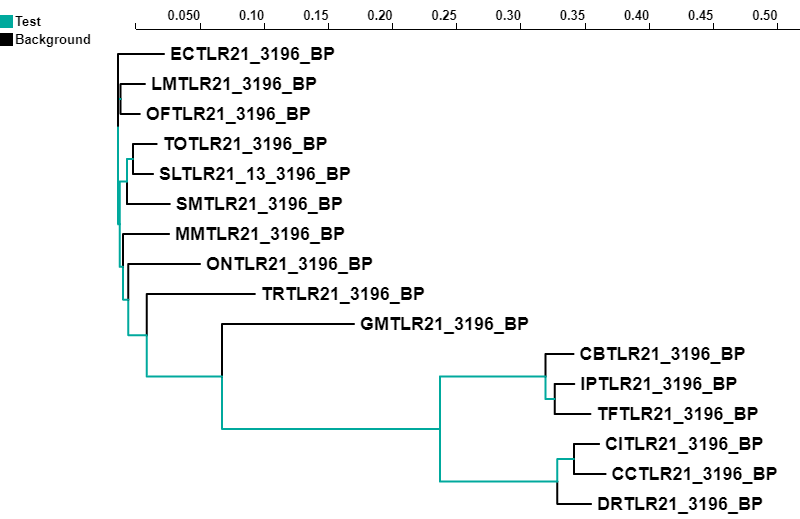

Supplement: Supplementary file 33 — Supplementary Information 33. [file 41598_2020_78347_MOESM33_ESM.zip › T21/busted/tree.png]

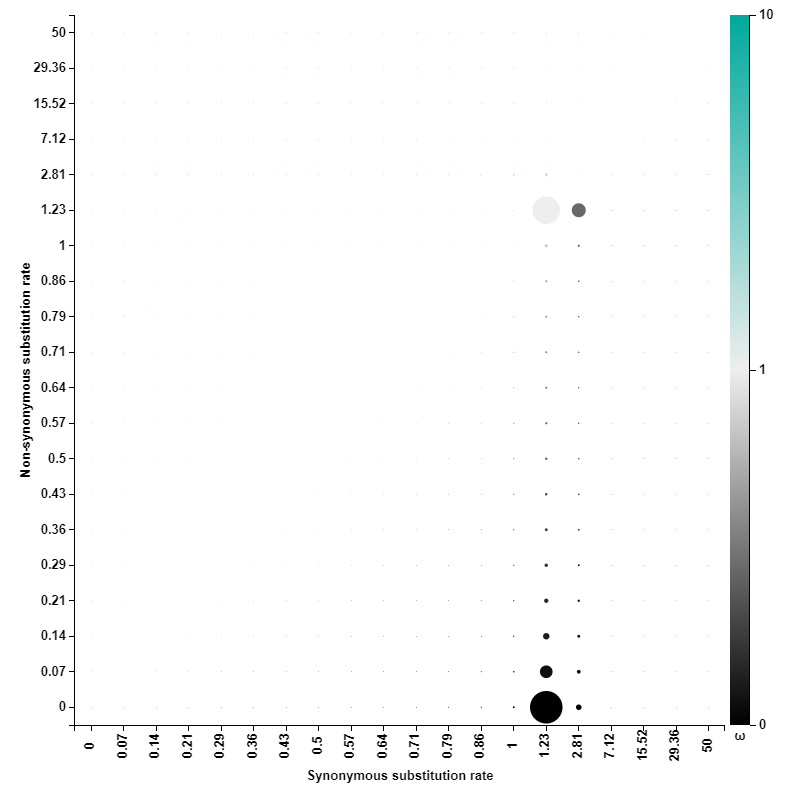

Supplement: Supplementary file 33 — Supplementary Information 33. [file 41598_2020_78347_MOESM33_ESM.zip › T21/fubar/datamonkey-chart.png]

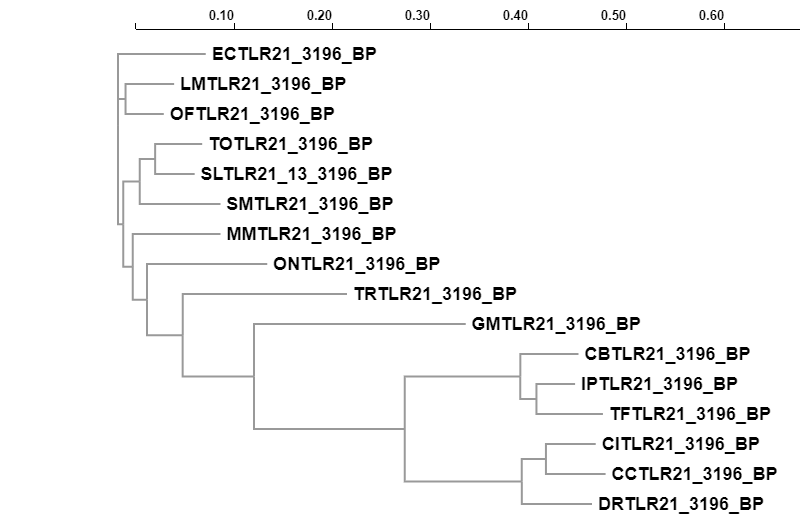

Supplement: Supplementary file 33 — Supplementary Information 33. [file 41598_2020_78347_MOESM33_ESM.zip › T21/fubar/tree.png]

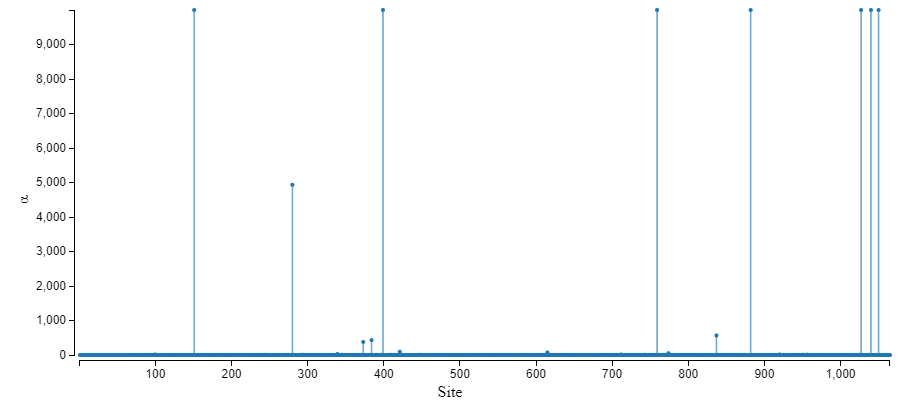

Supplement: Supplementary file 33 — Supplementary Information 33. [file 41598_2020_78347_MOESM33_ESM.zip › T21/meme/datamonkey-chart.png]

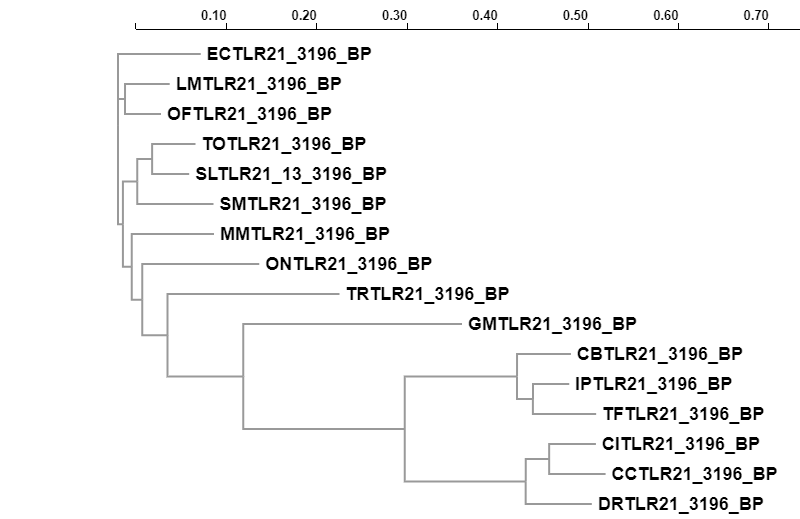

Supplement: Supplementary file 33 — Supplementary Information 33. [file 41598_2020_78347_MOESM33_ESM.zip › T21/meme/tree.png]

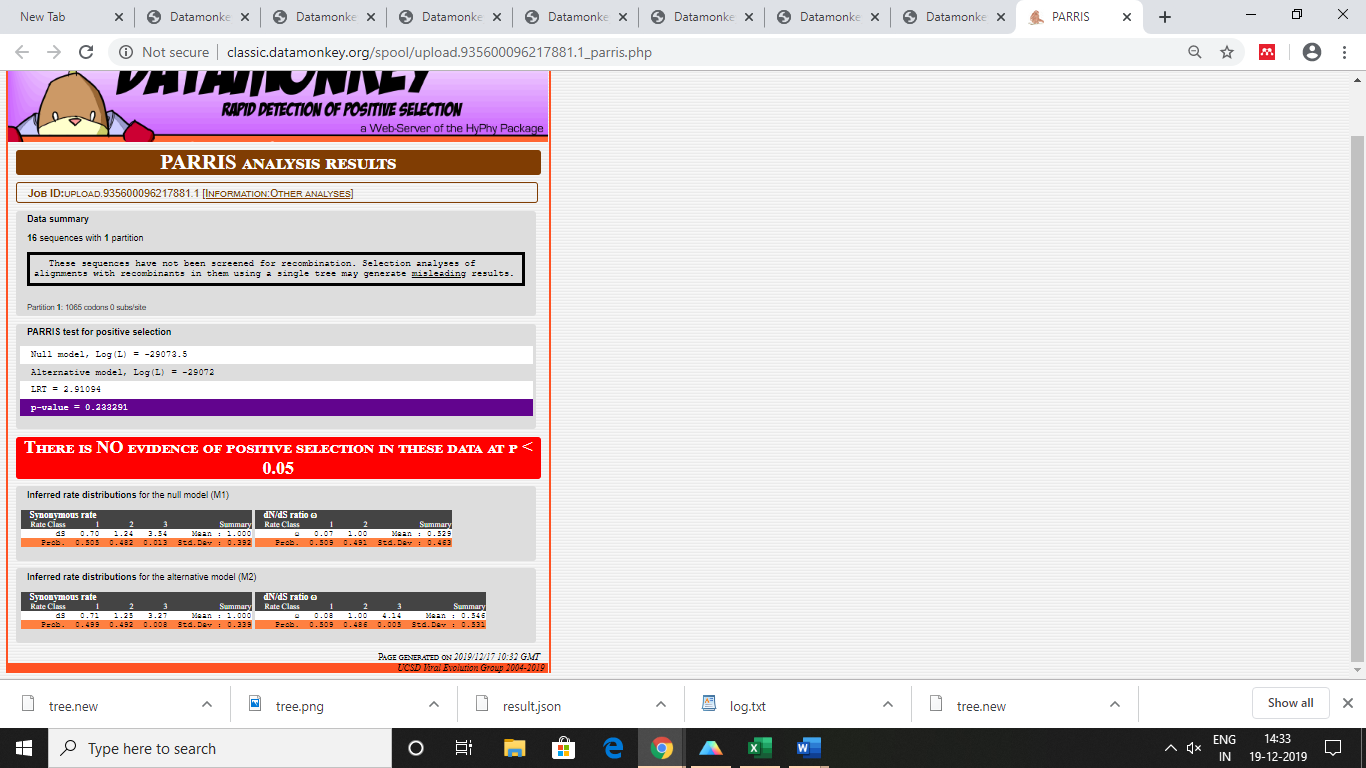

Supplement: Supplementary file 33 — Supplementary Information 33. [file 41598_2020_78347_MOESM33_ESM.zip › T21/parris.docx]

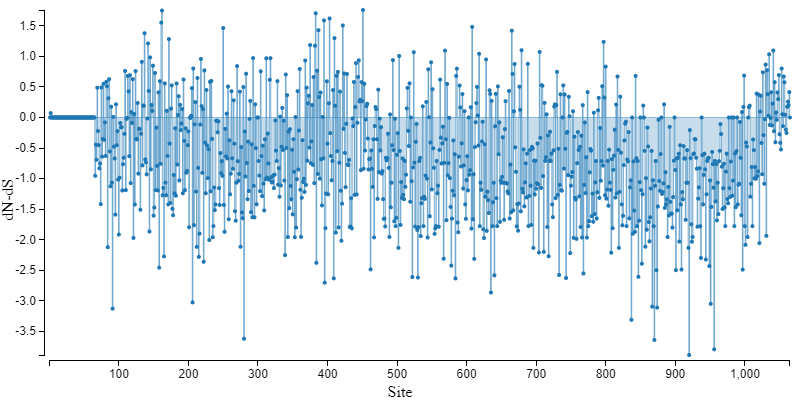

Supplement: Supplementary file 33 — Supplementary Information 33. [file 41598_2020_78347_MOESM33_ESM.zip › T21/slac/datamonkey-chart.png]

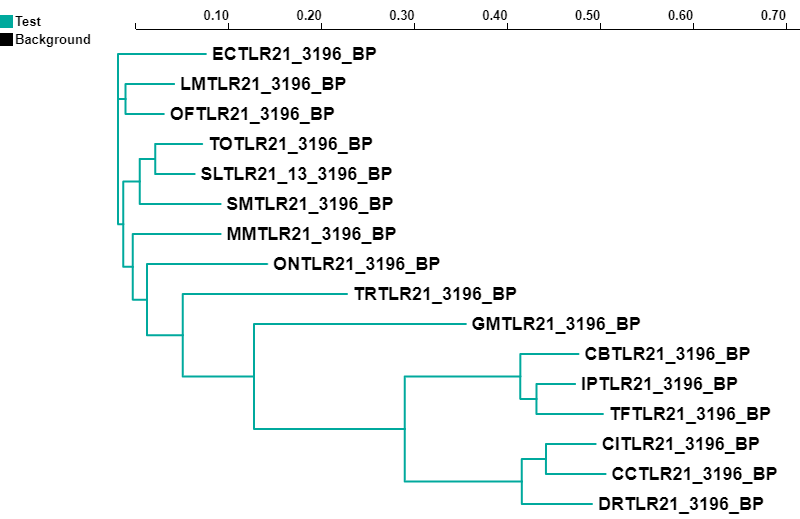

Supplement: Supplementary file 33 — Supplementary Information 33. [file 41598_2020_78347_MOESM33_ESM.zip › T21/slac/tree.png]
