# Supplementary material for: Variation in selection constraints on teleost TLRs with emphasis on their repertoire in the Walking catfish, Clarias batrachus
Source: Sci Rep. 2020 Dec 7;10:21394. doi: 10.1038/s41598-020-78347-6 (PMC7721727; doi:10.1038/s41598-020-78347-6)
Supplement: Supplementary file 30 — Supplementary Information 30. [file 41598_2020_78347_MOESM30_ESM.zip › T8/bis2/summary/PF00000-NONREDUNDANT-5DD-dim0-table.html]

BIS cluster table


Clusters with env. score >= 0.5 and sym. score >= 0.5 :

| Dim | Cluster | Sym | Env | Pvalue | Hit patterns and blocks |
| --- | --- | --- | --- | --- | --- |
| 0 | 3 | 1 | 1 | 1.675311e-07 | Hit patterns:   |  |  |  | | --- | --- | --- | | Positions: | 532 | 558 | | 15 sequences: | F | L | | 4 sequences: | L | P | | 3 sequences: | H | F |  All positions in cluster: 532 558-560 |
| 0 | 8 | 1 | 1 | 3.797372e-06 | Hit patterns:   |  |  |  |  | | --- | --- | --- | --- | | Positions: | 98 | 116 | 119 | | 17 sequences: | S | L | L | | 3 sequences: | - | - | - | | 2 sequences: | A | V | I |  All positions in cluster: 98 116 119 |
| 0 | 9 | 1 | 1 | 0.0001367054 | Hit patterns:   |  |  |  |  |  | | --- | --- | --- | --- | --- | | Positions: | 619 | 907 | 925 | 1053 | | 18 sequences: | N | K | V | A | | 4 sequences: | Y | T | I | S |  All positions in cluster: 619-620 907 924-925 1053-1054 |
| 0 | 5 | 1 | 1 | 0.0006493506 | Hit patterns:   |  |  |  |  |  |  |  |  |  |  |  | | --- | --- | --- | --- | --- | --- | --- | --- | --- | --- | --- | | Positions: | 203 | 216 | 227 | 230 | 232 | 235 | 241 | 244 | 245 | 253 | | 19 sequences: | N | I | L | L | L | N | P | L | P | L | | 3 sequences: | - | - | - | - | - | - | - | - | - | - |  All positions in cluster: 203 216 227 230 232 235 241 244-245 253 |
| 0 | 7 | 1 | 1 | 0.0006493506 | Hit patterns:   |  |  |  |  |  |  |  |  |  |  | | --- | --- | --- | --- | --- | --- | --- | --- | --- | --- | | Positions: | 76 | 81 | 85 | 91 | 93 | 100 | 102 | 121 | 143 | | 19 sequences: | C | L | P | N | T | N | I | L | F | | 3 sequences: | - | - | - | - | - | - | - | - | - |  All positions in cluster: 76 81 85 91 93 100 102 121 143 |
| 0 | 10 | 1 | 1 | 0.0006493506 | Hit patterns:   |  |  |  |  |  |  |  |  |  |  |  |  | | --- | --- | --- | --- | --- | --- | --- | --- | --- | --- | --- | --- | | Positions: | 554 | 566 | 580 | 628 | 832 | 926 | 991 | 1005 | 1016 | 1030 | 1031 | | 19 sequences: | F | L | D | M | I | T | Y | H | I | H | F | | 3 sequences: | I | F | Y | L | L | M | F | Q | M | R | I |  All positions in cluster: 554 566-567 580 626-629 832-833 926-929 991 1005-1008 1016-1017 1030-1031 |
| 0 | 2 | 1 | 1 | 0.004329004 | Hit patterns:   |  |  |  |  |  |  |  | | --- | --- | --- | --- | --- | --- | --- | | Positions: | 463 | 581 | 587 | 695 | 1027 | 1037 | | 20 sequences: | E | L | F | Y | Q | R | | 2 sequences: | D | M | L | F | R | C |  All positions in cluster: 463-464 581 586-587 695 1025-1027 1037-1039 |
| 0 | 4 | 1 | 1 | 0.004329004 | Hit patterns:   |  |  |  |  |  |  |  |  |  |  |  |  |  |  |  |  |  |  | | --- | --- | --- | --- | --- | --- | --- | --- | --- | --- | --- | --- | --- | --- | --- | --- | --- | --- | | Positions: | 303 | 308 | 311 | 314 | 317 | 321 | 322 | 323 | 331 | 338 | 341 | 343 | 346 | 348 | 372 | 374 | 376 | | 20 sequences: | I | F | L | L | L | G | N | S | W | L | L | L | N | L | D | S | N | | 2 sequences: | - | - | - | - | - | - | - | - | - | - | - | - | - | - | - | - | - |  All positions in cluster: 303 308 311 314 317 321-323 331 338 341 343 346 348 372 374 376 |
| 0 | 6 | 1 | 1 | 0.004329004 | Hit patterns:   |  |  |  |  |  |  |  |  | | --- | --- | --- | --- | --- | --- | --- | --- | | Positions: | 149 | 152 | 154 | 163 | 167 | 178 | 256 | | 20 sequences: | L | L | L | P | P | N | N | | 2 sequences: | - | - | - | - | - | - | - |  All positions in cluster: 149 152 154 163 167 178 256 |
| 0 | 1 | 1 | 1 | 1 | All positions in cluster: 464 520 523 528-529 546-550 552 559-560 562-563 567 569 571-575 577 586 590 593 596-597 599 601 603-605 609 616 620 623 626-627 629 631 633 636 643 645 648-649 651 654 656 660 671 676 680-681 683 685 699 710 712 717 731 734 751 754 756 759 761 778 783 806 809-810 813 815 820 823 833 835 841 861 867 889 891-894 897 904 924 927-929 938 942 946-947 957-958 961-963 966 973 976 980 982-983 985-988 995 1000 1006-1008 1014 1017 1021 1023 1025-1026 1034-1035 1038-1039 1042 1046-1047 1054 1057-1059 1067 |

Table created with bis2html version 8.
