# Supplementary material for: Variation in selection constraints on teleost TLRs with emphasis on their repertoire in the Walking catfish, Clarias batrachus
Source: Sci Rep. 2020 Dec 7;10:21394. doi: 10.1038/s41598-020-78347-6 (PMC7721727; doi:10.1038/s41598-020-78347-6)
Supplement: Supplementary file 30 — Supplementary Information 30. [file 41598_2020_78347_MOESM30_ESM.zip › T8/bis2/summary/PF00000-NONREDUNDANT-5DD-dim1.html]

Alignment and BIS clusters


|  |  |  |  |  |  |  |  |  |  |  |  |  |  |  |  |  |  |  |  |  |  |  |  |  |  |  |  |  |  |  |  |  |  |  |  |  |  |  |  |  |  |  |  |  |  |  |  |  |  |  |  |  |  |  |  |  |  |  |  |  |  |  |  |  |  |  |  |  |  |  |  |  |  |  |  |  |  |  |  |  |  |  |  |  |  |  |  |  |  |  |  |  |  |  |  |  |  |  |  |  |  |  |  |  |  |  |  |  |  |  |  |  |  |  |  |  |  |  |  |  |  |  |  |  |  |  |  |  |  |  |  |  |  |  |  |  |  |  |  |  |  |  |  |  |  |  |  |  |  |  |  |  |  |  |  |  |  |  |  |  |  |  |  |  |  |  |  |  |  |  |  |  |  |  |  |  |  |  |  |  |  |  |  |  |  |  |  |  |  |  |  |  |  |  |  |  |  |  |  |  |  |  |  |  |  |  |  |  |  |  |  |  |  |  |  |  |  |  |  |  |  |  |  |  |  |  |  |  |  |  |  |  |  |  |  |  |  |  |  |  |  |  |  |  |  |  |  |  |  |  |  |  |  |  |  |  |  |  |  |  |  |  |  |  |  |  |  |  |  |  |  |  |  |  |  |  |  |  |  |  |  |  |  |  |  |  |  |  |  |  |  |  |  |  |  |  |  |  |  |  |  |  |  |  |  |  |  |  |  |  |  |  |  |  |  |  |  |  |  |  |  |  |  |  |  |  |  |  |  |  |  |  |  |  |  |  |  |  |  |  |  |  |  |  |  |  |  |  |  |  |  |  |  |  |  |  |  |  |  |  |  |  |  |  |  |  |  |  |  |  |  |  |  |  |  |  |  |  |  |  |  |  |  |  |  |  |  |  |  |  |  |  |  |  |  |  |  |  |  |  |  |  |  |  |  |  |  |  |  |  |  |  |  |  |  |  |  |  |  |  |  |  |  |  |  |  |  |  |  |  |  |  |  |  |  |  |  |  |  |  |  |  |  |  |  |  |  |  |  |  |  |  |  |  |  |  |  |  |  |  |  |  |  |  |  |  |  |  |  |  |  |  |  |  |  |  |  |  |  |  |  |  |  |  |  |  |  |  |  |  |  |  |  |  |  |  |  |  |  |  |  |  |  |  |  |  |  |  |  |  |  |  |  |  |  |  |  |  |  |  |  |  |  |  |  |  |  |  |  |  |  |  |  |  |  |  |  |  |  |  |  |  |  |  |  |  |  |  |  |  |  |  |  |  |  |  |  |  |  |  |  |  |  |  |  |  |  |  |  |  |  |  |  |  |  |  |  |  |  |  |  |  |  |  |  |  |  |  |  |  |  |  |  |  |  |  |  |  |  |  |  |  |  |  |  |  |  |  |  |  |  |  |  |  |  |  |  |  |  |  |  |  |  |  |  |  |  |  |  |  |  |  |  |  |  |  |  |  |  |  |  |  |  |  |  |  |  |  |  |  |  |  |  |  |  |  |  |  |  |  |  |  |  |  |  |  |  |  |  |  |  |  |  |  |  |  |  |  |  |  |  |  |  |  |  |  |  |  |  |  |  |  |  |  |  |  |  |  |  |  |  |  |  |  |  |  |  |  |  |  |  |  |  |  |  |  |  |  |  |  |  |  |  |  |  |  |  |  |  |  |  |  |  |  |  |  |  |  |  |  |  |  |  |  |  |  |  |  |  |  |  |  |  |  |  |  |  |  |  |  |  |  |  |  |  |  |  |  |  |  |  |  |  |  |  |  |  |  |  |  |  |  |  |  |  |  |  |  |  |  |  |  |  |  |  |  |  |  |  |  |  |  |  |  |  |  |  |  |  |  |  |  |  |  |  |  |  |  |  |  |  |  |  |  |  |  |  |  |  |  |  |  |  |  |  |  |  |  |  |  |  |  |  |  |  |  |  |  |  |  |  |  |  |  |  |  |  |  |  |  |  |  |  |  |  |  |  |  |  |  |  |  |  |  |  |  |  |  |  |  |  |  |  |  |  |  |  |  |  |  |  |  |  |  |  |  |  |  |  |  |  |  |  |  |  |  |  |  |  |  |  |  |  |  |  |  |  |  |  |  |  |  |  |  |  |  |  |  |  |  |  |  |  |  |  |  |  |  |  |  |  |  |  |  |  |  |  |  |  |  |  |  |  |  |  |  |  |  |  |  |  |  |  |  |  |  |  |  |  |  |  |  |  |  |  |  |  |  |  |  |  |  |  |  |  |  |  |  |  |  |  |  |  |  |  |  |  |  |  |  |  |  |  |  |  |  |  |  |  |  |  |  |  |  |  |  |  |  |  |  |  |  |  |  |  |  |  |  |  |  |  |  |  |  |  |  |  |  |  |  |  |  |  |  |  |  |  |  |  |  |  |  |  |  |  |  |  |  |  |  |  |  |  |  |  |  |  |  |  |  |  |  |  |  |  |  |  |  |  |  |  |  |  |  |  |  |  |  |  |  |  |  |  |  |  |  |  |  |  |  |  |  |  |  |  |  |  |  |  |  |  |  |  |  |  |  |  |  |  |  |  |  |  |  |  |  |  |  |  |  |  |  |  |  |  |  |  |  |  |  |  |  |  |  |  |  |  |  |  |  |  |  |  |  |  |  |  |  |  |  |  |  |  |  |  |  |  |  |  |  |  |  |  |  |  |  |  |  |  |  |  |  |  |  |  |  |  |  |  |  |  |  |  |  |  |  |  |  |  |  |  |  |  |  |  |  |  |  |  |  |  |  |  |  |  |  |  |  |  |  |  |  |  |  |  |  |  |  |  |  |  |  |  |  |  |  |  |  |  |  |  |  |  |  |  |  |  |  |  |  |  |  |  |  |  |  |  |  |  |  |  |  |  |  |  |  |  |  |  |  |  |  |  |  |  |  |  |  |  |  |  |  |  |  |  |  |  |  |  |  |  |  |  |  |  |  |  |  |  |  |  |  |  |  |  |  |  |  |  |  |  |  |  |  |  |  |  |  |  |  |  |  |  |  |  |  |  |  |  |  |  |  |  |  |  |  |  |  |  |  |  |  |  |  |  |  |  |  |  |  |  |  |  |  |  |  |  |  |  |  |  |  |  |  |  |  |  |  |  |  |  |  |  |  |  |  |  |  |  |  |  |  |  |  |  |  |  |  |  |  |  |  |  |  |  |  |  |  |  |  |  |  |  |  |  |  |  |  |  |  |  |  |  |  |  |  |  |  |  |  |  |  |  |  |  |  |  |  |  |  |  |  |  |
| --- | --- | --- | --- | --- | --- | --- | --- | --- | --- | --- | --- | --- | --- | --- | --- | --- | --- | --- | --- | --- | --- | --- | --- | --- | --- | --- | --- | --- | --- | --- | --- | --- | --- | --- | --- | --- | --- | --- | --- | --- | --- | --- | --- | --- | --- | --- | --- | --- | --- | --- | --- | --- | --- | --- | --- | --- | --- | --- | --- | --- | --- | --- | --- | --- | --- | --- | --- | --- | --- | --- | --- | --- | --- | --- | --- | --- | --- | --- | --- | --- | --- | --- | --- | --- | --- | --- | --- | --- | --- | --- | --- | --- | --- | --- | --- | --- | --- | --- | --- | --- | --- | --- | --- | --- | --- | --- | --- | --- | --- | --- | --- | --- | --- | --- | --- | --- | --- | --- | --- | --- | --- | --- | --- | --- | --- | --- | --- | --- | --- | --- | --- | --- | --- | --- | --- | --- | --- | --- | --- | --- | --- | --- | --- | --- | --- | --- | --- | --- | --- | --- | --- | --- | --- | --- | --- | --- | --- | --- | --- | --- | --- | --- | --- | --- | --- | --- | --- | --- | --- | --- | --- | --- | --- | --- | --- | --- | --- | --- | --- | --- | --- | --- | --- | --- | --- | --- | --- | --- | --- | --- | --- | --- | --- | --- | --- | --- | --- | --- | --- | --- | --- | --- | --- | --- | --- | --- | --- | --- | --- | --- | --- | --- | --- | --- | --- | --- | --- | --- | --- | --- | --- | --- | --- | --- | --- | --- | --- | --- | --- | --- | --- | --- | --- | --- | --- | --- | --- | --- | --- | --- | --- | --- | --- | --- | --- | --- | --- | --- | --- | --- | --- | --- | --- | --- | --- | --- | --- | --- | --- | --- | --- | --- | --- | --- | --- | --- | --- | --- | --- | --- | --- | --- | --- | --- | --- | --- | --- | --- | --- | --- | --- | --- | --- | --- | --- | --- | --- | --- | --- | --- | --- | --- | --- | --- | --- | --- | --- | --- | --- | --- | --- | --- | --- | --- | --- | --- | --- | --- | --- | --- | --- | --- | --- | --- | --- | --- | --- | --- | --- | --- | --- | --- | --- | --- | --- | --- | --- | --- | --- | --- | --- | --- | --- | --- | --- | --- | --- | --- | --- | --- | --- | --- | --- | --- | --- | --- | --- | --- | --- | --- | --- | --- | --- | --- | --- | --- | --- | --- | --- | --- | --- | --- | --- | --- | --- | --- | --- | --- | --- | --- | --- | --- | --- | --- | --- | --- | --- | --- | --- | --- | --- | --- | --- | --- | --- | --- | --- | --- | --- | --- | --- | --- | --- | --- | --- | --- | --- | --- | --- | --- | --- | --- | --- | --- | --- | --- | --- | --- | --- | --- | --- | --- | --- | --- | --- | --- | --- | --- | --- | --- | --- | --- | --- | --- | --- | --- | --- | --- | --- | --- | --- | --- | --- | --- | --- | --- | --- | --- | --- | --- | --- | --- | --- | --- | --- | --- | --- | --- | --- | --- | --- | --- | --- | --- | --- | --- | --- | --- | --- | --- | --- | --- | --- | --- | --- | --- | --- | --- | --- | --- | --- | --- | --- | --- | --- | --- | --- | --- | --- | --- | --- | --- | --- | --- | --- | --- | --- | --- | --- | --- | --- | --- | --- | --- | --- | --- | --- | --- | --- | --- | --- | --- | --- | --- | --- | --- | --- | --- | --- | --- | --- | --- | --- | --- | --- | --- | --- | --- | --- | --- | --- | --- | --- | --- | --- | --- | --- | --- | --- | --- | --- | --- | --- | --- | --- | --- | --- | --- | --- | --- | --- | --- | --- | --- | --- | --- | --- | --- | --- | --- | --- | --- | --- | --- | --- | --- | --- | --- | --- | --- | --- | --- | --- | --- | --- | --- | --- | --- | --- | --- | --- | --- | --- | --- | --- | --- | --- | --- | --- | --- | --- | --- | --- | --- | --- | --- | --- | --- | --- | --- | --- | --- | --- | --- | --- | --- | --- | --- | --- | --- | --- | --- | --- | --- | --- | --- | --- | --- | --- | --- | --- | --- | --- | --- | --- | --- | --- | --- | --- | --- | --- | --- | --- | --- | --- | --- | --- | --- | --- | --- | --- | --- | --- | --- | --- | --- | --- | --- | --- | --- | --- | --- | --- | --- | --- | --- | --- | --- | --- | --- | --- | --- | --- | --- | --- | --- | --- | --- | --- | --- | --- | --- | --- | --- | --- | --- | --- | --- | --- | --- | --- | --- | --- | --- | --- | --- | --- | --- | --- | --- | --- | --- | --- | --- | --- | --- | --- | --- | --- | --- | --- | --- | --- | --- | --- | --- | --- | --- | --- | --- | --- | --- | --- | --- | --- | --- | --- | --- | --- | --- | --- | --- | --- | --- | --- | --- | --- | --- | --- | --- | --- | --- | --- | --- | --- | --- | --- | --- | --- | --- | --- | --- | --- | --- | --- | --- | --- | --- | --- | --- | --- | --- | --- | --- | --- | --- | --- | --- | --- | --- | --- | --- | --- | --- | --- | --- | --- | --- | --- | --- | --- | --- | --- | --- | --- | --- | --- | --- | --- | --- | --- | --- | --- | --- | --- | --- | --- | --- | --- | --- | --- | --- | --- | --- | --- | --- | --- | --- | --- | --- | --- | --- | --- | --- | --- | --- | --- | --- | --- | --- | --- | --- | --- | --- | --- | --- | --- | --- | --- | --- | --- | --- | --- | --- | --- | --- | --- | --- | --- | --- | --- | --- | --- | --- | --- | --- | --- | --- | --- | --- | --- | --- | --- | --- | --- | --- | --- | --- | --- | --- | --- | --- | --- | --- | --- | --- | --- | --- | --- | --- | --- | --- | --- | --- | --- | --- | --- | --- | --- | --- | --- | --- | --- | --- | --- | --- | --- | --- | --- | --- | --- | --- | --- | --- | --- | --- | --- | --- | --- | --- | --- | --- | --- | --- | --- | --- | --- | --- | --- | --- | --- | --- | --- | --- | --- | --- | --- | --- | --- | --- | --- | --- | --- | --- | --- | --- | --- | --- | --- | --- | --- | --- | --- | --- | --- | --- | --- | --- | --- | --- | --- | --- | --- | --- | --- | --- | --- | --- | --- | --- | --- | --- | --- | --- | --- | --- | --- | --- | --- | --- | --- | --- | --- | --- | --- | --- | --- | --- | --- | --- | --- | --- | --- | --- | --- | --- | --- | --- | --- | --- | --- | --- | --- | --- | --- | --- | --- | --- | --- | --- | --- | --- | --- | --- | --- | --- | --- | --- | --- | --- | --- | --- | --- | --- | --- | --- | --- | --- | --- | --- | --- | --- | --- | --- | --- | --- | --- | --- | --- | --- | --- | --- | --- | --- | --- | --- | --- | --- | --- | --- | --- | --- | --- | --- | --- | --- | --- | --- | --- | --- | --- | --- | --- | --- | --- | --- | --- | --- | --- | --- | --- | --- | --- | --- | --- | --- | --- | --- | --- | --- | --- | --- | --- | --- | --- | --- | --- | --- | --- | --- | --- | --- | --- | --- | --- | --- | --- | --- | --- | --- | --- | --- | --- | --- | --- | --- | --- | --- | --- | --- | --- | --- | --- | --- | --- | --- | --- | --- | --- | --- | --- | --- | --- | --- | --- | --- | --- | --- | --- | --- | --- | --- | --- | --- | --- | --- | --- | --- | --- | --- | --- | --- | --- | --- | --- | --- | --- | --- | --- | --- | --- | --- | --- | --- | --- | --- | --- | --- | --- | --- | --- | --- | --- | --- | --- | --- | --- | --- | --- | --- | --- | --- | --- | --- | --- | --- | --- | --- | --- | --- | --- | --- | --- | --- | --- | --- | --- | --- | --- | --- | --- | --- | --- | --- | --- | --- | --- | --- | --- | --- | --- | --- | --- | --- | --- | --- | --- | --- | --- | --- | --- | --- | --- | --- | --- | --- | --- | --- | --- | --- | --- | --- | --- | --- | --- | --- | --- | --- | --- | --- | --- | --- | --- | --- | --- | --- | --- | --- | --- | --- | --- | --- | --- | --- | --- | --- | --- | --- | --- | --- | --- | --- | --- | --- | --- | --- | --- | --- | --- | --- | --- | --- | --- | --- | --- | --- | --- | --- | --- | --- | --- | --- | --- | --- | --- | --- | --- | --- | --- | --- | --- | --- | --- | --- | --- | --- | --- | --- | --- | --- | --- | --- | --- | --- | --- | --- | --- | --- | --- | --- | --- | --- | --- | --- | --- | --- | --- | --- | --- | --- | --- | --- | --- | --- | --- | --- | --- | --- | --- | --- | --- | --- | --- | --- | --- | --- | --- | --- | --- | --- | --- | --- | --- | --- | --- | --- | --- | --- | --- | --- | --- | --- | --- | --- | --- | --- | --- | --- | --- | --- | --- | --- | --- | --- | --- | --- | --- | --- | --- | --- | --- | --- | --- | --- | --- | --- | --- | --- | --- | --- | --- | --- | --- | --- | --- | --- | --- | --- | --- | --- | --- | --- | --- | --- | --- | --- | --- | --- | --- | --- | --- | --- | --- | --- | --- | --- | --- | --- | --- | --- | --- | --- | --- | --- | --- | --- | --- | --- | --- | --- | --- | --- | --- | --- | --- | --- | --- | --- | --- | --- | --- | --- | --- | --- | --- | --- | --- | --- | --- | --- | --- | --- | --- | --- | --- | --- | --- | --- | --- | --- | --- | --- | --- | --- | --- | --- | --- | --- | --- | --- | --- | --- | --- | --- | --- | --- | --- | --- | --- | --- | --- | --- | --- | --- | --- | --- | --- | --- | --- | --- | --- | --- | --- |
|  |  |  |  |  |  |  |  | 1 | 0 |  |  |  |  |  |  |  |  | 2 | 0 |  |  |  |  |  |  |  |  | 3 | 0 |  |  |  |  |  |  |  |  | 4 | 0 |  |  |  |  |  |  |  |  | 5 | 0 |  |  |  |  |  |  |  |  | 6 | 0 |  |  |  |  |  |  |  |  | 7 | 0 |  |  |  |  |  |  |  |  | 8 | 0 |  |  |  |  |  |  |  |  | 9 | 0 |  |  |  |  |  |  |  | 1 | 0 | 0 |  |  |  |  |  |  |  | 1 | 1 | 0 |  |  |  |  |  |  |  | 1 | 2 | 0 |  |  |  |  |  |  |  | 1 | 3 | 0 |  |  |  |  |  |  |  | 1 | 4 | 0 |  |  |  |  |  |  |  | 1 | 5 | 0 |  |  |  |  |  |  |  | 1 | 6 | 0 |  |  |  |  |  |  |  | 1 | 7 | 0 |  |  |  |  |  |  |  | 1 | 8 | 0 |  |  |  |  |  |  |  | 1 | 9 | 0 |  |  |  |  |  |  |  | 2 | 0 | 0 |  |  |  |  |  |  |  | 2 | 1 | 0 |  |  |  |  |  |  |  | 2 | 2 | 0 |  |  |  |  |  |  |  | 2 | 3 | 0 |  |  |  |  |  |  |  | 2 | 4 | 0 |  |  |  |  |  |  |  | 2 | 5 | 0 |  |  |  |  |  |  |  | 2 | 6 | 0 |  |  |  |  |  |  |  | 2 | 7 | 0 |  |  |  |  |  |  |  | 2 | 8 | 0 |  |  |  |  |  |  |  | 2 | 9 | 0 |  |  |  |  |  |  |  | 3 | 0 | 0 |  |  |  |  |  |  |  | 3 | 1 | 0 |  |  |  |  |  |  |  | 3 | 2 | 0 |  |  |  |  |  |  |  | 3 | 3 | 0 |  |  |  |  |  |  |  | 3 | 4 | 0 |  |  |  |  |  |  |  | 3 | 5 | 0 |  |  |  |  |  |  |  | 3 | 6 | 0 |  |  |  |  |  |  |  | 3 | 7 | 0 |  |  |  |  |  |  |  | 3 | 8 | 0 |  |  |  |  |  |  |  | 3 | 9 | 0 |  |  |  |  |  |  |  | 4 | 0 | 0 |  |  |  |  |  |  |  | 4 | 1 | 0 |  |  |  |  |  |  |  | 4 | 2 | 0 |  |  |  |  |  |  |  | 4 | 3 | 0 |  |  |  |  |  |  |  | 4 | 4 | 0 |  |  |  |  |  |  |  | 4 | 5 | 0 |  |  |  |  |  |  |  | 4 | 6 | 0 |  |  |  |  |  |  |  | 4 | 7 | 0 |  |  |  |  |  |  |  | 4 | 8 | 0 |  |  |  |  |  |  |  | 4 | 9 | 0 |  |  |  |  |  |  |  | 5 | 0 | 0 |  |  |  |  |  |  |  | 5 | 1 | 0 |  |  |  |  |  |  |  | 5 | 2 | 0 |  |  |  |  |  |  |  | 5 | 3 | 0 |  |  |  |  |  |  |  | 5 | 4 | 0 |  |  |  |  |  |  |  | 5 | 5 | 0 |  |  |  |  |  |  |  | 5 | 6 | 0 |  |  |  |  |  |  |  | 5 | 7 | 0 |  |  |  |  |  |  |  | 5 | 8 | 0 |  |  |  |  |  |  |  | 5 | 9 | 0 |  |  |  |  |  |  |  | 6 | 0 | 0 |  |  |  |  |  |  |  | 6 | 1 | 0 |  |  |  |  |  |  |  | 6 | 2 | 0 |  |  |  |  |  |  |  | 6 | 3 | 0 |  |  |  |  |  |  |  | 6 | 4 | 0 |  |  |  |  |  |  |  | 6 | 5 | 0 |  |  |  |  |  |  |  | 6 | 6 | 0 |  |  |  |  |  |  |  | 6 | 7 | 0 |  |  |  |  |  |  |  | 6 | 8 | 0 |  |  |  |  |  |  |  | 6 | 9 | 0 |  |  |  |  |  |  |  | 7 | 0 | 0 |  |  |  |  |  |  |  | 7 | 1 | 0 |  |  |  |  |  |  |  | 7 | 2 | 0 |  |  |  |  |  |  |  | 7 | 3 | 0 |  |  |  |  |  |  |  | 7 | 4 | 0 |  |  |  |  |  |  |  | 7 | 5 | 0 |  |  |  |  |  |  |  | 7 | 6 | 0 |  |  |  |  |  |  |  | 7 | 7 | 0 |  |  |  |  |  |  |  | 7 | 8 | 0 |  |  |  |  |  |  |  | 7 | 9 | 0 |  |  |  |  |  |  |  | 8 | 0 | 0 |  |  |  |  |  |  |  | 8 | 1 | 0 |  |  |  |  |  |  |  | 8 | 2 | 0 |  |  |  |  |  |  |  | 8 | 3 | 0 |  |  |  |  |  |  |  | 8 | 4 | 0 |  |  |  |  |  |  |  | 8 | 5 | 0 |  |  |  |  |  |  |  | 8 | 6 | 0 |  |  |  |  |  |  |  | 8 | 7 | 0 |  |  |  |  |  |  |  | 8 | 8 | 0 |  |  |  |  |  |  |  | 8 | 9 | 0 |  |  |  |  |  |  |  | 9 | 0 | 0 |  |  |  |  |  |  |  | 9 | 1 | 0 |  |  |  |  |  |  |  | 9 | 2 | 0 |  |  |  |  |  |  |  | 9 | 3 | 0 |  |  |  |  |  |  |  | 9 | 4 | 0 |  |  |  |  |  |  |  | 9 | 5 | 0 |  |  |  |  |  |  |  | 9 | 6 | 0 |  |  |  |  |  |  |  | 9 | 7 | 0 |  |  |  |  |  |  |  | 9 | 8 | 0 |  |  |  |  |  |  |  | 9 | 9 | 0 |  |  |  |  |  |  | 1 | 0 | 0 | 0 |  |  |  |  |  |  | 1 | 0 | 1 | 0 |  |  |  |  |  |  | 1 | 0 | 2 | 0 |  |  |  |  |  |  | 1 | 0 | 3 | 0 |  |  |  |  |  |  | 1 | 0 | 4 | 0 |  |  |  |  |  |  | 1 | 0 | 5 | 0 |  |  |  |  |  |  | 1 | 0 | 6 | 0 |  |  |  |  |  |  | 1 | 0 | 7 | 0 |  |  |  |  |  |  | 1 | 0 | 8 | 0 |  |  |  |  |  |  | 1 | 0 | 9 | 0 |  |  |  |  |  |  | 1 | 1 | 0 | 0 |  |  |  |  |  |  | 1 | 1 | 1 | 0 |  |  |  |  |  |  | 1 | 1 | 2 | 0 |  |  |  |  |  |  | 1 | 1 | 3 | 0 |  |  |  |  |  |  | 1 | 1 | 4 | 0 |  |  |  |  |  |  | 1 | 1 | 5 | 0 |  |  |  |  |  |  | 1 | 1 | 6 | 0 |  |  |  |  |  |  | 1 | 1 | 7 | 0 |  |  |  |  |  |  | 1 | 1 | 8 | 0 |  |  |  |  |  |  | 1 | 1 | 9 | 0 |  |  |  |  |  |  | 1 | 2 | 0 | 0 |  |  |  |  |  |  | 1 | 2 | 1 | 0 |  |  |  |  |  |  | 1 | 2 | 2 | 0 |  |  |  |  |  |  | 1 | 2 | 3 | 0 |  |  |  |  |  |  | 1 | 2 | 4 | 0 |  |  |  |  |  |  | 1 | 2 | 5 | 0 |  |  |  |  |  |  | 1 | 2 | 6 | 0 |  |  |  |  |  |  | 1 | 2 | 7 | 0 |  |  |  |  |  |  | 1 | 2 | 8 | 0 |  |  |  |  |  |  | 1 | 2 | 9 | 0 |  |  |  |  |  |  | 1 | 3 | 0 | 0 |  |  |  |  |  |  | 1 | 3 | 1 | 0 |  |  |  |  |  |  | 1 | 3 | 2 | 0 |  |  |  |  |  |  | 1 | 3 | 3 | 0 |  |  |  |  |  |  | 1 | 3 | 4 | 0 |  |  |  |  |  |  | 1 | 3 | 5 | 0 |  |  |  |  |  |  | 1 | 3 | 6 | 0 |  |  |  |  |  |  | 1 | 3 | 7 | 0 |  |  |  |  |  |  | 1 | 3 | 8 | 0 |  |  |  |  |  |  | 1 | 3 | 9 | 0 |  |  |  |  |  |  | 1 | 4 | 0 | 0 |  |  |  |  |  |  | 1 | 4 | 1 | 0 |  |  |  |  |  |  | 1 | 4 | 2 | 0 |  |  |  |  |  |  | 1 | 4 | 3 | 0 |  |  |  |  |
|  |  |  |  |  |  |  |  |  | | |  |  |  |  |  |  |  |  |  | | |  |  |  |  |  |  |  |  |  | | |  |  |  |  |  |  |  |  |  | | |  |  |  |  |  |  |  |  |  | | |  |  |  |  |  |  |  |  |  | | |  |  |  |  |  |  |  |  |  | | |  |  |  |  |  |  |  |  |  | | |  |  |  |  |  |  |  |  |  | | |  |  |  |  |  |  |  |  |  | | |  |  |  |  |  |  |  |  |  | | |  |  |  |  |  |  |  |  |  | | |  |  |  |  |  |  |  |  |  | | |  |  |  |  |  |  |  |  |  | | |  |  |  |  |  |  |  |  |  | | |  |  |  |  |  |  |  |  |  | | |  |  |  |  |  |  |  |  |  | | |  |  |  |  |  |  |  |  |  | | |  |  |  |  |  |  |  |  |  | | |  |  |  |  |  |  |  |  |  | | |  |  |  |  |  |  |  |  |  | | |  |  |  |  |  |  |  |  |  | | |  |  |  |  |  |  |  |  |  | | |  |  |  |  |  |  |  |  |  | | |  |  |  |  |  |  |  |  |  | | |  |  |  |  |  |  |  |  |  | | |  |  |  |  |  |  |  |  |  | | |  |  |  |  |  |  |  |  |  | | |  |  |  |  |  |  |  |  |  | | |  |  |  |  |  |  |  |  |  | | |  |  |  |  |  |  |  |  |  | | |  |  |  |  |  |  |  |  |  | | |  |  |  |  |  |  |  |  |  | | |  |  |  |  |  |  |  |  |  | | |  |  |  |  |  |  |  |  |  | | |  |  |  |  |  |  |  |  |  | | |  |  |  |  |  |  |  |  |  | | |  |  |  |  |  |  |  |  |  | | |  |  |  |  |  |  |  |  |  | | |  |  |  |  |  |  |  |  |  | | |  |  |  |  |  |  |  |  |  | | |  |  |  |  |  |  |  |  |  | | |  |  |  |  |  |  |  |  |  | | |  |  |  |  |  |  |  |  |  | | |  |  |  |  |  |  |  |  |  | | |  |  |  |  |  |  |  |  |  | | |  |  |  |  |  |  |  |  |  | | |  |  |  |  |  |  |  |  |  | | |  |  |  |  |  |  |  |  |  | | |  |  |  |  |  |  |  |  |  | | |  |  |  |  |  |  |  |  |  | | |  |  |  |  |  |  |  |  |  | | |  |  |  |  |  |  |  |  |  | | |  |  |  |  |  |  |  |  |  | | |  |  |  |  |  |  |  |  |  | | |  |  |  |  |  |  |  |  |  | | |  |  |  |  |  |  |  |  |  | | |  |  |  |  |  |  |  |  |  | | |  |  |  |  |  |  |  |  |  | | |  |  |  |  |  |  |  |  |  | | |  |  |  |  |  |  |  |  |  | | |  |  |  |  |  |  |  |  |  | | |  |  |  |  |  |  |  |  |  | | |  |  |  |  |  |  |  |  |  | | |  |  |  |  |  |  |  |  |  | | |  |  |  |  |  |  |  |  |  | | |  |  |  |  |  |  |  |  |  | | |  |  |  |  |  |  |  |  |  | | |  |  |  |  |  |  |  |  |  | | |  |  |  |  |  |  |  |  |  | | |  |  |  |  |  |  |  |  |  | | |  |  |  |  |  |  |  |  |  | | |  |  |  |  |  |  |  |  |  | | |  |  |  |  |  |  |  |  |  | | |  |  |  |  |  |  |  |  |  | | |  |  |  |  |  |  |  |  |  | | |  |  |  |  |  |  |  |  |  | | |  |  |  |  |  |  |  |  |  | | |  |  |  |  |  |  |  |  |  | | |  |  |  |  |  |  |  |  |  | | |  |  |  |  |  |  |  |  |  | | |  |  |  |  |  |  |  |  |  | | |  |  |  |  |  |  |  |  |  | | |  |  |  |  |  |  |  |  |  | | |  |  |  |  |  |  |  |  |  | | |  |  |  |  |  |  |  |  |  | | |  |  |  |  |  |  |  |  |  | | |  |  |  |  |  |  |  |  |  | | |  |  |  |  |  |  |  |  |  | | |  |  |  |  |  |  |  |  |  | | |  |  |  |  |  |  |  |  |  | | |  |  |  |  |  |  |  |  |  | | |  |  |  |  |  |  |  |  |  | | |  |  |  |  |  |  |  |  |  | | |  |  |  |  |  |  |  |  |  | | |  |  |  |  |  |  |  |  |  | | |  |  |  |  |  |  |  |  |  | | |  |  |  |  |  |  |  |  |  | | |  |  |  |  |  |  |  |  |  | | |  |  |  |  |  |  |  |  |  | | |  |  |  |  |  |  |  |  |  | | |  |  |  |  |  |  |  |  |  | | |  |  |  |  |  |  |  |  |  | | |  |  |  |  |  |  |  |  |  | | |  |  |  |  |  |  |  |  |  | | |  |  |  |  |  |  |  |  |  | | |  |  |  |  |  |  |  |  |  | | |  |  |  |  |  |  |  |  |  | | |  |  |  |  |  |  |  |  |  | | |  |  |  |  |  |  |  |  |  | | |  |  |  |  |  |  |  |  |  | | |  |  |  |  |  |  |  |  |  | | |  |  |  |  |  |  |  |  |  | | |  |  |  |  |  |  |  |  |  | | |  |  |  |  |  |  |  |  |  | | |  |  |  |  |  |  |  |  |  | | |  |  |  |  |  |  |  |  |  | | |  |  |  |  |  |  |  |  |  | | |  |  |  |  |  |  |  |  |  | | |  |  |  |  |  |  |  |  |  | | |  |  |  |  |  |  |  |  |  | | |  |  |  |  |  |  |  |  |  | | |  |  |  |  |  |  |  |  |  | | |  |  |  |  |  |  |  |  |  | | |  |  |  |  |  |  |  |  |  | | |  |  |  |  |  |  |  |  |  | | |  |  |  |  |  |  |  |  |  | | |  |  |  |  |  |  |  |  |  | | |  |  |  |  |  |  |  |  |  | | |  |  |  |  |  |  |  |  |  | | |  |  |  |  |  |  |  |  |  | | |  |  |  |  |  |  |  |  |  | | |  |  |  |  |  |  |  |  |  | | |  |  |  |  |  |  |  |  |  | | |  |  |  |  |  |  |  |  |  | | |  |  |  |  |  |  |  |  |  | | |  |  |  |  |  |  |  |  |  | | |  |  |  |  |  |  |  |  |  | | |  |  |  |  |  |  |  |  |  | | |  |  |  |  |  |  |  |  |  | | |  |  |  |  |  |  |  |  |  | | |  |  |  |  |  |  |  |  |  | | |  |  |  |  |  |  |  |  |  | | |  |  |  |  |
| OMTLR8 | - | - | - | - | - | - | - | - | - | - | - | - | M | K | E | H | W | F | P | L | S | P | A | F | Q | R | L | L | F | I | L | V | L | L | C | L | - | - | - | - | - | - | - | S | L | S | C | S | G | R | T | W | A | Q | R | K | L | P | C | D | V | I | S | L | - | - | N | Q | - | - | S | E | S | F | D | C | S | D | R | K | L | L | Q | V | P | Q | D | I | S | R | N | A | T | R | L | D | L | S | S | N | R | I | K | K | L | S | G | I | S | F | R | N | L | R | N | L | T | R | L | D | L | S | N | N | F | H | P | Q | K | - | - | - | - | H | P | L | T | I | E | N | Q | T | F | S | M | L | D | Q | L | K | E | L | L | L | D | S | N | G | L | S | A | V | P | S | A | L | P | S | K | L | R | F | L | S | L | K | F | N | C | I | K | T | I | - | R | P | N | D | F | M | H | V | R | L | L | E | T | I | H | L | I | G | N | C | D | Y | E | S | V | C | N | G | - | L | V | I | E | N | R | T | F | S | H | L | T | N | L | A | S | L | S | L | S | N | N | R | L | I | G | V | P | L | F | L | P | P | S | L | Q | Q | L | K | L | K | Q | N | T | I | T | Y | I | H | Q | H | D | L | S | G | L | T | S | L | R | L | L | G | L | S | G | N | C | P | V | C | S | N | T | P | F | F | C | S | P | C | N | T | P | N | G | A | L | Q | I | H | P | Y | A | F | R | E | L | S | K | L | Q | E | L | R | L | S | G | N | S | L | D | H | L | Q | S | S | W | F | E | N | L | T | N | L | R | Y | L | Y | L | S | F | N | R | L | V | G | E | I | A | - | - | - | - | N | G | D | F | L | S | V | L | P | G | V | E | V | M | D | L | S | Y | N | H | P | G | Q | S | S | Y | S | - | - | - | - | H | N | L | K | L | S | R | N | F | S | N | L | T | S | L | K | T | L | H | L | E | N | Y | V | F | S | T | L | H | K | N | D | L | N | P | L | Y | N | L | S | H | L | S | V | L | N | L | G | T | N | F | I | Y | Q | T | N | L | S | L | F | G | M | F | H | - | - | N | L | S | S | V | G | L | S | E | N | K | F | A | F | L | S | K | K | E | D | V | L | S | T | C | - | G | C | S | C | G | Y | E | L | N | R | D | R | F | G | P | Y | V | H | - | K | D | R | L | F | R | - | Q | H | L | P | N | I | K | P | K | C | I | A | Y | G | S | V | L | D | L | S | R | N | N | I | F | H | I | N | P | D | M | L | Q | G | L | D | N | T | A | C | L | N | L | S | S | N | S | I | G | D | M | F | N | G | S | E | F | V | H | F | P | K | L | K | Y | L | D | L | S | R | N | K | I | Y | L | H | Y | E | H | A | F | S | E | L | T | E | L | E | V | L | D | L | S | H | N | N | H | Y | F | V | V | A | G | L | N | H | S | L | A | F | M | E | N | L | S | S | L | K | V | L | N | L | S | W | N | E | I | S | T | L | T | H | N | N | I | S | S | S | S | L | Q | E | L | D | F | Q | G | N | R | L | D | I | M | W | K | I | - | C | Q | N | Y | E | - | L | F | R | A | L | Q | N | L | T | S | L | D | L | S | Y | N | K | L | H | R | V | P | P | E | V | Y | K | H | F | P | K | T | L | R | R | L | S | L | S | K | N | R | L | K | V | F | D | W | E | R | L | T | Q | L | P | H | L | E | E | L | D | L | S | K | N | K | L | E | Q | V | A | C | A | L | - | - | - | T | S | - | S | L | K | F | L | D | L | S | H | N | R | I | S | Q | L | A | P | G | F | L | R | G | A | R | S | L | Y | V | L | D | L | S | F | N | L | L | E | L | I | N | Q | T | T | F | E | S | G | A | E | N | H | L | Q | Q | L | S | L | Q | G | N | P | L | H | C | T | C | D | L | L | D | F | H | L | W | M | R | - | S | N | E | V | E | L | P | L | L | A | T | E | V | T | C | D | M | P | V | E | R | R | G | K | S | V | L | S | Y | D | I | E | - | E | C | V | N | D | E | N | A | M | A | F | C | I | I | T | S | F | L | I | I | L | T | L | L | V | S | L | T | A | H | L | F | Y | W | D | L | S | Y | I | L | D | Y | C | G | A | K | M | K | H | H | R | R | L | V | P | - | - | T | D | F | I | Y | D | A | F | V | M | Y | D | T | A | D | P | L | A | S | D | W | V | L | N | H | L | R | V | E | L | E | E | R | G | E | R | A | R | Y | L | C | L | E | E | R | D | W | S | L | G | M | P | V | M | D | N | L | S | N | S | V | R | Q | S | R | K | T | V | F | V | L | T | D | G | F | L | L | R | G | V | V | K | M | A | A | L | L | V | Q | Q | R | L | V | E | E | G | V | D | S | M | V | L | L | L | L | Q | P | Q | V | L | Q | H | S | R | I | L | H | L | R | R | R | L | C | R | R | S | V | L | E | W | P | A | D | A | S | P | A | A | Q | R | W | F | W | H | H | L | K | R | T | I | R | K | D | Q | I | G | T | H | T | S | L | H | S | T | Y | F | T | G | R | X | X | X | X | X | X | X | X | - | - | - | - | - | - | - | - | - | - | - | - | - | - | - | - | - | - | - | - | - | - | - | - | - | - | - | - | - | - | - | - | - | - | - | - | - | - | - | - | - | - | - | - | - | - | - | - | - | - | - | - | - | - | - | - | - | - | - | - | - | - | - | - | - | - | - | - | - | - | - | - | - | - | - | - | - | - | - | - | - | - | - | - | - | - | - | - | - | - | - | - | - | - | - | - | - | - | - | - | - | - | - | - | - | - | - | - | - | - | - | - | - | - | - | - | - | - | - | - | - | - | - | - | - | - | - | - | - | - | - | - | - | - | - | - | - | - | - | - | - | - | - | - | - | - | - | - | - | - | - | - | - | - | - | - | - | - | - | - | - | - | - | - | - | - | - | - | - | - | - | - | - | - | - | - | - | - | - | - | - | - | - | - | - | - | - | - | - | - | - | - | - | - | - | - | - | - | - | - | - | - | - | - | - | - | - | - | - | - | - | - | - | - | - | - | - | - | - | - | - | - | - | - | - | - | - | - | - | - | - | - | - | - | - | - | - | - | - | - | - | - | - | - | - | - | - | - | - | - | - | - | - | - | - | - | - | - | - | - | - | - | - | - | - | - | - | - | - | - | - | - | - | - | - | - | - | - | - | - | - | - | - | - | - | - | - | - | - | - | - | - | - | - | - | - | - | - | - | - | - | - | - | - | - | - | - | - | - | - | - | - | - | - | - | - | - | - | - | - | - | - | - | - | - | - | - | - | - | - | - | - | - | - | - | - | - | - | - | - | - |
| DRTLR8B | - | - | - | - | - | - | - | - | - | - | - | - | - | - | - | - | - | - | - | - | - | - | - | - | - | - | M | I | V | A | F | F | L | L | C | T | - | - | - | - | - | - | - | V | H | S | S | E | G | Y | N | W | A | W | R | K | L | P | C | D | V | N | L | S | - | - | N | T | - | - | S | V | T | L | D | C | S | E | R | Y | L | K | K | I | P | K | N | L | I | W | N | T | T | N | L | N | L | A | N | N | K | I | H | N | I | S | K | D | A | F | W | N | L | N | N | V | T | W | I | D | L | R | R | N | Q | I | E | K | - | - | - | - | - | - | C | H | E | K | D | N | G | V | F | S | R | L | T | N | L | K | T | L | L | L | D | N | N | K | I | S | V | L | P | K | N | L | P | A | G | L | Q | W | L | S | L | N | S | N | H | I | K | S | I | - | E | Q | S | D | F | K | G | M | T | K | L | T | V | L | K | L | N | K | N | C | - | Y | H | N | I | S | A | E | - | L | T | I | Q | N | E | T | F | Q | H | L | Q | - | L | T | E | L | Q | L | S | K | N | G | L | H | N | V | P | F | A | L | P | R | T | L | H | N | L | S | L | L | L | N | R | I | D | H | V | H | E | S | D | L | N | H | L | T | R | L | K | V | L | D | L | S | G | N | C | P | I | C | F | T | T | P | F | P | C | T | S | C | Q | T | N | N | N | A | L | Q | I | H | P | N | A | F | S | K | L | S | Q | L | Q | D | L | R | L | S | G | N | S | L | Q | S | I | N | S | M | W | F | Q | N | L | T | N | L | K | Y | L | Y | L | S | F | N | S | L | I | S | E | F | E | - | - | - | - | S | G | Q | F | F | S | V | L | P | Q | V | E | V | V | D | I | S | Y | N | N | P | S | E | R | I | Y | P | - | - | - | - | - | R | L | K | L | S | E | G | F | S | R | L | E | S | L | Q | T | L | H | L | E | G | Y | I | F | H | K | L | S | E | D | D | L | R | P | L | F | S | L | R | N | L | S | V | L | N | L | A | V | N | F | L | Q | Q | V | N | L | S | V | F | R | N | F | H | - | - | N | L | S | L | I | S | L | I | D | N | R | L | T | F | S | S | P | I | R | R | W | E | G | Q | S | K | S | G | F | K | D | D | N | Q | G | D | H | R | E | G | P | Y | I | H | - | T | N | E | E | F | R | - | H | Y | P | P | F | T | K | A | E | C | L | A | T | G | P | V | L | D | L | S | R | N | N | I | Y | H | V | N | P | P | L | F | T | G | A | E | N | I | T | C | L | N | L | S | S | N | F | I | V | S | Y | F | N | G | T | E | F | A | H | F | P | K | L | K | Y | L | D | L | S | H | N | R | I | Y | M | H | S | D | S | A | L | S | E | L | K | A | L | E | V | L | D | L | S | H | N | Q | H | Y | F | E | V | A | G | V | R | N | C | L | T | F | L | E | N | L | Q | F | L | K | V | L | N | L | S | W | N | E | I | N | M | L | T | N | K | T | L | Q | S | D | S | L | N | E | L | Q | F | Q | G | N | R | L | D | I | M | W | K | K | - | Q | R | G | Y | Q | S | L | F | K | S | L | S | N | L | T | Y | L | D | I | S | Y | N | K | L | S | E | I | P | D | D | I | F | D | Y | F | P | K | T | L | R | Y | I | S | M | S | R | N | T | L | T | D | F | A | W | E | Q | L | Q | S | L | P | Q | L | E | T | L | D | L | S | K | N | K | L | R | V | V | P | R | K | L | S | K | H | T | R | - | S | L | K | V | L | D | L | S | H | N | Q | I | S | K | L | R | Y | S | F | L | E | N | V | K | S | L | Q | I | L | N | F | A | N | N | K | L | K | H | L | G | A | S | S | F | T | T | G | S | N | H | Q | L | Q | I | L | D | L | Q | R | N | P | I | H | C | T | C | N | L | L | D | F | I | L | W | L | E | - | K | S | D | T | I | L | P | R | L | A | T | D | V | L | C | D | L | P | E | S | K | R | G | H | P | M | V | S | L | D | F | K | N | A | C | I | N | N | S | I | A | E | I | L | Y | I | L | T | S | S | V | I | T | L | V | M | C | T | T | I | G | I | H | V | F | Y | W | D | I | S | Y | A | Y | N | F | C | M | A | R | F | K | S | Y | - | Y | L | K | T | - | - | N | D | C | I | Y | D | A | F | V | M | Y | D | T | K | D | P | M | V | A | E | W | V | L | N | H | L | R | L | E | L | E | D | R | G | R | H | V | R | P | L | C | L | E | E | R | D | W | T | P | G | I | P | I | M | D | N | L | N | L | S | V | H | R | S | R | K | T | I | F | V | L | T | E | G | F | V | H | S | G | I | F | K | M | A | A | F | L | A | Q | Q | R | L | L | E | E | G | V | D | V | M | V | L | V | L | L | E | P | - | V | L | R | Q | S | R | I | L | N | L | R | R | C | L | C | G | H | S | V | L | E | W | P | R | N | - | - | P | A | A | E | G | W | F | W | Q | S | L | R | N | A | V | R | F | E | S | Q | G | V | Q | S | K | M | F | K | N | Y | F | N | G | X | X | X | X | X | X | X | X | X | X | X | X | X | X | X | X | X | X | X | X | X | X | X | X | X | X | - | - | - | - | - | - | - | - | - | - | - | - | - | - | - | - | - | - | - | - | - | - | - | - | - | - | - | - | - | - | - | - | - | - | - | - | - | - | - | - | - | - | - | - | - | - | - | - | - | - | - | - | - | - | - | - | - | - | - | - | - | - | - | - | - | - | - | - | - | - | - | - | - | - | - | - | - | - | - | - | - | - | - | - | - | - | - | - | - | - | - | - | - | - | - | - | - | - | - | - | - | - | - | - | - | - | - | - | - | - | - | - | - | - | - | - | - | - | - | - | - | - | - | - | - | - | - | - | - | - | - | - | - | - | - | - | - | - | - | - | - | - | - | - | - | - | - | - | - | - | - | - | - | - | - | - | - | - | - | - | - | - | - | - | - | - | - | - | - | - | - | - | - | - | - | - | - | - | - | - | - | - | - | - | - | - | - | - | - | - | - | - | - | - | - | - | - | - | - | - | - | - | - | - | - | - | - | - | - | - | - | - | - | - | - | - | - | - | - | - | - | - | - | - | - | - | - | - | - | - | - | - | - | - | - | - | - | - | - | - | - | - | - | - | - | - | - | - | - | - | - | - | - | - | - | - | - | - | - | - | - | - | - | - | - | - | - | - | - | - | - | - | - | - | - | - | - | - | - | - | - | - | - | - | - | - | - | - | - | - | - | - | - | - | - | - | - | - | - | - | - | - | - | - | - | - | - | - | - | - | - | - | - | - | - | - | - | - | - | - | - | - | - | - |
| CITLR8 | M | G | K | T | Y | H | F | H | T | N | S | T | K | P | K | V | Q | L | R | S | L | H | N | H | K | K | M | I | F | V | F | I | L | I | C | T | - | - | - | - | - | - | - | A | H | S | S | Q | G | L | I | W | D | W | R | K | L | P | C | D | V | T | L | S | - | - | N | S | - | - | S | V | V | L | D | C | S | E | R | S | L | K | K | I | P | K | N | L | I | W | N | T | T | E | L | D | F | A | N | N | E | I | R | Y | L | P | K | D | A | F | W | N | L | T | N | V | T | R | I | N | L | R | K | N | R | I | E | K | - | - | - | - | - | - | C | L | E | K | D | R | G | I | F | S | R | L | A | N | L | E | T | L | L | L | D | N | N | K | I | S | A | I | P | R | D | L | P | P | G | L | Q | Q | L | S | L | N | S | N | R | I | K | N | I | - | G | P | S | D | F | E | R | T | R | K | L | K | V | L | K | L | N | K | N | C | - | Y | H | N | V | S | I | E | - | L | I | I | H | K | E | T | F | Q | H | L | Q | - | L | T | E | L | E | L | S | R | N | G | I | Q | K | I | P | S | A | L | P | G | S | L | N | T | L | S | L | L | L | N | R | I | E | R | V | H | A | S | D | L | N | N | L | T | N | L | T | V | L | D | L | S | G | N | C | P | F | C | F | T | A | P | F | P | C | T | T | C | R | T | K | N | H | A | L | H | I | H | P | D | A | F | N | E | L | S | Q | L | Q | D | L | R | L | S | G | N | S | L | K | T | I | K | S | S | W | F | R | N | L | T | K | L | K | Y | L | Y | L | S | F | N | S | L | I | S | E | F | Q | - | - | - | - | S | G | Q | F | F | S | V | L | P | Q | V | E | V | V | D | F | S | Y | N | N | P | Y | Q | T | F | Y | Q | - | - | - | - | - | R | M | N | L | S | K | G | F | G | S | L | K | S | L | H | T | L | H | I | E | G | Y | N | F | H | N | L | C | E | E | D | L | R | P | L | F | G | L | R | N | L | S | V | L | N | L | G | V | N | F | L | Q | H | I | N | I | S | V | F | R | N | F | H | - | - | N | L | S | L | I | S | L | I | D | N | R | L | T | L | T | S | P | L | W | R | R | E | C | R | N | - | E | I | L | A | D | E | D | E | G | D | R | R | E | D | P | Y | I | H | - | T | N | E | Q | F | R | - | N | Y | P | P | F | T | K | A | E | C | L | A | T | G | P | V | L | D | L | S | R | N | N | I | Y | H | I | K | Q | T | Y | F | T | G | A | E | N | T | T | C | L | N | L | S | S | N | F | I | A | S | Y | F | N | G | T | E | F | V | H | F | P | K | L | K | Y | L | D | L | S | H | N | R | V | Y | M | R | S | D | S | A | L | N | E | L | K | E | L | E | V | L | D | L | S | H | N | K | H | Y | F | E | V | A | G | V | R | S | T | L | T | F | L | E | N | L | Q | F | L | K | V | L | N | L | S | W | N | E | I | N | T | L | T | N | K | T | L | Q | S | D | S | L | K | E | L | Q | F | Q | G | N | R | L | D | I | M | W | K | K | - | G | R | G | F | K | G | L | F | K | S | L | S | N | L | T | H | L | D | I | S | Y | N | K | L | A | E | I | P | D | D | I | F | S | Y | F | P | C | T | L | K | Y | L | C | M | S | R | N | T | L | A | D | F | K | W | E | Q | L | K | Y | L | P | H | L | E | T | L | D | L | S | M | N | K | L | S | K | V | T | R | N | L | S | K | H | T | N | - | S | L | R | V | L | D | L | S | H | N | L | I | V | K | L | N | H | S | F | L | K | D | V | K | S | L | M | I | L | N | L | A | F | N | Y | L | K | H | L | S | D | A | S | F | Q | T | S | S | D | H | S | L | R | L | L | N | L | Q | R | N | P | I | H | C | S | C | D | L | L | D | F | I | L | W | L | E | - | K | S | D | T | V | L | P | Y | L | A | T | D | I | L | C | D | L | P | E | S | K | R | G | H | P | M | I | N | L | D | F | K | N | A | C | I | N | N | S | I | A | Q | I | L | Y | V | L | T | S | S | I | I | I | V | M | M | S | T | T | I | A | I | H | V | F | Y | W | D | I | S | Y | I | Y | N | F | W | T | A | K | I | K | S | Y | - | Y | L | K | T | - | - | T | D | C | T | Y | D | A | F | V | M | Y | D | T | N | D | P | M | A | E | E | W | V | L | N | H | L | R | F | E | L | E | D | R | G | K | R | V | R | P | L | C | L | A | E | R | D | W | T | P | G | T | P | I | M | D | N | L | N | Q | S | I | H | R | S | R | K | T | I | F | V | L | T | E | G | F | V | H | S | G | I | F | K | M | A | A | F | L | A | Q | Q | R | L | L | E | E | G | V | D | V | M | V | L | V | L | L | E | P | - | V | L | R | Q | S | R | I | L | N | L | R | R | C | L | C | G | H | S | V | L | E | W | P | R | N | - | - | P | A | A | E | G | W | F | W | Q | S | L | R | N | A | V | R | F | E | S | Q | G | G | Q | S | K | M | F | T | N | Y | F | S | G | R | - | - | - | - | - | - | - | - | - | - | - | - | - | - | - | - | - | - | - | - | - | - | - | - | - | - | - | - | - | - | - | - | - | - | - | - | - | - | - | - | - | - | - | - | - | - | - | - | - | - | - | - | - | - | - | - | - | - | - | - | - | - | - | - | - | - | - | - | - | - | - | - | - | - | - | - | - | - | - | - | - | - | - | - | - | - | - | - | - | - | - | - | - | - | - | - | - | - | - | - | - | - | - | - | - | - | - | - | - | - | - | - | - | - | - | - | - | - | - | - | - | - | - | - | - | - | - | - | - | - | - | - | - | - | - | - | - | - | - | - | - | - | - | - | - | - | - | - | - | - | - | - | - | - | - | - | - | - | - | - | - | - | - | - | - | - | - | - | - | - | - | - | - | - | - | - | - | - | - | - | - | - | - | - | - | - | - | - | - | - | - | - | - | - | - | - | - | - | - | - | - | - | - | - | - | - | - | - | - | - | - | - | - | - | - | - | - | - | - | - | - | - | - | - | - | - | - | - | - | - | - | - | - | - | - | - | - | - | - | - | - | - | - | - | - | - | - | - | - | - | - | - | - | - | - | - | - | - | - | - | - | - | - | - | - | - | - | - | - | - | - | - | - | - | - | - | - | - | - | - | - | - | - | - | - | - | - | - | - | - | - | - | - | - | - | - | - | - | - | - | - | - | - | - | - | - | - | - | - | - | - | - | - | - | - | - | - | - | - | - | - | - | - | - | - | - | - | - | - | - | - | - | - | - | - | - | - | - | - | - | - | - | - | - | - | - | - | - | - |
| DRTLR8A | - | - | - | - | - | - | - | - | - | - | - | - | - | - | - | M | V | K | N | L | - | D | I | G | L | I | L | I | L | K | L | T | - | - | - | - | - | - | - | - | - | - | - | L | A | L | S | E | L | N | V | R | I | L | K | T | Q | P | C | D | I | H | E | N | - | - | I | T | A | E | T | V | V | V | N | C | R | G | R | K | L | K | V | L | P | Q | - | F | L | A | N | T | T | Y | I | D | L | S | E | N | Y | I | K | N | L | T | V | Q | S | F | H | G | L | E | N | L | T | L | L | N | L | N | W | L | N | Q | N | R | - | - | - | - | - | - | E | V | V | I | A | K | G | V | F | S | N | L | T | K | L | R | V | L | D | L | N | G | I | K | L | K | Y | I | P | K | D | I | P | K | N | L | E | K | L | S | L | V | E | N | K | I | T | W | I | - | N | L | T | T | F | E | H | V | K | N | L | S | V | L | Y | L | S | N | N | C | Y | Y | W | N | P | C | S | R | R | Y | R | I | E | K | G | S | L | S | Y | L | V | N | L | K | R | L | T | L | S | F | N | N | L | T | Q | V | P | I | G | L | P | V | S | L | E | R | L | E | L | G | S | N | S | L | T | Y | I | G | E | H | D | F | R | G | L | F | N | L | T | V | L | K | I | Q | G | N | C | P | R | C | H | T | A | P | Y | P | C | I | P | C | K | - | - | N | T | S | I | E | I | H | P | Q | A | F | S | D | L | R | N | L | H | I | L | H | L | A | G | N | S | I | K | S | I | N | P | A | W | F | A | N | L | S | N | L | Q | Q | L | F | L | S | F | N | L | L | F | S | A | I | T | D | P | A | D | - | T | V | F | L | G | N | L | P | L | L | T | K | L | D | L | S | Y | N | F | A | F | K | - | T | Y | P | - | - | - | - | L | T | V | V | L | S | P | G | F | A | N | L | T | S | L | R | S | L | H | L | R | G | L | V | F | R | K | I | Q | K | D | T | F | K | S | L | F | D | L | Q | H | L | N | V | L | D | V | G | V | N | F | I | V | F | A | T | S | Y | I | - | - | - | F | Q | - | - | H | V | R | L | L | Y | L | A | E | N | R | L | Y | P | V | T | V | N | G | E | L | S | K | A | T | F | V | G | S | N | H | R | S | I | M | P | L | M | A | E | P | L | - | - | - | - | - | D | T | F | D | - | A | P | K | N | L | V | K | S | E | C | Y | N | A | G | R | V | L | D | L | S | R | N | N | L | F | L | I | S | P | E | Q | F | D | T | Y | G | N | I | S | C | L | N | L | S | R | N | G | F | S | T | A | P | N | G | S | E | F | T | S | L | P | N | L | K | Y | L | D | L | S | F | N | K | V | D | L | A | Y | D | N | A | F | R | E | L | Q | S | L | E | V | L | D | I | S | Y | N | S | H | Y | F | T | V | A | G | V | T | H | N | M | M | F | L | Q | Y | L | P | S | L | K | V | L | N | M | S | F | N | S | I | N | T | L | T | T | K | T | M | S | S | K | S | L | R | E | L | Q | F | R | G | N | K | L | G | R | M | W | R | D | K | D | N | T | Y | V | M | I | F | K | N | F | T | N | L | I | H | L | D | I | S | N | N | S | I | G | K | I | P | Y | T | V | Y | T | Q | L | P | I | T | I | Q | R | L | Q | M | S | H | N | Q | L | A | N | I | N | W | T | M | L | R | R | F | Q | N | L | R | E | L | I | L | H | D | N | N | I | I | E | I | A | S | N | L | S | V | D | V | P | - | S | L | E | L | L | N | L | Q | H | N | R | I | S | K | L | A | I | G | F | L | Q | G | V | V | N | L | K | E | L | D | L | S | Y | N | Y | L | I | T | V | N | Q | S | T | F | P | T | E | S | - | - | D | L | K | M | L | W | L | H | G | N | P | F | H | C | T | C | N | L | L | E | F | V | L | W | I | L | - | D | T | N | V | K | I | P | R | L | V | T | G | V | T | C | T | M | P | E | E | R | K | G | L | A | V | I | K | F | D | I | Q | - | E | C | I | D | D | Q | L | A | F | V | A | Y | F | I | S | A | A | C | I | I | C | T | T | F | A | A | I | T | M | H | L | F | Y | W | D | V | S | Y | L | Y | Y | Y | L | K | A | R | F | T | G | Y | Q | Q | L | S | S | - | - | E | S | C | I | Y | D | A | F | I | T | Y | D | T | K | D | P | Q | V | S | D | W | V | L | N | H | L | R | V | Q | L | E | E | R | S | E | L | F | L | P | I | C | L | E | E | R | D | W | I | P | G | S | P | V | L | D | S | L | T | Q | S | I | Q | N | S | R | K | T | V | F | V | L | T | E | G | Y | V | N | S | G | S | F | K | L | A | V | F | L | A | H | Q | R | L | L | E | E | N | E | D | V | I | V | L | L | L | L | E | P | - | V | L | Q | H | S | H | F | V | R | L | R | R | R | L | C | A | R | S | I | L | E | W | P | H | S | - | - | S | S | A | E | A | W | F | W | Q | S | L | R | N | A | I | R | V | D | N | Q | A | L | Y | S | E | L | Y | S | R | Y | F | T | T | K | X | X | X | X | X | X | X | X | X | X | X | X | X | X | X | X | X | X | X | X | X | X | - | - | - | - | - | - | - | - | - | - | - | - | - | - | - | - | - | - | - | - | - | - | - | - | - | - | - | - | - | - | - | - | - | - | - | - | - | - | - | - | - | - | - | - | - | - | - | - | - | - | - | - | - | - | - | - | - | - | - | - | - | - | - | - | - | - | - | - | - | - | - | - | - | - | - | - | - | - | - | - | - | - | - | - | - | - | - | - | - | - | - | - | - | - | - | - | - | - | - | - | - | - | - | - | - | - | - | - | - | - | - | - | - | - | - | - | - | - | - | - | - | - | - | - | - | - | - | - | - | - | - | - | - | - | - | - | - | - | - | - | - | - | - | - | - | - | - | - | - | - | - | - | - | - | - | - | - | - | - | - | - | - | - | - | - | - | - | - | - | - | - | - | - | - | - | - | - | - | - | - | - | - | - | - | - | - | - | - | - | - | - | - | - | - | - | - | - | - | - | - | - | - | - | - | - | - | - | - | - | - | - | - | - | - | - | - | - | - | - | - | - | - | - | - | - | - | - | - | - | - | - | - | - | - | - | - | - | - | - | - | - | - | - | - | - | - | - | - | - | - | - | - | - | - | - | - | - | - | - | - | - | - | - | - | - | - | - | - | - | - | - | - | - | - | - | - | - | - | - | - | - | - | - | - | - | - | - | - | - | - | - | - | - | - | - | - | - | - | - | - | - | - | - | - | - | - | - | - | - | - | - | - | - | - | - | - | - | - | - | - | - | - | - | - | - | - | - |
| MATLR8 | - | - | - | - | - | - | - | - | - | - | - | - | - | - | - | - | - | - | - | - | - | - | - | - | - | - | - | - | - | - | - | - | - | - | - | - | - | - | - | - | - | - | - | - | - | - | - | - | - | - | - | - | - | - | - | - | - | - | - | - | - | - | - | - | - | - | - | - | - | M | T | I | V | V | N | C | G | G | R | G | L | K | V | M | P | T | - | L | Q | R | N | T | T | S | L | D | L | S | E | N | R | I | K | N | L | T | S | G | V | F | R | Y | L | E | N | L | T | A | L | N | L | N | W | L | N | K | N | Q | - | - | - | - | - | - | K | V | V | V | G | D | E | E | F | V | N | L | T | K | L | R | T | L | E | L | D | G | F | R | L | K | N | V | P | K | K | L | P | K | N | L | Q | E | L | M | L | V | V | N | N | I | I | W | L | - | N | S | T | T | F | A | H | V | P | N | L | T | L | L | F | L | S | K | N | C | F | Y | W | N | P | C | L | R | D | Y | H | I | E | D | G | S | L | S | V | L | T | R | L | K | H | L | T | L | S | Y | N | N | I | T | R | V | P | R | G | L | P | V | S | L | R | T | L | E | L | A | S | N | R | I | A | Y | V | G | E | H | D | F | Q | G | L | D | N | L | T | T | L | K | I | Q | G | N | C | P | R | C | N | N | A | P | Y | P | C | V | P | C | K | - | - | N | V | S | I | E | I | H | P | R | A | F | S | N | L | K | K | L | Q | I | L | H | L | A | G | N | S | L | T | S | V | D | P | G | W | L | A | N | I | L | E | L | K | K | L | Y | L | S | F | N | Y | L | F | T | A | V | Q | - | - | - | E | - | N | V | F | L | G | N | L | P | L | L | T | K | L | D | L | S | F | N | F | A | L | K | - | T | Y | P | - | - | - | - | P | T | V | T | L | S | P | S | F | A | N | L | T | S | L | R | I | L | N | L | R | G | L | V | F | Q | E | I | H | D | D | T | F | R | S | L | Y | G | L | R | N | L | S | V | L | D | V | G | I | N | F | I | V | R | A | N | S | N | I | F | E | K | F | Q | - | - | H | V | K | L | L | Y | L | S | E | N | R | L | Y | P | V | I | - | N | R | N | L | R | K | D | T | G | V | G | L | K | S | S | F | A | M | P | S | V | S | D | P | Y | P | K | - | - | D | H | S | Y | D | - | V | P | K | N | L | V | K | P | E | C | Y | A | T | G | R | V | L | D | L | S | R | N | N | L | F | L | I | S | P | A | Q | F | E | S | Y | G | N | I | S | C | L | N | L | S | M | N | G | F | S | T | A | P | N | G | S | E | F | T | S | L | P | G | L | K | Y | L | D | L | S | Y | N | K | I | D | L | A | Y | D | H | A | F | Q | E | L | Q | S | L | E | V | L | D | L | S | Y | N | P | H | Y | F | T | V | Q | G | V | T | H | N | L | N | F | L | Q | Y | L | P | S | L | K | V | L | N | M | S | Y | N | Y | I | F | T | L | T | T | K | T | I | S | S | A | S | I | Q | E | L | Q | F | I | H | N | S | L | G | R | M | W | R | D | R | D | R | T | Y | D | R | L | F | A | N | L | T | N | L | T | Y | L | D | I | S | C | N | D | I | E | K | I | P | F | R | V | Y | T | L | L | P | R | S | I | Q | K | L | R | L | S | Q | N | D | L | V | V | F | N | W | T | M | L | R | N | F | P | H | L | Q | E | L | I | L | N | N | N | K | I | Y | Q | I | S | S | N | L | A | S | D | A | P | - | S | L | Q | F | L | N | L | Q | Y | N | R | I | S | K | L | S | D | G | F | L | K | G | A | V | N | L | E | A | L | D | L | S | H | N | Y | L | I | T | I | N | Q | S | T | F | P | P | E | T | E | S | Y | L | K | K | L | W | L | N | G | N | P | F | H | C | T | C | D | L | L | E | F | I | L | W | I | S | - | K | T | N | V | K | I | P | K | L | V | T | S | V | L | C | A | M | P | E | E | R | K | G | Q | S | V | I | K | F | K | T | E | - | E | C | N | N | D | Q | L | A | F | L | A | C | F | L | S | L | V | C | I | I | C | T | T | F | V | V | I | A | M | H | L | F | Y | W | D | A | S | Y | L | F | Y | Y | L | K | A | R | F | T | G | Y | Q | H | L | S | S | - | - | D | S | C | I | Y | D | A | F | I | T | Y | D | T | K | D | V | Q | V | S | D | W | V | L | N | H | L | R | V | Q | L | E | E | R | G | E | R | F | L | P | I | C | L | E | E | R | D | W | I | P | G | S | P | V | L | D | S | L | T | Q | S | I | Q | H | S | R | K | T | V | F | V | L | T | E | A | Y | V | N | S | G | S | F | K | L | A | V | F | L | A | H | Q | R | L | V | E | D | N | E | D | V | I | V | L | L | L | L | E | P | - | V | L | Q | Y | S | H | F | L | R | L | R | R | R | L | C | A | R | S | I | L | E | W | P | R | S | - | - | S | S | A | E | A | W | F | W | Q | S | L | R | N | A | I | R | V | D | N | Q | A | M | Y | S | E | L | Y | S | R | Y | F | T | T | K | X | X | X | X | X | X | X | X | X | X | X | X | X | X | X | X | X | X | X | X | X | X | X | X | X | X | X | X | X | X | X | X | X | X | X | X | X | X | X | X | X | X | X | X | X | X | - | X | - | - | X | X | X | - | - | - | X | X | - | X | X | X | X | X | - | X | - | - | - | - | - | - | - | - | - | - | - | - | - | - | - | - | - | - | - | - | - | - | - | - | - | - | - | - | - | - | - | - | - | - | - | - | - | - | - | - | - | - | - | - | - | - | - | - | - | - | - | - | - | - | - | - | - | - | - | - | - | - | - | - | - | - | - | - | - | - | - | - | - | - | - | - | - | - | - | - | - | - | - | - | - | - | - | - | - | - | - | - | - | - | - | - | - | - | - | - | - | - | - | - | - | - | - | - | - | - | - | - | - | - | - | - | - | - | - | - | - | - | - | - | - | - | - | - | - | - | - | - | - | - | - | - | - | - | - | - | - | - | - | - | - | - | - | - | - | - | - | - | - | - | - | - | - | - | - | - | - | - | - | - | - | - | - | - | - | - | - | - | - | - | - | - | - | - | - | - | - | - | - | - | - | - | - | - | - | - | - | - | - | - | - | - | - | - | - | - | - | - | - | - | - | - | - | - | - | - | - | - | - | - | - | - | - | - | - | - | - | - | - | - | - | - | - | - | - | - | - | - | - | - | - | - | - | - | - | - | - | - | - | - | - | - | - | - | - | - | - | - | - | - | - | - | - | - | - | - | - | - | - | - | - | - | - | - | - | - | - | - | - | - | - | - | - | - | - | - | - | - | - |
| CATLR8 | - | - | - | - | - | - | - | - | - | - | - | - | - | - | - | M | M | T | E | L | - | D | L | W | L | I | M | T | L | K | I | T | - | - | - | - | - | - | - | - | - | - | - | L | T | L | C | K | T | E | M | A | N | P | K | N | Q | P | C | D | I | Y | D | N | - | - | V | T | A | M | T | V | K | V | D | C | H | G | R | R | L | Q | V | F | P | K | - | F | L | P | N | T | T | D | L | D | L | S | E | N | R | I | K | N | I | T | A | E | N | F | Q | N | L | A | N | L | T | H | L | N | L | N | W | L | N | D | K | Y | - | - | - | - | - | - | K | V | L | V | G | A | G | V | F | S | N | M | T | K | L | R | K | L | E | L | N | G | I | G | L | K | D | V | P | K | D | I | P | K | N | L | Q | E | L | K | L | V | E | N | K | I | T | R | L | - | N | S | A | S | F | G | H | L | R | N | L | S | L | I | Y | L | S | R | N | C | Y | Y | W | N | P | C | S | R | - | Y | H | I | E | D | G | S | F | S | A | L | S | K | L | K | N | L | T | L | S | F | N | N | L | T | Q | V | P | K | G | L | P | V | S | L | M | K | L | E | L | A | S | N | R | I | S | Y | V | G | E | H | D | F | Q | G | L | D | N | L | I | S | L | N | I | Q | G | N | C | P | R | C | H | T | A | P | Y | P | C | I | P | C | K | - | - | N | V | S | I | E | I | H | P | R | A | F | A | G | L | G | K | L | R | I | L | D | L | A | G | N | S | I | E | K | I | D | P | T | W | F | A | N | I | S | K | L | E | Q | L | Y | L | S | F | N | F | L | F | E | A | V | Q | - | - | - | N | - | N | V | F | L | N | N | L | P | L | L | R | K | L | D | L | S | F | N | Y | A | L | K | - | I | Y | P | - | - | - | - | T | T | V | K | I | S | P | S | F | A | N | L | T | S | L | R | T | L | H | L | R | G | L | V | F | Q | E | I | Q | E | E | T | F | S | S | L | Y | G | L | R | N | L | S | V | L | D | V | G | V | N | F | I | V | R | A | K | A | E | I | F | E | K | F | Q | - | - | H | V | K | L | I | Y | L | S | E | N | R | L | Y | P | - | - | - | - | - | E | L | R | K | D | A | G | V | G | L | K | S | S | S | V | M | T | S | M | S | D | P | V | P | K | - | - | G | N | S | F | D | - | I | P | E | D | L | V | K | P | E | C | S | A | T | G | R | V | L | D | L | S | R | N | N | L | F | L | I | T | P | E | Q | F | Q | P | F | G | N | I | S | C | L | N | L | S | G | N | G | F | A | T | A | P | N | G | S | E | F | T | S | L | P | D | L | K | Y | L | D | L | S | S | N | R | I | D | L | A | Y | D | Y | A | F | R | E | L | Q | S | L | E | V | L | D | L | S | D | N | S | H | Y | F | T | V | E | G | V | T | L | N | L | N | F | L | K | Y | L | P | S | L | K | V | L | N | M | S | N | N | R | I | Y | T | L | T | T | K | T | M | S | S | K | S | L | Q | E | L | Q | F | Q | K | N | K | L | G | R | M | W | R | L | K | D | Q | T | Y | D | R | L | F | E | D | L | T | N | V | T | H | L | D | I | S | L | N | N | I | K | T | I | P | F | R | I | Y | T | Y | L | P | K | C | I | Q | K | L | V | L | S | E | N | R | L | T | S | I | S | W | E | L | L | R | Q | F | P | H | L | R | E | L | I | L | S | K | N | N | I | L | R | I | S | S | N | L | S | S | D | V | P | - | S | L | Q | F | L | N | L | W | H | N | R | I | S | E | L | S | S | G | F | L | Q | G | A | I | N | L | E | R | L | D | L | G | H | N | K | L | I | N | I | N | Q | S | T | F | P | S | E | N | E | I | H | L | K | T | L | W | L | N | G | N | P | F | H | C | T | C | N | M | F | D | F | V | L | W | I | R | - | K | T | K | V | K | I | P | K | L | V | T | A | V | T | C | A | M | P | E | E | R | Q | G | I | A | V | I | Q | F | D | I | T | - | E | C | N | D | D | Q | L | A | F | L | A | Y | F | F | S | T | F | C | I | I | C | T | T | F | V | A | V | A | M | H | L | F | Y | W | D | V | S | Y | L | F | Y | Y | V | K | A | I | F | T | G | Y | Q | K | L | S | S | - | - | D | S | C | I | Y | D | A | F | I | T | Y | D | T | K | D | T | Q | V | S | E | W | V | I | N | H | L | R | V | Q | L | E | E | Q | G | E | R | F | L | P | I | C | L | E | E | R | D | W | I | P | G | S | P | V | L | D | S | L | T | Q | S | I | Q | H | S | R | K | T | V | F | V | L | T | E | R | Y | V | N | S | G | S | F | K | M | A | I | F | L | A | H | Q | R | L | L | E | D | N | E | D | V | I | V | L | L | L | L | E | P | - | V | L | Q | H | S | H | F | M | R | L | R | R | R | L | C | G | R | S | V | L | E | W | P | H | S | - | - | P | S | A | E | A | W | F | W | Q | S | L | R | N | A | I | R | V | D | N | Q | A | M | Y | S | D | L | Y | S | R | Y | L | T | T | K | X | X | X | X | X | X | X | X | X | X | X | X | X | X | X | X | X | X | X | X | X | X | X | - | - | - | - | - | - | - | - | - | - | - | - | - | - | - | - | - | - | - | - | - | - | - | - | - | - | - | - | - | - | - | - | - | - | - | - | - | - | - | - | - | - | - | - | - | - | - | - | - | - | - | - | - | - | - | - | - | - | - | - | - | - | - | - | - | - | - | - | - | - | - | - | - | - | - | - | - | - | - | - | - | - | - | - | - | - | - | - | - | - | - | - | - | - | - | - | - | - | - | - | - | - | - | - | - | - | - | - | - | - | - | - | - | - | - | - | - | - | - | - | - | - | - | - | - | - | - | - | - | - | - | - | - | - | - | - | - | - | - | - | - | - | - | - | - | - | - | - | - | - | - | - | - | - | - | - | - | - | - | - | - | - | - | - | - | - | - | - | - | - | - | - | - | - | - | - | - | - | - | - | - | - | - | - | - | - | - | - | - | - | - | - | - | - | - | - | - | - | - | - | - | - | - | - | - | - | - | - | - | - | - | - | - | - | - | - | - | - | - | - | - | - | - | - | - | - | - | - | - | - | - | - | - | - | - | - | - | - | - | - | - | - | - | - | - | - | - | - | - | - | - | - | - | - | - | - | - | - | - | - | - | - | - | - | - | - | - | - | - | - | - | - | - | - | - | - | - | - | - | - | - | - | - | - | - | - | - | - | - | - | - | - | - | - | - | - | - | - | - | - | - | - | - | - | - | - | - | - | - | - | - | - | - | - | - | - | - | - | - | - | - | - | - | - | - | - |
| CCTLR8 | - | - | - | - | - | - | - | - | - | - | - | - | - | - | - | M | M | I | K | L | - | D | L | W | L | I | M | T | L | Q | I | T | - | - | - | - | - | - | - | - | - | - | - | L | T | L | C | Q | M | D | K | A | S | L | K | T | Q | P | C | D | I | H | E | N | - | - | V | T | A | M | T | V | V | V | N | C | R | G | R | A | L | K | K | V | P | M | - | L | L | P | N | T | T | Y | L | D | L | S | E | N | K | I | K | N | I | T | A | G | D | F | R | D | L | A | N | L | T | N | L | N | L | N | W | L | N | N | N | Q | - | - | - | - | - | - | K | V | L | I | G | A | G | V | F | S | N | M | T | M | L | R | T | L | E | L | N | G | I | G | L | K | D | V | P | K | D | I | P | K | N | L | R | E | L | K | L | V | E | N | K | I | T | R | L | - | N | S | T | S | F | R | H | V | Q | N | L | S | L | I | Y | L | S | K | N | C | Y | Y | W | N | P | C | S | S | F | Y | H | I | E | G | G | S | F | S | V | L | T | K | L | K | H | L | T | L | S | Y | N | N | I | T | Q | V | P | K | G | L | P | A | S | L | R | T | L | E | L | A | S | N | R | I | S | Y | V | G | E | H | D | F | Q | G | L | D | N | L | T | I | L | K | I | Q | G | N | C | P | R | C | H | N | A | P | Y | P | C | I | P | C | K | - | - | N | V | S | I | E | I | H | P | R | A | F | A | G | L | E | K | L | R | L | L | H | L | A | G | N | S | I | E | K | I | D | P | T | W | F | A | N | L | S | K | L | E | E | L | Y | L | S | F | N | F | L | F | N | A | V | A | - | - | - | N | - | N | V | F | L | N | N | L | P | L | L | T | K | L | D | L | S | F | N | Y | A | L | K | - | C | Y | P | - | - | - | - | N | T | V | T | L | S | P | S | F | A | N | L | T | S | L | R | N | L | H | L | R | G | L | V | F | R | E | I | Q | E | E | T | L | S | P | L | F | G | L | R | N | L | S | V | L | D | V | G | I | N | F | I | V | R | A | K | L | D | I | F | E | K | L | Q | - | - | H | V | K | L | I | Y | L | S | E | N | R | F | Y | P | V | T | V | N | G | E | L | R | K | D | V | G | I | G | L | K | S | S | S | M | M | P | S | M | S | E | P | Y | P | K | - | - | D | N | V | F | D | - | I | P | K | N | L | V | K | P | E | C | Y | A | A | G | R | V | L | D | L | S | R | N | N | L | F | L | I | S | P | K | Q | F | E | T | Y | G | K | I | S | C | L | N | L | S | G | N | G | F | A | T | A | P | N | G | S | E | F | T | S | L | P | D | L | K | Y | L | D | L | S | F | N | K | I | D | L | A | Y | D | Y | A | F | R | E | L | Q | S | L | E | V | L | D | L | S | Y | N | P | H | Y | F | T | V | Q | G | V | T | H | N | L | N | F | L | K | Y | L | P | S | L | K | V | L | N | M | S | Y | N | S | I | Y | T | L | T | T | K | T | M | S | S | K | S | L | Q | E | L | Q | F | Q | H | N | S | L | G | K | M | W | R | V | K | D | R | T | Y | D | R | L | F | E | D | L | T | N | L | T | Y | L | D | I | S | Y | N | Y | I | E | K | I | P | L | R | I | Y | K | H | L | P | R | S | I | Q | R | L | R | I | S | H | N | H | L | A | N | V | S | W | E | I | L | R | Q | F | P | H | L | R | E | L | I | L | S | N | N | N | I | L | K | I | S | G | N | L | S | S | D | V | P | - | S | L | E | F | L | D | L | R | H | N | L | I | S | E | L | S | G | G | F | L | L | G | A | V | N | L | K | N | L | D | L | S | H | N | H | L | I | T | I | N | Q | S | T | F | P | S | E | T | E | S | Y | L | K | T | L | S | L | N | G | N | P | F | H | C | T | C | N | L | L | E | F | I | L | W | I | R | - | K | T | N | V | K | I | P | R | L | V | T | A | V | T | C | A | M | P | E | E | R | K | G | L | A | V | V | H | F | D | I | K | - | E | C | I | D | D | Q | L | A | F | L | A | Y | F | F | S | I | V | C | I | I | C | T | T | F | V | A | V | S | M | H | L | F | Y | W | D | V | S | Y | L | F | Y | Y | L | K | A | R | F | T | G | Y | Q | H | L | S | S | - | - | D | S | C | I | Y | D | A | F | I | T | Y | D | T | K | D | T | Q | V | S | D | W | V | L | N | H | L | R | V | Q | L | E | E | Q | G | E | R | F | L | P | I | C | L | E | E | R | D | W | I | P | G | S | A | V | L | D | S | L | T | Q | S | I | Q | H | S | R | K | T | V | F | V | L | T | E | R | Y | V | N | S | G | S | F | K | L | A | V | F | L | A | H | Q | R | L | L | E | E | N | E | D | V | I | V | L | L | L | L | E | P | - | V | L | Q | H | S | H | F | L | R | L | R | R | R | L | C | G | R | S | I | L | E | W | P | H | S | - | - | P | S | A | E | A | W | F | W | Q | S | L | R | N | A | I | R | V | D | N | Q | A | M | Y | S | E | L | Y | S | R | Y | F | T | T | K | X | X | X | X | X | X | X | X | X | X | X | X | X | X | X | X | X | - | - | - | - | - | - | - | - | - | - | - | - | - | - | - | - | - | - | - | - | - | - | - | - | - | - | - | - | - | - | - | - | - | - | - | - | - | - | - | - | - | - | - | - | - | - | - | - | - | - | - | - | - | - | - | - | - | - | - | - | - | - | - | - | - | - | - | - | - | - | - | - | - | - | - | - | - | - | - | - | - | - | - | - | - | - | - | - | - | - | - | - | - | - | - | - | - | - | - | - | - | - | - | - | - | - | - | - | - | - | - | - | - | - | - | - | - | - | - | - | - | - | - | - | - | - | - | - | - | - | - | - | - | - | - | - | - | - | - | - | - | - | - | - | - | - | - | - | - | - | - | - | - | - | - | - | - | - | - | - | - | - | - | - | - | - | - | - | - | - | - | - | - | - | - | - | - | - | - | - | - | - | - | - | - | - | - | - | - | - | - | - | - | - | - | - | - | - | - | - | - | - | - | - | - | - | - | - | - | - | - | - | - | - | - | - | - | - | - | - | - | - | - | - | - | - | - | - | - | - | - | - | - | - | - | - | - | - | - | - | - | - | - | - | - | - | - | - | - | - | - | - | - | - | - | - | - | - | - | - | - | - | - | - | - | - | - | - | - | - | - | - | - | - | - | - | - | - | - | - | - | - | - | - | - | - | - | - | - | - | - | - | - | - | - | - | - | - | - | - | - | - | - | - | - | - | - | - | - | - | - | - | - | - | - | - | - | - | - | - | - | - | - | - | - | - | - | - | - | - | - | - |
| CBTLR8 | - | - | - | - | - | - | - | - | - | - | - | - | - | - | - | - | - | - | - | - | - | - | - | - | - | - | - | - | - | - | - | - | - | - | - | - | - | - | - | - | - | - | - | - | - | - | - | - | - | - | - | - | - | - | - | - | - | - | - | - | - | - | - | - | - | - | - | - | - | - | - | - | - | - | - | - | - | - | - | - | - | - | - | - | - | - | - | - | - | - | - | - | - | - | - | - | - | - | - | - | - | - | - | - | - | - | - | - | - | - | - | - | - | - | - | - | - | - | - | - | - | - | - | - | - | - | - | - | - | - | - | - | - | - | - | - | - | - | - | - | - | - | - | - | - | - | - | - | - | - | - | - | - | - | - | - | - | - | - | - | - | - | - | - | - | - | - | - | - | - | - | - | - | - | - | - | - | - | - | - | - | - | - | - | - | - | - | - | - | - | - | - | - | - | - | - | - | - | - | - | - | - | - | - | - | - | - | - | - | - | - | - | - | - | - | - | - | - | - | - | - | - | - | - | - | - | - | - | - | - | - | - | - | - | - | - | - | - | - | - | - | - | - | - | - | - | - | - | - | - | - | - | - | - | - | - | M | I | S | S | I | G | E | D | D | F | K | E | L | S | N | L | K | T | L | K | I | Q | G | N | C | P | R | C | H | N | A | P | Y | P | C | V | P | C | A | - | - | N | G | S | I | S | I | H | I | R | A | F | D | H | L | R | N | L | T | L | L | H | L | A | G | N | S | I | S | V | M | Q | K | D | W | F | G | N | L | T | N | L | R | E | L | Y | L | S | Y | N | F | L | S | D | Q | I | E | - | - | - | H | - | G | N | F | L | S | N | L | W | F | L | R | K | L | D | L | S | Y | N | Y | A | L | Q | - | S | Y | P | - | - | - | - | E | T | V | S | L | S | P | S | F | A | N | L | S | S | L | R | T | L | H | M | Q | G | L | V | F | R | K | I | E | K | D | S | L | K | H | L | Y | G | L | Q | N | L | S | T | L | D | L | G | I | N | F | I | V | A | V | D | P | D | V | F | K | Q | F | H | - | - | H | L | K | L | L | Y | L | S | E | N | R | L | Y | P | I | T | N | S | E | V | R | R | A | D | N | S | I | - | K | P | P | R | F | N | L | P | G | L | M | D | S | S | P | Q | R | Q | Q | E | P | F | T | K | P | I | P | R | L | I | K | P | E | C | Y | A | A | G | R | V | L | D | L | S | R | N | N | L | F | F | I | S | P | K | Q | F | E | G | Y | E | N | I | S | C | L | N | L | S | R | N | G | F | A | S | A | L | N | G | T | E | F | T | T | L | P | N | L | K | Y | L | D | L | S | C | N | K | I | D | L | A | Y | N | Y | A | F | E | E | L | K | E | L | E | V | L | D | L | S | S | N | S | H | Y | F | R | V | P | G | V | T | H | N | L | F | F | I | Q | N | L | P | K | L | R | V | L | N | M | S | S | N | S | I | F | T | L | T | T | R | Y | M | C | S | D | S | L | A | E | L | Q | F | Q | D | N | Q | L | G | K | L | W | K | - | - | D | E | K | Y | V | K | L | F | W | N | L | T | N | L | T | N | L | D | I | S | S | N | S | I | W | T | M | H | E | N | V | Y | K | Y | L | P | V | T | L | K | R | F | R | L | S | H | N | N | L | K | S | L | N | W | T | L | M | R | N | F | I | Q | L | E | E | L | I | L | S | H | N | S | M | T | Y | V | S | G | N | I | T | L | N | I | P | - | S | L | R | Y | L | D | L | S | H | N | K | I | S | Y | L | Q | D | R | F | L | E | N | A | L | N | L | K | R | L | D | L | S | Y | N | K | I | S | T | I | H | N | S | T | F | P | S | N | E | A | L | H | L | E | T | L | C | L | H | N | N | P | Y | H | C | T | C | G | M | L | D | F | I | L | W | I | K | - | N | S | K | V | K | I | P | K | L | Y | T | S | L | K | C | S | V | P | E | A | I | K | G | N | S | L | I | T | F | D | I | K | - | Q | C | L | D | P | N | V | A | W | - | I | Y | I | V | T | T | L | F | I | V | C | V | T | F | V | A | T | L | M | H | L | F | Y | W | D | L | S | Y | V | F | Y | F | A | K | A | K | V | K | G | Y | Q | H | L | S | S | - | - | R | D | N | V | Y | D | A | F | V | T | Y | D | T | K | D | P | Q | V | S | D | W | V | L | N | H | L | R | V | Q | L | E | E | Q | G | D | H | F | L | P | V | C | L | E | E | R | D | W | L | P | G | C | P | I | L | D | S | L | T | R | S | I | R | Q | S | R | K | T | V | F | V | L | T | H | S | Y | V | N | S | G | S | F | K | M | A | M | Y | L | A | H | Q | R | L | L | D | E | S | D | D | V | I | V | L | L | L | L | E | P | - | V | L | Q | N | S | H | F | L | R | L | R | R | R | L | C | S | H | S | V | L | E | W | P | R | T | - | - | P | A | A | E | P | W | F | W | Q | C | L | R | N | A | I | R | V | E | N | K | V | V | Y | N | N | I | Y | S | R | Y | F | T | I | K | K | R | P | R | E | K | X | X | X | X | X | X | X | X | X | X | X | X | X | X | X | X | X | X | X | X | X | X | X | X | X | X | X | X | X | X | X | X | X | X | X | X | X | X | X | X | - | X | - | - | X | X | X | X | - | X | X | X | X | X | X | X | X | X | X | X | X | X | - | X | X | X | X | X | X | X | - | X | X | X | X | X | X | X | - | X | X | X | X | X | X | X | X | X | X | X | X | X | X | X | X | X | X | X | X | X | X | X | - | X | X | X | X | X | X | X | X | X | X | X | X | X | X | X | X | X | X | X | X | X | X | - | X | X | X | X | X | X | X | X | X | X | X | X | - | - | - | X | X | X | X | X | X | X | X | X | X | X | X | X | X | X | X | X | X | X | X | X | X | X | X | X | X | X | X | X | X | X | X | X | X | X | X | X | X | X | X | X | - | X | X | X | X | X | X | X | X | X | X | X | X | X | - | X | X | X | X | X | X | X | X | X | X | X | X | - | X | X | X | X | X | X | X | X | X | X | X | X | X | X | X | X | X | - | X | X | X | X | X | - | X | X | X | X | - | - | X | X | X | X | - | - | X | X | X | - | - | X | X | - | - | - | - | X | - | - | - | - | - | - | - | - | - | - | - | - | - | - | - | - | - | - | - | - | - | - | - | - | - | - | - | - | - | - | - | - | - | - | - | - | - | - | - | - | - | - | - | - | - | - | - | - | - | - | - | - | - | - | - | - | - | - | - | - | - | - | - | - | - | - | - | - | - | - | - | - | - | - | - | - | - | - | - | - | - | - | - | - | - |
| TFTLR8 | - | - | - | - | - | - | - | - | - | - | M | E | A | S | K | H | W | L | M | L | - | I | C | W | V | I | I | T | F | W | I | S | - | - | - | - | - | - | - | - | - | - | - | L | V | A | C | K | P | N | N | E | P | L | M | T | R | R | C | D | I | Q | N | N | - | - | T | N | - | - | G | T | V | F | D | C | H | G | R | R | L | R | V | M | P | Q | - | V | Y | L | N | T | T | S | L | D | A | S | E | N | E | I | R | N | L | T | A | D | S | L | K | D | L | Q | K | L | K | Y | L | R | L | N | W | M | N | H | R | Q | - | - | - | - | - | - | K | V | S | V | S | R | G | V | F | T | N | L | S | N | L | E | I | L | E | L | N | G | V | G | V | S | E | V | P | A | H | L | P | I | S | L | R | Q | L | R | L | D | E | N | N | I | S | F | L | - | S | R | Q | N | L | S | Q | L | K | N | L | T | H | L | Y | L | S | K | N | C | Y | Y | G | R | P | C | Q | R | R | F | E | I | V | N | G | T | F | S | D | L | D | Q | L | K | H | L | S | L | S | Y | N | N | I | T | H | V | P | R | Y | L | P | V | S | L | V | T | F | E | L | A | S | N | V | I | S | F | I | G | E | D | D | F | K | G | L | P | N | L | K | T | L | K | I | Q | G | N | C | P | R | C | H | N | A | P | Y | P | C | T | P | C | E | - | - | N | G | S | I | D | I | H | E | Q | A | F | G | N | L | K | N | L | T | L | L | H | L | A | G | N | S | I | S | L | I | K | K | A | W | F | E | N | L | S | Q | L | Q | E | L | Y | L | S | Y | N | F | L | A | S | Q | I | E | - | - | - | D | - | G | A | F | L | S | N | L | R | L | L | K | K | L | D | L | S | Y | N | Y | H | L | Q | - | A | Y | P | - | - | - | - | D | T | V | R | L | S | P | T | F | A | N | L | Q | S | L | R | I | L | H | I | Q | G | L | V | F | K | K | I | Q | N | D | S | L | A | P | L | Y | G | L | Q | N | L | S | V | L | D | I | G | V | N | F | I | V | A | V | D | S | N | I | F | N | Q | F | S | - | - | N | L | Q | L | L | Y | L | S | E | N | R | L | Y | P | I | S | N | S | S | V | R | N | P | D | N | N | M | - | M | P | S | H | F | V | Q | P | E | L | T | D | S | - | - | Q | K | T | L | E | P | Y | Q | - | V | N | K | Q | L | V | K | P | E | C | V | A | A | G | R | V | L | D | L | S | R | N | N | L | F | F | I | S | P | K | Q | F | V | D | Y | K | N | I | S | C | L | N | L | S | R | N | G | F | S | S | A | L | N | G | T | E | F | T | S | L | P | N | L | K | Y | L | D | L | S | F | N | R | I | D | L | A | Y | D | Y | A | F | K | E | L | Q | E | L | E | V | L | D | L | S | F | N | P | H | Y | F | I | V | P | G | V | T | H | N | L | Q | F | L | Q | N | L | P | K | L | R | V | L | N | M | S | S | N | S | I | F | T | L | T | S | K | R | M | Y | S | K | S | L | A | E | L | Q | F | Q | D | N | N | L | A | R | I | W | R | - | - | D | R | K | Y | V | Q | L | F | W | N | L | T | N | L | T | Y | L | D | I | S | H | N | Y | I | Q | N | I | P | K | D | V | Y | K | Y | L | P | L | T | L | K | T | F | R | L | N | G | N | I | L | T | S | L | N | W | T | L | M | R | A | F | T | Q | L | E | E | L | S | L | S | Y | N | H | L | T | H | V | S | Q | N | I | T | Q | N | I | P | - | S | L | R | Y | L | D | L | S | N | N | R | I | S | Q | L | A | T | S | L | L | N | G | A | F | N | L | K | M | L | D | L | S | Y | N | K | L | S | T | V | N | Q | S | T | F | P | A | N | G | E | T | N | L | S | T | L | W | L | H | R | N | P | F | H | C | T | C | D | I | L | E | F | A | R | W | I | S | - | G | T | K | V | K | I | P | Y | L | Y | T | L | V | T | C | T | A | P | F | S | N | K | G | K | L | V | V | D | F | D | L | Q | - | E | C | L | D | G | Y | V | A | F | L | A | Y | F | F | T | T | F | F | I | V | G | V | T | F | V | T | T | L | M | H | I | F | Y | W | D | V | S | Y | V | F | Y | Y | V | K | A | K | F | K | G | Y | Q | H | L | S | S | - | - | G | D | N | V | Y | D | A | F | V | T | Y | D | T | K | D | P | Q | V | S | E | W | V | L | N | H | L | R | V | Q | L | E | E | E | G | D | R | F | L | P | V | C | L | E | E | R | D | W | L | P | G | C | P | I | L | D | S | L | T | Q | S | I | R | Q | S | R | K | T | I | F | V | L | T | Q | S | Y | V | N | S | G | S | F | K | M | A | I | Y | L | A | H | Q | R | L | L | D | E | N | E | D | V | I | V | L | L | L | L | E | P | - | V | L | Q | N | S | H | F | L | R | L | R | R | R | L | C | S | H | S | V | L | E | W | P | Q | T | - | - | P | A | A | E | P | W | F | W | Q | C | V | R | N | A | I | R | L | E | N | K | V | M | Y | S | S | I | Y | S | R | Y | F | T | I | K | K | R | P | R | E | K | X | X | X | X | X | X | X | X | X | X | X | - | - | - | - | - | - | - | - | - | - | - | - | - | - | - | - | - | - | - | - | - | - | - | - | - | - | - | - | - | - | - | - | - | - | - | - | - | - | - | - | - | - | - | - | - | - | - | - | - | - | - | - | - | - | - | - | - | - | - | - | - | - | - | - | - | - | - | - | - | - | - | - | - | - | - | - | - | - | - | - | - | - | - | - | - | - | - | - | - | - | - | - | - | - | - | - | - | - | - | - | - | - | - | - | - | - | - | - | - | - | - | - | - | - | - | - | - | - | - | - | - | - | - | - | - | - | - | - | - | - | - | - | - | - | - | - | - | - | - | - | - | - | - | - | - | - | - | - | - | - | - | - | - | - | - | - | - | - | - | - | - | - | - | - | - | - | - | - | - | - | - | - | - | - | - | - | - | - | - | - | - | - | - | - | - | - | - | - | - | - | - | - | - | - | - | - | - | - | - | - | - | - | - | - | - | - | - | - | - | - | - | - | - | - | - | - | - | - | - | - | - | - | - | - | - | - | - | - | - | - | - | - | - | - | - | - | - | - | - | - | - | - | - | - | - | - | - | - | - | - | - | - | - | - | - | - | - | - | - | - | - | - | - | - | - | - | - | - | - | - | - | - | - | - | - | - | - | - | - | - | - | - | - | - | - | - | - | - | - | - | - | - | - | - | - | - | - | - | - | - | - | - | - | - | - | - | - | - | - | - | - | - | - | - | - | - | - | - | - | - | - | - | - | - | - | - | - | - | - | - | - | - |
| IPTLR8 | - | - | - | - | - | - | - | - | - | - | M | E | D | A | K | Y | R | L | M | L | - | V | C | W | V | I | I | T | F | I | F | G | - | - | - | - | - | - | - | - | - | - | - | L | A | V | C | S | L | D | I | K | F | L | R | T | Q | P | C | D | I | H | Q | N | - | - | T | T | T | G | T | V | E | F | D | C | R | G | R | Q | L | R | V | V | P | L | - | V | F | E | N | T | T | S | L | D | L | S | E | N | E | I | K | N | L | T | L | D | S | L | K | G | L | Q | K | L | I | N | L | N | L | N | W | M | N | H | N | Q | - | - | - | - | - | - | H | V | N | I | T | E | G | V | F | A | N | L | T | N | L | E | H | L | R | L | N | G | L | G | L | P | E | V | P | A | H | L | P | K | S | L | K | E | L | R | L | D | E | N | K | I | F | S | L | - | S | P | R | N | F | S | Q | L | K | N | L | T | H | I | Y | L | S | K | N | C | Y | Y | W | N | P | C | L | I | N | F | E | I | E | I | G | T | F | S | G | L | H | K | L | Q | H | L | S | L | S | Y | N | N | L | S | H | V | P | R | D | L | P | V | S | L | V | T | L | E | L | A | S | N | N | I | S | F | I | G | E | D | D | F | K | E | L | R | N | L | K | T | L | K | I | Q | G | N | C | P | R | C | H | N | A | P | Y | P | C | I | P | C | S | - | - | N | G | S | I | D | I | H | E | R | A | F | N | Q | L | R | K | L | E | L | L | H | L | A | G | N | S | I | S | I | I | K | K | V | W | F | E | K | T | L | Y | L | R | E | L | Y | L | S | F | N | F | L | T | G | Q | I | K | - | - | - | D | - | G | A | F | L | S | N | L | R | L | L | E | K | L | D | L | S | F | N | Y | G | L | Q | - | A | Y | P | - | - | - | - | E | T | V | H | L | A | P | T | F | A | N | L | F | S | L | R | T | L | H | I | Q | G | L | V | F | K | K | I | Q | N | N | S | L | A | S | L | Y | G | L | K | N | L | S | T | L | D | L | G | V | N | F | I | V | S | V | D | S | G | I | F | N | K | F | H | - | - | N | L | K | L | L | Y | L | S | E | N | R | L | Y | P | I | T | N | S | D | V | S | K | P | D | N | N | I | - | K | P | P | L | F | N | L | P | Q | L | T | Y | S | S | S | K | R | K | L | D | P | Y | Q | - | L | N | K | G | L | I | K | P | E | C | F | A | A | G | H | V | L | D | L | S | R | N | N | L | F | F | I | S | P | K | Q | F | E | G | H | K | N | I | S | C | L | N | L | S | R | N | A | F | A | S | A | L | N | G | T | E | F | T | S | L | P | S | L | K | Y | L | D | L | S | F | N | K | I | D | L | A | Y | D | Y | A | F | N | E | L | I | E | L | E | V | L | D | L | S | F | N | E | H | Y | F | R | V | P | G | V | T | H | N | L | K | F | I | K | N | L | P | K | L | R | V | L | N | M | S | S | N | S | I | F | T | L | T | T | K | H | M | C | S | D | S | L | A | E | L | Q | F | H | H | N | Q | L | G | R | L | W | K | E | K | D | E | T | Y | F | K | L | F | W | N | L | T | N | L | T | H | L | D | I | S | Y | N | N | I | K | N | I | P | T | E | V | Y | K | Y | L | P | I | T | I | K | R | F | R | L | N | G | N | A | L | T | S | L | N | W | T | L | M | K | N | F | T | Q | L | E | E | L | I | L | G | Y | N | S | L | T | Y | A | S | R | N | I | T | Q | N | I | P | - | S | L | L | Y | L | D | L | S | H | N | R | I | S | Q | L | A | T | G | F | I | Q | G | A | V | N | L | K | M | L | D | L | S | N | N | K | L | S | T | I | N | Q | S | T | F | P | S | K | D | T | N | H | L | N | T | L | W | L | H | N | N | P | Y | R | C | T | C | D | I | M | D | F | I | L | W | I | F | - | E | S | D | V | K | I | P | H | L | V | T | M | V | K | C | N | V | P | E | A | T | K | G | K | G | V | I | F | F | D | I | R | - | E | C | I | D | G | Q | V | A | F | L | A | Y | F | F | S | T | L | L | I | V | G | V | T | F | V | A | T | L | M | H | M | F | Y | W | D | F | S | Y | V | F | Y | Y | V | K | A | K | F | K | G | Y | Q | H | F | S | S | - | - | G | D | N | V | Y | G | A | F | V | T | Y | D | T | K | D | S | Q | V | S | E | W | V | L | N | H | L | R | A | Q | L | E | E | Q | G | D | H | F | L | P | V | C | L | E | E | R | D | W | L | P | G | C | P | I | L | D | S | L | T | Q | S | I | R | Q | S | R | K | T | V | F | V | L | T | H | S | Y | V | K | S | G | S | F | K | M | A | M | Y | L | A | H | Q | R | L | L | D | E | S | E | D | V | I | V | L | L | L | L | D | P | - | V | L | Q | N | S | H | F | L | R | L | R | R | R | L | C | S | H | S | V | L | E | W | P | R | T | - | - | P | A | A | E | P | W | F | W | Q | C | L | R | N | A | I | R | V | E | N | K | V | M | Y | S | T | I | Y | S | R | Y | V | P | I | K | N | R | P | R | E | K | E | - | - | - | X | X | X | X | - | - | - | - | - | - | - | - | - | - | - | - | - | - | - | - | - | - | - | - | - | - | - | - | - | - | - | - | - | - | - | - | - | - | - | - | - | - | - | - | - | - | - | - | - | - | - | - | - | - | - | - | - | - | - | - | - | - | - | - | - | - | - | - | - | - | - | - | - | - | - | - | - | - | - | - | - | - | - | - | - | - | - | - | - | - | - | - | - | - | - | - | - | - | - | - | - | - | - | - | - | - | - | - | - | - | - | - | - | - | - | - | - | - | - | - | - | - | - | - | - | - | - | - | - | - | - | - | - | - | - | - | - | - | - | - | - | - | - | - | - | - | - | - | - | - | - | - | - | - | - | - | - | - | - | - | - | - | - | - | - | - | - | - | - | - | - | - | - | - | - | - | - | - | - | - | - | - | - | - | - | - | - | - | - | - | - | - | - | - | - | - | - | - | - | - | - | - | - | - | - | - | - | - | - | - | - | - | - | - | - | - | - | - | - | - | - | - | - | - | - | - | - | - | - | - | - | - | - | - | - | - | - | - | - | - | - | - | - | - | - | - | - | - | - | - | - | - | - | - | - | - | - | - | - | - | - | - | - | - | - | - | - | - | - | - | - | - | - | - | - | - | - | - | - | - | - | - | - | - | - | - | - | - | - | - | - | - | - | - | - | - | - | - | - | - | - | - | - | - | - | - | - | - | - | - | - | - | - | - | - | - | - | - | - | - | - | - | - | - | - | - | - | - | - | - | - | - | - | - | - | - | - | - | - |
| PHTLR8 | - | - | - | - | - | - | - | - | - | - | M | E | A | S | K | H | W | L | M | L | - | V | W | W | V | L | I | T | F | L | I | G | - | - | - | - | - | - | - | - | - | - | - | L | V | L | C | T | L | D | I | K | Y | L | R | T | Q | P | C | D | V | H | N | - | - | - | Y | P | N | G | T | V | E | F | N | C | R | G | R | R | L | R | D | M | P | V | - | I | F | E | N | T | T | S | L | D | A | S | E | N | E | I | K | N | L | T | V | E | S | L | K | G | R | Q | K | L | I | N | L | N | L | N | W | M | N | R | Y | H | - | - | - | - | - | - | N | L | N | I | T | E | G | V | F | A | N | L | T | K | L | K | S | L | R | L | N | G | L | G | L | P | E | I | P | A | Q | L | P | V | S | L | K | E | L | R | L | D | E | N | N | I | F | S | L | - | S | P | R | N | F | S | Q | L | K | N | L | T | H | L | Y | L | S | K | N | C | Y | Y | W | N | R | C | F | M | G | F | K | I | D | N | G | T | F | S | S | L | H | K | L | R | H | L | S | L | S | Y | N | N | L | T | H | V | P | R | A | L | P | V | S | L | V | T | L | E | L | A | S | N | N | I | S | F | I | G | E | D | D | F | T | G | L | P | N | L | K | T | L | K | I | Q | G | N | C | P | R | C | H | N | A | P | Y | P | C | T | P | C | A | - | - | N | G | S | I | D | I | H | E | R | A | F | D | H | L | R | N | L | R | I | L | H | L | A | G | N | S | I | S | V | I | K | K | A | W | F | E | N | I | S | Q | L | Q | Q | L | Y | L | S | F | N | F | L | T | G | P | I | T | - | - | - | D | - | G | A | F | L | S | N | L | R | F | L | E | K | L | D | L | S | F | N | Y | N | L | Q | - | A | Y | P | - | - | - | - | E | T | V | H | L | A | P | T | F | A | K | L | Y | S | L | R | T | L | H | I | Q | G | L | V | F | K | K | I | Q | N | D | S | L | A | P | L | Y | G | L | Q | N | L | S | T | L | D | I | G | V | N | F | I | V | H | V | D | P | D | I | F | N | K | F | V | - | - | N | L | K | L | L | Y | L | S | E | N | R | L | Y | P | V | T | N | S | A | D | R | G | P | D | N | N | I | - | K | P | P | R | F | N | L | P | G | L | T | D | S | S | S | K | R | K | L | D | P | Y | Q | - | P | I | R | R | L | V | K | P | E | C | F | A | A | G | H | V | L | D | L | S | R | N | N | L | F | F | I | S | A | K | Q | F | E | G | Y | K | N | I | A | C | L | N | L | S | R | N | G | F | A | S | A | L | N | G | T | E | F | T | S | L | P | N | L | K | Y | L | D | L | S | F | N | K | I | D | L | A | Y | D | Y | A | F | E | E | L | Q | E | L | E | V | L | D | L | S | Y | N | E | H | Y | F | L | V | P | G | V | T | H | N | L | K | F | I | K | N | L | P | K | L | R | V | L | N | M | S | S | N | N | I | F | T | L | T | T | K | H | M | C | S | H | S | L | A | E | L | Q | F | Q | Q | N | Q | L | G | R | L | W | K | - | - | D | E | T | Y | V | K | L | F | W | N | L | T | N | L | T | H | L | D | I | S | H | N | S | I | K | K | I | P | T | K | V | Y | K | Y | L | P | V | T | I | K | R | F | R | L | N | D | N | F | L | T | S | L | N | W | T | L | M | R | T | F | T | Q | L | E | E | L | I | L | G | Y | N | S | L | T | F | V | S | K | T | I | T | Q | N | I | P | - | S | L | R | Y | L | D | L | S | H | N | K | I | S | Q | L | A | N | G | F | L | D | G | A | V | N | L | N | K | L | D | L | S | Y | N | K | L | S | I | L | N | R | S | T | F | P | S | K | E | T | N | H | L | D | T | L | W | L | H | K | N | P | Y | R | C | T | C | D | I | M | D | F | I | L | W | I | F | - | E | S | E | V | K | I | P | N | L | V | T | S | V | K | C | N | V | P | D | A | T | K | G | K | G | V | V | F | F | D | I | R | - | E | C | V | D | D | H | V | A | S | L | I | Y | F | F | T | T | L | L | V | V | G | V | T | F | V | A | T | L | M | H | M | F | Y | W | D | V | S | Y | V | F | Y | Y | M | K | A | K | F | K | G | Y | Q | H | L | S | S | - | - | G | D | N | V | Y | D | A | F | V | T | Y | D | T | K | N | P | Q | V | S | E | W | V | L | N | H | L | R | V | Q | L | E | E | Q | G | N | H | F | L | P | V | C | L | E | E | R | D | W | L | P | G | C | P | V | L | D | S | L | T | Q | S | I | R | Q | S | R | K | T | V | F | V | L | T | H | S | Y | V | K | S | G | S | F | K | M | A | M | Y | L | A | H | Q | R | L | L | D | E | S | E | D | V | I | V | L | L | L | L | E | P | - | V | L | Q | N | T | H | F | L | R | L | R | R | R | L | C | S | H | S | V | L | E | W | P | R | T | - | - | P | A | A | E | P | W | F | W | Q | C | L | R | N | A | I | R | V | E | N | K | A | I | M | A | T | R | C | R | S | R | A | R | C | Q | G | R | W | L | A | V | F | S | L | A | V | C | K | X | - | - | - | - | - | - | - | - | - | - | - | - | - | - | - | - | - | - | - | - | - | - | - | - | - | - | - | - | - | - | - | - | - | - | - | - | - | - | - | - | - | - | - | - | - | - | - | - | - | - | - | - | - | - | - | - | - | - | - | - | - | - | - | - | - | - | - | - | - | - | - | - | - | - | - | - | - | - | - | - | - | - | - | - | - | - | - | - | - | - | - | - | - | - | - | - | - | - | - | - | - | - | - | - | - | - | - | - | - | - | - | - | - | - | - | - | - | - | - | - | - | - | - | - | - | - | - | - | - | - | - | - | - | - | - | - | - | - | - | - | - | - | - | - | - | - | - | - | - | - | - | - | - | - | - | - | - | - | - | - | - | - | - | - | - | - | - | - | - | - | - | - | - | - | - | - | - | - | - | - | - | - | - | - | - | - | - | - | - | - | - | - | - | - | - | - | - | - | - | - | - | - | - | - | - | - | - | - | - | - | - | - | - | - | - | - | - | - | - | - | - | - | - | - | - | - | - | - | - | - | - | - | - | - | - | - | - | - | - | - | - | - | - | - | - | - | - | - | - | - | - | - | - | - | - | - | - | - | - | - | - | - | - | - | - | - | - | - | - | - | - | - | - | - | - | - | - | - | - | - | - | - | - | - | - | - | - | - | - | - | - | - | - | - | - | - | - | - | - | - | - | - | - | - | - | - | - | - | - | - | - | - | - | - | - | - | - | - | - | - | - | - | - | - | - | - | - | - | - | - | - | - | - | - | - |
| GMTLR8 | - | - | - | - | - | - | - | - | - | - | - | - | - | - | - | - | - | - | - | - | - | - | - | - | - | - | - | - | - | - | - | - | - | - | - | - | - | - | - | - | - | - | - | - | - | - | - | - | - | - | - | - | - | - | - | - | - | - | - | - | - | - | - | - | - | - | - | - | - | - | - | - | - | - | - | - | - | - | - | - | - | - | - | - | - | - | - | - | - | - | - | - | - | - | - | - | - | - | - | - | - | - | - | - | - | - | - | - | - | - | - | - | - | - | - | - | - | - | - | - | - | - | - | - | - | - | - | - | - | - | - | - | - | - | - | - | - | - | - | - | - | - | - | M | N | L | T | N | L | K | I | L | Y | L | G | Y | N | R | L | T | R | V | P | G | I | L | P | H | S | L | T | T | I | W | L | N | N | N | N | I | L | V | I | - | N | N | M | S | F | S | G | I | R | N | V | T | H | L | F | L | Q | R | N | G | C | S | S | N | K | C | S | N | P | V | N | I | S | D | D | S | F | P | V | L | T | K | L | K | V | L | D | L | S | S | N | R | L | R | R | V | P | K | G | L | P | T | T | L | T | K | L | A | L | S | N | N | Q | I | H | Y | I | S | E | E | D | F | K | E | L | H | H | L | K | A | L | T | I | H | G | N | C | P | R | C | G | N | A | P | F | P | C | V | A | C | P | - | - | N | I | S | L | G | I | H | P | K | V | F | I | D | L | T | E | L | Q | T | L | D | L | A | G | N | S | L | T | Q | L | N | D | F | W | F | E | N | L | Q | N | L | T | K | L | S | L | S | F | N | L | L | Q | N | A | I | Q | G | E | L | K | - | - | - | C | F | S | S | L | S | K | L | E | H | L | D | L | S | F | N | Y | G | R | N | - | S | Y | P | - | - | - | - | K | T | V | Q | L | S | K | G | F | S | K | L | R | S | L | Q | V | L | H | L | V | G | L | V | F | R | E | I | E | F | D | T | L | K | P | L | V | S | L | K | N | L | T | V | L | N | L | G | T | N | F | I | V | H | S | N | S | T | I | F | K | E | L | H | - | - | Q | L | K | G | I | F | L | S | E | N | R | L | Y | P | V | S | V | S | T | P | N | P | P | G | Q | G | G | N | V | E | A | D | L | T | F | S | P | H | L | V | - | - | A | D | T | L | N | F | - | - | - | - | G | S | R | I | L | A | N | P | E | C | I | K | A | G | R | A | L | S | F | S | S | N | N | L | F | F | I | T | P | E | Q | F | E | G | Y | D | N | I | S | C | L | N | L | S | G | N | G | F | S | S | A | L | N | G | T | E | F | E | S | L | P | N | L | T | Y | L | D | L | S | F | N | K | I | D | L | A | Y | R | H | A | F | Q | E | L | Q | K | L | K | V | L | D | I | S | F | N | D | H | Y | F | K | A | Y | G | I | T | H | N | L | D | F | V | K | N | L | P | V | L | E | I | L | N | M | S | N | N | C | I | H | T | L | T | T | K | M | L | Q | S | E | S | L | N | E | L | R | F | G | H | N | E | L | G | T | L | W | K | - | - | D | D | S | Y | F | N | L | F | T | N | L | T | N | L | K | I | L | D | I | S | S | N | N | I | A | E | I | P | D | N | I | Y | V | L | L | P | R | Q | L | T | T | L | F | I | N | Y | N | K | L | S | D | F | N | W | T | K | L | E | H | F | N | N | L | Q | N | L | D | L | S | H | N | Y | I | T | Y | V | K | I | V | T | S | - | - | - | - | Q | T | L | R | T | L | N | L | A | H | N | Q | I | A | Q | L | S | N | G | F | L | E | S | A | V | S | L | T | T | L | S | L | N | H | N | M | L | T | V | I | N | Q | T | N | F | L | T | K | P | K | N | Y | L | Q | T | L | S | L | H | R | N | P | F | Q | C | S | C | D | T | L | D | F | I | L | W | I | E | - | D | N | D | V | T | I | P | N | L | T | T | D | V | Y | C | Y | V | S | Y | G | - | H | R | Q | V | M | I | Y | F | E | F | D | - | Q | C | V | N | N | T | L | A | L | L | I | S | V | L | T | T | T | F | I | V | V | T | T | S | M | A | T | V | A | H | I | F | Y | W | D | T | S | Y | V | L | R | Y | L | K | A | K | W | K | G | Y | R | S | S | A | S | - | - | Q | D | N | V | Y | E | V | F | V | T | Y | D | T | K | D | P | W | V | S | E | W | V | L | E | S | L | R | E | K | L | E | V | E | G | E | K | V | L | R | L | C | L | E | E | R | D | W | P | L | G | V | P | V | I | D | N | L | T | Q | S | I | R | Y | S | R | K | T | L | F | V | L | T | K | A | Y | A | K | T | G | V | F | R | L | A | M | Y | L | A | H | Q | R | L | L | D | E | N | L | D | V | I | V | V | L | M | L | E | P | - | V | L | Q | N | S | H | F | L | R | L | R | R | R | L | C | G | E | S | V | L | E | W | P | R | T | - | - | A | A | A | E | P | W | F | W | Q | N | L | R | N | V | V | R | V | D | N | Q | T | M | Y | P | S | T | Y | S | Q | Y | F | T | C | S | R | E | R | D | X | X | X | X | X | X | X | X | X | X | X | X | X | X | X | X | X | X | X | X | X | X | X | X | X | X | X | X | X | X | X | X | X | X | X | X | X | X | X | X | X | X | X | X | - | - | X | - | - | - | - | X | X | X | X | X | X | X | X | X | X | X | X | X | X | X | X | X | X | X | X | X | X | X | X | X | X | X | X | X | X | X | X | X | X | X | X | X | - | X | X | X | X | X | X | X | X | X | X | X | X | X | X | X | X | X | X | X | X | X | X | X | - | X | X | X | X | X | X | X | X | X | X | X | X | X | - | - | X | X | X | X | X | - | - | X | X | X | - | - | - | - | - | X | X | - | - | - | - | - | - | - | - | - | - | - | - | - | - | - | - | - | - | - | - | - | - | - | - | - | - | - | - | - | - | - | - | - | - | - | - | - | - | - | - | - | - | - | - | - | - | - | - | - | - | - | - | - | - | - | - | - | - | - | - | - | - | - | - | - | - | - | - | - | - | - | - | - | - | - | - | - | - | - | - | - | - | - | - | - | - | - | - | - | - | - | - | - | - | - | - | - | - | - | - | - | - | - | - | - | - | - | - | - | - | - | - | - | - | - | - | - | - | - | - | - | - | - | - | - | - | - | - | - | - | - | - | - | - | - | - | - | - | - | - | - | - | - | - | - | - | - | - | - | - | - | - | - | - | - | - | - | - | - | - | - | - | - | - | - | - | - | - | - | - | - | - | - | - | - | - | - | - | - | - | - | - | - | - | - | - | - | - | - | - | - | - | - | - | - | - | - | - | - | - |
| LCTLR8 | - | - | - | - | - | - | - | - | - | - | - | - | - | - | - | - | - | - | - | - | - | - | - | - | - | - | - | - | - | - | - | - | - | - | - | - | - | - | - | - | - | - | - | - | - | - | - | - | - | - | - | - | - | - | - | - | - | - | - | - | - | - | - | - | - | - | - | - | - | - | - | - | - | - | - | - | - | - | - | - | - | - | - | - | - | - | - | - | - | - | - | - | - | - | - | - | - | - | - | - | - | - | - | - | - | - | - | - | - | - | - | - | - | - | - | - | - | - | - | - | - | - | - | - | - | - | - | - | - | - | - | - | - | - | - | - | - | - | - | - | - | - | - | - | - | - | - | - | - | - | - | - | - | - | - | - | - | - | - | - | - | - | - | - | - | - | - | - | - | - | - | - | - | - | - | - | - | - | - | - | - | - | - | - | - | - | - | - | - | - | - | - | - | - | - | - | - | - | - | - | - | - | - | - | - | - | - | - | - | - | - | - | - | - | - | - | - | - | - | - | - | - | - | - | - | - | - | - | - | - | - | - | - | - | - | - | - | - | - | - | - | - | - | - | - | - | - | - | - | - | - | - | - | - | - | - | - | - | - | - | - | - | - | - | - | - | - | - | - | - | - | - | - | - | - | - | - | - | - | - | - | - | - | - | - | - | - | - | - | - | - | - | - | - | - | - | - | - | - | - | - | - | - | - | - | - | - | - | - | - | - | - | - | - | - | - | - | - | - | - | - | - | - | - | - | - | - | - | - | - | - | - | - | - | - | - | - | - | - | - | - | - | - | - | - | - | - | - | - | - | - | - | - | - | - | - | - | - | - | - | - | - | - | - | - | - | - | - | - | - | - | - | - | - | - | - | - | - | M | S | - | S | Y | S | S | S | T | L | Q | H | Y | I | F | Q | K | T | L | Q | K | L | Q | S | L | R | T | L | H | L | E | G | L | V | F | Q | T | I | V | Q | D | T | L | S | P | L | Y | G | L | R | N | L | S | A | L | N | L | G | T | N | F | I | I | K | S | D | S | K | I | F | S | K | F | Q | - | - | Q | L | K | M | I | Y | L | A | E | N | R | L | Y | P | S | P | V | K | S | I | A | N | P | S | D | G | F | H | Q | R | L | D | L | S | I | S | P | Y | I | K | - | - | P | - | H | T | D | F | A | Y | D | - | V | S | H | S | L | I | K | Q | E | C | F | D | S | G | R | V | L | I | L | S | S | N | N | L | F | F | I | S | P | K | Q | F | E | G | Y | G | N | I | A | C | L | N | L | S | G | N | G | F | S | A | A | L | N | G | T | E | F | S | S | L | P | N | L | T | Y | L | D | L | S | F | N | K | I | D | L | A | Y | D | N | A | F | I | E | L | Q | K | L | K | V | L | D | L | S | Y | N | P | H | Y | F | K | S | T | G | V | T | H | N | L | N | F | M | K | N | L | P | A | L | R | V | L | N | M | S | H | N | A | I | S | I | L | T | T | K | Q | M | H | S | K | S | L | S | E | L | Q | F | T | D | N | N | L | G | T | L | W | K | E | R | D | G | S | Y | E | M | L | F | T | N | L | T | N | L | T | I | L | D | I | S | Q | N | R | I | T | K | I | P | D | N | V | Y | E | R | L | P | H | N | L | T | K | L | R | I | S | G | N | S | L | T | D | F | K | W | N | R | L | K | C | F | H | Q | L | Q | I | L | D | L | S | F | N | S | L | S | H | V | K | G | I | N | S | N | I | T | Q | - | S | L | T | F | L | D | L | S | H | N | H | I | F | H | L | D | D | G | F | I | K | G | P | K | S | L | T | T | L | S | L | S | N | N | K | L | T | T | I | N | Q | S | T | L | L | S | I | S | N | N | Q | I | K | T | L | F | L | Q | Q | N | P | F | Q | C | T | C | Y | S | F | E | F | I | L | W | I | E | - | N | S | N | V | K | I | P | R | L | T | T | K | V | K | C | A | T | P | E | N | Q | K | G | R | A | L | I | Y | F | D | I | N | - | Q | C | V | N | D | S | Q | A | F | Q | I | Y | I | L | I | T | S | F | I | F | A | F | M | F | V | T | T | V | A | H | L | F | Y | W | D | A | S | Y | V | V | H | F | M | K | A | R | L | K | G | Y | R | S | L | N | S | - | - | P | E | S | V | Y | D | V | F | V | T | Y | D | T | R | D | P | H | V | S | E | W | V | M | K | N | L | R | V | K | L | E | E | E | G | E | T | H | L | P | L | C | L | E | E | R | D | W | P | L | G | V | P | L | V | D | N | L | T | Q | S | I | Q | Y | S | R | K | T | L | F | V | L | T | E | G | Y | V | K | T | G | V | F | K | L | A | M | Y | L | A | H | Q | R | L | L | D | E | N | V | D | V | I | V | L | L | M | L | E | P | - | V | L | Q | H | S | H | F | L | R | L | R | R | R | L | C | E | K | S | V | V | E | W | P | R | T | - | - | A | A | A | E | A | W | F | W | Q | N | L | R | S | V | V | R | V | D | N | Q | I | M | Y | N | K | T | Y | S | K | Y | F | T | N | K | X | X | X | X | X | X | X | X | X | X | X | X | X | X | X | X | X | X | X | X | X | X | X | X | X | X | X | X | X | X | X | X | X | X | X | X | X | X | X | X | X | X | X | X | X | X | X | X | X | X | X | X | X | X | X | X | X | X | X | X | X | X | X | X | X | X | X | X | X | X | X | X | X | X | X | X | X | X | X | X | X | X | X | X | X | X | X | X | X | X | X | X | X | X | X | X | X | X | X | X | X | X | X | X | X | X | X | X | X | X | X | X | X | X | X | X | X | X | X | X | X | X | X | X | X | X | X | X | X | X | X | X | X | X | X | X | X | X | X | X | X | X | X | X | X | X | X | X | X | X | X | X | X | X | X | X | X | X | X | X | X | X | X | X | X | X | X | X | X | X | X | X | X | X | X | X | X | X | X | X | X | X | X | X | X | X | X | X | X | X | X | X | X | X | X | X | X | X | X | X | X | X | X | X | X | X | X | X | X | X | X | X | X | X | X | X | X | X | X | X | X | X | X | X | X | X | X | X | X | X | X | X | X | X | X | X | X | X | X | X | X | X | X | X | X | X | X | X | X | X | X | X | X | X | X | X | X | X | X | X | X | X | X | X | X | X | X | X | X | X | X | X | X | X | X | X | X | X | X | X | X | X | X | X | X | X | X | X | X | X | X | X | X | X | X | X | X | X | X | X | X | X | X | X | X | X | X | X | X | X | X | X | X | X | X | X | X | X | X | X | X | X | X | X | X | X | X | X | X | X | X | X | X | X | X | X | X | X | X | X | X | X | X | X | X | X | X | X | X |
| SSTLR8 | - | - | - | - | - | - | - | - | - | - | - | - | - | - | - | - | M | D | A | T | - | T | C | W | L | Q | L | A | S | L | F | - | - | L | C | H | L | L | - | - | - | - | V | P | T | S | C | - | - | N | - | L | M | S | R | Q | F | P | C | D | I | T | V | N | T | T | N | T | T | N | D | I | I | F | N | C | E | E | R | R | L | K | N | V | P | V | G | I | T | R | N | V | T | V | L | D | L | S | E | N | N | I | R | N | V | S | L | G | A | F | S | N | L | E | N | L | T | L | L | N | L | N | C | V | N | K | E | G | - | - | - | - | - | - | D | T | H | I | A | E | G | A | F | K | N | L | T | N | L | Q | D | L | R | L | N | G | N | G | L | V | K | I | P | Q | N | L | P | L | I | L | D | K | L | M | L | D | N | N | K | I | N | F | S | - | N | M | S | N | L | V | G | I | Q | H | V | K | E | L | Y | L | S | K | N | C | Y | Y | W | N | P | C | E | T | D | F | T | I | G | N | G | T | F | S | V | L | T | N | L | K | V | L | M | L | A | Y | N | N | L | T | H | V | P | K | G | L | P | N | S | I | Q | E | L | H | L | D | S | N | K | I | Q | Q | I | A | E | D | D | F | L | G | L | T | H | L | T | I | L | E | L | Q | G | N | C | P | R | C | S | N | A | P | Y | P | C | V | P | C | P | - | - | N | D | S | L | D | I | H | P | H | A | F | H | G | L | T | K | L | Q | T | L | H | L | A | G | N | S | L | R | F | I | N | N | S | W | F | E | S | L | N | N | L | T | H | L | F | L | S | Y | N | Y | L | T | N | T | I | T | N | G | - | - | - | - | T | F | F | S | Y | L | P | K | L | E | K | I | D | L | S | F | N | Y | D | L | L | - | A | Y | P | - | - | - | - | E | T | L | Q | L | S | K | N | F | S | Q | L | V | S | L | T | T | L | H | I | M | G | F | V | F | R | E | I | H | L | E | T | L | R | P | L | Y | E | L | K | H | L | S | V | L | N | I | G | T | N | F | I | V | R | S | D | S | H | I | F | N | K | F | S | N | S | S | L | K | V | I | Y | L | A | E | N | R | L | Y | P | I | S | V | N | E | S | P | G | C | G | T | G | S | N | L | K | S | V | L | Y | T | S | P | L | T | G | Y | Y | S | N | S | R | D | F | S | Y | G | - | I | N | H | N | L | V | K | P | E | C | F | I | S | G | R | V | L | V | L | S | S | N | N | L | F | F | I | S | P | K | Q | F | E | G | Y | G | D | I | A | C | L | N | L | S | R | N | G | F | S | A | A | L | N | G | T | E | F | T | S | L | P | D | L | K | Y | L | D | L | S | F | N | K | I | D | L | A | Y | D | N | A | F | K | E | L | K | K | L | E | I | L | D | L | S | Y | N | S | H | Y | F | E | V | S | G | V | T | H | N | L | N | F | L | K | N | L | P | T | L | K | V | L | N | M | S | H | N | N | I | F | T | L | T | T | K | Q | M | T | S | T | S | L | K | E | L | Q | F | Q | H | N | L | L | A | T | L | W | K | E | G | D | D | S | Y | N | S | L | F | K | M | L | T | N | L | T | Y | L | D | I | S | F | N | S | I | E | K | I | P | S | K | V | Y | E | N | I | P | Y | T | L | Q | K | L | C | I | S | H | N | S | L | G | H | F | D | W | D | K | L | A | G | F | Q | Q | L | S | L | L | D | L | S | Y | N | S | L | F | H | V | S | A | N | L | S | N | F | T | D | - | T | L | Q | I | L | D | L | S | H | N | Q | I | F | Q | L | S | R | E | F | L | W | G | A | Q | S | L | Q | I | L | D | L | S | Y | N | Q | L | T | N | I | N | E | T | T | F | L | S | G | P | E | N | Y | M | K | T | L | S | L | Q | G | N | P | F | Q | C | T | C | D | L | L | E | F | I | Q | W | I | E | E | N | K | N | V | K | I | P | R | L | A | S | E | V | T | C | N | M | P | A | E | M | R | G | Q | P | I | I | L | F | H | I | A | - | E | C | I | N | D | D | I | A | F | L | I | Y | S | L | S | T | S | L | I | M | F | T | V | V | I | T | M | A | A | H | V | F | Y | W | D | A | S | Y | I | L | Y | Y | L | R | A | K | L | K | G | Y | H | S | L | R | S | T | T | T | D | N | L | Y | D | A | F | V | T | Y | D | T | R | D | P | L | V | S | D | W | V | L | N | Q | L | R | V | Q | L | E | E | R | G | E | R | H | L | P | L | C | L | E | E | R | D | W | A | P | G | V | P | L | I | D | N | L | F | Q | S | I | R | Q | S | R | K | T | V | F | V | L | T | K | A | Y | I | R | T | G | N | F | R | M | A | V | Y | L | A | H | Q | R | L | L | D | E | N | I | D | V | I | V | L | V | L | L | E | P | - | V | L | Q | H | S | H | F | L | L | L | R | R | R | L | C | G | R | S | V | L | E | W | P | Q | T | - | - | A | A | A | E | T | W | F | W | Q | H | L | R | N | A | V | R | L | D | N | Q | V | M | Y | N | K | I | Y | S | R | Y | F | T | S | K | X | X | X | X | X | X | - | - | - | - | - | - | - | - | - | - | - | - | - | - | - | - | - | - | - | - | - | - | - | - | - | - | - | - | - | - | - | - | - | - | - | - | - | - | - | - | - | - | - | - | - | - | - | - | - | - | - | - | - | - | - | - | - | - | - | - | - | - | - | - | - | - | - | - | - | - | - | - | - | - | - | - | - | - | - | - | - | - | - | - | - | - | - | - | - | - | - | - | - | - | - | - | - | - | - | - | - | - | - | - | - | - | - | - | - | - | - | - | - | - | - | - | - | - | - | - | - | - | - | - | - | - | - | - | - | - | - | - | - | - | - | - | - | - | - | - | - | - | - | - | - | - | - | - | - | - | - | - | - | - | - | - | - | - | - | - | - | - | - | - | - | - | - | - | - | - | - | - | - | - | - | - | - | - | - | - | - | - | - | - | - | - | - | - | - | - | - | - | - | - | - | - | - | - | - | - | - | - | - | - | - | - | - | - | - | - | - | - | - | - | - | - | - | - | - | - | - | - | - | - | - | - | - | - | - | - | - | - | - | - | - | - | - | - | - | - | - | - | - | - | - | - | - | - | - | - | - | - | - | - | - | - | - | - | - | - | - | - | - | - | - | - | - | - | - | - | - | - | - | - | - | - | - | - | - | - | - | - | - | - | - | - | - | - | - | - | - | - | - | - | - | - | - | - | - | - | - | - | - | - | - | - | - | - | - | - | - | - | - | - | - | - | - | - | - | - | - | - | - | - | - | - | - | - | - | - | - | - | - | - | - | - | - | - | - | - | - | - | - |
| SLTLR8 | - | - | - | - | - | - | - | - | - | - | - | - | - | - | - | - | - | - | - | - | - | - | - | - | - | - | - | - | - | - | - | - | - | - | - | - | - | - | - | - | - | - | - | - | - | - | - | - | - | - | - | - | M | L | P | Q | F | P | C | D | V | K | A | - | - | C | N | T | S | - | E | V | V | F | D | C | K | G | R | H | L | H | A | V | P | D | K | I | A | S | N | A | T | E | L | D | L | S | E | N | Y | I | K | N | I | S | V | N | S | F | S | K | L | L | N | L | T | K | L | D | L | K | W | A | N | K | K | E | - | - | - | - | - | - | G | L | I | I | A | A | N | T | F | K | N | L | T | Q | L | N | H | L | I | L | T | G | N | C | L | T | Q | I | P | S | N | L | P | L | S | V | E | I | L | E | L | N | T | N | N | I | T | L | L | - | D | N | R | S | V | A | G | L | T | N | V | T | Q | L | W | L | C | - | - | - | - | - | - | - | - | - | - | - | - | - | - | - | - | - | - | - | - | - | - | - | - | - | - | - | - | - | - | - | - | - | - | - | - | - | - | - | - | - | - | - | - | - | - | - | - | - | - | - | - | - | S | N | Q | I | Q | Y | I | S | E | F | D | F | F | G | L | Y | N | L | K | T | L | K | I | Q | G | N | C | P | R | C | Q | T | A | P | Y | P | C | V | P | C | P | - | - | N | I | S | L | - | - | - | - | - | - | - | - | - | - | - | - | - | - | - | - | - | - | - | - | - | - | - | - | - | - | - | - | - | - | - | - | - | - | - | - | - | - | - | - | - | - | - | - | - | - | - | - | - | - | - | - | - | - | - | - | - | - | - | - | - | - | - | - | - | - | - | - | - | - | - | - | - | - | - | - | - | - | - | - | - | - | - | - | - | - | - | - | - | - | - | - | - | - | - | - | - | - | - | - | - | - | - | - | - | - | - | - | - | - | - | - | - | - | - | - | - | - | - | - | - | - | - | - | - | - | - | - | - | - | - | - | - | - | - | - | - | - | - | - | - | - | - | - | - | - | - | - | - | - | - | - | - | - | - | - | - | - | - | - | - | E | N | R | L | H | P | I | P | V | E | H | P | P | S | P | S | D | G | Y | N | Q | R | P | D | L | S | I | S | P | L | R | K | - | - | P | Q | P | K | D | F | A | F | E | - | I | S | H | S | L | I | K | E | E | C | F | E | L | G | R | V | L | I | L | S | S | N | N | L | F | F | I | S | P | E | Q | F | N | G | Y | G | D | I | A | C | L | N | L | S | R | N | G | F | S | A | A | L | N | G | T | E | F | S | L | L | P | N | L | T | Y | L | D | L | S | F | N | R | I | D | L | A | Y | D | N | A | F | K | E | L | K | K | L | Q | V | L | D | L | S | Y | N | E | H | Y | F | K | A | Y | G | I | T | H | N | L | N | F | T | K | N | L | P | V | L | R | V | L | N | M | S | H | N | G | I | S | T | L | T | T | K | Q | M | Y | S | E | S | L | S | E | L | Q | F | T | D | N | N | L | G | T | L | W | K | E | R | D | G | S | Y | K | M | L | F | T | N | L | I | N | L | T | I | L | D | I | S | R | N | R | I | A | K | I | P | I | D | V | Y | K | Y | L | P | R | N | L | T | K | L | R | I | S | H | N | G | L | A | D | F | R | W | D | S | L | R | Y | F | H | Q | L | Q | I | L | D | L | S | F | N | S | L | S | N | V | T | G | I | N | S | N | I | T | Q | - | T | L | T | F | L | D | L | N | H | N | C | I | F | Q | L | D | N | G | F | I | K | G | P | K | S | L | K | T | L | S | L | S | N | N | K | L | T | T | I | N | Q | S | T | F | Q | S | R | P | D | N | Q | I | Q | T | L | F | L | Q | G | N | P | F | Q | C | T | C | D | L | L | D | F | I | L | W | I | E | - | K | S | E | V | N | I | P | R | L | T | T | M | V | T | C | H | T | P | A | N | Q | K | G | Q | A | L | I | Y | F | D | I | N | - | Q | C | V | N | D | S | Q | A | F | L | V | Y | I | V | T | T | S | F | I | I | A | F | M | I | V | A | T | V | A | H | L | F | Y | W | D | A | S | Y | V | I | H | Y | M | K | A | K | L | K | G | Y | R | S | L | S | S | - | - | P | D | S | V | Y | D | V | F | V | T | Y | D | T | R | D | P | R | V | S | E | W | V | M | R | N | L | R | L | K | L | E | D | E | G | E | K | H | L | P | L | C | L | E | E | R | D | W | P | P | G | V | P | L | V | E | N | L | T | Q | S | I | R | Y | S | R | K | T | L | F | V | L | T | E | G | Y | V | K | T | G | V | F | K | L | A | M | Y | L | A | H | Q | R | L | L | D | E | N | L | D | V | I | V | L | L | M | L | E | P | - | V | L | Q | H | S | H | F | L | R | L | R | R | R | L | C | G | K | S | V | V | E | W | P | R | T | - | - | A | A | A | E | P | W | F | W | Q | N | L | R | N | V | V | R | V | D | N | Q | V | I | Y | N | K | T | Y | S | K | Y | F | S | S | R | X | X | X | X | X | X | X | X | X | X | X | X | X | X | X | X | X | X | X | X | X | X | X | X | X | X | X | X | X | X | X | X | X | X | X | X | X | X | X | X | X | X | X | X | X | X | X | X | X | X | X | X | X | - | - | X | X | X | X | X | X | - | X | X | X | X | X | X | - | X | X | X | X | X | X | X | - | X | X | X | X | X | X | X | - | X | X | X | X | X | X | X | - | X | X | X | X | X | X | X | - | X | X | X | X | X | X | X | - | X | X | X | X | X | X | X | - | X | X | X | X | X | X | - | X | X | X | X | X | X | X | - | X | X | X | X | X | X | - | X | X | X | X | X | X | X | - | X | X | X | X | X | X | - | X | X | X | X | X | X | - | X | X | X | X | X | X | - | X | X | X | X | X | X | - | X | X | X | X | X | X | - | X | X | X | X | X | X | - | X | X | X | X | X | X | - | X | X | X | X | X | X | - | X | X | X | X | X | - | X | X | X | X | X | X | - | X | X | X | X | X | - | X | X | X | X | X | - | X | X | X | X | X | - | X | X | X | X | X | - | X | X | X | X | X | - | X | X | X | X | X | - | X | X | X | X | - | X | X | X | X | X | - | X | X | X | X | X | - | X | X | X | X | - | X | X | X | X | X | - | - | - | - | - | - | - | - | - | - | - | - | - | - | - | - | - | - | - | - | - | - | - | - | - | - | - | - | - | - | - | - | - | - | - | - | - | - | - | - | - | - | - | - | - | - | - | - | - | - | - | - | - | - | - | - | - | - | - | - | - | - | - | - | - | - | - | - | - | - |
| ONTLR8 | - | - | - | - | - | - | - | - | - | - | - | - | - | - | - | - | - | M | I | T | - | K | C | W | - | - | - | M | L | L | F | C | L | C | C | R | H | G | V | - | - | - | Q | L | A | A | H | K | P | F | - | W | M | T | R | Q | F | P | C | D | V | I | S | - | - | N | N | A | S | N | T | V | K | F | D | C | K | G | R | H | L | E | K | V | P | K | G | I | T | T | N | A | T | D | L | D | L | S | E | N | V | I | R | S | I | K | A | D | S | L | S | H | L | L | N | L | A | H | L | N | L | N | W | A | N | N | T | K | T | D | Y | G | C | G | A | L | K | I | A | E | N | A | F | K | N | L | T | K | L | K | Q | L | R | L | S | G | N | C | L | T | E | I | P | R | N | L | P | H | S | V | E | R | L | E | L | N | I | N | K | V | S | M | D | K | L | N | N | S | F | I | G | L | K | N | M | R | M | L | L | L | S | K | N | C | Y | F | R | N | P | C | G | K | S | V | A | I | N | E | N | T | F | A | L | L | K | K | L | K | Y | L | D | L | S | Y | N | N | L | T | Q | V | P | K | G | L | P | Q | S | I | T | I | L | R | L | D | A | N | K | I | E | F | I | S | K | D | D | F | Q | G | L | E | N | V | K | F | L | D | I | Q | G | N | C | P | R | C | H | N | A | R | Y | P | C | V | P | C | P | - | - | N | G | S | I | D | I | H | P | D | A | F | Q | R | L | T | Q | L | E | T | L | N | L | G | G | N | S | L | N | Y | L | D | P | S | W | F | Q | S | L | T | N | L | T | Q | L | F | L | E | F | N | F | L | Q | K | A | V | T | G | E | D | T | P | V | K | A | F | S | Y | L | H | R | L | E | K | L | D | L | S | F | N | Y | A | L | G | - | L | Y | P | - | - | - | - | E | K | L | T | L | S | K | D | F | S | Q | L | R | S | L | K | T | L | H | L | N | A | L | V | F | Q | S | I | G | P | D | T | L | R | P | L | Y | N | L | K | N | L | S | H | L | Y | L | S | T | N | F | I | I | H | S | D | P | T | V | F | S | K | L | S | - | - | H | L | R | M | I | Y | L | G | E | N | R | L | Y | P | T | T | V | E | S | L | P | T | L | R | D | R | N | N | E | N | S | E | V | S | M | S | P | F | V | K | - | - | L | T | P | K | D | S | V | Y | E | - | V | L | H | R | N | T | K | Q | E | C | A | N | S | G | R | V | L | I | L | S | S | N | N | L | F | F | I | S | P | K | Q | F | E | G | Y | G | N | I | S | C | L | N | L | S | R | N | G | F | S | Q | A | L | N | G | T | E | F | K | M | L | P | N | L | T | Y | L | D | L | S | F | N | R | V | D | L | A | Y | N | N | A | F | K | E | L | K | K | L | Q | V | L | D | L | S | Y | N | S | H | Y | F | E | A | F | G | V | T | L | N | L | N | F | T | Q | N | L | P | V | L | R | V | L | N | M | S | H | N | E | I | S | T | L | T | T | K | E | M | Y | S | K | S | L | A | E | L | I | F | A | Q | N | H | L | G | K | L | W | K | D | R | D | G | S | Y | K | K | L | F | T | N | L | S | N | L | T | V | L | D | I | S | S | N | G | I | A | K | I | P | D | D | V | Y | E | Y | L | P | H | N | L | T | T | L | R | I | S | H | N | L | L | S | D | V | N | W | D | K | L | P | - | F | H | Q | L | Q | I | M | D | L | S | Y | N | S | L | S | T | L | T | T | I | N | S | N | M | A | Q | - | T | L | T | F | L | D | L | S | H | N | H | I | F | H | A | D | N | C | F | L | K | S | L | K | S | L | M | T | L | S | L | S | N | N | K | L | T | I | V | N | E | T | T | F | K | S | G | P | A | N | - | - | L | T | L | F | L | Q | G | N | P | F | E | C | T | C | D | L | L | D | F | V | L | W | I | E | - | Q | S | D | V | K | I | P | R | L | T | T | Q | V | T | C | N | T | P | V | N | L | K | G | E | G | L | I | H | F | S | I | H | - | Q | C | V | N | P | S | Q | A | F | Q | I | Y | T | L | T | T | S | I | I | I | L | F | M | I | V | S | T | V | A | H | L | F | Y | W | D | A | S | Y | V | L | H | Y | I | K | A | K | L | K | G | Y | T | S | F | N | S | - | - | P | D | T | I | Y | D | V | F | V | T | Y | D | T | K | D | P | Q | V | S | E | W | V | M | S | N | L | R | V | Q | L | E | E | E | G | D | K | Y | H | P | L | C | L | E | E | R | D | W | P | P | G | V | P | L | V | D | N | L | T | Q | S | I | Q | Y | S | R | K | T | L | F | V | L | T | K | G | Y | V | K | T | G | A | F | K | L | A | M | Y | M | A | H | Q | R | L | L | D | E | N | V | D | V | I | V | L | L | M | L | E | P | - | V | L | Q | H | S | H | F | L | R | L | R | K | R | L | C | E | S | S | V | V | E | W | P | R | T | - | - | A | A | A | E | P | W | F | W | Q | N | L | R | S | V | I | R | V | D | N | Q | V | M | Y | N | K | T | Y | S | K | Y | F | T | T | K | X | X | X | X | X | X | - | - | - | - | - | - | - | - | - | - | - | - | - | - | - | - | - | - | - | - | - | - | - | - | - | - | - | - | - | - | - | - | - | - | - | - | - | - | - | - | - | - | - | - | - | - | - | - | - | - | - | - | - | - | - | - | - | - | - | - | - | - | - | - | - | - | - | - | - | - | - | - | - | - | - | - | - | - | - | - | - | - | - | - | - | - | - | - | - | - | - | - | - | - | - | - | - | - | - | - | - | - | - | - | - | - | - | - | - | - | - | - | - | - | - | - | - | - | - | - | - | - | - | - | - | - | - | - | - | - | - | - | - | - | - | - | - | - | - | - | - | - | - | - | - | - | - | - | - | - | - | - | - | - | - | - | - | - | - | - | - | - | - | - | - | - | - | - | - | - | - | - | - | - | - | - | - | - | - | - | - | - | - | - | - | - | - | - | - | - | - | - | - | - | - | - | - | - | - | - | - | - | - | - | - | - | - | - | - | - | - | - | - | - | - | - | - | - | - | - | - | - | - | - | - | - | - | - | - | - | - | - | - | - | - | - | - | - | - | - | - | - | - | - | - | - | - | - | - | - | - | - | - | - | - | - | - | - | - | - | - | - | - | - | - | - | - | - | - | - | - | - | - | - | - | - | - | - | - | - | - | - | - | - | - | - | - | - | - | - | - | - | - | - | - | - | - | - | - | - | - | - | - | - | - | - | - | - | - | - | - | - | - | - | - | - | - | - | - | - | - | - | - | - | - | - | - | - | - | - | - | - | - | - | - | - | - | - | - | - | - | - | - |
| TRTLR8 | - | - | - | - | - | - | - | - | - | - | - | - | - | - | - | - | - | M | T | L | - | A | W | - | - | - | - | - | - | - | M | L | L | S | C | L | S | W | L | S | E | L | H | H | A | A | C | K | P | T | - | W | L | L | P | Q | F | P | C | Q | V | T | G | - | - | Y | N | S | S | - | S | V | F | F | D | C | S | R | R | H | L | H | Q | V | P | K | G | I | T | S | N | V | T | D | L | N | L | S | E | N | S | I | K | E | I | S | P | L | A | F | S | T | M | W | N | L | T | K | L | N | L | S | W | A | N | K | K | T | - | - | - | - | - | - | G | V | N | I | S | N | N | L | F | R | N | L | T | K | L | Q | H | L | R | L | S | G | N | G | M | Y | R | I | P | G | N | I | P | V | S | V | R | I | L | K | M | G | N | N | K | I | T | S | L | - | D | A | H | S | L | A | G | L | K | N | V | T | H | F | S | L | S | R | N | C | Y | R | W | N | P | C | G | K | H | V | S | I | E | K | D | S | F | S | S | M | S | N | L | V | F | L | D | L | S | F | N | N | L | S | Q | V | P | K | G | L | P | Q | S | L | Q | F | L | K | L | D | N | N | K | I | Q | N | I | Y | S | D | D | F | Y | G | L | Q | N | L | K | E | L | K | L | E | G | N | C | P | R | C | E | N | A | P | F | P | C | D | T | C | Q | - | - | N | I | S | V | R | I | H | P | N | A | F | Y | N | L | T | N | L | E | T | L | N | L | G | G | N | S | L | K | E | L | N | P | S | W | F | E | R | L | Y | K | L | K | Q | L | F | L | A | F | N | F | L | L | K | P | I | T | E | Q | G | A | - | - | - | F | L | K | Y | I | P | N | I | K | K | M | D | L | S | F | N | Y | N | L | R | - | S | Y | P | - | - | - | - | K | T | I | N | L | S | P | E | F | S | N | L | A | A | L | Q | T | L | H | L | E | G | L | V | F | Q | N | I | G | P | D | T | L | R | P | L | Y | Q | L | K | N | L | S | V | L | N | L | G | T | N | F | I | I | Q | A | D | S | S | I | F | K | K | L | S | - | - | H | L | K | M | I | Y | L | G | E | N | R | L | Y | P | I | P | E | K | N | M | P | S | L | S | R | Q | Y | N | Q | R | L | D | F | S | V | S | S | Y | M | K | - | - | P | H | G | R | D | Y | F | F | K | - | I | S | Q | T | L | I | K | Q | E | C | F | D | A | G | K | V | L | S | L | N | S | N | N | F | L | F | I | S | T | K | E | F | E | G | - | - | D | I | K | C | L | N | L | S | R | N | G | F | A | P | A | L | N | G | T | E | F | S | F | L | P | N | L | T | Y | L | D | L | S | F | N | K | I | D | L | A | Y | S | L | A | F | N | D | L | K | K | L | Q | V | L | D | L | S | Y | N | P | H | Y | F | N | V | Q | G | I | T | H | K | V | N | F | L | R | N | L | P | V | L | R | V | L | N | M | S | H | N | D | I | S | T | L | T | T | K | Y | M | E | S | K | S | L | A | E | L | R | F | T | H | N | Y | L | G | T | L | W | K | E | N | D | L | S | Y | K | K | L | F | T | K | L | T | N | L | T | I | L | D | I | S | F | N | Q | I | I | K | I | P | D | E | M | Y | K | H | L | P | Q | N | L | T | T | L | I | I | S | H | N | F | L | T | D | F | K | W | N | K | L | I | F | L | P | Q | I | K | V | L | D | L | S | F | N | R | L | T | N | V | T | G | I | H | I | A | - | - | - | H | T | L | T | L | L | N | L | K | H | N | G | I | S | H | L | D | D | G | F | L | M | G | A | K | R | L | Q | V | L | N | L | K | S | N | Q | L | T | T | I | N | E | S | T | F | Q | P | R | P | E | N | Q | F | Q | T | L | Y | L | E | E | N | P | F | Q | C | T | C | D | L | L | D | F | I | L | W | I | E | - | N | S | D | V | K | I | P | G | L | A | T | D | V | K | C | D | A | P | A | N | Q | K | G | R | V | L | I | K | F | D | I | T | - | Q | C | V | N | N | S | E | A | F | L | I | Y | I | L | T | S | S | F | V | I | G | F | M | L | V | T | T | V | A | H | L | F | Y | W | D | A | T | Y | V | L | H | Y | M | K | A | K | L | K | G | Y | S | S | L | N | S | - | - | S | E | I | L | Y | G | A | F | V | T | Y | D | T | R | D | P | H | V | S | E | W | V | M | K | N | L | L | V | K | L | E | E | E | G | E | K | N | L | P | L | C | L | E | E | R | D | W | T | P | G | V | P | L | V | D | N | L | T | Q | S | I | R | Y | S | R | K | T | L | F | V | L | T | Q | D | Y | I | K | T | G | I | F | K | M | A | M | Y | L | A | H | Q | R | L | L | D | E | N | V | D | V | I | V | L | L | L | L | E | P | - | V | L | Q | H | S | H | F | L | R | L | R | R | R | L | C | G | E | S | V | V | D | W | P | R | T | - | - | A | A | A | E | P | W | F | W | Q | N | L | R | N | V | V | R | V | E | N | Q | V | M | Y | N | K | N | Y | S | K | Y | F | T | S | K | X | X | X | X | X | X | X | X | X | X | X | X | X | X | X | X | X | X | X | - | - | - | - | - | - | - | - | - | - | - | - | - | - | - | - | - | - | - | - | - | - | - | - | - | - | - | - | - | - | - | - | - | - | - | - | - | - | - | - | - | - | - | - | - | - | - | - | - | - | - | - | - | - | - | - | - | - | - | - | - | - | - | - | - | - | - | - | - | - | - | - | - | - | - | - | - | - | - | - | - | - | - | - | - | - | - | - | - | - | - | - | - | - | - | - | - | - | - | - | - | - | - | - | - | - | - | - | - | - | - | - | - | - | - | - | - | - | - | - | - | - | - | - | - | - | - | - | - | - | - | - | - | - | - | - | - | - | - | - | - | - | - | - | - | - | - | - | - | - | - | - | - | - | - | - | - | - | - | - | - | - | - | - | - | - | - | - | - | - | - | - | - | - | - | - | - | - | - | - | - | - | - | - | - | - | - | - | - | - | - | - | - | - | - | - | - | - | - | - | - | - | - | - | - | - | - | - | - | - | - | - | - | - | - | - | - | - | - | - | - | - | - | - | - | - | - | - | - | - | - | - | - | - | - | - | - | - | - | - | - | - | - | - | - | - | - | - | - | - | - | - | - | - | - | - | - | - | - | - | - | - | - | - | - | - | - | - | - | - | - | - | - | - | - | - | - | - | - | - | - | - | - | - | - | - | - | - | - | - | - | - | - | - | - | - | - | - | - | - | - | - | - | - | - | - | - | - | - | - | - | - | - | - | - | - | - | - | - | - | - | - | - | - | - | - | - | - | - | - |
| ATTLR8 | - | - | - | - | - | - | - | - | - | - | - | - | - | - | - | - | - | - | - | - | - | - | - | - | - | - | - | M | L | L | F | C | L | C | C | H | Y | E | V | - | - | - | Q | H | A | A | C | K | L | V | - | W | W | S | P | Q | F | P | C | D | V | T | S | - | - | - | N | D | S | - | N | V | I | F | D | C | R | G | R | H | L | E | L | V | P | H | G | I | T | S | N | A | T | W | L | D | L | S | E | N | L | I | K | N | I | S | I | D | S | F | S | H | L | S | N | L | T | T | L | D | L | S | W | A | N | N | K | K | R | - | - | - | - | Y | P | L | K | I | D | A | K | A | F | Q | N | L | T | K | L | S | H | L | K | L | T | G | N | H | L | N | E | I | P | R | N | I | P | V | S | V | K | I | L | E | L | N | I | N | H | I | V | L | L | - | D | N | R | S | F | A | G | L | A | N | L | T | E | L | W | I | S | K | N | C | Y | T | G | N | P | C | N | K | P | V | T | I | M | E | G | S | F | A | I | M | L | K | L | K | V | L | D | L | S | F | N | N | L | T | Q | V | P | K | G | L | P | K | S | I | I | V | L | K | L | G | S | N | K | I | E | H | I | S | E | V | D | F | L | G | L | H | Y | L | E | I | L | T | I | Q | R | N | C | P | R | C | E | I | A | P | Y | P | C | V | P | C | P | - | - | N | R | S | V | T | I | Q | P | N | A | F | D | H | L | T | K | L | Q | V | L | H | L | G | G | N | S | L | D | H | L | N | P | S | W | F | Q | K | L | K | N | L | K | E | L | Y | L | S | F | N | F | L | L | K | A | I | T | E | E | A | T | - | - | - | F | L | N | Y | L | P | K | L | E | I | L | D | L | S | F | N | Y | E | L | K | - | I | Y | P | - | - | - | - | T | T | L | N | L | S | Q | E | F | S | K | L | Q | S | L | R | T | L | N | L | Q | G | L | V | F | Q | N | I | E | Q | S | T | F | K | P | L | Y | E | L | K | N | L | S | A | L | N | L | G | T | N | F | I | I | H | S | D | A | T | I | F | S | R | L | P | - | - | H | L | K | M | I | Y | L | A | E | N | R | L | Y | P | F | P | E | K | N | P | P | Q | L | C | A | G | N | I | Q | R | S | D | F | S | I | A | P | L | L | K | - | - | P | Y | P | N | D | F | V | Y | E | - | I | S | H | N | L | I | K | K | E | C | I | D | S | G | K | M | L | I | L | S | S | N | N | L | F | F | I | S | P | K | L | F | E | G | Y | G | N | V | A | C | L | N | L | S | G | N | G | F | S | A | A | L | N | G | T | E | F | S | S | L | P | N | L | T | Y | L | D | L | S | N | N | K | L | D | L | A | F | D | N | A | F | K | E | L | K | K | L | Q | V | L | D | L | S | Y | N | S | H | Y | F | K | A | Y | G | L | L | H | N | L | N | F | L | K | N | L | P | N | L | R | V | L | N | M | S | H | N | F | I | S | T | L | L | T | K | Q | M | H | S | R | S | L | A | E | L | Q | F | T | N | N | N | L | G | N | L | W | K | H | R | D | G | S | Y | K | M | L | F | T | N | L | I | N | L | T | I | L | D | I | S | H | N | S | I | A | K | I | P | D | N | V | Y | G | H | L | P | R | N | L | T | L | L | R | I | G | H | N | S | L | T | D | F | N | W | G | Q | L | K | G | F | H | Q | L | Q | I | L | D | L | S | F | N | K | L | S | N | V | T | G | M | N | L | N | I | I | H | - | T | L | T | F | L | D | L | S | H | N | T | I | F | H | L | D | N | G | F | I | N | G | L | G | S | L | K | T | L | S | L | S | S | N | K | L | T | I | I | D | Q | S | T | N | A | S | Q | P | N | P | H | I | Q | T | L | F | L | Q | K | N | P | F | E | C | T | C | V | S | V | E | F | I | M | W | L | E | - | S | C | G | I | K | I | P | R | L | A | T | D | V | T | C | D | T | P | A | N | N | R | G | Q | L | L | I | T | F | D | I | N | - | Q | C | V | N | D | S | K | A | F | M | G | Y | T | L | T | T | S | F | I | I | I | F | M | F | V | T | T | V | A | H | L | F | Y | W | D | A | S | Y | L | I | H | Y | T | K | A | K | L | K | G | Y | K | S | L | K | P | - | - | L | D | N | I | H | N | V | F | V | T | Y | D | T | G | D | P | H | V | S | E | W | V | M | K | N | L | R | V | K | L | E | E | E | G | - | - | G | L | P | L | C | L | E | E | R | D | W | S | P | G | I | P | L | V | D | N | L | T | Q | S | I | R | Y | S | R | K | T | L | F | V | L | T | E | G | Y | V | K | T | G | V | F | K | L | A | M | Y | L | A | H | Q | R | L | L | D | E | N | V | D | V | I | V | L | L | M | L | D | P | - | V | L | Q | H | S | H | F | L | R | L | R | R | R | L | C | G | K | S | V | V | E | W | P | R | T | - | - | A | A | A | Q | R | W | F | W | Q | N | L | R | N | V | V | R | V | D | N | Q | V | M | Y | N | K | T | Y | S | K | Y | F | T | S | K | X | X | X | X | X | X | X | X | X | X | X | X | X | X | X | X | X | X | X | X | X | - | - | - | - | - | - | - | - | - | - | - | - | - | - | - | - | - | - | - | - | - | - | - | - | - | - | - | - | - | - | - | - | - | - | - | - | - | - | - | - | - | - | - | - | - | - | - | - | - | - | - | - | - | - | - | - | - | - | - | - | - | - | - | - | - | - | - | - | - | - | - | - | - | - | - | - | - | - | - | - | - | - | - | - | - | - | - | - | - | - | - | - | - | - | - | - | - | - | - | - | - | - | - | - | - | - | - | - | - | - | - | - | - | - | - | - | - | - | - | - | - | - | - | - | - | - | - | - | - | - | - | - | - | - | - | - | - | - | - | - | - | - | - | - | - | - | - | - | - | - | - | - | - | - | - | - | - | - | - | - | - | - | - | - | - | - | - | - | - | - | - | - | - | - | - | - | - | - | - | - | - | - | - | - | - | - | - | - | - | - | - | - | - | - | - | - | - | - | - | - | - | - | - | - | - | - | - | - | - | - | - | - | - | - | - | - | - | - | - | - | - | - | - | - | - | - | - | - | - | - | - | - | - | - | - | - | - | - | - | - | - | - | - | - | - | - | - | - | - | - | - | - | - | - | - | - | - | - | - | - | - | - | - | - | - | - | - | - | - | - | - | - | - | - | - | - | - | - | - | - | - | - | - | - | - | - | - | - | - | - | - | - | - | - | - | - | - | - | - | - | - | - | - | - | - | - | - | - | - | - | - | - | - | - | - | - | - | - | - | - | - | - | - | - | - | - | - | - |
| SMTLR8 | - | - | - | - | - | - | - | - | - | - | - | - | - | - | - | - | - | M | K | A | - | T | W | W | L | Q | L | L | L | L | L | C | L | Y | C | H | C | E | I | Q | P | F | Q | P | A | A | C | K | P | V | - | W | M | S | P | R | F | P | C | D | V | T | A | - | - | C | N | T | S | - | E | V | R | F | D | C | R | R | F | H | L | H | R | V | P | Y | G | I | T | G | N | A | T | E | L | N | L | S | E | N | Y | I | T | N | I | S | L | H | S | F | S | N | L | L | N | L | T | K | L | I | L | N | W | V | N | K | N | K | - | - | - | - | - | - | G | L | L | I | D | D | D | L | F | K | N | L | T | K | L | N | H | L | G | L | A | G | N | R | L | K | K | I | P | T | N | L | P | L | S | V | E | I | L | E | L | N | N | N | K | I | K | - | L | - | D | N | M | T | L | D | S | I | P | N | V | T | K | L | W | L | S | K | N | C | Y | T | W | N | P | C | K | R | N | V | T | I | K | E | G | T | F | A | V | L | N | K | L | Q | V | L | D | L | S | F | N | N | L | T | H | V | P | K | G | L | P | P | S | L | T | R | L | Q | L | G | S | N | K | I | Q | Y | I | S | E | D | D | F | V | G | L | H | D | L | K | I | L | R | I | Q | G | N | C | P | R | C | Q | N | A | P | Y | P | C | V | P | C | Q | - | - | N | I | S | L | G | I | H | P | N | A | F | N | S | L | T | R | L | E | T | L | H | L | G | G | N | S | L | Q | L | L | D | P | S | W | F | K | R | L | K | K | L | R | E | L | F | L | S | F | N | F | L | L | K | A | I | T | G | T | G | T | - | - | - | F | L | R | Y | L | P | R | L | E | K | L | D | L | S | F | N | F | A | L | K | - | L | Y | P | - | - | - | - | T | T | L | N | L | S | H | E | F | S | N | L | V | S | L | R | T | L | H | L | E | G | L | V | F | Q | N | I | G | P | D | T | L | R | P | L | Y | E | L | R | N | L | S | A | L | N | L | G | T | N | F | I | I | H | S | D | L | T | L | F | H | R | F | P | - | - | N | L | K | M | L | Y | L | A | E | N | R | L | Y | P | I | P | V | K | S | P | P | K | L | S | G | G | H | N | Q | V | S | D | F | S | I | S | P | Y | I | N | - | - | P | H | T | K | D | F | A | Y | E | - | V | S | H | K | L | V | K | Q | E | C | F | D | S | G | Q | M | L | I | L | S | S | N | N | L | F | F | I | S | P | K | Q | F | D | G | Y | E | N | I | A | C | L | N | L | S | G | N | G | F | S | A | A | L | N | G | T | E | F | S | L | L | P | N | L | T | Y | L | D | L | S | F | N | K | I | D | L | A | Y | D | H | A | F | K | E | L | E | K | L | Q | V | L | D | L | S | Y | N | S | H | Y | F | Q | A | Y | G | V | T | H | N | L | N | F | L | K | N | L | P | A | L | R | V | L | N | M | S | H | N | A | I | S | T | L | T | T | K | Q | L | Y | S | T | S | L | T | E | L | Q | F | T | N | N | Y | L | G | T | L | W | K | V | R | D | A | T | Y | T | M | L | F | T | N | L | T | N | L | T | F | L | D | I | S | Q | N | K | L | Q | N | I | P | D | E | V | Y | E | Y | F | P | R | N | L | T | K | L | C | I | S | R | N | L | L | T | D | F | K | W | E | A | L | K | F | F | H | Q | L | Q | I | L | D | L | S | F | N | S | I | S | A | V | A | A | I | N | S | S | I | T | K | - | T | L | T | L | L | D | L | S | H | N | Q | I | F | H | L | D | D | G | F | I | K | G | P | K | S | F | K | T | L | I | L | S | F | N | L | L | N | T | I | N | Q | T | T | F | Q | T | R | S | E | D | S | I | Q | T | L | F | L | Q | G | N | P | F | Q | C | I | C | D | S | F | D | F | I | L | W | I | E | - | N | S | Q | I | K | I | P | R | L | T | A | E | V | T | C | D | T | P | A | N | Q | K | D | Q | P | L | I | Y | F | D | I | T | - | Q | C | V | N | D | S | Q | A | F | H | V | Y | I | L | T | T | S | F | I | I | A | F | M | F | V | A | T | V | A | H | L | F | Y | W | D | A | S | Y | V | L | H | Y | M | R | A | K | L | K | G | Y | R | S | L | N | S | - | - | P | D | S | F | Y | N | L | F | V | T | Y | D | T | R | D | P | H | V | S | E | W | V | M | R | N | L | L | V | K | L | E | E | E | G | E | K | H | I | P | L | C | L | E | E | R | D | W | T | P | G | V | P | V | M | D | N | L | T | H | S | I | Q | Y | S | S | K | T | L | F | V | L | T | E | G | Y | V | K | T | G | V | F | K | L | A | M | Y | L | A | H | Q | R | L | L | D | E | N | V | D | V | I | V | L | L | M | L | E | P | - | V | L | Q | H | S | H | F | L | R | L | R | R | R | L | C | G | R | S | V | V | E | W | P | R | T | - | - | A | A | A | E | P | W | F | W | Q | N | L | R | N | V | V | R | V | D | N | Q | V | M | Y | N | K | T | Y | S | K | Y | I | T | A | G | E | V | L | X | X | X | X | X | X | - | - | - | - | - | - | - | - | - | - | - | - | - | - | - | - | - | - | - | - | - | - | - | - | - | - | - | - | - | - | - | - | - | - | - | - | - | - | - | - | - | - | - | - | - | - | - | - | - | - | - | - | - | - | - | - | - | - | - | - | - | - | - | - | - | - | - | - | - | - | - | - | - | - | - | - | - | - | - | - | - | - | - | - | - | - | - | - | - | - | - | - | - | - | - | - | - | - | - | - | - | - | - | - | - | - | - | - | - | - | - | - | - | - | - | - | - | - | - | - | - | - | - | - | - | - | - | - | - | - | - | - | - | - | - | - | - | - | - | - | - | - | - | - | - | - | - | - | - | - | - | - | - | - | - | - | - | - | - | - | - | - | - | - | - | - | - | - | - | - | - | - | - | - | - | - | - | - | - | - | - | - | - | - | - | - | - | - | - | - | - | - | - | - | - | - | - | - | - | - | - | - | - | - | - | - | - | - | - | - | - | - | - | - | - | - | - | - | - | - | - | - | - | - | - | - | - | - | - | - | - | - | - | - | - | - | - | - | - | - | - | - | - | - | - | - | - | - | - | - | - | - | - | - | - | - | - | - | - | - | - | - | - | - | - | - | - | - | - | - | - | - | - | - | - | - | - | - | - | - | - | - | - | - | - | - | - | - | - | - | - | - | - | - | - | - | - | - | - | - | - | - | - | - | - | - | - | - | - | - | - | - | - | - | - | - | - | - | - | - | - | - | - | - | - | - | - | - | - | - | - | - | - | - | - | - | - | - | - | - |
| MMTLR8 | - | - | - | - | - | - | - | - | - | - | - | - | - | - | - | - | - | - | - | - | - | - | - | - | - | M | H | F | L | L | L | S | L | W | C | H | Y | D | I | - | - | - | Q | P | A | A | C | K | P | V | - | W | M | M | P | Q | F | P | C | D | V | E | A | - | - | Y | N | T | S | - | N | I | L | F | D | C | K | G | R | R | L | H | R | V | P | Y | G | I | T | H | N | A | T | E | L | D | L | S | E | N | F | I | H | N | I | S | F | H | S | F | P | E | L | L | N | L | T | L | L | N | L | S | W | A | N | K | K | K | - | - | - | - | - | - | P | V | I | I | D | V | N | A | F | K | N | L | T | K | L | Y | Q | L | R | L | D | G | N | C | L | T | R | I | P | D | N | L | P | R | S | V | R | K | L | G | L | N | N | N | K | I | M | L | V | - | D | N | R | S | L | A | G | I | T | N | M | T | H | L | W | L | S | K | N | C | Y | M | W | N | P | C | Q | K | S | V | E | I | M | D | N | S | F | V | V | M | T | K | L | R | V | L | D | L | S | Y | N | N | I | T | K | V | P | K | G | L | P | K | S | L | E | V | L | M | L | G | S | N | K | I | Q | D | I | F | E | D | D | F | H | R | L | T | L | L | K | V | L | K | I | Q | G | N | C | P | R | C | L | N | A | P | F | P | C | V | P | C | K | - | - | N | I | S | L | G | I | H | P | N | A | F | H | N | L | T | K | L | E | S | L | D | M | G | G | N | S | L | N | Q | V | N | P | S | W | F | E | R | L | T | N | L | K | Q | L | F | L | A | F | N | F | L | L | K | P | I | T | E | E | A | K | - | - | - | F | L | S | Y | L | P | H | L | E | K | L | D | L | S | F | N | F | A | L | K | - | Y | Y | P | - | - | - | - | K | T | I | N | L | S | K | E | F | S | K | L | V | S | L | K | T | L | H | L | E | G | L | V | F | Q | S | I | G | P | D | T | L | S | P | L | Y | E | L | K | N | L | S | V | L | N | L | G | T | N | F | I | I | Q | S | N | S | T | V | F | S | K | F | S | - | - | K | M | K | M | I | Y | L | A | E | N | R | L | Y | P | I | P | V | K | N | P | P | H | P | S | E | G | Y | N | Q | R | S | D | L | S | I | S | P | L | M | K | - | - | P | R | T | K | D | F | S | F | K | - | V | S | H | S | F | V | K | A | E | C | F | N | A | G | R | V | L | I | L | S | S | N | N | L | F | F | I | S | P | K | Q | F | D | G | Y | G | D | I | A | C | L | N | L | S | G | N | G | F | S | S | A | L | N | G | T | E | F | S | L | L | P | N | L | T | Y | L | D | L | S | C | N | K | I | D | L | A | Y | D | H | A | F | T | E | L | K | K | L | E | V | L | D | L | S | H | N | S | H | Y | F | E | A | F | G | V | T | N | N | L | N | F | T | K | N | L | P | V | L | R | V | L | N | M | S | H | N | S | I | S | R | L | T | T | K | Q | M | Y | S | K | S | L | T | E | L | R | F | S | N | N | K | L | G | I | L | W | K | E | K | D | G | S | Y | K | M | L | F | T | N | L | T | N | L | T | I | L | D | I | S | Y | N | G | I | S | K | I | P | D | N | T | Y | K | Y | L | P | R | N | L | T | K | L | Y | I | N | H | N | S | L | T | D | F | K | W | D | Y | L | K | F | F | H | Q | L | Q | T | L | D | L | S | F | N | S | L | R | H | V | R | G | I | N | S | N | F | T | H | T | N | L | T | F | L | D | L | S | H | N | N | I | F | H | L | D | N | G | L | F | K | H | L | K | N | L | K | A | L | S | L | S | Y | N | K | L | S | I | I | N | Q | S | T | F | Q | L | G | P | E | N | Q | I | K | T | L | Y | L | Q | R | N | P | F | Q | C | T | C | D | S | L | D | F | I | L | W | I | E | - | N | S | D | I | K | I | P | R | L | T | T | E | V | I | C | I | T | P | E | N | Q | R | G | Q | A | L | I | G | F | D | I | N | - | Q | C | V | N | D | S | E | A | L | L | I | Y | I | L | T | H | A | C | I | G | I | F | M | F | V | T | T | V | A | H | L | F | Y | W | D | A | S | Y | V | L | H | Y | M | K | A | K | M | K | G | Y | S | P | L | S | S | - | - | P | D | S | A | Y | D | V | F | V | T | Y | D | T | T | D | P | H | V | S | E | W | V | M | R | N | L | R | V | K | L | E | E | E | G | E | K | H | L | P | L | C | L | E | E | R | D | W | P | P | G | V | P | M | V | D | N | L | T | Q | S | I | R | Y | S | H | K | T | L | F | V | L | T | E | G | Y | V | K | T | G | V | F | K | M | A | M | Y | L | A | H | Q | R | L | L | D | E | N | V | D | V | I | V | L | L | M | L | E | P | - | V | L | Q | H | S | H | F | L | R | L | R | R | R | L | C | G | K | S | V | V | E | W | P | R | T | - | - | A | A | A | E | P | W | F | W | Q | N | L | R | N | V | V | R | V | D | N | Q | A | M | Y | N | K | A | Y | S | K | Y | F | T | S | K | X | X | X | X | X | X | X | X | X | X | X | X | X | X | X | X | X | - | - | - | - | - | - | - | - | - | - | - | - | - | - | - | - | - | - | - | - | - | - | - | - | - | - | - | - | - | - | - | - | - | - | - | - | - | - | - | - | - | - | - | - | - | - | - | - | - | - | - | - | - | - | - | - | - | - | - | - | - | - | - | - | - | - | - | - | - | - | - | - | - | - | - | - | - | - | - | - | - | - | - | - | - | - | - | - | - | - | - | - | - | - | - | - | - | - | - | - | - | - | - | - | - | - | - | - | - | - | - | - | - | - | - | - | - | - | - | - | - | - | - | - | - | - | - | - | - | - | - | - | - | - | - | - | - | - | - | - | - | - | - | - | - | - | - | - | - | - | - | - | - | - | - | - | - | - | - | - | - | - | - | - | - | - | - | - | - | - | - | - | - | - | - | - | - | - | - | - | - | - | - | - | - | - | - | - | - | - | - | - | - | - | - | - | - | - | - | - | - | - | - | - | - | - | - | - | - | - | - | - | - | - | - | - | - | - | - | - | - | - | - | - | - | - | - | - | - | - | - | - | - | - | - | - | - | - | - | - | - | - | - | - | - | - | - | - | - | - | - | - | - | - | - | - | - | - | - | - | - | - | - | - | - | - | - | - | - | - | - | - | - | - | - | - | - | - | - | - | - | - | - | - | - | - | - | - | - | - | - | - | - | - | - | - | - | - | - | - | - | - | - | - | - | - | - | - | - | - | - | - | - | - | - | - | - | - | - | - | - | - | - | - | - | - | - | - | - | - | - | - |
| LMTLR8 | - | - | - | - | - | - | - | - | - | - | - | - | - | - | - | - | - | - | - | - | - | - | - | - | - | M | H | L | L | L | F | C | L | C | C | H | Y | E | I | - | - | - | Q | P | A | A | C | K | P | T | - | W | M | S | P | Q | F | P | C | D | V | T | A | - | - | Y | N | T | S | - | T | V | K | F | D | C | K | G | R | H | L | L | K | V | P | D | G | I | T | S | N | A | T | E | L | N | L | S | E | N | Y | I | A | N | I | S | V | N | A | L | S | E | L | L | N | L | T | Q | L | N | L | S | W | A | N | K | N | N | - | - | - | - | - | - | G | L | I | I | A | T | N | A | F | K | N | L | T | K | L | R | E | L | K | L | T | G | N | C | L | N | E | I | P | G | N | I | P | L | S | V | E | I | L | E | L | N | N | N | K | I | M | L | L | - | D | N | T | S | L | A | G | L | T | N | V | T | H | L | W | L | T | K | N | C | Y | F | W | N | P | C | G | K | S | V | T | I | M | D | D | S | F | A | V | M | T | K | L | Q | D | L | N | L | S | Y | N | N | L | T | E | V | P | K | G | L | P | N | S | L | I | M | L | Q | L | G | S | N | R | I | Q | Y | I | S | E | D | D | F | L | G | L | Q | N | L | N | I | L | K | I | Q | G | N | C | P | R | C | Q | N | A | P | Y | P | C | V | P | C | Q | - | - | N | I | S | I | G | I | H | P | N | A | F | H | S | L | T | Q | L | E | T | L | N | L | G | G | N | S | L | N | H | L | N | P | S | W | F | E | K | L | N | K | L | K | Q | L | F | L | A | F | N | F | L | L | K | P | I | T | E | E | A | T | - | - | - | F | L | R | Y | L | P | R | L | E | K | V | D | L | S | F | N | F | A | L | K | - | S | Y | P | - | - | - | - | T | T | L | S | L | S | K | E | F | S | N | L | V | S | L | R | T | L | H | M | E | G | L | V | F | Q | N | I | E | P | D | T | L | K | S | L | Y | G | L | K | N | L | S | A | L | N | L | G | T | N | F | I | I | H | S | D | S | N | I | F | R | K | F | S | - | - | H | L | K | M | I | Y | L | A | E | N | R | L | Y | P | V | S | V | T | S | P | H | A | P | S | E | G | Y | N | Q | G | S | D | L | S | I | S | P | F | I | K | - | - | A | H | P | K | D | F | A | Y | E | - | I | S | H | S | L | I | K | Q | E | C | F | D | S | G | R | V | L | I | L | S | S | N | N | L | F | F | I | S | P | K | Q | F | E | G | Y | G | N | I | A | C | L | N | L | S | G | N | G | F | S | A | A | L | N | G | T | E | F | S | L | L | P | N | L | T | Y | L | D | L | S | F | N | K | I | D | L | A | Y | D | N | A | F | K | E | L | K | K | L | Q | V | L | D | L | S | H | N | P | H | Y | F | E | A | F | G | V | T | H | N | L | N | F | T | K | N | L | P | A | L | R | V | L | N | M | S | H | N | S | I | S | T | L | T | T | K | V | M | H | S | K | S | L | A | E | L | Q | F | T | N | N | H | L | G | T | L | W | K | E | R | D | G | S | Y | K | M | L | F | T | N | L | T | N | L | T | I | L | D | I | S | H | N | R | I | S | K | I | P | D | N | V | Y | E | Y | L | P | R | N | L | T | I | L | R | I | S | H | N | S | L | T | D | F | E | W | D | K | L | R | C | F | H | Q | L | Q | I | L | D | L | S | F | N | S | L | S | N | M | T | G | I | N | S | N | F | S | N | - | T | L | T | F | L | D | L | S | H | N | G | I | F | H | L | D | N | G | F | L | K | G | A | T | T | L | K | T | L | S | L | S | Y | N | K | L | T | I | I | N | Q | S | T | F | E | P | T | S | E | N | Q | I | E | T | L | F | L | Q | R | N | P | F | Q | C | T | C | D | S | L | D | F | I | V | W | I | E | - | N | S | N | V | K | I | P | R | L | T | T | E | V | T | C | G | T | P | E | N | E | K | G | Q | A | L | I | Y | F | D | I | N | - | Q | C | V | N | D | S | Q | A | F | L | I | Y | I | L | T | N | S | F | I | V | I | F | M | F | V | T | T | V | A | H | L | F | Y | W | D | A | S | Y | V | L | H | F | M | K | A | K | L | K | G | Y | R | S | L | N | S | - | - | P | D | S | V | Y | D | V | F | V | T | Y | D | T | G | D | P | H | V | S | E | W | V | M | R | N | L | R | V | K | L | E | E | E | G | E | K | H | L | P | L | C | L | E | D | R | D | W | P | P | G | V | P | L | V | D | N | L | T | Q | S | I | R | Y | S | R | K | T | L | F | V | L | T | E | G | Y | V | K | T | G | V | F | K | L | A | M | Y | L | A | H | Q | R | L | L | D | E | N | V | D | V | I | V | L | L | M | L | E | P | - | V | L | Q | H | S | H | F | L | R | L | R | R | R | L | C | G | Q | S | I | V | E | W | P | R | T | - | - | A | A | A | E | P | W | F | W | Q | N | L | R | N | V | V | R | V | D | N | Q | V | M | Y | N | K | T | Y | S | K | Y | F | T | S | N | X | X | X | X | X | X | X | X | X | X | X | X | X | X | X | X | X | X | - | - | - | - | - | - | - | - | - | - | - | - | - | - | - | - | - | - | - | - | - | - | - | - | - | - | - | - | - | - | - | - | - | - | - | - | - | - | - | - | - | - | - | - | - | - | - | - | - | - | - | - | - | - | - | - | - | - | - | - | - | - | - | - | - | - | - | - | - | - | - | - | - | - | - | - | - | - | - | - | - | - | - | - | - | - | - | - | - | - | - | - | - | - | - | - | - | - | - | - | - | - | - | - | - | - | - | - | - | - | - | - | - | - | - | - | - | - | - | - | - | - | - | - | - | - | - | - | - | - | - | - | - | - | - | - | - | - | - | - | - | - | - | - | - | - | - | - | - | - | - | - | - | - | - | - | - | - | - | - | - | - | - | - | - | - | - | - | - | - | - | - | - | - | - | - | - | - | - | - | - | - | - | - | - | - | - | - | - | - | - | - | - | - | - | - | - | - | - | - | - | - | - | - | - | - | - | - | - | - | - | - | - | - | - | - | - | - | - | - | - | - | - | - | - | - | - | - | - | - | - | - | - | - | - | - | - | - | - | - | - | - | - | - | - | - | - | - | - | - | - | - | - | - | - | - | - | - | - | - | - | - | - | - | - | - | - | - | - | - | - | - | - | - | - | - | - | - | - | - | - | - | - | - | - | - | - | - | - | - | - | - | - | - | - | - | - | - | - | - | - | - | - | - | - | - | - | - | - | - | - | - | - | - | - | - | - | - | - | - | - | - | - | - | - | - | - | - | - | - | - |
| TOTLR8 | - | - | - | - | - | - | - | - | - | - | - | - | - | - | - | - | - | - | - | - | - | - | - | - | - | M | H | L | L | L | F | C | L | Y | C | H | Y | E | L | - | - | - | Q | P | A | A | C | K | P | G | - | W | M | L | P | Q | F | P | C | D | V | K | A | - | - | D | N | T | S | - | V | V | I | F | D | C | K | G | R | R | L | H | A | V | P | D | E | I | T | S | N | A | T | E | L | D | L | S | E | N | Y | I | N | N | I | S | V | N | S | F | S | K | L | V | N | L | T | K | L | N | L | S | W | A | N | K | K | E | - | - | - | - | - | - | A | V | T | I | A | A | N | A | F | K | N | L | T | M | L | N | H | L | I | L | T | G | N | C | L | T | Q | I | P | S | N | L | P | P | S | V | G | I | L | E | L | S | N | N | N | I | T | L | L | - | D | N | R | S | L | V | G | L | T | N | V | T | Q | L | W | L | S | K | N | C | Y | T | W | N | P | C | R | K | S | V | T | I | M | E | G | I | F | A | I | M | A | K | L | E | A | L | D | L | S | F | N | N | L | T | H | V | P | K | G | L | P | Q | S | L | R | V | L | K | L | G | S | N | K | I | Q | Y | I | S | E | E | D | F | R | G | L | Y | S | L | K | T | L | K | I | Q | G | N | C | P | R | C | Q | T | A | P | Y | P | C | V | P | C | Q | - | - | N | I | S | L | G | I | H | P | N | A | F | D | S | L | A | Q | L | E | T | L | H | L | G | G | N | S | L | D | H | L | K | P | S | W | F | E | K | L | K | N | L | K | Q | L | F | L | S | F | N | F | L | V | K | A | I | T | G | E | A | K | - | - | - | F | L | K | Y | L | P | R | L | E | K | F | D | L | S | F | N | F | A | L | K | - | Y | Y | P | - | - | - | - | T | T | L | N | L | S | K | D | F | S | N | L | V | S | L | R | T | L | H | L | Q | G | L | V | F | Q | S | I | G | P | G | T | L | K | P | L | Y | E | L | K | N | L | S | A | L | N | L | G | T | N | F | I | I | Q | S | N | S | S | I | F | S | R | F | P | - | - | R | L | K | M | I | Y | L | G | E | N | R | L | Y | P | I | P | V | K | N | P | P | L | P | N | D | G | Y | N | Q | R | P | G | L | S | I | P | P | F | I | K | - | - | P | H | P | K | D | F | A | F | E | - | I | S | H | L | L | I | K | Q | E | R | F | Q | S | G | R | V | L | I | L | S | S | N | N | L | F | F | I | S | P | K | Q | F | D | G | Y | G | D | I | A | C | L | N | L | S | R | N | G | F | S | A | A | L | N | G | T | E | F | S | L | L | P | N | L | T | Y | L | D | L | S | F | N | K | I | D | L | A | Y | D | N | A | F | K | E | L | Q | K | L | Q | V | L | D | L | S | Y | N | E | H | Y | F | K | A | Y | G | I | T | H | N | L | N | F | I | K | N | L | P | V | L | R | V | L | N | M | S | Y | N | A | I | S | T | L | T | T | K | Q | M | Y | S | K | S | L | S | E | L | Q | F | T | D | N | N | L | G | T | L | W | R | E | N | D | G | S | Y | N | M | L | F | T | N | L | I | N | L | T | I | L | D | I | S | R | N | K | I | T | K | I | P | I | D | V | Y | K | Y | L | P | H | N | L | T | K | L | R | I | S | Q | N | L | L | T | D | F | Q | W | D | R | L | R | Y | F | H | Q | L | Q | I | L | D | L | S | S | N | S | L | S | K | V | T | G | I | N | S | N | I | T | Q | - | T | L | T | F | L | N | L | N | H | N | C | I | F | Q | L | D | S | G | F | I | K | G | P | K | S | L | T | T | L | S | L | S | N | N | K | L | T | T | I | N | Q | S | T | F | Q | S | G | P | D | N | Q | I | H | T | S | Y | L | Q | G | N | P | F | Q | C | T | C | D | I | L | D | F | I | L | W | I | E | - | N | S | E | I | N | I | P | R | L | T | T | E | V | T | C | H | T | P | E | N | Q | K | G | Q | A | L | I | Y | F | D | I | N | - | Q | C | V | N | D | I | Q | A | F | L | M | Y | I | V | T | T | F | F | I | I | A | F | M | I | V | A | T | V | A | H | L | F | Y | W | D | A | S | Y | V | I | H | Y | M | K | A | K | L | K | G | Y | S | S | L | N | S | - | - | S | D | S | V | Y | N | V | F | V | T | Y | D | T | R | D | P | H | V | S | E | S | V | M | R | N | L | R | V | K | L | E | E | E | G | E | K | H | L | P | L | C | L | E | E | R | D | W | P | P | G | V | P | L | V | E | N | L | T | Q | S | I | R | Y | S | H | K | T | L | F | V | L | T | E | A | Y | V | K | T | G | V | F | K | L | A | M | Y | L | A | H | Q | R | L | L | D | E | N | V | D | V | I | V | L | L | M | L | E | P | - | V | L | Q | H | S | H | F | L | R | L | R | R | R | L | C | G | K | S | V | V | E | W | P | R | T | - | - | A | A | A | E | P | W | F | W | Q | N | L | R | N | V | V | R | V | D | N | Q | V | I | Y | N | K | T | Y | L | K | Y | F | T | S | R | X | X | X | X | X | X | X | X | X | X | X | X | X | X | X | X | X | X | - | - | - | - | - | - | - | - | - | - | - | - | - | - | - | - | - | - | - | - | - | - | - | - | - | - | - | - | - | - | - | - | - | - | - | - | - | - | - | - | - | - | - | - | - | - | - | - | - | - | - | - | - | - | - | - | - | - | - | - | - | - | - | - | - | - | - | - | - | - | - | - | - | - | - | - | - | - | - | - | - | - | - | - | - | - | - | - | - | - | - | - | - | - | - | - | - | - | - | - | - | - | - | - | - | - | - | - | - | - | - | - | - | - | - | - | - | - | - | - | - | - | - | - | - | - | - | - | - | - | - | - | - | - | - | - | - | - | - | - | - | - | - | - | - | - | - | - | - | - | - | - | - | - | - | - | - | - | - | - | - | - | - | - | - | - | - | - | - | - | - | - | - | - | - | - | - | - | - | - | - | - | - | - | - | - | - | - | - | - | - | - | - | - | - | - | - | - | - | - | - | - | - | - | - | - | - | - | - | - | - | - | - | - | - | - | - | - | - | - | - | - | - | - | - | - | - | - | - | - | - | - | - | - | - | - | - | - | - | - | - | - | - | - | - | - | - | - | - | - | - | - | - | - | - | - | - | - | - | - | - | - | - | - | - | - | - | - | - | - | - | - | - | - | - | - | - | - | - | - | - | - | - | - | - | - | - | - | - | - | - | - | - | - | - | - | - | - | - | - | - | - | - | - | - | - | - | - | - | - | - | - | - | - | - | - | - | - | - | - | - | - | - | - | - | - | - | - | - | - | - |
| conservation |  |  |  |  |  |  |  |  |  |  |  |  |  |  |  |  |  |  |  |  |  |  |  |  |  |  |  |  |  |  |  |  |  |  |  |  |  |  |  |  |  |  |  |  |  |  |  |  |  |  |  |  |  |  |  |  |  |  |  |  |  |  |  |  |  |  |  |  |  |  |  |  |  |  |  |  |  |  |  |  |  |  |  |  |  |  |  |  |  |  |  |  |  |  |  |  |  |  |  |  |  |  |  |  |  |  |  |  |  |  |  |  |  |  |  |  |  |  |  |  |  |  |  |  |  |  |  |  |  |  |  |  |  |  |  |  |  |  |  |  |  |  |  |  |  |  |  |  |  |  |  |  |  |  |  |  |  |  |  |  |  |  |  |  |  |  |  |  |  |  |  |  |  |  |  |  |  |  |  |  |  |  |  |  |  |  |  |  |  |  |  |  |  |  |  |  |  |  |  |  |  |  |  |  |  |  |  |  |  |  |  |  |  |  |  |  |  |  |  |  |  |  |  |  |  |  |  |  |  |  |  |  |  |  |  |  |  |  |  |  |  |  |  |  |  |  |  |  |  |  |  |  |  |  |  |  |  |  |  |  |  |  |  |  |  |  |  |  |  |  |  |  |  |  |  |  |  |  |  |  |  |  |  |  |  |  |  |  |  |  |  |  |  |  |  |  |  |  |  |  |  |  |  |  |  |  |  |  |  |  |  |  |  |  |  |  |  |  |  |  |  |  |  |  |  |  |  |  |  |  |  |  |  |  |  |  |  |  |  |  |  |  |  |  |  |  |  |  |  |  |  |  |  |  |  |  |  |  |  |  |  |  |  |  |  |  |  |  |  |  |  |  |  |  |  |  |  |  |  |  |  |  |  |  |  |  |  |  |  |  |  |  |  |  |  |  |  |  |  |  |  |  |  |  |  |  |  |  |  |  |  |  |  |  |  |  |  |  |  |  |  |  |  |  |  |  |  |  |  |  |  |  |  |  |  |  |  |  |  |  |  |  |  |  |  |  |  |  |  |  |  |  |  |  |  |  |  |  |  |  |  |  |  |  |  |  |  |  |  |  |  |  |  |  |  |  |  |  |  |  |  |  |  |  |  |  |  |  |  |  |  |  |  |  |  |  |  |  |  |  |  |  |  |  |  |  |  |  |  |  |  |  |  |  |  |  |  |  |  |  |  |  |  |  |  |  |  |  |  |  |  |  |  |  |  |  |  |  |  |  |  |  |  |  |  |  |  |  |  |  |  |  |  |  |  |  |  |  |  |  |  |  |  |  |  |  |  |  |  |  |  |  |  |  |  |  |  |  |  |  |  |  |  |  |  |  |  |  |  |  |  |  |  |  |  |  |  |  |  |  |  |  |  |  |  |  |  |  |  |  |  |  |  |  |  |  |  |  |  |  |  |  |  |  |  |  |  |  |  |  |  |  |  |  |  |  |  |  |  |  |  |  |  |  |  |  |  |  |  |  |  |  |  |  |  |  |  |  |  |  |  |  |  |  |  |  |  |  |  |  |  |  |  |  |  |  |  |  |  |  |  |  |  |  |  |  |  |  |  |  |  |  |  |  |  |  |  |  |  |  |  |  |  |  |  |  |  |  |  |  |  |  |  |  |  |  |  |  |  |  |  |  |  |  |  |  |  |  |  |  |  |  |  |  |  |  |  |  |  |  |  |  |  |  |  |  |  |  |  |  |  |  |  |  |  |  |  |  |  |  |  |  |  |  |  |  |  |  |  |  |  |  |  |  |  |  |  |  |  |  |  |  |  |  |  |  |  |  |  |  |  |  |  |  |  |  |  |  |  |  |  |  |  |  |  |  |  |  |  |  |  |  |  |  |  |  |  |  |  |  |  |  |  |  |  |  |  |  |  |  |  |  |  |  |  |  |  |  |  |  |  |  |  |  |  |  |  |  |  |  |  |  |  |  |  |  |  |  |  |  |  |  |  |  |  |  |  |  |  |  |  |  |  |  |  |  |  |  |  |  |  |  |  |  |  |  |  |  |  |  |  |  |  |  |  |  |  |  |  |  |  |  |  |  |  |  |  |  |  |  |  |  |  |  |  |  |  |  |  |  |  |  |  |  |  |  |  |  |  |  |  |  |  |  |  |  |  |  |  |  |  |  |  |  |  |  |  |  |  |  |  |  |  |  |  |  |  |  |  |  |  |  |  |  |  |  |  |  |  |  |  |  |  |  |  |  |  |  |  |  |  |  |  |  |  |  |  |  |  |  |  |  |  |  |  |  |  |  |  |  |  |  |  |  |  |  |  |  |  |  |  |  |  |  |  |  |  |  |  |  |  |  |  |  |  |  |  |  |  |  |  |  |  |  |  |  |  |  |  |  |  |  |  |  |  |  |  |  |  |  |  |  |  |  |  |  |  |  |  |  |  |  |  |  |  |  |  |  |  |  |  |  |  |  |  |  |  |  |  |  |  |  |  |  |  |  |  |  |  |  |  |  |  |  |  |  |  |  |  |  |  |  |  |  |  |  |  |  |  |  |  |  |  |  |  |  |  |  |  |  |  |  |  |  |  |  |  |  |  |  |  |  |  |  |  |  |  |  |  |  |  |  |  |  |  |  |  |  |  |  |  |  |  |  |  |  |  |  |  |  |  |  |  |  |  |  |  |  |  |  |  |  |  |  |  |  |  |  |  |  |  |  |  |  |  |  |  |  |  |  |  |  |  |  |  |  |  |  |  |  |  |  |  |  |  |  |  |  |  |  |  |  |  |  |  |  |  |  |  |  |  |  |  |  |  |  |  |  |  |  |  |  |  |  |  |  |  |  |  |  |  |  |  |  |  |  |  |  |  |  |  |  |  |  |  |  |  |  |  |  |  |  |  |  |  |  |  |  |  |  |  |  |  |  |  |  |  |  |  |  |  |  |  |  |  |  |  |  |  |  |  |  |  |  |  |  |  |  |  |  |  |  |  |  |  |  |  |  |  |  |  |  |  |  |  |  |  |  |  |  |  |  |  |  |  |  |  |  |  |  |  |  |  |  |  |  |  |  |  |  |  |  |  |  |  |  |  |  |  |  |  |  |  |  |  |  |  |  |  |  |  |  |  |  |  |  |  |  |  |  |  |  |  |  |  |  |  |  |  |  |  |  |  |  |  |  |  |  |  |  |  |  |  |  |  |  |  |  |  |  |  |  |  |  |  |  |  |  |  |  |  |  |  |  |  |  |  |  |  |  |  |  |  |  |  |  |  |  |  |  |  |  |  |  |
|  |  |  |  |  |  |  |  |  |  |  |  |  |  |  |  |  |  |  |  |  |  |  |  |  |  |  |  |  |  |  |  |  |  |  |  |  |  |  |  |  |  |  |  |  |  |  |  |  |  |  |  |  |  |  |  |  |  |  |  |  |  |  |  |  |  |  |  |  |  |  |  |  |  |  |  |  |  |  |  |  |  |  |  |  |  |  |  |  |  |  |  |  |  |  |  |  |  |  |  |  |  |  |  |  |  |  |  |  |  |  |  |  |  |  |  |  |  |  |  |  |  |  |  |  |  |  |  |  |  |  |  |  |  |  |  |  |  |  |  |  |  |  |  |  |  |  |  |  |  |  |  |  |  |  |  |  |  |  |  |  |  |  |  |  |  |  |  |  |  |  |  |  |  |  |  |  |  |  |  |  |  |  |  |  |  |  |  |  |  |  |  |  |  |  |  |  |  |  |  |  |  |  |  |  |  |  |  |  |  |  |  |  |  |  |  |  |  |  |  |  |  |  |  |  |  |  |  |  |  |  |  |  |  |  |  |  |  |  |  |  |  |  |  |  |  |  |  |  |  |  |  |  |  |  |  |  |  |  |  |  |  |  |  |  |  |  |  |  |  |  |  |  |  |  |  |  |  |  |  |  |  |  |  |  |  |  |  |  |  |  |  |  |  |  |  |  |  |  |  |  |  |  |  |  |  |  |  |  |  |  |  |  |  |  |  |  |  |  |  |  |  |  |  |  |  |  |  |  |  |  |  |  |  |  |  |  |  |  |  |  |  |  |  |  |  |  |  |  |  |  |  |  |  |  |  |  |  |  |  |  |  |  |  |  |  |  |  |  |  |  |  |  |  |  |  |  |  |  |  |  |  |  |  |  |  |  |  |  |  |  |  |  |  |  |  |  |  |  |  |  |  |  |  |  |  |  |  |  |  |  |  |  |  |  |  |  |  |  |  |  |  |  |  |  |  |  |  |  |  |  |  |  |  |  |  |  |  |  |  |  |  |  |  |  |  |  |  |  |  |  |  |  |  |  |  |  |  |  |  |  |  |  |  |  |  |  |  |  |  |  |  |  |  |  |  |  |  |  |  |  |  |  |  |  |  |  |  |  |  |  |  |  |  |  |  |  |  |  |  |  |  |  |  |  |  |  |  |  |  |  |  |  |  |  |  |  |  |  |  |  |  |  |  |  |  |  |  |  |  |  |  |  |  |  |  |  |  |  |  |  |  |  |  |  |  |  |  |  |  |  |  |  |  |  |  |  |  |  |  |  |  |  |  |  |  |  |  |  |  |  |  |  |  |  |  |  |  |  |  |  |  |  |  |  |  |  |  |  |  |  |  |  |  |  |  |  |  |  |  |  |  |  |  |  |  |  |  |  |  |  |  |  |  |  |  |  |  |  |  |  |  |  |  |  |  |  |  |  |  |  |  |  |  |  |  |  |  |  |  |  |  |  |  |  |  |  |  |  |  |  |  |  |  |  |  |  |  |  |  |  |  |  |  |  |  |  |  |  |  |  |  |  |  |  |  |  |  |  |  |  |  |  |  |  |  |  |  |  |  |  |  |  |  |  |  |  |  |  |  |  |  |  |  |  |  |  |  |  |  |  |  |  |  |  |  |  |  |  |  |  |  |  |  |  |  |  |  |  |  |  |  |  |  |  |  |  |  |  |  |  |  |  |  |  |  |  |  |  |  |  |  |  |  |  |  |  |  |  |  |  |  |  |  |  |  |  |  |  |  |  |  |  |  |  |  |  |  |  |  |  |  |  |  |  |  |  |  |  |  |  |  |  |  |  |  |  |  |  |  |  |  |  |  |  |  |  |  |  |  |  |  |  |  |  |  |  |  |  |  |  |  |  |  |  |  |  |  |  |  |  |  |  |  |  |  |  |  |  |  |  |  |  |  |  |  |  |  |  |  |  |  |  |  |  |  |  |  |  |  |  |  |  |  |  |  |  |  |  |  |  |  |  |  |  |  |  |  |  |  |  |  |  |  |  |  |  |  |  |  |  |  |  |  |  |  |  |  |  |  |  |  |  |  |  |  |  |  |  |  |  |  |  |  |  |  |  |  |  |  |  |  |  |  |  |  |  |  |  |  |  |  |  |  |  |  |  |  |  |  |  |  |  |  |  |  |  |  |  |  |  |  |  |  |  |  |  |  |  |  |  |  |  |  |  |  |  |  |  |  |  |  |  |  |  |  |  |  |  |  |  |  |  |  |  |  |  |  |  |  |  |  |  |  |  |  |  |  |  |  |  |  |  |  |  |  |  |  |  |  |  |  |  |  |  |  |  |  |  |  |  |  |  |  |  |  |  |  |  |  |  |  |  |  |  |  |  |  |  |  |  |  |  |  |  |  |  |  |  |  |  |  |  |  |  |  |  |  |  |  |  |  |  |  |  |  |  |  |  |  |  |  |  |  |  |  |  |  |  |  |  |  |  |  |  |  |  |  |  |  |  |  |  |  |  |  |  |  |  |  |  |  |  |  |  |  |  |  |  |  |  |  |  |  |  |  |  |  |  |  |  |  |  |  |  |  |  |  |  |  |  |  |  |  |  |  |  |  |  |  |  |  |  |  |  |  |  |  |  |  |  |  |  |  |  |  |  |  |  |  |  |  |  |  |  |  |  |  |  |  |  |  |  |  |  |  |  |  |  |  |  |  |  |  |  |  |  |  |  |  |  |  |  |  |  |  |  |  |  |  |  |  |  |  |  |  |  |  |  |  |  |  |  |  |  |  |  |  |  |  |  |  |  |  |  |  |  |  |  |  |  |  |  |  |  |  |  |  |  |  |  |  |  |  |  |  |  |  |  |  |  |  |  |  |  |  |  |  |  |  |  |  |  |  |  |  |  |  |  |  |  |  |  |  |  |  |  |  |  |  |  |  |  |  |  |  |  |  |  |  |  |  |  |  |  |  |  |  |  |  |  |  |  |  |  |  |  |  |  |  |  |  |  |  |  |  |  |  |  |  |  |  |  |  |  |  |  |  |  |  |  |  |  |  |  |  |  |  |  |  |  |  |  |  |  |  |  |  |  |  |  |  |  |  |  |  |  |  |  |  |  |  |  |  |  |  |  |  |  |  |  |  |  |  |  |  |  |  |  |  |  |  |  |  |  |  |  |  |  |  |  |  |  |  |  |  |  |  |  |  |  |  |  |  |  |  |  |  |  |  |  |  |  |  |  |  |  |  |  |  |  |  |  |  |  |  |  |  |  |  |  |  |  |  |  |
|  |  |  |  |  |  |  |  |  |  |  |  |  |  |  |  |  |  |  |  |  |  |  |  |  |  |  |  |  |  |  |  |  |  |  |  |  |  |  |  |  |  |  |  |  |  |  |  |  |  |  |  |  |  |  |  |  |  |  |  |  |  |  |  |  |  |  |  |  |  |  |  |  |  |  |  |  |  |  |  |  |  |  |  |  |  |  |  |  |  |  |  |  |  |  |  |  |  |  |  |  |  |  |  |  |  |  |  |  |  |  |  |  |  |  |  |  |  |  |  |  |  |  |  |  |  |  |  |  |  |  |  |  |  |  |  |  |  |  |  |  |  |  |  |  |  |  |  |  |  |  |  |  |  |  |  |  |  |  |  |  |  |  |  |  |  |  |  |  |  |  |  |  |  |  |  |  |  |  |  |  |  |  |  |  |  |  |  |  |  |  |  |  |  |  |  |  |  |  |  |  |  |  |  |  |  |  |  |  |  |  |  |  |  |  |  |  |  |  |  |  |  |  |  |  |  |  |  |  |  |  |  |  |  |  |  |  |  |  |  |  |  |  |  |  |  |  |  |  |  |  |  |  |  |  |  |  |  |  |  |  |  |  |  |  |  |  |  |  |  |  |  |  |  |  |  |  |  |  |  |  |  |  |  |  |  |  |  |  |  |  |  |  |  |  |  |  |  |  |  |  |  |  |  |  |  |  |  |  |  |  |  |  |  |  |  |  |  |  |  |  |  |  |  |  |  |  |  |  |  |  |  |  |  |  |  |  |  |  |  |  |  |  |  |  |  |  |  |  |  |  |  |  |  |  |  |  |  |  |  |  |  |  |  |  |  |  |  |  |  |  |  |  |  |  |  |  |  |  |  |  |  |  |  |  |  |  |  |  |  |  |  |  |  |  |  |  |  |  |  |  |  |  |  |  |  |  |  |  |  |  |  |  |  |  |  |  |  |  |  |  |  |  |  |  |  |  |  |  |  |  |  |  |  |  |  |  |  |  |  |  |  |  |  |  |  |  |  |  |  |  |  |  |  |  |  |  |  |  |  |  |  |  |  |  |  |  |  |  |  |  |  |  |  |  |  |  |  |  |  |  |  |  |  |  |  |  |  |  |  |  |  |  |  |  |  |  |  |  |  |  |  |  |  |  |  |  |  |  |  |  |  |  |  |  |  |  |  |  |  |  |  |  |  |  |  |  |  |  |  |  |  |  |  |  |  |  |  |  |  |  |  |  |  |  |  |  |  |  |  |  |  |  |  |  |  |  |  |  |  |  |  |  |  |  |  |  |  |  |  |  |  |  |  |  |  |  |  |  |  |  |  |  |  |  |  |  |  |  |  |  |  |  |  |  |  |  |  |  |  |  |  |  |  |  |  |  |  |  |  |  |  |  |  |  |  |  |  |  |  |  |  |  |  |  |  |  |  |  |  |  |  |  |  |  |  |  |  |  |  |  |  |  |  |  |  |  |  |  |  |  |  |  |  |  |  |  |  |  |  |  |  |  |  |  |  |  |  |  |  |  |  |  |  |  |  |  |  |  |  |  |  |  |  |  |  |  |  |  |  |  |  |  |  |  |  |  |  |  |  |  |  |  |  |  |  |  |  |  |  |  |  |  |  |  |  |  |  |  |  |  |  |  |  |  |  |  |  |  |  |  |  |  |  |  |  |  |  |  |  |  |  |  |  |  |  |  |  |  |  |  |  |  |  |  |  |  |  |  |  |  |  |  |  |  |  |  |  |  |  |  |  |  |  |  |  |  |  |  |  |  |  |  |  |  |  |  |  |  |  |  |  |  |  |  |  |  |  |  |  |  |  |  |  |  |  |  |  |  |  |  |  |  |  |  |  |  |  |  |  |  |  |  |  |  |  |  |  |  |  |  |  |  |  |  |  |  |  |  |  |  |  |  |  |  |  |  |  |  |  |  |  |  |  |  |  |  |  |  |  |  |  |  |  |  |  |  |  |  |  |  |  |  |  |  |  |  |  |  |  |  |  |  |  |  |  |  |  |  |  |  |  |  |  |  |  |  |  |  |  |  |  |  |  |  |  |  |  |  |  |  |  |  |  |  |  |  |  |  |  |  |  |  |  |  |  |  |  |  |  |  |  |  |  |  |  |  |  |  |  |  |  |  |  |  |  |  |  |  |  |  |  |  |  |  |  |  |  |  |  |  |  |  |  |  |  |  |  |  |  |  |  |  |  |  |  |  |  |  |  |  |  |  |  |  |  |  |  |  |  |  |  |  |  |  |  |  |  |  |  |  |  |  |  |  |  |  |  |  |  |  |  |  |  |  |  |  |  |  |  |  |  |  |  |  |  |  |  |  |  |  |  |  |  |  |  |  |  |  |  |  |  |  |  |  |  |  |  |  |  |  |  |  |  |  |  |  |  |  |  |  |  |  |  |  |  |  |  |  |  |  |  |  |  |  |  |  |  |  |  |  |  |  |  |  |  |  |  |  |  |  |  |  |  |  |  |  |  |  |  |  |  |  |  |  |  |  |  |  |  |  |  |  |  |  |  |  |  |  |  |  |  |  |  |  |  |  |  |  |  |  |  |  |  |  |  |  |  |  |  |  |  |  |  |  |  |  |  |  |  |  |  |  |  |  |  |  |  |  |  |  |  |  |  |  |  |  |  |  |  |  |  |  |  |  |  |  |  |  |  |  |  |  |  |  |  |  |  |  |  |  |  |  |  |  |  |  |  |  |  |  |  |  |  |  |  |  |  |  |  |  |  |  |  |  |  |  |  |  |  |  |  |  |  |  |  |  |  |  |  |  |  |  |  |  |  |  |  |  |  |  |  |  |  |  |  |  |  |  |  |  |  |  |  |  |  |  |  |  |  |  |  |  |  |  |  |  |  |  |  |  |  |  |  |  |  |  |  |  |  |  |  |  |  |  |  |  |  |  |  |  |  |  |  |  |  |  |  |  |  |  |  |  |  |  |  |  |  |  |  |  |  |  |  |  |  |  |  |  |  |  |  |  |  |  |  |  |  |  |  |  |  |  |  |  |  |  |  |  |  |  |  |  |  |  |  |  |  |  |  |  |  |  |  |  |  |  |  |  |  |  |  |  |  |  |  |  |  |  |  |  |  |  |  |  |  |  |  |  |  |  |  |  |  |  |  |  |  |  |  |  |  |  |  |  |  |  |  |  |  |  |  |  |  |  |  |  |  |  |  |  |  |  |  |  |  |  |  |  |  |  |  |  |  |  |  |  |  |  |  |  |  |  |  |  |
|  |  |  |  |  |  |  |  |  |  |  |  |  |  |  |  |  |  |  |  |  |  |  |  |  |  |  |  |  |  |  |  |  |  |  |  |  |  |  |  |  |  |  |  |  |  |  |  |  |  |  |  |  |  |  |  |  |  |  |  |  |  |  |  |  |  |  |  |  |  |  |  |  |  |  |  |  |  |  |  |  |  |  |  |  |  |  |  |  |  |  |  |  |  |  |  |  |  |  |  |  |  |  |  |  |  |  |  |  |  |  |  |  |  |  |  |  |  |  |  |  |  |  |  |  |  |  |  |  |  |  |  |  |  |  |  |  |  |  |  |  |  |  |  |  |  |  |  |  |  |  |  |  |  |  |  |  |  |  |  |  |  |  |  |  |  |  |  |  |  |  |  |  |  |  |  |  |  |  |  |  |  |  |  |  |  |  |  |  |  |  |  |  |  |  |  |  |  |  |  |  |  |  |  |  |  |  |  |  |  |  |  |  |  |  |  |  |  |  |  |  |  |  |  |  |  |  |  |  |  |  |  |  |  |  |  |  |  |  |  |  |  |  |  |  |  |  |  |  |  |  |  |  |  |  |  |  |  |  |  |  |  |  |  |  |  |  |  |  |  |  |  |  |  |  |  |  |  |  |  |  |  |  |  |  |  |  |  |  |  |  |  |  |  |  |  |  |  |  |  |  |  |  |  |  |  |  |  |  |  |  |  |  |  |  |  |  |  |  |  |  |  |  |  |  |  |  |  |  |  |  |  |  |  |  |  |  |  |  |  |  |  |  |  |  |  |  |  |  |  |  |  |  |  |  |  |  |  |  |  |  |  |  |  |  |  |  |  |  |  |  |  |  |  |  |  |  |  |  |  |  |  |  |  |  |  |  |  |  |  |  |  |  |  |  |  |  |  |  |  |  |  |  |  |  |  |  |  |  |  |  |  |  |  |  |  |  |  |  |  |  |  |  |  |  |  |  |  |  |  |  |  |  |  |  |  |  |  |  |  |  |  |  |  |  |  |  |  |  |  |  |  |  |  |  |  |  |  |  |  |  |  |  |  |  |  |  |  |  |  |  |  |  |  |  |  |  |  |  |  |  |  |  |  |  |  |  |  |  |  |  |  |  |  |  |  |  |  |  |  |  |  |  |  |  |  |  |  |  |  |  |  |  |  |  |  |  |  |  |  |  |  |  |  |  |  |  |  |  |  |  |  |  |  |  |  |  |  |  |  |  |  |  |  |  |  |  |  |  |  |  |  |  |  |  |  |  |  |  |  |  |  |  |  |  |  |  |  |  |  |  |  |  |  |  |  |  |  |  |  |  |  |  |  |  |  |  |  |  |  |  |  |  |  |  |  |  |  |  |  |  |  |  |  |  |  |  |  |  |  |  |  |  |  |  |  |  |  |  |  |  |  |  |  |  |  |  |  |  |  |  |  |  |  |  |  |  |  |  |  |  |  |  |  |  |  |  |  |  |  |  |  |  |  |  |  |  |  |  |  |  |  |  |  |  |  |  |  |  |  |  |  |  |  |  |  |  |  |  |  |  |  |  |  |  |  |  |  |  |  |  |  |  |  |  |  |  |  |  |  |  |  |  |  |  |  |  |  |  |  |  |  |  |  |  |  |  |  |  |  |  |  |  |  |  |  |  |  |  |  |  |  |  |  |  |  |  |  |  |  |  |  |  |  |  |  |  |  |  |  |  |  |  |  |  |  |  |  |  |  |  |  |  |  |  |  |  |  |  |  |  |  |  |  |  |  |  |  |  |  |  |  |  |  |  |  |  |  |  |  |  |  |  |  |  |  |  |  |  |  |  |  |  |  |  |  |  |  |  |  |  |  |  |  |  |  |  |  |  |  |  |  |  |  |  |  |  |  |  |  |  |  |  |  |  |  |  |  |  |  |  |  |  |  |  |  |  |  |  |  |  |  |  |  |  |  |  |  |  |  |  |  |  |  |  |  |  |  |  |  |  |  |  |  |  |  |  |  |  |  |  |  |  |  |  |  |  |  |  |  |  |  |  |  |  |  |  |  |  |  |  |  |  |  |  |  |  |  |  |  |  |  |  |  |  |  |  |  |  |  |  |  |  |  |  |  |  |  |  |  |  |  |  |  |  |  |  |  |  |  |  |  |  |  |  |  |  |  |  |  |  |  |  |  |  |  |  |  |  |  |  |  |  |  |  |  |  |  |  |  |  |  |  |  |  |  |  |  |  |  |  |  |  |  |  |  |  |  |  |  |  |  |  |  |  |  |  |  |  |  |  |  |  |  |  |  |  |  |  |  |  |  |  |  |  |  |  |  |  |  |  |  |  |  |  |  |  |  |  |  |  |  |  |  |  |  |  |  |  |  |  |  |  |  |  |  |  |  |  |  |  |  |  |  |  |  |  |  |  |  |  |  |  |  |  |  |  |  |  |  |  |  |  |  |  |  |  |  |  |  |  |  |  |  |  |  |  |  |  |  |  |  |  |  |  |  |  |  |  |  |  |  |  |  |  |  |  |  |  |  |  |  |  |  |  |  |  |  |  |  |  |  |  |  |  |  |  |  |  |  |  |  |  |  |  |  |  |  |  |  |  |  |  |  |  |  |  |  |  |  |  |  |  |  |  |  |  |  |  |  |  |  |  |  |  |  |  |  |  |  |  |  |  |  |  |  |  |  |  |  |  |  |  |  |  |  |  |  |  |  |  |  |  |  |  |  |  |  |  |  |  |  |  |  |  |  |  |  |  |  |  |  |  |  |  |  |  |  |  |  |  |  |  |  |  |  |  |  |  |  |  |  |  |  |  |  |  |  |  |  |  |  |  |  |  |  |  |  |  |  |  |  |  |  |  |  |  |  |  |  |  |  |  |  |  |  |  |  |  |  |  |  |  |  |  |  |  |  |  |  |  |  |  |  |  |  |  |  |  |  |  |  |  |  |  |  |  |  |  |  |  |  |  |  |  |  |  |  |  |  |  |  |  |  |  |  |  |  |  |  |  |  |  |  |  |  |  |  |  |  |  |  |  |  |  |  |  |  |  |  |  |  |  |  |  |  |  |  |  |  |  |  |  |  |  |  |  |  |  |  |  |  |  |  |  |  |  |  |  |  |  |  |  |  |  |  |  |  |  |  |  |  |  |  |  |  |  |  |  |  |  |  |  |  |  |  |  |  |  |  |  |  |  |  |  |  |  |  |  |  |  |  |  |  |  |  |  |  |  |  |  |  |  |  |  |  |  |  |  |  |  |  |  |  |  |
|  |  |  |  |  |  |  |  |  |  |  |  |  |  |  |  |  |  |  |  |  |  |  |  |  |  |  |  |  |  |  |  |  |  |  |  |  |  |  |  |  |  |  |  |  |  |  |  |  |  |  |  |  |  |  |  |  |  |  |  |  |  |  |  |  |  |  |  |  |  |  |  |  |  |  |  |  |  |  |  |  |  |  |  |  |  |  |  |  |  |  |  |  |  |  |  |  |  |  |  |  |  |  |  |  |  |  |  |  |  |  |  |  |  |  |  |  |  |  |  |  |  |  |  |  |  |  |  |  |  |  |  |  |  |  |  |  |  |  |  |  |  |  |  |  |  |  |  |  |  |  |  |  |  |  |  |  |  |  |  |  |  |  |  |  |  |  |  |  |  |  |  |  |  |  |  |  |  |  |  |  |  |  |  |  |  |  |  |  |  |  |  |  |  |  |  |  |  |  |  |  |  |  |  |  |  |  |  |  |  |  |  |  |  |  |  |  |  |  |  |  |  |  |  |  |  |  |  |  |  |  |  |  |  |  |  |  |  |  |  |  |  |  |  |  |  |  |  |  |  |  |  |  |  |  |  |  |  |  |  |  |  |  |  |  |  |  |  |  |  |  |  |  |  |  |  |  |  |  |  |  |  |  |  |  |  |  |  |  |  |  |  |  |  |  |  |  |  |  |  |  |  |  |  |  |  |  |  |  |  |  |  |  |  |  |  |  |  |  |  |  |  |  |  |  |  |  |  |  |  |  |  |  |  |  |  |  |  |  |  |  |  |  |  |  |  |  |  |  |  |  |  |  |  |  |  |  |  |  |  |  |  |  |  |  |  |  |  |  |  |  |  |  |  |  |  |  |  |  |  |  |  |  |  |  |  |  |  |  |  |  |  |  |  |  |  |  |  |  |  |  |  |  |  |  |  |  |  |  |  |  |  |  |  |  |  |  |  |  |  |  |  |  |  |  |  |  |  |  |  |  |  |  |  |  |  |  |  |  |  |  |  |  |  |  |  |  |  |  |  |  |  |  |  |  |  |  |  |  |  |  |  |  |  |  |  |  |  |  |  |  |  |  |  |  |  |  |  |  |  |  |  |  |  |  |  |  |  |  |  |  |  |  |  |  |  |  |  |  |  |  |  |  |  |  |  |  |  |  |  |  |  |  |  |  |  |  |  |  |  |  |  |  |  |  |  |  |  |  |  |  |  |  |  |  |  |  |  |  |  |  |  |  |  |  |  |  |  |  |  |  |  |  |  |  |  |  |  |  |  |  |  |  |  |  |  |  |  |  |  |  |  |  |  |  |  |  |  |  |  |  |  |  |  |  |  |  |  |  |  |  |  |  |  |  |  |  |  |  |  |  |  |  |  |  |  |  |  |  |  |  |  |  |  |  |  |  |  |  |  |  |  |  |  |  |  |  |  |  |  |  |  |  |  |  |  |  |  |  |  |  |  |  |  |  |  |  |  |  |  |  |  |  |  |  |  |  |  |  |  |  |  |  |  |  |  |  |  |  |  |  |  |  |  |  |  |  |  |  |  |  |  |  |  |  |  |  |  |  |  |  |  |  |  |  |  |  |  |  |  |  |  |  |  |  |  |  |  |  |  |  |  |  |  |  |  |  |  |  |  |  |  |  |  |  |  |  |  |  |  |  |  |  |  |  |  |  |  |  |  |  |  |  |  |  |  |  |  |  |  |  |  |  |  |  |  |  |  |  |  |  |  |  |  |  |  |  |  |  |  |  |  |  |  |  |  |  |  |  |  |  |  |  |  |  |  |  |  |  |  |  |  |  |  |  |  |  |  |  |  |  |  |  |  |  |  |  |  |  |  |  |  |  |  |  |  |  |  |  |  |  |  |  |  |  |  |  |  |  |  |  |  |  |  |  |  |  |  |  |  |  |  |  |  |  |  |  |  |  |  |  |  |  |  |  |  |  |  |  |  |  |  |  |  |  |  |  |  |  |  |  |  |  |  |  |  |  |  |  |  |  |  |  |  |  |  |  |  |  |  |  |  |  |  |  |  |  |  |  |  |  |  |  |  |  |  |  |  |  |  |  |  |  |  |  |  |  |  |  |  |  |  |  |  |  |  |  |  |  |  |  |  |  |  |  |  |  |  |  |  |  |  |  |  |  |  |  |  |  |  |  |  |  |  |  |  |  |  |  |  |  |  |  |  |  |  |  |  |  |  |  |  |  |  |  |  |  |  |  |  |  |  |  |  |  |  |  |  |  |  |  |  |  |  |  |  |  |  |  |  |  |  |  |  |  |  |  |  |  |  |  |  |  |  |  |  |  |  |  |  |  |  |  |  |  |  |  |  |  |  |  |  |  |  |  |  |  |  |  |  |  |  |  |  |  |  |  |  |  |  |  |  |  |  |  |  |  |  |  |  |  |  |  |  |  |  |  |  |  |  |  |  |  |  |  |  |  |  |  |  |  |  |  |  |  |  |  |  |  |  |  |  |  |  |  |  |  |  |  |  |  |  |  |  |  |  |  |  |  |  |  |  |  |  |  |  |  |  |  |  |  |  |  |  |  |  |  |  |  |  |  |  |  |  |  |  |  |  |  |  |  |  |  |  |  |  |  |  |  |  |  |  |  |  |  |  |  |  |  |  |  |  |  |  |  |  |  |  |  |  |  |  |  |  |  |  |  |  |  |  |  |  |  |  |  |  |  |  |  |  |  |  |  |  |  |  |  |  |  |  |  |  |  |  |  |  |  |  |  |  |  |  |  |  |  |  |  |  |  |  |  |  |  |  |  |  |  |  |  |  |  |  |  |  |  |  |  |  |  |  |  |  |  |  |  |  |  |  |  |  |  |  |  |  |  |  |  |  |  |  |  |  |  |  |  |  |  |  |  |  |  |  |  |  |  |  |  |  |  |  |  |  |  |  |  |  |  |  |  |  |  |  |  |  |  |  |  |  |  |  |  |  |  |  |  |  |  |  |  |  |  |  |  |  |  |  |  |  |  |  |  |  |  |  |  |  |  |  |  |  |  |  |  |  |  |  |  |  |  |  |  |  |  |  |  |  |  |  |  |  |  |  |  |  |  |  |  |  |  |  |  |  |  |  |  |  |  |  |  |  |  |  |  |  |  |  |  |  |  |  |  |  |  |  |  |  |  |  |  |  |  |  |  |  |  |  |  |  |  |  |  |  |  |  |  |  |  |  |  |  |  |  |  |  |  |  |  |  |  |  |  |  |  |  |  |  |  |  |  |  |  |  |  |  |  |
|  |  |  |  |  |  |  |  |  |  |  |  |  |  |  |  |  |  |  |  |  |  |  |  |  |  |  |  |  |  |  |  |  |  |  |  |  |  |  |  |  |  |  |  |  |  |  |  |  |  |  |  |  |  |  |  |  |  |  |  |  |  |  |  |  |  |  |  |  |  |  |  |  |  |  |  |  |  |  |  |  |  |  |  |  |  |  |  |  |  |  |  |  |  |  |  |  |  |  |  |  |  |  |  |  |  |  |  |  |  |  |  |  |  |  |  |  |  |  |  |  |  |  |  |  |  |  |  |  |  |  |  |  |  |  |  |  |  |  |  |  |  |  |  |  |  |  |  |  |  |  |  |  |  |  |  |  |  |  |  |  |  |  |  |  |  |  |  |  |  |  |  |  |  |  |  |  |  |  |  |  |  |  |  |  |  |  |  |  |  |  |  |  |  |  |  |  |  |  |  |  |  |  |  |  |  |  |  |  |  |  |  |  |  |  |  |  |  |  |  |  |  |  |  |  |  |  |  |  |  |  |  |  |  |  |  |  |  |  |  |  |  |  |  |  |  |  |  |  |  |  |  |  |  |  |  |  |  |  |  |  |  |  |  |  |  |  |  |  |  |  |  |  |  |  |  |  |  |  |  |  |  |  |  |  |  |  |  |  |  |  |  |  |  |  |  |  |  |  |  |  |  |  |  |  |  |  |  |  |  |  |  |  |  |  |  |  |  |  |  |  |  |  |  |  |  |  |  |  |  |  |  |  |  |  |  |  |  |  |  |  |  |  |  |  |  |  |  |  |  |  |  |  |  |  |  |  |  |  |  |  |  |  |  |  |  |  |  |  |  |  |  |  |  |  |  |  |  |  |  |  |  |  |  |  |  |  |  |  |  |  |  |  |  |  |  |  |  |  |  |  |  |  |  |  |  |  |  |  |  |  |  |  |  |  |  |  |  |  |  |  |  |  |  |  |  |  |  |  |  |  |  |  |  |  |  |  |  |  |  |  |  |  |  |  |  |  |  |  |  |  |  |  |  |  |  |  |  |  |  |  |  |  |  |  |  |  |  |  |  |  |  |  |  |  |  |  |  |  |  |  |  |  |  |  |  |  |  |  |  |  |  |  |  |  |  |  |  |  |  |  |  |  |  |  |  |  |  |  |  |  |  |  |  |  |  |  |  |  |  |  |  |  |  |  |  |  |  |  |  |  |  |  |  |  |  |  |  |  |  |  |  |  |  |  |  |  |  |  |  |  |  |  |  |  |  |  |  |  |  |  |  |  |  |  |  |  |  |  |  |  |  |  |  |  |  |  |  |  |  |  |  |  |  |  |  |  |  |  |  |  |  |  |  |  |  |  |  |  |  |  |  |  |  |  |  |  |  |  |  |  |  |  |  |  |  |  |  |  |  |  |  |  |  |  |  |  |  |  |  |  |  |  |  |  |  |  |  |  |  |  |  |  |  |  |  |  |  |  |  |  |  |  |  |  |  |  |  |  |  |  |  |  |  |  |  |  |  |  |  |  |  |  |  |  |  |  |  |  |  |  |  |  |  |  |  |  |  |  |  |  |  |  |  |  |  |  |  |  |  |  |  |  |  |  |  |  |  |  |  |  |  |  |  |  |  |  |  |  |  |  |  |  |  |  |  |  |  |  |  |  |  |  |  |  |  |  |  |  |  |  |  |  |  |  |  |  |  |  |  |  |  |  |  |  |  |  |  |  |  |  |  |  |  |  |  |  |  |  |  |  |  |  |  |  |  |  |  |  |  |  |  |  |  |  |  |  |  |  |  |  |  |  |  |  |  |  |  |  |  |  |  |  |  |  |  |  |  |  |  |  |  |  |  |  |  |  |  |  |  |  |  |  |  |  |  |  |  |  |  |  |  |  |  |  |  |  |  |  |  |  |  |  |  |  |  |  |  |  |  |  |  |  |  |  |  |  |  |  |  |  |  |  |  |  |  |  |  |  |  |  |  |  |  |  |  |  |  |  |  |  |  |  |  |  |  |  |  |  |  |  |  |  |  |  |  |  |  |  |  |  |  |  |  |  |  |  |  |  |  |  |  |  |  |  |  |  |  |  |  |  |  |  |  |  |  |  |  |  |  |  |  |  |  |  |  |  |  |  |  |  |  |  |  |  |  |  |  |  |  |  |  |  |  |  |  |  |  |  |  |  |  |  |  |  |  |  |  |  |  |  |  |  |  |  |  |  |  |  |  |  |  |  |  |  |  |  |  |  |  |  |  |  |  |  |  |  |  |  |  |  |  |  |  |  |  |  |  |  |  |  |  |  |  |  |  |  |  |  |  |  |  |  |  |  |  |  |  |  |  |  |  |  |  |  |  |  |  |  |  |  |  |  |  |  |  |  |  |  |  |  |  |  |  |  |  |  |  |  |  |  |  |  |  |  |  |  |  |  |  |  |  |  |  |  |  |  |  |  |  |  |  |  |  |  |  |  |  |  |  |  |  |  |  |  |  |  |  |  |  |  |  |  |  |  |  |  |  |  |  |  |  |  |  |  |  |  |  |  |  |  |  |  |  |  |  |  |  |  |  |  |  |  |  |  |  |  |  |  |  |  |  |  |  |  |  |  |  |  |  |  |  |  |  |  |  |  |  |  |  |  |  |  |  |  |  |  |  |  |  |  |  |  |  |  |  |  |  |  |  |  |  |  |  |  |  |  |  |  |  |  |  |  |  |  |  |  |  |  |  |  |  |  |  |  |  |  |  |  |  |  |  |  |  |  |  |  |  |  |  |  |  |  |  |  |  |  |  |  |  |  |  |  |  |  |  |  |  |  |  |  |  |  |  |  |  |  |  |  |  |  |  |  |  |  |  |  |  |  |  |  |  |  |  |  |  |  |  |  |  |  |  |  |  |  |  |  |  |  |  |  |  |  |  |  |  |  |  |  |  |  |  |  |  |  |  |  |  |  |  |  |  |  |  |  |  |  |  |  |  |  |  |  |  |  |  |  |  |  |  |  |  |  |  |  |  |  |  |  |  |  |  |  |  |  |  |  |  |  |  |  |  |  |  |  |  |  |  |  |  |  |  |  |  |  |  |  |  |  |  |  |  |  |  |  |  |  |  |  |  |  |  |  |  |  |  |  |  |  |  |  |  |  |  |  |  |  |  |  |  |  |  |  |  |  |  |  |  |  |  |  |  |  |  |  |  |  |  |  |  |  |  |  |  |  |  |  |  |  |  |  |  |  |  |  |  |  |  |  |  |  |  |  |  |  |
|  |  |  |  |  |  |  |  |  |  |  |  |  |  |  |  |  |  |  |  |  |  |  |  |  |  |  |  |  |  |  |  |  |  |  |  |  |  |  |  |  |  |  |  |  |  |  |  |  |  |  |  |  |  |  |  |  |  |  |  |  |  |  |  |  |  |  |  |  |  |  |  |  |  |  |  |  |  |  |  |  |  |  |  |  |  |  |  |  |  |  |  |  |  |  |  |  |  |  |  |  |  |  |  |  |  |  |  |  |  |  |  |  |  |  |  |  |  |  |  |  |  |  |  |  |  |  |  |  |  |  |  |  |  |  |  |  |  |  |  |  |  |  |  |  |  |  |  |  |  |  |  |  |  |  |  |  |  |  |  |  |  |  |  |  |  |  |  |  |  |  |  |  |  |  |  |  |  |  |  |  |  |  |  |  |  |  |  |  |  |  |  |  |  |  |  |  |  |  |  |  |  |  |  |  |  |  |  |  |  |  |  |  |  |  |  |  |  |  |  |  |  |  |  |  |  |  |  |  |  |  |  |  |  |  |  |  |  |  |  |  |  |  |  |  |  |  |  |  |  |  |  |  |  |  |  |  |  |  |  |  |  |  |  |  |  |  |  |  |  |  |  |  |  |  |  |  |  |  |  |  |  |  |  |  |  |  |  |  |  |  |  |  |  |  |  |  |  |  |  |  |  |  |  |  |  |  |  |  |  |  |  |  |  |  |  |  |  |  |  |  |  |  |  |  |  |  |  |  |  |  |  |  |  |  |  |  |  |  |  |  |  |  |  |  |  |  |  |  |  |  |  |  |  |  |  |  |  |  |  |  |  |  |  |  |  |  |  |  |  |  |  |  |  |  |  |  |  |  |  |  |  |  |  |  |  |  |  |  |  |  |  |  |  |  |  |  |  |  |  |  |  |  |  |  |  |  |  |  |  |  |  |  |  |  |  |  |  |  |  |  |  |  |  |  |  |  |  |  |  |  |  |  |  |  |  |  |  |  |  |  |  |  |  |  |  |  |  |  |  |  |  |  |  |  |  |  |  |  |  |  |  |  |  |  |  |  |  |  |  |  |  |  |  |  |  |  |  |  |  |  |  |  |  |  |  |  |  |  |  |  |  |  |  |  |  |  |  |  |  |  |  |  |  |  |  |  |  |  |  |  |  |  |  |  |  |  |  |  |  |  |  |  |  |  |  |  |  |  |  |  |  |  |  |  |  |  |  |  |  |  |  |  |  |  |  |  |  |  |  |  |  |  |  |  |  |  |  |  |  |  |  |  |  |  |  |  |  |  |  |  |  |  |  |  |  |  |  |  |  |  |  |  |  |  |  |  |  |  |  |  |  |  |  |  |  |  |  |  |  |  |  |  |  |  |  |  |  |  |  |  |  |  |  |  |  |  |  |  |  |  |  |  |  |  |  |  |  |  |  |  |  |  |  |  |  |  |  |  |  |  |  |  |  |  |  |  |  |  |  |  |  |  |  |  |  |  |  |  |  |  |  |  |  |  |  |  |  |  |  |  |  |  |  |  |  |  |  |  |  |  |  |  |  |  |  |  |  |  |  |  |  |  |  |  |  |  |  |  |  |  |  |  |  |  |  |  |  |  |  |  |  |  |  |  |  |  |  |  |  |  |  |  |  |  |  |  |  |  |  |  |  |  |  |  |  |  |  |  |  |  |  |  |  |  |  |  |  |  |  |  |  |  |  |  |  |  |  |  |  |  |  |  |  |  |  |  |  |  |  |  |  |  |  |  |  |  |  |  |  |  |  |  |  |  |  |  |  |  |  |  |  |  |  |  |  |  |  |  |  |  |  |  |  |  |  |  |  |  |  |  |  |  |  |  |  |  |  |  |  |  |  |  |  |  |  |  |  |  |  |  |  |  |  |  |  |  |  |  |  |  |  |  |  |  |  |  |  |  |  |  |  |  |  |  |  |  |  |  |  |  |  |  |  |  |  |  |  |  |  |  |  |  |  |  |  |  |  |  |  |  |  |  |  |  |  |  |  |  |  |  |  |  |  |  |  |  |  |  |  |  |  |  |  |  |  |  |  |  |  |  |  |  |  |  |  |  |  |  |  |  |  |  |  |  |  |  |  |  |  |  |  |  |  |  |  |  |  |  |  |  |  |  |  |  |  |  |  |  |  |  |  |  |  |  |  |  |  |  |  |  |  |  |  |  |  |  |  |  |  |  |  |  |  |  |  |  |  |  |  |  |  |  |  |  |  |  |  |  |  |  |  |  |  |  |  |  |  |  |  |  |  |  |  |  |  |  |  |  |  |  |  |  |  |  |  |  |  |  |  |  |  |  |  |  |  |  |  |  |  |  |  |  |  |  |  |  |  |  |  |  |  |  |  |  |  |  |  |  |  |  |  |  |  |  |  |  |  |  |  |  |  |  |  |  |  |  |  |  |  |  |  |  |  |  |  |  |  |  |  |  |  |  |  |  |  |  |  |  |  |  |  |  |  |  |  |  |  |  |  |  |  |  |  |  |  |  |  |  |  |  |  |  |  |  |  |  |  |  |  |  |  |  |  |  |  |  |  |  |  |  |  |  |  |  |  |  |  |  |  |  |  |  |  |  |  |  |  |  |  |  |  |  |  |  |  |  |  |  |  |  |  |  |  |  |  |  |  |  |  |  |  |  |  |  |  |  |  |  |  |  |  |  |  |  |  |  |  |  |  |  |  |  |  |  |  |  |  |  |  |  |  |  |  |  |  |  |  |  |  |  |  |  |  |  |  |  |  |  |  |  |  |  |  |  |  |  |  |  |  |  |  |  |  |  |  |  |  |  |  |  |  |  |  |  |  |  |  |  |  |  |  |  |  |  |  |  |  |  |  |  |  |  |  |  |  |  |  |  |  |  |  |  |  |  |  |  |  |  |  |  |  |  |  |  |  |  |  |  |  |  |  |  |  |  |  |  |  |  |  |  |  |  |  |  |  |  |  |  |  |  |  |  |  |  |  |  |  |  |  |  |  |  |  |  |  |  |  |  |  |  |  |  |  |  |  |  |  |  |  |  |  |  |  |  |  |  |  |  |  |  |  |  |  |  |  |  |  |  |  |  |  |  |  |  |  |  |  |  |  |  |  |  |  |  |  |  |  |  |  |  |  |  |  |  |  |  |  |  |  |  |  |  |  |  |  |  |  |  |  |  |  |  |  |  |  |  |  |  |  |  |  |  |  |  |  |  |  |  |  |  |  |  |  |  |  |  |  |  |  |  |  |  |  |  |
|  |  |  |  |  |  |  |  |  |  |  |  |  |  |  |  |  |  |  |  |  |  |  |  |  |  |  |  |  |  |  |  |  |  |  |  |  |  |  |  |  |  |  |  |  |  |  |  |  |  |  |  |  |  |  |  |  |  |  |  |  |  |  |  |  |  |  |  |  |  |  |  |  |  |  |  |  |  |  |  |  |  |  |  |  |  |  |  |  |  |  |  |  |  |  |  |  |  |  |  |  |  |  |  |  |  |  |  |  |  |  |  |  |  |  |  |  |  |  |  |  |  |  |  |  |  |  |  |  |  |  |  |  |  |  |  |  |  |  |  |  |  |  |  |  |  |  |  |  |  |  |  |  |  |  |  |  |  |  |  |  |  |  |  |  |  |  |  |  |  |  |  |  |  |  |  |  |  |  |  |  |  |  |  |  |  |  |  |  |  |  |  |  |  |  |  |  |  |  |  |  |  |  |  |  |  |  |  |  |  |  |  |  |  |  |  |  |  |  |  |  |  |  |  |  |  |  |  |  |  |  |  |  |  |  |  |  |  |  |  |  |  |  |  |  |  |  |  |  |  |  |  |  |  |  |  |  |  |  |  |  |  |  |  |  |  |  |  |  |  |  |  |  |  |  |  |  |  |  |  |  |  |  |  |  |  |  |  |  |  |  |  |  |  |  |  |  |  |  |  |  |  |  |  |  |  |  |  |  |  |  |  |  |  |  |  |  |  |  |  |  |  |  |  |  |  |  |  |  |  |  |  |  |  |  |  |  |  |  |  |  |  |  |  |  |  |  |  |  |  |  |  |  |  |  |  |  |  |  |  |  |  |  |  |  |  |  |  |  |  |  |  |  |  |  |  |  |  |  |  |  |  |  |  |  |  |  |  |  |  |  |  |  |  |  |  |  |  |  |  |  |  |  |  |  |  |  |  |  |  |  |  |  |  |  |  |  |  |  |  |  |  |  |  |  |  |  |  |  |  |  |  |  |  |  |  |  |  |  |  |  |  |  |  |  |  |  |  |  |  |  |  |  |  |  |  |  |  |  |  |  |  |  |  |  |  |  |  |  |  |  |  |  |  |  |  |  |  |  |  |  |  |  |  |  |  |  |  |  |  |  |  |  |  |  |  |  |  |  |  |  |  |  |  |  |  |  |  |  |  |  |  |  |  |  |  |  |  |  |  |  |  |  |  |  |  |  |  |  |  |  |  |  |  |  |  |  |  |  |  |  |  |  |  |  |  |  |  |  |  |  |  |  |  |  |  |  |  |  |  |  |  |  |  |  |  |  |  |  |  |  |  |  |  |  |  |  |  |  |  |  |  |  |  |  |  |  |  |  |  |  |  |  |  |  |  |  |  |  |  |  |  |  |  |  |  |  |  |  |  |  |  |  |  |  |  |  |  |  |  |  |  |  |  |  |  |  |  |  |  |  |  |  |  |  |  |  |  |  |  |  |  |  |  |  |  |  |  |  |  |  |  |  |  |  |  |  |  |  |  |  |  |  |  |  |  |  |  |  |  |  |  |  |  |  |  |  |  |  |  |  |  |  |  |  |  |  |  |  |  |  |  |  |  |  |  |  |  |  |  |  |  |  |  |  |  |  |  |  |  |  |  |  |  |  |  |  |  |  |  |  |  |  |  |  |  |  |  |  |  |  |  |  |  |  |  |  |  |  |  |  |  |  |  |  |  |  |  |  |  |  |  |  |  |  |  |  |  |  |  |  |  |  |  |  |  |  |  |  |  |  |  |  |  |  |  |  |  |  |  |  |  |  |  |  |  |  |  |  |  |  |  |  |  |  |  |  |  |  |  |  |  |  |  |  |  |  |  |  |  |  |  |  |  |  |  |  |  |  |  |  |  |  |  |  |  |  |  |  |  |  |  |  |  |  |  |  |  |  |  |  |  |  |  |  |  |  |  |  |  |  |  |  |  |  |  |  |  |  |  |  |  |  |  |  |  |  |  |  |  |  |  |  |  |  |  |  |  |  |  |  |  |  |  |  |  |  |  |  |  |  |  |  |  |  |  |  |  |  |  |  |  |  |  |  |  |  |  |  |  |  |  |  |  |  |  |  |  |  |  |  |  |  |  |  |  |  |  |  |  |  |  |  |  |  |  |  |  |  |  |  |  |  |  |  |  |  |  |  |  |  |  |  |  |  |  |  |  |  |  |  |  |  |  |  |  |  |  |  |  |  |  |  |  |  |  |  |  |  |  |  |  |  |  |  |  |  |  |  |  |  |  |  |  |  |  |  |  |  |  |  |  |  |  |  |  |  |  |  |  |  |  |  |  |  |  |  |  |  |  |  |  |  |  |  |  |  |  |  |  |  |  |  |  |  |  |  |  |  |  |  |  |  |  |  |  |  |  |  |  |  |  |  |  |  |  |  |  |  |  |  |  |  |  |  |  |  |  |  |  |  |  |  |  |  |  |  |  |  |  |  |  |  |  |  |  |  |  |  |  |  |  |  |  |  |  |  |  |  |  |  |  |  |  |  |  |  |  |  |  |  |  |  |  |  |  |  |  |  |  |  |  |  |  |  |  |  |  |  |  |  |  |  |  |  |  |  |  |  |  |  |  |  |  |  |  |  |  |  |  |  |  |  |  |  |  |  |  |  |  |  |  |  |  |  |  |  |  |  |  |  |  |  |  |  |  |  |  |  |  |  |  |  |  |  |  |  |  |  |  |  |  |  |  |  |  |  |  |  |  |  |  |  |  |  |  |  |  |  |  |  |  |  |  |  |  |  |  |  |  |  |  |  |  |  |  |  |  |  |  |  |  |  |  |  |  |  |  |  |  |  |  |  |  |  |  |  |  |  |  |  |  |  |  |  |  |  |  |  |  |  |  |  |  |  |  |  |  |  |  |  |  |  |  |  |  |  |  |  |  |  |  |  |  |  |  |  |  |  |  |  |  |  |  |  |  |  |  |  |  |  |  |  |  |  |  |  |  |  |  |  |  |  |  |  |  |  |  |  |  |  |  |  |  |  |  |  |  |  |  |  |  |  |  |  |  |  |  |  |  |  |  |  |  |  |  |  |  |  |  |  |  |  |  |  |  |  |  |  |  |  |  |  |  |  |  |  |  |  |  |  |  |  |  |  |  |  |  |  |  |  |  |  |  |  |  |  |  |  |  |  |  |  |  |  |  |  |  |  |  |  |  |  |  |  |  |  |  |  |  |  |  |  |  |  |  |  |  |  |  |  |  |  |  |  |  |  |  |  |  |  |  |  |  |  |
|  |  |  |  |  |  |  |  |  |  |  |  |  |  |  |  |  |  |  |  |  |  |  |  |  |  |  |  |  |  |  |  |  |  |  |  |  |  |  |  |  |  |  |  |  |  |  |  |  |  |  |  |  |  |  |  |  |  |  |  |  |  |  |  |  |  |  |  |  |  |  |  |  |  |  |  |  |  |  |  |  |  |  |  |  |  |  |  |  |  |  |  |  |  |  |  |  |  |  |  |  |  |  |  |  |  |  |  |  |  |  |  |  |  |  |  |  |  |  |  |  |  |  |  |  |  |  |  |  |  |  |  |  |  |  |  |  |  |  |  |  |  |  |  |  |  |  |  |  |  |  |  |  |  |  |  |  |  |  |  |  |  |  |  |  |  |  |  |  |  |  |  |  |  |  |  |  |  |  |  |  |  |  |  |  |  |  |  |  |  |  |  |  |  |  |  |  |  |  |  |  |  |  |  |  |  |  |  |  |  |  |  |  |  |  |  |  |  |  |  |  |  |  |  |  |  |  |  |  |  |  |  |  |  |  |  |  |  |  |  |  |  |  |  |  |  |  |  |  |  |  |  |  |  |  |  |  |  |  |  |  |  |  |  |  |  |  |  |  |  |  |  |  |  |  |  |  |  |  |  |  |  |  |  |  |  |  |  |  |  |  |  |  |  |  |  |  |  |  |  |  |  |  |  |  |  |  |  |  |  |  |  |  |  |  |  |  |  |  |  |  |  |  |  |  |  |  |  |  |  |  |  |  |  |  |  |  |  |  |  |  |  |  |  |  |  |  |  |  |  |  |  |  |  |  |  |  |  |  |  |  |  |  |  |  |  |  |  |  |  |  |  |  |  |  |  |  |  |  |  |  |  |  |  |  |  |  |  |  |  |  |  |  |  |  |  |  |  |  |  |  |  |  |  |  |  |  |  |  |  |  |  |  |  |  |  |  |  |  |  |  |  |  |  |  |  |  |  |  |  |  |  |  |  |  |  |  |  |  |  |  |  |  |  |  |  |  |  |  |  |  |  |  |  |  |  |  |  |  |  |  |  |  |  |  |  |  |  |  |  |  |  |  |  |  |  |  |  |  |  |  |  |  |  |  |  |  |  |  |  |  |  |  |  |  |  |  |  |  |  |  |  |  |  |  |  |  |  |  |  |  |  |  |  |  |  |  |  |  |  |  |  |  |  |  |  |  |  |  |  |  |  |  |  |  |  |  |  |  |  |  |  |  |  |  |  |  |  |  |  |  |  |  |  |  |  |  |  |  |  |  |  |  |  |  |  |  |  |  |  |  |  |  |  |  |  |  |  |  |  |  |  |  |  |  |  |  |  |  |  |  |  |  |  |  |  |  |  |  |  |  |  |  |  |  |  |  |  |  |  |  |  |  |  |  |  |  |  |  |  |  |  |  |  |  |  |  |  |  |  |  |  |  |  |  |  |  |  |  |  |  |  |  |  |  |  |  |  |  |  |  |  |  |  |  |  |  |  |  |  |  |  |  |  |  |  |  |  |  |  |  |  |  |  |  |  |  |  |  |  |  |  |  |  |  |  |  |  |  |  |  |  |  |  |  |  |  |  |  |  |  |  |  |  |  |  |  |  |  |  |  |  |  |  |  |  |  |  |  |  |  |  |  |  |  |  |  |  |  |  |  |  |  |  |  |  |  |  |  |  |  |  |  |  |  |  |  |  |  |  |  |  |  |  |  |  |  |  |  |  |  |  |  |  |  |  |  |  |  |  |  |  |  |  |  |  |  |  |  |  |  |  |  |  |  |  |  |  |  |  |  |  |  |  |  |  |  |  |  |  |  |  |  |  |  |  |  |  |  |  |  |  |  |  |  |  |  |  |  |  |  |  |  |  |  |  |  |  |  |  |  |  |  |  |  |  |  |  |  |  |  |  |  |  |  |  |  |  |  |  |  |  |  |  |  |  |  |  |  |  |  |  |  |  |  |  |  |  |  |  |  |  |  |  |  |  |  |  |  |  |  |  |  |  |  |  |  |  |  |  |  |  |  |  |  |  |  |  |  |  |  |  |  |  |  |  |  |  |  |  |  |  |  |  |  |  |  |  |  |  |  |  |  |  |  |  |  |  |  |  |  |  |  |  |  |  |  |  |  |  |  |  |  |  |  |  |  |  |  |  |  |  |  |  |  |  |  |  |  |  |  |  |  |  |  |  |  |  |  |  |  |  |  |  |  |  |  |  |  |  |  |  |  |  |  |  |  |  |  |  |  |  |  |  |  |  |  |  |  |  |  |  |  |  |  |  |  |  |  |  |  |  |  |  |  |  |  |  |  |  |  |  |  |  |  |  |  |  |  |  |  |  |  |  |  |  |  |  |  |  |  |  |  |  |  |  |  |  |  |  |  |  |  |  |  |  |  |  |  |  |  |  |  |  |  |  |  |  |  |  |  |  |  |  |  |  |  |  |  |  |  |  |  |  |  |  |  |  |  |  |  |  |  |  |  |  |  |  |  |  |  |  |  |  |  |  |  |  |  |  |  |  |  |  |  |  |  |  |  |  |  |  |  |  |  |  |  |  |  |  |  |  |  |  |  |  |  |  |  |  |  |  |  |  |  |  |  |  |  |  |  |  |  |  |  |  |  |  |  |  |  |  |  |  |  |  |  |  |  |  |  |  |  |  |  |  |  |  |  |  |  |  |  |  |  |  |  |  |  |  |  |  |  |  |  |  |  |  |  |  |  |  |  |  |  |  |  |  |  |  |  |  |  |  |  |  |  |  |  |  |  |  |  |  |  |  |  |  |  |  |  |  |  |  |  |  |  |  |  |  |  |  |  |  |  |  |  |  |  |  |  |  |  |  |  |  |  |  |  |  |  |  |  |  |  |  |  |  |  |  |  |  |  |  |  |  |  |  |  |  |  |  |  |  |  |  |  |  |  |  |  |  |  |  |  |  |  |  |  |  |  |  |  |  |  |  |  |  |  |  |  |  |  |  |  |  |  |  |  |  |  |  |  |  |  |  |  |  |  |  |  |  |  |  |  |  |  |  |  |  |  |  |  |  |  |  |  |  |  |  |  |  |  |  |  |  |  |  |  |  |  |  |  |  |  |  |  |  |  |  |  |  |  |  |  |  |  |  |  |  |  |  |  |  |  |  |  |  |  |  |  |  |  |  |  |  |  |  |  |  |  |  |  |  |  |  |  |  |  |  |  |  |  |  |  |  |  |  |  |  |  |  |  |  |  |  |  |  |  |  |  |  |  |  |  |
|  |  |  |  |  |  |  |  |  |  |  |  |  |  |  |  |  |  |  |  |  |  |  |  |  |  |  |  |  |  |  |  |  |  |  |  |  |  |  |  |  |  |  |  |  |  |  |  |  |  |  |  |  |  |  |  |  |  |  |  |  |  |  |  |  |  |  |  |  |  |  |  |  |  |  |  |  |  |  |  |  |  |  |  |  |  |  |  |  |  |  |  |  |  |  |  |  |  |  |  |  |  |  |  |  |  |  |  |  |  |  |  |  |  |  |  |  |  |  |  |  |  |  |  |  |  |  |  |  |  |  |  |  |  |  |  |  |  |  |  |  |  |  |  |  |  |  |  |  |  |  |  |  |  |  |  |  |  |  |  |  |  |  |  |  |  |  |  |  |  |  |  |  |  |  |  |  |  |  |  |  |  |  |  |  |  |  |  |  |  |  |  |  |  |  |  |  |  |  |  |  |  |  |  |  |  |  |  |  |  |  |  |  |  |  |  |  |  |  |  |  |  |  |  |  |  |  |  |  |  |  |  |  |  |  |  |  |  |  |  |  |  |  |  |  |  |  |  |  |  |  |  |  |  |  |  |  |  |  |  |  |  |  |  |  |  |  |  |  |  |  |  |  |  |  |  |  |  |  |  |  |  |  |  |  |  |  |  |  |  |  |  |  |  |  |  |  |  |  |  |  |  |  |  |  |  |  |  |  |  |  |  |  |  |  |  |  |  |  |  |  |  |  |  |  |  |  |  |  |  |  |  |  |  |  |  |  |  |  |  |  |  |  |  |  |  |  |  |  |  |  |  |  |  |  |  |  |  |  |  |  |  |  |  |  |  |  |  |  |  |  |  |  |  |  |  |  |  |  |  |  |  |  |  |  |  |  |  |  |  |  |  |  |  |  |  |  |  |  |  |  |  |  |  |  |  |  |  |  |  |  |  |  |  |  |  |  |  |  |  |  |  |  |  |  |  |  |  |  |  |  |  |  |  |  |  |  |  |  |  |  |  |  |  |  |  |  |  |  |  |  |  |  |  |  |  |  |  |  |  |  |  |  |  |  |  |  |  |  |  |  |  |  |  |  |  |  |  |  |  |  |  |  |  |  |  |  |  |  |  |  |  |  |  |  |  |  |  |  |  |  |  |  |  |  |  |  |  |  |  |  |  |  |  |  |  |  |  |  |  |  |  |  |  |  |  |  |  |  |  |  |  |  |  |  |  |  |  |  |  |  |  |  |  |  |  |  |  |  |  |  |  |  |  |  |  |  |  |  |  |  |  |  |  |  |  |  |  |  |  |  |  |  |  |  |  |  |  |  |  |  |  |  |  |  |  |  |  |  |  |  |  |  |  |  |  |  |  |  |  |  |  |  |  |  |  |  |  |  |  |  |  |  |  |  |  |  |  |  |  |  |  |  |  |  |  |  |  |  |  |  |  |  |  |  |  |  |  |  |  |  |  |  |  |  |  |  |  |  |  |  |  |  |  |  |  |  |  |  |  |  |  |  |  |  |  |  |  |  |  |  |  |  |  |  |  |  |  |  |  |  |  |  |  |  |  |  |  |  |  |  |  |  |  |  |  |  |  |  |  |  |  |  |  |  |  |  |  |  |  |  |  |  |  |  |  |  |  |  |  |  |  |  |  |  |  |  |  |  |  |  |  |  |  |  |  |  |  |  |  |  |  |  |  |  |  |  |  |  |  |  |  |  |  |  |  |  |  |  |  |  |  |  |  |  |  |  |  |  |  |  |  |  |  |  |  |  |  |  |  |  |  |  |  |  |  |  |  |  |  |  |  |  |  |  |  |  |  |  |  |  |  |  |  |  |  |  |  |  |  |  |  |  |  |  |  |  |  |  |  |  |  |  |  |  |  |  |  |  |  |  |  |  |  |  |  |  |  |  |  |  |  |  |  |  |  |  |  |  |  |  |  |  |  |  |  |  |  |  |  |  |  |  |  |  |  |  |  |  |  |  |  |  |  |  |  |  |  |  |  |  |  |  |  |  |  |  |  |  |  |  |  |  |  |  |  |  |  |  |  |  |  |  |  |  |  |  |  |  |  |  |  |  |  |  |  |  |  |  |  |  |  |  |  |  |  |  |  |  |  |  |  |  |  |  |  |  |  |  |  |  |  |  |  |  |  |  |  |  |  |  |  |  |  |  |  |  |  |  |  |  |  |  |  |  |  |  |  |  |  |  |  |  |  |  |  |  |  |  |  |  |  |  |  |  |  |  |  |  |  |  |  |  |  |  |  |  |  |  |  |  |  |  |  |  |  |  |  |  |  |  |  |  |  |  |  |  |  |  |  |  |  |  |  |  |  |  |  |  |  |  |  |  |  |  |  |  |  |  |  |  |  |  |  |  |  |  |  |  |  |  |  |  |  |  |  |  |  |  |  |  |  |  |  |  |  |  |  |  |  |  |  |  |  |  |  |  |  |  |  |  |  |  |  |  |  |  |  |  |  |  |  |  |  |  |  |  |  |  |  |  |  |  |  |  |  |  |  |  |  |  |  |  |  |  |  |  |  |  |  |  |  |  |  |  |  |  |  |  |  |  |  |  |  |  |  |  |  |  |  |  |  |  |  |  |  |  |  |  |  |  |  |  |  |  |  |  |  |  |  |  |  |  |  |  |  |  |  |  |  |  |  |  |  |  |  |  |  |  |  |  |  |  |  |  |  |  |  |  |  |  |  |  |  |  |  |  |  |  |  |  |  |  |  |  |  |  |  |  |  |  |  |  |  |  |  |  |  |  |  |  |  |  |  |  |  |  |  |  |  |  |  |  |  |  |  |  |  |  |  |  |  |  |  |  |  |  |  |  |  |  |  |  |  |  |  |  |  |  |  |  |  |  |  |  |  |  |  |  |  |  |  |  |  |  |  |  |  |  |  |  |  |  |  |  |  |  |  |  |  |  |  |  |  |  |  |  |  |  |  |  |  |  |  |  |  |  |  |  |  |  |  |  |  |  |  |  |  |  |  |  |  |  |  |  |  |  |  |  |  |  |  |  |  |  |  |  |  |  |  |  |  |  |  |  |  |  |  |  |  |  |  |  |  |  |  |  |  |  |  |  |  |  |  |  |  |  |  |  |  |  |  |  |  |  |  |  |  |  |  |  |  |  |  |  |  |  |  |  |  |  |  |  |  |  |  |  |  |  |  |  |  |  |  |  |  |  |  |  |  |  |  |  |  |  |  |  |  |  |  |  |  |  |  |  |  |  |  |  |  |  |  |  |  |  |
|  |  |  |  |  |  |  |  |  |  |  |  |  |  |  |  |  |  |  |  |  |  |  |  |  |  |  |  |  |  |  |  |  |  |  |  |  |  |  |  |  |  |  |  |  |  |  |  |  |  |  |  |  |  |  |  |  |  |  |  |  |  |  |  |  |  |  |  |  |  |  |  |  |  |  |  |  |  |  |  |  |  |  |  |  |  |  |  |  |  |  |  |  |  |  |  |  |  |  |  |  |  |  |  |  |  |  |  |  |  |  |  |  |  |  |  |  |  |  |  |  |  |  |  |  |  |  |  |  |  |  |  |  |  |  |  |  |  |  |  |  |  |  |  |  |  |  |  |  |  |  |  |  |  |  |  |  |  |  |  |  |  |  |  |  |  |  |  |  |  |  |  |  |  |  |  |  |  |  |  |  |  |  |  |  |  |  |  |  |  |  |  |  |  |  |  |  |  |  |  |  |  |  |  |  |  |  |  |  |  |  |  |  |  |  |  |  |  |  |  |  |  |  |  |  |  |  |  |  |  |  |  |  |  |  |  |  |  |  |  |  |  |  |  |  |  |  |  |  |  |  |  |  |  |  |  |  |  |  |  |  |  |  |  |  |  |  |  |  |  |  |  |  |  |  |  |  |  |  |  |  |  |  |  |  |  |  |  |  |  |  |  |  |  |  |  |  |  |  |  |  |  |  |  |  |  |  |  |  |  |  |  |  |  |  |  |  |  |  |  |  |  |  |  |  |  |  |  |  |  |  |  |  |  |  |  |  |  |  |  |  |  |  |  |  |  |  |  |  |  |  |  |  |  |  |  |  |  |  |  |  |  |  |  |  |  |  |  |  |  |  |  |  |  |  |  |  |  |  |  |  |  |  |  |  |  |  |  |  |  |  |  |  |  |  |  |  |  |  |  |  |  |  |  |  |  |  |  |  |  |  |  |  |  |  |  |  |  |  |  |  |  |  |  |  |  |  |  |  |  |  |  |  |  |  |  |  |  |  |  |  |  |  |  |  |  |  |  |  |  |  |  |  |  |  |  |  |  |  |  |  |  |  |  |  |  |  |  |  |  |  |  |  |  |  |  |  |  |  |  |  |  |  |  |  |  |  |  |  |  |  |  |  |  |  |  |  |  |  |  |  |  |  |  |  |  |  |  |  |  |  |  |  |  |  |  |  |  |  |  |  |  |  |  |  |  |  |  |  |  |  |  |  |  |  |  |  |  |  |  |  |  |  |  |  |  |  |  |  |  |  |  |  |  |  |  |  |  |  |  |  |  |  |  |  |  |  |  |  |  |  |  |  |  |  |  |  |  |  |  |  |  |  |  |  |  |  |  |  |  |  |  |  |  |  |  |  |  |  |  |  |  |  |  |  |  |  |  |  |  |  |  |  |  |  |  |  |  |  |  |  |  |  |  |  |  |  |  |  |  |  |  |  |  |  |  |  |  |  |  |  |  |  |  |  |  |  |  |  |  |  |  |  |  |  |  |  |  |  |  |  |  |  |  |  |  |  |  |  |  |  |  |  |  |  |  |  |  |  |  |  |  |  |  |  |  |  |  |  |  |  |  |  |  |  |  |  |  |  |  |  |  |  |  |  |  |  |  |  |  |  |  |  |  |  |  |  |  |  |  |  |  |  |  |  |  |  |  |  |  |  |  |  |  |  |  |  |  |  |  |  |  |  |  |  |  |  |  |  |  |  |  |  |  |  |  |  |  |  |  |  |  |  |  |  |  |  |  |  |  |  |  |  |  |  |  |  |  |  |  |  |  |  |  |  |  |  |  |  |  |  |  |  |  |  |  |  |  |  |  |  |  |  |  |  |  |  |  |  |  |  |  |  |  |  |  |  |  |  |  |  |  |  |  |  |  |  |  |  |  |  |  |  |  |  |  |  |  |  |  |  |  |  |  |  |  |  |  |  |  |  |  |  |  |  |  |  |  |  |  |  |  |  |  |  |  |  |  |  |  |  |  |  |  |  |  |  |  |  |  |  |  |  |  |  |  |  |  |  |  |  |  |  |  |  |  |  |  |  |  |  |  |  |  |  |  |  |  |  |  |  |  |  |  |  |  |  |  |  |  |  |  |  |  |  |  |  |  |  |  |  |  |  |  |  |  |  |  |  |  |  |  |  |  |  |  |  |  |  |  |  |  |  |  |  |  |  |  |  |  |  |  |  |  |  |  |  |  |  |  |  |  |  |  |  |  |  |  |  |  |  |  |  |  |  |  |  |  |  |  |  |  |  |  |  |  |  |  |  |  |  |  |  |  |  |  |  |  |  |  |  |  |  |  |  |  |  |  |  |  |  |  |  |  |  |  |  |  |  |  |  |  |  |  |  |  |  |  |  |  |  |  |  |  |  |  |  |  |  |  |  |  |  |  |  |  |  |  |  |  |  |  |  |  |  |  |  |  |  |  |  |  |  |  |  |  |  |  |  |  |  |  |  |  |  |  |  |  |  |  |  |  |  |  |  |  |  |  |  |  |  |  |  |  |  |  |  |  |  |  |  |  |  |  |  |  |  |  |  |  |  |  |  |  |  |  |  |  |  |  |  |  |  |  |  |  |  |  |  |  |  |  |  |  |  |  |  |  |  |  |  |  |  |  |  |  |  |  |  |  |  |  |  |  |  |  |  |  |  |  |  |  |  |  |  |  |  |  |  |  |  |  |  |  |  |  |  |  |  |  |  |  |  |  |  |  |  |  |  |  |  |  |  |  |  |  |  |  |  |  |  |  |  |  |  |  |  |  |  |  |  |  |  |  |  |  |  |  |  |  |  |  |  |  |  |  |  |  |  |  |  |  |  |  |  |  |  |  |  |  |  |  |  |  |  |  |  |  |  |  |  |  |  |  |  |  |  |  |  |  |  |  |  |  |  |  |  |  |  |  |  |  |  |  |  |  |  |  |  |  |  |  |  |  |  |  |  |  |  |  |  |  |  |  |  |  |  |  |  |  |  |  |  |  |  |  |  |  |  |  |  |  |  |  |  |  |  |  |  |  |  |  |  |  |  |  |  |  |  |  |  |  |  |  |  |  |  |  |  |  |  |  |  |  |  |  |  |  |  |  |  |  |  |  |  |  |  |  |  |  |  |  |  |  |  |  |  |  |  |  |  |  |  |  |  |  |  |  |  |  |  |  |  |  |  |  |  |  |  |  |  |  |  |  |  |  |  |  |  |  |  |  |  |  |  |  |  |  |  |  |  |  |  |  |  |  |  |  |  |  |  |  |  |  |  |
|  |  |  |  |  |  |  |  |  |  |  |  |  |  |  |  |  |  |  |  |  |  |  |  |  |  |  |  |  |  |  |  |  |  |  |  |  |  |  |  |  |  |  |  |  |  |  |  |  |  |  |  |  |  |  |  |  |  |  |  |  |  |  |  |  |  |  |  |  |  |  |  |  |  |  |  |  |  |  |  |  |  |  |  |  |  |  |  |  |  |  |  |  |  |  |  |  |  |  |  |  |  |  |  |  |  |  |  |  |  |  |  |  |  |  |  |  |  |  |  |  |  |  |  |  |  |  |  |  |  |  |  |  |  |  |  |  |  |  |  |  |  |  |  |  |  |  |  |  |  |  |  |  |  |  |  |  |  |  |  |  |  |  |  |  |  |  |  |  |  |  |  |  |  |  |  |  |  |  |  |  |  |  |  |  |  |  |  |  |  |  |  |  |  |  |  |  |  |  |  |  |  |  |  |  |  |  |  |  |  |  |  |  |  |  |  |  |  |  |  |  |  |  |  |  |  |  |  |  |  |  |  |  |  |  |  |  |  |  |  |  |  |  |  |  |  |  |  |  |  |  |  |  |  |  |  |  |  |  |  |  |  |  |  |  |  |  |  |  |  |  |  |  |  |  |  |  |  |  |  |  |  |  |  |  |  |  |  |  |  |  |  |  |  |  |  |  |  |  |  |  |  |  |  |  |  |  |  |  |  |  |  |  |  |  |  |  |  |  |  |  |  |  |  |  |  |  |  |  |  |  |  |  |  |  |  |  |  |  |  |  |  |  |  |  |  |  |  |  |  |  |  |  |  |  |  |  |  |  |  |  |  |  |  |  |  |  |  |  |  |  |  |  |  |  |  |  |  |  |  |  |  |  |  |  |  |  |  |  |  |  |  |  |  |  |  |  |  |  |  |  |  |  |  |  |  |  |  |  |  |  |  |  |  |  |  |  |  |  |  |  |  |  |  |  |  |  |  |  |  |  |  |  |  |  |  |  |  |  |  |  |  |  |  |  |  |  |  |  |  |  |  |  |  |  |  |  |  |  |  |  |  |  |  |  |  |  |  |  |  |  |  |  |  |  |  |  |  |  |  |  |  |  |  |  |  |  |  |  |  |  |  |  |  |  |  |  |  |  |  |  |  |  |  |  |  |  |  |  |  |  |  |  |  |  |  |  |  |  |  |  |  |  |  |  |  |  |  |  |  |  |  |  |  |  |  |  |  |  |  |  |  |  |  |  |  |  |  |  |  |  |  |  |  |  |  |  |  |  |  |  |  |  |  |  |  |  |  |  |  |  |  |  |  |  |  |  |  |  |  |  |  |  |  |  |  |  |  |  |  |  |  |  |  |  |  |  |  |  |  |  |  |  |  |  |  |  |  |  |  |  |  |  |  |  |  |  |  |  |  |  |  |  |  |  |  |  |  |  |  |  |  |  |  |  |  |  |  |  |  |  |  |  |  |  |  |  |  |  |  |  |  |  |  |  |  |  |  |  |  |  |  |  |  |  |  |  |  |  |  |  |  |  |  |  |  |  |  |  |  |  |  |  |  |  |  |  |  |  |  |  |  |  |  |  |  |  |  |  |  |  |  |  |  |  |  |  |  |  |  |  |  |  |  |  |  |  |  |  |  |  |  |  |  |  |  |  |  |  |  |  |  |  |  |  |  |  |  |  |  |  |  |  |  |  |  |  |  |  |  |  |  |  |  |  |  |  |  |  |  |  |  |  |  |  |  |  |  |  |  |  |  |  |  |  |  |  |  |  |  |  |  |  |  |  |  |  |  |  |  |  |  |  |  |  |  |  |  |  |  |  |  |  |  |  |  |  |  |  |  |  |  |  |  |  |  |  |  |  |  |  |  |  |  |  |  |  |  |  |  |  |  |  |  |  |  |  |  |  |  |  |  |  |  |  |  |  |  |  |  |  |  |  |  |  |  |  |  |  |  |  |  |  |  |  |  |  |  |  |  |  |  |  |  |  |  |  |  |  |  |  |  |  |  |  |  |  |  |  |  |  |  |  |  |  |  |  |  |  |  |  |  |  |  |  |  |  |  |  |  |  |  |  |  |  |  |  |  |  |  |  |  |  |  |  |  |  |  |  |  |  |  |  |  |  |  |  |  |  |  |  |  |  |  |  |  |  |  |  |  |  |  |  |  |  |  |  |  |  |  |  |  |  |  |  |  |  |  |  |  |  |  |  |  |  |  |  |  |  |  |  |  |  |  |  |  |  |  |  |  |  |  |  |  |  |  |  |  |  |  |  |  |  |  |  |  |  |  |  |  |  |  |  |  |  |  |  |  |  |  |  |  |  |  |  |  |  |  |  |  |  |  |  |  |  |  |  |  |  |  |  |  |  |  |  |  |  |  |  |  |  |  |  |  |  |  |  |  |  |  |  |  |  |  |  |  |  |  |  |  |  |  |  |  |  |  |  |  |  |  |  |  |  |  |  |  |  |  |  |  |  |  |  |  |  |  |  |  |  |  |  |  |  |  |  |  |  |  |  |  |  |  |  |  |  |  |  |  |  |  |  |  |  |  |  |  |  |  |  |  |  |  |  |  |  |  |  |  |  |  |  |  |  |  |  |  |  |  |  |  |  |  |  |  |  |  |  |  |  |  |  |  |  |  |  |  |  |  |  |  |  |  |  |  |  |  |  |  |  |  |  |  |  |  |  |  |  |  |  |  |  |  |  |  |  |  |  |  |  |  |  |  |  |  |  |  |  |  |  |  |  |  |  |  |  |  |  |  |  |  |  |  |  |  |  |  |  |  |  |  |  |  |  |  |  |  |  |  |  |  |  |  |  |  |  |  |  |  |  |  |  |  |  |  |  |  |  |  |  |  |  |  |  |  |  |  |  |  |  |  |  |  |  |  |  |  |  |  |  |  |  |  |  |  |  |  |  |  |  |  |  |  |  |  |  |  |  |  |  |  |  |  |  |  |  |  |  |  |  |  |  |  |  |  |  |  |  |  |  |  |  |  |  |  |  |  |  |  |  |  |  |  |  |  |  |  |  |  |  |  |  |  |  |  |  |  |  |  |  |  |  |  |  |  |  |  |  |  |  |  |  |  |  |  |  |  |  |  |  |  |  |  |  |  |  |  |  |  |  |  |  |  |  |  |  |  |  |  |  |  |  |  |  |  |  |  |  |  |  |  |  |  |  |  |  |  |  |  |  |  |  |  |  |  |  |  |  |  |  |  |  |  |  |  |  |  |  |  |  |  |  |  |  |  |  |
|  |  |  |  |  |  |  |  |  |  |  |  |  |  |  |  |  |  |  |  |  |  |  |  |  |  |  |  |  |  |  |  |  |  |  |  |  |  |  |  |  |  |  |  |  |  |  |  |  |  |  |  |  |  |  |  |  |  |  |  |  |  |  |  |  |  |  |  |  |  |  |  |  |  |  |  |  |  |  |  |  |  |  |  |  |  |  |  |  |  |  |  |  |  |  |  |  |  |  |  |  |  |  |  |  |  |  |  |  |  |  |  |  |  |  |  |  |  |  |  |  |  |  |  |  |  |  |  |  |  |  |  |  |  |  |  |  |  |  |  |  |  |  |  |  |  |  |  |  |  |  |  |  |  |  |  |  |  |  |  |  |  |  |  |  |  |  |  |  |  |  |  |  |  |  |  |  |  |  |  |  |  |  |  |  |  |  |  |  |  |  |  |  |  |  |  |  |  |  |  |  |  |  |  |  |  |  |  |  |  |  |  |  |  |  |  |  |  |  |  |  |  |  |  |  |  |  |  |  |  |  |  |  |  |  |  |  |  |  |  |  |  |  |  |  |  |  |  |  |  |  |  |  |  |  |  |  |  |  |  |  |  |  |  |  |  |  |  |  |  |  |  |  |  |  |  |  |  |  |  |  |  |  |  |  |  |  |  |  |  |  |  |  |  |  |  |  |  |  |  |  |  |  |  |  |  |  |  |  |  |  |  |  |  |  |  |  |  |  |  |  |  |  |  |  |  |  |  |  |  |  |  |  |  |  |  |  |  |  |  |  |  |  |  |  |  |  |  |  |  |  |  |  |  |  |  |  |  |  |  |  |  |  |  |  |  |  |  |  |  |  |  |  |  |  |  |  |  |  |  |  |  |  |  |  |  |  |  |  |  |  |  |  |  |  |  |  |  |  |  |  |  |  |  |  |  |  |  |  |  |  |  |  |  |  |  |  |  |  |  |  |  |  |  |  |  |  |  |  |  |  |  |  |  |  |  |  |  |  |  |  |  |  |  |  |  |  |  |  |  |  |  |  |  |  |  |  |  |  |  |  |  |  |  |  |  |  |  |  |  |  |  |  |  |  |  |  |  |  |  |  |  |  |  |  |  |  |  |  |  |  |  |  |  |  |  |  |  |  |  |  |  |  |  |  |  |  |  |  |  |  |  |  |  |  |  |  |  |  |  |  |  |  |  |  |  |  |  |  |  |  |  |  |  |  |  |  |  |  |  |  |  |  |  |  |  |  |  |  |  |  |  |  |  |  |  |  |  |  |  |  |  |  |  |  |  |  |  |  |  |  |  |  |  |  |  |  |  |  |  |  |  |  |  |  |  |  |  |  |  |  |  |  |  |  |  |  |  |  |  |  |  |  |  |  |  |  |  |  |  |  |  |  |  |  |  |  |  |  |  |  |  |  |  |  |  |  |  |  |  |  |  |  |  |  |  |  |  |  |  |  |  |  |  |  |  |  |  |  |  |  |  |  |  |  |  |  |  |  |  |  |  |  |  |  |  |  |  |  |  |  |  |  |  |  |  |  |  |  |  |  |  |  |  |  |  |  |  |  |  |  |  |  |  |  |  |  |  |  |  |  |  |  |  |  |  |  |  |  |  |  |  |  |  |  |  |  |  |  |  |  |  |  |  |  |  |  |  |  |  |  |  |  |  |  |  |  |  |  |  |  |  |  |  |  |  |  |  |  |  |  |  |  |  |  |  |  |  |  |  |  |  |  |  |  |  |  |  |  |  |  |  |  |  |  |  |  |  |  |  |  |  |  |  |  |  |  |  |  |  |  |  |  |  |  |  |  |  |  |  |  |  |  |  |  |  |  |  |  |  |  |  |  |  |  |  |  |  |  |  |  |  |  |  |  |  |  |  |  |  |  |  |  |  |  |  |  |  |  |  |  |  |  |  |  |  |  |  |  |  |  |  |  |  |  |  |  |  |  |  |  |  |  |  |  |  |  |  |  |  |  |  |  |  |  |  |  |  |  |  |  |  |  |  |  |  |  |  |  |  |  |  |  |  |  |  |  |  |  |  |  |  |  |  |  |  |  |  |  |  |  |  |  |  |  |  |  |  |  |  |  |  |  |  |  |  |  |  |  |  |  |  |  |  |  |  |  |  |  |  |  |  |  |  |  |  |  |  |  |  |  |  |  |  |  |  |  |  |  |  |  |  |  |  |  |  |  |  |  |  |  |  |  |  |  |  |  |  |  |  |  |  |  |  |  |  |  |  |  |  |  |  |  |  |  |  |  |  |  |  |  |  |  |  |  |  |  |  |  |  |  |  |  |  |  |  |  |  |  |  |  |  |  |  |  |  |  |  |  |  |  |  |  |  |  |  |  |  |  |  |  |  |  |  |  |  |  |  |  |  |  |  |  |  |  |  |  |  |  |  |  |  |  |  |  |  |  |  |  |  |  |  |  |  |  |  |  |  |  |  |  |  |  |  |  |  |  |  |  |  |  |  |  |  |  |  |  |  |  |  |  |  |  |  |  |  |  |  |  |  |  |  |  |  |  |  |  |  |  |  |  |  |  |  |  |  |  |  |  |  |  |  |  |  |  |  |  |  |  |  |  |  |  |  |  |  |  |  |  |  |  |  |  |  |  |  |  |  |  |  |  |  |  |  |  |  |  |  |  |  |  |  |  |  |  |  |  |  |  |  |  |  |  |  |  |  |  |  |  |  |  |  |  |  |  |  |  |  |  |  |  |  |  |  |  |  |  |  |  |  |  |  |  |  |  |  |  |  |  |  |  |  |  |  |  |  |  |  |  |  |  |  |  |  |  |  |  |  |  |  |  |  |  |  |  |  |  |  |  |  |  |  |  |  |  |  |  |  |  |  |  |  |  |  |  |  |  |  |  |  |  |  |  |  |  |  |  |  |  |  |  |  |  |  |  |  |  |  |  |  |  |  |  |  |  |  |  |  |  |  |  |  |  |  |  |  |  |  |  |  |  |  |  |  |  |  |  |  |  |  |  |  |  |  |  |  |  |  |  |  |  |  |  |  |  |  |  |  |  |  |  |  |  |  |  |  |  |  |  |  |  |  |  |  |  |  |  |  |  |  |  |  |  |  |  |  |  |  |  |  |  |  |  |  |  |  |  |  |  |  |  |  |  |  |  |  |  |  |  |  |  |  |  |  |  |  |  |  |  |  |  |  |  |  |  |  |  |  |  |  |  |  |  |  |  |  |  |  |  |  |  |  |  |  |  |  |  |  |  |  |  |  |  |  |  |
|  |  |  |  |  |  |  |  |  |  |  |  |  |  |  |  |  |  |  |  |  |  |  |  |  |  |  |  |  |  |  |  |  |  |  |  |  |  |  |  |  |  |  |  |  |  |  |  |  |  |  |  |  |  |  |  |  |  |  |  |  |  |  |  |  |  |  |  |  |  |  |  |  |  |  |  |  |  |  |  |  |  |  |  |  |  |  |  |  |  |  |  |  |  |  |  |  |  |  |  |  |  |  |  |  |  |  |  |  |  |  |  |  |  |  |  |  |  |  |  |  |  |  |  |  |  |  |  |  |  |  |  |  |  |  |  |  |  |  |  |  |  |  |  |  |  |  |  |  |  |  |  |  |  |  |  |  |  |  |  |  |  |  |  |  |  |  |  |  |  |  |  |  |  |  |  |  |  |  |  |  |  |  |  |  |  |  |  |  |  |  |  |  |  |  |  |  |  |  |  |  |  |  |  |  |  |  |  |  |  |  |  |  |  |  |  |  |  |  |  |  |  |  |  |  |  |  |  |  |  |  |  |  |  |  |  |  |  |  |  |  |  |  |  |  |  |  |  |  |  |  |  |  |  |  |  |  |  |  |  |  |  |  |  |  |  |  |  |  |  |  |  |  |  |  |  |  |  |  |  |  |  |  |  |  |  |  |  |  |  |  |  |  |  |  |  |  |  |  |  |  |  |  |  |  |  |  |  |  |  |  |  |  |  |  |  |  |  |  |  |  |  |  |  |  |  |  |  |  |  |  |  |  |  |  |  |  |  |  |  |  |  |  |  |  |  |  |  |  |  |  |  |  |  |  |  |  |  |  |  |  |  |  |  |  |  |  |  |  |  |  |  |  |  |  |  |  |  |  |  |  |  |  |  |  |  |  |  |  |  |  |  |  |  |  |  |  |  |  |  |  |  |  |  |  |  |  |  |  |  |  |  |  |  |  |  |  |  |  |  |  |  |  |  |  |  |  |  |  |  |  |  |  |  |  |  |  |  |  |  |  |  |  |  |  |  |  |  |  |  |  |  |  |  |  |  |  |  |  |  |  |  |  |  |  |  |  |  |  |  |  |  |  |  |  |  |  |  |  |  |  |  |  |  |  |  |  |  |  |  |  |  |  |  |  |  |  |  |  |  |  |  |  |  |  |  |  |  |  |  |  |  |  |  |  |  |  |  |  |  |  |  |  |  |  |  |  |  |  |  |  |  |  |  |  |  |  |  |  |  |  |  |  |  |  |  |  |  |  |  |  |  |  |  |  |  |  |  |  |  |  |  |  |  |  |  |  |  |  |  |  |  |  |  |  |  |  |  |  |  |  |  |  |  |  |  |  |  |  |  |  |  |  |  |  |  |  |  |  |  |  |  |  |  |  |  |  |  |  |  |  |  |  |  |  |  |  |  |  |  |  |  |  |  |  |  |  |  |  |  |  |  |  |  |  |  |  |  |  |  |  |  |  |  |  |  |  |  |  |  |  |  |  |  |  |  |  |  |  |  |  |  |  |  |  |  |  |  |  |  |  |  |  |  |  |  |  |  |  |  |  |  |  |  |  |  |  |  |  |  |  |  |  |  |  |  |  |  |  |  |  |  |  |  |  |  |  |  |  |  |  |  |  |  |  |  |  |  |  |  |  |  |  |  |  |  |  |  |  |  |  |  |  |  |  |  |  |  |  |  |  |  |  |  |  |  |  |  |  |  |  |  |  |  |  |  |  |  |  |  |  |  |  |  |  |  |  |  |  |  |  |  |  |  |  |  |  |  |  |  |  |  |  |  |  |  |  |  |  |  |  |  |  |  |  |  |  |  |  |  |  |  |  |  |  |  |  |  |  |  |  |  |  |  |  |  |  |  |  |  |  |  |  |  |  |  |  |  |  |  |  |  |  |  |  |  |  |  |  |  |  |  |  |  |  |  |  |  |  |  |  |  |  |  |  |  |  |  |  |  |  |  |  |  |  |  |  |  |  |  |  |  |  |  |  |  |  |  |  |  |  |  |  |  |  |  |  |  |  |  |  |  |  |  |  |  |  |  |  |  |  |  |  |  |  |  |  |  |  |  |  |  |  |  |  |  |  |  |  |  |  |  |  |  |  |  |  |  |  |  |  |  |  |  |  |  |  |  |  |  |  |  |  |  |  |  |  |  |  |  |  |  |  |  |  |  |  |  |  |  |  |  |  |  |  |  |  |  |  |  |  |  |  |  |  |  |  |  |  |  |  |  |  |  |  |  |  |  |  |  |  |  |  |  |  |  |  |  |  |  |  |  |  |  |  |  |  |  |  |  |  |  |  |  |  |  |  |  |  |  |  |  |  |  |  |  |  |  |  |  |  |  |  |  |  |  |  |  |  |  |  |  |  |  |  |  |  |  |  |  |  |  |  |  |  |  |  |  |  |  |  |  |  |  |  |  |  |  |  |  |  |  |  |  |  |  |  |  |  |  |  |  |  |  |  |  |  |  |  |  |  |  |  |  |  |  |  |  |  |  |  |  |  |  |  |  |  |  |  |  |  |  |  |  |  |  |  |  |  |  |  |  |  |  |  |  |  |  |  |  |  |  |  |  |  |  |  |  |  |  |  |  |  |  |  |  |  |  |  |  |  |  |  |  |  |  |  |  |  |  |  |  |  |  |  |  |  |  |  |  |  |  |  |  |  |  |  |  |  |  |  |  |  |  |  |  |  |  |  |  |  |  |  |  |  |  |  |  |  |  |  |  |  |  |  |  |  |  |  |  |  |  |  |  |  |  |  |  |  |  |  |  |  |  |  |  |  |  |  |  |  |  |  |  |  |  |  |  |  |  |  |  |  |  |  |  |  |  |  |  |  |  |  |  |  |  |  |  |  |  |  |  |  |  |  |  |  |  |  |  |  |  |  |  |  |  |  |  |  |  |  |  |  |  |  |  |  |  |  |  |  |  |  |  |  |  |  |  |  |  |  |  |  |  |  |  |  |  |  |  |  |  |  |  |  |  |  |  |  |  |  |  |  |  |  |  |  |  |  |  |  |  |  |  |  |  |  |  |  |  |  |  |  |  |  |  |  |  |  |  |  |  |  |  |  |  |  |  |  |  |  |  |  |  |  |  |  |  |  |  |  |  |  |  |  |  |  |  |  |  |  |  |  |  |  |  |  |  |  |  |  |  |  |  |  |  |  |  |  |  |  |  |  |  |  |  |  |  |  |  |  |  |  |  |  |  |  |  |  |  |  |  |  |  |  |  |  |  |  |  |  |  |  |  |  |
|  |  |  |  |  |  |  |  |  |  |  |  |  |  |  |  |  |  |  |  |  |  |  |  |  |  |  |  |  |  |  |  |  |  |  |  |  |  |  |  |  |  |  |  |  |  |  |  |  |  |  |  |  |  |  |  |  |  |  |  |  |  |  |  |  |  |  |  |  |  |  |  |  |  |  |  |  |  |  |  |  |  |  |  |  |  |  |  |  |  |  |  |  |  |  |  |  |  |  |  |  |  |  |  |  |  |  |  |  |  |  |  |  |  |  |  |  |  |  |  |  |  |  |  |  |  |  |  |  |  |  |  |  |  |  |  |  |  |  |  |  |  |  |  |  |  |  |  |  |  |  |  |  |  |  |  |  |  |  |  |  |  |  |  |  |  |  |  |  |  |  |  |  |  |  |  |  |  |  |  |  |  |  |  |  |  |  |  |  |  |  |  |  |  |  |  |  |  |  |  |  |  |  |  |  |  |  |  |  |  |  |  |  |  |  |  |  |  |  |  |  |  |  |  |  |  |  |  |  |  |  |  |  |  |  |  |  |  |  |  |  |  |  |  |  |  |  |  |  |  |  |  |  |  |  |  |  |  |  |  |  |  |  |  |  |  |  |  |  |  |  |  |  |  |  |  |  |  |  |  |  |  |  |  |  |  |  |  |  |  |  |  |  |  |  |  |  |  |  |  |  |  |  |  |  |  |  |  |  |  |  |  |  |  |  |  |  |  |  |  |  |  |  |  |  |  |  |  |  |  |  |  |  |  |  |  |  |  |  |  |  |  |  |  |  |  |  |  |  |  |  |  |  |  |  |  |  |  |  |  |  |  |  |  |  |  |  |  |  |  |  |  |  |  |  |  |  |  |  |  |  |  |  |  |  |  |  |  |  |  |  |  |  |  |  |  |  |  |  |  |  |  |  |  |  |  |  |  |  |  |  |  |  |  |  |  |  |  |  |  |  |  |  |  |  |  |  |  |  |  |  |  |  |  |  |  |  |  |  |  |  |  |  |  |  |  |  |  |  |  |  |  |  |  |  |  |  |  |  |  |  |  |  |  |  |  |  |  |  |  |  |  |  |  |  |  |  |  |  |  |  |  |  |  |  |  |  |  |  |  |  |  |  |  |  |  |  |  |  |  |  |  |  |  |  |  |  |  |  |  |  |  |  |  |  |  |  |  |  |  |  |  |  |  |  |  |  |  |  |  |  |  |  |  |  |  |  |  |  |  |  |  |  |  |  |  |  |  |  |  |  |  |  |  |  |  |  |  |  |  |  |  |  |  |  |  |  |  |  |  |  |  |  |  |  |  |  |  |  |  |  |  |  |  |  |  |  |  |  |  |  |  |  |  |  |  |  |  |  |  |  |  |  |  |  |  |  |  |  |  |  |  |  |  |  |  |  |  |  |  |  |  |  |  |  |  |  |  |  |  |  |  |  |  |  |  |  |  |  |  |  |  |  |  |  |  |  |  |  |  |  |  |  |  |  |  |  |  |  |  |  |  |  |  |  |  |  |  |  |  |  |  |  |  |  |  |  |  |  |  |  |  |  |  |  |  |  |  |  |  |  |  |  |  |  |  |  |  |  |  |  |  |  |  |  |  |  |  |  |  |  |  |  |  |  |  |  |  |  |  |  |  |  |  |  |  |  |  |  |  |  |  |  |  |  |  |  |  |  |  |  |  |  |  |  |  |  |  |  |  |  |  |  |  |  |  |  |  |  |  |  |  |  |  |  |  |  |  |  |  |  |  |  |  |  |  |  |  |  |  |  |  |  |  |  |  |  |  |  |  |  |  |  |  |  |  |  |  |  |  |  |  |  |  |  |  |  |  |  |  |  |  |  |  |  |  |  |  |  |  |  |  |  |  |  |  |  |  |  |  |  |  |  |  |  |  |  |  |  |  |  |  |  |  |  |  |  |  |  |  |  |  |  |  |  |  |  |  |  |  |  |  |  |  |  |  |  |  |  |  |  |  |  |  |  |  |  |  |  |  |  |  |  |  |  |  |  |  |  |  |  |  |  |  |  |  |  |  |  |  |  |  |  |  |  |  |  |  |  |  |  |  |  |  |  |  |  |  |  |  |  |  |  |  |  |  |  |  |  |  |  |  |  |  |  |  |  |  |  |  |  |  |  |  |  |  |  |  |  |  |  |  |  |  |  |  |  |  |  |  |  |  |  |  |  |  |  |  |  |  |  |  |  |  |  |  |  |  |  |  |  |  |  |  |  |  |  |  |  |  |  |  |  |  |  |  |  |  |  |  |  |  |  |  |  |  |  |  |  |  |  |  |  |  |  |  |  |  |  |  |  |  |  |  |  |  |  |  |  |  |  |  |  |  |  |  |  |  |  |  |  |  |  |  |  |  |  |  |  |  |  |  |  |  |  |  |  |  |  |  |  |  |  |  |  |  |  |  |  |  |  |  |  |  |  |  |  |  |  |  |  |  |  |  |  |  |  |  |  |  |  |  |  |  |  |  |  |  |  |  |  |  |  |  |  |  |  |  |  |  |  |  |  |  |  |  |  |  |  |  |  |  |  |  |  |  |  |  |  |  |  |  |  |  |  |  |  |  |  |  |  |  |  |  |  |  |  |  |  |  |  |  |  |  |  |  |  |  |  |  |  |  |  |  |  |  |  |  |  |  |  |  |  |  |  |  |  |  |  |  |  |  |  |  |  |  |  |  |  |  |  |  |  |  |  |  |  |  |  |  |  |  |  |  |  |  |  |  |  |  |  |  |  |  |  |  |  |  |  |  |  |  |  |  |  |  |  |  |  |  |  |  |  |  |  |  |  |  |  |  |  |  |  |  |  |  |  |  |  |  |  |  |  |  |  |  |  |  |  |  |  |  |  |  |  |  |  |  |  |  |  |  |  |  |  |  |  |  |  |  |  |  |  |  |  |  |  |  |  |  |  |  |  |  |  |  |  |  |  |  |  |  |  |  |  |  |  |  |  |  |  |  |  |  |  |  |  |  |  |  |  |  |  |  |  |  |  |  |  |  |  |  |  |  |  |  |  |  |  |  |  |  |  |  |  |  |  |  |  |  |  |  |  |  |  |  |  |  |  |  |  |  |  |  |  |  |  |  |  |  |  |  |  |  |  |  |  |  |  |  |  |  |  |  |  |  |  |  |  |  |  |  |  |  |  |  |  |  |  |  |  |  |  |  |  |  |  |  |  |  |  |  |  |  |  |  |  |  |  |  |  |  |  |  |  |  |  |  |  |  |  |  |  |  |  |
|  |  |  |  |  |  |  |  |  |  |  |  |  |  |  |  |  |  |  |  |  |  |  |  |  |  |  |  |  |  |  |  |  |  |  |  |  |  |  |  |  |  |  |  |  |  |  |  |  |  |  |  |  |  |  |  |  |  |  |  |  |  |  |  |  |  |  |  |  |  |  |  |  |  |  |  |  |  |  |  |  |  |  |  |  |  |  |  |  |  |  |  |  |  |  |  |  |  |  |  |  |  |  |  |  |  |  |  |  |  |  |  |  |  |  |  |  |  |  |  |  |  |  |  |  |  |  |  |  |  |  |  |  |  |  |  |  |  |  |  |  |  |  |  |  |  |  |  |  |  |  |  |  |  |  |  |  |  |  |  |  |  |  |  |  |  |  |  |  |  |  |  |  |  |  |  |  |  |  |  |  |  |  |  |  |  |  |  |  |  |  |  |  |  |  |  |  |  |  |  |  |  |  |  |  |  |  |  |  |  |  |  |  |  |  |  |  |  |  |  |  |  |  |  |  |  |  |  |  |  |  |  |  |  |  |  |  |  |  |  |  |  |  |  |  |  |  |  |  |  |  |  |  |  |  |  |  |  |  |  |  |  |  |  |  |  |  |  |  |  |  |  |  |  |  |  |  |  |  |  |  |  |  |  |  |  |  |  |  |  |  |  |  |  |  |  |  |  |  |  |  |  |  |  |  |  |  |  |  |  |  |  |  |  |  |  |  |  |  |  |  |  |  |  |  |  |  |  |  |  |  |  |  |  |  |  |  |  |  |  |  |  |  |  |  |  |  |  |  |  |  |  |  |  |  |  |  |  |  |  |  |  |  |  |  |  |  |  |  |  |  |  |  |  |  |  |  |  |  |  |  |  |  |  |  |  |  |  |  |  |  |  |  |  |  |  |  |  |  |  |  |  |  |  |  |  |  |  |  |  |  |  |  |  |  |  |  |  |  |  |  |  |  |  |  |  |  |  |  |  |  |  |  |  |  |  |  |  |  |  |  |  |  |  |  |  |  |  |  |  |  |  |  |  |  |  |  |  |  |  |  |  |  |  |  |  |  |  |  |  |  |  |  |  |  |  |  |  |  |  |  |  |  |  |  |  |  |  |  |  |  |  |  |  |  |  |  |  |  |  |  |  |  |  |  |  |  |  |  |  |  |  |  |  |  |  |  |  |  |  |  |  |  |  |  |  |  |  |  |  |  |  |  |  |  |  |  |  |  |  |  |  |  |  |  |  |  |  |  |  |  |  |  |  |  |  |  |  |  |  |  |  |  |  |  |  |  |  |  |  |  |  |  |  |  |  |  |  |  |  |  |  |  |  |  |  |  |  |  |  |  |  |  |  |  |  |  |  |  |  |  |  |  |  |  |  |  |  |  |  |  |  |  |  |  |  |  |  |  |  |  |  |  |  |  |  |  |  |  |  |  |  |  |  |  |  |  |  |  |  |  |  |  |  |  |  |  |  |  |  |  |  |  |  |  |  |  |  |  |  |  |  |  |  |  |  |  |  |  |  |  |  |  |  |  |  |  |  |  |  |  |  |  |  |  |  |  |  |  |  |  |  |  |  |  |  |  |  |  |  |  |  |  |  |  |  |  |  |  |  |  |  |  |  |  |  |  |  |  |  |  |  |  |  |  |  |  |  |  |  |  |  |  |  |  |  |  |  |  |  |  |  |  |  |  |  |  |  |  |  |  |  |  |  |  |  |  |  |  |  |  |  |  |  |  |  |  |  |  |  |  |  |  |  |  |  |  |  |  |  |  |  |  |  |  |  |  |  |  |  |  |  |  |  |  |  |  |  |  |  |  |  |  |  |  |  |  |  |  |  |  |  |  |  |  |  |  |  |  |  |  |  |  |  |  |  |  |  |  |  |  |  |  |  |  |  |  |  |  |  |  |  |  |  |  |  |  |  |  |  |  |  |  |  |  |  |  |  |  |  |  |  |  |  |  |  |  |  |  |  |  |  |  |  |  |  |  |  |  |  |  |  |  |  |  |  |  |  |  |  |  |  |  |  |  |  |  |  |  |  |  |  |  |  |  |  |  |  |  |  |  |  |  |  |  |  |  |  |  |  |  |  |  |  |  |  |  |  |  |  |  |  |  |  |  |  |  |  |  |  |  |  |  |  |  |  |  |  |  |  |  |  |  |  |  |  |  |  |  |  |  |  |  |  |  |  |  |  |  |  |  |  |  |  |  |  |  |  |  |  |  |  |  |  |  |  |  |  |  |  |  |  |  |  |  |  |  |  |  |  |  |  |  |  |  |  |  |  |  |  |  |  |  |  |  |  |  |  |  |  |  |  |  |  |  |  |  |  |  |  |  |  |  |  |  |  |  |  |  |  |  |  |  |  |  |  |  |  |  |  |  |  |  |  |  |  |  |  |  |  |  |  |  |  |  |  |  |  |  |  |  |  |  |  |  |  |  |  |  |  |  |  |  |  |  |  |  |  |  |  |  |  |  |  |  |  |  |  |  |  |  |  |  |  |  |  |  |  |  |  |  |  |  |  |  |  |  |  |  |  |  |  |  |  |  |  |  |  |  |  |  |  |  |  |  |  |  |  |  |  |  |  |  |  |  |  |  |  |  |  |  |  |  |  |  |  |  |  |  |  |  |  |  |  |  |  |  |  |  |  |  |  |  |  |  |  |  |  |  |  |  |  |  |  |  |  |  |  |  |  |  |  |  |  |  |  |  |  |  |  |  |  |  |  |  |  |  |  |  |  |  |  |  |  |  |  |  |  |  |  |  |  |  |  |  |  |  |  |  |  |  |  |  |  |  |  |  |  |  |  |  |  |  |  |  |  |  |  |  |  |  |  |  |  |  |  |  |  |  |  |  |  |  |  |  |  |  |  |  |  |  |  |  |  |  |  |  |  |  |  |  |  |  |  |  |  |  |  |  |  |  |  |  |  |  |  |  |  |  |  |  |  |  |  |  |  |  |  |  |  |  |  |  |  |  |  |  |  |  |  |  |  |  |  |  |  |  |  |  |  |  |  |  |  |  |  |  |  |  |  |  |  |  |  |  |  |  |  |  |  |  |  |  |  |  |  |  |  |  |  |  |  |  |  |  |  |  |  |  |  |  |  |  |  |  |  |  |  |  |  |  |  |  |  |  |  |  |  |  |  |  |  |  |  |  |  |  |  |  |  |  |  |  |  |  |  |  |  |  |  |  |  |  |  |  |  |  |  |  |  |  |  |  |  |  |  |  |  |  |  |  |  |  |  |  |
|  |  |  |  |  |  |  |  |  |  |  |  |  |  |  |  |  |  |  |  |  |  |  |  |  |  |  |  |  |  |  |  |  |  |  |  |  |  |  |  |  |  |  |  |  |  |  |  |  |  |  |  |  |  |  |  |  |  |  |  |  |  |  |  |  |  |  |  |  |  |  |  |  |  |  |  |  |  |  |  |  |  |  |  |  |  |  |  |  |  |  |  |  |  |  |  |  |  |  |  |  |  |  |  |  |  |  |  |  |  |  |  |  |  |  |  |  |  |  |  |  |  |  |  |  |  |  |  |  |  |  |  |  |  |  |  |  |  |  |  |  |  |  |  |  |  |  |  |  |  |  |  |  |  |  |  |  |  |  |  |  |  |  |  |  |  |  |  |  |  |  |  |  |  |  |  |  |  |  |  |  |  |  |  |  |  |  |  |  |  |  |  |  |  |  |  |  |  |  |  |  |  |  |  |  |  |  |  |  |  |  |  |  |  |  |  |  |  |  |  |  |  |  |  |  |  |  |  |  |  |  |  |  |  |  |  |  |  |  |  |  |  |  |  |  |  |  |  |  |  |  |  |  |  |  |  |  |  |  |  |  |  |  |  |  |  |  |  |  |  |  |  |  |  |  |  |  |  |  |  |  |  |  |  |  |  |  |  |  |  |  |  |  |  |  |  |  |  |  |  |  |  |  |  |  |  |  |  |  |  |  |  |  |  |  |  |  |  |  |  |  |  |  |  |  |  |  |  |  |  |  |  |  |  |  |  |  |  |  |  |  |  |  |  |  |  |  |  |  |  |  |  |  |  |  |  |  |  |  |  |  |  |  |  |  |  |  |  |  |  |  |  |  |  |  |  |  |  |  |  |  |  |  |  |  |  |  |  |  |  |  |  |  |  |  |  |  |  |  |  |  |  |  |  |  |  |  |  |  |  |  |  |  |  |  |  |  |  |  |  |  |  |  |  |  |  |  |  |  |  |  |  |  |  |  |  |  |  |  |  |  |  |  |  |  |  |  |  |  |  |  |  |  |  |  |  |  |  |  |  |  |  |  |  |  |  |  |  |  |  |  |  |  |  |  |  |  |  |  |  |  |  |  |  |  |  |  |  |  |  |  |  |  |  |  |  |  |  |  |  |  |  |  |  |  |  |  |  |  |  |  |  |  |  |  |  |  |  |  |  |  |  |  |  |  |  |  |  |  |  |  |  |  |  |  |  |  |  |  |  |  |  |  |  |  |  |  |  |  |  |  |  |  |  |  |  |  |  |  |  |  |  |  |  |  |  |  |  |  |  |  |  |  |  |  |  |  |  |  |  |  |  |  |  |  |  |  |  |  |  |  |  |  |  |  |  |  |  |  |  |  |  |  |  |  |  |  |  |  |  |  |  |  |  |  |  |  |  |  |  |  |  |  |  |  |  |  |  |  |  |  |  |  |  |  |  |  |  |  |  |  |  |  |  |  |  |  |  |  |  |  |  |  |  |  |  |  |  |  |  |  |  |  |  |  |  |  |  |  |  |  |  |  |  |  |  |  |  |  |  |  |  |  |  |  |  |  |  |  |  |  |  |  |  |  |  |  |  |  |  |  |  |  |  |  |  |  |  |  |  |  |  |  |  |  |  |  |  |  |  |  |  |  |  |  |  |  |  |  |  |  |  |  |  |  |  |  |  |  |  |  |  |  |  |  |  |  |  |  |  |  |  |  |  |  |  |  |  |  |  |  |  |  |  |  |  |  |  |  |  |  |  |  |  |  |  |  |  |  |  |  |  |  |  |  |  |  |  |  |  |  |  |  |  |  |  |  |  |  |  |  |  |  |  |  |  |  |  |  |  |  |  |  |  |  |  |  |  |  |  |  |  |  |  |  |  |  |  |  |  |  |  |  |  |  |  |  |  |  |  |  |  |  |  |  |  |  |  |  |  |  |  |  |  |  |  |  |  |  |  |  |  |  |  |  |  |  |  |  |  |  |  |  |  |  |  |  |  |  |  |  |  |  |  |  |  |  |  |  |  |  |  |  |  |  |  |  |  |  |  |  |  |  |  |  |  |  |  |  |  |  |  |  |  |  |  |  |  |  |  |  |  |  |  |  |  |  |  |  |  |  |  |  |  |  |  |  |  |  |  |  |  |  |  |  |  |  |  |  |  |  |  |  |  |  |  |  |  |  |  |  |  |  |  |  |  |  |  |  |  |  |  |  |  |  |  |  |  |  |  |  |  |  |  |  |  |  |  |  |  |  |  |  |  |  |  |  |  |  |  |  |  |  |  |  |  |  |  |  |  |  |  |  |  |  |  |  |  |  |  |  |  |  |  |  |  |  |  |  |  |  |  |  |  |  |  |  |  |  |  |  |  |  |  |  |  |  |  |  |  |  |  |  |  |  |  |  |  |  |  |  |  |  |  |  |  |  |  |  |  |  |  |  |  |  |  |  |  |  |  |  |  |  |  |  |  |  |  |  |  |  |  |  |  |  |  |  |  |  |  |  |  |  |  |  |  |  |  |  |  |  |  |  |  |  |  |  |  |  |  |  |  |  |  |  |  |  |  |  |  |  |  |  |  |  |  |  |  |  |  |  |  |  |  |  |  |  |  |  |  |  |  |  |  |  |  |  |  |  |  |  |  |  |  |  |  |  |  |  |  |  |  |  |  |  |  |  |  |  |  |  |  |  |  |  |  |  |  |  |  |  |  |  |  |  |  |  |  |  |  |  |  |  |  |  |  |  |  |  |  |  |  |  |  |  |  |  |  |  |  |  |  |  |  |  |  |  |  |  |  |  |  |  |  |  |  |  |  |  |  |  |  |  |  |  |  |  |  |  |  |  |  |  |  |  |  |  |  |  |  |  |  |  |  |  |  |  |  |  |  |  |  |  |  |  |  |  |  |  |  |  |  |  |  |  |  |  |  |  |  |  |  |  |  |  |  |  |  |  |  |  |  |  |  |  |  |  |  |  |  |  |  |  |  |  |  |  |  |  |  |  |  |  |  |  |  |  |  |  |  |  |  |  |  |  |  |  |  |  |  |  |  |  |  |  |  |  |  |  |  |  |  |  |  |  |  |  |  |  |  |  |  |  |  |  |  |  |  |  |  |  |  |  |  |  |  |  |  |  |  |  |  |  |  |  |  |  |  |  |  |  |  |  |  |  |  |  |  |  |  |  |  |  |  |  |  |  |  |  |  |  |  |  |  |  |  |  |  |  |  |  |  |  |  |  |  |  |  |  |  |  |  |  |  |  |
|  |  |  |  |  |  |  |  |  |  |  |  |  |  |  |  |  |  |  |  |  |  |  |  |  |  |  |  |  |  |  |  |  |  |  |  |  |  |  |  |  |  |  |  |  |  |  |  |  |  |  |  |  |  |  |  |  |  |  |  |  |  |  |  |  |  |  |  |  |  |  |  |  |  |  |  |  |  |  |  |  |  |  |  |  |  |  |  |  |  |  |  |  |  |  |  |  |  |  |  |  |  |  |  |  |  |  |  |  |  |  |  |  |  |  |  |  |  |  |  |  |  |  |  |  |  |  |  |  |  |  |  |  |  |  |  |  |  |  |  |  |  |  |  |  |  |  |  |  |  |  |  |  |  |  |  |  |  |  |  |  |  |  |  |  |  |  |  |  |  |  |  |  |  |  |  |  |  |  |  |  |  |  |  |  |  |  |  |  |  |  |  |  |  |  |  |  |  |  |  |  |  |  |  |  |  |  |  |  |  |  |  |  |  |  |  |  |  |  |  |  |  |  |  |  |  |  |  |  |  |  |  |  |  |  |  |  |  |  |  |  |  |  |  |  |  |  |  |  |  |  |  |  |  |  |  |  |  |  |  |  |  |  |  |  |  |  |  |  |  |  |  |  |  |  |  |  |  |  |  |  |  |  |  |  |  |  |  |  |  |  |  |  |  |  |  |  |  |  |  |  |  |  |  |  |  |  |  |  |  |  |  |  |  |  |  |  |  |  |  |  |  |  |  |  |  |  |  |  |  |  |  |  |  |  |  |  |  |  |  |  |  |  |  |  |  |  |  |  |  |  |  |  |  |  |  |  |  |  |  |  |  |  |  |  |  |  |  |  |  |  |  |  |  |  |  |  |  |  |  |  |  |  |  |  |  |  |  |  |  |  |  |  |  |  |  |  |  |  |  |  |  |  |  |  |  |  |  |  |  |  |  |  |  |  |  |  |  |  |  |  |  |  |  |  |  |  |  |  |  |  |  |  |  |  |  |  |  |  |  |  |  |  |  |  |  |  |  |  |  |  |  |  |  |  |  |  |  |  |  |  |  |  |  |  |  |  |  |  |  |  |  |  |  |  |  |  |  |  |  |  |  |  |  |  |  |  |  |  |  |  |  |  |  |  |  |  |  |  |  |  |  |  |  |  |  |  |  |  |  |  |  |  |  |  |  |  |  |  |  |  |  |  |  |  |  |  |  |  |  |  |  |  |  |  |  |  |  |  |  |  |  |  |  |  |  |  |  |  |  |  |  |  |  |  |  |  |  |  |  |  |  |  |  |  |  |  |  |  |  |  |  |  |  |  |  |  |  |  |  |  |  |  |  |  |  |  |  |  |  |  |  |  |  |  |  |  |  |  |  |  |  |  |  |  |  |  |  |  |  |  |  |  |  |  |  |  |  |  |  |  |  |  |  |  |  |  |  |  |  |  |  |  |  |  |  |  |  |  |  |  |  |  |  |  |  |  |  |  |  |  |  |  |  |  |  |  |  |  |  |  |  |  |  |  |  |  |  |  |  |  |  |  |  |  |  |  |  |  |  |  |  |  |  |  |  |  |  |  |  |  |  |  |  |  |  |  |  |  |  |  |  |  |  |  |  |  |  |  |  |  |  |  |  |  |  |  |  |  |  |  |  |  |  |  |  |  |  |  |  |  |  |  |  |  |  |  |  |  |  |  |  |  |  |  |  |  |  |  |  |  |  |  |  |  |  |  |  |  |  |  |  |  |  |  |  |  |  |  |  |  |  |  |  |  |  |  |  |  |  |  |  |  |  |  |  |  |  |  |  |  |  |  |  |  |  |  |  |  |  |  |  |  |  |  |  |  |  |  |  |  |  |  |  |  |  |  |  |  |  |  |  |  |  |  |  |  |  |  |  |  |  |  |  |  |  |  |  |  |  |  |  |  |  |  |  |  |  |  |  |  |  |  |  |  |  |  |  |  |  |  |  |  |  |  |  |  |  |  |  |  |  |  |  |  |  |  |  |  |  |  |  |  |  |  |  |  |  |  |  |  |  |  |  |  |  |  |  |  |  |  |  |  |  |  |  |  |  |  |  |  |  |  |  |  |  |  |  |  |  |  |  |  |  |  |  |  |  |  |  |  |  |  |  |  |  |  |  |  |  |  |  |  |  |  |  |  |  |  |  |  |  |  |  |  |  |  |  |  |  |  |  |  |  |  |  |  |  |  |  |  |  |  |  |  |  |  |  |  |  |  |  |  |  |  |  |  |  |  |  |  |  |  |  |  |  |  |  |  |  |  |  |  |  |  |  |  |  |  |  |  |  |  |  |  |  |  |  |  |  |  |  |  |  |  |  |  |  |  |  |  |  |  |  |  |  |  |  |  |  |  |  |  |  |  |  |  |  |  |  |  |  |  |  |  |  |  |  |  |  |  |  |  |  |  |  |  |  |  |  |  |  |  |  |  |  |  |  |  |  |  |  |  |  |  |  |  |  |  |  |  |  |  |  |  |  |  |  |  |  |  |  |  |  |  |  |  |  |  |  |  |  |  |  |  |  |  |  |  |  |  |  |  |  |  |  |  |  |  |  |  |  |  |  |  |  |  |  |  |  |  |  |  |  |  |  |  |  |  |  |  |  |  |  |  |  |  |  |  |  |  |  |  |  |  |  |  |  |  |  |  |  |  |  |  |  |  |  |  |  |  |  |  |  |  |  |  |  |  |  |  |  |  |  |  |  |  |  |  |  |  |  |  |  |  |  |  |  |  |  |  |  |  |  |  |  |  |  |  |  |  |  |  |  |  |  |  |  |  |  |  |  |  |  |  |  |  |  |  |  |  |  |  |  |  |  |  |  |  |  |  |  |  |  |  |  |  |  |  |  |  |  |  |  |  |  |  |  |  |  |  |  |  |  |  |  |  |  |  |  |  |  |  |  |  |  |  |  |  |  |  |  |  |  |  |  |  |  |  |  |  |  |  |  |  |  |  |  |  |  |  |  |  |  |  |  |  |  |  |  |  |  |  |  |  |  |  |  |  |  |  |  |  |  |  |  |  |  |  |  |  |  |  |  |  |  |  |  |  |  |  |  |  |  |  |  |  |  |  |  |  |  |  |  |  |  |  |  |  |  |  |  |  |  |  |  |  |  |  |  |  |  |  |  |  |  |  |  |  |  |  |  |  |  |  |  |  |  |  |  |  |  |  |  |  |  |  |  |  |  |  |  |  |  |  |  |  |  |  |  |  |  |  |  |  |  |  |  |  |  |  |  |  |  |  |
|  |  |  |  |  |  |  |  |  |  |  |  |  |  |  |  |  |  |  |  |  |  |  |  |  |  |  |  |  |  |  |  |  |  |  |  |  |  |  |  |  |  |  |  |  |  |  |  |  |  |  |  |  |  |  |  |  |  |  |  |  |  |  |  |  |  |  |  |  |  |  |  |  |  |  |  |  |  |  |  |  |  |  |  |  |  |  |  |  |  |  |  |  |  |  |  |  |  |  |  |  |  |  |  |  |  |  |  |  |  |  |  |  |  |  |  |  |  |  |  |  |  |  |  |  |  |  |  |  |  |  |  |  |  |  |  |  |  |  |  |  |  |  |  |  |  |  |  |  |  |  |  |  |  |  |  |  |  |  |  |  |  |  |  |  |  |  |  |  |  |  |  |  |  |  |  |  |  |  |  |  |  |  |  |  |  |  |  |  |  |  |  |  |  |  |  |  |  |  |  |  |  |  |  |  |  |  |  |  |  |  |  |  |  |  |  |  |  |  |  |  |  |  |  |  |  |  |  |  |  |  |  |  |  |  |  |  |  |  |  |  |  |  |  |  |  |  |  |  |  |  |  |  |  |  |  |  |  |  |  |  |  |  |  |  |  |  |  |  |  |  |  |  |  |  |  |  |  |  |  |  |  |  |  |  |  |  |  |  |  |  |  |  |  |  |  |  |  |  |  |  |  |  |  |  |  |  |  |  |  |  |  |  |  |  |  |  |  |  |  |  |  |  |  |  |  |  |  |  |  |  |  |  |  |  |  |  |  |  |  |  |  |  |  |  |  |  |  |  |  |  |  |  |  |  |  |  |  |  |  |  |  |  |  |  |  |  |  |  |  |  |  |  |  |  |  |  |  |  |  |  |  |  |  |  |  |  |  |  |  |  |  |  |  |  |  |  |  |  |  |  |  |  |  |  |  |  |  |  |  |  |  |  |  |  |  |  |  |  |  |  |  |  |  |  |  |  |  |  |  |  |  |  |  |  |  |  |  |  |  |  |  |  |  |  |  |  |  |  |  |  |  |  |  |  |  |  |  |  |  |  |  |  |  |  |  |  |  |  |  |  |  |  |  |  |  |  |  |  |  |  |  |  |  |  |  |  |  |  |  |  |  |  |  |  |  |  |  |  |  |  |  |  |  |  |  |  |  |  |  |  |  |  |  |  |  |  |  |  |  |  |  |  |  |  |  |  |  |  |  |  |  |  |  |  |  |  |  |  |  |  |  |  |  |  |  |  |  |  |  |  |  |  |  |  |  |  |  |  |  |  |  |  |  |  |  |  |  |  |  |  |  |  |  |  |  |  |  |  |  |  |  |  |  |  |  |  |  |  |  |  |  |  |  |  |  |  |  |  |  |  |  |  |  |  |  |  |  |  |  |  |  |  |  |  |  |  |  |  |  |  |  |  |  |  |  |  |  |  |  |  |  |  |  |  |  |  |  |  |  |  |  |  |  |  |  |  |  |  |  |  |  |  |  |  |  |  |  |  |  |  |  |  |  |  |  |  |  |  |  |  |  |  |  |  |  |  |  |  |  |  |  |  |  |  |  |  |  |  |  |  |  |  |  |  |  |  |  |  |  |  |  |  |  |  |  |  |  |  |  |  |  |  |  |  |  |  |  |  |  |  |  |  |  |  |  |  |  |  |  |  |  |  |  |  |  |  |  |  |  |  |  |  |  |  |  |  |  |  |  |  |  |  |  |  |  |  |  |  |  |  |  |  |  |  |  |  |  |  |  |  |  |  |  |  |  |  |  |  |  |  |  |  |  |  |  |  |  |  |  |  |  |  |  |  |  |  |  |  |  |  |  |  |  |  |  |  |  |  |  |  |  |  |  |  |  |  |  |  |  |  |  |  |  |  |  |  |  |  |  |  |  |  |  |  |  |  |  |  |  |  |  |  |  |  |  |  |  |  |  |  |  |  |  |  |  |  |  |  |  |  |  |  |  |  |  |  |  |  |  |  |  |  |  |  |  |  |  |  |  |  |  |  |  |  |  |  |  |  |  |  |  |  |  |  |  |  |  |  |  |  |  |  |  |  |  |  |  |  |  |  |  |  |  |  |  |  |  |  |  |  |  |  |  |  |  |  |  |  |  |  |  |  |  |  |  |  |  |  |  |  |  |  |  |  |  |  |  |  |  |  |  |  |  |  |  |  |  |  |  |  |  |  |  |  |  |  |  |  |  |  |  |  |  |  |  |  |  |  |  |  |  |  |  |  |  |  |  |  |  |  |  |  |  |  |  |  |  |  |  |  |  |  |  |  |  |  |  |  |  |  |  |  |  |  |  |  |  |  |  |  |  |  |  |  |  |  |  |  |  |  |  |  |  |  |  |  |  |  |  |  |  |  |  |  |  |  |  |  |  |  |  |  |  |  |  |  |  |  |  |  |  |  |  |  |  |  |  |  |  |  |  |  |  |  |  |  |  |  |  |  |  |  |  |  |  |  |  |  |  |  |  |  |  |  |  |  |  |  |  |  |  |  |  |  |  |  |  |  |  |  |  |  |  |  |  |  |  |  |  |  |  |  |  |  |  |  |  |  |  |  |  |  |  |  |  |  |  |  |  |  |  |  |  |  |  |  |  |  |  |  |  |  |  |  |  |  |  |  |  |  |  |  |  |  |  |  |  |  |  |  |  |  |  |  |  |  |  |  |  |  |  |  |  |  |  |  |  |  |  |  |  |  |  |  |  |  |  |  |  |  |  |  |  |  |  |  |  |  |  |  |  |  |  |  |  |  |  |  |  |  |  |  |  |  |  |  |  |  |  |  |  |  |  |  |  |  |  |  |  |  |  |  |  |  |  |  |  |  |  |  |  |  |  |  |  |  |  |  |  |  |  |  |  |  |  |  |  |  |  |  |  |  |  |  |  |  |  |  |  |  |  |  |  |  |  |  |  |  |  |  |  |  |  |  |  |  |  |  |  |  |  |  |  |  |  |  |  |  |  |  |  |  |  |  |  |  |  |  |  |  |  |  |  |  |  |  |  |  |  |  |  |  |  |  |  |  |  |  |  |  |  |  |  |  |  |  |  |  |  |  |  |  |  |  |  |  |  |  |  |  |  |  |  |  |  |  |  |  |  |  |  |  |  |  |  |  |  |  |  |  |  |  |  |  |  |  |  |  |  |  |  |  |  |  |  |  |  |  |  |  |  |  |  |  |  |  |  |  |  |  |  |  |  |  |  |  |  |  |  |  |  |  |  |  |  |  |  |  |  |  |  |  |  |  |
|  |  |  |  |  |  |  |  |  |  |  |  |  |  |  |  |  |  |  |  |  |  |  |  |  |  |  |  |  |  |  |  |  |  |  |  |  |  |  |  |  |  |  |  |  |  |  |  |  |  |  |  |  |  |  |  |  |  |  |  |  |  |  |  |  |  |  |  |  |  |  |  |  |  |  |  |  |  |  |  |  |  |  |  |  |  |  |  |  |  |  |  |  |  |  |  |  |  |  |  |  |  |  |  |  |  |  |  |  |  |  |  |  |  |  |  |  |  |  |  |  |  |  |  |  |  |  |  |  |  |  |  |  |  |  |  |  |  |  |  |  |  |  |  |  |  |  |  |  |  |  |  |  |  |  |  |  |  |  |  |  |  |  |  |  |  |  |  |  |  |  |  |  |  |  |  |  |  |  |  |  |  |  |  |  |  |  |  |  |  |  |  |  |  |  |  |  |  |  |  |  |  |  |  |  |  |  |  |  |  |  |  |  |  |  |  |  |  |  |  |  |  |  |  |  |  |  |  |  |  |  |  |  |  |  |  |  |  |  |  |  |  |  |  |  |  |  |  |  |  |  |  |  |  |  |  |  |  |  |  |  |  |  |  |  |  |  |  |  |  |  |  |  |  |  |  |  |  |  |  |  |  |  |  |  |  |  |  |  |  |  |  |  |  |  |  |  |  |  |  |  |  |  |  |  |  |  |  |  |  |  |  |  |  |  |  |  |  |  |  |  |  |  |  |  |  |  |  |  |  |  |  |  |  |  |  |  |  |  |  |  |  |  |  |  |  |  |  |  |  |  |  |  |  |  |  |  |  |  |  |  |  |  |  |  |  |  |  |  |  |  |  |  |  |  |  |  |  |  |  |  |  |  |  |  |  |  |  |  |  |  |  |  |  |  |  |  |  |  |  |  |  |  |  |  |  |  |  |  |  |  |  |  |  |  |  |  |  |  |  |  |  |  |  |  |  |  |  |  |  |  |  |  |  |  |  |  |  |  |  |  |  |  |  |  |  |  |  |  |  |  |  |  |  |  |  |  |  |  |  |  |  |  |  |  |  |  |  |  |  |  |  |  |  |  |  |  |  |  |  |  |  |  |  |  |  |  |  |  |  |  |  |  |  |  |  |  |  |  |  |  |  |  |  |  |  |  |  |  |  |  |  |  |  |  |  |  |  |  |  |  |  |  |  |  |  |  |  |  |  |  |  |  |  |  |  |  |  |  |  |  |  |  |  |  |  |  |  |  |  |  |  |  |  |  |  |  |  |  |  |  |  |  |  |  |  |  |  |  |  |  |  |  |  |  |  |  |  |  |  |  |  |  |  |  |  |  |  |  |  |  |  |  |  |  |  |  |  |  |  |  |  |  |  |  |  |  |  |  |  |  |  |  |  |  |  |  |  |  |  |  |  |  |  |  |  |  |  |  |  |  |  |  |  |  |  |  |  |  |  |  |  |  |  |  |  |  |  |  |  |  |  |  |  |  |  |  |  |  |  |  |  |  |  |  |  |  |  |  |  |  |  |  |  |  |  |  |  |  |  |  |  |  |  |  |  |  |  |  |  |  |  |  |  |  |  |  |  |  |  |  |  |  |  |  |  |  |  |  |  |  |  |  |  |  |  |  |  |  |  |  |  |  |  |  |  |  |  |  |  |  |  |  |  |  |  |  |  |  |  |  |  |  |  |  |  |  |  |  |  |  |  |  |  |  |  |  |  |  |  |  |  |  |  |  |  |  |  |  |  |  |  |  |  |  |  |  |  |  |  |  |  |  |  |  |  |  |  |  |  |  |  |  |  |  |  |  |  |  |  |  |  |  |  |  |  |  |  |  |  |  |  |  |  |  |  |  |  |  |  |  |  |  |  |  |  |  |  |  |  |  |  |  |  |  |  |  |  |  |  |  |  |  |  |  |  |  |  |  |  |  |  |  |  |  |  |  |  |  |  |  |  |  |  |  |  |  |  |  |  |  |  |  |  |  |  |  |  |  |  |  |  |  |  |  |  |  |  |  |  |  |  |  |  |  |  |  |  |  |  |  |  |  |  |  |  |  |  |  |  |  |  |  |  |  |  |  |  |  |  |  |  |  |  |  |  |  |  |  |  |  |  |  |  |  |  |  |  |  |  |  |  |  |  |  |  |  |  |  |  |  |  |  |  |  |  |  |  |  |  |  |  |  |  |  |  |  |  |  |  |  |  |  |  |  |  |  |  |  |  |  |  |  |  |  |  |  |  |  |  |  |  |  |  |  |  |  |  |  |  |  |  |  |  |  |  |  |  |  |  |  |  |  |  |  |  |  |  |  |  |  |  |  |  |  |  |  |  |  |  |  |  |  |  |  |  |  |  |  |  |  |  |  |  |  |  |  |  |  |  |  |  |  |  |  |  |  |  |  |  |  |  |  |  |  |  |  |  |  |  |  |  |  |  |  |  |  |  |  |  |  |  |  |  |  |  |  |  |  |  |  |  |  |  |  |  |  |  |  |  |  |  |  |  |  |  |  |  |  |  |  |  |  |  |  |  |  |  |  |  |  |  |  |  |  |  |  |  |  |  |  |  |  |  |  |  |  |  |  |  |  |  |  |  |  |  |  |  |  |  |  |  |  |  |  |  |  |  |  |  |  |  |  |  |  |  |  |  |  |  |  |  |  |  |  |  |  |  |  |  |  |  |  |  |  |  |  |  |  |  |  |  |  |  |  |  |  |  |  |  |  |  |  |  |  |  |  |  |  |  |  |  |  |  |  |  |  |  |  |  |  |  |  |  |  |  |  |  |  |  |  |  |  |  |  |  |  |  |  |  |  |  |  |  |  |  |  |  |  |  |  |  |  |  |  |  |  |  |  |  |  |  |  |  |  |  |  |  |  |  |  |  |  |  |  |  |  |  |  |  |  |  |  |  |  |  |  |  |  |  |  |  |  |  |  |  |  |  |  |  |  |  |  |  |  |  |  |  |  |  |  |  |  |  |  |  |  |  |  |  |  |  |  |  |  |  |  |  |  |  |  |  |  |  |  |  |  |  |  |  |  |  |  |  |  |  |  |  |  |  |  |  |  |  |  |  |  |  |  |  |  |  |  |  |  |  |  |  |  |  |  |  |  |  |  |  |  |  |  |  |  |  |  |  |  |  |  |  |  |  |  |  |  |  |  |  |  |  |  |  |  |  |  |  |  |  |  |  |  |  |  |  |  |  |  |  |  |  |  |  |  |  |  |  |  |  |  |  |  |  |  |  |  |  |  |
|  |  |  |  |  |  |  |  |  |  |  |  |  |  |  |  |  |  |  |  |  |  |  |  |  |  |  |  |  |  |  |  |  |  |  |  |  |  |  |  |  |  |  |  |  |  |  |  |  |  |  |  |  |  |  |  |  |  |  |  |  |  |  |  |  |  |  |  |  |  |  |  |  |  |  |  |  |  |  |  |  |  |  |  |  |  |  |  |  |  |  |  |  |  |  |  |  |  |  |  |  |  |  |  |  |  |  |  |  |  |  |  |  |  |  |  |  |  |  |  |  |  |  |  |  |  |  |  |  |  |  |  |  |  |  |  |  |  |  |  |  |  |  |  |  |  |  |  |  |  |  |  |  |  |  |  |  |  |  |  |  |  |  |  |  |  |  |  |  |  |  |  |  |  |  |  |  |  |  |  |  |  |  |  |  |  |  |  |  |  |  |  |  |  |  |  |  |  |  |  |  |  |  |  |  |  |  |  |  |  |  |  |  |  |  |  |  |  |  |  |  |  |  |  |  |  |  |  |  |  |  |  |  |  |  |  |  |  |  |  |  |  |  |  |  |  |  |  |  |  |  |  |  |  |  |  |  |  |  |  |  |  |  |  |  |  |  |  |  |  |  |  |  |  |  |  |  |  |  |  |  |  |  |  |  |  |  |  |  |  |  |  |  |  |  |  |  |  |  |  |  |  |  |  |  |  |  |  |  |  |  |  |  |  |  |  |  |  |  |  |  |  |  |  |  |  |  |  |  |  |  |  |  |  |  |  |  |  |  |  |  |  |  |  |  |  |  |  |  |  |  |  |  |  |  |  |  |  |  |  |  |  |  |  |  |  |  |  |  |  |  |  |  |  |  |  |  |  |  |  |  |  |  |  |  |  |  |  |  |  |  |  |  |  |  |  |  |  |  |  |  |  |  |  |  |  |  |  |  |  |  |  |  |  |  |  |  |  |  |  |  |  |  |  |  |  |  |  |  |  |  |  |  |  |  |  |  |  |  |  |  |  |  |  |  |  |  |  |  |  |  |  |  |  |  |  |  |  |  |  |  |  |  |  |  |  |  |  |  |  |  |  |  |  |  |  |  |  |  |  |  |  |  |  |  |  |  |  |  |  |  |  |  |  |  |  |  |  |  |  |  |  |  |  |  |  |  |  |  |  |  |  |  |  |  |  |  |  |  |  |  |  |  |  |  |  |  |  |  |  |  |  |  |  |  |  |  |  |  |  |  |  |  |  |  |  |  |  |  |  |  |  |  |  |  |  |  |  |  |  |  |  |  |  |  |  |  |  |  |  |  |  |  |  |  |  |  |  |  |  |  |  |  |  |  |  |  |  |  |  |  |  |  |  |  |  |  |  |  |  |  |  |  |  |  |  |  |  |  |  |  |  |  |  |  |  |  |  |  |  |  |  |  |  |  |  |  |  |  |  |  |  |  |  |  |  |  |  |  |  |  |  |  |  |  |  |  |  |  |  |  |  |  |  |  |  |  |  |  |  |  |  |  |  |  |  |  |  |  |  |  |  |  |  |  |  |  |  |  |  |  |  |  |  |  |  |  |  |  |  |  |  |  |  |  |  |  |  |  |  |  |  |  |  |  |  |  |  |  |  |  |  |  |  |  |  |  |  |  |  |  |  |  |  |  |  |  |  |  |  |  |  |  |  |  |  |  |  |  |  |  |  |  |  |  |  |  |  |  |  |  |  |  |  |  |  |  |  |  |  |  |  |  |  |  |  |  |  |  |  |  |  |  |  |  |  |  |  |  |  |  |  |  |  |  |  |  |  |  |  |  |  |  |  |  |  |  |  |  |  |  |  |  |  |  |  |  |  |  |  |  |  |  |  |  |  |  |  |  |  |  |  |  |  |  |  |  |  |  |  |  |  |  |  |  |  |  |  |  |  |  |  |  |  |  |  |  |  |  |  |  |  |  |  |  |  |  |  |  |  |  |  |  |  |  |  |  |  |  |  |  |  |  |  |  |  |  |  |  |  |  |  |  |  |  |  |  |  |  |  |  |  |  |  |  |  |  |  |  |  |  |  |  |  |  |  |  |  |  |  |  |  |  |  |  |  |  |  |  |  |  |  |  |  |  |  |  |  |  |  |  |  |  |  |  |  |  |  |  |  |  |  |  |  |  |  |  |  |  |  |  |  |  |  |  |  |  |  |  |  |  |  |  |  |  |  |  |  |  |  |  |  |  |  |  |  |  |  |  |  |  |  |  |  |  |  |  |  |  |  |  |  |  |  |  |  |  |  |  |  |  |  |  |  |  |  |  |  |  |  |  |  |  |  |  |  |  |  |  |  |  |  |  |  |  |  |  |  |  |  |  |  |  |  |  |  |  |  |  |  |  |  |  |  |  |  |  |  |  |  |  |  |  |  |  |  |  |  |  |  |  |  |  |  |  |  |  |  |  |  |  |  |  |  |  |  |  |  |  |  |  |  |  |  |  |  |  |  |  |  |  |  |  |  |  |  |  |  |  |  |  |  |  |  |  |  |  |  |  |  |  |  |  |  |  |  |  |  |  |  |  |  |  |  |  |  |  |  |  |  |  |  |  |  |  |  |  |  |  |  |  |  |  |  |  |  |  |  |  |  |  |  |  |  |  |  |  |  |  |  |  |  |  |  |  |  |  |  |  |  |  |  |  |  |  |  |  |  |  |  |  |  |  |  |  |  |  |  |  |  |  |  |  |  |  |  |  |  |  |  |  |  |  |  |  |  |  |  |  |  |  |  |  |  |  |  |  |  |  |  |  |  |  |  |  |  |  |  |  |  |  |  |  |  |  |  |  |  |  |  |  |  |  |  |  |  |  |  |  |  |  |  |  |  |  |  |  |  |  |  |  |  |  |  |  |  |  |  |  |  |  |  |  |  |  |  |  |  |  |  |  |  |  |  |  |  |  |  |  |  |  |  |  |  |  |  |  |  |  |  |  |  |  |  |  |  |  |  |  |  |  |  |  |  |  |  |  |  |  |  |  |  |  |  |  |  |  |  |  |  |  |  |  |  |  |  |  |  |  |  |  |  |  |  |  |  |  |  |  |  |  |  |  |  |  |  |  |  |  |  |  |  |  |  |  |  |  |  |  |  |  |  |  |  |  |  |  |  |  |  |  |  |  |  |  |  |  |  |  |  |  |  |  |  |  |  |  |  |  |  |  |  |  |  |  |  |  |  |  |  |  |  |  |  |  |  |  |  |  |  |  |  |  |  |  |  |  |  |  |  |  |  |  |  |  |
