# Supplementary material for: Variation in selection constraints on teleost TLRs with emphasis on their repertoire in the Walking catfish, Clarias batrachus
Source: Sci Rep. 2020 Dec 7;10:21394. doi: 10.1038/s41598-020-78347-6 (PMC7721727; doi:10.1038/s41598-020-78347-6)
Supplement: Supplementary file 31 — Supplementary Information 31. [file 41598_2020_78347_MOESM31_ESM.zip › T9/BIS2/summary/PF00000-NONREDUNDANT-5DD-dim0-table.html]

BIS cluster table


Clusters with env. score >= 0.5 and sym. score >= 0.5 :

| Dim | Cluster | Sym | Env | Pvalue | Hit patterns and blocks |
| --- | --- | --- | --- | --- | --- |
| 0 | 7 | 1 | 1 | 6.069968e-09 | Hit patterns:   |  |  |  | | --- | --- | --- | | Positions: | 462 | 619 | | 15 sequences: | N | Y | | 5 sequences: | E | S | | 4 sequences: | D | F |  All positions in cluster: 462 618-619 |
| 0 | 5 | 1 | 1 | 4.006179e-07 | Hit patterns:   |  |  |  |  | | --- | --- | --- | --- | | Positions: | 611 | 863 | 1035 | | 13 sequences: | S | D | S | | 11 sequences: | A | S | T |  All positions in cluster: 611 862-864 1032-1035 |
| 0 | 1 | 1 | 1 | 7.64816e-07 | Hit patterns:   |  |  |  |  |  |  | | --- | --- | --- | --- | --- | --- | | Positions: | 912 | 985 | 999 | 1058 | 1088 | | 15 sequences: | Y | V | R | M | L | | 9 sequences: | F | I | W | L | M |  All positions in cluster: 912-913 983-986 999 1058-1066 1084-1089 |
| 0 | 3 | 1 | 1 | 2.35272e-05 | Hit patterns:   |  |  |  |  | | --- | --- | --- | --- | | Positions: | 950 | 955 | 987 | | 19 sequences: | A | Y | N | | 5 sequences: | T | H | K |  All positions in cluster: 948-950 954-955 986-988 |
| 0 | 2 | 1 | 1 | 9.410879e-05 | Hit patterns:   |  |  |  |  | | --- | --- | --- | --- | | Positions: | 647 | 968 | 1129 | | 20 sequences: | M | Y | N | | 4 sequences: | I | H | K |  All positions in cluster: 647-649 968-971 1129 |
| 0 | 4 | 1 | 1 | 0.003623188 | Hit patterns:   |  |  |  |  |  | | --- | --- | --- | --- | --- | | Positions: | 402 | 666 | 667 | 881 | | 22 sequences: | L | A | N | P | | 2 sequences: | F | S | D | L |  All positions in cluster: 402 666-668 881 |
| 0 | 6 | 1 | 1 | 0.003623188 | Hit patterns:   |  |  |  | | --- | --- | --- | | Positions: | 629 | 869 | | 22 sequences: | T | F | | 2 sequences: | S | L |  All positions in cluster: 629-631 869 |
| 0 | 8 | 1 | 1 | 1 | All positions in cluster: 128 131 134 139 141-144 150 153 159 161 164 176 178 183 186-187 191-193 199 207 210 265 274 325-328 330 332 340-341 345 353 356-357 359 366 368-369 378 381 383-385 387-388 392 408 410 413-414 429 434-437 553 560 562 564 576-577 582 585-586 588-589 592 594 599 605 608 613-614 616 618 630-631 633-634 638 641-642 644 648-649 653 660 663 668 673 682 688 690-691 693 697 704 708 711 714 717-720 722 724 736 742 749 752 754 756 763 766 768 771 776-777 790-793 795 801 804 814 816 819 822 830 853 855-856 858-860 862 864 867 873 883 885 889 892-894 904 907-909 913 931-932 935-940 942-943 946 948-949 954 969-971 973-976 980-981 983-984 986 988 994 1003-1010 1012-1013 1016-1017 1019-1021 1024 1027 1029-1030 1032-1034 1050 1054 1056 1059-1066 1069 1071 1077 1080-1082 1084-1087 1089 1091-1093 1095 1097 1100-1101 1104 1107-1112 1118 1121 1126 1132 1134 |

Table created with bis2html version 8.
