# Supplementary material for: Variation in selection constraints on teleost TLRs with emphasis on their repertoire in the Walking catfish, Clarias batrachus
Source: Sci Rep. 2020 Dec 7;10:21394. doi: 10.1038/s41598-020-78347-6 (PMC7721727; doi:10.1038/s41598-020-78347-6)
Supplement: Supplementary file 31 — Supplementary Information 31. [file 41598_2020_78347_MOESM31_ESM.zip › T9/BIS2/summary/PF00000-NONREDUNDANT-5DD-dim1.html]

Alignment and BIS clusters


|  |  |  |  |  |  |  |  |  |  |  |  |  |  |  |  |  |  |  |  |  |  |  |  |  |  |  |  |  |  |  |  |  |  |  |  |  |  |  |  |  |  |  |  |  |  |  |  |  |  |  |  |  |  |  |  |  |  |  |  |  |  |  |  |  |  |  |  |  |  |  |  |  |  |  |  |  |  |  |  |  |  |  |  |  |  |  |  |  |  |  |  |  |  |  |  |  |  |  |  |  |  |  |  |  |  |  |  |  |  |  |  |  |  |  |  |  |  |  |  |  |  |  |  |  |  |  |  |  |  |  |  |  |  |  |  |  |  |  |  |  |  |  |  |  |  |  |  |  |  |  |  |  |  |  |  |  |  |  |  |  |  |  |  |  |  |  |  |  |  |  |  |  |  |  |  |  |  |  |  |  |  |  |  |  |  |  |  |  |  |  |  |  |  |  |  |  |  |  |  |  |  |  |  |  |  |  |  |  |  |  |  |  |  |  |  |  |  |  |  |  |  |  |  |  |  |  |  |  |  |  |  |  |  |  |  |  |  |  |  |  |  |  |  |  |  |  |  |  |  |  |  |  |  |  |  |  |  |  |  |  |  |  |  |  |  |  |  |  |  |  |  |  |  |  |  |  |  |  |  |  |  |  |  |  |  |  |  |  |  |  |  |  |  |  |  |  |  |  |  |  |  |  |  |  |  |  |  |  |  |  |  |  |  |  |  |  |  |  |  |  |  |  |  |  |  |  |  |  |  |  |  |  |  |  |  |  |  |  |  |  |  |  |  |  |  |  |  |  |  |  |  |  |  |  |  |  |  |  |  |  |  |  |  |  |  |  |  |  |  |  |  |  |  |  |  |  |  |  |  |  |  |  |  |  |  |  |  |  |  |  |  |  |  |  |  |  |  |  |  |  |  |  |  |  |  |  |  |  |  |  |  |  |  |  |  |  |  |  |  |  |  |  |  |  |  |  |  |  |  |  |  |  |  |  |  |  |  |  |  |  |  |  |  |  |  |  |  |  |  |  |  |  |  |  |  |  |  |  |  |  |  |  |  |  |  |  |  |  |  |  |  |  |  |  |  |  |  |  |  |  |  |  |  |  |  |  |  |  |  |  |  |  |  |  |  |  |  |  |  |  |  |  |  |  |  |  |  |  |  |  |  |  |  |  |  |  |  |  |  |  |  |  |  |  |  |  |  |  |  |  |  |  |  |  |  |  |  |  |  |  |  |  |  |  |  |  |  |  |  |  |  |  |  |  |  |  |  |  |  |  |  |  |  |  |  |  |  |  |  |  |  |  |  |  |  |  |  |  |  |  |  |  |  |  |  |  |  |  |  |  |  |  |  |  |  |  |  |  |  |  |  |  |  |  |  |  |  |  |  |  |  |  |  |  |  |  |  |  |  |  |  |  |  |  |  |  |  |  |  |  |  |  |  |  |  |  |  |  |  |  |  |  |  |  |  |  |  |  |  |  |  |  |  |  |  |  |  |  |  |  |  |  |  |  |  |  |  |  |  |  |  |  |  |  |  |  |  |  |  |  |  |  |  |  |  |  |  |  |  |  |  |  |  |  |  |  |  |  |  |  |  |  |  |  |  |  |  |  |  |  |  |  |  |  |  |  |  |  |  |  |  |  |  |  |  |  |  |  |  |  |  |  |  |  |  |  |  |  |  |  |  |  |  |  |  |  |  |  |  |  |  |  |  |  |  |  |  |  |  |  |  |  |  |  |  |  |  |  |  |  |  |  |  |  |  |  |  |  |  |  |  |  |  |  |  |  |  |  |  |  |  |  |  |  |  |  |  |  |  |  |  |  |  |  |  |  |  |  |  |  |  |  |  |  |  |  |  |  |  |  |  |  |  |  |  |  |  |  |  |  |  |  |  |  |  |  |  |  |  |  |  |  |  |  |  |  |  |  |  |  |  |  |  |  |  |  |  |  |  |  |  |  |  |  |  |  |  |  |  |  |  |  |  |  |  |  |  |  |  |  |  |  |  |  |  |  |  |  |  |  |  |  |  |  |  |  |  |  |  |  |  |  |  |  |  |  |  |  |  |  |  |  |  |  |  |  |  |  |  |  |  |  |  |  |  |  |  |  |  |  |  |  |  |  |  |  |  |  |  |  |  |  |  |  |  |  |  |  |  |  |  |  |  |  |  |  |  |  |  |  |  |  |  |  |  |  |  |  |  |  |  |  |  |  |  |  |  |  |  |  |  |  |  |  |  |  |  |  |  |  |  |  |  |  |  |  |  |  |  |  |  |  |  |  |  |  |  |  |  |  |  |  |  |  |  |  |  |  |  |  |  |  |  |  |  |  |  |  |  |  |  |  |  |  |  |  |  |  |  |  |  |  |  |  |  |  |  |  |  |  |  |  |  |  |  |  |  |  |  |  |  |  |  |  |  |  |  |  |  |  |  |  |  |  |  |  |  |  |  |  |  |  |  |  |  |  |  |  |  |  |  |  |  |  |  |  |  |  |  |  |  |  |  |  |  |  |  |  |  |  |  |  |  |  |  |  |  |  |  |  |  |  |  |  |  |  |  |  |  |  |  |  |  |  |  |  |  |  |  |  |  |  |  |  |  |  |  |  |  |  |  |  |  |  |  |  |  |  |  |  |  |  |  |  |  |  |  |  |  |  |  |  |  |  |  |  |  |  |  |  |  |  |  |  |  |  |  |  |  |  |  |  |  |  |  |  |  |  |  |  |  |  |  |  |  |  |  |  |  |  |  |  |  |  |  |  |  |  |  |  |  |  |  |  |  |  |  |  |  |  |  |  |  |  |  |  |  |  |  |  |  |  |  |  |  |  |  |  |  |  |  |  |  |  |  |  |  |  |  |  |  |  |  |  |  |  |  |  |  |  |  |  |  |  |  |  |  |  |  |  |  |  |  |  |  |  |  |  |  |  |  |  |  |  |  |  |  |  |  |  |  |  |  |  |  |  |  |  |  |  |  |  |  |  |  |  |  |  |  |  |  |  |  |  |  |  |  |  |  |  |  |  |  |  |  |  |  |  |  |  |  |  |  |  |  |  |
| --- | --- | --- | --- | --- | --- | --- | --- | --- | --- | --- | --- | --- | --- | --- | --- | --- | --- | --- | --- | --- | --- | --- | --- | --- | --- | --- | --- | --- | --- | --- | --- | --- | --- | --- | --- | --- | --- | --- | --- | --- | --- | --- | --- | --- | --- | --- | --- | --- | --- | --- | --- | --- | --- | --- | --- | --- | --- | --- | --- | --- | --- | --- | --- | --- | --- | --- | --- | --- | --- | --- | --- | --- | --- | --- | --- | --- | --- | --- | --- | --- | --- | --- | --- | --- | --- | --- | --- | --- | --- | --- | --- | --- | --- | --- | --- | --- | --- | --- | --- | --- | --- | --- | --- | --- | --- | --- | --- | --- | --- | --- | --- | --- | --- | --- | --- | --- | --- | --- | --- | --- | --- | --- | --- | --- | --- | --- | --- | --- | --- | --- | --- | --- | --- | --- | --- | --- | --- | --- | --- | --- | --- | --- | --- | --- | --- | --- | --- | --- | --- | --- | --- | --- | --- | --- | --- | --- | --- | --- | --- | --- | --- | --- | --- | --- | --- | --- | --- | --- | --- | --- | --- | --- | --- | --- | --- | --- | --- | --- | --- | --- | --- | --- | --- | --- | --- | --- | --- | --- | --- | --- | --- | --- | --- | --- | --- | --- | --- | --- | --- | --- | --- | --- | --- | --- | --- | --- | --- | --- | --- | --- | --- | --- | --- | --- | --- | --- | --- | --- | --- | --- | --- | --- | --- | --- | --- | --- | --- | --- | --- | --- | --- | --- | --- | --- | --- | --- | --- | --- | --- | --- | --- | --- | --- | --- | --- | --- | --- | --- | --- | --- | --- | --- | --- | --- | --- | --- | --- | --- | --- | --- | --- | --- | --- | --- | --- | --- | --- | --- | --- | --- | --- | --- | --- | --- | --- | --- | --- | --- | --- | --- | --- | --- | --- | --- | --- | --- | --- | --- | --- | --- | --- | --- | --- | --- | --- | --- | --- | --- | --- | --- | --- | --- | --- | --- | --- | --- | --- | --- | --- | --- | --- | --- | --- | --- | --- | --- | --- | --- | --- | --- | --- | --- | --- | --- | --- | --- | --- | --- | --- | --- | --- | --- | --- | --- | --- | --- | --- | --- | --- | --- | --- | --- | --- | --- | --- | --- | --- | --- | --- | --- | --- | --- | --- | --- | --- | --- | --- | --- | --- | --- | --- | --- | --- | --- | --- | --- | --- | --- | --- | --- | --- | --- | --- | --- | --- | --- | --- | --- | --- | --- | --- | --- | --- | --- | --- | --- | --- | --- | --- | --- | --- | --- | --- | --- | --- | --- | --- | --- | --- | --- | --- | --- | --- | --- | --- | --- | --- | --- | --- | --- | --- | --- | --- | --- | --- | --- | --- | --- | --- | --- | --- | --- | --- | --- | --- | --- | --- | --- | --- | --- | --- | --- | --- | --- | --- | --- | --- | --- | --- | --- | --- | --- | --- | --- | --- | --- | --- | --- | --- | --- | --- | --- | --- | --- | --- | --- | --- | --- | --- | --- | --- | --- | --- | --- | --- | --- | --- | --- | --- | --- | --- | --- | --- | --- | --- | --- | --- | --- | --- | --- | --- | --- | --- | --- | --- | --- | --- | --- | --- | --- | --- | --- | --- | --- | --- | --- | --- | --- | --- | --- | --- | --- | --- | --- | --- | --- | --- | --- | --- | --- | --- | --- | --- | --- | --- | --- | --- | --- | --- | --- | --- | --- | --- | --- | --- | --- | --- | --- | --- | --- | --- | --- | --- | --- | --- | --- | --- | --- | --- | --- | --- | --- | --- | --- | --- | --- | --- | --- | --- | --- | --- | --- | --- | --- | --- | --- | --- | --- | --- | --- | --- | --- | --- | --- | --- | --- | --- | --- | --- | --- | --- | --- | --- | --- | --- | --- | --- | --- | --- | --- | --- | --- | --- | --- | --- | --- | --- | --- | --- | --- | --- | --- | --- | --- | --- | --- | --- | --- | --- | --- | --- | --- | --- | --- | --- | --- | --- | --- | --- | --- | --- | --- | --- | --- | --- | --- | --- | --- | --- | --- | --- | --- | --- | --- | --- | --- | --- | --- | --- | --- | --- | --- | --- | --- | --- | --- | --- | --- | --- | --- | --- | --- | --- | --- | --- | --- | --- | --- | --- | --- | --- | --- | --- | --- | --- | --- | --- | --- | --- | --- | --- | --- | --- | --- | --- | --- | --- | --- | --- | --- | --- | --- | --- | --- | --- | --- | --- | --- | --- | --- | --- | --- | --- | --- | --- | --- | --- | --- | --- | --- | --- | --- | --- | --- | --- | --- | --- | --- | --- | --- | --- | --- | --- | --- | --- | --- | --- | --- | --- | --- | --- | --- | --- | --- | --- | --- | --- | --- | --- | --- | --- | --- | --- | --- | --- | --- | --- | --- | --- | --- | --- | --- | --- | --- | --- | --- | --- | --- | --- | --- | --- | --- | --- | --- | --- | --- | --- | --- | --- | --- | --- | --- | --- | --- | --- | --- | --- | --- | --- | --- | --- | --- | --- | --- | --- | --- | --- | --- | --- | --- | --- | --- | --- | --- | --- | --- | --- | --- | --- | --- | --- | --- | --- | --- | --- | --- | --- | --- | --- | --- | --- | --- | --- | --- | --- | --- | --- | --- | --- | --- | --- | --- | --- | --- | --- | --- | --- | --- | --- | --- | --- | --- | --- | --- | --- | --- | --- | --- | --- | --- | --- | --- | --- | --- | --- | --- | --- | --- | --- | --- | --- | --- | --- | --- | --- | --- | --- | --- | --- | --- | --- | --- | --- | --- | --- | --- | --- | --- | --- | --- | --- | --- | --- | --- | --- | --- | --- | --- | --- | --- | --- | --- | --- | --- | --- | --- | --- | --- | --- | --- | --- | --- | --- | --- | --- | --- | --- | --- | --- | --- | --- | --- | --- | --- | --- | --- | --- | --- | --- | --- | --- | --- | --- | --- | --- | --- | --- | --- | --- | --- | --- | --- | --- | --- | --- | --- | --- | --- | --- | --- | --- | --- | --- | --- | --- | --- | --- | --- | --- | --- | --- | --- | --- | --- | --- | --- | --- | --- | --- | --- | --- | --- | --- | --- | --- | --- | --- | --- | --- | --- | --- | --- | --- | --- | --- | --- | --- | --- | --- | --- | --- | --- | --- | --- | --- | --- | --- | --- | --- | --- | --- | --- | --- | --- | --- | --- | --- | --- | --- | --- | --- | --- | --- | --- | --- | --- | --- | --- | --- | --- | --- | --- | --- | --- | --- | --- | --- | --- | --- | --- | --- | --- | --- | --- | --- | --- | --- | --- | --- | --- | --- | --- | --- | --- | --- | --- | --- | --- | --- | --- | --- | --- | --- | --- | --- | --- | --- | --- | --- | --- | --- | --- | --- | --- | --- | --- | --- | --- | --- | --- | --- | --- | --- | --- | --- | --- | --- | --- | --- | --- | --- | --- | --- | --- | --- | --- | --- | --- | --- | --- | --- | --- | --- | --- | --- | --- | --- | --- | --- | --- | --- | --- | --- | --- | --- | --- | --- | --- | --- | --- | --- | --- | --- | --- | --- | --- | --- | --- | --- | --- | --- | --- | --- | --- | --- | --- | --- | --- | --- | --- | --- | --- | --- | --- | --- | --- | --- | --- | --- | --- | --- | --- | --- | --- | --- | --- | --- | --- | --- | --- | --- | --- | --- | --- | --- | --- | --- | --- | --- | --- | --- | --- | --- | --- | --- | --- | --- | --- | --- | --- | --- | --- | --- | --- | --- | --- | --- | --- | --- | --- | --- | --- | --- | --- | --- | --- | --- | --- | --- | --- | --- | --- | --- | --- | --- | --- | --- | --- | --- | --- | --- | --- | --- | --- | --- | --- | --- | --- | --- | --- | --- | --- | --- | --- | --- | --- | --- | --- | --- | --- | --- | --- | --- | --- | --- | --- | --- | --- | --- | --- | --- | --- | --- | --- | --- | --- | --- | --- | --- | --- | --- | --- | --- | --- | --- | --- | --- | --- | --- | --- | --- | --- | --- | --- | --- | --- | --- | --- | --- | --- | --- | --- | --- | --- | --- | --- | --- | --- | --- | --- | --- | --- | --- | --- | --- | --- | --- | --- | --- | --- | --- | --- | --- | --- | --- | --- | --- | --- | --- | --- | --- | --- | --- | --- | --- | --- | --- | --- | --- | --- | --- | --- | --- | --- | --- | --- | --- | --- | --- | --- | --- | --- | --- | --- | --- | --- | --- | --- | --- | --- | --- | --- | --- | --- | --- | --- | --- | --- | --- | --- | --- | --- | --- | --- | --- | --- | --- | --- | --- | --- | --- | --- | --- | --- | --- | --- | --- | --- | --- | --- | --- | --- | --- | --- | --- | --- | --- | --- | --- | --- | --- | --- | --- | --- | --- | --- | --- | --- | --- | --- | --- | --- | --- | --- | --- | --- | --- | --- | --- | --- | --- | --- | --- | --- | --- | --- |
|  |  |  |  |  |  |  |  | 1 | 0 |  |  |  |  |  |  |  |  | 2 | 0 |  |  |  |  |  |  |  |  | 3 | 0 |  |  |  |  |  |  |  |  | 4 | 0 |  |  |  |  |  |  |  |  | 5 | 0 |  |  |  |  |  |  |  |  | 6 | 0 |  |  |  |  |  |  |  |  | 7 | 0 |  |  |  |  |  |  |  |  | 8 | 0 |  |  |  |  |  |  |  |  | 9 | 0 |  |  |  |  |  |  |  | 1 | 0 | 0 |  |  |  |  |  |  |  | 1 | 1 | 0 |  |  |  |  |  |  |  | 1 | 2 | 0 |  |  |  |  |  |  |  | 1 | 3 | 0 |  |  |  |  |  |  |  | 1 | 4 | 0 |  |  |  |  |  |  |  | 1 | 5 | 0 |  |  |  |  |  |  |  | 1 | 6 | 0 |  |  |  |  |  |  |  | 1 | 7 | 0 |  |  |  |  |  |  |  | 1 | 8 | 0 |  |  |  |  |  |  |  | 1 | 9 | 0 |  |  |  |  |  |  |  | 2 | 0 | 0 |  |  |  |  |  |  |  | 2 | 1 | 0 |  |  |  |  |  |  |  | 2 | 2 | 0 |  |  |  |  |  |  |  | 2 | 3 | 0 |  |  |  |  |  |  |  | 2 | 4 | 0 |  |  |  |  |  |  |  | 2 | 5 | 0 |  |  |  |  |  |  |  | 2 | 6 | 0 |  |  |  |  |  |  |  | 2 | 7 | 0 |  |  |  |  |  |  |  | 2 | 8 | 0 |  |  |  |  |  |  |  | 2 | 9 | 0 |  |  |  |  |  |  |  | 3 | 0 | 0 |  |  |  |  |  |  |  | 3 | 1 | 0 |  |  |  |  |  |  |  | 3 | 2 | 0 |  |  |  |  |  |  |  | 3 | 3 | 0 |  |  |  |  |  |  |  | 3 | 4 | 0 |  |  |  |  |  |  |  | 3 | 5 | 0 |  |  |  |  |  |  |  | 3 | 6 | 0 |  |  |  |  |  |  |  | 3 | 7 | 0 |  |  |  |  |  |  |  | 3 | 8 | 0 |  |  |  |  |  |  |  | 3 | 9 | 0 |  |  |  |  |  |  |  | 4 | 0 | 0 |  |  |  |  |  |  |  | 4 | 1 | 0 |  |  |  |  |  |  |  | 4 | 2 | 0 |  |  |  |  |  |  |  | 4 | 3 | 0 |  |  |  |  |  |  |  | 4 | 4 | 0 |  |  |  |  |  |  |  | 4 | 5 | 0 |  |  |  |  |  |  |  | 4 | 6 | 0 |  |  |  |  |  |  |  | 4 | 7 | 0 |  |  |  |  |  |  |  | 4 | 8 | 0 |  |  |  |  |  |  |  | 4 | 9 | 0 |  |  |  |  |  |  |  | 5 | 0 | 0 |  |  |  |  |  |  |  | 5 | 1 | 0 |  |  |  |  |  |  |  | 5 | 2 | 0 |  |  |  |  |  |  |  | 5 | 3 | 0 |  |  |  |  |  |  |  | 5 | 4 | 0 |  |  |  |  |  |  |  | 5 | 5 | 0 |  |  |  |  |  |  |  | 5 | 6 | 0 |  |  |  |  |  |  |  | 5 | 7 | 0 |  |  |  |  |  |  |  | 5 | 8 | 0 |  |  |  |  |  |  |  | 5 | 9 | 0 |  |  |  |  |  |  |  | 6 | 0 | 0 |  |  |  |  |  |  |  | 6 | 1 | 0 |  |  |  |  |  |  |  | 6 | 2 | 0 |  |  |  |  |  |  |  | 6 | 3 | 0 |  |  |  |  |  |  |  | 6 | 4 | 0 |  |  |  |  |  |  |  | 6 | 5 | 0 |  |  |  |  |  |  |  | 6 | 6 | 0 |  |  |  |  |  |  |  | 6 | 7 | 0 |  |  |  |  |  |  |  | 6 | 8 | 0 |  |  |  |  |  |  |  | 6 | 9 | 0 |  |  |  |  |  |  |  | 7 | 0 | 0 |  |  |  |  |  |  |  | 7 | 1 | 0 |  |  |  |  |  |  |  | 7 | 2 | 0 |  |  |  |  |  |  |  | 7 | 3 | 0 |  |  |  |  |  |  |  | 7 | 4 | 0 |  |  |  |  |  |  |  | 7 | 5 | 0 |  |  |  |  |  |  |  | 7 | 6 | 0 |  |  |  |  |  |  |  | 7 | 7 | 0 |  |  |  |  |  |  |  | 7 | 8 | 0 |  |  |  |  |  |  |  | 7 | 9 | 0 |  |  |  |  |  |  |  | 8 | 0 | 0 |  |  |  |  |  |  |  | 8 | 1 | 0 |  |  |  |  |  |  |  | 8 | 2 | 0 |  |  |  |  |  |  |  | 8 | 3 | 0 |  |  |  |  |  |  |  | 8 | 4 | 0 |  |  |  |  |  |  |  | 8 | 5 | 0 |  |  |  |  |  |  |  | 8 | 6 | 0 |  |  |  |  |  |  |  | 8 | 7 | 0 |  |  |  |  |  |  |  | 8 | 8 | 0 |  |  |  |  |  |  |  | 8 | 9 | 0 |  |  |  |  |  |  |  | 9 | 0 | 0 |  |  |  |  |  |  |  | 9 | 1 | 0 |  |  |  |  |  |  |  | 9 | 2 | 0 |  |  |  |  |  |  |  | 9 | 3 | 0 |  |  |  |  |  |  |  | 9 | 4 | 0 |  |  |  |  |  |  |  | 9 | 5 | 0 |  |  |  |  |  |  |  | 9 | 6 | 0 |  |  |  |  |  |  |  | 9 | 7 | 0 |  |  |  |  |  |  |  | 9 | 8 | 0 |  |  |  |  |  |  |  | 9 | 9 | 0 |  |  |  |  |  |  | 1 | 0 | 0 | 0 |  |  |  |  |  |  | 1 | 0 | 1 | 0 |  |  |  |  |  |  | 1 | 0 | 2 | 0 |  |  |  |  |  |  | 1 | 0 | 3 | 0 |  |  |  |  |  |  | 1 | 0 | 4 | 0 |  |  |  |  |  |  | 1 | 0 | 5 | 0 |  |  |  |  |  |  | 1 | 0 | 6 | 0 |  |  |  |  |  |  | 1 | 0 | 7 | 0 |  |  |  |  |  |  | 1 | 0 | 8 | 0 |  |  |  |  |  |  | 1 | 0 | 9 | 0 |  |  |  |  |  |  | 1 | 1 | 0 | 0 |  |  |  |  |  |  | 1 | 1 | 1 | 0 |  |  |  |  |  |  | 1 | 1 | 2 | 0 |  |  |  |  |  |  | 1 | 1 | 3 | 0 |  |  |  |  |  |  | 1 | 1 | 4 | 0 |  |  |  |  |  |  | 1 | 1 | 5 | 0 |  |  |  |  |  |  | 1 | 1 | 6 | 0 |  |  |  |  |  |  | 1 | 1 | 7 | 0 |  |  |  |  |  |  | 1 | 1 | 8 | 0 |  |  |  |  |  |  | 1 | 1 | 9 | 0 |  |  |  |  |  |  | 1 | 2 | 0 | 0 |  |  |  |  |  |  | 1 | 2 | 1 | 0 |  |  |  |  |  |  | 1 | 2 | 2 | 0 |  |  |  |  |  |  | 1 | 2 | 3 | 0 |  |  |  |  |  |  | 1 | 2 | 4 | 0 |  |  |  |  |  |  | 1 | 2 | 5 | 0 |  |  |  |  |  |  | 1 | 2 | 6 | 0 |  |  |  |  |  |  | 1 | 2 | 7 | 0 |  |  |  |  |  |  | 1 | 2 | 8 | 0 |  |  |  |  |  |  | 1 | 2 | 9 | 0 |  |  |  |  |  |  | 1 | 3 | 0 | 0 |  |  |  |  |  |  | 1 | 3 | 1 | 0 |  |  |  |  |  |  | 1 | 3 | 2 | 0 |  |  |  |  |  |  | 1 | 3 | 3 | 0 |  |  |  |  |  |  | 1 | 3 | 4 | 0 |  |  |  |  |  |  |  |
|  |  |  |  |  |  |  |  |  | | |  |  |  |  |  |  |  |  |  | | |  |  |  |  |  |  |  |  |  | | |  |  |  |  |  |  |  |  |  | | |  |  |  |  |  |  |  |  |  | | |  |  |  |  |  |  |  |  |  | | |  |  |  |  |  |  |  |  |  | | |  |  |  |  |  |  |  |  |  | | |  |  |  |  |  |  |  |  |  | | |  |  |  |  |  |  |  |  |  | | |  |  |  |  |  |  |  |  |  | | |  |  |  |  |  |  |  |  |  | | |  |  |  |  |  |  |  |  |  | | |  |  |  |  |  |  |  |  |  | | |  |  |  |  |  |  |  |  |  | | |  |  |  |  |  |  |  |  |  | | |  |  |  |  |  |  |  |  |  | | |  |  |  |  |  |  |  |  |  | | |  |  |  |  |  |  |  |  |  | | |  |  |  |  |  |  |  |  |  | | |  |  |  |  |  |  |  |  |  | | |  |  |  |  |  |  |  |  |  | | |  |  |  |  |  |  |  |  |  | | |  |  |  |  |  |  |  |  |  | | |  |  |  |  |  |  |  |  |  | | |  |  |  |  |  |  |  |  |  | | |  |  |  |  |  |  |  |  |  | | |  |  |  |  |  |  |  |  |  | | |  |  |  |  |  |  |  |  |  | | |  |  |  |  |  |  |  |  |  | | |  |  |  |  |  |  |  |  |  | | |  |  |  |  |  |  |  |  |  | | |  |  |  |  |  |  |  |  |  | | |  |  |  |  |  |  |  |  |  | | |  |  |  |  |  |  |  |  |  | | |  |  |  |  |  |  |  |  |  | | |  |  |  |  |  |  |  |  |  | | |  |  |  |  |  |  |  |  |  | | |  |  |  |  |  |  |  |  |  | | |  |  |  |  |  |  |  |  |  | | |  |  |  |  |  |  |  |  |  | | |  |  |  |  |  |  |  |  |  | | |  |  |  |  |  |  |  |  |  | | |  |  |  |  |  |  |  |  |  | | |  |  |  |  |  |  |  |  |  | | |  |  |  |  |  |  |  |  |  | | |  |  |  |  |  |  |  |  |  | | |  |  |  |  |  |  |  |  |  | | |  |  |  |  |  |  |  |  |  | | |  |  |  |  |  |  |  |  |  | | |  |  |  |  |  |  |  |  |  | | |  |  |  |  |  |  |  |  |  | | |  |  |  |  |  |  |  |  |  | | |  |  |  |  |  |  |  |  |  | | |  |  |  |  |  |  |  |  |  | | |  |  |  |  |  |  |  |  |  | | |  |  |  |  |  |  |  |  |  | | |  |  |  |  |  |  |  |  |  | | |  |  |  |  |  |  |  |  |  | | |  |  |  |  |  |  |  |  |  | | |  |  |  |  |  |  |  |  |  | | |  |  |  |  |  |  |  |  |  | | |  |  |  |  |  |  |  |  |  | | |  |  |  |  |  |  |  |  |  | | |  |  |  |  |  |  |  |  |  | | |  |  |  |  |  |  |  |  |  | | |  |  |  |  |  |  |  |  |  | | |  |  |  |  |  |  |  |  |  | | |  |  |  |  |  |  |  |  |  | | |  |  |  |  |  |  |  |  |  | | |  |  |  |  |  |  |  |  |  | | |  |  |  |  |  |  |  |  |  | | |  |  |  |  |  |  |  |  |  | | |  |  |  |  |  |  |  |  |  | | |  |  |  |  |  |  |  |  |  | | |  |  |  |  |  |  |  |  |  | | |  |  |  |  |  |  |  |  |  | | |  |  |  |  |  |  |  |  |  | | |  |  |  |  |  |  |  |  |  | | |  |  |  |  |  |  |  |  |  | | |  |  |  |  |  |  |  |  |  | | |  |  |  |  |  |  |  |  |  | | |  |  |  |  |  |  |  |  |  | | |  |  |  |  |  |  |  |  |  | | |  |  |  |  |  |  |  |  |  | | |  |  |  |  |  |  |  |  |  | | |  |  |  |  |  |  |  |  |  | | |  |  |  |  |  |  |  |  |  | | |  |  |  |  |  |  |  |  |  | | |  |  |  |  |  |  |  |  |  | | |  |  |  |  |  |  |  |  |  | | |  |  |  |  |  |  |  |  |  | | |  |  |  |  |  |  |  |  |  | | |  |  |  |  |  |  |  |  |  | | |  |  |  |  |  |  |  |  |  | | |  |  |  |  |  |  |  |  |  | | |  |  |  |  |  |  |  |  |  | | |  |  |  |  |  |  |  |  |  | | |  |  |  |  |  |  |  |  |  | | |  |  |  |  |  |  |  |  |  | | |  |  |  |  |  |  |  |  |  | | |  |  |  |  |  |  |  |  |  | | |  |  |  |  |  |  |  |  |  | | |  |  |  |  |  |  |  |  |  | | |  |  |  |  |  |  |  |  |  | | |  |  |  |  |  |  |  |  |  | | |  |  |  |  |  |  |  |  |  | | |  |  |  |  |  |  |  |  |  | | |  |  |  |  |  |  |  |  |  | | |  |  |  |  |  |  |  |  |  | | |  |  |  |  |  |  |  |  |  | | |  |  |  |  |  |  |  |  |  | | |  |  |  |  |  |  |  |  |  | | |  |  |  |  |  |  |  |  |  | | |  |  |  |  |  |  |  |  |  | | |  |  |  |  |  |  |  |  |  | | |  |  |  |  |  |  |  |  |  | | |  |  |  |  |  |  |  |  |  | | |  |  |  |  |  |  |  |  |  | | |  |  |  |  |  |  |  |  |  | | |  |  |  |  |  |  |  |  |  | | |  |  |  |  |  |  |  |  |  | | |  |  |  |  |  |  |  |  |  | | |  |  |  |  |  |  |  |  |  | | |  |  |  |  |  |  |  |  |  | | |  |  |  |  |  |  |  |  |  | | |  |  |  |  |  |  |  |  |  | | |  |  |  |  |  |  |  |  |  | | |  |  |  |  |  |  |  |  |  | | |  |  |  |  |  |  |  |  |  | | |  |  |  |  |  |  |  |  |  | | |  |  |  |  |  |  |  |  |  | | |  |  |  |  |  |  |  |  |  | | |  |  |  |  |  |  |  |  |  | | |  |  |  |  |  |  |  |
| CBTLR9 | - | - | - | - | - | - | - | - | - | - | - | - | - | - | M | F | L | P | G | L | Y | L | F | F | A | L | D | L | I | K | L | V | X | T | M | N | P | I | F | Y | P | C | D | N | N | T | I | S | N | G | T | I | H | V | D | C | K | R | R | N | L | T | S | I | P | K | F | T | S | L | S | I | S | S | L | N | L | D | E | N | H | V | H | Q | V | K | N | D | D | F | S | N | I | P | N | L | Q | H | L | S | L | M | - | W | N | C | L | P | G | R | L | K | A | L | K | L | P | S | C | Q | V | L | I | D | X | X | A | F | V | N | L | K | X | L | N | S | L | Q | L | A | G | N | S | L | K | - | - | T | I | P | L | L | P | Q | N | L | E | V | L | G | L | E | F | N | N | I | L | R | I | E | Q | P | L | G | T | P | F | L | K | Q | L | L | L | A | K | N | C | Y | Y | A | N | P | C | N | E | S | F | F | I | D | Q | R | V | F | Q | G | L | P | K | L | Q | N | L | T | L | G | F | N | N | M | S | S | I | P | T | Q | L | P | P | S | L | K | S | L | D | L | G | E | N | R | I | T | E | I | Q | E | G | S | F | T | N | L | T | K | L | F | S | L | N | L | N | W | N | C | Q | R | C | D | N | A | A | H | P | C | F | P | C | P | N | N | A | S | L | G | L | P | N | Q | A | F | S | E | Q | R | D | S | L | V | I | L | Y | L | R | G | N | S | I | R | S | I | P | K | N | L | F | A | Q | L | R | K | - | L | K | V | L | D | L | S | D | N | F | L | A | Y | S | I | R | N | G | T | F | Y | E | E | L | Q | G | V | E | F | L | R | L | L | N | N | Y | Q | P | L | K | T | F | S | E | L | I | L | S | P | S | I | S | K | M | R | S | L | R | K | L | Y | L | S | G | L | F | F | H | Q | L | S | N | Y | S | L | M | H | L | S | N | L | P | Q | L | E | Y | L | D | L | R | M | N | F | M | N | Y | C | N | V | T | A | F | K | R | L | R | S | L | R | T | V | V | L | S | Q | N | M | L | T | I | N | P | Y | Y | T | S | S | A | N | E | A | R | R | L | - | - | - | - | - | - | D | R | Y | Q | M | D | - | D | H | L | D | L | Q | D | M | P | A | F | E | - | - | S | - | - | - | - | - | - | - | - | - | - | - | - | - | - | - | - | - | - | - | - | - | - | E | L | Q | M | N | Y | L | H | C | T | E | K | E | C | - | - | - | - | - | - | - | - | - | - | - | - | - | - | - | - | - | - | - | - | P | K | S | Y | S | M | E | N | F | Q | K | F | F | C | K | K | E | L | Y | F | D | L | S | Q | N | N | I | P | W | L | N | A | Y | T | F | K | G | M | E | K | T | V | C | L | D | L | S | Y | N | Y | M | S | Q | A | L | N | G | Q | Q | F | L | P | L | T | K | L | V | F | L | N | M | A | Y | N | R | I | D | L | Y | F | N | E | A | F | Q | E | I | R | S | T | L | K | V | L | D | L | S | N | N | E | F | H | F | Q | M | K | Q | I | G | H | R | L | D | F | I | K | N | L | T | S | L | E | V | L | S | L | A | N | N | K | I | G | L | R | I | S | N | T | L | Y | S | A | S | L | K | C | L | L | F | S | G | N | H | L | D | I | M | W | D | T | K | G | D | Q | Y | I | R | F | F | Q | G | L | T | N | L | T | Y | L | D | I | S | D | N | Q | L | K | S | C | S | P | A | A | I | I | N | L | P | P | S | I | R | V | L | R | M | D | S | N | F | L | D | Y | F | P | W | V | N | I | S | V | L | S | Q | L | C | Y | L | N | L | S | G | N | F | L | F | N | L | P | D | T | A | I | Q | F | S | I | K | L | K | S | L | D | L | S | H | N | R | L | S | A | I | P | E | S | F | F | S | Q | A | T | G | L | E | E | L | M | L | N | H | N | Q | L | K | I | I | E | M | H | A | F | P | P | L | L | Q | K | G | N | T | I | C | A | T | - | - | - | - | A | K | T | S | C | R | L | T | L | H | A | N | P | F | T | C | S | C | A | T | S | W | F | T | E | F | L | R | S | S | P | V | D | I | P | H | L | T | T | D | V | R | C | G | F | P | E | S | Q | K | G | V | N | V | L | S | N | D | P | R | S | C | Q | E | I | F | G | N | V | S | F | L | F | T | L | L | L | T | I | A | A | T | X | V | P | L | L | K | H | L | Y | G | W | D | L | W | Y | C | F | Q | I | L | W | A | G | H | K | G | Y | T | S | L | X | E | N | - | S | M | N | N | E | H | D | A | F | V | V | F | D | T | K | N | K | A | V | S | D | W | I | Y | N | E | M | L | I | N | L | E | G | R | G | R | W | K | L | R | L | C | L | E | E | R | D | W | V | P | G | L | S | C | I | E | N | L | H | X | A | V | H | N | S | R | K | T | V | F | V | L | T | N | Q | G | - | - | - | - | - | G | S | A | S | V | N | G | V | I | K | Q | A | F | L | L | V | Q | Q | R | L | L | D | E | R | V | D | V | A | I | V | V | L | L | D | P | L | F | P | K | F | K | Y | L | Q | M | R | K | R | L | C | K | K | S | V | L | S | W | P | K | N | P | R | A | Q | P | L | F | W | N | D | L | H | I | A | L | A | S | D | N | V | R | S | Y | D | K | K | V | T | E | S | F | L | S | N | E | L | L | X | X | X | X | X | X | X | X | X | X | X | X | X | X | X | X | X | - | - | - | - | - | - | - | - | - | - | - | - | - | - | - | - | - | - | - | - | - | - | - | - | - | - | - | - | - | - | - | - | - | - | - | - | - | - | - | - | - | - | - | - | - | - | - | - | - | - | - | - | - | - | - | - | - | - | - | - | - | - | - | - | - | - | - | - | - | - | - | - | - | - | - | - | - | - | - | - | - | - | - | - | - | - | - | - | - | - | - | - | - | - | - | - | - | - | - | - | - | - | - | - | - | - | - | - | - | - | - | - | - | - | - | - | - | - | - | - | - | - | - | - | - | - | - | - | - | - | - | - | - | - | - | - | - | - | - | - | - | - | - | - | - | - | - | - | - | - | - | - | - | - | - | - | - | - | - | - | - | - | - | - | - | - | - | - | - | - | - | - | - | - | - | - | - | - | - | - | - | - | - | - | - | - | - | - | - | - |
| TFTLR9 | - | - | - | - | - | - | - | - | M | Q | M | L | W | V | M | F | G | T | G | L | Y | L | F | F | V | L | D | Q | L | C | L | V | K | S | M | N | P | K | F | Y | P | C | D | N | N | T | D | S | D | G | N | I | H | L | D | C | K | H | R | D | L | T | G | I | P | K | F | K | S | P | F | I | I | S | L | N | L | D | E | N | R | I | H | Q | V | K | S | D | D | F | S | G | I | P | N | L | Q | H | L | S | L | M | - | W | N | C | L | P | G | R | L | K | A | L | K | L | P | S | C | Q | V | I | I | D | H | D | A | F | V | N | L | K | N | L | A | F | L | Q | L | A | G | N | S | L | K | - | - | T | I | P | L | L | P | K | H | L | E | V | L | G | L | E | F | N | N | I | F | R | I | E | Q | P | L | G | T | P | F | L | K | Q | L | L | L | A | K | N | C | Y | Y | A | N | P | C | N | G | S | F | F | I | D | Q | R | V | F | Q | D | L | T | K | L | Q | N | L | T | I | G | F | N | N | I | T | S | I | P | M | Q | L | P | P | S | L | E | S | L | D | L | K | E | N | M | I | T | E | I | Q | E | H | S | F | S | N | L | T | K | L | S | F | L | N | L | E | W | N | C | Q | R | C | D | H | A | A | N | P | C | I | P | C | P | N | N | S | S | L | D | L | H | H | K | A | F | F | D | Q | R | D | S | L | V | N | L | S | L | R | G | N | S | I | Y | S | I | P | K | N | L | F | A | H | L | K | K | - | L | K | T | L | D | L | S | E | N | L | L | A | F | S | I | Q | N | G | T | F | F | E | E | L | Q | G | V | V | S | L | N | L | L | Y | N | Y | I | P | Q | K | I | F | S | E | L | I | L | S | P | S | I | S | K | M | K | S | L | R | K | L | H | L | S | G | L | F | F | H | R | L | S | N | N | S | L | E | P | L | F | N | L | P | L | L | E | Y | L | D | L | S | L | N | F | I | N | S | C | N | I | T | A | F | N | Q | V | R | S | L | K | K | V | V | L | S | Q | N | M | L | T | I | N | P | Y | Y | T | E | S | S | N | I | A | L | T | P | - | - | - | - | - | - | D | G | Y | E | N | Y | - | D | H | Q | Q | L | Q | D | M | P | A | F | E | - | - | S | - | - | - | - | - | - | - | - | - | - | - | - | - | - | - | - | - | - | - | - | - | - | K | L | Q | M | N | L | L | H | C | T | D | E | E | C | - | - | - | - | - | - | - | - | - | - | - | - | - | - | - | - | - | - | - | - | P | K | H | F | S | M | W | Y | F | Q | K | C | H | C | Y | R | K | L | F | F | D | L | S | K | N | N | I | P | F | L | N | V | S | T | F | K | G | M | E | K | T | V | C | L | D | L | S | Y | N | Y | M | S | Q | A | L | N | G | Q | Q | F | H | P | L | N | K | L | V | Y | L | N | M | A | H | N | R | I | D | L | Y | F | K | E | A | F | E | E | L | R | S | T | L | K | V | L | D | L | S | N | N | E | F | H | F | H | M | R | G | I | G | H | R | F | N | F | I | K | N | L | T | S | L | E | V | L | S | L | A | N | N | N | I | G | M | R | I | S | S | I | I | N | S | T | S | L | K | C | L | L | F | S | G | N | R | L | D | I | M | W | D | T | R | G | D | Q | Y | I | R | F | F | Q | G | L | T | N | L | T | Y | L | D | I | S | D | N | Q | L | R | S | C | S | P | Q | A | I | V | N | L | P | L | S | I | R | V | L | R | M | D | S | N | L | L | N | Y | F | P | W | P | N | I | S | V | L | S | H | L | C | Y | L | N | L | S | G | N | Y | L | S | N | L | P | D | I | V | I | H | F | G | N | K | L | R | S | L | D | L | S | H | N | R | L | S | A | I | P | E | S | F | F | S | Q | A | T | G | L | K | E | L | M | L | N | H | N | Q | L | K | I | L | V | M | H | A | V | P | P | L | L | Q | N | G | N | T | N | C | T | N | - | - | - | - | A | K | T | S | C | K | L | T | L | H | A | N | P | F | T | C | S | C | A | T | S | W | F | T | E | F | L | R | K | S | A | V | D | I | P | H | L | T | T | D | V | R | C | G | F | P | E | S | L | T | G | V | N | V | L | T | I | D | P | R | S | C | Q | E | I | F | G | S | V | S | F | I | F | T | F | L | I | T | I | A | A | T | A | I | P | L | L | R | H | L | Y | G | W | D | L | W | Y | C | F | Q | I | L | W | A | G | H | K | G | Y | T | P | L | Q | G | N | - | N | M | S | N | Q | H | D | A | F | V | V | F | D | T | K | N | K | A | V | R | D | W | I | Y | N | E | M | L | I | N | L | E | S | R | G | R | W | K | F | R | L | C | L | E | E | R | D | W | V | P | G | V | P | C | I | E | N | L | H | N | S | V | Y | N | S | R | K | T | V | F | V | L | T | N | Q | G | - | - | - | - | - | G | C | A | S | L | N | G | V | I | K | Q | A | F | L | L | V | Q | Q | R | L | L | D | E | K | V | D | V | A | I | V | V | L | L | D | P | L | F | P | K | L | K | Y | L | Q | M | R | K | R | L | C | R | K | S | V | L | S | W | P | K | N | P | R | A | Q | P | L | F | W | N | N | L | H | I | A | L | A | S | D | I | V | R | S | Y | N | K | K | V | T | E | S | F | L | S | N | D | I | L | X | X | X | X | X | X | X | X | X | X | X | - | - | - | - | - | - | - | - | - | - | - | - | - | - | - | - | - | - | - | - | - | - | - | - | - | - | - | - | - | - | - | - | - | - | - | - | - | - | - | - | - | - | - | - | - | - | - | - | - | - | - | - | - | - | - | - | - | - | - | - | - | - | - | - | - | - | - | - | - | - | - | - | - | - | - | - | - | - | - | - | - | - | - | - | - | - | - | - | - | - | - | - | - | - | - | - | - | - | - | - | - | - | - | - | - | - | - | - | - | - | - | - | - | - | - | - | - | - | - | - | - | - | - | - | - | - | - | - | - | - | - | - | - | - | - | - | - | - | - | - | - | - | - | - | - | - | - | - | - | - | - | - | - | - | - | - | - | - | - | - | - | - | - | - | - | - | - | - | - | - | - | - | - | - | - | - | - | - | - | - | - | - | - | - | - | - | - | - | - | - | - | - | - | - | - | - |
| IPTLR9 | - | - | - | - | - | - | - | - | M | Q | M | L | L | F | M | F | G | P | G | L | Y | L | F | F | V | L | D | Q | L | N | L | V | K | T | M | N | P | K | F | Y | P | C | D | S | N | T | N | S | N | G | N | I | H | L | D | C | K | R | R | D | L | M | S | I | P | K | F | M | S | F | S | I | I | S | L | N | L | D | E | N | R | I | H | Q | V | K | R | D | D | F | S | G | I | P | N | L | Q | S | L | S | L | M | - | W | N | C | L | P | G | R | L | K | A | L | K | L | P | S | C | Q | V | I | I | D | P | E | A | F | V | N | L | K | N | L | T | F | L | Q | L | A | G | N | S | L | K | - | - | T | I | P | L | L | P | Q | H | L | E | V | L | G | L | E | F | N | N | I | F | R | I | E | Q | H | L | G | T | P | F | L | K | Q | L | L | L | A | K | N | C | Y | Y | A | N | P | C | N | E | S | F | F | I | D | Q | M | V | F | Q | D | L | P | E | L | Q | N | L | T | L | G | F | N | N | M | S | S | V | P | K | Q | L | P | S | S | L | E | S | L | D | L | T | E | N | R | I | T | E | I | W | E | S | S | F | T | N | L | T | K | L | S | F | L | N | L | Q | W | N | C | Q | R | C | D | H | A | A | Q | P | C | F | P | C | P | N | N | S | S | L | G | L | H | D | N | A | F | F | D | Q | R | E | S | L | V | I | L | S | L | R | G | N | S | I | Y | S | I | P | K | N | L | F | A | Q | L | K | K | - | L | K | T | L | D | L | S | E | N | L | L | A | Y | S | I | R | N | G | T | F | Y | E | E | L | Q | G | V | V | S | L | N | L | L | Y | N | Y | Q | P | L | K | T | F | S | E | L | I | L | S | P | S | I | S | R | M | K | S | L | R | N | L | H | L | S | G | L | F | F | H | Q | L | S | N | N | S | L | R | P | L | F | N | L | T | Q | L | E | Y | L | D | L | R | M | N | F | I | S | S | C | N | V | T | A | F | N | Q | L | R | S | L | R | M | V | V | L | S | Q | N | M | L | T | I | N | P | Y | Y | T | A | S | S | N | N | A | M | T | L | - | - | - | - | - | - | D | R | Y | Q | R | D | - | D | H | L | N | L | Q | D | M | P | A | F | E | - | - | S | - | - | - | - | - | - | - | - | - | - | - | - | - | - | - | - | - | - | - | - | - | - | K | L | Q | M | N | Y | L | H | C | T | D | K | E | C | - | - | - | - | - | - | - | - | - | - | - | - | - | - | - | - | - | - | - | - | P | K | S | Y | S | M | M | N | F | Q | K | C | F | C | Y | R | K | L | F | F | D | L | S | Q | N | N | I | P | W | L | N | A | S | T | F | K | G | M | E | K | T | V | C | L | D | L | S | Y | N | Y | M | S | Q | S | L | N | G | Q | Q | F | Y | P | L | T | E | L | V | Y | L | N | M | A | H | N | R | I | D | L | Y | F | N | E | A | F | Q | E | L | R | R | T | L | K | A | L | D | L | S | N | N | E | F | H | F | Q | M | R | G | I | G | H | R | F | T | F | I | K | N | L | T | S | L | E | V | L | S | L | A | N | N | N | I | G | M | R | I | S | N | T | L | Y | S | T | S | L | K | C | L | L | F | S | G | N | R | L | D | I | M | W | D | T | S | G | D | Q | Y | I | R | F | F | Q | G | L | T | N | L | T | Y | L | D | I | S | D | N | K | L | R | S | C | S | P | E | A | I | V | N | L | P | P | S | I | H | V | L | K | M | D | S | N | L | L | N | Y | F | P | W | N | N | I | S | V | L | S | Q | L | C | Y | L | N | L | S | G | N | Y | L | F | N | L | P | D | S | V | I | Y | F | G | I | K | L | R | S | L | D | L | S | H | N | R | L | S | A | I | P | E | S | F | F | S | Q | A | T | G | L | K | E | L | M | L | N | H | N | Q | L | K | I | L | E | M | H | A | L | P | P | S | L | Q | K | G | N | T | I | C | I | T | - | - | - | - | S | K | I | S | C | K | L | T | L | H | A | N | P | F | T | C | S | C | A | T | S | W | F | T | D | F | L | R | T | S | P | V | D | I | P | H | L | T | T | D | V | R | C | G | F | P | E | S | Q | T | G | V | N | V | L | S | I | D | P | R | S | C | Q | E | I | F | G | S | V | S | F | L | F | T | F | L | L | T | I | A | A | T | A | V | P | L | L | R | H | L | Y | G | W | D | L | W | Y | C | F | Q | I | L | W | A | G | H | K | G | Y | M | P | L | H | G | N | - | N | M | N | N | Q | H | D | A | F | V | V | F | D | T | K | N | K | A | V | R | D | W | I | Y | N | E | M | L | I | N | L | E | S | R | G | R | W | K | F | R | L | C | L | E | E | R | D | W | I | P | G | V | S | C | I | E | N | L | H | N | A | V | Y | N | S | R | K | T | V | F | V | L | T | N | Q | G | - | - | - | - | - | G | C | A | S | L | N | G | V | I | K | Q | A | F | I | L | V | Q | Q | R | L | L | D | E | K | V | D | V | A | I | V | V | L | L | D | P | L | F | P | K | L | K | Y | L | Q | M | R | K | R | L | C | R | K | S | V | L | S | W | P | K | N | P | R | A | Q | P | L | F | W | N | N | L | H | I | A | L | A | S | D | N | V | R | S | Y | D | K | K | V | T | E | S | F | L | S | N | E | L | I | X | X | X | X | X | X | X | X | X | X | X | - | - | - | - | - | - | - | - | - | - | - | - | - | - | - | - | - | - | - | - | - | - | - | - | - | - | - | - | - | - | - | - | - | - | - | - | - | - | - | - | - | - | - | - | - | - | - | - | - | - | - | - | - | - | - | - | - | - | - | - | - | - | - | - | - | - | - | - | - | - | - | - | - | - | - | - | - | - | - | - | - | - | - | - | - | - | - | - | - | - | - | - | - | - | - | - | - | - | - | - | - | - | - | - | - | - | - | - | - | - | - | - | - | - | - | - | - | - | - | - | - | - | - | - | - | - | - | - | - | - | - | - | - | - | - | - | - | - | - | - | - | - | - | - | - | - | - | - | - | - | - | - | - | - | - | - | - | - | - | - | - | - | - | - | - | - | - | - | - | - | - | - | - | - | - | - | - | - | - | - | - | - | - | - | - | - | - | - | - | - | - | - | - | - | - | - |
| PHTLR9 | - | - | - | - | - | - | - | - | M | Q | M | L | L | V | M | F | G | P | G | L | Y | L | F | L | A | L | D | Q | L | S | L | V | K | T | M | N | P | K | F | Y | P | C | D | N | R | T | S | S | D | G | N | I | H | L | N | C | K | H | R | D | L | T | S | I | P | K | F | M | S | L | F | I | I | S | L | N | L | D | E | N | R | I | H | H | I | K | S | D | D | F | S | D | I | P | N | L | Q | V | I | S | L | M | - | W | N | C | L | P | G | R | L | K | A | L | K | L | P | S | C | Q | V | I | I | E | R | D | A | F | V | N | L | K | N | L | T | F | L | Q | L | A | G | N | S | L | K | - | - | T | I | P | P | L | P | Q | H | L | E | V | L | G | L | E | F | N | N | I | F | R | I | E | R | P | L | G | T | P | F | L | K | Q | L | L | L | A | K | N | C | Y | Y | A | N | P | C | N | E | S | F | F | I | D | Q | R | V | F | Q | D | L | P | M | L | Q | N | L | T | L | G | C | N | N | I | T | S | I | P | T | Q | L | P | P | S | L | E | S | L | D | L | R | E | N | S | I | T | E | I | R | E | G | S | F | T | N | L | T | K | L | S | F | L | N | L | E | W | N | C | Q | R | C | D | H | A | A | Q | P | C | F | P | C | P | N | N | S | S | L | G | L | H | D | K | A | F | F | E | Q | R | D | S | L | V | N | L | S | L | R | G | N | S | I | H | S | I | P | K | N | L | F | A | Q | L | K | K | - | L | K | K | L | D | L | S | D | N | F | L | A | Y | S | I | Q | N | G | T | F | Y | E | E | L | Q | G | V | V | S | L | N | L | L | Y | N | Y | Q | P | L | K | T | F | S | E | L | I | L | S | P | S | I | S | R | M | K | S | L | R | K | L | Q | L | S | G | L | F | F | H | Q | L | S | N | N | S | L | R | P | L | F | N | L | P | Q | L | E | Y | L | D | L | R | M | N | F | I | N | S | C | N | V | T | V | F | N | Q | L | R | S | L | R | M | V | V | L | S | Q | N | M | L | T | L | N | P | Y | N | T | A | S | S | S | K | A | L | T | L | - | - | - | - | - | - | D | R | Y | Q | R | D | - | D | H | L | Q | L | Q | D | M | S | A | F | E | - | - | S | - | - | - | - | - | - | - | - | - | - | - | - | - | - | - | - | - | - | - | - | - | - | K | L | Q | R | N | Y | F | H | C | T | D | K | E | C | - | - | - | - | - | - | - | - | - | - | - | - | - | - | - | - | - | - | - | - | P | K | G | Y | S | M | W | N | L | R | R | C | F | C | Y | G | K | L | L | F | D | L | S | Q | N | N | I | P | W | L | N | A | N | T | F | K | G | M | E | Q | T | V | C | L | D | L | S | Y | N | Y | M | S | Q | A | L | N | G | Q | Q | F | Y | P | L | T | K | L | V | Y | L | N | M | A | H | N | R | I | D | L | Y | F | N | E | A | F | Q | E | I | R | R | T | L | K | I | L | D | L | S | N | N | E | F | H | F | H | M | R | G | I | G | H | R | F | T | F | I | K | N | L | T | S | L | E | V | L | S | L | A | N | N | N | I | G | M | R | I | T | N | I | L | Y | S | T | S | L | K | C | L | L | F | S | G | N | R | L | D | I | M | W | D | S | S | G | D | Q | Y | I | R | F | F | Q | G | L | T | N | L | T | Y | L | D | I | S | D | N | Q | L | R | S | C | S | P | E | A | I | A | N | P | P | P | S | I | R | V | L | R | M | D | S | N | L | L | N | Y | F | P | W | Q | N | I | S | V | L | S | L | L | C | Y | L | N | L | S | G | N | Y | L | S | N | L | P | D | S | V | I | Y | F | G | I | K | L | R | S | L | D | L | S | H | N | R | L | S | A | I | P | E | S | F | F | S | Q | A | T | G | L | K | E | L | M | L | N | H | N | Q | L | K | I | L | E | M | H | A | L | P | P | A | L | Q | K | G | S | T | I | C | T | T | - | - | - | - | S | E | T | S | C | K | L | T | L | H | A | N | P | F | T | C | S | C | A | T | S | W | F | T | D | F | L | R | K | S | P | V | D | I | P | H | L | T | T | D | V | R | C | G | F | P | E | S | Q | M | G | V | N | V | L | S | I | D | P | R | S | C | Q | E | I | F | G | S | V | S | F | L | F | T | L | F | L | T | I | A | A | T | A | I | P | L | L | R | H | L | Y | G | W | D | L | W | Y | C | F | Q | I | L | W | A | G | H | K | G | Y | T | P | I | Q | G | N | - | N | M | N | N | Q | H | D | A | F | V | V | F | D | T | K | N | K | A | V | S | D | W | I | Y | N | E | M | L | I | N | L | E | R | T | G | R | W | K | F | R | L | C | L | E | E | R | D | W | V | P | G | V | S | C | I | E | N | L | H | N | A | V | Y | N | S | R | K | T | V | F | V | L | T | N | Q | G | - | - | - | - | - | G | C | A | S | V | N | G | V | I | K | Q | A | F | L | L | V | Q | Q | R | L | L | D | E | K | V | D | V | A | I | V | V | L | L | D | P | L | F | P | K | L | K | Y | L | Q | M | R | K | R | L | C | R | K | S | V | L | S | W | P | K | N | P | R | A | Q | P | L | F | W | N | D | L | N | I | A | L | A | S | D | N | V | K | S | Y | D | K | K | V | T | E | S | F | L | S | N | E | I | L | X | X | X | X | X | X | X | X | X | X | X | - | - | - | - | - | - | - | - | - | - | - | - | - | - | - | - | - | - | - | - | - | - | - | - | - | - | - | - | - | - | - | - | - | - | - | - | - | - | - | - | - | - | - | - | - | - | - | - | - | - | - | - | - | - | - | - | - | - | - | - | - | - | - | - | - | - | - | - | - | - | - | - | - | - | - | - | - | - | - | - | - | - | - | - | - | - | - | - | - | - | - | - | - | - | - | - | - | - | - | - | - | - | - | - | - | - | - | - | - | - | - | - | - | - | - | - | - | - | - | - | - | - | - | - | - | - | - | - | - | - | - | - | - | - | - | - | - | - | - | - | - | - | - | - | - | - | - | - | - | - | - | - | - | - | - | - | - | - | - | - | - | - | - | - | - | - | - | - | - | - | - | - | - | - | - | - | - | - | - | - | - | - | - | - | - | - | - | - | - | - | - | - | - | - | - | - |
| DRTLR9 | - | - | - | - | - | - | - | - | - | - | - | - | - | - | M | F | G | P | M | V | S | L | I | L | L | L | N | Q | F | Q | L | F | A | A | S | H | P | Q | F | Y | P | C | E | S | H | S | T | K | D | G | H | I | N | V | D | C | Q | H | R | R | L | S | K | V | P | R | F | T | S | P | S | V | I | S | L | N | L | N | N | N | H | I | H | R | I | K | G | D | A | F | S | G | L | P | N | L | K | Y | L | S | L | M | - | W | N | C | I | S | D | R | L | K | E | A | R | W | P | L | C | S | V | N | I | D | P | D | A | F | V | G | L | K | N | L | T | S | L | Q | L | A | G | N | S | L | K | - | - | M | I | P | P | L | P | K | Q | L | E | V | L | G | L | E | F | N | N | I | F | N | I | V | K | P | L | G | T | P | Q | L | K | Q | L | L | L | S | K | N | C | F | Y | A | N | P | C | H | Q | P | Y | F | I | N | S | S | V | F | Q | D | L | P | E | L | L | N | L | T | L | S | Y | N | N | L | T | A | I | P | S | Y | L | P | G | S | L | E | S | L | D | L | R | E | N | T | I | D | H | I | N | K | E | S | F | A | N | L | R | N | L | R | H | L | N | L | G | W | N | C | Q | R | C | D | H | A | S | D | P | C | F | P | C | P | N | N | Q | S | L | D | L | H | Q | D | A | F | L | D | Q | R | D | S | L | V | S | L | H | L | Q | G | N | S | L | R | T | L | P | R | H | L | F | I | N | L | H | K | - | L | Q | E | L | D | L | S | S | N | F | L | A | F | T | I | Q | N | G | T | F | Y | E | E | L | Q | N | V | V | I | L | N | L | L | Y | N | Y | E | P | L | K | T | F | P | E | L | N | L | S | P | Y | I | E | K | M | A | S | L | R | E | L | Y | L | S | G | F | F | F | K | K | L | S | N | R | S | I | A | P | L | V | K | L | P | R | L | E | V | L | D | L | R | M | N | F | I | C | D | I | S | I | D | G | L | S | Q | L | R | T | L | R | R | V | D | L | S | Q | N | M | L | A | F | S | S | C | F | S | T | C | T | S | E | A | E | H | Q | - | - | - | - | I | P | E | R | Y | G | N | E | Q | F | N | L | Q | M | Q | E | L | P | I | L | N | - | - | A | - | - | - | - | - | - | - | - | - | - | - | - | - | - | - | - | - | - | - | - | - | - | E | - | - | - | - | T | Q | G | S | K | P | D | Y | C | - | - | - | - | - | - | - | - | - | - | - | - | - | - | - | - | - | - | - | - | S | F | Y | F | S | M | W | H | F | K | R | Q | I | C | S | K | S | L | Y | F | D | L | S | Q | N | N | I | P | W | L | N | A | S | T | F | R | G | M | D | R | V | A | C | V | D | L | S | Y | N | Y | I | S | Q | T | L | N | G | H | Q | F | S | H | L | S | K | L | S | Y | L | N | M | A | Y | N | R | I | D | L | Y | S | D | K | A | F | Q | E | V | S | G | T | L | K | A | L | D | L | S | N | N | E | F | H | F | I | M | K | G | M | G | H | Q | F | T | F | L | T | H | M | S | S | L | I | I | L | S | L | A | N | N | H | I | G | L | R | I | S | N | I | L | T | S | A | S | L | K | Y | L | I | F | S | G | N | R | L | D | I | L | W | D | S | W | R | N | Q | Y | I | N | L | F | Q | G | L | T | N | L | T | H | L | D | I | S | E | N | Q | L | K | S | L | S | P | E | V | I | V | N | L | P | L | S | L | Q | V | L | R | V | D | F | N | M | L | T | Y | F | P | W | A | N | I | S | V | L | Q | K | L | C | Y | L | N | L | S | S | N | M | L | S | Y | L | P | N | - | - | I | N | F | E | L | R | L | T | G | L | D | L | S | H | N | R | L | V | A | I | P | K | V | F | L | S | Q | A | A | N | L | K | N | L | N | L | N | N | N | Q | L | K | I | L | D | V | Q | A | L | P | L | P | F | H | K | G | C | T | F | I | P | G | G | Q | H | K | N | R | S | S | C | K | L | V | L | H | A | N | P | F | T | C | S | C | V | I | S | G | F | A | K | F | L | R | E | T | D | L | D | V | P | H | L | T | T | Q | V | H | C | G | F | P | E | S | L | A | G | V | N | V | L | S | V | D | L | R | S | C | Q | E | I | F | G | G | V | A | F | L | C | T | S | L | L | T | L | A | A | T | C | V | P | L | L | K | H | L | Y | G | W | D | L | W | Y | L | I | Q | I | L | W | T | G | H | R | G | H | T | P | A | N | G | - | N | P | T | D | T | Q | Y | D | A | F | V | V | F | D | T | S | N | K | A | V | R | D | W | I | Y | K | E | M | L | V | R | L | E | N | R | G | R | W | R | F | Q | L | C | L | E | E | R | D | W | I | P | G | V | S | C | I | E | N | L | H | K | S | V | Y | S | S | R | K | T | V | F | V | L | T | S | P | G | - | - | - | - | - | G | Y | S | D | A | S | G | I | V | R | Q | A | F | L | L | V | Q | Q | R | L | L | D | E | K | V | D | V | A | V | L | V | L | L | D | F | L | F | P | K | F | K | Y | L | Q | M | R | K | R | L | C | K | K | S | V | L | S | W | P | R | N | P | R | V | Q | P | L | F | W | N | D | L | R | V | A | L | V | S | D | N | V | R | A | Y | N | K | N | V | T | E | S | F | F | X | X | X | X | X | X | X | X | X | X | X | X | X | X | X | X | X | X | X | X | X | - | - | - | - | - | - | - | - | - | - | - | - | - | - | - | - | - | - | - | - | - | - | - | - | - | - | - | - | - | - | - | - | - | - | - | - | - | - | - | - | - | - | - | - | - | - | - | - | - | - | - | - | - | - | - | - | - | - | - | - | - | - | - | - | - | - | - | - | - | - | - | - | - | - | - | - | - | - | - | - | - | - | - | - | - | - | - | - | - | - | - | - | - | - | - | - | - | - | - | - | - | - | - | - | - | - | - | - | - | - | - | - | - | - | - | - | - | - | - | - | - | - | - | - | - | - | - | - | - | - | - | - | - | - | - | - | - | - | - | - | - | - | - | - | - | - | - | - | - | - | - | - | - | - | - | - | - | - | - | - | - | - | - | - | - | - | - | - | - | - | - | - | - | - | - | - | - | - | - | - | - | - | - | - | - | - | - | - | - | - | - |
| CITLR9 | - | - | - | - | - | - | - | - | - | - | - | - | - | - | M | F | G | H | - | V | Y | L | I | L | I | L | N | Q | F | H | L | F | A | T | K | H | P | E | F | Y | P | C | E | S | R | T | S | K | D | G | H | I | N | I | D | C | Q | H | R | R | L | S | K | I | P | K | F | I | S | L | S | V | V | S | L | N | L | N | Q | N | H | I | H | Y | I | K | G | D | A | F | S | D | L | P | N | L | K | H | L | S | L | M | - | W | N | C | V | P | D | P | L | K | E | Q | R | W | P | S | C | N | L | N | V | D | P | E | A | F | V | G | L | K | N | L | T | S | L | Q | L | A | G | N | S | L | K | - | - | T | I | P | P | L | P | K | Q | L | L | V | L | G | L | E | F | N | N | I | F | H | I | V | K | P | L | G | T | P | L | L | K | Q | L | L | L | N | K | N | C | F | Y | A | N | P | C | Y | R | P | Y | F | I | D | P | R | V | F | Q | E | L | P | E | L | L | N | L | T | L | S | Y | N | N | V | T | N | V | P | L | H | L | P | P | S | L | E | S | L | D | L | G | E | N | K | I | T | G | I | T | K | E | S | F | A | N | L | T | R | L | R | H | L | N | L | G | W | N | C | Q | R | C | D | H | A | S | E | P | C | F | P | C | P | N | N | Q | S | L | R | L | H | Q | D | A | F | L | D | Q | R | D | S | L | X | S | L | S | L | R | G | N | S | L | R | T | L | P | Q | Y | L | F | M | H | L | H | N | - | L | K | E | L | D | L | S | D | N | F | L | A | Y | T | I | Q | N | G | T | F | Y | E | E | L | Q | N | V | V | S | L | S | L | L | Y | N | Y | E | P | L | K | S | F | P | E | L | I | L | S | P | A | I | E | K | M | T | A | L | R | E | L | Y | L | S | G | F | F | F | K | E | L | S | N | H | S | L | A | P | L | I | K | L | P | R | L | E | S | L | E | L | R | M | N | F | I | C | F | I | S | I | D | S | I | S | Q | L | R | T | L | K | R | V | G | L | S | Q | N | M | L | A | F | S | P | C | Y | S | T | C | N | S | E | A | I | L | Q | - | Y | - | Q | T | L | E | K | R | H | D | Y | - | - | - | E | Q | M | H | E | V | P | A | P | N | - | - | S | - | - | - | - | - | - | - | - | - | - | - | - | - | - | - | - | - | - | - | - | - | - | K | L | N | - | N | M | Q | S | S | E | E | D | Y | C | - | - | - | - | - | - | - | - | - | - | - | - | - | - | - | - | - | - | - | - | P | F | Y | Y | S | M | W | H | F | K | K | Q | I | C | S | K | S | L | F | F | D | L | S | Q | N | S | I | P | W | L | N | A | S | T | F | R | G | M | E | N | V | V | C | I | D | L | S | Y | N | Y | I | S | Q | S | L | N | G | R | Q | F | S | H | L | S | K | L | A | F | L | N | M | A | H | N | R | I | D | L | Y | S | E | K | A | F | H | E | V | S | G | T | L | K | A | L | D | L | S | N | N | E | F | H | F | V | M | K | G | M | G | H | R | F | T | F | L | S | H | L | T | S | L | K | V | L | S | L | A | N | N | H | I | G | L | R | I | S | N | T | L | N | S | T | S | L | K | Y | L | A | F | S | G | N | R | L | D | I | M | W | D | S | R | R | N | Q | Y | I | H | F | F | Q | G | L | T | N | L | T | H | L | D | I | S | E | N | Q | L | K | S | F | P | P | E | V | I | V | N | L | P | P | S | L | Q | V | L | R | V | D | S | N | M | L | S | Y | F | P | W | A | N | I | S | V | L | R | Q | L | C | Y | L | N | L | S | S | N | M | L | L | F | L | P | N | - | - | I | H | F | E | L | H | L | V | S | L | D | L | S | H | N | R | L | V | T | I | P | K | A | F | L | S | Q | A | V | H | L | K | N | L | M | L | N | H | N | Q | L | K | I | L | D | V | Q | A | L | P | L | L | F | H | K | G | H | T | F | C | P | A | G | Q | N | K | N | R | S | S | C | K | L | V | L | H | S | N | P | F | I | C | S | C | V | I | S | G | F | A | K | F | L | R | E | T | E | L | D | I | P | H | L | T | T | Q | V | H | C | A | Y | P | E | S | L | S | G | V | N | V | L | S | I | D | L | R | S | C | Q | E | I | F | G | G | F | A | L | L | C | T | L | L | L | T | L | A | A | T | I | I | P | L | L | K | H | L | Y | G | W | D | L | W | Y | C | I | Q | I | L | W | T | G | H | R | G | H | T | P | A | N | G | - | N | M | T | D | D | Q | Y | D | A | F | V | V | F | D | T | S | N | K | A | V | R | D | W | I | Y | K | E | M | I | V | R | L | E | N | R | G | R | W | R | F | R | L | C | L | E | E | R | D | W | L | P | G | V | S | C | I | E | N | L | H | K | A | V | Y | S | S | R | K | T | V | F | V | L | T | S | P | S | - | - | - | - | - | G | Y | S | Q | S | S | G | V | V | R | Q | A | F | L | L | V | Q | Q | R | L | L | D | E | K | V | D | V | A | V | L | V | L | L | D | F | L | F | P | K | F | K | Y | L | Q | M | R | K | R | L | C | K | K | S | V | L | S | W | P | K | N | P | R | V | Q | P | L | F | W | N | N | L | R | V | A | L | V | S | D | N | V | K | A | Y | N | K | N | V | T | E | S | F | F | X | X | X | X | X | X | X | X | X | X | X | X | X | X | X | X | X | X | X | X | - | - | - | - | - | - | - | - | - | - | - | - | - | - | - | - | - | - | - | - | - | - | - | - | - | - | - | - | - | - | - | - | - | - | - | - | - | - | - | - | - | - | - | - | - | - | - | - | - | - | - | - | - | - | - | - | - | - | - | - | - | - | - | - | - | - | - | - | - | - | - | - | - | - | - | - | - | - | - | - | - | - | - | - | - | - | - | - | - | - | - | - | - | - | - | - | - | - | - | - | - | - | - | - | - | - | - | - | - | - | - | - | - | - | - | - | - | - | - | - | - | - | - | - | - | - | - | - | - | - | - | - | - | - | - | - | - | - | - | - | - | - | - | - | - | - | - | - | - | - | - | - | - | - | - | - | - | - | - | - | - | - | - | - | - | - | - | - | - | - | - | - | - | - | - | - | - | - | - | - | - | - | - | - | - | - | - | - | - | - | - | - |
| MATLR9 | - | - | - | - | - | - | - | - | - | - | - | - | - | - | M | F | G | Y | - | V | Y | L | I | L | I | L | N | Q | F | H | L | F | A | T | K | H | P | E | F | Y | P | C | E | S | H | T | S | K | D | G | H | I | N | I | D | C | Q | H | R | R | L | S | K | I | P | K | F | I | S | L | S | V | V | S | L | N | L | N | Q | N | H | I | H | Y | I | K | G | D | A | F | S | G | L | P | N | L | K | H | L | S | L | M | - | W | N | C | V | P | D | P | L | K | E | Q | R | W | P | S | C | N | L | N | V | D | P | E | A | F | V | G | L | K | N | L | T | S | L | Q | L | A | G | N | S | L | K | - | - | T | I | P | P | L | P | K | Q | L | L | V | L | G | L | E | F | N | K | I | F | H | I | V | K | P | L | G | T | P | L | L | K | Q | L | L | L | N | K | N | C | F | Y | A | N | P | C | Y | E | S | Y | F | I | D | P | R | V | F | Q | D | L | P | E | L | L | N | L | T | L | S | Y | N | N | V | T | F | V | P | P | H | L | P | P | S | L | E | S | L | D | L | G | E | N | K | I | T | G | I | T | K | E | S | F | A | N | L | T | R | L | R | H | L | N | L | G | W | N | C | Q | R | C | D | H | A | S | E | P | C | F | P | C | P | N | N | Q | S | L | R | L | H | Q | D | A | F | L | D | Q | R | D | S | L | I | S | L | S | L | R | G | N | S | L | R | T | L | P | Q | Y | L | F | T | Q | L | H | N | - | L | K | E | L | D | L | S | D | N | F | L | A | Y | V | I | Q | N | G | T | F | Y | E | E | L | Q | N | V | V | S | L | S | L | L | Y | N | Y | E | P | L | K | S | F | P | E | L | I | L | S | P | A | I | E | K | M | T | A | L | R | E | L | Y | L | S | G | F | F | F | K | E | L | S | N | H | S | L | A | P | L | M | K | L | P | R | L | E | S | L | E | L | R | M | N | F | I | C | F | I | S | I | D | S | I | S | Q | L | R | T | L | K | R | V | G | L | S | Q | N | M | L | A | F | S | P | C | Y | S | T | C | N | S | E | A | I | S | Q | - | Y | - | Q | T | L | E | K | R | H | D | Y | - | - | - | E | Q | I | H | E | V | P | A | P | D | - | - | S | - | - | - | - | - | - | - | - | - | - | - | - | - | - | - | - | - | - | - | - | - | - | K | L | N | - | N | M | Q | S | S | E | G | D | Y | C | - | - | - | - | - | - | - | - | - | - | - | - | - | - | - | - | - | - | - | - | P | F | Y | Y | S | M | W | H | F | K | K | Q | I | C | S | K | S | L | F | F | D | L | S | Q | N | S | I | P | W | L | N | A | S | T | F | R | G | M | D | N | V | V | C | I | D | L | S | Y | N | Y | I | S | Q | S | L | N | G | R | Q | F | S | H | L | S | K | L | A | F | L | N | M | A | H | N | R | I | D | L | Y | S | E | K | A | F | R | E | V | S | K | T | L | K | A | L | D | L | S | N | N | E | F | H | F | V | M | K | G | M | G | H | R | F | T | F | L | S | H | L | S | S | L | K | V | L | S | L | A | N | N | H | I | G | L | R | I | S | N | T | L | N | S | T | S | L | K | Y | L | A | F | S | G | N | R | L | D | I | M | W | D | S | R | R | N | Q | Y | I | H | F | F | Q | G | L | T | N | L | T | H | L | D | I | S | E | N | Q | L | K | S | F | P | P | E | A | I | V | N | L | P | L | S | L | Q | V | L | R | V | D | S | N | M | L | S | Y | F | P | W | A | N | I | S | V | L | K | Q | L | C | H | L | N | L | S | S | N | M | L | S | F | L | P | N | - | - | I | H | F | E | L | R | L | V | S | L | D | L | S | H | N | R | L | V | A | I | P | K | A | F | L | S | Q | A | V | H | L | K | N | L | M | L | N | H | N | Q | L | K | I | L | D | V | Q | A | L | P | L | L | F | H | K | G | H | T | F | C | P | A | G | Q | D | T | N | R | S | S | C | K | L | V | L | H | S | N | P | F | I | C | S | C | V | I | S | G | F | A | K | F | L | R | E | T | E | L | D | I | P | H | L | T | T | Q | V | H | C | A | Y | P | E | S | L | S | G | V | N | V | L | S | I | D | L | R | S | C | Q | E | I | F | G | G | F | A | L | L | C | T | L | L | L | T | L | A | A | T | I | I | P | L | L | K | H | L | Y | G | W | D | L | W | Y | C | I | Q | I | L | W | T | G | H | K | G | H | T | P | A | N | G | - | N | M | T | E | D | Q | Y | D | A | F | V | V | F | D | T | S | N | K | A | V | R | D | W | I | Y | K | E | M | I | V | R | L | E | K | R | G | R | W | R | F | R | L | C | L | E | E | R | D | W | L | P | G | E | S | C | I | E | N | L | H | K | A | V | Y | S | S | R | K | T | V | F | V | L | T | S | P | S | - | - | - | - | - | G | Y | S | Q | S | S | G | V | V | R | Q | A | F | L | L | V | Q | Q | R | L | L | D | E | K | V | D | V | A | V | L | V | L | L | D | F | L | F | P | K | F | K | Y | L | Q | M | R | K | R | L | C | K | K | S | V | L | S | W | P | K | N | P | R | V | Q | P | L | F | W | N | N | L | R | V | A | L | V | S | D | N | V | K | A | Y | N | K | N | V | I | E | S | F | F | X | X | X | X | X | X | X | X | X | X | X | X | X | X | X | X | X | X | X | X | - | - | - | - | - | - | - | - | - | - | - | - | - | - | - | - | - | - | - | - | - | - | - | - | - | - | - | - | - | - | - | - | - | - | - | - | - | - | - | - | - | - | - | - | - | - | - | - | - | - | - | - | - | - | - | - | - | - | - | - | - | - | - | - | - | - | - | - | - | - | - | - | - | - | - | - | - | - | - | - | - | - | - | - | - | - | - | - | - | - | - | - | - | - | - | - | - | - | - | - | - | - | - | - | - | - | - | - | - | - | - | - | - | - | - | - | - | - | - | - | - | - | - | - | - | - | - | - | - | - | - | - | - | - | - | - | - | - | - | - | - | - | - | - | - | - | - | - | - | - | - | - | - | - | - | - | - | - | - | - | - | - | - | - | - | - | - | - | - | - | - | - | - | - | - | - | - | - | - | - | - | - | - | - | - | - | - | - | - | - | - | - |
| CCTLR9 | - | - | - | - | - | - | - | - | - | - | - | - | - | - | M | F | G | H | M | L | Y | L | A | L | I | L | N | Q | F | N | H | F | A | T | L | H | P | E | F | Y | P | C | E | I | H | T | T | K | D | G | D | I | N | V | D | C | Q | R | R | H | L | A | N | V | P | K | F | T | S | L | S | V | I | S | L | N | L | N | E | N | H | I | H | H | I | K | G | A | T | F | S | G | L | A | N | L | K | H | L | S | L | M | - | W | N | C | I | P | D | R | F | K | E | L | R | W | P | S | C | S | L | K | I | D | P | N | A | F | S | G | L | K | N | L | T | S | L | Q | L | A | G | N | S | L | K | - | - | T | I | P | P | L | P | K | Q | L | E | I | L | G | L | E | F | N | H | I | F | Q | I | V | K | P | L | G | T | P | L | L | K | Q | L | L | L | N | K | N | C | F | Y | A | N | P | C | Y | Q | S | Y | F | I | D | P | R | V | F | Q | D | L | P | E | L | L | N | L | T | L | S | Y | N | N | V | T | I | V | P | P | Y | L | P | L | S | L | E | S | L | D | L | G | E | N | K | I | T | H | I | N | K | E | S | F | A | N | L | K | N | L | R | H | L | N | L | G | W | N | C | Q | R | C | D | H | A | S | E | P | C | F | P | C | P | N | N | Q | S | L | N | L | H | Q | D | A | F | L | D | Q | R | D | S | L | I | S | L | S | L | R | G | N | S | L | H | T | I | P | Q | H | L | F | I | R | L | H | K | - | L | Q | E | L | D | L | S | D | N | F | L | A | Y | A | I | Q | N | G | T | F | Y | E | E | L | R | N | V | V | S | L | S | L | L | Y | N | Y | E | P | L | T | S | F | S | E | L | I | L | S | P | S | I | E | K | M | T | A | L | R | E | L | H | L | S | G | L | F | F | R | V | L | S | N | H | S | L | A | P | L | V | K | L | P | R | F | E | F | L | E | L | R | M | N | F | I | C | S | V | S | M | D | A | I | S | Q | L | R | T | L | R | W | V | G | L | S | Q | N | M | I | A | F | S | S | C | F | S | T | C | T | S | E | A | I | P | N | N | Y | - | R | T | L | E | K | R | D | N | G | Q | L | N | L | Q | T | Q | K | V | I | L | P | T | - | - | S | - | - | - | - | - | - | - | - | - | - | - | - | - | - | - | - | - | - | - | - | - | - | E | L | N | - | T | M | Q | A | S | G | E | D | H | C | - | - | - | - | - | - | - | - | - | - | - | - | - | - | - | - | - | - | - | - | F | F | Y | Y | S | I | W | H | F | K | K | Q | I | C | S | K | K | L | F | F | D | L | S | Q | N | N | I | P | W | L | N | A | S | T | F | R | G | M | E | K | V | V | C | I | D | L | S | Y | N | Y | I | S | Q | T | L | N | G | Q | Q | F | T | H | L | S | K | L | A | Y | L | N | M | A | N | N | R | I | D | L | Y | S | D | K | A | F | Q | E | I | S | G | T | L | K | A | L | D | L | S | N | N | E | F | H | F | V | M | K | G | M | G | H | R | F | T | F | L | P | H | L | S | S | L | K | I | L | S | L | A | N | N | H | I | G | L | R | I | S | N | I | L | N | S | T | S | L | K | Y | L | D | F | S | G | N | R | L | D | I | M | W | D | S | R | R | N | Q | Y | L | H | F | F | Q | G | L | T | N | L | T | H | L | D | I | S | E | N | Q | L | K | S | F | P | P | E | V | I | V | N | L | P | S | S | L | Q | M | L | R | M | D | S | N | V | L | S | Y | F | P | W | G | N | I | S | V | L | K | Q | L | C | H | L | N | L | S | S | N | M | L | S | F | L | P | N | - | - | M | H | F | E | L | R | L | V | S | L | D | L | S | H | N | R | L | V | V | I | P | K | A | F | L | S | Q | A | I | N | L | K | N | L | M | L | N | H | N | Q | L | K | I | L | D | V | Q | A | L | P | L | S | F | H | K | G | Y | T | F | C | P | A | G | P | H | K | N | K | S | S | C | K | L | V | L | H | A | N | P | F | T | C | S | C | V | I | S | G | F | A | K | F | L | R | E | T | Y | L | D | I | P | H | L | T | T | E | V | H | C | G | Y | P | E | S | L | A | G | V | N | V | L | S | I | D | L | H | S | C | Q | E | I | F | G | S | V | A | F | L | C | T | L | W | L | T | L | A | A | T | S | I | P | L | L | K | H | L | Y | G | W | D | L | W | Y | C | I | Q | I | L | W | T | G | Q | K | G | H | T | P | V | N | G | G | S | M | M | D | N | Q | Y | D | A | F | V | V | F | D | T | S | N | K | A | V | R | D | W | I | Y | K | E | M | V | V | R | L | E | N | R | G | R | W | R | F | R | L | C | L | E | E | R | D | W | M | P | G | V | S | C | I | E | N | L | H | K | A | V | Y | N | S | R | K | T | V | F | V | L | T | S | P | S | - | - | - | - | - | G | C | S | H | E | S | G | V | V | R | Q | A | F | L | L | V | Q | Q | R | L | L | D | E | K | V | D | V | A | V | L | V | L | L | D | L | L | F | P | K | F | K | Y | L | Q | M | R | K | R | L | C | K | K | S | V | L | S | W | P | K | N | P | R | V | Q | P | L | F | W | N | N | L | R | V | A | L | V | S | D | N | V | K | A | Y | N | K | N | V | T | E | S | F | F | X | X | X | X | X | X | X | X | X | X | X | X | X | X | - | - | - | - | - | - | - | - | - | - | - | - | - | - | - | - | - | - | - | - | - | - | - | - | - | - | - | - | - | - | - | - | - | - | - | - | - | - | - | - | - | - | - | - | - | - | - | - | - | - | - | - | - | - | - | - | - | - | - | - | - | - | - | - | - | - | - | - | - | - | - | - | - | - | - | - | - | - | - | - | - | - | - | - | - | - | - | - | - | - | - | - | - | - | - | - | - | - | - | - | - | - | - | - | - | - | - | - | - | - | - | - | - | - | - | - | - | - | - | - | - | - | - | - | - | - | - | - | - | - | - | - | - | - | - | - | - | - | - | - | - | - | - | - | - | - | - | - | - | - | - | - | - | - | - | - | - | - | - | - | - | - | - | - | - | - | - | - | - | - | - | - | - | - | - | - | - | - | - | - | - | - | - | - | - | - | - | - | - | - | - | - | - | - | - | - | - | - |
| CATLR9 | - | - | - | - | - | - | - | - | - | - | - | - | - | - | M | F | G | H | M | F | Y | L | A | F | I | L | N | Q | F | H | L | F | A | T | L | H | P | E | F | Y | P | C | E | I | H | T | T | K | D | G | H | I | N | V | D | C | Q | R | R | R | L | A | N | V | P | K | F | T | S | L | S | V | I | S | L | N | L | N | E | N | H | I | H | H | I | K | G | A | T | F | S | G | L | A | N | L | K | H | L | S | L | M | - | W | N | C | V | P | D | Y | L | K | E | L | R | W | P | S | C | S | L | K | I | D | P | N | A | F | S | G | L | K | N | L | S | S | L | Q | L | A | G | N | S | L | K | - | - | T | I | P | P | L | P | K | Q | L | E | I | L | G | L | E | F | N | H | I | F | H | I | V | E | P | L | G | T | P | L | L | K | Q | L | L | L | N | K | N | C | F | Y | A | N | P | C | Y | Q | S | Y | F | I | D | P | R | V | F | Q | G | L | P | E | L | L | N | L | T | L | S | Y | N | N | V | T | I | V | P | P | Y | L | P | L | S | L | E | S | L | D | L | G | E | N | K | I | T | H | I | N | K | E | S | F | A | N | L | K | N | L | R | H | L | N | L | R | W | N | C | Q | R | C | D | H | A | S | E | P | C | F | P | C | P | N | N | Q | S | L | N | L | H | P | D | A | F | L | D | Q | R | D | S | L | I | S | L | S | L | R | G | N | S | L | H | T | I | P | Q | H | L | F | A | R | L | H | K | - | L | Q | V | L | D | L | S | D | N | F | L | A | Y | A | I | Q | N | G | T | F | Y | E | E | L | Q | N | V | V | S | L | S | L | H | Y | N | Y | E | P | L | Q | S | F | S | E | L | I | L | S | P | S | I | E | K | M | T | A | L | R | E | L | Y | L | S | G | L | F | F | R | E | L | S | N | Y | S | L | A | P | L | I | K | L | P | R | L | E | S | L | E | L | R | M | N | F | I | C | S | V | S | M | D | A | I | S | Q | L | R | T | L | R | W | V | D | L | S | Q | N | M | L | A | F | S | S | C | F | S | T | C | T | A | E | D | I | S | N | N | Y | - | Q | T | L | E | K | R | G | N | G | Q | L | N | L | Q | T | Q | K | V | I | L | P | S | - | - | S | - | - | - | - | - | - | - | - | - | - | - | - | - | - | - | - | - | - | - | - | - | - | E | L | N | - | T | M | Q | D | P | G | E | N | Q | C | - | - | - | - | - | - | - | - | - | - | - | - | - | - | - | - | - | - | - | - | F | F | N | Y | S | M | W | H | F | K | K | L | I | C | S | K | N | L | Y | F | D | L | S | Q | N | N | I | P | W | L | N | A | S | T | F | R | G | M | E | K | V | V | C | I | D | L | S | Y | N | Y | I | S | Q | T | L | N | G | Q | Q | F | T | D | L | S | K | L | A | Y | L | N | M | A | N | N | R | I | D | L | Y | S | E | K | A | F | Q | E | I | S | G | T | L | K | A | L | D | L | S | K | N | E | F | H | F | V | M | K | G | M | G | H | R | F | T | F | L | P | H | L | T | S | L | Q | I | L | S | L | A | N | N | H | I | G | L | R | I | S | N | I | L | N | S | T | S | L | K | Y | L | D | F | S | G | N | R | L | D | I | M | W | D | S | R | R | N | Q | Y | L | H | F | F | Q | G | L | T | K | L | T | H | L | D | I | S | E | N | Q | L | K | S | F | P | P | E | V | I | V | N | L | P | P | S | L | Q | M | L | R | L | D | S | N | V | L | S | Y | F | P | W | G | N | I | S | V | L | E | K | L | C | H | L | N | L | S | S | N | R | L | S | F | L | P | N | - | - | I | H | F | E | L | R | L | V | S | L | D | L | S | H | N | R | L | V | A | I | P | K | A | F | L | S | Q | A | I | N | L | K | N | L | M | L | N | H | N | Q | L | K | I | L | D | V | Q | A | L | P | L | S | F | H | T | G | Y | T | F | C | P | A | G | P | H | K | N | K | S | S | C | K | L | V | L | H | A | N | P | F | T | C | S | C | V | I | S | G | F | A | K | F | L | R | E | T | S | L | D | I | P | H | L | T | T | E | V | H | C | G | F | P | E | S | L | A | G | V | N | V | L | S | I | D | L | H | S | C | Q | E | I | F | G | S | V | A | F | F | C | T | L | W | L | T | L | A | A | T | S | I | P | L | L | K | H | L | Y | G | W | D | L | W | Y | C | I | Q | I | L | W | T | G | H | K | G | H | T | P | V | N | G | G | S | M | M | D | N | Q | Y | D | A | F | V | V | F | D | T | S | N | K | A | V | R | D | W | I | Y | K | E | M | V | V | R | L | E | N | R | G | R | W | R | F | R | L | C | L | E | E | R | D | W | L | P | G | V | S | C | I | E | N | L | H | K | A | V | Y | N | S | R | K | T | V | F | V | L | T | S | P | N | - | - | - | - | - | G | C | S | H | E | S | G | V | V | R | Q | A | F | L | L | V | Q | Q | R | L | L | D | E | K | V | D | V | A | V | L | V | L | L | D | F | L | F | P | K | F | K | Y | L | Q | M | R | K | R | L | C | K | K | S | V | I | S | W | P | K | N | P | R | V | Q | P | L | F | W | N | N | L | R | V | A | L | V | S | D | N | V | R | A | Y | N | K | N | V | T | E | S | F | F | X | X | X | X | X | X | X | X | X | X | X | X | X | X | - | - | - | - | - | - | - | - | - | - | - | - | - | - | - | - | - | - | - | - | - | - | - | - | - | - | - | - | - | - | - | - | - | - | - | - | - | - | - | - | - | - | - | - | - | - | - | - | - | - | - | - | - | - | - | - | - | - | - | - | - | - | - | - | - | - | - | - | - | - | - | - | - | - | - | - | - | - | - | - | - | - | - | - | - | - | - | - | - | - | - | - | - | - | - | - | - | - | - | - | - | - | - | - | - | - | - | - | - | - | - | - | - | - | - | - | - | - | - | - | - | - | - | - | - | - | - | - | - | - | - | - | - | - | - | - | - | - | - | - | - | - | - | - | - | - | - | - | - | - | - | - | - | - | - | - | - | - | - | - | - | - | - | - | - | - | - | - | - | - | - | - | - | - | - | - | - | - | - | - | - | - | - | - | - | - | - | - | - | - | - | - | - | - | - | - | - | - |
| GMTLR9 | - | - | - | - | - | - | - | - | - | - | - | - | - | - | M | G | I | F | Q | K | V | V | I | L | L | L | S | Q | L | P | L | L | R | G | R | I | P | L | F | F | P | C | D | N | N | E | K | - | - | - | G | T | E | V | Y | C | T | H | R | P | L | D | D | I | P | F | F | T | S | V | N | V | T | F | L | D | L | S | H | T | K | I | R | E | V | G | Q | H | Q | F | S | G | I | P | N | L | I | T | L | N | M | T | - | G | N | C | D | P | N | I | I | R | K | I | N | N | L | P | C | G | V | R | V | H | E | D | A | F | R | S | L | H | N | L | T | Y | L | H | L | A | G | N | S | L | T | - | - | M | L | P | W | L | P | E | S | I | K | V | L | D | L | E | S | N | C | I | F | N | I | I | I | P | F | G | T | P | H | L | E | K | L | F | L | T | M | N | C | Y | N | E | N | P | C | H | Q | S | F | Y | I | H | E | D | V | Y | K | E | L | S | H | L | K | N | L | T | L | G | Y | N | N | L | T | S | I | P | L | G | L | P | A | S | L | M | T | L | D | L | K | E | N | T | I | T | E | I | P | E | G | A | F | A | N | F | S | N | L | E | N | L | N | L | E | W | N | C | Q | R | C | D | H | A | A | R | P | C | F | P | C | P | N | N | A | S | L | Q | L | H | P | K | S | F | N | I | K | N | S | S | I | R | F | L | S | L | R | G | N | S | L | N | S | I | P | K | G | I | F | L | P | L | T | N | - | L | R | R | L | D | L | S | D | N | Y | L | A | Y | T | I | R | N | G | T | F | F | T | E | L | K | Q | L | T | W | I | S | L | I | Y | N | Y | Q | P | Y | K | T | F | H | E | L | I | L | S | E | H | L | G | N | V | S | N | L | E | T | L | L | L | S | G | N | F | F | H | T | V | S | E | Q | S | L | V | V | L | S | R | L | K | Q | L | K | M | L | E | L | R | M | N | F | I | S | Y | F | N | M | T | T | V | G | M | L | T | S | L | N | K | L | V | L | S | Q | N | A | L | S | Y | N | P | C | N | V | S | S | G | N | S | L | D | Q | - | - | G | S | Q | D | P | N | V | F | K | Y | - | - | - | - | - | - | F | S | D | P | F | M | P | P | I | T | A | H | V | Y | Q | T | V | P | E | S | D | M | C | I | T | N | - | - | - | - | - | - | - | - | - | - | - | - | - | - | - | - | - | - | - | - | - | - | - | - | - | - | - | - | - | - | - | - | - | - | - | - | - | - | - | - | - | N | I | M | F | G | M | S | N | F | T | Q | R | F | C | G | N | N | L | T | I | D | L | S | Q | N | N | L | M | F | L | H | E | H | V | F | D | G | M | E | N | V | L | C | L | N | L | S | Y | N | Y | A | S | Q | A | L | E | G | G | L | F | S | R | L | K | S | L | V | Y | L | D | M | S | F | N | R | I | D | L | Y | Y | K | E | A | F | S | E | L | N | R | S | L | K | V | L | D | L | S | H | N | E | Y | H | F | R | M | N | G | M | G | H | R | L | E | F | I | E | N | L | I | N | L | E | V | L | N | L | A | N | N | G | M | G | M | R | I | D | P | R | L | R | S | N | S | V | K | Y | M | Y | F | S | G | N | R | L | D | I | M | W | D | I | E | G | Q | T | Y | I | N | F | F | Q | E | L | K | E | L | I | Y | L | D | I | S | Q | N | M | L | R | S | F | E | Q | R | I | L | F | N | L | P | V | S | I | Q | A | L | S | L | S | N | N | Y | L | L | D | F | P | W | Q | N | I | T | F | F | S | N | L | T | H | L | S | L | S | G | N | H | L | S | V | L | P | N | N | V | T | K | F | P | P | Q | F | S | L | L | D | L | S | G | N | Q | I | T | H | L | P | D | K | F | F | S | L | A | N | V | L | Q | C | L | Y | L | N | R | N | S | L | K | V | L | N | L | Q | S | F | P | H | L | L | K | N | G | T | S | H | - | - | - | - | - | - | - | - | - | - | - | Q | L | L | T | L | H | D | N | P | F | I | C | D | C | H | N | S | G | L | H | E | F | L | R | T | S | N | T | R | I | P | Y | L | T | T | A | V | Q | C | E | F | P | E | S | L | Q | G | Q | S | V | L | T | V | D | Q | R | S | C | Q | E | I | Y | G | G | L | G | F | L | L | S | T | L | F | T | C | A | F | I | A | L | P | L | F | R | H | L | Y | G | W | D | V | W | Y | C | L | Q | I | L | W | A | G | C | K | G | Y | L | H | P | S | A | S | - | C | S | P | K | H | Y | D | A | F | M | V | F | D | T | N | N | Q | A | V | R | D | W | V | Y | N | E | L | V | I | R | L | E | S | S | G | H | R | R | F | S | L | C | L | E | E | R | D | W | I | P | G | L | S | C | I | E | N | L | H | Y | A | V | H | S | S | E | K | T | V | F | V | L | S | N | C | T | R | N | - | D | A | G | E | A | T | V | N | G | V | I | Q | Q | T | F | F | M | V | Q | Q | R | L | L | D | E | K | V | D | V | A | V | V | I | L | L | D | K | M | F | P | K | L | K | Y | L | Q | L | R | T | R | L | C | R | K | S | V | M | S | W | P | R | N | P | K | A | Q | P | L | F | W | N | Q | I | R | T | A | L | S | S | D | N | L | Q | I | Y | D | N | N | F | S | E | G | F | I | X | X | X | X | X | X | X | X | X | X | X | X | X | X | X | X | X | X | X | X | X | X | X | X | X | X | - | - | - | - | - | - | - | - | - | - | - | - | - | - | - | - | - | - | - | - | - | - | - | - | - | - | - | - | - | - | - | - | - | - | - | - | - | - | - | - | - | - | - | - | - | - | - | - | - | - | - | - | - | - | - | - | - | - | - | - | - | - | - | - | - | - | - | - | - | - | - | - | - | - | - | - | - | - | - | - | - | - | - | - | - | - | - | - | - | - | - | - | - | - | - | - | - | - | - | - | - | - | - | - | - | - | - | - | - | - | - | - | - | - | - | - | - | - | - | - | - | - | - | - | - | - | - | - | - | - | - | - | - | - | - | - | - | - | - | - | - | - | - | - | - | - | - | - | - | - | - | - | - | - | - | - | - | - | - | - | - | - | - | - | - | - | - | - | - | - | - | - | - | - | - | - | - | - | - | - | - | - | - | - | - | - |
| SSTLR9 | - | - | - | - | - | - | - | - | - | - | - | - | - | - | M | Q | G | Y | - | G | V | L | L | C | L | C | L | L | T | P | A | V | R | T | T | N | P | K | F | F | P | C | D | S | N | A | D | - | - | - | S | S | V | V | N | C | S | R | R | A | L | T | R | I | P | V | I | C | S | A | S | V | L | S | L | D | L | S | Y | T | G | I | Q | Q | V | G | K | D | A | F | S | G | V | P | N | L | Q | K | L | S | M | T | - | R | N | C | L | P | S | H | M | R | A | L | G | S | P | S | C | H | V | E | I | H | K | D | A | F | M | C | L | K | N | L | S | T | L | L | L | E | G | N | S | L | T | S | - | P | L | P | R | L | P | D | S | L | E | V | L | N | L | E | S | N | H | L | Y | N | I | T | Q | P | L | G | T | P | N | L | K | Q | L | L | L | T | M | N | C | Y | Y | G | N | P | C | N | Q | S | F | H | I | D | E | A | V | F | R | E | L | T | Q | L | Q | Y | L | T | L | G | F | N | N | V | T | S | I | P | P | G | M | P | P | S | L | K | R | L | D | L | N | E | N | K | I | S | V | L | N | E | W | A | F | A | N | L | T | Q | L | C | Q | L | N | L | G | W | N | C | Q | R | C | D | H | A | A | Q | P | C | F | P | C | F | N | N | T | P | L | D | I | N | P | R | A | F | Y | A | Q | N | N | S | L | K | F | L | S | L | R | G | N | S | L | R | N | I | P | E | G | L | F | S | P | L | V | H | - | L | E | R | L | D | L | S | D | N | L | L | A | I | A | I | R | T | G | T | F | F | I | E | L | K | R | L | T | W | I | S | L | I | Y | N | Y | E | P | M | K | T | F | K | E | L | V | L | S | P | N | L | T | Q | M | S | G | L | K | T | L | L | L | S | G | N | F | F | H | M | V | S | E | E | T | V | A | V | L | A | K | F | R | W | L | E | V | L | E | L | R | M | N | F | I | R | S | C | N | L | S | A | L | A | Q | L | P | A | L | V | R | V | G | L | S | Q | N | M | L | E | F | F | P | S | F | S | T | Q | S | N | M | C | K | S | F | K | S | F | Q | N | Q | N | R | Y | D | T | - | - | - | - | V | E | L | Q | N | P | P | M | L | L | S | D | R | K | - | - | - | A | V | P | - | D | E | P | W | H | H | P | G | P | V | V | T | A | A | E | P | G | P | N | T | L | A | I | L | E | D | E | C | T | Q | - | - | - | - | - | - | - | - | - | - | - | - | - | - | - | - | - | - | - | - | N | P | T | I | F | S | F | K | N | N | L | C | N | G | A | M | S | F | D | L | S | Q | N | N | I | L | A | L | N | G | S | L | F | L | G | M | E | K | A | V | C | L | D | L | S | Y | N | Y | M | S | Q | S | L | N | G | K | Q | F | L | H | L | D | N | L | A | Y | L | S | M | A | H | N | R | I | D | L | Y | Y | G | D | A | F | K | E | L | N | A | T | L | K | A | L | D | L | S | N | N | E | F | H | F | L | M | R | G | M | G | H | R | F | E | F | I | K | N | L | P | N | L | E | V | L | S | L | S | D | N | S | I | G | M | R | I | D | H | T | L | Y | S | D | S | L | R | Y | L | Y | F | S | G | N | N | L | D | I | M | W | D | T | R | S | N | D | Y | I | T | F | F | Q | N | L | T | N | L | I | Y | L | D | I | S | R | N | Q | L | R | S | L | S | P | A | A | F | C | N | L | P | V | S | L | Q | V | L | R | V | S | D | N | K | L | N | Y | F | P | W | E | N | I | T | A | L | G | R | L | C | H | L | N | L | S | L | N | Y | L | S | E | L | P | D | K | V | I | H | F | Q | A | K | L | T | L | L | D | L | S | H | N | Q | I | S | F | L | P | E | D | F | F | S | Q | A | L | A | L | R | F | L | Y | L | N | H | N | K | L | K | L | L | D | R | Q | S | L | P | A | P | L | R | Y | G | S | A | L | - | - | - | - | - | - | - | - | - | - | - | Q | L | L | T | L | H | A | N | P | F | D | C | S | C | D | T | S | W | F | A | D | F | L | R | A | S | Q | V | E | I | L | L | L | T | T | A | V | H | C | G | F | P | E | S | Q | Q | G | A | S | V | L | S | M | D | P | R | S | C | Q | E | I | Y | G | S | L | T | F | L | S | M | T | F | L | T | L | V | F | T | A | L | P | L | L | R | H | L | Y | G | W | D | V | W | Y | C | F | Q | V | L | W | A | G | H | K | G | Y | S | Q | L | P | G | G | - | N | F | Q | S | Q | Y | D | A | F | V | V | F | D | T | G | N | H | A | V | R | D | W | V | Y | N | E | L | L | I | H | L | E | N | V | G | R | R | R | F | C | L | C | L | E | E | R | D | W | V | P | G | L | S | C | I | E | N | L | H | S | A | V | Y | S | S | M | K | T | V | F | V | L | T | S | G | A | S | G | G | G | A | R | V | S | R | V | D | G | V | T | R | Q | A | F | Y | M | V | Q | Q | R | L | L | D | E | K | V | D | V | A | V | L | V | L | L | D | E | V | F | P | K | L | K | Y | L | Q | L | R | K | R | L | C | R | K | S | V | L | S | W | P | R | N | P | Q | A | Q | P | L | F | W | N | N | M | K | T | A | L | A | S | D | N | I | R | S | Y | D | S | N | V | S | E | S | F | I | X | X | X | X | - | - | - | - | - | - | - | - | - | - | - | - | - | - | - | - | - | - | - | - | - | - | - | - | - | - | - | - | - | - | - | - | - | - | - | - | - | - | - | - | - | - | - | - | - | - | - | - | - | - | - | - | - | - | - | - | - | - | - | - | - | - | - | - | - | - | - | - | - | - | - | - | - | - | - | - | - | - | - | - | - | - | - | - | - | - | - | - | - | - | - | - | - | - | - | - | - | - | - | - | - | - | - | - | - | - | - | - | - | - | - | - | - | - | - | - | - | - | - | - | - | - | - | - | - | - | - | - | - | - | - | - | - | - | - | - | - | - | - | - | - | - | - | - | - | - | - | - | - | - | - | - | - | - | - | - | - | - | - | - | - | - | - | - | - | - | - | - | - | - | - | - | - | - | - | - | - | - | - | - | - | - | - | - | - | - | - | - | - | - | - | - | - | - | - | - | - | - | - | - | - | - | - | - | - | - | - | - |
| OMTLR9 | - | - | - | - | - | - | - | - | - | - | - | - | - | - | M | Q | G | Y | - | G | V | L | L | C | L | C | L | L | P | P | A | V | R | T | T | N | P | K | F | F | P | C | D | S | N | A | D | - | - | - | S | S | V | V | N | C | S | R | R | A | L | T | R | I | P | V | I | C | S | A | S | V | L | S | L | D | L | S | Y | T | G | I | Q | Q | V | G | K | D | A | F | S | G | V | P | N | L | Q | K | L | S | M | T | - | R | N | C | L | P | S | H | M | R | A | L | G | S | P | S | C | H | V | E | I | H | K | D | A | F | M | C | L | K | N | L | S | T | L | L | L | E | G | N | S | L | T | S | - | P | L | P | R | L | P | D | S | L | E | V | L | N | L | E | S | N | H | L | Y | N | I | T | Q | P | L | G | T | P | N | L | K | Q | L | L | L | T | M | N | C | Y | Y | G | N | P | C | N | Q | S | F | H | I | D | E | A | V | F | R | E | L | T | Q | L | Q | T | L | T | L | G | F | N | N | V | T | S | I | P | P | G | M | P | P | S | L | K | S | L | D | L | N | E | N | K | I | S | V | L | D | E | W | A | F | A | N | L | T | Q | L | C | K | L | N | L | G | W | N | C | Q | R | C | D | H | A | A | Q | P | C | F | P | C | F | N | N | T | P | L | D | I | N | P | H | A | F | Y | A | Q | N | N | S | L | K | F | L | S | L | R | G | N | S | L | R | N | I | P | E | G | L | F | S | P | L | V | H | - | L | E | R | L | D | L | S | D | N | L | L | A | I | A | I | R | T | G | T | F | F | I | E | L | K | R | L | T | W | I | S | L | I | Y | N | Y | E | P | M | T | T | F | K | E | L | V | L | S | P | N | V | T | Q | M | S | G | L | K | T | L | L | L | S | G | N | F | F | H | M | V | S | E | E | S | V | A | V | L | A | K | F | R | W | L | E | V | L | E | L | R | M | N | F | I | R | S | C | N | L | S | A | L | A | Q | L | P | A | L | V | R | V | D | L | S | Q | N | M | L | E | F | L | P | S | F | S | T | Q | S | N | V | C | K | S | F | K | S | F | Q | N | Q | N | R | Y | A | T | - | - | - | - | V | E | L | Q | N | P | P | M | L | L | S | D | R | K | - | - | - | A | V | P | - | D | E | P | W | H | H | P | G | P | V | V | T | A | A | E | P | G | P | N | T | L | A | I | L | E | D | E | C | T | Q | - | - | - | - | - | - | - | - | - | - | - | - | - | - | - | - | - | - | - | - | N | P | T | I | L | S | F | K | N | N | L | C | N | G | A | M | S | F | D | L | S | Q | N | N | I | L | T | L | N | S | S | L | F | L | G | M | E | K | A | V | C | L | D | L | S | Y | N | Y | M | S | Q | S | L | N | G | K | Q | F | L | H | L | D | N | L | A | Y | L | S | M | A | H | N | R | I | D | L | Y | Y | G | D | A | F | K | E | L | S | A | T | L | K | T | L | D | L | S | N | N | E | F | H | F | L | M | R | G | M | G | H | R | F | E | F | I | K | N | L | P | N | L | E | A | L | S | L | S | D | N | S | I | G | M | R | I | D | H | T | L | Y | S | D | S | L | R | Y | L | Y | F | S | G | N | N | L | D | I | M | W | D | T | R | S | N | D | Y | I | T | F | F | Q | N | L | T | N | L | I | Y | L | D | I | S | R | N | Q | L | R | S | L | S | P | A | A | F | C | N | L | P | V | S | L | K | V | L | R | V | S | N | N | K | L | N | Y | F | P | W | E | N | I | T | A | L | G | Q | L | C | H | L | N | L | S | V | N | Y | L | S | E | L | P | D | K | V | I | P | F | Q | A | N | L | T | L | L | D | L | S | H | N | Q | I | S | F | L | P | E | D | F | F | S | Q | A | L | A | L | R | F | L | Y | L | N | H | N | K | L | K | L | L | D | R | Q | S | L | P | A | P | L | R | N | G | S | A | L | - | - | - | - | - | - | - | - | - | - | - | Q | L | L | T | L | H | A | N | P | F | D | C | S | C | D | T | S | W | F | A | D | F | L | R | A | G | Q | V | E | I | L | L | L | T | T | G | V | H | C | G | F | P | E | S | Q | Q | G | A | S | V | L | S | M | D | P | R | S | C | Q | E | I | Y | G | S | L | A | F | L | S | I | T | F | L | T | L | V | F | T | A | L | P | L | L | R | H | L | Y | G | W | D | V | W | Y | C | F | Q | V | L | W | A | G | H | K | G | Y | S | Q | L | P | G | A | - | N | F | Q | S | Q | Y | D | A | F | V | V | F | D | T | G | N | H | A | V | R | D | W | V | Y | N | E | L | L | V | H | L | E | N | V | G | R | R | R | F | C | L | C | L | E | E | R | D | W | V | P | G | L | S | C | I | E | N | L | H | S | A | V | Y | S | S | M | K | T | V | F | V | L | T | S | R | A | S | G | E | G | G | R | V | S | I | V | N | G | V | T | R | Q | A | F | Y | M | V | Q | Q | R | L | L | D | E | K | V | D | V | A | V | L | V | L | L | D | E | V | F | P | K | L | K | Y | L | Q | L | R | K | R | L | C | R | K | F | V | L | S | W | P | R | N | P | Q | A | Q | P | L | F | W | N | H | M | K | T | A | L | A | S | D | S | I | R | S | Y | D | S | N | V | N | E | S | F | I | X | X | X | X | - | - | - | - | - | - | - | - | - | - | - | - | - | - | - | - | - | - | - | - | - | - | - | - | - | - | - | - | - | - | - | - | - | - | - | - | - | - | - | - | - | - | - | - | - | - | - | - | - | - | - | - | - | - | - | - | - | - | - | - | - | - | - | - | - | - | - | - | - | - | - | - | - | - | - | - | - | - | - | - | - | - | - | - | - | - | - | - | - | - | - | - | - | - | - | - | - | - | - | - | - | - | - | - | - | - | - | - | - | - | - | - | - | - | - | - | - | - | - | - | - | - | - | - | - | - | - | - | - | - | - | - | - | - | - | - | - | - | - | - | - | - | - | - | - | - | - | - | - | - | - | - | - | - | - | - | - | - | - | - | - | - | - | - | - | - | - | - | - | - | - | - | - | - | - | - | - | - | - | - | - | - | - | - | - | - | - | - | - | - | - | - | - | - | - | - | - | - | - | - | - | - | - | - | - | - | - | - |
| LCTLR9 | - | - | - | - | - | - | - | - | - | - | - | - | - | - | - | - | - | - | - | - | - | - | - | - | - | - | - | - | - | - | - | - | - | - | - | - | - | - | - | - | - | - | - | - | - | - | - | - | - | - | - | - | - | - | - | - | - | - | - | - | - | - | - | - | - | - | - | - | - | - | - | - | - | - | - | - | - | - | - | - | - | - | - | - | - | - | - | - | - | - | - | - | - | - | - | - | - | - | - | - | - | - | - | - | - | - | - | - | - | - | - | - | - | - | - | - | - | - | - | - | M | E | I | H | H | Y | A | F | K | S | L | L | K | L | E | Y | L | H | L | S | G | N | S | L | T | - | - | S | I | P | W | L | P | E | T | L | R | V | L | D | L | Q | N | N | R | I | F | H | I | V | Q | P | L | N | T | P | N | L | E | N | L | F | L | T | R | N | C | F | Y | A | N | P | C | N | Q | S | F | Y | I | K | Q | L | H | - | - | H | L | P | K | L | E | R | - | - | - | - | - | - | - | - | - | - | - | - | - | - | - | - | - | - | - | - | - | - | - | - | - | - | - | - | - | - | - | - | - | - | - | - | - | - | - | - | - | - | - | - | - | - | - | - | - | - | - | - | Q | L | S | E | N | I | S | R | G | C | F | - | - | - | - | - | - | - | - | - | - | - | - | - | - | - | - | - | - | - | - | - | - | - | - | - | - | - | - | - | - | - | - | - | - | - | - | - | - | - | - | Q | T | F | E | E | F | K | E | X | L | D | L | S | D | N | L | L | A | Y | T | M | Q | N | G | T | F | F | T | E | L | K | G | L | T | W | I | S | L | I | Y | N | Y | E | P | L | K | T | F | P | E | L | I | L | S | P | H | I | V | D | I | S | G | L | Q | Y | L | L | L | S | G | N | F | F | H | Q | L | S | N | Q | S | L | V | V | L | S | K | L | K | N | L | R | K | L | E | L | R | M | N | F | I | N | T | C | N | L | T | D | L | K | Q | L | T | H | L | I | N | I | D | L | S | Q | N | M | L | N | F | L | P | C | C | S | S | L | S | N | E | I | V | A | Q | K | S | C | Q | N | Q | N | L | Y | T | H | - | - | - | - | D | - | L | R | D | Q | P | F | I | L | R | D | R | D | - | - | - | V | T | S | G | S | E | I | W | E | S | N | - | - | - | - | - | - | - | - | - | Q | S | N | V | Y | G | M | L | E | D | S | A | P | R | F | - | - | - | - | - | - | - | - | - | - | - | - | - | - | - | - | - | - | - | - | P | S | L | W | D | F | R | N | N | F | C | R | H | K | L | T | F | D | L | S | Q | N | D | I | L | S | L | N | K | N | V | F | V | G | M | E | N | A | V | C | L | D | L | S | F | N | Y | M | S | Q | A | L | R | G | G | E | F | D | S | V | K | N | L | V | F | L | D | L | S | Y | N | R | L | D | F | Y | Y | S | N | A | F | S | E | L | K | T | T | L | K | V | L | D | V | S | N | N | D | F | H | F | K | M | K | G | M | G | H | H | F | D | F | L | H | N | L | T | N | L | E | V | L | S | L | A | N | N | G | I | E | M | R | I | D | Q | R | L | I | S | S | S | V | K | Y | L | Y | F | Y | G | N | H | L | C | T | M | W | E | S | D | N | N | K | Y | T | H | F | F | Q | N | L | T | N | L | T | Y | L | D | I | S | N | N | D | L | I | S | I | S | P | E | V | L | C | N | F | P | G | N | L | E | T | L | S | I | S | N | N | L | L | N | Y | F | P | W | Q | N | I | S | A | L | S | N | L | C | H | L | D | L | S | Q | N | H | L | S | Y | L | P | D | R | V | I | E | F | G | A | N | F | S | L | L | D | L | S | H | N | H | L | S | F | V | P | E | N | F | F | R | K | V | K | S | L | K | Y | L | Y | L | S | H | N | Q | I | K | E | L | N | H | Q | Y | F | P | A | P | F | K | N | G | S | A | L | - | - | - | - | - | - | - | - | - | - | - | Q | Q | L | T | L | H | A | N | P | F | K | C | D | C | D | T | S | W | F | A | D | F | L | R | T | T | P | V | K | I | P | Y | L | T | T | Y | I | H | C | E | F | P | E | S | Q | Q | G | E | S | I | L | S | M | D | Q | R | S | C | Q | D | I | Y | G | S | L | A | F | L | I | C | S | F | M | A | V | T | F | T | V | L | P | L | L | K | H | L | Y | G | W | D | M | W | Y | C | L | Q | V | L | W | A | G | H | K | G | Y | S | Q | L | A | G | T | - | D | S | Q | Y | H | Y | D | A | F | V | V | F | D | T | R | N | Q | A | V | R | D | W | V | Y | N | E | L | T | V | N | L | E | K | L | G | H | R | R | F | C | L | C | L | E | E | R | D | W | I | P | G | L | S | C | I | E | N | L | H | S | A | V | Y | N | S | V | K | T | V | F | V | L | S | S | G | F | N | G | G | E | - | - | - | - | M | V | N | G | V | I | R | Q | A | F | F | M | V | Q | Q | R | L | L | D | E | K | V | D | A | A | V | L | V | L | L | D | E | M | F | P | K | L | K | Y | L | Q | L | R | K | R | L | C | R | K | S | V | L | S | W | P | R | N | P | R | V | Q | P | L | F | W | N | R | M | R | M | V | L | S | S | D | N | L | K | F | Y | D | N | N | M | S | E | S | F | I | X | X | X | X | X | X | X | X | X | X | X | X | X | X | X | X | X | X | X | X | X | X | X | X | X | X | X | X | X | X | X | X | X | X | X | X | X | X | X | X | X | X | X | X | X | X | X | X | X | X | X | X | X | X | X | X | X | X | X | X | X | X | X | X | X | X | X | X | X | X | X | X | X | X | X | X | X | X | X | X | X | X | X | X | X | X | X | X | X | X | X | X | X | X | X | X | X | X | X | X | X | X | X | X | X | X | X | X | X | X | X | X | X | X | X | X | X | X | X | X | X | X | X | X | X | X | X | X | X | X | X | X | X | X | X | X | X | X | X | X | X | X | X | X | X | X | X | X | X | X | X | X | X | X | X | X | X | X | X | X | X | X | X | X | X | X | X | X | X | X | X | X | X | X | X | X | X | X | X | X | X | X | X | X | X | X | X | X | X | X | X | X | X | X | X | X | X | X | X | X | X | X | X | X | X | X | X | X | X | X | X | X |
| TRTLR9 | - | - | - | - | - | - | - | - | - | - | - | - | - | - | - | - | - | - | - | M | R | F | L | I | L | C | Q | L | L | P | I | V | S | S | I | N | P | I | F | L | P | C | D | T | D | A | N | - | - | - | T | S | R | V | D | C | S | D | R | P | L | R | R | V | P | L | I | K | S | D | T | V | T | S | L | R | L | S | R | T | K | I | R | H | L | W | Q | Y | D | L | S | G | V | P | N | L | R | A | F | T | M | A | - | D | N | C | Q | P | S | S | M | K | A | - | S | L | H | D | C | E | V | K | I | D | H | G | V | F | R | N | L | S | L | L | Q | V | L | N | L | S | G | N | S | L | T | - | - | S | L | P | K | L | P | A | N | L | R | V | L | N | L | Q | R | N | H | L | F | N | I | V | E | P | L | K | T | P | H | L | Q | E | L | Y | L | T | K | N | C | F | Y | A | N | P | C | Q | Q | S | L | E | I | S | K | T | V | F | R | E | L | S | R | L | K | T | L | T | L | A | Y | N | N | L | T | S | V | P | E | G | L | P | S | S | L | E | I | L | D | L | S | E | N | T | I | T | E | V | L | E | G | A | F | A | D | L | T | H | L | R | E | L | N | L | E | W | N | C | Q | R | C | D | R | A | A | R | P | C | F | P | C | P | R | H | L | P | L | Q | L | H | S | K | S | F | Y | A | N | K | S | T | L | T | Y | L | N | L | R | G | N | S | L | R | T | F | P | K | G | L | F | Q | P | L | K | N | - | L | K | M | L | D | L | S | D | N | F | L | S | Y | A | I | Q | N | G | T | F | F | A | E | L | T | S | L | A | W | I | S | L | I | Y | N | Y | E | P | L | K | M | F | P | E | L | F | L | S | P | H | I | G | D | I | S | G | L | Q | Y | L | L | L | S | G | N | F | F | H | S | L | S | G | Q | S | F | E | V | L | S | K | L | R | N | L | K | K | L | E | L | R | M | N | F | I | D | N | I | N | M | K | A | L | H | Q | V | P | S | L | V | H | I | D | L | S | Q | N | R | L | S | F | I | P | Q | C | S | V | S | P | A | E | R | - | - | - | E | S | H | H | N | Q | N | V | R | D | L | - | - | - | - | S | - | F | S | Q | P | F | L | M | E | T | K | P | N | - | - | - | V | T | F | G | F | N | F | L | D | - | - | - | - | - | - | - | - | - | - | - | Q | S | H | R | L | E | T | S | Q | S | F | P | T | Q | - | - | - | - | - | - | - | - | - | - | - | - | - | - | - | - | - | - | - | - | S | S | P | L | W | E | - | - | - | T | F | C | K | N | K | V | T | F | D | L | S | Q | N | D | I | M | S | V | N | Q | E | V | L | A | G | M | E | N | A | V | C | L | D | L | S | F | N | Y | M | S | Q | A | L | K | Y | G | M | F | S | S | M | K | H | L | V | F | L | N | L | S | Y | N | R | L | D | F | Y | Y | N | E | S | F | S | E | L | N | N | T | L | K | L | L | D | L | S | N | N | D | F | H | F | K | M | R | G | M | G | H | R | L | T | F | I | K | N | L | V | N | L | E | V | L | S | L | A | N | N | A | I | A | M | R | I | D | Q | R | L | V | S | A | S | L | K | Q | L | S | F | N | G | N | D | L | N | T | M | W | S | S | E | N | N | Q | Y | V | N | F | F | H | N | L | T | S | L | T | Y | L | D | I | S | D | N | K | L | R | L | V | S | P | E | V | L | C | N | L | P | R | S | L | Q | N | L | S | L | S | N | N | R | L | N | Y | F | P | W | E | N | I | S | V | L | S | N | L | R | H | L | D | L | S | Q | N | F | I | S | F | L | P | Y | T | V | V | H | A | A | E | P | L | S | L | L | D | L | S | H | N | R | I | G | Y | V | P | R | S | F | F | L | A | M | N | S | L | Q | R | L | Y | L | S | H | N | Q | L | K | Q | L | N | Q | H | F | L | P | A | P | F | K | N | G | S | G | P | - | - | - | - | - | - | - | - | - | - | - | R | T | L | S | L | H | V | N | P | F | K | C | D | C | D | A | S | W | F | A | D | F | L | R | N | T | P | I | E | I | P | H | L | T | T | N | V | H | C | E | Y | P | E | S | Q | R | G | K | T | I | L | S | M | D | Q | R | S | C | Q | D | I | Y | G | N | L | A | S | V | V | C | S | F | L | V | I | G | F | T | V | L | P | L | L | K | H | L | Y | G | W | D | L | W | Y | S | L | Q | V | L | W | A | G | F | K | G | Y | S | Q | L | T | G | Q | - | D | T | K | Y | N | Y | D | A | F | V | V | F | D | T | S | D | L | A | V | R | D | W | V | Y | N | E | L | L | V | N | L | E | N | S | A | H | R | M | F | C | L | C | L | E | E | R | D | W | V | P | G | F | S | C | I | E | N | L | H | N | A | V | S | S | S | V | K | T | M | F | V | L | S | T | G | T | I | - | - | E | - | - | - | - | S | M | S | G | M | I | R | Q | A | F | F | M | V | Q | Q | R | L | L | D | E | K | V | D | T | A | V | L | V | L | L | D | E | M | F | P | K | L | K | Y | L | Q | L | R | K | R | L | C | R | K | S | V | L | S | W | P | R | N | P | K | A | Q | P | L | F | W | N | Q | L | R | M | A | L | S | S | D | N | L | S | F | Y | D | N | N | M | S | E | S | F | I | X | X | X | X | X | X | X | X | X | X | X | X | X | X | X | X | X | X | X | X | X | X | X | X | X | X | X | X | X | X | X | X | X | - | - | - | - | - | - | - | - | - | - | - | - | - | - | - | - | - | - | - | - | - | - | - | - | - | - | - | - | - | - | - | - | - | - | - | - | - | - | - | - | - | - | - | - | - | - | - | - | - | - | - | - | - | - | - | - | - | - | - | - | - | - | - | - | - | - | - | - | - | - | - | - | - | - | - | - | - | - | - | - | - | - | - | - | - | - | - | - | - | - | - | - | - | - | - | - | - | - | - | - | - | - | - | - | - | - | - | - | - | - | - | - | - | - | - | - | - | - | - | - | - | - | - | - | - | - | - | - | - | - | - | - | - | - | - | - | - | - | - | - | - | - | - | - | - | - | - | - | - | - | - | - | - | - | - | - | - | - | - | - | - | - | - | - | - | - | - | - | - | - | - | - | - | - | - | - | - | - | - |
| ONTLR9 | - | - | - | - | - | - | - | - | M | H | S | S | C | A | V | A | L | V | - | K | N | I | L | I | L | S | Q | L | F | P | L | V | K | T | I | N | T | K | F | L | P | C | D | T | N | L | N | - | - | - | E | T | K | V | D | C | S | D | R | P | L | K | H | V | P | F | I | K | A | T | S | V | A | Y | I | D | L | S | R | T | K | I | N | R | V | W | P | H | A | F | T | G | V | P | N | L | H | T | L | K | L | S | - | G | N | C | Q | P | G | S | P | R | A | L | E | D | R | S | C | R | V | K | I | S | R | Y | A | F | K | P | L | L | T | L | K | H | L | D | L | S | G | N | S | L | T | - | - | Y | I | P | W | L | P | E | S | L | E | V | L | H | L | E | N | N | Q | I | F | N | V | I | Y | P | L | K | T | P | H | L | E | K | L | F | L | S | K | N | C | F | Y | A | N | P | C | N | Q | S | F | Y | I | S | E | K | V | F | Q | E | L | P | K | L | K | N | L | T | L | G | Y | N | N | L | T | A | I | P | K | G | L | P | L | S | L | G | S | L | D | L | R | E | N | T | I | T | E | V | L | E | K | A | F | F | K | M | T | A | L | E | D | L | N | L | E | W | N | C | Q | R | C | D | H | A | A | R | P | C | F | P | C | P | H | N | Q | S | L | K | L | H | S | N | S | F | Y | S | E | N | S | S | I | T | I | L | S | L | R | G | N | S | L | R | T | F | P | K | G | L | F | R | P | L | K | K | - | L | K | I | L | D | L | S | D | N | L | L | A | Y | A | M | Q | N | G | T | F | F | K | E | L | T | G | V | E | W | L | S | L | I | Y | N | Y | E | P | L | K | T | F | P | E | L | N | L | S | P | Y | F | N | N | M | S | D | L | Q | C | L | L | L | S | G | N | F | F | L | K | F | S | N | E | S | I | E | V | L | S | K | L | K | S | L | K | V | L | E | L | R | M | N | F | I | N | T | F | N | M | T | F | L | K | Q | L | P | S | L | T | S | V | D | L | S | Q | N | M | L | N | F | L | Q | C | C | S | I | S | R | S | E | I | V | - | - | - | - | - | - | D | E | N | L | Y | A | G | - | - | - | - | T | - | L | H | N | Q | P | L | T | L | M | A | K | E | - | - | - | V | T | S | R | N | S | V | W | E | T | E | - | - | - | - | - | - | - | - | - | Q | L | N | A | L | E | I | L | E | D | S | V | T | D | I | P | T | L | L | D | F | Q | L | R | A | D | V | L | D | K | R | F | P | V | I | T | S | L | W | D | L | R | N | F | H | C | K | N | K | L | T | F | D | L | S | Q | N | D | I | V | Y | L | N | K | H | V | V | S | G | M | E | N | A | V | C | L | D | L | S | F | N | Y | M | N | Q | A | L | R | G | G | V | F | E | G | M | T | H | L | E | F | L | N | L | S | Y | N | R | L | D | L | Y | Y | N | D | A | F | R | E | L | N | S | S | L | K | I | L | D | I | S | N | N | E | F | H | F | N | M | R | G | M | G | H | R | F | E | F | L | E | N | L | N | Q | L | E | A | L | S | L | A | N | N | A | I | G | M | R | I | N | Q | R | L | I | C | N | S | L | K | Y | F | D | F | S | G | N | N | L | N | I | M | W | D | S | D | N | N | K | Y | T | N | F | F | Q | N | L | A | N | L | T | Y | L | D | I | S | N | N | H | L | K | S | L | S | S | E | V | L | C | N | L | P | S | S | L | R | A | L | I | I | S | S | N | S | L | N | Y | F | P | W | L | N | I | S | A | L | T | S | L | H | Y | L | N | L | N | H | N | F | L | Y | K | L | P | N | Y | A | V | I | F | G | V | N | F | T | F | L | D | L | S | H | N | R | I | S | Y | I | P | E | D | F | F | N | N | A | K | S | L | K | Y | L | Y | L | S | H | N | Q | I | K | E | L | N | R | E | H | L | P | V | L | F | K | N | D | T | R | L | - | - | - | - | - | - | - | - | - | - | - | E | N | L | T | L | H | A | N | P | F | K | C | D | C | N | T | S | W | L | A | D | F | L | S | T | T | P | V | H | I | P | Y | L | T | T | D | V | R | C | E | F | P | E | S | Q | Q | G | E | S | I | L | S | M | D | Q | R | S | C | Q | D | I | Y | G | S | V | A | F | V | A | C | S | F | M | T | L | V | F | T | V | L | P | L | L | K | H | L | Y | G | W | D | M | W | Y | C | L | Q | V | L | W | A | G | H | K | G | Y | S | Q | I | A | G | T | - | D | S | H | Y | Q | Y | D | A | F | V | V | F | D | T | S | N | Q | A | V | R | D | W | V | Y | N | E | L | T | V | N | L | E | N | S | G | H | R | R | F | C | L | C | L | E | E | R | D | W | I | P | G | L | S | C | I | E | N | L | H | N | A | V | Y | R | S | V | K | T | V | F | V | L | S | R | G | V | N | G | - | - | - | - | - | - | N | V | N | G | V | I | R | Q | A | F | F | M | V | Q | Q | R | L | L | D | E | K | V | D | V | A | V | L | V | L | M | D | K | M | F | P | K | L | K | Y | L | Q | L | R | K | R | L | C | R | K | S | V | L | S | W | P | H | H | P | C | A | Q | P | L | F | W | N | Q | M | R | M | A | L | S | S | D | N | L | K | F | Y | D | N | N | M | S | E | S | F | I | - | - | - | - | - | - | - | - | - | - | - | - | - | - | - | - | - | - | - | - | - | - | - | - | - | - | - | - | - | - | - | - | - | - | - | - | - | - | - | - | - | - | - | - | - | - | - | - | - | - | - | - | - | - | - | - | - | - | - | - | - | - | - | - | - | - | - | - | - | - | - | - | - | - | - | - | - | - | - | - | - | - | - | - | - | - | - | - | - | - | - | - | - | - | - | - | - | - | - | - | - | - | - | - | - | - | - | - | - | - | - | - | - | - | - | - | - | - | - | - | - | - | - | - | - | - | - | - | - | - | - | - | - | - | - | - | - | - | - | - | - | - | - | - | - | - | - | - | - | - | - | - | - | - | - | - | - | - | - | - | - | - | - | - | - | - | - | - | - | - | - | - | - | - | - | - | - | - | - | - | - | - | - | - | - | - | - | - | - | - | - | - | - | - | - | - | - | - | - | - | - | - | - | - | - | - | - | - | - | - | - | - |
| SMTLR9 | - | - | - | - | - | M | N | I | I | C | A | L | Q | A | M | A | M | L | - | R | N | I | L | I | I | C | Q | L | I | P | F | A | R | T | R | N | I | K | F | F | P | C | E | T | D | E | N | - | - | - | V | T | T | V | D | C | Y | E | R | P | I | R | N | I | P | V | I | T | F | T | T | V | V | S | L | N | L | N | P | M | K | I | R | Q | V | G | Q | H | A | F | A | D | A | P | N | L | L | T | L | K | I | M | - | G | T | C | Q | P | G | Q | L | R | A | I | K | D | Q | S | C | K | M | K | I | H | P | H | A | F | K | S | L | L | S | L | Q | S | L | Y | L | S | G | N | S | L | T | - | - | S | I | P | W | L | P | E | T | L | R | V | L | D | L | Q | N | N | C | I | F | H | I | T | D | P | L | N | T | P | N | L | E | M | L | F | L | T | K | N | C | F | Y | A | N | P | C | N | Q | S | F | Y | I | S | E | R | V | F | R | E | L | R | N | L | K | N | L | T | L | G | Y | N | N | L | T | A | V | P | N | G | L | P | P | S | L | E | S | L | D | L | R | E | N | R | I | T | V | V | S | D | G | A | F | A | N | L | T | M | L | K | K | L | N | L | E | W | N | C | Q | R | C | D | H | A | S | R | P | C | F | P | C | P | N | N | H | S | L | Y | L | H | P | N | S | F | Y | A | E | N | S | S | I | T | F | L | S | L | R | G | N | S | L | K | T | F | P | E | G | L | F | R | P | L | T | N | - | L | K | K | L | D | L | S | D | N | L | L | A | Y | A | I | Q | N | G | T | F | F | T | E | L | R | G | L | T | W | M | S | L | I | Y | N | Y | E | P | L | K | T | F | Q | E | L | S | L | S | S | H | I | G | N | I | S | G | L | Q | Q | L | L | L | S | G | N | F | F | H | S | L | S | P | Q | S | L | S | V | L | S | K | L | R | H | L | K | T | L | E | L | R | M | N | F | I | R | N | C | N | L | T | A | L | K | Q | L | P | S | L | I | E | I | D | L | S | Q | N | M | L | S | F | L | P | C | - | - | - | P | S | S | E | I | L | A | Q | H | R | C | Q | N | Q | N | L | Y | T | N | - | - | - | - | D | - | F | C | D | Q | P | I | I | V | V | D | R | E | - | - | - | V | T | S | G | D | G | I | W | E | P | N | - | - | - | - | - | - | - | - | - | Q | S | K | L | L | E | T | L | K | D | N | V | S | P | F | - | - | - | - | - | - | - | - | - | - | - | - | - | - | - | - | - | - | - | - | P | S | L | W | D | F | R | T | Y | F | C | Q | N | N | L | T | F | D | L | S | Q | N | D | I | L | S | L | N | K | H | V | F | L | G | M | E | N | A | V | C | L | D | L | S | F | N | Y | M | N | Q | A | L | R | R | G | Q | F | S | S | T | K | N | L | V | F | L | N | L | S | Y | N | R | F | D | L | Y | Y | T | D | A | F | S | E | L | K | T | T | L | K | V | L | D | V | S | N | N | D | F | H | F | R | M | R | G | M | G | H | R | F | E | F | L | Q | N | L | T | N | L | E | V | L | S | L | A | N | N | G | I | G | M | R | I | D | Q | M | L | I | S | S | S | V | K | Y | L | Y | F | Y | G | N | H | L | N | I | M | W | E | S | G | N | N | Q | Y | T | K | F | F | Q | N | L | T | N | L | I | Y | L | D | I | S | D | N | D | L | T | S | I | T | P | D | V | L | C | H | L | P | A | S | I | E | S | L | S | I | S | N | N | L | L | N | Y | F | P | W | Q | N | M | S | A | L | S | N | L | C | H | L | N | L | S | Q | N | Y | L | Y | Y | L | P | Y | R | V | I | E | F | G | A | N | F | S | L | L | D | L | S | H | N | R | F | S | V | I | P | E | N | F | F | R | K | A | K | S | M | Q | Y | L | Y | L | S | N | N | Q | I | K | E | L | D | H | Q | Y | V | P | T | P | F | K | N | G | S | A | L | - | - | - | - | - | - | - | - | - | - | - | K | I | L | T | L | H | A | N | P | F | K | C | D | C | S | T | S | W | F | A | D | F | L | S | T | T | P | V | N | I | P | H | L | T | T | R | I | H | C | E | Y | P | E | S | K | Q | G | Q | S | I | L | S | M | D | Q | K | S | C | Q | D | V | Y | G | S | L | A | F | L | V | C | S | F | L | A | L | T | I | T | A | L | P | L | L | K | H | L | Y | G | W | D | M | W | Y | C | I | Q | V | L | W | A | G | H | K | G | Y | S | Q | L | A | G | S | - | D | S | H | N | H | Y | D | A | F | V | V | F | D | T | T | N | Q | A | V | R | D | W | V | Y | N | E | L | T | V | N | L | E | N | F | G | P | R | R | F | C | L | C | L | E | E | R | D | W | T | P | G | L | S | C | I | E | N | L | H | S | A | V | Y | N | S | V | K | T | V | F | V | L | S | S | C | L | N | G | G | E | - | - | - | - | T | A | N | G | V | I | R | Q | A | F | Y | M | V | Q | Q | R | L | L | D | E | K | M | D | A | A | V | L | V | L | L | D | E | M | F | P | K | L | K | Y | L | Q | L | R | K | R | L | C | R | K | S | V | L | T | W | P | R | N | P | K | A | Q | P | L | F | W | N | R | M | R | M | A | L | S | S | D | N | L | K | L | Y | D | N | N | M | S | E | S | F | I | X | X | X | X | X | X | X | X | X | X | X | X | - | - | - | - | - | - | - | - | - | - | - | - | - | - | - | - | - | - | - | - | - | - | - | - | - | - | - | - | - | - | - | - | - | - | - | - | - | - | - | - | - | - | - | - | - | - | - | - | - | - | - | - | - | - | - | - | - | - | - | - | - | - | - | - | - | - | - | - | - | - | - | - | - | - | - | - | - | - | - | - | - | - | - | - | - | - | - | - | - | - | - | - | - | - | - | - | - | - | - | - | - | - | - | - | - | - | - | - | - | - | - | - | - | - | - | - | - | - | - | - | - | - | - | - | - | - | - | - | - | - | - | - | - | - | - | - | - | - | - | - | - | - | - | - | - | - | - | - | - | - | - | - | - | - | - | - | - | - | - | - | - | - | - | - | - | - | - | - | - | - | - | - | - | - | - | - | - | - | - | - | - | - | - | - | - | - | - | - | - | - | - | - | - | - | - | - | - | - | - | - |
| SATLR9 | - | - | - | - | - | - | - | - | M | H | A | I | S | T | M | A | M | L | - | N | T | I | V | I | L | C | Q | L | L | Q | L | T | R | T | I | N | T | S | F | F | P | C | D | T | D | M | N | - | - | - | T | T | Y | V | D | C | S | D | R | P | L | K | R | V | P | I | I | K | S | E | S | V | L | S | L | S | L | S | W | T | K | I | Q | Q | V | G | S | D | D | L | S | G | L | K | N | L | R | T | L | E | I | V | - | G | N | C | L | P | G | R | L | R | D | Y | S | D | R | S | C | K | M | E | I | H | D | D | A | F | R | S | L | W | N | L | T | F | A | N | L | S | G | N | S | L | T | - | - | R | I | P | W | L | P | E | S | L | K | I | L | D | L | Q | E | N | C | I | S | H | I | I | Q | P | L | K | T | P | N | L | E | A | L | Y | L | S | K | N | C | F | Y | A | N | P | C | G | Q | P | F | N | I | S | E | E | V | F | K | G | L | P | K | L | K | N | L | T | L | G | Y | N | N | L | T | A | I | P | I | G | L | P | R | S | L | E | L | L | D | L | R | E | N | T | I | T | E | V | L | E | G | A | F | A | N | L | T | F | L | H | H | L | I | L | E | W | N | C | Q | R | C | D | H | A | A | R | P | C | F | P | C | P | D | N | R | P | L | K | L | H | S | K | S | F | Y | A | E | N | S | S | V | T | L | L | S | L | R | G | N | S | L | R | T | F | P | E | G | L | F | R | P | L | K | N | - | L | K | G | L | D | L | S | D | N | L | L | A | Y | D | I | R | N | G | T | F | F | A | D | L | R | G | L | T | W | I | S | L | I | Y | N | Y | E | P | L | K | T | F | A | E | L | V | L | S | P | H | I | G | N | I | S | G | L | K | T | L | L | L | S | G | N | F | F | H | I | V | S | N | E | S | L | D | V | L | S | K | L | K | N | L | K | K | L | E | L | R | M | N | F | I | N | T | C | S | L | K | A | L | T | Q | L | P | S | L | I | D | I | D | L | S | Q | N | I | L | S | F | L | P | G | C | W | S | P | S | S | E | I | A | A | Q | E | S | C | Q | R | Q | N | L | Y | T | H | - | - | - | - | D | - | F | T | A | P | P | L | M | L | I | D | R | K | - | - | - | I | T | P | R | S | E | V | L | E | S | N | - | - | - | - | - | - | - | - | - | R | L | N | G | P | E | L | L | E | D | A | G | S | K | S | - | - | - | - | - | - | - | - | - | - | - | - | - | - | - | - | - | - | - | - | P | S | Q | W | R | - | - | - | S | Y | C | R | N | N | L | T | F | D | L | S | Q | N | D | I | M | S | L | H | K | E | V | F | V | G | M | E | N | A | V | C | L | D | L | S | F | N | Y | M | S | Q | A | L | R | G | G | V | F | D | T | M | K | D | L | V | F | L | N | L | S | Y | N | R | L | D | F | Y | Y | N | E | S | F | S | E | L | N | A | T | L | K | V | L | D | L | S | N | N | E | F | H | F | K | M | R | G | M | G | H | R | L | V | F | I | Q | G | L | A | N | L | E | V | L | S | L | A | N | N | G | I | G | M | R | I | D | E | Q | L | V | S | S | S | L | K | Y | L | Y | F | N | G | N | N | L | N | I | M | W | G | Y | D | N | N | R | Y | T | H | F | F | Q | N | L | T | S | L | M | Y | L | D | I | S | T | N | E | L | N | S | I | S | P | E | V | L | C | N | L | P | R | S | I | E | T | L | I | I | S | N | N | K | L | N | Y | F | P | W | Q | N | I | S | A | L | R | N | L | R | H | L | D | L | S | Q | N | R | L | S | Y | L | P | P | E | V | T | E | F | G | E | F | L | S | L | L | D | L | S | H | N | Y | F | S | F | I | P | Q | K | F | F | N | Q | A | G | S | L | R | Y | L | Y | L | S | H | N | Q | I | K | E | L | N | Q | Q | F | L | P | A | P | F | K | K | G | S | A | L | - | - | - | - | - | - | - | - | - | - | - | Q | K | L | T | L | H | A | N | P | F | K | C | D | C | V | T | S | W | F | A | D | F | L | R | S | T | P | V | K | I | P | H | L | T | T | L | V | H | C | Q | F | P | E | S | Q | Q | G | E | S | I | L | S | M | D | Q | R | S | C | Q | D | I | Y | G | G | L | A | F | L | V | C | S | F | L | A | V | A | F | T | V | L | P | L | L | K | H | L | Y | G | W | D | L | W | Y | C | L | Q | V | L | W | A | E | H | K | G | Y | T | Q | L | A | G | N | - | D | S | N | N | H | Y | D | A | F | V | V | F | D | T | S | N | N | A | V | R | D | W | V | Y | N | E | L | M | V | N | L | E | N | S | D | H | R | R | F | C | L | C | L | E | E | R | D | W | I | P | G | L | S | C | I | E | N | L | H | N | A | V | Y | S | S | V | K | T | V | F | V | L | S | S | A | A | A | G | S | E | - | - | - | - | T | V | N | G | V | I | R | Q | A | F | F | M | V | Q | Q | R | L | L | D | E | K | V | D | A | A | V | L | V | L | L | D | E | M | F | P | K | L | K | Y | L | Q | L | R | R | R | L | C | R | K | S | V | L | S | W | P | R | N | P | R | A | Q | P | L | F | W | N | R | V | R | M | A | L | S | S | D | N | L | K | F | Y | D | N | N | M | S | E | S | F | M | X | X | X | X | X | X | X | X | X | X | X | X | X | X | X | - | - | - | - | - | - | - | - | - | - | - | - | - | - | - | - | - | - | - | - | - | - | - | - | - | - | - | - | - | - | - | - | - | - | - | - | - | - | - | - | - | - | - | - | - | - | - | - | - | - | - | - | - | - | - | - | - | - | - | - | - | - | - | - | - | - | - | - | - | - | - | - | - | - | - | - | - | - | - | - | - | - | - | - | - | - | - | - | - | - | - | - | - | - | - | - | - | - | - | - | - | - | - | - | - | - | - | - | - | - | - | - | - | - | - | - | - | - | - | - | - | - | - | - | - | - | - | - | - | - | - | - | - | - | - | - | - | - | - | - | - | - | - | - | - | - | - | - | - | - | - | - | - | - | - | - | - | - | - | - | - | - | - | - | - | - | - | - | - | - | - | - | - | - | - | - | - | - | - | - | - | - | - | - | - | - | - | - | - | - | - | - | - | - | - | - | - |
| ATTLR9 | - | - | - | - | - | - | - | - | - | - | - | - | - | - | M | A | L | L | - | K | S | I | I | I | L | A | H | L | L | L | L | V | R | S | I | N | I | F | F | F | P | C | D | T | D | V | N | - | - | - | A | T | V | V | D | C | F | K | R | P | I | K | H | V | P | V | I | N | S | V | K | V | V | S | L | N | L | N | Q | T | K | I | R | K | V | E | Q | H | D | F | A | G | A | P | N | L | H | T | L | K | I | M | - | D | N | C | Q | P | G | R | L | R | S | L | Q | D | R | S | C | K | M | E | I | H | P | Y | A | F | K | D | L | L | R | L | T | F | L | Y | L | S | G | N | S | L | T | - | - | S | I | P | W | L | P | E | S | L | R | L | L | D | L | Q | N | N | R | I | F | H | I | V | H | P | L | K | T | P | N | L | E | V | F | Y | L | S | R | N | C | F | Y | A | N | P | C | N | Q | S | F | Y | I | S | E | G | V | F | R | E | L | P | K | L | K | N | L | S | L | G | Y | D | N | F | T | A | V | P | K | G | L | P | P | S | L | E | M | L | D | L | R | E | N | T | I | P | E | I | P | E | R | A | F | A | N | L | T | V | L | K | Y | L | N | L | E | W | N | C | Q | R | C | D | H | A | A | R | P | C | F | P | C | P | H | N | H | P | L | Y | L | H | S | D | S | L | Y | A | E | N | S | S | I | T | F | L | S | L | R | G | N | S | L | T | K | F | P | K | G | L | F | R | P | L | K | N | - | L | K | G | L | D | L | S | D | N | L | L | T | N | A | I | Q | H | G | T | F | F | S | E | L | T | G | L | T | W | M | S | L | I | Y | N | Y | E | P | L | K | T | Y | K | E | L | I | L | S | P | H | I | G | N | I | S | G | L | Q | Y | L | L | L | S | G | N | F | F | R | K | L | S | T | E | S | L | H | V | L | S | K | L | R | N | L | I | R | L | E | L | R | M | N | F | I | N | T | C | N | V | T | A | L | K | Q | L | P | S | L | T | Y | V | D | L | S | Q | N | M | L | N | F | L | P | C | C | A | G | P | S | S | D | I | A | A | Q | G | S | C | K | S | Q | N | L | Y | T | H | - | - | - | - | H | - | F | R | D | Q | P | L | I | E | I | D | R | E | - | - | - | V | T | S | A | S | N | T | W | A | S | N | - | - | - | - | - | - | - | - | - | P | S | N | M | L | E | M | S | V | D | D | A | S | Y | F | - | - | - | - | - | - | - | - | - | - | - | - | - | - | - | - | - | - | - | - | P | F | P | W | H | F | R | E | R | F | C | Q | H | K | L | T | F | D | L | S | Q | N | D | I | L | S | L | K | K | D | V | F | V | G | M | E | N | A | V | C | I | D | L | S | Y | N | Y | M | N | Q | A | L | R | G | G | L | F | D | D | M | K | R | L | V | L | L | N | L | S | Y | N | R | L | D | L | Y | Y | K | D | A | L | N | D | L | K | T | T | L | K | V | L | D | I | S | N | N | D | F | H | F | K | M | R | G | M | G | H | R | F | E | F | L | Q | N | L | T | N | L | E | V | L | S | L | A | N | N | G | I | G | M | R | I | D | Q | R | L | V | S | S | S | L | R | Y | L | Y | F | Y | G | N | Q | L | N | I | M | W | D | S | D | N | N | K | Y | T | H | F | F | Q | N | L | T | N | L | I | Y | L | D | I | S | D | N | D | L | G | S | I | S | P | E | V | L | I | N | L | P | G | T | L | K | A | L | S | I | S | R | N | V | L | N | Y | F | A | W | Q | N | I | S | A | L | R | Q | L | R | H | L | D | L | S | D | N | H | L | F | Y | L | P | S | N | V | I | D | F | G | A | N | F | S | F | L | D | L | S | R | N | R | I | N | G | I | P | Q | D | F | F | G | K | A | K | S | L | K | Y | L | Y | L | S | H | N | Q | I | K | E | L | D | H | Q | N | L | P | P | P | L | K | N | G | S | A | L | - | - | - | - | - | - | - | - | - | - | - | R | K | L | T | L | H | A | N | P | F | K | C | D | C | D | T | S | W | F | A | D | F | L | R | T | T | P | I | E | I | P | Y | L | T | T | S | V | H | C | D | Y | P | E | S | Q | Q | G | E | S | I | L | S | M | D | K | R | S | C | Q | D | I | Y | G | S | L | A | F | L | V | C | S | F | L | T | L | T | L | T | V | L | P | L | L | K | H | L | Y | G | W | D | M | W | Y | C | L | Q | V | L | W | A | G | H | K | G | Y | S | Q | L | P | G | T | - | D | S | P | H | H | Y | D | A | F | V | V | F | D | T | R | N | Q | A | V | R | D | W | V | Y | N | E | F | T | V | N | M | E | N | M | G | H | R | K | F | S | L | C | L | E | E | R | D | W | I | P | G | L | S | C | I | E | N | L | H | N | A | V | Y | D | S | V | K | T | V | F | V | L | S | S | G | S | N | G | G | E | - | - | - | - | T | V | N | G | V | I | R | Q | A | F | F | M | V | Q | Q | R | L | L | D | E | K | V | D | T | A | V | L | V | L | L | D | E | L | F | P | K | L | K | Y | L | Q | L | R | K | R | L | C | K | K | S | V | L | S | W | P | R | N | P | R | A | Q | P | L | F | W | N | R | M | R | M | A | L | S | L | D | N | L | K | F | Y | D | S | N | M | S | E | S | F | M | X | X | X | X | X | X | X | X | X | X | X | X | X | X | X | X | X | X | - | - | - | - | - | - | - | - | - | - | - | - | - | - | - | - | - | - | - | - | - | - | - | - | - | - | - | - | - | - | - | - | - | - | - | - | - | - | - | - | - | - | - | - | - | - | - | - | - | - | - | - | - | - | - | - | - | - | - | - | - | - | - | - | - | - | - | - | - | - | - | - | - | - | - | - | - | - | - | - | - | - | - | - | - | - | - | - | - | - | - | - | - | - | - | - | - | - | - | - | - | - | - | - | - | - | - | - | - | - | - | - | - | - | - | - | - | - | - | - | - | - | - | - | - | - | - | - | - | - | - | - | - | - | - | - | - | - | - | - | - | - | - | - | - | - | - | - | - | - | - | - | - | - | - | - | - | - | - | - | - | - | - | - | - | - | - | - | - | - | - | - | - | - | - | - | - | - | - | - | - | - | - | - | - | - | - | - | - | - | - | - | - | - |
| MMTLR9 | M | H | Y | Q | H | T | T | E | I | K | F | L | F | F | Q | A | M | L | - | K | N | I | F | I | L | C | Q | F | L | P | L | V | R | N | M | N | I | I | F | F | P | C | D | T | D | V | N | - | - | - | A | T | I | V | D | C | S | R | R | P | L | K | H | V | P | F | I | K | S | T | T | V | V | S | L | D | L | S | Q | T | K | I | Q | Q | V | E | Q | H | A | L | S | G | L | P | N | L | Q | T | L | K | I | M | - | G | N | C | Q | P | G | R | L | R | A | L | E | D | R | S | C | K | M | E | I | H | Y | Y | A | F | K | G | L | L | N | L | Q | S | L | Y | L | S | G | N | S | L | T | T | K | D | L | P | W | L | P | E | S | L | R | L | L | D | L | Q | N | N | H | I | F | H | I | I | H | P | F | K | T | P | C | L | E | E | L | Y | L | S | K | N | C | F | Y | T | N | P | C | N | Q | S | F | Y | I | S | E | R | V | F | K | G | L | P | K | L | T | N | L | T | L | G | Y | D | N | L | T | A | V | P | R | G | L | P | P | S | L | K | S | L | D | L | R | E | N | T | I | T | E | V | L | E | G | A | F | A | N | L | T | V | L | N | R | L | I | L | E | W | N | C | Q | R | C | D | H | A | A | R | P | C | F | P | C | P | H | N | L | P | L | H | L | L | P | N | S | L | Y | A | E | N | S | S | I | T | F | L | S | L | R | G | N | S | L | K | T | F | P | K | G | L | F | Q | P | L | K | N | - | L | Q | R | L | D | L | S | D | N | L | L | A | Y | A | I | R | D | G | T | F | F | A | E | L | K | G | L | T | W | I | S | L | I | Y | N | Y | E | P | L | K | T | F | P | E | L | I | L | S | P | D | I | G | N | I | S | G | L | E | Y | L | L | L | S | G | N | F | F | H | T | L | S | N | Q | S | L | N | V | L | S | K | L | Q | N | L | K | K | L | E | L | R | M | N | F | I | N | T | F | D | L | K | A | L | K | Q | I | P | S | L | I | D | I | N | L | S | Q | N | I | L | N | F | L | P | C | C | W | S | P | S | S | E | I | V | A | Q | E | S | C | Q | N | Q | N | L | Y | T | H | - | - | - | - | N | - | Y | P | G | Q | P | L | M | L | V | D | R | D | - | - | - | I | T | S | G | S | H | I | S | E | S | N | - | - | - | - | - | - | - | - | - | Q | L | N | R | V | E | M | P | K | D | S | V | S | P | P | - | - | - | - | - | - | - | - | - | - | - | - | - | - | - | - | - | - | - | - | S | S | L | W | D | - | - | - | K | F | C | R | H | N | L | T | F | D | L | S | Q | N | D | I | M | F | L | K | K | E | M | F | V | G | M | E | N | A | V | C | L | D | L | S | F | N | Y | M | S | Q | A | L | R | S | G | V | F | D | N | L | K | N | L | V | F | L | N | L | S | Y | N | R | L | D | F | Y | Y | R | E | A | F | I | E | L | N | S | T | L | K | V | L | D | V | S | N | N | E | F | H | F | K | M | R | G | M | G | H | R | L | E | F | I | Q | N | L | T | N | L | E | V | L | S | L | A | N | N | G | I | G | M | R | I | D | Q | K | L | V | S | R | S | L | K | Y | L | Y | F | Y | G | N | N | L | D | I | M | W | G | S | D | N | N | K | Y | T | H | F | F | Q | D | L | S | K | L | I | Y | L | D | I | S | H | N | D | L | R | S | I | S | P | E | V | L | C | N | L | P | E | S | L | Q | N | L | S | I | S | N | N | Q | L | S | Y | F | P | W | Q | N | I | S | A | L | I | N | L | Q | H | L | N | L | S | Q | N | H | F | S | Y | L | P | P | K | A | I | E | F | G | E | Y | F | Y | L | L | D | L | S | H | N | R | I | S | F | I | P | E | D | F | L | S | Q | A | D | V | L | Q | Y | L | Y | L | N | H | N | Q | I | K | E | F | N | H | Q | F | L | P | A | P | F | K | N | G | S | A | F | - | - | - | - | - | - | - | - | - | - | - | V | K | L | T | L | H | A | N | P | F | K | C | D | C | D | T | S | W | F | A | D | L | L | R | T | T | S | V | E | I | P | Y | L | T | T | Q | M | H | C | E | Y | P | E | S | Q | Q | G | Q | S | I | L | S | M | D | Q | R | S | C | Q | D | I | Y | G | S | L | A | S | V | I | C | F | F | L | V | V | T | F | T | V | L | P | L | L | K | H | L | Y | G | W | D | L | W | Y | C | L | Q | V | L | W | A | G | H | K | G | Y | S | Q | L | A | G | G | - | N | S | H | Q | H | Y | D | A | F | V | V | F | D | T | S | N | Q | A | V | R | D | W | V | Y | N | E | L | T | V | N | L | E | N | A | G | H | R | R | F | C | L | C | L | E | E | R | D | W | I | P | G | L | S | C | I | E | N | L | H | N | A | V | N | S | S | V | K | T | V | F | V | L | S | S | G | A | T | G | S | E | - | - | - | - | T | V | N | G | V | I | R | Q | A | F | F | M | V | Q | Q | R | L | L | D | E | K | V | D | A | A | V | L | I | L | L | D | E | M | F | P | K | L | K | Y | L | Q | L | R | K | R | L | C | R | K | S | V | L | S | W | P | K | N | P | R | A | Q | P | L | F | W | N | R | V | R | M | A | L | S | L | D | N | L | N | F | Y | D | N | N | M | S | E | S | F | I | X | X | X | X | X | - | - | - | - | - | - | - | - | - | - | - | - | - | - | - | - | - | - | - | - | - | - | - | - | - | - | - | - | - | - | - | - | - | - | - | - | - | - | - | - | - | - | - | - | - | - | - | - | - | - | - | - | - | - | - | - | - | - | - | - | - | - | - | - | - | - | - | - | - | - | - | - | - | - | - | - | - | - | - | - | - | - | - | - | - | - | - | - | - | - | - | - | - | - | - | - | - | - | - | - | - | - | - | - | - | - | - | - | - | - | - | - | - | - | - | - | - | - | - | - | - | - | - | - | - | - | - | - | - | - | - | - | - | - | - | - | - | - | - | - | - | - | - | - | - | - | - | - | - | - | - | - | - | - | - | - | - | - | - | - | - | - | - | - | - | - | - | - | - | - | - | - | - | - | - | - | - | - | - | - | - | - | - | - | - | - | - | - | - | - | - | - | - | - | - | - | - | - | - | - | - | - | - | - | - | - | - |
| SLTLR9 | - | - | - | - | - | - | - | M | W | F | L | V | F | P | Q | A | L | L | - | K | N | I | L | I | L | G | Q | L | L | P | L | V | R | T | I | N | I | I | F | L | P | C | D | T | D | V | N | - | - | - | A | T | T | V | D | C | S | D | R | P | L | R | H | V | P | F | I | Q | S | T | T | V | V | S | L | N | L | S | R | T | K | I | Q | Q | V | G | K | H | A | F | A | G | V | P | N | L | H | T | L | K | I | M | - | G | N | C | Q | P | R | R | A | - | - | L | E | D | R | S | C | K | M | Y | I | H | H | Y | A | F | K | N | L | L | K | L | T | F | L | H | L | S | G | N | S | L | T | - | - | S | I | P | W | L | P | E | T | L | R | F | L | D | L | Q | N | N | H | I | F | H | I | T | H | L | S | R | T | P | N | L | E | E | L | F | L | S | M | N | C | F | Y | A | N | P | C | N | Q | S | F | Y | I | S | E | R | V | F | S | G | L | D | K | L | K | N | L | T | L | G | F | N | N | L | T | S | V | P | R | G | L | P | P | S | L | E | S | L | D | L | K | E | N | T | I | T | E | V | L | D | R | A | F | S | S | L | T | M | L | K | S | L | N | L | E | W | N | C | Q | R | C | D | H | A | A | R | P | C | F | P | C | P | Q | N | L | P | L | K | L | Y | P | N | S | F | Y | A | K | N | S | S | I | M | Y | L | S | L | R | G | N | S | L | K | T | F | P | E | G | L | F | K | P | L | K | N | - | L | K | R | L | D | L | S | D | N | L | L | A | Y | A | I | R | N | G | T | F | F | S | E | L | R | G | L | T | W | I | S | L | I | Y | N | Y | E | P | L | R | T | F | K | D | L | I | L | S | P | H | I | G | N | I | S | G | L | Q | Y | L | L | L | S | G | N | F | F | H | K | L | S | I | Q | S | L | S | V | L | Y | K | L | K | N | L | M | K | L | E | L | R | M | N | F | I | N | T | C | N | L | T | D | L | K | Q | L | P | S | L | I | Y | I | D | L | S | Q | N | M | L | N | F | L | P | S | C | C | N | S | I | P | E | N | M | A | Q | T | S | C | H | N | E | N | L | Y | T | Q | - | - | - | - | H | - | F | P | D | Q | P | R | I | V | R | D | R | E | - | - | - | V | T | S | V | N | E | I | R | E | S | N | - | - | - | - | - | - | - | - | - | K | L | N | V | M | E | L | V | E | H | N | A | S | Q | F | - | - | - | - | - | - | - | - | - | - | - | - | - | - | - | - | - | - | - | - | P | S | L | L | D | F | R | N | H | F | C | R | H | K | L | T | F | D | L | S | Q | N | D | I | L | S | L | N | K | N | V | F | V | G | M | E | N | A | V | C | L | D | L | S | F | N | Y | M | S | Q | A | L | K | S | G | Q | F | D | G | L | K | N | L | V | L | L | N | L | S | Y | N | R | L | D | L | Y | Y | N | A | A | F | S | E | L | K | T | T | L | K | V | L | D | V | S | N | N | E | F | H | F | K | M | R | G | M | G | H | R | L | E | F | L | Q | N | L | T | N | L | E | V | L | S | L | A | N | N | D | I | G | M | R | I | D | Q | R | L | I | S | S | S | M | K | Y | L | Y | F | N | G | N | H | L | D | I | M | W | E | S | D | N | N | K | Y | T | H | F | F | Q | N | L | T | N | L | I | Y | L | D | I | S | N | N | G | L | K | S | V | S | P | E | V | L | C | N | L | P | R | S | I | Q | A | L | S | I | S | N | N | L | L | N | Y | F | P | W | K | N | I | S | A | L | S | N | L | R | H | L | D | L | S | Q | N | Y | L | S | Y | L | P | Y | K | F | I | E | F | G | A | N | F | S | L | L | D | L | S | H | N | R | L | S | F | V | P | A | D | F | F | R | K | A | K | S | L | Q | Y | L | Y | L | S | H | N | Q | I | K | E | L | K | H | Q | Y | L | P | A | P | F | K | N | G | - | A | L | - | - | - | - | - | - | - | - | - | - | - | Q | E | L | T | L | H | A | N | P | F | K | C | D | C | D | T | S | W | F | A | D | F | L | S | S | T | P | V | H | I | P | Y | L | T | T | H | V | H | C | D | Y | P | E | S | Q | Q | G | H | S | I | L | S | I | D | Q | R | S | C | Q | D | I | Y | G | S | L | A | F | L | V | C | S | F | L | A | L | T | F | T | V | L | P | L | L | K | H | L | Y | G | W | D | M | W | Y | C | L | Q | V | L | W | A | G | H | K | G | Y | S | Q | L | A | G | T | - | D | S | E | Y | H | Y | D | A | F | V | V | F | D | T | R | N | T | A | V | R | D | W | V | Y | N | E | L | I | V | N | L | E | N | S | D | H | R | R | F | C | L | C | L | E | E | R | D | W | I | P | G | L | S | C | I | E | N | L | H | S | A | V | Y | N | S | V | K | T | V | F | V | L | S | S | G | S | N | G | G | E | - | - | - | - | T | V | N | G | V | I | R | Q | A | F | F | M | V | Q | Q | R | L | L | D | E | K | V | D | A | A | V | L | V | L | L | D | E | M | F | P | K | L | K | Y | L | Q | L | R | K | R | L | C | K | K | S | V | L | S | W | P | R | N | P | R | A | Q | P | L | F | W | N | K | M | R | M | A | L | S | S | D | N | L | K | F | Y | D | N | N | I | S | E | S | F | V | X | X | X | X | X | X | X | X | X | X | X | X | X | X | - | - | - | - | - | - | - | - | - | - | - | - | - | - | - | - | - | - | - | - | - | - | - | - | - | - | - | - | - | - | - | - | - | - | - | - | - | - | - | - | - | - | - | - | - | - | - | - | - | - | - | - | - | - | - | - | - | - | - | - | - | - | - | - | - | - | - | - | - | - | - | - | - | - | - | - | - | - | - | - | - | - | - | - | - | - | - | - | - | - | - | - | - | - | - | - | - | - | - | - | - | - | - | - | - | - | - | - | - | - | - | - | - | - | - | - | - | - | - | - | - | - | - | - | - | - | - | - | - | - | - | - | - | - | - | - | - | - | - | - | - | - | - | - | - | - | - | - | - | - | - | - | - | - | - | - | - | - | - | - | - | - | - | - | - | - | - | - | - | - | - | - | - | - | - | - | - | - | - | - | - | - | - | - | - | - | - | - | - | - | - | - | - | - | - | - | - | - |
| TOTLR9 | - | - | - | - | - | - | - | - | - | - | - | - | - | - | - | - | M | L | - | K | N | I | F | I | L | G | Q | L | L | P | L | V | R | T | I | N | T | S | F | F | P | C | D | S | D | E | N | - | - | - | G | T | T | V | D | C | F | D | R | P | L | R | H | V | P | F | I | K | S | T | P | V | V | S | L | N | L | S | R | T | K | I | Q | Q | V | W | Q | H | A | F | A | G | V | P | D | L | H | T | L | K | I | M | - | G | S | C | Q | P | G | R | L | - | - | R | E | E | H | T | C | K | M | E | I | H | Y | Y | A | F | R | S | L | L | K | L | Q | F | L | N | L | S | G | N | S | L | T | - | - | S | I | P | W | I | P | E | T | L | R | V | L | D | L | Q | N | N | H | I | F | H | I | T | H | L | S | R | A | P | N | L | E | G | L | F | L | S | R | N | C | F | Y | A | N | P | C | N | Q | S | F | H | I | R | E | S | I | F | W | E | L | H | K | L | K | N | L | T | L | G | Y | N | N | L | T | S | V | P | K | G | L | P | P | S | L | E | S | L | D | L | K | E | N | T | I | T | E | V | L | E | G | A | F | A | N | L | T | M | L | K | S | L | N | L | E | W | N | C | Q | R | C | D | R | A | A | R | P | C | F | P | C | P | Q | N | L | P | L | K | L | H | P | N | S | F | Y | A | K | Y | S | S | I | K | Y | L | S | L | R | G | N | S | L | K | T | F | P | E | G | L | F | R | H | L | K | N | - | L | T | R | L | D | L | S | D | N | L | L | A | Y | A | I | R | N | D | T | F | F | S | E | L | K | G | L | T | W | I | S | L | I | Y | N | Y | E | P | L | R | T | F | P | E | L | I | L | S | P | H | I | G | D | I | S | G | L | Q | H | L | L | L | S | G | N | F | F | H | K | L | S | I | Q | S | L | S | V | L | S | K | L | K | S | L | K | R | L | E | L | R | M | N | F | I | N | T | C | D | L | T | Y | L | K | Q | L | P | S | L | I | D | I | D | L | S | Q | N | M | L | N | F | L | P | C | C | S | N | S | L | T | E | N | V | A | Q | K | N | C | H | N | Q | N | L | Y | A | H | - | - | - | - | N | - | L | R | D | Q | P | L | I | V | R | D | R | E | - | - | - | V | T | S | V | N | E | I | S | E | S | N | - | - | - | - | - | - | - | - | - | Q | L | N | V | M | E | L | A | E | D | N | A | S | Q | F | - | - | - | - | - | - | - | - | - | - | - | - | - | - | - | - | - | - | - | - | L | S | L | W | D | F | R | N | H | F | C | Q | Y | K | L | T | F | D | L | S | Q | N | D | I | L | S | L | N | K | H | V | F | V | G | M | E | K | A | V | C | L | D | L | S | F | N | Y | M | S | Q | A | L | K | G | G | H | F | D | G | L | E | N | L | V | L | L | N | L | S | Y | N | R | L | D | L | Y | Y | D | S | A | F | S | E | L | K | T | T | L | K | V | L | D | I | S | N | N | E | F | H | F | K | M | R | G | M | G | H | R | L | Q | F | L | Q | N | L | T | N | L | E | V | L | S | L | A | N | N | A | I | G | T | R | V | D | Q | R | L | I | S | S | S | V | K | Y | L | Y | F | N | G | N | H | L | D | I | M | W | E | T | D | N | N | K | Y | T | H | F | F | Q | N | L | T | N | L | I | Y | L | D | I | S | N | N | G | L | M | S | V | T | P | E | I | L | Y | N | L | P | G | R | I | E | A | L | S | I | S | H | N | L | L | N | Y | F | P | W | K | N | I | S | G | L | R | N | L | R | H | L | D | L | S | Q | N | Y | L | S | Y | L | P | F | K | A | I | D | F | G | A | N | F | S | L | L | D | L | S | H | N | R | L | S | F | I | P | E | I | F | F | R | K | A | K | S | L | Q | Y | L | Y | L | S | H | N | Q | I | K | E | L | N | H | Q | Y | L | P | A | P | F | K | N | G | S | A | L | - | - | - | - | - | - | - | - | - | - | - | Q | K | L | T | L | H | A | N | P | F | K | C | D | C | G | T | S | W | F | A | D | F | L | R | S | T | P | I | Q | I | P | F | L | S | T | H | I | H | C | E | Y | P | E | S | Q | L | G | H | S | V | L | S | I | D | Q | R | S | C | Q | D | I | Y | G | S | L | A | F | L | V | S | S | F | L | A | V | T | F | T | V | L | P | L | L | K | H | L | Y | G | W | D | M | W | Y | C | L | Q | V | L | W | A | G | H | K | G | Y | S | Q | L | A | G | T | - | D | S | Q | N | H | Y | D | A | F | V | V | F | D | T | R | N | Q | A | V | R | D | W | V | Y | N | E | L | I | V | N | L | E | N | S | N | H | R | R | F | Y | L | C | L | E | E | R | D | W | I | P | G | L | S | C | I | E | N | L | H | N | A | V | Y | N | S | V | K | T | V | F | V | L | S | S | G | S | N | G | G | E | - | - | - | - | T | V | N | G | V | I | R | Q | A | F | F | M | V | Q | Q | R | L | L | D | E | K | V | D | V | A | V | L | V | L | L | D | E | M | F | P | K | L | K | Y | L | Q | L | R | K | R | L | C | R | K | S | V | L | S | W | P | R | N | P | R | A | Q | P | L | F | W | N | K | M | R | M | A | L | S | S | D | N | L | T | F | Y | D | N | N | M | S | E | S | F | M | X | X | X | X | X | X | X | X | X | X | X | X | X | X | X | X | X | X | X | X | X | X | - | - | - | - | - | - | - | - | - | - | - | - | - | - | - | - | - | - | - | - | - | - | - | - | - | - | - | - | - | - | - | - | - | - | - | - | - | - | - | - | - | - | - | - | - | - | - | - | - | - | - | - | - | - | - | - | - | - | - | - | - | - | - | - | - | - | - | - | - | - | - | - | - | - | - | - | - | - | - | - | - | - | - | - | - | - | - | - | - | - | - | - | - | - | - | - | - | - | - | - | - | - | - | - | - | - | - | - | - | - | - | - | - | - | - | - | - | - | - | - | - | - | - | - | - | - | - | - | - | - | - | - | - | - | - | - | - | - | - | - | - | - | - | - | - | - | - | - | - | - | - | - | - | - | - | - | - | - | - | - | - | - | - | - | - | - | - | - | - | - | - | - | - | - | - | - | - | - | - | - | - | - | - | - | - | - | - | - | - | - |
| ECTLR9 | - | - | - | - | - | - | - | - | - | - | - | - | - | - | M | A | M | L | - | K | S | I | L | I | L | C | Q | F | L | P | L | V | M | T | I | N | T | I | F | F | P | C | D | T | D | K | N | - | - | - | T | T | E | V | D | C | S | D | R | P | L | K | R | V | P | S | I | K | S | T | T | V | E | S | L | D | L | S | R | T | K | I | Q | Y | V | G | V | R | A | F | S | G | V | P | N | L | R | T | L | K | M | I | N | R | N | C | Q | P | G | Q | L | G | S | L | E | D | R | L | C | K | L | E | I | H | H | D | A | F | K | C | L | S | K | L | N | F | L | N | L | S | G | N | S | L | I | - | - | S | I | P | Q | L | P | E | N | L | T | V | L | D | L | R | N | N | R | I | F | Q | I | N | Q | P | L | N | T | P | H | L | K | E | L | Y | L | S | K | N | C | F | Y | A | N | P | C | G | Q | S | F | Y | I | N | Q | S | V | F | R | E | L | S | E | L | K | S | L | I | L | G | Y | N | N | F | T | A | I | P | K | G | L | P | L | S | L | E | R | L | D | L | R | E | N | T | I | T | E | V | L | D | G | A | F | A | N | L | T | L | L | K | Y | L | N | L | E | W | N | C | Q | R | C | D | H | A | A | R | P | C | F | P | C | P | N | N | K | P | L | Q | L | H | S | N | S | F | Y | A | E | N | S | S | I | T | F | L | S | L | R | G | N | S | L | R | T | F | P | M | G | I | F | Q | P | L | K | N | - | L | K | G | L | D | L | S | D | N | F | L | A | H | A | L | H | N | G | T | F | F | A | E | L | E | G | L | T | W | I | S | L | I | Y | N | Y | E | P | L | T | T | F | P | K | L | S | L | S | P | H | I | G | K | M | T | H | L | R | Y | L | L | L | S | G | N | F | F | H | E | L | S | S | K | S | F | N | T | L | S | K | L | Q | N | L | R | T | L | E | L | R | M | N | F | I | N | T | F | N | L | T | S | L | K | R | L | P | F | L | T | N | I | D | L | S | Q | N | M | L | N | F | L | P | C | C | S | G | P | S | A | E | F | V | A | Q | E | S | C | Q | N | Q | N | L | F | T | H | - | - | - | - | D | - | F | S | N | L | H | V | M | S | I | D | R | K | - | - | - | A | T | S | G | N | D | I | W | E | S | N | - | - | - | - | - | - | - | - | - | Q | S | N | R | L | E | M | G | E | D | N | V | L | Q | F | - | - | - | - | - | - | - | - | - | - | - | - | - | - | - | - | - | - | - | - | K | S | L | L | D | F | K | N | D | F | C | S | R | K | L | T | F | D | L | S | Q | N | D | I | L | S | L | N | K | E | V | F | V | G | M | E | D | V | V | C | L | D | L | S | F | N | Y | M | S | Q | A | L | K | G | G | L | F | A | S | M | K | K | L | V | F | L | N | L | S | Y | N | R | L | D | F | Y | Y | R | E | A | F | S | E | L | N | S | T | L | K | V | L | D | V | G | N | N | E | F | H | F | K | M | K | G | M | G | H | R | F | E | F | L | Q | N | L | T | N | L | E | V | L | S | L | A | N | N | N | I | G | V | R | I | D | K | Q | L | I | S | S | S | L | K | Y | L | Y | F | Y | G | N | N | L | D | I | M | W | M | S | D | N | N | R | Y | T | Q | F | F | Q | N | L | T | A | L | T | Y | L | D | I | S | D | N | N | L | M | S | I | S | P | E | V | F | C | N | F | P | E | S | L | E | T | L | I | I | S | D | D | Q | L | K | Y | F | P | W | Q | N | I | S | V | L | S | N | L | C | H | L | N | L | S | Q | N | K | L | Y | Y | L | P | N | K | V | I | G | F | G | A | N | F | S | L | L | D | L | S | Y | N | R | F | S | V | I | P | E | M | F | F | S | K | V | E | S | L | R | Y | L | Y | L | S | H | N | Q | I | K | V | L | S | R | Q | F | L | P | A | P | F | K | D | G | S | A | L | - | - | - | - | - | - | - | - | - | - | - | Q | K | L | T | L | H | A | N | P | F | K | C | D | C | N | T | S | W | F | A | D | F | L | R | N | T | S | I | Q | I | P | Y | L | T | T | H | I | H | C | D | Y | P | E | S | Q | Q | G | M | S | I | L | S | M | D | Q | H | S | C | Q | D | I | Y | G | S | L | A | F | L | I | C | S | F | L | A | V | M | F | T | V | L | P | L | L | K | H | L | Y | G | W | D | L | W | Y | C | L | Q | V | L | W | A | G | H | K | G | Y | S | Q | L | A | G | S | - | D | S | Q | H | H | Y | D | A | F | V | V | F | D | T | S | N | Q | A | V | R | D | W | V | Y | N | E | L | T | V | N | L | E | N | S | G | H | R | R | F | C | L | C | L | E | E | R | D | W | V | P | G | V | S | C | I | D | N | L | H | N | A | V | Y | S | S | V | K | T | V | F | V | L | S | S | G | A | T | G | G | E | - | - | - | - | T | V | N | G | V | I | R | Q | A | F | F | M | V | Q | Q | R | L | L | D | E | K | V | D | A | A | M | L | V | L | L | D | E | M | F | P | K | L | K | Y | L | Q | L | R | K | R | L | C | K | K | S | V | L | S | W | P | K | N | P | R | A | Q | P | L | F | W | N | R | M | R | M | A | L | S | S | D | N | L | K | F | Y | D | N | N | M | S | E | S | F | I | X | X | X | X | X | X | X | X | X | X | X | X | X | X | X | X | X | - | - | - | - | - | - | - | - | - | - | - | - | - | - | - | - | - | - | - | - | - | - | - | - | - | - | - | - | - | - | - | - | - | - | - | - | - | - | - | - | - | - | - | - | - | - | - | - | - | - | - | - | - | - | - | - | - | - | - | - | - | - | - | - | - | - | - | - | - | - | - | - | - | - | - | - | - | - | - | - | - | - | - | - | - | - | - | - | - | - | - | - | - | - | - | - | - | - | - | - | - | - | - | - | - | - | - | - | - | - | - | - | - | - | - | - | - | - | - | - | - | - | - | - | - | - | - | - | - | - | - | - | - | - | - | - | - | - | - | - | - | - | - | - | - | - | - | - | - | - | - | - | - | - | - | - | - | - | - | - | - | - | - | - | - | - | - | - | - | - | - | - | - | - | - | - | - | - | - | - | - | - | - | - | - | - | - | - | - | - | - | - | - | - | - |
| LMTLR9 | - | - | - | - | - | - | - | - | - | - | - | - | - | - | M | A | V | L | - | R | N | I | L | I | L | C | Q | L | L | P | L | V | R | T | I | N | T | I | F | F | P | C | D | T | D | V | N | - | - | - | T | T | T | V | D | C | Y | D | R | P | L | K | H | V | P | F | I | K | S | T | A | V | V | S | L | N | L | S | R | T | K | I | Q | Q | V | G | Q | H | A | L | S | G | V | P | N | L | H | T | L | K | M | M | - | G | N | C | Q | P | G | R | L | R | D | L | E | D | R | S | C | K | M | E | I | H | Y | Y | A | F | K | S | L | L | K | L | Q | F | L | Y | L | S | G | N | S | L | T | - | - | S | I | P | W | L | P | K | S | L | R | V | L | D | L | Q | N | N | R | I | F | H | I | I | Y | P | L | K | T | P | H | L | E | E | L | F | L | S | R | N | C | F | Y | A | N | P | C | N | H | S | F | Y | I | S | E | M | V | F | R | E | L | P | K | L | K | N | L | T | L | G | Y | D | N | F | T | A | I | P | K | G | L | P | P | S | L | E | S | L | D | L | R | E | N | T | I | T | E | V | L | E | G | A | F | A | N | L | T | V | L | K | Y | L | N | L | E | W | N | C | Q | R | C | D | H | A | A | R | P | C | F | P | C | P | H | N | L | P | L | R | L | H | S | N | S | L | Y | I | E | N | S | S | I | T | F | L | S | L | R | G | N | S | L | K | T | F | P | E | G | L | F | R | P | L | K | N | - | L | K | G | L | D | L | S | D | N | L | L | A | Y | A | I | Q | N | G | T | F | F | S | E | L | K | G | L | T | W | I | S | L | I | Y | N | Y | E | P | L | K | T | F | P | E | L | I | L | S | P | H | I | G | N | I | S | G | L | Q | N | L | L | L | S | G | N | F | F | H | V | L | S | N | E | S | L | D | V | L | S | K | L | K | N | L | K | K | L | E | L | R | M | N | F | I | N | T | C | N | L | T | A | L | K | Q | L | P | S | L | M | D | I | N | L | S | Q | N | M | L | N | F | L | P | H | C | P | N | M | S | C | E | I | V | A | K | E | S | C | K | N | Q | N | L | Y | T | Q | - | - | - | - | N | - | F | P | D | P | P | L | M | Q | I | D | R | E | - | - | - | V | P | S | G | S | D | M | W | E | S | S | - | - | - | - | - | - | - | - | - | Q | S | G | K | L | E | M | L | E | D | N | V | S | Q | F | - | - | - | - | - | - | - | - | - | - | - | - | - | - | - | - | - | - | - | - | T | S | L | W | D | F | K | N | H | F | C | R | N | N | I | T | F | D | M | S | Q | N | D | I | L | S | L | R | K | E | V | F | V | G | M | E | K | A | V | C | L | D | L | S | F | N | Y | M | S | Q | A | L | R | R | G | V | F | A | G | M | K | K | L | V | F | L | D | L | S | Y | N | R | L | D | F | Y | Y | K | D | A | F | S | E | L | K | A | T | L | K | V | L | D | I | S | N | N | E | F | H | F | K | M | R | G | M | G | H | R | L | E | F | L | Q | N | L | T | N | L | E | V | L | N | M | A | N | N | G | I | G | M | R | I | D | Q | R | L | I | S | S | S | L | K | Y | L | Y | F | Y | G | N | H | L | D | I | M | W | D | S | D | N | N | K | Y | T | H | F | F | Q | N | L | T | N | L | T | F | L | D | I | S | M | N | E | L | K | S | I | S | P | E | V | L | C | N | L | P | R | S | I | K | N | L | S | I | S | N | N | L | L | N | Y | F | P | W | Q | N | I | S | A | L | S | N | L | C | H | L | D | L | S | Q | N | F | L | S | Y | L | P | H | K | V | I | E | F | G | A | F | F | S | S | L | D | L | S | H | N | R | L | S | L | I | P | K | D | F | F | S | K | A | K | S | L | K | Y | L | Y | L | S | H | N | Q | I | K | E | L | N | H | Q | F | F | P | A | P | F | K | N | G | S | A | L | - | - | - | - | - | - | - | - | - | - | - | Q | K | L | T | L | H | A | N | P | F | K | C | D | C | D | T | S | W | F | A | D | F | L | R | T | T | P | V | H | I | P | Y | L | T | T | H | I | H | C | E | Y | P | E | S | Q | Q | G | R | S | I | M | Y | M | D | Q | R | S | C | Q | D | I | Y | G | S | L | A | S | F | V | C | S | F | L | V | V | T | F | T | V | L | P | L | L | K | H | L | Y | G | W | D | L | W | Y | C | L | Q | L | L | W | A | G | H | K | G | Y | S | Q | L | A | G | S | - | D | S | H | Y | H | Y | D | A | F | V | V | F | D | T | E | N | Q | A | V | R | D | W | V | Y | N | E | L | I | A | N | L | E | N | S | G | H | R | K | F | C | L | C | L | E | E | R | D | W | T | P | G | L | S | C | I | E | N | L | H | N | A | V | Y | N | S | M | K | T | V | F | V | L | S | S | G | A | A | G | S | E | - | - | - | - | T | V | N | G | V | I | R | Q | S | F | F | M | V | Q | Q | R | L | L | D | E | K | V | D | A | A | V | L | V | L | L | D | E | L | F | P | K | L | K | Y | L | Q | L | R | K | R | L | C | R | K | S | V | L | S | W | P | R | N | P | R | A | Q | P | L | F | W | N | Q | V | R | M | A | L | S | S | D | N | L | K | F | Y | D | N | N | M | S | E | S | F | I | X | X | X | X | X | X | X | X | X | X | X | X | X | X | X | X | X | X | - | - | - | - | - | - | - | - | - | - | - | - | - | - | - | - | - | - | - | - | - | - | - | - | - | - | - | - | - | - | - | - | - | - | - | - | - | - | - | - | - | - | - | - | - | - | - | - | - | - | - | - | - | - | - | - | - | - | - | - | - | - | - | - | - | - | - | - | - | - | - | - | - | - | - | - | - | - | - | - | - | - | - | - | - | - | - | - | - | - | - | - | - | - | - | - | - | - | - | - | - | - | - | - | - | - | - | - | - | - | - | - | - | - | - | - | - | - | - | - | - | - | - | - | - | - | - | - | - | - | - | - | - | - | - | - | - | - | - | - | - | - | - | - | - | - | - | - | - | - | - | - | - | - | - | - | - | - | - | - | - | - | - | - | - | - | - | - | - | - | - | - | - | - | - | - | - | - | - | - | - | - | - | - | - | - | - | - | - | - | - | - | - | - |
| PFTLR9 | - | - | - | - | - | M | L | R | Y | D | A | I | P | I | M | A | M | L | - | K | N | I | L | I | L | C | Q | L | L | P | L | V | R | T | I | N | T | I | F | F | P | C | D | T | D | V | N | - | - | - | T | T | T | V | D | C | H | D | R | S | L | K | H | V | L | L | I | K | S | T | T | V | V | S | I | N | L | S | R | T | K | I | Q | Q | V | G | Q | H | A | F | L | G | V | P | N | L | H | T | L | K | I | M | - | G | N | C | Q | P | G | S | L | R | A | L | E | D | R | S | C | K | M | K | I | H | N | D | A | F | K | N | L | S | K | L | T | F | L | Y | L | S | G | N | S | L | T | - | - | S | I | P | R | L | P | K | N | L | T | V | L | D | L | Q | N | N | C | I | F | N | I | A | E | P | L | N | T | P | H | L | K | E | L | Y | L | S | K | N | C | F | Y | A | N | P | C | G | H | S | F | Y | I | N | E | S | V | F | R | E | L | S | E | L | K | N | L | T | L | G | Y | N | N | F | T | A | I | P | K | G | L | P | P | S | L | E | S | L | D | L | R | E | N | T | I | T | E | V | L | E | G | A | F | A | N | L | T | L | L | K | N | L | N | L | E | W | N | C | Q | R | C | D | H | A | A | R | P | C | F | P | C | P | H | N | L | P | L | H | L | H | P | N | S | F | Y | A | E | N | S | S | I | D | Y | L | S | L | R | G | N | S | L | K | T | F | P | E | G | L | F | R | P | L | K | N | - | L | K | R | L | D | L | S | D | N | L | L | A | Y | A | I | R | N | G | T | F | F | A | E | L | E | G | L | T | W | I | S | L | I | Y | N | Y | E | P | L | K | T | F | P | Y | L | I | L | S | P | Y | I | G | N | M | S | R | L | H | Y | L | L | L | S | G | N | F | F | H | K | L | S | S | E | S | L | N | V | V | S | K | L | Q | N | L | K | K | L | E | L | R | M | N | F | I | N | T | F | N | S | T | F | L | K | R | L | P | F | L | L | D | V | D | L | S | Q | N | M | L | N | F | I | P | R | C | S | C | L | S | S | D | I | V | T | Q | E | S | C | Q | N | Q | N | L | H | T | H | - | - | - | - | N | - | F | P | D | P | - | L | M | L | I | D | R | K | - | - | - | A | A | S | G | S | D | M | W | E | S | T | - | - | - | - | - | - | - | - | - | Q | T | N | R | L | D | I | V | K | D | N | V | P | Q | F | - | - | - | - | - | - | - | - | - | - | - | - | - | - | - | - | - | - | - | - | K | S | L | F | D | F | K | N | Y | F | C | R | G | K | L | T | F | D | L | S | Q | N | D | I | L | S | L | T | K | E | V | F | V | G | M | E | N | A | V | C | L | D | L | S | F | N | Y | M | S | Q | A | L | K | S | G | L | F | D | R | M | K | K | L | V | F | L | N | M | S | Y | N | R | L | D | F | Y | Y | G | G | A | F | S | E | L | N | T | T | L | K | V | L | D | I | S | N | N | E | F | H | F | K | M | K | G | M | G | H | S | F | E | F | L | K | N | L | T | N | L | E | V | L | S | L | A | N | N | D | I | G | M | R | I | D | E | R | L | I | S | S | S | L | K | Y | L | Y | F | Y | G | N | H | L | D | I | M | W | E | S | D | N | N | R | Y | T | N | F | F | Q | N | L | T | N | L | T | Y | L | D | I | S | N | N | N | L | K | S | I | Q | P | E | L | L | C | N | L | P | E | S | M | Q | T | L | S | I | G | N | N | Q | L | N | Y | F | P | W | Q | N | I | S | A | L | S | N | L | C | H | L | D | L | S | Q | N | F | L | F | Y | L | P | N | K | V | I | E | F | G | A | N | F | S | F | L | D | L | S | H | N | R | L | S | I | I | P | E | D | F | F | S | M | A | K | S | L | H | Y | L | Y | L | S | H | N | Q | I | K | A | L | N | H | Q | F | L | P | V | L | F | K | N | G | S | A | L | - | - | - | - | - | - | - | - | - | - | - | Q | K | L | T | L | H | N | N | P | F | K | C | D | C | D | T | S | W | F | A | D | F | L | R | S | T | P | V | Q | I | P | Y | L | T | T | K | V | H | C | D | Y | P | E | S | Q | Q | S | M | S | V | L | S | M | D | Q | R | S | C | Q | D | I | Y | G | S | L | A | F | L | V | C | S | F | L | A | V | T | F | T | V | L | P | L | L | K | H | L | Y | G | W | D | L | W | Y | C | L | Q | V | L | W | A | G | H | K | G | Y | S | Q | L | A | G | S | - | D | S | H | Y | H | Y | D | A | F | V | V | F | D | T | M | N | E | A | V | R | D | W | V | Y | N | E | L | M | V | N | L | E | N | S | G | H | R | R | F | C | L | C | L | E | E | R | D | W | I | P | G | L | S | C | I | E | N | L | H | N | A | V | Y | S | S | M | K | T | V | F | V | L | S | S | G | A | T | D | G | K | - | - | - | - | T | V | N | G | V | I | R | Q | A | F | F | M | V | Q | Q | R | L | L | D | E | K | V | D | S | A | V | L | V | V | L | D | E | M | F | P | K | L | K | Y | L | Q | L | R | K | R | L | C | K | K | S | V | L | S | W | P | R | N | P | R | V | Q | P | L | F | W | N | R | M | R | M | A | L | S | S | D | N | L | K | F | Y | D | N | N | M | S | E | S | F | V | X | X | X | X | X | X | X | X | X | X | - | - | - | - | - | - | - | - | - | - | - | - | - | - | - | - | - | - | - | - | - | - | - | - | - | - | - | - | - | - | - | - | - | - | - | - | - | - | - | - | - | - | - | - | - | - | - | - | - | - | - | - | - | - | - | - | - | - | - | - | - | - | - | - | - | - | - | - | - | - | - | - | - | - | - | - | - | - | - | - | - | - | - | - | - | - | - | - | - | - | - | - | - | - | - | - | - | - | - | - | - | - | - | - | - | - | - | - | - | - | - | - | - | - | - | - | - | - | - | - | - | - | - | - | - | - | - | - | - | - | - | - | - | - | - | - | - | - | - | - | - | - | - | - | - | - | - | - | - | - | - | - | - | - | - | - | - | - | - | - | - | - | - | - | - | - | - | - | - | - | - | - | - | - | - | - | - | - | - | - | - | - | - | - | - | - | - | - | - | - | - | - | - | - | - | - | - | - | - | - | - | - |
| conservation |  |  |  |  |  |  |  |  |  |  |  |  |  |  |  |  |  |  |  |  |  |  |  |  |  |  |  |  |  |  |  |  |  |  |  |  |  |  |  |  |  |  |  |  |  |  |  |  |  |  |  |  |  |  |  |  |  |  |  |  |  |  |  |  |  |  |  |  |  |  |  |  |  |  |  |  |  |  |  |  |  |  |  |  |  |  |  |  |  |  |  |  |  |  |  |  |  |  |  |  |  |  |  |  |  |  |  |  |  |  |  |  |  |  |  |  |  |  |  |  |  |  |  |  |  |  |  |  |  |  |  |  |  |  |  |  |  |  |  |  |  |  |  |  |  |  |  |  |  |  |  |  |  |  |  |  |  |  |  |  |  |  |  |  |  |  |  |  |  |  |  |  |  |  |  |  |  |  |  |  |  |  |  |  |  |  |  |  |  |  |  |  |  |  |  |  |  |  |  |  |  |  |  |  |  |  |  |  |  |  |  |  |  |  |  |  |  |  |  |  |  |  |  |  |  |  |  |  |  |  |  |  |  |  |  |  |  |  |  |  |  |  |  |  |  |  |  |  |  |  |  |  |  |  |  |  |  |  |  |  |  |  |  |  |  |  |  |  |  |  |  |  |  |  |  |  |  |  |  |  |  |  |  |  |  |  |  |  |  |  |  |  |  |  |  |  |  |  |  |  |  |  |  |  |  |  |  |  |  |  |  |  |  |  |  |  |  |  |  |  |  |  |  |  |  |  |  |  |  |  |  |  |  |  |  |  |  |  |  |  |  |  |  |  |  |  |  |  |  |  |  |  |  |  |  |  |  |  |  |  |  |  |  |  |  |  |  |  |  |  |  |  |  |  |  |  |  |  |  |  |  |  |  |  |  |  |  |  |  |  |  |  |  |  |  |  |  |  |  |  |  |  |  |  |  |  |  |  |  |  |  |  |  |  |  |  |  |  |  |  |  |  |  |  |  |  |  |  |  |  |  |  |  |  |  |  |  |  |  |  |  |  |  |  |  |  |  |  |  |  |  |  |  |  |  |  |  |  |  |  |  |  |  |  |  |  |  |  |  |  |  |  |  |  |  |  |  |  |  |  |  |  |  |  |  |  |  |  |  |  |  |  |  |  |  |  |  |  |  |  |  |  |  |  |  |  |  |  |  |  |  |  |  |  |  |  |  |  |  |  |  |  |  |  |  |  |  |  |  |  |  |  |  |  |  |  |  |  |  |  |  |  |  |  |  |  |  |  |  |  |  |  |  |  |  |  |  |  |  |  |  |  |  |  |  |  |  |  |  |  |  |  |  |  |  |  |  |  |  |  |  |  |  |  |  |  |  |  |  |  |  |  |  |  |  |  |  |  |  |  |  |  |  |  |  |  |  |  |  |  |  |  |  |  |  |  |  |  |  |  |  |  |  |  |  |  |  |  |  |  |  |  |  |  |  |  |  |  |  |  |  |  |  |  |  |  |  |  |  |  |  |  |  |  |  |  |  |  |  |  |  |  |  |  |  |  |  |  |  |  |  |  |  |  |  |  |  |  |  |  |  |  |  |  |  |  |  |  |  |  |  |  |  |  |  |  |  |  |  |  |  |  |  |  |  |  |  |  |  |  |  |  |  |  |  |  |  |  |  |  |  |  |  |  |  |  |  |  |  |  |  |  |  |  |  |  |  |  |  |  |  |  |  |  |  |  |  |  |  |  |  |  |  |  |  |  |  |  |  |  |  |  |  |  |  |  |  |  |  |  |  |  |  |  |  |  |  |  |  |  |  |  |  |  |  |  |  |  |  |  |  |  |  |  |  |  |  |  |  |  |  |  |  |  |  |  |  |  |  |  |  |  |  |  |  |  |  |  |  |  |  |  |  |  |  |  |  |  |  |  |  |  |  |  |  |  |  |  |  |  |  |  |  |  |  |  |  |  |  |  |  |  |  |  |  |  |  |  |  |  |  |  |  |  |  |  |  |  |  |  |  |  |  |  |  |  |  |  |  |  |  |  |  |  |  |  |  |  |  |  |  |  |  |  |  |  |  |  |  |  |  |  |  |  |  |  |  |  |  |  |  |  |  |  |  |  |  |  |  |  |  |  |  |  |  |  |  |  |  |  |  |  |  |  |  |  |  |  |  |  |  |  |  |  |  |  |  |  |  |  |  |  |  |  |  |  |  |  |  |  |  |  |  |  |  |  |  |  |  |  |  |  |  |  |  |  |  |  |  |  |  |  |  |  |  |  |  |  |  |  |  |  |  |  |  |  |  |  |  |  |  |  |  |  |  |  |  |  |  |  |  |  |  |  |  |  |  |  |  |  |  |  |  |  |  |  |  |  |  |  |  |  |  |  |  |  |  |  |  |  |  |  |  |  |  |  |  |  |  |  |  |  |  |  |  |  |  |  |  |  |  |  |  |  |  |  |  |  |  |  |  |  |  |  |  |  |  |  |  |  |  |  |  |  |  |  |  |  |  |  |  |  |  |  |  |  |  |  |  |  |  |  |  |  |  |  |  |  |  |  |  |  |  |  |  |  |  |  |  |  |  |  |  |  |  |  |  |  |  |  |  |  |  |  |  |  |  |  |  |  |  |  |  |  |  |  |  |  |  |  |  |  |  |  |  |  |  |  |  |  |  |  |  |  |  |  |  |  |  |  |  |  |  |  |  |  |  |  |  |  |  |  |  |  |  |  |  |  |  |  |  |  |  |  |  |  |  |  |  |  |  |  |  |  |  |  |  |  |  |  |  |  |  |  |  |  |  |  |  |  |  |  |  |  |  |  |  |  |  |  |  |  |  |  |  |  |  |  |  |  |  |  |  |  |  |  |  |  |  |  |  |  |  |  |  |  |  |  |  |  |  |  |  |  |  |  |  |  |  |  |  |  |  |  |  |  |  |  |  |  |  |  |  |  |  |  |  |  |  |  |  |  |  |  |  |  |  |  |  |  |  |  |  |  |  |  |  |  |  |  |  |  |  |  |  |  |  |  |  |  |  |  |  |  |  |  |  |  |  |  |  |  |  |  |  |  |  |  |  |  |  |  |  |  |  |  |  |
|  |  |  |  |  |  |  |  |  |  |  |  |  |  |  |  |  |  |  |  |  |  |  |  |  |  |  |  |  |  |  |  |  |  |  |  |  |  |  |  |  |  |  |  |  |  |  |  |  |  |  |  |  |  |  |  |  |  |  |  |  |  |  |  |  |  |  |  |  |  |  |  |  |  |  |  |  |  |  |  |  |  |  |  |  |  |  |  |  |  |  |  |  |  |  |  |  |  |  |  |  |  |  |  |  |  |  |  |  |  |  |  |  |  |  |  |  |  |  |  |  |  |  |  |  |  |  |  |  |  |  |  |  |  |  |  |  |  |  |  |  |  |  |  |  |  |  |  |  |  |  |  |  |  |  |  |  |  |  |  |  |  |  |  |  |  |  |  |  |  |  |  |  |  |  |  |  |  |  |  |  |  |  |  |  |  |  |  |  |  |  |  |  |  |  |  |  |  |  |  |  |  |  |  |  |  |  |  |  |  |  |  |  |  |  |  |  |  |  |  |  |  |  |  |  |  |  |  |  |  |  |  |  |  |  |  |  |  |  |  |  |  |  |  |  |  |  |  |  |  |  |  |  |  |  |  |  |  |  |  |  |  |  |  |  |  |  |  |  |  |  |  |  |  |  |  |  |  |  |  |  |  |  |  |  |  |  |  |  |  |  |  |  |  |  |  |  |  |  |  |  |  |  |  |  |  |  |  |  |  |  |  |  |  |  |  |  |  |  |  |  |  |  |  |  |  |  |  |  |  |  |  |  |  |  |  |  |  |  |  |  |  |  |  |  |  |  |  |  |  |  |  |  |  |  |  |  |  |  |  |  |  |  |  |  |  |  |  |  |  |  |  |  |  |  |  |  |  |  |  |  |  |  |  |  |  |  |  |  |  |  |  |  |  |  |  |  |  |  |  |  |  |  |  |  |  |  |  |  |  |  |  |  |  |  |  |  |  |  |  |  |  |  |  |  |  |  |  |  |  |  |  |  |  |  |  |  |  |  |  |  |  |  |  |  |  |  |  |  |  |  |  |  |  |  |  |  |  |  |  |  |  |  |  |  |  |  |  |  |  |  |  |  |  |  |  |  |  |  |  |  |  |  |  |  |  |  |  |  |  |  |  |  |  |  |  |  |  |  |  |  |  |  |  |  |  |  |  |  |  |  |  |  |  |  |  |  |  |  |  |  |  |  |  |  |  |  |  |  |  |  |  |  |  |  |  |  |  |  |  |  |  |  |  |  |  |  |  |  |  |  |  |  |  |  |  |  |  |  |  |  |  |  |  |  |  |  |  |  |  |  |  |  |  |  |  |  |  |  |  |  |  |  |  |  |  |  |  |  |  |  |  |  |  |  |  |  |  |  |  |  |  |  |  |  |  |  |  |  |  |  |  |  |  |  |  |  |  |  |  |  |  |  |  |  |  |  |  |  |  |  |  |  |  |  |  |  |  |  |  |  |  |  |  |  |  |  |  |  |  |  |  |  |  |  |  |  |  |  |  |  |  |  |  |  |  |  |  |  |  |  |  |  |  |  |  |  |  |  |  |  |  |  |  |  |  |  |  |  |  |  |  |  |  |  |  |  |  |  |  |  |  |  |  |  |  |  |  |  |  |  |  |  |  |  |  |  |  |  |  |  |  |  |  |  |  |  |  |  |  |  |  |  |  |  |  |  |  |  |  |  |  |  |  |  |  |  |  |  |  |  |  |  |  |  |  |  |  |  |  |  |  |  |  |  |  |  |  |  |  |  |  |  |  |  |  |  |  |  |  |  |  |  |  |  |  |  |  |  |  |  |  |  |  |  |  |  |  |  |  |  |  |  |  |  |  |  |  |  |  |  |  |  |  |  |  |  |  |  |  |  |  |  |  |  |  |  |  |  |  |  |  |  |  |  |  |  |  |  |  |  |  |  |  |  |  |  |  |  |  |  |  |  |  |  |  |  |  |  |  |  |  |  |  |  |  |  |  |  |  |  |  |  |  |  |  |  |  |  |  |  |  |  |  |  |  |  |  |  |  |  |  |  |  |  |  |  |  |  |  |  |  |  |  |  |  |  |  |  |  |  |  |  |  |  |  |  |  |  |  |  |  |  |  |  |  |  |  |  |  |  |  |  |  |  |  |  |  |  |  |  |  |  |  |  |  |  |  |  |  |  |  |  |  |  |  |  |  |  |  |  |  |  |  |  |  |  |  |  |  |  |  |  |  |  |  |  |  |  |  |  |  |  |  |  |  |  |  |  |  |  |  |  |  |  |  |  |  |  |  |  |  |  |  |  |  |  |  |  |  |  |  |  |  |  |  |  |  |  |  |  |  |  |  |  |  |  |  |  |  |  |  |  |  |  |  |  |  |  |  |  |  |  |  |  |  |  |  |  |  |  |  |  |  |  |  |  |  |  |  |  |  |  |  |  |  |  |  |  |  |  |  |  |  |  |  |  |  |  |  |  |  |  |  |  |  |  |  |  |  |  |  |  |  |  |  |  |  |  |  |  |  |  |  |  |  |  |  |  |  |  |  |  |  |  |  |  |  |  |  |  |  |  |  |  |  |  |  |  |  |  |  |  |  |  |  |  |  |  |  |  |  |  |  |  |  |  |  |  |  |  |  |  |  |  |  |  |  |  |  |  |  |  |  |  |  |  |  |  |  |  |  |  |  |  |  |  |  |  |  |  |  |  |  |  |  |  |  |  |  |  |  |  |  |  |  |  |  |  |  |  |  |  |  |  |  |  |  |  |  |  |  |  |  |  |  |  |  |  |  |  |  |  |  |  |  |  |  |  |  |  |  |  |  |  |  |  |  |  |  |  |  |  |  |  |  |  |  |  |  |  |  |  |  |  |  |  |  |  |  |  |  |  |  |  |  |  |  |  |  |  |  |  |  |  |  |  |  |  |  |  |  |  |  |  |  |  |  |  |  |  |  |  |  |  |  |  |  |  |  |  |  |  |  |  |  |  |  |  |  |  |  |  |  |  |  |  |  |  |  |  |  |  |  |  |  |  |  |  |  |  |  |  |  |  |  |  |  |  |  |  |  |  |  |  |  |  |  |
|  |  |  |  |  |  |  |  |  |  |  |  |  |  |  |  |  |  |  |  |  |  |  |  |  |  |  |  |  |  |  |  |  |  |  |  |  |  |  |  |  |  |  |  |  |  |  |  |  |  |  |  |  |  |  |  |  |  |  |  |  |  |  |  |  |  |  |  |  |  |  |  |  |  |  |  |  |  |  |  |  |  |  |  |  |  |  |  |  |  |  |  |  |  |  |  |  |  |  |  |  |  |  |  |  |  |  |  |  |  |  |  |  |  |  |  |  |  |  |  |  |  |  |  |  |  |  |  |  |  |  |  |  |  |  |  |  |  |  |  |  |  |  |  |  |  |  |  |  |  |  |  |  |  |  |  |  |  |  |  |  |  |  |  |  |  |  |  |  |  |  |  |  |  |  |  |  |  |  |  |  |  |  |  |  |  |  |  |  |  |  |  |  |  |  |  |  |  |  |  |  |  |  |  |  |  |  |  |  |  |  |  |  |  |  |  |  |  |  |  |  |  |  |  |  |  |  |  |  |  |  |  |  |  |  |  |  |  |  |  |  |  |  |  |  |  |  |  |  |  |  |  |  |  |  |  |  |  |  |  |  |  |  |  |  |  |  |  |  |  |  |  |  |  |  |  |  |  |  |  |  |  |  |  |  |  |  |  |  |  |  |  |  |  |  |  |  |  |  |  |  |  |  |  |  |  |  |  |  |  |  |  |  |  |  |  |  |  |  |  |  |  |  |  |  |  |  |  |  |  |  |  |  |  |  |  |  |  |  |  |  |  |  |  |  |  |  |  |  |  |  |  |  |  |  |  |  |  |  |  |  |  |  |  |  |  |  |  |  |  |  |  |  |  |  |  |  |  |  |  |  |  |  |  |  |  |  |  |  |  |  |  |  |  |  |  |  |  |  |  |  |  |  |  |  |  |  |  |  |  |  |  |  |  |  |  |  |  |  |  |  |  |  |  |  |  |  |  |  |  |  |  |  |  |  |  |  |  |  |  |  |  |  |  |  |  |  |  |  |  |  |  |  |  |  |  |  |  |  |  |  |  |  |  |  |  |  |  |  |  |  |  |  |  |  |  |  |  |  |  |  |  |  |  |  |  |  |  |  |  |  |  |  |  |  |  |  |  |  |  |  |  |  |  |  |  |  |  |  |  |  |  |  |  |  |  |  |  |  |  |  |  |  |  |  |  |  |  |  |  |  |  |  |  |  |  |  |  |  |  |  |  |  |  |  |  |  |  |  |  |  |  |  |  |  |  |  |  |  |  |  |  |  |  |  |  |  |  |  |  |  |  |  |  |  |  |  |  |  |  |  |  |  |  |  |  |  |  |  |  |  |  |  |  |  |  |  |  |  |  |  |  |  |  |  |  |  |  |  |  |  |  |  |  |  |  |  |  |  |  |  |  |  |  |  |  |  |  |  |  |  |  |  |  |  |  |  |  |  |  |  |  |  |  |  |  |  |  |  |  |  |  |  |  |  |  |  |  |  |  |  |  |  |  |  |  |  |  |  |  |  |  |  |  |  |  |  |  |  |  |  |  |  |  |  |  |  |  |  |  |  |  |  |  |  |  |  |  |  |  |  |  |  |  |  |  |  |  |  |  |  |  |  |  |  |  |  |  |  |  |  |  |  |  |  |  |  |  |  |  |  |  |  |  |  |  |  |  |  |  |  |  |  |  |  |  |  |  |  |  |  |  |  |  |  |  |  |  |  |  |  |  |  |  |  |  |  |  |  |  |  |  |  |  |  |  |  |  |  |  |  |  |  |  |  |  |  |  |  |  |  |  |  |  |  |  |  |  |  |  |  |  |  |  |  |  |  |  |  |  |  |  |  |  |  |  |  |  |  |  |  |  |  |  |  |  |  |  |  |  |  |  |  |  |  |  |  |  |  |  |  |  |  |  |  |  |  |  |  |  |  |  |  |  |  |  |  |  |  |  |  |  |  |  |  |  |  |  |  |  |  |  |  |  |  |  |  |  |  |  |  |  |  |  |  |  |  |  |  |  |  |  |  |  |  |  |  |  |  |  |  |  |  |  |  |  |  |  |  |  |  |  |  |  |  |  |  |  |  |  |  |  |  |  |  |  |  |  |  |  |  |  |  |  |  |  |  |  |  |  |  |  |  |  |  |  |  |  |  |  |  |  |  |  |  |  |  |  |  |  |  |  |  |  |  |  |  |  |  |  |  |  |  |  |  |  |  |  |  |  |  |  |  |  |  |  |  |  |  |  |  |  |  |  |  |  |  |  |  |  |  |  |  |  |  |  |  |  |  |  |  |  |  |  |  |  |  |  |  |  |  |  |  |  |  |  |  |  |  |  |  |  |  |  |  |  |  |  |  |  |  |  |  |  |  |  |  |  |  |  |  |  |  |  |  |  |  |  |  |  |  |  |  |  |  |  |  |  |  |  |  |  |  |  |  |  |  |  |  |  |  |  |  |  |  |  |  |  |  |  |  |  |  |  |  |  |  |  |  |  |  |  |  |  |  |  |  |  |  |  |  |  |  |  |  |  |  |  |  |  |  |  |  |  |  |  |  |  |  |  |  |  |  |  |  |  |  |  |  |  |  |  |  |  |  |  |  |  |  |  |  |  |  |  |  |  |  |  |  |  |  |  |  |  |  |  |  |  |  |  |  |  |  |  |  |  |  |  |  |  |  |  |  |  |  |  |  |  |  |  |  |  |  |  |  |  |  |  |  |  |  |  |  |  |  |  |  |  |  |  |  |  |  |  |  |  |  |  |  |  |  |  |  |  |  |  |  |  |  |  |  |  |  |  |  |  |  |  |  |  |  |  |  |  |  |  |  |  |  |  |  |  |  |  |  |  |  |  |  |  |  |  |  |  |  |  |  |  |  |  |  |  |  |  |  |  |  |  |  |  |  |  |  |  |  |  |  |  |  |  |  |  |  |  |  |  |  |  |  |  |  |  |  |  |  |  |  |  |  |  |  |  |  |  |  |  |  |  |  |  |  |  |  |  |  |  |  |  |  |  |  |  |  |  |  |  |  |  |  |  |  |  |  |  |  |  |  |  |
|  |  |  |  |  |  |  |  |  |  |  |  |  |  |  |  |  |  |  |  |  |  |  |  |  |  |  |  |  |  |  |  |  |  |  |  |  |  |  |  |  |  |  |  |  |  |  |  |  |  |  |  |  |  |  |  |  |  |  |  |  |  |  |  |  |  |  |  |  |  |  |  |  |  |  |  |  |  |  |  |  |  |  |  |  |  |  |  |  |  |  |  |  |  |  |  |  |  |  |  |  |  |  |  |  |  |  |  |  |  |  |  |  |  |  |  |  |  |  |  |  |  |  |  |  |  |  |  |  |  |  |  |  |  |  |  |  |  |  |  |  |  |  |  |  |  |  |  |  |  |  |  |  |  |  |  |  |  |  |  |  |  |  |  |  |  |  |  |  |  |  |  |  |  |  |  |  |  |  |  |  |  |  |  |  |  |  |  |  |  |  |  |  |  |  |  |  |  |  |  |  |  |  |  |  |  |  |  |  |  |  |  |  |  |  |  |  |  |  |  |  |  |  |  |  |  |  |  |  |  |  |  |  |  |  |  |  |  |  |  |  |  |  |  |  |  |  |  |  |  |  |  |  |  |  |  |  |  |  |  |  |  |  |  |  |  |  |  |  |  |  |  |  |  |  |  |  |  |  |  |  |  |  |  |  |  |  |  |  |  |  |  |  |  |  |  |  |  |  |  |  |  |  |  |  |  |  |  |  |  |  |  |  |  |  |  |  |  |  |  |  |  |  |  |  |  |  |  |  |  |  |  |  |  |  |  |  |  |  |  |  |  |  |  |  |  |  |  |  |  |  |  |  |  |  |  |  |  |  |  |  |  |  |  |  |  |  |  |  |  |  |  |  |  |  |  |  |  |  |  |  |  |  |  |  |  |  |  |  |  |  |  |  |  |  |  |  |  |  |  |  |  |  |  |  |  |  |  |  |  |  |  |  |  |  |  |  |  |  |  |  |  |  |  |  |  |  |  |  |  |  |  |  |  |  |  |  |  |  |  |  |  |  |  |  |  |  |  |  |  |  |  |  |  |  |  |  |  |  |  |  |  |  |  |  |  |  |  |  |  |  |  |  |  |  |  |  |  |  |  |  |  |  |  |  |  |  |  |  |  |  |  |  |  |  |  |  |  |  |  |  |  |  |  |  |  |  |  |  |  |  |  |  |  |  |  |  |  |  |  |  |  |  |  |  |  |  |  |  |  |  |  |  |  |  |  |  |  |  |  |  |  |  |  |  |  |  |  |  |  |  |  |  |  |  |  |  |  |  |  |  |  |  |  |  |  |  |  |  |  |  |  |  |  |  |  |  |  |  |  |  |  |  |  |  |  |  |  |  |  |  |  |  |  |  |  |  |  |  |  |  |  |  |  |  |  |  |  |  |  |  |  |  |  |  |  |  |  |  |  |  |  |  |  |  |  |  |  |  |  |  |  |  |  |  |  |  |  |  |  |  |  |  |  |  |  |  |  |  |  |  |  |  |  |  |  |  |  |  |  |  |  |  |  |  |  |  |  |  |  |  |  |  |  |  |  |  |  |  |  |  |  |  |  |  |  |  |  |  |  |  |  |  |  |  |  |  |  |  |  |  |  |  |  |  |  |  |  |  |  |  |  |  |  |  |  |  |  |  |  |  |  |  |  |  |  |  |  |  |  |  |  |  |  |  |  |  |  |  |  |  |  |  |  |  |  |  |  |  |  |  |  |  |  |  |  |  |  |  |  |  |  |  |  |  |  |  |  |  |  |  |  |  |  |  |  |  |  |  |  |  |  |  |  |  |  |  |  |  |  |  |  |  |  |  |  |  |  |  |  |  |  |  |  |  |  |  |  |  |  |  |  |  |  |  |  |  |  |  |  |  |  |  |  |  |  |  |  |  |  |  |  |  |  |  |  |  |  |  |  |  |  |  |  |  |  |  |  |  |  |  |  |  |  |  |  |  |  |  |  |  |  |  |  |  |  |  |  |  |  |  |  |  |  |  |  |  |  |  |  |  |  |  |  |  |  |  |  |  |  |  |  |  |  |  |  |  |  |  |  |  |  |  |  |  |  |  |  |  |  |  |  |  |  |  |  |  |  |  |  |  |  |  |  |  |  |  |  |  |  |  |  |  |  |  |  |  |  |  |  |  |  |  |  |  |  |  |  |  |  |  |  |  |  |  |  |  |  |  |  |  |  |  |  |  |  |  |  |  |  |  |  |  |  |  |  |  |  |  |  |  |  |  |  |  |  |  |  |  |  |  |  |  |  |  |  |  |  |  |  |  |  |  |  |  |  |  |  |  |  |  |  |  |  |  |  |  |  |  |  |  |  |  |  |  |  |  |  |  |  |  |  |  |  |  |  |  |  |  |  |  |  |  |  |  |  |  |  |  |  |  |  |  |  |  |  |  |  |  |  |  |  |  |  |  |  |  |  |  |  |  |  |  |  |  |  |  |  |  |  |  |  |  |  |  |  |  |  |  |  |  |  |  |  |  |  |  |  |  |  |  |  |  |  |  |  |  |  |  |  |  |  |  |  |  |  |  |  |  |  |  |  |  |  |  |  |  |  |  |  |  |  |  |  |  |  |  |  |  |  |  |  |  |  |  |  |  |  |  |  |  |  |  |  |  |  |  |  |  |  |  |  |  |  |  |  |  |  |  |  |  |  |  |  |  |  |  |  |  |  |  |  |  |  |  |  |  |  |  |  |  |  |  |  |  |  |  |  |  |  |  |  |  |  |  |  |  |  |  |  |  |  |  |  |  |  |  |  |  |  |  |  |  |  |  |  |  |  |  |  |  |  |  |  |  |  |  |  |  |  |  |  |  |  |  |  |  |  |  |  |  |  |  |  |  |  |  |  |  |  |  |  |  |  |  |  |  |  |  |  |  |  |  |  |  |  |  |  |  |  |  |  |  |  |  |  |  |  |  |  |  |  |  |  |  |  |  |  |  |  |  |  |  |  |  |  |  |  |  |  |  |  |  |  |  |  |  |  |  |  |  |  |  |  |  |  |  |  |  |  |  |  |  |  |  |  |  |  |  |  |  |  |  |  |  |  |  |  |
|  |  |  |  |  |  |  |  |  |  |  |  |  |  |  |  |  |  |  |  |  |  |  |  |  |  |  |  |  |  |  |  |  |  |  |  |  |  |  |  |  |  |  |  |  |  |  |  |  |  |  |  |  |  |  |  |  |  |  |  |  |  |  |  |  |  |  |  |  |  |  |  |  |  |  |  |  |  |  |  |  |  |  |  |  |  |  |  |  |  |  |  |  |  |  |  |  |  |  |  |  |  |  |  |  |  |  |  |  |  |  |  |  |  |  |  |  |  |  |  |  |  |  |  |  |  |  |  |  |  |  |  |  |  |  |  |  |  |  |  |  |  |  |  |  |  |  |  |  |  |  |  |  |  |  |  |  |  |  |  |  |  |  |  |  |  |  |  |  |  |  |  |  |  |  |  |  |  |  |  |  |  |  |  |  |  |  |  |  |  |  |  |  |  |  |  |  |  |  |  |  |  |  |  |  |  |  |  |  |  |  |  |  |  |  |  |  |  |  |  |  |  |  |  |  |  |  |  |  |  |  |  |  |  |  |  |  |  |  |  |  |  |  |  |  |  |  |  |  |  |  |  |  |  |  |  |  |  |  |  |  |  |  |  |  |  |  |  |  |  |  |  |  |  |  |  |  |  |  |  |  |  |  |  |  |  |  |  |  |  |  |  |  |  |  |  |  |  |  |  |  |  |  |  |  |  |  |  |  |  |  |  |  |  |  |  |  |  |  |  |  |  |  |  |  |  |  |  |  |  |  |  |  |  |  |  |  |  |  |  |  |  |  |  |  |  |  |  |  |  |  |  |  |  |  |  |  |  |  |  |  |  |  |  |  |  |  |  |  |  |  |  |  |  |  |  |  |  |  |  |  |  |  |  |  |  |  |  |  |  |  |  |  |  |  |  |  |  |  |  |  |  |  |  |  |  |  |  |  |  |  |  |  |  |  |  |  |  |  |  |  |  |  |  |  |  |  |  |  |  |  |  |  |  |  |  |  |  |  |  |  |  |  |  |  |  |  |  |  |  |  |  |  |  |  |  |  |  |  |  |  |  |  |  |  |  |  |  |  |  |  |  |  |  |  |  |  |  |  |  |  |  |  |  |  |  |  |  |  |  |  |  |  |  |  |  |  |  |  |  |  |  |  |  |  |  |  |  |  |  |  |  |  |  |  |  |  |  |  |  |  |  |  |  |  |  |  |  |  |  |  |  |  |  |  |  |  |  |  |  |  |  |  |  |  |  |  |  |  |  |  |  |  |  |  |  |  |  |  |  |  |  |  |  |  |  |  |  |  |  |  |  |  |  |  |  |  |  |  |  |  |  |  |  |  |  |  |  |  |  |  |  |  |  |  |  |  |  |  |  |  |  |  |  |  |  |  |  |  |  |  |  |  |  |  |  |  |  |  |  |  |  |  |  |  |  |  |  |  |  |  |  |  |  |  |  |  |  |  |  |  |  |  |  |  |  |  |  |  |  |  |  |  |  |  |  |  |  |  |  |  |  |  |  |  |  |  |  |  |  |  |  |  |  |  |  |  |  |  |  |  |  |  |  |  |  |  |  |  |  |  |  |  |  |  |  |  |  |  |  |  |  |  |  |  |  |  |  |  |  |  |  |  |  |  |  |  |  |  |  |  |  |  |  |  |  |  |  |  |  |  |  |  |  |  |  |  |  |  |  |  |  |  |  |  |  |  |  |  |  |  |  |  |  |  |  |  |  |  |  |  |  |  |  |  |  |  |  |  |  |  |  |  |  |  |  |  |  |  |  |  |  |  |  |  |  |  |  |  |  |  |  |  |  |  |  |  |  |  |  |  |  |  |  |  |  |  |  |  |  |  |  |  |  |  |  |  |  |  |  |  |  |  |  |  |  |  |  |  |  |  |  |  |  |  |  |  |  |  |  |  |  |  |  |  |  |  |  |  |  |  |  |  |  |  |  |  |  |  |  |  |  |  |  |  |  |  |  |  |  |  |  |  |  |  |  |  |  |  |  |  |  |  |  |  |  |  |  |  |  |  |  |  |  |  |  |  |  |  |  |  |  |  |  |  |  |  |  |  |  |  |  |  |  |  |  |  |  |  |  |  |  |  |  |  |  |  |  |  |  |  |  |  |  |  |  |  |  |  |  |  |  |  |  |  |  |  |  |  |  |  |  |  |  |  |  |  |  |  |  |  |  |  |  |  |  |  |  |  |  |  |  |  |  |  |  |  |  |  |  |  |  |  |  |  |  |  |  |  |  |  |  |  |  |  |  |  |  |  |  |  |  |  |  |  |  |  |  |  |  |  |  |  |  |  |  |  |  |  |  |  |  |  |  |  |  |  |  |  |  |  |  |  |  |  |  |  |  |  |  |  |  |  |  |  |  |  |  |  |  |  |  |  |  |  |  |  |  |  |  |  |  |  |  |  |  |  |  |  |  |  |  |  |  |  |  |  |  |  |  |  |  |  |  |  |  |  |  |  |  |  |  |  |  |  |  |  |  |  |  |  |  |  |  |  |  |  |  |  |  |  |  |  |  |  |  |  |  |  |  |  |  |  |  |  |  |  |  |  |  |  |  |  |  |  |  |  |  |  |  |  |  |  |  |  |  |  |  |  |  |  |  |  |  |  |  |  |  |  |  |  |  |  |  |  |  |  |  |  |  |  |  |  |  |  |  |  |  |  |  |  |  |  |  |  |  |  |  |  |  |  |  |  |  |  |  |  |  |  |  |  |  |  |  |  |  |  |  |  |  |  |  |  |  |  |  |  |  |  |  |  |  |  |  |  |  |  |  |  |  |  |  |  |  |  |  |  |  |  |  |  |  |  |  |  |  |  |  |  |  |  |  |  |  |  |  |  |  |  |  |  |  |  |  |  |  |  |  |  |  |  |  |  |  |  |  |  |  |  |  |  |  |  |  |  |  |  |  |  |  |  |  |  |  |  |  |  |  |  |  |  |  |  |  |  |  |  |  |  |  |  |  |  |  |  |  |  |  |  |  |  |  |  |  |  |  |  |  |  |  |  |  |  |  |  |  |  |  |  |  |  |  |  |  |  |  |  |  |
|  |  |  |  |  |  |  |  |  |  |  |  |  |  |  |  |  |  |  |  |  |  |  |  |  |  |  |  |  |  |  |  |  |  |  |  |  |  |  |  |  |  |  |  |  |  |  |  |  |  |  |  |  |  |  |  |  |  |  |  |  |  |  |  |  |  |  |  |  |  |  |  |  |  |  |  |  |  |  |  |  |  |  |  |  |  |  |  |  |  |  |  |  |  |  |  |  |  |  |  |  |  |  |  |  |  |  |  |  |  |  |  |  |  |  |  |  |  |  |  |  |  |  |  |  |  |  |  |  |  |  |  |  |  |  |  |  |  |  |  |  |  |  |  |  |  |  |  |  |  |  |  |  |  |  |  |  |  |  |  |  |  |  |  |  |  |  |  |  |  |  |  |  |  |  |  |  |  |  |  |  |  |  |  |  |  |  |  |  |  |  |  |  |  |  |  |  |  |  |  |  |  |  |  |  |  |  |  |  |  |  |  |  |  |  |  |  |  |  |  |  |  |  |  |  |  |  |  |  |  |  |  |  |  |  |  |  |  |  |  |  |  |  |  |  |  |  |  |  |  |  |  |  |  |  |  |  |  |  |  |  |  |  |  |  |  |  |  |  |  |  |  |  |  |  |  |  |  |  |  |  |  |  |  |  |  |  |  |  |  |  |  |  |  |  |  |  |  |  |  |  |  |  |  |  |  |  |  |  |  |  |  |  |  |  |  |  |  |  |  |  |  |  |  |  |  |  |  |  |  |  |  |  |  |  |  |  |  |  |  |  |  |  |  |  |  |  |  |  |  |  |  |  |  |  |  |  |  |  |  |  |  |  |  |  |  |  |  |  |  |  |  |  |  |  |  |  |  |  |  |  |  |  |  |  |  |  |  |  |  |  |  |  |  |  |  |  |  |  |  |  |  |  |  |  |  |  |  |  |  |  |  |  |  |  |  |  |  |  |  |  |  |  |  |  |  |  |  |  |  |  |  |  |  |  |  |  |  |  |  |  |  |  |  |  |  |  |  |  |  |  |  |  |  |  |  |  |  |  |  |  |  |  |  |  |  |  |  |  |  |  |  |  |  |  |  |  |  |  |  |  |  |  |  |  |  |  |  |  |  |  |  |  |  |  |  |  |  |  |  |  |  |  |  |  |  |  |  |  |  |  |  |  |  |  |  |  |  |  |  |  |  |  |  |  |  |  |  |  |  |  |  |  |  |  |  |  |  |  |  |  |  |  |  |  |  |  |  |  |  |  |  |  |  |  |  |  |  |  |  |  |  |  |  |  |  |  |  |  |  |  |  |  |  |  |  |  |  |  |  |  |  |  |  |  |  |  |  |  |  |  |  |  |  |  |  |  |  |  |  |  |  |  |  |  |  |  |  |  |  |  |  |  |  |  |  |  |  |  |  |  |  |  |  |  |  |  |  |  |  |  |  |  |  |  |  |  |  |  |  |  |  |  |  |  |  |  |  |  |  |  |  |  |  |  |  |  |  |  |  |  |  |  |  |  |  |  |  |  |  |  |  |  |  |  |  |  |  |  |  |  |  |  |  |  |  |  |  |  |  |  |  |  |  |  |  |  |  |  |  |  |  |  |  |  |  |  |  |  |  |  |  |  |  |  |  |  |  |  |  |  |  |  |  |  |  |  |  |  |  |  |  |  |  |  |  |  |  |  |  |  |  |  |  |  |  |  |  |  |  |  |  |  |  |  |  |  |  |  |  |  |  |  |  |  |  |  |  |  |  |  |  |  |  |  |  |  |  |  |  |  |  |  |  |  |  |  |  |  |  |  |  |  |  |  |  |  |  |  |  |  |  |  |  |  |  |  |  |  |  |  |  |  |  |  |  |  |  |  |  |  |  |  |  |  |  |  |  |  |  |  |  |  |  |  |  |  |  |  |  |  |  |  |  |  |  |  |  |  |  |  |  |  |  |  |  |  |  |  |  |  |  |  |  |  |  |  |  |  |  |  |  |  |  |  |  |  |  |  |  |  |  |  |  |  |  |  |  |  |  |  |  |  |  |  |  |  |  |  |  |  |  |  |  |  |  |  |  |  |  |  |  |  |  |  |  |  |  |  |  |  |  |  |  |  |  |  |  |  |  |  |  |  |  |  |  |  |  |  |  |  |  |  |  |  |  |  |  |  |  |  |  |  |  |  |  |  |  |  |  |  |  |  |  |  |  |  |  |  |  |  |  |  |  |  |  |  |  |  |  |  |  |  |  |  |  |  |  |  |  |  |  |  |  |  |  |  |  |  |  |  |  |  |  |  |  |  |  |  |  |  |  |  |  |  |  |  |  |  |  |  |  |  |  |  |  |  |  |  |  |  |  |  |  |  |  |  |  |  |  |  |  |  |  |  |  |  |  |  |  |  |  |  |  |  |  |  |  |  |  |  |  |  |  |  |  |  |  |  |  |  |  |  |  |  |  |  |  |  |  |  |  |  |  |  |  |  |  |  |  |  |  |  |  |  |  |  |  |  |  |  |  |  |  |  |  |  |  |  |  |  |  |  |  |  |  |  |  |  |  |  |  |  |  |  |  |  |  |  |  |  |  |  |  |  |  |  |  |  |  |  |  |  |  |  |  |  |  |  |  |  |  |  |  |  |  |  |  |  |  |  |  |  |  |  |  |  |  |  |  |  |  |  |  |  |  |  |  |  |  |  |  |  |  |  |  |  |  |  |  |  |  |  |  |  |  |  |  |  |  |  |  |  |  |  |  |  |  |  |  |  |  |  |  |  |  |  |  |  |  |  |  |  |  |  |  |  |  |  |  |  |  |  |  |  |  |  |  |  |  |  |  |  |  |  |  |  |  |  |  |  |  |  |  |  |  |  |  |  |  |  |  |  |  |  |  |  |  |  |  |  |  |  |  |  |  |  |  |  |  |  |  |  |  |  |  |  |  |  |  |  |  |  |  |  |  |  |  |  |  |  |  |  |  |  |  |  |  |  |  |  |  |  |  |  |  |  |  |  |  |  |  |  |  |  |  |  |  |  |  |  |  |  |  |  |  |  |  |  |  |  |  |  |  |  |  |  |  |
|  |  |  |  |  |  |  |  |  |  |  |  |  |  |  |  |  |  |  |  |  |  |  |  |  |  |  |  |  |  |  |  |  |  |  |  |  |  |  |  |  |  |  |  |  |  |  |  |  |  |  |  |  |  |  |  |  |  |  |  |  |  |  |  |  |  |  |  |  |  |  |  |  |  |  |  |  |  |  |  |  |  |  |  |  |  |  |  |  |  |  |  |  |  |  |  |  |  |  |  |  |  |  |  |  |  |  |  |  |  |  |  |  |  |  |  |  |  |  |  |  |  |  |  |  |  |  |  |  |  |  |  |  |  |  |  |  |  |  |  |  |  |  |  |  |  |  |  |  |  |  |  |  |  |  |  |  |  |  |  |  |  |  |  |  |  |  |  |  |  |  |  |  |  |  |  |  |  |  |  |  |  |  |  |  |  |  |  |  |  |  |  |  |  |  |  |  |  |  |  |  |  |  |  |  |  |  |  |  |  |  |  |  |  |  |  |  |  |  |  |  |  |  |  |  |  |  |  |  |  |  |  |  |  |  |  |  |  |  |  |  |  |  |  |  |  |  |  |  |  |  |  |  |  |  |  |  |  |  |  |  |  |  |  |  |  |  |  |  |  |  |  |  |  |  |  |  |  |  |  |  |  |  |  |  |  |  |  |  |  |  |  |  |  |  |  |  |  |  |  |  |  |  |  |  |  |  |  |  |  |  |  |  |  |  |  |  |  |  |  |  |  |  |  |  |  |  |  |  |  |  |  |  |  |  |  |  |  |  |  |  |  |  |  |  |  |  |  |  |  |  |  |  |  |  |  |  |  |  |  |  |  |  |  |  |  |  |  |  |  |  |  |  |  |  |  |  |  |  |  |  |  |  |  |  |  |  |  |  |  |  |  |  |  |  |  |  |  |  |  |  |  |  |  |  |  |  |  |  |  |  |  |  |  |  |  |  |  |  |  |  |  |  |  |  |  |  |  |  |  |  |  |  |  |  |  |  |  |  |  |  |  |  |  |  |  |  |  |  |  |  |  |  |  |  |  |  |  |  |  |  |  |  |  |  |  |  |  |  |  |  |  |  |  |  |  |  |  |  |  |  |  |  |  |  |  |  |  |  |  |  |  |  |  |  |  |  |  |  |  |  |  |  |  |  |  |  |  |  |  |  |  |  |  |  |  |  |  |  |  |  |  |  |  |  |  |  |  |  |  |  |  |  |  |  |  |  |  |  |  |  |  |  |  |  |  |  |  |  |  |  |  |  |  |  |  |  |  |  |  |  |  |  |  |  |  |  |  |  |  |  |  |  |  |  |  |  |  |  |  |  |  |  |  |  |  |  |  |  |  |  |  |  |  |  |  |  |  |  |  |  |  |  |  |  |  |  |  |  |  |  |  |  |  |  |  |  |  |  |  |  |  |  |  |  |  |  |  |  |  |  |  |  |  |  |  |  |  |  |  |  |  |  |  |  |  |  |  |  |  |  |  |  |  |  |  |  |  |  |  |  |  |  |  |  |  |  |  |  |  |  |  |  |  |  |  |  |  |  |  |  |  |  |  |  |  |  |  |  |  |  |  |  |  |  |  |  |  |  |  |  |  |  |  |  |  |  |  |  |  |  |  |  |  |  |  |  |  |  |  |  |  |  |  |  |  |  |  |  |  |  |  |  |  |  |  |  |  |  |  |  |  |  |  |  |  |  |  |  |  |  |  |  |  |  |  |  |  |  |  |  |  |  |  |  |  |  |  |  |  |  |  |  |  |  |  |  |  |  |  |  |  |  |  |  |  |  |  |  |  |  |  |  |  |  |  |  |  |  |  |  |  |  |  |  |  |  |  |  |  |  |  |  |  |  |  |  |  |  |  |  |  |  |  |  |  |  |  |  |  |  |  |  |  |  |  |  |  |  |  |  |  |  |  |  |  |  |  |  |  |  |  |  |  |  |  |  |  |  |  |  |  |  |  |  |  |  |  |  |  |  |  |  |  |  |  |  |  |  |  |  |  |  |  |  |  |  |  |  |  |  |  |  |  |  |  |  |  |  |  |  |  |  |  |  |  |  |  |  |  |  |  |  |  |  |  |  |  |  |  |  |  |  |  |  |  |  |  |  |  |  |  |  |  |  |  |  |  |  |  |  |  |  |  |  |  |  |  |  |  |  |  |  |  |  |  |  |  |  |  |  |  |  |  |  |  |  |  |  |  |  |  |  |  |  |  |  |  |  |  |  |  |  |  |  |  |  |  |  |  |  |  |  |  |  |  |  |  |  |  |  |  |  |  |  |  |  |  |  |  |  |  |  |  |  |  |  |  |  |  |  |  |  |  |  |  |  |  |  |  |  |  |  |  |  |  |  |  |  |  |  |  |  |  |  |  |  |  |  |  |  |  |  |  |  |  |  |  |  |  |  |  |  |  |  |  |  |  |  |  |  |  |  |  |  |  |  |  |  |  |  |  |  |  |  |  |  |  |  |  |  |  |  |  |  |  |  |  |  |  |  |  |  |  |  |  |  |  |  |  |  |  |  |  |  |  |  |  |  |  |  |  |  |  |  |  |  |  |  |  |  |  |  |  |  |  |  |  |  |  |  |  |  |  |  |  |  |  |  |  |  |  |  |  |  |  |  |  |  |  |  |  |  |  |  |  |  |  |  |  |  |  |  |  |  |  |  |  |  |  |  |  |  |  |  |  |  |  |  |  |  |  |  |  |  |  |  |  |  |  |  |  |  |  |  |  |  |  |  |  |  |  |  |  |  |  |  |  |  |  |  |  |  |  |  |  |  |  |  |  |  |  |  |  |  |  |  |  |  |  |  |  |  |  |  |  |  |  |  |  |  |  |  |  |  |  |  |  |  |  |  |  |  |  |  |  |  |  |  |  |  |  |  |  |  |  |  |  |  |  |  |  |  |  |  |  |  |  |  |  |  |  |  |  |  |  |  |  |  |  |  |  |  |  |  |  |  |  |  |  |  |  |  |  |  |  |  |  |  |  |  |  |  |  |  |  |  |  |  |  |  |  |  |  |  |  |  |  |  |  |  |  |  |  |  |  |  |  |
|  |  |  |  |  |  |  |  |  |  |  |  |  |  |  |  |  |  |  |  |  |  |  |  |  |  |  |  |  |  |  |  |  |  |  |  |  |  |  |  |  |  |  |  |  |  |  |  |  |  |  |  |  |  |  |  |  |  |  |  |  |  |  |  |  |  |  |  |  |  |  |  |  |  |  |  |  |  |  |  |  |  |  |  |  |  |  |  |  |  |  |  |  |  |  |  |  |  |  |  |  |  |  |  |  |  |  |  |  |  |  |  |  |  |  |  |  |  |  |  |  |  |  |  |  |  |  |  |  |  |  |  |  |  |  |  |  |  |  |  |  |  |  |  |  |  |  |  |  |  |  |  |  |  |  |  |  |  |  |  |  |  |  |  |  |  |  |  |  |  |  |  |  |  |  |  |  |  |  |  |  |  |  |  |  |  |  |  |  |  |  |  |  |  |  |  |  |  |  |  |  |  |  |  |  |  |  |  |  |  |  |  |  |  |  |  |  |  |  |  |  |  |  |  |  |  |  |  |  |  |  |  |  |  |  |  |  |  |  |  |  |  |  |  |  |  |  |  |  |  |  |  |  |  |  |  |  |  |  |  |  |  |  |  |  |  |  |  |  |  |  |  |  |  |  |  |  |  |  |  |  |  |  |  |  |  |  |  |  |  |  |  |  |  |  |  |  |  |  |  |  |  |  |  |  |  |  |  |  |  |  |  |  |  |  |  |  |  |  |  |  |  |  |  |  |  |  |  |  |  |  |  |  |  |  |  |  |  |  |  |  |  |  |  |  |  |  |  |  |  |  |  |  |  |  |  |  |  |  |  |  |  |  |  |  |  |  |  |  |  |  |  |  |  |  |  |  |  |  |  |  |  |  |  |  |  |  |  |  |  |  |  |  |  |  |  |  |  |  |  |  |  |  |  |  |  |  |  |  |  |  |  |  |  |  |  |  |  |  |  |  |  |  |  |  |  |  |  |  |  |  |  |  |  |  |  |  |  |  |  |  |  |  |  |  |  |  |  |  |  |  |  |  |  |  |  |  |  |  |  |  |  |  |  |  |  |  |  |  |  |  |  |  |  |  |  |  |  |  |  |  |  |  |  |  |  |  |  |  |  |  |  |  |  |  |  |  |  |  |  |  |  |  |  |  |  |  |  |  |  |  |  |  |  |  |  |  |  |  |  |  |  |  |  |  |  |  |  |  |  |  |  |  |  |  |  |  |  |  |  |  |  |  |  |  |  |  |  |  |  |  |  |  |  |  |  |  |  |  |  |  |  |  |  |  |  |  |  |  |  |  |  |  |  |  |  |  |  |  |  |  |  |  |  |  |  |  |  |  |  |  |  |  |  |  |  |  |  |  |  |  |  |  |  |  |  |  |  |  |  |  |  |  |  |  |  |  |  |  |  |  |  |  |  |  |  |  |  |  |  |  |  |  |  |  |  |  |  |  |  |  |  |  |  |  |  |  |  |  |  |  |  |  |  |  |  |  |  |  |  |  |  |  |  |  |  |  |  |  |  |  |  |  |  |  |  |  |  |  |  |  |  |  |  |  |  |  |  |  |  |  |  |  |  |  |  |  |  |  |  |  |  |  |  |  |  |  |  |  |  |  |  |  |  |  |  |  |  |  |  |  |  |  |  |  |  |  |  |  |  |  |  |  |  |  |  |  |  |  |  |  |  |  |  |  |  |  |  |  |  |  |  |  |  |  |  |  |  |  |  |  |  |  |  |  |  |  |  |  |  |  |  |  |  |  |  |  |  |  |  |  |  |  |  |  |  |  |  |  |  |  |  |  |  |  |  |  |  |  |  |  |  |  |  |  |  |  |  |  |  |  |  |  |  |  |  |  |  |  |  |  |  |  |  |  |  |  |  |  |  |  |  |  |  |  |  |  |  |  |  |  |  |  |  |  |  |  |  |  |  |  |  |  |  |  |  |  |  |  |  |  |  |  |  |  |  |  |  |  |  |  |  |  |  |  |  |  |  |  |  |  |  |  |  |  |  |  |  |  |  |  |  |  |  |  |  |  |  |  |  |  |  |  |  |  |  |  |  |  |  |  |  |  |  |  |  |  |  |  |  |  |  |  |  |  |  |  |  |  |  |  |  |  |  |  |  |  |  |  |  |  |  |  |  |  |  |  |  |  |  |  |  |  |  |  |  |  |  |  |  |  |  |  |  |  |  |  |  |  |  |  |  |  |  |  |  |  |  |  |  |  |  |  |  |  |  |  |  |  |  |  |  |  |  |  |  |  |  |  |  |  |  |  |  |  |  |  |  |  |  |  |  |  |  |  |  |  |  |  |  |  |  |  |  |  |  |  |  |  |  |  |  |  |  |  |  |  |  |  |  |  |  |  |  |  |  |  |  |  |  |  |  |  |  |  |  |  |  |  |  |  |  |  |  |  |  |  |  |  |  |  |  |  |  |  |  |  |  |  |  |  |  |  |  |  |  |  |  |  |  |  |  |  |  |  |  |  |  |  |  |  |  |  |  |  |  |  |  |  |  |  |  |  |  |  |  |  |  |  |  |  |  |  |  |  |  |  |  |  |  |  |  |  |  |  |  |  |  |  |  |  |  |  |  |  |  |  |  |  |  |  |  |  |  |  |  |  |  |  |  |  |  |  |  |  |  |  |  |  |  |  |  |  |  |  |  |  |  |  |  |  |  |  |  |  |  |  |  |  |  |  |  |  |  |  |  |  |  |  |  |  |  |  |  |  |  |  |  |  |  |  |  |  |  |  |  |  |  |  |  |  |  |  |  |  |  |  |  |  |  |  |  |  |  |  |  |  |  |  |  |  |  |  |  |  |  |  |  |  |  |  |  |  |  |  |  |  |  |  |  |  |  |  |  |  |  |  |  |  |  |  |  |  |  |  |  |  |  |  |  |  |  |  |  |  |  |  |  |  |  |  |  |  |  |  |  |  |  |  |  |  |  |  |  |  |  |  |  |  |  |  |  |  |  |  |  |  |  |  |  |  |  |  |  |  |  |  |  |  |  |  |  |  |  |  |  |  |  |  |  |  |  |  |  |  |  |  |  |
|  |  |  |  |  |  |  |  |  |  |  |  |  |  |  |  |  |  |  |  |  |  |  |  |  |  |  |  |  |  |  |  |  |  |  |  |  |  |  |  |  |  |  |  |  |  |  |  |  |  |  |  |  |  |  |  |  |  |  |  |  |  |  |  |  |  |  |  |  |  |  |  |  |  |  |  |  |  |  |  |  |  |  |  |  |  |  |  |  |  |  |  |  |  |  |  |  |  |  |  |  |  |  |  |  |  |  |  |  |  |  |  |  |  |  |  |  |  |  |  |  |  |  |  |  |  |  |  |  |  |  |  |  |  |  |  |  |  |  |  |  |  |  |  |  |  |  |  |  |  |  |  |  |  |  |  |  |  |  |  |  |  |  |  |  |  |  |  |  |  |  |  |  |  |  |  |  |  |  |  |  |  |  |  |  |  |  |  |  |  |  |  |  |  |  |  |  |  |  |  |  |  |  |  |  |  |  |  |  |  |  |  |  |  |  |  |  |  |  |  |  |  |  |  |  |  |  |  |  |  |  |  |  |  |  |  |  |  |  |  |  |  |  |  |  |  |  |  |  |  |  |  |  |  |  |  |  |  |  |  |  |  |  |  |  |  |  |  |  |  |  |  |  |  |  |  |  |  |  |  |  |  |  |  |  |  |  |  |  |  |  |  |  |  |  |  |  |  |  |  |  |  |  |  |  |  |  |  |  |  |  |  |  |  |  |  |  |  |  |  |  |  |  |  |  |  |  |  |  |  |  |  |  |  |  |  |  |  |  |  |  |  |  |  |  |  |  |  |  |  |  |  |  |  |  |  |  |  |  |  |  |  |  |  |  |  |  |  |  |  |  |  |  |  |  |  |  |  |  |  |  |  |  |  |  |  |  |  |  |  |  |  |  |  |  |  |  |  |  |  |  |  |  |  |  |  |  |  |  |  |  |  |  |  |  |  |  |  |  |  |  |  |  |  |  |  |  |  |  |  |  |  |  |  |  |  |  |  |  |  |  |  |  |  |  |  |  |  |  |  |  |  |  |  |  |  |  |  |  |  |  |  |  |  |  |  |  |  |  |  |  |  |  |  |  |  |  |  |  |  |  |  |  |  |  |  |  |  |  |  |  |  |  |  |  |  |  |  |  |  |  |  |  |  |  |  |  |  |  |  |  |  |  |  |  |  |  |  |  |  |  |  |  |  |  |  |  |  |  |  |  |  |  |  |  |  |  |  |  |  |  |  |  |  |  |  |  |  |  |  |  |  |  |  |  |  |  |  |  |  |  |  |  |  |  |  |  |  |  |  |  |  |  |  |  |  |  |  |  |  |  |  |  |  |  |  |  |  |  |  |  |  |  |  |  |  |  |  |  |  |  |  |  |  |  |  |  |  |  |  |  |  |  |  |  |  |  |  |  |  |  |  |  |  |  |  |  |  |  |  |  |  |  |  |  |  |  |  |  |  |  |  |  |  |  |  |  |  |  |  |  |  |  |  |  |  |  |  |  |  |  |  |  |  |  |  |  |  |  |  |  |  |  |  |  |  |  |  |  |  |  |  |  |  |  |  |  |  |  |  |  |  |  |  |  |  |  |  |  |  |  |  |  |  |  |  |  |  |  |  |  |  |  |  |  |  |  |  |  |  |  |  |  |  |  |  |  |  |  |  |  |  |  |  |  |  |  |  |  |  |  |  |  |  |  |  |  |  |  |  |  |  |  |  |  |  |  |  |  |  |  |  |  |  |  |  |  |  |  |  |  |  |  |  |  |  |  |  |  |  |  |  |  |  |  |  |  |  |  |  |  |  |  |  |  |  |  |  |  |  |  |  |  |  |  |  |  |  |  |  |  |  |  |  |  |  |  |  |  |  |  |  |  |  |  |  |  |  |  |  |  |  |  |  |  |  |  |  |  |  |  |  |  |  |  |  |  |  |  |  |  |  |  |  |  |  |  |  |  |  |  |  |  |  |  |  |  |  |  |  |  |  |  |  |  |  |  |  |  |  |  |  |  |  |  |  |  |  |  |  |  |  |  |  |  |  |  |  |  |  |  |  |  |  |  |  |  |  |  |  |  |  |  |  |  |  |  |  |  |  |  |  |  |  |  |  |  |  |  |  |  |  |  |  |  |  |  |  |  |  |  |  |  |  |  |  |  |  |  |  |  |  |  |  |  |  |  |  |  |  |  |  |  |  |  |  |  |  |  |  |  |  |  |  |  |  |  |  |  |  |  |  |  |  |  |  |  |  |  |  |  |  |  |  |  |  |  |  |  |  |  |  |  |  |  |  |  |  |  |  |  |  |  |  |  |  |  |  |  |  |  |  |  |  |  |  |  |  |  |  |  |  |  |  |  |  |  |  |  |  |  |  |  |  |  |  |  |  |  |  |  |  |  |  |  |  |  |  |  |  |  |  |  |  |  |  |  |  |  |  |  |  |  |  |  |  |  |  |  |  |  |  |  |  |  |  |  |  |  |  |  |  |  |  |  |  |  |  |  |  |  |  |  |  |  |  |  |  |  |  |  |  |  |  |  |  |  |  |  |  |  |  |  |  |  |  |  |  |  |  |  |  |  |  |  |  |  |  |  |  |  |  |  |  |  |  |  |  |  |  |  |  |  |  |  |  |  |  |  |  |  |  |  |  |  |  |  |  |  |  |  |  |  |  |  |  |  |  |  |  |  |  |  |  |  |  |  |  |  |  |  |  |  |  |  |  |  |  |  |  |  |  |  |  |  |  |  |  |  |  |  |  |  |  |  |  |  |  |  |  |  |  |  |  |  |  |  |  |  |  |  |  |  |  |  |  |  |  |  |  |  |  |  |  |  |  |  |  |  |  |  |  |  |  |  |  |  |  |  |  |  |  |  |  |  |  |  |  |  |  |  |  |  |  |  |  |  |  |  |  |  |  |  |  |  |  |  |  |  |  |  |  |  |  |  |  |  |  |  |  |  |  |  |  |  |  |  |  |  |  |  |  |  |  |  |  |  |  |  |  |  |  |  |  |  |  |  |  |  |  |  |  |  |  |  |  |  |  |  |  |  |  |  |  |  |  |  |  |
|  |  |  |  |  |  |  |  |  |  |  |  |  |  |  |  |  |  |  |  |  |  |  |  |  |  |  |  |  |  |  |  |  |  |  |  |  |  |  |  |  |  |  |  |  |  |  |  |  |  |  |  |  |  |  |  |  |  |  |  |  |  |  |  |  |  |  |  |  |  |  |  |  |  |  |  |  |  |  |  |  |  |  |  |  |  |  |  |  |  |  |  |  |  |  |  |  |  |  |  |  |  |  |  |  |  |  |  |  |  |  |  |  |  |  |  |  |  |  |  |  |  |  |  |  |  |  |  |  |  |  |  |  |  |  |  |  |  |  |  |  |  |  |  |  |  |  |  |  |  |  |  |  |  |  |  |  |  |  |  |  |  |  |  |  |  |  |  |  |  |  |  |  |  |  |  |  |  |  |  |  |  |  |  |  |  |  |  |  |  |  |  |  |  |  |  |  |  |  |  |  |  |  |  |  |  |  |  |  |  |  |  |  |  |  |  |  |  |  |  |  |  |  |  |  |  |  |  |  |  |  |  |  |  |  |  |  |  |  |  |  |  |  |  |  |  |  |  |  |  |  |  |  |  |  |  |  |  |  |  |  |  |  |  |  |  |  |  |  |  |  |  |  |  |  |  |  |  |  |  |  |  |  |  |  |  |  |  |  |  |  |  |  |  |  |  |  |  |  |  |  |  |  |  |  |  |  |  |  |  |  |  |  |  |  |  |  |  |  |  |  |  |  |  |  |  |  |  |  |  |  |  |  |  |  |  |  |  |  |  |  |  |  |  |  |  |  |  |  |  |  |  |  |  |  |  |  |  |  |  |  |  |  |  |  |  |  |  |  |  |  |  |  |  |  |  |  |  |  |  |  |  |  |  |  |  |  |  |  |  |  |  |  |  |  |  |  |  |  |  |  |  |  |  |  |  |  |  |  |  |  |  |  |  |  |  |  |  |  |  |  |  |  |  |  |  |  |  |  |  |  |  |  |  |  |  |  |  |  |  |  |  |  |  |  |  |  |  |  |  |  |  |  |  |  |  |  |  |  |  |  |  |  |  |  |  |  |  |  |  |  |  |  |  |  |  |  |  |  |  |  |  |  |  |  |  |  |  |  |  |  |  |  |  |  |  |  |  |  |  |  |  |  |  |  |  |  |  |  |  |  |  |  |  |  |  |  |  |  |  |  |  |  |  |  |  |  |  |  |  |  |  |  |  |  |  |  |  |  |  |  |  |  |  |  |  |  |  |  |  |  |  |  |  |  |  |  |  |  |  |  |  |  |  |  |  |  |  |  |  |  |  |  |  |  |  |  |  |  |  |  |  |  |  |  |  |  |  |  |  |  |  |  |  |  |  |  |  |  |  |  |  |  |  |  |  |  |  |  |  |  |  |  |  |  |  |  |  |  |  |  |  |  |  |  |  |  |  |  |  |  |  |  |  |  |  |  |  |  |  |  |  |  |  |  |  |  |  |  |  |  |  |  |  |  |  |  |  |  |  |  |  |  |  |  |  |  |  |  |  |  |  |  |  |  |  |  |  |  |  |  |  |  |  |  |  |  |  |  |  |  |  |  |  |  |  |  |  |  |  |  |  |  |  |  |  |  |  |  |  |  |  |  |  |  |  |  |  |  |  |  |  |  |  |  |  |  |  |  |  |  |  |  |  |  |  |  |  |  |  |  |  |  |  |  |  |  |  |  |  |  |  |  |  |  |  |  |  |  |  |  |  |  |  |  |  |  |  |  |  |  |  |  |  |  |  |  |  |  |  |  |  |  |  |  |  |  |  |  |  |  |  |  |  |  |  |  |  |  |  |  |  |  |  |  |  |  |  |  |  |  |  |  |  |  |  |  |  |  |  |  |  |  |  |  |  |  |  |  |  |  |  |  |  |  |  |  |  |  |  |  |  |  |  |  |  |  |  |  |  |  |  |  |  |  |  |  |  |  |  |  |  |  |  |  |  |  |  |  |  |  |  |  |  |  |  |  |  |  |  |  |  |  |  |  |  |  |  |  |  |  |  |  |  |  |  |  |  |  |  |  |  |  |  |  |  |  |  |  |  |  |  |  |  |  |  |  |  |  |  |  |  |  |  |  |  |  |  |  |  |  |  |  |  |  |  |  |  |  |  |  |  |  |  |  |  |  |  |  |  |  |  |  |  |  |  |  |  |  |  |  |  |  |  |  |  |  |  |  |  |  |  |  |  |  |  |  |  |  |  |  |  |  |  |  |  |  |  |  |  |  |  |  |  |  |  |  |  |  |  |  |  |  |  |  |  |  |  |  |  |  |  |  |  |  |  |  |  |  |  |  |  |  |  |  |  |  |  |  |  |  |  |  |  |  |  |  |  |  |  |  |  |  |  |  |  |  |  |  |  |  |  |  |  |  |  |  |  |  |  |  |  |  |  |  |  |  |  |  |  |  |  |  |  |  |  |  |  |  |  |  |  |  |  |  |  |  |  |  |  |  |  |  |  |  |  |  |  |  |  |  |  |  |  |  |  |  |  |  |  |  |  |  |  |  |  |  |  |  |  |  |  |  |  |  |  |  |  |  |  |  |  |  |  |  |  |  |  |  |  |  |  |  |  |  |  |  |  |  |  |  |  |  |  |  |  |  |  |  |  |  |  |  |  |  |  |  |  |  |  |  |  |  |  |  |  |  |  |  |  |  |  |  |  |  |  |  |  |  |  |  |  |  |  |  |  |  |  |  |  |  |  |  |  |  |  |  |  |  |  |  |  |  |  |  |  |  |  |  |  |  |  |  |  |  |  |  |  |  |  |  |  |  |  |  |  |  |  |  |  |  |  |  |  |  |  |  |  |  |  |  |  |  |  |  |  |  |  |  |  |  |  |  |  |  |  |  |  |  |  |  |  |  |  |  |  |  |  |  |  |  |  |  |  |  |  |  |  |  |  |  |  |  |  |  |  |  |  |  |  |  |  |  |  |  |  |  |  |  |  |  |  |  |  |  |  |  |  |  |  |  |  |  |  |  |  |  |  |  |  |  |  |  |  |  |  |  |  |  |  |  |  |  |  |  |  |  |  |
|  |  |  |  |  |  |  |  |  |  |  |  |  |  |  |  |  |  |  |  |  |  |  |  |  |  |  |  |  |  |  |  |  |  |  |  |  |  |  |  |  |  |  |  |  |  |  |  |  |  |  |  |  |  |  |  |  |  |  |  |  |  |  |  |  |  |  |  |  |  |  |  |  |  |  |  |  |  |  |  |  |  |  |  |  |  |  |  |  |  |  |  |  |  |  |  |  |  |  |  |  |  |  |  |  |  |  |  |  |  |  |  |  |  |  |  |  |  |  |  |  |  |  |  |  |  |  |  |  |  |  |  |  |  |  |  |  |  |  |  |  |  |  |  |  |  |  |  |  |  |  |  |  |  |  |  |  |  |  |  |  |  |  |  |  |  |  |  |  |  |  |  |  |  |  |  |  |  |  |  |  |  |  |  |  |  |  |  |  |  |  |  |  |  |  |  |  |  |  |  |  |  |  |  |  |  |  |  |  |  |  |  |  |  |  |  |  |  |  |  |  |  |  |  |  |  |  |  |  |  |  |  |  |  |  |  |  |  |  |  |  |  |  |  |  |  |  |  |  |  |  |  |  |  |  |  |  |  |  |  |  |  |  |  |  |  |  |  |  |  |  |  |  |  |  |  |  |  |  |  |  |  |  |  |  |  |  |  |  |  |  |  |  |  |  |  |  |  |  |  |  |  |  |  |  |  |  |  |  |  |  |  |  |  |  |  |  |  |  |  |  |  |  |  |  |  |  |  |  |  |  |  |  |  |  |  |  |  |  |  |  |  |  |  |  |  |  |  |  |  |  |  |  |  |  |  |  |  |  |  |  |  |  |  |  |  |  |  |  |  |  |  |  |  |  |  |  |  |  |  |  |  |  |  |  |  |  |  |  |  |  |  |  |  |  |  |  |  |  |  |  |  |  |  |  |  |  |  |  |  |  |  |  |  |  |  |  |  |  |  |  |  |  |  |  |  |  |  |  |  |  |  |  |  |  |  |  |  |  |  |  |  |  |  |  |  |  |  |  |  |  |  |  |  |  |  |  |  |  |  |  |  |  |  |  |  |  |  |  |  |  |  |  |  |  |  |  |  |  |  |  |  |  |  |  |  |  |  |  |  |  |  |  |  |  |  |  |  |  |  |  |  |  |  |  |  |  |  |  |  |  |  |  |  |  |  |  |  |  |  |  |  |  |  |  |  |  |  |  |  |  |  |  |  |  |  |  |  |  |  |  |  |  |  |  |  |  |  |  |  |  |  |  |  |  |  |  |  |  |  |  |  |  |  |  |  |  |  |  |  |  |  |  |  |  |  |  |  |  |  |  |  |  |  |  |  |  |  |  |  |  |  |  |  |  |  |  |  |  |  |  |  |  |  |  |  |  |  |  |  |  |  |  |  |  |  |  |  |  |  |  |  |  |  |  |  |  |  |  |  |  |  |  |  |  |  |  |  |  |  |  |  |  |  |  |  |  |  |  |  |  |  |  |  |  |  |  |  |  |  |  |  |  |  |  |  |  |  |  |  |  |  |  |  |  |  |  |  |  |  |  |  |  |  |  |  |  |  |  |  |  |  |  |  |  |  |  |  |  |  |  |  |  |  |  |  |  |  |  |  |  |  |  |  |  |  |  |  |  |  |  |  |  |  |  |  |  |  |  |  |  |  |  |  |  |  |  |  |  |  |  |  |  |  |  |  |  |  |  |  |  |  |  |  |  |  |  |  |  |  |  |  |  |  |  |  |  |  |  |  |  |  |  |  |  |  |  |  |  |  |  |  |  |  |  |  |  |  |  |  |  |  |  |  |  |  |  |  |  |  |  |  |  |  |  |  |  |  |  |  |  |  |  |  |  |  |  |  |  |  |  |  |  |  |  |  |  |  |  |  |  |  |  |  |  |  |  |  |  |  |  |  |  |  |  |  |  |  |  |  |  |  |  |  |  |  |  |  |  |  |  |  |  |  |  |  |  |  |  |  |  |  |  |  |  |  |  |  |  |  |  |  |  |  |  |  |  |  |  |  |  |  |  |  |  |  |  |  |  |  |  |  |  |  |  |  |  |  |  |  |  |  |  |  |  |  |  |  |  |  |  |  |  |  |  |  |  |  |  |  |  |  |  |  |  |  |  |  |  |  |  |  |  |  |  |  |  |  |  |  |  |  |  |  |  |  |  |  |  |  |  |  |  |  |  |  |  |  |  |  |  |  |  |  |  |  |  |  |  |  |  |  |  |  |  |  |  |  |  |  |  |  |  |  |  |  |  |  |  |  |  |  |  |  |  |  |  |  |  |  |  |  |  |  |  |  |  |  |  |  |  |  |  |  |  |  |  |  |  |  |  |  |  |  |  |  |  |  |  |  |  |  |  |  |  |  |  |  |  |  |  |  |  |  |  |  |  |  |  |  |  |  |  |  |  |  |  |  |  |  |  |  |  |  |  |  |  |  |  |  |  |  |  |  |  |  |  |  |  |  |  |  |  |  |  |  |  |  |  |  |  |  |  |  |  |  |  |  |  |  |  |  |  |  |  |  |  |  |  |  |  |  |  |  |  |  |  |  |  |  |  |  |  |  |  |  |  |  |  |  |  |  |  |  |  |  |  |  |  |  |  |  |  |  |  |  |  |  |  |  |  |  |  |  |  |  |  |  |  |  |  |  |  |  |  |  |  |  |  |  |  |  |  |  |  |  |  |  |  |  |  |  |  |  |  |  |  |  |  |  |  |  |  |  |  |  |  |  |  |  |  |  |  |  |  |  |  |  |  |  |  |  |  |  |  |  |  |  |  |  |  |  |  |  |  |  |  |  |  |  |  |  |  |  |  |  |  |  |  |  |  |  |  |  |  |  |  |  |  |  |  |  |  |  |  |  |  |  |  |  |  |  |  |  |  |  |  |  |  |  |  |  |  |  |  |  |  |  |  |  |  |  |  |  |  |  |  |  |  |  |  |  |  |  |  |  |  |  |  |  |  |  |  |  |  |  |  |  |  |  |  |  |  |  |  |  |  |  |  |  |  |  |  |  |  |  |  |  |  |  |  |  |  |  |  |  |  |  |
|  |  |  |  |  |  |  |  |  |  |  |  |  |  |  |  |  |  |  |  |  |  |  |  |  |  |  |  |  |  |  |  |  |  |  |  |  |  |  |  |  |  |  |  |  |  |  |  |  |  |  |  |  |  |  |  |  |  |  |  |  |  |  |  |  |  |  |  |  |  |  |  |  |  |  |  |  |  |  |  |  |  |  |  |  |  |  |  |  |  |  |  |  |  |  |  |  |  |  |  |  |  |  |  |  |  |  |  |  |  |  |  |  |  |  |  |  |  |  |  |  |  |  |  |  |  |  |  |  |  |  |  |  |  |  |  |  |  |  |  |  |  |  |  |  |  |  |  |  |  |  |  |  |  |  |  |  |  |  |  |  |  |  |  |  |  |  |  |  |  |  |  |  |  |  |  |  |  |  |  |  |  |  |  |  |  |  |  |  |  |  |  |  |  |  |  |  |  |  |  |  |  |  |  |  |  |  |  |  |  |  |  |  |  |  |  |  |  |  |  |  |  |  |  |  |  |  |  |  |  |  |  |  |  |  |  |  |  |  |  |  |  |  |  |  |  |  |  |  |  |  |  |  |  |  |  |  |  |  |  |  |  |  |  |  |  |  |  |  |  |  |  |  |  |  |  |  |  |  |  |  |  |  |  |  |  |  |  |  |  |  |  |  |  |  |  |  |  |  |  |  |  |  |  |  |  |  |  |  |  |  |  |  |  |  |  |  |  |  |  |  |  |  |  |  |  |  |  |  |  |  |  |  |  |  |  |  |  |  |  |  |  |  |  |  |  |  |  |  |  |  |  |  |  |  |  |  |  |  |  |  |  |  |  |  |  |  |  |  |  |  |  |  |  |  |  |  |  |  |  |  |  |  |  |  |  |  |  |  |  |  |  |  |  |  |  |  |  |  |  |  |  |  |  |  |  |  |  |  |  |  |  |  |  |  |  |  |  |  |  |  |  |  |  |  |  |  |  |  |  |  |  |  |  |  |  |  |  |  |  |  |  |  |  |  |  |  |  |  |  |  |  |  |  |  |  |  |  |  |  |  |  |  |  |  |  |  |  |  |  |  |  |  |  |  |  |  |  |  |  |  |  |  |  |  |  |  |  |  |  |  |  |  |  |  |  |  |  |  |  |  |  |  |  |  |  |  |  |  |  |  |  |  |  |  |  |  |  |  |  |  |  |  |  |  |  |  |  |  |  |  |  |  |  |  |  |  |  |  |  |  |  |  |  |  |  |  |  |  |  |  |  |  |  |  |  |  |  |  |  |  |  |  |  |  |  |  |  |  |  |  |  |  |  |  |  |  |  |  |  |  |  |  |  |  |  |  |  |  |  |  |  |  |  |  |  |  |  |  |  |  |  |  |  |  |  |  |  |  |  |  |  |  |  |  |  |  |  |  |  |  |  |  |  |  |  |  |  |  |  |  |  |  |  |  |  |  |  |  |  |  |  |  |  |  |  |  |  |  |  |  |  |  |  |  |  |  |  |  |  |  |  |  |  |  |  |  |  |  |  |  |  |  |  |  |  |  |  |  |  |  |  |  |  |  |  |  |  |  |  |  |  |  |  |  |  |  |  |  |  |  |  |  |  |  |  |  |  |  |  |  |  |  |  |  |  |  |  |  |  |  |  |  |  |  |  |  |  |  |  |  |  |  |  |  |  |  |  |  |  |  |  |  |  |  |  |  |  |  |  |  |  |  |  |  |  |  |  |  |  |  |  |  |  |  |  |  |  |  |  |  |  |  |  |  |  |  |  |  |  |  |  |  |  |  |  |  |  |  |  |  |  |  |  |  |  |  |  |  |  |  |  |  |  |  |  |  |  |  |  |  |  |  |  |  |  |  |  |  |  |  |  |  |  |  |  |  |  |  |  |  |  |  |  |  |  |  |  |  |  |  |  |  |  |  |  |  |  |  |  |  |  |  |  |  |  |  |  |  |  |  |  |  |  |  |  |  |  |  |  |  |  |  |  |  |  |  |  |  |  |  |  |  |  |  |  |  |  |  |  |  |  |  |  |  |  |  |  |  |  |  |  |  |  |  |  |  |  |  |  |  |  |  |  |  |  |  |  |  |  |  |  |  |  |  |  |  |  |  |  |  |  |  |  |  |  |  |  |  |  |  |  |  |  |  |  |  |  |  |  |  |  |  |  |  |  |  |  |  |  |  |  |  |  |  |  |  |  |  |  |  |  |  |  |  |  |  |  |  |  |  |  |  |  |  |  |  |  |  |  |  |  |  |  |  |  |  |  |  |  |  |  |  |  |  |  |  |  |  |  |  |  |  |  |  |  |  |  |  |  |  |  |  |  |  |  |  |  |  |  |  |  |  |  |  |  |  |  |  |  |  |  |  |  |  |  |  |  |  |  |  |  |  |  |  |  |  |  |  |  |  |  |  |  |  |  |  |  |  |  |  |  |  |  |  |  |  |  |  |  |  |  |  |  |  |  |  |  |  |  |  |  |  |  |  |  |  |  |  |  |  |  |  |  |  |  |  |  |  |  |  |  |  |  |  |  |  |  |  |  |  |  |  |  |  |  |  |  |  |  |  |  |  |  |  |  |  |  |  |  |  |  |  |  |  |  |  |  |  |  |  |  |  |  |  |  |  |  |  |  |  |  |  |  |  |  |  |  |  |  |  |  |  |  |  |  |  |  |  |  |  |  |  |  |  |  |  |  |  |  |  |  |  |  |  |  |  |  |  |  |  |  |  |  |  |  |  |  |  |  |  |  |  |  |  |  |  |  |  |  |  |  |  |  |  |  |  |  |  |  |  |  |  |  |  |  |  |  |  |  |  |  |  |  |  |  |  |  |  |  |  |  |  |  |  |  |  |  |  |  |  |  |  |  |  |  |  |  |  |  |  |  |  |  |  |  |  |  |  |  |  |  |  |  |  |  |  |  |  |  |  |  |  |  |  |  |  |  |  |  |  |  |  |  |  |  |  |  |  |  |  |  |  |  |  |  |  |  |  |  |  |  |  |  |  |  |  |  |  |  |  |  |  |  |  |  |  |  |  |  |  |  |  |  |  |  |  |  |
|  |  |  |  |  |  |  |  |  |  |  |  |  |  |  |  |  |  |  |  |  |  |  |  |  |  |  |  |  |  |  |  |  |  |  |  |  |  |  |  |  |  |  |  |  |  |  |  |  |  |  |  |  |  |  |  |  |  |  |  |  |  |  |  |  |  |  |  |  |  |  |  |  |  |  |  |  |  |  |  |  |  |  |  |  |  |  |  |  |  |  |  |  |  |  |  |  |  |  |  |  |  |  |  |  |  |  |  |  |  |  |  |  |  |  |  |  |  |  |  |  |  |  |  |  |  |  |  |  |  |  |  |  |  |  |  |  |  |  |  |  |  |  |  |  |  |  |  |  |  |  |  |  |  |  |  |  |  |  |  |  |  |  |  |  |  |  |  |  |  |  |  |  |  |  |  |  |  |  |  |  |  |  |  |  |  |  |  |  |  |  |  |  |  |  |  |  |  |  |  |  |  |  |  |  |  |  |  |  |  |  |  |  |  |  |  |  |  |  |  |  |  |  |  |  |  |  |  |  |  |  |  |  |  |  |  |  |  |  |  |  |  |  |  |  |  |  |  |  |  |  |  |  |  |  |  |  |  |  |  |  |  |  |  |  |  |  |  |  |  |  |  |  |  |  |  |  |  |  |  |  |  |  |  |  |  |  |  |  |  |  |  |  |  |  |  |  |  |  |  |  |  |  |  |  |  |  |  |  |  |  |  |  |  |  |  |  |  |  |  |  |  |  |  |  |  |  |  |  |  |  |  |  |  |  |  |  |  |  |  |  |  |  |  |  |  |  |  |  |  |  |  |  |  |  |  |  |  |  |  |  |  |  |  |  |  |  |  |  |  |  |  |  |  |  |  |  |  |  |  |  |  |  |  |  |  |  |  |  |  |  |  |  |  |  |  |  |  |  |  |  |  |  |  |  |  |  |  |  |  |  |  |  |  |  |  |  |  |  |  |  |  |  |  |  |  |  |  |  |  |  |  |  |  |  |  |  |  |  |  |  |  |  |  |  |  |  |  |  |  |  |  |  |  |  |  |  |  |  |  |  |  |  |  |  |  |  |  |  |  |  |  |  |  |  |  |  |  |  |  |  |  |  |  |  |  |  |  |  |  |  |  |  |  |  |  |  |  |  |  |  |  |  |  |  |  |  |  |  |  |  |  |  |  |  |  |  |  |  |  |  |  |  |  |  |  |  |  |  |  |  |  |  |  |  |  |  |  |  |  |  |  |  |  |  |  |  |  |  |  |  |  |  |  |  |  |  |  |  |  |  |  |  |  |  |  |  |  |  |  |  |  |  |  |  |  |  |  |  |  |  |  |  |  |  |  |  |  |  |  |  |  |  |  |  |  |  |  |  |  |  |  |  |  |  |  |  |  |  |  |  |  |  |  |  |  |  |  |  |  |  |  |  |  |  |  |  |  |  |  |  |  |  |  |  |  |  |  |  |  |  |  |  |  |  |  |  |  |  |  |  |  |  |  |  |  |  |  |  |  |  |  |  |  |  |  |  |  |  |  |  |  |  |  |  |  |  |  |  |  |  |  |  |  |  |  |  |  |  |  |  |  |  |  |  |  |  |  |  |  |  |  |  |  |  |  |  |  |  |  |  |  |  |  |  |  |  |  |  |  |  |  |  |  |  |  |  |  |  |  |  |  |  |  |  |  |  |  |  |  |  |  |  |  |  |  |  |  |  |  |  |  |  |  |  |  |  |  |  |  |  |  |  |  |  |  |  |  |  |  |  |  |  |  |  |  |  |  |  |  |  |  |  |  |  |  |  |  |  |  |  |  |  |  |  |  |  |  |  |  |  |  |  |  |  |  |  |  |  |  |  |  |  |  |  |  |  |  |  |  |  |  |  |  |  |  |  |  |  |  |  |  |  |  |  |  |  |  |  |  |  |  |  |  |  |  |  |  |  |  |  |  |  |  |  |  |  |  |  |  |  |  |  |  |  |  |  |  |  |  |  |  |  |  |  |  |  |  |  |  |  |  |  |  |  |  |  |  |  |  |  |  |  |  |  |  |  |  |  |  |  |  |  |  |  |  |  |  |  |  |  |  |  |  |  |  |  |  |  |  |  |  |  |  |  |  |  |  |  |  |  |  |  |  |  |  |  |  |  |  |  |  |  |  |  |  |  |  |  |  |  |  |  |  |  |  |  |  |  |  |  |  |  |  |  |  |  |  |  |  |  |  |  |  |  |  |  |  |  |  |  |  |  |  |  |  |  |  |  |  |  |  |  |  |  |  |  |  |  |  |  |  |  |  |  |  |  |  |  |  |  |  |  |  |  |  |  |  |  |  |  |  |  |  |  |  |  |  |  |  |  |  |  |  |  |  |  |  |  |  |  |  |  |  |  |  |  |  |  |  |  |  |  |  |  |  |  |  |  |  |  |  |  |  |  |  |  |  |  |  |  |  |  |  |  |  |  |  |  |  |  |  |  |  |  |  |  |  |  |  |  |  |  |  |  |  |  |  |  |  |  |  |  |  |  |  |  |  |  |  |  |  |  |  |  |  |  |  |  |  |  |  |  |  |  |  |  |  |  |  |  |  |  |  |  |  |  |  |  |  |  |  |  |  |  |  |  |  |  |  |  |  |  |  |  |  |  |  |  |  |  |  |  |  |  |  |  |  |  |  |  |  |  |  |  |  |  |  |  |  |  |  |  |  |  |  |  |  |  |  |  |  |  |  |  |  |  |  |  |  |  |  |  |  |  |  |  |  |  |  |  |  |  |  |  |  |  |  |  |  |  |  |  |  |  |  |  |  |  |  |  |  |  |  |  |  |  |  |  |  |  |  |  |  |  |  |  |  |  |  |  |  |  |  |  |  |  |  |  |  |  |  |  |  |  |  |  |  |  |  |  |  |  |  |  |  |  |  |  |  |  |  |  |  |  |  |  |  |  |  |  |  |  |  |  |  |  |  |  |  |  |  |  |  |  |  |  |  |  |  |  |  |  |  |  |  |  |  |  |  |  |  |  |  |  |  |  |  |  |  |  |  |  |  |  |  |  |  |  |  |  |  |  |  |  |  |  |  |
|  |  |  |  |  |  |  |  |  |  |  |  |  |  |  |  |  |  |  |  |  |  |  |  |  |  |  |  |  |  |  |  |  |  |  |  |  |  |  |  |  |  |  |  |  |  |  |  |  |  |  |  |  |  |  |  |  |  |  |  |  |  |  |  |  |  |  |  |  |  |  |  |  |  |  |  |  |  |  |  |  |  |  |  |  |  |  |  |  |  |  |  |  |  |  |  |  |  |  |  |  |  |  |  |  |  |  |  |  |  |  |  |  |  |  |  |  |  |  |  |  |  |  |  |  |  |  |  |  |  |  |  |  |  |  |  |  |  |  |  |  |  |  |  |  |  |  |  |  |  |  |  |  |  |  |  |  |  |  |  |  |  |  |  |  |  |  |  |  |  |  |  |  |  |  |  |  |  |  |  |  |  |  |  |  |  |  |  |  |  |  |  |  |  |  |  |  |  |  |  |  |  |  |  |  |  |  |  |  |  |  |  |  |  |  |  |  |  |  |  |  |  |  |  |  |  |  |  |  |  |  |  |  |  |  |  |  |  |  |  |  |  |  |  |  |  |  |  |  |  |  |  |  |  |  |  |  |  |  |  |  |  |  |  |  |  |  |  |  |  |  |  |  |  |  |  |  |  |  |  |  |  |  |  |  |  |  |  |  |  |  |  |  |  |  |  |  |  |  |  |  |  |  |  |  |  |  |  |  |  |  |  |  |  |  |  |  |  |  |  |  |  |  |  |  |  |  |  |  |  |  |  |  |  |  |  |  |  |  |  |  |  |  |  |  |  |  |  |  |  |  |  |  |  |  |  |  |  |  |  |  |  |  |  |  |  |  |  |  |  |  |  |  |  |  |  |  |  |  |  |  |  |  |  |  |  |  |  |  |  |  |  |  |  |  |  |  |  |  |  |  |  |  |  |  |  |  |  |  |  |  |  |  |  |  |  |  |  |  |  |  |  |  |  |  |  |  |  |  |  |  |  |  |  |  |  |  |  |  |  |  |  |  |  |  |  |  |  |  |  |  |  |  |  |  |  |  |  |  |  |  |  |  |  |  |  |  |  |  |  |  |  |  |  |  |  |  |  |  |  |  |  |  |  |  |  |  |  |  |  |  |  |  |  |  |  |  |  |  |  |  |  |  |  |  |  |  |  |  |  |  |  |  |  |  |  |  |  |  |  |  |  |  |  |  |  |  |  |  |  |  |  |  |  |  |  |  |  |  |  |  |  |  |  |  |  |  |  |  |  |  |  |  |  |  |  |  |  |  |  |  |  |  |  |  |  |  |  |  |  |  |  |  |  |  |  |  |  |  |  |  |  |  |  |  |  |  |  |  |  |  |  |  |  |  |  |  |  |  |  |  |  |  |  |  |  |  |  |  |  |  |  |  |  |  |  |  |  |  |  |  |  |  |  |  |  |  |  |  |  |  |  |  |  |  |  |  |  |  |  |  |  |  |  |  |  |  |  |  |  |  |  |  |  |  |  |  |  |  |  |  |  |  |  |  |  |  |  |  |  |  |  |  |  |  |  |  |  |  |  |  |  |  |  |  |  |  |  |  |  |  |  |  |  |  |  |  |  |  |  |  |  |  |  |  |  |  |  |  |  |  |  |  |  |  |  |  |  |  |  |  |  |  |  |  |  |  |  |  |  |  |  |  |  |  |  |  |  |  |  |  |  |  |  |  |  |  |  |  |  |  |  |  |  |  |  |  |  |  |  |  |  |  |  |  |  |  |  |  |  |  |  |  |  |  |  |  |  |  |  |  |  |  |  |  |  |  |  |  |  |  |  |  |  |  |  |  |  |  |  |  |  |  |  |  |  |  |  |  |  |  |  |  |  |  |  |  |  |  |  |  |  |  |  |  |  |  |  |  |  |  |  |  |  |  |  |  |  |  |  |  |  |  |  |  |  |  |  |  |  |  |  |  |  |  |  |  |  |  |  |  |  |  |  |  |  |  |  |  |  |  |  |  |  |  |  |  |  |  |  |  |  |  |  |  |  |  |  |  |  |  |  |  |  |  |  |  |  |  |  |  |  |  |  |  |  |  |  |  |  |  |  |  |  |  |  |  |  |  |  |  |  |  |  |  |  |  |  |  |  |  |  |  |  |  |  |  |  |  |  |  |  |  |  |  |  |  |  |  |  |  |  |  |  |  |  |  |  |  |  |  |  |  |  |  |  |  |  |  |  |  |  |  |  |  |  |  |  |  |  |  |  |  |  |  |  |  |  |  |  |  |  |  |  |  |  |  |  |  |  |  |  |  |  |  |  |  |  |  |  |  |  |  |  |  |  |  |  |  |  |  |  |  |  |  |  |  |  |  |  |  |  |  |  |  |  |  |  |  |  |  |  |  |  |  |  |  |  |  |  |  |  |  |  |  |  |  |  |  |  |  |  |  |  |  |  |  |  |  |  |  |  |  |  |  |  |  |  |  |  |  |  |  |  |  |  |  |  |  |  |  |  |  |  |  |  |  |  |  |  |  |  |  |  |  |  |  |  |  |  |  |  |  |  |  |  |  |  |  |  |  |  |  |  |  |  |  |  |  |  |  |  |  |  |  |  |  |  |  |  |  |  |  |  |  |  |  |  |  |  |  |  |  |  |  |  |  |  |  |  |  |  |  |  |  |  |  |  |  |  |  |  |  |  |  |  |  |  |  |  |  |  |  |  |  |  |  |  |  |  |  |  |  |  |  |  |  |  |  |  |  |  |  |  |  |  |  |  |  |  |  |  |  |  |  |  |  |  |  |  |  |  |  |  |  |  |  |  |  |  |  |  |  |  |  |  |  |  |  |  |  |  |  |  |  |  |  |  |  |  |  |  |  |  |  |  |  |  |  |  |  |  |  |  |  |  |  |  |  |  |  |  |  |  |  |  |  |  |  |  |  |  |  |  |  |  |  |  |  |  |  |  |  |  |  |  |  |  |  |  |  |  |  |  |  |  |  |  |  |  |  |  |  |  |  |  |  |  |  |  |  |  |  |  |  |  |  |  |  |  |  |  |  |  |  |  |  |  |  |  |  |  |  |  |  |  |  |  |
|  |  |  |  |  |  |  |  |  |  |  |  |  |  |  |  |  |  |  |  |  |  |  |  |  |  |  |  |  |  |  |  |  |  |  |  |  |  |  |  |  |  |  |  |  |  |  |  |  |  |  |  |  |  |  |  |  |  |  |  |  |  |  |  |  |  |  |  |  |  |  |  |  |  |  |  |  |  |  |  |  |  |  |  |  |  |  |  |  |  |  |  |  |  |  |  |  |  |  |  |  |  |  |  |  |  |  |  |  |  |  |  |  |  |  |  |  |  |  |  |  |  |  |  |  |  |  |  |  |  |  |  |  |  |  |  |  |  |  |  |  |  |  |  |  |  |  |  |  |  |  |  |  |  |  |  |  |  |  |  |  |  |  |  |  |  |  |  |  |  |  |  |  |  |  |  |  |  |  |  |  |  |  |  |  |  |  |  |  |  |  |  |  |  |  |  |  |  |  |  |  |  |  |  |  |  |  |  |  |  |  |  |  |  |  |  |  |  |  |  |  |  |  |  |  |  |  |  |  |  |  |  |  |  |  |  |  |  |  |  |  |  |  |  |  |  |  |  |  |  |  |  |  |  |  |  |  |  |  |  |  |  |  |  |  |  |  |  |  |  |  |  |  |  |  |  |  |  |  |  |  |  |  |  |  |  |  |  |  |  |  |  |  |  |  |  |  |  |  |  |  |  |  |  |  |  |  |  |  |  |  |  |  |  |  |  |  |  |  |  |  |  |  |  |  |  |  |  |  |  |  |  |  |  |  |  |  |  |  |  |  |  |  |  |  |  |  |  |  |  |  |  |  |  |  |  |  |  |  |  |  |  |  |  |  |  |  |  |  |  |  |  |  |  |  |  |  |  |  |  |  |  |  |  |  |  |  |  |  |  |  |  |  |  |  |  |  |  |  |  |  |  |  |  |  |  |  |  |  |  |  |  |  |  |  |  |  |  |  |  |  |  |  |  |  |  |  |  |  |  |  |  |  |  |  |  |  |  |  |  |  |  |  |  |  |  |  |  |  |  |  |  |  |  |  |  |  |  |  |  |  |  |  |  |  |  |  |  |  |  |  |  |  |  |  |  |  |  |  |  |  |  |  |  |  |  |  |  |  |  |  |  |  |  |  |  |  |  |  |  |  |  |  |  |  |  |  |  |  |  |  |  |  |  |  |  |  |  |  |  |  |  |  |  |  |  |  |  |  |  |  |  |  |  |  |  |  |  |  |  |  |  |  |  |  |  |  |  |  |  |  |  |  |  |  |  |  |  |  |  |  |  |  |  |  |  |  |  |  |  |  |  |  |  |  |  |  |  |  |  |  |  |  |  |  |  |  |  |  |  |  |  |  |  |  |  |  |  |  |  |  |  |  |  |  |  |  |  |  |  |  |  |  |  |  |  |  |  |  |  |  |  |  |  |  |  |  |  |  |  |  |  |  |  |  |  |  |  |  |  |  |  |  |  |  |  |  |  |  |  |  |  |  |  |  |  |  |  |  |  |  |  |  |  |  |  |  |  |  |  |  |  |  |  |  |  |  |  |  |  |  |  |  |  |  |  |  |  |  |  |  |  |  |  |  |  |  |  |  |  |  |  |  |  |  |  |  |  |  |  |  |  |  |  |  |  |  |  |  |  |  |  |  |  |  |  |  |  |  |  |  |  |  |  |  |  |  |  |  |  |  |  |  |  |  |  |  |  |  |  |  |  |  |  |  |  |  |  |  |  |  |  |  |  |  |  |  |  |  |  |  |  |  |  |  |  |  |  |  |  |  |  |  |  |  |  |  |  |  |  |  |  |  |  |  |  |  |  |  |  |  |  |  |  |  |  |  |  |  |  |  |  |  |  |  |  |  |  |  |  |  |  |  |  |  |  |  |  |  |  |  |  |  |  |  |  |  |  |  |  |  |  |  |  |  |  |  |  |  |  |  |  |  |  |  |  |  |  |  |  |  |  |  |  |  |  |  |  |  |  |  |  |  |  |  |  |  |  |  |  |  |  |  |  |  |  |  |  |  |  |  |  |  |  |  |  |  |  |  |  |  |  |  |  |  |  |  |  |  |  |  |  |  |  |  |  |  |  |  |  |  |  |  |  |  |  |  |  |  |  |  |  |  |  |  |  |  |  |  |  |  |  |  |  |  |  |  |  |  |  |  |  |  |  |  |  |  |  |  |  |  |  |  |  |  |  |  |  |  |  |  |  |  |  |  |  |  |  |  |  |  |  |  |  |  |  |  |  |  |  |  |  |  |  |  |  |  |  |  |  |  |  |  |  |  |  |  |  |  |  |  |  |  |  |  |  |  |  |  |  |  |  |  |  |  |  |  |  |  |  |  |  |  |  |  |  |  |  |  |  |  |  |  |  |  |  |  |  |  |  |  |  |  |  |  |  |  |  |  |  |  |  |  |  |  |  |  |  |  |  |  |  |  |  |  |  |  |  |  |  |  |  |  |  |  |  |  |  |  |  |  |  |  |  |  |  |  |  |  |  |  |  |  |  |  |  |  |  |  |  |  |  |  |  |  |  |  |  |  |  |  |  |  |  |  |  |  |  |  |  |  |  |  |  |  |  |  |  |  |  |  |  |  |  |  |  |  |  |  |  |  |  |  |  |  |  |  |  |  |  |  |  |  |  |  |  |  |  |  |  |  |  |  |  |  |  |  |  |  |  |  |  |  |  |  |  |  |  |  |  |  |  |  |  |  |  |  |  |  |  |  |  |  |  |  |  |  |  |  |  |  |  |  |  |  |  |  |  |  |  |  |  |  |  |  |  |  |  |  |  |  |  |  |  |  |  |  |  |  |  |  |  |  |  |  |  |  |  |  |  |  |  |  |  |  |  |  |  |  |  |  |  |  |  |  |  |  |  |  |  |  |  |  |  |  |  |  |  |  |  |  |  |  |  |  |  |  |  |  |  |  |  |  |  |  |  |  |  |  |  |  |  |  |  |  |  |  |  |  |  |  |  |  |  |  |  |  |  |  |  |  |  |  |  |  |  |  |  |  |  |  |  |  |  |  |  |  |  |  |  |  |  |  |  |  |  |  |  |
|  |  |  |  |  |  |  |  |  |  |  |  |  |  |  |  |  |  |  |  |  |  |  |  |  |  |  |  |  |  |  |  |  |  |  |  |  |  |  |  |  |  |  |  |  |  |  |  |  |  |  |  |  |  |  |  |  |  |  |  |  |  |  |  |  |  |  |  |  |  |  |  |  |  |  |  |  |  |  |  |  |  |  |  |  |  |  |  |  |  |  |  |  |  |  |  |  |  |  |  |  |  |  |  |  |  |  |  |  |  |  |  |  |  |  |  |  |  |  |  |  |  |  |  |  |  |  |  |  |  |  |  |  |  |  |  |  |  |  |  |  |  |  |  |  |  |  |  |  |  |  |  |  |  |  |  |  |  |  |  |  |  |  |  |  |  |  |  |  |  |  |  |  |  |  |  |  |  |  |  |  |  |  |  |  |  |  |  |  |  |  |  |  |  |  |  |  |  |  |  |  |  |  |  |  |  |  |  |  |  |  |  |  |  |  |  |  |  |  |  |  |  |  |  |  |  |  |  |  |  |  |  |  |  |  |  |  |  |  |  |  |  |  |  |  |  |  |  |  |  |  |  |  |  |  |  |  |  |  |  |  |  |  |  |  |  |  |  |  |  |  |  |  |  |  |  |  |  |  |  |  |  |  |  |  |  |  |  |  |  |  |  |  |  |  |  |  |  |  |  |  |  |  |  |  |  |  |  |  |  |  |  |  |  |  |  |  |  |  |  |  |  |  |  |  |  |  |  |  |  |  |  |  |  |  |  |  |  |  |  |  |  |  |  |  |  |  |  |  |  |  |  |  |  |  |  |  |  |  |  |  |  |  |  |  |  |  |  |  |  |  |  |  |  |  |  |  |  |  |  |  |  |  |  |  |  |  |  |  |  |  |  |  |  |  |  |  |  |  |  |  |  |  |  |  |  |  |  |  |  |  |  |  |  |  |  |  |  |  |  |  |  |  |  |  |  |  |  |  |  |  |  |  |  |  |  |  |  |  |  |  |  |  |  |  |  |  |  |  |  |  |  |  |  |  |  |  |  |  |  |  |  |  |  |  |  |  |  |  |  |  |  |  |  |  |  |  |  |  |  |  |  |  |  |  |  |  |  |  |  |  |  |  |  |  |  |  |  |  |  |  |  |  |  |  |  |  |  |  |  |  |  |  |  |  |  |  |  |  |  |  |  |  |  |  |  |  |  |  |  |  |  |  |  |  |  |  |  |  |  |  |  |  |  |  |  |  |  |  |  |  |  |  |  |  |  |  |  |  |  |  |  |  |  |  |  |  |  |  |  |  |  |  |  |  |  |  |  |  |  |  |  |  |  |  |  |  |  |  |  |  |  |  |  |  |  |  |  |  |  |  |  |  |  |  |  |  |  |  |  |  |  |  |  |  |  |  |  |  |  |  |  |  |  |  |  |  |  |  |  |  |  |  |  |  |  |  |  |  |  |  |  |  |  |  |  |  |  |  |  |  |  |  |  |  |  |  |  |  |  |  |  |  |  |  |  |  |  |  |  |  |  |  |  |  |  |  |  |  |  |  |  |  |  |  |  |  |  |  |  |  |  |  |  |  |  |  |  |  |  |  |  |  |  |  |  |  |  |  |  |  |  |  |  |  |  |  |  |  |  |  |  |  |  |  |  |  |  |  |  |  |  |  |  |  |  |  |  |  |  |  |  |  |  |  |  |  |  |  |  |  |  |  |  |  |  |  |  |  |  |  |  |  |  |  |  |  |  |  |  |  |  |  |  |  |  |  |  |  |  |  |  |  |  |  |  |  |  |  |  |  |  |  |  |  |  |  |  |  |  |  |  |  |  |  |  |  |  |  |  |  |  |  |  |  |  |  |  |  |  |  |  |  |  |  |  |  |  |  |  |  |  |  |  |  |  |  |  |  |  |  |  |  |  |  |  |  |  |  |  |  |  |  |  |  |  |  |  |  |  |  |  |  |  |  |  |  |  |  |  |  |  |  |  |  |  |  |  |  |  |  |  |  |  |  |  |  |  |  |  |  |  |  |  |  |  |  |  |  |  |  |  |  |  |  |  |  |  |  |  |  |  |  |  |  |  |  |  |  |  |  |  |  |  |  |  |  |  |  |  |  |  |  |  |  |  |  |  |  |  |  |  |  |  |  |  |  |  |  |  |  |  |  |  |  |  |  |  |  |  |  |  |  |  |  |  |  |  |  |  |  |  |  |  |  |  |  |  |  |  |  |  |  |  |  |  |  |  |  |  |  |  |  |  |  |  |  |  |  |  |  |  |  |  |  |  |  |  |  |  |  |  |  |  |  |  |  |  |  |  |  |  |  |  |  |  |  |  |  |  |  |  |  |  |  |  |  |  |  |  |  |  |  |  |  |  |  |  |  |  |  |  |  |  |  |  |  |  |  |  |  |  |  |  |  |  |  |  |  |  |  |  |  |  |  |  |  |  |  |  |  |  |  |  |  |  |  |  |  |  |  |  |  |  |  |  |  |  |  |  |  |  |  |  |  |  |  |  |  |  |  |  |  |  |  |  |  |  |  |  |  |  |  |  |  |  |  |  |  |  |  |  |  |  |  |  |  |  |  |  |  |  |  |  |  |  |  |  |  |  |  |  |  |  |  |  |  |  |  |  |  |  |  |  |  |  |  |  |  |  |  |  |  |  |  |  |  |  |  |  |  |  |  |  |  |  |  |  |  |  |  |  |  |  |  |  |  |  |  |  |  |  |  |  |  |  |  |  |  |  |  |  |  |  |  |  |  |  |  |  |  |  |  |  |  |  |  |  |  |  |  |  |  |  |  |  |  |  |  |  |  |  |  |  |  |  |  |  |  |  |  |  |  |  |  |  |  |  |  |  |  |  |  |  |  |  |  |  |  |  |  |  |  |  |  |  |  |  |  |  |  |  |  |  |  |  |  |  |  |  |  |  |  |  |  |  |  |  |  |  |  |  |  |  |  |  |  |  |  |  |  |  |  |  |  |  |  |  |  |  |  |  |  |  |  |  |  |  |  |  |  |  |  |  |  |  |  |  |  |  |  |  |  |  |  |  |  |  |
|  |  |  |  |  |  |  |  |  |  |  |  |  |  |  |  |  |  |  |  |  |  |  |  |  |  |  |  |  |  |  |  |  |  |  |  |  |  |  |  |  |  |  |  |  |  |  |  |  |  |  |  |  |  |  |  |  |  |  |  |  |  |  |  |  |  |  |  |  |  |  |  |  |  |  |  |  |  |  |  |  |  |  |  |  |  |  |  |  |  |  |  |  |  |  |  |  |  |  |  |  |  |  |  |  |  |  |  |  |  |  |  |  |  |  |  |  |  |  |  |  |  |  |  |  |  |  |  |  |  |  |  |  |  |  |  |  |  |  |  |  |  |  |  |  |  |  |  |  |  |  |  |  |  |  |  |  |  |  |  |  |  |  |  |  |  |  |  |  |  |  |  |  |  |  |  |  |  |  |  |  |  |  |  |  |  |  |  |  |  |  |  |  |  |  |  |  |  |  |  |  |  |  |  |  |  |  |  |  |  |  |  |  |  |  |  |  |  |  |  |  |  |  |  |  |  |  |  |  |  |  |  |  |  |  |  |  |  |  |  |  |  |  |  |  |  |  |  |  |  |  |  |  |  |  |  |  |  |  |  |  |  |  |  |  |  |  |  |  |  |  |  |  |  |  |  |  |  |  |  |  |  |  |  |  |  |  |  |  |  |  |  |  |  |  |  |  |  |  |  |  |  |  |  |  |  |  |  |  |  |  |  |  |  |  |  |  |  |  |  |  |  |  |  |  |  |  |  |  |  |  |  |  |  |  |  |  |  |  |  |  |  |  |  |  |  |  |  |  |  |  |  |  |  |  |  |  |  |  |  |  |  |  |  |  |  |  |  |  |  |  |  |  |  |  |  |  |  |  |  |  |  |  |  |  |  |  |  |  |  |  |  |  |  |  |  |  |  |  |  |  |  |  |  |  |  |  |  |  |  |  |  |  |  |  |  |  |  |  |  |  |  |  |  |  |  |  |  |  |  |  |  |  |  |  |  |  |  |  |  |  |  |  |  |  |  |  |  |  |  |  |  |  |  |  |  |  |  |  |  |  |  |  |  |  |  |  |  |  |  |  |  |  |  |  |  |  |  |  |  |  |  |  |  |  |  |  |  |  |  |  |  |  |  |  |  |  |  |  |  |  |  |  |  |  |  |  |  |  |  |  |  |  |  |  |  |  |  |  |  |  |  |  |  |  |  |  |  |  |  |  |  |  |  |  |  |  |  |  |  |  |  |  |  |  |  |  |  |  |  |  |  |  |  |  |  |  |  |  |  |  |  |  |  |  |  |  |  |  |  |  |  |  |  |  |  |  |  |  |  |  |  |  |  |  |  |  |  |  |  |  |  |  |  |  |  |  |  |  |  |  |  |  |  |  |  |  |  |  |  |  |  |  |  |  |  |  |  |  |  |  |  |  |  |  |  |  |  |  |  |  |  |  |  |  |  |  |  |  |  |  |  |  |  |  |  |  |  |  |  |  |  |  |  |  |  |  |  |  |  |  |  |  |  |  |  |  |  |  |  |  |  |  |  |  |  |  |  |  |  |  |  |  |  |  |  |  |  |  |  |  |  |  |  |  |  |  |  |  |  |  |  |  |  |  |  |  |  |  |  |  |  |  |  |  |  |  |  |  |  |  |  |  |  |  |  |  |  |  |  |  |  |  |  |  |  |  |  |  |  |  |  |  |  |  |  |  |  |  |  |  |  |  |  |  |  |  |  |  |  |  |  |  |  |  |  |  |  |  |  |  |  |  |  |  |  |  |  |  |  |  |  |  |  |  |  |  |  |  |  |  |  |  |  |  |  |  |  |  |  |  |  |  |  |  |  |  |  |  |  |  |  |  |  |  |  |  |  |  |  |  |  |  |  |  |  |  |  |  |  |  |  |  |  |  |  |  |  |  |  |  |  |  |  |  |  |  |  |  |  |  |  |  |  |  |  |  |  |  |  |  |  |  |  |  |  |  |  |  |  |  |  |  |  |  |  |  |  |  |  |  |  |  |  |  |  |  |  |  |  |  |  |  |  |  |  |  |  |  |  |  |  |  |  |  |  |  |  |  |  |  |  |  |  |  |  |  |  |  |  |  |  |  |  |  |  |  |  |  |  |  |  |  |  |  |  |  |  |  |  |  |  |  |  |  |  |  |  |  |  |  |  |  |  |  |  |  |  |  |  |  |  |  |  |  |  |  |  |  |  |  |  |  |  |  |  |  |  |  |  |  |  |  |  |  |  |  |  |  |  |  |  |  |  |  |  |  |  |  |  |  |  |  |  |  |  |  |  |  |  |  |  |  |  |  |  |  |  |  |  |  |  |  |  |  |  |  |  |  |  |  |  |  |  |  |  |  |  |  |  |  |  |  |  |  |  |  |  |  |  |  |  |  |  |  |  |  |  |  |  |  |  |  |  |  |  |  |  |  |  |  |  |  |  |  |  |  |  |  |  |  |  |  |  |  |  |  |  |  |  |  |  |  |  |  |  |  |  |  |  |  |  |  |  |  |  |  |  |  |  |  |  |  |  |  |  |  |  |  |  |  |  |  |  |  |  |  |  |  |  |  |  |  |  |  |  |  |  |  |  |  |  |  |  |  |  |  |  |  |  |  |  |  |  |  |  |  |  |  |  |  |  |  |  |  |  |  |  |  |  |  |  |  |  |  |  |  |  |  |  |  |  |  |  |  |  |  |  |  |  |  |  |  |  |  |  |  |  |  |  |  |  |  |  |  |  |  |  |  |  |  |  |  |  |  |  |  |  |  |  |  |  |  |  |  |  |  |  |  |  |  |  |  |  |  |  |  |  |  |  |  |  |  |  |  |  |  |  |  |  |  |  |  |  |  |  |  |  |  |  |  |  |  |  |  |  |  |  |  |  |  |  |  |  |  |  |  |  |  |  |  |  |  |  |  |  |  |  |  |  |  |  |  |  |  |  |  |  |  |  |  |  |  |  |  |  |  |  |  |  |  |  |  |  |  |  |  |  |  |  |  |  |  |  |  |  |  |  |  |  |  |  |  |  |  |  |  |  |  |  |  |  |  |  |  |  |  |  |
|  |  |  |  |  |  |  |  |  |  |  |  |  |  |  |  |  |  |  |  |  |  |  |  |  |  |  |  |  |  |  |  |  |  |  |  |  |  |  |  |  |  |  |  |  |  |  |  |  |  |  |  |  |  |  |  |  |  |  |  |  |  |  |  |  |  |  |  |  |  |  |  |  |  |  |  |  |  |  |  |  |  |  |  |  |  |  |  |  |  |  |  |  |  |  |  |  |  |  |  |  |  |  |  |  |  |  |  |  |  |  |  |  |  |  |  |  |  |  |  |  |  |  |  |  |  |  |  |  |  |  |  |  |  |  |  |  |  |  |  |  |  |  |  |  |  |  |  |  |  |  |  |  |  |  |  |  |  |  |  |  |  |  |  |  |  |  |  |  |  |  |  |  |  |  |  |  |  |  |  |  |  |  |  |  |  |  |  |  |  |  |  |  |  |  |  |  |  |  |  |  |  |  |  |  |  |  |  |  |  |  |  |  |  |  |  |  |  |  |  |  |  |  |  |  |  |  |  |  |  |  |  |  |  |  |  |  |  |  |  |  |  |  |  |  |  |  |  |  |  |  |  |  |  |  |  |  |  |  |  |  |  |  |  |  |  |  |  |  |  |  |  |  |  |  |  |  |  |  |  |  |  |  |  |  |  |  |  |  |  |  |  |  |  |  |  |  |  |  |  |  |  |  |  |  |  |  |  |  |  |  |  |  |  |  |  |  |  |  |  |  |  |  |  |  |  |  |  |  |  |  |  |  |  |  |  |  |  |  |  |  |  |  |  |  |  |  |  |  |  |  |  |  |  |  |  |  |  |  |  |  |  |  |  |  |  |  |  |  |  |  |  |  |  |  |  |  |  |  |  |  |  |  |  |  |  |  |  |  |  |  |  |  |  |  |  |  |  |  |  |  |  |  |  |  |  |  |  |  |  |  |  |  |  |  |  |  |  |  |  |  |  |  |  |  |  |  |  |  |  |  |  |  |  |  |  |  |  |  |  |  |  |  |  |  |  |  |  |  |  |  |  |  |  |  |  |  |  |  |  |  |  |  |  |  |  |  |  |  |  |  |  |  |  |  |  |  |  |  |  |  |  |  |  |  |  |  |  |  |  |  |  |  |  |  |  |  |  |  |  |  |  |  |  |  |  |  |  |  |  |  |  |  |  |  |  |  |  |  |  |  |  |  |  |  |  |  |  |  |  |  |  |  |  |  |  |  |  |  |  |  |  |  |  |  |  |  |  |  |  |  |  |  |  |  |  |  |  |  |  |  |  |  |  |  |  |  |  |  |  |  |  |  |  |  |  |  |  |  |  |  |  |  |  |  |  |  |  |  |  |  |  |  |  |  |  |  |  |  |  |  |  |  |  |  |  |  |  |  |  |  |  |  |  |  |  |  |  |  |  |  |  |  |  |  |  |  |  |  |  |  |  |  |  |  |  |  |  |  |  |  |  |  |  |  |  |  |  |  |  |  |  |  |  |  |  |  |  |  |  |  |  |  |  |  |  |  |  |  |  |  |  |  |  |  |  |  |  |  |  |  |  |  |  |  |  |  |  |  |  |  |  |  |  |  |  |  |  |  |  |  |  |  |  |  |  |  |  |  |  |  |  |  |  |  |  |  |  |  |  |  |  |  |  |  |  |  |  |  |  |  |  |  |  |  |  |  |  |  |  |  |  |  |  |  |  |  |  |  |  |  |  |  |  |  |  |  |  |  |  |  |  |  |  |  |  |  |  |  |  |  |  |  |  |  |  |  |  |  |  |  |  |  |  |  |  |  |  |  |  |  |  |  |  |  |  |  |  |  |  |  |  |  |  |  |  |  |  |  |  |  |  |  |  |  |  |  |  |  |  |  |  |  |  |  |  |  |  |  |  |  |  |  |  |  |  |  |  |  |  |  |  |  |  |  |  |  |  |  |  |  |  |  |  |  |  |  |  |  |  |  |  |  |  |  |  |  |  |  |  |  |  |  |  |  |  |  |  |  |  |  |  |  |  |  |  |  |  |  |  |  |  |  |  |  |  |  |  |  |  |  |  |  |  |  |  |  |  |  |  |  |  |  |  |  |  |  |  |  |  |  |  |  |  |  |  |  |  |  |  |  |  |  |  |  |  |  |  |  |  |  |  |  |  |  |  |  |  |  |  |  |  |  |  |  |  |  |  |  |  |  |  |  |  |  |  |  |  |  |  |  |  |  |  |  |  |  |  |  |  |  |  |  |  |  |  |  |  |  |  |  |  |  |  |  |  |  |  |  |  |  |  |  |  |  |  |  |  |  |  |  |  |  |  |  |  |  |  |  |  |  |  |  |  |  |  |  |  |  |  |  |  |  |  |  |  |  |  |  |  |  |  |  |  |  |  |  |  |  |  |  |  |  |  |  |  |  |  |  |  |  |  |  |  |  |  |  |  |  |  |  |  |  |  |  |  |  |  |  |  |  |  |  |  |  |  |  |  |  |  |  |  |  |  |  |  |  |  |  |  |  |  |  |  |  |  |  |  |  |  |  |  |  |  |  |  |  |  |  |  |  |  |  |  |  |  |  |  |  |  |  |  |  |  |  |  |  |  |  |  |  |  |  |  |  |  |  |  |  |  |  |  |  |  |  |  |  |  |  |  |  |  |  |  |  |  |  |  |  |  |  |  |  |  |  |  |  |  |  |  |  |  |  |  |  |  |  |  |  |  |  |  |  |  |  |  |  |  |  |  |  |  |  |  |  |  |  |  |  |  |  |  |  |  |  |  |  |  |  |  |  |  |  |  |  |  |  |  |  |  |  |  |  |  |  |  |  |  |  |  |  |  |  |  |  |  |  |  |  |  |  |  |  |  |  |  |  |  |  |  |  |  |  |  |  |  |  |  |  |  |  |  |  |  |  |  |  |  |  |  |  |  |  |  |  |  |  |  |  |  |  |  |  |  |  |  |  |  |  |  |  |  |  |  |  |  |  |  |  |  |  |  |  |  |  |  |  |  |  |  |  |  |  |  |  |  |  |  |  |  |  |  |  |  |  |  |  |  |  |  |  |  |  |  |  |  |  |  |
|  |  |  |  |  |  |  |  |  |  |  |  |  |  |  |  |  |  |  |  |  |  |  |  |  |  |  |  |  |  |  |  |  |  |  |  |  |  |  |  |  |  |  |  |  |  |  |  |  |  |  |  |  |  |  |  |  |  |  |  |  |  |  |  |  |  |  |  |  |  |  |  |  |  |  |  |  |  |  |  |  |  |  |  |  |  |  |  |  |  |  |  |  |  |  |  |  |  |  |  |  |  |  |  |  |  |  |  |  |  |  |  |  |  |  |  |  |  |  |  |  |  |  |  |  |  |  |  |  |  |  |  |  |  |  |  |  |  |  |  |  |  |  |  |  |  |  |  |  |  |  |  |  |  |  |  |  |  |  |  |  |  |  |  |  |  |  |  |  |  |  |  |  |  |  |  |  |  |  |  |  |  |  |  |  |  |  |  |  |  |  |  |  |  |  |  |  |  |  |  |  |  |  |  |  |  |  |  |  |  |  |  |  |  |  |  |  |  |  |  |  |  |  |  |  |  |  |  |  |  |  |  |  |  |  |  |  |  |  |  |  |  |  |  |  |  |  |  |  |  |  |  |  |  |  |  |  |  |  |  |  |  |  |  |  |  |  |  |  |  |  |  |  |  |  |  |  |  |  |  |  |  |  |  |  |  |  |  |  |  |  |  |  |  |  |  |  |  |  |  |  |  |  |  |  |  |  |  |  |  |  |  |  |  |  |  |  |  |  |  |  |  |  |  |  |  |  |  |  |  |  |  |  |  |  |  |  |  |  |  |  |  |  |  |  |  |  |  |  |  |  |  |  |  |  |  |  |  |  |  |  |  |  |  |  |  |  |  |  |  |  |  |  |  |  |  |  |  |  |  |  |  |  |  |  |  |  |  |  |  |  |  |  |  |  |  |  |  |  |  |  |  |  |  |  |  |  |  |  |  |  |  |  |  |  |  |  |  |  |  |  |  |  |  |  |  |  |  |  |  |  |  |  |  |  |  |  |  |  |  |  |  |  |  |  |  |  |  |  |  |  |  |  |  |  |  |  |  |  |  |  |  |  |  |  |  |  |  |  |  |  |  |  |  |  |  |  |  |  |  |  |  |  |  |  |  |  |  |  |  |  |  |  |  |  |  |  |  |  |  |  |  |  |  |  |  |  |  |  |  |  |  |  |  |  |  |  |  |  |  |  |  |  |  |  |  |  |  |  |  |  |  |  |  |  |  |  |  |  |  |  |  |  |  |  |  |  |  |  |  |  |  |  |  |  |  |  |  |  |  |  |  |  |  |  |  |  |  |  |  |  |  |  |  |  |  |  |  |  |  |  |  |  |  |  |  |  |  |  |  |  |  |  |  |  |  |  |  |  |  |  |  |  |  |  |  |  |  |  |  |  |  |  |  |  |  |  |  |  |  |  |  |  |  |  |  |  |  |  |  |  |  |  |  |  |  |  |  |  |  |  |  |  |  |  |  |  |  |  |  |  |  |  |  |  |  |  |  |  |  |  |  |  |  |  |  |  |  |  |  |  |  |  |  |  |  |  |  |  |  |  |  |  |  |  |  |  |  |  |  |  |  |  |  |  |  |  |  |  |  |  |  |  |  |  |  |  |  |  |  |  |  |  |  |  |  |  |  |  |  |  |  |  |  |  |  |  |  |  |  |  |  |  |  |  |  |  |  |  |  |  |  |  |  |  |  |  |  |  |  |  |  |  |  |  |  |  |  |  |  |  |  |  |  |  |  |  |  |  |  |  |  |  |  |  |  |  |  |  |  |  |  |  |  |  |  |  |  |  |  |  |  |  |  |  |  |  |  |  |  |  |  |  |  |  |  |  |  |  |  |  |  |  |  |  |  |  |  |  |  |  |  |  |  |  |  |  |  |  |  |  |  |  |  |  |  |  |  |  |  |  |  |  |  |  |  |  |  |  |  |  |  |  |  |  |  |  |  |  |  |  |  |  |  |  |  |  |  |  |  |  |  |  |  |  |  |  |  |  |  |  |  |  |  |  |  |  |  |  |  |  |  |  |  |  |  |  |  |  |  |  |  |  |  |  |  |  |  |  |  |  |  |  |  |  |  |  |  |  |  |  |  |  |  |  |  |  |  |  |  |  |  |  |  |  |  |  |  |  |  |  |  |  |  |  |  |  |  |  |  |  |  |  |  |  |  |  |  |  |  |  |  |  |  |  |  |  |  |  |  |  |  |  |  |  |  |  |  |  |  |  |  |  |  |  |  |  |  |  |  |  |  |  |  |  |  |  |  |  |  |  |  |  |  |  |  |  |  |  |  |  |  |  |  |  |  |  |  |  |  |  |  |  |  |  |  |  |  |  |  |  |  |  |  |  |  |  |  |  |  |  |  |  |  |  |  |  |  |  |  |  |  |  |  |  |  |  |  |  |  |  |  |  |  |  |  |  |  |  |  |  |  |  |  |  |  |  |  |  |  |  |  |  |  |  |  |  |  |  |  |  |  |  |  |  |  |  |  |  |  |  |  |  |  |  |  |  |  |  |  |  |  |  |  |  |  |  |  |  |  |  |  |  |  |  |  |  |  |  |  |  |  |  |  |  |  |  |  |  |  |  |  |  |  |  |  |  |  |  |  |  |  |  |  |  |  |  |  |  |  |  |  |  |  |  |  |  |  |  |  |  |  |  |  |  |  |  |  |  |  |  |  |  |  |  |  |  |  |  |  |  |  |  |  |  |  |  |  |  |  |  |  |  |  |  |  |  |  |  |  |  |  |  |  |  |  |  |  |  |  |  |  |  |  |  |  |  |  |  |  |  |  |  |  |  |  |  |  |  |  |  |  |  |  |  |  |  |  |  |  |  |  |  |  |  |  |  |  |  |  |  |  |  |  |  |  |  |  |  |  |  |  |  |  |  |  |  |  |  |  |  |  |  |  |  |  |  |  |  |  |  |  |  |  |  |  |  |  |  |  |  |  |  |  |  |  |  |  |  |  |  |  |  |  |  |  |  |  |  |  |  |  |  |  |  |  |  |  |  |  |  |  |  |  |  |  |  |  |  |  |  |  |  |  |  |  |  |  |
|  |  |  |  |  |  |  |  |  |  |  |  |  |  |  |  |  |  |  |  |  |  |  |  |  |  |  |  |  |  |  |  |  |  |  |  |  |  |  |  |  |  |  |  |  |  |  |  |  |  |  |  |  |  |  |  |  |  |  |  |  |  |  |  |  |  |  |  |  |  |  |  |  |  |  |  |  |  |  |  |  |  |  |  |  |  |  |  |  |  |  |  |  |  |  |  |  |  |  |  |  |  |  |  |  |  |  |  |  |  |  |  |  |  |  |  |  |  |  |  |  |  |  |  |  |  |  |  |  |  |  |  |  |  |  |  |  |  |  |  |  |  |  |  |  |  |  |  |  |  |  |  |  |  |  |  |  |  |  |  |  |  |  |  |  |  |  |  |  |  |  |  |  |  |  |  |  |  |  |  |  |  |  |  |  |  |  |  |  |  |  |  |  |  |  |  |  |  |  |  |  |  |  |  |  |  |  |  |  |  |  |  |  |  |  |  |  |  |  |  |  |  |  |  |  |  |  |  |  |  |  |  |  |  |  |  |  |  |  |  |  |  |  |  |  |  |  |  |  |  |  |  |  |  |  |  |  |  |  |  |  |  |  |  |  |  |  |  |  |  |  |  |  |  |  |  |  |  |  |  |  |  |  |  |  |  |  |  |  |  |  |  |  |  |  |  |  |  |  |  |  |  |  |  |  |  |  |  |  |  |  |  |  |  |  |  |  |  |  |  |  |  |  |  |  |  |  |  |  |  |  |  |  |  |  |  |  |  |  |  |  |  |  |  |  |  |  |  |  |  |  |  |  |  |  |  |  |  |  |  |  |  |  |  |  |  |  |  |  |  |  |  |  |  |  |  |  |  |  |  |  |  |  |  |  |  |  |  |  |  |  |  |  |  |  |  |  |  |  |  |  |  |  |  |  |  |  |  |  |  |  |  |  |  |  |  |  |  |  |  |  |  |  |  |  |  |  |  |  |  |  |  |  |  |  |  |  |  |  |  |  |  |  |  |  |  |  |  |  |  |  |  |  |  |  |  |  |  |  |  |  |  |  |  |  |  |  |  |  |  |  |  |  |  |  |  |  |  |  |  |  |  |  |  |  |  |  |  |  |  |  |  |  |  |  |  |  |  |  |  |  |  |  |  |  |  |  |  |  |  |  |  |  |  |  |  |  |  |  |  |  |  |  |  |  |  |  |  |  |  |  |  |  |  |  |  |  |  |  |  |  |  |  |  |  |  |  |  |  |  |  |  |  |  |  |  |  |  |  |  |  |  |  |  |  |  |  |  |  |  |  |  |  |  |  |  |  |  |  |  |  |  |  |  |  |  |  |  |  |  |  |  |  |  |  |  |  |  |  |  |  |  |  |  |  |  |  |  |  |  |  |  |  |  |  |  |  |  |  |  |  |  |  |  |  |  |  |  |  |  |  |  |  |  |  |  |  |  |  |  |  |  |  |  |  |  |  |  |  |  |  |  |  |  |  |  |  |  |  |  |  |  |  |  |  |  |  |  |  |  |  |  |  |  |  |  |  |  |  |  |  |  |  |  |  |  |  |  |  |  |  |  |  |  |  |  |  |  |  |  |  |  |  |  |  |  |  |  |  |  |  |  |  |  |  |  |  |  |  |  |  |  |  |  |  |  |  |  |  |  |  |  |  |  |  |  |  |  |  |  |  |  |  |  |  |  |  |  |  |  |  |  |  |  |  |  |  |  |  |  |  |  |  |  |  |  |  |  |  |  |  |  |  |  |  |  |  |  |  |  |  |  |  |  |  |  |  |  |  |  |  |  |  |  |  |  |  |  |  |  |  |  |  |  |  |  |  |  |  |  |  |  |  |  |  |  |  |  |  |  |  |  |  |  |  |  |  |  |  |  |  |  |  |  |  |  |  |  |  |  |  |  |  |  |  |  |  |  |  |  |  |  |  |  |  |  |  |  |  |  |  |  |  |  |  |  |  |  |  |  |  |  |  |  |  |  |  |  |  |  |  |  |  |  |  |  |  |  |  |  |  |  |  |  |  |  |  |  |  |  |  |  |  |  |  |  |  |  |  |  |  |  |  |  |  |  |  |  |  |  |  |  |  |  |  |  |  |  |  |  |  |  |  |  |  |  |  |  |  |  |  |  |  |  |  |  |  |  |  |  |  |  |  |  |  |  |  |  |  |  |  |  |  |  |  |  |  |  |  |  |  |  |  |  |  |  |  |  |  |  |  |  |  |  |  |  |  |  |  |  |  |  |  |  |  |  |  |  |  |  |  |  |  |  |  |  |  |  |  |  |  |  |  |  |  |  |  |  |  |  |  |  |  |  |  |  |  |  |  |  |  |  |  |  |  |  |  |  |  |  |  |  |  |  |  |  |  |  |  |  |  |  |  |  |  |  |  |  |  |  |  |  |  |  |  |  |  |  |  |  |  |  |  |  |  |  |  |  |  |  |  |  |  |  |  |  |  |  |  |  |  |  |  |  |  |  |  |  |  |  |  |  |  |  |  |  |  |  |  |  |  |  |  |  |  |  |  |  |  |  |  |  |  |  |  |  |  |  |  |  |  |  |  |  |  |  |  |  |  |  |  |  |  |  |  |  |  |  |  |  |  |  |  |  |  |  |  |  |  |  |  |  |  |  |  |  |  |  |  |  |  |  |  |  |  |  |  |  |  |  |  |  |  |  |  |  |  |  |  |  |  |  |  |  |  |  |  |  |  |  |  |  |  |  |  |  |  |  |  |  |  |  |  |  |  |  |  |  |  |  |  |  |  |  |  |  |  |  |  |  |  |  |  |  |  |  |  |  |  |  |  |  |  |  |  |  |  |  |  |  |  |  |  |  |  |  |  |  |  |  |  |  |  |  |  |  |  |  |  |  |  |  |  |  |  |  |  |  |  |  |  |  |  |  |  |  |  |  |  |  |  |  |  |  |  |  |  |  |  |  |  |  |  |  |  |  |  |  |  |  |  |  |  |  |  |  |  |  |  |  |  |  |  |  |  |  |  |  |  |  |  |  |  |  |  |  |  |  |  |  |  |  |  |  |  |  |  |  |
|  |  |  |  |  |  |  |  |  |  |  |  |  |  |  |  |  |  |  |  |  |  |  |  |  |  |  |  |  |  |  |  |  |  |  |  |  |  |  |  |  |  |  |  |  |  |  |  |  |  |  |  |  |  |  |  |  |  |  |  |  |  |  |  |  |  |  |  |  |  |  |  |  |  |  |  |  |  |  |  |  |  |  |  |  |  |  |  |  |  |  |  |  |  |  |  |  |  |  |  |  |  |  |  |  |  |  |  |  |  |  |  |  |  |  |  |  |  |  |  |  |  |  |  |  |  |  |  |  |  |  |  |  |  |  |  |  |  |  |  |  |  |  |  |  |  |  |  |  |  |  |  |  |  |  |  |  |  |  |  |  |  |  |  |  |  |  |  |  |  |  |  |  |  |  |  |  |  |  |  |  |  |  |  |  |  |  |  |  |  |  |  |  |  |  |  |  |  |  |  |  |  |  |  |  |  |  |  |  |  |  |  |  |  |  |  |  |  |  |  |  |  |  |  |  |  |  |  |  |  |  |  |  |  |  |  |  |  |  |  |  |  |  |  |  |  |  |  |  |  |  |  |  |  |  |  |  |  |  |  |  |  |  |  |  |  |  |  |  |  |  |  |  |  |  |  |  |  |  |  |  |  |  |  |  |  |  |  |  |  |  |  |  |  |  |  |  |  |  |  |  |  |  |  |  |  |  |  |  |  |  |  |  |  |  |  |  |  |  |  |  |  |  |  |  |  |  |  |  |  |  |  |  |  |  |  |  |  |  |  |  |  |  |  |  |  |  |  |  |  |  |  |  |  |  |  |  |  |  |  |  |  |  |  |  |  |  |  |  |  |  |  |  |  |  |  |  |  |  |  |  |  |  |  |  |  |  |  |  |  |  |  |  |  |  |  |  |  |  |  |  |  |  |  |  |  |  |  |  |  |  |  |  |  |  |  |  |  |  |  |  |  |  |  |  |  |  |  |  |  |  |  |  |  |  |  |  |  |  |  |  |  |  |  |  |  |  |  |  |  |  |  |  |  |  |  |  |  |  |  |  |  |  |  |  |  |  |  |  |  |  |  |  |  |  |  |  |  |  |  |  |  |  |  |  |  |  |  |  |  |  |  |  |  |  |  |  |  |  |  |  |  |  |  |  |  |  |  |  |  |  |  |  |  |  |  |  |  |  |  |  |  |  |  |  |  |  |  |  |  |  |  |  |  |  |  |  |  |  |  |  |  |  |  |  |  |  |  |  |  |  |  |  |  |  |  |  |  |  |  |  |  |  |  |  |  |  |  |  |  |  |  |  |  |  |  |  |  |  |  |  |  |  |  |  |  |  |  |  |  |  |  |  |  |  |  |  |  |  |  |  |  |  |  |  |  |  |  |  |  |  |  |  |  |  |  |  |  |  |  |  |  |  |  |  |  |  |  |  |  |  |  |  |  |  |  |  |  |  |  |  |  |  |  |  |  |  |  |  |  |  |  |  |  |  |  |  |  |  |  |  |  |  |  |  |  |  |  |  |  |  |  |  |  |  |  |  |  |  |  |  |  |  |  |  |  |  |  |  |  |  |  |  |  |  |  |  |  |  |  |  |  |  |  |  |  |  |  |  |  |  |  |  |  |  |  |  |  |  |  |  |  |  |  |  |  |  |  |  |  |  |  |  |  |  |  |  |  |  |  |  |  |  |  |  |  |  |  |  |  |  |  |  |  |  |  |  |  |  |  |  |  |  |  |  |  |  |  |  |  |  |  |  |  |  |  |  |  |  |  |  |  |  |  |  |  |  |  |  |  |  |  |  |  |  |  |  |  |  |  |  |  |  |  |  |  |  |  |  |  |  |  |  |  |  |  |  |  |  |  |  |  |  |  |  |  |  |  |  |  |  |  |  |  |  |  |  |  |  |  |  |  |  |  |  |  |  |  |  |  |  |  |  |  |  |  |  |  |  |  |  |  |  |  |  |  |  |  |  |  |  |  |  |  |  |  |  |  |  |  |  |  |  |  |  |  |  |  |  |  |  |  |  |  |  |  |  |  |  |  |  |  |  |  |  |  |  |  |  |  |  |  |  |  |  |  |  |  |  |  |  |  |  |  |  |  |  |  |  |  |  |  |  |  |  |  |  |  |  |  |  |  |  |  |  |  |  |  |  |  |  |  |  |  |  |  |  |  |  |  |  |  |  |  |  |  |  |  |  |  |  |  |  |  |  |  |  |  |  |  |  |  |  |  |  |  |  |  |  |  |  |  |  |  |  |  |  |  |  |  |  |  |  |  |  |  |  |  |  |  |  |  |  |  |  |  |  |  |  |  |  |  |  |  |  |  |  |  |  |  |  |  |  |  |  |  |  |  |  |  |  |  |  |  |  |  |  |  |  |  |  |  |  |  |  |  |  |  |  |  |  |  |  |  |  |  |  |  |  |  |  |  |  |  |  |  |  |  |  |  |  |  |  |  |  |  |  |  |  |  |  |  |  |  |  |  |  |  |  |  |  |  |  |  |  |  |  |  |  |  |  |  |  |  |  |  |  |  |  |  |  |  |  |  |  |  |  |  |  |  |  |  |  |  |  |  |  |  |  |  |  |  |  |  |  |  |  |  |  |  |  |  |  |  |  |  |  |  |  |  |  |  |  |  |  |  |  |  |  |  |  |  |  |  |  |  |  |  |  |  |  |  |  |  |  |  |  |  |  |  |  |  |  |  |  |  |  |  |  |  |  |  |  |  |  |  |  |  |  |  |  |  |  |  |  |  |  |  |  |  |  |  |  |  |  |  |  |  |  |  |  |  |  |  |  |  |  |  |  |  |  |  |  |  |  |  |  |  |  |  |  |  |  |  |  |  |  |  |  |  |  |  |  |  |  |  |  |  |  |  |  |  |  |  |  |  |  |  |  |  |  |  |  |  |  |  |  |  |  |  |  |  |  |  |  |  |  |  |  |  |  |  |  |  |  |  |  |  |  |  |  |  |  |  |  |  |  |  |  |  |  |  |  |  |  |  |  |  |  |  |  |  |  |  |  |  |  |  |  |  |  |  |  |  |  |  |  |  |
