# Supplementary material for: Variation in selection constraints on teleost TLRs with emphasis on their repertoire in the Walking catfish, Clarias batrachus
Source: Sci Rep. 2020 Dec 7;10:21394. doi: 10.1038/s41598-020-78347-6 (PMC7721727; doi:10.1038/s41598-020-78347-6)
Supplement: Supplementary file 31 — Supplementary Information 31. [file 41598_2020_78347_MOESM31_ESM.zip › T9/struct/S514930_results/index.html]

I-TASSER results


[Home]
[Server]
[Queue]
[About]
[Remove]
[Statistics]
  

|  |
| --- |
| I-TASSER results for job id S514930 (Click on S514930\_results.tar.bz2 to download the tarball file including all modeling results listed on this page. Click on Annotation of I-TASSER Output to read the instructions for how to interpret the results on this page. Model results are kept on the server for 60 days, there is no way to retrieve the modeling data older than 2 months) |
| |  | | --- | | Submitted Sequence in FASTA format | |
| |  | | --- | | Predicted Secondary Structure |   |  | | --- | | **Sequence** | |

 20                  40                  60                  80                 100                 120                 140                 160                 180                 200                 220                 240                 260                 280                 300                 320                 340                 360                 380                 400                 420                 440                 460                 480                 500                 520                 540                 560                 580                 600                 620                 640                 660                 680                 700                 720                 740                 760                 780                 800                 820                 840                 860                 880                 900                 920                 940                 960                 980                1000                1020                1040                1060                    |                   |                   |                   |                   |                   |                   |                   |                   |                   |                   |                   |                   |                   |                   |                   |                   |                   |                   |                   |                   |                   |                   |                   |                   |                   |                   |                   |                   |                   |                   |                   |                   |                   |                   |                   |                   |                   |                   |                   |                   |                   |                   |                   |                   |                   |                   |                   |                   |                   |                   |                   |                   |  MFLPGLYLFFALDLIKLVKTMNPIFYPCDNNTISNGTIHVDCKRRNLTSIPKFTSLSISSLNLDENHVHQVKNDDFSNIPNLQHLSLMWNCLPGRLKALKLPSCQVLIDHDAFVNLKCLNSLQLAGNSLKTIPLLPQNLEVLGLEFNNILRIEQPLGTPFLKQLLLAKNCYYANPCNESFFIDQRVFQGLPKLQNLTLGFNNMSSIPTQLPPSLKSLDLGENRITEIQEGSFTNLTKLFSLNLNWNCQRCDNAAHPCFPCPNNASLGLPNQAFSEQRDSLVILYLRGNSIRSIPKNLFAQLRKLKVLDLSDNFLAYSIRNGTFYEELQGVEFLRLLNNYQPLKTFSELILSPSISKMRSLRKLYLSGLFFHQLSNYSLMHLSNLPQLEYLDLRMNFMNYCNVTAFKRLRSLRTVVLSQNMLTINPYYTSSANEARRLDRYQMDDHLDLQDMPAFESELQMNYLHCTEKECPKSYSMENFQKFFCKKELYFDLSQNNIPWLNAYTFKGMEKTVCLDLSYNYMSQALNGQQFLPLTKLVFLNMAYNRIDLYFNEAFQEIRSTLKVLDLSNNEFHFQMKQIGHRLDFIKNLTSLEVLSLANNKIGLRISNTLYSASLKCLLFSGNHLDIMWDTKGDQYIRFFQGLTNLTYLDISDNQLKSCSPAAIINLPPSIRVLRMDSNFLDYFPWVNISVLSQLCYLNLSGNFLFNLPDTAIQFSIKLKSLDLSHNRLSAIPESFFSQATGLEELMLNHNQLKIIEMHAFPPLLQKGNTICATAKTSCRLTLHANPFTCSCATSWFTEFLRSSPVDIPHLTTDVRCGFPESQKGVNVLSNDPRSCQEIFGNVSFLFTLLLTIAATCVPLLKHLYGWDLWYCFQILWAGHKGYTSLCENSMNNEHDAFVVFDTKNKAVSDWIYNEMLINLEGRGRWKLRLCLEERDWVPGLSCIENLHNAVHNSRKTVFVLTNQGGSASVNGVIKQAFLLVQQRLLDERVDVAIVVLLDPLFPKFKYLQMRKRLCKKSVLSWPKNPRAQPLFWNDLHIALASDNVRSYDKKVTESFLSNELL || **Prediction** | CCCHHHHHHHHHHHHHHHHCCCCCCCCCCCCCCCCCCSSSSSCCCCCCCCCCCCCCCCCSSSCCCCCCCCCCHHHHCCCCCCCSSSCCCCCCCCCCCCCCCCCCCSSCCHHHHCCCCCCCSSSCCCCCCCCCCCCCCCCCSSSCCCCCCCCCCCCCCCCCCCSSSCCCCCCCCCCCCCCCCCCHHHHCCCCCCCSSSCCCCCCCCCCCCCCCCCCSSSCCCCCCCCCCHHHHHCCCCCCSSSCCCCCCCCCCCHHHHCCCCCCCCCCCCHHHHCCCCCCCCSSSCCCCCCCCCCHHHHHCCCCCCSSSCCCCCCCCCCCCHHHHCCCCCCCSSSCCCCCCCCCCCCHHCCCCCHHHCCCCCCCCCCCCCCCCCCCCCHHHHCCCCCCCSSSCCCCCCCCCCCCHHCCCCCCCSSSCCCCCCCCCCCCCCCCCCCCCHHHCCCCCCCCCCCCCCCCCCCCCCSSSCCCCCCCCCCCCCCCCCCCCCCCCSSSCCCCCCCCCCHHHHCCCCCCCSSSCCCCCCCCCCCHHHHCCCCCCCSSSCCCCCCCCCCHHHHHCCCCCCSSSSCCCCSSCCCCCCCCCCCHHHCCCCCCCSSSCCCCCCCCCCCHHHCCCCHHHHHHHCCCCCCCCCCCCCCCCHHHCCCCCCCSSSCCCCCCCCCCCHHHHCCCCCCCSSSCCCCCCCCCCHHHHCCCCCCCSSSCCCCCCCCCCHHHHCCCCCCCSSSCCCCCCCCCCHHHHCCCCCCCSSSCCCCCCCCCCHHHHHHHHHCCCHHHHHCCCCCCCCCCCCCCCCCCCHHHHHHHHHCCCCCCCCCHHHSSSCCCHHCCCCSSSCCCCCCCCCCCHHHHHHHHHHHHHHHHHHHHHHHHHHHHHHHHHHHHHHHHCCCCCCCCCCCCCCCCSSSSSSCCCCCHHHHHHHHHHHHHHCCCCCCSSSSSSCCCCCCCCCHHHHHHHHHHHCCCSSSSSCCHHHCCCCCHHHHHHHHHHHHHHHHHCCCSSSSSSSCCCCCCCCCHHHHHHHCCCCSSSCCCCCCCCHHHHHHHHHHHCCCCCCCCCCCHHCCHHHHCCC || **Conf.Score** | 94579999999999997514699776878676768985998409998836998778876878968969997967716899998898948978875442114455403386774689999989897998875567667899877888988887765677678987898983788875212535988865899999898989978866810158989898989867985814450577887877877755566650644267788615577134317777799898958737786915762699999897808968770587577445888888989788776656500024567533148865562677666777889356447876567888888568878311027898898989587348777663232445400021476765667785323444444114356666777775423233567642898978833886966755777676798988736871483661688888589791785586173566537333479955784313666421235212316777668889797148757155404305554310344422345557867221068887898989898468868166615576885898979967876956746788887798989968997936654888799789979978988978875799999898989978998844400134212211110245531135458623551007789886237642378666505447510069721004730014410234425999999999999999998999999999999998646755343444576787998627885457899999499883679997258874146867715999999999978868999873786486434999999999999986489749999937665332569999982799789799987544799999999718898666672210011341149 |
|  | H:Helix; S:Strand; C:Coil ||

| |  | | --- | | Predicted Solvent Accessibility |   |  |  | | --- | --- | | **Sequence** | 20                  40                  60                  80                 100                 120                 140                 160                 180                 200                 220                 240                 260                 280                 300                 320                 340                 360                 380                 400                 420                 440                 460                 480                 500                 520                 540                 560                 580                 600                 620                 640                 660                 680                 700                 720                 740                 760                 780                 800                 820                 840                 860                 880                 900                 920                 940                 960                 980                1000                1020                1040                1060                    |                   |                   |                   |                   |                   |                   |                   |                   |                   |                   |                   |                   |                   |                   |                   |                   |                   |                   |                   |                   |                   |                   |                   |                   |                   |                   |                   |                   |                   |                   |                   |                   |                   |                   |                   |                   |                   |                   |                   |                   |                   |                   |                   |                   |                   |                   |                   |                   |                   |                   |                   |                   |  MFLPGLYLFFALDLIKLVKTMNPIFYPCDNNTISNGTIHVDCKRRNLTSIPKFTSLSISSLNLDENHVHQVKNDDFSNIPNLQHLSLMWNCLPGRLKALKLPSCQVLIDHDAFVNLKCLNSLQLAGNSLKTIPLLPQNLEVLGLEFNNILRIEQPLGTPFLKQLLLAKNCYYANPCNESFFIDQRVFQGLPKLQNLTLGFNNMSSIPTQLPPSLKSLDLGENRITEIQEGSFTNLTKLFSLNLNWNCQRCDNAAHPCFPCPNNASLGLPNQAFSEQRDSLVILYLRGNSIRSIPKNLFAQLRKLKVLDLSDNFLAYSIRNGTFYEELQGVEFLRLLNNYQPLKTFSELILSPSISKMRSLRKLYLSGLFFHQLSNYSLMHLSNLPQLEYLDLRMNFMNYCNVTAFKRLRSLRTVVLSQNMLTINPYYTSSANEARRLDRYQMDDHLDLQDMPAFESELQMNYLHCTEKECPKSYSMENFQKFFCKKELYFDLSQNNIPWLNAYTFKGMEKTVCLDLSYNYMSQALNGQQFLPLTKLVFLNMAYNRIDLYFNEAFQEIRSTLKVLDLSNNEFHFQMKQIGHRLDFIKNLTSLEVLSLANNKIGLRISNTLYSASLKCLLFSGNHLDIMWDTKGDQYIRFFQGLTNLTYLDISDNQLKSCSPAAIINLPPSIRVLRMDSNFLDYFPWVNISVLSQLCYLNLSGNFLFNLPDTAIQFSIKLKSLDLSHNRLSAIPESFFSQATGLEELMLNHNQLKIIEMHAFPPLLQKGNTICATAKTSCRLTLHANPFTCSCATSWFTEFLRSSPVDIPHLTTDVRCGFPESQKGVNVLSNDPRSCQEIFGNVSFLFTLLLTIAATCVPLLKHLYGWDLWYCFQILWAGHKGYTSLCENSMNNEHDAFVVFDTKNKAVSDWIYNEMLINLEGRGRWKLRLCLEERDWVPGLSCIENLHNAVHNSRKTVFVLTNQGGSASVNGVIKQAFLLVQQRLLDERVDVAIVVLLDPLFPKFKYLQMRKRLCKKSVLSWPKNPRAQPLFWNDLHIALASDNVRSYDKKVTESFLSNELL | | **Prediction** | 33100010000000010021311100003133234201200223132433253024424342313133430334034313423141012020333043231331312024300331464323122412012013104302201012020330344220410220101301023032331330344004304333302433020230266105534353024030440345104303301101012020323303211030330321303441034204303312244030330243004303433322114030333034431043033022010120313232033012312033033022010030113303413141023033212012130203303341033031242323230202223332221230230230313221323323302311202202023031341030430122203301101013030430343104203301100002010112034310230330110101302033033300430221022010120101110330012031032031001010020201110332020320120101301031023434321130033033032010030203303341012003203322033020330234104304413312124030330133004303301101012020220143004304302201013020330344104302333321232133202010330101010203102400332213023113201012133224220230234303200000000000011220100000111100000000000000021234246456522000000000303433300242013303644333000000200030022000000200220000000003200201222001000100001003442200000001403644212301200342000203544554610242012004364433444423422344325 |||  |  | | --- | --- | |  | Values range from **0** (buried residue) to **9** (highly exposed residue) || |
| |  | | --- | | Predicted normalized B-factor |    (B-factor is a value to indicate the extent of the inherent thermal mobility of residues/atoms in proteins. In I-TASSER, this value is deduced from threading template proteins from the PDB in combination with the sequence profiles derived from sequence databases. The reported B-factor profile in the figure below corresponds to the normalized B-factor of the target protein, defined by B=(B'-u)/s, where B' is the raw B-factor value, u and s are respectively the mean and standard deviation of the raw B-factors along the sequence. Click here to read more about predicted normalized B-factor)     |  | | --- | |  | |
| |  | | --- | | Top 10 threading templates used by I-TASSER |    (I-TASSER modeling starts from the structure templates identified by LOMETS from the PDB library. LOMETS is a meta-server threading approach containing multiple threading programs, where each threading program can generate tens of thousands of template alignments. I-TASSER only uses the templates of the highest significance in the threading alignments, the significance of which are measured by the Z-score, i.e. the difference between the raw and average scores in the unit of standard deviation. The templates in this section are the 10 best templates selected from the LOMETS threading programs. Usually, one template of the highest Z-score is selected from each threading program, where the threading programs are sorted by the average performance in the large-scale benchmark test experiments.)     |  |  |  |  |  |  |  |  |  | | --- | --- | --- | --- | --- | --- | --- | --- | --- | | Rank | PDB Hit | Iden1 | Iden2 | Cov | Norm. Z-score | Download Align. |  | 20                  40                  60                  80                 100                 120                 140                 160                 180                 200                 220                 240                 260                 280                 300                 320                 340                 360                 380                 400                 420                 440                 460                 480                 500                 520                 540                 560                 580                 600                 620                 640                 660                 680                 700                 720                 740                 760                 780                 800                 820                 840                 860                 880                 900                 920                 940                 960                 980                1000                1020                1040                1060                    |                   |                   |                   |                   |                   |                   |                   |                   |                   |                   |                   |                   |                   |                   |                   |                   |                   |                   |                   |                   |                   |                   |                   |                   |                   |                   |                   |                   |                   |                   |                   |                   |                   |                   |                   |                   |                   |                   |                   |                   |                   |                   |                   |                   |                   |                   |                   |                   |                   |                   |                   |                   | | |  | | | | | | | Sec.Str Seq | CCCHHHHHHHHHHHHHHHHCCCCCCCCCCCCCCCCCCSSSSSCCCCCCCCCCCCCCCCCSSSCCCCCCCCCCHHHHCCCCCCCSSSCCCCCCCCCCCCCCCCCCCSSCCHHHHCCCCCCCSSSCCCCCCCCCCCCCCCCCSSSCCCCCCCCCCCCCCCCCCCSSSCCCCCCCCCCCCCCCCCCHHHHCCCCCCCSSSCCCCCCCCCCCCCCCCCCSSSCCCCCCCCCCHHHHHCCCCCCSSSCCCCCCCCCCCHHHHCCCCCCCCCCCCHHHHCCCCCCCCSSSCCCCCCCCCCHHHHHCCCCCCSSSCCCCCCCCCCCCHHHHCCCCCCCSSSCCCCCCCCCCCCHHCCCCCHHHCCCCCCCCCCCCCCCCCCCCCHHHHCCCCCCCSSSCCCCCCCCCCCCHHCCCCCCCSSSCCCCCCCCCCCCCCCCCCCCCHHHCCCCCCCCCCCCCCCCCCCCCCSSSCCCCCCCCCCCCCCCCCCCCCCCCSSSCCCCCCCCCCHHHHCCCCCCCSSSCCCCCCCCCCCHHHHCCCCCCCSSSCCCCCCCCCCHHHHHCCCCCCSSSSCCCCSSCCCCCCCCCCCHHHCCCCCCCSSSCCCCCCCCCCCHHHCCCCHHHHHHHCCCCCCCCCCCCCCCCHHHCCCCCCCSSSCCCCCCCCCCCHHHHCCCCCCCSSSCCCCCCCCCCHHHHCCCCCCCSSSCCCCCCCCCCHHHHCCCCCCCSSSCCCCCCCCCCHHHHCCCCCCCSSSCCCCCCCCCCHHHHHHHHHCCCHHHHHCCCCCCCCCCCCCCCCCCCHHHHHHHHHCCCCCCCCCHHHSSSCCCHHCCCCSSSCCCCCCCCCCCHHHHHHHHHHHHHHHHHHHHHHHHHHHHHHHHHHHHHHHHCCCCCCCCCCCCCCCCSSSSSSCCCCCHHHHHHHHHHHHHHCCCCCCSSSSSSCCCCCCCCCHHHHHHHHHHHCCCSSSSSCCHHHCCCCCHHHHHHHHHHHHHHHHHCCCSSSSSSSCCCCCCCCCHHHHHHHCCCCSSSCCCCCCCCHHHHHHHHHHHCCCCCCCCCCCHHCCHHHHCCC MFLPGLYLFFALDLIKLVKTMNPIFYPCDNNTISNGTIHVDCKRRNLTSIPKFTSLSISSLNLDENHVHQVKNDDFSNIPNLQHLSLMWNCLPGRLKALKLPSCQVLIDHDAFVNLKCLNSLQLAGNSLKTIPLLPQNLEVLGLEFNNILRIEQPLGTPFLKQLLLAKNCYYANPCNESFFIDQRVFQGLPKLQNLTLGFNNMSSIPTQLPPSLKSLDLGENRITEIQEGSFTNLTKLFSLNLNWNCQRCDNAAHPCFPCPNNASLGLPNQAFSEQRDSLVILYLRGNSIRSIPKNLFAQLRKLKVLDLSDNFLAYSIRNGTFYEELQGVEFLRLLNNYQPLKTFSELILSPSISKMRSLRKLYLSGLFFHQLSNYSLMHLSNLPQLEYLDLRMNFMNYCNVTAFKRLRSLRTVVLSQNMLTINPYYTSSANEARRLDRYQMDDHLDLQDMPAFESELQMNYLHCTEKECPKSYSMENFQKFFCKKELYFDLSQNNIPWLNAYTFKGMEKTVCLDLSYNYMSQALNGQQFLPLTKLVFLNMAYNRIDLYFNEAFQEIRSTLKVLDLSNNEFHFQMKQIGHRLDFIKNLTSLEVLSLANNKIGLRISNTLYSASLKCLLFSGNHLDIMWDTKGDQYIRFFQGLTNLTYLDISDNQLKSCSPAAIINLPPSIRVLRMDSNFLDYFPWVNISVLSQLCYLNLSGNFLFNLPDTAIQFSIKLKSLDLSHNRLSAIPESFFSQATGLEELMLNHNQLKIIEMHAFPPLLQKGNTICATAKTSCRLTLHANPFTCSCATSWFTEFLRSSPVDIPHLTTDVRCGFPESQKGVNVLSNDPRSCQEIFGNVSFLFTLLLTIAATCVPLLKHLYGWDLWYCFQILWAGHKGYTSLCENSMNNEHDAFVVFDTKNKAVSDWIYNEMLINLEGRGRWKLRLCLEERDWVPGLSCIENLHNAVHNSRKTVFVLTNQGGSASVNGVIKQAFLLVQQRLLDERVDVAIVVLLDPLFPKFKYLQMRKRLCKKSVLSWPKNPRAQPLFWNDLHIALASDNVRSYDKKVTESFLSNELL | | 1 | 5gmfA | 0.36 | 0.27 | 0.73 | 3.64 | Download |  | ---------------SPWARWFPKTLPCDVLDVSKNHVIVDCTDKHLTEIPGGIPTNTTNLTLTINHIPDISPASFHRLVHLVEIDFRCNCVPIRLGSKNMCPRRLQIKPRSFSGLTYLKSLYLDGNQLLEIPQGPPSLQLLSLEANNIFSIRKEQELANIEILYLGQNCYYRNPCYVSYSIEKDAFLNLTKLKVLSLKDNNVTTVPTVLPSTLTELYLYNNMIAEIQEDDFNNLNQLQILDLSGNCPRCYNAPFPCTPCKNNSPLQIPVNAF-DALTELKVLRLHSNSLQHVPPRWFKNINNLQELDLSQNFLAKEIGDAKFLHFLPNLIQLDLSFNFELQVYRASMNLSQAFSSLKSLKILRIRGYVFKELKSFQLSPLHNLQNLEVLDLGTNFIKIANLSMFKQFKRLKVIDLSVNKISPVLEQLYY-----------FRYDKYARSCRF---------------------------KSCYKYGQTLDLSKNSIFFIKSSDFQHLSFLKCLNLSGNLISQTLNGSEFQPLAELRYLDFSNNRLDLLHSTAFEELRK-LEVLDISSNSHYFQSEGITHMLNFTKNLKVLQKLMMNDNDISSSTSRTMESESLRTLEFRGNHLDVLWRDGDNRYLQLFKNLLKLEELDISKNSLSFLPSGVFDGMPPNLKNLSLAKNGLKSFIWEKLRYLKNLETLDLSHNQLTTVPERLSNCSRSLKNLILKNNQIRSLTKYFLQDAFQLRYLDLSSNKIQMIQKTSFPENVLNN---------LKMLLLHHNRFLCTCDAVWFVWWVQHTEVTIPYLATDVTCVGPGAHKGQSVISLDLYTC---------------------------------------------------------------------------------------------------------------------------------------------------------------------------------------------------------------------------------- | | 2 | 3j0aA | 0.23 | 0.21 | 0.72 | 4.92 | Download |  | ---------------------------------SFDGRIAFYRFCNLTQVPQVL-NTTERLLLSFNYIRTVTASSFPFLEQLQLLELGSQYTPL------------TIDKEAFRNLPNLRILDLGSSKIYFLHQGLFHLFELRLYFCGLSDAVYFRNLKALTRLDLSKNQIRSLYLH-------PSFGKLNSLKSIDFSSNQIFLVCEHELKTLSFFSLAANSLYSRVSVDWGKCMVLEILDVSGNGWTVDITGNFSNAISKSQAFSLI------LAHHIMGAGFGFHNIKDPDQNTFAGLAR------------------------SSVRHLDLSHG---------------------------------FVFSLNSRVFETLKDLKVLNLAYNKINKIADEAFYGLDNLQVLNLSYNLLGELYSSNFY----------------------------------------------------GLPKVAYIDLQKNHIAIIQDQTFKFLEKLQTLDLRDNALTT------IHFIPSIPDIFLSGNKLVTLPK-----------------------------------INLTANLIHLSENRLENLDILYF-----------------------------LLRVPHLQILILNQNRFSSCSGDQTPSENPSLEQLFLGENMLQELCWDVFEGLSHLQVLYLNHNYLNSLPPGVFSHLTALRGLSLNSNRLTVLSHNDL--PANLEILDISRNQLLAPNPDVFVSLSVL--------------DITHNKFICECELSTFINWLNHTNVTIAGPPADIYCVYPDSFSGVSLFSLSTEGC---------IVCTVTLTLFLMTILTVTKFR----------------DHPQGTEPDMYKYDAYLCFSSKD---FTWVQNALLKHLDTQDQNRFNLCFEERDFVPGENRIANIQDAIWNSRKIVCLVSRHFLRDGW---CLEAFSYAQGRCLSDLNSALIMVVVGSLSQYQKHQSIRGFVQKQQYLRWPEDFQDVGWFLHKLSQQILKKEKEKKK------------- | | 3 | 5gmfA | 0.35 | 0.27 | 0.73 | 4.54 | Download |  | ---------------SPWARWFPKTLPCDVTLDVKNHVIVDCTDKHLTEIPGGIPTNTTNLTLTINHIPDISPASFHRLVHLVEIDFRCNCVPIRLGSKSNMCRRLQIKPRSFSGLTYLKSLYLDGNQLLEIPQGPPSLQLLSLEANNIFSIRKLTELANIEILYLGQNCYYRNPCYVSYSIEKDAFLNLTKLKVLSLKDNNVTTVPTVLPSTLTELYLYNNMIAEIQEDDFNNLNQLQILDLSGNCPRCYNAPFPCTPCKNNSPLQIPVNAF-DALTELKVLRLHSNSLQHVPPRWFKNINNLQELDLSQNFLAKEIGDAKFLHFLPNLIQLDLSFNFELQVYRASMNLSQAFSSLKSLKILRIRGYVFKELKSFQLSPLHNLQNLEVLDLGTNFIKIANLSMFKQFKRLKVIDLSVNKISPVLEQLYYFRY--------DKYARSCRFKSCYKYGQ------------------------------TLDLSKNSIFFIKSSDFQHLSFLKCLNLSGNLISQTLNGSEFQPLAELRYLDFSNNRLDLLHSTAFEELRK-LEVLDISSNSHYFQSEGITHMLNFTKNLKVLQKLMMNDNDISSSTSRTMESESLRTLEFRGNHLDVLWRDGDNRYLQLFKNLLKLEELDISKNSLSFLPSGVFDGMPPNLKNLSLAKNGLKSFIWEKLRYLKNLETLDLSHNQLTTVPERLSNCSRSLKNLILKNNQIRSLTKYFLQDAFQLRYLDLSSNKIQMIQKTSFPE---------NVLNNLKMLLLHHNRFLCTCDAVWFVWWVQHTEVTIPYLATDVTCVGPGAHKGQSVISLDLYTC---------------------------------------------------------------------------------------------------------------------------------------------------------------------------------------------------------------------------------- | | 4 | 3j0a | 0.24 | 0.21 | 0.73 | 2.21 | Download |  | --------------------------SCSFD-----GRIAFYRFCNLTQVPQ-VLNTTERLLLSFNYIRTVTASSFPFLEQLQLLELGSQYT-------------PTIDKEAFRNLPNLRILDLGSSKIYFLHPD------------------AFQGLFHLFELRLYFCGLSD------AVLKDGYFRNLKALTRLDLSKNQIRSLYLHPLNSLKSIDFSSNQIFLVCEHELEPLKTLSFFSLAAN------------------------------------------SLYSVDWGCMNPFRNMEILDVSGNGWTVDIT-GNFSNAISKSQAF-------------------SLILAHHIMGAGFGFHNIKDPDQNTFA---GLSSVRHLDLSHGFVFSLNSRVFETLKDLKVLNLAYNKINKIADEAFYG----------------------------------------------------LDNLQVLNLSYNLLGELYSSNFYGLPKVAYIDLQKNHIAII-QDQTFKFLEKLQTLDLRDNALTTIH--FI---P-SIPDIFLSGNK------------------------------LVTLP--KINLTAN-LIHLSENRLENLDI-----LYFLL-RVPHLQILILNQNRFSSSGDQTPSEN-PSLEQLFLGENMLQLAWEDVFEGLSHLQVLYLNHNYLNSLPPGVFSHLTALRGLSLNSNRLTVLSHNDLP--ANLEILDISRNQLLAPNPDVFV-------S-------LSVLDITHNKFICECELSTFINWLNHTNVTIAGPPADIYCVYPDSFSGVSLFSLSTEGCF-----SLFIVCTVTLTLFLMTILTVTKFRD------H------P-QGT--E-PDMYKYDAYLCFSSKDF---TWVQNALLKHLDTQYQNRFNLCFEERDFVPGENRIANIQDAIWNSRKIVCLVSRHF---LRDGWC-LEFSYAQGRCLSDLNSALIMVVVGSLSQYQLMQSIRGFVQKQQYLRWPEDFQDVGWFLHKLSQQILKKEKEKKK------------- | | 5 | 3j0a | 0.23 | 0.21 | 0.72 | 1.59 | Download |  | --------------------------SCSF-----DGRIAFYRFCNLTQVPQ-VLNTTERLLLSFNYIRTVTASSFPFLEQLQLLELGSQYTPL------------TIDKEAFRNLPNLRILDLGSSKIYFLHPDLFHLFELRLYFCGLSDLKDFRNLKALTRLDLSKNQIRS--------LYLHPFGKLNSLKSIDFSSNQIFLVCEHELKTLSFFSLAANSLYSVDWGCMNPFRNMEILDVSGN------------------------------------------------------------------GWTVDI-TGNFSNAISKSQAFS----------------------LILAHHIMGAGFGFHNIKDPDQNTFAGLSSVRHLDLSHGFVFSLNSRVFETLKDLKVLNLAYNKINKIADEAF----------------------------------------------------YGLDNLQVLNLSYNLLGELYSSNFYGLPKVAYIDLQKNHIAI-IQDQTFKFLEKLQTLDLRDNALTTIH--FI----PSIPDIFLSGNKLVTLPK---------INLTAN-LIHLSENRLENLD----------------------------ILYFLL-RVPHLQILILNQNRFSSSGDQTP-SENPSLEQLFLGENMLQLAWEDVFEGLSHLQVLYLNHNYLNSLPPGVFSHLTALRGLSLNSNRLTVLSHNDLP--ANLEILDISRNQLLAPNPDVF--------------VSLSVLDITHNKFICECELSTFINWLNHTNVTIAGPPADIYCVYPDSFSGVSLFSLSTEGCF-----SLFIVCTVTLTLFLMTILTVTKFRD------------HP---QGTE-PDMYKYDAYLCFSSKDF---TWVQNALLKHLDTQYQNRFNLCFEERDFVPGENRIANIQDAIWNSRKIVCLVSRHFLRDG---WC-LEFSYAQGRCLSDLNSALIMVVVGSLSQYQKHQSIRGFVQKQQYLRWPEDFQDVGWFLHKLSQQILKKEKEKKK------------- | | 6 | 3j0a | 0.23 | 0.21 | 0.72 | 2.52 | Download |  | --------------------------SCSFD-----GRIAFYRFCNLTQVPQ-VLNTTERLLLSFNYIRTVTASSFPFLEQLQLLELGSQYTP------------LTIDKEAFRNLPNLRILDLGSSKIYFLHPDLFHLFELRLYFCGLSDLKDFRNLKALTRLDLSKNQIR--------SLYLHPFGKLNSLKSIDFSSNQIFLVCEHELKTLSFFSLAANSLYSVDWGCMNPFRNMEILDVSGNGWTVDITGNFSNAISKSQ---AFS--L-ILAHHIMGAGFGFHNIKDPDQNTFAGLA---------------------------------------------------------------------------------RSSVRHLDLSHGFVFSLNSRVFETLKDLKVLNLAYNKINKIADEAF--YGLD--------------------------------------------------NLQVLNLSYNLLGELYSSNFYG-------------------------LPKVAYIDLQKNHIAIIQDQTFKFL-EKLQTLDLRDNALTTIH-----------FIPSIPDIFLSGNKLVTLPK--INLTAN-LIHLSENRLENL-----DILYFLL-RVPHLQILILNQNRFSSSGDQTPSE-NPSLEQLFLGENMLQLAWEDVFEGLSHLQVLYLNHNYLNSLPPGVFSHLTALRGLSLNSNRLTVLSHNDLP--ANLEILDISRNQLLAPNPDVF--------------VSLSVLDITHNKFICECELSTFINWLNHTNVTIAGPPADIYCVYPDSFSGVSLFSLSTEGCF-----SLFIVCTVTLTLFLMTILTVTKFRD---------------HPQGTE-PDMYKYDAYLCFSSKDF---TWVQNALLKHLDTQYQNRFNLCFEERDFVPGENRIANIQDAIWNSRKIVCLVSRHFLRDG---WC-LEFSYAQGRCLSDLNSALIMVVVGSLSQYMKHQSIRGFVQKQQYLRWPEDFQDVGWFLHKLSQQILKKEKEK--------------- | | 7 | 5gmfA | 0.36 | 0.27 | 0.72 | 5.98 | Download |  | ---------------------FPKTLPCDVTLDVKNHVIVDCTDKHLTEIPGGIPTNTTNLTLTINHIPDISPASFHRLVHLVEIDFRCNCVPIRLGSKSNMCRRLQIKPRSFSGLTYLKSLYLDGNQLLEIPQGLPSLQLLSLEANNIFSIRKEQELANIEILYLGQNCYYRNPCYVSYSIEKDAFLNLTKLKVLSLKDNNVTTVPTVLPSTLTELYLYNNMIAEIQEDDFNNLNQLQILDLSGNCPRCYNAPFPCTPCKNNSPLQIPVNAFDA-LTELKVLRLHSNSLQHVPPRWFKNINNLQELDLSQNFLAKEIGDAKFLHFLPNLIQLDLSFNFELQVYRASMNLSQAFSSLKSLKILRIRGYVFKELKSFQLSPLHNLQNLEVLDLGTNFIKIANLSMFKQFKRLKVIDLSVNKISPVLEQLYYFRYDKY--------------------------------------ARSCRFKSCYKYGQTLDLSKNSIFFIKSSDFQHLSFLKCLNLSGNLISQTLNGSEFQPLAELRYLDFSNNRLDLLHSTAFEELRK-LEVLDISSNSHYFQSEGITHMLNFTKNLKVLQKLMMNDNDISSSTSRTMESESLRTLEFRGNHLDVLWRDGDNRYLQLFKNLLKLEELDISKNSLSFLPSGVFDGMPPNLKNLSLAKNGLKSFIWEKLRYLKNLETLDLSHNQLTTVPERLSNCSRSLKNLILKNNQIRSLTKYFLQDAFQLRYLDLSSNKIQMIQKTSFP---------ENVLNNLKMLLLHHNRFLCTCDAVWFVWWVQHTEVTIPYLATDVTCVGPGAHKGQSVISLDLYTC---------------------------------------------------------------------------------------------------------------------------------------------------------------------------------------------------------------------------------- | | 8 | 3j0aA | 0.25 | 0.21 | 0.71 | 21.06 | Download |  | -------------------------------CSF-DGRIAFYRFCNLTQVPQVLN-TTERLLLSFNYIRTVTASSFPFLEQLQLLELGSQYTL-------------TIDKEAFRNLPNLRILDLGSSKIYFLHP------------------DAFQGLFHLFELRLYFCGLSD------AVLKDGYFRNLKALTRLDLSKNQIRSLYLPSFNSLKSIDFSSNQIFLVCEHELEPL------------------------------------------QTLSFFSLAANSLYSRVSVDWGKCMVLEILDVSGNGWTV--------------------------------DITGNFSNAISIMGAGFGFHNIKDPDQ---NTFAGLSSVRHLDLSHGFVFSLNSRVF----------------------------------------------------------------------------ETLKDLKVLNLAYNKINKIADEAFYGLDNLQVLNLSYNLLGE-LYSSNFYGLPKVAYIDLQKNHIAIIQDQTFKFLE-KLQTLDLRDNALTTIH-----------FIPSIPDIFLSGNKLVTLPK---INLTANLIHLSENRLENLIL-------YFLLRVPHLQILILNQNRFSSCSGQTPSEN-PSLEQLFLGENMLQLAWWDVFEGLSHLQVLYLNHNYLNSLPPGVFSHLTALRGLSLNSNRLTVLSHNDLPA--NLEILDISRNQLLAPNPDVF--------------VSLSVLDITHNKFICECELSTFINWLNHTNVTIAGPPADIYCVYPDSFSGVSLFSLSTEGCFSL------------------FIVCTVTLTLFLMTILTVTKFRDHPQGTEPD---MYKYDAYLCFSSKD---FTWVQNALLKHLDTQDQNRFNLCFEERDFVPGENRIANIQDAIWNSRKIVCLVSRHFLR---DGWCLEAFSYAQGRCLSDLNSALIMVVVGSLSQLMKHQSIRGFVQKQQYLRWPEDFQDVGWFLHKLSQQILKKEKEK--------------- | | 9 | 3wpcA | 0.37 | 0.27 | 0.69 | 12.25 | Download |  | -------------------T-LPPFLPCQPHG------LVNCNWLFLKSVPHFSAANVTSLSLLSNRIHHLHDSDFAQLSNLQKLNLKWNCPPAGLSPMH-FPCHMTIEPNTFLAVPTLEELNLSYNGITTVPALPSSLVSLILSRTNILDPTSLTGLHALRFLYMDGNCYYKNPCGRALEVAPGALLGLGNLTHLSLKYNNLTTVPRSLPPSLEYLLLSYNHIVTLAPEDLANLTALRVLDVGGNCRRCDHARNPCVECPHKDTFS--------HLSRLEGLVLKDSSLYQLNPRWFRGLGNLTVLDLSENFLYDCITKTKAFQGLAQLRRLNLSFNYHKKVSFAHLTLAPSFGSLLSLQELDMHGIFFRSLSQKTLQPLARLPMLQRLYLQMNFINQAQLGIFKDFPGLRYIDLSDNRISGAVEEDFMPS---------------------------------------------------CKNLSTLDLSRNNLVTVQPEMFAQLSRLQCLRLSHNSISQAVNGSQFVPLTSLQVLDLSHNKLDLYHGRSFTELPR-LEALDLSYNSQPFSMRGVGHNLSFVAQLPTLRYLSLAHNGIHSRVSQQLCSTSLWALDFSGNSLSQMWAE-GDLYLRFFQGLRSLIRLDLSQNRLHTLLPCTLGNLPKSLQLLRLRNNYLAFFNWSSLTLLPNLETLDLAGNQLKALSNGSLPSGTQLQRLDVSRNSIIFVVPGFFALATRLRELNLSANALRTVEPSWFG-------------FLAGSLEVSANPLHCACA--AFVDFLLQVQAAVPGLPSRVKCGSPGQLQGRSIFAQDL------------------------------------------------------------------------------------------------------------------------------------------------------------------------------------------------------------------------------------- | | 10 | 3j0aA | 0.26 | 0.15 | 0.32 | 2.17 | Download |  | -------------------------------------------------------------------------------------------------------------------------------------------------------------------------------------------------------------------------------------------------------------------------------------------------------------------------------------------------------------------------------------------------------------------------------------------------------------------------------------------------------------------------------------------------------------------------------------------------------------------------------------------------------------------------------------------PSLEQLFLGENMLQLAWEDVFEGLSHLQVLYLNHNYLNSLPPGVFSHLTALRGLSLNSNRLTVLSHNDLP--ANLEILDISRNQLLAPNPDVF--------------VSLSVLDITHNKFICECELSTFINWLNHTNVTIAGPPADIYCVYPDSFSGVSLFSLSTEGCF-----SLFIVCTVTLTLFLMTILTVTKFRD-------------H--PQGTE-PDMYKYDAYLCFSSKDF---TWVQNALLKHLDTQYQNRFNLCFEERDFVPGENRIANIQDAIWNSRKIVCLVSRHFLRDG---WC-LEFSYAQGRCLSDLNSALIMVVVGSLSQYMKHQSIRGFVQKQQYLRWPEDFQDVGWFLHKLSQQILKKEKEK--------------- | | |  |  | | --- | --- | | (a) | All the residues are colored in black; however, those residues in template which are identical to the residue in the query sequence are highlighted in color. Coloring scheme is based on the property of amino acids, where polar are brightly coloured while non-polar residues are colored in dark shade. (more about the colors used) | | (b) | Rank of templates represents the top ten threading templates used by I-TASSER. | | (c) | Ident1 is the percentage sequence identity of the templates in the threading aligned region with the query sequence. | | (d) | Ident2 is the percentage sequence identity of the whole template chains with query sequence. | | (e) | Cov represents the coverage of the threading alignment and is equal to the number of aligned residues divided by the length of query protein. | | (f) | Norm. Z-score is the normalized Z-score of the threading alignments. Alignment with a Normalized Z-score >1 mean a good alignment and vice versa. | | (g) | Download Align. provides the 3D structure of the aligned regions of the threading templates. | | (h) | The top 10 alignments reported above (in order of their ranking) are from the following threading programs: | |  | 1: MUSTER   2: FFAS-3D   3: SPARKS-X   4: HHSEARCH2   5: HHSEARCH I   6: HHSEARCH   7: Neff-PPAS   8: PRC   9: pGenTHREADER   10: HHSEARCH | | | | | | | | | | |
| |  | | --- | | Top 5 final models predicted by I-TASSER |    (For each target, I-TASSER simulations generate a large ensemble of structural conformations, called decoys. To select the final models, I-TASSER uses the SPICKER program to cluster all the decoys based on the pair-wise structure similarity, and reports up to five models which corresponds to the five largest structure clusters. The confidence of each model is quantitatively measured by C-score that is calculated based on the significance of threading template alignments and the convergence parameters of the structure assembly simulations. C-score is typically in the range of [-5, 2], where a C-score of a higher value signifies a model with a higher confidence and vice-versa. TM-score and RMSD are estimated based on C-score and protein length following the correlation observed between these qualities. Since the top 5 models are ranked by the cluster size, it is possible that the lower-rank models have a higher C-score in rare cases. Although the first model has a better quality in most cases, it is also possible that the lower-rank models have a better quality than the higher-rank models as seen in our benchmark tests. If the I-TASSER simulations converge, it is possible to have less than 5 clusters generated; this is usually an indication that the models have a good quality because of the converged simulations.)  - More about C-score- Local structure accuracy profile of the top five models   |  |  |  |  |  | | --- | --- | --- | --- | --- | | (By right-click on the images, you can export image file or change the configurations, e.g. modifying the background color or stopping the spin of your models) | | | | | |  |  |  |  |  | | - Download Model 1- C-score=-1.54 (Read more about C-score)- Estimated TM-score = 0.53±0.15- Estimated RMSD = 12.9±4.2Å | - Download Model 2- C-score = -1.69 | - Download Model 3- C-score = -1.13 | - Download Model 4- C-score = -1.76 | - Download Model 5- C-score = -2.60 | |
|  | 0.510 | 3.01 | 0.193 | 0.544 | Download |
|  | 3 | 2z64A | 0.498 | 3.48 | 0.177 | 0.543 | Download |
|  | 4 | 4z0cA | 0.496 | 4.42 | 0.171 | 0.567 | Download |
|  | 5 | 2a0zA | 0.483 | 5.29 | 0.148 | 0.580 | Download |
|  | 6 | 3wpbA | 0.470 | 5.74 | 0.108 | 0.578 | Download |
|  | 7 | 5gmfA | 0.464 | 5.91 | 0.103 | 0.576 | Download |
|  | 8 | 3ciyB | 0.461 | 5.02 | 0.146 | 0.542 | Download |
|  | 9 | 3rg1A | 0.457 | 4.57 | 0.162 | 0.521 | Download |
|  | 10 | 3w3gA | 0.453 | 6.34 | 0.122 | 0.581 | Download |
  


|  |
