# Supplementary figures and images for: Variation in selection constraints on teleost TLRs with emphasis on their repertoire in the Walking catfish, Clarias batrachus (part 3 of 3)
Source: Sci Rep. 2020 Dec 7;10:21394. doi: 10.1038/s41598-020-78347-6 (PMC7721727; doi:10.1038/s41598-020-78347-6)

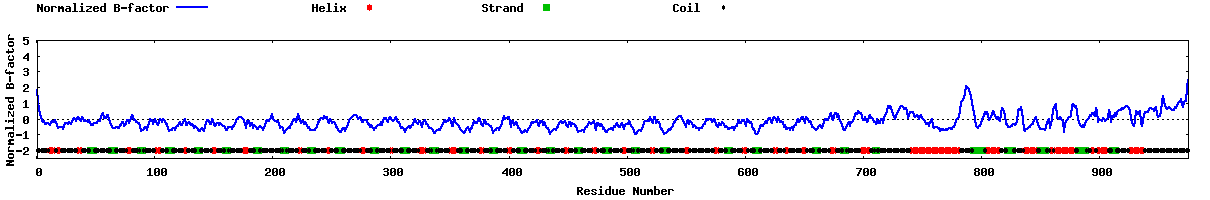

Supplement: Supplementary file 33 — Supplementary Information 33. [file 41598_2020_78347_MOESM33_ESM.zip › T21/struct/S511005_results/BFP.png]

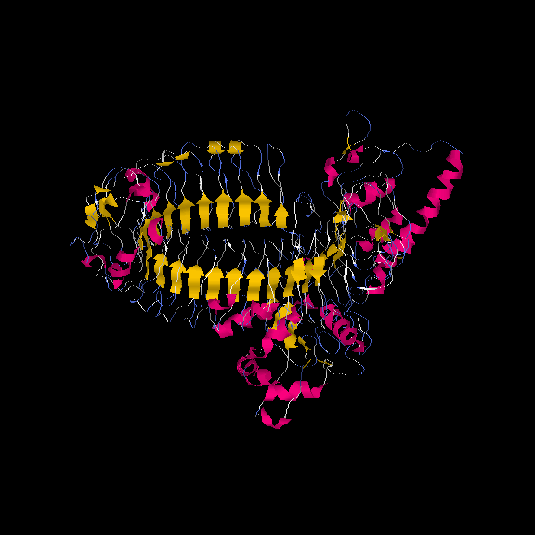

Supplement: Supplementary file 33 — Supplementary Information 33. [file 41598_2020_78347_MOESM33_ESM.zip › T21/struct/S511005_results/model1.gif]

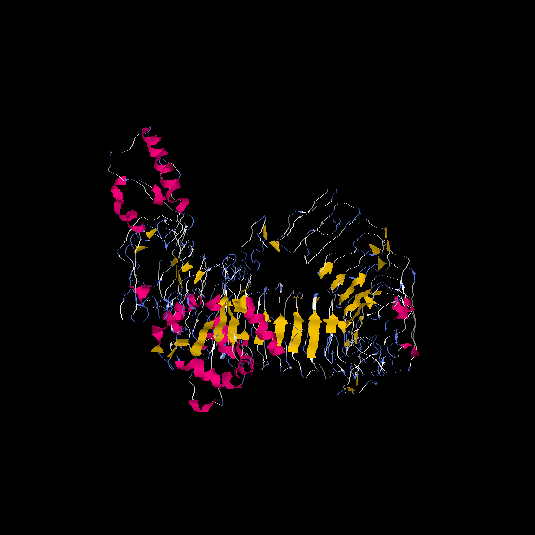

Supplement: Supplementary file 33 — Supplementary Information 33. [file 41598_2020_78347_MOESM33_ESM.zip › T21/struct/S511005_results/model2.gif]

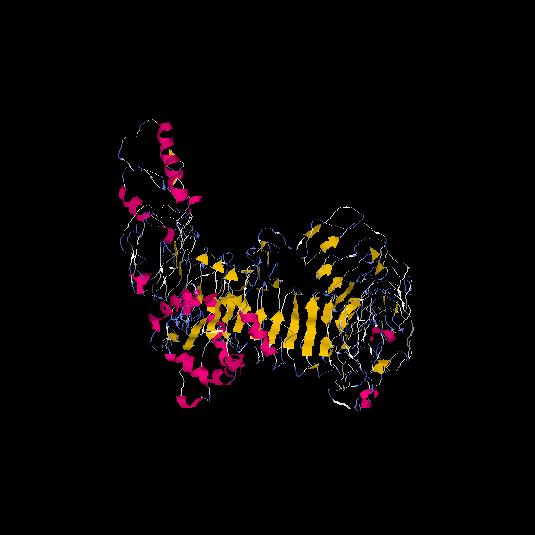

Supplement: Supplementary file 33 — Supplementary Information 33. [file 41598_2020_78347_MOESM33_ESM.zip › T21/struct/S511005_results/model3.gif]

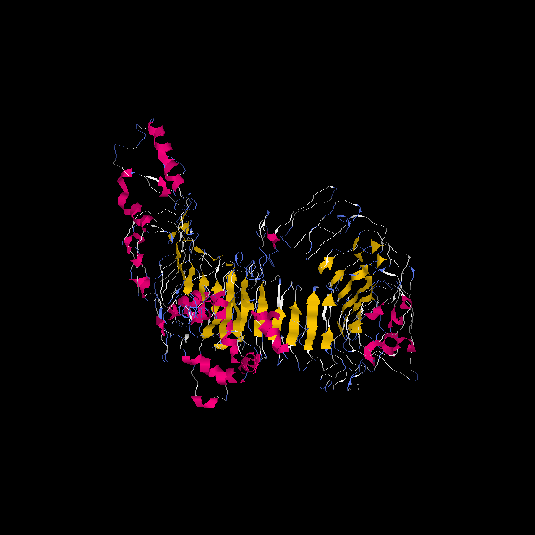

Supplement: Supplementary file 33 — Supplementary Information 33. [file 41598_2020_78347_MOESM33_ESM.zip › T21/struct/S511005_results/model4.gif]

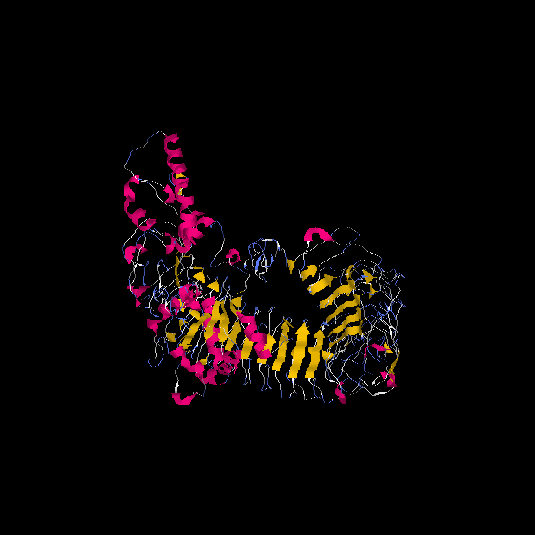

Supplement: Supplementary file 33 — Supplementary Information 33. [file 41598_2020_78347_MOESM33_ESM.zip › T21/struct/S511005_results/model5.gif]

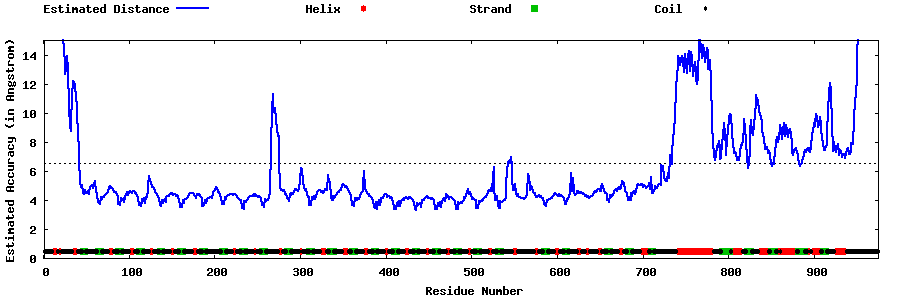

Supplement: Supplementary file 33 — Supplementary Information 33. [file 41598_2020_78347_MOESM33_ESM.zip › T21/struct/S511005_results/RSQ_1.png]

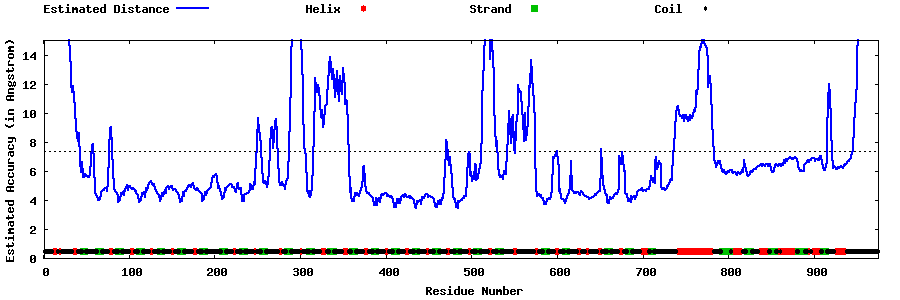

Supplement: Supplementary file 33 — Supplementary Information 33. [file 41598_2020_78347_MOESM33_ESM.zip › T21/struct/S511005_results/RSQ_2.png]

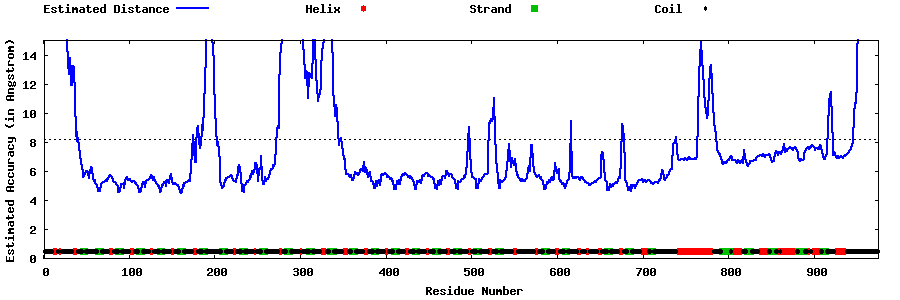

Supplement: Supplementary file 33 — Supplementary Information 33. [file 41598_2020_78347_MOESM33_ESM.zip › T21/struct/S511005_results/RSQ_3.png]

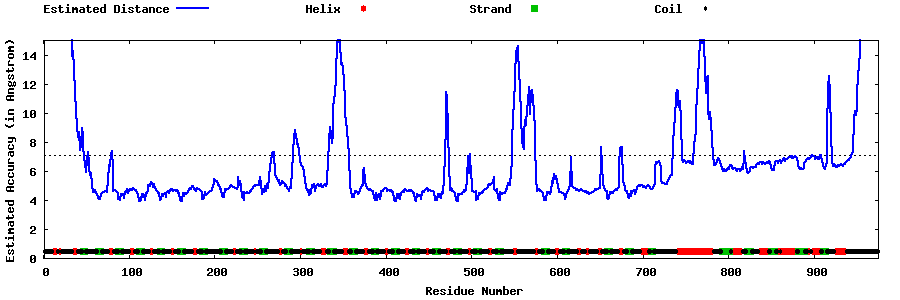

Supplement: Supplementary file 33 — Supplementary Information 33. [file 41598_2020_78347_MOESM33_ESM.zip › T21/struct/S511005_results/RSQ_4.png]

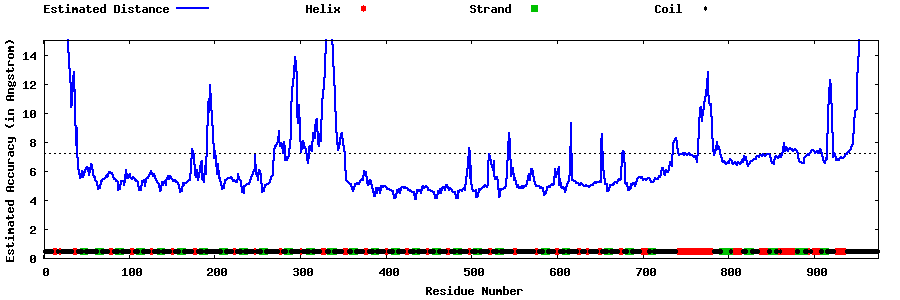

Supplement: Supplementary file 33 — Supplementary Information 33. [file 41598_2020_78347_MOESM33_ESM.zip › T21/struct/S511005_results/RSQ_5.png]

Tree scale: 0.1

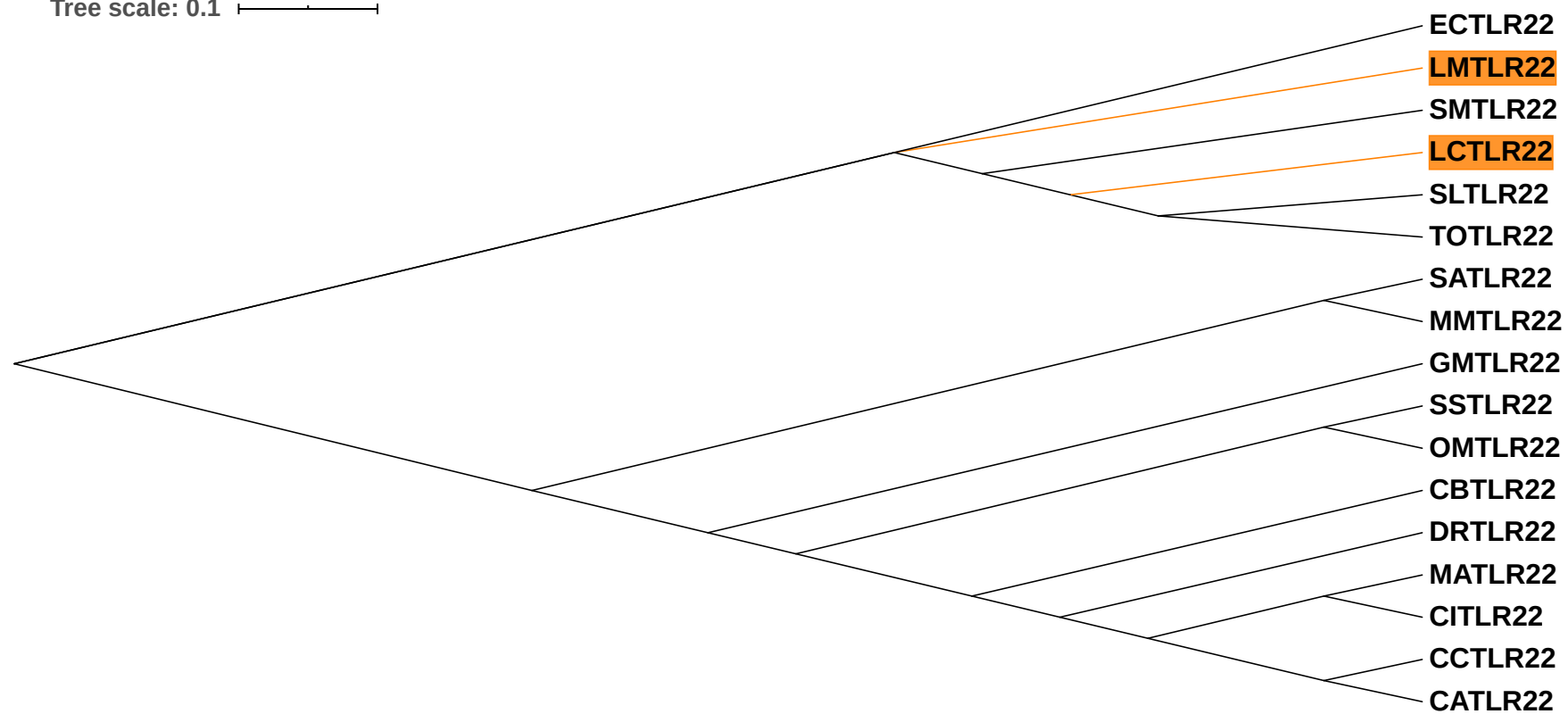

Supplement: Supplementary file 34 — Supplementary Information 34. [file 41598_2020_78347_MOESM34_ESM.zip › T22/absrel/labelledtree.pdf]

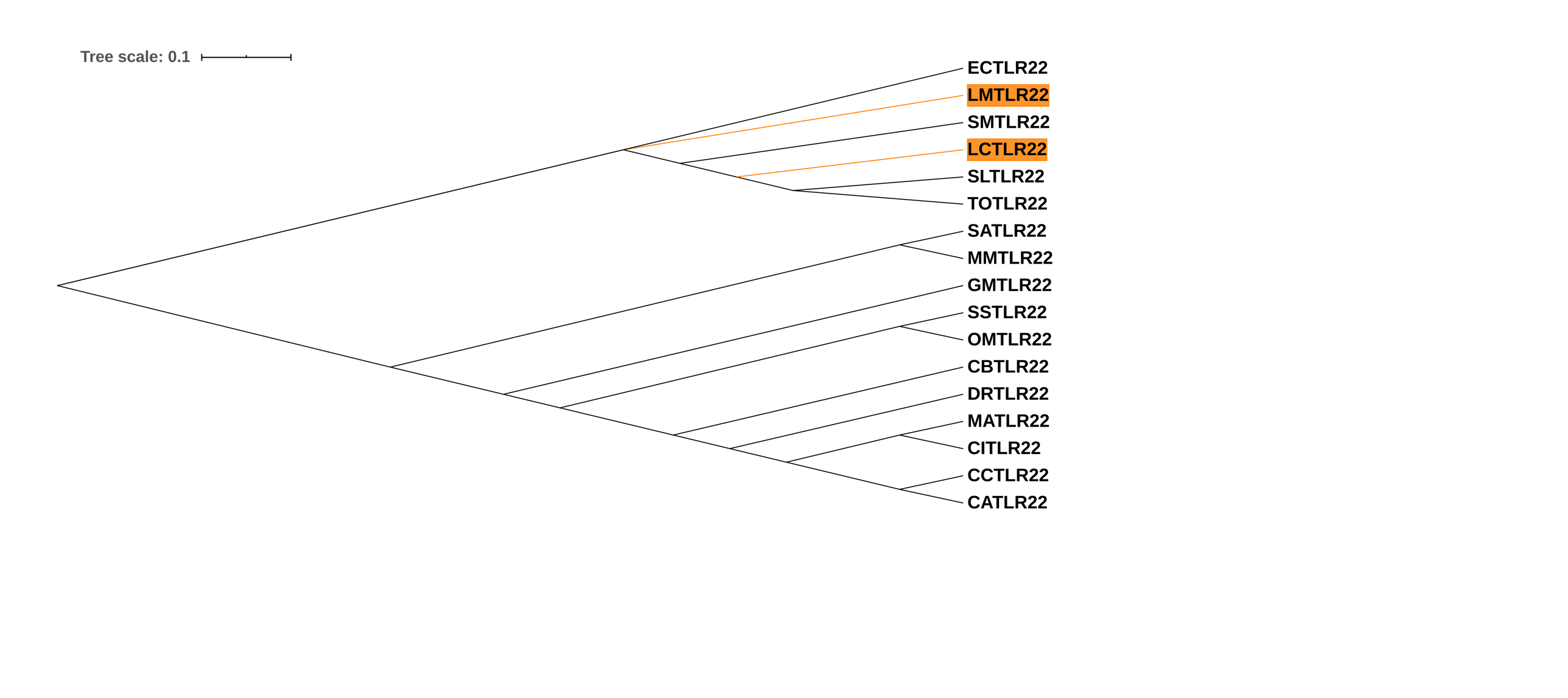

Supplement: Supplementary file 34 — Supplementary Information 34. [file 41598_2020_78347_MOESM34_ESM.zip › T22/absrel/labelledtree.png]

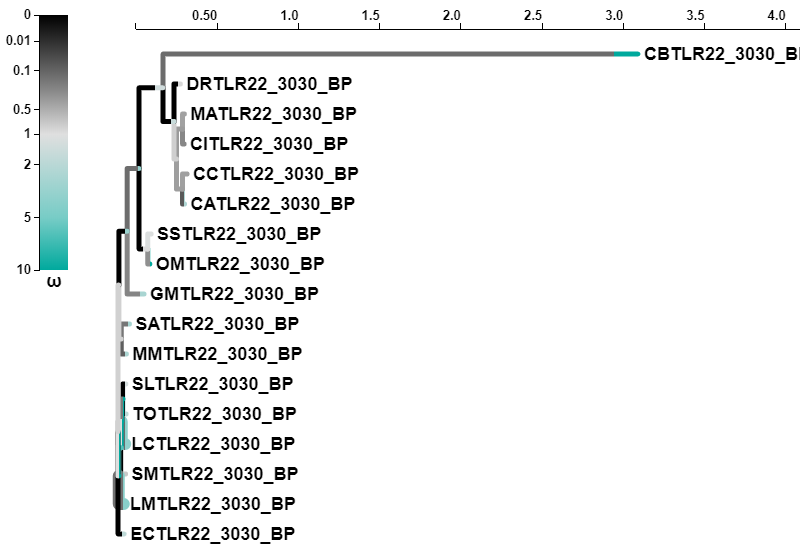

Supplement: Supplementary file 34 — Supplementary Information 34. [file 41598_2020_78347_MOESM34_ESM.zip › T22/absrel/tree.png]

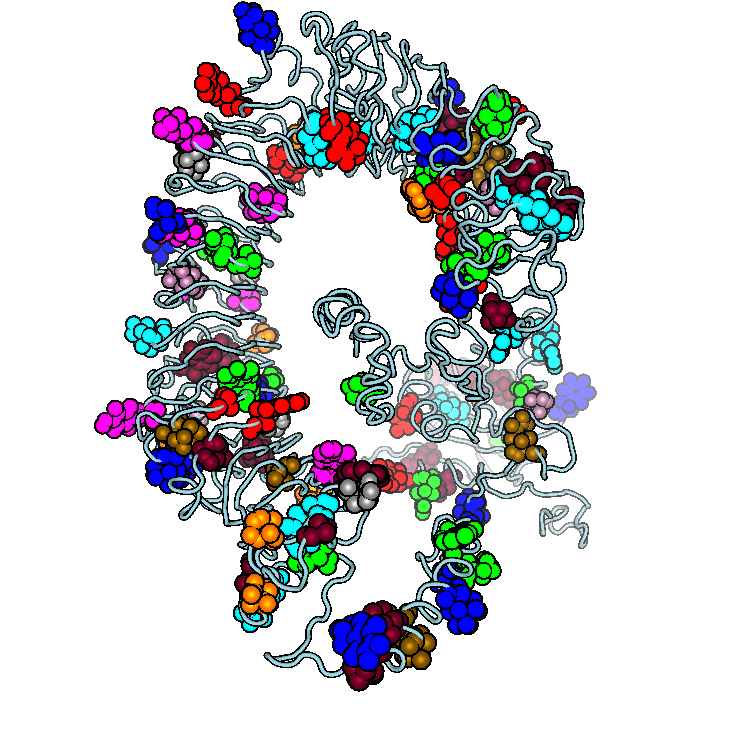

Supplement: Supplementary file 34 — Supplementary Information 34. [file 41598_2020_78347_MOESM34_ESM.zip › T22/bis2/download.png]

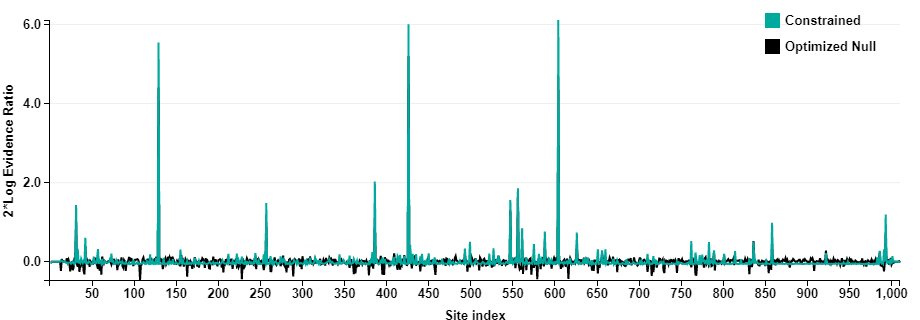

Supplement: Supplementary file 34 — Supplementary Information 34. [file 41598_2020_78347_MOESM34_ESM.zip › T22/busted/busted-chart (1).png]

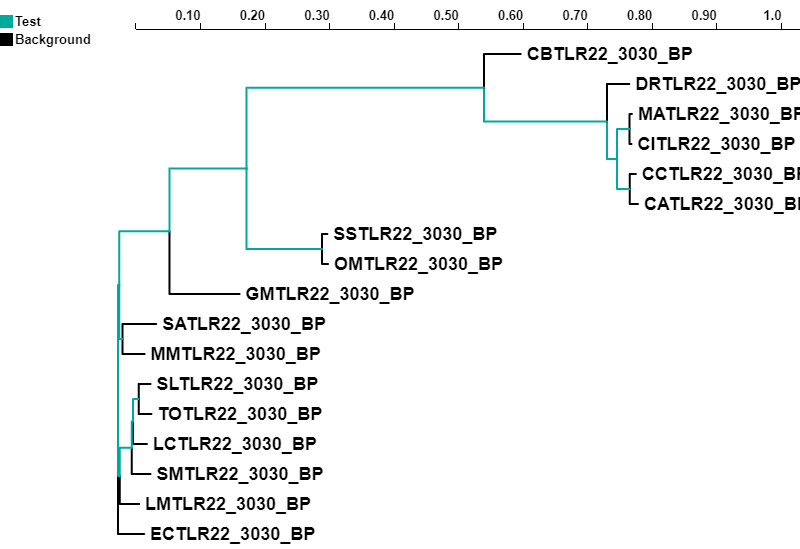

Supplement: Supplementary file 34 — Supplementary Information 34. [file 41598_2020_78347_MOESM34_ESM.zip › T22/busted/tree.png]

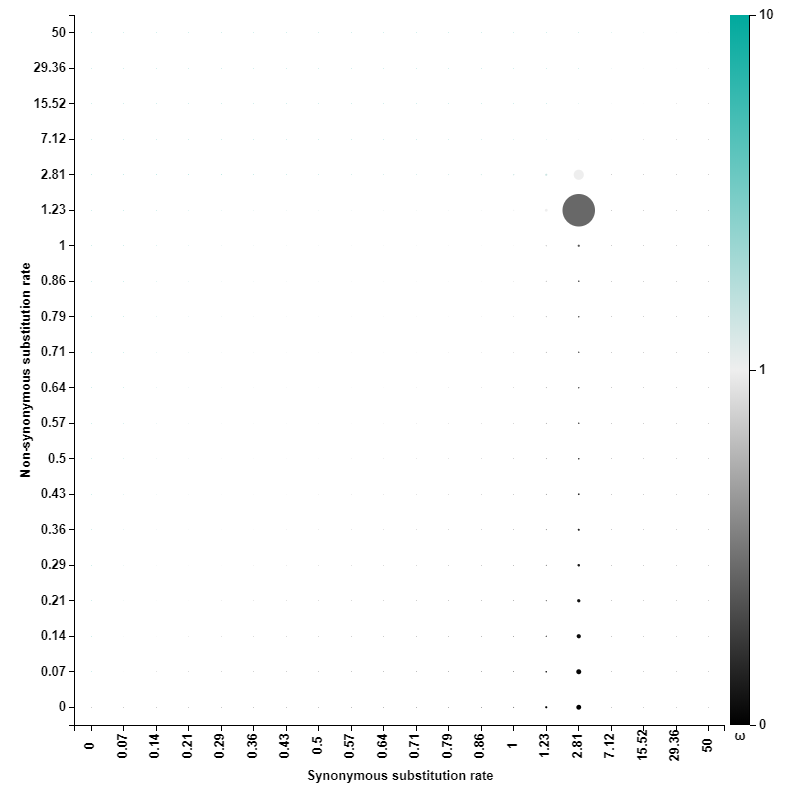

Supplement: Supplementary file 34 — Supplementary Information 34. [file 41598_2020_78347_MOESM34_ESM.zip › T22/fubar/datamonkey-chart.png]

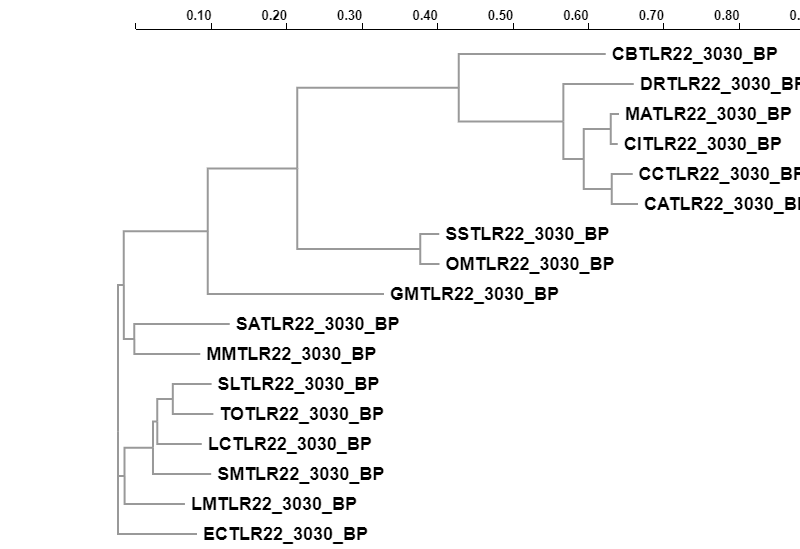

Supplement: Supplementary file 34 — Supplementary Information 34. [file 41598_2020_78347_MOESM34_ESM.zip › T22/fubar/tree.png]

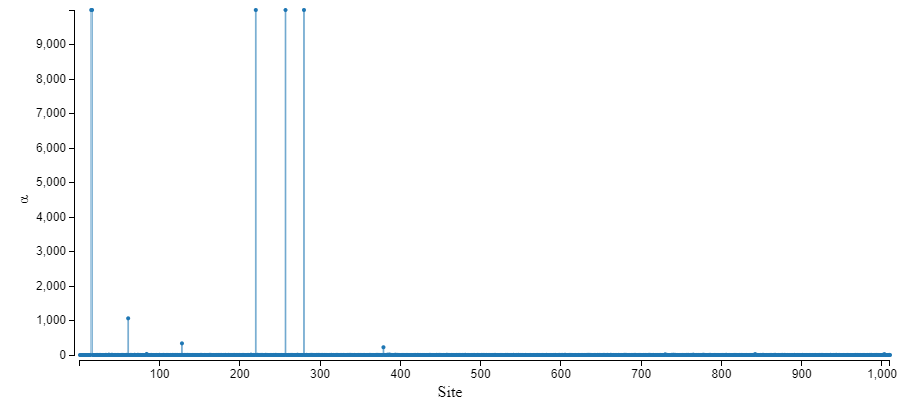

Supplement: Supplementary file 34 — Supplementary Information 34. [file 41598_2020_78347_MOESM34_ESM.zip › T22/meme/datamonkey-chart.png]

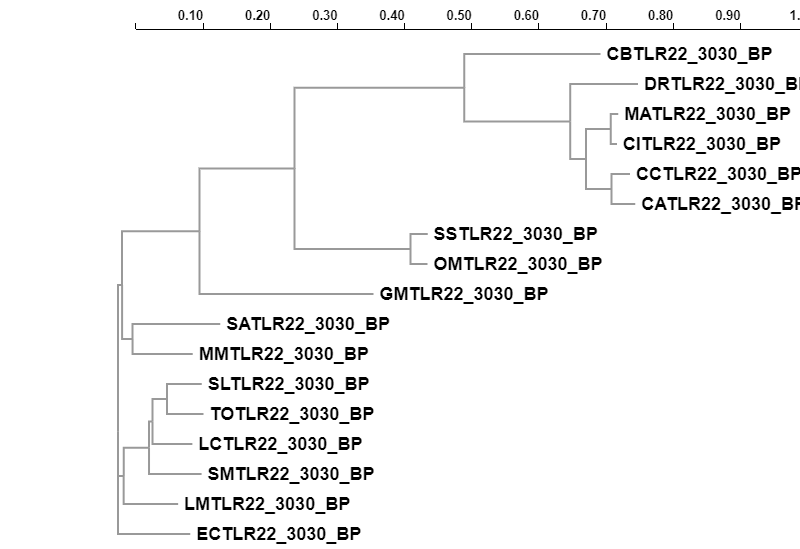

Supplement: Supplementary file 34 — Supplementary Information 34. [file 41598_2020_78347_MOESM34_ESM.zip › T22/meme/tree.png]

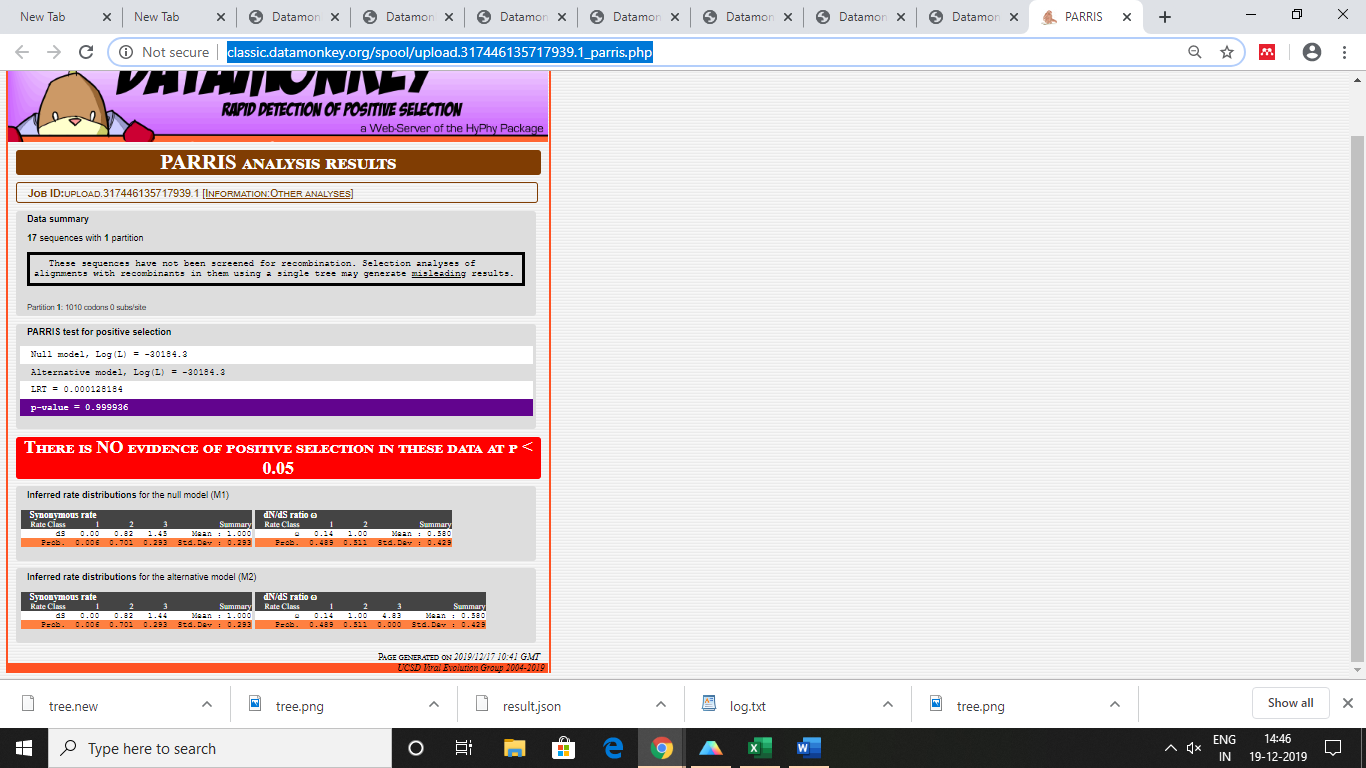

Supplement: Supplementary file 34 — Supplementary Information 34. [file 41598_2020_78347_MOESM34_ESM.zip › T22/parris.docx]

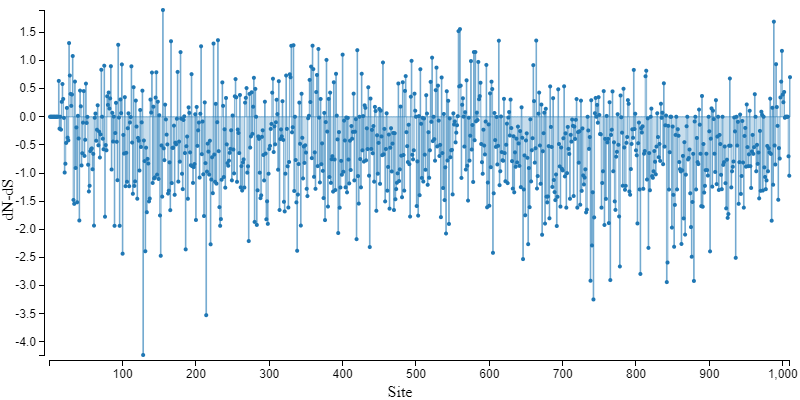

Supplement: Supplementary file 34 — Supplementary Information 34. [file 41598_2020_78347_MOESM34_ESM.zip › T22/slac/datamonkey-chart.png]

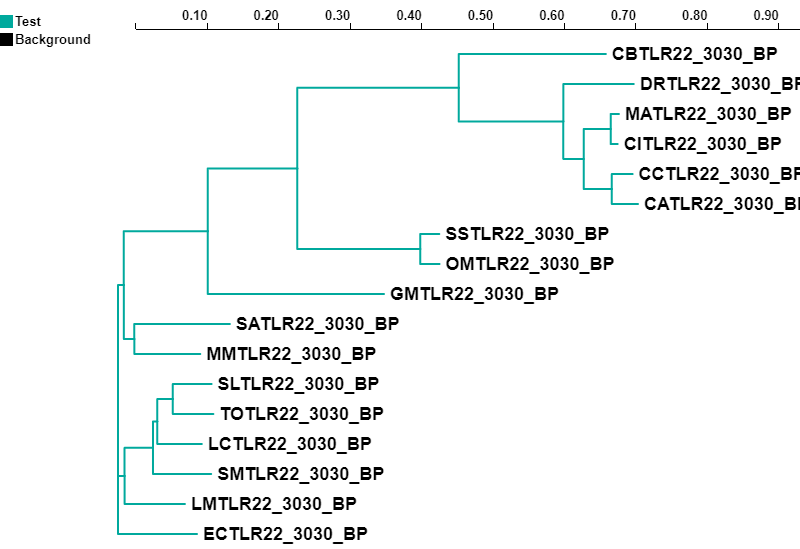

Supplement: Supplementary file 34 — Supplementary Information 34. [file 41598_2020_78347_MOESM34_ESM.zip › T22/slac/tree.png]

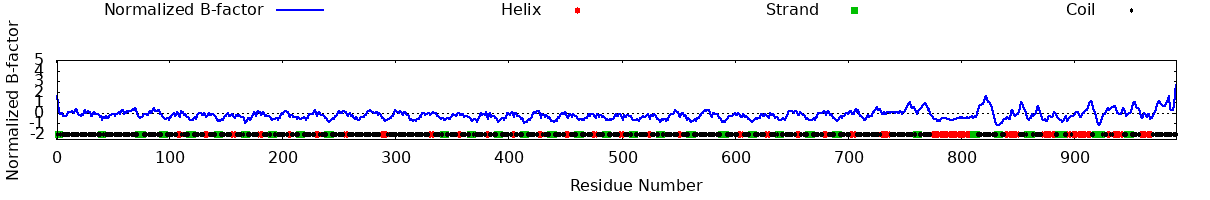

Supplement: Supplementary file 34 — Supplementary Information 34. [file 41598_2020_78347_MOESM34_ESM.zip › T22/struct/S533571_results/BFP.png]

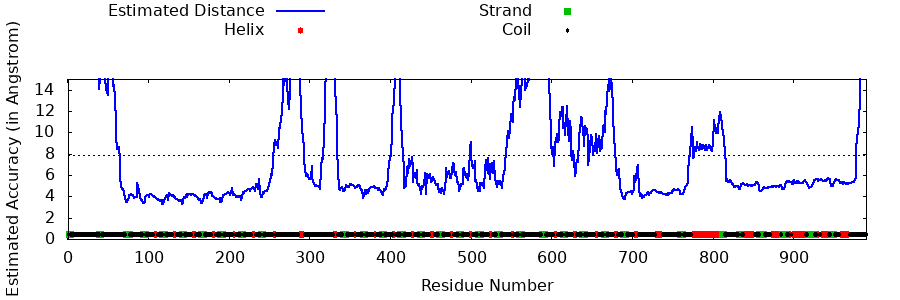

Supplement: Supplementary file 34 — Supplementary Information 34. [file 41598_2020_78347_MOESM34_ESM.zip › T22/struct/S533571_results/RSQ_1.png]

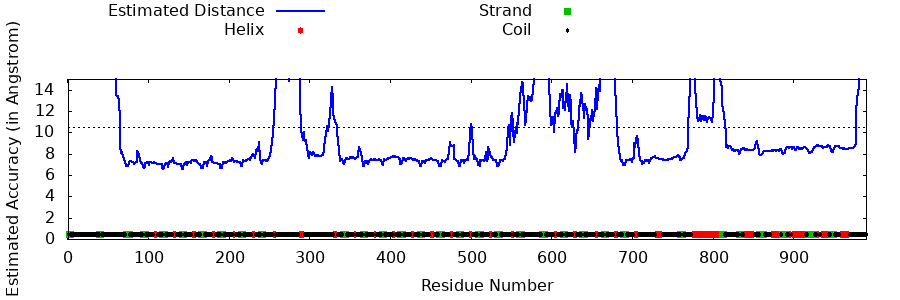

Supplement: Supplementary file 34 — Supplementary Information 34. [file 41598_2020_78347_MOESM34_ESM.zip › T22/struct/S533571_results/RSQ_2.png]

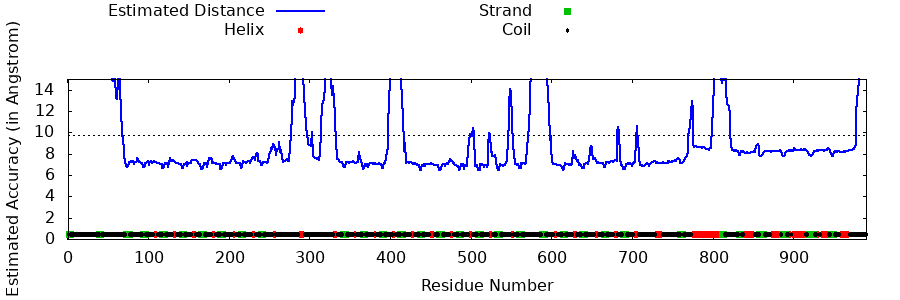

Supplement: Supplementary file 34 — Supplementary Information 34. [file 41598_2020_78347_MOESM34_ESM.zip › T22/struct/S533571_results/RSQ_3.png]

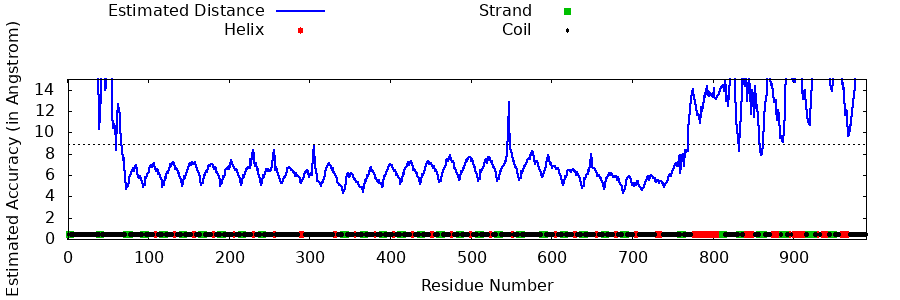

Supplement: Supplementary file 34 — Supplementary Information 34. [file 41598_2020_78347_MOESM34_ESM.zip › T22/struct/S533571_results/RSQ_4.png]

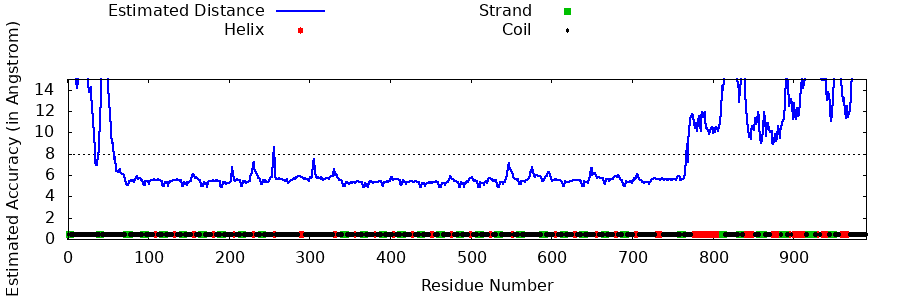

Supplement: Supplementary file 34 — Supplementary Information 34. [file 41598_2020_78347_MOESM34_ESM.zip › T22/struct/S533571_results/RSQ_5.png]

Tree scale: 0.1

Colored ranges

TLR1 family

TLR3 family

TLR5 family

TLR7 family

TLR11/13 family

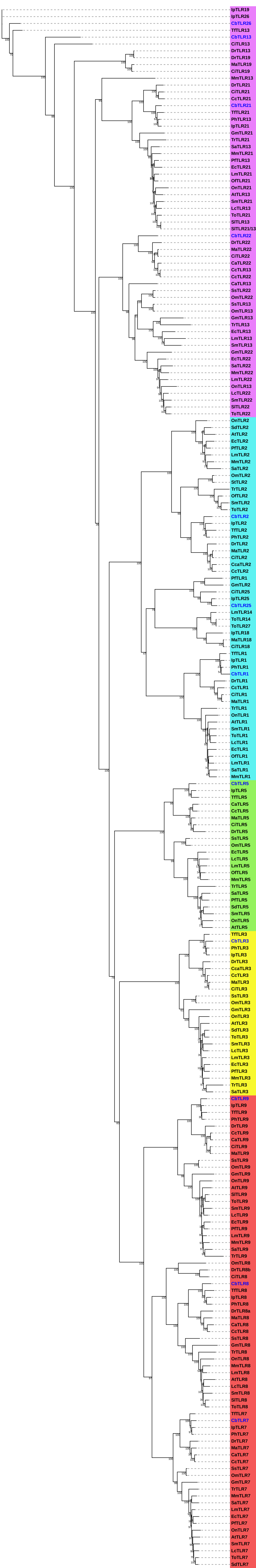

Supplement: Supplementary file 36 — Supplementary Information 36. [file 41598_2020_78347_MOESM36_ESM.zip › Supp. Data27 (Phylogenetic trees)/ML tree.pdf]

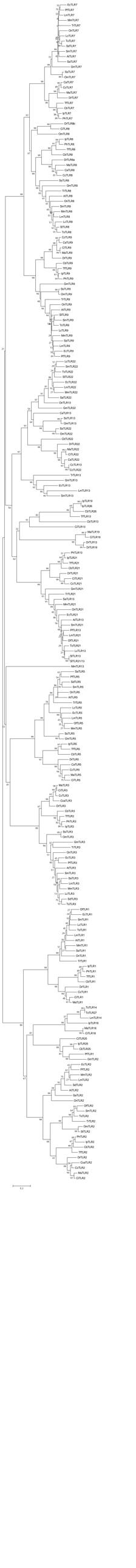

Supplement: Supplementary file 36 — Supplementary Information 36. [file 41598_2020_78347_MOESM36_ESM.zip › Supp. Data27 (Phylogenetic trees)/NJ tree.PDF]
