# Supplementary material for: Variation in selection constraints on teleost TLRs with emphasis on their repertoire in the Walking catfish, Clarias batrachus
Source: Sci Rep. 2020 Dec 7;10:21394. doi: 10.1038/s41598-020-78347-6 (PMC7721727; doi:10.1038/s41598-020-78347-6)
Supplement: Supplementary file 37 — Supplementary Information 37. [file 41598_2020_78347_MOESM37_ESM.docx]

***SUPPORTING DATA***

Variation in selection constraints on teleost TLRs with emphasis on their repertoire in the Walking catfish, *Clarias batrachus*

Manisha Priyam^a^, Sanjay K. Gupta^a,^*, Biplab Sarkar^a^, T.R. Sharma^a^, A. Pattanayak^a^

^a^ICAR- Indian Institute of Agricultural Biotechnology, Ranchi, Jharkhand, 834 010, India

*Corresponding Author

Sanjay K. Gupta

School of Molecular Diagnostics and Prophylactics

ICAR-Indian Institute of Agricultural Biotechnology

Namkum, Ranchi-834010, India,

Tel: +651 2261122, Fax:+6512261123

Email-[sanfish111@gmail.com](mailto:sanfish111@gmail.com) , [sanjay.gupta@icar.gov.in](mailto:sanjay.gupta@icar.gov.in)

**INDEX OF CONTENT IN SUPPORTING DATA FILES**

| **No.** | **File Name** | | **Contents** |
| --- | --- | --- | --- |
|  | Supp. Data1 | | Listing of TLRs from the selected species used in the study |
|  | Supp. Data2 (TLR1seq) | | Listing of TLR1 fasta sequences accessed from NCBI in the study |
|  | Supp. Data3 (TLR2seq) | | Listing of TLR2 fasta sequences accessed from NCBI in the study |
|  | Supp. Data4 (TLR3seq) | | Listing of TLR3 fasta sequences accessed from NCBI in the study |
|  | Supp. Data5 (TLR5seq) | | Listing of TLR5 fasta sequences accessed from NCBI in the study |
|  | Supp. Data6 (TLR7seq) | | Listing of TLR7 fasta sequences accessed from NCBI in the study |
|  | Supp. Data7 (TLR8seq) | | Listing of TLR8 fasta sequences accessed from NCBI in the study |
|  | Supp. Data8 (TLR9seq) | | Listing of TLR9 fasta sequences accessed from NCBI in the study |
|  | Supp. Data9 (TLR13seq) | | Listing of TLR13 fasta sequences accessed from NCBI in the study |
|  | Supp. Data10 (TLR21seq) | | Listing of TLR21 fasta sequences accessed from NCBI in the study |
|  | Supp. Data11 (TLR22seq) | | Listing of TLR22 fasta sequences accessed from NCBI in the study |
|  | Supp. Data12 (Misc. TLRseq) | | Listing of fasta sequences of fish-specific TLRs accessed from NCBI in the study |
|  | Supp. Data13 (CbTLR1) | | Source scaffold sequence, predicted orfs – nucleotide and amino acid sequences of *Cb* TLR1 |
|  | Supp. Data14 (CbTLR2) | | Source scaffold sequence, predicted orfs – nucleotide and amino acid sequences of *Cb* TLR2 |
|  | Supp. Data15 (CbTLR3) | | Source scaffold sequence, predicted orfs – nucleotide and amino acid sequences of *Cb* TLR3 |
|  | Supp. Data16 (CbTLR5) | | Source scaffold sequence, predicted orfs – nucleotide and amino acid sequences of *Cb* TLR5 |
|  | Supp. Data17 (CbTLR7) | | Source scaffold sequence, predicted orfs – nucleotide and amino acid sequences of *Cb* TLR7 |
|  | Supp. Data18 (CbTLR8) | | Source scaffold sequence, predicted orfs – nucleotide and amino acid sequences of *Cb* TLR8 |
|  | Supp. Data19 (CbTLR9) | | Source scaffold sequence, predicted orfs – nucleotide and amino acid sequences of *Cb* TLR9 |
|  | Supp. Data20 (CbTLR13) | | Source scaffold sequence, predicted orfs – nucleotide and amino acid sequences of *Cb* TLR13 |
|  | Supp. Data21 (CbTLR21) | | Complete cds of *Cb* TLR21 accessed from NCBI in the study |
|  | Supp. Data22 (CbTLR22) | | Source scaffold sequence, predicted orfs – nucleotide and amino acid sequences of *Cb* TLR22 |
|  | Supp. Data23 (CbTLR25) | | Source scaffold sequence, predicted orfs – nucleotide and amino acid sequences of *Cb* TLR25 |
|  | Supp. Data24 (CbTLR26) | | Source scaffold sequence, predicted orfs – nucleotide and amino acid sequences of *Cb* TLR26 |
|  | Supp. Data25(Raw Data-Selection tests, BIS2)  - Subfolders | *Subfolder* | *Contents of Subfolder* |
|  |  | TLR1 | Raw Data of results obtained from sequence analysis of TLR1 using ABSREL, BUSTED, FUBAR, MEME, SLAC and BIS2 |
|  |  | TLR2 | Raw Data of results obtained from sequence analysis of TLR2 using ABSREL, BUSTED, FUBAR, MEME, SLAC and BIS2 |
|  |  | TLR3 | Raw Data of results obtained from sequence analysis of TLR3 using ABSREL, BUSTED, FUBAR, MEME, SLAC and BIS2 |
|  |  | TLR5 | Raw Data of results obtained from sequence analysis of TLR5 using ABSREL, BUSTED, FUBAR, MEME, SLAC and BIS2 |
|  |  | TLR7 | Raw Data of results obtained from sequence analysis of TLR7 using ABSREL, BUSTED, FUBAR, MEME, SLAC and BIS2 |
|  |  | TLR8 | Raw Data of results obtained from sequence analysis of TLR8 using ABSREL, BUSTED, FUBAR, MEME, SLAC and BIS2 |
|  |  | TLR9 | Raw Data of results obtained from sequence analysis of TLR9 using ABSREL, BUSTED, FUBAR, MEME, SLAC and BIS2 |
|  |  | TLR13 | Raw Data of results obtained from sequence analysis of TLR13 using ABSREL, BUSTED, FUBAR, MEME, SLAC and BIS2 |
|  |  | TLR21 | Raw Data of results obtained from sequence analysis of TLR21 using ABSREL, BUSTED, FUBAR, MEME, SLAC and BIS2 |
|  |  | TLR22 | Raw Data of results obtained from sequence analysis of TLR22 using ABSREL, BUSTED, FUBAR, MEME, SLAC and BIS2 |
|  | Supp. Data26 (coevolved sites and selection sites) | | Listing of sites under negative and positive selection and co-evolved sites mapped on *Cb* TLR sequences |
|  | Supp. Data27 (Phylogenetic trees) | | Phylogenetic trees deduced by both NJ and ML methods. |
