# Supplementary material for: Variation in selection constraints on teleost TLRs with emphasis on their repertoire in the Walking catfish, Clarias batrachus
Source: Sci Rep. 2020 Dec 7;10:21394. doi: 10.1038/s41598-020-78347-6 (PMC7721727; doi:10.1038/s41598-020-78347-6)
Supplement: Supplementary file 28 — Supplementary Information 28. [file 41598_2020_78347_MOESM28_ESM.zip › T5/BIS2/summary.tgz!/summary/PF00000-NONREDUNDANT-5DD-dim0-table.html]

BIS cluster table


Clusters with env. score >= 0.5 and sym. score >= 0.5 :

| Dim | Cluster | Sym | Env | Pvalue | Hit patterns and blocks |
| --- | --- | --- | --- | --- | --- |
| 0 | 7 | 1 | 1 | 5.584371e-08 | Hit patterns: Positions: \| 267 \| 525; 14 sequences: \| I \| L; 5 sequences: \| L \| V; 3 sequences: \| F \| I All positions in cluster: 267 525 |
| 0 | 6 | 1 | 1 | 3.127248e-06 | Hit patterns: Positions: \| 420 \| 663; 14 sequences: \| L \| P; 8 sequences: \| X \| V All positions in cluster: 420 663 |
| 0 | 5 | 1 | 1 | 3.797372e-05 | Hit patterns: Positions: \| 433 \| 639 \| 722; 17 sequences: \| L \| T \| L; 5 sequences: \| F \| K \| V All positions in cluster: 433 639 722 |
| 0 | 2 | 1 | 1 | 0.0006493506 | Hit patterns: Positions: \| 369 \| 435 \| 479 \| 614; 19 sequences: \| G \| G \| G \| L; 3 sequences: \| D \| D \| E \| I All positions in cluster: 369 435 479 614 |
| 0 | 1 | 1 | 1 | 0.004329004 | Hit patterns: Positions: \| 448 \| 555 \| 653; 20 sequences: \| L \| G \| L; 2 sequences: \| I \| N \| V All positions in cluster: 448 555 653 |
| 0 | 3 | 1 | 1 | 0.004329004 | Hit patterns: Positions: \| 402 \| 462; 20 sequences: \| I \| G; 2 sequences: \| L \| M All positions in cluster: 402 462 |
| 0 | 4 | 1 | 1 | 1 | All positions in cluster: 169 173 182 184 187 196-197 199-200 208 211 218 223 226 228-229 232 234 237 244 248 262 268 271-272 275 277-278 280 300 303-307 315 317-318 321 331 337 339 362 367 370 373 379-381 385 388 410 412 417 419 424 427-429 432 442 451-454 456 458 461 466 469 472 475 477 480 494-495 505 530 556 561 578 580 589 592 594 598 607 612 617-618 622 625 627 632-633 636 638 647 655 658 665 679 681 683 687 693 711 714 |

Table created with bis2html version 8.
