# Supplementary material for: Variation in selection constraints on teleost TLRs with emphasis on their repertoire in the Walking catfish, Clarias batrachus
Source: Sci Rep. 2020 Dec 7;10:21394. doi: 10.1038/s41598-020-78347-6 (PMC7721727; doi:10.1038/s41598-020-78347-6)
Supplement: Supplementary file 28 — Supplementary Information 28. [file 41598_2020_78347_MOESM28_ESM.zip › T5/BIS2/summary.tgz!/summary/PF00000-NONREDUNDANT-5DD-dim1-table.html]

BIS cluster table


Clusters with env. score >= 0.5 and sym. score >= 0.5 :

| Dim | Cluster | Sym | Env | Pvalue | Hit patterns and blocks |
| --- | --- | --- | --- | --- | --- |
| 1 | 13 | 0.5909091 | 0.8166667 | 9.922998e-07 | Hit patterns: Positions: \| 403 \| 548; 12 sequences: \| D \| L; 7 sequences: \| T \| I; 1 sequence: \| S \| V; 1 sequence: \| S \| I; 1 sequence: \| L \| I All positions in cluster: 403 548 |
| 1 | 10 | 1 | 1 | 7.594744e-06 | Hit patterns: Positions: \| 119 \| 150; 17 sequences: \| L \| L; 4 sequences: \| H \| Y; 1 sequence: \| X \| X All positions in cluster: 119 150 |
| 1 | 12 | 0.5 | 0.6555556 | 0.0003267974 | Hit patterns: Positions: \| 299 \| 670 \| 691 \| 713; 14 sequences: \| D \| S \| L \| F; 4 sequences: \| G \| T \| Q \| S; 1 sequence: \| G \| N \| Q \| Y; 1 sequence: \| G \| T \| Q \| Y; 1 sequence: \| S \| T \| Q \| Y; 1 sequence: \| G \| T \| K \| S All positions in cluster: 299 670 691 713 |
| 1 | 1 | 1 | 1 | 0.04545455 | All positions in cluster: 690 697 |
| 1 | 2 | 1 | 1 | 0.04545455 | All positions in cluster: 179 668 |
| 1 | 3 | 1 | 1 | 0.04545455 | All positions in cluster: 166 190 316 650 676 |
| 1 | 4 | 1 | 1 | 0.04545455 | All positions in cluster: 430 502 |
| 1 | 5 | 1 | 1 | 0.04545455 | All positions in cluster: 416 500 |
| 1 | 6 | 1 | 1 | 0.04545455 | All positions in cluster: 393 640 |
| 1 | 7 | 1 | 1 | 0.04545455 | All positions in cluster: 206 225 283 351 358 609 |
| 1 | 8 | 1 | 1 | 0.04545455 | All positions in cluster: 203 285 |
| 1 | 9 | 1 | 1 | 0.04545455 | All positions in cluster: 105 112 114 122-123 137 139 141 146 153 161 386 |
| 1 | 11 | 0.5 | 0.8166667 | 0.05 | Hit patterns: Positions: \| 287 \| 402 \| 462; 19 sequences: \| S \| I \| G; 1 sequence: \| N \| L \| M; 1 sequence: \| D \| I \| G; 1 sequence: \| D \| L \| M All positions in cluster: 287 402 462 |

Table created with bis2html version 8.
