# Supplementary material for: Variation in selection constraints on teleost TLRs with emphasis on their repertoire in the Walking catfish, Clarias batrachus
Source: Sci Rep. 2020 Dec 7;10:21394. doi: 10.1038/s41598-020-78347-6 (PMC7721727; doi:10.1038/s41598-020-78347-6)
Supplement: Supplementary file 28 — Supplementary Information 28. [file 41598_2020_78347_MOESM28_ESM.zip › T5/BIS2/summary.tgz!/summary/PF00000-NONREDUNDANT-5DD-dim2-table.html]

BIS cluster table


Clusters with env. score >= 0.5 and sym. score >= 0.5 :

| Dim | Cluster | Sym | Env | Pvalue | Hit patterns and blocks |
| --- | --- | --- | --- | --- | --- |
| 2 | 1 | 0.5217391 | 0.9875 | 1 | All positions in cluster: 445 690 697 |

Table created with bis2html version 8.
