# Supplementary material for: Variation in selection constraints on teleost TLRs with emphasis on their repertoire in the Walking catfish, Clarias batrachus
Source: Sci Rep. 2020 Dec 7;10:21394. doi: 10.1038/s41598-020-78347-6 (PMC7721727; doi:10.1038/s41598-020-78347-6)
Supplement: Supplementary file 28 — Supplementary Information 28. [file 41598_2020_78347_MOESM28_ESM.zip › T5/BIS2/summary.tgz!/summary/PF00000-NONREDUNDANT-5DD-dim2.html]

Alignment and BIS clusters


|  |  |  |  |  |  |  |  |  |  |  |  |  |  |  |  |  |  |  |  |  |  |  |  |  |  |  |  |  |  |  |  |  |  |  |  |  |  |  |  |  |  |  |  |  |  |  |  |  |  |  |  |  |  |  |  |  |  |  |  |  |  |  |  |  |  |  |  |  |  |  |  |  |  |  |  |  |  |  |  |  |  |  |  |  |  |  |  |  |  |  |  |  |  |  |  |  |  |  |  |  |  |  |  |  |  |  |  |  |  |  |  |  |  |  |  |  |  |  |  |  |  |  |  |  |  |  |  |  |  |  |  |  |  |  |  |  |  |  |  |  |  |  |  |  |  |  |  |  |  |  |  |  |  |  |  |  |  |  |  |  |  |  |  |  |  |  |  |  |  |  |  |  |  |  |  |  |  |  |  |  |  |  |  |  |  |  |  |  |  |  |  |  |  |  |  |  |  |  |  |  |  |  |  |  |  |  |  |  |  |  |  |  |  |  |  |  |  |  |  |  |  |  |  |  |  |  |  |  |  |  |  |  |  |  |  |  |  |  |  |  |  |  |  |  |  |  |  |  |  |  |  |  |  |  |  |  |  |  |  |  |  |  |  |  |  |  |  |  |  |  |  |  |  |  |  |  |  |  |  |  |  |  |  |  |  |  |  |  |  |  |  |  |  |  |  |  |  |  |  |  |  |  |  |  |  |  |  |  |  |  |  |  |  |  |  |  |  |  |  |  |  |  |  |  |  |  |  |  |  |  |  |  |  |  |  |  |  |  |  |  |  |  |  |  |  |  |  |  |  |  |  |  |  |  |  |  |  |  |  |  |  |  |  |  |  |  |  |  |  |  |  |  |  |  |  |  |  |  |  |  |  |  |  |  |  |  |  |  |  |  |  |  |  |  |  |  |  |  |  |  |  |  |  |  |  |  |  |  |  |  |  |  |  |  |  |  |  |  |  |  |  |  |  |  |  |  |  |  |  |  |  |  |  |  |  |  |  |  |  |  |  |  |  |  |  |  |  |  |  |  |  |  |  |  |  |  |  |  |  |  |  |  |  |  |  |  |  |  |  |  |  |  |  |  |  |  |  |  |  |  |  |  |  |  |  |  |  |  |  |  |  |  |  |  |  |  |  |  |  |  |  |  |  |  |  |  |  |  |  |  |  |  |  |  |  |  |  |  |  |  |  |  |  |  |  |  |  |  |  |  |  |  |  |  |  |  |  |  |  |  |  |  |  |  |  |  |  |  |  |  |  |  |  |  |  |  |  |  |  |  |  |  |  |  |  |  |  |  |  |  |  |  |  |  |  |  |  |  |  |  |  |  |  |  |  |  |  |  |  |  |  |  |  |  |  |  |  |  |  |  |  |  |  |  |  |  |  |  |  |  |  |  |  |  |  |  |  |  |  |  |  |  |  |  |  |  |  |  |  |  |  |  |  |  |  |  |  |  |  |  |  |  |  |  |  |  |  |  |  |  |  |  |  |  |  |  |  |  |  |  |  |  |  |  |  |  |  |  |  |  |  |  |  |  |  |  |  |  |  |  |  |  |  |  |  |  |  |  |  |  |  |  |  |  |  |  |  |  |  |  |  |  |  |  |  |  |  |  |  |  |  |  |  |  |  |  |  |  |  |  |  |  |  |  |  |  |  |  |  |  |  |  |  |  |  |  |  |  |  |  |  |  |  |  |  |  |  |  |  |  |  |  |  |  |  |  |  |  |  |  |  |  |  |  |  |  |  |  |  |  |  |  |  |  |  |  |  |  |  |  |  |  |  |  |  |  |  |  |  |  |  |  |  |  |  |  |  |  |  |  |  |  |  |  |  |  |  |  |  |  |  |  |  |  |  |  |  |  |  |  |  |  |  |  |  |  |  |  |  |  |  |  |  |  |  |  |  |  |  |  |  |  |  |  |  |  |  |  |  |  |  |  |  |  |  |  |  |  |  |  |  |  |  |  |  |  |  |  |  |  |  |  |  |  |  |  |  |  |  |  |  |  |  |  |  |  |  |  |  |  |  |  |  |  |  |  |  |  |  |  |  |  |  |  |  |  |  |  |  |  |  |  |  |  |  |  |  |  |  |  |  |  |  |  |  |  |  |  |  |  |  |  |  |  |  |  |  |  |  |  |  |  |  |  |  |  |  |  |  |  |  |  |  |  |  |  |  |  |  |  |  |  |  |  |  |  |  |  |  |  |  |  |  |  |  |  |  |  |  |  |  |  |  |  |  |  |  |  |  |  |  |  |  |  |  |  |  |  |
| --- | --- | --- | --- | --- | --- | --- | --- | --- | --- | --- | --- | --- | --- | --- | --- | --- | --- | --- | --- | --- | --- | --- | --- | --- | --- | --- | --- | --- | --- | --- | --- | --- | --- | --- | --- | --- | --- | --- | --- | --- | --- | --- | --- | --- | --- | --- | --- | --- | --- | --- | --- | --- | --- | --- | --- | --- | --- | --- | --- | --- | --- | --- | --- | --- | --- | --- | --- | --- | --- | --- | --- | --- | --- | --- | --- | --- | --- | --- | --- | --- | --- | --- | --- | --- | --- | --- | --- | --- | --- | --- | --- | --- | --- | --- | --- | --- | --- | --- | --- | --- | --- | --- | --- | --- | --- | --- | --- | --- | --- | --- | --- | --- | --- | --- | --- | --- | --- | --- | --- | --- | --- | --- | --- | --- | --- | --- | --- | --- | --- | --- | --- | --- | --- | --- | --- | --- | --- | --- | --- | --- | --- | --- | --- | --- | --- | --- | --- | --- | --- | --- | --- | --- | --- | --- | --- | --- | --- | --- | --- | --- | --- | --- | --- | --- | --- | --- | --- | --- | --- | --- | --- | --- | --- | --- | --- | --- | --- | --- | --- | --- | --- | --- | --- | --- | --- | --- | --- | --- | --- | --- | --- | --- | --- | --- | --- | --- | --- | --- | --- | --- | --- | --- | --- | --- | --- | --- | --- | --- | --- | --- | --- | --- | --- | --- | --- | --- | --- | --- | --- | --- | --- | --- | --- | --- | --- | --- | --- | --- | --- | --- | --- | --- | --- | --- | --- | --- | --- | --- | --- | --- | --- | --- | --- | --- | --- | --- | --- | --- | --- | --- | --- | --- | --- | --- | --- | --- | --- | --- | --- | --- | --- | --- | --- | --- | --- | --- | --- | --- | --- | --- | --- | --- | --- | --- | --- | --- | --- | --- | --- | --- | --- | --- | --- | --- | --- | --- | --- | --- | --- | --- | --- | --- | --- | --- | --- | --- | --- | --- | --- | --- | --- | --- | --- | --- | --- | --- | --- | --- | --- | --- | --- | --- | --- | --- | --- | --- | --- | --- | --- | --- | --- | --- | --- | --- | --- | --- | --- | --- | --- | --- | --- | --- | --- | --- | --- | --- | --- | --- | --- | --- | --- | --- | --- | --- | --- | --- | --- | --- | --- | --- | --- | --- | --- | --- | --- | --- | --- | --- | --- | --- | --- | --- | --- | --- | --- | --- | --- | --- | --- | --- | --- | --- | --- | --- | --- | --- | --- | --- | --- | --- | --- | --- | --- | --- | --- | --- | --- | --- | --- | --- | --- | --- | --- | --- | --- | --- | --- | --- | --- | --- | --- | --- | --- | --- | --- | --- | --- | --- | --- | --- | --- | --- | --- | --- | --- | --- | --- | --- | --- | --- | --- | --- | --- | --- | --- | --- | --- | --- | --- | --- | --- | --- | --- | --- | --- | --- | --- | --- | --- | --- | --- | --- | --- | --- | --- | --- | --- | --- | --- | --- | --- | --- | --- | --- | --- | --- | --- | --- | --- | --- | --- | --- | --- | --- | --- | --- | --- | --- | --- | --- | --- | --- | --- | --- | --- | --- | --- | --- | --- | --- | --- | --- | --- | --- | --- | --- | --- | --- | --- | --- | --- | --- | --- | --- | --- | --- | --- | --- | --- | --- | --- | --- | --- | --- | --- | --- | --- | --- | --- | --- | --- | --- | --- | --- | --- | --- | --- | --- | --- | --- | --- | --- | --- | --- | --- | --- | --- | --- | --- | --- | --- | --- | --- | --- | --- | --- | --- | --- | --- | --- | --- | --- | --- | --- | --- | --- | --- | --- | --- | --- | --- | --- | --- | --- | --- | --- | --- | --- | --- | --- | --- | --- | --- | --- | --- | --- | --- | --- | --- | --- | --- | --- | --- | --- | --- | --- | --- | --- | --- | --- | --- | --- | --- | --- | --- | --- | --- | --- | --- | --- | --- | --- | --- | --- | --- | --- | --- | --- | --- | --- | --- | --- | --- | --- | --- | --- | --- | --- | --- | --- | --- | --- | --- | --- | --- | --- | --- | --- | --- | --- | --- | --- | --- | --- | --- | --- | --- | --- | --- | --- | --- | --- | --- | --- | --- | --- | --- | --- | --- | --- | --- | --- | --- | --- | --- | --- | --- | --- | --- | --- | --- | --- | --- | --- | --- | --- | --- | --- | --- | --- | --- | --- | --- | --- | --- | --- | --- | --- | --- | --- | --- | --- | --- | --- | --- | --- | --- | --- | --- | --- | --- | --- | --- | --- | --- | --- | --- | --- | --- | --- | --- | --- | --- | --- | --- | --- | --- | --- | --- | --- | --- | --- | --- | --- | --- | --- | --- | --- | --- | --- | --- | --- | --- | --- | --- | --- | --- | --- | --- | --- | --- | --- | --- | --- | --- | --- | --- | --- | --- | --- | --- | --- | --- | --- | --- | --- | --- | --- | --- | --- | --- | --- | --- | --- | --- | --- | --- | --- | --- | --- | --- | --- | --- | --- | --- | --- | --- | --- | --- | --- | --- | --- | --- | --- | --- | --- | --- | --- | --- | --- | --- | --- | --- | --- | --- | --- | --- | --- | --- | --- | --- | --- | --- | --- | --- | --- | --- | --- | --- | --- | --- | --- | --- | --- | --- | --- | --- | --- | --- | --- | --- | --- | --- | --- | --- | --- | --- | --- | --- | --- | --- | --- | --- | --- | --- | --- | --- | --- | --- | --- | --- | --- | --- | --- | --- | --- | --- | --- | --- | --- | --- | --- | --- | --- | --- | --- | --- | --- | --- | --- | --- | --- | --- | --- | --- | --- | --- | --- | --- | --- | --- | --- | --- | --- | --- | --- | --- | --- | --- | --- | --- | --- | --- | --- | --- | --- | --- | --- | --- | --- | --- | --- | --- | --- | --- | --- | --- | --- | --- | --- | --- | --- | --- | --- | --- | --- | --- | --- | --- | --- | --- | --- | --- | --- | --- | --- | --- | --- | --- | --- | --- | --- | --- | --- | --- | --- | --- | --- | --- | --- | --- | --- | --- | --- | --- | --- | --- | --- | --- | --- | --- | --- | --- | --- | --- | --- | --- | --- | --- | --- | --- | --- | --- | --- | --- | --- | --- | --- | --- | --- | --- | --- | --- | --- | --- | --- | --- | --- | --- | --- | --- | --- | --- | --- | --- | --- | --- | --- | --- | --- | --- | --- | --- | --- | --- | --- | --- | --- | --- | --- | --- | --- | --- | --- | --- | --- | --- | --- | --- | --- | --- | --- | --- | --- | --- | --- | --- | --- | --- | --- | --- | --- | --- | --- | --- | --- | --- | --- |
|  |  |  |  |  |  |  |  | 1 | 0 |  |  |  |  |  |  |  |  | 2 | 0 |  |  |  |  |  |  |  |  | 3 | 0 |  |  |  |  |  |  |  |  | 4 | 0 |  |  |  |  |  |  |  |  | 5 | 0 |  |  |  |  |  |  |  |  | 6 | 0 |  |  |  |  |  |  |  |  | 7 | 0 |  |  |  |  |  |  |  |  | 8 | 0 |  |  |  |  |  |  |  |  | 9 | 0 |  |  |  |  |  |  |  | 1 | 0 | 0 |  |  |  |  |  |  |  | 1 | 1 | 0 |  |  |  |  |  |  |  | 1 | 2 | 0 |  |  |  |  |  |  |  | 1 | 3 | 0 |  |  |  |  |  |  |  | 1 | 4 | 0 |  |  |  |  |  |  |  | 1 | 5 | 0 |  |  |  |  |  |  |  | 1 | 6 | 0 |  |  |  |  |  |  |  | 1 | 7 | 0 |  |  |  |  |  |  |  | 1 | 8 | 0 |  |  |  |  |  |  |  | 1 | 9 | 0 |  |  |  |  |  |  |  | 2 | 0 | 0 |  |  |  |  |  |  |  | 2 | 1 | 0 |  |  |  |  |  |  |  | 2 | 2 | 0 |  |  |  |  |  |  |  | 2 | 3 | 0 |  |  |  |  |  |  |  | 2 | 4 | 0 |  |  |  |  |  |  |  | 2 | 5 | 0 |  |  |  |  |  |  |  | 2 | 6 | 0 |  |  |  |  |  |  |  | 2 | 7 | 0 |  |  |  |  |  |  |  | 2 | 8 | 0 |  |  |  |  |  |  |  | 2 | 9 | 0 |  |  |  |  |  |  |  | 3 | 0 | 0 |  |  |  |  |  |  |  | 3 | 1 | 0 |  |  |  |  |  |  |  | 3 | 2 | 0 |  |  |  |  |  |  |  | 3 | 3 | 0 |  |  |  |  |  |  |  | 3 | 4 | 0 |  |  |  |  |  |  |  | 3 | 5 | 0 |  |  |  |  |  |  |  | 3 | 6 | 0 |  |  |  |  |  |  |  | 3 | 7 | 0 |  |  |  |  |  |  |  | 3 | 8 | 0 |  |  |  |  |  |  |  | 3 | 9 | 0 |  |  |  |  |  |  |  | 4 | 0 | 0 |  |  |  |  |  |  |  | 4 | 1 | 0 |  |  |  |  |  |  |  | 4 | 2 | 0 |  |  |  |  |  |  |  | 4 | 3 | 0 |  |  |  |  |  |  |  | 4 | 4 | 0 |  |  |  |  |  |  |  | 4 | 5 | 0 |  |  |  |  |  |  |  | 4 | 6 | 0 |  |  |  |  |  |  |  | 4 | 7 | 0 |  |  |  |  |  |  |  | 4 | 8 | 0 |  |  |  |  |  |  |  | 4 | 9 | 0 |  |  |  |  |  |  |  | 5 | 0 | 0 |  |  |  |  |  |  |  | 5 | 1 | 0 |  |  |  |  |  |  |  | 5 | 2 | 0 |  |  |  |  |  |  |  | 5 | 3 | 0 |  |  |  |  |  |  |  | 5 | 4 | 0 |  |  |  |  |  |  |  | 5 | 5 | 0 |  |  |  |  |  |  |  | 5 | 6 | 0 |  |  |  |  |  |  |  | 5 | 7 | 0 |  |  |  |  |  |  |  | 5 | 8 | 0 |  |  |  |  |  |  |  | 5 | 9 | 0 |  |  |  |  |  |  |  | 6 | 0 | 0 |  |  |  |  |  |  |  | 6 | 1 | 0 |  |  |  |  |  |  |  | 6 | 2 | 0 |  |  |  |  |  |  |  | 6 | 3 | 0 |  |  |  |  |  |  |  | 6 | 4 | 0 |  |  |  |  |  |  |  | 6 | 5 | 0 |  |  |  |  |  |  |  | 6 | 6 | 0 |  |  |  |  |  |  |  | 6 | 7 | 0 |  |  |  |  |  |  |  | 6 | 8 | 0 |  |  |  |  |  |  |  | 6 | 9 | 0 |  |  |  |  |  |  |  | 7 | 0 | 0 |  |  |  |  |  |  |  | 7 | 1 | 0 |  |  |  |  |  |  |  | 7 | 2 | 0 |  |  |  |  |  |  |  | 7 | 3 | 0 |  |  |  |  |  |  |  | 7 | 4 | 0 |  |  |  |  |  |  |  | 7 | 5 | 0 |  |  |  |  |  |  |  | 7 | 6 | 0 |  |  |  |  |  |  |  | 7 | 7 | 0 |  |  |  |  |  |  |  | 7 | 8 | 0 |  |  |  |  |  |  |  | 7 | 9 | 0 |  |  |  |  |  |  |  | 8 | 0 | 0 |  |  |  |  |  |  |  | 8 | 1 | 0 |  |  |  |  |  |  |  | 8 | 2 | 0 |  |  |  |  |  |  |  | 8 | 3 | 0 |  |  |  |  |  |  |  | 8 | 4 | 0 |  |  |  |  |  |  |  | 8 | 5 | 0 |  |  |  |  |  |  |  | 8 | 6 | 0 |  |  |  |  |  |  |  | 8 | 7 | 0 |  |  |  |  |  |  |  | 8 | 8 | 0 |  |  |  |  |  |  |  | 8 | 9 | 0 |  |  |  |  |  |  |  | 9 | 0 | 0 |  |  |  |  |  |  |  | 9 | 1 | 0 |  |  |  |  |  |  |  | 9 | 2 | 0 |  |  |  |  |  |  |  | 9 | 3 | 0 |  |  |  |  |  |  |  | 9 | 4 | 0 |  |  |  |  |  |  |  | 9 | 5 | 0 |  |  |  |  |  |  |  | 9 | 6 | 0 |  |  |  |  |  |  |  | 9 | 7 | 0 |  |  |  |  |  |  |  | 9 | 8 | 0 |  |  |  |  |  |  |  | 9 | 9 | 0 |  |  |  |  |  |  |  |  |  |
|  |  |  |  |  |  |  |  |  | \| |  |  |  |  |  |  |  |  |  | \| |  |  |  |  |  |  |  |  |  | \| |  |  |  |  |  |  |  |  |  | \| |  |  |  |  |  |  |  |  |  | \| |  |  |  |  |  |  |  |  |  | \| |  |  |  |  |  |  |  |  |  | \| |  |  |  |  |  |  |  |  |  | \| |  |  |  |  |  |  |  |  |  | \| |  |  |  |  |  |  |  |  |  | \| |  |  |  |  |  |  |  |  |  | \| |  |  |  |  |  |  |  |  |  | \| |  |  |  |  |  |  |  |  |  | \| |  |  |  |  |  |  |  |  |  | \| |  |  |  |  |  |  |  |  |  | \| |  |  |  |  |  |  |  |  |  | \| |  |  |  |  |  |  |  |  |  | \| |  |  |  |  |  |  |  |  |  | \| |  |  |  |  |  |  |  |  |  | \| |  |  |  |  |  |  |  |  |  | \| |  |  |  |  |  |  |  |  |  | \| |  |  |  |  |  |  |  |  |  | \| |  |  |  |  |  |  |  |  |  | \| |  |  |  |  |  |  |  |  |  | \| |  |  |  |  |  |  |  |  |  | \| |  |  |  |  |  |  |  |  |  | \| |  |  |  |  |  |  |  |  |  | \| |  |  |  |  |  |  |  |  |  | \| |  |  |  |  |  |  |  |  |  | \| |  |  |  |  |  |  |  |  |  | \| |  |  |  |  |  |  |  |  |  | \| |  |  |  |  |  |  |  |  |  | \| |  |  |  |  |  |  |  |  |  | \| |  |  |  |  |  |  |  |  |  | \| |  |  |  |  |  |  |  |  |  | \| |  |  |  |  |  |  |  |  |  | \| |  |  |  |  |  |  |  |  |  | \| |  |  |  |  |  |  |  |  |  | \| |  |  |  |  |  |  |  |  |  | \| |  |  |  |  |  |  |  |  |  | \| |  |  |  |  |  |  |  |  |  | \| |  |  |  |  |  |  |  |  |  | \| |  |  |  |  |  |  |  |  |  | \| |  |  |  |  |  |  |  |  |  | \| |  |  |  |  |  |  |  |  |  | \| |  |  |  |  |  |  |  |  |  | \| |  |  |  |  |  |  |  |  |  | \| |  |  |  |  |  |  |  |  |  | \| |  |  |  |  |  |  |  |  |  | \| |  |  |  |  |  |  |  |  |  | \| |  |  |  |  |  |  |  |  |  | \| |  |  |  |  |  |  |  |  |  | \| |  |  |  |  |  |  |  |  |  | \| |  |  |  |  |  |  |  |  |  | \| |  |  |  |  |  |  |  |  |  | \| |  |  |  |  |  |  |  |  |  | \| |  |  |  |  |  |  |  |  |  | \| |  |  |  |  |  |  |  |  |  | \| |  |  |  |  |  |  |  |  |  | \| |  |  |  |  |  |  |  |  |  | \| |  |  |  |  |  |  |  |  |  | \| |  |  |  |  |  |  |  |  |  | \| |  |  |  |  |  |  |  |  |  | \| |  |  |  |  |  |  |  |  |  | \| |  |  |  |  |  |  |  |  |  | \| |  |  |  |  |  |  |  |  |  | \| |  |  |  |  |  |  |  |  |  | \| |  |  |  |  |  |  |  |  |  | \| |  |  |  |  |  |  |  |  |  | \| |  |  |  |  |  |  |  |  |  | \| |  |  |  |  |  |  |  |  |  | \| |  |  |  |  |  |  |  |  |  | \| |  |  |  |  |  |  |  |  |  | \| |  |  |  |  |  |  |  |  |  | \| |  |  |  |  |  |  |  |  |  | \| |  |  |  |  |  |  |  |  |  | \| |  |  |  |  |  |  |  |  |  | \| |  |  |  |  |  |  |  |  |  | \| |  |  |  |  |  |  |  |  |  | \| |  |  |  |  |  |  |  |  |  | \| |  |  |  |  |  |  |  |  |  | \| |  |  |  |  |  |  |  |  |  | \| |  |  |  |  |  |  |  |  |  | \| |  |  |  |  |  |  |  |  |  | \| |  |  |  |  |  |  |  |  |  | \| |  |  |  |  |  |  |  |  |  | \| |  |  |  |  |  |  |  |  |  | \| |  |  |  |  |  |  |  |  |  | \| |  |  |  |  |  |  |  |  |  | \| |  |  |  |  |  |  |  |  |  | \| |  |  |  |  |  |  |  |  |  | \| |  |  |  |  |  |  |  |  |  | \| |  |  |  |  |  |  |  |  |  | \| |  |  |  |  |  |  |  |  |  | \| |  |  |  |  |  |  |  |  |  | \| |  |  |  |  |  |  |  |  |  | \| |  |  |  |  |  |  |  |  |  | \| |  |  |  |  |  |  |  |  |  | \| |  |  |  |  |  |  |  |  |  | \| |  |  |  |  |  |  |  |  |  |
| CBTLR5\_2938\_BP | - | X | X | X | X | X | X | X | X | X | X | X | X | X | X | X | X | X | X | X | X | X | X | X | X | X | X | X | X | X | X | X | X | X | X | X | X | X | X | X | X | X | - | X | X | X | X | X | X | X | X | X | X | X | X | X | X | X | X | X | X | X | X | X | X | X | X | X | X | X | X | X | X | X | X | X | M | T | D | R | I | R | F | I | L | P | L | L | Q | F | - | - | C | I | C | L | L | V | A | E | S | T | P | K | C | L | I | K | D | E | K | A | Y | C | A | L | R | N | L | Y | E | V | P | A | L | P | P | Y | I | T | Y | L | D | L | T | L | N | Y | I | S | E | I | S | E | E | S | F | S | G | L | E | A | L | Q | X | L | X | X | Q | Q | Q | X | X | X | X | L | T | L | R | N | N | S | F | I | G | L | S | S | L | T | T | L | D | L | A | Y | N | M | N | L | Q | V | D | P | G | A | F | N | G | L | I | N | L | Q | I | L | N | L | T | E | C | R | L | S | D | S | I | L | S | G | D | Y | L | R | P | L | V | S | L | E | Q | L | S | L | P | G | N | N | I | H | K | L | R | P | A | S | F | F | I | N | M | S | K | L | H | T | V | D | V | S | H | N | W | I | N | S | F | C | E | E | D | L | F | H | F | Q | G | K | H | F | T | R | L | K | L | H | N | I | R | M | S | D | M | N | P | Y | W | T | G | W | D | K | C | G | N | P | F | K | D | M | S | M | T | V | L | D | L | S | L | N | G | F | S | V | D | V | A | V | R | F | F | K | A | I | R | G | T | K | I | Y | I | L | I | L | N | S | S | G | S | M | G | K | G | V | W | Y | T | N | L | K | D | P | D | R | D | T | F | K | D | L | A | E | S | G | I | K | A | L | D | L | S | K | V | S | I | F | T | L | K | N | S | V | F | S | Y | M | P | D | L | E | E | L | S | L | A | N | N | V | I | N | Q | I | E | S | D | A | F | N | G | X | X | D | K | L | Q | K | L | N | L | N | H | N | L | L | D | K | I | D | S | S | M | F | N | N | L | P | S | L | K | T | L | D | L | S | N | N | N | I | R | L | L | M | Y | Q | S | F | Q | G | L | P | N | L | A | N | L | F | L | S | E | N | S | L | Q | S | L | X | X | X | H | T | L | A | N | L | P | S | L | K | G | L | Y | V | D | N | N | K | X | X | X | T | S | L | Y | G | L | P | S | Q | V | R | N | I | T | T | I | D | L | R | Y | N | R | L | S | N | A | E | S | F | Y | T | I | L | V | E | F | P | N | V | E | E | I | F | L | G | G | N | V | F | S | S | C | F | L | N | S | K | Y | S | V | A | P | L | N | N | V | H | V | L | D | L | Q | M | T | G | L | Q | K | F | W | S | Q | G | Q | C | L | T | M | F | D | H | L | P | Q | L | Q | T | L | I | L | K | N | N | F | I | Q | S | L | P | K | N | I | F | N | R | L | T | S | L | G | Y | L | D | L | S | M | N | S | L | T | Y | I | P | N | G | I | F | P | R | S | L | K | T | L | N | L | A | F | N | R | L | G | S | V | D | P | Q | A | F | S | N | L | T | T | L | E | L | S | G | N | F | F | L | C | D | C | S | L | R | D | F | Q | R | W | L | T | Q | T | N | V | T | L | L | T | S | T | E | X | N | L | R | C | E | Y | P | K | K | Q | Q | G | K | P | L | L | H | A | X | X | L | C | R | X | X | D | R | X | X | X | X | V | X | X | X | X | X | - | - | - | X | X | X | X | X | X | X | X | X | X | X | X | X | X | X | X | X | X | X | X | X | X | X | X | X | X | X | X | X | X | X | X | X | X | X | X | X | X | X | X | X | X | X | X | X | X | X | - | - | X | X | X | X | X | X | X | X | X | X | X | X | X | X | X | X | X | X | X | X | X | X | X | X | X | X | X | X | X | X | X | X | X | X | X | X | X | X | X | X | X | X | X | X | X | X | X | X | X | X | X | X | X | X | X | X | X | X | X | X | X | X | X | X | X | X | X | X | X | X | X | X | X | X | X | X | X | X | X | X | X | X | X | X | X | X | X | X | X | X | X | X | X | X | X | X | X | X | X | X | X | X | X | X | X | X | X | X | X | X | X | X | X | X | X | X | X | X | X | X | X | X | X | X | X | X | X | X | X | X | X | X | X | X | X | X | X | X | X | X | X | X | X | X | X | X | X | X | X | X | X | X | X | X | X | X | X | X | X | X | X | X | X | X | X | X | X | X | X | X | X | X | X | - | - | - | X | X | - | - | - | - | - | - | - | X | X | X | X | X | X | X | X | X | X | X | X | X | X | X | X | X |
| IPTLR5\_2938\_BP | - | X | X | X | X | X | X | X | X | X | X | X | X | X | X | X | X | X | X | X | X | X | X | X | X | X | X | X | X | X | X | X | X | X | X | X | X | X | X | X | X | X | - | X | X | X | X | X | X | X | X | X | X | X | X | X | X | X | X | X | X | X | X | X | X | X | X | X | X | X | X | X | X | X | X | X | M | A | S | G | P | S | L | I | L | A | L | L | N | L | - | - | C | I | C | F | L | L | A | E | S | T | S | K | C | F | I | K | G | D | E | A | Y | C | A | L | K | N | L | Y | T | V | P | V | L | P | P | Y | I | A | Y | L | D | L | T | L | N | Y | I | S | E | I | H | E | K | S | F | Y | G | L | E | A | L | Q | I | L | L | I | Q | Q | Q | E | G | X | X | L | V | L | R | N | N | A | F | S | G | L | S | N | L | I | R | L | D | L | A | Y | N | K | D | L | Q | V | D | P | G | A | F | N | G | L | F | N | L | R | V | L | N | L | T | E | C | K | L | N | G | S | I | L | S | G | D | Y | L | R | P | L | V | S | L | E | Q | L | S | L | S | G | N | N | I | P | K | I | R | P | A | S | F | F | V | N | M | S | K | L | H | I | V | D | V | S | R | N | G | I | Y | S | F | C | E | E | D | L | F | H | F | Q | G | K | H | F | T | L | L | K | L | Q | D | I | K | M | T | D | M | T | P | Y | W | S | G | W | N | K | C | G | N | P | F | K | N | M | S | M | T | L | L | D | L | S | Q | N | G | F | S | V | D | V | A | V | L | F | F | K | A | I | R | G | T | K | I | H | T | L | I | L | N | H | S | G | S | M | G | K | G | V | W | Y | N | N | L | K | D | P | D | Q | N | T | F | T | D | L | S | E | S | G | V | K | A | L | D | L | S | N | A | R | I | F | A | L | R | N | S | V | F | R | H | M | P | D | L | E | E | I | S | L | S | A | N | L | I | N | Q | I | E | R | D | A | F | Y | G | X | X | D | N | L | R | T | L | N | L | S | H | N | L | L | D | K | I | D | S | G | T | F | K | N | V | R | S | L | E | T | L | D | L | S | N | N | N | I | R | I | L | G | S | E | S | F | Q | G | L | P | N | L | V | H | L | S | L | S | E | N | S | L | Q | Y | V | X | X | X | H | T | L | A | R | L | P | Q | L | K | K | L | F | L | D | S | N | K | X | X | X | T | S | L | Y | G | L | P | R | Q | A | R | N | V | T | T | I | D | L | R | N | N | R | L | R | N | A | E | D | V | Y | T | I | L | V | E | F | P | N | I | E | T | I | F | L | G | G | N | V | F | S | W | C | V | L | N | A | K | Y | S | V | S | P | L | N | K | V | Q | F | L | D | L | H | M | T | G | L | Q | N | L | W | L | Q | G | R | C | L | D | M | F | D | H | L | H | Q | L | Q | T | L | L | L | Q | Q | N | M | I | H | S | L | P | K | D | I | F | K | G | L | T | S | L | N | T | L | D | L | S | F | N | S | L | T | Y | I | P | N | D | I | F | P | R | S | L | R | I | L | K | L | A | H | N | H | L | G | S | V | D | P | Q | A | F | S | T | L | T | T | L | E | L | F | G | N | R | F | L | C | D | C | T | L | R | D | F | Q | R | W | L | S | Q | T | N | V | K | M | F | T | P | A | G | X | K | L | T | C | A | Y | P | E | Q | Q | R | G | K | S | L | L | H | A | X | X | L | C | K | X | X | D | K | X | X | X | X | V | X | X | X | X | X | - | - | - | X | X | X | X | X | X | X | X | X | X | X | X | X | X | X | X | X | X | X | X | X | X | X | X | X | X | X | X | X | X | X | X | X | X | X | X | X | X | X | X | X | X | X | X | X | X | X | - | - | X | X | X | X | X | X | X | X | X | X | X | X | X | X | X | X | X | X | X | X | X | X | X | X | X | X | X | X | X | X | X | X | X | X | X | X | X | X | X | X | X | X | X | X | X | X | X | X | X | X | X | X | X | X | X | X | X | X | X | X | X | X | X | X | X | X | X | X | X | X | X | X | X | X | X | X | X | X | X | X | X | X | X | X | X | X | X | X | X | X | X | X | X | X | X | X | X | X | X | X | X | X | X | X | X | X | X | X | X | X | X | X | X | X | X | X | X | X | X | X | X | X | X | X | X | X | X | X | X | X | X | X | X | X | X | X | X | X | X | X | X | X | X | X | X | X | X | X | X | X | X | X | X | X | X | X | X | X | X | X | X | X | X | X | X | X | X | X | X | X | X | X | X | - | - | - | X | X | - | - | - | - | - | - | - | X | X | X | X | X | X | X | X | X | X | X | X | X | X | X | X | X |
| TFTLR5\_2938\_BP | - | X | X | X | X | X | X | X | X | X | X | X | X | X | X | X | X | X | X | X | X | X | X | X | X | X | X | X | X | X | X | X | X | X | X | X | X | X | X | X | X | X | - | X | X | X | X | X | X | X | X | X | X | X | X | X | X | X | X | X | X | X | X | X | X | X | X | X | X | X | X | X | X | X | X | M | M | K | S | R | P | S | F | I | L | P | F | L | Q | S | - | - | W | I | C | L | L | L | A | E | S | P | S | N | C | F | I | R | D | D | K | A | Y | C | A | L | R | N | L | Y | E | V | P | V | L | P | P | N | I | T | Y | L | D | L | T | L | N | Y | I | S | E | I | H | E | K | S | F | Y | G | L | E | K | L | Q | I | L | V | I | Q | Q | Q | E | V | X | X | L | V | L | R | N | N | A | F | S | G | L | S | N | L | I | K | L | D | L | A | Y | N | T | N | L | R | V | D | P | G | A | F | N | G | L | F | N | L | Q | I | L | N | L | T | E | C | K | L | Y | D | S | I | L | S | G | D | Y | L | R | P | L | V | S | L | E | Q | L | S | L | P | G | N | N | I | R | K | I | R | P | A | P | F | F | V | N | M | N | K | L | H | I | V | D | V | S | H | N | W | I | N | S | F | C | E | E | D | L | V | H | F | Q | G | K | H | F | T | L | L | K | L | R | D | I | K | M | S | D | M | N | P | Y | W | G | S | W | N | K | C | G | N | P | F | K | N | M | S | M | T | V | L | D | L | S | L | N | G | F | S | V | N | M | A | V | L | F | F | R | A | I | R | G | T | K | I | H | N | L | I | L | E | Q | S | G | S | M | G | K | G | V | W | Y | N | N | F | K | D | P | D | R | D | T | F | M | D | L | S | E | S | D | I | K | A | L | D | L | S | K | A | S | I | F | A | L | E | N | S | V | F | G | Y | M | S | D | L | E | E | L | L | L | A | G | N | S | I | N | L | I | E | K | D | A | F | Y | G | X | X | D | N | L | R | T | L | N | L | S | H | N | L | L | D | K | I | Y | S | G | T | F | K | N | L | P | S | L | E | T | L | D | L | S | N | N | N | I | R | M | L | M | S | Q | S | F | H | G | L | S | N | L | V | H | L | S | L | S | E | N | S | L | Q | Y | V | X | X | X | H | T | L | A | H | L | P | R | L | K | K | L | R | L | D | D | N | R | X | X | X | T | S | L | H | G | L | P | S | K | A | R | N | V | T | T | I | D | L | R | H | N | K | L | S | N | A | E | S | F | Y | T | V | L | V | E | F | P | Q | I | E | K | I | Y | L | G | G | N | R | F | S | W | C | F | L | N | S | H | Y | S | V | S | P | L | N | N | V | R | F | L | D | L | H | M | T | G | L | Q | T | L | W | Q | Q | G | K | C | L | H | M | F | D | H | L | H | Q | L | Q | T | L | L | L | Q | Q | N | S | I | H | S | L | P | K | D | I | F | K | G | L | T | S | L | S | A | L | D | L | S | V | N | S | L | T | Y | I | S | N | D | V | F | P | K | S | L | S | T | L | K | L | A | H | N | H | L | G | S | V | D | P | R | A | F | S | T | L | T | E | L | D | L | S | A | N | R | F | L | C | D | C | S | L | R | D | L | Q | T | W | L | S | Q | K | R | V | K | M | L | T | A | A | E | X | E | L | T | C | E | Y | P | E | Q | Q | R | G | K | S | L | L | Q | A | X | X | L | C | K | X | X | D | K | X | X | X | X | L | X | X | X | X | X | - | - | - | X | X | X | X | X | X | X | X | X | X | X | X | X | X | X | X | X | X | X | X | X | X | X | X | X | X | X | X | X | X | X | X | X | X | X | X | X | X | X | X | X | X | X | X | X | X | X | - | - | X | X | X | X | X | X | X | X | X | X | X | X | X | X | X | X | X | X | X | X | X | X | X | X | X | X | X | X | X | X | X | X | X | X | X | X | X | X | X | X | X | X | X | X | X | X | X | X | X | X | X | X | X | X | X | X | X | X | X | X | X | X | X | X | X | X | X | X | X | X | X | X | X | X | X | X | X | X | X | X | X | X | X | X | X | X | X | X | X | X | X | X | X | X | X | X | X | X | X | X | X | X | X | X | X | X | X | X | X | X | X | X | X | X | X | X | X | X | X | X | X | X | X | X | X | X | X | X | X | X | X | X | X | X | X | X | X | X | X | X | X | X | X | X | X | X | X | X | X | X | X | X | X | X | X | X | X | X | X | X | X | X | X | X | X | X | X | X | X | X | X | X | X | - | - | - | X | X | - | - | - | - | - | - | - | X | X | X | X | X | X | X | X | X | X | X | X | X | X | X | X | X |
| SMTLR5\_2938\_BP | M | Y | M | Y | F | P | S | L | C | K | S | G | F | C | K | E | R | Q | M | E | I | - | P | L | S | V | M | F | A | V | Q | G | Q | R | T | P | R | V | T | Q | G | L | - | T | Y | K | N | S | Q | Q | K | S | L | Q | N | Q | R | P | V | G | C | E | A | G | E | T | G | S | L | F | Y | L | K | K | R | R | F | M | T | M | W | M | P | F | L | Q | L | V | A | I | - | - | C | I | F | L | Q | V | P | G | C | F | P | S | C | L | I | R | G | S | V | A | N | C | A | F | R | G | L | V | S | V | P | A | L | P | A | H | V | T | H | L | Y | L | E | M | N | R | I | G | E | I | N | S | T | S | L | S | G | L | E | Q | L | Q | E | L | D | L | G | R | Q | Y | V | X | X | L | V | V | R | N | D | A | F | S | G | Q | S | R | M | R | K | L | T | L | G | F | N | V | G | L | R | L | E | P | Q | A | F | V | G | L | S | G | L | Q | E | L | H | L | D | Y | C | S | L | D | D | S | V | L | K | E | N | I | L | Q | P | L | S | S | L | E | T | L | D | L | F | G | N | Q | I | K | T | L | Q | P | S | R | F | F | X | X | I | S | N | L | T | D | L | N | L | K | L | N | K | I | D | K | I | C | E | S | D | L | A | G | F | R | G | K | H | F | K | V | L | N | L | D | S | V | N | L | K | A | M | S | K | D | S | F | D | W | Q | K | C | G | N | P | F | R | G | M | S | F | Q | T | L | D | L | S | S | N | G | L | S | V | A | R | S | K | Q | F | F | R | A | I | E | G | T | K | I | A | H | L | K | L | S | X | X | X | H | M | G | K | G | F | S | F | N | N | L | P | D | P | D | R | T | T | F | D | G | L | K | N | S | S | V | L | T | L | D | L | S | K | N | R | I | F | A | L | Q | H | G | V | F | S | P | L | Q | E | V | V | H | I | D | I | S | Q | N | R | V | N | Q | I | H | R | N | A | F | E | G | L | Q | G | H | L | K | L | L | N | L | S | H | N | L | L | G | E | I | Y | S | H | T | F | A | P | L | A | N | L | Q | V | L | D | L | S | H | N | H | I | G | A | L | G | Y | D | S | F | S | G | L | H | L | L | R | A | L | N | L | K | G | N | S | L | R | E | L | X | X | X | G | F | P | A | S | L | P | S | L | N | D | L | Q | L | S | D | N | R | L | T | P | S | S | V | S | S | I | T | R | F | A | S | N | I | V | H | L | D | I | R | D | N | R | L | T | N | L | A | D | V | H | T | F | G | T | H | L | K | R | L | K | H | L | A | F | G | G | N | T | I | R | G | C | T | L | S | A | R | V | P | S | T | G | L | N | H | L | Q | V | L | D | L | H | S | S | S | L | Q | S | L | W | S | Q | G | K | C | L | N | L | F | D | S | L | E | H | L | V | V | L | N | L | S | F | N | A | L | R | T | L | P | P | G | V | F | K | G | L | A | S | A | V | E | M | D | L | S | S | N | A | L | T | Y | L | Q | P | D | V | L | P | K | S | L | K | V | L | V | L | S | D | N | F | V | A | S | P | D | P | A | A | L | R | S | L | R | L | L | D | L | G | M | N | R | F | R | C | D | A | N | L | G | S | F | L | T | W | L | N | E | T | D | V | T | F | L | G | P | V | E | X | E | L | R | C | E | F | P | A | G | F | Y | R | V | P | L | L | N | Y | S | V | Q | V | T | Q | Q | S | N | E | L | Y | V | H | E | M | R | G | E | - | - | - | A | D | V | F | S | S | S | Y | S | R | G | R | P | Q | P | S | Q | E | T | D | C | C | P | S | C | C | S | L | S | S | E | A | S | S | S | S | I | I | N | Q | T | L | Q | Q | Q | Q | L | - | - | R | E | R | I | Q | T | N | D | L | S | G | D | I | V | E | M | X | X | X | X | X | X | X | X | X | X | X | X | X | X | X | X | X | X | X | X | X | X | X | X | X | X | X | X | X | X | X | X | X | X | X | X | X | X | X | X | X | X | X | X | X | X | X | X | X | X | X | X | X | X | X | X | X | X | X | X | X | X | X | X | X | X | X | X | X | X | X | X | X | X | X | X | X | X | X | X | X | X | X | X | X | X | X | X | X | X | X | X | X | X | X | X | X | X | X | X | X | X | X | X | X | X | X | X | X | X | X | X | X | X | X | X | X | X | X | X | X | X | X | X | X | X | X | X | X | X | X | X | X | X | X | X | X | X | X | X | X | X | X | X | X | X | X | X | X | X | X | X | X | X | X | X | X | - | - | - | X | X | - | - | - | - | - | - | - | X | X | X | X | X | X | X | X | X | X | X | X | X | X | X | X | X |
| ONTLR5\_2938\_BP | - | - | M | Y | L | P | A | H | V | T | Q | T | L | T | D | K | N | A | A | A | K | S | S | A | E | F | R | F | L | L | S | T | G | R | I | G | V | F | S | S | K | T | E | D | S | K | R | X | X | X | X | X | X | X | X | X | X | X | X | X | X | X | X | X | X | X | X | X | X | F | F | X | X | X | X | R | S | R | M | M | W | T | V | G | L | Q | A | V | A | I | - | - | C | V | F | L | Q | V | S | G | C | F | S | S | C | L | I | K | G | S | V | A | Y | C | G | F | Q | N | L | R | S | V | P | P | L | P | P | D | I | T | H | L | Y | L | E | M | N | H | I | S | E | I | N | S | T | S | L | S | G | L | E | E | L | Q | V | L | D | L | G | R | Q | H | A | X | X | L | V | I | R | N | H | A | F | S | R | Q | K | H | L | R | R | L | S | L | D | F | N | I | G | L | Q | L | E | P | Q | A | F | E | G | L | A | S | L | Q | I | L | N | L | D | Y | C | S | L | K | E | S | I | L | R | D | K | Y | L | E | P | L | L | S | L | Q | T | L | S | L | F | G | N | D | I | K | R | L | Q | P | S | P | F | F | V | N | M | T | H | L | K | D | L | N | L | K | L | N | K | I | D | Q | I | C | E | A | D | L | A | A | F | Q | G | K | T | F | T | V | L | N | L | N | S | V | Y | L | R | E | M | S | S | G | K | F | D | W | K | K | C | G | N | P | F | R | D | L | T | F | Q | T | L | D | L | S | S | N | G | F | G | V | G | Q | C | T | Q | F | F | K | A | I | E | G | T | K | I | S | H | L | K | M | S | X | X | X | H | M | G | R | G | F | S | F | N | N | L | P | D | P | D | R | S | T | F | E | G | L | H | N | S | S | V | L | S | L | D | L | S | K | S | R | I | F | A | L | Q | Q | G | V | F | S | A | L | T | E | V | T | I | I | D | I | S | Q | N | K | V | N | Q | I | H | R | N | A | F | E | G | L | Q | D | H | L | K | V | L | N | L | S | H | N | L | L | G | E | I | Y | S | Y | T | F | A | S | L | T | N | L | Q | V | L | D | L | S | H | N | H | I | G | A | L | G | Y | Q | S | F | S | R | L | P | N | L | K | L | L | Y | L | T | G | N | S | L | R | D | L | X | X | X | G | F | P | A | P | L | P | S | L | D | F | L | L | L | N | D | N | K | L | A | P | S | S | V | S | S | I | S | Q | F | A | S | N | L | M | H | L | D | L | Q | D | N | R | L | T | D | L | D | D | V | S | T | I | L | T | G | L | K | R | L | R | H | L | F | F | G | G | N | R | I | R | S | C | S | F | S | R | R | X | X | X | X | G | L | N | N | L | T | V | L | D | L | H | G | S | S | L | Q | S | V | W | T | Q | G | K | C | L | N | L | F | D | N | F | G | H | V | A | G | L | N | L | S | F | N | A | L | Q | S | L | P | Q | G | V | F | K | G | L | T | S | V | R | E | M | D | L | S | Y | N | A | L | T | Y | I | Q | P | D | A | F | P | K | S | L | K | A | L | Y | L | S | N | N | F | I | A | S | P | N | P | A | T | F | R | S | L | S | F | L | D | L | R | M | N | R | F | H | C | N | T | D | L | K | S | F | L | T | W | L | N | E | T | N | V | R | L | L | S | P | V | E | X | E | L | R | C | E | F | P | A | S | V | F | K | V | P | L | I | E | F | S | A | M | V | S | Q | Q | X | X | X | X | X | X | X | X | X | X | X | X | - | - | - | X | X | X | X | X | X | X | X | X | X | X | X | X | X | X | X | X | X | X | X | X | X | X | X | X | X | X | X | X | X | X | X | X | X | X | X | X | X | X | X | X | X | X | X | X | X | X | - | - | X | X | X | X | X | X | X | X | X | X | X | X | X | X | X | X | X | X | X | X | X | X | X | X | X | X | X | X | X | X | X | X | X | X | X | X | X | X | X | X | X | X | X | X | X | X | X | X | X | X | X | X | X | X | X | X | X | X | X | X | X | X | X | X | X | X | X | X | X | X | X | X | X | X | X | X | X | X | X | X | X | X | X | X | X | X | X | X | X | X | X | X | X | X | X | X | X | X | X | X | X | X | X | X | X | X | X | X | X | X | X | X | X | X | X | X | X | X | X | X | X | X | X | X | X | X | X | X | X | X | X | X | X | X | X | X | X | X | X | X | X | X | X | X | X | X | X | X | X | X | X | X | X | X | X | X | X | X | X | X | X | X | X | X | X | X | X | X | X | X | X | X | X | - | - | - | X | X | - | - | - | - | - | - | - | X | X | X | X | X | X | X | X | X | X | X | X | X | X | X | X | X |
| TRTLR5\_2938\_BP | - | X | X | X | X | X | X | X | X | X | X | X | X | X | X | X | X | X | X | X | X | X | - | X | X | X | X | X | X | X | X | X | X | X | X | X | X | X | X | X | X | X | X | X | X | X | X | X | X | X | X | X | X | X | X | X | X | X | X | X | X | X | X | X | X | X | X | X | X | X | X | X | X | X | X | X | X | X | X | M | X | X | L | L | A | P | A | S | L | V | - | - | C | V | L | L | Q | L | P | G | A | F | P | S | C | L | S | L | G | S | A | A | L | C | S | S | R | S | L | D | R | V | P | P | L | P | P | H | V | T | H | L | Y | L | E | M | N | R | I | A | E | I | N | S | S | S | L | S | G | L | E | Q | L | Q | V | L | D | L | G | S | Q | M | V | X | X | L | V | I | R | N | E | A | F | S | R | L | R | H | L | R | S | L | V | L | G | F | N | R | G | L | Q | L | E | P | R | A | F | V | G | L | S | R | V | K | V | L | H | L | D | Y | C | S | L | N | D | S | I | L | T | E | N | F | L | E | P | L | A | S | L | E | T | L | N | L | F | G | N | K | I | K | R | I | R | P | S | A | F | F | A | N | M | T | K | L | K | F | I | N | L | K | L | N | S | I | D | R | I | C | E | P | D | L | E | S | F | Q | G | K | H | F | G | A | L | D | V | S | S | A | R | F | L | G | L | S | S | K | G | F | D | W | K | T | C | G | N | P | F | K | G | M | S | F | H | T | L | D | L | S | H | N | G | F | N | A | A | K | S | Q | Q | F | F | R | A | V | Q | G | T | K | I | S | H | L | K | L | A | X | X | X | Y | Q | G | K | G | F | S | F | N | N | L | P | D | P | D | S | S | T | F | E | G | L | R | N | S | S | V | L | T | L | D | L | S | Q | G | R | I | F | A | L | R | Q | G | V | F | S | W | L | T | V | A | T | I | I | D | V | S | Q | N | R | V | N | Q | I | H | R | N | A | F | E | G | L | E | G | R | L | K | M | L | N | L | S | H | N | L | L | G | E | V | Y | S | H | T | F | A | S | L | T | H | L | K | V | L | D | L | S | H | N | H | I | G | V | L | G | Y | G | S | F | R | G | L | P | N | L | E | A | L | F | L | T | G | N | S | L | S | R | L | X | X | X | G | F | P | S | P | L | P | K | L | Q | Y | L | H | L | R | D | N | K | X | X | X | S | S | A | S | S | L | A | Q | F | A | W | N | I | K | Y | L | N | I | K | E | N | H | L | T | N | L | E | D | V | H | V | F | L | D | Q | L | R | E | L | Q | I | L | L | Y | G | G | N | T | V | R | W | C | T | L | S | Q | N | G | S | A | A | V | W | N | K | L | Q | V | L | D | L | H | G | S | S | L | Q | A | V | W | A | Q | G | Q | C | L | N | L | F | D | G | L | G | H | V | A | D | L | N | L | S | S | N | Q | L | Q | S | L | P | Q | G | V | F | Q | G | L | A | S | V | V | T | M | D | L | S | S | N | A | L | T | Y | L | Q | P | D | V | L | P | H | S | L | R | K | L | Y | L | S | N | N | F | I | A | A | P | D | P | A | A | F | R | S | L | S | H | L | D | L | S | S | N | R | F | H | C | T | R | N | L | R | G | F | L | T | W | L | N | Q | T | E | V | T | F | L | S | P | V | H | X | K | L | R | C | E | F | P | L | G | W | Y | K | V | P | L | L | E | Y | A | A | Q | F | A | Q | Q | X | X | X | X | X | X | X | X | X | X | X | X | - | - | - | X | X | X | X | X | X | X | X | X | X | X | X | X | X | X | X | X | X | X | X | X | X | X | X | X | X | X | X | X | X | X | X | X | X | X | X | X | X | X | X | X | X | X | X | X | X | X | - | - | X | X | X | X | X | X | X | X | X | X | X | X | X | X | X | X | X | X | X | X | X | X | X | X | X | X | X | X | X | X | X | X | X | X | X | X | X | X | X | X | X | X | X | X | X | X | X | X | X | X | X | X | X | X | X | X | X | X | X | X | X | X | X | X | X | X | X | X | X | X | X | X | X | X | X | X | X | X | X | X | X | X | X | X | X | X | X | X | X | X | X | X | X | X | X | X | X | X | X | X | X | X | X | X | X | X | X | X | X | X | X | X | X | X | X | X | X | X | X | X | X | X | X | X | X | X | X | X | X | X | X | X | X | X | X | X | X | X | X | X | X | X | X | X | X | X | X | X | X | X | X | X | X | X | X | X | X | X | X | X | X | X | X | X | X | X | X | X | X | X | X | X | X | - | - | - | X | X | - | - | - | - | - | - | - | X | X | X | X | X | X | X | X | X | X | X | X | X | X | X | X | X |
| ATTLR5\_2938\_BP | - | X | X | X | X | X | X | X | X | X | X | X | X | X | X | X | X | X | X | X | X | X | - | X | X | X | X | X | X | X | X | X | X | X | X | X | X | X | X | X | X | X | X | X | X | X | X | X | X | X | X | X | X | X | X | X | X | X | X | X | X | X | X | X | X | X | X | X | X | X | X | X | X | X | X | X | X | X | X | M | W | T | L | G | V | Q | V | V | I | V | - | - | C | V | F | L | Q | V | P | G | C | F | P | S | C | L | I | F | G | S | V | A | D | C | A | S | R | R | L | S | S | V | P | A | L | P | P | N | I | T | H | L | F | L | D | R | N | Y | I | S | E | I | N | S | T | S | L | S | G | L | E | Q | L | Q | D | L | D | L | G | L | Q | H | V | X | X | L | V | I | R | K | Y | S | F | R | T | Q | R | H | L | R | R | L | V | L | G | S | N | I | G | L | Q | L | E | P | Q | A | F | V | G | L | S | S | L | Q | K | L | D | L | D | F | C | S | L | H | D | S | I | L | T | E | N | Y | L | E | P | L | S | S | L | E | T | L | N | L | F | G | N | Q | I | R | R | L | Q | P | S | M | F | F | A | N | K | T | N | L | K | D | L | N | L | K | L | N | K | I | D | R | I | C | E | S | D | L | A | G | F | Q | G | K | H | F | K | V | L | N | L | Q | S | I | Q | L | X | X | X | Y | N | K | D | F | D | W | Q | K | C | G | N | P | F | R | G | M | S | F | D | T | L | D | L | S | N | N | G | L | S | I | A | T | S | K | Q | F | F | K | A | I | E | G | T | K | I | S | H | L | K | L | S | X | X | X | L | M | G | K | G | F | S | F | N | N | F | P | D | P | D | H | S | T | F | E | G | L | K | S | S | S | V | K | T | L | D | L | S | K | S | R | I | F | A | L | K | Q | G | V | F | S | A | L | K | E | V | V | N | I | D | V | S | Q | N | K | V | N | Q | I | D | R | N | A | F | E | G | L | Q | D | H | L | K | L | L | N | L | S | H | N | L | L | G | E | I | H | S | H | A | F | A | S | L | K | T | L | R | V | L | D | L | S | H | N | H | I | G | V | L | G | Y | G | S | F | Q | G | L | P | H | L | T | W | L | D | L | R | G | N | S | L | R | D | L | X | X | X | G | F | P | A | S | L | P | S | L | D | Y | L | L | L | R | D | N | K | X | X | X | S | S | V | S | S | I | P | S | M | A | S | N | I | M | H | L | D | V | Q | D | N | R | L | T | N | L | N | D | V | N | T | L | L | T | S | L | K | R | L | K | H | L | F | F | G | G | N | T | I | R | W | C | T | L | S | X | X | X | S | A | T | A | L | D | S | L | Q | I | L | D | L | H | S | S | S | L | Q | E | I | W | S | Q | G | R | C | L | S | L | F | D | D | L | G | H | V | L | G | L | N | L | S | F | N | A | L | Q | T | L | P | R | G | I | F | K | G | L | T | S | L | T | E | M | D | L | S | S | N | A | L | T | Y | L | Q | P | G | V | L | P | K | S | L | K | L | L | H | L | A | N | N | F | I | A | S | P | N | P | D | A | F | G | S | V | K | F | L | D | L | R | M | N | R | F | H | C | N | S | N | L | K | S | F | L | T | W | M | T | K | T | N | V | T | F | L | S | P | A | E | X | Q | L | Q | C | E | F | P | S | R | F | R | N | V | P | L | L | D | F | Y | S | R | I | T | H | T | S | K | X | X | X | X | Q | F | K | X | X | X | - | - | - | X | X | X | X | X | X | X | X | X | X | X | X | X | X | X | X | X | X | X | X | X | X | X | X | X | X | X | X | X | X | X | X | X | X | X | X | X | X | X | X | X | X | X | X | X | X | X | - | - | X | X | X | X | X | X | X | X | X | X | X | X | X | X | X | X | X | X | X | X | X | X | X | X | X | X | X | X | X | X | X | X | X | X | X | X | X | X | X | X | X | X | X | X | X | X | X | X | X | X | X | X | X | X | X | X | X | X | X | X | X | X | X | X | X | X | X | X | X | X | X | X | X | X | X | X | X | X | X | X | X | X | X | X | X | X | X | X | X | X | X | X | X | X | X | X | X | X | X | X | X | X | X | X | X | X | X | X | X | X | X | X | X | X | X | X | X | X | X | X | X | X | X | X | X | X | X | X | X | X | X | X | X | X | X | X | X | X | X | X | X | X | X | X | X | X | X | X | X | X | X | X | X | X | X | X | X | X | X | X | X | X | X | X | X | X | X | X | X | X | X | X | X | - | - | - | X | X | - | - | - | - | - | - | - | X | X | X | X | X | X | X | X | X | X | X | X | X | X | X | X | X |
| PFTLR5\_2938\_BP | - | X | X | X | X | X | X | X | X | X | X | X | X | X | X | X | X | X | X | X | X | X | - | X | X | X | X | X | X | X | X | X | X | X | X | X | X | X | X | X | X | X | X | X | X | X | X | X | X | X | X | X | X | X | X | X | X | X | X | X | X | X | X | X | X | X | X | X | X | X | X | X | X | X | X | X | X | X | X | M | W | T | L | G | L | H | V | V | V | I | - | - | C | V | F | L | Q | V | P | G | C | F | P | S | C | L | I | I | G | S | V | A | N | C | G | S | M | N | L | R | W | V | P | P | L | P | P | H | I | T | H | L | Y | L | D | L | N | H | I | S | E | I | N | S | T | S | L | T | G | L | E | E | L | Q | E | L | D | L | G | G | Q | R | V | X | X | L | V | I | R | N | N | A | F | S | G | Q | R | R | L | R | K | L | V | L | G | F | N | V | G | L | Q | L | E | P | Q | A | F | A | G | L | S | G | L | Q | N | L | Y | L | D | Y | C | S | L | H | E | S | I | L | Q | E | N | Y | L | E | P | L | S | S | L | E | T | L | D | L | F | G | N | Q | I | R | R | L | Q | P | S | M | F | F | V | N | M | T | K | F | T | V | L | N | L | K | L | N | N | I | D | R | I | C | E | S | D | L | V | G | F | Q | G | K | N | F | K | V | L | N | L | D | S | V | H | L | N | L | M | S | D | E | H | F | D | W | Q | K | C | G | N | P | F | S | G | M | S | F | Q | T | L | D | L | S | S | I | G | F | N | V | G | H | I | K | Q | L | F | K | A | I | K | G | T | K | I | S | H | L | K | L | S | X | X | X | H | I | G | K | G | F | S | F | N | N | L | P | D | P | D | R | S | M | F | E | G | L | N | D | S | S | V | H | T | L | D | L | S | K | N | R | I | F | A | L | Q | Q | G | F | F | S | L | L | K | D | V | A | I | I | D | V | S | Q | N | K | V | N | Q | I | H | R | N | A | F | E | G | L | Q | G | H | L | Q | T | L | N | L | S | H | N | L | L | G | E | I | R | S | Y | T | F | A | S | L | I | N | L | R | V | L | D | L | S | Y | N | H | I | G | V | L | G | V | G | S | F | S | G | L | P | K | L | K | A | L | Y | L | T | G | N | S | L | Q | N | I | X | X | X | G | F | P | A | S | L | P | S | L | D | Y | L | L | L | N | D | N | K | X | X | X | S | S | V | S | G | L | T | T | F | X | X | H | I | T | H | L | N | I | Q | D | N | R | L | T | N | L | G | D | L | Y | T | I | L | F | L | L | G | H | L | Q | H | L | S | Y | G | G | N | A | V | R | R | C | W | L | S | G | Q | P | S | K | L | G | L | N | N | V | Q | T | L | D | L | H | S | C | S | L | Q | S | V | W | S | Q | G | R | C | L | N | L | F | D | N | L | G | H | V | I | S | L | N | L | S | F | N | A | L | K | S | L | P | P | G | I | F | K | S | L | T | S | V | V | E | M | D | L | S | S | N | S | L | T | Y | L | Q | P | D | V | L | P | K | C | L | K | V | L | N | L | S | N | N | F | I | G | S | P | D | P | S | A | F | R | S | L | S | A | L | D | L | K | M | N | R | F | H | C | N | S | Y | L | K | S | F | L | T | W | L | N | K | T | N | V | T | F | L | S | P | V | E | X | E | L | R | C | E | F | P | P | A | F | Y | N | V | P | L | V | R | F | V | A | H | X | X | X | X | X | X | X | X | X | X | X | X | X | X | X | X | - | - | - | X | X | X | X | X | X | X | X | X | X | X | X | X | X | X | X | X | X | X | X | X | X | X | X | X | X | X | X | X | X | X | X | X | X | X | X | X | X | X | X | X | X | X | X | X | X | X | - | - | X | X | X | X | X | X | X | X | X | X | X | X | X | X | X | X | X | X | X | X | X | X | X | X | X | X | X | X | X | X | X | X | X | X | X | X | X | X | X | X | X | X | X | X | X | X | X | X | X | X | X | X | X | X | X | X | X | X | X | X | X | X | X | X | X | X | X | X | X | X | X | X | X | X | X | X | X | X | X | X | X | X | X | X | X | X | X | X | X | X | X | X | X | X | X | X | X | X | X | X | X | X | X | X | X | X | X | X | X | X | X | X | X | X | X | X | X | X | X | X | X | X | X | X | X | X | X | X | X | X | X | X | X | X | X | X | X | X | X | X | X | X | X | X | X | X | X | X | X | X | X | X | X | X | X | X | X | X | X | X | X | X | X | X | X | X | X | X | X | X | X | X | X | - | - | - | X | X | - | - | - | - | - | - | - | X | X | X | X | X | X | X | X | X | X | X | X | X | X | X | X | X |
| SATLR5\_2938\_BP | - | X | X | X | X | X | X | X | X | X | X | X | X | X | X | X | X | X | X | X | X | X | - | X | X | X | X | X | X | X | X | X | X | X | X | X | X | X | X | X | X | X | X | X | X | X | X | X | X | X | X | X | X | X | X | X | X | X | X | X | X | X | X | X | X | X | X | X | X | X | X | X | X | X | X | X | X | X | X | M | L | T | L | G | L | Q | V | I | V | I | - | - | C | V | F | L | Q | V | P | G | C | F | P | S | C | L | I | V | G | S | V | A | N | C | A | S | R | N | L | R | S | V | P | P | L | P | P | N | I | T | H | L | Y | L | E | M | N | R | I | A | E | I | N | S | T | S | L | S | G | L | K | E | L | Q | E | L | D | L | G | Y | Q | Y | V | P | A | F | V | I | R | N | N | A | F | S | G | Q | R | R | L | R | R | L | V | L | G | Y | N | K | G | I | R | L | E | P | K | A | F | E | G | L | S | G | L | K | N | L | Y | L | D | Y | C | S | L | E | D | D | I | L | M | E | N | Y | L | E | P | L | S | S | L | E | T | L | D | L | F | G | N | K | I | K | K | L | Q | P | A | M | F | F | A | N | M | T | N | L | K | D | V | N | L | K | L | N | L | I | D | K | I | C | E | S | D | L | V | G | F | R | G | K | H | F | E | V | L | N | L | D | S | V | L | L | K | A | M | S | S | K | G | F | D | W | Q | T | C | G | N | P | F | R | G | M | S | F | Q | T | L | Y | L | S | N | S | G | L | S | V | H | R | L | K | Q | L | F | R | A | I | E | G | T | K | I | S | C | L | Q | L | S | X | X | X | H | I | G | R | G | F | S | F | N | N | F | P | D | P | D | R | S | T | F | E | G | L | K | N | S | S | I | L | T | L | D | L | S | K | N | R | I | F | S | L | Q | Q | G | V | F | S | Q | L | K | E | V | A | T | I | D | I | S | Q | N | S | L | N | Q | I | H | R | D | A | F | E | G | L | Q | G | H | L | K | M | L | N | L | S | H | N | L | L | G | E | I | R | S | Y | T | F | A | S | L | T | N | L | K | V | L | D | L | S | H | N | H | I | G | V | L | G | F | H | S | F | D | G | L | N | N | L | K | S | L | I | L | T | G | N | S | L | R | D | L | X | X | X | G | F | P | A | S | L | P | S | L | D | Y | L | L | L | N | D | N | K | L | T | S | S | S | L | F | S | L | T | R | F | A | D | N | A | M | H | L | D | I | R | D | N | R | L | T | D | L | G | D | V | Y | T | I | L | A | R | L | E | R | L | Q | H | L | F | Y | G | G | N | T | I | R | E | C | K | L | N | R | Q | V | S | A | A | D | L | N | N | F | Q | T | L | D | L | H | S | S | S | L | Q | L | I | W | S | Q | G | K | C | L | N | L | F | D | Y | L | R | N | V | T | V | L | N | L | S | S | N | A | L | Q | S | L | P | Q | D | V | F | K | G | L | R | S | V | V | T | M | D | L | S | F | N | A | L | T | Y | L | Q | P | D | I | L | P | K | S | I | Q | V | L | N | L | S | N | N | F | I | T | S | P | D | P | T | A | F | R | S | L | S | S | L | D | I | S | V | N | R | F | R | C | D | P | N | L | K | S | F | L | T | W | L | S | N | T | N | V | T | F | L | S | P | V | K | X | K | L | R | C | E | F | P | S | S | F | Y | N | V | R | L | L | E | Y | S | A | Q | V | S | H | Q | X | X | X | X | X | X | X | X | X | X | X | X | - | - | - | X | X | X | X | X | X | X | X | X | X | X | X | X | X | X | X | X | X | X | X | X | X | X | X | X | X | X | X | X | X | X | X | X | X | X | X | X | X | X | X | X | X | X | X | X | X | X | - | - | X | X | X | X | X | X | X | X | X | X | X | X | X | X | X | X | X | X | X | X | X | X | X | X | X | X | X | X | X | X | X | X | X | X | X | X | X | X | X | X | X | X | X | X | X | X | X | X | X | X | X | X | X | X | X | X | X | X | X | X | X | X | X | X | X | X | X | X | X | X | X | X | X | X | X | X | X | X | X | X | X | X | X | X | X | X | X | X | X | X | X | X | X | X | X | X | X | X | X | X | X | X | X | X | X | X | X | X | X | X | X | X | X | X | X | X | X | X | X | X | X | X | X | X | X | X | X | X | X | X | X | X | X | X | X | X | X | X | X | X | X | X | X | X | X | X | X | X | X | X | X | X | X | X | X | X | X | X | X | X | X | X | X | X | X | X | X | X | X | X | X | X | X | - | - | - | X | X | - | - | - | - | - | - | - | X | X | X | X | X | X | X | X | X | X | X | X | X | X | X | X | X |
| SDTLR5\_2938\_BP | - | X | X | X | X | X | X | X | X | X | X | X | X | X | X | X | X | X | X | X | X | X | - | X | X | X | X | X | X | X | X | X | X | X | X | X | X | X | X | X | X | X | X | X | X | M | W | X | X | X | X | X | X | X | X | X | X | X | X | X | X | X | X | X | X | X | X | X | X | F | F | X | X | X | X | S | S | A | R | M | W | L | L | G | L | Q | V | V | A | I | - | - | C | V | F | L | Q | V | P | G | C | F | P | S | C | I | I | S | G | S | V | A | N | C | A | F | Q | N | L | V | S | V | P | A | L | P | S | H | I | T | H | L | Y | L | E | M | N | R | I | R | E | I | N | S | I | S | L | S | G | L | E | Q | L | Q | E | L | D | L | G | Q | Q | R | V | X | X | L | V | I | R | D | N | A | F | S | R | Q | S | R | L | R | K | L | I | L | G | F | N | I | G | L | R | L | E | P | Q | A | F | V | G | L | S | S | L | Q | Q | L | H | L | D | Y | C | S | L | Q | E | S | I | L | T | D | N | F | L | Q | P | L | S | S | L | E | T | L | D | L | F | G | N | Q | I | K | R | L | Q | P | S | M | F | F | A | N | L | T | N | L | R | D | L | N | L | K | L | N | K | I | D | K | I | C | G | S | D | L | A | G | F | Q | G | K | H | F | Q | V | L | N | L | D | S | V | H | I | K | A | M | S | D | K | G | F | D | W | Q | K | C | G | N | P | F | R | G | I | S | F | Q | T | L | D | L | S | Y | N | G | F | S | V | G | K | A | K | Q | F | F | R | A | I | E | G | T | K | I | S | H | L | K | L | S | X | X | X | F | M | G | K | G | F | S | F | N | N | F | P | D | P | D | N | S | T | F | C | G | L | N | N | S | S | V | H | I | L | D | L | S | K | N | R | I | F | S | L | R | Q | G | V | F | R | P | L | K | E | V | V | S | I | D | V | S | Q | N | R | V | N | Q | I | H | R | N | A | F | E | G | L | Q | G | H | L | K | M | L | N | L | S | H | N | L | L | G | E | I | Y | S | Y | T | F | A | S | L | T | N | L | Q | V | L | D | L | S | Y | N | H | I | G | V | L | G | F | G | S | F | S | G | L | P | N | L | K | A | L | Y | L | T | G | N | A | L | R | E | L | X | X | X | G | F | P | A | S | L | P | R | L | N | Y | L | L | L | N | D | N | K | L | T | S | S | S | V | G | A | I | T | Q | F | A | S | N | V | M | H | L | N | V | N | D | N | R | F | F | N | F | A | D | L | Y | T | F | L | T | R | L | K | R | L | Q | H | L | F | Y | G | G | N | T | I | R | W | C | T | L | S | R | Q | X | X | X | I | G | M | N | N | L | Q | V | L | D | L | H | S | S | S | L | Q | S | I | W | S | Q | G | K | C | L | N | L | F | D | N | L | G | H | V | V | S | L | N | L | S | F | N | A | L | Q | T | L | P | Q | G | I | F | K | G | L | T | S | V | V | Q | M | D | L | S | S | N | A | L | T | Y | L | Q | P | D | V | L | P | K | S | L | E | V | L | N | L | S | N | N | F | I | A | S | P | D | P | A | A | F | H | S | L | S | L | L | D | L | K | M | N | R | F | H | C | D | T | N | L | K | S | F | L | T | W | L | N | Q | T | N | A | T | F | L | S | P | V | Q | X | E | L | R | C | E | F | P | S | G | F | Y | M | V | P | L | L | T | Y | S | A | Q | V | T | Q | Q | X | X | X | X | X | X | X | X | X | X | X | X | - | - | - | X | X | X | X | X | X | X | X | X | X | X | X | X | X | X | X | X | X | X | X | X | X | X | X | X | X | X | X | X | X | X | X | X | X | X | X | X | X | X | X | X | X | X | X | X | X | X | - | - | X | X | X | X | X | X | X | X | X | X | X | X | X | X | X | X | X | X | X | X | X | X | X | X | X | X | X | X | X | X | X | X | X | X | X | X | X | X | X | X | X | X | X | X | X | X | X | X | X | X | X | X | X | X | X | X | X | X | X | X | X | X | X | X | X | X | X | X | X | X | X | X | X | X | X | X | X | X | X | X | X | X | X | X | X | X | X | X | X | X | X | X | X | X | X | X | X | X | X | X | X | X | X | X | X | X | X | X | X | X | X | X | X | X | X | X | X | X | X | X | X | X | X | X | X | X | X | X | X | X | X | X | X | X | X | X | X | X | X | X | X | X | X | X | X | X | X | X | X | X | X | X | X | X | X | X | X | X | X | X | X | X | X | X | X | X | X | X | X | X | X | X | X | - | - | - | X | X | - | - | - | - | - | - | - | X | X | X | X | X | X | X | X | X | X | X | X | X | X | X | X | X |
| DRTLR5\_2938\_BP | - | X | X | X | X | X | X | X | X | X | X | X | X | X | X | X | X | X | X | X | X | X | X | X | X | X | X | X | X | X | - | X | X | - | - | X | X | X | X | X | X | X | X | X | X | X | X | X | X | X | X | X | X | X | X | X | X | X | X | X | X | X | X | X | X | X | X | X | X | X | X | X | X | X | X | X | X | X | X | X | M | A | A | T | Y | T | L | F | L | M | L | L | F | I | W | T | P | T | V | K | S | T | S | V | C | S | T | D | G | Y | F | A | F | C | M | D | R | G | L | Q | E | V | P | K | I | S | T | Y | I | T | N | V | D | L | S | K | N | R | I | A | E | L | N | E | T | S | F | S | H | L | E | A | L | E | V | L | I | L | M | H | Q | T | P | X | X | L | V | I | R | R | N | S | F | M | R | L | S | N | L | T | S | L | Q | L | D | Y | N | H | H | L | R | I | D | A | G | A | F | N | G | L | S | D | L | K | N | L | T | L | T | Q | C | G | L | D | D | S | L | L | S | G | N | F | L | K | P | L | M | S | L | E | M | L | D | L | S | R | N | N | I | R | R | V | Q | P | A | S | F | F | L | N | M | R | R | F | H | V | L | D | L | T | F | N | K | V | K | S | I | C | E | E | D | L | L | N | F | Q | G | K | H | F | T | L | L | R | L | S | S | I | T | L | Q | D | M | N | E | Y | W | L | G | W | E | K | C | G | N | P | F | K | N | T | S | I | T | T | L | D | L | S | G | N | G | F | N | V | D | M | A | K | R | F | F | D | A | I | S | S | T | K | I | Q | S | L | I | I | S | N | T | Y | R | I | G | K | S | S | X | X | D | N | S | K | D | P | D | K | S | T | F | S | G | L | K | S | S | G | I | K | S | F | D | L | S | N | S | S | I | F | S | L | T | Y | S | V | F | S | Y | L | S | D | L | E | Q | I | T | L | A | E | S | Q | I | N | K | I | E | N | N | A | F | L | G | X | X | T | N | L | L | K | L | N | L | S | K | N | F | L | G | Y | I | D | F | R | T | F | Q | N | L | K | G | L | E | V | L | D | L | S | Y | N | H | I | W | R | L | G | S | Q | S | F | Q | G | L | P | N | L | L | S | L | N | L | T | G | N | S | L | G | H | V | X | X | X | Y | T | F | A | T | L | P | K | L | E | K | L | Y | L | G | D | N | N | X | X | X | Q | Y | V | Y | E | I | P | N | I | A | K | H | L | K | T | L | D | L | Q | F | N | Q | I | A | S | M | S | E | L | Y | T | I | L | D | E | F | P | Q | I | E | E | V | V | F | R | G | N | Q | L | L | Y | C | P | Q | D | N | H | X | X | X | V | L | S | Q | N | I | Q | V | L | D | L | S | F | A | G | L | Q | V | I | W | S | E | G | K | C | L | N | V | F | D | D | L | H | Q | L | E | V | L | H | L | S | S | N | L | L | Q | S | L | P | K | D | I | F | K | D | L | T | S | L | L | I | L | D | L | S | F | N | S | L | K | Y | L | P | T | D | V | F | P | K | T | L | Q | V | L | H | L | D | Y | N | S | I | Y | S | V | D | P | N | L | F | S | T | L | S | Y | L | S | L | M | N | N | D | F | R | C | D | C | D | L | K | D | F | Q | T | W | L | N | Q | T | N | I | T | F | V | H | S | I | E | X | D | V | T | C | A | S | P | E | D | Q | Y | M | V | P | V | V | S | S | S | I | Q | C | E | X | X | D | E | X | X | X | X | G | G | E | K | N | X | K | T | K | T | C | A | F | Y | F | L | H | C | I | D | Y | I | I | Q | R | Q | H | H | C | L | H | Q | S | - | - | - | - | - | - | - | - | - | - | T | R | C | H | L | Q | D | I | Q | K | T | N | W | Q | I | X | Q | X | T | S | R | G | T | X | C | K | W | I | F | V | R | C | V | P | V | F | Q | F | Q | R | H | E | V | G | R | K | S | V | V | E | E | A | G | L | S | V | F | R | A | Q | H | T | P | L | L | L | R | G | A | R | L | H | P | R | G | G | P | S | Y | Q | Y | E | K | C | H | P | A | X | S | K | N | H | L | R | G | V | X | T | L | S | E | G | R | L | V | S | R | N | I | H | P | G | T | M | X | D | A | S | G | A | X | G | H | S | G | G | A | G | C | R | K | H | T | A | V | Q | V | I | K | V | X | T | T | E | I | L | H | X | E | Q | K | L | P | R | V | A | X | X | R | S | R | L | G | V | V | L | X | P | T | S | A | Q | D | N | Q | K | H | Q | G | X | T | X | X | X | X | X | X | Q | - | - | - | X | X | X | R | K | R | Q | G | G | X | K | Q | S | X | S | S | R | C | S | S | R | H | C | S | - | - |
| MATLR5\_2938\_BP | - | X | X | X | X | X | X | X | X | X | X | X | X | X | X | X | X | X | X | X | X | X | X | X | X | X | X | X | X | X | - | X | X | - | - | X | X | X | X | X | X | X | X | X | X | X | X | X | X | X | X | X | X | X | X | X | X | X | X | X | X | X | X | X | X | X | X | X | X | X | X | X | X | X | X | X | X | X | X | X | M | G | F | T | F | I | L | I | L | F | G | L | C | I | N | T | E | V | V | K | C | T | S | V | C | L | V | N | G | Y | A | A | F | C | A | S | R | G | L | H | Q | V | P | E | L | P | T | Y | V | N | Y | V | D | L | N | F | N | S | I | A | E | L | N | E | T | S | F | S | R | L | E | G | L | Q | V | L | K | L | E | Q | Q | T | T | X | X | L | V | I | R | N | N | T | F | R | R | L | S | N | L | I | L | L | Q | L | D | Y | N | R | F | L | Q | I | E | T | G | A | F | N | G | L | S | N | L | E | I | L | T | L | T | Q | C | S | L | D | D | A | I | L | S | G | D | F | L | K | P | L | V | S | L | E | M | L | V | L | R | E | N | N | I | K | R | I | Q | P | A | S | F | F | L | N | M | K | K | F | H | V | L | N | L | S | R | N | K | V | K | S | I | C | E | E | D | L | L | S | F | Q | G | K | H | F | T | L | L | K | L | S | S | V | T | L | Q | D | M | N | E | Y | W | L | G | W | E | K | C | G | N | P | F | K | N | M | S | I | T | I | L | D | L | S | G | N | G | F | N | V | N | M | A | K | R | F | F | D | A | I | T | G | T | K | I | Q | S | L | I | L | S | N | S | Y | S | M | G | S | S | F | S | Y | N | N | F | K | D | P | D | K | F | T | F | K | G | L | G | D | S | G | I | E | I | F | D | L | S | K | S | K | I | F | A | L | S | S | S | V | F | S | H | F | Q | D | L | E | Q | I | T | L | A | E | N | E | I | N | I | I | E | I | D | S | F | W | G | X | X | T | N | L | L | K | L | N | L | S | K | N | F | L | G | S | I | D | S | N | T | F | Q | N | L | E | K | L | E | V | L | D | L | S | Y | N | H | I | R | V | L | G | D | K | S | F | Q | G | L | P | S | L | L | N | L | N | L | T | G | N | A | L | E | S | V | X | X | X | H | E | F | A | T | L | P | N | L | K | I | I | Y | L | G | D | N | K | X | X | X | S | S | L | S | S | L | P | N | I | A | K | I | L | T | T | L | D | L | D | S | N | Q | L | S | S | M | S | D | L | Y | A | I | L | Q | E | F | P | Q | I | E | E | I | F | L | E | G | N | E | L | I | N | C | P | N | D | A | H | X | X | X | V | L | S | Q | K | V | Q | I | L | D | L | A | S | A | G | L | E | V | I | W | S | E | G | K | C | L | N | V | F | N | N | L | H | Q | L | E | N | L | S | L | S | H | N | L | L | R | S | L | P | K | D | I | F | K | D | L | N | S | L | Y | H | L | D | L | S | F | N | S | L | K | Y | L | P | N | G | I | F | P | E | S | L | Q | V | L | Y | L | A | Y | N | S | I | Y | S | V | D | P | N | L | F | S | T | L | S | Y | L | S | L | E | K | N | D | F | R | C | D | C | N | L | R | D | F | K | T | W | L | N | Q | T | N | V | I | F | A | H | P | I | E | X | D | V | I | C | A | S | P | E | D | Q | Y | M | V | P | V | V | R | S | I | I | Q | C | E | X | X | D | E | X | X | X | X | G | G | E | K | C | X | K | T | E | A | C | A | F | Y | F | L | Y | R | I | Y | H | S | T | H | C | X | H | H | H | L | C | P | S | - | - | - | - | - | - | - | - | - | - | T | W | L | H | L | Q | A | L | Q | K | T | H | W | Q | T | C | E | W | K | A | R | G | A | X | S | X | T | I | L | I | X | R | V | S | L | F | X | F | Q | X | Y | X | M | G | R | K | S | A | A | E | Q | T | R | L | S | V | L | R | A | E | H | T | P | L | L | L | X | G | A | R | L | H | T | R | G | G | P | S | Y | Q | H | A | K | C | Y | P | E | X | S | X | N | P | L | C | G | V | X | T | F | P | E | G | R | L | V | P | R | N | L | H | P | G | T | V | Q | D | A | S | G | A | K | G | H | S | G | G | A | G | C | R | E | H | T | A | V | Q | A | T | E | V | R | S | T | E | I | L | H | X | E | Q | K | I | P | S | V | A | R | X | Q | P | G | L | E | L | V | L | X | P | T | S | A | X | N | K | K | K | H | X | G | X | T | X | X | X | X | X | X | Q | - | - | - | X | X | X | X | X | S | X | X | G | X | K | K | S | X | R | S | K | S | A | S | R | H | C | S | - | - |
| CITLR5\_2938\_BP | - | X | X | X | X | X | X | X | X | X | X | X | X | X | X | X | X | X | X | X | X | X | X | X | X | X | X | X | X | X | - | X | X | - | - | X | X | X | X | X | X | X | X | X | X | X | X | X | X | X | X | X | X | X | X | X | X | X | X | X | X | X | X | X | X | X | X | X | X | X | X | X | X | X | X | X | X | X | M | A | T | I | H | T | L | S | L | I | L | L | G | L | C | V | S | T | Q | I | V | K | C | T | S | V | C | S | V | G | A | S | V | A | L | C | I | D | K | G | L | Q | D | V | P | E | L | P | P | Y | V | N | K | V | D | L | S | K | N | N | I | A | E | L | N | E | T | S | F | S | H | L | E | G | L | Q | V | L | I | L | M | H | Q | T | T | X | X | L | V | I | R | N | N | T | F | R | R | L | S | N | L | T | S | L | Q | L | D | Y | N | N | F | L | R | M | D | T | G | A | F | N | G | L | S | N | L | K | N | L | T | L | T | Q | C | S | L | E | D | T | I | L | S | G | D | F | L | K | P | L | V | S | L | E | M | L | V | L | R | E | N | N | I | K | R | I | Q | P | A | L | L | F | L | N | M | R | R | F | H | V | L | D | L | S | R | N | K | V | K | S | I | C | E | E | D | L | L | S | F | Q | G | K | H | F | T | L | L | K | L | S | S | V | T | L | Q | D | M | N | E | Y | W | L | G | W | E | K | C | G | N | P | F | K | N | M | S | V | S | V | L | D | L | S | G | N | G | F | N | D | N | N | A | K | L | F | F | D | A | I | T | G | T | K | I | Q | S | L | I | L | S | N | S | H | S | M | G | S | S | S | X | X | N | N | S | K | D | P | N | K | F | T | F | K | G | L | E | A | S | G | I | K | I | F | D | L | S | N | S | S | I | F | A | L | S | Y | S | V | F | S | C | L | S | D | L | E | Q | I | T | L | A | E | S | R | I | N | K | I | E | K | S | A | F | L | G | X | X | A | N | L | L | K | L | N | L | S | K | N | F | L | G | N | I | D | S | N | T | F | Q | N | L | E | K | L | E | V | L | D | L | S | Y | N | H | I | W | M | L | G | Y | E | S | F | R | G | L | P | N | L | L | N | L | N | L | T | G | N | A | L | K | H | L | X | X | X | H | A | F | A | T | L | P | R | L | E | K | L | Y | L | G | D | N | K | X | X | X | L | S | V | F | Y | L | I | K | I | S | K | Y | L | T | T | L | Y | L | E | H | N | I | L | S | S | L | S | D | L | F | T | I | L | E | E | F | P | Q | I | E | E | I | V | F | R | G | N | E | L | L | Y | C | P | N | E | R | H | X | X | X | V | L | S | Q | K | I | Q | I | L | D | L | A | F | A | G | L | E | V | I | W | S | E | G | K | C | L | N | V | F | N | N | L | H | Q | L | K | Q | L | S | L | S | H | N | L | L | Q | S | L | P | K | D | I | F | K | D | L | T | S | L | Y | F | L | D | L | S | F | N | S | L | K | Y | L | P | N | G | I | F | P | E | S | L | Q | I | L | N | L | E | Y | N | S | I | Y | S | V | D | P | N | L | F | S | T | L | S | Y | L | S | L | I | K | N | D | F | R | C | D | C | K | L | R | D | F | Q | T | W | L | N | Q | T | N | V | I | I | S | H | S | I | E | X | D | V | I | C | A | S | P | E | D | Q | Y | M | V | P | V | V | R | S | S | I | Q | C | E | X | X | D | E | X | X | X | X | G | R | E | K | R | X | K | T | E | A | C | A | F | Y | F | L | Y | R | T | Y | H | V | T | H | C | X | R | H | H | L | C | P | S | - | - | - | - | - | - | - | - | - | - | T | W | L | H | L | Q | A | L | Q | K | T | H | W | Q | T | C | G | W | K | A | R | G | A | X | S | X | S | I | L | V | X | C | V | S | L | F | X | F | Q | X | Y | X | V | G | R | K | S | T | A | E | Q | A | R | L | S | V | L | R | A | E | H | T | P | L | L | L | X | G | A | R | L | H | T | R | G | G | Q | S | Y | Q | H | A | K | C | Y | P | E | X | S | X | N | P | L | C | G | V | X | A | F | P | E | G | R | L | V | P | R | N | L | H | P | G | T | V | Q | D | A | S | G | A | K | G | H | S | G | G | A | G | C | R | E | H | T | A | V | Q | A | T | E | V | R | T | T | E | I | L | H | X | E | Q | K | I | P | S | V | A | R | X | Q | P | G | L | G | V | V | L | X | P | T | S | A | X | N | X | K | K | H | Q | G | X | T | X | X | X | X | X | X | Q | - | - | - | X | X | X | X | X | S | Q | X | G | X | K | K | S | X | R | S | K | C | S | S | R | H | C | S | - | - |
| CATLR5\_2938\_BP | - | X | X | X | X | X | X | X | X | X | X | X | X | X | X | X | X | X | X | X | X | X | X | X | X | X | X | X | X | X | - | X | X | - | - | X | X | X | X | X | X | X | X | X | X | X | X | X | X | X | X | X | X | X | X | X | X | X | X | X | X | X | X | X | X | X | X | X | X | X | X | X | X | X | X | X | X | X | X | X | M | E | F | I | F | I | L | I | L | F | G | L | C | I | N | T | E | V | V | K | C | T | S | V | C | S | V | Y | G | Y | A | A | Y | C | I | N | R | G | L | H | H | V | P | E | V | P | S | H | V | N | L | M | D | L | S | L | N | S | I | A | E | L | N | E | T | S | F | S | R | L | E | A | L | Q | V | L | K | V | E | Q | Q | T | P | X | X | L | V | I | R | N | N | T | F | R | R | L | S | N | L | I | L | L | K | L | D | Y | N | L | F | L | Q | I | E | T | G | A | F | N | G | L | Y | N | L | E | F | L | T | L | T | Q | C | S | L | D | A | S | V | L | S | G | D | V | L | K | P | L | V | S | L | E | M | L | V | L | R | D | N | N | I | H | R | I | Q | P | A | S | F | F | L | N | M | R | R | F | H | V | L | D | L | S | H | N | K | V | K | S | I | C | E | E | D | L | V | S | F | Q | G | K | H | F | T | L | L | Q | L | S | S | V | T | L | Q | D | M | N | E | N | W | L | G | W | D | K | C | G | N | P | F | K | N | M | S | I | N | V | L | D | L | S | G | N | G | F | Y | V | P | M | A | K | R | F | F | D | T | F | T | S | T | K | I | L | G | L | I | L | S | K | S | Y | N | M | G | S | S | F | G | H | N | N | F | P | D | P | D | K | F | T | F | K | G | L | E | V | S | G | V | K | V | F | D | L | S | R | S | H | I | F | A | L | S | D | S | V | F | S | H | F | P | D | L | E | Q | I | T | L | A | Q | N | E | I | N | I | I | E | N | N | A | F | W | G | X | X | N | N | L | Q | K | L | N | L | S | N | N | F | L | G | S | I | D | S | K | T | F | H | N | L | E | K | L | E | V | L | D | L | S | Y | N | H | I | W | R | L | G | Y | Q | S | F | Q | G | L | P | N | L | L | I | L | N | L | T | G | N | S | I | K | H | L | X | X | X | D | T | F | A | T | L | P | S | M | E | K | L | Y | L | G | D | N | E | X | X | X | T | H | A | F | S | L | L | N | I | G | T | N | L | K | T | L | Y | L | Q | F | N | K | L | S | S | M | S | E | L | Y | M | I | L | E | K | F | P | Q | I | E | E | I | V | F | R | G | N | E | L | V | Y | C | P | Y | D | E | H | X | X | X | V | L | S | Q | K | I | K | I | L | D | L | A | I | S | G | L | E | V | V | W | L | E | G | T | C | L | N | I | F | D | E | L | H | Q | L | E | A | L | F | L | N | S | N | R | L | Q | S | L | P | K | D | V | F | K | D | L | T | S | L | I | F | L | D | L | S | S | N | S | L | K | Y | L | P | N | G | I | F | P | K | S | L | Q | Y | L | N | L | E | F | N | S | V | Y | S | V | D | P | N | L | F | S | T | L | S | Y | I | S | L | L | G | N | D | F | N | C | D | C | N | L | R | D | F | Q | T | W | L | N | Q | T | N | I | T | F | L | H | P | I | E | X | D | V | T | C | A | S | P | E | D | Q | Y | M | V | S | V | V | K | S | S | I | Q | C | E | X | X | D | E | X | X | X | X | G | X | E | E | R | X | K | P | E | T | C | A | F | H | F | L | F | H | T | D | H | I | I | H | C | Q | R | H | H | L | C | L | S | - | - | - | - | - | - | - | - | - | - | T | W | L | H | L | Q | A | L | Q | K | N | H | C | X | T | C | G | W | K | A | T | G | A | R | S | X | S | I | L | V | R | R | V | S | L | F | Q | F | Q | X | Y | X | V | G | G | K | S | A | V | E | E | A | R | L | S | V | L | R | A | E | Y | T | P | L | L | L | X | G | A | R | L | H | T | R | R | G | P | S | Y | Q | H | A | K | C | Y | P | E | X | S | K | T | H | L | C | G | V | X | T | F | P | E | G | W | L | V | L | G | N | L | Q | P | G | S | V | Q | N | A | S | G | T | X | R | H | S | G | G | A | C | C | R | E | H | T | A | V | Q | A | T | E | V | R | T | T | E | I | L | H | X | D | Q | E | I | P | S | V | A | R | R | Q | P | G | F | G | V | V | L | X | P | T | S | A | X | N | K | K | Q | H | Q | G | Y | T | X | X | X | X | X | X | Q | - | - | - | X | X | X | R | K | R | Q | R | G | K | K | X | S | G | S | S | K | C | V | C | R | H | G | S | - | - |
| CCTLR5\_2938\_BP | - | X | X | X | X | X | X | X | X | X | X | X | X | X | X | X | X | X | X | X | X | X | X | X | X | X | X | X | X | X | - | X | X | - | - | X | X | X | X | X | X | X | X | X | X | X | X | X | X | X | X | X | X | X | X | X | X | X | X | X | X | X | X | X | X | X | X | X | X | X | X | X | X | X | X | X | X | X | X | X | M | E | F | I | F | I | L | I | L | L | G | L | C | I | N | T | E | V | V | K | S | T | S | V | C | S | V | I | G | Y | A | A | Y | C | I | D | R | G | L | H | Y | V | P | E | V | P | T | H | V | N | Q | M | D | L | S | L | N | S | I | A | E | L | N | Q | T | S | F | S | S | L | E | G | L | Q | V | L | K | V | E | Q | Q | T | P | X | X | L | V | I | R | N | N | T | F | R | R | L | S | N | L | I | L | L | K | L | D | Y | N | X | X | L | Q | I | E | T | E | A | F | N | G | L | S | N | L | E | I | L | T | L | T | Q | C | S | L | D | G | S | V | L | S | G | D | V | L | K | P | L | V | S | L | Q | M | L | V | L | R | N | N | N | I | H | R | I | Q | P | A | S | F | F | L | N | M | R | R | F | H | V | L | D | L | S | H | N | K | V | K | S | I | C | E | E | D | L | V | S | F | Q | G | K | H | F | T | L | L | Q | L | A | S | V | T | L | Q | D | M | N | E | Y | W | L | G | W | D | K | C | G | N | P | F | K | N | M | S | V | T | V | L | D | L | S | G | N | G | F | Q | V | P | M | A | K | R | F | F | D | A | I | T | G | T | K | I | L | G | L | I | L | S | K | S | Y | S | M | G | S | S | F | S | H | N | N | F | P | D | P | D | K | F | T | F | K | G | L | E | V | S | G | V | K | I | F | D | L | S | Q | S | N | I | F | A | L | S | N | S | V | F | S | H | F | S | D | L | E | Q | I | T | L | A | Q | N | E | I | N | I | I | E | N | N | A | F | L | G | X | X | T | N | L | Q | K | L | N | L | S | N | N | F | L | G | S | V | D | S | K | T | F | Q | N | L | E | K | L | E | V | L | D | L | S | Y | N | H | I | R | V | L | G | D | Q | S | F | R | G | L | P | S | L | L | E | L | N | L | T | G | N | S | L | E | S | V | X | X | X | H | E | F | A | T | L | P | N | L | K | K | L | Y | L | G | E | N | R | X | X | X | L | S | L | S | S | L | P | N | I | A | K | N | I | T | T | L | D | L | E | F | N | R | L | K | D | L | S | D | L | Y | T | I | L | R | E | F | P | Q | I | E | K | I | F | L | R | G | N | M | F | S | S | C | Y | N | Q | N | Q | X | X | X | V | V | S | D | K | L | Q | L | L | N | L | E | F | S | T | M | Q | L | I | W | S | E | G | K | C | F | N | V | F | N | N | L | H | Q | L | E | Q | L | S | L | A | S | N | G | L | Q | S | L | P | K | E | I | F | K | D | L | T | S | L | F | F | L | D | L | S | F | N | S | L | K | Y | L | P | N | G | I | F | P | K | S | L | Q | I | L | N | L | E | Y | N | S | I | Y | S | V | D | P | N | L | F | S | T | L | S | Y | L | S | L | L | K | N | Q | F | R | C | D | C | N | L | R | D | F | Q | T | W | L | N | Q | T | N | V | T | I | F | H | P | I | E | X | D | V | T | C | A | S | P | E | D | Q | Y | M | V | P | V | V | R | S | S | I | Q | C | E | X | X | D | E | X | X | X | X | G | X | D | E | R | X | K | T | E | A | C | A | F | H | F | L | F | H | T | D | H | N | I | H | C | Q | R | H | H | L | C | P | S | - | - | - | - | - | - | - | - | - | - | T | W | L | H | L | Q | A | L | Q | K | T | Y | Y | X | T | C | G | W | K | A | T | G | A | R | S | X | S | I | L | V | R | R | V | S | L | F | Q | F | Q | X | Y | X | V | G | R | K | S | A | F | E | E | A | R | L | S | V | L | R | T | E | Y | T | P | L | L | L | X | G | A | R | L | H | T | R | R | G | P | S | Y | Q | H | A | K | C | Y | P | E | X | S | K | N | P | L | C | G | V | X | T | F | P | E | G | W | L | V | L | G | N | L | Q | L | G | T | K | K | D | A | S | G | P | X | R | H | S | G | G | I | G | C | R | E | H | T | T | V | Q | V | T | E | V | Q | T | S | E | I | L | H | X | E | Q | K | I | P | S | V | A | R | X | Q | P | G | L | G | V | V | L | X | P | T | F | A | X | N | X | K | R | H | Q | G | X | A | X | X | X | X | X | X | Q | - | - | - | I | N | Q | L | N | K | X | R | X | X | X | X | X | X | C | Y | K | C | P | C | K | H | R | S | - | - |
| LMTLR5\_2938\_BP | - | X | X | X | X | X | X | X | X | X | X | X | X | X | X | X | X | X | X | X | X | X | X | X | X | X | X | X | X | X | - | X | X | X | X | X | X | X | X | X | X | X | X | X | X | X | X | X | X | X | X | X | X | X | X | X | X | X | X | X | X | X | X | X | X | X | X | X | X | X | X | X | X | X | X | X | X | X | X | X | X | X | X | X | X | X | X | X | X | X | - | - | X | X | X | X | X | X | X | X | X | X | X | X | X | X | X | X | X | X | X | X | X | X | X | X | X | X | X | X | X | X | X | X | X | X | X | X | X | X | X | X | X | X | X | X | X | X | X | X | X | X | X | X | X | X | X | X | X | X | X | X | X | X | X | X | X | X | M | Q | L | Q | G | X | X | L | V | I | R | N | N | T | F | L | Q | Q | R | K | L | T | Q | L | V | L | G | Y | N | I | G | L | H | L | E | L | G | A | F | A | G | L | V | N | L | R | H | L | F | L | D | Y | C | N | L | E | D | S | I | L | R | D | T | Y | L | Q | P | L | L | S | L | E | T | L | D | L | F | G | N | K | I | V | R | L | Q | P | G | L | F | F | S | N | L | T | N | F | T | Q | L | R | L | K | L | N | Q | I | E | Q | L | C | E | K | D | L | V | G | F | R | G | K | N | F | K | K | L | D | L | N | S | N | H | L | Y | R | K | D | T | G | N | F | D | W | E | S | C | G | N | P | F | R | G | M | T | F | N | V | L | D | L | S | S | N | G | F | H | E | S | K | S | K | Q | F | F | K | A | I | K | G | T | P | I | S | H | L | V | F | S | X | X | X | Y | M | G | K | G | F | S | H | N | N | F | K | D | P | D | K | N | T | F | E | G | L | V | N | S | A | I | N | I | L | D | L | S | N | N | C | I | Y | A | L | Q | M | D | V | F | S | P | L | K | D | T | I | V | I | D | I | S | K | N | K | I | N | Q | I | N | R | N | A | F | N | G | L | Q | G | H | L | R | M | L | N | L | S | S | N | L | L | G | E | V | Y | S | H | T | F | T | S | L | T | D | L | W | V | L | D | L | S | Y | N | H | I | G | A | L | G | Y | D | A | F | S | G | L | P | K | L | Q | V | L | Y | L | T | G | N | S | L | R | N | L | X | X | X | G | F | P | V | S | L | P | N | L | G | Y | L | F | L | R | D | N | K | X | X | X | T | S | L | S | G | I | I | K | L | A | V | N | S | I | H | V | D | V | A | D | N | R | L | T | N | L | D | N | F | Y | I | I | L | S | H | F | K | H | L | Q | T | F | L | F | G | G | N | L | I | M | W | C | T | L | S | P | N | I | A | V | P | H | N | N | S | L | Q | V | L | D | L | H | G | S | S | L | Q | V | I | W | A | Q | G | R | C | L | D | L | F | N | H | L | E | N | L | L | G | L | N | L | S | L | N | S | L | A | A | L | P | Q | G | I | F | S | G | L | S | S | I | V | E | L | D | L | S | F | N | A | L | T | Y | L | Q | S | D | V | F | P | V | S | L | K | K | L | Y | L | S | S | N | F | I | A | N | P | D | P | M | T | F | R | S | L | S | F | L | T | L | A | G | N | R | F | H | C | D | C | K | L | E | S | F | L | K | W | M | N | V | T | N | V | T | F | L | S | P | V | Q | X | E | Y | T | C | E | F | P | T | A | L | R | N | L | P | L | L | D | Y | Y | T | V | V | E | P | C | E | G | X | X | X | X | R | R | E | G | H | P | X | S | X | V | G | S | L | R | L | L | C | S | P | H | H | H | A | H | P | R | W | D | C | I | R | P | S | - | - | - | - | - | - | - | - | - | - | P | R | T | H | I | H | H | L | Q | K | D | R | R | X | G | S | R | G | P | K | T | G | T | S | C | G | R | A | A | V | R | C | L | P | L | L | X | R | Q | X | L | Q | V | G | G | G | C | F | A | E | E | T | G | X | P | V | F | R | G | E | P | L | S | L | L | F | R | G | Q | R | L | P | A | R | X | G | S | P | F | Q | H | Q | R | C | H | L | G | Q | Q | E | D | C | V | H | R | L | Q | G | I | P | X | R | W | L | V | L | G | G | V | H | S | G | P | G | P | D | A | G | G | A | D | K | R | P | D | Y | V | G | G | R | E | G | G | S | L | P | A | N | E | V | Q | R | S | P | S | F | C | P | E | E | G | V | P | N | L | A | G | G | P | S | G | P | G | V | V | L | X | A | T | C | L | K | D | A | Q | R | H | Q | G | E | K | V | C | R | G | Q | T | R | A | C | T | A | X | H | S | T | S | E | X | G | R | H | R | A | X | E | R | E | S | S | C | H | - | - | - | - | - |
| ECTLR5\_2938\_BP | - | X | X | X | X | X | X | X | X | X | X | X | X | X | X | X | X | X | X | X | X | X | X | X | X | X | X | X | X | X | - | X | X | X | X | X | X | X | X | X | X | X | X | X | X | X | X | X | X | X | X | X | X | X | X | X | X | X | X | X | X | X | X | X | X | X | X | X | X | X | X | X | X | X | X | X | X | X | X | M | W | T | L | A | F | H | M | V | F | I | - | - | G | S | Y | L | Q | G | I | A | C | Y | P | S | C | T | L | Y | G | I | V | A | N | C | A | M | Q | H | H | Y | W | V | P | A | L | P | P | N | I | T | D | L | Y | L | E | M | N | Y | I | G | E | I | N | S | S | S | L | R | R | Y | D | Q | L | Q | Q | V | D | L | G | S | Q | K | V | X | X | L | V | I | R | N | N | A | F | L | R | Q | R | K | L | I | K | L | V | L | X | X | N | T | Q | L | Q | L | E | P | R | A | F | A | G | L | F | N | L | Q | N | L | F | L | D | H | C | N | L | A | D | S | I | L | A | E | S | Y | L | Q | P | L | L | S | L | E | K | L | D | L | S | G | N | K | I | V | R | L | R | P | G | L | F | F | S | K | L | T | K | F | T | Q | L | N | L | K | L | N | R | I | E | S | L | C | E | E | D | L | V | G | F | Q | G | K | Y | F | T | L | L | N | L | Q | S | N | Y | V | X | X | R | Y | S | D | D | F | D | W | E | R | C | G | N | P | F | R | G | M | A | F | N | T | L | D | L | S | S | N | G | F | S | V | T | I | A | R | N | F | F | K | A | I | Q | G | T | P | I | A | H | V | I | L | S | X | X | X | H | M | G | K | G | F | S | F | N | N | L | P | D | P | D | K | N | T | F | E | G | L | M | N | S | S | V | T | I | L | D | L | S | K | N | Y | I | F | T | L | Q | K | A | F | L | S | P | L | K | D | A | K | I | I | D | I | S | M | N | K | I | N | Q | I | N | R | N | A | F | D | G | L | Q | D | H | L | R | L | L | N | L | S | S | N | L | L | G | E | I | Y | S | H | T | F | T | N | L | T | D | L | R | V | L | D | L | S | Y | N | Y | I | G | A | L | G | Y | E | A | F | S | G | L | P | K | L | R | A | L | Y | L | T | G | N | S | L | R | V | L | X | X | X | G | S | P | A | P | L | P | N | L | D | F | L | L | L | G | D | N | R | X | X | X | T | S | I | Y | N | I | N | K | L | G | N | N | S | I | Y | V | D | V | A | D | N | K | L | T | N | L | D | D | I | F | V | I | L | T | Q | F | R | H | L | Q | T | F | F | Y | G | G | N | L | I | K | W | C | X | X | S | Q | N | V | T | V | P | H | N | N | S | L | R | V | L | D | L | H | D | S | S | L | H | I | I | W | G | R | G | E | C | L | N | L | F | D | H | L | E | N | L | Q | G | L | N | L | S | L | N | S | L | A | A | L | P | R | G | I | F | N | G | L | G | S | I | T | E | I | D | L | S | S | N | A | L | T | F | L | Q | P | D | I | F | P | V | S | L | K | R | L | D | L | S | N | N | F | L | A | T | P | D | P | R | T | F | Q | S | I | S | F | L | S | L | A | A | N | R | F | H | C | D | C | N | L | E | S | F | L | K | W | L | K | V | T | H | V | N | F | F | S | P | I | D | X | E | F | R | C | E | F | P | A | A | L | H | K | H | S | L | L | E | Y | S | T | N | V | K | P | C | E | X | X | X | X | X | X | X | X | X | R | P | R | P | X | D | C | A | F | H | P | L | C | S | P | H | H | C | F | H | P | R | W | D | C | L | R | P | S | - | - | - | - | - | - | - | - | - | - | P | R | A | H | I | H | Y | L | Q | K | D | H | Q | X | G | C | Q | G | P | K | T | D | N | L | C | X | R | S | A | V | X | R | L | P | L | L | X | Q | Q | X | L | W | V | G | R | G | C | I | A | X | E | A | G | X | P | V | F | K | G | E | H | P | P | L | L | F | R | G | Q | R | L | P | A | R | X | G | S | P | F | Q | H | Q | R | F | H | L | G | Q | X | E | D | C | V | R | R | L | Q | R | V | P | Q | R | W | L | V | L | G | G | I | L | I | G | P | G | P | D | A | G | G | A | D | E | H | P | D | Y | V | G | G | R | E | G | V | Y | K | A | A | X | X | X | Y | L | I | Y | T | H | S | L | G | V | S | K | S | H | I | X | X | X | X | X | X | X | X | X | X | X | X | X | X | X | X | X | X | X | X | X | X | X | X | X | X | X | X | X | X | X | X | X | X | X | X | X | X | X | X | X | X | X | X | X | X | X | X | X | X | X | X | X | X | X | X | X | - | - | - | - | - |
| LCTLR5\_2938\_BP | - | X | X | X | X | X | X | X | X | X | X | X | X | X | X | X | X | X | X | X | X | X | X | X | X | X | X | X | X | X | - | X | X | X | X | X | X | X | X | X | X | X | X | X | X | X | X | X | X | X | X | X | X | X | X | X | X | X | X | X | X | X | X | X | X | X | X | X | X | X | X | X | X | X | X | X | X | X | X | M | W | T | L | A | L | Q | L | V | F | T | - | - | G | L | H | I | Q | V | T | T | C | Y | P | S | C | T | L | Y | G | L | V | A | A | C | A | S | Q | H | H | Y | W | V | P | A | L | P | C | N | I | T | H | L | Y | L | E | R | N | Y | I | S | E | I | N | S | T | S | L | R | D | Y | D | Q | L | Q | Q | L | D | L | G | M | Q | N | V | X | X | L | I | I | R | N | N | A | F | L | R | Q | R | K | L | T | R | L | V | L | G | Y | N | I | G | L | Q | L | E | P | R | A | F | A | G | L | F | N | L | Q | H | F | F | L | D | K | C | S | L | N | D | S | I | L | S | E | S | Y | L | Q | H | L | F | S | L | E | L | L | D | L | S | Y | N | E | L | A | R | L | R | P | G | L | F | F | S | K | L | T | R | F | T | K | L | N | L | K | L | N | Q | I | R | G | L | C | E | E | D | L | V | G | F | R | G | K | Y | F | T | Y | M | N | L | T | S | N | L | F | G | R | M | Y | E | G | N | F | D | W | E | T | C | G | N | P | F | R | G | M | A | F | N | I | L | D | L | S | S | Y | G | F | N | I | I | T | T | R | N | F | F | N | A | I | Q | G | T | Q | I | D | H | L | I | F | S | X | X | X | N | L | S | K | D | L | S | Y | N | K | L | A | D | P | D | Q | S | T | F | E | G | L | M | N | S | K | I | K | H | F | D | L | S | N | S | Y | I | F | A | L | Q | R | A | V | F | S | S | L | K | A | A | I | I | I | D | I | S | G | N | K | I | N | Q | I | E | R | N | A | F | S | G | L | Q | G | H | L | Q | L | L | N | L | S | S | N | L | L | G | E | I | V | Y | H | T | F | T | N | L | A | E | L | R | V | L | D | L | S | Y | N | H | I | G | V | L | G | H | K | A | F | S | G | L | P | R | L | Q | T | L | N | L | T | G | N | S | L | R | I | L | X | X | X | G | S | P | A | S | L | P | N | L | D | Y | L | L | L | N | D | N | K | X | X | X | T | S | L | Y | H | I | T | D | M | G | M | N | S | L | H | V | D | V | S | D | N | R | L | T | N | L | E | D | V | Y | L | I | V | T | N | F | K | R | L | Q | N | F | F | Y | G | G | N | L | I | K | W | C | T | L | N | Q | I | F | I | I | P | Y | N | N | S | L | Q | K | L | D | L | H | D | S | S | L | Q | I | I | W | E | Q | G | K | C | F | D | L | F | D | H | L | E | N | L | L | S | L | S | I | N | F | N | S | L | T | T | L | P | Q | G | I | F | R | G | L | S | S | I | I | E | L | D | L | S | H | N | S | L | T | Y | L | Q | P | G | V | F | P | V | S | L | K | K | L | D | L | S | N | N | Y | L | A | S | P | D | P | M | T | F | Q | S | L | I | F | L | D | L | A | A | N | R | F | H | C | D | C | S | L | E | K | F | L | K | W | L | N | E | T | N | V | T | F | L | S | P | T | E | X | D | F | I | C | E | F | P | A | A | L | H | N | L | P | L | L | N | Y | T | T | S | T | E | P | C | E | E | X | X | X | X | X | X | K | G | C | S | W | S | X | V | C | S | L | H | P | L | C | S | P | R | H | C | C | H | P | Q | W | D | C | L | R | P | F | - | - | - | - | - | - | - | - | - | - | P | R | A | C | I | H | H | I | Q | K | N | Y | W | Q | G | S | X | G | P | E | T | G | G | Y | F | G | R | G | A | I | R | C | L | P | L | L | Q | Q | Q | X | L | X | V | G | G | S | C | F | A | E | X | A | X | X | P | V | F | R | G | E | H | L | P | L | L | F | X | G | Q | R | L | S | A | R | X | G | S | P | F | K | H | Q | R | S | N | L | G | Q | Q | E | D | C | V | Y | H | L | Q | G | V | P | X | R | R | L | V | L | G | G | V | H | F | G | T | L | P | D | A | G | G | A | D | K | H | L | D | Y | V | G | S | R | E | G | G | S | L | P | A | D | E | V | Q | C | N | Q | S | F | H | S | E | E | S | I | P | H | L | A | R | G | P | S | G | P | G | V | V | L | X | A | T | D | L | T | D | P | Q | R | H | Q | S | E | K | V | S | R | G | Q | T | S | A | C | T | A | X | H | P | T | S | I | X | G | Q | C | S | A | G | E | H | Q | D | K | C | H | - | - | - | - | - |
| OFTLR5\_2938\_BP | - | X | X | X | X | X | X | X | X | X | X | X | X | X | X | X | X | X | X | X | X | X | X | X | X | X | X | X | X | X | - | X | X | X | X | X | X | X | X | X | X | X | X | X | X | X | X | X | X | X | X | X | X | X | X | X | X | X | X | X | X | X | X | X | X | X | X | X | X | X | X | X | X | X | X | X | X | X | X | M | W | T | L | A | L | Q | L | V | F | T | - | - | G | L | Y | L | Q | V | S | M | C | Y | P | S | C | T | L | Y | G | L | V | A | A | C | A | S | Q | N | H | R | W | V | P | A | L | P | P | N | I | T | H | L | Y | L | E | M | N | Y | I | S | E | I | N | S | T | S | L | R | A | Y | D | Q | L | Q | E | L | D | F | G | L | Q | K | R | X | X | L | V | I | R | T | N | A | F | L | R | Q | R | K | L | T | R | L | V | L | G | Y | N | L | G | L | Q | L | E | P | R | A | F | A | G | L | F | N | L | Q | L | L | F | L | D | H | C | N | L | M | D | S | I | L | A | E | S | Y | L | Q | P | L | W | S | L | E | K | L | D | L | L | G | N | K | I | V | R | L | Q | P | G | L | F | F | S | K | L | T | K | F | T | E | L | N | L | K | L | N | Q | I | E | R | L | C | E | E | D | L | V | G | F | Q | G | K | Y | F | K | L | L | N | L | E | S | N | H | L | Y | I | T | H | S | G | D | F | D | W | E | R | C | G | N | P | F | R | G | M | A | F | D | T | L | D | L | S | S | N | G | L | S | I | N | T | S | R | Q | F | F | K | A | I | E | G | T | P | I | A | H | L | I | L | S | X | X | X | Y | W | G | K | D | F | S | H | D | N | L | P | E | P | D | E | S | T | F | E | G | L | G | N | S | K | V | N | I | F | D | L | S | S | N | R | I | F | A | L | Q | R | A | V | F | S | P | L | K | D | A | V | I | I | D | I | S | K | N | K | I | N | E | I | N | R | N | A | F | S | G | L | Q | G | H | L | R | M | L | N | L | S | S | N | L | L | G | E | I | Y | S | H | T | F | T | S | L | T | E | L | R | V | L | D | L | S | Y | N | H | I | G | A | L | G | Y | K | A | F | S | G | L | P | K | L | R | G | L | Y | L | T | G | N | S | L | R | D | L | X | X | X | G | F | P | E | S | L | P | N | L | D | I | L | L | L | G | D | N | K | X | X | X | K | S | L | S | H | I | I | D | L | G | M | N | S | A | H | V | D | I | T | E | N | R | L | T | N | L | E | D | V | Y | F | I | L | S | H | F | N | R | L | Q | N | F | F | Y | G | G | N | F | I | K | W | C | V | L | S | P | N | V | I | V | P | H | N | N | S | L | Q | V | L | D | L | H | D | S | S | L | Q | M | I | W | A | Q | G | K | C | L | D | L | F | D | R | L | E | N | L | L | G | L | N | L | S | F | N | S | L | A | T | L | P | Q | G | I | F | G | S | L | S | S | I | I | E | I | D | L | S | S | N | A | L | T | Y | L | Q | P | D | V | F | P | V | S | L | K | R | L | D | L | S | N | N | F | L | A | S | P | D | P | M | T | F | R | S | L | I | F | F | S | L | A | A | N | R | F | H | C | N | C | H | L | E | S | F | L | K | W | L | N | G | T | N | V | T | S | M | S | P | V | E | X | E | Y | R | C | E | F | P | A | A | L | H | N | L | P | L | L | N | Y | S | A | I | I | E | P | C | E | X | X | X | X | X | X | X | K | G | R | P | A | F | X | V | C | S | F | R | L | L | C | S | P | H | H | H | C | H | P | Q | W | H | C | L | R | P | S | - | - | - | - | - | - | - | - | - | - | T | G | A | H | I | H | H | L | Q | K | D | H | Q | X | G | S | R | G | T | K | T | D | T | P | C | G | R | G | A | V | R | C | L | P | L | L | X | R | Q | X | L | P | V | G | G | S | C | F | A | E | E | A | G | X | P | V | F | R | G | E | H | L | P | L | L | F | X | G | Q | R | L | P | A | R | X | G | S | P | V | Q | H | Q | R | C | H | L | G | Q | Q | E | D | R | V | C | R | L | Q | G | V | P | X | R | W | L | V | L | G | G | I | H | F | G | P | G | P | D | V | G | G | A | D | T | R | P | D | Y | V | G | G | R | T | G | G | S | L | R | A | D | E | V | Q | C | S | Q | S | F | R | P | E | E | R | V | P | D | L | A | R | G | P | S | G | P | G | V | V | L | X | A | A | R | L | A | D | A | Q | R | H | Q | G | E | N | V | C | R | G | Q | T | X | A | R | T | A | X | R | S | I | X | E | X | G | R | Y | P | A | S | E | H | Q | S | N | C | H | - | - | - | - | - |
| MMTLR5\_2938\_BP | - | X | X | X | X | X | X | X | X | X | X | X | X | X | X | X | X | X | X | X | X | X | X | X | X | X | X | X | X | X | - | X | X | X | X | X | X | X | X | X | X | X | X | X | X | X | X | X | X | X | X | X | X | X | X | X | X | X | X | X | X | X | X | X | X | X | X | X | X | X | X | X | X | X | X | X | X | X | X | M | W | T | L | A | L | Q | L | V | F | I | - | - | G | F | Y | L | Q | V | S | T | C | Y | Q | S | C | T | L | Y | N | L | V | A | A | C | G | Q | K | H | H | Y | W | V | P | A | L | P | F | N | I | T | H | L | Y | L | E | M | N | Y | I | S | E | I | N | R | T | S | L | K | N | Y | D | Q | L | I | E | L | D | L | G | M | Q | Y | S | R | I | L | V | I | R | N | N | A | F | L | R | Q | R | K | L | T | R | L | V | L | G | S | N | I | N | L | Q | L | E | P | R | A | F | A | G | L | Y | N | L | Q | N | L | Y | L | D | Y | C | N | F | T | D | S | I | L | S | G | G | F | L | Q | P | L | L | S | L | E | M | L | D | L | F | G | N | N | I | V | R | L | R | P | G | L | F | F | S | K | L | T | K | F | T | K | L | N | L | K | L | N | Q | I | Q | T | L | C | E | E | D | L | I | G | F | R | G | K | Y | F | T | L | L | N | L | N | S | N | D | L | Y | L | E | X | X | X | X | X | D | W | Q | R | C | G | N | P | F | R | E | M | A | F | D | T | L | D | L | S | S | N | G | F | S | L | N | K | S | Q | Q | F | F | K | A | I | E | G | T | P | I | A | H | L | I | F | S | X | X | X | Y | I | G | K | G | F | S | H | E | N | L | L | D | P | D | E | S | T | F | D | G | L | V | N | S | A | V | N | H | L | D | L | S | K | N | K | I | F | A | L | Q | R | A | V | F | S | P | L | K | D | A | I | I | I | D | I | S | Q | N | K | I | N | Q | I | H | E | N | A | F | S | G | L | Q | G | H | L | R | L | L | N | L | S | S | N | L | I | G | E | I | Y | S | Q | T | F | T | D | L | T | D | L | R | V | L | D | L | S | N | N | H | I | G | A | L | G | Y | K | A | F | S | G | L | P | N | L | R | G | L | Y | L | T | G | N | S | L | R | D | L | X | X | X | G | F | P | A | S | L | P | N | L | D | L | L | L | L | A | D | N | K | X | X | X | K | S | I | A | G | I | S | D | W | G | K | N | S | H | Y | V | N | V | V | D | N | R | L | T | N | L | K | D | L | F | V | I | L | S | D | F | T | R | L | K | T | F | F | Y | G | G | N | T | I | K | W | C | T | P | X | X | N | V | I | V | P | H | N | N | S | L | Q | V | L | D | L | H | G | S | S | L | Q | I | I | W | T | Q | G | Q | C | P | N | L | F | D | H | F | E | N | L | L | G | L | N | L | S | F | N | S | L | A | T | L | P | Q | G | I | F | N | G | L | H | S | V | I | E | I | D | L | S | F | N | A | L | T | Y | L | Q | L | G | I | F | P | V | S | L | K | R | L | D | L | S | N | N | F | L | A | S | P | D | P | L | T | F | S | S | L | S | F | L | S | L | A | V | N | R | F | H | C | D | C | H | L | E | N | F | L | K | W | L | N | V | T | N | L | T | F | L | S | P | V | E | X | E | Y | K | C | E | F | P | A | A | L | H | N | L | P | L | L | N | Y | S | T | I | I | E | P | C | E | E | X | X | X | X | X | X | K | G | C | S | R | T | E | V | L | S | L | H | L | I | R | S | P | H | H | H | G | C | P | Q | W | Y | S | L | R | P | S | - | - | - | - | - | - | - | - | - | - | S | R | A | H | I | H | H | L | Q | K | D | H | W | X | G | S | X | G | P | K | T | D | A | S | C | G | X | G | T | V | X | C | F | P | L | L | X | X | Q | X | L | W | V | G | G | G | C | T | A | E | K | A | G | X | S | V | F | R | G | E | H | V | P | L | L | F | X | G | X | R | L | S | A | R | R | G | S | S | F | Q | H | Q | R | C | H | L | G | Q | X | E | D | C | L | H | C | L | Q | R | V | P | X | R | W | M | V | L | G | G | V | H | F | G | P | W | S | D | A | G | G | A | N | K | R | P | D | Y | V | G | G | R | E | G | G | S | L | P | A | D | E | V | Q | C | S | Q | S | F | R | P | E | E | G | V | S | S | L | A | R | G | P | S | G | H | G | V | V | L | X | A | A | R | L | T | D | T | Q | G | H | X | G | E | K | D | C | R | G | X | P | X | T | C | T | V | X | H | S | S | S | G | R | G | R | Y | X | A | X | X | H | R | S | N | C | R | - | - | - | - | - |
| SSTLR5\_2938\_BP | - | X | X | X | X | X | X | X | X | X | X | X | X | X | X | X | X | X | X | X | X | X | X | X | X | X | X | X | X | X | - | X | X | X | X | X | X | X | X | X | X | X | X | X | X | X | X | X | X | X | X | X | X | X | X | X | X | X | X | X | X | X | X | X | X | X | X | X | X | X | X | X | X | X | X | X | X | X | X | M | M | R | N | L | I | L | L | A | I | F | - | - | G | E | Y | L | Q | V | V | K | C | T | P | K | C | P | I | Y | G | S | I | A | A | C | T | S | Q | S | L | H | Q | V | P | A | L | P | P | Y | I | T | I | V | Y | M | R | D | N | Y | I | S | E | I | N | E | T | S | F | S | G | L | E | G | L | T | E | L | D | L | S | W | Q | Q | V | N | G | L | I | I | R | T | N | T | F | Q | R | L | E | N | L | A | V | L | Y | L | G | Y | N | T | G | L | Q | I | E | P | D | A | F | V | G | L | S | N | L | R | A | L | S | L | Y | R | C | D | L | T | E | S | I | L | Q | G | D | Y | L | R | P | L | V | S | L | E | T | L | D | L | Y | G | N | Q | V | K | R | I | Q | P | A | L | F | F | V | N | I | T | D | F | H | E | L | N | V | S | L | N | Q | M | E | Y | I | C | E | E | D | L | L | G | F | Q | G | K | H | F | R | L | L | K | L | N | S | L | Y | L | N | S | M | T | Q | Y | G | F | D | W | K | R | C | G | N | P | F | R | N | M | S | M | E | T | L | D | L | S | S | N | G | F | D | V | D | K | A | K | L | F | F | N | A | I | R | G | T | K | I | H | H | L | I | L | E | H | X | X | T | M | G | K | S | F | G | F | S | N | V | K | D | P | D | R | Q | T | F | E | G | L | K | N | S | S | V | K | I | L | D | L | S | K | C | F | I | F | T | L | Q | Y | A | V | F | S | P | L | R | E | V | E | D | I | T | I | A | Q | N | K | I | N | Q | I | D | R | E | A | F | F | G | L | Q | X | X | L | Q | K | L | N | L | S | H | N | L | I | G | E | I | Y | S | Y | T | F | D | K | L | P | N | I | L | E | L | D | L | S | Y | N | H | I | G | A | L | G | Y | H | A | F | K | G | L | P | N | L | Q | F | L | D | L | T | G | N | S | I | R | E | L | G | T | Y | G | S | L | A | P | L | P | N | I | Q | H | L | R | L | A | D | N | K | X | X | X | T | S | L | E | G | L | S | V | F | A | N | S | T | I | I | L | N | V | Q | N | N | R | L | T | N | L | E | D | V | Y | T | V | L | A | K | L | M | H | I | E | V | L | W | Y | G | N | N | F | I | K | W | C | T | L | N | N | N | I | S | V | P | A | V | N | S | L | K | L | L | E | L | R | N | I | A | L | Q | M | I | W | A | R | G | E | C | M | H | V | F | E | N | L | G | K | L | L | S | L | D | L | S | F | N | S | L | Q | T | L | P | D | G | I | F | K | G | L | I | S | L | E | E | M | D | L | H | F | N | S | L | T | Y | L | K | P | D | I | F | P | E | S | L | K | T | V | D | L | S | Y | N | F | L | A | S | P | D | P | V | A | F | H | S | L | S | W | I | N | L | Y | R | N | Q | F | H | C | D | C | G | L | E | D | F | L | T | W | L | K | G | T | N | V | T | F | P | D | P | G | V | N | E | F | S | C | E | F | P | S | D | L | H | G | I | S | L | L | N | Y | S | S | X | V | I | S | C | E | E | X | X | X | X | X | R | E | A | G | S | G | A | E | A | L | A | L | H | R | L | H | S | A | H | H | P | D | H | G | R | N | N | R | F | R | S | S | - | - | - | - | - | - | - | - | - | - | P | W | I | P | L | Q | S | L | Q | K | V | D | R | Q | D | P | X | G | P | X | E | G | S | F | C | W | W | A | S | V | R | R | L | L | M | L | Q | Q | Q | R | L | Q | V | G | R | N | R | P | V | E | Q | T | R | L | S | V | L | G | A | K | R | P | P | L | L | L | R | G | Q | R | L | H | P | R | X | R | S | P | V | Q | Y | Q | G | R | H | M | G | E | Q | E | D | G | L | Y | C | V | X | R | V | P | Q | G | R | L | V | P | G | G | L | Y | P | G | S | E | Q | D | A | G | G | A | Q | R | C | S | H | H | G | G | C | G | E | C | P | P | L | Q | T | D | E | T | X | R | H | X | D | L | C | P | E | E | G | V | P | A | V | A | G | G | H | T | G | Y | S | M | V | L | X | E | A | H | V | Q | D | S | X | G | Q | K | X | X | X | X | X | X | X | X | - | - | - | X | X | X | X | X | X | X | X | H | R | Q | R | X | Q | W | G | Y | H | - | - | T | C | K | Y | D | S | W | N |
| OMTLR5\_2938\_BP | - | X | X | X | X | X | X | X | X | X | X | X | X | X | X | X | X | X | X | X | X | X | X | X | X | X | X | X | X | X | - | X | X | X | X | X | X | X | X | X | X | X | X | X | X | X | X | X | X | X | X | X | X | X | X | X | X | X | X | X | X | X | X | X | X | X | X | X | X | X | X | M | S | A | H | H | F | R | G | M | M | R | N | C | I | L | L | V | I | F | - | - | G | V | Y | L | Q | V | V | K | C | T | P | R | C | P | L | Y | G | Y | I | A | V | C | T | N | L | S | L | Y | Q | V | P | A | L | P | P | Y | I | T | H | V | Y | M | R | D | N | Y | I | S | E | I | N | E | T | S | F | S | G | L | E | G | L | K | E | L | D | L | S | W | Q | R | V | N | G | L | T | I | R | T | N | T | F | Q | R | L | A | N | L | A | V | L | Y | L | G | H | N | R | G | L | K | I | E | P | G | A | F | V | G | L | S | N | L | R | T | L | S | L | Y | V | C | D | L | T | E | S | I | L | Q | G | D | Y | L | R | P | L | V | S | L | K | T | L | D | L | Y | G | N | Q | V | K | R | I | Q | P | S | P | F | F | V | N | M | T | D | F | Q | E | L | N | I | T | L | N | Q | M | E | S | I | C | E | E | D | L | L | G | F | C | G | K | H | F | R | L | L | K | L | N | S | V | S | L | Y | G | M | T | Q | N | G | F | D | W | K | R | C | G | N | P | F | R | N | M | S | I | E | T | L | D | L | S | S | N | G | F | N | V | D | K | A | K | L | F | F | N | A | I | Q | G | T | K | I | H | H | I | I | L | E | H | X | X | T | M | G | K | S | F | G | F | S | N | F | K | D | P | N | K | K | T | F | N | G | L | K | N | S | G | I | K | I | L | D | L | S | K | C | F | I | F | A | L | Q | Y | A | V | F | S | P | L | R | E | V | E | D | I | T | L | A | Q | N | K | I | N | Q | I | D | R | G | A | F | W | G | L | E | X | X | L | Q | R | L | N | L | S | H | N | L | I | G | E | I | Y | S | Y | T | F | D | N | L | P | N | I | L | E | L | D | L | S | Y | N | H | I | G | A | L | G | Y | Q | A | F | T | G | L | P | N | L | Q | I | L | D | L | T | G | N | S | I | R | Q | L | G | T | Y | G | Y | L | A | P | L | P | N | L | Q | L | L | N | L | A | D | N | K | X | X | X | T | S | L | E | G | L | L | G | F | A | N | S | T | I | I | L | N | V | Q | N | N | R | L | T | N | L | E | D | V | Y | I | V | L | A | K | F | M | R | I | E | R | I | W | Y | G | N | N | N | I | K | W | C | I | F | S | S | N | I | S | V | P | A | V | N | S | L | K | L | L | E | L | R | N | I | A | L | Q | I | L | W | G | H | G | V | C | L | D | V | F | E | N | L | I | K | L | F | S | L | D | L | S | F | N | S | L | R | A | L | P | D | G | I | F | K | G | L | V | S | L | E | E | M | D | L | S | F | N | S | L | T | Y | L | Q | P | D | I | F | P | A | S | L | K | T | V | D | L | S | Y | N | F | L | S | S | P | D | P | A | A | F | S | S | L | S | W | I | N | L | Y | R | N | R | F | H | C | D | G | G | L | K | D | F | L | T | W | M | N | R | T | N | V | T | F | P | D | P | G | V | A | E | F | S | C | E | F | P | S | D | L | H | G | V | S | L | L | N | Y | S | K | X | V | I | T | E | K | X | X | X | X | X | X | X | X | X | X | X | X | X | X | X | X | X | X | X | X | X | X | X | X | X | X | X | X | X | X | X | X | X | X | X | X | X | X | - | - | - | - | - | - | - | - | - | - | X | X | X | X | X | X | X | X | X | X | X | X | X | X | X | X | X | X | X | X | X | X | X | X | X | X | X | X | X | X | X | X | X | X | X | X | X | X | X | X | X | X | X | X | X | X | X | X | X | X | X | X | X | X | X | X | X | X | X | X | X | X | X | X | X | X | X | X | X | X | X | X | X | X | X | X | X | X | X | X | X | X | X | X | X | X | X | X | X | X | X | X | X | X | X | X | X | X | X | X | X | X | X | X | X | X | X | X | X | X | X | X | X | X | X | X | X | X | X | X | X | X | X | X | X | X | X | X | X | X | X | X | X | X | X | X | X | X | X | X | X | X | X | X | X | X | X | X | X | X | X | X | X | X | X | X | X | X | X | X | X | X | X | X | X | X | X | X | X | X | X | Q | N | H | L | X | Q | K | X | X | X | X | X | X | X | X | - | - | - | X | X | X | X | X | X | X | X | X | X | X | X | X | X | X | X | X | X | - | - | X | X | X | X | X | X | X | X |
| conservation |  |  |  |  |  |  |  |  |  |  |  |  |  |  |  |  |  |  |  |  |  |  |  |  |  |  |  |  |  |  |  |  |  |  |  |  |  |  |  |  |  |  |  |  |  |  |  |  |  |  |  |  |  |  |  |  |  |  |  |  |  |  |  |  |  |  |  |  |  |  |  |  |  |  |  |  |  |  |  |  |  |  |  |  |  |  |  |  |  |  |  |  |  |  |  |  |  |  |  |  |  |  |  |  |  |  |  |  |  |  |  |  |  |  |  |  |  |  |  |  |  |  |  |  |  |  |  |  |  |  |  |  |  |  |  |  |  |  |  |  |  |  |  |  |  |  |  |  |  |  |  |  |  |  |  |  |  |  |  |  |  |  |  |  |  |  |  |  |  |  |  |  |  |  |  |  |  |  |  |  |  |  |  |  |  |  |  |  |  |  |  |  |  |  |  |  |  |  |  |  |  |  |  |  |  |  |  |  |  |  |  |  |  |  |  |  |  |  |  |  |  |  |  |  |  |  |  |  |  |  |  |  |  |  |  |  |  |  |  |  |  |  |  |  |  |  |  |  |  |  |  |  |  |  |  |  |  |  |  |  |  |  |  |  |  |  |  |  |  |  |  |  |  |  |  |  |  |  |  |  |  |  |  |  |  |  |  |  |  |  |  |  |  |  |  |  |  |  |  |  |  |  |  |  |  |  |  |  |  |  |  |  |  |  |  |  |  |  |  |  |  |  |  |  |  |  |  |  |  |  |  |  |  |  |  |  |  |  |  |  |  |  |  |  |  |  |  |  |  |  |  |  |  |  |  |  |  |  |  |  |  |  |  |  |  |  |  |  |  |  |  |  |  |  |  |  |  |  |  |  |  |  |  |  |  |  |  |  |  |  |  |  |  |  |  |  |  |  |  |  |  |  |  |  |  |  |  |  |  |  |  |  |  |  |  |  |  |  |  |  |  |  |  |  |  |  |  |  |  |  |  |  |  |  |  |  |  |  |  |  |  |  |  |  |  |  |  |  |  |  |  |  |  |  |  |  |  |  |  |  |  |  |  |  |  |  |  |  |  |  |  |  |  |  |  |  |  |  |  |  |  |  |  |  |  |  |  |  |  |  |  |  |  |  |  |  |  |  |  |  |  |  |  |  |  |  |  |  |  |  |  |  |  |  |  |  |  |  |  |  |  |  |  |  |  |  |  |  |  |  |  |  |  |  |  |  |  |  |  |  |  |  |  |  |  |  |  |  |  |  |  |  |  |  |  |  |  |  |  |  |  |  |  |  |  |  |  |  |  |  |  |  |  |  |  |  |  |  |  |  |  |  |  |  |  |  |  |  |  |  |  |  |  |  |  |  |  |  |  |  |  |  |  |  |  |  |  |  |  |  |  |  |  |  |  |  |  |  |  |  |  |  |  |  |  |  |  |  |  |  |  |  |  |  |  |  |  |  |  |  |  |  |  |  |  |  |  |  |  |  |  |  |  |  |  |  |  |  |  |  |  |  |  |  |  |  |  |  |  |  |  |  |  |  |  |  |  |  |  |  |  |  |  |  |  |  |  |  |  |  |  |  |  |  |  |  |  |  |  |  |  |  |  |  |  |  |  |  |  |  |  |  |  |  |  |  |  |  |  |  |  |  |  |  |  |  |  |  |  |  |  |  |  |  |  |  |  |  |  |  |  |  |  |  |  |  |  |  |  |  |  |  |  |  |  |  |  |  |  |  |  |  |  |  |  |  |  |  |  |  |  |  |  |  |  |  |  |  |  |  |  |  |  |  |  |  |  |  |  |  |  |  |  |  |  |  |  |  |  |  |  |  |  |  |  |  |  |  |  |  |  |  |  |  |  |  |  |  |  |  |  |  |  |  |  |  |  |  |  |  |  |  |  |  |  |  |  |  |  |  |  |  |  |  |  |  |  |  |  |  |  |  |  |  |  |  |  |  |  |  |  |  |  |  |  |  |  |  |  |  |  |  |  |  |  |  |  |  |  |  |  |  |  |  |  |  |  |  |  |  |  |  |  |  |  |  |  |  |  |  |  |  |  |  |  |  |  |  |  |  |  |  |  |  |  |  |  |  |  |  |  |  |  |  |  |  |  |  |  |  |  |  |  |  |  |  |  |  |  |  |  |  |  |  |  |  |  |  |  |  |  |  |  |  |  |  |  |  |  |  |  |  |  |  |  |  |  |  |  |  |  |  |  |  |  |  |  |  |  |  |  |  |  |  |  |  |  |  |  |  |  |  |  |  |  |  |  |  |
|  |  |  |  |  |  |  |  |  |  |  |  |  |  |  |  |  |  |  |  |  |  |  |  |  |  |  |  |  |  |  |  |  |  |  |  |  |  |  |  |  |  |  |  |  |  |  |  |  |  |  |  |  |  |  |  |  |  |  |  |  |  |  |  |  |  |  |  |  |  |  |  |  |  |  |  |  |  |  |  |  |  |  |  |  |  |  |  |  |  |  |  |  |  |  |  |  |  |  |  |  |  |  |  |  |  |  |  |  |  |  |  |  |  |  |  |  |  |  |  |  |  |  |  |  |  |  |  |  |  |  |  |  |  |  |  |  |  |  |  |  |  |  |  |  |  |  |  |  |  |  |  |  |  |  |  |  |  |  |  |  |  |  |  |  |  |  |  |  |  |  |  |  |  |  |  |  |  |  |  |  |  |  |  |  |  |  |  |  |  |  |  |  |  |  |  |  |  |  |  |  |  |  |  |  |  |  |  |  |  |  |  |  |  |  |  |  |  |  |  |  |  |  |  |  |  |  |  |  |  |  |  |  |  |  |  |  |  |  |  |  |  |  |  |  |  |  |  |  |  |  |  |  |  |  |  |  |  |  |  |  |  |  |  |  |  |  |  |  |  |  |  |  |  |  |  |  |  |  |  |  |  |  |  |  |  |  |  |  |  |  |  |  |  |  |  |  |  |  |  |  |  |  |  |  |  |  |  |  |  |  |  |  |  |  |  |  |  |  |  |  |  |  |  |  |  |  |  |  |  |  |  |  |  |  |  |  |  |  |  |  |  |  |  |  |  |  |  |  |  |  |  |  |  |  |  |  |  |  |  |  |  |  |  |  |  |  |  |  |  |  |  |  |  |  |  |  |  |  |  |  |  |  |  |  |  |  |  |  |  |  |  |  |  |  |  |  |  |  |  |  |  |  |  |  |  |  |  |  |  |  |  |  |  |  |  |  |  |  |  |  |  |  |  |  |  |  |  |  |  |  |  |  |  |  |  |  |  |  |  |  |  |  |  |  |  |  |  |  |  |  |  |  |  |  |  |  |  |  |  |  |  |  |  |  |  |  |  |  |  |  |  |  |  |  |  |  |  |  |  |  |  |  |  |  |  |  |  |  |  |  |  |  |  |  |  |  |  |  |  |  |  |  |  |  |  |  |  |  |  |  |  |  |  |  |  |  |  |  |  |  |  |  |  |  |  |  |  |  |  |  |  |  |  |  |  |  |  |  |  |  |  |  |  |  |  |  |  |  |  |  |  |  |  |  |  |  |  |  |  |  |  |  |  |  |  |  |  |  |  |  |  |  |  |  |  |  |  |  |  |  |  |  |  |  |  |  |  |  |  |  |  |  |  |  |  |  |  |  |  |  |  |  |  |  |  |  |  |  |  |  |  |  |  |  |  |  |  |  |  |  |  |  |  |  |  |  |  |  |  |  |  |  |  |  |  |  |  |  |  |  |  |  |  |  |  |  |  |  |  |  |  |  |  |  |  |  |  |  |  |  |  |  |  |  |  |  |  |  |  |  |  |  |  |  |  |  |  |  |  |  |  |  |  |  |  |  |  |  |  |  |  |  |  |  |  |  |  |  |  |  |  |  |  |  |  |  |  |  |  |  |  |  |  |  |  |  |  |  |  |  |  |  |  |  |  |  |  |  |  |  |  |  |  |  |  |  |  |  |  |  |  |  |  |  |  |  |  |  |  |  |  |  |  |  |  |  |  |  |  |  |  |  |  |  |  |  |  |  |  |  |  |  |  |  |  |  |  |  |  |  |  |  |  |  |  |  |  |  |  |  |  |  |  |  |  |  |  |  |  |  |  |  |  |  |  |  |  |  |  |  |  |  |  |  |  |  |  |  |  |  |  |  |  |  |  |  |  |  |  |  |  |  |  |  |  |  |  |  |  |  |  |  |  |  |  |  |  |  |  |  |  |  |  |  |  |  |  |  |  |  |  |  |  |  |  |  |  |  |  |  |  |  |  |  |  |  |  |  |  |  |  |  |  |  |  |  |  |  |  |  |  |  |  |  |  |  |  |  |  |  |  |  |  |  |  |  |  |  |  |  |  |  |  |  |  |  |  |  |  |  |  |  |  |  |  |  |  |  |  |  |  |  |  |  |  |  |  |  |  |  |  |  |  |  |  |  |  |  |  |  |  |  |  |  |  |  |  |  |  |  |  |  |  |  |  |  |  |  |  |  |  |  |  |  |  |  |  |  |  |  |  |  |  |  |  |  |  |  |  |  |  |  |  |  |  |  |  |  |
|  |  |  |  |  |  |  |  |  |  |  |  |  |  |  |  |  |  |  |  |  |  |  |  |  |  |  |  |  |  |  |  |  |  |  |  |  |  |  |  |  |  |  |  |  |  |  |  |  |  |  |  |  |  |  |  |  |  |  |  |  |  |  |  |  |  |  |  |  |  |  |  |  |  |  |  |  |  |  |  |  |  |  |  |  |  |  |  |  |  |  |  |  |  |  |  |  |  |  |  |  |  |  |  |  |  |  |  |  |  |  |  |  |  |  |  |  |  |  |  |  |  |  |  |  |  |  |  |  |  |  |  |  |  |  |  |  |  |  |  |  |  |  |  |  |  |  |  |  |  |  |  |  |  |  |  |  |  |  |  |  |  |  |  |  |  |  |  |  |  |  |  |  |  |  |  |  |  |  |  |  |  |  |  |  |  |  |  |  |  |  |  |  |  |  |  |  |  |  |  |  |  |  |  |  |  |  |  |  |  |  |  |  |  |  |  |  |  |  |  |  |  |  |  |  |  |  |  |  |  |  |  |  |  |  |  |  |  |  |  |  |  |  |  |  |  |  |  |  |  |  |  |  |  |  |  |  |  |  |  |  |  |  |  |  |  |  |  |  |  |  |  |  |  |  |  |  |  |  |  |  |  |  |  |  |  |  |  |  |  |  |  |  |  |  |  |  |  |  |  |  |  |  |  |  |  |  |  |  |  |  |  |  |  |  |  |  |  |  |  |  |  |  |  |  |  |  |  |  |  |  |  |  |  |  |  |  |  |  |  |  |  |  |  |  |  |  |  |  |  |  |  |  |  |  |  |  |  |  |  |  |  |  |  |  |  |  |  |  |  |  |  |  |  |  |  |  |  |  |  |  |  |  |  |  |  |  |  |  |  |  |  |  |  |  |  |  |  |  |  |  |  |  |  |  |  |  |  |  |  |  |  |  |  |  |  |  |  |  |  |  |  |  |  |  |  |  |  |  |  |  |  |  |  |  |  |  |  |  |  |  |  |  |  |  |  |  |  |  |  |  |  |  |  |  |  |  |  |  |  |  |  |  |  |  |  |  |  |  |  |  |  |  |  |  |  |  |  |  |  |  |  |  |  |  |  |  |  |  |  |  |  |  |  |  |  |  |  |  |  |  |  |  |  |  |  |  |  |  |  |  |  |  |  |  |  |  |  |  |  |  |  |  |  |  |  |  |  |  |  |  |  |  |  |  |  |  |  |  |  |  |  |  |  |  |  |  |  |  |  |  |  |  |  |  |  |  |  |  |  |  |  |  |  |  |  |  |  |  |  |  |  |  |  |  |  |  |  |  |  |  |  |  |  |  |  |  |  |  |  |  |  |  |  |  |  |  |  |  |  |  |  |  |  |  |  |  |  |  |  |  |  |  |  |  |  |  |  |  |  |  |  |  |  |  |  |  |  |  |  |  |  |  |  |  |  |  |  |  |  |  |  |  |  |  |  |  |  |  |  |  |  |  |  |  |  |  |  |  |  |  |  |  |  |  |  |  |  |  |  |  |  |  |  |  |  |  |  |  |  |  |  |  |  |  |  |  |  |  |  |  |  |  |  |  |  |  |  |  |  |  |  |  |  |  |  |  |  |  |  |  |  |  |  |  |  |  |  |  |  |  |  |  |  |  |  |  |  |  |  |  |  |  |  |  |  |  |  |  |  |  |  |  |  |  |  |  |  |  |  |  |  |  |  |  |  |  |  |  |  |  |  |  |  |  |  |  |  |  |  |  |  |  |  |  |  |  |  |  |  |  |  |  |  |  |  |  |  |  |  |  |  |  |  |  |  |  |  |  |  |  |  |  |  |  |  |  |  |  |  |  |  |  |  |  |  |  |  |  |  |  |  |  |  |  |  |  |  |  |  |  |  |  |  |  |  |  |  |  |  |  |  |  |  |  |  |  |  |  |  |  |  |  |  |  |  |  |  |  |  |  |  |  |  |  |  |  |  |  |  |  |  |  |  |  |  |  |  |  |  |  |  |  |  |  |  |  |  |  |  |  |  |  |  |  |  |  |  |  |  |  |  |  |  |  |  |  |  |  |  |  |  |  |  |  |  |  |  |  |  |  |  |  |  |  |  |  |  |  |  |  |  |  |  |  |  |  |  |  |  |  |  |  |  |  |  |  |  |  |  |  |  |  |  |  |  |  |  |  |  |  |  |  |  |  |  |  |  |  |  |  |  |  |  |  |  |  |  |  |  |  |  |  |  |  |  |  |  |  |  |  |  |  |  |  |  |  |  |  |
|  |  |  |  |  |  |  |  |  |  |  |  |  |  |  |  |  |  |  |  |  |  |  |  |  |  |  |  |  |  |  |  |  |  |  |  |  |  |  |  |  |  |  |  |  |  |  |  |  |  |  |  |  |  |  |  |  |  |  |  |  |  |  |  |  |  |  |  |  |  |  |  |  |  |  |  |  |  |  |  |  |  |  |  |  |  |  |  |  |  |  |  |  |  |  |  |  |  |  |  |  |  |  |  |  |  |  |  |  |  |  |  |  |  |  |  |  |  |  |  |  |  |  |  |  |  |  |  |  |  |  |  |  |  |  |  |  |  |  |  |  |  |  |  |  |  |  |  |  |  |  |  |  |  |  |  |  |  |  |  |  |  |  |  |  |  |  |  |  |  |  |  |  |  |  |  |  |  |  |  |  |  |  |  |  |  |  |  |  |  |  |  |  |  |  |  |  |  |  |  |  |  |  |  |  |  |  |  |  |  |  |  |  |  |  |  |  |  |  |  |  |  |  |  |  |  |  |  |  |  |  |  |  |  |  |  |  |  |  |  |  |  |  |  |  |  |  |  |  |  |  |  |  |  |  |  |  |  |  |  |  |  |  |  |  |  |  |  |  |  |  |  |  |  |  |  |  |  |  |  |  |  |  |  |  |  |  |  |  |  |  |  |  |  |  |  |  |  |  |  |  |  |  |  |  |  |  |  |  |  |  |  |  |  |  |  |  |  |  |  |  |  |  |  |  |  |  |  |  |  |  |  |  |  |  |  |  |  |  |  |  |  |  |  |  |  |  |  |  |  |  |  |  |  |  |  |  |  |  |  |  |  |  |  |  |  |  |  |  |  |  |  |  |  |  |  |  |  |  |  |  |  |  |  |  |  |  |  |  |  |  |  |  |  |  |  |  |  |  |  |  |  |  |  |  |  |  |  |  |  |  |  |  |  |  |  |  |  |  |  |  |  |  |  |  |  |  |  |  |  |  |  |  |  |  |  |  |  |  |  |  |  |  |  |  |  |  |  |  |  |  |  |  |  |  |  |  |  |  |  |  |  |  |  |  |  |  |  |  |  |  |  |  |  |  |  |  |  |  |  |  |  |  |  |  |  |  |  |  |  |  |  |  |  |  |  |  |  |  |  |  |  |  |  |  |  |  |  |  |  |  |  |  |  |  |  |  |  |  |  |  |  |  |  |  |  |  |  |  |  |  |  |  |  |  |  |  |  |  |  |  |  |  |  |  |  |  |  |  |  |  |  |  |  |  |  |  |  |  |  |  |  |  |  |  |  |  |  |  |  |  |  |  |  |  |  |  |  |  |  |  |  |  |  |  |  |  |  |  |  |  |  |  |  |  |  |  |  |  |  |  |  |  |  |  |  |  |  |  |  |  |  |  |  |  |  |  |  |  |  |  |  |  |  |  |  |  |  |  |  |  |  |  |  |  |  |  |  |  |  |  |  |  |  |  |  |  |  |  |  |  |  |  |  |  |  |  |  |  |  |  |  |  |  |  |  |  |  |  |  |  |  |  |  |  |  |  |  |  |  |  |  |  |  |  |  |  |  |  |  |  |  |  |  |  |  |  |  |  |  |  |  |  |  |  |  |  |  |  |  |  |  |  |  |  |  |  |  |  |  |  |  |  |  |  |  |  |  |  |  |  |  |  |  |  |  |  |  |  |  |  |  |  |  |  |  |  |  |  |  |  |  |  |  |  |  |  |  |  |  |  |  |  |  |  |  |  |  |  |  |  |  |  |  |  |  |  |  |  |  |  |  |  |  |  |  |  |  |  |  |  |  |  |  |  |  |  |  |  |  |  |  |  |  |  |  |  |  |  |  |  |  |  |  |  |  |  |  |  |  |  |  |  |  |  |  |  |  |  |  |  |  |  |  |  |  |  |  |  |  |  |  |  |  |  |  |  |  |  |  |  |  |  |  |  |  |  |  |  |  |  |  |  |  |  |  |  |  |  |  |  |  |  |  |  |  |  |  |  |  |  |  |  |  |  |  |  |  |  |  |  |  |  |  |  |  |  |  |  |  |  |  |  |  |  |  |  |  |  |  |  |  |  |  |  |  |  |  |  |  |  |  |  |  |  |  |  |  |  |  |  |  |  |  |  |  |  |  |  |  |  |  |  |  |  |  |  |  |  |  |  |  |  |  |  |  |  |  |  |  |  |  |  |  |  |  |  |  |  |  |  |  |  |  |  |  |  |  |  |  |  |  |  |  |  |  |  |  |  |  |  |  |  |  |  |  |  |  |  |
|  |  |  |  |  |  |  |  |  |  |  |  |  |  |  |  |  |  |  |  |  |  |  |  |  |  |  |  |  |  |  |  |  |  |  |  |  |  |  |  |  |  |  |  |  |  |  |  |  |  |  |  |  |  |  |  |  |  |  |  |  |  |  |  |  |  |  |  |  |  |  |  |  |  |  |  |  |  |  |  |  |  |  |  |  |  |  |  |  |  |  |  |  |  |  |  |  |  |  |  |  |  |  |  |  |  |  |  |  |  |  |  |  |  |  |  |  |  |  |  |  |  |  |  |  |  |  |  |  |  |  |  |  |  |  |  |  |  |  |  |  |  |  |  |  |  |  |  |  |  |  |  |  |  |  |  |  |  |  |  |  |  |  |  |  |  |  |  |  |  |  |  |  |  |  |  |  |  |  |  |  |  |  |  |  |  |  |  |  |  |  |  |  |  |  |  |  |  |  |  |  |  |  |  |  |  |  |  |  |  |  |  |  |  |  |  |  |  |  |  |  |  |  |  |  |  |  |  |  |  |  |  |  |  |  |  |  |  |  |  |  |  |  |  |  |  |  |  |  |  |  |  |  |  |  |  |  |  |  |  |  |  |  |  |  |  |  |  |  |  |  |  |  |  |  |  |  |  |  |  |  |  |  |  |  |  |  |  |  |  |  |  |  |  |  |  |  |  |  |  |  |  |  |  |  |  |  |  |  |  |  |  |  |  |  |  |  |  |  |  |  |  |  |  |  |  |  |  |  |  |  |  |  |  |  |  |  |  |  |  |  |  |  |  |  |  |  |  |  |  |  |  |  |  |  |  |  |  |  |  |  |  |  |  |  |  |  |  |  |  |  |  |  |  |  |  |  |  |  |  |  |  |  |  |  |  |  |  |  |  |  |  |  |  |  |  |  |  |  |  |  |  |  |  |  |  |  |  |  |  |  |  |  |  |  |  |  |  |  |  |  |  |  |  |  |  |  |  |  |  |  |  |  |  |  |  |  |  |  |  |  |  |  |  |  |  |  |  |  |  |  |  |  |  |  |  |  |  |  |  |  |  |  |  |  |  |  |  |  |  |  |  |  |  |  |  |  |  |  |  |  |  |  |  |  |  |  |  |  |  |  |  |  |  |  |  |  |  |  |  |  |  |  |  |  |  |  |  |  |  |  |  |  |  |  |  |  |  |  |  |  |  |  |  |  |  |  |  |  |  |  |  |  |  |  |  |  |  |  |  |  |  |  |  |  |  |  |  |  |  |  |  |  |  |  |  |  |  |  |  |  |  |  |  |  |  |  |  |  |  |  |  |  |  |  |  |  |  |  |  |  |  |  |  |  |  |  |  |  |  |  |  |  |  |  |  |  |  |  |  |  |  |  |  |  |  |  |  |  |  |  |  |  |  |  |  |  |  |  |  |  |  |  |  |  |  |  |  |  |  |  |  |  |  |  |  |  |  |  |  |  |  |  |  |  |  |  |  |  |  |  |  |  |  |  |  |  |  |  |  |  |  |  |  |  |  |  |  |  |  |  |  |  |  |  |  |  |  |  |  |  |  |  |  |  |  |  |  |  |  |  |  |  |  |  |  |  |  |  |  |  |  |  |  |  |  |  |  |  |  |  |  |  |  |  |  |  |  |  |  |  |  |  |  |  |  |  |  |  |  |  |  |  |  |  |  |  |  |  |  |  |  |  |  |  |  |  |  |  |  |  |  |  |  |  |  |  |  |  |  |  |  |  |  |  |  |  |  |  |  |  |  |  |  |  |  |  |  |  |  |  |  |  |  |  |  |  |  |  |  |  |  |  |  |  |  |  |  |  |  |  |  |  |  |  |  |  |  |  |  |  |  |  |  |  |  |  |  |  |  |  |  |  |  |  |  |  |  |  |  |  |  |  |  |  |  |  |  |  |  |  |  |  |  |  |  |  |  |  |  |  |  |  |  |  |  |  |  |  |  |  |  |  |  |  |  |  |  |  |  |  |  |  |  |  |  |  |  |  |  |  |  |  |  |  |  |  |  |  |  |  |  |  |  |  |  |  |  |  |  |  |  |  |  |  |  |  |  |  |  |  |  |  |  |  |  |  |  |  |  |  |  |  |  |  |  |  |  |  |  |  |  |  |  |  |  |  |  |  |  |  |  |  |  |  |  |  |  |  |  |  |  |  |  |  |  |  |  |  |  |  |  |  |  |  |  |  |  |  |  |  |  |  |  |  |  |  |  |  |  |  |  |  |  |  |  |  |  |  |  |  |  |  |  |  |  |  |  |  |
|  |  |  |  |  |  |  |  |  |  |  |  |  |  |  |  |  |  |  |  |  |  |  |  |  |  |  |  |  |  |  |  |  |  |  |  |  |  |  |  |  |  |  |  |  |  |  |  |  |  |  |  |  |  |  |  |  |  |  |  |  |  |  |  |  |  |  |  |  |  |  |  |  |  |  |  |  |  |  |  |  |  |  |  |  |  |  |  |  |  |  |  |  |  |  |  |  |  |  |  |  |  |  |  |  |  |  |  |  |  |  |  |  |  |  |  |  |  |  |  |  |  |  |  |  |  |  |  |  |  |  |  |  |  |  |  |  |  |  |  |  |  |  |  |  |  |  |  |  |  |  |  |  |  |  |  |  |  |  |  |  |  |  |  |  |  |  |  |  |  |  |  |  |  |  |  |  |  |  |  |  |  |  |  |  |  |  |  |  |  |  |  |  |  |  |  |  |  |  |  |  |  |  |  |  |  |  |  |  |  |  |  |  |  |  |  |  |  |  |  |  |  |  |  |  |  |  |  |  |  |  |  |  |  |  |  |  |  |  |  |  |  |  |  |  |  |  |  |  |  |  |  |  |  |  |  |  |  |  |  |  |  |  |  |  |  |  |  |  |  |  |  |  |  |  |  |  |  |  |  |  |  |  |  |  |  |  |  |  |  |  |  |  |  |  |  |  |  |  |  |  |  |  |  |  |  |  |  |  |  |  |  |  |  |  |  |  |  |  |  |  |  |  |  |  |  |  |  |  |  |  |  |  |  |  |  |  |  |  |  |  |  |  |  |  |  |  |  |  |  |  |  |  |  |  |  |  |  |  |  |  |  |  |  |  |  |  |  |  |  |  |  |  |  |  |  |  |  |  |  |  |  |  |  |  |  |  |  |  |  |  |  |  |  |  |  |  |  |  |  |  |  |  |  |  |  |  |  |  |  |  |  |  |  |  |  |  |  |  |  |  |  |  |  |  |  |  |  |  |  |  |  |  |  |  |  |  |  |  |  |  |  |  |  |  |  |  |  |  |  |  |  |  |  |  |  |  |  |  |  |  |  |  |  |  |  |  |  |  |  |  |  |  |  |  |  |  |  |  |  |  |  |  |  |  |  |  |  |  |  |  |  |  |  |  |  |  |  |  |  |  |  |  |  |  |  |  |  |  |  |  |  |  |  |  |  |  |  |  |  |  |  |  |  |  |  |  |  |  |  |  |  |  |  |  |  |  |  |  |  |  |  |  |  |  |  |  |  |  |  |  |  |  |  |  |  |  |  |  |  |  |  |  |  |  |  |  |  |  |  |  |  |  |  |  |  |  |  |  |  |  |  |  |  |  |  |  |  |  |  |  |  |  |  |  |  |  |  |  |  |  |  |  |  |  |  |  |  |  |  |  |  |  |  |  |  |  |  |  |  |  |  |  |  |  |  |  |  |  |  |  |  |  |  |  |  |  |  |  |  |  |  |  |  |  |  |  |  |  |  |  |  |  |  |  |  |  |  |  |  |  |  |  |  |  |  |  |  |  |  |  |  |  |  |  |  |  |  |  |  |  |  |  |  |  |  |  |  |  |  |  |  |  |  |  |  |  |  |  |  |  |  |  |  |  |  |  |  |  |  |  |  |  |  |  |  |  |  |  |  |  |  |  |  |  |  |  |  |  |  |  |  |  |  |  |  |  |  |  |  |  |  |  |  |  |  |  |  |  |  |  |  |  |  |  |  |  |  |  |  |  |  |  |  |  |  |  |  |  |  |  |  |  |  |  |  |  |  |  |  |  |  |  |  |  |  |  |  |  |  |  |  |  |  |  |  |  |  |  |  |  |  |  |  |  |  |  |  |  |  |  |  |  |  |  |  |  |  |  |  |  |  |  |  |  |  |  |  |  |  |  |  |  |  |  |  |  |  |  |  |  |  |  |  |  |  |  |  |  |  |  |  |  |  |  |  |  |  |  |  |  |  |  |  |  |  |  |  |  |  |  |  |  |  |  |  |  |  |  |  |  |  |  |  |  |  |  |  |  |  |  |  |  |  |  |  |  |  |  |  |  |  |  |  |  |  |  |  |  |  |  |  |  |  |  |  |  |  |  |  |  |  |  |  |  |  |  |  |  |  |  |  |  |  |  |  |  |  |  |  |  |  |  |  |  |  |  |  |  |  |  |  |  |  |  |  |  |  |  |  |  |  |  |  |  |  |  |  |  |  |  |  |  |  |  |  |  |  |  |  |  |  |  |  |  |  |  |  |  |  |  |  |  |  |  |  |  |  |  |
|  |  |  |  |  |  |  |  |  |  |  |  |  |  |  |  |  |  |  |  |  |  |  |  |  |  |  |  |  |  |  |  |  |  |  |  |  |  |  |  |  |  |  |  |  |  |  |  |  |  |  |  |  |  |  |  |  |  |  |  |  |  |  |  |  |  |  |  |  |  |  |  |  |  |  |  |  |  |  |  |  |  |  |  |  |  |  |  |  |  |  |  |  |  |  |  |  |  |  |  |  |  |  |  |  |  |  |  |  |  |  |  |  |  |  |  |  |  |  |  |  |  |  |  |  |  |  |  |  |  |  |  |  |  |  |  |  |  |  |  |  |  |  |  |  |  |  |  |  |  |  |  |  |  |  |  |  |  |  |  |  |  |  |  |  |  |  |  |  |  |  |  |  |  |  |  |  |  |  |  |  |  |  |  |  |  |  |  |  |  |  |  |  |  |  |  |  |  |  |  |  |  |  |  |  |  |  |  |  |  |  |  |  |  |  |  |  |  |  |  |  |  |  |  |  |  |  |  |  |  |  |  |  |  |  |  |  |  |  |  |  |  |  |  |  |  |  |  |  |  |  |  |  |  |  |  |  |  |  |  |  |  |  |  |  |  |  |  |  |  |  |  |  |  |  |  |  |  |  |  |  |  |  |  |  |  |  |  |  |  |  |  |  |  |  |  |  |  |  |  |  |  |  |  |  |  |  |  |  |  |  |  |  |  |  |  |  |  |  |  |  |  |  |  |  |  |  |  |  |  |  |  |  |  |  |  |  |  |  |  |  |  |  |  |  |  |  |  |  |  |  |  |  |  |  |  |  |  |  |  |  |  |  |  |  |  |  |  |  |  |  |  |  |  |  |  |  |  |  |  |  |  |  |  |  |  |  |  |  |  |  |  |  |  |  |  |  |  |  |  |  |  |  |  |  |  |  |  |  |  |  |  |  |  |  |  |  |  |  |  |  |  |  |  |  |  |  |  |  |  |  |  |  |  |  |  |  |  |  |  |  |  |  |  |  |  |  |  |  |  |  |  |  |  |  |  |  |  |  |  |  |  |  |  |  |  |  |  |  |  |  |  |  |  |  |  |  |  |  |  |  |  |  |  |  |  |  |  |  |  |  |  |  |  |  |  |  |  |  |  |  |  |  |  |  |  |  |  |  |  |  |  |  |  |  |  |  |  |  |  |  |  |  |  |  |  |  |  |  |  |  |  |  |  |  |  |  |  |  |  |  |  |  |  |  |  |  |  |  |  |  |  |  |  |  |  |  |  |  |  |  |  |  |  |  |  |  |  |  |  |  |  |  |  |  |  |  |  |  |  |  |  |  |  |  |  |  |  |  |  |  |  |  |  |  |  |  |  |  |  |  |  |  |  |  |  |  |  |  |  |  |  |  |  |  |  |  |  |  |  |  |  |  |  |  |  |  |  |  |  |  |  |  |  |  |  |  |  |  |  |  |  |  |  |  |  |  |  |  |  |  |  |  |  |  |  |  |  |  |  |  |  |  |  |  |  |  |  |  |  |  |  |  |  |  |  |  |  |  |  |  |  |  |  |  |  |  |  |  |  |  |  |  |  |  |  |  |  |  |  |  |  |  |  |  |  |  |  |  |  |  |  |  |  |  |  |  |  |  |  |  |  |  |  |  |  |  |  |  |  |  |  |  |  |  |  |  |  |  |  |  |  |  |  |  |  |  |  |  |  |  |  |  |  |  |  |  |  |  |  |  |  |  |  |  |  |  |  |  |  |  |  |  |  |  |  |  |  |  |  |  |  |  |  |  |  |  |  |  |  |  |  |  |  |  |  |  |  |  |  |  |  |  |  |  |  |  |  |  |  |  |  |  |  |  |  |  |  |  |  |  |  |  |  |  |  |  |  |  |  |  |  |  |  |  |  |  |  |  |  |  |  |  |  |  |  |  |  |  |  |  |  |  |  |  |  |  |  |  |  |  |  |  |  |  |  |  |  |  |  |  |  |  |  |  |  |  |  |  |  |  |  |  |  |  |  |  |  |  |  |  |  |  |  |  |  |  |  |  |  |  |  |  |  |  |  |  |  |  |  |  |  |  |  |  |  |  |  |  |  |  |  |  |  |  |  |  |  |  |  |  |  |  |  |  |  |  |  |  |  |  |  |  |  |  |  |  |  |  |  |  |  |  |  |  |  |  |  |  |  |  |  |  |  |  |  |  |  |  |  |  |  |  |  |  |  |  |  |  |  |  |  |  |  |  |  |  |  |  |  |  |  |  |  |  |  |  |  |  |
|  |  |  |  |  |  |  |  |  |  |  |  |  |  |  |  |  |  |  |  |  |  |  |  |  |  |  |  |  |  |  |  |  |  |  |  |  |  |  |  |  |  |  |  |  |  |  |  |  |  |  |  |  |  |  |  |  |  |  |  |  |  |  |  |  |  |  |  |  |  |  |  |  |  |  |  |  |  |  |  |  |  |  |  |  |  |  |  |  |  |  |  |  |  |  |  |  |  |  |  |  |  |  |  |  |  |  |  |  |  |  |  |  |  |  |  |  |  |  |  |  |  |  |  |  |  |  |  |  |  |  |  |  |  |  |  |  |  |  |  |  |  |  |  |  |  |  |  |  |  |  |  |  |  |  |  |  |  |  |  |  |  |  |  |  |  |  |  |  |  |  |  |  |  |  |  |  |  |  |  |  |  |  |  |  |  |  |  |  |  |  |  |  |  |  |  |  |  |  |  |  |  |  |  |  |  |  |  |  |  |  |  |  |  |  |  |  |  |  |  |  |  |  |  |  |  |  |  |  |  |  |  |  |  |  |  |  |  |  |  |  |  |  |  |  |  |  |  |  |  |  |  |  |  |  |  |  |  |  |  |  |  |  |  |  |  |  |  |  |  |  |  |  |  |  |  |  |  |  |  |  |  |  |  |  |  |  |  |  |  |  |  |  |  |  |  |  |  |  |  |  |  |  |  |  |  |  |  |  |  |  |  |  |  |  |  |  |  |  |  |  |  |  |  |  |  |  |  |  |  |  |  |  |  |  |  |  |  |  |  |  |  |  |  |  |  |  |  |  |  |  |  |  |  |  |  |  |  |  |  |  |  |  |  |  |  |  |  |  |  |  |  |  |  |  |  |  |  |  |  |  |  |  |  |  |  |  |  |  |  |  |  |  |  |  |  |  |  |  |  |  |  |  |  |  |  |  |  |  |  |  |  |  |  |  |  |  |  |  |  |  |  |  |  |  |  |  |  |  |  |  |  |  |  |  |  |  |  |  |  |  |  |  |  |  |  |  |  |  |  |  |  |  |  |  |  |  |  |  |  |  |  |  |  |  |  |  |  |  |  |  |  |  |  |  |  |  |  |  |  |  |  |  |  |  |  |  |  |  |  |  |  |  |  |  |  |  |  |  |  |  |  |  |  |  |  |  |  |  |  |  |  |  |  |  |  |  |  |  |  |  |  |  |  |  |  |  |  |  |  |  |  |  |  |  |  |  |  |  |  |  |  |  |  |  |  |  |  |  |  |  |  |  |  |  |  |  |  |  |  |  |  |  |  |  |  |  |  |  |  |  |  |  |  |  |  |  |  |  |  |  |  |  |  |  |  |  |  |  |  |  |  |  |  |  |  |  |  |  |  |  |  |  |  |  |  |  |  |  |  |  |  |  |  |  |  |  |  |  |  |  |  |  |  |  |  |  |  |  |  |  |  |  |  |  |  |  |  |  |  |  |  |  |  |  |  |  |  |  |  |  |  |  |  |  |  |  |  |  |  |  |  |  |  |  |  |  |  |  |  |  |  |  |  |  |  |  |  |  |  |  |  |  |  |  |  |  |  |  |  |  |  |  |  |  |  |  |  |  |  |  |  |  |  |  |  |  |  |  |  |  |  |  |  |  |  |  |  |  |  |  |  |  |  |  |  |  |  |  |  |  |  |  |  |  |  |  |  |  |  |  |  |  |  |  |  |  |  |  |  |  |  |  |  |  |  |  |  |  |  |  |  |  |  |  |  |  |  |  |  |  |  |  |  |  |  |  |  |  |  |  |  |  |  |  |  |  |  |  |  |  |  |  |  |  |  |  |  |  |  |  |  |  |  |  |  |  |  |  |  |  |  |  |  |  |  |  |  |  |  |  |  |  |  |  |  |  |  |  |  |  |  |  |  |  |  |  |  |  |  |  |  |  |  |  |  |  |  |  |  |  |  |  |  |  |  |  |  |  |  |  |  |  |  |  |  |  |  |  |  |  |  |  |  |  |  |  |  |  |  |  |  |  |  |  |  |  |  |  |  |  |  |  |  |  |  |  |  |  |  |  |  |  |  |  |  |  |  |  |  |  |  |  |  |  |  |  |  |  |  |  |  |  |  |  |  |  |  |  |  |  |  |  |  |  |  |  |  |  |  |  |  |  |  |  |  |  |  |  |  |  |  |  |  |  |  |  |  |  |  |  |  |  |  |  |  |  |  |  |  |  |  |  |  |  |  |  |  |  |  |  |  |  |  |  |  |  |  |  |  |  |  |  |  |  |  |  |  |  |
|  |  |  |  |  |  |  |  |  |  |  |  |  |  |  |  |  |  |  |  |  |  |  |  |  |  |  |  |  |  |  |  |  |  |  |  |  |  |  |  |  |  |  |  |  |  |  |  |  |  |  |  |  |  |  |  |  |  |  |  |  |  |  |  |  |  |  |  |  |  |  |  |  |  |  |  |  |  |  |  |  |  |  |  |  |  |  |  |  |  |  |  |  |  |  |  |  |  |  |  |  |  |  |  |  |  |  |  |  |  |  |  |  |  |  |  |  |  |  |  |  |  |  |  |  |  |  |  |  |  |  |  |  |  |  |  |  |  |  |  |  |  |  |  |  |  |  |  |  |  |  |  |  |  |  |  |  |  |  |  |  |  |  |  |  |  |  |  |  |  |  |  |  |  |  |  |  |  |  |  |  |  |  |  |  |  |  |  |  |  |  |  |  |  |  |  |  |  |  |  |  |  |  |  |  |  |  |  |  |  |  |  |  |  |  |  |  |  |  |  |  |  |  |  |  |  |  |  |  |  |  |  |  |  |  |  |  |  |  |  |  |  |  |  |  |  |  |  |  |  |  |  |  |  |  |  |  |  |  |  |  |  |  |  |  |  |  |  |  |  |  |  |  |  |  |  |  |  |  |  |  |  |  |  |  |  |  |  |  |  |  |  |  |  |  |  |  |  |  |  |  |  |  |  |  |  |  |  |  |  |  |  |  |  |  |  |  |  |  |  |  |  |  |  |  |  |  |  |  |  |  |  |  |  |  |  |  |  |  |  |  |  |  |  |  |  |  |  |  |  |  |  |  |  |  |  |  |  |  |  |  |  |  |  |  |  |  |  |  |  |  |  |  |  |  |  |  |  |  |  |  |  |  |  |  |  |  |  |  |  |  |  |  |  |  |  |  |  |  |  |  |  |  |  |  |  |  |  |  |  |  |  |  |  |  |  |  |  |  |  |  |  |  |  |  |  |  |  |  |  |  |  |  |  |  |  |  |  |  |  |  |  |  |  |  |  |  |  |  |  |  |  |  |  |  |  |  |  |  |  |  |  |  |  |  |  |  |  |  |  |  |  |  |  |  |  |  |  |  |  |  |  |  |  |  |  |  |  |  |  |  |  |  |  |  |  |  |  |  |  |  |  |  |  |  |  |  |  |  |  |  |  |  |  |  |  |  |  |  |  |  |  |  |  |  |  |  |  |  |  |  |  |  |  |  |  |  |  |  |  |  |  |  |  |  |  |  |  |  |  |  |  |  |  |  |  |  |  |  |  |  |  |  |  |  |  |  |  |  |  |  |  |  |  |  |  |  |  |  |  |  |  |  |  |  |  |  |  |  |  |  |  |  |  |  |  |  |  |  |  |  |  |  |  |  |  |  |  |  |  |  |  |  |  |  |  |  |  |  |  |  |  |  |  |  |  |  |  |  |  |  |  |  |  |  |  |  |  |  |  |  |  |  |  |  |  |  |  |  |  |  |  |  |  |  |  |  |  |  |  |  |  |  |  |  |  |  |  |  |  |  |  |  |  |  |  |  |  |  |  |  |  |  |  |  |  |  |  |  |  |  |  |  |  |  |  |  |  |  |  |  |  |  |  |  |  |  |  |  |  |  |  |  |  |  |  |  |  |  |  |  |  |  |  |  |  |  |  |  |  |  |  |  |  |  |  |  |  |  |  |  |  |  |  |  |  |  |  |  |  |  |  |  |  |  |  |  |  |  |  |  |  |  |  |  |  |  |  |  |  |  |  |  |  |  |  |  |  |  |  |  |  |  |  |  |  |  |  |  |  |  |  |  |  |  |  |  |  |  |  |  |  |  |  |  |  |  |  |  |  |  |  |  |  |  |  |  |  |  |  |  |  |  |  |  |  |  |  |  |  |  |  |  |  |  |  |  |  |  |  |  |  |  |  |  |  |  |  |  |  |  |  |  |  |  |  |  |  |  |  |  |  |  |  |  |  |  |  |  |  |  |  |  |  |  |  |  |  |  |  |  |  |  |  |  |  |  |  |  |  |  |  |  |  |  |  |  |  |  |  |  |  |  |  |  |  |  |  |  |  |  |  |  |  |  |  |  |  |  |  |  |  |  |  |  |  |  |  |  |  |  |  |  |  |  |  |  |  |  |  |  |  |  |  |  |  |  |  |  |  |  |  |  |  |  |  |  |  |  |  |  |  |  |  |  |  |  |  |  |  |  |  |  |  |  |  |  |  |  |  |  |  |  |  |  |  |  |  |  |  |  |  |  |  |  |  |  |  |  |
|  |  |  |  |  |  |  |  |  |  |  |  |  |  |  |  |  |  |  |  |  |  |  |  |  |  |  |  |  |  |  |  |  |  |  |  |  |  |  |  |  |  |  |  |  |  |  |  |  |  |  |  |  |  |  |  |  |  |  |  |  |  |  |  |  |  |  |  |  |  |  |  |  |  |  |  |  |  |  |  |  |  |  |  |  |  |  |  |  |  |  |  |  |  |  |  |  |  |  |  |  |  |  |  |  |  |  |  |  |  |  |  |  |  |  |  |  |  |  |  |  |  |  |  |  |  |  |  |  |  |  |  |  |  |  |  |  |  |  |  |  |  |  |  |  |  |  |  |  |  |  |  |  |  |  |  |  |  |  |  |  |  |  |  |  |  |  |  |  |  |  |  |  |  |  |  |  |  |  |  |  |  |  |  |  |  |  |  |  |  |  |  |  |  |  |  |  |  |  |  |  |  |  |  |  |  |  |  |  |  |  |  |  |  |  |  |  |  |  |  |  |  |  |  |  |  |  |  |  |  |  |  |  |  |  |  |  |  |  |  |  |  |  |  |  |  |  |  |  |  |  |  |  |  |  |  |  |  |  |  |  |  |  |  |  |  |  |  |  |  |  |  |  |  |  |  |  |  |  |  |  |  |  |  |  |  |  |  |  |  |  |  |  |  |  |  |  |  |  |  |  |  |  |  |  |  |  |  |  |  |  |  |  |  |  |  |  |  |  |  |  |  |  |  |  |  |  |  |  |  |  |  |  |  |  |  |  |  |  |  |  |  |  |  |  |  |  |  |  |  |  |  |  |  |  |  |  |  |  |  |  |  |  |  |  |  |  |  |  |  |  |  |  |  |  |  |  |  |  |  |  |  |  |  |  |  |  |  |  |  |  |  |  |  |  |  |  |  |  |  |  |  |  |  |  |  |  |  |  |  |  |  |  |  |  |  |  |  |  |  |  |  |  |  |  |  |  |  |  |  |  |  |  |  |  |  |  |  |  |  |  |  |  |  |  |  |  |  |  |  |  |  |  |  |  |  |  |  |  |  |  |  |  |  |  |  |  |  |  |  |  |  |  |  |  |  |  |  |  |  |  |  |  |  |  |  |  |  |  |  |  |  |  |  |  |  |  |  |  |  |  |  |  |  |  |  |  |  |  |  |  |  |  |  |  |  |  |  |  |  |  |  |  |  |  |  |  |  |  |  |  |  |  |  |  |  |  |  |  |  |  |  |  |  |  |  |  |  |  |  |  |  |  |  |  |  |  |  |  |  |  |  |  |  |  |  |  |  |  |  |  |  |  |  |  |  |  |  |  |  |  |  |  |  |  |  |  |  |  |  |  |  |  |  |  |  |  |  |  |  |  |  |  |  |  |  |  |  |  |  |  |  |  |  |  |  |  |  |  |  |  |  |  |  |  |  |  |  |  |  |  |  |  |  |  |  |  |  |  |  |  |  |  |  |  |  |  |  |  |  |  |  |  |  |  |  |  |  |  |  |  |  |  |  |  |  |  |  |  |  |  |  |  |  |  |  |  |  |  |  |  |  |  |  |  |  |  |  |  |  |  |  |  |  |  |  |  |  |  |  |  |  |  |  |  |  |  |  |  |  |  |  |  |  |  |  |  |  |  |  |  |  |  |  |  |  |  |  |  |  |  |  |  |  |  |  |  |  |  |  |  |  |  |  |  |  |  |  |  |  |  |  |  |  |  |  |  |  |  |  |  |  |  |  |  |  |  |  |  |  |  |  |  |  |  |  |  |  |  |  |  |  |  |  |  |  |  |  |  |  |  |  |  |  |  |  |  |  |  |  |  |  |  |  |  |  |  |  |  |  |  |  |  |  |  |  |  |  |  |  |  |  |  |  |  |  |  |  |  |  |  |  |  |  |  |  |  |  |  |  |  |  |  |  |  |  |  |  |  |  |  |  |  |  |  |  |  |  |  |  |  |  |  |  |  |  |  |  |  |  |  |  |  |  |  |  |  |  |  |  |  |  |  |  |  |  |  |  |  |  |  |  |  |  |  |  |  |  |  |  |  |  |  |  |  |  |  |  |  |  |  |  |  |  |  |  |  |  |  |  |  |  |  |  |  |  |  |  |  |  |  |  |  |  |  |  |  |  |  |  |  |  |  |  |  |  |  |  |  |  |  |  |  |  |  |  |  |  |  |  |  |  |  |  |  |  |  |  |  |  |  |  |  |  |  |  |  |  |  |  |  |  |  |  |  |  |  |  |  |  |  |  |  |  |  |  |  |  |  |
|  |  |  |  |  |  |  |  |  |  |  |  |  |  |  |  |  |  |  |  |  |  |  |  |  |  |  |  |  |  |  |  |  |  |  |  |  |  |  |  |  |  |  |  |  |  |  |  |  |  |  |  |  |  |  |  |  |  |  |  |  |  |  |  |  |  |  |  |  |  |  |  |  |  |  |  |  |  |  |  |  |  |  |  |  |  |  |  |  |  |  |  |  |  |  |  |  |  |  |  |  |  |  |  |  |  |  |  |  |  |  |  |  |  |  |  |  |  |  |  |  |  |  |  |  |  |  |  |  |  |  |  |  |  |  |  |  |  |  |  |  |  |  |  |  |  |  |  |  |  |  |  |  |  |  |  |  |  |  |  |  |  |  |  |  |  |  |  |  |  |  |  |  |  |  |  |  |  |  |  |  |  |  |  |  |  |  |  |  |  |  |  |  |  |  |  |  |  |  |  |  |  |  |  |  |  |  |  |  |  |  |  |  |  |  |  |  |  |  |  |  |  |  |  |  |  |  |  |  |  |  |  |  |  |  |  |  |  |  |  |  |  |  |  |  |  |  |  |  |  |  |  |  |  |  |  |  |  |  |  |  |  |  |  |  |  |  |  |  |  |  |  |  |  |  |  |  |  |  |  |  |  |  |  |  |  |  |  |  |  |  |  |  |  |  |  |  |  |  |  |  |  |  |  |  |  |  |  |  |  |  |  |  |  |  |  |  |  |  |  |  |  |  |  |  |  |  |  |  |  |  |  |  |  |  |  |  |  |  |  |  |  |  |  |  |  |  |  |  |  |  |  |  |  |  |  |  |  |  |  |  |  |  |  |  |  |  |  |  |  |  |  |  |  |  |  |  |  |  |  |  |  |  |  |  |  |  |  |  |  |  |  |  |  |  |  |  |  |  |  |  |  |  |  |  |  |  |  |  |  |  |  |  |  |  |  |  |  |  |  |  |  |  |  |  |  |  |  |  |  |  |  |  |  |  |  |  |  |  |  |  |  |  |  |  |  |  |  |  |  |  |  |  |  |  |  |  |  |  |  |  |  |  |  |  |  |  |  |  |  |  |  |  |  |  |  |  |  |  |  |  |  |  |  |  |  |  |  |  |  |  |  |  |  |  |  |  |  |  |  |  |  |  |  |  |  |  |  |  |  |  |  |  |  |  |  |  |  |  |  |  |  |  |  |  |  |  |  |  |  |  |  |  |  |  |  |  |  |  |  |  |  |  |  |  |  |  |  |  |  |  |  |  |  |  |  |  |  |  |  |  |  |  |  |  |  |  |  |  |  |  |  |  |  |  |  |  |  |  |  |  |  |  |  |  |  |  |  |  |  |  |  |  |  |  |  |  |  |  |  |  |  |  |  |  |  |  |  |  |  |  |  |  |  |  |  |  |  |  |  |  |  |  |  |  |  |  |  |  |  |  |  |  |  |  |  |  |  |  |  |  |  |  |  |  |  |  |  |  |  |  |  |  |  |  |  |  |  |  |  |  |  |  |  |  |  |  |  |  |  |  |  |  |  |  |  |  |  |  |  |  |  |  |  |  |  |  |  |  |  |  |  |  |  |  |  |  |  |  |  |  |  |  |  |  |  |  |  |  |  |  |  |  |  |  |  |  |  |  |  |  |  |  |  |  |  |  |  |  |  |  |  |  |  |  |  |  |  |  |  |  |  |  |  |  |  |  |  |  |  |  |  |  |  |  |  |  |  |  |  |  |  |  |  |  |  |  |  |  |  |  |  |  |  |  |  |  |  |  |  |  |  |  |  |  |  |  |  |  |  |  |  |  |  |  |  |  |  |  |  |  |  |  |  |  |  |  |  |  |  |  |  |  |  |  |  |  |  |  |  |  |  |  |  |  |  |  |  |  |  |  |  |  |  |  |  |  |  |  |  |  |  |  |  |  |  |  |  |  |  |  |  |  |  |  |  |  |  |  |  |  |  |  |  |  |  |  |  |  |  |  |  |  |  |  |  |  |  |  |  |  |  |  |  |  |  |  |  |  |  |  |  |  |  |  |  |  |  |  |  |  |  |  |  |  |  |  |  |  |  |  |  |  |  |  |  |  |  |  |  |  |  |  |  |  |  |  |  |  |  |  |  |  |  |  |  |  |  |  |  |  |  |  |  |  |  |  |  |  |  |  |  |  |  |  |  |  |  |  |  |  |  |  |  |  |  |  |  |  |  |  |  |  |  |  |  |  |  |  |  |  |  |  |  |  |  |  |  |  |  |  |  |  |  |  |  |  |  |  |
|  |  |  |  |  |  |  |  |  |  |  |  |  |  |  |  |  |  |  |  |  |  |  |  |  |  |  |  |  |  |  |  |  |  |  |  |  |  |  |  |  |  |  |  |  |  |  |  |  |  |  |  |  |  |  |  |  |  |  |  |  |  |  |  |  |  |  |  |  |  |  |  |  |  |  |  |  |  |  |  |  |  |  |  |  |  |  |  |  |  |  |  |  |  |  |  |  |  |  |  |  |  |  |  |  |  |  |  |  |  |  |  |  |  |  |  |  |  |  |  |  |  |  |  |  |  |  |  |  |  |  |  |  |  |  |  |  |  |  |  |  |  |  |  |  |  |  |  |  |  |  |  |  |  |  |  |  |  |  |  |  |  |  |  |  |  |  |  |  |  |  |  |  |  |  |  |  |  |  |  |  |  |  |  |  |  |  |  |  |  |  |  |  |  |  |  |  |  |  |  |  |  |  |  |  |  |  |  |  |  |  |  |  |  |  |  |  |  |  |  |  |  |  |  |  |  |  |  |  |  |  |  |  |  |  |  |  |  |  |  |  |  |  |  |  |  |  |  |  |  |  |  |  |  |  |  |  |  |  |  |  |  |  |  |  |  |  |  |  |  |  |  |  |  |  |  |  |  |  |  |  |  |  |  |  |  |  |  |  |  |  |  |  |  |  |  |  |  |  |  |  |  |  |  |  |  |  |  |  |  |  |  |  |  |  |  |  |  |  |  |  |  |  |  |  |  |  |  |  |  |  |  |  |  |  |  |  |  |  |  |  |  |  |  |  |  |  |  |  |  |  |  |  |  |  |  |  |  |  |  |  |  |  |  |  |  |  |  |  |  |  |  |  |  |  |  |  |  |  |  |  |  |  |  |  |  |  |  |  |  |  |  |  |  |  |  |  |  |  |  |  |  |  |  |  |  |  |  |  |  |  |  |  |  |  |  |  |  |  |  |  |  |  |  |  |  |  |  |  |  |  |  |  |  |  |  |  |  |  |  |  |  |  |  |  |  |  |  |  |  |  |  |  |  |  |  |  |  |  |  |  |  |  |  |  |  |  |  |  |  |  |  |  |  |  |  |  |  |  |  |  |  |  |  |  |  |  |  |  |  |  |  |  |  |  |  |  |  |  |  |  |  |  |  |  |  |  |  |  |  |  |  |  |  |  |  |  |  |  |  |  |  |  |  |  |  |  |  |  |  |  |  |  |  |  |  |  |  |  |  |  |  |  |  |  |  |  |  |  |  |  |  |  |  |  |  |  |  |  |  |  |  |  |  |  |  |  |  |  |  |  |  |  |  |  |  |  |  |  |  |  |  |  |  |  |  |  |  |  |  |  |  |  |  |  |  |  |  |  |  |  |  |  |  |  |  |  |  |  |  |  |  |  |  |  |  |  |  |  |  |  |  |  |  |  |  |  |  |  |  |  |  |  |  |  |  |  |  |  |  |  |  |  |  |  |  |  |  |  |  |  |  |  |  |  |  |  |  |  |  |  |  |  |  |  |  |  |  |  |  |  |  |  |  |  |  |  |  |  |  |  |  |  |  |  |  |  |  |  |  |  |  |  |  |  |  |  |  |  |  |  |  |  |  |  |  |  |  |  |  |  |  |  |  |  |  |  |  |  |  |  |  |  |  |  |  |  |  |  |  |  |  |  |  |  |  |  |  |  |  |  |  |  |  |  |  |  |  |  |  |  |  |  |  |  |  |  |  |  |  |  |  |  |  |  |  |  |  |  |  |  |  |  |  |  |  |  |  |  |  |  |  |  |  |  |  |  |  |  |  |  |  |  |  |  |  |  |  |  |  |  |  |  |  |  |  |  |  |  |  |  |  |  |  |  |  |  |  |  |  |  |  |  |  |  |  |  |  |  |  |  |  |  |  |  |  |  |  |  |  |  |  |  |  |  |  |  |  |  |  |  |  |  |  |  |  |  |  |  |  |  |  |  |  |  |  |  |  |  |  |  |  |  |  |  |  |  |  |  |  |  |  |  |  |  |  |  |  |  |  |  |  |  |  |  |  |  |  |  |  |  |  |  |  |  |  |  |  |  |  |  |  |  |  |  |  |  |  |  |  |  |  |  |  |  |  |  |  |  |  |  |  |  |  |  |  |  |  |  |  |  |  |  |  |  |  |  |  |  |  |  |  |  |  |  |  |  |  |  |  |  |  |  |  |  |  |  |  |  |  |  |  |  |  |  |  |  |  |  |  |  |  |  |  |  |  |  |  |  |  |  |  |  |  |  |  |  |  |  |
|  |  |  |  |  |  |  |  |  |  |  |  |  |  |  |  |  |  |  |  |  |  |  |  |  |  |  |  |  |  |  |  |  |  |  |  |  |  |  |  |  |  |  |  |  |  |  |  |  |  |  |  |  |  |  |  |  |  |  |  |  |  |  |  |  |  |  |  |  |  |  |  |  |  |  |  |  |  |  |  |  |  |  |  |  |  |  |  |  |  |  |  |  |  |  |  |  |  |  |  |  |  |  |  |  |  |  |  |  |  |  |  |  |  |  |  |  |  |  |  |  |  |  |  |  |  |  |  |  |  |  |  |  |  |  |  |  |  |  |  |  |  |  |  |  |  |  |  |  |  |  |  |  |  |  |  |  |  |  |  |  |  |  |  |  |  |  |  |  |  |  |  |  |  |  |  |  |  |  |  |  |  |  |  |  |  |  |  |  |  |  |  |  |  |  |  |  |  |  |  |  |  |  |  |  |  |  |  |  |  |  |  |  |  |  |  |  |  |  |  |  |  |  |  |  |  |  |  |  |  |  |  |  |  |  |  |  |  |  |  |  |  |  |  |  |  |  |  |  |  |  |  |  |  |  |  |  |  |  |  |  |  |  |  |  |  |  |  |  |  |  |  |  |  |  |  |  |  |  |  |  |  |  |  |  |  |  |  |  |  |  |  |  |  |  |  |  |  |  |  |  |  |  |  |  |  |  |  |  |  |  |  |  |  |  |  |  |  |  |  |  |  |  |  |  |  |  |  |  |  |  |  |  |  |  |  |  |  |  |  |  |  |  |  |  |  |  |  |  |  |  |  |  |  |  |  |  |  |  |  |  |  |  |  |  |  |  |  |  |  |  |  |  |  |  |  |  |  |  |  |  |  |  |  |  |  |  |  |  |  |  |  |  |  |  |  |  |  |  |  |  |  |  |  |  |  |  |  |  |  |  |  |  |  |  |  |  |  |  |  |  |  |  |  |  |  |  |  |  |  |  |  |  |  |  |  |  |  |  |  |  |  |  |  |  |  |  |  |  |  |  |  |  |  |  |  |  |  |  |  |  |  |  |  |  |  |  |  |  |  |  |  |  |  |  |  |  |  |  |  |  |  |  |  |  |  |  |  |  |  |  |  |  |  |  |  |  |  |  |  |  |  |  |  |  |  |  |  |  |  |  |  |  |  |  |  |  |  |  |  |  |  |  |  |  |  |  |  |  |  |  |  |  |  |  |  |  |  |  |  |  |  |  |  |  |  |  |  |  |  |  |  |  |  |  |  |  |  |  |  |  |  |  |  |  |  |  |  |  |  |  |  |  |  |  |  |  |  |  |  |  |  |  |  |  |  |  |  |  |  |  |  |  |  |  |  |  |  |  |  |  |  |  |  |  |  |  |  |  |  |  |  |  |  |  |  |  |  |  |  |  |  |  |  |  |  |  |  |  |  |  |  |  |  |  |  |  |  |  |  |  |  |  |  |  |  |  |  |  |  |  |  |  |  |  |  |  |  |  |  |  |  |  |  |  |  |  |  |  |  |  |  |  |  |  |  |  |  |  |  |  |  |  |  |  |  |  |  |  |  |  |  |  |  |  |  |  |  |  |  |  |  |  |  |  |  |  |  |  |  |  |  |  |  |  |  |  |  |  |  |  |  |  |  |  |  |  |  |  |  |  |  |  |  |  |  |  |  |  |  |  |  |  |  |  |  |  |  |  |  |  |  |  |  |  |  |  |  |  |  |  |  |  |  |  |  |  |  |  |  |  |  |  |  |  |  |  |  |  |  |  |  |  |  |  |  |  |  |  |  |  |  |  |  |  |  |  |  |  |  |  |  |  |  |  |  |  |  |  |  |  |  |  |  |  |  |  |  |  |  |  |  |  |  |  |  |  |  |  |  |  |  |  |  |  |  |  |  |  |  |  |  |  |  |  |  |  |  |  |  |  |  |  |  |  |  |  |  |  |  |  |  |  |  |  |  |  |  |  |  |  |  |  |  |  |  |  |  |  |  |  |  |  |  |  |  |  |  |  |  |  |  |  |  |  |  |  |  |  |  |  |  |  |  |  |  |  |  |  |  |  |  |  |  |  |  |  |  |  |  |  |  |  |  |  |  |  |  |  |  |  |  |  |  |  |  |  |  |  |  |  |  |  |  |  |  |  |  |  |  |  |  |  |  |  |  |  |  |  |  |  |  |  |  |  |  |  |  |  |  |  |  |  |  |  |  |  |  |  |  |  |  |  |  |  |  |  |  |  |  |  |  |  |  |  |  |  |  |  |
|  |  |  |  |  |  |  |  |  |  |  |  |  |  |  |  |  |  |  |  |  |  |  |  |  |  |  |  |  |  |  |  |  |  |  |  |  |  |  |  |  |  |  |  |  |  |  |  |  |  |  |  |  |  |  |  |  |  |  |  |  |  |  |  |  |  |  |  |  |  |  |  |  |  |  |  |  |  |  |  |  |  |  |  |  |  |  |  |  |  |  |  |  |  |  |  |  |  |  |  |  |  |  |  |  |  |  |  |  |  |  |  |  |  |  |  |  |  |  |  |  |  |  |  |  |  |  |  |  |  |  |  |  |  |  |  |  |  |  |  |  |  |  |  |  |  |  |  |  |  |  |  |  |  |  |  |  |  |  |  |  |  |  |  |  |  |  |  |  |  |  |  |  |  |  |  |  |  |  |  |  |  |  |  |  |  |  |  |  |  |  |  |  |  |  |  |  |  |  |  |  |  |  |  |  |  |  |  |  |  |  |  |  |  |  |  |  |  |  |  |  |  |  |  |  |  |  |  |  |  |  |  |  |  |  |  |  |  |  |  |  |  |  |  |  |  |  |  |  |  |  |  |  |  |  |  |  |  |  |  |  |  |  |  |  |  |  |  |  |  |  |  |  |  |  |  |  |  |  |  |  |  |  |  |  |  |  |  |  |  |  |  |  |  |  |  |  |  |  |  |  |  |  |  |  |  |  |  |  |  |  |  |  |  |  |  |  |  |  |  |  |  |  |  |  |  |  |  |  |  |  |  |  |  |  |  |  |  |  |  |  |  |  |  |  |  |  |  |  |  |  |  |  |  |  |  |  |  |  |  |  |  |  |  |  |  |  |  |  |  |  |  |  |  |  |  |  |  |  |  |  |  |  |  |  |  |  |  |  |  |  |  |  |  |  |  |  |  |  |  |  |  |  |  |  |  |  |  |  |  |  |  |  |  |  |  |  |  |  |  |  |  |  |  |  |  |  |  |  |  |  |  |  |  |  |  |  |  |  |  |  |  |  |  |  |  |  |  |  |  |  |  |  |  |  |  |  |  |  |  |  |  |  |  |  |  |  |  |  |  |  |  |  |  |  |  |  |  |  |  |  |  |  |  |  |  |  |  |  |  |  |  |  |  |  |  |  |  |  |  |  |  |  |  |  |  |  |  |  |  |  |  |  |  |  |  |  |  |  |  |  |  |  |  |  |  |  |  |  |  |  |  |  |  |  |  |  |  |  |  |  |  |  |  |  |  |  |  |  |  |  |  |  |  |  |  |  |  |  |  |  |  |  |  |  |  |  |  |  |  |  |  |  |  |  |  |  |  |  |  |  |  |  |  |  |  |  |  |  |  |  |  |  |  |  |  |  |  |  |  |  |  |  |  |  |  |  |  |  |  |  |  |  |  |  |  |  |  |  |  |  |  |  |  |  |  |  |  |  |  |  |  |  |  |  |  |  |  |  |  |  |  |  |  |  |  |  |  |  |  |  |  |  |  |  |  |  |  |  |  |  |  |  |  |  |  |  |  |  |  |  |  |  |  |  |  |  |  |  |  |  |  |  |  |  |  |  |  |  |  |  |  |  |  |  |  |  |  |  |  |  |  |  |  |  |  |  |  |  |  |  |  |  |  |  |  |  |  |  |  |  |  |  |  |  |  |  |  |  |  |  |  |  |  |  |  |  |  |  |  |  |  |  |  |  |  |  |  |  |  |  |  |  |  |  |  |  |  |  |  |  |  |  |  |  |  |  |  |  |  |  |  |  |  |  |  |  |  |  |  |  |  |  |  |  |  |  |  |  |  |  |  |  |  |  |  |  |  |  |  |  |  |  |  |  |  |  |  |  |  |  |  |  |  |  |  |  |  |  |  |  |  |  |  |  |  |  |  |  |  |  |  |  |  |  |  |  |  |  |  |  |  |  |  |  |  |  |  |  |  |  |  |  |  |  |  |  |  |  |  |  |  |  |  |  |  |  |  |  |  |  |  |  |  |  |  |  |  |  |  |  |  |  |  |  |  |  |  |  |  |  |  |  |  |  |  |  |  |  |  |  |  |  |  |  |  |  |  |  |  |  |  |  |  |  |  |  |  |  |  |  |  |  |  |  |  |  |  |  |  |  |  |  |  |  |  |  |  |  |  |  |  |  |  |  |  |  |  |  |  |  |  |  |  |  |  |  |  |  |  |  |  |  |  |  |  |  |  |  |  |  |  |  |  |  |  |  |  |  |  |  |  |  |  |  |  |  |  |  |  |  |  |  |  |  |  |  |  |  |
|  |  |  |  |  |  |  |  |  |  |  |  |  |  |  |  |  |  |  |  |  |  |  |  |  |  |  |  |  |  |  |  |  |  |  |  |  |  |  |  |  |  |  |  |  |  |  |  |  |  |  |  |  |  |  |  |  |  |  |  |  |  |  |  |  |  |  |  |  |  |  |  |  |  |  |  |  |  |  |  |  |  |  |  |  |  |  |  |  |  |  |  |  |  |  |  |  |  |  |  |  |  |  |  |  |  |  |  |  |  |  |  |  |  |  |  |  |  |  |  |  |  |  |  |  |  |  |  |  |  |  |  |  |  |  |  |  |  |  |  |  |  |  |  |  |  |  |  |  |  |  |  |  |  |  |  |  |  |  |  |  |  |  |  |  |  |  |  |  |  |  |  |  |  |  |  |  |  |  |  |  |  |  |  |  |  |  |  |  |  |  |  |  |  |  |  |  |  |  |  |  |  |  |  |  |  |  |  |  |  |  |  |  |  |  |  |  |  |  |  |  |  |  |  |  |  |  |  |  |  |  |  |  |  |  |  |  |  |  |  |  |  |  |  |  |  |  |  |  |  |  |  |  |  |  |  |  |  |  |  |  |  |  |  |  |  |  |  |  |  |  |  |  |  |  |  |  |  |  |  |  |  |  |  |  |  |  |  |  |  |  |  |  |  |  |  |  |  |  |  |  |  |  |  |  |  |  |  |  |  |  |  |  |  |  |  |  |  |  |  |  |  |  |  |  |  |  |  |  |  |  |  |  |  |  |  |  |  |  |  |  |  |  |  |  |  |  |  |  |  |  |  |  |  |  |  |  |  |  |  |  |  |  |  |  |  |  |  |  |  |  |  |  |  |  |  |  |  |  |  |  |  |  |  |  |  |  |  |  |  |  |  |  |  |  |  |  |  |  |  |  |  |  |  |  |  |  |  |  |  |  |  |  |  |  |  |  |  |  |  |  |  |  |  |  |  |  |  |  |  |  |  |  |  |  |  |  |  |  |  |  |  |  |  |  |  |  |  |  |  |  |  |  |  |  |  |  |  |  |  |  |  |  |  |  |  |  |  |  |  |  |  |  |  |  |  |  |  |  |  |  |  |  |  |  |  |  |  |  |  |  |  |  |  |  |  |  |  |  |  |  |  |  |  |  |  |  |  |  |  |  |  |  |  |  |  |  |  |  |  |  |  |  |  |  |  |  |  |  |  |  |  |  |  |  |  |  |  |  |  |  |  |  |  |  |  |  |  |  |  |  |  |  |  |  |  |  |  |  |  |  |  |  |  |  |  |  |  |  |  |  |  |  |  |  |  |  |  |  |  |  |  |  |  |  |  |  |  |  |  |  |  |  |  |  |  |  |  |  |  |  |  |  |  |  |  |  |  |  |  |  |  |  |  |  |  |  |  |  |  |  |  |  |  |  |  |  |  |  |  |  |  |  |  |  |  |  |  |  |  |  |  |  |  |  |  |  |  |  |  |  |  |  |  |  |  |  |  |  |  |  |  |  |  |  |  |  |  |  |  |  |  |  |  |  |  |  |  |  |  |  |  |  |  |  |  |  |  |  |  |  |  |  |  |  |  |  |  |  |  |  |  |  |  |  |  |  |  |  |  |  |  |  |  |  |  |  |  |  |  |  |  |  |  |  |  |  |  |  |  |  |  |  |  |  |  |  |  |  |  |  |  |  |  |  |  |  |  |  |  |  |  |  |  |  |  |  |  |  |  |  |  |  |  |  |  |  |  |  |  |  |  |  |  |  |  |  |  |  |  |  |  |  |  |  |  |  |  |  |  |  |  |  |  |  |  |  |  |  |  |  |  |  |  |  |  |  |  |  |  |  |  |  |  |  |  |  |  |  |  |  |  |  |  |  |  |  |  |  |  |  |  |  |  |  |  |  |  |  |  |  |  |  |  |  |  |  |  |  |  |  |  |  |  |  |  |  |  |  |  |  |  |  |  |  |  |  |  |  |  |  |  |  |  |  |  |  |  |  |  |  |  |  |  |  |  |  |  |  |  |  |  |  |  |  |  |  |  |  |  |  |  |  |  |  |  |  |  |  |  |  |  |  |  |  |  |  |  |  |  |  |  |  |  |  |  |  |  |  |  |  |  |  |  |  |  |  |  |  |  |  |  |  |  |  |  |  |  |  |  |  |  |  |  |  |  |  |  |  |  |  |  |  |  |  |  |  |  |  |  |  |  |  |  |  |  |  |  |  |  |  |  |  |  |  |  |  |  |  |  |  |  |  |  |  |  |  |  |  |
|  |  |  |  |  |  |  |  |  |  |  |  |  |  |  |  |  |  |  |  |  |  |  |  |  |  |  |  |  |  |  |  |  |  |  |  |  |  |  |  |  |  |  |  |  |  |  |  |  |  |  |  |  |  |  |  |  |  |  |  |  |  |  |  |  |  |  |  |  |  |  |  |  |  |  |  |  |  |  |  |  |  |  |  |  |  |  |  |  |  |  |  |  |  |  |  |  |  |  |  |  |  |  |  |  |  |  |  |  |  |  |  |  |  |  |  |  |  |  |  |  |  |  |  |  |  |  |  |  |  |  |  |  |  |  |  |  |  |  |  |  |  |  |  |  |  |  |  |  |  |  |  |  |  |  |  |  |  |  |  |  |  |  |  |  |  |  |  |  |  |  |  |  |  |  |  |  |  |  |  |  |  |  |  |  |  |  |  |  |  |  |  |  |  |  |  |  |  |  |  |  |  |  |  |  |  |  |  |  |  |  |  |  |  |  |  |  |  |  |  |  |  |  |  |  |  |  |  |  |  |  |  |  |  |  |  |  |  |  |  |  |  |  |  |  |  |  |  |  |  |  |  |  |  |  |  |  |  |  |  |  |  |  |  |  |  |  |  |  |  |  |  |  |  |  |  |  |  |  |  |  |  |  |  |  |  |  |  |  |  |  |  |  |  |  |  |  |  |  |  |  |  |  |  |  |  |  |  |  |  |  |  |  |  |  |  |  |  |  |  |  |  |  |  |  |  |  |  |  |  |  |  |  |  |  |  |  |  |  |  |  |  |  |  |  |  |  |  |  |  |  |  |  |  |  |  |  |  |  |  |  |  |  |  |  |  |  |  |  |  |  |  |  |  |  |  |  |  |  |  |  |  |  |  |  |  |  |  |  |  |  |  |  |  |  |  |  |  |  |  |  |  |  |  |  |  |  |  |  |  |  |  |  |  |  |  |  |  |  |  |  |  |  |  |  |  |  |  |  |  |  |  |  |  |  |  |  |  |  |  |  |  |  |  |  |  |  |  |  |  |  |  |  |  |  |  |  |  |  |  |  |  |  |  |  |  |  |  |  |  |  |  |  |  |  |  |  |  |  |  |  |  |  |  |  |  |  |  |  |  |  |  |  |  |  |  |  |  |  |  |  |  |  |  |  |  |  |  |  |  |  |  |  |  |  |  |  |  |  |  |  |  |  |  |  |  |  |  |  |  |  |  |  |  |  |  |  |  |  |  |  |  |  |  |  |  |  |  |  |  |  |  |  |  |  |  |  |  |  |  |  |  |  |  |  |  |  |  |  |  |  |  |  |  |  |  |  |  |  |  |  |  |  |  |  |  |  |  |  |  |  |  |  |  |  |  |  |  |  |  |  |  |  |  |  |  |  |  |  |  |  |  |  |  |  |  |  |  |  |  |  |  |  |  |  |  |  |  |  |  |  |  |  |  |  |  |  |  |  |  |  |  |  |  |  |  |  |  |  |  |  |  |  |  |  |  |  |  |  |  |  |  |  |  |  |  |  |  |  |  |  |  |  |  |  |  |  |  |  |  |  |  |  |  |  |  |  |  |  |  |  |  |  |  |  |  |  |  |  |  |  |  |  |  |  |  |  |  |  |  |  |  |  |  |  |  |  |  |  |  |  |  |  |  |  |  |  |  |  |  |  |  |  |  |  |  |  |  |  |  |  |  |  |  |  |  |  |  |  |  |  |  |  |  |  |  |  |  |  |  |  |  |  |  |  |  |  |  |  |  |  |  |  |  |  |  |  |  |  |  |  |  |  |  |  |  |  |  |  |  |  |  |  |  |  |  |  |  |  |  |  |  |  |  |  |  |  |  |  |  |  |  |  |  |  |  |  |  |  |  |  |  |  |  |  |  |  |  |  |  |  |  |  |  |  |  |  |  |  |  |  |  |  |  |  |  |  |  |  |  |  |  |  |  |  |  |  |  |  |  |  |  |  |  |  |  |  |  |  |  |  |  |  |  |  |  |  |  |  |  |  |  |  |  |  |  |  |  |  |  |  |  |  |  |  |  |  |  |  |  |  |  |  |  |  |  |  |  |  |  |  |  |  |  |  |  |  |  |  |  |  |  |  |  |  |  |  |  |  |  |  |  |  |  |  |  |  |  |  |  |  |  |  |  |  |  |  |  |  |  |  |  |  |  |  |  |  |  |  |  |  |  |  |  |  |  |  |  |  |  |  |  |  |  |  |  |  |  |  |  |  |  |  |  |  |  |  |  |  |  |  |  |  |  |  |  |  |  |  |
|  |  |  |  |  |  |  |  |  |  |  |  |  |  |  |  |  |  |  |  |  |  |  |  |  |  |  |  |  |  |  |  |  |  |  |  |  |  |  |  |  |  |  |  |  |  |  |  |  |  |  |  |  |  |  |  |  |  |  |  |  |  |  |  |  |  |  |  |  |  |  |  |  |  |  |  |  |  |  |  |  |  |  |  |  |  |  |  |  |  |  |  |  |  |  |  |  |  |  |  |  |  |  |  |  |  |  |  |  |  |  |  |  |  |  |  |  |  |  |  |  |  |  |  |  |  |  |  |  |  |  |  |  |  |  |  |  |  |  |  |  |  |  |  |  |  |  |  |  |  |  |  |  |  |  |  |  |  |  |  |  |  |  |  |  |  |  |  |  |  |  |  |  |  |  |  |  |  |  |  |  |  |  |  |  |  |  |  |  |  |  |  |  |  |  |  |  |  |  |  |  |  |  |  |  |  |  |  |  |  |  |  |  |  |  |  |  |  |  |  |  |  |  |  |  |  |  |  |  |  |  |  |  |  |  |  |  |  |  |  |  |  |  |  |  |  |  |  |  |  |  |  |  |  |  |  |  |  |  |  |  |  |  |  |  |  |  |  |  |  |  |  |  |  |  |  |  |  |  |  |  |  |  |  |  |  |  |  |  |  |  |  |  |  |  |  |  |  |  |  |  |  |  |  |  |  |  |  |  |  |  |  |  |  |  |  |  |  |  |  |  |  |  |  |  |  |  |  |  |  |  |  |  |  |  |  |  |  |  |  |  |  |  |  |  |  |  |  |  |  |  |  |  |  |  |  |  |  |  |  |  |  |  |  |  |  |  |  |  |  |  |  |  |  |  |  |  |  |  |  |  |  |  |  |  |  |  |  |  |  |  |  |  |  |  |  |  |  |  |  |  |  |  |  |  |  |  |  |  |  |  |  |  |  |  |  |  |  |  |  |  |  |  |  |  |  |  |  |  |  |  |  |  |  |  |  |  |  |  |  |  |  |  |  |  |  |  |  |  |  |  |  |  |  |  |  |  |  |  |  |  |  |  |  |  |  |  |  |  |  |  |  |  |  |  |  |  |  |  |  |  |  |  |  |  |  |  |  |  |  |  |  |  |  |  |  |  |  |  |  |  |  |  |  |  |  |  |  |  |  |  |  |  |  |  |  |  |  |  |  |  |  |  |  |  |  |  |  |  |  |  |  |  |  |  |  |  |  |  |  |  |  |  |  |  |  |  |  |  |  |  |  |  |  |  |  |  |  |  |  |  |  |  |  |  |  |  |  |  |  |  |  |  |  |  |  |  |  |  |  |  |  |  |  |  |  |  |  |  |  |  |  |  |  |  |  |  |  |  |  |  |  |  |  |  |  |  |  |  |  |  |  |  |  |  |  |  |  |  |  |  |  |  |  |  |  |  |  |  |  |  |  |  |  |  |  |  |  |  |  |  |  |  |  |  |  |  |  |  |  |  |  |  |  |  |  |  |  |  |  |  |  |  |  |  |  |  |  |  |  |  |  |  |  |  |  |  |  |  |  |  |  |  |  |  |  |  |  |  |  |  |  |  |  |  |  |  |  |  |  |  |  |  |  |  |  |  |  |  |  |  |  |  |  |  |  |  |  |  |  |  |  |  |  |  |  |  |  |  |  |  |  |  |  |  |  |  |  |  |  |  |  |  |  |  |  |  |  |  |  |  |  |  |  |  |  |  |  |  |  |  |  |  |  |  |  |  |  |  |  |  |  |  |  |  |  |  |  |  |  |  |  |  |  |  |  |  |  |  |  |  |  |  |  |  |  |  |  |  |  |  |  |  |  |  |  |  |  |  |  |  |  |  |  |  |  |  |  |  |  |  |  |  |  |  |  |  |  |  |  |  |  |  |  |  |  |  |  |  |  |  |  |  |  |  |  |  |  |  |  |  |  |  |  |  |  |  |  |  |  |  |  |  |  |  |  |  |  |  |  |  |  |  |  |  |  |  |  |  |  |  |  |  |  |  |  |  |  |  |  |  |  |  |  |  |  |  |  |  |  |  |  |  |  |  |  |  |  |  |  |  |  |  |  |  |  |  |  |  |  |  |  |  |  |  |  |  |  |  |  |  |  |  |  |  |  |  |  |  |  |  |  |  |  |  |  |  |  |  |  |  |  |  |  |  |  |  |  |  |  |  |  |  |  |  |  |  |  |  |  |  |  |  |  |  |  |  |  |  |  |  |  |  |  |  |  |  |  |  |  |  |  |  |  |  |  |  |  |  |
|  |  |  |  |  |  |  |  |  |  |  |  |  |  |  |  |  |  |  |  |  |  |  |  |  |  |  |  |  |  |  |  |  |  |  |  |  |  |  |  |  |  |  |  |  |  |  |  |  |  |  |  |  |  |  |  |  |  |  |  |  |  |  |  |  |  |  |  |  |  |  |  |  |  |  |  |  |  |  |  |  |  |  |  |  |  |  |  |  |  |  |  |  |  |  |  |  |  |  |  |  |  |  |  |  |  |  |  |  |  |  |  |  |  |  |  |  |  |  |  |  |  |  |  |  |  |  |  |  |  |  |  |  |  |  |  |  |  |  |  |  |  |  |  |  |  |  |  |  |  |  |  |  |  |  |  |  |  |  |  |  |  |  |  |  |  |  |  |  |  |  |  |  |  |  |  |  |  |  |  |  |  |  |  |  |  |  |  |  |  |  |  |  |  |  |  |  |  |  |  |  |  |  |  |  |  |  |  |  |  |  |  |  |  |  |  |  |  |  |  |  |  |  |  |  |  |  |  |  |  |  |  |  |  |  |  |  |  |  |  |  |  |  |  |  |  |  |  |  |  |  |  |  |  |  |  |  |  |  |  |  |  |  |  |  |  |  |  |  |  |  |  |  |  |  |  |  |  |  |  |  |  |  |  |  |  |  |  |  |  |  |  |  |  |  |  |  |  |  |  |  |  |  |  |  |  |  |  |  |  |  |  |  |  |  |  |  |  |  |  |  |  |  |  |  |  |  |  |  |  |  |  |  |  |  |  |  |  |  |  |  |  |  |  |  |  |  |  |  |  |  |  |  |  |  |  |  |  |  |  |  |  |  |  |  |  |  |  |  |  |  |  |  |  |  |  |  |  |  |  |  |  |  |  |  |  |  |  |  |  |  |  |  |  |  |  |  |  |  |  |  |  |  |  |  |  |  |  |  |  |  |  |  |  |  |  |  |  |  |  |  |  |  |  |  |  |  |  |  |  |  |  |  |  |  |  |  |  |  |  |  |  |  |  |  |  |  |  |  |  |  |  |  |  |  |  |  |  |  |  |  |  |  |  |  |  |  |  |  |  |  |  |  |  |  |  |  |  |  |  |  |  |  |  |  |  |  |  |  |  |  |  |  |  |  |  |  |  |  |  |  |  |  |  |  |  |  |  |  |  |  |  |  |  |  |  |  |  |  |  |  |  |  |  |  |  |  |  |  |  |  |  |  |  |  |  |  |  |  |  |  |  |  |  |  |  |  |  |  |  |  |  |  |  |  |  |  |  |  |  |  |  |  |  |  |  |  |  |  |  |  |  |  |  |  |  |  |  |  |  |  |  |  |  |  |  |  |  |  |  |  |  |  |  |  |  |  |  |  |  |  |  |  |  |  |  |  |  |  |  |  |  |  |  |  |  |  |  |  |  |  |  |  |  |  |  |  |  |  |  |  |  |  |  |  |  |  |  |  |  |  |  |  |  |  |  |  |  |  |  |  |  |  |  |  |  |  |  |  |  |  |  |  |  |  |  |  |  |  |  |  |  |  |  |  |  |  |  |  |  |  |  |  |  |  |  |  |  |  |  |  |  |  |  |  |  |  |  |  |  |  |  |  |  |  |  |  |  |  |  |  |  |  |  |  |  |  |  |  |  |  |  |  |  |  |  |  |  |  |  |  |  |  |  |  |  |  |  |  |  |  |  |  |  |  |  |  |  |  |  |  |  |  |  |  |  |  |  |  |  |  |  |  |  |  |  |  |  |  |  |  |  |  |  |  |  |  |  |  |  |  |  |  |  |  |  |  |  |  |  |  |  |  |  |  |  |  |  |  |  |  |  |  |  |  |  |  |  |  |  |  |  |  |  |  |  |  |  |  |  |  |  |  |  |  |  |  |  |  |  |  |  |  |  |  |  |  |  |  |  |  |  |  |  |  |  |  |  |  |  |  |  |  |  |  |  |  |  |  |  |  |  |  |  |  |  |  |  |  |  |  |  |  |  |  |  |  |  |  |  |  |  |  |  |  |  |  |  |  |  |  |  |  |  |  |  |  |  |  |  |  |  |  |  |  |  |  |  |  |  |  |  |  |  |  |  |  |  |  |  |  |  |  |  |  |  |  |  |  |  |  |  |  |  |  |  |  |  |  |  |  |  |  |  |  |  |  |  |  |  |  |  |  |  |  |  |  |  |  |  |  |  |  |  |  |  |  |  |  |  |  |  |  |  |  |  |  |  |  |  |  |  |  |  |  |  |  |  |  |  |  |  |  |  |  |  |  |  |  |
|  |  |  |  |  |  |  |  |  |  |  |  |  |  |  |  |  |  |  |  |  |  |  |  |  |  |  |  |  |  |  |  |  |  |  |  |  |  |  |  |  |  |  |  |  |  |  |  |  |  |  |  |  |  |  |  |  |  |  |  |  |  |  |  |  |  |  |  |  |  |  |  |  |  |  |  |  |  |  |  |  |  |  |  |  |  |  |  |  |  |  |  |  |  |  |  |  |  |  |  |  |  |  |  |  |  |  |  |  |  |  |  |  |  |  |  |  |  |  |  |  |  |  |  |  |  |  |  |  |  |  |  |  |  |  |  |  |  |  |  |  |  |  |  |  |  |  |  |  |  |  |  |  |  |  |  |  |  |  |  |  |  |  |  |  |  |  |  |  |  |  |  |  |  |  |  |  |  |  |  |  |  |  |  |  |  |  |  |  |  |  |  |  |  |  |  |  |  |  |  |  |  |  |  |  |  |  |  |  |  |  |  |  |  |  |  |  |  |  |  |  |  |  |  |  |  |  |  |  |  |  |  |  |  |  |  |  |  |  |  |  |  |  |  |  |  |  |  |  |  |  |  |  |  |  |  |  |  |  |  |  |  |  |  |  |  |  |  |  |  |  |  |  |  |  |  |  |  |  |  |  |  |  |  |  |  |  |  |  |  |  |  |  |  |  |  |  |  |  |  |  |  |  |  |  |  |  |  |  |  |  |  |  |  |  |  |  |  |  |  |  |  |  |  |  |  |  |  |  |  |  |  |  |  |  |  |  |  |  |  |  |  |  |  |  |  |  |  |  |  |  |  |  |  |  |  |  |  |  |  |  |  |  |  |  |  |  |  |  |  |  |  |  |  |  |  |  |  |  |  |  |  |  |  |  |  |  |  |  |  |  |  |  |  |  |  |  |  |  |  |  |  |  |  |  |  |  |  |  |  |  |  |  |  |  |  |  |  |  |  |  |  |  |  |  |  |  |  |  |  |  |  |  |  |  |  |  |  |  |  |  |  |  |  |  |  |  |  |  |  |  |  |  |  |  |  |  |  |  |  |  |  |  |  |  |  |  |  |  |  |  |  |  |  |  |  |  |  |  |  |  |  |  |  |  |  |  |  |  |  |  |  |  |  |  |  |  |  |  |  |  |  |  |  |  |  |  |  |  |  |  |  |  |  |  |  |  |  |  |  |  |  |  |  |  |  |  |  |  |  |  |  |  |  |  |  |  |  |  |  |  |  |  |  |  |  |  |  |  |  |  |  |  |  |  |  |  |  |  |  |  |  |  |  |  |  |  |  |  |  |  |  |  |  |  |  |  |  |  |  |  |  |  |  |  |  |  |  |  |  |  |  |  |  |  |  |  |  |  |  |  |  |  |  |  |  |  |  |  |  |  |  |  |  |  |  |  |  |  |  |  |  |  |  |  |  |  |  |  |  |  |  |  |  |  |  |  |  |  |  |  |  |  |  |  |  |  |  |  |  |  |  |  |  |  |  |  |  |  |  |  |  |  |  |  |  |  |  |  |  |  |  |  |  |  |  |  |  |  |  |  |  |  |  |  |  |  |  |  |  |  |  |  |  |  |  |  |  |  |  |  |  |  |  |  |  |  |  |  |  |  |  |  |  |  |  |  |  |  |  |  |  |  |  |  |  |  |  |  |  |  |  |  |  |  |  |  |  |  |  |  |  |  |  |  |  |  |  |  |  |  |  |  |  |  |  |  |  |  |  |  |  |  |  |  |  |  |  |  |  |  |  |  |  |  |  |  |  |  |  |  |  |  |  |  |  |  |  |  |  |  |  |  |  |  |  |  |  |  |  |  |  |  |  |  |  |  |  |  |  |  |  |  |  |  |  |  |  |  |  |  |  |  |  |  |  |  |  |  |  |  |  |  |  |  |  |  |  |  |  |  |  |  |  |  |  |  |  |  |  |  |  |  |  |  |  |  |  |  |  |  |  |  |  |  |  |  |  |  |  |  |  |  |  |  |  |  |  |  |  |  |  |  |  |  |  |  |  |  |  |  |  |  |  |  |  |  |  |  |  |  |  |  |  |  |  |  |  |  |  |  |  |  |  |  |  |  |  |  |  |  |  |  |  |  |  |  |  |  |  |  |  |  |  |  |  |  |  |  |  |  |  |  |  |  |  |  |  |  |  |  |  |  |  |  |  |  |  |  |  |  |  |  |  |  |  |  |  |  |  |  |  |  |  |  |  |  |  |  |  |  |  |  |  |  |  |  |  |  |  |  |  |  |  |  |  |  |  |  |
|  |  |  |  |  |  |  |  |  |  |  |  |  |  |  |  |  |  |  |  |  |  |  |  |  |  |  |  |  |  |  |  |  |  |  |  |  |  |  |  |  |  |  |  |  |  |  |  |  |  |  |  |  |  |  |  |  |  |  |  |  |  |  |  |  |  |  |  |  |  |  |  |  |  |  |  |  |  |  |  |  |  |  |  |  |  |  |  |  |  |  |  |  |  |  |  |  |  |  |  |  |  |  |  |  |  |  |  |  |  |  |  |  |  |  |  |  |  |  |  |  |  |  |  |  |  |  |  |  |  |  |  |  |  |  |  |  |  |  |  |  |  |  |  |  |  |  |  |  |  |  |  |  |  |  |  |  |  |  |  |  |  |  |  |  |  |  |  |  |  |  |  |  |  |  |  |  |  |  |  |  |  |  |  |  |  |  |  |  |  |  |  |  |  |  |  |  |  |  |  |  |  |  |  |  |  |  |  |  |  |  |  |  |  |  |  |  |  |  |  |  |  |  |  |  |  |  |  |  |  |  |  |  |  |  |  |  |  |  |  |  |  |  |  |  |  |  |  |  |  |  |  |  |  |  |  |  |  |  |  |  |  |  |  |  |  |  |  |  |  |  |  |  |  |  |  |  |  |  |  |  |  |  |  |  |  |  |  |  |  |  |  |  |  |  |  |  |  |  |  |  |  |  |  |  |  |  |  |  |  |  |  |  |  |  |  |  |  |  |  |  |  |  |  |  |  |  |  |  |  |  |  |  |  |  |  |  |  |  |  |  |  |  |  |  |  |  |  |  |  |  |  |  |  |  |  |  |  |  |  |  |  |  |  |  |  |  |  |  |  |  |  |  |  |  |  |  |  |  |  |  |  |  |  |  |  |  |  |  |  |  |  |  |  |  |  |  |  |  |  |  |  |  |  |  |  |  |  |  |  |  |  |  |  |  |  |  |  |  |  |  |  |  |  |  |  |  |  |  |  |  |  |  |  |  |  |  |  |  |  |  |  |  |  |  |  |  |  |  |  |  |  |  |  |  |  |  |  |  |  |  |  |  |  |  |  |  |  |  |  |  |  |  |  |  |  |  |  |  |  |  |  |  |  |  |  |  |  |  |  |  |  |  |  |  |  |  |  |  |  |  |  |  |  |  |  |  |  |  |  |  |  |  |  |  |  |  |  |  |  |  |  |  |  |  |  |  |  |  |  |  |  |  |  |  |  |  |  |  |  |  |  |  |  |  |  |  |  |  |  |  |  |  |  |  |  |  |  |  |  |  |  |  |  |  |  |  |  |  |  |  |  |  |  |  |  |  |  |  |  |  |  |  |  |  |  |  |  |  |  |  |  |  |  |  |  |  |  |  |  |  |  |  |  |  |  |  |  |  |  |  |  |  |  |  |  |  |  |  |  |  |  |  |  |  |  |  |  |  |  |  |  |  |  |  |  |  |  |  |  |  |  |  |  |  |  |  |  |  |  |  |  |  |  |  |  |  |  |  |  |  |  |  |  |  |  |  |  |  |  |  |  |  |  |  |  |  |  |  |  |  |  |  |  |  |  |  |  |  |  |  |  |  |  |  |  |  |  |  |  |  |  |  |  |  |  |  |  |  |  |  |  |  |  |  |  |  |  |  |  |  |  |  |  |  |  |  |  |  |  |  |  |  |  |  |  |  |  |  |  |  |  |  |  |  |  |  |  |  |  |  |  |  |  |  |  |  |  |  |  |  |  |  |  |  |  |  |  |  |  |  |  |  |  |  |  |  |  |  |  |  |  |  |  |  |  |  |  |  |  |  |  |  |  |  |  |  |  |  |  |  |  |  |  |  |  |  |  |  |  |  |  |  |  |  |  |  |  |  |  |  |  |  |  |  |  |  |  |  |  |  |  |  |  |  |  |  |  |  |  |  |  |  |  |  |  |  |  |  |  |  |  |  |  |  |  |  |  |  |  |  |  |  |  |  |  |  |  |  |  |  |  |  |  |  |  |  |  |  |  |  |  |  |  |  |  |  |  |  |  |  |  |  |  |  |  |  |  |  |  |  |  |  |  |  |  |  |  |  |  |  |  |  |  |  |  |  |  |  |  |  |  |  |  |  |  |  |  |  |  |  |  |  |  |  |  |  |  |  |  |  |  |  |  |  |  |  |  |  |  |  |  |  |  |  |  |  |  |  |  |  |  |  |  |  |  |  |  |  |  |  |  |  |  |  |  |  |  |  |  |  |  |  |  |  |  |  |  |  |  |  |  |  |  |  |  |  |  |  |
|  |  |  |  |  |  |  |  |  |  |  |  |  |  |  |  |  |  |  |  |  |  |  |  |  |  |  |  |  |  |  |  |  |  |  |  |  |  |  |  |  |  |  |  |  |  |  |  |  |  |  |  |  |  |  |  |  |  |  |  |  |  |  |  |  |  |  |  |  |  |  |  |  |  |  |  |  |  |  |  |  |  |  |  |  |  |  |  |  |  |  |  |  |  |  |  |  |  |  |  |  |  |  |  |  |  |  |  |  |  |  |  |  |  |  |  |  |  |  |  |  |  |  |  |  |  |  |  |  |  |  |  |  |  |  |  |  |  |  |  |  |  |  |  |  |  |  |  |  |  |  |  |  |  |  |  |  |  |  |  |  |  |  |  |  |  |  |  |  |  |  |  |  |  |  |  |  |  |  |  |  |  |  |  |  |  |  |  |  |  |  |  |  |  |  |  |  |  |  |  |  |  |  |  |  |  |  |  |  |  |  |  |  |  |  |  |  |  |  |  |  |  |  |  |  |  |  |  |  |  |  |  |  |  |  |  |  |  |  |  |  |  |  |  |  |  |  |  |  |  |  |  |  |  |  |  |  |  |  |  |  |  |  |  |  |  |  |  |  |  |  |  |  |  |  |  |  |  |  |  |  |  |  |  |  |  |  |  |  |  |  |  |  |  |  |  |  |  |  |  |  |  |  |  |  |  |  |  |  |  |  |  |  |  |  |  |  |  |  |  |  |  |  |  |  |  |  |  |  |  |  |  |  |  |  |  |  |  |  |  |  |  |  |  |  |  |  |  |  |  |  |  |  |  |  |  |  |  |  |  |  |  |  |  |  |  |  |  |  |  |  |  |  |  |  |  |  |  |  |  |  |  |  |  |  |  |  |  |  |  |  |  |  |  |  |  |  |  |  |  |  |  |  |  |  |  |  |  |  |  |  |  |  |  |  |  |  |  |  |  |  |  |  |  |  |  |  |  |  |  |  |  |  |  |  |  |  |  |  |  |  |  |  |  |  |  |  |  |  |  |  |  |  |  |  |  |  |  |  |  |  |  |  |  |  |  |  |  |  |  |  |  |  |  |  |  |  |  |  |  |  |  |  |  |  |  |  |  |  |  |  |  |  |  |  |  |  |  |  |  |  |  |  |  |  |  |  |  |  |  |  |  |  |  |  |  |  |  |  |  |  |  |  |  |  |  |  |  |  |  |  |  |  |  |  |  |  |  |  |  |  |  |  |  |  |  |  |  |  |  |  |  |  |  |  |  |  |  |  |  |  |  |  |  |  |  |  |  |  |  |  |  |  |  |  |  |  |  |  |  |  |  |  |  |  |  |  |  |  |  |  |  |  |  |  |  |  |  |  |  |  |  |  |  |  |  |  |  |  |  |  |  |  |  |  |  |  |  |  |  |  |  |  |  |  |  |  |  |  |  |  |  |  |  |  |  |  |  |  |  |  |  |  |  |  |  |  |  |  |  |  |  |  |  |  |  |  |  |  |  |  |  |  |  |  |  |  |  |  |  |  |  |  |  |  |  |  |  |  |  |  |  |  |  |  |  |  |  |  |  |  |  |  |  |  |  |  |  |  |  |  |  |  |  |  |  |  |  |  |  |  |  |  |  |  |  |  |  |  |  |  |  |  |  |  |  |  |  |  |  |  |  |  |  |  |  |  |  |  |  |  |  |  |  |  |  |  |  |  |  |  |  |  |  |  |  |  |  |  |  |  |  |  |  |  |  |  |  |  |  |  |  |  |  |  |  |  |  |  |  |  |  |  |  |  |  |  |  |  |  |  |  |  |  |  |  |  |  |  |  |  |  |  |  |  |  |  |  |  |  |  |  |  |  |  |  |  |  |  |  |  |  |  |  |  |  |  |  |  |  |  |  |  |  |  |  |  |  |  |  |  |  |  |  |  |  |  |  |  |  |  |  |  |  |  |  |  |  |  |  |  |  |  |  |  |  |  |  |  |  |  |  |  |  |  |  |  |  |  |  |  |  |  |  |  |  |  |  |  |  |  |  |  |  |  |  |  |  |  |  |  |  |  |  |  |  |  |  |  |  |  |  |  |  |  |  |  |  |  |  |  |  |  |  |  |  |  |  |  |  |  |  |  |  |  |  |  |  |  |  |  |  |  |  |  |  |  |  |  |  |  |  |  |  |  |  |  |  |  |  |  |  |  |  |  |  |  |  |  |  |  |  |  |  |  |  |  |  |  |  |  |  |  |  |  |  |  |  |  |  |  |  |  |  |  |  |  |  |  |
| BIS d=2 cl.1 sym=0.52 env=0.99 | - | - | - | - | - | - | - | - | - | - | - | - | - | - | - | - | - | - | - | - | - | - | - | - | - | - | - | - | - | - | - | - | - | - | - | - | - | - | - | - | - | - | - | - | - | - | - | - | - | - | - | - | - | - | - | - | - | - | - | - | - | - | - | - | - | - | - | - | - | - | - | - | - | - | - | - | - | - | - | - | - | - | - | - | - | - | - | - | - | - | - | - | - | - | - | - | - | - | - | - | - | - | - | - | - | - | - | - | - | - | - | - | - | - | - | - | - | - | - | - | - | - | - | - | - | - | - | - | - | - | - | - | - | - | - | - | - | - | - | - | - | - | - | - | - | - | - | - | - | - | - | - | - | - | - | - | - | - | - | - | - | - | - | - | - | - | - | - | - | - | - | - | - | - | - | - | - | - | - | - | - | - | - | - | - | - | - | - | - | - | - | - | - | - | - | - | - | - | - | - | - | - | - | - | - | - | - | - | - | - | - | - | - | - | - | - | - | - | - | - | - | - | - | - | - | - | - | - | - | - | - | - | - | - | - | - | - | - | - | - | - | - | - | - | - | - | - | - | - | - | - | - | - | - | - | - | - | - | - | - | - | - | - | - | - | - | - | - | - | - | - | - | - | - | - | - | - | - | - | - | - | - | - | - | - | - | - | - | - | - | - | - | - | - | - | - | - | - | - | - | - | - | - | - | - | - | - | - | - | - | - | - | - | - | - | - | - | - | - | - | - | - | - | - | - | - | - | - | - | - | - | - | - | - | - | - | - | - | - | - | - | - | - | - | - | - | - | - | - | - | - | - | - | - | - | - | - | - | - | - | - | - | - | - | - | - | - | - | - | - | - | - | - | - | - | - | - | - | - | - | - | - | - | - | - | - | - | - | - | - | - | - | - | - | - | - | - | - | - | - | - | - | - | - | - | - | - | - | - | - | - | - | - | - | - | - | - | - | - | - | - | - | - | - | - | - | - | - | - | - | - | - | - | - | - | - | - | - | - | - | - | - | - | - | H | x | x | x | x | x | x | x | x | x | x | x | x | x | x | x | x | x | x | x | x | x | x | x | x | x | x | x | x | x | x | x | x | x | x | x | x | x | x | x | x | x | x | x | x | x | x | x | x | x | x | x | x | x | x | x | x | x | x | x | x | x | x | x | x | x | x | x | x | x | x | x | x | x | x | x | x | x | x | x | x | x | x | x | x | x | x | x | x | x | x | x | x | x | x | x | x | x | x | x | x | x | x | x | x | x | x | x | x | x | x | x | x | x | x | x | x | x | x | x | x | x | x | x | x | x | x | x | x | x | x | x | x | x | x | x | x | x | x | x | x | x | x | x | x | x | x | x | x | x | x | x | x | x | x | x | x | x | x | x | x | x | x | x | x | x | x | x | x | x | x | x | x | x | x | x | x | x | x | x | x | x | x | x | x | x | x | x | x | x | x | x | x | x | x | x | x | x | x | x | x | x | x | x | x | x | x | x | x | x | x | x | x | x | x | x | x | x | x | x | x | x | x | x | x | x | x | x | x | x | x | x | x | x | x | x | x | x | x | x | x | x | x | x | x | H | x | x | x | x | x | x | H | - | - | - | - | - | - | - | - | - | - | - | - | - | - | - | - | - | - | - | - | - | - | - | - | - | - | - | - | - | - | - | - | - | - | - | - | - | - | - | - | - | - | - | - | - | - | - | - | - | - | - | - | - | - | - | - | - | - | - | - | - | - | - | - | - | - | - | - | - | - | - | - | - | - | - | - | - | - | - | - | - | - | - | - | - | - | - | - | - | - | - | - | - | - | - | - | - | - | - | - | - | - | - | - | - | - | - | - | - | - | - | - | - | - | - | - | - | - | - | - | - | - | - | - | - | - | - | - | - | - | - | - | - | - | - | - | - | - | - | - | - | - | - | - | - | - | - | - | - | - | - | - | - | - | - | - | - | - | - | - | - | - | - | - | - | - | - | - | - | - | - | - | - | - | - | - | - | - | - | - | - | - | - | - | - | - | - | - | - | - | - | - | - | - | - | - | - | - | - | - | - | - | - | - | - | - | - | - | - | - | - | - | - | - | - | - | - | - | - | - | - | - | - | - | - | - | - | - | - | - | - | - | - | - | - | - | - | - | - | - | - | - | - | - | - | - | - | - | - | - | - | - | - | - | - | - | - | - | - | - | - | - | - | - | - | - | - | - | - | - | - | - | - | - | - | - | - | - | - | - | - | - | - | - | - | - | - | - | - | - | - | - | - | - | - | - | - | - | - | - | - |
