# Supplementary material for: Variation in selection constraints on teleost TLRs with emphasis on their repertoire in the Walking catfish, Clarias batrachus
Source: Sci Rep. 2020 Dec 7;10:21394. doi: 10.1038/s41598-020-78347-6 (PMC7721727; doi:10.1038/s41598-020-78347-6)
Supplement: Supplementary file 29 — Supplementary Information 29. [file 41598_2020_78347_MOESM29_ESM.zip › T7/bis2/summary.tgz!/summary/PF00000-NONREDUNDANT-5DD-dim0-table.html]

BIS cluster table


Clusters with env. score >= 0.5 and sym. score >= 0.5 :

| Dim | Cluster | Sym | Env | Pvalue | Hit patterns and blocks |
| --- | --- | --- | --- | --- | --- |
| 0 | 6 | 1 | 1 | 0 | Hit patterns: Positions: \| 780 \| 818 \| 929; 13 sequences: \| D \| L \| Q; 4 sequences: \| N \| Y \| N; 4 sequences: \| E \| F \| E; 2 sequences: \| Y \| V \| P All positions in cluster: 780 818 929 |
| 0 | 4 | 1 | 1 | 8.740754e-07 | Hit patterns: Positions: \| 280 \| 596 \| 766 \| 820 \| 867; 13 sequences: \| I \| T \| K \| H \| V; 10 sequences: \| L \| S \| T \| N \| L All positions in cluster: 280 596 766 820 867 |
| 0 | 5 | 1 | 1 | 2.039509e-06 | Hit patterns: Positions: \| 524 \| 593 \| 618 \| 764 \| 814 \| 868 \| 983; 15 sequences: \| V \| L \| S \| L \| M \| D \| D; 8 sequences: \| - \| M \| A \| I \| L \| N \| E All positions in cluster: 524 593 618 764 814 868 983 |
| 0 | 12 | 1 | 1 | 9.906188e-06 | Hit patterns: Positions: \| 230 \| 1024; 17 sequences: \| L \| K; 6 sequences: \| M \| R All positions in cluster: 230 1024 |
| 0 | 3 | 1 | 1 | 0.0001129305 | Hit patterns: Positions: \| 568 \| 858 \| 963; 19 sequences: \| S \| A \| D; 4 sequences: \| A \| T \| E All positions in cluster: 568 858 963 |
| 0 | 9 | 1 | 1 | 0.0001129305 | Hit patterns: Positions: \| 264 \| 329 \| 496 \| 497 \| 502 \| 518 \| 613 \| 617 \| 1010; 19 sequences: \| V \| V \| R \| E \| R \| G \| N \| E \| K; 4 sequences: \| I \| I \| K \| D \| L \| W \| S \| V \| R All positions in cluster: 264 329 496-497 502 518 613 617 1010 |
| 0 | 7 | 1 | 1 | 0.0005646527 | Hit patterns: Positions: \| 716 \| 948; 20 sequences: \| L \| E; 3 sequences: \| F \| D All positions in cluster: 716 948 |
| 0 | 11 | 1 | 1 | 0.0005646527 | Hit patterns: Positions: \| 204 \| 234; 20 sequences: \| G \| S; 3 sequences: \| S \| C All positions in cluster: 204 234 |
| 0 | 1 | 1 | 1 | 0.003952569 | Hit patterns: Positions: \| 403 \| 749 \| 804 \| 911 \| 915 \| 945; 21 sequences: \| G \| L \| S \| I \| C \| A; 2 sequences: \| A \| I \| T \| L \| L \| E All positions in cluster: 403 749 804 911 915 945 |
| 0 | 2 | 1 | 1 | 0.003952569 | Hit patterns: Positions: \| 506 \| 886; 21 sequences: \| Y \| L; 2 sequences: \| F \| F All positions in cluster: 506 886 |
| 0 | 8 | 1 | 1 | 0.003952569 | Hit patterns: Positions: \| 416 \| 848; 21 sequences: \| P \| A; 2 sequences: \| H \| G All positions in cluster: 416 848 |
| 0 | 13 | 1 | 1 | 0.003952569 | Hit patterns: Positions: \| 892 \| 1017; 21 sequences: \| L \| H; 2 sequences: \| I \| N All positions in cluster: 892 1017 |
| 0 | 10 | 1 | 1 | 1 | All positions in cluster: 176 178-180 182 184 191 201-203 206-208 210-213 217 220 224 232-233 236-241 244 247-248 251 253-259 268 272-273 275-276 278-279 281-287 289 291 293-294 296-297 310 313 316 319-320 322 333-334 340 343-346 348 350 360 363 366 368 370-374 377-378 380-381 384 386 390 393 396 400 405-407 409 417 420 423 427-433 441 444-445 447 449-450 452-453 455 457 460-461 482-484 499-501 503-505 507-511 515-516 528-529 531-536 538-546 549-550 552 555 557-563 565-567 569-571 573-574 577 580 582-585 588-592 597-598 600-601 604 607-610 612 616 619-628 631 637 640 642 644-649 651 654 657-658 661-662 665-668 671-674 678 682 685 688 691-694 696-698 721 728 730 739 742-744 747 752-753 763 769 772 774 777 781-782 785 788 791-794 796 807 817 819 822 824 826 828 836 838 840 844-845 847 851 853 856 859 864 873-874 879 900-910 912 914 917-918 920 922 928 933-936 939 943 946 949-950 953 958-959 968-971 973-975 978 980 984-985 988-989 992 994-995 997 999-1000 1007 1009 1012-1014 1016 1018-1023 1026-1028 1030 1032-1034 1041 1043-1044 1046-1047 1049-1050 1069 |

Table created with bis2html version 8.
