# Supplementary material for: Variation in selection constraints on teleost TLRs with emphasis on their repertoire in the Walking catfish, Clarias batrachus
Source: Sci Rep. 2020 Dec 7;10:21394. doi: 10.1038/s41598-020-78347-6 (PMC7721727; doi:10.1038/s41598-020-78347-6)
Supplement: Supplementary file 29 — Supplementary Information 29. [file 41598_2020_78347_MOESM29_ESM.zip › T7/bis2/summary.tgz!/summary/PF00000-NONREDUNDANT-5DD-dim1-table.html]

BIS cluster table


Clusters with env. score >= 0.5 and sym. score >= 0.5 :

| Dim | Cluster | Sym | Env | Pvalue | Hit patterns and blocks |
| --- | --- | --- | --- | --- | --- |
| 1 | 12 | 1 | 1 | 6.723657e-08 | Hit patterns: Positions: \| 364 \| 413; 12 sequences: \| G \| S; 10 sequences: \| P \| D; 1 sequence: \| C \| G All positions in cluster: 364 413 |
| 1 | 16 | 1 | 1 | 2.549387e-07 | Hit patterns: Positions: \| 57 \| 115; 15 sequences: \| T \| L; 7 sequences: \| S \| I; 1 sequence: \| - \| - All positions in cluster: 57 115 |
| 1 | 18 | 1 | 1 | 5.943713e-06 | Hit patterns: Positions: \| 760 \| 1053; 18 sequences: \| C \| R; 4 sequences: \| Y \| K; 1 sequence: \| V \| Q All positions in cluster: 760 1053 |
| 1 | 1 | 1 | 1 | 0.04347826 | All positions in cluster: 214 216 303 987 1004 |
| 1 | 2 | 1 | 1 | 0.04347826 | All positions in cluster: 235 357 359 434 494 733 801 881 899 925 940 982 |
| 1 | 3 | 1 | 1 | 0.04347826 | All positions in cluster: 288 634 701 930 977 1011 |
| 1 | 4 | 1 | 1 | 0.04347826 | All positions in cluster: 880 897 921 |
| 1 | 5 | 1 | 1 | 0.04347826 | All positions in cluster: 376 495 513 523 677 767 861 |
| 1 | 6 | 1 | 1 | 0.04347826 | All positions in cluster: 349 367 454 456 702 718 829 924 979 1048 |
| 1 | 7 | 1 | 1 | 0.04347826 | All positions in cluster: 323-326 681 710 |
| 1 | 8 | 1 | 1 | 0.04347826 | All positions in cluster: 572 650 |
| 1 | 9 | 1 | 1 | 0.04347826 | All positions in cluster: 399 1006 |
| 1 | 10 | 1 | 1 | 0.04347826 | All positions in cluster: 375 605 615 723 |
| 1 | 11 | 1 | 1 | 0.04347826 | All positions in cluster: 192 369 888 1031 |
| 1 | 13 | 1 | 1 | 0.04347826 | All positions in cluster: 292 547 670 |
| 1 | 14 | 1 | 1 | 0.04347826 | All positions in cluster: 177 197 438 458 756 |
| 1 | 15 | 1 | 1 | 0.04347826 | All positions in cluster: 54-56 58 60 62 67 71 73 75-76 80 84 87-88 92-101 108-109 112 117-119 122-126 128 130-133 136 142 147 153 156 158-162 166-167 169-171 269 439 660 |
| 1 | 17 | 1 | 1 | 0.04347826 | All positions in cluster: 181 277 586 745 833 993 1051 1054 1058-1059 1061 1064 1067-1068 1070 1073 1084 1088 1090-1092 |

Table created with bis2html version 8.
