# Supplementary material for: Variation in selection constraints on teleost TLRs with emphasis on their repertoire in the Walking catfish, Clarias batrachus
Source: Sci Rep. 2020 Dec 7;10:21394. doi: 10.1038/s41598-020-78347-6 (PMC7721727; doi:10.1038/s41598-020-78347-6)
Supplement: Supplementary file 29 — Supplementary Information 29. [file 41598_2020_78347_MOESM29_ESM.zip › T7/bis2/summary.tgz!/summary/PF00000-NONREDUNDANT-5DD-dim2-table.html]

BIS cluster table


Clusters with env. score >= 0.5 and sym. score >= 0.5 :

| Dim | Cluster | Sym | Env | Pvalue | Hit patterns and blocks |
| --- | --- | --- | --- | --- | --- |
| 2 | 1 | 1 | 1 | 0 | Hit patterns: Positions: \| 636 \| 976; 13 sequences: \| I \| I; 8 sequences: \| K \| V; 1 sequence: \| T \| L; 1 sequence: \| V \| M All positions in cluster: 636 976 |
| 2 | 7 | 1 | 1 | 0 | Hit patterns: Positions: \| 1086 \| 1089; 14 sequences: \| N \| K; 7 sequences: \| K \| Q; 1 sequence: \| D \| R; 1 sequence: \| X \| X All positions in cluster: 1086 1089 |
| 2 | 2 | 1 | 1 | 0.003952569 | All positions in cluster: 706 882 |
| 2 | 3 | 1 | 1 | 0.003952569 | All positions in cluster: 290 700 |
| 2 | 4 | 1 | 1 | 0.003952569 | All positions in cluster: 656 1075 |
| 2 | 5 | 1 | 1 | 0.003952569 | All positions in cluster: 150 163 |
| 2 | 6 | 1 | 1 | 0.003952569 | All positions in cluster: 114 395 |
| 2 | 8 | 1 | 1 | 0.003952569 | All positions in cluster: 1065 1072 |

Table created with bis2html version 8.
