# Supplementary material for: Variation in selection constraints on teleost TLRs with emphasis on their repertoire in the Walking catfish, Clarias batrachus
Source: Sci Rep. 2020 Dec 7;10:21394. doi: 10.1038/s41598-020-78347-6 (PMC7721727; doi:10.1038/s41598-020-78347-6)
Supplement: Supplementary file 29 — Supplementary Information 29. [file 41598_2020_78347_MOESM29_ESM.zip › T7/bis2/summary.tgz!/summary/PF00000-NONREDUNDANT-5DD-dim2.html]

Alignment and BIS clusters


|  |  |  |  |  |  |  |  |  |  |  |  |  |  |  |  |  |  |  |  |  |  |  |  |  |  |  |  |  |  |  |  |  |  |  |  |  |  |  |  |  |  |  |  |  |  |  |  |  |  |  |  |  |  |  |  |  |  |  |  |  |  |  |  |  |  |  |  |  |  |  |  |  |  |  |  |  |  |  |  |  |  |  |  |  |  |  |  |  |  |  |  |  |  |  |  |  |  |  |  |  |  |  |  |  |  |  |  |  |  |  |  |  |  |  |  |  |  |  |  |  |  |  |  |  |  |  |  |  |  |  |  |  |  |  |  |  |  |  |  |  |  |  |  |  |  |  |  |  |  |  |  |  |  |  |  |  |  |  |  |  |  |  |  |  |  |  |  |  |  |  |  |  |  |  |  |  |  |  |  |  |  |  |  |  |  |  |  |  |  |  |  |  |  |  |  |  |  |  |  |  |  |  |  |  |  |  |  |  |  |  |  |  |  |  |  |  |  |  |  |  |  |  |  |  |  |  |  |  |  |  |  |  |  |  |  |  |  |  |  |  |  |  |  |  |  |  |  |  |  |  |  |  |  |  |  |  |  |  |  |  |  |  |  |  |  |  |  |  |  |  |  |  |  |  |  |  |  |  |  |  |  |  |  |  |  |  |  |  |  |  |  |  |  |  |  |  |  |  |  |  |  |  |  |  |  |  |  |  |  |  |  |  |  |  |  |  |  |  |  |  |  |  |  |  |  |  |  |  |  |  |  |  |  |  |  |  |  |  |  |  |  |  |  |  |  |  |  |  |  |  |  |  |  |  |  |  |  |  |  |  |  |  |  |  |  |  |  |  |  |  |  |  |  |  |  |  |  |  |  |  |  |  |  |  |  |  |  |  |  |  |  |  |  |  |  |  |  |  |  |  |  |  |  |  |  |  |  |  |  |  |  |  |  |  |  |  |  |  |  |  |  |  |  |  |  |  |  |  |  |  |  |  |  |  |  |  |  |  |  |  |  |  |  |  |  |  |  |  |  |  |  |  |  |  |  |  |  |  |  |  |  |  |  |  |  |  |  |  |  |  |  |  |  |  |  |  |  |  |  |  |  |  |  |  |  |  |  |  |  |  |  |  |  |  |  |  |  |  |  |  |  |  |  |  |  |  |  |  |  |  |  |  |  |  |  |  |  |  |  |  |  |  |  |  |  |  |  |  |  |  |  |  |  |  |  |  |  |  |  |  |  |  |  |  |  |  |  |  |  |  |  |  |  |  |  |  |  |  |  |  |  |  |  |  |  |  |  |  |  |  |  |  |  |  |  |  |  |  |  |  |  |  |  |  |  |  |  |  |  |  |  |  |  |  |  |  |  |  |  |  |  |  |  |  |  |  |  |  |  |  |  |  |  |  |  |  |  |  |  |  |  |  |  |  |  |  |  |  |  |  |  |  |  |  |  |  |  |  |  |  |  |  |  |  |  |  |  |  |  |  |  |  |  |  |  |  |  |  |  |  |  |  |  |  |  |  |  |  |  |  |  |  |  |  |  |  |  |  |  |  |  |  |  |  |  |  |  |  |  |  |  |  |  |  |  |  |  |  |  |  |  |  |  |  |  |  |  |  |  |  |  |  |  |  |  |  |  |  |  |  |  |  |  |  |  |  |  |  |  |  |  |  |  |  |  |  |  |  |  |  |  |  |  |  |  |  |  |  |  |  |  |  |  |  |  |  |  |  |  |  |  |  |  |  |  |  |  |  |  |  |  |  |  |  |  |  |  |  |  |  |  |  |  |  |  |  |  |  |  |  |  |  |  |  |  |  |  |  |  |  |  |  |  |  |  |  |  |  |  |  |  |  |  |  |  |  |  |  |  |  |  |  |  |  |  |  |  |  |  |  |  |  |  |  |  |  |  |  |  |  |  |  |  |  |  |  |  |  |  |  |  |  |  |  |  |  |  |  |  |  |  |  |  |  |  |  |  |  |  |  |  |  |  |  |  |  |  |  |  |  |  |  |  |  |  |  |  |  |  |  |  |  |  |  |  |  |  |  |  |  |  |  |  |  |  |  |  |  |  |  |  |  |  |  |  |  |  |  |  |  |  |  |  |  |  |  |  |  |  |  |  |  |  |  |  |  |  |  |  |  |  |  |  |  |  |  |  |  |  |  |  |  |  |  |  |  |  |  |  |  |  |  |  |  |  |  |  |  |  |  |  |  |  |  |  |  |  |  |  |  |  |  |  |  |  |  |  |  |  |  |  |  |  |  |  |  |  |  |  |  |  |  |  |  |  |  |  |  |  |  |  |  |  |  |  |  |  |  |  |  |  |  |  |  |  |  |  |  |  |  |  |  |  |  |  |  |  |  |  |  |  |  |  |  |  |  |  |  |  |  |  |  |  |  |  |  |  |  |  |  |  |  |  |  |  |  |  |  |  |  |  |  |  |  |  |  |  |  |  |  |  |  |  |  |  |  |  |  |  |  |  |  |  |  |  |  |  |  |  |  |  |  |  |  |  |  |  |  |  |  |  |  |  |  |  |  |  |  |  |  |  |  |  |  |  |  |  |  |  |  |  |  |  |  |  |  |  |  |  |  |  |  |  |  |  |  |  |  |  |  |  |  |  |  |  |  |  |  |  |  |  |  |  |  |  |  |  |  |  |  |  |  |  |  |  |  |  |  |  |  |  |  |  |  |  |  |  |  |  |  |  |  |  |  |  |  |  |  |  |  |  |  |  |  |  |  |  |  |  |  |  |  |  |  |  |  |  |  |  |  |  |  |  |  |  |  |  |  |  |  |  |  |  |  |  |  |  |  |  |  |  |  |  |  |  |  |  |  |  |  |  |  |  |  |  |  |  |  |  |
| --- | --- | --- | --- | --- | --- | --- | --- | --- | --- | --- | --- | --- | --- | --- | --- | --- | --- | --- | --- | --- | --- | --- | --- | --- | --- | --- | --- | --- | --- | --- | --- | --- | --- | --- | --- | --- | --- | --- | --- | --- | --- | --- | --- | --- | --- | --- | --- | --- | --- | --- | --- | --- | --- | --- | --- | --- | --- | --- | --- | --- | --- | --- | --- | --- | --- | --- | --- | --- | --- | --- | --- | --- | --- | --- | --- | --- | --- | --- | --- | --- | --- | --- | --- | --- | --- | --- | --- | --- | --- | --- | --- | --- | --- | --- | --- | --- | --- | --- | --- | --- | --- | --- | --- | --- | --- | --- | --- | --- | --- | --- | --- | --- | --- | --- | --- | --- | --- | --- | --- | --- | --- | --- | --- | --- | --- | --- | --- | --- | --- | --- | --- | --- | --- | --- | --- | --- | --- | --- | --- | --- | --- | --- | --- | --- | --- | --- | --- | --- | --- | --- | --- | --- | --- | --- | --- | --- | --- | --- | --- | --- | --- | --- | --- | --- | --- | --- | --- | --- | --- | --- | --- | --- | --- | --- | --- | --- | --- | --- | --- | --- | --- | --- | --- | --- | --- | --- | --- | --- | --- | --- | --- | --- | --- | --- | --- | --- | --- | --- | --- | --- | --- | --- | --- | --- | --- | --- | --- | --- | --- | --- | --- | --- | --- | --- | --- | --- | --- | --- | --- | --- | --- | --- | --- | --- | --- | --- | --- | --- | --- | --- | --- | --- | --- | --- | --- | --- | --- | --- | --- | --- | --- | --- | --- | --- | --- | --- | --- | --- | --- | --- | --- | --- | --- | --- | --- | --- | --- | --- | --- | --- | --- | --- | --- | --- | --- | --- | --- | --- | --- | --- | --- | --- | --- | --- | --- | --- | --- | --- | --- | --- | --- | --- | --- | --- | --- | --- | --- | --- | --- | --- | --- | --- | --- | --- | --- | --- | --- | --- | --- | --- | --- | --- | --- | --- | --- | --- | --- | --- | --- | --- | --- | --- | --- | --- | --- | --- | --- | --- | --- | --- | --- | --- | --- | --- | --- | --- | --- | --- | --- | --- | --- | --- | --- | --- | --- | --- | --- | --- | --- | --- | --- | --- | --- | --- | --- | --- | --- | --- | --- | --- | --- | --- | --- | --- | --- | --- | --- | --- | --- | --- | --- | --- | --- | --- | --- | --- | --- | --- | --- | --- | --- | --- | --- | --- | --- | --- | --- | --- | --- | --- | --- | --- | --- | --- | --- | --- | --- | --- | --- | --- | --- | --- | --- | --- | --- | --- | --- | --- | --- | --- | --- | --- | --- | --- | --- | --- | --- | --- | --- | --- | --- | --- | --- | --- | --- | --- | --- | --- | --- | --- | --- | --- | --- | --- | --- | --- | --- | --- | --- | --- | --- | --- | --- | --- | --- | --- | --- | --- | --- | --- | --- | --- | --- | --- | --- | --- | --- | --- | --- | --- | --- | --- | --- | --- | --- | --- | --- | --- | --- | --- | --- | --- | --- | --- | --- | --- | --- | --- | --- | --- | --- | --- | --- | --- | --- | --- | --- | --- | --- | --- | --- | --- | --- | --- | --- | --- | --- | --- | --- | --- | --- | --- | --- | --- | --- | --- | --- | --- | --- | --- | --- | --- | --- | --- | --- | --- | --- | --- | --- | --- | --- | --- | --- | --- | --- | --- | --- | --- | --- | --- | --- | --- | --- | --- | --- | --- | --- | --- | --- | --- | --- | --- | --- | --- | --- | --- | --- | --- | --- | --- | --- | --- | --- | --- | --- | --- | --- | --- | --- | --- | --- | --- | --- | --- | --- | --- | --- | --- | --- | --- | --- | --- | --- | --- | --- | --- | --- | --- | --- | --- | --- | --- | --- | --- | --- | --- | --- | --- | --- | --- | --- | --- | --- | --- | --- | --- | --- | --- | --- | --- | --- | --- | --- | --- | --- | --- | --- | --- | --- | --- | --- | --- | --- | --- | --- | --- | --- | --- | --- | --- | --- | --- | --- | --- | --- | --- | --- | --- | --- | --- | --- | --- | --- | --- | --- | --- | --- | --- | --- | --- | --- | --- | --- | --- | --- | --- | --- | --- | --- | --- | --- | --- | --- | --- | --- | --- | --- | --- | --- | --- | --- | --- | --- | --- | --- | --- | --- | --- | --- | --- | --- | --- | --- | --- | --- | --- | --- | --- | --- | --- | --- | --- | --- | --- | --- | --- | --- | --- | --- | --- | --- | --- | --- | --- | --- | --- | --- | --- | --- | --- | --- | --- | --- | --- | --- | --- | --- | --- | --- | --- | --- | --- | --- | --- | --- | --- | --- | --- | --- | --- | --- | --- | --- | --- | --- | --- | --- | --- | --- | --- | --- | --- | --- | --- | --- | --- | --- | --- | --- | --- | --- | --- | --- | --- | --- | --- | --- | --- | --- | --- | --- | --- | --- | --- | --- | --- | --- | --- | --- | --- | --- | --- | --- | --- | --- | --- | --- | --- | --- | --- | --- | --- | --- | --- | --- | --- | --- | --- | --- | --- | --- | --- | --- | --- | --- | --- | --- | --- | --- | --- | --- | --- | --- | --- | --- | --- | --- | --- | --- | --- | --- | --- | --- | --- | --- | --- | --- | --- | --- | --- | --- | --- | --- | --- | --- | --- | --- | --- | --- | --- | --- | --- | --- | --- | --- | --- | --- | --- | --- | --- | --- | --- | --- | --- | --- | --- | --- | --- | --- | --- | --- | --- | --- | --- | --- | --- | --- | --- | --- | --- | --- | --- | --- | --- | --- | --- | --- | --- | --- | --- | --- | --- | --- | --- | --- | --- | --- | --- | --- | --- | --- | --- | --- | --- | --- | --- | --- | --- | --- | --- | --- | --- | --- | --- | --- | --- | --- | --- | --- | --- | --- | --- | --- | --- | --- | --- | --- | --- | --- | --- | --- | --- | --- | --- | --- | --- | --- | --- | --- | --- | --- | --- | --- | --- | --- | --- | --- | --- | --- | --- | --- | --- | --- | --- | --- | --- | --- | --- | --- | --- | --- | --- | --- | --- | --- | --- | --- | --- | --- | --- | --- | --- | --- | --- | --- | --- | --- | --- | --- | --- | --- | --- | --- | --- | --- | --- | --- | --- | --- | --- | --- | --- | --- | --- | --- | --- | --- | --- | --- | --- | --- | --- | --- | --- | --- | --- | --- | --- | --- | --- | --- | --- | --- | --- | --- | --- | --- | --- | --- | --- | --- | --- | --- | --- | --- | --- | --- | --- | --- | --- | --- | --- | --- | --- | --- | --- | --- | --- | --- | --- | --- | --- | --- | --- | --- | --- | --- | --- | --- | --- | --- | --- | --- | --- | --- | --- | --- | --- | --- | --- | --- | --- | --- | --- | --- | --- | --- | --- | --- | --- | --- | --- | --- | --- | --- | --- | --- | --- | --- | --- | --- | --- | --- | --- | --- | --- | --- | --- | --- | --- | --- | --- | --- | --- | --- | --- | --- | --- | --- | --- | --- | --- | --- | --- | --- | --- | --- | --- | --- | --- | --- | --- | --- | --- | --- | --- | --- | --- | --- | --- | --- | --- | --- | --- | --- | --- | --- | --- | --- | --- | --- | --- | --- | --- | --- | --- | --- | --- | --- | --- | --- | --- | --- | --- | --- | --- | --- | --- | --- | --- | --- | --- | --- | --- | --- | --- | --- | --- | --- | --- | --- | --- | --- | --- | --- | --- | --- | --- | --- | --- | --- | --- | --- | --- | --- | --- | --- | --- | --- | --- | --- | --- | --- | --- | --- | --- | --- | --- | --- | --- | --- | --- | --- | --- | --- | --- | --- | --- | --- | --- | --- | --- | --- | --- | --- | --- | --- | --- | --- | --- | --- | --- | --- | --- | --- | --- | --- | --- | --- | --- | --- | --- | --- | --- | --- | --- | --- | --- | --- | --- | --- | --- | --- | --- | --- | --- | --- | --- | --- | --- | --- | --- | --- | --- | --- | --- | --- | --- | --- | --- | --- | --- | --- | --- | --- | --- | --- | --- | --- | --- | --- | --- | --- | --- | --- | --- | --- | --- | --- | --- | --- | --- | --- | --- | --- | --- | --- | --- | --- | --- | --- | --- | --- | --- | --- | --- | --- | --- | --- | --- | --- | --- | --- | --- | --- | --- | --- | --- | --- |
|  |  |  |  |  |  |  |  | 1 | 0 |  |  |  |  |  |  |  |  | 2 | 0 |  |  |  |  |  |  |  |  | 3 | 0 |  |  |  |  |  |  |  |  | 4 | 0 |  |  |  |  |  |  |  |  | 5 | 0 |  |  |  |  |  |  |  |  | 6 | 0 |  |  |  |  |  |  |  |  | 7 | 0 |  |  |  |  |  |  |  |  | 8 | 0 |  |  |  |  |  |  |  |  | 9 | 0 |  |  |  |  |  |  |  | 1 | 0 | 0 |  |  |  |  |  |  |  | 1 | 1 | 0 |  |  |  |  |  |  |  | 1 | 2 | 0 |  |  |  |  |  |  |  | 1 | 3 | 0 |  |  |  |  |  |  |  | 1 | 4 | 0 |  |  |  |  |  |  |  | 1 | 5 | 0 |  |  |  |  |  |  |  | 1 | 6 | 0 |  |  |  |  |  |  |  | 1 | 7 | 0 |  |  |  |  |  |  |  | 1 | 8 | 0 |  |  |  |  |  |  |  | 1 | 9 | 0 |  |  |  |  |  |  |  | 2 | 0 | 0 |  |  |  |  |  |  |  | 2 | 1 | 0 |  |  |  |  |  |  |  | 2 | 2 | 0 |  |  |  |  |  |  |  | 2 | 3 | 0 |  |  |  |  |  |  |  | 2 | 4 | 0 |  |  |  |  |  |  |  | 2 | 5 | 0 |  |  |  |  |  |  |  | 2 | 6 | 0 |  |  |  |  |  |  |  | 2 | 7 | 0 |  |  |  |  |  |  |  | 2 | 8 | 0 |  |  |  |  |  |  |  | 2 | 9 | 0 |  |  |  |  |  |  |  | 3 | 0 | 0 |  |  |  |  |  |  |  | 3 | 1 | 0 |  |  |  |  |  |  |  | 3 | 2 | 0 |  |  |  |  |  |  |  | 3 | 3 | 0 |  |  |  |  |  |  |  | 3 | 4 | 0 |  |  |  |  |  |  |  | 3 | 5 | 0 |  |  |  |  |  |  |  | 3 | 6 | 0 |  |  |  |  |  |  |  | 3 | 7 | 0 |  |  |  |  |  |  |  | 3 | 8 | 0 |  |  |  |  |  |  |  | 3 | 9 | 0 |  |  |  |  |  |  |  | 4 | 0 | 0 |  |  |  |  |  |  |  | 4 | 1 | 0 |  |  |  |  |  |  |  | 4 | 2 | 0 |  |  |  |  |  |  |  | 4 | 3 | 0 |  |  |  |  |  |  |  | 4 | 4 | 0 |  |  |  |  |  |  |  | 4 | 5 | 0 |  |  |  |  |  |  |  | 4 | 6 | 0 |  |  |  |  |  |  |  | 4 | 7 | 0 |  |  |  |  |  |  |  | 4 | 8 | 0 |  |  |  |  |  |  |  | 4 | 9 | 0 |  |  |  |  |  |  |  | 5 | 0 | 0 |  |  |  |  |  |  |  | 5 | 1 | 0 |  |  |  |  |  |  |  | 5 | 2 | 0 |  |  |  |  |  |  |  | 5 | 3 | 0 |  |  |  |  |  |  |  | 5 | 4 | 0 |  |  |  |  |  |  |  | 5 | 5 | 0 |  |  |  |  |  |  |  | 5 | 6 | 0 |  |  |  |  |  |  |  | 5 | 7 | 0 |  |  |  |  |  |  |  | 5 | 8 | 0 |  |  |  |  |  |  |  | 5 | 9 | 0 |  |  |  |  |  |  |  | 6 | 0 | 0 |  |  |  |  |  |  |  | 6 | 1 | 0 |  |  |  |  |  |  |  | 6 | 2 | 0 |  |  |  |  |  |  |  | 6 | 3 | 0 |  |  |  |  |  |  |  | 6 | 4 | 0 |  |  |  |  |  |  |  | 6 | 5 | 0 |  |  |  |  |  |  |  | 6 | 6 | 0 |  |  |  |  |  |  |  | 6 | 7 | 0 |  |  |  |  |  |  |  | 6 | 8 | 0 |  |  |  |  |  |  |  | 6 | 9 | 0 |  |  |  |  |  |  |  | 7 | 0 | 0 |  |  |  |  |  |  |  | 7 | 1 | 0 |  |  |  |  |  |  |  | 7 | 2 | 0 |  |  |  |  |  |  |  | 7 | 3 | 0 |  |  |  |  |  |  |  | 7 | 4 | 0 |  |  |  |  |  |  |  | 7 | 5 | 0 |  |  |  |  |  |  |  | 7 | 6 | 0 |  |  |  |  |  |  |  | 7 | 7 | 0 |  |  |  |  |  |  |  | 7 | 8 | 0 |  |  |  |  |  |  |  | 7 | 9 | 0 |  |  |  |  |  |  |  | 8 | 0 | 0 |  |  |  |  |  |  |  | 8 | 1 | 0 |  |  |  |  |  |  |  | 8 | 2 | 0 |  |  |  |  |  |  |  | 8 | 3 | 0 |  |  |  |  |  |  |  | 8 | 4 | 0 |  |  |  |  |  |  |  | 8 | 5 | 0 |  |  |  |  |  |  |  | 8 | 6 | 0 |  |  |  |  |  |  |  | 8 | 7 | 0 |  |  |  |  |  |  |  | 8 | 8 | 0 |  |  |  |  |  |  |  | 8 | 9 | 0 |  |  |  |  |  |  |  | 9 | 0 | 0 |  |  |  |  |  |  |  | 9 | 1 | 0 |  |  |  |  |  |  |  | 9 | 2 | 0 |  |  |  |  |  |  |  | 9 | 3 | 0 |  |  |  |  |  |  |  | 9 | 4 | 0 |  |  |  |  |  |  |  | 9 | 5 | 0 |  |  |  |  |  |  |  | 9 | 6 | 0 |  |  |  |  |  |  |  | 9 | 7 | 0 |  |  |  |  |  |  |  | 9 | 8 | 0 |  |  |  |  |  |  |  | 9 | 9 | 0 |  |  |  |  |  |  | 1 | 0 | 0 | 0 |  |  |  |  |  |  | 1 | 0 | 1 | 0 |  |  |  |  |  |  | 1 | 0 | 2 | 0 |  |  |  |  |  |  | 1 | 0 | 3 | 0 |  |  |  |  |  |  | 1 | 0 | 4 | 0 |  |  |  |  |  |  | 1 | 0 | 5 | 0 |  |  |  |  |  |  | 1 | 0 | 6 | 0 |  |  |  |  |  |  | 1 | 0 | 7 | 0 |  |  |  |  |  |  | 1 | 0 | 8 | 0 |  |  |  |  |  |  | 1 | 0 | 9 | 0 |  |  |  |  |  |  | 1 | 1 | 0 | 0 |  |  |  |  |  |  | 1 | 1 | 1 | 0 |  |  |  |  |  |  | 1 | 1 | 2 | 0 |  |  |  |  |  |  | 1 | 1 | 3 | 0 |  |  |  |  |  |  | 1 | 1 | 4 | 0 |  |  |  |  |  |  | 1 | 1 | 5 | 0 |  |  |  |  |  |  | 1 | 1 | 6 | 0 |  |  |  |  |  |  | 1 | 1 | 7 | 0 |  |  |  |  |  |  | 1 | 1 | 8 | 0 |  |  |  |  |  |  | 1 | 1 | 9 | 0 |  |  |  |  |  |  | 1 | 2 | 0 | 0 |  |  |  |  |  |  | 1 | 2 | 1 | 0 |  |  |  |  |  |  | 1 | 2 | 2 | 0 |  |  |  |  |  |  | 1 | 2 | 3 | 0 |  |  |  |  |  |  | 1 | 2 | 4 | 0 |  |  |  |  |  |  | 1 | 2 | 5 | 0 |  |  |  |  |  |  | 1 | 2 | 6 | 0 |
|  |  |  |  |  |  |  |  |  | \| |  |  |  |  |  |  |  |  |  | \| |  |  |  |  |  |  |  |  |  | \| |  |  |  |  |  |  |  |  |  | \| |  |  |  |  |  |  |  |  |  | \| |  |  |  |  |  |  |  |  |  | \| |  |  |  |  |  |  |  |  |  | \| |  |  |  |  |  |  |  |  |  | \| |  |  |  |  |  |  |  |  |  | \| |  |  |  |  |  |  |  |  |  | \| |  |  |  |  |  |  |  |  |  | \| |  |  |  |  |  |  |  |  |  | \| |  |  |  |  |  |  |  |  |  | \| |  |  |  |  |  |  |  |  |  | \| |  |  |  |  |  |  |  |  |  | \| |  |  |  |  |  |  |  |  |  | \| |  |  |  |  |  |  |  |  |  | \| |  |  |  |  |  |  |  |  |  | \| |  |  |  |  |  |  |  |  |  | \| |  |  |  |  |  |  |  |  |  | \| |  |  |  |  |  |  |  |  |  | \| |  |  |  |  |  |  |  |  |  | \| |  |  |  |  |  |  |  |  |  | \| |  |  |  |  |  |  |  |  |  | \| |  |  |  |  |  |  |  |  |  | \| |  |  |  |  |  |  |  |  |  | \| |  |  |  |  |  |  |  |  |  | \| |  |  |  |  |  |  |  |  |  | \| |  |  |  |  |  |  |  |  |  | \| |  |  |  |  |  |  |  |  |  | \| |  |  |  |  |  |  |  |  |  | \| |  |  |  |  |  |  |  |  |  | \| |  |  |  |  |  |  |  |  |  | \| |  |  |  |  |  |  |  |  |  | \| |  |  |  |  |  |  |  |  |  | \| |  |  |  |  |  |  |  |  |  | \| |  |  |  |  |  |  |  |  |  | \| |  |  |  |  |  |  |  |  |  | \| |  |  |  |  |  |  |  |  |  | \| |  |  |  |  |  |  |  |  |  | \| |  |  |  |  |  |  |  |  |  | \| |  |  |  |  |  |  |  |  |  | \| |  |  |  |  |  |  |  |  |  | \| |  |  |  |  |  |  |  |  |  | \| |  |  |  |  |  |  |  |  |  | \| |  |  |  |  |  |  |  |  |  | \| |  |  |  |  |  |  |  |  |  | \| |  |  |  |  |  |  |  |  |  | \| |  |  |  |  |  |  |  |  |  | \| |  |  |  |  |  |  |  |  |  | \| |  |  |  |  |  |  |  |  |  | \| |  |  |  |  |  |  |  |  |  | \| |  |  |  |  |  |  |  |  |  | \| |  |  |  |  |  |  |  |  |  | \| |  |  |  |  |  |  |  |  |  | \| |  |  |  |  |  |  |  |  |  | \| |  |  |  |  |  |  |  |  |  | \| |  |  |  |  |  |  |  |  |  | \| |  |  |  |  |  |  |  |  |  | \| |  |  |  |  |  |  |  |  |  | \| |  |  |  |  |  |  |  |  |  | \| |  |  |  |  |  |  |  |  |  | \| |  |  |  |  |  |  |  |  |  | \| |  |  |  |  |  |  |  |  |  | \| |  |  |  |  |  |  |  |  |  | \| |  |  |  |  |  |  |  |  |  | \| |  |  |  |  |  |  |  |  |  | \| |  |  |  |  |  |  |  |  |  | \| |  |  |  |  |  |  |  |  |  | \| |  |  |  |  |  |  |  |  |  | \| |  |  |  |  |  |  |  |  |  | \| |  |  |  |  |  |  |  |  |  | \| |  |  |  |  |  |  |  |  |  | \| |  |  |  |  |  |  |  |  |  | \| |  |  |  |  |  |  |  |  |  | \| |  |  |  |  |  |  |  |  |  | \| |  |  |  |  |  |  |  |  |  | \| |  |  |  |  |  |  |  |  |  | \| |  |  |  |  |  |  |  |  |  | \| |  |  |  |  |  |  |  |  |  | \| |  |  |  |  |  |  |  |  |  | \| |  |  |  |  |  |  |  |  |  | \| |  |  |  |  |  |  |  |  |  | \| |  |  |  |  |  |  |  |  |  | \| |  |  |  |  |  |  |  |  |  | \| |  |  |  |  |  |  |  |  |  | \| |  |  |  |  |  |  |  |  |  | \| |  |  |  |  |  |  |  |  |  | \| |  |  |  |  |  |  |  |  |  | \| |  |  |  |  |  |  |  |  |  | \| |  |  |  |  |  |  |  |  |  | \| |  |  |  |  |  |  |  |  |  | \| |  |  |  |  |  |  |  |  |  | \| |  |  |  |  |  |  |  |  |  | \| |  |  |  |  |  |  |  |  |  | \| |  |  |  |  |  |  |  |  |  | \| |  |  |  |  |  |  |  |  |  | \| |  |  |  |  |  |  |  |  |  | \| |  |  |  |  |  |  |  |  |  | \| |  |  |  |  |  |  |  |  |  | \| |  |  |  |  |  |  |  |  |  | \| |  |  |  |  |  |  |  |  |  | \| |  |  |  |  |  |  |  |  |  | \| |  |  |  |  |  |  |  |  |  | \| |  |  |  |  |  |  |  |  |  | \| |  |  |  |  |  |  |  |  |  | \| |  |  |  |  |  |  |  |  |  | \| |  |  |  |  |  |  |  |  |  | \| |  |  |  |  |  |  |  |  |  | \| |  |  |  |  |  |  |  |  |  | \| |  |  |  |  |  |  |  |  |  | \| |  |  |  |  |  |  |  |  |  | \| |  |  |  |  |  |  |  |  |  | \| |  |  |  |  |  |  |  |  |  | \| |  |  |  |  |  |  |  |  |  | \| |  |  |  |  |  |  |  |  |  | \| |  |  |  |  |  |  |  |  |  | \| |  |  |  |  |  |  |  |  |  | \| |  |  |  |  |  |  |  |  |  | \| |  |  |  |  |  |  |  |  |  | \| |  |  |  |  |  |  |  |  |  | \| |  |  |  |  |  |  |  |  |  | \| |  |  |  |  |  |  |  |  |  | \| |  |  |  |  |  |  |  |  |  | \| |  |  |  |  |  |  |  |  |  | \| |  |  |  |  |  |  |  |  |  | \| |
| GMTLR7 | - | - | - | - | - | - | - | - | - | - | - | - | - | - | - | - | - | - | - | - | - | M | D | L | E | K | G | G | V | F | I | L | L | C | L | Y | M | P | S | L | R | P | L | S | L | D | A | S | T | S | M | R | W | Y | P | K | T | L | P | C | N | V | S | L | A | S | N | G | S | A | V | M | V | D | C | T | E | K | S | L | T | S | I | P | S | G | I | P | G | N | A | T | N | L | T | L | T | I | N | H | I | P | E | L | N | P | T | S | F | R | G | L | E | N | L | T | E | I | D | M | R | C | N | C | V | P | M | K | I | G | P | K | D | R | V | C | T | K | G | L | T | I | K | E | D | T | F | S | D | L | K | K | L | Q | A | L | Y | L | D | G | N | Q | L | E | G | I | P | R | G | L | P | R | N | L | R | L | L | S | L | E | V | N | N | I | F | Y | I | S | K | E | N | L | S | E | I | P | N | V | E | I | L | Y | L | G | Q | N | C | Y | F | R | N | P | C | N | V | S | Y | S | I | E | A | G | A | F | L | Q | L | S | N | L | T | L | L | S | V | K | S | N | N | L | S | Y | I | P | S | L | L | P | L | S | L | R | E | L | Y | L | Y | N | N | N | I | E | K | V | T | E | K | D | F | K | N | L | T | N | L | E | I | L | D | I | S | G | N | C | P | R | C | Y | N | A | P | F | P | C | D | P | C | P | N | N | S | - | L | K | L | H | K | N | A | F | K | T | L | A | K | L | K | I | L | R | M | H | S | N | S | L | T | T | V | L | P | E | W | F | A | N | L | K | E | L | K | V | L | D | L | S | S | N | F | L | A | G | E | V | A | H | F | N | L | P | K | A | L | C | N | L | E | E | L | D | L | S | F | N | Y | E | L | Q | K | Y | P | A | T | L | N | L | N | Q | S | F | S | V | L | Q | S | L | K | V | L | R | L | R | G | F | V | F | Q | S | L | T | I | Q | G | I | Q | P | L | M | S | L | P | H | L | E | V | L | D | L | G | T | N | F | I | Q | M | A | N | L | S | V | L | M | E | L | K | S | F | K | I | I | N | L | S | D | N | K | I | S | S | P | S | D | G | P | E | P | Y | N | S | V | S | A | H | - | - | - | Q | A | R | R | W | S | P | M | T | G | A | Q | Q | F | G | D | E | G | V | R | E | I | H | Y | F | R | Y | D | E | Y | A | R | S | C | K | H | K | D | K | E | L | G | V | F | N | S | F | V | N | A | Q | C | S | H | F | G | K | T | L | D | V | S | R | N | N | I | F | F | L | H | S | K | F | L | N | L | G | D | L | K | C | L | N | L | S | G | N | A | M | S | Q | S | L | N | G | S | E | F | T | Y | L | K | N | L | E | Y | L | D | F | S | A | N | R | L | D | L | L | H | P | T | A | F | Q | E | L | Q | N | L | V | I | L | D | I | S | L | N | N | H | Y | F | E | S | E | G | L | T | H | M | L | N | F | T | K | H | L | P | N | L | K | I | L | L | M | N | H | N | K | I | S | T | S | T | N | T | E | M | S | S | F | S | L | E | R | L | E | F | I | D | N | R | L | D | M | L | W | R | D | G | D | T | R | Y | I | G | Y | F | K | H | L | V | N | L | R | I | L | D | I | S | E | N | N | L | N | F | I | P | R | E | V | L | H | N | L | P | E | G | L | S | E | L | Y | I | K | K | N | K | L | T | - | - | K | F | F | W | G | D | I | D | K | L | P | L | L | K | I | L | D | L | S | G | N | R | L | T | R | V | P | M | V | L | S | - | N | C | T | I | S | L | Q | K | L | I | L | T | G | N | Q | I | E | Y | L | S | P | D | F | L | K | D | A | F | S | L | K | Y | L | D | L | S | F | N | K | L | K | Y | M | E | H | S | S | L | P | D | N | I | V | N | N | M | D | M | L | L | L | H | N | N | I | F | T | C | T | C | N | T | T | W | F | I | K | W | L | N | S | T | T | A | T | I | P | L | L | A | T | D | V | T | C | D | T | P | V | A | Q | R | G | R | L | V | L | T | V | D | - | - | M | L | A | C | Q | Y | H | Y | L | S | L | V | L | Y | I | L | L | T | S | T | L | L | V | F | V | T | L | A | V | S | S | H | L | F | L | W | D | V | W | Y | I | Y | H | F | C | V | A | K | L | K | G | Y | G | R | Q | T | S | S | Q | S | C | P | Y | D | A | F | V | V | Y | E | K | K | D | P | A | V | S | E | W | V | M | N | E | L | C | V | Q | L | E | E | R | G | D | R | P | L | R | L | C | L | E | E | R | D | W | I | P | G | C | P | V | V | D | N | L | C | Q | S | I | H | Q | S | K | R | T | V | F | V | L | T | N | R | D | I | K | S | G | N | F | K | T | A | F | Y | M | A | H | Q | R | L | M | D | E | K | N | D | V | I | V | L | I | F | L | E | K | A | A | - | S | N | S | K | Y | L | R | L | R | K | R | L | Y | R | R | S | V | L | E | W | P | T | N | P | Q | A | Q | P | Y | F | W | F | G | L | R | S | V | L | A | T | E | S | N | K | Q | Y | S | D | M | F | R | E | T | L | X | X | X | X | X | X | X | X | X | X | X | X | X | X | X | X | X | X | - | - | - | - | - | - | - | - | - | - | - | - | - | - | - | - | - | - | - | - | - | - | - | - | - | - | - | - | - | - | - | - | - | - | - | - | - | - | - | - | - | - | - | - | - | - | - | - | - | - | - | - | - | - | - | - | - | - | - | - | - | - | - | - | - | - | - | - | - | - | - | - | - | - | - | - | - | - | - | - | - | - | - | - | - | - | - | - | - | - | - | - | - | - | - | - | - | - | - | - | - | - | - | - | - | - | - | - | - | - | - | - | - | - | - | - | - | - | - | - | - | - | - | - | - | - | - | - | - | - | - | - | - | - | - | - | - | - | - | - | - | - | - | - | - | - | - | - | - | - |
| SSTLR7 | - | - | - | - | - | - | - | - | - | - | - | - | - | - | - | - | - | M | L | S | R | M | T | R | S | E | C | A | S | F | H | V | C | G | V | I | L | L | G | L | W | - | C | S | S | - | V | L | A | A | G | S | W | Y | P | K | T | L | P | C | D | V | T | L | D | S | N | D | T | M | V | N | V | D | C | T | E | R | G | L | L | E | V | P | K | D | I | P | R | N | T | T | N | L | T | L | T | I | N | H | I | P | H | I | N | S | T | S | F | Q | G | L | E | N | L | T | E | I | D | M | R | C | N | C | V | P | I | K | I | G | P | K | D | R | M | C | T | E | S | V | T | I | K | T | N | T | F | K | D | L | R | N | L | K | A | L | Y | L | D | G | N | Q | L | S | S | I | P | K | G | L | P | P | N | L | I | L | L | S | L | E | V | N | K | I | Y | T | I | L | K | R | N | L | S | D | I | T | N | V | Q | I | L | Y | L | G | Q | N | C | Y | Y | R | N | P | C | N | V | S | Y | Q | I | E | E | G | A | F | L | Q | L | G | N | M | T | L | L | S | L | K | S | N | N | L | S | Y | I | P | P | R | L | P | T | S | L | R | E | L | Y | L | Y | N | N | K | I | E | M | V | T | D | K | D | F | H | N | L | T | Q | L | E | I | L | D | L | S | G | N | C | P | R | C | Y | N | A | P | F | P | C | I | P | C | P | N | N | S | - | L | Q | I | D | P | S | T | F | K | T | L | T | K | L | R | I | L | R | L | H | S | N | S | L | T | Y | V | L | R | E | W | F | Q | N | C | K | E | L | R | V | L | D | L | S | T | N | F | L | A | R | E | I | A | I | T | Y | F | P | R | A | L | P | N | L | E | E | L | D | L | S | F | N | Y | E | L | Q | R | Y | P | A | T | L | H | L | S | P | S | F | S | S | L | K | S | L | K | V | L | R | I | R | A | F | V | F | Q | Q | L | T | L | E | D | I | S | P | L | I | H | L | K | N | L | E | V | I | D | L | G | T | N | F | I | K | I | T | N | L | S | I | L | M | E | L | K | S | F | K | I | I | N | L | S | D | N | K | I | S | S | P | S | E | S | G | Q | S | V | A | F | S | G | - | G | - | - | - | E | A | I | H | G | S | P | M | S | D | A | G | H | N | R | N | G | E | V | R | E | I | H | Y | F | R | Y | D | E | Y | A | R | S | C | K | Y | K | D | K | E | D | G | T | L | N | S | F | V | N | T | Q | C | S | K | F | G | K | T | L | D | I | S | R | N | N | I | F | F | L | H | S | R | F | L | N | L | A | D | L | R | C | L | N | L | S | G | N | A | M | S | Q | S | L | N | G | S | E | F | T | F | L | T | K | L | Q | Y | L | D | F | S | F | N | R | L | D | L | L | Y | S | S | A | F | Q | E | L | E | N | L | V | I | L | D | I | S | N | N | N | H | Y | F | E | S | E | G | L | T | H | M | L | N | F | T | K | N | L | T | K | L | K | I | L | L | M | N | Y | N | K | I | S | T | S | T | N | T | E | L | E | S | R | S | L | E | K | L | E | F | K | G | N | R | L | D | M | L | W | R | D | G | D | T | R | Y | I | D | Y | F | K | K | L | L | N | L | R | V | L | D | I | S | H | N | N | L | N | F | I | P | Q | Q | V | F | Q | G | L | P | D | K | L | T | N | L | Y | I | N | N | N | R | L | K | - | - | I | F | S | W | E | K | L | I | I | L | Q | Y | L | E | V | L | D | L | S | S | N | S | I | S | T | V | P | P | E | L | S | - | N | C | T | K | S | L | K | T | L | L | L | R | R | N | Q | I | S | K | L | S | A | Y | F | L | K | D | A | F | S | L | K | Y | L | D | L | S | F | N | H | I | Q | N | I | E | Q | T | S | I | P | D | D | V | V | D | Q | M | D | T | L | V | L | N | N | N | K | F | M | C | N | C | N | A | L | M | F | V | M | W | L | N | R | T | M | V | N | I | P | R | L | A | T | A | V | V | C | A | A | P | G | A | Q | R | G | H | P | V | I | S | L | D | L | E | L | Q | A | C | Q | H | N | Y | L | S | I | I | L | Y | I | L | L | T | S | L | V | L | S | F | V | T | L | P | I | S | S | H | L | F | L | W | D | V | W | Y | L | Y | H | F | L | L | A | K | F | K | G | Y | R | R | L | - | S | S | P | S | A | A | Y | D | A | F | V | V | Y | D | K | K | D | P | E | V | S | E | W | V | L | K | E | L | L | V | Q | L | E | E | H | G | D | H | P | L | Q | L | C | L | E | E | R | D | W | I | P | G | C | P | L | I | D | N | L | S | Q | S | I | H | Q | S | K | R | T | V | F | I | L | T | N | K | Y | I | K | S | G | D | F | K | T | A | F | Y | M | A | H | Q | R | L | M | D | E | R | D | D | V | I | V | L | I | F | L | E | K | V | P | - | S | H | S | K | Y | L | R | L | R | K | R | L | Y | R | R | S | V | I | E | W | P | T | N | P | Q | A | Q | Q | Y | F | W | F | S | L | R | S | V | L | V | T | D | S | Q | K | Q | Y | S | N | L | F | K | E | T | L | X | X | X | X | X | X | X | X | X | X | X | X | X | X | X | X | - | - | - | - | - | - | - | - | - | - | - | - | - | - | - | - | - | - | - | - | - | - | - | - | - | - | - | - | - | - | - | - | - | - | - | - | - | - | - | - | - | - | - | - | - | - | - | - | - | - | - | - | - | - | - | - | - | - | - | - | - | - | - | - | - | - | - | - | - | - | - | - | - | - | - | - | - | - | - | - | - | - | - | - | - | - | - | - | - | - | - | - | - | - | - | - | - | - | - | - | - | - | - | - | - | - | - | - | - | - | - | - | - | - | - | - | - | - | - | - | - | - | - | - | - | - | - | - | - | - | - | - | - | - | - | - | - | - | - | - | - | - | - | - | - | - | - | - | - | - | - | - |
| OMTLR7 | - | - | - | - | - | - | - | - | - | - | - | - | - | - | - | - | - | - | - | - | - | M | M | R | S | E | C | A | S | F | H | M | C | G | V | I | L | L | G | L | W | - | C | S | S | - | L | L | E | A | G | S | W | Y | P | K | T | L | P | C | D | V | T | L | A | S | N | N | T | M | V | N | V | D | C | T | E | R | G | L | L | E | V | P | K | D | I | P | R | N | T | T | N | L | T | L | T | I | N | H | I | P | H | I | N | S | T | S | F | Q | G | L | E | N | L | T | E | I | D | M | R | C | N | C | V | P | I | K | I | G | P | K | D | R | M | C | T | E | S | V | T | I | K | T | N | T | F | K | D | L | R | N | L | K | A | L | Y | L | D | G | N | Q | L | S | T | I | P | K | G | L | P | P | N | L | I | L | L | S | L | E | V | N | K | I | Y | T | I | L | K | T | N | L | S | D | I | T | N | V | Q | I | L | Y | L | G | Q | N | C | Y | Y | R | N | P | C | N | V | S | Y | Q | I | E | E | G | A | F | L | Q | L | G | N | M | T | L | L | S | L | K | S | N | N | L | S | Y | I | P | P | R | L | P | T | S | L | R | E | L | Y | L | Y | N | N | K | I | E | M | V | T | D | K | D | F | H | N | L | T | Q | L | E | I | L | D | L | S | G | N | C | P | R | C | Y | N | A | P | F | P | C | I | P | C | P | N | N | S | - | L | Q | I | D | K | N | T | F | K | T | L | T | K | L | R | I | L | R | L | H | S | N | S | L | T | Y | V | L | R | E | W | F | Q | N | C | K | E | L | R | V | L | D | L | S | T | N | F | L | A | R | E | I | A | F | T | Y | F | P | R | A | L | P | N | L | E | E | L | D | L | S | F | N | Y | E | L | Q | R | Y | P | A | T | L | H | L | S | S | S | F | S | S | L | K | S | L | K | V | L | R | I | R | A | F | V | F | Q | Q | L | T | L | E | D | I | S | P | L | I | H | L | K | N | L | E | V | I | D | L | G | T | N | F | I | K | I | T | N | L | S | I | L | M | E | L | K | S | F | K | I | I | N | L | S | D | N | K | I | S | S | P | S | E | S | G | Q | S | V | A | F | S | G | - | G | - | - | - | E | A | I | H | G | S | P | M | S | D | S | G | H | N | S | N | G | E | V | R | E | I | H | Y | F | R | Y | D | E | Y | A | R | S | C | K | Y | K | D | K | E | D | G | T | L | N | S | F | V | K | T | Q | C | S | Q | F | G | K | T | L | D | I | S | R | N | N | I | F | F | L | H | S | R | F | L | N | L | A | D | L | R | C | L | N | L | S | G | N | A | M | S | Q | S | L | N | G | S | E | F | T | F | L | T | N | L | Q | Y | L | D | F | S | F | N | R | L | D | L | L | Y | S | S | A | F | Q | E | L | R | S | L | V | I | L | D | I | S | N | N | N | H | Y | F | E | S | E | G | L | T | H | M | L | N | F | T | K | N | L | T | N | L | K | I | L | L | M | N | Y | N | K | I | S | T | S | T | N | T | E | L | E | S | L | S | L | E | K | L | E | F | K | G | N | R | L | D | M | L | W | R | D | G | D | T | R | Y | I | D | Y | F | K | K | L | L | N | L | R | V | L | D | I | S | H | N | N | L | N | F | I | P | Q | Q | V | F | Q | G | L | P | D | K | L | T | N | L | Y | I | N | N | N | R | L | K | - | - | I | F | S | W | E | K | L | I | I | L | Q | Y | L | E | V | L | D | L | S | S | N | S | I | S | T | V | P | P | E | L | S | - | N | C | T | K | S | L | K | T | L | L | L | R | K | N | Q | I | S | K | L | S | T | Y | F | L | K | D | A | F | S | L | K | N | L | D | L | S | F | N | N | I | Q | N | I | E | Q | T | S | I | P | D | D | V | V | D | Q | M | D | T | L | V | L | N | N | N | K | F | M | C | N | C | N | A | L | M | F | V | M | W | L | N | R | T | M | V | N | I | P | R | L | A | T | A | V | T | C | A | A | P | G | A | Q | R | G | H | P | V | I | S | L | D | L | E | L | Q | A | C | Q | H | N | Y | L | S | I | I | L | Y | I | L | L | T | S | L | V | L | S | F | I | A | L | S | I | S | S | H | L | F | L | W | D | V | W | Y | L | Y | H | F | L | Q | A | K | F | K | G | Y | R | R | L | - | S | S | P | S | T | A | Y | D | A | F | V | V | Y | D | K | K | D | P | E | V | S | E | W | V | L | K | E | L | L | V | Q | L | E | N | Q | G | D | H | P | L | Q | L | C | L | E | E | R | D | W | I | P | G | C | P | L | I | D | N | L | S | Q | S | I | H | Q | S | K | R | T | V | F | I | L | T | N | K | Y | I | K | S | G | D | F | K | T | A | F | Y | M | A | H | Q | R | L | M | D | E | R | D | D | V | I | V | L | I | F | L | E | K | V | P | - | S | H | S | K | Y | L | R | L | R | K | R | L | Y | R | R | S | V | M | E | W | P | T | N | P | Q | A | Q | Q | Y | F | W | F | S | L | R | S | V | L | V | T | D | S | Q | K | Q | Y | S | N | L | F | K | E | T | L | X | X | X | X | X | X | X | X | X | X | X | X | X | X | X | X | X | X | X | X | - | - | - | - | - | - | - | - | - | - | - | - | - | - | - | - | - | - | - | - | - | - | - | - | - | - | - | - | - | - | - | - | - | - | - | - | - | - | - | - | - | - | - | - | - | - | - | - | - | - | - | - | - | - | - | - | - | - | - | - | - | - | - | - | - | - | - | - | - | - | - | - | - | - | - | - | - | - | - | - | - | - | - | - | - | - | - | - | - | - | - | - | - | - | - | - | - | - | - | - | - | - | - | - | - | - | - | - | - | - | - | - | - | - | - | - | - | - | - | - | - | - | - | - | - | - | - | - | - | - | - | - | - | - | - | - | - | - | - | - | - | - | - | - | - | - | - | - |
| SATLR7 | M | F | F | K | H | L | I | Q | L | D | S | R | T | H | S | R | T | S | L | P | - | - | S | P | E | R | T | M | F | F | H | L | M | C | V | S | L | L | G | L | W | - | C | F | L | P | L | S | T | A | K | V | S | Y | P | K | T | L | P | C | D | V | I | E | T | N | N | G | S | M | V | T | V | D | C | T | E | K | S | L | K | N | I | P | S | D | I | P | R | D | T | T | N | L | T | L | T | I | N | H | I | H | E | L | N | S | T | S | F | R | G | L | E | N | L | T | E | I | D | M | R | C | N | C | V | P | I | K | I | G | P | K | D | R | M | C | L | Q | S | V | T | I | E | D | N | T | F | T | S | L | R | N | L | R | A | L | F | L | D | G | N | Q | L | E | S | I | P | K | G | L | P | S | N | L | M | L | L | S | L | E | V | N | H | I | S | S | I | S | K | A | N | L | S | E | I | R | N | V | Q | I | L | Y | L | G | Q | N | C | Y | F | R | N | P | C | N | Y | S | Y | S | I | E | E | G | A | F | L | Q | L | S | N | L | T | L | L | S | L | K | S | N | N | L | S | F | I | P | P | H | L | P | T | S | L | K | E | L | Y | L | Y | N | N | N | I | L | K | V | T | D | E | D | F | K | N | L | T | N | L | E | I | L | D | I | S | G | N | C | P | R | C | Y | N | A | P | F | P | C | N | P | C | P | N | D | S | P | L | N | V | S | K | N | A | F | K | M | L | T | K | L | K | T | L | R | M | H | S | N | S | L | T | F | V | L | A | E | W | F | A | S | T | T | E | L | K | Q | L | D | L | S | S | N | F | L | G | R | A | I | G | D | T | T | F | P | R | Y | L | G | K | L | E | E | L | D | L | S | F | N | Y | D | L | Q | R | Y | P | Q | T | L | R | L | S | C | S | F | S | D | L | N | S | L | K | I | L | R | L | R | G | F | V | F | Q | Q | L | Q | P | E | S | I | E | H | L | K | P | L | R | G | L | E | V | L | D | L | G | T | N | F | I | K | M | A | N | L | S | S | L | M | E | L | K | S | F | K | I | I | S | L | S | D | N | K | I | S | S | P | S | D | G | E | V | P | V | G | V | S | G | G | G | - | - | - | E | P | L | D | W | S | P | M | S | A | A | D | Q | Y | Q | N | G | E | A | R | E | I | H | Y | F | R | Y | D | E | Y | A | R | S | C | K | Y | R | D | K | E | L | G | V | I | T | T | L | V | K | G | K | C | S | H | F | G | K | T | L | D | V | S | R | N | N | I | F | F | L | H | S | R | F | L | N | L | G | E | L | R | C | L | N | L | S | G | N | A | M | S | Q | S | L | N | G | S | E | F | T | Y | L | T | N | L | K | Y | L | D | F | S | S | N | R | L | D | L | L | H | S | T | A | F | Q | E | L | K | S | L | V | V | L | D | I | S | N | N | N | H | Y | F | E | S | E | G | L | T | H | M | L | N | F | T | R | N | L | K | N | L | K | I | L | L | M | N | N | N | K | I | S | T | S | T | N | T | E | L | E | S | Q | S | L | E | R | L | E | F | I | D | N | R | L | D | I | L | W | R | D | G | N | D | K | Y | F | N | Y | F | K | K | L | I | N | L | S | V | L | D | I | S | S | N | N | L | N | F | I | P | K | D | V | F | N | G | F | P | D | K | L | S | E | L | Y | I | K | D | N | R | L | N | - | - | Y | F | S | W | G | Q | L | Q | L | L | H | S | L | K | V | L | D | L | S | G | N | C | L | T | T | V | P | R | M | L | S | - | N | C | T | T | S | L | E | K | F | I | L | H | N | N | H | I | L | K | L | T | P | D | F | L | K | D | A | F | N | L | K | H | L | D | L | S | N | N | I | I | K | H | I | E | K | S | S | F | P | D | N | V | V | N | K | M | D | T | L | L | L | H | K | N | A | F | Q | C | N | C | N | A | T | W | F | V | M | W | L | N | S | T | N | V | T | I | P | R | L | G | T | E | V | T | C | A | S | P | G | A | Q | K | D | R | P | V | V | S | V | D | - | - | L | L | A | C | Q | H | N | Y | L | S | I | I | F | C | I | L | M | T | S | L | V | L | G | F | L | T | L | S | I | S | S | H | L | F | L | W | D | V | W | Y | I | Y | Y | F | C | R | A | K | L | K | G | Y | I | R | L | - | Q | S | Q | S | S | I | Y | D | A | F | V | I | Y | D | K | E | D | T | A | V | T | E | W | V | M | K | E | M | C | V | H | L | E | E | R | G | D | R | P | L | T | L | C | L | E | E | R | D | W | I | P | G | C | P | L | I | D | N | L | C | Q | S | I | H | R | S | K | R | T | V | F | I | L | T | S | R | Y | I | K | S | G | N | F | K | T | A | F | Y | M | A | H | Q | R | L | M | D | E | K | D | D | V | I | V | L | I | F | L | E | K | V | Q | - | C | N | S | K | Y | L | R | L | R | K | R | L | Y | K | R | S | V | L | E | W | P | T | N | P | L | A | Q | P | Y | F | W | F | S | L | R | S | V | L | A | T | E | S | H | K | Q | Y | N | N | L | F | K | E | T | L | - | - | - | - | - | - | - | - | - | - | - | - | - | - | - | - | - | - | - | - | - | - | - | - | - | - | - | - | - | - | - | - | - | - | - | - | - | - | - | - | - | - | - | - | - | - | - | - | - | - | - | - | - | - | - | - | - | - | - | - | - | - | - | - | - | - | - | - | - | - | - | - | - | - | - | - | - | - | - | - | - | - | - | - | - | - | - | - | - | - | - | - | - | - | - | - | - | - | - | - | - | - | - | - | - | - | - | - | - | - | - | - | - | - | - | - | - | - | - | - | - | - | - | - | - | - | - | - | - | - | - | - | - | - | - | - | - | - | - | - | - | - | - | - | - | - | - | - | - | - | - | - | - | - | - | - | - | - | - | - | - | - | - | - | - | - | - | - |
| LCTLR7 | - | - | - | - | - | - | - | - | - | - | - | - | - | - | - | - | - | M | L | Y | - | - | H | L | R | T | A | C | V | S | V | Q | M | C | V | A | L | L | V | L | W | - | C | C | R | C | I | S | T | A | D | V | S | Y | P | K | T | L | P | C | D | V | S | E | E | N | N | G | S | V | V | K | V | D | C | T | E | R | S | L | K | E | I | P | Y | G | I | P | R | D | T | T | N | L | T | L | T | I | N | H | I | P | E | L | N | S | S | S | F | H | G | L | E | N | L | T | E | I | D | M | R | C | N | C | V | P | I | K | I | G | P | K | D | H | M | C | T | Q | S | V | T | I | E | E | N | T | F | T | S | L | K | S | L | R | A | L | Y | L | D | G | N | Q | L | I | S | I | P | K | G | L | P | S | N | L | I | L | L | S | L | E | V | N | H | I | Y | N | I | S | K | A | N | L | S | E | L | R | N | V | G | L | L | Y | L | G | Q | N | C | Y | Y | R | N | P | C | N | N | S | Y | S | I | E | N | G | T | F | S | Q | L | I | N | L | T | L | L | S | L | K | S | N | N | L | S | F | I | P | P | Q | L | P | T | S | L | K | E | L | Y | L | Y | N | N | N | I | Q | N | V | T | N | E | D | F | K | S | L | T | N | L | E | I | L | D | I | S | G | N | C | P | R | C | Y | N | A | P | F | P | C | T | P | C | P | N | N | S | P | L | K | I | S | K | M | A | F | K | M | L | T | K | L | K | T | L | R | L | H | S | N | S | L | T | F | V | Q | S | E | W | F | T | N | T | K | E | L | K | V | L | D | L | S | T | N | F | L | A | S | D | I | G | D | T | R | F | P | H | F | L | G | K | L | E | E | L | D | L | S | F | N | Y | E | L | Q | R | Y | P | Q | T | L | T | L | S | R | S | F | S | S | L | R | S | L | K | I | L | R | L | K | G | F | V | F | Q | Q | L | R | P | E | S | I | D | P | L | K | P | L | P | N | L | E | V | L | D | L | G | T | N | F | I | K | M | A | N | L | S | I | L | M | E | L | K | S | F | K | I | I | S | L | S | D | N | K | I | S | S | P | S | D | G | Q | D | T | V | G | F | S | G | - | R | - | - | - | E | P | L | D | W | S | P | M | S | A | A | T | Q | Y | Q | - | R | E | V | R | E | I | H | Y | F | R | Y | D | E | Y | A | R | S | C | K | Y | K | D | K | E | L | G | V | I | T | S | F | V | K | R | K | C | S | R | F | G | K | T | L | D | V | S | R | N | N | I | F | F | L | H | S | R | F | L | N | L | R | E | L | R | C | L | N | L | S | G | N | L | M | S | Q | S | L | N | G | S | E | F | T | Y | L | T | N | L | K | Y | L | D | F | S | S | N | R | L | D | L | L | Y | S | T | A | F | Q | E | L | K | S | L | V | I | L | D | I | S | N | N | N | H | Y | F | E | S | E | G | L | T | H | M | L | N | F | T | R | N | L | K | N | L | K | I | L | L | M | N | H | N | K | I | S | T | S | T | N | T | E | L | E | S | Q | S | L | E | R | L | E | F | K | D | N | R | L | D | V | L | W | R | D | G | D | T | R | Y | F | N | Y | F | K | K | L | F | N | L | T | F | L | D | I | S | Q | N | N | L | N | F | I | P | E | E | V | F | S | S | L | P | D | K | L | S | E | L | H | I | K | N | N | K | L | K | - | - | S | F | H | W | E | K | L | Q | F | L | H | S | L | K | I | L | D | L | S | G | N | S | L | T | T | V | P | E | K | L | S | - | N | C | T | K | S | L | K | K | L | I | L | H | R | N | H | I | L | K | L | A | P | D | F | L | T | D | A | N | N | L | K | Y | L | D | L | S | F | N | D | I | Q | Y | I | E | K | S | S | I | P | D | N | I | D | E | N | M | K | K | L | L | L | H | N | N | K | F | L | C | T | C | N | A | T | W | F | V | T | W | L | N | K | T | N | I | T | I | P | R | L | A | T | D | V | T | C | D | A | P | G | A | Q | K | G | H | P | V | I | S | V | D | - | - | L | L | A | C | Q | H | N | Y | L | S | I | I | L | Y | I | L | M | T | S | L | I | L | S | L | L | T | L | S | I | S | S | H | L | F | L | W | D | V | W | Y | I | Y | H | F | C | R | A | K | L | K | G | Y | N | R | L | - | Y | S | Q | S | C | V | Y | D | A | F | V | I | Y | D | K | E | D | P | A | V | S | E | W | V | M | K | E | M | C | V | H | L | E | D | R | E | D | H | C | L | R | L | C | L | E | E | R | D | W | I | P | G | C | P | L | I | D | N | L | S | Q | S | I | H | R | S | K | R | T | V | F | I | L | T | K | K | Y | I | K | S | G | N | F | K | T | A | F | Y | I | A | H | Q | R | L | M | D | E | K | N | D | V | I | V | L | I | F | L | E | K | V | P | - | S | N | S | K | Y | L | K | L | R | K | R | L | Y | K | R | S | V | L | E | W | P | T | N | P | Q | A | Q | P | Y | F | W | F | S | L | R | N | V | L | A | T | E | G | H | K | Q | Y | S | N | L | F | K | E | T | L | X | X | X | X | X | X | X | X | X | X | X | X | X | X | X | X | X | X | X | - | - | - | - | - | - | - | - | - | - | - | - | - | - | - | - | - | - | - | - | - | - | - | - | - | - | - | - | - | - | - | - | - | - | - | - | - | - | - | - | - | - | - | - | - | - | - | - | - | - | - | - | - | - | - | - | - | - | - | - | - | - | - | - | - | - | - | - | - | - | - | - | - | - | - | - | - | - | - | - | - | - | - | - | - | - | - | - | - | - | - | - | - | - | - | - | - | - | - | - | - | - | - | - | - | - | - | - | - | - | - | - | - | - | - | - | - | - | - | - | - | - | - | - | - | - | - | - | - | - | - | - | - | - | - | - | - | - | - | - | - | - | - | - | - | - | - | - | - |
| TOTLR7 | - | - | - | - | - | - | - | - | - | - | - | - | - | - | - | - | - | - | - | - | - | - | - | - | - | - | - | - | - | - | - | - | M | C | A | A | L | L | A | L | W | - | C | C | L | C | I | S | T | A | T | I | S | Y | P | K | T | L | P | C | D | V | S | E | V | T | N | G | S | V | V | K | V | D | C | T | E | R | S | L | K | E | V | P | L | G | I | P | R | E | T | T | N | L | T | L | T | I | N | H | I | P | K | L | N | S | S | S | F | H | G | L | E | N | L | T | E | I | D | M | R | C | N | C | V | P | I | K | I | G | P | K | D | R | M | C | T | E | S | V | T | I | E | E | N | T | F | T | S | L | R | S | L | R | A | L | Y | L | D | G | N | Q | L | Y | S | I | P | K | G | L | P | S | N | L | V | L | L | S | L | E | V | N | H | I | C | S | I | S | K | A | N | L | S | V | L | R | N | V | E | M | L | Y | L | G | Q | N | C | Y | Y | R | N | P | C | N | N | S | Y | G | I | E | N | G | A | F | S | D | L | N | N | L | T | L | L | S | L | K | S | N | N | L | S | F | I | P | H | Q | L | P | T | C | L | K | E | L | Y | L | Y | N | N | N | I | E | K | V | T | D | E | D | F | K | N | L | T | N | L | E | I | L | D | I | S | G | N | C | P | R | C | Y | N | A | P | F | P | C | T | P | C | P | N | N | S | P | L | K | I | S | E | A | A | F | K | M | L | S | K | L | K | T | L | R | L | H | R | T | - | T | E | C | V | S | S | E | W | F | T | N | T | T | E | L | R | V | L | D | L | S | S | N | F | L | A | K | E | I | G | A | T | S | F | P | H | F | L | G | K | L | E | E | L | D | L | S | F | N | Y | E | L | Q | Q | Y | P | R | T | L | T | L | N | R | S | F | S | D | L | K | S | L | K | I | L | R | L | K | G | F | V | F | Q | E | L | K | L | G | S | I | A | P | L | K | P | L | K | H | L | E | V | L | D | L | G | T | N | F | I | K | I | T | N | L | S | I | L | M | E | L | K | S | F | K | I | I | S | L | S | D | N | K | I | S | S | P | S | E | N | Q | D | A | V | G | F | S | G | - | G | - | - | - | E | P | L | N | W | S | P | M | S | A | A | A | E | Y | Q | S | K | E | V | R | E | I | H | Y | F | R | Y | D | E | Y | A | R | S | C | K | Y | K | D | K | E | R | G | V | V | T | S | F | V | K | R | Q | C | S | H | F | G | K | T | L | D | V | S | R | N | N | I | F | F | L | H | S | R | F | L | N | L | R | E | L | R | C | L | N | L | S | G | N | L | M | S | Q | S | L | N | G | S | E | F | T | H | L | S | N | L | K | Y | L | D | F | S | S | N | R | L | D | L | L | Y | S | T | A | F | K | E | L | T | S | L | V | I | L | D | I | S | N | N | N | H | Y | F | E | S | E | G | L | T | H | M | L | N | F | T | R | N | L | N | N | L | K | I | L | M | M | N | H | N | K | I | S | T | S | T | N | T | E | L | E | S | Q | S | L | E | K | L | E | F | R | D | N | R | L | D | V | L | W | R | D | G | D | T | R | Y | F | N | F | F | K | K | L | L | N | L | T | V | L | D | I | S | H | N | N | L | H | F | I | P | A | E | V | S | A | V | - | C | Q | T | A | H | R | L | Y | I | K | N | N | K | L | T | - | - | S | F | K | W | E | K | L | Q | L | L | R | S | L | E | V | L | D | L | S | G | N | S | L | N | T | V | P | P | M | L | S | - | D | C | S | K | S | L | K | K | L | I | L | H | K | N | Q | I | L | K | L | E | P | D | F | L | K | G | A | Y | N | L | K | Y | L | D | L | S | F | N | Y | I | K | H | I | E | Q | S | N | F | P | D | D | V | V | N | Q | M | E | K | L | L | L | H | K | N | S | F | S | C | T | C | N | A | T | W | F | V | T | W | L | N | R | T | T | V | T | I | P | R | L | A | T | D | V | T | C | A | T | P | G | A | Q | I | G | H | P | V | I | S | V | D | - | - | L | L | A | C | Q | H | H | Y | L | S | I | F | L | Y | T | L | M | T | S | I | V | I | S | F | L | T | L | S | I | S | S | H | L | F | L | W | D | V | W | Y | I | Y | Y | F | C | R | A | K | L | K | G | Y | S | R | L | - | Y | S | Q | S | S | V | Y | D | A | F | V | I | Y | D | K | E | D | T | A | V | S | E | W | V | M | N | E | M | C | I | H | L | E | D | R | G | D | H | C | F | T | L | C | L | E | E | R | D | W | I | P | G | C | P | L | I | D | N | L | S | Q | S | I | H | R | S | K | R | T | V | F | I | L | T | N | K | Y | I | K | S | G | N | F | K | T | A | F | Y | M | A | N | Q | R | L | M | D | E | K | N | D | V | I | I | L | I | F | L | E | K | V | R | - | C | N | S | K | Y | L | R | L | R | K | R | L | Y | K | R | S | V | L | E | W | P | T | N | P | Q | A | Q | P | Y | F | W | F | S | L | R | S | V | L | A | T | E | S | H | K | Q | Y | N | N | L | F | K | E | T | L | X | X | X | X | X | X | X | X | X | X | X | X | X | X | X | X | X | X | X | X | X | X | X | X | X | X | - | - | X | X | X | X | X | X | X | - | - | - | - | - | - | - | - | - | - | - | - | - | - | - | - | - | - | - | - | - | - | - | - | - | - | - | - | - | - | - | - | - | - | - | - | - | - | - | - | - | - | - | - | - | - | - | - | - | - | - | - | - | - | - | - | - | - | - | - | - | - | - | - | - | - | - | - | - | - | - | - | - | - | - | - | - | - | - | - | - | - | - | - | - | - | - | - | - | - | - | - | - | - | - | - | - | - | - | - | - | - | - | - | - | - | - | - | - | - | - | - | - | - | - | - | - | - | - | - | - | - | - | - | - | - | - | - | - | - | - | - | - |
| SDTLR7 | - | - | - | - | - | - | - | - | - | - | - | - | - | - | - | - | - | - | - | - | - | - | - | - | - | - | - | - | - | - | - | - | M | C | V | A | L | L | A | L | W | - | C | C | L | C | I | S | T | A | T | V | S | Y | P | K | T | L | P | C | D | V | S | D | L | - | N | G | S | A | V | I | V | D | C | T | E | R | S | L | K | E | I | P | G | G | I | P | R | E | T | T | N | L | T | L | T | I | N | H | I | P | G | L | N | S | S | S | F | H | G | L | E | N | L | T | E | I | D | M | R | C | N | C | V | P | I | K | I | G | P | K | D | H | V | C | T | K | S | V | T | I | K | E | N | T | F | T | S | L | R | S | L | R | A | L | Y | L | D | G | N | Q | L | S | S | I | P | K | G | L | P | S | N | L | V | L | L | S | L | E | V | N | N | I | C | T | I | F | K | A | N | L | S | E | L | R | N | V | E | A | L | Y | L | G | Q | N | C | Y | Y | R | N | P | C | N | K | S | Y | E | I | E | N | G | T | F | S | H | L | Y | N | L | T | L | L | S | L | K | S | N | N | L | S | F | I | P | P | Q | L | P | T | S | L | K | E | L | Y | L | Y | N | N | N | I | Q | K | V | T | D | E | D | F | K | N | L | T | N | L | E | I | L | D | I | S | G | N | C | P | R | C | Y | N | A | P | F | P | C | N | P | C | P | N | N | S | P | L | K | I | S | E | A | A | F | K | M | L | A | K | L | R | T | L | R | L | H | S | N | S | L | R | C | V | S | S | E | W | F | T | N | T | T | E | L | R | V | L | D | L | S | S | N | F | L | G | K | E | I | G | T | T | S | F | P | L | F | L | G | K | L | E | E | L | D | L | S | F | N | Y | E | L | Q | E | Y | P | R | T | L | I | L | N | R | S | F | S | N | L | R | S | L | K | I | L | R | L | K | G | F | V | F | Q | E | L | K | P | G | S | I | A | P | L | K | P | L | A | H | L | E | V | L | D | L | G | T | N | F | I | K | I | T | N | L | S | I | L | M | E | L | K | S | F | K | I | I | S | L | S | D | N | K | I | S | S | P | S | E | G | Q | D | A | V | G | F | S | G | - | A | - | - | - | E | P | L | N | W | S | P | M | S | A | A | A | E | Y | Q | S | K | E | V | R | E | I | H | Y | F | R | Y | D | E | Y | A | R | S | C | K | H | K | D | K | E | L | G | V | V | T | S | F | V | K | R | Q | C | S | H | F | G | K | T | L | D | V | S | R | N | N | I | F | F | L | H | S | R | F | L | N | L | G | E | L | R | C | L | N | L | S | G | N | L | M | S | Q | S | L | N | G | S | E | F | T | Y | L | T | N | L | K | Y | L | D | F | S | W | N | R | L | D | L | L | Y | S | T | A | F | Q | E | L | K | S | L | V | V | L | D | I | S | N | N | N | H | Y | F | E | S | E | G | L | T | H | M | L | N | F | T | R | N | L | K | N | L | K | I | L | L | M | N | H | N | K | I | S | T | S | T | N | T | E | L | E | S | Q | S | L | E | R | L | E | F | R | D | N | R | L | D | I | L | W | R | D | G | D | K | R | Y | Y | N | Y | F | K | K | L | L | N | L | T | V | L | D | I | S | Q | N | N | L | N | F | I | P | E | E | V | F | S | S | L | P | D | N | L | A | E | L | H | I | K | N | N | K | L | K | - | - | L | F | K | W | E | K | L | Q | L | I | H | S | L | K | V | L | D | L | S | G | N | S | L | T | T | V | P | H | M | L | S | - | D | C | S | K | S | L | E | K | L | I | L | H | K | N | Q | I | L | K | L | E | Q | D | F | L | K | G | A | Y | N | L | K | Y | L | D | L | S | F | N | Y | I | R | H | I | E | Q | S | N | F | P | D | D | V | V | N | Q | M | D | K | L | L | L | H | N | N | R | F | S | C | T | C | N | A | T | W | F | V | T | W | L | N | R | T | T | V | T | I | P | R | L | A | T | D | V | T | C | A | A | P | G | A | Q | I | G | N | P | V | I | S | V | D | - | - | L | L | A | C | Q | H | H | Y | L | T | I | I | L | Y | I | L | M | T | S | L | V | I | S | F | L | T | V | S | I | S | S | H | L | F | L | W | D | V | W | Y | I | Y | H | F | C | R | A | K | L | K | N | Y | S | R | L | - | Y | S | Q | S | S | V | Y | D | A | F | V | I | Y | D | K | E | D | P | A | V | S | E | W | V | M | N | E | M | C | V | H | L | E | D | R | G | D | H | C | L | T | L | C | L | E | E | R | D | W | I | P | G | C | P | L | I | D | N | L | S | Q | S | I | H | R | S | K | R | T | L | F | I | L | T | N | K | Y | I | K | S | G | N | F | K | T | A | F | Y | I | A | N | Q | R | L | M | D | E | K | N | D | V | I | V | L | I | F | L | E | K | V | P | - | C | N | S | K | Y | L | R | L | R | K | R | L | Y | K | R | S | V | L | E | W | P | T | N | P | Q | A | Q | P | Y | F | W | F | S | L | R | S | V | L | A | T | E | S | H | K | Q | Y | N | N | L | F | K | E | T | L | X | X | X | X | X | X | X | X | X | X | X | X | X | X | X | X | X | X | X | X | X | X | X | X | X | - | - | - | X | X | X | X | X | X | X | - | - | - | - | - | - | - | - | - | - | - | - | - | - | - | - | - | - | - | - | - | - | - | - | - | - | - | - | - | - | - | - | - | - | - | - | - | - | - | - | - | - | - | - | - | - | - | - | - | - | - | - | - | - | - | - | - | - | - | - | - | - | - | - | - | - | - | - | - | - | - | - | - | - | - | - | - | - | - | - | - | - | - | - | - | - | - | - | - | - | - | - | - | - | - | - | - | - | - | - | - | - | - | - | - | - | - | - | - | - | - | - | - | - | - | - | - | - | - | - | - | - | - | - | - | - | - | - | - | - | - | - | - |
| TRTLR7 | - | - | - | - | - | - | - | - | - | - | - | - | - | - | - | - | - | - | - | - | - | - | - | - | - | - | - | - | - | - | - | M | F | L | L | L | I | L | G | L | W | - | C | P | L | C | I | L | T | A | N | V | S | Y | P | K | T | L | P | C | D | V | T | - | - | N | N | G | S | V | V | N | V | D | C | T | E | R | S | L | K | G | V | P | V | G | I | P | R | D | V | T | N | L | T | L | T | I | N | H | I | P | N | F | N | S | S | S | F | Q | G | L | D | N | L | R | E | I | D | M | R | C | N | C | V | P | V | K | I | G | P | K | D | R | I | C | T | E | S | V | T | I | E | E | N | T | F | T | S | L | R | N | L | Q | S | L | Y | L | D | G | N | Q | L | Y | I | I | P | E | G | L | P | S | S | L | I | L | L | S | L | E | V | N | H | I | Y | Y | I | S | K | A | N | L | S | E | I | R | N | I | E | S | L | Y | L | G | Q | N | C | Y | Y | R | N | P | C | N | F | S | Y | G | I | E | E | G | A | F | L | E | L | H | N | L | K | L | L | S | L | K | S | N | N | L | S | F | I | P | H | H | L | P | S | S | L | K | E | L | Y | L | Y | N | N | N | F | Q | Q | V | T | D | E | D | F | K | N | L | T | N | L | E | I | L | D | I | S | G | N | C | P | R | C | Y | N | V | P | F | P | C | N | P | C | P | N | N | S | P | L | Q | I | S | K | S | A | F | K | T | L | T | K | L | K | T | L | R | M | H | S | N | S | L | R | Y | V | V | P | E | W | F | A | S | T | K | E | L | R | V | L | D | L | S | S | N | H | L | A | R | D | I | G | V | T | L | F | P | H | H | L | G | K | L | Q | E | L | D | L | S | F | N | Y | E | L | Q | R | Y | P | R | T | L | R | L | S | C | S | F | K | S | L | N | S | L | R | I | L | R | L | Q | G | F | V | F | Q | Q | L | K | P | E | S | I | T | P | L | K | S | L | T | N | L | E | V | L | D | L | G | T | N | F | I | K | M | T | N | L | S | I | L | M | E | L | K | S | F | K | I | I | N | L | S | N | N | R | I | S | S | P | S | D | G | Q | A | A | V | G | F | S | G | - | G | - | - | - | Q | A | W | E | W | S | P | M | L | P | A | D | Q | Y | Q | N | E | E | V | R | E | I | H | Y | F | R | Y | D | E | Y | A | R | S | C | K | Y | K | D | K | E | L | G | V | I | A | S | F | V | K | R | E | C | S | Q | F | G | K | T | L | D | V | S | R | N | N | I | F | F | L | H | S | R | F | L | N | L | R | E | L | K | C | L | N | L | S | G | N | A | M | S | Q | S | L | N | G | S | E | F | T | Y | L | T | G | L | Q | Y | L | D | F | S | S | N | R | L | D | L | L | Y | S | T | A | F | Q | E | L | K | N | L | V | I | L | D | I | S | N | N | N | H | Y | F | E | S | E | G | L | T | H | M | L | N | F | T | Q | N | L | K | N | L | K | I | L | M | M | N | H | N | K | I | S | T | S | T | N | T | E | L | E | S | Q | S | L | E | R | L | E | F | R | G | N | R | L | D | M | L | W | R | D | G | N | T | R | Y | L | N | Y | F | K | K | L | S | N | L | A | V | L | D | I | S | H | N | N | L | N | S | I | Q | Q | Q | V | F | S | G | L | P | D | T | L | S | E | L | Y | V | N | N | N | K | L | K | - | - | S | F | A | W | G | E | L | S | L | L | P | S | L | L | V | L | D | L | S | G | N | Y | L | T | T | V | P | H | M | L | S | - | N | C | S | K | S | L | K | K | L | I | L | H | K | N | E | I | L | K | L | T | P | D | F | L | K | Y | A | Y | N | L | K | Y | L | D | L | S | F | N | R | I | G | Q | I | E | K | S | N | F | P | D | D | V | V | N | G | M | D | M | L | L | L | H | K | N | R | F | Q | C | S | C | S | A | I | W | F | V | M | W | L | N | Q | T | T | V | T | I | P | R | L | A | T | D | V | T | C | A | S | P | G | A | Q | R | G | H | P | V | I | S | V | D | - | - | L | L | A | C | Q | H | N | F | L | S | I | I | L | Y | F | L | M | T | S | F | T | L | I | F | F | T | L | A | I | S | S | H | L | F | L | W | D | V | W | Y | I | Y | H | F | C | R | A | K | L | K | G | Y | S | Q | L | - | S | S | Q | S | S | V | Y | D | A | F | V | M | Y | D | K | E | D | S | A | V | S | E | W | V | M | K | E | M | C | V | Q | L | E | G | R | E | D | R | R | L | T | L | C | L | E | E | R | D | W | I | P | G | T | P | V | I | D | N | L | S | Q | S | I | H | R | S | K | R | T | V | F | I | L | T | N | T | Y | I | K | S | G | S | F | K | T | A | F | Y | M | A | H | Q | R | L | M | D | E | K | N | D | V | I | V | L | I | F | L | E | N | I | P | - | Y | N | S | R | Y | L | R | L | R | R | R | L | Y | R | R | S | V | L | E | W | P | T | N | P | Q | A | R | P | Y | F | W | F | S | V | R | S | V | L | A | T | E | S | H | K | Q | Y | N | N | L | F | K | E | T | L | X | X | X | X | X | X | X | X | X | X | X | X | X | X | X | X | X | X | X | X | X | X | X | X | X | X | - | - | X | X | X | X | X | X | - | - | - | - | - | - | - | - | - | - | - | - | - | - | - | - | - | - | - | - | - | - | - | - | - | - | - | - | - | - | - | - | - | - | - | - | - | - | - | - | - | - | - | - | - | - | - | - | - | - | - | - | - | - | - | - | - | - | - | - | - | - | - | - | - | - | - | - | - | - | - | - | - | - | - | - | - | - | - | - | - | - | - | - | - | - | - | - | - | - | - | - | - | - | - | - | - | - | - | - | - | - | - | - | - | - | - | - | - | - | - | - | - | - | - | - | - | - | - | - | - | - | - | - | - | - | - | - | - | - | - | - | - | - |
| SMTLR7 | - | - | - | - | - | - | - | - | - | - | - | - | - | - | - | - | - | - | - | - | - | - | - | - | - | - | - | - | - | - | - | - | M | C | A | A | A | L | A | L | W | - | C | C | L | C | A | S | T | G | S | I | S | Y | P | K | T | L | P | C | D | V | S | E | A | N | N | G | S | M | V | K | V | D | C | T | D | R | S | L | K | E | I | P | Q | G | I | P | R | D | T | T | N | L | T | L | T | I | N | H | I | P | N | L | N | S | S | S | F | Y | G | L | K | N | L | T | E | I | D | M | R | C | N | C | V | P | I | K | I | G | P | K | D | H | M | C | T | K | S | V | T | I | E | E | N | T | F | T | S | M | R | G | L | R | A | L | Y | L | D | G | N | Q | I | Y | T | I | P | K | G | L | P | S | N | L | I | L | V | S | L | E | V | N | H | I | Y | T | I | S | K | A | N | L | F | G | P | N | L | H | R | M | L | Y | L | G | Q | N | C | Y | Y | R | N | P | C | N | M | S | Y | E | I | E | N | G | S | F | S | Q | L | N | N | L | T | L | L | S | L | K | S | N | N | L | S | F | I | P | H | H | L | P | T | S | L | K | E | L | Y | L | Y | N | N | N | I | Q | E | V | T | D | E | D | F | K | N | L | T | N | L | E | I | L | D | I | S | G | N | C | P | R | C | Y | N | A | P | F | P | C | N | P | C | P | H | N | S | Q | L | K | I | S | K | K | A | F | Q | M | L | T | K | L | K | T | L | R | L | H | S | N | S | L | T | R | V | P | Y | E | W | F | A | N | T | T | E | L | K | V | L | D | L | S | S | N | F | L | A | R | E | I | G | V | T | R | F | P | S | F | L | G | K | L | E | E | L | D | L | S | F | N | Y | E | L | Q | R | Y | P | Q | T | L | R | L | S | Q | T | F | S | S | L | K | S | L | K | I | L | R | L | K | G | Y | V | F | Q | Q | L | K | A | A | S | I | A | P | L | I | S | L | A | Y | L | E | V | V | D | L | G | T | N | F | I | K | M | T | N | F | S | I | L | M | E | L | K | S | F | K | I | I | S | L | S | D | N | K | I | T | N | P | S | D | G | Q | D | T | V | G | F | T | G | - | G | - | - | - | E | P | L | H | W | S | P | M | S | A | A | A | Q | Y | E | S | K | E | V | R | E | I | H | Y | F | R | Y | D | E | Y | A | R | S | C | K | H | K | D | K | E | L | G | V | V | T | S | F | V | K | T | E | C | S | Q | F | G | K | T | L | D | V | S | R | N | N | I | F | F | L | H | S | K | F | L | N | L | G | E | L | R | C | L | N | L | S | G | N | A | M | S | Q | S | L | N | G | S | E | F | T | F | L | T | N | L | Q | Y | L | D | F | S | M | N | R | L | D | L | L | Y | S | T | A | F | Q | E | L | R | S | L | V | V | L | D | I | S | H | N | N | H | Y | F | E | S | E | G | L | T | H | M | L | N | F | T | R | N | L | K | N | L | K | T | L | L | M | N | H | N | K | I | S | T | S | T | N | T | E | L | E | S | Q | S | L | E | R | L | E | F | R | D | N | R | L | D | M | L | W | R | D | G | D | N | R | Y | L | N | Y | F | R | K | L | H | N | L | T | V | L | D | I | S | H | N | N | L | N | F | I | P | D | D | V | F | S | S | L | P | D | K | L | S | E | L | Y | I | K | N | N | R | L | K | - | - | S | F | A | W | E | N | L | Q | L | L | R | S | L | Q | V | L | D | L | S | G | N | S | L | D | T | V | P | V | L | - | - | S | H | C | T | K | S | L | K | K | L | I | L | H | K | N | Q | I | V | K | L | A | Q | D | F | L | R | D | A | S | N | L | K | Y | L | D | L | S | F | N | R | I | Q | H | I | E | K | S | S | F | P | N | D | I | V | D | Q | M | D | M | L | L | L | H | N | N | R | F | M | C | T | C | N | A | T | W | F | V | K | W | L | N | E | T | R | V | I | I | P | R | L | A | T | D | V | T | C | A | A | P | G | A | Q | R | G | H | P | V | I | S | V | D | - | - | L | L | A | C | Q | H | N | Y | L | S | I | I | L | Y | T | L | M | T | S | L | I | L | S | F | L | I | L | F | I | S | S | H | L | F | L | W | D | V | W | Y | I | Y | H | F | C | R | A | K | V | K | G | Y | S | R | L | - | Y | S | Q | S | S | V | Y | D | A | F | V | I | Y | D | K | E | D | H | A | V | S | E | W | V | L | K | E | M | C | V | H | L | E | A | R | G | D | H | L | L | T | L | C | L | E | E | R | D | W | L | P | G | C | P | L | I | D | N | L | C | Q | S | I | H | R | S | K | R | T | V | F | I | L | T | D | K | Y | I | K | S | G | N | F | K | T | A | F | Y | M | A | H | Q | R | L | M | D | E | K | N | D | V | I | V | L | I | F | L | E | K | V | P | - | C | N | S | R | Y | L | R | L | R | K | R | L | Y | K | R | S | V | L | E | W | P | T | N | A | Q | A | Q | P | Y | F | W | F | S | L | R | S | V | L | A | T | E | S | H | K | Q | Y | N | N | L | F | K | E | T | L | X | X | X | X | X | X | X | X | X | X | X | X | X | X | X | X | X | X | X | X | X | X | X | X | X | X | - | - | X | X | X | X | X | X | - | - | - | - | - | - | - | - | - | - | - | - | - | - | - | - | - | - | - | - | - | - | - | - | - | - | - | - | - | - | - | - | - | - | - | - | - | - | - | - | - | - | - | - | - | - | - | - | - | - | - | - | - | - | - | - | - | - | - | - | - | - | - | - | - | - | - | - | - | - | - | - | - | - | - | - | - | - | - | - | - | - | - | - | - | - | - | - | - | - | - | - | - | - | - | - | - | - | - | - | - | - | - | - | - | - | - | - | - | - | - | - | - | - | - | - | - | - | - | - | - | - | - | - | - | - | - | - | - | - | - | - | - | - |
| ECTLR7 | - | - | - | - | - | - | - | - | - | - | - | - | - | - | - | - | - | - | - | - | - | - | - | - | - | - | - | M | F | F | H | Q | I | Y | V | A | L | L | G | L | W | - | C | C | L | S | T | S | T | A | S | V | S | Y | P | K | T | L | P | C | D | V | S | E | A | N | N | G | S | M | V | T | V | D | C | T | E | R | S | L | K | D | I | P | R | G | I | P | G | D | T | T | N | L | T | L | T | I | N | H | I | P | K | L | N | S | T | S | F | H | G | L | E | N | L | T | E | I | D | M | G | C | N | C | V | P | I | K | I | G | P | K | D | R | M | C | N | E | S | V | I | I | E | E | N | T | F | T | S | L | T | N | L | R | A | L | Y | L | D | G | N | Q | L | S | S | I | P | K | G | L | P | P | N | L | I | L | L | S | L | E | V | N | H | I | Y | N | I | S | K | A | N | L | S | D | I | R | N | I | E | V | L | Y | L | G | Q | N | C | Y | Y | R | N | P | C | D | N | N | Y | T | I | E | D | D | A | F | L | Q | L | N | N | L | K | L | L | S | L | K | S | N | N | L | S | F | I | P | H | Q | L | P | T | S | L | K | E | L | Y | L | Y | N | N | K | I | Q | E | V | T | D | E | D | F | K | N | L | T | N | L | E | I | L | D | I | S | G | N | C | P | R | C | Y | N | A | P | F | P | C | I | P | C | P | N | D | S | P | M | K | I | S | R | D | A | F | K | M | L | T | K | L | K | T | L | R | L | H | S | N | S | L | T | Y | V | L | P | K | W | F | I | N | M | T | D | L | R | V | L | D | L | S | S | N | F | L | A | T | D | I | G | Y | T | I | F | P | Q | F | L | G | K | L | E | E | L | D | L | S | F | N | Y | E | L | Q | R | Y | P | Q | T | L | R | L | S | C | S | F | S | L | L | K | S | L | R | I | L | R | L | K | G | Y | V | F | Q | Q | L | K | P | E | S | I | A | P | L | I | K | L | T | K | L | E | V | V | D | L | G | T | N | F | I | K | M | A | N | L | S | I | L | M | E | L | K | S | F | Q | I | I | S | L | S | D | N | K | I | S | S | P | S | D | S | Q | D | A | V | G | F | S | G | - | G | - | - | - | E | P | L | H | W | S | P | M | S | A | A | A | Q | Y | G | N | K | E | V | R | E | I | H | Y | F | R | Y | D | E | Y | A | R | S | C | K | Y | K | Y | K | E | L | G | T | V | T | S | F | V | N | K | K | C | S | E | F | G | K | T | L | D | V | S | R | N | N | I | F | F | L | H | S | R | F | L | N | L | A | E | L | R | C | L | N | L | S | G | N | A | M | S | Q | S | L | N | G | S | E | F | T | Y | L | T | N | L | N | Y | L | D | F | S | W | N | R | L | D | L | L | Y | S | T | A | F | Q | E | L | K | N | L | V | V | L | D | I | S | N | N | N | H | Y | F | E | S | E | G | L | T | H | M | L | N | F | T | S | N | L | K | K | L | K | I | L | R | M | N | H | N | K | I | S | T | S | T | N | T | E | L | E | S | Q | S | L | E | R | L | E | F | R | D | N | R | L | D | M | L | W | R | D | G | D | T | R | Y | I | N | Y | F | K | K | L | I | N | L | S | V | L | D | I | S | H | N | N | L | N | F | I | P | P | G | V | F | S | G | L | P | D | K | L | S | E | L | Y | I | K | N | N | K | L | K | D | G | S | F | D | W | K | K | L | Q | L | I | Q | S | L | E | V | L | D | L | S | G | N | I | L | T | T | V | P | A | M | L | S | T | T | C | P | K | S | L | K | K | L | L | L | Q | K | N | Q | I | V | E | L | T | P | D | F | L | K | D | A | F | H | L | K | H | L | D | L | S | F | N | H | I | Q | H | I | V | Q | S | S | F | P | D | D | V | I | D | Q | M | D | M | L | L | L | H | N | N | P | F | L | C | T | C | N | A | T | W | F | I | K | W | L | N | S | T | T | V | T | I | P | K | L | A | T | D | V | T | C | A | S | P | E | A | Q | K | G | N | L | V | I | S | V | D | - | - | L | L | A | C | Q | Y | N | Y | L | S | I | I | L | Y | T | L | M | T | T | L | V | L | S | F | L | T | L | S | I | S | S | H | L | F | L | W | D | V | W | Y | I | Y | H | F | C | R | A | K | L | K | G | Y | G | R | L | - | Y | S | Q | S | S | A | Y | D | A | F | V | I | Y | D | K | E | D | A | A | V | S | E | W | V | M | K | E | M | C | P | H | L | E | D | S | G | D | R | Q | L | T | L | C | L | E | E | R | D | W | I | P | G | C | P | L | I | D | N | L | S | R | S | I | N | N | S | K | R | T | V | F | I | L | T | N | R | Y | L | K | S | G | N | F | K | T | A | F | Y | M | A | H | Q | R | L | M | D | E | K | D | D | V | I | I | L | I | F | L | E | K | V | P | - | C | N | S | K | Y | L | R | L | R | K | R | L | Y | K | R | S | V | L | D | W | P | T | N | P | Q | A | Q | L | Y | F | W | F | S | L | R | S | V | L | A | T | E | S | H | K | Q | Y | N | N | L | F | K | E | T | L | X | X | X | X | X | X | X | X | X | X | X | X | X | X | X | X | X | X | X | X | X | X | X | - | - | - | - | - | - | - | - | - | - | - | - | - | - | - | - | - | - | - | - | - | - | - | - | - | - | - | - | - | - | - | - | - | - | - | - | - | - | - | - | - | - | - | - | - | - | - | - | - | - | - | - | - | - | - | - | - | - | - | - | - | - | - | - | - | - | - | - | - | - | - | - | - | - | - | - | - | - | - | - | - | - | - | - | - | - | - | - | - | - | - | - | - | - | - | - | - | - | - | - | - | - | - | - | - | - | - | - | - | - | - | - | - | - | - | - | - | - | - | - | - | - | - | - | - | - | - | - | - | - | - | - | - | - | - | - | - | - | - | - | - | - | - | - | - |
| MMTLR7 | - | - | - | - | - | - | - | - | - | - | - | - | - | - | - | - | - | - | - | - | - | - | - | - | - | - | - | - | - | - | - | - | M | C | V | P | L | L | G | L | W | - | C | F | L | S | I | S | T | A | S | I | S | Y | P | K | T | L | P | C | D | V | S | E | E | S | N | G | T | E | V | K | V | N | C | T | E | R | N | L | K | N | I | P | N | G | I | P | R | D | T | T | N | L | T | L | T | I | N | H | I | P | Q | L | N | S | T | S | F | R | D | L | V | N | L | T | E | I | D | M | R | C | N | C | V | P | V | K | I | G | P | K | D | R | I | C | T | K | S | V | T | I | E | E | H | A | F | A | S | L | K | N | L | R | A | L | Y | L | D | G | N | Q | L | D | S | I | P | K | G | L | P | S | N | L | I | L | L | S | L | E | V | N | N | I | F | H | I | S | K | A | N | L | S | G | I | R | N | V | E | M | L | Y | L | S | Q | N | C | Y | Y | R | N | P | C | N | Y | S | Y | Y | I | E | E | G | A | F | L | Q | L | K | N | L | T | L | L | C | L | K | S | N | N | L | S | Y | I | P | H | Q | L | P | T | S | L | K | E | L | Y | L | Y | N | N | N | I | E | E | V | T | D | E | D | F | K | N | L | T | N | L | E | I | L | D | I | S | G | N | C | P | R | C | Y | N | S | P | Y | P | C | N | P | C | P | N | N | S | P | L | K | I | S | K | T | A | F | K | M | L | T | K | L | K | T | L | R | M | H | S | N | S | L | V | S | V | P | S | D | W | F | A | N | T | R | E | L | R | V | L | D | L | S | S | N | F | L | A | R | E | M | G | V | T | A | F | P | R | F | L | G | K | L | E | E | L | D | L | S | F | N | Y | E | L | Q | K | Y | P | K | T | L | R | L | N | C | S | F | T | S | L | K | S | L | K | I | L | R | L | K | G | Y | V | F | Q | L | L | K | P | E | S | I | A | P | L | K | A | L | P | D | L | Q | V | V | D | L | G | T | N | F | I | K | M | A | N | L | S | I | L | M | E | L | K | S | F | Q | I | I | S | L | S | D | N | K | I | S | S | P | S | D | G | Q | D | A | F | G | F | S | G | - | G | - | - | - | E | L | F | Q | W | S | P | M | S | A | A | A | P | Y | Q | N | K | E | V | R | E | I | H | Y | F | R | Y | D | E | Y | A | R | S | C | K | Y | K | S | K | E | L | G | V | I | T | S | F | V | K | R | E | C | S | Q | F | G | K | T | L | D | V | S | R | N | N | I | F | F | L | H | P | S | F | L | N | L | G | E | L | R | C | L | N | L | S | G | N | A | M | S | Q | S | L | N | G | S | E | F | T | H | L | T | N | L | Q | Y | L | D | F | S | S | N | R | L | D | L | L | Y | S | T | A | F | Q | E | L | S | N | L | V | I | L | D | I | S | K | N | N | Y | Y | F | E | S | E | G | L | T | H | M | L | N | F | T | Q | N | L | K | K | L | K | I | L | M | M | N | H | N | K | I | S | T | S | T | N | T | E | L | E | S | E | S | L | E | R | L | E | F | R | D | N | R | L | D | V | M | W | R | D | G | D | S | R | Y | F | N | Y | F | K | K | L | F | N | L | T | V | L | D | I | S | E | N | N | L | D | N | I | P | K | P | V | F | S | G | L | P | D | K | L | S | E | L | Y | I | R | K | N | R | L | H | - | - | S | F | Y | W | G | E | L | Q | F | I | H | S | L | R | V | L | D | L | S | G | N | S | L | T | T | V | P | R | M | L | S | - | N | C | T | K | S | L | E | K | L | V | L | H | H | N | Q | I | L | K | L | T | P | D | F | L | K | D | A | Y | N | L | R | Y | L | D | L | S | Y | N | R | I | K | N | I | D | K | S | S | F | P | D | D | V | V | G | Q | M | Q | M | L | L | L | H | N | N | R | F | V | C | S | C | N | T | T | F | F | V | M | W | L | N | R | T | T | V | P | I | P | R | L | A | T | D | V | T | C | A | S | P | G | A | Q | R | G | K | P | V | V | S | V | D | - | - | L | Q | A | C | Q | H | S | Y | L | S | I | I | L | Y | T | L | M | T | S | L | T | L | S | F | L | A | L | S | I | S | S | H | L | F | L | W | D | V | W | Y | I | Y | H | F | C | R | A | K | L | K | G | Y | G | R | L | - | Y | S | Q | S | S | V | Y | D | A | F | V | M | Y | D | K | E | D | P | A | V | T | E | W | V | M | K | E | M | C | F | H | L | E | E | R | G | D | R | P | L | T | L | C | L | E | E | R | D | W | I | P | G | C | P | L | I | D | N | L | S | Q | S | I | H | K | S | K | R | T | M | F | I | L | T | S | R | Y | I | K | S | G | N | F | K | T | A | F | Y | M | A | H | Q | R | L | M | D | E | K | N | D | V | I | V | L | I | F | L | E | K | V | P | - | C | N | S | K | Y | L | R | L | R | K | R | L | Y | K | R | S | V | L | E | W | P | T | N | P | Q | A | Q | P | Y | F | W | F | S | L | R | S | V | L | A | T | E | S | H | K | Q | Y | S | N | L | F | K | E | T | L | X | X | X | X | X | X | X | X | X | X | X | X | X | X | X | X | X | X | X | X | X | X | X | X | X | X | - | - | X | X | X | X | X | - | - | - | - | - | - | - | - | - | - | - | - | - | - | - | - | - | - | - | - | - | - | - | - | - | - | - | - | - | - | - | - | - | - | - | - | - | - | - | - | - | - | - | - | - | - | - | - | - | - | - | - | - | - | - | - | - | - | - | - | - | - | - | - | - | - | - | - | - | - | - | - | - | - | - | - | - | - | - | - | - | - | - | - | - | - | - | - | - | - | - | - | - | - | - | - | - | - | - | - | - | - | - | - | - | - | - | - | - | - | - | - | - | - | - | - | - | - | - | - | - | - | - | - | - | - | - | - | - | - | - | - | - | - | - |
| ATTLR7 | - | - | - | - | - | - | - | - | - | - | - | - | - | - | - | - | - | - | - | - | - | - | - | - | - | - | - | M | L | S | N | L | M | C | V | I | L | L | A | V | W | - | C | S | H | S | M | L | T | A | T | I | F | Y | P | K | T | L | P | C | D | V | S | D | D | I | N | G | S | V | V | K | V | D | C | T | E | R | N | L | K | E | I | P | S | G | I | P | K | E | T | T | N | L | T | L | T | I | N | H | I | P | Q | L | N | S | S | S | F | L | G | L | E | Y | L | T | E | I | D | M | R | C | N | C | V | P | I | K | I | G | P | K | D | R | M | C | T | E | S | V | T | I | Q | E | N | T | F | T | S | L | R | S | L | R | A | L | Y | L | D | G | N | Q | L | S | S | I | P | K | G | L | P | S | Q | L | I | L | L | S | L | E | V | N | H | I | F | Y | L | S | K | A | N | F | S | E | I | R | N | I | E | M | L | Y | L | G | Q | N | C | Y | Y | R | N | P | C | N | V | S | Y | A | I | E | N | G | A | F | L | Q | L | N | S | L | R | L | L | S | L | K | S | N | N | L | S | F | I | P | H | Q | L | P | T | S | L | K | E | L | Y | L | Y | N | N | N | F | Q | K | V | T | D | E | D | F | K | N | L | T | N | L | E | I | L | D | I | S | G | N | C | P | R | C | Y | N | A | P | F | P | C | I | P | C | P | N | N | S | P | L | K | I | S | K | M | A | F | K | M | L | T | K | L | K | T | L | R | L | H | S | N | S | L | T | Y | V | P | P | E | W | F | T | N | T | K | E | L | R | V | L | D | L | S | S | N | F | L | A | R | E | I | G | V | T | S | F | P | Q | F | L | G | K | L | E | E | V | D | L | S | F | N | Y | E | L | Q | R | Y | P | Q | T | L | R | L | S | H | S | F | S | F | L | K | A | L | K | I | L | R | L | K | G | F | V | F | Q | Q | L | E | P | E | S | I | A | P | L | T | S | L | K | N | L | E | V | V | D | L | G | T | N | F | I | K | M | T | N | L | S | I | L | M | Q | L | K | S | F | K | I | I | S | L | S | D | N | K | I | S | S | P | S | D | G | Q | G | A | V | G | F | S | G | - | R | - | - | - | E | L | S | G | W | S | P | M | S | G | S | D | E | Y | K | H | K | E | V | R | E | I | H | Y | F | R | Y | D | E | Y | A | R | S | C | K | Y | K | D | K | E | L | G | V | I | T | S | F | V | N | R | K | C | S | Q | F | G | K | T | L | D | V | S | R | N | N | I | F | F | L | H | S | R | F | L | N | L | G | E | L | K | C | L | N | L | S | G | N | A | M | S | Q | S | L | N | G | S | E | F | T | Y | L | T | K | L | K | Y | L | D | F | S | S | N | R | L | D | L | L | Y | P | T | A | F | Q | E | L | K | S | L | V | I | L | D | I | S | N | N | N | H | Y | F | E | S | E | G | L | T | H | M | L | N | F | T | R | N | L | K | N | L | K | I | L | K | M | N | H | N | K | I | S | T | S | T | N | T | E | M | E | S | D | S | L | E | R | L | E | F | K | D | N | R | L | D | M | L | W | R | D | G | D | T | R | Y | V | N | Y | F | K | K | L | R | N | L | V | V | L | D | I | S | H | N | N | L | N | F | I | P | E | E | V | F | S | S | M | P | D | K | L | S | E | L | Y | I | R | N | N | K | L | K | - | - | S | F | I | W | A | K | L | I | F | L | H | S | L | Q | V | L | D | L | S | G | N | S | L | S | S | V | P | H | K | L | S | - | N | V | A | K | S | L | K | K | L | I | L | H | N | N | Q | I | L | K | L | T | P | D | F | L | K | G | A | Y | N | L | K | Y | L | D | L | S | F | N | N | I | Q | H | I | E | K | S | S | F | P | D | D | V | V | N | Q | M | D | M | L | L | L | H | N | N | K | F | L | C | T | C | N | A | T | W | F | V | T | W | L | N | S | T | T | V | T | I | P | R | L | A | T | D | V | T | C | A | A | P | G | A | Q | K | G | H | A | V | I | S | V | D | - | - | L | Q | A | C | Q | H | H | Y | L | S | I | I | L | Y | T | L | M | N | S | L | V | L | C | F | I | T | L | S | I | S | S | H | L | F | L | W | D | V | W | Y | I | Y | H | F | C | R | A | K | L | K | G | Y | R | R | L | - | Y | S | Q | S | S | A | Y | D | A | F | V | I | Y | D | K | D | D | S | A | V | S | E | W | V | M | K | E | M | C | V | H | L | E | D | S | G | D | R | R | L | T | L | C | L | E | E | R | D | W | I | P | G | C | P | L | I | D | N | L | C | Q | S | I | H | K | S | K | R | T | V | F | I | L | T | N | K | Y | I | K | S | G | N | F | K | T | A | F | Y | I | A | H | Q | R | L | M | D | E | K | N | D | V | I | V | L | L | F | L | E | K | V | S | - | C | S | S | K | Y | L | R | L | R | K | R | L | Y | K | Q | S | V | L | E | W | P | T | N | P | Q | A | Q | P | Y | F | W | F | S | L | R | S | V | L | A | T | E | S | H | K | Q | Y | N | N | L | F | K | E | T | L | X | X | X | X | X | X | X | X | X | X | X | X | X | X | X | X | X | X | X | X | X | X | X | X | X | X | - | - | - | - | - | - | - | - | - | - | - | - | - | - | - | - | - | - | - | - | - | - | - | - | - | - | - | - | - | - | - | - | - | - | - | - | - | - | - | - | - | - | - | - | - | - | - | - | - | - | - | - | - | - | - | - | - | - | - | - | - | - | - | - | - | - | - | - | - | - | - | - | - | - | - | - | - | - | - | - | - | - | - | - | - | - | - | - | - | - | - | - | - | - | - | - | - | - | - | - | - | - | - | - | - | - | - | - | - | - | - | - | - | - | - | - | - | - | - | - | - | - | - | - | - | - | - | - | - | - | - | - | - | - | - | - | - | - | - | - | - | - |
| ONTLR7 | - | - | - | - | - | - | - | - | - | - | - | - | - | - | - | - | - | - | - | - | - | - | - | - | - | - | - | M | F | L | H | L | M | C | V | A | L | L | T | L | W | - | S | C | L | P | I | L | A | T | G | I | Y | Y | P | K | T | L | P | C | D | V | N | E | I | N | N | G | S | E | V | R | V | D | C | T | E | R | N | L | K | K | I | P | H | G | I | P | R | D | T | T | N | L | T | L | T | I | N | H | I | P | S | L | N | S | T | S | F | H | G | L | E | N | L | T | E | I | D | M | R | C | N | C | V | P | I | K | I | G | P | K | D | K | M | C | L | K | S | V | T | I | E | E | N | T | F | S | R | L | S | N | L | R | A | L | Y | L | D | G | N | Q | L | Y | S | I | P | K | G | L | P | S | N | L | I | L | L | S | L | E | V | N | H | I | Y | Y | L | S | K | A | N | L | S | E | L | R | N | V | E | V | L | Y | L | G | Q | N | C | Y | F | R | N | P | C | N | D | S | Y | D | I | E | D | D | A | F | L | Q | F | N | N | L | K | L | L | S | L | K | S | N | N | L | S | F | I | P | H | Q | L | P | T | T | L | K | E | L | Y | L | Y | N | N | N | I | Q | E | V | T | D | E | D | F | K | N | L | T | N | L | E | I | L | D | I | S | G | N | C | P | R | C | Y | N | A | P | F | P | C | S | P | C | P | N | N | A | P | L | K | I | S | K | T | A | F | K | M | L | T | K | L | K | T | L | R | L | H | S | N | S | L | T | S | V | P | S | E | W | F | D | T | T | T | E | L | R | V | L | D | L | S | S | N | F | L | A | R | E | L | E | I | T | A | F | P | K | F | L | G | K | L | E | E | L | D | L | S | F | N | Y | E | L | Q | K | Y | P | Q | T | L | R | L | S | C | S | F | S | S | L | K | S | L | K | I | F | R | M | K | G | F | V | F | Q | Q | L | T | T | E | S | I | A | H | L | K | P | L | P | N | L | E | V | V | D | L | G | T | N | F | I | K | M | T | K | L | S | I | L | M | E | L | K | S | F | K | I | I | S | L | S | D | N | K | I | S | S | P | S | D | G | R | D | A | L | G | L | S | G | - | G | - | - | - | E | T | S | D | W | S | P | M | S | S | A | A | E | Y | Q | N | K | E | V | R | E | I | H | Y | F | R | Y | D | E | Y | A | R | S | C | K | Y | K | D | K | E | L | G | V | I | T | S | F | V | K | K | E | C | S | Q | F | G | K | T | L | D | V | S | R | N | N | I | F | F | L | H | S | R | F | L | N | L | T | E | L | R | C | L | N | L | S | G | N | A | M | S | Q | S | L | N | G | S | E | F | T | Y | L | A | N | L | Q | Y | L | D | F | S | W | N | R | L | D | L | L | Y | S | T | A | F | Q | E | L | K | N | L | V | I | L | D | I | S | N | N | N | H | Y | F | E | S | E | G | L | T | H | M | L | N | F | T | R | N | L | K | N | L | K | V | L | L | M | N | H | N | K | I | S | T | S | T | N | T | E | L | E | S | Q | S | L | E | R | L | E | F | R | D | N | R | L | D | V | L | W | R | D | G | N | T | R | Y | V | N | Y | F | K | N | L | V | N | L | T | V | L | D | I | S | H | N | N | L | P | F | I | P | S | D | V | F | S | G | L | P | E | K | L | S | E | L | Y | I | K | N | N | K | L | K | - | - | S | F | N | W | N | K | L | Q | L | L | H | S | L | R | V | L | D | L | S | G | N | S | L | T | T | V | P | H | V | L | S | - | N | C | T | T | S | L | E | K | L | I | L | H | K | N | Q | I | L | K | L | T | P | D | F | L | K | G | A | Y | H | L | K | Y | L | D | L | S | F | N | H | I | Q | Y | I | E | K | S | S | L | P | D | D | V | L | N | H | M | D | M | L | L | L | H | K | N | R | F | L | C | T | C | N | A | T | W | F | V | T | W | L | N | R | T | T | V | T | I | P | K | L | G | T | D | V | T | C | V | A | P | G | A | Q | R | G | R | P | V | V | S | V | D | - | - | L | L | A | C | Q | H | H | Y | L | S | I | I | L | S | T | L | M | T | S | L | V | L | I | L | V | T | L | S | I | S | S | H | L | F | L | W | D | V | W | Y | I | Y | H | F | C | R | A | K | L | K | G | Y | R | R | L | - | Y | S | Q | S | A | V | Y | D | A | F | V | I | Y | D | K | E | D | P | A | V | S | E | W | V | T | K | E | M | C | A | H | L | E | E | C | G | D | R | R | L | T | L | C | L | E | D | R | D | W | M | P | G | C | P | L | I | D | N | L | S | Q | S | I | H | R | S | K | R | T | V | F | I | L | T | K | K | Y | I | K | G | G | N | F | K | T | A | F | Y | M | A | H | Q | R | L | M | D | E | K | N | D | V | I | V | L | I | F | L | E | K | V | P | - | C | N | S | K | Y | L | R | L | R | K | R | L | Y | K | R | S | V | L | E | W | P | T | N | P | Q | A | Q | P | Y | F | W | F | S | L | R | S | V | L | A | T | E | G | H | K | Q | Y | N | N | L | F | K | E | T | L | X | X | X | X | X | X | X | X | X | X | X | X | X | X | X | X | X | X | X | X | X | X | X | X | X | X | - | - | - | - | - | - | - | - | - | - | - | - | - | - | - | - | - | - | - | - | - | - | - | - | - | - | - | - | - | - | - | - | - | - | - | - | - | - | - | - | - | - | - | - | - | - | - | - | - | - | - | - | - | - | - | - | - | - | - | - | - | - | - | - | - | - | - | - | - | - | - | - | - | - | - | - | - | - | - | - | - | - | - | - | - | - | - | - | - | - | - | - | - | - | - | - | - | - | - | - | - | - | - | - | - | - | - | - | - | - | - | - | - | - | - | - | - | - | - | - | - | - | - | - | - | - | - | - | - | - | - | - | - | - | - | - | - | - | - | - | - | - |
| LMTLR7 | - | - | - | - | - | - | - | - | - | - | - | - | - | - | - | - | - | - | - | - | - | - | - | - | - | - | - | M | F | F | N | L | M | C | V | A | L | L | G | L | W | - | C | G | R | S | I | S | T | A | T | I | S | Y | P | K | T | L | P | C | D | V | S | E | V | D | N | G | S | G | V | K | V | D | C | T | E | R | S | L | K | D | I | P | R | G | I | P | R | E | T | T | N | L | T | L | T | I | N | H | I | P | Q | L | N | S | T | S | F | H | G | L | G | N | L | T | E | I | D | M | R | C | N | C | V | P | I | K | I | G | P | K | D | R | M | C | T | E | S | V | T | I | E | E | N | T | F | S | S | L | R | N | L | R | A | L | Y | L | D | G | N | Q | L | Y | S | I | P | K | G | L | P | S | N | L | I | L | L | S | L | E | V | N | H | I | Y | Y | I | S | K | A | N | L | S | E | I | R | N | V | E | M | L | Y | L | S | S | N | C | Y | Y | R | N | P | C | N | V | S | Y | E | I | E | E | G | A | F | L | Q | L | K | N | L | T | L | L | C | L | K | S | N | N | L | S | Y | I | P | H | Q | L | P | T | S | L | K | E | L | Y | L | Y | N | N | N | I | Q | E | V | T | D | E | D | F | K | N | L | T | N | L | E | I | L | D | I | S | G | N | C | P | R | C | Y | N | A | P | F | P | C | N | P | C | P | N | N | S | P | L | K | I | S | K | T | A | F | K | M | L | T | K | L | K | T | L | R | L | H | S | N | S | L | T | C | V | P | A | E | W | F | A | N | T | T | E | L | R | V | L | D | L | S | S | N | F | L | A | R | E | I | G | V | T | T | F | P | H | F | L | G | K | L | E | E | L | D | L | S | F | N | Y | E | L | Q | R | Y | P | Q | T | L | R | L | S | C | S | F | S | S | L | K | S | L | K | V | L | R | L | K | G | F | V | F | Q | Q | L | K | P | E | S | I | A | P | L | K | P | L | T | N | L | E | V | V | D | L | G | T | N | F | I | K | M | T | N | L | S | I | L | M | E | L | K | S | F | K | I | I | S | L | S | D | N | K | I | S | S | P | S | D | G | Q | D | S | V | G | F | S | G | - | G | - | - | - | E | P | L | Y | W | S | P | M | T | S | A | A | Q | Y | Q | N | K | E | V | R | E | I | H | Y | F | R | Y | D | E | Y | A | R | S | C | K | Y | K | D | K | E | L | G | T | I | T | S | F | V | N | K | E | C | S | Q | F | G | K | T | L | D | V | S | R | N | N | I | F | F | L | H | S | R | F | L | N | L | G | E | L | R | C | L | N | L | S | G | N | A | M | S | Q | S | L | N | G | S | E | F | T | Y | L | T | N | L | Q | Y | L | D | F | S | S | N | R | L | D | L | L | Y | S | T | A | F | Q | E | L | K | N | L | V | I | L | D | I | S | N | N | N | H | Y | F | E | S | E | G | L | T | H | M | L | N | F | T | R | N | L | K | K | L | K | I | L | L | M | N | H | N | K | I | S | T | S | T | N | T | E | L | E | S | Q | S | L | E | R | L | E | F | R | D | N | R | L | D | M | L | W | R | D | G | D | T | R | Y | V | N | Y | F | K | K | L | L | N | L | T | V | L | D | I | S | H | N | N | L | N | F | I | P | L | Q | L | F | S | G | L | P | D | K | L | S | E | L | Y | I | K | N | N | K | L | K | - | - | S | F | S | W | G | K | L | Q | L | L | Q | S | L | Q | V | L | D | L | S | G | N | S | L | T | T | V | P | R | M | L | S | - | N | C | T | I | S | L | K | K | L | I | L | H | K | N | N | I | L | K | L | T | P | D | F | L | K | D | A | N | N | L | K | Y | L | D | L | S | F | N | H | L | Q | T | I | E | K | S | S | F | P | D | D | V | V | N | Q | M | E | M | L | L | L | H | K | N | R | F | L | C | T | C | N | A | T | W | F | I | T | W | L | N | K | T | K | V | T | I | P | R | L | A | T | D | V | T | C | A | T | P | V | A | Q | R | G | H | P | V | I | S | V | D | - | - | L | L | A | C | Q | H | S | Y | L | S | I | I | L | Y | I | L | M | T | S | L | V | L | S | F | L | T | L | S | I | S | S | H | L | F | L | W | D | V | W | Y | I | Y | H | F | C | R | A | K | L | K | G | Y | G | R | L | - | Y | S | Q | S | S | V | Y | D | A | F | V | I | Y | D | K | E | D | P | A | V | S | E | W | V | I | K | E | M | C | A | H | L | E | D | C | G | D | R | C | L | T | L | C | L | E | E | R | D | W | I | P | G | C | P | L | I | D | N | L | S | Q | S | I | H | K | S | K | R | T | V | F | I | L | T | S | K | Y | I | K | S | G | N | F | K | T | A | F | Y | M | A | H | Q | R | L | M | D | E | K | N | D | V | I | V | L | I | F | L | E | K | L | P | - | C | N | S | R | Y | L | R | L | R | K | R | L | Y | K | R | S | L | L | E | W | P | T | N | P | Q | A | Q | P | Y | F | W | F | S | L | R | S | V | L | A | T | E | S | H | K | Q | Y | N | N | L | F | K | E | T | L | X | X | X | X | X | X | X | X | X | X | X | X | X | X | X | X | X | X | X | X | X | X | X | X | X | X | - | - | - | - | - | - | - | - | - | - | - | - | - | - | - | - | - | - | - | - | - | - | - | - | - | - | - | - | - | - | - | - | - | - | - | - | - | - | - | - | - | - | - | - | - | - | - | - | - | - | - | - | - | - | - | - | - | - | - | - | - | - | - | - | - | - | - | - | - | - | - | - | - | - | - | - | - | - | - | - | - | - | - | - | - | - | - | - | - | - | - | - | - | - | - | - | - | - | - | - | - | - | - | - | - | - | - | - | - | - | - | - | - | - | - | - | - | - | - | - | - | - | - | - | - | - | - | - | - | - | - | - | - | - | - | - | - | - | - | - | - | - |
| PFTLR7 | - | - | - | - | - | - | - | - | - | - | - | - | - | - | - | - | - | - | - | - | - | - | - | - | - | - | - | M | F | F | H | L | M | C | V | A | L | L | G | L | W | - | C | C | L | T | T | S | T | A | S | V | S | Y | P | K | T | L | P | C | E | V | R | E | T | D | N | G | S | V | V | T | V | D | C | T | E | R | S | L | K | D | I | P | L | G | I | P | R | D | T | T | N | L | T | L | T | I | N | H | I | P | N | L | N | S | T | S | F | H | G | L | E | N | L | T | E | I | D | M | R | C | N | C | V | P | I | K | I | G | P | K | D | R | M | C | T | K | S | V | I | I | E | D | N | T | F | S | S | L | R | N | L | R | A | L | Y | L | D | G | N | Q | L | Y | S | I | P | K | G | L | P | P | N | L | I | L | L | S | L | E | V | N | H | I | F | Y | I | S | S | A | N | L | S | E | I | R | N | I | E | M | L | Y | L | S | Q | N | C | Y | F | R | N | P | C | N | V | S | Y | E | I | E | D | G | A | F | L | Q | L | N | N | L | T | L | L | C | L | K | S | N | N | L | S | F | I | P | H | R | L | P | T | S | L | K | E | L | Y | L | Y | N | N | N | I | Q | E | V | T | D | E | D | F | K | N | L | T | N | L | E | I | L | D | I | S | G | N | C | P | R | C | Y | N | A | P | F | P | C | N | P | C | P | N | N | S | P | L | K | I | S | K | T | A | F | K | M | L | T | K | L | K | T | L | R | L | H | S | N | S | L | T | C | V | P | S | E | W | F | A | S | T | T | E | L | R | V | L | D | L | S | S | N | F | L | A | R | E | I | G | V | T | H | F | P | H | F | L | G | K | L | E | E | L | D | L | S | F | N | Y | E | L | Q | R | Y | P | E | T | L | R | L | S | R | N | F | S | S | L | K | S | L | R | I | L | R | L | K | G | F | V | F | Q | Q | L | K | P | E | S | I | A | P | L | K | P | L | T | N | L | E | V | V | D | L | G | T | N | F | I | K | M | A | N | L | S | I | L | M | E | L | K | S | F | K | I | I | S | L | S | D | N | K | I | S | S | P | S | D | G | Q | E | A | V | G | F | S | G | - | G | - | - | - | E | P | L | Y | W | S | P | M | S | P | A | D | Q | Y | Q | S | K | E | V | R | E | I | H | Y | F | R | Y | D | E | Y | A | R | S | C | K | Y | K | D | K | E | L | G | T | I | T | S | F | V | N | K | N | C | S | Q | F | G | K | T | L | D | V | S | R | N | N | I | F | F | L | H | S | R | F | L | N | L | G | E | L | R | C | L | N | L | S | G | N | A | M | S | Q | S | L | N | G | S | E | F | S | Y | L | A | N | L | Q | Y | L | D | F | S | S | N | R | L | D | L | L | Y | S | T | A | F | Q | E | L | K | S | L | V | I | L | D | I | S | Y | N | N | H | Y | F | E | S | E | G | L | T | H | M | L | N | F | T | R | N | L | K | N | L | K | I | L | L | M | N | H | N | Q | I | S | T | S | T | N | T | E | L | E | S | E | S | L | E | R | L | E | F | T | G | N | R | L | D | M | L | W | R | D | G | D | I | R | Y | V | N | Y | F | K | K | L | H | N | L | T | V | L | D | I | S | H | N | N | L | N | F | I | P | Q | E | V | F | S | G | L | P | D | K | L | S | E | L | Y | I | K | N | N | K | L | K | D | N | S | F | D | W | E | K | L | K | L | L | S | S | L | Q | V | L | D | L | S | G | N | S | L | T | T | V | P | P | M | L | S | - | I | C | T | K | S | L | K | K | L | L | L | H | K | N | Q | I | L | K | L | T | P | D | F | L | K | D | A | Y | N | L | Q | Y | L | D | L | S | F | N | H | I | Q | Y | I | D | K | S | S | F | P | D | D | V | V | N | K | M | K | M | L | L | L | H | K | N | R | F | V | C | T | C | N | A | T | W | F | V | T | W | L | N | R | T | T | V | P | I | P | R | L | A | T | D | V | T | C | A | S | P | G | A | Q | R | G | H | P | V | L | S | V | D | - | - | L | L | A | C | Q | H | S | Y | L | S | I | I | L | Y | T | L | M | T | S | L | V | L | S | F | L | T | L | S | I | S | S | H | L | F | L | W | D | V | W | Y | I | Y | H | F | C | R | A | K | L | K | G | Y | G | R | L | - | N | S | Q | S | S | T | Y | D | A | F | V | I | Y | D | K | E | D | L | A | V | T | E | W | V | M | K | E | M | V | A | H | L | E | D | H | G | D | C | R | L | T | L | C | L | E | E | R | D | W | I | P | G | C | P | L | I | D | N | L | S | Q | S | I | H | N | S | K | R | T | V | F | I | L | T | S | K | Y | I | K | S | G | N | F | K | T | A | F | Y | M | A | H | Q | R | L | M | D | E | K | D | D | V | I | V | L | I | F | L | E | K | V | P | - | C | D | S | K | Y | L | R | L | R | K | R | L | Y | K | R | S | V | M | E | W | P | T | N | P | Q | A | Q | F | Y | F | W | F | S | L | R | S | V | L | A | T | E | S | H | K | Q | Y | N | N | L | F | K | E | T | L | X | X | X | X | X | X | X | X | X | X | X | X | X | X | X | X | X | X | X | X | X | - | - | X | X | X | - | - | - | - | - | - | - | - | - | - | - | - | - | - | - | - | - | - | - | - | - | - | - | - | - | - | - | - | - | - | - | - | - | - | - | - | - | - | - | - | - | - | - | - | - | - | - | - | - | - | - | - | - | - | - | - | - | - | - | - | - | - | - | - | - | - | - | - | - | - | - | - | - | - | - | - | - | - | - | - | - | - | - | - | - | - | - | - | - | - | - | - | - | - | - | - | - | - | - | - | - | - | - | - | - | - | - | - | - | - | - | - | - | - | - | - | - | - | - | - | - | - | - | - | - | - | - | - | - | - | - | - | - | - | - | - | - | - | - | - | - | - |
| DRTLR7 | - | - | - | - | - | - | - | - | - | - | - | - | - | - | - | - | - | - | - | - | - | - | - | - | - | - | - | - | - | - | - | - | M | T | E | K | T | M | I | I | F | A | S | F | I | S | L | L | V | A | A | E | W | Y | P | K | S | L | K | C | D | V | S | L | A | S | N | G | T | E | V | S | V | D | C | T | E | R | S | L | T | E | V | P | L | G | I | P | T | N | T | T | N | L | T | L | T | I | N | H | I | P | H | V | M | N | N | S | F | D | N | L | H | N | I | T | E | I | D | L | R | C | N | C | V | P | V | K | V | G | P | K | D | R | V | C | S | Q | S | V | S | I | D | N | G | T | F | W | K | L | K | N | L | K | S | L | Y | L | D | G | N | Q | L | S | S | I | P | K | G | L | P | A | N | I | V | L | L | S | L | E | I | N | S | I | Y | S | I | L | Q | E | N | L | T | E | L | T | N | I | R | T | L | Y | L | G | Q | N | C | Y | F | R | N | P | C | N | Q | S | Y | Y | I | E | K | D | A | F | M | L | L | D | K | M | T | L | L | S | L | K | S | N | N | L | S | Y | I | P | N | Q | L | P | S | S | L | K | E | L | Y | L | Y | N | N | N | I | E | K | I | T | E | N | D | F | C | N | L | T | E | L | E | V | L | D | L | S | G | N | C | P | R | C | Y | N | A | P | F | P | C | I | P | C | P | N | N | A | P | L | Q | I | H | P | N | S | F | K | T | L | R | N | L | K | T | L | R | L | H | S | N | S | L | T | N | I | P | P | E | W | F | Q | S | L | A | D | L | T | L | L | D | L | S | S | N | F | L | A | K | E | I | T | C | T | S | F | P | S | L | L | P | K | L | E | E | L | D | L | S | F | N | Y | E | L | Q | V | Y | P | A | S | L | S | L | S | E | S | F | S | Q | L | K | S | L | R | V | L | R | I | R | G | Y | V | F | Q | E | L | K | L | Q | D | I | Q | P | L | T | N | L | T | Y | L | E | F | L | D | L | G | T | N | F | I | K | I | A | Q | L | S | I | L | K | N | L | K | N | F | K | I | I | N | L | S | D | N | K | I | S | V | P | S | E | G | E | F | S | - | - | F | S | N | H | R | - | - | - | E | A | Y | Y | G | S | P | M | S | Q | G | A | Q | Y | H | N | G | E | V | K | D | M | H | Y | F | L | Y | D | E | F | A | R | S | C | K | Y | K | D | K | E | L | W | I | P | S | P | F | - | N | N | D | C | S | S | F | G | K | T | L | D | I | S | R | N | N | I | F | F | L | H | S | K | F | L | N | L | G | E | L | R | C | L | N | L | S | G | N | A | M | S | Q | S | L | N | G | S | E | F | V | Q | L | T | N | L | Q | Y | L | D | F | T | D | N | R | L | D | L | M | Y | P | S | A | F | Q | E | L | S | N | L | V | V | L | D | I | S | R | N | S | H | Y | F | V | A | E | G | L | T | H | M | L | N | F | T | E | N | L | S | K | L | R | K | L | I | M | N | D | N | Q | I | S | T | S | T | N | T | E | M | K | S | Y | K | L | E | H | L | E | F | K | G | N | R | L | D | M | L | W | R | D | G | D | T | R | Y | V | N | Y | F | K | N | L | M | S | L | K | T | L | D | I | S | R | N | N | L | N | F | I | P | L | V | V | F | Q | G | L | P | N | T | L | T | K | L | Y | I | T | D | N | K | L | K | - | - | L | F | K | W | E | G | L | V | Y | L | K | S | L | L | L | L | D | L | T | G | N | L | L | T | E | V | P | S | C | L | S | - | N | Y | T | K | S | I | Q | T | L | V | L | S | K | N | K | I | V | K | L | S | P | N | F | L | K | D | A | F | S | L | K | I | L | D | L | S | Y | N | S | I | Q | F | I | D | E | S | S | F | P | E | N | V | I | D | H | L | Q | T | L | Y | L | N | N | N | M | F | V | C | S | C | N | A | T | W | L | V | R | W | I | N | R | T | S | V | N | I | P | R | L | A | S | D | V | T | C | A | S | P | S | A | Q | K | G | Q | S | V | I | F | L | N | - | - | L | Q | A | C | Q | H | N | S | L | S | I | I | L | C | I | F | Q | T | T | L | I | L | T | I | L | T | L | T | I | S | S | H | L | F | L | W | D | V | W | Y | I | Y | H | F | C | L | A | K | L | K | G | Y | R | R | L | - | S | S | N | S | A | V | Y | D | A | F | V | I | Y | D | T | T | D | P | A | V | Q | E | W | V | M | Q | E | L | R | V | H | L | E | D | K | G | D | P | R | M | N | L | C | L | E | E | R | D | W | V | P | G | C | P | L | I | E | N | L | S | Q | S | I | Q | L | S | Q | R | T | V | F | I | L | T | E | R | Y | I | R | S | G | S | F | R | T | A | F | Y | L | A | H | Q | R | L | M | D | E | R | N | D | V | I | V | L | I | F | L | E | R | M | P | - | C | H | S | K | Y | L | R | L | R | K | R | L | C | I | K | E | L | K | G | T | S | G | S | V | - | - | S | G | A | L | W | R | L | R | V | N | I | I | H | S | S | R | - | R | V | L | X | X | X | X | X | X | X | X | X | X | X | X | X | X | X | X | X | X | X | X | X | X | X | X | X | X | X | X | X | X | X | X | X | X | X | X | X | X | X | X | X | X | X | - | - | - | - | - | - | - | - | - | - | - | - | - | - | - | - | - | - | - | - | - | - | - | - | - | - | - | - | - | - | - | - | - | - | - | - | - | - | - | - | - | - | - | - | - | - | - | - | - | - | - | - | - | - | - | - | - | - | - | - | - | - | - | - | - | - | - | - | - | - | - | - | - | - | - | - | - | - | - | - | - | - | - | - | - | - | - | - | - | - | - | - | - | - | - | - | - | - | - | - | - | - | - | - | - | - | - | - | - | - | - | - | - | - | - | - | - | - | - | - | - | - | - | - | - | - | - | - | - | - | - | - | - |
| MATLR7 | - | - | - | - | - | - | - | - | - | - | - | - | - | - | - | - | - | - | - | - | - | - | - | - | - | - | - | M | F | L | F | Q | V | T | E | K | I | L | I | I | F | A | G | L | V | S | L | R | D | A | A | E | W | Y | P | K | S | L | K | C | D | V | S | L | A | S | N | G | T | E | V | S | V | N | C | T | E | R | D | L | T | E | V | P | L | G | I | P | M | N | T | T | N | L | T | L | T | I | N | H | I | P | H | I | M | N | D | S | F | H | E | L | H | N | I | T | E | I | D | L | R | C | N | C | V | P | I | K | I | G | P | K | D | R | V | C | T | Q | S | V | T | I | D | N | G | T | F | W | K | L | K | N | L | K | S | L | Y | L | D | G | N | Q | L | S | S | I | P | K | G | L | P | P | N | I | V | L | L | S | L | E | V | N | S | I | Y | S | I | L | K | E | N | L | T | E | L | T | N | I | Q | R | L | Y | L | G | Q | N | C | Y | F | R | N | P | C | N | K | S | Y | Y | I | E | K | D | A | F | V | Q | L | N | K | M | T | L | L | S | L | K | S | N | N | L | S | Y | V | P | R | Q | L | P | S | S | L | K | E | L | Y | L | Y | N | N | N | I | Q | T | I | T | E | E | D | F | H | N | L | T | E | L | E | I | L | D | L | S | G | N | C | P | R | C | Y | N | A | P | F | P | C | N | P | C | P | N | N | A | P | L | Q | I | H | A | D | S | F | N | T | L | K | N | L | K | T | L | R | L | H | S | N | S | L | T | N | I | P | L | K | W | F | Q | N | L | T | G | L | S | L | L | D | L | S | S | N | F | L | G | K | E | I | T | C | T | N | F | P | F | K | L | P | N | L | E | E | L | D | L | S | F | N | F | E | L | Q | V | Y | P | T | S | L | N | L | S | S | S | F | S | S | L | K | S | L | R | V | L | R | I | R | G | Y | V | F | Q | K | L | T | E | Q | D | L | K | P | L | I | N | L | S | N | L | E | F | I | D | L | G | T | N | F | I | K | I | A | D | L | S | L | L | K | E | L | K | S | F | R | I | I | N | L | S | D | N | K | I | S | M | P | S | E | G | G | Y | A | T | W | H | S | N | H | R | - | - | - | E | K | H | Y | G | S | P | M | S | Q | G | A | Q | Y | H | N | G | E | V | K | D | V | H | Y | F | L | Y | D | E | F | A | R | S | C | K | Y | K | D | K | E | L | W | I | T | S | P | F | - | N | N | E | C | S | S | F | G | K | T | L | D | I | S | R | N | N | I | F | F | L | H | S | R | F | L | D | L | G | E | L | R | C | L | N | L | S | G | N | A | M | S | Q | S | L | N | G | S | E | F | V | Q | L | K | N | L | Q | Y | L | D | F | S | D | N | R | L | D | L | M | Y | S | S | A | F | Q | E | L | S | S | L | I | V | L | D | I | S | R | N | S | H | F | F | V | A | E | G | L | T | H | M | L | N | F | T | K | S | L | N | K | L | R | K | L | I | M | N | D | N | Q | I | S | T | S | T | N | T | E | M | E | S | F | T | L | E | H | L | E | F | R | G | N | R | L | D | M | L | W | R | D | G | D | T | R | Y | V | S | Y | F | K | K | L | V | A | L | K | T | L | D | I | S | Q | N | N | L | N | F | I | P | Q | G | V | F | E | G | L | P | D | T | L | T | E | L | Y | I | T | N | N | K | F | K | - | - | S | F | K | W | E | G | L | V | N | L | K | N | L | Q | L | L | D | L | S | E | N | Y | L | T | E | V | P | T | C | L | T | - | N | Y | T | K | S | I | Q | T | L | V | L | H | K | N | Y | I | A | K | L | T | P | N | F | L | K | D | A | F | S | L | K | V | L | D | L | S | Y | N | L | I | Q | F | I | D | E | S | S | F | P | E | N | V | V | D | H | L | E | T | L | Y | L | N | N | N | R | F | V | C | S | C | N | A | T | W | F | V | R | W | I | N | R | T | S | V | N | I | P | R | L | A | T | D | V | T | C | S | S | P | S | A | Q | K | G | Q | S | V | I | F | L | N | - | - | V | Q | A | C | Q | H | N | S | L | S | I | I | L | C | I | F | L | T | S | V | I | L | S | I | L | T | L | T | I | S | S | H | L | F | L | W | D | V | W | Y | I | Y | H | F | C | L | A | K | L | K | G | Y | R | R | L | - | S | S | N | S | T | V | Y | D | A | F | V | V | Y | D | T | A | D | P | A | V | H | E | W | V | M | Q | E | L | R | V | H | L | E | D | R | G | D | P | R | M | Q | L | C | L | E | E | R | D | W | V | P | G | C | P | L | I | E | N | L | S | Q | S | I | Q | L | S | K | R | T | V | F | I | L | T | G | Q | Y | I | K | S | G | S | F | R | T | A | F | Y | L | A | H | Q | R | L | M | D | E | R | N | D | V | I | V | L | I | F | L | E | K | M | P | - | C | H | S | K | Y | L | K | L | R | K | R | L | Y | K | K | S | I | L | E | W | P | R | N | P | Q | A | Q | R | Y | F | W | F | S | L | R | G | L | M | S | T | D | S | - | R | Q | Y | N | K | L | F | Q | E | T | L | X | X | X | X | X | X | X | X | X | X | X | X | X | X | X | X | X | X | X | X | X | X | X | X | X | X | - | - | - | - | - | - | - | - | - | - | - | - | - | - | - | - | - | - | - | - | - | - | - | - | - | - | - | - | - | - | - | - | - | - | - | - | - | - | - | - | - | - | - | - | - | - | - | - | - | - | - | - | - | - | - | - | - | - | - | - | - | - | - | - | - | - | - | - | - | - | - | - | - | - | - | - | - | - | - | - | - | - | - | - | - | - | - | - | - | - | - | - | - | - | - | - | - | - | - | - | - | - | - | - | - | - | - | - | - | - | - | - | - | - | - | - | - | - | - | - | - | - | - | - | - | - | - | - | - | - | - | - | - | - | - | - | - | - | - | - | - | - |
| CATLR7 | - | - | - | - | - | - | - | - | - | - | - | - | - | - | - | - | - | - | - | - | - | - | - | - | - | - | - | - | - | M | K | M | M | T | E | K | K | F | I | I | F | A | G | L | I | F | L | L | D | A | A | E | W | Y | P | K | S | L | K | C | D | V | S | L | A | S | N | G | S | E | V | T | V | N | C | T | E | R | E | L | T | E | I | P | L | G | I | P | A | N | T | T | N | L | T | L | T | I | N | H | I | P | H | I | T | N | D | S | F | H | K | L | Q | N | I | T | E | I | D | L | R | C | N | C | V | P | I | K | V | G | P | K | D | R | V | C | T | K | S | L | T | I | D | N | G | T | F | W | K | L | K | N | L | K | S | L | Y | L | D | G | N | Q | L | S | S | I | P | K | G | L | P | S | N | I | V | L | L | S | L | E | V | N | S | I | Y | S | I | L | K | E | N | L | T | E | L | T | N | I | Q | K | L | Y | L | G | Q | N | C | Y | F | R | N | P | C | N | T | S | Y | Y | I | E | K | D | A | F | L | K | L | D | K | M | T | L | L | S | L | K | S | N | N | L | S | Y | I | P | H | Q | L | P | S | S | L | K | E | L | Y | L | Y | N | N | N | I | Q | T | I | T | E | M | D | F | H | N | L | T | E | L | E | I | L | D | L | S | G | N | C | P | R | C | Y | N | A | P | F | P | C | V | P | C | P | N | N | A | P | L | Q | I | H | P | N | S | F | K | T | L | K | N | L | N | T | L | R | L | H | S | N | S | L | T | H | I | P | S | E | W | F | Q | N | L | T | Q | L | I | L | L | D | L | S | S | N | F | L | A | K | E | I | T | C | T | S | F | P | S | L | L | P | N | L | E | E | L | D | L | S | F | N | Y | E | L | Q | V | Y | P | T | A | L | N | L | S | R | S | F | H | H | L | K | S | L | K | V | L | R | I | R | G | Y | V | F | Q | E | L | K | Q | E | D | I | Q | P | L | A | K | L | N | K | L | E | L | I | D | L | G | T | N | F | I | K | I | A | H | L | S | V | L | R | T | L | K | N | F | K | I | I | N | L | S | D | N | K | I | S | M | P | S | Q | G | E | - | - | T | L | L | A | N | H | R | - | - | - | V | N | H | Y | G | S | P | M | T | Q | G | A | Q | Y | H | S | G | E | V | K | D | M | H | Y | F | L | Y | D | E | Y | A | R | S | C | K | Y | K | D | K | E | L | W | I | S | S | P | F | - | N | N | E | C | S | S | F | G | K | T | L | D | I | S | R | N | N | I | F | F | L | H | S | R | F | L | D | L | D | E | L | S | C | L | N | L | S | G | N | A | M | S | Q | S | L | N | G | S | E | F | V | H | L | K | N | L | Q | Y | L | D | F | S | D | N | R | L | D | L | M | F | S | S | A | F | Q | E | L | S | S | L | V | V | L | D | I | S | R | N | S | H | Y | F | V | A | E | G | L | T | H | M | L | N | F | T | K | N | L | G | K | L | N | K | L | I | L | N | D | N | Q | I | S | T | S | T | N | T | E | M | E | S | F | S | L | E | H | L | E | F | K | G | N | R | L | D | M | L | W | R | D | G | D | I | R | Y | A | N | Y | F | K | K | L | L | S | L | K | S | L | D | I | S | R | N | N | L | N | F | I | P | L | E | V | F | E | G | L | P | D | T | L | T | D | L | Y | I | T | N | N | R | L | K | - | - | S | F | N | W | E | G | L | A | N | L | K | N | L | K | L | L | D | L | S | G | N | Q | L | T | D | V | P | E | C | L | S | - | N | Y | T | K | S | I | Q | T | L | V | L | H | K | N | N | I | V | K | L | T | P | N | F | L | K | D | A | F | S | L | K | V | L | D | L | S | Y | N | R | I | Q | F | I | D | E | S | S | F | P | A | N | V | I | D | Q | L | Q | T | L | Y | L | N | N | N | R | F | V | C | S | C | N | A | T | W | F | V | R | W | I | N | R | T | S | V | N | I | P | R | L | A | M | D | V | T | C | A | S | P | S | A | Q | K | G | Q | S | V | L | F | L | N | - | - | I | Q | A | C | Q | H | N | S | L | S | I | I | L | C | I | L | M | T | S | V | I | L | S | I | L | T | L | T | I | S | S | H | L | F | L | W | D | V | W | Y | I | Y | H | F | C | L | A | K | L | K | G | Y | R | R | L | - | S | S | N | S | T | V | Y | D | A | F | V | V | Y | D | M | T | D | P | A | V | H | E | W | V | M | Q | E | L | R | V | Q | L | E | D | Q | G | D | P | G | M | Q | L | C | L | E | E | R | D | W | V | P | G | C | P | L | I | E | N | L | S | Q | S | I | Q | L | S | K | R | T | V | F | I | L | T | N | R | Y | I | R | S | G | S | F | R | T | A | F | Y | L | A | H | Q | R | L | M | D | E | R | D | D | V | I | V | L | I | F | L | E | K | M | P | - | C | H | S | K | Y | L | R | L | R | K | R | L | Y | K | K | S | V | L | E | W | P | R | N | P | Q | A | Q | R | Y | F | W | F | S | L | R | S | V | M | S | A | G | S | - | Q | Q | Y | N | K | L | F | Q | E | T | L | X | X | X | X | X | X | X | X | X | X | X | X | X | X | X | X | X | X | X | X | X | X | X | X | X | X | X | X | X | - | - | - | - | - | X | - | - | - | - | - | - | - | - | - | - | - | - | - | - | - | - | - | - | - | - | - | - | - | - | - | - | - | - | - | - | - | - | - | - | - | - | - | - | - | - | - | - | - | - | - | - | - | - | - | - | - | - | - | - | - | - | - | - | - | - | - | - | - | - | - | - | - | - | - | - | - | - | - | - | - | - | - | - | - | - | - | - | - | - | - | - | - | - | - | - | - | - | - | - | - | - | - | - | - | - | - | - | - | - | - | - | - | - | - | - | - | - | - | - | - | - | - | - | - | - | - | - | - | - | - | - | - | - | - | - | - | - | - |
| CCTLR7 | - | - | - | - | - | - | - | - | - | - | - | - | - | - | - | - | - | - | - | - | - | - | - | - | - | - | - | - | - | M | K | M | M | T | E | K | T | F | I | I | F | A | G | L | I | S | L | L | D | A | A | E | W | Y | P | K | S | L | K | C | D | V | S | L | A | S | N | G | S | E | V | T | V | N | C | T | E | R | E | L | I | E | I | P | L | G | I | P | T | N | T | T | N | L | T | L | T | I | N | H | I | P | H | I | T | N | D | S | F | H | K | L | Q | N | I | T | E | I | D | L | R | C | N | C | V | P | I | K | V | G | P | K | D | R | V | C | T | Q | S | L | M | I | D | N | G | T | F | W | K | L | K | N | L | K | S | L | Y | L | D | G | N | Q | L | S | S | I | P | K | G | L | P | P | N | I | V | L | L | S | L | E | V | N | S | I | Y | S | I | L | K | E | N | L | T | E | L | T | N | I | Q | T | L | Y | L | G | Q | N | C | Y | F | R | N | P | C | N | T | S | Y | Y | I | E | K | D | A | F | V | Q | L | D | K | M | T | L | L | S | L | K | S | N | N | L | S | Y | I | P | H | Q | L | P | S | S | L | K | E | L | Y | L | Y | N | N | N | I | Q | T | I | T | E | M | D | F | H | N | L | T | E | L | E | I | L | D | L | S | G | N | C | P | R | C | Y | N | A | P | F | P | C | V | P | C | P | N | N | A | P | L | Q | I | H | P | N | S | F | K | T | L | K | N | L | K | T | L | R | L | H | S | N | S | L | T | H | I | P | S | E | W | F | Q | N | L | T | Q | L | G | L | L | D | L | S | S | N | F | L | A | K | E | I | T | C | T | S | F | P | S | L | L | P | N | L | E | E | L | D | L | S | F | N | Y | E | L | Q | V | Y | P | T | S | L | N | L | S | R | S | F | R | H | L | K | S | L | K | V | L | R | I | R | G | Y | V | F | Q | E | L | K | Q | E | D | I | Q | P | L | A | K | L | N | K | L | E | L | I | D | L | G | T | N | F | I | K | I | A | H | L | S | I | L | R | N | L | K | N | F | K | I | I | N | L | S | D | N | K | I | S | M | P | S | Q | G | E | - | - | T | L | L | S | N | H | R | - | - | - | E | S | H | Y | S | S | P | M | S | Q | G | A | Q | Y | H | N | G | E | V | K | D | M | H | Y | F | L | Y | D | E | Y | A | R | S | C | K | Y | K | D | K | E | L | W | I | S | S | P | F | - | N | N | E | C | S | S | F | G | K | T | L | D | I | S | R | N | N | I | F | F | L | H | S | R | F | L | D | L | D | E | L | S | C | L | N | L | S | G | N | A | M | S | Q | S | L | N | G | S | E | F | V | Q | L | K | N | L | Q | Y | L | D | F | S | D | N | R | L | D | L | M | F | S | S | A | F | Q | E | L | S | S | L | V | V | L | D | I | S | R | N | S | H | Y | F | V | A | E | G | L | T | H | M | L | N | F | T | K | N | L | G | K | L | S | K | L | I | L | N | D | N | Q | I | S | T | S | T | N | T | E | M | E | S | F | S | L | E | H | L | E | F | K | G | N | R | L | D | M | L | W | R | D | G | D | T | R | Y | A | N | Y | F | K | K | L | L | A | L | K | S | L | D | I | S | R | N | N | L | N | F | I | P | L | E | V | F | E | G | L | P | D | T | L | T | D | L | Y | I | T | N | N | Q | L | K | - | - | S | F | H | W | E | G | L | V | N | L | X | N | L | K | L | L | D | L | S | G | N | H | L | T | D | V | P | E | C | L | S | - | N | Y | T | K | S | I | Q | T | L | V | L | H | K | N | N | I | V | K | L | T | P | N | F | L | K | D | A | F | S | L | K | V | L | D | L | S | Y | N | R | I | Q | F | I | D | E | S | S | F | P | G | N | V | I | D | Q | L | Q | T | L | Y | L | N | N | N | R | F | V | C | S | C | N | A | T | W | F | V | R | W | I | N | R | T | S | V | N | I | P | R | L | A | M | D | V | T | C | A | S | P | S | A | Q | K | G | Q | S | V | I | F | L | N | - | - | I | Q | A | C | Q | H | N | S | L | S | I | I | L | C | I | L | L | T | S | V | I | L | S | I | L | T | L | T | I | S | S | H | L | F | L | W | D | V | W | Y | I | Y | H | F | C | L | A | K | L | K | G | Y | R | R | L | - | S | S | N | S | T | V | Y | D | A | F | V | V | Y | D | T | T | D | P | A | V | H | E | W | V | M | Q | E | L | R | V | H | L | E | D | K | G | D | P | R | M | Q | L | C | L | E | E | R | D | W | V | P | G | C | P | L | I | E | N | L | S | Q | S | I | Q | L | S | K | R | T | V | F | I | L | T | G | R | Y | I | R | S | G | S | F | R | T | A | F | Y | L | A | H | Q | R | L | M | D | E | R | N | D | V | I | V | L | I | F | L | E | K | M | P | - | C | H | S | K | Y | L | R | L | R | K | R | L | Y | K | K | S | V | L | E | W | P | R | N | P | Q | A | Q | R | Y | F | W | F | S | L | R | S | V | M | A | T | G | S | - | Q | Q | Y | N | K | L | F | Q | E | T | L | X | X | X | X | X | X | X | X | X | X | X | X | X | X | X | X | X | X | X | X | X | X | X | X | X | X | X | X | X | - | - | - | - | - | X | - | - | - | - | - | - | - | - | - | - | - | - | - | - | - | - | - | - | - | - | - | - | - | - | - | - | - | - | - | - | - | - | - | - | - | - | - | - | - | - | - | - | - | - | - | - | - | - | - | - | - | - | - | - | - | - | - | - | - | - | - | - | - | - | - | - | - | - | - | - | - | - | - | - | - | - | - | - | - | - | - | - | - | - | - | - | - | - | - | - | - | - | - | - | - | - | - | - | - | - | - | - | - | - | - | - | - | - | - | - | - | - | - | - | - | - | - | - | - | - | - | - | - | - | - | - | - | - | - | - | - | - | - |
| CBTLR7 | - | - | - | - | - | - | - | - | - | - | - | - | - | - | - | - | - | - | - | - | - | - | - | - | - | - | - | - | - | - | - | - | - | - | - | - | - | - | - | - | - | - | - | - | - | - | - | - | - | - | - | - | - | - | - | - | - | - | - | - | - | - | - | - | - | - | - | - | - | - | - | - | - | - | - | - | - | - | - | - | - | - | - | - | - | - | - | - | - | - | - | - | - | - | - | - | - | - | - | - | - | - | - | - | - | - | - | - | - | - | - | - | - | - | - | - | - | - | - | - | - | - | - | - | - | - | - | - | - | - | - | - | - | - | - | - | - | - | - | - | - | - | - | - | - | - | - | - | - | - | - | - | - | - | - | - | - | - | - | - | - | - | - | - | - | - | - | - | - | - | - | - | - | - | M | L | L | S | L | E | V | N | S | I | S | S | I | L | K | E | N | L | T | E | L | T | N | I | Q | I | L | Y | L | G | Q | N | C | Y | F | R | N | P | C | N | R | S | Y | H | V | E | E | D | A | F | L | Q | L | D | K | L | T | L | L | S | L | K | S | N | N | L | S | Y | V | P | H | K | L | P | S | S | L | K | E | L | Y | L | Y | N | N | N | I | Q | K | V | T | P | E | D | L | N | N | L | T | E | L | E | I | L | D | L | S | G | N | C | P | R | C | Y | N | A | P | F | P | C | V | P | C | P | N | N | S | P | L | Q | I | D | E | N | T | F | L | S | L | T | K | L | K | I | L | R | L | H | S | N | S | L | T | T | V | Q | P | K | W | F | Q | G | C | N | N | L | Q | V | L | D | L | S | S | N | F | L | A | K | D | I | T | H | T | S | F | P | F | H | L | P | L | L | E | E | L | D | L | S | F | N | Y | E | L | Q | R | Y | P | S | S | L | N | L | S | S | A | F | S | N | L | T | N | L | R | I | L | R | I | R | G | F | V | F | Q | E | L | K | Q | E | D | V | N | P | L | T | D | L | N | N | L | E | M | I | D | L | G | T | N | F | I | K | L | T | N | L | G | I | L | K | E | L | K | S | F | H | I | I | N | L | S | D | N | K | I | S | S | P | S | E | A | E | H | A | A | A | L | S | M | A | R | G | F | P | K | Q | D | D | T | S | P | M | S | R | G | A | Q | Y | Q | S | G | E | V | R | E | I | H | Y | F | R | Y | D | E | Y | A | R | S | C | K | S | K | D | K | E | L | G | I | L | S | P | F | - | N | T | Q | C | S | S | F | G | K | T | L | D | L | S | R | N | N | I | F | F | L | H | S | S | F | L | N | L | C | E | L | R | C | L | N | L | S | G | N | A | M | S | Q | A | L | N | G | S | E | F | I | Y | L | K | N | L | K | Y | L | D | F | S | H | N | R | L | D | L | M | F | S | S | A | F | N | E | L | S | N | L | V | V | L | D | I | S | H | N | N | Y | Y | F | E | A | E | G | L | T | H | M | L | N | F | T | K | N | L | N | K | L | T | K | L | V | M | N | H | N | Q | I | S | T | S | T | N | T | E | M | Q | S | L | S | L | E | Y | V | E | F | K | G | N | R | L | D | L | L | W | R | D | G | D | T | R | Y | H | S | Y | F | Q | K | L | R | A | L | K | S | L | D | I | S | H | N | N | L | N | F | I | P | K | Q | V | F | Q | N | L | P | K | T | L | T | E | F | N | I | T | N | N | K | L | R | - | - | S | F | Y | W | D | G | L | R | F | L | P | N | L | M | V | L | D | L | S | G | N | Q | L | T | S | V | P | L | K | L | S | - | N | C | S | K | S | I | E | T | L | V | L | R | K | N | Q | I | S | R | L | S | P | E | F | L | Q | D | A | F | S | L | K | K | L | D | L | S | Y | N | Q | I | K | Y | I | E | E | S | S | F | P | E | A | I | V | D | N | L | E | V | L | F | L | N | N | N | R | F | V | C | S | C | N | A | T | W | F | V | R | W | I | N | R | T | S | V | N | I | P | R | L | A | T | D | V | T | C | A | S | P | G | T | Q | R | G | Q | S | V | I | S | L | N | - | - | L | Q | A | C | Q | H | N | S | L | S | I | I | L | S | I | L | G | T | S | V | I | L | S | V | L | T | L | S | I | S | S | H | L | F | L | W | D | V | W | Y | I | Y | H | F | C | L | A | K | I | K | G | Y | R | R | L | - | I | S | E | S | M | A | Y | D | A | F | I | V | Y | D | K | T | D | S | A | V | S | D | W | V | M | Q | E | L | R | V | Q | L | E | D | R | G | E | P | R | L | N | L | C | L | E | D | R | D | W | V | P | G | C | P | L | I | E | N | L | S | Q | S | I | Q | L | S | K | R | T | I | F | I | L | T | T | H | F | I | R | S | G | H | F | K | T | A | F | Y | L | A | H | Q | R | L | M | D | E | K | D | D | V | I | I | L | I | F | L | E | R | F | S | S | S | H | S | K | Y | L | R | L | R | K | R | L | Y | R | R | S | V | L | E | W | P | R | N | P | Q | A | Q | Q | Y | F | W | F | S | L | R | S | V | M | A | T | E | S | Q | Q | H | Y | N | K | L | F | Q | E | T | L | X | X | X | X | X | X | X | X | X | X | X | X | X | X | X | X | X | X | X | X | X | X | X | X | X | X | X | X | X | X | X | X | X | X | X | X | X | X | X | X | X | X | X | X | X | X | X | X | X | X | X | X | X | X | X | X | X | X | X | X | X | X | X | X | X | X | X | X | X | X | X | X | X | X | X | X | X | X | X | X | X | X | X | X | X | X | X | X | X | X | X | X | X | X | X | X | X | X | X | X | X | X | X | X | X | X | X | X | X | X | X | X | X | X | X | X | X | X | X | X | X | X | X | X | X | X | X | X | X | X | X | X | X | X | X | X | X | X | X | X | X | X | X | X | X | X | X | X | X | X | X | X | X | X | X | X | X | X | X | X | X | X | X | X | X | X | X | X |
| TFTLR7 | - | - | - | - | - | - | - | - | - | - | - | - | - | - | - | - | - | - | - | - | M | S | K | P | D | K | K | M | A | Q | R | I | V | I | L | K | I | A | V | L | L | I | F | C | P | S | V | C | S | E | S | K | W | Y | P | K | S | L | Q | C | D | V | S | R | I | S | N | D | T | E | V | K | V | D | C | T | S | R | G | L | K | S | V | P | P | G | I | P | S | N | T | T | N | L | T | L | T | I | N | H | I | S | H | I | N | N | T | S | F | L | G | L | G | N | I | T | E | I | D | M | R | C | N | C | V | P | I | K | I | G | P | K | D | H | V | C | M | Q | S | V | Q | I | D | N | G | S | F | W | E | L | K | T | L | K | S | L | Y | L | D | G | N | Q | L | S | S | I | P | K | G | L | P | Q | N | V | V | L | L | S | L | E | V | N | S | I | S | S | I | L | K | E | N | L | T | E | L | T | N | I | Q | I | L | Y | L | G | Q | N | C | Y | F | R | N | P | C | N | R | S | Y | Y | I | E | P | D | A | F | L | Q | L | D | K | L | T | L | L | S | L | K | S | N | N | L | S | Y | V | P | H | R | L | P | S | S | L | K | E | L | Y | L | Y | N | N | N | I | Q | K | V | T | E | E | D | F | N | N | L | T | S | L | E | I | L | D | L | S | G | N | C | P | R | C | H | N | A | P | F | P | C | V | P | C | A | N | N | A | P | L | Q | I | H | N | L | S | F | L | N | L | N | R | L | K | V | L | R | L | H | S | N | S | L | T | T | V | N | P | L | W | F | Q | G | C | K | E | L | Q | V | L | D | L | S | S | N | F | L | A | K | D | I | T | H | T | S | F | P | H | S | L | P | F | L | E | E | L | D | L | S | F | N | Y | E | L | Q | R | Y | P | S | S | L | N | L | S | R | A | F | S | S | L | K | S | L | R | I | L | R | I | R | G | F | V | F | Q | E | L | K | Q | E | D | I | Q | P | L | T | F | L | G | K | L | Q | V | I | D | L | G | T | N | F | I | K | L | T | N | L | S | I | L | K | G | L | K | N | F | H | I | I | N | L | S | D | N | K | I | S | S | P | S | E | A | E | H | A | A | A | L | S | M | A | R | G | F | P | K | Q | D | D | T | S | P | M | S | R | G | A | Q | Y | R | S | D | E | V | R | E | I | H | Y | F | R | Y | D | E | Y | A | R | S | C | K | N | K | D | K | E | L | G | T | L | S | P | F | - | N | T | K | C | S | S | F | G | K | T | L | D | L | S | R | N | N | I | F | F | L | H | S | R | F | L | N | L | G | E | L | R | C | L | N | L | S | G | N | A | M | S | Q | A | L | N | G | S | E | F | V | H | L | K | N | L | K | Y | L | D | F | S | N | N | R | L | D | L | M | F | S | S | A | F | K | E | L | S | N | L | V | V | L | D | I | S | H | N | N | Y | Y | F | E | A | E | G | L | T | H | M | L | N | F | T | K | N | L | H | N | M | K | K | L | I | M | N | H | N | Q | I | S | T | S | T | N | T | E | M | E | S | S | S | L | E | Y | L | E | F | K | N | N | R | L | D | M | L | W | R | D | G | D | T | R | Y | H | D | Y | F | K | K | L | E | K | L | K | S | L | D | I | S | H | N | N | L | N | F | F | P | E | P | I | F | Q | K | L | P | K | T | I | R | E | F | N | I | T | N | N | K | L | K | - | - | S | F | Y | W | E | G | L | R | F | L | P | N | L | E | V | L | D | L | S | A | N | L | L | T | S | V | P | P | K | L | S | - | N | C | S | T | S | I | T | T | L | V | L | R | K | N | Q | I | F | R | L | S | P | E | F | L | Q | D | A | F | S | L | K | K | L | D | L | S | Y | N | Q | I | K | Y | I | E | E | S | S | F | P | E | D | V | I | D | K | L | E | I | L | F | L | N | N | N | R | F | V | C | S | C | N | A | T | W | F | I | R | W | A | N | R | T | S | V | Y | I | P | R | L | A | T | D | V | T | C | A | S | P | G | T | Q | R | G | Q | S | V | I | S | L | N | - | - | L | Q | A | C | Q | H | N | S | L | S | I | I | F | S | I | L | T | T | S | V | T | L | S | V | L | T | L | S | I | S | S | H | L | F | L | W | D | V | W | Y | I | Y | H | F | C | L | A | K | L | K | G | Y | R | R | L | - | L | S | E | N | T | A | Y | D | A | F | I | V | Y | D | K | T | D | T | A | V | S | D | W | V | L | Q | E | L | R | A | Q | L | E | D | C | G | E | P | R | L | H | L | C | L | E | D | R | D | W | V | V | G | C | P | I | I | E | N | L | S | Q | S | I | Q | L | S | K | R | T | V | F | I | L | T | A | H | F | I | Q | S | G | Y | F | K | M | A | F | Y | L | A | H | Q | R | L | M | D | E | K | N | D | V | I | V | L | I | F | L | E | R | F | S | S | S | H | S | K | Y | L | R | L | R | K | R | L | Y | K | R | S | V | L | E | W | P | K | N | P | Q | A | Q | Q | Y | F | W | F | C | L | R | S | V | M | A | T | E | S | Q | R | H | Y | N | K | L | F | Q | E | T | L | X | X | X | X | X | X | X | X | X | X | X | X | X | X | - | - | - | - | - | - | - | - | - | - | - | - | - | - | - | - | - | - | - | - | - | - | - | - | - | - | - | - | - | - | - | - | - | - | - | - | - | - | - | - | - | - | - | - | - | - | - | - | - | - | - | - | - | - | - | - | - | - | - | - | - | - | - | - | - | - | - | - | - | - | - | - | - | - | - | - | - | - | - | - | - | - | - | - | - | - | - | - | - | - | - | - | - | - | - | - | - | - | - | - | - | - | - | - | - | - | - | - | - | - | - | - | - | - | - | - | - | - | - | - | - | - | - | - | - | - | - | - | - | - | - | - | - | - | - | - | - | - | - | - | - | - | - | - | - | - | - | - | - | - | - | - | - | - |
| IPTLR7 | - | - | - | - | - | - | - | - | - | - | - | - | - | - | - | - | - | - | - | - | - | - | - | - | - | - | - | M | A | G | R | I | I | I | L | K | I | A | F | L | L | L | F | C | P | S | V | C | S | E | T | E | W | Y | P | K | S | L | Q | C | D | V | S | R | I | S | N | D | T | E | V | K | V | D | C | T | S | R | G | L | T | S | I | P | P | G | I | P | S | N | T | T | N | L | T | L | T | I | N | H | I | P | H | I | N | E | T | S | F | L | G | L | N | N | I | T | E | I | D | M | R | C | N | C | V | P | I | K | I | G | P | K | D | H | V | C | A | K | S | V | Q | I | D | N | G | S | F | W | Q | L | K | T | L | K | S | L | Y | L | D | G | N | Q | L | S | S | I | P | K | G | L | P | P | N | I | V | L | L | S | L | E | V | N | S | I | S | S | I | L | K | E | N | L | T | E | L | T | N | I | E | I | L | Y | L | G | Q | N | C | Y | F | R | N | P | C | N | R | S | Y | H | V | E | E | D | A | F | L | Q | F | G | K | L | T | L | L | S | L | K | S | N | N | L | S | Y | V | P | H | K | L | P | S | S | L | K | E | L | Y | L | Y | N | N | N | I | Q | K | V | A | P | E | D | F | H | N | L | T | K | L | E | I | L | D | L | S | G | N | C | P | R | C | Y | N | A | P | F | P | C | V | P | C | A | D | G | A | P | L | Q | I | H | E | N | S | F | L | N | L | T | K | L | K | I | L | R | L | H | S | N | S | L | T | T | V | Q | P | Q | W | F | Q | G | C | R | E | L | Q | V | L | D | L | S | S | N | F | L | A | N | D | I | T | H | T | S | F | P | H | S | L | P | L | L | E | E | L | D | L | S | F | N | Y | E | L | Q | R | Y | P | S | S | L | N | L | S | P | S | F | S | S | L | K | S | L | R | I | L | R | I | R | G | F | V | F | Q | E | L | K | Q | Q | D | I | Q | P | L | T | C | L | E | N | L | E | M | I | D | L | G | T | N | F | I | K | L | T | N | L | S | I | L | T | G | L | K | S | F | R | I | I | N | L | S | D | N | K | I | S | S | P | S | E | A | E | Y | A | A | A | M | A | T | A | R | G | V | L | K | Q | D | G | T | S | P | M | S | R | G | T | Q | Y | Q | S | G | E | V | R | E | I | H | Y | F | R | Y | D | E | Y | A | R | S | C | K | S | K | D | K | E | L | G | T | L | S | P | F | - | N | T | Q | C | S | S | F | G | K | T | L | D | L | S | R | N | N | I | F | F | L | H | S | R | F | L | N | L | S | E | L | R | C | L | N | L | S | G | N | A | M | S | Q | A | L | N | G | S | E | F | V | C | L | K | N | L | K | Y | L | D | F | S | H | N | R | L | D | L | M | F | S | S | A | F | K | E | L | S | N | L | V | V | L | D | I | S | H | N | N | Y | Y | F | E | A | E | G | L | T | H | M | L | N | F | T | N | N | L | N | K | L | T | K | L | I | M | N | H | N | Q | I | S | T | S | T | N | T | E | I | Q | S | L | S | L | E | H | L | E | F | K | G | N | R | L | D | M | L | W | R | D | G | D | N | R | Y | H | N | Y | F | R | K | L | M | V | L | K | S | L | D | I | S | H | N | N | L | N | F | I | P | K | Q | V | F | Q | S | L | P | E | T | L | T | E | L | N | I | T | N | N | K | L | K | - | - | S | F | Y | W | D | G | L | R | F | L | P | N | L | E | V | L | D | L | S | G | N | L | L | T | S | V | P | P | K | L | S | - | N | C | S | K | S | I | V | T | L | V | L | R | K | N | Q | I | S | R | L | S | P | E | F | L | Q | D | A | F | S | L | K | K | L | D | L | S | Y | N | Q | I | K | Y | I | E | E | S | S | F | P | E | D | V | I | D | K | L | E | V | L | F | L | N | N | N | R | F | V | C | S | C | N | A | T | W | F | V | R | W | I | N | R | T | S | V | Y | I | P | R | L | A | T | D | V | T | C | A | S | P | G | T | Q | R | G | Q | S | V | I | S | L | N | - | - | L | Q | A | C | L | H | N | S | L | S | I | I | L | S | I | L | T | T | S | V | I | L | S | V | L | T | L | S | I | S | S | H | L | F | L | W | D | V | W | Y | I | Y | H | F | C | L | A | K | I | K | G | Y | R | R | L | - | L | S | E | S | T | P | Y | D | A | F | V | V | Y | D | R | T | D | T | A | V | S | E | W | V | L | K | E | L | R | V | Q | L | E | D | R | G | E | S | R | L | H | L | C | L | E | D | R | D | W | V | P | G | C | P | L | I | E | N | L | S | Q | S | I | Q | L | S | K | R | T | V | F | I | L | T | T | R | F | I | R | S | G | H | F | K | T | A | F | Y | L | A | H | Q | R | L | M | D | E | K | D | D | V | I | I | L | I | F | L | E | R | F | S | S | S | H | S | K | Y | L | R | L | R | K | R | L | Y | K | R | S | V | L | E | W | P | R | N | P | Q | A | Q | Q | Y | F | W | F | C | L | R | S | V | M | A | T | E | S | Q | K | H | Y | N | K | L | F | Q | E | T | L | X | X | X | X | X | X | X | X | X | X | X | X | X | X | X | X | X | X | X | X | X | - | - | - | - | - | - | - | - | - | - | - | - | - | - | - | - | - | - | - | - | - | - | - | - | - | - | - | - | - | - | - | - | - | - | - | - | - | - | - | - | - | - | - | - | - | - | - | - | - | - | - | - | - | - | - | - | - | - | - | - | - | - | - | - | - | - | - | - | - | - | - | - | - | - | - | - | - | - | - | - | - | - | - | - | - | - | - | - | - | - | - | - | - | - | - | - | - | - | - | - | - | - | - | - | - | - | - | - | - | - | - | - | - | - | - | - | - | - | - | - | - | - | - | - | - | - | - | - | - | - | - | - | - | - | - | - | - | - | - | - | - | - | - | - | - | - | - |
| PHTLR7 | - | - | - | - | - | - | - | - | - | - | - | - | - | - | - | - | - | - | - | - | M | P | E | P | N | K | K | M | A | R | R | I | I | V | L | K | I | A | I | L | L | L | F | C | P | S | A | C | S | E | T | E | W | Y | P | K | S | L | Q | C | D | V | S | R | L | S | N | D | S | E | V | K | V | D | C | T | S | R | G | L | T | S | I | P | P | G | I | P | S | N | T | T | N | L | T | L | T | I | N | H | I | P | H | I | N | G | T | S | F | L | G | L | S | N | I | T | E | I | D | M | R | C | N | C | V | P | I | K | I | G | P | K | D | H | V | C | T | K | S | V | Q | I | D | N | G | S | F | W | Q | L | K | T | L | K | S | L | Y | L | D | G | N | Q | L | F | S | I | P | K | G | L | P | P | N | V | V | L | L | S | L | E | V | N | S | I | S | S | I | L | K | E | N | L | T | E | L | T | N | I | E | I | L | Y | L | G | Q | N | C | Y | F | R | N | P | C | N | R | S | Y | Y | V | E | E | D | A | F | L | Q | L | D | K | L | T | L | L | S | L | K | S | N | N | L | S | Y | V | P | H | K | L | P | S | S | L | R | E | L | Y | L | Y | N | N | N | I | Q | K | V | V | A | E | D | F | H | N | L | T | E | L | E | I | L | D | L | S | G | N | C | P | R | C | Y | N | A | P | F | P | C | V | P | C | A | N | D | A | P | L | Q | I | H | E | H | S | F | L | N | L | T | K | L | K | I | L | R | L | H | S | N | S | L | T | T | V | Q | S | Q | W | F | Q | G | C | K | E | L | Q | V | L | D | L | S | S | N | F | L | A | K | D | I | T | H | T | S | F | P | H | S | L | P | F | L | E | E | L | D | L | S | F | N | Y | E | L | Q | R | Y | P | S | S | L | N | L | S | Q | S | F | S | S | L | K | S | L | R | I | L | R | I | R | G | F | V | F | Q | E | L | K | Q | Q | D | I | Q | P | L | T | F | L | E | N | L | E | M | I | D | L | G | T | N | F | I | K | L | T | N | L | S | I | L | K | G | L | K | N | F | R | I | I | N | L | S | D | N | K | I | S | S | P | S | E | A | E | H | A | A | A | L | A | M | A | R | G | F | P | K | Q | D | D | T | S | P | M | S | R | G | A | Q | Y | R | S | G | E | V | R | E | I | H | Y | F | R | Y | D | E | Y | A | R | S | C | K | S | K | D | K | E | L | G | T | L | S | P | F | - | N | T | Q | C | S | S | F | G | K | T | L | D | L | S | R | N | N | I | F | F | L | H | S | R | F | L | N | L | G | E | L | R | C | L | N | L | S | G | N | A | M | S | Q | A | L | N | G | T | E | F | I | Y | L | K | N | L | K | Y | L | D | F | S | H | N | R | L | D | L | M | F | S | S | A | F | K | E | L | S | N | L | V | V | L | D | I | S | H | N | N | Y | Y | F | E | A | E | G | L | T | H | M | L | N | F | T | K | N | L | H | K | L | T | K | L | I | M | N | H | N | Q | I | S | T | S | T | N | M | E | M | Q | S | L | S | L | E | Y | L | E | F | K | G | N | R | L | D | L | L | W | R | D | G | D | T | R | Y | H | S | Y | F | R | K | L | M | V | L | K | S | L | D | I | S | H | N | N | L | N | F | I | P | K | Q | V | F | Q | S | L | P | E | T | L | T | E | F | H | I | T | N | N | I | L | K | - | - | S | F | Y | W | D | G | L | R | F | L | P | N | L | E | L | L | D | L | S | G | N | H | L | T | I | V | P | P | K | L | S | - | N | C | S | K | S | I | A | T | L | V | L | R | K | N | Q | I | L | R | L | S | P | E | F | L | Q | D | A | F | S | L | T | R | L | D | L | S | Y | N | Q | I | K | Y | I | E | E | S | S | F | P | E | N | V | I | D | K | L | E | V | L | F | L | N | N | N | R | F | V | C | S | C | N | A | T | W | F | V | R | W | I | N | R | T | S | V | Y | I | P | R | L | A | T | D | V | T | C | A | S | P | G | T | Q | R | G | Q | S | V | I | S | L | N | - | - | L | Q | A | C | Q | H | N | S | L | S | I | I | L | S | I | L | T | T | S | V | I | L | S | V | L | I | L | S | I | S | S | H | L | F | L | W | D | V | W | Y | I | Y | H | F | C | L | A | K | I | K | G | Y | R | R | L | - | L | S | E | S | T | A | Y | D | A | F | V | V | Y | D | K | T | D | T | A | V | S | D | W | V | L | Q | E | L | R | V | Q | L | E | D | R | G | E | P | R | L | H | L | C | L | E | E | R | D | W | V | P | G | C | P | L | I | E | N | L | S | Q | S | I | Q | L | S | K | R | T | I | F | L | L | T | T | H | F | I | R | S | G | H | F | K | T | A | F | Y | L | A | H | Q | R | L | M | D | E | K | D | D | V | I | I | L | I | F | L | E | R | F | S | S | S | H | S | K | Y | L | R | L | R | K | R | L | Y | K | R | S | V | L | E | W | P | R | N | P | Q | A | Q | Q | Y | F | W | F | C | L | R | S | V | M | A | T | E | S | Q | Q | H | Y | N | K | L | F | Q | E | T | L | X | X | X | X | X | X | X | X | X | X | X | X | X | X | - | - | - | - | - | - | - | - | - | - | - | - | - | - | - | - | - | - | - | - | - | - | - | - | - | - | - | - | - | - | - | - | - | - | - | - | - | - | - | - | - | - | - | - | - | - | - | - | - | - | - | - | - | - | - | - | - | - | - | - | - | - | - | - | - | - | - | - | - | - | - | - | - | - | - | - | - | - | - | - | - | - | - | - | - | - | - | - | - | - | - | - | - | - | - | - | - | - | - | - | - | - | - | - | - | - | - | - | - | - | - | - | - | - | - | - | - | - | - | - | - | - | - | - | - | - | - | - | - | - | - | - | - | - | - | - | - | - | - | - | - | - | - | - | - | - | - | - | - | - | - | - | - | - |
| conservation |  |  |  |  |  |  |  |  |  |  |  |  |  |  |  |  |  |  |  |  |  |  |  |  |  |  |  |  |  |  |  |  |  |  |  |  |  |  |  |  |  |  |  |  |  |  |  |  |  |  |  |  |  |  |  |  |  |  |  |  |  |  |  |  |  |  |  |  |  |  |  |  |  |  |  |  |  |  |  |  |  |  |  |  |  |  |  |  |  |  |  |  |  |  |  |  |  |  |  |  |  |  |  |  |  |  |  |  |  |  |  |  |  |  |  |  |  |  |  |  |  |  |  |  |  |  |  |  |  |  |  |  |  |  |  |  |  |  |  |  |  |  |  |  |  |  |  |  |  |  |  |  |  |  |  |  |  |  |  |  |  |  |  |  |  |  |  |  |  |  |  |  |  |  |  |  |  |  |  |  |  |  |  |  |  |  |  |  |  |  |  |  |  |  |  |  |  |  |  |  |  |  |  |  |  |  |  |  |  |  |  |  |  |  |  |  |  |  |  |  |  |  |  |  |  |  |  |  |  |  |  |  |  |  |  |  |  |  |  |  |  |  |  |  |  |  |  |  |  |  |  |  |  |  |  |  |  |  |  |  |  |  |  |  |  |  |  |  |  |  |  |  |  |  |  |  |  |  |  |  |  |  |  |  |  |  |  |  |  |  |  |  |  |  |  |  |  |  |  |  |  |  |  |  |  |  |  |  |  |  |  |  |  |  |  |  |  |  |  |  |  |  |  |  |  |  |  |  |  |  |  |  |  |  |  |  |  |  |  |  |  |  |  |  |  |  |  |  |  |  |  |  |  |  |  |  |  |  |  |  |  |  |  |  |  |  |  |  |  |  |  |  |  |  |  |  |  |  |  |  |  |  |  |  |  |  |  |  |  |  |  |  |  |  |  |  |  |  |  |  |  |  |  |  |  |  |  |  |  |  |  |  |  |  |  |  |  |  |  |  |  |  |  |  |  |  |  |  |  |  |  |  |  |  |  |  |  |  |  |  |  |  |  |  |  |  |  |  |  |  |  |  |  |  |  |  |  |  |  |  |  |  |  |  |  |  |  |  |  |  |  |  |  |  |  |  |  |  |  |  |  |  |  |  |  |  |  |  |  |  |  |  |  |  |  |  |  |  |  |  |  |  |  |  |  |  |  |  |  |  |  |  |  |  |  |  |  |  |  |  |  |  |  |  |  |  |  |  |  |  |  |  |  |  |  |  |  |  |  |  |  |  |  |  |  |  |  |  |  |  |  |  |  |  |  |  |  |  |  |  |  |  |  |  |  |  |  |  |  |  |  |  |  |  |  |  |  |  |  |  |  |  |  |  |  |  |  |  |  |  |  |  |  |  |  |  |  |  |  |  |  |  |  |  |  |  |  |  |  |  |  |  |  |  |  |  |  |  |  |  |  |  |  |  |  |  |  |  |  |  |  |  |  |  |  |  |  |  |  |  |  |  |  |  |  |  |  |  |  |  |  |  |  |  |  |  |  |  |  |  |  |  |  |  |  |  |  |  |  |  |  |  |  |  |  |  |  |  |  |  |  |  |  |  |  |  |  |  |  |  |  |  |  |  |  |  |  |  |  |  |  |  |  |  |  |  |  |  |  |  |  |  |  |  |  |  |  |  |  |  |  |  |  |  |  |  |  |  |  |  |  |  |  |  |  |  |  |  |  |  |  |  |  |  |  |  |  |  |  |  |  |  |  |  |  |  |  |  |  |  |  |  |  |  |  |  |  |  |  |  |  |  |  |  |  |  |  |  |  |  |  |  |  |  |  |  |  |  |  |  |  |  |  |  |  |  |  |  |  |  |  |  |  |  |  |  |  |  |  |  |  |  |  |  |  |  |  |  |  |  |  |  |  |  |  |  |  |  |  |  |  |  |  |  |  |  |  |  |  |  |  |  |  |  |  |  |  |  |  |  |  |  |  |  |  |  |  |  |  |  |  |  |  |  |  |  |  |  |  |  |  |  |  |  |  |  |  |  |  |  |  |  |  |  |  |  |  |  |  |  |  |  |  |  |  |  |  |  |  |  |  |  |  |  |  |  |  |  |  |  |  |  |  |  |  |  |  |  |  |  |  |  |  |  |  |  |  |  |  |  |  |  |  |  |  |  |  |  |  |  |  |  |  |  |  |  |  |  |  |  |  |  |  |  |  |  |  |  |  |  |  |  |  |  |  |  |  |  |  |  |  |  |  |  |  |  |  |  |  |  |  |  |  |  |  |  |  |  |  |  |  |  |  |  |  |  |  |  |  |  |  |  |  |  |  |  |  |  |  |  |  |  |  |  |  |  |  |  |  |  |  |  |  |  |  |  |  |  |  |  |  |  |  |  |  |  |  |  |  |  |  |  |  |  |  |  |  |  |  |  |  |  |  |  |  |  |  |  |  |  |  |  |  |  |  |  |  |  |  |  |  |  |  |  |  |  |  |  |  |  |  |  |  |  |  |  |  |  |  |  |  |  |  |  |  |  |  |  |  |  |  |  |  |  |  |  |  |  |  |  |  |  |  |  |  |  |  |  |  |  |  |  |  |  |  |  |  |  |  |  |  |  |  |  |  |  |  |  |  |  |  |  |  |  |  |  |  |  |  |  |  |  |  |  |  |  |  |  |  |  |  |  |  |  |  |  |  |  |  |  |  |  |  |  |  |  |  |  |  |  |  |  |  |  |  |  |  |  |  |  |  |  |  |  |  |  |  |  |  |  |  |  |  |  |  |  |  |  |  |  |  |  |  |  |  |  |  |  |  |  |  |  |  |  |  |  |  |  |  |  |  |  |  |  |  |  |  |  |  |  |  |  |  |  |  |  |  |  |  |  |  |  |  |  |  |  |  |  |  |  |
|  |  |  |  |  |  |  |  |  |  |  |  |  |  |  |  |  |  |  |  |  |  |  |  |  |  |  |  |  |  |  |  |  |  |  |  |  |  |  |  |  |  |  |  |  |  |  |  |  |  |  |  |  |  |  |  |  |  |  |  |  |  |  |  |  |  |  |  |  |  |  |  |  |  |  |  |  |  |  |  |  |  |  |  |  |  |  |  |  |  |  |  |  |  |  |  |  |  |  |  |  |  |  |  |  |  |  |  |  |  |  |  |  |  |  |  |  |  |  |  |  |  |  |  |  |  |  |  |  |  |  |  |  |  |  |  |  |  |  |  |  |  |  |  |  |  |  |  |  |  |  |  |  |  |  |  |  |  |  |  |  |  |  |  |  |  |  |  |  |  |  |  |  |  |  |  |  |  |  |  |  |  |  |  |  |  |  |  |  |  |  |  |  |  |  |  |  |  |  |  |  |  |  |  |  |  |  |  |  |  |  |  |  |  |  |  |  |  |  |  |  |  |  |  |  |  |  |  |  |  |  |  |  |  |  |  |  |  |  |  |  |  |  |  |  |  |  |  |  |  |  |  |  |  |  |  |  |  |  |  |  |  |  |  |  |  |  |  |  |  |  |  |  |  |  |  |  |  |  |  |  |  |  |  |  |  |  |  |  |  |  |  |  |  |  |  |  |  |  |  |  |  |  |  |  |  |  |  |  |  |  |  |  |  |  |  |  |  |  |  |  |  |  |  |  |  |  |  |  |  |  |  |  |  |  |  |  |  |  |  |  |  |  |  |  |  |  |  |  |  |  |  |  |  |  |  |  |  |  |  |  |  |  |  |  |  |  |  |  |  |  |  |  |  |  |  |  |  |  |  |  |  |  |  |  |  |  |  |  |  |  |  |  |  |  |  |  |  |  |  |  |  |  |  |  |  |  |  |  |  |  |  |  |  |  |  |  |  |  |  |  |  |  |  |  |  |  |  |  |  |  |  |  |  |  |  |  |  |  |  |  |  |  |  |  |  |  |  |  |  |  |  |  |  |  |  |  |  |  |  |  |  |  |  |  |  |  |  |  |  |  |  |  |  |  |  |  |  |  |  |  |  |  |  |  |  |  |  |  |  |  |  |  |  |  |  |  |  |  |  |  |  |  |  |  |  |  |  |  |  |  |  |  |  |  |  |  |  |  |  |  |  |  |  |  |  |  |  |  |  |  |  |  |  |  |  |  |  |  |  |  |  |  |  |  |  |  |  |  |  |  |  |  |  |  |  |  |  |  |  |  |  |  |  |  |  |  |  |  |  |  |  |  |  |  |  |  |  |  |  |  |  |  |  |  |  |  |  |  |  |  |  |  |  |  |  |  |  |  |  |  |  |  |  |  |  |  |  |  |  |  |  |  |  |  |  |  |  |  |  |  |  |  |  |  |  |  |  |  |  |  |  |  |  |  |  |  |  |  |  |  |  |  |  |  |  |  |  |  |  |  |  |  |  |  |  |  |  |  |  |  |  |  |  |  |  |  |  |  |  |  |  |  |  |  |  |  |  |  |  |  |  |  |  |  |  |  |  |  |  |  |  |  |  |  |  |  |  |  |  |  |  |  |  |  |  |  |  |  |  |  |  |  |  |  |  |  |  |  |  |  |  |  |  |  |  |  |  |  |  |  |  |  |  |  |  |  |  |  |  |  |  |  |  |  |  |  |  |  |  |  |  |  |  |  |  |  |  |  |  |  |  |  |  |  |  |  |  |  |  |  |  |  |  |  |  |  |  |  |  |  |  |  |  |  |  |  |  |  |  |  |  |  |  |  |  |  |  |  |  |  |  |  |  |  |  |  |  |  |  |  |  |  |  |  |  |  |  |  |  |  |  |  |  |  |  |  |  |  |  |  |  |  |  |  |  |  |  |  |  |  |  |  |  |  |  |  |  |  |  |  |  |  |  |  |  |  |  |  |  |  |  |  |  |  |  |  |  |  |  |  |  |  |  |  |  |  |  |  |  |  |  |  |  |  |  |  |  |  |  |  |  |  |  |  |  |  |  |  |  |  |  |  |  |  |  |  |  |  |  |  |  |  |  |  |  |  |  |  |  |  |  |  |  |  |  |  |  |  |  |  |  |  |  |  |  |  |  |  |  |  |  |  |  |  |  |  |  |  |  |  |  |  |  |  |  |  |  |  |  |  |  |  |  |  |  |  |  |  |  |  |  |  |  |  |  |  |  |  |  |  |  |  |  |  |  |  |  |  |  |  |  |  |  |  |  |  |  |  |  |  |  |  |  |  |  |  |  |  |  |  |  |  |  |  |  |  |  |  |  |  |  |  |  |  |  |  |  |  |  |  |  |  |  |  |  |  |  |  |  |  |  |  |  |  |  |  |  |  |  |  |  |  |  |  |  |  |  |  |  |  |  |  |  |  |  |  |  |  |  |  |  |  |  |  |  |  |  |  |  |  |  |  |  |  |  |  |  |  |  |  |  |  |  |  |  |  |  |  |  |  |  |  |  |  |  |  |  |  |  |  |  |  |  |  |  |  |  |  |  |  |  |  |  |  |  |  |  |  |  |  |  |  |  |  |  |  |  |  |  |  |  |  |  |  |  |  |  |  |  |  |  |  |  |  |  |  |  |  |  |  |  |  |  |  |  |  |  |  |  |  |  |  |  |  |  |  |  |  |  |  |  |  |  |  |  |  |  |  |  |  |  |  |  |  |  |  |  |  |  |  |  |  |  |  |  |  |  |  |  |  |  |  |  |  |  |  |  |  |  |  |  |  |  |  |  |  |  |  |  |  |  |  |  |  |  |  |  |  |  |  |  |  |  |  |  |  |  |  |  |  |  |  |  |  |  |  |  |  |  |  |
|  |  |  |  |  |  |  |  |  |  |  |  |  |  |  |  |  |  |  |  |  |  |  |  |  |  |  |  |  |  |  |  |  |  |  |  |  |  |  |  |  |  |  |  |  |  |  |  |  |  |  |  |  |  |  |  |  |  |  |  |  |  |  |  |  |  |  |  |  |  |  |  |  |  |  |  |  |  |  |  |  |  |  |  |  |  |  |  |  |  |  |  |  |  |  |  |  |  |  |  |  |  |  |  |  |  |  |  |  |  |  |  |  |  |  |  |  |  |  |  |  |  |  |  |  |  |  |  |  |  |  |  |  |  |  |  |  |  |  |  |  |  |  |  |  |  |  |  |  |  |  |  |  |  |  |  |  |  |  |  |  |  |  |  |  |  |  |  |  |  |  |  |  |  |  |  |  |  |  |  |  |  |  |  |  |  |  |  |  |  |  |  |  |  |  |  |  |  |  |  |  |  |  |  |  |  |  |  |  |  |  |  |  |  |  |  |  |  |  |  |  |  |  |  |  |  |  |  |  |  |  |  |  |  |  |  |  |  |  |  |  |  |  |  |  |  |  |  |  |  |  |  |  |  |  |  |  |  |  |  |  |  |  |  |  |  |  |  |  |  |  |  |  |  |  |  |  |  |  |  |  |  |  |  |  |  |  |  |  |  |  |  |  |  |  |  |  |  |  |  |  |  |  |  |  |  |  |  |  |  |  |  |  |  |  |  |  |  |  |  |  |  |  |  |  |  |  |  |  |  |  |  |  |  |  |  |  |  |  |  |  |  |  |  |  |  |  |  |  |  |  |  |  |  |  |  |  |  |  |  |  |  |  |  |  |  |  |  |  |  |  |  |  |  |  |  |  |  |  |  |  |  |  |  |  |  |  |  |  |  |  |  |  |  |  |  |  |  |  |  |  |  |  |  |  |  |  |  |  |  |  |  |  |  |  |  |  |  |  |  |  |  |  |  |  |  |  |  |  |  |  |  |  |  |  |  |  |  |  |  |  |  |  |  |  |  |  |  |  |  |  |  |  |  |  |  |  |  |  |  |  |  |  |  |  |  |  |  |  |  |  |  |  |  |  |  |  |  |  |  |  |  |  |  |  |  |  |  |  |  |  |  |  |  |  |  |  |  |  |  |  |  |  |  |  |  |  |  |  |  |  |  |  |  |  |  |  |  |  |  |  |  |  |  |  |  |  |  |  |  |  |  |  |  |  |  |  |  |  |  |  |  |  |  |  |  |  |  |  |  |  |  |  |  |  |  |  |  |  |  |  |  |  |  |  |  |  |  |  |  |  |  |  |  |  |  |  |  |  |  |  |  |  |  |  |  |  |  |  |  |  |  |  |  |  |  |  |  |  |  |  |  |  |  |  |  |  |  |  |  |  |  |  |  |  |  |  |  |  |  |  |  |  |  |  |  |  |  |  |  |  |  |  |  |  |  |  |  |  |  |  |  |  |  |  |  |  |  |  |  |  |  |  |  |  |  |  |  |  |  |  |  |  |  |  |  |  |  |  |  |  |  |  |  |  |  |  |  |  |  |  |  |  |  |  |  |  |  |  |  |  |  |  |  |  |  |  |  |  |  |  |  |  |  |  |  |  |  |  |  |  |  |  |  |  |  |  |  |  |  |  |  |  |  |  |  |  |  |  |  |  |  |  |  |  |  |  |  |  |  |  |  |  |  |  |  |  |  |  |  |  |  |  |  |  |  |  |  |  |  |  |  |  |  |  |  |  |  |  |  |  |  |  |  |  |  |  |  |  |  |  |  |  |  |  |  |  |  |  |  |  |  |  |  |  |  |  |  |  |  |  |  |  |  |  |  |  |  |  |  |  |  |  |  |  |  |  |  |  |  |  |  |  |  |  |  |  |  |  |  |  |  |  |  |  |  |  |  |  |  |  |  |  |  |  |  |  |  |  |  |  |  |  |  |  |  |  |  |  |  |  |  |  |  |  |  |  |  |  |  |  |  |  |  |  |  |  |  |  |  |  |  |  |  |  |  |  |  |  |  |  |  |  |  |  |  |  |  |  |  |  |  |  |  |  |  |  |  |  |  |  |  |  |  |  |  |  |  |  |  |  |  |  |  |  |  |  |  |  |  |  |  |  |  |  |  |  |  |  |  |  |  |  |  |  |  |  |  |  |  |  |  |  |  |  |  |  |  |  |  |  |  |  |  |  |  |  |  |  |  |  |  |  |  |  |  |  |  |  |  |  |  |  |  |  |  |  |  |  |  |  |  |  |  |  |  |  |  |  |  |  |  |  |  |  |  |  |  |  |  |  |  |  |  |  |  |  |  |  |  |  |  |  |  |  |  |  |  |  |  |  |  |  |  |  |  |  |  |  |  |  |  |  |  |  |  |  |  |  |  |  |  |  |  |  |  |  |  |  |  |  |  |  |  |  |  |  |  |  |  |  |  |  |  |  |  |  |  |  |  |  |  |  |  |  |  |  |  |  |  |  |  |  |  |  |  |  |  |  |  |  |  |  |  |  |  |  |  |  |  |  |  |  |  |  |  |  |  |  |  |  |  |  |  |  |  |  |  |  |  |  |  |  |  |  |  |  |  |  |  |  |  |  |  |  |  |  |  |  |  |  |  |  |  |  |  |  |  |  |  |  |  |  |  |  |  |  |  |  |  |  |  |  |  |  |  |  |  |  |  |  |  |  |  |  |  |  |  |  |  |  |  |  |  |  |  |  |  |  |  |  |  |  |  |  |  |  |  |  |  |  |  |  |  |  |  |  |  |  |  |  |  |  |  |  |  |  |  |  |  |  |  |  |  |  |  |  |  |  |  |  |  |  |  |  |  |  |  |  |  |  |  |  |  |  |  |  |  |  |  |  |
|  |  |  |  |  |  |  |  |  |  |  |  |  |  |  |  |  |  |  |  |  |  |  |  |  |  |  |  |  |  |  |  |  |  |  |  |  |  |  |  |  |  |  |  |  |  |  |  |  |  |  |  |  |  |  |  |  |  |  |  |  |  |  |  |  |  |  |  |  |  |  |  |  |  |  |  |  |  |  |  |  |  |  |  |  |  |  |  |  |  |  |  |  |  |  |  |  |  |  |  |  |  |  |  |  |  |  |  |  |  |  |  |  |  |  |  |  |  |  |  |  |  |  |  |  |  |  |  |  |  |  |  |  |  |  |  |  |  |  |  |  |  |  |  |  |  |  |  |  |  |  |  |  |  |  |  |  |  |  |  |  |  |  |  |  |  |  |  |  |  |  |  |  |  |  |  |  |  |  |  |  |  |  |  |  |  |  |  |  |  |  |  |  |  |  |  |  |  |  |  |  |  |  |  |  |  |  |  |  |  |  |  |  |  |  |  |  |  |  |  |  |  |  |  |  |  |  |  |  |  |  |  |  |  |  |  |  |  |  |  |  |  |  |  |  |  |  |  |  |  |  |  |  |  |  |  |  |  |  |  |  |  |  |  |  |  |  |  |  |  |  |  |  |  |  |  |  |  |  |  |  |  |  |  |  |  |  |  |  |  |  |  |  |  |  |  |  |  |  |  |  |  |  |  |  |  |  |  |  |  |  |  |  |  |  |  |  |  |  |  |  |  |  |  |  |  |  |  |  |  |  |  |  |  |  |  |  |  |  |  |  |  |  |  |  |  |  |  |  |  |  |  |  |  |  |  |  |  |  |  |  |  |  |  |  |  |  |  |  |  |  |  |  |  |  |  |  |  |  |  |  |  |  |  |  |  |  |  |  |  |  |  |  |  |  |  |  |  |  |  |  |  |  |  |  |  |  |  |  |  |  |  |  |  |  |  |  |  |  |  |  |  |  |  |  |  |  |  |  |  |  |  |  |  |  |  |  |  |  |  |  |  |  |  |  |  |  |  |  |  |  |  |  |  |  |  |  |  |  |  |  |  |  |  |  |  |  |  |  |  |  |  |  |  |  |  |  |  |  |  |  |  |  |  |  |  |  |  |  |  |  |  |  |  |  |  |  |  |  |  |  |  |  |  |  |  |  |  |  |  |  |  |  |  |  |  |  |  |  |  |  |  |  |  |  |  |  |  |  |  |  |  |  |  |  |  |  |  |  |  |  |  |  |  |  |  |  |  |  |  |  |  |  |  |  |  |  |  |  |  |  |  |  |  |  |  |  |  |  |  |  |  |  |  |  |  |  |  |  |  |  |  |  |  |  |  |  |  |  |  |  |  |  |  |  |  |  |  |  |  |  |  |  |  |  |  |  |  |  |  |  |  |  |  |  |  |  |  |  |  |  |  |  |  |  |  |  |  |  |  |  |  |  |  |  |  |  |  |  |  |  |  |  |  |  |  |  |  |  |  |  |  |  |  |  |  |  |  |  |  |  |  |  |  |  |  |  |  |  |  |  |  |  |  |  |  |  |  |  |  |  |  |  |  |  |  |  |  |  |  |  |  |  |  |  |  |  |  |  |  |  |  |  |  |  |  |  |  |  |  |  |  |  |  |  |  |  |  |  |  |  |  |  |  |  |  |  |  |  |  |  |  |  |  |  |  |  |  |  |  |  |  |  |  |  |  |  |  |  |  |  |  |  |  |  |  |  |  |  |  |  |  |  |  |  |  |  |  |  |  |  |  |  |  |  |  |  |  |  |  |  |  |  |  |  |  |  |  |  |  |  |  |  |  |  |  |  |  |  |  |  |  |  |  |  |  |  |  |  |  |  |  |  |  |  |  |  |  |  |  |  |  |  |  |  |  |  |  |  |  |  |  |  |  |  |  |  |  |  |  |  |  |  |  |  |  |  |  |  |  |  |  |  |  |  |  |  |  |  |  |  |  |  |  |  |  |  |  |  |  |  |  |  |  |  |  |  |  |  |  |  |  |  |  |  |  |  |  |  |  |  |  |  |  |  |  |  |  |  |  |  |  |  |  |  |  |  |  |  |  |  |  |  |  |  |  |  |  |  |  |  |  |  |  |  |  |  |  |  |  |  |  |  |  |  |  |  |  |  |  |  |  |  |  |  |  |  |  |  |  |  |  |  |  |  |  |  |  |  |  |  |  |  |  |  |  |  |  |  |  |  |  |  |  |  |  |  |  |  |  |  |  |  |  |  |  |  |  |  |  |  |  |  |  |  |  |  |  |  |  |  |  |  |  |  |  |  |  |  |  |  |  |  |  |  |  |  |  |  |  |  |  |  |  |  |  |  |  |  |  |  |  |  |  |  |  |  |  |  |  |  |  |  |  |  |  |  |  |  |  |  |  |  |  |  |  |  |  |  |  |  |  |  |  |  |  |  |  |  |  |  |  |  |  |  |  |  |  |  |  |  |  |  |  |  |  |  |  |  |  |  |  |  |  |  |  |  |  |  |  |  |  |  |  |  |  |  |  |  |  |  |  |  |  |  |  |  |  |  |  |  |  |  |  |  |  |  |  |  |  |  |  |  |  |  |  |  |  |  |  |  |  |  |  |  |  |  |  |  |  |  |  |  |  |  |  |  |  |  |  |  |  |  |  |  |  |  |  |  |  |  |  |  |  |  |  |  |  |  |  |  |  |  |  |  |  |  |  |  |  |  |  |  |  |  |  |  |  |  |  |  |  |  |  |  |  |  |  |  |  |  |  |  |  |  |  |  |  |  |  |  |  |  |  |  |  |  |  |  |  |  |  |  |  |  |  |  |  |  |  |  |  |  |  |  |  |  |  |  |  |  |  |  |  |  |  |  |  |  |  |  |
|  |  |  |  |  |  |  |  |  |  |  |  |  |  |  |  |  |  |  |  |  |  |  |  |  |  |  |  |  |  |  |  |  |  |  |  |  |  |  |  |  |  |  |  |  |  |  |  |  |  |  |  |  |  |  |  |  |  |  |  |  |  |  |  |  |  |  |  |  |  |  |  |  |  |  |  |  |  |  |  |  |  |  |  |  |  |  |  |  |  |  |  |  |  |  |  |  |  |  |  |  |  |  |  |  |  |  |  |  |  |  |  |  |  |  |  |  |  |  |  |  |  |  |  |  |  |  |  |  |  |  |  |  |  |  |  |  |  |  |  |  |  |  |  |  |  |  |  |  |  |  |  |  |  |  |  |  |  |  |  |  |  |  |  |  |  |  |  |  |  |  |  |  |  |  |  |  |  |  |  |  |  |  |  |  |  |  |  |  |  |  |  |  |  |  |  |  |  |  |  |  |  |  |  |  |  |  |  |  |  |  |  |  |  |  |  |  |  |  |  |  |  |  |  |  |  |  |  |  |  |  |  |  |  |  |  |  |  |  |  |  |  |  |  |  |  |  |  |  |  |  |  |  |  |  |  |  |  |  |  |  |  |  |  |  |  |  |  |  |  |  |  |  |  |  |  |  |  |  |  |  |  |  |  |  |  |  |  |  |  |  |  |  |  |  |  |  |  |  |  |  |  |  |  |  |  |  |  |  |  |  |  |  |  |  |  |  |  |  |  |  |  |  |  |  |  |  |  |  |  |  |  |  |  |  |  |  |  |  |  |  |  |  |  |  |  |  |  |  |  |  |  |  |  |  |  |  |  |  |  |  |  |  |  |  |  |  |  |  |  |  |  |  |  |  |  |  |  |  |  |  |  |  |  |  |  |  |  |  |  |  |  |  |  |  |  |  |  |  |  |  |  |  |  |  |  |  |  |  |  |  |  |  |  |  |  |  |  |  |  |  |  |  |  |  |  |  |  |  |  |  |  |  |  |  |  |  |  |  |  |  |  |  |  |  |  |  |  |  |  |  |  |  |  |  |  |  |  |  |  |  |  |  |  |  |  |  |  |  |  |  |  |  |  |  |  |  |  |  |  |  |  |  |  |  |  |  |  |  |  |  |  |  |  |  |  |  |  |  |  |  |  |  |  |  |  |  |  |  |  |  |  |  |  |  |  |  |  |  |  |  |  |  |  |  |  |  |  |  |  |  |  |  |  |  |  |  |  |  |  |  |  |  |  |  |  |  |  |  |  |  |  |  |  |  |  |  |  |  |  |  |  |  |  |  |  |  |  |  |  |  |  |  |  |  |  |  |  |  |  |  |  |  |  |  |  |  |  |  |  |  |  |  |  |  |  |  |  |  |  |  |  |  |  |  |  |  |  |  |  |  |  |  |  |  |  |  |  |  |  |  |  |  |  |  |  |  |  |  |  |  |  |  |  |  |  |  |  |  |  |  |  |  |  |  |  |  |  |  |  |  |  |  |  |  |  |  |  |  |  |  |  |  |  |  |  |  |  |  |  |  |  |  |  |  |  |  |  |  |  |  |  |  |  |  |  |  |  |  |  |  |  |  |  |  |  |  |  |  |  |  |  |  |  |  |  |  |  |  |  |  |  |  |  |  |  |  |  |  |  |  |  |  |  |  |  |  |  |  |  |  |  |  |  |  |  |  |  |  |  |  |  |  |  |  |  |  |  |  |  |  |  |  |  |  |  |  |  |  |  |  |  |  |  |  |  |  |  |  |  |  |  |  |  |  |  |  |  |  |  |  |  |  |  |  |  |  |  |  |  |  |  |  |  |  |  |  |  |  |  |  |  |  |  |  |  |  |  |  |  |  |  |  |  |  |  |  |  |  |  |  |  |  |  |  |  |  |  |  |  |  |  |  |  |  |  |  |  |  |  |  |  |  |  |  |  |  |  |  |  |  |  |  |  |  |  |  |  |  |  |  |  |  |  |  |  |  |  |  |  |  |  |  |  |  |  |  |  |  |  |  |  |  |  |  |  |  |  |  |  |  |  |  |  |  |  |  |  |  |  |  |  |  |  |  |  |  |  |  |  |  |  |  |  |  |  |  |  |  |  |  |  |  |  |  |  |  |  |  |  |  |  |  |  |  |  |  |  |  |  |  |  |  |  |  |  |  |  |  |  |  |  |  |  |  |  |  |  |  |  |  |  |  |  |  |  |  |  |  |  |  |  |  |  |  |  |  |  |  |  |  |  |  |  |  |  |  |  |  |  |  |  |  |  |  |  |  |  |  |  |  |  |  |  |  |  |  |  |  |  |  |  |  |  |  |  |  |  |  |  |  |  |  |  |  |  |  |  |  |  |  |  |  |  |  |  |  |  |  |  |  |  |  |  |  |  |  |  |  |  |  |  |  |  |  |  |  |  |  |  |  |  |  |  |  |  |  |  |  |  |  |  |  |  |  |  |  |  |  |  |  |  |  |  |  |  |  |  |  |  |  |  |  |  |  |  |  |  |  |  |  |  |  |  |  |  |  |  |  |  |  |  |  |  |  |  |  |  |  |  |  |  |  |  |  |  |  |  |  |  |  |  |  |  |  |  |  |  |  |  |  |  |  |  |  |  |  |  |  |  |  |  |  |  |  |  |  |  |  |  |  |  |  |  |  |  |  |  |  |  |  |  |  |  |  |  |  |  |  |  |  |  |  |  |  |  |  |  |  |  |  |  |  |  |  |  |  |  |  |  |  |  |  |  |  |  |  |  |  |  |  |  |  |  |  |  |  |  |  |  |  |  |  |  |  |  |  |  |  |  |  |  |  |  |  |  |  |  |  |  |  |  |  |  |  |  |  |  |  |  |  |  |  |  |  |  |  |  |  |  |  |
|  |  |  |  |  |  |  |  |  |  |  |  |  |  |  |  |  |  |  |  |  |  |  |  |  |  |  |  |  |  |  |  |  |  |  |  |  |  |  |  |  |  |  |  |  |  |  |  |  |  |  |  |  |  |  |  |  |  |  |  |  |  |  |  |  |  |  |  |  |  |  |  |  |  |  |  |  |  |  |  |  |  |  |  |  |  |  |  |  |  |  |  |  |  |  |  |  |  |  |  |  |  |  |  |  |  |  |  |  |  |  |  |  |  |  |  |  |  |  |  |  |  |  |  |  |  |  |  |  |  |  |  |  |  |  |  |  |  |  |  |  |  |  |  |  |  |  |  |  |  |  |  |  |  |  |  |  |  |  |  |  |  |  |  |  |  |  |  |  |  |  |  |  |  |  |  |  |  |  |  |  |  |  |  |  |  |  |  |  |  |  |  |  |  |  |  |  |  |  |  |  |  |  |  |  |  |  |  |  |  |  |  |  |  |  |  |  |  |  |  |  |  |  |  |  |  |  |  |  |  |  |  |  |  |  |  |  |  |  |  |  |  |  |  |  |  |  |  |  |  |  |  |  |  |  |  |  |  |  |  |  |  |  |  |  |  |  |  |  |  |  |  |  |  |  |  |  |  |  |  |  |  |  |  |  |  |  |  |  |  |  |  |  |  |  |  |  |  |  |  |  |  |  |  |  |  |  |  |  |  |  |  |  |  |  |  |  |  |  |  |  |  |  |  |  |  |  |  |  |  |  |  |  |  |  |  |  |  |  |  |  |  |  |  |  |  |  |  |  |  |  |  |  |  |  |  |  |  |  |  |  |  |  |  |  |  |  |  |  |  |  |  |  |  |  |  |  |  |  |  |  |  |  |  |  |  |  |  |  |  |  |  |  |  |  |  |  |  |  |  |  |  |  |  |  |  |  |  |  |  |  |  |  |  |  |  |  |  |  |  |  |  |  |  |  |  |  |  |  |  |  |  |  |  |  |  |  |  |  |  |  |  |  |  |  |  |  |  |  |  |  |  |  |  |  |  |  |  |  |  |  |  |  |  |  |  |  |  |  |  |  |  |  |  |  |  |  |  |  |  |  |  |  |  |  |  |  |  |  |  |  |  |  |  |  |  |  |  |  |  |  |  |  |  |  |  |  |  |  |  |  |  |  |  |  |  |  |  |  |  |  |  |  |  |  |  |  |  |  |  |  |  |  |  |  |  |  |  |  |  |  |  |  |  |  |  |  |  |  |  |  |  |  |  |  |  |  |  |  |  |  |  |  |  |  |  |  |  |  |  |  |  |  |  |  |  |  |  |  |  |  |  |  |  |  |  |  |  |  |  |  |  |  |  |  |  |  |  |  |  |  |  |  |  |  |  |  |  |  |  |  |  |  |  |  |  |  |  |  |  |  |  |  |  |  |  |  |  |  |  |  |  |  |  |  |  |  |  |  |  |  |  |  |  |  |  |  |  |  |  |  |  |  |  |  |  |  |  |  |  |  |  |  |  |  |  |  |  |  |  |  |  |  |  |  |  |  |  |  |  |  |  |  |  |  |  |  |  |  |  |  |  |  |  |  |  |  |  |  |  |  |  |  |  |  |  |  |  |  |  |  |  |  |  |  |  |  |  |  |  |  |  |  |  |  |  |  |  |  |  |  |  |  |  |  |  |  |  |  |  |  |  |  |  |  |  |  |  |  |  |  |  |  |  |  |  |  |  |  |  |  |  |  |  |  |  |  |  |  |  |  |  |  |  |  |  |  |  |  |  |  |  |  |  |  |  |  |  |  |  |  |  |  |  |  |  |  |  |  |  |  |  |  |  |  |  |  |  |  |  |  |  |  |  |  |  |  |  |  |  |  |  |  |  |  |  |  |  |  |  |  |  |  |  |  |  |  |  |  |  |  |  |  |  |  |  |  |  |  |  |  |  |  |  |  |  |  |  |  |  |  |  |  |  |  |  |  |  |  |  |  |  |  |  |  |  |  |  |  |  |  |  |  |  |  |  |  |  |  |  |  |  |  |  |  |  |  |  |  |  |  |  |  |  |  |  |  |  |  |  |  |  |  |  |  |  |  |  |  |  |  |  |  |  |  |  |  |  |  |  |  |  |  |  |  |  |  |  |  |  |  |  |  |  |  |  |  |  |  |  |  |  |  |  |  |  |  |  |  |  |  |  |  |  |  |  |  |  |  |  |  |  |  |  |  |  |  |  |  |  |  |  |  |  |  |  |  |  |  |  |  |  |  |  |  |  |  |  |  |  |  |  |  |  |  |  |  |  |  |  |  |  |  |  |  |  |  |  |  |  |  |  |  |  |  |  |  |  |  |  |  |  |  |  |  |  |  |  |  |  |  |  |  |  |  |  |  |  |  |  |  |  |  |  |  |  |  |  |  |  |  |  |  |  |  |  |  |  |  |  |  |  |  |  |  |  |  |  |  |  |  |  |  |  |  |  |  |  |  |  |  |  |  |  |  |  |  |  |  |  |  |  |  |  |  |  |  |  |  |  |  |  |  |  |  |  |  |  |  |  |  |  |  |  |  |  |  |  |  |  |  |  |  |  |  |  |  |  |  |  |  |  |  |  |  |  |  |  |  |  |  |  |  |  |  |  |  |  |  |  |  |  |  |  |  |  |  |  |  |  |  |  |  |  |  |  |  |  |  |  |  |  |  |  |  |  |  |  |  |  |  |  |  |  |  |  |  |  |  |  |  |  |  |  |  |  |  |  |  |  |  |  |  |  |  |  |  |  |  |  |  |  |  |  |  |  |  |  |  |  |  |  |  |  |  |  |  |  |  |  |  |  |  |  |  |  |  |  |  |  |  |  |  |  |  |  |  |  |  |  |  |
|  |  |  |  |  |  |  |  |  |  |  |  |  |  |  |  |  |  |  |  |  |  |  |  |  |  |  |  |  |  |  |  |  |  |  |  |  |  |  |  |  |  |  |  |  |  |  |  |  |  |  |  |  |  |  |  |  |  |  |  |  |  |  |  |  |  |  |  |  |  |  |  |  |  |  |  |  |  |  |  |  |  |  |  |  |  |  |  |  |  |  |  |  |  |  |  |  |  |  |  |  |  |  |  |  |  |  |  |  |  |  |  |  |  |  |  |  |  |  |  |  |  |  |  |  |  |  |  |  |  |  |  |  |  |  |  |  |  |  |  |  |  |  |  |  |  |  |  |  |  |  |  |  |  |  |  |  |  |  |  |  |  |  |  |  |  |  |  |  |  |  |  |  |  |  |  |  |  |  |  |  |  |  |  |  |  |  |  |  |  |  |  |  |  |  |  |  |  |  |  |  |  |  |  |  |  |  |  |  |  |  |  |  |  |  |  |  |  |  |  |  |  |  |  |  |  |  |  |  |  |  |  |  |  |  |  |  |  |  |  |  |  |  |  |  |  |  |  |  |  |  |  |  |  |  |  |  |  |  |  |  |  |  |  |  |  |  |  |  |  |  |  |  |  |  |  |  |  |  |  |  |  |  |  |  |  |  |  |  |  |  |  |  |  |  |  |  |  |  |  |  |  |  |  |  |  |  |  |  |  |  |  |  |  |  |  |  |  |  |  |  |  |  |  |  |  |  |  |  |  |  |  |  |  |  |  |  |  |  |  |  |  |  |  |  |  |  |  |  |  |  |  |  |  |  |  |  |  |  |  |  |  |  |  |  |  |  |  |  |  |  |  |  |  |  |  |  |  |  |  |  |  |  |  |  |  |  |  |  |  |  |  |  |  |  |  |  |  |  |  |  |  |  |  |  |  |  |  |  |  |  |  |  |  |  |  |  |  |  |  |  |  |  |  |  |  |  |  |  |  |  |  |  |  |  |  |  |  |  |  |  |  |  |  |  |  |  |  |  |  |  |  |  |  |  |  |  |  |  |  |  |  |  |  |  |  |  |  |  |  |  |  |  |  |  |  |  |  |  |  |  |  |  |  |  |  |  |  |  |  |  |  |  |  |  |  |  |  |  |  |  |  |  |  |  |  |  |  |  |  |  |  |  |  |  |  |  |  |  |  |  |  |  |  |  |  |  |  |  |  |  |  |  |  |  |  |  |  |  |  |  |  |  |  |  |  |  |  |  |  |  |  |  |  |  |  |  |  |  |  |  |  |  |  |  |  |  |  |  |  |  |  |  |  |  |  |  |  |  |  |  |  |  |  |  |  |  |  |  |  |  |  |  |  |  |  |  |  |  |  |  |  |  |  |  |  |  |  |  |  |  |  |  |  |  |  |  |  |  |  |  |  |  |  |  |  |  |  |  |  |  |  |  |  |  |  |  |  |  |  |  |  |  |  |  |  |  |  |  |  |  |  |  |  |  |  |  |  |  |  |  |  |  |  |  |  |  |  |  |  |  |  |  |  |  |  |  |  |  |  |  |  |  |  |  |  |  |  |  |  |  |  |  |  |  |  |  |  |  |  |  |  |  |  |  |  |  |  |  |  |  |  |  |  |  |  |  |  |  |  |  |  |  |  |  |  |  |  |  |  |  |  |  |  |  |  |  |  |  |  |  |  |  |  |  |  |  |  |  |  |  |  |  |  |  |  |  |  |  |  |  |  |  |  |  |  |  |  |  |  |  |  |  |  |  |  |  |  |  |  |  |  |  |  |  |  |  |  |  |  |  |  |  |  |  |  |  |  |  |  |  |  |  |  |  |  |  |  |  |  |  |  |  |  |  |  |  |  |  |  |  |  |  |  |  |  |  |  |  |  |  |  |  |  |  |  |  |  |  |  |  |  |  |  |  |  |  |  |  |  |  |  |  |  |  |  |  |  |  |  |  |  |  |  |  |  |  |  |  |  |  |  |  |  |  |  |  |  |  |  |  |  |  |  |  |  |  |  |  |  |  |  |  |  |  |  |  |  |  |  |  |  |  |  |  |  |  |  |  |  |  |  |  |  |  |  |  |  |  |  |  |  |  |  |  |  |  |  |  |  |  |  |  |  |  |  |  |  |  |  |  |  |  |  |  |  |  |  |  |  |  |  |  |  |  |  |  |  |  |  |  |  |  |  |  |  |  |  |  |  |  |  |  |  |  |  |  |  |  |  |  |  |  |  |  |  |  |  |  |  |  |  |  |  |  |  |  |  |  |  |  |  |  |  |  |  |  |  |  |  |  |  |  |  |  |  |  |  |  |  |  |  |  |  |  |  |  |  |  |  |  |  |  |  |  |  |  |  |  |  |  |  |  |  |  |  |  |  |  |  |  |  |  |  |  |  |  |  |  |  |  |  |  |  |  |  |  |  |  |  |  |  |  |  |  |  |  |  |  |  |  |  |  |  |  |  |  |  |  |  |  |  |  |  |  |  |  |  |  |  |  |  |  |  |  |  |  |  |  |  |  |  |  |  |  |  |  |  |  |  |  |  |  |  |  |  |  |  |  |  |  |  |  |  |  |  |  |  |  |  |  |  |  |  |  |  |  |  |  |  |  |  |  |  |  |  |  |  |  |  |  |  |  |  |  |  |  |  |  |  |  |  |  |  |  |  |  |  |  |  |  |  |  |  |  |  |  |  |  |  |  |  |  |  |  |  |  |  |  |  |  |  |  |  |  |  |  |  |  |  |  |  |  |  |  |  |  |  |  |  |  |  |  |  |  |  |  |  |  |  |  |  |  |  |  |  |  |  |  |  |  |  |  |  |  |  |  |  |  |  |  |  |  |  |  |  |  |  |  |  |  |
|  |  |  |  |  |  |  |  |  |  |  |  |  |  |  |  |  |  |  |  |  |  |  |  |  |  |  |  |  |  |  |  |  |  |  |  |  |  |  |  |  |  |  |  |  |  |  |  |  |  |  |  |  |  |  |  |  |  |  |  |  |  |  |  |  |  |  |  |  |  |  |  |  |  |  |  |  |  |  |  |  |  |  |  |  |  |  |  |  |  |  |  |  |  |  |  |  |  |  |  |  |  |  |  |  |  |  |  |  |  |  |  |  |  |  |  |  |  |  |  |  |  |  |  |  |  |  |  |  |  |  |  |  |  |  |  |  |  |  |  |  |  |  |  |  |  |  |  |  |  |  |  |  |  |  |  |  |  |  |  |  |  |  |  |  |  |  |  |  |  |  |  |  |  |  |  |  |  |  |  |  |  |  |  |  |  |  |  |  |  |  |  |  |  |  |  |  |  |  |  |  |  |  |  |  |  |  |  |  |  |  |  |  |  |  |  |  |  |  |  |  |  |  |  |  |  |  |  |  |  |  |  |  |  |  |  |  |  |  |  |  |  |  |  |  |  |  |  |  |  |  |  |  |  |  |  |  |  |  |  |  |  |  |  |  |  |  |  |  |  |  |  |  |  |  |  |  |  |  |  |  |  |  |  |  |  |  |  |  |  |  |  |  |  |  |  |  |  |  |  |  |  |  |  |  |  |  |  |  |  |  |  |  |  |  |  |  |  |  |  |  |  |  |  |  |  |  |  |  |  |  |  |  |  |  |  |  |  |  |  |  |  |  |  |  |  |  |  |  |  |  |  |  |  |  |  |  |  |  |  |  |  |  |  |  |  |  |  |  |  |  |  |  |  |  |  |  |  |  |  |  |  |  |  |  |  |  |  |  |  |  |  |  |  |  |  |  |  |  |  |  |  |  |  |  |  |  |  |  |  |  |  |  |  |  |  |  |  |  |  |  |  |  |  |  |  |  |  |  |  |  |  |  |  |  |  |  |  |  |  |  |  |  |  |  |  |  |  |  |  |  |  |  |  |  |  |  |  |  |  |  |  |  |  |  |  |  |  |  |  |  |  |  |  |  |  |  |  |  |  |  |  |  |  |  |  |  |  |  |  |  |  |  |  |  |  |  |  |  |  |  |  |  |  |  |  |  |  |  |  |  |  |  |  |  |  |  |  |  |  |  |  |  |  |  |  |  |  |  |  |  |  |  |  |  |  |  |  |  |  |  |  |  |  |  |  |  |  |  |  |  |  |  |  |  |  |  |  |  |  |  |  |  |  |  |  |  |  |  |  |  |  |  |  |  |  |  |  |  |  |  |  |  |  |  |  |  |  |  |  |  |  |  |  |  |  |  |  |  |  |  |  |  |  |  |  |  |  |  |  |  |  |  |  |  |  |  |  |  |  |  |  |  |  |  |  |  |  |  |  |  |  |  |  |  |  |  |  |  |  |  |  |  |  |  |  |  |  |  |  |  |  |  |  |  |  |  |  |  |  |  |  |  |  |  |  |  |  |  |  |  |  |  |  |  |  |  |  |  |  |  |  |  |  |  |  |  |  |  |  |  |  |  |  |  |  |  |  |  |  |  |  |  |  |  |  |  |  |  |  |  |  |  |  |  |  |  |  |  |  |  |  |  |  |  |  |  |  |  |  |  |  |  |  |  |  |  |  |  |  |  |  |  |  |  |  |  |  |  |  |  |  |  |  |  |  |  |  |  |  |  |  |  |  |  |  |  |  |  |  |  |  |  |  |  |  |  |  |  |  |  |  |  |  |  |  |  |  |  |  |  |  |  |  |  |  |  |  |  |  |  |  |  |  |  |  |  |  |  |  |  |  |  |  |  |  |  |  |  |  |  |  |  |  |  |  |  |  |  |  |  |  |  |  |  |  |  |  |  |  |  |  |  |  |  |  |  |  |  |  |  |  |  |  |  |  |  |  |  |  |  |  |  |  |  |  |  |  |  |  |  |  |  |  |  |  |  |  |  |  |  |  |  |  |  |  |  |  |  |  |  |  |  |  |  |  |  |  |  |  |  |  |  |  |  |  |  |  |  |  |  |  |  |  |  |  |  |  |  |  |  |  |  |  |  |  |  |  |  |  |  |  |  |  |  |  |  |  |  |  |  |  |  |  |  |  |  |  |  |  |  |  |  |  |  |  |  |  |  |  |  |  |  |  |  |  |  |  |  |  |  |  |  |  |  |  |  |  |  |  |  |  |  |  |  |  |  |  |  |  |  |  |  |  |  |  |  |  |  |  |  |  |  |  |  |  |  |  |  |  |  |  |  |  |  |  |  |  |  |  |  |  |  |  |  |  |  |  |  |  |  |  |  |  |  |  |  |  |  |  |  |  |  |  |  |  |  |  |  |  |  |  |  |  |  |  |  |  |  |  |  |  |  |  |  |  |  |  |  |  |  |  |  |  |  |  |  |  |  |  |  |  |  |  |  |  |  |  |  |  |  |  |  |  |  |  |  |  |  |  |  |  |  |  |  |  |  |  |  |  |  |  |  |  |  |  |  |  |  |  |  |  |  |  |  |  |  |  |  |  |  |  |  |  |  |  |  |  |  |  |  |  |  |  |  |  |  |  |  |  |  |  |  |  |  |  |  |  |  |  |  |  |  |  |  |  |  |  |  |  |  |  |  |  |  |  |  |  |  |  |  |  |  |  |  |  |  |  |  |  |  |  |  |  |  |  |  |  |  |  |  |  |  |  |  |  |  |  |  |  |  |  |  |  |  |  |  |  |  |  |  |  |  |  |  |  |  |  |  |  |  |  |  |  |  |  |  |  |  |  |  |  |  |  |  |  |  |  |  |  |  |  |  |  |  |  |  |  |  |  |  |
|  |  |  |  |  |  |  |  |  |  |  |  |  |  |  |  |  |  |  |  |  |  |  |  |  |  |  |  |  |  |  |  |  |  |  |  |  |  |  |  |  |  |  |  |  |  |  |  |  |  |  |  |  |  |  |  |  |  |  |  |  |  |  |  |  |  |  |  |  |  |  |  |  |  |  |  |  |  |  |  |  |  |  |  |  |  |  |  |  |  |  |  |  |  |  |  |  |  |  |  |  |  |  |  |  |  |  |  |  |  |  |  |  |  |  |  |  |  |  |  |  |  |  |  |  |  |  |  |  |  |  |  |  |  |  |  |  |  |  |  |  |  |  |  |  |  |  |  |  |  |  |  |  |  |  |  |  |  |  |  |  |  |  |  |  |  |  |  |  |  |  |  |  |  |  |  |  |  |  |  |  |  |  |  |  |  |  |  |  |  |  |  |  |  |  |  |  |  |  |  |  |  |  |  |  |  |  |  |  |  |  |  |  |  |  |  |  |  |  |  |  |  |  |  |  |  |  |  |  |  |  |  |  |  |  |  |  |  |  |  |  |  |  |  |  |  |  |  |  |  |  |  |  |  |  |  |  |  |  |  |  |  |  |  |  |  |  |  |  |  |  |  |  |  |  |  |  |  |  |  |  |  |  |  |  |  |  |  |  |  |  |  |  |  |  |  |  |  |  |  |  |  |  |  |  |  |  |  |  |  |  |  |  |  |  |  |  |  |  |  |  |  |  |  |  |  |  |  |  |  |  |  |  |  |  |  |  |  |  |  |  |  |  |  |  |  |  |  |  |  |  |  |  |  |  |  |  |  |  |  |  |  |  |  |  |  |  |  |  |  |  |  |  |  |  |  |  |  |  |  |  |  |  |  |  |  |  |  |  |  |  |  |  |  |  |  |  |  |  |  |  |  |  |  |  |  |  |  |  |  |  |  |  |  |  |  |  |  |  |  |  |  |  |  |  |  |  |  |  |  |  |  |  |  |  |  |  |  |  |  |  |  |  |  |  |  |  |  |  |  |  |  |  |  |  |  |  |  |  |  |  |  |  |  |  |  |  |  |  |  |  |  |  |  |  |  |  |  |  |  |  |  |  |  |  |  |  |  |  |  |  |  |  |  |  |  |  |  |  |  |  |  |  |  |  |  |  |  |  |  |  |  |  |  |  |  |  |  |  |  |  |  |  |  |  |  |  |  |  |  |  |  |  |  |  |  |  |  |  |  |  |  |  |  |  |  |  |  |  |  |  |  |  |  |  |  |  |  |  |  |  |  |  |  |  |  |  |  |  |  |  |  |  |  |  |  |  |  |  |  |  |  |  |  |  |  |  |  |  |  |  |  |  |  |  |  |  |  |  |  |  |  |  |  |  |  |  |  |  |  |  |  |  |  |  |  |  |  |  |  |  |  |  |  |  |  |  |  |  |  |  |  |  |  |  |  |  |  |  |  |  |  |  |  |  |  |  |  |  |  |  |  |  |  |  |  |  |  |  |  |  |  |  |  |  |  |  |  |  |  |  |  |  |  |  |  |  |  |  |  |  |  |  |  |  |  |  |  |  |  |  |  |  |  |  |  |  |  |  |  |  |  |  |  |  |  |  |  |  |  |  |  |  |  |  |  |  |  |  |  |  |  |  |  |  |  |  |  |  |  |  |  |  |  |  |  |  |  |  |  |  |  |  |  |  |  |  |  |  |  |  |  |  |  |  |  |  |  |  |  |  |  |  |  |  |  |  |  |  |  |  |  |  |  |  |  |  |  |  |  |  |  |  |  |  |  |  |  |  |  |  |  |  |  |  |  |  |  |  |  |  |  |  |  |  |  |  |  |  |  |  |  |  |  |  |  |  |  |  |  |  |  |  |  |  |  |  |  |  |  |  |  |  |  |  |  |  |  |  |  |  |  |  |  |  |  |  |  |  |  |  |  |  |  |  |  |  |  |  |  |  |  |  |  |  |  |  |  |  |  |  |  |  |  |  |  |  |  |  |  |  |  |  |  |  |  |  |  |  |  |  |  |  |  |  |  |  |  |  |  |  |  |  |  |  |  |  |  |  |  |  |  |  |  |  |  |  |  |  |  |  |  |  |  |  |  |  |  |  |  |  |  |  |  |  |  |  |  |  |  |  |  |  |  |  |  |  |  |  |  |  |  |  |  |  |  |  |  |  |  |  |  |  |  |  |  |  |  |  |  |  |  |  |  |  |  |  |  |  |  |  |  |  |  |  |  |  |  |  |  |  |  |  |  |  |  |  |  |  |  |  |  |  |  |  |  |  |  |  |  |  |  |  |  |  |  |  |  |  |  |  |  |  |  |  |  |  |  |  |  |  |  |  |  |  |  |  |  |  |  |  |  |  |  |  |  |  |  |  |  |  |  |  |  |  |  |  |  |  |  |  |  |  |  |  |  |  |  |  |  |  |  |  |  |  |  |  |  |  |  |  |  |  |  |  |  |  |  |  |  |  |  |  |  |  |  |  |  |  |  |  |  |  |  |  |  |  |  |  |  |  |  |  |  |  |  |  |  |  |  |  |  |  |  |  |  |  |  |  |  |  |  |  |  |  |  |  |  |  |  |  |  |  |  |  |  |  |  |  |  |  |  |  |  |  |  |  |  |  |  |  |  |  |  |  |  |  |  |  |  |  |  |  |  |  |  |  |  |  |  |  |  |  |  |  |  |  |  |  |  |  |  |  |  |  |  |  |  |  |  |  |  |  |  |  |  |  |  |  |  |  |  |  |  |  |  |  |  |  |  |  |  |  |  |  |  |  |  |  |  |  |  |  |  |  |  |  |  |  |  |  |  |  |  |  |  |  |  |  |  |  |  |  |  |  |  |  |  |  |  |  |
|  |  |  |  |  |  |  |  |  |  |  |  |  |  |  |  |  |  |  |  |  |  |  |  |  |  |  |  |  |  |  |  |  |  |  |  |  |  |  |  |  |  |  |  |  |  |  |  |  |  |  |  |  |  |  |  |  |  |  |  |  |  |  |  |  |  |  |  |  |  |  |  |  |  |  |  |  |  |  |  |  |  |  |  |  |  |  |  |  |  |  |  |  |  |  |  |  |  |  |  |  |  |  |  |  |  |  |  |  |  |  |  |  |  |  |  |  |  |  |  |  |  |  |  |  |  |  |  |  |  |  |  |  |  |  |  |  |  |  |  |  |  |  |  |  |  |  |  |  |  |  |  |  |  |  |  |  |  |  |  |  |  |  |  |  |  |  |  |  |  |  |  |  |  |  |  |  |  |  |  |  |  |  |  |  |  |  |  |  |  |  |  |  |  |  |  |  |  |  |  |  |  |  |  |  |  |  |  |  |  |  |  |  |  |  |  |  |  |  |  |  |  |  |  |  |  |  |  |  |  |  |  |  |  |  |  |  |  |  |  |  |  |  |  |  |  |  |  |  |  |  |  |  |  |  |  |  |  |  |  |  |  |  |  |  |  |  |  |  |  |  |  |  |  |  |  |  |  |  |  |  |  |  |  |  |  |  |  |  |  |  |  |  |  |  |  |  |  |  |  |  |  |  |  |  |  |  |  |  |  |  |  |  |  |  |  |  |  |  |  |  |  |  |  |  |  |  |  |  |  |  |  |  |  |  |  |  |  |  |  |  |  |  |  |  |  |  |  |  |  |  |  |  |  |  |  |  |  |  |  |  |  |  |  |  |  |  |  |  |  |  |  |  |  |  |  |  |  |  |  |  |  |  |  |  |  |  |  |  |  |  |  |  |  |  |  |  |  |  |  |  |  |  |  |  |  |  |  |  |  |  |  |  |  |  |  |  |  |  |  |  |  |  |  |  |  |  |  |  |  |  |  |  |  |  |  |  |  |  |  |  |  |  |  |  |  |  |  |  |  |  |  |  |  |  |  |  |  |  |  |  |  |  |  |  |  |  |  |  |  |  |  |  |  |  |  |  |  |  |  |  |  |  |  |  |  |  |  |  |  |  |  |  |  |  |  |  |  |  |  |  |  |  |  |  |  |  |  |  |  |  |  |  |  |  |  |  |  |  |  |  |  |  |  |  |  |  |  |  |  |  |  |  |  |  |  |  |  |  |  |  |  |  |  |  |  |  |  |  |  |  |  |  |  |  |  |  |  |  |  |  |  |  |  |  |  |  |  |  |  |  |  |  |  |  |  |  |  |  |  |  |  |  |  |  |  |  |  |  |  |  |  |  |  |  |  |  |  |  |  |  |  |  |  |  |  |  |  |  |  |  |  |  |  |  |  |  |  |  |  |  |  |  |  |  |  |  |  |  |  |  |  |  |  |  |  |  |  |  |  |  |  |  |  |  |  |  |  |  |  |  |  |  |  |  |  |  |  |  |  |  |  |  |  |  |  |  |  |  |  |  |  |  |  |  |  |  |  |  |  |  |  |  |  |  |  |  |  |  |  |  |  |  |  |  |  |  |  |  |  |  |  |  |  |  |  |  |  |  |  |  |  |  |  |  |  |  |  |  |  |  |  |  |  |  |  |  |  |  |  |  |  |  |  |  |  |  |  |  |  |  |  |  |  |  |  |  |  |  |  |  |  |  |  |  |  |  |  |  |  |  |  |  |  |  |  |  |  |  |  |  |  |  |  |  |  |  |  |  |  |  |  |  |  |  |  |  |  |  |  |  |  |  |  |  |  |  |  |  |  |  |  |  |  |  |  |  |  |  |  |  |  |  |  |  |  |  |  |  |  |  |  |  |  |  |  |  |  |  |  |  |  |  |  |  |  |  |  |  |  |  |  |  |  |  |  |  |  |  |  |  |  |  |  |  |  |  |  |  |  |  |  |  |  |  |  |  |  |  |  |  |  |  |  |  |  |  |  |  |  |  |  |  |  |  |  |  |  |  |  |  |  |  |  |  |  |  |  |  |  |  |  |  |  |  |  |  |  |  |  |  |  |  |  |  |  |  |  |  |  |  |  |  |  |  |  |  |  |  |  |  |  |  |  |  |  |  |  |  |  |  |  |  |  |  |  |  |  |  |  |  |  |  |  |  |  |  |  |  |  |  |  |  |  |  |  |  |  |  |  |  |  |  |  |  |  |  |  |  |  |  |  |  |  |  |  |  |  |  |  |  |  |  |  |  |  |  |  |  |  |  |  |  |  |  |  |  |  |  |  |  |  |  |  |  |  |  |  |  |  |  |  |  |  |  |  |  |  |  |  |  |  |  |  |  |  |  |  |  |  |  |  |  |  |  |  |  |  |  |  |  |  |  |  |  |  |  |  |  |  |  |  |  |  |  |  |  |  |  |  |  |  |  |  |  |  |  |  |  |  |  |  |  |  |  |  |  |  |  |  |  |  |  |  |  |  |  |  |  |  |  |  |  |  |  |  |  |  |  |  |  |  |  |  |  |  |  |  |  |  |  |  |  |  |  |  |  |  |  |  |  |  |  |  |  |  |  |  |  |  |  |  |  |  |  |  |  |  |  |  |  |  |  |  |  |  |  |  |  |  |  |  |  |  |  |  |  |  |  |  |  |  |  |  |  |  |  |  |  |  |  |  |  |  |  |  |  |  |  |  |  |  |  |  |  |  |  |  |  |  |  |  |  |  |  |  |  |  |  |  |  |  |  |  |  |  |  |  |  |  |  |  |  |  |  |  |  |  |  |  |  |  |  |  |  |  |  |  |  |  |  |  |  |  |  |  |  |  |  |  |  |  |  |  |  |  |  |  |  |  |  |
|  |  |  |  |  |  |  |  |  |  |  |  |  |  |  |  |  |  |  |  |  |  |  |  |  |  |  |  |  |  |  |  |  |  |  |  |  |  |  |  |  |  |  |  |  |  |  |  |  |  |  |  |  |  |  |  |  |  |  |  |  |  |  |  |  |  |  |  |  |  |  |  |  |  |  |  |  |  |  |  |  |  |  |  |  |  |  |  |  |  |  |  |  |  |  |  |  |  |  |  |  |  |  |  |  |  |  |  |  |  |  |  |  |  |  |  |  |  |  |  |  |  |  |  |  |  |  |  |  |  |  |  |  |  |  |  |  |  |  |  |  |  |  |  |  |  |  |  |  |  |  |  |  |  |  |  |  |  |  |  |  |  |  |  |  |  |  |  |  |  |  |  |  |  |  |  |  |  |  |  |  |  |  |  |  |  |  |  |  |  |  |  |  |  |  |  |  |  |  |  |  |  |  |  |  |  |  |  |  |  |  |  |  |  |  |  |  |  |  |  |  |  |  |  |  |  |  |  |  |  |  |  |  |  |  |  |  |  |  |  |  |  |  |  |  |  |  |  |  |  |  |  |  |  |  |  |  |  |  |  |  |  |  |  |  |  |  |  |  |  |  |  |  |  |  |  |  |  |  |  |  |  |  |  |  |  |  |  |  |  |  |  |  |  |  |  |  |  |  |  |  |  |  |  |  |  |  |  |  |  |  |  |  |  |  |  |  |  |  |  |  |  |  |  |  |  |  |  |  |  |  |  |  |  |  |  |  |  |  |  |  |  |  |  |  |  |  |  |  |  |  |  |  |  |  |  |  |  |  |  |  |  |  |  |  |  |  |  |  |  |  |  |  |  |  |  |  |  |  |  |  |  |  |  |  |  |  |  |  |  |  |  |  |  |  |  |  |  |  |  |  |  |  |  |  |  |  |  |  |  |  |  |  |  |  |  |  |  |  |  |  |  |  |  |  |  |  |  |  |  |  |  |  |  |  |  |  |  |  |  |  |  |  |  |  |  |  |  |  |  |  |  |  |  |  |  |  |  |  |  |  |  |  |  |  |  |  |  |  |  |  |  |  |  |  |  |  |  |  |  |  |  |  |  |  |  |  |  |  |  |  |  |  |  |  |  |  |  |  |  |  |  |  |  |  |  |  |  |  |  |  |  |  |  |  |  |  |  |  |  |  |  |  |  |  |  |  |  |  |  |  |  |  |  |  |  |  |  |  |  |  |  |  |  |  |  |  |  |  |  |  |  |  |  |  |  |  |  |  |  |  |  |  |  |  |  |  |  |  |  |  |  |  |  |  |  |  |  |  |  |  |  |  |  |  |  |  |  |  |  |  |  |  |  |  |  |  |  |  |  |  |  |  |  |  |  |  |  |  |  |  |  |  |  |  |  |  |  |  |  |  |  |  |  |  |  |  |  |  |  |  |  |  |  |  |  |  |  |  |  |  |  |  |  |  |  |  |  |  |  |  |  |  |  |  |  |  |  |  |  |  |  |  |  |  |  |  |  |  |  |  |  |  |  |  |  |  |  |  |  |  |  |  |  |  |  |  |  |  |  |  |  |  |  |  |  |  |  |  |  |  |  |  |  |  |  |  |  |  |  |  |  |  |  |  |  |  |  |  |  |  |  |  |  |  |  |  |  |  |  |  |  |  |  |  |  |  |  |  |  |  |  |  |  |  |  |  |  |  |  |  |  |  |  |  |  |  |  |  |  |  |  |  |  |  |  |  |  |  |  |  |  |  |  |  |  |  |  |  |  |  |  |  |  |  |  |  |  |  |  |  |  |  |  |  |  |  |  |  |  |  |  |  |  |  |  |  |  |  |  |  |  |  |  |  |  |  |  |  |  |  |  |  |  |  |  |  |  |  |  |  |  |  |  |  |  |  |  |  |  |  |  |  |  |  |  |  |  |  |  |  |  |  |  |  |  |  |  |  |  |  |  |  |  |  |  |  |  |  |  |  |  |  |  |  |  |  |  |  |  |  |  |  |  |  |  |  |  |  |  |  |  |  |  |  |  |  |  |  |  |  |  |  |  |  |  |  |  |  |  |  |  |  |  |  |  |  |  |  |  |  |  |  |  |  |  |  |  |  |  |  |  |  |  |  |  |  |  |  |  |  |  |  |  |  |  |  |  |  |  |  |  |  |  |  |  |  |  |  |  |  |  |  |  |  |  |  |  |  |  |  |  |  |  |  |  |  |  |  |  |  |  |  |  |  |  |  |  |  |  |  |  |  |  |  |  |  |  |  |  |  |  |  |  |  |  |  |  |  |  |  |  |  |  |  |  |  |  |  |  |  |  |  |  |  |  |  |  |  |  |  |  |  |  |  |  |  |  |  |  |  |  |  |  |  |  |  |  |  |  |  |  |  |  |  |  |  |  |  |  |  |  |  |  |  |  |  |  |  |  |  |  |  |  |  |  |  |  |  |  |  |  |  |  |  |  |  |  |  |  |  |  |  |  |  |  |  |  |  |  |  |  |  |  |  |  |  |  |  |  |  |  |  |  |  |  |  |  |  |  |  |  |  |  |  |  |  |  |  |  |  |  |  |  |  |  |  |  |  |  |  |  |  |  |  |  |  |  |  |  |  |  |  |  |  |  |  |  |  |  |  |  |  |  |  |  |  |  |  |  |  |  |  |  |  |  |  |  |  |  |  |  |  |  |  |  |  |  |  |  |  |  |  |  |  |  |  |  |  |  |  |  |  |  |  |  |  |  |  |  |  |  |  |  |  |  |  |  |  |  |  |  |  |  |  |  |  |  |  |  |  |  |  |  |  |  |  |  |  |  |  |  |  |  |  |  |  |  |  |  |  |  |  |  |  |  |  |  |  |  |  |
|  |  |  |  |  |  |  |  |  |  |  |  |  |  |  |  |  |  |  |  |  |  |  |  |  |  |  |  |  |  |  |  |  |  |  |  |  |  |  |  |  |  |  |  |  |  |  |  |  |  |  |  |  |  |  |  |  |  |  |  |  |  |  |  |  |  |  |  |  |  |  |  |  |  |  |  |  |  |  |  |  |  |  |  |  |  |  |  |  |  |  |  |  |  |  |  |  |  |  |  |  |  |  |  |  |  |  |  |  |  |  |  |  |  |  |  |  |  |  |  |  |  |  |  |  |  |  |  |  |  |  |  |  |  |  |  |  |  |  |  |  |  |  |  |  |  |  |  |  |  |  |  |  |  |  |  |  |  |  |  |  |  |  |  |  |  |  |  |  |  |  |  |  |  |  |  |  |  |  |  |  |  |  |  |  |  |  |  |  |  |  |  |  |  |  |  |  |  |  |  |  |  |  |  |  |  |  |  |  |  |  |  |  |  |  |  |  |  |  |  |  |  |  |  |  |  |  |  |  |  |  |  |  |  |  |  |  |  |  |  |  |  |  |  |  |  |  |  |  |  |  |  |  |  |  |  |  |  |  |  |  |  |  |  |  |  |  |  |  |  |  |  |  |  |  |  |  |  |  |  |  |  |  |  |  |  |  |  |  |  |  |  |  |  |  |  |  |  |  |  |  |  |  |  |  |  |  |  |  |  |  |  |  |  |  |  |  |  |  |  |  |  |  |  |  |  |  |  |  |  |  |  |  |  |  |  |  |  |  |  |  |  |  |  |  |  |  |  |  |  |  |  |  |  |  |  |  |  |  |  |  |  |  |  |  |  |  |  |  |  |  |  |  |  |  |  |  |  |  |  |  |  |  |  |  |  |  |  |  |  |  |  |  |  |  |  |  |  |  |  |  |  |  |  |  |  |  |  |  |  |  |  |  |  |  |  |  |  |  |  |  |  |  |  |  |  |  |  |  |  |  |  |  |  |  |  |  |  |  |  |  |  |  |  |  |  |  |  |  |  |  |  |  |  |  |  |  |  |  |  |  |  |  |  |  |  |  |  |  |  |  |  |  |  |  |  |  |  |  |  |  |  |  |  |  |  |  |  |  |  |  |  |  |  |  |  |  |  |  |  |  |  |  |  |  |  |  |  |  |  |  |  |  |  |  |  |  |  |  |  |  |  |  |  |  |  |  |  |  |  |  |  |  |  |  |  |  |  |  |  |  |  |  |  |  |  |  |  |  |  |  |  |  |  |  |  |  |  |  |  |  |  |  |  |  |  |  |  |  |  |  |  |  |  |  |  |  |  |  |  |  |  |  |  |  |  |  |  |  |  |  |  |  |  |  |  |  |  |  |  |  |  |  |  |  |  |  |  |  |  |  |  |  |  |  |  |  |  |  |  |  |  |  |  |  |  |  |  |  |  |  |  |  |  |  |  |  |  |  |  |  |  |  |  |  |  |  |  |  |  |  |  |  |  |  |  |  |  |  |  |  |  |  |  |  |  |  |  |  |  |  |  |  |  |  |  |  |  |  |  |  |  |  |  |  |  |  |  |  |  |  |  |  |  |  |  |  |  |  |  |  |  |  |  |  |  |  |  |  |  |  |  |  |  |  |  |  |  |  |  |  |  |  |  |  |  |  |  |  |  |  |  |  |  |  |  |  |  |  |  |  |  |  |  |  |  |  |  |  |  |  |  |  |  |  |  |  |  |  |  |  |  |  |  |  |  |  |  |  |  |  |  |  |  |  |  |  |  |  |  |  |  |  |  |  |  |  |  |  |  |  |  |  |  |  |  |  |  |  |  |  |  |  |  |  |  |  |  |  |  |  |  |  |  |  |  |  |  |  |  |  |  |  |  |  |  |  |  |  |  |  |  |  |  |  |  |  |  |  |  |  |  |  |  |  |  |  |  |  |  |  |  |  |  |  |  |  |  |  |  |  |  |  |  |  |  |  |  |  |  |  |  |  |  |  |  |  |  |  |  |  |  |  |  |  |  |  |  |  |  |  |  |  |  |  |  |  |  |  |  |  |  |  |  |  |  |  |  |  |  |  |  |  |  |  |  |  |  |  |  |  |  |  |  |  |  |  |  |  |  |  |  |  |  |  |  |  |  |  |  |  |  |  |  |  |  |  |  |  |  |  |  |  |  |  |  |  |  |  |  |  |  |  |  |  |  |  |  |  |  |  |  |  |  |  |  |  |  |  |  |  |  |  |  |  |  |  |  |  |  |  |  |  |  |  |  |  |  |  |  |  |  |  |  |  |  |  |  |  |  |  |  |  |  |  |  |  |  |  |  |  |  |  |  |  |  |  |  |  |  |  |  |  |  |  |  |  |  |  |  |  |  |  |  |  |  |  |  |  |  |  |  |  |  |  |  |  |  |  |  |  |  |  |  |  |  |  |  |  |  |  |  |  |  |  |  |  |  |  |  |  |  |  |  |  |  |  |  |  |  |  |  |  |  |  |  |  |  |  |  |  |  |  |  |  |  |  |  |  |  |  |  |  |  |  |  |  |  |  |  |  |  |  |  |  |  |  |  |  |  |  |  |  |  |  |  |  |  |  |  |  |  |  |  |  |  |  |  |  |  |  |  |  |  |  |  |  |  |  |  |  |  |  |  |  |  |  |  |  |  |  |  |  |  |  |  |  |  |  |  |  |  |  |  |  |  |  |  |  |  |  |  |  |  |  |  |  |  |  |  |  |  |  |  |  |  |  |  |  |  |  |  |  |  |  |  |  |  |  |  |  |  |  |  |  |  |  |  |  |  |  |  |  |  |  |  |  |  |  |  |  |  |  |  |  |  |  |  |  |  |  |  |  |  |  |  |  |  |  |  |  |
|  |  |  |  |  |  |  |  |  |  |  |  |  |  |  |  |  |  |  |  |  |  |  |  |  |  |  |  |  |  |  |  |  |  |  |  |  |  |  |  |  |  |  |  |  |  |  |  |  |  |  |  |  |  |  |  |  |  |  |  |  |  |  |  |  |  |  |  |  |  |  |  |  |  |  |  |  |  |  |  |  |  |  |  |  |  |  |  |  |  |  |  |  |  |  |  |  |  |  |  |  |  |  |  |  |  |  |  |  |  |  |  |  |  |  |  |  |  |  |  |  |  |  |  |  |  |  |  |  |  |  |  |  |  |  |  |  |  |  |  |  |  |  |  |  |  |  |  |  |  |  |  |  |  |  |  |  |  |  |  |  |  |  |  |  |  |  |  |  |  |  |  |  |  |  |  |  |  |  |  |  |  |  |  |  |  |  |  |  |  |  |  |  |  |  |  |  |  |  |  |  |  |  |  |  |  |  |  |  |  |  |  |  |  |  |  |  |  |  |  |  |  |  |  |  |  |  |  |  |  |  |  |  |  |  |  |  |  |  |  |  |  |  |  |  |  |  |  |  |  |  |  |  |  |  |  |  |  |  |  |  |  |  |  |  |  |  |  |  |  |  |  |  |  |  |  |  |  |  |  |  |  |  |  |  |  |  |  |  |  |  |  |  |  |  |  |  |  |  |  |  |  |  |  |  |  |  |  |  |  |  |  |  |  |  |  |  |  |  |  |  |  |  |  |  |  |  |  |  |  |  |  |  |  |  |  |  |  |  |  |  |  |  |  |  |  |  |  |  |  |  |  |  |  |  |  |  |  |  |  |  |  |  |  |  |  |  |  |  |  |  |  |  |  |  |  |  |  |  |  |  |  |  |  |  |  |  |  |  |  |  |  |  |  |  |  |  |  |  |  |  |  |  |  |  |  |  |  |  |  |  |  |  |  |  |  |  |  |  |  |  |  |  |  |  |  |  |  |  |  |  |  |  |  |  |  |  |  |  |  |  |  |  |  |  |  |  |  |  |  |  |  |  |  |  |  |  |  |  |  |  |  |  |  |  |  |  |  |  |  |  |  |  |  |  |  |  |  |  |  |  |  |  |  |  |  |  |  |  |  |  |  |  |  |  |  |  |  |  |  |  |  |  |  |  |  |  |  |  |  |  |  |  |  |  |  |  |  |  |  |  |  |  |  |  |  |  |  |  |  |  |  |  |  |  |  |  |  |  |  |  |  |  |  |  |  |  |  |  |  |  |  |  |  |  |  |  |  |  |  |  |  |  |  |  |  |  |  |  |  |  |  |  |  |  |  |  |  |  |  |  |  |  |  |  |  |  |  |  |  |  |  |  |  |  |  |  |  |  |  |  |  |  |  |  |  |  |  |  |  |  |  |  |  |  |  |  |  |  |  |  |  |  |  |  |  |  |  |  |  |  |  |  |  |  |  |  |  |  |  |  |  |  |  |  |  |  |  |  |  |  |  |  |  |  |  |  |  |  |  |  |  |  |  |  |  |  |  |  |  |  |  |  |  |  |  |  |  |  |  |  |  |  |  |  |  |  |  |  |  |  |  |  |  |  |  |  |  |  |  |  |  |  |  |  |  |  |  |  |  |  |  |  |  |  |  |  |  |  |  |  |  |  |  |  |  |  |  |  |  |  |  |  |  |  |  |  |  |  |  |  |  |  |  |  |  |  |  |  |  |  |  |  |  |  |  |  |  |  |  |  |  |  |  |  |  |  |  |  |  |  |  |  |  |  |  |  |  |  |  |  |  |  |  |  |  |  |  |  |  |  |  |  |  |  |  |  |  |  |  |  |  |  |  |  |  |  |  |  |  |  |  |  |  |  |  |  |  |  |  |  |  |  |  |  |  |  |  |  |  |  |  |  |  |  |  |  |  |  |  |  |  |  |  |  |  |  |  |  |  |  |  |  |  |  |  |  |  |  |  |  |  |  |  |  |  |  |  |  |  |  |  |  |  |  |  |  |  |  |  |  |  |  |  |  |  |  |  |  |  |  |  |  |  |  |  |  |  |  |  |  |  |  |  |  |  |  |  |  |  |  |  |  |  |  |  |  |  |  |  |  |  |  |  |  |  |  |  |  |  |  |  |  |  |  |  |  |  |  |  |  |  |  |  |  |  |  |  |  |  |  |  |  |  |  |  |  |  |  |  |  |  |  |  |  |  |  |  |  |  |  |  |  |  |  |  |  |  |  |  |  |  |  |  |  |  |  |  |  |  |  |  |  |  |  |  |  |  |  |  |  |  |  |  |  |  |  |  |  |  |  |  |  |  |  |  |  |  |  |  |  |  |  |  |  |  |  |  |  |  |  |  |  |  |  |  |  |  |  |  |  |  |  |  |  |  |  |  |  |  |  |  |  |  |  |  |  |  |  |  |  |  |  |  |  |  |  |  |  |  |  |  |  |  |  |  |  |  |  |  |  |  |  |  |  |  |  |  |  |  |  |  |  |  |  |  |  |  |  |  |  |  |  |  |  |  |  |  |  |  |  |  |  |  |  |  |  |  |  |  |  |  |  |  |  |  |  |  |  |  |  |  |  |  |  |  |  |  |  |  |  |  |  |  |  |  |  |  |  |  |  |  |  |  |  |  |  |  |  |  |  |  |  |  |  |  |  |  |  |  |  |  |  |  |  |  |  |  |  |  |  |  |  |  |  |  |  |  |  |  |  |  |  |  |  |  |  |  |  |  |  |  |  |  |  |  |  |  |  |  |  |  |  |  |  |  |  |  |  |  |  |  |  |  |  |  |  |  |  |  |  |  |  |  |  |  |  |  |  |  |  |  |  |  |  |  |  |  |  |  |  |  |  |  |  |  |  |  |  |  |  |
|  |  |  |  |  |  |  |  |  |  |  |  |  |  |  |  |  |  |  |  |  |  |  |  |  |  |  |  |  |  |  |  |  |  |  |  |  |  |  |  |  |  |  |  |  |  |  |  |  |  |  |  |  |  |  |  |  |  |  |  |  |  |  |  |  |  |  |  |  |  |  |  |  |  |  |  |  |  |  |  |  |  |  |  |  |  |  |  |  |  |  |  |  |  |  |  |  |  |  |  |  |  |  |  |  |  |  |  |  |  |  |  |  |  |  |  |  |  |  |  |  |  |  |  |  |  |  |  |  |  |  |  |  |  |  |  |  |  |  |  |  |  |  |  |  |  |  |  |  |  |  |  |  |  |  |  |  |  |  |  |  |  |  |  |  |  |  |  |  |  |  |  |  |  |  |  |  |  |  |  |  |  |  |  |  |  |  |  |  |  |  |  |  |  |  |  |  |  |  |  |  |  |  |  |  |  |  |  |  |  |  |  |  |  |  |  |  |  |  |  |  |  |  |  |  |  |  |  |  |  |  |  |  |  |  |  |  |  |  |  |  |  |  |  |  |  |  |  |  |  |  |  |  |  |  |  |  |  |  |  |  |  |  |  |  |  |  |  |  |  |  |  |  |  |  |  |  |  |  |  |  |  |  |  |  |  |  |  |  |  |  |  |  |  |  |  |  |  |  |  |  |  |  |  |  |  |  |  |  |  |  |  |  |  |  |  |  |  |  |  |  |  |  |  |  |  |  |  |  |  |  |  |  |  |  |  |  |  |  |  |  |  |  |  |  |  |  |  |  |  |  |  |  |  |  |  |  |  |  |  |  |  |  |  |  |  |  |  |  |  |  |  |  |  |  |  |  |  |  |  |  |  |  |  |  |  |  |  |  |  |  |  |  |  |  |  |  |  |  |  |  |  |  |  |  |  |  |  |  |  |  |  |  |  |  |  |  |  |  |  |  |  |  |  |  |  |  |  |  |  |  |  |  |  |  |  |  |  |  |  |  |  |  |  |  |  |  |  |  |  |  |  |  |  |  |  |  |  |  |  |  |  |  |  |  |  |  |  |  |  |  |  |  |  |  |  |  |  |  |  |  |  |  |  |  |  |  |  |  |  |  |  |  |  |  |  |  |  |  |  |  |  |  |  |  |  |  |  |  |  |  |  |  |  |  |  |  |  |  |  |  |  |  |  |  |  |  |  |  |  |  |  |  |  |  |  |  |  |  |  |  |  |  |  |  |  |  |  |  |  |  |  |  |  |  |  |  |  |  |  |  |  |  |  |  |  |  |  |  |  |  |  |  |  |  |  |  |  |  |  |  |  |  |  |  |  |  |  |  |  |  |  |  |  |  |  |  |  |  |  |  |  |  |  |  |  |  |  |  |  |  |  |  |  |  |  |  |  |  |  |  |  |  |  |  |  |  |  |  |  |  |  |  |  |  |  |  |  |  |  |  |  |  |  |  |  |  |  |  |  |  |  |  |  |  |  |  |  |  |  |  |  |  |  |  |  |  |  |  |  |  |  |  |  |  |  |  |  |  |  |  |  |  |  |  |  |  |  |  |  |  |  |  |  |  |  |  |  |  |  |  |  |  |  |  |  |  |  |  |  |  |  |  |  |  |  |  |  |  |  |  |  |  |  |  |  |  |  |  |  |  |  |  |  |  |  |  |  |  |  |  |  |  |  |  |  |  |  |  |  |  |  |  |  |  |  |  |  |  |  |  |  |  |  |  |  |  |  |  |  |  |  |  |  |  |  |  |  |  |  |  |  |  |  |  |  |  |  |  |  |  |  |  |  |  |  |  |  |  |  |  |  |  |  |  |  |  |  |  |  |  |  |  |  |  |  |  |  |  |  |  |  |  |  |  |  |  |  |  |  |  |  |  |  |  |  |  |  |  |  |  |  |  |  |  |  |  |  |  |  |  |  |  |  |  |  |  |  |  |  |  |  |  |  |  |  |  |  |  |  |  |  |  |  |  |  |  |  |  |  |  |  |  |  |  |  |  |  |  |  |  |  |  |  |  |  |  |  |  |  |  |  |  |  |  |  |  |  |  |  |  |  |  |  |  |  |  |  |  |  |  |  |  |  |  |  |  |  |  |  |  |  |  |  |  |  |  |  |  |  |  |  |  |  |  |  |  |  |  |  |  |  |  |  |  |  |  |  |  |  |  |  |  |  |  |  |  |  |  |  |  |  |  |  |  |  |  |  |  |  |  |  |  |  |  |  |  |  |  |  |  |  |  |  |  |  |  |  |  |  |  |  |  |  |  |  |  |  |  |  |  |  |  |  |  |  |  |  |  |  |  |  |  |  |  |  |  |  |  |  |  |  |  |  |  |  |  |  |  |  |  |  |  |  |  |  |  |  |  |  |  |  |  |  |  |  |  |  |  |  |  |  |  |  |  |  |  |  |  |  |  |  |  |  |  |  |  |  |  |  |  |  |  |  |  |  |  |  |  |  |  |  |  |  |  |  |  |  |  |  |  |  |  |  |  |  |  |  |  |  |  |  |  |  |  |  |  |  |  |  |  |  |  |  |  |  |  |  |  |  |  |  |  |  |  |  |  |  |  |  |  |  |  |  |  |  |  |  |  |  |  |  |  |  |  |  |  |  |  |  |  |  |  |  |  |  |  |  |  |  |  |  |  |  |  |  |  |  |  |  |  |  |  |  |  |  |  |  |  |  |  |  |  |  |  |  |  |  |  |  |  |  |  |  |  |  |  |  |  |  |  |  |  |  |  |  |  |  |  |  |  |  |  |  |  |  |  |  |  |  |  |  |  |  |  |  |  |  |  |  |  |  |  |  |  |  |  |  |  |  |  |  |  |  |  |  |  |  |  |  |  |
|  |  |  |  |  |  |  |  |  |  |  |  |  |  |  |  |  |  |  |  |  |  |  |  |  |  |  |  |  |  |  |  |  |  |  |  |  |  |  |  |  |  |  |  |  |  |  |  |  |  |  |  |  |  |  |  |  |  |  |  |  |  |  |  |  |  |  |  |  |  |  |  |  |  |  |  |  |  |  |  |  |  |  |  |  |  |  |  |  |  |  |  |  |  |  |  |  |  |  |  |  |  |  |  |  |  |  |  |  |  |  |  |  |  |  |  |  |  |  |  |  |  |  |  |  |  |  |  |  |  |  |  |  |  |  |  |  |  |  |  |  |  |  |  |  |  |  |  |  |  |  |  |  |  |  |  |  |  |  |  |  |  |  |  |  |  |  |  |  |  |  |  |  |  |  |  |  |  |  |  |  |  |  |  |  |  |  |  |  |  |  |  |  |  |  |  |  |  |  |  |  |  |  |  |  |  |  |  |  |  |  |  |  |  |  |  |  |  |  |  |  |  |  |  |  |  |  |  |  |  |  |  |  |  |  |  |  |  |  |  |  |  |  |  |  |  |  |  |  |  |  |  |  |  |  |  |  |  |  |  |  |  |  |  |  |  |  |  |  |  |  |  |  |  |  |  |  |  |  |  |  |  |  |  |  |  |  |  |  |  |  |  |  |  |  |  |  |  |  |  |  |  |  |  |  |  |  |  |  |  |  |  |  |  |  |  |  |  |  |  |  |  |  |  |  |  |  |  |  |  |  |  |  |  |  |  |  |  |  |  |  |  |  |  |  |  |  |  |  |  |  |  |  |  |  |  |  |  |  |  |  |  |  |  |  |  |  |  |  |  |  |  |  |  |  |  |  |  |  |  |  |  |  |  |  |  |  |  |  |  |  |  |  |  |  |  |  |  |  |  |  |  |  |  |  |  |  |  |  |  |  |  |  |  |  |  |  |  |  |  |  |  |  |  |  |  |  |  |  |  |  |  |  |  |  |  |  |  |  |  |  |  |  |  |  |  |  |  |  |  |  |  |  |  |  |  |  |  |  |  |  |  |  |  |  |  |  |  |  |  |  |  |  |  |  |  |  |  |  |  |  |  |  |  |  |  |  |  |  |  |  |  |  |  |  |  |  |  |  |  |  |  |  |  |  |  |  |  |  |  |  |  |  |  |  |  |  |  |  |  |  |  |  |  |  |  |  |  |  |  |  |  |  |  |  |  |  |  |  |  |  |  |  |  |  |  |  |  |  |  |  |  |  |  |  |  |  |  |  |  |  |  |  |  |  |  |  |  |  |  |  |  |  |  |  |  |  |  |  |  |  |  |  |  |  |  |  |  |  |  |  |  |  |  |  |  |  |  |  |  |  |  |  |  |  |  |  |  |  |  |  |  |  |  |  |  |  |  |  |  |  |  |  |  |  |  |  |  |  |  |  |  |  |  |  |  |  |  |  |  |  |  |  |  |  |  |  |  |  |  |  |  |  |  |  |  |  |  |  |  |  |  |  |  |  |  |  |  |  |  |  |  |  |  |  |  |  |  |  |  |  |  |  |  |  |  |  |  |  |  |  |  |  |  |  |  |  |  |  |  |  |  |  |  |  |  |  |  |  |  |  |  |  |  |  |  |  |  |  |  |  |  |  |  |  |  |  |  |  |  |  |  |  |  |  |  |  |  |  |  |  |  |  |  |  |  |  |  |  |  |  |  |  |  |  |  |  |  |  |  |  |  |  |  |  |  |  |  |  |  |  |  |  |  |  |  |  |  |  |  |  |  |  |  |  |  |  |  |  |  |  |  |  |  |  |  |  |  |  |  |  |  |  |  |  |  |  |  |  |  |  |  |  |  |  |  |  |  |  |  |  |  |  |  |  |  |  |  |  |  |  |  |  |  |  |  |  |  |  |  |  |  |  |  |  |  |  |  |  |  |  |  |  |  |  |  |  |  |  |  |  |  |  |  |  |  |  |  |  |  |  |  |  |  |  |  |  |  |  |  |  |  |  |  |  |  |  |  |  |  |  |  |  |  |  |  |  |  |  |  |  |  |  |  |  |  |  |  |  |  |  |  |  |  |  |  |  |  |  |  |  |  |  |  |  |  |  |  |  |  |  |  |  |  |  |  |  |  |  |  |  |  |  |  |  |  |  |  |  |  |  |  |  |  |  |  |  |  |  |  |  |  |  |  |  |  |  |  |  |  |  |  |  |  |  |  |  |  |  |  |  |  |  |  |  |  |  |  |  |  |  |  |  |  |  |  |  |  |  |  |  |  |  |  |  |  |  |  |  |  |  |  |  |  |  |  |  |  |  |  |  |  |  |  |  |  |  |  |  |  |  |  |  |  |  |  |  |  |  |  |  |  |  |  |  |  |  |  |  |  |  |  |  |  |  |  |  |  |  |  |  |  |  |  |  |  |  |  |  |  |  |  |  |  |  |  |  |  |  |  |  |  |  |  |  |  |  |  |  |  |  |  |  |  |  |  |  |  |  |  |  |  |  |  |  |  |  |  |  |  |  |  |  |  |  |  |  |  |  |  |  |  |  |  |  |  |  |  |  |  |  |  |  |  |  |  |  |  |  |  |  |  |  |  |  |  |  |  |  |  |  |  |  |  |  |  |  |  |  |  |  |  |  |  |  |  |  |  |  |  |  |  |  |  |  |  |  |  |  |  |  |  |  |  |  |  |  |  |  |  |  |  |  |  |  |  |  |  |  |  |  |  |  |  |  |  |  |  |  |  |  |  |  |  |  |  |  |  |  |  |  |  |  |  |  |  |  |  |  |  |  |  |  |  |  |  |  |  |  |  |  |  |  |  |  |  |  |  |  |  |  |  |  |  |  |  |  |  |  |  |  |
|  |  |  |  |  |  |  |  |  |  |  |  |  |  |  |  |  |  |  |  |  |  |  |  |  |  |  |  |  |  |  |  |  |  |  |  |  |  |  |  |  |  |  |  |  |  |  |  |  |  |  |  |  |  |  |  |  |  |  |  |  |  |  |  |  |  |  |  |  |  |  |  |  |  |  |  |  |  |  |  |  |  |  |  |  |  |  |  |  |  |  |  |  |  |  |  |  |  |  |  |  |  |  |  |  |  |  |  |  |  |  |  |  |  |  |  |  |  |  |  |  |  |  |  |  |  |  |  |  |  |  |  |  |  |  |  |  |  |  |  |  |  |  |  |  |  |  |  |  |  |  |  |  |  |  |  |  |  |  |  |  |  |  |  |  |  |  |  |  |  |  |  |  |  |  |  |  |  |  |  |  |  |  |  |  |  |  |  |  |  |  |  |  |  |  |  |  |  |  |  |  |  |  |  |  |  |  |  |  |  |  |  |  |  |  |  |  |  |  |  |  |  |  |  |  |  |  |  |  |  |  |  |  |  |  |  |  |  |  |  |  |  |  |  |  |  |  |  |  |  |  |  |  |  |  |  |  |  |  |  |  |  |  |  |  |  |  |  |  |  |  |  |  |  |  |  |  |  |  |  |  |  |  |  |  |  |  |  |  |  |  |  |  |  |  |  |  |  |  |  |  |  |  |  |  |  |  |  |  |  |  |  |  |  |  |  |  |  |  |  |  |  |  |  |  |  |  |  |  |  |  |  |  |  |  |  |  |  |  |  |  |  |  |  |  |  |  |  |  |  |  |  |  |  |  |  |  |  |  |  |  |  |  |  |  |  |  |  |  |  |  |  |  |  |  |  |  |  |  |  |  |  |  |  |  |  |  |  |  |  |  |  |  |  |  |  |  |  |  |  |  |  |  |  |  |  |  |  |  |  |  |  |  |  |  |  |  |  |  |  |  |  |  |  |  |  |  |  |  |  |  |  |  |  |  |  |  |  |  |  |  |  |  |  |  |  |  |  |  |  |  |  |  |  |  |  |  |  |  |  |  |  |  |  |  |  |  |  |  |  |  |  |  |  |  |  |  |  |  |  |  |  |  |  |  |  |  |  |  |  |  |  |  |  |  |  |  |  |  |  |  |  |  |  |  |  |  |  |  |  |  |  |  |  |  |  |  |  |  |  |  |  |  |  |  |  |  |  |  |  |  |  |  |  |  |  |  |  |  |  |  |  |  |  |  |  |  |  |  |  |  |  |  |  |  |  |  |  |  |  |  |  |  |  |  |  |  |  |  |  |  |  |  |  |  |  |  |  |  |  |  |  |  |  |  |  |  |  |  |  |  |  |  |  |  |  |  |  |  |  |  |  |  |  |  |  |  |  |  |  |  |  |  |  |  |  |  |  |  |  |  |  |  |  |  |  |  |  |  |  |  |  |  |  |  |  |  |  |  |  |  |  |  |  |  |  |  |  |  |  |  |  |  |  |  |  |  |  |  |  |  |  |  |  |  |  |  |  |  |  |  |  |  |  |  |  |  |  |  |  |  |  |  |  |  |  |  |  |  |  |  |  |  |  |  |  |  |  |  |  |  |  |  |  |  |  |  |  |  |  |  |  |  |  |  |  |  |  |  |  |  |  |  |  |  |  |  |  |  |  |  |  |  |  |  |  |  |  |  |  |  |  |  |  |  |  |  |  |  |  |  |  |  |  |  |  |  |  |  |  |  |  |  |  |  |  |  |  |  |  |  |  |  |  |  |  |  |  |  |  |  |  |  |  |  |  |  |  |  |  |  |  |  |  |  |  |  |  |  |  |  |  |  |  |  |  |  |  |  |  |  |  |  |  |  |  |  |  |  |  |  |  |  |  |  |  |  |  |  |  |  |  |  |  |  |  |  |  |  |  |  |  |  |  |  |  |  |  |  |  |  |  |  |  |  |  |  |  |  |  |  |  |  |  |  |  |  |  |  |  |  |  |  |  |  |  |  |  |  |  |  |  |  |  |  |  |  |  |  |  |  |  |  |  |  |  |  |  |  |  |  |  |  |  |  |  |  |  |  |  |  |  |  |  |  |  |  |  |  |  |  |  |  |  |  |  |  |  |  |  |  |  |  |  |  |  |  |  |  |  |  |  |  |  |  |  |  |  |  |  |  |  |  |  |  |  |  |  |  |  |  |  |  |  |  |  |  |  |  |  |  |  |  |  |  |  |  |  |  |  |  |  |  |  |  |  |  |  |  |  |  |  |  |  |  |  |  |  |  |  |  |  |  |  |  |  |  |  |  |  |  |  |  |  |  |  |  |  |  |  |  |  |  |  |  |  |  |  |  |  |  |  |  |  |  |  |  |  |  |  |  |  |  |  |  |  |  |  |  |  |  |  |  |  |  |  |  |  |  |  |  |  |  |  |  |  |  |  |  |  |  |  |  |  |  |  |  |  |  |  |  |  |  |  |  |  |  |  |  |  |  |  |  |  |  |  |  |  |  |  |  |  |  |  |  |  |  |  |  |  |  |  |  |  |  |  |  |  |  |  |  |  |  |  |  |  |  |  |  |  |  |  |  |  |  |  |  |  |  |  |  |  |  |  |  |  |  |  |  |  |  |  |  |  |  |  |  |  |  |  |  |  |  |  |  |  |  |  |  |  |  |  |  |  |  |  |  |  |  |  |  |  |  |  |  |  |  |  |  |  |  |  |  |  |  |  |  |  |  |  |  |  |  |  |  |  |  |  |  |  |  |  |  |  |  |  |  |  |  |  |  |  |  |  |  |  |  |  |  |  |  |  |  |  |  |  |  |  |  |  |  |  |  |  |  |  |  |  |  |  |  |  |  |  |  |  |  |  |  |  |  |
|  |  |  |  |  |  |  |  |  |  |  |  |  |  |  |  |  |  |  |  |  |  |  |  |  |  |  |  |  |  |  |  |  |  |  |  |  |  |  |  |  |  |  |  |  |  |  |  |  |  |  |  |  |  |  |  |  |  |  |  |  |  |  |  |  |  |  |  |  |  |  |  |  |  |  |  |  |  |  |  |  |  |  |  |  |  |  |  |  |  |  |  |  |  |  |  |  |  |  |  |  |  |  |  |  |  |  |  |  |  |  |  |  |  |  |  |  |  |  |  |  |  |  |  |  |  |  |  |  |  |  |  |  |  |  |  |  |  |  |  |  |  |  |  |  |  |  |  |  |  |  |  |  |  |  |  |  |  |  |  |  |  |  |  |  |  |  |  |  |  |  |  |  |  |  |  |  |  |  |  |  |  |  |  |  |  |  |  |  |  |  |  |  |  |  |  |  |  |  |  |  |  |  |  |  |  |  |  |  |  |  |  |  |  |  |  |  |  |  |  |  |  |  |  |  |  |  |  |  |  |  |  |  |  |  |  |  |  |  |  |  |  |  |  |  |  |  |  |  |  |  |  |  |  |  |  |  |  |  |  |  |  |  |  |  |  |  |  |  |  |  |  |  |  |  |  |  |  |  |  |  |  |  |  |  |  |  |  |  |  |  |  |  |  |  |  |  |  |  |  |  |  |  |  |  |  |  |  |  |  |  |  |  |  |  |  |  |  |  |  |  |  |  |  |  |  |  |  |  |  |  |  |  |  |  |  |  |  |  |  |  |  |  |  |  |  |  |  |  |  |  |  |  |  |  |  |  |  |  |  |  |  |  |  |  |  |  |  |  |  |  |  |  |  |  |  |  |  |  |  |  |  |  |  |  |  |  |  |  |  |  |  |  |  |  |  |  |  |  |  |  |  |  |  |  |  |  |  |  |  |  |  |  |  |  |  |  |  |  |  |  |  |  |  |  |  |  |  |  |  |  |  |  |  |  |  |  |  |  |  |  |  |  |  |  |  |  |  |  |  |  |  |  |  |  |  |  |  |  |  |  |  |  |  |  |  |  |  |  |  |  |  |  |  |  |  |  |  |  |  |  |  |  |  |  |  |  |  |  |  |  |  |  |  |  |  |  |  |  |  |  |  |  |  |  |  |  |  |  |  |  |  |  |  |  |  |  |  |  |  |  |  |  |  |  |  |  |  |  |  |  |  |  |  |  |  |  |  |  |  |  |  |  |  |  |  |  |  |  |  |  |  |  |  |  |  |  |  |  |  |  |  |  |  |  |  |  |  |  |  |  |  |  |  |  |  |  |  |  |  |  |  |  |  |  |  |  |  |  |  |  |  |  |  |  |  |  |  |  |  |  |  |  |  |  |  |  |  |  |  |  |  |  |  |  |  |  |  |  |  |  |  |  |  |  |  |  |  |  |  |  |  |  |  |  |  |  |  |  |  |  |  |  |  |  |  |  |  |  |  |  |  |  |  |  |  |  |  |  |  |  |  |  |  |  |  |  |  |  |  |  |  |  |  |  |  |  |  |  |  |  |  |  |  |  |  |  |  |  |  |  |  |  |  |  |  |  |  |  |  |  |  |  |  |  |  |  |  |  |  |  |  |  |  |  |  |  |  |  |  |  |  |  |  |  |  |  |  |  |  |  |  |  |  |  |  |  |  |  |  |  |  |  |  |  |  |  |  |  |  |  |  |  |  |  |  |  |  |  |  |  |  |  |  |  |  |  |  |  |  |  |  |  |  |  |  |  |  |  |  |  |  |  |  |  |  |  |  |  |  |  |  |  |  |  |  |  |  |  |  |  |  |  |  |  |  |  |  |  |  |  |  |  |  |  |  |  |  |  |  |  |  |  |  |  |  |  |  |  |  |  |  |  |  |  |  |  |  |  |  |  |  |  |  |  |  |  |  |  |  |  |  |  |  |  |  |  |  |  |  |  |  |  |  |  |  |  |  |  |  |  |  |  |  |  |  |  |  |  |  |  |  |  |  |  |  |  |  |  |  |  |  |  |  |  |  |  |  |  |  |  |  |  |  |  |  |  |  |  |  |  |  |  |  |  |  |  |  |  |  |  |  |  |  |  |  |  |  |  |  |  |  |  |  |  |  |  |  |  |  |  |  |  |  |  |  |  |  |  |  |  |  |  |  |  |  |  |  |  |  |  |  |  |  |  |  |  |  |  |  |  |  |  |  |  |  |  |  |  |  |  |  |  |  |  |  |  |  |  |  |  |  |  |  |  |  |  |  |  |  |  |  |  |  |  |  |  |  |  |  |  |  |  |  |  |  |  |  |  |  |  |  |  |  |  |  |  |  |  |  |  |  |  |  |  |  |  |  |  |  |  |  |  |  |  |  |  |  |  |  |  |  |  |  |  |  |  |  |  |  |  |  |  |  |  |  |  |  |  |  |  |  |  |  |  |  |  |  |  |  |  |  |  |  |  |  |  |  |  |  |  |  |  |  |  |  |  |  |  |  |  |  |  |  |  |  |  |  |  |  |  |  |  |  |  |  |  |  |  |  |  |  |  |  |  |  |  |  |  |  |  |  |  |  |  |  |  |  |  |  |  |  |  |  |  |  |  |  |  |  |  |  |  |  |  |  |  |  |  |  |  |  |  |  |  |  |  |  |  |  |  |  |  |  |  |  |  |  |  |  |  |  |  |  |  |  |  |  |  |  |  |  |  |  |  |  |  |  |  |  |  |  |  |  |  |  |  |  |  |  |  |  |  |  |  |  |  |  |  |  |  |  |  |  |  |  |  |  |  |  |  |  |  |  |  |  |  |  |  |  |  |  |  |  |  |  |  |  |  |  |  |  |  |  |  |  |  |  |  |  |  |
|  |  |  |  |  |  |  |  |  |  |  |  |  |  |  |  |  |  |  |  |  |  |  |  |  |  |  |  |  |  |  |  |  |  |  |  |  |  |  |  |  |  |  |  |  |  |  |  |  |  |  |  |  |  |  |  |  |  |  |  |  |  |  |  |  |  |  |  |  |  |  |  |  |  |  |  |  |  |  |  |  |  |  |  |  |  |  |  |  |  |  |  |  |  |  |  |  |  |  |  |  |  |  |  |  |  |  |  |  |  |  |  |  |  |  |  |  |  |  |  |  |  |  |  |  |  |  |  |  |  |  |  |  |  |  |  |  |  |  |  |  |  |  |  |  |  |  |  |  |  |  |  |  |  |  |  |  |  |  |  |  |  |  |  |  |  |  |  |  |  |  |  |  |  |  |  |  |  |  |  |  |  |  |  |  |  |  |  |  |  |  |  |  |  |  |  |  |  |  |  |  |  |  |  |  |  |  |  |  |  |  |  |  |  |  |  |  |  |  |  |  |  |  |  |  |  |  |  |  |  |  |  |  |  |  |  |  |  |  |  |  |  |  |  |  |  |  |  |  |  |  |  |  |  |  |  |  |  |  |  |  |  |  |  |  |  |  |  |  |  |  |  |  |  |  |  |  |  |  |  |  |  |  |  |  |  |  |  |  |  |  |  |  |  |  |  |  |  |  |  |  |  |  |  |  |  |  |  |  |  |  |  |  |  |  |  |  |  |  |  |  |  |  |  |  |  |  |  |  |  |  |  |  |  |  |  |  |  |  |  |  |  |  |  |  |  |  |  |  |  |  |  |  |  |  |  |  |  |  |  |  |  |  |  |  |  |  |  |  |  |  |  |  |  |  |  |  |  |  |  |  |  |  |  |  |  |  |  |  |  |  |  |  |  |  |  |  |  |  |  |  |  |  |  |  |  |  |  |  |  |  |  |  |  |  |  |  |  |  |  |  |  |  |  |  |  |  |  |  |  |  |  |  |  |  |  |  |  |  |  |  |  |  |  |  |  |  |  |  |  |  |  |  |  |  |  |  |  |  |  |  |  |  |  |  |  |  |  |  |  |  |  |  |  |  |  |  |  |  |  |  |  |  |  |  |  |  |  |  |  |  |  |  |  |  |  |  |  |  |  |  |  |  |  |  |  |  |  |  |  |  |  |  |  |  |  |  |  |  |  |  |  |  |  |  |  |  |  |  |  |  |  |  |  |  |  |  |  |  |  |  |  |  |  |  |  |  |  |  |  |  |  |  |  |  |  |  |  |  |  |  |  |  |  |  |  |  |  |  |  |  |  |  |  |  |  |  |  |  |  |  |  |  |  |  |  |  |  |  |  |  |  |  |  |  |  |  |  |  |  |  |  |  |  |  |  |  |  |  |  |  |  |  |  |  |  |  |  |  |  |  |  |  |  |  |  |  |  |  |  |  |  |  |  |  |  |  |  |  |  |  |  |  |  |  |  |  |  |  |  |  |  |  |  |  |  |  |  |  |  |  |  |  |  |  |  |  |  |  |  |  |  |  |  |  |  |  |  |  |  |  |  |  |  |  |  |  |  |  |  |  |  |  |  |  |  |  |  |  |  |  |  |  |  |  |  |  |  |  |  |  |  |  |  |  |  |  |  |  |  |  |  |  |  |  |  |  |  |  |  |  |  |  |  |  |  |  |  |  |  |  |  |  |  |  |  |  |  |  |  |  |  |  |  |  |  |  |  |  |  |  |  |  |  |  |  |  |  |  |  |  |  |  |  |  |  |  |  |  |  |  |  |  |  |  |  |  |  |  |  |  |  |  |  |  |  |  |  |  |  |  |  |  |  |  |  |  |  |  |  |  |  |  |  |  |  |  |  |  |  |  |  |  |  |  |  |  |  |  |  |  |  |  |  |  |  |  |  |  |  |  |  |  |  |  |  |  |  |  |  |  |  |  |  |  |  |  |  |  |  |  |  |  |  |  |  |  |  |  |  |  |  |  |  |  |  |  |  |  |  |  |  |  |  |  |  |  |  |  |  |  |  |  |  |  |  |  |  |  |  |  |  |  |  |  |  |  |  |  |  |  |  |  |  |  |  |  |  |  |  |  |  |  |  |  |  |  |  |  |  |  |  |  |  |  |  |  |  |  |  |  |  |  |  |  |  |  |  |  |  |  |  |  |  |  |  |  |  |  |  |  |  |  |  |  |  |  |  |  |  |  |  |  |  |  |  |  |  |  |  |  |  |  |  |  |  |  |  |  |  |  |  |  |  |  |  |  |  |  |  |  |  |  |  |  |  |  |  |  |  |  |  |  |  |  |  |  |  |  |  |  |  |  |  |  |  |  |  |  |  |  |  |  |  |  |  |  |  |  |  |  |  |  |  |  |  |  |  |  |  |  |  |  |  |  |  |  |  |  |  |  |  |  |  |  |  |  |  |  |  |  |  |  |  |  |  |  |  |  |  |  |  |  |  |  |  |  |  |  |  |  |  |  |  |  |  |  |  |  |  |  |  |  |  |  |  |  |  |  |  |  |  |  |  |  |  |  |  |  |  |  |  |  |  |  |  |  |  |  |  |  |  |  |  |  |  |  |  |  |  |  |  |  |  |  |  |  |  |  |  |  |  |  |  |  |  |  |  |  |  |  |  |  |  |  |  |  |  |  |  |  |  |  |  |  |  |  |  |  |  |  |  |  |  |  |  |  |  |  |  |  |  |  |  |  |  |  |  |  |  |  |  |  |  |  |  |  |  |  |  |  |  |  |  |  |  |  |  |  |  |  |  |  |  |  |  |  |  |  |  |  |  |  |  |  |  |  |  |  |  |  |  |  |  |  |  |  |  |  |  |  |  |  |  |  |  |  |  |  |  |  |
|  |  |  |  |  |  |  |  |  |  |  |  |  |  |  |  |  |  |  |  |  |  |  |  |  |  |  |  |  |  |  |  |  |  |  |  |  |  |  |  |  |  |  |  |  |  |  |  |  |  |  |  |  |  |  |  |  |  |  |  |  |  |  |  |  |  |  |  |  |  |  |  |  |  |  |  |  |  |  |  |  |  |  |  |  |  |  |  |  |  |  |  |  |  |  |  |  |  |  |  |  |  |  |  |  |  |  |  |  |  |  |  |  |  |  |  |  |  |  |  |  |  |  |  |  |  |  |  |  |  |  |  |  |  |  |  |  |  |  |  |  |  |  |  |  |  |  |  |  |  |  |  |  |  |  |  |  |  |  |  |  |  |  |  |  |  |  |  |  |  |  |  |  |  |  |  |  |  |  |  |  |  |  |  |  |  |  |  |  |  |  |  |  |  |  |  |  |  |  |  |  |  |  |  |  |  |  |  |  |  |  |  |  |  |  |  |  |  |  |  |  |  |  |  |  |  |  |  |  |  |  |  |  |  |  |  |  |  |  |  |  |  |  |  |  |  |  |  |  |  |  |  |  |  |  |  |  |  |  |  |  |  |  |  |  |  |  |  |  |  |  |  |  |  |  |  |  |  |  |  |  |  |  |  |  |  |  |  |  |  |  |  |  |  |  |  |  |  |  |  |  |  |  |  |  |  |  |  |  |  |  |  |  |  |  |  |  |  |  |  |  |  |  |  |  |  |  |  |  |  |  |  |  |  |  |  |  |  |  |  |  |  |  |  |  |  |  |  |  |  |  |  |  |  |  |  |  |  |  |  |  |  |  |  |  |  |  |  |  |  |  |  |  |  |  |  |  |  |  |  |  |  |  |  |  |  |  |  |  |  |  |  |  |  |  |  |  |  |  |  |  |  |  |  |  |  |  |  |  |  |  |  |  |  |  |  |  |  |  |  |  |  |  |  |  |  |  |  |  |  |  |  |  |  |  |  |  |  |  |  |  |  |  |  |  |  |  |  |  |  |  |  |  |  |  |  |  |  |  |  |  |  |  |  |  |  |  |  |  |  |  |  |  |  |  |  |  |  |  |  |  |  |  |  |  |  |  |  |  |  |  |  |  |  |  |  |  |  |  |  |  |  |  |  |  |  |  |  |  |  |  |  |  |  |  |  |  |  |  |  |  |  |  |  |  |  |  |  |  |  |  |  |  |  |  |  |  |  |  |  |  |  |  |  |  |  |  |  |  |  |  |  |  |  |  |  |  |  |  |  |  |  |  |  |  |  |  |  |  |  |  |  |  |  |  |  |  |  |  |  |  |  |  |  |  |  |  |  |  |  |  |  |  |  |  |  |  |  |  |  |  |  |  |  |  |  |  |  |  |  |  |  |  |  |  |  |  |  |  |  |  |  |  |  |  |  |  |  |  |  |  |  |  |  |  |  |  |  |  |  |  |  |  |  |  |  |  |  |  |  |  |  |  |  |  |  |  |  |  |  |  |  |  |  |  |  |  |  |  |  |  |  |  |  |  |  |  |  |  |  |  |  |  |  |  |  |  |  |  |  |  |  |  |  |  |  |  |  |  |  |  |  |  |  |  |  |  |  |  |  |  |  |  |  |  |  |  |  |  |  |  |  |  |  |  |  |  |  |  |  |  |  |  |  |  |  |  |  |  |  |  |  |  |  |  |  |  |  |  |  |  |  |  |  |  |  |  |  |  |  |  |  |  |  |  |  |  |  |  |  |  |  |  |  |  |  |  |  |  |  |  |  |  |  |  |  |  |  |  |  |  |  |  |  |  |  |  |  |  |  |  |  |  |  |  |  |  |  |  |  |  |  |  |  |  |  |  |  |  |  |  |  |  |  |  |  |  |  |  |  |  |  |  |  |  |  |  |  |  |  |  |  |  |  |  |  |  |  |  |  |  |  |  |  |  |  |  |  |  |  |  |  |  |  |  |  |  |  |  |  |  |  |  |  |  |  |  |  |  |  |  |  |  |  |  |  |  |  |  |  |  |  |  |  |  |  |  |  |  |  |  |  |  |  |  |  |  |  |  |  |  |  |  |  |  |  |  |  |  |  |  |  |  |  |  |  |  |  |  |  |  |  |  |  |  |  |  |  |  |  |  |  |  |  |  |  |  |  |  |  |  |  |  |  |  |  |  |  |  |  |  |  |  |  |  |  |  |  |  |  |  |  |  |  |  |  |  |  |  |  |  |  |  |  |  |  |  |  |  |  |  |  |  |  |  |  |  |  |  |  |  |  |  |  |  |  |  |  |  |  |  |  |  |  |  |  |  |  |  |  |  |  |  |  |  |  |  |  |  |  |  |  |  |  |  |  |  |  |  |  |  |  |  |  |  |  |  |  |  |  |  |  |  |  |  |  |  |  |  |  |  |  |  |  |  |  |  |  |  |  |  |  |  |  |  |  |  |  |  |  |  |  |  |  |  |  |  |  |  |  |  |  |  |  |  |  |  |  |  |  |  |  |  |  |  |  |  |  |  |  |  |  |  |  |  |  |  |  |  |  |  |  |  |  |  |  |  |  |  |  |  |  |  |  |  |  |  |  |  |  |  |  |  |  |  |  |  |  |  |  |  |  |  |  |  |  |  |  |  |  |  |  |  |  |  |  |  |  |  |  |  |  |  |  |  |  |  |  |  |  |  |  |  |  |  |  |  |  |  |  |  |  |  |  |  |  |  |  |  |  |  |  |  |  |  |  |  |  |  |  |  |  |  |  |  |  |  |  |  |  |  |  |  |  |  |  |  |  |  |  |  |  |  |  |  |  |  |  |  |  |  |  |  |  |  |  |  |  |  |  |  |  |  |  |  |  |  |  |  |  |  |
|  |  |  |  |  |  |  |  |  |  |  |  |  |  |  |  |  |  |  |  |  |  |  |  |  |  |  |  |  |  |  |  |  |  |  |  |  |  |  |  |  |  |  |  |  |  |  |  |  |  |  |  |  |  |  |  |  |  |  |  |  |  |  |  |  |  |  |  |  |  |  |  |  |  |  |  |  |  |  |  |  |  |  |  |  |  |  |  |  |  |  |  |  |  |  |  |  |  |  |  |  |  |  |  |  |  |  |  |  |  |  |  |  |  |  |  |  |  |  |  |  |  |  |  |  |  |  |  |  |  |  |  |  |  |  |  |  |  |  |  |  |  |  |  |  |  |  |  |  |  |  |  |  |  |  |  |  |  |  |  |  |  |  |  |  |  |  |  |  |  |  |  |  |  |  |  |  |  |  |  |  |  |  |  |  |  |  |  |  |  |  |  |  |  |  |  |  |  |  |  |  |  |  |  |  |  |  |  |  |  |  |  |  |  |  |  |  |  |  |  |  |  |  |  |  |  |  |  |  |  |  |  |  |  |  |  |  |  |  |  |  |  |  |  |  |  |  |  |  |  |  |  |  |  |  |  |  |  |  |  |  |  |  |  |  |  |  |  |  |  |  |  |  |  |  |  |  |  |  |  |  |  |  |  |  |  |  |  |  |  |  |  |  |  |  |  |  |  |  |  |  |  |  |  |  |  |  |  |  |  |  |  |  |  |  |  |  |  |  |  |  |  |  |  |  |  |  |  |  |  |  |  |  |  |  |  |  |  |  |  |  |  |  |  |  |  |  |  |  |  |  |  |  |  |  |  |  |  |  |  |  |  |  |  |  |  |  |  |  |  |  |  |  |  |  |  |  |  |  |  |  |  |  |  |  |  |  |  |  |  |  |  |  |  |  |  |  |  |  |  |  |  |  |  |  |  |  |  |  |  |  |  |  |  |  |  |  |  |  |  |  |  |  |  |  |  |  |  |  |  |  |  |  |  |  |  |  |  |  |  |  |  |  |  |  |  |  |  |  |  |  |  |  |  |  |  |  |  |  |  |  |  |  |  |  |  |  |  |  |  |  |  |  |  |  |  |  |  |  |  |  |  |  |  |  |  |  |  |  |  |  |  |  |  |  |  |  |  |  |  |  |  |  |  |  |  |  |  |  |  |  |  |  |  |  |  |  |  |  |  |  |  |  |  |  |  |  |  |  |  |  |  |  |  |  |  |  |  |  |  |  |  |  |  |  |  |  |  |  |  |  |  |  |  |  |  |  |  |  |  |  |  |  |  |  |  |  |  |  |  |  |  |  |  |  |  |  |  |  |  |  |  |  |  |  |  |  |  |  |  |  |  |  |  |  |  |  |  |  |  |  |  |  |  |  |  |  |  |  |  |  |  |  |  |  |  |  |  |  |  |  |  |  |  |  |  |  |  |  |  |  |  |  |  |  |  |  |  |  |  |  |  |  |  |  |  |  |  |  |  |  |  |  |  |  |  |  |  |  |  |  |  |  |  |  |  |  |  |  |  |  |  |  |  |  |  |  |  |  |  |  |  |  |  |  |  |  |  |  |  |  |  |  |  |  |  |  |  |  |  |  |  |  |  |  |  |  |  |  |  |  |  |  |  |  |  |  |  |  |  |  |  |  |  |  |  |  |  |  |  |  |  |  |  |  |  |  |  |  |  |  |  |  |  |  |  |  |  |  |  |  |  |  |  |  |  |  |  |  |  |  |  |  |  |  |  |  |  |  |  |  |  |  |  |  |  |  |  |  |  |  |  |  |  |  |  |  |  |  |  |  |  |  |  |  |  |  |  |  |  |  |  |  |  |  |  |  |  |  |  |  |  |  |  |  |  |  |  |  |  |  |  |  |  |  |  |  |  |  |  |  |  |  |  |  |  |  |  |  |  |  |  |  |  |  |  |  |  |  |  |  |  |  |  |  |  |  |  |  |  |  |  |  |  |  |  |  |  |  |  |  |  |  |  |  |  |  |  |  |  |  |  |  |  |  |  |  |  |  |  |  |  |  |  |  |  |  |  |  |  |  |  |  |  |  |  |  |  |  |  |  |  |  |  |  |  |  |  |  |  |  |  |  |  |  |  |  |  |  |  |  |  |  |  |  |  |  |  |  |  |  |  |  |  |  |  |  |  |  |  |  |  |  |  |  |  |  |  |  |  |  |  |  |  |  |  |  |  |  |  |  |  |  |  |  |  |  |  |  |  |  |  |  |  |  |  |  |  |  |  |  |  |  |  |  |  |  |  |  |  |  |  |  |  |  |  |  |  |  |  |  |  |  |  |  |  |  |  |  |  |  |  |  |  |  |  |  |  |  |  |  |  |  |  |  |  |  |  |  |  |  |  |  |  |  |  |  |  |  |  |  |  |  |  |  |  |  |  |  |  |  |  |  |  |  |  |  |  |  |  |  |  |  |  |  |  |  |  |  |  |  |  |  |  |  |  |  |  |  |  |  |  |  |  |  |  |  |  |  |  |  |  |  |  |  |  |  |  |  |  |  |  |  |  |  |  |  |  |  |  |  |  |  |  |  |  |  |  |  |  |  |  |  |  |  |  |  |  |  |  |  |  |  |  |  |  |  |  |  |  |  |  |  |  |  |  |  |  |  |  |  |  |  |  |  |  |  |  |  |  |  |  |  |  |  |  |  |  |  |  |  |  |  |  |  |  |  |  |  |  |  |  |  |  |  |  |  |  |  |  |  |  |  |  |  |  |  |  |  |  |  |  |  |  |  |  |  |  |  |  |  |  |  |  |  |  |  |  |  |  |  |  |  |  |  |  |  |  |  |  |  |  |  |  |  |  |  |  |  |  |  |  |  |  |  |  |  |  |  |  |  |
|  |  |  |  |  |  |  |  |  |  |  |  |  |  |  |  |  |  |  |  |  |  |  |  |  |  |  |  |  |  |  |  |  |  |  |  |  |  |  |  |  |  |  |  |  |  |  |  |  |  |  |  |  |  |  |  |  |  |  |  |  |  |  |  |  |  |  |  |  |  |  |  |  |  |  |  |  |  |  |  |  |  |  |  |  |  |  |  |  |  |  |  |  |  |  |  |  |  |  |  |  |  |  |  |  |  |  |  |  |  |  |  |  |  |  |  |  |  |  |  |  |  |  |  |  |  |  |  |  |  |  |  |  |  |  |  |  |  |  |  |  |  |  |  |  |  |  |  |  |  |  |  |  |  |  |  |  |  |  |  |  |  |  |  |  |  |  |  |  |  |  |  |  |  |  |  |  |  |  |  |  |  |  |  |  |  |  |  |  |  |  |  |  |  |  |  |  |  |  |  |  |  |  |  |  |  |  |  |  |  |  |  |  |  |  |  |  |  |  |  |  |  |  |  |  |  |  |  |  |  |  |  |  |  |  |  |  |  |  |  |  |  |  |  |  |  |  |  |  |  |  |  |  |  |  |  |  |  |  |  |  |  |  |  |  |  |  |  |  |  |  |  |  |  |  |  |  |  |  |  |  |  |  |  |  |  |  |  |  |  |  |  |  |  |  |  |  |  |  |  |  |  |  |  |  |  |  |  |  |  |  |  |  |  |  |  |  |  |  |  |  |  |  |  |  |  |  |  |  |  |  |  |  |  |  |  |  |  |  |  |  |  |  |  |  |  |  |  |  |  |  |  |  |  |  |  |  |  |  |  |  |  |  |  |  |  |  |  |  |  |  |  |  |  |  |  |  |  |  |  |  |  |  |  |  |  |  |  |  |  |  |  |  |  |  |  |  |  |  |  |  |  |  |  |  |  |  |  |  |  |  |  |  |  |  |  |  |  |  |  |  |  |  |  |  |  |  |  |  |  |  |  |  |  |  |  |  |  |  |  |  |  |  |  |  |  |  |  |  |  |  |  |  |  |  |  |  |  |  |  |  |  |  |  |  |  |  |  |  |  |  |  |  |  |  |  |  |  |  |  |  |  |  |  |  |  |  |  |  |  |  |  |  |  |  |  |  |  |  |  |  |  |  |  |  |  |  |  |  |  |  |  |  |  |  |  |  |  |  |  |  |  |  |  |  |  |  |  |  |  |  |  |  |  |  |  |  |  |  |  |  |  |  |  |  |  |  |  |  |  |  |  |  |  |  |  |  |  |  |  |  |  |  |  |  |  |  |  |  |  |  |  |  |  |  |  |  |  |  |  |  |  |  |  |  |  |  |  |  |  |  |  |  |  |  |  |  |  |  |  |  |  |  |  |  |  |  |  |  |  |  |  |  |  |  |  |  |  |  |  |  |  |  |  |  |  |  |  |  |  |  |  |  |  |  |  |  |  |  |  |  |  |  |  |  |  |  |  |  |  |  |  |  |  |  |  |  |  |  |  |  |  |  |  |  |  |  |  |  |  |  |  |  |  |  |  |  |  |  |  |  |  |  |  |  |  |  |  |  |  |  |  |  |  |  |  |  |  |  |  |  |  |  |  |  |  |  |  |  |  |  |  |  |  |  |  |  |  |  |  |  |  |  |  |  |  |  |  |  |  |  |  |  |  |  |  |  |  |  |  |  |  |  |  |  |  |  |  |  |  |  |  |  |  |  |  |  |  |  |  |  |  |  |  |  |  |  |  |  |  |  |  |  |  |  |  |  |  |  |  |  |  |  |  |  |  |  |  |  |  |  |  |  |  |  |  |  |  |  |  |  |  |  |  |  |  |  |  |  |  |  |  |  |  |  |  |  |  |  |  |  |  |  |  |  |  |  |  |  |  |  |  |  |  |  |  |  |  |  |  |  |  |  |  |  |  |  |  |  |  |  |  |  |  |  |  |  |  |  |  |  |  |  |  |  |  |  |  |  |  |  |  |  |  |  |  |  |  |  |  |  |  |  |  |  |  |  |  |  |  |  |  |  |  |  |  |  |  |  |  |  |  |  |  |  |  |  |  |  |  |  |  |  |  |  |  |  |  |  |  |  |  |  |  |  |  |  |  |  |  |  |  |  |  |  |  |  |  |  |  |  |  |  |  |  |  |  |  |  |  |  |  |  |  |  |  |  |  |  |  |  |  |  |  |  |  |  |  |  |  |  |  |  |  |  |  |  |  |  |  |  |  |  |  |  |  |  |  |  |  |  |  |  |  |  |  |  |  |  |  |  |  |  |  |  |  |  |  |  |  |  |  |  |  |  |  |  |  |  |  |  |  |  |  |  |  |  |  |  |  |  |  |  |  |  |  |  |  |  |  |  |  |  |  |  |  |  |  |  |  |  |  |  |  |  |  |  |  |  |  |  |  |  |  |  |  |  |  |  |  |  |  |  |  |  |  |  |  |  |  |  |  |  |  |  |  |  |  |  |  |  |  |  |  |  |  |  |  |  |  |  |  |  |  |  |  |  |  |  |  |  |  |  |  |  |  |  |  |  |  |  |  |  |  |  |  |  |  |  |  |  |  |  |  |  |  |  |  |  |  |  |  |  |  |  |  |  |  |  |  |  |  |  |  |  |  |  |  |  |  |  |  |  |  |  |  |  |  |  |  |  |  |  |  |  |  |  |  |  |  |  |  |  |  |  |  |  |  |  |  |  |  |  |  |  |  |  |  |  |  |  |  |  |  |  |  |  |  |  |  |  |  |  |  |  |  |  |  |  |  |  |  |  |  |  |  |  |  |  |  |  |  |  |  |  |  |  |  |  |  |  |  |  |  |  |  |  |  |  |  |  |  |  |  |  |  |  |  |  |  |  |
| BIS d=2 cl.1 sym=1 env=1 | - | - | - | - | - | - | - | - | - | - | - | - | - | - | - | - | - | - | - | - | - | - | - | - | - | - | - | - | - | - | - | - | - | - | - | - | - | - | - | - | - | - | - | - | - | - | - | - | - | - | - | - | - | - | - | - | - | - | - | - | - | - | - | - | - | - | - | - | - | - | - | - | - | - | - | - | - | - | - | - | - | - | - | - | - | - | - | - | - | - | - | - | - | - | - | - | - | - | - | - | - | - | - | - | - | - | - | - | - | - | - | - | - | - | - | - | - | - | - | - | - | - | - | - | - | - | - | - | - | - | - | - | - | - | - | - | - | - | - | - | - | - | - | - | - | - | - | - | - | - | - | - | - | - | - | - | - | - | - | - | - | - | - | - | - | - | - | - | - | - | - | - | - | - | - | - | - | - | - | - | - | - | - | - | - | - | - | - | - | - | - | - | - | - | - | - | - | - | - | - | - | - | - | - | - | - | - | - | - | - | - | - | - | - | - | - | - | - | - | - | - | - | - | - | - | - | - | - | - | - | - | - | - | - | - | - | - | - | - | - | - | - | - | - | - | - | - | - | - | - | - | - | - | - | - | - | - | - | - | - | - | - | - | - | - | - | - | - | - | - | - | - | - | - | - | - | - | - | - | - | - | - | - | - | - | - | - | - | - | - | - | - | - | - | - | - | - | - | - | - | - | - | - | - | - | - | - | - | - | - | - | - | - | - | - | - | - | - | - | - | - | - | - | - | - | - | - | - | - | - | - | - | - | - | - | - | - | - | - | - | - | - | - | - | - | - | - | - | - | - | - | - | - | - | - | - | - | - | - | - | - | - | - | - | - | - | - | - | - | - | - | - | - | - | - | - | - | - | - | - | - | - | - | - | - | - | - | - | - | - | - | - | - | - | - | - | - | - | - | - | - | - | - | - | - | - | - | - | - | - | - | - | - | - | - | - | - | - | - | - | - | - | - | - | - | - | - | - | - | - | - | - | - | - | - | - | - | - | - | - | - | - | - | - | - | - | - | - | - | - | - | - | - | - | - | - | - | - | - | - | - | - | - | - | - | - | - | - | - | - | - | - | - | - | - | - | - | - | - | - | - | - | - | - | - | - | - | - | - | - | - | - | - | - | - | - | - | - | - | - | - | - | - | - | - | - | - | - | - | - | - | - | - | - | - | - | - | - | - | - | - | - | - | - | - | - | - | - | - | - | - | - | - | - | - | - | - | - | - | - | - | - | - | - | - | - | - | - | - | - | - | - | - | - | - | - | - | - | - | - | - | - | - | - | - | - | - | - | - | - | - | - | - | - | - | - | - | - | - | - | - | - | - | - | - | - | - | - | - | - | - | - | - | - | - | - | - | - | - | - | - | - | - | - | - | - | - | - | - | - | - | - | - | - | - | - | - | - | - | - | - | - | - | - | - | - | - | - | - | - | - | - | - | - | - | H | x | x | x | x | x | x | x | x | x | x | x | x | x | x | x | x | x | x | x | x | x | x | x | x | x | x | x | x | x | x | x | x | x | x | x | x | x | x | x | x | x | x | x | x | x | x | x | x | x | x | x | x | x | x | x | x | x | x | x | x | x | x | x | x | x | x | x | x | x | x | x | x | x | x | x | x | x | x | x | x | x | x | x | x | x | x | x | x | x | x | x | x | x | x | x | x | x | x | x | x | x | x | x | x | x | x | x | x | x | x | x | x | x | x | x | x | x | x | x | x | x | x | x | x | x | x | x | x | x | x | x | x | x | x | x | x | x | x | x | x | x | x | x | x | x | x | x | x | x | x | x | x | x | x | x | x | x | x | x | x | x | x | x | x | x | x | x | x | x | x | x | x | x | x | x | x | x | x | x | x | x | x | x | x | x | x | x | x | x | x | x | x | x | x | x | x | x | x | x | x | x | x | x | x | x | x | x | x | x | x | x | x | x | x | x | x | x | x | x | x | x | x | x | x | x | x | x | x | x | x | x | x | x | x | x | x | x | x | x | x | x | x | x | x | x | x | x | x | x | x | x | x | x | x | x | x | x | x | x | x | x | x | x | x | x | x | x | x | x | x | x | x | x | x | x | x | x | x | x | x | x | x | x | x | x | x | x | x | x | x | x | x | x | x | x | x | x | x | x | x | x | x | x | x | x | x | x | x | x | x | x | x | x | x | x | x | x | x | x | x | x | x | x | x | x | x | x | x | x | x | x | x | x | x | x | x | x | x | x | H | - | - | - | - | - | - | - | - | - | - | - | - | - | - | - | - | - | - | - | - | - | - | - | - | - | - | - | - | - | - | - | - | - | - | - | - | - | - | - | - | - | - | - | - | - | - | - | - | - | - | - | - | - | - | - | - | - | - | - | - | - | - | - | - | - | - | - | - | - | - | - | - | - | - | - | - | - | - | - | - | - | - | - | - | - | - | - | - | - | - | - | - | - | - | - | - | - | - | - | - | - | - | - | - | - | - | - | - | - | - | - | - | - | - | - | - | - | - | - | - | - | - | - | - | - | - | - | - | - | - | - | - | - | - | - | - | - | - | - | - | - | - | - | - | - | - | - | - | - | - | - | - | - | - | - | - | - | - | - | - | - | - | - | - | - | - | - | - | - | - | - | - | - | - | - | - | - | - | - | - | - | - | - | - | - | - | - | - | - | - | - | - | - | - | - | - | - | - | - | - | - | - | - | - | - | - | - | - | - | - | - | - | - | - | - | - | - | - | - | - | - | - | - | - | - | - | - | - | - | - | - | - | - | - | - | - | - | - | - | - | - | - | - | - | - | - | - | - | - | - | - | - | - | - | - | - | - | - | - | - | - | - | - | - | - | - | - | - | - | - | - | - | - | - | - | - | - | - | - | - | - | - | - | - |
| BIS d=2 cl.7 sym=1 env=1 | - | - | - | - | - | - | - | - | - | - | - | - | - | - | - | - | - | - | - | - | - | - | - | - | - | - | - | - | - | - | - | - | - | - | - | - | - | - | - | - | - | - | - | - | - | - | - | - | - | - | - | - | - | - | - | - | - | - | - | - | - | - | - | - | - | - | - | - | - | - | - | - | - | - | - | - | - | - | - | - | - | - | - | - | - | - | - | - | - | - | - | - | - | - | - | - | - | - | - | - | - | - | - | - | - | - | - | - | - | - | - | - | - | - | - | - | - | - | - | - | - | - | - | - | - | - | - | - | - | - | - | - | - | - | - | - | - | - | - | - | - | - | - | - | - | - | - | - | - | - | - | - | - | - | - | - | - | - | - | - | - | - | - | - | - | - | - | - | - | - | - | - | - | - | - | - | - | - | - | - | - | - | - | - | - | - | - | - | - | - | - | - | - | - | - | - | - | - | - | - | - | - | - | - | - | - | - | - | - | - | - | - | - | - | - | - | - | - | - | - | - | - | - | - | - | - | - | - | - | - | - | - | - | - | - | - | - | - | - | - | - | - | - | - | - | - | - | - | - | - | - | - | - | - | - | - | - | - | - | - | - | - | - | - | - | - | - | - | - | - | - | - | - | - | - | - | - | - | - | - | - | - | - | - | - | - | - | - | - | - | - | - | - | - | - | - | - | - | - | - | - | - | - | - | - | - | - | - | - | - | - | - | - | - | - | - | - | - | - | - | - | - | - | - | - | - | - | - | - | - | - | - | - | - | - | - | - | - | - | - | - | - | - | - | - | - | - | - | - | - | - | - | - | - | - | - | - | - | - | - | - | - | - | - | - | - | - | - | - | - | - | - | - | - | - | - | - | - | - | - | - | - | - | - | - | - | - | - | - | - | - | - | - | - | - | - | - | - | - | - | - | - | - | - | - | - | - | - | - | - | - | - | - | - | - | - | - | - | - | - | - | - | - | - | - | - | - | - | - | - | - | - | - | - | - | - | - | - | - | - | - | - | - | - | - | - | - | - | - | - | - | - | - | - | - | - | - | - | - | - | - | - | - | - | - | - | - | - | - | - | - | - | - | - | - | - | - | - | - | - | - | - | - | - | - | - | - | - | - | - | - | - | - | - | - | - | - | - | - | - | - | - | - | - | - | - | - | - | - | - | - | - | - | - | - | - | - | - | - | - | - | - | - | - | - | - | - | - | - | - | - | - | - | - | - | - | - | - | - | - | - | - | - | - | - | - | - | - | - | - | - | - | - | - | - | - | - | - | - | - | - | - | - | - | - | - | - | - | - | - | - | - | - | - | - | - | - | - | - | - | - | - | - | - | - | - | - | - | - | - | - | - | - | - | - | - | - | - | - | - | - | - | - | - | - | - | - | - | - | - | - | - | - | - | - | - | - | - | - | - | - | - | - | - | - | - | - | - | - | - | - | - | - | - | - | - | - | - | - | - | - | - | - | - | - | - | - | - | - | - | - | - | - | - | - | - | - | - | - | - | - | - | - | - | - | - | - | - | - | - | - | - | - | - | - | - | - | - | - | - | - | - | - | - | - | - | - | - | - | - | - | - | - | - | - | - | - | - | - | - | - | - | - | - | - | - | - | - | - | - | - | - | - | - | - | - | - | - | - | - | - | - | - | - | - | - | - | - | - | - | - | - | - | - | - | - | - | - | - | - | - | - | - | - | - | - | - | - | - | - | - | - | - | - | - | - | - | - | - | - | - | - | - | - | - | - | - | - | - | - | - | - | - | - | - | - | - | - | - | - | - | - | - | - | - | - | - | - | - | - | - | - | - | - | - | - | - | - | - | - | - | - | - | - | - | - | - | - | - | - | - | - | - | - | - | - | - | - | - | - | - | - | - | - | - | - | - | - | - | - | - | - | - | - | - | - | - | - | - | - | - | - | - | - | - | - | - | - | - | - | - | - | - | - | - | - | - | - | - | - | - | - | - | - | - | - | - | - | - | - | - | - | - | - | - | - | - | - | - | - | - | - | - | - | - | - | - | - | - | - | - | - | - | - | - | - | - | - | - | - | - | - | - | - | - | - | - | - | - | - | - | - | - | - | - | - | - | - | - | - | - | - | - | - | - | - | - | - | - | - | - | - | - | - | - | - | - | - | - | - | - | - | - | - | - | - | - | - | - | - | - | - | - | - | - | - | - | - | - | - | - | - | - | - | - | - | - | - | - | - | - | - | - | - | - | - | - | - | - | - | - | - | - | - | - | - | - | - | - | - | - | - | - | - | - | - | - | - | - | - | - | - | - | - | - | - | - | - | - | - | - | - | - | - | - | - | - | - | - | - | - | - | - | - | - | - | - | - | - | - | - | - | - | - | - | - | - | - | - | - | - | - | - | - | - | - | - | - | - | - | - | - | - | - | - | - | - | - | - | - | - | - | - | - | - | - | - | - | - | - | - | - | - | - | - | - | - | - | - | - | - | - | - | - | - | H | x | x | H | - | - | - | - | - | - | - | - | - | - | - | - | - | - | - | - | - | - | - | - | - | - | - | - | - | - | - | - | - | - | - | - | - | - | - | - | - | - | - | - | - | - | - | - | - | - | - | - | - | - | - | - | - | - | - | - | - | - | - | - | - | - | - | - | - | - | - | - | - | - | - | - | - | - | - | - | - | - | - | - | - | - | - | - | - | - | - | - | - | - | - | - | - | - | - | - | - | - | - | - | - | - | - | - | - | - | - | - | - | - | - | - | - | - | - | - | - | - | - | - | - | - | - | - | - | - | - | - | - | - | - | - | - | - | - | - | - | - | - | - | - | - | - | - | - | - | - | - | - | - | - | - | - | - | - | - | - | - | - | - | - | - | - | - | - | - | - | - | - | - | - |
| BIS d=2 cl.2 sym=1 env=1 | - | - | - | - | - | - | - | - | - | - | - | - | - | - | - | - | - | - | - | - | - | - | - | - | - | - | - | - | - | - | - | - | - | - | - | - | - | - | - | - | - | - | - | - | - | - | - | - | - | - | - | - | - | - | - | - | - | - | - | - | - | - | - | - | - | - | - | - | - | - | - | - | - | - | - | - | - | - | - | - | - | - | - | - | - | - | - | - | - | - | - | - | - | - | - | - | - | - | - | - | - | - | - | - | - | - | - | - | - | - | - | - | - | - | - | - | - | - | - | - | - | - | - | - | - | - | - | - | - | - | - | - | - | - | - | - | - | - | - | - | - | - | - | - | - | - | - | - | - | - | - | - | - | - | - | - | - | - | - | - | - | - | - | - | - | - | - | - | - | - | - | - | - | - | - | - | - | - | - | - | - | - | - | - | - | - | - | - | - | - | - | - | - | - | - | - | - | - | - | - | - | - | - | - | - | - | - | - | - | - | - | - | - | - | - | - | - | - | - | - | - | - | - | - | - | - | - | - | - | - | - | - | - | - | - | - | - | - | - | - | - | - | - | - | - | - | - | - | - | - | - | - | - | - | - | - | - | - | - | - | - | - | - | - | - | - | - | - | - | - | - | - | - | - | - | - | - | - | - | - | - | - | - | - | - | - | - | - | - | - | - | - | - | - | - | - | - | - | - | - | - | - | - | - | - | - | - | - | - | - | - | - | - | - | - | - | - | - | - | - | - | - | - | - | - | - | - | - | - | - | - | - | - | - | - | - | - | - | - | - | - | - | - | - | - | - | - | - | - | - | - | - | - | - | - | - | - | - | - | - | - | - | - | - | - | - | - | - | - | - | - | - | - | - | - | - | - | - | - | - | - | - | - | - | - | - | - | - | - | - | - | - | - | - | - | - | - | - | - | - | - | - | - | - | - | - | - | - | - | - | - | - | - | - | - | - | - | - | - | - | - | - | - | - | - | - | - | - | - | - | - | - | - | - | - | - | - | - | - | - | - | - | - | - | - | - | - | - | - | - | - | - | - | - | - | - | - | - | - | - | - | - | - | - | - | - | - | - | - | - | - | - | - | - | - | - | - | - | - | - | - | - | - | - | - | - | - | - | - | - | - | - | - | - | - | - | - | - | - | - | - | - | - | - | - | - | - | - | - | - | - | - | - | - | - | - | - | - | - | - | - | - | - | - | - | - | - | - | - | - | - | - | - | - | - | - | - | - | - | - | - | - | - | - | - | - | - | - | - | - | - | - | - | - | - | - | - | - | - | - | - | - | - | - | - | - | - | - | - | - | - | - | - | - | - | - | - | - | - | - | - | - | - | - | - | - | - | - | - | - | - | - | - | - | - | - | - | - | - | - | - | - | - | - | - | - | - | - | - | - | - | - | - | - | - | - | - | - | - | - | - | - | - | - | - | - | - | - | - | - | - | - | - | - | - | - | - | - | - | - | - | - | - | - | - | - | - | - | - | - | - | - | - | - | - | - | - | - | - | - | - | - | - | - | - | - | - | - | - | - | - | - | - | - | - | - | - | - | - | - | - | - | - | - | - | - | - | - | - | - | - | - | - | - | - | - | - | - | - | - | - | - | - | - | - | H | x | x | x | x | x | x | x | x | x | x | x | x | x | x | x | x | x | x | x | x | x | x | x | x | x | x | x | x | x | x | x | x | x | x | x | x | x | x | x | x | x | x | x | x | x | x | x | x | x | x | x | x | x | x | x | x | x | x | x | x | x | x | x | x | x | x | x | x | x | x | x | x | x | x | x | x | x | x | x | x | x | x | x | x | x | x | x | x | x | x | x | x | x | x | x | x | x | x | x | x | x | x | x | x | x | x | x | x | x | x | x | x | x | x | x | x | x | x | x | x | x | x | x | x | x | x | x | x | x | x | x | x | x | x | x | x | x | x | x | x | x | x | x | x | x | x | x | x | x | x | x | x | x | x | x | x | x | x | x | x | x | x | x | x | x | x | x | x | x | x | x | x | x | x | x | H | - | - | - | - | - | - | - | - | - | - | - | - | - | - | - | - | - | - | - | - | - | - | - | - | - | - | - | - | - | - | - | - | - | - | - | - | - | - | - | - | - | - | - | - | - | - | - | - | - | - | - | - | - | - | - | - | - | - | - | - | - | - | - | - | - | - | - | - | - | - | - | - | - | - | - | - | - | - | - | - | - | - | - | - | - | - | - | - | - | - | - | - | - | - | - | - | - | - | - | - | - | - | - | - | - | - | - | - | - | - | - | - | - | - | - | - | - | - | - | - | - | - | - | - | - | - | - | - | - | - | - | - | - | - | - | - | - | - | - | - | - | - | - | - | - | - | - | - | - | - | - | - | - | - | - | - | - | - | - | - | - | - | - | - | - | - | - | - | - | - | - | - | - | - | - | - | - | - | - | - | - | - | - | - | - | - | - | - | - | - | - | - | - | - | - | - | - | - | - | - | - | - | - | - | - | - | - | - | - | - | - | - | - | - | - | - | - | - | - | - | - | - | - | - | - | - | - | - | - | - | - | - | - | - | - | - | - | - | - | - | - | - | - | - | - | - | - | - | - | - | - | - | - | - | - | - | - | - | - | - | - | - | - | - | - | - | - | - | - | - | - | - | - | - | - | - | - | - | - | - | - | - | - | - | - | - | - | - | - | - | - | - | - | - | - | - | - | - | - | - | - | - | - | - | - | - | - | - | - | - | - | - | - | - | - | - | - | - | - | - | - | - | - | - | - | - | - | - | - | - | - | - | - | - | - | - | - | - | - | - | - | - | - | - | - | - | - | - | - | - | - | - | - | - | - | - | - | - | - | - | - | - | - | - | - | - | - | - | - | - | - | - | - | - | - | - | - | - |
| BIS d=2 cl.3 sym=1 env=1 | - | - | - | - | - | - | - | - | - | - | - | - | - | - | - | - | - | - | - | - | - | - | - | - | - | - | - | - | - | - | - | - | - | - | - | - | - | - | - | - | - | - | - | - | - | - | - | - | - | - | - | - | - | - | - | - | - | - | - | - | - | - | - | - | - | - | - | - | - | - | - | - | - | - | - | - | - | - | - | - | - | - | - | - | - | - | - | - | - | - | - | - | - | - | - | - | - | - | - | - | - | - | - | - | - | - | - | - | - | - | - | - | - | - | - | - | - | - | - | - | - | - | - | - | - | - | - | - | - | - | - | - | - | - | - | - | - | - | - | - | - | - | - | - | - | - | - | - | - | - | - | - | - | - | - | - | - | - | - | - | - | - | - | - | - | - | - | - | - | - | - | - | - | - | - | - | - | - | - | - | - | - | - | - | - | - | - | - | - | - | - | - | - | - | - | - | - | - | - | - | - | - | - | - | - | - | - | - | - | - | - | - | - | - | - | - | - | - | - | - | - | - | - | - | - | - | - | - | - | - | - | - | - | - | - | - | - | - | - | - | - | - | - | - | - | - | - | - | - | - | - | - | - | - | - | - | - | - | - | - | - | - | - | - | - | - | - | - | - | - | - | - | - | - | - | - | - | - | - | - | - | - | - | - | - | - | - | - | - | H | x | x | x | x | x | x | x | x | x | x | x | x | x | x | x | x | x | x | x | x | x | x | x | x | x | x | x | x | x | x | x | x | x | x | x | x | x | x | x | x | x | x | x | x | x | x | x | x | x | x | x | x | x | x | x | x | x | x | x | x | x | x | x | x | x | x | x | x | x | x | x | x | x | x | x | x | x | x | x | x | x | x | x | x | x | x | x | x | x | x | x | x | x | x | x | x | x | x | x | x | x | x | x | x | x | x | x | x | x | x | x | x | x | x | x | x | x | x | x | x | x | x | x | x | x | x | x | x | x | x | x | x | x | x | x | x | x | x | x | x | x | x | x | x | x | x | x | x | x | x | x | x | x | x | x | x | x | x | x | x | x | x | x | x | x | x | x | x | x | x | x | x | x | x | x | x | x | x | x | x | x | x | x | x | x | x | x | x | x | x | x | x | x | x | x | x | x | x | x | x | x | x | x | x | x | x | x | x | x | x | x | x | x | x | x | x | x | x | x | x | x | x | x | x | x | x | x | x | x | x | x | x | x | x | x | x | x | x | x | x | x | x | x | x | x | x | x | x | x | x | x | x | x | x | x | x | x | x | x | x | x | x | x | x | x | x | x | x | x | x | x | x | x | x | x | x | x | x | x | x | x | x | x | x | x | x | x | x | x | x | x | x | x | x | x | x | x | x | x | x | x | x | x | x | x | x | x | x | x | x | x | x | x | x | x | x | x | x | x | x | x | x | x | x | x | x | x | x | x | x | x | x | x | x | x | x | x | x | x | x | x | x | x | x | x | x | x | x | x | x | x | x | x | x | x | x | x | x | x | x | x | x | x | x | x | x | x | x | x | x | x | x | x | x | x | x | x | x | x | x | x | x | x | x | x | x | x | x | x | x | x | x | x | x | x | x | x | x | x | x | x | x | x | x | x | x | x | x | x | H | - | - | - | - | - | - | - | - | - | - | - | - | - | - | - | - | - | - | - | - | - | - | - | - | - | - | - | - | - | - | - | - | - | - | - | - | - | - | - | - | - | - | - | - | - | - | - | - | - | - | - | - | - | - | - | - | - | - | - | - | - | - | - | - | - | - | - | - | - | - | - | - | - | - | - | - | - | - | - | - | - | - | - | - | - | - | - | - | - | - | - | - | - | - | - | - | - | - | - | - | - | - | - | - | - | - | - | - | - | - | - | - | - | - | - | - | - | - | - | - | - | - | - | - | - | - | - | - | - | - | - | - | - | - | - | - | - | - | - | - | - | - | - | - | - | - | - | - | - | - | - | - | - | - | - | - | - | - | - | - | - | - | - | - | - | - | - | - | - | - | - | - | - | - | - | - | - | - | - | - | - | - | - | - | - | - | - | - | - | - | - | - | - | - | - | - | - | - | - | - | - | - | - | - | - | - | - | - | - | - | - | - | - | - | - | - | - | - | - | - | - | - | - | - | - | - | - | - | - | - | - | - | - | - | - | - | - | - | - | - | - | - | - | - | - | - | - | - | - | - | - | - | - | - | - | - | - | - | - | - | - | - | - | - | - | - | - | - | - | - | - | - | - | - | - | - | - | - | - | - | - | - | - | - | - | - | - | - | - | - | - | - | - | - | - | - | - | - | - | - | - | - | - | - | - | - | - | - | - | - | - | - | - | - | - | - | - | - | - | - | - | - | - | - | - | - | - | - | - | - | - | - | - | - | - | - | - | - | - | - | - | - | - | - | - | - | - | - | - | - | - | - | - | - | - | - | - | - | - | - | - | - | - | - | - | - | - | - | - | - | - | - | - | - | - | - | - | - | - | - | - | - | - | - | - | - | - | - | - | - | - | - | - | - | - | - | - | - | - | - | - | - | - | - | - | - | - | - | - | - | - | - | - | - | - | - | - | - | - | - | - | - | - | - | - | - | - | - | - | - | - | - | - | - | - | - | - | - | - | - | - | - | - | - | - | - | - | - | - | - | - | - | - | - | - | - | - | - | - | - | - | - | - | - | - | - | - | - | - | - | - | - | - | - | - | - | - | - | - | - | - | - | - | - | - | - | - | - | - | - | - | - | - | - | - | - | - | - | - | - | - | - | - | - | - | - | - | - | - | - | - | - | - | - | - | - | - | - | - | - | - | - | - | - | - | - | - | - | - | - | - | - | - | - | - | - | - | - | - | - | - | - | - | - | - | - | - | - | - | - | - | - | - | - | - | - | - | - | - | - |
| BIS d=2 cl.4 sym=1 env=1 | - | - | - | - | - | - | - | - | - | - | - | - | - | - | - | - | - | - | - | - | - | - | - | - | - | - | - | - | - | - | - | - | - | - | - | - | - | - | - | - | - | - | - | - | - | - | - | - | - | - | - | - | - | - | - | - | - | - | - | - | - | - | - | - | - | - | - | - | - | - | - | - | - | - | - | - | - | - | - | - | - | - | - | - | - | - | - | - | - | - | - | - | - | - | - | - | - | - | - | - | - | - | - | - | - | - | - | - | - | - | - | - | - | - | - | - | - | - | - | - | - | - | - | - | - | - | - | - | - | - | - | - | - | - | - | - | - | - | - | - | - | - | - | - | - | - | - | - | - | - | - | - | - | - | - | - | - | - | - | - | - | - | - | - | - | - | - | - | - | - | - | - | - | - | - | - | - | - | - | - | - | - | - | - | - | - | - | - | - | - | - | - | - | - | - | - | - | - | - | - | - | - | - | - | - | - | - | - | - | - | - | - | - | - | - | - | - | - | - | - | - | - | - | - | - | - | - | - | - | - | - | - | - | - | - | - | - | - | - | - | - | - | - | - | - | - | - | - | - | - | - | - | - | - | - | - | - | - | - | - | - | - | - | - | - | - | - | - | - | - | - | - | - | - | - | - | - | - | - | - | - | - | - | - | - | - | - | - | - | - | - | - | - | - | - | - | - | - | - | - | - | - | - | - | - | - | - | - | - | - | - | - | - | - | - | - | - | - | - | - | - | - | - | - | - | - | - | - | - | - | - | - | - | - | - | - | - | - | - | - | - | - | - | - | - | - | - | - | - | - | - | - | - | - | - | - | - | - | - | - | - | - | - | - | - | - | - | - | - | - | - | - | - | - | - | - | - | - | - | - | - | - | - | - | - | - | - | - | - | - | - | - | - | - | - | - | - | - | - | - | - | - | - | - | - | - | - | - | - | - | - | - | - | - | - | - | - | - | - | - | - | - | - | - | - | - | - | - | - | - | - | - | - | - | - | - | - | - | - | - | - | - | - | - | - | - | - | - | - | - | - | - | - | - | - | - | - | - | - | - | - | - | - | - | - | - | - | - | - | - | - | - | - | - | - | - | - | - | - | - | - | - | - | - | - | - | - | - | - | - | - | - | - | - | - | - | - | - | - | - | - | - | - | - | - | - | - | - | - | - | - | - | - | - | - | - | - | - | - | - | - | - | - | - | - | - | - | - | - | - | - | - | - | - | - | - | - | - | - | - | - | - | - | - | - | - | - | - | - | - | - | - | - | - | - | - | - | - | - | - | - | - | - | - | - | - | - | - | - | - | - | - | - | - | - | - | - | - | - | - | - | - | - | - | - | - | - | - | - | - | - | - | - | - | - | - | - | - | - | - | - | - | - | - | - | - | - | - | - | - | - | - | - | - | - | - | - | - | - | - | - | - | - | - | - | - | - | - | - | - | - | - | - | - | - | - | - | - | - | - | - | - | - | - | - | - | - | - | - | - | - | - | - | - | - | H | x | x | x | x | x | x | x | x | x | x | x | x | x | x | x | x | x | x | x | x | x | x | x | x | x | x | x | x | x | x | x | x | x | x | x | x | x | x | x | x | x | x | x | x | x | x | x | x | x | x | x | x | x | x | x | x | x | x | x | x | x | x | x | x | x | x | x | x | x | x | x | x | x | x | x | x | x | x | x | x | x | x | x | x | x | x | x | x | x | x | x | x | x | x | x | x | x | x | x | x | x | x | x | x | x | x | x | x | x | x | x | x | x | x | x | x | x | x | x | x | x | x | x | x | x | x | x | x | x | x | x | x | x | x | x | x | x | x | x | x | x | x | x | x | x | x | x | x | x | x | x | x | x | x | x | x | x | x | x | x | x | x | x | x | x | x | x | x | x | x | x | x | x | x | x | x | x | x | x | x | x | x | x | x | x | x | x | x | x | x | x | x | x | x | x | x | x | x | x | x | x | x | x | x | x | x | x | x | x | x | x | x | x | x | x | x | x | x | x | x | x | x | x | x | x | x | x | x | x | x | x | x | x | x | x | x | x | x | x | x | x | x | x | x | x | x | x | x | x | x | x | x | x | x | x | x | x | x | x | x | x | x | x | x | x | x | x | x | x | x | x | x | x | x | x | x | x | x | x | x | x | x | x | x | x | x | x | x | x | x | x | x | x | x | x | x | x | x | x | x | x | x | x | x | x | x | x | x | x | x | x | x | x | x | x | x | x | x | x | x | x | x | x | x | x | x | x | x | x | x | x | x | x | x | x | x | x | x | x | x | x | x | x | x | x | x | x | x | x | x | x | x | x | x | x | x | x | x | x | x | x | x | x | x | x | x | x | x | x | x | x | x | x | x | x | x | x | x | x | x | x | x | x | x | x | x | x | x | x | x | x | x | x | x | x | x | x | x | x | x | x | x | x | x | x | x | x | x | x | x | x | x | x | x | x | x | x | x | H | - | - | - | - | - | - | - | - | - | - | - | - | - | - | - | - | - | - | - | - | - | - | - | - | - | - | - | - | - | - | - | - | - | - | - | - | - | - | - | - | - | - | - | - | - | - | - | - | - | - | - | - | - | - | - | - | - | - | - | - | - | - | - | - | - | - | - | - | - | - | - | - | - | - | - | - | - | - | - | - | - | - | - | - | - | - | - | - | - | - | - | - | - | - | - | - | - | - | - | - | - | - | - | - | - | - | - | - | - | - | - | - | - | - | - | - | - | - | - | - | - | - | - | - | - | - | - | - | - | - | - | - | - | - | - | - | - | - | - | - | - | - | - | - | - | - | - | - | - | - | - | - | - | - | - | - | - | - | - | - | - | - | - | - | - | - | - | - | - | - | - | - | - | - | - | - | - | - | - | - | - | - | - | - | - |
| BIS d=2 cl.5 sym=1 env=1 | - | - | - | - | - | - | - | - | - | - | - | - | - | - | - | - | - | - | - | - | - | - | - | - | - | - | - | - | - | - | - | - | - | - | - | - | - | - | - | - | - | - | - | - | - | - | - | - | - | - | - | - | - | - | - | - | - | - | - | - | - | - | - | - | - | - | - | - | - | - | - | - | - | - | - | - | - | - | - | - | - | - | - | - | - | - | - | - | - | - | - | - | - | - | - | - | - | - | - | - | - | - | - | - | - | - | - | - | - | - | - | - | - | - | - | - | - | - | - | - | - | - | - | - | - | - | - | - | - | - | - | - | - | - | - | - | - | - | - | - | - | - | - | - | - | - | - | - | - | H | x | x | x | x | x | x | x | x | x | x | x | x | H | - | - | - | - | - | - | - | - | - | - | - | - | - | - | - | - | - | - | - | - | - | - | - | - | - | - | - | - | - | - | - | - | - | - | - | - | - | - | - | - | - | - | - | - | - | - | - | - | - | - | - | - | - | - | - | - | - | - | - | - | - | - | - | - | - | - | - | - | - | - | - | - | - | - | - | - | - | - | - | - | - | - | - | - | - | - | - | - | - | - | - | - | - | - | - | - | - | - | - | - | - | - | - | - | - | - | - | - | - | - | - | - | - | - | - | - | - | - | - | - | - | - | - | - | - | - | - | - | - | - | - | - | - | - | - | - | - | - | - | - | - | - | - | - | - | - | - | - | - | - | - | - | - | - | - | - | - | - | - | - | - | - | - | - | - | - | - | - | - | - | - | - | - | - | - | - | - | - | - | - | - | - | - | - | - | - | - | - | - | - | - | - | - | - | - | - | - | - | - | - | - | - | - | - | - | - | - | - | - | - | - | - | - | - | - | - | - | - | - | - | - | - | - | - | - | - | - | - | - | - | - | - | - | - | - | - | - | - | - | - | - | - | - | - | - | - | - | - | - | - | - | - | - | - | - | - | - | - | - | - | - | - | - | - | - | - | - | - | - | - | - | - | - | - | - | - | - | - | - | - | - | - | - | - | - | - | - | - | - | - | - | - | - | - | - | - | - | - | - | - | - | - | - | - | - | - | - | - | - | - | - | - | - | - | - | - | - | - | - | - | - | - | - | - | - | - | - | - | - | - | - | - | - | - | - | - | - | - | - | - | - | - | - | - | - | - | - | - | - | - | - | - | - | - | - | - | - | - | - | - | - | - | - | - | - | - | - | - | - | - | - | - | - | - | - | - | - | - | - | - | - | - | - | - | - | - | - | - | - | - | - | - | - | - | - | - | - | - | - | - | - | - | - | - | - | - | - | - | - | - | - | - | - | - | - | - | - | - | - | - | - | - | - | - | - | - | - | - | - | - | - | - | - | - | - | - | - | - | - | - | - | - | - | - | - | - | - | - | - | - | - | - | - | - | - | - | - | - | - | - | - | - | - | - | - | - | - | - | - | - | - | - | - | - | - | - | - | - | - | - | - | - | - | - | - | - | - | - | - | - | - | - | - | - | - | - | - | - | - | - | - | - | - | - | - | - | - | - | - | - | - | - | - | - | - | - | - | - | - | - | - | - | - | - | - | - | - | - | - | - | - | - | - | - | - | - | - | - | - | - | - | - | - | - | - | - | - | - | - | - | - | - | - | - | - | - | - | - | - | - | - | - | - | - | - | - | - | - | - | - | - | - | - | - | - | - | - | - | - | - | - | - | - | - | - | - | - | - | - | - | - | - | - | - | - | - | - | - | - | - | - | - | - | - | - | - | - | - | - | - | - | - | - | - | - | - | - | - | - | - | - | - | - | - | - | - | - | - | - | - | - | - | - | - | - | - | - | - | - | - | - | - | - | - | - | - | - | - | - | - | - | - | - | - | - | - | - | - | - | - | - | - | - | - | - | - | - | - | - | - | - | - | - | - | - | - | - | - | - | - | - | - | - | - | - | - | - | - | - | - | - | - | - | - | - | - | - | - | - | - | - | - | - | - | - | - | - | - | - | - | - | - | - | - | - | - | - | - | - | - | - | - | - | - | - | - | - | - | - | - | - | - | - | - | - | - | - | - | - | - | - | - | - | - | - | - | - | - | - | - | - | - | - | - | - | - | - | - | - | - | - | - | - | - | - | - | - | - | - | - | - | - | - | - | - | - | - | - | - | - | - | - | - | - | - | - | - | - | - | - | - | - | - | - | - | - | - | - | - | - | - | - | - | - | - | - | - | - | - | - | - | - | - | - | - | - | - | - | - | - | - | - | - | - | - | - | - | - | - | - | - | - | - | - | - | - | - | - | - | - | - | - | - | - | - | - | - | - | - | - | - | - | - | - | - | - | - | - | - | - | - | - | - | - | - | - | - | - | - | - | - | - | - | - | - | - | - | - | - | - | - | - | - | - | - | - | - | - | - | - | - | - | - | - | - | - | - | - | - | - | - | - | - | - | - | - | - | - | - | - | - | - | - | - | - | - | - | - | - | - | - | - | - | - | - | - | - | - | - | - | - | - | - | - | - | - | - | - | - | - | - | - | - | - | - | - | - | - | - | - | - | - | - | - | - | - | - | - | - | - | - | - | - | - | - | - | - | - | - | - | - | - | - | - | - | - | - | - | - | - | - | - | - | - | - | - | - | - | - | - | - | - | - | - | - | - | - | - | - | - | - | - | - | - | - | - | - | - | - | - | - | - | - | - | - | - | - | - | - | - | - | - | - | - | - | - | - | - | - | - | - | - | - | - | - | - | - | - | - | - | - | - | - | - | - | - | - | - | - | - | - | - | - | - | - | - | - | - | - | - | - | - | - | - | - | - | - | - | - | - | - | - | - | - | - | - | - | - | - | - | - | - | - | - | - | - | - | - | - | - | - | - | - | - | - | - | - |
| BIS d=2 cl.6 sym=1 env=1 | - | - | - | - | - | - | - | - | - | - | - | - | - | - | - | - | - | - | - | - | - | - | - | - | - | - | - | - | - | - | - | - | - | - | - | - | - | - | - | - | - | - | - | - | - | - | - | - | - | - | - | - | - | - | - | - | - | - | - | - | - | - | - | - | - | - | - | - | - | - | - | - | - | - | - | - | - | - | - | - | - | - | - | - | - | - | - | - | - | - | - | - | - | - | - | - | - | - | - | - | - | - | - | - | - | - | - | - | - | - | - | - | - | H | x | x | x | x | x | x | x | x | x | x | x | x | x | x | x | x | x | x | x | x | x | x | x | x | x | x | x | x | x | x | x | x | x | x | x | x | x | x | x | x | x | x | x | x | x | x | x | x | x | x | x | x | x | x | x | x | x | x | x | x | x | x | x | x | x | x | x | x | x | x | x | x | x | x | x | x | x | x | x | x | x | x | x | x | x | x | x | x | x | x | x | x | x | x | x | x | x | x | x | x | x | x | x | x | x | x | x | x | x | x | x | x | x | x | x | x | x | x | x | x | x | x | x | x | x | x | x | x | x | x | x | x | x | x | x | x | x | x | x | x | x | x | x | x | x | x | x | x | x | x | x | x | x | x | x | x | x | x | x | x | x | x | x | x | x | x | x | x | x | x | x | x | x | x | x | x | x | x | x | x | x | x | x | x | x | x | x | x | x | x | x | x | x | x | x | x | x | x | x | x | x | x | x | x | x | x | x | x | x | x | x | x | x | x | x | x | x | x | x | x | x | x | x | x | x | x | x | x | x | x | x | x | x | x | x | x | x | x | x | x | x | x | x | x | x | x | x | x | x | x | x | x | x | x | x | x | x | x | x | x | x | x | x | x | x | x | x | x | x | x | x | x | x | x | x | x | x | x | x | x | H | - | - | - | - | - | - | - | - | - | - | - | - | - | - | - | - | - | - | - | - | - | - | - | - | - | - | - | - | - | - | - | - | - | - | - | - | - | - | - | - | - | - | - | - | - | - | - | - | - | - | - | - | - | - | - | - | - | - | - | - | - | - | - | - | - | - | - | - | - | - | - | - | - | - | - | - | - | - | - | - | - | - | - | - | - | - | - | - | - | - | - | - | - | - | - | - | - | - | - | - | - | - | - | - | - | - | - | - | - | - | - | - | - | - | - | - | - | - | - | - | - | - | - | - | - | - | - | - | - | - | - | - | - | - | - | - | - | - | - | - | - | - | - | - | - | - | - | - | - | - | - | - | - | - | - | - | - | - | - | - | - | - | - | - | - | - | - | - | - | - | - | - | - | - | - | - | - | - | - | - | - | - | - | - | - | - | - | - | - | - | - | - | - | - | - | - | - | - | - | - | - | - | - | - | - | - | - | - | - | - | - | - | - | - | - | - | - | - | - | - | - | - | - | - | - | - | - | - | - | - | - | - | - | - | - | - | - | - | - | - | - | - | - | - | - | - | - | - | - | - | - | - | - | - | - | - | - | - | - | - | - | - | - | - | - | - | - | - | - | - | - | - | - | - | - | - | - | - | - | - | - | - | - | - | - | - | - | - | - | - | - | - | - | - | - | - | - | - | - | - | - | - | - | - | - | - | - | - | - | - | - | - | - | - | - | - | - | - | - | - | - | - | - | - | - | - | - | - | - | - | - | - | - | - | - | - | - | - | - | - | - | - | - | - | - | - | - | - | - | - | - | - | - | - | - | - | - | - | - | - | - | - | - | - | - | - | - | - | - | - | - | - | - | - | - | - | - | - | - | - | - | - | - | - | - | - | - | - | - | - | - | - | - | - | - | - | - | - | - | - | - | - | - | - | - | - | - | - | - | - | - | - | - | - | - | - | - | - | - | - | - | - | - | - | - | - | - | - | - | - | - | - | - | - | - | - | - | - | - | - | - | - | - | - | - | - | - | - | - | - | - | - | - | - | - | - | - | - | - | - | - | - | - | - | - | - | - | - | - | - | - | - | - | - | - | - | - | - | - | - | - | - | - | - | - | - | - | - | - | - | - | - | - | - | - | - | - | - | - | - | - | - | - | - | - | - | - | - | - | - | - | - | - | - | - | - | - | - | - | - | - | - | - | - | - | - | - | - | - | - | - | - | - | - | - | - | - | - | - | - | - | - | - | - | - | - | - | - | - | - | - | - | - | - | - | - | - | - | - | - | - | - | - | - | - | - | - | - | - | - | - | - | - | - | - | - | - | - | - | - | - | - | - | - | - | - | - | - | - | - | - | - | - | - | - | - | - | - | - | - | - | - | - | - | - | - | - | - | - | - | - | - | - | - | - | - | - | - | - | - | - | - | - | - | - | - | - | - | - | - | - | - | - | - | - | - | - | - | - | - | - | - | - | - | - | - | - | - | - | - | - | - | - | - | - | - | - | - | - | - | - | - | - | - | - | - | - | - | - | - | - | - | - | - | - | - | - | - | - | - | - | - | - | - | - | - | - | - | - | - | - | - | - | - | - | - | - | - | - | - | - | - | - | - | - | - | - | - | - | - | - | - | - | - | - | - | - | - | - | - | - | - | - | - | - | - | - | - | - | - | - | - | - | - | - | - | - | - | - | - | - | - | - | - | - | - | - | - | - | - | - | - | - | - | - | - | - | - | - | - | - | - | - | - | - | - | - | - | - | - | - | - | - | - | - | - | - | - | - | - | - | - | - | - | - | - | - | - | - | - | - | - | - | - | - | - | - | - | - | - | - | - | - | - | - | - | - | - | - | - | - | - | - | - | - | - | - | - | - | - | - | - | - | - | - | - | - | - | - | - | - | - | - | - | - | - | - | - | - | - | - | - | - | - | - | - | - | - | - | - | - | - | - | - | - | - | - | - | - | - | - | - | - | - | - |
| BIS d=2 cl.8 sym=1 env=1 | - | - | - | - | - | - | - | - | - | - | - | - | - | - | - | - | - | - | - | - | - | - | - | - | - | - | - | - | - | - | - | - | - | - | - | - | - | - | - | - | - | - | - | - | - | - | - | - | - | - | - | - | - | - | - | - | - | - | - | - | - | - | - | - | - | - | - | - | - | - | - | - | - | - | - | - | - | - | - | - | - | - | - | - | - | - | - | - | - | - | - | - | - | - | - | - | - | - | - | - | - | - | - | - | - | - | - | - | - | - | - | - | - | - | - | - | - | - | - | - | - | - | - | - | - | - | - | - | - | - | - | - | - | - | - | - | - | - | - | - | - | - | - | - | - | - | - | - | - | - | - | - | - | - | - | - | - | - | - | - | - | - | - | - | - | - | - | - | - | - | - | - | - | - | - | - | - | - | - | - | - | - | - | - | - | - | - | - | - | - | - | - | - | - | - | - | - | - | - | - | - | - | - | - | - | - | - | - | - | - | - | - | - | - | - | - | - | - | - | - | - | - | - | - | - | - | - | - | - | - | - | - | - | - | - | - | - | - | - | - | - | - | - | - | - | - | - | - | - | - | - | - | - | - | - | - | - | - | - | - | - | - | - | - | - | - | - | - | - | - | - | - | - | - | - | - | - | - | - | - | - | - | - | - | - | - | - | - | - | - | - | - | - | - | - | - | - | - | - | - | - | - | - | - | - | - | - | - | - | - | - | - | - | - | - | - | - | - | - | - | - | - | - | - | - | - | - | - | - | - | - | - | - | - | - | - | - | - | - | - | - | - | - | - | - | - | - | - | - | - | - | - | - | - | - | - | - | - | - | - | - | - | - | - | - | - | - | - | - | - | - | - | - | - | - | - | - | - | - | - | - | - | - | - | - | - | - | - | - | - | - | - | - | - | - | - | - | - | - | - | - | - | - | - | - | - | - | - | - | - | - | - | - | - | - | - | - | - | - | - | - | - | - | - | - | - | - | - | - | - | - | - | - | - | - | - | - | - | - | - | - | - | - | - | - | - | - | - | - | - | - | - | - | - | - | - | - | - | - | - | - | - | - | - | - | - | - | - | - | - | - | - | - | - | - | - | - | - | - | - | - | - | - | - | - | - | - | - | - | - | - | - | - | - | - | - | - | - | - | - | - | - | - | - | - | - | - | - | - | - | - | - | - | - | - | - | - | - | - | - | - | - | - | - | - | - | - | - | - | - | - | - | - | - | - | - | - | - | - | - | - | - | - | - | - | - | - | - | - | - | - | - | - | - | - | - | - | - | - | - | - | - | - | - | - | - | - | - | - | - | - | - | - | - | - | - | - | - | - | - | - | - | - | - | - | - | - | - | - | - | - | - | - | - | - | - | - | - | - | - | - | - | - | - | - | - | - | - | - | - | - | - | - | - | - | - | - | - | - | - | - | - | - | - | - | - | - | - | - | - | - | - | - | - | - | - | - | - | - | - | - | - | - | - | - | - | - | - | - | - | - | - | - | - | - | - | - | - | - | - | - | - | - | - | - | - | - | - | - | - | - | - | - | - | - | - | - | - | - | - | - | - | - | - | - | - | - | - | - | - | - | - | - | - | - | - | - | - | - | - | - | - | - | - | - | - | - | - | - | - | - | - | - | - | - | - | - | - | - | - | - | - | - | - | - | - | - | - | - | - | - | - | - | - | - | - | - | - | - | - | - | - | - | - | - | - | - | - | - | - | - | - | - | - | - | - | - | - | - | - | - | - | - | - | - | - | - | - | - | - | - | - | - | - | - | - | - | - | - | - | - | - | - | - | - | - | - | - | - | - | - | - | - | - | - | - | - | - | - | - | - | - | - | - | - | - | - | - | - | - | - | - | - | - | - | - | - | - | - | - | - | - | - | - | - | - | - | - | - | - | - | - | - | - | - | - | - | - | - | - | - | - | - | - | - | - | - | - | - | - | - | - | - | - | - | - | - | - | - | - | - | - | - | - | - | - | - | - | - | - | - | - | - | - | - | - | - | - | - | - | - | - | - | - | - | - | - | - | - | - | - | - | - | - | - | - | - | - | - | - | - | - | - | - | - | - | - | - | - | - | - | - | - | - | - | - | - | - | - | - | - | - | - | - | - | - | - | - | - | - | - | - | - | - | - | - | - | - | - | - | - | - | - | - | - | - | - | - | - | - | - | - | - | - | - | - | - | - | - | - | - | - | - | - | - | - | - | - | - | - | - | - | - | - | - | - | - | - | - | - | - | - | - | - | - | - | - | - | - | - | - | - | - | - | - | - | - | - | - | - | - | - | - | - | - | - | - | - | - | - | - | - | - | - | - | - | - | - | - | - | - | - | - | - | - | - | - | - | - | - | - | - | - | - | - | - | - | - | - | - | - | - | - | - | - | - | - | - | - | - | - | - | - | - | - | - | - | - | - | - | - | - | - | - | H | x | x | x | x | x | x | H | - | - | - | - | - | - | - | - | - | - | - | - | - | - | - | - | - | - | - | - | - | - | - | - | - | - | - | - | - | - | - | - | - | - | - | - | - | - | - | - | - | - | - | - | - | - | - | - | - | - | - | - | - | - | - | - | - | - | - | - | - | - | - | - | - | - | - | - | - | - | - | - | - | - | - | - | - | - | - | - | - | - | - | - | - | - | - | - | - | - | - | - | - | - | - | - | - | - | - | - | - | - | - | - | - | - | - | - | - | - | - | - | - | - | - | - | - | - | - | - | - | - | - | - | - | - | - | - | - | - | - | - | - | - | - | - | - | - | - | - | - | - | - | - | - | - | - | - | - | - | - | - | - | - | - | - | - | - | - | - | - | - | - | - | - | - | - | - | - | - | - | - | - | - | - | - | - | - | - | - | - | - | - | - | - | - | - | - |
