# Supplementary material for: Variation in selection constraints on teleost TLRs with emphasis on their repertoire in the Walking catfish, Clarias batrachus
Source: Sci Rep. 2020 Dec 7;10:21394. doi: 10.1038/s41598-020-78347-6 (PMC7721727; doi:10.1038/s41598-020-78347-6)
Supplement: Supplementary file 32 — Supplementary Information 32. [file 41598_2020_78347_MOESM32_ESM.zip › T13/BIS2/summary.tgz!/summary/PF00000-NONREDUNDANT-5DD-dim0.html]

Alignment and BIS clusters


|  |  |  |  |  |  |  |  |  |  |  |  |  |  |  |  |  |  |  |  |  |  |  |  |  |  |  |  |  |  |  |  |  |  |  |  |  |  |  |  |  |  |  |  |  |  |  |  |  |  |  |  |  |  |  |  |  |  |  |  |  |  |  |  |  |  |  |  |  |  |  |  |  |  |  |  |  |  |  |  |  |  |  |  |  |  |  |  |  |  |  |  |  |  |  |  |  |  |  |  |  |  |  |  |  |  |  |  |  |  |  |  |  |  |  |  |  |  |  |  |  |  |  |  |  |  |  |  |  |  |  |  |  |  |  |  |  |  |  |  |  |  |  |  |  |  |  |  |  |  |  |  |  |  |  |  |  |  |  |  |  |  |  |  |  |  |  |  |  |  |  |  |  |  |  |  |  |  |  |  |  |  |  |  |  |  |  |  |  |  |  |  |  |  |  |  |  |  |  |  |  |  |  |  |  |  |  |  |  |  |  |  |  |  |  |  |  |  |  |  |  |  |  |  |  |  |  |  |  |  |  |  |  |  |  |  |  |  |  |  |  |  |  |  |  |  |  |  |  |  |  |  |  |  |  |  |  |  |  |  |  |  |  |  |  |  |  |  |  |  |  |  |  |  |  |  |  |  |  |  |  |  |  |  |  |  |  |  |  |  |  |  |  |  |  |  |  |  |  |  |  |  |  |  |  |  |  |  |  |  |  |  |  |  |  |  |  |  |  |  |  |  |  |  |  |  |  |  |  |  |  |  |  |  |  |  |  |  |  |  |  |  |  |  |  |  |  |  |  |  |  |  |  |  |  |  |  |  |  |  |  |  |  |  |  |  |  |  |  |  |  |  |  |  |  |  |  |  |  |  |  |  |  |  |  |  |  |  |  |  |  |  |  |  |  |  |  |  |  |  |  |  |  |  |  |  |  |  |  |  |  |  |  |  |  |  |  |  |  |  |  |  |  |  |  |  |  |  |  |  |  |  |  |  |  |  |  |  |  |  |  |  |  |  |  |  |  |  |  |  |  |  |  |  |  |  |  |  |  |  |  |  |  |  |  |  |  |  |  |  |  |  |  |  |  |  |  |  |  |  |  |  |  |  |  |  |  |  |  |  |  |  |  |  |  |  |  |  |  |  |  |  |  |  |  |  |  |  |  |  |  |  |  |  |  |  |  |  |  |  |  |  |  |  |  |  |  |  |  |  |  |  |  |  |  |  |  |  |  |  |  |  |  |  |  |  |  |  |  |  |  |  |  |  |  |  |  |  |  |  |  |  |  |  |  |  |  |  |  |  |  |  |  |  |  |  |  |  |  |  |  |  |  |  |  |  |  |  |  |  |  |  |  |  |  |  |  |  |  |  |  |  |  |  |  |  |  |  |  |  |  |  |  |  |  |  |  |  |  |  |  |  |  |  |  |  |  |  |  |  |  |  |  |  |  |  |  |  |  |  |  |  |  |  |  |  |  |  |  |  |  |  |  |  |  |  |  |  |  |  |  |  |  |  |  |  |  |  |  |  |  |  |  |  |  |  |  |  |  |  |  |  |  |  |  |  |  |  |  |  |  |  |  |  |  |  |  |  |  |  |  |  |  |  |  |  |  |  |  |  |  |  |  |  |  |  |  |  |  |  |  |  |  |  |  |  |  |  |  |  |  |  |  |  |  |  |  |  |  |  |  |  |  |  |  |  |  |  |  |  |  |  |  |  |  |  |  |  |  |  |  |  |  |  |  |  |  |  |  |  |  |  |  |  |  |  |  |  |  |  |  |  |  |  |  |  |  |  |  |  |  |  |  |  |  |  |  |  |  |  |  |  |  |  |  |  |  |  |  |  |  |  |  |  |  |  |  |  |  |  |  |  |  |  |  |  |  |  |  |  |  |  |  |  |  |  |  |  |  |  |  |  |  |  |  |  |  |  |  |  |  |  |  |  |  |  |  |  |  |  |  |  |  |  |  |  |  |  |  |  |  |  |  |  |  |  |  |  |  |  |  |  |  |  |  |  |  |  |  |  |  |  |  |  |  |  |  |  |  |  |  |  |  |  |  |  |  |  |  |  |  |  |  |  |  |  |  |  |  |  |  |  |  |  |  |  |  |  |  |  |  |  |  |  |  |  |  |  |  |  |  |  |  |  |  |  |  |  |  |  |  |  |  |  |  |  |  |  |  |  |  |  |  |  |  |  |  |  |  |  |  |  |  |  |  |  |  |  |  |  |  |  |  |  |  |  |  |  |  |  |  |  |  |  |  |  |  |  |  |  |  |  |  |  |  |  |  |  |  |  |  |  |  |  |  |  |  |  |  |  |  |  |  |  |  |  |  |  |  |  |  |  |  |  |  |  |  |  |  |  |  |  |  |  |  |  |  |  |  |  |  |  |  |  |  |  |  |  |  |  |  |  |  |  |  |  |  |  |  |  |  |  |  |  |  |  |  |  |  |  |  |  |  |  |  |  |  |  |  |  |  |  |  |  |  |  |  |  |  |  |  |  |  |  |  |  |  |  |  |  |  |  |  |  |  |  |  |  |  |  |  |  |  |  |  |  |  |  |  |  |  |  |  |  |  |  |  |  |  |  |  |  |  |  |  |  |  |  |  |  |  |  |  |  |  |  |  |  |  |  |  |  |  |  |  |  |  |  |  |  |  |  |  |  |  |  |  |  |  |  |  |  |  |  |  |  |  |  |  |  |  |  |  |  |  |  |  |  |  |  |  |  |  |  |  |  |  |  |  |  |  |  |  |  |  |  |  |  |  |  |  |  |  |  |  |  |  |  |  |  |  |  |  |  |  |  |  |  |  |  |  |  |  |  |  |  |  |  |  |  |  |  |  |  |  |  |  |  |  |  |  |  |  |  |  |  |  |  |  |  |  |  |  |  |  |  |  |  |  |  |  |  |  |  |  |  |  |  |  |  |  |  |  |  |  |  |  |  |  |  |  |  |  |  |  |  |  |  |  |  |  |  |  |  |  |  |  |  |  |  |  |  |  |  |  |  |  |  |  |  |  |  |  |  |  |  |  |  |  |  |  |  |  |  |  |  |  |  |  |  |  |  |  |  |  |  |  |  |  |  |  |  |  |  |  |  |  |  |  |  |  |  |  |  |  |  |  |  |  |  |  |  |  |  |  |  |  |  |  |  |  |  |  |  |  |  |  |  |  |  |  |  |  |  |  |  |  |  |  |  |  |  |  |  |  |  |  |  |  |  |  |  |  |  |  |  |  |  |  |  |  |  |  |  |  |  |  |  |  |  |  |  |  |  |  |  |  |  |  |  |  |  |  |  |  |  |  |  |  |  |  |  |  |  |  |  |  |  |  |  |  |  |  |  |  |  |  |  |  |  |  |  |  |  |  |  |  |  |  |  |  |  |  |  |  |  |  |  |  |  |  |  |  |  |  |  |  |  |  |  |  |  |  |  |  |  |  |  |  |  |  |  |  |  |  |  |  |  |  |  |  |  |  |  |  |  |  |  |  |  |  |  |  |  |  |  |  |  |  |  |  |  |  |  |  |  |  |  |  |  |  |  |  |  |  |  |  |  |  |  |  |  |  |  |  |  |  |  |  |  |  |  |  |  |  |  |  |  |  |  |  |  |  |  |  |  |  |  |  |  |  |  |  |  |  |  |  |  |  |  |  |  |  |  |  |  |  |  |  |  |  |  |  |  |  |  |  |  |  |  |  |  |  |  |  |  |  |  |  |  |  |  |  |  |  |  |  |  |  |  |  |  |  |  |  |  |  |  |  |  |  |  |  |  |  |  |  |  |  |  |  |  |  |  |  |  |  |  |  |  |
| --- | --- | --- | --- | --- | --- | --- | --- | --- | --- | --- | --- | --- | --- | --- | --- | --- | --- | --- | --- | --- | --- | --- | --- | --- | --- | --- | --- | --- | --- | --- | --- | --- | --- | --- | --- | --- | --- | --- | --- | --- | --- | --- | --- | --- | --- | --- | --- | --- | --- | --- | --- | --- | --- | --- | --- | --- | --- | --- | --- | --- | --- | --- | --- | --- | --- | --- | --- | --- | --- | --- | --- | --- | --- | --- | --- | --- | --- | --- | --- | --- | --- | --- | --- | --- | --- | --- | --- | --- | --- | --- | --- | --- | --- | --- | --- | --- | --- | --- | --- | --- | --- | --- | --- | --- | --- | --- | --- | --- | --- | --- | --- | --- | --- | --- | --- | --- | --- | --- | --- | --- | --- | --- | --- | --- | --- | --- | --- | --- | --- | --- | --- | --- | --- | --- | --- | --- | --- | --- | --- | --- | --- | --- | --- | --- | --- | --- | --- | --- | --- | --- | --- | --- | --- | --- | --- | --- | --- | --- | --- | --- | --- | --- | --- | --- | --- | --- | --- | --- | --- | --- | --- | --- | --- | --- | --- | --- | --- | --- | --- | --- | --- | --- | --- | --- | --- | --- | --- | --- | --- | --- | --- | --- | --- | --- | --- | --- | --- | --- | --- | --- | --- | --- | --- | --- | --- | --- | --- | --- | --- | --- | --- | --- | --- | --- | --- | --- | --- | --- | --- | --- | --- | --- | --- | --- | --- | --- | --- | --- | --- | --- | --- | --- | --- | --- | --- | --- | --- | --- | --- | --- | --- | --- | --- | --- | --- | --- | --- | --- | --- | --- | --- | --- | --- | --- | --- | --- | --- | --- | --- | --- | --- | --- | --- | --- | --- | --- | --- | --- | --- | --- | --- | --- | --- | --- | --- | --- | --- | --- | --- | --- | --- | --- | --- | --- | --- | --- | --- | --- | --- | --- | --- | --- | --- | --- | --- | --- | --- | --- | --- | --- | --- | --- | --- | --- | --- | --- | --- | --- | --- | --- | --- | --- | --- | --- | --- | --- | --- | --- | --- | --- | --- | --- | --- | --- | --- | --- | --- | --- | --- | --- | --- | --- | --- | --- | --- | --- | --- | --- | --- | --- | --- | --- | --- | --- | --- | --- | --- | --- | --- | --- | --- | --- | --- | --- | --- | --- | --- | --- | --- | --- | --- | --- | --- | --- | --- | --- | --- | --- | --- | --- | --- | --- | --- | --- | --- | --- | --- | --- | --- | --- | --- | --- | --- | --- | --- | --- | --- | --- | --- | --- | --- | --- | --- | --- | --- | --- | --- | --- | --- | --- | --- | --- | --- | --- | --- | --- | --- | --- | --- | --- | --- | --- | --- | --- | --- | --- | --- | --- | --- | --- | --- | --- | --- | --- | --- | --- | --- | --- | --- | --- | --- | --- | --- | --- | --- | --- | --- | --- | --- | --- | --- | --- | --- | --- | --- | --- | --- | --- | --- | --- | --- | --- | --- | --- | --- | --- | --- | --- | --- | --- | --- | --- | --- | --- | --- | --- | --- | --- | --- | --- | --- | --- | --- | --- | --- | --- | --- | --- | --- | --- | --- | --- | --- | --- | --- | --- | --- | --- | --- | --- | --- | --- | --- | --- | --- | --- | --- | --- | --- | --- | --- | --- | --- | --- | --- | --- | --- | --- | --- | --- | --- | --- | --- | --- | --- | --- | --- | --- | --- | --- | --- | --- | --- | --- | --- | --- | --- | --- | --- | --- | --- | --- | --- | --- | --- | --- | --- | --- | --- | --- | --- | --- | --- | --- | --- | --- | --- | --- | --- | --- | --- | --- | --- | --- | --- | --- | --- | --- | --- | --- | --- | --- | --- | --- | --- | --- | --- | --- | --- | --- | --- | --- | --- | --- | --- | --- | --- | --- | --- | --- | --- | --- | --- | --- | --- | --- | --- | --- | --- | --- | --- | --- | --- | --- | --- | --- | --- | --- | --- | --- | --- | --- | --- | --- | --- | --- | --- | --- | --- | --- | --- | --- | --- | --- | --- | --- | --- | --- | --- | --- | --- | --- | --- | --- | --- | --- | --- | --- | --- | --- | --- | --- | --- | --- | --- | --- | --- | --- | --- | --- | --- | --- | --- | --- | --- | --- | --- | --- | --- | --- | --- | --- | --- | --- | --- | --- | --- | --- | --- | --- | --- | --- | --- | --- | --- | --- | --- | --- | --- | --- | --- | --- | --- | --- | --- | --- | --- | --- | --- | --- | --- | --- | --- | --- | --- | --- | --- | --- | --- | --- | --- | --- | --- | --- | --- | --- | --- | --- | --- | --- | --- | --- | --- | --- | --- | --- | --- | --- | --- | --- | --- | --- | --- | --- | --- | --- | --- | --- | --- | --- | --- | --- | --- | --- | --- | --- | --- | --- | --- | --- | --- | --- | --- | --- | --- | --- | --- | --- | --- | --- | --- | --- | --- | --- | --- | --- | --- | --- | --- | --- | --- | --- | --- | --- | --- | --- | --- | --- | --- | --- | --- | --- | --- | --- | --- | --- | --- | --- | --- | --- | --- | --- | --- | --- | --- | --- | --- | --- | --- | --- | --- | --- | --- | --- | --- | --- | --- | --- | --- | --- | --- | --- | --- | --- | --- | --- | --- | --- | --- | --- | --- | --- | --- | --- | --- | --- | --- | --- | --- | --- | --- | --- | --- | --- | --- | --- | --- | --- | --- | --- | --- | --- | --- | --- | --- | --- | --- | --- | --- | --- | --- | --- | --- | --- | --- | --- | --- | --- | --- | --- | --- | --- | --- | --- | --- | --- | --- | --- | --- | --- | --- | --- | --- | --- | --- | --- | --- | --- | --- | --- | --- | --- | --- | --- | --- | --- | --- | --- | --- | --- | --- | --- | --- | --- | --- | --- | --- | --- | --- | --- | --- | --- | --- | --- | --- | --- | --- | --- | --- | --- | --- | --- | --- | --- | --- | --- | --- | --- | --- | --- | --- | --- | --- | --- | --- | --- | --- | --- | --- | --- | --- | --- | --- | --- | --- | --- | --- | --- | --- | --- | --- | --- | --- | --- | --- | --- | --- | --- | --- | --- | --- | --- | --- | --- | --- | --- | --- | --- | --- | --- | --- | --- | --- | --- | --- | --- | --- | --- | --- | --- | --- | --- | --- | --- | --- | --- | --- | --- | --- | --- | --- | --- | --- | --- | --- | --- | --- | --- | --- | --- | --- | --- | --- | --- | --- | --- | --- | --- | --- | --- | --- | --- | --- | --- | --- | --- | --- | --- | --- | --- | --- | --- | --- | --- | --- | --- | --- | --- | --- | --- | --- | --- | --- | --- | --- | --- | --- | --- | --- | --- | --- | --- | --- | --- | --- | --- | --- | --- | --- | --- | --- | --- | --- | --- | --- | --- | --- | --- | --- | --- | --- | --- | --- | --- | --- | --- | --- | --- | --- | --- | --- | --- | --- | --- | --- | --- | --- | --- | --- | --- | --- | --- | --- | --- | --- | --- | --- | --- | --- | --- | --- | --- | --- | --- | --- | --- | --- | --- | --- | --- | --- | --- | --- | --- | --- | --- | --- | --- | --- | --- | --- | --- | --- | --- | --- | --- | --- | --- | --- | --- | --- | --- | --- | --- | --- | --- | --- | --- | --- | --- | --- | --- | --- | --- | --- | --- | --- | --- | --- | --- | --- | --- | --- | --- | --- | --- | --- | --- | --- | --- | --- | --- | --- | --- | --- | --- | --- | --- | --- | --- | --- | --- | --- | --- | --- | --- | --- | --- | --- | --- | --- | --- | --- | --- | --- | --- | --- | --- | --- | --- | --- | --- | --- | --- | --- | --- | --- | --- | --- | --- | --- | --- | --- | --- | --- | --- | --- | --- | --- | --- | --- | --- | --- | --- | --- | --- | --- | --- | --- | --- | --- | --- | --- | --- | --- | --- | --- | --- | --- | --- | --- | --- | --- | --- | --- | --- | --- | --- | --- | --- | --- | --- | --- | --- | --- | --- | --- | --- | --- | --- | --- | --- | --- | --- | --- | --- | --- | --- | --- | --- | --- | --- | --- | --- | --- | --- | --- | --- | --- | --- | --- | --- | --- | --- | --- | --- | --- | --- | --- | --- | --- | --- | --- | --- | --- | --- | --- | --- | --- | --- | --- | --- | --- | --- | --- | --- | --- | --- | --- | --- | --- | --- | --- | --- | --- | --- | --- | --- | --- | --- | --- | --- | --- | --- | --- | --- | --- | --- | --- | --- | --- | --- | --- | --- | --- | --- | --- | --- | --- | --- | --- | --- | --- | --- | --- | --- | --- | --- | --- | --- | --- | --- | --- | --- | --- | --- | --- | --- | --- | --- | --- | --- | --- | --- | --- | --- | --- | --- | --- | --- | --- | --- | --- | --- | --- | --- | --- | --- | --- | --- | --- | --- | --- | --- | --- | --- | --- | --- | --- | --- | --- | --- | --- | --- | --- | --- | --- | --- | --- | --- | --- | --- | --- | --- | --- | --- | --- | --- | --- | --- | --- | --- | --- | --- | --- | --- | --- | --- | --- | --- | --- | --- | --- | --- | --- | --- | --- | --- | --- | --- | --- | --- | --- | --- | --- | --- | --- | --- | --- | --- | --- | --- | --- | --- | --- | --- | --- | --- | --- | --- | --- | --- | --- | --- | --- | --- | --- | --- | --- | --- | --- | --- | --- | --- | --- | --- | --- | --- | --- | --- | --- | --- | --- | --- | --- | --- | --- | --- | --- | --- | --- | --- | --- | --- | --- | --- | --- | --- | --- | --- | --- | --- | --- | --- | --- | --- | --- | --- | --- | --- | --- | --- | --- | --- | --- | --- | --- | --- | --- | --- | --- | --- | --- | --- | --- | --- | --- | --- | --- | --- | --- | --- | --- | --- | --- | --- | --- | --- | --- | --- | --- | --- | --- | --- | --- | --- | --- | --- | --- | --- | --- | --- | --- | --- | --- | --- | --- | --- | --- | --- | --- | --- | --- | --- | --- | --- | --- | --- | --- | --- | --- | --- | --- | --- | --- | --- | --- | --- | --- | --- | --- | --- | --- | --- | --- | --- | --- | --- | --- | --- | --- | --- | --- | --- | --- | --- | --- | --- | --- | --- | --- | --- | --- | --- | --- | --- | --- | --- | --- | --- | --- | --- | --- | --- | --- | --- | --- | --- | --- | --- | --- | --- | --- | --- | --- | --- | --- | --- | --- | --- | --- | --- | --- | --- | --- | --- | --- | --- | --- | --- | --- | --- | --- | --- | --- | --- | --- | --- | --- | --- | --- | --- | --- | --- | --- | --- | --- | --- | --- | --- | --- | --- | --- | --- | --- | --- | --- | --- | --- | --- | --- | --- | --- | --- | --- | --- | --- | --- | --- | --- | --- | --- | --- | --- | --- | --- | --- | --- | --- | --- | --- | --- | --- | --- | --- | --- | --- | --- | --- | --- | --- | --- | --- | --- | --- | --- | --- | --- | --- | --- | --- | --- | --- | --- | --- | --- | --- | --- | --- | --- | --- | --- | --- | --- | --- | --- | --- | --- | --- | --- | --- | --- | --- | --- | --- | --- | --- | --- | --- |
|  |  |  |  |  |  |  |  | 1 | 0 |  |  |  |  |  |  |  |  | 2 | 0 |  |  |  |  |  |  |  |  | 3 | 0 |  |  |  |  |  |  |  |  | 4 | 0 |  |  |  |  |  |  |  |  | 5 | 0 |  |  |  |  |  |  |  |  | 6 | 0 |  |  |  |  |  |  |  |  | 7 | 0 |  |  |  |  |  |  |  |  | 8 | 0 |  |  |  |  |  |  |  |  | 9 | 0 |  |  |  |  |  |  |  | 1 | 0 | 0 |  |  |  |  |  |  |  | 1 | 1 | 0 |  |  |  |  |  |  |  | 1 | 2 | 0 |  |  |  |  |  |  |  | 1 | 3 | 0 |  |  |  |  |  |  |  | 1 | 4 | 0 |  |  |  |  |  |  |  | 1 | 5 | 0 |  |  |  |  |  |  |  | 1 | 6 | 0 |  |  |  |  |  |  |  | 1 | 7 | 0 |  |  |  |  |  |  |  | 1 | 8 | 0 |  |  |  |  |  |  |  | 1 | 9 | 0 |  |  |  |  |  |  |  | 2 | 0 | 0 |  |  |  |  |  |  |  | 2 | 1 | 0 |  |  |  |  |  |  |  | 2 | 2 | 0 |  |  |  |  |  |  |  | 2 | 3 | 0 |  |  |  |  |  |  |  | 2 | 4 | 0 |  |  |  |  |  |  |  | 2 | 5 | 0 |  |  |  |  |  |  |  | 2 | 6 | 0 |  |  |  |  |  |  |  | 2 | 7 | 0 |  |  |  |  |  |  |  | 2 | 8 | 0 |  |  |  |  |  |  |  | 2 | 9 | 0 |  |  |  |  |  |  |  | 3 | 0 | 0 |  |  |  |  |  |  |  | 3 | 1 | 0 |  |  |  |  |  |  |  | 3 | 2 | 0 |  |  |  |  |  |  |  | 3 | 3 | 0 |  |  |  |  |  |  |  | 3 | 4 | 0 |  |  |  |  |  |  |  | 3 | 5 | 0 |  |  |  |  |  |  |  | 3 | 6 | 0 |  |  |  |  |  |  |  | 3 | 7 | 0 |  |  |  |  |  |  |  | 3 | 8 | 0 |  |  |  |  |  |  |  | 3 | 9 | 0 |  |  |  |  |  |  |  | 4 | 0 | 0 |  |  |  |  |  |  |  | 4 | 1 | 0 |  |  |  |  |  |  |  | 4 | 2 | 0 |  |  |  |  |  |  |  | 4 | 3 | 0 |  |  |  |  |  |  |  | 4 | 4 | 0 |  |  |  |  |  |  |  | 4 | 5 | 0 |  |  |  |  |  |  |  | 4 | 6 | 0 |  |  |  |  |  |  |  | 4 | 7 | 0 |  |  |  |  |  |  |  | 4 | 8 | 0 |  |  |  |  |  |  |  | 4 | 9 | 0 |  |  |  |  |  |  |  | 5 | 0 | 0 |  |  |  |  |  |  |  | 5 | 1 | 0 |  |  |  |  |  |  |  | 5 | 2 | 0 |  |  |  |  |  |  |  | 5 | 3 | 0 |  |  |  |  |  |  |  | 5 | 4 | 0 |  |  |  |  |  |  |  | 5 | 5 | 0 |  |  |  |  |  |  |  | 5 | 6 | 0 |  |  |  |  |  |  |  | 5 | 7 | 0 |  |  |  |  |  |  |  | 5 | 8 | 0 |  |  |  |  |  |  |  | 5 | 9 | 0 |  |  |  |  |  |  |  | 6 | 0 | 0 |  |  |  |  |  |  |  | 6 | 1 | 0 |  |  |  |  |  |  |  | 6 | 2 | 0 |  |  |  |  |  |  |  | 6 | 3 | 0 |  |  |  |  |  |  |  | 6 | 4 | 0 |  |  |  |  |  |  |  | 6 | 5 | 0 |  |  |  |  |  |  |  | 6 | 6 | 0 |  |  |  |  |  |  |  | 6 | 7 | 0 |  |  |  |  |  |  |  | 6 | 8 | 0 |  |  |  |  |  |  |  | 6 | 9 | 0 |  |  |  |  |  |  |  | 7 | 0 | 0 |  |  |  |  |  |  |  | 7 | 1 | 0 |  |  |  |  |  |  |  | 7 | 2 | 0 |  |  |  |  |  |  |  | 7 | 3 | 0 |  |  |  |  |  |  |  | 7 | 4 | 0 |  |  |  |  |  |  |  | 7 | 5 | 0 |  |  |  |  |  |  |  | 7 | 6 | 0 |  |  |  |  |  |  |  | 7 | 7 | 0 |  |  |  |  |  |  |  | 7 | 8 | 0 |  |  |  |  |  |  |  | 7 | 9 | 0 |  |  |  |  |  |  |  | 8 | 0 | 0 |  |  |  |  |  |  |  | 8 | 1 | 0 |  |  |  |  |  |  |  | 8 | 2 | 0 |  |  |  |  |  |  |  | 8 | 3 | 0 |  |  |  |  |  |  |  | 8 | 4 | 0 |  |  |  |  |  |  |  | 8 | 5 | 0 |  |  |  |  |  |  |  | 8 | 6 | 0 |  |  |  |  |  |  |  | 8 | 7 | 0 |  |  |  |  |  |  |  | 8 | 8 | 0 |  |  |  |  |  |  |  | 8 | 9 | 0 |  |  |  |  |  |  |  | 9 | 0 | 0 |  |  |  |  |  |  |  | 9 | 1 | 0 |  |  |  |  |  |  |  | 9 | 2 | 0 |  |  |  |  |  |  |  | 9 | 3 | 0 |  |  |  |  |  |  |  | 9 | 4 | 0 |  |  |  |  |  |  |  | 9 | 5 | 0 |  |  |  |  |  |  |  | 9 | 6 | 0 |  |  |  |  |  |  |  | 9 | 7 | 0 |  |  |  |  |  |  |  | 9 | 8 | 0 |  |  |  |  |  |  |  | 9 | 9 | 0 |  |  |  |  |  |  | 1 | 0 | 0 | 0 |  |  |  |  |  |  | 1 | 0 | 1 | 0 |  |  |  |  |  |  | 1 | 0 | 2 | 0 |  |  |  |  |  |  | 1 | 0 | 3 | 0 |  |  |  |  |  |  | 1 | 0 | 4 | 0 |  |  |  |  |  |  | 1 | 0 | 5 | 0 |  |  |  |  |  |  | 1 | 0 | 6 | 0 |  |  |  |  |  |  | 1 | 0 | 7 | 0 |  |  |  |  |  |  | 1 | 0 | 8 | 0 |  |  |  |  |  |  | 1 | 0 | 9 | 0 |  |  |  |  |  |  | 1 | 1 | 0 | 0 |  |  |  |  |  |  | 1 | 1 | 1 | 0 |  |  |  |  |  |  | 1 | 1 | 2 | 0 |  |  |  |  |  |  | 1 | 1 | 3 | 0 |  |  |  |  |  |  | 1 | 1 | 4 | 0 |  |  |  |  |  |  | 1 | 1 | 5 | 0 |  |  |  |  |  |  | 1 | 1 | 6 | 0 |  |  |  |  |  |  | 1 | 1 | 7 | 0 |  |  |  |  |  |  | 1 | 1 | 8 | 0 |  |  |  |  |  |  | 1 | 1 | 9 | 0 |  |  |  |  |  |  | 1 | 2 | 0 | 0 |  |  |  |  |  |  | 1 | 2 | 1 | 0 |  |  |  |  |  |  | 1 | 2 | 2 | 0 |  |  |  |  |  |  | 1 | 2 | 3 | 0 |  |  |  |  |  |  | 1 | 2 | 4 | 0 |  |  |  |  |  |  | 1 | 2 | 5 | 0 |  |  |  |  |  |  | 1 | 2 | 6 | 0 |  |  |  |  |  |  | 1 | 2 | 7 | 0 |  |  |  |  |  |  | 1 | 2 | 8 | 0 |  |  |  |  |  |  | 1 | 2 | 9 | 0 |  |  |  |  |  |  | 1 | 3 | 0 | 0 |  |  |  |  |  |  | 1 | 3 | 1 | 0 |  |  |  |  |  |  | 1 | 3 | 2 | 0 |  |  |  |  |  |  | 1 | 3 | 3 | 0 |  |  |  |  |  |  | 1 | 3 | 4 | 0 |  |  |  |  |  |  | 1 | 3 | 5 | 0 |  |  |  |  |  |  | 1 | 3 | 6 | 0 |  |  |  |  |  |  | 1 | 3 | 7 | 0 |  |  |  |  |  |  | 1 | 3 | 8 | 0 |  |  |  |  |  |  | 1 | 3 | 9 | 0 |  |  |  |  |  |  | 1 | 4 | 0 | 0 |  |  |  |  |  |  | 1 | 4 | 1 | 0 |  |  |  |  |  |  | 1 | 4 | 2 | 0 |  |  |  |  |  |  | 1 | 4 | 3 | 0 |  |  |  |  |  |  | 1 | 4 | 4 | 0 |  |  |  |  |  |  | 1 | 4 | 5 | 0 |  |  |  |  |  |  | 1 | 4 | 6 | 0 |  |  |  |  |  |  | 1 | 4 | 7 | 0 |  |  |  |  |  |  | 1 | 4 | 8 | 0 |  |  |  |  |  |  | 1 | 4 | 9 | 0 |  |  |  |  |  |  | 1 | 5 | 0 | 0 |  |  |  |  |  |  | 1 | 5 | 1 | 0 |  |  |  |  |  |  | 1 | 5 | 2 | 0 |  |  |  |  |  |  | 1 | 5 | 3 | 0 |  |  |  |  |  |  | 1 | 5 | 4 | 0 |  |  |  |  |  |  | 1 | 5 | 5 | 0 |  |  |  |  |  |  | 1 | 5 | 6 | 0 |  |  |  |  |  |  | 1 | 5 | 7 | 0 |  |  |  |  |  |  | 1 | 5 | 8 | 0 |  |  |  |  |  |  | 1 | 5 | 9 | 0 |  |  |  |  |  |  | 1 | 6 | 0 | 0 |  |  |  |  |  |  | 1 | 6 | 1 | 0 |  |  |  |  |  |  | 1 | 6 | 2 | 0 |  |  |  |  |  |  | 1 | 6 | 3 | 0 |  |  |  |  |  |  | 1 | 6 | 4 | 0 |  |  |  |  |  |  | 1 | 6 | 5 | 0 |  |  |  |  |  |  | 1 | 6 | 6 | 0 |  |  |  |  |  |  | 1 | 6 | 7 | 0 |  |  |  |  |  |  | 1 | 6 | 8 | 0 |
|  |  |  |  |  |  |  |  |  | \| |  |  |  |  |  |  |  |  |  | \| |  |  |  |  |  |  |  |  |  | \| |  |  |  |  |  |  |  |  |  | \| |  |  |  |  |  |  |  |  |  | \| |  |  |  |  |  |  |  |  |  | \| |  |  |  |  |  |  |  |  |  | \| |  |  |  |  |  |  |  |  |  | \| |  |  |  |  |  |  |  |  |  | \| |  |  |  |  |  |  |  |  |  | \| |  |  |  |  |  |  |  |  |  | \| |  |  |  |  |  |  |  |  |  | \| |  |  |  |  |  |  |  |  |  | \| |  |  |  |  |  |  |  |  |  | \| |  |  |  |  |  |  |  |  |  | \| |  |  |  |  |  |  |  |  |  | \| |  |  |  |  |  |  |  |  |  | \| |  |  |  |  |  |  |  |  |  | \| |  |  |  |  |  |  |  |  |  | \| |  |  |  |  |  |  |  |  |  | \| |  |  |  |  |  |  |  |  |  | \| |  |  |  |  |  |  |  |  |  | \| |  |  |  |  |  |  |  |  |  | \| |  |  |  |  |  |  |  |  |  | \| |  |  |  |  |  |  |  |  |  | \| |  |  |  |  |  |  |  |  |  | \| |  |  |  |  |  |  |  |  |  | \| |  |  |  |  |  |  |  |  |  | \| |  |  |  |  |  |  |  |  |  | \| |  |  |  |  |  |  |  |  |  | \| |  |  |  |  |  |  |  |  |  | \| |  |  |  |  |  |  |  |  |  | \| |  |  |  |  |  |  |  |  |  | \| |  |  |  |  |  |  |  |  |  | \| |  |  |  |  |  |  |  |  |  | \| |  |  |  |  |  |  |  |  |  | \| |  |  |  |  |  |  |  |  |  | \| |  |  |  |  |  |  |  |  |  | \| |  |  |  |  |  |  |  |  |  | \| |  |  |  |  |  |  |  |  |  | \| |  |  |  |  |  |  |  |  |  | \| |  |  |  |  |  |  |  |  |  | \| |  |  |  |  |  |  |  |  |  | \| |  |  |  |  |  |  |  |  |  | \| |  |  |  |  |  |  |  |  |  | \| |  |  |  |  |  |  |  |  |  | \| |  |  |  |  |  |  |  |  |  | \| |  |  |  |  |  |  |  |  |  | \| |  |  |  |  |  |  |  |  |  | \| |  |  |  |  |  |  |  |  |  | \| |  |  |  |  |  |  |  |  |  | \| |  |  |  |  |  |  |  |  |  | \| |  |  |  |  |  |  |  |  |  | \| |  |  |  |  |  |  |  |  |  | \| |  |  |  |  |  |  |  |  |  | \| |  |  |  |  |  |  |  |  |  | \| |  |  |  |  |  |  |  |  |  | \| |  |  |  |  |  |  |  |  |  | \| |  |  |  |  |  |  |  |  |  | \| |  |  |  |  |  |  |  |  |  | \| |  |  |  |  |  |  |  |  |  | \| |  |  |  |  |  |  |  |  |  | \| |  |  |  |  |  |  |  |  |  | \| |  |  |  |  |  |  |  |  |  | \| |  |  |  |  |  |  |  |  |  | \| |  |  |  |  |  |  |  |  |  | \| |  |  |  |  |  |  |  |  |  | \| |  |  |  |  |  |  |  |  |  | \| |  |  |  |  |  |  |  |  |  | \| |  |  |  |  |  |  |  |  |  | \| |  |  |  |  |  |  |  |  |  | \| |  |  |  |  |  |  |  |  |  | \| |  |  |  |  |  |  |  |  |  | \| |  |  |  |  |  |  |  |  |  | \| |  |  |  |  |  |  |  |  |  | \| |  |  |  |  |  |  |  |  |  | \| |  |  |  |  |  |  |  |  |  | \| |  |  |  |  |  |  |  |  |  | \| |  |  |  |  |  |  |  |  |  | \| |  |  |  |  |  |  |  |  |  | \| |  |  |  |  |  |  |  |  |  | \| |  |  |  |  |  |  |  |  |  | \| |  |  |  |  |  |  |  |  |  | \| |  |  |  |  |  |  |  |  |  | \| |  |  |  |  |  |  |  |  |  | \| |  |  |  |  |  |  |  |  |  | \| |  |  |  |  |  |  |  |  |  | \| |  |  |  |  |  |  |  |  |  | \| |  |  |  |  |  |  |  |  |  | \| |  |  |  |  |  |  |  |  |  | \| |  |  |  |  |  |  |  |  |  | \| |  |  |  |  |  |  |  |  |  | \| |  |  |  |  |  |  |  |  |  | \| |  |  |  |  |  |  |  |  |  | \| |  |  |  |  |  |  |  |  |  | \| |  |  |  |  |  |  |  |  |  | \| |  |  |  |  |  |  |  |  |  | \| |  |  |  |  |  |  |  |  |  | \| |  |  |  |  |  |  |  |  |  | \| |  |  |  |  |  |  |  |  |  | \| |  |  |  |  |  |  |  |  |  | \| |  |  |  |  |  |  |  |  |  | \| |  |  |  |  |  |  |  |  |  | \| |  |  |  |  |  |  |  |  |  | \| |  |  |  |  |  |  |  |  |  | \| |  |  |  |  |  |  |  |  |  | \| |  |  |  |  |  |  |  |  |  | \| |  |  |  |  |  |  |  |  |  | \| |  |  |  |  |  |  |  |  |  | \| |  |  |  |  |  |  |  |  |  | \| |  |  |  |  |  |  |  |  |  | \| |  |  |  |  |  |  |  |  |  | \| |  |  |  |  |  |  |  |  |  | \| |  |  |  |  |  |  |  |  |  | \| |  |  |  |  |  |  |  |  |  | \| |  |  |  |  |  |  |  |  |  | \| |  |  |  |  |  |  |  |  |  | \| |  |  |  |  |  |  |  |  |  | \| |  |  |  |  |  |  |  |  |  | \| |  |  |  |  |  |  |  |  |  | \| |  |  |  |  |  |  |  |  |  | \| |  |  |  |  |  |  |  |  |  | \| |  |  |  |  |  |  |  |  |  | \| |  |  |  |  |  |  |  |  |  | \| |  |  |  |  |  |  |  |  |  | \| |  |  |  |  |  |  |  |  |  | \| |  |  |  |  |  |  |  |  |  | \| |  |  |  |  |  |  |  |  |  | \| |  |  |  |  |  |  |  |  |  | \| |  |  |  |  |  |  |  |  |  | \| |  |  |  |  |  |  |  |  |  | \| |  |  |  |  |  |  |  |  |  | \| |  |  |  |  |  |  |  |  |  | \| |  |  |  |  |  |  |  |  |  | \| |  |  |  |  |  |  |  |  |  | \| |  |  |  |  |  |  |  |  |  | \| |  |  |  |  |  |  |  |  |  | \| |  |  |  |  |  |  |  |  |  | \| |  |  |  |  |  |  |  |  |  | \| |  |  |  |  |  |  |  |  |  | \| |  |  |  |  |  |  |  |  |  | \| |  |  |  |  |  |  |  |  |  | \| |  |  |  |  |  |  |  |  |  | \| |  |  |  |  |  |  |  |  |  | \| |  |  |  |  |  |  |  |  |  | \| |  |  |  |  |  |  |  |  |  | \| |  |  |  |  |  |  |  |  |  | \| |  |  |  |  |  |  |  |  |  | \| |  |  |  |  |  |  |  |  |  | \| |  |  |  |  |  |  |  |  |  | \| |  |  |  |  |  |  |  |  |  | \| |  |  |  |  |  |  |  |  |  | \| |  |  |  |  |  |  |  |  |  | \| |  |  |  |  |  |  |  |  |  | \| |  |  |  |  |  |  |  |  |  | \| |  |  |  |  |  |  |  |  |  | \| |  |  |  |  |  |  |  |  |  | \| |  |  |  |  |  |  |  |  |  | \| |  |  |  |  |  |  |  |  |  | \| |  |  |  |  |  |  |  |  |  | \| |  |  |  |  |  |  |  |  |  | \| |  |  |  |  |  |  |  |  |  | \| |  |  |  |  |  |  |  |  |  | \| |  |  |  |  |  |  |  |  |  | \| |  |  |  |  |  |  |  |  |  | \| |  |  |  |  |  |  |  |  |  | \| |  |  |  |  |  |  |  |  |  | \| |  |  |  |  |  |  |  |  |  | \| |
| MMTLR13\_3900\_BP | - | - | - | - | - | - | - | X | X | X | X | X | X | G | A | N | X | X | X | X | X | X | X | X | X | X | X | X | X | X | X | X | X | X | X | X | X | X | X | X | X | X | X | - | - | - | - | X | X | X | X | X | X | X | X | X | X | X | X | X | X | X | X | X | X | X | X | X | X | X | X | X | X | X | X | X | X | X | - | - | - | - | - | X | X | X | X | X | X | X | X | X | X | X | X | X | X | X | X | X | X | X | X | X | X | X | X | X | F | C | - | S | C | F | C | S | T | P | R | - | - | - | - | - | - | - | - | - | - | - | P | A | V | V | L | A | - | - | - | - | - | X | X | X | X | X | X | X | X | X | K | G | C | S | X | X | X | X | X | - | - | - | E | L | P | C | S | X | X | X | X | X | X | X | X | X | - | - | - | - | - | - | - | - | - | - | - | - | X | X | N | I | M | W | C | X | X | X | X | X | X | X | X | X | Q | Q | E | - | H | R | X | P | H | X | C | R | X | H | A | P | R | X | P | N | R | N | Q | P | V | Q | E | Q | D | S | S | X | X | X | P | - | - | - | - | - | - | - | - | - | - | - | - | - | - | - | T | W | I | I | - | Q | S | G | T | W | T | K | K | P | G | L | K | P | E | P | V | G | L | S | K | R | R | R | I | Q | R | S | A | C | X | X | P | - | - | - | - | - | - | G | F | S | E | P | N | - | - | - | - | - | - | - | - | I | X | X | X | X | X | Q | H | L | T | X | X | X | S | T | P | M | L | L | M | - | D | S | S | V | Y | K | P | C | F | X | T | I | T | C | F | K | H | S | L | Q | I | Y | L | I | P | S | Q | R | L | K | Q | S | I | Y | L | X | T | G | S | K | L | - | - | - | S | - | - | - | - | - | - | - | - | - | - | - | X | X | L | W | T | V | W | W | X | - | - | - | - | X | L | H | S | H | R | P | R | S | F | S | K | Q | H | P | E | N | X | C | E | L | F | P | C | P | X | X | V | H | P | A | V | X | X | X | X | X | X | X | X | X | K | X | V | Q | A | A | G | X | X | X | X | X | X | X | X | X | X | X | X | X | X | X | X | - | - | - | X | X | X | X | - | - | - | - | - | - | - | X | X | X | X | X | X | X | X | X | X | X | X | X | X | R | C | F | C | - | - | - | - | - | - | - | - | - | - | F | Q | P | Q | A | E | E | S | A | L | S | X | X | X | X | X | X | X | X | X | - | - | - | X | X | X | X | - | - | - | - | - | X | X | X | X | E | L | K | Q | K | - | - | C | X | X | X | X | - | - | G | T | L | C | G | D | K | - | - | - | - | - | - | - | - | - | - | - | - | - | E | E | S | - | - | - | - | - | - | - | - | - | X | X | X | X | X | X | X | E | S | G | I | F | - | - | - | - | - | - | - | - | - | - | - | - | - | - | - | - | - | - | - | - | - | - | - | - | - | - | - | - | - | - | - | - | - | - | - | - | - | - | - | - | - | - | - | - | - | - | - | - | - | - | - | - | - | - | - | - | - | - | - | - | - | - | - | - | - | - | - | - | - | - | - | - | - | - | - | - | - | - | - | S | V | L | G | E | I | S | I | Y | Y | L | X | L | A | K | R | N | - | G | P | T | X | E | N | R | G | X | L | E | R | I | X | X | X | X | S | Y | L | R | L | T | P | A | C | X | X | X | - | - | X | V | L | H | H | L | W | L | L | S | X | X | X | X | X | X | M | Q | T | L | G | M | X | X | X | X | X | X | X | X | X | X | X | X | X | X | X | C | P | V | V | T | R | E | G | X | Y | Q | Q | T | - | - | L | S | Q | Q | L | W | F | E | A | D | I | S | Y | Y | I | X | L | P | W | I | X | X | X | X | X | N | - | - | - | - | - | - | S | A | D | E | X | K | X | G | H | H | S | A | - | - | - | - | G | H | I | Q | K | X | X | X | X | P | E | Q | P | Q | L | F | R | P | X | Q | E | Q | T | S | X | H | X | P | X | - | - | L | V | H | P | - | - | - | - | - | - | - | - | - | - | - | - | - | - | - | - | - | - | - | - | - | - | - | - | - | - | - | - | - | F | X | X | X | X | X | X | E | A | D | S | H | E | S | H | E | K | X | N | H | X | V | X | X | X | X | T | K | G | I | Q | - | - | - | - | - | C | P | Y | P | T | X | G | A | L | P | A | V | Q | S | A | E | I | H | H | Q | E | A | L | X | X | A | A | E | A | K | E | A | Q | P | Q | F | K | H | H | X | F | H | X | R | R | H | - | - | - | - | L | X | R | P | E | K | A | Q | L | - | - | - | - | - | - | - | - | - | - | - | - | - | - | - | - | - | - | - | - | - | P | G | - | - | - | - | - | - | F | K | W | E | P | H | Q | E | A | D | T | - | - | S | Y | S | V | R | P | G | Q | F | E | A | X | X | X | X | X | X | X | X | X | X | X | L | S | C | T | T | T | A | F | X | X | X | X | X | X | X | X | X | X | X | X | X | X | X | X | X | X | X | - | - | - | X | X | X | X | - | - | - | - | - | - | - | - | - | - | - | X | X | X | X | F | X | I | X | X | X | X | X | X | X | X | X | X | X | X | X | X | X | X | X | X | X | X | X | X | E | S | P | F | I | N | X | X | L | T | S | L | E | Y | V | E | M | N | Y | Q | G | P | X | X | G | R | G | I | G | X | X | T | I | G | P | R | F | F | Q | S | Q | H | R | L | T | A | I | A | I | G | H | T | I | I | L | N | F | H | P | D | A | F | V | P | X | X | L | V | N | L | K | T | L | Y | I | A | G | V | D | M | K | T | X | X | X | N | L | S | A | L | - | - | - | F | S | P | L | K | R | L | K | K | L | T | L | Y | R | T | D | L | X | X | X | N | L | P | X | X | X | X | X | X | N | L | M | P | P | D | N | T | L | E | I | L | K | V | T | S | N | H | F | H | T | V | D | K | A | M | L | D | A | F | P | R | L | R | V | L | D | I | T | E | N | P | L | T | C | T | C | D | N | A | W | F | K | T | W | A | I | H | D | T | Q | T | Q | V | X | S | Y | L | Y | X | X | X | X | X | M | Q | C | D | N | X | X | D | Q | R | - | - | F | P | Y | L | W | Q | F | D | D | K | A | C | S | F | E | V | A | S | F | T | L | F | I | T | C | S | V | X | X | X | X | L | D | V | L | F | M | C | V | C | V | L | W | Q | T | Q | G | P | A | L | R | F | L | M | L | I | L | R | A | K | L | R | X | X | X | X | X | R | X | X | R | R | S | R | G | Q | X | X | X | X | X | X | X | X | X | X | Q | Y | D | A | F | V | S | Y | S | S | K | D | E | A | W | V | M | - | - | - | - | - | - | - | - | - | - | - | - | - | - | - | - | - | - | - | - | - | E | Q | L | V | P | N | L | E | K | P | A | - | - | - | - | - | - | - | - | - | - | - | - | - | - | A | G | A | P | R | L | K | L | C | L | H | H | R | D | F | R | P | G | A | A | V | L | E | N | I | E | A | A | I | Y | N | S | R | H | T | I | C | V | V | T | R | H | F | L | Q | S | E | W | C | S | V | E | F | Q | L | A | S | L | R | L | L | F | D | G | S | D | V | L | V | L | V | F | L | E | E | I | P | E | - | - | - | - | H | C | L | S | P | Y | T | R | L | R | K | S | I | R | K | K | T | Y | L | L | W | P | E | K | P | Q | E | X | X | X | X | X | E | D | A | F | W | I | R | L | I | D | A | L | K | D | N | R | E | E | X | X | X | X | X | X | X | X | X | X | X | X | X | X | X | X | X | X | X | X | X | X | X | X | X | X | X | X | - | - | X | X | X | X | X | X | X | E | E | E | G | G | R | E | D | E | L | A | R | L | I | G | X | X | - | - | - | - | - | - | - | - | - | - | - |
| PHTLR13\_3900\_BP | - | - | X | X | X | X | X | X | G | F | I | X | X | X | X | X | X | X | X | X | K | - | - | - | P | S | T | D | L | X | X | X | X | X | X | X | X | X | X | X | X | X | X | X | X | X | X | X | X | X | X | - | X | - | - | X | X | X | X | X | X | X | X | X | X | X | X | X | X | X | X | X | X | X | X | X | X | X | X | X | X | X | X | X | X | X | X | X | X | X | X | X | X | D | D | N | R | F | X | X | X | X | X | X | - | - | - | - | - | X | X | X | C | F | - | - | S | - | - | - | - | - | - | - | - | - | - | - | - | - | S | A | X | A | T | V | - | - | - | - | - | X | X | X | X | X | X | X | X | X | R | N | C | I | E | X | X | X | X | - | - | - | S | R | L | K | P | X | X | X | X | X | X | X | X | X | - | - | X | X | T | T | F | K | - | - | - | - | - | - | - | - | - | - | C | X | X | X | X | X | X | X | X | X | X | T | P | S | S | D | S | Y | C | H | R | G | R | Y | T | T | F | C | N | K | H | F | D | L | L | L | Q | T | C | E | X | X | - | X | S | - | - | - | - | - | - | - | - | - | - | - | - | - | - | - | S | T | K | L | - | Q | S | P | - | - | A | K | A | Q | C | S | A | T | K | Q | - | - | Q | S | H | K | E | Y | X | Q | R | C | L | C | X | S | T | I | X | X | S | S | H | - | - | T | E | P | V | - | - | - | - | - | - | - | - | Q | X | X | X | X | X | X | H | S | L | X | X | X | S | I | L | P | F | S | M | - | T | S | G | T | S | A | L | X | S | W | L | T | T | D | X | Q | I | Y | L | L | T | Y | F | L | V | C | L | N | X | R | F | W | T | Y | V | G | T | G | X | W | - | - | - | I | S | Q | Q | W | C | T | P | Y | - | Q | X | X | X | K | G | X | R | P | W | X | X | - | - | - | - | X | X | X | X | X | X | - | - | X | F | - | L | X | Q | - | - | - | T | X | H | F | A | T | L | S | Q | P | A | X | X | X | X | X | X | X | X | X | X | X | X | - | - | - | - | - | - | X | X | F | P | S | L | S | W | X | X | X | X | X | X | X | X | X | X | X | X | X | N | M | L | T | - | - | - | - | - | - | - | T | X | X | X | X | X | - | X | X | X | X | E | M | Q | E | X | F | S | - | - | - | - | - | - | - | - | - | - | Q | - | X | R | Q | S | A | G | S | L | L | X | X | X | X | X | X | X | X | X | - | - | - | X | X | X | X | X | - | - | - | X | X | X | E | V | F | Q | P | R | L | - | - | - | - | - | - | - | - | - | X | X | X | X | W | S | E | - | - | - | - | - | - | - | - | - | - | - | - | - | X | X | X | - | - | - | - | - | - | - | X | X | X | X | X | X | X | X | X | X | X | E | Q | F | - | - | - | - | - | - | - | - | - | - | D | I | P | V | - | - | - | P | A | F | H | - | - | - | - | - | - | - | - | - | - | - | - | - | - | - | - | - | - | - | - | - | - | Q | H | F | N | N | N | A | X | X | X | X | L | N | R | - | - | - | - | - | - | - | - | - | - | - | - | - | - | - | - | - | - | - | - | - | T | N | V | M | P | W | H | V | D | F | X | X | X | Q | V | W | D | X | - | - | - | - | - | - | - | - | - | - | - | - | - | - | - | - | - | - | - | - | - | - | Q | S | P | K | C | X | F | H | - | - | C | V | N | N | C | X | X | X | L | F | K | X | K | X | X | X | I | E | K | M | L | L | Q | S | N | D | L | K | V | L | N | N | K | T | L | S | D | C | P | P | I | T | H | X | X | X | X | X | X | F | G | P | F | I | Q | S | A | X | - | - | - | - | - | X | R | A | X | A | V | C | N | S | - | - | - | - | L | K | - | - | - | D | X | K | S | S | C | E | T | Q | S | X | T | X | P | P | Y | P | S | N | K | L | Q | K | S | H | K | I | S | X | X | - | - | X | L | E | R | T | K | L | P | L | Q | S | H | S | X | S | E | F | L | C | L | Q | S | - | - | - | - | - | - | - | - | - | - | - | - | - | - | - | - | - | - | - | - | - | - | - | - | - | - | - | - | - | Y | X | X | X | X | X | X | K | S | D | N | S | S | T | E | Y | K | H | N | C | L | L | X | X | X | X | S | Q | S | T | E | W | T | X | R | - | P | C | Y | T | P | P | G | Q | Q | S | S | D | X | S | L | X | X | X | L | X | G | - | - | - | - | P | S | Q | S | S | D | T | X | L | T | Q | X | P | N | F | C | H | I | Q | Q | H | - | - | - | - | L | L | L | P | Q | X | A | - | - | - | - | - | - | - | - | - | - | - | - | - | - | - | - | - | - | - | - | - | E | H | F | R | - | - | - | - | - | - | F | G | W | E | Q | D | N | T | F | N | A | - | - | P | S | I | X | R | A | G | Q | L | E | X | X | X | X | X | X | X | X | X | X | X | X | F | T | X | T | A | I | I | X | L | K | L | X | X | X | G | H | L | I | G | S | L | H | R | X | X | X | X | X | X | X | X | X | X | X | - | - | - | - | - | - | - | - | - | - | - | X | L | Q | V | L | D | L | H | G | N | F | I | H | Y | F | X | X | E | H | V | Y | - | - | - | X | X | X | X | X | X | X | P | F | V | T | X | X | X | X | X | L | T | K | L | T | D | L | K | L | D | G | Q | M | P | Y | G | I | T | X | X | L | L | P | H | A | F | F | R | G | L | T | S | L | K | S | L | Y | L | S | N | N | H | I | S | S | L | S | T | D | T | F | D | D | L | R | N | L | K | F | L | T | L | D | D | S | C | V | G | V | T | Q | X | X | X | X | K | P | G | V | - | - | - | F | K | N | L | H | K | L | E | I | L | M | V | E | N | M | G | I | X | X | X | S | F | S | K | E | V | X | F | G | N | X | X | X | X | X | T | G | L | K | V | L | H | L | N | R | N | A | M | Q | T | L | D | V | D | L | L | E | N | L | T | N | L | Q | Y | L | D | V | R | N | T | P | L | S | C | N | C | P | N | S | E | L | Q | N | W | T | K | N | N | Q | R | V | Q | F | X | I | Y | L | Y | X | X | X | X | X | L | T | C | P | D | X | X | X | V | K | - | - | G | S | N | F | Y | N | F | D | T | H | V | C | X | X | D | F | G | V | Y | W | F | A | S | T | Y | I | V | T | L | L | V | T | L | L | P | L | L | Y | I | K | L | Y | W | K | X | X | X | X | X | I | W | L | L | X | X | V | F | C | S | W | F | G | E | Q | W | R | R | L | X | X | G | R | G | R | E | X | X | X | X | X | X | X | X | X | X | X | T | Y | D | A | F | I | S | Y | N | S | A | D | E | E | W | V | M | - | - | - | - | - | - | - | - | - | - | - | - | - | - | - | - | - | - | - | - | - | E | Q | L | L | P | N | L | E | G | S | G | - | - | - | - | - | - | - | - | - | - | - | - | - | - | X | X | X | X | X | F | R | L | C | L | H | H | R | D | F | E | P | G | R | N | I | V | D | N | I | V | A | A | V | Y | N | S | R | K | T | V | C | V | V | S | H | N | F | L | R | S | E | W | C | S | L | E | I | Q | L | A | S | Y | R | L | F | D | E | M | Q | D | V | L | L | L | V | F | L | E | Y | I | H | E | - | - | - | - | R | Q | L | S | A | Y | H | R | M | R | K | V | M | L | K | K | T | Y | L | Q | W | P | G | P | D | C | T | D | P | V | K | S | Q | E | L | F | W | K | Q | L | K | R | A | L | R | - | - | - | - | - | S | S | N | S | R | C | Q | D | E | E | Q | M | E | E | N | V | Q | N | G | Q | R | K | G | E | A | R | A | E | - | - | E | R | E | Y | F | M | N | Q | A | Q | M | D | D | X | X | X | X | P | Y | Y | L | M | P | X | X | X | X | X | X | - | - | - | - | - | - |
| SATLR13\_3900\_BP | - | X | X | X | X | X | X | X | X | E | X | X | X | X | - | - | X | X | X | X | X | - | - | - | N | S | S | A | V | V | X | X | X | X | X | X | X | X | X | X | X | X | X | X | X | X | X | X | X | X | X | X | X | - | - | X | X | X | X | X | X | X | X | X | X | X | X | X | X | X | X | X | X | X | X | X | X | X | X | X | X | X | X | X | X | X | X | X | X | X | X | X | X | D | S | C | P | W | X | X | X | X | X | X | - | - | - | - | - | X | X | X | Q | L | - | - | N | - | - | - | - | - | - | - | - | - | - | - | - | - | L | S | V | A | T | A | - | - | - | - | - | X | X | X | X | X | X | X | X | X | Q | N | C | I | E | X | X | X | X | - | - | - | P | I | L | P | W | X | X | X | X | X | X | X | X | X | - | - | X | X | K | S | F | N | - | - | - | - | - | - | - | - | - | - | C | X | X | X | X | X | X | X | X | X | S | S | K | E | Q | D | H | E | G | S | N | R | R | P | A | G | N | C | H | Q | S | H | H | H | C | X | - | S | C | V | A | X | X | X | S | - | - | - | - | - | - | - | - | - | - | - | - | - | - | - | Q | K | Q | L | - | C | Q | T | - | - | T | K | P | S | K | P | Q | T | R | - | - | - | - | S | K | X | I | E | G | H | R | T | I | C | F | P | K | I | E | S | X | X | S | Q | V | S | X | Y | V | - | - | - | - | - | - | - | - | R | X | X | X | X | X | Q | Y | I | R | X | X | X | S | T | L | L | C | F | R | - | T | C | T | T | S | P | F | S | F | X | Q | T | T | V | X | S | R | S | L | R | A | F | S | P | M | F | - | - | - | S | I | S | G | L | X | P | X | G | K | M | Y | X | X | I | S | Q | G | L | L | S | L | C | - | H | X | X | X | - | N | T | X | P | C | X | X | - | - | - | - | X | X | X | X | X | X | - | - | X | X | - | L | L | X | - | - | - | Q | - | - | - | F | D | F | S | X | X | L | K | R | V | T | T | Q | X | X | X | X | X | X | X | X | X | X | X | X | H | Y | V | I | H | M | P | X | - | - | X | X | X | X | X | X | X | X | X | X | X | N | N | L | L | - | - | - | - | - | - | - | T | X | X | X | X | X | - | X | X | X | X | G | M | X | A | F | I | S | - | - | - | - | - | - | - | - | - | - | K | - | V | H | S | G | A | G | F | F | I | Q | X | X | X | X | X | X | X | X | - | - | - | - | - | - | - | - | - | - | - | - | X | X | X | X | X | X | X | X | L | - | - | - | - | - | - | - | - | - | G | S | L | R | R | L | X | - | - | - | - | - | - | - | - | - | - | - | - | - | X | X | X | R | C | G | X | X | X | - | X | X | X | X | X | X | X | X | X | X | X | K | P | Y | - | - | - | - | - | - | - | - | - | - | Q | L | S | A | - | - | - | F | A | F | X | - | - | - | - | - | - | - | - | - | - | - | - | - | - | - | - | - | - | - | - | - | - | X | C | Q | D | I | R | V | X | X | X | X | L | N | I | - | - | - | - | - | - | - | - | - | - | - | - | - | - | - | - | - | - | - | - | - | S | N | V | P | V | N | H | V | D | F | X | X | X | Q | A | Q | V | X | - | - | - | - | - | - | - | - | - | - | - | - | - | - | - | - | - | - | - | - | - | - | T | M | T | P | Y | S | W | S | - | - | Y | V | X | X | X | X | X | X | L | L | X | X | K | A | K | W | I | K | D | L | N | L | D | S | N | G | I | R | N | Q | T | F | D | I | L | R | Y | C | P | E | I | T | R | X | X | X | X | X | X | F | E | S | I | T | Q | X | P | X | - | - | - | - | - | X | K | A | X | V | V | S | N | F | S | C | G | X | D | T | - | - | - | Y | X | R | H | H | C | R | A | X | P | F | Y | L | P | P | V | L | X | X | - | - | - | - | X | E | K | G | L | F | Q | - | - | R | S | G | R | S | E | L | S | L | Q | P | H | P | L | S | Q | L | S | C | F | L | S | - | - | - | - | - | - | - | - | - | - | - | - | - | - | - | - | - | - | - | - | - | - | - | - | - | - | - | - | - | Y | X | X | X | X | X | X | K | S | Q | V | A | K | T | X | H | K | H | D | C | I | S | X | X | X | X | S | X | S | S | Q | R | A | K | K | - | A | X | D | T | S | P | G | Q | Q | P | L | N | R | L | V | Q | X | H | L | X | R | - | - | - | - | P | F | Q | S | A | N | S | X | P | A | Q | Q | S | H | C | C | H | F | X | W | D | - | - | - | - | L | P | Q | S | R | E | S | - | - | - | - | - | - | - | - | - | - | - | - | - | - | - | - | - | - | - | - | - | D | Y | T | X | - | - | - | - | - | - | P | R | R | X | Q | D | N | S | Y | S | T | - | - | I | W | P | X | G | T | E | K | S | V | K | X | X | X | X | X | X | X | X | X | X | X | S | I | X | M | E | T | I | S | S | R | L | X | X | X | T | T | L | H | S | V | F | Q | D | X | X | X | X | X | X | X | X | X | X | X | - | - | - | - | - | - | - | - | - | - | - | X | L | T | T | L | D | L | Q | R | N | Q | I | C | F | L | X | X | E | D | V | S | - | - | - | Y | X | X | X | X | X | S | P | F | M | N | X | X | X | X | X | L | S | R | L | S | D | L | K | L | D | G | Q | Q | P | Y | G | L | N | X | X | I | L | P | R | N | L | F | R | G | L | H | S | L | K | S | L | Y | L | T | N | N | E | I | P | D | L | S | P | D | V | F | D | D | L | T | G | L | K | F | L | T | L | D | N | C | C | L | G | V | A | H | X | X | X | X | K | P | G | V | - | - | - | F | K | N | L | R | N | L | T | K | L | V | L | E | N | M | R | M | X | X | X | N | F | S | E | E | V | X | F | R | N | X | X | X | X | X | T | Q | L | S | Q | L | Q | L | N | H | N | A | I | Q | S | I | T | L | D | A | L | D | S | L | H | K | L | S | Y | L | D | M | R | G | S | S | L | S | C | T | C | K | N | T | L | L | Q | N | W | T | V | N | N | T | K | V | Q | V | X | V | Y | L | Y | X | X | X | X | X | L | T | C | P | H | X | X | X | D | P | - | - | K | Q | K | F | Y | N | F | H | T | N | V | C | Y | I | D | L | G | E | Y | L | F | L | S | T | A | L | V | I | F | L | F | T | L | I | P | V | L | Y | V | K | L | Y | W | R | X | X | X | X | E | V | Q | L | L | X | X | V | F | R | S | W | F | S | E | R | W | R | R | L | X | X | R | G | G | G | K | X | X | X | X | X | X | X | X | X | X | X | K | Y | D | A | F | V | S | Y | N | S | S | D | E | Q | W | I | M | - | - | - | - | - | - | - | - | - | - | - | - | - | - | - | - | - | - | - | - | - | D | Q | L | L | P | N | L | E | G | K | G | - | - | - | - | - | - | - | - | - | - | - | - | - | - | X | X | X | S | S | F | K | L | C | L | H | H | R | D | F | E | P | G | R | Y | I | V | D | N | I | V | S | A | V | Y | G | S | R | K | T | I | C | V | V | S | K | N | F | L | R | S | E | W | C | S | L | E | I | Q | L | A | S | Y | R | L | F | D | E | L | R | D | V | L | L | L | V | F | L | E | P | I | S | E | - | - | - | - | R | Q | L | S | S | Y | H | R | M | R | K | V | M | L | K | K | T | Y | L | Q | W | P | G | P | D | C | I | D | P | T | Q | A | Q | E | L | F | W | N | Q | L | R | R | A | I | G | - | - | - | - | - | T | G | S | R | L | X | X | X | X | X | X | X | X | E | T | E | E | N | Y | M | S | K | G | H | A | X | X | E | - | - | S | N | E | T | E | H | C | E | T | Y | T | S | D | X | X | X | X | N | F | Y | L | L | P | X | X | X | X | X | X | - | - | - | - | - | - |
| ATTLR13\_3900\_BP | X | X | X | X | X | X | X | X | K | X | - | X | X | X | - | - | X | X | X | X | X | - | - | - | N | X | S | A | V | V | X | X | X | X | X | X | X | X | X | X | X | X | X | X | X | X | X | X | X | X | X | X | X | - | - | X | X | X | X | X | X | X | X | X | X | X | X | X | X | X | X | X | X | X | X | X | X | X | X | X | X | X | X | X | X | X | X | X | X | X | X | X | X | Y | S | C | S | W | X | X | X | X | X | X | - | - | - | - | - | X | X | X | L | L | - | - | S | - | - | - | - | - | - | - | - | - | - | - | - | - | S | S | V | A | T | A | - | - | - | - | - | X | X | X | X | X | X | X | X | X | K | N | C | I | A | X | X | X | X | - | - | - | R | I | L | K | R | X | X | X | X | X | X | X | X | X | - | - | X | X | K | S | F | T | - | - | - | - | - | - | - | - | - | - | C | X | X | X | X | X | X | X | X | X | P | S | K | A | K | Q | H | V | C | Y | C | K | X | P | A | A | F | C | H | Q | S | H | H | L | H | X | - | S | S | E | S | X | X | X | S | - | - | - | - | - | - | - | - | - | - | - | - | - | - | - | Q | Q | Q | L | - | C | X | S | - | - | A | E | S | P | A | P | Q | N | R | - | - | - | - | S | X | P | L | D | G | H | Q | X | A | C | F | R | Q | V | V | S | X | X | A | E | V | F | K | F | V | - | - | - | - | - | - | - | - | L | X | X | X | X | X | L | H | I | K | X | X | X | S | A | L | L | C | L | T | - | T | C | T | T | S | P | F | S | P | X | P | T | T | Q | L | S | S | S | T | G | T | F | S | S | L | L | - | - | - | L | I | W | R | L | Y | Y | X | G | K | I | F | X | V | I | S | Q | R | L | L | S | L | C | - | H | X | X | X | - | E | T | X | R | L | X | X | - | - | - | - | X | X | X | X | X | X | - | - | X | X | - | L | L | Q | - | - | - | Q | - | - | - | F | D | L | S | X | P | L | K | C | V | T | T | Q | X | X | X | X | X | X | X | X | X | X | X | X | Y | N | F | I | S | M | X | X | - | - | X | X | X | X | X | X | X | X | X | X | X | N | N | L | F | - | - | - | - | - | - | - | T | X | X | X | X | X | - | X | X | X | X | R | M | X | T | F | I | S | - | - | - | - | - | - | - | - | - | - | Q | - | V | H | S | N | P | R | F | V | V | Q | X | X | X | X | X | X | X | X | - | - | - | - | - | - | - | - | - | - | - | - | X | X | X | X | X | X | X | X | L | - | - | - | - | - | - | - | - | - | G | S | P | R | W | L | X | - | - | - | - | - | - | - | - | - | - | - | - | - | X | X | X | R | S | G | X | X | X | - | X | X | X | X | X | X | X | X | X | X | X | G | T | N | - | - | - | - | - | - | - | - | - | - | K | L | S | A | - | - | - | L | A | F | N | - | - | - | - | - | - | - | - | - | - | - | - | - | - | - | - | - | - | - | - | - | - | R | C | Q | D | S | G | V | X | X | X | X | L | N | I | - | - | - | - | - | - | - | - | - | - | - | - | - | - | - | - | - | - | - | - | - | S | N | I | N | A | N | H | V | D | F | X | X | X | L | A | Q | V | L | - | - | - | - | - | - | - | - | - | - | - | - | - | - | - | - | - | - | - | - | - | - | K | M | T | T | C | S | Q | S | - | - | F | A | X | X | X | X | X | X | M | L | K | A | K | V | K | R | I | K | N | L | D | L | G | N | N | R | I | R | N | L | T | K | Y | T | L | F | R | C | P | D | I | T | G | X | X | X | X | X | X | S | G | F | I | P | Q | X | T | X | - | - | - | - | - | X | K | T | Q | G | V | L | I | F | X | X | D | X | D | K | - | - | - | Y | K | K | L | Q | C | R | A | X | P | S | H | L | P | P | R | L | X | N | - | - | - | - | K | K | Y | G | F | F | L | - | - | X | T | E | R | N | E | L | S | L | Q | P | H | P | L | S | Q | F | L | C | F | L | S | - | - | - | - | - | - | - | - | - | - | - | - | - | - | - | - | - | - | - | - | - | - | - | - | - | - | - | - | - | Y | X | X | X | X | X | X | K | Y | N | G | A | K | T | X | H | K | H | N | C | F | S | X | X | X | X | P | X | S | S | Q | R | A | K | K | - | A | X | D | T | P | F | R | Q | X | P | P | N | X | F | V | Q | X | H | L | X | R | - | - | - | - | X | C | K | P | E | N | P | Q | L | T | Q | Q | S | N | F | C | H | F | Q | W | D | - | - | - | - | L | P | Q | A | H | K | T | - | - | - | - | - | - | - | - | - | - | - | - | - | - | - | - | - | - | - | - | - | N | N | T | G | - | - | - | - | - | - | P | W | R | Q | X | D | N | S | L | X | A | - | - | L | R | S | R | W | T | K | T | P | I | Q | X | X | X | X | X | X | X | X | X | X | X | S | I | X | T | E | T | T | S | N | K | L | X | X | X | S | S | L | S | H | V | F | Q | D | X | X | X | X | X | X | X | X | X | X | X | - | - | - | - | - | - | - | - | - | - | - | X | L | T | V | L | D | L | A | G | N | Q | I | R | F | L | X | X | E | N | I | K | - | - | - | S | X | X | X | X | X | P | P | F | V | N | X | X | X | X | X | L | S | K | L | S | D | L | K | L | D | A | Q | R | P | F | G | L | T | X | X | V | L | P | R | T | L | F | R | G | L | R | S | L | K | S | L | Y | L | T | N | N | Y | I | N | H | L | P | P | D | A | F | D | D | L | T | G | L | H | F | L | T | L | D | N | C | C | V | G | V | I | D | X | X | X | X | Q | P | G | V | - | - | - | F | K | N | L | R | N | V | T | K | L | I | V | E | N | M | G | I | X | X | X | N | F | S | K | E | I | X | F | E | N | X | X | X | X | X | T | R | L | H | T | L | Q | L | N | R | N | V | M | Q | S | I | N | V | D | A | L | E | S | L | P | K | L | Q | Y | L | D | V | R | N | I | P | L | S | C | T | C | L | N | Q | G | L | Q | D | W | T | L | K | N | V | N | V | Q | V | X | V | Y | L | Y | X | X | X | X | X | L | S | C | Q | N | X | X | X | D | E | - | - | N | Y | K | F | Y | N | F | D | T | K | V | C | Y | I | D | L | G | E | S | L | F | F | S | T | A | A | V | I | F | L | F | T | V | I | P | I | L | Y | V | K | L | Y | W | K | X | X | X | X | E | V | Q | L | L | X | X | V | F | R | S | W | F | G | E | Q | W | R | R | L | X | X | R | G | G | G | K | X | X | X | X | X | X | X | X | X | X | X | K | F | D | A | F | I | S | Y | N | S | S | D | E | Q | W | V | M | - | - | - | - | - | - | - | - | - | - | - | - | - | - | - | - | - | - | - | - | - | D | Q | L | L | P | N | L | E | G | N | G | - | - | - | - | - | - | - | - | - | - | - | - | - | - | X | X | X | S | S | F | K | L | C | L | H | H | R | D | F | E | P | G | R | Y | I | V | D | N | I | V | S | A | V | Y | S | S | R | K | T | I | C | V | V | S | R | N | F | L | Q | S | E | W | C | S | L | E | I | Q | L | A | S | Y | R | L | F | D | E | H | R | D | V | L | L | L | V | F | L | E | P | I | S | E | - | - | - | - | R | Q | M | S | S | Y | H | R | M | R | K | V | M | L | K | K | T | Y | L | Q | W | P | G | S | D | C | D | D | P | E | P | A | Q | D | L | F | W | N | K | L | R | R | A | M | R | - | - | - | - | - | T | G | S | R | V | X | X | X | X | X | X | X | X | K | T | E | E | S | D | K | S | E | G | C | V | T | D | E | - | - | S | K | D | N | E | I | S | X | X | X | X | X | D | X | X | X | X | N | Y | Y | L | L | P | X | X | X | X | X | X | - | - | - | - | - | - |
| PFTLR13\_3900\_BP | X | X | X | X | X | X | X | X | K | X | - | X | X | X | - | - | X | X | X | X | X | - | - | - | N | L | S | A | V | V | X | X | X | X | X | X | X | X | X | X | X | X | X | X | X | X | X | X | X | X | X | X | X | - | - | X | X | X | X | X | X | X | X | X | X | X | X | X | X | X | X | X | X | X | X | X | X | X | X | X | X | X | X | X | X | X | X | X | X | X | X | X | X | Y | S | F | S | W | X | X | X | X | X | X | - | - | - | - | - | X | X | X | L | F | - | - | N | - | - | - | - | - | - | - | - | - | - | - | - | - | S | S | V | G | T | A | - | - | - | - | - | X | X | X | X | X | X | X | X | X | N | N | C | I | E | X | X | X | X | - | - | - | P | I | L | R | R | X | X | X | X | X | X | X | X | X | - | - | X | X | Q | S | F | N | - | - | - | - | - | - | - | - | - | - | C | X | X | X | X | X | X | X | X | X | P | S | E | G | K | E | H | V | C | Y | Y | K | G | S | A | A | I | C | C | X | P | Q | H | L | L | X | - | S | S | V | A | X | X | X | S | - | - | - | - | - | - | - | - | - | - | - | - | - | - | - | I | Q | E | L | - | C | S | S | - | - | T | K | P | S | T | P | X | N | R | - | - | - | - | X | Q | S | L | E | D | H | R | S | V | C | F | P | K | L | K | S | X | X | T | Q | V | S | E | F | V | - | - | - | - | - | - | - | - | L | X | X | X | X | X | Q | H | L | R | X | X | X | S | S | L | L | C | F | R | - | T | C | T | T | S | P | I | S | R | X | Q | T | I | H | X | S | S | S | P | R | A | F | S | P | L | F | - | - | - | S | T | W | T | R | X | X | X | D | K | T | F | X | Q | T | S | Q | V | L | L | S | L | F | - | H | X | X | X | - | Q | T | X | K | Y | X | X | - | - | - | - | X | X | X | X | X | X | - | - | X | X | - | L | L | X | - | - | - | Q | - | - | - | F | N | L | S | Q | P | L | K | C | V | T | T | R | X | X | X | X | X | X | X | X | X | X | X | X | H | Y | I | I | F | M | X | X | - | - | X | X | X | X | X | X | X | X | X | X | X | N | N | L | L | - | - | - | - | - | - | - | T | X | X | X | X | X | - | X | X | X | X | R | M | X | A | F | V | S | - | - | - | - | - | - | - | - | - | - | R | - | F | H | P | A | A | R | F | V | I | Q | X | X | X | X | X | X | X | X | - | - | - | - | - | - | - | - | - | - | - | - | X | X | X | X | X | X | X | X | L | - | - | - | - | - | - | - | - | - | G | S | P | R | W | L | X | - | - | - | - | - | - | - | - | - | - | - | - | - | X | X | X | R | S | E | X | X | X | - | X | X | X | X | X | X | X | X | X | X | X | E | S | Y | - | - | - | - | - | - | - | - | - | - | K | L | S | T | - | - | - | F | A | F | N | - | - | - | - | - | - | - | - | - | - | - | - | - | - | - | - | - | - | - | - | - | - | K | C | Q | C | S | G | V | X | X | X | X | L | N | I | - | - | - | - | - | - | - | - | - | - | - | - | - | - | - | - | - | - | - | - | - | S | N | V | N | T | G | H | I | D | F | X | X | X | L | A | R | V | X | - | - | - | - | - | - | - | - | - | - | - | - | - | - | - | - | - | - | - | - | - | - | K | M | S | T | C | S | R | S | - | - | Y | A | X | X | X | X | X | X | S | L | K | R | K | V | K | K | I | K | K | L | G | L | G | N | N | G | I | K | N | L | T | K | N | A | L | S | Y | C | P | N | I | T | E | X | X | X | X | X | X | F | G | S | V | P | Q | Q | P | X | - | - | - | - | - | X | K | A | Q | V | V | L | T | F | S | I | N | X | D | T | - | - | - | N | K | K | L | Q | C | R | A | X | P | S | H | Q | T | P | I | L | X | N | - | - | - | - | T | K | Y | G | X | F | R | - | - | T | S | G | R | A | E | L | S | L | Q | P | H | P | L | S | Q | L | L | C | F | L | X | - | - | - | - | - | - | - | - | - | - | - | - | - | - | - | - | - | - | - | - | - | - | - | - | - | - | - | - | - | Y | X | X | X | X | X | X | K | Y | Q | D | A | K | A | X | H | K | H | N | C | F | P | X | X | X | X | S | K | S | S | Q | R | A | X | T | - | A | N | N | S | P | I | G | X | X | P | F | N | X | F | V | H | X | H | V | X | R | - | - | - | - | Q | C | Q | P | A | N | P | X | P | T | Q | Q | S | N | F | C | H | F | X | R | D | - | - | - | - | L | P | Q | S | Q | Q | I | - | - | - | - | - | - | - | - | - | - | - | - | - | - | - | - | - | - | - | - | - | D | Y | T | R | - | - | - | - | - | - | L | R | R | X | Q | D | H | V | F | P | A | - | - | I | W | P | X | W | T | K | K | S | V | Q | X | X | X | X | X | X | X | X | X | X | X | S | I | X | M | E | T | T | S | N | R | L | X | X | X | T | S | F | Y | H | V | F | Q | D | X | X | X | X | X | X | X | X | X | X | X | - | - | - | - | - | - | - | - | - | - | - | X | L | T | V | L | D | L | Q | S | N | Q | I | R | F | L | X | X | A | E | I | R | - | - | - | X | X | X | X | X | X | X | P | F | M | N | X | X | X | X | X | L | S | K | L | T | D | L | K | L | D | G | Q | R | P | H | G | L | T | X | X | V | L | P | R | N | F | F | H | G | L | H | S | L | K | S | L | Y | L | T | N | N | M | I | N | Y | L | A | P | D | V | F | D | D | L | T | G | L | H | F | L | T | L | D | N | C | C | V | G | V | T | Q | X | X | X | X | H | P | G | V | - | - | - | F | K | N | L | K | N | L | T | K | L | I | V | E | N | M | G | I | X | X | X | N | I | S | K | E | V | X | F | G | N | X | X | X | X | X | T | Q | L | H | T | L | Q | L | N | R | N | V | M | Q | S | I | D | V | D | A | L | E | T | L | P | K | L | H | Y | L | D | I | R | N | I | P | L | S | C | T | C | K | N | S | L | L | Q | N | W | T | V | H | N | P | H | V | Q | V | X | V | Y | L | H | X | X | X | X | X | L | R | C | P | H | X | X | X | D | E | - | - | K | V | K | F | Y | N | F | D | T | K | V | C | Y | K | D | L | G | E | Y | M | F | I | S | T | A | V | V | I | F | L | F | T | V | T | P | L | L | H | V | K | L | Y | W | K | X | X | X | X | X | V | R | L | L | X | X | V | F | R | S | W | F | S | E | R | W | R | R | L | X | X | R | R | G | G | K | X | X | X | X | X | X | X | X | X | X | X | K | Y | D | A | F | I | S | Y | N | S | S | D | E | K | W | V | M | - | - | - | - | - | - | - | - | - | - | - | - | - | - | - | - | - | - | - | - | - | E | E | L | V | P | N | L | E | G | K | E | - | - | - | - | - | - | - | - | - | - | - | - | - | - | X | X | X | S | S | F | K | L | C | L | H | H | R | D | F | E | L | G | R | D | I | V | D | N | I | V | S | A | V | Y | S | S | R | K | T | I | C | V | V | S | R | N | F | L | R | S | E | W | C | S | L | E | I | Q | L | A | S | Y | R | L | F | D | E | H | R | D | I | L | L | L | V | F | L | E | P | I | S | E | - | - | - | - | R | Q | V | S | S | Y | H | R | M | R | K | V | M | L | K | K | T | Y | L | Q | W | P | G | S | D | C | T | N | P | T | Q | A | Q | E | L | F | W | Y | H | L | R | R | A | M | R | - | - | - | - | - | T | G | S | R | L | X | X | X | X | X | X | X | X | N | T | E | E | N | Y | K | S | K | G | Q | X | X | X | X | - | - | X | X | G | T | E | H | F | E | T | Q | L | S | D | X | X | X | X | N | Y | Y | L | Q | P | X | X | X | X | X | X | - | - | - | - | - | - |
| LCTLR13\_3900\_BP | - | X | X | X | X | X | X | X | X | W | X | X | X | X | - | - | X | X | X | X | X | - | - | - | N | S | S | V | V | V | X | X | X | X | X | X | X | X | X | X | X | X | X | X | X | X | X | X | X | X | X | X | X | - | - | X | X | X | X | X | X | X | X | X | X | X | X | X | X | X | X | X | X | X | X | X | X | X | X | X | X | X | X | X | X | X | X | X | X | X | X | X | X | Y | S | S | S | C | X | X | X | X | X | X | - | - | - | - | - | X | X | X | P | F | - | - | N | - | - | - | - | - | - | - | - | - | - | - | - | - | S | S | V | V | T | A | - | - | - | - | - | X | X | X | X | X | X | X | X | X | D | N | C | I | E | X | X | X | X | - | - | - | P | V | L | K | C | X | X | X | X | X | X | X | X | X | - | - | X | X | T | C | F | N | - | - | - | - | - | - | - | - | - | - | C | X | X | X | X | X | X | X | X | X | Q | S | K | E | K | E | H | V | Y | H | Y | T | X | P | A | A | I | C | H | Q | S | H | Y | L | H | E | - | X | G | G | V | X | X | X | S | - | - | - | - | - | - | - | - | - | - | - | - | - | - | - | Q | R | Q | L | - | C | S | S | - | - | T | R | A | S | I | P | Q | T | X | - | - | - | - | P | K | P | L | S | D | H | R | S | I | C | F | Q | N | V | A | S | X | X | T | S | V | S | K | F | V | - | - | - | - | - | - | - | - | C | X | X | X | X | X | Q | H | N | R | X | X | X | S | T | L | L | C | F | R | - | T | F | T | T | S | P | F | S | H | W | Q | T | I | N | X | S | S | F | L | W | A | F | S | L | P | L | - | - | - | P | N | X | T | P | X | S | X | G | K | T | F | X | Q | I | S | Q | G | S | L | S | L | C | - | H | X | X | Y | - | X | I | X | G | Y | X | X | - | - | - | - | X | X | X | X | X | X | - | - | X | X | - | L | L | X | - | - | - | Q | - | - | - | F | G | L | P | X | S | L | K | C | V | A | T | Q | X | X | X | X | X | X | X | X | X | X | X | X | H | Y | F | I | Y | M | X | X | - | - | X | X | X | X | X | X | X | X | X | X | X | N | N | L | S | - | - | - | - | - | - | - | T | X | X | X | X | X | - | X | X | X | X | R | M | R | T | F | I | S | - | - | - | - | - | - | - | - | - | - | W | - | I | P | Q | G | A | X | F | V | R | Q | X | X | X | X | X | X | X | X | - | - | - | - | - | - | - | - | - | - | - | - | X | X | X | X | X | X | X | X | L | - | - | - | - | - | - | - | - | - | G | S | L | R | W | L | X | - | - | - | - | - | - | - | - | - | - | - | - | - | X | X | X | R | S | G | X | X | X | - | X | X | X | X | X | X | X | X | X | X | X | E | T | Y | - | - | - | - | - | - | - | - | - | - | K | L | S | A | - | - | - | F | A | F | D | - | - | - | - | - | - | - | - | - | - | - | - | - | - | - | - | - | - | - | - | - | - | K | C | Q | G | R | G | V | X | X | X | X | L | N | I | - | - | - | - | - | - | - | - | - | - | - | - | - | - | - | - | - | - | - | - | - | S | N | V | K | A | G | A | V | D | F | X | X | X | L | A | R | V | X | - | - | - | - | - | - | - | - | - | - | - | - | - | - | - | - | - | - | - | - | - | - | K | T | T | A | C | X | W | S | - | - | Y | A | X | X | X | X | X | X | L | L | K | K | K | V | R | W | I | K | N | L | H | L | G | M | N | G | I | E | K | L | T | N | Y | T | L | Y | Y | C | P | K | I | K | G | X | X | X | X | X | X | S | G | S | I | P | Q | X | T | X | - | - | - | - | - | X | K | A | X | V | V | L | T | F | S | I | D | X | D | R | - | - | - | Y | K | K | L | Q | C | R | T | X | P | S | H | L | P | P | I | L | X | N | - | - | - | - | T | K | Y | G | X | F | T | - | - | K | S | G | R | A | V | L | S | L | Q | P | H | P | L | S | G | S | S | C | F | L | S | - | - | - | - | - | - | - | - | - | - | - | - | - | - | - | - | - | - | - | - | - | - | - | - | - | - | - | - | - | Y | X | X | X | X | X | X | K | C | Q | D | T | K | T | X | H | K | L | N | S | L | S | X | X | X | X | S | R | S | S | Q | R | A | K | K | - | A | X | D | T | P | V | G | Q | X | P | L | D | X | F | V | P | X | N | L | X | R | - | - | - | - | K | F | X | T | A | K | P | X | P | T | Q | Q | S | C | F | C | H | F | Q | X | D | - | - | - | - | L | P | Q | S | X | Q | S | - | - | - | - | - | - | - | - | - | - | - | - | - | - | - | - | - | - | - | - | - | E | C | I | G | - | - | - | - | - | - | P | R | R | X | X | D | H | S | H | X | A | - | - | I | R | P | X | W | T | K | K | S | V | Q | X | X | X | X | X | X | X | X | X | X | X | S | I | X | M | E | T | T | S | N | R | S | X | X | X | S | S | H | Y | H | V | F | Q | D | X | X | X | X | X | X | X | X | X | X | X | - | - | - | - | - | - | - | - | - | - | - | X | L | T | V | L | D | L | Q | S | N | K | I | S | F | L | X | X | E | N | M | E | - | - | - | X | X | X | X | X | X | X | P | F | M | N | X | X | X | X | X | L | S | K | L | S | D | L | K | L | D | G | Q | R | P | H | G | M | T | X | X | I | L | P | R | T | L | F | R | G | L | Y | S | L | K | F | L | Y | L | T | N | N | K | I | F | Y | L | A | P | D | T | F | D | D | L | T | G | L | R | F | L | T | L | D | T | C | C | V | G | V | T | Q | X | X | X | X | Q | P | G | V | - | - | - | F | K | N | L | R | N | L | T | A | L | A | A | E | N | M | G | I | X | X | X | N | F | S | K | E | V | X | F | G | N | X | X | X | X | X | T | Q | L | R | K | L | Q | L | N | R | N | A | L | Q | S | M | D | V | D | A | L | Q | S | L | P | K | L | L | Y | L | D | I | R | S | L | P | L | S | C | T | C | Q | N | S | L | L | Q | N | W | T | R | N | N | Q | N | V | Q | V | X | V | Y | L | Y | X | X | X | X | X | L | P | C | Q | H | X | X | X | N | A | - | - | K | S | N | F | Y | N | F | D | T | K | V | C | Y | I | D | L | G | E | Y | L | F | F | S | T | A | V | V | I | F | L | F | T | V | T | P | L | L | Y | V | K | L | Y | W | K | X | X | X | X | E | V | Q | L | L | X | X | V | F | R | S | W | F | S | E | Q | W | R | R | L | X | X | R | R | G | G | K | X | X | X | X | X | X | X | X | X | X | X | K | Y | D | A | F | I | S | Y | N | S | S | D | E | Q | W | V | M | - | - | - | - | - | - | - | - | - | - | - | - | - | - | - | - | - | - | - | - | - | D | Q | L | V | P | N | M | E | G | N | X | - | - | - | - | - | - | - | - | - | - | - | - | - | - | X | X | X | X | S | F | K | L | C | L | H | H | R | D | F | E | L | G | R | D | I | V | D | N | I | V | S | A | V | Y | S | S | R | K | T | I | C | V | V | S | R | N | F | L | Q | S | E | W | C | S | L | E | I | Q | L | A | S | Y | R | L | F | D | E | H | R | D | V | L | L | L | V | F | L | E | P | I | S | E | - | - | - | - | R | Q | L | S | S | Y | H | R | M | R | K | V | M | L | K | K | T | Y | L | Q | W | P | G | S | D | C | T | D | P | V | Q | A | Q | E | L | F | W | N | Q | L | H | R | A | M | R | - | - | - | - | - | T | G | T | R | V | X | X | X | X | X | X | X | X | X | I | E | G | N | V | I | S | K | D | C | V | T | T | K | - | - | S | E | E | T | E | H | F | E | X | X | X | S | D | X | X | X | X | N | Y | Y | L | L | P | X | X | X | X | X | X | - | - | - | - | - | - |
| SLTLR13\_3900\_BP | - | X | X | X | X | X | X | X | X | W | X | X | X | X | - | - | X | X | X | X | X | - | - | - | T | S | S | A | V | V | X | X | X | X | X | X | X | X | X | X | X | X | X | X | X | X | X | X | X | X | X | X | X | - | - | X | X | X | X | X | X | X | X | X | X | X | X | X | X | X | X | X | X | X | X | X | X | X | X | X | X | X | X | X | X | X | X | X | X | X | X | X | X | Y | S | C | S | W | X | X | X | X | X | X | - | - | - | - | - | X | X | X | L | F | - | - | N | - | - | - | - | - | - | - | - | - | - | - | - | - | S | X | L | V | T | A | - | - | - | - | - | X | X | X | X | X | X | X | X | X | K | N | C | I | A | X | X | X | X | - | - | - | P | T | L | K | W | X | X | X | X | X | X | X | X | X | - | - | X | X | S | V | F | N | - | - | - | - | - | - | - | - | - | - | C | X | X | X | X | X | X | X | X | X | Q | S | K | G | R | E | D | V | C | Y | F | T | X | P | A | N | I | C | H | P | S | H | H | L | Q | D | - | S | C | E | A | X | X | X | L | - | - | - | - | - | - | - | - | - | - | - | - | - | - | - | Q | L | Q | L | - | C | S | S | - | - | T | K | A | X | I | P | K | N | R | - | - | - | - | R | X | P | R | E | Y | H | R | S | I | C | F | P | K | L | A | S | X | X | T | S | V | S | K | F | I | - | - | - | - | - | - | - | - | C | X | X | X | X | X | Q | H | I | R | X | X | X | X | T | L | I | C | L | R | - | N | F | T | I | S | P | F | C | C | C | Q | T | I | N | X | S | S | S | L | R | A | F | S | L | P | L | - | - | - | S | I | X | T | L | X | T | X | G | K | T | F | X | Q | I | S | Q | R | S | L | S | L | C | - | H | X | X | X | - | X | I | X | K | Y | X | X | - | - | - | - | X | X | X | X | X | X | - | - | X | X | - | L | L | X | - | - | - | Q | - | - | - | F | D | L | S | Q | P | L | K | C | V | A | T | Q | X | X | X | X | X | X | X | X | X | X | X | X | H | Y | F | I | Y | M | X | X | - | - | X | X | X | X | X | X | X | X | X | X | X | N | N | L | F | - | - | - | - | - | - | - | T | X | X | X | X | X | - | X | X | X | X | R | M | X | X | F | I | S | - | - | - | - | - | - | - | - | - | - | H | - | V | H | Q | H | T | R | F | V | I | X | X | X | X | X | X | X | X | X | - | - | - | - | - | - | - | - | - | - | - | - | X | X | X | X | X | X | X | X | L | - | - | - | - | - | - | - | - | - | G | S | L | R | Q | L | X | - | - | - | - | - | - | - | - | - | - | - | - | - | X | X | X | R | S | G | X | X | X | - | X | X | X | X | X | X | X | X | X | X | X | E | T | Y | - | - | - | - | - | - | - | - | - | - | K | L | S | A | - | - | - | F | A | F | N | - | - | - | - | - | - | - | - | - | - | - | - | - | - | - | - | - | - | - | - | - | - | K | C | Q | R | S | G | V | X | X | X | X | L | N | I | - | - | - | - | - | - | - | - | - | - | - | - | - | - | - | - | - | - | - | - | - | S | N | V | K | V | S | Q | V | D | F | X | X | X | L | A | W | V | X | - | - | - | - | - | - | - | - | - | - | - | - | - | - | - | - | - | - | - | - | - | - | K | M | T | A | C | S | W | S | - | - | Y | A | X | X | X | X | X | X | L | L | M | R | K | V | K | W | I | K | N | L | N | L | S | D | N | G | I | G | S | L | T | N | Y | T | L | Y | Y | C | P | N | I | T | G | X | X | X | X | X | X | S | G | S | I | P | Q | Q | T | X | - | - | - | - | - | X | K | A | Q | V | V | L | A | F | S | E | D | X | G | A | - | - | - | D | K | K | L | Q | R | R | A | X | P | S | H | L | P | P | I | L | X | Q | - | - | - | - | T | N | Q | G | L | F | K | - | - | K | S | G | R | A | E | L | S | L | Q | P | H | S | L | S | E | F | L | C | F | L | S | - | - | - | - | - | - | - | - | - | - | - | - | - | - | - | - | - | - | - | - | - | - | - | - | - | - | - | - | - | Y | X | X | X | X | X | X | K | Y | Q | D | A | T | T | X | H | K | H | N | C | F | S | X | X | X | X | S | X | S | S | Q | R | A | E | K | - | A | C | E | T | P | F | G | Q | X | P | L | N | X | F | V | Q | X | D | F | X | R | - | - | - | - | Q | X | Q | T | T | S | P | X | L | T | Q | Q | S | Y | F | C | H | F | Q | W | D | - | - | - | - | L | P | H | A | S | E | S | - | - | - | - | - | - | - | - | - | - | - | - | - | - | - | - | - | - | - | - | - | D | N | I | G | - | - | - | - | - | - | P | G | R | X | X | D | H | S | F | S | A | - | - | I | R | F | X | W | T | K | K | S | V | Q | X | X | X | X | X | X | X | X | X | X | X | S | I | X | M | E | T | T | S | N | T | L | X | X | X | T | S | I | Y | R | V | F | Q | D | X | X | X | X | X | X | X | X | X | X | X | - | - | - | - | - | - | - | - | - | - | - | X | L | T | V | L | D | L | Q | S | N | K | I | R | F | L | X | X | E | N | N | S | - | - | - | X | X | X | X | X | X | X | P | F | M | N | X | X | X | X | X | L | S | K | L | S | D | L | K | L | D | G | Q | R | P | Y | G | M | T | X | X | V | L | P | H | T | F | F | R | G | L | Y | S | L | K | S | L | Y | L | T | N | N | N | I | F | H | L | P | P | D | V | F | D | D | L | T | S | L | Q | F | L | T | L | D | K | C | C | V | G | V | T | Q | X | X | X | X | V | P | G | V | - | - | - | F | K | N | L | R | N | L | T | K | L | T | V | E | S | M | G | I | X | X | X | N | F | S | K | E | V | X | F | G | N | X | X | X | X | X | I | Q | L | S | S | L | Q | L | N | R | N | V | M | Q | S | I | H | V | D | A | L | Q | S | L | P | K | L | R | Y | L | D | I | R | N | I | P | L | S | C | T | C | K | N | S | L | L | Q | N | W | T | I | Y | N | K | N | V | Q | V | X | V | Y | L | Y | X | X | X | X | X | L | Q | C | Q | N | X | X | X | D | A | - | - | N | Y | K | F | H | N | F | K | T | N | V | C | Y | I | D | L | G | E | Y | L | F | F | S | T | A | V | V | I | F | L | F | T | V | T | P | L | L | Y | V | K | L | Y | W | K | X | X | X | X | E | V | Q | L | L | X | X | V | F | R | S | W | F | S | D | Q | W | R | R | L | X | X | G | G | G | G | K | X | X | X | X | X | X | X | X | X | X | X | K | Y | D | A | F | I | S | Y | N | S | S | D | D | Q | W | V | M | - | - | - | - | - | - | - | - | - | - | - | - | - | - | - | - | - | - | - | - | - | D | Q | L | L | P | N | L | E | G | N | G | - | - | - | - | - | - | - | - | - | - | - | - | - | - | X | X | X | S | S | F | K | L | C | L | H | H | R | D | F | E | L | G | R | D | I | V | D | N | I | V | S | A | V | Y | S | S | R | K | T | I | C | V | V | S | R | N | F | L | Q | S | E | W | C | S | L | E | I | Q | L | A | S | Y | R | L | F | D | E | H | R | D | V | L | L | L | V | F | L | E | P | I | S | E | - | - | - | - | R | Q | L | S | A | Y | H | R | M | R | K | V | M | L | K | K | T | Y | L | Q | W | P | D | S | D | C | T | N | P | M | Q | A | Q | E | L | F | W | N | Q | L | R | R | A | M | R | - | - | - | - | - | M | G | S | R | L | X | X | X | X | X | X | X | X | K | M | E | E | N | I | N | N | E | G | C | V | N | A | E | - | - | S | E | E | T | E | P | F | E | X | X | X | S | D | X | X | X | X | E | Y | Y | L | L | P | X | X | X | X | X | X | - | - | - | - | - | - |
| SLTLR21\_13\_3900\_BP | - | X | X | X | X | X | X | X | X | W | X | X | X | X | - | - | X | X | X | X | X | - | - | - | T | S | S | A | V | V | X | X | X | X | X | X | X | X | X | X | X | X | X | X | X | X | X | X | X | X | X | X | X | - | - | X | X | X | X | X | X | X | X | X | X | X | X | X | X | X | X | X | X | X | X | X | X | X | X | X | X | X | X | X | X | X | X | X | X | X | X | X | X | Y | S | C | S | W | X | X | X | X | X | X | - | - | - | - | - | X | X | X | L | F | - | - | N | - | - | - | - | - | - | - | - | - | - | - | - | - | S | X | L | V | T | A | - | - | - | - | - | X | X | X | X | X | X | X | X | X | K | N | C | I | A | X | X | X | X | - | - | - | P | T | L | K | W | X | X | X | X | X | X | X | X | X | - | - | X | X | S | L | F | N | - | - | - | - | - | - | - | - | - | - | C | X | X | X | X | X | X | X | X | X | Q | S | K | G | R | E | D | V | C | Y | F | T | X | P | A | N | I | C | H | P | S | H | H | L | Q | D | - | S | C | E | A | X | X | X | L | - | - | - | - | - | - | - | - | - | - | - | - | - | - | - | Q | L | Q | L | - | C | S | S | - | - | T | K | A | X | I | P | K | N | R | - | - | - | - | R | X | P | R | E | Y | H | R | S | I | C | F | P | K | L | A | S | X | X | T | S | V | S | K | F | I | - | - | - | - | - | - | - | - | C | X | X | X | X | X | Q | H | I | R | X | X | X | X | T | L | I | C | L | R | - | N | F | T | I | S | P | F | C | C | C | Q | T | I | N | X | S | S | S | L | R | A | F | S | L | P | L | - | - | - | S | I | X | T | L | X | T | X | G | K | T | F | X | Q | I | S | Q | R | S | L | S | L | C | - | H | X | X | X | - | X | I | X | K | Y | X | X | - | - | - | - | X | X | X | X | X | X | - | - | X | X | - | L | L | X | - | - | - | Q | - | - | - | F | D | L | S | Q | P | L | K | C | V | A | T | Q | X | X | X | X | X | X | X | X | X | X | X | X | H | Y | F | I | Y | M | X | X | - | - | X | X | X | X | X | X | X | X | X | X | X | N | N | L | F | - | - | - | - | - | - | - | T | X | X | X | X | X | - | X | X | X | X | R | M | X | R | F | I | S | - | - | - | - | - | - | - | - | - | - | H | - | V | H | Q | H | T | R | F | V | I | X | X | X | X | X | X | X | X | X | - | - | - | - | - | - | - | - | - | - | - | - | X | X | X | X | X | X | X | X | L | - | - | - | - | - | - | - | - | - | G | S | L | R | Q | L | X | - | - | - | - | - | - | - | - | - | - | - | - | - | X | X | X | R | S | G | X | X | X | - | X | X | X | X | X | X | X | X | X | X | X | E | T | Y | - | - | - | - | - | - | - | - | - | - | K | L | S | A | - | - | - | F | A | F | N | - | - | - | - | - | - | - | - | - | - | - | - | - | - | - | - | - | - | - | - | - | - | K | C | Q | R | S | G | V | X | X | X | X | L | N | I | - | - | - | - | - | - | - | - | - | - | - | - | - | - | - | - | - | - | - | - | - | S | N | V | K | V | S | Q | V | D | F | X | X | X | L | A | W | V | X | - | - | - | - | - | - | - | - | - | - | - | - | - | - | - | - | - | - | - | - | - | - | K | M | T | A | C | S | W | S | - | - | Y | A | X | X | X | X | X | X | L | L | M | R | K | V | K | W | I | K | N | L | N | L | S | D | N | G | I | G | S | L | T | N | Y | T | L | Y | Y | C | P | N | I | T | G | X | X | X | X | X | X | S | G | S | I | P | Q | Q | T | X | - | - | - | - | - | X | K | A | Q | I | V | L | A | F | S | E | D | X | G | A | - | - | - | D | K | K | L | Q | R | R | A | X | P | S | H | L | P | P | I | L | X | Q | - | - | - | - | T | N | Q | G | L | F | K | - | - | K | S | G | R | A | E | L | S | L | Q | P | H | S | L | S | E | F | L | C | F | L | S | - | - | - | - | - | - | - | - | - | - | - | - | - | - | - | - | - | - | - | - | - | - | - | - | - | - | - | - | - | Y | X | X | X | X | X | X | K | Y | Q | D | A | T | T | X | H | K | H | N | C | F | S | X | X | X | X | S | X | S | S | Q | R | A | E | K | - | A | C | E | T | P | F | G | Q | X | P | L | N | X | F | V | Q | X | D | F | X | R | - | - | - | - | Q | X | Q | T | T | S | P | X | L | T | Q | Q | S | Y | F | C | H | F | Q | W | D | - | - | - | - | L | P | H | A | S | E | S | - | - | - | - | - | - | - | - | - | - | - | - | - | - | - | - | - | - | - | - | - | D | N | I | G | - | - | - | - | - | - | P | G | R | X | X | D | H | S | F | S | A | - | - | I | R | F | X | W | T | K | K | S | V | Q | X | X | X | X | X | X | X | X | X | X | X | S | I | X | M | E | T | T | S | N | T | L | X | X | X | T | S | I | Y | R | V | F | Q | D | X | X | X | X | X | X | X | X | X | X | X | - | - | - | - | - | - | - | - | - | - | - | X | L | T | V | L | D | L | Q | S | N | K | I | R | F | L | X | X | E | N | N | S | - | - | - | X | X | X | X | X | X | X | P | F | M | N | X | X | X | X | X | L | S | K | L | S | D | L | K | L | D | G | Q | R | P | Y | G | M | T | X | X | V | L | P | H | T | F | F | R | G | L | Y | S | L | K | S | L | Y | L | T | N | N | N | I | F | H | L | P | P | D | V | F | D | D | L | T | S | L | Q | F | L | T | L | D | K | C | C | V | G | V | T | Q | X | X | X | X | V | P | G | V | - | - | - | F | K | N | L | R | N | L | T | K | L | T | V | E | S | M | G | I | X | X | X | N | F | S | K | E | V | X | F | G | N | X | X | X | X | X | I | Q | L | S | S | L | Q | L | N | R | N | V | M | Q | S | I | H | V | D | A | L | Q | S | L | P | K | L | R | Y | L | D | I | R | N | I | P | L | S | C | T | C | K | N | S | L | L | Q | N | W | T | I | Y | N | K | N | V | Q | V | X | V | Y | L | Y | X | X | X | X | X | L | Q | C | Q | N | X | X | X | D | A | - | - | N | Y | K | F | H | N | F | K | T | N | V | C | Y | I | D | L | G | E | Y | L | F | F | S | T | A | V | V | I | F | L | F | T | V | T | P | L | L | Y | V | K | L | Y | W | K | X | X | X | X | E | V | Q | L | L | X | X | V | F | R | S | W | F | S | D | Q | W | R | R | L | X | X | G | G | G | G | K | X | X | X | X | X | X | X | X | X | X | X | K | Y | D | A | F | I | S | Y | N | S | S | D | D | Q | W | V | M | - | - | - | - | - | - | - | - | - | - | - | - | - | - | - | - | - | - | - | - | - | D | Q | L | L | P | N | L | E | G | N | G | - | - | - | - | - | - | - | - | - | - | - | - | - | - | X | X | X | S | S | F | K | L | C | L | H | H | R | D | F | E | L | G | R | D | I | V | D | N | I | V | S | A | V | Y | S | S | R | K | T | I | C | V | V | S | R | N | F | L | Q | S | E | W | C | S | L | E | I | Q | L | A | S | Y | R | L | F | D | E | H | R | D | V | L | L | L | V | F | L | E | P | I | S | E | - | - | - | - | R | Q | L | S | A | Y | H | R | M | R | K | V | M | L | K | K | T | Y | L | Q | W | P | D | S | D | C | T | N | P | M | Q | A | Q | E | L | F | W | N | Q | L | R | R | A | M | R | - | - | - | - | - | M | G | S | R | L | X | X | X | X | X | X | X | X | K | M | E | E | N | I | N | N | E | G | C | V | N | A | E | - | - | S | E | E | T | E | P | F | E | X | X | X | S | D | X | X | X | X | E | Y | Y | L | L | P | X | X | X | X | X | X | - | - | - | - | - | - |
| LMTLR13\_3900\_BP | - | - | - | X | X | X | X | X | C | W | Q | R | R | R | G | D | Q | G | C | R | E | L | V | Q | F | T | X | - | A | V | Q | V | R | S | K | P | G | D | Q | A | N | K | S | E | K | V | Q | N | - | - | - | - | - | - | - | R | L | F | R | K | N | R | I | Q | Y | - | - | - | - | A | G | Q | N | T | Q | D | N | V | A | E | X | L | W | T | N | S | K | K | L | R | Q | G | R | S | X | S | S | P | C | Y | V | T | P | A | S | P | P | S | F | C | L | C | C | F | I | L | T | - | - | - | - | - | - | - | - | - | - | - | - | - | P | H | W | L | T | H | - | - | - | - | - | X | X | X | X | X | X | X | X | X | K | N | C | T | V | X | X | X | X | - | - | - | X | V | F | C | X | X | X | X | X | X | X | X | X | X | X | X | V | A | S | V | S | X | X | X | X | X | X | X | X | X | Y | M | H | X | S | X | P | H | - | - | - | - | - | - | - | - | - | - | - | - | C | H | S | X | X | R | S | X | K | C | D | F | S | R | S | Q | L | Q | X | P | F | K | X | X | - | X | Q | - | - | - | - | - | - | - | - | - | - | - | - | - | - | - | Q | N | R | F | - | K | R | V | - | - | I | K | A | D | L | F | R | N | - | - | - | - | R | I | K | F | N | F | S | H | X | X | W | S | F | C | R | L | G | X | X | X | V | N | R | P | X | Y | T | - | - | - | - | - | - | - | - | L | X | X | X | X | X | X | P | H | K | X | X | X | X | Q | T | T | C | F | R | - | D | C | P | N | X | X | G | C | L | X | A | G | T | K | S | Q | T | S | P | R | W | P | S | S | P | S | S | A | Y | R | K | X | N | X | T | X | T | T | C | I | - | - | T | - | L | L | I | L | C | L | Y | Y | - | X | X | X | Y | Q | T | Y | T | N | X | X | X | - | - | - | - | X | X | X | X | X | X | - | - | X | G | - | I | K | H | - | - | - | V | H | I | F | X | X | L | G | R | P | A | V | X | X | X | X | X | - | - | X | X | X | X | X | X | K | P | Q | N | A | S | V | X | P | X | X | X | - | - | - | - | X | X | X | X | X | X | X | X | X | N | P | L | R | - | - | - | - | - | - | - | K | X | X | X | X | X | X | X | X | X | Q | H | H | E | R | R | L | S | - | - | - | - | - | - | - | - | - | - | - | - | P | S | S | V | Y | G | F | V | Q | X | X | X | X | X | X | X | X | X | - | - | - | X | - | - | X | X | - | - | - | - | - | - | - | X | X | X | X | X | X | - | - | X | M | Q | L | W | - | L | X | X | X | X | W | D | V | - | - | - | - | - | - | - | - | - | - | - | - | - | X | X | X | R | X | D | L | X | X | - | X | X | X | X | X | - | K | E | L | D | X | F | V | F | - | - | - | - | - | - | - | - | - | - | K | W | N | X | - | - | - | N | K | F | X | - | - | - | - | - | - | - | - | - | - | - | - | - | - | - | - | - | - | - | - | - | - | N | V | Q | N | D | A | S | E | - | - | - | - | - | - | - | - | - | - | - | - | - | - | - | - | - | - | - | - | - | - | - | - | - | - | - | L | G | V | C | G | V | S | L | A | E | L | X | X | X | X | X | K | N | - | G | S | I | - | - | - | - | - | - | - | - | - | - | - | - | - | - | - | - | - | - | - | - | K | V | X | S | T | S | - | - | P | V | R | F | L | X | X | X | X | X | X | X | X | X | X | X | L | T | S | L | D | L | T | F | N | N | I | Y | V | L | N | D | T | L | L | Q | P | C | S | K | I | T | E | X | X | X | X | X | X | X | G | F | S | X | X | X | X | X | - | - | - | - | - | X | D | D | R | P | V | X | E | F | T | Q | T | I | E | S | - | - | - | A | R | A | T | E | I | T | S | Q | L | S | T | Q | S | A | - | - | - | - | A | C | H | Q | M | X | X | X | X | P | L | - | - | H | T | X | S | P | L | P | E | L | Q | X | Y | W | R | V | R | L | L | X | L | H | E | - | - | - | - | - | - | - | - | - | - | - | - | - | - | - | - | - | - | - | - | - | - | - | - | - | - | - | - | - | F | X | X | X | X | X | X | K | I | N | A | T | L | S | R | Q | Q | S | H | F | K | T | X | X | X | X | R | M | C | F | S | R | L | E | E | - | F | K | I | T | L | C | X | R | K | S | N | F | X | I | V | X | Y | L | Q | C | K | - | - | - | - | F | A | E | P | P | H | F | E | Y | E | Q | E | X | F | X | G | T | P | E | R | R | - | - | - | - | L | W | D | S | V | F | P | L | D | F | - | - | - | - | - | - | - | - | - | - | - | - | - | - | - | - | - | - | - | - | - | G | - | - | - | - | - | - | F | R | V | T | V | R | L | P | S | G | X | - | - | W | D | F | X | G | T | X | S | S | P | N | X | X | X | X | X | X | X | X | X | X | X | L | S | F | H | H | T | L | S | G | K | T | X | X | X | X | X | X | F | R | G | L | P | H | X | X | X | X | X | X | X | X | X | X | X | - | - | - | - | - | - | - | - | - | - | - | X | L | E | S | L | T | L | Y | L | P | T | F | T | N | X | X | X | X | X | L | S | - | - | - | H | Q | I | N | N | E | P | P | F | L | N | X | X | V | P | S | L | R | K | L | M | I | I | N | N | X | X | X | X | X | K | W | A | M | X | X | V | I | S | S | D | L | L | R | G | L | K | Y | L | E | Q | F | A | A | E | K | F | F | T | G | T | P | H | P | D | T | F | R | Y | X | X | T | P | R | L | T | S | L | Q | I | I | S | S | N | V | S | Y | X | X | X | X | K | A | D | L | - | - | - | F | E | P | I | P | N | L | Q | A | L | D | L | S | K | N | R | L | R | S | L | D | F | L | T | Q | X | X | X | X | N | X | X | X | X | X | S | A | L | S | W | L | T | L | R | Q | N | E | L | T | V | I | N | E | T | V | F | Q | S | L | P | A | L | T | Y | L | D | L | Y | V | T | G | C | L | H | S | C | S | X | D | E | S | V | V | R | X | X | X | X | X | X | X | Q | S | X | V | P | G | C | X | X | X | X | X | X | X | X | X | X | X | X | X | X | X | X | A | V | S | E | Q | L | G | S | D | T | X | - | - | R | M | X | X | X | X | X | X | X | X | X | X | X | X | X | X | X | X | X | X | X | X | X | - | - | - | - | - | - | X | X | X | X | X | X | X | X | X | X | X | X | X | N | L | - | - | - | - | - | - | - | - | - | - | - | - | - | - | - | - | - | - | - | - | - | - | - | - | - | - | - | - | - | X | S | R | X | X | F | V | S | G | X | P | H | X | X | X | X | X | X | X | X | X | X | X | X | X | X | X | X | X | X | X | X | X | X | X | X | X | X | X | D | X | X | X | X | X | X | X | G | K | X | X | X | X | X | X | X | X | X | X | X | X | X | X | X | X | X | X | X | X | X | N | F | I | L | G | Y | T | D | D | E | V | H | K | S | I | L | X | X | X | X | X | X | X | X | X | X | X | X | X | X | X | X | V | N | R | E | - | - | - | - | - | - | - | - | - | - | - | - | - | - | - | - | - | L | L | D | E | N | H | S | V | I | Q | T | Q | X | X | X | X | X | X | X | X | X | X | X | I | I | M | D | Q | G | F | K | M | X | X | X | X | X | X | X | X | X | X | X | X | X | X | X | X | X | X | X | X | X | X | X | X | X | X | X | X | X | X | X | X | X | X | X | X | X | X | X | X | X | X | X | X | X | X | X | X | X | X | X | X | X | X | X | X | X | X | X | X | X | X | X | X | X | X | X | X | X | X | X | X | X | X | X | X | X | X | X | X | X | X | X | X | X | X | X | X | X | X | X | X | X | X | X | X | X | X | X | X | X | X | X | X | X | X |
| TRTLR13\_3900\_BP | - | - | - | X | X | X | X | X | X | X | X | X | X | X | X | X | X | X | X | X | X | X | - | - | - | - | - | - | X | X | X | X | X | X | X | X | X | X | X | X | X | X | X | X | X | X | X | X | X | X | X | X | X | X | X | X | X | X | X | X | X | X | X | X | X | X | X | X | X | X | X | X | X | X | X | X | X | - | - | - | - | - | - | X | X | X | X | X | X | X | X | X | X | X | X | X | X | X | X | X | G | N | S | A | C | T | T | X | X | X | X | X | X | X | X | X | D | - | - | - | - | - | - | - | - | - | - | - | - | - | F | Q | P | V | S | L | - | - | - | - | - | X | X | X | X | X | X | X | X | X | K | N | C | T | V | X | X | X | X | - | - | - | X | R | R | W | R | X | X | X | X | X | X | X | X | X | X | X | S | A | E | V | F | X | X | X | X | X | X | X | X | X | G | L | C | X | P | X | T | L | - | - | - | - | - | - | - | - | - | - | - | - | D | C | S | R | R | C | S | P | X | R | H | H | S | K | T | L | L | Q | P | S | D | S | X | X | - | X | E | - | - | - | - | - | - | - | - | - | - | - | - | - | - | - | E | E | R | C | - | X | T | S | - | - | D | Q | S | E | V | S | G | P | - | - | - | - | A | I | K | R | N | R | P | H | X | R | W | I | L | P | P | H | E | V | X | X | S | D | E | A | A | V | V | - | - | - | - | - | - | - | - | K | X | X | X | X | X | X | A | K | R | X | X | X | X | Q | L | S | C | F | R | - | A | S | P | T | S | P | I | L | I | C | R | A | T | S | S | R | S | S | I | P | P | P | S | R | T | C | Q | A | C | R | R | W | - | F | W | M | R | T | G | X | - | - | R | R | W | R | T | S | D | R | C | S | - | X | X | X | Y | Q | S | X | G | T | X | X | X | - | - | - | - | X | X | X | X | X | X | - | - | - | X | - | Q | Q | Q | - | - | - | S | V | H | L | V | P | K | Q | R | P | S | A | G | P | X | - | X | X | X | X | X | X | X | X | X | Q | P | E | G | S | G | C | V | V | X | X | X | - | - | - | - | X | X | X | X | X | X | X | X | X | S | I | F | E | - | - | - | - | - | - | - | A | X | X | X | X | X | X | X | X | X | Q | H | L | S | R | H | L | P | - | - | - | - | - | - | - | - | - | - | S | - | P | G | D | D | R | P | F | S | X | X | X | X | X | X | X | X | - | X | - | - | - | X | - | - | X | X | - | - | - | X | X | X | X | X | X | X | X | X | X | - | - | C | L | - | - | - | - | - | X | X | X | X | W | N | I | - | - | - | - | - | - | - | - | - | - | - | - | - | X | X | X | X | Q | N | F | X | X | - | X | X | X | X | X | - | A | E | H | H | S | A | - | F | - | - | - | - | - | - | - | - | - | - | L | Q | S | Y | - | - | E | S | F | I | P | - | - | - | - | - | - | - | - | - | - | - | - | - | - | - | - | - | - | - | - | - | - | T | D | P | G | D | P | A | E | - | - | - | - | - | - | - | - | - | - | - | - | - | - | - | - | - | - | - | - | - | - | - | - | - | - | - | H | G | F | T | Q | A | S | Q | D | E | L | X | X | X | X | S | M | S | - | G | S | A | - | - | - | - | - | - | - | - | - | - | - | - | - | - | - | - | - | - | - | - | K | V | C | X | Q | Q | - | - | S | A | G | S | P | X | X | X | X | X | X | X | X | X | X | X | L | R | V | L | E | L | Y | Y | N | K | V | P | N | L | S | A | X | X | V | E | F | C | S | E | L | V | Q | X | X | X | X | X | X | X | G | L | V | L | L | R | X | X | - | - | - | - | - | X | R | V | G | G | S | T | W | E | F | P | A | D | E | T | - | - | - | T | E | A | P | E | P | G | G | Q | P | S | H | Q | G | P | - | - | - | - | R | G | H | Q | E | X | X | X | X | H | L | - | - | L | S | A | G | P | L | P | Q | X | Q | P | D | H | X | G | G | L | X | R | V | L | Q | - | - | - | - | - | - | - | - | - | - | - | - | - | - | - | - | - | - | - | - | - | - | - | - | - | - | - | - | - | H | X | X | X | X | X | X | R | S | G | G | A | L | P | G | L | Q | S | D | H | V | P | X | X | X | X | T | V | Q | L | X | E | P | A | Q | - | V | K | D | F | G | F | E | Q | Q | P | S | V | E | D | X | R | C | L | Q | P | G | - | - | - | - | P | S | E | A | A | A | L | R | S | E | Q | K | L | G | V | V | V | R | X | W | I | - | - | - | - | F | P | E | S | G | M | V | D | A | S | - | - | - | - | - | - | - | - | - | - | - | - | - | - | - | - | - | - | - | - | - | G | - | - | - | - | - | - | R | V | F | R | X | G | G | P | S | D | A | - | - | W | S | F | C | W | S | T | Q | T | E | E | X | X | X | X | X | X | X | X | X | X | X | F | M | S | A | S | R | S | I | T | N | V | X | X | X | X | X | X | F | R | G | L | K | Q | X | X | X | X | X | X | X | X | X | X | X | - | - | - | - | - | - | - | - | - | - | - | X | L | E | N | L | T | I | S | I | X | X | X | X | X | X | X | X | X | X | I | S | D | T | R | S | P | P | K | Y | S | Q | A | X | X | L | F | H | L | K | S | L | R | S | F | H | V | A | C | Q | G | F | H | Y | - | - | - | G | F | P | L | D | V | P | L | S | M | L | S | S | M | T | Q | L | E | E | F | T | A | D | N | I | Y | L | S | A | P | D | P | E | T | F | R | S | X | X | N | S | R | L | R | S | L | K | I | S | Q | T | D | L | S | D | X | X | X | X | D | P | E | M | - | - | - | F | R | P | I | P | D | L | Q | S | L | D | L | S | G | T | Q | I | S | S | L | E | F | L | L | Q | X | X | X | X | D | X | X | X | X | X | S | S | L | R | D | L | R | L | C | D | N | D | I | T | S | I | N | H | T | L | F | Q | F | L | P | S | L | T | L | L | D | L | T | N | N | P | L | T | C | D | C | L | N | A | G | F | I | L | W | V | M | D | N | N | Q | T | Q | V | X | I | N | G | H | X | X | X | X | X | Y | S | C | S | F | P | V | A | K | K | - | - | G | T | N | L | L | D | F | E | V | Q | F | C | W | I | D | V | D | F | L | C | F | L | S | S | T | C | L | V | V | L | T | L | L | T | S | F | T | Y | H | F | L | R | W | Q | X | X | X | X | T | L | C | L | P | X | X | L | F | L | A | F | I | Y | X | X | X | X | X | Q | X | X | G | T | G | S | S | R | I | L | X | X | X | X | X | X | X | X | P | Y | D | A | F | V | S | Y | N | V | E | D | E | L | W | V | Y | - | - | - | - | - | - | - | - | - | - | - | - | - | - | - | - | - | - | - | - | - | E | E | M | L | P | A | L | E | D | Q | Q | - | - | - | - | - | - | - | - | - | - | - | - | - | - | X | X | X | X | G | W | K | L | C | L | H | H | R | D | F | Q | P | G | K | P | I | M | E | N | I | T | D | A | I | Y | N | S | R | K | T | I | C | V | I | S | R | S | Y | L | Q | S | E | W | C | S | R | E | I | Q | M | A | S | F | R | L | F | D | E | Q | K | D | V | L | I | L | L | F | L | E | E | I | P | A | - | - | - | - | H | Q | L | S | P | Y | H | R | M | R | K | L | L | K | R | Q | T | Y | L | S | W | T | Q | A | G | R | H | X | X | X | X | X | A | G | V | F | W | Q | N | V | Q | R | A | L | E | - | - | - | - | - | S | G | D | A | P | X | X | X | X | X | X | X | X | X | X | X | X | X | X | X | X | X | X | X | X | X | X | X | X | X | X | X | X | X | X | X | X | X | X | X | H | X | X | X | X | Q | V | D | P | L | T | G | P | A | E | P | X | - | - | - | - | - | - | - | - |
| GMTLR13\_3900\_BP | - | - | - | M | S | L | V | G | C | C | I | X | X | X | X | X | X | X | X | X | X | X | - | - | - | - | - | - | X | L | Q | N | V | X | X | X | X | X | X | X | X | X | X | X | X | X | X | X | X | X | X | X | X | X | X | X | X | X | X | X | X | X | X | X | X | X | X | X | X | X | X | X | X | X | X | X | X | - | - | - | - | - | - | X | X | X | X | X | X | X | X | X | X | X | X | X | X | X | X | X | X | X | X | X | X | X | X | S | F | F | F | P | C | F | I | L | I | - | - | - | - | - | - | - | - | - | - | - | - | - | L | Q | F | L | T | C | - | - | - | - | - | X | X | X | X | X | X | X | X | X | K | N | C | T | I | P | X | S | L | - | - | - | R | I | C | X | X | X | X | X | X | X | X | X | X | X | X | X | S | A | C | I | N | X | X | X | X | X | X | X | X | X | X | L | F | S | K | K | T | V | - | - | - | - | - | - | - | - | - | - | - | - | S | G | T | R | W | H | S | Q | R | C | D | T | V | I | H | L | S | X | S | N | P | S | X | X | - | X | S | - | - | - | - | - | - | - | - | - | - | - | - | - | - | - | Q | E | X | L | - | P | V | F | - | - | V | K | P | Y | S | V | K | P | - | - | - | - | S | H | E | S | N | C | S | Y | X | R | W | I | F | Y | X | F | A | R | X | X | A | K | G | T | X | H | S | - | - | - | - | - | - | - | - | Y | X | X | X | X | X | Q | T | D | Q | X | X | X | S | Q | T | T | P | L | K | - | D | C | G | S | S | Q | C | W | T | L | V | G | T | E | S | E | S | F | P | P | L | H | L | G | Q | C | P | A | C | I | G | X | - | I | W | I | K | T | C | X | - | - | Q | K | X | L | T | F | S | T | Y | Y | - | X | X | X | Y | H | T | L | I | I | S | X | X | - | - | - | - | X | X | X | X | X | X | - | - | X | X | - | X | X | P | - | - | - | L | Q | L | F | X | X | - | - | D | X | T | F | A | Q | X | X | X | X | X | X | X | X | V | X | L | E | S | Q | R | F | R | I | V | F | X | X | X | - | - | - | - | X | X | X | X | X | X | X | X | X | T | V | L | T | - | - | - | - | - | - | - | I | X | X | X | X | X | X | X | X | X | Q | H | H | N | T | C | L | S | - | - | - | - | - | - | - | - | - | - | - | - | P | S | X | E | V | K | L | X | W | X | X | X | X | X | X | X | X | X | - | - | - | X | - | - | X | X | - | - | - | X | X | X | X | X | X | L | P | K | S | - | - | C | Y | X | X | X | - | - | - | - | - | X | V | D | I | - | - | - | - | - | - | - | - | - | - | - | - | - | X | X | X | R | A | S | F | X | X | - | X | X | X | X | X | - | Q | D | C | N | S | S | S | I | - | - | - | - | - | - | - | - | - | - | F | N | S | L | H | L | F | Q | R | H | E | - | - | - | - | - | - | - | - | - | - | - | - | - | - | - | - | - | - | - | - | - | - | E | A | P | R | E | S | G | - | - | - | - | - | - | - | - | - | - | - | - | - | - | - | - | - | - | - | - | - | - | - | - | - | - | - | - | - | - | L | T | D | A | S | X | A | F | T | X | X | X | X | L | R | N | - | G | S | T | - | - | - | - | - | - | - | - | - | - | - | - | - | - | - | - | - | - | - | - | E | V | S | X | R | Q | - | - | P | A | A | S | P | X | X | X | X | X | X | X | X | X | X | X | L | R | S | L | D | L | H | Y | N | S | V | V | N | I | D | P | K | L | A | X | X | C | S | Q | I | T | K | X | X | X | X | X | X | X | G | P | I | L | Q | S | X | X | - | - | - | - | - | X | H | L | Q | S | L | Q | S | F | N | T | I | N | E | A | - | - | - | I | E | I | L | G | F | G | R | X | L | S | L | Q | R | A | - | - | - | - | S | X | P | Y | Q | X | X | X | X | S | L | - | - | V | S | X | N | P | I | S | V | S | Q | P | H | C | X | G | R | L | L | R | F | L | Q | - | - | - | - | - | - | - | - | - | - | - | - | - | - | - | - | - | - | - | - | - | - | - | - | - | - | - | - | - | F | X | X | X | X | X | X | K | T | Q | R | T | I | S | R | I | Q | L | H | X | X | T | X | X | X | X | W | L | C | V | S | I | S | K | G | - | S | K | G | F | G | F | K | Q | X | L | V | V | G | I | G | R | Y | F | X | D | R | - | - | - | - | S | K | E | S | R | V | L | R | F | E | Y | K | P | N | X | A | F | W | N | G | R | - | - | - | - | F | Y | K | F | G | I | A | Q | L | F | - | - | - | - | - | - | - | - | - | - | - | - | - | - | - | - | - | - | - | - | - | K | - | - | - | - | - | - | I | X | X | X | Y | N | X | R | C | T | Q | - | - | X | G | F | X | T | I | V | H | S | X | S | X | X | X | X | X | X | X | X | X | X | X | L | F | Y | Y | R | X | X | X | X | X | X | X | X | X | D | N | I | F | S | G | L | Q | L | X | X | X | X | X | X | X | X | X | X | X | - | - | - | - | - | - | - | - | - | - | - | X | L | R | N | L | T | V | Y | L | N | I | P | E | G | Y | K | G | V | D | L | N | - | - | - | S | Y | Q | X | X | X | X | X | L | I | N | X | X | L | P | S | L | R | Y | L | D | I | I | C | R | G | M | H | X | C | S | K | P | F | X | X | H | V | P | I | H | F | L | K | T | M | K | N | L | E | H | L | T | A | E | N | F | Q | L | V | E | P | D | K | D | T | F | I | Y | X | X | T | P | K | L | K | S | L | A | I | T | E | S | S | L | S | D | X | X | X | X | N | P | E | L | - | - | - | F | Q | P | I | S | N | L | E | A | L | D | L | S | R | N | T | L | K | N | L | D | F | L | A | Q | X | X | X | X | N | X | X | X | X | X | T | S | L | R | C | L | K | L | C | E | N | D | L | T | V | I | D | H | N | I | F | Q | F | L | P | A | L | T | Y | L | D | L | S | V | N | P | L | T | C | D | C | S | N | S | G | F | L | L | W | A | L | S | S | D | Q | T | Q | I | X | V | N | V | D | X | X | X | X | X | Y | V | C | T | Y | P | L | S | E | R | - | - | G | T | M | F | V | D | I | D | V | Q | S | C | W | M | D | A | S | F | L | C | Y | I | S | A | T | S | L | V | L | L | T | L | L | T | A | F | G | Y | H | F | L | H | W | R | X | X | X | X | G | L | C | L | L | X | X | R | L | M | A | L | L | Y | X | X | X | X | X | H | X | X | E | R | G | R | E | T | I | R | X | X | X | X | X | X | X | X | C | Y | D | A | F | I | S | Y | N | I | Q | D | E | A | W | V | H | - | - | - | - | - | - | - | - | - | - | - | - | - | - | - | - | - | - | - | - | - | R | E | M | L | P | A | L | E | D | E | R | - | - | - | - | - | - | - | - | - | - | - | - | - | - | X | X | X | X | G | W | R | L | C | L | H | H | R | D | F | Q | P | G | T | S | I | M | E | N | I | T | E | A | I | Y | G | S | R | K | T | I | C | V | I | S | R | H | Y | L | E | S | E | W | C | S | R | E | I | Q | M | A | S | Y | R | L | L | D | E | K | K | D | V | L | I | L | L | F | L | E | E | L | P | E | - | - | - | - | K | Q | L | S | P | Y | Y | R | M | R | K | L | V | R | R | S | T | Y | L | S | W | P | Q | A | A | R | H | X | X | X | X | X | P | G | L | F | W | Q | N | V | H | R | A | L | E | - | - | - | - | - | S | G | D | S | H | X | X | X | X | X | X | X | X | X | X | X | X | X | X | X | X | X | X | X | X | X | X | X | X | X | X | X | X | X | X | X | X | X | X | X | D | X | X | X | X | N | T | H | F | L | S | G | P | A | L | X | X | - | - | - | - | - | - | - | - |
| SMTLR13\_3900\_BP | - | - | - | M | Q | A | Q | G | S | W | X | X | X | X | X | X | X | X | X | X | X | X | - | - | - | - | - | - | X | X | X | X | X | X | X | X | X | X | X | X | X | X | X | X | X | X | X | X | X | X | X | X | X | X | X | X | X | X | X | X | X | X | X | X | X | X | X | X | X | X | X | X | X | X | X | X | X | - | - | - | - | - | - | X | X | X | X | X | X | X | X | X | X | X | X | X | X | X | X | X | X | X | L | C | C | L | S | H | F | C | F | P | C | F | M | L | I | - | - | - | - | - | - | - | - | - | - | - | - | - | P | H | W | L | S | H | - | - | - | - | - | X | X | X | X | X | X | X | X | X | K | N | C | S | I | X | X | X | X | - | - | - | X | L | F | X | Q | X | X | X | X | X | X | X | X | X | X | X | A | T | R | V | F | X | X | X | X | X | X | X | X | X | Y | L | P | A | S | F | P | V | - | - | - | - | - | - | - | - | - | - | - | - | R | H | S | R | R | H | S | P | K | C | R | R | F | G | S | R | L | Q | X | H | P | T | X | X | - | X | Q | - | - | - | - | - | - | - | - | - | - | - | - | - | - | - | Q | I | G | F | - | K | R | S | - | - | I | K | A | Q | I | C | T | S | - | - | - | - | C | M | Q | F | D | F | T | C | X | Q | W | S | F | Y | G | L | D | R | X | X | V | K | K | S | A | H | G | - | - | - | - | - | - | - | - | X | X | X | X | X | X | S | T | H | K | X | X | X | S | Q | T | T | R | F | R | - | D | C | P | S | X | K | H | S | L | X | T | A | T | T | S | H | I | S | P | L | W | P | F | S | P | W | S | T | X | T | Q | S | S | S | A | L | T | L | C | I | - | - | A | - | L | H | L | L | H | H | F | X | - | X | X | X | Y | Q | H | Y | T | F | X | X | X | - | - | - | - | X | X | X | X | X | X | - | - | X | W | - | E | Q | Q | - | - | - | V | Y | H | F | X | X | I | R | X | L | A | F | Q | X | X | X | - | - | X | X | X | X | X | X | X | E | P | Q | V | T | G | A | K | F | X | X | X | - | - | - | - | X | X | X | X | X | X | X | X | X | N | P | L | R | - | - | - | - | - | - | - | K | X | X | X | X | X | X | X | X | X | Q | H | Y | E | G | R | L | S | - | - | - | - | - | - | - | - | - | - | - | - | L | F | A | V | S | R | L | V | P | X | X | X | X | X | X | X | - | - | - | - | - | - | - | - | X | X | - | - | - | X | - | - | X | X | X | X | X | X | X | - | - | X | X | L | W | X | R | H | X | X | X | X | W | D | V | - | - | - | - | - | - | - | - | - | - | - | - | - | X | X | X | K | Q | D | L | X | X | - | X | - | - | - | X | - | X | X | X | E | E | P | Y | Y | - | - | - | - | - | - | - | - | - | - | F | V | P | Q | W | K | L | R | X | X | X | - | - | - | - | - | - | - | - | - | - | - | - | - | - | - | - | - | - | - | - | - | - | E | L | H | G | D | A | A | D | - | - | - | - | - | - | - | - | - | - | - | - | - | - | - | - | - | - | - | - | - | - | - | - | - | - | - | H | R | L | G | K | N | N | L | A | D | I | X | X | X | X | W | R | T | - | W | L | P | - | - | - | - | - | - | - | - | - | - | - | - | - | - | - | - | - | - | - | - | K | A | S | X | T | S | - | - | L | A | E | F | L | X | X | X | X | X | X | X | X | X | X | X | L | R | S | L | A | V | T | N | S | R | I | G | I | I | D | E | N | L | L | Q | S | C | S | Q | L | T | E | X | X | X | X | X | X | X | H | F | I | C | X | S | X | X | - | - | - | - | - | X | A | Y | X | A | F | X | T | V | V | Q | I | N | N | A | - | - | - | A | Q | A | F | E | T | G | Q | X | R | T | V | E | S | A | - | - | - | - | T | R | T | P | R | X | X | X | X | T | F | - | - | H | S | G | G | P | G | P | A | V | K | L | H | K | X | A | X | L | P | R | F | P | A | - | - | - | - | - | - | - | - | - | - | - | - | - | - | - | - | - | - | - | - | - | - | - | - | - | - | - | - | - | F | X | X | X | X | X | X | X | V | N | R | P | X | S | Q | P | K | S | H | L | K | T | X | X | X | X | R | L | S | F | S | K | F | E | X | - | F | K | S | P | G | Y | W | R | Q | R | P | F | Y | F | X | R | Y | L | Q | G | X | - | - | - | - | F | G | E | T | R | I | F | E | F | A | Q | Q | W | S | F | T | A | N | A | R | R | - | - | - | - | L | X | E | S | F | F | T | H | F | F | - | - | - | - | - | - | - | - | - | - | - | - | - | - | - | - | - | - | - | - | - | R | - | - | - | - | - | - | F | R | F | R | H | I | L | X | C | N | X | - | - | W | G | F | R | R | T | R | X | S | P | N | X | X | X | X | X | X | X | X | X | X | X | S | F | C | Q | L | I | N | I | K | T | I | X | X | X | X | X | X | F | R | G | L | Q | H | X | X | X | X | X | X | X | X | X | X | X | - | - | - | - | - | - | - | - | - | - | - | X | L | E | N | L | T | L | H | L | T | F | N | W | H | X | X | X | X | X | M | S | - | - | - | P | Q | H | N | V | E | P | P | F | G | N | X | X | L | P | N | L | K | K | X | X | X | V | L | Q | V | D | E | K | Y | X | X | X | V | X | X | D | S | S | P | D | M | L | G | G | L | K | S | L | E | Y | F | M | A | D | R | L | F | M | K | I | I | H | P | D | T | F | K | Y | X | X | T | P | Q | L | K | G | L | Q | I | I | N | S | N | L | I | E | X | X | X | X | D | P | E | L | - | - | - | F | R | P | I | P | N | L | Q | T | L | D | L | S | N | N | K | L | R | S | L | D | F | L | A | Q | X | X | X | X | N | X | X | X | X | X | Q | A | L | V | W | L | T | L | N | K | N | E | L | S | I | I | N | E | T | V | L | Q | S | L | P | A | L | T | Y | L | V | L | N | G | N | P | L | T | C | E | C | S | N | Y | G | F | N | Q | W | V | L | S | N | N | Q | T | Q | V | X | V | D | A | Y | X | X | X | X | X | Y | T | C | A | F | P | V | S | Q | Q | - | - | G | N | K | F | L | D | F | D | I | H | S | C | W | M | N | T | S | F | I | C | F | I | S | S | T | S | L | I | V | L | T | L | L | A | S | F | V | Y | H | F | L | R | W | H | X | X | X | X | S | L | R | L | L | X | X | L | F | L | A | Y | L | Y | X | X | X | X | X | X | X | X | G | G | G | R | L | A | L | L | X | X | X | X | X | X | X | X | H | Y | D | A | F | V | S | Y | N | V | H | D | E | A | W | V | Y | - | - | - | - | - | - | - | - | - | - | - | - | - | - | - | - | - | - | - | - | - | N | E | L | V | P | V | L | E | E | Q | Q | - | - | - | - | - | - | - | - | - | - | - | - | - | - | X | X | X | X | G | W | R | V | C | L | H | H | R | D | F | E | P | G | K | P | I | V | E | N | I | T | D | A | I | Y | G | S | R | K | T | I | C | V | I | S | R | N | Y | L | Q | S | E | W | C | S | R | E | I | Q | M | A | S | C | R | L | F | D | E | Q | E | D | V | L | I | L | L | F | L | E | D | I | P | A | - | - | - | - | R | Q | L | S | P | Y | Y | R | M | K | S | L | V | K | S | C | T | Y | L | S | W | A | K | A | G | Q | H | X | X | X | X | X | T | E | A | F | W | M | N | L | W | R | A | L | E | - | - | - | - | - | T | A | G | R | P | X | X | X | X | X | X | X | X | X | X | X | X | X | X | X | X | X | X | X | X | X | X | X | X | X | X | X | X | X | X | X | X | X | X | X | A | D | D | E | H | T | R | H | L | L | T | G | H | P | S | D | V | - | - | - | - | - | - | - | - |
| ECTLR13\_3900\_BP | - | - | - | M | S | V | M | G | S | W | X | X | X | X | X | X | X | X | X | X | X | X | - | - | - | - | - | - | X | L | C | S | F | X | X | X | X | X | X | X | X | X | X | X | X | X | X | X | X | X | X | X | X | X | X | X | X | X | X | X | X | X | X | X | X | X | X | X | X | X | X | X | X | X | X | X | X | - | - | - | - | - | - | X | X | X | X | X | X | X | X | X | X | X | X | X | X | X | X | X | R | A | S | S | S | X | X | Y | F | R | L | P | C | F | L | L | T | - | - | - | - | - | - | - | - | - | - | - | - | - | P | C | W | L | S | H | - | - | - | - | - | X | X | X | X | X | X | X | X | X | K | N | C | T | V | X | X | X | X | - | - | - | X | X | X | X | Q | X | X | X | X | X | X | X | X | X | X | X | V | T | W | X | X | X | X | X | X | X | X | X | X | X | R | M | R | R | S | X | P | H | - | - | - | - | - | - | - | - | - | - | - | - | W | G | S | X | X | H | S | H | N | C | G | N | S | R | S | Q | L | Q | S | H | L | K | X | X | - | X | K | - | - | - | - | - | - | - | - | - | - | - | - | - | - | - | Q | D | R | F | - | Q | P | F | - | - | F | K | A | W | V | F | K | N | - | - | - | - | C | K | X | L | D | F | T | C | R | X | W | S | F | C | R | V | G | G | X | X | V | N | R | A | X | H | G | - | - | - | - | - | - | - | - | F | X | X | X | X | X | Q | P | H | K | X | X | X | X | Q | T | I | C | F | R | - | A | C | G | S | X | F | F | C | Q | W | R | K | T | I | S | H | T | S | P | L | W | P | F | S | P | X | S | A | Y | R | R | Y | S | X | H | I | T | T | Y | I | - | - | K | - | X | P | T | L | C | P | S | Y | - | X | X | X | Y | Q | T | X | M | I | C | X | X | - | - | - | - | X | X | X | X | X | X | - | - | X | X | - | F | X | Q | - | - | - | S | H | F | L | X | X | V | R | X | P | A | F | X | X | X | X | - | - | X | X | X | X | X | X | X | K | P | Q | D | T | M | A | M | X | X | X | X | - | - | - | - | X | X | X | X | X | X | X | X | X | D | S | M | K | - | - | - | - | - | - | - | K | X | X | X | X | X | X | X | X | X | Q | H | Y | K | R | R | F | S | - | - | - | - | - | - | - | - | - | - | - | - | P | S | S | V | Y | P | F | X | X | X | X | X | X | X | X | X | - | - | - | - | - | - | - | - | X | X | - | - | - | X | X | X | X | X | X | X | X | X | X | - | - | C | X | H | W | L | - | - | X | X | X | X | W | D | V | - | - | - | - | - | - | - | - | - | - | - | - | - | X | X | X | R | P | N | V | X | X | - | X | - | - | - | X | - | X | X | X | E | E | P | D | Y | - | - | - | - | - | - | - | - | - | - | F | G | V | I | C | I | X | Q | X | L | X | - | - | - | - | - | - | - | - | - | - | - | - | - | - | - | - | - | - | - | - | - | - | D | V | P | S | D | A | E | E | - | - | - | - | - | - | - | - | - | - | - | - | - | - | - | - | - | - | - | - | - | - | - | - | - | - | - | C | R | V | S | A | G | T | V | T | P | L | L | A | P | X | P | R | X | - | X | X | X | - | - | - | - | - | - | - | - | - | - | - | - | - | - | - | - | - | - | - | - | E | I | W | X | T | S | - | - | P | A | K | X | L | X | X | X | X | X | X | X | X | X | X | X | L | T | S | L | H | L | S | G | I | S | F | V | S | I | N | K | T | F | L | Q | S | C | T | E | I | T | E | X | X | X | X | X | X | X | X | F | I | I | X | L | X | X | - | - | - | - | - | X | F | G | R | A | V | R | V | F | P | R | I | N | K | T | - | - | - | A | Q | T | P | G | L | S | E | K | L | P | V | X | S | A | - | - | - | - | T | G | C | Q | R | X | X | X | X | P | L | - | - | H | T | X | N | P | R | S | E | C | E | F | H | Q | X | V | R | L | L | X | L | S | K | - | - | - | - | - | - | - | - | - | - | - | - | - | - | - | - | - | - | - | - | - | - | - | - | - | - | - | - | - | F | X | X | X | X | X | X | K | T | S | R | T | Q | S | Q | P | K | S | H | F | N | T | X | X | X | X | G | M | C | F | S | R | S | E | X | - | F | E | S | P | E | R | C | R | K | W | S | S | F | T | C | X | F | L | Q | I | E | - | - | - | - | F | T | E | T | R | S | F | G | F | E | H | E | Y | F | D | A | A | Q | N | R | X | - | - | - | - | L | X | E | Y | V | F | P | Q | V | S | - | - | - | - | - | - | - | - | - | - | - | - | - | - | - | - | - | - | - | - | - | V | - | - | - | - | - | - | F | R | I | R | Y | I | L | R | C | P | X | - | - | G | G | F | X | R | T | G | Q | S | S | K | S | F | S | X | X | X | X | X | X | T | P | F | F | G | E | M | D | F | I | T | K | X | X | X | X | X | X | X | F | T | G | L | Q | H | X | X | X | X | X | X | X | X | X | X | X | - | - | - | - | - | - | - | - | - | - | - | X | L | Q | N | L | K | I | H | L | T | S | S | Y | D | X | X | X | X | X | K | S | - | - | - | S | C | Q | K | N | E | T | H | L | S | I | F | P | L | P | F | L | K | S | X | X | X | L | I | Q | N | Y | D | W | Y | X | X | X | X | X | X | E | I | S | P | D | F | L | T | G | L | N | S | L | D | Y | F | G | A | E | K | L | F | T | E | S | P | H | P | D | T | F | R | Y | X | X | T | P | L | L | T | S | L | H | I | F | Q | S | D | L | Q | V | X | X | X | X | N | P | E | V | - | - | - | L | Q | P | I | L | N | L | Q | A | L | D | L | S | K | N | K | L | R | S | L | D | F | L | A | Q | X | X | X | X | N | X | X | X | X | X | S | A | L | R | V | L | T | V | R | D | N | E | L | T | V | I | N | D | M | V | F | Q | S | L | P | A | L | T | Y | L | D | L | T | G | N | P | F | T | C | N | C | S | N | T | G | F | I | Q | W | V | K | N | N | N | Q | T | Q | V | X | V | N | A | Y | X | X | X | X | X | Y | T | C | T | F | P | V | A | K | Q | - | - | G | T | K | L | L | D | F | D | V | Q | S | C | W | M | D | V | S | F | L | C | F | I | S | S | F | C | L | T | L | M | I | L | L | T | S | F | I | Y | H | F | L | R | W | Q | X | X | X | X | S | L | H | L | L | X | X | L | F | L | A | F | L | Y | X | X | X | X | X | Q | X | X | G | R | G | R | R | A | L | L | X | X | X | X | X | X | X | X | H | Y | D | A | F | I | S | Y | N | V | H | D | E | D | W | V | Y | - | - | - | - | - | - | - | - | - | - | - | - | - | - | - | - | - | - | - | - | - | R | E | M | L | P | V | L | E | G | E | Q | - | - | - | - | - | - | - | - | - | - | - | - | - | - | X | X | X | X | G | W | R | L | C | L | H | H | R | D | F | Q | P | G | K | P | I | I | D | N | I | T | D | A | I | Y | G | S | R | K | T | I | C | V | I | T | R | R | Y | L | Q | S | E | W | C | S | R | E | I | Q | M | A | S | F | R | L | F | D | E | Q | K | D | V | L | I | L | L | F | L | E | E | I | P | A | - | - | - | - | Y | Q | L | S | P | Y | H | R | M | R | K | L | V | K | R | H | T | Y | L | S | W | P | Q | A | G | Q | H | X | X | X | X | X | T | G | V | F | W | Q | N | V | W | R | A | L | E | - | - | - | - | - | T | G | E | A | P | X | X | X | X | X | X | X | X | X | X | X | X | X | X | X | X | X | X | X | X | X | X | X | X | X | X | X | X | X | X | X | X | X | X | X | I | X | X | X | X | T | N | N | L | L | T | G | C | X | X | X | X | - | - | - | - | - | - | - | - |
| CCTLR13\_3900\_BP | - | - | - | - | - | - | X | X | X | W | E | H | X | N | K | X | X | X | X | X | - | - | - | X | X | X | X | X | T | L | Y | V | A | X | X | X | X | X | X | X | X | X | X | X | X | X | X | X | X | X | X | X | X | X | - | X | X | X | X | X | X | X | X | X | X | X | X | X | X | X | X | X | X | X | X | X | X | X | X | X | X | X | X | X | X | X | X | X | X | X | X | X | X | X | X | C | S | F | I | S | X | X | X | X | X | X | X | X | X | X | X | X | H | L | V | - | H | - | - | - | - | - | - | - | - | - | - | - | - | - | F | P | - | - | - | - | - | - | - | - | - | X | X | X | X | X | X | X | X | X | N | N | C | T | I | X | X | X | X | - | - | - | X | T | P | L | K | X | X | X | X | X | X | X | X | X | X | X | T | - | S | S | - | - | - | X | K | X | X | X | X | X | X | X | X | X | T | L | L | Q | N | G | L | F | Q | D | S | V | X | X | T | X | K | Y | A | - | - | - | - | - | - | - | - | D | F | R | Y | F | L | Q | C | L | C | T | X | X | - | X | P | - | - | - | - | - | - | - | - | - | - | - | - | - | - | - | N | W | R | L | - | Q | S | F | - | - | V | K | L | A | G | L | E | H | I | Q | Q | X | D | L | A | D | S | R | G | C | P | X | Q | S | F | Q | - | - | - | - | X | X | L | I | P | S | Q | S | G | - | - | - | - | - | - | - | - | Q | X | X | X | X | X | Q | T | T | R | X | X | X | S | R | E | V | C | Y | M | - | V | X | P | T | C | W | C | Y | V | W | I | T | I | I | L | K | T | L | T | X | Q | L | S | A | R | F | R | I | X | K | C | X | T | X | Q | K | T | I | S | I | - | - | I | - | X | R | K | S | S | R | F | L | - | X | X | X | P | H | I | W | R | N | C | X | X | - | - | - | - | X | X | X | X | X | X | - | - | X | C | - | K | Q | P | - | - | - | F | X | C | F | X | X | V | I | X | N | V | N | K | X | X | X | X | X | X | - | - | X | X | X | X | V | I | K | N | T | X | S | F | Q | X | X | X | - | - | - | - | X | X | X | X | X | X | X | X | X | N | P | F | A | - | - | - | - | - | - | - | T | X | X | X | X | X | X | X | X | X | S | A | N | X | Q | Y | I | S | - | - | - | - | - | - | - | - | - | - | I | - | S | X | S | S | X | F | I | L | X | X | X | X | X | X | X | X | X | - | - | - | - | - | - | - | - | - | - | - | - | - | - | X | X | X | X | X | X | X | X | - | - | X | W | S | K | W | N | Y | X | X | X | X | W | N | I | - | - | - | - | - | - | - | - | - | - | - | - | - | X | X | X | R | K | D | I | X | X | - | X | X | X | X | X | - | F | L | S | K | N | S | I | L | - | - | - | - | - | - | - | - | - | - | H | G | C | S | - | - | - | Y | F | S | P | - | - | - | - | - | - | - | - | - | - | - | - | - | - | - | - | - | - | - | - | - | - | E | C | C | X | C | A | S | E | - | - | - | - | - | - | - | - | - | - | - | - | - | - | - | - | - | - | - | - | - | - | - | - | - | - | - | L | P | K | F | V | E | X | - | - | - | - | N | Q | A | X | W | X | X | - | X | T | L | - | - | - | - | - | - | - | - | - | - | - | - | - | - | - | - | S | X | T | R | L | T | S | C | X | T | - | - | H | V | X | L | Q | X | X | X | X | X | X | X | X | X | X | X | L | Q | V | L | R | L | K | A | N | K | I | K | H | L | E | E | H | M | F | D | P | C | S | N | L | T | E | X | X | X | X | X | X | X | R | L | R | R | X | - | X | X | - | - | - | - | - | X | X | N | I | P | V | M | A | X | Y | L | H | R | I | Y | - | - | T | T | K | N | V | A | S | A | N | Q | X | T | D | S | N | N | - | - | - | - | K | L | L | S | N | X | X | X | X | A | L | - | - | H | S | X | V | H | R | S | Q | X | K | Q | H | R | H | P | H | L | X | X | F | C | Q | - | - | - | - | - | - | - | - | - | - | - | - | - | - | - | - | - | - | - | - | - | - | - | - | - | - | - | - | - | F | X | X | X | X | X | X | T | V | E | K | S | L | L | V | Q | X | Q | N | L | N | H | X | X | X | X | I | L | F | V | Q | G | P | E | E | - | P | X | G | P | Q | A | W | N | K | Q | S | V | K | D | R | X | C | F | Q | Q | W | - | - | - | - | A | T | F | P | G | G | A | A | T | Y | F | X | X | A | K | Q | N | R | K | V | H | - | - | - | - | F | X | K | C | V | T | A | E | Q | F | I | L | T | R | Q | S | D | F | R | D | R | K | P | C | I | R | R | T | E | E | - | - | - | - | - | - | - | - | - | - | - | - | - | - | P | D | L | X | X | - | - | - | - | - | - | - | - | - | - | - | - | - | - | - | X | X | X | X | X | X | X | X | L | L | S | S | N | K | I | S | A | K | T | L | T | K | N | Q | D | V | F | S | G | M | P | N | X | X | X | X | X | X | X | X | X | X | X | - | - | - | - | - | - | - | - | - | - | - | X | L | K | S | L | G | L | D | T | N | S | I | S | F | X | X | X | X | X | M | I | - | - | - | S | X | N | X | X | X | P | P | F | K | D | X | X | X | X | X | L | K | H | L | R | V | L | A | L | H | S | X | X | R | R | G | I | G | X | X | K | I | P | S | N | L | L | Q | G | L | S | S | L | E | M | F | Y | A | G | N | I | N | L | A | H | L | N | P | D | T | F | K | F | X | X | S | P | Q | L | W | F | L | D | L | S | K | N | A | L | S | E | D | H | S | I | P | A | E | L | - | - | - | F | H | P | I | S | R | L | T | K | L | I | L | S | R | T | Q | L | R | S | L | N | F | L | L | N | X | X | X | X | N | X | X | X | X | X | S | R | L | L | S | L | K | A | S | G | N | E | I | D | T | I | N | K | T | L | I | Q | S | L | P | R | L | E | V | L | D | L | G | K | N | T | F | T | C | D | C | T | N | E | F | F | I | D | W | A | K | N | S | N | S | T | Q | V | X | L | Y | L | N | X | X | X | X | X | Y | M | C | S | Y | P | S | S | L | R | - | - | G | M | S | L | S | A | F | N | T | E | S | C | T | L | N | I | D | F | I | C | F | L | C | S | S | I | V | V | I | L | T | L | L | L | S | F | V | W | H | F | L | R | F | Q | X | X | X | X | G | L | C | I | L | X | X | L | F | I | A | F | L | Y | X | X | X | X | X | Q | X | X | R | R | S | R | A | V | R | L | X | X | X | X | X | X | X | X | Q | Y | D | A | F | I | S | Y | N | A | K | D | E | P | W | V | M | - | - | - | - | - | - | - | - | - | - | - | - | - | - | - | - | - | - | - | - | - | E | E | L | I | P | K | L | E | G | E | Q | - | - | - | - | - | - | - | - | - | - | - | - | - | - | X | X | X | X | D | W | R | L | C | L | H | H | R | D | F | E | P | G | R | P | I | I | D | N | I | I | D | G | I | Y | S | S | R | K | T | I | C | L | I | T | R | N | Y | L | K | S | N | W | C | S | S | E | V | Q | V | A | S | F | R | L | F | D | E | Q | K | D | V | L | I | L | V | F | L | E | D | I | P | T | - | - | - | - | H | Q | L | S | P | Y | H | R | M | R | K | L | V | K | K | R | T | Y | L | R | W | P | K | P | G | E | D | X | X | X | X | X | T | K | V | F | W | Q | K | L | K | M | A | L | E | - | - | - | - | - | V | K | E | G | H | X | X | X | X | X | X | X | X | X | X | X | X | X | X | X | X | X | X | X | X | X | X | X | X | X | X | X | X | X | X | - | - | X | X | X | X | X | X | X | X | X | X | I | R | F | F | N | S | X | X | X | X | X | X | - | - | - | - | - | - |
| ONTLR13\_3900\_BP | - | - | - | X | X | X | X | G | S | W | I | G | R | G | T | N | Q | T | R | K | X | X | E | Q | N | V | A | X | X | X | X | X | X | X | X | X | X | X | X | X | X | X | X | - | - | - | - | X | X | X | X | X | - | - | - | X | X | X | X | X | X | X | X | X | X | X | X | X | X | X | X | X | X | X | X | X | X | X | X | X | X | X | X | X | X | X | X | X | X | X | X | X | X | X | X | H | A | L | F | F | X | X | T | C | C | A | S | S | I | S | L | H | Q | L | M | - | D | - | - | - | - | - | - | - | - | - | - | - | - | - | F | P | - | - | - | - | - | - | - | - | - | X | X | X | X | X | X | X | X | X | K | T | C | K | I | X | X | X | X | - | - | - | X | X | X | X | X | X | X | X | X | X | X | X | X | X | X | X | X | X | Y | S | T | X | X | X | X | X | X | X | X | X | H | M | H | R | E | X | T | Q | S | C | S | W | R | H | S | L | - | - | - | - | D | C | D | R | P | X | F | I | F | X | Q | N | F | N | X | X | X | T | C | I | R | L | Q | - | - | - | - | - | - | - | - | - | - | - | - | - | - | - | - | - | - | - | K | S | S | K | F | D | X | V | - | - | R | P | X | Y | Q | Q | H | F | T | D | R | K | C | - | - | - | - | C | V | C | Q | - | - | S | K | F | - | - | - | - | X | X | S | X | D | I | K | S | K | - | - | - | - | - | - | - | - | X | X | X | X | X | X | Q | T | C | - | Y | X | X | X | W | E | K | M | F | L | M | A | X | V | A | S | L | S | X | E | L | I | K | I | T | L | S | K | W | H | P | H | L | L | S | P | X | Q | V | X | N | F | W | T | F | L | I | T | N | C | T | - | - | T | - | L | Q | N | F | N | X | Y | C | - | N | X | X | C | H | I | Y | G | S | W | X | X | - | - | - | - | X | X | X | X | X | X | - | - | X | K | - | Q | Q | X | - | - | - | F | I | H | F | X | X | I | I | X | T | D | K | Q | X | X | X | X | X | X | - | - | X | X | X | X | R | T | S | V | P | X | F | V | E | X | X | X | - | - | - | - | X | X | X | X | X | X | X | X | X | N | P | L | A | - | - | - | - | - | - | - | V | X | X | X | X | X | X | X | X | X | Q | D | H | C | X | C | F | S | - | - | - | - | - | - | - | - | - | - | K | - | S | H | L | V | X | N | W | E | X | X | X | X | X | X | X | X | X | - | - | - | - | - | - | - | - | - | - | - | - | - | - | X | X | X | X | X | X | X | X | - | - | X | S | Q | K | E | T | N | X | X | X | X | W | D | V | - | - | - | - | - | - | - | - | - | - | - | - | - | X | X | X | K | Q | D | I | X | X | - | X | X | X | X | X | - | K | S | C | Y | Y | S | X | H | - | - | - | - | - | - | - | - | - | - | X | X | A | S | - | - | - | F | G | S | W | - | - | - | - | - | - | - | - | - | - | - | - | - | - | - | - | - | - | - | - | - | - | G | H | G | K | V | V | R | V | - | - | - | - | - | - | - | - | - | - | - | - | - | - | - | - | - | - | - | - | - | - | - | - | - | - | - | S | E | L | I | I | S | Y | S | E | N | E | X | X | - | - | - | - | X | - | X | Q | X | - | - | - | - | - | - | - | - | - | - | - | - | - | - | - | - | H | I | T | X | P | H | X | S | T | F | - | - | P | A | P | S | Q | X | X | X | X | X | X | X | X | X | X | X | M | R | T | L | Q | L | R | S | N | K | L | S | F | V | N | A | Y | L | F | K | L | C | I | N | V | T | E | X | X | X | X | X | X | X | S | F | D | R | Q | Q | X | X | - | - | - | - | - | X | N | K | I | H | P | X | X | C | F | Q | I | P | X | K | - | - | - | F | K | D | F | N | A | E | X | K | Q | A | S | I | S | S | - | - | - | - | S | C | N | K | E | X | X | X | X | S | T | - | - | K | S | F | R | A | G | S | Q | H | Q | X | H | Q | H | T | G | L | P | X | F | H | Q | - | - | - | - | - | - | - | - | - | - | - | - | - | - | - | - | - | - | - | - | - | - | - | - | - | - | - | - | - | S | X | X | X | X | X | X | K | A | Q | K | A | L | S | L | S | E | F | N | H | S | S | X | X | X | X | T | V | S | F | Q | G | F | K | R | - | I | R | N | S | K | I | T | D | K | P | N | N | X | I | E | R | C | F | Q | R | T | - | - | - | - | F | A | K | T | Y | T | A | A | F | E | W | K | Q | T | H | C | D | Q | K | G | C | - | - | - | - | I | X | R | L | T | F | P | P | E | L | V | I | T | P | K | R | N | K | S | - | - | - | - | - | - | T | X | K | X | E | F | Q | - | - | - | - | - | - | W | T | D | K | S | D | P | X | X | - | - | - | - | - | - | - | - | - | - | - | - | - | - | - | - | - | X | X | X | X | X | X | X | X | X | L | I | Y | K | I | M | I | F | H | K | M | X | L | N | K | D | V | F | S | P | L | I | N | X | X | X | X | X | X | X | X | X | X | X | - | - | - | - | - | - | - | - | - | - | - | X | L | R | R | L | D | L | S | E | N | H | I | N | Y | K | X | X | X | X | V | E | - | - | - | I | C | L | X | X | X | P | P | F | S | Q | X | X | L | S | H | L | E | E | L | L | V | F | S | Q | H | H | R | L | H | X | X | X | X | X | X | Y | L | P | V | N | F | L | Q | G | L | T | N | L | L | S | F | K | A | R | N | I | R | I | A | S | L | D | K | D | T | F | V | Y | X | X | T | P | Q | L | Q | L | L | E | L | S | S | N | E | L | K | E | X | X | X | X | P | P | D | L | - | - | - | F | F | P | I | P | N | L | K | S | L | Y | I | S | R | T | G | L | Y | S | L | D | F | L | I | H | X | X | X | X | N | X | X | X | X | X | T | K | L | E | F | L | Q | A | R | Q | N | L | Y | S | V | I | H | E | E | D | I | Q | S | L | P | A | L | T | Y | L | D | L | Q | G | N | G | F | T | C | N | C | D | N | S | W | F | L | S | W | A | I | N | D | S | K | T | Q | V | X | Y | D | A | Y | X | X | X | X | X | F | T | C | N | Y | P | K | E | F | K | - | - | S | K | K | L | L | D | L | D | T | S | S | C | L | D | N | T | D | F | I | C | F | V | L | T | T | C | T | I | L | C | F | M | V | V | S | F | T | Y | H | F | M | G | L | Q | X | X | X | X | G | L | C | L | L | X | X | L | F | I | A | W | L | F | X | X | X | X | X | X | X | X | S | T | K | T | R | K | L | L | X | X | X | X | X | X | X | X | Q | Y | D | A | F | I | S | Y | N | T | H | D | E | P | W | V | I | - | - | - | - | - | - | - | - | - | - | - | - | - | - | - | - | - | - | - | - | - | D | K | L | L | P | K | L | E | G | E | Q | - | - | - | - | - | - | - | - | - | - | - | - | - | - | X | X | X | X | G | W | R | L | C | L | H | H | R | D | F | E | P | G | R | P | I | I | D | N | I | T | D | A | I | Y | G | S | R | K | T | I | C | V | I | S | R | K | Y | L | E | S | E | W | C | S | R | E | I | Q | A | A | S | F | R | L | F | D | E | Q | T | D | V | L | I | L | V | F | M | E | E | I | P | S | - | - | - | - | Y | L | L | S | P | Y | H | R | M | R | K | M | L | K | K | K | T | Y | L | S | W | P | R | A | G | E | H | X | X | X | X | X | T | E | L | F | W | E | K | L | C | Q | A | L | E | - | - | - | - | - | T | R | E | D | P | X | X | X | X | X | X | X | X | X | X | X | X | X | X | X | X | X | X | X | X | X | X | X | X | X | X | X | X | X | X | X | X | X | X | X | E | X | X | X | X | K | T | L | L | L | T | V | P | T | M | P | X | - | - | - | - | - | - | - | - |
| CATLR13\_3900\_BP | - | - | - | - | - | - | - | - | - | M | S | K | R | E | H | X | X | X | X | X | X | X | X | X | X | X | X | X | X | X | C | A | - | S | X | X | X | X | X | X | X | X | X | X | X | X | X | X | X | X | X | X | X | X | X | X | X | X | X | X | X | X | X | X | X | X | X | X | X | X | X | X | X | X | X | X | X | X | X | X | X | X | X | X | X | X | X | X | X | X | X | X | X | X | X | N | T | V | V | X | P | H | G | C | S | T | A | V | F | S | S | V | L | X | M | - | D | - | - | - | - | - | - | - | - | - | - | - | - | - | T | Q | - | - | - | - | - | - | - | - | - | X | X | X | X | X | X | X | X | X | K | N | C | T | V | X | X | X | X | - | - | - | R | V | F | E | I | X | X | X | X | X | X | X | X | X | X | X | A | Q | N | I | - | - | - | - | - | - | - | - | N | X | X | X | X | X | X | X | X | X | V | L | X | X | E | V | P | A | F | R | T | K | E | Y | T | G | Q | S | H | I | F | G | H | - | - | - | - | F | K | X | C | H | T | E | - | X | - | X | X | - | - | - | - | - | - | - | - | - | - | - | - | - | - | - | N | Q | G | V | F | L | S | T | - | - | V | X | S | X | K | S | K | C | T | T | Q | X | D | - | - | - | - | P | G | S | G | R | R | S | I | Q | Q | F | G | G | X | X | F | A | G | V | K | S | G | - | - | - | - | - | - | - | - | L | X | X | X | X | X | Q | A | D | K | X | X | X | S | W | E | E | C | F | R | - | I | W | E | I | S | L | S | Y | I | X | M | K | T | R | S | Q | I | F | T | S | L | H | F | H | P | W | S | V | X | E | K | L | I | C | P | E | I | I | X | R | - | - | K | - | S | T | K | F | S | L | S | F | - | X | X | X | Y | H | N | Y | R | N | C | X | X | - | - | - | - | X | X | X | X | X | X | - | - | X | W | - | E | X | W | - | - | - | L | H | L | F | X | X | D | L | G | A | V | Q | H | X | X | X | X | X | X | - | - | X | X | X | X | E | T | T | S | S | G | F | I | X | X | X | X | - | - | - | - | X | X | X | X | X | X | X | X | X | N | P | L | K | - | - | - | - | - | - | - | V | X | X | X | X | X | X | X | X | X | Q | Y | H | C | R | Y | F | P | - | - | - | - | - | - | - | - | - | - | S | - | P | X | X | F | G | L | G | F | X | X | X | X | X | X | X | X | X | - | - | - | - | - | - | - | - | - | - | - | - | - | - | X | X | X | X | X | X | X | X | - | - | X | X | Y | Y | W | K | H | X | X | X | X | W | D | V | - | - | - | - | - | - | - | - | - | - | - | - | - | X | X | X | G | Q | K | I | X | X | - | X | X | X | X | X | - | T | K | C | E | T | S | K | F | - | - | - | - | - | - | - | - | - | - | K | W | N | T | - | - | - | I | A | L | G | - | - | - | - | - | - | - | - | - | - | - | - | - | - | - | - | - | - | - | - | - | - | E | D | Q | R | S | H | T | N | - | - | - | - | - | - | - | - | - | - | - | - | - | - | - | - | - | - | - | - | - | - | - | - | - | - | - | L | Q | L | F | I | G | Q | P | E | V | T | L | X | X | X | W | G | I | - | X | K | X | - | - | - | - | - | - | - | - | - | - | - | - | - | - | - | - | E | L | X | X | E | - | - | - | - | T | - | - | S | A | X | F | H | X | X | X | X | X | X | X | X | X | X | X | L | K | F | L | G | L | Q | W | N | N | I | T | S | I | S | K | D | E | M | Q | S | C | T | Q | L | T | E | X | X | X | X | X | X | X | X | S | L | X | X | Q | X | X | - | - | - | - | - | X | X | X | X | X | X | X | X | X | X | X | X | X | X | X | - | - | - | X | X | X | X | X | X | X | X | X | X | T | I | Q | C | P | - | - | - | - | C | C | H | Q | Q | X | X | X | X | P | T | - | - | R | S | S | F | F | G | P | H | L | Q | Q | Y | K | E | H | K | X | C | X | F | C | X | - | - | - | - | - | - | - | - | - | - | - | - | - | - | - | - | - | - | - | - | - | - | - | - | - | - | - | - | - | L | X | X | X | X | X | X | P | I | T | D | P | S | D | L | X | Q | X | N | I | F | Y | X | X | X | X | G | I | C | F | X | T | S | E | K | - | H | N | N | S | N | D | W | I | X | Q | T | L | X | X | Q | Q | I | F | X | K | R | - | - | - | - | S | S | K | T | X | D | F | G | S | S | K | X | Q | A | G | L | C | P | X | G | T | - | - | - | - | F | X | L | L | E | F | T | X | E | F | V | S | T | R | Q | S | N | I | I | Y | R | S | R | V | L | X | X | T | D | Q | F | N | - | - | - | - | - | - | F | X | X | X | X | X | - | - | - | - | - | - | - | - | - | - | - | - | - | - | - | - | - | - | - | - | - | - | - | X | X | X | X | X | X | X | I | C | N | Q | T | S | S | L | S | V | X | I | K | A | Y | V | F | S | K | L | P | N | X | X | X | X | X | X | X | X | X | X | X | - | - | - | - | - | - | - | - | - | - | - | X | L | K | T | L | L | L | N | N | N | Y | I | S | Y | N | X | X | X | X | K | S | - | - | - | L | L | N | X | X | X | P | P | F | Q | Y | X | X | X | X | X | L | K | K | L | K | T | L | N | I | F | S | Q | G | H | K | G | M | L | X | X | Y | L | P | S | N | L | F | K | G | L | W | H | L | E | E | L | Q | A | E | N | L | N | I | N | S | L | H | P | D | T | F | T | Y | X | X | T | P | Y | L | W | V | L | D | L | S | R | N | D | F | I | L | X | X | X | X | T | T | K | I | - | - | - | F | L | P | L | H | V | L | K | T | L | H | L | S | K | V | G | L | Q | S | L | D | F | L | I | N | X | X | X | X | N | X | X | X | X | X | S | K | L | H | L | L | H | V | S | K | N | A | L | S | V | I | N | E | T | V | I | Q | S | F | P | T | L | I | Y | L | D | L | R | G | N | Y | L | S | C | D | C | T | N | A | W | F | V | N | W | T | I | S | S | N | D | T | E | V | X | E | S | A | Y | X | X | X | X | X | L | T | C | N | Y | P | D | K | L | K | - | - | G | H | K | L | M | D | L | D | I | G | S | C | N | V | D | V | G | F | F | C | F | I | S | T | A | T | L | V | S | V | T | L | L | S | T | F | L | F | H | F | L | K | W | Q | X | X | X | X | Y | L | C | L | L | X | X | L | F | L | A | L | L | H | X | X | X | X | X | X | X | X | N | S | R | E | S | P | M | V | X | X | X | X | X | X | X | X | Q | Y | D | A | F | I | S | Y | N | V | H | D | E | L | W | V | M | - | - | - | - | - | - | - | - | - | - | - | - | - | - | - | - | - | - | - | - | - | R | E | L | L | P | Q | L | E | G | E | Q | - | - | - | - | - | - | - | - | - | - | - | - | - | - | X | X | X | X | G | W | K | L | C | L | H | H | R | D | F | E | P | G | K | P | I | M | D | N | I | V | D | G | I | Y | S | S | R | K | T | I | C | V | I | S | R | H | Y | L | E | S | E | W | C | S | R | E | I | Q | I | A | S | F | R | L | F | D | E | K | K | D | V | L | I | L | V | F | L | E | N | I | P | S | - | - | - | - | H | Q | L | S | P | Y | Y | R | M | K | R | L | I | Q | R | K | T | Y | L | S | W | P | K | P | G | E | D | X | X | X | X | X | T | R | V | F | W | Q | K | L | R | I | A | L | E | - | - | - | - | - | T | K | G | X | T | X | X | X | X | X | X | X | X | X | X | X | X | X | X | X | X | X | X | X | X | X | X | X | X | X | X | X | X | X | X | X | X | - | - | - | - | - | X | X | X | Q | X | X | X | X | W | S | L | T | L | A | G | I | X | - | G | - | - | S | X |
| SSTLR13\_3900\_BP | - | - | - | - | - | - | - | - | - | M | S | G | K | G | H | G | X | X | X | X | X | X | X | X | X | X | X | X | X | L | H | I | L | X | X | X | X | X | X | X | X | X | X | X | X | X | X | X | X | X | X | X | X | X | X | X | X | X | X | X | X | X | X | X | X | X | X | X | X | X | X | X | X | X | X | X | X | X | X | X | X | X | X | X | X | X | X | X | X | X | X | X | X | X | X | V | S | F | Y | X | X | F | F | C | I | S | G | F | - | Y | L | I | Q | X | M | - | V | - | - | - | - | - | - | - | - | - | - | - | - | - | T | Q | - | - | - | - | - | - | - | - | - | X | X | X | X | X | X | X | X | X | K | H | C | R | I | X | X | X | X | - | - | - | G | I | S | X | X | X | X | X | X | X | X | X | X | X | X | X | X | X | N | T | - | - | - | - | - | - | - | - | S | X | X | X | X | X | X | X | X | X | A | M | X | W | Q | E | P | Q | C | R | T | S | - | - | - | - | R | H | P | I | I | G | E | N | C | K | H | S | K | E | X | H | F | T | X | X | - | X | K | - | - | - | - | - | - | - | - | - | - | - | - | - | - | - | K | X | G | L | Q | R | S | N | - | - | V | P | Q | - | N | V | N | H | V | Q | Q | Q | D | - | - | - | - | L | S | C | G | E | W | V | F | F | R | P | G | G | X | X | S | R | A | A | E | S | V | - | - | - | - | - | - | - | - | L | X | X | X | X | X | Q | T | H | F | X | X | X | S | Q | T | K | C | L | R | - | A | W | I | T | S | Q | Y | Y | T | W | I | T | T | T | S | Q | V | X | X | H | H | L | F | S | Y | S | S | A | X | R | Y | X | T | X | P | A | T | N | C | I | - | - | V | - | S | R | S | F | S | T | F | Y | - | X | X | X | Y | Q | A | C | X | S | C | X | X | - | - | - | - | X | X | X | X | X | X | - | - | X | W | - | E | Q | Q | - | - | - | L | D | L | F | X | X | V | T | G | N | I | K | X | X | X | X | X | X | X | - | - | X | X | X | X | R | A | D | R | A | G | S | I | Q | X | X | X | - | - | - | - | X | X | X | X | X | X | X | X | X | N | P | L | E | - | - | - | - | - | - | - | I | X | X | X | X | X | X | X | X | X | Q | C | H | S | R | Y | F | S | - | - | - | - | - | - | - | - | - | - | L | - | S | X | E | F | R | P | L | L | X | X | X | X | X | X | X | X | X | - | - | - | - | - | - | - | - | - | - | - | - | - | - | X | X | X | X | X | X | X | X | - | - | X | W | H | X | W | K | H | X | X | X | X | W | D | V | - | - | - | - | - | - | - | - | - | - | - | - | - | X | X | X | R | Q | V | F | X | X | - | X | X | X | X | X | - | E | E | C | E | Q | S | R | L | - | - | - | - | - | - | - | - | - | - | E | W | H | S | - | - | - | H | V | F | T | - | - | - | - | - | - | - | - | - | - | - | - | - | - | - | - | - | - | - | - | - | - | G | D | X | F | S | A | P | E | - | - | - | - | - | - | - | - | - | - | - | - | - | - | - | - | - | - | - | - | - | - | - | - | - | - | - | H | Q | P | F | F | G | I | S | G | X | X | X | X | X | X | W | V | K | - | X | E | E | - | - | - | - | - | - | - | - | - | - | - | - | - | - | - | - | I | I | S | L | M | - | - | - | - | X | - | - | P | A | T | Y | L | X | X | X | X | X | X | X | X | X | X | X | L | K | T | L | R | L | Q | C | N | N | F | S | T | V | S | D | K | L | L | Q | S | C | T | Q | V | T | D | X | X | X | X | X | X | X | G | H | I | R | X | X | X | X | - | - | - | - | - | X | Y | Y | X | P | V | R | S | R | F | S | I | N | X | R | - | - | - | S | X | N | S | D | T | R | Q | X | R | P | L | F | C | A | - | - | - | - | E | C | Y | K | E | X | X | X | X | S | P | - | - | H | I | E | K | I | G | S | Q | L | Q | H | H | Q | N | F | R | L | L | G | F | L | Q | - | - | - | - | - | - | - | - | - | - | - | - | - | - | - | - | - | - | - | - | - | - | - | - | - | - | - | - | - | S | X | X | X | X | X | X | K | T | R | K | T | L | S | X | Q | Q | F | N | L | K | P | X | X | X | X | R | L | C | F | P | G | F | K | G | - | L | K | X | P | D | T | X | K | E | P | H | P | K | L | R | X | C | F | H | K | G | - | - | - | - | - | - | - | - | - | - | - | - | - | - | - | - | - | - | - | - | - | - | F | A | T | - | - | - | - | F | K | E | L | G | F | R | I | Q | X | T | N | X | H | Q | N | K | V | L | Q | K | - | - | - | L | K | I | T | X | G | F | G | - | - | - | - | - | - | F | I | X | Q | S | N | K | D | T | G | R | - | - | W | G | L | C | G | F | G | Q | T | X | K | X | X | X | X | X | X | X | X | X | X | X | L | F | C | X | T | M | L | X | V | K | V | X | I | R | G | A | V | F | K | G | L | R | S | X | X | X | X | X | X | X | X | X | X | X | - | - | - | - | - | - | - | - | - | - | - | X | L | K | T | L | N | M | A | S | N | A | I | K | Y | E | X | X | X | X | M | N | - | - | - | Y | S | N | X | X | X | P | P | F | T | D | X | X | L | S | S | L | E | N | L | Y | I | F | S | Q | H | Y | S | K | Y | K | R | Q | S | X | X | Y | L | P | S | N | F | L | E | G | L | K | M | L | S | E | F | Q | A | G | N | L | H | I | M | S | L | H | P | D | V | F | I | Y | X | X | T | P | Q | L | L | S | L | D | I | S | Q | N | W | F | K | A | X | X | X | X | S | P | E | L | - | - | - | F | H | P | I | P | K | L | K | R | L | T | I | S | K | T | E | L | E | S | L | D | F | L | I | E | X | X | X | X | N | X | X | X | X | X | T | Q | V | H | F | L | K | V | R | Q | N | K | I | T | W | T | N | E | T | V | L | H | S | L | P | A | L | T | L | L | D | M | Q | R | N | P | F | I | C | D | C | R | N | A | W | F | V | H | W | A | E | S | N | N | Q | T | Q | V | X | A | Y | A | H | X | X | X | X | X | Y | P | C | N | Y | P | D | D | L | K | - | - | N | T | K | L | L | D | L | D | T | H | S | C | S | V | D | L | G | V | F | Y | F | I | S | T | T | S | L | V | L | L | T | L | L | G | S | F | I | Y | H | H | L | R | W | Q | X | X | X | X | G | I | C | L | L | X | X | L | F | L | A | Y | L | H | X | X | X | X | X | H | X | X | S | G | G | T | D | K | L | L | X | X | X | X | X | X | X | X | Q | Y | D | A | F | I | S | Y | N | T | H | D | E | S | W | V | V | - | - | - | - | - | - | - | - | - | - | - | - | - | - | - | - | - | - | - | - | - | R | E | L | L | P | E | L | E | G | E | Q | - | - | - | - | - | - | - | - | - | - | - | - | - | - | X | X | X | X | G | W | K | L | C | L | H | H | R | D | F | Q | P | G | K | P | I | I | E | N | I | T | D | A | I | Y | R | S | R | K | T | I | C | V | I | T | H | Q | Y | L | E | S | E | W | C | S | R | E | I | Q | V | A | S | F | R | L | F | D | E | Q | K | D | V | L | I | L | V | F | L | E | E | I | P | D | - | - | - | - | H | Q | L | S | P | Y | H | R | M | R | R | L | V | K | R | R | T | Y | L | S | W | P | R | A | G | E | D | X | X | X | X | X | T | R | V | F | W | Q | K | L | Q | V | A | L | E | - | - | - | - | - | T | R | D | L | P | X | X | X | X | X | X | X | X | X | X | X | X | X | X | X | X | X | X | X | X | X | X | X | X | X | X | X | X | X | X | X | X | - | - | - | - | - | X | X | X | A | X | X | X | X | E | N | P | I | L | T | G | V | E | - | R | - | - | Q | X |
| OMTLR13\_3900\_BP | - | - | - | - | - | - | - | - | - | L | S | G | K | G | N | G | X | X | X | X | X | X | X | X | X | X | X | X | X | L | H | I | L | X | X | X | X | X | X | X | X | X | X | X | X | X | X | X | X | X | X | X | X | X | X | X | X | X | X | X | X | X | X | X | X | X | X | X | X | X | X | X | X | X | X | X | X | X | X | X | X | X | X | X | X | X | X | X | X | X | X | X | X | X | X | V | S | F | Y | X | X | F | F | C | I | S | G | F | - | Y | L | I | Q | X | M | - | A | - | - | - | - | - | - | - | - | - | - | - | - | - | T | Q | - | - | - | - | - | - | - | - | - | X | X | X | X | X | X | X | X | X | K | H | C | R | I | X | X | X | X | - | - | - | G | I | S | X | X | X | X | X | X | X | X | X | X | X | X | X | X | X | N | T | - | - | - | - | - | - | - | - | S | X | X | X | X | X | X | X | X | X | A | M | X | W | Q | E | P | Q | C | R | T | S | - | - | - | - | R | H | P | I | I | G | E | I | C | K | H | S | K | E | X | H | F | T | X | X | - | X | K | - | - | - | - | - | - | - | - | - | - | - | - | - | - | - | K | G | G | L | Q | R | S | N | - | - | N | P | Q | - | N | V | N | H | V | Q | Q | Q | D | - | - | - | - | F | S | C | X | E | W | V | F | F | R | P | G | G | X | X | S | R | A | A | E | S | G | - | - | - | - | - | - | - | - | L | X | X | X | X | X | Q | T | H | I | X | X | X | S | Q | T | K | C | L | R | - | A | W | T | T | S | Q | Y | Y | N | W | R | T | T | T | S | Q | V | L | P | P | H | L | F | S | Y | S | S | A | X | R | Y | X | T | X | P | A | T | N | Y | I | - | - | V | - | S | R | S | X | S | T | F | Y | - | X | X | X | C | Q | A | C | X | S | C | X | X | - | - | - | - | X | X | X | X | X | X | - | - | X | W | - | E | Q | Q | - | - | - | L | E | L | F | X | X | V | T | G | N | I | K | X | X | X | X | X | X | X | - | - | X | X | X | X | R | A | D | R | A | G | S | I | Q | X | X | X | - | - | - | - | X | X | X | X | X | X | X | X | X | N | P | L | E | - | - | - | - | - | - | - | I | X | X | X | X | X | X | X | X | X | Q | C | H | S | R | Y | F | S | - | - | - | - | - | - | - | - | - | - | L | - | S | X | E | T | R | P | L | L | X | X | X | X | X | X | X | X | X | - | - | - | - | - | - | - | - | - | - | - | - | - | - | X | X | X | X | X | X | X | X | - | - | X | W | H | X | W | K | H | X | X | X | X | W | D | V | - | - | - | - | - | - | - | - | - | - | - | - | - | X | X | X | G | Q | V | F | X | X | - | X | X | X | X | X | - | E | X | C | E | Q | P | R | L | - | - | - | - | - | - | - | - | - | - | E | W | H | S | - | - | - | Y | V | F | T | - | - | - | - | - | - | - | - | - | - | - | - | - | - | - | - | - | - | - | - | - | - | G | D | W | F | G | A | P | E | - | - | - | - | - | - | - | - | - | - | - | - | - | - | - | - | - | - | - | - | - | - | - | - | - | - | - | H | Q | L | F | V | G | I | S | G | X | X | X | X | X | X | W | I | K | - | X | E | K | - | - | - | - | - | - | - | - | - | - | - | - | - | - | - | - | I | T | X | L | M | - | - | - | - | X | - | - | P | A | T | Y | L | X | X | X | X | X | X | X | X | X | X | X | L | K | T | L | R | L | Q | C | N | N | F | S | S | V | S | D | K | M | L | Q | S | C | T | Q | V | T | D | X | X | X | X | X | X | X | G | H | I | R | X | X | X | X | - | - | - | - | - | X | Y | Y | X | P | V | R | S | C | F | S | I | N | K | R | - | - | - | S | X | N | S | E | T | R | L | X | W | P | L | F | C | A | - | - | - | - | E | C | Y | K | E | X | X | X | X | S | P | - | - | H | I | K | K | I | G | S | Q | P | Q | H | H | Q | N | F | R | L | L | G | F | L | Q | - | - | - | - | - | - | - | - | - | - | - | - | - | - | - | - | - | - | - | - | - | - | - | - | - | - | - | - | - | S | X | X | X | X | X | X | K | T | X | K | T | L | S | X | Q | Q | F | N | L | K | T | X | X | X | X | R | V | C | F | P | G | F | K | G | - | L | K | X | P | D | T | X | K | E | Q | N | P | K | R | G | R | R | F | H | K | G | - | - | - | - | - | - | - | - | - | - | - | - | - | - | - | - | - | - | - | - | - | - | F | A | T | - | - | - | - | F | K | E | L | G | F | R | I | Q | X | T | N | Q | H | Q | N | K | G | L | Q | K | - | - | - | L | K | I | T | X | G | F | G | - | - | - | - | - | - | F | I | X | Q | X | N | K | D | T | G | X | - | - | W | G | L | C | G | F | G | Q | T | X | K | X | X | X | X | X | X | X | X | X | X | X | L | S | C | X | T | M | L | X | V | K | V | X | I | R | G | A | V | F | K | G | L | R | S | X | X | X | X | X | X | X | X | X | X | X | - | - | - | - | - | - | - | - | - | - | - | X | L | K | T | L | N | M | A | S | N | A | I | K | Y | E | X | X | X | X | M | N | - | - | - | N | Y | N | X | X | X | P | P | F | T | D | X | X | L | S | S | L | E | N | L | Y | I | F | S | Q | H | Y | S | K | Y | M | R | Q | S | X | X | C | L | P | S | N | F | L | E | G | L | N | M | L | S | E | F | Q | A | G | N | L | H | I | V | T | L | H | P | D | T | F | I | H | X | X | T | P | Q | L | L | S | L | D | I | S | Q | N | F | F | H | A | X | X | X | X | S | P | E | L | - | - | - | F | H | P | I | P | K | L | K | R | L | T | I | S | K | T | E | L | E | S | L | D | F | L | I | E | X | X | X | X | N | X | X | X | X | X | T | Q | V | R | F | L | K | V | R | Q | N | Q | I | K | W | T | N | E | T | V | L | H | S | L | P | A | L | T | L | L | D | M | Q | R | N | P | F | I | C | D | C | R | N | A | W | F | V | Y | W | A | E | S | N | N | Q | T | Q | V | X | A | Y | A | H | X | X | X | X | X | Y | P | C | S | Y | P | D | D | L | K | - | - | D | T | K | L | L | D | L | D | T | H | S | C | S | V | D | L | G | I | F | Y | F | I | S | T | T | S | L | V | L | L | T | L | L | G | S | F | I | Y | H | L | L | R | W | Q | X | X | X | X | G | I | C | L | L | X | X | L | F | L | A | Y | L | H | X | X | X | X | X | L | X | X | S | G | G | T | D | K | L | L | X | X | X | X | X | X | X | X | Q | Y | D | A | F | V | S | Y | N | T | H | D | E | C | W | V | V | - | - | - | - | - | - | - | - | - | - | - | - | - | - | - | - | - | - | - | - | - | R | E | L | L | P | E | L | E | G | E | Q | - | - | - | - | - | - | - | - | - | - | - | - | - | - | X | X | X | X | G | W | K | L | C | L | H | H | R | D | F | Q | P | G | K | P | I | I | E | N | I | T | D | A | I | Y | R | S | R | K | T | I | C | V | I | T | H | R | Y | L | Q | S | E | W | C | S | R | E | I | Q | V | A | S | F | R | L | F | D | E | Q | K | D | V | L | I | L | V | F | L | E | E | I | P | D | - | - | - | - | H | Q | L | S | P | Y | H | R | M | R | R | L | V | K | R | R | T | Y | L | S | W | P | R | V | G | E | D | X | X | X | X | X | T | R | V | F | W | Q | K | L | Q | V | A | L | E | - | - | - | - | - | T | R | D | W | P | X | X | X | X | X | X | X | X | X | X | X | X | X | X | X | X | X | X | X | X | X | X | X | X | X | X | X | X | X | X | X | X | - | - | - | - | - | X | X | X | A | X | X | X | X | E | N | P | I | L | T | G | V | E | - | R | - | - | Q | X |
| DRTLR13\_3900\_BP | - | - | - | - | - | - | - | - | - | - | - | X | X | X | X | X | X | X | X | X | X | - | - | X | - | X | X | X | X | X | X | X | X | X | X | X | X | X | X | X | X | X | X | X | X | X | - | X | X | X | X | X | X | X | - | X | X | X | X | X | X | X | X | - | - | X | - | - | - | X | X | X | X | X | X | X | X | X | X | X | X | X | X | X | X | X | X | X | X | X | X | X | X | X | X | X | X | X | X | X | X | X | X | X | X | X | X | M | F | A | F | - | - | - | - | - | - | - | - | - | - | - | A | T | N | S | N | F | L | R | F | F | W | R | I | A | L | G | L | S | I | C | N | V | V | L | T | R | I | T | K | R | C | I | T | I | E | E | H | L | L | K | Q | M | P | W | T | P | - | - | - | - | - | - | - | - | - | - | - | - | - | - | - | - | - | - | - | - | - | H | C | L | H | Y | P | G | K | G | P | Y | - | - | - | - | - | - | - | - | X | X | X | X | X | X | X | - | - | - | X | X | K | L | Q | Y | H | R | P | E | H | X | S | V | T | G | R | L | X | G | X | N | P | L | C | L | W | R | X | X | Y | E | X | X | X | S | C | X | S | L | F | T | L | T | - | - | F | S | G | G | - | - | - | - | - | T | S | H | R | W | N | T | S | G | E | S | S | G | W | C | L | X | R | T | P | Q | X | X | S | X | V | S | - | - | - | - | V | N | A | F | Q | X | X | S | - | L | Q | I | G | Q | X | X | X | W | I | I | G | H | L | L | V | X | I | - | I | W | - | - | - | R | S | C | H | X | Q | V | X | C | C | S | M | D | P | V | V | Y | L | T | H | W | L | V | X | S | D | X | T | L | S | E | L | A | H | K | - | - | - | T | L | V | K | F | S | V | - | - | - | - | - | - | - | - | - | - | - | - | - | - | - | - | - | - | - | - | - | - | - | - | - | - | - | - | - | - | - | - | - | - | - | - | - | - | - | - | - | - | - | - | - | - | - | - | - | - | - | - | - | - | - | - | - | - | - | - | - | - | - | - | - | - | - | - | - | - | - | - | - | - | - | V | F | R | M | E | X | P | G | X | D | T | X | X | X | X | X | X | X | X | X | X | R | R | Q | X | D | F | N | N | X | X | E | Q | R | M | C | R | G | L | N | D | L | A | A | Y | C | P | - | - | F | W | G | S | K | S | V | S | N | W | E | Q | Y | X | I | Y | S | S | X | X | X | X | N | S | L | N | I | F | Q | N | L | S | S | L | F | L | E | F | E | G | K | - | - | S | L | G | S | I | W | E | S | G | V | - | G | X | X | X | X | X | X | X | X | X | X | X | X | X | X | X | X | X | X | X | S | Q | X | F | D | F | X | X | X | X | G | K | V | X | X | X | K | K | I | L | - | - | - | - | - | - | - | - | X | X | X | X | X | X | X | N | K | L | Q | R | F | V | S | S | - | - | - | - | - | - | - | - | - | - | - | - | - | - | - | - | - | - | - | - | - | C | X | X | X | X | X | X | - | X | X | X | - | - | - | - | - | - | - | - | - | - | - | - | - | - | - | - | - | X | X | X | X | X | X | R | V | F | I | R | N | H | S | A | L | F | I | H | Q | X | T | D | X | L | L | R | T | X | R | T | V | G | L | X | X | X | X | X | X | X | X | X | - | - | - | - | - | - | - | - | - | - | - | - | - | - | - | - | - | - | - | - | - | X | X | X | X | X | L | T | - | - | - | K | L | L | I | Q | N | S | K | I | D | N | L | D | X | X | X | X | X | X | X | X | - | - | - | - | - | - | - | - | - | - | - | - | X | X | - | - | - | - | - | - | X | - | - | - | - | - | - | - | - | - | - | - | X | X | X | X | X | X | X | X | X | V | L | D | Q | X | T | X | X | X | X | X | - | - | - | - | - | - | - | - | - | - | - | - | - | - | X | X | X | X | X | X | X | X | X | X | X | N | A | G | P | S | N | G | L | H | E | V | D | R | C | S | L | L | F | C | C | E | Q | H | X | N | V | V | F | N | L | T | G | P | H | W | K | L | Y | H | Q | Y | X | W | R | S | V | C | M | Y | X | X | X | X | X | X | F | S | X | A | A | L | S | E | P | E | C | H | X | N | P | X | X | X | X | A | A | C | I | S | R | F | A | - | S | T | Q | N | P | T | T | X | L | K | Q | D | L | - | - | T | A | F | C | Q | X | F | - | - | - | - | - | Q | K | P | S | C | - | - | - | - | - | - | - | - | - | - | - | - | - | - | - | - | - | - | - | - | - | F | R | G | S | A | D | - | - | - | - | X | R | X | H | H | X | D | Y | R | D | R | N | I | L | G | P | A | G | A | S | X | T | X | X | X | X | X | X | X | X | X | X | X | X | X | X | I | W | X | T | G | I | C | L | X | X | X | X | X | X | X | X | X | X | X | X | C | I | X | R | T | X | X | X | X | - | - | - | - | - | X | X | X | X | X | X | X | X | F | F | E | F | P | P | K | L | Q | R | L | S | I | D | A | X | X | X | L | W | N | S | Y | L | - | - | - | - | H | W | K | C | I | T | T | Q | W | N | V | C | S | G | T | K | W | X | X | K | L | - | - | - | S | F | A | D | C | K | S | - | - | - | - | - | - | - | D | V | L | M | A | V | R | E | L | K | V | N | C | T | F | F | L | C | E | N | X | X | L | F | M | A | P | Y | F | L | N | L | E | S | F | E | I | X | X | X | G | G | A | E | R | A | P | L | N | Y | A | T | I | N | N | X | X | L | H | H | L | K | H | L | K | L | A | G | L | N | F | P | N | X | X | X | X | X | T | E | S | R | S | A | F | W | N | L | T | Q | L | Q | T | L | V | M | V | N | C | - | - | - | Y | L | S | F | L | T | K | S | M | F | R | D | X | X | X | X | X | T | S | L | Q | L | L | R | L | Y | S | D | S | P | L | I | L | T | D | G | V | F | G | V | L | P | V | L | K | A | F | I | L | D | R | V | D | F | Q | C | S | C | E | N | G | W | L | L | D | W | A | D | S | T | E | K | V | Q | V | X | I | Y | L | Q | X | X | X | X | X | Q | Q | C | V | W | X | X | H | Y | Q | - | - | K | L | N | F | L | A | T | M | E | K | L | C | Q | T | D | A | Q | Y | I | C | Y | V | A | T | A | S | S | I | S | L | L | L | S | A | A | V | G | Y | R | F | A | R | W | P | X | X | X | X | T | C | P | L | L | X | X | R | L | K | G | W | M | E | R | R | F | G | R | R | W | N | R | R | R | R | R | M | E | D | D | Y | G | E | I | E | E | M | Q | Y | D | A | F | V | S | F | C | S | Q | D | E | A | W | V | L | - | - | - | - | - | - | - | - | - | - | - | - | - | - | - | - | - | - | - | - | - | G | E | M | A | P | R | L | E | D | Q | G | - | - | - | - | - | - | - | - | - | - | - | - | - | - | X | X | N | P | R | L | R | L | C | L | H | N | R | D | F | E | V | G | K | D | I | M | D | N | I | T | D | S | I | H | N | S | Q | C | T | V | C | L | I | S | R | R | Y | L | R | S | D | W | C | G | L | E | M | R | V | A | T | H | R | Q | L | E | E | Q | K | H | R | L | I | L | I | F | L | Q | H | I | S | P | - | - | - | - | F | E | L | S | A | F | H | R | L | A | K | L | V | R | S | R | T | Y | L | D | W | P | E | D | E | G | D | X | X | X | X | X | R | E | H | F | W | D | R | L | R | R | N | I | A | E | D | S | E | A | S | X | X | X | X | X | X | X | X | X | X | X | X | X | X | X | X | X | X | X | X | X | X | X | X | X | X | X | - | - | - | - | - | - | - | X | X | X | X | X | X | X | X | X | X | X | X | X | X | X | X | X | X | X | X | X | X | X | X | - | - | - | - | - | - |
| TFTLR13\_3900\_BP | - | - | - | - | - | - | - | - | - | - | - | X | X | X | X | X | X | X | X | X | X | X | X | X | X | X | X | X | X | X | X | X | X | X | X | X | X | X | X | X | X | X | X | X | X | X | - | X | X | X | X | X | X | X | - | X | X | X | X | X | X | X | X | - | - | X | - | - | - | X | X | X | X | X | X | X | X | X | X | X | X | X | X | X | X | X | X | X | X | X | X | X | X | X | X | X | X | X | X | X | X | X | X | X | X | X | X | X | X | C | F | G | - | - | - | - | - | - | - | - | - | - | S | S | P | C | S | Y | X | H | Q | L | Q | A | G | C | X | X | X | - | - | - | - | - | X | X | X | X | X | X | K | K | C | F | V | X | T | K | M | - | - | - | K | L | S | K | T | X | X | X | X | X | X | X | X | - | - | - | - | - | - | - | - | - | - | X | X | X | P | D | Y | S | V | L | P | E | G | K | P | N | - | - | - | - | - | - | - | - | S | K | L | F | G | C | Q | X | R | E | A | G | S | C | H | A | S | S | S | S | X | Q | S | L | P | G | S | S | T | G | T | G | X | X | X | - | - | - | - | - | - | - | - | - | - | - | T | S | K | X | F | X | A | I | - | - | L | H | S | X | T | T | A | H | X | P | - | - | I | L | - | - | S | Q | G | H | S | F | W | S | F | C | R | V | A | K | S | A | S | D | - | - | - | - | - | - | - | - | I | I | X | R | T | F | X | X | X | K | K | Q | P | V | A | G | L | L | F | Q | Q | M | L | L | G | - | S | Y | P | A | X | A | S | F | F | F | I | T | T | I | C | Q | Q | Y | Q | Q | M | Y | L | V | E | X | K | N | Y | K | P | C | T | X | X | R | A | V | A | S | - | - | - | K | S | X | I | L | P | V | K | L | L | N | S | H | N | Q | X | C | - | P | C | X | X | X | X | X | X | X | X | X | R | H | R | X | H | G | C | P | E | K | S | - | - | - | - | - | - | - | - | - | A | L | S | S | I | R | G | R | I | D | G | X | X | N | I | S | K | P | X | K | G | S | V | L | Y | - | - | - | - | - | - | - | - | - | F | R | V | X | N | P | W | G | K | A | S | S | S | T | - | - | - | - | - | - | - | - | - | - | - | F | K | I | - | F | L | Y | X | T | C | P | X | I | - | R | - | F | S | R | C | S | C | C | S | L | E | Y | K | R | F | R | R | W | R | Q | N | W | - | - | - | N | K | V | V | - | - | - | L | G | L | F | V | - | - | - | I | L | Y | T | I | F | Q | L | K | A | X | N | X | K | T | I | F | P | - | - | - | - | - | - | - | F | - | - | - | - | - | - | - | - | - | - | - | - | V | R | - | - | - | - | - | - | - | - | I | S | S | S | K | D | V | R | D | C | E | K | Y | Y | L | X | A | L | T | C | K | I | S | Y | V | S | L | G | L | X | K | I | Y | K | F | X | X | X | X | - | X | X | X | X | X | X | X | X | X | X | X | - | - | - | - | - | - | - | - | - | - | - | - | - | W | I | - | X | D | S | N | F | C | H | S | Y | S | I | - | - | - | - | - | - | - | - | - | - | - | - | I | F | V | T | H | L | I | F | C | S | X | X | X | X | L | Q | K | F | S | L | N | - | - | - | - | - | - | - | F | S | L | L | S | R | I | T | R | R | Q | F | Y | C | L | Q | N | L | Q | T | X | X | X | X | X | X | X | X | S | R | L | X | - | X | X | X | X | N | L | C | D | X | R | F | C | I | C | R | I | X | X | V | A | R | F | L | H | E | G | H | G | F | A | E | - | - | - | - | - | - | - | - | - | - | - | - | X | X | S | D | F | X | W | X | X | X | X | - | - | - | - | S | P | L | F | G | I | F | N | F | E | K | X | X | N | L | V | N | X | L | Y | C | I | X | R | P | - | - | - | - | - | - | - | - | - | - | - | - | - | - | X | X | X | X | - | - | X | X | K | F | E | S | L | G | P | S | R | Q | P | D | Y | S | D | R | C | E | T | F | D | S | V | S | G | T | - | - | - | - | - | - | - | - | - | - | - | - | - | - | - | - | - | - | - | - | - | - | - | - | - | - | - | - | - | - | G | E | F | G | K | F | R | - | - | - | - | - | - | - | - | - | - | - | - | - | - | - | L | E | I | X | - | - | - | - | - | - | - | - | - | - | - | - | - | - | Q | N | H | V | - | R | R | X | L | R | I | Q | T | T | E | K | F | - | K | D | F | N | - | - | F | G | X | Q | Q | H | F | X | N | X | Q | V | H | L | L | W | S | G | W | F | R | V | I | D | D | X | I | X | C | - | - | - | - | - | - | - | - | - | - | S | X | L | Y | R | A | L | C | P | E | - | - | - | - | - | - | - | - | - | - | - | - | - | - | - | - | - | - | - | Q | F | I | C | F | - | - | - | - | A | E | X | X | X | S | V | G | C | L | K | H | P | S | S | M | T | A | E | V | E | I | N | L | X | X | X | X | L | W | F | G | R | I | P | I | N | L | T | E | L | I | I | S | S | C | S | R | P | M | S | I | V | I | G | S | E | S | A | P | K | P | X | X | L | Q | L | E | I | F | G | X | X | - | - | - | - | E | S | - | - | - | V | S | E | T | X | E | K | - | - | - | - | - | - | - | P | F | F | L | S | V | V | G | L | K | A | I | V | K | Q | L | V | C | G | S | X | X | R | F | A | G | K | Y | F | K | F | L | E | S | F | E | L | S | S | X | X | X | V | M | T | T | F | D | D | L | V | D | L | N | T | X | X | L | L | H | L | R | Y | L | K | L | T | C | I | D | L | F | D | X | X | X | X | X | P | H | L | G | I | L | L | H | N | L | T | A | L | Q | F | F | G | L | Y | R | S | - | - | - | R | I | P | S | I | S | E | D | L | T | K | D | X | X | X | X | X | K | S | L | K | Y | L | Y | F | S | L | Y | N | D | L | N | V | I | D | N | F | A | K | P | L | L | N | L | R | Y | I | V | M | I | N | M | M | L | F | C | S | C | D | S | A | W | F | N | Q | W | S | T | D | N | R | Q | V | Q | V | L | T | W | L | Y | P | T | R | H | A | L | E | C | R | N | M | N | G | I | Q | - | - | X | X | X | F | A | K | Y | S | V | S | S | C | S | L | H | V | G | F | I | L | Y | I | S | T | S | L | G | I | L | L | F | M | L | V | V | L | V | H | N | L | A | G | D | Y | X | X | X | X | A | G | V | Y | I | X | X | I | A | Q | G | W | M | D | X | X | X | X | X | P | X | X | R | G | S | A | K | R | X | A | X | X | X | X | X | X | X | X | Q | Y | D | V | F | V | S | Y | S | G | T | D | E | S | W | V | V | - | - | - | - | - | - | - | - | - | - | - | - | - | - | - | - | - | - | - | - | - | D | E | L | L | P | N | L | E | R | R | G | - | - | - | - | - | - | - | - | - | - | - | - | - | - | X | X | P | P | F | L | Q | L | C | L | H | S | R | D | F | Q | L | G | V | D | I | A | E | N | I | T | G | S | L | Y | R | S | R | H | T | L | C | L | L | S | R | Q | Y | L | R | S | K | W | C | S | L | E | M | K | L | A | T | D | Y | L | I | A | E | H | R | D | I | L | I | V | V | F | L | E | K | I | S | P | - | - | - | - | K | L | L | S | A | H | H | R | L | A | R | F | V | K | R | K | T | Y | L | D | W | P | Q | D | P | Q | Q | X | X | X | X | X | Q | V | A | F | W | D | R | L | W | A | K | L | A | P | K | P | F | X | X | X | X | X | X | X | X | X | X | X | X | X | X | X | X | X | X | X | X | X | X | X | X | X | X | X | X | X | - | - | - | - | - | - | - | X | X | X | X | X | X | X | X | X | X | X | X | X | X | X | X | X | X | X | X | X | X | X | X | - | - | - | - | - | - |
| CBTLR13\_3900\_BP | - | X | X | X | X | X | X | X | X | X | X | X | X | X | X | X | - | - | - | - | - | - | - | - | - | - | - | - | - | X | X | X | X | X | X | X | X | X | X | X | X | X | X | X | X | X | - | X | X | X | X | X | X | X | - | X | X | X | X | X | X | X | X | - | - | X | - | - | - | X | X | X | X | X | X | X | X | X | X | X | X | X | X | X | X | X | X | X | X | X | X | X | X | X | X | X | X | X | X | X | X | X | X | X | X | X | X | X | X | C | I | F | S | L | Q | L | F | S | R | - | - | - | - | - | - | - | A | F | X | R | V | R | C | V | G | C | X | X | X | - | - | - | - | - | X | X | X | X | X | X | E | K | C | F | V | X | X | X | L | - | - | - | K | I | T | W | M | X | X | X | X | X | X | X | X | - | - | - | - | - | - | - | - | - | - | X | X | X | K | H | N | R | X | M | S | K | K | X | P | V | - | - | - | - | - | - | - | - | R | N | L | X | R | C | L | Q | C | G | T | R | S | C | W | D | S | I | S | S | R | G | P | L | F | A | L | F | K | G | F | V | X | X | X | - | - | - | - | - | - | - | - | - | - | - | A | T | X | L | F | L | K | V | - | - | Y | K | A | H | T | L | N | N | X | H | R | I | C | L | N | - | - | - | - | C | P | S | W | S | F | X | K | S | - | - | - | - | F | E | X | X | S | E | N | T | G | N | Y | L | X | X | X | X | X | X | N | H | X | - | - | - | - | I | F | P | F | H | L | R | F | S | V | - | T | F | R | T | S | K | H | F | X | S | S | T | A | S | S | H | P | X | Q | X | T | C | S | K | G | W | X | S | X | K | I | S | H | C | X | X | T | V | X | R | - | - | - | T | F | L | S | C | C | A | - | - | - | - | - | D | X | H | L | C | - | H | - | - | - | - | - | - | - | - | - | - | - | - | H | H | X | T | I | X | X | - | - | - | - | - | - | - | - | - | - | - | - | - | - | - | - | - | - | - | - | - | - | - | - | - | - | - | - | - | - | - | - | - | - | - | - | - | - | - | - | - | - | - | - | - | - | - | - | - | - | - | - | - | - | - | X | X | X | X | - | - | - | - | - | - | - | - | - | - | - | X | X | X | - | X | - | - | - | I | C | X | E | F | S | X | F | Y | N | T | N | L | C | - | - | F | F | X | R | D | F | F | X | X | X | L | S | F | M | N | L | G | P | S | I | - | - | - | L | T | L | L | R | - | - | - | L | M | X | X | I | K | Q | X | X | N | I | S | P | K | X | X | X | X | X | X | X | - | X | X | D | I | - | - | - | - | - | - | - | - | - | - | - | - | L | K | I | H | S | T | S | F | E | V | L | K | S | E | L | T | N | I | D | X | X | X | X | X | X | X | X | X | X | F | R | Y | T | F | L | Q | L | X | V | X | Q | L | X | R | N | L | X | S | H | T | X | P | F | X | X | X | X | X | X | X | D | W | H | H | C | V | I | P | K | C | N | X | X | S | F | V | - | P | D | L | E | R | C | M | - | - | - | - | - | - | - | - | - | - | - | - | - | - | - | - | G | L | E | H | L | F | I | Y | C | E | D | Q | X | X | V | Q | Q | X | I | X | H | S | - | - | - | - | - | - | - | S | M | X | X | X | X | X | X | X | X | - | - | - | - | - | - | - | - | - | X | X | - | - | X | X | X | X | X | X | X | X | - | X | X | L | K | N | L | T | - | - | - | L | L | L | I | Q | W | K | X | X | X | X | X | X | X | X | - | - | X | X | X | X | - | - | - | - | - | - | - | - | - | - | - | - | X | X | X | X | X | X | X | X | X | X | Y | - | - | - | - | S | R | E | Y | X | X | X | X | X | X | X | X | X | - | - | - | X | X | X | X | X | X | K | R | Q | - | - | - | - | - | - | - | - | - | - | - | - | - | - | G | I | G | T | V | X | K | S | E | X | S | X | X | X | X | X | X | X | X | X | X | X | X | X | C | H | K | T | Q | N | S | K | P | F | - | - | - | - | - | Y | - | - | K | Q | F | Q | K | N | X | L | Q | T | - | - | - | - | - | - | - | - | - | - | - | - | - | - | - | - | - | F | X | - | - | - | - | - | - | - | - | - | - | - | - | - | - | - | L | P | X | X | X | X | X | X | R | T | R | G | T | A | L | D | L | X | X | D | X | T | Y | X | X | X | X | R | F | Y | - | I | X | Q | Y | K | F | A | X | N | - | - | F | G | - | P | Q | S | X | X | N | I | S | A | H | K | L | Y | L | L | R | V | I | X | P | K | N | S | P | T | S | R | K | X | A | A | Y | D | X | D | G | D | - | - | F | T | S | F | X | X | X | X | X | - | - | - | - | X | X | X | X | X | X | X | X | X | X | X | X | X | X | X | I | C | R | V | C | K | F | G | S | X | X | X | X | X | X | X | X | Q | Y | S | S | S | E | P | S | K | I | Q | I | N | L | X | X | X | X | X | X | X | X | X | L | P | E | N | L | T | K | L | S | I | S | S | G | I | R | P | M | T | L | N | L | S | K | S | K | K | S | Q | V | X | X | L | S | L | N | V | C | G | X | X | - | - | - | - | L | X | - | - | - | L | F | R | I | X | D | N | - | - | - | - | - | - | - | T | L | F | K | X | L | X | X | L | T | A | E | T | E | Q | L | L | C | G | Q | X | X | S | F | P | G | Q | F | L | K | S | L | R | H | L | M | I | K | P | X | X | X | R | K | T | T | Q | L | D | L | T | D | L | N | Q | X | X | M | V | N | L | K | S | L | I | L | F | N | V | D | L | S | R | X | X | X | X | X | S | G | L | D | V | I | F | R | N | L | S | N | L | E | Y | L | Y | M | S | F | X | - | - | - | S | X | P | L | L | E | K | D | L | S | R | D | X | X | X | X | X | K | S | L | K | V | M | F | L | H | A | D | D | V | F | S | V | M | E | N | F | V | E | P | L | K | N | L | H | Y | L | I | L | K | D | A | L | L | H | C | S | C | D | N | A | W | I | I | N | W | A | K | Y | V | Q | N | V | Q | V | X | N | F | R | D | S | A | L | E | S | L | S | C | K | T | A | D | E | T | K | - | - | X | X | X | L | Q | K | Y | A | Q | E | N | C | F | N | N | V | D | F | L | L | F | V | S | T | S | L | G | L | V | F | F | M | L | V | V | L | L | H | Q | L | F | G | D | Y | X | X | X | X | A | G | V | L | S | X | X | I | A | R | A | W | V | E | X | X | X | X | X | P | X | X | R | A | N | R | K | G | X | I | X | X | X | X | X | X | X | X | H | F | D | V | F | V | S | Y | S | G | K | D | E | R | W | V | M | - | - | - | - | - | - | - | - | - | - | - | - | - | - | - | - | - | - | - | - | - | D | E | L | L | P | N | L | G | K | R | G | - | - | - | - | - | - | - | - | - | - | - | - | - | - | X | X | P | P | S | L | K | L | C | L | H | S | R | D | F | E | L | G | K | D | I | V | E | N | I | X | D | S | L | Y | R | S | R | H | T | L | C | L | V | S | R | N | Y | L | R | S | T | W | C | S | L | E | M | R | L | A | T | Y | R | L | L | A | E | H | R | D | V | L | V | L | V | F | L | E | K | V | P | H | - | - | - | - | Q | L | L | N | V | H | H | R | L | S | R | L | V | K | T | R | T | Y | M | D | W | P | H | D | P | A | L | X | X | X | X | X | H | N | A | F | W | D | K | L | W | R | K | L | A | P | E | S | A | N | X | X | X | X | X | X | X | X | X | X | X | X | X | X | X | X | X | X | X | X | X | X | X | X | X | X | X | X | - | - | - | - | - | - | - | X | X | X | X | X | X | X | X | X | X | X | X | X | X | X | X | X | X | X | X | X | X | X | X | - | - | - | - | - | - |
| CITLR13\_3900\_BP | - | X | X | X | X | X | X | X | X | X | X | X | X | X | X | X | X | X | X | X | X | X | X | X | X | X | X | X | X | X | X | X | X | X | X | X | X | X | X | X | X | X | X | X | X | X | - | X | X | X | X | X | X | X | - | X | X | X | X | X | X | X | X | - | - | X | - | - | - | X | X | X | X | X | X | X | X | X | X | X | X | X | X | X | X | X | X | X | X | X | X | X | X | X | G | A | S | V | L | I | S | N | T | I | S | E | D | I | M | C | F | F | L | V | C | W | X | X | X | X | X | X | X | X | X | N | V | F | S | T | V | M | W | K | S | F | L | L | L | - | - | - | - | - | X | X | X | X | X | X | X | X | X | X | X | X | X | X | X | - | - | - | N | L | C | W | K | N | K | R | C | L | L | P | S | - | - | - | - | - | - | - | - | - | - | C | H | X | - | H | X | R | - | - | - | - | - | R | P | A | - | - | - | - | - | - | - | - | - | - | - | - | - | - | - | - | X | T | S | I | K | L | A | E | S | L | H | S | R | G | P | R | L | P | R | C | L | G | S | R | L | I | - | - | - | - | - | - | - | - | - | - | - | - | - | - | - | - | - | - | - | - | - | - | - | - | - | - | - | - | - | - | - | R | T | F | C | F | S | G | L | P | X | N | X | X | W | M | F | F | R | D | P | F | G | S | F | X | W | F | D | K | X | X | C | N | F | I | Q | I | N | E | X | X | X | X | X | R | L | Q | Q | A | V | V | R | X | L | L | I | S | V | - | A | F | P | H | L | T | H | C | I | F | Q | T | I | D | Y | H | Y | W | H | Q | M | F | L | K | X | F | L | T | C | K | N | Y | T | Y | S | K | C | V | X | R | - | - | - | I | Y | L | R | F | C | V | - | - | - | - | - | D | W | Q | X | X | - | X | - | - | - | - | - | - | - | - | - | - | - | - | N | H | X | R | C | F | X | X | X | - | - | - | - | - | - | - | - | - | X | X | - | - | - | - | X | X | X | X | K | X | T | N | I | K | X | T | S | V | P | K | L | F | S | F | Q | H | L | S | W | X | H | I | Y | C | V | K | H | X | R | G | R | I | V | S | X | E | - | - | - | - | - | - | - | - | - | - | - | Y | X | S | - | M | - | - | - | X | M | K | E | L | W | K | F | L | E | I | C | L | C | - | - | F | T | Y | A | - | - | - | - | - | - | - | - | - | - | - | - | C | P | L | I | - | - | - | Y | S | E | F | F | - | - | - | L | X | - | - | - | - | - | - | - | - | - | - | - | - | - | - | - | - | - | - | - | - | - | - | - | - | - | - | - | - | - | - | - | - | - | - | - | - | - | - | - | - | - | - | - | L | E | Y | I | K | S | I | X | X | X | F | D | X | X | X | X | X | X | X | X | X | X | S | W | W | M | Y | L | K | L | M | I | X | X | X | X | X | X | X | X | X | X | X | X | X | X | X | X | X | X | X | X | X | V | Y | S | S | K | V | I | F | D | X | V | F | S | S | F | L | - | X | D | X | X | X | X | X | X | X | X | X | X | X | X | X | X | X | X | X | X | X | X | X | X | X | X | X | X | X | L | T | C | H | X | L | L | L | I | S | Q | M | X | X | X | X | X | X | X | X | X | X | V | K | M | L | R | R | X | X | X | X | X | X | X | X | X | X | X | X | X | X | X | X | - | - | X | X | X | X | X | X | X | X | - | X | X | X | X | N | L | K | - | - | - | K | L | L | L | P | V | N | L | - | - | - | - | - | - | - | - | - | - | - | - | - | - | - | - | - | - | - | - | - | - | - | - | - | - | - | - | - | - | - | - | L | D | V | Y | - | - | - | - | S | - | - | - | - | F | F | Q | X | X | X | X | X | - | - | - | X | X | X | X | X | X | X | - | X | - | - | - | - | - | - | - | - | - | - | - | - | - | - | X | X | X | X | X | X | X | X | X | X | X | X | X | X | X | F | Q | E | P | H | F | G | X | H | C | X | A | X | A | X | T | K | X | F | - | - | - | - | - | P | I | S | L | C | F | R | S | T | Y | S | Q | T | A | F | L | Y | V | S | K | L | K | X | D | R | X | D | X | X | X | X | F | - | - | - | - | - | - | - | - | - | - | - | - | - | - | - | I | P | I | Y | V | F | - | R | R | S | X | N | S | R | F | I | H | R | S | H | F | X | H | R | R | F | C | F | L | W | - | I | E | Q | F | E | R | A | I | S | - | - | L | X | - | Q | Q | V | I | L | H | L | S | T | H | I | Q | W | F | I | W | T | N | G | - | - | - | - | P | R | S | P | R | K | S | Y | Y | S | Y | R | I | X | F | I | W | T | S | Y | X | P | X | H | X | - | - | - | - | - | - | - | - | - | - | - | - | - | - | - | - | - | - | - | - | - | X | X | X | L | L | G | D | - | - | - | - | - | - | - | - | L | N | F | P | P | D | M | S | P | I | K | L | H | L | X | X | X | X | D | I | F | G | I | I | P | H | N | L | S | N | I | L | I | S | S | G | L | R | P | M | Q | L | V | I | G | S | N | N | T | L | D | Q | X | X | L | N | L | H | I | K | G | X | X | - | - | - | - | M | X | - | - | - | L | L | R | T | X | N | S | - | - | - | - | - | - | - | S | L | L | T | S | V | V | T | L | Q | I | D | A | A | Y | V | S | C | G | N | X | X | E | F | I | G | K | Y | V | R | S | V | V | N | L | D | F | N | S | V | F | S | D | D | I | G | X | X | D | L | A | V | I | N | Q | X | X | L | V | H | L | K | T | L | K | L | E | N | I | E | L | T | K | X | X | X | X | X | P | N | V | A | V | M | F | H | N | L | T | K | L | Q | T | L | I | L | A | N | C | - | - | - | R | F | F | F | L | D | G | S | L | T | K | D | X | X | X | X | X | K | A | L | T | G | L | V | L | I | L | K | D | N | V | N | V | L | Q | N | L | V | E | H | L | T | S | L | K | Y | L | C | L | Q | G | L | G | L | Y | C | S | C | D | N | A | W | L | V | S | W | V | K | D | N | S | K | V | Q | V | X | D | M | F | N | P | T | M | K | E | L | Q | C | L | S | D | N | G | I | D | - | - | H | L | N | F | I | G | Y | A | K | E | N | C | S | F | E | I | E | F | V | F | F | A | S | T | S | A | F | L | C | I | F | I | I | V | V | L | S | Y | K | F | A | G | Q | Y | X | X | X | X | C | A | F | L | S | X | X | I | A | S | G | W | F | R | X | X | X | X | X | L | X | X | R | V | N | G | K | H | X | S | X | X | X | X | X | X | X | X | R | Y | D | V | F | V | S | Y | S | A | K | D | E | H | W | V | M | - | - | - | - | - | - | - | - | - | - | - | - | - | - | - | - | - | - | - | - | - | E | E | L | L | P | N | L | E | R | R | G | - | - | - | - | - | - | - | - | - | - | - | - | - | - | X | X | P | P | F | L | R | L | C | L | H | S | R | D | F | Q | L | G | E | D | I | V | E | N | I | T | D | S | I | Y | A | S | C | Q | T | L | C | L | I | S | R | N | Y | L | G | S | N | W | C | S | L | E | M | Q | L | A | T | Y | R | L | Q | V | E | H | R | D | I | L | I | L | V | F | L | E | M | I | P | S | - | - | - | - | R | L | L | S | S | H | H | R | L | A | R | L | V | K | T | R | T | Y | L | D | W | P | Q | D | P | E | M | X | X | X | X | X | H | E | A | F | W | D | R | L | W | C | K | L | S | S | N | K | A | N | X | X | X | X | X | X | X | X | X | X | X | X | X | X | X | X | X | X | X | X | X | X | X | X | X | X | X | X | - | - | - | - | - | - | - | X | X | X | X | X | X | X | X | X | X | X | X | X | X | X | X | X | X | X | X | X | X | X | X | - | - | - | - | - | - |
| conservation |  |  |  |  |  |  |  |  |  |  |  |  |  |  |  |  |  |  |  |  |  |  |  |  |  |  |  |  |  |  |  |  |  |  |  |  |  |  |  |  |  |  |  |  |  |  |  |  |  |  |  |  |  |  |  |  |  |  |  |  |  |  |  |  |  |  |  |  |  |  |  |  |  |  |  |  |  |  |  |  |  |  |  |  |  |  |  |  |  |  |  |  |  |  |  |  |  |  |  |  |  |  |  |  |  |  |  |  |  |  |  |  |  |  |  |  |  |  |  |  |  |  |  |  |  |  |  |  |  |  |  |  |  |  |  |  |  |  |  |  |  |  |  |  |  |  |  |  |  |  |  |  |  |  |  |  |  |  |  |  |  |  |  |  |  |  |  |  |  |  |  |  |  |  |  |  |  |  |  |  |  |  |  |  |  |  |  |  |  |  |  |  |  |  |  |  |  |  |  |  |  |  |  |  |  |  |  |  |  |  |  |  |  |  |  |  |  |  |  |  |  |  |  |  |  |  |  |  |  |  |  |  |  |  |  |  |  |  |  |  |  |  |  |  |  |  |  |  |  |  |  |  |  |  |  |  |  |  |  |  |  |  |  |  |  |  |  |  |  |  |  |  |  |  |  |  |  |  |  |  |  |  |  |  |  |  |  |  |  |  |  |  |  |  |  |  |  |  |  |  |  |  |  |  |  |  |  |  |  |  |  |  |  |  |  |  |  |  |  |  |  |  |  |  |  |  |  |  |  |  |  |  |  |  |  |  |  |  |  |  |  |  |  |  |  |  |  |  |  |  |  |  |  |  |  |  |  |  |  |  |  |  |  |  |  |  |  |  |  |  |  |  |  |  |  |  |  |  |  |  |  |  |  |  |  |  |  |  |  |  |  |  |  |  |  |  |  |  |  |  |  |  |  |  |  |  |  |  |  |  |  |  |  |  |  |  |  |  |  |  |  |  |  |  |  |  |  |  |  |  |  |  |  |  |  |  |  |  |  |  |  |  |  |  |  |  |  |  |  |  |  |  |  |  |  |  |  |  |  |  |  |  |  |  |  |  |  |  |  |  |  |  |  |  |  |  |  |  |  |  |  |  |  |  |  |  |  |  |  |  |  |  |  |  |  |  |  |  |  |  |  |  |  |  |  |  |  |  |  |  |  |  |  |  |  |  |  |  |  |  |  |  |  |  |  |  |  |  |  |  |  |  |  |  |  |  |  |  |  |  |  |  |  |  |  |  |  |  |  |  |  |  |  |  |  |  |  |  |  |  |  |  |  |  |  |  |  |  |  |  |  |  |  |  |  |  |  |  |  |  |  |  |  |  |  |  |  |  |  |  |  |  |  |  |  |  |  |  |  |  |  |  |  |  |  |  |  |  |  |  |  |  |  |  |  |  |  |  |  |  |  |  |  |  |  |  |  |  |  |  |  |  |  |  |  |  |  |  |  |  |  |  |  |  |  |  |  |  |  |  |  |  |  |  |  |  |  |  |  |  |  |  |  |  |  |  |  |  |  |  |  |  |  |  |  |  |  |  |  |  |  |  |  |  |  |  |  |  |  |  |  |  |  |  |  |  |  |  |  |  |  |  |  |  |  |  |  |  |  |  |  |  |  |  |  |  |  |  |  |  |  |  |  |  |  |  |  |  |  |  |  |  |  |  |  |  |  |  |  |  |  |  |  |  |  |  |  |  |  |  |  |  |  |  |  |  |  |  |  |  |  |  |  |  |  |  |  |  |  |  |  |  |  |  |  |  |  |  |  |  |  |  |  |  |  |  |  |  |  |  |  |  |  |  |  |  |  |  |  |  |  |  |  |  |  |  |  |  |  |  |  |  |  |  |  |  |  |  |  |  |  |  |  |  |  |  |  |  |  |  |  |  |  |  |  |  |  |  |  |  |  |  |  |  |  |  |  |  |  |  |  |  |  |  |  |  |  |  |  |  |  |  |  |  |  |  |  |  |  |  |  |  |  |  |  |  |  |  |  |  |  |  |  |  |  |  |  |  |  |  |  |  |  |  |  |  |  |  |  |  |  |  |  |  |  |  |  |  |  |  |  |  |  |  |  |  |  |  |  |  |  |  |  |  |  |  |  |  |  |  |  |  |  |  |  |  |  |  |  |  |  |  |  |  |  |  |  |  |  |  |  |  |  |  |  |  |  |  |  |  |  |  |  |  |  |  |  |  |  |  |  |  |  |  |  |  |  |  |  |  |  |  |  |  |  |  |  |  |  |  |  |  |  |  |  |  |  |  |  |  |  |  |  |  |  |  |  |  |  |  |  |  |  |  |  |  |  |  |  |  |  |  |  |  |  |  |  |  |  |  |  |  |  |  |  |  |  |  |  |  |  |  |  |  |  |  |  |  |  |  |  |  |  |  |  |  |  |  |  |  |  |  |  |  |  |  |  |  |  |  |  |  |  |  |  |  |  |  |  |  |  |  |  |  |  |  |  |  |  |  |  |  |  |  |  |  |  |  |  |  |  |  |  |  |  |  |  |  |  |  |  |  |  |  |  |  |  |  |  |  |  |  |  |  |  |  |  |  |  |  |  |  |  |  |  |  |  |  |  |  |  |  |  |  |  |  |  |  |  |  |  |  |  |  |  |  |  |  |  |  |  |  |  |  |  |  |  |  |  |  |  |  |  |  |  |  |  |  |  |  |  |  |  |  |  |  |  |  |  |  |  |  |  |  |  |  |  |  |  |  |  |  |  |  |  |  |  |  |  |  |  |  |  |  |  |  |  |  |  |  |  |  |  |  |  |  |  |  |  |  |  |  |  |  |  |  |  |  |  |  |  |  |  |  |  |  |  |  |  |  |  |  |  |  |  |  |  |  |  |  |  |  |  |  |  |  |  |  |  |  |  |  |  |  |  |  |  |  |  |  |  |  |  |  |  |  |  |  |  |  |  |  |  |  |  |  |  |  |  |  |  |  |  |  |  |  |  |  |  |  |  |  |  |  |  |  |  |  |  |  |  |  |  |  |  |  |  |  |  |  |  |  |  |  |  |  |  |  |  |  |  |  |  |  |  |  |  |  |  |  |  |  |  |  |  |  |  |  |  |  |  |  |  |  |  |  |  |  |  |  |  |  |  |  |  |  |  |  |  |  |  |  |  |  |  |  |  |  |  |  |  |  |  |  |  |  |  |  |  |  |  |  |  |  |  |  |  |  |  |  |  |  |  |  |  |  |  |  |  |  |  |  |  |  |  |  |  |  |  |  |  |  |  |  |  |  |  |  |  |  |  |  |  |  |  |  |  |  |  |  |  |  |  |  |  |  |  |  |  |  |  |  |  |  |  |  |  |  |  |  |  |  |  |  |  |  |  |  |  |  |  |  |  |  |  |  |  |  |  |  |  |  |  |  |  |  |  |  |  |  |  |  |  |  |  |  |  |  |  |  |  |  |  |  |  |  |  |  |  |  |  |  |  |  |  |  |  |  |  |  |  |  |  |  |  |  |  |  |  |  |  |  |  |  |  |  |  |  |  |  |  |  |  |  |  |  |  |  |  |  |  |  |  |  |  |  |  |  |  |  |  |  |  |  |  |  |  |  |  |  |  |  |  |  |  |  |  |  |  |  |  |  |  |  |  |  |  |  |  |  |  |  |  |  |  |  |  |  |  |  |  |  |  |  |  |  |  |  |  |  |  |  |  |  |  |  |  |  |  |  |  |  |  |  |  |  |  |  |  |  |  |  |  |  |  |  |  |  |  |  |  |  |  |  |  |  |  |  |  |  |  |  |  |  |  |  |  |  |  |  |  |  |  |  |  |  |  |  |  |  |
|  |  |  |  |  |  |  |  |  |  |  |  |  |  |  |  |  |  |  |  |  |  |  |  |  |  |  |  |  |  |  |  |  |  |  |  |  |  |  |  |  |  |  |  |  |  |  |  |  |  |  |  |  |  |  |  |  |  |  |  |  |  |  |  |  |  |  |  |  |  |  |  |  |  |  |  |  |  |  |  |  |  |  |  |  |  |  |  |  |  |  |  |  |  |  |  |  |  |  |  |  |  |  |  |  |  |  |  |  |  |  |  |  |  |  |  |  |  |  |  |  |  |  |  |  |  |  |  |  |  |  |  |  |  |  |  |  |  |  |  |  |  |  |  |  |  |  |  |  |  |  |  |  |  |  |  |  |  |  |  |  |  |  |  |  |  |  |  |  |  |  |  |  |  |  |  |  |  |  |  |  |  |  |  |  |  |  |  |  |  |  |  |  |  |  |  |  |  |  |  |  |  |  |  |  |  |  |  |  |  |  |  |  |  |  |  |  |  |  |  |  |  |  |  |  |  |  |  |  |  |  |  |  |  |  |  |  |  |  |  |  |  |  |  |  |  |  |  |  |  |  |  |  |  |  |  |  |  |  |  |  |  |  |  |  |  |  |  |  |  |  |  |  |  |  |  |  |  |  |  |  |  |  |  |  |  |  |  |  |  |  |  |  |  |  |  |  |  |  |  |  |  |  |  |  |  |  |  |  |  |  |  |  |  |  |  |  |  |  |  |  |  |  |  |  |  |  |  |  |  |  |  |  |  |  |  |  |  |  |  |  |  |  |  |  |  |  |  |  |  |  |  |  |  |  |  |  |  |  |  |  |  |  |  |  |  |  |  |  |  |  |  |  |  |  |  |  |  |  |  |  |  |  |  |  |  |  |  |  |  |  |  |  |  |  |  |  |  |  |  |  |  |  |  |  |  |  |  |  |  |  |  |  |  |  |  |  |  |  |  |  |  |  |  |  |  |  |  |  |  |  |  |  |  |  |  |  |  |  |  |  |  |  |  |  |  |  |  |  |  |  |  |  |  |  |  |  |  |  |  |  |  |  |  |  |  |  |  |  |  |  |  |  |  |  |  |  |  |  |  |  |  |  |  |  |  |  |  |  |  |  |  |  |  |  |  |  |  |  |  |  |  |  |  |  |  |  |  |  |  |  |  |  |  |  |  |  |  |  |  |  |  |  |  |  |  |  |  |  |  |  |  |  |  |  |  |  |  |  |  |  |  |  |  |  |  |  |  |  |  |  |  |  |  |  |  |  |  |  |  |  |  |  |  |  |  |  |  |  |  |  |  |  |  |  |  |  |  |  |  |  |  |  |  |  |  |  |  |  |  |  |  |  |  |  |  |  |  |  |  |  |  |  |  |  |  |  |  |  |  |  |  |  |  |  |  |  |  |  |  |  |  |  |  |  |  |  |  |  |  |  |  |  |  |  |  |  |  |  |  |  |  |  |  |  |  |  |  |  |  |  |  |  |  |  |  |  |  |  |  |  |  |  |  |  |  |  |  |  |  |  |  |  |  |  |  |  |  |  |  |  |  |  |  |  |  |  |  |  |  |  |  |  |  |  |  |  |  |  |  |  |  |  |  |  |  |  |  |  |  |  |  |  |  |  |  |  |  |  |  |  |  |  |  |  |  |  |  |  |  |  |  |  |  |  |  |  |  |  |  |  |  |  |  |  |  |  |  |  |  |  |  |  |  |  |  |  |  |  |  |  |  |  |  |  |  |  |  |  |  |  |  |  |  |  |  |  |  |  |  |  |  |  |  |  |  |  |  |  |  |  |  |  |  |  |  |  |  |  |  |  |  |  |  |  |  |  |  |  |  |  |  |  |  |  |  |  |  |  |  |  |  |  |  |  |  |  |  |  |  |  |  |  |  |  |  |  |  |  |  |  |  |  |  |  |  |  |  |  |  |  |  |  |  |  |  |  |  |  |  |  |  |  |  |  |  |  |  |  |  |  |  |  |  |  |  |  |  |  |  |  |  |  |  |  |  |  |  |  |  |  |  |  |  |  |  |  |  |  |  |  |  |  |  |  |  |  |  |  |  |  |  |  |  |  |  |  |  |  |  |  |  |  |  |  |  |  |  |  |  |  |  |  |  |  |  |  |  |  |  |  |  |  |  |  |  |  |  |  |  |  |  |  |  |  |  |  |  |  |  |  |  |  |  |  |  |  |  |  |  |  |  |  |  |  |  |  |  |  |  |  |  |  |  |  |  |  |  |  |  |  |  |  |  |  |  |  |  |  |  |  |  |  |  |  |  |  |  |  |  |  |  |  |  |  |  |  |  |  |  |  |  |  |  |  |  |  |  |  |  |  |  |  |  |  |  |  |  |  |  |  |  |  |  |  |  |  |  |  |  |  |  |  |  |  |  |  |  |  |  |  |  |  |  |  |  |  |  |  |  |  |  |  |  |  |  |  |  |  |  |  |  |  |  |  |  |  |  |  |  |  |  |  |  |  |  |  |  |  |  |  |  |  |  |  |  |  |  |  |  |  |  |  |  |  |  |  |  |  |  |  |  |  |  |  |  |  |  |  |  |  |  |  |  |  |  |  |  |  |  |  |  |  |  |  |  |  |  |  |  |  |  |  |  |  |  |  |  |  |  |  |  |  |  |  |  |  |  |  |  |  |  |  |  |  |  |  |  |  |  |  |  |  |  |  |  |  |  |  |  |  |  |  |  |  |  |  |  |  |  |  |  |  |  |  |  |  |  |  |  |  |  |  |  |  |  |  |  |  |  |  |  |  |  |  |  |  |  |  |  |  |  |  |  |  |  |  |  |  |  |  |  |  |  |  |  |  |  |  |  |  |  |  |  |  |  |  |  |  |  |  |  |  |  |  |  |  |  |  |  |  |  |  |  |  |  |  |  |  |  |  |  |  |  |  |  |  |  |  |  |  |  |  |  |  |  |  |  |  |  |  |  |  |  |  |  |  |  |  |  |  |  |  |  |  |  |  |  |  |  |  |  |  |  |  |  |  |  |  |  |  |  |  |  |  |  |  |  |  |  |  |  |  |  |  |  |  |  |  |  |  |  |  |  |  |  |  |  |  |  |  |  |  |  |  |  |  |  |  |  |  |  |  |  |  |  |  |  |  |  |  |  |  |  |  |  |  |  |  |  |  |  |  |  |  |  |  |  |  |  |  |  |  |  |  |  |  |  |  |  |  |  |  |  |  |  |  |  |  |  |  |  |  |  |  |  |  |  |  |  |  |  |  |  |  |  |  |  |  |  |  |  |  |  |  |  |  |  |  |  |  |  |  |  |  |  |  |  |  |  |  |  |  |  |  |  |  |  |  |  |  |  |  |  |  |  |  |  |  |  |  |  |  |  |  |  |  |  |  |  |  |  |  |  |  |  |  |  |  |  |  |  |  |  |  |  |  |  |  |  |  |  |  |  |  |  |  |  |  |  |  |  |  |  |  |  |  |  |  |  |  |  |  |  |  |  |  |  |  |  |  |  |  |  |  |  |  |  |  |  |  |  |  |  |  |  |  |  |  |  |  |  |  |  |  |  |  |  |  |  |  |  |  |  |  |  |  |  |  |  |  |  |  |  |  |  |  |  |  |  |  |  |  |  |  |  |  |  |  |  |  |  |  |  |  |  |  |  |  |  |  |  |  |  |  |  |  |  |  |  |  |  |  |  |  |  |  |  |  |  |  |  |  |  |  |  |  |  |  |  |  |  |  |  |  |  |  |  |  |  |  |  |  |  |  |  |  |  |  |  |  |  |  |  |  |  |  |  |  |  |  |  |  |  |  |  |  |  |  |  |  |
|  |  |  |  |  |  |  |  |  |  |  |  |  |  |  |  |  |  |  |  |  |  |  |  |  |  |  |  |  |  |  |  |  |  |  |  |  |  |  |  |  |  |  |  |  |  |  |  |  |  |  |  |  |  |  |  |  |  |  |  |  |  |  |  |  |  |  |  |  |  |  |  |  |  |  |  |  |  |  |  |  |  |  |  |  |  |  |  |  |  |  |  |  |  |  |  |  |  |  |  |  |  |  |  |  |  |  |  |  |  |  |  |  |  |  |  |  |  |  |  |  |  |  |  |  |  |  |  |  |  |  |  |  |  |  |  |  |  |  |  |  |  |  |  |  |  |  |  |  |  |  |  |  |  |  |  |  |  |  |  |  |  |  |  |  |  |  |  |  |  |  |  |  |  |  |  |  |  |  |  |  |  |  |  |  |  |  |  |  |  |  |  |  |  |  |  |  |  |  |  |  |  |  |  |  |  |  |  |  |  |  |  |  |  |  |  |  |  |  |  |  |  |  |  |  |  |  |  |  |  |  |  |  |  |  |  |  |  |  |  |  |  |  |  |  |  |  |  |  |  |  |  |  |  |  |  |  |  |  |  |  |  |  |  |  |  |  |  |  |  |  |  |  |  |  |  |  |  |  |  |  |  |  |  |  |  |  |  |  |  |  |  |  |  |  |  |  |  |  |  |  |  |  |  |  |  |  |  |  |  |  |  |  |  |  |  |  |  |  |  |  |  |  |  |  |  |  |  |  |  |  |  |  |  |  |  |  |  |  |  |  |  |  |  |  |  |  |  |  |  |  |  |  |  |  |  |  |  |  |  |  |  |  |  |  |  |  |  |  |  |  |  |  |  |  |  |  |  |  |  |  |  |  |  |  |  |  |  |  |  |  |  |  |  |  |  |  |  |  |  |  |  |  |  |  |  |  |  |  |  |  |  |  |  |  |  |  |  |  |  |  |  |  |  |  |  |  |  |  |  |  |  |  |  |  |  |  |  |  |  |  |  |  |  |  |  |  |  |  |  |  |  |  |  |  |  |  |  |  |  |  |  |  |  |  |  |  |  |  |  |  |  |  |  |  |  |  |  |  |  |  |  |  |  |  |  |  |  |  |  |  |  |  |  |  |  |  |  |  |  |  |  |  |  |  |  |  |  |  |  |  |  |  |  |  |  |  |  |  |  |  |  |  |  |  |  |  |  |  |  |  |  |  |  |  |  |  |  |  |  |  |  |  |  |  |  |  |  |  |  |  |  |  |  |  |  |  |  |  |  |  |  |  |  |  |  |  |  |  |  |  |  |  |  |  |  |  |  |  |  |  |  |  |  |  |  |  |  |  |  |  |  |  |  |  |  |  |  |  |  |  |  |  |  |  |  |  |  |  |  |  |  |  |  |  |  |  |  |  |  |  |  |  |  |  |  |  |  |  |  |  |  |  |  |  |  |  |  |  |  |  |  |  |  |  |  |  |  |  |  |  |  |  |  |  |  |  |  |  |  |  |  |  |  |  |  |  |  |  |  |  |  |  |  |  |  |  |  |  |  |  |  |  |  |  |  |  |  |  |  |  |  |  |  |  |  |  |  |  |  |  |  |  |  |  |  |  |  |  |  |  |  |  |  |  |  |  |  |  |  |  |  |  |  |  |  |  |  |  |  |  |  |  |  |  |  |  |  |  |  |  |  |  |  |  |  |  |  |  |  |  |  |  |  |  |  |  |  |  |  |  |  |  |  |  |  |  |  |  |  |  |  |  |  |  |  |  |  |  |  |  |  |  |  |  |  |  |  |  |  |  |  |  |  |  |  |  |  |  |  |  |  |  |  |  |  |  |  |  |  |  |  |  |  |  |  |  |  |  |  |  |  |  |  |  |  |  |  |  |  |  |  |  |  |  |  |  |  |  |  |  |  |  |  |  |  |  |  |  |  |  |  |  |  |  |  |  |  |  |  |  |  |  |  |  |  |  |  |  |  |  |  |  |  |  |  |  |  |  |  |  |  |  |  |  |  |  |  |  |  |  |  |  |  |  |  |  |  |  |  |  |  |  |  |  |  |  |  |  |  |  |  |  |  |  |  |  |  |  |  |  |  |  |  |  |  |  |  |  |  |  |  |  |  |  |  |  |  |  |  |  |  |  |  |  |  |  |  |  |  |  |  |  |  |  |  |  |  |  |  |  |  |  |  |  |  |  |  |  |  |  |  |  |  |  |  |  |  |  |  |  |  |  |  |  |  |  |  |  |  |  |  |  |  |  |  |  |  |  |  |  |  |  |  |  |  |  |  |  |  |  |  |  |  |  |  |  |  |  |  |  |  |  |  |  |  |  |  |  |  |  |  |  |  |  |  |  |  |  |  |  |  |  |  |  |  |  |  |  |  |  |  |  |  |  |  |  |  |  |  |  |  |  |  |  |  |  |  |  |  |  |  |  |  |  |  |  |  |  |  |  |  |  |  |  |  |  |  |  |  |  |  |  |  |  |  |  |  |  |  |  |  |  |  |  |  |  |  |  |  |  |  |  |  |  |  |  |  |  |  |  |  |  |  |  |  |  |  |  |  |  |  |  |  |  |  |  |  |  |  |  |  |  |  |  |  |  |  |  |  |  |  |  |  |  |  |  |  |  |  |  |  |  |  |  |  |  |  |  |  |  |  |  |  |  |  |  |  |  |  |  |  |  |  |  |  |  |  |  |  |  |  |  |  |  |  |  |  |  |  |  |  |  |  |  |  |  |  |  |  |  |  |  |  |  |  |  |  |  |  |  |  |  |  |  |  |  |  |  |  |  |  |  |  |  |  |  |  |  |  |  |  |  |  |  |  |  |  |  |  |  |  |  |  |  |  |  |  |  |  |  |  |  |  |  |  |  |  |  |  |  |  |  |  |  |  |  |  |  |  |  |  |  |  |  |  |  |  |  |  |  |  |  |  |  |  |  |  |  |  |  |  |  |  |  |  |  |  |  |  |  |  |  |  |  |  |  |  |  |  |  |  |  |  |  |  |  |  |  |  |  |  |  |  |  |  |  |  |  |  |  |  |  |  |  |  |  |  |  |  |  |  |  |  |  |  |  |  |  |  |  |  |  |  |  |  |  |  |  |  |  |  |  |  |  |  |  |  |  |  |  |  |  |  |  |  |  |  |  |  |  |  |  |  |  |  |  |  |  |  |  |  |  |  |  |  |  |  |  |  |  |  |  |  |  |  |  |  |  |  |  |  |  |  |  |  |  |  |  |  |  |  |  |  |  |  |  |  |  |  |  |  |  |  |  |  |  |  |  |  |  |  |  |  |  |  |  |  |  |  |  |  |  |  |  |  |  |  |  |  |  |  |  |  |  |  |  |  |  |  |  |  |  |  |  |  |  |  |  |  |  |  |  |  |  |  |  |  |  |  |  |  |  |  |  |  |  |  |  |  |  |  |  |  |  |  |  |  |  |  |  |  |  |  |  |  |  |  |  |  |  |  |  |  |  |  |  |  |  |  |  |  |  |  |  |  |  |  |  |  |  |  |  |  |  |  |  |  |  |  |  |  |  |  |  |  |  |  |  |  |  |  |  |  |  |  |  |  |  |  |  |  |  |  |  |  |  |  |  |  |  |  |  |  |  |  |  |  |  |  |  |  |  |  |  |  |  |  |  |  |  |  |  |  |  |  |  |  |  |  |  |  |  |  |  |  |  |  |  |  |  |  |  |  |  |  |  |  |  |  |  |  |  |  |  |  |  |  |  |  |  |  |  |  |  |  |  |  |  |  |  |  |  |  |  |  |  |  |  |  |  |  |  |  |  |  |  |  |  |  |  |
|  |  |  |  |  |  |  |  |  |  |  |  |  |  |  |  |  |  |  |  |  |  |  |  |  |  |  |  |  |  |  |  |  |  |  |  |  |  |  |  |  |  |  |  |  |  |  |  |  |  |  |  |  |  |  |  |  |  |  |  |  |  |  |  |  |  |  |  |  |  |  |  |  |  |  |  |  |  |  |  |  |  |  |  |  |  |  |  |  |  |  |  |  |  |  |  |  |  |  |  |  |  |  |  |  |  |  |  |  |  |  |  |  |  |  |  |  |  |  |  |  |  |  |  |  |  |  |  |  |  |  |  |  |  |  |  |  |  |  |  |  |  |  |  |  |  |  |  |  |  |  |  |  |  |  |  |  |  |  |  |  |  |  |  |  |  |  |  |  |  |  |  |  |  |  |  |  |  |  |  |  |  |  |  |  |  |  |  |  |  |  |  |  |  |  |  |  |  |  |  |  |  |  |  |  |  |  |  |  |  |  |  |  |  |  |  |  |  |  |  |  |  |  |  |  |  |  |  |  |  |  |  |  |  |  |  |  |  |  |  |  |  |  |  |  |  |  |  |  |  |  |  |  |  |  |  |  |  |  |  |  |  |  |  |  |  |  |  |  |  |  |  |  |  |  |  |  |  |  |  |  |  |  |  |  |  |  |  |  |  |  |  |  |  |  |  |  |  |  |  |  |  |  |  |  |  |  |  |  |  |  |  |  |  |  |  |  |  |  |  |  |  |  |  |  |  |  |  |  |  |  |  |  |  |  |  |  |  |  |  |  |  |  |  |  |  |  |  |  |  |  |  |  |  |  |  |  |  |  |  |  |  |  |  |  |  |  |  |  |  |  |  |  |  |  |  |  |  |  |  |  |  |  |  |  |  |  |  |  |  |  |  |  |  |  |  |  |  |  |  |  |  |  |  |  |  |  |  |  |  |  |  |  |  |  |  |  |  |  |  |  |  |  |  |  |  |  |  |  |  |  |  |  |  |  |  |  |  |  |  |  |  |  |  |  |  |  |  |  |  |  |  |  |  |  |  |  |  |  |  |  |  |  |  |  |  |  |  |  |  |  |  |  |  |  |  |  |  |  |  |  |  |  |  |  |  |  |  |  |  |  |  |  |  |  |  |  |  |  |  |  |  |  |  |  |  |  |  |  |  |  |  |  |  |  |  |  |  |  |  |  |  |  |  |  |  |  |  |  |  |  |  |  |  |  |  |  |  |  |  |  |  |  |  |  |  |  |  |  |  |  |  |  |  |  |  |  |  |  |  |  |  |  |  |  |  |  |  |  |  |  |  |  |  |  |  |  |  |  |  |  |  |  |  |  |  |  |  |  |  |  |  |  |  |  |  |  |  |  |  |  |  |  |  |  |  |  |  |  |  |  |  |  |  |  |  |  |  |  |  |  |  |  |  |  |  |  |  |  |  |  |  |  |  |  |  |  |  |  |  |  |  |  |  |  |  |  |  |  |  |  |  |  |  |  |  |  |  |  |  |  |  |  |  |  |  |  |  |  |  |  |  |  |  |  |  |  |  |  |  |  |  |  |  |  |  |  |  |  |  |  |  |  |  |  |  |  |  |  |  |  |  |  |  |  |  |  |  |  |  |  |  |  |  |  |  |  |  |  |  |  |  |  |  |  |  |  |  |  |  |  |  |  |  |  |  |  |  |  |  |  |  |  |  |  |  |  |  |  |  |  |  |  |  |  |  |  |  |  |  |  |  |  |  |  |  |  |  |  |  |  |  |  |  |  |  |  |  |  |  |  |  |  |  |  |  |  |  |  |  |  |  |  |  |  |  |  |  |  |  |  |  |  |  |  |  |  |  |  |  |  |  |  |  |  |  |  |  |  |  |  |  |  |  |  |  |  |  |  |  |  |  |  |  |  |  |  |  |  |  |  |  |  |  |  |  |  |  |  |  |  |  |  |  |  |  |  |  |  |  |  |  |  |  |  |  |  |  |  |  |  |  |  |  |  |  |  |  |  |  |  |  |  |  |  |  |  |  |  |  |  |  |  |  |  |  |  |  |  |  |  |  |  |  |  |  |  |  |  |  |  |  |  |  |  |  |  |  |  |  |  |  |  |  |  |  |  |  |  |  |  |  |  |  |  |  |  |  |  |  |  |  |  |  |  |  |  |  |  |  |  |  |  |  |  |  |  |  |  |  |  |  |  |  |  |  |  |  |  |  |  |  |  |  |  |  |  |  |  |  |  |  |  |  |  |  |  |  |  |  |  |  |  |  |  |  |  |  |  |  |  |  |  |  |  |  |  |  |  |  |  |  |  |  |  |  |  |  |  |  |  |  |  |  |  |  |  |  |  |  |  |  |  |  |  |  |  |  |  |  |  |  |  |  |  |  |  |  |  |  |  |  |  |  |  |  |  |  |  |  |  |  |  |  |  |  |  |  |  |  |  |  |  |  |  |  |  |  |  |  |  |  |  |  |  |  |  |  |  |  |  |  |  |  |  |  |  |  |  |  |  |  |  |  |  |  |  |  |  |  |  |  |  |  |  |  |  |  |  |  |  |  |  |  |  |  |  |  |  |  |  |  |  |  |  |  |  |  |  |  |  |  |  |  |  |  |  |  |  |  |  |  |  |  |  |  |  |  |  |  |  |  |  |  |  |  |  |  |  |  |  |  |  |  |  |  |  |  |  |  |  |  |  |  |  |  |  |  |  |  |  |  |  |  |  |  |  |  |  |  |  |  |  |  |  |  |  |  |  |  |  |  |  |  |  |  |  |  |  |  |  |  |  |  |  |  |  |  |  |  |  |  |  |  |  |  |  |  |  |  |  |  |  |  |  |  |  |  |  |  |  |  |  |  |  |  |  |  |  |  |  |  |  |  |  |  |  |  |  |  |  |  |  |  |  |  |  |  |  |  |  |  |  |  |  |  |  |  |  |  |  |  |  |  |  |  |  |  |  |  |  |  |  |  |  |  |  |  |  |  |  |  |  |  |  |  |  |  |  |  |  |  |  |  |  |  |  |  |  |  |  |  |  |  |  |  |  |  |  |  |  |  |  |  |  |  |  |  |  |  |  |  |  |  |  |  |  |  |  |  |  |  |  |  |  |  |  |  |  |  |  |  |  |  |  |  |  |  |  |  |  |  |  |  |  |  |  |  |  |  |  |  |  |  |  |  |  |  |  |  |  |  |  |  |  |  |  |  |  |  |  |  |  |  |  |  |  |  |  |  |  |  |  |  |  |  |  |  |  |  |  |  |  |  |  |  |  |  |  |  |  |  |  |  |  |  |  |  |  |  |  |  |  |  |  |  |  |  |  |  |  |  |  |  |  |  |  |  |  |  |  |  |  |  |  |  |  |  |  |  |  |  |  |  |  |  |  |  |  |  |  |  |  |  |  |  |  |  |  |  |  |  |  |  |  |  |  |  |  |  |  |  |  |  |  |  |  |  |  |  |  |  |  |  |  |  |  |  |  |  |  |  |  |  |  |  |  |  |  |  |  |  |  |  |  |  |  |  |  |  |  |  |  |  |  |  |  |  |  |  |  |  |  |  |  |  |  |  |  |  |  |  |  |  |  |  |  |  |  |  |  |  |  |  |  |  |  |  |  |  |  |  |  |  |  |  |  |  |  |  |  |  |  |  |  |  |  |  |  |  |  |  |  |  |  |  |  |  |  |  |  |  |  |  |  |  |  |  |  |  |  |  |  |  |  |  |  |  |  |  |  |  |  |  |  |  |  |  |  |  |  |  |  |  |  |  |  |  |  |  |  |  |  |  |  |  |  |  |  |  |  |  |  |  |  |  |  |  |  |  |  |
|  |  |  |  |  |  |  |  |  |  |  |  |  |  |  |  |  |  |  |  |  |  |  |  |  |  |  |  |  |  |  |  |  |  |  |  |  |  |  |  |  |  |  |  |  |  |  |  |  |  |  |  |  |  |  |  |  |  |  |  |  |  |  |  |  |  |  |  |  |  |  |  |  |  |  |  |  |  |  |  |  |  |  |  |  |  |  |  |  |  |  |  |  |  |  |  |  |  |  |  |  |  |  |  |  |  |  |  |  |  |  |  |  |  |  |  |  |  |  |  |  |  |  |  |  |  |  |  |  |  |  |  |  |  |  |  |  |  |  |  |  |  |  |  |  |  |  |  |  |  |  |  |  |  |  |  |  |  |  |  |  |  |  |  |  |  |  |  |  |  |  |  |  |  |  |  |  |  |  |  |  |  |  |  |  |  |  |  |  |  |  |  |  |  |  |  |  |  |  |  |  |  |  |  |  |  |  |  |  |  |  |  |  |  |  |  |  |  |  |  |  |  |  |  |  |  |  |  |  |  |  |  |  |  |  |  |  |  |  |  |  |  |  |  |  |  |  |  |  |  |  |  |  |  |  |  |  |  |  |  |  |  |  |  |  |  |  |  |  |  |  |  |  |  |  |  |  |  |  |  |  |  |  |  |  |  |  |  |  |  |  |  |  |  |  |  |  |  |  |  |  |  |  |  |  |  |  |  |  |  |  |  |  |  |  |  |  |  |  |  |  |  |  |  |  |  |  |  |  |  |  |  |  |  |  |  |  |  |  |  |  |  |  |  |  |  |  |  |  |  |  |  |  |  |  |  |  |  |  |  |  |  |  |  |  |  |  |  |  |  |  |  |  |  |  |  |  |  |  |  |  |  |  |  |  |  |  |  |  |  |  |  |  |  |  |  |  |  |  |  |  |  |  |  |  |  |  |  |  |  |  |  |  |  |  |  |  |  |  |  |  |  |  |  |  |  |  |  |  |  |  |  |  |  |  |  |  |  |  |  |  |  |  |  |  |  |  |  |  |  |  |  |  |  |  |  |  |  |  |  |  |  |  |  |  |  |  |  |  |  |  |  |  |  |  |  |  |  |  |  |  |  |  |  |  |  |  |  |  |  |  |  |  |  |  |  |  |  |  |  |  |  |  |  |  |  |  |  |  |  |  |  |  |  |  |  |  |  |  |  |  |  |  |  |  |  |  |  |  |  |  |  |  |  |  |  |  |  |  |  |  |  |  |  |  |  |  |  |  |  |  |  |  |  |  |  |  |  |  |  |  |  |  |  |  |  |  |  |  |  |  |  |  |  |  |  |  |  |  |  |  |  |  |  |  |  |  |  |  |  |  |  |  |  |  |  |  |  |  |  |  |  |  |  |  |  |  |  |  |  |  |  |  |  |  |  |  |  |  |  |  |  |  |  |  |  |  |  |  |  |  |  |  |  |  |  |  |  |  |  |  |  |  |  |  |  |  |  |  |  |  |  |  |  |  |  |  |  |  |  |  |  |  |  |  |  |  |  |  |  |  |  |  |  |  |  |  |  |  |  |  |  |  |  |  |  |  |  |  |  |  |  |  |  |  |  |  |  |  |  |  |  |  |  |  |  |  |  |  |  |  |  |  |  |  |  |  |  |  |  |  |  |  |  |  |  |  |  |  |  |  |  |  |  |  |  |  |  |  |  |  |  |  |  |  |  |  |  |  |  |  |  |  |  |  |  |  |  |  |  |  |  |  |  |  |  |  |  |  |  |  |  |  |  |  |  |  |  |  |  |  |  |  |  |  |  |  |  |  |  |  |  |  |  |  |  |  |  |  |  |  |  |  |  |  |  |  |  |  |  |  |  |  |  |  |  |  |  |  |  |  |  |  |  |  |  |  |  |  |  |  |  |  |  |  |  |  |  |  |  |  |  |  |  |  |  |  |  |  |  |  |  |  |  |  |  |  |  |  |  |  |  |  |  |  |  |  |  |  |  |  |  |  |  |  |  |  |  |  |  |  |  |  |  |  |  |  |  |  |  |  |  |  |  |  |  |  |  |  |  |  |  |  |  |  |  |  |  |  |  |  |  |  |  |  |  |  |  |  |  |  |  |  |  |  |  |  |  |  |  |  |  |  |  |  |  |  |  |  |  |  |  |  |  |  |  |  |  |  |  |  |  |  |  |  |  |  |  |  |  |  |  |  |  |  |  |  |  |  |  |  |  |  |  |  |  |  |  |  |  |  |  |  |  |  |  |  |  |  |  |  |  |  |  |  |  |  |  |  |  |  |  |  |  |  |  |  |  |  |  |  |  |  |  |  |  |  |  |  |  |  |  |  |  |  |  |  |  |  |  |  |  |  |  |  |  |  |  |  |  |  |  |  |  |  |  |  |  |  |  |  |  |  |  |  |  |  |  |  |  |  |  |  |  |  |  |  |  |  |  |  |  |  |  |  |  |  |  |  |  |  |  |  |  |  |  |  |  |  |  |  |  |  |  |  |  |  |  |  |  |  |  |  |  |  |  |  |  |  |  |  |  |  |  |  |  |  |  |  |  |  |  |  |  |  |  |  |  |  |  |  |  |  |  |  |  |  |  |  |  |  |  |  |  |  |  |  |  |  |  |  |  |  |  |  |  |  |  |  |  |  |  |  |  |  |  |  |  |  |  |  |  |  |  |  |  |  |  |  |  |  |  |  |  |  |  |  |  |  |  |  |  |  |  |  |  |  |  |  |  |  |  |  |  |  |  |  |  |  |  |  |  |  |  |  |  |  |  |  |  |  |  |  |  |  |  |  |  |  |  |  |  |  |  |  |  |  |  |  |  |  |  |  |  |  |  |  |  |  |  |  |  |  |  |  |  |  |  |  |  |  |  |  |  |  |  |  |  |  |  |  |  |  |  |  |  |  |  |  |  |  |  |  |  |  |  |  |  |  |  |  |  |  |  |  |  |  |  |  |  |  |  |  |  |  |  |  |  |  |  |  |  |  |  |  |  |  |  |  |  |  |  |  |  |  |  |  |  |  |  |  |  |  |  |  |  |  |  |  |  |  |  |  |  |  |  |  |  |  |  |  |  |  |  |  |  |  |  |  |  |  |  |  |  |  |  |  |  |  |  |  |  |  |  |  |  |  |  |  |  |  |  |  |  |  |  |  |  |  |  |  |  |  |  |  |  |  |  |  |  |  |  |  |  |  |  |  |  |  |  |  |  |  |  |  |  |  |  |  |  |  |  |  |  |  |  |  |  |  |  |  |  |  |  |  |  |  |  |  |  |  |  |  |  |  |  |  |  |  |  |  |  |  |  |  |  |  |  |  |  |  |  |  |  |  |  |  |  |  |  |  |  |  |  |  |  |  |  |  |  |  |  |  |  |  |  |  |  |  |  |  |  |  |  |  |  |  |  |  |  |  |  |  |  |  |  |  |  |  |  |  |  |  |  |  |  |  |  |  |  |  |  |  |  |  |  |  |  |  |  |  |  |  |  |  |  |  |  |  |  |  |  |  |  |  |  |  |  |  |  |  |  |  |  |  |  |  |  |  |  |  |  |  |  |  |  |  |  |  |  |  |  |  |  |  |  |  |  |  |  |  |  |  |  |  |  |  |  |  |  |  |  |  |  |  |  |  |  |  |  |  |  |  |  |  |  |  |  |  |  |  |  |  |  |  |  |  |  |  |  |  |  |  |  |  |  |  |  |  |  |  |  |  |  |  |  |  |  |  |  |  |  |  |  |  |  |  |  |  |  |  |  |  |  |  |  |  |  |  |  |  |  |  |  |  |  |  |  |  |  |  |  |  |  |  |  |  |  |  |  |  |
|  |  |  |  |  |  |  |  |  |  |  |  |  |  |  |  |  |  |  |  |  |  |  |  |  |  |  |  |  |  |  |  |  |  |  |  |  |  |  |  |  |  |  |  |  |  |  |  |  |  |  |  |  |  |  |  |  |  |  |  |  |  |  |  |  |  |  |  |  |  |  |  |  |  |  |  |  |  |  |  |  |  |  |  |  |  |  |  |  |  |  |  |  |  |  |  |  |  |  |  |  |  |  |  |  |  |  |  |  |  |  |  |  |  |  |  |  |  |  |  |  |  |  |  |  |  |  |  |  |  |  |  |  |  |  |  |  |  |  |  |  |  |  |  |  |  |  |  |  |  |  |  |  |  |  |  |  |  |  |  |  |  |  |  |  |  |  |  |  |  |  |  |  |  |  |  |  |  |  |  |  |  |  |  |  |  |  |  |  |  |  |  |  |  |  |  |  |  |  |  |  |  |  |  |  |  |  |  |  |  |  |  |  |  |  |  |  |  |  |  |  |  |  |  |  |  |  |  |  |  |  |  |  |  |  |  |  |  |  |  |  |  |  |  |  |  |  |  |  |  |  |  |  |  |  |  |  |  |  |  |  |  |  |  |  |  |  |  |  |  |  |  |  |  |  |  |  |  |  |  |  |  |  |  |  |  |  |  |  |  |  |  |  |  |  |  |  |  |  |  |  |  |  |  |  |  |  |  |  |  |  |  |  |  |  |  |  |  |  |  |  |  |  |  |  |  |  |  |  |  |  |  |  |  |  |  |  |  |  |  |  |  |  |  |  |  |  |  |  |  |  |  |  |  |  |  |  |  |  |  |  |  |  |  |  |  |  |  |  |  |  |  |  |  |  |  |  |  |  |  |  |  |  |  |  |  |  |  |  |  |  |  |  |  |  |  |  |  |  |  |  |  |  |  |  |  |  |  |  |  |  |  |  |  |  |  |  |  |  |  |  |  |  |  |  |  |  |  |  |  |  |  |  |  |  |  |  |  |  |  |  |  |  |  |  |  |  |  |  |  |  |  |  |  |  |  |  |  |  |  |  |  |  |  |  |  |  |  |  |  |  |  |  |  |  |  |  |  |  |  |  |  |  |  |  |  |  |  |  |  |  |  |  |  |  |  |  |  |  |  |  |  |  |  |  |  |  |  |  |  |  |  |  |  |  |  |  |  |  |  |  |  |  |  |  |  |  |  |  |  |  |  |  |  |  |  |  |  |  |  |  |  |  |  |  |  |  |  |  |  |  |  |  |  |  |  |  |  |  |  |  |  |  |  |  |  |  |  |  |  |  |  |  |  |  |  |  |  |  |  |  |  |  |  |  |  |  |  |  |  |  |  |  |  |  |  |  |  |  |  |  |  |  |  |  |  |  |  |  |  |  |  |  |  |  |  |  |  |  |  |  |  |  |  |  |  |  |  |  |  |  |  |  |  |  |  |  |  |  |  |  |  |  |  |  |  |  |  |  |  |  |  |  |  |  |  |  |  |  |  |  |  |  |  |  |  |  |  |  |  |  |  |  |  |  |  |  |  |  |  |  |  |  |  |  |  |  |  |  |  |  |  |  |  |  |  |  |  |  |  |  |  |  |  |  |  |  |  |  |  |  |  |  |  |  |  |  |  |  |  |  |  |  |  |  |  |  |  |  |  |  |  |  |  |  |  |  |  |  |  |  |  |  |  |  |  |  |  |  |  |  |  |  |  |  |  |  |  |  |  |  |  |  |  |  |  |  |  |  |  |  |  |  |  |  |  |  |  |  |  |  |  |  |  |  |  |  |  |  |  |  |  |  |  |  |  |  |  |  |  |  |  |  |  |  |  |  |  |  |  |  |  |  |  |  |  |  |  |  |  |  |  |  |  |  |  |  |  |  |  |  |  |  |  |  |  |  |  |  |  |  |  |  |  |  |  |  |  |  |  |  |  |  |  |  |  |  |  |  |  |  |  |  |  |  |  |  |  |  |  |  |  |  |  |  |  |  |  |  |  |  |  |  |  |  |  |  |  |  |  |  |  |  |  |  |  |  |  |  |  |  |  |  |  |  |  |  |  |  |  |  |  |  |  |  |  |  |  |  |  |  |  |  |  |  |  |  |  |  |  |  |  |  |  |  |  |  |  |  |  |  |  |  |  |  |  |  |  |  |  |  |  |  |  |  |  |  |  |  |  |  |  |  |  |  |  |  |  |  |  |  |  |  |  |  |  |  |  |  |  |  |  |  |  |  |  |  |  |  |  |  |  |  |  |  |  |  |  |  |  |  |  |  |  |  |  |  |  |  |  |  |  |  |  |  |  |  |  |  |  |  |  |  |  |  |  |  |  |  |  |  |  |  |  |  |  |  |  |  |  |  |  |  |  |  |  |  |  |  |  |  |  |  |  |  |  |  |  |  |  |  |  |  |  |  |  |  |  |  |  |  |  |  |  |  |  |  |  |  |  |  |  |  |  |  |  |  |  |  |  |  |  |  |  |  |  |  |  |  |  |  |  |  |  |  |  |  |  |  |  |  |  |  |  |  |  |  |  |  |  |  |  |  |  |  |  |  |  |  |  |  |  |  |  |  |  |  |  |  |  |  |  |  |  |  |  |  |  |  |  |  |  |  |  |  |  |  |  |  |  |  |  |  |  |  |  |  |  |  |  |  |  |  |  |  |  |  |  |  |  |  |  |  |  |  |  |  |  |  |  |  |  |  |  |  |  |  |  |  |  |  |  |  |  |  |  |  |  |  |  |  |  |  |  |  |  |  |  |  |  |  |  |  |  |  |  |  |  |  |  |  |  |  |  |  |  |  |  |  |  |  |  |  |  |  |  |  |  |  |  |  |  |  |  |  |  |  |  |  |  |  |  |  |  |  |  |  |  |  |  |  |  |  |  |  |  |  |  |  |  |  |  |  |  |  |  |  |  |  |  |  |  |  |  |  |  |  |  |  |  |  |  |  |  |  |  |  |  |  |  |  |  |  |  |  |  |  |  |  |  |  |  |  |  |  |  |  |  |  |  |  |  |  |  |  |  |  |  |  |  |  |  |  |  |  |  |  |  |  |  |  |  |  |  |  |  |  |  |  |  |  |  |  |  |  |  |  |  |  |  |  |  |  |  |  |  |  |  |  |  |  |  |  |  |  |  |  |  |  |  |  |  |  |  |  |  |  |  |  |  |  |  |  |  |  |  |  |  |  |  |  |  |  |  |  |  |  |  |  |  |  |  |  |  |  |  |  |  |  |  |  |  |  |  |  |  |  |  |  |  |  |  |  |  |  |  |  |  |  |  |  |  |  |  |  |  |  |  |  |  |  |  |  |  |  |  |  |  |  |  |  |  |  |  |  |  |  |  |  |  |  |  |  |  |  |  |  |  |  |  |  |  |  |  |  |  |  |  |  |  |  |  |  |  |  |  |  |  |  |  |  |  |  |  |  |  |  |  |  |  |  |  |  |  |  |  |  |  |  |  |  |  |  |  |  |  |  |  |  |  |  |  |  |  |  |  |  |  |  |  |  |  |  |  |  |  |  |  |  |  |  |  |  |  |  |  |  |  |  |  |  |  |  |  |  |  |  |  |  |  |  |  |  |  |  |  |  |  |  |  |  |  |  |  |  |  |  |  |  |  |  |  |  |  |  |  |  |  |  |  |  |  |  |  |  |  |  |  |  |  |  |  |  |  |  |  |  |  |  |  |  |  |  |  |  |  |  |  |  |  |  |  |  |  |  |  |  |  |  |  |  |  |  |  |  |  |  |  |  |  |  |  |  |  |  |  |  |  |  |  |  |  |  |  |  |  |  |  |  |  |  |
|  |  |  |  |  |  |  |  |  |  |  |  |  |  |  |  |  |  |  |  |  |  |  |  |  |  |  |  |  |  |  |  |  |  |  |  |  |  |  |  |  |  |  |  |  |  |  |  |  |  |  |  |  |  |  |  |  |  |  |  |  |  |  |  |  |  |  |  |  |  |  |  |  |  |  |  |  |  |  |  |  |  |  |  |  |  |  |  |  |  |  |  |  |  |  |  |  |  |  |  |  |  |  |  |  |  |  |  |  |  |  |  |  |  |  |  |  |  |  |  |  |  |  |  |  |  |  |  |  |  |  |  |  |  |  |  |  |  |  |  |  |  |  |  |  |  |  |  |  |  |  |  |  |  |  |  |  |  |  |  |  |  |  |  |  |  |  |  |  |  |  |  |  |  |  |  |  |  |  |  |  |  |  |  |  |  |  |  |  |  |  |  |  |  |  |  |  |  |  |  |  |  |  |  |  |  |  |  |  |  |  |  |  |  |  |  |  |  |  |  |  |  |  |  |  |  |  |  |  |  |  |  |  |  |  |  |  |  |  |  |  |  |  |  |  |  |  |  |  |  |  |  |  |  |  |  |  |  |  |  |  |  |  |  |  |  |  |  |  |  |  |  |  |  |  |  |  |  |  |  |  |  |  |  |  |  |  |  |  |  |  |  |  |  |  |  |  |  |  |  |  |  |  |  |  |  |  |  |  |  |  |  |  |  |  |  |  |  |  |  |  |  |  |  |  |  |  |  |  |  |  |  |  |  |  |  |  |  |  |  |  |  |  |  |  |  |  |  |  |  |  |  |  |  |  |  |  |  |  |  |  |  |  |  |  |  |  |  |  |  |  |  |  |  |  |  |  |  |  |  |  |  |  |  |  |  |  |  |  |  |  |  |  |  |  |  |  |  |  |  |  |  |  |  |  |  |  |  |  |  |  |  |  |  |  |  |  |  |  |  |  |  |  |  |  |  |  |  |  |  |  |  |  |  |  |  |  |  |  |  |  |  |  |  |  |  |  |  |  |  |  |  |  |  |  |  |  |  |  |  |  |  |  |  |  |  |  |  |  |  |  |  |  |  |  |  |  |  |  |  |  |  |  |  |  |  |  |  |  |  |  |  |  |  |  |  |  |  |  |  |  |  |  |  |  |  |  |  |  |  |  |  |  |  |  |  |  |  |  |  |  |  |  |  |  |  |  |  |  |  |  |  |  |  |  |  |  |  |  |  |  |  |  |  |  |  |  |  |  |  |  |  |  |  |  |  |  |  |  |  |  |  |  |  |  |  |  |  |  |  |  |  |  |  |  |  |  |  |  |  |  |  |  |  |  |  |  |  |  |  |  |  |  |  |  |  |  |  |  |  |  |  |  |  |  |  |  |  |  |  |  |  |  |  |  |  |  |  |  |  |  |  |  |  |  |  |  |  |  |  |  |  |  |  |  |  |  |  |  |  |  |  |  |  |  |  |  |  |  |  |  |  |  |  |  |  |  |  |  |  |  |  |  |  |  |  |  |  |  |  |  |  |  |  |  |  |  |  |  |  |  |  |  |  |  |  |  |  |  |  |  |  |  |  |  |  |  |  |  |  |  |  |  |  |  |  |  |  |  |  |  |  |  |  |  |  |  |  |  |  |  |  |  |  |  |  |  |  |  |  |  |  |  |  |  |  |  |  |  |  |  |  |  |  |  |  |  |  |  |  |  |  |  |  |  |  |  |  |  |  |  |  |  |  |  |  |  |  |  |  |  |  |  |  |  |  |  |  |  |  |  |  |  |  |  |  |  |  |  |  |  |  |  |  |  |  |  |  |  |  |  |  |  |  |  |  |  |  |  |  |  |  |  |  |  |  |  |  |  |  |  |  |  |  |  |  |  |  |  |  |  |  |  |  |  |  |  |  |  |  |  |  |  |  |  |  |  |  |  |  |  |  |  |  |  |  |  |  |  |  |  |  |  |  |  |  |  |  |  |  |  |  |  |  |  |  |  |  |  |  |  |  |  |  |  |  |  |  |  |  |  |  |  |  |  |  |  |  |  |  |  |  |  |  |  |  |  |  |  |  |  |  |  |  |  |  |  |  |  |  |  |  |  |  |  |  |  |  |  |  |  |  |  |  |  |  |  |  |  |  |  |  |  |  |  |  |  |  |  |  |  |  |  |  |  |  |  |  |  |  |  |  |  |  |  |  |  |  |  |  |  |  |  |  |  |  |  |  |  |  |  |  |  |  |  |  |  |  |  |  |  |  |  |  |  |  |  |  |  |  |  |  |  |  |  |  |  |  |  |  |  |  |  |  |  |  |  |  |  |  |  |  |  |  |  |  |  |  |  |  |  |  |  |  |  |  |  |  |  |  |  |  |  |  |  |  |  |  |  |  |  |  |  |  |  |  |  |  |  |  |  |  |  |  |  |  |  |  |  |  |  |  |  |  |  |  |  |  |  |  |  |  |  |  |  |  |  |  |  |  |  |  |  |  |  |  |  |  |  |  |  |  |  |  |  |  |  |  |  |  |  |  |  |  |  |  |  |  |  |  |  |  |  |  |  |  |  |  |  |  |  |  |  |  |  |  |  |  |  |  |  |  |  |  |  |  |  |  |  |  |  |  |  |  |  |  |  |  |  |  |  |  |  |  |  |  |  |  |  |  |  |  |  |  |  |  |  |  |  |  |  |  |  |  |  |  |  |  |  |  |  |  |  |  |  |  |  |  |  |  |  |  |  |  |  |  |  |  |  |  |  |  |  |  |  |  |  |  |  |  |  |  |  |  |  |  |  |  |  |  |  |  |  |  |  |  |  |  |  |  |  |  |  |  |  |  |  |  |  |  |  |  |  |  |  |  |  |  |  |  |  |  |  |  |  |  |  |  |  |  |  |  |  |  |  |  |  |  |  |  |  |  |  |  |  |  |  |  |  |  |  |  |  |  |  |  |  |  |  |  |  |  |  |  |  |  |  |  |  |  |  |  |  |  |  |  |  |  |  |  |  |  |  |  |  |  |  |  |  |  |  |  |  |  |  |  |  |  |  |  |  |  |  |  |  |  |  |  |  |  |  |  |  |  |  |  |  |  |  |  |  |  |  |  |  |  |  |  |  |  |  |  |  |  |  |  |  |  |  |  |  |  |  |  |  |  |  |  |  |  |  |  |  |  |  |  |  |  |  |  |  |  |  |  |  |  |  |  |  |  |  |  |  |  |  |  |  |  |  |  |  |  |  |  |  |  |  |  |  |  |  |  |  |  |  |  |  |  |  |  |  |  |  |  |  |  |  |  |  |  |  |  |  |  |  |  |  |  |  |  |  |  |  |  |  |  |  |  |  |  |  |  |  |  |  |  |  |  |  |  |  |  |  |  |  |  |  |  |  |  |  |  |  |  |  |  |  |  |  |  |  |  |  |  |  |  |  |  |  |  |  |  |  |  |  |  |  |  |  |  |  |  |  |  |  |  |  |  |  |  |  |  |  |  |  |  |  |  |  |  |  |  |  |  |  |  |  |  |  |  |  |  |  |  |  |  |  |  |  |  |  |  |  |  |  |  |  |  |  |  |  |  |  |  |  |  |  |  |  |  |  |  |  |  |  |  |  |  |  |  |  |  |  |  |  |  |  |  |  |  |  |  |  |  |  |  |  |  |  |  |  |  |  |  |  |  |  |  |  |  |  |  |  |  |  |  |  |  |  |  |  |  |  |  |  |  |  |  |  |  |  |  |  |  |  |  |  |  |  |  |  |  |  |  |  |  |  |  |  |  |  |  |  |  |  |  |  |  |  |  |  |  |  |  |  |  |  |  |  |  |  |
|  |  |  |  |  |  |  |  |  |  |  |  |  |  |  |  |  |  |  |  |  |  |  |  |  |  |  |  |  |  |  |  |  |  |  |  |  |  |  |  |  |  |  |  |  |  |  |  |  |  |  |  |  |  |  |  |  |  |  |  |  |  |  |  |  |  |  |  |  |  |  |  |  |  |  |  |  |  |  |  |  |  |  |  |  |  |  |  |  |  |  |  |  |  |  |  |  |  |  |  |  |  |  |  |  |  |  |  |  |  |  |  |  |  |  |  |  |  |  |  |  |  |  |  |  |  |  |  |  |  |  |  |  |  |  |  |  |  |  |  |  |  |  |  |  |  |  |  |  |  |  |  |  |  |  |  |  |  |  |  |  |  |  |  |  |  |  |  |  |  |  |  |  |  |  |  |  |  |  |  |  |  |  |  |  |  |  |  |  |  |  |  |  |  |  |  |  |  |  |  |  |  |  |  |  |  |  |  |  |  |  |  |  |  |  |  |  |  |  |  |  |  |  |  |  |  |  |  |  |  |  |  |  |  |  |  |  |  |  |  |  |  |  |  |  |  |  |  |  |  |  |  |  |  |  |  |  |  |  |  |  |  |  |  |  |  |  |  |  |  |  |  |  |  |  |  |  |  |  |  |  |  |  |  |  |  |  |  |  |  |  |  |  |  |  |  |  |  |  |  |  |  |  |  |  |  |  |  |  |  |  |  |  |  |  |  |  |  |  |  |  |  |  |  |  |  |  |  |  |  |  |  |  |  |  |  |  |  |  |  |  |  |  |  |  |  |  |  |  |  |  |  |  |  |  |  |  |  |  |  |  |  |  |  |  |  |  |  |  |  |  |  |  |  |  |  |  |  |  |  |  |  |  |  |  |  |  |  |  |  |  |  |  |  |  |  |  |  |  |  |  |  |  |  |  |  |  |  |  |  |  |  |  |  |  |  |  |  |  |  |  |  |  |  |  |  |  |  |  |  |  |  |  |  |  |  |  |  |  |  |  |  |  |  |  |  |  |  |  |  |  |  |  |  |  |  |  |  |  |  |  |  |  |  |  |  |  |  |  |  |  |  |  |  |  |  |  |  |  |  |  |  |  |  |  |  |  |  |  |  |  |  |  |  |  |  |  |  |  |  |  |  |  |  |  |  |  |  |  |  |  |  |  |  |  |  |  |  |  |  |  |  |  |  |  |  |  |  |  |  |  |  |  |  |  |  |  |  |  |  |  |  |  |  |  |  |  |  |  |  |  |  |  |  |  |  |  |  |  |  |  |  |  |  |  |  |  |  |  |  |  |  |  |  |  |  |  |  |  |  |  |  |  |  |  |  |  |  |  |  |  |  |  |  |  |  |  |  |  |  |  |  |  |  |  |  |  |  |  |  |  |  |  |  |  |  |  |  |  |  |  |  |  |  |  |  |  |  |  |  |  |  |  |  |  |  |  |  |  |  |  |  |  |  |  |  |  |  |  |  |  |  |  |  |  |  |  |  |  |  |  |  |  |  |  |  |  |  |  |  |  |  |  |  |  |  |  |  |  |  |  |  |  |  |  |  |  |  |  |  |  |  |  |  |  |  |  |  |  |  |  |  |  |  |  |  |  |  |  |  |  |  |  |  |  |  |  |  |  |  |  |  |  |  |  |  |  |  |  |  |  |  |  |  |  |  |  |  |  |  |  |  |  |  |  |  |  |  |  |  |  |  |  |  |  |  |  |  |  |  |  |  |  |  |  |  |  |  |  |  |  |  |  |  |  |  |  |  |  |  |  |  |  |  |  |  |  |  |  |  |  |  |  |  |  |  |  |  |  |  |  |  |  |  |  |  |  |  |  |  |  |  |  |  |  |  |  |  |  |  |  |  |  |  |  |  |  |  |  |  |  |  |  |  |  |  |  |  |  |  |  |  |  |  |  |  |  |  |  |  |  |  |  |  |  |  |  |  |  |  |  |  |  |  |  |  |  |  |  |  |  |  |  |  |  |  |  |  |  |  |  |  |  |  |  |  |  |  |  |  |  |  |  |  |  |  |  |  |  |  |  |  |  |  |  |  |  |  |  |  |  |  |  |  |  |  |  |  |  |  |  |  |  |  |  |  |  |  |  |  |  |  |  |  |  |  |  |  |  |  |  |  |  |  |  |  |  |  |  |  |  |  |  |  |  |  |  |  |  |  |  |  |  |  |  |  |  |  |  |  |  |  |  |  |  |  |  |  |  |  |  |  |  |  |  |  |  |  |  |  |  |  |  |  |  |  |  |  |  |  |  |  |  |  |  |  |  |  |  |  |  |  |  |  |  |  |  |  |  |  |  |  |  |  |  |  |  |  |  |  |  |  |  |  |  |  |  |  |  |  |  |  |  |  |  |  |  |  |  |  |  |  |  |  |  |  |  |  |  |  |  |  |  |  |  |  |  |  |  |  |  |  |  |  |  |  |  |  |  |  |  |  |  |  |  |  |  |  |  |  |  |  |  |  |  |  |  |  |  |  |  |  |  |  |  |  |  |  |  |  |  |  |  |  |  |  |  |  |  |  |  |  |  |  |  |  |  |  |  |  |  |  |  |  |  |  |  |  |  |  |  |  |  |  |  |  |  |  |  |  |  |  |  |  |  |  |  |  |  |  |  |  |  |  |  |  |  |  |  |  |  |  |  |  |  |  |  |  |  |  |  |  |  |  |  |  |  |  |  |  |  |  |  |  |  |  |  |  |  |  |  |  |  |  |  |  |  |  |  |  |  |  |  |  |  |  |  |  |  |  |  |  |  |  |  |  |  |  |  |  |  |  |  |  |  |  |  |  |  |  |  |  |  |  |  |  |  |  |  |  |  |  |  |  |  |  |  |  |  |  |  |  |  |  |  |  |  |  |  |  |  |  |  |  |  |  |  |  |  |  |  |  |  |  |  |  |  |  |  |  |  |  |  |  |  |  |  |  |  |  |  |  |  |  |  |  |  |  |  |  |  |  |  |  |  |  |  |  |  |  |  |  |  |  |  |  |  |  |  |  |  |  |  |  |  |  |  |  |  |  |  |  |  |  |  |  |  |  |  |  |  |  |  |  |  |  |  |  |  |  |  |  |  |  |  |  |  |  |  |  |  |  |  |  |  |  |  |  |  |  |  |  |  |  |  |  |  |  |  |  |  |  |  |  |  |  |  |  |  |  |  |  |  |  |  |  |  |  |  |  |  |  |  |  |  |  |  |  |  |  |  |  |  |  |  |  |  |  |  |  |  |  |  |  |  |  |  |  |  |  |  |  |  |  |  |  |  |  |  |  |  |  |  |  |  |  |  |  |  |  |  |  |  |  |  |  |  |  |  |  |  |  |  |  |  |  |  |  |  |  |  |  |  |  |  |  |  |  |  |  |  |  |  |  |  |  |  |  |  |  |  |  |  |  |  |  |  |  |  |  |  |  |  |  |  |  |  |  |  |  |  |  |  |  |  |  |  |  |  |  |  |  |  |  |  |  |  |  |  |  |  |  |  |  |  |  |  |  |  |  |  |  |  |  |  |  |  |  |  |  |  |  |  |  |  |  |  |  |  |  |  |  |  |  |  |  |  |  |  |  |  |  |  |  |  |  |  |  |  |  |  |  |  |  |  |  |  |  |  |  |  |  |  |  |  |  |  |  |  |  |  |  |  |  |  |  |  |  |  |  |  |  |  |  |  |  |  |  |  |  |  |  |  |  |  |  |  |  |  |  |  |  |  |  |  |  |  |  |  |  |  |  |  |  |  |  |  |  |  |  |  |  |  |  |  |  |  |  |  |  |  |  |  |  |  |  |  |  |  |  |  |
|  |  |  |  |  |  |  |  |  |  |  |  |  |  |  |  |  |  |  |  |  |  |  |  |  |  |  |  |  |  |  |  |  |  |  |  |  |  |  |  |  |  |  |  |  |  |  |  |  |  |  |  |  |  |  |  |  |  |  |  |  |  |  |  |  |  |  |  |  |  |  |  |  |  |  |  |  |  |  |  |  |  |  |  |  |  |  |  |  |  |  |  |  |  |  |  |  |  |  |  |  |  |  |  |  |  |  |  |  |  |  |  |  |  |  |  |  |  |  |  |  |  |  |  |  |  |  |  |  |  |  |  |  |  |  |  |  |  |  |  |  |  |  |  |  |  |  |  |  |  |  |  |  |  |  |  |  |  |  |  |  |  |  |  |  |  |  |  |  |  |  |  |  |  |  |  |  |  |  |  |  |  |  |  |  |  |  |  |  |  |  |  |  |  |  |  |  |  |  |  |  |  |  |  |  |  |  |  |  |  |  |  |  |  |  |  |  |  |  |  |  |  |  |  |  |  |  |  |  |  |  |  |  |  |  |  |  |  |  |  |  |  |  |  |  |  |  |  |  |  |  |  |  |  |  |  |  |  |  |  |  |  |  |  |  |  |  |  |  |  |  |  |  |  |  |  |  |  |  |  |  |  |  |  |  |  |  |  |  |  |  |  |  |  |  |  |  |  |  |  |  |  |  |  |  |  |  |  |  |  |  |  |  |  |  |  |  |  |  |  |  |  |  |  |  |  |  |  |  |  |  |  |  |  |  |  |  |  |  |  |  |  |  |  |  |  |  |  |  |  |  |  |  |  |  |  |  |  |  |  |  |  |  |  |  |  |  |  |  |  |  |  |  |  |  |  |  |  |  |  |  |  |  |  |  |  |  |  |  |  |  |  |  |  |  |  |  |  |  |  |  |  |  |  |  |  |  |  |  |  |  |  |  |  |  |  |  |  |  |  |  |  |  |  |  |  |  |  |  |  |  |  |  |  |  |  |  |  |  |  |  |  |  |  |  |  |  |  |  |  |  |  |  |  |  |  |  |  |  |  |  |  |  |  |  |  |  |  |  |  |  |  |  |  |  |  |  |  |  |  |  |  |  |  |  |  |  |  |  |  |  |  |  |  |  |  |  |  |  |  |  |  |  |  |  |  |  |  |  |  |  |  |  |  |  |  |  |  |  |  |  |  |  |  |  |  |  |  |  |  |  |  |  |  |  |  |  |  |  |  |  |  |  |  |  |  |  |  |  |  |  |  |  |  |  |  |  |  |  |  |  |  |  |  |  |  |  |  |  |  |  |  |  |  |  |  |  |  |  |  |  |  |  |  |  |  |  |  |  |  |  |  |  |  |  |  |  |  |  |  |  |  |  |  |  |  |  |  |  |  |  |  |  |  |  |  |  |  |  |  |  |  |  |  |  |  |  |  |  |  |  |  |  |  |  |  |  |  |  |  |  |  |  |  |  |  |  |  |  |  |  |  |  |  |  |  |  |  |  |  |  |  |  |  |  |  |  |  |  |  |  |  |  |  |  |  |  |  |  |  |  |  |  |  |  |  |  |  |  |  |  |  |  |  |  |  |  |  |  |  |  |  |  |  |  |  |  |  |  |  |  |  |  |  |  |  |  |  |  |  |  |  |  |  |  |  |  |  |  |  |  |  |  |  |  |  |  |  |  |  |  |  |  |  |  |  |  |  |  |  |  |  |  |  |  |  |  |  |  |  |  |  |  |  |  |  |  |  |  |  |  |  |  |  |  |  |  |  |  |  |  |  |  |  |  |  |  |  |  |  |  |  |  |  |  |  |  |  |  |  |  |  |  |  |  |  |  |  |  |  |  |  |  |  |  |  |  |  |  |  |  |  |  |  |  |  |  |  |  |  |  |  |  |  |  |  |  |  |  |  |  |  |  |  |  |  |  |  |  |  |  |  |  |  |  |  |  |  |  |  |  |  |  |  |  |  |  |  |  |  |  |  |  |  |  |  |  |  |  |  |  |  |  |  |  |  |  |  |  |  |  |  |  |  |  |  |  |  |  |  |  |  |  |  |  |  |  |  |  |  |  |  |  |  |  |  |  |  |  |  |  |  |  |  |  |  |  |  |  |  |  |  |  |  |  |  |  |  |  |  |  |  |  |  |  |  |  |  |  |  |  |  |  |  |  |  |  |  |  |  |  |  |  |  |  |  |  |  |  |  |  |  |  |  |  |  |  |  |  |  |  |  |  |  |  |  |  |  |  |  |  |  |  |  |  |  |  |  |  |  |  |  |  |  |  |  |  |  |  |  |  |  |  |  |  |  |  |  |  |  |  |  |  |  |  |  |  |  |  |  |  |  |  |  |  |  |  |  |  |  |  |  |  |  |  |  |  |  |  |  |  |  |  |  |  |  |  |  |  |  |  |  |  |  |  |  |  |  |  |  |  |  |  |  |  |  |  |  |  |  |  |  |  |  |  |  |  |  |  |  |  |  |  |  |  |  |  |  |  |  |  |  |  |  |  |  |  |  |  |  |  |  |  |  |  |  |  |  |  |  |  |  |  |  |  |  |  |  |  |  |  |  |  |  |  |  |  |  |  |  |  |  |  |  |  |  |  |  |  |  |  |  |  |  |  |  |  |  |  |  |  |  |  |  |  |  |  |  |  |  |  |  |  |  |  |  |  |  |  |  |  |  |  |  |  |  |  |  |  |  |  |  |  |  |  |  |  |  |  |  |  |  |  |  |  |  |  |  |  |  |  |  |  |  |  |  |  |  |  |  |  |  |  |  |  |  |  |  |  |  |  |  |  |  |  |  |  |  |  |  |  |  |  |  |  |  |  |  |  |  |  |  |  |  |  |  |  |  |  |  |  |  |  |  |  |  |  |  |  |  |  |  |  |  |  |  |  |  |  |  |  |  |  |  |  |  |  |  |  |  |  |  |  |  |  |  |  |  |  |  |  |  |  |  |  |  |  |  |  |  |  |  |  |  |  |  |  |  |  |  |  |  |  |  |  |  |  |  |  |  |  |  |  |  |  |  |  |  |  |  |  |  |  |  |  |  |  |  |  |  |  |  |  |  |  |  |  |  |  |  |  |  |  |  |  |  |  |  |  |  |  |  |  |  |  |  |  |  |  |  |  |  |  |  |  |  |  |  |  |  |  |  |  |  |  |  |  |  |  |  |  |  |  |  |  |  |  |  |  |  |  |  |  |  |  |  |  |  |  |  |  |  |  |  |  |  |  |  |  |  |  |  |  |  |  |  |  |  |  |  |  |  |  |  |  |  |  |  |  |  |  |  |  |  |  |  |  |  |  |  |  |  |  |  |  |  |  |  |  |  |  |  |  |  |  |  |  |  |  |  |  |  |  |  |  |  |  |  |  |  |  |  |  |  |  |  |  |  |  |  |  |  |  |  |  |  |  |  |  |  |  |  |  |  |  |  |  |  |  |  |  |  |  |  |  |  |  |  |  |  |  |  |  |  |  |  |  |  |  |  |  |  |  |  |  |  |  |  |  |  |  |  |  |  |  |  |  |  |  |  |  |  |  |  |  |  |  |  |  |  |  |  |  |  |  |  |  |  |  |  |  |  |  |  |  |  |  |  |  |  |  |  |  |  |  |  |  |  |  |  |  |  |  |  |  |  |  |  |  |  |  |  |  |  |  |  |  |  |  |  |  |  |  |  |  |  |  |  |  |  |  |  |  |  |  |  |  |  |  |  |  |  |  |  |  |  |  |  |  |  |  |  |  |  |  |  |  |  |  |  |  |  |  |  |  |  |  |  |  |  |  |  |  |  |  |  |  |  |  |  |  |
|  |  |  |  |  |  |  |  |  |  |  |  |  |  |  |  |  |  |  |  |  |  |  |  |  |  |  |  |  |  |  |  |  |  |  |  |  |  |  |  |  |  |  |  |  |  |  |  |  |  |  |  |  |  |  |  |  |  |  |  |  |  |  |  |  |  |  |  |  |  |  |  |  |  |  |  |  |  |  |  |  |  |  |  |  |  |  |  |  |  |  |  |  |  |  |  |  |  |  |  |  |  |  |  |  |  |  |  |  |  |  |  |  |  |  |  |  |  |  |  |  |  |  |  |  |  |  |  |  |  |  |  |  |  |  |  |  |  |  |  |  |  |  |  |  |  |  |  |  |  |  |  |  |  |  |  |  |  |  |  |  |  |  |  |  |  |  |  |  |  |  |  |  |  |  |  |  |  |  |  |  |  |  |  |  |  |  |  |  |  |  |  |  |  |  |  |  |  |  |  |  |  |  |  |  |  |  |  |  |  |  |  |  |  |  |  |  |  |  |  |  |  |  |  |  |  |  |  |  |  |  |  |  |  |  |  |  |  |  |  |  |  |  |  |  |  |  |  |  |  |  |  |  |  |  |  |  |  |  |  |  |  |  |  |  |  |  |  |  |  |  |  |  |  |  |  |  |  |  |  |  |  |  |  |  |  |  |  |  |  |  |  |  |  |  |  |  |  |  |  |  |  |  |  |  |  |  |  |  |  |  |  |  |  |  |  |  |  |  |  |  |  |  |  |  |  |  |  |  |  |  |  |  |  |  |  |  |  |  |  |  |  |  |  |  |  |  |  |  |  |  |  |  |  |  |  |  |  |  |  |  |  |  |  |  |  |  |  |  |  |  |  |  |  |  |  |  |  |  |  |  |  |  |  |  |  |  |  |  |  |  |  |  |  |  |  |  |  |  |  |  |  |  |  |  |  |  |  |  |  |  |  |  |  |  |  |  |  |  |  |  |  |  |  |  |  |  |  |  |  |  |  |  |  |  |  |  |  |  |  |  |  |  |  |  |  |  |  |  |  |  |  |  |  |  |  |  |  |  |  |  |  |  |  |  |  |  |  |  |  |  |  |  |  |  |  |  |  |  |  |  |  |  |  |  |  |  |  |  |  |  |  |  |  |  |  |  |  |  |  |  |  |  |  |  |  |  |  |  |  |  |  |  |  |  |  |  |  |  |  |  |  |  |  |  |  |  |  |  |  |  |  |  |  |  |  |  |  |  |  |  |  |  |  |  |  |  |  |  |  |  |  |  |  |  |  |  |  |  |  |  |  |  |  |  |  |  |  |  |  |  |  |  |  |  |  |  |  |  |  |  |  |  |  |  |  |  |  |  |  |  |  |  |  |  |  |  |  |  |  |  |  |  |  |  |  |  |  |  |  |  |  |  |  |  |  |  |  |  |  |  |  |  |  |  |  |  |  |  |  |  |  |  |  |  |  |  |  |  |  |  |  |  |  |  |  |  |  |  |  |  |  |  |  |  |  |  |  |  |  |  |  |  |  |  |  |  |  |  |  |  |  |  |  |  |  |  |  |  |  |  |  |  |  |  |  |  |  |  |  |  |  |  |  |  |  |  |  |  |  |  |  |  |  |  |  |  |  |  |  |  |  |  |  |  |  |  |  |  |  |  |  |  |  |  |  |  |  |  |  |  |  |  |  |  |  |  |  |  |  |  |  |  |  |  |  |  |  |  |  |  |  |  |  |  |  |  |  |  |  |  |  |  |  |  |  |  |  |  |  |  |  |  |  |  |  |  |  |  |  |  |  |  |  |  |  |  |  |  |  |  |  |  |  |  |  |  |  |  |  |  |  |  |  |  |  |  |  |  |  |  |  |  |  |  |  |  |  |  |  |  |  |  |  |  |  |  |  |  |  |  |  |  |  |  |  |  |  |  |  |  |  |  |  |  |  |  |  |  |  |  |  |  |  |  |  |  |  |  |  |  |  |  |  |  |  |  |  |  |  |  |  |  |  |  |  |  |  |  |  |  |  |  |  |  |  |  |  |  |  |  |  |  |  |  |  |  |  |  |  |  |  |  |  |  |  |  |  |  |  |  |  |  |  |  |  |  |  |  |  |  |  |  |  |  |  |  |  |  |  |  |  |  |  |  |  |  |  |  |  |  |  |  |  |  |  |  |  |  |  |  |  |  |  |  |  |  |  |  |  |  |  |  |  |  |  |  |  |  |  |  |  |  |  |  |  |  |  |  |  |  |  |  |  |  |  |  |  |  |  |  |  |  |  |  |  |  |  |  |  |  |  |  |  |  |  |  |  |  |  |  |  |  |  |  |  |  |  |  |  |  |  |  |  |  |  |  |  |  |  |  |  |  |  |  |  |  |  |  |  |  |  |  |  |  |  |  |  |  |  |  |  |  |  |  |  |  |  |  |  |  |  |  |  |  |  |  |  |  |  |  |  |  |  |  |  |  |  |  |  |  |  |  |  |  |  |  |  |  |  |  |  |  |  |  |  |  |  |  |  |  |  |  |  |  |  |  |  |  |  |  |  |  |  |  |  |  |  |  |  |  |  |  |  |  |  |  |  |  |  |  |  |  |  |  |  |  |  |  |  |  |  |  |  |  |  |  |  |  |  |  |  |  |  |  |  |  |  |  |  |  |  |  |  |  |  |  |  |  |  |  |  |  |  |  |  |  |  |  |  |  |  |  |  |  |  |  |  |  |  |  |  |  |  |  |  |  |  |  |  |  |  |  |  |  |  |  |  |  |  |  |  |  |  |  |  |  |  |  |  |  |  |  |  |  |  |  |  |  |  |  |  |  |  |  |  |  |  |  |  |  |  |  |  |  |  |  |  |  |  |  |  |  |  |  |  |  |  |  |  |  |  |  |  |  |  |  |  |  |  |  |  |  |  |  |  |  |  |  |  |  |  |  |  |  |  |  |  |  |  |  |  |  |  |  |  |  |  |  |  |  |  |  |  |  |  |  |  |  |  |  |  |  |  |  |  |  |  |  |  |  |  |  |  |  |  |  |  |  |  |  |  |  |  |  |  |  |  |  |  |  |  |  |  |  |  |  |  |  |  |  |  |  |  |  |  |  |  |  |  |  |  |  |  |  |  |  |  |  |  |  |  |  |  |  |  |  |  |  |  |  |  |  |  |  |  |  |  |  |  |  |  |  |  |  |  |  |  |  |  |  |  |  |  |  |  |  |  |  |  |  |  |  |  |  |  |  |  |  |  |  |  |  |  |  |  |  |  |  |  |  |  |  |  |  |  |  |  |  |  |  |  |  |  |  |  |  |  |  |  |  |  |  |  |  |  |  |  |  |  |  |  |  |  |  |  |  |  |  |  |  |  |  |  |  |  |  |  |  |  |  |  |  |  |  |  |  |  |  |  |  |  |  |  |  |  |  |  |  |  |  |  |  |  |  |  |  |  |  |  |  |  |  |  |  |  |  |  |  |  |  |  |  |  |  |  |  |  |  |  |  |  |  |  |  |  |  |  |  |  |  |  |  |  |  |  |  |  |  |  |  |  |  |  |  |  |  |  |  |  |  |  |  |  |  |  |  |  |  |  |  |  |  |  |  |  |  |  |  |  |  |  |  |  |  |  |  |  |  |  |  |  |  |  |  |  |  |  |  |  |  |  |  |  |  |  |  |  |  |  |  |  |  |  |  |  |  |  |  |  |  |  |  |  |  |  |  |  |  |  |  |  |  |  |  |  |  |  |  |  |  |  |  |  |  |  |  |  |  |  |  |  |  |  |  |  |  |  |  |  |  |  |  |  |  |  |  |  |  |  |  |  |  |  |  |  |  |  |  |  |  |
|  |  |  |  |  |  |  |  |  |  |  |  |  |  |  |  |  |  |  |  |  |  |  |  |  |  |  |  |  |  |  |  |  |  |  |  |  |  |  |  |  |  |  |  |  |  |  |  |  |  |  |  |  |  |  |  |  |  |  |  |  |  |  |  |  |  |  |  |  |  |  |  |  |  |  |  |  |  |  |  |  |  |  |  |  |  |  |  |  |  |  |  |  |  |  |  |  |  |  |  |  |  |  |  |  |  |  |  |  |  |  |  |  |  |  |  |  |  |  |  |  |  |  |  |  |  |  |  |  |  |  |  |  |  |  |  |  |  |  |  |  |  |  |  |  |  |  |  |  |  |  |  |  |  |  |  |  |  |  |  |  |  |  |  |  |  |  |  |  |  |  |  |  |  |  |  |  |  |  |  |  |  |  |  |  |  |  |  |  |  |  |  |  |  |  |  |  |  |  |  |  |  |  |  |  |  |  |  |  |  |  |  |  |  |  |  |  |  |  |  |  |  |  |  |  |  |  |  |  |  |  |  |  |  |  |  |  |  |  |  |  |  |  |  |  |  |  |  |  |  |  |  |  |  |  |  |  |  |  |  |  |  |  |  |  |  |  |  |  |  |  |  |  |  |  |  |  |  |  |  |  |  |  |  |  |  |  |  |  |  |  |  |  |  |  |  |  |  |  |  |  |  |  |  |  |  |  |  |  |  |  |  |  |  |  |  |  |  |  |  |  |  |  |  |  |  |  |  |  |  |  |  |  |  |  |  |  |  |  |  |  |  |  |  |  |  |  |  |  |  |  |  |  |  |  |  |  |  |  |  |  |  |  |  |  |  |  |  |  |  |  |  |  |  |  |  |  |  |  |  |  |  |  |  |  |  |  |  |  |  |  |  |  |  |  |  |  |  |  |  |  |  |  |  |  |  |  |  |  |  |  |  |  |  |  |  |  |  |  |  |  |  |  |  |  |  |  |  |  |  |  |  |  |  |  |  |  |  |  |  |  |  |  |  |  |  |  |  |  |  |  |  |  |  |  |  |  |  |  |  |  |  |  |  |  |  |  |  |  |  |  |  |  |  |  |  |  |  |  |  |  |  |  |  |  |  |  |  |  |  |  |  |  |  |  |  |  |  |  |  |  |  |  |  |  |  |  |  |  |  |  |  |  |  |  |  |  |  |  |  |  |  |  |  |  |  |  |  |  |  |  |  |  |  |  |  |  |  |  |  |  |  |  |  |  |  |  |  |  |  |  |  |  |  |  |  |  |  |  |  |  |  |  |  |  |  |  |  |  |  |  |  |  |  |  |  |  |  |  |  |  |  |  |  |  |  |  |  |  |  |  |  |  |  |  |  |  |  |  |  |  |  |  |  |  |  |  |  |  |  |  |  |  |  |  |  |  |  |  |  |  |  |  |  |  |  |  |  |  |  |  |  |  |  |  |  |  |  |  |  |  |  |  |  |  |  |  |  |  |  |  |  |  |  |  |  |  |  |  |  |  |  |  |  |  |  |  |  |  |  |  |  |  |  |  |  |  |  |  |  |  |  |  |  |  |  |  |  |  |  |  |  |  |  |  |  |  |  |  |  |  |  |  |  |  |  |  |  |  |  |  |  |  |  |  |  |  |  |  |  |  |  |  |  |  |  |  |  |  |  |  |  |  |  |  |  |  |  |  |  |  |  |  |  |  |  |  |  |  |  |  |  |  |  |  |  |  |  |  |  |  |  |  |  |  |  |  |  |  |  |  |  |  |  |  |  |  |  |  |  |  |  |  |  |  |  |  |  |  |  |  |  |  |  |  |  |  |  |  |  |  |  |  |  |  |  |  |  |  |  |  |  |  |  |  |  |  |  |  |  |  |  |  |  |  |  |  |  |  |  |  |  |  |  |  |  |  |  |  |  |  |  |  |  |  |  |  |  |  |  |  |  |  |  |  |  |  |  |  |  |  |  |  |  |  |  |  |  |  |  |  |  |  |  |  |  |  |  |  |  |  |  |  |  |  |  |  |  |  |  |  |  |  |  |  |  |  |  |  |  |  |  |  |  |  |  |  |  |  |  |  |  |  |  |  |  |  |  |  |  |  |  |  |  |  |  |  |  |  |  |  |  |  |  |  |  |  |  |  |  |  |  |  |  |  |  |  |  |  |  |  |  |  |  |  |  |  |  |  |  |  |  |  |  |  |  |  |  |  |  |  |  |  |  |  |  |  |  |  |  |  |  |  |  |  |  |  |  |  |  |  |  |  |  |  |  |  |  |  |  |  |  |  |  |  |  |  |  |  |  |  |  |  |  |  |  |  |  |  |  |  |  |  |  |  |  |  |  |  |  |  |  |  |  |  |  |  |  |  |  |  |  |  |  |  |  |  |  |  |  |  |  |  |  |  |  |  |  |  |  |  |  |  |  |  |  |  |  |  |  |  |  |  |  |  |  |  |  |  |  |  |  |  |  |  |  |  |  |  |  |  |  |  |  |  |  |  |  |  |  |  |  |  |  |  |  |  |  |  |  |  |  |  |  |  |  |  |  |  |  |  |  |  |  |  |  |  |  |  |  |  |  |  |  |  |  |  |  |  |  |  |  |  |  |  |  |  |  |  |  |  |  |  |  |  |  |  |  |  |  |  |  |  |  |  |  |  |  |  |  |  |  |  |  |  |  |  |  |  |  |  |  |  |  |  |  |  |  |  |  |  |  |  |  |  |  |  |  |  |  |  |  |  |  |  |  |  |  |  |  |  |  |  |  |  |  |  |  |  |  |  |  |  |  |  |  |  |  |  |  |  |  |  |  |  |  |  |  |  |  |  |  |  |  |  |  |  |  |  |  |  |  |  |  |  |  |  |  |  |  |  |  |  |  |  |  |  |  |  |  |  |  |  |  |  |  |  |  |  |  |  |  |  |  |  |  |  |  |  |  |  |  |  |  |  |  |  |  |  |  |  |  |  |  |  |  |  |  |  |  |  |  |  |  |  |  |  |  |  |  |  |  |  |  |  |  |  |  |  |  |  |  |  |  |  |  |  |  |  |  |  |  |  |  |  |  |  |  |  |  |  |  |  |  |  |  |  |  |  |  |  |  |  |  |  |  |  |  |  |  |  |  |  |  |  |  |  |  |  |  |  |  |  |  |  |  |  |  |  |  |  |  |  |  |  |  |  |  |  |  |  |  |  |  |  |  |  |  |  |  |  |  |  |  |  |  |  |  |  |  |  |  |  |  |  |  |  |  |  |  |  |  |  |  |  |  |  |  |  |  |  |  |  |  |  |  |  |  |  |  |  |  |  |  |  |  |  |  |  |  |  |  |  |  |  |  |  |  |  |  |  |  |  |  |  |  |  |  |  |  |  |  |  |  |  |  |  |  |  |  |  |  |  |  |  |  |  |  |  |  |  |  |  |  |  |  |  |  |  |  |  |  |  |  |  |  |  |  |  |  |  |  |  |  |  |  |  |  |  |  |  |  |  |  |  |  |  |  |  |  |  |  |  |  |  |  |  |  |  |  |  |  |  |  |  |  |  |  |  |  |  |  |  |  |  |  |  |  |  |  |  |  |  |  |  |  |  |  |  |  |  |  |  |  |  |  |  |  |  |  |  |  |  |  |  |  |  |  |  |  |  |  |  |  |  |  |  |  |  |  |  |  |  |  |  |  |  |  |  |  |  |  |  |  |  |  |  |  |  |  |  |  |  |  |  |  |  |  |  |  |  |  |  |  |  |  |  |  |  |  |  |  |  |  |  |  |  |  |  |  |  |  |  |  |  |  |  |  |  |  |  |  |  |  |  |  |  |  |  |  |  |
|  |  |  |  |  |  |  |  |  |  |  |  |  |  |  |  |  |  |  |  |  |  |  |  |  |  |  |  |  |  |  |  |  |  |  |  |  |  |  |  |  |  |  |  |  |  |  |  |  |  |  |  |  |  |  |  |  |  |  |  |  |  |  |  |  |  |  |  |  |  |  |  |  |  |  |  |  |  |  |  |  |  |  |  |  |  |  |  |  |  |  |  |  |  |  |  |  |  |  |  |  |  |  |  |  |  |  |  |  |  |  |  |  |  |  |  |  |  |  |  |  |  |  |  |  |  |  |  |  |  |  |  |  |  |  |  |  |  |  |  |  |  |  |  |  |  |  |  |  |  |  |  |  |  |  |  |  |  |  |  |  |  |  |  |  |  |  |  |  |  |  |  |  |  |  |  |  |  |  |  |  |  |  |  |  |  |  |  |  |  |  |  |  |  |  |  |  |  |  |  |  |  |  |  |  |  |  |  |  |  |  |  |  |  |  |  |  |  |  |  |  |  |  |  |  |  |  |  |  |  |  |  |  |  |  |  |  |  |  |  |  |  |  |  |  |  |  |  |  |  |  |  |  |  |  |  |  |  |  |  |  |  |  |  |  |  |  |  |  |  |  |  |  |  |  |  |  |  |  |  |  |  |  |  |  |  |  |  |  |  |  |  |  |  |  |  |  |  |  |  |  |  |  |  |  |  |  |  |  |  |  |  |  |  |  |  |  |  |  |  |  |  |  |  |  |  |  |  |  |  |  |  |  |  |  |  |  |  |  |  |  |  |  |  |  |  |  |  |  |  |  |  |  |  |  |  |  |  |  |  |  |  |  |  |  |  |  |  |  |  |  |  |  |  |  |  |  |  |  |  |  |  |  |  |  |  |  |  |  |  |  |  |  |  |  |  |  |  |  |  |  |  |  |  |  |  |  |  |  |  |  |  |  |  |  |  |  |  |  |  |  |  |  |  |  |  |  |  |  |  |  |  |  |  |  |  |  |  |  |  |  |  |  |  |  |  |  |  |  |  |  |  |  |  |  |  |  |  |  |  |  |  |  |  |  |  |  |  |  |  |  |  |  |  |  |  |  |  |  |  |  |  |  |  |  |  |  |  |  |  |  |  |  |  |  |  |  |  |  |  |  |  |  |  |  |  |  |  |  |  |  |  |  |  |  |  |  |  |  |  |  |  |  |  |  |  |  |  |  |  |  |  |  |  |  |  |  |  |  |  |  |  |  |  |  |  |  |  |  |  |  |  |  |  |  |  |  |  |  |  |  |  |  |  |  |  |  |  |  |  |  |  |  |  |  |  |  |  |  |  |  |  |  |  |  |  |  |  |  |  |  |  |  |  |  |  |  |  |  |  |  |  |  |  |  |  |  |  |  |  |  |  |  |  |  |  |  |  |  |  |  |  |  |  |  |  |  |  |  |  |  |  |  |  |  |  |  |  |  |  |  |  |  |  |  |  |  |  |  |  |  |  |  |  |  |  |  |  |  |  |  |  |  |  |  |  |  |  |  |  |  |  |  |  |  |  |  |  |  |  |  |  |  |  |  |  |  |  |  |  |  |  |  |  |  |  |  |  |  |  |  |  |  |  |  |  |  |  |  |  |  |  |  |  |  |  |  |  |  |  |  |  |  |  |  |  |  |  |  |  |  |  |  |  |  |  |  |  |  |  |  |  |  |  |  |  |  |  |  |  |  |  |  |  |  |  |  |  |  |  |  |  |  |  |  |  |  |  |  |  |  |  |  |  |  |  |  |  |  |  |  |  |  |  |  |  |  |  |  |  |  |  |  |  |  |  |  |  |  |  |  |  |  |  |  |  |  |  |  |  |  |  |  |  |  |  |  |  |  |  |  |  |  |  |  |  |  |  |  |  |  |  |  |  |  |  |  |  |  |  |  |  |  |  |  |  |  |  |  |  |  |  |  |  |  |  |  |  |  |  |  |  |  |  |  |  |  |  |  |  |  |  |  |  |  |  |  |  |  |  |  |  |  |  |  |  |  |  |  |  |  |  |  |  |  |  |  |  |  |  |  |  |  |  |  |  |  |  |  |  |  |  |  |  |  |  |  |  |  |  |  |  |  |  |  |  |  |  |  |  |  |  |  |  |  |  |  |  |  |  |  |  |  |  |  |  |  |  |  |  |  |  |  |  |  |  |  |  |  |  |  |  |  |  |  |  |  |  |  |  |  |  |  |  |  |  |  |  |  |  |  |  |  |  |  |  |  |  |  |  |  |  |  |  |  |  |  |  |  |  |  |  |  |  |  |  |  |  |  |  |  |  |  |  |  |  |  |  |  |  |  |  |  |  |  |  |  |  |  |  |  |  |  |  |  |  |  |  |  |  |  |  |  |  |  |  |  |  |  |  |  |  |  |  |  |  |  |  |  |  |  |  |  |  |  |  |  |  |  |  |  |  |  |  |  |  |  |  |  |  |  |  |  |  |  |  |  |  |  |  |  |  |  |  |  |  |  |  |  |  |  |  |  |  |  |  |  |  |  |  |  |  |  |  |  |  |  |  |  |  |  |  |  |  |  |  |  |  |  |  |  |  |  |  |  |  |  |  |  |  |  |  |  |  |  |  |  |  |  |  |  |  |  |  |  |  |  |  |  |  |  |  |  |  |  |  |  |  |  |  |  |  |  |  |  |  |  |  |  |  |  |  |  |  |  |  |  |  |  |  |  |  |  |  |  |  |  |  |  |  |  |  |  |  |  |  |  |  |  |  |  |  |  |  |  |  |  |  |  |  |  |  |  |  |  |  |  |  |  |  |  |  |  |  |  |  |  |  |  |  |  |  |  |  |  |  |  |  |  |  |  |  |  |  |  |  |  |  |  |  |  |  |  |  |  |  |  |  |  |  |  |  |  |  |  |  |  |  |  |  |  |  |  |  |  |  |  |  |  |  |  |  |  |  |  |  |  |  |  |  |  |  |  |  |  |  |  |  |  |  |  |  |  |  |  |  |  |  |  |  |  |  |  |  |  |  |  |  |  |  |  |  |  |  |  |  |  |  |  |  |  |  |  |  |  |  |  |  |  |  |  |  |  |  |  |  |  |  |  |  |  |  |  |  |  |  |  |  |  |  |  |  |  |  |  |  |  |  |  |  |  |  |  |  |  |  |  |  |  |  |  |  |  |  |  |  |  |  |  |  |  |  |  |  |  |  |  |  |  |  |  |  |  |  |  |  |  |  |  |  |  |  |  |  |  |  |  |  |  |  |  |  |  |  |  |  |  |  |  |  |  |  |  |  |  |  |  |  |  |  |  |  |  |  |  |  |  |  |  |  |  |  |  |  |  |  |  |  |  |  |  |  |  |  |  |  |  |  |  |  |  |  |  |  |  |  |  |  |  |  |  |  |  |  |  |  |  |  |  |  |  |  |  |  |  |  |  |  |  |  |  |  |  |  |  |  |  |  |  |  |  |  |  |  |  |  |  |  |  |  |  |  |  |  |  |  |  |  |  |  |  |  |  |  |  |  |  |  |  |  |  |  |  |  |  |  |  |  |  |  |  |  |  |  |  |  |  |  |  |  |  |  |  |  |  |  |  |  |  |  |  |  |  |  |  |  |  |  |  |  |  |  |  |  |  |  |  |  |  |  |  |  |  |  |  |  |  |  |  |  |  |  |  |  |  |  |  |  |  |  |  |  |  |  |  |  |  |  |  |  |  |  |  |  |  |  |  |  |  |  |  |  |  |  |  |  |  |  |  |  |  |  |  |  |  |  |  |  |  |  |  |  |  |  |  |  |  |  |  |  |  |  |  |  |  |  |  |  |  |  |  |
|  |  |  |  |  |  |  |  |  |  |  |  |  |  |  |  |  |  |  |  |  |  |  |  |  |  |  |  |  |  |  |  |  |  |  |  |  |  |  |  |  |  |  |  |  |  |  |  |  |  |  |  |  |  |  |  |  |  |  |  |  |  |  |  |  |  |  |  |  |  |  |  |  |  |  |  |  |  |  |  |  |  |  |  |  |  |  |  |  |  |  |  |  |  |  |  |  |  |  |  |  |  |  |  |  |  |  |  |  |  |  |  |  |  |  |  |  |  |  |  |  |  |  |  |  |  |  |  |  |  |  |  |  |  |  |  |  |  |  |  |  |  |  |  |  |  |  |  |  |  |  |  |  |  |  |  |  |  |  |  |  |  |  |  |  |  |  |  |  |  |  |  |  |  |  |  |  |  |  |  |  |  |  |  |  |  |  |  |  |  |  |  |  |  |  |  |  |  |  |  |  |  |  |  |  |  |  |  |  |  |  |  |  |  |  |  |  |  |  |  |  |  |  |  |  |  |  |  |  |  |  |  |  |  |  |  |  |  |  |  |  |  |  |  |  |  |  |  |  |  |  |  |  |  |  |  |  |  |  |  |  |  |  |  |  |  |  |  |  |  |  |  |  |  |  |  |  |  |  |  |  |  |  |  |  |  |  |  |  |  |  |  |  |  |  |  |  |  |  |  |  |  |  |  |  |  |  |  |  |  |  |  |  |  |  |  |  |  |  |  |  |  |  |  |  |  |  |  |  |  |  |  |  |  |  |  |  |  |  |  |  |  |  |  |  |  |  |  |  |  |  |  |  |  |  |  |  |  |  |  |  |  |  |  |  |  |  |  |  |  |  |  |  |  |  |  |  |  |  |  |  |  |  |  |  |  |  |  |  |  |  |  |  |  |  |  |  |  |  |  |  |  |  |  |  |  |  |  |  |  |  |  |  |  |  |  |  |  |  |  |  |  |  |  |  |  |  |  |  |  |  |  |  |  |  |  |  |  |  |  |  |  |  |  |  |  |  |  |  |  |  |  |  |  |  |  |  |  |  |  |  |  |  |  |  |  |  |  |  |  |  |  |  |  |  |  |  |  |  |  |  |  |  |  |  |  |  |  |  |  |  |  |  |  |  |  |  |  |  |  |  |  |  |  |  |  |  |  |  |  |  |  |  |  |  |  |  |  |  |  |  |  |  |  |  |  |  |  |  |  |  |  |  |  |  |  |  |  |  |  |  |  |  |  |  |  |  |  |  |  |  |  |  |  |  |  |  |  |  |  |  |  |  |  |  |  |  |  |  |  |  |  |  |  |  |  |  |  |  |  |  |  |  |  |  |  |  |  |  |  |  |  |  |  |  |  |  |  |  |  |  |  |  |  |  |  |  |  |  |  |  |  |  |  |  |  |  |  |  |  |  |  |  |  |  |  |  |  |  |  |  |  |  |  |  |  |  |  |  |  |  |  |  |  |  |  |  |  |  |  |  |  |  |  |  |  |  |  |  |  |  |  |  |  |  |  |  |  |  |  |  |  |  |  |  |  |  |  |  |  |  |  |  |  |  |  |  |  |  |  |  |  |  |  |  |  |  |  |  |  |  |  |  |  |  |  |  |  |  |  |  |  |  |  |  |  |  |  |  |  |  |  |  |  |  |  |  |  |  |  |  |  |  |  |  |  |  |  |  |  |  |  |  |  |  |  |  |  |  |  |  |  |  |  |  |  |  |  |  |  |  |  |  |  |  |  |  |  |  |  |  |  |  |  |  |  |  |  |  |  |  |  |  |  |  |  |  |  |  |  |  |  |  |  |  |  |  |  |  |  |  |  |  |  |  |  |  |  |  |  |  |  |  |  |  |  |  |  |  |  |  |  |  |  |  |  |  |  |  |  |  |  |  |  |  |  |  |  |  |  |  |  |  |  |  |  |  |  |  |  |  |  |  |  |  |  |  |  |  |  |  |  |  |  |  |  |  |  |  |  |  |  |  |  |  |  |  |  |  |  |  |  |  |  |  |  |  |  |  |  |  |  |  |  |  |  |  |  |  |  |  |  |  |  |  |  |  |  |  |  |  |  |  |  |  |  |  |  |  |  |  |  |  |  |  |  |  |  |  |  |  |  |  |  |  |  |  |  |  |  |  |  |  |  |  |  |  |  |  |  |  |  |  |  |  |  |  |  |  |  |  |  |  |  |  |  |  |  |  |  |  |  |  |  |  |  |  |  |  |  |  |  |  |  |  |  |  |  |  |  |  |  |  |  |  |  |  |  |  |  |  |  |  |  |  |  |  |  |  |  |  |  |  |  |  |  |  |  |  |  |  |  |  |  |  |  |  |  |  |  |  |  |  |  |  |  |  |  |  |  |  |  |  |  |  |  |  |  |  |  |  |  |  |  |  |  |  |  |  |  |  |  |  |  |  |  |  |  |  |  |  |  |  |  |  |  |  |  |  |  |  |  |  |  |  |  |  |  |  |  |  |  |  |  |  |  |  |  |  |  |  |  |  |  |  |  |  |  |  |  |  |  |  |  |  |  |  |  |  |  |  |  |  |  |  |  |  |  |  |  |  |  |  |  |  |  |  |  |  |  |  |  |  |  |  |  |  |  |  |  |  |  |  |  |  |  |  |  |  |  |  |  |  |  |  |  |  |  |  |  |  |  |  |  |  |  |  |  |  |  |  |  |  |  |  |  |  |  |  |  |  |  |  |  |  |  |  |  |  |  |  |  |  |  |  |  |  |  |  |  |  |  |  |  |  |  |  |  |  |  |  |  |  |  |  |  |  |  |  |  |  |  |  |  |  |  |  |  |  |  |  |  |  |  |  |  |  |  |  |  |  |  |  |  |  |  |  |  |  |  |  |  |  |  |  |  |  |  |  |  |  |  |  |  |  |  |  |  |  |  |  |  |  |  |  |  |  |  |  |  |  |  |  |  |  |  |  |  |  |  |  |  |  |  |  |  |  |  |  |  |  |  |  |  |  |  |  |  |  |  |  |  |  |  |  |  |  |  |  |  |  |  |  |  |  |  |  |  |  |  |  |  |  |  |  |  |  |  |  |  |  |  |  |  |  |  |  |  |  |  |  |  |  |  |  |  |  |  |  |  |  |  |  |  |  |  |  |  |  |  |  |  |  |  |  |  |  |  |  |  |  |  |  |  |  |  |  |  |  |  |  |  |  |  |  |  |  |  |  |  |  |  |  |  |  |  |  |  |  |  |  |  |  |  |  |  |  |  |  |  |  |  |  |  |  |  |  |  |  |  |  |  |  |  |  |  |  |  |  |  |  |  |  |  |  |  |  |  |  |  |  |  |  |  |  |  |  |  |  |  |  |  |  |  |  |  |  |  |  |  |  |  |  |  |  |  |  |  |  |  |  |  |  |  |  |  |  |  |  |  |  |  |  |  |  |  |  |  |  |  |  |  |  |  |  |  |  |  |  |  |  |  |  |  |  |  |  |  |  |  |  |  |  |  |  |  |  |  |  |  |  |  |  |  |  |  |  |  |  |  |  |  |  |  |  |  |  |  |  |  |  |  |  |  |  |  |  |  |  |  |  |  |  |  |  |  |  |  |  |  |  |  |  |  |  |  |  |  |  |  |  |  |  |  |  |  |  |  |  |  |  |  |  |  |  |  |  |  |  |  |  |  |  |  |  |  |  |  |  |  |  |  |  |  |  |  |  |  |  |  |  |  |  |  |  |  |  |  |  |  |  |  |  |  |  |  |  |  |  |  |  |  |  |  |  |  |  |  |  |  |  |  |  |  |  |  |  |  |  |  |  |  |  |  |  |  |  |  |  |  |  |
|  |  |  |  |  |  |  |  |  |  |  |  |  |  |  |  |  |  |  |  |  |  |  |  |  |  |  |  |  |  |  |  |  |  |  |  |  |  |  |  |  |  |  |  |  |  |  |  |  |  |  |  |  |  |  |  |  |  |  |  |  |  |  |  |  |  |  |  |  |  |  |  |  |  |  |  |  |  |  |  |  |  |  |  |  |  |  |  |  |  |  |  |  |  |  |  |  |  |  |  |  |  |  |  |  |  |  |  |  |  |  |  |  |  |  |  |  |  |  |  |  |  |  |  |  |  |  |  |  |  |  |  |  |  |  |  |  |  |  |  |  |  |  |  |  |  |  |  |  |  |  |  |  |  |  |  |  |  |  |  |  |  |  |  |  |  |  |  |  |  |  |  |  |  |  |  |  |  |  |  |  |  |  |  |  |  |  |  |  |  |  |  |  |  |  |  |  |  |  |  |  |  |  |  |  |  |  |  |  |  |  |  |  |  |  |  |  |  |  |  |  |  |  |  |  |  |  |  |  |  |  |  |  |  |  |  |  |  |  |  |  |  |  |  |  |  |  |  |  |  |  |  |  |  |  |  |  |  |  |  |  |  |  |  |  |  |  |  |  |  |  |  |  |  |  |  |  |  |  |  |  |  |  |  |  |  |  |  |  |  |  |  |  |  |  |  |  |  |  |  |  |  |  |  |  |  |  |  |  |  |  |  |  |  |  |  |  |  |  |  |  |  |  |  |  |  |  |  |  |  |  |  |  |  |  |  |  |  |  |  |  |  |  |  |  |  |  |  |  |  |  |  |  |  |  |  |  |  |  |  |  |  |  |  |  |  |  |  |  |  |  |  |  |  |  |  |  |  |  |  |  |  |  |  |  |  |  |  |  |  |  |  |  |  |  |  |  |  |  |  |  |  |  |  |  |  |  |  |  |  |  |  |  |  |  |  |  |  |  |  |  |  |  |  |  |  |  |  |  |  |  |  |  |  |  |  |  |  |  |  |  |  |  |  |  |  |  |  |  |  |  |  |  |  |  |  |  |  |  |  |  |  |  |  |  |  |  |  |  |  |  |  |  |  |  |  |  |  |  |  |  |  |  |  |  |  |  |  |  |  |  |  |  |  |  |  |  |  |  |  |  |  |  |  |  |  |  |  |  |  |  |  |  |  |  |  |  |  |  |  |  |  |  |  |  |  |  |  |  |  |  |  |  |  |  |  |  |  |  |  |  |  |  |  |  |  |  |  |  |  |  |  |  |  |  |  |  |  |  |  |  |  |  |  |  |  |  |  |  |  |  |  |  |  |  |  |  |  |  |  |  |  |  |  |  |  |  |  |  |  |  |  |  |  |  |  |  |  |  |  |  |  |  |  |  |  |  |  |  |  |  |  |  |  |  |  |  |  |  |  |  |  |  |  |  |  |  |  |  |  |  |  |  |  |  |  |  |  |  |  |  |  |  |  |  |  |  |  |  |  |  |  |  |  |  |  |  |  |  |  |  |  |  |  |  |  |  |  |  |  |  |  |  |  |  |  |  |  |  |  |  |  |  |  |  |  |  |  |  |  |  |  |  |  |  |  |  |  |  |  |  |  |  |  |  |  |  |  |  |  |  |  |  |  |  |  |  |  |  |  |  |  |  |  |  |  |  |  |  |  |  |  |  |  |  |  |  |  |  |  |  |  |  |  |  |  |  |  |  |  |  |  |  |  |  |  |  |  |  |  |  |  |  |  |  |  |  |  |  |  |  |  |  |  |  |  |  |  |  |  |  |  |  |  |  |  |  |  |  |  |  |  |  |  |  |  |  |  |  |  |  |  |  |  |  |  |  |  |  |  |  |  |  |  |  |  |  |  |  |  |  |  |  |  |  |  |  |  |  |  |  |  |  |  |  |  |  |  |  |  |  |  |  |  |  |  |  |  |  |  |  |  |  |  |  |  |  |  |  |  |  |  |  |  |  |  |  |  |  |  |  |  |  |  |  |  |  |  |  |  |  |  |  |  |  |  |  |  |  |  |  |  |  |  |  |  |  |  |  |  |  |  |  |  |  |  |  |  |  |  |  |  |  |  |  |  |  |  |  |  |  |  |  |  |  |  |  |  |  |  |  |  |  |  |  |  |  |  |  |  |  |  |  |  |  |  |  |  |  |  |  |  |  |  |  |  |  |  |  |  |  |  |  |  |  |  |  |  |  |  |  |  |  |  |  |  |  |  |  |  |  |  |  |  |  |  |  |  |  |  |  |  |  |  |  |  |  |  |  |  |  |  |  |  |  |  |  |  |  |  |  |  |  |  |  |  |  |  |  |  |  |  |  |  |  |  |  |  |  |  |  |  |  |  |  |  |  |  |  |  |  |  |  |  |  |  |  |  |  |  |  |  |  |  |  |  |  |  |  |  |  |  |  |  |  |  |  |  |  |  |  |  |  |  |  |  |  |  |  |  |  |  |  |  |  |  |  |  |  |  |  |  |  |  |  |  |  |  |  |  |  |  |  |  |  |  |  |  |  |  |  |  |  |  |  |  |  |  |  |  |  |  |  |  |  |  |  |  |  |  |  |  |  |  |  |  |  |  |  |  |  |  |  |  |  |  |  |  |  |  |  |  |  |  |  |  |  |  |  |  |  |  |  |  |  |  |  |  |  |  |  |  |  |  |  |  |  |  |  |  |  |  |  |  |  |  |  |  |  |  |  |  |  |  |  |  |  |  |  |  |  |  |  |  |  |  |  |  |  |  |  |  |  |  |  |  |  |  |  |  |  |  |  |  |  |  |  |  |  |  |  |  |  |  |  |  |  |  |  |  |  |  |  |  |  |  |  |  |  |  |  |  |  |  |  |  |  |  |  |  |  |  |  |  |  |  |  |  |  |  |  |  |  |  |  |  |  |  |  |  |  |  |  |  |  |  |  |  |  |  |  |  |  |  |  |  |  |  |  |  |  |  |  |  |  |  |  |  |  |  |  |  |  |  |  |  |  |  |  |  |  |  |  |  |  |  |  |  |  |  |  |  |  |  |  |  |  |  |  |  |  |  |  |  |  |  |  |  |  |  |  |  |  |  |  |  |  |  |  |  |  |  |  |  |  |  |  |  |  |  |  |  |  |  |  |  |  |  |  |  |  |  |  |  |  |  |  |  |  |  |  |  |  |  |  |  |  |  |  |  |  |  |  |  |  |  |  |  |  |  |  |  |  |  |  |  |  |  |  |  |  |  |  |  |  |  |  |  |  |  |  |  |  |  |  |  |  |  |  |  |  |  |  |  |  |  |  |  |  |  |  |  |  |  |  |  |  |  |  |  |  |  |  |  |  |  |  |  |  |  |  |  |  |  |  |  |  |  |  |  |  |  |  |  |  |  |  |  |  |  |  |  |  |  |  |  |  |  |  |  |  |  |  |  |  |  |  |  |  |  |  |  |  |  |  |  |  |  |  |  |  |  |  |  |  |  |  |  |  |  |  |  |  |  |  |  |  |  |  |  |  |  |  |  |  |  |  |  |  |  |  |  |  |  |  |  |  |  |  |  |  |  |  |  |  |  |  |  |  |  |  |  |  |  |  |  |  |  |  |  |  |  |  |  |  |  |  |  |  |  |  |  |  |  |  |  |  |  |  |  |  |  |  |  |  |  |  |  |  |  |  |  |  |  |  |  |  |  |  |  |  |  |  |  |  |  |  |  |  |  |  |  |  |  |  |  |  |  |  |  |  |  |  |  |  |  |  |  |  |  |  |  |  |  |  |  |  |  |  |  |  |  |  |  |  |  |  |  |  |  |  |  |  |  |  |  |  |  |  |  |  |
|  |  |  |  |  |  |  |  |  |  |  |  |  |  |  |  |  |  |  |  |  |  |  |  |  |  |  |  |  |  |  |  |  |  |  |  |  |  |  |  |  |  |  |  |  |  |  |  |  |  |  |  |  |  |  |  |  |  |  |  |  |  |  |  |  |  |  |  |  |  |  |  |  |  |  |  |  |  |  |  |  |  |  |  |  |  |  |  |  |  |  |  |  |  |  |  |  |  |  |  |  |  |  |  |  |  |  |  |  |  |  |  |  |  |  |  |  |  |  |  |  |  |  |  |  |  |  |  |  |  |  |  |  |  |  |  |  |  |  |  |  |  |  |  |  |  |  |  |  |  |  |  |  |  |  |  |  |  |  |  |  |  |  |  |  |  |  |  |  |  |  |  |  |  |  |  |  |  |  |  |  |  |  |  |  |  |  |  |  |  |  |  |  |  |  |  |  |  |  |  |  |  |  |  |  |  |  |  |  |  |  |  |  |  |  |  |  |  |  |  |  |  |  |  |  |  |  |  |  |  |  |  |  |  |  |  |  |  |  |  |  |  |  |  |  |  |  |  |  |  |  |  |  |  |  |  |  |  |  |  |  |  |  |  |  |  |  |  |  |  |  |  |  |  |  |  |  |  |  |  |  |  |  |  |  |  |  |  |  |  |  |  |  |  |  |  |  |  |  |  |  |  |  |  |  |  |  |  |  |  |  |  |  |  |  |  |  |  |  |  |  |  |  |  |  |  |  |  |  |  |  |  |  |  |  |  |  |  |  |  |  |  |  |  |  |  |  |  |  |  |  |  |  |  |  |  |  |  |  |  |  |  |  |  |  |  |  |  |  |  |  |  |  |  |  |  |  |  |  |  |  |  |  |  |  |  |  |  |  |  |  |  |  |  |  |  |  |  |  |  |  |  |  |  |  |  |  |  |  |  |  |  |  |  |  |  |  |  |  |  |  |  |  |  |  |  |  |  |  |  |  |  |  |  |  |  |  |  |  |  |  |  |  |  |  |  |  |  |  |  |  |  |  |  |  |  |  |  |  |  |  |  |  |  |  |  |  |  |  |  |  |  |  |  |  |  |  |  |  |  |  |  |  |  |  |  |  |  |  |  |  |  |  |  |  |  |  |  |  |  |  |  |  |  |  |  |  |  |  |  |  |  |  |  |  |  |  |  |  |  |  |  |  |  |  |  |  |  |  |  |  |  |  |  |  |  |  |  |  |  |  |  |  |  |  |  |  |  |  |  |  |  |  |  |  |  |  |  |  |  |  |  |  |  |  |  |  |  |  |  |  |  |  |  |  |  |  |  |  |  |  |  |  |  |  |  |  |  |  |  |  |  |  |  |  |  |  |  |  |  |  |  |  |  |  |  |  |  |  |  |  |  |  |  |  |  |  |  |  |  |  |  |  |  |  |  |  |  |  |  |  |  |  |  |  |  |  |  |  |  |  |  |  |  |  |  |  |  |  |  |  |  |  |  |  |  |  |  |  |  |  |  |  |  |  |  |  |  |  |  |  |  |  |  |  |  |  |  |  |  |  |  |  |  |  |  |  |  |  |  |  |  |  |  |  |  |  |  |  |  |  |  |  |  |  |  |  |  |  |  |  |  |  |  |  |  |  |  |  |  |  |  |  |  |  |  |  |  |  |  |  |  |  |  |  |  |  |  |  |  |  |  |  |  |  |  |  |  |  |  |  |  |  |  |  |  |  |  |  |  |  |  |  |  |  |  |  |  |  |  |  |  |  |  |  |  |  |  |  |  |  |  |  |  |  |  |  |  |  |  |  |  |  |  |  |  |  |  |  |  |  |  |  |  |  |  |  |  |  |  |  |  |  |  |  |  |  |  |  |  |  |  |  |  |  |  |  |  |  |  |  |  |  |  |  |  |  |  |  |  |  |  |  |  |  |  |  |  |  |  |  |  |  |  |  |  |  |  |  |  |  |  |  |  |  |  |  |  |  |  |  |  |  |  |  |  |  |  |  |  |  |  |  |  |  |  |  |  |  |  |  |  |  |  |  |  |  |  |  |  |  |  |  |  |  |  |  |  |  |  |  |  |  |  |  |  |  |  |  |  |  |  |  |  |  |  |  |  |  |  |  |  |  |  |  |  |  |  |  |  |  |  |  |  |  |  |  |  |  |  |  |  |  |  |  |  |  |  |  |  |  |  |  |  |  |  |  |  |  |  |  |  |  |  |  |  |  |  |  |  |  |  |  |  |  |  |  |  |  |  |  |  |  |  |  |  |  |  |  |  |  |  |  |  |  |  |  |  |  |  |  |  |  |  |  |  |  |  |  |  |  |  |  |  |  |  |  |  |  |  |  |  |  |  |  |  |  |  |  |  |  |  |  |  |  |  |  |  |  |  |  |  |  |  |  |  |  |  |  |  |  |  |  |  |  |  |  |  |  |  |  |  |  |  |  |  |  |  |  |  |  |  |  |  |  |  |  |  |  |  |  |  |  |  |  |  |  |  |  |  |  |  |  |  |  |  |  |  |  |  |  |  |  |  |  |  |  |  |  |  |  |  |  |  |  |  |  |  |  |  |  |  |  |  |  |  |  |  |  |  |  |  |  |  |  |  |  |  |  |  |  |  |  |  |  |  |  |  |  |  |  |  |  |  |  |  |  |  |  |  |  |  |  |  |  |  |  |  |  |  |  |  |  |  |  |  |  |  |  |  |  |  |  |  |  |  |  |  |  |  |  |  |  |  |  |  |  |  |  |  |  |  |  |  |  |  |  |  |  |  |  |  |  |  |  |  |  |  |  |  |  |  |  |  |  |  |  |  |  |  |  |  |  |  |  |  |  |  |  |  |  |  |  |  |  |  |  |  |  |  |  |  |  |  |  |  |  |  |  |  |  |  |  |  |  |  |  |  |  |  |  |  |  |  |  |  |  |  |  |  |  |  |  |  |  |  |  |  |  |  |  |  |  |  |  |  |  |  |  |  |  |  |  |  |  |  |  |  |  |  |  |  |  |  |  |  |  |  |  |  |  |  |  |  |  |  |  |  |  |  |  |  |  |  |  |  |  |  |  |  |  |  |  |  |  |  |  |  |  |  |  |  |  |  |  |  |  |  |  |  |  |  |  |  |  |  |  |  |  |  |  |  |  |  |  |  |  |  |  |  |  |  |  |  |  |  |  |  |  |  |  |  |  |  |  |  |  |  |  |  |  |  |  |  |  |  |  |  |  |  |  |  |  |  |  |  |  |  |  |  |  |  |  |  |  |  |  |  |  |  |  |  |  |  |  |  |  |  |  |  |  |  |  |  |  |  |  |  |  |  |  |  |  |  |  |  |  |  |  |  |  |  |  |  |  |  |  |  |  |  |  |  |  |  |  |  |  |  |  |  |  |  |  |  |  |  |  |  |  |  |  |  |  |  |  |  |  |  |  |  |  |  |  |  |  |  |  |  |  |  |  |  |  |  |  |  |  |  |  |  |  |  |  |  |  |  |  |  |  |  |  |  |  |  |  |  |  |  |  |  |  |  |  |  |  |  |  |  |  |  |  |  |  |  |  |  |  |  |  |  |  |  |  |  |  |  |  |  |  |  |  |  |  |  |  |  |  |  |  |  |  |  |  |  |  |  |  |  |  |  |  |  |  |  |  |  |  |  |  |  |  |  |  |  |  |  |  |  |  |  |  |  |  |  |  |  |  |  |  |  |  |  |  |  |  |  |  |  |  |  |  |  |  |  |  |  |  |  |  |  |  |  |  |  |  |  |  |  |  |  |  |  |  |  |  |  |  |  |  |  |  |  |  |  |  |  |  |  |  |  |  |
|  |  |  |  |  |  |  |  |  |  |  |  |  |  |  |  |  |  |  |  |  |  |  |  |  |  |  |  |  |  |  |  |  |  |  |  |  |  |  |  |  |  |  |  |  |  |  |  |  |  |  |  |  |  |  |  |  |  |  |  |  |  |  |  |  |  |  |  |  |  |  |  |  |  |  |  |  |  |  |  |  |  |  |  |  |  |  |  |  |  |  |  |  |  |  |  |  |  |  |  |  |  |  |  |  |  |  |  |  |  |  |  |  |  |  |  |  |  |  |  |  |  |  |  |  |  |  |  |  |  |  |  |  |  |  |  |  |  |  |  |  |  |  |  |  |  |  |  |  |  |  |  |  |  |  |  |  |  |  |  |  |  |  |  |  |  |  |  |  |  |  |  |  |  |  |  |  |  |  |  |  |  |  |  |  |  |  |  |  |  |  |  |  |  |  |  |  |  |  |  |  |  |  |  |  |  |  |  |  |  |  |  |  |  |  |  |  |  |  |  |  |  |  |  |  |  |  |  |  |  |  |  |  |  |  |  |  |  |  |  |  |  |  |  |  |  |  |  |  |  |  |  |  |  |  |  |  |  |  |  |  |  |  |  |  |  |  |  |  |  |  |  |  |  |  |  |  |  |  |  |  |  |  |  |  |  |  |  |  |  |  |  |  |  |  |  |  |  |  |  |  |  |  |  |  |  |  |  |  |  |  |  |  |  |  |  |  |  |  |  |  |  |  |  |  |  |  |  |  |  |  |  |  |  |  |  |  |  |  |  |  |  |  |  |  |  |  |  |  |  |  |  |  |  |  |  |  |  |  |  |  |  |  |  |  |  |  |  |  |  |  |  |  |  |  |  |  |  |  |  |  |  |  |  |  |  |  |  |  |  |  |  |  |  |  |  |  |  |  |  |  |  |  |  |  |  |  |  |  |  |  |  |  |  |  |  |  |  |  |  |  |  |  |  |  |  |  |  |  |  |  |  |  |  |  |  |  |  |  |  |  |  |  |  |  |  |  |  |  |  |  |  |  |  |  |  |  |  |  |  |  |  |  |  |  |  |  |  |  |  |  |  |  |  |  |  |  |  |  |  |  |  |  |  |  |  |  |  |  |  |  |  |  |  |  |  |  |  |  |  |  |  |  |  |  |  |  |  |  |  |  |  |  |  |  |  |  |  |  |  |  |  |  |  |  |  |  |  |  |  |  |  |  |  |  |  |  |  |  |  |  |  |  |  |  |  |  |  |  |  |  |  |  |  |  |  |  |  |  |  |  |  |  |  |  |  |  |  |  |  |  |  |  |  |  |  |  |  |  |  |  |  |  |  |  |  |  |  |  |  |  |  |  |  |  |  |  |  |  |  |  |  |  |  |  |  |  |  |  |  |  |  |  |  |  |  |  |  |  |  |  |  |  |  |  |  |  |  |  |  |  |  |  |  |  |  |  |  |  |  |  |  |  |  |  |  |  |  |  |  |  |  |  |  |  |  |  |  |  |  |  |  |  |  |  |  |  |  |  |  |  |  |  |  |  |  |  |  |  |  |  |  |  |  |  |  |  |  |  |  |  |  |  |  |  |  |  |  |  |  |  |  |  |  |  |  |  |  |  |  |  |  |  |  |  |  |  |  |  |  |  |  |  |  |  |  |  |  |  |  |  |  |  |  |  |  |  |  |  |  |  |  |  |  |  |  |  |  |  |  |  |  |  |  |  |  |  |  |  |  |  |  |  |  |  |  |  |  |  |  |  |  |  |  |  |  |  |  |  |  |  |  |  |  |  |  |  |  |  |  |  |  |  |  |  |  |  |  |  |  |  |  |  |  |  |  |  |  |  |  |  |  |  |  |  |  |  |  |  |  |  |  |  |  |  |  |  |  |  |  |  |  |  |  |  |  |  |  |  |  |  |  |  |  |  |  |  |  |  |  |  |  |  |  |  |  |  |  |  |  |  |  |  |  |  |  |  |  |  |  |  |  |  |  |  |  |  |  |  |  |  |  |  |  |  |  |  |  |  |  |  |  |  |  |  |  |  |  |  |  |  |  |  |  |  |  |  |  |  |  |  |  |  |  |  |  |  |  |  |  |  |  |  |  |  |  |  |  |  |  |  |  |  |  |  |  |  |  |  |  |  |  |  |  |  |  |  |  |  |  |  |  |  |  |  |  |  |  |  |  |  |  |  |  |  |  |  |  |  |  |  |  |  |  |  |  |  |  |  |  |  |  |  |  |  |  |  |  |  |  |  |  |  |  |  |  |  |  |  |  |  |  |  |  |  |  |  |  |  |  |  |  |  |  |  |  |  |  |  |  |  |  |  |  |  |  |  |  |  |  |  |  |  |  |  |  |  |  |  |  |  |  |  |  |  |  |  |  |  |  |  |  |  |  |  |  |  |  |  |  |  |  |  |  |  |  |  |  |  |  |  |  |  |  |  |  |  |  |  |  |  |  |  |  |  |  |  |  |  |  |  |  |  |  |  |  |  |  |  |  |  |  |  |  |  |  |  |  |  |  |  |  |  |  |  |  |  |  |  |  |  |  |  |  |  |  |  |  |  |  |  |  |  |  |  |  |  |  |  |  |  |  |  |  |  |  |  |  |  |  |  |  |  |  |  |  |  |  |  |  |  |  |  |  |  |  |  |  |  |  |  |  |  |  |  |  |  |  |  |  |  |  |  |  |  |  |  |  |  |  |  |  |  |  |  |  |  |  |  |  |  |  |  |  |  |  |  |  |  |  |  |  |  |  |  |  |  |  |  |  |  |  |  |  |  |  |  |  |  |  |  |  |  |  |  |  |  |  |  |  |  |  |  |  |  |  |  |  |  |  |  |  |  |  |  |  |  |  |  |  |  |  |  |  |  |  |  |  |  |  |  |  |  |  |  |  |  |  |  |  |  |  |  |  |  |  |  |  |  |  |  |  |  |  |  |  |  |  |  |  |  |  |  |  |  |  |  |  |  |  |  |  |  |  |  |  |  |  |  |  |  |  |  |  |  |  |  |  |  |  |  |  |  |  |  |  |  |  |  |  |  |  |  |  |  |  |  |  |  |  |  |  |  |  |  |  |  |  |  |  |  |  |  |  |  |  |  |  |  |  |  |  |  |  |  |  |  |  |  |  |  |  |  |  |  |  |  |  |  |  |  |  |  |  |  |  |  |  |  |  |  |  |  |  |  |  |  |  |  |  |  |  |  |  |  |  |  |  |  |  |  |  |  |  |  |  |  |  |  |  |  |  |  |  |  |  |  |  |  |  |  |  |  |  |  |  |  |  |  |  |  |  |  |  |  |  |  |  |  |  |  |  |  |  |  |  |  |  |  |  |  |  |  |  |  |  |  |  |  |  |  |  |  |  |  |  |  |  |  |  |  |  |  |  |  |  |  |  |  |  |  |  |  |  |  |  |  |  |  |  |  |  |  |  |  |  |  |  |  |  |  |  |  |  |  |  |  |  |  |  |  |  |  |  |  |  |  |  |  |  |  |  |  |  |  |  |  |  |  |  |  |  |  |  |  |  |  |  |  |  |  |  |  |  |  |  |  |  |  |  |  |  |  |  |  |  |  |  |  |  |  |  |  |  |  |  |  |  |  |  |  |  |  |  |  |  |  |  |  |  |  |  |  |  |  |  |  |  |  |  |  |  |  |  |  |  |  |  |  |  |  |  |  |  |  |  |  |  |  |  |  |  |  |  |  |  |  |  |  |  |  |  |  |  |  |  |  |  |  |  |  |  |  |  |  |  |  |  |  |  |  |  |  |  |  |  |  |  |  |  |  |  |  |  |  |  |  |  |  |  |  |
|  |  |  |  |  |  |  |  |  |  |  |  |  |  |  |  |  |  |  |  |  |  |  |  |  |  |  |  |  |  |  |  |  |  |  |  |  |  |  |  |  |  |  |  |  |  |  |  |  |  |  |  |  |  |  |  |  |  |  |  |  |  |  |  |  |  |  |  |  |  |  |  |  |  |  |  |  |  |  |  |  |  |  |  |  |  |  |  |  |  |  |  |  |  |  |  |  |  |  |  |  |  |  |  |  |  |  |  |  |  |  |  |  |  |  |  |  |  |  |  |  |  |  |  |  |  |  |  |  |  |  |  |  |  |  |  |  |  |  |  |  |  |  |  |  |  |  |  |  |  |  |  |  |  |  |  |  |  |  |  |  |  |  |  |  |  |  |  |  |  |  |  |  |  |  |  |  |  |  |  |  |  |  |  |  |  |  |  |  |  |  |  |  |  |  |  |  |  |  |  |  |  |  |  |  |  |  |  |  |  |  |  |  |  |  |  |  |  |  |  |  |  |  |  |  |  |  |  |  |  |  |  |  |  |  |  |  |  |  |  |  |  |  |  |  |  |  |  |  |  |  |  |  |  |  |  |  |  |  |  |  |  |  |  |  |  |  |  |  |  |  |  |  |  |  |  |  |  |  |  |  |  |  |  |  |  |  |  |  |  |  |  |  |  |  |  |  |  |  |  |  |  |  |  |  |  |  |  |  |  |  |  |  |  |  |  |  |  |  |  |  |  |  |  |  |  |  |  |  |  |  |  |  |  |  |  |  |  |  |  |  |  |  |  |  |  |  |  |  |  |  |  |  |  |  |  |  |  |  |  |  |  |  |  |  |  |  |  |  |  |  |  |  |  |  |  |  |  |  |  |  |  |  |  |  |  |  |  |  |  |  |  |  |  |  |  |  |  |  |  |  |  |  |  |  |  |  |  |  |  |  |  |  |  |  |  |  |  |  |  |  |  |  |  |  |  |  |  |  |  |  |  |  |  |  |  |  |  |  |  |  |  |  |  |  |  |  |  |  |  |  |  |  |  |  |  |  |  |  |  |  |  |  |  |  |  |  |  |  |  |  |  |  |  |  |  |  |  |  |  |  |  |  |  |  |  |  |  |  |  |  |  |  |  |  |  |  |  |  |  |  |  |  |  |  |  |  |  |  |  |  |  |  |  |  |  |  |  |  |  |  |  |  |  |  |  |  |  |  |  |  |  |  |  |  |  |  |  |  |  |  |  |  |  |  |  |  |  |  |  |  |  |  |  |  |  |  |  |  |  |  |  |  |  |  |  |  |  |  |  |  |  |  |  |  |  |  |  |  |  |  |  |  |  |  |  |  |  |  |  |  |  |  |  |  |  |  |  |  |  |  |  |  |  |  |  |  |  |  |  |  |  |  |  |  |  |  |  |  |  |  |  |  |  |  |  |  |  |  |  |  |  |  |  |  |  |  |  |  |  |  |  |  |  |  |  |  |  |  |  |  |  |  |  |  |  |  |  |  |  |  |  |  |  |  |  |  |  |  |  |  |  |  |  |  |  |  |  |  |  |  |  |  |  |  |  |  |  |  |  |  |  |  |  |  |  |  |  |  |  |  |  |  |  |  |  |  |  |  |  |  |  |  |  |  |  |  |  |  |  |  |  |  |  |  |  |  |  |  |  |  |  |  |  |  |  |  |  |  |  |  |  |  |  |  |  |  |  |  |  |  |  |  |  |  |  |  |  |  |  |  |  |  |  |  |  |  |  |  |  |  |  |  |  |  |  |  |  |  |  |  |  |  |  |  |  |  |  |  |  |  |  |  |  |  |  |  |  |  |  |  |  |  |  |  |  |  |  |  |  |  |  |  |  |  |  |  |  |  |  |  |  |  |  |  |  |  |  |  |  |  |  |  |  |  |  |  |  |  |  |  |  |  |  |  |  |  |  |  |  |  |  |  |  |  |  |  |  |  |  |  |  |  |  |  |  |  |  |  |  |  |  |  |  |  |  |  |  |  |  |  |  |  |  |  |  |  |  |  |  |  |  |  |  |  |  |  |  |  |  |  |  |  |  |  |  |  |  |  |  |  |  |  |  |  |  |  |  |  |  |  |  |  |  |  |  |  |  |  |  |  |  |  |  |  |  |  |  |  |  |  |  |  |  |  |  |  |  |  |  |  |  |  |  |  |  |  |  |  |  |  |  |  |  |  |  |  |  |  |  |  |  |  |  |  |  |  |  |  |  |  |  |  |  |  |  |  |  |  |  |  |  |  |  |  |  |  |  |  |  |  |  |  |  |  |  |  |  |  |  |  |  |  |  |  |  |  |  |  |  |  |  |  |  |  |  |  |  |  |  |  |  |  |  |  |  |  |  |  |  |  |  |  |  |  |  |  |  |  |  |  |  |  |  |  |  |  |  |  |  |  |  |  |  |  |  |  |  |  |  |  |  |  |  |  |  |  |  |  |  |  |  |  |  |  |  |  |  |  |  |  |  |  |  |  |  |  |  |  |  |  |  |  |  |  |  |  |  |  |  |  |  |  |  |  |  |  |  |  |  |  |  |  |  |  |  |  |  |  |  |  |  |  |  |  |  |  |  |  |  |  |  |  |  |  |  |  |  |  |  |  |  |  |  |  |  |  |  |  |  |  |  |  |  |  |  |  |  |  |  |  |  |  |  |  |  |  |  |  |  |  |  |  |  |  |  |  |  |  |  |  |  |  |  |  |  |  |  |  |  |  |  |  |  |  |  |  |  |  |  |  |  |  |  |  |  |  |  |  |  |  |  |  |  |  |  |  |  |  |  |  |  |  |  |  |  |  |  |  |  |  |  |  |  |  |  |  |  |  |  |  |  |  |  |  |  |  |  |  |  |  |  |  |  |  |  |  |  |  |  |  |  |  |  |  |  |  |  |  |  |  |  |  |  |  |  |  |  |  |  |  |  |  |  |  |  |  |  |  |  |  |  |  |  |  |  |  |  |  |  |  |  |  |  |  |  |  |  |  |  |  |  |  |  |  |  |  |  |  |  |  |  |  |  |  |  |  |  |  |  |  |  |  |  |  |  |  |  |  |  |  |  |  |  |  |  |  |  |  |  |  |  |  |  |  |  |  |  |  |  |  |  |  |  |  |  |  |  |  |  |  |  |  |  |  |  |  |  |  |  |  |  |  |  |  |  |  |  |  |  |  |  |  |  |  |  |  |  |  |  |  |  |  |  |  |  |  |  |  |  |  |  |  |  |  |  |  |  |  |  |  |  |  |  |  |  |  |  |  |  |  |  |  |  |  |  |  |  |  |  |  |  |  |  |  |  |  |  |  |  |  |  |  |  |  |  |  |  |  |  |  |  |  |  |  |  |  |  |  |  |  |  |  |  |  |  |  |  |  |  |  |  |  |  |  |  |  |  |  |  |  |  |  |  |  |  |  |  |  |  |  |  |  |  |  |  |  |  |  |  |  |  |  |  |  |  |  |  |  |  |  |  |  |  |  |  |  |  |  |  |  |  |  |  |  |  |  |  |  |  |  |  |  |  |  |  |  |  |  |  |  |  |  |  |  |  |  |  |  |  |  |  |  |  |  |  |  |  |  |  |  |  |  |  |  |  |  |  |  |  |  |  |  |  |  |  |  |  |  |  |  |  |  |  |  |  |  |  |  |  |  |  |  |  |  |  |  |  |  |  |  |  |  |  |  |  |  |  |  |  |  |  |  |  |  |  |  |  |  |  |  |  |  |  |  |  |  |  |  |  |  |  |  |  |  |  |  |  |  |  |  |  |  |  |  |  |  |  |  |  |  |  |  |  |  |  |  |  |  |  |  |
|  |  |  |  |  |  |  |  |  |  |  |  |  |  |  |  |  |  |  |  |  |  |  |  |  |  |  |  |  |  |  |  |  |  |  |  |  |  |  |  |  |  |  |  |  |  |  |  |  |  |  |  |  |  |  |  |  |  |  |  |  |  |  |  |  |  |  |  |  |  |  |  |  |  |  |  |  |  |  |  |  |  |  |  |  |  |  |  |  |  |  |  |  |  |  |  |  |  |  |  |  |  |  |  |  |  |  |  |  |  |  |  |  |  |  |  |  |  |  |  |  |  |  |  |  |  |  |  |  |  |  |  |  |  |  |  |  |  |  |  |  |  |  |  |  |  |  |  |  |  |  |  |  |  |  |  |  |  |  |  |  |  |  |  |  |  |  |  |  |  |  |  |  |  |  |  |  |  |  |  |  |  |  |  |  |  |  |  |  |  |  |  |  |  |  |  |  |  |  |  |  |  |  |  |  |  |  |  |  |  |  |  |  |  |  |  |  |  |  |  |  |  |  |  |  |  |  |  |  |  |  |  |  |  |  |  |  |  |  |  |  |  |  |  |  |  |  |  |  |  |  |  |  |  |  |  |  |  |  |  |  |  |  |  |  |  |  |  |  |  |  |  |  |  |  |  |  |  |  |  |  |  |  |  |  |  |  |  |  |  |  |  |  |  |  |  |  |  |  |  |  |  |  |  |  |  |  |  |  |  |  |  |  |  |  |  |  |  |  |  |  |  |  |  |  |  |  |  |  |  |  |  |  |  |  |  |  |  |  |  |  |  |  |  |  |  |  |  |  |  |  |  |  |  |  |  |  |  |  |  |  |  |  |  |  |  |  |  |  |  |  |  |  |  |  |  |  |  |  |  |  |  |  |  |  |  |  |  |  |  |  |  |  |  |  |  |  |  |  |  |  |  |  |  |  |  |  |  |  |  |  |  |  |  |  |  |  |  |  |  |  |  |  |  |  |  |  |  |  |  |  |  |  |  |  |  |  |  |  |  |  |  |  |  |  |  |  |  |  |  |  |  |  |  |  |  |  |  |  |  |  |  |  |  |  |  |  |  |  |  |  |  |  |  |  |  |  |  |  |  |  |  |  |  |  |  |  |  |  |  |  |  |  |  |  |  |  |  |  |  |  |  |  |  |  |  |  |  |  |  |  |  |  |  |  |  |  |  |  |  |  |  |  |  |  |  |  |  |  |  |  |  |  |  |  |  |  |  |  |  |  |  |  |  |  |  |  |  |  |  |  |  |  |  |  |  |  |  |  |  |  |  |  |  |  |  |  |  |  |  |  |  |  |  |  |  |  |  |  |  |  |  |  |  |  |  |  |  |  |  |  |  |  |  |  |  |  |  |  |  |  |  |  |  |  |  |  |  |  |  |  |  |  |  |  |  |  |  |  |  |  |  |  |  |  |  |  |  |  |  |  |  |  |  |  |  |  |  |  |  |  |  |  |  |  |  |  |  |  |  |  |  |  |  |  |  |  |  |  |  |  |  |  |  |  |  |  |  |  |  |  |  |  |  |  |  |  |  |  |  |  |  |  |  |  |  |  |  |  |  |  |  |  |  |  |  |  |  |  |  |  |  |  |  |  |  |  |  |  |  |  |  |  |  |  |  |  |  |  |  |  |  |  |  |  |  |  |  |  |  |  |  |  |  |  |  |  |  |  |  |  |  |  |  |  |  |  |  |  |  |  |  |  |  |  |  |  |  |  |  |  |  |  |  |  |  |  |  |  |  |  |  |  |  |  |  |  |  |  |  |  |  |  |  |  |  |  |  |  |  |  |  |  |  |  |  |  |  |  |  |  |  |  |  |  |  |  |  |  |  |  |  |  |  |  |  |  |  |  |  |  |  |  |  |  |  |  |  |  |  |  |  |  |  |  |  |  |  |  |  |  |  |  |  |  |  |  |  |  |  |  |  |  |  |  |  |  |  |  |  |  |  |  |  |  |  |  |  |  |  |  |  |  |  |  |  |  |  |  |  |  |  |  |  |  |  |  |  |  |  |  |  |  |  |  |  |  |  |  |  |  |  |  |  |  |  |  |  |  |  |  |  |  |  |  |  |  |  |  |  |  |  |  |  |  |  |  |  |  |  |  |  |  |  |  |  |  |  |  |  |  |  |  |  |  |  |  |  |  |  |  |  |  |  |  |  |  |  |  |  |  |  |  |  |  |  |  |  |  |  |  |  |  |  |  |  |  |  |  |  |  |  |  |  |  |  |  |  |  |  |  |  |  |  |  |  |  |  |  |  |  |  |  |  |  |  |  |  |  |  |  |  |  |  |  |  |  |  |  |  |  |  |  |  |  |  |  |  |  |  |  |  |  |  |  |  |  |  |  |  |  |  |  |  |  |  |  |  |  |  |  |  |  |  |  |  |  |  |  |  |  |  |  |  |  |  |  |  |  |  |  |  |  |  |  |  |  |  |  |  |  |  |  |  |  |  |  |  |  |  |  |  |  |  |  |  |  |  |  |  |  |  |  |  |  |  |  |  |  |  |  |  |  |  |  |  |  |  |  |  |  |  |  |  |  |  |  |  |  |  |  |  |  |  |  |  |  |  |  |  |  |  |  |  |  |  |  |  |  |  |  |  |  |  |  |  |  |  |  |  |  |  |  |  |  |  |  |  |  |  |  |  |  |  |  |  |  |  |  |  |  |  |  |  |  |  |  |  |  |  |  |  |  |  |  |  |  |  |  |  |  |  |  |  |  |  |  |  |  |  |  |  |  |  |  |  |  |  |  |  |  |  |  |  |  |  |  |  |  |  |  |  |  |  |  |  |  |  |  |  |  |  |  |  |  |  |  |  |  |  |  |  |  |  |  |  |  |  |  |  |  |  |  |  |  |  |  |  |  |  |  |  |  |  |  |  |  |  |  |  |  |  |  |  |  |  |  |  |  |  |  |  |  |  |  |  |  |  |  |  |  |  |  |  |  |  |  |  |  |  |  |  |  |  |  |  |  |  |  |  |  |  |  |  |  |  |  |  |  |  |  |  |  |  |  |  |  |  |  |  |  |  |  |  |  |  |  |  |  |  |  |  |  |  |  |  |  |  |  |  |  |  |  |  |  |  |  |  |  |  |  |  |  |  |  |  |  |  |  |  |  |  |  |  |  |  |  |  |  |  |  |  |  |  |  |  |  |  |  |  |  |  |  |  |  |  |  |  |  |  |  |  |  |  |  |  |  |  |  |  |  |  |  |  |  |  |  |  |  |  |  |  |  |  |  |  |  |  |  |  |  |  |  |  |  |  |  |  |  |  |  |  |  |  |  |  |  |  |  |  |  |  |  |  |  |  |  |  |  |  |  |  |  |  |  |  |  |  |  |  |  |  |  |  |  |  |  |  |  |  |  |  |  |  |  |  |  |  |  |  |  |  |  |  |  |  |  |  |  |  |  |  |  |  |  |  |  |  |  |  |  |  |  |  |  |  |  |  |  |  |  |  |  |  |  |  |  |  |  |  |  |  |  |  |  |  |  |  |  |  |  |  |  |  |  |  |  |  |  |  |  |  |  |  |  |  |  |  |  |  |  |  |  |  |  |  |  |  |  |  |  |  |  |  |  |  |  |  |  |  |  |  |  |  |  |  |  |  |  |  |  |  |  |  |  |  |  |  |  |  |  |  |  |  |  |  |  |  |  |  |  |  |  |  |  |  |  |  |  |  |  |  |  |  |  |  |  |  |  |  |  |  |  |  |  |  |  |  |  |  |  |  |  |  |  |  |  |  |  |  |  |  |  |  |  |  |  |  |  |  |  |  |  |  |  |  |  |  |  |  |  |
|  |  |  |  |  |  |  |  |  |  |  |  |  |  |  |  |  |  |  |  |  |  |  |  |  |  |  |  |  |  |  |  |  |  |  |  |  |  |  |  |  |  |  |  |  |  |  |  |  |  |  |  |  |  |  |  |  |  |  |  |  |  |  |  |  |  |  |  |  |  |  |  |  |  |  |  |  |  |  |  |  |  |  |  |  |  |  |  |  |  |  |  |  |  |  |  |  |  |  |  |  |  |  |  |  |  |  |  |  |  |  |  |  |  |  |  |  |  |  |  |  |  |  |  |  |  |  |  |  |  |  |  |  |  |  |  |  |  |  |  |  |  |  |  |  |  |  |  |  |  |  |  |  |  |  |  |  |  |  |  |  |  |  |  |  |  |  |  |  |  |  |  |  |  |  |  |  |  |  |  |  |  |  |  |  |  |  |  |  |  |  |  |  |  |  |  |  |  |  |  |  |  |  |  |  |  |  |  |  |  |  |  |  |  |  |  |  |  |  |  |  |  |  |  |  |  |  |  |  |  |  |  |  |  |  |  |  |  |  |  |  |  |  |  |  |  |  |  |  |  |  |  |  |  |  |  |  |  |  |  |  |  |  |  |  |  |  |  |  |  |  |  |  |  |  |  |  |  |  |  |  |  |  |  |  |  |  |  |  |  |  |  |  |  |  |  |  |  |  |  |  |  |  |  |  |  |  |  |  |  |  |  |  |  |  |  |  |  |  |  |  |  |  |  |  |  |  |  |  |  |  |  |  |  |  |  |  |  |  |  |  |  |  |  |  |  |  |  |  |  |  |  |  |  |  |  |  |  |  |  |  |  |  |  |  |  |  |  |  |  |  |  |  |  |  |  |  |  |  |  |  |  |  |  |  |  |  |  |  |  |  |  |  |  |  |  |  |  |  |  |  |  |  |  |  |  |  |  |  |  |  |  |  |  |  |  |  |  |  |  |  |  |  |  |  |  |  |  |  |  |  |  |  |  |  |  |  |  |  |  |  |  |  |  |  |  |  |  |  |  |  |  |  |  |  |  |  |  |  |  |  |  |  |  |  |  |  |  |  |  |  |  |  |  |  |  |  |  |  |  |  |  |  |  |  |  |  |  |  |  |  |  |  |  |  |  |  |  |  |  |  |  |  |  |  |  |  |  |  |  |  |  |  |  |  |  |  |  |  |  |  |  |  |  |  |  |  |  |  |  |  |  |  |  |  |  |  |  |  |  |  |  |  |  |  |  |  |  |  |  |  |  |  |  |  |  |  |  |  |  |  |  |  |  |  |  |  |  |  |  |  |  |  |  |  |  |  |  |  |  |  |  |  |  |  |  |  |  |  |  |  |  |  |  |  |  |  |  |  |  |  |  |  |  |  |  |  |  |  |  |  |  |  |  |  |  |  |  |  |  |  |  |  |  |  |  |  |  |  |  |  |  |  |  |  |  |  |  |  |  |  |  |  |  |  |  |  |  |  |  |  |  |  |  |  |  |  |  |  |  |  |  |  |  |  |  |  |  |  |  |  |  |  |  |  |  |  |  |  |  |  |  |  |  |  |  |  |  |  |  |  |  |  |  |  |  |  |  |  |  |  |  |  |  |  |  |  |  |  |  |  |  |  |  |  |  |  |  |  |  |  |  |  |  |  |  |  |  |  |  |  |  |  |  |  |  |  |  |  |  |  |  |  |  |  |  |  |  |  |  |  |  |  |  |  |  |  |  |  |  |  |  |  |  |  |  |  |  |  |  |  |  |  |  |  |  |  |  |  |  |  |  |  |  |  |  |  |  |  |  |  |  |  |  |  |  |  |  |  |  |  |  |  |  |  |  |  |  |  |  |  |  |  |  |  |  |  |  |  |  |  |  |  |  |  |  |  |  |  |  |  |  |  |  |  |  |  |  |  |  |  |  |  |  |  |  |  |  |  |  |  |  |  |  |  |  |  |  |  |  |  |  |  |  |  |  |  |  |  |  |  |  |  |  |  |  |  |  |  |  |  |  |  |  |  |  |  |  |  |  |  |  |  |  |  |  |  |  |  |  |  |  |  |  |  |  |  |  |  |  |  |  |  |  |  |  |  |  |  |  |  |  |  |  |  |  |  |  |  |  |  |  |  |  |  |  |  |  |  |  |  |  |  |  |  |  |  |  |  |  |  |  |  |  |  |  |  |  |  |  |  |  |  |  |  |  |  |  |  |  |  |  |  |  |  |  |  |  |  |  |  |  |  |  |  |  |  |  |  |  |  |  |  |  |  |  |  |  |  |  |  |  |  |  |  |  |  |  |  |  |  |  |  |  |  |  |  |  |  |  |  |  |  |  |  |  |  |  |  |  |  |  |  |  |  |  |  |  |  |  |  |  |  |  |  |  |  |  |  |  |  |  |  |  |  |  |  |  |  |  |  |  |  |  |  |  |  |  |  |  |  |  |  |  |  |  |  |  |  |  |  |  |  |  |  |  |  |  |  |  |  |  |  |  |  |  |  |  |  |  |  |  |  |  |  |  |  |  |  |  |  |  |  |  |  |  |  |  |  |  |  |  |  |  |  |  |  |  |  |  |  |  |  |  |  |  |  |  |  |  |  |  |  |  |  |  |  |  |  |  |  |  |  |  |  |  |  |  |  |  |  |  |  |  |  |  |  |  |  |  |  |  |  |  |  |  |  |  |  |  |  |  |  |  |  |  |  |  |  |  |  |  |  |  |  |  |  |  |  |  |  |  |  |  |  |  |  |  |  |  |  |  |  |  |  |  |  |  |  |  |  |  |  |  |  |  |  |  |  |  |  |  |  |  |  |  |  |  |  |  |  |  |  |  |  |  |  |  |  |  |  |  |  |  |  |  |  |  |  |  |  |  |  |  |  |  |  |  |  |  |  |  |  |  |  |  |  |  |  |  |  |  |  |  |  |  |  |  |  |  |  |  |  |  |  |  |  |  |  |  |  |  |  |  |  |  |  |  |  |  |  |  |  |  |  |  |  |  |  |  |  |  |  |  |  |  |  |  |  |  |  |  |  |  |  |  |  |  |  |  |  |  |  |  |  |  |  |  |  |  |  |  |  |  |  |  |  |  |  |  |  |  |  |  |  |  |  |  |  |  |  |  |  |  |  |  |  |  |  |  |  |  |  |  |  |  |  |  |  |  |  |  |  |  |  |  |  |  |  |  |  |  |  |  |  |  |  |  |  |  |  |  |  |  |  |  |  |  |  |  |  |  |  |  |  |  |  |  |  |  |  |  |  |  |  |  |  |  |  |  |  |  |  |  |  |  |  |  |  |  |  |  |  |  |  |  |  |  |  |  |  |  |  |  |  |  |  |  |  |  |  |  |  |  |  |  |  |  |  |  |  |  |  |  |  |  |  |  |  |  |  |  |  |  |  |  |  |  |  |  |  |  |  |  |  |  |  |  |  |  |  |  |  |  |  |  |  |  |  |  |  |  |  |  |  |  |  |  |  |  |  |  |  |  |  |  |  |  |  |  |  |  |  |  |  |  |  |  |  |  |  |  |  |  |  |  |  |  |  |  |  |  |  |  |  |  |  |  |  |  |  |  |  |  |  |  |  |  |  |  |  |  |  |  |  |  |  |  |  |  |  |  |  |  |  |  |  |  |  |  |  |  |  |  |  |  |  |  |  |  |  |  |  |  |  |  |  |  |  |  |  |  |  |  |  |  |  |  |  |  |  |  |  |  |  |  |  |  |  |  |  |  |  |  |  |  |  |  |  |  |  |  |  |  |  |  |  |  |  |  |  |  |  |  |  |  |  |  |  |  |  |  |  |  |  |  |  |  |  |  |  |
|  |  |  |  |  |  |  |  |  |  |  |  |  |  |  |  |  |  |  |  |  |  |  |  |  |  |  |  |  |  |  |  |  |  |  |  |  |  |  |  |  |  |  |  |  |  |  |  |  |  |  |  |  |  |  |  |  |  |  |  |  |  |  |  |  |  |  |  |  |  |  |  |  |  |  |  |  |  |  |  |  |  |  |  |  |  |  |  |  |  |  |  |  |  |  |  |  |  |  |  |  |  |  |  |  |  |  |  |  |  |  |  |  |  |  |  |  |  |  |  |  |  |  |  |  |  |  |  |  |  |  |  |  |  |  |  |  |  |  |  |  |  |  |  |  |  |  |  |  |  |  |  |  |  |  |  |  |  |  |  |  |  |  |  |  |  |  |  |  |  |  |  |  |  |  |  |  |  |  |  |  |  |  |  |  |  |  |  |  |  |  |  |  |  |  |  |  |  |  |  |  |  |  |  |  |  |  |  |  |  |  |  |  |  |  |  |  |  |  |  |  |  |  |  |  |  |  |  |  |  |  |  |  |  |  |  |  |  |  |  |  |  |  |  |  |  |  |  |  |  |  |  |  |  |  |  |  |  |  |  |  |  |  |  |  |  |  |  |  |  |  |  |  |  |  |  |  |  |  |  |  |  |  |  |  |  |  |  |  |  |  |  |  |  |  |  |  |  |  |  |  |  |  |  |  |  |  |  |  |  |  |  |  |  |  |  |  |  |  |  |  |  |  |  |  |  |  |  |  |  |  |  |  |  |  |  |  |  |  |  |  |  |  |  |  |  |  |  |  |  |  |  |  |  |  |  |  |  |  |  |  |  |  |  |  |  |  |  |  |  |  |  |  |  |  |  |  |  |  |  |  |  |  |  |  |  |  |  |  |  |  |  |  |  |  |  |  |  |  |  |  |  |  |  |  |  |  |  |  |  |  |  |  |  |  |  |  |  |  |  |  |  |  |  |  |  |  |  |  |  |  |  |  |  |  |  |  |  |  |  |  |  |  |  |  |  |  |  |  |  |  |  |  |  |  |  |  |  |  |  |  |  |  |  |  |  |  |  |  |  |  |  |  |  |  |  |  |  |  |  |  |  |  |  |  |  |  |  |  |  |  |  |  |  |  |  |  |  |  |  |  |  |  |  |  |  |  |  |  |  |  |  |  |  |  |  |  |  |  |  |  |  |  |  |  |  |  |  |  |  |  |  |  |  |  |  |  |  |  |  |  |  |  |  |  |  |  |  |  |  |  |  |  |  |  |  |  |  |  |  |  |  |  |  |  |  |  |  |  |  |  |  |  |  |  |  |  |  |  |  |  |  |  |  |  |  |  |  |  |  |  |  |  |  |  |  |  |  |  |  |  |  |  |  |  |  |  |  |  |  |  |  |  |  |  |  |  |  |  |  |  |  |  |  |  |  |  |  |  |  |  |  |  |  |  |  |  |  |  |  |  |  |  |  |  |  |  |  |  |  |  |  |  |  |  |  |  |  |  |  |  |  |  |  |  |  |  |  |  |  |  |  |  |  |  |  |  |  |  |  |  |  |  |  |  |  |  |  |  |  |  |  |  |  |  |  |  |  |  |  |  |  |  |  |  |  |  |  |  |  |  |  |  |  |  |  |  |  |  |  |  |  |  |  |  |  |  |  |  |  |  |  |  |  |  |  |  |  |  |  |  |  |  |  |  |  |  |  |  |  |  |  |  |  |  |  |  |  |  |  |  |  |  |  |  |  |  |  |  |  |  |  |  |  |  |  |  |  |  |  |  |  |  |  |  |  |  |  |  |  |  |  |  |  |  |  |  |  |  |  |  |  |  |  |  |  |  |  |  |  |  |  |  |  |  |  |  |  |  |  |  |  |  |  |  |  |  |  |  |  |  |  |  |  |  |  |  |  |  |  |  |  |  |  |  |  |  |  |  |  |  |  |  |  |  |  |  |  |  |  |  |  |  |  |  |  |  |  |  |  |  |  |  |  |  |  |  |  |  |  |  |  |  |  |  |  |  |  |  |  |  |  |  |  |  |  |  |  |  |  |  |  |  |  |  |  |  |  |  |  |  |  |  |  |  |  |  |  |  |  |  |  |  |  |  |  |  |  |  |  |  |  |  |  |  |  |  |  |  |  |  |  |  |  |  |  |  |  |  |  |  |  |  |  |  |  |  |  |  |  |  |  |  |  |  |  |  |  |  |  |  |  |  |  |  |  |  |  |  |  |  |  |  |  |  |  |  |  |  |  |  |  |  |  |  |  |  |  |  |  |  |  |  |  |  |  |  |  |  |  |  |  |  |  |  |  |  |  |  |  |  |  |  |  |  |  |  |  |  |  |  |  |  |  |  |  |  |  |  |  |  |  |  |  |  |  |  |  |  |  |  |  |  |  |  |  |  |  |  |  |  |  |  |  |  |  |  |  |  |  |  |  |  |  |  |  |  |  |  |  |  |  |  |  |  |  |  |  |  |  |  |  |  |  |  |  |  |  |  |  |  |  |  |  |  |  |  |  |  |  |  |  |  |  |  |  |  |  |  |  |  |  |  |  |  |  |  |  |  |  |  |  |  |  |  |  |  |  |  |  |  |  |  |  |  |  |  |  |  |  |  |  |  |  |  |  |  |  |  |  |  |  |  |  |  |  |  |  |  |  |  |  |  |  |  |  |  |  |  |  |  |  |  |  |  |  |  |  |  |  |  |  |  |  |  |  |  |  |  |  |  |  |  |  |  |  |  |  |  |  |  |  |  |  |  |  |  |  |  |  |  |  |  |  |  |  |  |  |  |  |  |  |  |  |  |  |  |  |  |  |  |  |  |  |  |  |  |  |  |  |  |  |  |  |  |  |  |  |  |  |  |  |  |  |  |  |  |  |  |  |  |  |  |  |  |  |  |  |  |  |  |  |  |  |  |  |  |  |  |  |  |  |  |  |  |  |  |  |  |  |  |  |  |  |  |  |  |  |  |  |  |  |  |  |  |  |  |  |  |  |  |  |  |  |  |  |  |  |  |  |  |  |  |  |  |  |  |  |  |  |  |  |  |  |  |  |  |  |  |  |  |  |  |  |  |  |  |  |  |  |  |  |  |  |  |  |  |  |  |  |  |  |  |  |  |  |  |  |  |  |  |  |  |  |  |  |  |  |  |  |  |  |  |  |  |  |  |  |  |  |  |  |  |  |  |  |  |  |  |  |  |  |  |  |  |  |  |  |  |  |  |  |  |  |  |  |  |  |  |  |  |  |  |  |  |  |  |  |  |  |  |  |  |  |  |  |  |  |  |  |  |  |  |  |  |  |  |  |  |  |  |  |  |  |  |  |  |  |  |  |  |  |  |  |  |  |  |  |  |  |  |  |  |  |  |  |  |  |  |  |  |  |  |  |  |  |  |  |  |  |  |  |  |  |  |  |  |  |  |  |  |  |  |  |  |  |  |  |  |  |  |  |  |  |  |  |  |  |  |  |  |  |  |  |  |  |  |  |  |  |  |  |  |  |  |  |  |  |  |  |  |  |  |  |  |  |  |  |  |  |  |  |  |  |  |  |  |  |  |  |  |  |  |  |  |  |  |  |  |  |  |  |  |  |  |  |  |  |  |  |  |  |  |  |  |  |  |  |  |  |  |  |  |  |  |  |  |  |  |  |  |  |  |  |  |  |  |  |  |  |  |  |  |  |  |  |  |  |  |  |  |  |  |  |  |  |  |  |  |  |  |  |  |  |  |  |  |  |  |  |  |  |  |  |  |  |  |  |  |  |  |  |  |  |  |  |  |  |  |  |  |  |  |  |  |  |  |  |  |  |  |
|  |  |  |  |  |  |  |  |  |  |  |  |  |  |  |  |  |  |  |  |  |  |  |  |  |  |  |  |  |  |  |  |  |  |  |  |  |  |  |  |  |  |  |  |  |  |  |  |  |  |  |  |  |  |  |  |  |  |  |  |  |  |  |  |  |  |  |  |  |  |  |  |  |  |  |  |  |  |  |  |  |  |  |  |  |  |  |  |  |  |  |  |  |  |  |  |  |  |  |  |  |  |  |  |  |  |  |  |  |  |  |  |  |  |  |  |  |  |  |  |  |  |  |  |  |  |  |  |  |  |  |  |  |  |  |  |  |  |  |  |  |  |  |  |  |  |  |  |  |  |  |  |  |  |  |  |  |  |  |  |  |  |  |  |  |  |  |  |  |  |  |  |  |  |  |  |  |  |  |  |  |  |  |  |  |  |  |  |  |  |  |  |  |  |  |  |  |  |  |  |  |  |  |  |  |  |  |  |  |  |  |  |  |  |  |  |  |  |  |  |  |  |  |  |  |  |  |  |  |  |  |  |  |  |  |  |  |  |  |  |  |  |  |  |  |  |  |  |  |  |  |  |  |  |  |  |  |  |  |  |  |  |  |  |  |  |  |  |  |  |  |  |  |  |  |  |  |  |  |  |  |  |  |  |  |  |  |  |  |  |  |  |  |  |  |  |  |  |  |  |  |  |  |  |  |  |  |  |  |  |  |  |  |  |  |  |  |  |  |  |  |  |  |  |  |  |  |  |  |  |  |  |  |  |  |  |  |  |  |  |  |  |  |  |  |  |  |  |  |  |  |  |  |  |  |  |  |  |  |  |  |  |  |  |  |  |  |  |  |  |  |  |  |  |  |  |  |  |  |  |  |  |  |  |  |  |  |  |  |  |  |  |  |  |  |  |  |  |  |  |  |  |  |  |  |  |  |  |  |  |  |  |  |  |  |  |  |  |  |  |  |  |  |  |  |  |  |  |  |  |  |  |  |  |  |  |  |  |  |  |  |  |  |  |  |  |  |  |  |  |  |  |  |  |  |  |  |  |  |  |  |  |  |  |  |  |  |  |  |  |  |  |  |  |  |  |  |  |  |  |  |  |  |  |  |  |  |  |  |  |  |  |  |  |  |  |  |  |  |  |  |  |  |  |  |  |  |  |  |  |  |  |  |  |  |  |  |  |  |  |  |  |  |  |  |  |  |  |  |  |  |  |  |  |  |  |  |  |  |  |  |  |  |  |  |  |  |  |  |  |  |  |  |  |  |  |  |  |  |  |  |  |  |  |  |  |  |  |  |  |  |  |  |  |  |  |  |  |  |  |  |  |  |  |  |  |  |  |  |  |  |  |  |  |  |  |  |  |  |  |  |  |  |  |  |  |  |  |  |  |  |  |  |  |  |  |  |  |  |  |  |  |  |  |  |  |  |  |  |  |  |  |  |  |  |  |  |  |  |  |  |  |  |  |  |  |  |  |  |  |  |  |  |  |  |  |  |  |  |  |  |  |  |  |  |  |  |  |  |  |  |  |  |  |  |  |  |  |  |  |  |  |  |  |  |  |  |  |  |  |  |  |  |  |  |  |  |  |  |  |  |  |  |  |  |  |  |  |  |  |  |  |  |  |  |  |  |  |  |  |  |  |  |  |  |  |  |  |  |  |  |  |  |  |  |  |  |  |  |  |  |  |  |  |  |  |  |  |  |  |  |  |  |  |  |  |  |  |  |  |  |  |  |  |  |  |  |  |  |  |  |  |  |  |  |  |  |  |  |  |  |  |  |  |  |  |  |  |  |  |  |  |  |  |  |  |  |  |  |  |  |  |  |  |  |  |  |  |  |  |  |  |  |  |  |  |  |  |  |  |  |  |  |  |  |  |  |  |  |  |  |  |  |  |  |  |  |  |  |  |  |  |  |  |  |  |  |  |  |  |  |  |  |  |  |  |  |  |  |  |  |  |  |  |  |  |  |  |  |  |  |  |  |  |  |  |  |  |  |  |  |  |  |  |  |  |  |  |  |  |  |  |  |  |  |  |  |  |  |  |  |  |  |  |  |  |  |  |  |  |  |  |  |  |  |  |  |  |  |  |  |  |  |  |  |  |  |  |  |  |  |  |  |  |  |  |  |  |  |  |  |  |  |  |  |  |  |  |  |  |  |  |  |  |  |  |  |  |  |  |  |  |  |  |  |  |  |  |  |  |  |  |  |  |  |  |  |  |  |  |  |  |  |  |  |  |  |  |  |  |  |  |  |  |  |  |  |  |  |  |  |  |  |  |  |  |  |  |  |  |  |  |  |  |  |  |  |  |  |  |  |  |  |  |  |  |  |  |  |  |  |  |  |  |  |  |  |  |  |  |  |  |  |  |  |  |  |  |  |  |  |  |  |  |  |  |  |  |  |  |  |  |  |  |  |  |  |  |  |  |  |  |  |  |  |  |  |  |  |  |  |  |  |  |  |  |  |  |  |  |  |  |  |  |  |  |  |  |  |  |  |  |  |  |  |  |  |  |  |  |  |  |  |  |  |  |  |  |  |  |  |  |  |  |  |  |  |  |  |  |  |  |  |  |  |  |  |  |  |  |  |  |  |  |  |  |  |  |  |  |  |  |  |  |  |  |  |  |  |  |  |  |  |  |  |  |  |  |  |  |  |  |  |  |  |  |  |  |  |  |  |  |  |  |  |  |  |  |  |  |  |  |  |  |  |  |  |  |  |  |  |  |  |  |  |  |  |  |  |  |  |  |  |  |  |  |  |  |  |  |  |  |  |  |  |  |  |  |  |  |  |  |  |  |  |  |  |  |  |  |  |  |  |  |  |  |  |  |  |  |  |  |  |  |  |  |  |  |  |  |  |  |  |  |  |  |  |  |  |  |  |  |  |  |  |  |  |  |  |  |  |  |  |  |  |  |  |  |  |  |  |  |  |  |  |  |  |  |  |  |  |  |  |  |  |  |  |  |  |  |  |  |  |  |  |  |  |  |  |  |  |  |  |  |  |  |  |  |  |  |  |  |  |  |  |  |  |  |  |  |  |  |  |  |  |  |  |  |  |  |  |  |  |  |  |  |  |  |  |  |  |  |  |  |  |  |  |  |  |  |  |  |  |  |  |  |  |  |  |  |  |  |  |  |  |  |  |  |  |  |  |  |  |  |  |  |  |  |  |  |  |  |  |  |  |  |  |  |  |  |  |  |  |  |  |  |  |  |  |  |  |  |  |  |  |  |  |  |  |  |  |  |  |  |  |  |  |  |  |  |  |  |  |  |  |  |  |  |  |  |  |  |  |  |  |  |  |  |  |  |  |  |  |  |  |  |  |  |  |  |  |  |  |  |  |  |  |  |  |  |  |  |  |  |  |  |  |  |  |  |  |  |  |  |  |  |  |  |  |  |  |  |  |  |  |  |  |  |  |  |  |  |  |  |  |  |  |  |  |  |  |  |  |  |  |  |  |  |  |  |  |  |  |  |  |  |  |  |  |  |  |  |  |  |  |  |  |  |  |  |  |  |  |  |  |  |  |  |  |  |  |  |  |  |  |  |  |  |  |  |  |  |  |  |  |  |  |  |  |  |  |  |  |  |  |  |  |  |  |  |  |  |  |  |  |  |  |  |  |  |  |  |  |  |  |  |  |  |  |  |  |  |  |  |  |  |  |  |  |  |  |  |  |  |  |  |  |  |  |  |  |  |  |  |  |  |  |  |  |  |  |  |  |  |  |  |  |  |  |  |  |  |  |  |  |  |  |  |  |  |  |  |  |  |  |  |  |  |  |  |  |  |  |  |  |  |  |  |  |  |  |
| BIS d=0 cl.1 sym=1 env=1 | - | - | - | - | - | - | - | - | - | - | - | - | - | - | - | - | - | - | - | - | - | - | - | - | - | - | - | - | - | - | - | - | - | - | - | - | - | - | - | - | - | - | - | - | - | - | - | - | - | - | - | - | - | - | - | - | - | - | - | - | - | - | - | - | - | - | - | - | - | - | - | - | - | - | - | - | - | - | - | - | - | - | - | - | - | - | - | - | - | - | - | - | - | - | - | - | - | - | - | - | - | - | - | - | - | - | - | - | - | - | - | - | - | - | - | - | - | - | - | - | - | - | - | - | - | - | - | - | - | - | - | - | - | - | - | - | - | - | - | - | - | - | - | - | - | - | - | - | - | - | - | - | - | - | - | - | - | - | - | - | - | - | - | - | - | - | - | - | - | - | - | - | - | - | - | - | - | - | - | - | - | - | - | - | - | - | - | - | - | - | - | - | - | - | - | - | - | - | - | - | - | - | - | - | - | - | - | - | - | - | - | - | - | - | - | - | - | - | - | - | - | - | - | - | - | - | - | - | - | - | - | - | - | - | - | - | - | - | - | - | - | - | - | - | - | - | - | - | - | - | - | - | - | - | - | - | - | - | - | - | - | - | - | - | - | - | - | - | - | - | - | - | - | - | - | - | - | - | - | - | - | - | - | - | - | - | - | - | - | - | - | - | - | - | - | - | - | - | - | - | - | - | - | - | - | - | - | - | - | - | - | - | - | - | - | - | - | - | - | - | - | - | - | - | - | - | - | - | - | - | - | - | - | - | - | - | - | - | - | - | - | - | - | - | - | - | - | - | - | - | - | - | - | - | - | - | - | - | - | - | - | - | - | - | - | - | - | - | - | - | - | - | - | - | - | - | - | - | - | - | - | - | - | - | - | - | - | - | - | - | - | - | - | - | - | - | - | - | - | - | - | - | - | - | - | - | - | - | - | - | - | - | - | - | - | - | - | - | - | - | - | - | - | - | - | - | - | - | - | - | - | - | - | - | - | - | - | - | - | - | - | - | - | - | - | - | - | - | - | - | - | - | - | - | - | - | - | - | - | - | - | - | - | - | - | - | - | - | - | - | - | - | - | - | - | - | - | - | - | - | - | - | - | - | - | - | - | - | - | - | - | - | - | - | - | - | - | - | - | - | - | - | - | - | - | - | - | - | - | - | - | - | - | - | - | - | - | - | - | - | - | - | - | - | - | - | - | - | - | - | - | - | - | - | - | - | - | - | - | - | - | - | - | - | - | - | - | - | - | - | - | - | - | - | - | - | - | - | - | - | - | - | - | - | - | - | - | - | - | - | - | - | - | - | - | - | - | - | - | - | - | - | - | - | - | - | - | - | - | - | - | - | - | - | - | - | - | - | - | - | - | - | - | - | - | - | - | - | - | - | - | - | - | - | - | - | - | - | - | - | - | - | - | - | - | - | - | - | - | - | - | - | - | - | - | - | - | - | - | - | - | - | - | - | - | - | - | - | - | - | - | - | - | - | - | - | - | - | - | - | - | - | - | - | - | - | - | - | - | - | - | - | - | - | - | - | - | - | - | - | - | - | - | - | - | - | - | - | - | - | - | - | - | - | - | - | - | - | - | - | - | - | - | - | - | - | - | - | - | - | - | - | - | - | - | - | - | - | - | - | - | - | - | - | - | - | - | - | - | - | - | - | - | - | - | - | - | - | - | - | - | - | - | - | - | - | - | - | - | - | - | - | - | - | - | - | - | - | - | - | - | - | - | - | - | - | - | - | - | - | - | - | - | - | - | - | - | - | - | - | - | - | - | - | - | - | - | - | - | - | - | - | - | - | - | - | - | - | - | - | - | - | - | - | - | - | - | - | - | - | - | - | - | - | - | - | - | - | - | - | - | - | - | - | - | - | - | - | - | - | - | - | - | - | - | - | - | - | - | - | - | - | - | - | - | - | - | - | - | - | - | - | - | - | - | - | - | - | - | - | - | - | - | - | - | - | - | - | - | - | - | - | - | - | - | - | - | - | - | - | - | - | - | - | - | - | - | - | - | - | - | - | - | - | - | - | - | - | - | - | - | - | - | - | - | - | - | - | - | - | - | - | - | - | - | - | - | - | - | - | - | - | - | - | - | - | - | - | - | - | - | - | - | - | - | - | - | - | - | - | - | - | - | - | - | - | - | - | - | - | - | - | - | - | - | - | - | - | - | - | - | - | - | - | - | - | - | - | - | - | - | - | - | - | - | - | - | - | - | - | - | - | - | - | - | - | - | - | - | - | - | - | - | - | - | - | - | - | - | - | - | - | - | - | - | - | - | - | - | - | - | - | - | - | - | - | - | - | - | - | - | - | - | - | - | - | - | - | - | - | - | - | - | - | - | - | - | - | - | - | - | - | - | - | - | - | - | - | - | - | - | - | - | - | - | - | - | - | - | - | - | - | - | - | - | - | - | - | - | - | - | - | - | - | - | - | - | - | - | - | - | - | - | - | - | - | - | - | - | - | - | - | - | - | - | - | - | - | - | - | - | - | - | - | - | - | - | - | - | - | - | - | - | - | - | - | - | - | - | - | - | - | - | - | - | - | - | - | - | - | - | - | - | - | - | - | - | - | - | - | - | - | - | - | - | - | - | - | - | - | - | - | - | - | - | - | - | - | - | - | - | - | - | - | - | - | - | - | - | - | - | - | - | - | - | - | - | - | - | - | - | - | - | - | - | - | - | - | - | - | - | - | - | - | - | - | - | - | H | x | x | x | x | x | x | x | x | x | x | x | x | x | x | x | x | x | x | x | x | x | x | x | x | x | x | x | x | x | x | x | x | x | x | x | x | x | x | x | x | x | x | x | x | x | x | x | x | x | x | x | x | x | x | x | x | x | x | x | x | x | x | x | x | x | x | x | x | x | x | x | x | x | x | x | x | x | x | x | x | x | H | x | x | x | x | x | x | x | x | x | x | x | x | x | H | x | x | x | x | x | x | x | x | x | x | x | x | x | x | x | x | x | x | x | x | x | x | x | x | x | x | x | x | x | x | x | x | x | x | x | x | x | x | x | x | x | x | x | x | x | x | x | x | x | x | x | x | x | x | x | x | x | x | x | x | x | x | x | x | x | x | x | x | x | x | x | x | x | x | x | x | x | x | x | x | x | x | x | x | x | x | x | x | x | x | x | x | x | x | x | x | x | x | x | x | x | x | x | x | x | x | x | x | x | x | x | x | x | x | x | x | x | x | x | x | x | x | x | x | x | x | x | x | x | H | x | H | x | x | x | x | x | x | x | x | x | x | x | x | x | x | x | x | x | x | x | x | x | x | x | x | x | x | x | x | x | x | x | x | x | x | x | x | x | x | x | x | x | x | x | x | x | x | x | x | x | x | x | x | x | x | x | x | x | x | x | x | x | x | x | x | x | H | x | x | x | H | - | - | - | - | - | - | - | - | - | - | - | - | - | - | - | - | - | - | - | - | - | - | - | - | - | - | - | - | - | - | - | - | - | - | - | - | - | - | - | - | - | - | - | - | - | - | - | - | - | - | - | - | - | - | - | - | - | - | - | - | - | - | - | - | - | - | - | - | - | - | - | - | - | - | - | - | - | - | - | - | - | - | - | - | - | - | - | - | - | - | - | - | - | - | - | - | - | - | - | - | - | - | - | - | - | - | - | - | - | - | - | - | - | - | - | - | - | - | - | - | - | - | - | - | - | - | - | - | - | - | - | - | - | - | - | - | - | - | - | - | - | - | - | - | - | - | - | - | - | - | - | - | - | - | - | - | - | - | - | - | - | - | - | - | - | - | - | - | - | - | - | - | - | - | - | - | - | - | - | - | - | - | - |
