# Supplementary material for: Variation in selection constraints on teleost TLRs with emphasis on their repertoire in the Walking catfish, Clarias batrachus
Source: Sci Rep. 2020 Dec 7;10:21394. doi: 10.1038/s41598-020-78347-6 (PMC7721727; doi:10.1038/s41598-020-78347-6)
Supplement: Supplementary file 32 — Supplementary Information 32. [file 41598_2020_78347_MOESM32_ESM.zip › T13/BIS2/summary.tgz!/summary/PF00000-NONREDUNDANT-5DD-dim1-table.html]

BIS cluster table


Clusters with env. score >= 0.5 and sym. score >= 0.5 :

| Dim | Cluster | Sym | Env | Pvalue | Hit patterns and blocks |
| --- | --- | --- | --- | --- | --- |
| 1 | 5 | 0.5652174 | 0.5978261 | 0.005263158 | Hit patterns: Positions: \| 767 \| 1188; 18 sequences: \| C \| F; 1 sequence: \| X \| I; 1 sequence: \| A \| L; 1 sequence: \| X \| L; 1 sequence: \| - \| I All positions in cluster: 767 1188 |
| 1 | 1 | 1 | 1 | 0.04545455 | All positions in cluster: 1293 1303 1325 1346 1423 1432 1435 1463 1492 1494 1496 1498 1501 1507-1508 1515 1518 1520 1527 1529 1531-1532 1535 1539 1551 1555 1573 1582-1583 1586 1601-1602 |
| 1 | 2 | 1 | 1 | 0.04545455 | All positions in cluster: 1504 1546 |
| 1 | 3 | 0.6956522 | 0.9130435 | 1 | Hit patterns: Positions: \| 1257 \| 1311; 19 sequences: \| L \| Q; 2 sequences: \| V \| Q; 1 sequence: \| L \| E All positions in cluster: 1257 1311 |
| 1 | 4 | 0.6086957 | 0.826087 | 1 | Hit patterns: Positions: \| 153 \| 1543; 20 sequences: \| C \| L; 1 sequence: \| C \| Q; 1 sequence: \| X \| L All positions in cluster: 153 1543 |

Table created with bis2html version 8.
