# Supplementary material for: Variation in selection constraints on teleost TLRs with emphasis on their repertoire in the Walking catfish, Clarias batrachus
Source: Sci Rep. 2020 Dec 7;10:21394. doi: 10.1038/s41598-020-78347-6 (PMC7721727; doi:10.1038/s41598-020-78347-6)
Supplement: Supplementary file 32 — Supplementary Information 32. [file 41598_2020_78347_MOESM32_ESM.zip › T13/BIS2/summary.tgz!/summary/PF00000-NONREDUNDANT-5DD-dim2-table.html]

BIS cluster table


Clusters with env. score >= 0.5 and sym. score >= 0.5 :

| Dim | Cluster | Sym | Env | Pvalue | Hit patterns and blocks |
| --- | --- | --- | --- | --- | --- |
| 2 | 3 | 0.6363636 | 0.505814 | 5.159959e-05 | Hit patterns: Positions: \| 1102 \| 1495 \| 1571 \| 1608; 16 sequences: \| L \| H \| Y \| A; 3 sequences: \| X \| S \| H \| K; 1 sequence: \| F \| H \| Y \| A; 1 sequence: \| L \| Y \| G \| X; 1 sequence: \| I \| N \| F \| N All positions in cluster: 1102 1495 1571 1608 |
| 2 | 1 | 1 | 1 | 0.004329004 | All positions in cluster: 1466 1569 1584 |
| 2 | 2 | 1 | 1 | 0.004329004 | All positions in cluster: 1297 1542 1553 |
| 2 | 5 | 0.8636364 | 0.7732558 | 0.05 | Hit patterns: Positions: \| 1491 \| 1556; 19 sequences: \| L \| L; 1 sequence: \| F \| X; 1 sequence: \| V \| L; 1 sequence: \| L \| M All positions in cluster: 1491 1556 |
| 2 | 7 | 0.6363636 | 0.5290698 | 0.05 | Hit patterns: Positions: \| 749 \| 1284; 19 sequences: \| L \| L; 1 sequence: \| M \| L; 1 sequence: \| L \| F; 1 sequence: \| C \| I All positions in cluster: 749 1284 |
| 2 | 4 | 0.5454545 | 0.5174419 | 0.05263158 | Hit patterns: Positions: \| 1428 \| 1436 \| 1465 \| 1513 \| 1533 \| 1549 \| 1557; 18 sequences: \| Y \| V \| L \| Y \| S \| D \| E; 1 sequence: \| Y \| I \| L \| Y \| S \| D \| E; 1 sequence: \| Y \| V \| M \| Y \| S \| D \| E; 1 sequence: \| G \| X \| X \| X \| - \| S \| X; 1 sequence: \| F \| V \| L \| H \| G \| H \| Q All positions in cluster: 1428 1436 1465 1513 1533 1549 1557 |
| 2 | 6 | 0.6363636 | 0.7267442 | 1 | Hit patterns: Positions: \| 153 \| 1229 \| 1260 \| 1543; 18 sequences: \| C \| L \| L \| L; 1 sequence: \| C \| L \| L \| Q; 1 sequence: \| C \| F \| L \| L; 1 sequence: \| C \| L \| M \| L; 1 sequence: \| X \| L \| L \| L All positions in cluster: 153 1229 1260 1543 |
| 2 | 8 | 0.5 | 0.5406977 | 1 | Hit patterns: Positions: \| 1138 \| 1220 \| 1226 \| 1278; 16 sequences: \| L \| F \| L \| L; 2 sequences: \| L \| F \| L \| F; 1 sequence: \| L \| F \| V \| L; 1 sequence: \| L \| L \| L \| L; 1 sequence: \| F \| L \| L \| L; 1 sequence: \| F \| F \| L \| L All positions in cluster: 1138 1220 1226 1278 |

Table created with bis2html version 8.
