# Supplementary material for: Variation in selection constraints on teleost TLRs with emphasis on their repertoire in the Walking catfish, Clarias batrachus
Source: Sci Rep. 2020 Dec 7;10:21394. doi: 10.1038/s41598-020-78347-6 (PMC7721727; doi:10.1038/s41598-020-78347-6)
Supplement: Supplementary file 33 — Supplementary Information 33. [file 41598_2020_78347_MOESM33_ESM.zip › T21/bis2/summary.tgz!/summary/PF00000-NONREDUNDANT-5DD-dim0-table.html]

BIS cluster table


Clusters with env. score >= 0.5 and sym. score >= 0.5 :

| Dim | Cluster | Sym | Env | Pvalue | Hit patterns and blocks |
| --- | --- | --- | --- | --- | --- |
| 0 | 8 | 1 | 1 | 1.248751e-05 | Hit patterns: Positions: \| 212 \| 765; 10 sequences: \| P \| T; 3 sequences: \| D \| G; 3 sequences: \| S \| N All positions in cluster: 212 765 |
| 0 | 5 | 1 | 1 | 0.0001248751 | Hit patterns: Positions: \| 267 \| 426 \| 567 \| 581 \| 595 \| 703 \| 823 \| 897 \| 917 \| 954 \| 955 \| 1009; 10 sequences: \| S \| I \| G \| G \| F \| N \| I \| K \| S \| H \| R \| R; 6 sequences: \| X \| L \| A \| R \| L \| K \| T \| R \| A \| M \| Q \| K All positions in cluster: 267 426 567 581 595 703 823 897 917 954-955 1009 |
| 0 | 1 | 1 | 1 | 0.0002289377 | Hit patterns: Positions: \| 864 \| 931; 11 sequences: \| N \| R; 5 sequences: \| K \| Q All positions in cluster: 864 931 |
| 0 | 3 | 1 | 1 | 0.001785714 | Hit patterns: Positions: \| 253 \| 894 \| 895; 13 sequences: \| L \| S \| S; 3 sequences: \| I \| X \| X All positions in cluster: 253 894-895 |
| 0 | 4 | 1 | 1 | 0.001785714 | Hit patterns: Positions: \| 93 \| 123 \| 394 \| 670 \| 781 \| 865 \| 893 \| 932 \| 952 \| 995; 13 sequences: \| N \| P \| L \| D \| V \| C \| G \| N \| D \| T; 3 sequences: \| S \| F \| F \| E \| L \| Y \| X \| S \| Q \| S All positions in cluster: 93 123 394 670 781 865 893 932 952 995 |
| 0 | 7 | 1 | 1 | 0.001785714 | Hit patterns: Positions: \| 346 \| 477; 13 sequences: \| V \| N; 3 sequences: \| I \| K All positions in cluster: 346 477 |
| 0 | 6 | 1 | 1 | 0.008333333 | Hit patterns: Positions: \| 459 \| 833; 14 sequences: \| Y \| Y; 2 sequences: \| F \| H All positions in cluster: 459 833 |
| 0 | 9 | 1 | 1 | 0.008333333 | Hit patterns: Positions: \| 131 \| 835; 14 sequences: \| I \| K; 2 sequences: \| V \| R All positions in cluster: 131 835 |
| 0 | 2 | 1 | 1 | 1 | All positions in cluster: 1 91 94 107 121 144 153 158 163 167-168 174 177 182 187 192 195 198 201 206 208 216 222 230 232 235-236 245 248 251-252 256 261 268-269 272 280 282 285 287 298-299 301 303 306 316 322 327 348 353 362-363 381 384 397 400 405 407 416 419-420 432-435 442 460-465 471-472 481 483-486 488 498-499 502 505-512 520 526 529-534 536 538-540 544 547 550 553-560 570-571 574 578-580 582 584 587-588 598-599 602-604 607 609 619-621 627 629-632 635 637 639 644 649-651 653-654 657 672 674-675 678 681 683 687 689 693 696 698-699 701 704 707 713 721-722 733 736 738 746 749 752 754 756 758 766 768 771 773 775 782 791 799 801-802 806-807 810 814 816 819 830 836-838 842 844-846 848 850-851 855 862-863 867 869-870 872-874 880 885 887-890 896 899-905 908-909 911 913-916 918-920 922-924 927-928 930 933 936-951 953 956-961 963-964 971-972 974-978 981-982 984-986 988-990 994 997 1000 1003-1005 1008 1010-1011 |

Table created with bis2html version 8.
