# Supplementary material for: Variation in selection constraints on teleost TLRs with emphasis on their repertoire in the Walking catfish, Clarias batrachus
Source: Sci Rep. 2020 Dec 7;10:21394. doi: 10.1038/s41598-020-78347-6 (PMC7721727; doi:10.1038/s41598-020-78347-6)
Supplement: Supplementary file 33 — Supplementary Information 33. [file 41598_2020_78347_MOESM33_ESM.zip › T21/bis2/summary.tgz!/summary/PF00000-NONREDUNDANT-5DD-dim1-table.html]

BIS cluster table


Clusters with env. score >= 0.5 and sym. score >= 0.5 :

| Dim | Cluster | Sym | Env | Pvalue | Hit patterns and blocks |
| --- | --- | --- | --- | --- | --- |
| 1 | 10 | 1 | 1 | 0 | Hit patterns: Positions: \| 369 \| 566 \| 999; 9 sequences: \| K \| S \| Q; 3 sequences: \| H \| H \| S; 3 sequences: \| Y \| L \| K; 1 sequence: \| S \| G \| M All positions in cluster: 369 566 999 |
| 1 | 17 | 1 | 1 | 1.248751e-05 | Hit patterns: Positions: \| 260 \| 716; 9 sequences: \| S \| N; 6 sequences: \| T \| S; 1 sequence: \| N \| T All positions in cluster: 260 716 |
| 1 | 18 | 1 | 1 | 1.248751e-05 | Hit patterns: Positions: \| 74 \| 254 \| 516; 9 sequences: \| L \| C \| F; 6 sequences: \| I \| S \| Y; 1 sequence: \| S \| G \| W All positions in cluster: 74 254 516 |
| 1 | 22 | 1 | 1 | 1.248751e-05 | Hit patterns: Positions: \| 314 \| 1016; 9 sequences: \| V \| S; 6 sequences: \| L \| N; 1 sequence: \| I \| T All positions in cluster: 314 1016 |
| 1 | 3 | 1 | 1 | 0.0001373626 | Hit patterns: Positions: \| 386 \| 926; 12 sequences: \| I \| I; 3 sequences: \| L \| V; 1 sequence: \| F \| L All positions in cluster: 386 926 |
| 1 | 5 | 1 | 1 | 0.0001373626 | Hit patterns: Positions: \| 404 \| 787; 12 sequences: \| L \| Y; 3 sequences: \| M \| F; 1 sequence: \| F \| H All positions in cluster: 404 787 |
| 1 | 13 | 1 | 1 | 0.0001373626 | Hit patterns: Positions: \| 290 \| 319; 12 sequences: \| S \| I; 3 sequences: \| D \| L; 1 sequence: \| L \| V All positions in cluster: 290 319 |
| 1 | 19 | 1 | 1 | 0.0005952381 | Hit patterns: Positions: \| 156 \| 259; 13 sequences: \| D \| T; 2 sequences: \| N \| I; 1 sequence: \| T \| V All positions in cluster: 156 259 |
| 1 | 20 | 1 | 1 | 0.0005952381 | Hit patterns: Positions: \| 139 \| 243; 13 sequences: \| I \| S; 2 sequences: \| V \| T; 1 sequence: \| L \| A All positions in cluster: 139 243 |
| 1 | 1 | 1 | 1 | 0.0625 | All positions in cluster: 291 723 847 987 |
| 1 | 2 | 1 | 1 | 0.0625 | All positions in cluster: 786 979 |
| 1 | 4 | 1 | 1 | 0.0625 | All positions in cluster: 347 728 858 |
| 1 | 6 | 1 | 1 | 0.0625 | All positions in cluster: 225 321 398 778 |
| 1 | 7 | 1 | 1 | 0.0625 | All positions in cluster: 203 412 454 648 658 762 774 832 841 856 |
| 1 | 8 | 1 | 1 | 0.0625 | All positions in cluster: 180 258 271 292 389 401 496 643 718 881 |
| 1 | 9 | 1 | 1 | 0.0625 | All positions in cluster: 489 684 |
| 1 | 11 | 1 | 1 | 0.0625 | All positions in cluster: 439 495 761 934 966 |
| 1 | 12 | 1 | 1 | 0.0625 | All positions in cluster: 90 171 178 264 324 406 514 543 662 714 724 734 834 857 877 906 |
| 1 | 14 | 1 | 1 | 0.0625 | All positions in cluster: 311 349 409 601 659 673 719 731 962 |
| 1 | 15 | 1 | 1 | 0.0625 | All positions in cluster: 300 968-969 |
| 1 | 16 | 1 | 1 | 0.0625 | All positions in cluster: 150 263 275 476 664 763 854 1007 |
| 1 | 21 | 1 | 1 | 0.0625 | All positions in cluster: 89 105 764 839 868 925 |

Table created with bis2html version 8.
