# Supplementary material for: Variation in selection constraints on teleost TLRs with emphasis on their repertoire in the Walking catfish, Clarias batrachus
Source: Sci Rep. 2020 Dec 7;10:21394. doi: 10.1038/s41598-020-78347-6 (PMC7721727; doi:10.1038/s41598-020-78347-6)
Supplement: Supplementary file 33 — Supplementary Information 33. [file 41598_2020_78347_MOESM33_ESM.zip › T21/bis2/summary.tgz!/summary/PF00000-NONREDUNDANT-5DD-dim2.html]

Alignment and BIS clusters


|  |  |  |  |  |  |  |  |  |  |  |  |  |  |  |  |  |  |  |  |  |  |  |  |  |  |  |  |  |  |  |  |  |  |  |  |  |  |  |  |  |  |  |  |  |  |  |  |  |  |  |  |  |  |  |  |  |  |  |  |  |  |  |  |  |  |  |  |  |  |  |  |  |  |  |  |  |  |  |  |  |  |  |  |  |  |  |  |  |  |  |  |  |  |  |  |  |  |  |  |  |  |  |  |  |  |  |  |  |  |  |  |  |  |  |  |  |  |  |  |  |  |  |  |  |  |  |  |  |  |  |  |  |  |  |  |  |  |  |  |  |  |  |  |  |  |  |  |  |  |  |  |  |  |  |  |  |  |  |  |  |  |  |  |  |  |  |  |  |  |  |  |  |  |  |  |  |  |  |  |  |  |  |  |  |  |  |  |  |  |  |  |  |  |  |  |  |  |  |  |  |  |  |  |  |  |  |  |  |  |  |  |  |  |  |  |  |  |  |  |  |  |  |  |  |  |  |  |  |  |  |  |  |  |  |  |  |  |  |  |  |  |  |  |  |  |  |  |  |  |  |  |  |  |  |  |  |  |  |  |  |  |  |  |  |  |  |  |  |  |  |  |  |  |  |  |  |  |  |  |  |  |  |  |  |  |  |  |  |  |  |  |  |  |  |  |  |  |  |  |  |  |  |  |  |  |  |  |  |  |  |  |  |  |  |  |  |  |  |  |  |  |  |  |  |  |  |  |  |  |  |  |  |  |  |  |  |  |  |  |  |  |  |  |  |  |  |  |  |  |  |  |  |  |  |  |  |  |  |  |  |  |  |  |  |  |  |  |  |  |  |  |  |  |  |  |  |  |  |  |  |  |  |  |  |  |  |  |  |  |  |  |  |  |  |  |  |  |  |  |  |  |  |  |  |  |  |  |  |  |  |  |  |  |  |  |  |  |  |  |  |  |  |  |  |  |  |  |  |  |  |  |  |  |  |  |  |  |  |  |  |  |  |  |  |  |  |  |  |  |  |  |  |  |  |  |  |  |  |  |  |  |  |  |  |  |  |  |  |  |  |  |  |  |  |  |  |  |  |  |  |  |  |  |  |  |  |  |  |  |  |  |  |  |  |  |  |  |  |  |  |  |  |  |  |  |  |  |  |  |  |  |  |  |  |  |  |  |  |  |  |  |  |  |  |  |  |  |  |  |  |  |  |  |  |  |  |  |  |  |  |  |  |  |  |  |  |  |  |  |  |  |  |  |  |  |  |  |  |  |  |  |  |  |  |  |  |  |  |  |  |  |  |  |  |  |  |  |  |  |  |  |  |  |  |  |  |  |  |  |  |  |  |  |  |  |  |  |  |  |  |  |  |  |  |  |  |  |  |  |  |  |  |  |  |  |  |  |  |  |  |  |  |  |  |  |  |  |  |  |  |  |  |  |  |  |  |  |  |  |  |  |  |  |  |  |  |  |  |  |  |  |  |  |  |  |  |  |  |  |  |  |  |  |  |  |  |  |  |  |  |  |  |  |  |  |  |  |  |  |  |  |  |  |  |  |  |  |  |  |  |  |  |  |  |  |  |  |  |  |  |  |  |  |  |  |  |  |  |  |  |  |  |  |  |  |  |  |  |  |  |  |  |  |  |  |  |  |  |  |  |  |  |  |  |  |  |  |  |  |  |  |  |  |  |  |  |  |  |  |  |  |  |  |  |  |  |  |  |  |  |  |  |  |  |  |  |  |  |  |  |  |  |  |  |  |  |  |  |  |  |  |  |  |  |  |  |  |  |  |  |  |  |  |  |  |  |  |  |  |  |  |  |  |  |  |  |  |  |  |  |  |  |  |  |  |  |  |  |  |  |  |  |  |  |  |  |  |  |  |  |  |  |  |  |  |  |  |  |  |  |  |  |  |  |  |  |  |  |  |  |  |  |  |  |  |  |  |  |  |  |  |  |  |  |  |  |  |  |  |  |  |  |  |  |  |  |  |  |  |  |  |  |  |  |  |  |  |  |  |  |  |  |  |  |  |  |  |  |  |  |  |  |  |  |  |  |  |  |  |  |  |  |  |  |  |  |  |  |  |  |  |  |  |  |  |  |  |  |  |  |  |  |  |  |  |  |  |  |  |  |  |  |  |  |  |  |  |  |  |  |  |  |  |  |  |  |  |  |  |  |  |  |  |  |  |  |  |  |  |  |  |  |  |  |  |  |  |  |  |  |  |  |  |  |  |  |  |  |  |  |  |  |  |  |  |  |  |  |  |  |  |  |  |  |  |  |  |  |  |  |  |  |  |  |  |  |  |  |  |  |  |  |  |  |  |  |  |  |  |  |  |  |  |  |  |  |  |  |  |  |  |  |  |  |  |  |  |  |  |  |  |  |  |  |  |  |  |  |  |  |  |  |  |  |  |  |  |  |  |  |  |  |  |  |
| --- | --- | --- | --- | --- | --- | --- | --- | --- | --- | --- | --- | --- | --- | --- | --- | --- | --- | --- | --- | --- | --- | --- | --- | --- | --- | --- | --- | --- | --- | --- | --- | --- | --- | --- | --- | --- | --- | --- | --- | --- | --- | --- | --- | --- | --- | --- | --- | --- | --- | --- | --- | --- | --- | --- | --- | --- | --- | --- | --- | --- | --- | --- | --- | --- | --- | --- | --- | --- | --- | --- | --- | --- | --- | --- | --- | --- | --- | --- | --- | --- | --- | --- | --- | --- | --- | --- | --- | --- | --- | --- | --- | --- | --- | --- | --- | --- | --- | --- | --- | --- | --- | --- | --- | --- | --- | --- | --- | --- | --- | --- | --- | --- | --- | --- | --- | --- | --- | --- | --- | --- | --- | --- | --- | --- | --- | --- | --- | --- | --- | --- | --- | --- | --- | --- | --- | --- | --- | --- | --- | --- | --- | --- | --- | --- | --- | --- | --- | --- | --- | --- | --- | --- | --- | --- | --- | --- | --- | --- | --- | --- | --- | --- | --- | --- | --- | --- | --- | --- | --- | --- | --- | --- | --- | --- | --- | --- | --- | --- | --- | --- | --- | --- | --- | --- | --- | --- | --- | --- | --- | --- | --- | --- | --- | --- | --- | --- | --- | --- | --- | --- | --- | --- | --- | --- | --- | --- | --- | --- | --- | --- | --- | --- | --- | --- | --- | --- | --- | --- | --- | --- | --- | --- | --- | --- | --- | --- | --- | --- | --- | --- | --- | --- | --- | --- | --- | --- | --- | --- | --- | --- | --- | --- | --- | --- | --- | --- | --- | --- | --- | --- | --- | --- | --- | --- | --- | --- | --- | --- | --- | --- | --- | --- | --- | --- | --- | --- | --- | --- | --- | --- | --- | --- | --- | --- | --- | --- | --- | --- | --- | --- | --- | --- | --- | --- | --- | --- | --- | --- | --- | --- | --- | --- | --- | --- | --- | --- | --- | --- | --- | --- | --- | --- | --- | --- | --- | --- | --- | --- | --- | --- | --- | --- | --- | --- | --- | --- | --- | --- | --- | --- | --- | --- | --- | --- | --- | --- | --- | --- | --- | --- | --- | --- | --- | --- | --- | --- | --- | --- | --- | --- | --- | --- | --- | --- | --- | --- | --- | --- | --- | --- | --- | --- | --- | --- | --- | --- | --- | --- | --- | --- | --- | --- | --- | --- | --- | --- | --- | --- | --- | --- | --- | --- | --- | --- | --- | --- | --- | --- | --- | --- | --- | --- | --- | --- | --- | --- | --- | --- | --- | --- | --- | --- | --- | --- | --- | --- | --- | --- | --- | --- | --- | --- | --- | --- | --- | --- | --- | --- | --- | --- | --- | --- | --- | --- | --- | --- | --- | --- | --- | --- | --- | --- | --- | --- | --- | --- | --- | --- | --- | --- | --- | --- | --- | --- | --- | --- | --- | --- | --- | --- | --- | --- | --- | --- | --- | --- | --- | --- | --- | --- | --- | --- | --- | --- | --- | --- | --- | --- | --- | --- | --- | --- | --- | --- | --- | --- | --- | --- | --- | --- | --- | --- | --- | --- | --- | --- | --- | --- | --- | --- | --- | --- | --- | --- | --- | --- | --- | --- | --- | --- | --- | --- | --- | --- | --- | --- | --- | --- | --- | --- | --- | --- | --- | --- | --- | --- | --- | --- | --- | --- | --- | --- | --- | --- | --- | --- | --- | --- | --- | --- | --- | --- | --- | --- | --- | --- | --- | --- | --- | --- | --- | --- | --- | --- | --- | --- | --- | --- | --- | --- | --- | --- | --- | --- | --- | --- | --- | --- | --- | --- | --- | --- | --- | --- | --- | --- | --- | --- | --- | --- | --- | --- | --- | --- | --- | --- | --- | --- | --- | --- | --- | --- | --- | --- | --- | --- | --- | --- | --- | --- | --- | --- | --- | --- | --- | --- | --- | --- | --- | --- | --- | --- | --- | --- | --- | --- | --- | --- | --- | --- | --- | --- | --- | --- | --- | --- | --- | --- | --- | --- | --- | --- | --- | --- | --- | --- | --- | --- | --- | --- | --- | --- | --- | --- | --- | --- | --- | --- | --- | --- | --- | --- | --- | --- | --- | --- | --- | --- | --- | --- | --- | --- | --- | --- | --- | --- | --- | --- | --- | --- | --- | --- | --- | --- | --- | --- | --- | --- | --- | --- | --- | --- | --- | --- | --- | --- | --- | --- | --- | --- | --- | --- | --- | --- | --- | --- | --- | --- | --- | --- | --- | --- | --- | --- | --- | --- | --- | --- | --- | --- | --- | --- | --- | --- | --- | --- | --- | --- | --- | --- | --- | --- | --- | --- | --- | --- | --- | --- | --- | --- | --- | --- | --- | --- | --- | --- | --- | --- | --- | --- | --- | --- | --- | --- | --- | --- | --- | --- | --- | --- | --- | --- | --- | --- | --- | --- | --- | --- | --- | --- | --- | --- | --- | --- | --- | --- | --- | --- | --- | --- | --- | --- | --- | --- | --- | --- | --- | --- | --- | --- | --- | --- | --- | --- | --- | --- | --- | --- | --- | --- | --- | --- | --- | --- | --- | --- | --- | --- | --- | --- | --- | --- | --- | --- | --- | --- | --- | --- | --- | --- | --- | --- | --- | --- | --- | --- | --- | --- | --- | --- | --- | --- | --- | --- | --- | --- | --- | --- | --- | --- | --- | --- | --- | --- | --- | --- | --- | --- | --- | --- | --- | --- | --- | --- | --- | --- | --- | --- | --- | --- | --- | --- | --- | --- | --- | --- | --- | --- | --- | --- | --- | --- | --- | --- | --- | --- | --- | --- | --- | --- | --- | --- | --- | --- | --- | --- | --- | --- | --- | --- | --- | --- | --- | --- | --- | --- | --- | --- | --- | --- | --- | --- | --- | --- | --- | --- | --- | --- | --- | --- | --- | --- | --- | --- | --- | --- | --- | --- | --- | --- | --- | --- | --- | --- | --- | --- | --- | --- | --- | --- | --- | --- | --- | --- | --- | --- | --- | --- | --- | --- | --- | --- | --- | --- | --- | --- | --- | --- | --- | --- | --- | --- | --- | --- | --- | --- | --- | --- | --- | --- | --- | --- | --- | --- | --- | --- | --- | --- | --- | --- | --- | --- | --- | --- | --- | --- | --- | --- | --- | --- | --- | --- | --- | --- | --- | --- | --- | --- | --- | --- | --- | --- | --- | --- | --- | --- | --- | --- | --- | --- | --- | --- | --- | --- | --- | --- | --- | --- | --- | --- | --- | --- | --- | --- | --- | --- | --- | --- | --- | --- | --- | --- | --- | --- | --- | --- | --- | --- | --- | --- | --- | --- | --- | --- | --- | --- | --- | --- | --- | --- | --- | --- | --- | --- | --- | --- | --- | --- | --- | --- | --- | --- | --- | --- | --- | --- | --- | --- | --- | --- | --- | --- | --- | --- | --- | --- | --- | --- | --- | --- | --- | --- | --- | --- | --- | --- | --- | --- | --- | --- | --- | --- | --- | --- | --- | --- | --- | --- | --- | --- | --- | --- | --- | --- | --- | --- | --- | --- | --- | --- | --- | --- | --- | --- |
|  |  |  |  |  |  |  |  | 1 | 0 |  |  |  |  |  |  |  |  | 2 | 0 |  |  |  |  |  |  |  |  | 3 | 0 |  |  |  |  |  |  |  |  | 4 | 0 |  |  |  |  |  |  |  |  | 5 | 0 |  |  |  |  |  |  |  |  | 6 | 0 |  |  |  |  |  |  |  |  | 7 | 0 |  |  |  |  |  |  |  |  | 8 | 0 |  |  |  |  |  |  |  |  | 9 | 0 |  |  |  |  |  |  |  | 1 | 0 | 0 |  |  |  |  |  |  |  | 1 | 1 | 0 |  |  |  |  |  |  |  | 1 | 2 | 0 |  |  |  |  |  |  |  | 1 | 3 | 0 |  |  |  |  |  |  |  | 1 | 4 | 0 |  |  |  |  |  |  |  | 1 | 5 | 0 |  |  |  |  |  |  |  | 1 | 6 | 0 |  |  |  |  |  |  |  | 1 | 7 | 0 |  |  |  |  |  |  |  | 1 | 8 | 0 |  |  |  |  |  |  |  | 1 | 9 | 0 |  |  |  |  |  |  |  | 2 | 0 | 0 |  |  |  |  |  |  |  | 2 | 1 | 0 |  |  |  |  |  |  |  | 2 | 2 | 0 |  |  |  |  |  |  |  | 2 | 3 | 0 |  |  |  |  |  |  |  | 2 | 4 | 0 |  |  |  |  |  |  |  | 2 | 5 | 0 |  |  |  |  |  |  |  | 2 | 6 | 0 |  |  |  |  |  |  |  | 2 | 7 | 0 |  |  |  |  |  |  |  | 2 | 8 | 0 |  |  |  |  |  |  |  | 2 | 9 | 0 |  |  |  |  |  |  |  | 3 | 0 | 0 |  |  |  |  |  |  |  | 3 | 1 | 0 |  |  |  |  |  |  |  | 3 | 2 | 0 |  |  |  |  |  |  |  | 3 | 3 | 0 |  |  |  |  |  |  |  | 3 | 4 | 0 |  |  |  |  |  |  |  | 3 | 5 | 0 |  |  |  |  |  |  |  | 3 | 6 | 0 |  |  |  |  |  |  |  | 3 | 7 | 0 |  |  |  |  |  |  |  | 3 | 8 | 0 |  |  |  |  |  |  |  | 3 | 9 | 0 |  |  |  |  |  |  |  | 4 | 0 | 0 |  |  |  |  |  |  |  | 4 | 1 | 0 |  |  |  |  |  |  |  | 4 | 2 | 0 |  |  |  |  |  |  |  | 4 | 3 | 0 |  |  |  |  |  |  |  | 4 | 4 | 0 |  |  |  |  |  |  |  | 4 | 5 | 0 |  |  |  |  |  |  |  | 4 | 6 | 0 |  |  |  |  |  |  |  | 4 | 7 | 0 |  |  |  |  |  |  |  | 4 | 8 | 0 |  |  |  |  |  |  |  | 4 | 9 | 0 |  |  |  |  |  |  |  | 5 | 0 | 0 |  |  |  |  |  |  |  | 5 | 1 | 0 |  |  |  |  |  |  |  | 5 | 2 | 0 |  |  |  |  |  |  |  | 5 | 3 | 0 |  |  |  |  |  |  |  | 5 | 4 | 0 |  |  |  |  |  |  |  | 5 | 5 | 0 |  |  |  |  |  |  |  | 5 | 6 | 0 |  |  |  |  |  |  |  | 5 | 7 | 0 |  |  |  |  |  |  |  | 5 | 8 | 0 |  |  |  |  |  |  |  | 5 | 9 | 0 |  |  |  |  |  |  |  | 6 | 0 | 0 |  |  |  |  |  |  |  | 6 | 1 | 0 |  |  |  |  |  |  |  | 6 | 2 | 0 |  |  |  |  |  |  |  | 6 | 3 | 0 |  |  |  |  |  |  |  | 6 | 4 | 0 |  |  |  |  |  |  |  | 6 | 5 | 0 |  |  |  |  |  |  |  | 6 | 6 | 0 |  |  |  |  |  |  |  | 6 | 7 | 0 |  |  |  |  |  |  |  | 6 | 8 | 0 |  |  |  |  |  |  |  | 6 | 9 | 0 |  |  |  |  |  |  |  | 7 | 0 | 0 |  |  |  |  |  |  |  | 7 | 1 | 0 |  |  |  |  |  |  |  | 7 | 2 | 0 |  |  |  |  |  |  |  | 7 | 3 | 0 |  |  |  |  |  |  |  | 7 | 4 | 0 |  |  |  |  |  |  |  | 7 | 5 | 0 |  |  |  |  |  |  |  | 7 | 6 | 0 |  |  |  |  |  |  |  | 7 | 7 | 0 |  |  |  |  |  |  |  | 7 | 8 | 0 |  |  |  |  |  |  |  | 7 | 9 | 0 |  |  |  |  |  |  |  | 8 | 0 | 0 |  |  |  |  |  |  |  | 8 | 1 | 0 |  |  |  |  |  |  |  | 8 | 2 | 0 |  |  |  |  |  |  |  | 8 | 3 | 0 |  |  |  |  |  |  |  | 8 | 4 | 0 |  |  |  |  |  |  |  | 8 | 5 | 0 |  |  |  |  |  |  |  | 8 | 6 | 0 |  |  |  |  |  |  |  | 8 | 7 | 0 |  |  |  |  |  |  |  | 8 | 8 | 0 |  |  |  |  |  |  |  | 8 | 9 | 0 |  |  |  |  |  |  |  | 9 | 0 | 0 |  |  |  |  |  |  |  | 9 | 1 | 0 |  |  |  |  |  |  |  | 9 | 2 | 0 |  |  |  |  |  |  |  | 9 | 3 | 0 |  |  |  |  |  |  |  | 9 | 4 | 0 |  |  |  |  |  |  |  | 9 | 5 | 0 |  |  |  |  |  |  |  | 9 | 6 | 0 |  |  |  |  |  |  |  | 9 | 7 | 0 |  |  |  |  |  |  |  | 9 | 8 | 0 |  |  |  |  |  |  |  | 9 | 9 | 0 |  |  |  |  |  |  | 1 | 0 | 0 | 0 |  |  |  |  |  |  | 1 | 0 | 1 | 0 |  |  |  |  |  |  | 1 | 0 | 2 | 0 |  |  |  |  |  |  | 1 | 0 | 3 | 0 |  |  |  |  |  |  | 1 | 0 | 4 | 0 |  |  |  |  |  |  | 1 | 0 | 5 | 0 |  |  |  |  |  |  | 1 | 0 | 6 | 0 |  |  |  |  |  |  | 1 | 0 | 7 | 0 |  |  |  |  |  |
|  |  |  |  |  |  |  |  |  | \| |  |  |  |  |  |  |  |  |  | \| |  |  |  |  |  |  |  |  |  | \| |  |  |  |  |  |  |  |  |  | \| |  |  |  |  |  |  |  |  |  | \| |  |  |  |  |  |  |  |  |  | \| |  |  |  |  |  |  |  |  |  | \| |  |  |  |  |  |  |  |  |  | \| |  |  |  |  |  |  |  |  |  | \| |  |  |  |  |  |  |  |  |  | \| |  |  |  |  |  |  |  |  |  | \| |  |  |  |  |  |  |  |  |  | \| |  |  |  |  |  |  |  |  |  | \| |  |  |  |  |  |  |  |  |  | \| |  |  |  |  |  |  |  |  |  | \| |  |  |  |  |  |  |  |  |  | \| |  |  |  |  |  |  |  |  |  | \| |  |  |  |  |  |  |  |  |  | \| |  |  |  |  |  |  |  |  |  | \| |  |  |  |  |  |  |  |  |  | \| |  |  |  |  |  |  |  |  |  | \| |  |  |  |  |  |  |  |  |  | \| |  |  |  |  |  |  |  |  |  | \| |  |  |  |  |  |  |  |  |  | \| |  |  |  |  |  |  |  |  |  | \| |  |  |  |  |  |  |  |  |  | \| |  |  |  |  |  |  |  |  |  | \| |  |  |  |  |  |  |  |  |  | \| |  |  |  |  |  |  |  |  |  | \| |  |  |  |  |  |  |  |  |  | \| |  |  |  |  |  |  |  |  |  | \| |  |  |  |  |  |  |  |  |  | \| |  |  |  |  |  |  |  |  |  | \| |  |  |  |  |  |  |  |  |  | \| |  |  |  |  |  |  |  |  |  | \| |  |  |  |  |  |  |  |  |  | \| |  |  |  |  |  |  |  |  |  | \| |  |  |  |  |  |  |  |  |  | \| |  |  |  |  |  |  |  |  |  | \| |  |  |  |  |  |  |  |  |  | \| |  |  |  |  |  |  |  |  |  | \| |  |  |  |  |  |  |  |  |  | \| |  |  |  |  |  |  |  |  |  | \| |  |  |  |  |  |  |  |  |  | \| |  |  |  |  |  |  |  |  |  | \| |  |  |  |  |  |  |  |  |  | \| |  |  |  |  |  |  |  |  |  | \| |  |  |  |  |  |  |  |  |  | \| |  |  |  |  |  |  |  |  |  | \| |  |  |  |  |  |  |  |  |  | \| |  |  |  |  |  |  |  |  |  | \| |  |  |  |  |  |  |  |  |  | \| |  |  |  |  |  |  |  |  |  | \| |  |  |  |  |  |  |  |  |  | \| |  |  |  |  |  |  |  |  |  | \| |  |  |  |  |  |  |  |  |  | \| |  |  |  |  |  |  |  |  |  | \| |  |  |  |  |  |  |  |  |  | \| |  |  |  |  |  |  |  |  |  | \| |  |  |  |  |  |  |  |  |  | \| |  |  |  |  |  |  |  |  |  | \| |  |  |  |  |  |  |  |  |  | \| |  |  |  |  |  |  |  |  |  | \| |  |  |  |  |  |  |  |  |  | \| |  |  |  |  |  |  |  |  |  | \| |  |  |  |  |  |  |  |  |  | \| |  |  |  |  |  |  |  |  |  | \| |  |  |  |  |  |  |  |  |  | \| |  |  |  |  |  |  |  |  |  | \| |  |  |  |  |  |  |  |  |  | \| |  |  |  |  |  |  |  |  |  | \| |  |  |  |  |  |  |  |  |  | \| |  |  |  |  |  |  |  |  |  | \| |  |  |  |  |  |  |  |  |  | \| |  |  |  |  |  |  |  |  |  | \| |  |  |  |  |  |  |  |  |  | \| |  |  |  |  |  |  |  |  |  | \| |  |  |  |  |  |  |  |  |  | \| |  |  |  |  |  |  |  |  |  | \| |  |  |  |  |  |  |  |  |  | \| |  |  |  |  |  |  |  |  |  | \| |  |  |  |  |  |  |  |  |  | \| |  |  |  |  |  |  |  |  |  | \| |  |  |  |  |  |  |  |  |  | \| |  |  |  |  |  |  |  |  |  | \| |  |  |  |  |  |  |  |  |  | \| |  |  |  |  |  |  |  |  |  | \| |  |  |  |  |  |  |  |  |  | \| |  |  |  |  |  |  |  |  |  | \| |  |  |  |  |  |  |  |  |  | \| |  |  |  |  |  |  |  |  |  | \| |  |  |  |  |  |  |  |  |  | \| |  |  |  |  |  |  |  |  |  | \| |  |  |  |  |  |  |  |  |  | \| |  |  |  |  |  |  |  |  |  | \| |  |  |  |  |  |  |  |  |  | \| |  |  |  |  |  |  |  |  |  | \| |  |  |  |  |  |  |  |  |  | \| |  |  |  |  |  |  |  |  |  | \| |  |  |  |  |  |  |  |  |  | \| |  |  |  |  |  |  |  |  |  | \| |  |  |  |  |  |  |  |  |  | \| |  |  |  |  |  |  |  |  |  | \| |  |  |  |  |  |  |  |  |  | \| |  |  |  |  |  |  |  |  |  | \| |  |  |  |  |  |  |  |  |  | \| |  |  |  |  |  |  |  |  |  | \| |  |  |  |  |  |
| GMTLR21\_3196\_BP | M | X | X | X | X | X | X | X | X | X | X | X | X | X | X | X | X | X | X | X | X | X | X | X | X | X | X | X | X | X | X | X | X | X | X | X | X | X | X | X | X | X | X | X | X | X | X | X | X | X | X | X | X | X | X | X | X | X | X | X | X | X | X | X | X | X | D | S | T | R | X | X | L | S | I | L | T | V | G | L | L | I | H | L | S | X | I | X | Y | G | F | K | N | C | I | G | D | Q | Y | S | N | N | L | T | F | T | C | S | Q | R | A | E | K | L | I | S | L | I | V | G | D | L | P | A | S | A | I | N | I | T | I | G | N | N | N | V | S | H | I | P | T | E | S | F | I | R | F | F | K | L | E | N | L | S | M | D | S | N | N | L | K | F | I | D | G | L | A | F | R | R | M | H | Q | L | R | T | L | I | L | S | S | N | R | L | S | K | L | N | C | S | V | F | Q | D | L | Y | N | L | T | Y | L | S | L | K | H | N | L | L | E | D | L | P | Q | S | L | F | T | S | T | P | Q | L | N | T | L | I | L | R | N | N | S | L | H | N | F | S | V | V | V | R | S | V | S | S | L | S | Q | L | K | T | L | D | L | G | F | N | F | L | T | S | L | T | H | L | N | S | S | L | P | K | S | L | T | E | L | F | V | C | G | N | K | L | L | S | L | A | C | A | D | S | F | L | T | N | I | R | L | L | D | L | S | K | N | V | E | L | S | T | T | A | F | K | G | V | D | L | A | Q | I | R | Y | L | Q | M | G | S | T | K | V | K | I | F | E | F | F | K | V | T | N | X | X | D | A | T | Y | V | D | F | S | D | M | G | L | T | D | P | S | Q | I | I | K | L | C | T | F | F | R | K | S | K | H | T | X | X | X | X | X | H | M | T | L | K | L | N | R | F | N | M | L | P | N | T | S | L | T | N | C | P | Q | I | T | G | T | F | D | I | S | S | N | N | L | K | Q | V | K | C | L | E | F | L | E | G | Q | K | H | I | K | S | S | K | I | E | H | N | H | I | K | R | L | L | S | C | K | G | T | K | Y | K | L | P | X | X | X | L | T | E | L | S | F | R | Y | N | R | I | L | S | V | K | S | D | A | F | A | H | T | P | N | V | T | I | L | N | L | N | I | N | S | I | A | S | L | H | F | G | A | L | R | G | L | T | K | L | Q | I | L | R | L | D | N | N | L | L | T | E | L | W | D | S | T | F | E | G | L | S | S | L | E | T | L | N | L | R | N | N | R | I | A | V | I | F | Y | G | R | F | Q | D | L | G | R | L | T | I | L | D | L | G | G | N | K | I | S | H | I | Y | E | G | G | L | R | G | L | K | R | L | R | N | L | Y | L | D | G | N | N | L | K | Q | I | D | S | I | E | F | A | P | F | Q | N | T | L | E | V | L | D | L | L | K | N | R | I | R | F | T | H | E | X | X | F | V | S | P | F | V | N | L | S | R | L | R | D | L | K | L | D | G | Q | Q | P | F | G | I | T | R | L | P | H | G | F | F | R | G | L | H | S | L | R | N | L | Y | L | T | N | S | R | I | D | H | L | A | S | D | T | F | D | D | L | T | G | L | A | Y | L | T | L | D | N | S | C | N | G | L | V | H | L | E | R | G | S | F | K | G | L | R | N | L | K | R | L | S | L | Q | N | M | G | L | Q | N | L | S | K | E | V | F | G | H | L | A | K | L | Q | G | L | Y | L | T | H | N | V | M | Q | H | I | D | V | E | V | L | N | S | L | S | S | L | S | Y | L | D | I | R | K | I | P | L | S | C | T | C | Q | N | L | E | L | Q | N | W | T | L | N | N | Y | K | V | Q | L | I | Y | L | H | S | L | R | C | K | E | S | I | G | N | F | F | F | N | F | D | T | K | V | C | F | V | D | L | E | K | Y | M | F | F | V | T | A | A | V | I | F | V | S | I | V | V | P | L | L | H | F | K | L | Y | W | K | M | K | Y | G | Y | Y | V | F | R | A | W | F | S | E | Q | W | R | K | L | R | E | D | E | E | N | C | E | Y | D | A | F | I | S | Y | N | F | N | N | E | A | W | V | L | E | Q | L | L | P | N | L | E | G | N | G | S | S | F | K | L | C | L | H | H | R | D | F | A | P | G | R | N | I | V | D | N | I | V | S | A | V | Y | G | S | R | K | T | L | C | V | V | S | R | N | F | L | Q | S | E | W | C | S | L | E | I | Q | L | A | S | Y | R | L | F | D | E | H | R | D | V | L | L | L | V | F | L | E | H | I | P | E | R | E | L | S | S | Y | H | R | M | R | K | V | M | L | R | K | T | Y | L | Q | W | P | G | S | E | C | T | D | P | A | M | A | Q | L | L | F | W | K | Q | L | R | R | A | L | R | T | G | S | R | M | E | D | E | D | A | A | G | E | K | K | S | S | G | T | A | E | G | D | Q | T | D | S | A | R | A | N | S | X | X | X | X | X | X | X | X | X | X | X | X | X | X | X | X | X | X | X | X | - | - | - | - | - | - | - | - | - | - |
| DRTLR21\_3196\_BP | M | X | X | X | X | X | X | X | X | X | X | X | X | X | X | X | X | X | X | X | X | X | X | X | X | X | X | X | X | X | X | X | X | X | X | X | X | X | X | X | X | X | X | X | X | X | X | X | X | X | X | X | X | X | X | X | X | X | X | X | X | X | X | X | X | X | H | S | A | C | H | K | L | I | L | K | A | T | F | I | C | L | I | K | L | A | C | S | Y | S | F | R | S | C | I | E | I | P | D | S | N | H | T | I | F | T | C | V | K | S | Y | E | R | D | I | T | A | I | V | S | D | V | F | P | T | A | L | N | L | T | I | S | H | S | N | I | E | Y | V | Q | S | Q | S | F | D | H | L | P | N | L | T | A | L | I | L | D | A | N | H | I | T | N | I | D | K | D | A | F | N | N | L | H | H | L | Q | T | L | N | L | S | C | N | N | I | S | F | L | H | R | D | V | F | S | D | L | H | N | L | T | Q | L | L | L | G | S | N | K | L | T | N | P | D | K | Y | L | F | S | S | L | T | N | L | K | I | L | D | L | R | R | N | H | L | Y | N | F | S | S | V | V | E | S | I | T | N | L | T | S | L | I | K | L | D | L | S | F | N | K | L | T | T | L | Q | H | S | T | X | X | L | P | E | S | L | A | L | L | Y | L | G | D | N | K | L | K | T | L | G | C | D | K | S | F | L | M | N | V | K | K | L | D | L | S | D | N | K | N | L | A | S | K | S | F | L | G | L | K | L | E | N | I | T | Y | L | R | L | R | F | T | N | I | S | I | F | K | L | L | T | Y | P | N | X | X | H | P | W | H | I | D | F | S | G | L | K | L | K | N | S | H | A | L | S | S | L | C | V | L | M | R | N | H | S | H | K | H | L | Q | I | P | K | M | L | L | Q | S | N | S | I | G | A | L | K | N | N | T | F | R | Y | C | P | S | I | T | R | E | M | D | L | S | R | N | E | L | K | S | A | G | C | L | Q | F | L | H | N | Q | T | Q | L | K | S | L | K | M | E | H | N | H | L | T | K | L | T | S | C | N | K | A | X | X | I | H | N | X | X | S | L | T | N | L | S | Y | R | Y | N | R | I | L | Q | I | S | G | Y | A | F | S | N | T | P | K | L | E | I | L | E | L | N | I | N | I | I | A | Y | M | D | H | K | A | L | S | G | L | K | D | L | K | T | L | R | L | D | N | N | L | L | S | D | I | Y | N | D | S | F | E | D | L | T | N | L | K | T | L | N | L | R | N | N | E | I | A | V | I | F | N | Y | T | F | Q | N | L | S | K | L | D | I | L | D | L | G | G | N | K | I | S | Q | L | K | P | H | A | F | D | G | L | Y | S | L | T | N | L | Y | L | D | R | N | H | L | K | M | I | D | V | V | L | F | G | K | L | S | G | T | L | Q | V | L | D | L | Q | G | N | M | I | M | Y | F | T | E | X | X | T | H | S | P | F | K | N | M | S | R | L | Y | D | L | K | L | N | G | Q | Q | P | N | G | I | N | L | L | P | R | A | F | F | R | G | L | T | S | L | K | S | L | Y | L | T | N | N | H | I | I | G | F | G | D | E | T | F | D | D | L | K | N | L | D | Y | L | T | L | D | N | S | C | A | G | V | A | Q | L | Q | S | G | I | F | K | S | L | Q | K | L | R | T | L | S | L | E | N | M | G | I | K | S | F | S | K | D | V | F | G | N | L | T | G | L | K | T | L | H | L | N | R | N | P | M | M | S | L | D | I | S | L | L | D | N | L | T | N | L | I | Y | I | D | M | R | S | C | P | L | S | C | G | C | H | N | S | D | L | Q | N | W | T | I | M | N | K | R | L | Q | F | P | H | L | F | N | I | T | C | P | D | H | P | G | S | Y | F | H | N | F | D | T | K | V | C | Y | L | D | I | E | L | Y | F | F | S | S | T | S | T | L | T | I | L | L | T | L | I | P | L | L | Y | V | K | L | Y | W | K | F | K | Y | G | Y | Y | V | F | R | S | W | F | G | E | Q | W | R | R | L | R | D | Q | E | E | K | Y | N | Y | D | A | F | V | S | Y | N | S | A | D | E | D | W | V | M | E | Q | L | L | P | N | L | E | X | X | X | S | S | F | R | L | C | L | H | H | R | D | F | E | L | G | R | D | I | V | D | N | I | V | A | A | V | Y | G | S | R | K | T | I | C | V | V | S | Q | S | F | L | R | S | E | W | C | S | L | E | I | Q | L | A | S | Y | R | L | F | Q | E | M | Q | D | V | L | L | L | V | F | L | E | P | I | P | E | R | Q | L | S | A | Y | H | R | M | R | K | V | M | L | K | K | T | Y | L | Q | W | P | G | S | N | C | S | D | P | N | S | A | K | E | L | F | W | N | Q | L | K | R | A | L | R | S | S | N | S | G | S | Q | D | X | A | E | D | G | X | Q | X | T | E | E | K | R | - | - | - | - | - | - | E | S | V | C | G | Q | G | G | K | R | I | F | C | K | S | D | T | N | R | R | X | N | I | L | P | D | A | X | X | X | X | - | - | - | - |
| CITLR21\_3196\_BP | M | X | X | X | X | X | X | X | X | X | X | X | X | X | X | X | X | X | X | X | X | X | X | X | X | X | X | X | X | X | X | X | X | X | X | X | X | X | X | X | X | X | X | X | X | X | X | X | X | X | X | X | X | X | X | X | X | X | X | X | X | X | X | X | X | X | D | S | A | C | R | E | L | I | L | K | A | V | F | M | C | L | L | K | L | A | F | S | Y | S | F | R | S | C | T | E | D | A | D | S | N | H | K | I | F | T | C | T | K | C | H | E | P | D | I | A | K | I | V | S | D | V | F | P | T | A | T | N | L | T | V | S | Y | S | N | T | T | Y | V | Q | G | R | S | F | Y | H | L | S | N | L | T | S | L | V | L | D | S | N | Y | I | S | K | I | H | K | D | A | F | N | N | L | Q | Q | L | Q | T | L | N | L | S | C | N | N | I | S | F | L | H | R | D | V | F | S | D | L | H | S | L | T | E | L | I | L | E | S | N | N | L | N | N | T | D | M | F | L | F | S | K | L | T | N | L | T | I | L | N | L | R | R | N | H | L | K | N | F | S | A | L | V | E | C | I | T | N | L | S | S | L | I | K | L | D | L | S | F | N | K | L | T | T | L | N | H | S | H | X | X | L | P | E | S | L | A | S | L | N | L | S | Y | N | N | L | Y | K | L | G | C | D | K | S | F | L | M | Y | V | K | V | L | D | F | S | E | N | K | X | L | S | S | K | T | F | Q | G | L | N | L | E | N | I | T | Y | L | R | L | R | Y | T | G | V | S | A | N | K | L | L | N | Y | T | N | X | X | R | P | W | S | V | D | F | S | G | L | K | L | K | D | S | N | A | L | S | S | L | C | N | L | L | R | H | H | H | H | K | T | Q | H | I | H | N | M | Y | L | Q | A | N | S | I | K | A | L | K | R | N | T | F | D | N | C | P | N | I | T | G | I | M | D | L | S | I | N | D | M | K | S | T | V | C | L | Q | F | L | H | G | Q | Y | Q | L | E | S | L | K | M | E | H | N | H | L | T | R | L | L | P | C | N | K | T | X | X | K | L | Y | X | X | S | L | R | S | L | S | Y | R | Y | N | R | I | L | Q | V | G | G | F | A | F | S | N | T | P | N | L | T | N | L | E | L | N | I | N | I | I | A | Y | M | H | H | K | A | L | H | G | L | K | D | L | V | T | L | R | L | D | N | N | L | L | T | D | V | Y | N | D | S | F | E | D | L | T | S | L | K | T | L | N | L | R | N | N | Q | I | S | V | I | F | N | Y | T | F | H | S | L | S | N | L | N | I | L | D | L | G | G | N | K | I | T | Q | L | K | P | H | A | F | D | G | L | H | S | L | A | N | L | Y | L | D | R | N | R | L | K | E | I | D | S | G | L | F | G | K | L | H | A | T | L | K | V | L | D | L | E | A | N | Q | I | V | Y | F | R | E | X | X | K | F | S | P | F | I | N | M | S | R | L | L | D | L | K | L | S | A | Q | K | P | N | G | I | N | L | L | P | R | A | F | F | R | G | L | T | S | L | K | R | L | Y | L | T | N | N | H | I | I | G | F | G | A | E | T | F | D | D | L | K | S | L | E | L | L | T | L | D | N | S | C | A | G | I | A | Q | L | N | P | G | I | F | K | N | L | R | K | L | N | L | L | S | V | E | N | M | G | I | Q | S | F | S | K | E | V | F | G | N | L | T | G | L | K | T | L | H | L | N | R | N | A | M | T | S | L | D | I | S | L | L | D | N | L | T | N | L | T | Y | I | D | M | R | S | C | P | L | S | C | G | C | Q | N | S | D | L | Q | N | W | T | L | T | N | K | K | L | Q | F | P | F | L | F | N | I | T | C | Q | D | H | P | G | S | Y | F | H | N | F | D | T | N | V | C | Y | L | D | I | E | L | Y | L | F | S | L | T | F | T | F | T | I | L | L | T | L | I | P | F | L | Y | V | R | L | Y | W | K | F | K | Y | G | Y | Y | V | F | R | S | W | F | G | E | Q | W | R | R | L | R | D | Q | E | E | K | Y | K | Y | D | A | F | V | S | Y | N | S | A | D | E | D | W | V | M | E | Q | L | L | P | N | L | E | X | X | X | S | S | F | R | L | C | L | H | H | R | D | F | E | L | G | R | D | I | V | D | N | I | V | A | A | V | Y | G | S | R | K | T | I | C | V | V | S | Q | S | F | L | R | S | E | W | C | S | L | E | I | Q | L | A | S | Y | R | L | F | Q | E | M | Q | D | V | L | L | L | V | F | L | E | P | I | P | K | Q | Q | L | S | T | Y | H | R | M | R | K | V | M | L | K | K | T | Y | L | Q | W | P | G | S | N | C | S | D | P | S | S | A | K | E | L | F | W | N | Q | L | K | R | A | L | R | S | S | N | T | G | N | Q | E | X | A | E | D | G | G | E | X | S | E | E | T | R | - | - | - | - | - | - | E | X | X | X | G | H | E | G | K | R | I | F | C | E | S | D | T | N | R | X | G | R | V | L | P | N | A | X | X | X | X | - | - | - | - |
| TFTLR21\_3196\_BP | M | X | X | X | X | X | X | X | X | X | X | X | X | X | X | X | X | X | X | X | X | X | X | X | X | X | X | X | X | X | X | X | X | X | X | X | X | X | X | X | X | X | X | X | X | X | X | X | X | X | X | X | X | X | X | X | X | X | X | X | X | X | X | X | X | X | R | Y | R | N | Q | A | L | I | S | V | T | I | I | I | S | V | F | Q | L | S | M | S | Y | S | F | M | N | C | I | E | N | A | G | S | N | N | T | T | F | K | C | I | N | R | Q | A | Q | R | V | A | D | F | V | G | D | I | P | Q | S | A | T | N | I | S | I | S | D | C | K | L | D | S | I | L | N | D | S | F | S | N | Q | P | Q | L | R | V | L | L | L | N | N | N | Q | I | K | R | I | D | K | D | A | F | G | N | L | Q | Y | L | H | T | L | N | L | S | S | N | Y | I | V | Y | L | D | S | P | I | F | Q | N | L | Q | N | L | S | T | L | L | L | A | N | N | R | L | T | N | I | S | S | E | L | F | S | S | L | S | K | L | E | I | L | D | L | Q | K | N | Q | L | K | D | F | S | A | V | V | N | S | I | S | N | L | K | V | L | K | K | L | D | I | S | S | N | R | L | I | T | L | L | H | S | A | X | X | L | P | Q | S | L | S | H | L | S | L | G | N | N | E | L | T | T | L | D | C | R | N | D | F | L | S | K | V | K | F | L | D | L | S | Y | N | R | G | L | S | A | Q | A | F | F | G | L | N | L | S | S | L | T | Y | L | G | L | H | F | T | N | I | S | I | I | T | L | L | N | H | T | N | X | X | I | P | W | H | V | N | F | S | G | L | A | L | T | E | P | Q | M | X | I | S | L | C | K | Q | L | S | Y | Y | P | N | K | X | X | X | I | K | K | M | V | L | Q | N | N | E | L | K | D | L | N | D | K | T | L | S | D | C | P | T | I | T | D | F | L | D | L | S | N | N | Q | L | K | S | I | G | C | F | Q | F | L | E | R | Q | K | H | L | A | R | L | R | L | E | H | N | H | F | S | E | L | L | S | C | K | H | G | X | X | K | F | L | X | X | H | L | K | E | L | S | F | R | Y | N | R | I | L | K | V | N | S | F | A | F | S | H | T | P | N | L | T | T | L | Q | L | N | I | N | I | I | A | Y | L | D | R | K | A | L | T | G | L | K | D | L | I | T | L | R | L | D | N | N | L | L | S | D | I | Y | N | E | S | F | E | D | L | Q | S | L | Q | I | L | N | L | R | N | N | Q | I | S | V | I | F | N | N | T | F | Y | S | L | S | Q | L | R | I | L | D | L | G | G | N | K | I | T | Q | L | M | P | L | A | F | V | G | L | D | S | L | N | N | L | Y | L | D | R | N | R | L | K | N | I | D | G | Q | L | I | G | S | L | H | R | T | L | H | V | L | D | L | H | G | N | L | I | H | Y | T | K | E | X | X | V | C | S | P | F | V | N | L | T | K | L | T | D | L | K | L | D | Q | Q | M | P | Y | G | I | T | L | L | P | R | A | F | F | R | G | L | T | S | L | K | S | L | Y | L | S | N | N | H | I | A | F | L | S | T | D | T | F | D | D | L | T | N | L | Q | F | L | T | L | D | D | S | C | V | G | I | T | Q | L | K | P | G | I | F | K | N | L | Q | K | L | E | I | L | M | L | E | N | M | G | I | E | S | F | S | K | E | V | F | G | N | L | T | G | V | K | V | L | H | L | N | R | N | A | M | R | T | L | D | V | D | L | L | K | N | L | T | N | L | Q | Y | L | D | V | R | N | T | P | L | S | C | N | C | P | N | S | E | L | Q | N | W | T | K | T | N | S | R | V | Q | L | I | Y | L | Y | N | L | T | C | P | G | V | N | D | S | N | F | Y | N | F | D | T | N | V | C | X | X | D | F | G | V | Y | F | F | S | S | T | Y | V | V | T | L | L | L | T | L | L | P | L | L | H | V | K | L | Y | W | K | F | K | Y | G | Y | Y | V | F | R | S | W | F | A | E | Q | W | R | R | I | R | E | E | E | E | K | C | T | Y | D | A | F | I | S | Y | N | S | A | D | E | E | W | V | M | E | E | L | L | P | N | L | E | G | S | G | X | X | F | R | V | C | L | H | H | R | D | F | E | P | G | R | N | I | V | D | N | I | V | A | A | V | Y | N | S | R | K | T | V | C | V | V | S | Q | N | F | L | R | S | E | W | C | S | L | E | I | Q | L | A | S | Y | R | L | F | D | E | M | Q | D | V | L | L | L | V | F | L | E | S | I | H | E | R | Q | L | S | A | Y | H | R | M | R | K | V | M | L | K | K | T | Y | L | Q | W | P | G | L | E | C | T | D | P | A | K | A | K | G | L | F | W | K | Q | L | K | R | A | L | R | S | S | N | C | R | G | Q | D | X | R | T | D | G | G | E | C | S | E | R | A | A | - | - | - | - | - | - | X | X | X | C | K | S | R | G | K | R | T | Y | Q | E | P | A | T | N | G | X | X | S | I | L | P | N | A | X | X | X | X | - | - | - | - |
| IPTLR21\_3196\_BP | M | X | X | X | X | X | X | X | X | X | X | X | X | X | X | X | X | X | X | X | X | X | X | X | X | X | X | X | X | X | X | X | X | X | X | X | X | X | X | X | X | X | X | X | X | X | X | X | X | X | X | X | X | X | X | X | X | X | X | X | X | X | X | X | X | X | S | Y | R | N | Q | A | L | I | S | V | S | I | I | A | S | M | F | Q | F | S | M | S | Y | S | F | R | N | C | I | E | S | P | G | S | N | H | T | T | F | K | C | T | K | R | Q | A | Q | T | V | T | A | I | V | S | D | I | P | H | S | A | T | N | I | S | I | S | Y | C | K | L | V | K | I | P | P | Q | S | F | S | H | L | P | K | L | S | D | L | Q | L | N | N | N | H | M | R | N | I | D | N | D | A | F | A | N | L | Q | Y | L | H | T | L | N | L | S | S | N | D | M | V | H | L | N | S | S | I | F | H | D | L | Q | N | L | R | T | L | I | L | A | D | N | R | L | T | N | I | S | S | D | L | F | S | N | L | S | K | L | E | I | L | D | L | R | M | N | Q | L | M | N | F | S | A | V | V | H | S | I | S | N | L | K | G | L | K | K | L | D | I | S | F | N | R | L | I | T | L | L | H | S | A | X | X | L | P | Q | S | L | S | Q | L | Y | L | G | N | N | L | L | T | T | L | E | C | Q | N | D | F | L | S | S | V | Q | V | L | D | L | S | Y | N | K | V | L | T | A | Q | A | F | F | G | L | N | L | S | N | L | E | Y | L | R | L | H | S | T | N | I | S | I | I | M | F | L | N | H | T | N | X | X | M | P | W | H | V | D | F | S | G | L | G | L | R | E | P | Q | M | L | V | S | L | C | K | Q | L | S | Y | Y | P | N | K | X | X | X | I | E | K | M | L | L | Q | S | N | D | L | N | G | L | N | D | K | T | L | S | H | C | P | P | I | T | N | V | L | D | L | S | Y | N | Q | L | K | S | I | S | C | L | Q | F | L | E | R | Q | N | H | L | A | K | L | R | V | E | H | N | H | L | T | H | L | I | S | C | K | N | H | X | X | K | F | P | X | X | H | L | K | E | L | S | Y | R | Y | N | R | I | L | E | V | N | S | F | A | F | S | H | T | P | N | L | T | T | L | Q | L | N | I | N | I | I | A | Y | L | D | R | K | A | L | N | G | L | K | Q | L | V | T | L | R | L | D | N | N | L | L | S | D | I | Y | K | E | S | F | E | N | L | H | S | L | Q | T | L | N | L | R | N | N | Q | I | S | V | I | F | N | N | T | F | Y | S | L | S | K | L | S | I | L | D | L | G | G | N | K | I | T | H | L | M | P | L | A | L | E | G | L | D | S | L | N | N | L | Y | L | D | R | N | H | L | A | E | I | D | G | H | L | I | G | K | L | H | R | T | L | Q | V | L | D | L | Q | G | N | F | I | H | Y | I | T | E | X | X | V | Y | S | P | F | I | T | L | S | K | L | T | D | L | K | L | D | G | Q | M | P | Y | G | I | T | L | L | P | H | A | F | F | R | G | L | T | S | L | K | S | L | Y | L | S | N | N | H | I | S | Y | F | S | T | D | T | F | D | D | L | K | N | L | T | F | L | T | L | D | D | S | C | V | G | V | T | Q | L | K | P | G | I | F | K | N | L | H | K | L | E | T | L | T | V | E | N | M | G | I | G | S | F | S | K | E | V | F | G | N | L | T | G | L | K | V | L | H | L | N | R | N | A | M | Q | T | L | D | V | D | L | L | E | T | L | T | N | L | Q | Y | L | D | V | R | N | T | P | L | S | C | N | C | P | N | S | E | L | Q | N | W | T | K | N | N | Q | R | V | Q | L | I | Y | L | Y | N | L | T | C | P | D | V | K | G | S | N | F | Y | N | F | D | T | H | V | C | X | X | D | F | G | V | Y | W | F | A | S | T | Y | I | V | T | L | L | V | T | L | L | P | L | L | Y | V | K | L | Y | W | K | F | K | Y | G | Y | Y | V | F | R | S | W | F | G | E | Q | W | R | R | L | R | E | E | E | E | N | C | T | Y | D | A | F | I | S | Y | N | S | A | D | E | E | W | V | M | E | Q | L | L | P | N | L | E | G | S | G | X | X | F | R | L | C | L | H | H | R | D | F | E | P | G | R | N | I | I | D | N | I | V | A | A | V | Y | S | S | R | K | T | V | C | V | I | S | R | N | F | L | C | S | E | W | C | S | L | E | I | Q | L | A | S | Y | R | L | F | D | E | M | Q | D | V | L | L | L | V | F | L | E | D | I | H | E | R | Q | L | S | A | Y | H | R | M | R | K | F | M | L | K | K | T | Y | L | Q | W | P | G | L | D | C | T | D | P | I | K | A | Q | E | L | F | W | T | Q | L | K | R | A | L | R | S | S | N | S | R | S | Q | D | X | R | T | N | V | G | E | C | S | G | W | A | A | - | - | - | - | - | - | D | R | X | C | K | S | R | G | K | R | I | F | H | E | P | G | T | N | G | X | W | A | I | L | P | N | T | X | X | X | X | - | - | - | - |
| CCTLR21\_3196\_BP | M | X | X | X | X | X | X | X | X | X | X | X | X | X | X | X | X | X | X | X | X | X | X | X | X | X | X | X | X | X | X | X | X | X | X | X | X | X | X | X | X | X | X | X | X | X | X | X | X | X | X | X | X | X | X | X | X | X | X | X | X | X | X | X | X | X | D | S | A | C | R | E | M | I | I | K | S | I | I | I | C | L | L | K | L | A | F | S | Y | S | F | R | S | C | V | E | I | P | D | S | N | H | K | I | F | R | C | V | K | C | H | E | S | D | I | T | T | I | V | S | D | V | F | P | T | A | T | N | L | T | V | S | R | N | N | I | T | Y | I | Q | G | Q | S | F | H | H | L | S | N | L | T | S | L | V | L | D | A | N | Y | I | V | N | I | H | K | D | A | F | N | N | L | Q | K | L | Q | T | L | N | L | S | W | N | Y | I | S | S | L | H | R | D | V | F | S | D | L | H | H | L | T | K | L | L | L | G | N | N | N | L | T | N | I | D | M | F | L | F | S | N | L | T | N | L | R | I | I | D | L | R | R | N | H | L | K | N | F | S | A | L | V | E | S | I | S | N | L | S | R | L | I | H | L | D | L | S | F | N | K | L | T | T | L | H | H | S | T | X | X | L | P | N | S | L | A | K | L | I | L | S | N | N | N | L | H | K | L | G | C | D | K | S | F | L | K | Y | V | E | V | L | D | L | S | K | N | T | L | L | S | S | K | T | F | Q | G | L | N | L | E | Y | I | K | N | L | H | L | S | F | T | K | V | S | A | G | K | L | L | N | Y | T | N | X | X | Q | P | R | H | I | D | F | S | G | L | N | L | K | D | X | X | A | L | T | L | L | C | F | H | L | H | D | H | X | X | X | X | X | X | I | P | K | M | I | L | Q | S | N | S | I | R | V | L | N | K | N | T | F | C | N | C | A | N | I | T | D | E | M | D | L | S | Q | N | A | M | K | T | T | G | C | L | K | F | L | H | C | Q | K | Q | L | E | T | L | M | I | E | H | N | H | L | T | K | L | K | S | C | K | K | S | X | X | K | V | Y | X | X | S | L | K | S | L | S | Y | R | Y | N | R | I | L | Q | V | N | G | Y | A | F | S | N | T | P | K | L | T | N | L | E | L | N | I | N | I | I | A | Y | L | D | H | K | A | L | S | G | L | K | D | L | V | T | L | R | L | D | N | N | L | L | T | D | I | Y | N | D | T | F | K | D | L | T | S | L | K | T | L | N | L | R | N | N | H | I | A | V | I | F | N | Y | T | F | Q | Y | L | S | N | L | T | I | L | D | L | G | G | N | K | I | T | Q | M | K | P | H | A | F | D | G | L | K | S | L | T | N | L | Y | L | D | R | N | R | L | K | E | I | D | G | M | L | F | G | K | L | H | A | T | L | Q | V | L | D | L | Q | A | N | H | I | M | Y | S | T | E | X | X | T | D | S | P | F | I | N | M | S | R | L | L | D | L | K | L | D | A | Q | E | P | N | G | I | N | L | L | P | H | A | F | F | R | G | L | I | S | L | N | S | L | Y | L | T | K | N | H | I | I | G | F | G | A | E | T | F | D | D | L | K | S | L | E | T | L | T | L | D | N | S | C | V | G | I | A | Q | L | K | P | G | I | F | K | N | L | R | K | L | K | T | L | S | M | V | N | M | G | I | T | S | F | S | K | E | V | F | G | N | V | T | G | L | K | L | L | H | L | N | R | N | A | M | M | S | L | D | I | S | L | L | D | N | L | T | N | L | T | Y | V | D | L | R | S | C | P | L | S | C | G | C | Q | N | S | E | L | Q | N | W | T | L | S | S | K | R | L | Q | F | P | Y | L | F | N | I | T | C | Q | D | H | P | G | S | F | F | H | N | F | D | T | N | V | C | Y | L | D | I | A | L | Y | L | F | S | S | T | F | T | F | T | I | L | L | S | L | I | P | L | L | Y | V | R | L | Y | W | K | F | K | Y | G | Y | Y | V | F | R | S | W | F | G | E | Q | W | R | R | L | K | D | Q | E | E | K | Y | K | Y | D | A | F | I | S | Y | N | S | A | D | E | D | W | V | M | E | Q | L | L | P | N | L | E | X | X | X | S | S | F | R | L | C | L | H | H | R | D | F | E | L | G | R | D | I | V | D | N | I | V | A | A | V | Y | G | S | R | K | T | I | C | V | A | S | Q | S | F | L | R | S | E | W | C | S | L | E | I | Q | L | A | S | Y | R | L | F | Q | E | M | Q | D | V | L | L | L | V | F | L | E | P | I | P | E | R | Q | L | S | T | Y | H | R | M | R | K | V | M | L | K | K | T | Y | L | Q | W | P | G | S | N | C | S | D | P | N | S | A | K | E | L | F | W | N | Q | L | K | R | A | L | R | S | S | N | T | R | S | Q | E | X | A | E | X | X | X | X | X | S | E | E | T | X | X | X | X | X | X | X | X | - | X | X | X | X | X | X | X | X | X | X | X | - | - | - | - | - | R | Q | R | G | A | L | I | T | A | R | S | S | F | - | - | - | - |
| CBTLR21\_3196\_BP | M | X | X | X | X | X | X | X | X | X | X | X | X | X | X | X | X | X | X | X | X | X | X | X | X | X | X | X | X | X | X | X | X | X | X | X | X | X | X | X | X | X | X | X | X | X | X | X | X | X | X | X | X | X | X | X | X | X | X | X | X | X | X | X | X | X | S | Y | R | K | Q | A | L | I | S | M | T | I | I | A | S | I | F | Q | F | G | V | S | Y | S | F | R | N | C | I | E | R | P | G | S | Y | H | R | N | F | T | C | I | K | R | Q | V | Q | T | V | T | A | I | V | G | D | I | P | A | S | A | T | T | L | T | I | S | D | C | T | L | V | N | I | P | P | Q | S | F | S | H | L | P | E | L | R | T | L | L | L | T | N | N | H | M | R | Y | I | H | E | D | A | F | A | N | L | P | Y | L | R | T | L | N | L | S | S | N | Y | I | V | S | L | N | S | S | M | F | H | D | L | Q | N | L | S | K | L | I | L | A | D | N | R | L | K | N | I | S | S | D | S | F | S | S | L | S | K | L | Q | F | L | D | L | R | K | N | E | L | M | N | F | S | A | V | V | Q | S | I | S | N | L | K | E | L | K | N | L | D | I | S | S | N | K | L | V | T | L | Y | H | S | D | X | X | L | P | Q | S | L | S | C | L | Y | L | G | N | N | M | L | T | T | L | Q | C | T | N | D | S | L | S | S | V | K | V | L | D | L | S | F | N | T | R | L | S | N | Q | A | F | F | G | L | N | L | S | N | L | T | Y | L | R | L | R | S | T | N | I | S | I | I | S | L | L | N | Q | T | K | G | M | M | T | W | Y | I | D | F | S | G | L | G | L | T | E | P | Q | M | V | V | S | L | C | K | Q | L | S | Y | Y | P | N | K | X | X | X | I | K | R | I | L | L | Q | K | N | K | L | K | G | L | N | N | N | T | L | S | D | C | P | R | I | T | N | L | L | D | L | S | F | N | E | L | K | S | T | N | C | L | Q | F | L | E | G | Q | T | Y | L | T | K | L | T | V | E | H | N | H | F | T | R | L | X | N | C | K | N | R | X | X | X | F | F | X | X | H | L | K | E | L | S | Y | R | Y | N | R | I | L | E | V | S | P | F | A | F | S | H | T | P | K | L | T | T | L | K | L | N | I | N | I | I | A | Y | L | D | H | K | A | L | N | G | L | K | D | L | I | I | L | R | L | D | N | N | L | L | T | D | L | Y | N | D | S | F | K | D | L | H | S | L | Q | T | L | N | L | R | N | N | Q | I | S | V | I | F | N | N | T | F | Y | F | L | R | N | L | S | I | L | D | L | G | G | N | K | I | A | H | L | M | P | L | A | F | E | G | L | D | S | L | K | N | L | Y | L | D | R | N | R | L | G | K | I | D | G | Q | L | I | G | S | L | H | R | T | L | Q | V | L | D | L | Q | G | N | F | I | R | Y | K | T | D | X | X | A | R | S | P | F | V | N | L | T | K | L | T | D | L | K | L | D | G | Q | R | P | Y | G | I | T | L | L | P | H | A | F | F | R | G | L | T | S | L | K | X | L | Y | L | T | N | N | R | I | S | S | L | S | S | D | T | F | D | D | L | K | N | L | T | F | L | T | L | D | D | S | C | V | G | V | A | Q | L | K | P | G | I | F | K | N | L | Q | K | L | E | I | L | T | A | E | N | M | G | I | G | S | F | S | K | E | V | F | X | N | L | T | G | L | K | K | L | N | L | N | K | N | T | M | Q | T | L | D | V | D | L | L | E | N | L | T | N | L | Q | Y | L | D | V | R | G | T | P | L | S | C | N | C | P | N | S | E | L | Q | N | W | T | K | X | N | E | R | V | Q | I | V | Y | L | Y | N | L | T | C | P | D | V | K | G | S | Y | F | Y | N | F | D | T | H | V | C | X | X | D | F | G | V | Y | W | F | A | S | T | Y | V | V | T | X | X | L | T | L | L | P | L | L | Y | V | K | L | Y | W | K | F | K | Y | G | Y | Y | V | X | R | S | W | F | G | E | Q | W | R | R | L | R | E | E | E | E | K | C | T | Y | D | A | F | I | S | Y | N | S | A | D | E | A | W | V | M | E | Q | L | L | P | N | L | E | G | S | G | X | X | F | R | L | C | L | H | H | R | D | F | E | P | G | R | N | I | V | D | N | I | V | A | A | V | Y | N | S | R | K | T | V | C | V | V | S | Q | N | F | L | R | S | E | W | C | S | L | E | I | Q | L | A | S | Y | R | L | F | D | E | M | Q | D | V | L | L | L | V | F | L | E | Y | I | Y | E | R | Q | L | S | A | Y | H | R | M | R | K | V | M | L | K | K | T | Y | I | Q | W | P | X | V | D | C | T | D | P | N | K | A | R | E | L | F | W | K | Q | L | K | R | A | L | R | S | S | N | S | K | S | Q | D | X | R | T | G | G | G | E | X | A | E | X | A | A | A | X | X | X | X | X | X | - | X | X | X | X | X | X | X | X | X | X | X | - | - | - | - | - | R | X | R | A | I | L | P | N | A | X | X | X | X | - | - | - | - |
| ONTLR21\_3196\_BP | M | X | X | X | X | X | X | X | X | X | X | X | X | X | X | X | X | X | X | X | X | X | X | X | X | X | X | X | X | X | X | X | X | X | X | X | X | X | X | X | X | X | X | X | X | X | X | X | X | X | X | X | X | X | X | X | X | X | X | X | X | X | X | X | X | X | N | L | T | H | X | X | L | L | S | V | T | I | V | L | A | A | V | Q | L | I | S | S | Y | S | F | R | N | C | I | A | E | A | D | S | D | G | K | T | F | N | C | I | H | R | N | A | M | N | I | F | D | I | V | S | D | L | P | P | S | A | I | N | L | T | I | L | R | T | R | L | T | H | L | P | N | N | S | F | S | N | L | A | D | L | Q | H | L | R | I | D | N | N | S | L | T | T | I | S | Q | L | A | F | Q | G | L | Y | Q | L | K | T | L | N | M | S | L | N | K | I | S | E | L | N | P | S | L | F | K | D | L | Q | N | L | T | S | L | F | M | A | N | N | A | L | K | Q | L | P | G | D | I | F | S | S | V | L | K | L | D | T | L | I | L | R | Q | N | S | L | S | D | F | S | G | I | A | K | S | V | A | H | L | T | N | L | T | T | L | D | L | C | F | N | N | L | T | S | L | R | H | S | N | V | S | L | P | Q | S | L | T | V | L | Y | V | C | K | N | N | L | L | T | L | G | C | Q | P | S | F | L | S | F | I | N | I | L | D | L | S | Y | N | S | R | L | P | T | K | A | F | D | Q | I | D | L | S | T | I | N | Y | L | L | L | H | S | T | N | V | T | I | V | E | F | L | D | I | S | N | X | X | N | A | S | H | V | D | F | S | G | T | G | L | N | N | D | S | M | L | K | E | L | C | I | L | L | K | K | K | V | K | E | X | X | N | L | K | N | M | N | L | G | N | N | G | I | R | I | L | T | N | S | T | L | S | Y | C | P | N | I | T | G | A | L | D | L | S | R | N | N | L | N | D | T | Y | C | L | E | F | L | R | R | H | V | Y | I | E | D | F | K | A | E | H | N | H | L | S | F | L | K | Y | C | K | T | N | X | X | M | F | Y | L | K | K | M | E | N | L | S | Y | R | Y | N | R | I | L | N | V | D | P | Y | A | F | Y | N | T | P | N | L | R | T | L | Q | L | N | I | N | S | I | A | F | L | D | Q | H | A | L | K | G | L | K | K | L | E | T | L | R | L | D | N | N | L | L | T | D | L | F | S | V | T | F | E | D | L | V | S | L | Q | I | L | N | L | R | N | N | R | I | S | V | I | F | K | R | T | F | F | N | L | K | F | L | N | T | L | D | L | G | G | N | K | I | T | H | F | Q | Q | S | G | L | D | G | L | K | S | L | S | K | L | Y | L | D | G | N | Q | L | K | H | I | D | P | V | L | Y | H | A | F | Q | D | T | L | T | V | L | D | L | Q | S | N | Q | I | R | Y | V | T | E | X | X | H | E | S | P | F | M | N | L | S | R | L | T | D | L | K | L | D | R | Q | R | P | N | G | L | T | V | L | P | R | T | L | L | R | G | L | Y | S | L | K | S | L | C | L | T | N | N | N | I | Y | H | L | A | P | D | T | F | D | D | L | T | D | L | S | F | L | T | L | D | N | C | C | V | G | V | T | K | L | E | P | G | V | F | K | N | L | R | N | L | S | R | L | A | A | E | N | M | G | I | Q | N | F | S | K | E | V | F | G | N | L | T | Q | L | R | S | L | Q | L | N | R | N | A | M | Q | T | L | S | V | D | A | L | M | G | L | P | M | L | R | Y | L | D | I | R | D | N | P | V | S | C | T | C | N | N | A | P | L | Q | N | Y | T | K | N | N | T | D | V | Q | V | V | Y | L | Y | N | L | S | C | Q | H | N | P | K | L | K | F | H | N | F | D | T | K | V | C | R | G | D | I | G | M | Y | L | F | F | S | T | S | A | A | I | F | L | F | T | I | I | P | L | F | Y | V | K | L | Y | W | K | M | R | Y | S | Y | Y | V | F | R | A | W | F | S | E | Q | W | S | R | L | H | E | E | E | E | N | C | K | Y | D | A | F | I | S | Y | N | S | S | D | E | Q | W | V | M | D | Q | L | L | P | N | L | E | G | G | G | S | S | F | K | L | C | L | H | H | R | D | F | E | L | G | R | D | I | V | D | N | I | V | S | A | V | Y | S | S | R | K | T | I | C | V | V | S | R | N | F | L | Q | S | E | W | C | S | L | E | I | Q | L | A | S | Y | R | L | F | D | E | H | R | D | V | L | L | L | V | F | L | E | P | I | S | E | R | Q | L | S | S | Y | H | R | M | R | K | V | M | L | K | K | T | Y | L | Q | W | P | G | S | D | C | T | N | P | T | Q | A | Q | E | L | F | W | N | Q | L | R | R | A | I | K | T | G | T | R | L | E | T | E | X | X | X | X | X | X | X | C | S | A | T | H | E | S | X | X | X | X | X | X | R | S | X | R | L | X | G | R | X | X | X | I | - | - | - | - | - | X | X | K | L | L | L | A | T | X | X | X | X | - | - | - | - | - |
| ECTLR21\_3196\_BP | M | X | X | X | X | X | X | X | X | X | X | X | X | X | X | X | X | X | X | X | X | X | X | X | X | X | X | X | X | X | X | X | X | X | X | X | X | X | X | X | X | X | X | X | X | X | X | X | X | X | X | X | X | X | X | X | X | X | X | X | X | X | X | X | X | X | S | L | T | Y | X | X | L | L | S | V | T | L | A | L | C | A | V | H | L | I | S | G | Y | S | F | R | N | C | I | E | D | P | Y | P | Y | G | Q | S | F | K | C | I | L | R | K | E | S | N | L | S | A | L | I | G | D | L | P | Q | T | A | V | N | L | T | I | T | I | N | P | V | W | H | I | P | S | M | S | F | V | N | V | T | N | L | E | Y | L | R | L | D | H | N | Y | L | R | K | I | D | P | F | A | F | Q | N | L | T | R | L | K | S | L | N | V | S | S | N | N | I | S | Q | L | N | P | Y | V | F | K | D | L | H | N | L | T | Y | L | S | L | T | N | N | S | L | K | Q | L | P | V | G | I | F | S | T | L | L | N | L | D | T | L | I | M | R | Q | N | L | L | T | N | F | S | G | I | A | E | S | V | S | H | L | P | K | L | R | V | L | D | L | C | F | N | N | L | T | N | L | K | H | S | N | A | S | L | P | K | S | L | T | T | L | Y | I | C | R | N | N | L | L | T | L | G | C | H | Q | S | F | L | G | F | I | Q | L | L | D | L | S | Y | N | P | R | L | P | T | M | A | F | Q | G | V | D | L | S | H | I | N | Y | L | R | L | R | S | T | S | V | K | V | V | D | F | L | N | I | S | N | X | X | N | A | G | S | V | D | F | S | G | M | G | L | K | N | D | I | L | L | T | E | L | C | T | S | L | K | G | K | V | K | S | X | X | X | X | K | R | M | G | L | S | N | N | G | I | K | N | L | T | N | N | A | L | Q | Y | C | P | T | I | T | G | S | L | D | L | S | R | N | N | L | K | S | T | G | C | F | K | F | L | D | K | H | T | Q | I | K | S | L | N | A | E | H | N | H | I | T | S | L | Q | S | C | K | T | Q | X | X | M | V | Y | L | N | H | L | E | E | L | S | Y | R | Y | N | R | I | L | S | V | N | A | Y | A | F | S | H | T | P | N | I | K | T | L | K | L | N | I | N | T | I | S | F | L | H | R | K | A | L | K | G | L | K | S | L | E | M | L | R | L | D | N | N | L | L | T | D | L | F | N | D | T | F | E | D | N | V | N | L | Q | T | L | N | L | R | N | N | R | I | A | V | I | F | N | G | T | F | L | S | L | R | N | L | T | T | L | D | L | G | G | N | K | I | T | H | F | E | Q | S | G | L | D | G | L | T | S | L | S | K | F | Y | L | D | G | N | N | L | K | E | I | D | T | S | L | Y | R | V | F | Q | D | T | L | T | V | L | D | L | K | S | N | Q | I | Y | F | F | H | K | X | X | T | S | S | P | F | M | N | L | S | K | L | T | D | L | K | L | D | G | Q | R | P | H | G | L | S | V | L | P | Q | N | F | F | R | G | L | H | S | L | K | S | L | Y | L | T | N | N | N | I | Y | Y | L | A | P | D | A | F | D | D | L | T | N | L | T | F | L | S | L | E | G | C | C | V | G | V | A | Q | L | Q | P | G | I | F | K | N | L | R | N | L | N | I | L | S | M | E | N | M | G | I | Q | T | F | S | K | E | V | F | G | N | L | T | K | L | H | K | L | Q | L | N | R | N | V | M | Q | S | I | H | Y | E | I | L | D | S | L | P | E | L | H | Y | L | D | L | R | D | T | P | L | S | C | T | C | K | N | H | L | L | Q | N | W | T | L | H | N | R | R | V | Q | V | V | Y | L | Y | N | M | K | C | Q | D | D | N | Q | H | N | F | Y | N | F | K | T | N | V | C | Y | I | D | L | G | E | Y | L | F | L | S | T | A | I | V | I | F | L | F | T | V | T | P | L | L | Y | V | K | L | Y | W | K | M | K | Y | G | Y | Y | V | F | R | S | W | F | S | D | Q | W | R | R | L | R | E | Q | E | E | N | C | K | Y | D | A | F | I | S | Y | N | S | S | D | E | Q | W | V | I | E | Q | L | M | P | N | L | E | G | N | G | S | S | F | K | L | C | L | H | H | R | D | F | E | L | G | R | D | I | V | D | N | I | V | S | A | V | Y | S | S | R | K | T | I | C | V | V | S | R | N | F | L | T | S | E | W | C | S | L | E | I | Q | L | A | S | Y | R | L | F | D | E | H | R | D | V | L | L | L | V | F | L | E | P | I | S | E | R | Q | L | S | S | Y | H | R | M | R | K | V | M | L | K | K | T | Y | L | Q | W | P | G | S | D | C | T | N | P | T | Q | A | Q | E | L | F | W | N | Q | L | R | R | A | M | R | T | G | S | R | F | E | T | E | X | X | N | X | C | S | E | G | R | E | K | E | G | R | X | X | X | X | X | X | R | T | C | X | C | S | X | X | X | X | X | R | - | - | - | - | - | R | X | K | L | L | L | A | S | X | X | X | X | - | - | - | - | - |
| SMTLR21\_3196\_BP | M | X | X | X | X | X | X | X | X | X | X | X | X | X | X | X | X | X | X | X | X | X | X | X | X | X | X | X | X | X | X | X | X | X | X | X | X | X | X | X | X | X | X | X | X | X | X | X | X | X | X | X | X | X | X | X | X | X | X | X | X | X | X | X | X | X | G | L | T | H | X | X | F | L | S | V | T | V | V | L | G | A | V | Q | L | I | N | G | Y | S | F | N | N | C | I | L | D | V | N | S | N | R | T | H | F | N | C | I | N | L | K | D | K | N | M | T | A | I | I | H | D | L | P | R | S | V | T | S | L | T | I | S | K | N | Q | V | W | H | I | P | N | N | S | F | A | H | L | P | W | M | Q | N | L | R | L | D | H | N | R | L | R | I | I | D | Q | L | A | F | Q | N | L | H | Q | L | Q | S | L | N | L | S | F | N | N | I | S | V | L | H | P | S | L | F | V | D | L | H | N | L | T | F | L | S | L | T | D | N | T | L | K | Q | L | P | G | G | I | F | S | A | T | L | N | L | N | T | L | N | M | S | Q | N | L | L | T | N | F | S | G | I | T | E | S | L | S | H | L | R | N | L | K | K | L | D | L | C | F | N | S | L | T | S | L | X | X | S | N | V | S | L | P | T | S | L | T | A | I | Y | L | C | R | N | N | L | S | T | L | G | C | E | H | S | F | L | G | L | I | E | V | L | D | L | S | Y | N | S | R | L | P | T | M | A | F | E | G | V | D | L | G | R | I | N | Y | L | R | L | R | S | T | S | V | K | V | V | A | F | L | N | I | S | N | X | X | N | A | G | S | V | D | F | S | G | T | G | L | K | N | D | R | L | L | I | E | L | C | R | L | L | K | T | K | V | K | W | X | X | X | X | K | N | L | H | L | G | I | N | G | I | K | N | L | T | T | D | T | L | N | Y | C | P | N | I | T | G | Y | L | D | L | S | H | N | E | L | K | F | T | N | C | L | H | F | L | K | R | Q | T | Y | I | K | G | F | N | A | E | H | N | H | L | T | T | L | Q | S | C | K | T | Q | T | Q | N | T | A | F | K | Y | L | E | E | L | T | Y | R | Y | N | R | I | L | S | V | S | S | H | A | F | Y | H | M | Q | N | I | K | T | L | Q | L | N | I | N | T | I | A | F | L | H | H | E | A | L | K | G | L | T | K | L | E | T | L | R | L | D | N | N | L | L | T | D | L | F | N | D | T | F | E | D | N | V | K | L | Q | T | L | N | L | R | N | N | R | I | A | V | I | F | N | G | T | F | H | R | L | G | K | L | I | T | L | D | L | G | G | N | K | I | T | H | F | Q | P | S | G | L | D | G | L | K | R | L | S | K | F | Y | L | D | G | N | N | L | K | Q | I | D | S | S | L | Y | R | V | F | Q | G | T | L | T | V | L | D | L | Q | S | N | M | I | R | F | F | N | G | T | E | I | M | S | P | F | Q | N | L | S | A | L | R | D | L | K | L | D | G | Q | R | P | H | G | L | T | L | L | P | R | A | F | F | R | G | L | H | S | L | K | S | L | Y | L | T | Q | N | N | L | I | A | L | A | A | D | A | F | D | D | L | T | G | L | R | F | L | T | L | D | S | C | C | V | G | V | T | K | L | Q | P | G | V | F | K | N | L | R | N | L | T | I | L | I | V | E | N | M | G | I | Q | N | F | S | K | E | V | F | G | N | L | T | R | L | S | V | L | H | L | N | R | N | I | M | Q | S | I | N | V | D | A | L | Q | N | L | T | K | L | Q | Y | L | D | I | R | G | I | P | L | T | C | T | C | Q | N | S | L | L | Q | N | W | T | R | N | N | P | K | V | Q | V | V | L | L | Y | N | V | P | C | Q | H | D | A | K | F | K | F | Y | N | F | D | S | K | V | C | Y | I | D | L | G | K | Y | L | F | L | S | T | A | V | V | I | F | L | F | T | L | S | P | I | L | Y | V | K | L | Y | W | K | M | K | Y | S | Y | Y | V | F | R | S | W | F | G | E | K | W | R | R | L | R | E | E | E | E | N | C | E | Y | D | A | F | I | S | Y | N | S | F | D | E | Q | W | V | M | E | Q | L | I | P | N | L | E | G | N | G | S | S | F | K | L | C | L | H | H | R | D | F | E | L | G | R | N | I | V | D | N | I | V | S | A | V | Y | S | S | R | K | T | I | C | V | V | S | R | N | F | L | Q | S | E | W | C | S | L | E | I | Q | L | A | S | Y | R | L | F | D | E | H | R | D | V | L | L | L | V | F | L | E | P | I | S | E | R | Q | L | S | S | Y | H | R | M | R | K | V | M | L | K | K | T | Y | L | Q | W | P | G | S | D | C | T | N | P | M | Q | A | Q | D | L | F | W | N | H | L | R | R | A | M | T | M | G | S | R | L | K | T | E | X | X | S | L | K | C | D | V | C | V | P | A | E | S | X | X | X | X | X | X | R | N | Q | T | F | X | X | X | X | X | X | I | - | - | - | - | - | R | X | K | R | L | L | A | A | K | R | K | X | - | - | - | - | - |
| MMTLR21\_3196\_BP | M | X | X | X | X | X | X | X | X | X | X | X | X | X | X | X | X | X | X | X | X | X | X | X | X | X | X | X | X | X | X | X | X | X | X | X | X | X | X | X | X | X | X | X | X | X | X | X | X | X | X | X | X | X | X | X | X | X | X | X | X | X | X | X | X | X | S | L | I | H | X | X | L | L | S | V | A | V | V | L | D | A | V | Q | L | V | S | G | Y | S | F | N | N | C | I | E | D | Q | N | X | K | E | K | T | F | K | C | I | E | R | R | T | K | N | I | S | A | I | V | D | D | L | P | S | S | T | I | N | L | S | I | S | F | S | R | V | S | H | I | P | N | M | A | F | V | K | L | P | N | L | Q | S | L | R | L | D | H | N | P | L | T | T | I | D | P | F | A | F | Q | N | L | T | Q | L | T | H | L | N | L | T | S | N | N | L | S | E | L | N | P | S | L | F | K | D | L | H | S | L | I | F | L | S | L | V | N | N | T | L | R | Q | L | P | E | G | I | F | L | T | V | P | N | L | D | T | L | L | I | R | Q | N | F | L | T | N | F | S | E | I | A | E | S | V | S | N | L | T | R | L | R | I | L | D | L | C | F | N | N | F | T | S | L | S | H | S | N | V | S | L | P | Q | N | L | T | T | L | Y | I | C | R | N | N | L | S | T | L | G | C | N | H | L | F | F | S | F | I | Q | L | L | D | L | S | Y | N | P | R | L | P | T | E | A | F | Q | G | V | D | L | R | N | V | K | Y | L | R | L | R | S | T | S | V | K | I | I | E | F | L | N | M | S | N | X | X | Y | A | G | H | V | D | F | S | N | T | G | L | K | N | D | S | L | L | M | G | L | C | R | L | L | K | R | K | V | K | V | X | X | X | X | K | K | L | N | L | L | S | N | E | I | Q | N | I | T | N | D | A | L | R | Y | C | P | K | I | K | V | A | L | D | L | S | R | N | E | L | K | S | V | N | C | L | N | F | L | K | N | Q | I | D | I | K | N | F | S | A | E | H | N | H | L | T | S | L | P | K | C | K | T | Q | X | X | M | V | Y | F | K | K | L | E | E | V | S | Y | R | Y | N | R | I | L | S | V | G | S | H | A | F | Y | H | T | P | N | I | K | T | L | K | L | N | I | N | T | I | A | Y | L | D | L | E | A | F | K | G | L | K | E | L | K | T | L | R | L | D | N | N | L | L | T | D | L | F | K | K | T | F | E | D | L | Y | S | L | Q | V | L | N | L | R | N | N | R | I | S | V | I | F | N | E | T | F | F | H | L | R | N | L | T | I | L | D | L | G | G | N | K | I | T | Y | F | Q | P | S | G | L | N | G | L | K | R | L | S | K | F | Y | L | D | G | N | N | L | K | T | I | D | T | S | H | Y | R | A | F | Q | N | T | L | T | V | L | D | L | K | G | N | Q | I | C | F | R | T | G | K | F | T | F | S | P | F | V | N | F | S | K | L | S | D | L | K | L | D | A | Q | Q | P | H | G | L | T | T | I | P | R | N | F | F | R | G | L | Y | S | L | K | S | L | Y | L | T | N | N | K | I | F | H | L | P | P | D | A | F | D | D | L | T | E | L | R | F | L | S | L | D | D | C | C | V | G | V | T | Q | L | E | P | G | V | F | K | N | L | R | N | L | T | K | L | A | V | E | N | I | G | I | Q | N | F | T | K | E | V | F | G | N | L | T | K | L | R | T | L | Q | L | N | H | N | V | M | Q | S | I | D | V | D | A | L | Q | S | L | T | K | L | H | Y | L | D | I | R | N | I | P | L | S | C | T | C | E | N | T | L | L | R | N | W | T | V | H | N | P | K | V | Q | V | V | Y | L | Y | S | L | S | C | P | H | N | P | G | H | K | F | Y | N | F | D | S | K | V | C | F | I | D | L | G | E | Y | L | F | L | S | T | A | I | V | I | F | L | F | T | V | T | P | L | L | Y | V | K | L | Y | W | K | M | K | Y | G | Y | Y | V | F | R | S | W | F | S | E | Q | W | R | R | L | R | D | E | E | E | N | C | K | Y | D | A | F | I | S | Y | N | S | S | D | E | Q | W | I | M | D | E | L | L | P | N | L | E | G | N | G | S | S | F | K | L | C | L | H | H | R | D | F | E | L | G | R | D | I | V | D | N | I | V | S | A | V | Y | G | S | R | K | T | I | C | V | V | S | R | N | F | L | K | S | E | W | C | S | L | E | I | Q | L | A | S | Y | R | L | F | D | E | H | R | D | V | L | L | L | V | F | L | E | P | I | S | E | R | H | L | S | S | Y | H | R | M | R | K | I | M | L | K | K | T | Y | L | Q | W | P | G | S | D | C | T | D | P | T | Q | A | Q | D | L | F | W | N | Q | L | R | R | A | I | R | T | G | S | R | L | E | T | V | X | X | T | Y | K | S | D | K | C | V | T | X | X | X | X | X | X | X | X | X | R | N | X | R | S | X | X | X | X | X | X | V | - | - | - | - | - | R | X | K | L | L | L | A | T | X | X | X | X | - | - | - | - | - |
| SLTLR21\_13\_3196\_BP | M | X | X | X | X | X | X | X | X | X | X | X | X | X | X | X | X | X | X | X | X | X | X | X | X | X | X | X | X | X | X | X | X | X | X | X | X | X | X | X | X | X | X | X | X | X | X | X | X | X | X | X | X | X | X | X | X | X | X | X | X | X | X | X | X | X | G | L | L | H | X | X | L | L | S | V | T | V | V | L | G | A | V | Q | L | I | T | G | Y | S | F | K | N | C | I | A | D | P | R | S | N | G | S | L | F | N | C | I | S | R | K | E | E | K | M | S | A | I | L | H | D | L | P | T | S | A | I | H | L | T | I | S | K | T | H | V | K | H | I | C | N | Y | S | F | V | H | L | P | K | L | K | Y | L | R | I | D | D | N | P | V | N | T | I | D | Q | S | A | F | Q | N | L | H | Q | L | Q | S | L | N | L | S | V | N | N | I | S | E | L | N | P | Y | L | F | K | E | L | H | N | L | T | F | L | L | L | S | D | N | K | L | K | Q | L | P | E | G | I | F | S | A | T | L | N | L | N | T | L | N | M | R | Q | N | L | L | T | N | F | S | E | I | A | E | S | V | S | H | L | M | N | L | K | V | L | D | L | C | Y | N | S | L | T | S | L | S | H | S | N | V | S | L | P | K | S | L | T | T | L | Y | I | C | R | N | N | L | F | T | L | G | C | K | D | S | F | L | T | F | I | N | I | L | D | L | S | Y | N | S | R | L | P | T | T | A | F | Q | G | V | D | L | R | R | I | N | Y | L | R | L | R | S | T | N | V | S | V | V | E | F | L | N | I | S | N | X | X | K | V | S | Q | V | D | F | S | G | L | G | L | K | N | D | S | L | L | M | E | L | C | K | L | L | M | R | K | V | K | W | X | X | X | X | K | N | L | N | L | S | D | N | G | I | G | S | L | T | N | Y | T | L | Y | Y | C | P | N | I | T | G | D | L | D | L | S | R | N | K | L | K | S | T | N | C | L | S | F | L | R | R | Q | A | Q | I | K | S | F | N | A | E | H | N | H | L | T | S | L | P | S | C | N | K | Q | X | X | K | V | C | L | K | S | L | E | E | L | S | Y | R | Y | N | R | I | L | S | V | N | S | F | A | F | F | H | T | P | N | I | K | T | L | Q | L | N | I | N | T | I | A | F | L | D | R | K | A | L | K | G | L | K | K | L | V | K | L | R | L | D | N | N | L | L | T | D | L | F | N | E | T | F | E | D | N | N | K | L | Q | V | L | N | L | R | N | N | R | I | S | V | I | F | N | G | T | F | L | M | L | R | N | L | T | T | L | D | L | G | G | N | K | I | T | H | F | Q | P | S | G | F | D | G | L | K | S | L | S | N | L | Y | L | D | G | N | N | L | K | H | I | D | T | S | I | Y | R | V | F | Q | D | T | L | T | V | L | D | L | Q | S | N | K | I | R | F | L | T | E | X | X | N | S | S | P | F | M | N | L | S | K | L | S | D | L | K | L | D | G | Q | R | P | Y | G | M | T | V | L | P | H | T | F | F | R | G | L | Y | S | L | K | S | L | Y | L | T | N | N | N | I | F | H | L | P | P | D | V | F | D | D | L | T | S | L | Q | F | L | T | L | D | K | C | C | V | G | V | T | Q | L | V | P | G | V | F | K | N | L | R | N | L | T | K | L | T | V | E | S | M | G | I | Q | N | F | S | K | E | V | F | G | N | L | I | Q | L | S | S | L | Q | L | N | R | N | V | M | Q | S | I | H | V | D | A | L | Q | S | L | P | K | L | R | Y | L | D | I | R | N | I | P | L | S | C | T | C | K | N | S | L | L | Q | N | W | T | I | Y | N | K | N | V | Q | V | V | Y | L | Y | N | L | Q | C | Q | N | D | A | N | Y | K | F | H | N | F | K | T | N | V | C | Y | I | D | L | G | E | Y | L | F | F | S | T | A | V | V | I | F | L | F | T | V | T | P | L | L | Y | V | K | L | Y | W | K | M | K | Y | S | Y | Y | V | F | R | S | W | F | S | D | Q | W | R | R | L | R | E | E | E | E | N | C | K | Y | D | A | F | I | S | Y | N | S | S | D | D | Q | W | V | M | D | Q | L | L | P | N | L | E | G | N | G | S | S | F | K | L | C | L | H | H | R | D | F | E | L | G | R | D | I | V | D | N | I | V | S | A | V | Y | S | S | R | K | T | I | C | V | V | S | R | N | F | L | Q | S | E | W | C | S | L | E | I | Q | L | A | S | Y | R | L | F | D | E | H | R | D | V | L | L | L | V | F | L | E | P | I | S | E | R | Q | L | S | A | Y | H | R | M | R | K | V | M | L | K | K | T | Y | L | Q | W | P | D | S | D | C | T | N | P | M | Q | A | Q | E | L | F | W | N | Q | L | R | R | A | M | R | M | G | S | R | L | K | M | E | X | X | N | I | N | N | E | G | C | V | N | A | E | S | X | X | X | X | X | X | R | N | X | T | F | X | X | X | X | X | X | I | - | - | - | - | - | R | X | R | I | L | L | A | T | X | X | X | X | - | - | - | - | - |
| TOTLR21\_3196\_BP | M | X | X | X | X | X | X | X | X | X | X | X | X | X | X | X | X | X | X | X | X | X | X | X | X | X | X | X | X | X | X | X | X | X | X | X | X | X | X | X | X | X | X | X | X | X | X | X | X | X | X | X | X | X | X | X | X | X | X | X | X | X | X | X | X | X | G | Q | I | H | X | X | L | L | S | V | T | F | A | L | G | A | V | Q | L | I | S | G | C | S | F | K | N | C | I | A | D | P | Y | S | N | G | T | I | Y | K | C | I | S | R | K | E | E | N | L | A | A | I | V | N | D | V | P | T | S | V | I | N | L | S | I | S | V | T | Y | V | T | H | I | R | N | Y | S | F | V | H | L | P | N | L | Q | Y | L | R | I | D | H | N | N | L | S | T | I | D | P | S | A | F | E | N | L | H | Q | L | Q | S | L | N | L | S | F | N | Y | I | S | E | L | H | S | S | L | F | K | D | L | H | N | L | T | F | L | S | L | T | N | N | K | L | K | Q | L | P | E | G | I | F | S | T | T | L | N | L | N | T | L | I | M | R | Q | N | H | L | T | N | F | S | G | I | A | E | S | V | S | H | L | M | N | L | K | V | L | D | L | C | F | N | S | L | T | S | L | N | H | S | N | V | S | L | P | E | S | L | T | T | L | Y | I | C | R | N | N | L | S | T | L | G | C | K | N | S | F | L | R | F | I | N | L | L | D | L | S | Y | N | S | R | L | P | A | M | A | F | Q | D | V | D | L | R | H | I | N | Y | L | R | L | R | S | T | D | V | R | V | V | E | F | L | N | I | S | N | X | X | N | A | G | H | V | D | F | S | G | T | G | L | K | N | D | N | L | L | M | E | L | C | R | L | L | K | R | K | V | K | W | X | X | X | X | K | N | L | H | L | S | D | N | G | I | E | N | L | T | N | Y | A | L | Y | D | C | P | K | I | T | G | H | L | D | L | S | R | N | K | L | K | T | T | S | C | L | N | F | L | K | E | Q | T | H | I | K | S | F | N | A | E | H | N | H | L | T | S | L | Q | S | C | K | T | Q | X | X | X | X | X | F | K | N | L | E | E | A | S | Y | R | Y | N | R | I | L | S | V | N | A | F | A | F | Q | H | M | P | N | I | Q | R | L | Q | L | N | I | N | T | I | A | F | L | H | R | K | A | L | K | G | L | K | R | L | V | T | L | R | L | D | N | N | L | L | T | D | L | F | N | E | T | F | E | D | N | F | K | L | Q | V | L | N | L | R | N | N | R | I | S | V | I | F | N | G | T | F | L | R | L | K | N | L | S | I | L | D | L | G | G | N | K | I | T | Y | F | Q | P | S | G | L | D | G | L | K | N | L | S | K | L | Y | L | D | G | N | N | L | K | Q | I | D | T | S | I | Y | H | A | F | Q | D | T | L | T | V | L | D | L | Q | S | N | Q | I | H | F | L | T | D | X | X | N | L | S | P | F | M | N | L | S | K | L | S | D | L | K | L | D | G | Q | R | P | Y | G | F | T | V | L | P | H | N | F | F | R | G | L | Y | S | L | K | S | L | Y | L | T | N | N | N | I | Y | H | L | A | P | D | V | F | D | D | L | T | S | L | R | F | L | T | L | D | R | C | C | V | G | V | A | H | L | E | P | G | V | F | K | N | L | R | N | L | K | K | L | T | I | E | S | M | G | I | Q | N | F | S | K | D | V | F | G | N | L | T | Q | L | Q | S | L | Q | L | N | R | N | V | M | Q | S | I | H | V | D | A | L | Q | S | L | P | K | L | L | Y | L | D | I | R | N | I | P | L | S | Y | T | C | Q | N | S | L | L | Q | N | W | T | K | N | N | P | D | V | Q | V | V | Y | L | Y | D | L | M | C | Q | H | D | A | K | F | K | F | Y | N | F | D | T | K | V | C | Y | I | D | L | G | E | Y | L | F | F | S | T | A | V | V | I | F | L | F | T | V | T | P | L | L | Y | V | K | L | Y | W | T | M | K | Y | S | Y | Y | V | F | R | S | W | F | S | N | Q | W | R | R | L | R | E | E | E | E | N | C | K | Y | G | A | F | I | S | Y | N | S | S | D | E | Q | W | V | M | N | H | L | L | P | N | L | E | E | N | G | S | S | F | K | L | C | L | H | H | R | D | F | E | L | G | R | D | I | V | D | N | I | V | S | A | V | Y | S | S | R | K | S | I | C | V | V | S | R | N | F | L | Q | S | E | W | C | S | L | E | I | Q | L | A | S | Y | R | L | F | D | E | H | R | D | V | L | L | L | V | F | L | E | P | I | F | E | R | Q | L | S | S | Y | H | R | M | R | K | V | M | L | K | K | T | Y | L | Q | W | P | D | P | D | C | T | D | P | M | Q | A | Q | E | L | F | W | N | Q | L | R | R | A | M | R | T | G | S | K | L | E | T | E | X | X | N | A | E | I | E | G | C | V | T | A | E | X | X | X | X | X | X | X | X | X | X | T | I | Q | V | - | - | - | - | - | - | - | - | - | - | X | X | X | X | A | V | G | R | G | S | F | N | I | X | X | X | X |
| OFTLR21\_3196\_BP | M | X | X | X | X | X | X | X | X | X | X | X | X | X | X | X | X | X | X | X | X | X | X | X | X | X | X | X | X | X | X | X | X | X | X | X | X | X | X | X | X | X | X | X | X | X | X | X | X | X | X | X | X | X | X | X | X | X | X | X | X | X | X | X | X | X | S | L | P | F | X | X | L | L | S | V | T | V | V | L | G | A | A | Q | L | I | S | G | Y | S | F | N | N | C | I | E | D | P | Y | S | K | G | X | S | F | N | C | I | N | R | K | E | K | K | I | S | A | V | I | N | D | L | P | P | S | A | I | N | L | T | I | S | K | N | H | L | W | H | I | P | S | K | C | F | A | H | L | P | N | L | R | H | L | R | I | D | D | N | K | L | S | I | I | E | Q | S | A | F | Q | N | L | H | Q | L | Q | C | L | N | L | S | I | N | N | I | S | E | L | K | P | S | L | F | A | D | L | H | N | L | N | F | L | L | L | T | D | N | K | L | K | K | L | P | E | G | I | F | S | T | V | L | K | L | N | T | L | I | M | R | Q | N | L | L | T | N | F | S | G | I | A | K | S | V | S | H | L | T | K | L | R | K | L | D | L | C | S | N | N | L | T | S | L | S | H | S | N | V | S | L | P | K | S | L | T | T | L | Y | I | C | R | N | N | L | F | T | L | G | C | E | H | S | F | L | R | F | I | Q | L | L | D | L | S | Y | N | S | R | L | P | T | T | A | F | Q | G | V | D | L | R | H | I | N | Y | L | R | L | R | S | T | S | V | K | V | V | E | L | L | N | I | S | N | X | X | N | A | E | Y | V | D | F | S | G | T | G | L | K | N | D | T | L | L | M | E | L | C | K | L | L | K | K | K | V | K | W | X | X | X | X | K | N | M | G | L | S | S | N | W | I | E | N | L | A | N | N | T | L | N | Y | C | P | K | I | T | G | T | L | D | L | S | H | N | Q | L | K | Y | T | N | C | L | N | F | L | H | G | Q | T | H | I | Q | N | F | S | A | E | H | N | H | L | T | S | L | P | S | C | K | T | Q | X | X | M | V | Y | L | K | N | L | E | E | L | S | Y | R | Y | N | R | I | L | S | V | N | S | Y | A | F | Y | H | T | P | N | I | K | T | L | Q | L | N | I | N | T | I | A | F | L | H | R | K | A | L | K | G | L | K | R | L | K | T | L | R | L | D | N | N | L | L | T | D | L | F | N | N | T | F | E | D | N | L | N | L | Q | I | L | N | L | R | N | N | R | I | S | V | I | F | N | G | T | F | L | S | L | R | N | L | T | I | L | D | L | G | G | N | K | I | T | H | F | Q | P | S | G | L | D | G | L | K | S | L | S | K | F | Y | L | D | G | N | N | L | R | Q | I | D | T | S | L | Y | R | V | F | Q | D | T | L | T | V | L | D | L | Q | S | N | Q | I | R | F | F | T | E | X | X | I | I | S | P | F | M | N | L | S | K | L | S | D | L | K | L | D | G | Q | Q | P | Y | G | I | T | L | L | P | R | N | F | F | R | G | L | Y | S | L | K | F | L | Y | L | T | N | N | K | I | S | H | L | A | P | D | T | F | D | D | L | T | G | L | Q | F | L | T | L | D | K | C | C | A | G | V | T | Q | L | E | P | G | V | F | K | N | L | R | N | L | N | R | L | I | V | E | D | I | G | I | Q | N | F | S | K | E | V | F | G | N | L | T | Q | L | S | A | L | Q | L | N | R | N | G | M | Q | T | I | E | V | D | A | L | E | S | L | P | E | L | H | Y | L | D | I | R | N | I | P | L | S | C | T | C | K | N | S | L | L | Q | N | W | T | L | N | N | P | N | V | Q | V | V | Y | F | Y | N | L | Q | C | P | H | D | A | K | H | K | F | Y | N | F | D | T | Q | V | C | Y | I | D | L | G | E | Y | L | F | F | S | T | A | V | V | I | F | L | F | T | V | T | P | L | L | Y | V | K | L | Y | W | K | M | K | Y | G | Y | Y | V | F | R | S | W | F | S | E | Q | W | R | R | L | R | D | E | E | E | N | C | K | Y | D | A | F | V | S | Y | N | S | S | D | E | Q | W | V | M | D | Q | L | L | P | N | L | E | G | N | G | S | S | F | K | L | C | L | H | H | R | D | F | E | L | G | R | D | I | V | D | N | I | V | S | A | V | Y | G | S | R | K | T | I | C | V | V | S | R | N | F | L | S | S | E | W | C | S | L | E | I | Q | L | A | S | Y | R | L | F | D | E | H | R | D | V | L | L | L | V | F | L | E | P | I | S | E | R | Q | L | S | S | Y | H | R | M | R | M | V | M | L | K | K | T | Y | L | Q | W | P | G | S | D | C | T | D | P | T | Q | A | Q | D | L | F | W | N | Q | L | R | R | A | M | R | T | G | S | R | L | V | T | E | X | X | N | X | E | R | E | G | P | X | X | X | X | X | X | X | X | X | X | X | R | N | X | T | F | X | X | X | X | X | X | V | - | - | - | - | - | R | X | K | P | L | L | A | T | X | X | X | X | - | - | - | - | - |
| LMTLR21\_3196\_BP | M | X | X | X | X | X | X | X | X | X | X | X | X | X | X | X | X | X | X | X | X | X | X | X | X | X | X | X | X | X | X | X | X | X | X | X | X | X | X | X | X | X | X | X | X | X | X | X | X | X | X | X | X | X | X | X | X | X | X | X | X | X | X | X | X | X | S | L | T | Y | X | X | L | L | S | I | T | V | V | L | G | A | A | Q | L | I | S | G | Y | S | F | N | N | C | I | E | E | P | Y | S | K | G | Q | I | F | N | C | L | N | R | K | N | K | N | M | S | A | I | I | S | D | L | P | P | S | A | I | N | L | T | I | S | I | N | H | L | W | H | I | P | N | K | S | F | V | H | L | P | K | L | Q | L | L | R | L | D | Q | N | N | L | R | S | I | D | Q | F | A | F | Q | N | L | H | Q | L | K | S | L | N | M | S | N | N | N | I | S | E | L | N | P | F | L | F | K | D | L | H | N | L | T | F | L | S | L | T | N | N | K | L | K | Q | L | P | E | G | I | F | L | P | L | L | N | L | T | T | L | I | M | R | Q | N | F | L | T | N | F | S | G | I | A | Q | S | V | S | F | L | K | K | L | R | E | L | D | L | C | F | N | N | L | T | S | L | S | H | S | N | V | S | L | P | K | S | L | T | V | L | Y | I | C | R | N | N | L | S | T | L | G | C | K | N | S | F | L | E | F | V | Q | L | L | D | L | S | Y | N | S | R | L | P | T | M | A | F | Q | G | V | D | L | S | H | I | N | Y | L | R | L | R | S | T | S | V | K | I | V | E | F | L | N | I | S | N | X | X | T | A | G | H | V | D | F | S | G | M | G | L | K | N | D | N | M | L | I | K | L | C | K | L | L | K | R | K | V | K | G | X | X | X | X | T | N | V | D | L | S | G | N | W | I | R | N | L | T | N | Y | T | L | C | Y | C | P | K | I | T | G | V | L | D | L | S | R | N | K | L | K | S | L | S | C | L | K | F | L | H | T | Q | K | H | I | K | S | L | R | V | E | H | N | H | L | T | S | I | K | S | C | K | T | Q | X | X | X | X | X | L | R | N | L | E | E | V | S | Y | R | Y | N | R | I | L | S | V | N | A | F | A | F | Y | H | T | P | N | I | K | T | L | E | L | N | I | N | T | I | A | F | L | H | R | K | G | L | K | G | L | K | R | L | E | T | L | R | L | D | N | N | L | L | T | D | L | F | N | N | T | F | E | D | H | F | N | L | K | I | L | N | L | R | N | N | R | I | S | V | I | F | N | G | T | F | L | S | L | R | N | L | T | T | L | D | L | G | G | N | K | I | T | H | F | E | P | S | G | L | D | G | L | E | S | L | S | K | F | Y | L | D | G | N | N | L | K | Q | I | D | T | S | L | Y | H | V | F | Q | D | T | L | T | V | L | D | L | Q | S | N | Q | I | R | F | F | T | E | X | X | I | G | S | P | F | M | N | L | S | K | L | S | D | L | K | L | D | G | Q | R | P | Y | G | L | V | I | L | P | R | T | L | F | R | G | L | H | S | L | K | S | L | Y | L | T | N | N | K | I | Y | H | L | D | S | D | T | F | D | D | L | T | S | L | N | F | L | T | L | D | N | C | C | V | G | V | T | Q | L | Q | P | G | V | F | K | N | L | R | N | L | T | K | L | I | V | E | N | M | G | I | Q | N | F | S | K | E | V | F | G | N | L | T | Q | L | N | T | L | Q | L | N | R | N | G | M | Q | S | I | Q | I | D | A | L | E | S | L | P | E | L | H | Y | L | D | I | R | N | I | A | L | S | C | T | C | K | N | S | L | L | Q | N | W | T | L | H | N | P | K | V | Q | V | V | Y | L | Y | N | L | Q | C | P | H | D | A | K | V | K | F | Y | N | F | D | T | K | V | C | Y | I | D | L | G | E | Y | L | F | L | S | T | A | A | V | I | L | L | F | T | V | T | P | L | L | Y | V | K | L | Y | W | K | I | K | Y | S | Y | Y | V | F | R | S | W | F | S | E | Q | W | R | R | L | R | D | E | E | E | N | C | K | Y | D | A | F | I | S | Y | N | F | S | D | E | Q | W | V | M | D | Q | L | L | P | N | L | E | G | N | G | S | S | F | K | L | C | L | H | H | R | D | F | E | L | G | R | D | I | V | D | N | I | V | S | A | V | Y | S | S | R | K | T | I | C | V | V | S | R | N | F | M | S | S | E | W | C | S | L | E | I | Q | L | A | S | Y | R | L | F | D | E | H | R | D | V | L | L | L | V | F | L | E | Q | M | S | E | R | Q | L | S | S | Y | H | R | M | R | K | V | M | L | Q | K | T | Y | L | Q | W | P | G | S | D | C | T | D | P | A | Q | A | Q | D | L | F | W | K | Q | L | R | R | A | V | K | T | G | S | R | L | E | K | E | X | X | K | X | E | Q | K | G | F | X | X | X | X | X | X | X | X | X | X | X | X | X | X | X | X | X | X | X | X | X | X | X | - | - | - | - | - | X | X | X | X | X | X | X | X | X | X | X | X | - | - | - | - | - |
| TRTLR21\_3196\_BP | M | F | L | F | Y | S | Q | G | L | F | Y | R | W | N | K | K | L | T | G | G | K | C | H | K | K | Y | A | V | D | T | G | Y | S | L | I | L | N | I | G | G | F | L | F | S | I | F | K | Q | A | A | K | A | L | S | N | T | S | C | G | R | R | R | N | K | M | W | S | L | S | F | X | X | L | L | S | V | T | V | L | L | C | A | T | R | L | V | G | G | Y | S | F | H | N | C | I | E | X | X | F | S | K | G | K | T | F | K | C | I | H | R | N | Q | Y | I | L | G | D | I | I | K | D | L | P | H | S | T | L | D | L | T | I | A | V | N | P | V | S | H | I | P | D | R | S | F | I | H | L | R | N | L | Q | Q | L | R | L | D | H | N | H | L | G | V | I | D | Q | F | A | F | Q | G | L | H | Q | L | K | S | L | N | L | S | F | N | Y | I | P | E | L | S | T | S | V | F | D | D | L | H | N | L | T | F | L | S | L | T | N | N | S | L | K | R | L | P | H | G | I | F | S | H | L | F | N | L | N | T | L | I | I | K | Q | N | Y | L | T | N | F | S | E | I | A | M | A | V | S | S | L | K | N | L | T | L | L | D | L | C | F | N | R | L | T | S | L | S | H | S | N | V | S | L | P | E | S | L | N | R | L | Y | L | C | R | N | N | L | S | T | L | G | C | E | P | S | F | L | G | S | I | E | I | L | D | L | S | Y | N | S | E | L | P | T | K | A | L | E | G | V | N | L | R | R | I | N | Y | L | R | L | R | S | T | K | V | N | I | V | E | F | I | Q | N | S | D | X | X | H | A | G | H | V | D | F | T | S | T | H | L | N | T | E | A | K | L | V | E | L | C | K | L | L | K | R | K | L | S | R | X | X | X | X | T | K | L | T | L | V | G | N | K | I | E | T | L | T | A | N | T | L | A | H | C | P | N | I | T | K | T | L | D | L | S | K | V | G | Q | K | K | S | D | C | L | Q | F | L | K | E | Q | R | Q | I | T | T | F | I | A | E | H | N | H | Y | S | S | L | P | T | C | E | X | X | X | X | E | D | P | F | R | Q | L | E | E | L | R | Y | R | Y | N | R | I | L | S | V | N | S | H | A | F | H | H | T | P | N | L | K | T | L | W | L | N | I | N | T | I | A | F | L | H | Q | K | A | L | S | G | L | R | Q | L | S | T | L | R | L | D | N | N | L | L | S | D | L | F | A | D | T | F | E | D | L | F | N | L | N | I | L | N | L | R | N | N | R | I | S | V | I | F | N | N | T | F | R | N | L | K | N | L | T | T | L | D | L | G | G | N | K | I | T | H | F | E | P | S | G | L | C | G | L | E | R | L | S | K | L | Y | L | D | G | N | N | L | Q | T | I | D | S | S | A | Y | H | I | F | Q | N | T | L | T | T | L | D | L | R | Q | N | M | I | H | F | E | E | D | X | X | N | F | S | P | F | V | N | L | T | K | L | E | D | L | K | L | D | E | Q | K | P | Y | G | L | H | I | L | P | R | T | L | F | R | G | L | Y | S | L | R | S | L | Y | V | K | N | N | M | I | S | Y | L | A | A | D | V | F | R | D | L | K | H | L | N | F | L | S | L | D | N | C | C | V | G | P | T | H | L | P | A | G | I | F | K | D | L | T | N | L | T | I | L | T | V | E | N | M | G | I | Q | N | L | S | T | E | V | F | G | N | I | S | Q | L | K | K | I | Q | L | N | H | N | V | M | Q | T | F | P | V | T | V | L | Q | S | L | T | K | L | Q | Y | L | D | I | R | N | V | P | L | S | C | T | C | E | N | S | L | L | R | N | W | T | V | N | N | Q | K | V | Q | M | I | Y | L | Y | S | L | P | C | P | H | D | P | K | V | K | F | F | N | F | D | T | S | V | C | N | I | D | L | G | Q | Y | L | F | F | C | T | A | P | W | I | F | L | F | T | V | W | P | L | L | Y | V | K | L | Y | W | K | I | K | Y | S | Y | Y | V | F | R | S | W | F | S | E | Q | W | R | R | L | R | E | K | E | E | N | C | K | Y | D | A | F | I | S | Y | N | S | S | D | E | L | W | V | M | N | E | L | L | P | N | L | E | G | N | G | S | S | F | K | I | C | L | H | H | R | D | F | E | P | G | R | Y | I | I | D | N | I | V | S | A | V | Y | S | S | R | K | T | I | C | V | V | S | R | N | F | L | S | S | E | W | C | S | L | E | I | Q | L | A | S | Y | R | L | F | D | E | H | R | D | V | L | L | L | V | L | L | E | P | I | S | E | R | Q | L | S | S | Y | H | R | M | R | K | V | M | L | K | K | T | Y | L | Q | W | P | G | S | E | C | T | N | P | P | Q | A | Q | G | L | F | W | S | Q | L | R | R | A | I | G | T | T | S | R | I | E | T | E | X | X | K | X | X | X | G | T | R | V | A | N | K | X | X | X | X | X | X | X | X | X | X | G | R | X | X | X | X | X | X | I | - | - | - | - | - | R | Q | S | X | L | I | X | X | X | X | X | X | - | - | - | - | - |
| conservation |  |  |  |  |  |  |  |  |  |  |  |  |  |  |  |  |  |  |  |  |  |  |  |  |  |  |  |  |  |  |  |  |  |  |  |  |  |  |  |  |  |  |  |  |  |  |  |  |  |  |  |  |  |  |  |  |  |  |  |  |  |  |  |  |  |  |  |  |  |  |  |  |  |  |  |  |  |  |  |  |  |  |  |  |  |  |  |  |  |  |  |  |  |  |  |  |  |  |  |  |  |  |  |  |  |  |  |  |  |  |  |  |  |  |  |  |  |  |  |  |  |  |  |  |  |  |  |  |  |  |  |  |  |  |  |  |  |  |  |  |  |  |  |  |  |  |  |  |  |  |  |  |  |  |  |  |  |  |  |  |  |  |  |  |  |  |  |  |  |  |  |  |  |  |  |  |  |  |  |  |  |  |  |  |  |  |  |  |  |  |  |  |  |  |  |  |  |  |  |  |  |  |  |  |  |  |  |  |  |  |  |  |  |  |  |  |  |  |  |  |  |  |  |  |  |  |  |  |  |  |  |  |  |  |  |  |  |  |  |  |  |  |  |  |  |  |  |  |  |  |  |  |  |  |  |  |  |  |  |  |  |  |  |  |  |  |  |  |  |  |  |  |  |  |  |  |  |  |  |  |  |  |  |  |  |  |  |  |  |  |  |  |  |  |  |  |  |  |  |  |  |  |  |  |  |  |  |  |  |  |  |  |  |  |  |  |  |  |  |  |  |  |  |  |  |  |  |  |  |  |  |  |  |  |  |  |  |  |  |  |  |  |  |  |  |  |  |  |  |  |  |  |  |  |  |  |  |  |  |  |  |  |  |  |  |  |  |  |  |  |  |  |  |  |  |  |  |  |  |  |  |  |  |  |  |  |  |  |  |  |  |  |  |  |  |  |  |  |  |  |  |  |  |  |  |  |  |  |  |  |  |  |  |  |  |  |  |  |  |  |  |  |  |  |  |  |  |  |  |  |  |  |  |  |  |  |  |  |  |  |  |  |  |  |  |  |  |  |  |  |  |  |  |  |  |  |  |  |  |  |  |  |  |  |  |  |  |  |  |  |  |  |  |  |  |  |  |  |  |  |  |  |  |  |  |  |  |  |  |  |  |  |  |  |  |  |  |  |  |  |  |  |  |  |  |  |  |  |  |  |  |  |  |  |  |  |  |  |  |  |  |  |  |  |  |  |  |  |  |  |  |  |  |  |  |  |  |  |  |  |  |  |  |  |  |  |  |  |  |  |  |  |  |  |  |  |  |  |  |  |  |  |  |  |  |  |  |  |  |  |  |  |  |  |  |  |  |  |  |  |  |  |  |  |  |  |  |  |  |  |  |  |  |  |  |  |  |  |  |  |  |  |  |  |  |  |  |  |  |  |  |  |  |  |  |  |  |  |  |  |  |  |  |  |  |  |  |  |  |  |  |  |  |  |  |  |  |  |  |  |  |  |  |  |  |  |  |  |  |  |  |  |  |  |  |  |  |  |  |  |  |  |  |  |  |  |  |  |  |  |  |  |  |  |  |  |  |  |  |  |  |  |  |  |  |  |  |  |  |  |  |  |  |  |  |  |  |  |  |  |  |  |  |  |  |  |  |  |  |  |  |  |  |  |  |  |  |  |  |  |  |  |  |  |  |  |  |  |  |  |  |  |  |  |  |  |  |  |  |  |  |  |  |  |  |  |  |  |  |  |  |  |  |  |  |  |  |  |  |  |  |  |  |  |  |  |  |  |  |  |  |  |  |  |  |  |  |  |  |  |  |  |  |  |  |  |  |  |  |  |  |  |  |  |  |  |  |  |  |  |  |  |  |  |  |  |  |  |  |  |  |  |  |  |  |  |  |  |  |  |  |  |  |  |  |  |  |  |  |  |  |  |  |  |  |  |  |  |  |  |  |  |  |  |  |  |  |  |  |  |  |  |  |  |  |  |  |  |  |  |  |  |  |  |  |  |  |  |  |  |  |  |  |  |  |  |  |  |  |  |  |  |  |  |  |  |  |  |  |  |  |  |  |  |  |  |  |  |  |  |  |  |  |  |  |  |  |  |  |  |  |  |  |  |  |  |  |  |  |  |  |  |  |  |  |  |  |  |  |  |  |  |  |  |  |  |  |  |  |  |  |  |  |  |  |  |  |  |  |  |  |  |  |  |  |  |  |  |  |  |  |  |  |  |  |  |  |  |  |  |  |  |  |  |  |  |  |  |  |  |  |  |  |  |  |  |  |  |  |  |  |  |  |  |  |  |  |  |  |  |  |  |  |  |  |  |  |  |  |  |  |  |  |  |  |  |  |  |  |  |  |  |  |  |  |  |  |  |  |  |  |  |  |  |  |  |  |  |  |  |  |  |  |  |  |  |  |  |  |  |  |  |  |  |  |  |  |  |  |  |  |  |  |  |  |  |  |  |  |  |  |  |  |  |  |
|  |  |  |  |  |  |  |  |  |  |  |  |  |  |  |  |  |  |  |  |  |  |  |  |  |  |  |  |  |  |  |  |  |  |  |  |  |  |  |  |  |  |  |  |  |  |  |  |  |  |  |  |  |  |  |  |  |  |  |  |  |  |  |  |  |  |  |  |  |  |  |  |  |  |  |  |  |  |  |  |  |  |  |  |  |  |  |  |  |  |  |  |  |  |  |  |  |  |  |  |  |  |  |  |  |  |  |  |  |  |  |  |  |  |  |  |  |  |  |  |  |  |  |  |  |  |  |  |  |  |  |  |  |  |  |  |  |  |  |  |  |  |  |  |  |  |  |  |  |  |  |  |  |  |  |  |  |  |  |  |  |  |  |  |  |  |  |  |  |  |  |  |  |  |  |  |  |  |  |  |  |  |  |  |  |  |  |  |  |  |  |  |  |  |  |  |  |  |  |  |  |  |  |  |  |  |  |  |  |  |  |  |  |  |  |  |  |  |  |  |  |  |  |  |  |  |  |  |  |  |  |  |  |  |  |  |  |  |  |  |  |  |  |  |  |  |  |  |  |  |  |  |  |  |  |  |  |  |  |  |  |  |  |  |  |  |  |  |  |  |  |  |  |  |  |  |  |  |  |  |  |  |  |  |  |  |  |  |  |  |  |  |  |  |  |  |  |  |  |  |  |  |  |  |  |  |  |  |  |  |  |  |  |  |  |  |  |  |  |  |  |  |  |  |  |  |  |  |  |  |  |  |  |  |  |  |  |  |  |  |  |  |  |  |  |  |  |  |  |  |  |  |  |  |  |  |  |  |  |  |  |  |  |  |  |  |  |  |  |  |  |  |  |  |  |  |  |  |  |  |  |  |  |  |  |  |  |  |  |  |  |  |  |  |  |  |  |  |  |  |  |  |  |  |  |  |  |  |  |  |  |  |  |  |  |  |  |  |  |  |  |  |  |  |  |  |  |  |  |  |  |  |  |  |  |  |  |  |  |  |  |  |  |  |  |  |  |  |  |  |  |  |  |  |  |  |  |  |  |  |  |  |  |  |  |  |  |  |  |  |  |  |  |  |  |  |  |  |  |  |  |  |  |  |  |  |  |  |  |  |  |  |  |  |  |  |  |  |  |  |  |  |  |  |  |  |  |  |  |  |  |  |  |  |  |  |  |  |  |  |  |  |  |  |  |  |  |  |  |  |  |  |  |  |  |  |  |  |  |  |  |  |  |  |  |  |  |  |  |  |  |  |  |  |  |  |  |  |  |  |  |  |  |  |  |  |  |  |  |  |  |  |  |  |  |  |  |  |  |  |  |  |  |  |  |  |  |  |  |  |  |  |  |  |  |  |  |  |  |  |  |  |  |  |  |  |  |  |  |  |  |  |  |  |  |  |  |  |  |  |  |  |  |  |  |  |  |  |  |  |  |  |  |  |  |  |  |  |  |  |  |  |  |  |  |  |  |  |  |  |  |  |  |  |  |  |  |  |  |  |  |  |  |  |  |  |  |  |  |  |  |  |  |  |  |  |  |  |  |  |  |  |  |  |  |  |  |  |  |  |  |  |  |  |  |  |  |  |  |  |  |  |  |  |  |  |  |  |  |  |  |  |  |  |  |  |  |  |  |  |  |  |  |  |  |  |  |  |  |  |  |  |  |  |  |  |  |  |  |  |  |  |  |  |  |  |  |  |  |  |  |  |  |  |  |  |  |  |  |  |  |  |  |  |  |  |  |  |  |  |  |  |  |  |  |  |  |  |  |  |  |  |  |  |  |  |  |  |  |  |  |  |  |  |  |  |  |  |  |  |  |  |  |  |  |  |  |  |  |  |  |  |  |  |  |  |  |  |  |  |  |  |  |  |  |  |  |  |  |  |  |  |  |  |  |  |  |  |  |  |  |  |  |  |  |  |  |  |  |  |  |  |  |  |  |  |  |  |  |  |  |  |  |  |  |  |  |  |  |  |  |  |  |  |  |  |  |  |  |  |  |  |  |  |  |  |  |  |  |  |  |  |  |  |  |  |  |  |  |  |  |  |  |  |  |  |  |  |  |  |  |  |  |  |  |  |  |  |  |  |  |  |  |  |  |  |  |  |  |  |  |  |  |  |  |  |  |  |  |  |  |  |  |  |  |  |  |  |  |  |  |  |  |  |  |  |  |  |  |  |  |  |  |  |  |  |  |  |  |  |  |  |  |  |  |  |  |  |  |  |  |  |  |  |  |  |  |  |  |  |  |  |  |  |  |  |  |  |  |  |  |  |  |  |  |  |  |  |  |  |  |  |  |  |  |  |  |  |  |  |  |  |  |  |  |  |  |  |  |  |  |  |  |  |  |  |  |  |  |  |  |  |  |  |  |  |  |  |  |  |  |  |  |  |  |  |  |  |  |  |  |  |  |  |  |  |  |  |  |  |  |  |  |  |  |  |
|  |  |  |  |  |  |  |  |  |  |  |  |  |  |  |  |  |  |  |  |  |  |  |  |  |  |  |  |  |  |  |  |  |  |  |  |  |  |  |  |  |  |  |  |  |  |  |  |  |  |  |  |  |  |  |  |  |  |  |  |  |  |  |  |  |  |  |  |  |  |  |  |  |  |  |  |  |  |  |  |  |  |  |  |  |  |  |  |  |  |  |  |  |  |  |  |  |  |  |  |  |  |  |  |  |  |  |  |  |  |  |  |  |  |  |  |  |  |  |  |  |  |  |  |  |  |  |  |  |  |  |  |  |  |  |  |  |  |  |  |  |  |  |  |  |  |  |  |  |  |  |  |  |  |  |  |  |  |  |  |  |  |  |  |  |  |  |  |  |  |  |  |  |  |  |  |  |  |  |  |  |  |  |  |  |  |  |  |  |  |  |  |  |  |  |  |  |  |  |  |  |  |  |  |  |  |  |  |  |  |  |  |  |  |  |  |  |  |  |  |  |  |  |  |  |  |  |  |  |  |  |  |  |  |  |  |  |  |  |  |  |  |  |  |  |  |  |  |  |  |  |  |  |  |  |  |  |  |  |  |  |  |  |  |  |  |  |  |  |  |  |  |  |  |  |  |  |  |  |  |  |  |  |  |  |  |  |  |  |  |  |  |  |  |  |  |  |  |  |  |  |  |  |  |  |  |  |  |  |  |  |  |  |  |  |  |  |  |  |  |  |  |  |  |  |  |  |  |  |  |  |  |  |  |  |  |  |  |  |  |  |  |  |  |  |  |  |  |  |  |  |  |  |  |  |  |  |  |  |  |  |  |  |  |  |  |  |  |  |  |  |  |  |  |  |  |  |  |  |  |  |  |  |  |  |  |  |  |  |  |  |  |  |  |  |  |  |  |  |  |  |  |  |  |  |  |  |  |  |  |  |  |  |  |  |  |  |  |  |  |  |  |  |  |  |  |  |  |  |  |  |  |  |  |  |  |  |  |  |  |  |  |  |  |  |  |  |  |  |  |  |  |  |  |  |  |  |  |  |  |  |  |  |  |  |  |  |  |  |  |  |  |  |  |  |  |  |  |  |  |  |  |  |  |  |  |  |  |  |  |  |  |  |  |  |  |  |  |  |  |  |  |  |  |  |  |  |  |  |  |  |  |  |  |  |  |  |  |  |  |  |  |  |  |  |  |  |  |  |  |  |  |  |  |  |  |  |  |  |  |  |  |  |  |  |  |  |  |  |  |  |  |  |  |  |  |  |  |  |  |  |  |  |  |  |  |  |  |  |  |  |  |  |  |  |  |  |  |  |  |  |  |  |  |  |  |  |  |  |  |  |  |  |  |  |  |  |  |  |  |  |  |  |  |  |  |  |  |  |  |  |  |  |  |  |  |  |  |  |  |  |  |  |  |  |  |  |  |  |  |  |  |  |  |  |  |  |  |  |  |  |  |  |  |  |  |  |  |  |  |  |  |  |  |  |  |  |  |  |  |  |  |  |  |  |  |  |  |  |  |  |  |  |  |  |  |  |  |  |  |  |  |  |  |  |  |  |  |  |  |  |  |  |  |  |  |  |  |  |  |  |  |  |  |  |  |  |  |  |  |  |  |  |  |  |  |  |  |  |  |  |  |  |  |  |  |  |  |  |  |  |  |  |  |  |  |  |  |  |  |  |  |  |  |  |  |  |  |  |  |  |  |  |  |  |  |  |  |  |  |  |  |  |  |  |  |  |  |  |  |  |  |  |  |  |  |  |  |  |  |  |  |  |  |  |  |  |  |  |  |  |  |  |  |  |  |  |  |  |  |  |  |  |  |  |  |  |  |  |  |  |  |  |  |  |  |  |  |  |  |  |  |  |  |  |  |  |  |  |  |  |  |  |  |  |  |  |  |  |  |  |  |  |  |  |  |  |  |  |  |  |  |  |  |  |  |  |  |  |  |  |  |  |  |  |  |  |  |  |  |  |  |  |  |  |  |  |  |  |  |  |  |  |  |  |  |  |  |  |  |  |  |  |  |  |  |  |  |  |  |  |  |  |  |  |  |  |  |  |  |  |  |  |  |  |  |  |  |  |  |  |  |  |  |  |  |  |  |  |  |  |  |  |  |  |  |  |  |  |  |  |  |  |  |  |  |  |  |  |  |  |  |  |  |  |  |  |  |  |  |  |  |  |  |  |  |  |  |  |  |  |  |  |  |  |  |  |  |  |  |  |  |  |  |  |  |  |  |  |  |  |  |  |  |  |  |  |  |  |  |  |  |  |  |  |  |  |  |  |  |  |  |  |  |  |  |  |  |  |  |  |  |  |  |  |  |  |  |  |  |  |  |  |  |  |  |  |  |  |  |  |  |  |  |  |  |  |  |  |  |  |  |  |  |  |  |  |  |  |  |  |  |  |  |  |  |  |  |  |  |  |  |  |  |  |  |
|  |  |  |  |  |  |  |  |  |  |  |  |  |  |  |  |  |  |  |  |  |  |  |  |  |  |  |  |  |  |  |  |  |  |  |  |  |  |  |  |  |  |  |  |  |  |  |  |  |  |  |  |  |  |  |  |  |  |  |  |  |  |  |  |  |  |  |  |  |  |  |  |  |  |  |  |  |  |  |  |  |  |  |  |  |  |  |  |  |  |  |  |  |  |  |  |  |  |  |  |  |  |  |  |  |  |  |  |  |  |  |  |  |  |  |  |  |  |  |  |  |  |  |  |  |  |  |  |  |  |  |  |  |  |  |  |  |  |  |  |  |  |  |  |  |  |  |  |  |  |  |  |  |  |  |  |  |  |  |  |  |  |  |  |  |  |  |  |  |  |  |  |  |  |  |  |  |  |  |  |  |  |  |  |  |  |  |  |  |  |  |  |  |  |  |  |  |  |  |  |  |  |  |  |  |  |  |  |  |  |  |  |  |  |  |  |  |  |  |  |  |  |  |  |  |  |  |  |  |  |  |  |  |  |  |  |  |  |  |  |  |  |  |  |  |  |  |  |  |  |  |  |  |  |  |  |  |  |  |  |  |  |  |  |  |  |  |  |  |  |  |  |  |  |  |  |  |  |  |  |  |  |  |  |  |  |  |  |  |  |  |  |  |  |  |  |  |  |  |  |  |  |  |  |  |  |  |  |  |  |  |  |  |  |  |  |  |  |  |  |  |  |  |  |  |  |  |  |  |  |  |  |  |  |  |  |  |  |  |  |  |  |  |  |  |  |  |  |  |  |  |  |  |  |  |  |  |  |  |  |  |  |  |  |  |  |  |  |  |  |  |  |  |  |  |  |  |  |  |  |  |  |  |  |  |  |  |  |  |  |  |  |  |  |  |  |  |  |  |  |  |  |  |  |  |  |  |  |  |  |  |  |  |  |  |  |  |  |  |  |  |  |  |  |  |  |  |  |  |  |  |  |  |  |  |  |  |  |  |  |  |  |  |  |  |  |  |  |  |  |  |  |  |  |  |  |  |  |  |  |  |  |  |  |  |  |  |  |  |  |  |  |  |  |  |  |  |  |  |  |  |  |  |  |  |  |  |  |  |  |  |  |  |  |  |  |  |  |  |  |  |  |  |  |  |  |  |  |  |  |  |  |  |  |  |  |  |  |  |  |  |  |  |  |  |  |  |  |  |  |  |  |  |  |  |  |  |  |  |  |  |  |  |  |  |  |  |  |  |  |  |  |  |  |  |  |  |  |  |  |  |  |  |  |  |  |  |  |  |  |  |  |  |  |  |  |  |  |  |  |  |  |  |  |  |  |  |  |  |  |  |  |  |  |  |  |  |  |  |  |  |  |  |  |  |  |  |  |  |  |  |  |  |  |  |  |  |  |  |  |  |  |  |  |  |  |  |  |  |  |  |  |  |  |  |  |  |  |  |  |  |  |  |  |  |  |  |  |  |  |  |  |  |  |  |  |  |  |  |  |  |  |  |  |  |  |  |  |  |  |  |  |  |  |  |  |  |  |  |  |  |  |  |  |  |  |  |  |  |  |  |  |  |  |  |  |  |  |  |  |  |  |  |  |  |  |  |  |  |  |  |  |  |  |  |  |  |  |  |  |  |  |  |  |  |  |  |  |  |  |  |  |  |  |  |  |  |  |  |  |  |  |  |  |  |  |  |  |  |  |  |  |  |  |  |  |  |  |  |  |  |  |  |  |  |  |  |  |  |  |  |  |  |  |  |  |  |  |  |  |  |  |  |  |  |  |  |  |  |  |  |  |  |  |  |  |  |  |  |  |  |  |  |  |  |  |  |  |  |  |  |  |  |  |  |  |  |  |  |  |  |  |  |  |  |  |  |  |  |  |  |  |  |  |  |  |  |  |  |  |  |  |  |  |  |  |  |  |  |  |  |  |  |  |  |  |  |  |  |  |  |  |  |  |  |  |  |  |  |  |  |  |  |  |  |  |  |  |  |  |  |  |  |  |  |  |  |  |  |  |  |  |  |  |  |  |  |  |  |  |  |  |  |  |  |  |  |  |  |  |  |  |  |  |  |  |  |  |  |  |  |  |  |  |  |  |  |  |  |  |  |  |  |  |  |  |  |  |  |  |  |  |  |  |  |  |  |  |  |  |  |  |  |  |  |  |  |  |  |  |  |  |  |  |  |  |  |  |  |  |  |  |  |  |  |  |  |  |  |  |  |  |  |  |  |  |  |  |  |  |  |  |  |  |  |  |  |  |  |  |  |  |  |  |  |  |  |  |  |  |  |  |  |  |  |  |  |  |  |  |  |  |  |  |  |  |  |  |  |  |  |  |  |  |  |  |  |  |  |  |  |  |  |  |  |  |  |  |  |  |  |  |  |  |  |  |  |  |  |  |  |  |  |  |  |  |  |  |  |  |  |  |  |  |  |  |
|  |  |  |  |  |  |  |  |  |  |  |  |  |  |  |  |  |  |  |  |  |  |  |  |  |  |  |  |  |  |  |  |  |  |  |  |  |  |  |  |  |  |  |  |  |  |  |  |  |  |  |  |  |  |  |  |  |  |  |  |  |  |  |  |  |  |  |  |  |  |  |  |  |  |  |  |  |  |  |  |  |  |  |  |  |  |  |  |  |  |  |  |  |  |  |  |  |  |  |  |  |  |  |  |  |  |  |  |  |  |  |  |  |  |  |  |  |  |  |  |  |  |  |  |  |  |  |  |  |  |  |  |  |  |  |  |  |  |  |  |  |  |  |  |  |  |  |  |  |  |  |  |  |  |  |  |  |  |  |  |  |  |  |  |  |  |  |  |  |  |  |  |  |  |  |  |  |  |  |  |  |  |  |  |  |  |  |  |  |  |  |  |  |  |  |  |  |  |  |  |  |  |  |  |  |  |  |  |  |  |  |  |  |  |  |  |  |  |  |  |  |  |  |  |  |  |  |  |  |  |  |  |  |  |  |  |  |  |  |  |  |  |  |  |  |  |  |  |  |  |  |  |  |  |  |  |  |  |  |  |  |  |  |  |  |  |  |  |  |  |  |  |  |  |  |  |  |  |  |  |  |  |  |  |  |  |  |  |  |  |  |  |  |  |  |  |  |  |  |  |  |  |  |  |  |  |  |  |  |  |  |  |  |  |  |  |  |  |  |  |  |  |  |  |  |  |  |  |  |  |  |  |  |  |  |  |  |  |  |  |  |  |  |  |  |  |  |  |  |  |  |  |  |  |  |  |  |  |  |  |  |  |  |  |  |  |  |  |  |  |  |  |  |  |  |  |  |  |  |  |  |  |  |  |  |  |  |  |  |  |  |  |  |  |  |  |  |  |  |  |  |  |  |  |  |  |  |  |  |  |  |  |  |  |  |  |  |  |  |  |  |  |  |  |  |  |  |  |  |  |  |  |  |  |  |  |  |  |  |  |  |  |  |  |  |  |  |  |  |  |  |  |  |  |  |  |  |  |  |  |  |  |  |  |  |  |  |  |  |  |  |  |  |  |  |  |  |  |  |  |  |  |  |  |  |  |  |  |  |  |  |  |  |  |  |  |  |  |  |  |  |  |  |  |  |  |  |  |  |  |  |  |  |  |  |  |  |  |  |  |  |  |  |  |  |  |  |  |  |  |  |  |  |  |  |  |  |  |  |  |  |  |  |  |  |  |  |  |  |  |  |  |  |  |  |  |  |  |  |  |  |  |  |  |  |  |  |  |  |  |  |  |  |  |  |  |  |  |  |  |  |  |  |  |  |  |  |  |  |  |  |  |  |  |  |  |  |  |  |  |  |  |  |  |  |  |  |  |  |  |  |  |  |  |  |  |  |  |  |  |  |  |  |  |  |  |  |  |  |  |  |  |  |  |  |  |  |  |  |  |  |  |  |  |  |  |  |  |  |  |  |  |  |  |  |  |  |  |  |  |  |  |  |  |  |  |  |  |  |  |  |  |  |  |  |  |  |  |  |  |  |  |  |  |  |  |  |  |  |  |  |  |  |  |  |  |  |  |  |  |  |  |  |  |  |  |  |  |  |  |  |  |  |  |  |  |  |  |  |  |  |  |  |  |  |  |  |  |  |  |  |  |  |  |  |  |  |  |  |  |  |  |  |  |  |  |  |  |  |  |  |  |  |  |  |  |  |  |  |  |  |  |  |  |  |  |  |  |  |  |  |  |  |  |  |  |  |  |  |  |  |  |  |  |  |  |  |  |  |  |  |  |  |  |  |  |  |  |  |  |  |  |  |  |  |  |  |  |  |  |  |  |  |  |  |  |  |  |  |  |  |  |  |  |  |  |  |  |  |  |  |  |  |  |  |  |  |  |  |  |  |  |  |  |  |  |  |  |  |  |  |  |  |  |  |  |  |  |  |  |  |  |  |  |  |  |  |  |  |  |  |  |  |  |  |  |  |  |  |  |  |  |  |  |  |  |  |  |  |  |  |  |  |  |  |  |  |  |  |  |  |  |  |  |  |  |  |  |  |  |  |  |  |  |  |  |  |  |  |  |  |  |  |  |  |  |  |  |  |  |  |  |  |  |  |  |  |  |  |  |  |  |  |  |  |  |  |  |  |  |  |  |  |  |  |  |  |  |  |  |  |  |  |  |  |  |  |  |  |  |  |  |  |  |  |  |  |  |  |  |  |  |  |  |  |  |  |  |  |  |  |  |  |  |  |  |  |  |  |  |  |  |  |  |  |  |  |  |  |  |  |  |  |  |  |  |  |  |  |  |  |  |  |  |  |  |  |  |  |  |  |  |  |  |  |  |  |  |  |  |  |  |  |  |  |  |  |  |  |  |  |  |  |  |  |  |  |  |  |  |  |  |  |  |  |  |  |  |  |  |  |  |  |  |  |  |
|  |  |  |  |  |  |  |  |  |  |  |  |  |  |  |  |  |  |  |  |  |  |  |  |  |  |  |  |  |  |  |  |  |  |  |  |  |  |  |  |  |  |  |  |  |  |  |  |  |  |  |  |  |  |  |  |  |  |  |  |  |  |  |  |  |  |  |  |  |  |  |  |  |  |  |  |  |  |  |  |  |  |  |  |  |  |  |  |  |  |  |  |  |  |  |  |  |  |  |  |  |  |  |  |  |  |  |  |  |  |  |  |  |  |  |  |  |  |  |  |  |  |  |  |  |  |  |  |  |  |  |  |  |  |  |  |  |  |  |  |  |  |  |  |  |  |  |  |  |  |  |  |  |  |  |  |  |  |  |  |  |  |  |  |  |  |  |  |  |  |  |  |  |  |  |  |  |  |  |  |  |  |  |  |  |  |  |  |  |  |  |  |  |  |  |  |  |  |  |  |  |  |  |  |  |  |  |  |  |  |  |  |  |  |  |  |  |  |  |  |  |  |  |  |  |  |  |  |  |  |  |  |  |  |  |  |  |  |  |  |  |  |  |  |  |  |  |  |  |  |  |  |  |  |  |  |  |  |  |  |  |  |  |  |  |  |  |  |  |  |  |  |  |  |  |  |  |  |  |  |  |  |  |  |  |  |  |  |  |  |  |  |  |  |  |  |  |  |  |  |  |  |  |  |  |  |  |  |  |  |  |  |  |  |  |  |  |  |  |  |  |  |  |  |  |  |  |  |  |  |  |  |  |  |  |  |  |  |  |  |  |  |  |  |  |  |  |  |  |  |  |  |  |  |  |  |  |  |  |  |  |  |  |  |  |  |  |  |  |  |  |  |  |  |  |  |  |  |  |  |  |  |  |  |  |  |  |  |  |  |  |  |  |  |  |  |  |  |  |  |  |  |  |  |  |  |  |  |  |  |  |  |  |  |  |  |  |  |  |  |  |  |  |  |  |  |  |  |  |  |  |  |  |  |  |  |  |  |  |  |  |  |  |  |  |  |  |  |  |  |  |  |  |  |  |  |  |  |  |  |  |  |  |  |  |  |  |  |  |  |  |  |  |  |  |  |  |  |  |  |  |  |  |  |  |  |  |  |  |  |  |  |  |  |  |  |  |  |  |  |  |  |  |  |  |  |  |  |  |  |  |  |  |  |  |  |  |  |  |  |  |  |  |  |  |  |  |  |  |  |  |  |  |  |  |  |  |  |  |  |  |  |  |  |  |  |  |  |  |  |  |  |  |  |  |  |  |  |  |  |  |  |  |  |  |  |  |  |  |  |  |  |  |  |  |  |  |  |  |  |  |  |  |  |  |  |  |  |  |  |  |  |  |  |  |  |  |  |  |  |  |  |  |  |  |  |  |  |  |  |  |  |  |  |  |  |  |  |  |  |  |  |  |  |  |  |  |  |  |  |  |  |  |  |  |  |  |  |  |  |  |  |  |  |  |  |  |  |  |  |  |  |  |  |  |  |  |  |  |  |  |  |  |  |  |  |  |  |  |  |  |  |  |  |  |  |  |  |  |  |  |  |  |  |  |  |  |  |  |  |  |  |  |  |  |  |  |  |  |  |  |  |  |  |  |  |  |  |  |  |  |  |  |  |  |  |  |  |  |  |  |  |  |  |  |  |  |  |  |  |  |  |  |  |  |  |  |  |  |  |  |  |  |  |  |  |  |  |  |  |  |  |  |  |  |  |  |  |  |  |  |  |  |  |  |  |  |  |  |  |  |  |  |  |  |  |  |  |  |  |  |  |  |  |  |  |  |  |  |  |  |  |  |  |  |  |  |  |  |  |  |  |  |  |  |  |  |  |  |  |  |  |  |  |  |  |  |  |  |  |  |  |  |  |  |  |  |  |  |  |  |  |  |  |  |  |  |  |  |  |  |  |  |  |  |  |  |  |  |  |  |  |  |  |  |  |  |  |  |  |  |  |  |  |  |  |  |  |  |  |  |  |  |  |  |  |  |  |  |  |  |  |  |  |  |  |  |  |  |  |  |  |  |  |  |  |  |  |  |  |  |  |  |  |  |  |  |  |  |  |  |  |  |  |  |  |  |  |  |  |  |  |  |  |  |  |  |  |  |  |  |  |  |  |  |  |  |  |  |  |  |  |  |  |  |  |  |  |  |  |  |  |  |  |  |  |  |  |  |  |  |  |  |  |  |  |  |  |  |  |  |  |  |  |  |  |  |  |  |  |  |  |  |  |  |  |  |  |  |  |  |  |  |  |  |  |  |  |  |  |  |  |  |  |  |  |  |  |  |  |  |  |  |  |  |  |  |  |  |  |  |  |  |  |  |  |  |  |  |  |  |  |  |  |  |  |  |  |  |  |  |  |  |  |  |  |  |  |  |  |  |  |  |  |  |  |  |  |  |  |  |  |  |  |  |  |  |  |  |  |  |  |  |  |  |  |
|  |  |  |  |  |  |  |  |  |  |  |  |  |  |  |  |  |  |  |  |  |  |  |  |  |  |  |  |  |  |  |  |  |  |  |  |  |  |  |  |  |  |  |  |  |  |  |  |  |  |  |  |  |  |  |  |  |  |  |  |  |  |  |  |  |  |  |  |  |  |  |  |  |  |  |  |  |  |  |  |  |  |  |  |  |  |  |  |  |  |  |  |  |  |  |  |  |  |  |  |  |  |  |  |  |  |  |  |  |  |  |  |  |  |  |  |  |  |  |  |  |  |  |  |  |  |  |  |  |  |  |  |  |  |  |  |  |  |  |  |  |  |  |  |  |  |  |  |  |  |  |  |  |  |  |  |  |  |  |  |  |  |  |  |  |  |  |  |  |  |  |  |  |  |  |  |  |  |  |  |  |  |  |  |  |  |  |  |  |  |  |  |  |  |  |  |  |  |  |  |  |  |  |  |  |  |  |  |  |  |  |  |  |  |  |  |  |  |  |  |  |  |  |  |  |  |  |  |  |  |  |  |  |  |  |  |  |  |  |  |  |  |  |  |  |  |  |  |  |  |  |  |  |  |  |  |  |  |  |  |  |  |  |  |  |  |  |  |  |  |  |  |  |  |  |  |  |  |  |  |  |  |  |  |  |  |  |  |  |  |  |  |  |  |  |  |  |  |  |  |  |  |  |  |  |  |  |  |  |  |  |  |  |  |  |  |  |  |  |  |  |  |  |  |  |  |  |  |  |  |  |  |  |  |  |  |  |  |  |  |  |  |  |  |  |  |  |  |  |  |  |  |  |  |  |  |  |  |  |  |  |  |  |  |  |  |  |  |  |  |  |  |  |  |  |  |  |  |  |  |  |  |  |  |  |  |  |  |  |  |  |  |  |  |  |  |  |  |  |  |  |  |  |  |  |  |  |  |  |  |  |  |  |  |  |  |  |  |  |  |  |  |  |  |  |  |  |  |  |  |  |  |  |  |  |  |  |  |  |  |  |  |  |  |  |  |  |  |  |  |  |  |  |  |  |  |  |  |  |  |  |  |  |  |  |  |  |  |  |  |  |  |  |  |  |  |  |  |  |  |  |  |  |  |  |  |  |  |  |  |  |  |  |  |  |  |  |  |  |  |  |  |  |  |  |  |  |  |  |  |  |  |  |  |  |  |  |  |  |  |  |  |  |  |  |  |  |  |  |  |  |  |  |  |  |  |  |  |  |  |  |  |  |  |  |  |  |  |  |  |  |  |  |  |  |  |  |  |  |  |  |  |  |  |  |  |  |  |  |  |  |  |  |  |  |  |  |  |  |  |  |  |  |  |  |  |  |  |  |  |  |  |  |  |  |  |  |  |  |  |  |  |  |  |  |  |  |  |  |  |  |  |  |  |  |  |  |  |  |  |  |  |  |  |  |  |  |  |  |  |  |  |  |  |  |  |  |  |  |  |  |  |  |  |  |  |  |  |  |  |  |  |  |  |  |  |  |  |  |  |  |  |  |  |  |  |  |  |  |  |  |  |  |  |  |  |  |  |  |  |  |  |  |  |  |  |  |  |  |  |  |  |  |  |  |  |  |  |  |  |  |  |  |  |  |  |  |  |  |  |  |  |  |  |  |  |  |  |  |  |  |  |  |  |  |  |  |  |  |  |  |  |  |  |  |  |  |  |  |  |  |  |  |  |  |  |  |  |  |  |  |  |  |  |  |  |  |  |  |  |  |  |  |  |  |  |  |  |  |  |  |  |  |  |  |  |  |  |  |  |  |  |  |  |  |  |  |  |  |  |  |  |  |  |  |  |  |  |  |  |  |  |  |  |  |  |  |  |  |  |  |  |  |  |  |  |  |  |  |  |  |  |  |  |  |  |  |  |  |  |  |  |  |  |  |  |  |  |  |  |  |  |  |  |  |  |  |  |  |  |  |  |  |  |  |  |  |  |  |  |  |  |  |  |  |  |  |  |  |  |  |  |  |  |  |  |  |  |  |  |  |  |  |  |  |  |  |  |  |  |  |  |  |  |  |  |  |  |  |  |  |  |  |  |  |  |  |  |  |  |  |  |  |  |  |  |  |  |  |  |  |  |  |  |  |  |  |  |  |  |  |  |  |  |  |  |  |  |  |  |  |  |  |  |  |  |  |  |  |  |  |  |  |  |  |  |  |  |  |  |  |  |  |  |  |  |  |  |  |  |  |  |  |  |  |  |  |  |  |  |  |  |  |  |  |  |  |  |  |  |  |  |  |  |  |  |  |  |  |  |  |  |  |  |  |  |  |  |  |  |  |  |  |  |  |  |  |  |  |  |  |  |  |  |  |  |  |  |  |  |  |  |  |  |  |  |  |  |  |  |  |  |  |  |  |  |  |  |  |  |  |  |  |  |  |  |  |  |  |  |  |  |  |  |  |  |  |  |  |  |  |  |  |  |  |  |
|  |  |  |  |  |  |  |  |  |  |  |  |  |  |  |  |  |  |  |  |  |  |  |  |  |  |  |  |  |  |  |  |  |  |  |  |  |  |  |  |  |  |  |  |  |  |  |  |  |  |  |  |  |  |  |  |  |  |  |  |  |  |  |  |  |  |  |  |  |  |  |  |  |  |  |  |  |  |  |  |  |  |  |  |  |  |  |  |  |  |  |  |  |  |  |  |  |  |  |  |  |  |  |  |  |  |  |  |  |  |  |  |  |  |  |  |  |  |  |  |  |  |  |  |  |  |  |  |  |  |  |  |  |  |  |  |  |  |  |  |  |  |  |  |  |  |  |  |  |  |  |  |  |  |  |  |  |  |  |  |  |  |  |  |  |  |  |  |  |  |  |  |  |  |  |  |  |  |  |  |  |  |  |  |  |  |  |  |  |  |  |  |  |  |  |  |  |  |  |  |  |  |  |  |  |  |  |  |  |  |  |  |  |  |  |  |  |  |  |  |  |  |  |  |  |  |  |  |  |  |  |  |  |  |  |  |  |  |  |  |  |  |  |  |  |  |  |  |  |  |  |  |  |  |  |  |  |  |  |  |  |  |  |  |  |  |  |  |  |  |  |  |  |  |  |  |  |  |  |  |  |  |  |  |  |  |  |  |  |  |  |  |  |  |  |  |  |  |  |  |  |  |  |  |  |  |  |  |  |  |  |  |  |  |  |  |  |  |  |  |  |  |  |  |  |  |  |  |  |  |  |  |  |  |  |  |  |  |  |  |  |  |  |  |  |  |  |  |  |  |  |  |  |  |  |  |  |  |  |  |  |  |  |  |  |  |  |  |  |  |  |  |  |  |  |  |  |  |  |  |  |  |  |  |  |  |  |  |  |  |  |  |  |  |  |  |  |  |  |  |  |  |  |  |  |  |  |  |  |  |  |  |  |  |  |  |  |  |  |  |  |  |  |  |  |  |  |  |  |  |  |  |  |  |  |  |  |  |  |  |  |  |  |  |  |  |  |  |  |  |  |  |  |  |  |  |  |  |  |  |  |  |  |  |  |  |  |  |  |  |  |  |  |  |  |  |  |  |  |  |  |  |  |  |  |  |  |  |  |  |  |  |  |  |  |  |  |  |  |  |  |  |  |  |  |  |  |  |  |  |  |  |  |  |  |  |  |  |  |  |  |  |  |  |  |  |  |  |  |  |  |  |  |  |  |  |  |  |  |  |  |  |  |  |  |  |  |  |  |  |  |  |  |  |  |  |  |  |  |  |  |  |  |  |  |  |  |  |  |  |  |  |  |  |  |  |  |  |  |  |  |  |  |  |  |  |  |  |  |  |  |  |  |  |  |  |  |  |  |  |  |  |  |  |  |  |  |  |  |  |  |  |  |  |  |  |  |  |  |  |  |  |  |  |  |  |  |  |  |  |  |  |  |  |  |  |  |  |  |  |  |  |  |  |  |  |  |  |  |  |  |  |  |  |  |  |  |  |  |  |  |  |  |  |  |  |  |  |  |  |  |  |  |  |  |  |  |  |  |  |  |  |  |  |  |  |  |  |  |  |  |  |  |  |  |  |  |  |  |  |  |  |  |  |  |  |  |  |  |  |  |  |  |  |  |  |  |  |  |  |  |  |  |  |  |  |  |  |  |  |  |  |  |  |  |  |  |  |  |  |  |  |  |  |  |  |  |  |  |  |  |  |  |  |  |  |  |  |  |  |  |  |  |  |  |  |  |  |  |  |  |  |  |  |  |  |  |  |  |  |  |  |  |  |  |  |  |  |  |  |  |  |  |  |  |  |  |  |  |  |  |  |  |  |  |  |  |  |  |  |  |  |  |  |  |  |  |  |  |  |  |  |  |  |  |  |  |  |  |  |  |  |  |  |  |  |  |  |  |  |  |  |  |  |  |  |  |  |  |  |  |  |  |  |  |  |  |  |  |  |  |  |  |  |  |  |  |  |  |  |  |  |  |  |  |  |  |  |  |  |  |  |  |  |  |  |  |  |  |  |  |  |  |  |  |  |  |  |  |  |  |  |  |  |  |  |  |  |  |  |  |  |  |  |  |  |  |  |  |  |  |  |  |  |  |  |  |  |  |  |  |  |  |  |  |  |  |  |  |  |  |  |  |  |  |  |  |  |  |  |  |  |  |  |  |  |  |  |  |  |  |  |  |  |  |  |  |  |  |  |  |  |  |  |  |  |  |  |  |  |  |  |  |  |  |  |  |  |  |  |  |  |  |  |  |  |  |  |  |  |  |  |  |  |  |  |  |  |  |  |  |  |  |  |  |  |  |  |  |  |  |  |  |  |  |  |  |  |  |  |  |  |  |  |  |  |  |  |  |  |  |  |  |  |  |  |  |  |  |  |  |  |  |  |  |  |  |  |  |  |  |  |  |  |  |  |  |  |  |  |  |  |  |  |  |  |
|  |  |  |  |  |  |  |  |  |  |  |  |  |  |  |  |  |  |  |  |  |  |  |  |  |  |  |  |  |  |  |  |  |  |  |  |  |  |  |  |  |  |  |  |  |  |  |  |  |  |  |  |  |  |  |  |  |  |  |  |  |  |  |  |  |  |  |  |  |  |  |  |  |  |  |  |  |  |  |  |  |  |  |  |  |  |  |  |  |  |  |  |  |  |  |  |  |  |  |  |  |  |  |  |  |  |  |  |  |  |  |  |  |  |  |  |  |  |  |  |  |  |  |  |  |  |  |  |  |  |  |  |  |  |  |  |  |  |  |  |  |  |  |  |  |  |  |  |  |  |  |  |  |  |  |  |  |  |  |  |  |  |  |  |  |  |  |  |  |  |  |  |  |  |  |  |  |  |  |  |  |  |  |  |  |  |  |  |  |  |  |  |  |  |  |  |  |  |  |  |  |  |  |  |  |  |  |  |  |  |  |  |  |  |  |  |  |  |  |  |  |  |  |  |  |  |  |  |  |  |  |  |  |  |  |  |  |  |  |  |  |  |  |  |  |  |  |  |  |  |  |  |  |  |  |  |  |  |  |  |  |  |  |  |  |  |  |  |  |  |  |  |  |  |  |  |  |  |  |  |  |  |  |  |  |  |  |  |  |  |  |  |  |  |  |  |  |  |  |  |  |  |  |  |  |  |  |  |  |  |  |  |  |  |  |  |  |  |  |  |  |  |  |  |  |  |  |  |  |  |  |  |  |  |  |  |  |  |  |  |  |  |  |  |  |  |  |  |  |  |  |  |  |  |  |  |  |  |  |  |  |  |  |  |  |  |  |  |  |  |  |  |  |  |  |  |  |  |  |  |  |  |  |  |  |  |  |  |  |  |  |  |  |  |  |  |  |  |  |  |  |  |  |  |  |  |  |  |  |  |  |  |  |  |  |  |  |  |  |  |  |  |  |  |  |  |  |  |  |  |  |  |  |  |  |  |  |  |  |  |  |  |  |  |  |  |  |  |  |  |  |  |  |  |  |  |  |  |  |  |  |  |  |  |  |  |  |  |  |  |  |  |  |  |  |  |  |  |  |  |  |  |  |  |  |  |  |  |  |  |  |  |  |  |  |  |  |  |  |  |  |  |  |  |  |  |  |  |  |  |  |  |  |  |  |  |  |  |  |  |  |  |  |  |  |  |  |  |  |  |  |  |  |  |  |  |  |  |  |  |  |  |  |  |  |  |  |  |  |  |  |  |  |  |  |  |  |  |  |  |  |  |  |  |  |  |  |  |  |  |  |  |  |  |  |  |  |  |  |  |  |  |  |  |  |  |  |  |  |  |  |  |  |  |  |  |  |  |  |  |  |  |  |  |  |  |  |  |  |  |  |  |  |  |  |  |  |  |  |  |  |  |  |  |  |  |  |  |  |  |  |  |  |  |  |  |  |  |  |  |  |  |  |  |  |  |  |  |  |  |  |  |  |  |  |  |  |  |  |  |  |  |  |  |  |  |  |  |  |  |  |  |  |  |  |  |  |  |  |  |  |  |  |  |  |  |  |  |  |  |  |  |  |  |  |  |  |  |  |  |  |  |  |  |  |  |  |  |  |  |  |  |  |  |  |  |  |  |  |  |  |  |  |  |  |  |  |  |  |  |  |  |  |  |  |  |  |  |  |  |  |  |  |  |  |  |  |  |  |  |  |  |  |  |  |  |  |  |  |  |  |  |  |  |  |  |  |  |  |  |  |  |  |  |  |  |  |  |  |  |  |  |  |  |  |  |  |  |  |  |  |  |  |  |  |  |  |  |  |  |  |  |  |  |  |  |  |  |  |  |  |  |  |  |  |  |  |  |  |  |  |  |  |  |  |  |  |  |  |  |  |  |  |  |  |  |  |  |  |  |  |  |  |  |  |  |  |  |  |  |  |  |  |  |  |  |  |  |  |  |  |  |  |  |  |  |  |  |  |  |  |  |  |  |  |  |  |  |  |  |  |  |  |  |  |  |  |  |  |  |  |  |  |  |  |  |  |  |  |  |  |  |  |  |  |  |  |  |  |  |  |  |  |  |  |  |  |  |  |  |  |  |  |  |  |  |  |  |  |  |  |  |  |  |  |  |  |  |  |  |  |  |  |  |  |  |  |  |  |  |  |  |  |  |  |  |  |  |  |  |  |  |  |  |  |  |  |  |  |  |  |  |  |  |  |  |  |  |  |  |  |  |  |  |  |  |  |  |  |  |  |  |  |  |  |  |  |  |  |  |  |  |  |  |  |  |  |  |  |  |  |  |  |  |  |  |  |  |  |  |  |  |  |  |  |  |  |  |  |  |  |  |  |  |  |  |  |  |  |  |  |  |  |  |  |  |  |  |  |  |  |  |  |  |  |  |  |  |  |  |  |  |  |  |  |  |  |  |  |  |  |  |  |  |  |  |
|  |  |  |  |  |  |  |  |  |  |  |  |  |  |  |  |  |  |  |  |  |  |  |  |  |  |  |  |  |  |  |  |  |  |  |  |  |  |  |  |  |  |  |  |  |  |  |  |  |  |  |  |  |  |  |  |  |  |  |  |  |  |  |  |  |  |  |  |  |  |  |  |  |  |  |  |  |  |  |  |  |  |  |  |  |  |  |  |  |  |  |  |  |  |  |  |  |  |  |  |  |  |  |  |  |  |  |  |  |  |  |  |  |  |  |  |  |  |  |  |  |  |  |  |  |  |  |  |  |  |  |  |  |  |  |  |  |  |  |  |  |  |  |  |  |  |  |  |  |  |  |  |  |  |  |  |  |  |  |  |  |  |  |  |  |  |  |  |  |  |  |  |  |  |  |  |  |  |  |  |  |  |  |  |  |  |  |  |  |  |  |  |  |  |  |  |  |  |  |  |  |  |  |  |  |  |  |  |  |  |  |  |  |  |  |  |  |  |  |  |  |  |  |  |  |  |  |  |  |  |  |  |  |  |  |  |  |  |  |  |  |  |  |  |  |  |  |  |  |  |  |  |  |  |  |  |  |  |  |  |  |  |  |  |  |  |  |  |  |  |  |  |  |  |  |  |  |  |  |  |  |  |  |  |  |  |  |  |  |  |  |  |  |  |  |  |  |  |  |  |  |  |  |  |  |  |  |  |  |  |  |  |  |  |  |  |  |  |  |  |  |  |  |  |  |  |  |  |  |  |  |  |  |  |  |  |  |  |  |  |  |  |  |  |  |  |  |  |  |  |  |  |  |  |  |  |  |  |  |  |  |  |  |  |  |  |  |  |  |  |  |  |  |  |  |  |  |  |  |  |  |  |  |  |  |  |  |  |  |  |  |  |  |  |  |  |  |  |  |  |  |  |  |  |  |  |  |  |  |  |  |  |  |  |  |  |  |  |  |  |  |  |  |  |  |  |  |  |  |  |  |  |  |  |  |  |  |  |  |  |  |  |  |  |  |  |  |  |  |  |  |  |  |  |  |  |  |  |  |  |  |  |  |  |  |  |  |  |  |  |  |  |  |  |  |  |  |  |  |  |  |  |  |  |  |  |  |  |  |  |  |  |  |  |  |  |  |  |  |  |  |  |  |  |  |  |  |  |  |  |  |  |  |  |  |  |  |  |  |  |  |  |  |  |  |  |  |  |  |  |  |  |  |  |  |  |  |  |  |  |  |  |  |  |  |  |  |  |  |  |  |  |  |  |  |  |  |  |  |  |  |  |  |  |  |  |  |  |  |  |  |  |  |  |  |  |  |  |  |  |  |  |  |  |  |  |  |  |  |  |  |  |  |  |  |  |  |  |  |  |  |  |  |  |  |  |  |  |  |  |  |  |  |  |  |  |  |  |  |  |  |  |  |  |  |  |  |  |  |  |  |  |  |  |  |  |  |  |  |  |  |  |  |  |  |  |  |  |  |  |  |  |  |  |  |  |  |  |  |  |  |  |  |  |  |  |  |  |  |  |  |  |  |  |  |  |  |  |  |  |  |  |  |  |  |  |  |  |  |  |  |  |  |  |  |  |  |  |  |  |  |  |  |  |  |  |  |  |  |  |  |  |  |  |  |  |  |  |  |  |  |  |  |  |  |  |  |  |  |  |  |  |  |  |  |  |  |  |  |  |  |  |  |  |  |  |  |  |  |  |  |  |  |  |  |  |  |  |  |  |  |  |  |  |  |  |  |  |  |  |  |  |  |  |  |  |  |  |  |  |  |  |  |  |  |  |  |  |  |  |  |  |  |  |  |  |  |  |  |  |  |  |  |  |  |  |  |  |  |  |  |  |  |  |  |  |  |  |  |  |  |  |  |  |  |  |  |  |  |  |  |  |  |  |  |  |  |  |  |  |  |  |  |  |  |  |  |  |  |  |  |  |  |  |  |  |  |  |  |  |  |  |  |  |  |  |  |  |  |  |  |  |  |  |  |  |  |  |  |  |  |  |  |  |  |  |  |  |  |  |  |  |  |  |  |  |  |  |  |  |  |  |  |  |  |  |  |  |  |  |  |  |  |  |  |  |  |  |  |  |  |  |  |  |  |  |  |  |  |  |  |  |  |  |  |  |  |  |  |  |  |  |  |  |  |  |  |  |  |  |  |  |  |  |  |  |  |  |  |  |  |  |  |  |  |  |  |  |  |  |  |  |  |  |  |  |  |  |  |  |  |  |  |  |  |  |  |  |  |  |  |  |  |  |  |  |  |  |  |  |  |  |  |  |  |  |  |  |  |  |  |  |  |  |  |  |  |  |  |  |  |  |  |  |  |  |  |  |  |  |  |  |  |  |  |  |  |  |  |  |  |  |  |  |  |  |  |  |  |  |  |  |  |  |  |  |  |  |  |  |  |  |  |  |  |  |  |  |  |  |  |  |  |  |  |  |
|  |  |  |  |  |  |  |  |  |  |  |  |  |  |  |  |  |  |  |  |  |  |  |  |  |  |  |  |  |  |  |  |  |  |  |  |  |  |  |  |  |  |  |  |  |  |  |  |  |  |  |  |  |  |  |  |  |  |  |  |  |  |  |  |  |  |  |  |  |  |  |  |  |  |  |  |  |  |  |  |  |  |  |  |  |  |  |  |  |  |  |  |  |  |  |  |  |  |  |  |  |  |  |  |  |  |  |  |  |  |  |  |  |  |  |  |  |  |  |  |  |  |  |  |  |  |  |  |  |  |  |  |  |  |  |  |  |  |  |  |  |  |  |  |  |  |  |  |  |  |  |  |  |  |  |  |  |  |  |  |  |  |  |  |  |  |  |  |  |  |  |  |  |  |  |  |  |  |  |  |  |  |  |  |  |  |  |  |  |  |  |  |  |  |  |  |  |  |  |  |  |  |  |  |  |  |  |  |  |  |  |  |  |  |  |  |  |  |  |  |  |  |  |  |  |  |  |  |  |  |  |  |  |  |  |  |  |  |  |  |  |  |  |  |  |  |  |  |  |  |  |  |  |  |  |  |  |  |  |  |  |  |  |  |  |  |  |  |  |  |  |  |  |  |  |  |  |  |  |  |  |  |  |  |  |  |  |  |  |  |  |  |  |  |  |  |  |  |  |  |  |  |  |  |  |  |  |  |  |  |  |  |  |  |  |  |  |  |  |  |  |  |  |  |  |  |  |  |  |  |  |  |  |  |  |  |  |  |  |  |  |  |  |  |  |  |  |  |  |  |  |  |  |  |  |  |  |  |  |  |  |  |  |  |  |  |  |  |  |  |  |  |  |  |  |  |  |  |  |  |  |  |  |  |  |  |  |  |  |  |  |  |  |  |  |  |  |  |  |  |  |  |  |  |  |  |  |  |  |  |  |  |  |  |  |  |  |  |  |  |  |  |  |  |  |  |  |  |  |  |  |  |  |  |  |  |  |  |  |  |  |  |  |  |  |  |  |  |  |  |  |  |  |  |  |  |  |  |  |  |  |  |  |  |  |  |  |  |  |  |  |  |  |  |  |  |  |  |  |  |  |  |  |  |  |  |  |  |  |  |  |  |  |  |  |  |  |  |  |  |  |  |  |  |  |  |  |  |  |  |  |  |  |  |  |  |  |  |  |  |  |  |  |  |  |  |  |  |  |  |  |  |  |  |  |  |  |  |  |  |  |  |  |  |  |  |  |  |  |  |  |  |  |  |  |  |  |  |  |  |  |  |  |  |  |  |  |  |  |  |  |  |  |  |  |  |  |  |  |  |  |  |  |  |  |  |  |  |  |  |  |  |  |  |  |  |  |  |  |  |  |  |  |  |  |  |  |  |  |  |  |  |  |  |  |  |  |  |  |  |  |  |  |  |  |  |  |  |  |  |  |  |  |  |  |  |  |  |  |  |  |  |  |  |  |  |  |  |  |  |  |  |  |  |  |  |  |  |  |  |  |  |  |  |  |  |  |  |  |  |  |  |  |  |  |  |  |  |  |  |  |  |  |  |  |  |  |  |  |  |  |  |  |  |  |  |  |  |  |  |  |  |  |  |  |  |  |  |  |  |  |  |  |  |  |  |  |  |  |  |  |  |  |  |  |  |  |  |  |  |  |  |  |  |  |  |  |  |  |  |  |  |  |  |  |  |  |  |  |  |  |  |  |  |  |  |  |  |  |  |  |  |  |  |  |  |  |  |  |  |  |  |  |  |  |  |  |  |  |  |  |  |  |  |  |  |  |  |  |  |  |  |  |  |  |  |  |  |  |  |  |  |  |  |  |  |  |  |  |  |  |  |  |  |  |  |  |  |  |  |  |  |  |  |  |  |  |  |  |  |  |  |  |  |  |  |  |  |  |  |  |  |  |  |  |  |  |  |  |  |  |  |  |  |  |  |  |  |  |  |  |  |  |  |  |  |  |  |  |  |  |  |  |  |  |  |  |  |  |  |  |  |  |  |  |  |  |  |  |  |  |  |  |  |  |  |  |  |  |  |  |  |  |  |  |  |  |  |  |  |  |  |  |  |  |  |  |  |  |  |  |  |  |  |  |  |  |  |  |  |  |  |  |  |  |  |  |  |  |  |  |  |  |  |  |  |  |  |  |  |  |  |  |  |  |  |  |  |  |  |  |  |  |  |  |  |  |  |  |  |  |  |  |  |  |  |  |  |  |  |  |  |  |  |  |  |  |  |  |  |  |  |  |  |  |  |  |  |  |  |  |  |  |  |  |  |  |  |  |  |  |  |  |  |  |  |  |  |  |  |  |  |  |  |  |  |  |  |  |  |  |  |  |  |  |  |  |  |  |  |  |  |  |  |  |  |  |  |  |  |  |  |  |  |  |  |  |  |  |  |  |  |  |  |  |  |  |  |  |  |  |  |  |  |  |  |
|  |  |  |  |  |  |  |  |  |  |  |  |  |  |  |  |  |  |  |  |  |  |  |  |  |  |  |  |  |  |  |  |  |  |  |  |  |  |  |  |  |  |  |  |  |  |  |  |  |  |  |  |  |  |  |  |  |  |  |  |  |  |  |  |  |  |  |  |  |  |  |  |  |  |  |  |  |  |  |  |  |  |  |  |  |  |  |  |  |  |  |  |  |  |  |  |  |  |  |  |  |  |  |  |  |  |  |  |  |  |  |  |  |  |  |  |  |  |  |  |  |  |  |  |  |  |  |  |  |  |  |  |  |  |  |  |  |  |  |  |  |  |  |  |  |  |  |  |  |  |  |  |  |  |  |  |  |  |  |  |  |  |  |  |  |  |  |  |  |  |  |  |  |  |  |  |  |  |  |  |  |  |  |  |  |  |  |  |  |  |  |  |  |  |  |  |  |  |  |  |  |  |  |  |  |  |  |  |  |  |  |  |  |  |  |  |  |  |  |  |  |  |  |  |  |  |  |  |  |  |  |  |  |  |  |  |  |  |  |  |  |  |  |  |  |  |  |  |  |  |  |  |  |  |  |  |  |  |  |  |  |  |  |  |  |  |  |  |  |  |  |  |  |  |  |  |  |  |  |  |  |  |  |  |  |  |  |  |  |  |  |  |  |  |  |  |  |  |  |  |  |  |  |  |  |  |  |  |  |  |  |  |  |  |  |  |  |  |  |  |  |  |  |  |  |  |  |  |  |  |  |  |  |  |  |  |  |  |  |  |  |  |  |  |  |  |  |  |  |  |  |  |  |  |  |  |  |  |  |  |  |  |  |  |  |  |  |  |  |  |  |  |  |  |  |  |  |  |  |  |  |  |  |  |  |  |  |  |  |  |  |  |  |  |  |  |  |  |  |  |  |  |  |  |  |  |  |  |  |  |  |  |  |  |  |  |  |  |  |  |  |  |  |  |  |  |  |  |  |  |  |  |  |  |  |  |  |  |  |  |  |  |  |  |  |  |  |  |  |  |  |  |  |  |  |  |  |  |  |  |  |  |  |  |  |  |  |  |  |  |  |  |  |  |  |  |  |  |  |  |  |  |  |  |  |  |  |  |  |  |  |  |  |  |  |  |  |  |  |  |  |  |  |  |  |  |  |  |  |  |  |  |  |  |  |  |  |  |  |  |  |  |  |  |  |  |  |  |  |  |  |  |  |  |  |  |  |  |  |  |  |  |  |  |  |  |  |  |  |  |  |  |  |  |  |  |  |  |  |  |  |  |  |  |  |  |  |  |  |  |  |  |  |  |  |  |  |  |  |  |  |  |  |  |  |  |  |  |  |  |  |  |  |  |  |  |  |  |  |  |  |  |  |  |  |  |  |  |  |  |  |  |  |  |  |  |  |  |  |  |  |  |  |  |  |  |  |  |  |  |  |  |  |  |  |  |  |  |  |  |  |  |  |  |  |  |  |  |  |  |  |  |  |  |  |  |  |  |  |  |  |  |  |  |  |  |  |  |  |  |  |  |  |  |  |  |  |  |  |  |  |  |  |  |  |  |  |  |  |  |  |  |  |  |  |  |  |  |  |  |  |  |  |  |  |  |  |  |  |  |  |  |  |  |  |  |  |  |  |  |  |  |  |  |  |  |  |  |  |  |  |  |  |  |  |  |  |  |  |  |  |  |  |  |  |  |  |  |  |  |  |  |  |  |  |  |  |  |  |  |  |  |  |  |  |  |  |  |  |  |  |  |  |  |  |  |  |  |  |  |  |  |  |  |  |  |  |  |  |  |  |  |  |  |  |  |  |  |  |  |  |  |  |  |  |  |  |  |  |  |  |  |  |  |  |  |  |  |  |  |  |  |  |  |  |  |  |  |  |  |  |  |  |  |  |  |  |  |  |  |  |  |  |  |  |  |  |  |  |  |  |  |  |  |  |  |  |  |  |  |  |  |  |  |  |  |  |  |  |  |  |  |  |  |  |  |  |  |  |  |  |  |  |  |  |  |  |  |  |  |  |  |  |  |  |  |  |  |  |  |  |  |  |  |  |  |  |  |  |  |  |  |  |  |  |  |  |  |  |  |  |  |  |  |  |  |  |  |  |  |  |  |  |  |  |  |  |  |  |  |  |  |  |  |  |  |  |  |  |  |  |  |  |  |  |  |  |  |  |  |  |  |  |  |  |  |  |  |  |  |  |  |  |  |  |  |  |  |  |  |  |  |  |  |  |  |  |  |  |  |  |  |  |  |  |  |  |  |  |  |  |  |  |  |  |  |  |  |  |  |  |  |  |  |  |  |  |  |  |  |  |  |  |  |  |  |  |  |  |  |  |  |  |  |  |  |  |  |  |  |  |  |  |  |  |  |  |  |  |  |  |  |  |  |  |  |  |  |  |  |  |  |  |  |  |  |  |  |  |  |  |  |  |  |  |  |
|  |  |  |  |  |  |  |  |  |  |  |  |  |  |  |  |  |  |  |  |  |  |  |  |  |  |  |  |  |  |  |  |  |  |  |  |  |  |  |  |  |  |  |  |  |  |  |  |  |  |  |  |  |  |  |  |  |  |  |  |  |  |  |  |  |  |  |  |  |  |  |  |  |  |  |  |  |  |  |  |  |  |  |  |  |  |  |  |  |  |  |  |  |  |  |  |  |  |  |  |  |  |  |  |  |  |  |  |  |  |  |  |  |  |  |  |  |  |  |  |  |  |  |  |  |  |  |  |  |  |  |  |  |  |  |  |  |  |  |  |  |  |  |  |  |  |  |  |  |  |  |  |  |  |  |  |  |  |  |  |  |  |  |  |  |  |  |  |  |  |  |  |  |  |  |  |  |  |  |  |  |  |  |  |  |  |  |  |  |  |  |  |  |  |  |  |  |  |  |  |  |  |  |  |  |  |  |  |  |  |  |  |  |  |  |  |  |  |  |  |  |  |  |  |  |  |  |  |  |  |  |  |  |  |  |  |  |  |  |  |  |  |  |  |  |  |  |  |  |  |  |  |  |  |  |  |  |  |  |  |  |  |  |  |  |  |  |  |  |  |  |  |  |  |  |  |  |  |  |  |  |  |  |  |  |  |  |  |  |  |  |  |  |  |  |  |  |  |  |  |  |  |  |  |  |  |  |  |  |  |  |  |  |  |  |  |  |  |  |  |  |  |  |  |  |  |  |  |  |  |  |  |  |  |  |  |  |  |  |  |  |  |  |  |  |  |  |  |  |  |  |  |  |  |  |  |  |  |  |  |  |  |  |  |  |  |  |  |  |  |  |  |  |  |  |  |  |  |  |  |  |  |  |  |  |  |  |  |  |  |  |  |  |  |  |  |  |  |  |  |  |  |  |  |  |  |  |  |  |  |  |  |  |  |  |  |  |  |  |  |  |  |  |  |  |  |  |  |  |  |  |  |  |  |  |  |  |  |  |  |  |  |  |  |  |  |  |  |  |  |  |  |  |  |  |  |  |  |  |  |  |  |  |  |  |  |  |  |  |  |  |  |  |  |  |  |  |  |  |  |  |  |  |  |  |  |  |  |  |  |  |  |  |  |  |  |  |  |  |  |  |  |  |  |  |  |  |  |  |  |  |  |  |  |  |  |  |  |  |  |  |  |  |  |  |  |  |  |  |  |  |  |  |  |  |  |  |  |  |  |  |  |  |  |  |  |  |  |  |  |  |  |  |  |  |  |  |  |  |  |  |  |  |  |  |  |  |  |  |  |  |  |  |  |  |  |  |  |  |  |  |  |  |  |  |  |  |  |  |  |  |  |  |  |  |  |  |  |  |  |  |  |  |  |  |  |  |  |  |  |  |  |  |  |  |  |  |  |  |  |  |  |  |  |  |  |  |  |  |  |  |  |  |  |  |  |  |  |  |  |  |  |  |  |  |  |  |  |  |  |  |  |  |  |  |  |  |  |  |  |  |  |  |  |  |  |  |  |  |  |  |  |  |  |  |  |  |  |  |  |  |  |  |  |  |  |  |  |  |  |  |  |  |  |  |  |  |  |  |  |  |  |  |  |  |  |  |  |  |  |  |  |  |  |  |  |  |  |  |  |  |  |  |  |  |  |  |  |  |  |  |  |  |  |  |  |  |  |  |  |  |  |  |  |  |  |  |  |  |  |  |  |  |  |  |  |  |  |  |  |  |  |  |  |  |  |  |  |  |  |  |  |  |  |  |  |  |  |  |  |  |  |  |  |  |  |  |  |  |  |  |  |  |  |  |  |  |  |  |  |  |  |  |  |  |  |  |  |  |  |  |  |  |  |  |  |  |  |  |  |  |  |  |  |  |  |  |  |  |  |  |  |  |  |  |  |  |  |  |  |  |  |  |  |  |  |  |  |  |  |  |  |  |  |  |  |  |  |  |  |  |  |  |  |  |  |  |  |  |  |  |  |  |  |  |  |  |  |  |  |  |  |  |  |  |  |  |  |  |  |  |  |  |  |  |  |  |  |  |  |  |  |  |  |  |  |  |  |  |  |  |  |  |  |  |  |  |  |  |  |  |  |  |  |  |  |  |  |  |  |  |  |  |  |  |  |  |  |  |  |  |  |  |  |  |  |  |  |  |  |  |  |  |  |  |  |  |  |  |  |  |  |  |  |  |  |  |  |  |  |  |  |  |  |  |  |  |  |  |  |  |  |  |  |  |  |  |  |  |  |  |  |  |  |  |  |  |  |  |  |  |  |  |  |  |  |  |  |  |  |  |  |  |  |  |  |  |  |  |  |  |  |  |  |  |  |  |  |  |  |  |  |  |  |  |  |  |  |  |  |  |  |  |  |  |  |  |  |  |  |  |  |  |  |  |  |  |  |  |  |  |  |  |  |  |  |  |  |  |  |  |  |  |  |  |  |
|  |  |  |  |  |  |  |  |  |  |  |  |  |  |  |  |  |  |  |  |  |  |  |  |  |  |  |  |  |  |  |  |  |  |  |  |  |  |  |  |  |  |  |  |  |  |  |  |  |  |  |  |  |  |  |  |  |  |  |  |  |  |  |  |  |  |  |  |  |  |  |  |  |  |  |  |  |  |  |  |  |  |  |  |  |  |  |  |  |  |  |  |  |  |  |  |  |  |  |  |  |  |  |  |  |  |  |  |  |  |  |  |  |  |  |  |  |  |  |  |  |  |  |  |  |  |  |  |  |  |  |  |  |  |  |  |  |  |  |  |  |  |  |  |  |  |  |  |  |  |  |  |  |  |  |  |  |  |  |  |  |  |  |  |  |  |  |  |  |  |  |  |  |  |  |  |  |  |  |  |  |  |  |  |  |  |  |  |  |  |  |  |  |  |  |  |  |  |  |  |  |  |  |  |  |  |  |  |  |  |  |  |  |  |  |  |  |  |  |  |  |  |  |  |  |  |  |  |  |  |  |  |  |  |  |  |  |  |  |  |  |  |  |  |  |  |  |  |  |  |  |  |  |  |  |  |  |  |  |  |  |  |  |  |  |  |  |  |  |  |  |  |  |  |  |  |  |  |  |  |  |  |  |  |  |  |  |  |  |  |  |  |  |  |  |  |  |  |  |  |  |  |  |  |  |  |  |  |  |  |  |  |  |  |  |  |  |  |  |  |  |  |  |  |  |  |  |  |  |  |  |  |  |  |  |  |  |  |  |  |  |  |  |  |  |  |  |  |  |  |  |  |  |  |  |  |  |  |  |  |  |  |  |  |  |  |  |  |  |  |  |  |  |  |  |  |  |  |  |  |  |  |  |  |  |  |  |  |  |  |  |  |  |  |  |  |  |  |  |  |  |  |  |  |  |  |  |  |  |  |  |  |  |  |  |  |  |  |  |  |  |  |  |  |  |  |  |  |  |  |  |  |  |  |  |  |  |  |  |  |  |  |  |  |  |  |  |  |  |  |  |  |  |  |  |  |  |  |  |  |  |  |  |  |  |  |  |  |  |  |  |  |  |  |  |  |  |  |  |  |  |  |  |  |  |  |  |  |  |  |  |  |  |  |  |  |  |  |  |  |  |  |  |  |  |  |  |  |  |  |  |  |  |  |  |  |  |  |  |  |  |  |  |  |  |  |  |  |  |  |  |  |  |  |  |  |  |  |  |  |  |  |  |  |  |  |  |  |  |  |  |  |  |  |  |  |  |  |  |  |  |  |  |  |  |  |  |  |  |  |  |  |  |  |  |  |  |  |  |  |  |  |  |  |  |  |  |  |  |  |  |  |  |  |  |  |  |  |  |  |  |  |  |  |  |  |  |  |  |  |  |  |  |  |  |  |  |  |  |  |  |  |  |  |  |  |  |  |  |  |  |  |  |  |  |  |  |  |  |  |  |  |  |  |  |  |  |  |  |  |  |  |  |  |  |  |  |  |  |  |  |  |  |  |  |  |  |  |  |  |  |  |  |  |  |  |  |  |  |  |  |  |  |  |  |  |  |  |  |  |  |  |  |  |  |  |  |  |  |  |  |  |  |  |  |  |  |  |  |  |  |  |  |  |  |  |  |  |  |  |  |  |  |  |  |  |  |  |  |  |  |  |  |  |  |  |  |  |  |  |  |  |  |  |  |  |  |  |  |  |  |  |  |  |  |  |  |  |  |  |  |  |  |  |  |  |  |  |  |  |  |  |  |  |  |  |  |  |  |  |  |  |  |  |  |  |  |  |  |  |  |  |  |  |  |  |  |  |  |  |  |  |  |  |  |  |  |  |  |  |  |  |  |  |  |  |  |  |  |  |  |  |  |  |  |  |  |  |  |  |  |  |  |  |  |  |  |  |  |  |  |  |  |  |  |  |  |  |  |  |  |  |  |  |  |  |  |  |  |  |  |  |  |  |  |  |  |  |  |  |  |  |  |  |  |  |  |  |  |  |  |  |  |  |  |  |  |  |  |  |  |  |  |  |  |  |  |  |  |  |  |  |  |  |  |  |  |  |  |  |  |  |  |  |  |  |  |  |  |  |  |  |  |  |  |  |  |  |  |  |  |  |  |  |  |  |  |  |  |  |  |  |  |  |  |  |  |  |  |  |  |  |  |  |  |  |  |  |  |  |  |  |  |  |  |  |  |  |  |  |  |  |  |  |  |  |  |  |  |  |  |  |  |  |  |  |  |  |  |  |  |  |  |  |  |  |  |  |  |  |  |  |  |  |  |  |  |  |  |  |  |  |  |  |  |  |  |  |  |  |  |  |  |  |  |  |  |  |  |  |  |  |  |  |  |  |  |  |  |  |  |  |  |  |  |  |  |  |  |  |  |  |  |  |  |  |  |  |  |  |  |  |  |  |  |  |  |  |  |  |  |  |  |  |  |  |
|  |  |  |  |  |  |  |  |  |  |  |  |  |  |  |  |  |  |  |  |  |  |  |  |  |  |  |  |  |  |  |  |  |  |  |  |  |  |  |  |  |  |  |  |  |  |  |  |  |  |  |  |  |  |  |  |  |  |  |  |  |  |  |  |  |  |  |  |  |  |  |  |  |  |  |  |  |  |  |  |  |  |  |  |  |  |  |  |  |  |  |  |  |  |  |  |  |  |  |  |  |  |  |  |  |  |  |  |  |  |  |  |  |  |  |  |  |  |  |  |  |  |  |  |  |  |  |  |  |  |  |  |  |  |  |  |  |  |  |  |  |  |  |  |  |  |  |  |  |  |  |  |  |  |  |  |  |  |  |  |  |  |  |  |  |  |  |  |  |  |  |  |  |  |  |  |  |  |  |  |  |  |  |  |  |  |  |  |  |  |  |  |  |  |  |  |  |  |  |  |  |  |  |  |  |  |  |  |  |  |  |  |  |  |  |  |  |  |  |  |  |  |  |  |  |  |  |  |  |  |  |  |  |  |  |  |  |  |  |  |  |  |  |  |  |  |  |  |  |  |  |  |  |  |  |  |  |  |  |  |  |  |  |  |  |  |  |  |  |  |  |  |  |  |  |  |  |  |  |  |  |  |  |  |  |  |  |  |  |  |  |  |  |  |  |  |  |  |  |  |  |  |  |  |  |  |  |  |  |  |  |  |  |  |  |  |  |  |  |  |  |  |  |  |  |  |  |  |  |  |  |  |  |  |  |  |  |  |  |  |  |  |  |  |  |  |  |  |  |  |  |  |  |  |  |  |  |  |  |  |  |  |  |  |  |  |  |  |  |  |  |  |  |  |  |  |  |  |  |  |  |  |  |  |  |  |  |  |  |  |  |  |  |  |  |  |  |  |  |  |  |  |  |  |  |  |  |  |  |  |  |  |  |  |  |  |  |  |  |  |  |  |  |  |  |  |  |  |  |  |  |  |  |  |  |  |  |  |  |  |  |  |  |  |  |  |  |  |  |  |  |  |  |  |  |  |  |  |  |  |  |  |  |  |  |  |  |  |  |  |  |  |  |  |  |  |  |  |  |  |  |  |  |  |  |  |  |  |  |  |  |  |  |  |  |  |  |  |  |  |  |  |  |  |  |  |  |  |  |  |  |  |  |  |  |  |  |  |  |  |  |  |  |  |  |  |  |  |  |  |  |  |  |  |  |  |  |  |  |  |  |  |  |  |  |  |  |  |  |  |  |  |  |  |  |  |  |  |  |  |  |  |  |  |  |  |  |  |  |  |  |  |  |  |  |  |  |  |  |  |  |  |  |  |  |  |  |  |  |  |  |  |  |  |  |  |  |  |  |  |  |  |  |  |  |  |  |  |  |  |  |  |  |  |  |  |  |  |  |  |  |  |  |  |  |  |  |  |  |  |  |  |  |  |  |  |  |  |  |  |  |  |  |  |  |  |  |  |  |  |  |  |  |  |  |  |  |  |  |  |  |  |  |  |  |  |  |  |  |  |  |  |  |  |  |  |  |  |  |  |  |  |  |  |  |  |  |  |  |  |  |  |  |  |  |  |  |  |  |  |  |  |  |  |  |  |  |  |  |  |  |  |  |  |  |  |  |  |  |  |  |  |  |  |  |  |  |  |  |  |  |  |  |  |  |  |  |  |  |  |  |  |  |  |  |  |  |  |  |  |  |  |  |  |  |  |  |  |  |  |  |  |  |  |  |  |  |  |  |  |  |  |  |  |  |  |  |  |  |  |  |  |  |  |  |  |  |  |  |  |  |  |  |  |  |  |  |  |  |  |  |  |  |  |  |  |  |  |  |  |  |  |  |  |  |  |  |  |  |  |  |  |  |  |  |  |  |  |  |  |  |  |  |  |  |  |  |  |  |  |  |  |  |  |  |  |  |  |  |  |  |  |  |  |  |  |  |  |  |  |  |  |  |  |  |  |  |  |  |  |  |  |  |  |  |  |  |  |  |  |  |  |  |  |  |  |  |  |  |  |  |  |  |  |  |  |  |  |  |  |  |  |  |  |  |  |  |  |  |  |  |  |  |  |  |  |  |  |  |  |  |  |  |  |  |  |  |  |  |  |  |  |  |  |  |  |  |  |  |  |  |  |  |  |  |  |  |  |  |  |  |  |  |  |  |  |  |  |  |  |  |  |  |  |  |  |  |  |  |  |  |  |  |  |  |  |  |  |  |  |  |  |  |  |  |  |  |  |  |  |  |  |  |  |  |  |  |  |  |  |  |  |  |  |  |  |  |  |  |  |  |  |  |  |  |  |  |  |  |  |  |  |  |  |  |  |  |  |  |  |  |  |  |  |  |  |  |  |  |  |  |  |  |  |  |  |  |  |  |  |  |  |  |  |  |  |  |  |  |  |  |  |  |  |  |  |  |  |  |  |  |  |  |  |  |  |
|  |  |  |  |  |  |  |  |  |  |  |  |  |  |  |  |  |  |  |  |  |  |  |  |  |  |  |  |  |  |  |  |  |  |  |  |  |  |  |  |  |  |  |  |  |  |  |  |  |  |  |  |  |  |  |  |  |  |  |  |  |  |  |  |  |  |  |  |  |  |  |  |  |  |  |  |  |  |  |  |  |  |  |  |  |  |  |  |  |  |  |  |  |  |  |  |  |  |  |  |  |  |  |  |  |  |  |  |  |  |  |  |  |  |  |  |  |  |  |  |  |  |  |  |  |  |  |  |  |  |  |  |  |  |  |  |  |  |  |  |  |  |  |  |  |  |  |  |  |  |  |  |  |  |  |  |  |  |  |  |  |  |  |  |  |  |  |  |  |  |  |  |  |  |  |  |  |  |  |  |  |  |  |  |  |  |  |  |  |  |  |  |  |  |  |  |  |  |  |  |  |  |  |  |  |  |  |  |  |  |  |  |  |  |  |  |  |  |  |  |  |  |  |  |  |  |  |  |  |  |  |  |  |  |  |  |  |  |  |  |  |  |  |  |  |  |  |  |  |  |  |  |  |  |  |  |  |  |  |  |  |  |  |  |  |  |  |  |  |  |  |  |  |  |  |  |  |  |  |  |  |  |  |  |  |  |  |  |  |  |  |  |  |  |  |  |  |  |  |  |  |  |  |  |  |  |  |  |  |  |  |  |  |  |  |  |  |  |  |  |  |  |  |  |  |  |  |  |  |  |  |  |  |  |  |  |  |  |  |  |  |  |  |  |  |  |  |  |  |  |  |  |  |  |  |  |  |  |  |  |  |  |  |  |  |  |  |  |  |  |  |  |  |  |  |  |  |  |  |  |  |  |  |  |  |  |  |  |  |  |  |  |  |  |  |  |  |  |  |  |  |  |  |  |  |  |  |  |  |  |  |  |  |  |  |  |  |  |  |  |  |  |  |  |  |  |  |  |  |  |  |  |  |  |  |  |  |  |  |  |  |  |  |  |  |  |  |  |  |  |  |  |  |  |  |  |  |  |  |  |  |  |  |  |  |  |  |  |  |  |  |  |  |  |  |  |  |  |  |  |  |  |  |  |  |  |  |  |  |  |  |  |  |  |  |  |  |  |  |  |  |  |  |  |  |  |  |  |  |  |  |  |  |  |  |  |  |  |  |  |  |  |  |  |  |  |  |  |  |  |  |  |  |  |  |  |  |  |  |  |  |  |  |  |  |  |  |  |  |  |  |  |  |  |  |  |  |  |  |  |  |  |  |  |  |  |  |  |  |  |  |  |  |  |  |  |  |  |  |  |  |  |  |  |  |  |  |  |  |  |  |  |  |  |  |  |  |  |  |  |  |  |  |  |  |  |  |  |  |  |  |  |  |  |  |  |  |  |  |  |  |  |  |  |  |  |  |  |  |  |  |  |  |  |  |  |  |  |  |  |  |  |  |  |  |  |  |  |  |  |  |  |  |  |  |  |  |  |  |  |  |  |  |  |  |  |  |  |  |  |  |  |  |  |  |  |  |  |  |  |  |  |  |  |  |  |  |  |  |  |  |  |  |  |  |  |  |  |  |  |  |  |  |  |  |  |  |  |  |  |  |  |  |  |  |  |  |  |  |  |  |  |  |  |  |  |  |  |  |  |  |  |  |  |  |  |  |  |  |  |  |  |  |  |  |  |  |  |  |  |  |  |  |  |  |  |  |  |  |  |  |  |  |  |  |  |  |  |  |  |  |  |  |  |  |  |  |  |  |  |  |  |  |  |  |  |  |  |  |  |  |  |  |  |  |  |  |  |  |  |  |  |  |  |  |  |  |  |  |  |  |  |  |  |  |  |  |  |  |  |  |  |  |  |  |  |  |  |  |  |  |  |  |  |  |  |  |  |  |  |  |  |  |  |  |  |  |  |  |  |  |  |  |  |  |  |  |  |  |  |  |  |  |  |  |  |  |  |  |  |  |  |  |  |  |  |  |  |  |  |  |  |  |  |  |  |  |  |  |  |  |  |  |  |  |  |  |  |  |  |  |  |  |  |  |  |  |  |  |  |  |  |  |  |  |  |  |  |  |  |  |  |  |  |  |  |  |  |  |  |  |  |  |  |  |  |  |  |  |  |  |  |  |  |  |  |  |  |  |  |  |  |  |  |  |  |  |  |  |  |  |  |  |  |  |  |  |  |  |  |  |  |  |  |  |  |  |  |  |  |  |  |  |  |  |  |  |  |  |  |  |  |  |  |  |  |  |  |  |  |  |  |  |  |  |  |  |  |  |  |  |  |  |  |  |  |  |  |  |  |  |  |  |  |  |  |  |  |  |  |  |  |  |  |  |  |  |  |  |  |  |  |  |  |  |  |  |  |  |  |  |  |  |  |  |  |  |  |  |  |  |  |  |  |  |  |  |  |  |  |  |  |  |  |  |  |
|  |  |  |  |  |  |  |  |  |  |  |  |  |  |  |  |  |  |  |  |  |  |  |  |  |  |  |  |  |  |  |  |  |  |  |  |  |  |  |  |  |  |  |  |  |  |  |  |  |  |  |  |  |  |  |  |  |  |  |  |  |  |  |  |  |  |  |  |  |  |  |  |  |  |  |  |  |  |  |  |  |  |  |  |  |  |  |  |  |  |  |  |  |  |  |  |  |  |  |  |  |  |  |  |  |  |  |  |  |  |  |  |  |  |  |  |  |  |  |  |  |  |  |  |  |  |  |  |  |  |  |  |  |  |  |  |  |  |  |  |  |  |  |  |  |  |  |  |  |  |  |  |  |  |  |  |  |  |  |  |  |  |  |  |  |  |  |  |  |  |  |  |  |  |  |  |  |  |  |  |  |  |  |  |  |  |  |  |  |  |  |  |  |  |  |  |  |  |  |  |  |  |  |  |  |  |  |  |  |  |  |  |  |  |  |  |  |  |  |  |  |  |  |  |  |  |  |  |  |  |  |  |  |  |  |  |  |  |  |  |  |  |  |  |  |  |  |  |  |  |  |  |  |  |  |  |  |  |  |  |  |  |  |  |  |  |  |  |  |  |  |  |  |  |  |  |  |  |  |  |  |  |  |  |  |  |  |  |  |  |  |  |  |  |  |  |  |  |  |  |  |  |  |  |  |  |  |  |  |  |  |  |  |  |  |  |  |  |  |  |  |  |  |  |  |  |  |  |  |  |  |  |  |  |  |  |  |  |  |  |  |  |  |  |  |  |  |  |  |  |  |  |  |  |  |  |  |  |  |  |  |  |  |  |  |  |  |  |  |  |  |  |  |  |  |  |  |  |  |  |  |  |  |  |  |  |  |  |  |  |  |  |  |  |  |  |  |  |  |  |  |  |  |  |  |  |  |  |  |  |  |  |  |  |  |  |  |  |  |  |  |  |  |  |  |  |  |  |  |  |  |  |  |  |  |  |  |  |  |  |  |  |  |  |  |  |  |  |  |  |  |  |  |  |  |  |  |  |  |  |  |  |  |  |  |  |  |  |  |  |  |  |  |  |  |  |  |  |  |  |  |  |  |  |  |  |  |  |  |  |  |  |  |  |  |  |  |  |  |  |  |  |  |  |  |  |  |  |  |  |  |  |  |  |  |  |  |  |  |  |  |  |  |  |  |  |  |  |  |  |  |  |  |  |  |  |  |  |  |  |  |  |  |  |  |  |  |  |  |  |  |  |  |  |  |  |  |  |  |  |  |  |  |  |  |  |  |  |  |  |  |  |  |  |  |  |  |  |  |  |  |  |  |  |  |  |  |  |  |  |  |  |  |  |  |  |  |  |  |  |  |  |  |  |  |  |  |  |  |  |  |  |  |  |  |  |  |  |  |  |  |  |  |  |  |  |  |  |  |  |  |  |  |  |  |  |  |  |  |  |  |  |  |  |  |  |  |  |  |  |  |  |  |  |  |  |  |  |  |  |  |  |  |  |  |  |  |  |  |  |  |  |  |  |  |  |  |  |  |  |  |  |  |  |  |  |  |  |  |  |  |  |  |  |  |  |  |  |  |  |  |  |  |  |  |  |  |  |  |  |  |  |  |  |  |  |  |  |  |  |  |  |  |  |  |  |  |  |  |  |  |  |  |  |  |  |  |  |  |  |  |  |  |  |  |  |  |  |  |  |  |  |  |  |  |  |  |  |  |  |  |  |  |  |  |  |  |  |  |  |  |  |  |  |  |  |  |  |  |  |  |  |  |  |  |  |  |  |  |  |  |  |  |  |  |  |  |  |  |  |  |  |  |  |  |  |  |  |  |  |  |  |  |  |  |  |  |  |  |  |  |  |  |  |  |  |  |  |  |  |  |  |  |  |  |  |  |  |  |  |  |  |  |  |  |  |  |  |  |  |  |  |  |  |  |  |  |  |  |  |  |  |  |  |  |  |  |  |  |  |  |  |  |  |  |  |  |  |  |  |  |  |  |  |  |  |  |  |  |  |  |  |  |  |  |  |  |  |  |  |  |  |  |  |  |  |  |  |  |  |  |  |  |  |  |  |  |  |  |  |  |  |  |  |  |  |  |  |  |  |  |  |  |  |  |  |  |  |  |  |  |  |  |  |  |  |  |  |  |  |  |  |  |  |  |  |  |  |  |  |  |  |  |  |  |  |  |  |  |  |  |  |  |  |  |  |  |  |  |  |  |  |  |  |  |  |  |  |  |  |  |  |  |  |  |  |  |  |  |  |  |  |  |  |  |  |  |  |  |  |  |  |  |  |  |  |  |  |  |  |  |  |  |  |  |  |  |  |  |  |  |  |  |  |  |  |  |  |  |  |  |  |  |  |  |  |  |  |  |  |  |  |  |  |  |  |  |  |  |  |  |  |  |  |  |  |  |  |  |  |  |  |  |  |  |  |
|  |  |  |  |  |  |  |  |  |  |  |  |  |  |  |  |  |  |  |  |  |  |  |  |  |  |  |  |  |  |  |  |  |  |  |  |  |  |  |  |  |  |  |  |  |  |  |  |  |  |  |  |  |  |  |  |  |  |  |  |  |  |  |  |  |  |  |  |  |  |  |  |  |  |  |  |  |  |  |  |  |  |  |  |  |  |  |  |  |  |  |  |  |  |  |  |  |  |  |  |  |  |  |  |  |  |  |  |  |  |  |  |  |  |  |  |  |  |  |  |  |  |  |  |  |  |  |  |  |  |  |  |  |  |  |  |  |  |  |  |  |  |  |  |  |  |  |  |  |  |  |  |  |  |  |  |  |  |  |  |  |  |  |  |  |  |  |  |  |  |  |  |  |  |  |  |  |  |  |  |  |  |  |  |  |  |  |  |  |  |  |  |  |  |  |  |  |  |  |  |  |  |  |  |  |  |  |  |  |  |  |  |  |  |  |  |  |  |  |  |  |  |  |  |  |  |  |  |  |  |  |  |  |  |  |  |  |  |  |  |  |  |  |  |  |  |  |  |  |  |  |  |  |  |  |  |  |  |  |  |  |  |  |  |  |  |  |  |  |  |  |  |  |  |  |  |  |  |  |  |  |  |  |  |  |  |  |  |  |  |  |  |  |  |  |  |  |  |  |  |  |  |  |  |  |  |  |  |  |  |  |  |  |  |  |  |  |  |  |  |  |  |  |  |  |  |  |  |  |  |  |  |  |  |  |  |  |  |  |  |  |  |  |  |  |  |  |  |  |  |  |  |  |  |  |  |  |  |  |  |  |  |  |  |  |  |  |  |  |  |  |  |  |  |  |  |  |  |  |  |  |  |  |  |  |  |  |  |  |  |  |  |  |  |  |  |  |  |  |  |  |  |  |  |  |  |  |  |  |  |  |  |  |  |  |  |  |  |  |  |  |  |  |  |  |  |  |  |  |  |  |  |  |  |  |  |  |  |  |  |  |  |  |  |  |  |  |  |  |  |  |  |  |  |  |  |  |  |  |  |  |  |  |  |  |  |  |  |  |  |  |  |  |  |  |  |  |  |  |  |  |  |  |  |  |  |  |  |  |  |  |  |  |  |  |  |  |  |  |  |  |  |  |  |  |  |  |  |  |  |  |  |  |  |  |  |  |  |  |  |  |  |  |  |  |  |  |  |  |  |  |  |  |  |  |  |  |  |  |  |  |  |  |  |  |  |  |  |  |  |  |  |  |  |  |  |  |  |  |  |  |  |  |  |  |  |  |  |  |  |  |  |  |  |  |  |  |  |  |  |  |  |  |  |  |  |  |  |  |  |  |  |  |  |  |  |  |  |  |  |  |  |  |  |  |  |  |  |  |  |  |  |  |  |  |  |  |  |  |  |  |  |  |  |  |  |  |  |  |  |  |  |  |  |  |  |  |  |  |  |  |  |  |  |  |  |  |  |  |  |  |  |  |  |  |  |  |  |  |  |  |  |  |  |  |  |  |  |  |  |  |  |  |  |  |  |  |  |  |  |  |  |  |  |  |  |  |  |  |  |  |  |  |  |  |  |  |  |  |  |  |  |  |  |  |  |  |  |  |  |  |  |  |  |  |  |  |  |  |  |  |  |  |  |  |  |  |  |  |  |  |  |  |  |  |  |  |  |  |  |  |  |  |  |  |  |  |  |  |  |  |  |  |  |  |  |  |  |  |  |  |  |  |  |  |  |  |  |  |  |  |  |  |  |  |  |  |  |  |  |  |  |  |  |  |  |  |  |  |  |  |  |  |  |  |  |  |  |  |  |  |  |  |  |  |  |  |  |  |  |  |  |  |  |  |  |  |  |  |  |  |  |  |  |  |  |  |  |  |  |  |  |  |  |  |  |  |  |  |  |  |  |  |  |  |  |  |  |  |  |  |  |  |  |  |  |  |  |  |  |  |  |  |  |  |  |  |  |  |  |  |  |  |  |  |  |  |  |  |  |  |  |  |  |  |  |  |  |  |  |  |  |  |  |  |  |  |  |  |  |  |  |  |  |  |  |  |  |  |  |  |  |  |  |  |  |  |  |  |  |  |  |  |  |  |  |  |  |  |  |  |  |  |  |  |  |  |  |  |  |  |  |  |  |  |  |  |  |  |  |  |  |  |  |  |  |  |  |  |  |  |  |  |  |  |  |  |  |  |  |  |  |  |  |  |  |  |  |  |  |  |  |  |  |  |  |  |  |  |  |  |  |  |  |  |  |  |  |  |  |  |  |  |  |  |  |  |  |  |  |  |  |  |  |  |  |  |  |  |  |  |  |  |  |  |  |  |  |  |  |  |  |  |  |  |  |  |  |  |  |  |  |  |  |  |  |  |  |  |  |  |  |  |  |  |  |  |  |  |  |  |  |  |  |  |  |  |  |  |  |  |  |  |  |  |  |
|  |  |  |  |  |  |  |  |  |  |  |  |  |  |  |  |  |  |  |  |  |  |  |  |  |  |  |  |  |  |  |  |  |  |  |  |  |  |  |  |  |  |  |  |  |  |  |  |  |  |  |  |  |  |  |  |  |  |  |  |  |  |  |  |  |  |  |  |  |  |  |  |  |  |  |  |  |  |  |  |  |  |  |  |  |  |  |  |  |  |  |  |  |  |  |  |  |  |  |  |  |  |  |  |  |  |  |  |  |  |  |  |  |  |  |  |  |  |  |  |  |  |  |  |  |  |  |  |  |  |  |  |  |  |  |  |  |  |  |  |  |  |  |  |  |  |  |  |  |  |  |  |  |  |  |  |  |  |  |  |  |  |  |  |  |  |  |  |  |  |  |  |  |  |  |  |  |  |  |  |  |  |  |  |  |  |  |  |  |  |  |  |  |  |  |  |  |  |  |  |  |  |  |  |  |  |  |  |  |  |  |  |  |  |  |  |  |  |  |  |  |  |  |  |  |  |  |  |  |  |  |  |  |  |  |  |  |  |  |  |  |  |  |  |  |  |  |  |  |  |  |  |  |  |  |  |  |  |  |  |  |  |  |  |  |  |  |  |  |  |  |  |  |  |  |  |  |  |  |  |  |  |  |  |  |  |  |  |  |  |  |  |  |  |  |  |  |  |  |  |  |  |  |  |  |  |  |  |  |  |  |  |  |  |  |  |  |  |  |  |  |  |  |  |  |  |  |  |  |  |  |  |  |  |  |  |  |  |  |  |  |  |  |  |  |  |  |  |  |  |  |  |  |  |  |  |  |  |  |  |  |  |  |  |  |  |  |  |  |  |  |  |  |  |  |  |  |  |  |  |  |  |  |  |  |  |  |  |  |  |  |  |  |  |  |  |  |  |  |  |  |  |  |  |  |  |  |  |  |  |  |  |  |  |  |  |  |  |  |  |  |  |  |  |  |  |  |  |  |  |  |  |  |  |  |  |  |  |  |  |  |  |  |  |  |  |  |  |  |  |  |  |  |  |  |  |  |  |  |  |  |  |  |  |  |  |  |  |  |  |  |  |  |  |  |  |  |  |  |  |  |  |  |  |  |  |  |  |  |  |  |  |  |  |  |  |  |  |  |  |  |  |  |  |  |  |  |  |  |  |  |  |  |  |  |  |  |  |  |  |  |  |  |  |  |  |  |  |  |  |  |  |  |  |  |  |  |  |  |  |  |  |  |  |  |  |  |  |  |  |  |  |  |  |  |  |  |  |  |  |  |  |  |  |  |  |  |  |  |  |  |  |  |  |  |  |  |  |  |  |  |  |  |  |  |  |  |  |  |  |  |  |  |  |  |  |  |  |  |  |  |  |  |  |  |  |  |  |  |  |  |  |  |  |  |  |  |  |  |  |  |  |  |  |  |  |  |  |  |  |  |  |  |  |  |  |  |  |  |  |  |  |  |  |  |  |  |  |  |  |  |  |  |  |  |  |  |  |  |  |  |  |  |  |  |  |  |  |  |  |  |  |  |  |  |  |  |  |  |  |  |  |  |  |  |  |  |  |  |  |  |  |  |  |  |  |  |  |  |  |  |  |  |  |  |  |  |  |  |  |  |  |  |  |  |  |  |  |  |  |  |  |  |  |  |  |  |  |  |  |  |  |  |  |  |  |  |  |  |  |  |  |  |  |  |  |  |  |  |  |  |  |  |  |  |  |  |  |  |  |  |  |  |  |  |  |  |  |  |  |  |  |  |  |  |  |  |  |  |  |  |  |  |  |  |  |  |  |  |  |  |  |  |  |  |  |  |  |  |  |  |  |  |  |  |  |  |  |  |  |  |  |  |  |  |  |  |  |  |  |  |  |  |  |  |  |  |  |  |  |  |  |  |  |  |  |  |  |  |  |  |  |  |  |  |  |  |  |  |  |  |  |  |  |  |  |  |  |  |  |  |  |  |  |  |  |  |  |  |  |  |  |  |  |  |  |  |  |  |  |  |  |  |  |  |  |  |  |  |  |  |  |  |  |  |  |  |  |  |  |  |  |  |  |  |  |  |  |  |  |  |  |  |  |  |  |  |  |  |  |  |  |  |  |  |  |  |  |  |  |  |  |  |  |  |  |  |  |  |  |  |  |  |  |  |  |  |  |  |  |  |  |  |  |  |  |  |  |  |  |  |  |  |  |  |  |  |  |  |  |  |  |  |  |  |  |  |  |  |  |  |  |  |  |  |  |  |  |  |  |  |  |  |  |  |  |  |  |  |  |  |  |  |  |  |  |  |  |  |  |  |  |  |  |  |  |  |  |  |  |  |  |  |  |  |  |  |  |  |  |  |  |  |  |  |  |  |  |  |  |  |  |  |  |  |  |  |  |  |  |  |  |  |  |  |  |  |  |  |  |  |  |  |  |  |  |  |  |  |  |  |  |  |  |  |  |
|  |  |  |  |  |  |  |  |  |  |  |  |  |  |  |  |  |  |  |  |  |  |  |  |  |  |  |  |  |  |  |  |  |  |  |  |  |  |  |  |  |  |  |  |  |  |  |  |  |  |  |  |  |  |  |  |  |  |  |  |  |  |  |  |  |  |  |  |  |  |  |  |  |  |  |  |  |  |  |  |  |  |  |  |  |  |  |  |  |  |  |  |  |  |  |  |  |  |  |  |  |  |  |  |  |  |  |  |  |  |  |  |  |  |  |  |  |  |  |  |  |  |  |  |  |  |  |  |  |  |  |  |  |  |  |  |  |  |  |  |  |  |  |  |  |  |  |  |  |  |  |  |  |  |  |  |  |  |  |  |  |  |  |  |  |  |  |  |  |  |  |  |  |  |  |  |  |  |  |  |  |  |  |  |  |  |  |  |  |  |  |  |  |  |  |  |  |  |  |  |  |  |  |  |  |  |  |  |  |  |  |  |  |  |  |  |  |  |  |  |  |  |  |  |  |  |  |  |  |  |  |  |  |  |  |  |  |  |  |  |  |  |  |  |  |  |  |  |  |  |  |  |  |  |  |  |  |  |  |  |  |  |  |  |  |  |  |  |  |  |  |  |  |  |  |  |  |  |  |  |  |  |  |  |  |  |  |  |  |  |  |  |  |  |  |  |  |  |  |  |  |  |  |  |  |  |  |  |  |  |  |  |  |  |  |  |  |  |  |  |  |  |  |  |  |  |  |  |  |  |  |  |  |  |  |  |  |  |  |  |  |  |  |  |  |  |  |  |  |  |  |  |  |  |  |  |  |  |  |  |  |  |  |  |  |  |  |  |  |  |  |  |  |  |  |  |  |  |  |  |  |  |  |  |  |  |  |  |  |  |  |  |  |  |  |  |  |  |  |  |  |  |  |  |  |  |  |  |  |  |  |  |  |  |  |  |  |  |  |  |  |  |  |  |  |  |  |  |  |  |  |  |  |  |  |  |  |  |  |  |  |  |  |  |  |  |  |  |  |  |  |  |  |  |  |  |  |  |  |  |  |  |  |  |  |  |  |  |  |  |  |  |  |  |  |  |  |  |  |  |  |  |  |  |  |  |  |  |  |  |  |  |  |  |  |  |  |  |  |  |  |  |  |  |  |  |  |  |  |  |  |  |  |  |  |  |  |  |  |  |  |  |  |  |  |  |  |  |  |  |  |  |  |  |  |  |  |  |  |  |  |  |  |  |  |  |  |  |  |  |  |  |  |  |  |  |  |  |  |  |  |  |  |  |  |  |  |  |  |  |  |  |  |  |  |  |  |  |  |  |  |  |  |  |  |  |  |  |  |  |  |  |  |  |  |  |  |  |  |  |  |  |  |  |  |  |  |  |  |  |  |  |  |  |  |  |  |  |  |  |  |  |  |  |  |  |  |  |  |  |  |  |  |  |  |  |  |  |  |  |  |  |  |  |  |  |  |  |  |  |  |  |  |  |  |  |  |  |  |  |  |  |  |  |  |  |  |  |  |  |  |  |  |  |  |  |  |  |  |  |  |  |  |  |  |  |  |  |  |  |  |  |  |  |  |  |  |  |  |  |  |  |  |  |  |  |  |  |  |  |  |  |  |  |  |  |  |  |  |  |  |  |  |  |  |  |  |  |  |  |  |  |  |  |  |  |  |  |  |  |  |  |  |  |  |  |  |  |  |  |  |  |  |  |  |  |  |  |  |  |  |  |  |  |  |  |  |  |  |  |  |  |  |  |  |  |  |  |  |  |  |  |  |  |  |  |  |  |  |  |  |  |  |  |  |  |  |  |  |  |  |  |  |  |  |  |  |  |  |  |  |  |  |  |  |  |  |  |  |  |  |  |  |  |  |  |  |  |  |  |  |  |  |  |  |  |  |  |  |  |  |  |  |  |  |  |  |  |  |  |  |  |  |  |  |  |  |  |  |  |  |  |  |  |  |  |  |  |  |  |  |  |  |  |  |  |  |  |  |  |  |  |  |  |  |  |  |  |  |  |  |  |  |  |  |  |  |  |  |  |  |  |  |  |  |  |  |  |  |  |  |  |  |  |  |  |  |  |  |  |  |  |  |  |  |  |  |  |  |  |  |  |  |  |  |  |  |  |  |  |  |  |  |  |  |  |  |  |  |  |  |  |  |  |  |  |  |  |  |  |  |  |  |  |  |  |  |  |  |  |  |  |  |  |  |  |  |  |  |  |  |  |  |  |  |  |  |  |  |  |  |  |  |  |  |  |  |  |  |  |  |  |  |  |  |  |  |  |  |  |  |  |  |  |  |  |  |  |  |  |  |  |  |  |  |  |  |  |  |  |  |  |  |  |  |  |  |  |  |  |  |  |  |  |  |  |  |  |  |  |  |  |  |  |  |  |  |  |  |  |  |  |  |  |  |  |  |  |  |  |  |  |  |  |  |  |
|  |  |  |  |  |  |  |  |  |  |  |  |  |  |  |  |  |  |  |  |  |  |  |  |  |  |  |  |  |  |  |  |  |  |  |  |  |  |  |  |  |  |  |  |  |  |  |  |  |  |  |  |  |  |  |  |  |  |  |  |  |  |  |  |  |  |  |  |  |  |  |  |  |  |  |  |  |  |  |  |  |  |  |  |  |  |  |  |  |  |  |  |  |  |  |  |  |  |  |  |  |  |  |  |  |  |  |  |  |  |  |  |  |  |  |  |  |  |  |  |  |  |  |  |  |  |  |  |  |  |  |  |  |  |  |  |  |  |  |  |  |  |  |  |  |  |  |  |  |  |  |  |  |  |  |  |  |  |  |  |  |  |  |  |  |  |  |  |  |  |  |  |  |  |  |  |  |  |  |  |  |  |  |  |  |  |  |  |  |  |  |  |  |  |  |  |  |  |  |  |  |  |  |  |  |  |  |  |  |  |  |  |  |  |  |  |  |  |  |  |  |  |  |  |  |  |  |  |  |  |  |  |  |  |  |  |  |  |  |  |  |  |  |  |  |  |  |  |  |  |  |  |  |  |  |  |  |  |  |  |  |  |  |  |  |  |  |  |  |  |  |  |  |  |  |  |  |  |  |  |  |  |  |  |  |  |  |  |  |  |  |  |  |  |  |  |  |  |  |  |  |  |  |  |  |  |  |  |  |  |  |  |  |  |  |  |  |  |  |  |  |  |  |  |  |  |  |  |  |  |  |  |  |  |  |  |  |  |  |  |  |  |  |  |  |  |  |  |  |  |  |  |  |  |  |  |  |  |  |  |  |  |  |  |  |  |  |  |  |  |  |  |  |  |  |  |  |  |  |  |  |  |  |  |  |  |  |  |  |  |  |  |  |  |  |  |  |  |  |  |  |  |  |  |  |  |  |  |  |  |  |  |  |  |  |  |  |  |  |  |  |  |  |  |  |  |  |  |  |  |  |  |  |  |  |  |  |  |  |  |  |  |  |  |  |  |  |  |  |  |  |  |  |  |  |  |  |  |  |  |  |  |  |  |  |  |  |  |  |  |  |  |  |  |  |  |  |  |  |  |  |  |  |  |  |  |  |  |  |  |  |  |  |  |  |  |  |  |  |  |  |  |  |  |  |  |  |  |  |  |  |  |  |  |  |  |  |  |  |  |  |  |  |  |  |  |  |  |  |  |  |  |  |  |  |  |  |  |  |  |  |  |  |  |  |  |  |  |  |  |  |  |  |  |  |  |  |  |  |  |  |  |  |  |  |  |  |  |  |  |  |  |  |  |  |  |  |  |  |  |  |  |  |  |  |  |  |  |  |  |  |  |  |  |  |  |  |  |  |  |  |  |  |  |  |  |  |  |  |  |  |  |  |  |  |  |  |  |  |  |  |  |  |  |  |  |  |  |  |  |  |  |  |  |  |  |  |  |  |  |  |  |  |  |  |  |  |  |  |  |  |  |  |  |  |  |  |  |  |  |  |  |  |  |  |  |  |  |  |  |  |  |  |  |  |  |  |  |  |  |  |  |  |  |  |  |  |  |  |  |  |  |  |  |  |  |  |  |  |  |  |  |  |  |  |  |  |  |  |  |  |  |  |  |  |  |  |  |  |  |  |  |  |  |  |  |  |  |  |  |  |  |  |  |  |  |  |  |  |  |  |  |  |  |  |  |  |  |  |  |  |  |  |  |  |  |  |  |  |  |  |  |  |  |  |  |  |  |  |  |  |  |  |  |  |  |  |  |  |  |  |  |  |  |  |  |  |  |  |  |  |  |  |  |  |  |  |  |  |  |  |  |  |  |  |  |  |  |  |  |  |  |  |  |  |  |  |  |  |  |  |  |  |  |  |  |  |  |  |  |  |  |  |  |  |  |  |  |  |  |  |  |  |  |  |  |  |  |  |  |  |  |  |  |  |  |  |  |  |  |  |  |  |  |  |  |  |  |  |  |  |  |  |  |  |  |  |  |  |  |  |  |  |  |  |  |  |  |  |  |  |  |  |  |  |  |  |  |  |  |  |  |  |  |  |  |  |  |  |  |  |  |  |  |  |  |  |  |  |  |  |  |  |  |  |  |  |  |  |  |  |  |  |  |  |  |  |  |  |  |  |  |  |  |  |  |  |  |  |  |  |  |  |  |  |  |  |  |  |  |  |  |  |  |  |  |  |  |  |  |  |  |  |  |  |  |  |  |  |  |  |  |  |  |  |  |  |  |  |  |  |  |  |  |  |  |  |  |  |  |  |  |  |  |  |  |  |  |  |  |  |  |  |  |  |  |  |  |  |  |  |  |  |  |  |  |  |  |  |  |  |  |  |  |  |  |  |  |  |  |  |  |  |  |  |  |  |  |  |  |  |  |  |  |  |  |  |  |  |  |  |  |  |  |  |  |  |  |  |  |  |  |  |  |  |  |
| BIS d=2 cl.1 sym=1 env=1 | - | - | - | - | - | - | - | - | - | - | - | - | - | - | - | - | - | - | - | - | - | - | - | - | - | - | - | - | - | - | - | - | - | - | - | - | - | - | - | - | - | - | - | - | - | - | - | - | - | - | - | - | - | - | - | - | - | - | - | - | - | - | - | - | - | - | - | - | - | - | - | - | - | - | - | - | - | - | - | - | - | - | - | - | - | - | - | - | - | - | - | - | - | - | - | - | - | - | - | - | - | - | - | - | - | - | - | - | - | - | - | - | - | - | - | - | - | - | - | - | - | - | - | - | - | - | - | - | - | - | - | - | - | - | - | - | - | - | - | - | - | - | - | - | - | - | - | - | - | - | - | - | - | - | - | - | - | - | - | - | - | - | - | - | - | - | - | - | - | - | - | - | - | - | - | - | - | - | - | - | - | - | - | - | - | - | - | - | - | - | - | - | - | - | - | - | - | - | - | - | - | - | - | - | - | - | - | - | - | - | - | - | - | - | - | - | - | - | - | - | - | - | - | - | - | - | - | - | - | - | - | - | - | - | - | - | - | - | - | - | - | - | - | - | - | - | - | - | - | - | - | - | - | - | - | - | - | - | - | - | - | - | - | - | - | - | - | - | - | - | - | - | - | - | - | - | - | - | - | - | - | - | - | - | - | - | - | - | - | - | - | - | - | - | - | - | - | - | - | - | - | - | - | - | - | - | - | - | - | - | - | - | - | - | - | - | - | - | - | - | - | - | - | - | - | - | - | - | - | - | - | - | - | - | - | - | - | - | - | - | - | - | - | - | - | - | - | - | - | - | - | - | - | - | - | - | - | - | - | - | - | - | - | - | - | - | - | - | - | - | - | - | - | - | - | - | - | - | - | - | - | - | - | - | - | - | - | - | - | - | - | - | - | - | - | - | - | - | - | - | - | - | - | - | - | - | - | - | - | - | - | - | - | - | - | - | - | - | - | - | - | - | - | - | - | - | - | - | - | - | - | - | - | - | - | - | - | - | - | - | - | - | - | - | - | - | - | - | - | - | - | - | - | - | - | - | - | - | - | - | - | - | - | - | - | - | - | - | - | - | - | - | - | - | - | - | - | - | - | - | - | - | - | - | - | - | - | - | - | - | - | - | - | - | - | - | - | - | - | - | - | - | - | - | - | - | - | - | - | - | - | - | - | - | - | - | - | - | - | - | - | - | - | - | - | - | - | - | - | - | - | - | - | - | - | - | - | - | - | - | - | - | - | - | - | - | - | - | - | - | - | - | - | - | - | - | - | - | - | - | - | - | - | - | - | - | - | - | - | - | - | - | - | - | - | - | - | - | - | - | - | - | - | - | - | - | - | - | - | - | - | - | - | - | - | - | - | - | - | - | - | - | - | - | - | - | - | - | - | - | - | - | - | - | - | - | - | - | - | - | - | - | - | - | - | - | - | - | - | - | - | - | - | - | - | - | - | - | - | - | - | - | - | - | - | - | - | - | - | - | - | - | - | - | - | - | - | - | - | - | - | - | - | - | - | - | - | - | - | - | - | - | - | - | - | - | - | - | - | - | - | - | - | - | - | - | - | - | - | - | - | - | - | - | - | - | - | - | - | - | - | - | - | - | - | - | - | - | - | H | x | x | x | x | x | x | x | x | x | x | x | x | x | x | x | x | x | x | x | x | x | x | x | x | x | x | x | x | x | x | x | x | x | x | x | x | x | x | x | x | x | x | x | x | x | x | x | x | x | x | x | x | x | x | x | x | x | x | x | x | x | x | x | x | x | x | x | x | x | x | x | x | x | x | x | x | x | x | x | x | x | x | x | x | x | x | x | x | x | x | x | x | x | x | x | x | x | x | x | x | x | x | x | x | x | x | x | x | x | x | x | x | x | x | x | x | H | - | - | - | - | - | - | - | - | - | - | - | - | - | - | - | - | - | - | - | - | - | - | - | - | - | - | - | - | - | - | - | - | - | - | - | - | - | - | - | - | - | - | - | - | - | - | - | - | - | - | - | - | - | - | - | - | - | - | - | - | - | - | - | - | - | - | - | - | - | - | - | - | - | - | - | - | - | - | - | - | - | - | - | - | - | - | - | - | - | - | - | - | - | - | - | - | - | - | - | - | - | - | - | - | - | - | - | - | - | - | - | - | - | - | - | - | - | - | - | - | - | - | - | - | - | - | - | - | - | - | - | - | - | - | - | - | - | - | - | - | - | - | - | - | - | - | - | - | - | - | - | - | - | - | - | - | - | - | - | - | - | - | - | - | - | - | - | - | - | - | - | - | - | - | - | - | - | - | - | - | - | - | - | - | - | - | - | - | - | - | - | - | - | - | - | - | - | - | - | - | - | - | - | - | - | - | - | - | - | - | - | - | - | - | - | - | - | - | - | - | - | - | - | - | - | - | - | - | - | - | - | - | - | - | - | - | - | - | - | - | - | - | - | - | - | - | - | - |
| BIS d=2 cl.2 sym=1 env=1 | - | - | - | - | - | - | - | - | - | - | - | - | - | - | - | - | - | - | - | - | - | - | - | - | - | - | - | - | - | - | - | - | - | - | - | - | - | - | - | - | - | - | - | - | - | - | - | - | - | - | - | - | - | - | - | - | - | - | - | - | - | - | - | - | - | - | - | - | - | - | - | - | H | x | x | x | x | x | x | x | x | x | x | x | x | x | x | x | x | x | x | x | x | x | x | x | x | x | x | x | x | x | x | x | x | x | x | x | x | x | x | x | x | x | x | x | x | x | x | x | x | x | x | x | x | x | x | x | x | x | x | x | x | x | x | x | x | x | x | x | x | x | x | x | x | x | x | x | x | x | x | x | x | x | x | x | x | x | x | x | x | x | x | x | x | x | x | x | x | x | x | x | x | x | x | x | x | x | x | x | x | x | x | x | x | x | x | x | x | x | x | x | x | x | x | x | x | x | x | x | x | x | x | x | x | x | x | x | x | x | x | x | x | x | x | x | x | x | x | x | x | x | x | x | x | x | x | x | x | x | x | x | x | x | x | x | x | x | x | x | x | x | x | x | x | x | x | x | x | x | x | x | x | x | x | x | x | x | x | x | x | x | x | x | x | x | x | x | x | x | x | x | x | x | x | x | x | x | x | x | x | x | x | x | x | x | x | x | x | x | x | x | x | x | x | x | x | x | x | x | x | x | x | x | x | x | x | x | x | x | x | x | x | x | x | x | x | x | x | x | x | x | x | x | x | x | x | x | x | x | x | x | x | x | x | x | x | x | x | x | x | x | x | x | x | x | x | x | x | x | x | x | x | x | x | x | x | x | x | x | x | x | x | x | x | x | x | x | x | x | x | x | x | x | x | x | x | x | x | x | x | x | x | x | x | x | x | x | x | x | x | x | x | x | x | x | x | x | x | x | x | x | x | x | x | x | x | x | x | x | x | x | x | x | x | x | x | x | x | x | x | x | x | x | x | x | x | x | x | x | x | x | x | x | x | x | x | x | x | x | x | x | x | x | x | x | x | x | x | x | x | x | x | x | x | x | x | x | x | x | x | x | x | x | x | x | x | x | x | x | x | x | x | x | x | x | x | x | x | x | x | x | x | x | x | x | x | x | x | x | x | x | x | x | x | x | x | x | x | x | x | x | x | x | x | x | x | x | x | x | x | x | x | x | x | x | x | x | x | x | x | x | x | x | x | x | x | x | x | x | x | x | x | x | x | x | x | x | x | x | x | x | x | x | x | x | x | x | x | x | x | x | x | x | x | x | x | x | x | x | x | x | x | x | x | x | x | x | x | x | x | x | x | x | x | x | x | x | x | x | x | x | x | x | x | x | x | x | x | x | x | x | x | x | x | x | x | x | x | x | x | x | x | x | x | x | x | x | x | x | x | x | x | x | x | x | x | x | x | x | x | x | x | x | x | x | x | x | x | x | x | x | x | x | x | x | x | x | x | x | x | x | x | x | x | x | x | x | x | x | x | x | x | x | x | x | x | x | x | x | H | - | - | - | - | - | - | - | - | - | - | - | - | - | - | - | - | - | - | - | - | - | - | - | - | - | - | - | - | - | - | - | - | - | - | - | - | - | - | - | - | - | - | - | - | - | - | - | - | - | - | - | - | - | - | - | - | - | - | - | - | - | - | - | - | - | - | - | - | - | - | - | - | - | - | - | - | - | - | - | - | - | - | - | - | - | - | - | - | - | - | - | - | - | - | - | - | - | - | - | - | - | - | - | - | - | - | - | - | - | - | - | - | - | - | - | - | - | - | - | - | - | - | - | - | - | - | - | - | - | - | - | - | - | - | - | - | - | - | - | - | - | - | - | - | - | - | - | - | - | - | - | - | - | - | - | - | - | - | - | - | - | - | - | - | - | - | - | - | - | - | - | - | - | - | - | - | - | - | - | - | - | - | - | - | - | - | - | - | - | - | - | - | - | - | - | - | - | - | - | - | - | - | - | - | - | - | - | - | - | - | - | - | - | - | - | - | - | - | - | - | - | - | - | - | - | - | - | - | - | - | - | - | - | - | - | - | - | - | - | - | - | - | - | - | - | - | - | - | - | - | - | - | - | - | - | - | - | - | - | - | - | - | - | - | - | - | - | - | - | - | - | - | - | - | - | - | - | - | - | - | - | - | - | - | - | - | - | - | - | - | - | - | - | - | - | - | - | - | - | - | - | - | - | - | - | - | - | - | - | - | - | - | - | - | - | - | - | - | - | - | - | - | - | - | - | - | - | - | - | - | - | - | - | - | - | - | - | - | - | - | - | - | - | - | - | - | - | - | - | - | - | - | - | - | - | - | - | - | - | - | - | - | - | - | - | - | - | - | - | - | - | - | - | - | - | - | - | - | - | - | - | - | - | - | - | - | - | - | - | - | - | - | - | - | - | - | - | - | - | - | - | - | - | - | - | - | - | - | - | - | - | - | - | - |
