# Supplementary material for: Variation in selection constraints on teleost TLRs with emphasis on their repertoire in the Walking catfish, Clarias batrachus
Source: Sci Rep. 2020 Dec 7;10:21394. doi: 10.1038/s41598-020-78347-6 (PMC7721727; doi:10.1038/s41598-020-78347-6)
Supplement: Supplementary file 34 — Supplementary Information 34. [file 41598_2020_78347_MOESM34_ESM.zip › T22/bis2/summary.tgz!/summary/PF00000-NONREDUNDANT-5DD-dim0-table.html]

BIS cluster table


Clusters with env. score >= 0.5 and sym. score >= 0.5 :

| Dim | Cluster | Sym | Env | Pvalue | Hit patterns and blocks |
| --- | --- | --- | --- | --- | --- |
| 0 | 14 | 1 | 1 | 7.345596e-07 | Hit patterns: Positions: \| 301 \| 302; 9 sequences: \| D \| I; 5 sequences: \| Y \| F; 3 sequences: \| N \| L All positions in cluster: 301-302 |
| 0 | 15 | 1 | 1 | 1.469119e-06 | Hit patterns: Positions: \| 147 \| 761; 9 sequences: \| S \| D; 6 sequences: \| N \| Y; 2 sequences: \| A \| G All positions in cluster: 147 761 |
| 0 | 9 | 1 | 1 | 4.113534e-05 | Hit patterns: Positions: \| 185 \| 200 \| 477 \| 628 \| 819 \| 896 \| 979; 9 sequences: \| V \| D \| Q \| N \| L \| A \| E; 8 sequences: \| I \| N \| G \| S \| V \| G \| Q All positions in cluster: 185 200 477 628 819 896 979 |
| 0 | 13 | 1 | 1 | 4.113534e-05 | Hit patterns: Positions: \| 80 \| 201 \| 241 \| 362 \| 644 \| 806 \| 831; 9 sequences: \| V \| L \| S \| I \| P \| L \| Y; 8 sequences: \| A \| I \| T \| L \| K \| M \| F All positions in cluster: 80 201 241 362 644 806 831 |
| 0 | 12 | 1 | 1 | 5.141917e-05 | Hit patterns: Positions: \| 376 \| 768; 10 sequences: \| V \| N; 7 sequences: \| L \| S All positions in cluster: 376 768 |
| 0 | 2 | 1 | 1 | 8.080155e-05 | Hit patterns: Positions: \| 110 \| 166 \| 349 \| 533 \| 746 \| 765 \| 887 \| 906 \| 908; 11 sequences: \| I \| F \| I \| L \| W \| F \| K \| V \| S; 6 sequences: \| F \| L \| X \| I \| F \| Y \| R \| L \| T All positions in cluster: 110 166 349 533 746 765 887 906 908 |
| 0 | 11 | 1 | 1 | 0.0001616031 | Hit patterns: Positions: \| 426 \| 460 \| 519 \| 584 \| 730; 12 sequences: \| L \| L \| S \| Y \| Y; 5 sequences: \| I \| I \| Q \| F \| V All positions in cluster: 426 460 519 584 730 |
| 0 | 6 | 1 | 1 | 0.0004201681 | Hit patterns: Positions: \| 413 \| 650; 13 sequences: \| V \| T; 4 sequences: \| I \| S All positions in cluster: 413 650 |
| 0 | 10 | 1 | 1 | 0.0004201681 | Hit patterns: Positions: \| 193 \| 593; 13 sequences: \| L \| P; 4 sequences: \| M \| A All positions in cluster: 193 593 |
| 0 | 5 | 1 | 1 | 0.001470588 | Hit patterns: Positions: \| 639 \| 709; 14 sequences: \| L \| A; 3 sequences: \| I \| V All positions in cluster: 639 709 |
| 0 | 3 | 1 | 1 | 0.007352941 | Hit patterns: Positions: \| 400 \| 863; 15 sequences: \| L \| E; 2 sequences: \| I \| Q All positions in cluster: 400 863 |
| 0 | 4 | 1 | 1 | 0.007352941 | Hit patterns: Positions: \| 137 \| 140 \| 199 \| 566 \| 677 \| 744 \| 775 \| 793 \| 980; 15 sequences: \| I \| G \| L \| F \| I \| N \| G \| I \| K; 2 sequences: \| V \| D \| V \| L \| T \| D \| D \| Y \| Q All positions in cluster: 137 140 199 566 677 744 775 793 980 |
| 0 | 7 | 1 | 1 | 0.007352941 | Hit patterns: Positions: \| 479 \| 632; 15 sequences: \| N \| F; 2 sequences: \| S \| L All positions in cluster: 479 632 |
| 0 | 8 | 1 | 1 | 0.007352941 | Hit patterns: Positions: \| 629 \| 926; 15 sequences: \| L \| F; 2 sequences: \| M \| Y All positions in cluster: 629 926 |
| 0 | 1 | 1 | 1 | 1 | All positions in cluster: 65 82 108 124 134 148 151 153 156 158 169 172 175 180 182 190 196 206 222 225 235 237 247 251 255 263 270 286 325 342 347 373 380 394 403 408 427 429 431 433 436 438 441 443 450 452 455 463 465 468 471 474 488 496 499 504 506 514 520 523 525 528 538 540-541 544 549 552 575 580 582 598 601 604 619 621 623 625-626 647 651 653 656 661 674 680 690 693 701 712 724 728 738 740 742 750 757-759 767 769-770 786 809 814-815 821 823-824 828 830 846-847 849 851-853 856-857 859-860 864 866 868-869 872 874 876-883 885-886 889 892-893 897-898 900-903 905 911-912 914 916-918 920 922 927-931 933-937 940-942 944 951 956 958 964 967 977-978 981 984 |

Table created with bis2html version 8.
