# Supplementary material for: Variation in selection constraints on teleost TLRs with emphasis on their repertoire in the Walking catfish, Clarias batrachus
Source: Sci Rep. 2020 Dec 7;10:21394. doi: 10.1038/s41598-020-78347-6 (PMC7721727; doi:10.1038/s41598-020-78347-6)
Supplement: Supplementary file 34 — Supplementary Information 34. [file 41598_2020_78347_MOESM34_ESM.zip › T22/bis2/summary.tgz!/summary/PF00000-NONREDUNDANT-5DD-dim0.html]

Alignment and BIS clusters


|  |  |  |  |  |  |  |  |  |  |  |  |  |  |  |  |  |  |  |  |  |  |  |  |  |  |  |  |  |  |  |  |  |  |  |  |  |  |  |  |  |  |  |  |  |  |  |  |  |  |  |  |  |  |  |  |  |  |  |  |  |  |  |  |  |  |  |  |  |  |  |  |  |  |  |  |  |  |  |  |  |  |  |  |  |  |  |  |  |  |  |  |  |  |  |  |  |  |  |  |  |  |  |  |  |  |  |  |  |  |  |  |  |  |  |  |  |  |  |  |  |  |  |  |  |  |  |  |  |  |  |  |  |  |  |  |  |  |  |  |  |  |  |  |  |  |  |  |  |  |  |  |  |  |  |  |  |  |  |  |  |  |  |  |  |  |  |  |  |  |  |  |  |  |  |  |  |  |  |  |  |  |  |  |  |  |  |  |  |  |  |  |  |  |  |  |  |  |  |  |  |  |  |  |  |  |  |  |  |  |  |  |  |  |  |  |  |  |  |  |  |  |  |  |  |  |  |  |  |  |  |  |  |  |  |  |  |  |  |  |  |  |  |  |  |  |  |  |  |  |  |  |  |  |  |  |  |  |  |  |  |  |  |  |  |  |  |  |  |  |  |  |  |  |  |  |  |  |  |  |  |  |  |  |  |  |  |  |  |  |  |  |  |  |  |  |  |  |  |  |  |  |  |  |  |  |  |  |  |  |  |  |  |  |  |  |  |  |  |  |  |  |  |  |  |  |  |  |  |  |  |  |  |  |  |  |  |  |  |  |  |  |  |  |  |  |  |  |  |  |  |  |  |  |  |  |  |  |  |  |  |  |  |  |  |  |  |  |  |  |  |  |  |  |  |  |  |  |  |  |  |  |  |  |  |  |  |  |  |  |  |  |  |  |  |  |  |  |  |  |  |  |  |  |  |  |  |  |  |  |  |  |  |  |  |  |  |  |  |  |  |  |  |  |  |  |  |  |  |  |  |  |  |  |  |  |  |  |  |  |  |  |  |  |  |  |  |  |  |  |  |  |  |  |  |  |  |  |  |  |  |  |  |  |  |  |  |  |  |  |  |  |  |  |  |  |  |  |  |  |  |  |  |  |  |  |  |  |  |  |  |  |  |  |  |  |  |  |  |  |  |  |  |  |  |  |  |  |  |  |  |  |  |  |  |  |  |  |  |  |  |  |  |  |  |  |  |  |  |  |  |  |  |  |  |  |  |  |  |  |  |  |  |  |  |  |  |  |  |  |  |  |  |  |  |  |  |  |  |  |  |  |  |  |  |  |  |  |  |  |  |  |  |  |  |  |  |  |  |  |  |  |  |  |  |  |  |  |  |  |  |  |  |  |  |  |  |  |  |  |  |  |  |  |  |  |  |  |  |  |  |  |  |  |  |  |  |  |  |  |  |  |  |  |  |  |  |  |  |  |  |  |  |  |  |  |  |  |  |  |  |  |  |  |  |  |  |  |  |  |  |  |  |  |  |  |  |  |  |  |  |  |  |  |  |  |  |  |  |  |  |  |  |  |  |  |  |  |  |  |  |  |  |  |  |  |  |  |  |  |  |  |  |  |  |  |  |  |  |  |  |  |  |  |  |  |  |  |  |  |  |  |  |  |  |  |  |  |  |  |  |  |  |  |  |  |  |  |  |  |  |  |  |  |  |  |  |  |  |  |  |  |  |  |  |  |  |  |  |  |  |  |  |  |  |  |  |  |  |  |  |  |  |  |  |  |  |  |  |  |  |  |  |  |  |  |  |  |  |  |  |  |  |  |  |  |  |  |  |  |  |  |  |  |  |  |  |  |  |  |  |  |  |  |  |  |  |  |  |  |  |  |  |  |  |  |  |  |  |  |  |  |  |  |  |  |  |  |  |  |  |  |  |  |  |  |  |  |  |  |  |  |  |  |  |  |  |  |  |  |  |  |  |  |  |  |  |  |  |  |  |  |  |  |  |  |  |  |  |  |  |  |  |  |  |  |  |  |  |  |  |  |  |  |  |  |  |  |  |  |  |  |  |  |  |  |  |  |  |  |  |  |  |  |  |  |  |  |  |  |  |  |  |  |  |  |  |  |  |  |  |  |  |  |  |  |  |  |  |  |  |  |  |  |  |  |  |  |  |  |  |  |  |  |  |  |  |  |  |  |  |  |  |  |  |  |  |  |  |  |  |  |  |  |  |  |  |  |  |  |  |  |  |  |  |  |  |  |  |  |  |  |  |  |  |  |  |  |  |  |  |  |  |  |  |  |  |  |  |  |  |  |  |  |  |  |  |  |  |  |  |  |  |  |  |
| --- | --- | --- | --- | --- | --- | --- | --- | --- | --- | --- | --- | --- | --- | --- | --- | --- | --- | --- | --- | --- | --- | --- | --- | --- | --- | --- | --- | --- | --- | --- | --- | --- | --- | --- | --- | --- | --- | --- | --- | --- | --- | --- | --- | --- | --- | --- | --- | --- | --- | --- | --- | --- | --- | --- | --- | --- | --- | --- | --- | --- | --- | --- | --- | --- | --- | --- | --- | --- | --- | --- | --- | --- | --- | --- | --- | --- | --- | --- | --- | --- | --- | --- | --- | --- | --- | --- | --- | --- | --- | --- | --- | --- | --- | --- | --- | --- | --- | --- | --- | --- | --- | --- | --- | --- | --- | --- | --- | --- | --- | --- | --- | --- | --- | --- | --- | --- | --- | --- | --- | --- | --- | --- | --- | --- | --- | --- | --- | --- | --- | --- | --- | --- | --- | --- | --- | --- | --- | --- | --- | --- | --- | --- | --- | --- | --- | --- | --- | --- | --- | --- | --- | --- | --- | --- | --- | --- | --- | --- | --- | --- | --- | --- | --- | --- | --- | --- | --- | --- | --- | --- | --- | --- | --- | --- | --- | --- | --- | --- | --- | --- | --- | --- | --- | --- | --- | --- | --- | --- | --- | --- | --- | --- | --- | --- | --- | --- | --- | --- | --- | --- | --- | --- | --- | --- | --- | --- | --- | --- | --- | --- | --- | --- | --- | --- | --- | --- | --- | --- | --- | --- | --- | --- | --- | --- | --- | --- | --- | --- | --- | --- | --- | --- | --- | --- | --- | --- | --- | --- | --- | --- | --- | --- | --- | --- | --- | --- | --- | --- | --- | --- | --- | --- | --- | --- | --- | --- | --- | --- | --- | --- | --- | --- | --- | --- | --- | --- | --- | --- | --- | --- | --- | --- | --- | --- | --- | --- | --- | --- | --- | --- | --- | --- | --- | --- | --- | --- | --- | --- | --- | --- | --- | --- | --- | --- | --- | --- | --- | --- | --- | --- | --- | --- | --- | --- | --- | --- | --- | --- | --- | --- | --- | --- | --- | --- | --- | --- | --- | --- | --- | --- | --- | --- | --- | --- | --- | --- | --- | --- | --- | --- | --- | --- | --- | --- | --- | --- | --- | --- | --- | --- | --- | --- | --- | --- | --- | --- | --- | --- | --- | --- | --- | --- | --- | --- | --- | --- | --- | --- | --- | --- | --- | --- | --- | --- | --- | --- | --- | --- | --- | --- | --- | --- | --- | --- | --- | --- | --- | --- | --- | --- | --- | --- | --- | --- | --- | --- | --- | --- | --- | --- | --- | --- | --- | --- | --- | --- | --- | --- | --- | --- | --- | --- | --- | --- | --- | --- | --- | --- | --- | --- | --- | --- | --- | --- | --- | --- | --- | --- | --- | --- | --- | --- | --- | --- | --- | --- | --- | --- | --- | --- | --- | --- | --- | --- | --- | --- | --- | --- | --- | --- | --- | --- | --- | --- | --- | --- | --- | --- | --- | --- | --- | --- | --- | --- | --- | --- | --- | --- | --- | --- | --- | --- | --- | --- | --- | --- | --- | --- | --- | --- | --- | --- | --- | --- | --- | --- | --- | --- | --- | --- | --- | --- | --- | --- | --- | --- | --- | --- | --- | --- | --- | --- | --- | --- | --- | --- | --- | --- | --- | --- | --- | --- | --- | --- | --- | --- | --- | --- | --- | --- | --- | --- | --- | --- | --- | --- | --- | --- | --- | --- | --- | --- | --- | --- | --- | --- | --- | --- | --- | --- | --- | --- | --- | --- | --- | --- | --- | --- | --- | --- | --- | --- | --- | --- | --- | --- | --- | --- | --- | --- | --- | --- | --- | --- | --- | --- | --- | --- | --- | --- | --- | --- | --- | --- | --- | --- | --- | --- | --- | --- | --- | --- | --- | --- | --- | --- | --- | --- | --- | --- | --- | --- | --- | --- | --- | --- | --- | --- | --- | --- | --- | --- | --- | --- | --- | --- | --- | --- | --- | --- | --- | --- | --- | --- | --- | --- | --- | --- | --- | --- | --- | --- | --- | --- | --- | --- | --- | --- | --- | --- | --- | --- | --- | --- | --- | --- | --- | --- | --- | --- | --- | --- | --- | --- | --- | --- | --- | --- | --- | --- | --- | --- | --- | --- | --- | --- | --- | --- | --- | --- | --- | --- | --- | --- | --- | --- | --- | --- | --- | --- | --- | --- | --- | --- | --- | --- | --- | --- | --- | --- | --- | --- | --- | --- | --- | --- | --- | --- | --- | --- | --- | --- | --- | --- | --- | --- | --- | --- | --- | --- | --- | --- | --- | --- | --- | --- | --- | --- | --- | --- | --- | --- | --- | --- | --- | --- | --- | --- | --- | --- | --- | --- | --- | --- | --- | --- | --- | --- | --- | --- | --- | --- | --- | --- | --- | --- | --- | --- | --- | --- | --- | --- | --- | --- | --- | --- | --- | --- | --- | --- | --- | --- | --- | --- | --- | --- | --- | --- | --- | --- | --- | --- | --- | --- | --- | --- | --- | --- | --- | --- | --- | --- | --- | --- | --- | --- | --- | --- | --- | --- | --- | --- | --- | --- | --- | --- | --- | --- | --- | --- | --- | --- | --- | --- | --- | --- | --- | --- | --- | --- | --- | --- | --- | --- | --- | --- | --- | --- | --- | --- | --- | --- | --- | --- | --- | --- | --- | --- | --- | --- | --- | --- | --- | --- | --- | --- | --- | --- | --- | --- | --- | --- | --- | --- | --- | --- | --- | --- | --- | --- | --- | --- | --- | --- | --- | --- | --- | --- | --- | --- | --- | --- | --- | --- | --- | --- | --- | --- | --- | --- | --- | --- | --- | --- | --- | --- | --- | --- | --- | --- | --- | --- | --- | --- | --- | --- | --- | --- | --- | --- | --- | --- | --- | --- | --- | --- | --- | --- | --- | --- | --- | --- | --- | --- | --- | --- | --- | --- | --- | --- | --- | --- | --- | --- | --- | --- | --- | --- | --- | --- | --- | --- | --- | --- | --- | --- | --- | --- | --- | --- | --- | --- | --- | --- | --- | --- | --- | --- | --- | --- | --- | --- | --- | --- | --- | --- | --- | --- | --- | --- | --- | --- | --- | --- | --- | --- | --- | --- | --- | --- | --- | --- | --- | --- | --- | --- | --- | --- | --- | --- | --- | --- | --- | --- | --- | --- | --- | --- | --- | --- | --- | --- | --- | --- | --- | --- | --- | --- | --- | --- | --- | --- | --- | --- | --- | --- | --- | --- | --- | --- | --- | --- | --- | --- | --- | --- | --- | --- | --- | --- | --- | --- | --- | --- | --- | --- | --- | --- | --- | --- | --- | --- | --- | --- | --- | --- | --- | --- | --- | --- | --- | --- | --- | --- |
|  |  |  |  |  |  |  |  | 1 | 0 |  |  |  |  |  |  |  |  | 2 | 0 |  |  |  |  |  |  |  |  | 3 | 0 |  |  |  |  |  |  |  |  | 4 | 0 |  |  |  |  |  |  |  |  | 5 | 0 |  |  |  |  |  |  |  |  | 6 | 0 |  |  |  |  |  |  |  |  | 7 | 0 |  |  |  |  |  |  |  |  | 8 | 0 |  |  |  |  |  |  |  |  | 9 | 0 |  |  |  |  |  |  |  | 1 | 0 | 0 |  |  |  |  |  |  |  | 1 | 1 | 0 |  |  |  |  |  |  |  | 1 | 2 | 0 |  |  |  |  |  |  |  | 1 | 3 | 0 |  |  |  |  |  |  |  | 1 | 4 | 0 |  |  |  |  |  |  |  | 1 | 5 | 0 |  |  |  |  |  |  |  | 1 | 6 | 0 |  |  |  |  |  |  |  | 1 | 7 | 0 |  |  |  |  |  |  |  | 1 | 8 | 0 |  |  |  |  |  |  |  | 1 | 9 | 0 |  |  |  |  |  |  |  | 2 | 0 | 0 |  |  |  |  |  |  |  | 2 | 1 | 0 |  |  |  |  |  |  |  | 2 | 2 | 0 |  |  |  |  |  |  |  | 2 | 3 | 0 |  |  |  |  |  |  |  | 2 | 4 | 0 |  |  |  |  |  |  |  | 2 | 5 | 0 |  |  |  |  |  |  |  | 2 | 6 | 0 |  |  |  |  |  |  |  | 2 | 7 | 0 |  |  |  |  |  |  |  | 2 | 8 | 0 |  |  |  |  |  |  |  | 2 | 9 | 0 |  |  |  |  |  |  |  | 3 | 0 | 0 |  |  |  |  |  |  |  | 3 | 1 | 0 |  |  |  |  |  |  |  | 3 | 2 | 0 |  |  |  |  |  |  |  | 3 | 3 | 0 |  |  |  |  |  |  |  | 3 | 4 | 0 |  |  |  |  |  |  |  | 3 | 5 | 0 |  |  |  |  |  |  |  | 3 | 6 | 0 |  |  |  |  |  |  |  | 3 | 7 | 0 |  |  |  |  |  |  |  | 3 | 8 | 0 |  |  |  |  |  |  |  | 3 | 9 | 0 |  |  |  |  |  |  |  | 4 | 0 | 0 |  |  |  |  |  |  |  | 4 | 1 | 0 |  |  |  |  |  |  |  | 4 | 2 | 0 |  |  |  |  |  |  |  | 4 | 3 | 0 |  |  |  |  |  |  |  | 4 | 4 | 0 |  |  |  |  |  |  |  | 4 | 5 | 0 |  |  |  |  |  |  |  | 4 | 6 | 0 |  |  |  |  |  |  |  | 4 | 7 | 0 |  |  |  |  |  |  |  | 4 | 8 | 0 |  |  |  |  |  |  |  | 4 | 9 | 0 |  |  |  |  |  |  |  | 5 | 0 | 0 |  |  |  |  |  |  |  | 5 | 1 | 0 |  |  |  |  |  |  |  | 5 | 2 | 0 |  |  |  |  |  |  |  | 5 | 3 | 0 |  |  |  |  |  |  |  | 5 | 4 | 0 |  |  |  |  |  |  |  | 5 | 5 | 0 |  |  |  |  |  |  |  | 5 | 6 | 0 |  |  |  |  |  |  |  | 5 | 7 | 0 |  |  |  |  |  |  |  | 5 | 8 | 0 |  |  |  |  |  |  |  | 5 | 9 | 0 |  |  |  |  |  |  |  | 6 | 0 | 0 |  |  |  |  |  |  |  | 6 | 1 | 0 |  |  |  |  |  |  |  | 6 | 2 | 0 |  |  |  |  |  |  |  | 6 | 3 | 0 |  |  |  |  |  |  |  | 6 | 4 | 0 |  |  |  |  |  |  |  | 6 | 5 | 0 |  |  |  |  |  |  |  | 6 | 6 | 0 |  |  |  |  |  |  |  | 6 | 7 | 0 |  |  |  |  |  |  |  | 6 | 8 | 0 |  |  |  |  |  |  |  | 6 | 9 | 0 |  |  |  |  |  |  |  | 7 | 0 | 0 |  |  |  |  |  |  |  | 7 | 1 | 0 |  |  |  |  |  |  |  | 7 | 2 | 0 |  |  |  |  |  |  |  | 7 | 3 | 0 |  |  |  |  |  |  |  | 7 | 4 | 0 |  |  |  |  |  |  |  | 7 | 5 | 0 |  |  |  |  |  |  |  | 7 | 6 | 0 |  |  |  |  |  |  |  | 7 | 7 | 0 |  |  |  |  |  |  |  | 7 | 8 | 0 |  |  |  |  |  |  |  | 7 | 9 | 0 |  |  |  |  |  |  |  | 8 | 0 | 0 |  |  |  |  |  |  |  | 8 | 1 | 0 |  |  |  |  |  |  |  | 8 | 2 | 0 |  |  |  |  |  |  |  | 8 | 3 | 0 |  |  |  |  |  |  |  | 8 | 4 | 0 |  |  |  |  |  |  |  | 8 | 5 | 0 |  |  |  |  |  |  |  | 8 | 6 | 0 |  |  |  |  |  |  |  | 8 | 7 | 0 |  |  |  |  |  |  |  | 8 | 8 | 0 |  |  |  |  |  |  |  | 8 | 9 | 0 |  |  |  |  |  |  |  | 9 | 0 | 0 |  |  |  |  |  |  |  | 9 | 1 | 0 |  |  |  |  |  |  |  | 9 | 2 | 0 |  |  |  |  |  |  |  | 9 | 3 | 0 |  |  |  |  |  |  |  | 9 | 4 | 0 |  |  |  |  |  |  |  | 9 | 5 | 0 |  |  |  |  |  |  |  | 9 | 6 | 0 |  |  |  |  |  |  |  | 9 | 7 | 0 |  |  |  |  |  |  |  | 9 | 8 | 0 |  |  |  |  |  |  |  | 9 | 9 | 0 |  |  |  |  |  |  | 1 | 0 | 0 | 0 |  |  |  |  |  |  | 1 | 0 | 1 | 0 |  |  |  |  |  |
|  |  |  |  |  |  |  |  |  | \| |  |  |  |  |  |  |  |  |  | \| |  |  |  |  |  |  |  |  |  | \| |  |  |  |  |  |  |  |  |  | \| |  |  |  |  |  |  |  |  |  | \| |  |  |  |  |  |  |  |  |  | \| |  |  |  |  |  |  |  |  |  | \| |  |  |  |  |  |  |  |  |  | \| |  |  |  |  |  |  |  |  |  | \| |  |  |  |  |  |  |  |  |  | \| |  |  |  |  |  |  |  |  |  | \| |  |  |  |  |  |  |  |  |  | \| |  |  |  |  |  |  |  |  |  | \| |  |  |  |  |  |  |  |  |  | \| |  |  |  |  |  |  |  |  |  | \| |  |  |  |  |  |  |  |  |  | \| |  |  |  |  |  |  |  |  |  | \| |  |  |  |  |  |  |  |  |  | \| |  |  |  |  |  |  |  |  |  | \| |  |  |  |  |  |  |  |  |  | \| |  |  |  |  |  |  |  |  |  | \| |  |  |  |  |  |  |  |  |  | \| |  |  |  |  |  |  |  |  |  | \| |  |  |  |  |  |  |  |  |  | \| |  |  |  |  |  |  |  |  |  | \| |  |  |  |  |  |  |  |  |  | \| |  |  |  |  |  |  |  |  |  | \| |  |  |  |  |  |  |  |  |  | \| |  |  |  |  |  |  |  |  |  | \| |  |  |  |  |  |  |  |  |  | \| |  |  |  |  |  |  |  |  |  | \| |  |  |  |  |  |  |  |  |  | \| |  |  |  |  |  |  |  |  |  | \| |  |  |  |  |  |  |  |  |  | \| |  |  |  |  |  |  |  |  |  | \| |  |  |  |  |  |  |  |  |  | \| |  |  |  |  |  |  |  |  |  | \| |  |  |  |  |  |  |  |  |  | \| |  |  |  |  |  |  |  |  |  | \| |  |  |  |  |  |  |  |  |  | \| |  |  |  |  |  |  |  |  |  | \| |  |  |  |  |  |  |  |  |  | \| |  |  |  |  |  |  |  |  |  | \| |  |  |  |  |  |  |  |  |  | \| |  |  |  |  |  |  |  |  |  | \| |  |  |  |  |  |  |  |  |  | \| |  |  |  |  |  |  |  |  |  | \| |  |  |  |  |  |  |  |  |  | \| |  |  |  |  |  |  |  |  |  | \| |  |  |  |  |  |  |  |  |  | \| |  |  |  |  |  |  |  |  |  | \| |  |  |  |  |  |  |  |  |  | \| |  |  |  |  |  |  |  |  |  | \| |  |  |  |  |  |  |  |  |  | \| |  |  |  |  |  |  |  |  |  | \| |  |  |  |  |  |  |  |  |  | \| |  |  |  |  |  |  |  |  |  | \| |  |  |  |  |  |  |  |  |  | \| |  |  |  |  |  |  |  |  |  | \| |  |  |  |  |  |  |  |  |  | \| |  |  |  |  |  |  |  |  |  | \| |  |  |  |  |  |  |  |  |  | \| |  |  |  |  |  |  |  |  |  | \| |  |  |  |  |  |  |  |  |  | \| |  |  |  |  |  |  |  |  |  | \| |  |  |  |  |  |  |  |  |  | \| |  |  |  |  |  |  |  |  |  | \| |  |  |  |  |  |  |  |  |  | \| |  |  |  |  |  |  |  |  |  | \| |  |  |  |  |  |  |  |  |  | \| |  |  |  |  |  |  |  |  |  | \| |  |  |  |  |  |  |  |  |  | \| |  |  |  |  |  |  |  |  |  | \| |  |  |  |  |  |  |  |  |  | \| |  |  |  |  |  |  |  |  |  | \| |  |  |  |  |  |  |  |  |  | \| |  |  |  |  |  |  |  |  |  | \| |  |  |  |  |  |  |  |  |  | \| |  |  |  |  |  |  |  |  |  | \| |  |  |  |  |  |  |  |  |  | \| |  |  |  |  |  |  |  |  |  | \| |  |  |  |  |  |  |  |  |  | \| |  |  |  |  |  |  |  |  |  | \| |  |  |  |  |  |  |  |  |  | \| |  |  |  |  |  |  |  |  |  | \| |  |  |  |  |  |  |  |  |  | \| |  |  |  |  |  |  |  |  |  | \| |  |  |  |  |  |  |  |  |  | \| |  |  |  |  |  |  |  |  |  | \| |  |  |  |  |  |  |  |  |  | \| |  |  |  |  |  |  |  |  |  | \| |  |  |  |  |  |  |  |  |  | \| |  |  |  |  |  |  |  |  |  | \| |  |  |  |  |  |  |  |  |  | \| |  |  |  |  |  |  |  |  |  | \| |  |  |  |  |  |  |  |  |  | \| |  |  |  |  |  |  |  |  |  | \| |  |  |  |  |  |  |  |  |  | \| |  |  |  |  |  |  |  |  |  | \| |  |  |  |  |  |  |  |  |  | \| |  |  |  |  |  |  |  |  |  | \| |  |  |  |  |  |
| CBTLR22\_3030\_BP | M | I | F | L | T | G | L | A | Q | F | Q | M | - | - | - | - | F | S | S | C | A | S | Q | L | N | N | S | S | T | N | L | K | M | V | C | F | Q | A | T | M | X | X | L | A | L | L | T | I | S | S | Y | I | F | S | F | Q | V | S | A | F | S | L | T | N | C | T | V | S | G | A | L | N | N | T | E | X | E | L | K | V | L | C | Y | K | M | G | X | X | F | Y | T | V | P | S | P | I | P | S | N | A | R | F | I | X | M | S | F | N | F | F | S | N | I | R | A | G | D | X | E | D | L | W | X | L | R | G | F | N | L | S | N | S | N | I | S | Q | I | E | D | G | T | A | K | H | F | P | N | L | I | K | L | N | L | A | Y | N | K | L | K | T | V | S | R | G | L | L | D | G | L | V | N | L | Q | V | L | R | L | D | G | N | L | I | E | S | I | D | E | F | A | F | N | T | L | R | S | L | I | I | L | N | L | T | E | N | N | L | R | Q | I | V | N | V | R | P | V | L | L | X | S | P | S | L | E | E | L | L | I | G | S | N | K | F | D | V | F | N | S | C | D | L | S | S | T | A | L | S | L | K | K | I | D | L | S | Y | N | P | L | T | K | F | Q | I | T | E | N | I | F | P | N | L | D | Y | L | D | L | S | H | C | G | R | N | X | T | M | L | W | N | I | T | D | K | T | F | L | N | S | V | K | A | L | N | L | T | A | I | Y | M | P | E | E | D | L | T | A | V | L | Q | N | I | T | W | X | X | S | L | F | K | L | R | L | S | E | L | K | W | M | K | P | X | G | T | L | L | Q | S | G | C | X | X | P | G | L | K | V | L | R | M | L | Q | N | G | I | S | K | L | T | A | Q | M | L | D | P | C | S | N | L | T | E | L | D | F | G | N | N | K | I | S | A | I | S | A | P | I | F | K | E | L | T | Q | L | K | T | L | Y | L | Q | V | N | K | L | T | K | V | N | N | S | F | K | N | L | L | M | L | E | Y | L | D | L | S | R | N | S | I | S | D | L | T | C | S | D | F | A | N | L | T | Q | L | N | T | L | Y | L | Y | S | N | K | I | S | K | L | V | P | C | V | F | K | Y | L | N | N | L | E | I | L | K | L | G | T | N | K | L | L | T | I | N | V | A | F | Q | Q | X | X | L | P | S | L | K | Y | L | Q | L | A | Y | N | K | L | S | K | I | S | K | E | S | F | K | S | L | S | S | L | R | T | L | D | L | G | D | N | Q | I | F | E | I | E | A | H | A | F | S | G | L | T | N | L | T | D | M | L | L | H | S | N | R | I | T | S | K | X | X | I | K | D | P | A | V | F | S | G | I | P | K | L | T | T | L | E | L | R | S | N | V | I | H | Y | E | S | D | T | X | L | Q | N | P | P | F | L | N | L | N | S | L | K | I | L | S | I | H | S | Q | R | X | X | X | G | F | G | K | I | P | S | N | L | L | Q | G | L | T | S | L | T | M | F | Y | G | G | R | M | N | L | N | N | L | H | P | D | X | F | K | S | T | P | N | L | W | F | L | D | L | S | K | N | V | F | T | D | D | D | S | I | T | A | E | V | F | H | P | I | P | K | L | S | R | L | I | I | S | S | A | Q | L | H | S | L | N | F | L | I | N | A | N | L | S | R | L | S | A | L | R | A | P | E | N | K | L | D | V | I | N | Q | T | L | I | Q | S | L | P | H | L | T | Y | L | D | L | Q | X | N | X | F | T | C | D | C | N | N | A | F | F | I | N | W | A | L | T | S | N | Y | T | Q | V | L | Y | L | N | R | Y | T | C | S | Y | P | L | S | L | R | G | K | S | I | L | D | M | N | V | E | S | C | N | V | N | I | D | F | I | M | F | V | V | S | S | V | V | V | M | L | T | L | L | G | S | F | I | Y | Q | F | L | H | W | Q | V | L | Y | A | Y | Y | L | F | L | A | Y | L | Y | D | S | K | R | K | Q | M | X | Q | Q | H | G | F | K | Y | D | A | F | I | S | Y | N | A | R | D | E | P | W | V | V | E | E | L | I | P | N | L | E | D | X | Q | G | W | R | L | C | L | H | H | R | D | F | E | P | G | R | S | I | I | D | N | I | M | D | G | I | Y | S | S | R | K | T | I | C | L | I | T | H | N | Y | L | R | S | X | W | C | S | K | E | I | Q | M | A | N | F | R | L | F | D | E | K | K | D | V | L | I | L | I | F | L | E | D | I | P | T | L | Q | L | S | P | Y | Y | X | M | R | X | L | V | K | K | K | T | Y | L | K | W | P | K | H | G | E | D | T | R | V | F | W | Q | K | L | R | M | A | L | E | T | K | E | G | P | E | D | E | K | A | L | L | S | G | H | G | K | R | D | L | L | X | X | X | X | X | X | X | - |
| DRTLR22\_3030\_BP | M | X | X | X | X | X | X | X | X | X | X | X | X | X | X | X | X | X | X | X | K | R | K | S | R | E | S | M | G | T | L | Q | - | - | - | - | Q | I | T | V | X | X | X | X | X | X | X | I | T | I | C | A | M | V | S | P | C | S | T | F | S | L | T | N | C | T | I | S | S | S | Q | K | D | I | K | T | Q | P | K | V | L | C | Y | K | M | N | X | X | F | F | T | I | X | X | R | V | P | N | N | T | W | I | L | D | I | S | F | N | S | F | A | Q | I | Q | I | E | D | L | N | I | L | L | N | L | Q | Y | L | N | V | S | N | N | K | I | S | K | I | Q | D | G | A | F | G | S | L | F | N | L | T | D | L | N | L | A | S | N | R | L | N | A | V | S | N | G | M | L | R | G | L | T | N | L | L | V | L | R | L | D | R | N | Y | I | S | V | I | K | E | S | A | F | S | T | L | H | S | L | Q | V | L | N | L | S | K | N | H | L | R | H | I | D | D | V | K | P | V | L | A | X | S | L | Y | L | E | E | L | Y | I | G | S | N | Y | F | N | V | F | N | S | S | E | L | S | T | K | P | L | S | L | K | R | L | D | L | S | N | N | R | F | A | A | F | Q | L | T | N | N | I | F | P | T | L | N | H | L | D | L | S | Y | C | G | H | N | G | T | M | A | W | N | V | T | E | K | S | Y | L | A | S | V | K | T | L | Y | F | M | D | V | N | M | S | I | Q | T | A | D | N | V | L | Q | S | F | N | X | X | X | T | L | N | K | I | R | L | N | G | N | V | R | L | N | K | X | T | S | L | L | L | S | V | C | X | X | P | M | L | R | V | L | R | L | I | A | T | K | I | K | H | L | T | N | H | M | F | D | P | C | S | E | L | S | E | L | D | L | G | G | N | E | I | S | N | L | S | Q | S | M | F | R | G | F | K | Q | L | K | K | L | Q | L | Q | I | N | K | L | T | R | V | T | N | S | F | Q | I | L | T | S | L | E | F | I | D | L | S | R | N | H | I | H | K | L | S | C | N | D | F | A | N | L | T | Q | V | K | T | L | Y | L | Y | S | N | K | I | S | S | I | R | S | C | L | F | K | D | L | K | S | L | E | V | L | R | L | G | T | N | N | L | L | K | I | D | D | V | F | S | N | X | X | P | Y | F | L | K | E | L | Q | L | S | Y | N | K | L | S | L | I | K | S | H | T | F | G | N | L | P | Q | L | N | N | L | S | L | E | D | N | Q | I | S | E | I | E | G | Y | A | F | G | G | L | E | N | L | T | S | L | F | L | S | S | N | K | I | T | G | K | T | L | T | T | H | P | N | V | F | S | G | M | P | N | L | Q | N | L | D | L | F | A | N | S | I | S | F | A | Y | D | K | X | L | K | Y | P | L | F | K | D | L | K | N | L | R | E | L | S | L | Y | S | Q | R | X | X | X | G | I | G | Q | L | P | S | N | L | L | E | G | L | S | S | L | Q | M | F | Y | I | G | N | T | N | L | N | Q | L | N | P | N | L | F | N | F | T | P | Q | L | W | F | L | D | L | S | K | N | A | L | R | E | D | D | A | I | P | P | E | L | F | H | P | I | P | N | L | T | K | L | I | I | S | R | T | Q | L | R | S | L | N | F | L | L | G | A | N | L | N | K | L | S | T | L | R | A | S | G | N | E | I | D | S | V | N | E | T | L | I | K | S | L | P | R | L | A | V | L | D | L | Q | N | N | T | F | T | C | D | C | N | N | A | F | F | I | D | W | A | K | K | N | E | S | T | Q | V | Y | Y | L | S | R | Y | T | C | S | Y | P | R | T | L | R | G | T | S | L | A | E | F | N | T | D | S | C | T | F | N | W | D | Y | I | C | F | V | C | S | S | I | L | V | I | L | T | L | L | L | S | F | I | W | N | F | L | R | Y | Q | V | V | Y | T | Y | Y | L | F | L | A | F | L | Y | D | S | R | R | N | Q | S | G | Q | T | X | X | X | Q | Y | D | A | F | I | S | Y | N | T | L | D | E | A | W | V | M | E | E | L | I | P | K | L | E | G | E | Q | G | W | R | L | C | L | H | H | R | D | F | E | P | G | R | P | I | I | D | N | I | V | D | G | I | Y | S | S | R | K | T | I | C | L | I | T | R | N | Y | L | K | S | N | W | C | S | S | E | V | Q | V | A | S | F | R | L | F | D | E | Q | K | D | V | L | I | L | V | F | L | E | D | I | P | T | H | Q | L | S | P | Y | H | R | L | R | K | L | V | K | K | R | T | Y | I | R | W | P | K | P | G | E | D | N | K | I | F | W | Q | K | L | K | M | A | L | E | T | K | D | S | H | K | S | E | N | C | I | L | X | X | X | X | X | X | X | X | X | X | X | X | X | X | X | X | - |
| MATLR22\_3030\_BP | X | X | X | X | X | X | X | X | X | X | X | X | X | X | X | X | X | X | X | X | X | X | X | X | X | X | X | M | G | K | L | Q | - | - | - | - | Q | I | T | L | X | X | X | X | X | X | X | I | V | L | F | G | F | I | S | P | C | S | A | F | S | L | Q | N | C | T | I | S | T | P | L | R | D | I | X | X | Q | P | K | V | L | C | Y | K | M | G | X | X | F | F | R | I | P | W | W | I | P | R | N | T | Q | I | L | D | I | S | F | N | A | F | A | Q | I | Q | I | G | D | F | R | H | L | S | N | L | Q | D | L | N | I | S | N | N | R | I | S | Q | I | Q | E | G | A | L | D | D | L | S | N | L | T | Y | L | N | L | A | S | N | R | L | K | A | V | S | S | G | M | L | H | G | L | S | N | L | L | V | L | R | L | D | D | N | N | I | K | D | I | E | E | S | A | F | S | T | L | Q | N | L | K | V | L | N | L | T | K | N | H | L | H | Y | I | D | K | V | K | P | V | L | A | X | S | P | L | L | E | E | L | Y | I | G | S | N | S | F | D | V | F | N | S | Y | E | M | S | T | K | P | L | S | L | K | K | L | D | F | S | N | N | P | L | A | T | F | Q | L | T | D | N | I | F | P | S | L | N | H | L | D | L | S | Y | C | G | Q | N | G | S | M | T | W | N | V | T | E | K | T | Y | F | S | S | V | Q | T | L | Y | F | M | D | V | N | M | S | P | Q | S | V | A | N | V | L | P | S | F | K | X | X | X | S | L | N | K | I | R | F | N | G | N | V | E | L | N | K | X | T | N | L | L | L | S | A | C | X | X | P | M | L | R | V | V | R | L | N | A | N | K | I | K | H | L | T | D | N | M | F | D | P | C | F | D | L | T | E | L | D | L | G | Q | N | E | I | S | K | L | S | Q | S | M | F | R | G | F | T | Q | L | K | K | L | L | L | Q | I | N | K | L | T | Q | I | T | N | S | F | Q | I | L | S | T | L | E | F | I | D | F | S | R | N | S | I | N | K | L | T | C | D | D | F | A | N | L | T | Q | V | K | T | L | Y | L | Y | G | N | K | I | S | L | I | R | S | C | L | F | K | D | L | K | S | L | E | V | L | K | L | G | T | N | D | L | L | R | I | D | D | A | F | S | N | X | X | P | H | S | L | K | D | L | Q | I | N | Y | N | K | L | S | K | I | E | K | Y | T | F | R | N | L | S | Q | L | N | S | L | T | L | N | D | N | Q | I | S | E | I | E | A | Q | A | F | E | G | L | K | N | L | T | S | L | F | L | S | S | N | K | I | T | A | K | T | L | T | R | Y | P | N | V | F | S | G | M | P | N | L | Q | N | L | D | L | Y | A | N | S | I | S | F | A | D | N | K | X | L | K | H | P | P | F | K | D | L | N | Q | L | R | V | L | T | L | H | S | Q | R | X | X | X | G | I | D | K | I | P | S | N | L | L | Q | G | L | S | S | M | E | M | F | Y | V | G | N | T | N | L | G | H | L | N | P | D | T | F | K | F | S | P | Q | L | W | F | L | D | L | S | K | N | A | L | S | E | D | N | S | I | P | A | E | L | F | H | P | I | S | G | L | T | K | L | I | L | S | R | T | Q | L | R | S | L | N | F | L | L | N | A | N | L | S | R | L | S | T | L | K | A | P | G | N | E | I | D | T | I | N | K | T | L | I | Q | S | L | P | R | L | E | V | L | D | L | Q | K | N | T | F | T | C | D | C | H | N | E | F | F | I | E | W | A | M | K | T | N | S | T | Q | V | F | Y | F | N | R | Y | T | C | S | Y | P | R | S | L | R | G | M | S | L | T | A | F | N | I | E | S | C | T | L | N | I | D | F | I | C | F | L | C | S | S | I | M | V | T | L | T | L | L | L | S | F | V | W | H | F | L | R | Y | Q | V | I | Y | A | Y | Y | L | F | L | A | Y | L | Y | D | S | K | K | K | Q | T | V | S | T | X | X | X | R | Y | D | A | F | I | S | Y | N | T | E | D | E | P | W | V | M | E | E | L | I | P | K | L | E | G | E | Q | S | W | R | L | C | L | H | H | R | D | F | E | P | G | R | P | I | I | D | N | I | I | D | G | I | Y | S | S | R | K | T | I | C | L | I | T | R | N | Y | L | K | S | N | W | C | S | S | E | V | Q | V | A | S | Y | R | L | F | D | E | Q | K | D | V | L | I | L | V | F | L | E | D | I | P | A | H | Q | L | S | P | H | H | R | M | R | K | L | V | K | K | R | T | Y | L | R | W | P | K | P | G | E | D | T | K | I | F | W | Q | K | L | K | M | A | L | E | T | R | E | G | H | N | P | E | S | S | I | L | X | X | X | X | X | X | X | X | X | X | X | X | X | X | X | X | - |
| CITLR22\_3030\_BP | M | X | X | X | X | X | X | X | X | X | X | X | X | X | X | X | X | X | X | X | K | K | E | S | R | K | S | M | G | K | L | Q | - | - | - | - | Q | I | T | L | X | X | X | X | X | X | X | I | V | L | C | G | F | I | S | A | C | S | A | F | S | L | K | N | C | T | I | S | T | P | L | R | D | I | X | X | Q | P | K | V | L | C | Y | N | M | G | X | X | F | F | R | I | P | W | W | I | P | R | N | T | R | I | L | D | I | S | F | N | D | F | A | Q | I | Q | I | G | D | F | R | H | L | S | N | L | Q | D | L | N | I | S | N | N | R | I | S | Q | I | Q | E | G | A | L | D | D | L | S | N | L | T | Y | L | N | L | A | S | N | R | L | K | A | V | S | S | G | M | L | H | G | L | S | N | L | L | V | L | R | L | D | E | N | N | I | K | D | I | E | E | S | A | F | S | T | L | Q | N | L | K | V | L | N | L | T | K | N | H | L | H | Y | I | D | K | V | K | P | V | L | A | X | S | P | L | L | E | E | L | Y | I | G | S | N | N | F | D | V | F | N | S | Y | E | M | S | T | K | P | L | S | L | K | K | L | D | F | S | N | N | P | L | A | T | F | Q | L | T | D | N | I | F | P | S | L | N | H | L | D | L | S | Y | C | G | Q | N | G | S | M | T | W | N | V | T | E | K | T | Y | F | S | S | V | Q | T | L | Y | F | M | D | V | N | M | S | P | Q | N | V | A | N | V | L | L | S | F | K | X | X | X | S | L | N | K | I | R | F | N | G | N | V | E | L | N | K | X | T | N | L | L | L | S | A | C | X | X | P | M | L | R | V | V | R | L | N | A | N | K | I | K | H | L | T | D | N | M | F | D | P | C | S | D | L | T | E | L | D | L | G | D | N | E | I | S | K | L | S | P | S | M | F | R | G | F | T | Q | L | K | K | L | L | L | Q | I | N | K | L | T | Q | I | T | N | S | F | Q | I | L | T | T | L | E | F | I | D | L | S | R | N | S | I | N | K | L | T | C | N | D | F | A | N | L | T | Q | V | K | T | L | Y | L | Y | G | N | K | I | S | L | I | R | S | C | L | F | K | D | L | K | S | L | E | V | L | K | L | G | T | N | D | L | L | R | I | D | D | A | F | S | N | X | X | P | H | S | L | K | D | L | Q | I | N | F | N | K | L | S | K | I | E | K | Y | T | F | R | N | L | S | Q | L | N | S | L | T | L | N | D | N | Q | I | S | E | I | E | A | Q | A | F | E | G | L | K | N | L | T | S | L | F | L | S | S | N | K | I | T | A | K | T | L | T | R | H | P | N | V | F | S | G | M | P | N | L | Q | N | L | D | L | Y | A | N | S | I | S | F | A | D | N | K | X | L | K | H | P | P | F | K | D | L | K | Q | L | R | V | L | T | L | H | S | Q | R | X | X | X | G | I | N | K | I | P | S | N | L | L | Q | G | L | S | S | M | E | M | F | Y | V | G | N | T | N | L | G | H | L | N | P | D | T | F | K | F | S | P | Q | L | W | F | L | D | L | S | K | N | A | L | S | E | D | N | S | I | P | A | E | L | F | H | P | I | S | R | L | T | K | L | I | L | S | R | T | Q | L | R | S | L | N | F | L | L | N | A | N | L | S | R | L | S | T | L | R | A | P | G | N | E | I | D | T | I | N | K | T | L | I | Q | S | L | P | R | L | E | V | L | D | L | Q | R | N | T | F | T | C | D | C | H | N | E | F | F | I | E | W | A | M | K | T | N | S | T | Q | V | F | Y | F | N | R | Y | T | C | S | Y | P | R | S | L | R | G | M | S | L | T | A | F | N | I | E | S | C | T | L | N | I | D | F | I | C | F | L | C | S | S | I | V | V | T | L | T | L | L | L | S | F | V | W | H | F | L | R | Y | Q | V | I | Y | A | Y | Y | L | F | L | A | F | L | Y | D | N | K | K | K | Q | T | V | S | T | X | X | X | R | Y | D | T | F | I | S | Y | N | T | E | D | E | P | W | V | M | E | E | L | V | P | K | L | E | G | E | Q | G | W | K | L | C | L | H | H | R | D | F | V | P | G | R | P | I | I | D | N | I | I | D | G | I | Y | S | S | R | K | T | I | C | L | I | T | R | N | Y | L | K | S | N | W | C | S | S | E | V | Q | V | A | S | Y | R | L | F | D | E | Q | K | D | V | L | I | L | V | F | L | E | D | I | P | A | H | Q | L | S | P | H | H | R | M | R | K | L | V | K | K | R | T | Y | L | R | W | P | K | P | G | E | D | T | K | I | F | W | Q | K | L | K | M | A | L | E | T | K | E | G | H | N | P | E | S | A | I | L | X | X | X | X | X | X | X | X | X | X | X | X | X | X | X | X | - |
| CCTLR22\_3030\_BP | X | X | X | X | X | X | X | X | X | X | X | X | X | X | X | X | X | X | X | X | X | X | X | X | X | X | X | M | G | T | L | K | - | - | - | - | Q | I | T | L | X | X | X | X | X | X | X | V | A | V | C | S | F | I | S | S | S | S | A | F | S | L | N | N | C | T | I | R | T | P | L | K | D | I | X | X | Q | P | K | V | L | C | Y | K | M | G | X | X | F | F | G | I | X | X | W | I | P | R | N | T | R | I | L | D | I | S | F | N | A | F | A | Q | I | Q | I | G | D | F | S | H | L | S | N | L | Q | D | L | N | I | S | N | N | K | I | S | Q | I | Q | E | G | A | L | D | N | L | S | N | L | S | R | L | N | L | A | S | N | R | L | R | E | I | S | R | G | M | L | H | G | L | T | N | L | L | V | L | R | L | D | N | N | Y | I | K | D | I | D | V | T | A | F | S | T | L | Q | N | L | K | V | L | N | L | T | K | N | H | L | H | Y | I | E | K | I | K | P | V | L | A | X | S | P | Y | L | E | E | L | Y | I | A | S | N | H | F | D | A | F | K | S | Y | E | M | S | T | N | P | L | S | L | K | I | L | D | L | S | K | N | P | F | A | T | F | Q | L | T | D | N | I | F | P | F | L | N | H | L | D | L | S | Y | C | G | Q | N | G | T | M | T | W | N | I | T | E | K | T | F | F | S | S | V | K | T | L | Y | F | M | D | V | H | I | S | A | Q | N | A | A | N | V | L | Q | S | F | Q | X | X | X | L | L | N | K | I | R | L | N | G | N | V | E | L | N | K | X | T | D | L | L | L | N | A | C | X | X | P | M | L | Q | V | L | R | L | K | A | N | K | I | K | H | L | T | E | H | M | F | D | P | C | S | N | L | T | E | V | D | L | G | D | N | E | I | S | R | L | W | P | S | I | F | I | G | F | T | Q | L | K | T | L | R | L | Q | I | N | K | L | T | Q | I | T | N | S | F | Q | M | L | S | T | L | E | F | I | D | L | S | R | N | S | I | D | T | L | T | C | N | D | F | A | N | L | T | Q | L | K | N | L | Y | L | Y | S | N | K | I | S | I | I | K | S | C | L | F | K | D | L | K | S | L | E | V | L | K | L | G | T | N | N | L | L | R | I | G | D | A | F | S | N | X | X | P | Y | S | L | V | E | L | Q | L | T | F | N | K | L | S | K | I | E | K | Y | T | F | R | N | V | S | Q | L | N | S | L | S | L | Q | D | N | Q | I | S | E | I | E | S | H | A | F | G | G | L | K | N | L | T | Y | L | L | L | S | S | N | K | I | S | A | K | T | L | T | K | N | Q | D | V | F | S | G | M | P | N | L | K | S | L | G | L | D | T | N | S | I | S | F | S | D | D | Q | X | L | K | F | P | P | F | K | D | L | K | H | L | R | V | L | A | L | H | S | Q | R | X | X | X | G | I | G | K | I | P | S | N | L | L | Q | G | L | S | S | L | E | M | F | Y | A | G | N | I | N | L | A | H | L | N | P | D | T | F | K | F | S | P | Q | L | W | F | L | D | L | S | K | N | A | L | S | E | D | H | S | T | P | A | E | L | F | H | P | I | S | R | L | T | K | L | I | L | S | R | T | Q | L | R | S | L | N | F | L | L | N | A | N | L | S | R | L | L | S | L | K | A | S | G | N | E | I | D | T | I | N | K | T | L | I | Q | S | L | P | R | L | E | V | L | D | L | G | K | N | T | F | T | C | D | C | T | N | E | F | F | I | D | W | A | K | N | S | N | S | T | Q | V | L | Y | L | N | K | Y | M | C | S | Y | P | S | S | L | R | G | M | S | L | S | A | F | N | T | E | S | C | T | L | N | I | D | F | I | C | F | L | C | S | S | I | V | V | I | L | T | L | L | L | S | F | V | W | H | F | L | R | F | Q | V | V | Y | A | Y | Y | L | F | I | A | F | L | Y | D | N | K | K | K | Q | S | G | S | T | X | X | X | Q | Y | D | A | F | I | S | Y | N | A | K | D | E | P | W | V | M | E | E | L | I | P | K | L | E | G | E | Q | D | W | R | L | C | L | H | H | R | D | F | E | P | G | R | P | I | I | D | N | I | I | D | G | I | Y | S | S | R | K | T | T | C | L | I | T | R | N | Y | L | K | S | N | W | C | S | S | E | V | Q | V | A | S | F | R | L | F | D | E | Q | K | D | V | L | I | L | V | F | L | E | D | I | P | T | H | Q | P | S | P | Y | H | R | M | R | K | L | V | K | K | R | T | Y | L | R | W | P | K | P | G | E | D | T | K | V | F | W | Q | K | L | K | M | A | L | E | V | K | E | G | H | K | S | D | F | L | I | P | X | X | X | X | X | X | X | X | X | X | X | X | X | X | X | X | - |
| CATLR22\_3030\_BP | X | X | X | X | X | X | X | X | X | X | X | X | X | X | X | X | X | X | X | X | X | X | X | X | X | X | X | M | G | T | L | K | - | - | - | - | Q | I | T | L | X | X | X | X | X | X | X | I | A | L | C | G | F | I | S | S | S | S | A | F | S | L | N | N | C | T | I | S | T | P | L | K | D | I | X | X | Q | L | K | V | L | C | Y | K | M | G | X | X | F | F | R | I | X | X | S | I | P | G | N | T | H | I | L | D | I | S | F | N | A | F | A | Q | I | Q | I | G | D | F | N | H | L | S | N | L | Q | D | L | N | I | S | H | N | K | I | S | Q | I | Q | E | G | A | F | D | S | L | S | N | L | Y | S | L | N | L | A | S | N | R | L | Q | A | I | S | R | G | M | L | H | G | L | T | N | L | L | V | L | R | L | D | N | N | Y | I | K | D | I | E | E | T | A | F | S | M | L | H | N | L | K | V | L | N | L | T | K | N | Q | L | H | Y | V | E | K | I | K | P | V | L | A | X | S | P | H | L | E | E | L | Y | I | G | S | N | H | L | N | A | F | K | S | Y | E | M | S | T | N | P | L | A | L | K | I | L | D | F | S | N | N | P | F | A | T | F | Q | L | T | D | N | I | F | Q | F | L | N | H | L | D | L | S | Y | C | G | Q | N | G | T | M | T | W | N | V | T | E | K | T | F | L | A | S | V | K | S | L | Y | F | M | D | V | H | M | S | V | Q | N | A | A | N | V | L | Q | S | F | Q | X | X | X | L | L | N | K | I | R | L | N | G | N | A | E | L | N | R | X | T | D | L | L | L | N | A | C | X | X | P | V | L | Q | V | L | R | L | K | T | N | K | I | K | H | L | T | E | H | M | F | D | L | C | S | N | L | T | E | I | D | L | G | D | N | E | I | S | N | I | P | P | G | M | F | S | R | F | T | Q | L | K | T | L | R | L | Q | I | N | K | F | T | R | I | T | N | F | F | Q | N | L | F | T | L | E | F | I | D | L | S | R | N | S | I | K | T | L | N | C | D | V | F | A | N | L | T | Q | L | K | M | L | Y | L | Y | S | N | K | I | S | I | I | E | S | C | S | F | K | D | L | K | S | L | E | V | L | R | L | G | T | N | N | L | L | R | I | G | D | A | F | R | N | X | X | P | Q | S | L | L | E | L | Q | L | S | F | N | K | L | S | K | I | E | K | H | T | F | R | N | M | S | Q | L | K | Y | L | H | L | Q | D | N | Q | I | S | E | I | E | S | Y | A | F | G | G | L | K | N | L | S | S | L | F | L | S | S | N | K | I | S | A | K | T | L | T | K | H | Q | D | V | F | S | G | M | P | N | L | E | N | L | G | L | D | A | N | I | I | L | F | T | D | D | Q | X | L | K | F | P | P | F | K | D | L | K | H | L | T | V | L | S | L | H | S | Q | R | X | X | X | G | I | G | Q | I | P | S | N | L | L | Q | G | L | S | S | L | E | M | F | Y | V | G | N | T | N | L | A | H | L | N | P | D | T | F | K | F | S | P | Q | L | W | F | L | D | L | S | K | N | A | L | S | E | D | H | S | I | P | A | E | L | F | H | P | I | S | R | L | T | K | L | L | I | S | R | T | Q | L | R | S | L | N | F | L | L | N | A | N | L | S | R | L | S | T | L | K | A | T | G | N | E | I | D | L | I | N | K | T | L | I | Q | S | L | P | R | L | E | V | L | D | L | Q | K | N | T | F | T | C | D | C | D | N | E | F | F | I | D | W | A | K | N | S | N | S | T | Q | V | V | Y | L | N | K | Y | M | C | S | Y | P | S | S | L | R | G | M | S | L | S | A | F | N | T | E | S | C | T | L | N | I | D | F | I | C | F | L | C | S | S | I | V | V | F | L | T | L | L | S | S | F | V | W | H | F | L | R | F | Q | V | V | Y | A | Y | Y | L | F | L | A | F | L | Y | D | N | K | K | K | Q | S | G | S | T | X | X | X | Q | Y | D | A | F | I | S | Y | N | A | Q | D | E | P | W | V | M | E | E | L | I | P | K | L | E | G | E | Q | S | W | R | L | C | L | H | H | R | D | F | E | P | G | R | P | I | I | D | N | I | V | D | G | I | Y | S | S | R | K | T | I | C | L | I | T | K | N | Y | L | K | S | N | W | C | S | S | E | V | Q | V | A | S | F | R | L | F | D | E | Q | K | D | V | L | I | L | V | F | L | E | D | I | P | T | H | Q | L | S | P | Y | H | R | M | R | K | L | V | K | K | R | T | Y | L | R | W | P | K | P | G | E | D | T | K | I | F | W | Q | K | L | K | L | A | L | E | A | K | E | G | H | K | S | D | L | L | I | P | X | X | X | X | X | X | X | X | X | X | X | X | X | X | X | X | - |
| SSTLR22\_3030\_BP | M | X | X | X | X | X | X | X | X | - | - | X | X | X | S | S | P | S | P | V | T | L | R | I | T | M | P | G | K | R | R | G | S | T | F | S | Q | L | R | L | I | L | C | F | L | - | - | L | Y | C | W | I | L | A | G | P | V | C | G | Y | F | L | K | N | C | P | I | R | G | N | L | S | D | N | F | X | N | M | K | V | L | C | Y | N | R | N | X | X | L | E | V | M | P | I | N | I | L | W | K | V | S | V | L | D | V | A | M | N | N | I | S | K | I | G | K | F | D | F | K | G | L | S | N | L | K | I | L | N | M | F | M | N | Q | I | S | Q | V | D | N | D | A | L | A | H | L | E | A | L | Q | E | L | Y | L | A | Y | N | R | L | T | T | L | S | D | H | L | F | Q | D | L | A | N | L | S | L | L | H | L | D | N | N | L | I | T | T | I | G | S | S | S | F | Q | L | L | S | S | L | K | T | V | N | L | T | K | N | N | L | H | N | M | K | E | V | Q | P | I | I | Q | X | L | P | H | L | R | E | L | Y | I | G | S | N | R | F | T | S | F | Q | S | Q | E | I | S | N | K | S | I | G | L | R | L | L | D | L | S | R | N | P | X | X | X | X | X | I | T | A | D | V | L | P | Y | L | E | V | L | D | I | A | Y | C | G | Q | L | G | R | M | E | W | D | V | L | D | R | S | F | L | S | N | I | K | G | L | N | L | S | G | I | E | M | S | F | E | R | M | A | M | E | L | Q | T | V | N | X | X | X | S | L | V | H | L | R | L | Y | D | I | G | E | E | R | V | X | K | A | L | T | D | I | A | C | H | I | P | T | L | S | L | L | R | L | H | H | N | N | I | S | S | L | S | E | E | F | L | Q | S | C | K | Q | V | T | E | V | D | L | E | N | N | N | I | I | Q | L | S | A | V | S | F | R | S | M | E | Q | L | S | T | L | R | L | G | H | N | M | L | S | S | V | P | D | A | T | R | N | V | S | T | L | K | F | L | D | L | S | F | N | I | I | L | K | L | G | C | S | D | F | A | N | L | T | G | L | T | Q | L | F | L | F | H | N | Q | I | S | N | L | P | G | C | V | F | K | D | L | K | E | L | R | I | L | K | L | G | S | N | K | I | L | T | L | N | D | D | F | M | S | X | X | L | Y | K | L | E | Y | L | S | M | S | Y | N | K | L | S | S | I | S | K | G | D | F | K | G | L | A | S | L | K | T | L | L | L | F | D | N | Q | I | A | S | L | E | D | G | A | F | E | G | L | V | N | L | T | E | L | R | L | E | S | N | K | I | T | Q | X | X | X | D | I | R | N | T | V | L | T | G | L | P | R | F | R | T | L | D | I | S | C | N | F | I | T | Y | V | N | N | D | K | L | D | P | P | P | F | S | H | L | T | S | L | E | N | L | H | I | H | S | Q | R | X | X | K | G | L | S | H | L | P | S | N | F | L | E | G | L | K | S | L | L | A | F | K | A | G | N | L | N | I | K | E | L | H | P | N | T | F | I | H | T | P | R | L | W | Y | L | D | I | S | K | N | A | F | T | X | X | X | X | L | K | P | E | L | F | H | P | T | P | R | L | N | R | M | Y | L | S | K | A | R | L | Q | A | L | D | F | L | I | G | A | N | L | S | R | V | T | F | L | Q | V | S | K | N | D | I | T | V | V | N | E | T | V | L | H | S | L | P | A | L | T | Y | L | D | M | Q | D | N | A | F | T | C | G | C | S | D | A | W | F | V | N | W | M | E | N | D | N | Q | T | Q | V | V | G | A | G | E | F | T | C | N | Y | P | A | D | L | K | D | T | K | L | L | D | V | E | L | Q | F | C | T | V | H | L | G | L | Y | Y | Y | I | S | T | T | C | L | V | L | L | T | L | V | A | S | F | A | Y | H | F | L | K | W | Q | V | I | Y | G | Y | Y | L | F | L | A | F | L | Y | D | T | K | Q | R | N | K | L | T | P | H | G | C | Q | Y | D | A | F | I | S | Y | N | A | H | D | E | P | W | V | L | R | E | L | L | P | E | L | E | G | E | Q | G | W | K | L | C | L | H | H | R | D | F | Q | P | G | K | P | I | I | D | N | I | M | E | G | I | Y | G | S | R | K | T | I | C | V | I | S | R | R | Y | L | E | S | E | W | C | S | R | E | I | Q | V | A | S | F | R | L | F | D | E | Q | K | D | V | L | I | L | V | F | L | E | E | I | P | T | H | Q | L | S | P | Y | H | R | M | R | K | L | V | K | R | R | T | Y | L | S | W | P | R | A | G | E | E | T | G | V | F | W | Q | Q | L | R | L | A | L | E | T | K | D | G | S | S | E | E | N | P | I | L | S | G | V | Q | A | L | X | X | X | X | X | X | X | X | X | X | - |
| OMTLR22\_3030\_BP | X | X | X | X | X | X | X | X | X | - | - | X | X | X | X | X | X | X | X | M | T | L | K | I | T | M | P | G | E | Q | S | G | S | T | F | S | Q | L | R | L | F | L | C | F | L | - | - | M | Y | C | W | V | L | A | D | P | V | C | G | Y | F | L | N | N | C | T | I | R | G | N | L | S | D | N | F | X | N | M | K | V | L | C | Y | N | R | N | X | X | I | E | V | M | P | I | N | I | P | C | K | V | S | V | L | D | V | A | M | N | N | I | S | K | I | R | R | L | D | F | K | G | L | S | N | L | K | I | L | N | M | S | R | N | Q | I | S | Q | V | D | N | D | A | L | L | P | L | K | A | L | R | E | L | N | L | A | H | N | R | L | T | T | L | S | D | H | L | F | Q | G | L | D | N | L | S | L | L | H | L | D | N | N | L | I | A | T | I | S | S | S | S | F | Q | P | L | S | S | L | K | T | V | N | L | T | K | N | N | L | H | N | M | K | E | V | Q | P | I | V | Q | X | L | P | H | L | Q | E | L | Y | I | G | S | N | R | F | T | S | F | Q | S | Q | E | I | S | N | M | S | I | E | L | R | L | L | D | L | S | R | N | P | L | G | V | F | R | I | T | T | D | V | L | P | Y | L | E | V | L | D | I | A | Y | C | G | Q | L | G | H | M | E | W | D | V | L | D | R | S | F | L | R | N | V | K | S | L | N | L | S | G | I | E | M | S | L | E | R | M | A | M | V | L | Q | T | V | N | X | X | X | S | L | V | H | L | R | L | Y | D | I | S | E | E | R | V | X | K | A | L | I | D | F | A | C | N | I | P | T | L | S | L | L | R | L | H | H | N | N | I | S | A | L | S | E | E | F | L | Q | S | C | K | Q | V | T | E | V | D | L | E | N | N | N | I | I | Q | L | S | E | V | S | F | R | S | M | E | Q | L | S | T | L | R | L | G | H | N | R | L | S | S | V | P | D | A | T | R | N | I | S | T | L | M | L | L | D | L | S | F | N | I | I | H | K | L | G | C | S | D | F | S | N | L | T | G | L | T | Q | L | F | L | F | H | N | Q | I | S | N | L | P | G | C | V | F | Q | D | L | K | D | L | R | I | L | K | L | G | S | N | K | I | L | T | L | N | D | D | F | M | S | X | X | L | H | K | L | E | F | L | S | M | S | Y | N | K | L | S | S | I | S | K | G | D | F | K | G | L | A | S | L | K | T | L | L | L | F | D | N | Q | I | A | S | L | E | D | G | A | F | E | G | L | V | N | L | T | E | L | R | L | Q | S | N | K | I | T | Q | X | X | X | D | I | R | N | T | V | L | T | G | L | P | H | L | R | T | L | D | I | S | C | N | Y | I | T | Y | V | N | D | D | K | L | D | P | P | P | L | S | H | L | T | S | L | E | N | L | L | I | F | S | Q | R | X | X | K | G | L | C | H | L | P | I | N | F | L | E | G | L | T | S | L | L | S | F | Q | A | G | S | L | N | I | K | D | L | H | P | D | T | F | I | H | T | P | Q | L | W | F | L | D | M | S | K | N | E | F | T | X | X | X | X | L | T | P | K | L | F | H | P | T | P | S | L | N | R | L | Y | L | P | K | A | R | L | Q | S | L | D | F | L | K | G | A | N | L | S | R | V | T | F | L | Q | V | R | K | N | D | I | T | A | V | N | E | T | V | L | Q | S | L | P | A | L | T | Y | L | D | M | Q | D | N | P | F | T | C | G | C | S | D | A | W | F | V | Q | W | M | E | S | N | N | Q | T | Q | V | V | G | A | G | E | F | T | C | N | Y | P | A | D | L | K | D | T | K | L | L | D | V | E | L | Q | F | C | T | V | D | L | G | L | Y | Y | Y | I | S | T | T | C | L | V | L | L | T | L | I | A | S | F | A | Y | H | F | L | K | W | E | V | I | Y | G | Y | Y | L | F | L | A | F | L | Y | D | T | K | Q | R | N | K | H | K | P | H | G | C | Q | Y | D | A | F | I | S | Y | N | A | H | D | E | P | W | V | L | R | E | L | L | P | E | L | E | G | E | Q | G | W | K | L | C | L | H | H | R | D | F | Q | P | G | K | P | I | I | D | N | I | M | D | G | I | Y | G | S | R | K | T | I | C | V | I | S | H | R | Y | L | E | S | E | W | C | S | R | E | I | Q | V | A | S | F | R | L | F | D | E | Q | K | D | V | L | I | L | V | F | L | E | E | I | P | T | H | Q | L | S | P | Y | H | R | M | R | K | L | V | K | R | R | S | Y | L | S | W | P | R | A | G | E | Q | T | G | V | F | W | Q | Q | L | R | L | A | L | E | S | K | D | G | S | A | E | E | N | P | I | L | S | X | V | E | A | L | X | X | X | X | X | X | X | X | E | P | - |
| GMTLR22\_3030\_BP | X | X | X | X | X | X | X | X | X | - | - | X | X | X | X | X | X | X | X | X | X | X | X | X | X | X | X | M | P | L | R | G | I | R | F | C | S | F | X | X | I | L | Y | L | F | - | - | L | X | X | N | S | F | V | V | P | T | A | S | Y | T | L | K | D | C | M | V | K | Q | X | X | X | X | X | X | X | N | T | Q | V | I | C | R | K | N | S | X | X | F | K | V | F | P | K | D | I | P | A | R | V | T | S | I | E | L | S | G | N | T | I | S | T | L | N | K | T | D | L | E | N | I | P | N | L | L | R | L | D | L | T | S | N | R | I | S | K | I | E | S | G | T | F | V | V | Q | L | S | L | E | V | L | I | L | K | N | N | S | L | Y | K | L | Q | E | G | M | F | D | G | L | V | N | L | K | E | L | Y | L | T | S | N | Q | I | Q | T | V | A | P | A | S | F | K | T | L | S | K | L | R | I | L | D | L | G | Y | N | K | L | R | H | L | T | N | I | X | X | X | X | Q | H | T | P | H | L | Q | T | L | Y | I | P | A | N | N | I | S | T | F | H | S | W | E | L | S | N | K | S | T | E | L | V | I | L | D | L | S | Q | N | E | L | R | F | F | R | L | T | A | G | I | F | P | K | L | K | T | L | N | L | R | X | X | G | I | K | N | G | I | V | W | E | V | N | D | T | S | Y | L | R | S | V | H | K | L | D | I | S | G | V | R | S | S | L | H | G | L | Q | E | V | I | E | T | F | N | S | S | S | S | L | I | Y | L | Q | L | N | N | I | S | N | X | X | L | X | Q | A | L | I | D | V | S | C | K | I | L | K | L | S | T | F | E | I | R | N | N | G | I | K | V | I | R | S | D | M | L | H | L | C | S | Y | L | K | I | L | D | L | G | M | N | E | I | T | N | I | S | E | N | S | F | Q | S | L | R | Q | L | D | T | L | N | I | K | N | N | C | L | K | S | V | P | N | A | V | R | K | T | Q | X | X | T | K | L | D | L | S | H | N | N | I | N | V | L | G | C | D | D | F | S | N | M | T | R | L | R | V | L | H | L | D | N | N | P | L | V | A | L | Q | D | C | V | F | K | D | L | V | N | L | N | E | L | I | F | Q | N | S | S | X | X | E | L | N | G | A | F | K | K | X | X | T | P | N | L | K | T | L | S | L | I | N | N | E | L | T | D | L | V | H | G | G | F | R | A | L | N | S | L | Q | Y | L | S | L | K | G | N | Q | L | Q | Q | L | K | D | G | T | F | F | G | L | S | S | L | T | H | L | D | L | E | S | N | K | I | X | X | X | X | X | E | I | S | N | C | T | F | V | E | L | K | A | L | K | T | L | N | L | R | R | N | C | I | Q | Y | A | S | V | E | P | I | L | N | P | P | F | A | E | L | S | K | L | D | T | L | Y | I | S | D | Q | I | R | S | H | K | R | T | N | F | P | Q | N | F | L | Q | G | L | T | N | L | S | I | L | Y | M | Q | T | N | N | I | K | F | V | H | P | H | T | F | N | Y | T | P | N | L | N | V | L | Y | L | S | D | N | F | L | T | X | X | X | X | I | P | D | N | L | F | C | S | I | Q | K | L | K | S | L | E | M | S | K | T | R | L | R | S | L | D | F | L | L | H | A | N | L | T | E | L | E | V | L | H | V | A | Y | N | P | F | S | V | I | R | E | P | V | M | L | S | L | S | A | L | T | Y | L | N | M | Q | G | N | S | F | T | C | N | C | D | N | A | W | F | H | Q | W | V | I | T | N | K | Q | T | Q | V | F | D | A | Y | N | F | E | C | S | Y | P | P | N | L | K | G | R | E | M | L | Q | I | D | F | S | S | C | T | V | D | T | D | F | I | C | Y | I | S | T | A | C | A | V | I | M | T | L | A | V | S | F | T | H | H | F | L | Q | W | H | L | V | Y | A | Y | Y | L | M | L | A | F | L | Y | N | S | K | N | K | D | K | R | A | X | X | X | X | Q | Y | D | A | F | V | S | Y | N | T | N | D | E | G | W | V | L | G | E | L | L | P | K | L | E | D | E | Q | G | W | R | L | C | L | H | H | R | D | F | Q | P | G | K | P | I | M | E | N | I | T | D | A | I | Y | G | S | R | K | T | I | C | V | V | S | R | D | Y | L | E | S | E | W | C | S | R | E | I | Q | V | A | S | F | R | L | F | D | E | Q | K | D | V | L | I | L | V | F | L | E | D | I | P | M | Q | Q | L | S | P | Y | Y | R | M | R | R | L | L | K | R | Q | T | Y | L | S | W | S | R | A | D | A | H | P | N | L | F | W | E | K | L | R | Q | A | L | E | T | Q | E | H | P | M | G | E | H | L | R | L | T | I | E | D | G | P | P | G | E | R | Q | D | Q | S | K | V | - |
| SATLR22\_3030\_BP | M | X | X | X | X | X | X | X | X | - | - | X | X | X | H | P | X | X | X | X | X | V | K | E | D | K | T | R | L | K | G | G | S | E | C | I | K | L | X | X | I | I | F | L | L | - | - | L | N | I | N | Y | L | V | V | P | V | T | G | Y | S | L | R | N | C | R | I | S | R | X | X | X | X | X | X | X | G | X | X | A | L | C | T | K | N | K | Q | Q | V | T | E | V | P | Q | D | I | P | S | T | V | E | G | I | D | L | S | S | N | K | I | S | K | I | H | V | S | D | F | K | N | F | P | I | L | T | T | L | D | L | S | S | N | K | I | T | Q | I | D | T | G | A | F | A | N | L | I | S | L | N | S | L | N | L | N | N | N | K | L | D | I | L | G | D | D | L | F | Y | G | L | S | X | L | T | E | L | R | I | H | G | N | K | I | K | S | V | A | P | T | S | F | K | S | L | T | S | L | T | F | L | D | I | S | N | S | K | L | H | Q | L | N | K | V | H | L | I | I | K | H | L | P | N | L | Q | V | L | S | L | Q | N | N | D | I | S | Y | F | N | S | W | E | L | T | N | S | S | L | G | L | T | S | L | D | L | S | K | N | P | I | K | V | F | N | I | T | A | D | I | F | P | N | L | T | W | F | N | I | G | Q | C | P | Q | T | K | P | T | K | W | D | V | R | N | K | T | F | L | S | R | V | S | T | L | D | I | S | R | L | H | L | K | M | D | D | M | K | M | L | L | E | S | V | N | X | X | X | S | L | T | T | L | R | M | A | Q | M | K | H | X | X | L | L | S | T | L | I | N | I | S | C | T | I | P | T | M | S | K | L | Q | L | K | Q | N | Q | L | S | V | I | Q | S | H | F | F | Q | L | C | V | N | V | T | E | L | D | L | S | Y | N | Q | I | Q | N | I | Q | E | G | A | F | R | S | T | Q | G | I | K | I | L | S | L | S | H | N | K | L | P | S | V | P | A | P | L | N | D | I | S | N | L | T | E | L | D | L | S | S | N | E | I | T | K | L | E | C | H | D | F | A | N | M | T | K | L | R | H | L | H | L | S | Y | N | S | F | P | A | L | Q | D | C | L | F | K | D | L | V | K | L | Q | V | L | K | L | Q | N | S | S | I | A | D | L | K | N | A | F | K | N | X | X | L | P | N | L | I | K | L | H | L | D | Q | N | K | L | T | A | I | K | H | G | D | F | M | G | L | Q | S | L | Q | N | L | S | L | H | D | N | Q | I | K | K | L | N | K | G | C | F | I | G | L | T | N | L | T | N | L | L | L | Q | A | N | R | L | E | E | X | X | X | S | I | K | H | Y | S | F | N | N | L | K | N | L | K | K | L | D | L | S | N | N | H | I | R | Y | K | K | S | S | A | L | H | P | P | P | F | F | N | L | S | R | L | E | T | L | A | F | Y | V | Q | R | X | X | R | E | R | A | Y | L | P | C | N | I | L | Q | G | L | T | N | L | L | V | L | D | M | R | N | L | H | L | P | S | F | P | K | D | V | F | K | Y | T | P | Q | L | Q | T | L | D | I | R | S | N | E | L | S | X | X | X | X | L | S | P | D | V | F | S | P | I | Q | S | L | E | K | L | Y | I | S | T | T | N | L | R | S | L | D | F | L | I | D | A | N | L | T | K | L | K | F | I | E | A | K | K | N | Q | L | H | V | I | R | E | E | L | I | N | S | L | P | A | L | V | Y | L | D | L | E | G | N | S | F | T | C | D | C | D | N | V | W | F | L | L | W | V | Q | K | S | N | Q | T | Q | V | F | D | A | Y | N | F | E | C | N | Y | P | A | V | M | L | G | K | K | L | M | D | F | D | P | Q | S | C | R | D | N | I | D | F | I | C | F | I | S | T | T | S | T | I | L | L | F | M | V | V | S | Y | T | Y | H | F | L | R | W | Q | L | A | Y | A | Y | Y | F | F | L | A | L | L | F | D | A | K | N | K | N | R | Q | T | H | X | X | X | Q | Y | D | A | F | I | S | Y | N | A | H | D | E | P | W | V | I | R | Q | L | L | P | K | L | E | D | E | Q | G | W | R | L | C | L | H | H | R | D | F | E | P | G | K | P | I | I | D | N | I | T | D | A | I | Y | G | S | R | K | T | I | C | V | I | S | R | K | Y | L | E | S | E | W | C | S | R | E | I | Q | V | A | S | F | R | L | F | D | E | Q | K | D | V | L | I | L | V | F | L | E | D | I | P | S | Y | Q | L | S | P | Y | Y | R | M | R | K | L | L | K | K | K | S | Y | L | S | W | P | R | A | G | E | H | T | E | L | F | W | E | K | L | R | Q | A | L | K | T | R | E | Q | P | E | D | D | K | F | L | L | N | V | M | D | R | P | X | X | X | X | X | X | X | X | X | X | - |
| MMTLR22\_3030\_BP | X | X | X | X | X | X | X | X | X | - | - | X | X | X | X | X | X | X | X | X | X | X | X | X | X | X | X | M | P | K | G | R | A | K | Y | F | K | L | X | X | I | I | F | L | L | - | - | L | N | I | N | N | F | V | V | P | V | T | G | F | F | L | R | S | C | R | I | S | Y | X | X | X | X | X | X | X | S | X | X | A | I | C | A | K | S | R | P | S | L | K | A | V | P | K | D | I | P | P | T | V | K | G | L | D | L | S | V | N | K | I | T | K | I | Q | G | S | D | F | E | N | L | P | L | L | T | Q | L | E | I | D | R | N | R | I | S | Q | I | D | T | G | A | F | A | N | L | I | S | L | K | K | L | N | L | N | N | N | K | L | H | E | L | R | A | D | L | F | D | G | L | R | N | L | T | E | L | R | I | R | S | N | G | I | K | V | V | A | S | T | S | F | K | S | L | T | S | L | K | L | L | D | I | S | F | N | R | L | N | H | L | T | N | V | H | A | I | I | Q | H | L | P | N | L | K | D | L | S | I | Y | K | N | G | F | T | T | F | N | S | W | E | L | T | N | G | S | L | G | L | T | S | L | D | V | S | Q | N | P | F | G | V | F | N | I | T | T | N | I | F | P | N | L | T | R | F | N | I | G | G | F | H | S | K | Q | H | M | K | W | E | V | R | N | T | T | F | P | S | R | V | S | T | L | D | I | S | G | L | E | L | A | F | G | H | M | K | T | F | L | E | S | V | N | X | X | X | S | L | I | S | M | R | M | N | A | M | K | Y | X | X | L | X | T | G | L | I | N | I | S | C | T | I | P | T | M | S | T | L | Q | L | R | H | N | K | L | H | F | I | R | S | N | F | F | K | L | C | I | N | V | T | E | L | D | L | A | E | S | R | I | K | S | I | Q | D | E | A | F | A | S | M | K | G | L | K | I | L | T | L | S | R | N | T | L | H | S | V | P | A | A | I | K | Y | I | P | T | L | E | E | L | D | L | S | K | N | N | I | T | T | L | G | C | Q | D | F | V | N | T | T | K | L | K | H | L | R | L | F | N | N | F | I | S | A | L | K | D | C | V | F | K | D | L | I | Q | L | Q | V | L | K | L | Q | G | N | H | I | C | T | F | G | G | A | F | K | K | P | N | L | P | N | L | T | Q | L | R | L | N | G | N | G | L | H | T | I | E | S | G | A | F | N | G | L | Q | S | L | Q | I | L | S | L | H | E | N | K | I | E | K | L | K | D | G | C | F | I | G | L | T | D | L | T | D | L | Q | L | Q | S | N | N | I | K | E | X | X | X | Y | I | K | Q | E | V | F | N | H | L | T | N | L | R | R | L | D | L | R | T | N | H | I | R | Y | N | D | S | S | A | M | H | N | P | P | F | S | K | L | S | H | L | Q | I | L | A | I | P | G | Q | H | X | X | R | G | K | S | L | L | P | S | N | I | L | Q | G | L | T | N | L | L | V | F | N | T | R | N | T | Q | L | L | S | L | S | K | D | M | F | N | H | T | P | Q | L | Q | T | L | D | I | S | S | N | D | I | V | X | X | X | X | L | S | P | E | L | F | F | P | I | Q | N | L | K | S | L | Y | I | S | R | T | S | L | R | S | L | D | F | L | I | D | A | G | L | T | K | M | E | F | M | Q | A | R | K | N | A | F | S | V | L | S | E | D | V | M | D | S | L | K | A | L | V | Y | V | D | L | Q | G | N | S | F | T | C | D | C | D | N | A | W | F | I | N | W | T | E | N | N | N | K | T | Q | V | F | D | A | Y | N | F | E | C | N | Y | P | P | D | L | K | G | M | K | L | M | D | F | D | I | Q | F | C | K | V | S | T | D | F | I | C | F | V | S | T | T | C | M | V | L | L | F | M | G | V | S | F | T | Y | H | F | L | R | W | Q | L | T | Y | A | Y | Y | Y | F | L | A | L | L | F | D | S | K | N | Q | N | R | Q | A | S | X | X | X | L | Y | D | A | F | I | S | Y | N | T | H | D | E | P | W | V | I | Q | E | L | L | P | K | L | E | G | E | Q | G | W | R | L | C | L | H | H | R | D | F | E | P | G | K | P | I | I | D | N | I | T | D | A | I | Y | A | S | R | K | T | I | C | V | I | S | R | R | Y | L | E | S | E | W | C | S | R | E | I | Q | V | A | S | F | R | L | F | D | E | R | K | D | V | L | I | L | V | F | L | E | E | I | P | T | Y | Q | M | S | P | Y | Y | R | M | R | K | L | L | K | K | Q | T | Y | L | S | W | P | Q | G | Q | E | H | T | E | V | F | W | E | K | L | R | K | A | L | R | A | E | E | K | L | Y | E | D | R | L | L | L | T | V | M | G | X | X | X | X | X | X | X | X | X | X | X | X | - |
| ECTLR22\_3030\_BP | M | X | X | X | X | X | X | X | X | - | - | X | X | X | G | P | X | X | X | X | X | L | K | E | E | Q | X | X | X | K | R | G | S | K | C | F | K | L | X | X | F | V | F | F | L | - | - | L | N | I | S | S | F | F | V | P | V | T | G | Y | V | L | R | S | C | H | I | S | Y | X | X | X | X | X | X | X | S | X | X | A | I | C | S | K | R | R | X | X | L | T | A | V | P | Q | D | I | P | S | T | V | K | G | L | D | L | Y | G | N | K | I | S | K | I | Q | V | S | D | F | K | K | L | S | N | L | T | E | L | N | L | K | W | N | I | I | S | E | I | D | T | G | S | F | T | N | L | N | S | L | K | M | L | N | L | N | D | N | K | L | T | G | L | G | D | N | V | F | D | G | L | G | S | L | T | H | L | R | M | N | R | N | Q | I | K | T | V | A | S | T | S | F | K | S | L | T | N | L | M | V | L | D | I | S | V | N | K | L | Y | R | I | T | N | V | H | T | I | L | Q | H | L | P | N | L | L | E | L | S | V | A | N | N | N | L | T | S | F | Q | S | W | E | L | T | N | S | S | L | K | L | G | Q | L | D | L | S | Q | N | P | I | G | D | F | R | I | T | A | D | V | F | P | N | L | T | W | L | N | T | G | G | S | S | K | R | H | H | M | K | W | D | V | R | N | K | T | F | L | R | R | V | S | T | F | D | I | S | G | L | Q | I | P | S | E | D | M | K | T | I | L | G | T | F | N | X | X | X | S | L | T | T | V | K | M | N | D | M | K | R | T | Y | L | X | R | A | L | I | N | I | S | C | S | I | P | T | V | S | S | L | Q | L | R | S | N | N | L | F | F | M | S | S | N | L | L | S | L | C | F | K | V | T | E | L | D | L | A | D | N | S | M | K | N | I | K | D | D | A | F | K | H | L | Q | G | I | K | V | L | A | L | N | H | N | R | L | S | S | V | P | A | A | I | R | N | L | P | A | L | E | E | L | D | L | S | K | N | N | I | T | T | L | G | C | Q | D | F | A | N | L | T | K | L | R | Q | L | S | L | H | Q | N | S | I | S | A | L | Q | G | C | V | F | K | D | L | I | H | L | E | V | L | K | L | Q | N | N | S | I | S | K | L | N | D | A | F | I | K | X | X | L | P | N | L | R | Q | L | H | L | N | S | N | K | L | T | A | I | K | H | G | E | F | R | G | L | R | S | L | Q | N | L | S | L | H | D | N | Q | I | T | V | L | E | K | E | C | F | T | G | L | R | N | L | M | A | I | Q | L | Q | M | N | H | I | K | R | X | X | X | K | D | Y | K | D | S | F | R | D | L | I | N | L | K | R | L | D | L | S | H | N | S | I | K | Y | I | E | S | S | A | L | P | D | P | P | F | S | H | L | S | N | L | E | R | L | A | F | T | G | N | H | X | X | R | V | K | S | Q | L | P | C | N | F | L | Q | G | L | T | N | L | L | D | F | T | G | R | N | L | Q | L | L | S | L | H | K | D | T | F | N | H | T | P | K | L | Q | K | L | D | I | S | S | N | E | L | M | X | X | X | X | L | S | S | E | L | F | Y | P | I | R | N | L | K | S | L | Y | I | S | R | I | N | L | G | S | L | D | F | F | K | D | A | N | L | T | K | L | E | F | L | Q | A | R | R | N | Q | F | S | V | F | S | E | E | V | L | E | S | M | P | A | L | A | Y | L | D | L | Q | G | N | S | F | T | C | D | C | D | N | A | W | F | L | K | W | T | E | N | N | Q | Q | T | Q | V | S | D | A | Y | N | F | V | C | N | Y | P | P | H | L | K | G | M | K | L | L | K | L | D | V | R | S | C | T | V | D | T | E | F | I | C | F | I | S | T | T | C | T | I | L | V | F | M | V | A | S | F | T | Y | H | F | L | R | W | Y | L | A | Y | A | Y | Y | I | F | L | A | F | L | F | D | T | K | H | K | N | K | Q | A | P | X | X | X | Q | Y | D | A | F | I | S | Y | N | T | H | D | E | P | W | V | I | R | Q | L | L | P | K | L | E | G | E | Q | G | W | R | L | C | L | H | H | R | D | F | E | P | G | K | P | I | I | D | N | I | T | D | A | I | Y | G | S | R | K | T | I | C | V | I | S | H | R | Y | L | E | S | E | W | C | S | R | E | I | Q | V | A | S | F | R | L | F | D | E | Q | K | D | V | L | I | L | V | F | L | E | D | I | P | T | T | Q | L | S | P | Y | Y | R | M | R | K | L | L | K | R | Q | T | Y | L | S | W | P | R | A | S | E | H | P | E | L | F | W | E | K | L | R | Q | A | M | K | T | R | E | D | L | G | E | D | E | L | N | L | T | V | T | D | K | P | X | X | X | X | X | X | X | X | X | X | - |
| LMTLR22\_3030\_BP | M | X | X | X | X | X | X | X | X | - | - | X | X | X | H | P | X | X | X | X | X | M | K | E | D | K | T | M | P | K | G | G | S | T | C | F | K | R | X | X | I | I | F | L | L | - | - | L | N | I | S | S | F | V | V | P | V | A | G | F | A | M | K | T | C | R | I | S | Y | X | X | X | X | X | X | X | D | X | X | A | I | C | D | Q | T | T | X | X | L | Y | A | V | P | Q | D | I | P | P | T | V | K | V | I | D | L | S | V | N | R | I | S | K | I | Q | L | L | D | F | K | N | R | T | V | L | T | E | L | N | L | K | R | N | I | I | S | Q | I | D | T | G | A | F | A | D | L | I | S | L | K | T | L | N | L | N | N | N | K | L | V | H | L | G | D | D | L | F | K | G | L | S | N | L | T | E | L | R | I | Y | G | N | Q | I | K | A | V | A | S | T | A | F | K | S | L | T | S | L | T | I | L | D | I | C | N | N | N | L | H | N | L | T | V | V | H | S | I | L | Q | H | L | P | N | L | Q | E | L | V | I | R | N | I | G | L | T | E | F | H | S | W | E | L | T | N | S | S | L | E | L | S | S | L | D | L | S | Q | N | L | I | A | D | F | R | I | T | A | D | V | F | P | K | L | T | W | L | K | I | G | G | X | X | X | K | Q | R | M | K | W | D | V | R | N | K | T | F | L | S | R | V | S | T | L | D | I | S | A | L | Q | M | A | L | H | D | M | E | T | L | I | E | S | F | N | X | X | X | S | L | I | S | L | R | M | N | T | M | K | Y | X | X | L | X | P | A | L | I | N | T | S | C | T | I | P | T | V | S | T | L | Q | L | R | S | N | K | L | W | F | V | D | S | K | L | F | H | L | C | I | N | V | K | E | V | D | L | K | G | N | R | I | K | V | I | Q | E | E | A | F | R | S | F | Q | N | L | R | I | L | S | L | S | H | N | K | L | L | S | V | P | A | A | T | R | N | L | P | A | L | A | E | L | D | L | S | S | N | A | I | S | T | L | E | C | Q | D | F | A | N | L | V | T | L | R | Q | L | S | L | Q | H | N | S | I | S | N | L | E | E | C | L | F | K | N | L | I | R | L | Q | V | L | E | L | Q | N | N | T | I | S | K | L | N | N | A | F | I | K | X | X | L | P | N | L | K | L | L | R | L | N | G | N | E | L | T | I | I | K | H | G | E | F | K | G | L | I | S | L | R | N | L | S | L | H | E | N | K | I | K | K | L | E | K | G | S | F | I | G | L | T | N | L | T | D | L | Q | L | L | S | N | N | I | E | K | X | X | X | G | L | I | K | G | C | F | N | G | L | I | N | L | K | R | L | D | M | R | Q | N | H | I | K | Y | K | K | N | V | A | L | R | D | P | P | F | S | E | L | S | N | L | E | T | L | A | I | L | S | Q | H | X | X | R | G | K | S | Q | L | P | R | N | L | L | Q | G | L | K | N | L | L | V | F | N | T | R | N | S | Q | L | L | S | L | H | K | D | M | F | N | Y | T | P | Q | L | Q | T | L | D | I | S | S | N | D | L | E | X | X | X | X | L | S | P | D | L | F | S | P | I | Q | S | L | K | S | L | Y | I | S | R | T | S | L | W | S | L | D | F | L | I | H | A | N | L | T | K | L | E | F | L | Q | A | R | K | N | E | Y | S | V | F | S | E | E | I | M | K | S | L | P | A | L | I | Y | V | D | L | Q | G | N | S | F | T | C | D | C | S | N | A | W | F | L | K | W | A | E | K | N | E | Q | T | Q | V | Y | D | A | Y | N | F | T | C | N | Y | P | P | S | L | K | G | M | K | L | L | E | L | D | V | R | S | C | L | V | E | T | D | F | I | C | F | I | S | T | T | C | A | I | L | L | F | M | V | V | S | F | T | Y | N | F | L | R | W | Q | L | A | Y | A | Y | Y | L | F | L | A | L | L | F | D | S | K | H | R | N | K | Q | A | P | X | X | X | Q | Y | D | A | F | I | S | Y | N | S | H | D | E | P | W | V | I | R | E | L | L | P | K | L | E | G | E | Q | G | W | R | L | C | L | H | H | R | D | F | E | P | G | K | P | I | I | E | N | I | T | E | A | I | Y | G | S | R | K | T | I | C | V | I | S | R | R | Y | L | E | S | E | W | C | S | R | E | I | Q | V | A | S | F | R | L | F | D | E | N | K | D | V | L | I | L | V | F | L | E | D | I | S | T | S | Q | L | S | P | Y | Y | R | I | R | K | L | L | K | R | Q | T | Y | L | S | W | P | Q | A | W | E | H | T | E | L | F | W | E | K | L | R | Q | A | L | K | T | R | E | D | L | Y | E | D | R | F | C | L | T | V | V | D | S | P | X | X | X | X | X | X | X | X | X | X | - |
| SMTLR22\_3030\_BP | M | X | X | X | X | X | X | X | X | - | - | X | X | X | D | P | X | X | X | X | X | V | K | D | D | K | T | R | E | T | G | G | R | N | S | F | P | L | X | X | L | F | F | C | L | - | - | L | N | I | G | G | L | L | V | P | V | T | G | F | A | L | K | T | C | R | V | S | Y | X | X | X | X | X | X | X | N | X | X | A | I | C | V | K | M | K | X | X | L | I | A | V | P | Q | D | I | P | S | T | V | K | G | F | D | L | S | A | N | I | I | A | R | V | Q | V | T | D | F | Q | N | F | K | I | L | T | Q | L | E | L | N | R | N | R | I | S | G | I | D | N | G | A | F | A | N | L | I | S | L | E | K | L | N | L | N | N | N | K | L | V | G | L | G | E | D | L | F | H | G | L | S | N | L | T | E | L | R | I | N | G | N | R | I | Q | D | V | A | S | S | S | F | R | D | M | K | S | L | T | F | L | D | I | S | H | N | K | L | H | Q | I | T | K | V | H | S | I | L | Q | H | L | P | H | L | R | E | L | N | I | K | N | N | G | L | T | T | F | C | S | W | E | L | T | N | S | S | V | E | L | K | Y | L | D | L | A | Q | N | P | I | A | N | F | S | I | T | A | D | V | F | P | N | L | T | W | F | N | I | G | G | S | P | G | K | Q | Q | M | I | W | D | V | H | N | K | T | F | F | N | R | V | S | T | L | D | I | S | G | L | Q | M | A | F | D | D | M | K | T | L | L | E | I | V | N | X | X | X | S | L | T | S | L | R | M | N | A | M | K | R | X | X | L | X | T | A | L | I | N | I | S | C | T | I | P | T | M | S | R | L | Q | V | R | R | N | K | L | S | Y | V | S | S | D | L | F | K | L | C | F | D | V | T | E | M | D | L | A | E | N | K | I | K | N | I | P | D | N | A | F | R | S | L | Q | S | L | R | I | L | S | L | S | R | N | R | L | S | S | V | P | A | A | T | R | K | L | P | S | L | T | E | L | D | L | S | F | N | N | I | T | K | L | G | C | D | D | F | A | N | Q | T | K | L | R | R | L | N | L | Y | G | N | S | I | S | A | L | Q | E | C | L | F | K | D | L | I | Q | L | Q | V | L | K | L | Q | N | N | R | I | S | N | L | N | S | A | F | Q | N | X | X | L | P | N | L | R | Q | L | L | L | N | G | N | E | L | T | E | I | I | H | G | E | F | S | G | L | K | S | L | Q | N | L | S | L | L | G | N | K | I | K | T | L | E | E | G | C | F | I | G | L | T | N | L | T | D | I | L | L | Q | N | N | H | I | T | E | X | X | X | E | I | N | K | S | C | F | N | D | L | I | N | L | R | R | L | D | L | R | E | N | H | I | K | Y | E | N | S | S | S | L | P | H | A | P | F | S | Q | L | S | R | L | E | S | L | A | I | P | S | Q | H | X | X | K | G | K | S | Q | L | P | R | N | F | L | Q | G | L | T | N | L | L | F | F | N | S | R | N | I | Q | L | L | A | L | H | K | D | M | F | N | D | T | P | Q | L | Q | T | L | D | I | S | S | N | E | L | M | X | X | X | X | L | S | P | D | L | F | S | P | I | Q | N | L | K | S | L | Y | I | S | R | T | S | L | R | S | L | D | Y | L | T | G | S | N | L | T | K | L | E | F | L | Q | A | R | K | N | E | Y | S | V | I | S | E | E | I | I | K | S | V | P | G | L | V | Y | A | D | F | Q | G | N | S | F | T | C | D | C | D | N | A | W | F | I | K | W | V | K | N | N | N | E | T | Q | V | F | D | A | Y | N | F | E | C | N | Y | P | V | N | L | K | G | M | K | L | L | E | L | D | I | R | S | C | S | V | D | V | E | F | I | F | F | I | S | S | T | C | S | I | L | F | F | M | V | V | S | F | T | Y | H | F | L | R | W | Q | L | T | Y | A | Y | Y | L | F | L | A | L | L | F | D | T | K | H | K | N | K | R | A | P | X | X | X | Q | Y | D | A | F | I | S | Y | N | T | H | D | E | A | W | V | I | R | E | L | L | P | K | L | E | G | E | Q | G | W | R | L | C | L | H | H | R | D | F | E | P | G | K | P | I | I | E | N | I | T | D | A | I | Y | G | S | R | K | T | I | C | V | I | S | R | R | Y | L | E | S | E | W | C | S | R | E | M | Q | V | A | S | F | R | L | F | D | E | Q | K | D | V | L | I | L | V | F | L | E | E | I | P | T | S | E | L | F | P | H | Y | R | M | R | K | L | P | N | K | R | T | Y | L | S | W | P | R | A | G | E | H | T | E | L | F | W | E | K | L | R | Q | A | L | Q | T | R | D | D | R | A | E | D | C | F | L | L | T | V | L | D | G | R | Q | X | X | X | X | X | X | X | X | X | - |
| LCTLR22\_3030\_BP | M | X | X | X | X | X | X | X | X | - | - | X | X | X | D | P | X | X | X | X | X | L | K | A | D | K | T | N | P | K | R | R | S | K | C | F | K | L | X | X | N | I | F | F | L | - | - | L | N | I | I | S | F | V | V | L | V | T | G | F | A | L | K | T | C | R | I | S | Y | X | X | X | X | X | X | X | N | X | X | A | I | C | V | R | N | D | X | X | L | R | A | V | P | Q | D | I | P | S | T | V | E | G | F | D | L | S | S | N | K | I | S | R | I | Q | V | S | D | F | K | H | L | P | V | L | T | Q | L | D | L | N | R | N | Y | I | S | Q | I | E | S | G | A | F | A | N | L | T | S | L | K | R | L | N | L | N | N | N | K | L | T | E | L | G | E | D | L | F | H | G | L | S | N | L | T | E | L | R | I | M | S | N | H | I | K | A | V | A | S | T | S | F | K | S | M | T | R | L | K | L | L | D | I | S | H | N | K | L | Q | H | I | T | K | V | H | S | I | I | Q | H | L | P | H | L | R | E | L | Y | I | K | N | N | N | L | T | T | F | H | S | W | E | L | T | N | S | S | L | E | L | K | Y | L | D | L | S | Q | N | P | I | S | V | F | S | V | T | A | D | V | F | P | N | L | T | W | F | N | I | G | G | S | P | R | K | Q | L | M | K | W | D | V | R | N | K | T | F | L | S | R | V | S | T | L | D | I | S | G | L | Q | M | T | F | D | D | M | K | S | L | L | E | T | V | N | X | X | X | S | L | T | S | L | R | M | N | A | M | K | Q | X | X | L | X | P | A | L | I | N | I | S | C | S | I | P | T | V | S | T | L | Q | L | R | Y | N | K | L | R | F | V | S | S | D | L | F | K | L | C | I | N | V | T | E | L | D | L | T | E | N | K | I | R | N | I | H | D | S | A | F | R | S | L | Q | R | L | R | I | L | S | L | S | H | N | R | L | S | S | V | P | A | A | T | R | N | L | P | T | L | R | E | L | D | L | S | S | N | N | I | S | K | L | G | C | D | D | F | T | N | Q | T | M | L | R | Q | L | S | L | S | E | N | S | V | S | A | L | S | K | C | V | F | K | D | L | I | R | L | Q | V | L | K | L | Q | T | N | R | I | S | N | L | N | G | A | F | Q | M | X | X | L | P | N | L | R | Q | L | L | L | N | G | N | E | L | T | T | I | K | H | G | E | F | R | G | L | Q | S | L | Q | N | L | S | L | H | D | N | Q | I | Q | D | L | K | N | G | C | F | I | G | L | T | N | L | T | D | I | L | L | Q | K | N | H | I | K | E | X | X | X | E | I | N | K | G | V | F | N | D | L | I | N | L | G | R | L | D | L | R | E | N | R | I | K | Y | D | D | C | S | A | L | P | Y | A | P | F | S | Q | L | S | R | L | E | T | L | A | I | P | S | Q | H | X | X | R | L | K | S | H | L | P | R | N | F | L | Q | G | L | T | N | L | S | E | F | N | A | R | N | I | Q | L | L | S | L | H | K | D | M | F | N | Y | T | P | Q | L | Q | T | L | D | I | S | S | N | D | L | R | X | X | X | X | L | S | P | E | L | F | S | P | I | Q | N | L | Q | S | L | Y | I | S | H | V | D | L | G | S | L | D | F | L | I | D | A | N | L | T | K | L | E | F | L | Q | A | R | K | N | Q | Y | S | V | I | S | E | E | I | I | Q | S | L | P | A | L | V | Y | V | D | L | F | G | D | S | F | T | C | D | C | D | N | A | W | L | L | K | W | I | E | N | N | N | Q | T | Q | V | F | D | A | Y | N | F | E | C | N | Y | P | V | N | L | K | G | K | K | L | L | D | L | S | I | Q | S | C | S | V | D | I | E | F | I | Y | F | I | S | T | T | C | T | I | L | L | S | M | V | V | S | F | T | Y | H | F | L | R | W | H | L | A | Y | A | Y | Y | L | F | L | A | F | L | F | D | T | K | H | K | N | K | Q | A | P | X | X | X | Q | Y | D | A | F | I | S | Y | N | T | H | D | E | P | W | V | I | R | E | L | L | P | K | L | E | G | E | Q | G | W | R | L | C | L | H | H | R | D | F | E | P | G | K | P | I | I | D | N | I | T | D | A | I | Y | G | S | R | K | T | I | C | V | I | S | R | R | Y | L | E | S | E | W | C | S | R | E | I | Q | V | A | S | F | R | L | F | D | E | Q | K | D | V | L | I | L | V | F | L | E | E | I | P | T | Y | H | L | S | P | Y | Y | R | M | R | K | L | L | K | K | R | T | Y | L | S | W | P | R | A | G | E | H | T | E | I | F | W | E | K | L | H | Q | A | L | K | T | R | A | D | C | X | X | D | S | F | L | F | N | V | V | D | R | Q | - | W | X | X | X | X | X | X | X | X | X |
| SLTLR22\_3030\_BP | M | X | X | X | X | X | X | X | X | - | - | X | X | X | G | P | X | X | X | X | X | V | K | E | N | E | T | T | A | T | E | G | R | K | Q | F | Q | L | X | X | I | F | L | C | L | - | - | L | N | I | S | S | F | V | V | P | V | R | G | F | A | M | K | N | C | K | I | S | Y | X | X | X | X | X | X | X | N | X | X | A | I | C | V | Q | S | K | X | X | L | R | A | V | P | Q | D | I | P | S | T | V | K | G | F | D | L | S | A | N | K | I | S | R | V | K | I | S | D | F | K | N | L | P | V | L | T | K | L | E | L | N | R | N | F | I | S | Q | I | D | N | G | A | F | A | N | L | I | S | L | K | K | L | N | L | N | N | N | K | L | V | E | L | R | E | D | L | F | N | G | L | S | N | L | T | E | L | R | I | N | G | N | R | I | R | A | V | A | S | T | S | F | K | S | M | I | S | L | T | F | L | D | I | S | Q | N | K | L | H | D | I | R | K | V | H | S | I | I | Q | H | L | P | Q | L | R | E | L | Y | I | Q | K | N | G | F | T | T | F | H | S | W | E | L | T | N | S | S | L | Q | L | K | Y | L | D | L | S | R | N | P | M | T | V | F | Q | I | T | A | D | V | L | P | N | L | T | W | F | N | V | G | G | S | S | X | X | Q | K | M | R | W | D | V | R | N | K | T | F | F | S | R | V | S | T | L | D | I | T | G | L | Q | M | S | L | N | D | M | K | T | L | L | E | S | V | N | X | X | X | S | L | T | C | L | R | M | N | A | M | N | H | X | X | F | X | A | A | L | I | N | I | S | C | S | I | P | T | L | S | K | L | Q | L | R | N | Y | K | L | S | S | V | S | S | D | L | I | K | L | C | I | N | V | T | E | L | D | L | A | K | N | Q | I | Q | N | I | S | D | N | T | F | R | S | I | Q | R | L | R | I | L | D | L | S | R | N | K | L | S | S | V | P | A | A | T | R | N | L | P | I | L | Q | E | L | D | L | S | S | N | L | I | S | K | L | E | C | D | D | F | A | N | Q | T | M | L | R | Q | L | N | L | Y | Q | N | S | I | S | A | L | K | G | C | V | F | K | D | L | I | R | L | Q | V | L | K | L | Q | S | N | Q | M | S | E | L | H | G | A | F | N | K | X | X | L | P | N | L | R | Q | L | L | L | N | Q | N | K | L | T | A | I | K | H | G | E | F | R | G | L | Q | S | L | Q | N | L | S | L | H | H | N | Q | I | H | Q | L | E | M | G | C | F | I | G | L | M | N | L | T | D | I | Q | L | Q | E | N | R | I | K | E | X | X | X | E | I | N | K | G | S | F | N | D | L | I | N | L | R | R | L | E | L | R | D | N | N | I | R | Y | G | N | N | S | T | L | P | N | A | P | F | S | Q | L | S | R | L | D | T | L | G | I | A | L | Q | H | X | X | K | G | K | S | Y | L | P | H | N | F | L | Q | G | L | T | N | L | R | V | F | N | T | R | N | I | Q | L | L | T | L | H | K | D | T | F | I | Y | T | P | Q | L | Q | E | L | D | I | S | T | N | E | F | R | X | X | X | X | L | S | P | E | L | F | S | P | I | P | N | L | K | S | L | Y | I | S | K | T | I | L | G | S | L | E | F | L | V | N | A | S | L | S | K | L | E | F | L | Q | A | R | K | N | Q | Y | S | V | I | S | E | E | I | I | K | S | V | P | A | L | V | Y | V | D | L | Q | G | N | S | F | R | C | D | C | D | N | A | W | F | L | Q | W | I | E | N | N | N | Q | T | Q | V | F | D | A | Y | N | F | T | C | N | Y | P | V | H | L | K | G | K | E | L | L | E | L | D | I | S | S | C | S | V | D | I | E | F | I | Y | F | I | S | T | T | C | T | V | L | L | F | M | V | V | S | F | T | Y | H | F | L | R | W | H | L | A | Y | A | Y | Y | L | F | L | A | L | L | F | D | T | K | H | K | N | K | Q | A | P | X | X | X | Q | Y | D | A | F | I | S | Y | N | T | H | D | E | P | W | V | V | Q | E | L | L | P | K | L | E | G | D | Q | G | W | K | L | C | L | H | H | R | D | F | E | P | G | K | P | I | I | D | N | I | T | D | A | I | Y | G | S | R | K | T | I | C | V | I | S | R | R | Y | L | E | S | E | W | C | S | R | E | I | Q | V | A | S | F | R | L | F | D | E | Q | K | D | V | L | I | M | V | F | L | E | D | I | P | T | H | E | L | S | P | Y | Y | R | M | R | K | L | L | K | K | R | T | Y | L | S | W | P | R | A | E | E | H | T | E | L | F | W | E | K | L | R | Q | A | L | K | T | R | E | D | P | G | E | D | I | F | Q | L | T | V | V | D | A | H | - | W | Q | X | X | X | X | X | X | X | X |
| TOTLR22\_3030\_BP | M | X | X | X | X | X | X | X | X | - | - | X | X | X | G | P | X | X | X | X | X | M | K | E | D | E | K | R | A | T | G | R | R | K | L | F | Q | L | X | X | I | F | L | C | L | - | - | L | N | I | S | S | F | V | V | P | V | T | G | F | A | L | K | T | C | R | I | S | Y | X | X | X | X | X | X | X | N | X | X | A | I | C | A | Q | S | K | X | X | L | R | A | V | P | Q | D | I | P | S | T | V | I | G | F | D | L | S | V | N | K | I | P | R | V | Q | V | S | A | F | K | N | L | P | L | L | T | Q | L | D | L | N | R | N | F | I | S | H | I | D | V | G | A | F | A | S | L | I | S | L | K | K | L | N | L | N | N | N | K | L | V | E | L | G | E | D | L | F | N | G | L | S | N | L | T | E | L | R | I | N | K | N | H | I | K | V | V | A | S | S | S | F | K | S | M | I | S | L | K | L | L | D | I | S | H | N | R | L | Q | D | I | R | K | V | H | S | I | I | Q | H | L | P | H | L | Q | E | L | H | I | Q | T | N | N | F | A | T | F | H | S | W | E | L | T | N | S | S | L | K | L | K | Y | L | D | L | S | Q | N | P | I | A | V | F | Q | I | T | A | D | V | L | P | N | L | T | W | L | N | I | G | N | S | H | I | K | X | X | X | X | W | D | V | R | N | K | T | X | L | S | Q | V | S | T | L | D | I | S | E | L | Q | M | G | F | D | D | M | K | T | L | L | E | T | V | N | X | X | X | S | L | T | T | L | R | M | N | A | I | K | H | X | X | L | X | A | A | L | I | N | I | S | C | S | I | P | T | M | S | R | L | Q | L | R | H | Y | K | L | S | F | V | S | S | D | L | F | K | L | C | I | N | V | T | E | L | D | L | R | K | N | K | I | Q | N | I | S | D | N | A | F | R | S | L | Q | S | L | R | I | L | S | L | K | Q | N | N | L | S | S | V | P | A | A | T | R | N | L | A | I | L | D | E | L | D | L | S | F | N | R | I | S | K | L | G | C | D | D | F | A | N | Q | T | M | L | R | H | L | Y | L | Y | N | N | S | I | S | T | L | K | G | C | V | F | K | D | L | I | R | L | Q | V | L | K | L | Q | T | N | R | I | L | N | L | N | D | A | F | K | K | X | X | L | P | N | L | R | Q | L | L | L | H | Q | N | E | L | T | A | I | K | Y | G | E | F | R | G | L | Q | S | L | Q | N | L | S | L | H | D | N | K | I | H | K | L | E | K | G | C | F | L | G | L | T | N | L | T | D | I | L | L | Q | K | N | L | I | Q | E | X | X | X | E | I | N | K | G | S | F | N | D | L | I | N | L | R | R | L | E | L | R | D | N | H | I | R | Y | E | N | N | S | A | L | L | H | A | P | F | S | Q | L | S | R | L | E | T | L | A | I | P | L | Q | H | X | X | K | G | K | S | R | L | P | H | N | F | L | Q | G | L | T | N | L | K | V | F | N | T | R | N | I | Q | L | L | S | L | H | K | D | T | F | I | Y | T | P | Q | L | Q | E | L | D | I | S | S | N | E | L | K | X | X | X | X | L | S | P | D | L | F | S | P | I | P | N | L | K | N | L | Y | I | S | R | T | I | L | Q | S | L | D | Y | L | T | Y | A | N | L | T | K | L | E | F | L | Q | A | R | K | N | Q | Y | S | V | I | S | E | E | I | I | K | S | V | P | A | L | V | Y | A | D | L | Q | G | N | S | F | T | C | D | C | D | N | A | W | F | L | Q | W | V | Q | N | N | N | Q | T | Q | V | F | D | A | Y | N | F | T | C | N | Y | P | V | H | L | K | G | K | K | L | L | D | L | D | V | R | L | C | S | V | D | I | E | F | I | Y | Y | I | S | T | T | C | A | I | L | L | F | M | V | V | S | S | T | Y | H | F | L | R | S | H | L | E | Y | A | Y | Y | L | F | L | A | L | L | F | D | R | K | H | K | N | K | Q | A | L | X | X | X | Q | Y | D | A | F | I | S | Y | N | T | H | D | E | P | W | V | V | Q | E | L | L | P | K | L | E | G | E | Q | G | W | R | L | C | L | H | H | R | D | F | E | P | G | K | P | I | I | E | N | I | T | D | A | I | Y | G | S | R | K | T | I | C | V | I | S | R | K | Y | L | E | S | E | W | C | S | R | E | I | Q | V | T | S | F | R | L | F | D | E | Q | K | D | V | L | I | L | V | F | L | E | E | I | P | T | C | Q | L | S | P | Y | H | R | M | R | K | L | L | K | R | R | T | Y | L | S | W | T | R | A | G | E | H | P | E | L | F | W | E | K | L | C | Q | A | L | I | T | G | E | D | H | G | E | D | I | F | Q | L | T | V | V | D | R | E | E | W | X | X | X | X | X | X | X | X | - |
| conservation |  |  |  |  |  |  |  |  |  |  |  |  |  |  |  |  |  |  |  |  |  |  |  |  |  |  |  |  |  |  |  |  |  |  |  |  |  |  |  |  |  |  |  |  |  |  |  |  |  |  |  |  |  |  |  |  |  |  |  |  |  |  |  |  |  |  |  |  |  |  |  |  |  |  |  |  |  |  |  |  |  |  |  |  |  |  |  |  |  |  |  |  |  |  |  |  |  |  |  |  |  |  |  |  |  |  |  |  |  |  |  |  |  |  |  |  |  |  |  |  |  |  |  |  |  |  |  |  |  |  |  |  |  |  |  |  |  |  |  |  |  |  |  |  |  |  |  |  |  |  |  |  |  |  |  |  |  |  |  |  |  |  |  |  |  |  |  |  |  |  |  |  |  |  |  |  |  |  |  |  |  |  |  |  |  |  |  |  |  |  |  |  |  |  |  |  |  |  |  |  |  |  |  |  |  |  |  |  |  |  |  |  |  |  |  |  |  |  |  |  |  |  |  |  |  |  |  |  |  |  |  |  |  |  |  |  |  |  |  |  |  |  |  |  |  |  |  |  |  |  |  |  |  |  |  |  |  |  |  |  |  |  |  |  |  |  |  |  |  |  |  |  |  |  |  |  |  |  |  |  |  |  |  |  |  |  |  |  |  |  |  |  |  |  |  |  |  |  |  |  |  |  |  |  |  |  |  |  |  |  |  |  |  |  |  |  |  |  |  |  |  |  |  |  |  |  |  |  |  |  |  |  |  |  |  |  |  |  |  |  |  |  |  |  |  |  |  |  |  |  |  |  |  |  |  |  |  |  |  |  |  |  |  |  |  |  |  |  |  |  |  |  |  |  |  |  |  |  |  |  |  |  |  |  |  |  |  |  |  |  |  |  |  |  |  |  |  |  |  |  |  |  |  |  |  |  |  |  |  |  |  |  |  |  |  |  |  |  |  |  |  |  |  |  |  |  |  |  |  |  |  |  |  |  |  |  |  |  |  |  |  |  |  |  |  |  |  |  |  |  |  |  |  |  |  |  |  |  |  |  |  |  |  |  |  |  |  |  |  |  |  |  |  |  |  |  |  |  |  |  |  |  |  |  |  |  |  |  |  |  |  |  |  |  |  |  |  |  |  |  |  |  |  |  |  |  |  |  |  |  |  |  |  |  |  |  |  |  |  |  |  |  |  |  |  |  |  |  |  |  |  |  |  |  |  |  |  |  |  |  |  |  |  |  |  |  |  |  |  |  |  |  |  |  |  |  |  |  |  |  |  |  |  |  |  |  |  |  |  |  |  |  |  |  |  |  |  |  |  |  |  |  |  |  |  |  |  |  |  |  |  |  |  |  |  |  |  |  |  |  |  |  |  |  |  |  |  |  |  |  |  |  |  |  |  |  |  |  |  |  |  |  |  |  |  |  |  |  |  |  |  |  |  |  |  |  |  |  |  |  |  |  |  |  |  |  |  |  |  |  |  |  |  |  |  |  |  |  |  |  |  |  |  |  |  |  |  |  |  |  |  |  |  |  |  |  |  |  |  |  |  |  |  |  |  |  |  |  |  |  |  |  |  |  |  |  |  |  |  |  |  |  |  |  |  |  |  |  |  |  |  |  |  |  |  |  |  |  |  |  |  |  |  |  |  |  |  |  |  |  |  |  |  |  |  |  |  |  |  |  |  |  |  |  |  |  |  |  |  |  |  |  |  |  |  |  |  |  |  |  |  |  |  |  |  |  |  |  |  |  |  |  |  |  |  |  |  |  |  |  |  |  |  |  |  |  |  |  |  |  |  |  |  |  |  |  |  |  |  |  |  |  |  |  |  |  |  |  |  |  |  |  |  |  |  |  |  |  |  |  |  |  |  |  |  |  |  |  |  |  |  |  |  |  |  |  |  |  |  |  |  |  |  |  |  |  |  |  |  |  |  |  |  |  |  |  |  |  |  |  |  |  |  |  |  |  |  |  |  |  |  |  |  |  |  |  |  |  |  |  |  |  |  |  |  |  |  |  |  |  |  |  |  |  |  |  |  |  |  |  |  |  |  |  |  |  |  |  |  |  |  |  |  |  |  |  |  |  |  |  |  |  |  |  |  |  |  |  |  |  |  |  |  |  |  |  |  |  |  |  |  |  |  |  |  |  |  |  |  |  |  |  |  |  |  |  |  |  |  |  |  |  |  |  |  |  |  |  |  |  |  |  |  |  |  |  |  |  |  |  |  |  |  |  |  |  |  |  |  |  |  |  |  |  |  |  |  |  |  |  |  |  |  |  |  |  |  |  |  |  |  |  |  |  |  |
|  |  |  |  |  |  |  |  |  |  |  |  |  |  |  |  |  |  |  |  |  |  |  |  |  |  |  |  |  |  |  |  |  |  |  |  |  |  |  |  |  |  |  |  |  |  |  |  |  |  |  |  |  |  |  |  |  |  |  |  |  |  |  |  |  |  |  |  |  |  |  |  |  |  |  |  |  |  |  |  |  |  |  |  |  |  |  |  |  |  |  |  |  |  |  |  |  |  |  |  |  |  |  |  |  |  |  |  |  |  |  |  |  |  |  |  |  |  |  |  |  |  |  |  |  |  |  |  |  |  |  |  |  |  |  |  |  |  |  |  |  |  |  |  |  |  |  |  |  |  |  |  |  |  |  |  |  |  |  |  |  |  |  |  |  |  |  |  |  |  |  |  |  |  |  |  |  |  |  |  |  |  |  |  |  |  |  |  |  |  |  |  |  |  |  |  |  |  |  |  |  |  |  |  |  |  |  |  |  |  |  |  |  |  |  |  |  |  |  |  |  |  |  |  |  |  |  |  |  |  |  |  |  |  |  |  |  |  |  |  |  |  |  |  |  |  |  |  |  |  |  |  |  |  |  |  |  |  |  |  |  |  |  |  |  |  |  |  |  |  |  |  |  |  |  |  |  |  |  |  |  |  |  |  |  |  |  |  |  |  |  |  |  |  |  |  |  |  |  |  |  |  |  |  |  |  |  |  |  |  |  |  |  |  |  |  |  |  |  |  |  |  |  |  |  |  |  |  |  |  |  |  |  |  |  |  |  |  |  |  |  |  |  |  |  |  |  |  |  |  |  |  |  |  |  |  |  |  |  |  |  |  |  |  |  |  |  |  |  |  |  |  |  |  |  |  |  |  |  |  |  |  |  |  |  |  |  |  |  |  |  |  |  |  |  |  |  |  |  |  |  |  |  |  |  |  |  |  |  |  |  |  |  |  |  |  |  |  |  |  |  |  |  |  |  |  |  |  |  |  |  |  |  |  |  |  |  |  |  |  |  |  |  |  |  |  |  |  |  |  |  |  |  |  |  |  |  |  |  |  |  |  |  |  |  |  |  |  |  |  |  |  |  |  |  |  |  |  |  |  |  |  |  |  |  |  |  |  |  |  |  |  |  |  |  |  |  |  |  |  |  |  |  |  |  |  |  |  |  |  |  |  |  |  |  |  |  |  |  |  |  |  |  |  |  |  |  |  |  |  |  |  |  |  |  |  |  |  |  |  |  |  |  |  |  |  |  |  |  |  |  |  |  |  |  |  |  |  |  |  |  |  |  |  |  |  |  |  |  |  |  |  |  |  |  |  |  |  |  |  |  |  |  |  |  |  |  |  |  |  |  |  |  |  |  |  |  |  |  |  |  |  |  |  |  |  |  |  |  |  |  |  |  |  |  |  |  |  |  |  |  |  |  |  |  |  |  |  |  |  |  |  |  |  |  |  |  |  |  |  |  |  |  |  |  |  |  |  |  |  |  |  |  |  |  |  |  |  |  |  |  |  |  |  |  |  |  |  |  |  |  |  |  |  |  |  |  |  |  |  |  |  |  |  |  |  |  |  |  |  |  |  |  |  |  |  |  |  |  |  |  |  |  |  |  |  |  |  |  |  |  |  |  |  |  |  |  |  |  |  |  |  |  |  |  |  |  |  |  |  |  |  |  |  |  |  |  |  |  |  |  |  |  |  |  |  |  |  |  |  |  |  |  |  |  |  |  |  |  |  |  |  |  |  |  |  |  |  |  |  |  |  |  |  |  |  |  |  |  |  |  |  |  |  |  |  |  |  |  |  |  |  |  |  |  |  |  |  |  |  |  |  |  |  |  |  |  |  |  |  |  |  |  |  |  |  |  |  |  |  |  |  |  |  |  |  |  |  |  |  |  |  |  |  |  |  |  |  |  |  |  |  |  |  |  |  |  |  |  |  |  |  |  |  |  |  |  |  |  |  |  |  |  |  |  |  |  |  |  |  |  |  |  |  |  |  |  |  |  |  |  |  |  |  |  |  |  |  |  |  |  |  |  |  |  |  |  |  |  |  |  |  |  |  |  |  |  |  |  |  |  |  |  |  |  |  |  |  |  |  |  |  |  |  |  |  |  |  |  |  |  |  |  |  |  |  |  |  |  |  |  |  |  |  |  |  |  |  |  |  |  |  |  |  |  |  |  |  |  |  |  |  |  |  |  |  |  |  |  |  |  |  |  |  |  |  |  |  |  |  |  |  |  |  |  |  |  |  |  |  |  |  |  |  |  |  |  |  |  |  |  |  |  |  |  |  |  |  |  |  |  |  |  |  |  |  |
|  |  |  |  |  |  |  |  |  |  |  |  |  |  |  |  |  |  |  |  |  |  |  |  |  |  |  |  |  |  |  |  |  |  |  |  |  |  |  |  |  |  |  |  |  |  |  |  |  |  |  |  |  |  |  |  |  |  |  |  |  |  |  |  |  |  |  |  |  |  |  |  |  |  |  |  |  |  |  |  |  |  |  |  |  |  |  |  |  |  |  |  |  |  |  |  |  |  |  |  |  |  |  |  |  |  |  |  |  |  |  |  |  |  |  |  |  |  |  |  |  |  |  |  |  |  |  |  |  |  |  |  |  |  |  |  |  |  |  |  |  |  |  |  |  |  |  |  |  |  |  |  |  |  |  |  |  |  |  |  |  |  |  |  |  |  |  |  |  |  |  |  |  |  |  |  |  |  |  |  |  |  |  |  |  |  |  |  |  |  |  |  |  |  |  |  |  |  |  |  |  |  |  |  |  |  |  |  |  |  |  |  |  |  |  |  |  |  |  |  |  |  |  |  |  |  |  |  |  |  |  |  |  |  |  |  |  |  |  |  |  |  |  |  |  |  |  |  |  |  |  |  |  |  |  |  |  |  |  |  |  |  |  |  |  |  |  |  |  |  |  |  |  |  |  |  |  |  |  |  |  |  |  |  |  |  |  |  |  |  |  |  |  |  |  |  |  |  |  |  |  |  |  |  |  |  |  |  |  |  |  |  |  |  |  |  |  |  |  |  |  |  |  |  |  |  |  |  |  |  |  |  |  |  |  |  |  |  |  |  |  |  |  |  |  |  |  |  |  |  |  |  |  |  |  |  |  |  |  |  |  |  |  |  |  |  |  |  |  |  |  |  |  |  |  |  |  |  |  |  |  |  |  |  |  |  |  |  |  |  |  |  |  |  |  |  |  |  |  |  |  |  |  |  |  |  |  |  |  |  |  |  |  |  |  |  |  |  |  |  |  |  |  |  |  |  |  |  |  |  |  |  |  |  |  |  |  |  |  |  |  |  |  |  |  |  |  |  |  |  |  |  |  |  |  |  |  |  |  |  |  |  |  |  |  |  |  |  |  |  |  |  |  |  |  |  |  |  |  |  |  |  |  |  |  |  |  |  |  |  |  |  |  |  |  |  |  |  |  |  |  |  |  |  |  |  |  |  |  |  |  |  |  |  |  |  |  |  |  |  |  |  |  |  |  |  |  |  |  |  |  |  |  |  |  |  |  |  |  |  |  |  |  |  |  |  |  |  |  |  |  |  |  |  |  |  |  |  |  |  |  |  |  |  |  |  |  |  |  |  |  |  |  |  |  |  |  |  |  |  |  |  |  |  |  |  |  |  |  |  |  |  |  |  |  |  |  |  |  |  |  |  |  |  |  |  |  |  |  |  |  |  |  |  |  |  |  |  |  |  |  |  |  |  |  |  |  |  |  |  |  |  |  |  |  |  |  |  |  |  |  |  |  |  |  |  |  |  |  |  |  |  |  |  |  |  |  |  |  |  |  |  |  |  |  |  |  |  |  |  |  |  |  |  |  |  |  |  |  |  |  |  |  |  |  |  |  |  |  |  |  |  |  |  |  |  |  |  |  |  |  |  |  |  |  |  |  |  |  |  |  |  |  |  |  |  |  |  |  |  |  |  |  |  |  |  |  |  |  |  |  |  |  |  |  |  |  |  |  |  |  |  |  |  |  |  |  |  |  |  |  |  |  |  |  |  |  |  |  |  |  |  |  |  |  |  |  |  |  |  |  |  |  |  |  |  |  |  |  |  |  |  |  |  |  |  |  |  |  |  |  |  |  |  |  |  |  |  |  |  |  |  |  |  |  |  |  |  |  |  |  |  |  |  |  |  |  |  |  |  |  |  |  |  |  |  |  |  |  |  |  |  |  |  |  |  |  |  |  |  |  |  |  |  |  |  |  |  |  |  |  |  |  |  |  |  |  |  |  |  |  |  |  |  |  |  |  |  |  |  |  |  |  |  |  |  |  |  |  |  |  |  |  |  |  |  |  |  |  |  |  |  |  |  |  |  |  |  |  |  |  |  |  |  |  |  |  |  |  |  |  |  |  |  |  |  |  |  |  |  |  |  |  |  |  |  |  |  |  |  |  |  |  |  |  |  |  |  |  |  |  |  |  |  |  |  |  |  |  |  |  |  |  |  |  |  |  |  |  |  |  |  |  |  |  |  |  |  |  |  |  |  |  |  |  |  |  |  |  |  |  |  |  |  |  |  |  |  |  |  |  |  |  |  |  |  |  |  |  |  |  |  |  |  |  |  |  |  |  |  |  |  |  |  |  |  |
|  |  |  |  |  |  |  |  |  |  |  |  |  |  |  |  |  |  |  |  |  |  |  |  |  |  |  |  |  |  |  |  |  |  |  |  |  |  |  |  |  |  |  |  |  |  |  |  |  |  |  |  |  |  |  |  |  |  |  |  |  |  |  |  |  |  |  |  |  |  |  |  |  |  |  |  |  |  |  |  |  |  |  |  |  |  |  |  |  |  |  |  |  |  |  |  |  |  |  |  |  |  |  |  |  |  |  |  |  |  |  |  |  |  |  |  |  |  |  |  |  |  |  |  |  |  |  |  |  |  |  |  |  |  |  |  |  |  |  |  |  |  |  |  |  |  |  |  |  |  |  |  |  |  |  |  |  |  |  |  |  |  |  |  |  |  |  |  |  |  |  |  |  |  |  |  |  |  |  |  |  |  |  |  |  |  |  |  |  |  |  |  |  |  |  |  |  |  |  |  |  |  |  |  |  |  |  |  |  |  |  |  |  |  |  |  |  |  |  |  |  |  |  |  |  |  |  |  |  |  |  |  |  |  |  |  |  |  |  |  |  |  |  |  |  |  |  |  |  |  |  |  |  |  |  |  |  |  |  |  |  |  |  |  |  |  |  |  |  |  |  |  |  |  |  |  |  |  |  |  |  |  |  |  |  |  |  |  |  |  |  |  |  |  |  |  |  |  |  |  |  |  |  |  |  |  |  |  |  |  |  |  |  |  |  |  |  |  |  |  |  |  |  |  |  |  |  |  |  |  |  |  |  |  |  |  |  |  |  |  |  |  |  |  |  |  |  |  |  |  |  |  |  |  |  |  |  |  |  |  |  |  |  |  |  |  |  |  |  |  |  |  |  |  |  |  |  |  |  |  |  |  |  |  |  |  |  |  |  |  |  |  |  |  |  |  |  |  |  |  |  |  |  |  |  |  |  |  |  |  |  |  |  |  |  |  |  |  |  |  |  |  |  |  |  |  |  |  |  |  |  |  |  |  |  |  |  |  |  |  |  |  |  |  |  |  |  |  |  |  |  |  |  |  |  |  |  |  |  |  |  |  |  |  |  |  |  |  |  |  |  |  |  |  |  |  |  |  |  |  |  |  |  |  |  |  |  |  |  |  |  |  |  |  |  |  |  |  |  |  |  |  |  |  |  |  |  |  |  |  |  |  |  |  |  |  |  |  |  |  |  |  |  |  |  |  |  |  |  |  |  |  |  |  |  |  |  |  |  |  |  |  |  |  |  |  |  |  |  |  |  |  |  |  |  |  |  |  |  |  |  |  |  |  |  |  |  |  |  |  |  |  |  |  |  |  |  |  |  |  |  |  |  |  |  |  |  |  |  |  |  |  |  |  |  |  |  |  |  |  |  |  |  |  |  |  |  |  |  |  |  |  |  |  |  |  |  |  |  |  |  |  |  |  |  |  |  |  |  |  |  |  |  |  |  |  |  |  |  |  |  |  |  |  |  |  |  |  |  |  |  |  |  |  |  |  |  |  |  |  |  |  |  |  |  |  |  |  |  |  |  |  |  |  |  |  |  |  |  |  |  |  |  |  |  |  |  |  |  |  |  |  |  |  |  |  |  |  |  |  |  |  |  |  |  |  |  |  |  |  |  |  |  |  |  |  |  |  |  |  |  |  |  |  |  |  |  |  |  |  |  |  |  |  |  |  |  |  |  |  |  |  |  |  |  |  |  |  |  |  |  |  |  |  |  |  |  |  |  |  |  |  |  |  |  |  |  |  |  |  |  |  |  |  |  |  |  |  |  |  |  |  |  |  |  |  |  |  |  |  |  |  |  |  |  |  |  |  |  |  |  |  |  |  |  |  |  |  |  |  |  |  |  |  |  |  |  |  |  |  |  |  |  |  |  |  |  |  |  |  |  |  |  |  |  |  |  |  |  |  |  |  |  |  |  |  |  |  |  |  |  |  |  |  |  |  |  |  |  |  |  |  |  |  |  |  |  |  |  |  |  |  |  |  |  |  |  |  |  |  |  |  |  |  |  |  |  |  |  |  |  |  |  |  |  |  |  |  |  |  |  |  |  |  |  |  |  |  |  |  |  |  |  |  |  |  |  |  |  |  |  |  |  |  |  |  |  |  |  |  |  |  |  |  |  |  |  |  |  |  |  |  |  |  |  |  |  |  |  |  |  |  |  |  |  |  |  |  |  |  |  |  |  |  |  |  |  |  |  |  |  |  |  |  |  |  |  |  |  |  |  |  |  |  |  |  |  |  |  |  |  |  |  |  |  |  |  |  |  |  |  |  |  |  |  |  |  |  |  |  |  |  |  |  |  |  |
|  |  |  |  |  |  |  |  |  |  |  |  |  |  |  |  |  |  |  |  |  |  |  |  |  |  |  |  |  |  |  |  |  |  |  |  |  |  |  |  |  |  |  |  |  |  |  |  |  |  |  |  |  |  |  |  |  |  |  |  |  |  |  |  |  |  |  |  |  |  |  |  |  |  |  |  |  |  |  |  |  |  |  |  |  |  |  |  |  |  |  |  |  |  |  |  |  |  |  |  |  |  |  |  |  |  |  |  |  |  |  |  |  |  |  |  |  |  |  |  |  |  |  |  |  |  |  |  |  |  |  |  |  |  |  |  |  |  |  |  |  |  |  |  |  |  |  |  |  |  |  |  |  |  |  |  |  |  |  |  |  |  |  |  |  |  |  |  |  |  |  |  |  |  |  |  |  |  |  |  |  |  |  |  |  |  |  |  |  |  |  |  |  |  |  |  |  |  |  |  |  |  |  |  |  |  |  |  |  |  |  |  |  |  |  |  |  |  |  |  |  |  |  |  |  |  |  |  |  |  |  |  |  |  |  |  |  |  |  |  |  |  |  |  |  |  |  |  |  |  |  |  |  |  |  |  |  |  |  |  |  |  |  |  |  |  |  |  |  |  |  |  |  |  |  |  |  |  |  |  |  |  |  |  |  |  |  |  |  |  |  |  |  |  |  |  |  |  |  |  |  |  |  |  |  |  |  |  |  |  |  |  |  |  |  |  |  |  |  |  |  |  |  |  |  |  |  |  |  |  |  |  |  |  |  |  |  |  |  |  |  |  |  |  |  |  |  |  |  |  |  |  |  |  |  |  |  |  |  |  |  |  |  |  |  |  |  |  |  |  |  |  |  |  |  |  |  |  |  |  |  |  |  |  |  |  |  |  |  |  |  |  |  |  |  |  |  |  |  |  |  |  |  |  |  |  |  |  |  |  |  |  |  |  |  |  |  |  |  |  |  |  |  |  |  |  |  |  |  |  |  |  |  |  |  |  |  |  |  |  |  |  |  |  |  |  |  |  |  |  |  |  |  |  |  |  |  |  |  |  |  |  |  |  |  |  |  |  |  |  |  |  |  |  |  |  |  |  |  |  |  |  |  |  |  |  |  |  |  |  |  |  |  |  |  |  |  |  |  |  |  |  |  |  |  |  |  |  |  |  |  |  |  |  |  |  |  |  |  |  |  |  |  |  |  |  |  |  |  |  |  |  |  |  |  |  |  |  |  |  |  |  |  |  |  |  |  |  |  |  |  |  |  |  |  |  |  |  |  |  |  |  |  |  |  |  |  |  |  |  |  |  |  |  |  |  |  |  |  |  |  |  |  |  |  |  |  |  |  |  |  |  |  |  |  |  |  |  |  |  |  |  |  |  |  |  |  |  |  |  |  |  |  |  |  |  |  |  |  |  |  |  |  |  |  |  |  |  |  |  |  |  |  |  |  |  |  |  |  |  |  |  |  |  |  |  |  |  |  |  |  |  |  |  |  |  |  |  |  |  |  |  |  |  |  |  |  |  |  |  |  |  |  |  |  |  |  |  |  |  |  |  |  |  |  |  |  |  |  |  |  |  |  |  |  |  |  |  |  |  |  |  |  |  |  |  |  |  |  |  |  |  |  |  |  |  |  |  |  |  |  |  |  |  |  |  |  |  |  |  |  |  |  |  |  |  |  |  |  |  |  |  |  |  |  |  |  |  |  |  |  |  |  |  |  |  |  |  |  |  |  |  |  |  |  |  |  |  |  |  |  |  |  |  |  |  |  |  |  |  |  |  |  |  |  |  |  |  |  |  |  |  |  |  |  |  |  |  |  |  |  |  |  |  |  |  |  |  |  |  |  |  |  |  |  |  |  |  |  |  |  |  |  |  |  |  |  |  |  |  |  |  |  |  |  |  |  |  |  |  |  |  |  |  |  |  |  |  |  |  |  |  |  |  |  |  |  |  |  |  |  |  |  |  |  |  |  |  |  |  |  |  |  |  |  |  |  |  |  |  |  |  |  |  |  |  |  |  |  |  |  |  |  |  |  |  |  |  |  |  |  |  |  |  |  |  |  |  |  |  |  |  |  |  |  |  |  |  |  |  |  |  |  |  |  |  |  |  |  |  |  |  |  |  |  |  |  |  |  |  |  |  |  |  |  |  |  |  |  |  |  |  |  |  |  |  |  |  |  |  |  |  |  |  |  |  |  |  |  |  |  |  |  |  |  |  |  |  |  |  |  |  |  |  |  |  |  |  |  |  |  |  |  |  |  |  |  |  |  |  |  |  |  |  |  |  |  |  |  |  |  |  |  |  |  |  |
|  |  |  |  |  |  |  |  |  |  |  |  |  |  |  |  |  |  |  |  |  |  |  |  |  |  |  |  |  |  |  |  |  |  |  |  |  |  |  |  |  |  |  |  |  |  |  |  |  |  |  |  |  |  |  |  |  |  |  |  |  |  |  |  |  |  |  |  |  |  |  |  |  |  |  |  |  |  |  |  |  |  |  |  |  |  |  |  |  |  |  |  |  |  |  |  |  |  |  |  |  |  |  |  |  |  |  |  |  |  |  |  |  |  |  |  |  |  |  |  |  |  |  |  |  |  |  |  |  |  |  |  |  |  |  |  |  |  |  |  |  |  |  |  |  |  |  |  |  |  |  |  |  |  |  |  |  |  |  |  |  |  |  |  |  |  |  |  |  |  |  |  |  |  |  |  |  |  |  |  |  |  |  |  |  |  |  |  |  |  |  |  |  |  |  |  |  |  |  |  |  |  |  |  |  |  |  |  |  |  |  |  |  |  |  |  |  |  |  |  |  |  |  |  |  |  |  |  |  |  |  |  |  |  |  |  |  |  |  |  |  |  |  |  |  |  |  |  |  |  |  |  |  |  |  |  |  |  |  |  |  |  |  |  |  |  |  |  |  |  |  |  |  |  |  |  |  |  |  |  |  |  |  |  |  |  |  |  |  |  |  |  |  |  |  |  |  |  |  |  |  |  |  |  |  |  |  |  |  |  |  |  |  |  |  |  |  |  |  |  |  |  |  |  |  |  |  |  |  |  |  |  |  |  |  |  |  |  |  |  |  |  |  |  |  |  |  |  |  |  |  |  |  |  |  |  |  |  |  |  |  |  |  |  |  |  |  |  |  |  |  |  |  |  |  |  |  |  |  |  |  |  |  |  |  |  |  |  |  |  |  |  |  |  |  |  |  |  |  |  |  |  |  |  |  |  |  |  |  |  |  |  |  |  |  |  |  |  |  |  |  |  |  |  |  |  |  |  |  |  |  |  |  |  |  |  |  |  |  |  |  |  |  |  |  |  |  |  |  |  |  |  |  |  |  |  |  |  |  |  |  |  |  |  |  |  |  |  |  |  |  |  |  |  |  |  |  |  |  |  |  |  |  |  |  |  |  |  |  |  |  |  |  |  |  |  |  |  |  |  |  |  |  |  |  |  |  |  |  |  |  |  |  |  |  |  |  |  |  |  |  |  |  |  |  |  |  |  |  |  |  |  |  |  |  |  |  |  |  |  |  |  |  |  |  |  |  |  |  |  |  |  |  |  |  |  |  |  |  |  |  |  |  |  |  |  |  |  |  |  |  |  |  |  |  |  |  |  |  |  |  |  |  |  |  |  |  |  |  |  |  |  |  |  |  |  |  |  |  |  |  |  |  |  |  |  |  |  |  |  |  |  |  |  |  |  |  |  |  |  |  |  |  |  |  |  |  |  |  |  |  |  |  |  |  |  |  |  |  |  |  |  |  |  |  |  |  |  |  |  |  |  |  |  |  |  |  |  |  |  |  |  |  |  |  |  |  |  |  |  |  |  |  |  |  |  |  |  |  |  |  |  |  |  |  |  |  |  |  |  |  |  |  |  |  |  |  |  |  |  |  |  |  |  |  |  |  |  |  |  |  |  |  |  |  |  |  |  |  |  |  |  |  |  |  |  |  |  |  |  |  |  |  |  |  |  |  |  |  |  |  |  |  |  |  |  |  |  |  |  |  |  |  |  |  |  |  |  |  |  |  |  |  |  |  |  |  |  |  |  |  |  |  |  |  |  |  |  |  |  |  |  |  |  |  |  |  |  |  |  |  |  |  |  |  |  |  |  |  |  |  |  |  |  |  |  |  |  |  |  |  |  |  |  |  |  |  |  |  |  |  |  |  |  |  |  |  |  |  |  |  |  |  |  |  |  |  |  |  |  |  |  |  |  |  |  |  |  |  |  |  |  |  |  |  |  |  |  |  |  |  |  |  |  |  |  |  |  |  |  |  |  |  |  |  |  |  |  |  |  |  |  |  |  |  |  |  |  |  |  |  |  |  |  |  |  |  |  |  |  |  |  |  |  |  |  |  |  |  |  |  |  |  |  |  |  |  |  |  |  |  |  |  |  |  |  |  |  |  |  |  |  |  |  |  |  |  |  |  |  |  |  |  |  |  |  |  |  |  |  |  |  |  |  |  |  |  |  |  |  |  |  |  |  |  |  |  |  |  |  |  |  |  |  |  |  |  |  |  |  |  |  |  |  |  |  |  |  |  |  |  |  |  |  |  |  |  |  |  |  |  |  |  |  |  |  |  |  |  |  |  |  |  |  |  |  |
|  |  |  |  |  |  |  |  |  |  |  |  |  |  |  |  |  |  |  |  |  |  |  |  |  |  |  |  |  |  |  |  |  |  |  |  |  |  |  |  |  |  |  |  |  |  |  |  |  |  |  |  |  |  |  |  |  |  |  |  |  |  |  |  |  |  |  |  |  |  |  |  |  |  |  |  |  |  |  |  |  |  |  |  |  |  |  |  |  |  |  |  |  |  |  |  |  |  |  |  |  |  |  |  |  |  |  |  |  |  |  |  |  |  |  |  |  |  |  |  |  |  |  |  |  |  |  |  |  |  |  |  |  |  |  |  |  |  |  |  |  |  |  |  |  |  |  |  |  |  |  |  |  |  |  |  |  |  |  |  |  |  |  |  |  |  |  |  |  |  |  |  |  |  |  |  |  |  |  |  |  |  |  |  |  |  |  |  |  |  |  |  |  |  |  |  |  |  |  |  |  |  |  |  |  |  |  |  |  |  |  |  |  |  |  |  |  |  |  |  |  |  |  |  |  |  |  |  |  |  |  |  |  |  |  |  |  |  |  |  |  |  |  |  |  |  |  |  |  |  |  |  |  |  |  |  |  |  |  |  |  |  |  |  |  |  |  |  |  |  |  |  |  |  |  |  |  |  |  |  |  |  |  |  |  |  |  |  |  |  |  |  |  |  |  |  |  |  |  |  |  |  |  |  |  |  |  |  |  |  |  |  |  |  |  |  |  |  |  |  |  |  |  |  |  |  |  |  |  |  |  |  |  |  |  |  |  |  |  |  |  |  |  |  |  |  |  |  |  |  |  |  |  |  |  |  |  |  |  |  |  |  |  |  |  |  |  |  |  |  |  |  |  |  |  |  |  |  |  |  |  |  |  |  |  |  |  |  |  |  |  |  |  |  |  |  |  |  |  |  |  |  |  |  |  |  |  |  |  |  |  |  |  |  |  |  |  |  |  |  |  |  |  |  |  |  |  |  |  |  |  |  |  |  |  |  |  |  |  |  |  |  |  |  |  |  |  |  |  |  |  |  |  |  |  |  |  |  |  |  |  |  |  |  |  |  |  |  |  |  |  |  |  |  |  |  |  |  |  |  |  |  |  |  |  |  |  |  |  |  |  |  |  |  |  |  |  |  |  |  |  |  |  |  |  |  |  |  |  |  |  |  |  |  |  |  |  |  |  |  |  |  |  |  |  |  |  |  |  |  |  |  |  |  |  |  |  |  |  |  |  |  |  |  |  |  |  |  |  |  |  |  |  |  |  |  |  |  |  |  |  |  |  |  |  |  |  |  |  |  |  |  |  |  |  |  |  |  |  |  |  |  |  |  |  |  |  |  |  |  |  |  |  |  |  |  |  |  |  |  |  |  |  |  |  |  |  |  |  |  |  |  |  |  |  |  |  |  |  |  |  |  |  |  |  |  |  |  |  |  |  |  |  |  |  |  |  |  |  |  |  |  |  |  |  |  |  |  |  |  |  |  |  |  |  |  |  |  |  |  |  |  |  |  |  |  |  |  |  |  |  |  |  |  |  |  |  |  |  |  |  |  |  |  |  |  |  |  |  |  |  |  |  |  |  |  |  |  |  |  |  |  |  |  |  |  |  |  |  |  |  |  |  |  |  |  |  |  |  |  |  |  |  |  |  |  |  |  |  |  |  |  |  |  |  |  |  |  |  |  |  |  |  |  |  |  |  |  |  |  |  |  |  |  |  |  |  |  |  |  |  |  |  |  |  |  |  |  |  |  |  |  |  |  |  |  |  |  |  |  |  |  |  |  |  |  |  |  |  |  |  |  |  |  |  |  |  |  |  |  |  |  |  |  |  |  |  |  |  |  |  |  |  |  |  |  |  |  |  |  |  |  |  |  |  |  |  |  |  |  |  |  |  |  |  |  |  |  |  |  |  |  |  |  |  |  |  |  |  |  |  |  |  |  |  |  |  |  |  |  |  |  |  |  |  |  |  |  |  |  |  |  |  |  |  |  |  |  |  |  |  |  |  |  |  |  |  |  |  |  |  |  |  |  |  |  |  |  |  |  |  |  |  |  |  |  |  |  |  |  |  |  |  |  |  |  |  |  |  |  |  |  |  |  |  |  |  |  |  |  |  |  |  |  |  |  |  |  |  |  |  |  |  |  |  |  |  |  |  |  |  |  |  |  |  |  |  |  |  |  |  |  |  |  |  |  |  |  |  |  |  |  |  |  |  |  |  |  |  |  |  |  |  |  |  |  |  |  |  |  |  |  |  |  |  |  |  |  |  |  |  |  |  |  |  |  |  |  |  |  |  |  |  |  |  |  |
|  |  |  |  |  |  |  |  |  |  |  |  |  |  |  |  |  |  |  |  |  |  |  |  |  |  |  |  |  |  |  |  |  |  |  |  |  |  |  |  |  |  |  |  |  |  |  |  |  |  |  |  |  |  |  |  |  |  |  |  |  |  |  |  |  |  |  |  |  |  |  |  |  |  |  |  |  |  |  |  |  |  |  |  |  |  |  |  |  |  |  |  |  |  |  |  |  |  |  |  |  |  |  |  |  |  |  |  |  |  |  |  |  |  |  |  |  |  |  |  |  |  |  |  |  |  |  |  |  |  |  |  |  |  |  |  |  |  |  |  |  |  |  |  |  |  |  |  |  |  |  |  |  |  |  |  |  |  |  |  |  |  |  |  |  |  |  |  |  |  |  |  |  |  |  |  |  |  |  |  |  |  |  |  |  |  |  |  |  |  |  |  |  |  |  |  |  |  |  |  |  |  |  |  |  |  |  |  |  |  |  |  |  |  |  |  |  |  |  |  |  |  |  |  |  |  |  |  |  |  |  |  |  |  |  |  |  |  |  |  |  |  |  |  |  |  |  |  |  |  |  |  |  |  |  |  |  |  |  |  |  |  |  |  |  |  |  |  |  |  |  |  |  |  |  |  |  |  |  |  |  |  |  |  |  |  |  |  |  |  |  |  |  |  |  |  |  |  |  |  |  |  |  |  |  |  |  |  |  |  |  |  |  |  |  |  |  |  |  |  |  |  |  |  |  |  |  |  |  |  |  |  |  |  |  |  |  |  |  |  |  |  |  |  |  |  |  |  |  |  |  |  |  |  |  |  |  |  |  |  |  |  |  |  |  |  |  |  |  |  |  |  |  |  |  |  |  |  |  |  |  |  |  |  |  |  |  |  |  |  |  |  |  |  |  |  |  |  |  |  |  |  |  |  |  |  |  |  |  |  |  |  |  |  |  |  |  |  |  |  |  |  |  |  |  |  |  |  |  |  |  |  |  |  |  |  |  |  |  |  |  |  |  |  |  |  |  |  |  |  |  |  |  |  |  |  |  |  |  |  |  |  |  |  |  |  |  |  |  |  |  |  |  |  |  |  |  |  |  |  |  |  |  |  |  |  |  |  |  |  |  |  |  |  |  |  |  |  |  |  |  |  |  |  |  |  |  |  |  |  |  |  |  |  |  |  |  |  |  |  |  |  |  |  |  |  |  |  |  |  |  |  |  |  |  |  |  |  |  |  |  |  |  |  |  |  |  |  |  |  |  |  |  |  |  |  |  |  |  |  |  |  |  |  |  |  |  |  |  |  |  |  |  |  |  |  |  |  |  |  |  |  |  |  |  |  |  |  |  |  |  |  |  |  |  |  |  |  |  |  |  |  |  |  |  |  |  |  |  |  |  |  |  |  |  |  |  |  |  |  |  |  |  |  |  |  |  |  |  |  |  |  |  |  |  |  |  |  |  |  |  |  |  |  |  |  |  |  |  |  |  |  |  |  |  |  |  |  |  |  |  |  |  |  |  |  |  |  |  |  |  |  |  |  |  |  |  |  |  |  |  |  |  |  |  |  |  |  |  |  |  |  |  |  |  |  |  |  |  |  |  |  |  |  |  |  |  |  |  |  |  |  |  |  |  |  |  |  |  |  |  |  |  |  |  |  |  |  |  |  |  |  |  |  |  |  |  |  |  |  |  |  |  |  |  |  |  |  |  |  |  |  |  |  |  |  |  |  |  |  |  |  |  |  |  |  |  |  |  |  |  |  |  |  |  |  |  |  |  |  |  |  |  |  |  |  |  |  |  |  |  |  |  |  |  |  |  |  |  |  |  |  |  |  |  |  |  |  |  |  |  |  |  |  |  |  |  |  |  |  |  |  |  |  |  |  |  |  |  |  |  |  |  |  |  |  |  |  |  |  |  |  |  |  |  |  |  |  |  |  |  |  |  |  |  |  |  |  |  |  |  |  |  |  |  |  |  |  |  |  |  |  |  |  |  |  |  |  |  |  |  |  |  |  |  |  |  |  |  |  |  |  |  |  |  |  |  |  |  |  |  |  |  |  |  |  |  |  |  |  |  |  |  |  |  |  |  |  |  |  |  |  |  |  |  |  |  |  |  |  |  |  |  |  |  |  |  |  |  |  |  |  |  |  |  |  |  |  |  |  |  |  |  |  |  |  |  |  |  |  |  |  |  |  |  |  |  |  |  |  |  |  |  |  |  |  |  |  |  |  |  |  |  |  |  |  |  |  |  |  |  |  |  |  |  |  |  |  |  |  |  |  |  |  |  |  |  |  |  |  |  |  |  |  |  |  |
|  |  |  |  |  |  |  |  |  |  |  |  |  |  |  |  |  |  |  |  |  |  |  |  |  |  |  |  |  |  |  |  |  |  |  |  |  |  |  |  |  |  |  |  |  |  |  |  |  |  |  |  |  |  |  |  |  |  |  |  |  |  |  |  |  |  |  |  |  |  |  |  |  |  |  |  |  |  |  |  |  |  |  |  |  |  |  |  |  |  |  |  |  |  |  |  |  |  |  |  |  |  |  |  |  |  |  |  |  |  |  |  |  |  |  |  |  |  |  |  |  |  |  |  |  |  |  |  |  |  |  |  |  |  |  |  |  |  |  |  |  |  |  |  |  |  |  |  |  |  |  |  |  |  |  |  |  |  |  |  |  |  |  |  |  |  |  |  |  |  |  |  |  |  |  |  |  |  |  |  |  |  |  |  |  |  |  |  |  |  |  |  |  |  |  |  |  |  |  |  |  |  |  |  |  |  |  |  |  |  |  |  |  |  |  |  |  |  |  |  |  |  |  |  |  |  |  |  |  |  |  |  |  |  |  |  |  |  |  |  |  |  |  |  |  |  |  |  |  |  |  |  |  |  |  |  |  |  |  |  |  |  |  |  |  |  |  |  |  |  |  |  |  |  |  |  |  |  |  |  |  |  |  |  |  |  |  |  |  |  |  |  |  |  |  |  |  |  |  |  |  |  |  |  |  |  |  |  |  |  |  |  |  |  |  |  |  |  |  |  |  |  |  |  |  |  |  |  |  |  |  |  |  |  |  |  |  |  |  |  |  |  |  |  |  |  |  |  |  |  |  |  |  |  |  |  |  |  |  |  |  |  |  |  |  |  |  |  |  |  |  |  |  |  |  |  |  |  |  |  |  |  |  |  |  |  |  |  |  |  |  |  |  |  |  |  |  |  |  |  |  |  |  |  |  |  |  |  |  |  |  |  |  |  |  |  |  |  |  |  |  |  |  |  |  |  |  |  |  |  |  |  |  |  |  |  |  |  |  |  |  |  |  |  |  |  |  |  |  |  |  |  |  |  |  |  |  |  |  |  |  |  |  |  |  |  |  |  |  |  |  |  |  |  |  |  |  |  |  |  |  |  |  |  |  |  |  |  |  |  |  |  |  |  |  |  |  |  |  |  |  |  |  |  |  |  |  |  |  |  |  |  |  |  |  |  |  |  |  |  |  |  |  |  |  |  |  |  |  |  |  |  |  |  |  |  |  |  |  |  |  |  |  |  |  |  |  |  |  |  |  |  |  |  |  |  |  |  |  |  |  |  |  |  |  |  |  |  |  |  |  |  |  |  |  |  |  |  |  |  |  |  |  |  |  |  |  |  |  |  |  |  |  |  |  |  |  |  |  |  |  |  |  |  |  |  |  |  |  |  |  |  |  |  |  |  |  |  |  |  |  |  |  |  |  |  |  |  |  |  |  |  |  |  |  |  |  |  |  |  |  |  |  |  |  |  |  |  |  |  |  |  |  |  |  |  |  |  |  |  |  |  |  |  |  |  |  |  |  |  |  |  |  |  |  |  |  |  |  |  |  |  |  |  |  |  |  |  |  |  |  |  |  |  |  |  |  |  |  |  |  |  |  |  |  |  |  |  |  |  |  |  |  |  |  |  |  |  |  |  |  |  |  |  |  |  |  |  |  |  |  |  |  |  |  |  |  |  |  |  |  |  |  |  |  |  |  |  |  |  |  |  |  |  |  |  |  |  |  |  |  |  |  |  |  |  |  |  |  |  |  |  |  |  |  |  |  |  |  |  |  |  |  |  |  |  |  |  |  |  |  |  |  |  |  |  |  |  |  |  |  |  |  |  |  |  |  |  |  |  |  |  |  |  |  |  |  |  |  |  |  |  |  |  |  |  |  |  |  |  |  |  |  |  |  |  |  |  |  |  |  |  |  |  |  |  |  |  |  |  |  |  |  |  |  |  |  |  |  |  |  |  |  |  |  |  |  |  |  |  |  |  |  |  |  |  |  |  |  |  |  |  |  |  |  |  |  |  |  |  |  |  |  |  |  |  |  |  |  |  |  |  |  |  |  |  |  |  |  |  |  |  |  |  |  |  |  |  |  |  |  |  |  |  |  |  |  |  |  |  |  |  |  |  |  |  |  |  |  |  |  |  |  |  |  |  |  |  |  |  |  |  |  |  |  |  |  |  |  |  |  |  |  |  |  |  |  |  |  |  |  |  |  |  |  |  |  |  |  |  |  |  |  |  |  |  |  |  |  |  |  |  |  |  |  |  |  |  |  |  |  |  |  |  |  |  |  |  |  |  |  |  |  |  |  |  |
|  |  |  |  |  |  |  |  |  |  |  |  |  |  |  |  |  |  |  |  |  |  |  |  |  |  |  |  |  |  |  |  |  |  |  |  |  |  |  |  |  |  |  |  |  |  |  |  |  |  |  |  |  |  |  |  |  |  |  |  |  |  |  |  |  |  |  |  |  |  |  |  |  |  |  |  |  |  |  |  |  |  |  |  |  |  |  |  |  |  |  |  |  |  |  |  |  |  |  |  |  |  |  |  |  |  |  |  |  |  |  |  |  |  |  |  |  |  |  |  |  |  |  |  |  |  |  |  |  |  |  |  |  |  |  |  |  |  |  |  |  |  |  |  |  |  |  |  |  |  |  |  |  |  |  |  |  |  |  |  |  |  |  |  |  |  |  |  |  |  |  |  |  |  |  |  |  |  |  |  |  |  |  |  |  |  |  |  |  |  |  |  |  |  |  |  |  |  |  |  |  |  |  |  |  |  |  |  |  |  |  |  |  |  |  |  |  |  |  |  |  |  |  |  |  |  |  |  |  |  |  |  |  |  |  |  |  |  |  |  |  |  |  |  |  |  |  |  |  |  |  |  |  |  |  |  |  |  |  |  |  |  |  |  |  |  |  |  |  |  |  |  |  |  |  |  |  |  |  |  |  |  |  |  |  |  |  |  |  |  |  |  |  |  |  |  |  |  |  |  |  |  |  |  |  |  |  |  |  |  |  |  |  |  |  |  |  |  |  |  |  |  |  |  |  |  |  |  |  |  |  |  |  |  |  |  |  |  |  |  |  |  |  |  |  |  |  |  |  |  |  |  |  |  |  |  |  |  |  |  |  |  |  |  |  |  |  |  |  |  |  |  |  |  |  |  |  |  |  |  |  |  |  |  |  |  |  |  |  |  |  |  |  |  |  |  |  |  |  |  |  |  |  |  |  |  |  |  |  |  |  |  |  |  |  |  |  |  |  |  |  |  |  |  |  |  |  |  |  |  |  |  |  |  |  |  |  |  |  |  |  |  |  |  |  |  |  |  |  |  |  |  |  |  |  |  |  |  |  |  |  |  |  |  |  |  |  |  |  |  |  |  |  |  |  |  |  |  |  |  |  |  |  |  |  |  |  |  |  |  |  |  |  |  |  |  |  |  |  |  |  |  |  |  |  |  |  |  |  |  |  |  |  |  |  |  |  |  |  |  |  |  |  |  |  |  |  |  |  |  |  |  |  |  |  |  |  |  |  |  |  |  |  |  |  |  |  |  |  |  |  |  |  |  |  |  |  |  |  |  |  |  |  |  |  |  |  |  |  |  |  |  |  |  |  |  |  |  |  |  |  |  |  |  |  |  |  |  |  |  |  |  |  |  |  |  |  |  |  |  |  |  |  |  |  |  |  |  |  |  |  |  |  |  |  |  |  |  |  |  |  |  |  |  |  |  |  |  |  |  |  |  |  |  |  |  |  |  |  |  |  |  |  |  |  |  |  |  |  |  |  |  |  |  |  |  |  |  |  |  |  |  |  |  |  |  |  |  |  |  |  |  |  |  |  |  |  |  |  |  |  |  |  |  |  |  |  |  |  |  |  |  |  |  |  |  |  |  |  |  |  |  |  |  |  |  |  |  |  |  |  |  |  |  |  |  |  |  |  |  |  |  |  |  |  |  |  |  |  |  |  |  |  |  |  |  |  |  |  |  |  |  |  |  |  |  |  |  |  |  |  |  |  |  |  |  |  |  |  |  |  |  |  |  |  |  |  |  |  |  |  |  |  |  |  |  |  |  |  |  |  |  |  |  |  |  |  |  |  |  |  |  |  |  |  |  |  |  |  |  |  |  |  |  |  |  |  |  |  |  |  |  |  |  |  |  |  |  |  |  |  |  |  |  |  |  |  |  |  |  |  |  |  |  |  |  |  |  |  |  |  |  |  |  |  |  |  |  |  |  |  |  |  |  |  |  |  |  |  |  |  |  |  |  |  |  |  |  |  |  |  |  |  |  |  |  |  |  |  |  |  |  |  |  |  |  |  |  |  |  |  |  |  |  |  |  |  |  |  |  |  |  |  |  |  |  |  |  |  |  |  |  |  |  |  |  |  |  |  |  |  |  |  |  |  |  |  |  |  |  |  |  |  |  |  |  |  |  |  |  |  |  |  |  |  |  |  |  |  |  |  |  |  |  |  |  |  |  |  |  |  |  |  |  |  |  |  |  |  |  |  |  |  |  |  |  |  |  |  |  |  |  |  |  |  |  |  |  |  |  |  |  |  |  |  |  |  |  |  |  |  |  |  |  |  |  |  |  |  |  |  |  |  |  |  |  |
|  |  |  |  |  |  |  |  |  |  |  |  |  |  |  |  |  |  |  |  |  |  |  |  |  |  |  |  |  |  |  |  |  |  |  |  |  |  |  |  |  |  |  |  |  |  |  |  |  |  |  |  |  |  |  |  |  |  |  |  |  |  |  |  |  |  |  |  |  |  |  |  |  |  |  |  |  |  |  |  |  |  |  |  |  |  |  |  |  |  |  |  |  |  |  |  |  |  |  |  |  |  |  |  |  |  |  |  |  |  |  |  |  |  |  |  |  |  |  |  |  |  |  |  |  |  |  |  |  |  |  |  |  |  |  |  |  |  |  |  |  |  |  |  |  |  |  |  |  |  |  |  |  |  |  |  |  |  |  |  |  |  |  |  |  |  |  |  |  |  |  |  |  |  |  |  |  |  |  |  |  |  |  |  |  |  |  |  |  |  |  |  |  |  |  |  |  |  |  |  |  |  |  |  |  |  |  |  |  |  |  |  |  |  |  |  |  |  |  |  |  |  |  |  |  |  |  |  |  |  |  |  |  |  |  |  |  |  |  |  |  |  |  |  |  |  |  |  |  |  |  |  |  |  |  |  |  |  |  |  |  |  |  |  |  |  |  |  |  |  |  |  |  |  |  |  |  |  |  |  |  |  |  |  |  |  |  |  |  |  |  |  |  |  |  |  |  |  |  |  |  |  |  |  |  |  |  |  |  |  |  |  |  |  |  |  |  |  |  |  |  |  |  |  |  |  |  |  |  |  |  |  |  |  |  |  |  |  |  |  |  |  |  |  |  |  |  |  |  |  |  |  |  |  |  |  |  |  |  |  |  |  |  |  |  |  |  |  |  |  |  |  |  |  |  |  |  |  |  |  |  |  |  |  |  |  |  |  |  |  |  |  |  |  |  |  |  |  |  |  |  |  |  |  |  |  |  |  |  |  |  |  |  |  |  |  |  |  |  |  |  |  |  |  |  |  |  |  |  |  |  |  |  |  |  |  |  |  |  |  |  |  |  |  |  |  |  |  |  |  |  |  |  |  |  |  |  |  |  |  |  |  |  |  |  |  |  |  |  |  |  |  |  |  |  |  |  |  |  |  |  |  |  |  |  |  |  |  |  |  |  |  |  |  |  |  |  |  |  |  |  |  |  |  |  |  |  |  |  |  |  |  |  |  |  |  |  |  |  |  |  |  |  |  |  |  |  |  |  |  |  |  |  |  |  |  |  |  |  |  |  |  |  |  |  |  |  |  |  |  |  |  |  |  |  |  |  |  |  |  |  |  |  |  |  |  |  |  |  |  |  |  |  |  |  |  |  |  |  |  |  |  |  |  |  |  |  |  |  |  |  |  |  |  |  |  |  |  |  |  |  |  |  |  |  |  |  |  |  |  |  |  |  |  |  |  |  |  |  |  |  |  |  |  |  |  |  |  |  |  |  |  |  |  |  |  |  |  |  |  |  |  |  |  |  |  |  |  |  |  |  |  |  |  |  |  |  |  |  |  |  |  |  |  |  |  |  |  |  |  |  |  |  |  |  |  |  |  |  |  |  |  |  |  |  |  |  |  |  |  |  |  |  |  |  |  |  |  |  |  |  |  |  |  |  |  |  |  |  |  |  |  |  |  |  |  |  |  |  |  |  |  |  |  |  |  |  |  |  |  |  |  |  |  |  |  |  |  |  |  |  |  |  |  |  |  |  |  |  |  |  |  |  |  |  |  |  |  |  |  |  |  |  |  |  |  |  |  |  |  |  |  |  |  |  |  |  |  |  |  |  |  |  |  |  |  |  |  |  |  |  |  |  |  |  |  |  |  |  |  |  |  |  |  |  |  |  |  |  |  |  |  |  |  |  |  |  |  |  |  |  |  |  |  |  |  |  |  |  |  |  |  |  |  |  |  |  |  |  |  |  |  |  |  |  |  |  |  |  |  |  |  |  |  |  |  |  |  |  |  |  |  |  |  |  |  |  |  |  |  |  |  |  |  |  |  |  |  |  |  |  |  |  |  |  |  |  |  |  |  |  |  |  |  |  |  |  |  |  |  |  |  |  |  |  |  |  |  |  |  |  |  |  |  |  |  |  |  |  |  |  |  |  |  |  |  |  |  |  |  |  |  |  |  |  |  |  |  |  |  |  |  |  |  |  |  |  |  |  |  |  |  |  |  |  |  |  |  |  |  |  |  |  |  |  |  |  |  |  |  |  |  |  |  |  |  |  |  |  |  |  |  |  |  |  |  |  |  |  |  |  |  |  |  |  |  |  |  |  |  |  |  |  |  |  |  |  |  |  |  |  |  |  |  |  |  |
|  |  |  |  |  |  |  |  |  |  |  |  |  |  |  |  |  |  |  |  |  |  |  |  |  |  |  |  |  |  |  |  |  |  |  |  |  |  |  |  |  |  |  |  |  |  |  |  |  |  |  |  |  |  |  |  |  |  |  |  |  |  |  |  |  |  |  |  |  |  |  |  |  |  |  |  |  |  |  |  |  |  |  |  |  |  |  |  |  |  |  |  |  |  |  |  |  |  |  |  |  |  |  |  |  |  |  |  |  |  |  |  |  |  |  |  |  |  |  |  |  |  |  |  |  |  |  |  |  |  |  |  |  |  |  |  |  |  |  |  |  |  |  |  |  |  |  |  |  |  |  |  |  |  |  |  |  |  |  |  |  |  |  |  |  |  |  |  |  |  |  |  |  |  |  |  |  |  |  |  |  |  |  |  |  |  |  |  |  |  |  |  |  |  |  |  |  |  |  |  |  |  |  |  |  |  |  |  |  |  |  |  |  |  |  |  |  |  |  |  |  |  |  |  |  |  |  |  |  |  |  |  |  |  |  |  |  |  |  |  |  |  |  |  |  |  |  |  |  |  |  |  |  |  |  |  |  |  |  |  |  |  |  |  |  |  |  |  |  |  |  |  |  |  |  |  |  |  |  |  |  |  |  |  |  |  |  |  |  |  |  |  |  |  |  |  |  |  |  |  |  |  |  |  |  |  |  |  |  |  |  |  |  |  |  |  |  |  |  |  |  |  |  |  |  |  |  |  |  |  |  |  |  |  |  |  |  |  |  |  |  |  |  |  |  |  |  |  |  |  |  |  |  |  |  |  |  |  |  |  |  |  |  |  |  |  |  |  |  |  |  |  |  |  |  |  |  |  |  |  |  |  |  |  |  |  |  |  |  |  |  |  |  |  |  |  |  |  |  |  |  |  |  |  |  |  |  |  |  |  |  |  |  |  |  |  |  |  |  |  |  |  |  |  |  |  |  |  |  |  |  |  |  |  |  |  |  |  |  |  |  |  |  |  |  |  |  |  |  |  |  |  |  |  |  |  |  |  |  |  |  |  |  |  |  |  |  |  |  |  |  |  |  |  |  |  |  |  |  |  |  |  |  |  |  |  |  |  |  |  |  |  |  |  |  |  |  |  |  |  |  |  |  |  |  |  |  |  |  |  |  |  |  |  |  |  |  |  |  |  |  |  |  |  |  |  |  |  |  |  |  |  |  |  |  |  |  |  |  |  |  |  |  |  |  |  |  |  |  |  |  |  |  |  |  |  |  |  |  |  |  |  |  |  |  |  |  |  |  |  |  |  |  |  |  |  |  |  |  |  |  |  |  |  |  |  |  |  |  |  |  |  |  |  |  |  |  |  |  |  |  |  |  |  |  |  |  |  |  |  |  |  |  |  |  |  |  |  |  |  |  |  |  |  |  |  |  |  |  |  |  |  |  |  |  |  |  |  |  |  |  |  |  |  |  |  |  |  |  |  |  |  |  |  |  |  |  |  |  |  |  |  |  |  |  |  |  |  |  |  |  |  |  |  |  |  |  |  |  |  |  |  |  |  |  |  |  |  |  |  |  |  |  |  |  |  |  |  |  |  |  |  |  |  |  |  |  |  |  |  |  |  |  |  |  |  |  |  |  |  |  |  |  |  |  |  |  |  |  |  |  |  |  |  |  |  |  |  |  |  |  |  |  |  |  |  |  |  |  |  |  |  |  |  |  |  |  |  |  |  |  |  |  |  |  |  |  |  |  |  |  |  |  |  |  |  |  |  |  |  |  |  |  |  |  |  |  |  |  |  |  |  |  |  |  |  |  |  |  |  |  |  |  |  |  |  |  |  |  |  |  |  |  |  |  |  |  |  |  |  |  |  |  |  |  |  |  |  |  |  |  |  |  |  |  |  |  |  |  |  |  |  |  |  |  |  |  |  |  |  |  |  |  |  |  |  |  |  |  |  |  |  |  |  |  |  |  |  |  |  |  |  |  |  |  |  |  |  |  |  |  |  |  |  |  |  |  |  |  |  |  |  |  |  |  |  |  |  |  |  |  |  |  |  |  |  |  |  |  |  |  |  |  |  |  |  |  |  |  |  |  |  |  |  |  |  |  |  |  |  |  |  |  |  |  |  |  |  |  |  |  |  |  |  |  |  |  |  |  |  |  |  |  |  |  |  |  |  |  |  |  |  |  |  |  |  |  |  |  |  |  |  |  |  |  |  |  |  |  |  |  |  |  |  |  |  |  |  |  |  |  |  |  |  |  |  |  |  |  |  |  |  |  |  |  |  |  |  |  |  |  |  |  |  |  |  |
|  |  |  |  |  |  |  |  |  |  |  |  |  |  |  |  |  |  |  |  |  |  |  |  |  |  |  |  |  |  |  |  |  |  |  |  |  |  |  |  |  |  |  |  |  |  |  |  |  |  |  |  |  |  |  |  |  |  |  |  |  |  |  |  |  |  |  |  |  |  |  |  |  |  |  |  |  |  |  |  |  |  |  |  |  |  |  |  |  |  |  |  |  |  |  |  |  |  |  |  |  |  |  |  |  |  |  |  |  |  |  |  |  |  |  |  |  |  |  |  |  |  |  |  |  |  |  |  |  |  |  |  |  |  |  |  |  |  |  |  |  |  |  |  |  |  |  |  |  |  |  |  |  |  |  |  |  |  |  |  |  |  |  |  |  |  |  |  |  |  |  |  |  |  |  |  |  |  |  |  |  |  |  |  |  |  |  |  |  |  |  |  |  |  |  |  |  |  |  |  |  |  |  |  |  |  |  |  |  |  |  |  |  |  |  |  |  |  |  |  |  |  |  |  |  |  |  |  |  |  |  |  |  |  |  |  |  |  |  |  |  |  |  |  |  |  |  |  |  |  |  |  |  |  |  |  |  |  |  |  |  |  |  |  |  |  |  |  |  |  |  |  |  |  |  |  |  |  |  |  |  |  |  |  |  |  |  |  |  |  |  |  |  |  |  |  |  |  |  |  |  |  |  |  |  |  |  |  |  |  |  |  |  |  |  |  |  |  |  |  |  |  |  |  |  |  |  |  |  |  |  |  |  |  |  |  |  |  |  |  |  |  |  |  |  |  |  |  |  |  |  |  |  |  |  |  |  |  |  |  |  |  |  |  |  |  |  |  |  |  |  |  |  |  |  |  |  |  |  |  |  |  |  |  |  |  |  |  |  |  |  |  |  |  |  |  |  |  |  |  |  |  |  |  |  |  |  |  |  |  |  |  |  |  |  |  |  |  |  |  |  |  |  |  |  |  |  |  |  |  |  |  |  |  |  |  |  |  |  |  |  |  |  |  |  |  |  |  |  |  |  |  |  |  |  |  |  |  |  |  |  |  |  |  |  |  |  |  |  |  |  |  |  |  |  |  |  |  |  |  |  |  |  |  |  |  |  |  |  |  |  |  |  |  |  |  |  |  |  |  |  |  |  |  |  |  |  |  |  |  |  |  |  |  |  |  |  |  |  |  |  |  |  |  |  |  |  |  |  |  |  |  |  |  |  |  |  |  |  |  |  |  |  |  |  |  |  |  |  |  |  |  |  |  |  |  |  |  |  |  |  |  |  |  |  |  |  |  |  |  |  |  |  |  |  |  |  |  |  |  |  |  |  |  |  |  |  |  |  |  |  |  |  |  |  |  |  |  |  |  |  |  |  |  |  |  |  |  |  |  |  |  |  |  |  |  |  |  |  |  |  |  |  |  |  |  |  |  |  |  |  |  |  |  |  |  |  |  |  |  |  |  |  |  |  |  |  |  |  |  |  |  |  |  |  |  |  |  |  |  |  |  |  |  |  |  |  |  |  |  |  |  |  |  |  |  |  |  |  |  |  |  |  |  |  |  |  |  |  |  |  |  |  |  |  |  |  |  |  |  |  |  |  |  |  |  |  |  |  |  |  |  |  |  |  |  |  |  |  |  |  |  |  |  |  |  |  |  |  |  |  |  |  |  |  |  |  |  |  |  |  |  |  |  |  |  |  |  |  |  |  |  |  |  |  |  |  |  |  |  |  |  |  |  |  |  |  |  |  |  |  |  |  |  |  |  |  |  |  |  |  |  |  |  |  |  |  |  |  |  |  |  |  |  |  |  |  |  |  |  |  |  |  |  |  |  |  |  |  |  |  |  |  |  |  |  |  |  |  |  |  |  |  |  |  |  |  |  |  |  |  |  |  |  |  |  |  |  |  |  |  |  |  |  |  |  |  |  |  |  |  |  |  |  |  |  |  |  |  |  |  |  |  |  |  |  |  |  |  |  |  |  |  |  |  |  |  |  |  |  |  |  |  |  |  |  |  |  |  |  |  |  |  |  |  |  |  |  |  |  |  |  |  |  |  |  |  |  |  |  |  |  |  |  |  |  |  |  |  |  |  |  |  |  |  |  |  |  |  |  |  |  |  |  |  |  |  |  |  |  |  |  |  |  |  |  |  |  |  |  |  |  |  |  |  |  |  |  |  |  |  |  |  |  |  |  |  |  |  |  |  |  |  |  |  |  |  |  |  |  |  |  |  |  |  |  |  |  |  |  |  |  |  |  |  |  |  |  |  |  |  |  |  |  |  |  |  |  |  |  |  |  |  |  |  |  |
|  |  |  |  |  |  |  |  |  |  |  |  |  |  |  |  |  |  |  |  |  |  |  |  |  |  |  |  |  |  |  |  |  |  |  |  |  |  |  |  |  |  |  |  |  |  |  |  |  |  |  |  |  |  |  |  |  |  |  |  |  |  |  |  |  |  |  |  |  |  |  |  |  |  |  |  |  |  |  |  |  |  |  |  |  |  |  |  |  |  |  |  |  |  |  |  |  |  |  |  |  |  |  |  |  |  |  |  |  |  |  |  |  |  |  |  |  |  |  |  |  |  |  |  |  |  |  |  |  |  |  |  |  |  |  |  |  |  |  |  |  |  |  |  |  |  |  |  |  |  |  |  |  |  |  |  |  |  |  |  |  |  |  |  |  |  |  |  |  |  |  |  |  |  |  |  |  |  |  |  |  |  |  |  |  |  |  |  |  |  |  |  |  |  |  |  |  |  |  |  |  |  |  |  |  |  |  |  |  |  |  |  |  |  |  |  |  |  |  |  |  |  |  |  |  |  |  |  |  |  |  |  |  |  |  |  |  |  |  |  |  |  |  |  |  |  |  |  |  |  |  |  |  |  |  |  |  |  |  |  |  |  |  |  |  |  |  |  |  |  |  |  |  |  |  |  |  |  |  |  |  |  |  |  |  |  |  |  |  |  |  |  |  |  |  |  |  |  |  |  |  |  |  |  |  |  |  |  |  |  |  |  |  |  |  |  |  |  |  |  |  |  |  |  |  |  |  |  |  |  |  |  |  |  |  |  |  |  |  |  |  |  |  |  |  |  |  |  |  |  |  |  |  |  |  |  |  |  |  |  |  |  |  |  |  |  |  |  |  |  |  |  |  |  |  |  |  |  |  |  |  |  |  |  |  |  |  |  |  |  |  |  |  |  |  |  |  |  |  |  |  |  |  |  |  |  |  |  |  |  |  |  |  |  |  |  |  |  |  |  |  |  |  |  |  |  |  |  |  |  |  |  |  |  |  |  |  |  |  |  |  |  |  |  |  |  |  |  |  |  |  |  |  |  |  |  |  |  |  |  |  |  |  |  |  |  |  |  |  |  |  |  |  |  |  |  |  |  |  |  |  |  |  |  |  |  |  |  |  |  |  |  |  |  |  |  |  |  |  |  |  |  |  |  |  |  |  |  |  |  |  |  |  |  |  |  |  |  |  |  |  |  |  |  |  |  |  |  |  |  |  |  |  |  |  |  |  |  |  |  |  |  |  |  |  |  |  |  |  |  |  |  |  |  |  |  |  |  |  |  |  |  |  |  |  |  |  |  |  |  |  |  |  |  |  |  |  |  |  |  |  |  |  |  |  |  |  |  |  |  |  |  |  |  |  |  |  |  |  |  |  |  |  |  |  |  |  |  |  |  |  |  |  |  |  |  |  |  |  |  |  |  |  |  |  |  |  |  |  |  |  |  |  |  |  |  |  |  |  |  |  |  |  |  |  |  |  |  |  |  |  |  |  |  |  |  |  |  |  |  |  |  |  |  |  |  |  |  |  |  |  |  |  |  |  |  |  |  |  |  |  |  |  |  |  |  |  |  |  |  |  |  |  |  |  |  |  |  |  |  |  |  |  |  |  |  |  |  |  |  |  |  |  |  |  |  |  |  |  |  |  |  |  |  |  |  |  |  |  |  |  |  |  |  |  |  |  |  |  |  |  |  |  |  |  |  |  |  |  |  |  |  |  |  |  |  |  |  |  |  |  |  |  |  |  |  |  |  |  |  |  |  |  |  |  |  |  |  |  |  |  |  |  |  |  |  |  |  |  |  |  |  |  |  |  |  |  |  |  |  |  |  |  |  |  |  |  |  |  |  |  |  |  |  |  |  |  |  |  |  |  |  |  |  |  |  |  |  |  |  |  |  |  |  |  |  |  |  |  |  |  |  |  |  |  |  |  |  |  |  |  |  |  |  |  |  |  |  |  |  |  |  |  |  |  |  |  |  |  |  |  |  |  |  |  |  |  |  |  |  |  |  |  |  |  |  |  |  |  |  |  |  |  |  |  |  |  |  |  |  |  |  |  |  |  |  |  |  |  |  |  |  |  |  |  |  |  |  |  |  |  |  |  |  |  |  |  |  |  |  |  |  |  |  |  |  |  |  |  |  |  |  |  |  |  |  |  |  |  |  |  |  |  |  |  |  |  |  |  |  |  |  |  |  |  |  |  |  |  |  |  |  |  |  |  |  |  |  |  |  |  |  |  |  |  |  |  |  |  |  |  |  |  |  |  |  |  |  |  |  |  |  |  |  |  |  |  |  |  |  |  |  |  |  |  |  |
|  |  |  |  |  |  |  |  |  |  |  |  |  |  |  |  |  |  |  |  |  |  |  |  |  |  |  |  |  |  |  |  |  |  |  |  |  |  |  |  |  |  |  |  |  |  |  |  |  |  |  |  |  |  |  |  |  |  |  |  |  |  |  |  |  |  |  |  |  |  |  |  |  |  |  |  |  |  |  |  |  |  |  |  |  |  |  |  |  |  |  |  |  |  |  |  |  |  |  |  |  |  |  |  |  |  |  |  |  |  |  |  |  |  |  |  |  |  |  |  |  |  |  |  |  |  |  |  |  |  |  |  |  |  |  |  |  |  |  |  |  |  |  |  |  |  |  |  |  |  |  |  |  |  |  |  |  |  |  |  |  |  |  |  |  |  |  |  |  |  |  |  |  |  |  |  |  |  |  |  |  |  |  |  |  |  |  |  |  |  |  |  |  |  |  |  |  |  |  |  |  |  |  |  |  |  |  |  |  |  |  |  |  |  |  |  |  |  |  |  |  |  |  |  |  |  |  |  |  |  |  |  |  |  |  |  |  |  |  |  |  |  |  |  |  |  |  |  |  |  |  |  |  |  |  |  |  |  |  |  |  |  |  |  |  |  |  |  |  |  |  |  |  |  |  |  |  |  |  |  |  |  |  |  |  |  |  |  |  |  |  |  |  |  |  |  |  |  |  |  |  |  |  |  |  |  |  |  |  |  |  |  |  |  |  |  |  |  |  |  |  |  |  |  |  |  |  |  |  |  |  |  |  |  |  |  |  |  |  |  |  |  |  |  |  |  |  |  |  |  |  |  |  |  |  |  |  |  |  |  |  |  |  |  |  |  |  |  |  |  |  |  |  |  |  |  |  |  |  |  |  |  |  |  |  |  |  |  |  |  |  |  |  |  |  |  |  |  |  |  |  |  |  |  |  |  |  |  |  |  |  |  |  |  |  |  |  |  |  |  |  |  |  |  |  |  |  |  |  |  |  |  |  |  |  |  |  |  |  |  |  |  |  |  |  |  |  |  |  |  |  |  |  |  |  |  |  |  |  |  |  |  |  |  |  |  |  |  |  |  |  |  |  |  |  |  |  |  |  |  |  |  |  |  |  |  |  |  |  |  |  |  |  |  |  |  |  |  |  |  |  |  |  |  |  |  |  |  |  |  |  |  |  |  |  |  |  |  |  |  |  |  |  |  |  |  |  |  |  |  |  |  |  |  |  |  |  |  |  |  |  |  |  |  |  |  |  |  |  |  |  |  |  |  |  |  |  |  |  |  |  |  |  |  |  |  |  |  |  |  |  |  |  |  |  |  |  |  |  |  |  |  |  |  |  |  |  |  |  |  |  |  |  |  |  |  |  |  |  |  |  |  |  |  |  |  |  |  |  |  |  |  |  |  |  |  |  |  |  |  |  |  |  |  |  |  |  |  |  |  |  |  |  |  |  |  |  |  |  |  |  |  |  |  |  |  |  |  |  |  |  |  |  |  |  |  |  |  |  |  |  |  |  |  |  |  |  |  |  |  |  |  |  |  |  |  |  |  |  |  |  |  |  |  |  |  |  |  |  |  |  |  |  |  |  |  |  |  |  |  |  |  |  |  |  |  |  |  |  |  |  |  |  |  |  |  |  |  |  |  |  |  |  |  |  |  |  |  |  |  |  |  |  |  |  |  |  |  |  |  |  |  |  |  |  |  |  |  |  |  |  |  |  |  |  |  |  |  |  |  |  |  |  |  |  |  |  |  |  |  |  |  |  |  |  |  |  |  |  |  |  |  |  |  |  |  |  |  |  |  |  |  |  |  |  |  |  |  |  |  |  |  |  |  |  |  |  |  |  |  |  |  |  |  |  |  |  |  |  |  |  |  |  |  |  |  |  |  |  |  |  |  |  |  |  |  |  |  |  |  |  |  |  |  |  |  |  |  |  |  |  |  |  |  |  |  |  |  |  |  |  |  |  |  |  |  |  |  |  |  |  |  |  |  |  |  |  |  |  |  |  |  |  |  |  |  |  |  |  |  |  |  |  |  |  |  |  |  |  |  |  |  |  |  |  |  |  |  |  |  |  |  |  |  |  |  |  |  |  |  |  |  |  |  |  |  |  |  |  |  |  |  |  |  |  |  |  |  |  |  |  |  |  |  |  |  |  |  |  |  |  |  |  |  |  |  |  |  |  |  |  |  |  |  |  |  |  |  |  |  |  |  |  |  |  |  |  |  |  |  |  |  |  |  |  |  |  |  |  |  |  |  |  |  |  |  |  |  |  |  |  |  |  |  |  |  |  |  |  |  |  |  |  |  |  |  |
|  |  |  |  |  |  |  |  |  |  |  |  |  |  |  |  |  |  |  |  |  |  |  |  |  |  |  |  |  |  |  |  |  |  |  |  |  |  |  |  |  |  |  |  |  |  |  |  |  |  |  |  |  |  |  |  |  |  |  |  |  |  |  |  |  |  |  |  |  |  |  |  |  |  |  |  |  |  |  |  |  |  |  |  |  |  |  |  |  |  |  |  |  |  |  |  |  |  |  |  |  |  |  |  |  |  |  |  |  |  |  |  |  |  |  |  |  |  |  |  |  |  |  |  |  |  |  |  |  |  |  |  |  |  |  |  |  |  |  |  |  |  |  |  |  |  |  |  |  |  |  |  |  |  |  |  |  |  |  |  |  |  |  |  |  |  |  |  |  |  |  |  |  |  |  |  |  |  |  |  |  |  |  |  |  |  |  |  |  |  |  |  |  |  |  |  |  |  |  |  |  |  |  |  |  |  |  |  |  |  |  |  |  |  |  |  |  |  |  |  |  |  |  |  |  |  |  |  |  |  |  |  |  |  |  |  |  |  |  |  |  |  |  |  |  |  |  |  |  |  |  |  |  |  |  |  |  |  |  |  |  |  |  |  |  |  |  |  |  |  |  |  |  |  |  |  |  |  |  |  |  |  |  |  |  |  |  |  |  |  |  |  |  |  |  |  |  |  |  |  |  |  |  |  |  |  |  |  |  |  |  |  |  |  |  |  |  |  |  |  |  |  |  |  |  |  |  |  |  |  |  |  |  |  |  |  |  |  |  |  |  |  |  |  |  |  |  |  |  |  |  |  |  |  |  |  |  |  |  |  |  |  |  |  |  |  |  |  |  |  |  |  |  |  |  |  |  |  |  |  |  |  |  |  |  |  |  |  |  |  |  |  |  |  |  |  |  |  |  |  |  |  |  |  |  |  |  |  |  |  |  |  |  |  |  |  |  |  |  |  |  |  |  |  |  |  |  |  |  |  |  |  |  |  |  |  |  |  |  |  |  |  |  |  |  |  |  |  |  |  |  |  |  |  |  |  |  |  |  |  |  |  |  |  |  |  |  |  |  |  |  |  |  |  |  |  |  |  |  |  |  |  |  |  |  |  |  |  |  |  |  |  |  |  |  |  |  |  |  |  |  |  |  |  |  |  |  |  |  |  |  |  |  |  |  |  |  |  |  |  |  |  |  |  |  |  |  |  |  |  |  |  |  |  |  |  |  |  |  |  |  |  |  |  |  |  |  |  |  |  |  |  |  |  |  |  |  |  |  |  |  |  |  |  |  |  |  |  |  |  |  |  |  |  |  |  |  |  |  |  |  |  |  |  |  |  |  |  |  |  |  |  |  |  |  |  |  |  |  |  |  |  |  |  |  |  |  |  |  |  |  |  |  |  |  |  |  |  |  |  |  |  |  |  |  |  |  |  |  |  |  |  |  |  |  |  |  |  |  |  |  |  |  |  |  |  |  |  |  |  |  |  |  |  |  |  |  |  |  |  |  |  |  |  |  |  |  |  |  |  |  |  |  |  |  |  |  |  |  |  |  |  |  |  |  |  |  |  |  |  |  |  |  |  |  |  |  |  |  |  |  |  |  |  |  |  |  |  |  |  |  |  |  |  |  |  |  |  |  |  |  |  |  |  |  |  |  |  |  |  |  |  |  |  |  |  |  |  |  |  |  |  |  |  |  |  |  |  |  |  |  |  |  |  |  |  |  |  |  |  |  |  |  |  |  |  |  |  |  |  |  |  |  |  |  |  |  |  |  |  |  |  |  |  |  |  |  |  |  |  |  |  |  |  |  |  |  |  |  |  |  |  |  |  |  |  |  |  |  |  |  |  |  |  |  |  |  |  |  |  |  |  |  |  |  |  |  |  |  |  |  |  |  |  |  |  |  |  |  |  |  |  |  |  |  |  |  |  |  |  |  |  |  |  |  |  |  |  |  |  |  |  |  |  |  |  |  |  |  |  |  |  |  |  |  |  |  |  |  |  |  |  |  |  |  |  |  |  |  |  |  |  |  |  |  |  |  |  |  |  |  |  |  |  |  |  |  |  |  |  |  |  |  |  |  |  |  |  |  |  |  |  |  |  |  |  |  |  |  |  |  |  |  |  |  |  |  |  |  |  |  |  |  |  |  |  |  |  |  |  |  |  |  |  |  |  |  |  |  |  |  |  |  |  |  |  |  |  |  |  |  |  |  |  |  |  |  |  |  |  |  |  |  |  |  |  |  |  |  |  |  |  |  |  |  |  |  |  |  |  |  |  |  |  |  |  |  |  |  |  |  |  |  |  |  |  |
|  |  |  |  |  |  |  |  |  |  |  |  |  |  |  |  |  |  |  |  |  |  |  |  |  |  |  |  |  |  |  |  |  |  |  |  |  |  |  |  |  |  |  |  |  |  |  |  |  |  |  |  |  |  |  |  |  |  |  |  |  |  |  |  |  |  |  |  |  |  |  |  |  |  |  |  |  |  |  |  |  |  |  |  |  |  |  |  |  |  |  |  |  |  |  |  |  |  |  |  |  |  |  |  |  |  |  |  |  |  |  |  |  |  |  |  |  |  |  |  |  |  |  |  |  |  |  |  |  |  |  |  |  |  |  |  |  |  |  |  |  |  |  |  |  |  |  |  |  |  |  |  |  |  |  |  |  |  |  |  |  |  |  |  |  |  |  |  |  |  |  |  |  |  |  |  |  |  |  |  |  |  |  |  |  |  |  |  |  |  |  |  |  |  |  |  |  |  |  |  |  |  |  |  |  |  |  |  |  |  |  |  |  |  |  |  |  |  |  |  |  |  |  |  |  |  |  |  |  |  |  |  |  |  |  |  |  |  |  |  |  |  |  |  |  |  |  |  |  |  |  |  |  |  |  |  |  |  |  |  |  |  |  |  |  |  |  |  |  |  |  |  |  |  |  |  |  |  |  |  |  |  |  |  |  |  |  |  |  |  |  |  |  |  |  |  |  |  |  |  |  |  |  |  |  |  |  |  |  |  |  |  |  |  |  |  |  |  |  |  |  |  |  |  |  |  |  |  |  |  |  |  |  |  |  |  |  |  |  |  |  |  |  |  |  |  |  |  |  |  |  |  |  |  |  |  |  |  |  |  |  |  |  |  |  |  |  |  |  |  |  |  |  |  |  |  |  |  |  |  |  |  |  |  |  |  |  |  |  |  |  |  |  |  |  |  |  |  |  |  |  |  |  |  |  |  |  |  |  |  |  |  |  |  |  |  |  |  |  |  |  |  |  |  |  |  |  |  |  |  |  |  |  |  |  |  |  |  |  |  |  |  |  |  |  |  |  |  |  |  |  |  |  |  |  |  |  |  |  |  |  |  |  |  |  |  |  |  |  |  |  |  |  |  |  |  |  |  |  |  |  |  |  |  |  |  |  |  |  |  |  |  |  |  |  |  |  |  |  |  |  |  |  |  |  |  |  |  |  |  |  |  |  |  |  |  |  |  |  |  |  |  |  |  |  |  |  |  |  |  |  |  |  |  |  |  |  |  |  |  |  |  |  |  |  |  |  |  |  |  |  |  |  |  |  |  |  |  |  |  |  |  |  |  |  |  |  |  |  |  |  |  |  |  |  |  |  |  |  |  |  |  |  |  |  |  |  |  |  |  |  |  |  |  |  |  |  |  |  |  |  |  |  |  |  |  |  |  |  |  |  |  |  |  |  |  |  |  |  |  |  |  |  |  |  |  |  |  |  |  |  |  |  |  |  |  |  |  |  |  |  |  |  |  |  |  |  |  |  |  |  |  |  |  |  |  |  |  |  |  |  |  |  |  |  |  |  |  |  |  |  |  |  |  |  |  |  |  |  |  |  |  |  |  |  |  |  |  |  |  |  |  |  |  |  |  |  |  |  |  |  |  |  |  |  |  |  |  |  |  |  |  |  |  |  |  |  |  |  |  |  |  |  |  |  |  |  |  |  |  |  |  |  |  |  |  |  |  |  |  |  |  |  |  |  |  |  |  |  |  |  |  |  |  |  |  |  |  |  |  |  |  |  |  |  |  |  |  |  |  |  |  |  |  |  |  |  |  |  |  |  |  |  |  |  |  |  |  |  |  |  |  |  |  |  |  |  |  |  |  |  |  |  |  |  |  |  |  |  |  |  |  |  |  |  |  |  |  |  |  |  |  |  |  |  |  |  |  |  |  |  |  |  |  |  |  |  |  |  |  |  |  |  |  |  |  |  |  |  |  |  |  |  |  |  |  |  |  |  |  |  |  |  |  |  |  |  |  |  |  |  |  |  |  |  |  |  |  |  |  |  |  |  |  |  |  |  |  |  |  |  |  |  |  |  |  |  |  |  |  |  |  |  |  |  |  |  |  |  |  |  |  |  |  |  |  |  |  |  |  |  |  |  |  |  |  |  |  |  |  |  |  |  |  |  |  |  |  |  |  |  |  |  |  |  |  |  |  |  |  |  |  |  |  |  |  |  |  |  |  |  |  |  |  |  |  |  |  |  |  |  |  |  |  |  |  |  |  |  |  |  |  |  |  |  |  |  |  |  |  |  |  |  |  |  |  |  |  |  |  |  |  |  |  |  |  |  |  |  |  |  |  |  |  |  |  |
|  |  |  |  |  |  |  |  |  |  |  |  |  |  |  |  |  |  |  |  |  |  |  |  |  |  |  |  |  |  |  |  |  |  |  |  |  |  |  |  |  |  |  |  |  |  |  |  |  |  |  |  |  |  |  |  |  |  |  |  |  |  |  |  |  |  |  |  |  |  |  |  |  |  |  |  |  |  |  |  |  |  |  |  |  |  |  |  |  |  |  |  |  |  |  |  |  |  |  |  |  |  |  |  |  |  |  |  |  |  |  |  |  |  |  |  |  |  |  |  |  |  |  |  |  |  |  |  |  |  |  |  |  |  |  |  |  |  |  |  |  |  |  |  |  |  |  |  |  |  |  |  |  |  |  |  |  |  |  |  |  |  |  |  |  |  |  |  |  |  |  |  |  |  |  |  |  |  |  |  |  |  |  |  |  |  |  |  |  |  |  |  |  |  |  |  |  |  |  |  |  |  |  |  |  |  |  |  |  |  |  |  |  |  |  |  |  |  |  |  |  |  |  |  |  |  |  |  |  |  |  |  |  |  |  |  |  |  |  |  |  |  |  |  |  |  |  |  |  |  |  |  |  |  |  |  |  |  |  |  |  |  |  |  |  |  |  |  |  |  |  |  |  |  |  |  |  |  |  |  |  |  |  |  |  |  |  |  |  |  |  |  |  |  |  |  |  |  |  |  |  |  |  |  |  |  |  |  |  |  |  |  |  |  |  |  |  |  |  |  |  |  |  |  |  |  |  |  |  |  |  |  |  |  |  |  |  |  |  |  |  |  |  |  |  |  |  |  |  |  |  |  |  |  |  |  |  |  |  |  |  |  |  |  |  |  |  |  |  |  |  |  |  |  |  |  |  |  |  |  |  |  |  |  |  |  |  |  |  |  |  |  |  |  |  |  |  |  |  |  |  |  |  |  |  |  |  |  |  |  |  |  |  |  |  |  |  |  |  |  |  |  |  |  |  |  |  |  |  |  |  |  |  |  |  |  |  |  |  |  |  |  |  |  |  |  |  |  |  |  |  |  |  |  |  |  |  |  |  |  |  |  |  |  |  |  |  |  |  |  |  |  |  |  |  |  |  |  |  |  |  |  |  |  |  |  |  |  |  |  |  |  |  |  |  |  |  |  |  |  |  |  |  |  |  |  |  |  |  |  |  |  |  |  |  |  |  |  |  |  |  |  |  |  |  |  |  |  |  |  |  |  |  |  |  |  |  |  |  |  |  |  |  |  |  |  |  |  |  |  |  |  |  |  |  |  |  |  |  |  |  |  |  |  |  |  |  |  |  |  |  |  |  |  |  |  |  |  |  |  |  |  |  |  |  |  |  |  |  |  |  |  |  |  |  |  |  |  |  |  |  |  |  |  |  |  |  |  |  |  |  |  |  |  |  |  |  |  |  |  |  |  |  |  |  |  |  |  |  |  |  |  |  |  |  |  |  |  |  |  |  |  |  |  |  |  |  |  |  |  |  |  |  |  |  |  |  |  |  |  |  |  |  |  |  |  |  |  |  |  |  |  |  |  |  |  |  |  |  |  |  |  |  |  |  |  |  |  |  |  |  |  |  |  |  |  |  |  |  |  |  |  |  |  |  |  |  |  |  |  |  |  |  |  |  |  |  |  |  |  |  |  |  |  |  |  |  |  |  |  |  |  |  |  |  |  |  |  |  |  |  |  |  |  |  |  |  |  |  |  |  |  |  |  |  |  |  |  |  |  |  |  |  |  |  |  |  |  |  |  |  |  |  |  |  |  |  |  |  |  |  |  |  |  |  |  |  |  |  |  |  |  |  |  |  |  |  |  |  |  |  |  |  |  |  |  |  |  |  |  |  |  |  |  |  |  |  |  |  |  |  |  |  |  |  |  |  |  |  |  |  |  |  |  |  |  |  |  |  |  |  |  |  |  |  |  |  |  |  |  |  |  |  |  |  |  |  |  |  |  |  |  |  |  |  |  |  |  |  |  |  |  |  |  |  |  |  |  |  |  |  |  |  |  |  |  |  |  |  |  |  |  |  |  |  |  |  |  |  |  |  |  |  |  |  |  |  |  |  |  |  |  |  |  |  |  |  |  |  |  |  |  |  |  |  |  |  |  |  |  |  |  |  |  |  |  |  |  |  |  |  |  |  |  |  |  |  |  |  |  |  |  |  |  |  |  |  |  |  |  |  |  |  |  |  |  |  |  |  |  |  |  |  |  |  |  |  |  |  |  |  |  |  |  |  |  |  |  |  |  |  |  |  |  |  |  |  |  |  |  |  |  |  |  |  |  |  |  |  |  |  |  |  |  |  |  |
|  |  |  |  |  |  |  |  |  |  |  |  |  |  |  |  |  |  |  |  |  |  |  |  |  |  |  |  |  |  |  |  |  |  |  |  |  |  |  |  |  |  |  |  |  |  |  |  |  |  |  |  |  |  |  |  |  |  |  |  |  |  |  |  |  |  |  |  |  |  |  |  |  |  |  |  |  |  |  |  |  |  |  |  |  |  |  |  |  |  |  |  |  |  |  |  |  |  |  |  |  |  |  |  |  |  |  |  |  |  |  |  |  |  |  |  |  |  |  |  |  |  |  |  |  |  |  |  |  |  |  |  |  |  |  |  |  |  |  |  |  |  |  |  |  |  |  |  |  |  |  |  |  |  |  |  |  |  |  |  |  |  |  |  |  |  |  |  |  |  |  |  |  |  |  |  |  |  |  |  |  |  |  |  |  |  |  |  |  |  |  |  |  |  |  |  |  |  |  |  |  |  |  |  |  |  |  |  |  |  |  |  |  |  |  |  |  |  |  |  |  |  |  |  |  |  |  |  |  |  |  |  |  |  |  |  |  |  |  |  |  |  |  |  |  |  |  |  |  |  |  |  |  |  |  |  |  |  |  |  |  |  |  |  |  |  |  |  |  |  |  |  |  |  |  |  |  |  |  |  |  |  |  |  |  |  |  |  |  |  |  |  |  |  |  |  |  |  |  |  |  |  |  |  |  |  |  |  |  |  |  |  |  |  |  |  |  |  |  |  |  |  |  |  |  |  |  |  |  |  |  |  |  |  |  |  |  |  |  |  |  |  |  |  |  |  |  |  |  |  |  |  |  |  |  |  |  |  |  |  |  |  |  |  |  |  |  |  |  |  |  |  |  |  |  |  |  |  |  |  |  |  |  |  |  |  |  |  |  |  |  |  |  |  |  |  |  |  |  |  |  |  |  |  |  |  |  |  |  |  |  |  |  |  |  |  |  |  |  |  |  |  |  |  |  |  |  |  |  |  |  |  |  |  |  |  |  |  |  |  |  |  |  |  |  |  |  |  |  |  |  |  |  |  |  |  |  |  |  |  |  |  |  |  |  |  |  |  |  |  |  |  |  |  |  |  |  |  |  |  |  |  |  |  |  |  |  |  |  |  |  |  |  |  |  |  |  |  |  |  |  |  |  |  |  |  |  |  |  |  |  |  |  |  |  |  |  |  |  |  |  |  |  |  |  |  |  |  |  |  |  |  |  |  |  |  |  |  |  |  |  |  |  |  |  |  |  |  |  |  |  |  |  |  |  |  |  |  |  |  |  |  |  |  |  |  |  |  |  |  |  |  |  |  |  |  |  |  |  |  |  |  |  |  |  |  |  |  |  |  |  |  |  |  |  |  |  |  |  |  |  |  |  |  |  |  |  |  |  |  |  |  |  |  |  |  |  |  |  |  |  |  |  |  |  |  |  |  |  |  |  |  |  |  |  |  |  |  |  |  |  |  |  |  |  |  |  |  |  |  |  |  |  |  |  |  |  |  |  |  |  |  |  |  |  |  |  |  |  |  |  |  |  |  |  |  |  |  |  |  |  |  |  |  |  |  |  |  |  |  |  |  |  |  |  |  |  |  |  |  |  |  |  |  |  |  |  |  |  |  |  |  |  |  |  |  |  |  |  |  |  |  |  |  |  |  |  |  |  |  |  |  |  |  |  |  |  |  |  |  |  |  |  |  |  |  |  |  |  |  |  |  |  |  |  |  |  |  |  |  |  |  |  |  |  |  |  |  |  |  |  |  |  |  |  |  |  |  |  |  |  |  |  |  |  |  |  |  |  |  |  |  |  |  |  |  |  |  |  |  |  |  |  |  |  |  |  |  |  |  |  |  |  |  |  |  |  |  |  |  |  |  |  |  |  |  |  |  |  |  |  |  |  |  |  |  |  |  |  |  |  |  |  |  |  |  |  |  |  |  |  |  |  |  |  |  |  |  |  |  |  |  |  |  |  |  |  |  |  |  |  |  |  |  |  |  |  |  |  |  |  |  |  |  |  |  |  |  |  |  |  |  |  |  |  |  |  |  |  |  |  |  |  |  |  |  |  |  |  |  |  |  |  |  |  |  |  |  |  |  |  |  |  |  |  |  |  |  |  |  |  |  |  |  |  |  |  |  |  |  |  |  |  |  |  |  |  |  |  |  |  |  |  |  |  |  |  |  |  |  |  |  |  |  |  |  |  |  |  |  |  |  |  |  |  |  |  |  |  |  |  |  |  |  |  |  |  |  |  |  |  |  |  |  |  |  |  |  |  |  |  |  |  |  |  |  |  |  |  |  |  |  |  |  |  |  |
|  |  |  |  |  |  |  |  |  |  |  |  |  |  |  |  |  |  |  |  |  |  |  |  |  |  |  |  |  |  |  |  |  |  |  |  |  |  |  |  |  |  |  |  |  |  |  |  |  |  |  |  |  |  |  |  |  |  |  |  |  |  |  |  |  |  |  |  |  |  |  |  |  |  |  |  |  |  |  |  |  |  |  |  |  |  |  |  |  |  |  |  |  |  |  |  |  |  |  |  |  |  |  |  |  |  |  |  |  |  |  |  |  |  |  |  |  |  |  |  |  |  |  |  |  |  |  |  |  |  |  |  |  |  |  |  |  |  |  |  |  |  |  |  |  |  |  |  |  |  |  |  |  |  |  |  |  |  |  |  |  |  |  |  |  |  |  |  |  |  |  |  |  |  |  |  |  |  |  |  |  |  |  |  |  |  |  |  |  |  |  |  |  |  |  |  |  |  |  |  |  |  |  |  |  |  |  |  |  |  |  |  |  |  |  |  |  |  |  |  |  |  |  |  |  |  |  |  |  |  |  |  |  |  |  |  |  |  |  |  |  |  |  |  |  |  |  |  |  |  |  |  |  |  |  |  |  |  |  |  |  |  |  |  |  |  |  |  |  |  |  |  |  |  |  |  |  |  |  |  |  |  |  |  |  |  |  |  |  |  |  |  |  |  |  |  |  |  |  |  |  |  |  |  |  |  |  |  |  |  |  |  |  |  |  |  |  |  |  |  |  |  |  |  |  |  |  |  |  |  |  |  |  |  |  |  |  |  |  |  |  |  |  |  |  |  |  |  |  |  |  |  |  |  |  |  |  |  |  |  |  |  |  |  |  |  |  |  |  |  |  |  |  |  |  |  |  |  |  |  |  |  |  |  |  |  |  |  |  |  |  |  |  |  |  |  |  |  |  |  |  |  |  |  |  |  |  |  |  |  |  |  |  |  |  |  |  |  |  |  |  |  |  |  |  |  |  |  |  |  |  |  |  |  |  |  |  |  |  |  |  |  |  |  |  |  |  |  |  |  |  |  |  |  |  |  |  |  |  |  |  |  |  |  |  |  |  |  |  |  |  |  |  |  |  |  |  |  |  |  |  |  |  |  |  |  |  |  |  |  |  |  |  |  |  |  |  |  |  |  |  |  |  |  |  |  |  |  |  |  |  |  |  |  |  |  |  |  |  |  |  |  |  |  |  |  |  |  |  |  |  |  |  |  |  |  |  |  |  |  |  |  |  |  |  |  |  |  |  |  |  |  |  |  |  |  |  |  |  |  |  |  |  |  |  |  |  |  |  |  |  |  |  |  |  |  |  |  |  |  |  |  |  |  |  |  |  |  |  |  |  |  |  |  |  |  |  |  |  |  |  |  |  |  |  |  |  |  |  |  |  |  |  |  |  |  |  |  |  |  |  |  |  |  |  |  |  |  |  |  |  |  |  |  |  |  |  |  |  |  |  |  |  |  |  |  |  |  |  |  |  |  |  |  |  |  |  |  |  |  |  |  |  |  |  |  |  |  |  |  |  |  |  |  |  |  |  |  |  |  |  |  |  |  |  |  |  |  |  |  |  |  |  |  |  |  |  |  |  |  |  |  |  |  |  |  |  |  |  |  |  |  |  |  |  |  |  |  |  |  |  |  |  |  |  |  |  |  |  |  |  |  |  |  |  |  |  |  |  |  |  |  |  |  |  |  |  |  |  |  |  |  |  |  |  |  |  |  |  |  |  |  |  |  |  |  |  |  |  |  |  |  |  |  |  |  |  |  |  |  |  |  |  |  |  |  |  |  |  |  |  |  |  |  |  |  |  |  |  |  |  |  |  |  |  |  |  |  |  |  |  |  |  |  |  |  |  |  |  |  |  |  |  |  |  |  |  |  |  |  |  |  |  |  |  |  |  |  |  |  |  |  |  |  |  |  |  |  |  |  |  |  |  |  |  |  |  |  |  |  |  |  |  |  |  |  |  |  |  |  |  |  |  |  |  |  |  |  |  |  |  |  |  |  |  |  |  |  |  |  |  |  |  |  |  |  |  |  |  |  |  |  |  |  |  |  |  |  |  |  |  |  |  |  |  |  |  |  |  |  |  |  |  |  |  |  |  |  |  |  |  |  |  |  |  |  |  |  |  |  |  |  |  |  |  |  |  |  |  |  |  |  |  |  |  |  |  |  |  |  |  |  |  |  |  |  |  |  |  |  |  |  |  |  |  |  |  |  |  |  |  |  |  |  |  |  |  |  |  |  |  |  |  |  |  |  |  |  |  |  |  |  |  |  |  |  |  |  |  |  |  |  |  |  |  |  |
|  |  |  |  |  |  |  |  |  |  |  |  |  |  |  |  |  |  |  |  |  |  |  |  |  |  |  |  |  |  |  |  |  |  |  |  |  |  |  |  |  |  |  |  |  |  |  |  |  |  |  |  |  |  |  |  |  |  |  |  |  |  |  |  |  |  |  |  |  |  |  |  |  |  |  |  |  |  |  |  |  |  |  |  |  |  |  |  |  |  |  |  |  |  |  |  |  |  |  |  |  |  |  |  |  |  |  |  |  |  |  |  |  |  |  |  |  |  |  |  |  |  |  |  |  |  |  |  |  |  |  |  |  |  |  |  |  |  |  |  |  |  |  |  |  |  |  |  |  |  |  |  |  |  |  |  |  |  |  |  |  |  |  |  |  |  |  |  |  |  |  |  |  |  |  |  |  |  |  |  |  |  |  |  |  |  |  |  |  |  |  |  |  |  |  |  |  |  |  |  |  |  |  |  |  |  |  |  |  |  |  |  |  |  |  |  |  |  |  |  |  |  |  |  |  |  |  |  |  |  |  |  |  |  |  |  |  |  |  |  |  |  |  |  |  |  |  |  |  |  |  |  |  |  |  |  |  |  |  |  |  |  |  |  |  |  |  |  |  |  |  |  |  |  |  |  |  |  |  |  |  |  |  |  |  |  |  |  |  |  |  |  |  |  |  |  |  |  |  |  |  |  |  |  |  |  |  |  |  |  |  |  |  |  |  |  |  |  |  |  |  |  |  |  |  |  |  |  |  |  |  |  |  |  |  |  |  |  |  |  |  |  |  |  |  |  |  |  |  |  |  |  |  |  |  |  |  |  |  |  |  |  |  |  |  |  |  |  |  |  |  |  |  |  |  |  |  |  |  |  |  |  |  |  |  |  |  |  |  |  |  |  |  |  |  |  |  |  |  |  |  |  |  |  |  |  |  |  |  |  |  |  |  |  |  |  |  |  |  |  |  |  |  |  |  |  |  |  |  |  |  |  |  |  |  |  |  |  |  |  |  |  |  |  |  |  |  |  |  |  |  |  |  |  |  |  |  |  |  |  |  |  |  |  |  |  |  |  |  |  |  |  |  |  |  |  |  |  |  |  |  |  |  |  |  |  |  |  |  |  |  |  |  |  |  |  |  |  |  |  |  |  |  |  |  |  |  |  |  |  |  |  |  |  |  |  |  |  |  |  |  |  |  |  |  |  |  |  |  |  |  |  |  |  |  |  |  |  |  |  |  |  |  |  |  |  |  |  |  |  |  |  |  |  |  |  |  |  |  |  |  |  |  |  |  |  |  |  |  |  |  |  |  |  |  |  |  |  |  |  |  |  |  |  |  |  |  |  |  |  |  |  |  |  |  |  |  |  |  |  |  |  |  |  |  |  |  |  |  |  |  |  |  |  |  |  |  |  |  |  |  |  |  |  |  |  |  |  |  |  |  |  |  |  |  |  |  |  |  |  |  |  |  |  |  |  |  |  |  |  |  |  |  |  |  |  |  |  |  |  |  |  |  |  |  |  |  |  |  |  |  |  |  |  |  |  |  |  |  |  |  |  |  |  |  |  |  |  |  |  |  |  |  |  |  |  |  |  |  |  |  |  |  |  |  |  |  |  |  |  |  |  |  |  |  |  |  |  |  |  |  |  |  |  |  |  |  |  |  |  |  |  |  |  |  |  |  |  |  |  |  |  |  |  |  |  |  |  |  |  |  |  |  |  |  |  |  |  |  |  |  |  |  |  |  |  |  |  |  |  |  |  |  |  |  |  |  |  |  |  |  |  |  |  |  |  |  |  |  |  |  |  |  |  |  |  |  |  |  |  |  |  |  |  |  |  |  |  |  |  |  |  |  |  |  |  |  |  |  |  |  |  |  |  |  |  |  |  |  |  |  |  |  |  |  |  |  |  |  |  |  |  |  |  |  |  |  |  |  |  |  |  |  |  |  |  |  |  |  |  |  |  |  |  |  |  |  |  |  |  |  |  |  |  |  |  |  |  |  |  |  |  |  |  |  |  |  |  |  |  |  |  |  |  |  |  |  |  |  |  |  |  |  |  |  |  |  |  |  |  |  |  |  |  |  |  |  |  |  |  |  |  |  |  |  |  |  |  |  |  |  |  |  |  |  |  |  |  |  |  |  |  |  |  |  |  |  |  |  |  |  |  |  |  |  |  |  |  |  |  |  |  |  |  |  |  |  |  |  |  |  |  |  |  |  |  |  |  |  |  |  |  |  |  |  |  |  |  |  |  |  |  |  |  |  |  |  |  |  |  |  |  |  |  |  |  |  |  |  |  |  |  |  |  |  |  |
| BIS d=0 cl.14 sym=1 env=1 | - | - | - | - | - | - | - | - | - | - | - | - | - | - | - | - | - | - | - | - | - | - | - | - | - | - | - | - | - | - | - | - | - | - | - | - | - | - | - | - | - | - | - | - | - | - | - | - | - | - | - | - | - | - | - | - | - | - | - | - | - | - | - | - | - | - | - | - | - | - | - | - | - | - | - | - | - | - | - | - | - | - | - | - | - | - | - | - | - | - | - | - | - | - | - | - | - | - | - | - | - | - | - | - | - | - | - | - | - | - | - | - | - | - | - | - | - | - | - | - | - | - | - | - | - | - | - | - | - | - | - | - | - | - | - | - | - | - | - | - | - | - | - | - | - | - | - | - | - | - | - | - | - | - | - | - | - | - | - | - | - | - | - | - | - | - | - | - | - | - | - | - | - | - | - | - | - | - | - | - | - | - | - | - | - | - | - | - | - | - | - | - | - | - | - | - | - | - | - | - | - | - | - | - | - | - | - | - | - | - | - | - | - | - | - | - | - | - | - | - | - | - | - | - | - | - | - | - | - | - | - | - | - | - | - | - | - | - | - | - | - | - | - | - | - | - | - | - | - | - | - | - | - | - | - | - | - | - | - | - | - | - | - | - | - | - | - | - | - | - | - | - | - | - | - | - | - | - | - | - | - | - | - | - | - | - | - | - | - | - | - | - | - | - | - | - | - | - | - | - | H | H | - | - | - | - | - | - | - | - | - | - | - | - | - | - | - | - | - | - | - | - | - | - | - | - | - | - | - | - | - | - | - | - | - | - | - | - | - | - | - | - | - | - | - | - | - | - | - | - | - | - | - | - | - | - | - | - | - | - | - | - | - | - | - | - | - | - | - | - | - | - | - | - | - | - | - | - | - | - | - | - | - | - | - | - | - | - | - | - | - | - | - | - | - | - | - | - | - | - | - | - | - | - | - | - | - | - | - | - | - | - | - | - | - | - | - | - | - | - | - | - | - | - | - | - | - | - | - | - | - | - | - | - | - | - | - | - | - | - | - | - | - | - | - | - | - | - | - | - | - | - | - | - | - | - | - | - | - | - | - | - | - | - | - | - | - | - | - | - | - | - | - | - | - | - | - | - | - | - | - | - | - | - | - | - | - | - | - | - | - | - | - | - | - | - | - | - | - | - | - | - | - | - | - | - | - | - | - | - | - | - | - | - | - | - | - | - | - | - | - | - | - | - | - | - | - | - | - | - | - | - | - | - | - | - | - | - | - | - | - | - | - | - | - | - | - | - | - | - | - | - | - | - | - | - | - | - | - | - | - | - | - | - | - | - | - | - | - | - | - | - | - | - | - | - | - | - | - | - | - | - | - | - | - | - | - | - | - | - | - | - | - | - | - | - | - | - | - | - | - | - | - | - | - | - | - | - | - | - | - | - | - | - | - | - | - | - | - | - | - | - | - | - | - | - | - | - | - | - | - | - | - | - | - | - | - | - | - | - | - | - | - | - | - | - | - | - | - | - | - | - | - | - | - | - | - | - | - | - | - | - | - | - | - | - | - | - | - | - | - | - | - | - | - | - | - | - | - | - | - | - | - | - | - | - | - | - | - | - | - | - | - | - | - | - | - | - | - | - | - | - | - | - | - | - | - | - | - | - | - | - | - | - | - | - | - | - | - | - | - | - | - | - | - | - | - | - | - | - | - | - | - | - | - | - | - | - | - | - | - | - | - | - | - | - | - | - | - | - | - | - | - | - | - | - | - | - | - | - | - | - | - | - | - | - | - | - | - | - | - | - | - | - | - | - | - | - | - | - | - | - | - | - | - | - | - | - | - | - | - | - | - | - | - | - | - | - | - | - | - | - | - | - | - | - | - | - | - | - | - | - | - | - | - | - | - | - | - | - | - | - | - | - | - | - | - | - | - | - | - | - | - | - | - | - | - | - | - | - | - | - | - | - | - | - | - | - | - | - | - | - | - | - | - | - | - | - | - | - | - | - | - | - | - | - | - | - | - | - | - | - | - | - | - | - | - | - | - | - | - | - | - | - | - | - | - | - | - | - | - | - | - | - | - | - | - | - | - | - | - | - | - | - | - | - | - | - | - | - | - | - | - | - | - | - | - | - | - | - | - | - | - | - | - | - | - | - | - | - | - | - | - | - | - | - | - | - | - | - | - | - | - | - | - | - | - | - | - | - | - | - | - | - | - | - | - | - | - | - | - | - | - | - | - | - | - | - | - | - | - | - | - | - | - | - | - | - | - | - | - | - | - | - | - | - | - | - | - | - | - | - | - | - | - | - | - | - | - | - | - | - | - | - | - | - | - | - | - | - | - | - | - | - | - |
| BIS d=0 cl.15 sym=1 env=1 | - | - | - | - | - | - | - | - | - | - | - | - | - | - | - | - | - | - | - | - | - | - | - | - | - | - | - | - | - | - | - | - | - | - | - | - | - | - | - | - | - | - | - | - | - | - | - | - | - | - | - | - | - | - | - | - | - | - | - | - | - | - | - | - | - | - | - | - | - | - | - | - | - | - | - | - | - | - | - | - | - | - | - | - | - | - | - | - | - | - | - | - | - | - | - | - | - | - | - | - | - | - | - | - | - | - | - | - | - | - | - | - | - | - | - | - | - | - | - | - | - | - | - | - | - | - | - | - | - | - | - | - | - | - | - | - | - | - | - | - | - | - | - | - | - | - | H | x | x | x | x | x | x | x | x | x | x | x | x | x | x | x | x | x | x | x | x | x | x | x | x | x | x | x | x | x | x | x | x | x | x | x | x | x | x | x | x | x | x | x | x | x | x | x | x | x | x | x | x | x | x | x | x | x | x | x | x | x | x | x | x | x | x | x | x | x | x | x | x | x | x | x | x | x | x | x | x | x | x | x | x | x | x | x | x | x | x | x | x | x | x | x | x | x | x | x | x | x | x | x | x | x | x | x | x | x | x | x | x | x | x | x | x | x | x | x | x | x | x | x | x | x | x | x | x | x | x | x | x | x | x | x | x | x | x | x | x | x | x | x | x | x | x | x | x | x | x | x | x | x | x | x | x | x | x | x | x | x | x | x | x | x | x | x | x | x | x | x | x | x | x | x | x | x | x | x | x | x | x | x | x | x | x | x | x | x | x | x | x | x | x | x | x | x | x | x | x | x | x | x | x | x | x | x | x | x | x | x | x | x | x | x | x | x | x | x | x | x | x | x | x | x | x | x | x | x | x | x | x | x | x | x | x | x | x | x | x | x | x | x | x | x | x | x | x | x | x | x | x | x | x | x | x | x | x | x | x | x | x | x | x | x | x | x | x | x | x | x | x | x | x | x | x | x | x | x | x | x | x | x | x | x | x | x | x | x | x | x | x | x | x | x | x | x | x | x | x | x | x | x | x | x | x | x | x | x | x | x | x | x | x | x | x | x | x | x | x | x | x | x | x | x | x | x | x | x | x | x | x | x | x | x | x | x | x | x | x | x | x | x | x | x | x | x | x | x | x | x | x | x | x | x | x | x | x | x | x | x | x | x | x | x | x | x | x | x | x | x | x | x | x | x | x | x | x | x | x | x | x | x | x | x | x | x | x | x | x | x | x | x | x | x | x | x | x | x | x | x | x | x | x | x | x | x | x | x | x | x | x | x | x | x | x | x | x | x | x | x | x | x | x | x | x | x | x | x | x | x | x | x | x | x | x | x | x | x | x | x | x | x | x | x | x | x | x | x | x | x | x | x | x | x | x | x | x | x | x | x | x | x | x | x | x | x | x | x | x | x | x | x | x | x | x | x | x | x | x | x | x | x | x | x | x | x | x | x | x | x | x | x | x | x | x | x | x | x | x | x | x | x | x | x | x | x | x | x | x | x | x | x | x | x | x | x | x | x | x | x | x | x | x | x | x | x | x | x | x | x | x | x | x | x | x | x | x | x | x | x | x | x | x | x | x | x | x | x | x | x | x | x | x | x | x | x | x | x | x | x | x | x | x | x | x | x | x | x | x | x | x | x | x | x | x | x | x | x | x | x | x | x | x | x | x | x | x | x | x | x | x | x | x | x | x | x | x | x | x | x | x | x | x | x | x | x | x | x | x | x | x | x | H | - | - | - | - | - | - | - | - | - | - | - | - | - | - | - | - | - | - | - | - | - | - | - | - | - | - | - | - | - | - | - | - | - | - | - | - | - | - | - | - | - | - | - | - | - | - | - | - | - | - | - | - | - | - | - | - | - | - | - | - | - | - | - | - | - | - | - | - | - | - | - | - | - | - | - | - | - | - | - | - | - | - | - | - | - | - | - | - | - | - | - | - | - | - | - | - | - | - | - | - | - | - | - | - | - | - | - | - | - | - | - | - | - | - | - | - | - | - | - | - | - | - | - | - | - | - | - | - | - | - | - | - | - | - | - | - | - | - | - | - | - | - | - | - | - | - | - | - | - | - | - | - | - | - | - | - | - | - | - | - | - | - | - | - | - | - | - | - | - | - | - | - | - | - | - | - | - | - | - | - | - | - | - | - | - | - | - | - | - | - | - | - | - | - | - | - | - | - | - | - | - | - | - | - | - | - | - | - | - | - | - | - | - | - | - | - | - | - | - | - | - | - | - | - | - | - | - | - | - | - | - | - | - | - | - | - | - | - | - | - | - | - | - | - | - | - | - | - | - | - | - | - | - | - |
| BIS d=0 cl.9 sym=1 env=1 | - | - | - | - | - | - | - | - | - | - | - | - | - | - | - | - | - | - | - | - | - | - | - | - | - | - | - | - | - | - | - | - | - | - | - | - | - | - | - | - | - | - | - | - | - | - | - | - | - | - | - | - | - | - | - | - | - | - | - | - | - | - | - | - | - | - | - | - | - | - | - | - | - | - | - | - | - | - | - | - | - | - | - | - | - | - | - | - | - | - | - | - | - | - | - | - | - | - | - | - | - | - | - | - | - | - | - | - | - | - | - | - | - | - | - | - | - | - | - | - | - | - | - | - | - | - | - | - | - | - | - | - | - | - | - | - | - | - | - | - | - | - | - | - | - | - | - | - | - | - | - | - | - | - | - | - | - | - | - | - | - | - | - | - | - | - | - | - | - | - | - | - | - | - | - | - | - | - | - | - | - | - | - | - | H | x | x | x | x | x | x | x | x | x | x | x | x | x | x | H | x | x | x | x | x | x | x | x | x | x | x | x | x | x | x | x | x | x | x | x | x | x | x | x | x | x | x | x | x | x | x | x | x | x | x | x | x | x | x | x | x | x | x | x | x | x | x | x | x | x | x | x | x | x | x | x | x | x | x | x | x | x | x | x | x | x | x | x | x | x | x | x | x | x | x | x | x | x | x | x | x | x | x | x | x | x | x | x | x | x | x | x | x | x | x | x | x | x | x | x | x | x | x | x | x | x | x | x | x | x | x | x | x | x | x | x | x | x | x | x | x | x | x | x | x | x | x | x | x | x | x | x | x | x | x | x | x | x | x | x | x | x | x | x | x | x | x | x | x | x | x | x | x | x | x | x | x | x | x | x | x | x | x | x | x | x | x | x | x | x | x | x | x | x | x | x | x | x | x | x | x | x | x | x | x | x | x | x | x | x | x | x | x | x | x | x | x | x | x | x | x | x | x | x | x | x | x | x | x | x | x | x | x | x | x | x | x | x | x | x | x | x | x | x | x | x | x | x | x | x | x | x | x | x | x | x | x | x | x | x | x | x | x | x | x | x | x | x | x | x | x | x | x | x | x | x | x | x | x | x | x | x | x | x | x | x | x | x | x | x | x | x | x | x | x | x | H | x | x | x | x | x | x | x | x | x | x | x | x | x | x | x | x | x | x | x | x | x | x | x | x | x | x | x | x | x | x | x | x | x | x | x | x | x | x | x | x | x | x | x | x | x | x | x | x | x | x | x | x | x | x | x | x | x | x | x | x | x | x | x | x | x | x | x | x | x | x | x | x | x | x | x | x | x | x | x | x | x | x | x | x | x | x | x | x | x | x | x | x | x | x | x | x | x | x | x | x | x | x | x | x | x | x | x | x | x | x | x | x | x | x | x | x | x | x | x | x | x | x | x | x | x | x | x | x | x | x | x | x | x | x | x | x | x | x | x | x | x | x | x | x | x | x | x | x | x | x | H | x | x | x | x | x | x | x | x | x | x | x | x | x | x | x | x | x | x | x | x | x | x | x | x | x | x | x | x | x | x | x | x | x | x | x | x | x | x | x | x | x | x | x | x | x | x | x | x | x | x | x | x | x | x | x | x | x | x | x | x | x | x | x | x | x | x | x | x | x | x | x | x | x | x | x | x | x | x | x | x | x | x | x | x | x | x | x | x | x | x | x | x | x | x | x | x | x | x | x | x | x | x | x | x | x | x | x | x | x | x | x | x | x | x | x | x | x | x | x | x | x | x | x | x | x | x | x | x | x | x | x | x | x | x | x | x | x | x | x | x | x | x | x | x | x | x | x | x | x | x | x | x | x | x | x | x | x | x | x | x | x | x | x | x | x | x | x | x | x | x | x | x | x | x | x | x | x | x | x | x | x | x | x | x | x | x | x | x | x | x | H | x | x | x | x | x | x | x | x | x | x | x | x | x | x | x | x | x | x | x | x | x | x | x | x | x | x | x | x | x | x | x | x | x | x | x | x | x | x | x | x | x | x | x | x | x | x | x | x | x | x | x | x | x | x | x | x | x | x | x | x | x | x | x | x | x | x | x | x | x | x | x | x | x | x | x | x | H | x | x | x | x | x | x | x | x | x | x | x | x | x | x | x | x | x | x | x | x | x | x | x | x | x | x | x | x | x | x | x | x | x | x | x | x | x | x | x | x | x | x | x | x | x | x | x | x | x | x | x | x | x | x | x | x | x | x | x | x | x | x | x | x | x | x | x | x | x | x | x | x | x | x | x | x | x | x | x | x | x | x | H | - | - | - | - | - | - | - | - | - | - | - | - | - | - | - | - | - | - | - | - | - | - | - | - | - | - | - | - | - | - | - | - | - | - | - | - |
| BIS d=0 cl.13 sym=1 env=1 | - | - | - | - | - | - | - | - | - | - | - | - | - | - | - | - | - | - | - | - | - | - | - | - | - | - | - | - | - | - | - | - | - | - | - | - | - | - | - | - | - | - | - | - | - | - | - | - | - | - | - | - | - | - | - | - | - | - | - | - | - | - | - | - | - | - | - | - | - | - | - | - | - | - | - | - | - | - | - | H | x | x | x | x | x | x | x | x | x | x | x | x | x | x | x | x | x | x | x | x | x | x | x | x | x | x | x | x | x | x | x | x | x | x | x | x | x | x | x | x | x | x | x | x | x | x | x | x | x | x | x | x | x | x | x | x | x | x | x | x | x | x | x | x | x | x | x | x | x | x | x | x | x | x | x | x | x | x | x | x | x | x | x | x | x | x | x | x | x | x | x | x | x | x | x | x | x | x | x | x | x | x | x | x | x | x | x | x | x | x | x | x | x | x | x | x | x | x | x | x | H | x | x | x | x | x | x | x | x | x | x | x | x | x | x | x | x | x | x | x | x | x | x | x | x | x | x | x | x | x | x | x | x | x | x | x | x | x | x | x | H | x | x | x | x | x | x | x | x | x | x | x | x | x | x | x | x | x | x | x | x | x | x | x | x | x | x | x | x | x | x | x | x | x | x | x | x | x | x | x | x | x | x | x | x | x | x | x | x | x | x | x | x | x | x | x | x | x | x | x | x | x | x | x | x | x | x | x | x | x | x | x | x | x | x | x | x | x | x | x | x | x | x | x | x | x | x | x | x | x | x | x | x | x | x | x | x | x | x | x | x | x | x | x | x | x | x | x | x | x | x | x | x | x | x | x | x | x | x | x | x | H | x | x | x | x | x | x | x | x | x | x | x | x | x | x | x | x | x | x | x | x | x | x | x | x | x | x | x | x | x | x | x | x | x | x | x | x | x | x | x | x | x | x | x | x | x | x | x | x | x | x | x | x | x | x | x | x | x | x | x | x | x | x | x | x | x | x | x | x | x | x | x | x | x | x | x | x | x | x | x | x | x | x | x | x | x | x | x | x | x | x | x | x | x | x | x | x | x | x | x | x | x | x | x | x | x | x | x | x | x | x | x | x | x | x | x | x | x | x | x | x | x | x | x | x | x | x | x | x | x | x | x | x | x | x | x | x | x | x | x | x | x | x | x | x | x | x | x | x | x | x | x | x | x | x | x | x | x | x | x | x | x | x | x | x | x | x | x | x | x | x | x | x | x | x | x | x | x | x | x | x | x | x | x | x | x | x | x | x | x | x | x | x | x | x | x | x | x | x | x | x | x | x | x | x | x | x | x | x | x | x | x | x | x | x | x | x | x | x | x | x | x | x | x | x | x | x | x | x | x | x | x | x | x | x | x | x | x | x | x | x | x | x | x | x | x | x | x | x | x | x | x | x | x | x | x | x | x | x | x | x | x | x | x | x | x | x | x | x | x | x | x | x | x | x | x | x | x | x | x | x | x | H | x | x | x | x | x | x | x | x | x | x | x | x | x | x | x | x | x | x | x | x | x | x | x | x | x | x | x | x | x | x | x | x | x | x | x | x | x | x | x | x | x | x | x | x | x | x | x | x | x | x | x | x | x | x | x | x | x | x | x | x | x | x | x | x | x | x | x | x | x | x | x | x | x | x | x | x | x | x | x | x | x | x | x | x | x | x | x | x | x | x | x | x | x | x | x | x | x | x | x | x | x | x | x | x | x | x | x | x | x | x | x | x | x | x | x | x | x | x | x | x | x | x | x | x | x | x | x | x | x | x | x | x | x | x | x | x | x | x | x | x | x | x | x | x | x | x | x | x | x | x | x | x | x | x | x | x | x | x | x | x | x | H | x | x | x | x | x | x | x | x | x | x | x | x | x | x | x | x | x | x | x | x | x | x | x | x | H | - | - | - | - | - | - | - | - | - | - | - | - | - | - | - | - | - | - | - | - | - | - | - | - | - | - | - | - | - | - | - | - | - | - | - | - | - | - | - | - | - | - | - | - | - | - | - | - | - | - | - | - | - | - | - | - | - | - | - | - | - | - | - | - | - | - | - | - | - | - | - | - | - | - | - | - | - | - | - | - | - | - | - | - | - | - | - | - | - | - | - | - | - | - | - | - | - | - | - | - | - | - | - | - | - | - | - | - | - | - | - | - | - | - | - | - | - | - | - | - | - | - | - | - | - | - | - | - | - | - | - | - | - | - | - | - | - | - | - | - | - | - | - | - | - | - | - | - | - | - | - | - | - | - | - | - | - | - | - | - | - | - | - | - | - | - | - | - | - | - | - | - | - | - | - | - | - | - | - | - | - | - | - | - |
| BIS d=0 cl.12 sym=1 env=1 | - | - | - | - | - | - | - | - | - | - | - | - | - | - | - | - | - | - | - | - | - | - | - | - | - | - | - | - | - | - | - | - | - | - | - | - | - | - | - | - | - | - | - | - | - | - | - | - | - | - | - | - | - | - | - | - | - | - | - | - | - | - | - | - | - | - | - | - | - | - | - | - | - | - | - | - | - | - | - | - | - | - | - | - | - | - | - | - | - | - | - | - | - | - | - | - | - | - | - | - | - | - | - | - | - | - | - | - | - | - | - | - | - | - | - | - | - | - | - | - | - | - | - | - | - | - | - | - | - | - | - | - | - | - | - | - | - | - | - | - | - | - | - | - | - | - | - | - | - | - | - | - | - | - | - | - | - | - | - | - | - | - | - | - | - | - | - | - | - | - | - | - | - | - | - | - | - | - | - | - | - | - | - | - | - | - | - | - | - | - | - | - | - | - | - | - | - | - | - | - | - | - | - | - | - | - | - | - | - | - | - | - | - | - | - | - | - | - | - | - | - | - | - | - | - | - | - | - | - | - | - | - | - | - | - | - | - | - | - | - | - | - | - | - | - | - | - | - | - | - | - | - | - | - | - | - | - | - | - | - | - | - | - | - | - | - | - | - | - | - | - | - | - | - | - | - | - | - | - | - | - | - | - | - | - | - | - | - | - | - | - | - | - | - | - | - | - | - | - | - | - | - | - | - | - | - | - | - | - | - | - | - | - | - | - | - | - | - | - | - | - | - | - | - | - | - | - | - | - | - | - | - | - | - | - | - | - | - | - | - | - | - | - | - | - | - | - | - | - | - | - | - | - | - | - | - | - | - | - | - | - | - | - | - | - | - | - | - | - | - | - | - | - | - | - | H | x | x | x | x | x | x | x | x | x | x | x | x | x | x | x | x | x | x | x | x | x | x | x | x | x | x | x | x | x | x | x | x | x | x | x | x | x | x | x | x | x | x | x | x | x | x | x | x | x | x | x | x | x | x | x | x | x | x | x | x | x | x | x | x | x | x | x | x | x | x | x | x | x | x | x | x | x | x | x | x | x | x | x | x | x | x | x | x | x | x | x | x | x | x | x | x | x | x | x | x | x | x | x | x | x | x | x | x | x | x | x | x | x | x | x | x | x | x | x | x | x | x | x | x | x | x | x | x | x | x | x | x | x | x | x | x | x | x | x | x | x | x | x | x | x | x | x | x | x | x | x | x | x | x | x | x | x | x | x | x | x | x | x | x | x | x | x | x | x | x | x | x | x | x | x | x | x | x | x | x | x | x | x | x | x | x | x | x | x | x | x | x | x | x | x | x | x | x | x | x | x | x | x | x | x | x | x | x | x | x | x | x | x | x | x | x | x | x | x | x | x | x | x | x | x | x | x | x | x | x | x | x | x | x | x | x | x | x | x | x | x | x | x | x | x | x | x | x | x | x | x | x | x | x | x | x | x | x | x | x | x | x | x | x | x | x | x | x | x | x | x | x | x | x | x | x | x | x | x | x | x | x | x | x | x | x | x | x | x | x | x | x | x | x | x | x | x | x | x | x | x | x | x | x | x | x | x | x | x | x | x | x | x | x | x | x | x | x | x | x | x | x | x | x | x | x | x | x | x | x | x | x | x | x | x | x | x | x | x | x | x | x | x | x | x | x | x | x | x | x | x | x | x | x | x | x | x | x | x | x | x | x | x | x | x | x | x | x | x | x | x | x | x | x | x | x | x | x | x | x | x | x | x | x | x | x | x | x | x | x | x | H | - | - | - | - | - | - | - | - | - | - | - | - | - | - | - | - | - | - | - | - | - | - | - | - | - | - | - | - | - | - | - | - | - | - | - | - | - | - | - | - | - | - | - | - | - | - | - | - | - | - | - | - | - | - | - | - | - | - | - | - | - | - | - | - | - | - | - | - | - | - | - | - | - | - | - | - | - | - | - | - | - | - | - | - | - | - | - | - | - | - | - | - | - | - | - | - | - | - | - | - | - | - | - | - | - | - | - | - | - | - | - | - | - | - | - | - | - | - | - | - | - | - | - | - | - | - | - | - | - | - | - | - | - | - | - | - | - | - | - | - | - | - | - | - | - | - | - | - | - | - | - | - | - | - | - | - | - | - | - | - | - | - | - | - | - | - | - | - | - | - | - | - | - | - | - | - | - | - | - | - | - | - | - | - | - | - | - | - | - | - | - | - | - | - | - | - | - | - | - | - | - | - | - | - | - | - | - | - | - | - | - | - | - | - | - | - | - | - | - | - | - | - | - | - | - | - | - | - | - | - | - | - | - | - | - | - | - | - | - | - | - | - | - | - | - | - | - |
| BIS d=0 cl.2 sym=1 env=1 | - | - | - | - | - | - | - | - | - | - | - | - | - | - | - | - | - | - | - | - | - | - | - | - | - | - | - | - | - | - | - | - | - | - | - | - | - | - | - | - | - | - | - | - | - | - | - | - | - | - | - | - | - | - | - | - | - | - | - | - | - | - | - | - | - | - | - | - | - | - | - | - | - | - | - | - | - | - | - | - | - | - | - | - | - | - | - | - | - | - | - | - | - | - | - | - | - | - | - | - | - | - | - | - | - | - | - | - | - | H | x | x | x | x | x | x | x | x | x | x | x | x | x | x | x | x | x | x | x | x | x | x | x | x | x | x | x | x | x | x | x | x | x | x | x | x | x | x | x | x | x | x | x | x | x | x | x | x | x | x | x | x | x | x | x | H | x | x | x | x | x | x | x | x | x | x | x | x | x | x | x | x | x | x | x | x | x | x | x | x | x | x | x | x | x | x | x | x | x | x | x | x | x | x | x | x | x | x | x | x | x | x | x | x | x | x | x | x | x | x | x | x | x | x | x | x | x | x | x | x | x | x | x | x | x | x | x | x | x | x | x | x | x | x | x | x | x | x | x | x | x | x | x | x | x | x | x | x | x | x | x | x | x | x | x | x | x | x | x | x | x | x | x | x | x | x | x | x | x | x | x | x | x | x | x | x | x | x | x | x | x | x | x | x | x | x | x | x | x | x | x | x | x | x | x | x | x | x | x | x | x | x | x | x | x | x | x | x | x | x | x | x | x | x | x | x | x | x | x | x | x | x | x | x | x | x | x | x | x | x | x | x | x | x | x | x | x | x | H | x | x | x | x | x | x | x | x | x | x | x | x | x | x | x | x | x | x | x | x | x | x | x | x | x | x | x | x | x | x | x | x | x | x | x | x | x | x | x | x | x | x | x | x | x | x | x | x | x | x | x | x | x | x | x | x | x | x | x | x | x | x | x | x | x | x | x | x | x | x | x | x | x | x | x | x | x | x | x | x | x | x | x | x | x | x | x | x | x | x | x | x | x | x | x | x | x | x | x | x | x | x | x | x | x | x | x | x | x | x | x | x | x | x | x | x | x | x | x | x | x | x | x | x | x | x | x | x | x | x | x | x | x | x | x | x | x | x | x | x | x | x | x | x | x | x | x | x | x | x | x | x | x | x | x | x | x | x | x | x | x | x | x | x | x | x | x | x | x | x | x | x | x | x | x | x | x | x | x | x | x | x | x | H | x | x | x | x | x | x | x | x | x | x | x | x | x | x | x | x | x | x | x | x | x | x | x | x | x | x | x | x | x | x | x | x | x | x | x | x | x | x | x | x | x | x | x | x | x | x | x | x | x | x | x | x | x | x | x | x | x | x | x | x | x | x | x | x | x | x | x | x | x | x | x | x | x | x | x | x | x | x | x | x | x | x | x | x | x | x | x | x | x | x | x | x | x | x | x | x | x | x | x | x | x | x | x | x | x | x | x | x | x | x | x | x | x | x | x | x | x | x | x | x | x | x | x | x | x | x | x | x | x | x | x | x | x | x | x | x | x | x | x | x | x | x | x | x | x | x | x | x | x | x | x | x | x | x | x | x | x | x | x | x | x | x | x | x | x | x | x | x | x | x | x | x | x | x | x | x | x | x | x | x | x | x | x | x | x | x | x | x | x | x | x | x | x | x | x | x | x | x | x | x | x | x | x | x | x | x | x | x | x | x | x | x | H | x | x | x | x | x | x | x | x | x | x | x | x | x | x | x | x | x | x | H | x | x | x | x | x | x | x | x | x | x | x | x | x | x | x | x | x | x | x | x | x | x | x | x | x | x | x | x | x | x | x | x | x | x | x | x | x | x | x | x | x | x | x | x | x | x | x | x | x | x | x | x | x | x | x | x | x | x | x | x | x | x | x | x | x | x | x | x | x | x | x | x | x | x | x | x | x | x | x | x | x | x | x | x | x | x | x | x | x | x | x | x | x | x | x | x | x | x | x | x | x | x | x | x | x | x | x | x | x | x | x | x | x | x | x | x | x | x | x | x | x | H | x | x | x | x | x | x | x | x | x | x | x | x | x | x | x | x | x | x | H | x | H | - | - | - | - | - | - | - | - | - | - | - | - | - | - | - | - | - | - | - | - | - | - | - | - | - | - | - | - | - | - | - | - | - | - | - | - | - | - | - | - | - | - | - | - | - | - | - | - | - | - | - | - | - | - | - | - | - | - | - | - | - | - | - | - | - | - | - | - | - | - | - | - | - | - | - | - | - | - | - | - | - | - | - | - | - | - | - | - | - | - | - | - | - | - | - | - | - | - | - | - | - | - | - | - | - | - | - |
| BIS d=0 cl.11 sym=1 env=1 | - | - | - | - | - | - | - | - | - | - | - | - | - | - | - | - | - | - | - | - | - | - | - | - | - | - | - | - | - | - | - | - | - | - | - | - | - | - | - | - | - | - | - | - | - | - | - | - | - | - | - | - | - | - | - | - | - | - | - | - | - | - | - | - | - | - | - | - | - | - | - | - | - | - | - | - | - | - | - | - | - | - | - | - | - | - | - | - | - | - | - | - | - | - | - | - | - | - | - | - | - | - | - | - | - | - | - | - | - | - | - | - | - | - | - | - | - | - | - | - | - | - | - | - | - | - | - | - | - | - | - | - | - | - | - | - | - | - | - | - | - | - | - | - | - | - | - | - | - | - | - | - | - | - | - | - | - | - | - | - | - | - | - | - | - | - | - | - | - | - | - | - | - | - | - | - | - | - | - | - | - | - | - | - | - | - | - | - | - | - | - | - | - | - | - | - | - | - | - | - | - | - | - | - | - | - | - | - | - | - | - | - | - | - | - | - | - | - | - | - | - | - | - | - | - | - | - | - | - | - | - | - | - | - | - | - | - | - | - | - | - | - | - | - | - | - | - | - | - | - | - | - | - | - | - | - | - | - | - | - | - | - | - | - | - | - | - | - | - | - | - | - | - | - | - | - | - | - | - | - | - | - | - | - | - | - | - | - | - | - | - | - | - | - | - | - | - | - | - | - | - | - | - | - | - | - | - | - | - | - | - | - | - | - | - | - | - | - | - | - | - | - | - | - | - | - | - | - | - | - | - | - | - | - | - | - | - | - | - | - | - | - | - | - | - | - | - | - | - | - | - | - | - | - | - | - | - | - | - | - | - | - | - | - | - | - | - | - | - | - | - | - | - | - | - | - | - | - | - | - | - | - | - | - | - | - | - | - | - | - | - | - | - | - | - | - | - | - | - | - | - | - | - | - | - | - | - | - | - | - | - | - | - | - | - | - | - | - | - | - | - | - | - | - | - | H | x | x | x | x | x | x | x | x | x | x | x | x | x | x | x | x | x | x | x | x | x | x | x | x | x | x | x | x | x | x | x | x | x | H | x | x | x | x | x | x | x | x | x | x | x | x | x | x | x | x | x | x | x | x | x | x | x | x | x | x | x | x | x | x | x | x | x | x | x | x | x | x | x | x | x | x | x | x | x | x | x | x | x | x | x | x | x | x | x | x | x | x | H | x | x | x | x | x | x | x | x | x | x | x | x | x | x | x | x | x | x | x | x | x | x | x | x | x | x | x | x | x | x | x | x | x | x | x | x | x | x | x | x | x | x | x | x | x | x | x | x | x | x | x | x | x | x | x | x | x | x | x | x | x | x | x | x | H | x | x | x | x | x | x | x | x | x | x | x | x | x | x | x | x | x | x | x | x | x | x | x | x | x | x | x | x | x | x | x | x | x | x | x | x | x | x | x | x | x | x | x | x | x | x | x | x | x | x | x | x | x | x | x | x | x | x | x | x | x | x | x | x | x | x | x | x | x | x | x | x | x | x | x | x | x | x | x | x | x | x | x | x | x | x | x | x | x | x | x | x | x | x | x | x | x | x | x | x | x | x | x | x | x | x | x | x | x | x | x | x | x | x | x | x | x | x | x | x | x | x | x | x | x | x | x | x | x | x | x | x | x | x | x | x | x | x | x | x | x | x | x | x | x | H | - | - | - | - | - | - | - | - | - | - | - | - | - | - | - | - | - | - | - | - | - | - | - | - | - | - | - | - | - | - | - | - | - | - | - | - | - | - | - | - | - | - | - | - | - | - | - | - | - | - | - | - | - | - | - | - | - | - | - | - | - | - | - | - | - | - | - | - | - | - | - | - | - | - | - | - | - | - | - | - | - | - | - | - | - | - | - | - | - | - | - | - | - | - | - | - | - | - | - | - | - | - | - | - | - | - | - | - | - | - | - | - | - | - | - | - | - | - | - | - | - | - | - | - | - | - | - | - | - | - | - | - | - | - | - | - | - | - | - | - | - | - | - | - | - | - | - | - | - | - | - | - | - | - | - | - | - | - | - | - | - | - | - | - | - | - | - | - | - | - | - | - | - | - | - | - | - | - | - | - | - | - | - | - | - | - | - | - | - | - | - | - | - | - | - | - | - | - | - | - | - | - | - | - | - | - | - | - | - | - | - | - | - | - | - | - | - | - | - | - | - | - | - | - | - | - | - | - | - | - | - | - | - | - | - | - | - | - | - | - | - | - | - | - | - | - | - | - | - | - | - | - | - | - | - | - | - | - | - | - | - | - | - | - | - | - | - | - | - | - | - | - | - | - | - | - | - | - | - | - | - | - | - | - | - |
| BIS d=0 cl.6 sym=1 env=1 | - | - | - | - | - | - | - | - | - | - | - | - | - | - | - | - | - | - | - | - | - | - | - | - | - | - | - | - | - | - | - | - | - | - | - | - | - | - | - | - | - | - | - | - | - | - | - | - | - | - | - | - | - | - | - | - | - | - | - | - | - | - | - | - | - | - | - | - | - | - | - | - | - | - | - | - | - | - | - | - | - | - | - | - | - | - | - | - | - | - | - | - | - | - | - | - | - | - | - | - | - | - | - | - | - | - | - | - | - | - | - | - | - | - | - | - | - | - | - | - | - | - | - | - | - | - | - | - | - | - | - | - | - | - | - | - | - | - | - | - | - | - | - | - | - | - | - | - | - | - | - | - | - | - | - | - | - | - | - | - | - | - | - | - | - | - | - | - | - | - | - | - | - | - | - | - | - | - | - | - | - | - | - | - | - | - | - | - | - | - | - | - | - | - | - | - | - | - | - | - | - | - | - | - | - | - | - | - | - | - | - | - | - | - | - | - | - | - | - | - | - | - | - | - | - | - | - | - | - | - | - | - | - | - | - | - | - | - | - | - | - | - | - | - | - | - | - | - | - | - | - | - | - | - | - | - | - | - | - | - | - | - | - | - | - | - | - | - | - | - | - | - | - | - | - | - | - | - | - | - | - | - | - | - | - | - | - | - | - | - | - | - | - | - | - | - | - | - | - | - | - | - | - | - | - | - | - | - | - | - | - | - | - | - | - | - | - | - | - | - | - | - | - | - | - | - | - | - | - | - | - | - | - | - | - | - | - | - | - | - | - | - | - | - | - | - | - | - | - | - | - | - | - | - | - | - | - | - | - | - | - | - | - | - | - | - | - | - | - | - | - | - | - | - | - | - | - | - | - | - | - | - | - | - | - | - | - | - | - | - | - | - | - | - | - | - | - | - | - | - | - | - | - | - | - | - | - | - | - | - | - | - | H | x | x | x | x | x | x | x | x | x | x | x | x | x | x | x | x | x | x | x | x | x | x | x | x | x | x | x | x | x | x | x | x | x | x | x | x | x | x | x | x | x | x | x | x | x | x | x | x | x | x | x | x | x | x | x | x | x | x | x | x | x | x | x | x | x | x | x | x | x | x | x | x | x | x | x | x | x | x | x | x | x | x | x | x | x | x | x | x | x | x | x | x | x | x | x | x | x | x | x | x | x | x | x | x | x | x | x | x | x | x | x | x | x | x | x | x | x | x | x | x | x | x | x | x | x | x | x | x | x | x | x | x | x | x | x | x | x | x | x | x | x | x | x | x | x | x | x | x | x | x | x | x | x | x | x | x | x | x | x | x | x | x | x | x | x | x | x | x | x | x | x | x | x | x | x | x | x | x | x | x | x | x | x | x | x | x | x | x | x | x | x | x | x | x | x | x | x | x | x | x | x | x | x | x | x | x | x | x | x | x | x | x | x | x | x | x | x | x | x | x | x | x | x | x | x | x | x | x | x | x | x | x | x | x | x | x | H | - | - | - | - | - | - | - | - | - | - | - | - | - | - | - | - | - | - | - | - | - | - | - | - | - | - | - | - | - | - | - | - | - | - | - | - | - | - | - | - | - | - | - | - | - | - | - | - | - | - | - | - | - | - | - | - | - | - | - | - | - | - | - | - | - | - | - | - | - | - | - | - | - | - | - | - | - | - | - | - | - | - | - | - | - | - | - | - | - | - | - | - | - | - | - | - | - | - | - | - | - | - | - | - | - | - | - | - | - | - | - | - | - | - | - | - | - | - | - | - | - | - | - | - | - | - | - | - | - | - | - | - | - | - | - | - | - | - | - | - | - | - | - | - | - | - | - | - | - | - | - | - | - | - | - | - | - | - | - | - | - | - | - | - | - | - | - | - | - | - | - | - | - | - | - | - | - | - | - | - | - | - | - | - | - | - | - | - | - | - | - | - | - | - | - | - | - | - | - | - | - | - | - | - | - | - | - | - | - | - | - | - | - | - | - | - | - | - | - | - | - | - | - | - | - | - | - | - | - | - | - | - | - | - | - | - | - | - | - | - | - | - | - | - | - | - | - | - | - | - | - | - | - | - | - | - | - | - | - | - | - | - | - | - | - | - | - | - | - | - | - | - | - | - | - | - | - | - | - | - | - | - | - | - | - | - | - | - | - | - | - | - | - | - | - | - | - | - | - | - | - | - | - | - | - | - | - | - | - | - | - | - | - | - | - | - | - | - | - | - | - | - | - | - | - | - | - | - | - | - | - | - | - | - | - | - | - | - | - | - | - | - | - | - | - | - | - | - | - | - | - | - | - | - | - | - | - | - | - | - | - | - | - | - | - |
| BIS d=0 cl.10 sym=1 env=1 | - | - | - | - | - | - | - | - | - | - | - | - | - | - | - | - | - | - | - | - | - | - | - | - | - | - | - | - | - | - | - | - | - | - | - | - | - | - | - | - | - | - | - | - | - | - | - | - | - | - | - | - | - | - | - | - | - | - | - | - | - | - | - | - | - | - | - | - | - | - | - | - | - | - | - | - | - | - | - | - | - | - | - | - | - | - | - | - | - | - | - | - | - | - | - | - | - | - | - | - | - | - | - | - | - | - | - | - | - | - | - | - | - | - | - | - | - | - | - | - | - | - | - | - | - | - | - | - | - | - | - | - | - | - | - | - | - | - | - | - | - | - | - | - | - | - | - | - | - | - | - | - | - | - | - | - | - | - | - | - | - | - | - | - | - | - | - | - | - | - | - | - | - | - | - | - | - | - | - | - | - | - | - | - | - | - | - | - | - | - | - | - | H | x | x | x | x | x | x | x | x | x | x | x | x | x | x | x | x | x | x | x | x | x | x | x | x | x | x | x | x | x | x | x | x | x | x | x | x | x | x | x | x | x | x | x | x | x | x | x | x | x | x | x | x | x | x | x | x | x | x | x | x | x | x | x | x | x | x | x | x | x | x | x | x | x | x | x | x | x | x | x | x | x | x | x | x | x | x | x | x | x | x | x | x | x | x | x | x | x | x | x | x | x | x | x | x | x | x | x | x | x | x | x | x | x | x | x | x | x | x | x | x | x | x | x | x | x | x | x | x | x | x | x | x | x | x | x | x | x | x | x | x | x | x | x | x | x | x | x | x | x | x | x | x | x | x | x | x | x | x | x | x | x | x | x | x | x | x | x | x | x | x | x | x | x | x | x | x | x | x | x | x | x | x | x | x | x | x | x | x | x | x | x | x | x | x | x | x | x | x | x | x | x | x | x | x | x | x | x | x | x | x | x | x | x | x | x | x | x | x | x | x | x | x | x | x | x | x | x | x | x | x | x | x | x | x | x | x | x | x | x | x | x | x | x | x | x | x | x | x | x | x | x | x | x | x | x | x | x | x | x | x | x | x | x | x | x | x | x | x | x | x | x | x | x | x | x | x | x | x | x | x | x | x | x | x | x | x | x | x | x | x | x | x | x | x | x | x | x | x | x | x | x | x | x | x | x | x | x | x | x | x | x | x | x | x | x | x | x | x | x | x | x | x | x | x | x | x | x | x | x | x | x | x | x | x | x | x | x | x | x | x | x | x | x | x | x | x | x | x | x | x | x | x | x | x | x | x | x | x | x | x | x | x | x | x | x | x | x | x | x | x | x | x | x | x | x | x | x | x | x | x | x | x | x | x | x | x | x | x | x | x | x | x | x | x | x | x | x | x | x | H | - | - | - | - | - | - | - | - | - | - | - | - | - | - | - | - | - | - | - | - | - | - | - | - | - | - | - | - | - | - | - | - | - | - | - | - | - | - | - | - | - | - | - | - | - | - | - | - | - | - | - | - | - | - | - | - | - | - | - | - | - | - | - | - | - | - | - | - | - | - | - | - | - | - | - | - | - | - | - | - | - | - | - | - | - | - | - | - | - | - | - | - | - | - | - | - | - | - | - | - | - | - | - | - | - | - | - | - | - | - | - | - | - | - | - | - | - | - | - | - | - | - | - | - | - | - | - | - | - | - | - | - | - | - | - | - | - | - | - | - | - | - | - | - | - | - | - | - | - | - | - | - | - | - | - | - | - | - | - | - | - | - | - | - | - | - | - | - | - | - | - | - | - | - | - | - | - | - | - | - | - | - | - | - | - | - | - | - | - | - | - | - | - | - | - | - | - | - | - | - | - | - | - | - | - | - | - | - | - | - | - | - | - | - | - | - | - | - | - | - | - | - | - | - | - | - | - | - | - | - | - | - | - | - | - | - | - | - | - | - | - | - | - | - | - | - | - | - | - | - | - | - | - | - | - | - | - | - | - | - | - | - | - | - | - | - | - | - | - | - | - | - | - | - | - | - | - | - | - | - | - | - | - | - | - | - | - | - | - | - | - | - | - | - | - | - | - | - | - | - | - | - | - | - | - | - | - | - | - | - | - | - | - | - | - | - | - | - | - | - | - | - | - | - | - | - | - | - | - | - | - | - | - | - | - | - | - | - | - | - | - | - | - | - | - | - | - | - | - | - | - | - | - | - | - | - | - | - | - | - | - | - | - | - | - | - | - | - | - | - | - | - | - | - | - | - | - | - | - | - | - | - | - | - | - | - | - | - | - | - | - | - | - | - | - | - | - | - | - | - | - | - | - | - | - | - | - | - | - | - | - | - | - | - | - | - | - | - | - | - | - | - |
| BIS d=0 cl.5 sym=1 env=1 | - | - | - | - | - | - | - | - | - | - | - | - | - | - | - | - | - | - | - | - | - | - | - | - | - | - | - | - | - | - | - | - | - | - | - | - | - | - | - | - | - | - | - | - | - | - | - | - | - | - | - | - | - | - | - | - | - | - | - | - | - | - | - | - | - | - | - | - | - | - | - | - | - | - | - | - | - | - | - | - | - | - | - | - | - | - | - | - | - | - | - | - | - | - | - | - | - | - | - | - | - | - | - | - | - | - | - | - | - | - | - | - | - | - | - | - | - | - | - | - | - | - | - | - | - | - | - | - | - | - | - | - | - | - | - | - | - | - | - | - | - | - | - | - | - | - | - | - | - | - | - | - | - | - | - | - | - | - | - | - | - | - | - | - | - | - | - | - | - | - | - | - | - | - | - | - | - | - | - | - | - | - | - | - | - | - | - | - | - | - | - | - | - | - | - | - | - | - | - | - | - | - | - | - | - | - | - | - | - | - | - | - | - | - | - | - | - | - | - | - | - | - | - | - | - | - | - | - | - | - | - | - | - | - | - | - | - | - | - | - | - | - | - | - | - | - | - | - | - | - | - | - | - | - | - | - | - | - | - | - | - | - | - | - | - | - | - | - | - | - | - | - | - | - | - | - | - | - | - | - | - | - | - | - | - | - | - | - | - | - | - | - | - | - | - | - | - | - | - | - | - | - | - | - | - | - | - | - | - | - | - | - | - | - | - | - | - | - | - | - | - | - | - | - | - | - | - | - | - | - | - | - | - | - | - | - | - | - | - | - | - | - | - | - | - | - | - | - | - | - | - | - | - | - | - | - | - | - | - | - | - | - | - | - | - | - | - | - | - | - | - | - | - | - | - | - | - | - | - | - | - | - | - | - | - | - | - | - | - | - | - | - | - | - | - | - | - | - | - | - | - | - | - | - | - | - | - | - | - | - | - | - | - | - | - | - | - | - | - | - | - | - | - | - | - | - | - | - | - | - | - | - | - | - | - | - | - | - | - | - | - | - | - | - | - | - | - | - | - | - | - | - | - | - | - | - | - | - | - | - | - | - | - | - | - | - | - | - | - | - | - | - | - | - | - | - | - | - | - | - | - | - | - | - | - | - | - | - | - | - | - | - | - | - | - | - | - | - | - | - | - | - | - | - | - | - | - | - | - | - | - | - | - | - | - | - | - | - | - | - | - | - | - | - | - | - | - | - | - | - | - | - | - | - | - | - | - | - | - | - | - | - | - | - | - | - | - | - | - | - | - | - | - | - | - | - | - | - | - | - | - | - | - | - | - | - | - | - | - | - | - | - | - | - | - | - | - | - | - | - | - | - | - | - | - | - | - | - | - | - | - | - | - | - | - | - | - | - | - | - | - | - | - | - | - | - | - | - | - | - | - | - | - | - | - | - | - | - | - | - | - | - | - | - | - | - | - | - | - | - | - | - | - | - | - | - | - | - | H | x | x | x | x | x | x | x | x | x | x | x | x | x | x | x | x | x | x | x | x | x | x | x | x | x | x | x | x | x | x | x | x | x | x | x | x | x | x | x | x | x | x | x | x | x | x | x | x | x | x | x | x | x | x | x | x | x | x | x | x | x | x | x | x | x | x | x | x | x | H | - | - | - | - | - | - | - | - | - | - | - | - | - | - | - | - | - | - | - | - | - | - | - | - | - | - | - | - | - | - | - | - | - | - | - | - | - | - | - | - | - | - | - | - | - | - | - | - | - | - | - | - | - | - | - | - | - | - | - | - | - | - | - | - | - | - | - | - | - | - | - | - | - | - | - | - | - | - | - | - | - | - | - | - | - | - | - | - | - | - | - | - | - | - | - | - | - | - | - | - | - | - | - | - | - | - | - | - | - | - | - | - | - | - | - | - | - | - | - | - | - | - | - | - | - | - | - | - | - | - | - | - | - | - | - | - | - | - | - | - | - | - | - | - | - | - | - | - | - | - | - | - | - | - | - | - | - | - | - | - | - | - | - | - | - | - | - | - | - | - | - | - | - | - | - | - | - | - | - | - | - | - | - | - | - | - | - | - | - | - | - | - | - | - | - | - | - | - | - | - | - | - | - | - | - | - | - | - | - | - | - | - | - | - | - | - | - | - | - | - | - | - | - | - | - | - | - | - | - | - | - | - | - | - | - | - | - | - | - | - | - | - | - | - | - | - | - | - | - | - | - | - | - | - | - | - | - | - | - | - | - | - | - | - | - | - | - | - | - | - | - | - | - | - | - | - | - | - | - | - | - | - | - | - | - | - | - | - | - | - | - | - | - | - | - | - | - | - | - | - | - | - | - | - | - | - |
| BIS d=0 cl.3 sym=1 env=1 | - | - | - | - | - | - | - | - | - | - | - | - | - | - | - | - | - | - | - | - | - | - | - | - | - | - | - | - | - | - | - | - | - | - | - | - | - | - | - | - | - | - | - | - | - | - | - | - | - | - | - | - | - | - | - | - | - | - | - | - | - | - | - | - | - | - | - | - | - | - | - | - | - | - | - | - | - | - | - | - | - | - | - | - | - | - | - | - | - | - | - | - | - | - | - | - | - | - | - | - | - | - | - | - | - | - | - | - | - | - | - | - | - | - | - | - | - | - | - | - | - | - | - | - | - | - | - | - | - | - | - | - | - | - | - | - | - | - | - | - | - | - | - | - | - | - | - | - | - | - | - | - | - | - | - | - | - | - | - | - | - | - | - | - | - | - | - | - | - | - | - | - | - | - | - | - | - | - | - | - | - | - | - | - | - | - | - | - | - | - | - | - | - | - | - | - | - | - | - | - | - | - | - | - | - | - | - | - | - | - | - | - | - | - | - | - | - | - | - | - | - | - | - | - | - | - | - | - | - | - | - | - | - | - | - | - | - | - | - | - | - | - | - | - | - | - | - | - | - | - | - | - | - | - | - | - | - | - | - | - | - | - | - | - | - | - | - | - | - | - | - | - | - | - | - | - | - | - | - | - | - | - | - | - | - | - | - | - | - | - | - | - | - | - | - | - | - | - | - | - | - | - | - | - | - | - | - | - | - | - | - | - | - | - | - | - | - | - | - | - | - | - | - | - | - | - | - | - | - | - | - | - | - | - | - | - | - | - | - | - | - | - | - | - | - | - | - | - | - | - | - | - | - | - | - | - | - | - | - | - | - | - | - | - | - | - | - | - | - | - | - | - | - | - | - | - | - | - | - | - | - | - | - | - | - | - | - | - | - | - | - | - | - | - | - | - | - | - | - | H | x | x | x | x | x | x | x | x | x | x | x | x | x | x | x | x | x | x | x | x | x | x | x | x | x | x | x | x | x | x | x | x | x | x | x | x | x | x | x | x | x | x | x | x | x | x | x | x | x | x | x | x | x | x | x | x | x | x | x | x | x | x | x | x | x | x | x | x | x | x | x | x | x | x | x | x | x | x | x | x | x | x | x | x | x | x | x | x | x | x | x | x | x | x | x | x | x | x | x | x | x | x | x | x | x | x | x | x | x | x | x | x | x | x | x | x | x | x | x | x | x | x | x | x | x | x | x | x | x | x | x | x | x | x | x | x | x | x | x | x | x | x | x | x | x | x | x | x | x | x | x | x | x | x | x | x | x | x | x | x | x | x | x | x | x | x | x | x | x | x | x | x | x | x | x | x | x | x | x | x | x | x | x | x | x | x | x | x | x | x | x | x | x | x | x | x | x | x | x | x | x | x | x | x | x | x | x | x | x | x | x | x | x | x | x | x | x | x | x | x | x | x | x | x | x | x | x | x | x | x | x | x | x | x | x | x | x | x | x | x | x | x | x | x | x | x | x | x | x | x | x | x | x | x | x | x | x | x | x | x | x | x | x | x | x | x | x | x | x | x | x | x | x | x | x | x | x | x | x | x | x | x | x | x | x | x | x | x | x | x | x | x | x | x | x | x | x | x | x | x | x | x | x | x | x | x | x | x | x | x | x | x | x | x | x | x | x | x | x | x | x | x | x | x | x | x | x | x | x | x | x | x | x | x | x | x | x | x | x | x | x | x | x | x | x | x | x | x | x | x | x | x | x | x | x | x | x | x | x | x | x | x | x | x | x | x | x | x | x | x | x | x | x | x | x | x | x | x | x | x | x | x | x | x | x | x | x | x | x | x | x | x | x | x | x | x | x | x | x | x | x | x | x | x | x | x | x | x | x | x | x | x | x | x | x | x | x | x | x | x | x | x | x | x | x | x | x | x | x | x | x | x | x | x | x | x | x | x | x | x | x | x | x | x | x | x | x | x | x | x | x | x | x | x | x | x | x | x | x | x | x | x | H | - | - | - | - | - | - | - | - | - | - | - | - | - | - | - | - | - | - | - | - | - | - | - | - | - | - | - | - | - | - | - | - | - | - | - | - | - | - | - | - | - | - | - | - | - | - | - | - | - | - | - | - | - | - | - | - | - | - | - | - | - | - | - | - | - | - | - | - | - | - | - | - | - | - | - | - | - | - | - | - | - | - | - | - | - | - | - | - | - | - | - | - | - | - | - | - | - | - | - | - | - | - | - | - | - | - | - | - | - | - | - | - | - | - | - | - | - | - | - | - | - | - | - | - | - | - | - | - | - | - | - | - | - | - | - | - | - | - | - | - | - | - | - | - | - | - | - | - | - | - | - | - |
| BIS d=0 cl.4 sym=1 env=1 | - | - | - | - | - | - | - | - | - | - | - | - | - | - | - | - | - | - | - | - | - | - | - | - | - | - | - | - | - | - | - | - | - | - | - | - | - | - | - | - | - | - | - | - | - | - | - | - | - | - | - | - | - | - | - | - | - | - | - | - | - | - | - | - | - | - | - | - | - | - | - | - | - | - | - | - | - | - | - | - | - | - | - | - | - | - | - | - | - | - | - | - | - | - | - | - | - | - | - | - | - | - | - | - | - | - | - | - | - | - | - | - | - | - | - | - | - | - | - | - | - | - | - | - | - | - | - | - | - | - | - | - | - | - | - | - | H | x | x | H | x | x | x | x | x | x | x | x | x | x | x | x | x | x | x | x | x | x | x | x | x | x | x | x | x | x | x | x | x | x | x | x | x | x | x | x | x | x | x | x | x | x | x | x | x | x | x | x | x | x | x | x | x | x | x | x | x | x | H | x | x | x | x | x | x | x | x | x | x | x | x | x | x | x | x | x | x | x | x | x | x | x | x | x | x | x | x | x | x | x | x | x | x | x | x | x | x | x | x | x | x | x | x | x | x | x | x | x | x | x | x | x | x | x | x | x | x | x | x | x | x | x | x | x | x | x | x | x | x | x | x | x | x | x | x | x | x | x | x | x | x | x | x | x | x | x | x | x | x | x | x | x | x | x | x | x | x | x | x | x | x | x | x | x | x | x | x | x | x | x | x | x | x | x | x | x | x | x | x | x | x | x | x | x | x | x | x | x | x | x | x | x | x | x | x | x | x | x | x | x | x | x | x | x | x | x | x | x | x | x | x | x | x | x | x | x | x | x | x | x | x | x | x | x | x | x | x | x | x | x | x | x | x | x | x | x | x | x | x | x | x | x | x | x | x | x | x | x | x | x | x | x | x | x | x | x | x | x | x | x | x | x | x | x | x | x | x | x | x | x | x | x | x | x | x | x | x | x | x | x | x | x | x | x | x | x | x | x | x | x | x | x | x | x | x | x | x | x | x | x | x | x | x | x | x | x | x | x | x | x | x | x | x | x | x | x | x | x | x | x | x | x | x | x | x | x | x | x | x | x | x | x | x | x | x | x | x | x | x | x | x | x | x | x | x | x | x | x | x | x | x | x | x | x | x | x | x | x | x | x | x | x | x | x | x | x | x | x | x | x | x | x | x | x | x | x | x | x | x | x | x | x | x | x | x | x | x | x | x | x | x | x | x | x | x | x | x | x | x | x | x | x | x | x | x | x | x | x | x | x | x | x | x | x | x | x | x | x | x | x | x | x | x | x | x | H | x | x | x | x | x | x | x | x | x | x | x | x | x | x | x | x | x | x | x | x | x | x | x | x | x | x | x | x | x | x | x | x | x | x | x | x | x | x | x | x | x | x | x | x | x | x | x | x | x | x | x | x | x | x | x | x | x | x | x | x | x | x | x | x | x | x | x | x | x | x | x | x | x | x | x | x | x | x | x | x | x | x | x | x | x | x | x | x | x | x | x | x | x | x | x | x | x | x | x | x | x | x | x | x | x | x | x | x | x | x | H | x | x | x | x | x | x | x | x | x | x | x | x | x | x | x | x | x | x | x | x | x | x | x | x | x | x | x | x | x | x | x | x | x | x | x | x | x | x | x | x | x | x | x | x | x | x | x | x | x | x | x | x | x | x | x | x | x | x | x | x | x | x | x | x | x | x | H | x | x | x | x | x | x | x | x | x | x | x | x | x | x | x | x | x | x | x | x | x | x | x | x | x | x | x | x | x | x | H | x | x | x | x | x | x | x | x | x | x | x | x | x | x | x | x | x | H | x | x | x | x | x | x | x | x | x | x | x | x | x | x | x | x | x | x | x | x | x | x | x | x | x | x | x | x | x | x | x | x | x | x | x | x | x | x | x | x | x | x | x | x | x | x | x | x | x | x | x | x | x | x | x | x | x | x | x | x | x | x | x | x | x | x | x | x | x | x | x | x | x | x | x | x | x | x | x | x | x | x | x | x | x | x | x | x | x | x | x | x | x | x | x | x | x | x | x | x | x | x | x | x | x | x | x | x | x | x | x | x | x | x | x | x | x | x | x | x | x | x | x | x | x | x | x | x | x | x | x | x | x | x | x | x | x | x | x | x | x | x | x | x | x | x | x | x | x | x | x | x | x | x | x | x | x | x | x | x | x | x | x | x | x | x | x | x | x | x | x | x | x | x | x | x | x | x | x | x | x | x | x | x | x | x | H | - | - | - | - | - | - | - | - | - | - | - | - | - | - | - | - | - | - | - | - | - | - | - | - | - | - | - | - | - | - | - | - | - | - | - |
| BIS d=0 cl.7 sym=1 env=1 | - | - | - | - | - | - | - | - | - | - | - | - | - | - | - | - | - | - | - | - | - | - | - | - | - | - | - | - | - | - | - | - | - | - | - | - | - | - | - | - | - | - | - | - | - | - | - | - | - | - | - | - | - | - | - | - | - | - | - | - | - | - | - | - | - | - | - | - | - | - | - | - | - | - | - | - | - | - | - | - | - | - | - | - | - | - | - | - | - | - | - | - | - | - | - | - | - | - | - | - | - | - | - | - | - | - | - | - | - | - | - | - | - | - | - | - | - | - | - | - | - | - | - | - | - | - | - | - | - | - | - | - | - | - | - | - | - | - | - | - | - | - | - | - | - | - | - | - | - | - | - | - | - | - | - | - | - | - | - | - | - | - | - | - | - | - | - | - | - | - | - | - | - | - | - | - | - | - | - | - | - | - | - | - | - | - | - | - | - | - | - | - | - | - | - | - | - | - | - | - | - | - | - | - | - | - | - | - | - | - | - | - | - | - | - | - | - | - | - | - | - | - | - | - | - | - | - | - | - | - | - | - | - | - | - | - | - | - | - | - | - | - | - | - | - | - | - | - | - | - | - | - | - | - | - | - | - | - | - | - | - | - | - | - | - | - | - | - | - | - | - | - | - | - | - | - | - | - | - | - | - | - | - | - | - | - | - | - | - | - | - | - | - | - | - | - | - | - | - | - | - | - | - | - | - | - | - | - | - | - | - | - | - | - | - | - | - | - | - | - | - | - | - | - | - | - | - | - | - | - | - | - | - | - | - | - | - | - | - | - | - | - | - | - | - | - | - | - | - | - | - | - | - | - | - | - | - | - | - | - | - | - | - | - | - | - | - | - | - | - | - | - | - | - | - | - | - | - | - | - | - | - | - | - | - | - | - | - | - | - | - | - | - | - | - | - | - | - | - | - | - | - | - | - | - | - | - | - | - | - | - | - | - | - | - | - | - | - | - | - | - | - | - | - | - | - | - | - | - | - | - | - | - | - | - | - | - | - | - | - | - | - | - | - | - | - | - | - | - | - | - | - | - | - | - | - | - | - | - | - | - | - | - | - | - | - | - | - | - | - | - | - | - | - | - | - | - | - | H | x | x | x | x | x | x | x | x | x | x | x | x | x | x | x | x | x | x | x | x | x | x | x | x | x | x | x | x | x | x | x | x | x | x | x | x | x | x | x | x | x | x | x | x | x | x | x | x | x | x | x | x | x | x | x | x | x | x | x | x | x | x | x | x | x | x | x | x | x | x | x | x | x | x | x | x | x | x | x | x | x | x | x | x | x | x | x | x | x | x | x | x | x | x | x | x | x | x | x | x | x | x | x | x | x | x | x | x | x | x | x | x | x | x | x | x | x | x | x | x | x | x | x | x | x | x | x | x | x | x | x | x | x | x | x | x | x | x | x | x | x | x | x | x | x | x | x | x | x | x | x | x | H | - | - | - | - | - | - | - | - | - | - | - | - | - | - | - | - | - | - | - | - | - | - | - | - | - | - | - | - | - | - | - | - | - | - | - | - | - | - | - | - | - | - | - | - | - | - | - | - | - | - | - | - | - | - | - | - | - | - | - | - | - | - | - | - | - | - | - | - | - | - | - | - | - | - | - | - | - | - | - | - | - | - | - | - | - | - | - | - | - | - | - | - | - | - | - | - | - | - | - | - | - | - | - | - | - | - | - | - | - | - | - | - | - | - | - | - | - | - | - | - | - | - | - | - | - | - | - | - | - | - | - | - | - | - | - | - | - | - | - | - | - | - | - | - | - | - | - | - | - | - | - | - | - | - | - | - | - | - | - | - | - | - | - | - | - | - | - | - | - | - | - | - | - | - | - | - | - | - | - | - | - | - | - | - | - | - | - | - | - | - | - | - | - | - | - | - | - | - | - | - | - | - | - | - | - | - | - | - | - | - | - | - | - | - | - | - | - | - | - | - | - | - | - | - | - | - | - | - | - | - | - | - | - | - | - | - | - | - | - | - | - | - | - | - | - | - | - | - | - | - | - | - | - | - | - | - | - | - | - | - | - | - | - | - | - | - | - | - | - | - | - | - | - | - | - | - | - | - | - | - | - | - | - | - | - | - | - | - | - | - | - | - | - | - | - | - | - | - | - | - | - | - | - | - | - | - | - | - | - | - | - | - | - | - | - | - | - | - | - | - | - | - | - | - | - | - | - | - | - | - | - | - | - | - | - | - | - | - | - | - | - | - | - | - | - | - | - | - | - | - | - | - | - | - | - | - | - | - | - | - | - | - | - | - | - | - | - | - | - | - | - | - | - | - | - | - | - | - | - | - | - | - | - |
| BIS d=0 cl.8 sym=1 env=1 | - | - | - | - | - | - | - | - | - | - | - | - | - | - | - | - | - | - | - | - | - | - | - | - | - | - | - | - | - | - | - | - | - | - | - | - | - | - | - | - | - | - | - | - | - | - | - | - | - | - | - | - | - | - | - | - | - | - | - | - | - | - | - | - | - | - | - | - | - | - | - | - | - | - | - | - | - | - | - | - | - | - | - | - | - | - | - | - | - | - | - | - | - | - | - | - | - | - | - | - | - | - | - | - | - | - | - | - | - | - | - | - | - | - | - | - | - | - | - | - | - | - | - | - | - | - | - | - | - | - | - | - | - | - | - | - | - | - | - | - | - | - | - | - | - | - | - | - | - | - | - | - | - | - | - | - | - | - | - | - | - | - | - | - | - | - | - | - | - | - | - | - | - | - | - | - | - | - | - | - | - | - | - | - | - | - | - | - | - | - | - | - | - | - | - | - | - | - | - | - | - | - | - | - | - | - | - | - | - | - | - | - | - | - | - | - | - | - | - | - | - | - | - | - | - | - | - | - | - | - | - | - | - | - | - | - | - | - | - | - | - | - | - | - | - | - | - | - | - | - | - | - | - | - | - | - | - | - | - | - | - | - | - | - | - | - | - | - | - | - | - | - | - | - | - | - | - | - | - | - | - | - | - | - | - | - | - | - | - | - | - | - | - | - | - | - | - | - | - | - | - | - | - | - | - | - | - | - | - | - | - | - | - | - | - | - | - | - | - | - | - | - | - | - | - | - | - | - | - | - | - | - | - | - | - | - | - | - | - | - | - | - | - | - | - | - | - | - | - | - | - | - | - | - | - | - | - | - | - | - | - | - | - | - | - | - | - | - | - | - | - | - | - | - | - | - | - | - | - | - | - | - | - | - | - | - | - | - | - | - | - | - | - | - | - | - | - | - | - | - | - | - | - | - | - | - | - | - | - | - | - | - | - | - | - | - | - | - | - | - | - | - | - | - | - | - | - | - | - | - | - | - | - | - | - | - | - | - | - | - | - | - | - | - | - | - | - | - | - | - | - | - | - | - | - | - | - | - | - | - | - | - | - | - | - | - | - | - | - | - | - | - | - | - | - | - | - | - | - | - | - | - | - | - | - | - | - | - | - | - | - | - | - | - | - | - | - | - | - | - | - | - | - | - | - | - | - | - | - | - | - | - | - | - | - | - | - | - | - | - | - | - | - | - | - | - | - | - | - | - | - | - | - | - | - | - | - | - | - | - | - | - | - | - | - | - | - | - | - | - | - | - | - | - | - | - | - | - | - | - | - | - | - | - | - | - | - | - | - | - | - | - | - | - | - | - | - | - | - | - | - | - | - | - | - | - | - | - | - | - | - | - | - | - | - | - | - | - | - | - | - | - | - | - | - | - | - | - | - | - | - | - | - | - | - | - | - | - | - | - | - | - | - | - | - | - | - | - | H | x | x | x | x | x | x | x | x | x | x | x | x | x | x | x | x | x | x | x | x | x | x | x | x | x | x | x | x | x | x | x | x | x | x | x | x | x | x | x | x | x | x | x | x | x | x | x | x | x | x | x | x | x | x | x | x | x | x | x | x | x | x | x | x | x | x | x | x | x | x | x | x | x | x | x | x | x | x | x | x | x | x | x | x | x | x | x | x | x | x | x | x | x | x | x | x | x | x | x | x | x | x | x | x | x | x | x | x | x | x | x | x | x | x | x | x | x | x | x | x | x | x | x | x | x | x | x | x | x | x | x | x | x | x | x | x | x | x | x | x | x | x | x | x | x | x | x | x | x | x | x | x | x | x | x | x | x | x | x | x | x | x | x | x | x | x | x | x | x | x | x | x | x | x | x | x | x | x | x | x | x | x | x | x | x | x | x | x | x | x | x | x | x | x | x | x | x | x | x | x | x | x | x | x | x | x | x | x | x | x | x | x | x | x | x | x | x | x | x | x | x | x | x | x | x | x | x | x | x | x | x | x | x | x | x | x | x | x | x | x | x | x | x | x | x | x | x | x | x | x | x | x | x | x | x | x | x | x | x | x | x | x | x | x | x | x | x | x | x | x | x | x | x | x | x | x | x | x | x | x | x | x | x | x | x | x | x | x | x | x | x | x | x | x | x | x | H | - | - | - | - | - | - | - | - | - | - | - | - | - | - | - | - | - | - | - | - | - | - | - | - | - | - | - | - | - | - | - | - | - | - | - | - | - | - | - | - | - | - | - | - | - | - | - | - | - | - | - | - | - | - | - | - | - | - | - | - | - | - | - | - | - | - | - | - | - | - | - | - | - | - | - | - | - | - | - | - | - | - | - | - | - | - | - | - | - |
| BIS d=0 cl.1 sym=1 env=1 | - | - | - | - | - | - | - | - | - | - | - | - | - | - | - | - | - | - | - | - | - | - | - | - | - | - | - | - | - | - | - | - | - | - | - | - | - | - | - | - | - | - | - | - | - | - | - | - | - | - | - | - | - | - | - | - | - | - | - | - | - | - | - | - | H | x | x | x | x | x | x | x | x | x | x | x | x | x | x | x | x | H | x | x | x | x | x | x | x | x | x | x | x | x | x | x | x | x | x | x | x | x | x | x | x | x | x | H | x | x | x | x | x | x | x | x | x | x | x | x | x | x | x | H | x | x | x | x | x | x | x | x | x | H | x | x | x | x | x | x | x | x | x | x | x | x | x | H | x | x | H | x | H | x | x | H | x | H | x | x | x | x | x | x | x | x | x | x | H | x | x | H | x | x | H | x | x | x | x | H | x | H | x | x | x | x | x | x | x | H | x | x | x | x | x | H | x | x | x | x | x | x | x | x | x | H | x | x | x | x | x | x | x | x | x | x | x | x | x | x | x | H | x | x | H | x | x | x | x | x | x | x | x | x | H | x | H | x | x | x | x | x | x | x | x | x | H | x | x | x | H | x | x | x | H | x | x | x | x | x | x | x | H | x | x | x | x | x | x | H | x | x | x | x | x | x | x | x | x | x | x | x | x | x | x | H | x | x | x | x | x | x | x | x | x | x | x | x | x | x | x | x | x | x | x | x | x | x | x | x | x | x | x | x | x | x | x | x | x | x | x | x | x | x | H | x | x | x | x | x | x | x | x | x | x | x | x | x | x | x | x | H | x | x | x | x | H | x | x | x | x | x | x | x | x | x | x | x | x | x | x | x | x | x | x | x | x | x | x | x | x | x | H | x | x | x | x | x | x | H | x | x | x | x | x | x | x | x | x | x | x | x | x | H | x | x | x | x | x | x | x | x | H | x | x | x | x | H | x | x | x | x | x | x | x | x | x | x | x | x | x | x | x | x | x | x | H | x | H | x | H | x | H | x | x | H | x | H | x | x | H | x | H | x | x | x | x | x | x | H | x | H | x | x | H | x | x | x | x | x | x | x | H | x | H | x | x | H | x | x | H | x | x | H | x | x | x | x | x | x | x | x | x | x | x | x | x | H | x | x | x | x | x | x | x | H | x | x | H | x | x | x | x | H | x | H | x | x | x | x | x | x | x | H | x | x | x | x | x | H | x | x | H | x | H | x | x | H | x | x | x | x | x | x | x | x | x | H | x | H | H | x | x | H | x | x | x | x | H | x | x | H | x | x | x | x | x | x | x | x | x | x | x | x | x | x | x | x | x | x | x | x | x | x | H | x | x | x | x | H | x | H | x | x | x | x | x | x | x | x | x | x | x | x | x | x | x | H | x | x | H | x | x | H | x | x | x | x | x | x | x | x | x | x | x | x | x | x | H | x | H | x | H | x | H | H | x | x | x | x | x | x | x | x | x | x | x | x | x | x | x | x | x | x | x | x | H | x | x | x | H | x | H | x | x | H | x | x | x | x | H | x | x | x | x | x | x | x | x | x | x | x | x | H | x | x | x | x | x | H | x | x | x | x | x | x | x | x | x | H | x | x | H | x | x | x | x | x | x | x | H | x | x | x | x | x | x | x | x | x | x | H | x | x | x | x | x | x | x | x | x | x | x | H | x | x | x | H | x | x | x | x | x | x | x | x | x | H | x | H | x | H | x | x | x | x | x | x | x | H | x | x | x | x | x | x | H | H | H | x | x | x | x | x | x | x | H | x | H | H | x | x | x | x | x | x | x | x | x | x | x | x | x | x | x | H | x | x | x | x | x | x | x | x | x | x | x | x | x | x | x | x | x | x | x | x | x | x | H | x | x | x | x | H | H | x | x | x | x | x | H | x | H | H | x | x | x | H | x | H | x | x | x | x | x | x | x | x | x | x | x | x | x | x | x | H | H | x | H | x | H | H | H | x | x | H | H | x | H | H | x | x | x | H | x | H | x | H | H | x | x | H | x | H | x | H | H | H | H | H | H | H | H | x | H | H | x | x | H | x | x | H | H | x | x | x | H | H | x | H | H | H | H | x | H | x | x | x | x | x | H | H | x | H | x | H | H | H | x | H | x | H | x | x | x | x | H | H | H | H | H | x | H | H | H | H | H | x | x | H | H | H | x | H | x | x | x | x | x | x | H | x | x | x | x | H | x | H | x | x | x | x | x | H | x | x | H | x | x | x | x | x | x | x | x | x | H | H | x | x | H | x | x | H | - | - | - | - | - | - | - | - | - | - | - | - | - | - | - | - | - | - | - | - | - | - | - | - | - | - | - | - | - | - | - |
