# Supplementary material for: Variation in selection constraints on teleost TLRs with emphasis on their repertoire in the Walking catfish, Clarias batrachus
Source: Sci Rep. 2020 Dec 7;10:21394. doi: 10.1038/s41598-020-78347-6 (PMC7721727; doi:10.1038/s41598-020-78347-6)
Supplement: Supplementary file 34 — Supplementary Information 34. [file 41598_2020_78347_MOESM34_ESM.zip › T22/bis2/summary.tgz!/summary/PF00000-NONREDUNDANT-5DD-dim1-table.html]

BIS cluster table


Clusters with env. score >= 0.5 and sym. score >= 0.5 :

| Dim | Cluster | Sym | Env | Pvalue | Hit patterns and blocks |
| --- | --- | --- | --- | --- | --- |
| 1 | 1 | 1 | 1 | 0 | Hit patterns: Positions: \| 99 \| 276; 8 sequences: \| T \| G; 6 sequences: \| N \| S; 2 sequences: \| K \| A; 1 sequence: \| R \| R All positions in cluster: 99 276 |
| 1 | 14 | 1 | 1 | 0 | Hit patterns: Positions: \| 412 \| 512; 11 sequences: \| S \| G; 3 sequences: \| Q \| Y; 2 sequences: \| R \| H; 1 sequence: \| K \| E All positions in cluster: 412 512 |
| 1 | 15 | 1 | 1 | 0 | Hit patterns: Positions: \| 495 \| 763; 9 sequences: \| N \| Y; 5 sequences: \| S \| N; 2 sequences: \| K \| G; 1 sequence: \| F \| S All positions in cluster: 495 763 |
| 1 | 16 | 1 | 1 | 7.345596e-06 | Hit patterns: Positions: \| 484 \| 899; 10 sequences: \| L \| G; 6 sequences: \| I \| S; 1 sequence: \| F \| A All positions in cluster: 484 899 |
| 1 | 21 | 1 | 1 | 7.345596e-06 | Hit patterns: Positions: \| 215 \| 219 \| 799 \| 837; 10 sequences: \| I \| L \| T \| N; 6 sequences: \| V \| S \| S \| Q; 1 sequence: \| X \| T \| A \| D All positions in cluster: 215 219 799 837 |
| 1 | 22 | 1 | 1 | 9.141186e-06 | Hit patterns: Positions: \| 154 \| 356 \| 635 \| 654; 8 sequences: \| A \| R \| G \| W; 8 sequences: \| N \| Q \| R \| Q; 1 sequence: \| K \| E \| Q \| N All positions in cluster: 154 356 635 654 |
| 1 | 4 | 1 | 1 | 1.346693e-05 | Hit patterns: Positions: \| 100 \| 242 \| 244 \| 414 \| 569 \| 797 \| 913 \| 915 \| 919 \| 966; 11 sequences: \| V \| N \| S \| P \| L \| S \| E \| E \| R \| S; 5 sequences: \| T \| T \| P \| T \| M \| C \| K \| N \| S \| R; 1 sequence: \| A \| S \| A \| N \| I \| V \| R \| X \| K \| K All positions in cluster: 100 242 244 414 569 797 913 915 919 966 |
| 1 | 17 | 1 | 1 | 1.346693e-05 | Hit patterns: Positions: \| 45 \| 493 \| 812; 11 sequences: \| L \| L \| Y; 5 sequences: \| X \| P \| W; 1 sequence: \| F \| T \| H All positions in cluster: 45 493 812 |
| 1 | 7 | 1 | 1 | 0.0004901961 | Hit patterns: Positions: \| 487 \| 792 \| 822; 14 sequences: \| A \| F \| A; 2 sequences: \| D \| L \| G; 1 sequence: \| V \| Y \| T All positions in cluster: 487 792 822 |
| 1 | 9 | 1 | 1 | 0.0004901961 | Hit patterns: Positions: \| 176 \| 741; 14 sequences: \| R \| D; 2 sequences: \| H \| G; 1 sequence: \| Y \| N All positions in cluster: 176 741 |
| 1 | 2 | 1 | 1 | 0.05882353 | All positions in cluster: 300 386 609 696 985 |
| 1 | 3 | 1 | 1 | 0.05882353 | All positions in cluster: 117 924 |
| 1 | 5 | 1 | 1 | 0.05882353 | All positions in cluster: 96 220 594 834 965 |
| 1 | 6 | 1 | 1 | 0.05882353 | All positions in cluster: 827 904 |
| 1 | 8 | 1 | 1 | 0.05882353 | All positions in cluster: 736 747 |
| 1 | 10 | 1 | 1 | 0.05882353 | All positions in cluster: 739 938 |
| 1 | 11 | 1 | 1 | 0.05882353 | All positions in cluster: 253 699 950 960 |
| 1 | 12 | 1 | 1 | 0.05882353 | All positions in cluster: 97 168 260 297 572 683 692 |
| 1 | 13 | 1 | 1 | 0.05882353 | All positions in cluster: 466 595 686 |
| 1 | 18 | 1 | 1 | 0.05882353 | All positions in cluster: 268 410 440 |
| 1 | 19 | 1 | 1 | 0.05882353 | All positions in cluster: 350 378 405 423 476 509 530 657 676 732 826 832 850 890 907 972 |
| 1 | 20 | 1 | 1 | 0.05882353 | All positions in cluster: 230 445 945 |
| 1 | 23 | 1 | 1 | 0.05882353 | All positions in cluster: 135 204 554 659 773 |
| 1 | 24 | 1 | 1 | 0.05882353 | All positions in cluster: 127 132 239 250 381 888 923 925 939 954 |

Table created with bis2html version 8.
