# Supplementary material for: Variation in selection constraints on teleost TLRs with emphasis on their repertoire in the Walking catfish, Clarias batrachus
Source: Sci Rep. 2020 Dec 7;10:21394. doi: 10.1038/s41598-020-78347-6 (PMC7721727; doi:10.1038/s41598-020-78347-6)
Supplement: Supplementary file 34 — Supplementary Information 34. [file 41598_2020_78347_MOESM34_ESM.zip › T22/bis2/summary.tgz!/summary/PF00000-NONREDUNDANT-5DD-dim2-table.html]

BIS cluster table


Clusters with env. score >= 0.5 and sym. score >= 0.5 :

| Dim | Cluster | Sym | Env | Pvalue | Hit patterns and blocks |
| --- | --- | --- | --- | --- | --- |
| 2 | 5 | 1 | 1 | 0 | Hit patterns: Positions: \| 217 \| 800; 10 sequences: \| Q \| C; 5 sequences: \| A \| I; 1 sequence: \| L \| V; 1 sequence: \| K \| S All positions in cluster: 217 800 |
| 2 | 6 | 1 | 1 | 0 | Hit patterns: Positions: \| 125 \| 173 \| 213 \| 271 \| 975; 8 sequences: \| T \| T \| H \| T \| E; 5 sequences: \| Q \| L \| K \| N \| K; 2 sequences: \| K \| S \| Q \| E \| G; 1 sequence: \| R \| Q \| R \| D \| R; 1 sequence: \| L \| K \| X \| K \| N All positions in cluster: 125 173 213 271 975 |
| 2 | 7 | 0.5714286 | 0.8627968 | 9.71251e-06 | Hit patterns: Positions: \| 415 \| 658; 8 sequences: \| A \| I; 7 sequences: \| N \| L; 1 sequence: \| D \| I; 1 sequence: \| D \| M All positions in cluster: 415 658 |
| 2 | 1 | 1 | 1 | 0.007352941 | All positions in cluster: 104 145 778 957 |
| 2 | 2 | 1 | 1 | 0.007352941 | All positions in cluster: 458 642 |
| 2 | 3 | 1 | 1 | 0.007352941 | All positions in cluster: 543 590 726 |
| 2 | 4 | 1 | 1 | 0.007352941 | All positions in cluster: 171 227 |

Table created with bis2html version 8.
